# Genetic Analysis of SARS-CoV-2 Variants in Mexico during the First Year of the COVID-19 Pandemic

**DOI:** 10.3390/v13112161

**Published:** 2021-10-26

**Authors:** Blanca Taboada, Selene Zárate, Pavel Iša, Celia Boukadida, Joel Armando Vazquez-Perez, José Esteban Muñoz-Medina, José Ernesto Ramírez-González, Andreu Comas-García, Concepción Grajales-Muñiz, Alma Rincón-Rubio, Margarita Matías-Florentino, Alejandro Sanchez-Flores, Edgar Mendieta-Condado, Jerome Verleyen, Gisela Barrera-Badillo, Lucía Hernández-Rivas, Fidencio Mejía-Nepomuceno, José Arturo Martínez-Orozco, Eduardo Becerril-Vargas, Susana López, Irma López-Martínez, Santiago Ávila-Ríos, Carlos F. Arias

**Affiliations:** 1 Departamento de Genética del Desarrollo y Fisiología Molecular, Instituto de Biotecnología, Universidad Nacional Autónoma de México, Cuernavaca 62210, Mexico; btaboada@ibt.unam.mx (B.T.); pavel@ibt.unam.mx (P.I.); susana@ibt.unam.mx (S.L.); 2 Posgrado en Ciencias Genómicas, Universidad Autónoma de la Ciudad de México, Mexico City 03100, Mexico; selene.zarate@uacm.edu.mx; 3 Centro de Investigación en Enfermedades Infecciosas, Instituto Nacional de Enfermedades Respiratorias Ismael Cosío Villegas, Mexico City 14080, Mexico; celia.boukadida@cieni.org.mx (C.B.); alma.rinconr@gmail.com (A.R.-R.); margarita.matias@cieni.org.mx (M.M.-F.); santiago.avila@cieni.org.mx (S.Á.-R.); 4 Instituto Nacional de Enfermedades Respiratorias Ismael Cosío Villegas, Mexico City 14080, Mexico; joevazpe@gmail.com (J.A.V.-P.); biolfimene@gmail.com (F.M.-N.); drjamoinfectologia@gmail.com (J.A.M.-O.); edobec.var@gmail.com (E.B.-V.); 5 División de Laboratorios de Vigilancia e Investigación Epidemiológica, Instituto Mexicano del Seguro Social, Mexico City 07760, Mexico; eban10@hotmail.com; 6 Instituto de Diagnóstico y Referencia Epidemiológicos, Dirección General de Epidemiología, Mexico City 01480, Mexico; ernesto.ramirez@salud.gob.mx (J.E.R.-G.); emendiet76@gmail.com (E.M.-C.); gisela.barrera20@yahoo.com.mx (G.B.-B.); lucia.hernandez@salud.gob.mx (L.H.-R.); lopezmi74@gmail.com (I.L.-M.); 7 Facultad de Medicina y Centro de Investigación en Ciencias de la Salud y Biomedicina, Universidad Autónoma de San Luis Potosí, San Luis Potosí 78120, Mexico; andreu.comas@uaslp.mx; 8 Coordinación de Control Técnico de Insumos, Instituto Mexicano del Seguro Social, Mexico City 07760, Mexico; concepcion.grajales@imss.gob.mx; 9 Unidad Universitaria de Secuenciación Masiva y Bioinformática, Instituto de Biotecnología, Universidad Nacional Autónoma de México, Cuernavaca 62210, Mexico; alexsf@ibt.unam.mx (A.S.-F.); jerome.verleyen@ibt.unam.mx (J.V.)

**Keywords:** viral diversity, SARS-CoV-2, genomic surveillance

## Abstract

During the first year of the SARS-CoV-2 pandemic in Mexico, more than two million people were infected. In this study, we analyzed full genome sequences from 27 February 2020 to 28 February 2021 to characterize the geographical and temporal distribution of SARS-CoV-2 lineages and identify the most common circulating lineages during this period. We defined six different geographical regions with particular dynamics of lineage circulation. The Northeast and Northwest regions were the ones that exhibited the highest lineage diversity, while the Central south and South/Southeast regions presented less diversity with predominance of a certain lineage. Additionally, by late February 2021, lineage B.1.1.519 represented more than 89% of all circulating lineages in the country.

## 1. Introduction

The Mexican government reported the first case of SARS-CoV-2 in Mexico on 28 February 2020 [1]; the first death was officially registered on 17 March 2020. One year after the first positive case was recorded, 2,084,128 cases and 185,257 deaths were reported, placing Mexico as one of the most severely affected countries in the world [2]. During the first year, the official cumulative incidence rate was 1646 cases/100,000 inhabitants, while the mortality rate was 146 deaths/100,000 inhabitants, for a lethality of 8.9% [3]. The median numbers of new daily reported infections and fatalities in Mexico during the first year were 5250 and 481, respectively. On 21 January 2021, the highest number of cases in one day (22,339) was reported, and on 16 January 2021, the highest number of daily deaths (2324) was attained (2). In Mexico, vaccinations started on 24 December 2020. By the end of February 2021, 1,889,672 inhabitants (1.5% of the population) had been vaccinated with the half vaccination scheme and 565,423 (0.4%) were fully vaccinated. During this period, the vaccines administered by the Mexican government were from Pfizer/BioNTech and AstraZeneca/Oxford pharmaceutical companies [4].

Genomic surveillance of SARS-CoV-2 has proven to be crucial to identify the appearance of mutations that change the phenotype of the virus, incrementing its transmissibility, immune escape, or reinfection risk. For instance, early in the pandemic, the D614G amino acid change identified in the spike protein that was described as spreading rapidly through Europe was later shown to increase transmissibility [5,6]. Most of the SARS-CoV-2 variants that circulate today descended from the lineage in which this mutation appeared. Other changes have been shown to increase transmissibility, including S:N501Y [7,8], which is present in most variants of concern. Additionally, changes close to the furin cleavage site, such as the S:P681H change, increase spike cleavage and possibly enhance virus infectivity [9]. In some lineages, these changes are coupled with mutations that permit the virus to escape from neutralizing antibodies (S:E484K, S:L452R) [10,11,12] elicited from previous infection or vaccination. These viruses are referred to as variants of concern (VOC) by the World Health Organization (WHO), posing an increased threat to global public health. The continuous monitoring of genetic changes is necessary to detect the appearance of such variants as early as possible.

We have previously described the first introductions of SARS-CoV-2 variants in Mexico and their phylogenomic characterization by whole genome sequencing [13]. We detected that there were two main introduction events and a low lineage diversity. In this work, we report the analysis of 3915 complete genome sequences of SARS-CoV-2, with collection dates between February 2020 and February 2021, from respiratory samples from all 32 states of Mexico; 1520 of them were obtained in this work. We describe the most common lineages circulating in the country during the first year of the pandemic and regional, temporal, and geographical differences found in the circulating variants. Understanding SARS-CoV-2 evolution is essential to determine its diversity, variation, and spread in the country and its effect on disease severity and potential impact on the effectiveness of diagnostics, vaccines, and therapeutics. Therefore, our analysis contributes to the national and global knowledge regarding SARS-CoV-2 and COVID-19 dynamics.

## 2. Materials and Methods

### 2.1. Ethical Statement and Sample Collection

The genome sequences used in this study and their associated metadata were obtained from residual RNA positive samples that are part of the national response to COVID-19, collected and processed under the Mexican Official NOM-017-SSA2-2012 for epidemiological surveillance of Viral Respiratory Disease emitted and approved by the CONAVE (National Counsel of Epidemiology Surveillance) of the Ministry of Health of the Government of Mexico, so neither Institutional Review Board (IRB) approval nor informed consent from patients were required.

Clinical specimens were collected in laboratories and hospitals belonging to the Ministry of Public Health of Mexico (Red Nacional de Laboratorios Estatales de Salud Publica (RNLSP); Instituto Nacional de Enfermedades Respiratorias (INER); and Instituto Mexicano del Seguro Social (IMSS) in all 32 states in Mexico from 27 February 2020, to 28 February 2021. Oropharyngeal or nasopharyngeal swabs and tracheal aspirates of PCR-confirmed SARS-CoV-2 cases were placed in virus transport medium following official procedures for further sample processing [14]. The procedure was performed using protocols validated by Instituto de Diagnóstico y Referencia Epidemiológicos (InDRE), Secretaria de Salud, Mexico, as approved by the World Health Organization [15]. In general, for RT-PCR, 5 μL of RNA were used in a 25 µL reaction using the Superscript III one-step RT-PCR system (Invitrogen, Darmstadt, Germany). Reverse transcription was performed at 55 °C:10 min, followed by a PCR reaction of 95 °C:3 min and then 95 °C:15 s—58 °C:30 s × 45 cycles. Samples selected for sequencing had a cycle threshold (Ct) of SARS-CoV-2 quantitative real-time (RT)-qPCR value of less than 26.

### 2.2. Sample Processing, Sequencing, and Viral Genome Assembly

Viral nucleic acid extraction was performed using a MagNa Pure LC 2-0 system (Roche, Indianapolis, OH, USA) or ExiPrep 96 Viral DNA/RNA Kit (LaunchWorks CDMO, Beverly, MA, USA). Total cDNA was synthesized by Superscript III Reverse Transcriptase (Thermo Fisher, Waltham, MA, USA) with primer 5′-GTTTCCCAGTAGGTCTCN9-3′. The second strand was generated by two rounds of synthesis with Sequenase 2.0 (Affymetrix, USB, Ohio, USA). The resulting product was used as input for an amplicon-based protocol with the POLAR method (https://www.protocols.io/view/pathogen-oriented-low-cost-assembly-amp-re-sequenc-bearjad6.html, accessed on 20 March 2020 and 5 December 2020). Later, the cDNA was separated into two pools for amplification using two sets of Artic v3 primers covering the whole SARS-CoV-2 genome. PCR reactions were performed on both pools as follows: 98 °C:30 s × 1 cycle; 98 °C:15 s; 65 °C:5 min × 30 cycles. PCR cleaning was performed using AmpPure (Beckman Coulter, Indianapolis, IN, USA) beads for quantification prior to Illumina library preparation. Amplicon libraries were pooled after normalization and loaded into an Illumina 300 cycles mid-output sequencing kit in the NextSeq500 sequencing platform (Illumina, San Diego, CA, USA) with a paired-end read configuration (2 × 150 bp reads).

Quality control, removal of duplicates, and off-target reads were performed as described previously [13]. These preprocessed reads were then mapped using Bowtie2 v2.3.4.3 (Johns Hopkins University, Baltimore, MD, USA) [16] against Wuhan-Hu-1 (MN908947) reference sequence, followed by consensus calling by iVar v1.3.1 (The Scripps Research Institute, La Jolla, CA, USA), with consensus minimum read depth of 20 [17]. In total, 1515 full virus genome sequences with a coverage ≥ 90% and a mean depth ≥ 1000×, were deposited in the Global Initiative on Sharing All Influenza Data (GISAID) platform [18] and GenBank (OK435170-OK436684; Supplementary Table S1).

### 2.3. Sequence Data Collation

In addition, 2400 complete Mexican genomes and their metadata, available in the GISAID platform, with collection dates between 17 February 2020 and 28 February 2021, were downloaded on 14 May 2021. The metadata of the 3915 Mexican sequences used in this work are reported in Supplementary Table S2.

### 2.4. Genomic Characterization

All 3915 Mexican sequences were aligned against the NCBI reference sequence from Wuhan-Hu-1 (NC_045512.2) using MAFFTv7 (option—addfragments) (Research Institute for Microbial Diseases, Osaka, Japan) [19]. An in-house python script was used to identify polymorphic sites and classify them as synonymous and nonsynonymous. Further, the number of sequences where these SNPs appeared was determined, discarding the SNPs presented in less than three sequences. A bar plot of the number of polymorphic sites in each ORF was built using R v.4.1.0 (R Developing Core Team) [20]. Additionally, a heatmap of nonsynonymous mutations through time was built for those with at least one frequency per month higher than 10%, using the package ComplexHeatmap in R.

### 2.5. Lineage Classification and Analysis

Phylogenetic Assignment of Named Global Outbreak LINeages (PANGOLIN v2.4.2 of 4 May 2021) software suite (https://github.com/cov-lineages/pangolin, accessed on 14 May 2021) was used to classify the 3915 Mexican sequences and identify the most common lineages of SARS-CoV-2 circulating during the first year of the pandemic. PANGO lineage and collection date were used to build a stacked density plot in R v4.1.0 with the package ggplot2 [21]. Lineages that represented less than 1% of the reported sequences were grouped and reported as rare. Additionally, in order to reduce sampling bias and limited sequencing depth in some states, the data were grouped by geographical regions, as follows: The Northwest (NW) includes Baja California, Baja California Sur, Chihuahua, Durango, Sonora, and Sinaloa states; Northeast (NE): Coahuila, Nuevo León, and Tamaulipas states; West (W): Colima, Jalisco, Michoacán, and Nayarit states; Central north (CN): Aguascalientes, Guanajuato, Querétaro, San Luis Potosi, and Zacatecas states; Central south (CS): Mexico City, Estado de México, Morelos, Hidalgo, Puebla, and Tlaxcala states; South/Southeast (S/SE): Guerrero, Oaxaca, Chiapas, Veracruz, Tabasco, Campeche, Yucatán, and Quintana Roo states (Figure S1). For each region, a stacked density plot was built as described above.

### 2.6. Phylogenetic Analyses

To build a tree containing the circulating lineages in Mexico during the pandemic’s first year, we carried out the following strategy. From the 3915 Mexican genomes, we selected 3030 complete genomes with high coverage, having less than 1% of Ns. An in-house python script (available upon request) was used to subset the sequences to evenly represent lineage diversity by region of Mexico and month. Lineages for which only one sequence was available were excluded. We used the following sampling strategy to select 200 sequences per region. Given that region W had fewer than 200 sequences, the remainder was evenly distributed between the rest. For each region, the sequences were divided evenly by month; if a month did not fill its quota, the rest was distributed among the others. Then, the available spots were distributed by lineage for each month, excluding lineages for which a single sequence was present and considering the frequency of each lineage. The sequences were then randomly selected from the pool of candidate sequences. A set of global worldwide reference sequences was constructed by sampling the oldest available sequence of the 750 most common PANGO lineages. An alignment of Mexican and reference sequences was carried out using MAFFT v7 [19], and then was manually edited to exclude the UTRs. A maximum-likelihood phylogeny was estimated in iqtree2 (IQ-TREE Development Team) [22] using the GTR + F + R2 substitution model [23]. The tree was later scaled using LSD2 and tip dates [24]. Tree visualization was performed in R using the packages ggtree and treeio [25,26].

To analyze the evolution of B.1.1.519 lineage, all high-coverage complete genome sequences for B.1.1.222 and B.1.1.519 lineages were downloaded from GISAID. An in-house python script (available upon request) was used to subset the sequences available from Mexico and the USA to no more than 20 per month per country for each lineage. All available sequences from other countries were also included in the analysis. The sequences were aligned using MAFFT as above, and the alignment was edited to eliminate the UTRs. The phylogeny was estimated as described above.

### 2.7. Statistical Analysis

Statistical analyses were conducted in Rv.4.1.0 statistical environment [20], using the Vegan package v.2.5.7 [27]. Richness, diversity (Invsimpson index), and Pielou’s evenness were calculated using the frequencies of SARS-CoV-2 variants. For beta diversity, Bray–Curtis distance metric was used. Then, frequencies were also used as input for a Canonical Correlation Analysis (CCA) [28] using Bray–Curtis distances. All results of metrics described above were used to compare geographical groups with a nonparametric multivariate permutation test (PERMANOVA), using the Adonis function with 999 permutations, and Mann–Whitney test for measures [28]; homogeneity variances between groups were verified in all comparisons. All statistics were considered significant if *p* < 0.05.

## 3. Results

### 3.1. Sample Distribution

**Figure 1:**
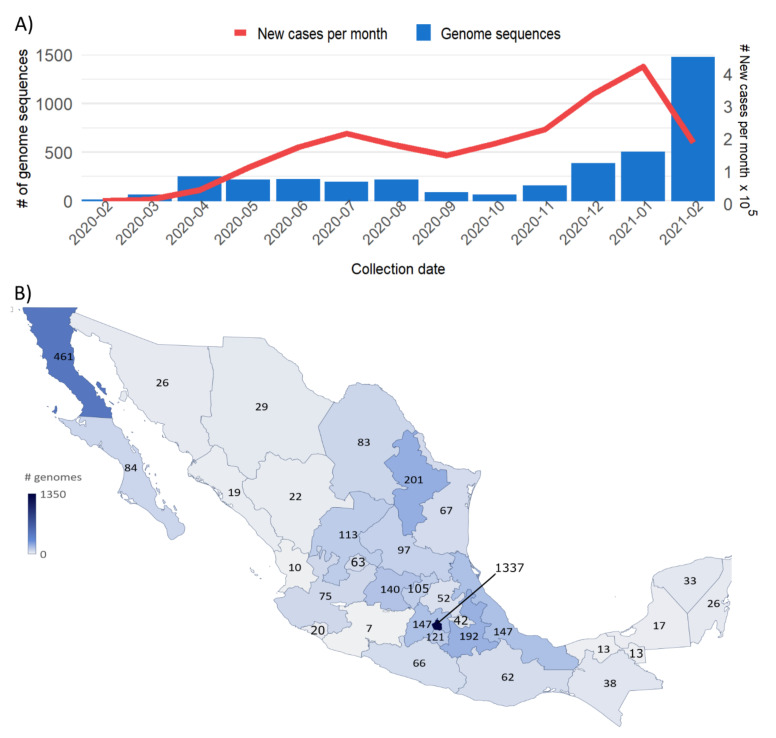
Geographical and monthly distribution of the 3915 Mexican SARS-CoV-2 genome sequences obtained from 27 February 2020 to 28 February 2021. (**A**) Number of national positive reported cases and SARS-CoV-2 genomes obtained per month. (**B**) Distribution of virus genomes by state. Adapted from [29] https://mapchart.net/mexico.html, accessed on 14 May 2021.

In total, 1515 sequences with a coverage higher than 90% of the SARS-CoV-2 genome were obtained in this study, with a mean depth of 2865×. These sequences were part of the 3915 sequences deposited in the GISAID database, collected in Mexico from 27 February 2020 to 28 February 2021 (Table S1). The temporal distribution of genomes correlated with the confirmed, positive COVID-19 cases (Spearman’s rho = 0.552, *p* = 0.05), suggesting that the number of sequences was proportional to the number of infections in Mexico during that time (Figure 1A). In agreement with international recommendations, these results excluded the last month because the genomes generated and deposited in GISAID in February 2021 increased significantly as a result of the implementation of national genomic surveillance programs. Regarding sampling, genome sequences were obtained from all 32 states in the country, although it was not homogeneous. Virus sequences from Baja California and Mexico City were significantly overrepresented (Figure 1B), while states such as Michoacán (7 genomes), Nayarit (10 genomes), Tabasco (13 genomes), Campeche (17 genomes), and Sinaloa (19 genomes) were poorly sampled.

### 3.2. Demographic Data Associated with the Sequences Analyzed

**Figure 2:**
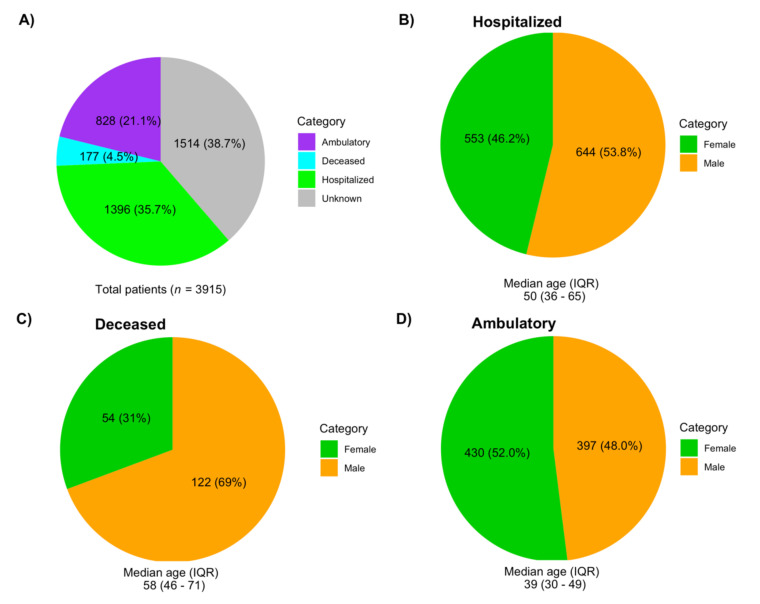
Demographic data of the 3915 patients whose genomes were included in this study. (**A**) Types of patients. (**B**) Gender composition of hospitalized patients. (**C**) Gender composition of deceased patients. (**D**) Gender composition of ambulatory patients.

Of the total samples analyzed, 42.4% were from females, 46.8% from males, and no information was available for 10.8% of the patients (Table S2). The median age was 44 years (IQR 32–58; minimum under 1 year of age and maximum 105 years). Regarding clinical status, 35.7% of the samples were obtained from hospitalized patients, 21.1% from ambulatory patients, and 4.5% were labeled as deceased; the clinical status for 38.7% of the patients was unknown (Figure 2A). The proportion of males was larger in hospitalized (chi squared test, *p* = 0.01) and deceased patients (chi squared test, *p* = 4.3 × 10^−7^), but not in ambulatory patients (Figure 2B–D).

### 3.3. SARS-CoV-2 Genomic Substitutions

The single-nucleotide polymorphisms (SNPs) in the genome of the 3915 analyzed viruses were determined using as reference the sequence of the original Wuhan-Hu-1 genome (Figure S2). ORF8, ORF3a, ORF7b, N, and S exhibited the largest proportion of nonsynonymous substitutions, and an excess of these substitutions compared with synonymous substitutions. Evidence for positive selection has been reported previously for ORF8 and ORF3a [30]. On the other hand, M and ORF7a showed an excess of synonymous substitutions. ORF1a and N had a similar frequency of both types of substitutions, although the frequency was four times higher in N. No SNPs were observed for ORF6 or E.

In total, 4278 different amino acid changes were found in the various ORFs of all lineages. Interestingly, only 65 changes were identified in more than 1% of the sequences and only 17 in more than 5%, implying a possible role for positive selection at these sites, although founder effects or hitchhiking events cannot be ruled out. As expected, the most frequent mutations corresponded to lineages that circulated commonly in the country during the first year of the pandemic. The two most frequent SNPs that led to an amino acid change were P314L in nsp12 protein (99%) and D614G in S (98%). Other frequent mutations in S were T732A (50%), P681H (41%), and T478K (38%). The high frequency observed for some of these mutations reflected their presence in viruses that circulated early after the pandemic started in the country—notably lineage B.1 and other lineages derived from it. In other cases, the predominance of a particular lineage that bore those mutations, such as lineage B.1.1.519 that contains the substitution S:T478K and is responsible for its high frequency, was as described in the following section.

**Figure 3:**
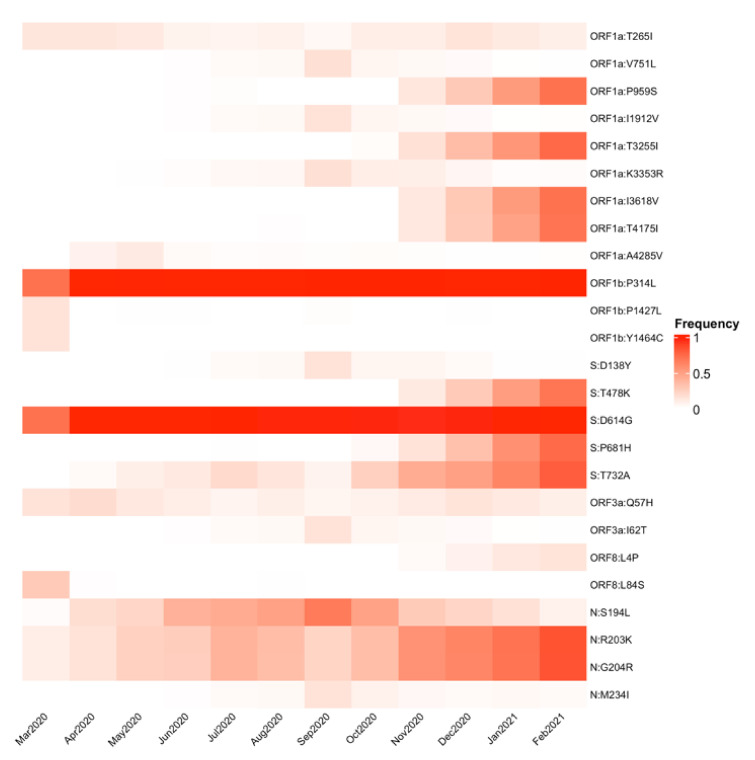
Heatmap of monthly amino acid mutation frequency. The heatmap shows the changes in mutation frequency from March 2020 to February 2021. Only nonsynonymous substitutions with a 10% frequency in at least one month are shown.

To understand the dynamics of these nonsynonymous mutations, we constructed a frequency heatmap. Figure 3 shows the monthly frequency of those nonsynonymous mutations that reached at least 10% in one month. Changes S:D614G and ORF1b:P314L were the most prevalent, starting in March 2020, congruent with their appearance in lineage B.1, from which many circulating lineages inherited them. Additionally, seven mutations jointly increased their frequency from November 2020, three of which were in the spike protein. These changes corresponded to lineage B.1.1.519, the most prevalent in Mexico, which was identified in November (see below). Two mutations in the N gene circulated throughout the year 2020 but increased their frequency after November. In general, Figure 3 shows temporal variations in the circulation of different substitutions and a steady increase in a group of mutations in the last months of the study.

### 3.4. Global Genetic Diversity of SARS-CoV-2 in Mexico

**Figure 4:**
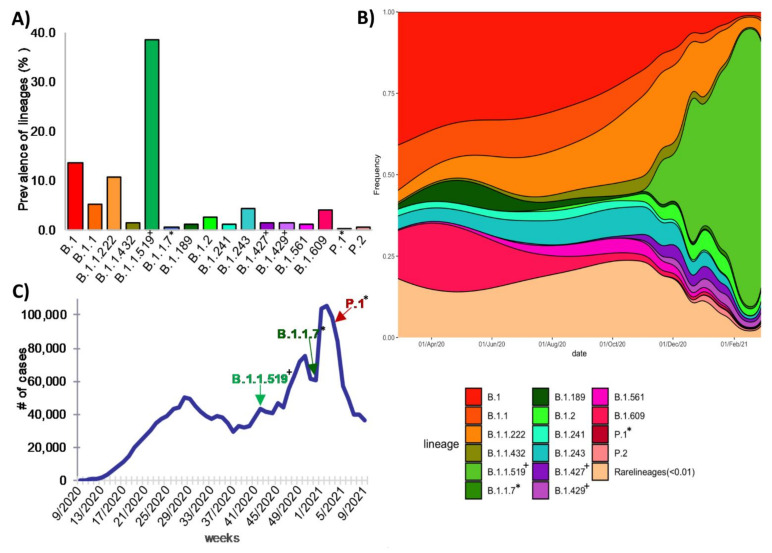
Circulation of SARS-CoV-2 lineages in Mexico during the first year of the pandemic. (**A**) Most frequent lineages. (**B**) Temporal distribution of lineages in Mexico between February 2020 and February 2021. (**C**) First detection of the B.1.1.519 lineage in the context of the COVID-19 epidemic curve, as well as Alpha (B.1.1.7) and Gamma (P.1) variants. * Variants of concern (VOCs): B.1.1.7 and P.1. ^+^ Variants under monitoring: B.1.1.9, B.1.427 and B.1.429.

To determine the genetic diversity of SARS-CoV-2 that circulated in the country during the first year of the pandemic, the sequences were first phylogenetically characterized with the global PANGO lineage tool. The selected genomes were assigned to 103 different global lineages, of which more than half (54 lineages) had only one or two associated genomes (Supplementary Table S3). In most cases, the low distribution of some lineages was not due to a sampling bias, because they were similar to a general tendency observed worldwide, being also at low abundance. Interestingly, we found that the worldwide prevalence of these low-frequency lineages was below 0.5%, and they were only identified for a limited period and then disappeared (Table S3), suggesting that the mutations they bear might reduce their ability to compete with other virus lineages; however, we cannot rule out the action of genetic drift resulting in the loss of these lineages by sampling effects independent of their fitness, given their low frequency. Regarding the variants most frequently identified in the country during the year of study, lineages B.1 (*n* = 530, 13.5%), B.1.1 (*n* = 204, 5.2%), B.1.1.222 (*n* = 424, 10.8%), B.1.1.519 (*n* = 1512, 38.6%), B.1.243 (*n* = 173, 4.4%) and B.1.609 (*n* = 158, 4.1%) were predominant. These six lineages represent more than 76.5% of the characterized genomes (Figure 4A).

We built a stacked density plot to visualize the proportion of the detected lineages along time (Figure 4B). Lineages whose frequency was less than 1% were grouped and labeled as rare lineages in the plot. An exception was made for lineages classified by the WHO as variants of concern (VOC), variants of interest (VOI), and variants under monitoring (VUM), given their importance. Although sequence sampling was neither spatially nor temporally homogeneous, some interesting trends could be seen (Figure 4B). In the first 3 months of the epidemic (March–May 2020), lineages B.1, B.1.1, B.1.609, and B.1.189 accounted for 75.4% of the genomic sequences. Around June 2020, all four lineages began to decline, representing only around 6% in February 2021. On the other hand, lineage B.1.1.222, which was not frequently identified during the first months (representing only 4.31% of the existent lineages), began to spread in May 2020 and was identified in 14% of sequences by November, and then declined to 11.8% during December 2020–February 2021. The same could be observed for B.1.241 and B.1.243 lineages, but they reached their highest prevalence in the second trimester of the pandemic and then started declining.

Few sequences were detected for VOC, VOI, and VUM other than B.1.1.519 during the first year. VOC B.1.1.7 (Alpha) and P.1 (Gamma), and VUM B.1.427, B.1.429, and P.2 were found in frequencies not higher than 2% (Table 1, Figure 4). B.1.427 and B.1.429 lineages were the earliest VUM identified in Mexico, in July 2020, and by February 2021 they had been detected in 19 different states. Variant Alpha was detected at the end of December for the first time and was found in six different states by February. Of particular interest is lineage B.1.1.519, which is considered a VUM; it was first detected in Mexico in November 2020, and then started to increase its frequency dramatically to become the dominant lineage by February 2021, representing 70% of the sequences at the national level, as previously reported [31]. Moreover, B.1.2 increased slowly, passing from less than 0.5% before December 2020 to more than 2% of all sequences in February 2021.

### 3.5. Regional Genetic Diversity

**Figure 5:**
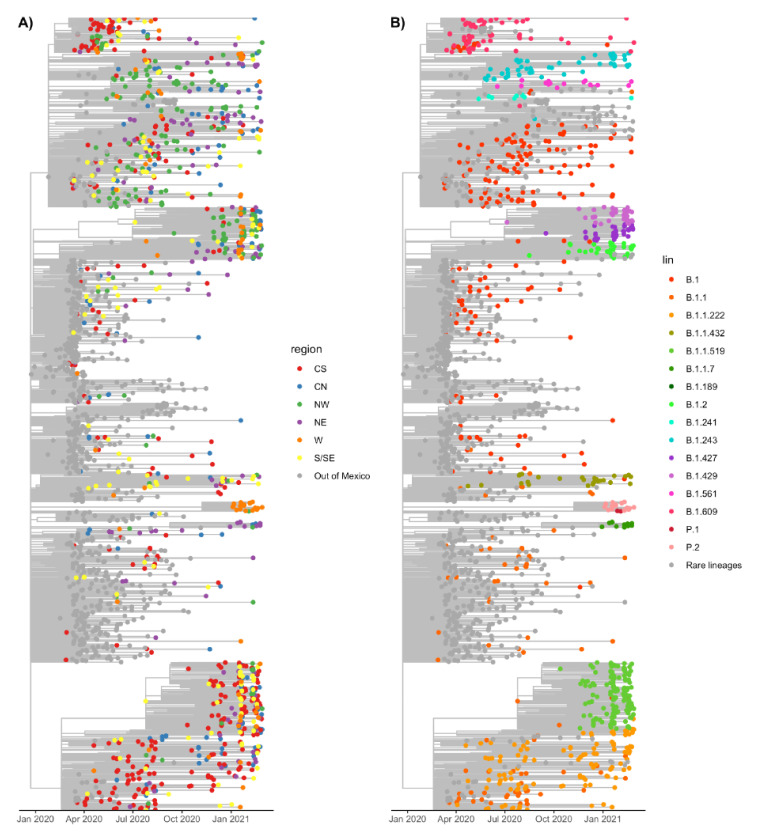
Maximum likelihood time-scaled phylogeny of Mexican and reference sequences. (**A**) The tips are colored by geographical region, while worldwide sequences are shown in grey. (**B**) Tips are colored by lineage. Lineages present in less than 1% of the sequences that do not correspond to VOC or VOM are shown in grey.

Given the sampling bias in some states, for further analysis we clustered the data into six geographical regions, as described in the Methods section (Figure S1). To determine the phylogenetic relationships and distribution of lineages in the country, we built a maximum likelihood scaled phylogenetic tree using a sample set of 1000 sequences from Mexico and, as reference, 700 sequences from the most common worldwide lineages. Figure 5A shows the tree with the tips colored by region, with the sequences sampled outside of Mexico colored in grey. In Figure 5B, the tips are colored by lineage; lineages whose frequencies in Mexico were less than 1% are shown in grey, except for VOC and VUM.

The phylogenetic reconstruction shown in Figure 5 revealed that there was no evident clustering of lineages by region, indicating a dynamic distribution and possibly multiple introductions into the country. However, exceptions to this observation were lineages B.1.1.222 and B.1.1.519, two of the most frequent in the country, which appeared first in the CS region, followed by their presence in S/SE, and spreading widely to other regions later. To analyze the regional lineage circulation in more detail, we built a stacked density plot for each region and evaluated their diversity and richness.

**Figure 6:**
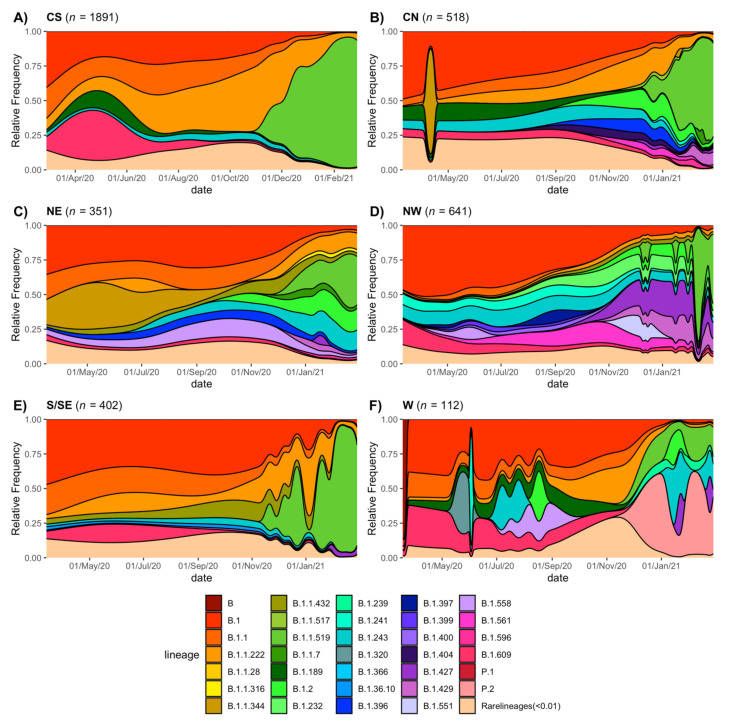
Circulation of SARS-CoV-2 lineages by region. Temporal distribution of lineages in each region (**A**) Central-South, (**B**) Central-North, (**C**) Northeast, (**D**) Northwest, (**E**) South/Southeast, and (**F**) West.

As shown in Figure 6 (panels A to F), lineage B.1 predominated early in the pandemic in the six regions, which also can be observed in Figure 5B, where many earlier tips correspond to B.1. Lineage B.1.609 was frequent in the CS, W, S/SE, and NW regions in the early months but sharply decreased around July 2020. This is also observed in the phylogenetic tree that shows B.1.609 as a tight cluster early in the pandemic. Additionally, B.1.1 was more abundant in CS, CN, NE, and S/SE, almost disappearing by January 2021. As stated before, lineage B.1.1.222 dominated the CS region from July to November 2020, but it was detected in all the other regions, albeit at lower frequencies or for limited periods of time. Lineage B.1.1.519 replaced B.1.1.222 in the CS (Figure 5 and Figure 6), rapidly rising to be the dominant lineage and representing 90% of all sampled genomes in this region by February 2021. The expansion of B.1.1.519 to other areas was delayed, increasing its frequency in January 2021 in the S/SE, CN, NE, and W regions. In contrast, in the NW region, the presence of this lineage was sporadic until February 2021.

Notably, VUM B.1.427 and B.1.429 were frequently sampled in the NW starting from December 2020, coinciding with their increase in California, USA. These results corroborated the same geographical and temporal patterns presented in the phylogenetic analysis. The VOC B.1.1.7 (Alpha) appeared predominantly in the NE region, whereas P2 was present only in the W area. The initial geographical detection of some of the variants mentioned above can be explained as a product of the international USA–Mexico border mobility of workers as well as the tourist flow (an introduction effect). Then, a local workflow between the larger cities can explain the spread of those variants to other regions, although multiple introductions cannot be ruled out.

To evaluate a possible sampling bias at the regional level over time in the interpretation of our results, we carried out Spearman correlation tests between the monthly distribution of genomes with positive cases in each region. We found that these correlations were strongly related to what is observed at the national level of cases-genomes of SARS-CoV-2; all correlation tests except those for regions NW and W had a rho larger than 0.59, with a *p* < 0.05. In the region W, low sampling could explain this absence of correlation, whereas in the NW area, the sampling was homogenous through time regardless of the number of cases. Additionally, a Kolmogorov–Smirnov two-sample test of lineage distribution was applied between the months preceding and following the low sampling period. We found that there was no statistically significant difference in lineage distribution in all regions, which we interpret as a low impact on sequencing bias in the lineage dynamics depicted in Figure 6.

**Figure 7:**
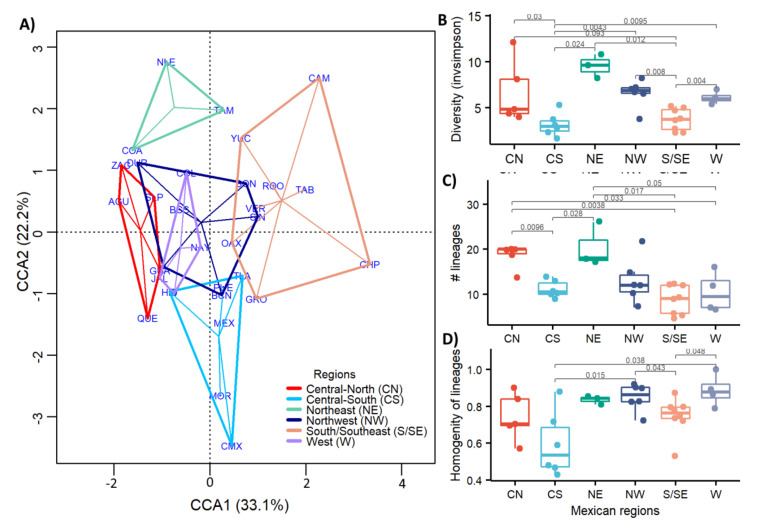
Diversity of lineage transmission comparison among the six regions along the year. (**A**) Canonical correlation analysis (CCA) of the different lineages. (**B**) Variant diversity index (H) by region. (**C**) Number of different lineages in each region. (**D**) Relative abundance (homogeneity) of lineages by region.

Overall, Figure 5 and Figure 6 reveal a difference in lineage diversity when comparing the six areas in their number, identity, and lineage replacement dynamics. The small number of sequences available for the W region may explain the intermittent detections of some lineages. The results show that although some lineages were widespread, they were present in different regions at different times, congruent with an observed abundance of rare lineages in some regions. Thus, we statistically evaluated the changes in the frequency of the different lineages among regions (Figure 7).

The canonical correlation analysis (Figure 7A) identified different patterns in population lineages between regions based on the genetic variation of the sequenced samples. Clearly, regions CS and S/SE and NE were significantly different (adonis with *p* < 0.5 and beta dispersion > 0.5 in all cases) from all other regions, as observed in Figure 7A. In the CS and S/SE regions, the lineage diversity was lower, as pointed out in the density plots of Figure 6. These regions were characterized by some dominant lineages, while in NE the diversity was the highest. Moreover, to understand better the diversity within and between regions, the frequency and abundance of the variants per region were evaluated and compared using InvSimpson’s diversity index (Figure 7B). The InvSimpson metric expresses effective diversity with less prone to biases introduced by incomplete or biased sampling. The lowest values obtained for the CS and S/SE regions (Figure 7B) confirmed, as shown in Figure 6A,B, a lower and dominant number of circulating lineages.

In the case of the CS region, the relative abundance or homogeneity of variants was also lower (Figure 7D), indicating that after one or more new variants appeared, one of them took over, reducing the variability. Moreover, the S/SE region had the lowest number of variants identified (Figure 7C). However, their relative abundances were high, suggesting that the low diversity value was probably due to a low sampling bias (Figure 1B) that resulted in a limited variant identification.

Conversely, NE was the region with the highest diversity and number of different lineages (Figure 7B,C), suggesting that different variants coexisted at a comparable relative frequency, followed by the NW and CN regions.

### 3.6. Lineages of National Interest

Lineages B.1.1.222 and B.1.1.519 are considered of national interest due their high circulation frequencies, especially in central Mexico. Both lineages have been reported to circulate mainly in the USA and Mexico, although they have been also described in Canada, South America, and Europe.

Lineage B.1.1.222 has five key nonsynonymous substitutions, some of which have been associated with changes in the virus phenotype: P314L in nsp12, D614G and T732A in the spike, and R203K and G204R in the nucleocapsid (Table 2) [5,32]. As shown in Table 3, lineage B.1.1.222 at its peak constituted about 14% of all sequenced genomes circulating in Mexico between July and November 2020. For the rest of the year, it was found at frequencies between 1.1% and 12%. Even though most reported sequences of B.1.1.222 are from the USA, it never accounted for more than 1% of the lineages reported in that country.

**Figure 8:**
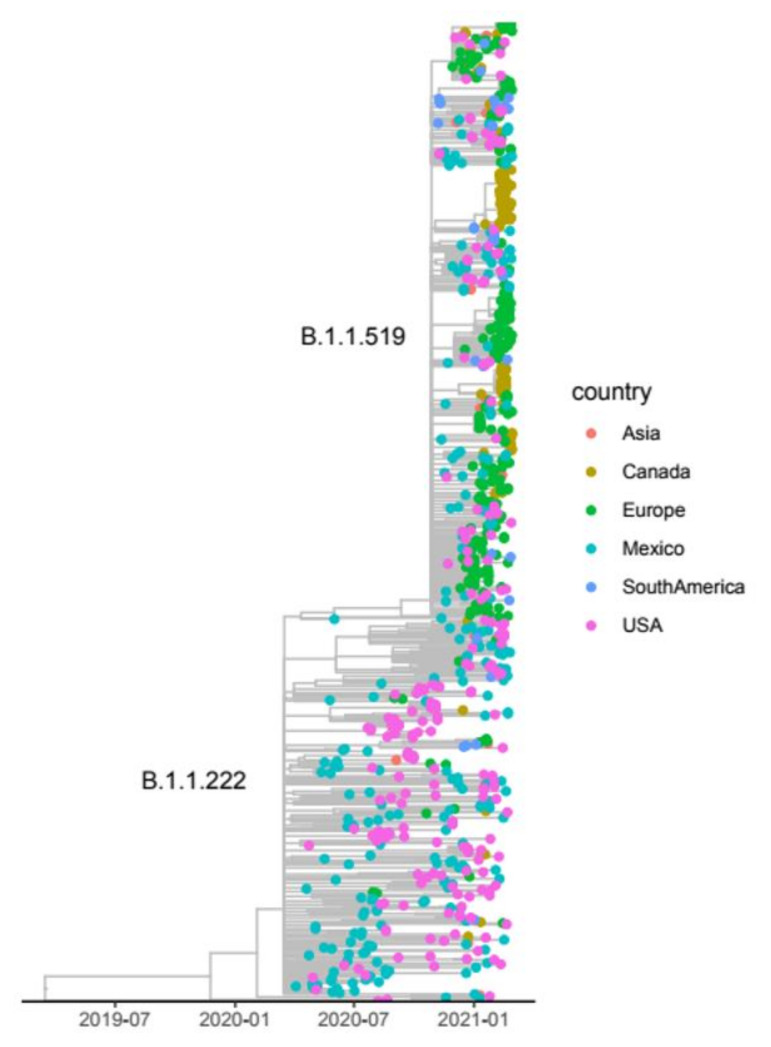
Maximum likelihood time-scaled phylogeny of lineages B.1.1.222 and B.1.1.519. The tips of the tree are colored by country/continent of sampling.

The evolution of lineage B.1.1.519 from B.1.1.222 and its rise to become the dominant lineage in various regions of Mexico has been reported previously [31], with the acquisition of six additional nonsynonymous substitutions (Table 2). Of these, two amino acid changes in S have been reported to have phenotypic effects. S:T478K was reported to increase receptor binding affinity and viral entry [10], and S:P681H [9] was reported to increase spike cleavage efficiency. To gain better insight into the evolution of B.1.1.519, we reconstructed a phylogeny using available sequences of lineages B.1.1.222 and B.1.1.519 from samples collected through February 2021. The phylogeny in Figure 8 shows that lineage B.1.1.222 circulated in Mexico and the USA and was rarely detected in other countries. Interestingly, while this lineage was the most frequently sampled in the center and south of Mexico until December 2020, it was not detected at high frequency in the north (Figure 6A–E), where the lineage diversity was the largest.

Lineage B.1.1.519 was first detected in Mexico in November 2020, and sequences from Argentina and the USA predate this earliest detection by 4 and 12 days, respectively. In November, B.1.1.519 accounted for 6.1% of the total reported sequences from Mexico, while it accounted for 4.1% in Argentina and only 0.02% in the USA (Table 3). The frequencies of B.1.1.519 rapidly increased in Mexico City, indicating a possibly undetected circulation in September and October 2020, because the number of sequences obtained in these 2 months was considerably lower (see Table 3). Most of the B.1.1.519 sequences from November and December 2020 reported in GISAID are from Mexico, suggesting that the origin of this lineage may be in central Mexico (see Figure 5A), where it spread rapidly afterwards, reaching the CN and S/SE regions in January 2021. Additionally, by February 2021, a sharp increase in frequency in the NW and NE regions was observed. The spread pattern could be explained by the workflow between the largest cities between regions. By February 2021, the overall frequency of this lineage in Mexico increased up to 62.3%, whereas it reached only 2.7% in the USA and 3.5% in Argentina.

## 4. Discussion

In this study, we aimed to characterize the genomic diversity of SARS-CoV-2 variants that circulated in Mexico during the first year of the pandemic. To this end, we analyzed 3915 genomes, of which 1515 were sequenced in this work. Metadata analysis revealed that a more significant burden of disease was observed in men, representing a larger proportion of hospitalized and deceased patients, as has been previously reported [35]. The median age of hospitalized patients was 50, and 58 years for the deceased, an age younger than expected in comparison to other countries [36]. The high prevalence of co-morbidities such as obesity and diabetes in the Mexican population may explain this younger age for severe disease [37]. However, it cannot be ruled out that the lower median age in Mexico (28 years) as compared to European countries (>40 years) and the USA (38 years) also impacted the observed ages for hospitalized and deceased patients.

The number of sequenced genomes throughout the year roughly corresponds with the incidence curve at the national level; however, a strong geographical bias is present, given that in some states the sampling was very limited. Therefore, we clustered sequences by geographical region to attain enough power to compare lineage diversity and homogeneity between regions. It is important to highlight that the W region had the lower number of sequences, and consequently, the results in this region should be interpreted with caution.

Interestingly, the CS region exhibited the lowest lineage diversity of SARS-CoV-2. This region includes the two states with the highest population density per km2, Mexico City, and the State of Mexico, with more than 26 million inhabitants (Supplementary Table S4). Additionally, Mexico City harbors the main international airport, being the busiest airport in Mexico and Latin America. However, land communication is the main route between cities and states; airports are mainly used for connections abroad and do not exceed road traffic. For example, in 2019, the number of passengers traveling by plane in Mexico was 149,178,000 and by land, 3,773,000,000 [38]. This observation suggests that initial entry points for SARS-CoV-2 lineages could be by air travelers; most likely, the dispersion between cities occurred by land. In addition, the other states in the CS region are very interconnected with Mexico City, contributing to the wide transmission of some variants and reducing the spread of novel lineages. This phenomenon completely contrasts with what has been reported in other countries, in which the regions with the highest diversity of SARS-CoV-2 lineages corresponded to the cities with the highest population, frequent local travelers, and where the main international airports are located [39,40,41,42,43]. Moreover, these reports showed a modest diversification of lineages in less densely populated areas, with less intense interconnections with other countries. In contrast, regions NE and NW, which have the lowest population density, exhibit the highest viral diversity in Mexico, probably due to low competition among the lineages, allowing the persistence of low fitness viruses. Even more critical is probably the fact that the states in the NW region have high connectivity with the USA, facilitating many viral introductions into these states. For instance, the most prominent lineages in these regions are those present in the USA border states, as is the case for lineages B.1.427 and B.1.429 that originated in California and were commonly detected in the NW of Mexico.

After one year of the pandemic in Mexico, we observed distinct patterns of lineage distribution and dissemination throughout the country. In particular, the larger number of sequences obtained from the central and north regions allowed us to determine their different dynamics. For example, successive lineages dominated the pandemic in central Mexico between February 2020 and February 2021, starting with B.1, B.1.1.222, and B.1.1.519. A striking observation was the emergence of the lineage B.1.1.519 that quickly displaced other lineages. The genome of B.1.1.519 has 11 characteristic nonsynonymous substitutions as compared to the original Wuhan-1 virus. Five of these substitutions in nsp12, S, and N were inherited from B.1.1.222, from which the B.1.1.519 lineage evolved. Substitutions nsp12:P314L and S:D614G were reported first in lineage B.1 and are part of the common background of all VOC and VOI circulating today. Of interest, changes N:R203K, and N:G204R in the nucleocapsid, which are also present in VOC B.1.1.7 and P.1, have been shown to increase viral replication and infectivity in vitro in a lung cell line, as well as in animal models [32]; these changes in the nucleocapsid seem also to be associated with an increase in disease severity [32]. The substitution S:T732A has not been associated with changes in phenotype.

B.1.1.519 harbors six additional amino acid substitutions, four in ORF1a and two in the spike protein. Change nsp3:T4175I has been identified as evolving under pervasive and episodic positive selection [44], but the phenotypic importance of this mutation remains to be established. The two amino acid changes in the spike protein seem to be of relevance. Substitution S:P681H, shared with VOC B.1.1.7 and B.1.351, has also been identified as evolving under strong pervasive and episodic positive selection and overlaps with a CTL epitope [44]. More importantly, this mutation has been shown to increase cleavage of the spike, but the impact on viral infectivity remains unclear [9]. Perhaps the most interesting change regarding the rapid spread of the virus is S:T478K, located in the receptor binding domain RBD. This amino acid change has been reported to have a significant increase in the binding free energy to ACE2 of around 1 kcal/mol according to a modeling prediction [45]; interestingly, in the same work the observed binding free energy for N501Y and L423R mutants increased only by 0.55 and 0.58 kcal/mol, respectively. The substitution T478S was also identified when RBD mutations with increased affinity for ACE2 were selected by in vitro evolution [46]; in that work the dominant mutations were E484K and N501Y. Additionally, amino acid substitution T478I showed a reduced neutralization by two monoclonal antibodies and by convalescent serum from two patients [47].

The combination of these amino acid substitutions, including the T478K mutation, as well as the potential ability of B.1.1.519 to evade the partial immunity of the population, may have influenced the rapid dispersal of the virus, eliminating other circulating variants. Despite becoming the dominant variant in Mexico and harboring mutations of interest, B.1.1.519 was not declared a VOC or VOI. However, on 2 June 2021, it was designated by the WHO as a VUM. Interestingly, lineage B.1.1519 did not become dominant in any other country. Given the apparent advantage in the spread of this lineage, it is puzzling that it never attained a frequency higher than 3% in the USA, which was the country with the second-highest prevalence. It is important to consider that viral fitness of a lineage is relative to other lineages circulating in the region. The diversity of lineages in the USA was probably different at the time of B.1.1.519′s introduction. Moreover, stochastic transmission events, especially while lineage frequency is low, also impact lineage fate, and high fitness variants can be fortuitously lost. However, characterization of the B.1.1.519 lineage in vitro and in animal models is needed to further explore its real relevance.

The dynamics of lineage diversity in the CN, NE, and NW regions was markedly different from that in the CS, since at almost all time points a large diversity of lineages was observed in these regions. Notably, starting in December 2020, a relatively high frequency of lineages B.1.427 and B.1.429 was found in the NW. This observation is not surprising given that these lineages were first detected in California, and most of the Mexican sequences in this region were from Baja California, particularly from the border city of Tijuana. Furthermore, lineages B.1.241, B.2.243, B.1.2, and B.1.561 that circulated at different times in the NW were reported concomitantly in California. In addition, the CN, NE, and NW regions share many lineages that seem to have circulated primarily in Mexico. One interesting example is lineage B.1.243, which started circulating in the CN region in April 2020 at frequencies around 30% until October, when it started to decline. This lineage appeared in the NE in September and has been maintained at intermediate frequencies of around 15%. Moreover, it was present in the NW throughout 2020 at frequencies that oscillated between 5% to 15%.

The limitation of our analysis, as mentioned before, is that the sequenced samples represent a small proportion of all confirmed cases in Mexico during the first year of the pandemic, which might not be representative of all circulating lineages. Thus, our findings on epidemiological patterns or the spread of variants should be interpreted with caution. However, our results highlight the importance of genomic surveillance to determine the geographic dissemination of virus variants, information that can guide public health policies at the regional and local levels. In the light of the several VOC and VOI that have been recently described, including the Alpha, Beta, Gamma, and Delta variants, it is of utmost importance to continue the genomic surveillance in Mexico as well as in all other countries, and to identify viral mutations that may be associated with differences in the epidemiological or clinical features of these viruses.

## Figures and Tables

**Table 1:** VOC/VUM variants identified in the first year of the pandemic in Mexico.

	VOC		VUM		
Variants	Alpha (B.1.1.7)	Gamma (P.1)	B.1.427 + B.1.429	B.1.1.519	P.2
Identified for the first time	UK	Japan	USA—California	Multiple	Brazil
First date identified in Mexico	31 December 2020	28 January 2021	20 July 2020	11 November 2021	4 January 2021
Number of samples	20	3	119	1512	23
Number of states where they were identified	6	3	19	30	2

**Table 2:** Reported mutations in lineages B.1.1.222 and B.1.1.519.

Mutation	Presence in B.1.1.222	Presence in B.1.1.519	Presence in VOCs and VOIs	Phenotypic Effect (If Known)
ORF1a:P959S	X	✓	No	No
ORF1a:T3255I	X	✓	Delta sublineages and Lambda	No
ORF1a:I3618V	X	✓	No	No
ORF1a:T4175I	X	✓	No	No
ORF1b:P314L	✓	✓	Present in all B.1 derived lineages	No
S:T478K	X	✓	Delta	Increased receptor binding, cell entry, and transmissibility [33,34]
S:D614G	✓	✓	Present in all B.1 derived lineages	Increase transmissibility [5,6]
S:P681H	X	✓	Alpha, Mu	Increased spike cleavage [1,9]
S:T732A	✓	✓	No	
N:R203K	✓	✓	Alpha, Gamma, Lambda	Increased infectivity and disease severity [32]
N:G204R	✓	✓	Alpha, Gamma, Lambda	Increased infectivity and disease severity [32]

**Table 3:** Monthly percentage of sequences classified as B.1.1.222 and B.1.1.519.

Month	B.1.1.222		B.1.1.519	
	Mexico	USA	Mexico	USA
2020-04	2.10	0.02	0.00	0.00
2020-05	6.60	0.01	0.00	0.00
2020-06	8.80	0.01	0.00	0.00
2020-07	13.90	0.07	0.00	0.00
2020-08	7.30	0.41	0.00	0.00
2020-09	1.30	0.67	0.00	0.00
2020-10	5.60	0.90	0.00	0.00
2020-11	12.10	0.97	6.10	0.02
2020-12	4.70	1.00	11.5	0.15
2021-01	6.70	0.65	36.50	1.11
2021-02	6.00	0.43	62.30	2.69

## Data Availability

The generated sequences of SARS-CoV-2 used in this study have been publicly shared through the Global Initiative on Sharing All Influenza Data (GISAID) repository and have also been deposited in the Genbank NCBI database. Accession numbers are listed in Supplementary Table S1.

## Supplementary Materials

The following are available online at https://www.mdpi.com/article/10.3390/v13112161/s1, Figure S1. The six regions of Mexico used in the analyses. Figure S2. Number of synonymous and nonsynonymous single nucleotide polymorphisms (SNPs) per site by ORF. Table S1. Description of full-length SARS-CoV-2 Mexican genomes generated and used in this study. Table S2. Patient demographic and clinical characteristics. Table S3. List of variants that appears one or two times throughout the year in Mexico. Table S4. Population density in each federal state of Mexico. Table S5. GISAID acknowledgements table.

## References

[1] Garcés-Ayala F., Araiza-Rodríguez A., Mendieta-Condado E., Rodríguez-Maldonado A.P., Wong-Arámbula C., Landa-Flores M., del Mazo-López J.C., González-Villa M., Escobar-Escamilla N., Fragoso-Fonseca D.E. (2020). Full Genome Sequence of the First SARS-CoV-2 Detected in Mexico. Arch. Virol..

[2] Johns Hopkins Coronavirus Resource Center Mortality Analyses -Johns Hopkins Coronavirus Resource Center. https://coronavirus.jhu.edu/data/mortality.

[3] Instituto Nacional de Salud Pública INSP Tablero Interactivo Sobre COVID 19. https://www.insp.mx/informacion-institucional-covid-19.html.

[4] Mathieu E., Ritchie H., Ortiz-Ospina E., Roser M., Hasell J., Appel C., Giattino C., Rodés-Guirao L. (2021). A Global Database of COVID-19 Vaccinations. Nat. Hum. Behav..

[5] Volz E., Hill V., McCrone J.T., Price A., Jorgensen D., O’Toole Á., Southgate J., Johnson R., Jackson B., Nascimento F.F. (2021). Evaluating the Effects of SARS-CoV-2 Spike Mutation D614G on Transmissibility and Pathogenicity. Cell.

[6] Hou Y.J., Chiba S., Halfmann P., Ehre C., Kuroda M., Dinnon K.H., Leist S.R., Schäfer A., Nakajima N., Takahashi K. (2020). SARS-CoV-2 D614G Variant Exhibits Efficient Replication Ex Vivo and Transmission in Vivo. Science.

[7] Tian F., Tong B., Sun L., Shi S., Zheng B., Wang Z., Dong X., Zheng P. (2021). N501Y Mutation of Spike Protein in SARS-CoV-2 Strengthens Its Binding to Receptor ACE2. eLife.

[8] Davies N.G., Abbott S., Barnard R.C., Jarvis C.I., Kucharski A.J., Munday J.D., Pearson C.A.B., Russell T.W., Tully D.C., Washburne A.D. (2021). Estimated Transmissibility and Impact of SARS-CoV-2 Lineage B.1.1.7 in England. Science.

[9] Lubinski B., Tang T., Daniel S., Jaimes J.A., Whittaker G.R. (2021). Functional Evaluation of Proteolytic Activation for the SARS-CoV-2 Variant B.1.1.7: Role of the P681H Mutation. bioRxiv.

[10] Wilhelm A., Toptan T., Pallas C., Wolf T., Goetsch U., Gottschalk R., Vehreschild M.J.G.T., Ciesek S., Widera M. (2021). Antibody-Mediated Neutralization of Authentic SARS-CoV-2 B.1.617 Variants Harboring L452R and T478K/E484Q. Viruses.

[11] Deng X., Garcia-Knight M.A., Khalid M.M., Servellita V., Wang C., Morris M.K., Sotomayor-González A., Glasner D.R., Reyes K.R., Gliwa A.S. (2021). Transmission, Infectivity, and Neutralization of a Spike L452R SARS-CoV-2 Variant. Cell.

[12] Nonaka C.K.V., Franco M.M., Gräf T., Barcia C.A.d.L., Mendonça R.N.d.Á., de Sousa K.A.F., Neiva L.M.C., Fosenca V., Mendes A.V.A., de Aguiar R.S. (2021). Genomic Evidence of SARS-CoV-2 Reinfection Involving E484K Spike Mutation, Brazil. Emerg. Infect. Dis..

[13] Taboada B., Vazquez-Perez J.A., Muñoz-Medina J.E., Ramos-Cervantes P., Escalera-Zamudio M., Boukadida C., Sanchez-Flores A., Isa P., Mendieta-Condado E., Martínez-Orozco J.A. (2020). Genomic Analysis of Early SARS-CoV-2 Variants Introduced in Mexico. J. Virol..

[14] Instituto de Diagnóstico y Referencia Epidemiológicos Lineamientos Para La Toma, Manejo y Envió de Muestras Para El Diagnóstico a La Red Nacional de Laboratorios de Salud Pública; 2020. https://www.gob.mx/cms/uploads/attachment/file/558702/Lineamientos_TMEM_2020_180620.pdf.

[15] Corman V.M., Landt O., Kaiser M., Molenkamp R., Meijer A., Chu D.K., Bleicker T., Brünink S., Schneider J., Schmidt M.L. (2020). Detection of 2019 Novel Coronavirus (2019-NCoV) by Real-Time RT-PCR. Eurosurveillance.

[16] Langmead B., Salzberg S.L. (2012). Fast Gapped-Read Alignment with Bowtie 2. Nat. Methods.

[17] Grubaugh N.D., Gangavarapu K., Quick J., Matteson N.L., De Jesus J.G., Main B.J., Tan A.L., Paul L.M., Brackney D.E., Grewal S. (2019). An Amplicon-Based Sequencing Framework for Accurately Measuring Intrahost Virus Diversity Using PrimalSeq and IVar. Genome Biol..

[18] Shu Y., McCauley J. (2017). GISAID: Global Initiative on Sharing All Influenza Data—from Vision to Reality. Euro Surveill.

[19] Katoh K., Standley D.M. (2013). MAFFT Multiple Sequence Alignment Software Version 7: Improvements in Performance and Usability. Mol. Biol. Evol..

[20] R Core Team R (2021). A Language and Environment for Statistical Computing.

[21] Wickham H. (2016). Ggplot2: Elegant Graphics for Data Analysis.

[22] Minh B.Q., Schmidt H.A., Chernomor O., Schrempf D., Woodhams M.D., von Haeseler A., Lanfear R. (2020). IQ-TREE 2: New Models and Efficient Methods for Phylogenetic Inference in the Genomic Era. Mol. Biol. Evol..

[23] Kalyaanamoorthy S., Minh B.Q., Wong T.K.F., Haeseler A., von Jermiin L.S. (2017). ModelFinder: Fast Model Selection for Accurate Phylogenetic Estimates. Nat. Methods.

[24] To T.-H., Jung M., Lycett S., Gascuel O. (2016). Fast Dating Using Least-Squares Criteria and Algorithms. Syst. Biol..

[25] Yu G., Smith D.K., Zhu H., Guan Y., Lam T.T.-Y. (2017). Ggtree: An r Package for Visualization and Annotation of Phylogenetic Trees with Their Covariates and Other Associated Data. Methods Ecol. Evol..

[26] Wang L.-G., Lam T.T.-Y., Xu S., Dai Z., Zhou L., Feng T., Guo P., Dunn C.W., Jones B.R., Bradley T. (2020). Treeio: An R Package for Phylogenetic Tree Input and Output with Richly Annotated and Associated Data. Mol. Biol. Evol..

[27] Oksanen J., Blanchet F.G., Kindt R., Legendre P., Minchin P.R., O’hara R.B., Simpson G.L., Solymos P., Stevens M.H.H., Wagner H. Package Vegan: Community Ecol. Package Version 2.5.7, 2014, R Package. https://cran.ism.ac.jp/web/packages/vegan/vegan.pdf.

[28] Anderson M.J. (2001). A New Method for Non-Parametric Multivariate Analysis of Variance. Austral Ecol..

[29] Mexico | MapChart. https://mapchart.net/mexico.html.

[30] Velazquez-Salinas L., Zarate S., Eberl S., Gladue D.P., Novella I., Borca M.V. (2020). Positive Selection of ORF1ab, ORF3a, and ORF8 Genes Drives the Early Evolutionary Trends of SARS-CoV-2 During the 2020 COVID-19 Pandemic. Front. Microbiol..

[31] Rodríguez-Maldonado A.P., Vázquez-Pérez J.A., Cedro-Tanda A., Taboada B., Boukadida C., Wong-Arámbula C., Nuñez-García T.E., Cruz-Ortiz N., Barrera-Badillo G., Hernández-Rivas L. (2021). Emergence and Spread of the Potential Variant of Interest (VOI) B.1.1.519 of SARS-CoV-2 Predominantly Present in Mexico. Arch. Virol..

[32] Wu H., Xing N., Meng K., Fu B., Xue W., Dong P., Xiao Y., Liu G., Luo H., Zhu W. (2021). Nucleocapsid Mutation R203K/G204R Increases the Infectivity, Fitness and Virulence of SARS-CoV-2. bioRxiv.

[33] Liu C., Ginn H.M., Dejnirattisai W., Supasa P., Wang B., Tuekprakhon A., Nutalai R., Zhou D., Mentzer A.J., Zhao Y. (2021). Reduced Neutralization of SARS-CoV-2 B.1.617 by Vaccine and Convalescent Serum. Cell.

[34] Ren W., Ju X., Gong M., Lan J., Yu Y., Long Q., Zhang Y., Zhong J., Zhong G., Wang X. (2021). Characterization of SARS-CoV-2 Variants B.1.617.1 (Kappa), B.1.617.2 (Delta) and B.1.618 on Cell Entry, Host Range, and Sensitivity to Convalescent Plasma and ACE2 Decoy Receptor. bioRxiv.

[35] Jin J.-M., Bai P., He W., Wu F., Liu X.-F., Han D.-M., Liu S., Yang J.-K. (2020). Gender Differences in Patients With COVID-19: Focus on Severity and Mortality. Front. Public Health.

[36] O’Driscoll M., Ribeiro Dos Santos G., Wang L., Cummings D.A.T., Azman A.S., Paireau J., Fontanet A., Cauchemez S., Salje H. (2021). Age-Specific Mortality and Immunity Patterns of SARS-CoV-2. Nature.

[37] Kammar-García A., Vidal-Mayo J.d.J., Vera-Zertuche J.M., Lazcano-Hernández M., Vera-López O., Segura-Badilla O., Aguilar-Alonso P., Navarro-Cruz A.R. (2020). Impact of Comorbidities in Mexican SARS-CoV-2-Positive Patients: A Retrospective Analysis in a National Cohort. Rev. Investig. Clin..

[38] Secretaria de Comunicacione y Transportes Estadística Mensual Del Sector de Comunicaciones y TRansportes. http://www.sct.gob.mx/fileadmin/DireccionesGrales/DGP/estadistica/Indicador-Mensual/INDI-2020/CI-ENERO_2020.pdf.

[39] Alteri C., Cento V., Piralla A., Costabile V., Tallarita M., Colagrossi L., Renica S., Giardina F., Novazzi F., Gaiarsa S. (2021). Genomic Epidemiology of SARS-CoV-2 Reveals Multiple Lineages and Early Spread of SARS-CoV-2 Infections in Lombardy, Italy. Nat. Commun..

[40] Franco D., Gonzalez C., Abrego L.E., Carrera J.-P., Diaz Y., Caicedo Y., Moreno A., Chavarria O., Gondola J., Castillo M. (2021). Early Transmission Dynamics, Spread, and Genomic Characterization of SARS-CoV-2 in Panama. Emerg. Infect. Dis..

[41] du Plessis L., McCrone J.T., Zarebski A.E., Hill V., Ruis C., Gutierrez B., Raghwani J., Ashworth J., Colquhoun R., Connor T.R. (2021). Establishment and Lineage Dynamics of the SARS-CoV-2 Epidemic in the UK. Science.

[42] Hodcroft E.B., Zuber M., Nadeau S., Vaughan T.G., Crawford K.H.D., Althaus C.L., Reichmuth M.L., Bowen J.E., Walls A.C., Corti D. (2021). Spread of a SARS-CoV-2 Variant through Europe in the Summer of 2020. Nature.

[43] Wells C.R., Sah P., Moghadas S.M., Pandey A., Shoukat A., Wang Y., Wang Z., Meyers L.A., Singer B.H., Galvani A.P. (2020). Impact of International Travel and Border Control Measures on the Global Spread of the Novel 2019 Coronavirus Outbreak. Proc. Natl. Acad. Sci. USA.

[44] Pond S. Natural Selection Analysis of Global SARS-CoV-2/COVID-19. https://observablehq.com/@spond/revised-sars-cov-2-analytics-page.

[45] Wang R., Chen J., Gao K., Wei G.W. (2021). Vaccine-Escape and Fast-Growing Mutations in the United Kingdom, the United States, Singapore, Spain, India, and Other COVID-19-Devastated Countries. Genomics.

[46] Zahradník J., Marciano S., Shemesh M., Zoler E., Chiaravalli J., Meyer B., Rudich Y., Dym O., Elad N., Schreiber G. (2021). SARS-CoV-2 RBD in Vitro Evolution Follows Contagious Mutation Spread, yet Generates an Able Infection Inhibitor. bioRxiv.

[47] Liu Z., VanBlargan L.A., Bloyet L.-M., Rothlauf P.W., Chen R.E., Stumpf S., Zhao H., Errico J.M., Theel E.S., Liebeskind M.J. (2021). Identification of SARS-CoV-2 Spike Mutations That Attenuate Monoclonal and Serum Antibody Neutralization. Cell Host Microbe.

