# Supplementary material for: Genetic Analysis of SARS-CoV-2 Variants in Mexico during the First Year of the COVID-19 Pandemic
Source: Viruses. 2021 Oct 26;13(11):2161. doi: 10.3390/v13112161 (PMC8622467; doi:10.3390/v13112161)
Supplement: Supplementary file 1 [file viruses-13-02161-s001.zip › Supplementary_Figures.pdf]

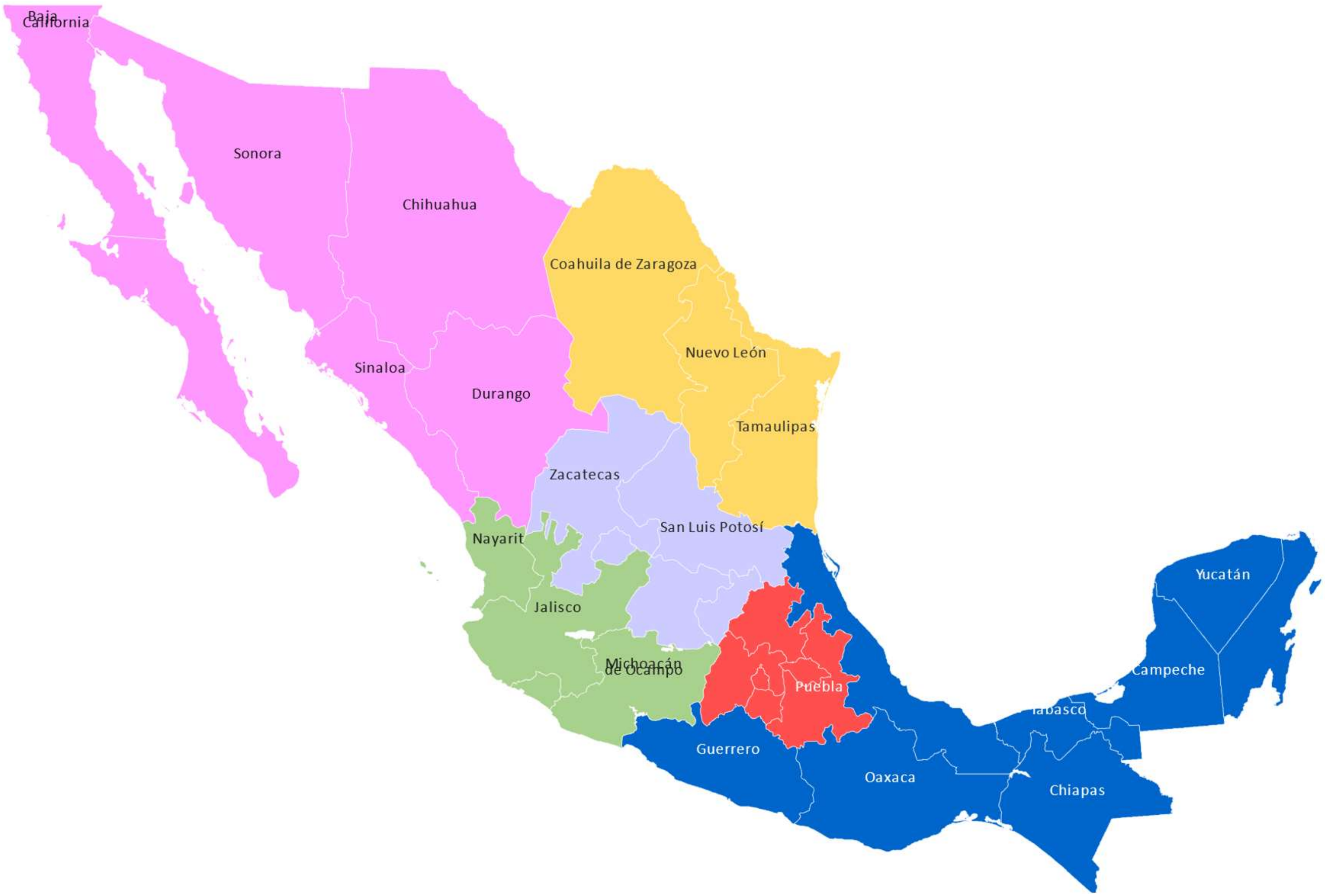

Supplementary Figure S1. The six regions of Mexico used in the analyises. Northwest (NW) in pink, Northeast (NE) in yellow, West (W) in green, Central north (CN) in lilac, Central south (CS) in red and South/Southeast (S/SE) in blue.

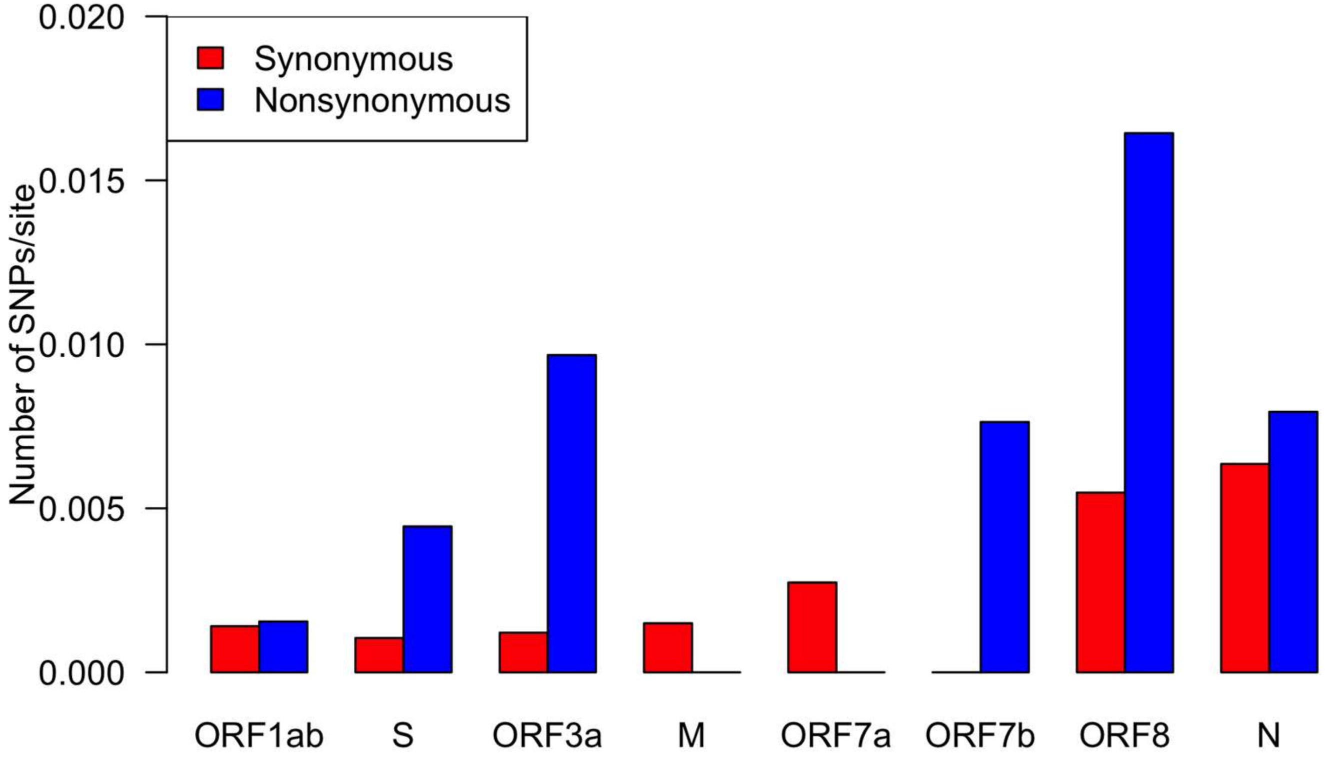

Supplementary Figure S2. Number of synonymous and nonsynonymous single nucleotide polymorphisms (SNPs) per site by ORF. Only SNPs presented in at least three of the 3915 analyzed sequences were considered.
