# Supplementary material for: Genetic Analysis of SARS-CoV-2 Variants in Mexico during the First Year of the COVID-19 Pandemic
Source: Viruses. 2021 Oct 26;13(11):2161. doi: 10.3390/v13112161 (PMC8622467; doi:10.3390/v13112161)
Supplement: Supplementary file 1 [file viruses-13-02161-s001.zip › SupplementaryTable_S1.pdf]

Table S1. Description of full-length SARS-CoV-2 Mexican genomes generated and used in this study. The 1520 genome sequences generated by us are shown in blue.

Virus name	Accession ID	Genbank Id	Nextstrain Clade	Pango lineage (v2.2.1, 21-02-06)	Changes compared to the reference genome of Wohan				
					# of nt	nt substitutions	# of aa	aa changes	Del
hCoV-19/Mexico/AGU-InDRE-IBT-2320/NC/2020	EPI_ISL_3463645	In process	20B	B.1.1.344	11	5'UTR:C241T, ORF1ab:C3037T, ORF1ab:T9172C, ORF1ab:A10948G, ORF1ab:C14408T, ORF1ab:T16950C, S:A23403G, S:G23608T, M:G26730T, N:G28881A, N:G28882A, N:G28883C,	5	M:V70F, N:R203K, N:G204R, ORF1b:P314L, S:D614G,	
hCoV-19/Mexico/AGU-InDRE-IBT-308408/NC/2020	EPI_ISL_3463440	In process	20G	B.1.2	21	5'UTR:C241T, ORF1ab:C1059T, ORF1ab:C3037T, ORF1ab:G6975T, ORF1ab:G6977A, ORF1ab:G7360T, ORF1ab:C10232T, ORF1ab:C10319T, ORF1ab:C14408T, ORF1ab:A18424G, ORF1ab:C18657T, ORF1ab:G20060T, S:G22017T, ORF3a:G25563T, ORF3a:G25907T, ORF8:C27964T, N:A28343G, N:C28472T, N:C28677T, N:C28869T, N:G29348T,ORF1ab:C17130T,	18	N:T24A, N:P67S, N:T135I, N:P199L, N:A359S, ORF1a:T265I, ORF1a:S2237I, ORF1a:V2238I, ORF1a:M2365I, ORF1a:R3323C, ORF1a:L3352F, ORF1b:P314L, ORF1b:N1653D, ORF1b:S2198I, ORF3a:Q57H, ORF3a:G172V, ORF8:S24L, S:W152L,	
hCoV-19/Mexico/AGU-InDRE-IBT-308412/NC/2020	EPI_ISL_3463601	In process	20B	B.1.1.222	15	5'UTR:C241T, ORF1ab:T1452A, ORF1ab:A2444G, ORF1ab:C3037T, ORF1ab:T3295C, ORF1ab:C10165T, ORF1ab:G12514T, ORF1ab:A13498G, ORF1ab:C14408T, ORF1ab:T19839C, S:G22331A, S:A23756G, ORF3a:C25886T, N:G28881A, N:G28882A, N:G28883C,	9	N:R203K, N:G204R, ORF1a:I396N, ORF1a:T727A, ORF1b:T11A, ORF1b:P314L, ORF3a:S165F, S:G257S, S:T732A,	ORF7b:27879-27891,
hCoV-19/Mexico/AGU-InDRE-IBT-308472/NC/2020	EPI_ISL_3463509	In process	20A	B.1.243	19	5'UTR:C241T, ORF1ab:G806A, ORF1ab:C3037T, ORF1ab:T3700C, ORF1ab:C5140A, ORF1ab:C6027T, ORF1ab:C6578T, ORF1ab:C12439T, ORF1ab:C14408T, ORF1ab:C18486T, ORF1ab:C18555T, ORF1ab:C19524T, ORF1ab:C21057T, S:A23403G, S:G23587C, S:T24076C, S:C24334T, E:C26455T, N:C28854T, N:G29543T,	9	E:P71S, N:S194L, ORF1a:A181T, ORF1a:D1625E, ORF1a:P1921L, ORF1a:L2105F, ORF1b:P314L, S:D614G, S:Q675H,	
hCoV-19/Mexico/AGU-InDRE-IBT-308483/NC/2020	EPI_ISL_3463485	In process	20A	B.1.561	22	5'UTR:C241T, ORF1ab:G1685A, ORF1ab:G2516T, ORF1ab:C3037T, ORF1ab:A5999G, ORF1ab:A10323G, ORF1ab:C10798T, ORF1ab:C11866T, ORF1ab:C14408T, ORF1ab:C14599T, ORF1ab:C18693T, S:G21974T, S:A23403G, ORF3a:T25577C, ORF3a:C25714T, ORF3a:T26123C, ORF7a:C27434T, ORF7a:C27741T, ORF8:T27941G, N:G28703C, N:C28854T, N:G28975T,N:C28511A,	16	N:D144H, N:S194L, N:M234I, ORF1a:A474T, ORF1a:V751L, ORF1a:I1912V, ORF1a:K3353R, ORF1b:P314L, ORF3a:I62T, ORF3a:L108F, ORF3a:V244A, ORF7a:T14I, ORF8:F16L, S:D138Y, S:D614G,N:P80T,	
hCoV-19/Mexico/AGU-InDRE-IBT-308486/NC/2020	EPI_ISL_3463377	In process	20G	B.1.2	19	5'UTR:C241T, ORF1ab:C1059T, ORF1ab:C3037T, ORF1ab:C3874T, ORF1ab:C10319T, ORF1ab:G13960A, ORF1ab:C14408T, ORF1ab:A18424G, ORF1ab:T19434C, ORF1ab:G20060T, S:C21727T, S:A23403G, S:G25354T, ORF3a:G25563T, ORF3a:G25785T, ORF3a:G25907T, ORF6:C27247T, ORF8:C27964T, N:C28472T. N:C28869T.	12	N:P67S, N:P199L, ORF1a:T265I, ORF1a:L3352F, ORF1b:V165I, ORF1b:P314L, ORF1b:N1653D, ORF1b:S2198I, ORF3a:Q57H, ORF3a:W131C, ORF3a:G172V, ORF8:S24L,	S:21665
hCoV-19/Mexico/AGU-InDRE-IBT-308487/NC/2020	EPI_ISL_3463497	In process	21C (Epsilon)	B.1.427	25	5'UTR:C186T, 5'UTR:C241T, ORF1ab:C1059T, ORF1ab:C3037T, ORF1ab:C3817T, ORF1ab:T6148C, ORF1ab:C8895T, ORF1ab:C8950T, ORF1ab:G9738C, ORF1ab:C13019T, ORF1ab:G13713A, ORF1ab:C14408T, ORF1ab:C16394T, ORF1ab:G17014T, S:G21600T, S:G22018T, S:G22335T, S:C22597T, S:T22917G, S:A23403G, ORF3a:G25563T, M:C26681T, ORF8:C28087T, ORF8:A28272T, N:C28887T, N:C29362T,	14	N:T205I, ORF1a:T265I, ORF1a:T2877I, ORF1a:S3158T, ORF1b:P314L, ORF1b:P976L, ORF1b:D1183Y, ORF3a:Q57H, ORF8:A65V, S:S13I, S:W152C, S:W258L, S:L452R, S:D614G,	
hCoV-19/Mexico/BCN-InDRE-IBT-31425/NC/2020	EPI_ISL_3463396	In process	20A	B.1	8	5'UTR:C241T, ORF1ab:C3037T, ORF1ab:C4331T, ORF1ab:C14408T, ORF1ab:C16041T, S:C22264T, S:A23403G, ORF6:G27204T, N:C28854T,	4	N:S194L, ORF1b:P314L, ORF6:M1I, S:D614G,	

hCoV-19/Mexico/CHH-InDRE-IBT-32583/NC/2020	EPI_ISL_3463574	In process	20A	B.1.1	8	5'UTR:C241T, ORF1ab:C3037T, ORF1ab:T4092C, ORF1ab:G9269A, ORF1ab:C14408T, S:C22314A, S:A23403G, ORF3a:A25756G,ORF1ab:G9267C,	7	ORF1a:I1276T, ORF1a:G3002S, ORF1b:P314L, ORF3a:R122G, S:P251H, S:D614G,ORF1a:S3001T,	
hCoV-19/Mexico/CHP-IBT-IMSS-18228/NC/2020	EPI_ISL_3463399	In process	20A	B.1	5	5'UTR:C241T, ORF1ab:C3037T, ORF1ab:T4092C, ORF1ab:C14408T, S:A23403G, N:G28378T,	4	ORF1a:I1276T, ORF1b:P314L, ORF9b:R32L, S:D614G,	
hCoV-19/Mexico/CHP-InDRE-IBT-24337/NC/2020	EPI_ISL_3463526	In process	20B	B.1.1	8	5'UTR:C241T, ORF1ab:C3037T, ORF1ab:C14408T, ORF1ab:T19839C, S:T22328C, S:A23403G, N:G28881A, N:G28882A, N:G28883C,	5	N:R203K, N:G204R, ORF1b:P314L, S:S256P, S:D614G,	
hCoV-19/Mexico/CHP-InDRE-IBT-308232/NC/2020	EPI_ISL_3463515	In process	20A	B.1.36.10	14	5'UTR:C241T, ORF1ab:T1877C, ORF1ab:C3037T, ORF1ab:C13372T, ORF1ab:C14408T, S:C21855T, S:G22225A, S:A23403G, ORF3a:A25910G, E:C26447T, ORF8:C27925T, ORF8:A27943G, N:C28647T, N:C28854T,ORF1ab:C6363T,	11	E:S68F, N:A125V, N:S194L, ORF1a:S538P, ORF1b:P314L, ORF3a:D173G, ORF8:T11I, ORF8:H17R, S:S98F, S:D614G,ORF1a:A2033V,	
hCoV-19/Mexico/CHP-InDRE-IBT-308281/NC/2020	EPI_ISL_3463442	In process	20B	B.1.1.519	23	5'UTR:T201C, 5'UTR:C203T, 5'UTR:C222T, 5'UTR:C241T, ORF1ab:T277C, ORF1ab:G1738T, ORF1ab:C3037T, ORF1ab:C3140T, ORF1ab:G7829T, ORF1ab:C10029T, ORF1ab:C10954T, ORF1ab:A11117G, ORF1ab:C12789T, ORF1ab:C14408T, ORF1ab:T19839C, ORF1ab:A19974G, S:C22995A, S:A23403G, S:C23604A, S:A23756G, N:G28881A, N:G28882A, N:G28883C, N:C29197T,	12	N:R203K, N:G204R, ORF1a:P959S, ORF1a:V2522F, ORF1a:T3255I, ORF1a:I3618V, ORF1a:T4175I, ORF1b:P314L, S:T478K, S:D614G, S:P681H, S:T732A,	
hCoV-19/Mexico/CHP-InDRE-IBT-308290/NC/2021	EPI_ISL_3463505	In process	20A	B.1.561	23	5'UTR:C222T, 5'UTR:C241T, ORF1ab:C527T, ORF1ab:G2516T, ORF1ab:C3037T, ORF1ab:C3510T, ORF1ab:A4636G, ORF1ab:A5999G, ORF1ab:A10323G, ORF1ab:C10798T, ORF1ab:C11866T, ORF1ab:C14408T, ORF1ab:G16917T, ORF1ab:C18693T, S:C21614T, S:G21974T, S:A23403G, S:C24378T, ORF3a:T25577C, ORF8:C27998T, N:C28854T, N:G28975T, N:C29144T,S:A24813G,	13	N:S194L, N:M234I, ORF1a:V751L, ORF1a:A1082V, ORF1a:I1912V, ORF1a:K3353R, ORF1b:P314L, ORF3a:I62T, S:L18F, S:D138Y, S:D614G, S:S939F,S:D1084G,	
hCoV-19/Mexico/CHP-InDRE-IBT-308298/NC/2020	EPI_ISL_3463432	In process	20B	B.1.1.222	13	5'UTR:C241T, ORF1ab:C1059T, ORF1ab:C3037T, ORF1ab:C6285T, ORF1ab:A13498G, ORF1ab:C14408T, ORF1ab:T19839C, S:G22331A, S:A23403G, S:A23756G, M:C26527T, N:G28881A, N:G28882A, N:G28883C,	10	M:A2V, N:R203K, N:G204R, ORF1a:T265I, ORF1a:T2007I, ORF1b:T11A, ORF1b:P314L, S:G257S, S:D614G, S:T732A,	ORF7b:27879-27891,
hCoV-19/Mexico/CHP-InDRE-IBT-308302/NC/2020	EPI_ISL_3463427	In process	20B	B.1.1.519	22	5'UTR:T201C, 5'UTR:C203T, 5'UTR:C222T, 5'UTR:C241T, ORF1ab:T277C, ORF1ab:G1738T, ORF1ab:C3037T, ORF1ab:C3140T, ORF1ab:G7829T, ORF1ab:C10029T, ORF1ab:C10954T, ORF1ab:A11117G, ORF1ab:C12789T, ORF1ab:C14408T, ORF1ab:T19839C, ORF1ab:A19974G, S:C22995A, S:C23604A, S:A23756G, N:G28881A, N:G28882A, N:G28883C, N:C29197T,	11	N:R203K, N:G204R, ORF1a:P959S, ORF1a:V2522F, ORF1a:T3255I, ORF1a:I3618V, ORF1a:T4175I, ORF1b:P314L, S:T478K, S:P681H, S:T732A,	
hCoV-19/Mexico/CHP-InDRE-IBT-308303/NC/2020	EPI_ISL_3463363	In process	20B	B.1.1.519	25	5'UTR:T201C, 5'UTR:C203T, 5'UTR:C222T, 5'UTR:C241T, ORF1ab:G1738T, ORF1ab:C3037T, ORF1ab:C3140T, ORF1ab:G7829T, ORF1ab:C10029T, ORF1ab:C10954T, ORF1ab:A11117G, ORF1ab:C12789T, ORF1ab:C14318T, ORF1ab:C14408T, ORF1ab:T16456C, ORF1ab:T19839C, ORF1ab:A19974G, S:C22995A, S:C23604A, S:A23756G, N:G28881A, N:G28882A, N:G28883C, N:C29197T,ORF1ab:A4453T, ORF1ab:T16029C,	14	N:R203K, N:G204R, ORF1a:P959S, ORF1a:V2522F, ORF1a:T3255I, ORF1a:I3618V, ORF1a:T4175I, ORF1b:T284I, ORF1b:P314L, ORF1b:S997P, S:T478K, S:P681H, S:T732A,ORF1a:K1396N,	
hCoV-19/Mexico/CHP-InDRE-IBT-308305/NC/2020	EPI_ISL_3463629	In process	20B	B.1.1.519	21	5'UTR:T201C, 5'UTR:C203T, 5'UTR:C222T, 5'UTR:C241T, ORF1ab:T277C, ORF1ab:G1738T, ORF1ab:C3037T, ORF1ab:C3140T, ORF1ab:G7829T, ORF1ab:C10029T, ORF1ab:C10954T, ORF1ab:A11117G, ORF1ab:C14408T, ORF1ab:T19839C, ORF1ab:A19974G, S:C22995A, S:C23604A, S:A23756G, N:G28881A, N:G28882A, N:G28883C, N:C29197T,	10	N:R203K, N:G204R, ORF1a:P959S, ORF1a:V2522F, ORF1a:T3255I, ORF1a:I3618V, ORF1b:P314L, S:T478K, S:P681H, S:T732A,	

hCoV-19/Mexico/CHP-InDRE-IBT-308309/NC/2020	EPI_ISL_3463533	In process	20A	B.1.404	16	5'UTR:C241T, ORF1ab:C3037T, ORF1ab:C4683T, ORF1ab:G5572T, ORF1ab:G7042T, ORF1ab:A10323G, ORF1ab:A12644G, ORF1ab:C14408T, ORF1ab:T16743C, ORF1ab:G17721T, ORF1ab:G19086T, S:G22992A, S:A23403G, S:C23589T, S:C24223T, E:G26416T, N:C28854T.	12	E:V58F, N:S194L, ORF1a:A1473V, ORF1a:M1769I, ORF1a:M2259I, ORF1a:K3353R, ORF1a:I4127V, ORF1b:P314L, ORF1b:K1873N, S:S477N, S:D614G, S:T676I,
hCoV-19/Mexico/CMX-IBT-IMSS-46932/NC/2021	EPI_ISL_3463476	In process	20B	B.1.1.519	17	5'UTR:C203T, 5'UTR:C222T, 5'UTR:C241T, ORF1ab:C3037T, ORF1ab:C3140T, ORF1ab:C10029T, ORF1ab:C10954T, ORF1ab:A11117G, ORF1ab:C12789T, ORF1ab:C14408T, ORF1ab:T19839C, S:C22995A, S:C23604A, S:A23756G, N:G28881A, N:G28882A, N:G28883C, N:C29197T.	10	N:R203K, N:G204R, ORF1a:P959S, ORF1a:T3255I, ORF1a:I3618V, ORF1a:T4175I, ORF1b:P314L, S:T478K, S:P681H, S:T732A,
hCoV-19/Mexico/CMX-IBT-IMSS-46934/NC/2021	EPI_ISL_3463524	In process	20B	B.1.1.519	19	5'UTR:C203T, 5'UTR:C222T, 5'UTR:C241T, ORF1ab:C3037T, ORF1ab:C3140T, ORF1ab:C10029T, ORF1ab:C10954T, ORF1ab:A11117G, ORF1ab:C12789T, ORF1ab:C14408T, ORF1ab:G18180A, ORF1ab:T19839C, S:C22995A, S:C23604A, S:A23756G, N:G28881A, N:G28882A, N:G28883C, N:C29197T, N:G29527T,	11	N:R203K, N:G204R, N:Q418H, ORF1a:P959S, ORF1a:T3255I, ORF1a:I3618V, ORF1a:T4175I, ORF1b:P314L, S:T478K, S:P681H, S:T732A,
hCoV-19/Mexico/CMX-IBT-IMSS-47488/NC/2021	EPI_ISL_3463499	In process	20B	B.1.1.519	24	5'UTR:C203T, 5'UTR:C222T, 5'UTR:C241T, ORF1ab:C843T, ORF1ab:C3037T, ORF1ab:C3140T, ORF1ab:C10029T, ORF1ab:C10954T, ORF1ab:A11117G, ORF1ab:C12789T, ORF1ab:C14408T, ORF1ab:G18255T, ORF1ab:T19839C, S:C22995A, S:A23403G, S:C23604A, S:C23673T, S:A23756G, ORF8:T27904C, ORF8:C28087T, N:G28881A, N:G28882A, N:G28883C, N:C29197T,ORF1ab:G3937T,	17	N:R203K, N:G204R, ORF1a:P193L, ORF1a:P959S, ORF1a:T3255I, ORF1a:I3618V, ORF1a:T4175I, ORF1b:P314L, ORF1b:M1596I, ORF8:L4P, ORF8:A65V, S:T478K, S:D614G, S:P681H, S:S704L, S:T732A,ORF1a:Q1224H,
hCoV-19/Mexico/CMX-IBT-IMSS-47872/NC/2021	EPI_ISL_3463564	In process	20B	B.1.1.519	23	5'UTR:C203T, 5'UTR:C222T, 5'UTR:C241T, ORF1ab:C3037T, ORF1ab:C3140T, ORF1ab:C5183T, ORF1ab:A5336T, ORF1ab:C10029T, ORF1ab:C10954T, ORF1ab:A11117G, ORF1ab:C12789T, ORF1ab:T12934C, ORF1ab:C14408T, ORF1ab:T17067C, ORF1ab:T19839C, S:C22995A, S:A23403G, S:C23604A, S:A23756G, ORF8:T27904C, N:G28881A, N:G28882A, N:G28883C, N:C29197T,	14	N:R203K, N:G204R, ORF1a:P959S, ORF1a:P1640S, ORF1a:N1691Y, ORF1a:T3255I, ORF1a:I3618V, ORF1a:T4175I, ORF1b:P314L, ORF8:L4P, S:T478K, S:D614G, S:P681H, S:T732A,
hCoV-19/Mexico/CMX-IBT-IMSS-47881/NC/2021	EPI_ISL_3463507	In process	20B	B.1.1.519	25	5'UTR:T201C, 5'UTR:C203T, 5'UTR:C222T, 5'UTR:C241T, ORF1ab:C936T, ORF1ab:G1738T, ORF1ab:C3037T, ORF1ab:C3140T, ORF1ab:C10029T, ORF1ab:C10954T, ORF1ab:A11117G, ORF1ab:C12789T, ORF1ab:C14408T, ORF1ab:T19839C, ORF1ab:A19974G, S:C21575T, S:C22995A, S:A23403G, S:C23604A, S:A23756G, S:G25290T, N:G28881A, N:G28882A, N:G28883C, N:C29197T, N:G29227T,	14	N:R203K, N:G204R, ORF1a:T224I, ORF1a:P959S, ORF1a:T3255I, ORF1a:I3618V, ORF1a:T4175I, ORF1b:P314L, S:L5F, S:T478K, S:D614G, S:P681H, S:T732A, S:C1243F,
hCoV-19/Mexico/CMX-IBT-IMSS-48574/NC/2021	EPI_ISL_3463537	In process	20B	B.1.1.519	23	5'UTR:C203T, 5'UTR:C222T, 5'UTR:C241T, ORF1ab:C683T, ORF1ab:C1912T, ORF1ab:C3037T, ORF1ab:A3039G, ORF1ab:C3140T, ORF1ab:C10029T, ORF1ab:C10954T, ORF1ab:A11117G, ORF1ab:C12789T, ORF1ab:C14408T, ORF1ab:T19839C, S:C22995A, S:A23403G, S:C23604A, S:A23756G, N:G28881A, N:G28882A, N:G28883C, N:C29197T, N:G29527T.ORF1ab:T13716C.	13	N:R203K, N:G204R, N:Q418H, ORF1a:Y925C, ORF1a:P959S, ORF1a:T3255I, ORF1a:I3618V, ORF1a:T4175I, ORF1b:P314L, S:T478K, S:D614G, S:P681H, S:T732A,

hCoV-19/Mexico/CMX-IBT-IMSS-48576/NC/2021	EPI_ISL_3463556	In process	20B	B.1.1.519	20	5'UTR:C203T, 5'UTR:C222T, 5'UTR:C241T, ORF1ab:C3037T, ORF1ab:C3140T, ORF1ab:C10029T, ORF1ab:C10954T, ORF1ab:A11117G, ORF1ab:C12789T, ORF1ab:C14408T, ORF1ab:T19839C, S:C22995A, S:A23403G, S:C23604A, S:A23756G, ORF8:T27904C, ORF8:C28087T, N:G28881A, N:G28882A, N:G28883C, N:C29197T.	13	N:R203K, N:G204R, ORF1a:P959S, ORF1a:T3255I, ORF1a:I3618V, ORF1a:T4175I, ORF1b:P314L, ORF8:L4P, ORF8:A65V, S:T478K, S:D614G, S:P681H, S:T732A,	
hCoV-19/Mexico/CMX-IBT-IMSS-48587/NC/2021	EPI_ISL_3463517	In process	20B	B.1.1.519	23	5'UTR:T201C, 5'UTR:C203T, 5'UTR:C222T, 5'UTR:C241T, ORF1ab:C745T, ORF1ab:G1738T, ORF1ab:C3037T, ORF1ab:C3140T, ORF1ab:C7162T, ORF1ab:C10029T, ORF1ab:C10954T, ORF1ab:A11117G, ORF1ab:C12789T, ORF1ab:C14408T, ORF1ab:A19821G, ORF1ab:T19839C, ORF1ab:A19974G, S:C22995A, S:C23604A, S:A23756G, N:G28881A, N:G28882A, N:G28883C, N:C29197T,	10	N:R203K, N:G204R, ORF1a:P959S, ORF1a:T3255I, ORF1a:I3618V, ORF1a:T4175I, ORF1b:P314L, S:T478K, S:P681H, S:T732A,	
hCoV-19/Mexico/CMX-InDRE-IBT-37541/NC/2020	EPI_ISL_3463367	In process	20B	B.1.1.222	13	5'UTR:C241T, ORF1ab:C1568T, ORF1ab:C3037T, ORF1ab:A8658G, ORF1ab:G9753A, ORF1ab:C14120T, ORF1ab:C14408T, S:A23403G, S:A23756G, ORF3a:C25703T, N:G28881A, N:G28882A, N:G28883C,ORF1ab:A7449T,	7	N:R203K, N:G204R, ORF1b:P218L, ORF1b:P314L, ORF3a:P104L, S:D614G, S:T732A,	
hCoV-19/Mexico/CMX-INER-IBT-10/NC/2020	EPI_ISL_3463405	In process	20B	B.1.1.222	16	5'UTR:C241T, ORF1ab:G487A, ORF1ab:C3037T, ORF1ab:T8053C, ORF1ab:C9120T, ORF1ab:C9615T, ORF1ab:C14408T, ORF1ab:C14802T, ORF1ab:T19839C, S:A23403G, S:A23756G, ORF7a:T27597C, ORF7a:C27741T, ORF8:C28253T, N:G28881A, N:G28882A, N:G28883C,ORF1ab:C19227T	7	N:R203K, N:G204R, ORF1a:T2952I, ORF1a:T3117I, ORF1b:P314L, S:D614G, S:T732A,	
hCoV-19/Mexico/CMX-INER-IBT-11/NC/2020	EPI_ISL_3463623	In process	20B	B.1.1.222	17	5'UTR:C241T, ORF1ab:A1422C, ORF1ab:T2533C, ORF1ab:C3037T, ORF1ab:C10336T, ORF1ab:C11094T, ORF1ab:G13858T, ORF1ab:C14408T, ORF1ab:C17304T, ORF1ab:G19174A, ORF1ab:T19839C, S:G23593T, S:A23756G, N:A28704G, N:G28881A, N:G28882A, N:G28883C,ORF1ab:A8132C,	11	N:D144G, N:R203K, N:G204R, ORF1a:E386A, ORF1a:A3610V, ORF1b:D131Y, ORF1b:P314L, ORF1b:D1903N, S:Q677H, S:T732A,ORF1a:N2623H,	
hCoV-19/Mexico/CMX-INER-IBT-12/NC/2020	EPI_ISL_3463398	In process	20B	B.1.1.222	13	5'UTR:C241T, ORF1ab:C3037T, ORF1ab:C10029T, ORF1ab:C14408T, ORF1ab:T19839C, S:A23756G, ORF3a:C25710T, ORF3a:G25912T, ORF8:A27921G, ORF8:G28001T, ORF8:G28237C, N:G28881A, N:G28882A, N:G28883C,	8	N:R203K, N:G204R, ORF1a:T3255I, ORF1b:P314L, ORF3a:G174C, ORF8:I10V, ORF8:R115P, S:T732A,	
hCoV-19/Mexico/CMX-INER-IBT-14/NC/2020	EPI_ISL_3463643	In process	20B	B.1.1.161	15	5'UTR:C190T, 5'UTR:C241T, ORF1ab:C2062T, ORF1ab:C3037T, ORF1ab:C12651T, ORF1ab:C14408T, ORF1ab:G19549T, ORF1ab:T19839C, S:C23604A, S:C25000T, ORF3a:G25522A, ORF7b:G27890A, N:G28881A, N:G28882A, N:G28883C, 3'UTR:G29751C,	7	N:R203K, N:G204R, ORF1a:T4129I, ORF1b:P314L, ORF1b:A2028S, ORF3a:G44R, S:P681H,	ORF8:28 254,
hCoV-19/Mexico/CMX-INER-IBT-15/NC/2020	EPI_ISL_3463617	In process	20B	B.1.1.161	17	5'UTR:C190T, 5'UTR:C241T, ORF1ab:C2062T, ORF1ab:C3037T, ORF1ab:C12651T, ORF1ab:C14408T, ORF1ab:G19549T, ORF1ab:T19839C, S:C23604A, S:C25000T, ORF3a:G25522A, ORF7b:G27890A, ORF8:T28251C, ORF8:C28253G, N:G28881A, N:G28882A, N:G28883C. 3'UTR:G29751C.	7	N:R203K, N:G204R, ORF1a:T4129I, ORF1b:P314L, ORF1b:A2028S, ORF3a:G44R, S:P681H,	
hCoV-19/Mexico/CMX-INER-IBT-157/NC/2020	EPI_ISL_3463560	In process	20A	B.1	8	5'UTR:C241T, ORF1ab:C379T, ORF1ab:A1839G, ORF1ab:C2650T, ORF1ab:C3037T, ORF1ab:C4582T, ORF1ab:C14408T, S:A23403G,S:A25374T,	3	ORF1a:E525G, S:D614G,S:H1271L,	ORF1ab: 20671-20672

hCoV-19/Mexico/CMX-INER-IBT-177/NC/2020	EPI_ISL_3463466	In process	20B	B.1.1.519	21	5'UTR:C203T, 5'UTR:C222T, 5'UTR:C241T, ORF1ab:C3037T, ORF1ab:C3140T, ORF1ab:C6541T, ORF1ab:C10029T, ORF1ab:T10738C, ORF1ab:C10954T, ORF1ab:A11117G, ORF1ab:C12789T, ORF1ab:C14408T, ORF1ab:T19839C, S:A22023C, S:A23403G, S:C23604A, S:A23756G, ORF8:T27904C, N:G28881A, N:G28882A, N:G28883C, N:C29197T,	12	N:R203K, N:G204R, ORF1a:P959S, ORF1a:T3255I, ORF1a:I3618V, ORF1a:T4175I, ORF1b:P314L, ORF8:L4P, S:E154A, S:D614G, S:P681H, S:T732A,
hCoV-19/Mexico/CMX-INER-IBT-183/NC/2020	EPI_ISL_3463359	In process	20B	B.1.1.222	14	5'UTR:C241T, ORF1ab:C3037T, ORF1ab:C10029T, ORF1ab:C14408T, ORF1ab:C17634T, ORF1ab:C19011A, ORF1ab:T19839C, S:A23403G, S:A23756G, ORF3a:G25912T, ORF8:A27921G, ORF8:G28001T, N:G28881A, N:G28882A, N:G28883C,	9	N:R203K, N:G204R, ORF1a:T3255I, ORF1b:P314L, ORF1b:D1848E, ORF3a:G174C, ORF8:I10V, S:D614G, S:T732A,
hCoV-19/Mexico/CMX-INER-IBT-185/NC/2020	EPI_ISL_3463401	In process	20B	B.1.1.519	18	5'UTR:C203T, 5'UTR:C222T, 5'UTR:C241T, ORF1ab:C3037T, ORF1ab:C3140T, ORF1ab:C10029T, ORF1ab:C10954T, ORF1ab:A11117G, ORF1ab:C12789T, ORF1ab:C14408T, ORF1ab:G19684T, ORF1ab:T19839C, S:A23403G, S:C23604A, S:A23756G, N:G28881A, N:G28882A, N:G28883C, N:C29197T,	11	N:R203K, N:G204R, ORF1a:P959S, ORF1a:T3255I, ORF1a:I3618V, ORF1a:T4175I, ORF1b:P314L, ORF1b:V2073L, S:D614G, S:P681H, S:T732A,
hCoV-19/Mexico/CMX-INER-IBT-2/NC/2020	EPI_ISL_3463619	In process	20B	B.1.1.432	13	5'UTR:C241T, ORF1ab:C3037T, ORF1ab:G4255A, ORF1ab:A6985T, ORF1ab:C9319T, ORF1ab:C12412T, ORF1ab:T14313C, ORF1ab:C14408T, ORF1ab:G17427T, ORF3a:C25613T, N:G28881A, N:G28882A, N:G28883C, N:T29317C,	4	N:R203K, N:G204R, ORF1b:P314L, ORF3a:S74F,
hCoV-19/Mexico/CMX-INER-IBT-21/NC/2020	EPI_ISL_3463541	In process	20B	B.1.1.222	22	5'UTR:C241T, ORF1ab:G2035T, ORF1ab:C3037T, ORF1ab:C3884T, ORF1ab:C4686T, ORF1ab:G6337A, ORF1ab:G11083A, ORF1ab:C14408T, ORF1ab:C18814T, ORF1ab:C18981T, ORF1ab:T19839C, S:A23403G, S:A23756G, S:C24382T, ORF3a:G25534T, ORF8:G27987T, ORF8:G28083T, ORF8:C28253T, N:G28881A, N:G28882A, N:G28883C, 3'UTR:G29736T, 3'UTR:G29764T,	11	N:R203K, N:G204R, ORF1a:L590F, ORF1a:P1207S, ORF1a:T1474I, ORF1b:P314L, ORF3a:V48F, ORF8:V32L, ORF8:E64*, S:D614G, S:T732A,
hCoV-19/Mexico/CMX-INER-IBT-227/NC/2021	EPI_ISL_3463425	In process	20B	B.1.1.222	22	5'UTR:C241T, ORF1ab:G1148T, ORF1ab:G2393A, ORF1ab:C3037T, ORF1ab:C10029T, ORF1ab:C14408T, ORF1ab:C16887T, ORF1ab:C17339T, ORF1ab:C19011A, ORF1ab:G19086T, ORF1ab:C19554T, ORF1ab:T19839C, S:A23403G, S:C23439T, S:A23756G, ORF3a:C25810T, ORF3a:G25912T, M:G27074T, ORF8:A27921G, ORF8:G28001T, N:G28881A, N:G28882A, N:G28883C,	15	N:R203K, N:G204R, ORF1a:G295C, ORF1a:V710I, ORF1a:T3255I, ORF1b:P314L, ORF1b:A1291V, ORF1b:D1848E, ORF1b:K1873N, ORF3a:L140F, ORF3a:G174C, ORF8:I10V, S:D614G, S:A626V, S:T732A,
hCoV-19/Mexico/CMX-INER-IBT-2321/NC/2020	EPI_ISL_3463416	In process	20B	B.1.1	16	5'UTR:T137C, 5'UTR:C241T, ORF1ab:C3037T, ORF1ab:C3176T, ORF1ab:C8956T, ORF1ab:C14120T, ORF1ab:C14408T, ORF1ab:G19788A, ORF1ab:T19839C, S:C21614T, S:C22050T, S:A23403G, ORF3a:G26196T, N:G28460T, N:G28881A, N:G28882A, N:G28883C,	10	N:D63Y, N:R203K, N:G204R, ORF1a:P971S, ORF1b:P218L, ORF1b:P314L, ORF9b:K59N, S:L18F, S:A163V, S:D614G,
hCoV-19/Mexico/CMX-INER-IBT-238/NC/2021	EPI_ISL_3463392	In process	20B	B.1.1.519	25	5'UTR:T201C, 5'UTR:C203T, 5'UTR:C222T, 5'UTR:C241T, ORF1ab:C936T, ORF1ab:G1738T, ORF1ab:C3037T, ORF1ab:C3140T, ORF1ab:C10029T, ORF1ab:C10954T, ORF1ab:A11117G, ORF1ab:C12789T, ORF1ab:C14408T, ORF1ab:T19839C, ORF1ab:A19974G, S:A23403G, S:C23604A, S:A23756G, M:C26801T, N:G28881A, N:G28882A, N:G28883C, N:C29197T, N:G29227T, 3'UTR:A29740G,ORF1ab:T3781C,	11	N:R203K, N:G204R, ORF1a:T224I, ORF1a:P959S, ORF1a:T3255I, ORF1a:I3618V, ORF1a:T4175I, ORF1b:P314L, S:D614G, S:P681H, S:T732A,
hCoV-19/Mexico/CMX-INER-IBT-2405/NC/2020	EPI_ISL_3463519	In process	20B	B.1.1.222	11	5'UTR:C241T, ORF1ab:C3037T, ORF1ab:A5989T, ORF1ab:C12669T, ORF1ab:C14408T, ORF1ab:C17825T, ORF1ab:T19839C, S:A23756G, M:A26612T, N:G28881A, N:G28882A, N:G28883C,	6	N:R203K, N:G204R, ORF1a:S4135F, ORF1b:P314L, ORF1b:T1453I, S:T732A,

hCoV-19/Mexico/CMX-INER-IBT-2417/NC/2020	EPI_ISL_3463535	In process	20B	B.1.1.222	8	5'UTR:C241T, ORF1ab:C3037T, ORF1ab:C14408T, ORF1ab:T19839C, S:A23403G, S:A23756G, N:G28881A, N:G28882A, N:G28883C,	5	N:R203K, N:G204R, ORF1b:P314L, S:D614G, S:T732A,	
hCoV-19/Mexico/CMX-INER-IBT-242/NC/2021	EPI_ISL_3463366	In process	20B	B.1.1.519	22	5'UTR:T201C, 5'UTR:C203T, 5'UTR:C222T, 5'UTR:C241T, ORF1ab:G1738T, ORF1ab:C3037T, ORF1ab:C3140T, ORF1ab:C10029T, ORF1ab:C10954T, ORF1ab:A11117G, ORF1ab:C12789T, ORF1ab:C14408T, ORF1ab:T19839C, ORF1ab:A19974G, S:C22995A, S:A23403G, S:C23604A, S:A23756G, ORF3a:C25553T, N:G28881A, N:G28882A, N:G28883C, N:C29197T,	12	N:R203K, N:G204R, ORF1a:P959S, ORF1a:T3255I, ORF1a:I3618V, ORF1a:T4175I, ORF1b:P314L, ORF3a:A54V, S:T478K, S:D614G, S:P681H, S:T732A,	
hCoV-19/Mexico/CMX-INER-IBT-245/NC/2020	EPI_ISL_3463599	In process	20A	B.1	9	5'UTR:C186T, 5'UTR:C241T, ORF1ab:C3037T, ORF1ab:C4582T, ORF1ab:C14408T, S:A23403G, S:T25123C, N:G28373T, N:G28378T,S:G23412C,	5	N:G34W, ORF1b:P314L, ORF9b:R32L, S:D614G,S:C617S,	E:26284-26286
hCoV-19/Mexico/CMX-INER-IBT-245/NC/2021	EPI_ISL_3463487	In process	20B	B.1.1.519	24	5'UTR:C203T, 5'UTR:C222T, 5'UTR:C241T, ORF1ab:C3037T, ORF1ab:C3140T, ORF1ab:C6016T, ORF1ab:A9707G, ORF1ab:C10029T, ORF1ab:C10954T, ORF1ab:A11117G, ORF1ab:G11365T, ORF1ab:C12789T, ORF1ab:C14408T, ORF1ab:T19839C, ORF1ab:C21058T, S:A23403G, S:C23604A, S:A23756G, N:G28881A, N:G28882A, N:G28883C, N:C29197T, N:G29527T,ORF1ab:A462G, N:A28675T,	14	N:R203K, N:G204R, N:Q418H, ORF1a:P959S, ORF1a:I3148V, ORF1a:T3255I, ORF1a:I3618V, ORF1a:T4175I, ORF1b:P314L, ORF1b:P2531S, S:D614G, S:P681H, S:T732A,ORF1a:Q66R,	
hCoV-19/Mexico/CMX-INER-IBT-2478/NC/2020	EPI_ISL_3463631	In process	20B	B.1.1.222	10	5'UTR:C241T, ORF1ab:A1193G, ORF1ab:C3037T, ORF1ab:C3987T, ORF1ab:C14408T, ORF1ab:T19839C, S:A23756G, N:G28881A, N:G28882A, N:G28883C,ORF1ab:G16696T,	7	N:R203K, N:G204R, ORF1a:N310D, ORF1a:T1241I, ORF1b:P314L, S:T732A,ORF1b:V1077L,	
hCoV-19/Mexico/CMX-INER-IBT-2538/NC/2020	EPI_ISL_3463641	In process	20B	B.1.1.161	10	5'UTR:C241T, ORF1ab:C3037T, ORF1ab:T10107C, ORF1ab:C14408T, ORF8:G28083T, ORF8:C28093T, N:G28881A, N:G28882A, N:G28883C, 3'UTR:A29700G,ORF1ab:C18582A,	6	N:R203K, N:G204R, ORF1a:V3281A, ORF1b:P314L, ORF8:E64*, ORF8:S67F,	
hCoV-19/Mexico/CMX-INER-IBT-2539/NC/2020	EPI_ISL_3463474	In process	20B	B.1.1.222	12	5'UTR:C241T, ORF1ab:C3037T, ORF1ab:C14408T, ORF1ab:T19839C, ORF1ab:C20133T, S:G22331A, S:C22993T, S:A23403G, S:A23756G, N:G28881A, N:G28882A, N:G28883C,ORF1ab:G13520A,	7	N:R203K, N:G204R, ORF1b:P314L, S:G257S, S:D614G, S:T732A,ORF1b:S18N,	
hCoV-19/Mexico/CMX-INER-IBT-2646/NC/2020	EPI_ISL_3463390	In process	20B	B.1.1.222	13	5'UTR:C241T, ORF1ab:C3037T, ORF1ab:C8655T, ORF1ab:C14120T, ORF1ab:C14408T, ORF1ab:T19839C, S:A23756G, M:A26612T, N:C28292A, N:C28453T, N:G28881A, N:G28882A, N:G28883C, ORF10:C29614T,	8	N:Q7K, N:R203K, N:G204R, ORF1a:S2797F, ORF1b:P218L, ORF1b:P314L, ORF9b:A57V, S:T732A,	
hCoV-19/Mexico/CMX-INER-IBT-2679/NC/2020	EPI_ISL_3463625	In process	20B	B.1.1.222	9	5'UTR:C241T, ORF1ab:C3037T, ORF1ab:A13498G, ORF1ab:C14408T, ORF1ab:T19839C, S:G22331A, S:A23756G, N:G28881A, N:G28882A, N:G28883C,	6	N:R203K, N:G204R, ORF1b:T11A, ORF1b:P314L, S:G257S, S:T732A,	ORF7b:27879-27891,
hCoV-19/Mexico/CMX-INER-IBT-2693/NC/2020	EPI_ISL_3463550	In process	20A	B.1	14	5'UTR:C241T, ORF1ab:T1237C, ORF1ab:C3037T, ORF1ab:C3768T, ORF1ab:T13929C, ORF1ab:C14408T, ORF1ab:C16329T, ORF1ab:T18632G, S:A23403G, S:G23587T, S:G24368T, N:G28655T, N:C28887T, 3'UTR:G29773T,ORF8:A27947C,	9	N:D128Y, N:T205I, ORF1a:T1168I, ORF1b:P314L, ORF1b:F1722C, ORF8:Q18H, S:D614G, S:Q675H, S:D936Y,	
hCoV-19/Mexico/CMX-INER-IBT-2728/NC/2020	EPI_ISL_3463381	In process	20A	B.1.561	21	5'UTR:C241T, ORF1ab:G2516T, ORF1ab:C3037T, ORF1ab:C4475T, ORF1ab:C4543T, ORF1ab:A5999G, ORF1ab:C7851T, ORF1ab:A10323G, ORF1ab:C10798T, ORF1ab:A11305G, ORF1ab:C11866T, ORF1ab:C14408T, ORF1ab:C18693T, S:G21974T, S:A23403G, S:G24812T, ORF3a:T25577C, ORF7b:G27827T, N:C28472T, N:C28854T, N:G28975T,S:A24389C,	15	N:P67S, N:S194L, N:M234I, ORF1a:V751L, ORF1a:R1404C, ORF1a:I1912V, ORF1a:A2529V, ORF1a:K3353R, ORF1b:P314L, ORF3a:I62T, ORF7b:M24I, S:D138Y, S:D614G, S:D1084Y,S:S943R,	
hCoV-19/Mexico/CMX-INER-IBT-2748/NC/2020	EPI_ISL_3463421	In process	20B	B.1.1.222	15	5'UTR:C241T, ORF1ab:C3037T, ORF1ab:T3295C, ORF1ab:C10165T, ORF1ab:G12514T, ORF1ab:A13498G, ORF1ab:C14408T, ORF1ab:T15786C, ORF1ab:T19839C, S:G22331A, S:A23403G, S:A23756G, N:G28881A, N:G28882A, N:G28883C, N:G28903T,	8	N:R203K, N:G204R, N:M210I, ORF1b:T11A, ORF1b:P314L, S:G257S, S:D614G, S:T732A,	ORF7b:27879-27891,

hCoV-19/Mexico/CMX-INER-IBT-2757/NC/2020	EPI_ISL_3463566	In process	20A	B.1	11	5'UTR:C241T, ORF1ab:C3037T, ORF1ab:G3496A, ORF1ab:G4504A, ORF1ab:A10323G, ORF1ab:C14408T, ORF1ab:T17160C, S:G22051T, ORF3a:A25472G, ORF8:G28038T, N:C28854T,M:G26660A,	5	N:S194L, ORF1a:K3353R, ORF1b:P314L, ORF3a:D27G, ORF8:V49L,	
hCoV-19/Mexico/CMX-INER-IBT-2797/NC/2020	EPI_ISL_3463495	In process	20B	B.1.1.222	14	5'UTR:C241T, ORF1ab:C3037T, ORF1ab:C4965T, ORF1ab:C10450T, ORF1ab:C14408T, ORF1ab:T19839C, ORF1ab:G19962A, S:C21721T, S:A23403G, S:A23756G, N:G28881A, N:G28882A, N:G28883C,ORF1ab:A339G, ORF1ab:A9873T,	8	N:R203K, N:G204R, ORF1a:T1567I, ORF1b:P314L, S:D614G, S:T732A,ORF1a:D25G, ORF1a:Q3203L,	
hCoV-19/Mexico/CMX-INER-IBT-2807/NC/2020	EPI_ISL_3463430	In process	20A	B.1	15	5'UTR:C241T, ORF1ab:C664T, ORF1ab:C2197T, ORF1ab:C2749T, ORF1ab:C3037T, ORF1ab:T6069C, ORF1ab:C9565T, ORF1ab:C14408T, ORF1ab:C15720T, S:G22017T, S:A23403G, M:T26897C, ORF7a:G27703T, N:C28706T, N:C28854T, 3'UTR:G29751C,	7	N:H145Y, N:S194L, ORF1a:I1935T, ORF1b:P314L, ORF7a:V104F, S:W152L, S:D614G,	
hCoV-19/Mexico/CMX-INER-IBT-2839/NC/2020	EPI_ISL_3463371	In process	20A	B.1	20	5'UTR:C241T, ORF1ab:T724C, ORF1ab:C1059T, ORF1ab:C1489T, ORF1ab:C3037T, ORF1ab:G5194T, ORF1ab:C12919T, ORF1ab:A13733G, ORF1ab:C14408T, ORF1ab:C16568T, ORF1ab:T17268C, ORF1ab:G19414T, ORF3a:C25452T, ORF3a:C25658T, ORF3a:C25777T, ORF3a:C26110T, ORF7b:T27826A, ORF8:G28109T, N:C28717T, N:G28851A, N:T28921C,	11	N:S193N, ORF1a:T265I, ORF1b:K89R, ORF1b:P314L, ORF1b:T1034I, ORF1b:V1983L, ORF3a:T89I, ORF3a:L129F, ORF3a:P240S, ORF7b:M24K, ORF8:Q72H,	
hCoV-19/Mexico/CMX-INER-IBT-303/NC/2020	EPI_ISL_3463370	In process	20A	B.1	5	5'UTR:C241T, ORF1ab:C3037T, ORF1ab:C4582T, ORF1ab:C14408T, S:A23403G, S:T25123C,	2	ORF1b:P314L, S:D614G,	
hCoV-19/Mexico/CMX-INER-IBT-32/NC/2020	EPI_ISL_3463383	In process	20B	B.1.1.222	22	5'UTR:C241T, ORF1ab:C593T, ORF1ab:A1558G, ORF1ab:C3037T, ORF1ab:A3916G, ORF1ab:C6543T, ORF1ab:C8606T, ORF1ab:G11222T, ORF1ab:C14216T, ORF1ab:C14408T, ORF1ab:T19839C, S:C21614T, S:G22026A, S:G22104T, S:A23403G, S:A23756G, ORF3a:C25587T, ORF3a:C25904T, ORF7a:G27415A, N:G28881A, N:G28882A, N:G28883C,ORF1ab:A14905T,	17	N:R203K, N:G204R, ORF1a:H110Y, ORF1a:I431M, ORF1a:T2093I, ORF1a:L2781F, ORF1a:V3653F, ORF1b:T250I, ORF1b:P314L, ORF3a:S171L, ORF7a:A8T, S:L18F, S:S155N, S:G181V, S:D614G, S:T732A,ORF1b:N480Y,	
hCoV-19/Mexico/CMX-INER-IBT-338/NC/2020	EPI_ISL_3463375	In process	20A	B.1.189	8	5'UTR:C241T, ORF1ab:C3037T, ORF1ab:C4582T, ORF1ab:G6404T, ORF1ab:C6726T, ORF1ab:C14408T, S:A23403G, S:T25123C, N:G28378T,	5	ORF1a:V2047F, ORF1a:T2154I, ORF1b:P314L, ORF9b:R32L, S:D614G,	
hCoV-19/Mexico/CMX-INER-IBT-34/NC/2020	EPI_ISL_3463587	In process	20A	B.1	7	5'UTR:C241T, ORF1ab:C3037T, ORF1ab:C4582T, ORF1ab:C14408T, S:A23403G, ORF10:G29573T,ORF1ab:T20969G, M:A26603T,	3	ORF1b:P314L, S:D614G,ORF1b:V2501G,	
hCoV-19/Mexico/CMX-INER-IBT-5/NC/2020	EPI_ISL_3463468	In process	20B	B.1.1.222	19	5'UTR:C241T, ORF1ab:G2035T, ORF1ab:C3037T, ORF1ab:C3884T, ORF1ab:C4686T, ORF1ab:G11083A, ORF1ab:C14408T, ORF1ab:C18814T, ORF1ab:C18981T, ORF1ab:T19839C, S:A23403G, S:A23756G, S:C24382T, S:C25339T, ORF3a:G25534T, ORF8:G28083T, ORF8:C28253T, N:G28881A, N:G28882A, N:G28883C,	10	N:R203K, N:G204R, ORF1a:L590F, ORF1a:P1207S, ORF1a:T1474I, ORF1b:P314L, ORF3a:V48F, ORF8:E64*, S:D614G, S:T732A,	
hCoV-19/Mexico/CMX-INER-IBT-6/NC/2020	EPI_ISL_3463492	In process	20B	B.1.1.222	14	5'UTR:C241T, ORF1ab:C3037T, ORF1ab:C7749T, ORF1ab:C8637T, ORF1ab:C9924T, ORF1ab:A13498G, ORF1ab:C14408T, ORF1ab:T19839C, S:G22331A, S:A23403G, S:A23756G, N:G28817T, N:G28881A, N:G28882A, N:G28883C,	11	N:A182S, N:R203K, N:G204R, ORF1a:T2495I, ORF1a:T2791I, ORF1a:A3220V, ORF1b:T11A, ORF1b:P314L, S:G257S, S:D614G, S:T732A,	ORF7b:27879-27891,
hCoV-19/Mexico/CMX-INER-IBT-659/NC/2020	EPI_ISL_3463615	In process	20A	B.1.1	3	5'UTR:C241T, ORF1ab:C3037T, ORF1ab:C14408T, S:A23403G,	2	ORF1b:P314L, S:D614G,	
hCoV-19/Mexico/CMX-INER-IBT-7/NC/2020	EPI_ISL_3463589	In process	20B	B.1.1.432	16	5'UTR:C241T, ORF1ab:C3037T, ORF1ab:A3372G, ORF1ab:A3722G, ORF1ab:C9112T, ORF1ab:C9319T, ORF1ab:C12412T, ORF1ab:T14313C, ORF1ab:C14408T, ORF1ab:G15652T, ORF1ab:T17664C, ORF3a:C25613T, N:G28396T, N:G28881A, N:G28882A, N:G28883C, N:T29317C,	8	N:R203K, N:G204R, ORF1a:D1036G, ORF1a:I1153V, ORF1b:P314L, ORF1b:D729Y, ORF3a:S74F, ORF9b:G38V,	

hCoV-19/Mexico/CMX-INER-IBT-710/NC/2020	EPI_ISL_3463493	In process	20B	B.1	11	5'UTR:C241T, ORF1ab:T1476C, ORF1ab:C2399T, ORF1ab:C3037T, ORF1ab:C14408T, ORF1ab:A20511G, S:A23403G, M:C26907T, ORF8:C28054G, N:G28881A, N:G28882A, N:G28883C,	6	N:R203K, N:G204R, ORF1a:I404T, ORF1a:H712Y, ORF8:S54*, S:D614G,	ORF1ab: 20140-20141
hCoV-19/Mexico/CMX-INER-IBT-8/NC/2020	EPI_ISL_3463385	In process	20B	B.1.1.222	12	5'UTR:C241T, ORF1ab:C3037T, ORF1ab:C10029T, ORF1ab:C14408T, ORF1ab:T19839C, S:A23756G, ORF3a:G25912T, ORF8:A27921G, ORF8:G28001T, ORF8:G28237C, N:G28881A, N:G28882A, N:G28883C,	8	N:R203K, N:G204R, ORF1a:T3255I, ORF1b:P314L, ORF3a:G174C, ORF8:I10V, ORF8:R115P, S:T732A,	
hCoV-19/Mexico/CMX-INER-IBT-9/NC/2020	EPI_ISL_3463612	In process	20B	B.1.1.316	23	5'UTR:C241T, ORF1ab:C2102T, ORF1ab:C3037T, ORF1ab:C3987T, ORF1ab:C9430T, ORF1ab:C13862T, ORF1ab:C14408T, ORF1ab:T19839C, S:C21757T, S:C22267T, S:C23188T, S:A23756T, ORF3a:G25563T, ORF3a:G25654T, E:C26447T, ORF7a:C27509T, N:G28881A, N:G28882A, N:G28883C, 3'UTR:G29736T, 3'UTR:G29751T,ORF1ab:A3911C, ORF1ab:A9433T, ORF3a:G25737A,	11	E:S68F, N:R203K, N:G204R, ORF1a:T1241I, ORF1b:T132I, ORF1b:P314L, ORF3a:Q57H, ORF3a:V88L, ORF7a:T39I, S:T732S,ORF1a:T1216P,	
hCoV-19/Mexico/CMX-INER-IBT-90/NC/2020	EPI_ISL_3463576	In process	20A	B.1.1	4	5'UTR:C241T, ORF1ab:C3037T, ORF1ab:C14408T, S:A23403G,ORF1ab:T12503C,	3	ORF1b:P314L, S:D614G,ORF1a:Y4080H,	
hCoV-19/Mexico/CMX-INER-IBT-975/NC/2020	EPI_ISL_3463613	In process	20A	B.1.189	6	5'UTR:C241T, ORF1ab:C3037T, ORF1ab:C4582T, ORF1ab:G6404T, ORF1ab:C14408T, S:A23403G, N:G28378T,	4	ORF1a:V2047F, ORF1b:P314L, ORF9b:R32L, S:D614G,	
hCoV-19/Mexico/COA-InDRE-IBT-28845/NC/2020	EPI_ISL_3463608	In process	20A	B.1.1	6	5'UTR:C241T, ORF1ab:C3037T, ORF1ab:C12119T, ORF1ab:C14408T, S:A23403G, S:T25123C,S:G22644C,	2	ORF1a:P3952S, ORF1b:P314L,	S:21665
hCoV-19/Mexico/COL-InDRE-IBT-20796/NC/2020	EPI_ISL_3463595	In process	20A	B.1.189	10	5'UTR:G174T, 5'UTR:C241T, ORF1ab:C3037T, ORF1ab:C4582T, ORF1ab:G6404T, ORF1ab:C8655T, ORF1ab:C14408T, ORF1ab:T19956C, S:A23403G, N:G28857T,ORF1ab:G21232T,	4	N:R195I, ORF1a:V2047F, ORF1a:S2797F, S:D614G,	ORF1ab: 21199
hCoV-19/Mexico/COL-InDRE-IBT-39994/NC/2020	EPI_ISL_3463555	In process	20A	B.1	14	5'UTR:G208T, 5'UTR:C241T, ORF1ab:T856C, ORF1ab:C3037T, ORF1ab:C3045T, ORF1ab:C12885T, ORF1ab:C14408T, ORF1ab:G16935T, ORF1ab:C17430T, ORF1ab:A20268G, S:A23403G, S:C24865T, S:G25244T, N:C28854T,ORF1ab:A5133G,	8	N:S194L, ORF1a:P927L, ORF1a:T4207I, ORF1b:P314L, ORF1b:M1156I, S:D614G, S:V1228L,ORF1a:N1623S,	
hCoV-19/Mexico/DUR-InDRE-IBT-34067/NC/2020	EPI_ISL_3463610	In process	20A	B.1.1	10	5'UTR:C241T, ORF1ab:T1237C, ORF1ab:C3037T, ORF1ab:T3493C, ORF1ab:G9269A, ORF1ab:C14408T, S:A23403G, ORF3a:C25685T, N:G28690T,5'UTR:C245A, ORF1ab:G9267C,	6	N:L139F, ORF1a:G3002S, ORF1b:P314L, ORF3a:A98V, S:D614G,ORF1a:S3001T,	
hCoV-19/Mexico/DUR-InDRE-IBT-34216/NC/2020	EPI_ISL_3463621	In process	20A	B.1.1	6	5'UTR:C241T, ORF1ab:C3037T, ORF1ab:C8655T, ORF1ab:A8658G, ORF1ab:C14184T, ORF1ab:C14408T, S:A23403G,	2	ORF1b:P314L, S:D614G,	ORF1ab: 2888
hCoV-19/Mexico/HID-InDRE-IBT-23549/NC/2020	EPI_ISL_3463591	In process	20B	B.1.1	14	5'UTR:C241T, ORF1ab:C3037T, ORF1ab:C5835T, ORF1ab:A11830G, ORF1ab:C14408T, ORF1ab:C19186T, ORF1ab:T19839C, ORF1ab:G21004T, S:A23403G, N:G28881A, N:G28882A, N:G28883C, 3'UTR:G29751A,ORF1ab:C21541T, ORF3a:T26123G,	8	N:R203K, N:G204R, ORF1a:S1857L, ORF1b:P314L, ORF1b:A2513S, S:D614G,ORF1b:L2692F, ORF3a:V244G,	
hCoV-19/Mexico/HID-InDRE-IBT-23555/NC/2020	EPI_ISL_3463418	In process	20A	B.1	6	5'UTR:C241T, ORF1ab:C3037T, ORF1ab:C14408T, S:A23403G,ORF1ab:A13692C, ORF1ab:A13696C, ORF1ab:G19106T,	5	ORF1b:P314L, S:D614G,ORF1b:E75D, ORF1b:I77L, ORF1b:C1880F,	
hCoV-19/Mexico/HID-InDRE-IBT-23556/NC/2020	EPI_ISL_3463414	In process	20B	B.1.1	10	5'UTR:C241T, ORF1ab:T1476C, ORF1ab:C3037T, ORF1ab:C5184A, ORF1ab:C14408T, S:A23403G, ORF8:T27922C, N:G28881A, N:G28882A, N:G28883C,ORF1ab:G9095A,	8	N:R203K, N:G204R, ORF1a:I404T, ORF1a:P1640H, ORF1b:P314L, ORF8:I10T, S:D614G,ORF1a:A2944T,	
hCoV-19/Mexico/JAL-InDRE-IBT-43035/NC/2020	EPI_ISL_3463583	In process	20B	B.1.1	10	5'UTR:C241T, ORF1ab:C313T, ORF1ab:C3037T, ORF1ab:C14408T, S:A23403G, ORF3a:A25756G, N:G28881A, N:G28882A, N:G28883C,ORF1ab:G8498A, ORF1ab:A19887T,	7	N:R203K, N:G204R, ORF1b:P314L, ORF3a:R122G, S:D614G,ORF1a:A2745T, ORF1b:K2140N,	

hCoV-19/Mexico/MEX-IBT-IMSS-46960/NC/2021	EPI_ISL_3463502	In process	20B	B.1.1.519	25	5'UTR:C203T, 5'UTR:C222T, 5'UTR:C241T, ORF1ab:C3037T, ORF1ab:C3140T, ORF1ab:T8104C, ORF1ab:C10029T, ORF1ab:T10093A, ORF1ab:A10829C, ORF1ab:C10954T, ORF1ab:A11117G, ORF1ab:C12789T, ORF1ab:C14408T, ORF1ab:T19839C, S:C22995A, S:A23403G, S:C23604A, S:A23756G, E:T26436C, ORF6:G27281T, ORF8:T27904C, ORF8:C28087T, N:G28881A, N:G28882A, N:G28883C, N:C29197T,	15	N:R203K, N:G204R, ORF1a:P959S, ORF1a:T3255I, ORF1a:I3522L, ORF1a:I3618V, ORF1a:T4175I, ORF1b:P314L, ORF6:W27L, ORF8:L4P, ORF8:A65V, S:T478K, S:D614G, S:P681H, S:T732A,	ORF3a:25432-25437
hCoV-19/Mexico/MEX-IBT-IMSS-46962/NC/2021	EPI_ISL_3463446	In process	20B	B.1.1.519	25	5'UTR:T201C, 5'UTR:C203T, 5'UTR:C222T, 5'UTR:C241T, ORF1ab:G1738T, ORF1ab:C3037T, ORF1ab:C3140T, ORF1ab:C10029T, ORF1ab:C10954T, ORF1ab:A11117G, ORF1ab:C12789T, ORF1ab:C14408T, ORF1ab:A16770G, ORF1ab:C17934T, ORF1ab:T19839C, ORF1ab:A19974G, S:C22995A, S:A23403G, S:C23604A, S:A23756G, S:C24642T, N:G28881A, N:G28882A, N:G28883C, N:C29197T, 3'UTR:G29734T,	12	N:R203K, N:G204R, ORF1a:P959S, ORF1a:T3255I, ORF1a:I3618V, ORF1a:T4175I, ORF1b:P314L, S:T478K, S:D614G, S:P681H, S:T732A, S:T1027I,	
hCoV-19/Mexico/MEX-IBT-IMSS-48296/NC/2021	EPI_ISL_3463491	In process	20B	B.1.1.519	23	5'UTR:C203T, 5'UTR:C222T, 5'UTR:C241T, ORF1ab:A1269G, ORF1ab:C3037T, ORF1ab:C3140T, ORF1ab:C7764T, ORF1ab:C10029T, ORF1ab:C10954T, ORF1ab:A11117G, ORF1ab:C12789T, ORF1ab:T13905C, ORF1ab:C14362T, ORF1ab:C14408T, ORF1ab:T19839C, S:C22995A, S:A23403G, S:C23604A, S:A23756G, ORF3a:G25906T, N:G28881A, N:G28882A, N:G28883C, N:C29197T,	13	N:R203K, N:G204R, ORF1a:D335G, ORF1a:P959S, ORF1a:S2500F, ORF1a:T3255I, ORF1a:I3618V, ORF1a:T4175I, ORF1b:P314L, S:T478K, S:D614G, S:P681H, S:T732A,	ORF3a:26161,
hCoV-19/Mexico/MEX-IBT-IMSS-48303/NC/2021	EPI_ISL_3463637	In process	20B	B.1.1.222	12	5'UTR:C241T, ORF1ab:C3037T, ORF1ab:T3295C, ORF1ab:C10165T, ORF1ab:A13498G, ORF1ab:C14408T, ORF1ab:T19839C, S:G22331A, S:A23756G, ORF3a:C25549T, N:G28881A, N:G28882A, N:G28883C,	7	N:R203K, N:G204R, ORF1b:T11A, ORF1b:P314L, ORF3a:L53F, S:G257S, S:T732A,	ORF7b:27879-27891,
hCoV-19/Mexico/MEX-IBT-IMSS-48308/NC/2021	EPI_ISL_3463521	In process	20B	B.1.1.519	29	5'UTR:T201C, 5'UTR:C203T, 5'UTR:C222T, 5'UTR:C241T, ORF1ab:C936T, ORF1ab:G1738T, ORF1ab:C3037T, ORF1ab:C3140T, ORF1ab:C5575T, ORF1ab:G5950T, ORF1ab:C10029T, ORF1ab:C10615T, ORF1ab:C10954T, ORF1ab:A11117G, ORF1ab:C12789T, ORF1ab:C14408T, ORF1ab:T19839C, ORF1ab:A19974G, S:C22995A, S:A23403G, S:C23604A, S:A23756G, ORF3a:G25455T, ORF3a:C25626T, N:G28817T, N:G28881A, N:G28882A, N:G28883C, N:C29197T, N:G29227T,	15	N:A182S, N:R203K, N:G204R, ORF1a:T224I, ORF1a:P959S, ORF1a:K1895N, ORF1a:T3255I, ORF1a:I3618V, ORF1a:T4175I, ORF1b:P314L, ORF3a:K21N, S:T478K, S:D614G, S:P681H, S:T732A,	
hCoV-19/Mexico/MEX-IBT-IMSS-48313/NC/2021	EPI_ISL_3463388	In process	20B	B.1.1.519	25	5'UTR:C203T, 5'UTR:C222T, 5'UTR:C241T, ORF1ab:C3037T, ORF1ab:C3140T, ORF1ab:C6573T, ORF1ab:C10029T, ORF1ab:C10165T, ORF1ab:C10954T, ORF1ab:A11117G, ORF1ab:C12789T, ORF1ab:C14408T, ORF1ab:T19839C, S:C22995A, S:C23604A, S:A23756G, ORF3a:T25713C, M:C26681T, ORF7a:G27441T, ORF8:T27904C, ORF8:C28087T, N:C28775T, N:G28881A, N:G28882A, N:G28883C, N:C29197T,	15	N:P168S, N:R203K, N:G204R, ORF1a:P959S, ORF1a:S2103F, ORF1a:T3255I, ORF1a:I3618V, ORF1a:T4175I, ORF1b:P314L, ORF7a:E16D, ORF8:L4P, ORF8:A65V, S:T478K, S:P681H, S:T732A,	
hCoV-19/Mexico/MEX-IBT-IMSS-48336/NC/2021	EPI_ISL_3463407	In process	20B	B.1.1.519	22	5'UTR:T201C, 5'UTR:C203T, 5'UTR:C222T, 5'UTR:C241T, ORF1ab:C936T, ORF1ab:G1738T, ORF1ab:C3037T, ORF1ab:C3140T, ORF1ab:C10029T, ORF1ab:C10954T, ORF1ab:A11117G, ORF1ab:C12789T, ORF1ab:C14408T, ORF1ab:T19839C, ORF1ab:A19974G, S:C22995A, S:C23604A, S:A23756G, N:G28881A, N:G28882A, N:G28883C, N:C29197T, N:G29227T,	11	N:R203K, N:G204R, ORF1a:T224I, ORF1a:P959S, ORF1a:T3255I, ORF1a:I3618V, ORF1a:T4175I, ORF1b:P314L, S:T478K, S:P681H, S:T732A,	

hCoV-19/Mexico/MOR-InDRE-IBT-307998/NC/2020	EPI_ISL_3463532	In process	20B	B.1.1.222	18	5'UTR:C241T, ORF1ab:C1912T, ORF1ab:C3037T, ORF1ab:C3884T, ORF1ab:C6730T, ORF1ab:C11941T, ORF1ab:C14408T, ORF1ab:T19839C, S:T21961C, S:A23403G, S:A23756G, ORF3a:T25969C, M:G26529T, ORF8:T28066C, ORF8:T28199C, N:G28881A, N:G28882A, N:G28883C,S:A23806G,	9	M:D3Y, N:R203K, N:G204R, ORF1a:P1207S, ORF1b:P314L, ORF3a:W193R, ORF8:I58T, S:D614G, S:T732A,	
hCoV-19/Mexico/MOR-InDRE-IBT-308004/NC/2020	EPI_ISL_3463409	In process	20B	B.1.1.222	17	5'UTR:C241T, ORF1ab:C3037T, ORF1ab:A6693G, ORF1ab:C8655T, ORF1ab:C9430T, ORF1ab:C10029T, ORF1ab:C10039T, ORF1ab:C14408T, ORF1ab:G19009A, ORF1ab:T19839C, S:A23756G, ORF3a:G25912T, ORF7b:A27756G, ORF8:A27921G, ORF8:G28001T, N:G28881A, N:G28882A, N:G28883C,	11	N:R203K, N:G204R, ORF1a:K2143R, ORF1a:S2797F, ORF1a:T3255I, ORF1b:P314L, ORF1b:D1848N, ORF3a:G174C, ORF7b:M1V, ORF8:I10V, S:T732A,	
hCoV-19/Mexico/MOR-InDRE-IBT-308212/NC/2020	EPI_ISL_3463568	In process	20B	B.1.1.519	24	5'UTR:T201C, 5'UTR:C203T, 5'UTR:C222T, 5'UTR:C241T, ORF1ab:C745T, ORF1ab:G1738T, ORF1ab:C3037T, ORF1ab:C3140T, ORF1ab:C10029T, ORF1ab:C10954T, ORF1ab:A11117G, ORF1ab:C12789T, ORF1ab:C14408T, ORF1ab:T19839C, ORF1ab:A19974G, S:C22995A, S:A23403G, S:C23604A, S:A23756G, S:G25297T, N:G28881A, N:G28882A, N:G28883C, N:C29197T, 3'UTR:C29741T,	12	N:R203K, N:G204R, ORF1a:P959S, ORF1a:T3255I, ORF1a:I3618V, ORF1a:T4175I, ORF1b:P314L, S:T478K, S:D614G, S:P681H, S:T732A, S:K1245N,	
hCoV-19/Mexico/MOR-InDRE-IBT-308214/NC/2020	EPI_ISL_3463552	In process	20B	B.1.1.133	24	5'UTR:C190T, ORF1ab:T380A, ORF1ab:G3004T, ORF1ab:C3037T, ORF1ab:C3961T, ORF1ab:C9112T, ORF1ab:G13193A, ORF1ab:C14408T, ORF1ab:A16011G, ORF1ab:C16329T, ORF1ab:C18687T, ORF1ab:T19839C, S:A23403G, S:G23426A, S:C23604A, ORF3a:G25522A, ORF3a:T25580C, ORF3a:C25844T, ORF7b:G27890A, ORF8:C28093T, N:G28881A, N:G28882A, N:G28883C, ORF10:T29650C, 3'UTR:G29747T,	13	N:R203K, N:G204R, ORF1a:L39I, ORF1a:E913D, ORF1a:V4310I, ORF1b:P314L, ORF3a:G44R, ORF3a:I63T, ORF3a:T151I, ORF8:S67F, S:D614G, S:V622I, S:P681H,	
hCoV-19/Mexico/MOR-InDRE-IBT-308234/NC/2020	EPI_ISL_3463411	In process	20B	B.1.1.519	23	5'UTR:T201C, 5'UTR:C203T, 5'UTR:C222T, 5'UTR:C241T, ORF1ab:G1738T, ORF1ab:C3037T, ORF1ab:C3140T, ORF1ab:C10029T, ORF1ab:C10954T, ORF1ab:A11117G, ORF1ab:C12789T, ORF1ab:C14408T, ORF1ab:C15324T, ORF1ab:T19839C, ORF1ab:A19974G, S:C22995A, S:C23604A, S:A23756G, S:G25244T, ORF7a:C27427T, N:G28881A, N:G28882A, N:G28883C, N:C29197T,	12	N:R203K, N:G204R, ORF1a:P959S, ORF1a:T3255I, ORF1a:I3618V, ORF1a:T4175I, ORF1b:P314L, ORF7a:L12F, S:T478K, S:P681H, S:T732A, S:V1228L,	
hCoV-19/Mexico/MOR-InDRE-IBT-308259/NC/2020	EPI_ISL_3463373	In process	20G	B.1.2	20	5'UTR:C241T, ORF1ab:C1059T, ORF1ab:C1593T, ORF1ab:C3037T, ORF1ab:G3340T, ORF1ab:C10319T, ORF1ab:C10615T, ORF1ab:C14408T, ORF1ab:C15328T, ORF1ab:G17686T, ORF1ab:A18424G, ORF1ab:A19416G, S:C22858T, S:C23635T, ORF3a:G25563T, ORF3a:G25907T, ORF8:C27964T, N:C28472T, N:C28869T,ORF1ab:T13177C, M:C26873A,	13	N:P67S, N:P199L, ORF1a:T265I, ORF1a:S443F, ORF1a:L3352F, ORF1b:P314L, ORF1b:L621F, ORF1b:V1407F, ORF1b:N1653D, ORF3a:Q57H, ORF3a:G172V, ORF8:S24L,M:N117K,	
hCoV-19/Mexico/MOR-InDRE-IBT-308329/NC/2020	EPI_ISL_3463597	In process	20B	B.1.1.519	21	5'UTR:C203T, 5'UTR:A223T, 5'UTR:C241T, ORF1ab:C3037T, ORF1ab:G3085C, ORF1ab:C3140T, ORF1ab:T3745C, ORF1ab:A8658G, ORF1ab:C10029T, ORF1ab:C10954T, ORF1ab:A11117G, ORF1ab:C14408T, ORF1ab:T19839C, S:C22995A, S:C23604A, S:A23756G, ORF3a:C25844T, N:G28881A, N:G28882A, N:G28883C, N:C29197T, 3'UTR:A29700G.	12	N:R203K, N:G204R, ORF1a:E940D, ORF1a:P959S, ORF1a:K2798R, ORF1a:T3255I, ORF1a:I3618V, ORF1b:P314L, ORF3a:T151I, S:T478K, S:P681H, S:T732A,	5'UTR:2 22,

hCoV-19/Mexico/MOR-InDRE-IBT-308342/NC/2020	EPI_ISL_3463558	In process	20B	B.1.1	26	5'UTR:C190T, 5'UTR:C241T, ORF1ab:C3037T, ORF1ab:C5548T, ORF1ab:G7829T, ORF1ab:C9195T, ORF1ab:G9805T, ORF1ab:C11020T, ORF1ab:C14408T, ORF1ab:T18168C, ORF1ab:G19567T, ORF1ab:T19839C, S:C22033T, S:C22224T, S:A23403G, S:C23604A, S:G24445C, ORF3a:G25522A, ORF7b:G27890A, N:A28877T, N:G28878C, N:G28881A, N:G28882A, N:G28883C, N:C29149T, N:G29402T,ORF1ab:G13765T,	12	N:R203K, N:G204R, N:D377Y, ORF1a:V2522F, ORF1a:T2977I, ORF1b:P314L, ORF1b:V2034F, ORF3a:G44R, S:S221L, S:D614G, S:P681H,ORF1b:D100Y,	
hCoV-19/Mexico/MOR-InDRE-IBT-308351/NC/2020	EPI_ISL_3463548	In process	20B	B.1.1	26	5'UTR:C190T, 5'UTR:C241T, ORF1ab:C3037T, ORF1ab:C5548T, ORF1ab:G7829T, ORF1ab:C9195T, ORF1ab:C11020T, ORF1ab:C14408T, ORF1ab:T18168C, ORF1ab:T19839C, ORF1ab:G20931T, S:C22033T, S:C22858T, S:A23403G, S:C23604A, S:G24445C, ORF3a:G25522A, ORF7b:G27890A, N:A28877T, N:G28878C, N:G28881A, N:G28882A, N:G28883C, N:C29149T, N:G29402T, N:G29543C,S:A22190T,	10	N:R203K, N:G204R, N:D377Y, ORF1a:V2522F, ORF1a:T2977I, ORF1b:P314L, ORF3a:G44R, S:D614G, S:P681H,S:I210F,	
hCoV-19/Mexico/NAY-InDRE-IBT-25291/NC/2020	EPI_ISL_3463627	In process	20C	B.1.320	12	5'UTR:C241T, ORF1ab:C379A, ORF1ab:C1059T, ORF1ab:T2153C, ORF1ab:C3037T, ORF1ab:G4657A, ORF1ab:C14408T, ORF1ab:T15465C, S:A23403G, ORF3a:C25487T, ORF3a:G25563T, N:C28657T, N:G28842T,	7	N:S190I, ORF1a:T265I, ORF1a:W630R, ORF1b:P314L, ORF3a:T32I, ORF3a:Q57H, S:D614G,	
hCoV-19/Mexico/NLE-InDRE-IBT-25241/NC/2020	EPI_ISL_3463633	In process	20A	B.1.1	6	5'UTR:C241T, ORF1ab:C3037T, ORF1ab:C6720T, ORF1ab:C13119T, ORF1ab:C14408T, S:A23403G,S:G24618T,	5	ORF1a:T2152I, ORF1a:A4285V, ORF1b:P314L, S:D614G,S:R1019I,	ORF1ab: 20407-20412
hCoV-19/Mexico/NLE-InDRE-IBT-25243/NC/2020	EPI_ISL_3463603	In process	20C	B.1	11	5'UTR:C241T, ORF1ab:C1059T, ORF1ab:C3037T, ORF1ab:C3393T, ORF1ab:C4331T, ORF1ab:C5173T, ORF1ab:A6693G, ORF1ab:C11916T, ORF1ab:C14408T, S:A23403G, ORF3a:G25563T, N:C29514T,	8	N:A414V, ORF1a:T265I, ORF1a:A1043V, ORF1a:K2143R, ORF1a:S3884L, ORF1b:P314L, ORF3a:Q57H, S:D614G,	
hCoV-19/Mexico/OAX-InDRE-IBT-27468/NC/2020	EPI_ISL_3463472	In process	20A	B.1	11	5'UTR:G174C, 5'UTR:C241T, ORF1ab:C2062T, ORF1ab:A2775G, ORF1ab:C3037T, ORF1ab:G6404T, ORF1ab:C14408T, S:A23403G, N:C28854T, N:A29350G,ORF1ab:T20694C, ORF1ab:A20695T,	6	N:S194L, ORF1a:K837R, ORF1a:V2047F, ORF1b:P314L, S:D614G,ORF1b:N2410Y,	
hCoV-19/Mexico/OAX-InDRE-IBT-27475/NC/2020	EPI_ISL_3463578	In process	20A	B.1	9	5'UTR:C241T, ORF1ab:C3037T, ORF1ab:G4561A, ORF1ab:C4582T, ORF1ab:G11745A, ORF1ab:C14408T, S:A23403G, S:T23842C, ORF3a:G25500T, N:G29179T,	2	ORF1a:G3827E, ORF1b:P314L,	S:21845
hCoV-19/Mexico/PUE-IBT-IMSS-47245/NC/2021	EPI_ISL_3463513	In process	20B	B.1.1.519	23	5'UTR:T201C, 5'UTR:C203T, 5'UTR:C222T, 5'UTR:C241T, ORF1ab:G1738T, ORF1ab:C3037T, ORF1ab:C3140T, ORF1ab:G4184A, ORF1ab:C10029T, ORF1ab:C10954T, ORF1ab:A11117G, ORF1ab:C12789T, ORF1ab:C14408T, ORF1ab:C19170T, ORF1ab:T19839C, ORF1ab:A19974G, S:C22995A, S:A23403G, S:C23604A, S:A23756G, N:G28881A, N:G28882A, N:G28883C, N:C29197T,	12	N:R203K, N:G204R, ORF1a:P959S, ORF1a:G1307S, ORF1a:T3255I, ORF1a:I3618V, ORF1a:T4175I, ORF1b:P314L, S:T478K, S:D614G, S:P681H, S:T732A,	
hCoV-19/Mexico/PUE-IBT-IMSS-48688/NC/2021	EPI_ISL_3463553	In process	20B	B.1.1.519	27	5'UTR:T201C, 5'UTR:C203T, 5'UTR:C222T, 5'UTR:C241T, ORF1ab:G1264T, ORF1ab:G1738T, ORF1ab:C3037T, ORF1ab:C3140T, ORF1ab:G6205A, ORF1ab:C10029T, ORF1ab:C10954T, ORF1ab:A11117G, ORF1ab:C12789T, ORF1ab:A12796C, ORF1ab:C14408T, ORF1ab:G14559T, ORF1ab:T19839C, ORF1ab:A19974G, S:C22995A, S:A23403G, S:C23604A, S:A23756G, ORF3a:G25699A, ORF3a:G26116T, N:G28881A, N:G28882A, N:G28883C, N:C29197T	13	N:R203K, N:G204R, ORF1a:P959S, ORF1a:T3255I, ORF1a:I3618V, ORF1a:T4175I, ORF1b:P314L, ORF3a:A103T, ORF3a:E242*, S:T478K, S:D614G, S:P681H, S:T732A,	

hCoV-19/Mexico/PUE-InDRE-IBT-28747/NC/2020	EPI_ISL_3463545	In process	20B	B.1.1.432	15	5'UTR:C241T, ORF1ab:C3037T, ORF1ab:C9319T, ORF1ab:C12412T, ORF1ab:T14313C, ORF1ab:C14408T, ORF1ab:A16427G, ORF1ab:C17999T, S:A23403G, ORF3a:C25613T, ORF3a:G25912T, M:G26918T, N:G28881A, N:G28882A, N:G28883C, N:T29317C,	8	N:R203K, N:G204R, ORF1b:P314L, ORF1b:Y987C, ORF1b:T1511I, ORF3a:S74F, ORF3a:G174C, S:D614G,	
hCoV-19/Mexico/PUE-InDRE-IBT-308002/NC/2020	EPI_ISL_3463420	In process	20B	B.1.1.222	14	5'UTR:C241T, ORF1ab:C3037T, ORF1ab:C6285T, ORF1ab:A13498G, ORF1ab:C14408T, ORF1ab:C15579T, ORF1ab:C16323T, ORF1ab:T19839C, S:G22331A, S:T22510C, S:A23403G, S:A23756G, N:G28881A, N:G28882A, N:G28883C,	8	N:R203K, N:G204R, ORF1a:T2007I, ORF1b:T11A, ORF1b:P314L, S:G257S, S:D614G, S:T732A,	ORF7b:27879-27891,
hCoV-19/Mexico/PUE-InDRE-IBT-308027/NC/2020	EPI_ISL_3463482	In process	20B	B.1.1.222	15	5'UTR:C241T, ORF1ab:C3037T, ORF1ab:C4788T, ORF1ab:C6285T, ORF1ab:A13498G, ORF1ab:C14408T, ORF1ab:C15579T, ORF1ab:C18312T, ORF1ab:T19839C, S:G22331A, S:A23403G, S:A23756G, ORF3a:C26111T, N:G28881A, N:G28882A, N:G28883C,	10	N:R203K, N:G204R, ORF1a:A1508V, ORF1a:T2007I, ORF1b:T11A, ORF1b:P314L, ORF3a:P240L, S:G257S, S:D614G, S:T732A,	ORF7b:27879-27891,
hCoV-19/Mexico/PUE-InDRE-IBT-308032/NC/2020	EPI_ISL_3463450	In process	20B	B.1.1.222	16	5'UTR:C241T, ORF1ab:C3037T, ORF1ab:C6285T, ORF1ab:A13498G, ORF1ab:C14408T, ORF1ab:C15579T, ORF1ab:T19839C, S:G22331A, S:A23403G, S:A23756G, ORF3a:C25708T, ORF3a:A26118G, N:G28881A, N:G28882A, N:G28883C,ORF1ab:G17170A, ORF1ab:C17560T,	11	N:R203K, N:G204R, ORF1a:T2007I, ORF1b:T11A, ORF1b:P314L, ORF3a:L106F, S:G257S, S:D614G, S:T732A,ORF1b:A1235T, ORF1b:R1365W,	ORF7b:27879-27891,
hCoV-19/Mexico/PUE-InDRE-IBT-308052/NC/2020	EPI_ISL_3463457	In process	20B	B.1.1.222	16	5'UTR:C241T, ORF1ab:C2910T, ORF1ab:C3037T, ORF1ab:C6285T, ORF1ab:A13498G, ORF1ab:C14408T, ORF1ab:C15579T, ORF1ab:C17746T, ORF1ab:T19839C, S:G22331A, S:T22510C, S:A23403G, S:C23575T, S:A23756G, N:G28881A, N:G28882A, N:G28883C,	10	N:R203K, N:G204R, ORF1a:T882I, ORF1a:T2007I, ORF1b:T11A, ORF1b:P314L, ORF1b:P1427S, S:G257S, S:D614G, S:T732A,	ORF7b:27879-27891,
hCoV-19/Mexico/PUE-InDRE-IBT-308062/NC/2020	EPI_ISL_3463543	In process	20B	B.1.1.222	21	5'UTR:C241T, ORF1ab:G1268A, ORF1ab:C2416T, ORF1ab:C3037T, ORF1ab:C5986T, ORF1ab:A13288G, ORF1ab:C14408T, ORF1ab:G14772T, ORF1ab:G17562T, ORF1ab:T19839C, ORF1ab:G20208T, S:A23403G, S:A23756G, S:C25046T, ORF3a:G26056T, N:C28435T, N:G28881A, N:G28882A, N:G28883C, N:C29409T, 3'UTR:G29773T,N:G29151A,	13	N:R203K, N:G204R, N:T379I, ORF1a:D335N, ORF1b:P314L, ORF1b:Q435H, ORF1b:Q2247H, ORF3a:D222Y, ORF9b:P51L, S:D614G, S:T732A, S:P1162S,N:R293K,	
hCoV-19/Mexico/PUE-InDRE-IBT-308100/NC/2020	EPI_ISL_3463423	In process	20B	B.1.1.222	16	5'UTR:C241T, ORF1ab:C3037T, ORF1ab:C6285T, ORF1ab:A13498G, ORF1ab:C13643T, ORF1ab:C14408T, ORF1ab:C15579T, ORF1ab:T19839C, S:G22331A, S:T22510C, S:A23403G, S:A23756G, ORF3a:G25429T, N:G28881A, N:G28882A, N:G28883C,ORF1ab:A14545C,	11	N:R203K, N:G204R, ORF1a:T2007I, ORF1b:T11A, ORF1b:S59F, ORF1b:P314L, ORF3a:V13L, S:G257S, S:D614G, S:T732A,ORF1b:K360Q,	ORF7b:27879-27891,
hCoV-19/Mexico/PUE-InDRE-IBT-308102/NC/2020	EPI_ISL_3463464	In process	20B	B.1.1	22	5'UTR:C190T, 5'UTR:C241T, ORF1ab:G3004T, ORF1ab:C3037T, ORF1ab:C4776A, ORF1ab:C6726T, ORF1ab:C14408T, ORF1ab:T19839C, S:C22993T, S:A23403G, S:G23426A, S:C23604A, ORF3a:G25522A, ORF3a:T25580C, ORF7b:G27890A, ORF8:C27972T, N:A28877T, N:G28878C, N:G28881A, N:G28882A, N:G28883C, N:G29254T, 3'UTR:G29747T,	12	N:R203K, N:G204R, ORF1a:E913D, ORF1a:T1504N, ORF1a:T2154I, ORF1b:P314L, ORF3a:G44R, ORF3a:I63T, ORF8:Q27*, S:D614G, S:V622I, S:P681H,	
hCoV-19/Mexico/PUE-InDRE-IBT-308107/NC/2020	EPI_ISL_3463530	In process	20B	B.1.1.519	21	5'UTR:C203T, 5'UTR:C222T, 5'UTR:C241T, ORF1ab:C1469T, ORF1ab:C3037T, ORF1ab:C3140T, ORF1ab:G3549A, ORF1ab:C10029T, ORF1ab:C10954T, ORF1ab:A11117G, ORF1ab:C12789T, ORF1ab:C14408T, ORF1ab:T19839C, S:C22995A, S:A23403G, S:C23604A, S:A23756G, ORF8:T27904C, N:G28881A, N:G28882A, N:G28883C, N:C29197T,	14	N:R203K, N:G204R, ORF1a:R402C, ORF1a:P959S, ORF1a:G1095E, ORF1a:T3255I, ORF1a:I3618V, ORF1a:T4175I, ORF1b:P314L, ORF8:L4P, S:T478K, S:D614G, S:P681H, S:T732A,	

hCoV-19/Mexico/PUE-InDRE-IBT-308128/NC/2020	EPI_ISL_3463435	In process	20B	B.1.1.222	13	5'UTR:C241T, ORF1ab:C3037T, ORF1ab:C6285T, ORF1ab:A13498G, ORF1ab:C14408T, ORF1ab:C15579T, ORF1ab:T19839C, S:G22331A, S:T22510C, S:A23403G, S:A23756G, N:G28881A, N:G28882A, N:G28883C,	8	N:R203K, N:G204R, ORF1a:T2007I, ORF1b:T11A, ORF1b:P314L, S:G257S, S:D614G, S:T732A,	ORF7b:27879-27891,
hCoV-19/Mexico/PUE-InDRE-IBT-308132/NC/2020	EPI_ISL_3463452	In process	20B	B.1.1.222	16	5'UTR:C241T, ORF1ab:C3037T, ORF1ab:C6285T, ORF1ab:A12759G, ORF1ab:A13498G, ORF1ab:C14408T, ORF1ab:C15579T, ORF1ab:T19839C, S:G22021T, S:G22331A, S:T22510C, S:A23403G, S:A23756G, N:G28881A, N:G28882A, N:G28883C,ORF1ab:G11548T,	11	N:R203K, N:G204R, ORF1a:T2007I, ORF1a:M3761I, ORF1a:D4165G, ORF1b:T11A, ORF1b:P314L, S:M153I, S:G257S, S:D614G, S:T732A,	ORF7b:27879-27891,
hCoV-19/Mexico/PUE-InDRE-IBT-308135/NC/2020	EPI_ISL_3463470	In process	20B	B.1.1.222	13	5'UTR:C241T, ORF1ab:C3037T, ORF1ab:C6285T, ORF1ab:A13498G, ORF1ab:C14408T, ORF1ab:C15579T, ORF1ab:C18312T, ORF1ab:T19839C, S:G22331A, S:A23403G, S:A23756G, N:G28881A, N:G28882A, N:G28883C,	8	N:R203K, N:G204R, ORF1a:T2007I, ORF1b:T11A, ORF1b:P314L, S:G257S, S:D614G, S:T732A,	ORF7b:27879-27891,
hCoV-19/Mexico/PUE-InDRE-IBT-308138/NC/2020	EPI_ISL_3463459	In process	20B	B.1.1.222	13	5'UTR:C241T, ORF1ab:C3037T, ORF1ab:C6285T, ORF1ab:A13498G, ORF1ab:C14408T, ORF1ab:C15579T, ORF1ab:T19839C, S:G22331A, S:T22510C, S:A23403G, S:A23756G, N:G28881A, N:G28882A, N:G28883C,	8	N:R203K, N:G204R, ORF1a:T2007I, ORF1b:T11A, ORF1b:P314L, S:G257S, S:D614G, S:T732A,	ORF7b:27879-27891,
hCoV-19/Mexico/PUE-InDRE-IBT-308154/NC/2020	EPI_ISL_3463501	In process	20B	B.1.1	21	5'UTR:C241T, ORF1ab:T1711C, ORF1ab:C3037T, ORF1ab:C4320T, ORF1ab:C6040T, ORF1ab:G6362A, ORF1ab:G9802T, ORF1ab:A11451G, ORF1ab:C12076T, ORF1ab:C14408T, ORF1ab:G17325T, ORF1ab:C17795T, S:A22765G, S:G22992A, S:A23403G, ORF3a:G25855T, ORF7a:T27597A, ORF7a:G27598A, ORF7a:C27679T, N:G28881A, N:G28882A, N:G28883C,	13	N:R203K, N:G204R, ORF1a:A1352V, ORF1a:A2033T, ORF1a:Q3729R, ORF1b:P314L, ORF1b:L1286F, ORF1b:A1443V, ORF3a:D155Y, ORF7a:D69N, ORF7a:L96F, S:S477N, S:D614G,	
hCoV-19/Mexico/PUE-InDRE-IBT-308173/NC/2020	EPI_ISL_3463454	In process	20B	B.1.1.222	13	5'UTR:C241T, ORF1ab:C829T, ORF1ab:C3037T, ORF1ab:C6285T, ORF1ab:A13498G, ORF1ab:C14408T, ORF1ab:C15579T, ORF1ab:T19839C, S:G22331A, S:A23403G, S:A23756G, N:G28881A, N:G28882A, N:G28883C,	8	N:R203K, N:G204R, ORF1a:T2007I, ORF1b:T11A, ORF1b:P314L, S:G257S, S:D614G, S:T732A,	ORF7b:27879-27891,
hCoV-19/Mexico/PUE-InDRE-IBT-308186/NC/2020	EPI_ISL_3463448	In process	20C	B.1	24	5'UTR:C241T, ORF1ab:C1059T, ORF1ab:C3037T, ORF1ab:G6734A, ORF1ab:C8139T, ORF1ab:C11916T, ORF1ab:C14408T, ORF1ab:C18657T, ORF1ab:C18998T, S:A23403G, S:C24157T, S:G24794T, ORF3a:G25563T, ORF3a:C25728T, N:G28851T, N:G28899T, N:G29540A, 3'UTR:C29835T,ORF1ab:G3287A, ORF1ab:G10753T, ORF1ab:C12116A, ORF1ab:A12796G, ORF1ab:T19230C, S:C25100A, N:G28280A,	15	N:S193I, N:R209I, ORF1a:T265I, ORF1a:V2157I, ORF1a:S2625F, ORF1a:S3884L, ORF1b:P314L, ORF1b:A1844V, ORF3a:Q57H, S:D614G, S:A1078S,N:D3N, ORF1a:V1008I, ORF1a:L3951I, S:Q1180K,	
hCoV-19/Mexico/PUE-InDRE-IBT-308366/NC/2020	EPI_ISL_3463572	In process	20B	B.1.1.519	24	5'UTR:C203T, 5'UTR:C222T, 5'UTR:C241T, ORF1ab:C3037T, ORF1ab:C3140T, ORF1ab:C5140T, ORF1ab:C10029T, ORF1ab:C10954T, ORF1ab:A11117G, ORF1ab:C12789T, ORF1ab:C14408T, ORF1ab:C14931T, ORF1ab:T15726A, ORF1ab:C17733T, ORF1ab:T19839C, S:C22995A, S:A23403G, S:C23604A, S:A23756G, M:C26833T, N:G28881A, N:G28882A, N:G28883C, N:C29197T, N:G29527T,	13	M:A104V, N:R203K, N:G204R, N:Q418H, ORF1a:P959S, ORF1a:T3255I, ORF1a:I3618V, ORF1a:T4175I, ORF1b:P314L, S:T478K, S:D614G, S:P681H, S:T732A,	
hCoV-19/Mexico/PUE-InDRE-IBT-308405/NC/2020	EPI_ISL_3463437	In process	20B	B.1.1.222	15	5'UTR:C241T, ORF1ab:C3037T, ORF1ab:C6285T, ORF1ab:A13498G, ORF1ab:C14408T, ORF1ab:C15579T, ORF1ab:C17746T, ORF1ab:T19839C, S:G22331A, S:T22510C, S:A23403G, S:C23575T, S:A23756G, N:G28881A, N:G28882A, N:G28883C,	9	N:R203K, N:G204R, ORF1a:T2007I, ORF1b:T11A, ORF1b:P314L, ORF1b:P1427S, S:G257S, S:D614G, S:T732A,	ORF7b:27879-27891,
hCoV-19/Mexico/PUE-InDRE-IBT-308415/NC/2020	EPI_ISL_3463494	In process	20B	B.1.1.222	14	5'UTR:C241T, ORF1ab:C3037T, ORF1ab:C6285T, ORF1ab:C9636T, ORF1ab:A13498G, ORF1ab:C14408T, ORF1ab:C15579T, ORF1ab:T19839C, S:G22331A, S:T22510C, S:A23403G, S:A23756G, N:G28881A, N:G28882A, N:G28883C,	9	N:R203K, N:G204R, ORF1a:T2007I, ORF1a:A3124V, ORF1b:T11A, ORF1b:P314L, S:G257S, S:D614G, S:T732A,	ORF7b:27879-27891,

hCoV-19/Mexico/PUE-InDRE-IBT-308424/NC/2020	EPI_ISL_3463511	In process	20A	B.1	10	5'UTR:C241T, ORF1ab:C3037T, ORF1ab:C9778T, ORF1ab:G10882T, ORF1ab:A11782G, ORF1ab:A12124T, ORF1ab:C14408T, ORF1ab:C18508T, S:A23403G, N:C28854T, N:G29239A,	6	N:S194L, N:M322I, ORF1a:M3539I, ORF1b:P314L, ORF1b:L1681F, S:D614G,	
hCoV-19/Mexico/PUE-InDRE-IBT-308426/NC/2020	EPI_ISL_3463444	In process	20B	B.1.1.222	20	5'UTR:C241T, ORF1ab:C3037T, ORF1ab:T4063C, ORF1ab:C10029T, ORF1ab:C14408T, ORF1ab:C17012T, ORF1ab:G17721T, ORF1ab:T19839C, S:A23403G, S:A23756G, ORF3a:G25567A, ORF3a:T25569A, ORF3a:T25570A, ORF3a:G25912T, ORF3a:A26108G, ORF8:A27921G, ORF8:G28001T, ORF8:C28087T, N:G28881A, N:G28882A, N:G28883C,	13	N:R203K, N:G204R, ORF1a:T3255I, ORF1b:P314L, ORF1b:S1182L, ORF3a:A59T, ORF3a:S60T, ORF3a:G174C, ORF3a:E239G, ORF8:I10V, ORF8:A65V, S:D614G, S:T732A,	
hCoV-19/Mexico/PUE-InDRE-IBT-308428/NC/2020	EPI_ISL_3463489	In process	20B	B.1.1.222	19	5'UTR:C203T, 5'UTR:C241T, ORF1ab:C3037T, ORF1ab:G3403T, ORF1ab:A4681G, ORF1ab:T5218C, ORF1ab:C12525T, ORF1ab:C14408T, ORF1ab:C14913T, ORF1ab:T19839C, S:C21622T, S:A23403G, S:A23756G, S:G25061A, M:G26660T, N:G28300T, N:G28881A, N:G28882A, N:G28883C, N:G29449T,	10	M:L46F, N:Q9H, N:R203K, N:G204R, ORF1a:T4087I, ORF1b:P314L, ORF9b:S6I, S:D614G, S:T732A, S:G1167S,	
hCoV-19/Mexico/PUE-InDRE-IBT-308441/NC/2020	EPI_ISL_3463480	In process	20A	B.1.561	25	5'UTR:C241T, ORF1ab:G1685A, ORF1ab:G2516T, ORF1ab:C3037T, ORF1ab:A5999G, ORF1ab:A10323G, ORF1ab:C10798T, ORF1ab:G11083T, ORF1ab:C11866T, ORF1ab:C14408T, ORF1ab:A17490G, ORF1ab:C18568T, ORF1ab:C18693T, S:C21636T, S:G21974T, S:A23403G, S:G25088T, ORF3a:T25577C, ORF3a:C25714T, ORF3a:T26123C, ORF7a:C27434T, ORF7a:C27741T, ORF8:T27941G, N:G28703C, N:C28854T, N:G28975T,	19	N:D144H, N:S194L, N:M234I, ORF1a:A474T, ORF1a:V751L, ORF1a:I1912V, ORF1a:K3353R, ORF1a:L3606F, ORF1b:P314L, ORF1b:L1701F, ORF3a:I62T, ORF3a:L108F, ORF3a:V244A, ORF7a:T14I, ORF8:F16L, S:P25L, S:D138Y, S:D614G, S:V1176F,	
hCoV-19/Mexico/PUE-InDRE-IBT-308454/NC/2020	EPI_ISL_3463434	In process	20B	B.1.1.222	16	5'UTR:C241T, ORF1ab:T1452A, ORF1ab:A2444G, ORF1ab:C3037T, ORF1ab:T3295C, ORF1ab:C10165T, ORF1ab:G12514T, ORF1ab:A13498G, ORF1ab:C14408T, ORF1ab:T19839C, S:G22331A, S:A23403G, S:A23756G, ORF3a:C25886T, N:G28881A, N:G28882A, N:G28883C,	10	N:R203K, N:G204R, ORF1a:I396N, ORF1a:T727A, ORF1b:T11A, ORF1b:P314L, ORF3a:S165F, S:G257S, S:D614G, S:T732A,	ORF7b:27879-27891,
hCoV-19/Mexico/PUE-InDRE-IBT-308466/NC/2020	EPI_ISL_3463462	In process	20B	B.1.1.222	16	5'UTR:C241T, ORF1ab:C3037T, ORF1ab:C6196T, ORF1ab:C6449T, ORF1ab:A13498G, ORF1ab:C14408T, ORF1ab:G17964T, ORF1ab:T19839C, S:G22331A, S:A23403G, S:A23756G, S:A24292G, N:G28881A, N:G28882A, N:G28883C, N:G29477T, 3'UTR:G29734T,	10	N:R203K, N:G204R, N:D402Y, ORF1a:L2062F, ORF1b:T11A, ORF1b:P314L, ORF1b:M1499I, S:G257S, S:D614G, S:T732A,	ORF7b:27879-27891,
hCoV-19/Mexico/QUE-InDRE-IBT-21063/NC/2020	EPI_ISL_3463605	In process	20A	B.1	10	5'UTR:C241T, ORF1ab:A2205G, ORF1ab:C3037T, ORF1ab:C4582T, ORF1ab:C14408T, ORF1ab:C18877T, S:A23403G, ORF1ab:A13692C, ORF1ab:A13696C, ORF3a:G25641A,	1	ORF1a:E647G,	ORF1ab: 19300-19301, S:21665
hCoV-19/Mexico/QUE-InDRE-IBT-21066/NC/2020	EPI_ISL_3463635	In process	20A	B.1	11	5'UTR:C241T, ORF1ab:C3037T, ORF1ab:C4582T, ORF1ab:C7262T, ORF1ab:T13521C, ORF1ab:C14408T, ORF1ab:C19151T, S:A23403G, S:T25123C, N:G28378T,ORF1ab:C4419G, ORF1ab:T10216C,	4	ORF1b:P314L, ORF1b:A1895V, ORF9b:R32L, S:D614G,	ORF1ab: 4031, ORF1ab: 6116
hCoV-19/Mexico/QUE-InDRE-IBT-21067/NC/2020	EPI_ISL_3463413	In process	20A	B.1.609	7	5'UTR:C241T, ORF1ab:C3037T, ORF1ab:C4582T, ORF1ab:C13119T, ORF1ab:C14408T, S:C22624T, S:G22661T, S:A23403G,	4	ORF1a:A4285V, ORF1b:P314L, S:V367F, S:D614G,	
hCoV-19/Mexico/SIN-InDRE-IBT-27557/NC/2020	EPI_ISL_3463593	In process	20A	B.1	5	5'UTR:C241T, ORF1ab:C3037T, ORF1ab:C4582T, ORF1ab:C7573T, ORF1ab:C14408T, S:A23403G,	2	ORF1b:P314L, S:D614G,	3'UTR:29854

hCoV-19/Mexico/SLP-IBT-IMSS-48517/NC/2021	EPI_ISL_3463403	In process	20A	B.1.396	21	5'UTR:C241T, ORF1ab:C1191T, ORF1ab:C3037T, ORF1ab:T6971C, ORF1ab:C9521A, ORF1ab:C11109T, ORF1ab:C14184T, ORF1ab:C14408T, ORF1ab:C17747T, S:A23403G, S:G24547A, S:C24904T, ORF3a:C26111T, ORF3a:G26158T, M:C26858T, ORF8:C28253T, ORF8:A28254C, N:C28854T, N:G29422T, ORF10:A29567G,ORF1ab:G1298T, ORF1ab:G14346T,	12	N:S194L, ORF1a:P309L, ORF1a:L3086I, ORF1a:A3615V, ORF1b:P314L, ORF1b:P1427L, ORF3a:P240L, ORF3a:V256F, ORF8:I121L, S:D614G,ORF1a:G345C, ORF1b:L293F,	
hCoV-19/Mexico/SLP-IBT-IMSS-48530/NC/2021	EPI_ISL_3463528	In process	20B	B.1.1.519	24	5'UTR:C203T, 5'UTR:C222T, 5'UTR:C241T, ORF1ab:C1710T, ORF1ab:C3037T, ORF1ab:C3140T, ORF1ab:G3145A, ORF1ab:C10029T, ORF1ab:C10954T, ORF1ab:A11117G, ORF1ab:C12789T, ORF1ab:C14408T, ORF1ab:T19839C, S:C21637T, S:C22995A, S:A23403G, S:C23604A, S:A23756G, ORF8:G28188T, N:G28881A, N:G28882A, N:G28883C, N:C29197T, N:G29440T, N:G29527T,	15	N:R203K, N:G204R, N:Q389H, N:Q418H, ORF1a:A482V, ORF1a:P959S, ORF1a:T3255I, ORF1a:I3618V, ORF1a:T4175I, ORF1b:P314L, ORF8:V99L, S:T478K, S:D614G, S:P681H, S:T732A,	
hCoV-19/Mexico/SLP-IBT-IMSS-48798/NC/2021	EPI_ISL_3463571	In process	20B	B.1.1.519	23	5'UTR:C203T, 5'UTR:C222T, 5'UTR:C241T, ORF1ab:C3037T, ORF1ab:C3140T, ORF1ab:G3692T, ORF1ab:G3871T, ORF1ab:C10029T, ORF1ab:C10954T, ORF1ab:A11117G, ORF1ab:C11824A, ORF1ab:C12789T, ORF1ab:C14408T, ORF1ab:T19839C, ORF1ab:C21431T, S:C22995A, S:A23403G, S:C23604A, S:A23756G, ORF8:T27904C, N:G28881A, N:G28882A, N:G28883C, N:C29197T,	15	N:R203K, N:G204R, ORF1a:P959S, ORF1a:V1143F, ORF1a:K1202N, ORF1a:T3255I, ORF1a:I3618V, ORF1a:T4175I, ORF1b:P314L, ORF1b:A2655V, ORF8:L4P, S:T478K, S:D614G, S:P681H, S:T732A,	
hCoV-19/Mexico/SLP-IBT-IMSS-48825/NC/2021	EPI_ISL_3463539	In process	20A	B.1.241	16	5'UTR:C241T, ORF1ab:G1757A, ORF1ab:C3037T, ORF1ab:G6884A, ORF1ab:C11575T, ORF1ab:C12053T, ORF1ab:C14408T, ORF1ab:A16840C, ORF1ab:C17733T, ORF1ab:C19029T, S:C21676T, S:T22222C, S:C22987T, S:A23403G, ORF7b:C27804A, ORF8:C28115T, N:C28854T,	8	N:S194L, ORF1a:A498T, ORF1a:G2207S, ORF1a:L3930F, ORF1b:P314L, ORF1b:K1125Q, ORF7b:L17M, S:D614G,	
hCoV-19/Mexico/SLP-InDRE-IBT-977/NC/2020	EPI_ISL_3463379	In process	20A	B.1	13	5'UTR:C241T, ORF1ab:C3037T, ORF1ab:C8655T, ORF1ab:A8658G, ORF1ab:C14408T, ORF1ab:C18877T, ORF1ab:C20233T, S:A23403G, ORF3a:G25563T, ORF3a:G25855T,ORF1ab:T1723C, ORF1ab:A5718T, S:A22556G, N:G28845T,	6	ORF1b:P314L, ORF3a:Q57H, ORF3a:D155Y, S:D614G,N:R191L, S:I332V,	ORF1ab: 4790
hCoV-19/Mexico/SLP-InDRE-IBT-978/NC/2020	EPI_ISL_3463639	In process	20A	B.1	8	5'UTR:C241T, ORF1ab:C3037T, ORF1ab:G13920T, ORF1ab:C14408T, ORF1ab:C18877T, S:A23403G, ORF3a:G25563T,ORF1ab:T1723C, N:G28845T,	5	ORF1b:K151N, ORF1b:P314L, ORF3a:Q57H, S:D614G,N:R191L,	
hCoV-19/Mexico/SON-InDRE-IBT-29407/NC/2020	EPI_ISL_3463563	In process	20A	B.1.558	9	5'UTR:C241T, ORF1ab:C1288T, ORF1ab:C3037T, ORF1ab:C8655T, ORF1ab:C11575T, ORF1ab:C14408T, ORF1ab:C18060T, S:G22487C, S:A23403G, N:C28854T,	5	N:S194L, ORF1a:S2797F, ORF1b:P314L, S:E309Q, S:D614G,	
hCoV-19/Mexico/SON-InDRE-IBT-29413/NC/2020	EPI_ISL_3463394	In process	20A	B.1	8	5'UTR:C241T, ORF1ab:C3037T, ORF1ab:C4331T, ORF1ab:C14408T, ORF1ab:C16041T, S:C22264T, S:A23403G, ORF6:G27204T, N:C28854T,	4	N:S194L, ORF1b:P314L, ORF6:M1I, S:D614G,	
hCoV-19/Mexico/TAB-InDRE-IBT-28657/NC/2020	EPI_ISL_3463581	In process	20B	B.1	14	5'UTR:C241T, ORF1ab:C3037T, ORF1ab:G9479T, ORF1ab:T11630C, ORF1ab:C14408T, ORF1ab:C15579T, ORF1ab:G20578T, S:A23403G, N:G28881A, N:G28882A, N:G28883C, 3'UTR:T29867C,ORF1ab:C8369T, ORF1ab:T17034A, ORF1ab:A18221C,	9	N:R203K, N:G204R, ORF1a:G3072C, ORF1a:F3789L, ORF1b:P314L, ORF1b:V2371L, S:D614G,ORF1a:Q2702*, ORF1b:K1585T,	
hCoV-19/Mexico/TLA-InDRE-IBT-24011/NC/2020	EPI_ISL_3463361	In process	20A	B.1.189	7	5'UTR:C241T, ORF1ab:C3037T, ORF1ab:C4582T, ORF1ab:G6404T, ORF1ab:C14408T, S:A23403G,ORF1ab:G6306C, ORF1ab:G16589T,	5	ORF1a:V2047F, ORF1b:P314L, S:D614G,ORF1a:W2014S, ORF1b:G1041V,	
hCoV-19/Mexico/TLA-InDRE-IBT-24014/NC/2020	EPI_ISL_3463580	In process	20A	B.1.1	7	5'UTR:C241T, ORF1ab:C3037T, ORF1ab:C14408T, ORF1ab:A20465G, S:A23403G, S:C23565T,ORF1ab:G13754T, ORF1ab:C18894A,	6	ORF1b:P314L, ORF1b:D2333G, S:D614G, S:A668V,ORF1b:R96I, ORF1b:C1809*,	

hCoV-19/Mexico/VER-IBT-IMSS-48502/NC/2021	EPI_ISL_3463478	In process	20B	B.1.1.222	14	5'UTR:C241T, ORF1ab:C3037T, ORF1ab:C3505T, ORF1ab:A13498G, ORF1ab:C14408T, ORF1ab:C16393T, ORF1ab:T19839C, S:G22331A, S:A23403G, S:A23756G, S:A24292G, ORF3a:C25614T, N:G28881A, N:G28882A, N:G28883C,	8	N:R203K, N:G204R, ORF1b:T11A, ORF1b:P314L, ORF1b:P976S, S:G257S, S:D614G, S:T732A,	
hCoV-19/Mexico/YUC-InDRE-IBT-22139/NC/2020	EPI_ISL_3463585	In process	20A	B.1.1	8	5'UTR:C241T, ORF1ab:C3037T, ORF1ab:C8655T, ORF1ab:C14408T, S:A23403G, M:C26996T, ORF8:C27945T,ORF1ab:A18923G, S:A23550G,	6	ORF1a:S2797F, ORF1b:P314L, ORF8:Q18*, S:D614G,ORF1b:E1819G, S:D663G,	
oV-19/Mexico/PUE_LANGEBIO_IMSS_81667-NC/20	EPI_ISL_2970015	In process	20B	B.1.1.519	28	5'UTR:C203T, 5'UTR:C222T, 5'UTR:C241T, ORF1ab:A642G, ORF1ab:C1121T, ORF1ab:G2527A, ORF1ab:C3037T, ORF1ab:C3140T, ORF1ab:T3745C, ORF1ab:A4926C, ORF1ab:C10029T, ORF1ab:C10954T, ORF1ab:A11117G, ORF1ab:C12789T, ORF1ab:C14408T, ORF1ab:G18213T, ORF1ab:C21306T, ORF1ab:A21312G, S:C22995A, S:A23403G, S:C23604A, S:A23756G, ORF3a:C25844T, ORF3a:C26228A, ORF7a:C27513T, N:G28881A, N:G28882A, N:G28883C, N:C29197T,	16	N:R203K, N:G204R, ORF1a:N126S, ORF1a:P286S, ORF1a:P959S, ORF1a:D1554A, ORF1a:T3255I, ORF1a:I3618V, ORF1a:T4175I, ORF1b:P314L, ORF1b:M1582I, ORF3a:T151I, S:T478K, S:D614G, S:P681H, S:T732A,	
oV-19/Mexico/CHH_LANGEBIO_IMSS_36471-NC/2	EPI_ISL_2969872	In process	20A	B.1.189	21	ORF1ab:T565C, ORF1ab:A2213G, ORF1ab:C3037T, ORF1ab:C4582T, ORF1ab:G6404T, ORF1ab:C9724T, ORF1ab:C10277T, ORF1ab:T10651C, ORF1ab:C12005T, ORF1ab:T12645C, ORF1ab:C14408T, ORF1ab:C17010T, ORF1ab:A20268G, S:A23403G, S:G23593T, S:C23604A, S:T25123C, ORF3a:T25689C, M:G26720T, ORF7b:C27879T, N:G28378T, N:G28881T,	11	N:R203M, ORF1a:K650E, ORF1a:V2047F, ORF1a:L3338F, ORF1a:I4127T, ORF1b:P314L, ORF7b:H42Y, ORF9b:R32L, S:D614G, S:Q677H, S:P681H,	
oV-19/Mexico/CHH_LANGEBIO_IMSS_36483-NC/2	Falta	In process	20B	B.1.1.519	22	ORF1ab:T1417C, ORF1ab:C1889T, ORF1ab:G2118T, ORF1ab:C3037T, ORF1ab:C3140T, ORF1ab:C10029T, ORF1ab:A10323G, ORF1ab:C10954T, ORF1ab:A11117G, ORF1ab:C12789T, ORF1ab:C14120T, ORF1ab:C14408T, ORF1ab:C17410T, ORF1ab:T19839C, ORF1ab:C21306T, S:A23403G, S:C23604A, S:A23756G, S:A25108C, N:G28881A, N:G28882A, N:G28883C, N:C29197T,	13	N:R203K, N:G204R, ORF1a:R542C, ORF1a:G618V, ORF1a:P959S, ORF1a:T3255I, ORF1a:K3353R, ORF1a:I3618V, ORF1a:T4175I, S:D614G, S:P681H, S:T732A, S:E1182D,	
oV-19/Mexico/CHH_LANGEBIO_IMSS_37309-NC/2	Falta	In process	20B	B.1.1.222	19	ORF1ab:C3037T, ORF1ab:C3140T, ORF1ab:C5622T, ORF1ab:C10029T, ORF1ab:C10954T, ORF1ab:A11117G, ORF1ab:C12789T, ORF1ab:C14408T, ORF1ab:T19839C, ORF1ab:C21306T, S:A23403G, S:C23604A, S:A23756G, S:C25000T, N:G28881A, N:G28882A, N:G28883C, N:C29197T, 3'UTR:C29870A.ORF8:A28012G.	12	N:R203K, N:G204R, ORF1a:P959S, ORF1a:P1786L, ORF1a:T3255I, ORF1a:I3618V, ORF1a:T4175I, ORF1b:P314L, S:D614G, S:P681H, S:T732A,ORF8:H40R,	ORF7a:2 7622
oV-19/Mexico/COA_LANGEBIO_IMSS_37339-NC/2	Falta	In process	20A	B.1.1	28	ORF1ab:C1288T, ORF1ab:G2305T, ORF1ab:A2526G, ORF1ab:C2902T, ORF1ab:C3037T, ORF1ab:G4444T, ORF1ab:G6476A, ORF1ab:C10335T, ORF1ab:C10525T, ORF1ab:T12751G, ORF1ab:C12778A, ORF1ab:C14408T, ORF1ab:C15081T, ORF1ab:A18038T, ORF1ab:A18039T, ORF1ab:C18060T, ORF1ab:A20268G, S:C21627T, S:G22147A, S:G22487C, S:A23403G, S:C23664T, ORF3a:G25455T, ORF3a:G25770T, ORF3a:T25807C, ORF7a:C27418T, N:C28854T, N:C28948T, N:G29543T,	12	N:S194L, ORF1a:K680N, ORF1a:E754G, ORF1a:V2071I, ORF1a:A3357V, ORF1a:Y4171*, ORF3a:K21N, ORF3a:R126S, S:T22I, S:E309Q, S:D614G, S:A701V,	

oV-19/Mexico/COA_LANGEBIO_IMSS_37994-NC/2	EPI_ISL_2969918	In process	20B	B.1.1.519	21	ORF1ab:G2118T, ORF1ab:A2956G, ORF1ab:C3037T, ORF1ab:C3140T, ORF1ab:C5986T, ORF1ab:C10029T, ORF1ab:C10954T, ORF1ab:A11117G, ORF1ab:C12789T, ORF1ab:C14408T, ORF1ab:G14707A, ORF1ab:T19839C, ORF1ab:C21306T, S:A23403G, S:C23604A, S:A23756G, M:G26526T, ORF7b:C27874T, N:G28881A, N:G28882A, N:G28883C, N:G29527T,	15	M:A2S, N:R203K, N:G204R, N:Q418H, ORF1a:G618V, ORF1a:P959S, ORF1a:T3255I, ORF1a:I3618V, ORF1a:T4175I, ORF1b:P314L, ORF1b:A414T, ORF7b:T40I, S:D614G, S:P681H, S:T732A,	
oV-19/Mexico/COA_LANGEBIO_IMSS_38056-NC/2	EPI_ISL_2969919	In process	20A	B.1.404	23	ORF1ab:G2118T, ORF1ab:C2156T, ORF1ab:G2173A, ORF1ab:C3037T, ORF1ab:G5572T, ORF1ab:C6543T, ORF1ab:G7042T, ORF1ab:A10323G, ORF1ab:C14408T, ORF1ab:T16743C, ORF1ab:C18395T, ORF1ab:G18462A, ORF1ab:C18885T, ORF1ab:G19086T, ORF1ab:A20268G, ORF1ab:A20450G, S:C21614T, S:A23403G, S:G25049T, ORF3a:G25552T, M:G26660T, ORF7a:C27450T, ORF8:C27998T, N:C28854T,	16	M:L46F, N:S194L, ORF1a:G618V, ORF1a:L631F, ORF1a:M1769I, ORF1a:T2093I, ORF1a:M2259I, ORF1a:K3353R, ORF1b:P314L, ORF1b:A1643V, ORF1b:K1873N, ORF1b:N2328S, ORF3a:A54S, S:L18F, S:D614G, S:D1163Y,	
oV-19/Mexico/COA_LANGEBIO_IMSS_38556-NC/2	Falta	In process	20B	B.1.1.222	21	ORF1ab:A623G, ORF1ab:G2118T, ORF1ab:G2632T, ORF1ab:C3037T, ORF1ab:C8637T, ORF1ab:C9924T, ORF1ab:C10186T, ORF1ab:A13498G, ORF1ab:C14408T, ORF1ab:G14707A, ORF1ab:A18038T, ORF1ab:A18039T, ORF1ab:T19839C, ORF1ab:G20208T, S:G22331A, S:A23403G, S:A23756G, S:G25249T, N:G28817T, N:G28881A, N:G28882A, N:G28883C,	17	N:A182S, N:R203K, N:G204R, ORF1a:K120E, ORF1a:G618V, ORF1a:M789I, ORF1a:T2791I, ORF1a:A3220V, ORF1b:T11A, ORF1b:P314L, ORF1b:A414T, ORF1b:Q1524L, ORF1b:Q2247H, S:G257S, S:D614G, S:T732A, S:M1229I,	ORF7b:27879-27891,
oV-19/Mexico/DUR_LANGEBIO_IMSS_36866-NC/2	EPI_ISL_2969927	In process	20B	B.1.1.222	18	ORF1ab:C346T, ORF1ab:C478T, ORF1ab:T682G, ORF1ab:C3037T, ORF1ab:C10029T, ORF1ab:C10036T, ORF1ab:C14408T, ORF1ab:C18326T, ORF1ab:C19011A, ORF1ab:T19839C, S:G22021T, S:A23403G, S:A23756G, S:C24237T, ORF3a:G25912T, ORF8:G28001T, N:G28881A, N:G28882A, N:G28883C,	12	N:R203K, N:G204R, ORF1a:D139E, ORF1a:T3255I, ORF1b:P314L, ORF1b:A1620V, ORF1b:D1848E, ORF3a:G174C, S:M153I, S:D614G, S:T732A, S:A892V,	
oV-19/Mexico/DUR_LANGEBIO_IMSS_37503-NC/2	EPI_ISL_2969928	In process	20B	B.1.1.519	25	ORF1ab:A866G, ORF1ab:C1009T, ORF1ab:C1170T, ORF1ab:T1618C, ORF1ab:C3037T, ORF1ab:C3140T, ORF1ab:C5183T, ORF1ab:G6753A, ORF1ab:C10029T, ORF1ab:C10954T, ORF1ab:A11117G, ORF1ab:C11916T, ORF1ab:C12789T, ORF1ab:C14408T, ORF1ab:T19839C, ORF1ab:C21306T, S:G22256A, S:A23403G, S:C23604A, S:A23756G, ORF8:T27904C, N:A28864T, N:G28881A, N:G28882A, N:G28883C, N:C29197T,	17	N:R203K, N:G204R, ORF1a:I201V, ORF1a:S302F, ORF1a:P959S, ORF1a:P1640S, ORF1a:R2163H, ORF1a:T3255I, ORF1a:I3618V, ORF1a:S3884L, ORF1a:T4175I, ORF1b:P314L, ORF8:L4P, S:G232S, S:D614G, S:P681H, S:T732A,	
oV-19/Mexico/DUR_LANGEBIO_IMSS_38016-NC/2	EPI_ISL_2969929	In process	20B	B.1.1.519	23	ORF1ab:C3037T, ORF1ab:C3140T, ORF1ab:C10029T, ORF1ab:C10954T, ORF1ab:A11117G, ORF1ab:C12789T, ORF1ab:C14408T, ORF1ab:G14831T, ORF1ab:C15720T, ORF1ab:A15812G, ORF1ab:T19839C, ORF1ab:A20540C, ORF1ab:C21161T, ORF1ab:C21306T, ORF1ab:G21422A, S:A23403G, S:C23604A, S:A23756G, S:C24919T, ORF3a:C25521T, N:G28881A, N:G28882A, N:G28883C, ORF10:C29614T,	15	N:R203K, N:G204R, ORF1a:P959S, ORF1a:T3255I, ORF1a:I3618V, ORF1a:T4175I, ORF1b:P314L, ORF1b:C455F, ORF1b:N782S, ORF1b:K2358T, ORF1b:A2565V, ORF1b:R2652K, S:D614G, S:P681H, S:T732A,	

oV-19/Mexico/DUR_LANGEBIO_IMSS_38025-NC/2(	EPI_ISL_2969930	In process	20A	B.1	26	ORF1ab:C1288T, ORF1ab:G1408T, ORF1ab:G2118T, ORF1ab:G2305T, ORF1ab:C2902T, ORF1ab:C3037T, ORF1ab:G6476A, ORF1ab:G6802T, ORF1ab:A9614G, ORF1ab:C10335T, ORF1ab:T12751G, ORF1ab:C14408T, ORF1ab:C16877T, ORF1ab:C17304T, ORF1ab:C17548A, ORF1ab:C18060T, ORF1ab:G18816C, ORF1ab:A20268G, S:C21846G, S:G22147A, S:G22487C, S:A23403G, S:C23664T, ORF3a:G25455T, ORF3a:G25770T, ORF3a:T25807C, 3'UTR:T29807C,	16	ORF1a:E381D, ORF1a:G618V, ORF1a:K680N, ORF1a:V2071I, ORF1a:L2179F, ORF1a:T3117A, ORF1a:A3357V, ORF1b:P314L, ORF1b:T1137I, ORF1b:L1361I, ORF3a:K21N, ORF3a:R126S, S:T95S, S:E309Q, S:D614G, S:A701V,
oV-19/Mexico/NLE_LANGEBIO_IMSS_34985-NC/2(	EPI_ISL_2969990	In process	20B	B.1.1.519	17	ORF1ab:C3037T, ORF1ab:C3140T, ORF1ab:T3745C, ORF1ab:C3768T, ORF1ab:C10029T, ORF1ab:C10954T, ORF1ab:A11117G, ORF1ab:C12789T, ORF1ab:C14408T, ORF1ab:C21306T, S:A23403G, S:C23604A, S:A23756G, ORF3a:C25844T, N:G28881A, N:G28882A, N:G28883C, N:C29197T,	12	N:R203K, N:G204R, ORF1a:P959S, ORF1a:T1168I, ORF1a:T3255I, ORF1a:I3618V, ORF1a:T4175I, ORF1b:P314L, ORF3a:T151I, S:D614G, S:P681H, S:T732A,
oV-19/Mexico/NLE_LANGEBIO_IMSS_35136-NC/2(	EPI_ISL_2969991	In process	20B	B.1.1.519	22	ORF1ab:G1738T, ORF1ab:C3037T, ORF1ab:C3140T, ORF1ab:C10029T, ORF1ab:C10954T, ORF1ab:A11117G, ORF1ab:C12789T, ORF1ab:C14408T, ORF1ab:G14707A, ORF1ab:A18038T, ORF1ab:A18039T, ORF1ab:T19839C, ORF1ab:A19974G, ORF1ab:T20958C, ORF1ab:C21306T, S:A23403G, S:C23604A, S:A23756G, ORF7a:C27600T, N:G28881A, N:G28882A, N:G28883C, N:C29197T,	12	N:R203K, N:G204R, ORF1a:P959S, ORF1a:T3255I, ORF1a:I3618V, ORF1a:T4175I, ORF1b:P314L, ORF1b:A414T, ORF1b:Q1524L, S:D614G, S:P681H, S:T732A,
oV-19/Mexico/NLE_LANGEBIO_IMSS_35312-NC/2(	EPI_ISL_2969992	In process	20B	B.1.1.519	21	ORF1ab:G2118T, ORF1ab:C3037T, ORF1ab:C3140T, ORF1ab:T3745C, ORF1ab:A8031G, ORF1ab:C10029T, ORF1ab:C10954T, ORF1ab:A11117G, ORF1ab:C12789T, ORF1ab:C14408T, ORF1ab:C16726T, ORF1ab:T19839C, ORF1ab:C21306T, S:A23403G, S:C23604A, S:A23756G, ORF3a:C25844T, N:G28881A, N:G28882A, N:G28883C, N:C29197T, N:G29315A,	15	N:R203K, N:G204R, N:D348N, ORF1a:G618V, ORF1a:P959S, ORF1a:K2589R, ORF1a:T3255I, ORF1a:I3618V, ORF1a:T4175I, ORF1b:P314L, ORF1b:H1087Y, ORF3a:T151I, S:D614G, S:P681H, S:T732A,
oV-19/Mexico/NLE_LANGEBIO_IMSS_35857-NC/2(	EPI_ISL_2969993	In process	20B	B.1.1.519	22	ORF1ab:G2118T, ORF1ab:C3037T, ORF1ab:C3140T, ORF1ab:C10029T, ORF1ab:C10954T, ORF1ab:A11117G, ORF1ab:A12174G, ORF1ab:C12789T, ORF1ab:C14408T, ORF1ab:G14707A, ORF1ab:C15738T, ORF1ab:T19839C, ORF1ab:C21306T, S:A23403G, S:C23604A, S:A23756G, M:C26882T, ORF8:T27904C, N:G28881A, N:G28882A, N:G28883C, N:C29197T, N:G29383T,	15	N:R203K, N:G204R, N:K370N, ORF1a:G618V, ORF1a:P959S, ORF1a:T3255I, ORF1a:I3618V, ORF1a:N3970S, ORF1a:T4175I, ORF1b:P314L, ORF1b:A414T, ORF8:L4P, S:D614G, S:P681H, S:T732A,
oV-19/Mexico/NLE_LANGEBIO_IMSS_37009-NC/2(	EPI_ISL_2969994	In process	20A	B.1.243	16	ORF1ab:G2118T, ORF1ab:C3037T, ORF1ab:T5077C, ORF1ab:G9575A, ORF1ab:C10741T, ORF1ab:C14408T, ORF1ab:C16887T, ORF1ab:C19097T, ORF1ab:A19137G, ORF1ab:A20268G, S:A23403G, S:C23604A, S:T24076C, ORF8:G28233A, N:C28603T, N:C28854T, 3'UTR:T29710C,	8	N:S194L, ORF1a:G618V, ORF1a:V3104I, ORF1b:P314L, ORF1b:A1877V, ORF8:V114I, S:D614G, S:P681H,
oV-19/Mexico/NLE_LANGEBIO_IMSS_37061-NC/2(	EPI_ISL_2969995	In process	20A	B.1.243	19	ORF1ab:A2071G, ORF1ab:C2749T, ORF1ab:C3037T, ORF1ab:G3875T, ORF1ab:C7420T, ORF1ab:C10741T, ORF1ab:A11474G, ORF1ab:C14408T, ORF1ab:G18040T, ORF1ab:C19097T, ORF1ab:A19137G, ORF1ab:A20268G, S:A23403G, S:C23604A, S:T24076C, S:G25244T, N:C28854T, N:C29119T, ORF10:G29587T, 3'UTR:T29710C,	9	N:S194L, ORF1a:A1204S, ORF1a:I3737V, ORF1b:P314L, ORF1b:A1525S, ORF1b:A1877V, S:D614G, S:P681H, S:V1228L,

oV-19/Mexico/NLE_LANGEBIO_IMSS_37456/NC/2	EPI_ISL_2969996	In process	20B	D.2	17	ORF1ab:C3037T, ORF1ab:C3140T, ORF1ab:C10029T, ORF1ab:C10954T, ORF1ab:A11117G, ORF1ab:C12789T, ORF1ab:C14408T, ORF1ab:T19839C, ORF1ab:C21306T, S:G23401A, S:A23403G, S:C23604A, S:A23756G, ORF3a:C25413T, ORF8:G28027T, N:G28881A, N:G28882A, N:G28883C,	11	N:R203K, N:G204R, ORF1a:P959S, ORF1a:T3255I, ORF1a:I3618V, ORF1a:T4175I, ORF1b:P314L, ORF8:W45L, S:D614G, S:P681H, S:T732A,
oV-19/Mexico/SON_LANGEBIO_IMSS_28900-NC/2	EPI_ISL_2970041	In process	20A	B.1.243	15	5'UTR:C241T, ORF1ab:T3016C, ORF1ab:C3037T, ORF1ab:C10741T, ORF1ab:C14408T, ORF1ab:G19542T, S:A23403G, S:C23604A, S:T24076C, S:A24774T, S:G25311T, N:C28854T, N:G29134T, N:C29236T, N:G29266A, 3'UTR:T29710C,	7	N:S194L, ORF1b:P314L, ORF1b:M2025I, S:D614G, S:P681H, S:Q1071L, S:C1250F,
oV-19/Mexico/YUC_LANGEBIO_IMSS_05338-NC/2	EPI_ISL_2970060	In process	20B	B.1.1.519	27	5'UTR:C203T, 5'UTR:C222T, 5'UTR:C241T, ORF1ab:C1513T, ORF1ab:A2497T, ORF1ab:C3037T, ORF1ab:C3140T, ORF1ab:C3464T, ORF1ab:T3745C, ORF1ab:C8344T, ORF1ab:C10029T, ORF1ab:C10954T, ORF1ab:A11117G, ORF1ab:C12789T, ORF1ab:C14408T, ORF1ab:T17877A, ORF1ab:C20703T, ORF1ab:C21306T, S:C22995A, S:A23403G, S:C23595T, S:C23604A, S:A23756G, ORF3a:C25844T, N:G28881A, N:G28882A, N:G28883C, N:C29197T,	14	N:R203K, N:G204R, ORF1a:P959S, ORF1a:H1067Y, ORF1a:T3255I, ORF1a:I3618V, ORF1a:T4175I, ORF1b:P314L, ORF3a:T151I, S:T478K, S:D614G, S:T678I, S:P681H, S:T732A,
hCoV-19/Mexico/AGU-IBT-IMSS-195/2021	EPI_ISL_1288392	In process	20G	B.1.596	19	5'UTR:C241T, ORF1ab:C1059T, ORF1ab:C1223T, ORF1ab:G2054A, ORF1ab:C3037T, ORF1ab:A9828G, ORF1ab:C10319T, ORF1ab:A11789G, ORF1ab:C14408T, ORF1ab:A18424G, ORF1ab:C21304T, S:A23403G, ORF3a:G25563T, ORF3a:G25907T, M:C26781T, ORF8:C27964T, N:G28307T, N:C28472T, N:C28869T, N:T28888C,	18	M:L87F, N:A12S, N:P67S, N:P199L, ORF1a:T265I, ORF1a:L320F, ORF1a:V597I, ORF1a:E3188G, ORF1a:L3352F, ORF1a:I3842V, ORF1b:P314L, ORF1b:N1653D, ORF1b:R2613C, ORF3a:Q57H, ORF3a:G172V, ORF8:S24L, ORF9b:M8I, S:D614G,
hCoV-19/Mexico/AGU-IBT-IMSS-196/2021	EPI_ISL_1288393	In process	20G	B.1.2	18	5'UTR:C241T, ORF1ab:C1059T, ORF1ab:C3037T, ORF1ab:C5907T, ORF1ab:C10319T, ORF1ab:C12663T, ORF1ab:C14408T, ORF1ab:A18424G, ORF1ab:G20060T, ORF1ab:T20346C, ORF1ab:C21304T, S:A22600T, S:A23403G, S:C24034T, ORF3a:G25563T, ORF3a:G25907T, ORF8:C27964T, N:C28472T, N:C28869T.	15	N:P67S, N:P199L, ORF1a:T265I, ORF1a:T1881I, ORF1a:L3352F, ORF1a:A4133V, ORF1b:P314L, ORF1b:N1653D, ORF1b:S2198I, ORF1b:R2613C, ORF3a:Q57H, ORF3a:G172V, ORF8:S24L, S:R346S, S:D614G,
hCoV-19/Mexico/AGU-IBT-IMSS-223/2021	EPI_ISL_1288417	In process	20B	B.1.1.519	25	5'UTR:C203T, 5'UTR:C222T, 5'UTR:C241T, ORF1ab:C3037T, ORF1ab:C3140T, ORF1ab:G9407A, ORF1ab:C10029T, ORF1ab:C10954T, ORF1ab:A11117G, ORF1ab:C12789T, ORF1ab:C14408T, ORF1ab:T19839C, ORF1ab:C21306T, S:G22936T, S:C22995A, S:C23086T, S:A23403G, S:C23604A, S:A23756G, ORF3a:C25782T, N:G28378T, N:G28881A, N:G28882A, N:G28883C, N:C29197T, 3'UTR:G29773T,	14	N:R203K, N:G204R, ORF1a:P959S, ORF1a:V3048I, ORF1a:T3255I, ORF1a:I3618V, ORF1a:T4175I, ORF1b:P314L, ORF9b:R32L, S:K458N, S:T478K, S:D614G, S:P681H, S:T732A,
hCoV-19/Mexico/AGU-IBT-IMSS-238/2021	EPI_ISL_1288154	In process	20G	B.1.2	23	5'UTR:C241T, ORF1ab:C1059T, ORF1ab:A1808G, ORF1ab:C3037T, ORF1ab:C3874T, ORF1ab:C10319T, ORF1ab:C13366T, ORF1ab:G13471A, ORF1ab:G13960A, ORF1ab:C14408T, ORF1ab:C15720T, ORF1ab:A18424G, ORF1ab:G20060T, ORF1ab:C21304T, S:C21727T, S:A23403G, S:G25354T, ORF3a:G25563T, ORF3a:G25785T, ORF3a:G25907T, ORF6:C27247T, ORF8:C27964T, N:C28472T, N:C28869T,	16	N:P67S, N:P199L, ORF1a:T265I, ORF1a:K515E, ORF1a:L3352F, ORF1b:V2I, ORF1b:V165I, ORF1b:P314L, ORF1b:N1653D, ORF1b:S2198I, ORF1b:R2613C, ORF3a:Q57H, ORF3a:W131C, ORF3a:G172V, ORF8:S24L, S:D614G,

hCoV-19/Mexico/AGU-IBT-IMSS-348/2021	EPI_ISL_1288155	In process	21C (Epsilon)	B.1.429	26	5'UTR:C241T, ORF1ab:C1059T, ORF1ab:C2395T, ORF1ab:T2597C, ORF1ab:C3037T, ORF1ab:C8326T, ORF1ab:C8947T, ORF1ab:G10523C, ORF1ab:C12100T, ORF1ab:A12878G, ORF1ab:C13554T, ORF1ab:C14408T, ORF1ab:G17014T, S:G21600T, S:G22018T, S:T22917G, S:A23403G, S:T24349C, ORF3a:G25563T, ORF3a:C25587T, M:C26681T, ORF7b:G27890T, ORF8:G28191T, ORF8:A28272T, N:C28887T, N:G28975T, N:C29362T,	13	N:T205I, N:M234I, ORF1a:T265I, ORF1a:V3420L, ORF1a:I4205V, ORF1b:P314L, ORF1b:D1183Y, ORF3a:Q57H, ORF8:V100L, S:S13I, S:W152C, S:L452R, S:D614G,
hCoV-19/Mexico/AGU-IBT-IMSS-446/2020	EPI_ISL_1301631	In process	20C	B.1	5	5'UTR:C241T, ORF1ab:C1059T, ORF1ab:C3037T, ORF1ab:C14408T, S:A23403G, ORF3a:G25563T,	4	ORF1a:T265I, ORF1b:P314L, ORF3a:Q57H, S:D614G,
hCoV-19/Mexico/AGU-IBT-IMSS-447/2020	EPI_ISL_1301525	In process	20B	B.1.1.344	12	5'UTR:C241T, ORF1ab:C3037T, ORF1ab:T9172C, ORF1ab:A10948G, ORF1ab:C14408T, ORF1ab:T16950C, ORF1ab:G18255T, S:A23403G, S:G23608T, M:G26730T, N:G28881A, N:G28882A, N:G28883C,	6	M:V70F, N:R203K, N:G204R, ORF1b:P314L, ORF1b:M1596I, S:D614G,
hCoV-19/Mexico/AGU-InDRE-IBT-201/2020	EPI_ISL_1302183	In process	20G	B.1.2	18	5'UTR:C241T, ORF1ab:C1059T, ORF1ab:C3037T, ORF1ab:C10319T, ORF1ab:C10969T, ORF1ab:C12663T, ORF1ab:C13965T, ORF1ab:C14408T, ORF1ab:A18424G, ORF1ab:G20060T, ORF1ab:C21110T, S:A22600T, S:A23403G, ORF3a:G25563T, ORF3a:G25907T, M:G26589C, ORF8:C27964T, N:C28472T, N:C28869T,	15	M:V23L, N:P67S, N:P199L, ORF1a:T265I, ORF1a:L3352F, ORF1a:A4133V, ORF1b:P314L, ORF1b:N1653D, ORF1b:S2198I, ORF1b:T2548I, ORF3a:Q57H, ORF3a:G172V, ORF8:S24L, S:R346S, S:D614G,
hCoV-19/Mexico/AGU-InDRE-IBT-206/2020	EPI_ISL_1302200	In process	20A	B.1.396	19	5'UTR:C241T, ORF1ab:T689C, ORF1ab:C1191T, ORF1ab:A1678G, ORF1ab:C3037T, ORF1ab:G4960T, ORF1ab:A5608G, ORF1ab:C9521A, ORF1ab:C14408T, ORF1ab:A20129C, S:A23403G, S:C24904T, ORF3a:G25835A, ORF7a:C27654T, ORF7b:C27893A, ORF8:G28077T, ORF8:G28248A, N:C28854T, N:G29422T, ORF10:A29567G,	10	N:S194L, ORF1a:S142P, ORF1a:P309L, ORF1a:L3086I, ORF1b:P314L, ORF1b:E2221A, ORF3a:C148Y, ORF8:V62L, ORF8:D119N, S:D614G,
hCoV-19/Mexico/AGU-InDRE-IBT-210/2020	EPI_ISL_1302177	In process	20B	B.1.1.519	20	5'UTR:C203T, 5'UTR:C222T, 5'UTR:C241T, ORF1ab:G2879A, ORF1ab:C3037T, ORF1ab:C3140T, ORF1ab:C10029T, ORF1ab:C10954T, ORF1ab:A11117G, ORF1ab:C12789T, ORF1ab:C14408T, ORF1ab:T19839C, S:C22995A, S:A23403G, S:C23604A, S:A23756G, N:C28519T, N:G28881A, N:G28882A, N:G28883C, N:C29197T,	13	N:R203K, N:G204R, ORF1a:A872T, ORF1a:P959S, ORF1a:T3255I, ORF1a:I3618V, ORF1a:T4175I, ORF1b:P314L, ORF9b:T79I, S:T478K, S:D614G, S:P681H, S:T732A,
hCoV-19/Mexico/AGU-InDRE-IBT-211/2020	EPI_ISL_1302201	In process	20G	B.1.2	17	5'UTR:C241T, ORF1ab:C1059T, ORF1ab:C1884T, ORF1ab:G2243A, ORF1ab:C3037T, ORF1ab:C4093T, ORF1ab:C5055T, ORF1ab:C10319T, ORF1ab:G11222T, ORF1ab:C14408T, ORF1ab:A18424G, ORF1ab:G20060T, S:A23403G, ORF3a:G25563T, ORF3a:G25907T, ORF8:C27964T, N:C28472T, N:C28869T,	15	N:P67S, N:P199L, ORF1a:T265I, ORF1a:A540V, ORF1a:V660I, ORF1a:T1597I, ORF1a:L3352F, ORF1a:V3653F, ORF1b:P314L, ORF1b:N1653D, ORF1b:S2198I, ORF3a:Q57H, ORF3a:G172V, ORF8:S24L, S:D614G,
hCoV-19/Mexico/AGU-InDRE-IBT-212/2020	EPI_ISL_1302337	In process	20B	B.1.1.519	24	5'UTR:C203T, 5'UTR:C222T, 5'UTR:C241T, ORF1ab:T1099A, ORF1ab:C3037T, ORF1ab:A3103C, ORF1ab:C3140T, ORF1ab:C10029T, ORF1ab:C10954T, ORF1ab:G11083T, ORF1ab:A11117G, ORF1ab:G11365T, ORF1ab:C12789T, ORF1ab:C14408T, ORF1ab:T19839C, S:C22995A, S:A23403G, S:C23604A, S:A23756G, ORF3a:G25687T, N:G28881A, N:G28882A, N:G28883C, N:C29197T, N:G29527T,	16	N:R203K, N:G204R, N:Q418H, ORF1a:N278K, ORF1a:Q946H, ORF1a:P959S, ORF1a:T3255I, ORF1a:L3606F, ORF1a:I3618V, ORF1a:T4175I, ORF1b:P314L, ORF3a:A99S, S:T478K, S:D614G, S:P681H, S:T732A,
hCoV-19/Mexico/AGU-InDRE-IBT-213/2020	EPI_ISL_1302166	In process	20A	B.1.36.10	16	5'UTR:C241T, ORF1ab:C3037T, ORF1ab:C5869T, ORF1ab:A8116C, ORF1ab:C11095T, ORF1ab:C14408T, ORF1ab:C15738T, ORF1ab:G16935T, ORF1ab:G17721T, ORF1ab:C18189T, S:A23403G, ORF3a:G25793T, ORF3a:T25942C, ORF3a:C26022T, N:A28627G, N:C28854T, 3'UTR:G29711T,	7	N:S194L, ORF1a:E2617D, ORF1b:P314L, ORF1b:M1156I, ORF3a:R134L, ORF3a:Y184H, S:D614G,

hCoV-19/Mexico/AGU-InDRE-IBT-214/2020	EPI_ISL_1302184	In process	20A	B.1.396	19	5'UTR:C241T, ORF1ab:T689C, ORF1ab:C1191T, ORF1ab:A1678G, ORF1ab:C3037T, ORF1ab:G4960T, ORF1ab:A5608G, ORF1ab:C9521A, ORF1ab:C14408T, ORF1ab:A20129C, S:A23403G, S:C24904T, ORF3a:G25835A, ORF7a:C27654T, ORF7b:C27893A, ORF8:G28077T, ORF8:G28248A, N:C28854T, N:G29422T, ORF10:A29567G,	10	N:S194L, ORF1a:S142P, ORF1a:P309L, ORF1a:L3086I, ORF1b:P314L, ORF1b:E2221A, ORF3a:C148Y, ORF8:V62L, ORF8:D119N, S:D614G,	
hCoV-19/Mexico/AGU-InDRE-IBT-215/2020	EPI_ISL_1302274	In process	20B	B.1.1.517	18	5'UTR:C241T, ORF1ab:G2246A, ORF1ab:A2255G, ORF1ab:C3037T, ORF1ab:C9967T, ORF1ab:C14408T, ORF1ab:C18388T, ORF1ab:T19839C, ORF1ab:G19891T, S:C22388T, S:A23403G, S:A23756G, ORF8:A27994G, ORF8:G28083T, N:C28610T, N:G28881A, N:G28882A, N:G28883C, N:G29227T,	10	N:R203K, N:G204R, ORF1a:G661S, ORF1a:I664V, ORF1b:P314L, ORF1b:D2142Y, ORF8:D34G, ORF8:E64*, S:D614G, S:T732A,	3'UTR:29835,
hCoV-19/Mexico/AGU-InDRE-IBT-216/2020	EPI_ISL_1302202	In process	20B	B.1.1.519	24	5'UTR:C203T, 5'UTR:C222T, 5'UTR:C241T, ORF1ab:C3037T, ORF1ab:C3140T, ORF1ab:C10029T, ORF1ab:C10954T, ORF1ab:A11117G, ORF1ab:C12789T, ORF1ab:C13019T, ORF1ab:C14408T, ORF1ab:G14829A, ORF1ab:G16117T, ORF1ab:T19839C, S:C22995A, S:A23403G, S:C23604A, S:A23756G, ORF3a:A25411G, ORF8:T27904C, ORF8:C28087T, N:G28881A, N:G28882A, N:G28883C, N:C29197T,	16	N:R203K, N:G204R, ORF1a:P959S, ORF1a:T3255I, ORF1a:I3618V, ORF1a:T4175I, ORF1b:P314L, ORF1b:M454I, ORF1b:D884Y, ORF3a:I7V, ORF8:L4P, ORF8:A65V, S:T478K, S:D614G, S:P681H, S:T732A,	
hCoV-19/Mexico/AGU-InDRE-IBT-42/2020	EPI_ISL_1301681	In process	20C	B.1	5	5'UTR:C241T, ORF1ab:C1059T, ORF1ab:C3037T, ORF1ab:C14408T, S:A23403G, ORF3a:G25563T,	4	ORF1a:T265I, ORF1b:P314L, ORF3a:Q57H, S:D614G,	
hCoV-19/Mexico/AGU-InDRE-IBT-43/2020	EPI_ISL_1301682	In process	20B	B.1.1.344	11	5'UTR:C241T, ORF1ab:C3037T, ORF1ab:T9172C, ORF1ab:A10948G, ORF1ab:C14408T, ORF1ab:T16374C, ORF1ab:T16950C, S:A23403G, M:G26730T, N:G28881A, N:G28882A, N:G28883C,	5	M:V70F, N:R203K, N:G204R, ORF1b:P314L, S:D614G,	
hCoV-19/Mexico/AGU-InDRE-IBT-44/2020	EPI_ISL_1301683	In process	20B	B.1.1.344	12	5'UTR:C241T, ORF1ab:C3037T, ORF1ab:T9172C, ORF1ab:A10948G, ORF1ab:C14408T, ORF1ab:T16950C, ORF1ab:A18366G, S:A23403G, S:G23608T, M:G26730T, N:G28881A, N:G28882A, N:G28883C,	5	M:V70F, N:R203K, N:G204R, ORF1b:P314L, S:D614G,	
hCoV-19/Mexico/AGU-InDRE-IBT-45/2020	EPI_ISL_1301684	In process	20A	B.1	5	5'UTR:C241T, ORF1ab:C3037T, ORF1ab:C14408T, ORF1ab:C18877T, S:A23403G, ORF3a:G25563T,	3	ORF1b:P314L, ORF3a:Q57H, S:D614G,	
hCoV-19/Mexico/AGU-InDRE-IBT-46/2020	EPI_ISL_1301685	In process	20C	B.1	6	5'UTR:C241T, ORF1ab:T754C, ORF1ab:C1059T, ORF1ab:C3037T, ORF1ab:C14408T, S:A23403G, ORF3a:G25563T,	4	ORF1a:T265I, ORF1b:P314L, ORF3a:Q57H, S:D614G,	
hCoV-19/Mexico/AGU-InDRE-IBT-47/2020	EPI_ISL_1301686	In process	20B	B.1.1.344	11	5'UTR:C241T, ORF1ab:C3037T, ORF1ab:T9172C, ORF1ab:A10948G, ORF1ab:C14408T, ORF1ab:T16950C, S:A23403G, S:G23608T, M:G26730T, N:G28881A, N:G28882A, N:G28883C,	5	M:V70F, N:R203K, N:G204R, ORF1b:P314L, S:D614G,	
hCoV-19/Mexico/AGU-INER-IMSS-00190/2021	EPI_ISL_1279461	In process	20B	B.1.1.519	25	5'UTR:C203T, 5'UTR:C222T, 5'UTR:C241T, ORF1ab:C3037T, ORF1ab:C3140T, ORF1ab:A9848G, ORF1ab:C10029T, ORF1ab:C10954T, ORF1ab:A11117G, ORF1ab:C12789T, ORF1ab:A12961G, ORF1ab:C14408T, ORF1ab:T19839C, ORF1ab:C21306T, S:C22995A, S:A23403G, S:C23604A, S:A23756G, S:G24697T, ORF3a:C25867T, ORF8:G28001T, ORF8:C28057T, N:G28881A, N:G28882A, N:G28883C, N:C29197T,	15	N:R203K, N:G204R, ORF1a:P959S, ORF1a:S3195G, ORF1a:T3255I, ORF1a:I3618V, ORF1a:T4175I, ORF1b:P314L, ORF3a:P159S, ORF8:A55V, S:T478K, S:D614G, S:P681H, S:T732A, S:K1045N,	
hCoV-19/Mexico/BCN-IBT-IMSS-17/2020	EPI_ISL_955240	In process	20A	B.1.609	8	5'UTR:C241T, ORF1ab:C2594T, ORF1ab:C3037T, ORF1ab:C4582T, ORF1ab:C11102T, ORF1ab:C14408T, ORF1ab:A16474G, ORF1ab:A20268G, S:A23403G,	5	ORF1a:P777S, ORF1a:P3613S, ORF1b:P314L, ORF1b:S1003G, S:D614G,	
hCoV-19/Mexico/BCN-IBT-IMSS-18/2020	EPI_ISL_955241	In process	20A	B.1.609	7	5'UTR:C241T, ORF1ab:C3037T, ORF1ab:C4582T, ORF1ab:C11102T, ORF1ab:C14408T, ORF1ab:A20268G, S:A23403G, 3'UTR:C29743T,	3	ORF1a:P3613S, ORF1b:P314L, S:D614G,	
hCoV-19/Mexico/BCN-IBT-IMSS-445/2020	EPI_ISL_1301630	In process	20A	B.1	7	5'UTR:C241T, ORF1ab:C3037T, ORF1ab:C9438T, ORF1ab:C10277T, ORF1ab:C14408T, ORF1ab:C18877T, S:A23403G, ORF3a:G25563T,	5	ORF1a:T3058I, ORF1a:L3338F, ORF1b:P314L, ORF3a:Q57H, S:D614G,	

hCoV-19/Mexico/BCN-IBT-IMSS-474/2020	EPI_ISL_1301655	In process	20C	B.1	7	5'UTR:C241T, ORF1ab:C1059T, ORF1ab:C3037T, ORF1ab:C14408T, ORF1ab:G17014T, S:A23403G, ORF3a:G25563T, ORF3a:G25855C,	6	ORF1a:T265I, ORF1b:P314L, ORF1b:D1183Y, ORF3a:Q57H, ORF3a:D155H, S:D614G,
hCoV-19/Mexico/BCN-IBT-IMSS-475/2020	EPI_ISL_1301656	In process	20A	B.1.36.10	6	5'UTR:C241T, ORF1ab:C3037T, ORF1ab:C14408T, ORF1ab:C19983T, S:G21786T, S:A23403G, N:C28854T,	4	N:S194L, ORF1b:P314L, S:G75V, S:D614G,
hCoV-19/Mexico/BCN-IBT-IMSS-476/2020	EPI_ISL_1301657	In process	20A	B.1	7	5'UTR:C241T, ORF1ab:C3037T, ORF1ab:C4456T, ORF1ab:C12513T, ORF1ab:C14408T, ORF1ab:A20268G, S:A23403G, N:C28854T,	4	N:S194L, ORF1a:T4083M, ORF1b:P314L, S:D614G,
hCoV-19/Mexico/BCN-IBT-IMSS-477/2020	EPI_ISL_1301658	In process	20A	B.1	8	5'UTR:C241T, ORF1ab:C3037T, ORF1ab:A9377C, ORF1ab:C9491T, ORF1ab:C14408T, ORF1ab:A20268G, S:A23403G, S:G24867T, N:C28854T,	6	N:S194L, ORF1a:I3038L, ORF1a:H3076Y, ORF1b:P314L, S:D614G, S:W1102L,
hCoV-19/Mexico/BCN-IBT-IMSS-478/2020	EPI_ISL_1301659	In process	20A	B.1	7	5'UTR:C241T, ORF1ab:C3037T, ORF1ab:C5467T, ORF1ab:C14408T, ORF1ab:A20268G, S:A23403G, N:G28326T, N:C28854T,	5	N:G18V, N:S194L, ORF1b:P314L, ORF9b:V15L, S:D614G,
hCoV-19/Mexico/BCN-IBT-IMSS-479/2020	EPI_ISL_1301530	In process	20A	B.1.36.10	6	5'UTR:C241T, ORF1ab:C3037T, ORF1ab:C4456T, ORF1ab:C14408T, ORF1ab:T19914C, S:A23403G, N:C28854T,	3	N:S194L, ORF1b:P314L, S:D614G,
hCoV-19/Mexico/BCN-IBT-IMSS-480/2020	EPI_ISL_1301660	In process	20A	B.1	5	5'UTR:C241T, ORF1ab:C1489T, ORF1ab:C3037T, ORF1ab:C14408T, ORF1ab:A20268G, S:A23403G,	2	ORF1b:P314L, S:D614G,
hCoV-19/Mexico/BCN-IBT-IMSS-481/2020	EPI_ISL_1301661	In process	20B	B.1.1.222	8	5'UTR:C241T, ORF1ab:C3037T, ORF1ab:C14408T, ORF1ab:T19839C, S:A23403G, S:A23756G, N:G28881A, N:G28882A, N:G28883C,	5	N:R203K, N:G204R, ORF1b:P314L, S:D614G, S:T732A,
hCoV-19/Mexico/BCN-IBT-IMSS-482/2020	EPI_ISL_1301538	In process	20A	B.1.241	9	5'UTR:C241T, ORF1ab:C3037T, ORF1ab:C7768T, ORF1ab:C11575T, ORF1ab:C14408T, S:C21575T, S:C21621T, S:A23403G, S:C23778T, N:C28854T,	6	N:S194L, ORF1b:P314L, S:L5F, S:T20I, S:D614G, S:T739I,
hCoV-19/Mexico/BCN-IBT-IMSS-483/2020	EPI_ISL_1301662	In process	20A	B.1.245	11	5'UTR:C241T, ORF1ab:A1096G, ORF1ab:C3037T, ORF1ab:C6494A, ORF1ab:C13011T, ORF1ab:C14408T, ORF1ab:G14874T, ORF1ab:T17995C, ORF1ab:C18877T, S:C21575T, S:A23403G, ORF3a:G25563T,	8	ORF1a:L2077I, ORF1a:T4249I, ORF1b:P314L, ORF1b:K469N, ORF1b:F1510L, ORF3a:Q57H, S:L5F, S:D614G,
hCoV-19/Mexico/BCN-IBT-IMSS-484/2020	EPI_ISL_1301663	In process	20A	B.1.243	6	5'UTR:C241T, ORF1ab:C3037T, ORF1ab:C14408T, S:A23403G, S:T24076C, ORF7b:C27879T, N:C28854T,	4	N:S194L, ORF1b:P314L, ORF7b:H42Y, S:D614G,
hCoV-19/Mexico/BCN-IBT-IMSS-486/2020	EPI_ISL_1301665	In process	20C	B.1.320	12	5'UTR:C241T, ORF1ab:C379A, ORF1ab:C1059T, ORF1ab:C3037T, ORF1ab:T3718C, ORF1ab:G4657A, ORF1ab:C14408T, ORF1ab:C15738T, ORF1ab:G17722T, S:G21578T, S:A23403G, ORF3a:G25563T, ORF3a:C25721T,	7	ORF1a:T265I, ORF1b:P314L, ORF1b:V1419L, ORF3a:Q57H, ORF3a:A110V, S:V6F, S:D614G,
hCoV-19/Mexico/BCN-IBT-IMSS-487/2020	EPI_ISL_1301666	In process	20A	B.1.609	9	5'UTR:C241T, ORF1ab:C3037T, ORF1ab:C4582T, ORF1ab:G6847A, ORF1ab:C7600T, ORF1ab:C8175T, ORF1ab:C14408T, ORF1ab:A20268G, S:A23403G, ORF7b:C27879T,	5	ORF1a:M2194I, ORF1a:A2637V, ORF1b:P314L, ORF7b:H42Y, S:D614G,
hCoV-19/Mexico/BCN-IBT-IMSS-488/2020	EPI_ISL_1301714	In process	20A	B.1.609	9	5'UTR:C241T, ORF1ab:C2594T, ORF1ab:C3037T, ORF1ab:C4582T, ORF1ab:C11102T, ORF1ab:C14408T, ORF1ab:A16474G, ORF1ab:A20268G, S:A23403G, N:G28580A,	6	N:D103N, ORF1a:P777S, ORF1a:P3613S, ORF1b:P314L, ORF1b:S1003G, S:D614G,
hCoV-19/Mexico/BCN-IBT-IMSS-489/2020	EPI_ISL_1301715	In process	20A	B.1	6	5'UTR:C241T, ORF1ab:C3037T, ORF1ab:C8655T, ORF1ab:C14408T, ORF1ab:A20268G, S:A23403G, N:C28854T,	4	N:S194L, ORF1a:S2797F, ORF1b:P314L, S:D614G,
hCoV-19/Mexico/BCN-IBT-IMSS-519/2021	EPI_ISL_1302220	In process	20B	B.1.1.519	27	5'UTR:T201C, 5'UTR:C203T, 5'UTR:C222T, 5'UTR:C241T, ORF1ab:C936T, ORF1ab:G1738T, ORF1ab:C3037T, ORF1ab:C3140T, ORF1ab:T8569C, ORF1ab:T8729C, ORF1ab:C9430T, ORF1ab:C10029T, ORF1ab:C10954T, ORF1ab:A11117G, ORF1ab:C12789T, ORF1ab:C14408T, ORF1ab:T17068A, ORF1ab:T19839C, ORF1ab:A19974G, S:C22995A, S:A23403G, S:C23604A, S:A23756G, N:G28881A, N:G28882A, N:G28883C, N:C29197T, N:G29227T,	14	N:R203K, N:G204R, ORF1a:T224I, ORF1a:P959S, ORF1a:S2822P, ORF1a:T3255I, ORF1a:I3618V, ORF1a:T4175I, ORF1b:P314L, ORF1b:S1201T, S:T478K, S:D614G, S:P681H, S:T732A,
hCoV-19/Mexico/BCN-InDRE-IBT-1/2020	EPI_ISL_1301495	In process	20A	B.1.243	11	5'UTR:C241T, ORF1ab:C3037T, ORF1ab:C4010T, ORF1ab:C4534T, ORF1ab:C6196T, ORF1ab:C8655T, ORF1ab:C14408T, ORF1ab:T15432C, ORF1ab:A20268G, S:A23403G, S:T24076C, N:C28854T,	5	N:S194L, ORF1a:L1249F, ORF1a:S2797F, ORF1b:P314L, S:D614G,

hCoV-19/Mexico/BCN-InDRE-IBT-144/2020	EPI_ISL_1302182	In process	20A	B.1.36.10	8	5'UTR:G61T, 5'UTR:C241T, ORF1ab:C3037T, ORF1ab:C14408T, S:A23403G, S:A24913G, N:C28854T, N:C29095T, 3'UTR:C29718T,	3	N:S194L, ORF1b:P314L, S:D614G,	
hCoV-19/Mexico/BCN-InDRE-IBT-2/2020	EPI_ISL_1301521	In process	20A	B.1.243	11	5'UTR:C241T, ORF1ab:A1047G, ORF1ab:C3037T, ORF1ab:C8605T, ORF1ab:C14408T, ORF1ab:T16857C, S:A23403G, S:T24076C, S:C24919T, ORF3a:G25660T, N:C28854T, ORF10:C29642T,	5	N:S194L, ORF1a:K261R, ORF1b:P314L, ORF3a:V90F, S:D614G,	
hCoV-19/Mexico/BCN-InDRE-IBT-3/2020	EPI_ISL_1301532	In process	20A	B.1.399	7	5'UTR:C241T, ORF1ab:C3037T, ORF1ab:C3117T, ORF1ab:C8326T, ORF1ab:C14408T, S:A23403G, N:C28854T, N:C28887T,	5	N:S194L, N:T205I, ORF1a:T951I, ORF1b:P314L, S:D614G,	
hCoV-19/Mexico/BCN-InDRE-IBT-4/2020	EPI_ISL_1301470	In process	20A	B.1.243	8	5'UTR:C241T, ORF1ab:T778G, ORF1ab:C3037T, ORF1ab:G10318T, ORF1ab:C14408T, ORF1ab:A20268G, S:A23403G, S:T24076C, N:C28854T,	4	N:S194L, ORF1a:K3351N, ORF1b:P314L, S:D614G,	
hCoV-19/Mexico/BCN-InDRE-IBT-5/2020	EPI_ISL_1301695	In process	20C	B.1.320	11	5'UTR:C241T, ORF1ab:C379A, ORF1ab:C1059T, ORF1ab:C3037T, ORF1ab:G4657A, ORF1ab:C14408T, S:G21578T, S:A23403G, S:G25290T, ORF3a:G25407T, ORF3a:G25563T, ORF3a:G26167T,	8	ORF1a:T265I, ORF1b:P314L, ORF3a:M5I, ORF3a:Q57H, ORF3a:V259L, S:V6F, S:D614G, S:C1243F,	
hCoV-19/Mexico/BCS_INER_IMSS_00482/2021	EPI_ISL_1595603	In process	20B	B.1.1.222	12	5'UTR:C241T, ORF1ab:C3037T, ORF1ab:C6285T, ORF1ab:A13498G, ORF1ab:C14408T, ORF1ab:T19839C, S:G22331A, S:A23403G, S:A23756G, ORF3a:G25527T, N:G28881A, N:G28882A, N:G28883C,	9	N:R203K, N:G204R, ORF1a:T2007I, ORF1b:T11A, ORF1b:P314L, ORF3a:W45C, S:G257S, S:D614G, S:T732A,	ORF7b:27879-27891, ORF8:27915-28225,
hCoV-19/Mexico/BCS-IBT-IMSS-09/2020	EPI_ISL_955250	In process	20A	B.1.609	5	5'UTR:C241T, ORF1ab:C3037T, ORF1ab:C4582T, ORF1ab:C14408T, ORF1ab:A20268G, S:A23403G,	2	ORF1b:P314L, S:D614G,	
hCoV-19/Mexico/BCS-IBT-IMSS-101/2021	EPI_ISL_1288312	In process	21C (Epsilon)	B.1.427	21	5'UTR:C241T, ORF1ab:C1059T, ORF1ab:G1210A, ORF1ab:C2485T, ORF1ab:C3037T, ORF1ab:G9738C, ORF1ab:G13713A, ORF1ab:C14408T, ORF1ab:C16394T, ORF1ab:G17014T, ORF1ab:G20931T, ORF1ab:T21147A, S:G21600T, S:G22018T, S:T22917G, S:A23403G, ORF3a:G25563T, ORF3a:C25624T, M:C26681T, ORF8:A28272T, N:C28887T, N:C29362T,	13	N:T205I, ORF1a:T265I, ORF1a:M315I, ORF1a:S3158T, ORF1b:P314L, ORF1b:P976L, ORF1b:D1183Y, ORF3a:Q57H, ORF3a:H78Y, S:S13I, S:W152C, S:L452R, S:D614G,	
hCoV-19/Mexico/BCS-IBT-IMSS-102/2021	EPI_ISL_1288313	In process	20B	B.1.1.519	30	5'UTR:T201C, 5'UTR:C203T, 5'UTR:C222T, 5'UTR:C241T, ORF1ab:C583T, ORF1ab:G1738T, ORF1ab:C3037T, ORF1ab:C3140T, ORF1ab:C3737T, ORF1ab:C10029T, ORF1ab:C10954T, ORF1ab:A11117G, ORF1ab:C12789T, ORF1ab:G13576T, ORF1ab:C14408T, ORF1ab:G14559T, ORF1ab:T18042C, ORF1ab:A19782G, ORF1ab:T19839C, ORF1ab:A19974G, ORF1ab:C21306T, S:C22995A, S:A23403G, S:C23604A, S:A23756G, ORF3a:G25699A, M:T26861C, N:G28881A, N:G28882A, N:G28883C, N:C29197T,	14	N:R203K, N:G204R, ORF1a:P959S, ORF1a:P1158S, ORF1a:T3255I, ORF1a:I3618V, ORF1a:T4175I, ORF1b:A37S, ORF1b:P314L, ORF3a:A103T, S:T478K, S:D614G, S:P681H, S:T732A,	
hCoV-19/Mexico/BCS-IBT-IMSS-104/2021	EPI_ISL_1288314	In process	20A	B.1.232	22	5'UTR:C241T, ORF1ab:C643T, ORF1ab:C829T, ORF1ab:C1594T, ORF1ab:G2293T, ORF1ab:C3037T, ORF1ab:T3265C, ORF1ab:C7564T, ORF1ab:C11758T, ORF1ab:C11941T, ORF1ab:C14408T, ORF1ab:A20268G, ORF1ab:C21145T, ORF1ab:C21304T, S:G22599A, S:A23403G, S:G23593T, S:C25282T, ORF3a:G25690T, M:C26681T, ORF8:G27987T, N:C28854T, N:C29272T,	10	N:S194L, ORF1a:Q676H, ORF1b:P314L, ORF1b:L2560F, ORF1b:R2613C, ORF3a:G100C, ORF8:V32L, S:R346K, S:D614G, S:Q677H,	

hCoV-19/Mexico/BCS-IBT-IMSS-106/2021	EPI_ISL_1288316	In process	20B	B.1.1.519	27	5'UTR:C203T, 5'UTR:G204T, 5'UTR:C222T, 5'UTR:C241T, ORF1ab:C3037T, ORF1ab:C3140T, ORF1ab:C4898A, ORF1ab:C8078T, ORF1ab:C10029T, ORF1ab:C10954T, ORF1ab:A11117G, ORF1ab:C14408T, ORF1ab:C16887T, ORF1ab:T19839C, ORF1ab:C21034T, ORF1ab:C21306T, S:C22995A, S:A23403G, S:C23604A, S:A23756G, ORF3a:G25423C, ORF3a:G25996T, ORF8:T27904C, ORF8:C28087T, N:G28881A, N:G28882A, N:G28883C, N:C29197T,	17	N:R203K, N:G204R, ORF1a:P959S, ORF1a:H1545N, ORF1a:P2605S, ORF1a:T3255I, ORF1a:I3618V, ORF1b:P314L, ORF1b:L2523F, ORF3a:G11R, ORF3a:V202L, ORF8:L4P, ORF8:A65V, S:T478K, S:D614G, S:P681H, S:T732A,	
hCoV-19/Mexico/BCS-IBT-IMSS-108/2021	EPI_ISL_1288317	In process	20A	B.1.243	27	5'UTR:C241T, ORF1ab:G806A, ORF1ab:C1514T, ORF1ab:C1551T, ORF1ab:G2458T, ORF1ab:C3037T, ORF1ab:G3483T, ORF1ab:C5140A, ORF1ab:A6223G, ORF1ab:C7072T, ORF1ab:T8473C, ORF1ab:G10396T, ORF1ab:C14408T, ORF1ab:T16976C, ORF1ab:G17280T, ORF1ab:G17814A, ORF1ab:C18795T, ORF1ab:T19149C, ORF1ab:A19667G, ORF1ab:A20268G, S:G21893A, S:A23403G, S:G23587C, S:T24076C, S:A25060G, ORF3a:T25899C, N:C28854T, N:G29543T,	13	N:S194L, ORF1a:A181T, ORF1a:H417Y, ORF1a:A429V, ORF1a:M731I, ORF1a:G1073V, ORF1a:D1625E, ORF1b:P314L, ORF1b:V1170A, ORF1b:D2067G, S:D111N, S:D614G, S:Q675H,	
hCoV-19/Mexico/BCS-IBT-IMSS-109/2021	EPI_ISL_1288318	In process	20B	B.1.1.519	24	5'UTR:C203T, 5'UTR:C222T, 5'UTR:C241T, ORF1ab:G443A, ORF1ab:C3037T, ORF1ab:C3140T, ORF1ab:C5869T, ORF1ab:C10029T, ORF1ab:C10954T, ORF1ab:A11117G, ORF1ab:C12789T, ORF1ab:C14408T, ORF1ab:T19839C, ORF1ab:C21306T, S:C22995A, S:A23403G, S:C23604A, S:A23756G, ORF8:T27904C, ORF8:C28087T, N:G28881A, N:G28882A, N:G28883C, N:C29197T, N:G29449T,	14	N:R203K, N:G204R, ORF1a:V60I, ORF1a:P959S, ORF1a:T3255I, ORF1a:I3618V, ORF1a:T4175I, ORF1b:P314L, ORF8:L4P, ORF8:A65V, S:T478K, S:D614G, S:P681H, S:T732A,	
hCoV-19/Mexico/BCS-IBT-IMSS-11/2020	EPI_ISL_955233	In process	20A	B.1.609	6	5'UTR:C241T, ORF1ab:C3037T, ORF1ab:C4582T, ORF1ab:C13458T, ORF1ab:C14408T, ORF1ab:A20268G, S:A23403G,	3	ORF1a:S4398L, ORF1b:P314L, S:D614G,	
hCoV-19/Mexico/BCS-IBT-IMSS-12/2020	EPI_ISL_955251	In process	20A	B.1.609	5	5'UTR:C241T, ORF1ab:C3037T, ORF1ab:C4582T, ORF1ab:C14408T, ORF1ab:A20268G, S:A23403G,	2	ORF1b:P314L, S:D614G,	
hCoV-19/Mexico/BCS-IBT-IMSS-13/2020	EPI_ISL_955252	In process	20A	B.1.609	5	5'UTR:C241T, ORF1ab:C3037T, ORF1ab:C4582T, ORF1ab:C14408T, ORF1ab:A20268G, S:A23403G,	2	ORF1b:P314L, S:D614G,	
hCoV-19/Mexico/BCS-IBT-IMSS-14/2020	EPI_ISL_955253	In process	20A	B.1.609	6	5'UTR:C241T, ORF1ab:C3037T, ORF1ab:C4582T, ORF1ab:C14408T, ORF1ab:C19524T, ORF1ab:A20268G, S:A23403G,	2	ORF1b:P314L, S:D614G,	
hCoV-19/Mexico/BCS-IBT-IMSS-15/2020	EPI_ISL_955242	In process	20A	B.1.609	5	5'UTR:C241T, ORF1ab:C3037T, ORF1ab:C4582T, ORF1ab:C14408T, ORF1ab:A20268G, S:A23403G,	2	ORF1b:P314L, S:D614G,	
hCoV-19/Mexico/BCS-IBT-IMSS-16/2020	EPI_ISL_955243	In process	20A	B.1.609	5	5'UTR:C241T, ORF1ab:C3037T, ORF1ab:C4582T, ORF1ab:C14408T, ORF1ab:A20268G, S:A23403G,	2	ORF1b:P314L, S:D614G,	
hCoV-19/Mexico/BCS-IBT-IMSS-20/2020	EPI_ISL_955255	In process	20A	B.1.609	5	5'UTR:C241T, ORF1ab:C3037T, ORF1ab:C4582T, ORF1ab:C14408T, ORF1ab:A20268G, S:A23403G,	2	ORF1b:P314L, S:D614G,	
hCoV-19/Mexico/BCS-IBT-IMSS-226/2021	EPI_ISL_1288179	In process	20B	B.1.1.222	17	5'UTR:G61T, 5'UTR:C241T, ORF1ab:C3037T, ORF1ab:C10029T, ORF1ab:C14408T, ORF1ab:C15237T, ORF1ab:T19839C, S:A23403G, S:A23756G, S:C23802T, ORF3a:G25912T, ORF3a:C26195T, ORF7b:A27756G, ORF8:A27921G, ORF8:G28001T, N:G28881A, N:G28882A, N:G28883C,	11	N:R203K, N:G204R, ORF1a:T3255I, ORF1b:P314L, ORF3a:G174C, ORF3a:T268M, ORF7b:M1V, ORF8:I10V, S:D614G, S:T732A, S:T747I,	3'UTR:29746-29762,

hCoV-19/Mexico/BCS-IBT-IMSS-230/2021	EPI_ISL_1288423	In process	20B	B.1.1.519	29	5'UTR:C203T, 5'UTR:C222T, 5'UTR:C241T, ORF1ab:G443A, ORF1ab:C2710T, ORF1ab:C3037T, ORF1ab:C3140T, ORF1ab:C5869T, ORF1ab:C7162T, ORF1ab:C10029T, ORF1ab:C10954T, ORF1ab:A11117G, ORF1ab:C12789T, ORF1ab:C14408T, ORF1ab:C17000T, ORF1ab:G17815A, ORF1ab:C18348T, ORF1ab:T19839C, ORF1ab:C21306T, S:C22995A, S:A23403G, S:C23604A, S:A23756G, ORF3a:G25816C, ORF8:T27904C, ORF8:C28087T, N:G28881A, N:G28882A, N:G28883C, N:C29197T,	17	N:R203K, N:G204R, ORF1a:V60I, ORF1a:P959S, ORF1a:T3255I, ORF1a:I3618V, ORF1a:T4175I, ORF1b:P314L, ORF1b:T1178I, ORF1b:G1450R, ORF3a:D142H, ORF8:L4P, ORF8:A65V, S:T478K, S:D614G, S:P681H, S:T732A,
hCoV-19/Mexico/BCS-IBT-IMSS-231/2021	EPI_ISL_1288424	In process	20B	B.1.1.519	25	5'UTR:C203T, 5'UTR:C222T, 5'UTR:C241T, ORF1ab:G443A, ORF1ab:C3037T, ORF1ab:C3140T, ORF1ab:C5869T, ORF1ab:C10029T, ORF1ab:C10954T, ORF1ab:A11117G, ORF1ab:C12789T, ORF1ab:C14408T, ORF1ab:C17000T, ORF1ab:G17815A, ORF1ab:T19839C, ORF1ab:C21306T, S:C22995A, S:A23403G, S:C23604A, S:A23756G, ORF8:T27904C, ORF8:C28087T, N:G28881A, N:G28882A, N:G28883C, N:C29197T,	16	N:R203K, N:G204R, ORF1a:V60I, ORF1a:P959S, ORF1a:T3255I, ORF1a:I3618V, ORF1a:T4175I, ORF1b:P314L, ORF1b:T1178I, ORF1b:G1450R, ORF8:L4P, ORF8:A65V, S:T478K, S:D614G, S:P681H, S:T732A,
hCoV-19/Mexico/BCS-IBT-IMSS-232/2021	EPI_ISL_1288425	In process	20B	B.1.1.519	33	5'UTR:C203T, 5'UTR:C222T, 5'UTR:C241T, ORF1ab:G443A, ORF1ab:C2459T, ORF1ab:G3004T, ORF1ab:C3037T, ORF1ab:C3140T, ORF1ab:C5869T, ORF1ab:C10029T, ORF1ab:C10954T, ORF1ab:A11117G, ORF1ab:C12789T, ORF1ab:C14408T, ORF1ab:C14676T, ORF1ab:G15594T, ORF1ab:C17000T, ORF1ab:G17815A, ORF1ab:T18018C, ORF1ab:C18705T, ORF1ab:T19839C, ORF1ab:C20719T, ORF1ab:C21306T, S:C22995A, S:A23403G, S:C23604A, S:A23756G, ORF8:T27904C, ORF8:C28087T, N:G28395T, N:G28881A, N:G28882A, N:G28883C, N:C29197T,	21	N:R41L, N:R203K, N:G204R, ORF1a:V60I, ORF1a:P732S, ORF1a:E913D, ORF1a:P959S, ORF1a:T3255I, ORF1a:I3618V, ORF1a:T4175I, ORF1b:P314L, ORF1b:K709N, ORF1b:T1178I, ORF1b:G1450R, ORF8:L4P, ORF8:A65V, ORF9b:G38C, S:T478K, S:D614G, S:P681H, S:T732A,
hCoV-19/Mexico/BCS-IBT-IMSS-233/2021	EPI_ISL_1288426	In process	20B	B.1.1.519	32	5'UTR:T201C, 5'UTR:C203T, 5'UTR:C222T, 5'UTR:C241T, ORF1ab:C583T, ORF1ab:G1738T, ORF1ab:C3037T, ORF1ab:C3140T, ORF1ab:T9322C, ORF1ab:C10029T, ORF1ab:C10954T, ORF1ab:A11117G, ORF1ab:C12789T, ORF1ab:G13576T, ORF1ab:C14408T, ORF1ab:G14559T, ORF1ab:A15671G, ORF1ab:C16616T, ORF1ab:T19839C, ORF1ab:A19974G, ORF1ab:C21306T, ORF1ab:G21438T, S:C22995A, S:A23403G, S:C23604A, S:A23756G, ORF3a:G25699A, M:T26861C, M:C27155T, N:G28881A, N:G28882A, N:G28883C, N:C29197T,	16	N:R203K, N:G204R, ORF1a:P959S, ORF1a:T3255I, ORF1a:I3618V, ORF1a:T4175I, ORF1b:A37S, ORF1b:P314L, ORF1b:E735G, ORF1b:T1050I, ORF1b:M2657I, ORF3a:A103T, S:T478K, S:D614G, S:P681H, S:T732A,
hCoV-19/Mexico/BCS-IBT-IMSS-235/2021	EPI_ISL_1288427	In process	21C (Epsilon)	B.1.429	25	5'UTR:C241T, ORF1ab:C1059T, ORF1ab:G2354A, ORF1ab:C2395T, ORF1ab:T2597C, ORF1ab:G2747T, ORF1ab:C3037T, ORF1ab:C8947T, ORF1ab:C12100T, ORF1ab:A12878G, ORF1ab:C14408T, ORF1ab:G17014T, S:G21600T, S:G22018T, S:T22917G, S:A23403G, S:T24349C, ORF3a:G25563T, ORF3a:C25614T, M:C26681T, M:C27012T, ORF7b:G27890T, ORF8:A28272T, N:C28603T, N:C28887T, N:C29362T,	12	N:T205I, ORF1a:T265I, ORF1a:G697R, ORF1a:D828Y, ORF1a:I4205V, ORF1b:P314L, ORF1b:D1183Y, ORF3a:Q57H, S:S13I, S:W152C, S:L452R, S:D614G,

hCoV-19/Mexico/BCS-IBT-IMSS-236/2021	EPI_ISL_1288428	In process	20B	B.1.1.519	29	5'UTR:C203T, 5'UTR:C222T, 5'UTR:C241T, ORF1ab:G443A, ORF1ab:C2459T, ORF1ab:G3004T, ORF1ab:C3037T, ORF1ab:C3140T, ORF1ab:C5869T, ORF1ab:C10029T, ORF1ab:C10954T, ORF1ab:A11117G, ORF1ab:C12789T, ORF1ab:C14408T, ORF1ab:C14676T, ORF1ab:G15594T, ORF1ab:C17000T, ORF1ab:G17815A, ORF1ab:T19839C, ORF1ab:C21306T, S:C22995A, S:A23403G, S:C23604A, S:A23756G, ORF8:T27904C, ORF8:C28087T, N:G28881A, N:G28882A, N:G28883C, N:C29197T,	19	N:R203K, N:G204R, ORF1a:V60I, ORF1a:P732S, ORF1a:E913D, ORF1a:P959S, ORF1a:T3255I, ORF1a:I3618V, ORF1a:T4175I, ORF1b:P314L, ORF1b:K709N, ORF1b:T1178I, ORF1b:G1450R, ORF8:L4P, ORF8:A65V, S:T478K, S:D614G, S:P681H, S:T732A,
hCoV-19/Mexico/BCS-IBT-IMSS-237/2021	EPI_ISL_1288180	In process	20B	B.1.1.432	22	5'UTR:C241T, ORF1ab:G1599T, ORF1ab:G2144T, ORF1ab:C3037T, ORF1ab:A3183G, ORF1ab:G4960T, ORF1ab:A6985T, ORF1ab:C9319T, ORF1ab:A10323G, ORF1ab:C12412T, ORF1ab:G14181C, ORF1ab:T14313C, ORF1ab:C14408T, ORF1ab:C14724T, S:A23403G, ORF3a:G25912T, M:G26828T, N:G28321A, N:G28371T, N:G28881A, N:G28882A, N:G28883C, N:T29317C,	13	N:S33I, N:R203K, N:G204R, ORF1a:G445V, ORF1a:V627F, ORF1a:E973G, ORF1a:K3353R, ORF1b:L238F, ORF1b:P314L, ORF3a:G174C, ORF9b:R13H, ORF9b:V30L, S:D614G,
hCoV-19/Mexico/BCS-IBT-IMSS-239/2021	EPI_ISL_1288429	In process	20B	B.1.1.519	32	5'UTR:T201C, 5'UTR:C203T, 5'UTR:C222T, 5'UTR:C241T, ORF1ab:C583T, ORF1ab:G1738T, ORF1ab:A2406T, ORF1ab:C3037T, ORF1ab:C3140T, ORF1ab:A3581G, ORF1ab:C10029T, ORF1ab:C10954T, ORF1ab:A11117G, ORF1ab:C12789T, ORF1ab:G13576T, ORF1ab:G13897T, ORF1ab:C14408T, ORF1ab:G14559T, ORF1ab:A17047G, ORF1ab:T19839C, ORF1ab:A19974G, ORF1ab:C21306T, S:C22995A, S:A23403G, S:C23604A, S:A23756G, S:C23996T, ORF3a:G25699A, M:T26861C, N:G28881A, N:G28882A, N:G28883C, N:C29197T,	18	N:R203K, N:G204R, ORF1a:K714M, ORF1a:P959S, ORF1a:S1106G, ORF1a:T3255I, ORF1a:I3618V, ORF1a:T4175I, ORF1b:A37S, ORF1b:D144Y, ORF1b:P314L, ORF1b:K1194E, ORF3a:A103T, S:T478K, S:D614G, S:P681H, S:T732A, S:P812S,
hCoV-19/Mexico/BCS-IBT-IMSS-240/2021	EPI_ISL_1288430	In process	20B	B.1.1.519	29	5'UTR:T201C, 5'UTR:C203T, 5'UTR:C222T, 5'UTR:C241T, ORF1ab:C583T, ORF1ab:G1738T, ORF1ab:C3037T, ORF1ab:C3140T, ORF1ab:C10029T, ORF1ab:C10954T, ORF1ab:A11117G, ORF1ab:C12789T, ORF1ab:G13576T, ORF1ab:C14408T, ORF1ab:G14559T, ORF1ab:T19839C, ORF1ab:A19974G, ORF1ab:C20844T, ORF1ab:C21306T, ORF1ab:G21438T, S:C22995A, S:A23403G, S:C23604A, S:A23756G, ORF3a:G25699A, M:T26861C, N:G28881A, N:G28882A, N:G28883C, N:C29197T	14	N:R203K, N:G204R, ORF1a:P959S, ORF1a:T3255I, ORF1a:I3618V, ORF1a:T4175I, ORF1b:A37S, ORF1b:P314L, ORF1b:M2657I, ORF3a:A103T, S:T478K, S:D614G, S:P681H, S:T732A,
hCoV-19/Mexico/BCS-IBT-IMSS-242/2021	EPI_ISL_1288431	In process	20B	B.1.1.519	29	5'UTR:C203T, 5'UTR:C222T, 5'UTR:C241T, ORF1ab:G443A, ORF1ab:C3037T, ORF1ab:C3140T, ORF1ab:C5869T, ORF1ab:C7162T, ORF1ab:C9810T, ORF1ab:C10029T, ORF1ab:C10954T, ORF1ab:A11117G, ORF1ab:A11991G, ORF1ab:C12789T, ORF1ab:C14408T, ORF1ab:C17000T, ORF1ab:G17815A, ORF1ab:T19839C, ORF1ab:C21306T, S:C22995A, S:A23403G, S:C23604A, S:A23756G, ORF8:T27904C, ORF8:C27911T, ORF8:C28087T, N:G28881A, N:G28882A, N:G28883C, N:C29197T,	18	N:R203K, N:G204R, ORF1a:V60I, ORF1a:P959S, ORF1a:T3182I, ORF1a:T3255I, ORF1a:I3618V, ORF1a:E3909G, ORF1a:T4175I, ORF1b:P314L, ORF1b:T1178I, ORF1b:G1450R, ORF8:L4P, ORF8:A65V, S:T478K, S:D614G, S:P681H, S:T732A,

hCoV-19/Mexico/BCS-IBT-IMSS-243/2021	EPI_ISL_1288432	In process	20G	B.1.2	25	5'UTR:C241T, ORF1ab:G707A, ORF1ab:C1059T, ORF1ab:C1627T, ORF1ab:T1927C, ORF1ab:C3037T, ORF1ab:C6196T, ORF1ab:A7535G, ORF1ab:C10319T, ORF1ab:A10724G, ORF1ab:C14408T, ORF1ab:G15766T, ORF1ab:A18424G, ORF1ab:G18538T, ORF1ab:C21304T, S:A22255T, S:G23120T, S:A23403G, ORF3a:G25563T, ORF3a:G25907T, ORF3a:T25930C, M:G26951T, ORF8:C27964T, N:C28472T, N:C28869T, N:A29439T,	19	N:P67S, N:P199L, N:Q389L, ORF1a:E148K, ORF1a:T265I, ORF1a:T2424A, ORF1a:L3352F, ORF1a:T3487A, ORF1b:P314L, ORF1b:V767L, ORF1b:N1653D, ORF1b:V1691L, ORF1b:R2613C, ORF3a:Q57H, ORF3a:G172V, ORF3a:S180P, ORF8:S24L, S:A520S, S:D614G,
hCoV-19/Mexico/BCS-IBT-IMSS-244/2021	EPI_ISL_1288433	In process	20A	B.1.243	19	5'UTR:C241T, ORF1ab:G806A, ORF1ab:T2584A, ORF1ab:C3037T, ORF1ab:C5140A, ORF1ab:C5339T, ORF1ab:C14408T, ORF1ab:T16200C, ORF1ab:T18024C, ORF1ab:A20268G, S:A23403G, S:G23587C, S:C23589T, S:T24076C, ORF3a:C25844T, ORF6:T27384C, N:C28854T, N:G29440T, N:G29543T, 3'UTR:G29692T,	10	N:S194L, N:Q389H, ORF1a:A181T, ORF1a:D1625E, ORF1a:P1692S, ORF1b:P314L, ORF3a:T151I, S:D614G, S:Q675H, S:T676I,
hCoV-19/Mexico/BCS-IBT-IMSS-245/2021	EPI_ISL_1288434	In process	20B	B.1.1.519	28	5'UTR:T201C, 5'UTR:C203T, 5'UTR:C222T, 5'UTR:C241T, ORF1ab:C583T, ORF1ab:G1738T, ORF1ab:C3037T, ORF1ab:C3140T, ORF1ab:G6802C, ORF1ab:C10029T, ORF1ab:C10954T, ORF1ab:A11117G, ORF1ab:C12789T, ORF1ab:G13576T, ORF1ab:C14408T, ORF1ab:G14559T, ORF1ab:T19839C, ORF1ab:A19974G, ORF1ab:G20679T, ORF1ab:C21306T, S:C22995A, S:A23403G, S:C23604A, S:A23756G, ORF3a:G25699A, N:G28881A, N:G28882A, N:G28883C, N:C29197T,	14	N:R203K, N:G204R, ORF1a:P959S, ORF1a:L2179F, ORF1a:T3255I, ORF1a:I3618V, ORF1a:T4175I, ORF1b:A37S, ORF1b:P314L, ORF3a:A103T, S:T478K, S:D614G, S:P681H, S:T732A,
hCoV-19/Mexico/BCS-IBT-IMSS-246/2021	EPI_ISL_1288435	In process	20B	B.1.1.519	31	5'UTR:T201C, 5'UTR:C203T, 5'UTR:C222T, 5'UTR:C241T, ORF1ab:C583T, ORF1ab:G1738T, ORF1ab:C3037T, ORF1ab:C3140T, ORF1ab:G4960T, ORF1ab:G8083A, ORF1ab:C10029T, ORF1ab:C10954T, ORF1ab:A11117G, ORF1ab:C12789T, ORF1ab:G13576T, ORF1ab:C14408T, ORF1ab:G14559T, ORF1ab:T19839C, ORF1ab:A19974G, ORF1ab:C21306T, S:C22995A, S:A23403G, S:C23604A, S:A23756G, ORF3a:T25662C, ORF3a:G25699A, M:T26861C, ORF6:C27213T, N:G28881A, N:G28882A, N:G28883C, N:C29197T,	14	N:R203K, N:G204R, ORF1a:P959S, ORF1a:M2606I, ORF1a:T3255I, ORF1a:I3618V, ORF1a:T4175I, ORF1b:A37S, ORF1b:P314L, ORF3a:A103T, S:T478K, S:D614G, S:P681H, S:T732A,
hCoV-19/Mexico/BCS-IBT-IMSS-247/2021	EPI_ISL_1288436	In process	20B	B.1.1.519	34	5'UTR:T201C, 5'UTR:C203T, 5'UTR:C222T, 5'UTR:C241T, ORF1ab:C583T, ORF1ab:G1738T, ORF1ab:C2523T, ORF1ab:C3037T, ORF1ab:C3140T, ORF1ab:T8426C, ORF1ab:C10029T, ORF1ab:C10156T, ORF1ab:G10324T, ORF1ab:C10954T, ORF1ab:A11088C, ORF1ab:A11117G, ORF1ab:C12789T, ORF1ab:G13576T, ORF1ab:C14408T, ORF1ab:G14559T, ORF1ab:G16377T, ORF1ab:T19839C, ORF1ab:A19974G, ORF1ab:C21306T, S:C22995A, S:A23403G, S:C23422T, S:C23604A, S:A23756G, ORF3a:G25699A, M:T26861C, N:G28881A, N:G28882A, N:G28883C, N:C29197T	16	N:R203K, N:G204R, ORF1a:T753I, ORF1a:P959S, ORF1a:T3255I, ORF1a:K3353N, ORF1a:E3608A, ORF1a:I3618V, ORF1a:T4175I, ORF1b:A37S, ORF1b:P314L, ORF3a:A103T, S:T478K, S:D614G, S:P681H, S:T732A,

hCoV-19/Mexico/BCS-IBT-IMSS-248/2021	EPI_ISL_1288437	In process	21C (Epsilon)	B.1.429	28	5'UTR:C241T, ORF1ab:C1059T, ORF1ab:C2395T, ORF1ab:T2597C, ORF1ab:C3037T, ORF1ab:C3225T, ORF1ab:C8947T, ORF1ab:C12100T, ORF1ab:A12878G, ORF1ab:C13452T, ORF1ab:C14408T, ORF1ab:G17014T, ORF1ab:G18186T, ORF1ab:G18589A, S:G21600T, S:G21624T, S:G22018T, S:T22917G, S:T23155C, S:A23403G, S:T24349C, ORF3a:G25563T, M:C26681T, ORF7b:G27890T, ORF8:G28191T, ORF8:A28272T, N:C28887T, N:G28975T, N:C29362T,	17	N:T205I, N:M234I, ORF1a:T265I, ORF1a:T987I, ORF1a:I4205V, ORF1a:A4396V, ORF1b:P314L, ORF1b:D1183Y, ORF1b:M1573I, ORF1b:V1708I, ORF3a:Q57H, ORF8:V100L, S:S13I, S:R21I, S:W152C, S:L452R, S:D614G,
hCoV-19/Mexico/BCS-IBT-IMSS-250/2021	EPI_ISL_1288438	In process	20B	B.1.1.519	34	5'UTR:C66T, 5'UTR:T201C, 5'UTR:C203T, 5'UTR:C222T, 5'UTR:C241T, ORF1ab:C583T, ORF1ab:C1263T, ORF1ab:C1437T, ORF1ab:G1738T, ORF1ab:C3037T, ORF1ab:C3140T, ORF1ab:C10029T, ORF1ab:G10384A, ORF1ab:C10954T, ORF1ab:A11117G, ORF1ab:C12100T, ORF1ab:C12789T, ORF1ab:G13576T, ORF1ab:C14408T, ORF1ab:G14559T, ORF1ab:C14625T, ORF1ab:T19839C, ORF1ab:A19974G, ORF1ab:C21306T, S:C22995A, S:A23403G, S:C23604A, S:A23756G, ORF3a:G25699A, N:G28881A, N:G28882A, N:G28883C, N:C29197T, 3'UTR:C29733T, 3'UTR:C29774T,	15	N:R203K, N:G204R, ORF1a:T333M, ORF1a:S391F, ORF1a:P959S, ORF1a:T3255I, ORF1a:I3618V, ORF1a:T4175I, ORF1b:A37S, ORF1b:P314L, ORF3a:A103T, S:T478K, S:D614G, S:P681H, S:T732A,
hCoV-19/Mexico/BCS-IBT-IMSS-251/2021	EPI_ISL_1288439	In process	20B	B.1.1.519	32	5'UTR:C66T, 5'UTR:T201C, 5'UTR:C203T, 5'UTR:C222T, 5'UTR:C241T, ORF1ab:C583T, ORF1ab:C1263T, ORF1ab:C1437T, ORF1ab:G1738T, ORF1ab:C3037T, ORF1ab:C3140T, ORF1ab:C10029T, ORF1ab:C10954T, ORF1ab:A11117G, ORF1ab:C12100T, ORF1ab:G13576T, ORF1ab:C14408T, ORF1ab:G14559T, ORF1ab:C14625T, ORF1ab:T19839C, ORF1ab:A19974G, ORF1ab:C21306T, S:C22995A, S:A23403G, S:C23604A, S:A23756G, ORF3a:G25699A, N:G28881A, N:G28882A, N:G28883C, N:C29197T, 3'UTR:C29733T, 3'UTR:C29774T,	14	N:R203K, N:G204R, ORF1a:T333M, ORF1a:S391F, ORF1a:P959S, ORF1a:T3255I, ORF1a:I3618V, ORF1b:A37S, ORF1b:P314L, ORF3a:A103T, S:T478K, S:D614G, S:P681H, S:T732A,
hCoV-19/Mexico/BCS-IBT-IMSS-252/2021	EPI_ISL_1288440	In process	20B	B.1.1.519	26	5'UTR:C203T, 5'UTR:C222T, 5'UTR:C241T, ORF1ab:G443A, ORF1ab:C3037T, ORF1ab:C3140T, ORF1ab:C5869T, ORF1ab:C7162T, ORF1ab:C10029T, ORF1ab:C10954T, ORF1ab:A11117G, ORF1ab:C12789T, ORF1ab:C14408T, ORF1ab:C17000T, ORF1ab:G17815A, ORF1ab:T19839C, ORF1ab:C21306T, S:C22995A, S:A23403G, S:C23604A, S:A23756G, ORF8:T27904C, ORF8:C28087T, N:G28881A, N:G28882A, N:G28883C, N:C29197T,	16	N:R203K, N:G204R, ORF1a:V60I, ORF1a:P959S, ORF1a:T3255I, ORF1a:I3618V, ORF1a:T4175I, ORF1b:P314L, ORF1b:T1178I, ORF1b:G1450R, ORF8:L4P, ORF8:A65V, S:T478K, S:D614G, S:P681H, S:T732A,
hCoV-19/Mexico/BCS-IBT-IMSS-260/2021	EPI_ISL_1288181	In process	21C (Epsilon)	B.1.429	30	5'UTR:C241T, ORF1ab:C1059T, ORF1ab:C2395T, ORF1ab:T2597C, ORF1ab:C3037T, ORF1ab:A3344G, ORF1ab:G4162T, ORF1ab:C8947T, ORF1ab:C12100T, ORF1ab:A12878G, ORF1ab:C14408T, ORF1ab:G17014T, ORF1ab:G19872T, ORF1ab:T20277C, ORF1ab:G21073A, S:G21600T, S:G22018T, S:T22917G, S:T23155C, S:A23403G, S:T24349C, S:G25177T, ORF3a:G25563T, M:C26681T, ORF7b:G27890T, ORF8:G28191T, ORF8:A28272T, N:C28887T, N:G28975T, N:C29362T, 3'UTR:G29773T,	15	N:T205I, N:M234I, ORF1a:T265I, ORF1a:S1027G, ORF1a:I4205V, ORF1b:P314L, ORF1b:D1183Y, ORF1b:V2536I, ORF3a:Q57H, ORF8:V100L, S:S13I, S:W152C, S:L452R, S:D614G, S:K1205N,

hCoV-19/Mexico/BCS-IBT-IMSS-282/2021	EPI_ISL_1288182	In process	20B	B.1.1.519	30	5'UTR:T201C, 5'UTR:C203T, 5'UTR:C222T, 5'UTR:C241T, ORF1ab:C583T, ORF1ab:G1738T, ORF1ab:C3037T, ORF1ab:C3140T, ORF1ab:C10029T, ORF1ab:C10954T, ORF1ab:A11117G, ORF1ab:C12789T, ORF1ab:G13576T, ORF1ab:C14408T, ORF1ab:G14559T, ORF1ab:T19839C, ORF1ab:A19974G, ORF1ab:C21306T, ORF1ab:G21438T, S:C22995A, S:A23403G, S:C23604A, S:A23756G, ORF3a:G25699A, M:T26861C, N:G28881A, N:G28882A, N:G28883C, N:C29197T, N:C29218T, 3'UTR:C29741T	14	N:R203K, N:G204R, ORF1a:P959S, ORF1a:T3255I, ORF1a:I3618V, ORF1a:T4175I, ORF1b:A37S, ORF1b:P314L, ORF1b:M2657I, ORF3a:A103T, S:T478K, S:D614G, S:P681H, S:T732A,
hCoV-19/Mexico/BCS-IBT-IMSS-308/2021	EPI_ISL_1288491	In process	20B	B.1.1.519	27	5'UTR:C203T, 5'UTR:C222T, 5'UTR:C241T, ORF1ab:C3037T, ORF1ab:C3140T, ORF1ab:C8078T, ORF1ab:C10029T, ORF1ab:C10954T, ORF1ab:A11117G, ORF1ab:C12789T, ORF1ab:C14408T, ORF1ab:C16887T, ORF1ab:A17655G, ORF1ab:T19839C, ORF1ab:C21034T, ORF1ab:C21306T, S:C22995A, S:A23403G, S:C23604A, S:A23756G, ORF3a:G25423C, ORF3a:G25996T, ORF8:T27904C, ORF8:C28087T, N:G28881A, N:G28882A, N:G28883C, N:C29197T,	17	N:R203K, N:G204R, ORF1a:P959S, ORF1a:P2605S, ORF1a:T3255I, ORF1a:I3618V, ORF1a:T4175I, ORF1b:P314L, ORF1b:L2523F, ORF3a:G11R, ORF3a:V202L, ORF8:L4P, ORF8:A65V, S:T478K, S:D614G, S:P681H, S:T732A,
hCoV-19/Mexico/BCS-IBT-IMSS-309/2021	EPI_ISL_1288492	In process	20B	B.1.1.519	27	5'UTR:C203T, 5'UTR:C222T, 5'UTR:C241T, ORF1ab:C3037T, ORF1ab:C3140T, ORF1ab:C6636T, ORF1ab:C10029T, ORF1ab:C10954T, ORF1ab:G11083T, ORF1ab:A11117G, ORF1ab:C12789T, ORF1ab:C14408T, ORF1ab:C14838T, ORF1ab:C16733T, ORF1ab:T19839C, ORF1ab:G20635C, ORF1ab:C21306T, S:C22995A, S:A23403G, S:C23604A, S:C23707T, S:A23756G, ORF3a:G26062T, M:C26912T, N:G28881A, N:G28882A, N:G28883C, N:C29197T	16	N:R203K, N:G204R, ORF1a:P959S, ORF1a:T2124I, ORF1a:T3255I, ORF1a:L3606F, ORF1a:I3618V, ORF1a:T4175I, ORF1b:P314L, ORF1b:S1089L, ORF1b:E2390Q, ORF3a:G224C, S:T478K, S:D614G, S:P681H, S:T732A,
hCoV-19/Mexico/BCS-IBT-IMSS-310/2021	EPI_ISL_1288493	In process	20A	B.1.243	16	5'UTR:C241T, ORF1ab:G806A, ORF1ab:T2584A, ORF1ab:C3037T, ORF1ab:C5140A, ORF1ab:C5239T, ORF1ab:T6516C, ORF1ab:G7059A, ORF1ab:C14408T, ORF1ab:A20268G, S:A23403G, S:G23587C, S:T24076C, ORF3a:C25844T, N:C28854T, N:C28887T, N:G29543T,	10	N:S194L, N:T205I, ORF1a:A181T, ORF1a:D1625E, ORF1a:L2084S, ORF1a:G2265D, ORF1b:P314L, ORF3a:T151I, S:D614G, S:Q675H,
hCoV-19/Mexico/BCS-IBT-IMSS-468/2020	EPI_ISL_1301593	In process	20A	B.1.609	8	5'UTR:C241T, ORF1ab:A1752G, ORF1ab:C3037T, ORF1ab:C4582T, ORF1ab:C14408T, ORF1ab:A15165G, ORF1ab:G19999T, ORF1ab:A20268G, S:A23403G,	4	ORF1a:Y496C, ORF1b:P314L, ORF1b:V2178F, S:D614G,
hCoV-19/Mexico/BCS-IBT-IMSS-469/2020	EPI_ISL_1301651	In process	20A	B.1.609	7	5'UTR:C241T, ORF1ab:C3037T, ORF1ab:C4582T, ORF1ab:C8175T, ORF1ab:C14408T, ORF1ab:G17808T, S:A23403G, ORF8:T27939C,	5	ORF1a:A2637V, ORF1b:P314L, ORF1b:K1447N, ORF8:F16L, S:D614G,
hCoV-19/Mexico/BCS-IBT-IMSS-470/2020	EPI_ISL_1301652	In process	20A	B.1	5	5'UTR:C241T, ORF1ab:C3037T, ORF1ab:C4582T, ORF1ab:C14408T, ORF1ab:G19999T, S:A23403G,	3	ORF1b:P314L, ORF1b:V2178F, S:D614G,
hCoV-19/Mexico/BCS-IBT-IMSS-473/2020	EPI_ISL_1301535	In process	20A	B.1	6	5'UTR:C241T, ORF1ab:C3037T, ORF1ab:C4582T, ORF1ab:C14408T, ORF1ab:G19999T, S:G21761T, S:A23403G,	4	ORF1b:P314L, ORF1b:V2178F, S:A67S, S:D614G,
hCoV-19/Mexico/BCS-IBT-IMSS-490/2020	EPI_ISL_1301594	In process	20A	B.1	6	5'UTR:C241T, ORF1ab:C3037T, ORF1ab:C4582T, ORF1ab:C14408T, ORF1ab:A18480G, S:A23403G, S:T23509C,	2	ORF1b:P314L, S:D614G,
hCoV-19/Mexico/BCS-IBT-IMSS-491/2020	EPI_ISL_1301667	In process	20A	B.1.609	5	5'UTR:C241T, ORF1ab:C3037T, ORF1ab:C4582T, ORF1ab:C14408T, ORF1ab:A20268G, S:A23403G,	2	ORF1b:P314L, S:D614G,
hCoV-19/Mexico/BCS-IBT-IMSS-493/2020	EPI_ISL_1301669	In process	20A	B.1.609	6	5'UTR:C241T, ORF1ab:C3037T, ORF1ab:C4582T, ORF1ab:C14408T, ORF1ab:G19999T, ORF1ab:A20268G, S:A23403G,	3	ORF1b:P314L, ORF1b:V2178F, S:D614G,
hCoV-19/Mexico/BCS-IBT-IMSS-494/2020	EPI_ISL_1301670	In process	20A	B.1	4	5'UTR:C241T, ORF1ab:C3037T, ORF1ab:C4582T, ORF1ab:C14408T, S:A23403G,	2	ORF1b:P314L, S:D614G,
hCoV-19/Mexico/BCS-IBT-IMSS-499/2020	EPI_ISL_1301596	In process	20A	B.1	6	5'UTR:C241T, ORF1ab:C3037T, ORF1ab:C4582T, ORF1ab:C14408T, ORF1ab:A18480G, S:A23403G, S:T23509C,	2	ORF1b:P314L, S:D614G,

hCoV-19/Mexico/BCS-IBT-IMSS-509/2020	EPI_ISL_1301597	In process	20A	B.1	5	5'UTR:C241T, ORF1ab:C3037T, ORF1ab:C14408T, ORF1ab:A20268G, S:A23403G, N:C28854T,	3	N:S194L, ORF1b:P314L, S:D614G,
hCoV-19/Mexico/BCS-IBT-IMSS-96/2021	EPI_ISL_1288308	In process	21C (Epsilon)	B.1.427	25	5'UTR:C241T, ORF1ab:C823T, ORF1ab:C1059T, ORF1ab:C3037T, ORF1ab:C5497T, ORF1ab:C9286T, ORF1ab:G9738C, ORF1ab:A11353G, ORF1ab:C12049T, ORF1ab:G13713A, ORF1ab:C14408T, ORF1ab:C15960T, ORF1ab:C16394T, ORF1ab:G17014T, S:G21600T, S:G22018T, S:T22917G, S:A23403G, S:T23560A, ORF3a:G25563T, ORF3a:A25968G, ORF3a:C26198A, M:C26681T, ORF8:A28272T, N:C28887T, N:C29362T,	12	N:T205I, ORF1a:T265I, ORF1a:S3158T, ORF1b:P314L, ORF1b:P976L, ORF1b:D1183Y, ORF3a:Q57H, ORF3a:T269K, S:S13I, S:W152C, S:L452R, S:D614G,
hCoV-19/Mexico/BCS-IBT-IMSS-97/2021	EPI_ISL_1288309	In process	20B	B.1.1.519	24	5'UTR:C203T, 5'UTR:C222T, 5'UTR:C241T, ORF1ab:C3037T, ORF1ab:C3140T, ORF1ab:C5183T, ORF1ab:G5917A, ORF1ab:C10029T, ORF1ab:G10870T, ORF1ab:C10954T, ORF1ab:A11117G, ORF1ab:C12789T, ORF1ab:C14408T, ORF1ab:T19839C, ORF1ab:C21306T, S:C22995A, S:A23403G, S:C23604A, S:A23756G, ORF8:C28253T, N:G28881A, N:G28882A, N:G28883C, N:G29072T, N:C29197T,	13	N:R203K, N:G204R, N:A267S, ORF1a:P959S, ORF1a:P1640S, ORF1a:T3255I, ORF1a:I3618V, ORF1a:T4175I, ORF1b:P314L, S:T478K, S:D614G, S:P681H, S:T732A,
hCoV-19/Mexico/BCS-IBT-IMSS-98/2021	EPI_ISL_1288310	In process	20B	B.1.1.432	26	5'UTR:C241T, ORF1ab:C3037T, ORF1ab:C4320T, ORF1ab:T5494C, ORF1ab:A6985T, ORF1ab:A8466G, ORF1ab:C9319T, ORF1ab:C10279T, ORF1ab:C12412T, ORF1ab:G14290T, ORF1ab:T14313C, ORF1ab:C14408T, ORF1ab:T19181C, ORF1ab:T19710C, S:A23403G, S:G24914T, ORF3a:C25613T, ORF3a:G25912T, ORF3a:G26062T, M:C26895T, N:G28881A, N:G28882A, N:G28883C, N:C29077T, N:T29317C, 3'UTR:T29728C, 3'UTR:G29744T,	13	M:H125Y, N:R203K, N:G204R, ORF1a:A1352V, ORF1a:K2734R, ORF1b:D275Y, ORF1b:P314L, ORF1b:V1905A, ORF3a:S74F, ORF3a:G174C, ORF3a:G224C, S:D614G, S:D1118Y,
hCoV-19/Mexico/BCS-InDRE-IBT-58/2020	EPI_ISL_1301567	In process	20A	B.1.36.10	4	5'UTR:C241T, ORF1ab:C3037T, ORF1ab:C14408T, S:A23403G, N:C28854T,	3	N:S194L, ORF1b:P314L, S:D614G,
hCoV-19/Mexico/BCS-InDRE-IBT-6/2020	EPI_ISL_1301460	In process	20A	B.1	10	5'UTR:C241T, ORF1ab:G520T, ORF1ab:C2536T, ORF1ab:C3037T, ORF1ab:C4582T, ORF1ab:C13436T, ORF1ab:C14408T, ORF1ab:C18981T, ORF1ab:T20571C, S:A23403G, 3'UTR:G29779T,	4	ORF1a:M85I, ORF1a:L4391F, ORF1b:P314L, S:D614G,
hCoV-19/Mexico/BCS-InDRE-IBT-60/2020	EPI_ISL_1301451	In process	20A	B.1	7	5'UTR:C241T, ORF1ab:C3037T, ORF1ab:C4582T, ORF1ab:C13436T, ORF1ab:C14408T, ORF1ab:C18981T, ORF1ab:T20571C, S:A23403G,	3	ORF1a:L4391F, ORF1b:P314L, S:D614G,
hCoV-19/Mexico/BCS-InDRE-IBT-61/2020	EPI_ISL_1301730	In process	20A	B.1.36.10	4	5'UTR:C241T, ORF1ab:C3037T, ORF1ab:C14408T, S:A23403G, N:C28854T,	3	N:S194L, ORF1b:P314L, S:D614G,
hCoV-19/Mexico/BCS-InDRE-IBT-62/2020	EPI_ISL_1301555	In process	20B	B.1.1	14	5'UTR:C241T, ORF1ab:C2113T, ORF1ab:C3037T, ORF1ab:T4996C, ORF1ab:C7834T, ORF1ab:C10228T, ORF1ab:C14408T, ORF1ab:C16457T, ORF1ab:T19839C, S:A23403G, ORF7a:T27687C, N:G28881A, N:G28882A, N:G28883C, N:G28884T,	5	N:R203K, N:G204L, ORF1b:P314L, ORF1b:S997L, S:D614G,
hCoV-19/Mexico/BCS-InDRE-IBT-63/2020	EPI_ISL_1301568	In process	20B	B.1.1	6	5'UTR:C241T, ORF1ab:C3037T, ORF1ab:C14408T, S:A23403G, N:G28881A, N:G28882A, N:G28883C,	4	N:R203K, N:G204R, ORF1b:P314L, S:D614G,
hCoV-19/Mexico/CAM-IBT-IMSS-426/2020	EPI_ISL_1301620	In process	20B	B.1.1	12	5'UTR:C241T, ORF1ab:C3037T, ORF1ab:G3685T, ORF1ab:C14408T, S:A23403G, ORF8:T27922C, ORF8:A28173G, ORF8:A28247G, N:A28296G, N:A28304G, N:G28881A, N:G28882A, N:G28883C,	10	N:N8S, N:N11D, N:R203K, N:G204R, ORF1a:Q1140H, ORF1b:P314L, ORF8:I10T, ORF8:K94E, ORF9b:I5V, S:D614G,
hCoV-19/Mexico/CAM-InDRE-IBT-33/2020	EPI_ISL_1301527	In process	20B	B.1.1.222	11	5'UTR:C241T, ORF1ab:C3037T, ORF1ab:C5826T, ORF1ab:C14408T, ORF1ab:T19839C, ORF1ab:G21055A, S:A23403G, S:A23756G, M:A26612T, N:G28881A, N:G28882A, N:G28883C,	7	N:R203K, N:G204R, ORF1a:T1854I, ORF1b:P314L, ORF1b:D2530N, S:D614G, S:T732A,
hCoV-19/Mexico/CAM-InDRE-IBT-34/2020	EPI_ISL_1301457	In process	20B	B.1.1.222	13	5'UTR:C241T, ORF1ab:C2595T, ORF1ab:C3037T, ORF1ab:C5826T, ORF1ab:C14408T, ORF1ab:C14925T, ORF1ab:T19839C, ORF1ab:G21055A, S:A23403G, S:A23756G, M:A26612T, N:G28881A, N:G28882A, N:G28883C,	8	N:R203K, N:G204R, ORF1a:P777L, ORF1a:T1854I, ORF1b:P314L, ORF1b:D2530N, S:D614G, S:T732A,

hCoV-19/Mexico/CAM-InDRE-IBT-35/2020	EPI_ISL_1301702	In process	20A	B.1	12	5'UTR:C241T, ORF1ab:C3037T, ORF1ab:C4582T, ORF1ab:C11941T, ORF1ab:C13860T, ORF1ab:C14408T, ORF1ab:G15109T, ORF1ab:C16952T, ORF1ab:G18756T, S:C21742T, S:G22627A, S:A23403G, S:C24861A,	5	ORF1b:P314L, ORF1b:V548L, ORF1b:T1162I, S:D614G, S:T1100K,	
hCoV-19/Mexico/CAM-InDRE-IBT-36/2020	EPI_ISL_1301703	In process	20A	B.1	11	5'UTR:C241T, ORF1ab:C3037T, ORF1ab:C4582T, ORF1ab:C11941T, ORF1ab:C13860T, ORF1ab:C14408T, ORF1ab:G15109T, ORF1ab:C16952T, S:C21742T, S:G22627A, S:A23403G, S:C24861A,	5	ORF1b:P314L, ORF1b:V548L, ORF1b:T1162I, S:D614G, S:T1100K,	
hCoV-19/Mexico/CAM-InDRE-IBT-37/2020	EPI_ISL_1301704	In process	20C	B.1.578	7	5'UTR:C241T, ORF1ab:C1059T, ORF1ab:C3037T, ORF1ab:C4534T, ORF1ab:C14408T, ORF1ab:A16011G, S:A23403G, ORF3a:G25563T,	4	ORF1a:T265I, ORF1b:P314L, ORF3a:Q57H, S:D614G,	
hCoV-19/Mexico/CAM-InDRE-IBT-48/2020	EPI_ISL_1301687	In process	20C	B.1	7	5'UTR:C241T, ORF1ab:C1059T, ORF1ab:C3037T, ORF1ab:G4868T, ORF1ab:C14408T, S:A23403G, ORF3a:G25563T, N:G28690T,	6	N:L139F, ORF1a:T265I, ORF1a:V1535L, ORF1b:P314L, ORF3a:Q57H, S:D614G,	
hCoV-19/Mexico/CAM-INER-IMSS-00368/2021	EPI_ISL_1279282	In process	20B	B.1.1.519	30	5'UTR:C203T, 5'UTR:C222T, 5'UTR:C241T, ORF1ab:A866G, ORF1ab:C1009T, ORF1ab:C3037T, ORF1ab:C3140T, ORF1ab:C5183T, ORF1ab:T7597C, ORF1ab:C10029T, ORF1ab:C10954T, ORF1ab:A11117G, ORF1ab:C11916T, ORF1ab:C12789T, ORF1ab:C13584T, ORF1ab:C14408T, ORF1ab:G15906T, ORF1ab:T19839C, ORF1ab:C21306T, S:C22995A, S:A23403G, S:C23604A, S:A23756G, ORF3a:G26199A, ORF8:T27904C, N:A28864T, N:G28881A, N:G28882A, N:G28883C, N:C29197T, 3'UTR:G29736T,	16	N:R203K, N:G204R, ORF1a:I201V, ORF1a:P959S, ORF1a:P1640S, ORF1a:T3255I, ORF1a:I3618V, ORF1a:S3884L, ORF1a:T4175I, ORF1b:P314L, ORF1b:Q813H, ORF8:L4P, S:T478K, S:D614G, S:P681H, S:T732A,	3'UTR:29750-29754,
hCoV-19/Mexico/CAM-INER-IMSS-00369/2021	EPI_ISL_1279294	In process	20B	B.1.1.222	18	5'UTR:C241T, ORF1ab:C3037T, ORF1ab:C8727T, ORF1ab:C10029T, ORF1ab:C14408T, ORF1ab:C18657T, ORF1ab:T19839C, ORF1ab:C20402T, S:A23403G, S:A23756G, ORF3a:G25481T, ORF3a:G25912T, ORF3a:C26195T, ORF7b:A27756G, ORF8:A27921G, ORF8:G28001T, N:G28881A, N:G28882A, N:G28883C,	13	N:R203K, N:G204R, ORF1a:A2821V, ORF1a:T3255I, ORF1b:P314L, ORF1b:S2312L, ORF3a:R30L, ORF3a:G174C, ORF3a:T268M, ORF7b:M1V, ORF8:I10V, S:D614G, S:T732A,	3'UTR:29746-29762,
hCoV-19/Mexico/CAM-INER-IMSS-00371/2021	EPI_ISL_1279614	In process	20B	B.1.1.519	27	5'UTR:C203T, 5'UTR:C222T, 5'UTR:C241T, ORF1ab:C3037T, ORF1ab:A3069G, ORF1ab:C3140T, ORF1ab:C6543T, ORF1ab:T9697C, ORF1ab:C10029T, ORF1ab:C10954T, ORF1ab:A11117G, ORF1ab:C12357T, ORF1ab:C12789T, ORF1ab:C14408T, ORF1ab:T18648C, ORF1ab:T19839C, ORF1ab:G20356T, ORF1ab:C21306T, S:C22995A, S:A23403G, S:C23604A, S:A23756G, ORF8:T27904C, ORF8:C28087T, N:G28881A, N:G28882A, N:G28883C, N:C29197T,	17	N:R203K, N:G204R, ORF1a:D935G, ORF1a:P959S, ORF1a:T2093I, ORF1a:T3255I, ORF1a:I3618V, ORF1a:T4031I, ORF1a:T4175I, ORF1b:P314L, ORF1b:G2297C, ORF8:L4P, ORF8:A65V, S:T478K, S:D614G, S:P681H, S:T732A,	
hCoV-19/Mexico/CHH_INER_IMSS_00375/2021	EPI_ISL_1585387	In process	20G	B.1.596	25	5'UTR:C222T, 5'UTR:C241T, ORF1ab:C1059T, ORF1ab:C3037T, ORF1ab:T5840C, ORF1ab:C10319T, ORF1ab:A11451G, ORF1ab:C14408T, ORF1ab:C16989T, ORF1ab:C17410T, ORF1ab:A18424G, ORF1ab:T18471C, ORF1ab:G20002T, ORF1ab:C21304T, S:A21643G, S:C21811A, S:A23403G, S:A23592C, ORF3a:G25563T, ORF3a:G25907T, E:C26456T, ORF8:C27964T, N:C28472T, N:C28869T, N:T29194C, N:T29377A,	17	E:P71L, N:P67S, N:P199L, ORF1a:T265I, ORF1a:Y1859H, ORF1a:L3352F, ORF1a:Q3729R, ORF1b:P314L, ORF1b:R1315C, ORF1b:N1653D, ORF1b:D2179Y, ORF1b:R2613C, ORF3a:Q57H, ORF3a:G172V, ORF8:S24L, S:D614G, S:Q677P,	
hCoV-19/Mexico/CHH_INER_IMSS_00376/2021	EPI_ISL_1595595	In process	20A	B.1.609	18	5'UTR:C241T, ORF1ab:C679T, ORF1ab:C2536T, ORF1ab:C3037T, ORF1ab:C4582T, ORF1ab:A8972G, ORF1ab:C11767T, ORF1ab:C14408T, ORF1ab:T19668C, ORF1ab:A20268G, S:G21786T, S:A23403G, S:C23604A, S:C23683T, S:A23898G, ORF3a:G25687T, N:G28975T, ORF10:G29645T, 3'UTR:C29738T,	8	N:M234I, ORF1a:I2903V, ORF1b:P314L, ORF3a:A99S, S:G75V, S:D614G, S:P681H, S:Q779R,	ORF7b:27786-27793

hCoV-19/Mexico/CHH-IBT-IMSS-439/2020	EPI_ISL_1301552	In process	20C	B.1	10	5'UTR:C241T, ORF1ab:T582C, ORF1ab:C1059T, ORF1ab:C3037T, ORF1ab:C10279T, ORF1ab:C14408T, S:A23403G, ORF3a:G25563T, ORF7b:G27758A, ORF7b:T27760A, ORF7b:T27761C,	7	ORF1a:V106A, ORF1a:T265I, ORF1b:P314L, ORF3a:Q57H, ORF7b:M1I, ORF7b:I2N, S:D614G,	ORF1ab: 686-694,
hCoV-19/Mexico/CHH-IBT-IMSS-497/2020	EPI_ISL_1301716	In process	20C	B.1	12	5'UTR:C241T, ORF1ab:T582C, ORF1ab:C1059T, ORF1ab:C3037T, ORF1ab:G3685T, ORF1ab:C8139T, ORF1ab:C9180T, ORF1ab:C10279T, ORF1ab:C14408T, ORF1ab:A18251G, S:A23403G, ORF3a:G25563T, 3'UTR:C29719T,	9	ORF1a:V106A, ORF1a:T265I, ORF1a:Q1140H, ORF1a:S2625F, ORF1a:S2972F, ORF1b:P314L, ORF1b:N1595S, ORF3a:Q57H, S:D614G,	
hCoV-19/Mexico/CHH-InDRE-IBT-104/2020	EPI_ISL_1301481	In process	20A	B.1.189	10	5'UTR:C241T, ORF1ab:C3037T, ORF1ab:C4202T, ORF1ab:C4582T, ORF1ab:G6404T, ORF1ab:C8655T, ORF1ab:C14408T, S:A23403G, S:T25123C, N:G28378T, N:G28881T,	6	N:R203M, ORF1a:V2047F, ORF1a:S2797F, ORF1b:P314L, ORF9b:R32L, S:D614G,	
hCoV-19/Mexico/CHH-InDRE-IBT-89/2020	EPI_ISL_1301705	In process	20C	B.1	13	5'UTR:C241T, ORF1ab:T582C, ORF1ab:C1059T, ORF1ab:C3037T, ORF1ab:T9519C, ORF1ab:C10279T, ORF1ab:C10626T, ORF1ab:C13824T, ORF1ab:C14408T, ORF1ab:C17747T, S:A23403G, ORF3a:G25563T, ORF7a:A27456G, 3'UTR:A29700G,	8	ORF1a:V106A, ORF1a:T265I, ORF1a:F3085S, ORF1a:A3454V, ORF1b:P314L, ORF1b:P1427L, ORF3a:Q57H, S:D614G,	
hCoV-19/Mexico/CHH-INER-IMSS-00195/2021	EPI_ISL_1279466	In process	20B	B.1.1.519	28	5'UTR:C203T, 5'UTR:C222T, 5'UTR:C241T, ORF1ab:C1960T, ORF1ab:C3037T, ORF1ab:C3140T, ORF1ab:C10029T, ORF1ab:G10384A, ORF1ab:C10954T, ORF1ab:A11117G, ORF1ab:C11653T, ORF1ab:C12789T, ORF1ab:C14408T, ORF1ab:T15726A, ORF1ab:T19590C, ORF1ab:T19839C, ORF1ab:C21306T, S:A22766G, S:C22995A, S:A23403G, S:C23604A, S:A23756G, M:C26833T, M:C27046T, N:G28881A, N:G28882A, N:G28883C, N:C29197T, N:G29527T,	15	M:A104V, M:T175M, N:R203K, N:G204R, N:Q418H, ORF1a:P959S, ORF1a:T3255I, ORF1a:I3618V, ORF1a:T4175I, ORF1b:P314L, S:I402V, S:T478K, S:D614G, S:P681H, S:T732A,	
hCoV-19/Mexico/CHH-INER-IMSS-00219/2021	EPI_ISL_1279485	In process	20G	B.1.596	25	5'UTR:C222T, 5'UTR:C241T, ORF1ab:C1059T, ORF1ab:C3037T, ORF1ab:T5840C, ORF1ab:C10319T, ORF1ab:A11451G, ORF1ab:C14408T, ORF1ab:C16989T, ORF1ab:C17410T, ORF1ab:A18424G, ORF1ab:T18471C, ORF1ab:G20002T, ORF1ab:C21304T, S:A21643G, S:C21811A, S:A23403G, S:A23592C, ORF3a:G25563T, ORF3a:G25907T, E:C26456T, ORF8:C27964T, N:C28472T, N:C28869T, N:T29194C, N:T29377A,	17	E:P71L, N:P67S, N:P199L, ORF1a:T265I, ORF1a:Y1859H, ORF1a:L3352F, ORF1a:Q3729R, ORF1b:P314L, ORF1b:R1315C, ORF1b:N1653D, ORF1b:D2179Y, ORF1b:R2613C, ORF3a:Q57H, ORF3a:G172V, ORF8:S24L, S:D614G, S:Q677P,	
hCoV-19/Mexico/CHH-INER-IMSS-00220/2021	EPI_ISL_1279266	In process	20I (Alpha, V1)	B.1.1.7	28	5'UTR:C241T, ORF1ab:C913T, ORF1ab:C3037T, ORF1ab:C3267T, ORF1ab:G5164T, ORF1ab:C5388A, ORF1ab:C5986T, ORF1ab:T6954C, ORF1ab:C14408T, ORF1ab:C14676T, ORF1ab:C15279T, ORF1ab:T16176C, S:A23063T, S:C23271A, S:A23403G, S:C23604A, S:C23709T, S:T24506G, S:G24914C, ORF8:C27972T, ORF8:G28048T, ORF8:A28111G, N:G28280C, N:A28281T, N:T28282A, N:G28881A, N:G28882A, N:G28883C, N:C28977T,	19	N:D3L, N:R203K, N:G204R, N:S235F, ORF1a:T1001I, ORF1a:E1633D, ORF1a:A1708D, ORF1a:I2230T, ORF1b:P314L, ORF8:Q27*, ORF8:R52I, ORF8:Y73C, S:N501Y, S:A570D, S:D614G, S:P681H, S:T716I, S:S982A, S:D1118H,	ORF1ab: 11288-11296, S:21765-21770, S:21992-21994, ORF8:28271,

hCoV-19/Mexico/CHH-INER-IMSS-00251/2021	EPI_ISL_1279513	In process	20G	B.1.2	24	5'UTR:C241T, ORF1ab:C583T, ORF1ab:C1059T, ORF1ab:C3037T, ORF1ab:C3832T, ORF1ab:C9474T, ORF1ab:C10319T, ORF1ab:C11109T, ORF1ab:G13094T, ORF1ab:T13216C, ORF1ab:G14187A, ORF1ab:C14408T, ORF1ab:C15738T, ORF1ab:A18424G, ORF1ab:C21304T, ORF1ab:A21390G, S:A23403G, S:C23604A, S:A24956G, ORF3a:G25563T, ORF3a:G25907T, ORF8:C27964T, N:C28472T, N:C28869T, N:C29363T,	17	N:P67S, N:P199L, N:P364S, ORF1a:T265I, ORF1a:A3070V, ORF1a:L3352F, ORF1a:A3615V, ORF1a:A4277S, ORF1b:P314L, ORF1b:N1653D, ORF1b:R2613C, ORF3a:Q57H, ORF3a:G172V, ORF8:S24L, S:D614G, S:P681H, S:I1132V,	
hCoV-19/Mexico/CHH-INER-IMSS-00260/2021	EPI_ISL_1279522	In process	20B	B.1.1.362	21	5'UTR:C186T, 5'UTR:C241T, ORF1ab:C280T, ORF1ab:G2246A, ORF1ab:A2375C, ORF1ab:C3037T, ORF1ab:A6424G, ORF1ab:C8917T, ORF1ab:C14408T, ORF1ab:C15656T, ORF1ab:C16887T, ORF1ab:A19062T, S:A23403G, S:T23878C, ORF3a:G25819T, N:G28881A, N:G28882A, N:G28883C, N:G28903T, N:C29249T, N:C29311T, 3'UTR:G29810T,	10	N:R203K, N:G204R, N:M210I, N:P326S, ORF1a:G661S, ORF1a:N704H, ORF1b:P314L, ORF1b:T730I, ORF3a:A143S, S:D614G,	
hCoV-19/Mexico/CHH-INER-IMSS-00261/2021	EPI_ISL_1279523	In process	20B	B.1.1.362	19	5'UTR:C186T, 5'UTR:C241T, ORF1ab:C280T, ORF1ab:G2246A, ORF1ab:A2375C, ORF1ab:C3037T, ORF1ab:C8917T, ORF1ab:C14408T, ORF1ab:C16887T, ORF1ab:A19062T, S:A23403G, S:T23878C, ORF3a:G25819T, N:G28881A, N:G28882A, N:G28883C, N:G28903T, N:C29249T, N:C29311T, 3'UTR:G29810T,	9	N:R203K, N:G204R, N:M210I, N:P326S, ORF1a:G661S, ORF1a:N704H, ORF1b:P314L, ORF3a:A143S, S:D614G,	
hCoV-19/Mexico/CHH-INER-IMSS-00262/2021	EPI_ISL_1279276	In process	:0J (Gamma, V3	P.1	31	5'UTR:C241T, ORF1ab:T733C, ORF1ab:C2749T, ORF1ab:C3037T, ORF1ab:C3828T, ORF1ab:A5648C, ORF1ab:A6319G, ORF1ab:A6613G, ORF1ab:C12778T, ORF1ab:C13860T, ORF1ab:C14408T, ORF1ab:G17259T, S:C21614T, S:C21621A, S:C21638T, S:G21974T, S:G22132T, S:A22812C, S:G23012A, S:A23063T, S:A23403G, S:C23525T, S:C24642T, S:G25088T, ORF3a:T26149C, ORF8:G28167A, N:C28512G, N:A28877T, N:G28878C, N:G28881A, N:G28882A, N:G28883C,	22	N:P80R, N:R203K, N:G204R, ORF1a:S1188L, ORF1a:K1795Q, ORF1b:P314L, ORF1b:E1264D, ORF3a:S253P, ORF8:E92K, ORF9b:Q77E, S:L18F, S:T20N, S:P26S, S:D138Y, S:R190S, S:K417T, S:E484K, S:N501Y, S:D614G, S:H655Y, S:T1027I, S:V1176F,	ORF1ab: 11288-11296,
hCoV-19/Mexico/CHH-INER-IMSS-00269/2021	EPI_ISL_1279528	In process	21C (Epsilon)	B.1.429	27	5'UTR:C241T, ORF1ab:C1059T, ORF1ab:C1419T, ORF1ab:C2395T, ORF1ab:T2597C, ORF1ab:C3037T, ORF1ab:C8320T, ORF1ab:C8947T, ORF1ab:C12100T, ORF1ab:A12878G, ORF1ab:C14408T, ORF1ab:G17014T, S:G21600T, S:G22018T, S:A22125G, S:C22127T, S:T22917G, S:A23403G, S:T24349C, ORF3a:G25563T, E:T26463C, M:C26681T, ORF7a:C27538T, ORF7b:G27890T, ORF8:A28272T, N:C28887T, N:T29233A, N:C29362T,	13	N:T205I, ORF1a:T265I, ORF1a:A385V, ORF1a:I4205V, ORF1b:P314L, ORF1b:D1183Y, ORF3a:Q57H, S:S13I, S:W152C, S:N188S, S:L189F, S:L452R, S:D614G,	
hCoV-19/Mexico/CHH-INER-IMSS-00272/2021	EPI_ISL_1279531	In process	20B	B.1.1.519	26	5'UTR:C203T, 5'UTR:C222T, 5'UTR:C241T, ORF1ab:T1417C, ORF1ab:C1889T, ORF1ab:C3037T, ORF1ab:C3140T, ORF1ab:C10029T, ORF1ab:A10323G, ORF1ab:C10954T, ORF1ab:A11117G, ORF1ab:C12789T, ORF1ab:C14120T, ORF1ab:C14408T, ORF1ab:C17410T, ORF1ab:T19839C, ORF1ab:C20451T, ORF1ab:C21306T, S:C22995A, S:A23403G, S:C23604A, S:A23756G, S:A25108C, N:G28881A, N:G28882A, N:G28883C, N:C29197T,	16	N:R203K, N:G204R, ORF1a:R542C, ORF1a:P959S, ORF1a:T3255I, ORF1a:K3353R, ORF1a:I3618V, ORF1a:T4175I, ORF1b:P218L, ORF1b:P314L, ORF1b:R1315C, S:T478K, S:D614G, S:P681H, S:T732A, S:E1182D,	

hCoV-19/Mexico/CHP-IBT-IMSS-118/2021	EPI_ISL_1288326	In process	20B	B.1.1.519	30	5'UTR:T201C, 5'UTR:C203T, 5'UTR:C222T, 5'UTR:C241T, ORF1ab:G323A, ORF1ab:G1738T, ORF1ab:C3037T, ORF1ab:C3140T, ORF1ab:T6329A, ORF1ab:C10029T, ORF1ab:C10954T, ORF1ab:A11117G, ORF1ab:C12789T, ORF1ab:C14408T, ORF1ab:A14527G, ORF1ab:G14559T, ORF1ab:G18538T, ORF1ab:T19839C, ORF1ab:A19974G, ORF1ab:C21306T, S:C22995A, S:A23403G, S:C23604A, S:A23756G, ORF3a:C25693T, ORF3a:G25699A, ORF7a:T27618C, N:G28881A, N:G28882A, N:G28883C, N:C29197T,	17	N:R203K, N:G204R, ORF1a:V20I, ORF1a:P959S, ORF1a:S2022T, ORF1a:T3255I, ORF1a:I3618V, ORF1a:T4175I, ORF1b:P314L, ORF1b:S354G, ORF1b:V1691L, ORF3a:L101F, ORF3a:A103T, S:T478K, S:D614G, S:P681H, S:T732A,	
hCoV-19/Mexico/CHP-IBT-IMSS-119/2021	EPI_ISL_1288327	In process	20B	B.1.1.519	29	5'UTR:T201C, 5'UTR:C203T, 5'UTR:C222T, 5'UTR:C241T, ORF1ab:T277C, ORF1ab:A1521G, ORF1ab:G1738T, ORF1ab:C3037T, ORF1ab:C3140T, ORF1ab:G7829T, ORF1ab:C10029T, ORF1ab:C10954T, ORF1ab:A11117G, ORF1ab:G11327A, ORF1ab:C12789T, ORF1ab:C14183T, ORF1ab:C14408T, ORF1ab:T19839C, ORF1ab:A19974G, ORF1ab:C21306T, S:T22897A, S:C22995A, S:A23403G, S:C23604A, S:A23756G, N:C28311T, N:G28881A, N:G28882A, N:G28883C, N:C29197T,	17	N:P13L, N:R203K, N:G204R, ORF1a:K419R, ORF1a:P959S, ORF1a:V2522F, ORF1a:T3255I, ORF1a:I3618V, ORF1a:A3688T, ORF1a:T4175I, ORF1b:T239I, ORF1b:P314L, ORF9b:P10S, S:T478K, S:D614G, S:P681H, S:T732A,	
hCoV-19/Mexico/CHP-IBT-IMSS-122/2021	EPI_ISL_1288329	In process	20B	B.1.1.519	23	5'UTR:C203T, 5'UTR:C222T, 5'UTR:C241T, ORF1ab:C3037T, ORF1ab:C3140T, ORF1ab:T5680C, ORF1ab:C10029T, ORF1ab:C10954T, ORF1ab:A11117G, ORF1ab:C12789T, ORF1ab:C14408T, ORF1ab:G17259T, ORF1ab:G19518T, ORF1ab:T19839C, ORF1ab:C21306T, S:C22995A, S:A23403G, S:C23604A, S:A23756G, ORF7b:G27806A, N:G28881A, N:G28882A, N:G28883C, N:C29197T,	13	N:R203K, N:G204R, ORF1a:P959S, ORF1a:T3255I, ORF1a:I3618V, ORF1a:T4175I, ORF1b:P314L, ORF1b:E1264D, ORF1b:L2017F, S:T478K, S:D614G, S:P681H, S:T732A,	
hCoV-19/Mexico/CHP-IBT-IMSS-123/2021	EPI_ISL_1288330	In process	20B	B.1.1.519	25	5'UTR:C203T, 5'UTR:C222T, 5'UTR:C241T, ORF1ab:C3037T, ORF1ab:C3140T, ORF1ab:T5680C, ORF1ab:C10029T, ORF1ab:C10954T, ORF1ab:A11117G, ORF1ab:C12789T, ORF1ab:C14408T, ORF1ab:G17259T, ORF1ab:G19518T, ORF1ab:T19839C, ORF1ab:C21306T, S:C21691T, S:C22995A, S:A23403G, S:C23604A, S:A23756G, ORF3a:C26165T, ORF7b:G27806A, N:G28881A, N:G28882A, N:G28883C, N:C29197T,	14	N:R203K, N:G204R, ORF1a:P959S, ORF1a:T3255I, ORF1a:I3618V, ORF1a:T4175I, ORF1b:P314L, ORF1b:E1264D, ORF1b:L2017F, ORF3a:P258L, S:T478K, S:D614G, S:P681H, S:T732A,	
hCoV-19/Mexico/CHP-IBT-IMSS-95/2021	EPI_ISL_1288307	In process	20B	B.1.1.519	28	5'UTR:T201C, 5'UTR:C203T, 5'UTR:C222T, 5'UTR:C241T, ORF1ab:T277C, ORF1ab:G1738T, ORF1ab:C3037T, ORF1ab:C3140T, ORF1ab:G7829T, ORF1ab:C10029T, ORF1ab:C10954T, ORF1ab:A11117G, ORF1ab:C12789T, ORF1ab:C14408T, ORF1ab:T19839C, ORF1ab:A19974G, ORF1ab:C21306T, S:C22995A, S:A23403G, S:C23604A, S:A23756G, S:C25069T, ORF8:G28001C, N:G28881A, N:G28882A, N:G28883C, N:G29081T, N:C29197T, N:C29249T,	14	N:R203K, N:G204R, N:V270L, N:P326S, ORF1a:P959S, ORF1a:V2522F, ORF1a:T3255I, ORF1a:I3618V, ORF1a:T4175I, ORF1b:P314L, S:T478K, S:D614G, S:P681H, S:T732A,	3'UTR:29722-29723
hCoV-19/Mexico/CHP-InDRE-IBT-159/2020	EPI_ISL_1302287	In process	20A	B.1	10	5'UTR:C241T, ORF1ab:C3037T, ORF1ab:C9778T, ORF1ab:G10882T, ORF1ab:A11782G, ORF1ab:A12124T, ORF1ab:C14408T, ORF1ab:C18508T, S:A23403G, N:C28854T, N:G29239A,	6	N:S194L, N:M322I, ORF1a:M3539I, ORF1b:P314L, ORF1b:L1681F, S:D614G,	

hCoV-19/Mexico/CHP-InDRE-IBT-194/2020	EPI_ISL_1302167	In process	20B	B.1.1.519	24	5'UTR:T201C, 5'UTR:C203T, 5'UTR:C222T, 5'UTR:C241T, ORF1ab:T277C, ORF1ab:G1738T, ORF1ab:C3037T, ORF1ab:C3140T, ORF1ab:G7829T, ORF1ab:C10029T, ORF1ab:C10954T, ORF1ab:A11117G, ORF1ab:C12789T, ORF1ab:C14408T, ORF1ab:T19839C, ORF1ab:A19974G, S:T22897A, S:C22995A, S:A23403G, S:C23604A, S:A23756G, N:G28881A, N:G28882A, N:G28883C, N:C29197T,	12	N:R203K, N:G204R, ORF1a:P959S, ORF1a:V2522F, ORF1a:T3255I, ORF1a:I3618V, ORF1a:T4175I, ORF1b:P314L, S:T478K, S:D614G, S:P681H, S:T732A,
hCoV-19/Mexico/CHP-InDRE-IBT-195/2020	EPI_ISL_1302300	In process	20B	B.1.1.519	23	5'UTR:T201C, 5'UTR:C203T, 5'UTR:C222T, 5'UTR:C241T, ORF1ab:T277C, ORF1ab:G1738T, ORF1ab:C3037T, ORF1ab:C3140T, ORF1ab:G7829T, ORF1ab:C10029T, ORF1ab:C10954T, ORF1ab:A11117G, ORF1ab:C12789T, ORF1ab:C14408T, ORF1ab:T19839C, ORF1ab:A19974G, S:C22995A, S:A23403G, S:C23604A, S:A23756G, N:G28881A, N:G28882A, N:G28883C, N:C29197T,	12	N:R203K, N:G204R, ORF1a:P959S, ORF1a:V2522F, ORF1a:T3255I, ORF1a:I3618V, ORF1a:T4175I, ORF1b:P314L, S:T478K, S:D614G, S:P681H, S:T732A,
hCoV-19/Mexico/CHP-InDRE-IBT-196/2020	EPI_ISL_1302310	In process	20B	B.1.1.519	22	5'UTR:C203T, 5'UTR:C222T, 5'UTR:C241T, ORF1ab:A1631G, ORF1ab:C3037T, ORF1ab:C3140T, ORF1ab:C10029T, ORF1ab:A10829C, ORF1ab:C10954T, ORF1ab:A11117G, ORF1ab:C12789T, ORF1ab:C14408T, ORF1ab:T19839C, S:C22995A, S:A23403G, S:C23604A, S:A23756G, ORF8:T27904C, ORF8:C28087T, N:G28881A, N:G28882A, N:G28883C, N:C29197T,	15	N:R203K, N:G204R, ORF1a:K456E, ORF1a:P959S, ORF1a:T3255I, ORF1a:I3522L, ORF1a:I3618V, ORF1a:T4175I, ORF1b:P314L, ORF8:L4P, ORF8:A65V, S:T478K, S:D614G, S:P681H, S:T732A,
hCoV-19/Mexico/CHP-InDRE-IBT-197/2020	EPI_ISL_1302197	In process	20B	B.1.1	15	5'UTR:C241T, ORF1ab:C3037T, ORF1ab:C5170T, ORF1ab:C7420T, ORF1ab:T10107C, ORF1ab:C14408T, ORF1ab:G18449T, ORF1ab:C19366T, S:A23403G, ORF8:G28083T, N:G28881A, N:G28882A, N:G28883C, N:C29197T, ORF10:C29635T, 3'UTR:A29700G,	8	N:R203K, N:G204R, ORF1a:V3281A, ORF1b:P314L, ORF1b:S1661I, ORF1b:P1967S, ORF8:E64*, S:D614G,
hCoV-19/Mexico/CHP-InDRE-IBT-83/2020	EPI_ISL_1301562	In process	20A	B.1	6	5'UTR:C241T, ORF1ab:C3037T, ORF1ab:C14408T, ORF1ab:G20709T, S:A23403G, N:G28907T, N:G29402T,	5	N:G212C, N:D377Y, ORF1b:P314L, ORF1b:M2414I, S:D614G,
hCoV-19/Mexico/CHP-InDRE-IBT-84/2020	EPI_ISL_1301570	In process	20A	B.1.36.10	5	5'UTR:C241T, ORF1ab:C3037T, ORF1ab:C14408T, S:A23403G, N:C28647T, N:C28854T,	4	N:A125V, N:S194L, ORF1b:P314L, S:D614G,
hCoV-19/Mexico/CHP-InDRE-IBT-85/2020	EPI_ISL_1301571	In process	20A	B.1.609	10	5'UTR:C241T, ORF1ab:C3037T, ORF1ab:T4092C, ORF1ab:C4582T, ORF1ab:C14184T, ORF1ab:C14408T, ORF1ab:G20134T, ORF1ab:A20268G, S:C22314A, S:A23403G, M:C26873T,	5	ORF1a:I1276T, ORF1b:P314L, ORF1b:V2223L, S:P251H, S:D614G,
hCoV-19/Mexico/CHP-InDRE-IBT-86/2020	EPI_ISL_1301572	In process	20A	B.1	5	5'UTR:C241T, ORF1ab:C3037T, ORF1ab:C14408T, ORF1ab:G18394T, S:A23403G, ORF3a:A25756G,	4	ORF1b:P314L, ORF1b:A1643S, ORF3a:R122G, S:D614G,
hCoV-19/Mexico/CHP-InDRE-IBT-87/2020	EPI_ISL_1301448	In process	20A	B.1	7	5'UTR:C241T, ORF1ab:G377A, ORF1ab:A2806C, ORF1ab:C3037T, ORF1ab:C14408T, ORF1ab:T17960C, S:A23403G, N:C28854T,	6	N:S194L, ORF1a:V38I, ORF1a:E847D, ORF1b:P314L, ORF1b:I1498T, S:D614G,
hCoV-19/Mexico/CMX_INER_IMSS_00677/2021	EPI_ISL_2091133	In process	20B	B.1.1.519	28	5'UTR:C203T, 5'UTR:C222T, 5'UTR:C241T, ORF1ab:C3037T, ORF1ab:C3140T, ORF1ab:C10029T, ORF1ab:C10954T, ORF1ab:A11117G, ORF1ab:T12349A, ORF1ab:C12789T, ORF1ab:C14408T, ORF1ab:T19839C, ORF1ab:G20578T, ORF1ab:C21306T, S:G21830T, S:C22995A, S:A23403G, S:C23604A, S:A23756G, M:C26826T, N:G28378T, N:A28837G, N:G28881A, N:G28882A, N:G28883C, N:C29137T, N:C29197T, N:G29527T, 3'UTR:G29747T,	15	N:R203K, N:G204R, N:Q418H, ORF1a:P959S, ORF1a:T3255I, ORF1a:I3618V, ORF1a:T4175I, ORF1b:P314L, ORF1b:V2371L, ORF9b:R32L, S:V90F, S:T478K, S:D614G, S:P681H, S:T732A,

hCoV-19/Mexico/CMX_INER_IMSS_00681/2021	EPI_ISL_2091137	In process	20B	B.1.1.519	27	5'UTR:T201C, 5'UTR:C203T, 5'UTR:C222T, 5'UTR:C241T, ORF1ab:G1274T, ORF1ab:G1738T, ORF1ab:C3037T, ORF1ab:C3140T, ORF1ab:C7504T, ORF1ab:C10029T, ORF1ab:C10954T, ORF1ab:A11117G, ORF1ab:C12789T, ORF1ab:C13115T, ORF1ab:C14408T, ORF1ab:T19839C, ORF1ab:A19974G, ORF1ab:C21306T, S:C22995A, S:A23403G, S:C23604A, S:A23756G, S:C24130T, ORF3a:G25913A, N:G28881A, N:G28882A, N:G28883C, N:C29197T,	13	N:R203K, N:G204R, ORF1a:V337F, ORF1a:P959S, ORF1a:T3255I, ORF1a:I3618V, ORF1a:T4175I, ORF1b:P314L, ORF3a:G174D, S:T478K, S:D614G, S:P681H, S:T732A,
hCoV-19/Mexico/CMX-IBT-IMSS-02/2020	EPI_ISL_955257	In process	20C	B.1	5	5'UTR:C241T, ORF1ab:C1059T, ORF1ab:C3037T, ORF1ab:C14408T, S:A23403G, ORF3a:G25563T,	4	ORF1a:T265I, ORF1b:P314L, ORF3a:Q57H, S:D614G,
hCoV-19/Mexico/CMX-IBT-IMSS-03/2020	EPI_ISL_955256	In process	20A	B.1	5	5'UTR:C241T, ORF1ab:C3037T, ORF1ab:C14408T, ORF1ab:A20268G, S:A23403G, N:G29348T,	3	N:A359S, ORF1b:P314L, S:D614G,
hCoV-19/Mexico/CMX-IBT-IMSS-05/2020	EPI_ISL_955248	In process	20B	B.1.1	9	5'UTR:C241T, ORF1ab:C3037T, ORF1ab:A4702G, ORF1ab:C6781T, ORF1ab:C14408T, ORF1ab:T19839C, S:A23403G, N:G28881A, N:G28882A, N:G28883C,	4	N:R203K, N:G204R, ORF1b:P314L, S:D614G,
hCoV-19/Mexico/CMX-IBT-IMSS-06/2020	EPI_ISL_955249	In process	20A	B.1	7	5'UTR:C241T, ORF1ab:C3037T, ORF1ab:T11220C, ORF1ab:C14408T, ORF1ab:C18877T, ORF1ab:G19872T, S:A23403G, ORF3a:G25563T,	4	ORF1a:M3652T, ORF1b:P314L, ORF3a:Q57H, S:D614G,
hCoV-19/Mexico/CMX-IBT-IMSS-07/2020	EPI_ISL_955254	In process	20A	B.1	7	5'UTR:C241T, ORF1ab:C3037T, ORF1ab:C12357T, ORF1ab:C14408T, ORF1ab:A20268G, S:A23403G, S:G24926T, N:G29000T,	5	N:G243C, ORF1a:T4031I, ORF1b:P314L, S:D614G, S:V1122L,
hCoV-19/Mexico/CMX-IBT-IMSS-147/2021	EPI_ISL_1288350	In process	20B	B.1.1.519	24	5'UTR:C203T, 5'UTR:C222T, 5'UTR:C241T, ORF1ab:C3037T, ORF1ab:C3140T, ORF1ab:A3523G, ORF1ab:C10029T, ORF1ab:C10954T, ORF1ab:A11117G, ORF1ab:C12789T, ORF1ab:C14408T, ORF1ab:G15921T, ORF1ab:A17971G, ORF1ab:T19839C, ORF1ab:G20464A, ORF1ab:C21306T, S:C22995A, S:A23403G, S:C23604A, S:C23635T, S:A23756G, N:G28881A, N:G28882A, N:G28883C, N:C29197T,	13	N:R203K, N:G204R, ORF1a:P959S, ORF1a:T3255I, ORF1a:I3618V, ORF1a:T4175I, ORF1b:P314L, ORF1b:R1502G, ORF1b:D2333N, S:T478K, S:D614G, S:P681H, S:T732A,
hCoV-19/Mexico/CMX-IBT-IMSS-21/2020	EPI_ISL_955239	In process	20B	B.1.1	9	5'UTR:C241T, ORF1ab:C3037T, ORF1ab:C6781T, ORF1ab:C14408T, ORF1ab:G18583A, ORF1ab:T19839C, S:A23403G, N:G28881A, N:G28882A, N:G28883C,	5	N:R203K, N:G204R, ORF1b:P314L, ORF1b:V1706I, S:D614G,
hCoV-19/Mexico/CMX-IBT-IMSS-216/2021	EPI_ISL_1288411	In process	20B	B.1.1.519	23	5'UTR:C203T, 5'UTR:C222T, 5'UTR:C241T, ORF1ab:C3037T, ORF1ab:C3140T, ORF1ab:A6084C, ORF1ab:C10029T, ORF1ab:C10954T, ORF1ab:A11117G, ORF1ab:C11575T, ORF1ab:C12789T, ORF1ab:C14408T, ORF1ab:C17550T, ORF1ab:T19839C, ORF1ab:C21306T, S:C22995A, S:A23403G, S:C23604A, S:A23756G, S:A25369G, N:G28881A, N:G28882A, N:G28883C, N:C29197T,	12	N:R203K, N:G204R, ORF1a:P959S, ORF1a:D1940A, ORF1a:T3255I, ORF1a:I3618V, ORF1a:T4175I, ORF1b:P314L, S:T478K, S:D614G, S:P681H, S:T732A,
hCoV-19/Mexico/CMX-IBT-IMSS-217/2021	EPI_ISL_1288412	In process	20B	B.1.1.519	20	5'UTR:C203T, 5'UTR:C222T, 5'UTR:C241T, ORF1ab:C3037T, ORF1ab:C3140T, ORF1ab:C10029T, ORF1ab:C10954T, ORF1ab:A11117G, ORF1ab:C12789T, ORF1ab:C14408T, ORF1ab:T19839C, ORF1ab:C21306T, S:C22995A, S:A23403G, S:C23604A, S:A23756G, N:G28881A, N:G28882A, N:G28883C, N:C29197T, N:G29527T,	12	N:R203K, N:G204R, N:Q418H, ORF1a:P959S, ORF1a:T3255I, ORF1a:I3618V, ORF1a:T4175I, ORF1b:P314L, S:T478K, S:D614G, S:P681H, S:T732A,
hCoV-19/Mexico/CMX-IBT-IMSS-219/2021	EPI_ISL_1288414	In process	20B	B.1.1.519	22	5'UTR:C203T, 5'UTR:C222T, 5'UTR:C241T, ORF1ab:C3037T, ORF1ab:C3140T, ORF1ab:C5365T, ORF1ab:C10029T, ORF1ab:C10954T, ORF1ab:G11083T, ORF1ab:A11117G, ORF1ab:C12789T, ORF1ab:C14408T, ORF1ab:T19839C, ORF1ab:C21306T, S:C22995A, S:A23403G, S:C23604A, S:A23756G, S:C23987T, N:G28881A, N:G28882A, N:G28883C, N:C29197T,	13	N:R203K, N:G204R, ORF1a:P959S, ORF1a:T3255I, ORF1a:L3606F, ORF1a:I3618V, ORF1a:T4175I, ORF1b:P314L, S:T478K, S:D614G, S:P681H, S:T732A, S:P809S,

hCoV-19/Mexico/CMX-IBT-IMSS-22/2020	EPI_ISL_955234	In process	20A	B.1.189	9	5'UTR:C241T, ORF1ab:C3037T, ORF1ab:C4582T, ORF1ab:G6404T, ORF1ab:C14408T, ORF1ab:A20268G, S:A23403G, S:T25123C, ORF7a:G27676T, N:G28378T,	5	ORF1a:V2047F, ORF1b:P314L, ORF7a:E95*, ORF9b:R32L, S:D614G,	
hCoV-19/Mexico/CMX-IBT-IMSS-221/2021	EPI_ISL_1288415	In process	20B	B.1.1.519	21	5'UTR:C203T, 5'UTR:C222T, 5'UTR:C241T, ORF1ab:C3037T, ORF1ab:C3140T, ORF1ab:C10029T, ORF1ab:C10954T, ORF1ab:A11117G, ORF1ab:C12789T, ORF1ab:C14408T, ORF1ab:T19839C, ORF1ab:C21306T, S:C22995A, S:A23403G, S:C23604A, S:A23756G, ORF8:T27904C, ORF8:C28087T, N:G28881A, N:G28882A, N:G28883C, N:C29197T,	13	N:R203K, N:G204R, ORF1a:P959S, ORF1a:T3255I, ORF1a:I3618V, ORF1a:T4175I, ORF1b:P314L, ORF8:L4P, ORF8:A65V, S:T478K, S:D614G, S:P681H, S:T732A,	
hCoV-19/Mexico/CMX-IBT-IMSS-224/2021	EPI_ISL_1288418	In process	20B	B.1.1.519	26	5'UTR:C203T, 5'UTR:C222T, 5'UTR:C241T, ORF1ab:C3037T, ORF1ab:C3140T, ORF1ab:T3745C, ORF1ab:G9176A, ORF1ab:C10029T, ORF1ab:C10954T, ORF1ab:A11117G, ORF1ab:C12789T, ORF1ab:C14134T, ORF1ab:C14408T, ORF1ab:T19839C, ORF1ab:C21306T, S:T22729C, S:C22995A, S:A23403G, S:C23604A, S:A23756G, ORF3a:C25844T, N:C28849T, N:G28881A, N:G28882A, N:G28883C, N:G28904T, N:C29197T,	15	N:R203K, N:G204R, N:A211S, ORF1a:P959S, ORF1a:G2971S, ORF1a:T3255I, ORF1a:I3618V, ORF1a:T4175I, ORF1b:P223S, ORF1b:P314L, ORF3a:T151I, S:T478K, S:D614G, S:P681H, S:T732A,	
hCoV-19/Mexico/CMX-IBT-IMSS-225/2021	EPI_ISL_1288419	In process	20B	B.1.1.519	30	5'UTR:C203T, 5'UTR:C222T, 5'UTR:C241T, ORF1ab:A866G, ORF1ab:C1009T, ORF1ab:C3037T, ORF1ab:C3140T, ORF1ab:C3743T, ORF1ab:C5183T, ORF1ab:C5812T, ORF1ab:C10029T, ORF1ab:C10954T, ORF1ab:G11083T, ORF1ab:A11117G, ORF1ab:A11623G, ORF1ab:C11916T, ORF1ab:C12789T, ORF1ab:C14408T, ORF1ab:T19839C, ORF1ab:C21306T, S:C21648T, S:C22995A, S:A23403G, S:C23604A, S:A23756G, ORF3a:C25490T, ORF8:T27904C, N:G28881A, N:G28882A, N:G28883C, N:C29197T,	19	N:R203K, N:G204R, ORF1a:I201V, ORF1a:P959S, ORF1a:H1160Y, ORF1a:P1640S, ORF1a:T3255I, ORF1a:L3606F, ORF1a:I3618V, ORF1a:S3884L, ORF1a:T4175I, ORF1b:P314L, ORF3a:A33V, ORF8:L4P, S:T29I, S:T478K, S:D614G, S:P681H, S:T732A,	
hCoV-19/Mexico/CMX-IBT-IMSS-227/2021	EPI_ISL_1288420	In process	20B	B.1.1.519	23	5'UTR:C203T, 5'UTR:C222T, 5'UTR:C241T, ORF1ab:C3037T, ORF1ab:C3140T, ORF1ab:G7037T, ORF1ab:C10029T, ORF1ab:C10954T, ORF1ab:A11117G, ORF1ab:C12789T, ORF1ab:C14408T, ORF1ab:C19145T, ORF1ab:T19839C, ORF1ab:C20823T, ORF1ab:C21306T, S:C22995A, S:A23403G, S:C23604A, S:A23756G, ORF3a:C25782T, N:G28881A, N:G28882A, N:G28883C, N:C29197T,	13	N:R203K, N:G204R, ORF1a:P959S, ORF1a:G2258C, ORF1a:T3255I, ORF1a:I3618V, ORF1a:T4175I, ORF1b:P314L, ORF1b:S1893F, S:T478K, S:D614G, S:P681H, S:T732A,	3'UTR:29819-29821,
hCoV-19/Mexico/CMX-IBT-IMSS-23/2020	EPI_ISL_955235	In process	20A	B.1.189	8	5'UTR:C241T, ORF1ab:C3037T, ORF1ab:C4582T, ORF1ab:G6404T, ORF1ab:C14408T, ORF1ab:A20268G, S:A23403G, S:T25123C, N:G28378T,	4	ORF1a:V2047F, ORF1b:P314L, ORF9b:R32L, S:D614G,	
hCoV-19/Mexico/CMX-IBT-IMSS-24/2020	EPI_ISL_955238	In process	20B	B.1.1	10	5'UTR:C241T, ORF1ab:C2399T, ORF1ab:C3037T, ORF1ab:C9438T, ORF1ab:T10660C, ORF1ab:G11083T, ORF1ab:C14408T, S:A23403G, N:G28881A, N:G28882A, N:G28883C,	7	N:R203K, N:G204R, ORF1a:H712Y, ORF1a:T3058I, ORF1a:L3606F, ORF1b:P314L, S:D614G,	
hCoV-19/Mexico/CMX-IBT-IMSS-284/2021	EPI_ISL_1288470	In process	20B	B.1.1.519	27	5'UTR:C203T, 5'UTR:C222T, 5'UTR:C241T, ORF1ab:A866G, ORF1ab:C1009T, ORF1ab:C1959A, ORF1ab:C3037T, ORF1ab:C3140T, ORF1ab:C3593T, ORF1ab:C5183T, ORF1ab:C10029T, ORF1ab:C10954T, ORF1ab:A11117G, ORF1ab:C11916T, ORF1ab:G12223T, ORF1ab:C12789T, ORF1ab:C14408T, ORF1ab:T19839C, ORF1ab:C21306T, S:C22995A, S:A23403G, S:C23604A, S:A23756G, ORF8:T27904C, N:G28881A, N:G28882A, N:G28883C, N:C29197T,	17	N:R203K, N:G204R, ORF1a:I201V, ORF1a:A565D, ORF1a:P959S, ORF1a:L1110F, ORF1a:P1640S, ORF1a:T3255I, ORF1a:I3618V, ORF1a:S3884L, ORF1a:T4175I, ORF1b:P314L, ORF8:L4P, S:T478K, S:D614G, S:P681H, S:T732A,	

hCoV-19/Mexico/CMX-IBT-IMSS-285/2021	EPI_ISL_1288471	In process	20B	B.1.1.519	30	5'UTR:T201C, 5'UTR:C203T, 5'UTR:C222T, 5'UTR:C241T, ORF1ab:T277C, ORF1ab:G487T, ORF1ab:G1738T, ORF1ab:C3037T, ORF1ab:C3140T, ORF1ab:C4540T, ORF1ab:G7829T, ORF1ab:C10029T, ORF1ab:C10954T, ORF1ab:A11117G, ORF1ab:C12789T, ORF1ab:C14408T, ORF1ab:C14805T, ORF1ab:T16728C, ORF1ab:T19839C, ORF1ab:A19974G, ORF1ab:C20091T, ORF1ab:C21306T, S:C22995A, S:A23403G, S:C23604A, S:A23756G, ORF3a:C26124T, N:G28881A, N:G28882A, N:G28883C, N:C29197T,	12	N:R203K, N:G204R, ORF1a:P959S, ORF1a:V2522F, ORF1a:T3255I, ORF1a:I3618V, ORF1a:T4175I, ORF1b:P314L, S:T478K, S:D614G, S:P681H, S:T732A,
hCoV-19/Mexico/CMX-IBT-IMSS-290/2021	EPI_ISL_1288476	In process	20B	B.1.1.519	25	5'UTR:C203T, 5'UTR:C222T, 5'UTR:C241T, ORF1ab:C3037T, ORF1ab:C3140T, ORF1ab:T3745C, ORF1ab:C5167T, ORF1ab:G6884A, ORF1ab:C10029T, ORF1ab:C10954T, ORF1ab:A11117G, ORF1ab:C12789T, ORF1ab:C14408T, ORF1ab:C18646T, ORF1ab:T19839C, ORF1ab:C21306T, S:C21846T, S:C22995A, S:A23403G, S:C23604A, S:A23756G, ORF3a:C25844T, N:G28881A, N:G28882A, N:G28883C, N:C29197T,	15	N:R203K, N:G204R, ORF1a:P959S, ORF1a:G2207S, ORF1a:T3255I, ORF1a:I3618V, ORF1a:T4175I, ORF1b:P314L, ORF1b:P1727S, ORF3a:T151I, S:T95I, S:T478K, S:D614G, S:P681H, S:T732A,
hCoV-19/Mexico/CMX-IBT-IMSS-291/2021	EPI_ISL_1288477	In process	20B	B.1.1.519	19	5'UTR:C203T, 5'UTR:C222T, 5'UTR:C241T, ORF1ab:C3037T, ORF1ab:C3140T, ORF1ab:C10029T, ORF1ab:C10954T, ORF1ab:A11117G, ORF1ab:C12789T, ORF1ab:C14408T, ORF1ab:T19839C, ORF1ab:C21306T, S:C22995A, S:A23403G, S:C23604A, S:A23756G, N:G28881A, N:G28882A, N:G28883C, N:C29197T,	11	N:R203K, N:G204R, ORF1a:P959S, ORF1a:T3255I, ORF1a:I3618V, ORF1a:T4175I, ORF1b:P314L, S:T478K, S:D614G, S:P681H, S:T732A,
hCoV-19/Mexico/CMX-IBT-IMSS-293/2021	EPI_ISL_1288478	In process	20B	B.1.1.519	24	5'UTR:C203T, 5'UTR:C222T, 5'UTR:C241T, ORF1ab:C629T, ORF1ab:C3037T, ORF1ab:C3140T, ORF1ab:C10029T, ORF1ab:C10954T, ORF1ab:A11117G, ORF1ab:C12789T, ORF1ab:C14408T, ORF1ab:C14621T, ORF1ab:C15108T, ORF1ab:C15933T, ORF1ab:T19839C, ORF1ab:C21306T, S:C22995A, S:A23403G, S:C23604A, S:A23756G, N:G28881A, N:G28882A, N:G28883C, N:C29197T, N:G29527T,	14	N:R203K, N:G204R, N:Q418H, ORF1a:L122F, ORF1a:P959S, ORF1a:T3255I, ORF1a:I3618V, ORF1a:T4175I, ORF1b:P314L, ORF1b:T385M, S:T478K, S:D614G, S:P681H, S:T732A,
hCoV-19/Mexico/CMX-IBT-IMSS-294/2021	EPI_ISL_1288479	In process	20B	B.1.1.519	26	5'UTR:C203T, 5'UTR:C222T, 5'UTR:C241T, ORF1ab:C3037T, ORF1ab:C3140T, ORF1ab:C4780T, ORF1ab:C10029T, ORF1ab:A10323G, ORF1ab:C10954T, ORF1ab:A11117G, ORF1ab:C12789T, ORF1ab:C13378T, ORF1ab:C14408T, ORF1ab:C14925T, ORF1ab:T19839C, ORF1ab:C21077T, ORF1ab:C21306T, S:C22995A, S:A23403G, S:C23604A, S:A23756G, ORF3a:G25906T, ORF8:C28253T, N:G28881A, N:G28882A, N:G28883C, N:C29197T	14	N:R203K, N:G204R, ORF1a:P959S, ORF1a:T3255I, ORF1a:K3353R, ORF1a:I3618V, ORF1a:T4175I, ORF1b:P314L, ORF1b:T2537I, ORF3a:G172C, S:T478K, S:D614G, S:P681H, S:T732A,
hCoV-19/Mexico/CMX-IBT-IMSS-296/2021	EPI_ISL_1288481	In process	20B	B.1.1.519	23	5'UTR:C203T, 5'UTR:C222T, 5'UTR:C241T, ORF1ab:A2410G, ORF1ab:C3037T, ORF1ab:C3140T, ORF1ab:T4339C, ORF1ab:C10029T, ORF1ab:C10954T, ORF1ab:A11117G, ORF1ab:C12789T, ORF1ab:C14408T, ORF1ab:T19839C, ORF1ab:C21306T, S:C22995A, S:A23403G, S:C23604A, S:A23756G, ORF3a:G25906T, ORF8:C28253T, N:G28881A, N:G28882A, N:G28883C, N:C29197T,	12	N:R203K, N:G204R, ORF1a:P959S, ORF1a:T3255I, ORF1a:I3618V, ORF1a:T4175I, ORF1b:P314L, ORF3a:G172C, S:T478K, S:D614G, S:P681H, S:T732A,

hCoV-19/Mexico/CMX-IBT-IMSS-297/2021	EPI_ISL_1288482	In process	20B	B.1.1.519	26	5'UTR:T201C, 5'UTR:C203T, 5'UTR:C222T, 5'UTR:C241T, ORF1ab:C745T, ORF1ab:G1738T, ORF1ab:C3037T, ORF1ab:C3140T, ORF1ab:C10029T, ORF1ab:C10156T, ORF1ab:C10954T, ORF1ab:A11117G, ORF1ab:C12789T, ORF1ab:C14408T, ORF1ab:T19839C, ORF1ab:A19974G, ORF1ab:C21306T, S:C22995A, S:A23403G, S:C23604A, S:A23756G, E:C26447T, ORF6:C27208T, N:G28881A, N:G28882A, N:G28883C, N:C29197T,	13	E:S68F, N:R203K, N:G204R, ORF1a:P959S, ORF1a:T3255I, ORF1a:I3618V, ORF1a:T4175I, ORF1b:P314L, ORF6:H3Y, S:T478K, S:D614G, S:P681H, S:T732A,
hCoV-19/Mexico/CMX-IBT-IMSS-298/2021	EPI_ISL_1288483	In process	20B	B.1.1.519	28	5'UTR:T201C, 5'UTR:C203T, 5'UTR:C222T, 5'UTR:C241T, ORF1ab:G1738T, ORF1ab:C3037T, ORF1ab:C3140T, ORF1ab:C3768T, ORF1ab:C10029T, ORF1ab:C10954T, ORF1ab:A11117G, ORF1ab:C12789T, ORF1ab:C14408T, ORF1ab:C16457T, ORF1ab:C19836T, ORF1ab:T19839C, ORF1ab:A19974G, ORF1ab:C21306T, S:C21575T, S:C22995A, S:A23235G, S:A23403G, S:C23604A, S:A23756G, N:G28881A, N:G28882A, N:G28883C, N:C29197T, N:G29260T,	15	N:R203K, N:G204R, ORF1a:P959S, ORF1a:T1168I, ORF1a:T3255I, ORF1a:I3618V, ORF1a:T4175I, ORF1b:P314L, ORF1b:S997L, S:L5F, S:T478K, S:K558R, S:D614G, S:P681H, S:T732A,
hCoV-19/Mexico/CMX-IBT-IMSS-300/2021	EPI_ISL_1288485	In process	20B	B.1.1.519	25	5'UTR:T201C, 5'UTR:C203T, 5'UTR:C222T, 5'UTR:C241T, ORF1ab:G1738T, ORF1ab:C3037T, ORF1ab:C3140T, ORF1ab:C3177T, ORF1ab:C5281T, ORF1ab:C10029T, ORF1ab:C10954T, ORF1ab:A11117G, ORF1ab:C12789T, ORF1ab:C14408T, ORF1ab:C19662T, ORF1ab:T19839C, ORF1ab:A19974G, ORF1ab:C21306T, S:C22995A, S:A23403G, S:C23604A, S:A23756G, N:G28881A, N:G28882A, N:G28883C, N:C29197T,	12	N:R203K, N:G204R, ORF1a:P959S, ORF1a:P971L, ORF1a:T3255I, ORF1a:I3618V, ORF1a:T4175I, ORF1b:P314L, S:T478K, S:D614G, S:P681H, S:T732A,
hCoV-19/Mexico/CMX-IBT-IMSS-301/2021	EPI_ISL_1288486	In process	20B	B.1.1.519	28	5'UTR:T201C, 5'UTR:C203T, 5'UTR:C222T, 5'UTR:C241T, ORF1ab:G1738T, ORF1ab:C2892T, ORF1ab:C3037T, ORF1ab:C3140T, ORF1ab:A5845C, ORF1ab:C6026T, ORF1ab:C10029T, ORF1ab:C10954T, ORF1ab:A11117G, ORF1ab:C12789T, ORF1ab:C14408T, ORF1ab:T19839C, ORF1ab:A19974G, ORF1ab:C21306T, S:C22995A, S:A23403G, S:C23604A, S:A23756G, ORF7b:C27804T, N:G28881A, N:G28882A, N:G28883C, N:C29197T, N:C29421T, ORF10:C29627T,	15	N:R203K, N:G204R, N:P383L, ORF1a:A876V, ORF1a:P959S, ORF1a:K1860N, ORF1a:P1921S, ORF1a:T3255I, ORF1a:I3618V, ORF1a:T4175I, ORF1b:P314L, S:T478K, S:D614G, S:P681H, S:T732A,
hCoV-19/Mexico/CMX-IBT-IMSS-317/2021	EPI_ISL_1288499	In process	20B	B.1.1.519	30	5'UTR:C203T, 5'UTR:C222T, 5'UTR:C241T, ORF1ab:C3037T, ORF1ab:C3140T, ORF1ab:G3483T, ORF1ab:C3784T, ORF1ab:T6853C, ORF1ab:C10029T, ORF1ab:C10954T, ORF1ab:A11117G, ORF1ab:C12789T, ORF1ab:A13063G, ORF1ab:C14408T, ORF1ab:C16726T, ORF1ab:T19839C, ORF1ab:C21306T, S:C22995A, S:A23403G, S:C23604A, S:A23756G, ORF3a:G25523T, ORF3a:G25947T, M:G27014T, ORF7a:A27617G, N:G28881A, N:G28882A, N:G28883C, N:C29197T, N:G29527T, 3'UTR:A29776G,	17	N:R203K, N:G204R, N:Q418H, ORF1a:P959S, ORF1a:G1073V, ORF1a:T3255I, ORF1a:I3618V, ORF1a:T4175I, ORF1b:P314L, ORF1b:H1087Y, ORF3a:G44V, ORF3a:Q185H, ORF7a:Y75C, S:T478K, S:D614G, S:P681H, S:T732A,
hCoV-19/Mexico/CMX-IBT-IMSS-326/2021	EPI_ISL_1288507	In process	20B	B.1.1.519	18	5'UTR:C203T, 5'UTR:C222T, 5'UTR:C241T, ORF1ab:C3037T, ORF1ab:C3140T, ORF1ab:C10029T, ORF1ab:C10954T, ORF1ab:A11117G, ORF1ab:C14408T, ORF1ab:T19839C, ORF1ab:C21306T, S:C22995A, S:A23403G, S:C23604A, S:A23756G, N:G28881A, N:G28882A, N:G28883C, N:C29197T,	10	N:R203K, N:G204R, ORF1a:P959S, ORF1a:T3255I, ORF1a:I3618V, ORF1b:P314L, S:T478K, S:D614G, S:P681H, S:T732A,

hCoV-19/Mexico/CMX-IBT-IMSS-327/2021	EPI_ISL_1288508	In process	20B	B.1.1.519	27	5'UTR:C203T, 5'UTR:C222T, 5'UTR:C241T, ORF1ab:G443A, ORF1ab:C3037T, ORF1ab:C3140T, ORF1ab:C5869T, ORF1ab:C10029T, ORF1ab:C10954T, ORF1ab:A11117G, ORF1ab:G12769T, ORF1ab:C12789T, ORF1ab:C13968T, ORF1ab:C14408T, ORF1ab:T19839C, ORF1ab:C21306T, S:C22995A, S:A23403G, S:C23604A, S:A23756G, ORF3a:G25595A, ORF8:T27904C, ORF8:C28087T, N:G28881A, N:G28882A, N:G28883C, N:C29197T, N:T29461C	15	N:R203K, N:G204R, ORF1a:V60I, ORF1a:P959S, ORF1a:T3255I, ORF1a:I3618V, ORF1a:T4175I, ORF1b:P314L, ORF3a:R68K, ORF8:L4P, ORF8:A65V, S:T478K, S:D614G, S:P681H, S:T732A,
hCoV-19/Mexico/CMX-IBT-IMSS-328/2021	EPI_ISL_1288509	In process	20B	B.1.1.519	34	5'UTR:T201C, 5'UTR:C203T, 5'UTR:C222T, 5'UTR:C241T, ORF1ab:C936T, ORF1ab:G1738T, ORF1ab:T2489C, ORF1ab:C3037T, ORF1ab:C3140T, ORF1ab:C5784T, ORF1ab:A5877G, ORF1ab:C10029T, ORF1ab:C10207T, ORF1ab:C10954T, ORF1ab:A11117G, ORF1ab:C12789T, ORF1ab:C14408T, ORF1ab:T19839C, ORF1ab:A19974G, ORF1ab:C21306T, S:C21627T, S:C21638T, S:C22995A, S:A23403G, S:G23593C, S:C23604A, S:A23756G, ORF7a:A27426G, ORF8:A28064T, N:G28881A, N:G28882A, N:G28883C, N:C29197T, N:G29227T, N:G29321T	19	N:R203K, N:G204R, N:V350F, ORF1a:T224I, ORF1a:P959S, ORF1a:T1840I, ORF1a:N1871S, ORF1a:T3255I, ORF1a:I3618V, ORF1a:T4175I, ORF1b:P314L, ORF8:L57F, S:T22I, S:P26S, S:T478K, S:D614G, S:Q677H, S:P681H, S:T732A,
hCoV-19/Mexico/CMX-IBT-IMSS-329/2021	EPI_ISL_1288510	In process	20B	B.1.1.519	28	5'UTR:C203T, 5'UTR:C222T, 5'UTR:C241T, ORF1ab:T712C, ORF1ab:G2849A, ORF1ab:C3037T, ORF1ab:C3140T, ORF1ab:C3695T, ORF1ab:C5183T, ORF1ab:T7045C, ORF1ab:C10029T, ORF1ab:C10954T, ORF1ab:A11117G, ORF1ab:C12789T, ORF1ab:C14408T, ORF1ab:T19839C, ORF1ab:C21306T, S:T21577C, S:C22995A, S:A23403G, S:C23604A, S:A23756G, S:G25135T, ORF8:T27904C, ORF8:G28195T, N:G28881A, N:G28882A, N:G28883C, N:C29197T,	16	N:R203K, N:G204R, ORF1a:V862I, ORF1a:P959S, ORF1a:P1640S, ORF1a:T3255I, ORF1a:I3618V, ORF1a:T4175I, ORF1b:P314L, ORF8:L4P, ORF8:R101L, S:T478K, S:D614G, S:P681H, S:T732A, S:K1191N,
hCoV-19/Mexico/CMX-IBT-IMSS-330/2021	EPI_ISL_1288511	In process	20B	B.1.1.519	27	5'UTR:C203T, 5'UTR:C222T, 5'UTR:C241T, ORF1ab:C3037T, ORF1ab:C3140T, ORF1ab:C10029T, ORF1ab:A10630G, ORF1ab:C10954T, ORF1ab:A11117G, ORF1ab:C12789T, ORF1ab:C14408T, ORF1ab:T19839C, ORF1ab:C21306T, ORF1ab:G21430T, S:C22995A, S:A23403G, S:C23604A, S:A23756G, S:G24914T, S:C25254T, ORF3a:C25642T, ORF3a:C25782T, ORF3a:G26063T, N:G28881A, N:G28882A, N:G28883C, N:C29197T, N:G29402T,	16	N:R203K, N:G204R, N:D377Y, ORF1a:P959S, ORF1a:T3255I, ORF1a:I3618V, ORF1a:T4175I, ORF1b:P314L, ORF1b:A2655S, ORF3a:G224V, S:T478K, S:D614G, S:P681H, S:T732A, S:D1118Y, S:T1231I,
hCoV-19/Mexico/CMX-IBT-IMSS-331/2021	EPI_ISL_1288512	In process	20B	B.1.1.519	26	5'UTR:C203T, 5'UTR:C222T, 5'UTR:C241T, ORF1ab:C3037T, ORF1ab:C3140T, ORF1ab:C5365T, ORF1ab:G9407A, ORF1ab:C10029T, ORF1ab:C10954T, ORF1ab:A11117G, ORF1ab:C12789T, ORF1ab:C14408T, ORF1ab:T19839C, ORF1ab:A20532C, ORF1ab:C21306T, S:C22995A, S:C23086T, S:A23403G, S:C23604A, S:A23756G, ORF3a:C25782T, ORF7a:C27600T, N:G28881A, N:G28882A, N:G28883C, N:G29050A, N:C29197T,	13	N:R203K, N:G204R, ORF1a:P959S, ORF1a:V3048I, ORF1a:T3255I, ORF1a:I3618V, ORF1a:T4175I, ORF1b:P314L, ORF1b:E2355D, S:T478K, S:D614G, S:P681H, S:T732A,
hCoV-19/Mexico/CMX-IBT-IMSS-334/2021	EPI_ISL_1288515	In process	20B	B.1.1.519	27	5'UTR:T201C, 5'UTR:C203T, 5'UTR:C222T, 5'UTR:C241T, ORF1ab:G1681T, ORF1ab:G1738T, ORF1ab:C2137A, ORF1ab:C3037T, ORF1ab:C3140T, ORF1ab:G5461T, ORF1ab:C10029T, ORF1ab:C10332T, ORF1ab:C10954T, ORF1ab:A11117G, ORF1ab:C12789T, ORF1ab:C14408T, ORF1ab:C16393T, ORF1ab:T19839C, ORF1ab:A19974G, ORF1ab:C21306T, S:C22995A, S:A23403G, S:C23604A, S:A23756G, N:G28881A, N:G28882A, N:G28883C, N:C29197T	15	N:R203K, N:G204R, ORF1a:E472D, ORF1a:P959S, ORF1a:M1732I, ORF1a:T3255I, ORF1a:T3356I, ORF1a:I3618V, ORF1a:T4175I, ORF1b:P314L, ORF1b:P976S, S:T478K, S:D614G, S:P681H, S:T732A,

hCoV-19/Mexico/CMX-IBT-IMSS-338/2021	EPI_ISL_1288517	In process	20A	B.1.551	14	5'UTR:C241T, ORF1ab:A513G, ORF1ab:C2455T, ORF1ab:C3037T, ORF1ab:C3738T, ORF1ab:C11036T, ORF1ab:C14408T, ORF1ab:C19524T, ORF1ab:A20268G, S:A23403G, S:C23604G, ORF7b:G27762C, N:C28854T, N:C29311T, N:C29466T,	9	N:S194L, N:A398V, ORF1a:H83R, ORF1a:P1158L, ORF1a:L3591F, ORF1b:P314L, ORF7b:E3Q, S:D614G, S:P681R,
hCoV-19/Mexico/CMX-IBT-IMSS-340/2021	EPI_ISL_1288519	In process	20B	B.1.1.519	27	5'UTR:C203T, 5'UTR:C222T, 5'UTR:C241T, ORF1ab:C583T, ORF1ab:C3037T, ORF1ab:C3140T, ORF1ab:C5467T, ORF1ab:C5784T, ORF1ab:C10029T, ORF1ab:C10712T, ORF1ab:C10954T, ORF1ab:A11117G, ORF1ab:G11365T, ORF1ab:C12073T, ORF1ab:C12789T, ORF1ab:C14408T, ORF1ab:T19839C, ORF1ab:C21306T, S:C22995A, S:A23403G, S:C23604A, S:A23756G, M:A26927G, N:G28881A, N:G28882A, N:G28883C, N:C29197T, N:G29527T,	14	N:R203K, N:G204R, N:Q418H, ORF1a:P959S, ORF1a:T1840I, ORF1a:T3255I, ORF1a:L3483F, ORF1a:I3618V, ORF1a:T4175I, ORF1b:P314L, S:T478K, S:D614G, S:P681H, S:T732A,
hCoV-19/Mexico/CMX-IBT-IMSS-341/2021	EPI_ISL_1288520	In process	20B	B.1.1.519	27	5'UTR:C203T, 5'UTR:C222T, 5'UTR:C241T, ORF1ab:C3037T, ORF1ab:C3140T, ORF1ab:C3736T, ORF1ab:C5654T, ORF1ab:G7037T, ORF1ab:C10029T, ORF1ab:C10954T, ORF1ab:A11117G, ORF1ab:G11596T, ORF1ab:C12789T, ORF1ab:C14408T, ORF1ab:T19839C, ORF1ab:C21306T, S:C22995A, S:A23403G, S:C23604A, S:A23756G, S:C24099T, ORF3a:C25782T, ORF8:C28093T, N:G28881A, N:G28882A, N:G28883C, N:C29197T, 3'UTR:G29759T,	15	N:R203K, N:G204R, ORF1a:P959S, ORF1a:G2258C, ORF1a:T3255I, ORF1a:I3618V, ORF1a:Q3777H, ORF1a:T4175I, ORF1b:P314L, ORF8:S67F, S:T478K, S:D614G, S:P681H, S:T732A, S:A846V,
hCoV-19/Mexico/CMX-IBT-IMSS-342/2021	EPI_ISL_1288521	In process	20B	B.1.1.519	27	5'UTR:T201C, 5'UTR:C203T, 5'UTR:C222T, 5'UTR:C241T, ORF1ab:G526T, ORF1ab:G1738T, ORF1ab:C3037T, ORF1ab:C3140T, ORF1ab:C10029T, ORF1ab:C10954T, ORF1ab:A11117G, ORF1ab:C14408T, ORF1ab:G18079T, ORF1ab:T19839C, ORF1ab:A19974G, ORF1ab:C21306T, S:G22899T, S:C22995A, S:A23403G, S:C23604A, S:A23756G, ORF3a:T25421G, M:C26907T, ORF8:A28175G, N:G28881A, N:G28882A, N:G28883C, N:C29197T,	14	N:R203K, N:G204R, ORF1a:E87D, ORF1a:P959S, ORF1a:T3255I, ORF1a:I3618V, ORF1b:P314L, ORF1b:V1538L, ORF3a:I10S, S:G446V, S:T478K, S:D614G, S:P681H, S:T732A,
hCoV-19/Mexico/CMX-IBT-IMSS-345/2021	EPI_ISL_1288524	In process	20B	B.1.1.519	25	5'UTR:C203T, 5'UTR:C222T, 5'UTR:C241T, ORF1ab:G351A, ORF1ab:C3037T, ORF1ab:C3140T, ORF1ab:C10029T, ORF1ab:C10954T, ORF1ab:A11117G, ORF1ab:C12789T, ORF1ab:C14408T, ORF1ab:T19839C, ORF1ab:C21077T, ORF1ab:C21306T, S:C22677T, S:C22995A, S:A23403G, S:C23604A, S:A23756G, ORF3a:G25906T, M:G26774T, ORF8:C28253T, N:G28881A, N:G28882A, N:G28883C, N:C29197T,	16	M:M84I, N:R203K, N:G204R, ORF1a:R29H, ORF1a:P959S, ORF1a:T3255I, ORF1a:I3618V, ORF1a:T4175I, ORF1b:P314L, ORF1b:T2537I, ORF3a:G172C, S:A372V, S:T478K, S:D614G, S:P681H, S:T732A,
hCoV-19/Mexico/CMX-IBT-IMSS-346/2021	EPI_ISL_1288525	In process	20B	B.1.1.519	26	5'UTR:C203T, 5'UTR:C222T, 5'UTR:C241T, ORF1ab:G351A, ORF1ab:G2780T, ORF1ab:C3037T, ORF1ab:C3140T, ORF1ab:C10029T, ORF1ab:C10954T, ORF1ab:A11117G, ORF1ab:C12789T, ORF1ab:C14408T, ORF1ab:T19839C, ORF1ab:C21077T, ORF1ab:C21306T, S:C22677T, S:C22995A, S:A23403G, S:C23604A, S:A23756G, ORF3a:G25906T, M:G26774T, ORF8:C28253T, N:G28881A, N:G28882A, N:G28883C, N:C29197T,	17	M:M84I, N:R203K, N:G204R, ORF1a:R29H, ORF1a:V839L, ORF1a:P959S, ORF1a:T3255I, ORF1a:I3618V, ORF1a:T4175I, ORF1b:P314L, ORF1b:T2537I, ORF3a:G172C, S:A372V, S:T478K, S:D614G, S:P681H, S:T732A,

hCoV-19/Mexico/CMX-IBT-IMSS-349/2021	EPI_ISL_1288526	In process	20B	B.1.1.222	25	5'UTR:C241T, ORF1ab:C3037T, ORF1ab:C6361T, ORF1ab:C6548T, ORF1ab:C7858T, ORF1ab:C10029T, ORF1ab:A10323G, ORF1ab:C14408T, ORF1ab:T15453C, ORF1ab:C17012T, ORF1ab:G17721T, ORF1ab:T19839C, S:A23403G, S:A23756G, ORF3a:G25567A, ORF3a:T25569A, ORF3a:T25570A, ORF3a:G25912T, ORF3a:A26108G, ORF8:A27921G, ORF8:C27945T, ORF8:G28001T, ORF8:C28087T, N:G28881A, N:G28882A, N:G28883C,	15	N:R203K, N:G204R, ORF1a:T3255I, ORF1a:K3353R, ORF1b:P314L, ORF1b:S1182L, ORF3a:A59T, ORF3a:S60T, ORF3a:G174C, ORF3a:E239G, ORF8:I10V, ORF8:Q18*, ORF8:A65V, S:D614G, S:T732A,	
hCoV-19/Mexico/CMX-IBT-IMSS-350/2021	EPI_ISL_1288527	In process	20B	B.1.1.519	22	5'UTR:C203T, 5'UTR:C222T, 5'UTR:C241T, ORF1ab:G625T, ORF1ab:C3037T, ORF1ab:C3140T, ORF1ab:C10029T, ORF1ab:C10954T, ORF1ab:A11117G, ORF1ab:C12789T, ORF1ab:C14408T, ORF1ab:T19839C, ORF1ab:C21306T, S:C21575T, S:C22995A, S:A23403G, S:C23604A, S:A23756G, ORF3a:C25469T, N:G28881A, N:G28882A, N:G28883C, N:C29197T,	14	N:R203K, N:G204R, ORF1a:K120N, ORF1a:P959S, ORF1a:T3255I, ORF1a:I3618V, ORF1a:T4175I, ORF1b:P314L, ORF3a:S26L, S:L5F, S:T478K, S:D614G, S:P681H, S:T732A,	
hCoV-19/Mexico/CMX-IBT-IMSS-351/2021	EPI_ISL_1288528	In process	20B	B.1.1.222	16	5'UTR:C241T, ORF1ab:C2005T, ORF1ab:C3037T, ORF1ab:C10029T, ORF1ab:C14408T, ORF1ab:C18501T, ORF1ab:C18652T, ORF1ab:C19610T, ORF1ab:T19839C, ORF1ab:C19875T, S:A23403G, S:A23756G, ORF3a:G25912T, ORF7b:A27756G, N:G28881A, N:G28882A, N:G28883C,	10	N:R203K, N:G204R, ORF1a:T3255I, ORF1b:P314L, ORF1b:R1729C, ORF1b:T2048I, ORF3a:G174C, ORF7b:M1V, S:D614G, S:T732A,	3'UTR:29746-29762,
hCoV-19/Mexico/CMX-IBT-IMSS-355/2021	EPI_ISL_1288532	In process	20B	B.1.1.222	24	5'UTR:C241T, ORF1ab:C1758T, ORF1ab:G2086T, ORF1ab:C2252A, ORF1ab:C3037T, ORF1ab:C10029T, ORF1ab:C12068T, ORF1ab:C14408T, ORF1ab:C15279T, ORF1ab:G19816T, ORF1ab:T19839C, S:C21575T, S:T22020C, S:A23403G, S:A23756G, ORF3a:G25912T, ORF3a:C26078T, ORF7b:A27756G, ORF8:A27921G, ORF8:G28001T, N:A28384T, N:G28881A, N:G28882A, N:G28883C, N:G29383A,	17	N:R203K, N:G204R, ORF1a:A498V, ORF1a:Q607H, ORF1a:Q663K, ORF1a:T3255I, ORF1b:P314L, ORF1b:V2117L, ORF3a:G174C, ORF3a:T229I, ORF7b:M1V, ORF8:I10V, ORF9b:Q34L, S:L5F, S:M153T, S:D614G, S:T732A,	
hCoV-19/Mexico/CMX-IBT-IMSS-359/2021	EPI_ISL_1288189	In process	20B	B.1.1.519	27	5'UTR:C203T, 5'UTR:C222T, 5'UTR:C241T, ORF1ab:C2650T, ORF1ab:C3037T, ORF1ab:C3140T, ORF1ab:G4311C, ORF1ab:G7027T, ORF1ab:C7735T, ORF1ab:C10029T, ORF1ab:C10954T, ORF1ab:A11117G, ORF1ab:C12789T, ORF1ab:C14408T, ORF1ab:G17721T, ORF1ab:T19839C, ORF1ab:C19884T, ORF1ab:C21306T, S:C22995A, S:A23403G, S:C23604A, S:A23756G, S:C25207T, M:A26708G, N:G28881A, N:G28882A, N:G28883C, N:C29197T,	13	N:R203K, N:G204R, ORF1a:P959S, ORF1a:C1349S, ORF1a:M2254I, ORF1a:T3255I, ORF1a:I3618V, ORF1a:T4175I, ORF1b:P314L, S:T478K, S:D614G, S:P681H, S:T732A,	
hCoV-19/Mexico/CMX-IBT-IMSS-361/2021	EPI_ISL_1288190	In process	20B	B.1.1.519	23	5'UTR:C203T, 5'UTR:C222T, 5'UTR:C241T, ORF1ab:C3037T, ORF1ab:C3140T, ORF1ab:T9519C, ORF1ab:C10029T, ORF1ab:C10954T, ORF1ab:A11117G, ORF1ab:C12789T, ORF1ab:C14408T, ORF1ab:T19839C, ORF1ab:C21306T, S:C22747T, S:C22995A, S:A23403G, S:C23604A, S:A23756G, ORF6:C27219T, ORF8:C27978T, N:G28881A, N:G28882A, N:G28883C, N:C29197T,	13	N:R203K, N:G204R, ORF1a:P959S, ORF1a:F3085S, ORF1a:T3255I, ORF1a:I3618V, ORF1a:T4175I, ORF1b:P314L, ORF8:Q29*, S:T478K, S:D614G, S:P681H, S:T732A,	
hCoV-19/Mexico/CMX-IBT-IMSS-362/2021	EPI_ISL_1288191	In process	20B	B.1.1.519	22	5'UTR:C203T, 5'UTR:C222T, 5'UTR:C241T, ORF1ab:G2095A, ORF1ab:C3037T, ORF1ab:C3140T, ORF1ab:C10029T, ORF1ab:C10954T, ORF1ab:A11117G, ORF1ab:C12789T, ORF1ab:A13712G, ORF1ab:C14408T, ORF1ab:T19839C, ORF1ab:C21306T, S:C22995A, S:A23403G, S:C23604A, S:A23756G, ORF3a:C25782T, N:G28881A, N:G28882A, N:G28883C, N:C29197T,	12	N:R203K, N:G204R, ORF1a:P959S, ORF1a:T3255I, ORF1a:I3618V, ORF1a:T4175I, ORF1b:K82R, ORF1b:P314L, S:T478K, S:D614G, S:P681H, S:T732A,	

hCoV-19/Mexico/CMX-IBT-IMSS-363/2021	EPI_ISL_1288192	In process	20B	B.1.1.519	23	5'UTR:C203T, 5'UTR:C222T, 5'UTR:C241T, ORF1ab:C3037T, ORF1ab:C3140T, ORF1ab:C8660T, ORF1ab:C10029T, ORF1ab:C10954T, ORF1ab:A11117G, ORF1ab:C12789T, ORF1ab:C14408T, ORF1ab:G15921T, ORF1ab:T19839C, ORF1ab:A20055G, ORF1ab:C21306T, S:C22995A, S:C23127T, S:A23403G, S:C23604A, S:A23756G, N:G28881A, N:G28882A, N:G28883C, N:C29197T,	13	N:R203K, N:G204R, ORF1a:P959S, ORF1a:H2799Y, ORF1a:T3255I, ORF1a:I3618V, ORF1a:T4175I, ORF1b:P314L, S:T478K, S:A522V, S:D614G, S:P681H, S:T732A,
hCoV-19/Mexico/CMX-IBT-IMSS-364/2021	EPI_ISL_1288193	In process	20B	B.1.1.519	29	5'UTR:C203T, 5'UTR:C222T, 5'UTR:C241T, ORF1ab:C920T, ORF1ab:C3037T, ORF1ab:C3140T, ORF1ab:G3692T, ORF1ab:G3871T, ORF1ab:C6312T, ORF1ab:A8446G, ORF1ab:C10029T, ORF1ab:C10954T, ORF1ab:A11117G, ORF1ab:C11824A, ORF1ab:C12789T, ORF1ab:C14408T, ORF1ab:C15120T, ORF1ab:C18171T, ORF1ab:T19839C, ORF1ab:C21306T, S:C22995A, S:A23403G, S:C23604A, S:A23756G, S:G24368T, ORF8:T27904C, N:G28881A, N:G28882A, N:G28883C, N:C29197T,	16	N:R203K, N:G204R, ORF1a:P959S, ORF1a:V1143F, ORF1a:K1202N, ORF1a:T2016I, ORF1a:T3255I, ORF1a:I3618V, ORF1a:T4175I, ORF1b:P314L, ORF8:L4P, S:T478K, S:D614G, S:P681H, S:T732A, S:D936Y,
hCoV-19/Mexico/CMX-IBT-IMSS-365/2021	EPI_ISL_1288194	In process	20B	B.1.1.519	25	5'UTR:C203T, 5'UTR:C222T, 5'UTR:C241T, ORF1ab:A1856G, ORF1ab:C3037T, ORF1ab:C3140T, ORF1ab:C3318T, ORF1ab:A4485G, ORF1ab:C10029T, ORF1ab:C10954T, ORF1ab:A11117G, ORF1ab:C12789T, ORF1ab:C14408T, ORF1ab:C18086T, ORF1ab:T19839C, ORF1ab:C21306T, S:C22995A, S:A23403G, S:C23604A, S:A23756G, S:C25184G, ORF7a:C27434T, N:G28881A, N:G28882A, N:G28883C, N:C29197T,	17	N:R203K, N:G204R, ORF1a:S531G, ORF1a:P959S, ORF1a:P1018L, ORF1a:K1407R, ORF1a:T3255I, ORF1a:I3618V, ORF1a:T4175I, ORF1b:P314L, ORF1b:T1540I, ORF7a:T14I, S:T478K, S:D614G, S:P681H, S:T732A, S:Q1208E,
hCoV-19/Mexico/CMX-IBT-IMSS-366/2021	EPI_ISL_1288195	In process	20B	B.1.1.519	25	5'UTR:C203T, 5'UTR:C222T, 5'UTR:C241T, ORF1ab:A1856G, ORF1ab:C3037T, ORF1ab:C3140T, ORF1ab:C3318T, ORF1ab:A4485G, ORF1ab:C10029T, ORF1ab:C10954T, ORF1ab:A11117G, ORF1ab:C12789T, ORF1ab:C14408T, ORF1ab:C18086T, ORF1ab:T19839C, ORF1ab:C21306T, S:C22995A, S:A23403G, S:C23604A, S:A23756G, S:C25184G, ORF7a:C27434T, N:G28881A, N:G28882A, N:G28883C, N:C29197T,	17	N:R203K, N:G204R, ORF1a:S531G, ORF1a:P959S, ORF1a:P1018L, ORF1a:K1407R, ORF1a:T3255I, ORF1a:I3618V, ORF1a:T4175I, ORF1b:P314L, ORF1b:T1540I, ORF7a:T14I, S:T478K, S:D614G, S:P681H, S:T732A, S:Q1208E,
hCoV-19/Mexico/CMX-IBT-IMSS-367/2021	EPI_ISL_1288196	In process	20B	B.1.1.519	23	5'UTR:C203T, 5'UTR:C222T, 5'UTR:C241T, ORF1ab:C3037T, ORF1ab:C3140T, ORF1ab:C6026T, ORF1ab:C6628T, ORF1ab:C10029T, ORF1ab:C10954T, ORF1ab:A11117G, ORF1ab:G11365T, ORF1ab:C12513T, ORF1ab:C14408T, ORF1ab:T19839C, ORF1ab:C21306T, S:C22995A, S:A23403G, S:C23604A, S:A23756G, N:G28881A, N:G28882A, N:G28883C, N:C29197T, N:G29527T,	13	N:R203K, N:G204R, N:Q418H, ORF1a:P959S, ORF1a:P1921S, ORF1a:T3255I, ORF1a:I3618V, ORF1a:T4083M, ORF1b:P314L, S:T478K, S:D614G, S:P681H, S:T732A,
hCoV-19/Mexico/CMX-IBT-IMSS-368/2021	EPI_ISL_1288197	In process	20B	B.1.1.519	25	5'UTR:T201C, 5'UTR:C203T, 5'UTR:C222T, 5'UTR:C241T, ORF1ab:C934T, ORF1ab:G1738T, ORF1ab:C3037T, ORF1ab:C3140T, ORF1ab:C3821T, ORF1ab:C10029T, ORF1ab:C10954T, ORF1ab:A11117G, ORF1ab:C12789T, ORF1ab:C14408T, ORF1ab:T19839C, ORF1ab:A19974G, ORF1ab:C21306T, S:C22995A, S:A23403G, S:C23604A, S:A23756G, S:C24034T, N:G28881A, N:G28882A, N:G28883C, N:C29197T,	12	N:R203K, N:G204R, ORF1a:P959S, ORF1a:L1186F, ORF1a:T3255I, ORF1a:I3618V, ORF1a:T4175I, ORF1b:P314L, S:T478K, S:D614G, S:P681H, S:T732A,

hCoV-19/Mexico/CMX-IBT-IMSS-372/2021	EPI_ISL_1288200	In process	20B	B.1.1.519	23	5'UTR:C203T, 5'UTR:C222T, 5'UTR:C241T, ORF1ab:T1303A, ORF1ab:C3037T, ORF1ab:C3140T, ORF1ab:C10029T, ORF1ab:C10954T, ORF1ab:A11117G, ORF1ab:C12786T, ORF1ab:C12789T, ORF1ab:C14408T, ORF1ab:C19017T, ORF1ab:T19839C, ORF1ab:C21306T, S:C22995A, S:A23403G, S:C23604A, S:A23756G, N:G28881A, N:G28882A, N:G28883C, N:C29197T, ORF10:T29661C,	12	N:R203K, N:G204R, ORF1a:P959S, ORF1a:T3255I, ORF1a:I3618V, ORF1a:T4174I, ORF1a:T4175I, ORF1b:P314L, S:T478K, S:D614G, S:P681H, S:T732A,
hCoV-19/Mexico/CMX-IBT-IMSS-373/2021	EPI_ISL_1288201	In process	20B	B.1.1.222	18	5'UTR:C241T, ORF1ab:C3037T, ORF1ab:C4897T, ORF1ab:C6628T, ORF1ab:C10029T, ORF1ab:A11430G, ORF1ab:C14408T, ORF1ab:C19011A, ORF1ab:T19839C, ORF1ab:A21137G, S:A23403G, S:A23756G, ORF3a:G25912T, ORF3a:A26115C, ORF8:A27921G, ORF8:G28001T, N:G28881A, N:G28882A, N:G28883C,	12	N:R203K, N:G204R, ORF1a:T3255I, ORF1a:Y3722C, ORF1b:P314L, ORF1b:D1848E, ORF1b:K2557R, ORF3a:G174C, ORF3a:E241D, ORF8:I10V, S:D614G, S:T732A,
hCoV-19/Mexico/CMX-IBT-IMSS-384/2021	EPI_ISL_1288211	In process	20B	B.1.1.519	25	5'UTR:C203T, 5'UTR:C222T, 5'UTR:C241T, ORF1ab:C3037T, ORF1ab:C3140T, ORF1ab:G3692T, ORF1ab:G3871T, ORF1ab:A5638G, ORF1ab:C10029T, ORF1ab:C10954T, ORF1ab:A11117G, ORF1ab:C11824A, ORF1ab:C12789T, ORF1ab:C14408T, ORF1ab:G18781A, ORF1ab:T19839C, ORF1ab:C21306T, S:C22995A, S:A23403G, S:C23604A, S:A23756G, ORF8:T27904C, N:G28881A, N:G28882A, N:G28883C, N:C29197T,	15	N:R203K, N:G204R, ORF1a:P959S, ORF1a:V1143F, ORF1a:K1202N, ORF1a:T3255I, ORF1a:I3618V, ORF1a:T4175I, ORF1b:P314L, ORF1b:G1772S, ORF8:L4P, S:T478K, S:D614G, S:P681H, S:T732A,
hCoV-19/Mexico/CMX-IBT-IMSS-385/2021	EPI_ISL_1288212	In process	20B	B.1.1.519	26	5'UTR:T201C, 5'UTR:C203T, 5'UTR:C222T, 5'UTR:C241T, ORF1ab:A655C, ORF1ab:C745T, ORF1ab:G1738T, ORF1ab:C3037T, ORF1ab:C3140T, ORF1ab:C10029T, ORF1ab:C10954T, ORF1ab:A11117G, ORF1ab:C14408T, ORF1ab:A19505G, ORF1ab:T19839C, ORF1ab:A19974G, ORF1ab:C21306T, S:C22995A, S:A23403G, S:C23604A, S:A23756G, ORF7b:G27806T, N:C28453T, N:G28881A, N:G28882A, N:G28883C, N:C29197T,	12	N:R203K, N:G204R, ORF1a:P959S, ORF1a:T3255I, ORF1a:I3618V, ORF1b:P314L, ORF1b:N2013S, ORF9b:A57V, S:T478K, S:D614G, S:P681H, S:T732A,
hCoV-19/Mexico/CMX-IBT-IMSS-492/2020	EPI_ISL_1301668	In process	20A	B.1.609	9	5'UTR:C241T, ORF1ab:C1403A, ORF1ab:C3037T, ORF1ab:C4582T, ORF1ab:C12049T, ORF1ab:C13119T, ORF1ab:C14408T, ORF1ab:A20268G, S:A23403G, ORF7a:C27476T,	5	ORF1a:P380T, ORF1a:A4285V, ORF1b:P314L, ORF7a:T28I, S:D614G,
hCoV-19/Mexico/CMX-IBT-IMSS-505/2020	EPI_ISL_1301672	In process	20C	B.1	6	5'UTR:C241T, ORF1ab:C1059T, ORF1ab:C3037T, ORF1ab:C4878T, ORF1ab:C14408T, S:A23403G, ORF3a:G25563T,	5	ORF1a:T265I, ORF1a:T1538I, ORF1b:P314L, ORF3a:Q57H, S:D614G,
hCoV-19/Mexico/CMX-IBT-IMSS-518/2021	EPI_ISL_1302272	In process	20B	B.1.1.519	27	5'UTR:C203T, 5'UTR:C222T, 5'UTR:C241T, ORF1ab:C1342T, ORF1ab:C2334T, ORF1ab:C3037T, ORF1ab:C3140T, ORF1ab:G3692T, ORF1ab:G3871T, ORF1ab:G5150A, ORF1ab:C10029T, ORF1ab:C10954T, ORF1ab:A11117G, ORF1ab:C11824A, ORF1ab:C12789T, ORF1ab:C14408T, ORF1ab:T19839C, ORF1ab:G21123T, S:C22995A, S:A23403G, S:C23604A, S:A23756G, ORF8:T27904C, N:C28453T, N:G28881A, N:G28882A, N:G28883C, N:C29197T,	17	N:R203K, N:G204R, ORF1a:A690V, ORF1a:P959S, ORF1a:V1143F, ORF1a:K1202N, ORF1a:V1629I, ORF1a:T3255I, ORF1a:I3618V, ORF1a:T4175I, ORF1b:P314L, ORF8:L4P, ORF9b:A57V, S:T478K, S:D614G, S:P681H, S:T732A,

hCoV-19/Mexico/CMX-IBT-IMSS-520/2021	EPI_ISL_1302355	In process	20B	B.1.1.519	28	5'UTR:T201C, 5'UTR:C203T, 5'UTR:C222T, 5'UTR:C241T, ORF1ab:C745T, ORF1ab:G1738T, ORF1ab:C3037T, ORF1ab:C3140T, ORF1ab:A8893G, ORF1ab:C10029T, ORF1ab:C10954T, ORF1ab:A11117G, ORF1ab:C12789T, ORF1ab:C14408T, ORF1ab:T15195A, ORF1ab:T19839C, ORF1ab:A19974G, S:C22995A, S:A23403G, S:C23604A, S:A23756G, ORF3a:C26032T, ORF8:C28253T, ORF8:A28254C, N:C28770T, N:G28881A, N:G28882A, N:G28883C, N:C29197T	13	N:T166I, N:R203K, N:G204R, ORF1a:P959S, ORF1a:T3255I, ORF1a:I3618V, ORF1a:T4175I, ORF1b:P314L, ORF8:I121L, S:T478K, S:D614G, S:P681H, S:T732A,
hCoV-19/Mexico/CMX-IBT-IMSS-521/2021	EPI_ISL_1302284	In process	20B	B.1.1.519	27	5'UTR:C203T, 5'UTR:C222T, 5'UTR:C241T, ORF1ab:C1342T, ORF1ab:C3037T, ORF1ab:C3140T, ORF1ab:G3692T, ORF1ab:G3871T, ORF1ab:C10029T, ORF1ab:C10954T, ORF1ab:A11117G, ORF1ab:C11824A, ORF1ab:C12789T, ORF1ab:C14408T, ORF1ab:G18255T, ORF1ab:T19839C, S:C22995A, S:A23403G, S:C23604A, S:A23756G, ORF3a:C25710T, ORF8:T27904C, N:T28516C, N:G28881A, N:G28882A, N:G28883C, N:C29197T, 3'UTR:G29777T,	16	N:R203K, N:G204R, ORF1a:P959S, ORF1a:V1143F, ORF1a:K1202N, ORF1a:T3255I, ORF1a:I3618V, ORF1a:T4175I, ORF1b:P314L, ORF1b:M1596I, ORF8:L4P, ORF9b:M78T, S:T478K, S:D614G, S:P681H, S:T732A,
hCoV-19/Mexico/CMX-IBT-IMSS-523/2021	EPI_ISL_1302203	In process	20B	B.1.1.519	24	5'UTR:T201C, 5'UTR:C203T, 5'UTR:C222T, 5'UTR:C241T, ORF1ab:C745T, ORF1ab:G1738T, ORF1ab:C3037T, ORF1ab:C3140T, ORF1ab:C7162T, ORF1ab:C10029T, ORF1ab:C10954T, ORF1ab:A11117G, ORF1ab:C12789T, ORF1ab:C14408T, ORF1ab:A19821G, ORF1ab:T19839C, ORF1ab:A19974G, S:C22995A, S:A23403G, S:C23604A, S:A23756G, N:G28881A, N:G28882A, N:G28883C, N:C29197T,	11	N:R203K, N:G204R, ORF1a:P959S, ORF1a:T3255I, ORF1a:I3618V, ORF1a:T4175I, ORF1b:P314L, S:T478K, S:D614G, S:P681H, S:T732A,
hCoV-19/Mexico/CMX-IBT-IMSS-524/2021	EPI_ISL_1302221	In process	20B	B.1.1.519	23	5'UTR:C203T, 5'UTR:C222T, 5'UTR:C241T, ORF1ab:C3037T, ORF1ab:C3140T, ORF1ab:C7039T, ORF1ab:C7083T, ORF1ab:C10029T, ORF1ab:C10954T, ORF1ab:A11117G, ORF1ab:C12789T, ORF1ab:C14408T, ORF1ab:T19839C, S:C22995A, S:A23403G, S:C23604A, S:A23756G, ORF3a:G25440A, ORF3a:G25785T, N:G28881A, N:G28882A, N:G28883C, N:C29197T, N:G29527T,	14	N:R203K, N:G204R, N:Q418H, ORF1a:P959S, ORF1a:S2273F, ORF1a:T3255I, ORF1a:I3618V, ORF1a:T4175I, ORF1b:P314L, ORF3a:W131C, S:T478K, S:D614G, S:P681H, S:T732A,
hCoV-19/Mexico/CMX-IBT-IMSS-526/2021	EPI_ISL_1302261	In process	20B	B.1.1.519	24	5'UTR:C203T, 5'UTR:C222T, 5'UTR:C241T, ORF1ab:G611T, ORF1ab:C842T, ORF1ab:C3037T, ORF1ab:C3140T, ORF1ab:C4999T, ORF1ab:C10029T, ORF1ab:C10954T, ORF1ab:A11117G, ORF1ab:G11417T, ORF1ab:C12789T, ORF1ab:C14408T, ORF1ab:T19839C, S:C22995A, S:A23403G, S:C23604A, S:A23756G, N:G28881A, N:G28882A, N:G28883C, N:C29197T, N:G29527T, 3'UTR:C29719T,	15	N:R203K, N:G204R, N:Q418H, ORF1a:V116L, ORF1a:P193S, ORF1a:P959S, ORF1a:T3255I, ORF1a:I3618V, ORF1a:V3718F, ORF1a:T4175I, ORF1b:P314L, S:T478K, S:D614G, S:P681H, S:T732A,
hCoV-19/Mexico/CMX-IBT-IMSS-528/2021	EPI_ISL_1302275	In process	20B	B.1.1.519	26	5'UTR:T201C, 5'UTR:C203T, 5'UTR:C222T, 5'UTR:C241T, ORF1ab:G1738T, ORF1ab:A2725T, ORF1ab:C3037T, ORF1ab:C3140T, ORF1ab:C10029T, ORF1ab:C10954T, ORF1ab:A11117G, ORF1ab:G12038A, ORF1ab:C12789T, ORF1ab:C14408T, ORF1ab:G14559T, ORF1ab:G18079T, ORF1ab:T19839C, ORF1ab:A19974G, S:C22995A, S:A23403G, S:C23604A, S:A23756G, ORF3a:G25699A, N:G28881A, N:G28882A, N:G28883C, N:C29197T,	14	N:R203K, N:G204R, ORF1a:P959S, ORF1a:T3255I, ORF1a:I3618V, ORF1a:V3925I, ORF1a:T4175I, ORF1b:P314L, ORF1b:V1538L, ORF3a:A103T, S:T478K, S:D614G, S:P681H, S:T732A,

hCoV-19/Mexico/CMX-IBT-IMSS-529/2021	EPI_ISL_1302222	In process	20B	B.1.1.519	23	5'UTR:C106T, 5'UTR:C203T, 5'UTR:C222T, 5'UTR:C241T, ORF1ab:G1126T, ORF1ab:C3037T, ORF1ab:C3140T, ORF1ab:C10029T, ORF1ab:C10954T, ORF1ab:A11117G, ORF1ab:G11365T, ORF1ab:C12789T, ORF1ab:C14408T, ORF1ab:T19839C, S:C22995A, S:A23403G, S:C23604A, S:A23756G, ORF3a:G25687T, N:G28881A, N:G28882A, N:G28883C, N:C29197T, N:G29527T,	14	N:R203K, N:G204R, N:Q418H, ORF1a:R287S, ORF1a:P959S, ORF1a:T3255I, ORF1a:I3618V, ORF1a:T4175I, ORF1b:P314L, ORF3a:A99S, S:T478K, S:D614G, S:P681H, S:T732A,	
hCoV-19/Mexico/CMX-IBT-IMSS-530/2021	EPI_ISL_1302204	In process	20B	B.1.1.519	23	5'UTR:C203T, 5'UTR:C222T, 5'UTR:C241T, ORF1ab:C2061T, ORF1ab:C3037T, ORF1ab:C3140T, ORF1ab:C10029T, ORF1ab:C10954T, ORF1ab:A11117G, ORF1ab:C12789T, ORF1ab:C14408T, ORF1ab:T19839C, S:C22995A, S:A23403G, S:C23604A, S:A23756G, M:G26526A, ORF6:C27393T, N:C28472T, N:G28881A, N:G28882A, N:G28883C, N:C29197T, N:G29527T,	15	M:A2T, N:P67S, N:R203K, N:G204R, N:Q418H, ORF1a:A599V, ORF1a:P959S, ORF1a:T3255I, ORF1a:I3618V, ORF1a:T4175I, ORF1b:P314L, S:T478K, S:D614G, S:P681H, S:T732A,	
hCoV-19/Mexico/CMX-IBT-IMSS-531/2021	EPI_ISL_1302294	In process	20B	B.1.1.519	26	5'UTR:C203T, 5'UTR:C222T, 5'UTR:C241T, ORF1ab:C920T, ORF1ab:C3037T, ORF1ab:C3140T, ORF1ab:G3692T, ORF1ab:G3871T, ORF1ab:A8446G, ORF1ab:C10029T, ORF1ab:C10954T, ORF1ab:A11117G, ORF1ab:C11824A, ORF1ab:C12789T, ORF1ab:C14408T, ORF1ab:C15120T, ORF1ab:C18171T, ORF1ab:T19839C, S:C22995A, S:A23403G, S:C23604A, S:A23756G, ORF8:T27904C, N:G28881A, N:G28882A, N:G28883C, N:C29197T,	14	N:R203K, N:G204R, ORF1a:P959S, ORF1a:V1143F, ORF1a:K1202N, ORF1a:T3255I, ORF1a:I3618V, ORF1a:T4175I, ORF1b:P314L, ORF8:L4P, S:T478K, S:D614G, S:P681H, S:T732A,	
hCoV-19/Mexico/CMX-IBT-IMSS-532/2021	EPI_ISL_1302240	In process	20B	B.1.1.222	23	5'UTR:G219T, 5'UTR:C241T, ORF1ab:C2091T, ORF1ab:C3037T, ORF1ab:G4124T, ORF1ab:C8293T, ORF1ab:A10323G, ORF1ab:A11782G, ORF1ab:C14408T, ORF1ab:G14772T, ORF1ab:G16365T, ORF1ab:C17333T, ORF1ab:T19839C, ORF1ab:T20976C, S:A23403G, S:A23756G, S:C23936A, S:G25135T, ORF3a:C25904T, M:C26681T, N:C28292A, N:G28881A, N:G28882A, N:G28883C,	15	N:Q7K, N:R203K, N:G204R, ORF1a:T609I, ORF1a:V1287L, ORF1a:K3353R, ORF1b:P314L, ORF1b:Q435H, ORF1b:L966F, ORF1b:T1289M, ORF3a:S171L, S:D614G, S:T732A, S:P792T, S:K1191N,	
hCoV-19/Mexico/CMX-IBT-IMSS-533/2021	EPI_ISL_1302315	In process	20B	B.1.1.519	22	5'UTR:T201C, 5'UTR:C203T, 5'UTR:C222T, 5'UTR:C241T, ORF1ab:C745T, ORF1ab:G1738T, ORF1ab:C3037T, ORF1ab:C3140T, ORF1ab:C10029T, ORF1ab:C10954T, ORF1ab:A11117G, ORF1ab:C12789T, ORF1ab:C14408T, ORF1ab:T19839C, ORF1ab:A19974G, S:C22995A, S:A23403G, S:C23604A, S:A23756G, N:G28881A, N:G28882A, N:G28883C, N:C29197T,	11	N:R203K, N:G204R, ORF1a:P959S, ORF1a:T3255I, ORF1a:I3618V, ORF1a:T4175I, ORF1b:P314L, S:T478K, S:D614G, S:P681H, S:T732A,	
hCoV-19/Mexico/CMX-IBT-IMSS-534/2021	EPI_ISL_1302378	In process	20B	B.1.1.519	21	5'UTR:C203T, 5'UTR:C222T, 5'UTR:C241T, ORF1ab:C3037T, ORF1ab:C3140T, ORF1ab:C10029T, ORF1ab:C10078T, ORF1ab:C10954T, ORF1ab:A11117G, ORF1ab:G11365T, ORF1ab:C12789T, ORF1ab:C14408T, ORF1ab:T19839C, S:C22995A, S:A23403G, S:C23604A, S:A23756G, N:G28881A, N:G28882A, N:G28883C, N:C29197T, N:G29527T,	12	N:R203K, N:G204R, N:Q418H, ORF1a:P959S, ORF1a:T3255I, ORF1a:I3618V, ORF1a:T4175I, ORF1b:P314L, S:T478K, S:D614G, S:P681H, S:T732A,	
hCoV-19/Mexico/CMX-IBT-IMSS-535/2021	EPI_ISL_1302165	In process	20B	B.1.1.519	21	5'UTR:C203T, 5'UTR:C222T, 5'UTR:C241T, ORF1ab:C3037T, ORF1ab:C3140T, ORF1ab:C10029T, ORF1ab:C10078T, ORF1ab:C10954T, ORF1ab:A11117G, ORF1ab:G11365T, ORF1ab:C12789T, ORF1ab:C14408T, ORF1ab:T19839C, S:C22995A, S:A23403G, S:C23604A, S:A23756G, N:G28881A, N:G28882A, N:G28883C, N:C29197T, N:G29527T,	12	N:R203K, N:G204R, N:Q418H, ORF1a:P959S, ORF1a:T3255I, ORF1a:I3618V, ORF1a:T4175I, ORF1b:P314L, S:T478K, S:D614G, S:P681H, S:T732A,	ORF7b:27795-27797,

hCoV-19/Mexico/CMX-IBT-IMSS-536/2021	EPI_ISL_1302223	In process	20B	B.1.1.519	23	5'UTR:T201C, 5'UTR:C203T, 5'UTR:C222T, 5'UTR:C241T, ORF1ab:G1738T, ORF1ab:C3037T, ORF1ab:C3140T, ORF1ab:C8841T, ORF1ab:C10029T, ORF1ab:G10283A, ORF1ab:C10954T, ORF1ab:A11117G, ORF1ab:C12789T, ORF1ab:C14408T, ORF1ab:T19839C, ORF1ab:A19974G, S:C22995A, S:A23403G, S:C23604A, S:A23756G, N:G28881A, N:G28882A, N:G28883C, N:C29197T,	13	N:R203K, N:G204R, ORF1a:P959S, ORF1a:T2859I, ORF1a:T3255I, ORF1a:V3340I, ORF1a:I3618V, ORF1a:T4175I, ORF1b:P314L, S:T478K, S:D614G, S:P681H, S:T732A,
hCoV-19/Mexico/CMX-IBT-IMSS-537/2021	EPI_ISL_1302347	In process	20B	B.1.1.519	25	5'UTR:C203T, 5'UTR:C222T, 5'UTR:C241T, ORF1ab:C3037T, ORF1ab:C3140T, ORF1ab:G3692T, ORF1ab:G3871T, ORF1ab:C10029T, ORF1ab:C10954T, ORF1ab:A11117G, ORF1ab:C11824A, ORF1ab:C12789T, ORF1ab:C14408T, ORF1ab:G15260T, ORF1ab:T19839C, ORF1ab:G19947T, S:C22971T, S:C22995A, S:A23403G, S:C23604A, S:A23756G, ORF8:T27904C, N:G28881A, N:G28882A, N:G28883C, N:C29197T,	17	N:R203K, N:G204R, ORF1a:P959S, ORF1a:V1143F, ORF1a:K1202N, ORF1a:T3255I, ORF1a:I3618V, ORF1a:T4175I, ORF1b:P314L, ORF1b:S598I, ORF1b:K2160N, ORF8:L4P, S:T470I, S:T478K, S:D614G, S:P681H, S:T732A,
hCoV-19/Mexico/CMX-IBT-IMSS-538/2021	EPI_ISL_1302178	In process	20B	B.1.1.519	27	5'UTR:C203T, 5'UTR:C222T, 5'UTR:C241T, ORF1ab:G793A, ORF1ab:C1342T, ORF1ab:C3037T, ORF1ab:C3140T, ORF1ab:G3692T, ORF1ab:G3871T, ORF1ab:C6573T, ORF1ab:C10029T, ORF1ab:C10954T, ORF1ab:A11117G, ORF1ab:C11824A, ORF1ab:C12756T, ORF1ab:C12789T, ORF1ab:C14408T, ORF1ab:T18488C, ORF1ab:T19839C, S:C22995A, S:A23403G, S:C23604A, S:A23756G, ORF8:T27904C, N:G28881A, N:G28882A, N:G28883C, N:C29197T,	17	N:R203K, N:G204R, ORF1a:P959S, ORF1a:V1143F, ORF1a:K1202N, ORF1a:S2103F, ORF1a:T3255I, ORF1a:I3618V, ORF1a:T4164I, ORF1a:T4175I, ORF1b:P314L, ORF1b:I1674T, ORF8:L4P, S:T478K, S:D614G, S:P681H, S:T732A,
hCoV-19/Mexico/CMX-IBT-IMSS-563/2021	EPI_ISL_1302224	In process	20B	B.1.1.519	22	5'UTR:T201C, 5'UTR:C203T, 5'UTR:C222T, 5'UTR:C241T, ORF1ab:G1738T, ORF1ab:C3037T, ORF1ab:C3140T, ORF1ab:G9783T, ORF1ab:C10029T, ORF1ab:C10954T, ORF1ab:A11117G, ORF1ab:C12789T, ORF1ab:C14408T, ORF1ab:T19839C, ORF1ab:A19974G, S:C22995A, S:A23403G, S:C23604A, S:A23756G, N:G28881A, N:G28882A, N:G28883C, N:C29197T,	12	N:R203K, N:G204R, ORF1a:P959S, ORF1a:S3173I, ORF1a:T3255I, ORF1a:I3618V, ORF1a:T4175I, ORF1b:P314L, S:T478K, S:D614G, S:P681H, S:T732A,
hCoV-19/Mexico/CMX-IBT-IMSS-564/2021	EPI_ISL_1302243	In process	20B	B.1.1.222	21	5'UTR:C241T, ORF1ab:C3037T, ORF1ab:C10029T, ORF1ab:G13993A, ORF1ab:C14408T, ORF1ab:G17721T, ORF1ab:T19839C, ORF1ab:C21034G, S:A23403G, S:G23522C, S:A23756G, ORF3a:G25567A, ORF3a:T25569A, ORF3a:T25570A, ORF3a:C25587T, ORF3a:G25912T, ORF3a:A26108G, ORF8:A27921G, ORF8:G28001T, N:G28881A, N:G28882A, N:G28883C,	14	N:R203K, N:G204R, ORF1a:T3255I, ORF1b:A176T, ORF1b:P314L, ORF1b:L2523V, ORF3a:A59T, ORF3a:S60T, ORF3a:G174C, ORF3a:E239G, ORF8:I10V, S:D614G, S:E654Q, S:T732A,
hCoV-19/Mexico/CMX-IBT-IMSS-565/2021	EPI_ISL_1302379	In process	20B	B.1.1.519	23	5'UTR:T201C, 5'UTR:C203T, 5'UTR:C222T, 5'UTR:C241T, ORF1ab:A1515G, ORF1ab:G1738T, ORF1ab:C3037T, ORF1ab:C3140T, ORF1ab:G9783T, ORF1ab:C10029T, ORF1ab:C10954T, ORF1ab:A11117G, ORF1ab:C12789T, ORF1ab:C14408T, ORF1ab:T19839C, ORF1ab:A19974G, S:C22995A, S:A23403G, S:C23604A, S:A23756G, N:G28881A, N:G28882A, N:G28883C, N:C29197T,	13	N:R203K, N:G204R, ORF1a:H417R, ORF1a:P959S, ORF1a:S3173I, ORF1a:T3255I, ORF1a:I3618V, ORF1a:T4175I, ORF1b:P314L, S:T478K, S:D614G, S:P681H, S:T732A,

hCoV-19/Mexico/CMX-IBT-IMSS-566/2021	EPI_ISL_1302392	In process	20B	B.1.1.519	25	5'UTR:T201C, 5'UTR:C203T, 5'UTR:C222T, 5'UTR:C241T, ORF1ab:C745T, ORF1ab:G1738T, ORF1ab:C3037T, ORF1ab:C3140T, ORF1ab:G6352T, ORF1ab:C10029T, ORF1ab:C10954T, ORF1ab:A11117G, ORF1ab:C12789T, ORF1ab:C13487T, ORF1ab:C14408T, ORF1ab:T19839C, ORF1ab:A19974G, S:C22450A, S:C22995A, S:A23403G, S:C23604A, S:A23756G, N:G28881A, N:G28882A, N:G28883C, N:C29197T,	13	N:R203K, N:G204R, ORF1a:P959S, ORF1a:K2029N, ORF1a:T3255I, ORF1a:I3618V, ORF1a:T4175I, ORF1b:A7V, ORF1b:P314L, S:T478K, S:D614G, S:P681H, S:T732A,
hCoV-19/Mexico/CMX-IBT-IMSS-567/2021	EPI_ISL_1302281	In process	20B	B.1.1.519	22	5'UTR:C203T, 5'UTR:C222T, 5'UTR:C241T, ORF1ab:T1012C, ORF1ab:C3037T, ORF1ab:C3140T, ORF1ab:C8140T, ORF1ab:C10029T, ORF1ab:C10954T, ORF1ab:G11083T, ORF1ab:A11117G, ORF1ab:C12789T, ORF1ab:C14408T, ORF1ab:T19839C, S:C22995A, S:A23403G, S:C23604A, S:A23756G, N:G28881A, N:G28882A, N:G28883C, N:C29197T, N:G29527T,	13	N:R203K, N:G204R, N:Q418H, ORF1a:P959S, ORF1a:T3255I, ORF1a:L3606F, ORF1a:I3618V, ORF1a:T4175I, ORF1b:P314L, S:T478K, S:D614G, S:P681H, S:T732A,
hCoV-19/Mexico/CMX-IBT-IMSS-568/2021	EPI_ISL_1302156	In process	20B	B.1.1.519	22	5'UTR:C203T, 5'UTR:C222T, 5'UTR:C241T, ORF1ab:T1012C, ORF1ab:C3037T, ORF1ab:C3140T, ORF1ab:C8140T, ORF1ab:C10029T, ORF1ab:C10954T, ORF1ab:G11083T, ORF1ab:A11117G, ORF1ab:C12789T, ORF1ab:C14408T, ORF1ab:T19839C, S:C22995A, S:A23403G, S:C23604A, S:A23756G, N:G28881A, N:G28882A, N:G28883C, N:C29197T, N:G29527T,	13	N:R203K, N:G204R, N:Q418H, ORF1a:P959S, ORF1a:T3255I, ORF1a:L3606F, ORF1a:I3618V, ORF1a:T4175I, ORF1b:P314L, S:T478K, S:D614G, S:P681H, S:T732A,
hCoV-19/Mexico/CMX-IBT-IMSS-569/2021	EPI_ISL_1302324	In process	20B	B.1.1.519	24	5'UTR:C203T, 5'UTR:C222T, 5'UTR:C241T, ORF1ab:C3037T, ORF1ab:C3140T, ORF1ab:C4746T, ORF1ab:C10029T, ORF1ab:C10954T, ORF1ab:A11117G, ORF1ab:G12028T, ORF1ab:C12789T, ORF1ab:C14408T, ORF1ab:G15921T, ORF1ab:T19839C, S:C22995A, S:A23403G, S:C23604A, S:A23756G, M:C26537T, N:G28881A, N:G28882A, N:G28883C, N:C29197T, N:C29274T, 3'UTR:G29810T,	14	N:R203K, N:G204R, N:T334I, ORF1a:P959S, ORF1a:S1494F, ORF1a:T3255I, ORF1a:I3618V, ORF1a:M3921I, ORF1a:T4175I, ORF1b:P314L, S:T478K, S:D614G, S:P681H, S:T732A,
hCoV-19/Mexico/CMX-IBT-IMSS-570/2021	EPI_ISL_1302372	In process	20B	B.1.1.519	27	5'UTR:C203T, 5'UTR:C222T, 5'UTR:C241T, ORF1ab:G443A, ORF1ab:C3037T, ORF1ab:C3140T, ORF1ab:C5869T, ORF1ab:C8419T, ORF1ab:C10029T, ORF1ab:C10954T, ORF1ab:A11117G, ORF1ab:C12789T, ORF1ab:G13385A, ORF1ab:C14408T, ORF1ab:T19839C, S:G21800T, S:C22995A, S:A23403G, S:C23604A, S:A23756G, ORF3a:G25595A, ORF8:T27904C, ORF8:C28087T, N:G28881A, N:G28882A, N:G28883C, N:C29197T, N:T29461C,	17	N:R203K, N:G204R, ORF1a:V60I, ORF1a:P959S, ORF1a:T3255I, ORF1a:I3618V, ORF1a:T4175I, ORF1a:G4374S, ORF1b:P314L, ORF3a:R68K, ORF8:L4P, ORF8:A65V, S:D80Y, S:T478K, S:D614G, S:P681H, S:T732A,
hCoV-19/Mexico/CMX-IBT-IMSS-571/2021	EPI_ISL_1302225	In process	20B	B.1.1.519	24	5'UTR:C203T, 5'UTR:C222T, 5'UTR:C241T, ORF1ab:C3037T, ORF1ab:C3140T, ORF1ab:C3787T, ORF1ab:A5128G, ORF1ab:C10029T, ORF1ab:C10954T, ORF1ab:A11117G, ORF1ab:C12225T, ORF1ab:C12789T, ORF1ab:C14408T, ORF1ab:T19839C, S:G22335T, S:G22778C, S:C22995A, S:A23403G, S:C23604A, S:A23756G, N:G28881A, N:G28882A, N:G28883C, N:C29197T, N:G29527T,	15	N:R203K, N:G204R, N:Q418H, ORF1a:P959S, ORF1a:T3255I, ORF1a:I3618V, ORF1a:A3987V, ORF1a:T4175I, ORF1b:P314L, S:W258L, S:E406Q, S:T478K, S:D614G, S:P681H, S:T732A,

hCoV-19/Mexico/CMX-IBT-IMSS-572/2021	EPI_ISL_1302190	In process	20B	B.1.1.519	25	5'UTR:T201C, 5'UTR:C203T, 5'UTR:C222T, 5'UTR:C241T, ORF1ab:G1738T, ORF1ab:C3037T, ORF1ab:C3140T, ORF1ab:C10029T, ORF1ab:C10954T, ORF1ab:A11117G, ORF1ab:C12789T, ORF1ab:A12796C, ORF1ab:C14408T, ORF1ab:G14559T, ORF1ab:T19839C, ORF1ab:A19974G, S:C22995A, S:A23403G, S:C23604A, S:A23756G, ORF3a:G25699A, N:A28856G, N:G28881A, N:G28882A, N:G28883C, N:C29197T,	13	N:R195G, N:R203K, N:G204R, ORF1a:P959S, ORF1a:T3255I, ORF1a:I3618V, ORF1a:T4175I, ORF1b:P314L, ORF3a:A103T, S:T478K, S:D614G, S:P681H, S:T732A,	
hCoV-19/Mexico/CMX-IBT-IMSS-573/2021	EPI_ISL_1302226	In process	20B	B.1.1.519	22	5'UTR:C203T, 5'UTR:C222T, 5'UTR:C241T, ORF1ab:C3037T, ORF1ab:C3140T, ORF1ab:C5878T, ORF1ab:C10029T, ORF1ab:C10954T, ORF1ab:A11117G, ORF1ab:C12789T, ORF1ab:C14408T, ORF1ab:T19839C, ORF1ab:C21058T, S:C22995A, S:A23403G, S:C23604A, S:A23756G, M:C26985T, M:G27074T, N:G28881A, N:G28882A, N:G28883C, N:C29197T,	13	M:H155Y, N:R203K, N:G204R, ORF1a:P959S, ORF1a:T3255I, ORF1a:I3618V, ORF1a:T4175I, ORF1b:P314L, ORF1b:P2531S, S:T478K, S:D614G, S:P681H, S:T732A,	
hCoV-19/Mexico/CMX-IBT-IMSS-586/2021	EPI_ISL_1302244	In process	20B	B.1.1.519	19	5'UTR:C203T, 5'UTR:C222T, 5'UTR:C241T, ORF1ab:C3037T, ORF1ab:C3140T, ORF1ab:C10029T, ORF1ab:C10954T, ORF1ab:A11117G, ORF1ab:C12789T, ORF1ab:C14408T, ORF1ab:G15921T, ORF1ab:T19839C, S:C22995A, S:A23403G, S:C23604A, S:A23756G, N:G28881A, N:G28882A, N:G28883C, N:C29197T,	11	N:R203K, N:G204R, ORF1a:P959S, ORF1a:T3255I, ORF1a:I3618V, ORF1a:T4175I, ORF1b:P314L, S:T478K, S:D614G, S:P681H, S:T732A,	
hCoV-19/Mexico/CMX-IMSS_K2/2020	EPI_ISL_1096985	In process	20B	B.1.1.70	37	5'UTR:C44T, 5'UTR:C241T, ORF1ab:T3380A, ORF1ab:A3669G, ORF1ab:T4591C, ORF1ab:G5739A, ORF1ab:T5860C, ORF1ab:A6863G, ORF1ab:A7025C, ORF1ab:T7276C, ORF1ab:A8734T, ORF1ab:T9833A, ORF1ab:A9834C, ORF1ab:G11087A, ORF1ab:A12421C, ORF1ab:C17474T, ORF1ab:A19062G, ORF1ab:A19326C, ORF1ab:G19327T, ORF1ab:G19918A, ORF1ab:T20633A, ORF1ab:C20879T, S:C22187T, S:G22638C, S:C22639T, S:A22908T, S:G23958A, ORF3a:G25793A, M:G26951T, ORF6:C27211G, ORF6:C27213A, ORF8:G28167A, N:C28344T, N:G28881A, N:G28882A, N:G28883C, N:A29190T, 3'UTR:G29751T,	10	N:T24I, N:R203K, N:G204R, N:Q306L, ORF6:L4V, ORF8:E92K, S:P209S, S:S359T, S:Y449F, S:G799D,	ORF1ab: 980, ORF1ab: 14512, ORF1ab: 15947, ORF3a:25412,ORF1ab:15955-16013, ORF1ab: 16039-16060
hCoV-19/Mexico/CMX-IMSS_K4/2020	EPI_ISL_1096141	In process	20A	B.1.609	26	5'UTR:C241T, ORF1ab:C281T, ORF1ab:G347A, ORF1ab:A2497G, ORF1ab:C3037T, ORF1ab:C4582T, ORF1ab:T7258C, ORF1ab:G12106T, ORF1ab:C13119T, ORF1ab:C14220A, ORF1ab:C14408T, ORF1ab:G16236A, ORF1ab:A17367G, S:G22225A, S:A24032G, ORF3a:G25690T, E:T26409C, ORF8:T27904G, ORF8:T27909G, ORF8:C27911T, ORF8:G27916A, ORF8:A27917T, ORF8:A27918T, ORF8:A27924T, ORF8:C27925A, ORF8:T27929G, N:G28899T,	9	N:R209I, ORF1a:P6S, ORF1a:V28I, ORF1a:E3947D, ORF1a:A4285V, ORF1b:D251E, ORF1b:P314L, ORF3a:G100C, S:N824D,	
hCoV-19/Mexico/CMX-InDRE-IBT-113/2020	EPI_ISL_1301581	In process	20B	B.1.1.222	9	5'UTR:C241T, ORF1ab:C3037T, ORF1ab:C14408T, ORF1ab:G18040T, ORF1ab:T19839C, S:A23403G, S:A23756G, N:G28881A, N:G28882A, N:G28883C,	6	N:R203K, N:G204R, ORF1b:P314L, ORF1b:A1525S, S:D614G, S:T732A,	
hCoV-19/Mexico/CMX-InDRE-IBT-114/2020	EPI_ISL_1301582	In process	20B	B.1.1.222	9	5'UTR:C241T, ORF1ab:C3037T, ORF1ab:C14408T, ORF1ab:G18040T, ORF1ab:T19839C, S:A23403G, S:A23756G, N:G28881A, N:G28882A, N:G28883C,	6	N:R203K, N:G204R, ORF1b:P314L, ORF1b:A1525S, S:D614G, S:T732A,	

hCoV-19/Mexico/CMX-InDRE-IBT-115/2020	EPI_ISL_1301583	In process	20A	B.1.609	12	5'UTR:C241T, ORF1ab:C337T, ORF1ab:G1181T, ORF1ab:C3037T, ORF1ab:T4092C, ORF1ab:C4582T, ORF1ab:C11345T, ORF1ab:C14184T, ORF1ab:C14408T, ORF1ab:A20268G, S:G22199T, S:A23403G, N:A29172G,	7	N:H300R, ORF1a:V306F, ORF1a:I1276T, ORF1a:L3694F, ORF1b:P314L, S:V213L, S:D614G,
hCoV-19/Mexico/CMX-InDRE-IBT-118/2020	EPI_ISL_1301580	In process	20B	B.1.1.222	9	5'UTR:C241T, ORF1ab:C3037T, ORF1ab:C14408T, ORF1ab:G18040T, ORF1ab:T19839C, S:A23403G, S:A23756G, N:G28881A, N:G28882A, N:G28883C,	6	N:R203K, N:G204R, ORF1b:P314L, ORF1b:A1525S, S:D614G, S:T732A,
hCoV-19/Mexico/CMX-InDRE-IBT-126/2020	EPI_ISL_1301586	In process	20B	B.1.1.222	14	5'UTR:C241T, ORF1ab:C1568T, ORF1ab:C3037T, ORF1ab:G6443T, ORF1ab:G9753A, ORF1ab:C14120T, ORF1ab:C14408T, ORF1ab:G15708T, ORF1ab:T19839C, S:A23403G, S:A23756G, N:G28881A, N:G28882A, N:G28883C, N:C29077T,	10	N:R203K, N:G204R, ORF1a:H435Y, ORF1a:D2060Y, ORF1a:R3163K, ORF1b:P218L, ORF1b:P314L, ORF1b:M747I, S:D614G, S:T732A,
hCoV-19/Mexico/CMX-InDRE-IBT-127/2020	EPI_ISL_1301587	In process	20B	B.1.1.222	13	5'UTR:C241T, ORF1ab:C3037T, ORF1ab:G13812T, ORF1ab:C14408T, ORF1ab:G15227T, ORF1ab:T19839C, S:A23403G, S:A23756G, S:A24560C, N:G28881A, N:G28882A, N:G28883C, N:C29253T, 3'UTR:G29751T,	8	N:R203K, N:G204R, N:S327L, ORF1b:M115I, ORF1b:P314L, ORF1b:G587V, S:D614G, S:T732A,
hCoV-19/Mexico/CMX-InDRE-IBT-128/2020	EPI_ISL_1301588	In process	20A	B.1	6	5'UTR:C241T, ORF1ab:C3037T, ORF1ab:C10537T, ORF1ab:C14408T, ORF1ab:A17861G, S:A23403G, N:C28854T,	4	N:S194L, ORF1b:P314L, ORF1b:D1465G, S:D614G,
hCoV-19/Mexico/CMX-InDRE-IBT-38/2020	EPI_ISL_1301497	In process	20C	B.1	8	5'UTR:C241T, ORF1ab:A929G, ORF1ab:C1059T, ORF1ab:C3037T, ORF1ab:A5570G, ORF1ab:C14408T, S:A23403G, ORF3a:G25494A, ORF3a:G25563T,	6	ORF1a:I222V, ORF1a:T265I, ORF1a:M1769V, ORF1b:P314L, ORF3a:Q57H, S:D614G,
hCoV-19/Mexico/CMX-InDRE-IBT-39/2020	EPI_ISL_1301477	In process	20A	B.1.243	10	5'UTR:C241T, ORF1ab:G806A, ORF1ab:C3037T, ORF1ab:G6266A, ORF1ab:C14408T, S:A23403G, S:T24076C, S:T24703C, ORF3a:C25513T, N:C28854T, N:G29543T,	6	N:S194L, ORF1a:A181T, ORF1a:A2001T, ORF1b:P314L, ORF3a:L41F, S:D614G,
hCoV-19/Mexico/CMX-InDRE-IBT-97/2020	EPI_ISL_1301501	In process	20B	B.1.1	9	5'UTR:C241T, ORF1ab:C3037T, ORF1ab:C14408T, ORF1ab:T19839C, S:A23403G, S:A23923G, ORF3a:G26209A, N:G28881A, N:G28882A, N:G28883C,	5	N:R203K, N:G204R, ORF1b:P314L, ORF3a:V273M, S:D614G,
hCoV-19/Mexico/CMX-INER-IBT-1/2020	EPI_ISL_1301717	In process	20A	B.1	5	5'UTR:C241T, ORF1ab:C3037T, ORF1ab:C4582T, ORF1ab:C14408T, S:A23403G, 3'UTR:C29750T,	2	ORF1b:P314L, S:D614G,
hCoV-19/Mexico/CMX-INER-IBT-10/2020	EPI_ISL_1301600	In process	20A	B.1	4	5'UTR:C241T, ORF1ab:C3037T, ORF1ab:C4582T, ORF1ab:C14408T, S:A23403G,	2	ORF1b:P314L, S:D614G,
hCoV-19/Mexico/CMX-INER-IBT-100/2020	EPI_ISL_1302391	In process	20B	B.1.1.519	21	5'UTR:C203T, 5'UTR:C222T, 5'UTR:C241T, ORF1ab:C3037T, ORF1ab:C3140T, ORF1ab:C10029T, ORF1ab:C10954T, ORF1ab:A11117G, ORF1ab:C12789T, ORF1ab:C14408T, ORF1ab:T19839C, ORF1ab:C21306T, S:C22995A, S:A23403G, S:C23604A, S:A23756G, M:G26526A, N:G28881A, N:G28882A, N:G28883C, N:C29197T, N:G29527T,	13	M:A2T, N:R203K, N:G204R, N:Q418H, ORF1a:P959S, ORF1a:T3255I, ORF1a:I3618V, ORF1a:T4175I, ORF1b:P314L, S:T478K, S:D614G, S:P681H, S:T732A,
hCoV-19/Mexico/CMX-INER-IBT-101/2020	EPI_ISL_1302364	In process	20B	B.1.1.222	16	5'UTR:C241T, ORF1ab:C3037T, ORF1ab:C5822T, ORF1ab:G8056A, ORF1ab:A10323G, ORF1ab:G11146T, ORF1ab:C14408T, ORF1ab:C17285T, ORF1ab:T19839C, S:G22567A, S:A23403G, S:A23756G, S:T24601C, ORF3a:T25679C, N:G28881A, N:G28882A, N:G28883C,	10	N:R203K, N:G204R, ORF1a:L1853F, ORF1a:K3353R, ORF1a:M3627I, ORF1b:P314L, ORF1b:S1273L, ORF3a:L96P, S:D614G, S:T732A,
hCoV-19/Mexico/CMX-INER-IBT-102/2020	EPI_ISL_1302389	In process	20B	B.1.1.519	21	5'UTR:C203T, 5'UTR:C222T, 5'UTR:C241T, ORF1ab:C3037T, ORF1ab:C3140T, ORF1ab:C5812T, ORF1ab:C10029T, ORF1ab:C10954T, ORF1ab:A11117G, ORF1ab:C12789T, ORF1ab:C14408T, ORF1ab:T19839C, ORF1ab:C21306T, S:C22995A, S:A23403G, S:C23604A, S:A23756G, N:G28881A, N:G28882A, N:G28883C, N:C29197T, N:C29555T,	11	N:R203K, N:G204R, ORF1a:P959S, ORF1a:T3255I, ORF1a:I3618V, ORF1a:T4175I, ORF1b:P314L, S:T478K, S:D614G, S:P681H, S:T732A,

hCoV-19/Mexico/CMX-INER-IBT-103/2020	EPI_ISL_1302375	In process	20B	B.1.1.222	20	5'UTR:G210T, 5'UTR:C241T, ORF1ab:C1415T, ORF1ab:C3037T, ORF1ab:C8293T, ORF1ab:G9130T, ORF1ab:A10323G, ORF1ab:C14408T, ORF1ab:G14772T, ORF1ab:T19839C, ORF1ab:T20976C, S:A23403G, S:A23756G, S:G25135T, E:C26299A, M:C26681T, ORF8:C28153T, N:C28292A, N:G28881A, N:G28882A, N:G28883C,	12	E:L19I, N:Q7K, N:R203K, N:G204R, ORF1a:L384F, ORF1a:K3353R, ORF1b:P314L, ORF1b:Q435H, ORF8:T87I, S:D614G, S:T732A, S:K1191N,
hCoV-19/Mexico/CMX-INER-IBT-104/2020	EPI_ISL_1302399	In process	20B	B.1.1.222	21	5'UTR:C241T, ORF1ab:G1999T, ORF1ab:C3037T, ORF1ab:G3242T, ORF1ab:C3510T, ORF1ab:C4980A, ORF1ab:C9344T, ORF1ab:C13297T, ORF1ab:C13517T, ORF1ab:C14408T, ORF1ab:T14472G, ORF1ab:G15906T, ORF1ab:T17065C, ORF1ab:G18186T, ORF1ab:T19839C, S:A23403G, S:A23756G, ORF8:C28253T, N:G28881A, N:G28882A, N:G28883C, 3'UTR:G29747C,	13	N:R203K, N:G204R, ORF1a:G993C, ORF1a:A1082V, ORF1a:T1572K, ORF1a:L3027F, ORF1b:T17I, ORF1b:P314L, ORF1b:Q813H, ORF1b:Y1200H, ORF1b:M1573I, S:D614G, S:T732A,
hCoV-19/Mexico/CMX-INER-IBT-105/2020	EPI_ISL_1302345	In process	20B	B.1.1.222	12	5'UTR:C241T, ORF1ab:C3037T, ORF1ab:C14408T, ORF1ab:G16246T, ORF1ab:T17023C, ORF1ab:T19839C, S:A23403G, S:A23756G, ORF3a:C25904T, N:G28881A, N:G28882A, N:G28883C, N:G29402T,	9	N:R203K, N:G204R, N:D377Y, ORF1b:P314L, ORF1b:A927S, ORF1b:S1186P, ORF3a:S171L, S:D614G, S:T732A,
hCoV-19/Mexico/CMX-INER-IBT-106/2020	EPI_ISL_1302377	In process	20A	B.1.239	13	5'UTR:C241T, 5'UTR:G256A, ORF1ab:C3037T, ORF1ab:C5239T, ORF1ab:C8733T, ORF1ab:G11150A, ORF1ab:G11417T, ORF1ab:C14408T, ORF1ab:C14925T, ORF1ab:G16741A, ORF1ab:T19773A, S:A23403G, N:C28854T, N:G29254T,	7	N:S194L, ORF1a:T2823I, ORF1a:V3629I, ORF1a:V3718F, ORF1b:P314L, ORF1b:V1092I, S:D614G,
hCoV-19/Mexico/CMX-INER-IBT-107/2020	EPI_ISL_1302191	In process	20A	B.1.243	15	5'UTR:C241T, ORF1ab:T1578C, ORF1ab:C3037T, ORF1ab:A3627G, ORF1ab:G6618A, ORF1ab:A7833C, ORF1ab:C10741T, ORF1ab:C14408T, S:G23383T, S:A23403G, S:C23604A, S:T24076C, ORF3a:G25855C, N:C28854T, N:G29266A, 3'UTR:T29710C,	10	N:S194L, ORF1a:V438A, ORF1a:N1121S, ORF1a:G2118D, ORF1a:N2523T, ORF1b:P314L, ORF3a:D155H, S:Q607H, S:D614G, S:P681H,
hCoV-19/Mexico/CMX-INER-IBT-108/2020	EPI_ISL_1302384	In process	20B	B.1.1.222	20	5'UTR:G213T, 5'UTR:C241T, ORF1ab:C3037T, ORF1ab:C4116T, ORF1ab:C4897T, ORF1ab:C10029T, ORF1ab:G13045A, ORF1ab:C14408T, ORF1ab:C19011A, ORF1ab:T19839C, ORF1ab:A21137G, S:A23403G, S:C23415T, S:A23756G, ORF3a:G25912T, ORF3a:A26115C, ORF8:A27921G, ORF8:G28001T, N:G28881A, N:G28882A, N:G28883C,	13	N:R203K, N:G204R, ORF1a:P1284L, ORF1a:T3255I, ORF1b:P314L, ORF1b:D1848E, ORF1b:K2557R, ORF3a:G174C, ORF3a:E241D, ORF8:I10V, S:D614G, S:T618I, S:T732A,
hCoV-19/Mexico/CMX-INER-IBT-109/2020	EPI_ISL_1302179	In process	20B	B.1.1.519	25	5'UTR:C203T, 5'UTR:C222T, 5'UTR:C241T, ORF1ab:C398T, ORF1ab:C3037T, ORF1ab:C3140T, ORF1ab:C5183T, ORF1ab:A5336T, ORF1ab:C10029T, ORF1ab:C10954T, ORF1ab:A11117G, ORF1ab:C11195T, ORF1ab:C12789T, ORF1ab:T12934C, ORF1ab:C14408T, ORF1ab:T17067C, ORF1ab:T19839C, S:C22995A, S:A23403G, S:C23604A, S:A23756G, ORF8:T27904C, N:G28881A, N:G28882A, N:G28883C, N:C29197T,	16	N:R203K, N:G204R, ORF1a:H45Y, ORF1a:P959S, ORF1a:P1640S, ORF1a:N1691Y, ORF1a:T3255I, ORF1a:I3618V, ORF1a:L3644F, ORF1a:T4175I, ORF1b:P314L, ORF8:L4P, S:T478K, S:D614G, S:P681H, S:T732A,
hCoV-19/Mexico/CMX-INER-IBT-11/2020	EPI_ISL_1301490	In process	20A	B.1	4	5'UTR:C241T, ORF1ab:C3037T, ORF1ab:C4582T, ORF1ab:C14408T, S:A23403G,	2	ORF1b:P314L, S:D614G,
hCoV-19/Mexico/CMX-INER-IBT-110/2020	EPI_ISL_1302334	In process	20B	B.1.1.519	21	5'UTR:C203T, 5'UTR:C222T, 5'UTR:C241T, ORF1ab:C1263T, ORF1ab:C3037T, ORF1ab:C3140T, ORF1ab:C10029T, ORF1ab:C10954T, ORF1ab:A11117G, ORF1ab:G11365T, ORF1ab:C12789T, ORF1ab:C14408T, ORF1ab:T19839C, S:C22995A, S:A23403G, S:C23604A, S:A23756G, N:G28881A, N:G28882A, N:G28883C, N:C29197T, N:G29527T,	13	N:R203K, N:G204R, N:Q418H, ORF1a:T333M, ORF1a:P959S, ORF1a:T3255I, ORF1a:I3618V, ORF1a:T4175I, ORF1b:P314L, S:T478K, S:D614G, S:P681H, S:T732A,

hCoV-19/Mexico/CMX-INER-IBT-111/2020	EPI_ISL_1302163	In process	20B	B.1.1.222	18	5'UTR:C241T, ORF1ab:G1805A, ORF1ab:C3037T, ORF1ab:G3563A, ORF1ab:T7157C, ORF1ab:C9223T, ORF1ab:C12924T, ORF1ab:C14408T, ORF1ab:C18747T, ORF1ab:T19839C, S:A23403G, S:A23756G, ORF3a:C25452T, N:G28881A, N:G28882A, N:G28883C, N:G29465T, N:G29468C, 3'UTR:C29708T,	10	N:R203K, N:G204R, N:A398S, N:D399H, ORF1a:G514R, ORF1a:G1100S, ORF1a:P4220L, ORF1b:P314L, S:D614G, S:T732A,
hCoV-19/Mexico/CMX-INER-IBT-112/2020	EPI_ISL_1302373	In process	20B	B.1.1.222	17	5'UTR:C241T, ORF1ab:C3037T, ORF1ab:C3093T, ORF1ab:C5826T, ORF1ab:G11596A, ORF1ab:C14408T, ORF1ab:T19839C, S:A23403G, S:A23756G, S:G24193T, ORF3a:T25518C, M:A26612T, N:C28292A, N:C28453T, N:G28881A, N:G28882A, N:G28883C, N:A29403G,	10	N:Q7K, N:R203K, N:G204R, N:D377G, ORF1a:P943L, ORF1a:T1854I, ORF1b:P314L, ORF9b:A57V, S:D614G, S:T732A,
hCoV-19/Mexico/CMX-INER-IBT-113/2020	EPI_ISL_1302266	In process	20C	B.1.499	28	5'UTR:C241T, ORF1ab:C1059T, ORF1ab:C3037T, ORF1ab:G4399T, ORF1ab:C5239T, ORF1ab:G8102A, ORF1ab:A8667G, ORF1ab:A9984C, ORF1ab:A10323G, ORF1ab:C11224T, ORF1ab:C11916T, ORF1ab:A12381G, ORF1ab:A12390G, ORF1ab:C13499T, ORF1ab:C14408T, ORF1ab:G15906T, ORF1ab:T16911C, ORF1ab:G18898T, ORF1ab:C18998T, ORF1ab:A19020G, ORF1ab:C19263T, ORF1ab:C19884T, S:A23403G, S:C23604G, S:C24319T, ORF3a:G25563T, ORF7a:T27460C, N:G28895T, N:G29540A,	19	N:A208S, ORF1a:T265I, ORF1a:M1378I, ORF1a:V2613I, ORF1a:D2801G, ORF1a:D3240A, ORF1a:K3353R, ORF1a:S3884L, ORF1a:K4039R, ORF1a:N4042S, ORF1b:T11I, ORF1b:P314L, ORF1b:Q813H, ORF1b:V1811F, ORF1b:A1844V, ORF3a:Q57H, ORF7a:C23R, S:D614G, S:P681R,
hCoV-19/Mexico/CMX-INER-IBT-114/2020	EPI_ISL_1302400	In process	20B	B.1.1.519	23	5'UTR:C203T, 5'UTR:C222T, 5'UTR:C241T, ORF1ab:C3037T, ORF1ab:C3140T, ORF1ab:C4084T, ORF1ab:C10029T, ORF1ab:C10954T, ORF1ab:A11117G, ORF1ab:T11362C, ORF1ab:C12789T, ORF1ab:C14408T, ORF1ab:T19839C, ORF1ab:C21306T, S:C22995A, S:A23403G, S:T23509C, S:C23604A, S:A23756G, ORF3a:G25726T, N:G28881A, N:G28882A, N:G28883C, N:C29197T,	12	N:R203K, N:G204R, ORF1a:P959S, ORF1a:T3255I, ORF1a:I3618V, ORF1a:T4175I, ORF1b:P314L, ORF3a:V112F, S:T478K, S:D614G, S:P681H, S:T732A,
hCoV-19/Mexico/CMX-INER-IBT-115/2020	EPI_ISL_1302356	In process	20B	B.1.1.519	23	5'UTR:C203T, 5'UTR:C222T, 5'UTR:C241T, ORF1ab:C3037T, ORF1ab:C3140T, ORF1ab:G3692T, ORF1ab:G3871T, ORF1ab:C10029T, ORF1ab:C10755T, ORF1ab:C10954T, ORF1ab:A11117G, ORF1ab:C11824A, ORF1ab:C12789T, ORF1ab:C14408T, ORF1ab:T19839C, S:C22995A, S:A23403G, S:C23604A, S:A23756G, ORF8:T27904C, N:G28881A, N:G28882A, N:G28883C, N:C29197T,	15	N:R203K, N:G204R, ORF1a:P959S, ORF1a:V1143F, ORF1a:K1202N, ORF1a:T3255I, ORF1a:A3497V, ORF1a:I3618V, ORF1a:T4175I, ORF1b:P314L, ORF8:L4P, S:T478K, S:D614G, S:P681H, S:T732A,
hCoV-19/Mexico/CMX-INER-IBT-116/2021	EPI_ISL_1302227	In process	20B	B.1.1.519	24	5'UTR:C203T, 5'UTR:C222T, 5'UTR:C241T, ORF1ab:C1076T, ORF1ab:C3037T, ORF1ab:C3140T, ORF1ab:T9456G, ORF1ab:C10029T, ORF1ab:C10954T, ORF1ab:A11117G, ORF1ab:C12789T, ORF1ab:C14408T, ORF1ab:T14429C, ORF1ab:T19839C, S:C22995A, S:A23403G, S:C23604A, S:A23756G, S:T23925C, S:C24919T, ORF3a:T25496C, N:G28881A, N:G28882A, N:G28883C, N:C29197T,	16	N:R203K, N:G204R, ORF1a:P271S, ORF1a:P959S, ORF1a:F3064C, ORF1a:T3255I, ORF1a:I3618V, ORF1a:T4175I, ORF1b:P314L, ORF1b:V321A, ORF3a:I35T, S:T478K, S:D614G, S:P681H, S:T732A, S:I788T,

hCoV-19/Mexico/CMX-INER-IBT-117/2021	EPI_ISL_1302327	In process	20B	B.1.1.519	26	5'UTR:T201C, 5'UTR:C203T, 5'UTR:C222T, 5'UTR:C241T, ORF1ab:G1738T, ORF1ab:C3037T, ORF1ab:C3140T, ORF1ab:G3753A, ORF1ab:A9280G, ORF1ab:C10029T, ORF1ab:C10954T, ORF1ab:A11117G, ORF1ab:C12789T, ORF1ab:C14408T, ORF1ab:A16026G, ORF1ab:G18020A, ORF1ab:T19839C, ORF1ab:A19974G, S:C22995A, S:A23403G, S:C23604A, S:A23756G, ORF3a:C25566A, N:G28881A, N:G28882A, N:G28883C, N:C29197T,	14	N:R203K, N:G204R, ORF1a:P959S, ORF1a:R1163K, ORF1a:T3255I, ORF1a:I3618V, ORF1a:T4175I, ORF1b:P314L, ORF1b:R1518K, ORF3a:S58R, S:T478K, S:D614G, S:P681H, S:T732A,
hCoV-19/Mexico/CMX-INER-IBT-118/2021	EPI_ISL_1302192	In process	20B	B.1.1.519	23	5'UTR:C203T, 5'UTR:G204T, 5'UTR:C222T, 5'UTR:C241T, ORF1ab:C3037T, ORF1ab:C3140T, ORF1ab:C10029T, ORF1ab:C10954T, ORF1ab:A11117G, ORF1ab:G11365T, ORF1ab:C12789T, ORF1ab:A12790G, ORF1ab:C14408T, ORF1ab:T19839C, S:C22995A, S:A23403G, S:C23604A, S:A23756G, ORF3a:G25650T, N:G28881A, N:G28882A, N:G28883C, N:C29197T, N:G29527T,	13	N:R203K, N:G204R, N:Q418H, ORF1a:P959S, ORF1a:T3255I, ORF1a:I3618V, ORF1a:T4175M, ORF1b:P314L, ORF3a:L86F, S:T478K, S:D614G, S:P681H, S:T732A,
hCoV-19/Mexico/CMX-INER-IBT-119/2021	EPI_ISL_1302316	In process	20B	B.1.1.519	23	5'UTR:T201C, 5'UTR:C203T, 5'UTR:C222T, 5'UTR:C241T, ORF1ab:G1738T, ORF1ab:C3037T, ORF1ab:C3140T, ORF1ab:C10029T, ORF1ab:C10954T, ORF1ab:A11117G, ORF1ab:C12789T, ORF1ab:C14408T, ORF1ab:T19839C, ORF1ab:A19974G, S:C22995A, S:A23403G, S:C23604A, S:A23756G, N:C28333T, N:G28881A, N:G28882A, N:G28883C, N:C29197T, ORF10:G29587T,	12	N:R203K, N:G204R, ORF1a:P959S, ORF1a:T3255I, ORF1a:I3618V, ORF1a:T4175I, ORF1b:P314L, ORF9b:P17L, S:T478K, S:D614G, S:P681H, S:T732A,
hCoV-19/Mexico/CMX-INER-IBT-12/2020	EPI_ISL_1301605	In process	20B	B.1.1	10	5'UTR:C241T, ORF1ab:C3037T, ORF1ab:C8655T, ORF1ab:C14408T, ORF1ab:C17676T, ORF1ab:T19839C, ORF1ab:A21137G, S:A23403G, N:G28881A, N:G28882A, N:G28883C,	6	N:R203K, N:G204R, ORF1a:S2797F, ORF1b:P314L, ORF1b:K2557R, S:D614G,
hCoV-19/Mexico/CMX-INER-IBT-120/2021	EPI_ISL_1302228	In process	20B	B.1.1.222	18	5'UTR:C241T, ORF1ab:C3037T, ORF1ab:C3093T, ORF1ab:C5826T, ORF1ab:G11596A, ORF1ab:C14408T, ORF1ab:T19839C, S:A23403G, S:A23756G, S:G24193T, ORF3a:T25518C, M:A26612T, ORF7a:C27630T, N:C28292A, N:C28453T, N:G28881A, N:G28882A, N:G28883C, N:A29403G,	10	N:Q7K, N:R203K, N:G204R, N:D377G, ORF1a:P943L, ORF1a:T1854I, ORF1b:P314L, ORF9b:A57V, S:D614G, S:T732A,
hCoV-19/Mexico/CMX-INER-IBT-121/2021	EPI_ISL_1302360	In process	20B	B.1.1.519	22	5'UTR:C203T, 5'UTR:C222T, 5'UTR:C241T, ORF1ab:C307T, ORF1ab:C3037T, ORF1ab:C3140T, ORF1ab:C10029T, ORF1ab:C10954T, ORF1ab:A11117G, ORF1ab:C12789T, ORF1ab:C14408T, ORF1ab:T19839C, S:A22798G, S:C22995A, S:A23403G, S:C23604A, S:A23756G, M:G26849T, ORF7b:C27849T, N:G28881A, N:G28882A, N:G28883C, N:C29197T,	13	M:M109I, N:R203K, N:G204R, ORF1a:P959S, ORF1a:T3255I, ORF1a:I3618V, ORF1a:T4175I, ORF1b:P314L, ORF7b:L32F, S:T478K, S:D614G, S:P681H, S:T732A,
hCoV-19/Mexico/CMX-INER-IBT-122/2021	EPI_ISL_1302278	In process	20B	B.1.1.519	26	5'UTR:T201C, 5'UTR:C203T, 5'UTR:C222T, 5'UTR:C241T, ORF1ab:G1738T, ORF1ab:C3037T, ORF1ab:C3140T, ORF1ab:G3753A, ORF1ab:A9280G, ORF1ab:C10029T, ORF1ab:C10954T, ORF1ab:A11117G, ORF1ab:C12789T, ORF1ab:C14408T, ORF1ab:A16026G, ORF1ab:G18020A, ORF1ab:T19839C, ORF1ab:A19974G, S:C22995A, S:A23403G, S:C23604A, S:A23756G, ORF3a:C25566A, N:G28881A, N:G28882A, N:G28883C, N:C29197T,	14	N:R203K, N:G204R, ORF1a:P959S, ORF1a:R1163K, ORF1a:T3255I, ORF1a:I3618V, ORF1a:T4175I, ORF1b:P314L, ORF1b:R1518K, ORF3a:S58R, S:T478K, S:D614G, S:P681H, S:T732A,

hCoV-19/Mexico/CMX-INER-IBT-123/2021	EPI_ISL_1302387	In process	20B	B.1.1.519	24	5'UTR:T201C, 5'UTR:C203T, 5'UTR:C222T, 5'UTR:C241T, ORF1ab:T1443C, ORF1ab:G1738T, ORF1ab:C3037T, ORF1ab:C3140T, ORF1ab:C10029T, ORF1ab:C10954T, ORF1ab:A11117G, ORF1ab:C12789T, ORF1ab:C14408T, ORF1ab:T19839C, ORF1ab:A19974G, ORF1ab:C21306T, S:C22995A, S:A23403G, S:C23604A, S:A23756G, ORF7a:C27600T, N:G28881A, N:G28882A, N:G28883C, N:C29197T,	12	N:R203K, N:G204R, ORF1a:L393S, ORF1a:P959S, ORF1a:T3255I, ORF1a:I3618V, ORF1a:T4175I, ORF1b:P314L, S:T478K, S:D614G, S:P681H, S:T732A,	
hCoV-19/Mexico/CMX-INER-IBT-124/2021	EPI_ISL_1302273	In process	20B	B.1.1.519	23	5'UTR:T201C, 5'UTR:C203T, 5'UTR:C222T, 5'UTR:C241T, ORF1ab:C936T, ORF1ab:G1738T, ORF1ab:C3037T, ORF1ab:C3140T, ORF1ab:C10029T, ORF1ab:C10954T, ORF1ab:A11117G, ORF1ab:C12789T, ORF1ab:C14408T, ORF1ab:T19839C, ORF1ab:A19974G, S:C22995A, S:A23403G, S:C23604A, S:A23756G, N:G28881A, N:G28882A, N:G28883C, N:C29197T, N:G29227T,	12	N:R203K, N:G204R, ORF1a:T224I, ORF1a:P959S, ORF1a:T3255I, ORF1a:I3618V, ORF1a:T4175I, ORF1b:P314L, S:T478K, S:D614G, S:P681H, S:T732A,	
hCoV-19/Mexico/CMX-INER-IBT-125/2021	EPI_ISL_1302267	In process	20B	B.1.1.222	18	5'UTR:C241T, ORF1ab:C3037T, ORF1ab:C3093T, ORF1ab:C5826T, ORF1ab:G11596A, ORF1ab:C14408T, ORF1ab:T19839C, S:A23403G, S:A23756G, S:G24193T, ORF3a:T25518C, M:A26612T, ORF7a:C27630T, N:C28292A, N:C28453T, N:G28881A, N:G28882A, N:G28883C, N:A29403G.	10	N:Q7K, N:R203K, N:G204R, N:D377G, ORF1a:P943L, ORF1a:T1854I, ORF1b:P314L, ORF9b:A57V, S:D614G, S:T732A,	
hCoV-19/Mexico/CMX-INER-IBT-127/2021	EPI_ISL_1302198	In process	20B	B.1.1.519	23	5'UTR:C203T, 5'UTR:C222T, 5'UTR:C241T, ORF1ab:C3037T, ORF1ab:C3140T, ORF1ab:T8104C, ORF1ab:C10029T, ORF1ab:A10829C, ORF1ab:C10954T, ORF1ab:A11117G, ORF1ab:C11152T, ORF1ab:C12789T, ORF1ab:C14408T, ORF1ab:T19839C, S:C22995A, S:A23403G, S:C23604A, S:A23756G, ORF8:T27904C, ORF8:C28087T, N:G28881A, N:G28882A, N:G28883C, N:C29197T,	14	N:R203K, N:G204R, ORF1a:P959S, ORF1a:T3255I, ORF1a:I3522L, ORF1a:I3618V, ORF1a:T4175I, ORF1b:P314L, ORF8:L4P, ORF8:A65V, S:T478K, S:D614G, S:P681H, S:T732A,	3'UTR:29756-29762
hCoV-19/Mexico/CMX-INER-IBT-128/2021	EPI_ISL_1302361	In process	20G	B.1.2	13	5'UTR:C241T, ORF1ab:C1059T, ORF1ab:C3037T, ORF1ab:C4455T, ORF1ab:C10319T, ORF1ab:C14408T, ORF1ab:A18424G, ORF1ab:C19881T, S:A23403G, ORF3a:G25563T, ORF3a:G25907T, ORF8:C27964T, N:C28472T, N:C28869T,	11	N:P67S, N:P199L, ORF1a:T265I, ORF1a:A1397V, ORF1a:L3352F, ORF1b:P314L, ORF1b:N1653D, ORF3a:Q57H, ORF3a:G172V, ORF8:S24L, S:D614G,	
hCoV-19/Mexico/CMX-INER-IBT-129/2021	EPI_ISL_1302376	In process	20B	B.1.1.519	23	5'UTR:C203T, 5'UTR:C222T, 5'UTR:C241T, ORF1ab:C307T, ORF1ab:C3037T, ORF1ab:C3140T, ORF1ab:C10029T, ORF1ab:C10954T, ORF1ab:A11117G, ORF1ab:C12789T, ORF1ab:C14408T, ORF1ab:T19839C, ORF1ab:C21306T, S:A22798G, S:C22995A, S:A23403G, S:C23604A, S:A23756G, M:G26849T, ORF7b:C27849T, N:G28881A, N:G28882A, N:G28883C, N:C29197T,	13	M:M109I, N:R203K, N:G204R, ORF1a:P959S, ORF1a:T3255I, ORF1a:I3618V, ORF1a:T4175I, ORF1b:P314L, ORF7b:L32F, S:T478K, S:D614G, S:P681H, S:T732A,	
hCoV-19/Mexico/CMX-INER-IBT-13/2020	EPI_ISL_1301465	In process	20C	B.1	8	5'UTR:C241T, ORF1ab:C1059T, ORF1ab:C3037T, ORF1ab:C8655T, ORF1ab:C14408T, S:A23403G, ORF3a:G25563T, M:G26959A, ORF8:C27964T,	7	M:R146H, ORF1a:T265I, ORF1a:S2797F, ORF1b:P314L, ORF3a:Q57H, ORF8:S24L, S:D614G,	
hCoV-19/Mexico/CMX-INER-IBT-130/2021	EPI_ISL_1302368	In process	20G	B.1.2	19	5'UTR:C241T, ORF1ab:C1059T, ORF1ab:A1236G, ORF1ab:C3037T, ORF1ab:A9259G, ORF1ab:C10319T, ORF1ab:C14408T, ORF1ab:C14768T, ORF1ab:T15417C, ORF1ab:C18118A, ORF1ab:A18424G, ORF1ab:G18546T, S:A23403G, ORF3a:T25548G, ORF3a:G25563T, ORF3a:G25907T, ORF8:C27923T, ORF8:C27964T, N:C28472T, N:C28869T,	14	N:P67S, N:P199L, ORF1a:T265I, ORF1a:D324G, ORF1a:L3352F, ORF1b:P314L, ORF1b:A434V, ORF1b:L1551I, ORF1b:N1653D, ORF1b:M1693I, ORF3a:Q57H, ORF3a:G172V, ORF8:S24L, S:D614G,	

hCoV-19/Mexico/CMX-INER-IBT-131/2021	EPI_ISL_1302245	In process	20B	B.1.1.519	21	5'UTR:C203T, 5'UTR:C222T, 5'UTR:C241T, ORF1ab:C3037T, ORF1ab:C3140T, ORF1ab:G3728T, ORF1ab:C6027T, ORF1ab:C10029T, ORF1ab:C10954T, ORF1ab:A11117G, ORF1ab:C12789T, ORF1ab:C14408T, ORF1ab:T19839C, S:C22995A, S:A23403G, S:C23604A, S:A23756G, ORF3a:C25528T, N:G28881A, N:G28882A, N:G28883C, N:C29197T,	14	N:R203K, N:G204R, ORF1a:P959S, ORF1a:G1155C, ORF1a:P1921L, ORF1a:T3255I, ORF1a:I3618V, ORF1a:T4175I, ORF1b:P314L, ORF3a:L46F, S:T478K, S:D614G, S:P681H, S:T732A,
hCoV-19/Mexico/CMX-INER-IBT-132/2021	EPI_ISL_1302306	In process	20B	B.1.1.519	24	5'UTR:C203T, 5'UTR:C222T, 5'UTR:C241T, ORF1ab:C1377T, ORF1ab:T1971C, ORF1ab:C3037T, ORF1ab:C3140T, ORF1ab:C10029T, ORF1ab:C10954T, ORF1ab:A11117G, ORF1ab:C12789T, ORF1ab:C14408T, ORF1ab:C17746T, ORF1ab:T19839C, S:C22747T, S:C22995A, S:A23403G, S:C23604A, S:A23756G, N:G28881A, N:G28882A, N:G28883C, N:C29197T, N:G29254T, 3'UTR:G29690T,	14	N:R203K, N:G204R, ORF1a:P371L, ORF1a:I569T, ORF1a:P959S, ORF1a:T3255I, ORF1a:I3618V, ORF1a:T4175I, ORF1b:P314L, ORF1b:P1427S, S:T478K, S:D614G, S:P681H, S:T732A,
hCoV-19/Mexico/CMX-INER-IBT-133/2021	EPI_ISL_1302374	In process	20B	B.1.1.519	23	5'UTR:T201C, 5'UTR:C203T, 5'UTR:C222T, 5'UTR:C241T, ORF1ab:G1738T, ORF1ab:C3037T, ORF1ab:C3140T, ORF1ab:C10029T, ORF1ab:C10954T, ORF1ab:A11117G, ORF1ab:C12789T, ORF1ab:C14408T, ORF1ab:T19839C, ORF1ab:A19974G, ORF1ab:C21306T, S:C22995A, S:A23403G, S:C23604A, S:A23756G, ORF3a:C25553T, N:G28881A, N:G28882A, N:G28883C, N:C29197T,	12	N:R203K, N:G204R, ORF1a:P959S, ORF1a:T3255I, ORF1a:I3618V, ORF1a:T4175I, ORF1b:P314L, ORF3a:A54V, S:T478K, S:D614G, S:P681H, S:T732A,
hCoV-19/Mexico/CMX-INER-IBT-134/2020	EPI_ISL_1302229	In process	20C	B.1.499	15	5'UTR:C241T, ORF1ab:C774T, ORF1ab:C1059T, ORF1ab:C3037T, ORF1ab:A3058G, ORF1ab:C5694T, ORF1ab:C11916T, ORF1ab:C14408T, ORF1ab:C18998T, S:A23403G, ORF3a:C25487T, ORF3a:G25563T, N:C28863T, N:C28948T, N:G29540A, 3'UTR:G29736T,	10	N:S197L, ORF1a:T170I, ORF1a:T265I, ORF1a:P1810L, ORF1a:S3884L, ORF1b:P314L, ORF1b:A1844V, ORF3a:T32I, ORF3a:Q57H, S:D614G,
hCoV-19/Mexico/CMX-INER-IBT-135/2020	EPI_ISL_1302328	In process	20A	B.1	7	5'UTR:C241T, ORF1ab:C3037T, ORF1ab:C11747T, ORF1ab:C14408T, S:A23403G, ORF3a:C25936T, ORF8:G27948C, N:C28854T,	5	N:S194L, ORF1b:P314L, ORF3a:H182Y, ORF8:E19Q, S:D614G,
hCoV-19/Mexico/CMX-INER-IBT-136/2020	EPI_ISL_1302280	In process	20B	B.1.1	13	5'UTR:C241T, ORF1ab:C2334T, ORF1ab:C3037T, ORF1ab:C8099T, ORF1ab:C14408T, ORF1ab:G19096A, ORF1ab:T19839C, S:A23403G, ORF3a:C25844T, ORF3a:G25991T, ORF8:C28253T, N:G28881A, N:G28882A, N:G28883C,	8	N:R203K, N:G204R, ORF1a:A690V, ORF1b:P314L, ORF1b:A1877T, ORF3a:T151I, ORF3a:C200F, S:D614G,
hCoV-19/Mexico/CMX-INER-IBT-137/2020	EPI_ISL_1302291	In process	20A	B.1.609	10	5'UTR:C241T, ORF1ab:C583T, ORF1ab:C3037T, ORF1ab:C4582T, ORF1ab:G11083T, ORF1ab:C13119T, ORF1ab:C14408T, ORF1ab:A18081G, S:C21621T, ORF7b:C27765T, N:G28396T,	6	ORF1a:L3606F, ORF1a:A4285V, ORF1b:P314L, ORF7b:L4F, ORF9b:G38V, S:T20I,
hCoV-19/Mexico/CMX-INER-IBT-138/2020	EPI_ISL_1302155	In process	20A	B.1	12	5'UTR:C241T, ORF1ab:T1237C, ORF1ab:C3037T, ORF1ab:C3768T, ORF1ab:A13614G, ORF1ab:C14408T, ORF1ab:C16329T, ORF1ab:A21137G, S:A23403G, S:G23587T, N:G28655T, N:C28887T, 3'UTR:G29773T,	7	N:D128Y, N:T205I, ORF1a:T1168I, ORF1b:P314L, ORF1b:K2557R, S:D614G, S:Q675H,
hCoV-19/Mexico/CMX-INER-IBT-139/2020	EPI_ISL_1302271	In process	20B	B.1.1.222	15	5'UTR:C241T, ORF1ab:C3037T, ORF1ab:C3736T, ORF1ab:A6716G, ORF1ab:T7835C, ORF1ab:C7926T, ORF1ab:C8626T, ORF1ab:A9713G, ORF1ab:C10279T, ORF1ab:C14408T, ORF1ab:T19839C, S:A23403G, S:A23756G, N:G28881A, N:G28882A, N:G28883C,	8	N:R203K, N:G204R, ORF1a:S2151G, ORF1a:A2554V, ORF1a:T3150A, ORF1b:P314L, S:D614G, S:T732A,
hCoV-19/Mexico/CMX-INER-IBT-14/2020	EPI_ISL_1301606	In process	20A	B.1.609	5	5'UTR:C241T, ORF1ab:C3037T, ORF1ab:C4582T, ORF1ab:C13119T, ORF1ab:C14408T, S:A23403G,	3	ORF1a:A4285V, ORF1b:P314L, S:D614G,
hCoV-19/Mexico/CMX-INER-IBT-140/2020	EPI_ISL_1302338	In process	20A	B.1.609	9	5'UTR:C241T, ORF1ab:C3037T, ORF1ab:C4582T, ORF1ab:C4784T, ORF1ab:C11563T, ORF1ab:C13119T, ORF1ab:C14408T, S:A23403G, M:C27145T, N:T28813G,	6	M:T208I, N:S180R, ORF1a:L1507F, ORF1a:A4285V, ORF1b:P314L, S:D614G,

hCoV-19/Mexico/CMX-INER-IBT-141/2020	EPI_ISL_1302160	In process	20A	B.1.243	11	5'UTR:C241T, ORF1ab:G806A, ORF1ab:C3037T, ORF1ab:C8389T, ORF1ab:C14408T, ORF1ab:G14580A, ORF1ab:C17373T, ORF1ab:C19961T, S:A23403G, S:T24076C, N:C28854T, N:G29543T,	6	N:S194L, ORF1a:A181T, ORF1b:P314L, ORF1b:M371I, ORF1b:T2165M, S:D614G,
hCoV-19/Mexico/CMX-INER-IBT-142/2020	EPI_ISL_1302185	In process	20B	B.1.1.222	12	5'UTR:C241T, ORF1ab:T2533C, ORF1ab:C3037T, ORF1ab:T11428C, ORF1ab:C14408T, ORF1ab:G14653T, ORF1ab:T19839C, S:C21762T, S:A23403G, S:A23756G, N:G28881A, N:G28882A, N:G28883C,	7	N:R203K, N:G204R, ORF1b:P314L, ORF1b:V396F, S:A67V, S:D614G, S:T732A,
hCoV-19/Mexico/CMX-INER-IBT-143/2020	EPI_ISL_1302312	In process	20A	B.1	12	5'UTR:C241T, ORF1ab:C3037T, ORF1ab:G3871T, ORF1ab:C14408T, ORF1ab:A16534G, ORF1ab:G17278T, ORF1ab:C18877T, S:A23403G, S:A24610G, ORF3a:G25563T, ORF3a:T26066C, ORF8:C28045T, ORF8:T28157C,	8	ORF1a:K1202N, ORF1b:P314L, ORF1b:S1023G, ORF1b:V1271L, ORF3a:Q57H, ORF3a:V225A, ORF8:A51V, S:D614G,
hCoV-19/Mexico/CMX-INER-IBT-144/2020	EPI_ISL_1302303	In process	20C	B.1	17	5'UTR:C241T, ORF1ab:C355T, ORF1ab:C1059T, ORF1ab:C3037T, ORF1ab:G4802A, ORF1ab:C11916T, ORF1ab:C14408T, ORF1ab:C18998T, S:G22708T, S:A23403G, S:C24157T, S:G24794T, ORF3a:A25531G, ORF3a:G25563T, N:C28819T, N:G28851T, N:A29439G, N:G29540A,	11	N:S193I, N:Q389R, ORF1a:T265I, ORF1a:D1513N, ORF1a:S3884L, ORF1b:P314L, ORF1b:A1844V, ORF3a:I47V, ORF3a:Q57H, S:D614G, S:A1078S,
hCoV-19/Mexico/CMX-INER-IBT-145/2020	EPI_ISL_1302277	In process	20B	B.1.1.222	17	5'UTR:C241T, ORF1ab:C3037T, ORF1ab:C3510T, ORF1ab:C4980A, ORF1ab:C9344T, ORF1ab:C13297T, ORF1ab:C13517T, ORF1ab:C14408T, ORF1ab:G15906T, ORF1ab:G18186T, ORF1ab:T19839C, S:A23403G, S:A23756G, ORF8:C28253T, N:G28881A, N:G28882A, N:G28883C, 3'UTR:G29747C,	11	N:R203K, N:G204R, ORF1a:A1082V, ORF1a:T1572K, ORF1a:L3027F, ORF1b:T17I, ORF1b:P314L, ORF1b:Q813H, ORF1b:M1573I, S:D614G, S:T732A,
hCoV-19/Mexico/CMX-INER-IBT-146/2020	EPI_ISL_1302381	In process	20B	B.1.1.222	14	5'UTR:C241T, ORF1ab:C1912T, ORF1ab:C3037T, ORF1ab:C3884T, ORF1ab:C14408T, ORF1ab:T19839C, ORF1ab:C19983T, S:A23403G, S:C23664T, S:A23756G, ORF8:T28199C, N:G28881A, N:G28882A, N:G28883C, N:A29533G,	7	N:R203K, N:G204R, ORF1a:P1207S, ORF1b:P314L, S:D614G, S:A701V, S:T732A,
hCoV-19/Mexico/CMX-INER-IBT-147/2020	EPI_ISL_1302257	In process	20A	B.1.396	13	5'UTR:C241T, ORF1ab:C1191T, ORF1ab:C3037T, ORF1ab:C9521A, ORF1ab:C14408T, ORF1ab:C15848T, ORF1ab:C18705T, S:A23403G, S:C24904T, ORF7a:C27654T, ORF7b:C27893A, N:C28854T, N:G29422T, ORF10:A29567G,	6	N:S194L, ORF1a:P309L, ORF1a:L3086I, ORF1b:P314L, ORF1b:T794I, S:D614G,
hCoV-19/Mexico/CMX-INER-IBT-148/2020	EPI_ISL_1302205	In process	20B	B.1.1.512	15	5'UTR:C241T, ORF1ab:C3037T, ORF1ab:G10265A, ORF1ab:C11235T, ORF1ab:G11417T, ORF1ab:C14408T, ORF1ab:G16014A, ORF1ab:A16474G, S:C21614T, S:A23403G, S:G24794T, S:A25256T, N:G28881A, N:G28882A, N:G28883C, N:G29449T,	11	N:R203K, N:G204R, ORF1a:G3334S, ORF1a:A3657V, ORF1a:V3718F, ORF1b:P314L, ORF1b:S1003G, S:L18F, S:D614G, S:A1078S, S:I1232F,
hCoV-19/Mexico/CMX-INER-IBT-149/2020	EPI_ISL_1302206	In process	20B	B.1.1	18	5'UTR:C241T, ORF1ab:C3037T, ORF1ab:G4399T, ORF1ab:C7165T, ORF1ab:G8137T, ORF1ab:T11137A, ORF1ab:A11585G, ORF1ab:C13792T, ORF1ab:C14408T, ORF1ab:C15738T, ORF1ab:C19547T, S:C22281T, S:A23403G, ORF7b:G27890T, ORF8:C28087T, N:G28881A, N:G28882A, N:G28883C, 3'UTR:G29757T,	11	N:R203K, N:G204R, ORF1a:M1378I, ORF1a:F3624L, ORF1a:N3774D, ORF1b:R109C, ORF1b:P314L, ORF1b:S2027L, ORF8:A65V, S:T240I, S:D614G,
hCoV-19/Mexico/CMX-INER-IBT-15/2020	EPI_ISL_1301607	In process	20A	B.1.609	6	5'UTR:C241T, ORF1ab:G1466A, ORF1ab:C3037T, ORF1ab:C4582T, ORF1ab:C14408T, ORF1ab:A20268G, S:A23403G,	3	ORF1a:G401S, ORF1b:P314L, S:D614G,
hCoV-19/Mexico/CMX-INER-IBT-150/2020	EPI_ISL_1302207	In process	20B	B.1.1	18	5'UTR:C241T, ORF1ab:C3037T, ORF1ab:G4399T, ORF1ab:C7165T, ORF1ab:G8137T, ORF1ab:T11137A, ORF1ab:A11585G, ORF1ab:C13792T, ORF1ab:C14408T, ORF1ab:C15738T, ORF1ab:C19547T, S:C22281T, S:A23403G, ORF7b:G27890T, ORF8:C28087T, N:G28881A, N:G28882A, N:G28883C, 3'UTR:G29757T,	11	N:R203K, N:G204R, ORF1a:M1378I, ORF1a:F3624L, ORF1a:N3774D, ORF1b:R109C, ORF1b:P314L, ORF1b:S2027L, ORF8:A65V, S:T240I, S:D614G,

hCoV-19/Mexico/CMX-INER-IBT-151/2020	EPI_ISL_1302255	In process	20A	B.1	15	5'UTR:C241T, ORF1ab:C3037T, ORF1ab:G10255A, ORF1ab:C11095A, ORF1ab:C14408T, ORF1ab:A16534G, ORF1ab:G16975T, ORF1ab:C18877T, S:A23403G, S:G25218T, ORF3a:G25563T, ORF3a:A25574G, ORF3a:T26066C, ORF8:T28157C, N:G28457A, N:A28959G,	10	N:E62K, N:Q229R, ORF1b:P314L, ORF1b:S1023G, ORF1b:V1170F, ORF3a:Q57H, ORF3a:K61R, ORF3a:V225A, S:D614G, S:G1219V,
hCoV-19/Mexico/CMX-INER-IBT-152/2020	EPI_ISL_1302249	In process	20B	B.1.1.432	17	5'UTR:C241T, ORF1ab:C3037T, ORF1ab:C4540T, ORF1ab:G5461T, ORF1ab:A6985T, ORF1ab:C9319T, ORF1ab:C11339T, ORF1ab:C12412T, ORF1ab:T14313C, ORF1ab:C14408T, S:A23403G, ORF3a:C25613T, ORF3a:G25912T, N:G28881A, N:G28882A, N:G28883C, N:T29317C, ORF10:G29645A,	7	N:R203K, N:G204R, ORF1a:M1732I, ORF1b:P314L, ORF3a:S74F, ORF3a:G174C, S:D614G,
hCoV-19/Mexico/CMX-INER-IBT-153/2020	EPI_ISL_1302319	In process	20A	B.1	15	5'UTR:C241T, ORF1ab:G1085T, ORF1ab:C3037T, ORF1ab:G4201T, ORF1ab:C14408T, ORF1ab:G15202T, ORF1ab:C15540T, ORF1ab:G16741A, ORF1ab:C17474T, ORF1ab:G19670A, ORF1ab:T21042G, ORF1ab:A21043T, ORF1ab:G21044C, S:A23403G, ORF3a:C25613T, N:C28854T,	11	N:S194L, ORF1a:V274L, ORF1a:M1312I, ORF1b:P314L, ORF1b:V579L, ORF1b:V1092I, ORF1b:T1336I, ORF1b:G2068E, ORF1b:I2525M, ORF3a:S74F, S:D614G,
hCoV-19/Mexico/CMX-INER-IBT-154/2020	EPI_ISL_1302247	In process	20A	B.1.243	11	5'UTR:C241T, ORF1ab:G806A, ORF1ab:C3037T, ORF1ab:C9857T, ORF1ab:C14408T, ORF1ab:T16074C, ORF1ab:T20850C, S:A23403G, S:T24076C, ORF3a:A25910G, N:C28854T, N:G29543T,	5	N:S194L, ORF1a:A181T, ORF1b:P314L, ORF3a:D173G, S:D614G,
hCoV-19/Mexico/CMX-INER-IBT-155/2020	EPI_ISL_1302180	In process	20B	B.1.1	12	5'UTR:C241T, ORF1ab:C3037T, ORF1ab:C8140T, ORF1ab:G10360A, ORF1ab:G11222T, ORF1ab:C14408T, ORF1ab:T18723C, S:C23191T, S:A23403G, ORF7a:G27613T, N:G28881A, N:G28882A, N:G28883C,	6	N:R203K, N:G204R, ORF1a:V3653F, ORF1b:P314L, ORF7a:V74F, S:D614G,
hCoV-19/Mexico/CMX-INER-IBT-156/2020	EPI_ISL_1302230	In process	20A	B.1.36.10	10	5'UTR:T209C, 5'UTR:C241T, ORF1ab:C678T, ORF1ab:C2706T, ORF1ab:C3037T, ORF1ab:C14408T, ORF1ab:G14653T, S:A23403G, S:T24640C, ORF8:C28076T, N:C28854T,	6	N:S194L, ORF1a:A138V, ORF1a:T814I, ORF1b:P314L, ORF1b:V396F, S:D614G,
hCoV-19/Mexico/CMX-INER-IBT-157/2020	EPI_ISL_1302349	In process	20A	B.1.609	12	5'UTR:C241T, ORF1ab:C3037T, ORF1ab:C4582T, ORF1ab:C4784T, ORF1ab:C8895T, ORF1ab:A11085G, ORF1ab:C11563T, ORF1ab:C13119T, ORF1ab:C14408T, ORF1ab:T18741C, ORF1ab:C19170T, S:A23403G, N:T28813G,	7	N:S180R, ORF1a:L1507F, ORF1a:T2877I, ORF1a:Y3607C, ORF1a:A4285V, ORF1b:P314L, S:D614G,
hCoV-19/Mexico/CMX-INER-IBT-158/2020	EPI_ISL_1302152	In process	20B	B.1.1.222	18	5'UTR:C241T, ORF1ab:C2453T, ORF1ab:C3037T, ORF1ab:C3924T, ORF1ab:C12970T, ORF1ab:C14408T, ORF1ab:C17597T, ORF1ab:T19839C, S:C21789T, S:C23520T, S:C23644T, S:A23756G, ORF7a:C27752T, N:C28290A, N:G28375A, N:G28881A, N:G28882A, N:G28883C, 3'UTR:G29751T,	13	N:P6H, N:R203K, N:G204R, ORF1a:L730F, ORF1a:P1220L, ORF1b:P314L, ORF1b:A1377V, ORF7a:T120I, ORF9b:P3T, ORF9b:G31E, S:T76I, S:A653V, S:T732A,
hCoV-19/Mexico/CMX-INER-IBT-159/2020	EPI_ISL_1302193	In process	20B	B.1.1.222	19	5'UTR:C241T, ORF1ab:C2334T, ORF1ab:G2879A, ORF1ab:C3037T, ORF1ab:T5218C, ORF1ab:A10323G, ORF1ab:G10565A, ORF1ab:C14408T, ORF1ab:T19839C, S:C21575T, S:C21904T, S:A23403G, S:A23756G, ORF3a:C26028T, M:C26858T, N:G28881A, N:G28882A, N:G28883C, ORF10:C29642T, 3'UTR:G29688C,	10	N:R203K, N:G204R, ORF1a:A690V, ORF1a:A872T, ORF1a:K3353R, ORF1a:V3434I, ORF1b:P314L, S:L5F, S:D614G, S:T732A,
hCoV-19/Mexico/CMX-INER-IBT-16/2020	EPI_ISL_1301608	In process	20A	B.1.609	6	5'UTR:C241T, ORF1ab:C3037T, ORF1ab:C4582T, ORF1ab:C8655T, ORF1ab:C13119T, ORF1ab:C14408T, S:A23403G,	4	ORF1a:S2797F, ORF1a:A4285V, ORF1b:P314L, S:D614G,
hCoV-19/Mexico/CMX-INER-IBT-160/2020	EPI_ISL_1302297	In process	20B	B.1.1	16	5'UTR:C241T, ORF1ab:C3037T, ORF1ab:C8074T, ORF1ab:A11144C, ORF1ab:C14408T, ORF1ab:C15656T, ORF1ab:C18981T, S:A23403G, ORF3a:G25690T, ORF6:T27384C, ORF8:C28253T, ORF8:A28254C, N:C28315T, N:G28881A, N:G28882A, N:G28883C, 3'UTR:A29700G,	9	N:R203K, N:G204R, ORF1a:M3627L, ORF1b:P314L, ORF1b:T730I, ORF3a:G100C, ORF8:I121L, ORF9b:A11V, S:D614G,

hCoV-19/Mexico/CMX-INER-IBT-161/2020	EPI_ISL_1302231	In process	20B	B.1.1.432	17	5'UTR:C241T, ORF1ab:C3037T, ORF1ab:C4540T, ORF1ab:G5461T, ORF1ab:A6985T, ORF1ab:C9319T, ORF1ab:C11339T, ORF1ab:C12412T, ORF1ab:T14313C, ORF1ab:C14408T, S:A23403G, ORF3a:C25613T, ORF3a:G25912T, N:G28881A, N:G28882A, N:G28883C, N:T29317C, ORF10:G29645A,	7	N:R203K, N:G204R, ORF1a:M1732I, ORF1b:P314L, ORF3a:S74F, ORF3a:G174C, S:D614G,	
hCoV-19/Mexico/CMX-INER-IBT-162/2020	EPI_ISL_1302326	In process	20B	B.1.1.222	14	5'UTR:C241T, ORF1ab:C3037T, ORF1ab:C6031T, ORF1ab:G7393T, ORF1ab:G8548A, ORF1ab:C14408T, ORF1ab:T19839C, ORF1ab:C19955T, S:A23403G, S:A23756G, ORF3a:T25830C, ORF7a:C27559T, N:G28881A, N:G28882A, N:G28883C,	6	N:R203K, N:G204R, ORF1b:P314L, ORF1b:T2163I, S:D614G, S:T732A,	
hCoV-19/Mexico/CMX-INER-IBT-163/2020	EPI_ISL_1302256	In process	20A	B.1	7	5'UTR:C241T, ORF1ab:C3037T, ORF1ab:T4741C, ORF1ab:C14408T, S:A23403G, M:C27005T, ORF8:C28253T, N:C28854T,	3	N:S194L, ORF1b:P314L, S:D614G,	
hCoV-19/Mexico/CMX-INER-IBT-164/2020	EPI_ISL_1302298	In process	20B	B.1.1.222	13	5'UTR:C241T, ORF1ab:C3037T, ORF1ab:C5850T, ORF1ab:C8320T, ORF1ab:C9988T, ORF1ab:C14408T, ORF1ab:T19839C, S:A23403G, S:A23756G, S:G25352T, ORF8:A27997T, N:G28881A, N:G28882A, N:G28883C,	8	N:R203K, N:G204R, ORF1a:P1862L, ORF1b:P314L, ORF8:D35V, S:D614G, S:T732A, S:V1264L,	
hCoV-19/Mexico/CMX-INER-IBT-165/2020	EPI_ISL_1302250	In process	20B	B.1.1.222	12	5'UTR:C241T, ORF1ab:C2268A, ORF1ab:C3037T, ORF1ab:C8092T, ORF1ab:G12070T, ORF1ab:C14408T, ORF1ab:T19839C, S:A23403G, S:A23756G, S:C24374T, N:G28881A, N:G28882A, N:G28883C,	7	N:R203K, N:G204R, ORF1a:A668E, ORF1b:P314L, S:D614G, S:T732A, S:L938F,	
hCoV-19/Mexico/CMX-INER-IBT-166/2020	EPI_ISL_1302170	In process	20B	B.1.1.222	16	5'UTR:C241T, ORF1ab:C1419T, ORF1ab:C3037T, ORF1ab:G9203A, ORF1ab:C11747T, ORF1ab:C14408T, ORF1ab:C15579T, ORF1ab:C16017T, ORF1ab:G19009A, ORF1ab:T19839C, S:A23403G, S:A23756G, ORF6:C27263T, N:G28881A, N:G28882A, N:G28883C, N:A29487G,	10	N:R203K, N:G204R, N:K405R, ORF1a:A385V, ORF1a:D2980N, ORF1b:P314L, ORF1b:D1848N, ORF6:T21I, S:D614G, S:T732A,	
hCoV-19/Mexico/CMX-INER-IBT-167/2020	EPI_ISL_1302194	In process	20B	B.1.1.222	15	5'UTR:C241T, ORF1ab:C3037T, ORF1ab:A6693G, ORF1ab:C9430T, ORF1ab:C10029T, ORF1ab:C14408T, ORF1ab:T19839C, S:A23403G, S:A23756G, ORF3a:G25912T, ORF7b:A27756G, ORF8:A27921G, ORF8:G28001T, N:G28881A, N:G28882A, N:G28883C,	10	N:R203K, N:G204R, ORF1a:K2143R, ORF1a:T3255I, ORF1b:P314L, ORF3a:G174C, ORF7b:M1V, ORF8:I10V, S:D614G, S:T732A,	
hCoV-19/Mexico/CMX-INER-IBT-168/2020	EPI_ISL_1302292	In process	20B	B.1.1.222	14	5'UTR:C241T, ORF1ab:C3037T, ORF1ab:C5850T, ORF1ab:C8320T, ORF1ab:C9988T, ORF1ab:C14408T, ORF1ab:T19839C, S:A23403G, S:A23756G, S:G25352T, ORF8:A27997T, N:G28881A, N:G28882A, N:G28883C, 3'UTR:G29810T,	8	N:R203K, N:G204R, ORF1a:P1862L, ORF1b:P314L, ORF8:D35V, S:D614G, S:T732A, S:V1264L,	
hCoV-19/Mexico/CMX-INER-IBT-169/2020	EPI_ISL_1302290	In process	20A	B.1	11	5'UTR:C241T, ORF1ab:C556T, ORF1ab:C1190T, ORF1ab:C3037T, ORF1ab:G11042T, ORF1ab:C14408T, ORF1ab:G16741A, ORF1ab:C19884T, S:C21952T, S:A23403G, N:C28854T, N:C29409T,	7	N:S194L, N:T379I, ORF1a:P309S, ORF1a:V3593F, ORF1b:P314L, ORF1b:V1092I, S:D614G,	ORF1ab: 9860-9862
hCoV-19/Mexico/CMX-INER-IBT-17/2020	EPI_ISL_1301719	In process	20A	B.1.189	8	5'UTR:C241T, ORF1ab:C788T, ORF1ab:C3037T, ORF1ab:C4582T, ORF1ab:G6404T, ORF1ab:C14408T, S:A23403G, S:T25123C, N:G28378T,	5	ORF1a:R175C, ORF1a:V2047F, ORF1b:P314L, ORF9b:R32L, S:D614G,	
hCoV-19/Mexico/CMX-INER-IBT-170/2020	EPI_ISL_1302246	In process	20A	B.1.36.10	10	5'UTR:C241T, ORF1ab:C3037T, ORF1ab:A10317G, ORF1ab:C12513T, ORF1ab:C14408T, S:C21859T, S:A23403G, ORF3a:G25996T, ORF8:G28193T, N:C28854T, ORF10:C29642T,	6	N:S194L, ORF1a:K3351R, ORF1a:T4083M, ORF1b:P314L, ORF3a:V202L, S:D614G,	
hCoV-19/Mexico/CMX-INER-IBT-171/2020	EPI_ISL_1302208	In process	20B	B.1.1	18	5'UTR:C241T, ORF1ab:C593T, ORF1ab:C3037T, ORF1ab:C6401T, ORF1ab:C6636T, ORF1ab:C7165T, ORF1ab:C9866T, ORF1ab:C10731T, ORF1ab:C14408T, ORF1ab:C14491T, ORF1ab:T16800C, ORF1ab:G16827A, ORF1ab:T19839C, S:A23403G, S:A23756G, ORF7a:C27434T, N:G28881A, N:G28882A, N:G28883C.	11	N:R203K, N:G204R, ORF1a:H110Y, ORF1a:P2046S, ORF1a:T2124I, ORF1a:L3201F, ORF1a:T3489I, ORF1b:P314L, ORF7a:T14I, S:D614G, S:T732A,	

hCoV-19/Mexico/CMX-INER-IBT-172/2020	EPI_ISL_1302329	In process	20B	B.1.1	15	5'UTR:C241T, ORF1ab:G370T, ORF1ab:C3037T, ORF1ab:A3916C, ORF1ab:A8413G, ORF1ab:G12769A, ORF1ab:C13965T, ORF1ab:C14408T, ORF1ab:T19839C, S:G21770T, S:C22624T, S:A23403G, N:C28603T, N:G28881A, N:G28882A, N:G28883C,	6	N:R203K, N:G204R, ORF1a:E1217D, ORF1b:P314L, S:V70F, S:D614G,
hCoV-19/Mexico/CMX-INER-IBT-173/2020	EPI_ISL_1302336	In process	20B	B.1.1.222	20	5'UTR:C241T, ORF1ab:C2334T, ORF1ab:G2879A, ORF1ab:C3037T, ORF1ab:T5218C, ORF1ab:A10323G, ORF1ab:G10565A, ORF1ab:C14408T, ORF1ab:C15738T, ORF1ab:T19839C, S:C21904T, S:A23403G, S:A23756G, ORF3a:C26028T, M:C26858T, N:G28881A, N:G28882A, N:G28883C, N:G28985T, ORF10:C29642T, 3'UTR:G29688C,	10	N:R203K, N:G204R, N:G238C, ORF1a:A690V, ORF1a:A872T, ORF1a:K3353R, ORF1a:V3434I, ORF1b:P314L, S:D614G, S:T732A,
hCoV-19/Mexico/CMX-INER-IBT-174/2020	EPI_ISL_1302186	In process	20A	B.1.1	11	5'UTR:C241T, ORF1ab:C3037T, ORF1ab:G6532T, ORF1ab:G9479T, ORF1ab:G12661A, ORF1ab:C14408T, ORF1ab:C15656T, S:A23403G, ORF8:C27981T, ORF8:G28077T, N:C28854T, ORF10:G29628T,	8	N:S194L, ORF1a:E2089D, ORF1a:G3072C, ORF1b:P314L, ORF1b:T730I, ORF8:P30S, ORF8:V62L, S:D614G,
hCoV-19/Mexico/CMX-INER-IBT-175/2020	EPI_ISL_1302339	In process	20A	B.1.243	14	5'UTR:C241T, ORF1ab:T1506C, ORF1ab:C1869T, ORF1ab:C3037T, ORF1ab:C5192T, ORF1ab:C11962G, ORF1ab:G12067T, ORF1ab:C14408T, S:C21911A, S:A23403G, S:G23628T, S:T24076C, N:C28854T, ORF10:C29642T, 3'UTR:A29700G,	8	N:S194L, ORF1a:V414A, ORF1a:A535V, ORF1a:M3934I, ORF1b:P314L, S:L117I, S:D614G, S:S689I,
hCoV-19/Mexico/CMX-INER-IBT-18/2020	EPI_ISL_1301609	In process	20A	B.1.609	5	5'UTR:C241T, ORF1ab:C3037T, ORF1ab:C4582T, ORF1ab:C13119T, ORF1ab:C14408T, S:A23403G,	3	ORF1a:A4285V, ORF1b:P314L, S:D614G,
hCoV-19/Mexico/CMX-INER-IBT-19/2020	EPI_ISL_1301503	In process	20A	B.1	5	5'UTR:C241T, ORF1ab:C3037T, ORF1ab:C4582T, ORF1ab:C8655T, ORF1ab:C14408T, S:A23403G,	3	ORF1a:S2797F, ORF1b:P314L, S:D614G,
hCoV-19/Mexico/CMX-INER-IBT-2/2020	EPI_ISL_1301718	In process	19B	A.1	4	ORF1ab:C17747T, ORF1ab:A17858G, ORF1ab:C18060T, S:A24694T, ORF8:T28144C,	3	ORF1b:P1427L, ORF1b:Y1464C, ORF8:L84S,
hCoV-19/Mexico/CMX-INER-IBT-20/2020	EPI_ISL_1301610	In process	20A	B.1.189	9	5'UTR:C241T, ORF1ab:C3037T, ORF1ab:C4582T, ORF1ab:G6404T, ORF1ab:G13370A, ORF1ab:C14408T, ORF1ab:A20268G, S:A23403G, S:T25123C, N:G28378T,	5	ORF1a:V2047F, ORF1a:V4369I, ORF1b:P314L, ORF9b:R32L, S:D614G,
hCoV-19/Mexico/CMX-INER-IBT-21/2020	EPI_ISL_1301611	In process	20A	B.1.609	5	5'UTR:C241T, ORF1ab:C3037T, ORF1ab:C4582T, ORF1ab:C13119T, ORF1ab:C14408T, S:A23403G,	3	ORF1a:A4285V, ORF1b:P314L, S:D614G,
hCoV-19/Mexico/CMX-INER-IBT-22/2020	EPI_ISL_1301499	In process	20B	B.1.1	11	5'UTR:C106T, 5'UTR:C241T, ORF1ab:C3037T, ORF1ab:G4859T, ORF1ab:G4904A, ORF1ab:C14408T, ORF1ab:T19839C, S:A23403G, ORF3a:G26167T, N:G28881A, N:G28882A, N:G28883C,	7	N:R203K, N:G204R, ORF1a:D1532Y, ORF1a:D1547N, ORF1b:P314L, ORF3a:V259L, S:D614G,
hCoV-19/Mexico/CMX-INER-IBT-23/2020	EPI_ISL_1301515	In process	20C	B.1	8	5'UTR:C241T, ORF1ab:C1059T, ORF1ab:C3037T, ORF1ab:C11152T, ORF1ab:C14408T, S:A23403G, ORF3a:G25563T, N:C28887T, 3'UTR:C29750T,	5	N:T205I, ORF1a:T265I, ORF1b:P314L, ORF3a:Q57H, S:D614G,
hCoV-19/Mexico/CMX-INER-IBT-24/2020	EPI_ISL_1301612	In process	20A	B.1.1	6	5'UTR:C241T, ORF1ab:C3037T, ORF1ab:C8655T, ORF1ab:C11653T, ORF1ab:C14408T, ORF1ab:T20609C, S:A23403G,	4	ORF1a:S2797F, ORF1b:P314L, ORF1b:M2381T, S:D614G,
hCoV-19/Mexico/CMX-INER-IBT-25/2020	EPI_ISL_1301613	In process	20A	B.1.609	5	5'UTR:C241T, ORF1ab:C3037T, ORF1ab:C4582T, ORF1ab:C13119T, ORF1ab:C14408T, S:A23403G,	3	ORF1a:A4285V, ORF1b:P314L, S:D614G,
hCoV-19/Mexico/CMX-INER-IBT-26/2020	EPI_ISL_1301522	In process	20A	B.1.609	5	5'UTR:C241T, ORF1ab:C3037T, ORF1ab:C4582T, ORF1ab:C13119T, ORF1ab:C14408T, S:A23403G,	3	ORF1a:A4285V, ORF1b:P314L, S:D614G,
hCoV-19/Mexico/CMX-INER-IBT-27/2020	EPI_ISL_1301720	In process	20B	B.1.1	8	5'UTR:C37G, 5'UTR:C241T, ORF1ab:C3037T, ORF1ab:C14408T, ORF1ab:T19839C, S:A23403G, N:G28881A, N:G28882A, N:G28883C,	4	N:R203K, N:G204R, ORF1b:P314L, S:D614G,
hCoV-19/Mexico/CMX-INER-IBT-28/2020	EPI_ISL_1301614	In process	20B	B.1.1	7	5'UTR:C241T, ORF1ab:C3037T, ORF1ab:C14408T, ORF1ab:T19839C, S:A23403G, N:G28881A, N:G28882A, N:G28883C,	4	N:R203K, N:G204R, ORF1b:P314L, S:D614G,
hCoV-19/Mexico/CMX-INER-IBT-29/2020	EPI_ISL_1301524	In process	20B	B.1.1.222	8	5'UTR:C241T, ORF1ab:C3037T, ORF1ab:C14408T, ORF1ab:T19839C, S:A23403G, S:A23756G, N:G28881A, N:G28882A, N:G28883C,	5	N:R203K, N:G204R, ORF1b:P314L, S:D614G, S:T732A,
hCoV-19/Mexico/CMX-INER-IBT-30/2020	EPI_ISL_1301472	In process	20A	B.1.189	11	5'UTR:C241T, ORF1ab:G373A, ORF1ab:C3037T, ORF1ab:C4582T, ORF1ab:G6404T, ORF1ab:C8655T, ORF1ab:C9693T, ORF1ab:C14408T, ORF1ab:A19155G, S:A23403G, S:T25123C, N:G28378T,	6	ORF1a:V2047F, ORF1a:S2797F, ORF1a:A3143V, ORF1b:P314L, ORF9b:R32L, S:D614G,

hCoV-19/Mexico/CMX-INER-IBT-31/2020	EPI_ISL_1301602	In process	20B	B.1.1.222	13	5'UTR:C241T, ORF1ab:C3037T, ORF1ab:C3264T, ORF1ab:G3838T, ORF1ab:T5218C, ORF1ab:C14408T, ORF1ab:C18747T, ORF1ab:T19839C, S:A23403G, S:A23756G, ORF3a:C26028T, N:G28881A, N:G28882A, N:G28883C,	7	N:R203K, N:G204R, ORF1a:T1000I, ORF1a:L1191F, ORF1b:P314L, S:D614G, S:T732A,	
hCoV-19/Mexico/CMX-INER-IBT-32/2020	EPI_ISL_1301603	In process	20B	B.1.1	11	5'UTR:C241T, ORF1ab:C3037T, ORF1ab:C14408T, ORF1ab:C14639T, ORF1ab:C19343T, ORF1ab:T19839C, S:G22028C, S:C22329T, S:A23403G, N:G28881A, N:G28882A, N:G28883C,	8	N:R203K, N:G204R, ORF1b:P314L, ORF1b:A391V, ORF1b:A1959V, S:E156Q, S:S256L, S:D614G,	
hCoV-19/Mexico/CMX-INER-IBT-33/2020	EPI_ISL_1301604	In process	20A	B.1	9	5'UTR:C241T, ORF1ab:C3037T, ORF1ab:C4582T, ORF1ab:T12436C, ORF1ab:C14408T, ORF1ab:C14805T, ORF1ab:G18525T, ORF1ab:G21448A, S:A23403G, ORF3a:G25906T,	4	ORF1b:P314L, ORF1b:E2661K, ORF3a:G172C, S:D614G,	
hCoV-19/Mexico/CMX-INER-IBT-34/2020	EPI_ISL_1301721	In process	20A	B.1.189	8	5'UTR:C241T, ORF1ab:C3037T, ORF1ab:C4582T, ORF1ab:G6404T, ORF1ab:C14408T, ORF1ab:A20268G, S:A23403G, S:T25123C, N:G28378T,	4	ORF1a:V2047F, ORF1b:P314L, ORF9b:R32L, S:D614G,	
hCoV-19/Mexico/CMX-INER-IBT-35/2020	EPI_ISL_1301550	In process	20A	B.1.189	11	5'UTR:C241T, ORF1ab:G2098T, ORF1ab:C3037T, ORF1ab:C4582T, ORF1ab:G6404T, ORF1ab:C14408T, ORF1ab:C17762T, ORF1ab:T19215C, S:A23403G, S:T25123C, ORF7a:G27676T, N:G28378T,	7	ORF1a:Q611H, ORF1a:V2047F, ORF1b:P314L, ORF1b:A1432V, ORF7a:E95*, ORF9b:R32L, S:D614G,	
hCoV-19/Mexico/CMX-INER-IBT-36/2020	EPI_ISL_1301722	In process	20B	B.1.1	10	5'UTR:C241T, ORF1ab:G1135T, ORF1ab:A2024G, ORF1ab:C3037T, ORF1ab:C9166T, ORF1ab:C14408T, ORF1ab:T19839C, S:A23403G, N:G28881A, N:G28882A, N:G28883C,	6	N:R203K, N:G204R, ORF1a:K290N, ORF1a:T587A, ORF1b:P314L, S:D614G,	
hCoV-19/Mexico/CMX-INER-IBT-37/2020	EPI_ISL_1301471	In process	20A	B.1.189	8	5'UTR:C241T, ORF1ab:C3037T, ORF1ab:C4582T, ORF1ab:G6404T, ORF1ab:C8655T, ORF1ab:C14408T, S:A23403G, S:T25123C, N:G28378T,	5	ORF1a:V2047F, ORF1a:S2797F, ORF1b:P314L, ORF9b:R32L, S:D614G,	
hCoV-19/Mexico/CMX-INER-IBT-38/2020	EPI_ISL_1301488	In process	20B	B.1.1.322	30	ORF1ab:T511C, ORF1ab:G515C, ORF1ab:T517C, ORF1ab:A518T, ORF1ab:A532G, ORF1ab:C534T, ORF1ab:C2523T, ORF1ab:C3037T, ORF1ab:A8658G, ORF1ab:C14408T, ORF1ab:T19839C, S:A23403G, S:A23756G, N:A28877T, N:G28878C, N:G28881A, N:G28882A, N:G28883C,ORF1ab:C506A, ORF1ab:A507C, ORF1ab:G509T, ORF1ab:G510T, ORF1ab:C512G, ORF1ab:G524T, ORF1ab:C527G, ORF1ab:G529C, ORF1ab:G530A, ORF1ab:G533T, ORF1ab:G536T, ORF1ab:A537G, ORF1ab:A538G,	13	N:R203K, N:G204R, ORF1a:T753I, ORF1a:K2798R, ORF1b:P314L, S:D614G, S:T732A,ORF1a:P80D, ORF1a:H81L, ORF1a:G82L, ORF1a:H83*, ORF1a:A90L, ORF1a:E91W,	ORF1ab: 521-523,
hCoV-19/Mexico/CMX-INER-IBT-39/2020	EPI_ISL_1301510	In process	20A	B.1.609	6	5'UTR:C241T, ORF1ab:C3037T, ORF1ab:C4582T, ORF1ab:C13119T, ORF1ab:C14408T, S:A23403G, N:C29095T,	3	ORF1a:A4285V, ORF1b:P314L, S:D614G,	
hCoV-19/Mexico/CMX-INER-IBT-4/2020	EPI_ISL_1301520	In process	19B	A.5	2	ORF1ab:C17470T, ORF3a:C26088T, ORF8:T28144C,	1	ORF8:L84S,	
hCoV-19/Mexico/CMX-INER-IBT-40/2020	EPI_ISL_1301445	In process	20A	B.1.189	11	5'UTR:C241T, ORF1ab:C3037T, ORF1ab:C4582T, ORF1ab:G6404T, ORF1ab:C11442T, ORF1ab:C14408T, ORF1ab:A19182G, S:C22855T, S:A23403G, S:T25123C, ORF7a:C27509T, N:G28378T,	6	ORF1a:V2047F, ORF1a:A3726V, ORF1b:P314L, ORF7a:T39I, ORF9b:R32L, S:D614G,	ORF1ab: 686-694,
hCoV-19/Mexico/CMX-INER-IBT-41/2020	EPI_ISL_1301533	In process	20C	B.1.564	7	5'UTR:C241T, ORF1ab:C1059T, ORF1ab:C3037T, ORF1ab:C14408T, ORF1ab:C16260T, S:A23403G, ORF3a:G25563T, N:C28821A,	5	N:S183Y, ORF1a:T265I, ORF1b:P314L, ORF3a:Q57H, S:D614G,	
hCoV-19/Mexico/CMX-INER-IBT-42/2020	EPI_ISL_1301539	In process	20A	B.1.189	8	5'UTR:C241T, ORF1ab:C3037T, ORF1ab:C4582T, ORF1ab:G6352T, ORF1ab:G6404T, ORF1ab:C14408T, S:A23403G, S:T25123C, N:G28378T,	5	ORF1a:K2029N, ORF1a:V2047F, ORF1b:P314L, ORF9b:R32L, S:D614G,	
hCoV-19/Mexico/CMX-INER-IBT-43/2020	EPI_ISL_1301453	In process	20C	B.1	7	5'UTR:C241T, ORF1ab:C1059T, ORF1ab:C3037T, ORF1ab:C14408T, S:A23403G, S:G24368T, ORF3a:G25563T, ORF6:T27384C,	5	ORF1a:T265I, ORF1b:P314L, ORF3a:Q57H, S:D614G, S:D936Y,	
hCoV-19/Mexico/CMX-INER-IBT-44/2020	EPI_ISL_1301615	In process	20A	B.1.609	6	5'UTR:C241T, ORF1ab:C3037T, ORF1ab:C4582T, ORF1ab:C13119T, ORF1ab:C14408T, S:A23403G, N:G28812T,	4	N:S180I, ORF1a:A4285V, ORF1b:P314L, S:D614G,	

hCoV-19/Mexico/CMX-INER-IBT-45/2020	EPI_ISL_1301616	In process	20A	B.1.609	6	5'UTR:C241T, ORF1ab:C3037T, ORF1ab:C4582T, ORF1ab:C8175T, ORF1ab:C8655T, ORF1ab:C14408T, S:A23403G,	4	ORF1a:A2637V, ORF1a:S2797F, ORF1b:P314L, S:D614G,	
hCoV-19/Mexico/CMX-INER-IBT-46/2020	EPI_ISL_1301504	In process	20A	B.1.189	8	5'UTR:C241T, ORF1ab:C3037T, ORF1ab:C4582T, ORF1ab:G6404T, ORF1ab:C14408T, ORF1ab:A20268G, S:A23403G, S:T25123C, N:G28378T,	4	ORF1a:V2047F, ORF1b:P314L, ORF9b:R32L, S:D614G,	
hCoV-19/Mexico/CMX-INER-IBT-47/2020	EPI_ISL_1301723	In process	20A	B.1.609	7	5'UTR:C241T, ORF1ab:C3037T, ORF1ab:C4582T, ORF1ab:C13119T, ORF1ab:C14408T, ORF1ab:A20268G, S:A23403G, N:G28812T,	4	N:S180I, ORF1a:A4285V, ORF1b:P314L, S:D614G,	
hCoV-19/Mexico/CMX-INER-IBT-48/2020	EPI_ISL_1301724	In process	20B	B.1.1.222	10	5'UTR:C241T, ORF1ab:C3037T, ORF1ab:C5826T, ORF1ab:C14408T, ORF1ab:T19839C, S:A23403G, S:A23756G, M:A26612T, N:G28881A, N:G28882A, N:G28883C,	6	N:R203K, N:G204R, ORF1a:T1854I, ORF1b:P314L, S:D614G, S:T732A,	
hCoV-19/Mexico/CMX-INER-IBT-49/2020	EPI_ISL_1301617	In process	20A	B.1	6	5'UTR:C241T, ORF1ab:C3037T, ORF1ab:C14408T, ORF1ab:T17007C, ORF1ab:G19633A, S:G21578A, S:A23403G,	4	ORF1b:P314L, ORF1b:V2056M, S:V6I, S:D614G,	
hCoV-19/Mexico/CMX-INER-IBT-5/2020	EPI_ISL_1301731	In process	20A	B.1	3	5'UTR:C241T, ORF1ab:C3037T, ORF1ab:C14408T, S:A23403G,	2	ORF1b:P314L, S:D614G,	
hCoV-19/Mexico/CMX-INER-IBT-50/2020	EPI_ISL_1301545	In process	20A	B.1.609	9	5'UTR:C241T, ORF1ab:C3037T, ORF1ab:C4582T, ORF1ab:T6023C, ORF1ab:C8655T, ORF1ab:A8658G, ORF1ab:C13119T, ORF1ab:C14408T, ORF1ab:A20268G, S:A23403G,	7	ORF1a:E1024K, ORF1a:Y1920H, ORF1a:S2797F, ORF1a:K2798R, ORF1a:A4285V, ORF1b:P314L, S:D614G,	ORF1ab: 3333-3335,
hCoV-19/Mexico/CMX-INER-IBT-51/2020	EPI_ISL_1301725	In process	20B	B.1.1.222	10	5'UTR:C241T, ORF1ab:C3037T, ORF1ab:C14408T, ORF1ab:T19839C, S:C21846T, S:A23403G, S:A23756G, M:C26895T, N:G28881A, N:G28882A, N:G28883C,	7	M:H125Y, N:R203K, N:G204R, ORF1b:P314L, S:T95I, S:D614G, S:T732A,	
hCoV-19/Mexico/CMX-INER-IBT-52/2020	EPI_ISL_1301618	In process	20A	B.1.243	10	5'UTR:C241T, ORF1ab:C3037T, ORF1ab:G4399T, ORF1ab:C8655T, ORF1ab:C13490T, ORF1ab:C14408T, S:A23403G, S:C24076C, M:C26537T, N:C28854T, 3'UTR:G29810T,	6	N:S194L, ORF1a:M1378I, ORF1a:S2797F, ORF1b:A8V, ORF1b:P314L, S:D614G,	
hCoV-19/Mexico/CMX-INER-IBT-53/2020	EPI_ISL_1301726	In process	20B	B.1.1	9	5'UTR:C241T, ORF1ab:C313T, ORF1ab:C3037T, ORF1ab:A8938T, ORF1ab:C14408T, S:G22599T, S:A23403G, N:G28881A, N:G28882A, N:G28883C,	5	N:R203K, N:G204R, ORF1b:P314L, S:R346I, S:D614G,	
hCoV-19/Mexico/CMX-INER-IBT-54/2020	EPI_ISL_1301507	In process	20B	B.1.1.222	8	5'UTR:C241T, ORF1ab:C3037T, ORF1ab:G16075T, ORF1ab:T19839C, S:A23403G, S:A23756G, N:G28881A, N:G28882A, N:G28883C,	5	N:R203K, N:G204R, ORF1b:D870Y, S:D614G, S:T732A,	
hCoV-19/Mexico/CMX-INER-IBT-55/2020	EPI_ISL_1301551	In process	20A	B.1.189	11	5'UTR:C241T, ORF1ab:C3037T, ORF1ab:C4582T, ORF1ab:G6404T, ORF1ab:C12119T, ORF1ab:C14408T, ORF1ab:G17122T, ORF1ab:A20268G, S:A23403G, S:T25123C, ORF7a:G27676T, N:G28378T,	7	ORF1a:V2047F, ORF1a:P3952S, ORF1b:P314L, ORF1b:A1219S, ORF7a:E95*, ORF9b:R32L, S:D614G,	
hCoV-19/Mexico/CMX-INER-IBT-56/2020	EPI_ISL_1301732	In process	20A	B.1.609	9	5'UTR:C241T, ORF1ab:C3037T, ORF1ab:G3231T, ORF1ab:C4582T, ORF1ab:C13119T, ORF1ab:C14408T, ORF1ab:A20268G, S:T22501C, S:A23403G, M:G26918T,	4	ORF1a:G989V, ORF1a:A4285V, ORF1b:P314L, S:D614G,	
hCoV-19/Mexico/CMX-INER-IBT-57/2020	EPI_ISL_1301733	In process	20A	B.1.189	9	5'UTR:C241T, ORF1ab:C3037T, ORF1ab:C4582T, ORF1ab:G6404T, ORF1ab:C14408T, ORF1ab:A19655G, ORF1ab:A20268G, S:A23403G, S:T25123C, N:G28378T,	5	ORF1a:V2047F, ORF1b:P314L, ORF1b:K2063R, ORF9b:R32L, S:D614G,	
hCoV-19/Mexico/CMX-INER-IBT-58/2020	EPI_ISL_1301727	In process	20A	B.1.609	8	5'UTR:C241T, ORF1ab:C3037T, ORF1ab:C4582T, ORF1ab:A12110G, ORF1ab:C14408T, ORF1ab:A20268G, S:A23403G, ORF3a:A25592G, N:A29487G,	5	N:K405R, ORF1a:S3949G, ORF1b:P314L, ORF3a:K67R, S:D614G,	
hCoV-19/Mexico/CMX-INER-IBT-59/2020	EPI_ISL_1301506	In process	20A	B.1	6	5'UTR:C241T, ORF1ab:C3037T, ORF1ab:C4582T, ORF1ab:C14408T, ORF1ab:G15732T, ORF1ab:T20592C, S:A23403G,	2	ORF1b:P314L, S:D614G,	
hCoV-19/Mexico/CMX-INER-IBT-6/2020	EPI_ISL_1301452	In process	20A	B.1	6	5'UTR:C241T, ORF1ab:C3037T, ORF1ab:C4582T, ORF1ab:C6190T, ORF1ab:C14408T, S:A23403G, 3'UTR:C29679T,	2	ORF1b:P314L, S:D614G,	
hCoV-19/Mexico/CMX-INER-IBT-60/2020	EPI_ISL_1301554	In process	20A	B.1.609	10	5'UTR:C241T, ORF1ab:C3037T, ORF1ab:C4582T, ORF1ab:T6023C, ORF1ab:C8655T, ORF1ab:A8658G, ORF1ab:C13119T, ORF1ab:C14053T, ORF1ab:C14408T, ORF1ab:A20268G, S:A23403G,	7	ORF1a:E1024K, ORF1a:Y1920H, ORF1a:S2797F, ORF1a:K2798R, ORF1a:A4285V, ORF1b:P314L, S:D614G,	ORF1ab: 3333-3335,

hCoV-19/Mexico/CMX-INER-IBT-61/2020	EPI_ISL_1301728	In process	20A	B.1.609	7	5'UTR:C241T, ORF1ab:C3037T, ORF1ab:C4582T, ORF1ab:C13119T, ORF1ab:G14250T, ORF1ab:C14408T, S:G21989T, S:A23403G,	5	ORF1a:A4285V, ORF1b:L261F, ORF1b:P314L, S:V143F, S:D614G,	
hCoV-19/Mexico/CMX-INER-IBT-62/2020	EPI_ISL_1301619	In process	20B	B.1.1	11	5'UTR:C241T, ORF1ab:C3037T, ORF1ab:G10870T, ORF1ab:C14408T, ORF1ab:C19186T, ORF1ab:T19839C, S:A23403G, M:G27147C, N:C28606T, N:G28881A, N:G28882A, N:G28883C,	5	M:D209H, N:R203K, N:G204R, ORF1b:P314L, S:D614G,	
hCoV-19/Mexico/CMX-INER-IBT-63/2020	EPI_ISL_1301729	In process	20A	B.1.610	8	5'UTR:C241T, ORF1ab:C414T, ORF1ab:C3037T, ORF1ab:C14408T, ORF1ab:A20268G, S:A23403G, S:G23868T, ORF3a:G25906T, N:C28868T,	6	N:P199S, ORF1a:T50I, ORF1b:P314L, ORF3a:G172C, S:D614G, S:G769V,	
hCoV-19/Mexico/CMX-INER-IBT-66/2020	EPI_ISL_1302385	In process	20A	B.1.609	15	5'UTR:C241T, ORF1ab:C1327T, ORF1ab:G2458T, ORF1ab:C3037T, ORF1ab:A3046T, ORF1ab:C4582T, ORF1ab:C10747T, ORF1ab:C13119T, ORF1ab:G13459T, ORF1ab:C14408T, ORF1ab:C17518T, S:G21850T, S:A23403G, S:G24928T, ORF3a:C25936T, N:G28975T,	8	N:M234I, ORF1a:M731I, ORF1a:A4285V, ORF1b:P314L, ORF1b:L1351F, ORF3a:H182Y, S:E96D, S:D614G,	
hCoV-19/Mexico/CMX-INER-IBT-67/2020	EPI_ISL_1302263	In process	20C	B.1	11	5'UTR:C241T, ORF1ab:C1059T, ORF1ab:C2070T, ORF1ab:C3037T, ORF1ab:C3884T, ORF1ab:C14358T, ORF1ab:C14408T, S:A23403G, ORF3a:G25563T, ORF3a:G25650T, ORF3a:C25904T, N:C28887T,	9	N:T205I, ORF1a:T265I, ORF1a:T602I, ORF1a:P1207S, ORF1b:P314L, ORF3a:Q57H, ORF3a:L86F, ORF3a:S171L, S:D614G,	3'UTR:29736-29759
hCoV-19/Mexico/CMX-INER-IBT-69/2020	EPI_ISL_1302365	In process	20B	B.1.1.222	17	5'UTR:C241T, ORF1ab:C3037T, ORF1ab:C5974T, ORF1ab:C8334T, ORF1ab:A10864G, ORF1ab:C14408T, ORF1ab:G14772T, ORF1ab:C18683T, ORF1ab:T19839C, S:C22879T, S:A23403G, S:A23756G, ORF8:T28157C, N:G28881A, N:G28882A, N:G28883C, N:G29543T, ORF10:T29652C,	8	N:R203K, N:G204R, ORF1a:A2690V, ORF1b:P314L, ORF1b:Q435H, ORF1b:T1739I, S:D614G, S:T732A,	
hCoV-19/Mexico/CMX-INER-IBT-7/2020	EPI_ISL_1301598	In process	20A	B.1	4	5'UTR:C241T, ORF1ab:C3037T, ORF1ab:C4582T, ORF1ab:C14408T, S:A23403G,	2	ORF1b:P314L, S:D614G,	
hCoV-19/Mexico/CMX-INER-IBT-70/2020	EPI_ISL_1302369	In process	20B	B.1.1.222	18	5'UTR:C241T, ORF1ab:C1684T, ORF1ab:C3037T, ORF1ab:T3694C, ORF1ab:G6369T, ORF1ab:T6427C, ORF1ab:A8658G, ORF1ab:C8668A, ORF1ab:C11653T, ORF1ab:C14408T, ORF1ab:C18981T, ORF1ab:T19839C, ORF1ab:A21213G, S:A23403G, S:A23756G, S:C25046T, N:G28881A, N:G28882A, N:G28883C,	9	N:R203K, N:G204R, ORF1a:G2035V, ORF1a:K2798R, ORF1a:D2801E, ORF1b:P314L, S:D614G, S:T732A, S:P1162S,	
hCoV-19/Mexico/CMX-INER-IBT-71/2020	EPI_ISL_1302301	In process	20B	B.1.1.519	25	5'UTR:C203T, 5'UTR:C222T, 5'UTR:C241T, ORF1ab:G922A, ORF1ab:C3037T, ORF1ab:C3140T, ORF1ab:C5183T, ORF1ab:C10029T, ORF1ab:C10954T, ORF1ab:A11117G, ORF1ab:C12789T, ORF1ab:C13862T, ORF1ab:C14408T, ORF1ab:T19839C, S:C21575T, S:C22995A, S:A23403G, S:C23604A, S:A23756G, S:G24834A, M:C26681T, ORF8:T27904C, N:G28881A, N:G28882A, N:G28883C, N:C29197T,	16	N:R203K, N:G204R, ORF1a:P959S, ORF1a:P1640S, ORF1a:T3255I, ORF1a:I3618V, ORF1a:T4175I, ORF1b:T132I, ORF1b:P314L, ORF8:L4P, S:L5F, S:T478K, S:D614G, S:P681H, S:T732A, S:R1091H,	
hCoV-19/Mexico/CMX-INER-IBT-72/2020	EPI_ISL_1302253	In process	20B	B.1.1.432	19	5'UTR:C241T, ORF1ab:C3037T, ORF1ab:A3372G, ORF1ab:A3722G, ORF1ab:A6985T, ORF1ab:C9112T, ORF1ab:C9319T, ORF1ab:C12412T, ORF1ab:T14313C, ORF1ab:C14408T, ORF1ab:G15652T, ORF1ab:T17664C, S:A23403G, ORF3a:C25613T, ORF3a:G25912T, N:G28396T, N:G28881A, N:G28882A, N:G28883C, N:T29317C,	10	N:R203K, N:G204R, ORF1a:D1036G, ORF1a:I1153V, ORF1b:P314L, ORF1b:D729Y, ORF3a:S74F, ORF3a:G174C, ORF9b:G38V, S:D614G,	
hCoV-19/Mexico/CMX-INER-IBT-74/2020	EPI_ISL_1302307	In process	20B	B.1.1.519	22	5'UTR:C203T, 5'UTR:C222T, 5'UTR:C241T, ORF1ab:C3037T, ORF1ab:C3140T, ORF1ab:C5183T, ORF1ab:C10029T, ORF1ab:C10954T, ORF1ab:A11117G, ORF1ab:C12789T, ORF1ab:C14408T, ORF1ab:T19839C, S:C22995A, S:A23403G, S:C23604A, S:A23756G, S:G25135T, ORF8:T27904C, ORF8:G28195T, N:G28881A, N:G28882A, N:G28883C, N:C29197T,	15	N:R203K, N:G204R, ORF1a:P959S, ORF1a:P1640S, ORF1a:T3255I, ORF1a:I3618V, ORF1a:T4175I, ORF1b:P314L, ORF8:L4P, ORF8:R101L, S:T478K, S:D614G, S:P681H, S:T732A, S:K1191N,	

hCoV-19/Mexico/CMX-INER-IBT-75/2020	EPI_ISL_1302325	In process	20B	B.1.1.222	18	5'UTR:C241T, ORF1ab:C3037T, ORF1ab:C10029T, ORF1ab:C14408T, ORF1ab:C15852T, ORF1ab:G18651T, ORF1ab:C19011A, ORF1ab:T19839C, S:G22021T, S:A22666G, S:A23403G, S:A23534T, S:A23756G, ORF3a:G25912T, ORF8:A27921G, ORF8:G28001T, N:G28881A, N:G28882A, N:G28883C.	12	N:R203K, N:G204R, ORF1a:T3255I, ORF1b:P314L, ORF1b:E1728D, ORF1b:D1848E, ORF3a:G174C, ORF8:I10V, S:M153I, S:D614G, S:N658Y, S:T732A,
hCoV-19/Mexico/CMX-INER-IBT-76/2020	EPI_ISL_1302357	In process	20B	B.1.1.222	18	5'UTR:C241T, ORF1ab:C3037T, ORF1ab:C10029T, ORF1ab:C14408T, ORF1ab:G17721T, ORF1ab:T19839C, S:A23403G, S:G23522C, S:A23756G, ORF3a:G25567A, ORF3a:T25569A, ORF3a:T25570A, ORF3a:G25912T, ORF3a:A26108G, ORF8:A27921G, ORF8:G28001T, N:G28881A, N:G28882A, N:G28883C.	12	N:R203K, N:G204R, ORF1a:T3255I, ORF1b:P314L, ORF3a:A59T, ORF3a:S60T, ORF3a:G174C, ORF3a:E239G, ORF8:I10V, S:D614G, S:E654Q, S:T732A,
hCoV-19/Mexico/CMX-INER-IBT-77/2020	EPI_ISL_1302299	In process	20B	B.1.1.222	22	5'UTR:C241T, ORF1ab:G1738T, ORF1ab:C3037T, ORF1ab:G6404T, ORF1ab:C11572T, ORF1ab:T11891C, ORF1ab:T12722C, ORF1ab:C13059T, ORF1ab:C13860T, ORF1ab:C14408T, ORF1ab:T19839C, ORF1ab:A21137G, S:G21800T, S:C22987T, S:A23403G, S:A23756G, ORF3a:C25714T, ORF3a:G25775T, N:G28881A, N:G28882A, N:G28883C, N:G29402T, 3'UTR:G29862A.	13	N:R203K, N:G204R, N:D377Y, ORF1a:V2047F, ORF1a:S4153P, ORF1a:T4265I, ORF1b:P314L, ORF1b:K2557R, ORF3a:L108F, ORF3a:W128L, S:D80Y, S:D614G, S:T732A,
hCoV-19/Mexico/CMX-INER-IBT-78/2020	EPI_ISL_1302171	In process	20B	B.1.1.222	19	5'UTR:C241T, ORF1ab:T2014C, ORF1ab:C3037T, ORF1ab:G4207T, ORF1ab:C10525T, ORF1ab:G11417T, ORF1ab:C14189T, ORF1ab:C14408T, ORF1ab:G14772T, ORF1ab:C16887T, ORF1ab:G18651T, ORF1ab:T19839C, S:G22216T, S:A23403G, S:A23756G, ORF7a:T27722C, N:G28881A, N:G28882A, N:G28883C, ORF10:T29652C.	11	N:R203K, N:G204R, ORF1a:V3718F, ORF1b:A241V, ORF1b:P314L, ORF1b:Q435H, ORF1b:E1728D, ORF7a:I110T, S:Q218H, S:D614G, S:T732A,
hCoV-19/Mexico/CMX-INER-IBT-79/2020	EPI_ISL_1302159	In process	20B	B.1.1.519	21	5'UTR:C203T, 5'UTR:C222T, 5'UTR:C241T, ORF1ab:C3037T, ORF1ab:C3140T, ORF1ab:C10029T, ORF1ab:C10954T, ORF1ab:A11117G, ORF1ab:C12789T, ORF1ab:C14408T, ORF1ab:C18131T, ORF1ab:T19839C, S:C22995A, S:A23403G, S:C23604A, S:A23756G, N:C28657T, N:G28881A, N:G28882A, N:G28883C, N:C29197T, N:G29527T.	13	N:R203K, N:G204R, N:Q418H, ORF1a:P959S, ORF1a:T3255I, ORF1a:I3618V, ORF1a:T4175I, ORF1b:P314L, ORF1b:T1555I, S:T478K, S:D614G, S:P681H, S:T732A,
hCoV-19/Mexico/CMX-INER-IBT-8/2020	EPI_ISL_1301489	In process	20A	B.1	5	5'UTR:C241T, ORF1ab:C3037T, ORF1ab:C4582T, ORF1ab:C8655T, ORF1ab:C14408T, S:A23403G,	3	ORF1a:S2797F, ORF1b:P314L, S:D614G,
hCoV-19/Mexico/CMX-INER-IBT-80/2020	EPI_ISL_1302346	In process	20B	B.1.1.222	19	5'UTR:C241T, ORF1ab:T1285C, ORF1ab:G2144A, ORF1ab:C3037T, ORF1ab:T3289C, ORF1ab:G3328T, ORF1ab:G3947T, ORF1ab:C4586T, ORF1ab:T13644C, ORF1ab:C14408T, ORF1ab:C14913T, ORF1ab:T19839C, ORF1ab:C21516T, S:C21846T, S:A23403G, S:A23756G, ORF7a:C27524T, N:G28881A, N:G28882A, N:G28883C,	10	N:R203K, N:G204R, ORF1a:V627I, ORF1a:Q1021H, ORF1a:D1228Y, ORF1b:P314L, ORF7a:S44L, S:T95I, S:D614G, S:T732A,
hCoV-19/Mexico/CMX-INER-IBT-81/2020	EPI_ISL_1302366	In process	20B	B.1.1.222	22	5'UTR:C241T, ORF1ab:C2299T, ORF1ab:C3037T, ORF1ab:G3242T, ORF1ab:C3510T, ORF1ab:C4980A, ORF1ab:C8352T, ORF1ab:C9344T, ORF1ab:C13297T, ORF1ab:C13517T, ORF1ab:C14408T, ORF1ab:T14472G, ORF1ab:G15906T, ORF1ab:T17065C, ORF1ab:G18186T, ORF1ab:T19839C, S:A23403G, S:A23756G, ORF8:C28253T, N:G28881A, N:G28882A, N:G28883C, 3'UTR:G29747C,	14	N:R203K, N:G204R, ORF1a:G993C, ORF1a:A1082V, ORF1a:T1572K, ORF1a:A2696V, ORF1a:L3027F, ORF1b:T17I, ORF1b:P314L, ORF1b:Q813H, ORF1b:Y1200H, ORF1b:M1573I, S:D614G, S:T732A,
hCoV-19/Mexico/CMX-INER-IBT-83/2020	EPI_ISL_1302317	In process	20A	B.1.243	17	5'UTR:C241T, ORF1ab:C751T, ORF1ab:G806A, ORF1ab:G1042A, ORF1ab:G1599T, ORF1ab:C3037T, ORF1ab:C8650T, ORF1ab:C14408T, ORF1ab:T16074C, S:G21578T, S:A23403G, S:T24076C, ORF3a:G25793T, ORF3a:A25910G, ORF3a:G26062C, N:C28854T, N:G29543T, ORF10:G29645T.	9	N:S194L, ORF1a:A181T, ORF1a:G445V, ORF1b:P314L, ORF3a:R134L, ORF3a:D173G, ORF3a:G224R, S:V6F, S:D614G,

hCoV-19/Mexico/CMX-INER-IBT-84/2020	EPI_ISL_1302395	In process	20B	B.1.1	17	5'UTR:C241T, ORF1ab:C313T, ORF1ab:G1439T, ORF1ab:C3037T, ORF1ab:G6032T, ORF1ab:C8809T, ORF1ab:G11335T, ORF1ab:C11671T, ORF1ab:C14408T, S:C21721T, S:A23403G, S:A25048G, S:G25135T, ORF7b:G27870T, N:G28881A, N:G28882A, N:G28883C, 3'UTR:G29717A.	8	N:R203K, N:G204R, ORF1a:G392C, ORF1a:A1923S, ORF1b:P314L, ORF7b:E39*, S:D614G, S:K1191N,
hCoV-19/Mexico/CMX-INER-IBT-85/2020	EPI_ISL_1302320	In process	20C	B.1	19	5'UTR:C241T, ORF1ab:C1059T, ORF1ab:C3037T, ORF1ab:G8017T, ORF1ab:C9521T, ORF1ab:G11243T, ORF1ab:G11521T, ORF1ab:C12459T, ORF1ab:C14408T, ORF1ab:T14949C, ORF1ab:C15960T, ORF1ab:C18814T, S:C21575T, S:A23403G, ORF3a:G25563T, ORF3a:C25844T, ORF8:A27898C, ORF8:G28085T, N:C28453T, N:G28899T,	14	N:R209I, ORF1a:T265I, ORF1a:L3086F, ORF1a:V3660L, ORF1a:M3752I, ORF1a:T4065I, ORF1b:P314L, ORF3a:Q57H, ORF3a:T151I, ORF8:K2T, ORF8:E64D, ORF9b:A57V, S:L5F, S:D614G,
hCoV-19/Mexico/CMX-INER-IBT-86/2020	EPI_ISL_1302302	In process	20B	B.1.1.222	18	5'UTR:G219T, 5'UTR:C241T, ORF1ab:C3037T, ORF1ab:A6693G, ORF1ab:T7402C, ORF1ab:C9430T, ORF1ab:C10029T, ORF1ab:C10039T, ORF1ab:C14408T, ORF1ab:T19839C, S:A23403G, S:A23756G, ORF3a:G25912T, ORF7b:A27756G, ORF8:A27921G, ORF8:G28001T, N:G28881A, N:G28882A, N:G28883C,	10	N:R203K, N:G204R, ORF1a:K2143R, ORF1a:T3255I, ORF1b:P314L, ORF3a:G174C, ORF7b:M1V, ORF8:I10V, S:D614G, S:T732A,
hCoV-19/Mexico/CMX-INER-IBT-87/2020	EPI_ISL_1302308	In process	20A	B.1.551	12	5'UTR:C241T, ORF1ab:C3037T, ORF1ab:C3738T, ORF1ab:G11521T, ORF1ab:C14119T, ORF1ab:C14408T, ORF1ab:G16852T, ORF1ab:G19891T, S:A23403G, S:C23604G, ORF3a:G25456C, N:C28854T, N:C29466T,	11	N:S194L, N:A398V, ORF1a:P1158L, ORF1a:M3752I, ORF1b:P218S, ORF1b:P314L, ORF1b:G1129C, ORF1b:D2142Y, ORF3a:D22H, S:D614G, S:P681R,
hCoV-19/Mexico/CMX-INER-IBT-88/2020	EPI_ISL_1302350	In process	20B	B.1.1.222	19	5'UTR:C241T, ORF1ab:C3037T, ORF1ab:C6026T, ORF1ab:A6315G, ORF1ab:G10907A, ORF1ab:C13994T, ORF1ab:C14408T, ORF1ab:C17883T, ORF1ab:G18803T, ORF1ab:T19839C, S:A23403G, S:A23756G, ORF3a:G25687T, N:G28881A, N:G28882A, N:G28883C, N:G29254T, 3'UTR:G29751T, 3'UTR:G29810T. 3'UTR:G29825T.	11	N:R203K, N:G204R, ORF1a:P1921S, ORF1a:K2017R, ORF1a:A3548T, ORF1b:A176V, ORF1b:P314L, ORF1b:S1779I, ORF3a:A99S, S:D614G, S:T732A,
hCoV-19/Mexico/CMX-INER-IBT-89/2020	EPI_ISL_1302258	In process	20B	B.1.1.222	21	5'UTR:C241T, ORF1ab:C3037T, ORF1ab:C3315A, ORF1ab:T5218C, ORF1ab:G5972T, ORF1ab:A6729G, ORF1ab:T9704C, ORF1ab:G9705T, ORF1ab:C13554T, ORF1ab:C14408T, ORF1ab:T19839C, ORF1ab:T19866C, S:T22873C, S:A23403G, S:A23756G, S:A24661G, S:C24745T, ORF3a:C26028T, N:G28378T, N:G28881A, N:G28882A, N:G28883C,	10	N:R203K, N:G204R, ORF1a:T1017K, ORF1a:D1903Y, ORF1a:N2155S, ORF1a:C3147L, ORF1b:P314L, ORF9b:R32L, S:D614G, S:T732A,
hCoV-19/Mexico/CMX-INER-IBT-90/2020	EPI_ISL_1302386	In process	20A	B.1.243	17	5'UTR:C241T, ORF1ab:C751T, ORF1ab:G806A, ORF1ab:G1042A, ORF1ab:G1599T, ORF1ab:C3037T, ORF1ab:C8650T, ORF1ab:C14408T, ORF1ab:T16074C, S:G21578T, S:A23403G, S:T24076C, ORF3a:G25793T, ORF3a:A25910G, ORF3a:G26062C, N:C28854T, N:G29543T, ORF10:G29645T.	9	N:S194L, ORF1a:A181T, ORF1a:G445V, ORF1b:P314L, ORF3a:R134L, ORF3a:D173G, ORF3a:G224R, S:V6F, S:D614G,
hCoV-19/Mexico/CMX-INER-IBT-91/2020	EPI_ISL_1302232	In process	20B	B.1.1.222	18	5'UTR:C241T, ORF1ab:C2252A, ORF1ab:C3037T, ORF1ab:C8782T, ORF1ab:C10029T, ORF1ab:C14408T, ORF1ab:C15279T, ORF1ab:C16457T, ORF1ab:G19816T, ORF1ab:T19839C, S:A23403G, S:A23756G, ORF3a:G25912T, ORF7b:A27756G, ORF8:A27921G, ORF8:G28001T, N:G28881A, N:G28882A, N:G28883C,	12	N:R203K, N:G204R, ORF1a:Q663K, ORF1a:T3255I, ORF1b:P314L, ORF1b:S997L, ORF1b:V2117L, ORF3a:G174C, ORF7b:M1V, ORF8:I10V, S:D614G, S:T732A,
hCoV-19/Mexico/CMX-INER-IBT-92/2020	EPI_ISL_1302330	In process	20A	B.1.551	14	5'UTR:C66T, 5'UTR:C241T, ORF1ab:C3037T, ORF1ab:C3738T, ORF1ab:C13274T, ORF1ab:C14408T, ORF1ab:A17841G, S:A23403G, S:C23604G, M:C26681T, M:C26885T, M:C27059T, N:C28854T, N:G29262T, 3'UTR:G29751C,	7	N:S194L, N:W330L, ORF1a:P1158L, ORF1a:P4337S, ORF1b:P314L, S:D614G, S:P681R,

hCoV-19/Mexico/CMX-INER-IBT-94/2020	EPI_ISL_1302394	In process	20B	B.1.1.519	24	5'UTR:T201C, 5'UTR:C203T, 5'UTR:C222T, 5'UTR:C241T, ORF1ab:C936T, ORF1ab:G1738T, ORF1ab:C3037T, ORF1ab:C3140T, ORF1ab:C10029T, ORF1ab:C10954T, ORF1ab:A11117G, ORF1ab:C12789T, ORF1ab:C14408T, ORF1ab:T19839C, ORF1ab:A19974G, ORF1ab:C21306T, S:C22995A, S:A23403G, S:C23604A, S:A23756G, N:G28881A, N:G28882A, N:G28883C, N:C29197T, N:G29227T,	12	N:R203K, N:G204R, ORF1a:T224I, ORF1a:P959S, ORF1a:T3255I, ORF1a:I3618V, ORF1a:T4175I, ORF1b:P314L, S:T478K, S:D614G, S:P681H, S:T732A,
hCoV-19/Mexico/CMX-INER-IBT-95/2020	EPI_ISL_1302233	In process	20B	B.1.1.222	20	5'UTR:C241T, ORF1ab:C3037T, ORF1ab:C6026T, ORF1ab:A6315G, ORF1ab:A9259G, ORF1ab:G10907A, ORF1ab:C13994T, ORF1ab:C14408T, ORF1ab:C17883T, ORF1ab:G18803T, ORF1ab:T19839C, S:A23403G, S:A23756G, ORF3a:G25687T, N:G28881A, N:G28882A, N:G28883C, N:G29254T, 3'UTR:G29751T, 3'UTR:G29810T, 3'UTR:G29825T,	11	N:R203K, N:G204R, ORF1a:P1921S, ORF1a:K2017R, ORF1a:A3548T, ORF1b:A176V, ORF1b:P314L, ORF1b:S1779I, ORF3a:A99S, S:D614G, S:T732A,
hCoV-19/Mexico/CMX-INER-IBT-96/2020	EPI_ISL_1302234	In process	20A	B.1.243	20	5'UTR:C241T, ORF1ab:T851C, ORF1ab:A1239G, ORF1ab:C1686T, ORF1ab:C2037T, ORF1ab:C2065T, ORF1ab:C3037T, ORF1ab:G5138T, ORF1ab:C5239T, ORF1ab:C6401T, ORF1ab:C7086T, ORF1ab:G13975T, ORF1ab:C14408T, ORF1ab:G19999T, S:C21575T, S:A23403G, S:T24076C, ORF7b:C27883T, ORF8:C28087T, N:C28854T, ORF10:C29585T,	15	N:S194L, ORF1a:Y196H, ORF1a:H325R, ORF1a:A474V, ORF1a:A591V, ORF1a:D1625Y, ORF1a:P2046S, ORF1a:T2274I, ORF1b:G170C, ORF1b:P314L, ORF1b:V2178F, ORF7b:A43V, ORF8:A65V, S:L5F, S:D614G,
hCoV-19/Mexico/CMX-INER-IBT-97/2020	EPI_ISL_1302304	In process	20B	B.1.1.222	18	5'UTR:C241T, ORF1ab:C3037T, ORF1ab:C4897T, ORF1ab:C10029T, ORF1ab:A11430G, ORF1ab:C14408T, ORF1ab:C19011A, ORF1ab:T19839C, ORF1ab:A21137G, S:A23403G, S:A23756G, ORF3a:G25912T, ORF3a:A26115C, M:A26555C, ORF8:A27921G, ORF8:G28001T, N:G28881A, N:G28882A, N:G28883C,	13	M:E11D, N:R203K, N:G204R, ORF1a:T3255I, ORF1a:Y3722C, ORF1b:P314L, ORF1b:D1848E, ORF1b:K2557R, ORF3a:G174C, ORF3a:E241D, ORF8:I10V, S:D614G, S:T732A,
hCoV-19/Mexico/CMX-INER-IBT-98/2020	EPI_ISL_1302383	In process	20B	B.1.1.519	24	5'UTR:T201C, 5'UTR:C203T, 5'UTR:C222T, 5'UTR:C241T, ORF1ab:C936T, ORF1ab:G1738T, ORF1ab:C3037T, ORF1ab:C3140T, ORF1ab:C10029T, ORF1ab:C10954T, ORF1ab:A11117G, ORF1ab:C12789T, ORF1ab:C14408T, ORF1ab:T19839C, ORF1ab:A19974G, ORF1ab:C21306T, S:C22995A, S:A23403G, S:C23604A, S:A23756G, N:G28881A, N:G28882A, N:G28883C, N:C29197T, N:G29227T,	12	N:R203K, N:G204R, ORF1a:T224I, ORF1a:P959S, ORF1a:T3255I, ORF1a:I3618V, ORF1a:T4175I, ORF1b:P314L, S:T478K, S:D614G, S:P681H, S:T732A,
hCoV-19/Mexico/CMX-INER-IMSS-00102/2021	EPI_ISL_1279386	In process	20B	B.1.1.519	30	5'UTR:T201C, 5'UTR:C203T, 5'UTR:C222T, 5'UTR:C241T, ORF1ab:C936T, ORF1ab:G1738T, ORF1ab:C3037T, ORF1ab:C3140T, ORF1ab:T9445C, ORF1ab:C10029T, ORF1ab:C10954T, ORF1ab:A11117G, ORF1ab:C12789T, ORF1ab:C14408T, ORF1ab:C14741A, ORF1ab:A18818G, ORF1ab:T19839C, ORF1ab:A19974G, ORF1ab:C21306T, S:C22995A, S:A23403G, S:C23604A, S:A23756G, ORF3a:C25626T, M:G26779T, N:G28817T, N:G28881A, N:G28882A, N:G28883C, N:C29197T, N:G29227T	16	M:C86F, N:A182S, N:R203K, N:G204R, ORF1a:T224I, ORF1a:P959S, ORF1a:T3255I, ORF1a:I3618V, ORF1a:T4175I, ORF1b:P314L, ORF1b:S425Y, ORF1b:Y1784C, S:T478K, S:D614G, S:P681H, S:T732A,
hCoV-19/Mexico/CMX-INER-IMSS-00178/2021	EPI_ISL_1279452	In process	20B	B.1.1.519	29	5'UTR:T201C, 5'UTR:C203T, 5'UTR:C222T, 5'UTR:C241T, ORF1ab:G1738T, ORF1ab:C2892T, ORF1ab:C3037T, ORF1ab:C3140T, ORF1ab:A5845C, ORF1ab:C6026T, ORF1ab:C10029T, ORF1ab:C10954T, ORF1ab:A11117G, ORF1ab:C12789T, ORF1ab:C14408T, ORF1ab:T19839C, ORF1ab:A19974G, ORF1ab:C21306T, S:C22995A, S:A23403G, S:C23604A, S:A23756G, ORF7a:T27522C, ORF7b:C27804T, N:G28881A, N:G28882A, N:G28883C, N:C29197T, N:C29421T, ORF10:C29627T	15	N:R203K, N:G204R, N:P383L, ORF1a:A876V, ORF1a:P959S, ORF1a:K1860N, ORF1a:P1921S, ORF1a:T3255I, ORF1a:I3618V, ORF1a:T4175I, ORF1b:P314L, S:T478K, S:D614G, S:P681H, S:T732A,

hCoV-19/Mexico/CMX-INER-IMSS-00179/2021	EPI_ISL_1279290	In process	20B	B.1.1.519	25	5'UTR:T201C, 5'UTR:C203T, 5'UTR:C222T, 5'UTR:C241T, ORF1ab:C745T, ORF1ab:G1738T, ORF1ab:C3037T, ORF1ab:C3140T, ORF1ab:C10029T, ORF1ab:C10954T, ORF1ab:A11117G, ORF1ab:C12789T, ORF1ab:C12952T, ORF1ab:C14408T, ORF1ab:T19839C, ORF1ab:A19974G, ORF1ab:C21306T, S:C22995A, S:A23403G, S:C23604A, S:A23756G, ORF8:G27987T, N:G28881A, N:G28882A, N:G28883C, N:C29197T,	12	N:R203K, N:G204R, ORF1a:P959S, ORF1a:T3255I, ORF1a:I3618V, ORF1a:T4175I, ORF1b:P314L, ORF8:V32L, S:T478K, S:D614G, S:P681H, S:T732A,	ORF7a:27704-27721,
hCoV-19/Mexico/CMX-INER-IMSS-00180/2021	EPI_ISL_1279291	In process	20B	B.1.1.519	25	5'UTR:T201C, 5'UTR:C203T, 5'UTR:C222T, 5'UTR:C241T, ORF1ab:C745T, ORF1ab:G1738T, ORF1ab:C3037T, ORF1ab:C3140T, ORF1ab:C10029T, ORF1ab:C10954T, ORF1ab:A11117G, ORF1ab:C12789T, ORF1ab:C12952T, ORF1ab:C14408T, ORF1ab:T19839C, ORF1ab:A19974G, ORF1ab:C21306T, S:C22995A, S:A23403G, S:C23604A, S:A23756G, ORF8:G27987T, N:G28881A, N:G28882A, N:G28883C, N:C29197T,	12	N:R203K, N:G204R, ORF1a:P959S, ORF1a:T3255I, ORF1a:I3618V, ORF1a:T4175I, ORF1b:P314L, ORF8:V32L, S:T478K, S:D614G, S:P681H, S:T732A,	ORF7a:27704-27721,
hCoV-19/Mexico/CMX-INER-IMSS-00181/2021	EPI_ISL_1279292	In process	20B	B.1.1.519	27	5'UTR:T201C, 5'UTR:C203T, 5'UTR:C222T, 5'UTR:C241T, ORF1ab:C745T, ORF1ab:G1738T, ORF1ab:C3037T, ORF1ab:C3140T, ORF1ab:C9746T, ORF1ab:C10029T, ORF1ab:C10954T, ORF1ab:A11117G, ORF1ab:C12789T, ORF1ab:C12952T, ORF1ab:C14408T, ORF1ab:A15002G, ORF1ab:T19839C, ORF1ab:A19974G, ORF1ab:C21306T, S:C22995A, S:A23403G, S:C23604A, S:A23756G, ORF8:G27987T, N:G28881A, N:G28882A, N:G28883C, N:C29197T,	13	N:R203K, N:G204R, ORF1a:P959S, ORF1a:T3255I, ORF1a:I3618V, ORF1a:T4175I, ORF1b:P314L, ORF1b:Y512C, ORF8:V32L, S:T478K, S:D614G, S:P681H, S:T732A,	ORF7a:27704-27721,
hCoV-19/Mexico/CMX-INER-IMSS-00184/2021	EPI_ISL_1279455	In process	20A	B.1	19	5'UTR:C241T, ORF1ab:C2508T, ORF1ab:C3037T, ORF1ab:C3738T, ORF1ab:C13274T, ORF1ab:C14408T, ORF1ab:T17190C, ORF1ab:A17841G, ORF1ab:A20268G, ORF1ab:A20575G, ORF1ab:C20762T, S:A23403G, S:C23430T, S:C23604G, S:T24506G, ORF3a:T25514C, ORF3a:T25570G, ORF8:C28253T, N:C28854T. 3'UTR:G29751C.	13	N:S194L, ORF1a:P748L, ORF1a:P1158L, ORF1a:P4337S, ORF1b:P314L, ORF1b:K2370E, ORF1b:T2432I, ORF3a:L41P, ORF3a:S60A, S:D614G, S:A623V, S:P681R, S:S982A,	
hCoV-19/Mexico/CMX-INER-IMSS-00185/2021	EPI_ISL_1279456	In process	20B	B.1.1.10	26	5'UTR:C190T, 5'UTR:C241T, ORF1ab:C758T, ORF1ab:C3037T, ORF1ab:G4486A, ORF1ab:A4797T, ORF1ab:C5548T, ORF1ab:G7829T, ORF1ab:C11020T, ORF1ab:G12243A, ORF1ab:C14408T, ORF1ab:T18168C, ORF1ab:T19839C, S:T23030C, S:A23403G, S:C23604A, S:G24445C, ORF3a:C25452T, ORF3a:G25522A, ORF6:T27292C, ORF7b:G27882A, ORF7b:G27890A, N:G28881A, N:G28882A, N:G28883C, N:C29149T, N:G29402T,	14	N:R203K, N:G204R, N:D377Y, ORF1a:H165Y, ORF1a:Y1511F, ORF1a:V2522F, ORF1a:R3993H, ORF1b:P314L, ORF3a:G44R, ORF6:Y31H, ORF7b:A43T, S:F490L, S:D614G, S:P681H,	
hCoV-19/Mexico/CMX-INER-IMSS-00186/2021	EPI_ISL_1279457	In process	20B	B.1.1.519	26	5'UTR:G94A, 5'UTR:C203T, 5'UTR:C222T, 5'UTR:C241T, ORF1ab:C3037T, ORF1ab:C3042T, ORF1ab:C3140T, ORF1ab:C5392T, ORF1ab:C10029T, ORF1ab:C10954T, ORF1ab:A11117G, ORF1ab:C12789T, ORF1ab:C14408T, ORF1ab:T19839C, ORF1ab:C21306T, S:C22995A, S:C23380T, S:A23403G, S:C23604A, S:A23756G, M:C26885T, ORF8:T27904C, ORF8:C28087T, N:G28881A, N:G28882A, N:G28883C, N:C29197T,	14	N:R203K, N:G204R, ORF1a:P926L, ORF1a:P959S, ORF1a:T3255I, ORF1a:I3618V, ORF1a:T4175I, ORF1b:P314L, ORF8:L4P, ORF8:A65V, S:T478K, S:D614G, S:P681H, S:T732A,	

hCoV-19/Mexico/CMX-INER-IMSS-00188/2021	EPI_ISL_1279459	In process	20B	B.1.1.519	29	5'UTR:C203T, 5'UTR:C222T, 5'UTR:C241T, ORF1ab:C936T, ORF1ab:C3037T, ORF1ab:C3140T, ORF1ab:C4423T, ORF1ab:C5183T, ORF1ab:T7045C, ORF1ab:C10029T, ORF1ab:C10954T, ORF1ab:A11117G, ORF1ab:C12789T, ORF1ab:C14408T, ORF1ab:T19839C, ORF1ab:G21204T, ORF1ab:C21306T, S:C21622T, S:C22995A, S:A23403G, S:C23604A, S:A23756G, S:C23997T, S:G25135T, ORF3a:T25969C, ORF8:T27904C, N:G28881A, N:G28882A, N:G28883C, N:C29197T	18	N:R203K, N:G204R, ORF1a:T224I, ORF1a:P959S, ORF1a:P1640S, ORF1a:T3255I, ORF1a:I3618V, ORF1a:T4175I, ORF1b:P314L, ORF1b:K2579N, ORF3a:W193R, ORF8:L4P, S:T478K, S:D614G, S:P681H, S:T732A, S:P812L, S:K1191N,
hCoV-19/Mexico/CMX-INER-IMSS-00189/2021	EPI_ISL_1279460	In process	20B	B.1.1.519	26	5'UTR:C203T, 5'UTR:C222T, 5'UTR:C241T, ORF1ab:C2005T, ORF1ab:C3037T, ORF1ab:C3140T, ORF1ab:T3745C, ORF1ab:C7296T, ORF1ab:C10029T, ORF1ab:C10954T, ORF1ab:A11117G, ORF1ab:C12789T, ORF1ab:C14408T, ORF1ab:T19839C, ORF1ab:C21306T, S:C22323T, S:C22995A, S:A23403G, S:C23604A, S:A23756G, ORF3a:C25844T, ORF3a:G25855T, N:G28307A, N:G28881A, N:G28882A, N:G28883C, N:C29197T,	17	N:A12T, N:R203K, N:G204R, ORF1a:P959S, ORF1a:A2344V, ORF1a:T3255I, ORF1a:I3618V, ORF1a:T4175I, ORF1b:P314L, ORF3a:T151I, ORF3a:D155Y, ORF9b:M8I, S:S254F, S:T478K, S:D614G, S:P681H, S:T732A,
hCoV-19/Mexico/CMX-INER-IMSS-00334/2021	EPI_ISL_1279582	In process	20B	B.1.1.519	28	5'UTR:T201C, 5'UTR:C203T, 5'UTR:C222T, 5'UTR:C241T, ORF1ab:G1738T, ORF1ab:C3037T, ORF1ab:C3140T, ORF1ab:T6508C, ORF1ab:C10029T, ORF1ab:C10954T, ORF1ab:A11117G, ORF1ab:C12789T, ORF1ab:C13384T, ORF1ab:C14408T, ORF1ab:C18646T, ORF1ab:T19839C, ORF1ab:C19884T, ORF1ab:A19974G, ORF1ab:C21306T, S:C22088T, S:C22995A, S:A23403G, S:C23604A, S:A23756G, N:C28377T, N:G28881A, N:G28882A, N:G28883C, N:C29197T	15	N:A35V, N:R203K, N:G204R, ORF1a:P959S, ORF1a:T3255I, ORF1a:I3618V, ORF1a:T4175I, ORF1b:P314L, ORF1b:P1727S, ORF9b:R32C, S:L176F, S:T478K, S:D614G, S:P681H, S:T732A,
hCoV-19/Mexico/CMX-INER-IMSS-00341/2021	EPI_ISL_1279587	In process	20B	B.1.1.519	22	5'UTR:C203T, 5'UTR:C222T, 5'UTR:C241T, ORF1ab:C3037T, ORF1ab:C3140T, ORF1ab:C10029T, ORF1ab:C10954T, ORF1ab:A11117G, ORF1ab:C11575T, ORF1ab:C12789T, ORF1ab:C14408T, ORF1ab:T19839C, ORF1ab:C21306T, S:C22995A, S:A23403G, S:C23604A, S:C23709T, S:A23756G, E:T26442A, N:G28881A, N:G28882A, N:G28883C, N:C29197T,	13	E:N66K, N:R203K, N:G204R, ORF1a:P959S, ORF1a:T3255I, ORF1a:I3618V, ORF1a:T4175I, ORF1b:P314L, S:T478K, S:D614G, S:P681H, S:T716I, S:T732A,
hCoV-19/Mexico/CMX-INER-IMSS-00342/2021	EPI_ISL_1279588	In process	20G	B.1.2	19	5'UTR:C241T, ORF1ab:C1059T, ORF1ab:C3037T, ORF1ab:G3122T, ORF1ab:C4084T, ORF1ab:A6308G, ORF1ab:G8264T, ORF1ab:C10319T, ORF1ab:C14408T, ORF1ab:T14598C, ORF1ab:T16656C, ORF1ab:A18424G, ORF1ab:C21304T, S:A23403G, ORF3a:G25563T, ORF3a:G25907T, ORF3a:C26060T, ORF8:C27964T, N:C28472T, N:C28869T,	15	N:P67S, N:P199L, ORF1a:T265I, ORF1a:D953Y, ORF1a:S2015G, ORF1a:G2667C, ORF1a:L3352F, ORF1b:P314L, ORF1b:N1653D, ORF1b:R2613C, ORF3a:Q57H, ORF3a:G172V, ORF3a:T223I, ORF8:S24L, S:D614G,
hCoV-19/Mexico/CMX-INER-IMSS-00349/2021	EPI_ISL_1279595	In process	20B	B.1.1.519	25	5'UTR:C203T, 5'UTR:C222T, 5'UTR:C241T, ORF1ab:C1057T, ORF1ab:C3037T, ORF1ab:C3140T, ORF1ab:C5183T, ORF1ab:C10029T, ORF1ab:C10369T, ORF1ab:C10954T, ORF1ab:A11117G, ORF1ab:C12789T, ORF1ab:C14408T, ORF1ab:T19839C, ORF1ab:C21306T, S:C22995A, S:A23403G, S:C23604A, S:A23688G, S:A23756G, ORF3a:C25513T, ORF8:T27904C, N:G28881A, N:G28882A, N:G28883C, N:C29197T,	15	N:R203K, N:G204R, ORF1a:P959S, ORF1a:P1640S, ORF1a:T3255I, ORF1a:I3618V, ORF1a:T4175I, ORF1b:P314L, ORF3a:L41F, ORF8:L4P, S:T478K, S:D614G, S:P681H, S:N709S, S:T732A,

hCoV-19/Mexico/CMX-INER-IMSS-00350/2021	EPI_ISL_1279596	In process	20B	B.1.1.519	28	5'UTR:C203T, 5'UTR:C222T, 5'UTR:C241T, ORF1ab:C3037T, ORF1ab:C3140T, ORF1ab:C9661T, ORF1ab:C10029T, ORF1ab:C10954T, ORF1ab:A11117G, ORF1ab:C12789T, ORF1ab:C12970T, ORF1ab:C14408T, ORF1ab:G15253A, ORF1ab:A18282G, ORF1ab:C19602T, ORF1ab:T19839C, ORF1ab:T20322C, ORF1ab:C21306T, S:C22995A, S:A23403G, S:C23604A, S:A23756G, S:C24797T, ORF3a:G25906T, ORF8:C28253T, N:G28881A, N:G28882A, N:G28883C, N:C29197T,	14	N:R203K, N:G204R, ORF1a:P959S, ORF1a:T3255I, ORF1a:I3618V, ORF1a:T4175I, ORF1b:P314L, ORF1b:V596I, ORF3a:G172C, S:T478K, S:D614G, S:P681H, S:T732A, S:P1079S,
hCoV-19/Mexico/CMX-INER-IMSS-00352/2021	EPI_ISL_1279598	In process	20B	B.1.1.519	22	5'UTR:C203T, 5'UTR:C222T, 5'UTR:C241T, ORF1ab:C3037T, ORF1ab:C3140T, ORF1ab:C6027T, ORF1ab:C10029T, ORF1ab:C10954T, ORF1ab:A11117G, ORF1ab:C12473T, ORF1ab:C12789T, ORF1ab:C14408T, ORF1ab:T19839C, ORF1ab:C21306T, S:C22995A, S:A23403G, S:C23604A, S:A23756G, ORF3a:C25528T, N:G28881A, N:G28882A, N:G28883C, N:C29197T,	13	N:R203K, N:G204R, ORF1a:P959S, ORF1a:P1921L, ORF1a:T3255I, ORF1a:I3618V, ORF1a:T4175I, ORF1b:P314L, ORF3a:L46F, S:T478K, S:D614G, S:P681H, S:T732A,
hCoV-19/Mexico/CMX-INER-IMSS-00355/2021	EPI_ISL_1279600	In process	20B	B.1.1.519	30	5'UTR:T201C, 5'UTR:C203T, 5'UTR:C222T, 5'UTR:C241T, ORF1ab:C745T, ORF1ab:G1738T, ORF1ab:C3037T, ORF1ab:C3140T, ORF1ab:G9329A, ORF1ab:C10029T, ORF1ab:C10954T, ORF1ab:A11117G, ORF1ab:G12224A, ORF1ab:C12789T, ORF1ab:C14408T, ORF1ab:T19839C, ORF1ab:A19974G, ORF1ab:C21306T, S:C21575T, S:C22995A, S:A23403G, S:C23604A, S:A23756G, M:G27074A, ORF8:G28167A, N:T28759C, N:G28881A, N:G28882A, N:G28883C, N:C29197T, ORF10:C29585T,	15	N:R203K, N:G204R, ORF1a:P959S, ORF1a:D3022N, ORF1a:T3255I, ORF1a:I3618V, ORF1a:A3987T, ORF1a:T4175I, ORF1b:P314L, ORF8:E92K, S:L5F, S:T478K, S:D614G, S:P681H, S:T732A,
hCoV-19/Mexico/COA_INER_IMSS_00679/2021	EPI_ISL_2091135	In process	20A	B.1.558	25	5'UTR:C241T, ORF1ab:C344T, ORF1ab:C1288T, ORF1ab:A2006G, ORF1ab:C3037T, ORF1ab:A4305G, ORF1ab:C8655T, ORF1ab:C9223T, ORF1ab:A9483G, ORF1ab:G11083T, ORF1ab:C12439T, ORF1ab:C13297T, ORF1ab:C14408T, ORF1ab:C19662T, ORF1ab:A20268G, ORF1ab:G20962T, S:C21646T, S:C23191T, S:A23403G, ORF3a:C26060T, ORF7a:C27630T, ORF7a:A27724G, ORF8:G28086T, N:C28333T, N:G28727T, N:C28854T,	15	N:A152S, N:S194L, ORF1a:L27F, ORF1a:I581V, ORF1a:K1347R, ORF1a:S2797F, ORF1a:E3073G, ORF1a:L3606F, ORF1b:P314L, ORF1b:D2499Y, ORF3a:T223I, ORF7a:T111A, ORF8:A65S, ORF9b:P17L, S:D614G,
hCoV-19/Mexico/COA-InDRE-IBT-105/2020	EPI_ISL_1301500	In process	20B	B.1.1.432	21	5'UTR:A29G, 5'UTR:C241T, ORF1ab:G1148T, ORF1ab:C3037T, ORF1ab:G4255T, ORF1ab:A6985T, ORF1ab:C8655T, ORF1ab:C9319T, ORF1ab:C9661T, ORF1ab:C12412T, ORF1ab:T14313C, ORF1ab:C14408T, ORF1ab:C16323T, S:A22005C, S:A23403G, S:G25273T, ORF3a:C25613T, ORF7a:C27425T, N:G28881A, N:G28882A, N:G28883C, 3'UTR:G29706A,	10	N:R203K, N:G204R, ORF1a:G295C, ORF1a:S2797F, ORF1b:P314L, ORF3a:S74F, ORF7a:T11I, S:N148T, S:D614G, S:M1237I,
hCoV-19/Mexico/COA-INER-IMSS-00193/2021	EPI_ISL_1279464	In process	20A	B.1.558	22	5'UTR:C241T, ORF1ab:C344T, ORF1ab:C1288T, ORF1ab:A2006G, ORF1ab:C3037T, ORF1ab:A4305G, ORF1ab:C9223T, ORF1ab:A9726G, ORF1ab:G11083T, ORF1ab:C12439T, ORF1ab:C13297T, ORF1ab:C14408T, ORF1ab:C19662T, ORF1ab:A20268G, ORF1ab:G20962T, S:C21646T, S:A23403G, ORF3a:C26060T, ORF7a:C27630T, ORF7a:A27724G, ORF8:G28086T, N:G28727T, N:C28854T,	13	N:A152S, N:S194L, ORF1a:L27F, ORF1a:I581V, ORF1a:K1347R, ORF1a:Y3154C, ORF1a:L3606F, ORF1b:P314L, ORF1b:D2499Y, ORF3a:T223I, ORF7a:T111A, ORF8:A65S, S:D614G,

hCoV-19/Mexico/COA-INER-IMSS-00199/2021	EPI_ISL_1279470	In process	20G	B.1.2	23	5'UTR:C222T, 5'UTR:C241T, ORF1ab:C799T, ORF1ab:C1059T, ORF1ab:C3037T, ORF1ab:G4300T, ORF1ab:G6476T, ORF1ab:C9438T, ORF1ab:C10319T, ORF1ab:G11083T, ORF1ab:C12049T, ORF1ab:C14408T, ORF1ab:C14425A, ORF1ab:C15237T, ORF1ab:G16846T, ORF1ab:A18424G, ORF1ab:T18471C, ORF1ab:C21304T, S:A23403G, ORF3a:G25563T, ORF3a:G25907T, ORF8:C27964T, N:C28472T, N:C28869T,	16	N:P67S, N:P199L, ORF1a:T265I, ORF1a:V2071F, ORF1a:T3058I, ORF1a:L3352F, ORF1a:L3606F, ORF1b:P314L, ORF1b:L320I, ORF1b:D1127Y, ORF1b:N1653D, ORF1b:R2613C, ORF3a:Q57H, ORF3a:G172V, ORF8:S24L, S:D614G,
hCoV-19/Mexico/COA-INER-IMSS-00207/2021	EPI_ISL_1287771	In process		B.1.1.222	0	,	1	,
hCoV-19/Mexico/COA-INER-IMSS-00211/2021	EPI_ISL_1279477	In process	20B	B.1.1.519	24	5'UTR:C203T, 5'UTR:C222T, 5'UTR:C241T, ORF1ab:C3037T, ORF1ab:C3140T, ORF1ab:T3745C, ORF1ab:A6900C, ORF1ab:C10029T, ORF1ab:C10954T, ORF1ab:A11117G, ORF1ab:C12789T, ORF1ab:C13105T, ORF1ab:C14408T, ORF1ab:T19839C, ORF1ab:C21306T, S:C22995A, S:A23403G, S:C23604A, S:A23756G, ORF3a:C25578T, ORF3a:C25844T, N:G28881A, N:G28882A, N:G28883C, N:C29197T,	13	N:R203K, N:G204R, ORF1a:P959S, ORF1a:E2212A, ORF1a:T3255I, ORF1a:I3618V, ORF1a:T4175I, ORF1b:P314L, ORF3a:T151I, S:T478K, S:D614G, S:P681H, S:T732A,
hCoV-19/Mexico/COA-INER-IMSS-00212/2021	EPI_ISL_1279478	In process	20G	B.1.2	23	5'UTR:C241T, ORF1ab:C1059T, ORF1ab:C2371T, ORF1ab:C3037T, ORF1ab:T3334C, ORF1ab:G5206A, ORF1ab:A6277G, ORF1ab:C10319T, ORF1ab:T12391C, ORF1ab:C12439T, ORF1ab:A12820G, ORF1ab:A13222G, ORF1ab:C14408T, ORF1ab:C16580T, ORF1ab:A18424G, ORF1ab:C21304T, S:A22629G, S:A23403G, ORF3a:G25563T, ORF3a:G25907T, ORF8:C27964T, N:C28472T, N:C28869T, 3'UTR:G29737A,	14	N:P67S, N:P199L, ORF1a:T265I, ORF1a:M1647I, ORF1a:L3352F, ORF1b:P314L, ORF1b:T1038I, ORF1b:N1653D, ORF1b:R2613C, ORF3a:Q57H, ORF3a:G172V, ORF8:S24L, S:K356R, S:D614G,
hCoV-19/Mexico/COA-INER-IMSS-00216/2021	EPI_ISL_1279482	In process	20A	B.1.558	21	5'UTR:C241T, ORF1ab:C344G, ORF1ab:C1288T, ORF1ab:C3037T, ORF1ab:G3955A, ORF1ab:T4111C, ORF1ab:C9223T, ORF1ab:C12439T, ORF1ab:C14408T, ORF1ab:C15273T, ORF1ab:G18449T, ORF1ab:C19662T, ORF1ab:A20268G, ORF1ab:G20962T, ORF1ab:G21523T, S:G21986A, S:A23403G, ORF3a:C25549T, ORF3a:C26060T, ORF7a:C27630T, N:C28854T, N:G29422T,	10	N:S194L, ORF1a:L27V, ORF1b:P314L, ORF1b:S1661I, ORF1b:D2499Y, ORF1b:V2686F, ORF3a:L53F, ORF3a:T223I, S:G142S, S:D614G,
hCoV-19/Mexico/COA-INER-IMSS-00217/2021	EPI_ISL_1279483	In process	20A	B.1.558	22	5'UTR:C241T, ORF1ab:C344T, ORF1ab:C1288T, ORF1ab:A2006G, ORF1ab:C3037T, ORF1ab:A4305G, ORF1ab:C9223T, ORF1ab:A9726G, ORF1ab:G11083T, ORF1ab:C12439T, ORF1ab:C13297T, ORF1ab:C14408T, ORF1ab:C19662T, ORF1ab:A20268G, ORF1ab:G20962T, S:C21646T, S:A23403G, ORF3a:C26060T, ORF7a:C27630T, ORF7a:A27724G, ORF8:G28086T, N:G28727T, N:C28854T,	13	N:A152S, N:S194L, ORF1a:L27F, ORF1a:I581V, ORF1a:K1347R, ORF1a:Y3154C, ORF1a:L3606F, ORF1b:P314L, ORF1b:D2499Y, ORF3a:T223I, ORF7a:T111A, ORF8:A65S, S:D614G,
hCoV-19/Mexico/COA-INER-IMSS-00238/2021	EPI_ISL_1279502	In process	20G	B.1.2	21	5'UTR:C241T, ORF1ab:C1059T, ORF1ab:C3037T, ORF1ab:T5338C, ORF1ab:C10319T, ORF1ab:G12243A, ORF1ab:C14408T, ORF1ab:A18424G, ORF1ab:G18756T, ORF1ab:C19881T, ORF1ab:C21304T, S:A21661G, S:G21985T, S:A23403G, S:T23590C, ORF3a:G25563T, ORF3a:T25651C, ORF3a:G25907T, ORF8:C27964T, N:C28472T, N:C28869T, 3'UTR:G29747T,	14	N:P67S, N:P199L, ORF1a:T265I, ORF1a:L3352F, ORF1a:R3993H, ORF1b:P314L, ORF1b:N1653D, ORF1b:R2613C, ORF3a:Q57H, ORF3a:F87L, ORF3a:G172V, ORF8:S24L, S:L141F, S:D614G,

hCoV-19/Mexico/COA-INER-IMSS-00239/2021	EPI_ISL_1279503	In process	20G	B.1.2	22	5'UTR:C241T, ORF1ab:C835T, ORF1ab:C1059T, ORF1ab:C3037T, ORF1ab:G8179A, ORF1ab:T9629C, ORF1ab:C10319T, ORF1ab:C14408T, ORF1ab:A18424G, ORF1ab:C21304T, ORF1ab:A21390G, S:C21736T, S:G22028C, S:G23282T, S:A23403G, ORF3a:G25563T, ORF3a:C25613T, ORF3a:C25904T, ORF3a:G25907T, ORF8:C27964T, N:C28472T, N:C28869T, 3'UTR:G29781T,	16	N:P67S, N:P199L, ORF1a:T265I, ORF1a:F3122L, ORF1a:L3352F, ORF1b:P314L, ORF1b:N1653D, ORF1b:R2613C, ORF3a:Q57H, ORF3a:S74F, ORF3a:S171L, ORF3a:G172V, ORF8:S24L, S:E156Q, S:D574Y, S:D614G,
hCoV-19/Mexico/COA-INER-IMSS-00240/2021	EPI_ISL_1279504	In process	20B	B.1.1.519	26	5'UTR:T154A, 5'UTR:T201C, 5'UTR:C203T, 5'UTR:C222T, 5'UTR:C241T, ORF1ab:C1218T, ORF1ab:G1738T, ORF1ab:C3037T, ORF1ab:C3140T, ORF1ab:G4907A, ORF1ab:C10029T, ORF1ab:C10954T, ORF1ab:A11117G, ORF1ab:C12789T, ORF1ab:C14408T, ORF1ab:T19839C, ORF1ab:A19974G, ORF1ab:C21306T, S:C21621T, S:C22995A, S:A23403G, S:C23604A, S:A23756G, N:G28881A, N:G28882A, N:G28883C, N:C29197T,	14	N:R203K, N:G204R, ORF1a:S318L, ORF1a:P959S, ORF1a:G1548S, ORF1a:T3255I, ORF1a:I3618V, ORF1a:T4175I, ORF1b:P314L, S:T20I, S:T478K, S:D614G, S:P681H, S:T732A,
hCoV-19/Mexico/COA-INER-IMSS-00241/2021	EPI_ISL_1279505	In process	20B	B.1.1	19	5'UTR:C241T, ORF1ab:C3037T, ORF1ab:G4218A, ORF1ab:C6629T, ORF1ab:C6648T, ORF1ab:C14408T, ORF1ab:T14853C, ORF1ab:C16293T, ORF1ab:T19839C, S:C21595T, S:G21800T, S:A23403G, S:T24079G, ORF7b:C27807T, ORF8:C28011T, N:A28792T, N:G28881A, N:G28882A, N:G28883C, N:C29067T,	11	N:R203K, N:G204R, N:T265I, ORF1a:R1318K, ORF1a:L2122F, ORF1a:A2128V, ORF1b:P314L, ORF8:H40Y, S:D80Y, S:D614G, S:D839E,
hCoV-19/Mexico/COA-INER-IMSS-00242/2021	EPI_ISL_1279267	In process	20A	B.1.609	22	5'UTR:C241T, ORF1ab:C882T, ORF1ab:C1931A, ORF1ab:C3037T, ORF1ab:C4582T, ORF1ab:C7173T, ORF1ab:C8175T, ORF1ab:C8937T, ORF1ab:G9237A, ORF1ab:C14408T, ORF1ab:C16017T, ORF1ab:C17733T, ORF1ab:A20268G, S:G23345A, S:A23403G, S:G24620T, S:T25144C, ORF3a:C25587T, M:G26690T, ORF8:A27938T, ORF8:C27972T, N:C28932T, 3'UTR:C29750T,	12	N:A220V, ORF1a:A206V, ORF1a:Q556K, ORF1a:S2303F, ORF1a:A2637V, ORF1a:A2891V, ORF1a:R2991K, ORF1b:P314L, ORF8:Q27*, S:V595I, S:D614G, S:A1020S,
hCoV-19/Mexico/COA-INER-IMSS-00243/2021	EPI_ISL_1279506	In process	20B	B.1.1.519	23	5'UTR:C203T, 5'UTR:C222T, 5'UTR:C241T, ORF1ab:C3037T, ORF1ab:C3140T, ORF1ab:C10029T, ORF1ab:C10954T, ORF1ab:A11117G, ORF1ab:C12789T, ORF1ab:C14408T, ORF1ab:C15738T, ORF1ab:T19839C, ORF1ab:C21306T, S:C22995A, S:A23403G, S:C23604A, S:A23756G, M:C26882T, ORF8:T27904C, N:G28881A, N:G28882A, N:G28883C, N:G28916A, N:C29197T,	13	N:R203K, N:G204R, N:G215S, ORF1a:P959S, ORF1a:T3255I, ORF1a:I3618V, ORF1a:T4175I, ORF1b:P314L, ORF8:L4P, S:T478K, S:D614G, S:P681H, S:T732A,
hCoV-19/Mexico/COA-INER-IMSS-00244/2021	EPI_ISL_1279507	In process	20G	B.1.2	22	5'UTR:C222T, 5'UTR:C241T, ORF1ab:C799T, ORF1ab:C1059T, ORF1ab:C3037T, ORF1ab:G4300T, ORF1ab:G6476T, ORF1ab:C9438T, ORF1ab:C10319T, ORF1ab:G11083T, ORF1ab:C12049T, ORF1ab:C14408T, ORF1ab:C14425A, ORF1ab:G16846T, ORF1ab:A18424G, ORF1ab:T18471C, ORF1ab:C21304T, S:A23403G, ORF3a:G25563T, ORF3a:G25907T, ORF8:C27964T, N:C28472T, N:C28869T,	16	N:P67S, N:P199L, ORF1a:T265I, ORF1a:V2071F, ORF1a:T3058I, ORF1a:L3352F, ORF1a:L3606F, ORF1b:P314L, ORF1b:L320I, ORF1b:D1127Y, ORF1b:N1653D, ORF1b:R2613C, ORF3a:Q57H, ORF3a:G172V, ORF8:S24L, S:D614G,
hCoV-19/Mexico/COA-INER-IMSS-00245/2021	EPI_ISL_1279508	In process	20G	B.1.2	19	5'UTR:C241T, ORF1ab:C1059T, ORF1ab:C3037T, ORF1ab:T5338C, ORF1ab:C10319T, ORF1ab:G12243A, ORF1ab:C14408T, ORF1ab:A18424G, ORF1ab:G18756T, ORF1ab:C19881T, ORF1ab:C21304T, S:A21661G, S:A23403G, S:T23590C, ORF3a:G25563T, ORF3a:G25907T, ORF8:C27964T, N:C28472T, N:C28869T, 3'UTR:G29747T,	12	N:P67S, N:P199L, ORF1a:T265I, ORF1a:L3352F, ORF1a:R3993H, ORF1b:P314L, ORF1b:N1653D, ORF1b:R2613C, ORF3a:Q57H, ORF3a:G172V, ORF8:S24L, S:D614G,

hCoV-19/Mexico/COA-INER-IMSS-00246/2021	EPI_ISL_1279509	In process	20B	B.1.1.519	31	5'UTR:T201C, 5'UTR:C203T, 5'UTR:C222T, 5'UTR:C241T, ORF1ab:C936T, ORF1ab:G1738T, ORF1ab:C3037T, ORF1ab:C3140T, ORF1ab:C9112T, ORF1ab:C10029T, ORF1ab:G10523C, ORF1ab:C10954T, ORF1ab:A11117G, ORF1ab:C12789T, ORF1ab:C14408T, ORF1ab:T19839C, ORF1ab:A19974G, ORF1ab:A20532C, ORF1ab:T20644C, ORF1ab:G21255A, ORF1ab:C21306T, S:C22995A, S:A23403G, S:C23604A, S:A23756G, S:C24382T, N:G28881A, N:G28882A, N:G28883C, N:C29197T, N:G29227T, 3'UTR:G29703T,	15	N:R203K, N:G204R, ORF1a:T224I, ORF1a:P959S, ORF1a:T3255I, ORF1a:V3420L, ORF1a:I3618V, ORF1a:T4175I, ORF1b:P314L, ORF1b:E2355D, ORF1b:Y2393H, S:T478K, S:D614G, S:P681H, S:T732A,
hCoV-19/Mexico/COA-INER-IMSS-00247/2021	EPI_ISL_1279510	In process	20G	B.1.2	25	5'UTR:C241T, ORF1ab:C920T, ORF1ab:C1059T, ORF1ab:C1376T, ORF1ab:T1927C, ORF1ab:C3037T, ORF1ab:C10319T, ORF1ab:T11329G, ORF1ab:C14408T, ORF1ab:C14928T, ORF1ab:G15766T, ORF1ab:A18424G, ORF1ab:G18538T, ORF1ab:G18583A, ORF1ab:C21304T, S:A22255T, S:C23315T, S:A23403G, ORF3a:G25563T, ORF3a:G25907T, ORF3a:T25930C, M:T27134C, ORF8:C27964T, N:C28472T, N:C28869T, N:A29439T,	18	N:P67S, N:P199L, N:Q389L, ORF1a:T265I, ORF1a:P371S, ORF1a:L3352F, ORF1b:P314L, ORF1b:V767L, ORF1b:N1653D, ORF1b:V1691L, ORF1b:V1706I, ORF1b:R2613C, ORF3a:Q57H, ORF3a:G172V, ORF3a:S180P, ORF8:S24L, S:L585F, S:D614G,
hCoV-19/Mexico/COA-INER-IMSS-00248/2021	EPI_ISL_1279511	In process	20B	B.1.1.316	19	5'UTR:C241T, ORF1ab:G2830A, ORF1ab:A2866G, ORF1ab:C3037T, ORF1ab:C3411T, ORF1ab:G3871T, ORF1ab:A5040G, ORF1ab:G7693A, ORF1ab:C8772T, ORF1ab:A12519G, ORF1ab:C14408T, ORF1ab:T19839C, S:C21757T, S:A23403G, S:G23593C, S:A23756T, N:G28881A, N:G28882A, N:G28883C, 3'UTR:G29751T,	11	N:R203K, N:G204R, ORF1a:A1049V, ORF1a:K1202N, ORF1a:Q1592R, ORF1a:T2836I, ORF1a:D4085G, ORF1b:P314L, S:D614G, S:Q677H, S:T732S,
hCoV-19/Mexico/COA-INER-IMSS-00256/2021	EPI_ISL_1279518	In process	20A	B.1.243	18	5'UTR:C241T, ORF1ab:C3037T, ORF1ab:C7162T, ORF1ab:C8318A, ORF1ab:A10323G, ORF1ab:C10741T, ORF1ab:C14408T, ORF1ab:A17969G, ORF1ab:C19097T, ORF1ab:A19137G, ORF1ab:C19263T, ORF1ab:A20268G, S:T22228A, S:A23403G, S:C23604A, S:T24076C, N:C28854T, N:C29367T, 3'UTR:T29710C.	9	N:S194L, N:P365L, ORF1a:P2685T, ORF1a:K3353R, ORF1b:P314L, ORF1b:D1501G, ORF1b:A1877V, S:D614G, S:P681H,
hCoV-19/Mexico/COA-INER-IMSS-00257/2021	EPI_ISL_1279519	In process	20A	B.1.558	23	5'UTR:C241T, ORF1ab:C344T, ORF1ab:C1288T, ORF1ab:A2006G, ORF1ab:C3037T, ORF1ab:A4305G, ORF1ab:C9223T, ORF1ab:C9474T, ORF1ab:G11083T, ORF1ab:C12439T, ORF1ab:C13297T, ORF1ab:C14408T, ORF1ab:C19662T, ORF1ab:A20268G, ORF1ab:G20962T, S:C21646T, S:C22597T, S:A23403G, ORF3a:C26060T, ORF7a:C27630T, ORF7a:A27724G, ORF8:G28086T, N:G28727T, N:C28854T,	13	N:A152S, N:S194L, ORF1a:L27F, ORF1a:I581V, ORF1a:K1347R, ORF1a:A3070V, ORF1a:L3606F, ORF1b:P314L, ORF1b:D2499Y, ORF3a:T223I, ORF7a:T111A, ORF8:A65S, S:D614G,
hCoV-19/Mexico/COA-INER-IMSS-00258/2021	EPI_ISL_1279520	In process	20B	B.1.1.222	24	5'UTR:C241T, ORF1ab:C1380T, ORF1ab:C3037T, ORF1ab:A6693G, ORF1ab:T7402C, ORF1ab:C9430T, ORF1ab:C10029T, ORF1ab:C10039T, ORF1ab:T12532C, ORF1ab:C14408T, ORF1ab:G19009A, ORF1ab:T19839C, ORF1ab:G21159T, S:A23403G, S:A23756G, ORF3a:G25912T, ORF3a:G26211T, ORF7b:A27756G, ORF8:A27921G, ORF8:C27923T, ORF8:G28001T, N:G28881A, N:G28882A, N:G28883C, N:C29253T,	13	N:R203K, N:G204R, N:S327L, ORF1a:A372V, ORF1a:K2143R, ORF1a:T3255I, ORF1b:P314L, ORF1b:D1848N, ORF3a:G174C, ORF7b:M1V, ORF8:I10V, S:D614G, S:T732A,
hCoV-19/Mexico/COL-IBT-IMSS-430/2020	EPI_ISL_1301450	In process	20A	B.1	5	5'UTR:G174C, 5'UTR:C241T, ORF1ab:C3037T, ORF1ab:C4582T, ORF1ab:C14408T, S:A23403G,	2	ORF1b:P314L, S:D614G,
hCoV-19/Mexico/COL-IBT-IMSS-458/2020	EPI_ISL_1301641	In process	20A	B.1	6	5'UTR:C241T, ORF1ab:C3037T, ORF1ab:C4582T, ORF1ab:C11653T, ORF1ab:C14408T, S:A23403G, N:C28708T,	2	ORF1b:P314L, S:D614G,

hCoV-19/Mexico/COL-IBT-IMSS-461/2020	EPI_ISL_1301644	In process	20A	B.1.609	10	5'UTR:G174C, 5'UTR:C241T, ORF1ab:A1003T, ORF1ab:C3037T, ORF1ab:C4582T, ORF1ab:G12721T, ORF1ab:C14408T, ORF1ab:G16935T, ORF1ab:A20268G, S:A23403G, ORF7a:C27476T,	6	ORF1a:E246D, ORF1a:M4152I, ORF1b:P314L, ORF1b:M1156I, ORF7a:T28I, S:D614G,	
hCoV-19/Mexico/COL-IBT-IMSS-463/2020	EPI_ISL_1301646	In process	20A	B.1.609	7	5'UTR:G174C, 5'UTR:C241T, ORF1ab:C2433T, ORF1ab:C3037T, ORF1ab:C4582T, ORF1ab:C14408T, ORF1ab:A20268G, S:A23403G,	3	ORF1a:S723F, ORF1b:P314L, S:D614G,	
hCoV-19/Mexico/COL-IBT-IMSS-500/2020	EPI_ISL_1301516	In process	20A	B.1	5	5'UTR:C241T, ORF1ab:C3037T, ORF1ab:C14408T, ORF1ab:C18326T, ORF1ab:A20268G, S:A23403G,	3	ORF1b:P314L, ORF1b:A1620V, S:D614G,	
hCoV-19/Mexico/COL-IBT-IMSS-501/2020	EPI_ISL_1301518	In process	20A	B.1.609	6	5'UTR:G174C, 5'UTR:C241T, ORF1ab:C3037T, ORF1ab:C4582T, ORF1ab:C14408T, ORF1ab:A20268G, S:A23403G,	2	ORF1b:P314L, S:D614G,	
hCoV-19/Mexico/COL-InDRE-IBT-136/2020	EPI_ISL_1301564	In process	20A	B.1.243	11	5'UTR:C241T, ORF1ab:C3037T, ORF1ab:C7113T, ORF1ab:T13354C, ORF1ab:C14408T, S:A23403G, S:T24076C, S:C24337T, S:G25266T, M:T26669C, M:G26690T, N:C28854T,	5	N:S194L, ORF1a:T2283I, ORF1b:P314L, S:D614G, S:C1235F,	
hCoV-19/Mexico/COL-InDRE-IBT-137/2020	EPI_ISL_1301482	In process	20A	B.1	6	5'UTR:C241T, ORF1ab:A2497G, ORF1ab:C3037T, ORF1ab:C8655T, ORF1ab:C14408T, S:A23403G, N:C28854T,	4	N:S194L, ORF1a:S2797F, ORF1b:P314L, S:D614G,	
hCoV-19/Mexico/DUR_INER_IMSS_00680/2021	EPI_ISL_2091136	In process	20B	B.1.1.432	47	5'UTR:C222T, 5'UTR:C241T, ORF1ab:G1135T, ORF1ab:C3037T, ORF1ab:C3695T, ORF1ab:G5194T, ORF1ab:A6985T, ORF1ab:T9167C, ORF1ab:C9319T, ORF1ab:G9928T, ORF1ab:C12412T, ORF1ab:C12924T, ORF1ab:T14313C, ORF1ab:C14408T, ORF1ab:C15720T, ORF1ab:A16339G, ORF1ab:A18057G, S:A21792G, S:A23403G, S:T24397C, ORF3a:C25587A, ORF3a:C25613T, ORF3a:G25912T, ORF3a:G25996T, N:G28881A, N:G28882A, N:G28883C, N:T29317C, 3'UTR:T29728G, 3'UTR:T29729G, 3'UTR:C29730G, 3'UTR:C29732G, 3'UTR:C29733A, 3'UTR:G29736T, 3'UTR:C29741T, 3'UTR:G29745A, 3'UTR:G29751C, 3'UTR:A29752C, 3'UTR:C29754A, 3'UTR:G29755A, 3'UTR:A29756T, 3'UTR:C29762A, 3'UTR:G29764A, 3'UTR:G29766T, 3'UTR:C29769T,3'UTR:A29735C, 3'UTR:T29748A, 3'UTR:A29749G,	13	N:R203K, N:G204R, ORF1a:K290N, ORF1a:Y2968H, ORF1a:M3221I, ORF1a:P4220L, ORF1b:P314L, ORF1b:I958V, ORF3a:S74F, ORF3a:G174C, ORF3a:V202L, S:K77R, S:D614G,	3'UTR:2 9743
hCoV-19/Mexico/DUR-InDRE-IBT-108/2020	EPI_ISL_1301475	In process	20A	B.1.558	11	5'UTR:C241T, ORF1ab:C1288T, ORF1ab:C3037T, ORF1ab:A9614G, ORF1ab:A10435C, ORF1ab:C14408T, ORF1ab:C19662T, S:A23403G, S:A25034G, M:C26688T, N:C28854T, N:G29315T,	7	N:S194L, N:D348Y, ORF1a:T3117A, ORF1a:Q3390H, ORF1b:P314L, S:D614G, S:N1158D,	
hCoV-19/Mexico/DUR-InDRE-IBT-109/2020	EPI_ISL_1301456	In process	20C	B.1.366	9	5'UTR:C241T, ORF1ab:C1059T, ORF1ab:C3037T, ORF1ab:T8041C, ORF1ab:C8655T, ORF1ab:C14408T, ORF1ab:C14805T, S:G22225T, S:A23403G, ORF3a:G25563T,	5	ORF1a:T265I, ORF1a:S2797F, ORF1b:P314L, ORF3a:Q57H, S:D614G,	
hCoV-19/Mexico/DUR-INER-IMSS-00194/2021	EPI_ISL_1279465	In process	20B	B.1.1.519	26	5'UTR:C203T, 5'UTR:C222T, 5'UTR:C241T, ORF1ab:C3037T, ORF1ab:C3140T, ORF1ab:C10029T, ORF1ab:C10954T, ORF1ab:A11117G, ORF1ab:C12789T, ORF1ab:C14408T, ORF1ab:T15030C, ORF1ab:C19839C, ORF1ab:C21077T, ORF1ab:C21306T, S:C22995A, S:A23403G, S:C23604A, S:A23756G, ORF3a:G25906T, M:G27047T, ORF7a:C27612T, ORF8:C28253T, ORF8:A28254C, N:G28881A, N:G28882A, N:G28883C, N:C29197T,	14	N:R203K, N:G204R, ORF1a:P959S, ORF1a:T3255I, ORF1a:I3618V, ORF1a:T4175I, ORF1b:P314L, ORF1b:T2537I, ORF3a:G172C, ORF8:I121L, S:T478K, S:D614G, S:P681H, S:T732A,	

hCoV-19/Mexico/DUR-INER-IMSS-00196/2021	EPI_ISL_1279467	In process	20G	B.1.2	24	5'UTR:C241T, ORF1ab:C1059T, ORF1ab:C1263T, ORF1ab:C2227A, ORF1ab:C3037T, ORF1ab:G3242A, ORF1ab:C10319T, ORF1ab:C10969T, ORF1ab:C11344T, ORF1ab:G14118T, ORF1ab:C14408T, ORF1ab:C15579T, ORF1ab:A18424G, ORF1ab:G20060T, ORF1ab:T20346C, ORF1ab:C21304T, S:T21799A, S:A23403G, ORF3a:G25563T, ORF3a:G25907T, ORF8:C27964T, N:A28343G, N:C28472T, N:C28677T, N:C28869T,	17	N:T24A, N:P67S, N:T135I, N:P199L, ORF1a:T265I, ORF1a:T333M, ORF1a:G993S, ORF1a:L3352F, ORF1b:P314L, ORF1b:N1653D, ORF1b:S2198I, ORF1b:R2613C, ORF3a:Q57H, ORF3a:G172V, ORF8:S24L, S:F79L, S:D614G,	
hCoV-19/Mexico/DUR-INER-IMSS-00197/2021	EPI_ISL_1279468	In process	20A	B.1.609	15	5'UTR:C241T, ORF1ab:G2144T, ORF1ab:C3037T, ORF1ab:C4582T, ORF1ab:C4999T, ORF1ab:C8106T, ORF1ab:C10039T, ORF1ab:A10874G, ORF1ab:C14408T, ORF1ab:C19862T, ORF1ab:A20268G, S:A23403G, S:C23683T, M:C26710T, N:G28280T, N:G28975T,	9	M:A63V, N:D3Y, N:M234I, ORF1a:V627F, ORF1a:A2614V, ORF1a:N3537D, ORF1b:P314L, ORF1b:A2132V, S:D614G,	
hCoV-19/Mexico/DUR-INER-IMSS-00198/2021	EPI_ISL_1279469	In process	20G	B.1.596	25	5'UTR:C203T, 5'UTR:C241T, ORF1ab:C1059T, ORF1ab:C3037T, ORF1ab:C4331T, ORF1ab:C10319T, ORF1ab:A11451G, ORF1ab:G13714C, ORF1ab:C14408T, ORF1ab:A18424G, ORF1ab:A19137G, ORF1ab:C21302T, ORF1ab:C21304A, ORF1ab:G21305A, S:C21811A, S:T23042C, S:A23403G, S:A23592C, ORF3a:G25563T, ORF3a:G25907T, ORF7a:C27612T, ORF8:C27964T, N:C28472T, N:C28869T, N:T29194C, N:T29377A,	16	N:P67S, N:P199L, ORF1a:T265I, ORF1a:L3352F, ORF1a:Q3729R, ORF1b:D83H, ORF1b:P314L, ORF1b:N1653D, ORF1b:P2612L, ORF1b:R2613N, ORF3a:Q57H, ORF3a:G172V, ORF8:S24L, S:S494P, S:D614G, S:Q677P,	
hCoV-19/Mexico/DUR-INER-IMSS-00228/2021	EPI_ISL_1279493	In process	20A	B.1	12	5'UTR:C241T, ORF1ab:C3037T, ORF1ab:C14408T, ORF1ab:C15763T, ORF1ab:A20268G, S:C21721T, S:C22286T, S:A23403G, S:G25266T, E:G26314A, E:G26428T, ORF8:A27926C, N:C28854T,	7	E:V24M, E:V62F, N:S194L, ORF1b:P314L, S:L242F, S:D614G, S:C1235F,	
hCoV-19/Mexico/DUR-INER-IMSS-00229/2021	EPI_ISL_1279494	In process	20A	B.1.609	19	5'UTR:C241T, ORF1ab:G2144T, ORF1ab:C3037T, ORF1ab:C4582T, ORF1ab:C5175T, ORF1ab:C8106T, ORF1ab:C10039T, ORF1ab:A10874G, ORF1ab:C12020T, ORF1ab:C14408T, ORF1ab:A15436G, ORF1ab:C18687T, ORF1ab:C19524T, ORF1ab:A20268G, S:G21786A, S:A23403G, S:C23683T, ORF7a:G27516A, N:G28280T, N:G28975T,	11	N:D3Y, N:M234I, ORF1a:V627F, ORF1a:T1637I, ORF1a:A2614V, ORF1a:N3537D, ORF1a:L3919F, ORF1b:P314L, ORF1b:M657V, S:G75D, S:D614G,	
hCoV-19/Mexico/DUR-INER-IMSS-00270/2021	EPI_ISL_1279529	In process	20A	B.1.609	18	5'UTR:C241T, ORF1ab:G2144T, ORF1ab:C3037T, ORF1ab:C4582T, ORF1ab:G6960T, ORF1ab:C8106T, ORF1ab:C10039T, ORF1ab:A10874G, ORF1ab:C14408T, ORF1ab:A20268G, S:T23338C, S:A23403G, S:C23683T, ORF3a:G25644T, N:G28280T, N:A28779G, N:G28975T, N:G29399T, N:G29468T,	11	N:D3Y, N:K169R, N:M234I, N:A376S, N:D399Y, ORF1a:V627F, ORF1a:W2232L, ORF1a:A2614V, ORF1a:N3537D, ORF1b:P314L, S:D614G,	
hCoV-19/Mexico/DUR-INER-IMSS-00271/2021	EPI_ISL_1279530	In process	20A	B.1.243	22	5'UTR:C241T, ORF1ab:G806A, ORF1ab:C1716T, ORF1ab:T2584A, ORF1ab:C3037T, ORF1ab:C5140A, ORF1ab:C6388T, ORF1ab:C8299T, ORF1ab:C14408T, ORF1ab:T16743A, ORF1ab:G16744A, ORF1ab:G16745A, ORF1ab:T16746C, ORF1ab:C19961T, ORF1ab:A20268G, S:A23403G, S:G23587C, S:T24076C, S:C24378T, ORF3a:C25844T, M:C26571T, N:C28854T, N:G29543T,	12	M:L17F, N:S194L, ORF1a:A181T, ORF1a:T484I, ORF1a:D1625E, ORF1b:P314L, ORF1b:G1093N, ORF1b:T2165M, ORF3a:T151I, S:D614G, S:Q675H, S:S939F,	
hCoV-19/Mexico/DUR-INER-IMSS-00273/2021	EPI_ISL_1287772	In process	20B	B.1.1.519	24	5'UTR:C203T, 5'UTR:C222T, 5'UTR:C241T, ORF1ab:C664T, ORF1ab:C3037T, ORF1ab:C3140T, ORF1ab:G8861A, ORF1ab:C10029T, ORF1ab:C10954T, ORF1ab:A11117G, ORF1ab:C12789T, ORF1ab:C14322T, ORF1ab:C14408T, ORF1ab:T19839C, ORF1ab:C21306T, S:C22995A, S:A23403G, S:C23604A, S:A23756G, S:A24302G, S:G24944A, N:G28881A, N:G28882A, N:G28883C, N:C29197T,	14	N:R203K, N:G204R, ORF1a:P959S, ORF1a:V2866M, ORF1a:T3255I, ORF1a:I3618V, ORF1a:T4175I, ORF1b:P314L, S:T478K, S:D614G, S:P681H, S:T732A, S:N914D, S:V1128I,	ORF3a:26158-26161,

hCoV-19/Mexico/DUR-INER-IMSS-00274/2021	EPI_ISL_1279532	In process	20B	B.1.1.519	24	5'UTR:C203T, 5'UTR:C222T, 5'UTR:C241T, ORF1ab:C3037T, ORF1ab:C3140T, ORF1ab:C10029T, ORF1ab:C10954T, ORF1ab:A11117G, ORF1ab:C12789T, ORF1ab:C14408T, ORF1ab:C14931T, ORF1ab:T15726A, ORF1ab:T19839C, ORF1ab:C21306T, S:T21802C, S:C22995A, S:A23403G, S:C23604A, S:A23756G, M:C26833T, N:G28881A, N:G28882A, N:G28883C, N:C29197T, N:G29527T,	13	M:A104V, N:R203K, N:G204R, N:Q418H, ORF1a:P959S, ORF1a:T3255I, ORF1a:I3618V, ORF1a:T4175I, ORF1b:P314L, S:T478K, S:D614G, S:P681H, S:T732A,
hCoV-19/Mexico/DUR-INER-IMSS-00275/2021	EPI_ISL_1279533	In process	20B	B.1.1.519	24	5'UTR:C203T, 5'UTR:C222T, 5'UTR:C241T, ORF1ab:G2849A, ORF1ab:C3037T, ORF1ab:C3140T, ORF1ab:C10029T, ORF1ab:C10954T, ORF1ab:A11117G, ORF1ab:C12789T, ORF1ab:C14408T, ORF1ab:G15921T, ORF1ab:T19839C, ORF1ab:A20055G, ORF1ab:C21306T, S:C22995A, S:A23403G, S:C23604A, S:A23756G, S:A23898G, ORF8:C28054T, N:G28881A, N:G28882A, N:G28883C, N:C29197T,	14	N:R203K, N:G204R, ORF1a:V862I, ORF1a:P959S, ORF1a:T3255I, ORF1a:I3618V, ORF1a:T4175I, ORF1b:P314L, ORF8:S54L, S:T478K, S:D614G, S:P681H, S:T732A, S:Q779R,
hCoV-19/Mexico/GRO-IBT-IMSS-08/2020	EPI_ISL_955244	In process	20B	B.1.1	7	5'UTR:C241T, ORF1ab:C3037T, ORF1ab:C14408T, ORF1ab:T19839C, S:A23403G, N:G28881A, N:G28882A, N:G28883C,	4	N:R203K, N:G204R, ORF1b:P314L, S:D614G,
hCoV-19/Mexico/GRO-IBT-IMSS-10/2020	EPI_ISL_955245	In process	20A	B.1	9	5'UTR:C21T, 5'UTR:C241T, ORF1ab:C414T, ORF1ab:C690T, ORF1ab:C3037T, ORF1ab:C11455T, ORF1ab:C14408T, ORF1ab:A20268G, S:A23403G, M:C27092T,	4	ORF1a:T50I, ORF1a:S142L, ORF1b:P314L, S:D614G,
hCoV-19/Mexico/GRO-IBT-IMSS-154/2021	EPI_ISL_1288356	In process	20B	B.1.1.519	28	5'UTR:T201C, 5'UTR:C203T, 5'UTR:C222T, 5'UTR:C241T, ORF1ab:G1738T, ORF1ab:C2119T, ORF1ab:C3037T, ORF1ab:C3140T, ORF1ab:C10029T, ORF1ab:C10954T, ORF1ab:G11083T, ORF1ab:A11117G, ORF1ab:C12789T, ORF1ab:C13487T, ORF1ab:C14408T, ORF1ab:T19839C, ORF1ab:A19974G, ORF1ab:C21306T, S:C22995A, S:A23403G, S:C23604A, S:A23756G, S:A23764G, N:C28844A, N:G28881A, N:G28882A, N:G28883C, N:C29197T, 3'UTR:G29690T,	14	N:R191S, N:R203K, N:G204R, ORF1a:P959S, ORF1a:T3255I, ORF1a:L3606F, ORF1a:I3618V, ORF1a:T4175I, ORF1b:A7V, ORF1b:P314L, S:T478K, S:D614G, S:P681H, S:T732A,
hCoV-19/Mexico/GRO-IBT-IMSS-155/2021	EPI_ISL_1288357	In process	20B	B.1.1.519	25	5'UTR:T201C, 5'UTR:C203T, 5'UTR:C222T, 5'UTR:C241T, ORF1ab:G1738T, ORF1ab:C3037T, ORF1ab:C3140T, ORF1ab:C5025T, ORF1ab:G6337A, ORF1ab:C10029T, ORF1ab:C10954T, ORF1ab:A11117G, ORF1ab:C12789T, ORF1ab:C14408T, ORF1ab:T19839C, ORF1ab:A19974G, ORF1ab:C21306T, S:C22995A, S:A23403G, S:C23604A, S:A23756G, ORF3a:C25553T, N:G28881A, N:G28882A, N:G28883C, N:C29197T,	13	N:R203K, N:G204R, ORF1a:P959S, ORF1a:S1587L, ORF1a:T3255I, ORF1a:I3618V, ORF1a:T4175I, ORF1b:P314L, ORF3a:A54V, S:T478K, S:D614G, S:P681H, S:T732A,
hCoV-19/Mexico/GRO-IBT-IMSS-161/2021	EPI_ISL_1288362	In process	20B	B.1.1.519	24	5'UTR:C203T, 5'UTR:C222T, 5'UTR:C241T, ORF1ab:C3037T, ORF1ab:C3140T, ORF1ab:A3643G, ORF1ab:C3811T, ORF1ab:C10029T, ORF1ab:C10954T, ORF1ab:A11117G, ORF1ab:G12749A, ORF1ab:C12789T, ORF1ab:C14408T, ORF1ab:C18647T, ORF1ab:C19718T, ORF1ab:T19839C, ORF1ab:C21306T, S:C22995A, S:A23403G, S:C23604A, S:A23756G, N:G28881A, N:G28882A, N:G28883C, N:C29197T,	14	N:R203K, N:G204R, ORF1a:P959S, ORF1a:T3255I, ORF1a:I3618V, ORF1a:A4162T, ORF1a:T4175I, ORF1b:P314L, ORF1b:P1727L, ORF1b:T2084I, S:T478K, S:D614G, S:P681H, S:T732A,
hCoV-19/Mexico/GRO-IBT-IMSS-182/2021	EPI_ISL_1288175	In process	20B	B.1.1.222	19	5'UTR:C241T, ORF1ab:G1696T, ORF1ab:C1758T, ORF1ab:C2252A, ORF1ab:C3037T, ORF1ab:C4524T, ORF1ab:C10029T, ORF1ab:C14408T, ORF1ab:C15279T, ORF1ab:G19816T, ORF1ab:T19839C, S:A23403G, S:A23756G, ORF3a:G25912T, ORF7b:A27756G, ORF8:A27921G, ORF8:G28001T, N:G28881A, N:G28882A, N:G28883C,	14	N:R203K, N:G204R, ORF1a:L477F, ORF1a:A498V, ORF1a:Q663K, ORF1a:A1420V, ORF1a:T3255I, ORF1b:P314L, ORF1b:V2117L, ORF3a:G174C, ORF7b:M1V, ORF8:I10V, S:D614G, S:T732A,

hCoV-19/Mexico/GRO-IBT-IMSS-193/2021	EPI_ISL_1288176	In process	20B	B.1.1.519	24	5'UTR:C203T, 5'UTR:C222T, 5'UTR:C241T, ORF1ab:C3037T, ORF1ab:C3140T, ORF1ab:T3745C, ORF1ab:C10029T, ORF1ab:C10954T, ORF1ab:A11117G, ORF1ab:C12789T, ORF1ab:C14408T, ORF1ab:C17747T, ORF1ab:T19839C, ORF1ab:C21077T, ORF1ab:C21306T, S:C22995A, S:A23403G, S:C23604A, S:A23756G, ORF3a:C25844T, N:C28849T, N:G28881A, N:G28882A, N:G28883C, N:C29197T,	14	N:R203K, N:G204R, ORF1a:P959S, ORF1a:T3255I, ORF1a:I3618V, ORF1a:T4175I, ORF1b:P314L, ORF1b:P1427L, ORF1b:T2537I, ORF3a:T151I, S:T478K, S:D614G, S:P681H, S:T732A,
hCoV-19/Mexico/GRO-IBT-IMSS-204/2021	EPI_ISL_1288177	In process	20B	B.1.1.519	24	5'UTR:C203T, 5'UTR:C222T, 5'UTR:C241T, ORF1ab:C3037T, ORF1ab:C3140T, ORF1ab:T3745C, ORF1ab:A6390G, ORF1ab:C10029T, ORF1ab:C10954T, ORF1ab:A11117G, ORF1ab:C12789T, ORF1ab:C14408T, ORF1ab:C16887T, ORF1ab:T19839C, ORF1ab:C20629T, ORF1ab:C21306T, S:C22995A, S:A23403G, S:C23604A, S:A23756G, ORF3a:C25844T, N:G28881A, N:G28882A, N:G28883C, N:C29197T,	14	N:R203K, N:G204R, ORF1a:P959S, ORF1a:E2042G, ORF1a:T3255I, ORF1a:I3618V, ORF1a:T4175I, ORF1b:P314L, ORF1b:H2388Y, ORF3a:T151I, S:T478K, S:D614G, S:P681H, S:T732A,
hCoV-19/Mexico/GRO-IBT-IMSS-215/2021	EPI_ISL_1288178	In process	20B	B.1.1.519	21	5'UTR:C203T, 5'UTR:C222T, 5'UTR:C241T, ORF1ab:C3037T, ORF1ab:C3140T, ORF1ab:C10029T, ORF1ab:C10954T, ORF1ab:A11117G, ORF1ab:C12789T, ORF1ab:C14408T, ORF1ab:T19839C, ORF1ab:C21306T, S:C22995A, S:A23403G, S:C23604A, S:A23756G, M:C26882T, ORF8:T27904C, N:G28881A, N:G28882A, N:G28883C, N:C29197T,	12	N:R203K, N:G204R, ORF1a:P959S, ORF1a:T3255I, ORF1a:I3618V, ORF1a:T4175I, ORF1b:P314L, ORF8:L4P, S:T478K, S:D614G, S:P681H, S:T732A,
hCoV-19/Mexico/GRO-IBT-IMSS-26/2020	EPI_ISL_955236	In process	20A	B.1	5	5'UTR:C241T, ORF1ab:C2509T, ORF1ab:C3037T, ORF1ab:C14408T, ORF1ab:A20268G, S:A23403G,	2	ORF1b:P314L, S:D614G,
hCoV-19/Mexico/GRO-IBT-IMSS-302/2021	EPI_ISL_1288487	In process	20B	B.1.1.222	19	5'UTR:C241T, ORF1ab:C3037T, ORF1ab:C10029T, ORF1ab:C14408T, ORF1ab:C15141T, ORF1ab:G17721T, ORF1ab:T19839C, S:A23403G, S:G23522C, S:A23756G, ORF3a:G25567A, ORF3a:T25569A, ORF3a:T25570A, ORF3a:G25912T, ORF3a:A26108G, ORF8:A27921G, ORF8:G28001T, N:G28881A, N:G28882A, N:G28883C,	12	N:R203K, N:G204R, ORF1a:T3255I, ORF1b:P314L, ORF3a:A59T, ORF3a:S60T, ORF3a:G174C, ORF3a:E239G, ORF8:I10V, S:D614G, S:E654Q, S:T732A,
hCoV-19/Mexico/GRO-IBT-IMSS-391/2021	EPI_ISL_1288218	In process	20B	B.1.1.519	27	5'UTR:C203T, 5'UTR:C222T, 5'UTR:C241T, ORF1ab:C3037T, ORF1ab:C3140T, ORF1ab:G5206T, ORF1ab:C6651T, ORF1ab:C10029T, ORF1ab:C10954T, ORF1ab:A11117G, ORF1ab:C12439T, ORF1ab:C12789T, ORF1ab:C13724T, ORF1ab:C14408T, ORF1ab:C14925T, ORF1ab:G18756T, ORF1ab:T19839C, ORF1ab:C21306T, S:C22995A, S:A23403G, S:C23604A, S:A23756G, ORF8:T27904C, N:C28697T, N:G28881A, N:G28882A, N:G28883C, N:C29197T,	16	N:P142S, N:R203K, N:G204R, ORF1a:P959S, ORF1a:M1647I, ORF1a:A2129V, ORF1a:T3255I, ORF1a:I3618V, ORF1a:T4175I, ORF1b:A86V, ORF1b:P314L, ORF8:L4P, S:T478K, S:D614G, S:P681H, S:T732A,
hCoV-19/Mexico/GRO-IBT-IMSS-393/2021	EPI_ISL_1288219	In process	20B	B.1.1.519	28	5'UTR:T201C, 5'UTR:C203T, 5'UTR:C222T, 5'UTR:C241T, ORF1ab:C936T, ORF1ab:G1738T, ORF1ab:C3037T, ORF1ab:C3140T, ORF1ab:C10029T, ORF1ab:C10954T, ORF1ab:A11117G, ORF1ab:C11941T, ORF1ab:C12789T, ORF1ab:C14408T, ORF1ab:T19839C, ORF1ab:A19974G, ORF1ab:C21306T, S:C22995A, S:A23403G, S:C23604A, S:A23756G, ORF3a:T25502C, ORF3a:A25524C, ORF3a:T25997C, N:G28881A, N:G28882A, N:G28883C, N:C29197T, N:G29227T	14	N:R203K, N:G204R, ORF1a:T224I, ORF1a:P959S, ORF1a:T3255I, ORF1a:I3618V, ORF1a:T4175I, ORF1b:P314L, ORF3a:I37T, ORF3a:V202A, S:T478K, S:D614G, S:P681H, S:T732A,

hCoV-19/Mexico/GRO-IBT-IMSS-394/2021	EPI_ISL_1288220	In process	20B	B.1.1.519	25	5'UTR:T201C, 5'UTR:C203T, 5'UTR:C222T, 5'UTR:C241T, ORF1ab:G1738T, ORF1ab:C3037T, ORF1ab:C3140T, ORF1ab:C10029T, ORF1ab:C10954T, ORF1ab:A11117G, ORF1ab:C12789T, ORF1ab:C14408T, ORF1ab:G14854T, ORF1ab:C16329T, ORF1ab:T19839C, ORF1ab:A19974G, ORF1ab:C21306T, S:C22995A, S:A23403G, S:C23604A, S:A23756G, N:G28881A, N:G28882A, N:G28883C, N:C29197T, N:G29392T,	13	N:R203K, N:G204R, N:K373N, ORF1a:P959S, ORF1a:T3255I, ORF1a:I3618V, ORF1a:T4175I, ORF1b:P314L, ORF1b:V463L, S:T478K, S:D614G, S:P681H, S:T732A,
hCoV-19/Mexico/GRO-IBT-IMSS-395/2021	EPI_ISL_1288221	In process	20B	B.1.1.519	25	5'UTR:C203T, 5'UTR:C222T, 5'UTR:C241T, ORF1ab:C3037T, ORF1ab:C3140T, ORF1ab:G5206T, ORF1ab:C6651T, ORF1ab:C10029T, ORF1ab:C10954T, ORF1ab:A11117G, ORF1ab:C12439T, ORF1ab:C13724T, ORF1ab:C14408T, ORF1ab:C14925T, ORF1ab:G18756T, ORF1ab:T19839C, S:C22995A, S:A23403G, S:C23604A, S:A23756G, ORF8:T27904C, N:C28697T, N:G28881A, N:G28882A, N:G28883C, N:C29197T,	15	N:P142S, N:R203K, N:G204R, ORF1a:P959S, ORF1a:M1647I, ORF1a:A2129V, ORF1a:T3255I, ORF1a:I3618V, ORF1b:A86V, ORF1b:P314L, ORF8:L4P, S:T478K, S:D614G, S:P681H, S:T732A,
hCoV-19/Mexico/GRO-IBT-IMSS-396/2021	EPI_ISL_1288222	In process	20B	B.1.1.519	22	5'UTR:C203T, 5'UTR:C222T, 5'UTR:C241T, ORF1ab:C3037T, ORF1ab:C3140T, ORF1ab:C10029T, ORF1ab:C10954T, ORF1ab:A11117G, ORF1ab:C12789T, ORF1ab:C14408T, ORF1ab:G16647T, ORF1ab:G18090A, ORF1ab:T19839C, ORF1ab:C21306T, S:C22995A, S:A23403G, S:C23604A, S:A23756G, N:G28881A, N:G28882A, N:G28883C, N:G28899A, N:C29197T,	12	N:R203K, N:G204R, N:R209K, ORF1a:P959S, ORF1a:T3255I, ORF1a:I3618V, ORF1a:T4175I, ORF1b:P314L, S:T478K, S:D614G, S:P681H, S:T732A,
hCoV-19/Mexico/GRO-IBT-IMSS-401/2021	EPI_ISL_1288225	In process	20B	B.1.1.322	24	5'UTR:C230T, 5'UTR:C241T, ORF1ab:C3037T, ORF1ab:C4540T, ORF1ab:C5175T, ORF1ab:T6895C, ORF1ab:C9891T, ORF1ab:C10507T, ORF1ab:G14131A, ORF1ab:G14245T, ORF1ab:C14408T, ORF1ab:G18960T, ORF1ab:T19839C, S:A23403G, S:G23587T, S:A23756G, ORF6:T27285C, ORF8:C27972T, N:A28877T, N:G28878C, N:G28881A, N:G28882A, N:G28883C, N:C29144T, 3'UTR:G29779T,	11	N:R203K, N:G204R, ORF1a:T1637I, ORF1a:A3209V, ORF1b:V222I, ORF1b:D260Y, ORF1b:P314L, ORF8:Q27*, S:D614G, S:Q675H, S:T732A,
hCoV-19/Mexico/GRO-IBT-IMSS-404/2021	EPI_ISL_1288226	In process	20B	B.1.1.519	23	5'UTR:C203T, 5'UTR:C222T, 5'UTR:C241T, ORF1ab:C3037T, ORF1ab:C3140T, ORF1ab:G3549A, ORF1ab:A9848G, ORF1ab:C10029T, ORF1ab:C10954T, ORF1ab:A11117G, ORF1ab:C11195T, ORF1ab:C12789T, ORF1ab:C14408T, ORF1ab:T19839C, ORF1ab:C21306T, S:C22995A, S:A23403G, S:C23604A, S:A23756G, ORF8:T27904C, N:G28881A, N:G28882A, N:G28883C, N:C29197T,	15	N:R203K, N:G204R, ORF1a:P959S, ORF1a:G1095E, ORF1a:S3195G, ORF1a:T3255I, ORF1a:I3618V, ORF1a:L3644F, ORF1a:T4175I, ORF1b:P314L, ORF8:L4P, S:T478K, S:D614G, S:P681H, S:T732A,
hCoV-19/Mexico/GRO-IBT-IMSS-405/2021	EPI_ISL_1288227	In process	20B	B.1.1.519	30	5'UTR:C203T, 5'UTR:C222T, 5'UTR:C241T, ORF1ab:C3037T, ORF1ab:C3140T, ORF1ab:C4423T, ORF1ab:C10029T, ORF1ab:G10318A, ORF1ab:G10540T, ORF1ab:C10954T, ORF1ab:C11103T, ORF1ab:A11117G, ORF1ab:C11455T, ORF1ab:C12789T, ORF1ab:C12890T, ORF1ab:C13536T, ORF1ab:C14408T, ORF1ab:T19839C, ORF1ab:C20233T, ORF1ab:C21306T, S:C22995A, S:A23403G, S:C23604A, S:A23756G, E:T26497C, ORF8:C28171T, N:G28881A, N:G28882A, N:G28883C, N:C29197T, N:G29315C,	16	N:R203K, N:G204R, N:D348H, ORF1a:P959S, ORF1a:T3255I, ORF1a:M3425I, ORF1a:P3613L, ORF1a:I3618V, ORF1a:T4175I, ORF1b:P314L, ORF1b:P2256S, ORF8:P93L, S:T478K, S:D614G, S:P681H, S:T732A,

hCoV-19/Mexico/GRO-IBT-IMSS-406/2021	EPI_ISL_1288228	In process	20B	B.1.1.519	27	5'UTR:T201C, 5'UTR:C203T, 5'UTR:C222T, 5'UTR:C241T, ORF1ab:G1738T, ORF1ab:C3037T, ORF1ab:C3140T, ORF1ab:T3710C, ORF1ab:C10029T, ORF1ab:C10954T, ORF1ab:A11117G, ORF1ab:C12789T, ORF1ab:G13654A, ORF1ab:C14408T, ORF1ab:T19839C, ORF1ab:A19974G, ORF1ab:C21306T, S:C22995A, S:A23403G, S:C23604A, S:A23756G, ORF6:G27261T, ORF7a:G27754T, N:G28881A, N:G28882A, N:G28883C, N:C29197T, 3'UTR:G29736T,	14	N:R203K, N:G204R, ORF1a:P959S, ORF1a:T3255I, ORF1a:I3618V, ORF1a:T4175I, ORF1b:V63I, ORF1b:P314L, ORF6:R20S, ORF7a:E121*, S:T478K, S:D614G, S:P681H, S:T732A,
hCoV-19/Mexico/GRO-IBT-IMSS-407/2021	EPI_ISL_1288229	In process	20G	B.1.2	24	5'UTR:C241T, ORF1ab:C1059T, ORF1ab:C1314T, ORF1ab:C1758T, ORF1ab:C3037T, ORF1ab:C10156T, ORF1ab:C10319T, ORF1ab:T11069G, ORF1ab:C14408T, ORF1ab:C14925T, ORF1ab:A18424G, ORF1ab:G20060T, ORF1ab:T20346C, ORF1ab:C21304T, S:A23403G, S:T23698A, S:A25105G, ORF3a:G25563T, ORF3a:G25907T, ORF8:C27964T, N:A28343G, N:C28472T, N:C28677T, N:C28869T, ORF10:G29628T,	17	N:T24A, N:P67S, N:T135I, N:P199L, ORF1a:T265I, ORF1a:T350I, ORF1a:A498V, ORF1a:L3352F, ORF1a:L3602V, ORF1b:P314L, ORF1b:N1653D, ORF1b:S2198I, ORF1b:R2613C, ORF3a:Q57H, ORF3a:G172V, ORF8:S24L, S:D614G,
hCoV-19/Mexico/GRO-IBT-IMSS-408/2021	EPI_ISL_1288230	In process	20B	B.1.1.222	23	5'UTR:C241T, ORF1ab:C3037T, ORF1ab:C3096T, ORF1ab:A6693G, ORF1ab:T7402C, ORF1ab:C9430T, ORF1ab:C10029T, ORF1ab:C13592T, ORF1ab:C14408T, ORF1ab:T17103C, ORF1ab:T19491C, ORF1ab:T19839C, S:G22017T, S:A23403G, S:A23756G, ORF3a:G25912T, ORF6:T27384C, ORF7b:A27756G, ORF8:A27921G, ORF8:G28001T, N:C28657T, N:G28881A, N:G28882A, N:G28883C,	13	N:R203K, N:G204R, ORF1a:S944L, ORF1a:K2143R, ORF1a:T3255I, ORF1b:T42I, ORF1b:P314L, ORF3a:G174C, ORF7b:M1V, ORF8:I10V, S:W152L, S:D614G, S:T732A,
hCoV-19/Mexico/GRO-IBT-IMSS-409/2021	EPI_ISL_1288231	In process	20B	B.1.1.432	24	5'UTR:C241T, ORF1ab:C664T, ORF1ab:C1205A, ORF1ab:C3037T, ORF1ab:C4276T, ORF1ab:A6985T, ORF1ab:A9204G, ORF1ab:C9319T, ORF1ab:G9856T, ORF1ab:C12412T, ORF1ab:T14313C, ORF1ab:C14408T, ORF1ab:A15596G, ORF1ab:C19875T, S:A23403G, S:C23595T, ORF3a:C25613T, ORF3a:G25912T, ORF7b:T27810C, ORF8:G28166A, N:G28881A, N:G28882A, N:G28883C, N:C28948T, N:T29317C,	11	N:R203K, N:G204R, ORF1a:Q314K, ORF1a:D2980G, ORF1b:P314L, ORF1b:Y710C, ORF3a:S74F, ORF3a:G174C, ORF7b:F19L, S:D614G, S:T678I,
hCoV-19/Mexico/GRO-IBT-IMSS-410/2021	EPI_ISL_1288232	In process	20B	B.1.301	17	5'UTR:C241T, ORF1ab:C2536T, ORF1ab:C3037T, ORF1ab:C3695T, ORF1ab:A6985T, ORF1ab:C9319T, ORF1ab:C12412T, ORF1ab:T14313C, ORF1ab:C14408T, ORF1ab:G16158T, ORF1ab:G20292A, S:C21952T, S:A23403G, ORF3a:G25440T, ORF3a:C25613T, ORF3a:G25912T, N:C28697T, N:T29317C,	7	N:P142S, ORF1b:P314L, ORF1b:M897I, ORF3a:K16N, ORF3a:S74F, ORF3a:G174C, S:D614G,
hCoV-19/Mexico/GRO-IBT-IMSS-411/2021	EPI_ISL_1288233	In process	20B	B.1.1.519	25	5'UTR:C203T, 5'UTR:C222T, 5'UTR:C241T, ORF1ab:C1150T, ORF1ab:C3037T, ORF1ab:C3140T, ORF1ab:C10029T, ORF1ab:C10954T, ORF1ab:A11117G, ORF1ab:C12789T, ORF1ab:C14408T, ORF1ab:C14585T, ORF1ab:T19839C, ORF1ab:C21306T, S:C22995A, S:A23403G, S:C23604A, S:A23756G, M:T26624G, ORF8:T27904C, ORF8:C28087T, ORF8:C28253T, N:G28881A, N:G28882A, N:G28883C, N:C29197T,	14	N:R203K, N:G204R, ORF1a:P959S, ORF1a:T3255I, ORF1a:I3618V, ORF1a:T4175I, ORF1b:P314L, ORF1b:A373V, ORF8:L4P, ORF8:A65V, S:T478K, S:D614G, S:P681H, S:T732A,

hCoV-19/Mexico/GRO-IBT-IMSS-412/2021	EPI_ISL_1288234	In process	20B	B.1.1.519	24	5'UTR:C203T, 5'UTR:C222T, 5'UTR:C241T, ORF1ab:C3037T, ORF1ab:C3140T, ORF1ab:G3231T, ORF1ab:T3745C, ORF1ab:C10029T, ORF1ab:C10954T, ORF1ab:G11083T, ORF1ab:A11117G, ORF1ab:C12789T, ORF1ab:C14408T, ORF1ab:T19839C, ORF1ab:C21306T, S:C22995A, S:A23403G, S:C23604A, S:A23756G, ORF3a:C25844T, ORF3a:A26105C, N:G28881A, N:G28882A, N:G28883C, N:C29197T,	15	N:R203K, N:G204R, ORF1a:P959S, ORF1a:G989V, ORF1a:T3255I, ORF1a:L3606F, ORF1a:I3618V, ORF1a:T4175I, ORF1b:P314L, ORF3a:T151I, ORF3a:D238A, S:T478K, S:D614G, S:P681H, S:T732A,	
hCoV-19/Mexico/GRO-IBT-IMSS-413/2021	EPI_ISL_1288235	In process	20B	B.1.1.519	28	5'UTR:T201C, 5'UTR:C203T, 5'UTR:C222T, 5'UTR:C241T, ORF1ab:C936T, ORF1ab:G1738T, ORF1ab:C3037T, ORF1ab:C3140T, ORF1ab:C10029T, ORF1ab:C10954T, ORF1ab:A11117G, ORF1ab:C11941T, ORF1ab:C12789T, ORF1ab:C14408T, ORF1ab:T19839C, ORF1ab:A19974G, ORF1ab:C21306T, S:C22995A, S:A23403G, S:C23604A, S:A23756G, ORF3a:T25502C, ORF3a:A25524C, ORF3a:T25997C, N:G28881A, N:G28882A, N:G28883C, N:C29197T, N:G29227T	14	N:R203K, N:G204R, ORF1a:T224I, ORF1a:P959S, ORF1a:T3255I, ORF1a:I3618V, ORF1a:T4175I, ORF1b:P314L, ORF3a:I37T, ORF3a:V202A, S:T478K, S:D614G, S:P681H, S:T732A,	
hCoV-19/Mexico/GRO-IBT-IMSS-415/2021	EPI_ISL_1288236	In process	20B	B.1.1.519	24	5'UTR:C203T, 5'UTR:C222T, 5'UTR:C241T, ORF1ab:A1445G, ORF1ab:C1684T, ORF1ab:C3037T, ORF1ab:C3140T, ORF1ab:T3745C, ORF1ab:A6390G, ORF1ab:C10029T, ORF1ab:C10954T, ORF1ab:A11117G, ORF1ab:C12789T, ORF1ab:C14408T, ORF1ab:T19839C, ORF1ab:C21306T, S:C22995A, S:A23403G, S:C23604A, S:A23756G, ORF3a:C25844T, N:G28881A, N:G28882A, N:G28883C, N:C29197T,	14	N:R203K, N:G204R, ORF1a:K394E, ORF1a:P959S, ORF1a:E2042G, ORF1a:T3255I, ORF1a:I3618V, ORF1a:T4175I, ORF1b:P314L, ORF3a:T151I, S:T478K, S:D614G, S:P681H, S:T732A,	
hCoV-19/Mexico/GRO-IBT-IMSS-416/2021	EPI_ISL_1288237	In process	20G	B.1.2	23	5'UTR:C241T, ORF1ab:C1059T, ORF1ab:C1314T, ORF1ab:C1758T, ORF1ab:C3037T, ORF1ab:C10319T, ORF1ab:T11069G, ORF1ab:C14408T, ORF1ab:C14925T, ORF1ab:A18424G, ORF1ab:G20060T, ORF1ab:T20346C, ORF1ab:C21304T, S:A23403G, S:T23698A, S:A25105G, ORF3a:G25563T, ORF3a:G25907T, ORF8:C27964T, N:A28343G, N:C28472T, N:C28677T, N:C28869T, ORF10:G29628T,	17	N:T24A, N:P67S, N:T135I, N:P199L, ORF1a:T265I, ORF1a:T350I, ORF1a:A498V, ORF1a:L3352F, ORF1a:L3602V, ORF1b:P314L, ORF1b:N1653D, ORF1b:S2198I, ORF1b:R2613C, ORF3a:Q57H, ORF3a:G172V, ORF8:S24L, S:D614G,	
hCoV-19/Mexico/GRO-IBT-IMSS-417/2021	EPI_ISL_1288238	In process	20B	B.1.1.519	27	5'UTR:C203T, 5'UTR:C222T, 5'UTR:C241T, ORF1ab:C3037T, ORF1ab:C3140T, ORF1ab:T5439C, ORF1ab:G6894A, ORF1ab:C8281T, ORF1ab:T8396C, ORF1ab:C10029T, ORF1ab:C10954T, ORF1ab:A11117G, ORF1ab:G11365T, ORF1ab:C12789T, ORF1ab:C14408T, ORF1ab:C19269T, ORF1ab:T19839C, ORF1ab:A20461G, ORF1ab:C21306T, S:C22995A, S:A23403G, S:C23604A, S:A23756G, N:G28881A, N:G28882A, N:G28883C, N:C29197T, N:G29527T,	15	N:R203K, N:G204R, N:Q418H, ORF1a:P959S, ORF1a:L1725S, ORF1a:C2210Y, ORF1a:T3255I, ORF1a:I3618V, ORF1a:T4175I, ORF1b:P314L, ORF1b:T2332A, S:T478K, S:D614G, S:P681H, S:T732A,	
hCoV-19/Mexico/GRO-IBT-IMSS-485/2020	EPI_ISL_1301664	In process	20A	B.1.610	8	5'UTR:C241T, ORF1ab:C414T, ORF1ab:C3037T, ORF1ab:C14408T, ORF1ab:C17036T, ORF1ab:G17280T, ORF1ab:A20268G, S:A23403G, N:G29540T,	4	ORF1a:T50I, ORF1b:P314L, ORF1b:A1190V, S:D614G,	
hCoV-19/Mexico/GRO-IBT-IMSS-502/2020	EPI_ISL_1301671	In process	20A	B.1.609	8	5'UTR:C241T, ORF1ab:C3037T, ORF1ab:C3365T, ORF1ab:C4582T, ORF1ab:C14408T, ORF1ab:A20268G, S:A23403G, ORF3a:G25595T, ORF6:C27294T,	4	ORF1a:L1034F, ORF1b:P314L, ORF3a:R68I, S:D614G,	
hCoV-19/Mexico/GRO-IBT-IMSS-503/2020	EPI_ISL_1301553	In process	20A	B.1.609	7	5'UTR:C241T, ORF1ab:T325C, ORF1ab:C3037T, ORF1ab:C4582T, ORF1ab:C13119T, ORF1ab:C14408T, ORF1ab:A20268G, S:A23403G,	4	ORF1a:A4285V, ORF1b:P314L, S:D614G,E:V75*,	E:26465-26467
hCoV-19/Mexico/GRO-IBT-IMSS-504/2020	EPI_ISL_1301536	In process	20A	B.1	5	5'UTR:C241T, ORF1ab:C414T, ORF1ab:C3037T, ORF1ab:C10198T, ORF1ab:C14408T, S:A23403G,	3	ORF1a:T50I, ORF1b:P314L, S:D614G,	

hCoV-19/Mexico/GRO-InDRE-IBT-107/2020	EPI_ISL_1301574	In process	20A	B.1	5	5'UTR:C241T, ORF1ab:C355T, ORF1ab:C3037T, ORF1ab:C14408T, S:A23403G, ORF3a:C25685T,	3	ORF1b:P314L, ORF3a:A98V, S:D614G,	
hCoV-19/Mexico/GRO-InDRE-IBT-110/2020	EPI_ISL_1301508	In process	20C	B.1.446	10	5'UTR:G90T, 5'UTR:C241T, ORF1ab:C1059T, ORF1ab:C3037T, ORF1ab:G6337T, ORF1ab:C14408T, S:A23403G, S:G24872T, ORF3a:G25563T, M:C26900T, ORF8:G28068T,	6	ORF1a:T265I, ORF1b:P314L, ORF3a:Q57H, ORF8:E59*, S:D614G, S:V1104L,	
hCoV-19/Mexico/GRO-InDRE-IBT-111/2020	EPI_ISL_1301455	In process	20B	B.1.1	8	5'UTR:C241T, ORF1ab:C3037T, ORF1ab:C14408T, ORF1ab:T19839C, ORF1ab:C20762T, S:A23403G, N:G28881A, N:G28882A, N:G28883C,	5	N:R203K, N:G204R, ORF1b:P314L, ORF1b:T2432I, S:D614G,	
hCoV-19/Mexico/GRO-InDRE-IBT-117/2020	EPI_ISL_1301585	In process	20A	B.1.609	9	5'UTR:C241T, ORF1ab:T325C, ORF1ab:C3037T, ORF1ab:C4582T, ORF1ab:C6258T, ORF1ab:C13119T, ORF1ab:C14408T, ORF1ab:A20268G, S:A23403G, S:G24933T,	5	ORF1a:T1998I, ORF1a:A4285V, ORF1b:P314L, S:D614G, S:G1124V,	
hCoV-19/Mexico/GRO-InDRE-IBT-129/2020	EPI_ISL_1301589	In process	20B	B.1.1.222	13	5'UTR:C241T, ORF1ab:C3037T, ORF1ab:C8655T, ORF1ab:C14408T, ORF1ab:T19839C, S:A23403G, S:A23756G, S:G25249T, ORF3a:C25515T, ORF3a:G25677T, M:A26612T, N:G28881A, N:G28882A, N:G28883C,	8	N:R203K, N:G204R, ORF1a:S2797F, ORF1b:P314L, ORF3a:L95F, S:D614G, S:T732A, S:M1229I,	
hCoV-19/Mexico/GRO-InDRE-IBT-130/2020	EPI_ISL_1301547	In process	20B	B.1.1.222	12	5'UTR:C241T, ORF1ab:C3037T, ORF1ab:C14408T, ORF1ab:T19839C, S:A23403G, S:A23756G, S:G25249T, ORF3a:C25515T, ORF3a:G25677T, M:A26612T, N:G28881A, N:G28882A, N:G28883C,	7	N:R203K, N:G204R, ORF1b:P314L, ORF3a:L95F, S:D614G, S:T732A, S:M1229I,	
hCoV-19/Mexico/GRO-InDRE-IBT-131/2020	EPI_ISL_1301590	In process	20B	B.1.1.322	14	5'UTR:G159A, 5'UTR:C241T, ORF1ab:C3037T, ORF1ab:C5284T, ORF1ab:G5470T, ORF1ab:C14408T, ORF1ab:T19839C, S:A23403G, S:A23756G, N:A28877T, N:G28878C, N:G28881A, N:G28882A, N:G28883C, N:C29144T,	6	N:R203K, N:G204R, ORF1a:L1735F, ORF1b:P314L, S:D614G, S:T732A,	
hCoV-19/Mexico/GRO-InDRE-IBT-132/2020	EPI_ISL_1301523	In process	20B	B.1.1.222	12	5'UTR:C241T, ORF1ab:C3037T, ORF1ab:G3473T, ORF1ab:C14408T, ORF1ab:T18849C, ORF1ab:T19839C, S:A23403G, S:A23756G, E:G26428T, N:G28881A, N:G28882A, N:G28883C, 3'UTR:G29751T,	7	E:V62F, N:R203K, N:G204R, ORF1a:G1070C, ORF1b:P314L, S:D614G, S:T732A,	
hCoV-19/Mexico/GRO-InDRE-IBT-153/2020	EPI_ISL_1302199	In process	20A	B.1	17	5'UTR:C241T, ORF1ab:T1237C, ORF1ab:C3037T, ORF1ab:A3481C, ORF1ab:C3768T, ORF1ab:C14408T, ORF1ab:C16329T, ORF1ab:C20844T, S:G23311T, S:A23403G, S:C23536T, S:G23587T, S:C23664T, S:G24038T, ORF6:C27213T, N:G28655T, N:C28887T, 3'UTR:G29773T,	9	N:D128Y, N:T205I, ORF1a:T1168I, ORF1b:P314L, S:E583D, S:D614G, S:Q675H, S:A701V, S:V826L,	ORF3a:26155-26157,
hCoV-19/Mexico/GRO-InDRE-IBT-94/2020	EPI_ISL_1301487	In process	20B	B.1.1	9	5'UTR:C241T, ORF1ab:C3037T, ORF1ab:C14408T, ORF1ab:T19839C, S:A23403G, S:A23923G, ORF3a:G26209A, N:G28881A, N:G28882A, N:G28883C,	5	N:R203K, N:G204R, ORF1b:P314L, ORF3a:V273M, S:D614G,	
hCoV-19/Mexico/GRO-InDRE-IBT-95/2020	EPI_ISL_1301458	In process	20A	B.1.1	6	5'UTR:C241T, ORF1ab:C414T, ORF1ab:C3037T, ORF1ab:C8655T, ORF1ab:C10198T, ORF1ab:C14408T, S:A23403G,	4	ORF1a:T50I, ORF1a:S2797F, ORF1b:P314L, S:D614G,	
hCoV-19/Mexico/GRO-INER-IMSS-00280/2021	EPI_ISL_1279537	In process	20B	B.1.1.519	28	5'UTR:C203T, 5'UTR:C222T, 5'UTR:C241T, ORF1ab:G2447T, ORF1ab:C3037T, ORF1ab:C3140T, ORF1ab:A3940G, ORF1ab:G5629A, ORF1ab:C10029T, ORF1ab:C10954T, ORF1ab:A11117G, ORF1ab:C12789T, ORF1ab:A13510G, ORF1ab:T13905C, ORF1ab:C14408T, ORF1ab:T19839C, ORF1ab:C21306T, S:C22995A, S:A23403G, S:C23604A, S:A23756G, ORF3a:C25708T, ORF3a:G25906T, ORF3a:C25916T, N:G28817T, N:G28881A, N:G28882A, N:G28883C, N:C29197T,	17	N:A182S, N:R203K, N:G204R, ORF1a:G728C, ORF1a:P959S, ORF1a:T3255I, ORF1a:I3618V, ORF1a:T4175I, ORF1b:T15A, ORF1b:P314L, ORF3a:L106F, ORF3a:G172C, ORF3a:T175I, S:T478K, S:D614G, S:P681H, S:T732A,	
hCoV-19/Mexico/GRO-INER-IMSS-00281/2021	EPI_ISL_1287774	In process	20B	B.1.1.222	25	5'UTR:C241T, ORF1ab:C3037T, ORF1ab:G3403T, ORF1ab:T5218C, ORF1ab:G10447A, ORF1ab:C12525T, ORF1ab:C14117T, ORF1ab:C14408T, ORF1ab:T19839C, S:T22549C, S:A23403G, S:T23689C, S:A23756G, S:C24642T, S:G24794T, S:C25100G, ORF3a:G25913T, ORF3a:G26153T, ORF6:C27294T, N:G28300T, N:G28881A, N:G28882A, N:G28883C, N:C28957T, N:G28975T, N:G29449T,	13	N:Q9H, N:R203K, N:G204R, N:M234I, ORF1a:T4087I, ORF1b:T217M, ORF1b:P314L, ORF9b:S6I, S:D614G, S:T732A, S:T1027I, S:A1078S, S:Q1180E,	ORF3a:26158-26161,

hCoV-19/Mexico/GRO-INER-IMSS-00304/2021	EPI_ISL_1279557	In process	20B	B.1.1.519	23	5'UTR:C203T, 5'UTR:C222T, 5'UTR:C241T, ORF1ab:C3037T, ORF1ab:C3140T, ORF1ab:T3745C, ORF1ab:A6390G, ORF1ab:C10029T, ORF1ab:C10954T, ORF1ab:A11117G, ORF1ab:C12789T, ORF1ab:C14408T, ORF1ab:C16887T, ORF1ab:T19839C, ORF1ab:C21306T, S:C22995A, S:A23403G, S:C23604A, S:A23756G, ORF3a:C25844T, N:G28881A, N:G28882A, N:G28883C, N:C29197T,	13	N:R203K, N:G204R, ORF1a:P959S, ORF1a:E2042G, ORF1a:T3255I, ORF1a:I3618V, ORF1a:T4175I, ORF1b:P314L, ORF3a:T151I, S:T478K, S:D614G, S:P681H, S:T732A,
hCoV-19/Mexico/GUA-IBT-IMSS-428/2020	EPI_ISL_1301621	In process	20A	B.1	4	5'UTR:C241T, ORF1ab:C3037T, ORF1ab:C14408T, S:A23403G, 3'UTR:G29734C,	2	ORF1b:P314L, S:D614G,
hCoV-19/Mexico/GUA-IBT-IMSS-437/2020	EPI_ISL_1301509	In process	20C	B.1.446	8	5'UTR:C241T, ORF1ab:C1059T, ORF1ab:C3037T, ORF1ab:G12514T, ORF1ab:C14408T, ORF1ab:C19781T, S:C23230T, S:A23403G, ORF3a:G25563T,	5	ORF1a:T265I, ORF1b:P314L, ORF1b:A2105V, ORF3a:Q57H, S:D614G,
hCoV-19/Mexico/GUA-IBT-IMSS-438/2020	EPI_ISL_1301466	In process	20A	B.1	4	5'UTR:C241T, ORF1ab:C3037T, ORF1ab:C14408T, ORF1ab:C15324T, S:A23403G,	2	ORF1b:P314L, S:D614G,
hCoV-19/Mexico/GUA-IBT-IMSS-498/2020	EPI_ISL_1301468	In process	20B	B.1	13	5'UTR:C241T, ORF1ab:C1514T, ORF1ab:C2399T, ORF1ab:C3037T, ORF1ab:G7954T, ORF1ab:C8655T, ORF1ab:C14408T, ORF1ab:A15945T, ORF1ab:G18255T, S:A23403G, ORF3a:G25785T, N:G28881A, N:G28882A, N:G28883C,	10	N:R203K, N:G204R, ORF1a:H417Y, ORF1a:H712Y, ORF1a:Q2563H, ORF1a:S2797F, ORF1b:P314L, ORF1b:M1596I, ORF3a:W131C, S:D614G,
hCoV-19/Mexico/GUA-InDRE-IBT-100/2020	EPI_ISL_1301707	In process	20B	B.1.1	13	5'UTR:C241T, ORF1ab:T1476C, ORF1ab:C2399T, ORF1ab:C3037T, ORF1ab:G5008T, ORF1ab:C13818T, ORF1ab:C13994T, ORF1ab:C14408T, ORF1ab:G15327T, S:A23403G, N:G28881A, N:G28882A, N:G28883C, 3'UTR:G29773T,	8	N:R203K, N:G204R, ORF1a:I404T, ORF1a:H712Y, ORF1b:A176V, ORF1b:P314L, ORF1b:M620I, S:D614G,
hCoV-19/Mexico/GUA-InDRE-IBT-101/2020	EPI_ISL_1301548	In process	20B	B.1	8	5'UTR:C241T, ORF1ab:C2399T, ORF1ab:C3037T, ORF1ab:C14408T, S:A23403G, S:G25234T, N:G28881A, N:G28882A, N:G28883C,	6	N:R203K, N:G204R, ORF1a:H712Y, ORF1b:P314L, S:D614G, S:L1224F,
hCoV-19/Mexico/GUA-InDRE-IBT-102/2020	EPI_ISL_1301708	In process	20A	B.1.243	11	5'UTR:C241T, ORF1ab:C3037T, ORF1ab:C7113T, ORF1ab:T13354C, ORF1ab:C14408T, S:A23403G, S:T24076C, S:C24337T, S:G25266T, M:T26669C, M:G26690T, N:C28854T,	5	N:S194L, ORF1a:T2283I, ORF1b:P314L, S:D614G, S:C1235F,
hCoV-19/Mexico/GUA-InDRE-IBT-103/2020	EPI_ISL_1301485	In process	20A	B.1.189	10	5'UTR:C241T, ORF1ab:C657T, ORF1ab:C3037T, ORF1ab:C3768T, ORF1ab:C4582T, ORF1ab:G6404T, ORF1ab:C14408T, ORF1ab:G17014T, S:A23403G, S:T25123C, N:G28378T,	7	ORF1a:A131V, ORF1a:T1168I, ORF1a:V2047F, ORF1b:P314L, ORF1b:D1183Y, ORF9b:R32L, S:D614G,
hCoV-19/Mexico/GUA-InDRE-IBT-99/2020	EPI_ISL_1301511	In process	20A	B.1	6	5'UTR:C241T, ORF1ab:C3037T, ORF1ab:C4582T, ORF1ab:C7011T, ORF1ab:G11426A, ORF1ab:C14408T, S:A23403G,	4	ORF1a:A2249V, ORF1a:V3721I, ORF1b:P314L, S:D614G,
hCoV-19/Mexico/HID-IBT-IMSS-254/2021	EPI_ISL_1288443	In process	20B	B.1.1.519	31	5'UTR:C203T, 5'UTR:C222T, 5'UTR:C241T, ORF1ab:C3037T, ORF1ab:C3140T, ORF1ab:G3692T, ORF1ab:G3871T, ORF1ab:C4455T, ORF1ab:G6653T, ORF1ab:T8200C, ORF1ab:C9520T, ORF1ab:C10029T, ORF1ab:C10954T, ORF1ab:A11117G, ORF1ab:C11824A, ORF1ab:C12789T, ORF1ab:C14408T, ORF1ab:G16795T, ORF1ab:T19839C, ORF1ab:C21306T, S:C22995A, S:C23248T, S:A23403G, S:C23604A, S:A23756G, ORF3a:A25601T, ORF3a:G25690T, ORF8:T27904C, N:G28881A, N:G28882A, N:G28883C, N:C29197T,	19	N:R203K, N:G204R, ORF1a:P959S, ORF1a:V1143F, ORF1a:K1202N, ORF1a:A1397V, ORF1a:V2130F, ORF1a:T3255I, ORF1a:I3618V, ORF1a:T4175I, ORF1b:P314L, ORF1b:V1110L, ORF3a:Q70L, ORF3a:G100C, ORF8:L4P, S:T478K, S:D614G, S:P681H, S:T732A,

hCoV-19/Mexico/HID-IBT-IMSS-265/2021	EPI_ISL_1288453	In process	20B	B.1.1.519	24	5'UTR:C203T, 5'UTR:C222T, 5'UTR:C241T, ORF1ab:C3037T, ORF1ab:C3140T, ORF1ab:C6541T, ORF1ab:C9039T, ORF1ab:C10029T, ORF1ab:C10954T, ORF1ab:A11117G, ORF1ab:C12789T, ORF1ab:C14408T, ORF1ab:A18434G, ORF1ab:T19839C, ORF1ab:C21306T, S:A22023C, S:C22995A, S:A23403G, S:C23604A, S:A23756G, ORF8:T27904C, N:G28881A, N:G28882A, N:G28883C, N:C29197T,	15	N:R203K, N:G204R, ORF1a:P959S, ORF1a:A2925V, ORF1a:T3255I, ORF1a:I3618V, ORF1a:T4175I, ORF1b:P314L, ORF1b:D1656G, ORF8:L4P, S:E154A, S:T478K, S:D614G, S:P681H, S:T732A,	ORF1ab: 516-518, 3'UTR:29738-29760,
hCoV-19/Mexico/HID-IBT-IMSS-28/2021	EPI_ISL_1288247	In process	20B	B.1.1.519	21	5'UTR:C203T, 5'UTR:C222T, 5'UTR:C241T, ORF1ab:C3037T, ORF1ab:C3140T, ORF1ab:C10029T, ORF1ab:C10954T, ORF1ab:A11117G, ORF1ab:C12789T, ORF1ab:C12885T, ORF1ab:C14408T, ORF1ab:T19839C, ORF1ab:C21306T, S:C22995A, S:A23403G, S:C23604A, S:A23756G, E:G26367T, N:G28881A, N:G28882A. N:G28883C. N:C29197T.	12	N:R203K, N:G204R, ORF1a:P959S, ORF1a:T3255I, ORF1a:I3618V, ORF1a:T4175I, ORF1a:T4207I, ORF1b:P314L, S:T478K, S:D614G, S:P681H, S:T732A,	
hCoV-19/Mexico/HID-IBT-IMSS-332/2021	EPI_ISL_1288513	In process	20B	B.1.1.519	24	5'UTR:C203T, 5'UTR:C222T, 5'UTR:C241T, ORF1ab:C3037T, ORF1ab:C3140T, ORF1ab:G3971T, ORF1ab:C10029T, ORF1ab:C10954T, ORF1ab:A11117G, ORF1ab:C12789T, ORF1ab:C14408T, ORF1ab:C17678T, ORF1ab:C18246T, ORF1ab:T19839C, ORF1ab:C21306T, S:C22995A, S:A23403G, S:C23604A, S:A23756G, N:G28881A, N:G28882A, N:G28883C, N:C29197T, N:C29518T, N:G29527A,	13	N:R203K, N:G204R, ORF1a:P959S, ORF1a:V1236F, ORF1a:T3255I, ORF1a:I3618V, ORF1a:T4175I, ORF1b:P314L, ORF1b:T1404M, S:T478K, S:D614G, S:P681H, S:T732A,	
hCoV-19/Mexico/HID-IBT-IMSS-333/2021	EPI_ISL_1288514	In process	20B	B.1.1.519	21	5'UTR:C203T, 5'UTR:C222T, 5'UTR:C241T, ORF1ab:C1170T, ORF1ab:C3037T, ORF1ab:C3140T, ORF1ab:C10029T, ORF1ab:C10954T, ORF1ab:A11117G, ORF1ab:C12789T, ORF1ab:C14408T, ORF1ab:T19839C, ORF1ab:C21306T, S:C22995A, S:A23403G, S:C23604A, S:A23756G, M:G26828T, N:G28881A, N:G28882A. N:G28883C. N:C29197T.	12	N:R203K, N:G204R, ORF1a:S302F, ORF1a:P959S, ORF1a:T3255I, ORF1a:I3618V, ORF1a:T4175I, ORF1b:P314L, S:T478K, S:D614G, S:P681H, S:T732A,	
hCoV-19/Mexico/HID-IBT-IMSS-506/2020	EPI_ISL_1301463	In process	20A	B.1.189	10	5'UTR:C241T, ORF1ab:C3037T, ORF1ab:C3987T, ORF1ab:C4582T, ORF1ab:G6404T, ORF1ab:C8655T, ORF1ab:C9207T, ORF1ab:C14408T, S:A23403G, S:T25123C, N:G28378T,	7	ORF1a:T1241I, ORF1a:V2047F, ORF1a:S2797F, ORF1a:S2981F, ORF1b:P314L, ORF9b:R32L, S:D614G,	
hCoV-19/Mexico/HID-IBT-IMSS-508/2020	EPI_ISL_1301674	In process	20A	B.1.189	12	5'UTR:C241T, ORF1ab:C3037T, ORF1ab:A3427G, ORF1ab:C3987T, ORF1ab:C4582T, ORF1ab:G6404T, ORF1ab:C9207T, ORF1ab:C14408T, ORF1ab:A20268G, S:A23403G, S:T25123C, ORF7a:C27494T, N:G28378T,	7	ORF1a:T1241I, ORF1a:V2047F, ORF1a:S2981F, ORF1b:P314L, ORF7a:P34L, ORF9b:R32L, S:D614G,	
hCoV-19/Mexico/HID-IBT-IMSS-514/2021	EPI_ISL_1302235	In process	20B	B.1.1.519	26	5'UTR:T201C, 5'UTR:C203T, 5'UTR:C222T, 5'UTR:C241T, ORF1ab:G1738T, ORF1ab:C3037T, ORF1ab:C3140T, ORF1ab:C6395T, ORF1ab:C10029T, ORF1ab:C10954T, ORF1ab:A11117G, ORF1ab:G11299A, ORF1ab:C12068T, ORF1ab:A12421T, ORF1ab:C12789T, ORF1ab:C14408T, ORF1ab:T19839C, ORF1ab:A19974G, S:C22995A, S:A23403G, S:C23604A, S:A23756G, ORF8:A28175G, N:G28881A, N:G28882A, N:G28883C, N:C29197T,	11	N:R203K, N:G204R, ORF1a:P959S, ORF1a:T3255I, ORF1a:I3618V, ORF1a:T4175I, ORF1b:P314L, S:T478K, S:D614G, S:P681H, S:T732A,	
hCoV-19/Mexico/HID-IBT-IMSS-522/2021	EPI_ISL_1302264	In process	20B	B.1.1.432	21	5'UTR:C241T, ORF1ab:A1732G, ORF1ab:C3037T, ORF1ab:C3695T, ORF1ab:G5194T, ORF1ab:A6659G, ORF1ab:A6985T, ORF1ab:C9319T, ORF1ab:C10626T, ORF1ab:C11379T, ORF1ab:C12412T, ORF1ab:T14313C, ORF1ab:C14408T, S:A23403G, ORF3a:C25613T, ORF3a:G25912T, ORF8:G28077T, N:C28744T, N:G28881A, N:G28882A, N:G28883C, N:T29317C,	10	N:R203K, N:G204R, ORF1a:S2132G, ORF1a:A3454V, ORF1a:A3705V, ORF1b:P314L, ORF3a:S74F, ORF3a:G174C, ORF8:V62L, S:D614G,	

hCoV-19/Mexico/HID-InDRE-IBT-65/2020	EPI_ISL_1301569	In process	20B	B.1.1	10	5'UTR:C241T, ORF1ab:G1135T, ORF1ab:A2024G, ORF1ab:C3037T, ORF1ab:C14408T, ORF1ab:T19839C, S:A23403G, S:C25069T, N:G28881A, N:G28882A, N:G28883C,	6	N:R203K, N:G204R, ORF1a:K290N, ORF1a:T587A, ORF1b:P314L, S:D614G,
hCoV-19/Mexico/HID-InDRE-IBT-66/2020	EPI_ISL_1301549	In process	20B	B.1.1	10	5'UTR:C241T, ORF1ab:G1942A, ORF1ab:C3037T, ORF1ab:G10360A, ORF1ab:C14408T, S:A23403G, M:C26996T, ORF8:C27945T, N:G28881A, N:G28882A, N:G28883C,	5	N:R203K, N:G204R, ORF1b:P314L, ORF8:Q18*, S:D614G,
hCoV-19/Mexico/HID-InDRE-IBT-67/2020	EPI_ISL_1301476	In process	20A	B.1.243	6	5'UTR:C241T, ORF1ab:C3037T, ORF1ab:C14408T, S:G22093T, S:A23403G, S:T24076C, N:C28854T,	4	N:S194L, ORF1b:P314L, S:M177I, S:D614G,
hCoV-19/Mexico/HID-INER-IMSS-00114/2021	EPI_ISL_1279397	In process	20B	B.1.1.519	24	5'UTR:C203T, 5'UTR:C222T, 5'UTR:C241T, ORF1ab:C835T, ORF1ab:C1377T, ORF1ab:C3037T, ORF1ab:C3140T, ORF1ab:C10029T, ORF1ab:C10954T, ORF1ab:A11117G, ORF1ab:C12789T, ORF1ab:C14408T, ORF1ab:G15921T, ORF1ab:T19839C, ORF1ab:A20055G, ORF1ab:C21306T, S:C21575T, S:C22995A, S:A23403G, S:C23604A, S:A23756G, N:G28881A, N:G28882A, N:G28883C, N:C29197T,	13	N:R203K, N:G204R, ORF1a:P371L, ORF1a:P959S, ORF1a:T3255I, ORF1a:I3618V, ORF1a:T4175I, ORF1b:P314L, S:L5F, S:T478K, S:D614G, S:P681H, S:T732A,
hCoV-19/Mexico/HID-INER-IMSS-00330/2021	EPI_ISL_1279578	In process	20B	B.1.1.519	23	5'UTR:C203T, 5'UTR:C222T, 5'UTR:C241T, ORF1ab:C3037T, ORF1ab:C3140T, ORF1ab:C9693T, ORF1ab:C10029T, ORF1ab:C10954T, ORF1ab:A11117G, ORF1ab:G11365T, ORF1ab:C12789T, ORF1ab:C14408T, ORF1ab:T19839C, ORF1ab:C21306T, S:C22995A, S:A23403G, S:C23604A, S:A23756G, M:T26861C, N:G28881A, N:G28882A, N:G28883C, N:C29197T, N:G29527T,	13	N:R203K, N:G204R, N:Q418H, ORF1a:P959S, ORF1a:A3143V, ORF1a:T3255I, ORF1a:I3618V, ORF1a:T4175I, ORF1b:P314L, S:T478K, S:D614G, S:P681H, S:T732A,
hCoV-19/Mexico/HID-INER-IMSS-00332/2021	EPI_ISL_1279580	In process	20B	B.1.1.519	25	5'UTR:C203T, 5'UTR:C222T, 5'UTR:C241T, ORF1ab:C3037T, ORF1ab:C3140T, ORF1ab:G3955T, ORF1ab:C10029T, ORF1ab:C10954T, ORF1ab:G11083T, ORF1ab:A11117G, ORF1ab:G11365T, ORF1ab:C12789T, ORF1ab:C14408T, ORF1ab:T16958C, ORF1ab:T19839C, ORF1ab:C21306T, S:A22259G, S:C22995A, S:A23403G, S:C23604A, S:A23756G, N:G28881A, N:G28882A, N:G28883C, N:C29197T, N:G29527T,	16	N:R203K, N:G204R, N:Q418H, ORF1a:P959S, ORF1a:K1230N, ORF1a:T3255I, ORF1a:L3606F, ORF1a:I3618V, ORF1a:T4175I, ORF1b:P314L, ORF1b:V1164A, S:I233V, S:T478K, S:D614G, S:P681H, S:T732A,
hCoV-19/Mexico/JAL-InDRE-IBT-138/2020	EPI_ISL_1301710	In process	20A	B.1.609	13	5'UTR:C241T, ORF1ab:C3037T, ORF1ab:C4582T, ORF1ab:G6364T, ORF1ab:C13119T, ORF1ab:G14250T, ORF1ab:C14408T, ORF1ab:A16076G, ORF1ab:A20036G, ORF1ab:A20268G, S:A23403G, ORF3a:C25721T, N:C28854T, 3'UTR:C29774T,	8	N:S194L, ORF1a:A4285V, ORF1b:L261F, ORF1b:P314L, ORF1b:D870G, ORF1b:N2190S, ORF3a:A110V, S:D614G,
hCoV-19/Mexico/JAL-InDRE-IBT-139/2020	EPI_ISL_1301711	In process	20B	B.1.1.222	14	5'UTR:C241T, ORF1ab:C3037T, ORF1ab:C3317T, ORF1ab:T5218C, ORF1ab:C8890T, ORF1ab:T10687C, ORF1ab:C14408T, ORF1ab:C15182T, ORF1ab:T19839C, S:A23403G, S:A23756G, N:G28881A, N:G28882A, N:G28883C, N:G29449T,	7	N:R203K, N:G204R, ORF1a:P1018S, ORF1b:P314L, ORF1b:A572V, S:D614G, S:T732A,
hCoV-19/Mexico/JAL-InDRE-IBT-140/2020	EPI_ISL_1301712	In process	20A	B.1	9	5'UTR:C241T, ORF1ab:G1548A, ORF1ab:C2509T, ORF1ab:C3037T, ORF1ab:A5723G, ORF1ab:C8986T, ORF1ab:C14408T, ORF1ab:A20268G, S:A23403G, N:G28808C,	5	N:G179R, ORF1a:S428N, ORF1a:T1820A, ORF1b:P314L, S:D614G,
hCoV-19/Mexico/JAL-InDRE-IBT-141/2020	EPI_ISL_1301454	In process	20A	B.1.189	16	5'UTR:C241T, ORF1ab:C3037T, ORF1ab:C4582T, ORF1ab:C5974T, ORF1ab:G6404T, ORF1ab:C7840T, ORF1ab:G11417T, ORF1ab:T11541G, ORF1ab:C14408T, ORF1ab:C14724T, ORF1ab:T16035C, S:A23403G, S:G24620T, S:T25123C, ORF3a:C25463A, N:G28300T, N:C28748T,	9	N:Q9H, ORF1a:V2047F, ORF1a:V3718F, ORF1a:V3759G, ORF1b:P314L, ORF3a:T24N, ORF9b:S6I, S:D614G, S:A1020S,
hCoV-19/Mexico/JAL-InDRE-IBT-142/2020	EPI_ISL_1301713	In process	20A	B.1.558	14	5'UTR:C241T, ORF1ab:C1288T, ORF1ab:C2445T, ORF1ab:C3037T, ORF1ab:C3096T, ORF1ab:A5999G, ORF1ab:C11575T, ORF1ab:A14013G, ORF1ab:C14408T, ORF1ab:C18060T, ORF1ab:T19791G, S:A23403G, M:T26729C, N:C28854T, N:G29402T,	7	N:S194L, N:D377Y, ORF1a:T727I, ORF1a:S944L, ORF1a:I1912V, ORF1b:P314L, S:D614G,

hCoV-19/Mexico/JAL-InDRE-IBT-143/2020	EPI_ISL_1301592	In process	20A	B.1	8	5'UTR:C241T, ORF1ab:C3037T, ORF1ab:G9802T, ORF1ab:C14408T, ORF1ab:A20268G, S:A23403G, S:C24138T, N:C28854T, 3'UTR:C29750T,	4	N:S194L, ORF1b:P314L, S:D614G, S:T859I,
hCoV-19/Mexico/MEX-IBT-IMSS-04/2020	EPI_ISL_955246	In process	20A	B.1	7	5'UTR:C241T, ORF1ab:A2205G, ORF1ab:C3037T, ORF1ab:T3493C, ORF1ab:A6659G, ORF1ab:C14408T, ORF1ab:A20268G, S:A23403G,	4	ORF1a:E647G, ORF1a:S2132G, ORF1b:P314L, S:D614G,
hCoV-19/Mexico/MEX-IBT-IMSS-164/2021	EPI_ISL_1288365	In process	20B	B.1.1.519	26	5'UTR:G61T, 5'UTR:C203T, 5'UTR:C222T, 5'UTR:C241T, ORF1ab:C3037T, ORF1ab:C3140T, ORF1ab:C8802T, ORF1ab:C10029T, ORF1ab:C10954T, ORF1ab:A11117G, ORF1ab:C12789T, ORF1ab:T13905C, ORF1ab:C14408T, ORF1ab:T19839C, ORF1ab:C21306T, S:C22995A, S:A23403G, S:C23604A, S:A23756G, ORF3a:G25906T, ORF3a:C25916T, M:G26526T, N:G28881A, N:G28882A, N:G28883C, N:C29197T, 3'UTR:C29769T,	15	M:A2S, N:R203K, N:G204R, ORF1a:P959S, ORF1a:T2846I, ORF1a:T3255I, ORF1a:I3618V, ORF1a:T4175I, ORF1b:P314L, ORF3a:G172C, ORF3a:T175I, S:T478K, S:D614G, S:P681H, S:T732A,
hCoV-19/Mexico/MEX-IBT-IMSS-165/2021	EPI_ISL_1288366	In process	20B	B.1.1.519	21	5'UTR:C203T, 5'UTR:C222T, 5'UTR:C241T, ORF1ab:G960A, ORF1ab:C3037T, ORF1ab:C3140T, ORF1ab:C3448T, ORF1ab:C10029T, ORF1ab:C10954T, ORF1ab:A11117G, ORF1ab:C12789T, ORF1ab:C14408T, ORF1ab:T19839C, ORF1ab:C21306T, S:C22995A, S:A23403G, S:C23604A, S:A23756G, N:G28881A, N:G28882A, N:G28883C, N:C29197T,	12	N:R203K, N:G204R, ORF1a:R232H, ORF1a:P959S, ORF1a:T3255I, ORF1a:I3618V, ORF1a:T4175I, ORF1b:P314L, S:T478K, S:D614G, S:P681H, S:T732A,
hCoV-19/Mexico/MEX-IBT-IMSS-166/2021	EPI_ISL_1288367	In process	20B	B.1.1.519	28	5'UTR:C203T, 5'UTR:C222T, 5'UTR:C241T, ORF1ab:C843A, ORF1ab:G942A, ORF1ab:C3037T, ORF1ab:C3140T, ORF1ab:C10029T, ORF1ab:C10138T, ORF1ab:C10954T, ORF1ab:A11117G, ORF1ab:C11124T, ORF1ab:C12789T, ORF1ab:C14408T, ORF1ab:G17562T, ORF1ab:T19839C, ORF1ab:A20676G, ORF1ab:C21306T, S:C21575T, S:C21772T, S:C22995A, S:A23403G, S:C23604A, S:A23756G, N:A28522G, N:G28881A, N:G28882A, N:G28883C, N:C29197T	16	N:R203K, N:G204R, ORF1a:P193H, ORF1a:R226K, ORF1a:P959S, ORF1a:T3255I, ORF1a:I3618V, ORF1a:A3620V, ORF1a:T4175I, ORF1b:P314L, ORF9b:K80R, S:L5F, S:T478K, S:D614G, S:P681H, S:T732A,
hCoV-19/Mexico/MEX-IBT-IMSS-167/2021	EPI_ISL_1288368	In process	20B	B.1.1.519	24	5'UTR:C203T, 5'UTR:C222T, 5'UTR:C241T, ORF1ab:C3037T, ORF1ab:C3140T, ORF1ab:C10029T, ORF1ab:C10954T, ORF1ab:A11117G, ORF1ab:C12789T, ORF1ab:C14408T, ORF1ab:C18086T, ORF1ab:C18348T, ORF1ab:T19839C, ORF1ab:C21306T, S:C22995A, S:A23403G, S:C23604A, S:A23756G, S:C24034T, N:A28850G, N:G28881A, N:G28882A, N:G28883C, N:C29197T, N:G29527T,	14	N:S193G, N:R203K, N:G204R, N:Q418H, ORF1a:P959S, ORF1a:T3255I, ORF1a:I3618V, ORF1a:T4175I, ORF1b:P314L, ORF1b:T1540I, S:T478K, S:D614G, S:P681H, S:T732A,
hCoV-19/Mexico/MEX-IBT-IMSS-169/2021	EPI_ISL_1288370	In process	20B	B.1.1.519	24	5'UTR:C203T, 5'UTR:C222T, 5'UTR:C241T, ORF1ab:C3037T, ORF1ab:C3140T, ORF1ab:C10029T, ORF1ab:C10954T, ORF1ab:A11117G, ORF1ab:G12031T, ORF1ab:C12789T, ORF1ab:T13905C, ORF1ab:C14408T, ORF1ab:T19839C, ORF1ab:C21306T, S:C22995A, S:A23403G, S:C23604A, S:A23756G, S:C24795T, ORF3a:G25906T, ORF3a:C25916T, N:G28881A, N:G28882A, N:G28883C, N:C29197T,	15	N:R203K, N:G204R, ORF1a:P959S, ORF1a:T3255I, ORF1a:I3618V, ORF1a:Q3922H, ORF1a:T4175I, ORF1b:P314L, ORF3a:G172C, ORF3a:T175I, S:T478K, S:D614G, S:P681H, S:T732A, S:A1078V,

hCoV-19/Mexico/MEX-IBT-IMSS-177/2021	EPI_ISL_1288376	In process	20B	B.1.1.519	33	5'UTR:T201C, 5'UTR:C203T, 5'UTR:C222T, 5'UTR:C241T, ORF1ab:G1738T, ORF1ab:A1809G, ORF1ab:C3037T, ORF1ab:C3140T, ORF1ab:T4159C, ORF1ab:C9438T, ORF1ab:C10029T, ORF1ab:C10954T, ORF1ab:A11117G, ORF1ab:G12038A, ORF1ab:C12789T, ORF1ab:C14408T, ORF1ab:G14559T, ORF1ab:G18079T, ORF1ab:T19839C, ORF1ab:A19974G, ORF1ab:C21306T, S:C22995A, S:A23403G, S:C23604A, S:A23756G, ORF3a:G25538T, ORF3a:G25699A, M:C27075A, ORF7a:G27506T, ORF7a:G27709T, N:G28881A, N:G28882A, N:G28883C, N:C29197T,	20	M:Q185K, N:R203K, N:G204R, ORF1a:K515R, ORF1a:P959S, ORF1a:T3058I, ORF1a:T3255I, ORF1a:I3618V, ORF1a:V3925I, ORF1a:T4175I, ORF1b:P314L, ORF1b:V1538L, ORF3a:G49V, ORF3a:A103T, ORF7a:G38V, ORF7a:A106S, S:T478K, S:D614G, S:P681H, S:T732A,	
hCoV-19/Mexico/MEX-IBT-IMSS-178/2021	EPI_ISL_1288377	In process	20B	B.1.1.519	26	5'UTR:C203T, 5'UTR:C222T, 5'UTR:C241T, ORF1ab:C3037T, ORF1ab:C3140T, ORF1ab:C4780T, ORF1ab:A6613G, ORF1ab:G8861A, ORF1ab:G9973A, ORF1ab:C10029T, ORF1ab:C10954T, ORF1ab:A11117G, ORF1ab:C12789T, ORF1ab:C14408T, ORF1ab:T19839C, ORF1ab:C21306T, S:C22995A, S:A23403G, S:C23604A, S:A23756G, S:C24795T, N:G28881A, N:G28882A, N:G28883C, N:C29197T, N:G29405C, N:G29527T,	15	N:R203K, N:G204R, N:E378Q, N:Q418H, ORF1a:P959S, ORF1a:V2866M, ORF1a:T3255I, ORF1a:I3618V, ORF1a:T4175I, ORF1b:P314L, S:T478K, S:D614G, S:P681H, S:T732A, S:A1078V,	
hCoV-19/Mexico/MEX-IBT-IMSS-179/2021	EPI_ISL_1288378	In process	20B	B.1.1.519	27	5'UTR:C203T, 5'UTR:C222T, 5'UTR:C241T, ORF1ab:C3037T, ORF1ab:C3140T, ORF1ab:T3745C, ORF1ab:C7594T, ORF1ab:T9421C, ORF1ab:C10029T, ORF1ab:C10954T, ORF1ab:A11117G, ORF1ab:C12789T, ORF1ab:C14408T, ORF1ab:T16419G, ORF1ab:C17012T, ORF1ab:T19839C, ORF1ab:C21306T, S:C22995A, S:A23403G, S:C23604A, S:A23756G, ORF3a:G25523T, ORF3a:C25844T, N:G28881A, N:G28882A, N:G28883C, N:C29197T, N:C29466T,	15	N:R203K, N:G204R, N:A398V, ORF1a:P959S, ORF1a:T3255I, ORF1a:I3618V, ORF1a:T4175I, ORF1b:P314L, ORF1b:S1182L, ORF3a:G44V, ORF3a:T151I, S:T478K, S:D614G, S:P681H, S:T732A,	
hCoV-19/Mexico/MEX-IBT-IMSS-180/2021	EPI_ISL_1288379	In process	20B	B.1.1.519	29	5'UTR:C203T, 5'UTR:C222T, 5'UTR:C241T, ORF1ab:C1122T, ORF1ab:C3037T, ORF1ab:C3140T, ORF1ab:C10029T, ORF1ab:C10954T, ORF1ab:A11117G, ORF1ab:C12789T, ORF1ab:A13098G, ORF1ab:C14408T, ORF1ab:G19174T, ORF1ab:C19263T, ORF1ab:T19839C, ORF1ab:C21306T, S:C22995A, S:A23403G, S:C23604A, S:A23756G, S:C24802T, S:G25244T, ORF3a:C25521T, ORF8:T27904C, ORF8:C28087T, N:G28881A, N:G28882A, N:G28883C, N:C29197T, 3'UTR:G29736T,	17	N:R203K, N:G204R, ORF1a:P286L, ORF1a:P959S, ORF1a:T3255I, ORF1a:I3618V, ORF1a:T4175I, ORF1a:K4278R, ORF1b:P314L, ORF1b:D1903Y, ORF8:L4P, ORF8:A65V, S:T478K, S:D614G, S:P681H, S:T732A, S:V1228L,	N:28766-28768,
hCoV-19/Mexico/MEX-IBT-IMSS-181/2021	EPI_ISL_1288380	In process	20B	B.1.1.519	29	5'UTR:C203T, 5'UTR:C222T, 5'UTR:C241T, ORF1ab:C3037T, ORF1ab:C3140T, ORF1ab:T3166A, ORF1ab:G9805T, ORF1ab:C10029T, ORF1ab:C10954T, ORF1ab:A11117G, ORF1ab:C12789T, ORF1ab:C14408T, ORF1ab:T19839C, ORF1ab:C21306T, S:C21622T, S:C22995A, S:A23403G, S:C23604A, S:A23756G, S:C24916T, M:T27080C, M:C27110T, ORF7a:G27601T, ORF8:T28002C, N:G28881A, N:G28882A, N:G28883C, N:T28906C, N:C29197T, ORF10:T29661C,	13	N:R203K, N:G204R, ORF1a:P959S, ORF1a:T3255I, ORF1a:I3618V, ORF1a:T4175I, ORF1b:P314L, ORF7a:G70C, ORF8:C37R, S:T478K, S:D614G, S:P681H, S:T732A,	

hCoV-19/Mexico/MEX-IBT-IMSS-183/2021	EPI_ISL_1288381	In process	20B	B.1.1.519	25	5'UTR:C203T, 5'UTR:C222T, 5'UTR:C241T, ORF1ab:C1218T, ORF1ab:A2219G, ORF1ab:C3037T, ORF1ab:C3140T, ORF1ab:C10029T, ORF1ab:C10078T, ORF1ab:C10954T, ORF1ab:A11117G, ORF1ab:G11365T, ORF1ab:C12789T, ORF1ab:C14408T, ORF1ab:T19839C, ORF1ab:G21004T, ORF1ab:C21306T, S:C22995A, S:A23403G, S:C23604A, S:A23756G, N:G28881A, N:G28882A, N:G28883C, N:C29197T, N:G29527T,	15	N:R203K, N:G204R, N:Q418H, ORF1a:S318L, ORF1a:I652V, ORF1a:P959S, ORF1a:T3255I, ORF1a:I3618V, ORF1a:T4175I, ORF1b:P314L, ORF1b:A2513S, S:T478K, S:D614G, S:P681H, S:T732A,
hCoV-19/Mexico/MEX-IBT-IMSS-184/2021	EPI_ISL_1288382	In process	20B	B.1.1.519	22	5'UTR:C203T, 5'UTR:C222T, 5'UTR:C241T, ORF1ab:A2730C, ORF1ab:C3037T, ORF1ab:C3140T, ORF1ab:C10029T, ORF1ab:C10954T, ORF1ab:A11117G, ORF1ab:C12789T, ORF1ab:C14408T, ORF1ab:T19839C, ORF1ab:G20419T, ORF1ab:C21306T, S:C22995A, S:A23403G, S:C23604A, S:A23756G, ORF3a:G25906T, N:G28881A, N:G28882A, N:G28883C, N:C29197T,	14	N:R203K, N:G204R, ORF1a:K822T, ORF1a:P959S, ORF1a:T3255I, ORF1a:I3618V, ORF1a:T4175I, ORF1b:P314L, ORF1b:D2318Y, ORF3a:G172C, S:T478K, S:D614G, S:P681H, S:T732A,
hCoV-19/Mexico/MEX-IBT-IMSS-185/2021	EPI_ISL_1288383	In process	20B	B.1.1.519	23	5'UTR:C203T, 5'UTR:C222T, 5'UTR:C241T, ORF1ab:C344T, ORF1ab:C3037T, ORF1ab:C3140T, ORF1ab:C10029T, ORF1ab:C10954T, ORF1ab:A11117G, ORF1ab:C12789T, ORF1ab:C14408T, ORF1ab:C16293T, ORF1ab:T19839C, ORF1ab:C21306T, S:C22995A, S:A23403G, S:C23604A, S:A23756G, ORF8:C28253T, N:G28881A, N:G28882A, N:G28883C, N:C29197T, N:G29527T,	13	N:R203K, N:G204R, N:Q418H, ORF1a:L27F, ORF1a:P959S, ORF1a:T3255I, ORF1a:I3618V, ORF1a:T4175I, ORF1b:P314L, S:T478K, S:D614G, S:P681H, S:T732A,
hCoV-19/Mexico/MEX-IBT-IMSS-186/2021	EPI_ISL_1288384	In process	20B	B.1.1.519	23	5'UTR:C203T, 5'UTR:C222T, 5'UTR:C241T, ORF1ab:C3037T, ORF1ab:C3140T, ORF1ab:C10029T, ORF1ab:C10954T, ORF1ab:A11117G, ORF1ab:C12789T, ORF1ab:C14408T, ORF1ab:C19602T, ORF1ab:T19839C, ORF1ab:C21306T, S:C22995A, S:A23403G, S:C23604A, S:A23756G, ORF3a:G25775T, ORF3a:G25906T, ORF8:C28253T, N:G28881A, N:G28882A, N:G28883C, N:C29197T,	13	N:R203K, N:G204R, ORF1a:P959S, ORF1a:T3255I, ORF1a:I3618V, ORF1a:T4175I, ORF1b:P314L, ORF3a:W128L, ORF3a:G172C, S:T478K, S:D614G, S:P681H, S:T732A,
hCoV-19/Mexico/MEX-IBT-IMSS-187/2021	EPI_ISL_1288385	In process	20B	B.1.1.222	22	5'UTR:C241T, ORF1ab:C2676T, ORF1ab:C3037T, ORF1ab:C8389T, ORF1ab:C10029T, ORF1ab:C14408T, ORF1ab:G17721T, ORF1ab:T19839C, S:A23403G, S:G23522C, S:A23756G, S:C24374T, ORF3a:G25567A, ORF3a:T25569A, ORF3a:T25570A, ORF3a:G25912T, ORF3a:A26108G, ORF8:A27921G, ORF8:G28001T, N:G28881A, N:G28882A, N:G28883C, N:C29167T,	14	N:R203K, N:G204R, ORF1a:P804L, ORF1a:T3255I, ORF1b:P314L, ORF3a:A59T, ORF3a:S60T, ORF3a:G174C, ORF3a:E239G, ORF8:I10V, S:D614G, S:E654Q, S:T732A, S:L938F,
hCoV-19/Mexico/MEX-IBT-IMSS-188/2021	EPI_ISL_1288386	In process	20B	B.1.1.519	25	5'UTR:C203T, 5'UTR:C222T, 5'UTR:C241T, ORF1ab:C3037T, ORF1ab:C3140T, ORF1ab:C3162T, ORF1ab:G7042T, ORF1ab:C10029T, ORF1ab:C10954T, ORF1ab:A11117G, ORF1ab:G12310A, ORF1ab:C12789T, ORF1ab:C14408T, ORF1ab:G15921T, ORF1ab:A17971G, ORF1ab:T19839C, ORF1ab:C21306T, S:C22995A, S:A23403G, S:C23604A, S:C23635T, S:A23756G, N:G28881A, N:G28882A, N:G28883C, N:C29197T,	14	N:R203K, N:G204R, ORF1a:P959S, ORF1a:S966F, ORF1a:M2259I, ORF1a:T3255I, ORF1a:I3618V, ORF1a:T4175I, ORF1b:P314L, ORF1b:R1502G, S:T478K, S:D614G, S:P681H, S:T732A,
hCoV-19/Mexico/MEX-IBT-IMSS-19/2020	EPI_ISL_955247	In process	20A	B.1	7	5'UTR:C241T, ORF1ab:C3037T, ORF1ab:C13329T, ORF1ab:C14408T, ORF1ab:A20268G, S:A23403G, ORF3a:C25413T, E:C26461T,	4	E:L73F, ORF1a:T4355I, ORF1b:P314L, S:D614G,

hCoV-19/Mexico/MEX-IBT-IMSS-197/2021	EPI_ISL_1288394	In process	20B	B.1.1.519	22	5'UTR:C203T, 5'UTR:C222T, 5'UTR:C241T, ORF1ab:C3037T, ORF1ab:C3140T, ORF1ab:C7860T, ORF1ab:C10029T, ORF1ab:C10954T, ORF1ab:A11117G, ORF1ab:G11417T, ORF1ab:C12789T, ORF1ab:C14408T, ORF1ab:T19839C, ORF1ab:C21306T, S:C22995A, S:A23403G, S:C23604A, S:A23756G, ORF7a:C27707T, N:G28881A, N:G28882A, N:G28883C, N:C29197T,	14	N:R203K, N:G204R, ORF1a:P959S, ORF1a:T2532I, ORF1a:T3255I, ORF1a:I3618V, ORF1a:V3718F, ORF1a:T4175I, ORF1b:P314L, ORF7a:A105V, S:T478K, S:D614G, S:P681H, S:T732A,
hCoV-19/Mexico/MEX-IBT-IMSS-198/2021	EPI_ISL_1288395	In process	20B	B.1.1.519	25	5'UTR:T201C, 5'UTR:C203T, 5'UTR:C222T, 5'UTR:C241T, ORF1ab:C3037T, ORF1ab:C3140T, ORF1ab:C10029T, ORF1ab:C10954T, ORF1ab:A11117G, ORF1ab:C12789T, ORF1ab:C14408T, ORF1ab:C14724T, ORF1ab:G17110T, ORF1ab:C18377T, ORF1ab:T19839C, ORF1ab:A19974G, ORF1ab:C21306T, S:C22995A, S:A23403G, S:C23604A, S:A23756G, N:C28732T, N:G28881A, N:G28882A, N:G28883C, N:C29197T,	13	N:R203K, N:G204R, ORF1a:P959S, ORF1a:T3255I, ORF1a:I3618V, ORF1a:T4175I, ORF1b:P314L, ORF1b:A1215S, ORF1b:T1637I, S:T478K, S:D614G, S:P681H, S:T732A,
hCoV-19/Mexico/MEX-IBT-IMSS-199/2021	EPI_ISL_1288396	In process	20B	B.1.1.519	25	5'UTR:C203T, 5'UTR:C222T, 5'UTR:C241T, ORF1ab:C2857T, ORF1ab:C3037T, ORF1ab:C3140T, ORF1ab:T5820C, ORF1ab:C10029T, ORF1ab:C10954T, ORF1ab:A11117G, ORF1ab:C12473T, ORF1ab:C12789T, ORF1ab:C14408T, ORF1ab:T19839C, ORF1ab:C21306T, S:C22995A, S:A23403G, S:C23604A, S:A23756G, ORF3a:C25528T, M:C26586T, N:G28881A, N:G28882A, N:G28883C, N:C29197T, N:C29353T,	13	N:R203K, N:G204R, ORF1a:P959S, ORF1a:L1852S, ORF1a:T3255I, ORF1a:I3618V, ORF1a:T4175I, ORF1b:P314L, ORF3a:L46F, S:T478K, S:D614G, S:P681H, S:T732A,
hCoV-19/Mexico/MEX-IBT-IMSS-200/2021	EPI_ISL_1288397	In process	20B	B.1.1.222	23	5'UTR:C241T, ORF1ab:C2919T, ORF1ab:C3037T, ORF1ab:C4897T, ORF1ab:C5259T, ORF1ab:C10029T, ORF1ab:A11430G, ORF1ab:C14408T, ORF1ab:C14649T, ORF1ab:G17259T, ORF1ab:C18329T, ORF1ab:C19011A, ORF1ab:G19446A, ORF1ab:T19839C, ORF1ab:A21137G, S:A23403G, S:A23756G, ORF3a:G25912T, ORF3a:A26115C, ORF8:A27921G, ORF8:G28001T, N:G28881A, N:G28882A, N:G28883C,	16	N:R203K, N:G204R, ORF1a:P885L, ORF1a:T1665I, ORF1a:T3255I, ORF1a:Y3722C, ORF1b:P314L, ORF1b:E1264D, ORF1b:T1621I, ORF1b:D1848E, ORF1b:K2557R, ORF3a:G174C, ORF3a:E241D, ORF8:I10V, S:D614G, S:T732A,
hCoV-19/Mexico/MEX-IBT-IMSS-201/2021	EPI_ISL_1288398	In process	20B	B.1.1.519	24	5'UTR:C203T, 5'UTR:C222T, 5'UTR:C241T, ORF1ab:C3037T, ORF1ab:C3140T, ORF1ab:T3745C, ORF1ab:A6948G, ORF1ab:C10029T, ORF1ab:C10954T, ORF1ab:A11117G, ORF1ab:C12789T, ORF1ab:C14408T, ORF1ab:G19816T, ORF1ab:T19839C, ORF1ab:C21306T, S:C22995A, S:A23403G, S:C23422T, S:C23604A, S:A23756G, ORF3a:C25844T, N:G28881A, N:G28882A, N:G28883C, N:C29197T,	14	N:R203K, N:G204R, ORF1a:P959S, ORF1a:N2228S, ORF1a:T3255I, ORF1a:I3618V, ORF1a:T4175I, ORF1b:P314L, ORF1b:V2117L, ORF3a:T151I, S:T478K, S:D614G, S:P681H, S:T732A,
hCoV-19/Mexico/MEX-IBT-IMSS-202/2021	EPI_ISL_1288399	In process	20B	B.1.1.519	23	5'UTR:C203T, 5'UTR:C222T, 5'UTR:C241T, ORF1ab:C3037T, ORF1ab:C3140T, ORF1ab:C9985T, ORF1ab:C10029T, ORF1ab:C10954T, ORF1ab:A11117G, ORF1ab:C12789T, ORF1ab:C14408T, ORF1ab:G17964T, ORF1ab:G19518T, ORF1ab:T19839C, ORF1ab:C21306T, S:C22995A, S:A23403G, S:C23604A, S:A23756G, N:G28881A, N:G28882A, N:G28883C, N:C29197T, N:G29527T,	14	N:R203K, N:G204R, N:Q418H, ORF1a:P959S, ORF1a:T3255I, ORF1a:I3618V, ORF1a:T4175I, ORF1b:P314L, ORF1b:M1499I, ORF1b:L2017F, S:T478K, S:D614G, S:P681H, S:T732A,

hCoV-19/Mexico/MEX-IBT-IMSS-203/2021	EPI_ISL_1288400	In process	20B	B.1.1.519	25	5'UTR:C203T, 5'UTR:C222T, 5'UTR:C241T, ORF1ab:C3037T, ORF1ab:C3140T, ORF1ab:C3787T, ORF1ab:A5128G, ORF1ab:C10029T, ORF1ab:C10954T, ORF1ab:A11117G, ORF1ab:C12225T, ORF1ab:C12789T, ORF1ab:C14408T, ORF1ab:T19839C, ORF1ab:C21306T, S:G22335T, S:G22778C, S:C22995A, S:A23403G, S:C23604A, S:A23756G, N:G28881A, N:G28882A, N:G28883C, N:C29197T, N:G29527T,	15	N:R203K, N:G204R, N:Q418H, ORF1a:P959S, ORF1a:T3255I, ORF1a:I3618V, ORF1a:A3987V, ORF1a:T4175I, ORF1b:P314L, S:W258L, S:E406Q, S:T478K, S:D614G, S:P681H, S:T732A,
hCoV-19/Mexico/MEX-IBT-IMSS-205/2021	EPI_ISL_1288401	In process	20B	B.1.1.519	23	5'UTR:C203T, 5'UTR:C222T, 5'UTR:C241T, ORF1ab:C3037T, ORF1ab:C3140T, ORF1ab:C3646T, ORF1ab:C10029T, ORF1ab:C10954T, ORF1ab:A11117G, ORF1ab:C12789T, ORF1ab:C14408T, ORF1ab:T19839C, ORF1ab:C21306T, S:C22995A, S:A23403G, S:C23604A, S:A23756G, ORF3a:C25587T, ORF8:C28253T, N:G28881A, N:G28882A, N:G28883C, N:C29197T, N:G29527T,	12	N:R203K, N:G204R, N:Q418H, ORF1a:P959S, ORF1a:T3255I, ORF1a:I3618V, ORF1a:T4175I, ORF1b:P314L, S:T478K, S:D614G, S:P681H, S:T732A,
hCoV-19/Mexico/MEX-IBT-IMSS-206/2021	EPI_ISL_1288402	In process	20B	B.1.1.519	22	5'UTR:C203T, 5'UTR:C222T, 5'UTR:C241T, ORF1ab:C3037T, ORF1ab:C3140T, ORF1ab:C10029T, ORF1ab:C10954T, ORF1ab:A11117G, ORF1ab:C12789T, ORF1ab:C14408T, ORF1ab:T19839C, ORF1ab:C21306T, S:C22995A, S:A23403G, S:C23604A, S:A23756G, ORF8:T27904C, ORF8:C28087T, N:G28328A, N:G28881A, N:G28882A, N:G28883C, N:C29197T,	14	N:G19R, N:R203K, N:G204R, ORF1a:P959S, ORF1a:T3255I, ORF1a:I3618V, ORF1a:T4175I, ORF1b:P314L, ORF8:L4P, ORF8:A65V, S:T478K, S:D614G, S:P681H, S:T732A,
hCoV-19/Mexico/MEX-IBT-IMSS-207/2021	EPI_ISL_1288403	In process	20B	B.1.1.519	25	5'UTR:T201C, 5'UTR:C203T, 5'UTR:C222T, 5'UTR:C241T, ORF1ab:C745T, ORF1ab:G1738T, ORF1ab:C3037T, ORF1ab:C3140T, ORF1ab:C10029T, ORF1ab:C10954T, ORF1ab:A11117G, ORF1ab:C12789T, ORF1ab:C14408T, ORF1ab:T19839C, ORF1ab:A19974G, ORF1ab:C21306T, S:C22995A, S:A23403G, S:C23604A, S:A23756G, ORF3a:C25413T, ORF8:G28077T, N:G28881A, N:G28882A, N:G28883C, N:C29197T,	12	N:R203K, N:G204R, ORF1a:P959S, ORF1a:T3255I, ORF1a:I3618V, ORF1a:T4175I, ORF1b:P314L, ORF8:V62L, S:T478K, S:D614G, S:P681H, S:T732A,
hCoV-19/Mexico/MEX-IBT-IMSS-208/2021	EPI_ISL_1288404	In process	20B	B.1.1.519	23	5'UTR:C203T, 5'UTR:C222T, 5'UTR:C241T, ORF1ab:C2388T, ORF1ab:C2925T, ORF1ab:C3037T, ORF1ab:C3140T, ORF1ab:G9973A, ORF1ab:C10029T, ORF1ab:C10954T, ORF1ab:A11117G, ORF1ab:C12789T, ORF1ab:C14408T, ORF1ab:T19839C, ORF1ab:C21306T, S:C22995A, S:C23191T, S:A23403G, S:C23604A, S:A23756G, N:G28881A, N:G28882A, N:G28883C, N:C29197T,	13	N:R203K, N:G204R, ORF1a:T708I, ORF1a:S887F, ORF1a:P959S, ORF1a:T3255I, ORF1a:I3618V, ORF1a:T4175I, ORF1b:P314L, S:T478K, S:D614G, S:P681H, S:T732A,
hCoV-19/Mexico/MEX-IBT-IMSS-209/2021	EPI_ISL_1288405	In process	20B	B.1.1.519	25	5'UTR:C203T, 5'UTR:C222T, 5'UTR:C241T, ORF1ab:G1928T, ORF1ab:C3037T, ORF1ab:C3140T, ORF1ab:C6633T, ORF1ab:C8092T, ORF1ab:C10029T, ORF1ab:C10954T, ORF1ab:A11117G, ORF1ab:C12789T, ORF1ab:C14408T, ORF1ab:G19684T, ORF1ab:T19839C, ORF1ab:C21306T, S:T21644C, S:C22995A, S:A23403G, S:C23604A, S:A23756G, ORF3a:C25528T, N:G28881A, N:G28882A, N:G28883C, N:C29197T,	16	N:R203K, N:G204R, ORF1a:A555S, ORF1a:P959S, ORF1a:A2123V, ORF1a:T3255I, ORF1a:I3618V, ORF1a:T4175I, ORF1b:P314L, ORF1b:V2073L, ORF3a:L46F, S:Y28H, S:T478K, S:D614G, S:P681H, S:T732A,

hCoV-19/Mexico/MEX-IBT-IMSS-210/2021	EPI_ISL_1288406	In process	20B	B.1.1.519	26	5'UTR:C203T, 5'UTR:C222T, 5'UTR:C241T, ORF1ab:C3037T, ORF1ab:C3045T, ORF1ab:C3140T, ORF1ab:C6445T, ORF1ab:C6982T, ORF1ab:C10029T, ORF1ab:C10954T, ORF1ab:A11117G, ORF1ab:C12789T, ORF1ab:A13579C, ORF1ab:T13593C, ORF1ab:C14408T, ORF1ab:G15921T, ORF1ab:T19839C, ORF1ab:C21306T, S:C22995A, S:A23403G, S:C23604A, S:A23756G, S:G24757T, N:G28881A, N:G28882A, N:G28883C, N:C29197T,	13	N:R203K, N:G204R, ORF1a:P927L, ORF1a:P959S, ORF1a:T3255I, ORF1a:I3618V, ORF1a:T4175I, ORF1b:K38Q, ORF1b:P314L, S:T478K, S:D614G, S:P681H, S:T732A,
hCoV-19/Mexico/MEX-IBT-IMSS-211/2021	EPI_ISL_1288407	In process	20B	B.1.1.519	24	5'UTR:T201C, 5'UTR:C203T, 5'UTR:C222T, 5'UTR:C241T, ORF1ab:G1738T, ORF1ab:C3037T, ORF1ab:C3140T, ORF1ab:C10029T, ORF1ab:C10954T, ORF1ab:A11117G, ORF1ab:C12119T, ORF1ab:C12789T, ORF1ab:C14408T, ORF1ab:T19839C, ORF1ab:A19974G, ORF1ab:C21306T, S:C22480T, S:C22995A, S:A23403G, S:C23604A, S:A23756G, N:G28881A, N:G28882A, N:G28883C, N:C29197T,	12	N:R203K, N:G204R, ORF1a:P959S, ORF1a:T3255I, ORF1a:I3618V, ORF1a:P3952S, ORF1a:T4175I, ORF1b:P314L, S:T478K, S:D614G, S:P681H, S:T732A,
hCoV-19/Mexico/MEX-IBT-IMSS-212/2021	EPI_ISL_1288408	In process	20B	B.1.1.519	25	5'UTR:C203T, 5'UTR:C222T, 5'UTR:C241T, ORF1ab:C3037T, ORF1ab:C3140T, ORF1ab:C6445T, ORF1ab:C6982T, ORF1ab:C10029T, ORF1ab:C10954T, ORF1ab:A11117G, ORF1ab:C12789T, ORF1ab:A13579C, ORF1ab:T13593C, ORF1ab:C14408T, ORF1ab:G15921T, ORF1ab:T19839C, ORF1ab:C21306T, S:C22995A, S:A23403G, S:C23604A, S:A23756G, S:G24757T, N:G28881A, N:G28882A, N:G28883C, N:C29197T,	12	N:R203K, N:G204R, ORF1a:P959S, ORF1a:T3255I, ORF1a:I3618V, ORF1a:T4175I, ORF1b:K38Q, ORF1b:P314L, S:T478K, S:D614G, S:P681H, S:T732A,
hCoV-19/Mexico/MEX-IBT-IMSS-213/2021	EPI_ISL_1288409	In process	20B	B.1.1.519	23	5'UTR:C203T, 5'UTR:C222T, 5'UTR:C241T, ORF1ab:C3037T, ORF1ab:C3140T, ORF1ab:T4339C, ORF1ab:C10029T, ORF1ab:C10954T, ORF1ab:A11117G, ORF1ab:C12789T, ORF1ab:C14408T, ORF1ab:T19839C, ORF1ab:C21306T, S:C22995A, S:A23403G, S:C23604A, S:A23756G, ORF3a:G25906T, ORF8:C28005T, ORF8:C28253T, N:G28881A, N:G28882A, N:G28883C, N:C29197T,	13	N:R203K, N:G204R, ORF1a:P959S, ORF1a:T3255I, ORF1a:I3618V, ORF1a:T4175I, ORF1b:P314L, ORF3a:G172C, ORF8:P38S, S:T478K, S:D614G, S:P681H, S:T732A,
hCoV-19/Mexico/MEX-IBT-IMSS-214/2021	EPI_ISL_1288410	In process	20B	B.1.1.519	24	5'UTR:C203T, 5'UTR:C222T, 5'UTR:C241T, ORF1ab:C3037T, ORF1ab:C3140T, ORF1ab:A9390G, ORF1ab:C10029T, ORF1ab:C10954T, ORF1ab:A11117G, ORF1ab:C12789T, ORF1ab:C14408T, ORF1ab:T19839C, ORF1ab:C21077T, ORF1ab:C21306T, S:C22995A, S:A23403G, S:C23604A, S:A23756G, ORF3a:G25906T, ORF8:C28253T, N:G28881A, N:G28882A, N:G28883C, N:C29197T, N:C29482T,	14	N:R203K, N:G204R, ORF1a:P959S, ORF1a:D3042G, ORF1a:T3255I, ORF1a:I3618V, ORF1a:T4175I, ORF1b:P314L, ORF1b:T2537I, ORF3a:G172C, S:T478K, S:D614G, S:P681H, S:T732A,
hCoV-19/Mexico/MEX-IBT-IMSS-218/2021	EPI_ISL_1288413	In process	20B	B.1.1.519	25	5'UTR:C203T, 5'UTR:C222T, 5'UTR:C241T, ORF1ab:C3037T, ORF1ab:C3140T, ORF1ab:C7600T, ORF1ab:C10029T, ORF1ab:C10954T, ORF1ab:G11083T, ORF1ab:A11117G, ORF1ab:C12789T, ORF1ab:C14408T, ORF1ab:C14708T, ORF1ab:A19837C, ORF1ab:T19839C, ORF1ab:C21306T, S:C22995A, S:A23403G, S:C23604A, S:A23756G, ORF8:T27904C, ORF8:C28087T, N:G28881A, N:G28882A, N:G28883C, N:C29197T,	16	N:R203K, N:G204R, ORF1a:P959S, ORF1a:T3255I, ORF1a:L3606F, ORF1a:I3618V, ORF1a:T4175I, ORF1b:P314L, ORF1b:A414V, ORF1b:N2124H, ORF8:L4P, ORF8:A65V, S:T478K, S:D614G, S:P681H, S:T732A,

hCoV-19/Mexico/MEX-IBT-IMSS-222/2021	EPI_ISL_1288416	In process	20B	B.1.1.519	28	5'UTR:C203T, 5'UTR:C222T, 5'UTR:C241T, ORF1ab:T931C, ORF1ab:C3037T, ORF1ab:C3140T, ORF1ab:C8299T, ORF1ab:C9474T, ORF1ab:A9782G, ORF1ab:C10029T, ORF1ab:C10186T, ORF1ab:C10954T, ORF1ab:A11117G, ORF1ab:C12789T, ORF1ab:C14408T, ORF1ab:G15906T, ORF1ab:A16770C, ORF1ab:T19839C, ORF1ab:C21306T, S:C22995A, S:A23403G, S:C23604A, S:A23756G, ORF3a:C26058T, ORF8:T27904C, N:G28881A, N:G28882A, N:G28883C, N:C29197T,	15	N:R203K, N:G204R, ORF1a:P959S, ORF1a:A3070V, ORF1a:S3173G, ORF1a:T3255I, ORF1a:I3618V, ORF1a:T4175I, ORF1b:P314L, ORF1b:Q813H, ORF8:L4P, S:T478K, S:D614G, S:P681H, S:T732A,
hCoV-19/Mexico/MEX-IBT-IMSS-25/2020	EPI_ISL_955237	In process	20A	B.1	6	5'UTR:C241T, ORF1ab:C1489T, ORF1ab:C3037T, ORF1ab:C14408T, ORF1ab:A20268G, S:A23403G, S:T23431C,	2	ORF1b:P314L, S:D614G,
hCoV-19/Mexico/MEX-IBT-IMSS-286/2021	EPI_ISL_1288472	In process	20B	B.1.1.519	24	5'UTR:C203T, 5'UTR:C222T, 5'UTR:C241T, ORF1ab:C3037T, ORF1ab:C3140T, ORF1ab:C10029T, ORF1ab:C10954T, ORF1ab:A11117G, ORF1ab:G11609A, ORF1ab:C12789T, ORF1ab:C14408T, ORF1ab:C16394T, ORF1ab:T19839C, ORF1ab:C21306T, S:C22747T, S:C22995A, S:A23403G, S:C23604A, S:A23756G, M:C26882T, N:G28881A, N:G28882A, N:G28883C, N:C29197T, N:G29212A,	13	N:R203K, N:G204R, ORF1a:P959S, ORF1a:T3255I, ORF1a:I3618V, ORF1a:V3782I, ORF1a:T4175I, ORF1b:P314L, ORF1b:P976L, S:T478K, S:D614G, S:P681H, S:T732A,
hCoV-19/Mexico/MEX-IBT-IMSS-29/2021	EPI_ISL_1288248	In process	20B	B.1.1.519	27	5'UTR:T201C, 5'UTR:C203T, 5'UTR:C222T, 5'UTR:C241T, ORF1ab:G1738T, ORF1ab:C2197T, ORF1ab:C3037T, ORF1ab:C3140T, ORF1ab:C10029T, ORF1ab:C10332T, ORF1ab:C10954T, ORF1ab:A11117G, ORF1ab:C12789T, ORF1ab:C14408T, ORF1ab:C14724T, ORF1ab:T19839C, ORF1ab:A19974G, ORF1ab:C19983T, ORF1ab:C21306T, S:C22995A, S:A23403G, S:C23604A, S:A23756G, ORF7a:C27629T, N:G28881A, N:G28882A, N:G28883C, N:C29197T,	13	N:R203K, N:G204R, ORF1a:P959S, ORF1a:T3255I, ORF1a:T3356I, ORF1a:I3618V, ORF1a:T4175I, ORF1b:P314L, ORF7a:A79V, S:T478K, S:D614G, S:P681H, S:T732A,
hCoV-19/Mexico/MEX-IBT-IMSS-295/2021	EPI_ISL_1288480	In process	20B	B.1.1.519	23	5'UTR:C203T, 5'UTR:C222T, 5'UTR:C241T, ORF1ab:C3037T, ORF1ab:C3140T, ORF1ab:T8104C, ORF1ab:C10029T, ORF1ab:A10829C, ORF1ab:C10954T, ORF1ab:A11117G, ORF1ab:C12789T, ORF1ab:C14408T, ORF1ab:T19839C, ORF1ab:C21306T, S:C22995A, S:A23403G, S:C23604A, S:A23756G, ORF8:T27904C, ORF8:C28087T, N:G28881A, N:G28882A, N:G28883C, N:C29197T,	14	N:R203K, N:G204R, ORF1a:P959S, ORF1a:T3255I, ORF1a:I3522L, ORF1a:I3618V, ORF1a:T4175I, ORF1b:P314L, ORF8:L4P, ORF8:A65V, S:T478K, S:D614G, S:P681H, S:T732A,
hCoV-19/Mexico/MEX-IBT-IMSS-299/2021	EPI_ISL_1288484	In process	20A	B.1.243	23	5'UTR:C241T, ORF1ab:C274T, ORF1ab:C478T, ORF1ab:G806A, ORF1ab:C3037T, ORF1ab:G3308A, ORF1ab:T5071G, ORF1ab:C5140A, ORF1ab:C6027T, ORF1ab:T9370C, ORF1ab:T10480C, ORF1ab:C14408T, ORF1ab:G16122A, ORF1ab:G16558T, ORF1ab:C18486T, ORF1ab:G19086T, ORF1ab:A20268G, S:G23120T, S:A23403G, S:G23587C, S:T24076C, E:C26455T, N:C28854T, N:G29543T,	12	E:P71S, N:S194L, ORF1a:A181T, ORF1a:E1015K, ORF1a:D1625E, ORF1a:P1921L, ORF1b:P314L, ORF1b:A1031S, ORF1b:K1873N, S:A520S, S:D614G, S:Q675H,
hCoV-19/Mexico/MEX-IBT-IMSS-30/2021	EPI_ISL_1288249	In process	20B	B.1.1.519	24	5'UTR:T201C, 5'UTR:C203T, 5'UTR:C222T, 5'UTR:C241T, ORF1ab:C745T, ORF1ab:G1738T, ORF1ab:C3037T, ORF1ab:C3140T, ORF1ab:C10029T, ORF1ab:C10954T, ORF1ab:A11117G, ORF1ab:C12789T, ORF1ab:C14408T, ORF1ab:T19839C, ORF1ab:A19974G, ORF1ab:C21306T, S:C22995A, S:A23403G, S:C23604A, S:A23756G, N:T28726C, N:G28881A, N:G28882A, N:G28883C, N:C29197T,	11	N:R203K, N:G204R, ORF1a:P959S, ORF1a:T3255I, ORF1a:I3618V, ORF1a:T4175I, ORF1b:P314L, S:T478K, S:D614G, S:P681H, S:T732A,

hCoV-19/Mexico/MEX-IBT-IMSS-311/2021	EPI_ISL_1288494	In process	20B	B.1.1.519	24	5'UTR:C203T, 5'UTR:C222T, 5'UTR:C241T, ORF1ab:C3037T, ORF1ab:C3140T, ORF1ab:G6881A, ORF1ab:C10029T, ORF1ab:C10228T, ORF1ab:C10954T, ORF1ab:A11117G, ORF1ab:C12789T, ORF1ab:C14408T, ORF1ab:C18377T, ORF1ab:T19839C, ORF1ab:C21306T, S:C22735T, S:C22995A, S:A23403G, S:C23604A, S:A23756G, N:G28881A, N:G28882A, N:G28883C, N:C29197T, N:G29527T,	14	N:R203K, N:G204R, N:Q418H, ORF1a:P959S, ORF1a:V2206I, ORF1a:T3255I, ORF1a:I3618V, ORF1a:T4175I, ORF1b:P314L, ORF1b:T1637I, S:T478K, S:D614G, S:P681H, S:T732A,
hCoV-19/Mexico/MEX-IBT-IMSS-312/2021	EPI_ISL_1288495	In process	20B	B.1.1.519	24	5'UTR:C203T, 5'UTR:C222T, 5'UTR:C241T, ORF1ab:T1971C, ORF1ab:C3037T, ORF1ab:C3140T, ORF1ab:C4410T, ORF1ab:C8299T, ORF1ab:C10029T, ORF1ab:C10954T, ORF1ab:A11117G, ORF1ab:C12789T, ORF1ab:C14408T, ORF1ab:T19839C, ORF1ab:C21306T, S:C22747T, S:C22995A, S:A23403G, S:C23604A, S:A23756G, N:A28761G, N:G28881A, N:G28882A, N:G28883C, N:C29197T,	14	N:Q163R, N:R203K, N:G204R, ORF1a:I569T, ORF1a:P959S, ORF1a:A1382V, ORF1a:T3255I, ORF1a:I3618V, ORF1a:T4175I, ORF1b:P314L, S:T478K, S:D614G, S:P681H, S:T732A,
hCoV-19/Mexico/MEX-IBT-IMSS-313/2021	EPI_ISL_1288496	In process	20B	B.1.1.519	24	5'UTR:T201C, 5'UTR:C203T, 5'UTR:C222T, 5'UTR:C241T, ORF1ab:G1738T, ORF1ab:C3037T, ORF1ab:C3140T, ORF1ab:C10029T, ORF1ab:C10954T, ORF1ab:A11117G, ORF1ab:C12789T, ORF1ab:C14408T, ORF1ab:T19839C, ORF1ab:A19974G, ORF1ab:C21306T, S:C22995A, S:A23403G, S:C23604A, S:G23612T, S:A23756G, N:G28881A, N:G28882A, N:G28883C, N:C29197T, 3'UTR:T29791C,	12	N:R203K, N:G204R, ORF1a:P959S, ORF1a:T3255I, ORF1a:I3618V, ORF1a:T4175I, ORF1b:P314L, S:T478K, S:D614G, S:P681H, S:A684S, S:T732A,
hCoV-19/Mexico/MEX-IBT-IMSS-315/2021	EPI_ISL_1288497	In process	20B	B.1.1.519	26	5'UTR:C203T, 5'UTR:C222T, 5'UTR:C241T, ORF1ab:A2692G, ORF1ab:C3037T, ORF1ab:C3140T, ORF1ab:G3692T, ORF1ab:G3871T, ORF1ab:C10029T, ORF1ab:C10954T, ORF1ab:A11117G, ORF1ab:A11782G, ORF1ab:C11824A, ORF1ab:C12789T, ORF1ab:C14408T, ORF1ab:T19839C, ORF1ab:C21306T, S:C22995A, S:A23403G, S:C23604A, S:A23756G, M:C27143T, ORF8:T27904C, N:G28881A, N:G28882A, N:G28883C, N:C29197T,	14	N:R203K, N:G204R, ORF1a:P959S, ORF1a:V1143F, ORF1a:K1202N, ORF1a:T3255I, ORF1a:I3618V, ORF1a:T4175I, ORF1b:P314L, ORF8:L4P, S:T478K, S:D614G, S:P681H, S:T732A,
hCoV-19/Mexico/MEX-IBT-IMSS-318/2021	EPI_ISL_1288500	In process	20B	B.1.1.519	25	5'UTR:C203T, 5'UTR:C222T, 5'UTR:C241T, ORF1ab:T950C, ORF1ab:C3037T, ORF1ab:C3140T, ORF1ab:T9585C, ORF1ab:C10029T, ORF1ab:C10954T, ORF1ab:A11117G, ORF1ab:C12488T, ORF1ab:C12789T, ORF1ab:C14408T, ORF1ab:C19602T, ORF1ab:T19839C, ORF1ab:C21306T, S:C22995A, S:A23403G, S:C23604A, S:A23756G, ORF3a:G25906T, ORF8:C28253T, N:G28881A, N:G28882A, N:G28883C, N:C29197T,	15	N:R203K, N:G204R, ORF1a:Y229H, ORF1a:P959S, ORF1a:V3107A, ORF1a:T3255I, ORF1a:I3618V, ORF1a:P4075S, ORF1a:T4175I, ORF1b:P314L, ORF3a:G172C, S:T478K, S:D614G, S:P681H, S:T732A,
hCoV-19/Mexico/MEX-IBT-IMSS-337/2021	EPI_ISL_1288516	In process	20B	B.1.1.519	28	5'UTR:C203T, 5'UTR:C222T, 5'UTR:C241T, ORF1ab:C3037T, ORF1ab:C3140T, ORF1ab:G3692T, ORF1ab:G3871T, ORF1ab:C4158T, ORF1ab:C9520T, ORF1ab:C10029T, ORF1ab:C10954T, ORF1ab:A11117G, ORF1ab:C11824A, ORF1ab:C12789T, ORF1ab:C14408T, ORF1ab:C18747T, ORF1ab:T19839C, ORF1ab:C21306T, S:T22327C, S:C22995A, S:A23403G, S:C23557T, S:C23604A, S:A23756G, ORF8:T27904C, N:G28881A, N:G28882A, N:G28883C, N:C29197T,	15	N:R203K, N:G204R, ORF1a:P959S, ORF1a:V1143F, ORF1a:K1202N, ORF1a:A1298V, ORF1a:T3255I, ORF1a:I3618V, ORF1a:T4175I, ORF1b:P314L, ORF8:L4P, S:T478K, S:D614G, S:P681H, S:T732A,

hCoV-19/Mexico/MEX-IBT-IMSS-339/2021	EPI_ISL_1288518	In process	20B	B.1.1.519	30	5'UTR:T201C, 5'UTR:C203T, 5'UTR:C222T, 5'UTR:C241T, ORF1ab:C1288T, ORF1ab:G1738T, ORF1ab:C3037T, ORF1ab:C3140T, ORF1ab:A4750G, ORF1ab:C10029T, ORF1ab:A10471G, ORF1ab:C10954T, ORF1ab:A11117G, ORF1ab:C12789T, ORF1ab:C14408T, ORF1ab:C15441T, ORF1ab:A16770G, ORF1ab:C17934T, ORF1ab:T19839C, ORF1ab:A19974G, ORF1ab:C21306T, S:C22995A, S:A23403G, S:C23604A, S:A23756G, S:C24642T, N:G28881A, N:G28882A, N:G28883C, N:C29197T, 3'UTR:G29734T,	12	N:R203K, N:G204R, ORF1a:P959S, ORF1a:T3255I, ORF1a:I3618V, ORF1a:T4175I, ORF1b:P314L, S:T478K, S:D614G, S:P681H, S:T732A, S:T1027I,
hCoV-19/Mexico/MEX-IBT-IMSS-343/2021	EPI_ISL_1288522	In process	20B	B.1.1.519	25	5'UTR:C203T, 5'UTR:C222T, 5'UTR:C241T, ORF1ab:A1038G, ORF1ab:C3037T, ORF1ab:C3140T, ORF1ab:A6393C, ORF1ab:C10029T, ORF1ab:C10954T, ORF1ab:A11117G, ORF1ab:C12789T, ORF1ab:C14408T, ORF1ab:C14805T, ORF1ab:C14937T, ORF1ab:T19839C, ORF1ab:C21306T, S:C22995A, S:A23403G, S:C23604A, S:A23756G, N:G28881A, N:G28882A, N:G28883C, N:T28894C, N:C29095T, N:C29197T,	13	N:R203K, N:G204R, ORF1a:K258R, ORF1a:P959S, ORF1a:D2043A, ORF1a:T3255I, ORF1a:I3618V, ORF1a:T4175I, ORF1b:P314L, S:T478K, S:D614G, S:P681H, S:T732A,
hCoV-19/Mexico/MEX-IBT-IMSS-344/2021	EPI_ISL_1288523	In process	20B	B.1.1.519	26	5'UTR:C203T, 5'UTR:C222T, 5'UTR:C241T, ORF1ab:T682C, ORF1ab:G2123A, ORF1ab:C3037T, ORF1ab:C3140T, ORF1ab:G7037T, ORF1ab:C10029T, ORF1ab:C10954T, ORF1ab:A11117G, ORF1ab:T13223G, ORF1ab:C14408T, ORF1ab:C17172T, ORF1ab:T19839C, ORF1ab:C21306T, S:C22938T, S:C22995A, S:A23403G, S:C23604A, S:A23756G, ORF3a:C25782T, ORF6:G27390T, N:G28881A, N:G28882A, N:G28883C, N:C29197T,	14	N:R203K, N:G204R, ORF1a:V620I, ORF1a:P959S, ORF1a:G2258C, ORF1a:T3255I, ORF1a:I3618V, ORF1a:S4320A, ORF1b:P314L, S:S459F, S:T478K, S:D614G, S:P681H, S:T732A,
hCoV-19/Mexico/MEX-IBT-IMSS-356/2021	EPI_ISL_1288533	In process	20B	B.1.1.519	22	5'UTR:C222T, ORF1ab:C3037T, ORF1ab:C3140T, ORF1ab:C10029T, ORF1ab:C10954T, ORF1ab:A11117G, ORF1ab:C12789T, ORF1ab:C13115T, ORF1ab:T13593C, ORF1ab:C14408T, ORF1ab:G15921T, ORF1ab:T19839C, ORF1ab:T20028C, ORF1ab:C21306T, S:C22995A, S:A23403G, S:C23604A, S:A23756G, S:G24757T, N:G28881A, N:G28882A, N:G28883C, N:C29197T,	11	N:R203K, N:G204R, ORF1a:P959S, ORF1a:T3255I, ORF1a:I3618V, ORF1a:T4175I, ORF1b:P314L, S:T478K, S:D614G, S:P681H, S:T732A,
hCoV-19/Mexico/MEX-IBT-IMSS-369/2021	EPI_ISL_1288198	In process	20B	B.1.1.519	23	5'UTR:T201C, 5'UTR:C203T, 5'UTR:C222T, 5'UTR:C241T, ORF1ab:G1738T, ORF1ab:C3037T, ORF1ab:C3140T, ORF1ab:C10029T, ORF1ab:C10954T, ORF1ab:A11117G, ORF1ab:C14408T, ORF1ab:C16092T, ORF1ab:T19839C, ORF1ab:A19974G, ORF1ab:C21306T, S:C22995A, S:A23403G, S:C23604A, S:A23756G, ORF3a:C25553T, N:G28881A, N:G28882A, N:G28883C, N:C29197T,	11	N:R203K, N:G204R, ORF1a:P959S, ORF1a:T3255I, ORF1a:I3618V, ORF1b:P314L, ORF3a:A54V, S:T478K, S:D614G, S:P681H, S:T732A,
hCoV-19/Mexico/MEX-IBT-IMSS-371/2021	EPI_ISL_1288199	In process	20B	B.1.1.519	27	5'UTR:C203T, 5'UTR:C222T, 5'UTR:C241T, ORF1ab:C3037T, ORF1ab:C3140T, ORF1ab:C10029T, ORF1ab:C10277T, ORF1ab:C10954T, ORF1ab:A11117G, ORF1ab:C11941T, ORF1ab:C12225T, ORF1ab:C12789T, ORF1ab:C14408T, ORF1ab:T19839C, ORF1ab:C21306T, S:G22778C, S:C22995A, S:A23403G, S:C23604A, S:A23756G, S:G24219T, S:G24220T, ORF3a:C25810T, N:G28881A, N:G28882A, N:G28883C, N:C29197T, N:G29527T,	17	N:R203K, N:G204R, N:Q418H, ORF1a:P959S, ORF1a:T3255I, ORF1a:L3338F, ORF1a:I3618V, ORF1a:A3987V, ORF1a:T4175I, ORF1b:P314L, ORF3a:L140F, S:E406Q, S:T478K, S:D614G, S:P681H, S:T732A, S:W886F,

hCoV-19/Mexico/MEX-IBT-IMSS-377/2021	EPI_ISL_1288205	In process	20B	B.1.1.519	27	5'UTR:T201C, 5'UTR:C203T, 5'UTR:C222T, 5'UTR:C241T, ORF1ab:C1218T, ORF1ab:G1738T, ORF1ab:C3037T, ORF1ab:C3140T, ORF1ab:G4907A, ORF1ab:C10029T, ORF1ab:C10954T, ORF1ab:A11117G, ORF1ab:C12789T, ORF1ab:C13059T, ORF1ab:C14408T, ORF1ab:T19839C, ORF1ab:A19974G, ORF1ab:C21306T, S:C21621T, S:C22995A, S:A23403G, S:C23604A, S:A23756G, ORF3a:C25889T, N:G28881A, N:G28882A, N:G28883C, N:C29197T	16	N:R203K, N:G204R, ORF1a:S318L, ORF1a:P959S, ORF1a:G1548S, ORF1a:T3255I, ORF1a:I3618V, ORF1a:T4175I, ORF1a:T4265I, ORF1b:P314L, ORF3a:S166L, S:T20I, S:T478K, S:D614G, S:P681H, S:T732A,
hCoV-19/Mexico/MEX-IBT-IMSS-378/2021	EPI_ISL_1288206	In process	20B	B.1.1.519	24	5'UTR:T201C, 5'UTR:C203T, 5'UTR:C222T, 5'UTR:C241T, ORF1ab:G1738T, ORF1ab:C3037T, ORF1ab:C3140T, ORF1ab:G4682T, ORF1ab:C10029T, ORF1ab:C10954T, ORF1ab:A11117G, ORF1ab:C12789T, ORF1ab:C13792T, ORF1ab:C14408T, ORF1ab:T19839C, ORF1ab:A19974G, ORF1ab:C21306T, S:C22995A, S:A23403G, S:C23604A, S:A23756G, N:G28881A, N:G28882A, N:G28883C, N:C29197T,	13	N:R203K, N:G204R, ORF1a:P959S, ORF1a:A1473S, ORF1a:T3255I, ORF1a:I3618V, ORF1a:T4175I, ORF1b:R109C, ORF1b:P314L, S:T478K, S:D614G, S:P681H, S:T732A,
hCoV-19/Mexico/MEX-IBT-IMSS-379/2021	EPI_ISL_1288207	In process	20B	B.1.1.519	33	5'UTR:T201C, 5'UTR:C203T, 5'UTR:C222T, 5'UTR:C241T, ORF1ab:C936T, ORF1ab:G1738T, ORF1ab:C3037T, ORF1ab:C3140T, ORF1ab:G9756A, ORF1ab:C10029T, ORF1ab:C10954T, ORF1ab:C11074T, ORF1ab:G11083T, ORF1ab:A11117G, ORF1ab:C12789T, ORF1ab:G13762A, ORF1ab:C14408T, ORF1ab:T19839C, ORF1ab:A19974G, ORF1ab:C21306T, S:T21835C, S:C22995A, S:A23403G, S:C23604A, S:A23756G, ORF3a:A25524C, M:C26801T, N:G28881A, N:G28882A, N:G28883C, N:G28968T, N:C29085T, N:C29197T, N:G29227T	17	N:R203K, N:G204R, N:S232I, N:T271I, ORF1a:T224I, ORF1a:P959S, ORF1a:R3164H, ORF1a:T3255I, ORF1a:L3606F, ORF1a:I3618V, ORF1a:T4175I, ORF1b:G99S, ORF1b:P314L, S:T478K, S:D614G, S:P681H, S:T732A,
hCoV-19/Mexico/MEX-IBT-IMSS-380/2021	EPI_ISL_1288208	In process	20B	B.1.1.519	23	5'UTR:C203T, 5'UTR:C222T, 5'UTR:C241T, ORF1ab:C3037T, ORF1ab:C3140T, ORF1ab:G7798C, ORF1ab:C10029T, ORF1ab:C10954T, ORF1ab:A11117G, ORF1ab:C12789T, ORF1ab:C13297T, ORF1ab:C14408T, ORF1ab:T19839C, ORF1ab:C21306T, S:C22995A, S:A23403G, S:C23604A, S:A23756G, ORF8:T27904C, ORF8:C28087T, N:G28881A, N:G28882A, N:G28883C, N:C29197T,	14	N:R203K, N:G204R, ORF1a:P959S, ORF1a:K2511N, ORF1a:T3255I, ORF1a:I3618V, ORF1a:T4175I, ORF1b:P314L, ORF8:L4P, ORF8:A65V, S:T478K, S:D614G, S:P681H, S:T732A,
hCoV-19/Mexico/MEX-IBT-IMSS-382/2021	EPI_ISL_1288209	In process	20B	B.1.1.519	27	5'UTR:C203T, 5'UTR:C222T, 5'UTR:C241T, ORF1ab:G443A, ORF1ab:C3037T, ORF1ab:C3140T, ORF1ab:C5869T, ORF1ab:C10029T, ORF1ab:C10954T, ORF1ab:A11117G, ORF1ab:C12789T, ORF1ab:C14408T, ORF1ab:C16616T, ORF1ab:T19839C, ORF1ab:C21306T, S:C22995A, S:A23403G, S:C23604A, S:A23756G, ORF3a:G25595A, ORF8:T27904C, ORF8:C28087T, N:G28881A, N:G28882A, N:G28883C, N:C29197T, N:T29461C, N:C29502T	17	N:R203K, N:G204R, N:S410F, ORF1a:V60I, ORF1a:P959S, ORF1a:T3255I, ORF1a:I3618V, ORF1a:T4175I, ORF1b:P314L, ORF1b:T1050I, ORF3a:R68K, ORF8:L4P, ORF8:A65V, S:T478K, S:D614G, S:P681H, S:T732A,
hCoV-19/Mexico/MEX-IBT-IMSS-383/2021	EPI_ISL_1288210	In process	20B	B.1.1.519	29	5'UTR:C203T, 5'UTR:C222T, 5'UTR:C241T, ORF1ab:A1162G, ORF1ab:C1427T, ORF1ab:C3037T, ORF1ab:C3140T, ORF1ab:G6266T, ORF1ab:C10029T, ORF1ab:C10954T, ORF1ab:A11117G, ORF1ab:G11893T, ORF1ab:C12789T, ORF1ab:C14408T, ORF1ab:T19839C, ORF1ab:G21255C, ORF1ab:C21306T, S:C22995A, S:C23248T, S:A23403G, S:C23604A, S:A23756G, S:T24709A, M:C26882T, ORF8:T27904C, N:G28881A, N:G28882A, N:G28883C, N:C29197T, N:G29543T,	15	N:R203K, N:G204R, ORF1a:H388Y, ORF1a:P959S, ORF1a:A2001S, ORF1a:T3255I, ORF1a:I3618V, ORF1a:L3876F, ORF1a:T4175I, ORF1b:P314L, ORF8:L4P, S:T478K, S:D614G, S:P681H, S:T732A,

hCoV-19/Mexico/MEX-IBT-IMSS-515/2021	EPI_ISL_1302236	In process	20B	B.1.1.519	27	5'UTR:C203T, 5'UTR:C222T, 5'UTR:C241T, ORF1ab:C3037T, ORF1ab:C3140T, ORF1ab:T3745C, ORF1ab:C6317T, ORF1ab:C10029T, ORF1ab:T10721C, ORF1ab:C10954T, ORF1ab:A11117G, ORF1ab:T11587C, ORF1ab:C12789T, ORF1ab:C14408T, ORF1ab:T19839C, S:C22995A, S:A23403G, S:C23604A, S:A23756G, S:C24023T, S:G24781T, ORF3a:C25844T, N:G28881A, N:G28882A, N:G28883C, N:C29171T, N:C29197T, ORF10:C29670T	16	N:R203K, N:G204R, N:H300Y, ORF1a:P959S, ORF1a:P2018S, ORF1a:T3255I, ORF1a:F3486L, ORF1a:I3618V, ORF1a:T4175I, ORF1b:P314L, ORF3a:T151I, S:T478K, S:D614G, S:P681H, S:T732A, S:K1073N,
hCoV-19/Mexico/MEX-IBT-IMSS-525/2021	EPI_ISL_1302196	In process	20B	B.1.1.519	26	5'UTR:C203T, 5'UTR:C222T, 5'UTR:C241T, ORF1ab:C1342T, ORF1ab:C3037T, ORF1ab:C3140T, ORF1ab:G3692T, ORF1ab:G3871T, ORF1ab:C10029T, ORF1ab:C10954T, ORF1ab:A11117G, ORF1ab:C11824A, ORF1ab:C12789T, ORF1ab:C14408T, ORF1ab:T19839C, S:C22995A, S:A23403G, S:C23604A, S:A23756G, ORF7b:G27777T, ORF8:T27904C, ORF8:G27915T, N:G28881A, N:G28882A, N:G28883C, N:C29197T, 3'UTR:G29777T,	16	N:R203K, N:G204R, ORF1a:P959S, ORF1a:V1143F, ORF1a:K1202N, ORF1a:T3255I, ORF1a:I3618V, ORF1a:T4175I, ORF1b:P314L, ORF7b:D8Y, ORF8:L4P, ORF8:G8*, S:T478K, S:D614G, S:P681H, S:T732A,
hCoV-19/Mexico/MEX-IBT-IMSS-539/2021	EPI_ISL_1302393	In process	20B	B.1.1.519	27	5'UTR:C203T, 5'UTR:C222T, 5'UTR:C241T, ORF1ab:C527T, ORF1ab:A866G, ORF1ab:C1009T, ORF1ab:C3037T, ORF1ab:C3140T, ORF1ab:C5183T, ORF1ab:C10029T, ORF1ab:C10954T, ORF1ab:A11117G, ORF1ab:C11916T, ORF1ab:C12789T, ORF1ab:C14408T, ORF1ab:T19839C, S:C22995A, S:A23403G, S:C23604A, S:A23756G, ORF8:T27904C, ORF8:G28221T, N:G28747T, N:G28881A, N:G28882A, N:G28883C, N:C29197T, N:G29425T,	17	N:R203K, N:G204R, N:Q384H, ORF1a:I201V, ORF1a:P959S, ORF1a:P1640S, ORF1a:T3255I, ORF1a:I3618V, ORF1a:S3884L, ORF1a:T4175I, ORF1b:P314L, ORF8:L4P, ORF8:E110*, S:T478K, S:D614G, S:P681H, S:T732A,
hCoV-19/Mexico/MEX-IBT-IMSS-540/2021	EPI_ISL_1302251	In process	20B	B.1.1.519	23	5'UTR:C203T, 5'UTR:C222T, 5'UTR:C241T, ORF1ab:C3037T, ORF1ab:C3140T, ORF1ab:C5869T, ORF1ab:C6568T, ORF1ab:C10029T, ORF1ab:C10954T, ORF1ab:A11117G, ORF1ab:C12789T, ORF1ab:C14408T, ORF1ab:T19839C, S:C22995A, S:A23403G, S:C23604A, S:A23756G, ORF8:T27904C, ORF8:G27987T, ORF8:C28087T, N:G28881A, N:G28882A, N:G28883C, N:C29197T,	14	N:R203K, N:G204R, ORF1a:P959S, ORF1a:T3255I, ORF1a:I3618V, ORF1a:T4175I, ORF1b:P314L, ORF8:L4P, ORF8:V32L, ORF8:A65V, S:T478K, S:D614G, S:P681H, S:T732A,
hCoV-19/Mexico/MEX-IBT-IMSS-541/2021	EPI_ISL_1302370	In process	20B	B.1.1.519	23	5'UTR:C203T, 5'UTR:C222T, 5'UTR:C241T, ORF1ab:C3037T, ORF1ab:C3140T, ORF1ab:C10029T, ORF1ab:C10954T, ORF1ab:A11117G, ORF1ab:C12789T, ORF1ab:C14408T, ORF1ab:T15726A, ORF1ab:T19839C, ORF1ab:C21306T, S:C22995A, S:A23403G, S:C23604A, S:A23756G, ORF3a:C25626T, M:C26833T, N:G28881A, N:G28882A, N:G28883C, N:C29197T, N:G29527T,	13	M:A104V, N:R203K, N:G204R, N:Q418H, ORF1a:P959S, ORF1a:T3255I, ORF1a:I3618V, ORF1a:T4175I, ORF1b:P314L, S:T478K, S:D614G, S:P681H, S:T732A,
hCoV-19/Mexico/MEX-IBT-IMSS-542/2021	EPI_ISL_1302248	In process	20B	B.1.1.519	21	5'UTR:C203T, 5'UTR:C222T, 5'UTR:C241T, ORF1ab:A1674G, ORF1ab:C3037T, ORF1ab:C3140T, ORF1ab:C5826T, ORF1ab:C10029T, ORF1ab:C10954T, ORF1ab:A11117G, ORF1ab:C12789T, ORF1ab:C14408T, ORF1ab:T19839C, S:C22995A, S:A23403G, S:C23604A, S:A23756G, N:G28881A, N:G28882A, N:G28883C, N:C29197T, N:G29527T,	14	N:R203K, N:G204R, N:Q418H, ORF1a:N470S, ORF1a:P959S, ORF1a:T1854I, ORF1a:T3255I, ORF1a:I3618V, ORF1a:T4175I, ORF1b:P314L, S:T478K, S:D614G, S:P681H, S:T732A,

hCoV-19/Mexico/MEX-IBT-IMSS-543/2021	EPI_ISL_1302348	In process	20B	B.1.1.519	27	5'UTR:C203T, 5'UTR:C222T, 5'UTR:C241T, ORF1ab:A866G, ORF1ab:C1009T, ORF1ab:C1457T, ORF1ab:C3037T, ORF1ab:C3140T, ORF1ab:C5183T, ORF1ab:C10029T, ORF1ab:C10954T, ORF1ab:A11117G, ORF1ab:C11916T, ORF1ab:C12789T, ORF1ab:C14408T, ORF1ab:T19839C, S:C22995A, S:A23403G, S:C23604A, S:A23756G, S:T24923C, ORF8:T27904C, N:G28747T, N:G28764T, N:G28881A, N:G28882A, N:G28883C, N:C29197T,	18	N:G164V, N:R203K, N:G204R, ORF1a:I201V, ORF1a:R398C, ORF1a:P959S, ORF1a:P1640S, ORF1a:T3255I, ORF1a:I3618V, ORF1a:S3884L, ORF1a:T4175I, ORF1b:P314L, ORF8:L4P, S:T478K, S:D614G, S:P681H, S:T732A, S:F1121L,
hCoV-19/Mexico/MEX-IBT-IMSS-544/2021	EPI_ISL_1302172	In process	20B	B.1.1.519	25	5'UTR:C203T, 5'UTR:C222T, 5'UTR:C241T, ORF1ab:C1912T, ORF1ab:C3037T, ORF1ab:C3140T, ORF1ab:T3745C, ORF1ab:T5840C, ORF1ab:C10029T, ORF1ab:C10954T, ORF1ab:A11117G, ORF1ab:C12789T, ORF1ab:C14408T, ORF1ab:T19839C, S:C22995A, S:A23403G, S:C23604A, S:A23756G, ORF3a:C25844T, N:G28539T, N:C28789T, N:G28881A, N:G28882A, N:G28883C, N:C29197T, 3'UTR:G29692T,	15	N:R89I, N:R203K, N:G204R, ORF1a:P959S, ORF1a:Y1859H, ORF1a:T3255I, ORF1a:I3618V, ORF1a:T4175I, ORF1b:P314L, ORF3a:T151I, ORF9b:E86*, S:T478K, S:D614G, S:P681H, S:T732A,
hCoV-19/Mexico/MEX-IBT-IMSS-545/2021	EPI_ISL_1302195	In process	20B	B.1.1.519	25	5'UTR:C203T, 5'UTR:C222T, 5'UTR:C241T, ORF1ab:C1342T, ORF1ab:C3037T, ORF1ab:C3140T, ORF1ab:G3692T, ORF1ab:G3871T, ORF1ab:C9733T, ORF1ab:C10029T, ORF1ab:C10954T, ORF1ab:A11117G, ORF1ab:C11824A, ORF1ab:C12789T, ORF1ab:C14408T, ORF1ab:T19839C, S:C22995A, S:A23403G, S:C23604A, S:T23698C, S:A23756G, ORF8:T27904C, N:G28881A, N:G28882A, N:G28883C, N:C29197T,	14	N:R203K, N:G204R, ORF1a:P959S, ORF1a:V1143F, ORF1a:K1202N, ORF1a:T3255I, ORF1a:I3618V, ORF1a:T4175I, ORF1b:P314L, ORF8:L4P, S:T478K, S:D614G, S:P681H, S:T732A,
hCoV-19/Mexico/MEX-IBT-IMSS-546/2021	EPI_ISL_1302169	In process	20B	B.1.1.519	25	5'UTR:C203T, 5'UTR:C222T, 5'UTR:C241T, ORF1ab:C3037T, ORF1ab:C3140T, ORF1ab:T3745C, ORF1ab:C9165T, ORF1ab:C10029T, ORF1ab:C10954T, ORF1ab:A11117G, ORF1ab:C12789T, ORF1ab:C14408T, ORF1ab:C18189T, ORF1ab:G18756A, ORF1ab:T19839C, ORF1ab:C21034T, S:C22995A, S:A23403G, S:C23604A, S:A23756G, ORF3a:C25844T, ORF7b:C27847T, N:G28881A, N:G28882A, N:G28883C, N:C29197T,	15	N:R203K, N:G204R, ORF1a:P959S, ORF1a:T2967I, ORF1a:T3255I, ORF1a:I3618V, ORF1a:T4175I, ORF1b:P314L, ORF1b:L2523F, ORF3a:T151I, ORF7b:S31L, S:T478K, S:D614G, S:P681H, S:T732A,
hCoV-19/Mexico/MEX-IBT-IMSS-547/2021	EPI_ISL_1302157	In process	20B	B.1.1.519	23	5'UTR:C203T, 5'UTR:C222T, 5'UTR:C241T, ORF1ab:G1886A, ORF1ab:C3037T, ORF1ab:C3140T, ORF1ab:C10029T, ORF1ab:C10954T, ORF1ab:A11117G, ORF1ab:C12789T, ORF1ab:C14408T, ORF1ab:C16329T, ORF1ab:T19839C, S:C22995A, S:A23403G, S:C23604A, S:A23756G, M:G26526A, N:G28881A, N:G28882A, N:G28883C, N:C29197T, N:G29527T, ORF10:A29567G,	14	M:A2T, N:R203K, N:G204R, N:Q418H, ORF1a:A541T, ORF1a:P959S, ORF1a:T3255I, ORF1a:I3618V, ORF1a:T4175I, ORF1b:P314L, S:T478K, S:D614G, S:P681H, S:T732A,
hCoV-19/Mexico/MEX-IBT-IMSS-549/2021	EPI_ISL_1302351	In process	20B	B.1.1.519	23	5'UTR:C203T, 5'UTR:C222T, 5'UTR:C241T, ORF1ab:C3037T, ORF1ab:C3140T, ORF1ab:C5031T, ORF1ab:C6037T, ORF1ab:C10029T, ORF1ab:C10954T, ORF1ab:G11083T, ORF1ab:A11117G, ORF1ab:C12789T, ORF1ab:C14408T, ORF1ab:T19839C, ORF1ab:C21306T, S:C22995A, S:C23604A, S:A23756G, ORF8:T27904C, ORF8:C28087T, N:G28881A, N:G28882A, N:G28883C, N:C29197T,	14	N:R203K, N:G204R, ORF1a:P959S, ORF1a:T1589I, ORF1a:T3255I, ORF1a:L3606F, ORF1a:I3618V, ORF1a:T4175I, ORF1b:P314L, ORF8:L4P, ORF8:A65V, S:T478K, S:P681H, S:T732A,

hCoV-19/Mexico/MEX-IBT-IMSS-550/2021	EPI_ISL_1302270	In process	20B	B.1.1.519	23	5'UTR:C203T, 5'UTR:C222T, 5'UTR:C241T, ORF1ab:C1346T, ORF1ab:C3037T, ORF1ab:C3140T, ORF1ab:T3745C, ORF1ab:C10029T, ORF1ab:C10954T, ORF1ab:A11117G, ORF1ab:C12789T, ORF1ab:C14408T, ORF1ab:T16494C, ORF1ab:T19839C, S:C22995A, S:A23403G, S:G23405A, S:C23604A, S:A23756G, ORF3a:C25844T, N:G28881A, N:G28882A, N:G28883C, N:C29197T,	14	N:R203K, N:G204R, ORF1a:P361S, ORF1a:P959S, ORF1a:T3255I, ORF1a:I3618V, ORF1a:T4175I, ORF1b:P314L, ORF3a:T151I, S:T478K, S:D614G, S:V615I, S:P681H, S:T732A,	
hCoV-19/Mexico/MEX-IBT-IMSS-551/2021	EPI_ISL_1302252	In process	20B	B.1.1.222	14	5'UTR:C241T, ORF1ab:C1594T, ORF1ab:C1968T, ORF1ab:C3037T, ORF1ab:C11455T, ORF1ab:C14408T, ORF1ab:G14653T, ORF1ab:T19839C, S:A23403G, S:T23427C, S:A23756G, S:T24869C, N:G28881A, N:G28882A, N:G28883C,	9	N:R203K, N:G204R, ORF1a:T568I, ORF1b:P314L, ORF1b:V396F, S:D614G, S:V622A, S:T732A, S:F1103L,	
hCoV-19/Mexico/MEX-IBT-IMSS-552/2021	EPI_ISL_1302359	In process	20B	B.1.1.519	23	5'UTR:T201C, 5'UTR:C203T, 5'UTR:C222T, 5'UTR:C241T, ORF1ab:G1738T, ORF1ab:C3037T, ORF1ab:C3140T, ORF1ab:C10029T, ORF1ab:G10283A, ORF1ab:C10954T, ORF1ab:A11117G, ORF1ab:C12789T, ORF1ab:C14408T, ORF1ab:T19839C, ORF1ab:A19974G, S:C22995A, S:A23403G, S:C23604A, S:A23756G, ORF7a:C27673T, N:G28881A, N:G28882A, N:G28883C, N:C29197T,	13	N:R203K, N:G204R, ORF1a:P959S, ORF1a:T3255I, ORF1a:V3340I, ORF1a:I3618V, ORF1a:T4175I, ORF1b:P314L, ORF7a:Q94*, S:T478K, S:D614G, S:P681H, S:T732A,	3'UTR:29749-29766,
hCoV-19/Mexico/MEX-IBT-IMSS-553/2021	EPI_ISL_1302158	In process	20B	B.1.1.519	22	5'UTR:C203T, 5'UTR:C222T, 5'UTR:C241T, ORF1ab:C3037T, ORF1ab:C3140T, ORF1ab:C9985T, ORF1ab:C10029T, ORF1ab:C10954T, ORF1ab:A11117G, ORF1ab:C12789T, ORF1ab:C14408T, ORF1ab:G17964T, ORF1ab:G19518T, ORF1ab:T19839C, S:C22995A, S:A23403G, S:C23604A, S:A23756G, N:G28881A, N:G28882A, N:G28883C, N:C29197T, N:G29527T,	14	N:R203K, N:G204R, N:Q418H, ORF1a:P959S, ORF1a:T3255I, ORF1a:I3618V, ORF1a:T4175I, ORF1b:P314L, ORF1b:M1499I, ORF1b:L2017F, S:T478K, S:D614G, S:P681H, S:T732A,	
hCoV-19/Mexico/MEX-InDRE-IBT-112/2020	EPI_ISL_1301447	In process	20B	B.1.1	9	5'UTR:C241T, ORF1ab:C3037T, ORF1ab:C8655T, ORF1ab:C14408T, ORF1ab:C19186T, ORF1ab:T19839C, S:A23403G, N:G28881A, N:G28882A, N:G28883C,	5	N:R203K, N:G204R, ORF1a:S2797F, ORF1b:P314L, S:D614G,	
hCoV-19/Mexico/MEX-InDRE-IBT-116/2020	EPI_ISL_1301584	In process	20A	B.1.609	6	5'UTR:C241T, ORF1ab:C3037T, ORF1ab:C4582T, ORF1ab:C13119T, ORF1ab:C14408T, S:A23403G, ORF3a:G25618A,	4	ORF1a:A4285V, ORF1b:P314L, ORF3a:G76S, S:D614G,	
hCoV-19/Mexico/MEX-InDRE-IBT-119/2020	EPI_ISL_1301575	In process	20B	B.1.1.222	9	5'UTR:C241T, ORF1ab:C3037T, ORF1ab:C8299T, ORF1ab:C14408T, ORF1ab:T19839C, S:A23403G, S:A23756G, N:G28881A, N:G28882A, N:G28883C,	5	N:R203K, N:G204R, ORF1b:P314L, S:D614G, S:T732A,	
hCoV-19/Mexico/MEX-InDRE-IBT-120/2020	EPI_ISL_1301576	In process	20A	B.1.609	8	5'UTR:C241T, ORF1ab:C3037T, ORF1ab:C4582T, ORF1ab:C11020T, ORF1ab:C14408T, ORF1ab:A20268G, S:A23403G, S:C24034T, 3'UTR:C29679T,	2	ORF1b:P314L, S:D614G,	
hCoV-19/Mexico/MEX-InDRE-IBT-121/2020	EPI_ISL_1301577	In process	20B	B.1.1	10	5'UTR:C241T, ORF1ab:G1135T, ORF1ab:A2024G, ORF1ab:C2695T, ORF1ab:C3037T, ORF1ab:C14408T, ORF1ab:T19839C, S:A23403G, N:G28881A, N:G28882A, N:G28883C,	6	N:R203K, N:G204R, ORF1a:K290N, ORF1a:T587A, ORF1b:P314L, S:D614G,	
hCoV-19/Mexico/MEX-InDRE-IBT-122/2020	EPI_ISL_1301578	In process	20A	B.1.243	9	5'UTR:C241T, ORF1ab:G806A, ORF1ab:C3037T, ORF1ab:C14408T, ORF1ab:C17373T, ORF1ab:A20268G, S:A23403G, S:T24076C, N:C28854T, N:G29543T,	4	N:S194L, ORF1a:A181T, ORF1b:P314L, S:D614G,	
hCoV-19/Mexico/MEX-InDRE-IBT-123/2020	EPI_ISL_1301579	In process	20B	B.1.1	8	5'UTR:C241T, ORF1ab:C3037T, ORF1ab:C14408T, ORF1ab:T19839C, S:A23403G, N:G28881A, N:G28882A, N:G28883C, N:C29545T,	4	N:R203K, N:G204R, ORF1b:P314L, S:D614G,	
hCoV-19/Mexico/MEX-InDRE-IBT-124/2020	EPI_ISL_1301494	In process	20A	B.1.243	6	5'UTR:C241T, ORF1ab:C3037T, ORF1ab:C14408T, S:A23403G, S:C24034T, S:T24076C, N:C28854T,	3	N:S194L, ORF1b:P314L, S:D614G,	
hCoV-19/Mexico/MEX-InDRE-IBT-125/2020	EPI_ISL_1301709	In process	20A	B.1.609	7	5'UTR:C241T, ORF1ab:C3037T, ORF1ab:C4582T, ORF1ab:C13119T, ORF1ab:C14408T, ORF1ab:A20268G, S:A23403G, 3'UTR:G29781T,	3	ORF1a:A4285V, ORF1b:P314L, S:D614G,	

hCoV-19/Mexico/MEX-INER-IMSS-00098/2021	EPI_ISL_1279383	In process	20B	B.1.1.519	29	5'UTR:C203T, 5'UTR:C222T, 5'UTR:C241T, ORF1ab:C3037T, ORF1ab:C3140T, ORF1ab:G3692T, ORF1ab:G3871T, ORF1ab:C4158T, ORF1ab:A4405G, ORF1ab:C9520T, ORF1ab:C10029T, ORF1ab:C10954T, ORF1ab:A11117G, ORF1ab:C11824A, ORF1ab:C12789T, ORF1ab:C14408T, ORF1ab:C14676T, ORF1ab:C18747T, ORF1ab:T19839C, ORF1ab:C21306T, S:G21724T, S:C22995A, S:A23403G, S:C23604A, S:A23756G, ORF8:T27904C, N:G28881A, N:G28882A, N:G28883C, N:C29197T,	16	N:R203K, N:G204R, ORF1a:P959S, ORF1a:V1143F, ORF1a:K1202N, ORF1a:A1298V, ORF1a:T3255I, ORF1a:I3618V, ORF1a:T4175I, ORF1b:P314L, ORF8:L4P, S:L54F, S:T478K, S:D614G, S:P681H, S:T732A,
hCoV-19/Mexico/MEX-INER-IMSS-00100/2021	EPI_ISL_1279384	In process	20B	B.1.1.519	25	5'UTR:C203T, 5'UTR:C222T, 5'UTR:C241T, ORF1ab:C337T, ORF1ab:C3037T, ORF1ab:C3140T, ORF1ab:C9559T, ORF1ab:C10029T, ORF1ab:A10829C, ORF1ab:C10954T, ORF1ab:A11117G, ORF1ab:C12789T, ORF1ab:C14408T, ORF1ab:T19839C, ORF1ab:C21306T, S:C22986T, S:C22995A, S:A23403G, S:C23604A, S:A23756G, ORF8:T27904C, ORF8:C28087T, N:G28881A, N:G28882A, N:G28883C, N:C29197T,	15	N:R203K, N:G204R, ORF1a:P959S, ORF1a:T3255I, ORF1a:I3522L, ORF1a:I3618V, ORF1a:T4175I, ORF1b:P314L, ORF8:L4P, ORF8:A65V, S:A475V, S:T478K, S:D614G, S:P681H, S:T732A,
hCoV-19/Mexico/MEX-INER-IMSS-00101/2021	EPI_ISL_1279385	In process	20B	B.1.1.519	28	5'UTR:T201C, 5'UTR:C203T, 5'UTR:C222T, 5'UTR:C241T, ORF1ab:C745T, ORF1ab:G1738T, ORF1ab:C1779T, ORF1ab:C3037T, ORF1ab:C3140T, ORF1ab:C10029T, ORF1ab:C10954T, ORF1ab:A11117G, ORF1ab:C11824T, ORF1ab:C12789T, ORF1ab:C14408T, ORF1ab:G18040T, ORF1ab:T19839C, ORF1ab:A19974G, ORF1ab:C21306T, S:G22017T, S:C22995A, S:A23403G, S:C23604A, S:A23756G, ORF8:G28086T, N:G28881A, N:G28882A, N:G28883C, N:C29197T,	15	N:R203K, N:G204R, ORF1a:S505F, ORF1a:P959S, ORF1a:T3255I, ORF1a:I3618V, ORF1a:T4175I, ORF1b:P314L, ORF1b:A1525S, ORF8:A65S, S:W152L, S:T478K, S:D614G, S:P681H, S:T732A,
hCoV-19/Mexico/MEX-INER-IMSS-00103/2021	EPI_ISL_1279387	In process	20B	B.1.1.519	25	5'UTR:C203T, 5'UTR:C222T, 5'UTR:C241T, ORF1ab:C629T, ORF1ab:C3037T, ORF1ab:C3140T, ORF1ab:C10029T, ORF1ab:C10954T, ORF1ab:A11117G, ORF1ab:C12669T, ORF1ab:C12789T, ORF1ab:C14408T, ORF1ab:C14621T, ORF1ab:C15108T, ORF1ab:C15933T, ORF1ab:T19839C, ORF1ab:C21306T, S:C22995A, S:A23403G, S:C23604A, S:A23756G, N:G28881A, N:G28882A, N:G28883C, N:C29197T, N:G29527T,	15	N:R203K, N:G204R, N:Q418H, ORF1a:L122F, ORF1a:P959S, ORF1a:T3255I, ORF1a:I3618V, ORF1a:S4135F, ORF1a:T4175I, ORF1b:P314L, ORF1b:T385M, S:T478K, S:D614G, S:P681H, S:T732A,
hCoV-19/Mexico/MEX-INER-IMSS-00104/2021	EPI_ISL_1279388	In process	20B	B.1.1.519	26	5'UTR:C203T, 5'UTR:C222T, 5'UTR:C241T, ORF1ab:C3037T, ORF1ab:C3140T, ORF1ab:G7037T, ORF1ab:C10029T, ORF1ab:C10186T, ORF1ab:C10954T, ORF1ab:A11117G, ORF1ab:G11417T, ORF1ab:C12789T, ORF1ab:C14408T, ORF1ab:G18394T, ORF1ab:T19839C, ORF1ab:C21306T, S:C21846T, S:C22995A, S:A23403G, S:C23604A, S:A23756G, S:T24856C, ORF3a:C25782T, N:G28881A, N:G28882A, N:G28883C, N:C29197T,	15	N:R203K, N:G204R, ORF1a:P959S, ORF1a:G2258C, ORF1a:T3255I, ORF1a:I3618V, ORF1a:V3718F, ORF1a:T4175I, ORF1b:P314L, ORF1b:A1643S, S:T95I, S:T478K, S:D614G, S:P681H, S:T732A,
hCoV-19/Mexico/MEX-INER-IMSS-00106/2021	EPI_ISL_1279390	In process	20B	B.1.1.519	27	5'UTR:T201C, 5'UTR:C203T, 5'UTR:C222T, 5'UTR:C241T, ORF1ab:G1738T, ORF1ab:C3037T, ORF1ab:C3140T, ORF1ab:G4476A, ORF1ab:C7267T, ORF1ab:C10029T, ORF1ab:G10283A, ORF1ab:C10954T, ORF1ab:A11117G, ORF1ab:C12789T, ORF1ab:C14408T, ORF1ab:T19839C, ORF1ab:A19974G, ORF1ab:C21306T, S:C22995A, S:A23403G, S:C23604A, S:A23756G, M:C26585T, ORF7a:G27604A, N:G28881A, N:G28882A, N:G28883C, N:C29197T	14	N:R203K, N:G204R, ORF1a:P959S, ORF1a:R1404H, ORF1a:T3255I, ORF1a:V3340I, ORF1a:I3618V, ORF1a:T4175I, ORF1b:P314L, ORF7a:V71I, S:T478K, S:D614G, S:P681H, S:T732A,

hCoV-19/Mexico/MEX-INER-IMSS-00107/2021	EPI_ISL_1279391	In process	20B	B.1.1.519	21	5'UTR:C203T, 5'UTR:C222T, 5'UTR:C241T, ORF1ab:C3037T, ORF1ab:C3140T, ORF1ab:C10029T, ORF1ab:C10954T, ORF1ab:A11117G, ORF1ab:C12789T, ORF1ab:C14408T, ORF1ab:T19839C, ORF1ab:C21306T, S:C22995A, S:A23403G, S:C23604A, S:A23756G, ORF8:T27904C, ORF8:C28087T, N:G28881A, N:G28882A, N:G28883C, N:C29197T,	13	N:R203K, N:G204R, ORF1a:P959S, ORF1a:T3255I, ORF1a:I3618V, ORF1a:T4175I, ORF1b:P314L, ORF8:L4P, ORF8:A65V, S:T478K, S:D614G, S:P681H, S:T732A,
hCoV-19/Mexico/MEX-INER-IMSS-00113/2021	EPI_ISL_1279396	In process	20B	B.1.1.519	26	5'UTR:C203T, 5'UTR:C222T, 5'UTR:C241T, ORF1ab:G942A, ORF1ab:C3037T, ORF1ab:C3140T, ORF1ab:C8814T, ORF1ab:C10029T, ORF1ab:C10954T, ORF1ab:A11117G, ORF1ab:C11124T, ORF1ab:C12789T, ORF1ab:C14408T, ORF1ab:T19839C, ORF1ab:A20676G, ORF1ab:C21306T, S:T22246C, S:C22995A, S:A23403G, S:C23604A, S:A23756G, ORF7a:T27722C, N:G28881A, N:G28882A, N:G28883C, N:C29197T, 3'UTR:G29759T,	15	N:R203K, N:G204R, ORF1a:R226K, ORF1a:P959S, ORF1a:A2850V, ORF1a:T3255I, ORF1a:I3618V, ORF1a:A3620V, ORF1a:T4175I, ORF1b:P314L, ORF7a:I110T, S:T478K, S:D614G, S:P681H, S:T732A,
hCoV-19/Mexico/MEX-INER-IMSS-00187/2021	EPI_ISL_1279458	In process	20B	B.1.1.519	27	5'UTR:C203T, 5'UTR:C222T, 5'UTR:C241T, ORF1ab:C3037T, ORF1ab:C3140T, ORF1ab:C10029T, ORF1ab:C10954T, ORF1ab:A11117G, ORF1ab:C12789T, ORF1ab:C13005T, ORF1ab:C14408T, ORF1ab:C17352T, ORF1ab:G17794A, ORF1ab:T19839C, ORF1ab:T21015C, ORF1ab:C21306T, S:C22281T, S:C22995A, S:A23403G, S:C23604A, S:A23756G, S:C25300T, S:G25302T, ORF8:G27915A, N:G28881A, N:G28882A, N:G28883C, N:C29197T,	16	N:R203K, N:G204R, ORF1a:P959S, ORF1a:T3255I, ORF1a:I3618V, ORF1a:T4175I, ORF1a:A4247V, ORF1b:P314L, ORF1b:A1443T, ORF8:G8R, S:T240I, S:T478K, S:D614G, S:P681H, S:T732A, S:C1247F,
hCoV-19/Mexico/MEX-INER-IMSS-00306/2021	EPI_ISL_1279559	In process	20B	B.1.1.519	27	5'UTR:C203T, 5'UTR:C222T, 5'UTR:C241T, ORF1ab:G2281A, ORF1ab:C3037T, ORF1ab:C3140T, ORF1ab:C3311T, ORF1ab:G5230T, ORF1ab:A8031G, ORF1ab:C10029T, ORF1ab:C10954T, ORF1ab:A11117G, ORF1ab:C12789T, ORF1ab:C14408T, ORF1ab:A19084C, ORF1ab:T19839C, ORF1ab:T20154C, ORF1ab:C21306T, S:C21998T, S:C22995A, S:A23403G, S:C23604A, S:A23756G, ORF3a:C25714T, N:G28881A, N:G28882A, N:G28883C, N:C29197T,	17	N:R203K, N:G204R, ORF1a:P959S, ORF1a:L1016F, ORF1a:K1655N, ORF1a:K2589R, ORF1a:T3255I, ORF1a:I3618V, ORF1a:T4175I, ORF1b:P314L, ORF1b:K1873Q, ORF3a:L108F, S:H146Y, S:T478K, S:D614G, S:P681H, S:T732A,
hCoV-19/Mexico/MEX-INER-IMSS-00308/2021	EPI_ISL_1279561	In process	20B	B.1.1.519	25	5'UTR:C203T, 5'UTR:C222T, 5'UTR:C241T, ORF1ab:A3021G, ORF1ab:C3037T, ORF1ab:C3140T, ORF1ab:C10029T, ORF1ab:A10829C, ORF1ab:C10954T, ORF1ab:A11117G, ORF1ab:C12789T, ORF1ab:C14408T, ORF1ab:T17307C, ORF1ab:T19839C, ORF1ab:C21306T, S:G22785T, S:C22995A, S:A23403G, S:C23604A, S:A23756G, ORF8:T27904C, ORF8:C28087T, N:G28881A, N:G28882A, N:G28883C, N:C29197T,	16	N:R203K, N:G204R, ORF1a:H919R, ORF1a:P959S, ORF1a:T3255I, ORF1a:I3522L, ORF1a:I3618V, ORF1a:T4175I, ORF1b:P314L, ORF8:L4P, ORF8:A65V, S:R408I, S:T478K, S:D614G, S:P681H, S:T732A,
hCoV-19/Mexico/MEX-INER-IMSS-00309/2021	EPI_ISL_1279562	In process	20B	B.1.1.519	31	5'UTR:T201C, 5'UTR:C203T, 5'UTR:C222T, 5'UTR:C241T, ORF1ab:C936T, ORF1ab:G1738T, ORF1ab:C3037T, ORF1ab:C3140T, ORF1ab:C5817T, ORF1ab:C10029T, ORF1ab:C10647T, ORF1ab:C10954T, ORF1ab:A11117G, ORF1ab:C12789T, ORF1ab:C14408T, ORF1ab:C14937T, ORF1ab:T19839C, ORF1ab:A19974G, ORF1ab:G21204T, ORF1ab:C21306T, S:C22995A, S:C23202T, S:A23403G, S:C23604A, S:A23756G, S:G23868T, ORF8:G28086T, N:G28881A, N:G28882A, N:G28883C, N:C29197T, N:G29227T	18	N:R203K, N:G204R, ORF1a:T224I, ORF1a:P959S, ORF1a:A1851V, ORF1a:T3255I, ORF1a:T3461I, ORF1a:I3618V, ORF1a:T4175I, ORF1b:P314L, ORF1b:K2579N, ORF8:A65S, S:T478K, S:T547I, S:D614G, S:P681H, S:T732A, S:G769V,

hCoV-19/Mexico/MEX-INER-IMSS-00310/2021	EPI_ISL_1279563	In process	20B	B.1.1.519	29	5'UTR:C203T, 5'UTR:C222T, 5'UTR:C241T, ORF1ab:C1314T, ORF1ab:C2189T, ORF1ab:C3037T, ORF1ab:C3140T, ORF1ab:G3692T, ORF1ab:G3871T, ORF1ab:G5558T, ORF1ab:A8962G, ORF1ab:C10029T, ORF1ab:C10954T, ORF1ab:A11117G, ORF1ab:C11824A, ORF1ab:C12789T, ORF1ab:C14408T, ORF1ab:G17686T, ORF1ab:T19839C, ORF1ab:C21306T, S:C22995A, S:A23403G, S:C23604A, S:A23756G, ORF8:T27904C, N:C28744T, N:G28881A, N:G28882A, N:G28883C, N:C29197T,	18	N:R203K, N:G204R, ORF1a:T350I, ORF1a:L642F, ORF1a:P959S, ORF1a:V1143F, ORF1a:K1202N, ORF1a:V1765L, ORF1a:T3255I, ORF1a:I3618V, ORF1a:T4175I, ORF1b:P314L, ORF1b:V1407F, ORF8:L4P, S:T478K, S:D614G, S:P681H, S:T732A,	
hCoV-19/Mexico/MEX-INER-IMSS-00320/2021	EPI_ISL_1279570	In process	20B	B.1.1.519	26	5'UTR:C203T, 5'UTR:C222T, 5'UTR:C241T, ORF1ab:C3037T, ORF1ab:C3140T, ORF1ab:C6629T, ORF1ab:C7926T, ORF1ab:T9070C, ORF1ab:C10029T, ORF1ab:C10954T, ORF1ab:A11117G, ORF1ab:C11173T, ORF1ab:C12789T, ORF1ab:C14408T, ORF1ab:T19839C, ORF1ab:C21306T, S:C21575T, S:C22995A, S:A23403G, S:C23604A, S:A23756G, N:G28337T, N:G28881A, N:G28882A, N:G28883C, N:C29197T, N:G29527T,	17	N:D22Y, N:R203K, N:G204R, N:Q418H, ORF1a:P959S, ORF1a:L2122F, ORF1a:A2554V, ORF1a:T3255I, ORF1a:I3618V, ORF1a:T4175I, ORF1b:P314L, ORF9b:Q18H, S:L5F, S:T478K, S:D614G, S:P681H, S:T732A,	
hCoV-19/Mexico/MEX-INER-IMSS-00321/2021	EPI_ISL_1279571	In process	20B	B.1.1.519	27	5'UTR:C203T, 5'UTR:C222T, 5'UTR:C241T, ORF1ab:C3037T, ORF1ab:C3140T, ORF1ab:C4002T, ORF1ab:C10029T, ORF1ab:C10954T, ORF1ab:A11117G, ORF1ab:C12789T, ORF1ab:T13345C, ORF1ab:C14408T, ORF1ab:C18264T, ORF1ab:C18283T, ORF1ab:T19839C, ORF1ab:C21306T, S:C22088T, S:C22995A, S:A23403G, S:C23604A, S:A23756G, E:A26435G, N:G28881A, N:G28882A, N:G28883C, N:C29197T, N:G29527T, 3'UTR:C29741T,	16	E:N64S, N:R203K, N:G204R, N:Q418H, ORF1a:P959S, ORF1a:T1246I, ORF1a:T3255I, ORF1a:I3618V, ORF1a:T4175I, ORF1b:P314L, ORF1b:H1606Y, S:L176F, S:T478K, S:D614G, S:P681H, S:T732A,	
hCoV-19/Mexico/MEX-INER-IMSS-00322/2021	EPI_ISL_1279572	In process	20B	B.1.1.519	28	5'UTR:T201C, 5'UTR:C203T, 5'UTR:C222T, 5'UTR:C241T, ORF1ab:C936T, ORF1ab:G1738T, ORF1ab:G2447T, ORF1ab:C3037T, ORF1ab:C3140T, ORF1ab:C9611T, ORF1ab:C10029T, ORF1ab:C10954T, ORF1ab:A11117G, ORF1ab:T11458C, ORF1ab:C12789T, ORF1ab:C14408T, ORF1ab:T19839C, ORF1ab:A19974G, ORF1ab:C21306T, S:C22995A, S:A23403G, S:C23604A, S:A23756G, S:T25316C, N:G28881A, N:G28882A, N:G28883C, N:C29197T, N:G29227T	15	N:R203K, N:G204R, ORF1a:T224I, ORF1a:G728C, ORF1a:P959S, ORF1a:L3116F, ORF1a:T3255I, ORF1a:I3618V, ORF1a:T4175I, ORF1b:P314L, S:T478K, S:D614G, S:P681H, S:T732A, S:S1252P,	
hCoV-19/Mexico/MEX-INER-IMSS-00323/2021	EPI_ISL_1279573	In process	20B	B.1.1.519	27	5'UTR:T201C, 5'UTR:C203T, 5'UTR:C222T, 5'UTR:C241T, ORF1ab:G1738T, ORF1ab:C3037T, ORF1ab:C3140T, ORF1ab:C10029T, ORF1ab:C10954T, ORF1ab:A11117G, ORF1ab:C12789T, ORF1ab:C14408T, ORF1ab:C16092T, ORF1ab:T19839C, ORF1ab:A19974G, ORF1ab:C21306T, S:G22241T, S:C22995A, S:A23403G, S:C23604A, S:A23756G, ORF3a:C25553T, E:C26408T, N:G28881A, N:G28882A, N:G28883C, N:C29197T, 3'UTR:G29755T,	14	E:S55F, N:R203K, N:G204R, ORF1a:P959S, ORF1a:T3255I, ORF1a:I3618V, ORF1a:T4175I, ORF1b:P314L, ORF3a:A54V, S:V227L, S:T478K, S:D614G, S:P681H, S:T732A,	
hCoV-19/Mexico/MEX-INER-IMSS-00324/2021	EPI_ISL_1279297	In process	20A	B.1	25	5'UTR:C241T, ORF1ab:A2010G, ORF1ab:C3037T, ORF1ab:A3904T, ORF1ab:C9344T, ORF1ab:C11020T, ORF1ab:A11430G, ORF1ab:C14408T, ORF1ab:C15960T, ORF1ab:A16534G, ORF1ab:T17673G, ORF1ab:G20060T, ORF1ab:A20268G, S:C21618T, S:C21707T, S:C22338G, S:T22912G, S:G22992A, S:A23403G, M:C26681T, M:G26709A, M:C26728T, ORF7a:C27494T, ORF8:A28273T, N:G28514T, N:G28917T,	18	M:A63T, M:A69V, N:D81Y, N:G215V, ORF1a:D582G, ORF1a:L3027F, ORF1a:Y3722C, ORF1b:P314L, ORF1b:S1023G, ORF1b:S2198I, ORF7a:P34L, ORF9b:Q77H, S:T19I, S:H49Y, S:T259R, S:N450K, S:S477N, S:D614G,	S:21983-21994, ORF3a:26155-26157,

hCoV-19/Mexico/MEX-INER-IMSS-00325/2021	EPI_ISL_1279574	In process	21C (Epsilon)	B.1.427	23	5'UTR:C241T, ORF1ab:C1059T, ORF1ab:C3037T, ORF1ab:G4207T, ORF1ab:G9738C, ORF1ab:C10714T, ORF1ab:C11345T, ORF1ab:G13713A, ORF1ab:C14408T, ORF1ab:C14877T, ORF1ab:C16394T, ORF1ab:G17014T, S:G21600T, S:G22018T, S:T22917G, S:A23403G, ORF3a:C25511T, ORF3a:G25563T, M:C26681T, M:C27016T, ORF8:A28272T, N:C28887T, N:C29362T, 3'UTR:G29690T.	14	M:P165L, N:T205I, ORF1a:T265I, ORF1a:S3158T, ORF1a:L3694F, ORF1b:P314L, ORF1b:P976L, ORF1b:D1183Y, ORF3a:S40L, ORF3a:Q57H, S:S13I, S:W152C, S:L452R, S:D614G,	
hCoV-19/Mexico/MEX-INER-IMSS-00326/2021	EPI_ISL_1279575	In process	20B	B.1.1.519	26	5'UTR:T201C, 5'UTR:C203T, 5'UTR:C222T, 5'UTR:C241T, ORF1ab:C936T, ORF1ab:G1738T, ORF1ab:C3037T, ORF1ab:C3140T, ORF1ab:C10029T, ORF1ab:C10954T, ORF1ab:A11117G, ORF1ab:C12789T, ORF1ab:C14408T, ORF1ab:T19839C, ORF1ab:A19974G, ORF1ab:G21157A, ORF1ab:C21306T, S:G22599A, S:C22995A, S:A23403G, S:C23604A, S:A23756G, N:G28881A, N:G28882A, N:G28883C, N:C29197T, N:G29227T.	14	N:R203K, N:G204R, ORF1a:T224I, ORF1a:P959S, ORF1a:T3255I, ORF1a:I3618V, ORF1a:T4175I, ORF1b:P314L, ORF1b:V2564M, S:R346K, S:T478K, S:D614G, S:P681H, S:T732A,	
hCoV-19/Mexico/MEX-INER-IMSS-00327/2021	EPI_ISL_1279576	In process	20B	B.1.1.519	26	5'UTR:T201C, 5'UTR:C203T, 5'UTR:C222T, 5'UTR:C241T, ORF1ab:G1738T, ORF1ab:C3037T, ORF1ab:C3140T, ORF1ab:C3177T, ORF1ab:C10029T, ORF1ab:C10954T, ORF1ab:A11117G, ORF1ab:C12789T, ORF1ab:C14408T, ORF1ab:T19839C, ORF1ab:A19974G, ORF1ab:C21306T, S:C22995A, S:A23403G, S:C23604A, S:G23608T, S:A23756G, S:T24271G, N:G28881A, N:G28882A, N:G28883C, N:C29197T, N:C29311T.	12	N:R203K, N:G204R, ORF1a:P959S, ORF1a:P971L, ORF1a:T3255I, ORF1a:I3618V, ORF1a:T4175I, ORF1b:P314L, S:T478K, S:D614G, S:P681H, S:T732A,	
hCoV-19/Mexico/MEX-INER-IMSS-00328/2021	EPI_ISL_1287777	In process	20B	B.1.1.519	26	5'UTR:C203T, 5'UTR:C222T, 5'UTR:C241T, ORF1ab:C3037T, ORF1ab:C3140T, ORF1ab:G3620T, ORF1ab:C10029T, ORF1ab:C10954T, ORF1ab:A11117G, ORF1ab:G12014T, ORF1ab:C12789T, ORF1ab:C14408T, ORF1ab:G15418T, ORF1ab:T19839C, ORF1ab:C21306T, S:C22995A, S:A23403G, S:C23604A, S:A23756G, ORF3a:T25473C, ORF3a:C25782T, E:C26256T, N:G28881A, N:G28882A, N:G28883C, N:C29197T, N:A29371C,	14	N:R203K, N:G204R, ORF1a:P959S, ORF1a:G1119C, ORF1a:T3255I, ORF1a:I3618V, ORF1a:V3917F, ORF1a:T4175I, ORF1b:P314L, ORF1b:A651S, S:T478K, S:D614G, S:P681H, S:T732A,	ORF7b:27850-27988
hCoV-19/Mexico/MEX-INER-IMSS-00329/2021	EPI_ISL_1279577	In process	20B	B.1.1.519	26	5'UTR:C203T, 5'UTR:C222T, 5'UTR:C241T, ORF1ab:A866G, ORF1ab:C1009T, ORF1ab:C3037T, ORF1ab:C3140T, ORF1ab:C5183T, ORF1ab:C10029T, ORF1ab:C10954T, ORF1ab:A11117G, ORF1ab:C11916T, ORF1ab:C12789T, ORF1ab:C14408T, ORF1ab:T19839C, ORF1ab:C21306T, S:C22995A, S:A23403G, S:C23604A, S:A23756G, ORF8:T27904C, ORF8:G28178A, N:G28747T, N:G28881A, N:G28882A, N:G28883C, N:C29197T,	15	N:R203K, N:G204R, ORF1a:I201V, ORF1a:P959S, ORF1a:P1640S, ORF1a:T3255I, ORF1a:I3618V, ORF1a:S3884L, ORF1a:T4175I, ORF1b:P314L, ORF8:L4P, S:T478K, S:D614G, S:P681H, S:T732A,	
hCoV-19/Mexico/MEX-INER-IMSS-00335/2021	EPI_ISL_1279583	In process	20B	B.1.1.519	23	5'UTR:C203T, 5'UTR:C222T, 5'UTR:C241T, ORF1ab:C3037T, ORF1ab:C3140T, ORF1ab:C7104T, ORF1ab:C10029T, ORF1ab:C10954T, ORF1ab:A11117G, ORF1ab:C12789T, ORF1ab:C14408T, ORF1ab:T19839C, ORF1ab:C21306T, S:C21638T, S:T22246C, S:C22995A, S:A23403G, S:C23604A, S:A23756G, ORF3a:C25528T, N:G28881A, N:G28882A, N:G28883C, N:C29197T.	14	N:R203K, N:G204R, ORF1a:P959S, ORF1a:T2280I, ORF1a:T3255I, ORF1a:I3618V, ORF1a:T4175I, ORF1b:P314L, ORF3a:L46F, S:P26S, S:T478K, S:D614G, S:P681H, S:T732A,	

hCoV-19/Mexico/MEX-INER-IMSS-00338/2021	EPI_ISL_1279585	In process	20B	B.1.1.519	24	5'UTR:C203T, 5'UTR:C222T, 5'UTR:C241T, ORF1ab:C3037T, ORF1ab:C3140T, ORF1ab:T3745C, ORF1ab:G4133A, ORF1ab:C10029T, ORF1ab:C10954T, ORF1ab:A11117G, ORF1ab:G12106A, ORF1ab:C12789T, ORF1ab:C14408T, ORF1ab:A19397C, ORF1ab:T19839C, ORF1ab:C21306T, S:C22995A, S:A23403G, S:C23604A, S:A23756G, ORF3a:C25844T, N:G28881A, N:G28882A, N:G28883C, N:C29197T,	14	N:R203K, N:G204R, ORF1a:P959S, ORF1a:V1290I, ORF1a:T3255I, ORF1a:I3618V, ORF1a:T4175I, ORF1b:P314L, ORF1b:E1977A, ORF3a:T151I, S:T478K, S:D614G, S:P681H, S:T732A,	
hCoV-19/Mexico/MEX-INER-IMSS-00339/2021	EPI_ISL_1279586	In process	20B	B.1.1.519	28	5'UTR:C203T, 5'UTR:C222T, 5'UTR:C241T, ORF1ab:C2005T, ORF1ab:C2037T, ORF1ab:C3037T, ORF1ab:C3140T, ORF1ab:T3745C, ORF1ab:C4345T, ORF1ab:C10029T, ORF1ab:A10323G, ORF1ab:C10954T, ORF1ab:A11117G, ORF1ab:C12789T, ORF1ab:T12934C, ORF1ab:A13904G, ORF1ab:C14408T, ORF1ab:C16407T, ORF1ab:T19839C, ORF1ab:C21306T, S:C22995A, S:A23403G, S:C23604A, S:A23756G, ORF3a:C25844T, N:G28881A, N:G28882A, N:G28883C, N:C29197T,	15	N:R203K, N:G204R, ORF1a:A591V, ORF1a:P959S, ORF1a:T3255I, ORF1a:K3353R, ORF1a:I3618V, ORF1a:T4175I, ORF1b:D146G, ORF1b:P314L, ORF3a:T151I, S:T478K, S:D614G, S:P681H, S:T732A,	
hCoV-19/Mexico/MEX-INER-IMSS-00340/2021	EPI_ISL_1279303	In process	20B	B.1.1.222	19	5'UTR:C241T, ORF1ab:C3037T, ORF1ab:C10449T, ORF1ab:C14408T, ORF1ab:A14601G, ORF1ab:C14805T, ORF1ab:C19554T, ORF1ab:T19839C, S:C21646T, S:G22344C, S:A23403G, S:A23756G, ORF3a:C26151T, ORF7a:C27707T, ORF8:C27925T, N:C28473T, N:G28881A, N:G28882A, N:G28883C, ORF10:T29577G.	11	N:P67L, N:R203K, N:G204R, ORF1a:P3395L, ORF1b:P314L, ORF7a:A105V, ORF8:T11I, ORF9b:L64F, S:G261A, S:D614G, S:T732A,	ORF8:28 271,
hCoV-19/Mexico/MEX-INER-IMSS-00351/2021	EPI_ISL_1279597	In process	20B	B.1.1.519	29	5'UTR:C106T, 5'UTR:C203T, 5'UTR:C222T, 5'UTR:C241T, ORF1ab:C3037T, ORF1ab:C3140T, ORF1ab:C6629T, ORF1ab:C7926T, ORF1ab:T9070C, ORF1ab:C10029T, ORF1ab:C10954T, ORF1ab:A11117G, ORF1ab:C11173T, ORF1ab:C12789T, ORF1ab:C14408T, ORF1ab:T19839C, ORF1ab:C21306T, S:C22747T, S:C22995A, S:T23042C, S:A23403G, S:C23604A, S:A23756G, S:A25050G, N:G28337T, N:G28881A, N:G28882A, N:G28883C, N:C29197T, N:G29527T,	18	N:D22Y, N:R203K, N:G204R, N:Q418H, ORF1a:P959S, ORF1a:L2122F, ORF1a:A2554V, ORF1a:T3255I, ORF1a:I3618V, ORF1a:T4175I, ORF1b:P314L, ORF9b:Q18H, S:T478K, S:S494P, S:D614G, S:P681H, S:T732A, S:D1163G,	
hCoV-19/Mexico/MEX-INER-IMSS-00356/2021	EPI_ISL_1279601	In process	20B	B.1.1.519	25	5'UTR:C203T, 5'UTR:C222T, 5'UTR:C241T, ORF1ab:C2005T, ORF1ab:C3037T, ORF1ab:C3140T, ORF1ab:T3745C, ORF1ab:C7296T, ORF1ab:C10029T, ORF1ab:C10954T, ORF1ab:A11117G, ORF1ab:C12789T, ORF1ab:C14408T, ORF1ab:T19839C, ORF1ab:C21306T, S:C22995A, S:A23403G, S:C23604A, S:A23756G, ORF3a:C25844T, ORF7a:G27459T, N:G28307A, N:G28881A, N:G28882A, N:G28883C, N:C29197T,	16	N:A12T, N:R203K, N:G204R, ORF1a:P959S, ORF1a:A2344V, ORF1a:T3255I, ORF1a:I3618V, ORF1a:T4175I, ORF1b:P314L, ORF3a:T151I, ORF7a:E22D, ORF9b:M8I, S:T478K, S:D614G, S:P681H, S:T732A,	
hCoV-19/Mexico/MIC-IBT-IMSS-459/2020	EPI_ISL_1301642	In process	20B	B.1.1	10	5'UTR:C241T, ORF1ab:G1135T, ORF1ab:A2024G, ORF1ab:C3037T, ORF1ab:C14408T, ORF1ab:T19839C, S:A23403G, ORF6:C27213T, N:G28881A, N:G28882A, N:G28883C,	6	N:R203K, N:G204R, ORF1a:K290N, ORF1a:T587A, ORF1b:P314L, S:D614G,	
hCoV-19/Mexico/MOR-IBT-IMSS-103/2021	EPI_ISL_1288167	In process	20B	B.1.1.322	16	5'UTR:C241T, ORF1ab:G1042T, ORF1ab:C1567T, ORF1ab:C3037T, ORF1ab:T8038C, ORF1ab:G11330T, ORF1ab:C14408T, ORF1ab:C18582T, ORF1ab:T19839C, ORF1ab:T21462C, S:A23403G, S:A23756G, N:A28877T, N:G28878C, N:G28881A, N:G28882A, N:G28883C,	7	N:R203K, N:G204R, ORF1a:L259F, ORF1a:V3689L, ORF1b:P314L, S:D614G, S:T732A,	

hCoV-19/Mexico/MOR-IBT-IMSS-168/2021	EPI_ISL_1288369	In process	20B	B.1.1.519	19	5'UTR:C203T, 5'UTR:C222T, 5'UTR:C241T, ORF1ab:C3037T, ORF1ab:C3140T, ORF1ab:C10029T, ORF1ab:C10954T, ORF1ab:A11117G, ORF1ab:C12789T, ORF1ab:C14408T, ORF1ab:T19839C, ORF1ab:C21306T, S:C22995A, S:A23403G, S:C23604A, S:A23756G, N:G28881A, N:G28882A, N:G28883C, N:C29197T,	11	N:R203K, N:G204R, ORF1a:P959S, ORF1a:T3255I, ORF1a:I3618V, ORF1a:T4175I, ORF1b:P314L, S:T478K, S:D614G, S:P681H, S:T732A,
hCoV-19/Mexico/MOR-IBT-IMSS-172/2021	EPI_ISL_1288371	In process	21C (Epsilon)	B.1.429	27	5'UTR:C241T, ORF1ab:C1059T, ORF1ab:C2395T, ORF1ab:T2597C, ORF1ab:C3037T, ORF1ab:C8947T, ORF1ab:C10335T, ORF1ab:C12100T, ORF1ab:A12878G, ORF1ab:G13425A, ORF1ab:C14408T, ORF1ab:G14559T, ORF1ab:G17014T, S:G21600T, S:G22018T, S:T22917G, S:A23403G, S:T24349C, ORF3a:G25563T, E:A26441G, M:C26681T, M:C27092A, ORF7b:G27890T, ORF8:A28272T, N:C28887T, N:T29047C, N:C29362T, N:C29445T	15	E:N66S, M:D190E, N:T205I, N:T391I, ORF1a:T265I, ORF1a:A3357V, ORF1a:I4205V, ORF1a:R4387H, ORF1b:P314L, ORF1b:D1183Y, ORF3a:Q57H, S:S13I, S:W152C, S:L452R, S:D614G,
hCoV-19/Mexico/MOR-IBT-IMSS-173/2021	EPI_ISL_1288372	In process	20B	B.1.1.519	30	5'UTR:T201C, 5'UTR:C203T, 5'UTR:C222T, 5'UTR:C241T, ORF1ab:C1218T, ORF1ab:G1738T, ORF1ab:C3037T, ORF1ab:C3140T, ORF1ab:G4907A, ORF1ab:C10029T, ORF1ab:C10954T, ORF1ab:A11117G, ORF1ab:C12789T, ORF1ab:C13517T, ORF1ab:C14408T, ORF1ab:C18457T, ORF1ab:C19586T, ORF1ab:T19839C, ORF1ab:A19974G, ORF1ab:C21306T, S:C21621T, S:G22801T, S:C22995A, S:A23403G, S:C23604A, S:A23756G, N:G28881A, N:G28882A, N:G28883C, N:C29197T, 3'UTR:G29800A	17	N:R203K, N:G204R, ORF1a:S318L, ORF1a:P959S, ORF1a:G1548S, ORF1a:T3255I, ORF1a:I3618V, ORF1a:T4175I, ORF1b:T17I, ORF1b:P314L, ORF1b:P1664S, ORF1b:T2040I, S:T20I, S:T478K, S:D614G, S:P681H, S:T732A,
hCoV-19/Mexico/MOR-IBT-IMSS-174/2021	EPI_ISL_1288373	In process	20B	B.1.1.519	29	5'UTR:C203T, 5'UTR:C222T, 5'UTR:C241T, ORF1ab:T2820C, ORF1ab:C3037T, ORF1ab:C3140T, ORF1ab:C10029T, ORF1ab:C10954T, ORF1ab:A11117G, ORF1ab:A12556C, ORF1ab:C12789T, ORF1ab:C14408T, ORF1ab:G15732T, ORF1ab:C18131T, ORF1ab:G18651T, ORF1ab:T19839C, ORF1ab:C20719T, ORF1ab:C21306T, S:A22320G, S:C22995A, S:A23403G, S:C23604A, S:A23756G, ORF7a:A27533T, N:G28881A, N:G28882A, N:G28883C, N:C29149T, N:C29197T, N:G29527T,	18	N:R203K, N:G204R, N:Q418H, ORF1a:V852A, ORF1a:P959S, ORF1a:T3255I, ORF1a:I3618V, ORF1a:E4097D, ORF1a:T4175I, ORF1b:P314L, ORF1b:T1555I, ORF1b:E1728D, ORF7a:H47L, S:D253G, S:T478K, S:D614G, S:P681H, S:T732A,
hCoV-19/Mexico/MOR-IBT-IMSS-175/2021	EPI_ISL_1288374	In process	21C (Epsilon)	B.1.429	26	5'UTR:C241T, ORF1ab:C1059T, ORF1ab:C2395T, ORF1ab:C3037T, ORF1ab:C8947T, ORF1ab:C10335T, ORF1ab:C12100T, ORF1ab:A12878G, ORF1ab:G13425A, ORF1ab:C14408T, ORF1ab:G14559T, ORF1ab:G17014T, S:G21600T, S:G22018T, S:T22917G, S:A23403G, S:T24349C, ORF3a:G25563T, E:A26441G, M:C26681T, M:C27092A, ORF7b:G27890T, ORF8:A28272T, N:C28887T, N:T29047C, N:C29362T, N:C29445T,	15	E:N66S, M:D190E, N:T205I, N:T391I, ORF1a:T265I, ORF1a:A3357V, ORF1a:I4205V, ORF1a:R4387H, ORF1b:P314L, ORF1b:D1183Y, ORF3a:Q57H, S:S13I, S:W152C, S:L452R, S:D614G,
hCoV-19/Mexico/MOR-IBT-IMSS-176/2021	EPI_ISL_1288375	In process	20B	B.1.1.519	26	5'UTR:C203T, 5'UTR:C222T, 5'UTR:C241T, ORF1ab:C3037T, ORF1ab:C3140T, ORF1ab:G6894A, ORF1ab:C10029T, ORF1ab:C10954T, ORF1ab:A11117G, ORF1ab:G11365T, ORF1ab:C12789T, ORF1ab:C14408T, ORF1ab:C14805T, ORF1ab:C14928T, ORF1ab:C16726T, ORF1ab:T19839C, ORF1ab:C21306T, S:C22995A, S:A23403G, S:C23604A, S:A23756G, N:T28360C, N:G28881A, N:G28882A, N:G28883C, N:C29197T, N:G29527T,	15	N:R203K, N:G204R, N:Q418H, ORF1a:P959S, ORF1a:C2210Y, ORF1a:T3255I, ORF1a:I3618V, ORF1a:T4175I, ORF1b:P314L, ORF1b:H1087Y, ORF9b:M26T, S:T478K, S:D614G, S:P681H, S:T732A,

hCoV-19/Mexico/MOR-IBT-IMSS-27/2021	EPI_ISL_1288246	In process	20B	B.1.1.519	23	5'UTR:C203T, 5'UTR:C222T, 5'UTR:C241T, ORF1ab:C3037T, ORF1ab:C3140T, ORF1ab:C10029T, ORF1ab:C10138T, ORF1ab:C10954T, ORF1ab:A11117G, ORF1ab:C11575T, ORF1ab:C12789T, ORF1ab:C14408T, ORF1ab:T19839C, ORF1ab:C21306T, S:A22330G, S:C22995A, S:A23403G, S:C23604A, S:A23756G, N:G28881A, N:G28882A, N:G28883C, N:C29197T, N:C29358T.	12	N:R203K, N:G204R, N:T362I, ORF1a:P959S, ORF1a:T3255I, ORF1a:I3618V, ORF1a:T4175I, ORF1b:P314L, S:T478K, S:D614G, S:P681H, S:T732A,
hCoV-19/Mexico/MOR-IBT-IMSS-319/2021	EPI_ISL_1288501	In process	20B	B.1.1.519	27	5'UTR:C203T, 5'UTR:C222T, 5'UTR:C241T, ORF1ab:C1457T, ORF1ab:C3037T, ORF1ab:C3140T, ORF1ab:C10029T, ORF1ab:C10954T, ORF1ab:A11117G, ORF1ab:C12741T, ORF1ab:C12789T, ORF1ab:C14408T, ORF1ab:T19839C, ORF1ab:C21306T, S:G22017T, S:C22995A, S:A23403G, S:C23604A, S:A23756G, S:G24914T, ORF3a:C25782T, ORF3a:G26063T, E:G26428T, N:C28377T, N:G28881A, N:G28882A, N:G28883C, N:C29197T,	19	E:V62F, N:A35V, N:R203K, N:G204R, ORF1a:R398C, ORF1a:P959S, ORF1a:T3255I, ORF1a:I3618V, ORF1a:T4159I, ORF1a:T4175I, ORF1b:P314L, ORF3a:G224V, ORF9b:R32C, S:W152L, S:T478K, S:D614G, S:P681H, S:T732A, S:D1118Y,
hCoV-19/Mexico/MOR-IBT-IMSS-320/2021	EPI_ISL_1288502	In process	20A	B.1.243	20	5'UTR:C241T, ORF1ab:C274T, ORF1ab:G806A, ORF1ab:C1302T, ORF1ab:C3037T, ORF1ab:C5140A, ORF1ab:C6027T, ORF1ab:A9992T, ORF1ab:C14408T, ORF1ab:G14701T, ORF1ab:C17642T, ORF1ab:C18486T, ORF1ab:A20268G, S:G22241T, S:A23403G, S:G23587C, S:T24076C, ORF3a:C25708T, E:C26455T, N:C28854T, N:G29543T,	14	E:P71S, N:S194L, ORF1a:A181T, ORF1a:T346I, ORF1a:D1625E, ORF1a:P1921L, ORF1a:N3243Y, ORF1b:P314L, ORF1b:D412Y, ORF1b:A1392V, ORF3a:L106F, S:V227L, S:D614G, S:Q675H,
hCoV-19/Mexico/MOR-IBT-IMSS-321/2021	EPI_ISL_1288503	In process	20B	B.1.1.519	27	5'UTR:C203T, 5'UTR:C222T, 5'UTR:C241T, ORF1ab:C900T, ORF1ab:C3037T, ORF1ab:C3140T, ORF1ab:C6092A, ORF1ab:C6541T, ORF1ab:C10029T, ORF1ab:C10954T, ORF1ab:A11117G, ORF1ab:C12789T, ORF1ab:G14118T, ORF1ab:C14408T, ORF1ab:A19831G, ORF1ab:T19839C, ORF1ab:C20133T, ORF1ab:C21306T, S:A22023C, S:C22995A, S:A23403G, S:C23604A, S:A23756G, ORF8:T27904C, N:G28881A, N:G28882A, N:G28883C, N:C29197T,	16	N:R203K, N:G204R, ORF1a:S212L, ORF1a:P959S, ORF1a:Q1943K, ORF1a:T3255I, ORF1a:I3618V, ORF1a:T4175I, ORF1b:P314L, ORF1b:I2122V, ORF8:L4P, S:E154A, S:T478K, S:D614G, S:P681H, S:T732A,
hCoV-19/Mexico/MOR-IBT-IMSS-322/2021	EPI_ISL_1288504	In process	20B	B.1.1.519	27	5'UTR:T201C, 5'UTR:C203T, 5'UTR:C222T, 5'UTR:C241T, ORF1ab:G1738T, ORF1ab:C3037T, ORF1ab:C3140T, ORF1ab:C8389T, ORF1ab:C10029T, ORF1ab:C10954T, ORF1ab:C11109T, ORF1ab:A11117G, ORF1ab:G11851T, ORF1ab:C12789T, ORF1ab:C14408T, ORF1ab:G19735T, ORF1ab:T19839C, ORF1ab:A19974G, ORF1ab:C21306T, S:C22995A, S:A23403G, S:C23604A, S:A23756G, S:G24368T, N:G28881A, N:G28882A, N:G28883C, N:C29197T,	15	N:R203K, N:G204R, ORF1a:P959S, ORF1a:T3255I, ORF1a:A3615V, ORF1a:I3618V, ORF1a:M3862I, ORF1a:T4175I, ORF1b:P314L, ORF1b:D2090Y, S:T478K, S:D614G, S:P681H, S:T732A, S:D936Y,
hCoV-19/Mexico/MOR-IBT-IMSS-323/2021	EPI_ISL_1288505	In process	20B	B.1.1.519	25	5'UTR:C203T, 5'UTR:C222T, 5'UTR:C241T, ORF1ab:G1820A, ORF1ab:C3037T, ORF1ab:C3140T, ORF1ab:C10029T, ORF1ab:C10954T, ORF1ab:A11117G, ORF1ab:C12513T, ORF1ab:T13905C, ORF1ab:C14408T, ORF1ab:A19631G, ORF1ab:T19839C, ORF1ab:C21306T, S:C22995A, S:A23403G, S:C23604A, S:A23756G, ORF3a:G25906T, ORF3a:C25916T, ORF7a:G27415A, N:G28881A, N:G28882A, N:G28883C, N:C29197T,	16	N:R203K, N:G204R, ORF1a:G519S, ORF1a:P959S, ORF1a:T3255I, ORF1a:I3618V, ORF1a:T4083M, ORF1b:P314L, ORF1b:N2055S, ORF3a:G172C, ORF3a:T175I, ORF7a:A8T, S:T478K, S:D614G, S:P681H, S:T732A,

hCoV-19/Mexico/MOR-IBT-IMSS-324/2021	EPI_ISL_1288506	In process	20B	B.1.1.519	25	5'UTR:T201C, 5'UTR:C203T, 5'UTR:C222T, 5'UTR:C241T, ORF1ab:G1738T, ORF1ab:C3037T, ORF1ab:C3140T, ORF1ab:C10029T, ORF1ab:C10954T, ORF1ab:A11117G, ORF1ab:C12789T, ORF1ab:C14408T, ORF1ab:C14694T, ORF1ab:A17342G, ORF1ab:T19839C, ORF1ab:A19974G, ORF1ab:C21306T, S:C22995A, S:A23403G, S:C23604A, S:A23756G, N:G28881A, N:G28882A, N:G28883C, N:G29041T, N:C29197T,	13	N:R203K, N:G204R, N:K256N, ORF1a:P959S, ORF1a:T3255I, ORF1a:I3618V, ORF1a:T4175I, ORF1b:P314L, ORF1b:D1292G, S:T478K, S:D614G, S:P681H, S:T732A,	
hCoV-19/Mexico/MOR-IBT-IMSS-370/2021	EPI_ISL_1288156	In process	20B	B.1.1.519	28	5'UTR:T201C, 5'UTR:C203T, 5'UTR:C222T, 5'UTR:C241T, ORF1ab:C745T, ORF1ab:G1738T, ORF1ab:C3037T, ORF1ab:C3140T, ORF1ab:G6254A, ORF1ab:C10029T, ORF1ab:C10954T, ORF1ab:A11117G, ORF1ab:A11887G, ORF1ab:C12789T, ORF1ab:C14408T, ORF1ab:T19839C, ORF1ab:A19974G, ORF1ab:C21306T, S:C22995A, S:A23403G, S:C23604A, S:A23756G, ORF3a:C26201T, ORF8:G28077T, ORF8:G28178T, N:G28881A, N:G28882A, N:G28883C, N:C29197T,	15	N:R203K, N:G204R, ORF1a:P959S, ORF1a:A1997T, ORF1a:T3255I, ORF1a:I3618V, ORF1a:T4175I, ORF1b:P314L, ORF3a:T270I, ORF8:V62L, ORF8:L95F, S:T478K, S:D614G, S:P681H, S:T732A,	
hCoV-19/Mexico/MOR-IBT-IMSS-381/2021	EPI_ISL_1288157	In process	20B	B.1.1.519	22	5'UTR:C203T, 5'UTR:C222T, 5'UTR:C241T, ORF1ab:C3037T, ORF1ab:C3140T, ORF1ab:G7037T, ORF1ab:C10029T, ORF1ab:C10954T, ORF1ab:A11117G, ORF1ab:C12789T, ORF1ab:C14408T, ORF1ab:T19839C, ORF1ab:C19875T, ORF1ab:C21306T, S:C22995A, S:A23403G, S:C23604A, S:A23756G, ORF3a:C25782T, N:G28881A, N:G28882A, N:G28883C, N:C29197T,	12	N:R203K, N:G204R, ORF1a:P959S, ORF1a:G2258C, ORF1a:T3255I, ORF1a:I3618V, ORF1a:T4175I, ORF1b:P314L, S:T478K, S:D614G, S:P681H, S:T732A,	3'UTR:29819-29821,
hCoV-19/Mexico/MOR-IBT-IMSS-386/2021	EPI_ISL_1288213	In process	20B	B.1.1.519	24	5'UTR:C203T, 5'UTR:C222T, 5'UTR:C241T, ORF1ab:C3037T, ORF1ab:C3140T, ORF1ab:C10029T, ORF1ab:T10210C, ORF1ab:C10954T, ORF1ab:A11117G, ORF1ab:C12789T, ORF1ab:C14408T, ORF1ab:T19839C, ORF1ab:C21306T, S:C21707T, S:G22205C, S:C22995A, S:A23403G, S:C23604A, S:C23638T, S:A23756G, S:C23887T, N:G28881A, N:G28882A, N:G28883C, N:C29197T,	13	N:R203K, N:G204R, ORF1a:P959S, ORF1a:T3255I, ORF1a:I3618V, ORF1a:T4175I, ORF1b:P314L, S:H49Y, S:D215H, S:T478K, S:D614G, S:P681H, S:T732A,	
hCoV-19/Mexico/MOR-IBT-IMSS-387/2021	EPI_ISL_1288214	In process	20B	B.1.1.519	22	5'UTR:C203T, 5'UTR:C222T, 5'UTR:C241T, ORF1ab:C3037T, ORF1ab:C3140T, ORF1ab:C10029T, ORF1ab:C10954T, ORF1ab:A11117G, ORF1ab:C12789T, ORF1ab:C14408T, ORF1ab:T19839C, ORF1ab:C21306T, S:C22995A, S:A23403G, S:C23604A, S:A23756G, M:G26690T, ORF8:T27904C, ORF8:C28087T, N:G28881A, N:G28882A, N:G28883C, N:C29197T,	13	N:R203K, N:G204R, ORF1a:P959S, ORF1a:T3255I, ORF1a:I3618V, ORF1a:T4175I, ORF1b:P314L, ORF8:L4P, ORF8:A65V, S:T478K, S:D614G, S:P681H, S:T732A,	
hCoV-19/Mexico/MOR-IBT-IMSS-388/2021	EPI_ISL_1288215	In process	20B	B.1.1.519	25	5'UTR:C203T, 5'UTR:C222T, 5'UTR:C241T, ORF1ab:C1102T, ORF1ab:C3037T, ORF1ab:C3140T, ORF1ab:C10029T, ORF1ab:C10954T, ORF1ab:C11094T, ORF1ab:A11117G, ORF1ab:T11419C, ORF1ab:C12789T, ORF1ab:C14408T, ORF1ab:T19839C, ORF1ab:C21306T, S:C22995A, S:A23403G, S:C23604A, S:A23756G, ORF3a:C25810T, ORF8:T27904C, N:G28827T, N:G28881A, N:G28882A, N:G28883C, N:C29197T,	16	N:R185L, N:R203K, N:G204R, ORF1a:P959S, ORF1a:T3255I, ORF1a:A3610V, ORF1a:I3618V, ORF1a:T4175I, ORF1b:P314L, ORF3a:L140F, ORF8:L4P, S:Y145D, S:T478K, S:D614G, S:P681H, S:T732A,	S:21987-21995,
hCoV-19/Mexico/MOR-IBT-IMSS-389/2021	EPI_ISL_1288216	In process	20B	B.1.1.519	22	5'UTR:T201C, 5'UTR:C203T, 5'UTR:C222T, 5'UTR:C241T, ORF1ab:G1738T, ORF1ab:C3037T, ORF1ab:C3140T, ORF1ab:C10029T, ORF1ab:C10954T, ORF1ab:A11117G, ORF1ab:C12789T, ORF1ab:C14408T, ORF1ab:T19839C, ORF1ab:A19974G, ORF1ab:C21306T, S:C22995A, S:A23403G, S:C23604A, S:A23756G, N:G28881A, N:G28882A, N:G28883C, N:C29197T,	11	N:R203K, N:G204R, ORF1a:P959S, ORF1a:T3255I, ORF1a:I3618V, ORF1a:T4175I, ORF1b:P314L, S:T478K, S:D614G, S:P681H, S:T732A,	

hCoV-19/Mexico/MOR-IBT-IMSS-390/2021	EPI_ISL_1288217	In process	20B	B.1.1.519	27	5'UTR:C203T, 5'UTR:C222T, 5'UTR:C241T, ORF1ab:C3037T, ORF1ab:C3140T, ORF1ab:G5206T, ORF1ab:C6651T, ORF1ab:C10029T, ORF1ab:C10954T, ORF1ab:A11117G, ORF1ab:C12439T, ORF1ab:C12789T, ORF1ab:C13724T, ORF1ab:C14408T, ORF1ab:C14925T, ORF1ab:G18756T, ORF1ab:T19839C, ORF1ab:C21306T, S:C22995A, S:A23403G, S:C23604A, S:A23756G, ORF8:T27904C, N:C28697T, N:G28881A, N:G28882A, N:G28883C, N:C29197T,	16	N:P142S, N:R203K, N:G204R, ORF1a:P959S, ORF1a:M1647I, ORF1a:A2129V, ORF1a:T3255I, ORF1a:I3618V, ORF1a:T4175I, ORF1b:A86V, ORF1b:P314L, ORF8:L4P, S:T478K, S:D614G, S:P681H, S:T732A,
hCoV-19/Mexico/MOR-IBT-IMSS-392/2021	EPI_ISL_1288158	In process	20B	B.1.1.519	25	5'UTR:C203T, 5'UTR:C222T, 5'UTR:C241T, ORF1ab:C3037T, ORF1ab:C3140T, ORF1ab:A3262G, ORF1ab:C6070T, ORF1ab:C10029T, ORF1ab:C10954T, ORF1ab:A11117G, ORF1ab:C14408T, ORF1ab:G15921T, ORF1ab:A17971G, ORF1ab:T19839C, ORF1ab:C21306T, S:C22995A, S:A23403G, S:C23604A, S:C23635T, S:A23756G, ORF3a:T25695C, ORF7a:C27406T, N:G28881A, N:G28882A, N:G28883C, N:C29197T,	12	N:R203K, N:G204R, ORF1a:P959S, ORF1a:T3255I, ORF1a:I3618V, ORF1b:P314L, ORF1b:R1502G, ORF7a:L5F, S:T478K, S:D614G, S:P681H, S:T732A,
hCoV-19/Mexico/MOR-IBT-IMSS-403/2021	EPI_ISL_1288159	In process	20B	B.1.1.519	22	5'UTR:C203T, 5'UTR:C222T, 5'UTR:C241T, ORF1ab:C3037T, ORF1ab:C3140T, ORF1ab:G3549A, ORF1ab:C10029T, ORF1ab:C10954T, ORF1ab:A11117G, ORF1ab:C14408T, ORF1ab:T19839C, ORF1ab:C21306T, S:C22995A, S:A23403G, S:C23604A, S:G23678T, S:A23756G, M:G26526T, ORF8:T27904C, N:G28881A, N:G28882A, N:G28883C, N:C29197T,	14	M:A2S, N:R203K, N:G204R, ORF1a:P959S, ORF1a:G1095E, ORF1a:T3255I, ORF1a:I3618V, ORF1b:P314L, ORF8:L4P, S:T478K, S:D614G, S:P681H, S:A706S, S:T732A,
hCoV-19/Mexico/MOR-IBT-IMSS-414/2021	EPI_ISL_1288160	In process	20B	B.1.1.519	24	5'UTR:C203T, 5'UTR:C222T, 5'UTR:C241T, ORF1ab:C3037T, ORF1ab:C3140T, ORF1ab:T3745C, ORF1ab:C10029T, ORF1ab:C10954T, ORF1ab:A11117G, ORF1ab:C14134T, ORF1ab:C14408T, ORF1ab:T19839C, ORF1ab:C21306T, S:T22729C, S:C22995A, S:A23403G, S:C23604A, S:A23756G, ORF3a:C25844T, N:C28849T, N:G28881A, N:G28882A, N:G28883C, N:C29197T, 3'UTR:G29734T,	12	N:R203K, N:G204R, ORF1a:P959S, ORF1a:T3255I, ORF1a:I3618V, ORF1b:P223S, ORF1b:P314L, ORF3a:T151I, S:T478K, S:D614G, S:P681H, S:T732A,
hCoV-19/Mexico/MOR-IBT-IMSS-453/2020	EPI_ISL_1301636	In process	20A	B.1.610	5	5'UTR:C241T, ORF1ab:C3037T, ORF1ab:C14408T, ORF1ab:A20268G, S:A23403G, N:G29300T,	3	N:D343Y, ORF1b:P314L, S:D614G,
hCoV-19/Mexico/MOR-IBT-IMSS-464/2020	EPI_ISL_1301647	In process	20A	B.1.609	5	5'UTR:C241T, ORF1ab:C3037T, ORF1ab:C4582T, ORF1ab:C13119T, ORF1ab:C14408T, S:A23403G,	3	ORF1a:A4285V, ORF1b:P314L, S:D614G,
hCoV-19/Mexico/MOR-IBT-IMSS-465/2020	EPI_ISL_1301648	In process	20A	B.1	5	5'UTR:C241T, ORF1ab:T1237C, ORF1ab:C3037T, ORF1ab:C14408T, S:A23403G, 3'UTR:G29773T,	2	ORF1b:P314L, S:D614G,
hCoV-19/Mexico/MOR-IBT-IMSS-467/2020	EPI_ISL_1301650	In process	20A	B.1.609	8	5'UTR:C241T, ORF1ab:C2334T, ORF1ab:C3037T, ORF1ab:C4582T, ORF1ab:T12230G, ORF1ab:C14408T, ORF1ab:C15017T, ORF1ab:A20268G, S:A23403G,	5	ORF1a:A690V, ORF1a:S3989A, ORF1b:P314L, ORF1b:A517V, S:D614G,
hCoV-19/Mexico/MOR-IBT-IMSS-471/2020	EPI_ISL_1301653	In process	20C	B.1	9	5'UTR:C241T, ORF1ab:A297G, ORF1ab:C1059T, ORF1ab:C3037T, ORF1ab:C14408T, S:A23403G, ORF3a:G25563T, M:C27167T, ORF8:G27987T, 3'UTR:C29750T,	6	ORF1a:K11R, ORF1a:T265I, ORF1b:P314L, ORF3a:Q57H, ORF8:V32L, S:D614G,
hCoV-19/Mexico/MOR-IBT-IMSS-472/2020	EPI_ISL_1301654	In process	20C	B.1	9	5'UTR:C241T, ORF1ab:C1059T, ORF1ab:C3037T, ORF1ab:C14408T, ORF1ab:C15952A, ORF1ab:C16616T, S:A23403G, S:G25074T, ORF3a:G25563T, 3'UTR:C29750T,	7	ORF1a:T265I, ORF1b:P314L, ORF1b:L829I, ORF1b:T1050I, ORF3a:Q57H, S:D614G, S:G1171V,
hCoV-19/Mexico/MOR-IBT-IMSS-496/2020	EPI_ISL_1301595	In process	20A	B.1	8	5'UTR:C241T, ORF1ab:T1237C, ORF1ab:C3037T, ORF1ab:C4158T, ORF1ab:C14408T, S:A23403G, S:A24118T, ORF7a:G27561T, 3'UTR:G29773T,	3	ORF1a:A1298V, ORF1b:P314L, S:D614G,

hCoV-19/Mexico/MOR-IBT-IMSS-81/2021	EPI_ISL_1288165	In process	20B	B.1.1.517	25	5'UTR:C241T, ORF1ab:G2890T, ORF1ab:C3037T, ORF1ab:A3148G, ORF1ab:C6541T, ORF1ab:C10029T, ORF1ab:C11919T, ORF1ab:C14408T, ORF1ab:C15738T, ORF1ab:G17721T, ORF1ab:T19839C, ORF1ab:G19891T, S:A23403G, S:A23756G, ORF3a:G25567A, ORF3a:T25569A, ORF3a:T25570A, ORF3a:G25912T, ORF3a:A26108G, ORF7a:G27670T, ORF8:A27921G, ORF8:G28001T, N:G28881A, N:G28882A, N:G28883C, 3'UTR:G29766C,	14	N:R203K, N:G204R, ORF1a:T3255I, ORF1a:S3885F, ORF1b:P314L, ORF1b:D2142Y, ORF3a:A59T, ORF3a:S60T, ORF3a:G174C, ORF3a:E239G, ORF7a:V93F, ORF8:I10V, S:D614G, S:T732A,	
hCoV-19/Mexico/MOR-IBT-IMSS-92/2021	EPI_ISL_1288166	In process	20B	B.1.1.222	18	5'UTR:C241T, ORF1ab:C3037T, ORF1ab:G3085T, ORF1ab:G3871T, ORF1ab:C5482T, ORF1ab:A6542G, ORF1ab:G12988T, ORF1ab:C14408T, ORF1ab:T19839C, S:G22225T, S:A23403G, S:A23756G, ORF3a:G25906T, ORF7b:C27828T, ORF8:G27916T, N:G28881A, N:G28882A, N:G28883C, N:C29445T.	13	N:R203K, N:G204R, N:T391I, ORF1a:E940D, ORF1a:K1202N, ORF1a:T2093A, ORF1a:M4241I, ORF1b:P314L, ORF3a:G172C, ORF7b:L25F, ORF8:G8V, S:D614G, S:T732A,	
hCoV-19/Mexico/MOR-InDRE-IBT-145/2020	EPI_ISL_1302265	In process	20B	B.1.1	22	5'UTR:C190T, 5'UTR:C241T, ORF1ab:C1513T, ORF1ab:C1594T, ORF1ab:G3004T, ORF1ab:C3037T, ORF1ab:G13993T, ORF1ab:C14408T, ORF1ab:C14919T, ORF1ab:T19839C, S:G22299T, S:A23403G, S:G23426A, S:C23604A, ORF3a:G25522A, ORF3a:T25580C, ORF7b:G27890A, ORF8:G28198T, N:G28881A, N:G28882A, N:G28883C, N:C29144T, 3'UTR:G29747T,	12	N:R203K, N:G204R, ORF1a:E913D, ORF1b:A176S, ORF1b:P314L, ORF3a:G44R, ORF3a:I63T, ORF8:C102F, S:R246I, S:D614G, S:V622I, S:P681H,	
hCoV-19/Mexico/MOR-InDRE-IBT-147/2020	EPI_ISL_1302162	In process	20B	B.1.1.519	20	5'UTR:C203T, 5'UTR:C222T, 5'UTR:C241T, ORF1ab:C3037T, ORF1ab:C3140T, ORF1ab:G7037T, ORF1ab:C10029T, ORF1ab:C10954T, ORF1ab:A11117G, ORF1ab:C12789T, ORF1ab:C14408T, ORF1ab:T19839C, S:C22995A, S:A23403G, S:C23604A, S:A23756G, ORF3a:C25782T, N:G28881A, N:G28882A, N:G28883C. N:C29197T.	12	N:R203K, N:G204R, ORF1a:P959S, ORF1a:G2258C, ORF1a:T3255I, ORF1a:I3618V, ORF1a:T4175I, ORF1b:P314L, S:T478K, S:D614G, S:P681H, S:T732A,	3'UTR:29819-29821,
hCoV-19/Mexico/MOR-InDRE-IBT-148/2020	EPI_ISL_1302382	In process	20B	B.1.1.519	25	5'UTR:T201C, 5'UTR:C203T, 5'UTR:C222T, 5'UTR:C241T, ORF1ab:G1738T, ORF1ab:C3037T, ORF1ab:C3140T, ORF1ab:C8389T, ORF1ab:C10029T, ORF1ab:C10954T, ORF1ab:A11117G, ORF1ab:C12789T, ORF1ab:C14408T, ORF1ab:T19839C, ORF1ab:A19974G, ORF1ab:C21306T, S:C22995A, S:C23188T, S:A23403G, S:C23604A, S:A23756G, S:G24368T, N:G28881A, N:G28882A, N:G28883C, N:C29197T,	12	N:R203K, N:G204R, ORF1a:P959S, ORF1a:T3255I, ORF1a:I3618V, ORF1a:T4175I, ORF1b:P314L, S:T478K, S:D614G, S:P681H, S:T732A, S:D936Y,	
hCoV-19/Mexico/MOR-InDRE-IBT-151/2020	EPI_ISL_1302276	In process	20B	B.1.1	24	5'UTR:C190T, 5'UTR:C241T, ORF1ab:C3037T, ORF1ab:C5548T, ORF1ab:G7829T, ORF1ab:C9195T, ORF1ab:G9805T, ORF1ab:C11020T, ORF1ab:C14408T, ORF1ab:G16914T, ORF1ab:T18168C, ORF1ab:T19839C, S:C22033T, S:A23403G, S:C23604A, S:G24445C, ORF3a:G25522A, ORF7b:G27890A, N:A28877T, N:G28878C, N:G28881A, N:G28882A, N:G28883C, N:C29149T, N:G29402T,	9	N:R203K, N:G204R, N:D377Y, ORF1a:V2522F, ORF1a:T2977I, ORF1b:P314L, ORF3a:G44R, S:D614G, S:P681H,	
hCoV-19/Mexico/MOR-InDRE-IBT-185/2020	EPI_ISL_1302358	In process	20G	B.1.2	22	5'UTR:C241T, ORF1ab:C1059T, ORF1ab:A1804G, ORF1ab:A2969G, ORF1ab:C3037T, ORF1ab:T4165C, ORF1ab:C4824T, ORF1ab:C5079T, ORF1ab:G9928T, ORF1ab:C10319T, ORF1ab:C14408T, ORF1ab:A18424G, S:A23403G, ORF3a:G25563T, ORF3a:A25768C, ORF3a:C25782T, ORF3a:G25907T, ORF3a:A26161C, E:C26408T, ORF8:C27964T, N:C28472T, N:C28869T, N:G29422T,	16	E:S55F, N:P67S, N:P199L, ORF1a:T265I, ORF1a:M902V, ORF1a:S1520F, ORF1a:T1605I, ORF1a:M3221I, ORF1a:L3352F, ORF1b:P314L, ORF1b:N1653D, ORF3a:Q57H, ORF3a:G172V, ORF3a:N257H, ORF8:S24L, S:D614G,	

hCoV-19/Mexico/MOR-InDRE-IBT-186/2020	EPI_ISL_1302363	In process	20B	B.1.1.133	24	5'UTR:C190T, ORF1ab:T380A, ORF1ab:G3004T, ORF1ab:C3037T, ORF1ab:C3961T, ORF1ab:C9112T, ORF1ab:G13193A, ORF1ab:C14408T, ORF1ab:A16011G, ORF1ab:C16329T, ORF1ab:C18687T, ORF1ab:T19839C, S:A23403G, S:G23426A, S:C23604A, ORF3a:G25522A, ORF3a:T25580C, ORF3a:C25844T, ORF7b:G27890A, ORF8:C28093T, N:G28881A, N:G28882A, N:G28883C, ORF10:T29650C, 3'UTR:G29747T,	13	N:R203K, N:G204R, ORF1a:L39I, ORF1a:E913D, ORF1a:V4310I, ORF1b:P314L, ORF3a:G44R, ORF3a:I63T, ORF3a:T151I, ORF8:S67F, S:D614G, S:V622I, S:P681H,	
hCoV-19/Mexico/MOR-InDRE-IBT-187/2020	EPI_ISL_1302175	In process	20B	B.1.1.519	22	5'UTR:C203T, 5'UTR:C222T, 5'UTR:C241T, ORF1ab:C3037T, ORF1ab:C3140T, ORF1ab:G3692T, ORF1ab:C10029T, ORF1ab:C10954T, ORF1ab:A11117G, ORF1ab:C12789T, ORF1ab:G12794A, ORF1ab:C13515T, ORF1ab:C14408T, ORF1ab:T19839C, S:C22995A, S:A23403G, S:T23443C, S:C23604A, S:A23756G, N:G28881A, N:G28882A, N:G28883C, N:C29197T,	13	N:R203K, N:G204R, ORF1a:P959S, ORF1a:V1143F, ORF1a:T3255I, ORF1a:I3618V, ORF1a:T4175I, ORF1a:G4177R, ORF1b:P314L, S:T478K, S:D614G, S:P681H, S:T732A,	
hCoV-19/Mexico/MOR-InDRE-IBT-188/2020	EPI_ISL_1302314	In process	20B	B.1.1.222	18	5'UTR:C241T, ORF1ab:C3037T, ORF1ab:G3085T, ORF1ab:G3871T, ORF1ab:C5482T, ORF1ab:A6542G, ORF1ab:C11620T, ORF1ab:G12988T, ORF1ab:C14408T, ORF1ab:T19839C, S:G22225T, S:A23403G, S:A23756G, ORF3a:G25906T, ORF8:G27916T, N:G28881A, N:G28882A, N:G28883C, N:C29445T,	12	N:R203K, N:G204R, N:T391I, ORF1a:E940D, ORF1a:K1202N, ORF1a:T2093A, ORF1a:M4241I, ORF1b:P314L, ORF3a:G172C, ORF8:G8V, S:D614G, S:T732A,	
hCoV-19/Mexico/MOR-InDRE-IBT-189/2020	EPI_ISL_1302340	In process	20B	B.1.1.519	24	5'UTR:C203T, 5'UTR:C222T, 5'UTR:C241T, ORF1ab:C3037T, ORF1ab:C3140T, ORF1ab:C5183T, ORF1ab:A5336T, ORF1ab:C10029T, ORF1ab:C10954T, ORF1ab:A11117G, ORF1ab:C12789T, ORF1ab:T12934C, ORF1ab:C14408T, ORF1ab:T17067C, ORF1ab:C19263T, ORF1ab:T19839C, S:C22995A, S:A23403G, S:C23604A, S:A23756G, ORF8:T27904C, N:G28881A, N:G28882A, N:G28883C, N:C29197T,	14	N:R203K, N:G204R, ORF1a:P959S, ORF1a:P1640S, ORF1a:N1691Y, ORF1a:T3255I, ORF1a:I3618V, ORF1a:T4175I, ORF1b:P314L, ORF8:L4P, S:T478K, S:D614G, S:P681H, S:T732A,	3'UTR:29726-29728,
hCoV-19/Mexico/MOR-InDRE-IBT-190/2020	EPI_ISL_1302209	In process	20B	B.1.1.222	19	5'UTR:C241T, ORF1ab:C3037T, ORF1ab:G3085T, ORF1ab:G3871T, ORF1ab:A6542G, ORF1ab:G12988T, ORF1ab:C14408T, ORF1ab:T19839C, S:G22225T, S:A23403G, S:A23756G, ORF3a:G25906T, ORF8:G27916T, ORF8:C28045T, N:G28881A, N:G28882A, N:G28883C, N:C29445T, 3'UTR:C29686T, 3'UTR:G29711T,	13	N:R203K, N:G204R, N:T391I, ORF1a:E940D, ORF1a:K1202N, ORF1a:T2093A, ORF1a:M4241I, ORF1b:P314L, ORF3a:G172C, ORF8:G8V, ORF8:A51V, S:D614G, S:T732A,	
hCoV-19/Mexico/MOR-InDRE-IBT-191/2020	EPI_ISL_1302396	In process	20B	B.1.1.519	28	5'UTR:T201C, 5'UTR:C203T, 5'UTR:C222T, 5'UTR:C241T, ORF1ab:C936T, ORF1ab:G1738T, ORF1ab:C3037T, ORF1ab:C3140T, ORF1ab:T7106C, ORF1ab:C10029T, ORF1ab:C10954T, ORF1ab:A11117G, ORF1ab:C12789T, ORF1ab:C14408T, ORF1ab:T19839C, ORF1ab:A19974G, ORF1ab:C21306T, S:C22995A, S:A23403G, S:C23604A, S:A23756G, ORF3a:C25626T, ORF7a:C27494T, N:G28881A, N:G28882A, N:G28883C, N:C29197T, N:G29227T, N:C29311T,	14	N:R203K, N:G204R, ORF1a:T224I, ORF1a:P959S, ORF1a:Y2281H, ORF1a:T3255I, ORF1a:I3618V, ORF1a:T4175I, ORF1b:P314L, ORF7a:P34L, S:T478K, S:D614G, S:P681H, S:T732A,	
hCoV-19/Mexico/MOR-InDRE-IBT-192/2020	EPI_ISL_1302295	In process	20B	B.1.1.519	25	5'UTR:T201C, 5'UTR:C203T, 5'UTR:C222T, 5'UTR:C241T, ORF1ab:G1738T, ORF1ab:C3037T, ORF1ab:C3140T, ORF1ab:C10029T, ORF1ab:C10954T, ORF1ab:A11117G, ORF1ab:C12789T, ORF1ab:C14408T, ORF1ab:C15324T, ORF1ab:T19839C, ORF1ab:A19974G, S:C22995A, S:A23403G, S:C23604A, S:A23756G, S:G25244T, M:C26895T, ORF7a:C27427T, N:G28881A, N:G28882A, N:G28883C, N:C29197T,	14	M:H125Y, N:R203K, N:G204R, ORF1a:P959S, ORF1a:T3255I, ORF1a:I3618V, ORF1a:T4175I, ORF1b:P314L, ORF7a:L12F, S:T478K, S:D614G, S:P681H, S:T732A, S:V1228L,	

hCoV-19/Mexico/MOR-InDRE-IBT-193/2020	EPI_ISL_1302168	In process	20B	B.1.1	22	5'UTR:C190T, 5'UTR:C241T, ORF1ab:C1594T, ORF1ab:G3004T, ORF1ab:C3037T, ORF1ab:G8368A, ORF1ab:G13993T, ORF1ab:C14408T, ORF1ab:C14919T, ORF1ab:T19839C, S:G22299T, S:A23403G, S:G23426A, S:C23604A, ORF3a:G25522A, ORF3a:T25580C, ORF7b:G27890A, ORF8:G28198T, N:G28881A, N:G28882A, N:G28883C, N:C29144T, 3'UTR:G29747T,	12	N:R203K, N:G204R, ORF1a:E913D, ORF1b:A176S, ORF1b:P314L, ORF3a:G44R, ORF3a:I63T, ORF8:C102F, S:R246I, S:D614G, S:V622I, S:P681H,
hCoV-19/Mexico/MOR-InDRE-IBT-198/2020	EPI_ISL_1302259	In process	20B	B.1.1	23	5'UTR:C190T, 5'UTR:C241T, ORF1ab:C1594T, ORF1ab:G3004T, ORF1ab:C3037T, ORF1ab:G13993T, ORF1ab:G14020A, ORF1ab:C14408T, ORF1ab:C14919T, ORF1ab:T19839C, S:G22299T, S:A23403G, S:G23426A, S:C23604A, ORF3a:G25522A, ORF3a:T25580C, ORF3a:C25941T, ORF7b:G27890A, ORF8:G28198T, N:G28881A, N:G28882A, N:G28883C, N:C29144T, 3'UTR:G29747T,	13	N:R203K, N:G204R, ORF1a:E913D, ORF1b:A176S, ORF1b:D185N, ORF1b:P314L, ORF3a:G44R, ORF3a:I63T, ORF8:C102F, S:R246I, S:D614G, S:V622I, S:P681H,
hCoV-19/Mexico/MOR-InDRE-IBT-199/2020	EPI_ISL_1302293	In process	20B	B.1.1.519	22	5'UTR:C203T, 5'UTR:C222T, 5'UTR:C241T, ORF1ab:C3037T, ORF1ab:C3140T, ORF1ab:T9456G, ORF1ab:C10029T, ORF1ab:C10954T, ORF1ab:A11117G, ORF1ab:C12789T, ORF1ab:C14408T, ORF1ab:T14429C, ORF1ab:T19839C, S:C22995A, S:A23403G, S:C23604A, S:A23756G, S:C24919T, ORF3a:T25496C, N:G28881A, N:G28882A, N:G28883C, N:C29197T,	14	N:R203K, N:G204R, ORF1a:P959S, ORF1a:F3064C, ORF1a:T3255I, ORF1a:I3618V, ORF1a:T4175I, ORF1b:P314L, ORF1b:V321A, ORF3a:I35T, S:T478K, S:D614G, S:P681H, S:T732A,
hCoV-19/Mexico/MOR-InDRE-IBT-7/2020	EPI_ISL_1301480	In process	20A	B.1.1	7	5'UTR:C241T, ORF1ab:C683T, ORF1ab:T1237C, ORF1ab:C3037T, ORF1ab:C14408T, S:A23403G, 3'UTR:G29773T, 3'UTR:A29815G,	2	ORF1b:P314L, S:D614G,
hCoV-19/Mexico/MOR-InDRE-IBT-98/2020	EPI_ISL_1301478	In process	20A	B.1.1	6	5'UTR:C241T, ORF1ab:C414T, ORF1ab:C3037T, ORF1ab:C8655T, ORF1ab:C10198T, ORF1ab:C14408T, S:A23403G,	4	ORF1a:T50I, ORF1a:S2797F, ORF1b:P314L, S:D614G,
hCoV-19/Mexico/MOR-INER-IMSS-00177/2021	EPI_ISL_1279451	In process	20B	B.1.1	26	5'UTR:C190T, 5'UTR:C241T, ORF1ab:T1885A, ORF1ab:A2786G, ORF1ab:G3004T, ORF1ab:C3037T, ORF1ab:C4776A, ORF1ab:C5407T, ORF1ab:G11801A, ORF1ab:T13972C, ORF1ab:C14408T, ORF1ab:G16795T, ORF1ab:T19839C, S:G22203T, S:A23013G, S:A23403G, S:G23426A, S:C23604A, ORF3a:G25522A, ORF3a:T25580C, ORF6:C27213T, ORF7b:G27890A, N:G28881A, N:G28882A, N:G28883C, N:G29254T, 3'UTR:G29747T,	15	N:R203K, N:G204R, ORF1a:I841V, ORF1a:E913D, ORF1a:T1504N, ORF1a:G3846S, ORF1b:P314L, ORF1b:V1110L, ORF3a:G44R, ORF3a:I63T, S:R214L, S:E484G, S:D614G, S:V622I, S:P681H,
hCoV-19/Mexico/MOR-INER-IMSS-00305/2021	EPI_ISL_1279558	In process	20B	B.1	25	5'UTR:C203T, 5'UTR:C222T, 5'UTR:C241T, ORF1ab:T1303A, ORF1ab:C3037T, ORF1ab:C3140T, ORF1ab:G4720T, ORF1ab:C10029T, ORF1ab:C10319T, ORF1ab:C10954T, ORF1ab:A11117G, ORF1ab:C12789T, ORF1ab:C14408T, ORF1ab:T19839C, ORF1ab:C20703T, ORF1ab:C21306T, S:C22995A, S:A23403G, S:C23604A, S:A23756G, N:G28817A, N:G28881A, N:G28882A, N:G28883C, N:C29197T, ORF10:T29661C,	13	N:A182T, N:R203K, N:G204R, ORF1a:P959S, ORF1a:T3255I, ORF1a:L3352F, ORF1a:I3618V, ORF1a:T4175I, ORF1b:P314L, S:T478K, S:D614G, S:P681H, S:T732A,
hCoV-19/Mexico/MOR-INER-IMSS-00307/2021	EPI_ISL_1279560	In process	20B	B.1.1.519	22	5'UTR:C203T, 5'UTR:C222T, 5'UTR:C241T, ORF1ab:C3037T, ORF1ab:C3140T, ORF1ab:C6539T, ORF1ab:C10029T, ORF1ab:C10954T, ORF1ab:A11117G, ORF1ab:C12789T, ORF1ab:C14408T, ORF1ab:T19839C, ORF1ab:C21306T, S:C22995A, S:A23403G, S:C23604A, S:A23756G, ORF3a:G25489T, ORF3a:C25904T, N:G28881A, N:G28882A, N:G28883C, N:C29197T,	14	N:R203K, N:G204R, ORF1a:P959S, ORF1a:H2092Y, ORF1a:T3255I, ORF1a:I3618V, ORF1a:T4175I, ORF1b:P314L, ORF3a:A33S, ORF3a:S171L, S:T478K, S:D614G, S:P681H, S:T732A,

hCoV-19/Mexico/MOR-INER-IMSS-00333/2021	EPI_ISL_1279581	In process	20B	B.1.1.519	24	5'UTR:C203T, 5'UTR:C222T, 5'UTR:C241T, ORF1ab:C3037T, ORF1ab:C3140T, ORF1ab:T8593C, ORF1ab:C10029T, ORF1ab:C10954T, ORF1ab:A11117G, ORF1ab:C11575T, ORF1ab:C12789T, ORF1ab:C14408T, ORF1ab:T19839C, ORF1ab:C21306T, S:A22330G, S:C22995A, S:A23403G, S:C23604A, S:A23756G, N:G28881A, N:G28882A, N:G28883C, N:C29197T, N:C29358T, N:T29458G,	12	N:R203K, N:G204R, N:T362I, ORF1a:P959S, ORF1a:T3255I, ORF1a:I3618V, ORF1a:T4175I, ORF1b:P314L, S:T478K, S:D614G, S:P681H, S:T732A,	
hCoV-19/Mexico/NAY-InDRE-IBT-77/2020	EPI_ISL_1301694	In process	20A	B.1.241	11	5'UTR:C241T, ORF1ab:C3037T, ORF1ab:C7768T, ORF1ab:C11575T, ORF1ab:C12068T, ORF1ab:C14408T, ORF1ab:A20268G, S:A23403G, ORF7a:T27484C, N:C28399T, N:G28683C, N:C28854T,	5	N:G137A, N:S194L, ORF1b:P314L, ORF9b:P39L, S:D614G,	
hCoV-19/Mexico/NAY-InDRE-IBT-79/2020	EPI_ISL_1301534	In process	20B	B.1	10	5'UTR:C241T, ORF1ab:G1068A, ORF1ab:C3037T, ORF1ab:C14408T, S:A23403G, ORF3a:G26227T, ORF7b:C27847T, N:G28881A, N:G28882A, N:G28883C, 3'UTR:A29700G,	6	N:R203K, N:G204R, ORF1a:G268E, ORF1b:P314L, ORF7b:S31L, S:D614G,	
hCoV-19/Mexico/NAY-InDRE-IBT-80/2020	EPI_ISL_1301559	In process	20A	B.1	6	5'UTR:C241T, ORF1ab:G487T, ORF1ab:C3037T, ORF1ab:C14408T, S:C22858T, S:A23403G, N:C28854T,	3	N:S194L, ORF1b:P314L, S:D614G,	
hCoV-19/Mexico/NAY-InDRE-IBT-81/2020	EPI_ISL_1301560	In process	20A	B.1.241	10	5'UTR:C241T, ORF1ab:C3037T, ORF1ab:C7768T, ORF1ab:C11575T, ORF1ab:C14408T, ORF1ab:A20268G, S:C22326T, S:A23403G, ORF7a:T27484C, N:C28399T, N:C28854T,	5	N:S194L, ORF1b:P314L, ORF9b:P39L, S:S255F, S:D614G,	
hCoV-19/Mexico/NAY-InDRE-IBT-82/2020	EPI_ISL_1301561	In process	20B	B.1.1.344	12	5'UTR:C241T, ORF1ab:C3037T, ORF1ab:T9172C, ORF1ab:A10948G, ORF1ab:C14119T, ORF1ab:C14408T, ORF1ab:T16950C, S:A23403G, M:G26730T, N:G28881A, N:G28882A, N:G28883C, 3'UTR:G29773T,	6	M:V70F, N:R203K, N:G204R, ORF1b:P218S, ORF1b:P314L, S:D614G,	
hCoV-19/Mexico/NLE-INER-IMSS-00192/2021	EPI_ISL_1279463	In process	20B	B.1.1.432	27	5'UTR:C241T, ORF1ab:C3037T, ORF1ab:G3122T, ORF1ab:A6985T, ORF1ab:G8990A, ORF1ab:C9319T, ORF1ab:C10369T, ORF1ab:C12412T, ORF1ab:C12784T, ORF1ab:T14313C, ORF1ab:C14408T, ORF1ab:C16394T, S:G21770T, S:C22127T, S:C23127T, S:A23403G, S:C24382T, ORF3a:C25613T, ORF3a:G25912T, ORF3a:G26062T, N:G28881A, N:G28882A, N:G28883C, N:C28948T, N:C29149T, N:C29167T, N:T29317C, ORF10:C29585T,	13	N:R203K, N:G204R, ORF1a:D953Y, ORF1a:A2909T, ORF1b:P314L, ORF1b:P976L, ORF3a:S74F, ORF3a:G174C, ORF3a:G224C, S:V70F, S:L189F, S:A522V, S:D614G,	
hCoV-19/Mexico/NLE-INER-IMSS-00201/2021	EPI_ISL_1279471	In process	20A	B.1.396	21	5'UTR:C241T, ORF1ab:C1191T, ORF1ab:C2048T, ORF1ab:C3037T, ORF1ab:C3743T, ORF1ab:C9521A, ORF1ab:G10870T, ORF1ab:G11055A, ORF1ab:C14184T, ORF1ab:C14408T, ORF1ab:A16842G, ORF1ab:C17012T, ORF1ab:A20268G, S:A23403G, S:A24696C, S:C24904T, ORF6:G27240T, ORF6:G27382C, N:C28854T, N:G29422T, ORF10:A29567G, 3'UTR:T29685C,	11	N:S194L, ORF1a:P309L, ORF1a:H1160Y, ORF1a:L3086I, ORF1a:S3597N, ORF1b:P314L, ORF1b:S1182L, ORF6:E13D, ORF6:D61H, S:D614G, S:K1045T,	
hCoV-19/Mexico/NLE-INER-IMSS-00202/2021	EPI_ISL_1279287	In process	20B	B.1.1	15	5'UTR:C241T, ORF1ab:C3037T, ORF1ab:T7912C, ORF1ab:C9565T, ORF1ab:C14408T, ORF1ab:A15471T, S:A23403G, S:C23575T, S:G24257T, ORF6:T27231C, ORF8:T28251G, ORF8:C28253T, N:A28741T, N:G28881A, N:G28882A, N:G28883C,	6	N:R203K, N:G204R, ORF1b:P314L, ORF8:F120V, S:D614G, S:A899S,	3'UTR:29718-29720
hCoV-19/Mexico/NLE-INER-IMSS-00203/2021	EPI_ISL_1279472	In process	20A	B.1	18	5'UTR:C241T, ORF1ab:C3037T, ORF1ab:G10882T, ORF1ab:G11521T, ORF1ab:A11782G, ORF1ab:A12124T, ORF1ab:C14408T, ORF1ab:C16386T, ORF1ab:C17883T, ORF1ab:C18115T, ORF1ab:C18508T, ORF1ab:G18745A, ORF1ab:G19518T, ORF1ab:A20268G, S:A23403G, S:C24023T, M:T26966C, ORF7a:G27506C, N:C28854T,	10	N:S194L, ORF1a:M3539I, ORF1a:M3752I, ORF1b:P314L, ORF1b:H1550Y, ORF1b:L1681F, ORF1b:V1760I, ORF1b:L2017F, ORF7a:G38A, S:D614G,	

hCoV-19/Mexico/NLE-INER-IMSS-00204/2021	EPI_ISL_1279298	In process	20G	B.1.2	21	5'UTR:C241T, ORF1ab:C1059T, ORF1ab:C3037T, ORF1ab:G3483A, ORF1ab:C7164T, ORF1ab:C7869T, ORF1ab:C10078T, ORF1ab:C10319T, ORF1ab:T10423C, ORF1ab:C12513T, ORF1ab:C14408T, ORF1ab:T17751G, ORF1ab:A18424G, ORF1ab:C21304T, S:A23403G, ORF3a:G25563T, ORF3a:G25907T, ORF3a:C26139T, ORF8:C27964T, N:C28472T, N:C28869T, 3'UTR:T29760C,	15	N:P67S, N:P199L, ORF1a:T265I, ORF1a:G1073E, ORF1a:T2300I, ORF1a:S2535L, ORF1a:L3352F, ORF1a:T4083M, ORF1b:P314L, ORF1b:N1653D, ORF1b:R2613C, ORF3a:Q57H, ORF3a:G172V, ORF8:S24L, S:D614G,	ORF1ab: 3332-3343
hCoV-19/Mexico/NLE-INER-IMSS-00205/2021	EPI_ISL_1279473	In process	20G	B.1.2	27	5'UTR:C241T, ORF1ab:C527T, ORF1ab:C1059T, ORF1ab:C3037T, ORF1ab:T6691A, ORF1ab:T7348A, ORF1ab:G8083A, ORF1ab:C10319T, ORF1ab:C10448T, ORF1ab:C12115A, ORF1ab:C13119T, ORF1ab:C14408T, ORF1ab:C14805T, ORF1ab:C17597T, ORF1ab:C18131T, ORF1ab:A18424G, ORF1ab:C21304T, S:C21575T, S:A23403G, S:G24348C, S:G24755C, ORF3a:G25563T, ORF3a:G25907T, ORF8:C27964T, ORF8:C28093T, N:C28472T, N:C28869T, 3'UTR:C29784T,	21	N:P67S, N:P199L, ORF1a:T265I, ORF1a:N2361K, ORF1a:M2606I, ORF1a:L3352F, ORF1a:P3395S, ORF1a:A4285V, ORF1b:P314L, ORF1b:A1377V, ORF1b:T1555I, ORF1b:N1653D, ORF1b:R2613C, ORF3a:Q57H, ORF3a:G172V, ORF8:S24L, ORF8:S67F, S:L5F, S:D614G, S:S929T, S:V1065L,	
hCoV-19/Mexico/NLE-INER-IMSS-00206/2021	EPI_ISL_1279474	In process	20A	B.1.396	22	5'UTR:C241T, ORF1ab:C615A, ORF1ab:C1191T, ORF1ab:C2091T, ORF1ab:C3037T, ORF1ab:T9124C, ORF1ab:C9521A, ORF1ab:G9928T, ORF1ab:A10693G, ORF1ab:G10996T, ORF1ab:C14408T, ORF1ab:A20268G, S:C21575T, S:A21634G, S:A23403G, S:C24904T, ORF3a:A25602G, ORF3a:G25644T, M:T27035C, N:C28854T, N:G29422T, ORF10:A29567G, 3'UTR:C29722T,	11	N:S194L, ORF1a:A117D, ORF1a:P309L, ORF1a:T609I, ORF1a:L3086I, ORF1a:M3221I, ORF1a:I3476M, ORF1a:K3577N, ORF1b:P314L, S:L5F, S:D614G,	
hCoV-19/Mexico/NLE-INER-IMSS-00213/2021	EPI_ISL_1279479	In process	20B	B.1.1.432	21	5'UTR:C241T, ORF1ab:C3037T, ORF1ab:C3695T, ORF1ab:G5194T, ORF1ab:A6985T, ORF1ab:G9242A, ORF1ab:C9319T, ORF1ab:C12412T, ORF1ab:T14313C, ORF1ab:C14408T, S:C22938T, S:A23403G, S:C23422T, ORF3a:C25613T, ORF3a:C25844T, ORF3a:G25912T, ORF8:T27909C, N:G28881A, N:G28882A, N:G28883C, N:T29317C, N:A29533G,	10	N:R203K, N:G204R, ORF1a:E2993K, ORF1b:P314L, ORF3a:S74F, ORF3a:T151I, ORF3a:G174C, ORF8:F6L, S:S459F, S:D614G,	
hCoV-19/Mexico/NLE-INER-IMSS-00214/2021	EPI_ISL_1279480	In process	20A	B.1.243	17	5'UTR:C241T, ORF1ab:C3037T, ORF1ab:C4410T, ORF1ab:T5077C, ORF1ab:G9575A, ORF1ab:C10741T, ORF1ab:C14408T, ORF1ab:C16887T, ORF1ab:C17850T, ORF1ab:C19097T, ORF1ab:A19137G, ORF1ab:A20268G, S:A23403G, S:C23604A, S:T24076C, N:C28603T, N:C28854T, 3'UTR:T29710C,	7	N:S194L, ORF1a:A1382V, ORF1a:V3104I, ORF1b:P314L, ORF1b:A1877V, S:D614G, S:P681H,	
hCoV-19/Mexico/NLE-INER-IMSS-00215/2021	EPI_ISL_1279481	In process	20A	B.1.243	18	5'UTR:C241T, ORF1ab:C1218T, ORF1ab:C3037T, ORF1ab:C3264T, ORF1ab:A4166G, ORF1ab:C10741T, ORF1ab:C12119T, ORF1ab:A12356G, ORF1ab:C14408T, ORF1ab:C19097T, ORF1ab:A20268G, S:A23403G, S:C23604A, S:T24076C, S:C24442T, ORF3a:G25595T, ORF3a:T25773C, N:C28854T, 3'UTR:T29710C,	11	N:S194L, ORF1a:S318L, ORF1a:T1000I, ORF1a:I1301V, ORF1a:P3952S, ORF1a:T4031A, ORF1b:P314L, ORF1b:A1877V, ORF3a:R68I, S:D614G, S:P681H,	
hCoV-19/Mexico/NLE-INER-IMSS-00221/2021	EPI_ISL_1279486	In process	20A	B.1.243	17	5'UTR:C241T, ORF1ab:T606C, ORF1ab:C1450T, ORF1ab:C3037T, ORF1ab:A3174T, ORF1ab:C4795T, ORF1ab:C10741T, ORF1ab:C14408T, ORF1ab:C19097T, ORF1ab:A19137G, ORF1ab:A20268G, S:G21800T, S:A23403G, S:C23604A, S:T24076C, S:G25261T, N:C28854T, 3'UTR:T29710C,	9	N:S194L, ORF1a:I114T, ORF1a:Q970L, ORF1b:P314L, ORF1b:A1877V, S:D80Y, S:D614G, S:P681H, S:M1233I,	

hCoV-19/Mexico/NLE-INER-IMSS-00222/2021	EPI_ISL_1279487	In process	20B	B.1.1.222	26	5'UTR:C241T, 5'UTR:G256A, ORF1ab:T2533C, ORF1ab:C3037T, ORF1ab:G6446T, ORF1ab:C8320T, ORF1ab:C8344T, ORF1ab:C9985A, ORF1ab:G11291A, ORF1ab:G11417T, ORF1ab:C12484T, ORF1ab:C14408T, ORF1ab:G17331T, ORF1ab:C17474T, ORF1ab:G18782T, ORF1ab:T19839C, ORF1ab:C20078T, S:C22264T, S:A23403G, S:A23756G, ORF8:C28153T, ORF8:C28253T, N:G28881A, N:G28882A, N:G28883C, N:G28985T, 3'UTR:C29743T	15	N:R203K, N:G204R, N:G238C, ORF1a:V2061F, ORF1a:D3240E, ORF1a:G3676S, ORF1a:V3718F, ORF1b:P314L, ORF1b:E1288D, ORF1b:T1336I, ORF1b:G1772V, ORF1b:P2204L, ORF8:T87I, S:D614G, S:T732A,	
hCoV-19/Mexico/NLE-INER-IMSS-00223/2021	EPI_ISL_1279488	In process	20A	B.1.243	19	5'UTR:C241T, ORF1ab:C1218T, ORF1ab:C3037T, ORF1ab:C3264T, ORF1ab:C10408T, ORF1ab:C10741T, ORF1ab:C12119T, ORF1ab:A12356G, ORF1ab:C14408T, ORF1ab:C19097T, ORF1ab:A20268G, S:A23403G, S:C23604A, S:T24076C, ORF3a:G25407T, ORF3a:G25595T, ORF3a:T25773C, N:C28854T, N:A29442T. 3'UTR:T29710C.	12	N:S194L, N:Q390L, ORF1a:S318L, ORF1a:T1000I, ORF1a:P3952S, ORF1a:T4031A, ORF1b:P314L, ORF1b:A1877V, ORF3a:M5I, ORF3a:R68I, S:D614G, S:P681H,	
hCoV-19/Mexico/NLE-INER-IMSS-00224/2021	EPI_ISL_1279489	In process	20G	B.1.2	21	5'UTR:C241T, ORF1ab:G680A, ORF1ab:C1059T, ORF1ab:C3037T, ORF1ab:A3262G, ORF1ab:C5826T, ORF1ab:C10319T, ORF1ab:C14408T, ORF1ab:C17555T, ORF1ab:A18273C, ORF1ab:A18424G, ORF1ab:C21304T, S:C21575T, S:C21682T, S:A23403G, S:C23604A, ORF3a:G25563T, ORF3a:G25907T, ORF8:C27964T, N:C28472T, N:C28869T, N:T29404C,	17	N:P67S, N:P199L, ORF1a:D139N, ORF1a:T265I, ORF1a:T1854I, ORF1a:L3352F, ORF1b:P314L, ORF1b:T1363I, ORF1b:E1602D, ORF1b:N1653D, ORF1b:R2613C, ORF3a:Q57H, ORF3a:G172V, ORF8:S24L, S:L5F, S:D614G, S:P681H,	
hCoV-19/Mexico/NLE-INER-IMSS-00225/2021	EPI_ISL_1279490	In process	20B	B.1.1.519	32	5'UTR:T201C, 5'UTR:C203T, 5'UTR:C222T, 5'UTR:C241T, ORF1ab:C337T, ORF1ab:C936T, ORF1ab:G1738T, ORF1ab:A3021T, ORF1ab:C3037T, ORF1ab:C3140T, ORF1ab:A8073G, ORF1ab:C10029T, ORF1ab:C10954T, ORF1ab:A11117G, ORF1ab:C12789T, ORF1ab:C14408T, ORF1ab:C17977T, ORF1ab:T19839C, ORF1ab:A19974G, ORF1ab:C21306T, S:C22995A, S:C23191T, S:A23324G, S:A23403G, S:C23604A, S:A23756G, N:C28838T, N:G28881A, N:G28882A, N:G28883C, N:C29197T, N:G29227T, 3'UTR:C29784T,	17	N:R189C, N:R203K, N:G204R, ORF1a:T224I, ORF1a:H919L, ORF1a:P959S, ORF1a:N2603S, ORF1a:T3255I, ORF1a:I3618V, ORF1a:T4175I, ORF1b:P314L, ORF1b:L1504F, S:T478K, S:T588A, S:D614G, S:P681H, S:T732A,	
hCoV-19/Mexico/NLE-INER-IMSS-00226/2021	EPI_ISL_1279491	In process	20A	B.1.243	19	5'UTR:C241T, ORF1ab:G2921T, ORF1ab:C3037T, ORF1ab:G3206T, ORF1ab:C4551T, ORF1ab:G9575A, ORF1ab:C10741T, ORF1ab:C14408T, ORF1ab:C14697T, ORF1ab:G16975T, ORF1ab:C17850T, ORF1ab:C19097T, ORF1ab:A19137G, ORF1ab:A20268G, S:A23403G, S:C23604A, S:T24076C, ORF7a:A27533G, N:C28854T, 3'UTR:T29710C,	11	N:S194L, ORF1a:V886L, ORF1a:D981Y, ORF1a:T1429I, ORF1a:V3104I, ORF1b:P314L, ORF1b:V1170F, ORF1b:A1877V, ORF7a:H47R, S:D614G, S:P681H,	
hCoV-19/Mexico/NLE-INER-IMSS-00230/2021	EPI_ISL_1279285	In process	20A	B.1	25	5'UTR:G61T, 5'UTR:C241T, ORF1ab:G370T, ORF1ab:C2450T, ORF1ab:C3037T, ORF1ab:G3562T, ORF1ab:G4184A, ORF1ab:C4920T, ORF1ab:A6360G, ORF1ab:C10525T, ORF1ab:A10646T, ORF1ab:G11083T, ORF1ab:C14408T, ORF1ab:G14500T, ORF1ab:G17561A, ORF1ab:G18225T, ORF1ab:A20268G, S:G22468A, S:G23120T, S:A23403G, S:T23569C, S:A25286G, ORF3a:C25546T, ORF8:C28000T, N:C28854T, N:G29402T,	16	N:S194L, N:D377Y, ORF1a:G1307S, ORF1a:T1552I, ORF1a:D2032G, ORF1a:T3461S, ORF1a:L3606F, ORF1b:P314L, ORF1b:V345L, ORF1b:R1365Q, ORF1b:M1586I, ORF3a:L52F, ORF8:P36L, S:A520S, S:D614G, S:S1242G,	3'UTR:29721-29760

hCoV-19/Mexico/NLE-INER-IMSS-00231/2021	EPI_ISL_1279495	In process	20B	B.1.1.222	22	5'UTR:C241T, ORF1ab:C1545T, ORF1ab:C3037T, ORF1ab:T5047A, ORF1ab:C9430T, ORF1ab:C10029T, ORF1ab:C14408T, ORF1ab:C16394T, ORF1ab:G18703T, ORF1ab:T19839C, S:A23403G, S:A23756G, ORF3a:G25567A, ORF3a:T25569A, ORF3a:T25570A, ORF3a:G25912T, ORF3a:C25919T, ORF8:A27921G, ORF8:G28001T, N:C28606T, N:G28881A, N:G28882A, N:G28883C,	15	N:R203K, N:G204R, ORF1a:A427V, ORF1a:F1594L, ORF1a:T3255I, ORF1b:P314L, ORF1b:P976L, ORF1b:D1746Y, ORF3a:A59T, ORF3a:S60T, ORF3a:G174C, ORF3a:T176I, ORF8:I10V, S:D614G, S:T732A,
hCoV-19/Mexico/NLE-INER-IMSS-00232/2021	EPI_ISL_1279496	In process	20B	B.1.1.519	28	5'UTR:T201C, 5'UTR:C203T, 5'UTR:C222T, 5'UTR:C241T, ORF1ab:C936T, ORF1ab:G1738T, ORF1ab:C2523T, ORF1ab:C3037T, ORF1ab:C3140T, ORF1ab:C10029T, ORF1ab:C10954T, ORF1ab:A11117G, ORF1ab:C12789T, ORF1ab:C13994T, ORF1ab:C14220T, ORF1ab:C14408T, ORF1ab:T19839C, ORF1ab:A19974G, ORF1ab:C21306T, S:C22995A, S:A23403G, S:C23604A, S:A23756G, N:G28881A, N:G28882A, N:G28883C, N:C29197T, N:G29227T, 3'UTR:C29738T,	14	N:R203K, N:G204R, ORF1a:T224I, ORF1a:T753I, ORF1a:P959S, ORF1a:T3255I, ORF1a:I3618V, ORF1a:T4175I, ORF1b:A176V, ORF1b:P314L, S:T478K, S:D614G, S:P681H, S:T732A,
hCoV-19/Mexico/NLE-INER-IMSS-00233/2021	EPI_ISL_1279497	In process	20B	B.1.1	20	5'UTR:C241T, ORF1ab:C3037T, ORF1ab:C3695T, ORF1ab:G5194T, ORF1ab:A6985T, ORF1ab:G9242A, ORF1ab:C9319T, ORF1ab:C12412T, ORF1ab:C13887T, ORF1ab:T14313C, ORF1ab:C14408T, S:C21812T, S:A23403G, ORF3a:C25613T, ORF3a:C25844T, ORF3a:G25912T, ORF8:T27909C, N:G28881A, N:G28882A, N:G28883C, N:T29317C,	9	N:R203K, N:G204R, ORF1a:E2993K, ORF1b:P314L, ORF3a:S74F, ORF3a:T151I, ORF3a:G174C, ORF8:F6L, S:D614G,
hCoV-19/Mexico/NLE-INER-IMSS-00234/2021	EPI_ISL_1279498	In process	20B	B.1.1.519	26	5'UTR:C203T, 5'UTR:C222T, 5'UTR:C241T, ORF1ab:C2453T, ORF1ab:C3037T, ORF1ab:C3140T, ORF1ab:C6336T, ORF1ab:C7712T, ORF1ab:C10029T, ORF1ab:C10954T, ORF1ab:A11117G, ORF1ab:C12789T, ORF1ab:C14408T, ORF1ab:C15848T, ORF1ab:T19839C, ORF1ab:C21306T, S:C22995A, S:A23403G, S:C23604A, S:A23756G, S:C24904T, ORF3a:A25472G, ORF8:C27998T, N:G28881A, N:G28882A, N:G28883C, N:C29197T,	16	N:R203K, N:G204R, ORF1a:L730F, ORF1a:P959S, ORF1a:S2024L, ORF1a:P2483S, ORF1a:T3255I, ORF1a:I3618V, ORF1a:T4175I, ORF1b:P314L, ORF1b:T794I, ORF3a:D27G, S:T478K, S:D614G, S:P681H, S:T732A,
hCoV-19/Mexico/NLE-INER-IMSS-00235/2021	EPI_ISL_1279499	In process	20A	B.1.243	14	5'UTR:C241T, ORF1ab:C3037T, ORF1ab:A4500G, ORF1ab:C10741T, ORF1ab:C14408T, ORF1ab:C19097T, ORF1ab:A19137G, ORF1ab:A20268G, S:A23403G, S:C23604A, S:T24076C, ORF3a:C25427T, ORF8:C27970T, N:C28854T, 3'UTR:T29710C,	8	N:S194L, ORF1a:Q1412R, ORF1b:P314L, ORF1b:A1877V, ORF3a:T12I, ORF8:T26I, S:D614G, S:P681H,
hCoV-19/Mexico/NLE-INER-IMSS-00236/2021	EPI_ISL_1279500	In process	20B	B.1.1.519	22	5'UTR:C203T, 5'UTR:C222T, 5'UTR:C241T, ORF1ab:C3037T, ORF1ab:C3140T, ORF1ab:T3745C, ORF1ab:C4890T, ORF1ab:C10029T, ORF1ab:C10954T, ORF1ab:A11117G, ORF1ab:C12789T, ORF1ab:C14408T, ORF1ab:T19839C, ORF1ab:C21306T, S:C22995A, S:A23403G, S:C23604A, S:A23756G, ORF3a:C25844T, N:G28881A, N:G28882A, N:G28883C, N:C29197T,	13	N:R203K, N:G204R, ORF1a:P959S, ORF1a:T1542I, ORF1a:T3255I, ORF1a:I3618V, ORF1a:T4175I, ORF1b:P314L, ORF3a:T151I, S:T478K, S:D614G, S:P681H, S:T732A,
hCoV-19/Mexico/NLE-INER-IMSS-00237/2021	EPI_ISL_1279501	In process	20B	B.1.1.207	23	5'UTR:C241T, ORF1ab:C3037T, ORF1ab:C4230T, ORF1ab:A5641C, ORF1ab:G6753A, ORF1ab:T7348A, ORF1ab:C7973T, ORF1ab:T9172C, ORF1ab:A10948G, ORF1ab:C10977T, ORF1ab:C13968A, ORF1ab:C14408T, ORF1ab:G16945T, ORF1ab:A20763G, S:A23403G, S:C23604A, S:A24874G, E:G26268A, M:G26730T, M:A26927G, N:G28881A, N:G28882A, N:G28883C, 3'UTR:C29769T,	12	M:V70F, N:R203K, N:G204R, ORF1a:T1322I, ORF1a:Q1792H, ORF1a:R2163H, ORF1a:N2361K, ORF1a:A3571V, ORF1b:P314L, ORF1b:A1160S, S:D614G, S:P681H,

hCoV-19/Mexico/NLE-INER-IMSS-00249/2021	EPI_ISL_1279302	In process	20B	B.1.1.519	24	5'UTR:C203T, 5'UTR:A223T, 5'UTR:C241T, ORF1ab:C3037T, ORF1ab:C3140T, ORF1ab:G5992A, ORF1ab:C10029T, ORF1ab:A10323G, ORF1ab:C10954T, ORF1ab:A11117G, ORF1ab:G11365T, ORF1ab:C12789T, ORF1ab:C14408T, ORF1ab:T19839C, ORF1ab:C21306T, S:G22894A, S:C22995A, S:A23403G, S:C23604A, S:A23756G, N:G28881A, N:G28882A, N:G28883C, N:C29197T, N:G29527T,	13	N:R203K, N:G204R, N:Q418H, ORF1a:P959S, ORF1a:T3255I, ORF1a:K3353R, ORF1a:I3618V, ORF1a:T4175I, ORF1b:P314L, S:T478K, S:D614G, S:P681H, S:T732A,	5'UTR:2 22,
hCoV-19/Mexico/NLE-INER-IMSS-00250/2021	EPI_ISL_1279512	In process	20G	B.1.2	19	5'UTR:C241T, ORF1ab:T445C, ORF1ab:C1059T, ORF1ab:C3037T, ORF1ab:C4543T, ORF1ab:C9430T, ORF1ab:C10319T, ORF1ab:T10795C, ORF1ab:C14408T, ORF1ab:C14670T, ORF1ab:T14808C, ORF1ab:A18424G, ORF1ab:C21304T, S:A23403G, ORF3a:G25563T, ORF3a:G25907T, ORF8:C27964T, N:C28472T, N:C28869T, N:G29402T,	12	N:P67S, N:P199L, N:D377Y, ORF1a:T265I, ORF1a:L3352F, ORF1b:P314L, ORF1b:N1653D, ORF1b:R2613C, ORF3a:Q57H, ORF3a:G172V, ORF8:S24L, S:D614G,	
hCoV-19/Mexico/NLE-INER-IMSS-00252/2021	EPI_ISL_1279514	In process	20B	B.1.1.519	23	5'UTR:C203T, 5'UTR:C222T, 5'UTR:C241T, ORF1ab:C3037T, ORF1ab:C3140T, ORF1ab:T3745C, ORF1ab:C10029T, ORF1ab:C10954T, ORF1ab:A11117G, ORF1ab:C12789T, ORF1ab:C14408T, ORF1ab:C16726T, ORF1ab:T19839C, ORF1ab:C21306T, S:C22995A, S:A23403G, S:C23604A, S:A23756G, ORF3a:C25844T, N:G28881A, N:G28882A, N:G28883C, N:C29197T, N:G29315A,	14	N:R203K, N:G204R, N:D348N, ORF1a:P959S, ORF1a:T3255I, ORF1a:I3618V, ORF1a:T4175I, ORF1b:P314L, ORF1b:H1087Y, ORF3a:T151I, S:T478K, S:D614G, S:P681H, S:T732A,	
hCoV-19/Mexico/NLE-INER-IMSS-00253/2021	EPI_ISL_1279515	In process	20G	B.1.2	24	5'UTR:C241T, ORF1ab:C1059T, ORF1ab:C3037T, ORF1ab:C3140T, ORF1ab:G8083A, ORF1ab:C10319T, ORF1ab:A12986T, ORF1ab:C13421T, ORF1ab:C14408T, ORF1ab:C14805T, ORF1ab:C15026T, ORF1ab:A18424G, ORF1ab:C18555T, ORF1ab:C19164T, ORF1ab:C21302T, ORF1ab:C21304A, ORF1ab:G21305A, S:A23403G, ORF3a:C25469T, ORF3a:G25563T, ORF3a:G25907T, M:G27074T, ORF8:C27964T, N:C28472T, N:C28869T,	18	N:P67S, N:P199L, ORF1a:T265I, ORF1a:P959S, ORF1a:M2606I, ORF1a:L3352F, ORF1a:M4241L, ORF1a:L4386F, ORF1b:P314L, ORF1b:A520V, ORF1b:N1653D, ORF1b:P2612L, ORF1b:R2613N, ORF3a:S26L, ORF3a:Q57H, ORF3a:G172V, ORF8:S24L, S:D614G,	
hCoV-19/Mexico/NLE-INER-IMSS-00263/2021	EPI_ISL_1279284	In process		B.1.243	0	,	1	,	
hCoV-19/Mexico/NLE-INER-IMSS-00264/2021	EPI_ISL_1279524	In process	20G	B.1.596	19	5'UTR:C241T, ORF1ab:C1059T, ORF1ab:C3037T, ORF1ab:T7001A, ORF1ab:C10319T, ORF1ab:A11451G, ORF1ab:C13405T, ORF1ab:C14408T, ORF1ab:A18424G, ORF1ab:C21304T, S:C21811A, S:A23403G, S:A23592C, ORF3a:G25563T, ORF3a:G25907T, ORF8:C27964T, N:C28472T, N:C28869T, N:T29194C, N:T29377A,	14	N:P67S, N:P199L, ORF1a:T265I, ORF1a:S2246T, ORF1a:L3352F, ORF1a:Q3729R, ORF1b:P314L, ORF1b:N1653D, ORF1b:R2613C, ORF3a:Q57H, ORF3a:G172V, ORF8:S24L, S:D614G, S:Q677P,	
hCoV-19/Mexico/NLE-INER-IMSS-00265/2021	EPI_ISL_1279525	In process	20A	B.1.243	23	5'UTR:C241T, ORF1ab:C1218T, ORF1ab:C3037T, ORF1ab:C3264T, ORF1ab:C3773T, ORF1ab:G4486T, ORF1ab:C4540T, ORF1ab:A5097G, ORF1ab:A6124G, ORF1ab:C10741T, ORF1ab:C12119T, ORF1ab:C14408T, ORF1ab:G14511T, ORF1ab:C19097T, ORF1ab:A20268G, S:C21575T, S:A23403G, S:C23604A, S:T24076C, ORF3a:C25635T, ORF3a:T25773C, ORF3a:G26211T, N:C28854T, 3'UTR:T29710C,	13	N:S194L, ORF1a:S318L, ORF1a:T1000I, ORF1a:R1170C, ORF1a:K1407N, ORF1a:N1611S, ORF1a:P3952S, ORF1b:P314L, ORF1b:Q348H, ORF1b:A1877V, S:L5F, S:D614G, S:P681H,	

hCoV-19/Mexico/NLE-INER-IMSS-00266/2021	EPI_ISL_1279275	In process	19B	A.2.5	30	ORF1ab:C275T, ORF1ab:C1567T, ORF1ab:C4543T, ORF1ab:A5234G, ORF1ab:A5462G, ORF1ab:A5488G, ORF1ab:C8782T, ORF1ab:T9477A, ORF1ab:C9724T, ORF1ab:C10029T, ORF1ab:C11005A, ORF1ab:C12815T, ORF1ab:C14805T, ORF1ab:C16466T, ORF1ab:T16857C, ORF1ab:T18417C, S:A22525G, S:T22917G, S:A23403G, S:C23638T, S:G24697A, ORF3a:C25613T, ORF3a:G25979T, M:C26681T, ORF8:T28144C, ORF8:A28272T, N:C28657T, N:C28863T, N:G28975T, N:C29366T, N:C29421T,	17	N:S197L, N:M234I, N:P365S, N:P383L, ORF1a:L4F, ORF1a:K1657E, ORF1a:S1733G, ORF1a:F3071Y, ORF1a:T3255I, ORF1a:H3580Q, ORF1b:P1000L, ORF3a:S74F, ORF3a:G196V, ORF8:L84S, S:D215Y, S:L452R, S:D614G,	S:21983-21991,
hCoV-19/Mexico/NLE-INER-IMSS-00267/2021	EPI_ISL_1279526	In process	20B	B.1.1.432	23	5'UTR:C241T, ORF1ab:C3037T, ORF1ab:C3695T, ORF1ab:G5194T, ORF1ab:A6985T, ORF1ab:G9242A, ORF1ab:C9319T, ORF1ab:C12412T, ORF1ab:T14313C, ORF1ab:C14408T, S:C22938T, S:A23403G, S:C23422T, ORF3a:C25613T, ORF3a:C25844T, ORF3a:G25912T, ORF8:T27909C, N:G28881A, N:G28882A, N:G28883C, N:T29317C, N:A29478G, N:T29479C, N:A29533G,	11	N:R203K, N:G204R, N:D402G, ORF1a:E2993K, ORF1b:P314L, ORF3a:S74F, ORF3a:T151I, ORF3a:G174C, ORF8:F6L, S:S459F, S:D614G,	
hCoV-19/Mexico/NLE-INER-IMSS-00279/2021	EPI_ISL_1279536	In process	20B	B.1.1	19	5'UTR:C241T, ORF1ab:T1481C, ORF1ab:C3037T, ORF1ab:C8829T, ORF1ab:C11450A, ORF1ab:G13617T, ORF1ab:C14408T, ORF1ab:C17004T, ORF1ab:C18695T, ORF1ab:C18747T, S:A23403G, S:G24842A, S:A24902G, ORF3a:G25720T, N:G28881A, N:G28882A, N:G28883C, N:C29303T, 3'UTR:A29700G, 3'UTR:G29779T,	13	N:R203K, N:G204R, N:P344S, ORF1a:F406L, ORF1a:A2855V, ORF1a:Q3729K, ORF1b:K50N, ORF1b:P314L, ORF1b:T1743I, ORF3a:A110S, S:D614G, S:V1094I, S:I1114V,	
hCoV-19/Mexico/OAX-IBT-IMSS-114/2021	EPI_ISL_1288168	In process	20B	B.1.1.519	27	5'UTR:T201C, 5'UTR:C203T, 5'UTR:C222T, 5'UTR:C241T, ORF1ab:C936T, ORF1ab:G1738T, ORF1ab:T2872C, ORF1ab:C3037T, ORF1ab:C3140T, ORF1ab:T5035C, ORF1ab:C10029T, ORF1ab:C10954T, ORF1ab:A11117G, ORF1ab:C12789T, ORF1ab:C14408T, ORF1ab:T19839C, ORF1ab:A19974G, ORF1ab:C21306T, S:C22995A, S:A23403G, S:C23604A, S:A23756G, ORF3a:G25429T, N:G28881A, N:G28882A, N:G28883C, N:C29197T, N:G29227T	13	N:R203K, N:G204R, ORF1a:T224I, ORF1a:P959S, ORF1a:T3255I, ORF1a:I3618V, ORF1a:T4175I, ORF1b:P314L, ORF3a:V13L, S:T478K, S:D614G, S:P681H, S:T732A,	
hCoV-19/Mexico/OAX-IBT-IMSS-125/2021	EPI_ISL_1288169	In process	20B	B.1.1.519	31	5'UTR:C203T, 5'UTR:C222T, 5'UTR:C241T, ORF1ab:C1213T, ORF1ab:C1342T, ORF1ab:C3037T, ORF1ab:C3140T, ORF1ab:G3692T, ORF1ab:G3871T, ORF1ab:C10029T, ORF1ab:C10954T, ORF1ab:A11117G, ORF1ab:C11824A, ORF1ab:C11916T, ORF1ab:C12789T, ORF1ab:C14408T, ORF1ab:C18395T, ORF1ab:T19839C, ORF1ab:C21306T, S:G21796A, S:C21952T, S:C22995A, S:A23403G, S:C23604A, S:A23756G, M:T26987C, ORF8:T27904C, N:G28881A, N:G28882A, N:G28883C, N:C29197T, N:G29402T,	17	N:R203K, N:G204R, N:D377Y, ORF1a:P959S, ORF1a:V1143F, ORF1a:K1202N, ORF1a:T3255I, ORF1a:I3618V, ORF1a:S3884L, ORF1a:T4175I, ORF1b:P314L, ORF1b:A1643V, ORF8:L4P, S:T478K, S:D614G, S:P681H, S:T732A,	

hCoV-19/Mexico/OAX-IBT-IMSS-136/2021	EPI_ISL_1288170	In process	20B	B.1.1.519	35	5'UTR:C203T, 5'UTR:C222T, 5'UTR:C241T, ORF1ab:C1213T, ORF1ab:C1342T, ORF1ab:C3037T, ORF1ab:C3140T, ORF1ab:C3411T, ORF1ab:G3692T, ORF1ab:G3871T, ORF1ab:C9510T, ORF1ab:C10029T, ORF1ab:C10954T, ORF1ab:A11117G, ORF1ab:C11824A, ORF1ab:C11916T, ORF1ab:C12789T, ORF1ab:C14408T, ORF1ab:C16428T, ORF1ab:C18395T, ORF1ab:T19839C, ORF1ab:C21306T, S:C21952T, S:C22995A, S:A23403G, S:C23604A, S:A23756G, S:C24621T, M:T26987C, ORF6:T27221A, ORF8:T27904C, N:G28881A, N:G28882A, N:G28883C, N:C29197T, N:G29402T,	21	N:R203K, N:G204R, N:D377Y, ORF1a:P959S, ORF1a:A1049V, ORF1a:V1143F, ORF1a:K1202N, ORF1a:T3082I, ORF1a:T3255I, ORF1a:I3618V, ORF1a:S3884L, ORF1a:T4175I, ORF1b:P314L, ORF1b:A1643V, ORF6:F7Y, ORF8:L4P, S:T478K, S:D614G, S:P681H, S:T732A, S:A1020V,
hCoV-19/Mexico/OAX-IBT-IMSS-148/2021	EPI_ISL_1288171	In process	20B	B.1.1.519	25	5'UTR:C203T, 5'UTR:C222T, 5'UTR:C241T, ORF1ab:C3037T, ORF1ab:C3140T, ORF1ab:T4339C, ORF1ab:C4890T, ORF1ab:C6539T, ORF1ab:C10029T, ORF1ab:C10954T, ORF1ab:A11117G, ORF1ab:C12789T, ORF1ab:C14408T, ORF1ab:T19839C, ORF1ab:C21306T, S:C22995A, S:A23403G, S:C23604A, S:A23756G, ORF3a:C25904T, ORF3a:G25906T, ORF8:C28253T, N:G28881A, N:G28882A, N:G28883C, N:C29197T,	15	N:R203K, N:G204R, ORF1a:P959S, ORF1a:T1542I, ORF1a:H2092Y, ORF1a:T3255I, ORF1a:I3618V, ORF1a:T4175I, ORF1b:P314L, ORF3a:S171L, ORF3a:G172C, S:T478K, S:D614G, S:P681H, S:T732A,
hCoV-19/Mexico/OAX-IBT-IMSS-152/2021	EPI_ISL_1288354	In process	20B	B.1.1.519	29	5'UTR:T201C, 5'UTR:C203T, 5'UTR:C222T, 5'UTR:C241T, ORF1ab:T277C, ORF1ab:G1738T, ORF1ab:G2782T, ORF1ab:C3037T, ORF1ab:C3140T, ORF1ab:G7829T, ORF1ab:C10029T, ORF1ab:C10954T, ORF1ab:A11117G, ORF1ab:A12417G, ORF1ab:C12789T, ORF1ab:C14408T, ORF1ab:T19839C, ORF1ab:A19974G, ORF1ab:C21306T, S:C22995A, S:G23270T, S:A23403G, S:C23604A, S:A23756G, S:G24390T, N:G28541T, N:G28881A, N:G28882A, N:G28883C, N:C29197T,	17	N:A90S, N:R203K, N:G204R, ORF1a:P959S, ORF1a:V2522F, ORF1a:T3255I, ORF1a:I3618V, ORF1a:N4051S, ORF1a:T4175I, ORF1b:P314L, ORF9b:E86D, S:T478K, S:A570S, S:D614G, S:P681H, S:T732A, S:S943I,
hCoV-19/Mexico/OAX-IBT-IMSS-159/2021	EPI_ISL_1288172	In process	20B	B.1.1.519	27	5'UTR:T201C, 5'UTR:C203T, 5'UTR:C222T, 5'UTR:C241T, ORF1ab:C936T, ORF1ab:G1738T, ORF1ab:T2872C, ORF1ab:C3037T, ORF1ab:C3140T, ORF1ab:T5035C, ORF1ab:C10029T, ORF1ab:C10954T, ORF1ab:A11117G, ORF1ab:C12789T, ORF1ab:C14408T, ORF1ab:T19839C, ORF1ab:A19974G, ORF1ab:C21306T, S:C22995A, S:A23403G, S:C23604A, S:A23756G, ORF3a:G25429T, N:G28881A, N:G28882A, N:G28883C, N:C29197T, N:G29227T	13	N:R203K, N:G204R, ORF1a:T224I, ORF1a:P959S, ORF1a:T3255I, ORF1a:I3618V, ORF1a:T4175I, ORF1b:P314L, ORF3a:V13L, S:T478K, S:D614G, S:P681H, S:T732A,
hCoV-19/Mexico/OAX-IBT-IMSS-170/2021	EPI_ISL_1288173	In process	20B	B.1.1.519	29	5'UTR:T201C, 5'UTR:C203T, 5'UTR:C222T, 5'UTR:C241T, ORF1ab:T277C, ORF1ab:G1738T, ORF1ab:C3037T, ORF1ab:C3140T, ORF1ab:G4474A, ORF1ab:C6730T, ORF1ab:G7829T, ORF1ab:C10029T, ORF1ab:C10954T, ORF1ab:A11117G, ORF1ab:C12789T, ORF1ab:C14408T, ORF1ab:T19839C, ORF1ab:A19974G, ORF1ab:C21306T, S:C22995A, S:C23086T, S:A23403G, S:C23604A, S:A23756G, S:G25302T, E:C26313T, N:G28881A, N:G28882A, N:G28883C, N:C29197T,	13	N:R203K, N:G204R, ORF1a:P959S, ORF1a:V2522F, ORF1a:T3255I, ORF1a:I3618V, ORF1a:T4175I, ORF1b:P314L, S:T478K, S:D614G, S:P681H, S:T732A, S:C1247F,

hCoV-19/Mexico/OAX-IBT-IMSS-171/2021	EPI_ISL_1288174	In process	20B	B.1.1.519	28	5'UTR:T201C, 5'UTR:C203T, 5'UTR:C222T, 5'UTR:C241T, ORF1ab:T277C, ORF1ab:G1738T, ORF1ab:C3037T, ORF1ab:C3140T, ORF1ab:G7829T, ORF1ab:C10029T, ORF1ab:C10954T, ORF1ab:A11117G, ORF1ab:A12417G, ORF1ab:C12789T, ORF1ab:C14408T, ORF1ab:T19839C, ORF1ab:A19974G, ORF1ab:C21306T, S:C22995A, S:G23270T, S:A23403G, S:C23604A, S:A23756G, S:G24390T, N:G28541T, N:G28881A, N:G28882A, N:G28883C, N:C29197T	17	N:A90S, N:R203K, N:G204R, ORF1a:P959S, ORF1a:V2522F, ORF1a:T3255I, ORF1a:I3618V, ORF1a:N4051S, ORF1a:T4175I, ORF1b:P314L, ORF9b:E86D, S:T478K, S:A570S, S:D614G, S:P681H, S:T732A, S:S943I,
hCoV-19/Mexico/OAX-IBT-IMSS-189/2021	EPI_ISL_1288387	In process	21C (Epsilon)	B.1.427	27	5'UTR:C241T, ORF1ab:C1059T, ORF1ab:C3037T, ORF1ab:C3817T, ORF1ab:C4543T, ORF1ab:G8368A, ORF1ab:G9738C, ORF1ab:C11202A, ORF1ab:C13019T, ORF1ab:G13713A, ORF1ab:C14408T, ORF1ab:C16394T, ORF1ab:G17014T, ORF1ab:G19525T, ORF1ab:C20233T, S:G21600T, S:G22018T, S:G22335T, S:C22597T, S:T22917G, S:A23403G, S:C23638T, ORF3a:G25563T, M:C26681T, ORF8:C28087T, ORF8:A28272T, N:C28887T, N:C29362T	16	N:T205I, ORF1a:T265I, ORF1a:S3158T, ORF1a:T3646N, ORF1b:P314L, ORF1b:P976L, ORF1b:D1183Y, ORF1b:D2020Y, ORF1b:P2256S, ORF3a:Q57H, ORF8:A65V, S:S13I, S:W152C, S:W258L, S:L452R, S:D614G,
hCoV-19/Mexico/OAX-IBT-IMSS-190/2021	EPI_ISL_1288388	In process	20B	B.1.1.432	26	5'UTR:C241T, ORF1ab:C1594T, ORF1ab:C3037T, ORF1ab:G4925T, ORF1ab:G6421T, ORF1ab:A6985T, ORF1ab:C8326T, ORF1ab:T8806C, ORF1ab:C9319T, ORF1ab:C9438T, ORF1ab:G11266T, ORF1ab:C12412T, ORF1ab:A13653G, ORF1ab:T14313C, ORF1ab:C14408T, ORF1ab:C15656T, S:A23403G, ORF3a:C25613T, ORF3a:C25721T, ORF3a:G25912T, ORF8:C27972T, ORF8:T28033C, N:G28881A, N:G28882A, N:G28883C, N:G28985T, N:T29317C,	14	N:R203K, N:G204R, N:G238C, ORF1a:D1554Y, ORF1a:T3058I, ORF1a:L3667F, ORF1b:P314L, ORF1b:T730I, ORF3a:S74F, ORF3a:A110V, ORF3a:G174C, ORF8:Q27*, ORF8:I47T, S:D614G,
hCoV-19/Mexico/OAX-IBT-IMSS-191/2021	EPI_ISL_1288389	In process	20B	B.1.1.222	18	5'UTR:C241T, ORF1ab:C3037T, ORF1ab:A4828G, ORF1ab:G5629C, ORF1ab:C6285T, ORF1ab:G12052T, ORF1ab:A13498G, ORF1ab:C14408T, ORF1ab:G18782T, ORF1ab:T19839C, S:G22331A, S:A23403G, S:C23657A, S:A23756G, ORF3a:C25521T, ORF3a:G26227T, N:G28881A, N:G28882A, N:G28883C.	11	N:R203K, N:G204R, ORF1a:T2007I, ORF1a:K3929N, ORF1b:T11A, ORF1b:P314L, ORF1b:G1772V, S:G257S, S:D614G, S:L699I, S:T732A,
hCoV-19/Mexico/OAX-IBT-IMSS-192/2021	EPI_ISL_1288390	In process	20B	B.1.1.222	22	5'UTR:C241T, ORF1ab:C1288T, ORF1ab:C2252A, ORF1ab:C2433T, ORF1ab:C3037T, ORF1ab:C10029T, ORF1ab:A11782G, ORF1ab:A12075G, ORF1ab:C14408T, ORF1ab:C15279T, ORF1ab:G19816T, ORF1ab:T19839C, S:A23403G, S:A23756G, ORF3a:G25907T, ORF3a:G25912T, ORF7a:G27613A, ORF7b:A27756G, ORF8:A27921G, ORF8:G28001T, N:G28881A, N:G28882A, N:G28883C,	15	N:R203K, N:G204R, ORF1a:Q663K, ORF1a:S723F, ORF1a:T3255I, ORF1a:N3937S, ORF1b:P314L, ORF1b:V2117L, ORF3a:G172V, ORF3a:G174C, ORF7a:V74I, ORF7b:M1V, ORF8:I10V, S:D614G, S:T732A,
hCoV-19/Mexico/OAX-IBT-IMSS-194/2021	EPI_ISL_1288391	In process	21C (Epsilon)	B.1.427	22	5'UTR:C241T, ORF1ab:C1059T, ORF1ab:C3037T, ORF1ab:G4207T, ORF1ab:G9738C, ORF1ab:C10714T, ORF1ab:C11345T, ORF1ab:G13713A, ORF1ab:C14408T, ORF1ab:C14877T, ORF1ab:C16394T, ORF1ab:G17014T, S:G21600T, S:G22018T, S:T22917G, S:A23403G, ORF3a:C25511T, ORF3a:G25563T, M:C26681T, ORF8:A28272T, N:C28887T, N:C29362T, 3'UTR:G29690T.	13	N:T205I, ORF1a:T265I, ORF1a:S3158T, ORF1a:L3694F, ORF1b:P314L, ORF1b:P976L, ORF1b:D1183Y, ORF3a:S40L, ORF3a:Q57H, S:S13I, S:W152C, S:L452R, S:D614G,

hCoV-19/Mexico/OAX-IBT-IMSS-304/2021	EPI_ISL_1288488	In process	20B	B.1.1.519	27	5'UTR:T201C, 5'UTR:C203T, 5'UTR:C222T, 5'UTR:C241T, ORF1ab:C936T, ORF1ab:G1738T, ORF1ab:T2872C, ORF1ab:C3037T, ORF1ab:C3140T, ORF1ab:T5035C, ORF1ab:C10029T, ORF1ab:C10954T, ORF1ab:A11117G, ORF1ab:C12789T, ORF1ab:C14408T, ORF1ab:T19839C, ORF1ab:A19974G, ORF1ab:C21306T, S:C22995A, S:A23403G, S:C23604A, S:A23756G, ORF3a:G25429T, N:G28881A, N:G28882A, N:G28883C, N:C29197T, N:G29227T	13	N:R203K, N:G204R, ORF1a:T224I, ORF1a:P959S, ORF1a:T3255I, ORF1a:I3618V, ORF1a:T4175I, ORF1b:P314L, ORF3a:V13L, S:T478K, S:D614G, S:P681H, S:T732A,
hCoV-19/Mexico/OAX-IBT-IMSS-305/2021	EPI_ISL_1288489	In process	20B	B.1.1.519	35	5'UTR:C203T, 5'UTR:C222T, 5'UTR:C241T, ORF1ab:C1213T, ORF1ab:C1342T, ORF1ab:C3037T, ORF1ab:C3140T, ORF1ab:C3411T, ORF1ab:G3692T, ORF1ab:G3871T, ORF1ab:C9510T, ORF1ab:C10029T, ORF1ab:C10954T, ORF1ab:A11117G, ORF1ab:C11824A, ORF1ab:C11916T, ORF1ab:C12789T, ORF1ab:C14408T, ORF1ab:C16428T, ORF1ab:C18395T, ORF1ab:T19839C, ORF1ab:C21306T, S:C21952T, S:C22995A, S:A23403G, S:C23604A, S:A23756G, S:C24621T, M:T26987C, ORF6:T27221A, ORF8:T27904C, N:G28881A, N:G28882A, N:G28883C, N:C29197T, N:G29402T,	21	N:R203K, N:G204R, N:D377Y, ORF1a:P959S, ORF1a:A1049V, ORF1a:V1143F, ORF1a:K1202N, ORF1a:T3082I, ORF1a:T3255I, ORF1a:I3618V, ORF1a:S3884L, ORF1a:T4175I, ORF1b:P314L, ORF1b:A1643V, ORF6:F7Y, ORF8:L4P, S:T478K, S:D614G, S:P681H, S:T732A, S:A1020V,
hCoV-19/Mexico/OAX-IBT-IMSS-306/2021	EPI_ISL_1288490	In process	20B	B.1.1.519	26	5'UTR:C203T, 5'UTR:C222T, 5'UTR:C241T, ORF1ab:C3037T, ORF1ab:C3140T, ORF1ab:C8860T, ORF1ab:T9723C, ORF1ab:C10029T, ORF1ab:C10954T, ORF1ab:A11117G, ORF1ab:C12789T, ORF1ab:C14408T, ORF1ab:G18180A, ORF1ab:G19150A, ORF1ab:T19839C, ORF1ab:C21306T, S:C22995A, S:A23403G, S:C23604A, S:A23756G, ORF3a:G25690A, ORF3a:G25767C, M:G26918T, N:G28881A, N:G28882A, N:G28883C, N:C29197T,	15	N:R203K, N:G204R, ORF1a:P959S, ORF1a:F3153S, ORF1a:T3255I, ORF1a:I3618V, ORF1a:T4175I, ORF1b:P314L, ORF1b:A1895T, ORF3a:G100S, ORF3a:M125I, S:T478K, S:D614G, S:P681H, S:T732A,
hCoV-19/Mexico/OAX-IBT-IMSS-316/2021	EPI_ISL_1288498	In process	20B	B.1.1.222	17	5'UTR:C241T, ORF1ab:C1437T, ORF1ab:C3037T, ORF1ab:C7749T, ORF1ab:C8637T, ORF1ab:A13498G, ORF1ab:C14408T, ORF1ab:T19839C, S:C21575T, S:G22331A, S:A23403G, S:G23593T, S:A23756G, ORF7b:G27777T, N:G28881A, N:G28882A, N:G28883C. N:G28907A.	14	N:R203K, N:G204R, N:G212S, ORF1a:S391F, ORF1a:T2495I, ORF1a:T2791I, ORF1b:T11A, ORF1b:P314L, ORF7b:D8Y, S:L5F, S:G257S, S:D614G, S:Q677H, S:T732A,
hCoV-19/Mexico/OAX-IBT-IMSS-37/2021	EPI_ISL_1288162	In process	21C (Epsilon)	B.1.427	16	5'UTR:C241T, ORF1ab:C1059T, ORF1ab:C3037T, ORF1ab:G9738C, ORF1ab:G13713A, ORF1ab:C14408T, ORF1ab:C16394T, ORF1ab:G17014T, S:G21600T, S:G22018T, S:T22917G, S:A23403G, ORF3a:G25563T, M:C26681T, ORF8:A28272T, N:C28887T, N:C29362T,	11	N:T205I, ORF1a:T265I, ORF1a:S3158T, ORF1b:P314L, ORF1b:P976L, ORF1b:D1183Y, ORF3a:Q57H, S:S13I, S:W152C, S:L452R, S:D614G,
hCoV-19/Mexico/OAX-IBT-IMSS-398/2021	EPI_ISL_1288223	In process	20B	B.1.1.432	27	5'UTR:C241T, ORF1ab:C1594T, ORF1ab:G2659T, ORF1ab:C3037T, ORF1ab:C4331T, ORF1ab:G4925T, ORF1ab:G6421T, ORF1ab:A6985T, ORF1ab:T8806C, ORF1ab:C9319T, ORF1ab:G11266T, ORF1ab:C12412T, ORF1ab:T14313C, ORF1ab:C14408T, ORF1ab:C15656T, ORF1ab:C15720T, ORF1ab:C18508T, ORF1ab:T20757C, S:A23403G, ORF3a:C25721T, ORF3a:G25912T, ORF8:C27972T, ORF8:T28033C, N:G28881A, N:G28882A, N:G28883C, N:G28985T, N:T29317C,	14	N:R203K, N:G204R, N:G238C, ORF1a:K798N, ORF1a:D1554Y, ORF1a:L3667F, ORF1b:P314L, ORF1b:T730I, ORF1b:L1681F, ORF3a:A110V, ORF3a:G174C, ORF8:Q27*, ORF8:I47T, S:D614G,

hCoV-19/Mexico/OAX-IBT-IMSS-399/2021	EPI_ISL_1288224	In process	20A	B.1	21	5'UTR:C241T, ORF1ab:A2596G, ORF1ab:G2782T, ORF1ab:C3037T, ORF1ab:C5784T, ORF1ab:C8760T, ORF1ab:G9856T, ORF1ab:C14408T, ORF1ab:T15825A, ORF1ab:C17304T, ORF1ab:A20055G, ORF1ab:A20127C, ORF1ab:A20268G, S:C21910T, S:G22532C, S:A23403G, S:T24814C, ORF7a:G27478T, ORF8:G28077T, N:C28854T, 3'UTR:G29742T, 3'UTR:A29752G.	8	N:S194L, ORF1a:T1840I, ORF1a:A2832V, ORF1b:P314L, ORF7a:V29L, ORF8:V62L, S:E324Q, S:D614G,
hCoV-19/Mexico/OAX-IBT-IMSS-425/2021	EPI_ISL_1288161	In process	21C (Epsilon)	B.1.429	25	5'UTR:C241T, ORF1ab:A544C, ORF1ab:C1059T, ORF1ab:C2395T, ORF1ab:T2597C, ORF1ab:G2747T, ORF1ab:C3037T, ORF1ab:C8947T, ORF1ab:C12100T, ORF1ab:C12513T, ORF1ab:A12878G, ORF1ab:C14408T, ORF1ab:G17014T, S:G21600T, S:G22018T, S:T22917G, S:A23403G, S:T24349C, ORF3a:G25563T, M:C26681T, M:C27012T, ORF7b:G27890T, ORF8:A28272T, N:C28887T, N:C29362T, ORF10:A29600G,	13	N:T205I, ORF1a:E93D, ORF1a:T265I, ORF1a:D828Y, ORF1a:T4083M, ORF1a:I4205V, ORF1b:P314L, ORF1b:D1183Y, ORF3a:Q57H, S:S13I, S:W152C, S:L452R, S:D614G,
hCoV-19/Mexico/OAX-IBT-IMSS-443/2020	EPI_ISL_1301446	In process	20A	B.1	5	5'UTR:C241T, ORF1ab:C3037T, ORF1ab:C10969T, ORF1ab:C14408T, S:A23403G, N:G28423A,	3	ORF1b:P314L, ORF9b:R47H, S:D614G,
hCoV-19/Mexico/OAX-IBT-IMSS-455/2020	EPI_ISL_1301638	In process	20A	B.1.36.10	4	5'UTR:C241T, ORF1ab:C3037T, ORF1ab:C14408T, S:A23403G, N:C28854T,	3	N:S194L, ORF1b:P314L, S:D614G,
hCoV-19/Mexico/OAX-IBT-IMSS-48/2021	EPI_ISL_1288163	In process	20G	B.1.2	24	5'UTR:C140T, 5'UTR:C241T, ORF1ab:C1059T, ORF1ab:C1191T, ORF1ab:C3037T, ORF1ab:G3179A, ORF1ab:C4788T, ORF1ab:G8083A, ORF1ab:A9085G, ORF1ab:C10319T, ORF1ab:C14408T, ORF1ab:C14805T, ORF1ab:A18424G, ORF1ab:C18828T, ORF1ab:C21304T, S:A23403G, S:G23593T, ORF3a:G25563T, ORF3a:T25827C, ORF3a:G25907T, ORF3a:A26148G, ORF8:C27964T, N:C28472T, N:C28869T, N:G29402T,	17	N:P67S, N:P199L, N:D377Y, ORF1a:T265I, ORF1a:P309L, ORF1a:E972K, ORF1a:A1508V, ORF1a:M2606I, ORF1a:L3352F, ORF1b:P314L, ORF1b:N1653D, ORF1b:R2613C, ORF3a:Q57H, ORF3a:G172V, ORF8:S24L, S:D614G, S:Q677H,
hCoV-19/Mexico/OAX-IBT-IMSS-59/2021	EPI_ISL_1288164	In process	20B	B.1.1.519	28	5'UTR:C203T, 5'UTR:C222T, 5'UTR:C241T, ORF1ab:G1462A, ORF1ab:C3037T, ORF1ab:C3140T, ORF1ab:C3992T, ORF1ab:C6145T, ORF1ab:A6393C, ORF1ab:C10029T, ORF1ab:C10954T, ORF1ab:A11117G, ORF1ab:G12052T, ORF1ab:C12789T, ORF1ab:C14408T, ORF1ab:C14805T, ORF1ab:C15981T, ORF1ab:T19839C, ORF1ab:C21306T, S:C22995A, S:A23403G, S:C23604A, S:A23756G, ORF8:C28253T, N:G28881A, N:G28882A, N:G28883C, N:C29095T, N:C29197T,	13	N:R203K, N:G204R, ORF1a:P959S, ORF1a:D2043A, ORF1a:T3255I, ORF1a:I3618V, ORF1a:K3929N, ORF1a:T4175I, ORF1b:P314L, S:T478K, S:D614G, S:P681H, S:T732A,
hCoV-19/Mexico/OAX-InDRE-IBT-15/2020	EPI_ISL_1301512	In process	20A	B.1.609	10	5'UTR:C241T, ORF1ab:C3037T, ORF1ab:C4582T, ORF1ab:C4633T, ORF1ab:C8175T, ORF1ab:T8263A, ORF1ab:C14408T, S:C23315T, S:A23403G, S:C24237T, S:C24370T,	5	ORF1a:A2637V, ORF1b:P314L, S:L585F, S:D614G, S:A892V,
hCoV-19/Mexico/OAX-InDRE-IBT-17/2020	EPI_ISL_1301697	In process	20A	B.1.36.10	6	5'UTR:C241T, ORF1ab:C3037T, ORF1ab:C14408T, S:A23403G, S:G24328T, ORF3a:G25775T, N:C28854T,	5	N:S194L, ORF1b:P314L, ORF3a:W128L, S:D614G, S:L922F,
hCoV-19/Mexico/OAX-InDRE-IBT-18/2020	EPI_ISL_1301529	In process	20A	B.1.609	9	5'UTR:C241T, ORF1ab:C3037T, ORF1ab:C4582T, ORF1ab:C4633T, ORF1ab:C8175T, ORF1ab:T8263A, ORF1ab:C14408T, S:A23403G, S:C24370T, ORF3a:T25999C,	3	ORF1a:A2637V, ORF1b:P314L, S:D614G,
hCoV-19/Mexico/OAX-InDRE-IBT-19/2020	EPI_ISL_1301698	In process	20C	B.1	11	5'UTR:C241T, ORF1ab:A929G, ORF1ab:C1059T, ORF1ab:C3037T, ORF1ab:C3737T, ORF1ab:A5570G, ORF1ab:C13356T, ORF1ab:C14408T, S:A23403G, ORF3a:G25471T, ORF3a:G25494A, ORF3a:G25563T,	9	ORF1a:I222V, ORF1a:T265I, ORF1a:P1158S, ORF1a:M1769V, ORF1a:T4364I, ORF1b:P314L, ORF3a:D27Y, ORF3a:Q57H, S:D614G,
hCoV-19/Mexico/OAX-InDRE-IBT-20/2020	EPI_ISL_1301492	In process	20B	B.1.1.222	13	5'UTR:C241T, ORF1ab:C3037T, ORF1ab:C6541T, ORF1ab:C10954T, ORF1ab:C12786T, ORF1ab:C14408T, ORF1ab:T19839C, S:A23403G, S:A23756G, ORF7b:G27877A, N:G28881A, N:G28882A, N:G28883C, 3'UTR:G29745T,	7	N:R203K, N:G204R, ORF1a:T4174I, ORF1b:P314L, ORF7b:C41Y, S:D614G, S:T732A,

hCoV-19/Mexico/OAX-InDRE-IBT-21/2020	EPI_ISL_1301502	In process	20B	B.1.1.222	11	5'UTR:C241T, ORF1ab:C3037T, ORF1ab:C14408T, ORF1ab:T19590C, ORF1ab:T19839C, S:T22888C, S:A23403G, S:A23756G, N:G28881A, N:G28882A, N:G28883C, 3'UTR:G29751T,	5	N:R203K, N:G204R, ORF1b:P314L, S:D614G, S:T732A,
hCoV-19/Mexico/OAX-InDRE-IBT-22/2020	EPI_ISL_1301474	In process	20A	B.1.189	13	5'UTR:C241T, ORF1ab:C3037T, ORF1ab:C4582T, ORF1ab:C4633T, ORF1ab:G5518A, ORF1ab:C5575T, ORF1ab:G6404T, ORF1ab:C8175T, ORF1ab:C14408T, ORF1ab:G19117T, S:G21796T, S:A23403G, S:G23587T, S:C24370T,	7	ORF1a:V2047F, ORF1a:A2637V, ORF1b:P314L, ORF1b:A1884S, S:R78S, S:D614G, S:Q675H,
hCoV-19/Mexico/OAX-INER-IMSS-00165/2021	EPI_ISL_1279441	In process	20B	B.1.1.519	27	5'UTR:C203T, 5'UTR:C222T, 5'UTR:C241T, ORF1ab:G2801A, ORF1ab:C3037T, ORF1ab:C3140T, ORF1ab:C6500T, ORF1ab:C8660T, ORF1ab:C10029T, ORF1ab:C10954T, ORF1ab:A11117G, ORF1ab:G11365T, ORF1ab:C12789T, ORF1ab:C14408T, ORF1ab:C18493T, ORF1ab:T19839C, ORF1ab:C21306T, S:C22995A, S:G23311A, S:A23403G, S:C23604A, S:A23756G, S:G24216A, N:G28881A, N:G28882A, N:G28883C, N:C29197T, N:G29527T,	17	N:R203K, N:G204R, N:Q418H, ORF1a:D846N, ORF1a:P959S, ORF1a:P2079S, ORF1a:H2799Y, ORF1a:T3255I, ORF1a:I3618V, ORF1a:T4175I, ORF1b:P314L, ORF1b:L1676F, S:T478K, S:D614G, S:P681H, S:T732A, S:G885D,
hCoV-19/Mexico/OAX-INER-IMSS-00166/2021	EPI_ISL_1279442	In process	20B	B.1.1.519	25	5'UTR:C203T, 5'UTR:C222T, 5'UTR:C241T, ORF1ab:C3037T, ORF1ab:C3140T, ORF1ab:C5144T, ORF1ab:C5812T, ORF1ab:T9757C, ORF1ab:C10029T, ORF1ab:C10954T, ORF1ab:A11117G, ORF1ab:C12789T, ORF1ab:C14408T, ORF1ab:C18457T, ORF1ab:T19839C, ORF1ab:C21306T, S:C22995A, S:A23403G, S:C23604A, S:A23756G, ORF8:C28153T, N:G28881A, N:G28882A, N:G28883C, N:C29197T, N:C29555T,	13	N:R203K, N:G204R, ORF1a:P959S, ORF1a:T3255I, ORF1a:I3618V, ORF1a:T4175I, ORF1b:P314L, ORF1b:P1664S, ORF8:T87I, S:T478K, S:D614G, S:P681H, S:T732A,
hCoV-19/Mexico/OAX-INER-IMSS-00167/2021	EPI_ISL_1279443	In process	20B	B.1.1.519	24	5'UTR:C203T, 5'UTR:C222T, 5'UTR:C241T, ORF1ab:C3037T, ORF1ab:C3140T, ORF1ab:C3768T, ORF1ab:C10029T, ORF1ab:C10039T, ORF1ab:C10954T, ORF1ab:A11117G, ORF1ab:C11379T, ORF1ab:C12789T, ORF1ab:C14408T, ORF1ab:T19839C, ORF1ab:C21306T, S:C22995A, S:A23403G, S:C23604A, S:A23756G, ORF3a:G25720A, ORF6:C27213T, N:G28881A, N:G28882A, N:G28883C, N:C29197T,	14	N:R203K, N:G204R, ORF1a:P959S, ORF1a:T1168I, ORF1a:T3255I, ORF1a:I3618V, ORF1a:A3705V, ORF1a:T4175I, ORF1b:P314L, ORF3a:A110T, S:T478K, S:D614G, S:P681H, S:T732A,
hCoV-19/Mexico/OAX-INER-IMSS-00182/2021	EPI_ISL_1279453	In process	20B	B.1.1.519	27	5'UTR:T201C, 5'UTR:C203T, 5'UTR:C222T, 5'UTR:C241T, ORF1ab:T277C, ORF1ab:G1738T, ORF1ab:G2305T, ORF1ab:C3037T, ORF1ab:C3140T, ORF1ab:C6730T, ORF1ab:G7829T, ORF1ab:C10029T, ORF1ab:C10954T, ORF1ab:A11117G, ORF1ab:C12789T, ORF1ab:C14408T, ORF1ab:T19839C, ORF1ab:A19974G, ORF1ab:C21306T, S:C22995A, S:A23403G, S:C23604A, S:A23756G, N:G28881A, N:G28882A, N:G28883C, N:C29197T, N:G29427A,	14	N:R203K, N:G204R, N:R385K, ORF1a:K680N, ORF1a:P959S, ORF1a:V2522F, ORF1a:T3255I, ORF1a:I3618V, ORF1a:T4175I, ORF1b:P314L, S:T478K, S:D614G, S:P681H, S:T732A,
hCoV-19/Mexico/OAX-INER-IMSS-00183/2021	EPI_ISL_1279454	In process	20B	B.1.1.519	28	5'UTR:C203T, 5'UTR:C222T, 5'UTR:C241T, ORF1ab:T358C, ORF1ab:C3037T, ORF1ab:C3140T, ORF1ab:T3833C, ORF1ab:A6946G, ORF1ab:C10029T, ORF1ab:C10954T, ORF1ab:A11117G, ORF1ab:C12789T, ORF1ab:C14408T, ORF1ab:C18744T, ORF1ab:T19839C, ORF1ab:G20580T, ORF1ab:C21306T, S:T21771C, S:C22995A, S:A23403G, S:C23604A, S:A23756G, M:C26882T, ORF8:T27904C, N:C28507T, N:G28881A, N:G28882A, N:G28883C, N:C29197T	16	N:R203K, N:G204R, ORF1a:P959S, ORF1a:F1190L, ORF1a:I2227M, ORF1a:T3255I, ORF1a:I3618V, ORF1a:T4175I, ORF1b:P314L, ORF8:L4P, ORF9b:A75V, S:V70A, S:T478K, S:D614G, S:P681H, S:T732A,

hCoV-19/Mexico/OAX-INER-IMSS-00312/2021	EPI_ISL_1279565	In process	21C (Epsilon)	B.1.427	22	5'UTR:C241T, ORF1ab:C1059T, ORF1ab:C3037T, ORF1ab:G4207T, ORF1ab:G9738C, ORF1ab:C10714T, ORF1ab:C11345T, ORF1ab:G13713A, ORF1ab:C14408T, ORF1ab:C14877T, ORF1ab:C16394T, ORF1ab:G17014T, S:G21600T, S:G22018T, S:T22917G, S:A23403G, ORF3a:C25511T, ORF3a:G25563T, M:C26681T, ORF8:A28272T, N:C28887T, N:C29362T, 3'UTR:G29690T,	13	N:T205I, ORF1a:T265I, ORF1a:S3158T, ORF1a:L3694F, ORF1b:P314L, ORF1b:P976L, ORF1b:D1183Y, ORF3a:S40L, ORF3a:Q57H, S:S13I, S:W152C, S:L452R, S:D614G,
hCoV-19/Mexico/OAX-INER-IMSS-00343/2021	EPI_ISL_1279589	In process	20B	B.1.1.519	23	5'UTR:C203T, 5'UTR:C222T, 5'UTR:C241T, ORF1ab:C3037T, ORF1ab:C3140T, ORF1ab:C6500T, ORF1ab:C8660T, ORF1ab:C10029T, ORF1ab:C10954T, ORF1ab:A11117G, ORF1ab:G11365T, ORF1ab:C12789T, ORF1ab:C14408T, ORF1ab:T19839C, ORF1ab:C21306T, S:C22995A, S:A23403G, S:C23604A, S:A23756G, N:G28881A, N:G28882A, N:G28883C, N:C29197T, N:G29527T,	14	N:R203K, N:G204R, N:Q418H, ORF1a:P959S, ORF1a:P2079S, ORF1a:H2799Y, ORF1a:T3255I, ORF1a:I3618V, ORF1a:T4175I, ORF1b:P314L, S:T478K, S:D614G, S:P681H, S:T732A,
hCoV-19/Mexico/OAX-INER-IMSS-00344/2021	EPI_ISL_1279590	In process	20B	B.1.1.519	26	5'UTR:T201C, 5'UTR:C203T, 5'UTR:C222T, 5'UTR:C241T, ORF1ab:C745T, ORF1ab:G1738T, ORF1ab:C3037T, ORF1ab:C3140T, ORF1ab:C6031T, ORF1ab:C10029T, ORF1ab:C10954T, ORF1ab:A11117G, ORF1ab:C12789T, ORF1ab:C14408T, ORF1ab:T16170C, ORF1ab:T19839C, ORF1ab:A19974G, ORF1ab:C21306T, S:C22995A, S:A23403G, S:C23604A, S:A23756G, N:G28881A, N:G28882A, N:G28883C, N:C29197T, 3'UTR:C29762T,	11	N:R203K, N:G204R, ORF1a:P959S, ORF1a:T3255I, ORF1a:I3618V, ORF1a:T4175I, ORF1b:P314L, S:T478K, S:D614G, S:P681H, S:T732A,
hCoV-19/Mexico/PUE_INER_IMSS_00678/2021	EPI_ISL_2091134	In process	20B	B.1.1.519	23	5'UTR:C203T, 5'UTR:C222T, 5'UTR:C241T, ORF1ab:C3037T, ORF1ab:C3140T, ORF1ab:T3745C, ORF1ab:C10029T, ORF1ab:C10954T, ORF1ab:A11117G, ORF1ab:C12789T, ORF1ab:C14408T, ORF1ab:C14805T, ORF1ab:T19839C, ORF1ab:C21306T, S:C22995A, S:A23403G, S:C23604A, S:A23756G, ORF3a:C25844T, ORF7b:C27889T, N:G28881A, N:G28882A, N:G28883C, N:C29197T,	12	N:R203K, N:G204R, ORF1a:P959S, ORF1a:T3255I, ORF1a:I3618V, ORF1a:T4175I, ORF1b:P314L, ORF3a:T151I, S:T478K, S:D614G, S:P681H, S:T732A,
hCoV-19/Mexico/PUE-IBT-IMSS-120/2021	EPI_ISL_1288328	In process	20B	B.1.1.519	24	5'UTR:C203T, 5'UTR:C222T, 5'UTR:C241T, ORF1ab:G2990A, ORF1ab:C3037T, ORF1ab:C3140T, ORF1ab:G3549A, ORF1ab:T4888C, ORF1ab:C8626T, ORF1ab:C10029T, ORF1ab:C10954T, ORF1ab:A11117G, ORF1ab:C12789T, ORF1ab:C14408T, ORF1ab:T19839C, ORF1ab:C21306T, S:C22995A, S:A23403G, S:C23604A, S:A23756G, ORF8:T27904C, N:G28881A, N:G28882A, N:G28883C, N:C29197T,	14	N:R203K, N:G204R, ORF1a:D909N, ORF1a:P959S, ORF1a:G1095E, ORF1a:T3255I, ORF1a:I3618V, ORF1a:T4175I, ORF1b:P314L, ORF8:L4P, S:T478K, S:D614G, S:P681H, S:T732A,
hCoV-19/Mexico/PUE-IBT-IMSS-124/2021	EPI_ISL_1288331	In process	20B	B.1.1.519	24	5'UTR:T201C, 5'UTR:C203T, 5'UTR:C222T, 5'UTR:C241T, ORF1ab:G1738T, ORF1ab:C3037T, ORF1ab:C3140T, ORF1ab:C10029T, ORF1ab:C10954T, ORF1ab:A11117G, ORF1ab:C12789T, ORF1ab:C14408T, ORF1ab:T19839C, ORF1ab:A19974G, ORF1ab:C21306T, S:C21638T, S:C22995A, S:A23403G, S:C23604A, S:A23756G, ORF3a:G25855T, N:G28881A, N:G28882A, N:G28883C, N:C29197T,	13	N:R203K, N:G204R, ORF1a:P959S, ORF1a:T3255I, ORF1a:I3618V, ORF1a:T4175I, ORF1b:P314L, ORF3a:D155Y, S:P26S, S:T478K, S:D614G, S:P681H, S:T732A,

hCoV-19/Mexico/PUE-IBT-IMSS-126/2021	EPI_ISL_1288332	In process	20B	B.1.1.519	24	5'UTR:C203T, 5'UTR:C222T, 5'UTR:C241T, ORF1ab:C1377T, ORF1ab:T1971C, ORF1ab:C3037T, ORF1ab:C3140T, ORF1ab:C10029T, ORF1ab:C10954T, ORF1ab:A11117G, ORF1ab:C12789T, ORF1ab:C14408T, ORF1ab:T19839C, ORF1ab:C21306T, S:C22747T, S:C22995A, S:A23403G, S:C23604A, S:A23756G, S:T24637A, N:A28761G, N:G28881A, N:G28882A, N:G28883C, N:C29197T,	14	N:Q163R, N:R203K, N:G204R, ORF1a:P371L, ORF1a:I569T, ORF1a:P959S, ORF1a:T3255I, ORF1a:I3618V, ORF1a:T4175I, ORF1b:P314L, S:T478K, S:D614G, S:P681H, S:T732A,
hCoV-19/Mexico/PUE-IBT-IMSS-127/2021	EPI_ISL_1288333	In process	20B	B.1.1.519	25	5'UTR:C203T, 5'UTR:C222T, 5'UTR:C241T, ORF1ab:A3019G, ORF1ab:C3037T, ORF1ab:C3140T, ORF1ab:C3487T, ORF1ab:G3549A, ORF1ab:C10029T, ORF1ab:C10954T, ORF1ab:A11117G, ORF1ab:C12789T, ORF1ab:T14257C, ORF1ab:C14408T, ORF1ab:T19839C, ORF1ab:C21306T, S:C22995A, S:A23403G, S:C23604A, S:A23756G, ORF3a:C25603T, ORF8:T27904C, N:G28881A, N:G28882A, N:G28883C, N:C29197T,	14	N:R203K, N:G204R, ORF1a:P959S, ORF1a:G1095E, ORF1a:T3255I, ORF1a:I3618V, ORF1a:T4175I, ORF1b:Y264H, ORF1b:P314L, ORF8:L4P, S:T478K, S:D614G, S:P681H, S:T732A,
hCoV-19/Mexico/PUE-IBT-IMSS-128/2021	EPI_ISL_1288334	In process	20B	B.1.1.519	27	5'UTR:C203T, 5'UTR:C222T, 5'UTR:C241T, ORF1ab:C3037T, ORF1ab:C3140T, ORF1ab:G3549A, ORF1ab:G9049T, ORF1ab:C10029T, ORF1ab:C10954T, ORF1ab:A11117G, ORF1ab:A11765G, ORF1ab:C12789T, ORF1ab:C13105T, ORF1ab:C14408T, ORF1ab:A17295G, ORF1ab:T19839C, ORF1ab:C21306T, S:C22995A, S:A23403G, S:C23604A, S:A23756G, ORF3a:T25520C, ORF3a:G25563C, ORF8:T27904C, N:G28881A, N:G28882A, N:G28883C, N:C29197T,	17	N:R203K, N:G204R, ORF1a:P959S, ORF1a:G1095E, ORF1a:K2928N, ORF1a:T3255I, ORF1a:I3618V, ORF1a:S3834G, ORF1a:T4175I, ORF1b:P314L, ORF3a:F43S, ORF3a:Q57H, ORF8:L4P, S:T478K, S:D614G, S:P681H, S:T732A,
hCoV-19/Mexico/PUE-IBT-IMSS-129/2021	EPI_ISL_1288335	In process	20B	B.1.1.519	27	5'UTR:T201C, 5'UTR:C203T, 5'UTR:C222T, 5'UTR:C241T, ORF1ab:C745T, ORF1ab:G1738T, ORF1ab:C3037T, ORF1ab:C3140T, ORF1ab:C7093T, ORF1ab:C10029T, ORF1ab:C10954T, ORF1ab:G11083T, ORF1ab:A11117G, ORF1ab:C11941T, ORF1ab:C12789T, ORF1ab:C14408T, ORF1ab:T19839C, ORF1ab:A19974G, ORF1ab:C21306T, S:C21575T, S:C22995A, S:A23403G, S:C23604A, S:A23756G, N:G28881A, N:G28882A, N:G28883C, N:C29197T,	13	N:R203K, N:G204R, ORF1a:P959S, ORF1a:T3255I, ORF1a:L3606F, ORF1a:I3618V, ORF1a:T4175I, ORF1b:P314L, S:L5F, S:T478K, S:D614G, S:P681H, S:T732A,
hCoV-19/Mexico/PUE-IBT-IMSS-130/2021	EPI_ISL_1288336	In process	20B	B.1.1.519	25	5'UTR:T201C, 5'UTR:C203T, 5'UTR:C222T, 5'UTR:C241T, ORF1ab:G1738T, ORF1ab:C3037T, ORF1ab:C3140T, ORF1ab:C3768T, ORF1ab:C3884T, ORF1ab:C10029T, ORF1ab:C10954T, ORF1ab:A11117G, ORF1ab:C12789T, ORF1ab:C14408T, ORF1ab:T19839C, ORF1ab:A19974G, ORF1ab:C21306T, S:C22995A, S:A23403G, S:C23604A, S:A23756G, ORF8:A28175G, N:G28881A, N:G28882A, N:G28883C, N:C29197T,	13	N:R203K, N:G204R, ORF1a:P959S, ORF1a:T1168I, ORF1a:P1207S, ORF1a:T3255I, ORF1a:I3618V, ORF1a:T4175I, ORF1b:P314L, S:T478K, S:D614G, S:P681H, S:T732A,
hCoV-19/Mexico/PUE-IBT-IMSS-131/2021	EPI_ISL_1288337	In process	20B	B.1.1.519	24	5'UTR:T201C, 5'UTR:C203T, 5'UTR:C222T, 5'UTR:C241T, ORF1ab:G1522A, ORF1ab:C3037T, ORF1ab:C3140T, ORF1ab:C10029T, ORF1ab:C10954T, ORF1ab:A11117G, ORF1ab:C12789T, ORF1ab:C14408T, ORF1ab:T19839C, ORF1ab:A19974G, ORF1ab:C21306T, S:C22995A, S:A23403G, S:C23604A, S:A23756G, ORF3a:G25538T, M:T27056C, N:G28881A, N:G28882A, N:G28883C, N:C29197T,	12	N:R203K, N:G204R, ORF1a:P959S, ORF1a:T3255I, ORF1a:I3618V, ORF1a:T4175I, ORF1b:P314L, ORF3a:G49V, S:T478K, S:D614G, S:P681H, S:T732A,

hCoV-19/Mexico/PUE-IBT-IMSS-132/2021	EPI_ISL_1288338	In process	20B	B.1.1.519	23	5'UTR:C203T, 5'UTR:C222T, 5'UTR:C241T, ORF1ab:C3037T, ORF1ab:C3140T, ORF1ab:C10029T, ORF1ab:C10954T, ORF1ab:A11117G, ORF1ab:C12789T, ORF1ab:A13237G, ORF1ab:C14408T, ORF1ab:T19839C, ORF1ab:C21306T, S:C22995A, S:A23403G, S:C23604A, S:A23756G, ORF7a:A27674G, ORF8:T27904C, ORF8:C28087T, N:G28881A, N:G28882A, N:G28883C, N:C29197T,	14	N:R203K, N:G204R, ORF1a:P959S, ORF1a:T3255I, ORF1a:I3618V, ORF1a:T4175I, ORF1b:P314L, ORF7a:Q94R, ORF8:L4P, ORF8:A65V, S:T478K, S:D614G, S:P681H, S:T732A,	
hCoV-19/Mexico/PUE-IBT-IMSS-133/2021	EPI_ISL_1288339	In process	20B	B.1.1.222	17	5'UTR:C241T, ORF1ab:C556T, ORF1ab:C3037T, ORF1ab:C7267T, ORF1ab:C10029T, ORF1ab:C14408T, ORF1ab:T19839C, S:A23403G, S:A23756G, ORF3a:G25912T, ORF7a:C27657T, ORF7a:C27661T, ORF7b:A27756G, ORF8:A27921G, ORF8:G28001T, N:G28881A, N:G28882A, N:G28883C,	10	N:R203K, N:G204R, ORF1a:T3255I, ORF1b:P314L, ORF3a:G174C, ORF7a:Q90*, ORF7b:M1V, ORF8:I10V, S:D614G, S:T732A,	3'UTR:29746-29762,
hCoV-19/Mexico/PUE-IBT-IMSS-134/2021	EPI_ISL_1288340	In process	20B	B.1.1.519	25	5'UTR:C203T, 5'UTR:C222T, 5'UTR:C241T, ORF1ab:C3037T, ORF1ab:C3140T, ORF1ab:T4597G, ORF1ab:G7037T, ORF1ab:C10029T, ORF1ab:C10954T, ORF1ab:A11117G, ORF1ab:C12789T, ORF1ab:A13196G, ORF1ab:C14408T, ORF1ab:T19839C, ORF1ab:C21306T, S:C21846T, S:C22995A, S:A23403G, S:C23525T, S:C23604A, S:A23756G, ORF3a:C25782T, N:G28881A, N:G28882A, N:G28883C, N:C29197T,	15	N:R203K, N:G204R, ORF1a:P959S, ORF1a:G2258C, ORF1a:T3255I, ORF1a:I3618V, ORF1a:T4175I, ORF1a:T4311A, ORF1b:P314L, S:T95I, S:T478K, S:D614G, S:H655Y, S:P681H, S:T732A,	
hCoV-19/Mexico/PUE-IBT-IMSS-138/2021	EPI_ISL_1288341	In process	20B	B.1.1.519	22	5'UTR:C203T, 5'UTR:C222T, 5'UTR:C241T, ORF1ab:C3037T, ORF1ab:C3140T, ORF1ab:C10029T, ORF1ab:C10954T, ORF1ab:G11083T, ORF1ab:A11117G, ORF1ab:A11814G, ORF1ab:C12789T, ORF1ab:C14408T, ORF1ab:T19839C, ORF1ab:C21306T, S:C22995A, S:A23403G, S:C23604A, S:A23756G, ORF3a:C25528T, N:G28881A, N:G28882A, N:G28883C, N:C29197T,	14	N:R203K, N:G204R, ORF1a:P959S, ORF1a:T3255I, ORF1a:L3606F, ORF1a:I3618V, ORF1a:K3850R, ORF1a:T4175I, ORF1b:P314L, ORF3a:L46F, S:T478K, S:D614G, S:P681H, S:T732A,	
hCoV-19/Mexico/PUE-IBT-IMSS-139/2021	EPI_ISL_1288342	In process	20B	B.1.1.519	24	5'UTR:G165T, 5'UTR:C203T, 5'UTR:C222T, 5'UTR:C241T, ORF1ab:C3037T, ORF1ab:C3140T, ORF1ab:G3549A, ORF1ab:C10029T, ORF1ab:C10954T, ORF1ab:A11117G, ORF1ab:C12789T, ORF1ab:C13119T, ORF1ab:C14408T, ORF1ab:T19839C, ORF1ab:C21306T, S:C22995A, S:A23403G, S:C23604A, S:A23756G, ORF3a:C25889T, ORF8:T27904C, N:G28881A, N:G28882A, N:G28883C, N:C29197T,	15	N:R203K, N:G204R, ORF1a:P959S, ORF1a:G1095E, ORF1a:T3255I, ORF1a:I3618V, ORF1a:T4175I, ORF1a:A4285V, ORF1b:P314L, ORF3a:S166L, ORF8:L4P, S:T478K, S:D614G, S:P681H, S:T732A,	
hCoV-19/Mexico/PUE-IBT-IMSS-140/2021	EPI_ISL_1288343	In process	20B	B.1.1.519	24	5'UTR:C203T, 5'UTR:C222T, 5'UTR:C241T, ORF1ab:C3037T, ORF1ab:C3140T, ORF1ab:C10029T, ORF1ab:C10954T, ORF1ab:A11117G, ORF1ab:C12789T, ORF1ab:T14133C, ORF1ab:C14408T, ORF1ab:G15921T, ORF1ab:A17971G, ORF1ab:T19839C, ORF1ab:C21306T, S:G22225T, S:C22995A, S:A23403G, S:C23604A, S:C23635T, S:A23756G, N:G28881A, N:G28882A, N:G28883C, N:C29197T,	12	N:R203K, N:G204R, ORF1a:P959S, ORF1a:T3255I, ORF1a:I3618V, ORF1a:T4175I, ORF1b:P314L, ORF1b:R1502G, S:T478K, S:D614G, S:P681H, S:T732A,	

hCoV-19/Mexico/PUE-IBT-IMSS-141/2021	EPI_ISL_1288344	In process	20B	B.1.1.519	33	5'UTR:C203T, 5'UTR:C222T, 5'UTR:C241T, ORF1ab:A866G, ORF1ab:C1009T, ORF1ab:C3037T, ORF1ab:C3140T, ORF1ab:C5183T, ORF1ab:C9561T, ORF1ab:C10029T, ORF1ab:C10954T, ORF1ab:A11117G, ORF1ab:C11916T, ORF1ab:C12789T, ORF1ab:C12853T, ORF1ab:C14408T, ORF1ab:G17632T, ORF1ab:C19662T, ORF1ab:T19839C, ORF1ab:C21306T, S:C21575T, S:C22995A, S:A23403G, S:C23604A, S:A23756G, ORF3a:A25470C, M:T27113C, ORF8:T27904C, N:G28747T, N:G28881A, N:G28882A, N:G28883C, N:C29197T, N:G29227T,	18	N:R203K, N:G204R, ORF1a:I201V, ORF1a:P959S, ORF1a:P1640S, ORF1a:S3099L, ORF1a:T3255I, ORF1a:I3618V, ORF1a:S3884L, ORF1a:T4175I, ORF1b:P314L, ORF1b:D1389Y, ORF8:L4P, S:L5F, S:T478K, S:D614G, S:P681H, S:T732A,	
hCoV-19/Mexico/PUE-IBT-IMSS-142/2021	EPI_ISL_1288345	In process	20B	B.1.1.519	27	5'UTR:C203T, 5'UTR:C222T, 5'UTR:C241T, ORF1ab:C3037T, ORF1ab:C3140T, ORF1ab:C6445T, ORF1ab:C10029T, ORF1ab:C10954T, ORF1ab:A11117G, ORF1ab:C11824T, ORF1ab:G12385A, ORF1ab:C12473T, ORF1ab:C12789T, ORF1ab:C14408T, ORF1ab:T19839C, ORF1ab:G20995T, ORF1ab:C21306T, S:C22995A, S:A23403G, S:C23604A, S:A23756G, M:G26526A, M:C26882T, N:G28881A, N:G28882A, N:G28883C, N:C29197T, N:G29527T	14	M:A2T, N:R203K, N:G204R, N:Q418H, ORF1a:P959S, ORF1a:T3255I, ORF1a:I3618V, ORF1a:T4175I, ORF1b:P314L, ORF1b:G2510C, S:T478K, S:D614G, S:P681H, S:T732A,	
hCoV-19/Mexico/PUE-IBT-IMSS-143/2021	EPI_ISL_1288346	In process	20A	B.1.243	19	5'UTR:C241T, ORF1ab:C274T, ORF1ab:G806A, ORF1ab:G1855T, ORF1ab:T2584A, ORF1ab:C3037T, ORF1ab:C5140A, ORF1ab:C6027T, ORF1ab:C6406T, ORF1ab:C10525T, ORF1ab:C14408T, ORF1ab:C18486T, ORF1ab:A20268G, S:G23120T, S:A23403G, S:G23587C, S:T24076C, E:C26455T, N:C28854T, N:G29543T,	9	E:P71S, N:S194L, ORF1a:A181T, ORF1a:D1625E, ORF1a:P1921L, ORF1b:P314L, S:A520S, S:D614G, S:Q675H,	
hCoV-19/Mexico/PUE-IBT-IMSS-144/2021	EPI_ISL_1288347	In process	20B	B.1.1.519	26	5'UTR:T201C, 5'UTR:C203T, 5'UTR:C222T, 5'UTR:C241T, ORF1ab:G1738T, ORF1ab:C3037T, ORF1ab:C3140T, ORF1ab:C5144A, ORF1ab:C10029T, ORF1ab:C10954T, ORF1ab:A11117G, ORF1ab:C12789T, ORF1ab:C14408T, ORF1ab:A17148C, ORF1ab:T19839C, ORF1ab:A19974G, ORF1ab:C21306T, S:C22995A, S:A23403G, S:C23604A, S:A23756G, N:C28310T, N:G28881A, N:G28882A, N:G28883C, N:C29197T, ORF10:C29614T,	13	N:P13S, N:R203K, N:G204R, ORF1a:P959S, ORF1a:L1627I, ORF1a:T3255I, ORF1a:I3618V, ORF1a:T4175I, ORF1b:P314L, S:T478K, S:D614G, S:P681H, S:T732A,	
hCoV-19/Mexico/PUE-IBT-IMSS-145/2021	EPI_ISL_1288348	In process	20B	B.1.1.222	18	5'UTR:C241T, ORF1ab:C3037T, ORF1ab:C3717A, ORF1ab:C4421T, ORF1ab:C6285T, ORF1ab:A13498G, ORF1ab:C14408T, ORF1ab:G15202T, ORF1ab:C15579T, ORF1ab:T19839C, S:G22225T, S:G22331A, S:T22510C, S:A23403G, S:A23756G, ORF3a:C25521T, N:G28881A, N:G28882A, N:G28883C.	11	N:R203K, N:G204R, ORF1a:A1151D, ORF1a:R1386C, ORF1a:T2007I, ORF1b:T11A, ORF1b:P314L, ORF1b:V579L, S:G257S, S:D614G, S:T732A,	
hCoV-19/Mexico/PUE-IBT-IMSS-146/2021	EPI_ISL_1288349	In process	20B	B.1.1.222	22	5'UTR:C241T, ORF1ab:C1415T, ORF1ab:T2943A, ORF1ab:C3037T, ORF1ab:C10029T, ORF1ab:C13423T, ORF1ab:C14408T, ORF1ab:C15738T, ORF1ab:G17721T, ORF1ab:T19839C, S:A23403G, S:A23756G, ORF3a:G25567A, ORF3a:T25569A, ORF3a:T25570A, ORF3a:G25912T, ORF3a:A26108G, ORF7a:G27670T, ORF8:A27921G, ORF8:G28001T, N:G28881A, N:G28882A, N:G28883C,	14	N:R203K, N:G204R, ORF1a:L384F, ORF1a:L893Q, ORF1a:T3255I, ORF1b:P314L, ORF3a:A59T, ORF3a:S60T, ORF3a:G174C, ORF3a:E239G, ORF7a:V93F, ORF8:I10V, S:D614G, S:T732A,	3'UTR:29722-29723
hCoV-19/Mexico/PUE-IBT-IMSS-149/2021	EPI_ISL_1288351	In process	20A	B.1.551	13	5'UTR:C241T, ORF1ab:C3037T, ORF1ab:C3738T, ORF1ab:C5974T, ORF1ab:T10600C, ORF1ab:C14408T, ORF1ab:A16471G, ORF1ab:A20268G, S:A23403G, S:C23604G, M:G26951T, N:C28473T, N:C28854T, N:C29466T,	9	N:P67L, N:S194L, N:A398V, ORF1a:P1158L, ORF1b:P314L, ORF1b:I1002V, ORF9b:L64F, S:D614G, S:P681R,	

hCoV-19/Mexico/PUE-IBT-IMSS-35/2021	EPI_ISL_1288254	In process	20B	B.1.1.519	26	5'UTR:T201C, 5'UTR:C203T, 5'UTR:C222T, 5'UTR:C241T, ORF1ab:G1738T, ORF1ab:C3037T, ORF1ab:C3140T, ORF1ab:C10029T, ORF1ab:C10954T, ORF1ab:A11117G, ORF1ab:G11335T, ORF1ab:C12789T, ORF1ab:C14408T, ORF1ab:T19839C, ORF1ab:A19974G, ORF1ab:C21306T, S:C22995A, S:A23403G, S:C23604A, S:A23756G, M:T26726C, M:T26972C, ORF8:A28175G, N:G28881A, N:G28882A, N:G28883C, N:C29197T,	11	N:R203K, N:G204R, ORF1a:P959S, ORF1a:T3255I, ORF1a:I3618V, ORF1a:T4175I, ORF1b:P314L, S:T478K, S:D614G, S:P681H, S:T732A,
hCoV-19/Mexico/PUE-IBT-IMSS-36/2021	EPI_ISL_1288255	In process	20B	B.1.1.519	28	5'UTR:C203T, 5'UTR:C222T, 5'UTR:C241T, ORF1ab:C3037T, ORF1ab:C3140T, ORF1ab:T5713C, ORF1ab:C10029T, ORF1ab:C10954T, ORF1ab:A11117G, ORF1ab:C12789T, ORF1ab:C13019T, ORF1ab:C14408T, ORF1ab:G14829A, ORF1ab:T19839C, ORF1ab:C21306T, S:C22995A, S:A23403G, S:C23604A, S:A23756G, S:C25317T, ORF3a:A25411G, ORF8:T27904C, ORF8:C28087T, ORF8:T28240C, ORF8:C28253T, N:G28881A, N:G28882A, N:G28883C, N:C29197T	17	N:R203K, N:G204R, ORF1a:P959S, ORF1a:T3255I, ORF1a:I3618V, ORF1a:T4175I, ORF1b:P314L, ORF1b:M454I, ORF3a:I7V, ORF8:L4P, ORF8:A65V, ORF8:V116A, S:T478K, S:D614G, S:P681H, S:T732A, S:S1252F,
hCoV-19/Mexico/PUE-IBT-IMSS-38/2021	EPI_ISL_1288256	In process	20B	B.1.1.519	29	5'UTR:C203T, 5'UTR:C222T, 5'UTR:C241T, ORF1ab:A866G, ORF1ab:C1009T, ORF1ab:C1314T, ORF1ab:C3037T, ORF1ab:C3140T, ORF1ab:C5183T, ORF1ab:C10029T, ORF1ab:C10702T, ORF1ab:C10954T, ORF1ab:A11117G, ORF1ab:C11916T, ORF1ab:G12223T, ORF1ab:C12789T, ORF1ab:C14408T, ORF1ab:T19839C, ORF1ab:C21306T, S:C22995A, S:C23191T, S:A23403G, S:C23604A, S:A23756G, ORF3a:G26167T, ORF8:T27904C, N:G28881A, N:G28882A, N:G28883C, N:C29197T,	17	N:R203K, N:G204R, ORF1a:I201V, ORF1a:T350I, ORF1a:P959S, ORF1a:P1640S, ORF1a:T3255I, ORF1a:I3618V, ORF1a:S3884L, ORF1a:T4175I, ORF1b:P314L, ORF3a:V259L, ORF8:L4P, S:T478K, S:D614G, S:P681H, S:T732A,
hCoV-19/Mexico/PUE-IBT-IMSS-39/2021	EPI_ISL_1288257	In process	20B	B.1.1.519	25	5'UTR:C203T, 5'UTR:C222T, 5'UTR:C241T, ORF1ab:C3037T, ORF1ab:C3140T, ORF1ab:G3549A, ORF1ab:C5183T, ORF1ab:C10029T, ORF1ab:C10954T, ORF1ab:A11117G, ORF1ab:C12789T, ORF1ab:C14408T, ORF1ab:C14724T, ORF1ab:A18184G, ORF1ab:T19839C, ORF1ab:C21306T, S:C22995A, S:A23403G, S:C23604A, S:A23756G, ORF8:T27904C, N:C28791T, N:G28881A, N:G28882A, N:G28883C, N:C29197T,	16	N:A173V, N:R203K, N:G204R, ORF1a:P959S, ORF1a:G1095E, ORF1a:P1640S, ORF1a:T3255I, ORF1a:I3618V, ORF1a:T4175I, ORF1b:P314L, ORF1b:M1573V, ORF8:L4P, S:T478K, S:D614G, S:P681H, S:T732A,
hCoV-19/Mexico/PUE-IBT-IMSS-40/2021	EPI_ISL_1288258	In process	20B	B.1.1.519	24	5'UTR:C203T, 5'UTR:C222T, 5'UTR:C241T, ORF1ab:A3019G, ORF1ab:C3037T, ORF1ab:C3140T, ORF1ab:C3487T, ORF1ab:G3549A, ORF1ab:C8386T, ORF1ab:C8655T, ORF1ab:C10029T, ORF1ab:C10954T, ORF1ab:A11117G, ORF1ab:C14408T, ORF1ab:T19839C, ORF1ab:C21306T, S:C22995A, S:A23403G, S:C23604A, S:A23756G, ORF8:T27904C, N:G28881A, N:G28882A, N:G28883C, N:C29197T,	13	N:R203K, N:G204R, ORF1a:P959S, ORF1a:G1095E, ORF1a:S2797F, ORF1a:T3255I, ORF1a:I3618V, ORF1b:P314L, ORF8:L4P, S:T478K, S:D614G, S:P681H, S:T732A,
hCoV-19/Mexico/PUE-IBT-IMSS-41/2021	EPI_ISL_1288259	In process	20B	B.1.1.519	25	5'UTR:C203T, 5'UTR:C222T, 5'UTR:C241T, ORF1ab:C3037T, ORF1ab:C3140T, ORF1ab:G6271T, ORF1ab:C6428T, ORF1ab:C6541T, ORF1ab:C10029T, ORF1ab:C10954T, ORF1ab:A11117G, ORF1ab:G11365T, ORF1ab:C12789T, ORF1ab:C14408T, ORF1ab:T19839C, ORF1ab:C21306T, S:T22849C, S:C22995A, S:A23403G, S:C23604A, S:A23756G, N:G28881A, N:G28882A, N:G28883C, N:C29197T, N:G29527T,	13	N:R203K, N:G204R, N:Q418H, ORF1a:P959S, ORF1a:P2055S, ORF1a:T3255I, ORF1a:I3618V, ORF1a:T4175I, ORF1b:P314L, S:T478K, S:D614G, S:P681H, S:T732A,

hCoV-19/Mexico/PUE-IBT-IMSS-42/2021	EPI_ISL_1288260	In process	20B	B.1.1.519	28	5'UTR:T201C, 5'UTR:C203T, 5'UTR:C222T, 5'UTR:C241T, ORF1ab:G1738T, ORF1ab:C3037T, ORF1ab:C3140T, ORF1ab:A5570G, ORF1ab:C6336T, ORF1ab:C10029T, ORF1ab:A10317G, ORF1ab:C10954T, ORF1ab:A11117G, ORF1ab:C12789T, ORF1ab:C14408T, ORF1ab:T19839C, ORF1ab:A19974G, ORF1ab:C21306T, S:C22329T, S:C22995A, S:A23403G, S:C23604A, S:A23756G, M:A26702G, ORF8:C28253T, N:G28881A, N:G28882A, N:G28883C, N:C29197T	15	N:R203K, N:G204R, ORF1a:P959S, ORF1a:M1769V, ORF1a:S2024L, ORF1a:T3255I, ORF1a:K3351R, ORF1a:I3618V, ORF1a:T4175I, ORF1b:P314L, S:S256L, S:T478K, S:D614G, S:P681H, S:T732A,
hCoV-19/Mexico/PUE-IBT-IMSS-43/2021	EPI_ISL_1288261	In process	20B	B.1.1.519	26	5'UTR:C203T, 5'UTR:C222T, 5'UTR:C241T, ORF1ab:C3037T, ORF1ab:C3140T, ORF1ab:C10029T, ORF1ab:C10954T, ORF1ab:A11117G, ORF1ab:C12789T, ORF1ab:C14408T, ORF1ab:C14931T, ORF1ab:T15726A, ORF1ab:T19839C, ORF1ab:C21306T, S:C22995A, S:A23403G, S:C23604A, S:A23756G, M:C26833T, M:C27110T, ORF7b:T27785C, N:G28881A, N:G28882A, N:G28883C, N:C29197T, N:C29347T, N:G29527T,	13	M:A104V, N:R203K, N:G204R, N:Q418H, ORF1a:P959S, ORF1a:T3255I, ORF1a:I3618V, ORF1a:T4175I, ORF1b:P314L, S:T478K, S:D614G, S:P681H, S:T732A,
hCoV-19/Mexico/PUE-IBT-IMSS-44/2021	EPI_ISL_1288262	In process	20B	B.1.1.222	21	5'UTR:C241T, ORF1ab:G428A, ORF1ab:C3037T, ORF1ab:C4897T, ORF1ab:C10029T, ORF1ab:A11430G, ORF1ab:C13517T, ORF1ab:C14408T, ORF1ab:C19011A, ORF1ab:T19839C, ORF1ab:A21137G, S:A23403G, S:G23478T, S:A23756G, ORF3a:G25912T, ORF3a:A26115C, M:A26555C, ORF8:A27921G, ORF8:G28001T, N:G28881A, N:G28882A, N:G28883C,	16	M:E11D, N:R203K, N:G204R, ORF1a:E55K, ORF1a:T3255I, ORF1a:Y3722C, ORF1b:T17I, ORF1b:P314L, ORF1b:D1848E, ORF1b:K2557R, ORF3a:G174C, ORF3a:E241D, ORF8:I10V, S:D614G, S:G639V, S:T732A,
hCoV-19/Mexico/PUE-IBT-IMSS-449/2020	EPI_ISL_1301632	In process	20A	B.1.609	7	5'UTR:C241T, ORF1ab:C2334T, ORF1ab:C3037T, ORF1ab:C4582T, ORF1ab:T12230G, ORF1ab:C14408T, ORF1ab:A20268G, S:A23403G,	4	ORF1a:A690V, ORF1a:S3989A, ORF1b:P314L, S:D614G,
hCoV-19/Mexico/PUE-IBT-IMSS-45/2021	EPI_ISL_1288263	In process	20B	B.1.1.519	27	5'UTR:T201C, 5'UTR:C203T, 5'UTR:C222T, 5'UTR:C241T, ORF1ab:G1738T, ORF1ab:C3037T, ORF1ab:C3140T, ORF1ab:C3768T, ORF1ab:C10029T, ORF1ab:C10954T, ORF1ab:A11117G, ORF1ab:C12513T, ORF1ab:C12789T, ORF1ab:C14408T, ORF1ab:C15810T, ORF1ab:T19839C, ORF1ab:A19974G, ORF1ab:C21306T, S:C22995A, S:A23403G, S:C23604A, S:A23756G, ORF3a:C25886T, ORF8:A28175G, N:G28881A, N:G28882A, N:G28883C, N:C29197T	14	N:R203K, N:G204R, ORF1a:P959S, ORF1a:T1168I, ORF1a:T3255I, ORF1a:I3618V, ORF1a:T4083M, ORF1a:T4175I, ORF1b:P314L, ORF3a:S165F, S:T478K, S:D614G, S:P681H, S:T732A,
hCoV-19/Mexico/PUE-IBT-IMSS-450/2020	EPI_ISL_1301633	In process	20A	B.1	6	5'UTR:C241T, ORF1ab:C2334T, ORF1ab:C3037T, ORF1ab:C4582T, ORF1ab:T12230G, ORF1ab:C14408T, S:A23403G,	4	ORF1a:A690V, ORF1a:S3989A, ORF1b:P314L, S:D614G,
hCoV-19/Mexico/PUE-IBT-IMSS-451/2020	EPI_ISL_1301634	In process	20C	B.1	8	5'UTR:C241T, ORF1ab:C1059T, ORF1ab:C3037T, ORF1ab:C4633T, ORF1ab:C6033T, ORF1ab:C9198T, ORF1ab:C14408T, S:A23403G, ORF3a:G25563T,	6	ORF1a:T265I, ORF1a:A1923V, ORF1a:T2978I, ORF1b:P314L, ORF3a:Q57H, S:D614G,
hCoV-19/Mexico/PUE-IBT-IMSS-452/2020	EPI_ISL_1301635	In process	20C	B.1	7	5'UTR:C241T, ORF1ab:C1059T, ORF1ab:C3037T, ORF1ab:C11916T, ORF1ab:C12043T, ORF1ab:C14408T, S:A23403G, ORF3a:G25563T,	5	ORF1a:T265I, ORF1a:S3884L, ORF1b:P314L, ORF3a:Q57H, S:D614G,
hCoV-19/Mexico/PUE-IBT-IMSS-454/2020	EPI_ISL_1301637	In process	20A	B.1.189	8	5'UTR:C241T, ORF1ab:C3037T, ORF1ab:C4582T, ORF1ab:G6404T, ORF1ab:C14408T, ORF1ab:A20268G, S:A23403G, S:T25123C, N:G28378T,	4	ORF1a:V2047F, ORF1b:P314L, ORF9b:R32L, S:D614G,
hCoV-19/Mexico/PUE-IBT-IMSS-456/2020	EPI_ISL_1301639	In process	20A	B.1.610	4	5'UTR:C241T, ORF1ab:C3037T, ORF1ab:C14408T, ORF1ab:A20268G, S:A23403G,	2	ORF1b:P314L, S:D614G,
hCoV-19/Mexico/PUE-IBT-IMSS-457/2020	EPI_ISL_1301640	In process	20A	B.1	7	5'UTR:C241T, ORF1ab:C3037T, ORF1ab:C4582T, ORF1ab:C12085T, ORF1ab:C14408T, S:A23403G, ORF7b:C27883T, N:A29487G,	4	N:K405R, ORF1b:P314L, ORF7b:A43V, S:D614G,

hCoV-19/Mexico/PUE-IBT-IMSS-46/2021	EPI_ISL_1288264	In process	20B	B.1.1.519	27	5'UTR:C203T, 5'UTR:C222T, 5'UTR:C241T, ORF1ab:C3037T, ORF1ab:C3140T, ORF1ab:G7331T, ORF1ab:C9763T, ORF1ab:C10029T, ORF1ab:C10183T, ORF1ab:C10954T, ORF1ab:A11117G, ORF1ab:C12789T, ORF1ab:C14408T, ORF1ab:G16853T, ORF1ab:T19839C, ORF1ab:C21306T, S:C22995A, S:A23403G, S:C23604A, S:A23756G, M:C26882T, M:A26927G, ORF8:T27904C, ORF8:G28195T, N:G28881A, N:G28882A, N:G28883C, N:C29197T,	15	N:R203K, N:G204R, ORF1a:P959S, ORF1a:V2356L, ORF1a:T3255I, ORF1a:I3618V, ORF1a:T4175I, ORF1b:P314L, ORF1b:G1129V, ORF8:L4P, ORF8:R101L, S:T478K, S:D614G, S:P681H, S:T732A,
hCoV-19/Mexico/PUE-IBT-IMSS-460/2020	EPI_ISL_1301643	In process	20A	B.1	7	5'UTR:T168C, 5'UTR:C241T, ORF1ab:C3037T, ORF1ab:G11083T, ORF1ab:C14408T, ORF1ab:A20268G, S:G21989T, S:A23403G,	4	ORF1a:L3606F, ORF1b:P314L, S:V143F, S:D614G,
hCoV-19/Mexico/PUE-IBT-IMSS-462/2020	EPI_ISL_1301645	In process	20C	B.1	8	5'UTR:C241T, ORF1ab:C1059T, ORF1ab:C3037T, ORF1ab:C14408T, S:C21914T, S:A23403G, S:C23589T, ORF3a:G25563T, 3'UTR:C29750T,	6	ORF1a:T265I, ORF1b:P314L, ORF3a:Q57H, S:L118F, S:D614G, S:T676I,
hCoV-19/Mexico/PUE-IBT-IMSS-466/2020	EPI_ISL_1301649	In process	20A	B.1	8	5'UTR:C241T, ORF1ab:C3037T, ORF1ab:G12079A, ORF1ab:C14408T, ORF1ab:A20268G, S:A23403G, S:G24757T, N:G28378T, 3'UTR:G29751T,	3	ORF1b:P314L, ORF9b:R32L, S:D614G,
hCoV-19/Mexico/PUE-IBT-IMSS-47/2021	EPI_ISL_1288265	In process	20B	B.1.1.519	30	5'UTR:C203T, 5'UTR:C222T, 5'UTR:C241T, ORF1ab:C3037T, ORF1ab:C3140T, ORF1ab:A4485G, ORF1ab:C6541T, ORF1ab:C8386T, ORF1ab:C9348T, ORF1ab:C10029T, ORF1ab:C10954T, ORF1ab:A11117G, ORF1ab:C12789T, ORF1ab:C13255T, ORF1ab:C14408T, ORF1ab:C15774T, ORF1ab:T19839C, ORF1ab:C21057T, ORF1ab:C21306T, S:A21631C, S:A22023C, S:C22995A, S:A23403G, S:C23604A, S:A23756G, S:G25244T, ORF8:T27904C, N:G28881A, N:G28882A, N:G28883C, N:C29197T,	17	N:R203K, N:G204R, ORF1a:P959S, ORF1a:K1407R, ORF1a:T3028I, ORF1a:T3255I, ORF1a:I3618V, ORF1a:T4175I, ORF1b:P314L, ORF8:L4P, S:Q23H, S:E154A, S:T478K, S:D614G, S:P681H, S:T732A, S:V1228L,
hCoV-19/Mexico/PUE-IBT-IMSS-49/2021	EPI_ISL_1288266	In process	20B	B.1.1.519	24	5'UTR:T201C, 5'UTR:C203T, 5'UTR:C222T, 5'UTR:C241T, ORF1ab:T577C, ORF1ab:G1738T, ORF1ab:C3037T, ORF1ab:C3140T, ORF1ab:C8772T, ORF1ab:C10029T, ORF1ab:C10954T, ORF1ab:A11117G, ORF1ab:C12789T, ORF1ab:C14408T, ORF1ab:T19839C, ORF1ab:A19974G, ORF1ab:C21306T, S:C22995A, S:A23403G, S:C23604A, S:A23756G, N:G28881A, N:G28882A, N:G28883C, N:C29197T,	12	N:R203K, N:G204R, ORF1a:P959S, ORF1a:T2836I, ORF1a:T3255I, ORF1a:I3618V, ORF1a:T4175I, ORF1b:P314L, S:T478K, S:D614G, S:P681H, S:T732A,
hCoV-19/Mexico/PUE-IBT-IMSS-50/2021	EPI_ISL_1288267	In process	20B	B.1.1.519	26	5'UTR:C203T, 5'UTR:C222T, 5'UTR:C241T, ORF1ab:C3037T, ORF1ab:C3140T, ORF1ab:C6445T, ORF1ab:C10029T, ORF1ab:C10954T, ORF1ab:A11117G, ORF1ab:C11824T, ORF1ab:G12385A, ORF1ab:C12789T, ORF1ab:C14408T, ORF1ab:T19839C, ORF1ab:G20995T, ORF1ab:C21306T, S:C22995A, S:A23403G, S:C23604A, S:A23756G, M:G26526A, M:C26882T, N:G28881A, N:G28882A, N:G28883C, N:C29197T, N:G29527T,	14	M:A2T, N:R203K, N:G204R, N:Q418H, ORF1a:P959S, ORF1a:T3255I, ORF1a:I3618V, ORF1a:T4175I, ORF1b:P314L, ORF1b:G2510C, S:T478K, S:D614G, S:P681H, S:T732A,
hCoV-19/Mexico/PUE-IBT-IMSS-51/2021	EPI_ISL_1288268	In process	20B	B.1.1.519	23	5'UTR:C203T, 5'UTR:C222T, 5'UTR:C241T, ORF1ab:A3019G, ORF1ab:C3037T, ORF1ab:C3140T, ORF1ab:C3487T, ORF1ab:G3549A, ORF1ab:C10029T, ORF1ab:C10954T, ORF1ab:A11117G, ORF1ab:C12789T, ORF1ab:C14408T, ORF1ab:T19839C, ORF1ab:C21306T, S:C22995A, S:A23403G, S:C23604A, S:A23756G, ORF8:T27904C, N:G28881A, N:G28882A, N:G28883C, N:C29197T,	13	N:R203K, N:G204R, ORF1a:P959S, ORF1a:G1095E, ORF1a:T3255I, ORF1a:I3618V, ORF1a:T4175I, ORF1b:P314L, ORF8:L4P, S:T478K, S:D614G, S:P681H, S:T732A,

hCoV-19/Mexico/PUE-IBT-IMSS-52/2021	EPI_ISL_1288269	In process	20B	B.1.1.519	23	5'UTR:T201C, 5'UTR:C203T, 5'UTR:C222T, 5'UTR:C241T, ORF1ab:G1738T, ORF1ab:C3037T, ORF1ab:C3140T, ORF1ab:A6659G, ORF1ab:C10029T, ORF1ab:C10954T, ORF1ab:A11117G, ORF1ab:C12789T, ORF1ab:C14408T, ORF1ab:T19839C, ORF1ab:A19974G, ORF1ab:C21306T, S:C22995A, S:A23403G, S:C23604A, S:A23756G, N:G28881A, N:G28882A, N:G28883C, N:C29197T,	12	N:R203K, N:G204R, ORF1a:P959S, ORF1a:S2132G, ORF1a:T3255I, ORF1a:I3618V, ORF1a:T4175I, ORF1b:P314L, S:T478K, S:D614G, S:P681H, S:T732A,
hCoV-19/Mexico/PUE-IBT-IMSS-53/2021	EPI_ISL_1288270	In process	20B	B.1.1.519	26	5'UTR:C203T, 5'UTR:C222T, 5'UTR:C241T, ORF1ab:C3037T, ORF1ab:C3140T, ORF1ab:C6445T, ORF1ab:C10029T, ORF1ab:C10954T, ORF1ab:A11117G, ORF1ab:C11824T, ORF1ab:G12385A, ORF1ab:C12789T, ORF1ab:C14408T, ORF1ab:T19839C, ORF1ab:G20995T, ORF1ab:C21306T, S:C22995A, S:A23403G, S:C23604A, S:A23756G, M:G26526A, M:C26882T, N:G28881A, N:G28882A, N:G28883C, N:C29197T, N:G29527T,	14	M:A2T, N:R203K, N:G204R, N:Q418H, ORF1a:P959S, ORF1a:T3255I, ORF1a:I3618V, ORF1a:T4175I, ORF1b:P314L, ORF1b:G2510C, S:T478K, S:D614G, S:P681H, S:T732A,
hCoV-19/Mexico/PUE-IBT-IMSS-54/2021	EPI_ISL_1288271	In process	20B	B.1.1.519	26	5'UTR:T191A, 5'UTR:T201C, 5'UTR:C203T, 5'UTR:C222T, 5'UTR:C241T, ORF1ab:G1738T, ORF1ab:C3037T, ORF1ab:C3140T, ORF1ab:C9474T, ORF1ab:C10029T, ORF1ab:C10954T, ORF1ab:A11117G, ORF1ab:C12789T, ORF1ab:C14408T, ORF1ab:T19839C, ORF1ab:A19974G, ORF1ab:C21306T, S:C22995A, S:A23403G, S:C23604A, S:A23756G, S:C24407A, ORF7b:A27888G, N:G28881A, N:G28882A, N:G28883C, N:C29197T,	13	N:R203K, N:G204R, ORF1a:P959S, ORF1a:A3070V, ORF1a:T3255I, ORF1a:I3618V, ORF1a:T4175I, ORF1b:P314L, S:T478K, S:D614G, S:P681H, S:T732A, S:Q949K,
hCoV-19/Mexico/PUE-IBT-IMSS-55/2021	EPI_ISL_1288272	In process	20B	B.1.1.519	28	5'UTR:C203T, 5'UTR:C222T, 5'UTR:C241T, ORF1ab:C3037T, ORF1ab:C3140T, ORF1ab:T5713C, ORF1ab:C10029T, ORF1ab:C10954T, ORF1ab:A11117G, ORF1ab:C12789T, ORF1ab:C13019T, ORF1ab:C14408T, ORF1ab:G14829A, ORF1ab:T19839C, ORF1ab:C21306T, S:C22995A, S:A23403G, S:C23604A, S:A23756G, S:C25317T, ORF3a:A25411G, ORF8:T27904C, ORF8:C28087T, ORF8:T28240C, ORF8:C28253T, N:G28881A, N:G28882A, N:G28883C, N:C29197T,	17	N:R203K, N:G204R, ORF1a:P959S, ORF1a:T3255I, ORF1a:I3618V, ORF1a:T4175I, ORF1b:P314L, ORF1b:M454I, ORF3a:I7V, ORF8:L4P, ORF8:A65V, ORF8:V116A, S:T478K, S:D614G, S:P681H, S:T732A, S:S1252F,
hCoV-19/Mexico/PUE-IBT-IMSS-56/2021	EPI_ISL_1288273	In process	20B	B.1.1.519	23	5'UTR:C203T, 5'UTR:C222T, 5'UTR:C241T, ORF1ab:C3037T, ORF1ab:C3140T, ORF1ab:C8318T, ORF1ab:C10029T, ORF1ab:C10078T, ORF1ab:C10954T, ORF1ab:A11117G, ORF1ab:G11365T, ORF1ab:C12789T, ORF1ab:C14408T, ORF1ab:T19839C, ORF1ab:C21306T, S:C22995A, S:A23403G, S:C23604A, S:A23756G, N:G28881A, N:G28882A, N:G28883C, N:C29197T, N:G29527T,	13	N:R203K, N:G204R, N:Q418H, ORF1a:P959S, ORF1a:P2685S, ORF1a:T3255I, ORF1a:I3618V, ORF1a:T4175I, ORF1b:P314L, S:T478K, S:D614G, S:P681H, S:T732A,
hCoV-19/Mexico/PUE-IBT-IMSS-57/2021	EPI_ISL_1288274	In process	20B	B.1.1.519	29	5'UTR:C203T, 5'UTR:C222T, 5'UTR:C241T, ORF1ab:C3037T, ORF1ab:C3140T, ORF1ab:A8763G, ORF1ab:C10029T, ORF1ab:C10954T, ORF1ab:A11117G, ORF1ab:T12015G, ORF1ab:C12789T, ORF1ab:C14408T, ORF1ab:C14931T, ORF1ab:T15726A, ORF1ab:T19839C, ORF1ab:C21306T, S:C22995A, S:A23403G, S:C23604A, S:A23756G, ORF3a:A25859G, M:C26833T, ORF7b:T27785C, N:G28881A, N:G28882A, N:G28883C, N:C29197T, N:C29347T, N:G29527T, 3'UTR:C29774T,	16	M:A104V, N:R203K, N:G204R, N:Q418H, ORF1a:P959S, ORF1a:D2833G, ORF1a:T3255I, ORF1a:I3618V, ORF1a:V3917G, ORF1a:T4175I, ORF1b:P314L, ORF3a:Y156C, S:T478K, S:D614G, S:P681H, S:T732A,

hCoV-19/Mexico/PUE-IBT-IMSS-574/2021	EPI_ISL_1302288	In process	20B	B.1.1.519	23	5'UTR:T201C, 5'UTR:C203T, 5'UTR:C222T, 5'UTR:C241T, ORF1ab:C1077T, ORF1ab:G1738T, ORF1ab:C3037T, ORF1ab:C3140T, ORF1ab:G5063A, ORF1ab:C10029T, ORF1ab:C10954T, ORF1ab:A11117G, ORF1ab:C12789T, ORF1ab:C14408T, ORF1ab:T19839C, ORF1ab:A19974G, S:C22995A, S:A23403G, S:C23604A, S:A23756G, N:G28881A, N:G28882A, N:G28883C, N:C29197T,	13	N:R203K, N:G204R, ORF1a:P271L, ORF1a:P959S, ORF1a:D1600N, ORF1a:T3255I, ORF1a:I3618V, ORF1a:T4175I, ORF1b:P314L, S:T478K, S:D614G, S:P681H, S:T732A,
hCoV-19/Mexico/PUE-IBT-IMSS-575/2021	EPI_ISL_1302268	In process	20B	B.1.1.519	24	5'UTR:C203T, 5'UTR:C222T, 5'UTR:C241T, ORF1ab:A3019G, ORF1ab:C3037T, ORF1ab:C3140T, ORF1ab:G3549A, ORF1ab:C4543T, ORF1ab:C10029T, ORF1ab:C10954T, ORF1ab:A11117G, ORF1ab:C12789T, ORF1ab:C14408T, ORF1ab:G18181A, ORF1ab:T19839C, S:C22995A, S:A23403G, S:C23604A, S:A23756G, ORF8:T27904C, N:G28881A, N:G28882A, N:G28883C, N:C29197T, N:C29253T,	15	N:R203K, N:G204R, N:S327L, ORF1a:P959S, ORF1a:G1095E, ORF1a:T3255I, ORF1a:I3618V, ORF1a:T4175I, ORF1b:P314L, ORF1b:D1572N, ORF8:L4P, S:T478K, S:D614G, S:P681H, S:T732A,
hCoV-19/Mexico/PUE-IBT-IMSS-576/2021	EPI_ISL_1302173	In process	20B	B.1.1.519	23	5'UTR:C203T, 5'UTR:C222T, 5'UTR:C241T, ORF1ab:C3037T, ORF1ab:C3140T, ORF1ab:C5183T, ORF1ab:C6628T, ORF1ab:C10029T, ORF1ab:C10954T, ORF1ab:A11117G, ORF1ab:G11365T, ORF1ab:C12789T, ORF1ab:C14408T, ORF1ab:T19839C, S:C22995A, S:A23403G, S:C23604A, S:A23756G, M:G26660T, N:G28881A, N:G28882A, N:G28883C, N:C29197T, N:G29527T,	14	M:L46F, N:R203K, N:G204R, N:Q418H, ORF1a:P959S, ORF1a:P1640S, ORF1a:T3255I, ORF1a:I3618V, ORF1a:T4175I, ORF1b:P314L, S:T478K, S:D614G, S:P681H, S:T732A,
hCoV-19/Mexico/PUE-IBT-IMSS-577/2021	EPI_ISL_1302390	In process	20B	B.1.1.519	28	5'UTR:T201C, 5'UTR:C203T, 5'UTR:C222T, 5'UTR:C241T, ORF1ab:G1738T, ORF1ab:C3037T, ORF1ab:C3140T, ORF1ab:T8791C, ORF1ab:C9214T, ORF1ab:C9430T, ORF1ab:C10029T, ORF1ab:C10954T, ORF1ab:A11117G, ORF1ab:C12789T, ORF1ab:C14408T, ORF1ab:T19839C, ORF1ab:A19974G, S:C22995A, S:A23403G, S:C23604A, S:A23756G, S:T23986C, S:C25047T, M:C26801T, ORF8:A28175G, N:G28881A, N:G28882A, N:G28883C, N:C29197T,	12	N:R203K, N:G204R, ORF1a:P959S, ORF1a:T3255I, ORF1a:I3618V, ORF1a:T4175I, ORF1b:P314L, S:T478K, S:D614G, S:P681H, S:T732A, S:P1162L,
hCoV-19/Mexico/PUE-IBT-IMSS-58/2021	EPI_ISL_1288275	In process	20B	B.1.1.519	27	5'UTR:C203T, 5'UTR:C222T, 5'UTR:C241T, ORF1ab:C3037T, ORF1ab:C3140T, ORF1ab:C10029T, ORF1ab:C10954T, ORF1ab:A11117G, ORF1ab:T11294C, ORF1ab:C12789T, ORF1ab:C13965T, ORF1ab:C14408T, ORF1ab:C18647T, ORF1ab:T19839C, ORF1ab:C21306T, S:A21849G, S:C22995A, S:A23403G, S:C23604A, S:A23756G, ORF3a:C25511T, M:G26730T, ORF8:T27904C, ORF8:C28087T, N:G28881A, N:G28882A, N:G28883C, N:C29197T,	18	M:V70F, N:R203K, N:G204R, ORF1a:P959S, ORF1a:T3255I, ORF1a:I3618V, ORF1a:F3677L, ORF1a:T4175I, ORF1b:P314L, ORF1b:P1727L, ORF3a:S40L, ORF8:L4P, ORF8:A65V, S:E96G, S:T478K, S:D614G, S:P681H, S:T732A,
hCoV-19/Mexico/PUE-IBT-IMSS-60/2021	EPI_ISL_1288276	In process	20B	B.1.1.519	25	5'UTR:T201C, 5'UTR:C203T, 5'UTR:C222T, 5'UTR:C241T, ORF1ab:G1738T, ORF1ab:C3037T, ORF1ab:C3096T, ORF1ab:C3140T, ORF1ab:C10029T, ORF1ab:C10954T, ORF1ab:A11117G, ORF1ab:C12789T, ORF1ab:C14408T, ORF1ab:C19170T, ORF1ab:T19839C, ORF1ab:A19974G, ORF1ab:C21306T, S:C22995A, S:A23403G, S:C23604A, S:A23756G, ORF3a:C26198T, N:G28881A, N:G28882A, N:G28883C, N:C29197T,	13	N:R203K, N:G204R, ORF1a:S944L, ORF1a:P959S, ORF1a:T3255I, ORF1a:I3618V, ORF1a:T4175I, ORF1b:P314L, ORF3a:T269M, S:T478K, S:D614G, S:P681H, S:T732A,

hCoV-19/Mexico/PUE-IBT-IMSS-61/2021	EPI_ISL_1288277	In process	20B	B.1.1.519	25	5'UTR:T201C, 5'UTR:C203T, 5'UTR:C222T, 5'UTR:C241T, ORF1ab:G1738T, ORF1ab:C3037T, ORF1ab:C3140T, ORF1ab:G4184A, ORF1ab:C10029T, ORF1ab:C10954T, ORF1ab:A11117G, ORF1ab:C12789T, ORF1ab:C14408T, ORF1ab:C18252T, ORF1ab:C19170T, ORF1ab:T19839C, ORF1ab:A19974G, ORF1ab:C21306T, S:C22995A, S:A23403G, S:C23604A, S:A23756G, N:G28881A, N:G28882A, N:G28883C, N:C29197T,	12	N:R203K, N:G204R, ORF1a:P959S, ORF1a:G1307S, ORF1a:T3255I, ORF1a:I3618V, ORF1a:T4175I, ORF1b:P314L, S:T478K, S:D614G, S:P681H, S:T732A,
hCoV-19/Mexico/PUE-InDRE-IBT-146/2020	EPI_ISL_1302187	In process	20B	B.1.1.222	19	5'UTR:C241T, ORF1ab:G347A, ORF1ab:G1544T, ORF1ab:C3037T, ORF1ab:C10029T, ORF1ab:C14408T, ORF1ab:C19011A, ORF1ab:T19839C, S:C21707T, S:C21855T, S:A23403G, S:A23756G, ORF3a:G25912T, ORF8:A27921G, ORF8:G28001T, N:C28791T, N:G28881A, N:G28882A, N:G28883C, 3'UTR:G29744A,	14	N:A173V, N:R203K, N:G204R, ORF1a:V28I, ORF1a:A427S, ORF1a:T3255I, ORF1b:P314L, ORF1b:D1848E, ORF3a:G174C, ORF8:I10V, S:H49Y, S:S98F, S:D614G, S:T732A,
hCoV-19/Mexico/PUE-InDRE-IBT-149/2020	EPI_ISL_1302397	In process	20B	B.1.1.519	21	5'UTR:C203T, 5'UTR:C222T, 5'UTR:C241T, ORF1ab:C3037T, ORF1ab:C3140T, ORF1ab:G3549A, ORF1ab:C10029T, ORF1ab:C10954T, ORF1ab:A11117G, ORF1ab:C12789T, ORF1ab:C14408T, ORF1ab:T19839C, ORF1ab:C21306T, S:C22995A, S:A23403G, S:C23604A, S:A23756G, ORF8:T27904C, N:G28881A, N:G28882A, N:G28883C, N:C29197T,	13	N:R203K, N:G204R, ORF1a:P959S, ORF1a:G1095E, ORF1a:T3255I, ORF1a:I3618V, ORF1a:T4175I, ORF1b:P314L, ORF8:L4P, S:T478K, S:D614G, S:P681H, S:T732A,
hCoV-19/Mexico/PUE-InDRE-IBT-150/2020	EPI_ISL_1302241	In process	20B	B.1.1	21	5'UTR:T221C, 5'UTR:C241T, ORF1ab:C2710T, ORF1ab:C3037T, ORF1ab:G3994T, ORF1ab:G4148T, ORF1ab:C5173T, ORF1ab:C6941T, ORF1ab:C7834T, ORF1ab:C9598T, ORF1ab:C9891T, ORF1ab:G11230T, ORF1ab:C14408T, ORF1ab:T19839C, S:A23403G, S:C24138T, S:G24620T, ORF3a:T25689C, ORF3a:G26062T, N:G28881A, N:G28882A, N:G28883C,	10	N:R203K, N:G204R, ORF1a:V1295F, ORF1a:A3209V, ORF1a:M3655I, ORF1b:P314L, ORF3a:G224C, S:D614G, S:T859I, S:A1020S,
hCoV-19/Mexico/PUE-InDRE-IBT-152/2020	EPI_ISL_1302313	In process	20B	B.1.1.519	22	5'UTR:C203T, 5'UTR:C222T, 5'UTR:C241T, ORF1ab:G942A, ORF1ab:C3037T, ORF1ab:C3140T, ORF1ab:T7984C, ORF1ab:C10029T, ORF1ab:C10277T, ORF1ab:C10954T, ORF1ab:A11117G, ORF1ab:C11124T, ORF1ab:C12789T, ORF1ab:C14408T, ORF1ab:T19839C, S:C22995A, S:A23403G, S:C23604A, S:A23756G, N:G28881A, N:G28882A, N:G28883C, N:C29197T,	14	N:R203K, N:G204R, ORF1a:R226K, ORF1a:P959S, ORF1a:T3255I, ORF1a:L3338F, ORF1a:I3618V, ORF1a:A3620V, ORF1a:T4175I, ORF1b:P314L, S:T478K, S:D614G, S:P681H, S:T732A,
hCoV-19/Mexico/PUE-InDRE-IBT-154/2020	EPI_ISL_1302210	In process	20B	B.1.1.519	26	5'UTR:T201C, 5'UTR:C203T, 5'UTR:C222T, 5'UTR:C241T, ORF1ab:G368T, ORF1ab:G598T, ORF1ab:G1738T, ORF1ab:C3037T, ORF1ab:C3140T, ORF1ab:C10029T, ORF1ab:C10954T, ORF1ab:A11117G, ORF1ab:C12789T, ORF1ab:C14408T, ORF1ab:G16647T, ORF1ab:C17304T, ORF1ab:T19839C, ORF1ab:A19974G, S:C22995A, S:A23403G, S:C23604A, S:A23756G, ORF8:A28175G, N:G28881A, N:G28882A, N:G28883C, N:C29197T,	12	N:R203K, N:G204R, ORF1a:V35L, ORF1a:P959S, ORF1a:T3255I, ORF1a:I3618V, ORF1a:T4175I, ORF1b:P314L, S:T478K, S:D614G, S:P681H, S:T732A,
hCoV-19/Mexico/PUE-InDRE-IBT-155/2020	EPI_ISL_1302242	In process	20B	B.1.1.222	20	5'UTR:C241T, ORF1ab:G1268A, ORF1ab:C3037T, ORF1ab:C5986T, ORF1ab:A13288G, ORF1ab:C14408T, ORF1ab:G14772T, ORF1ab:G17562T, ORF1ab:T19839C, ORF1ab:G20208T, S:A23403G, S:A23756G, S:C25046T, ORF3a:C26022T, ORF3a:G26056T, N:C28369T, N:G28881A, N:G28882A, N:G28883C, N:C29409T, 3'UTR:G29773T,	12	N:R203K, N:G204R, N:T379I, ORF1a:D335N, ORF1b:P314L, ORF1b:Q435H, ORF1b:Q2247H, ORF3a:D222Y, ORF9b:A29V, S:D614G, S:T732A, S:P1162S,

hCoV-19/Mexico/PUE-InDRE-IBT-156/2020	EPI_ISL_1302211	In process	20B	B.1.1.519	22	5'UTR:T201C, 5'UTR:C203T, 5'UTR:C222T, 5'UTR:C241T, ORF1ab:G1738T, ORF1ab:C3037T, ORF1ab:C3140T, ORF1ab:C10029T, ORF1ab:C10954T, ORF1ab:A11117G, ORF1ab:C12789T, ORF1ab:C14408T, ORF1ab:T19839C, ORF1ab:A19974G, S:C22995A, S:A23403G, S:C23604A, S:A23756G, ORF7a:C27527T, N:G28881A, N:G28882A, N:G28883C. N:C29197T.	12	N:R203K, N:G204R, ORF1a:P959S, ORF1a:T3255I, ORF1a:I3618V, ORF1a:T4175I, ORF1b:P314L, ORF7a:P45L, S:T478K, S:D614G, S:P681H, S:T732A,
hCoV-19/Mexico/PUE-InDRE-IBT-157/2020	EPI_ISL_1302174	In process	20B	B.1.1.519	21	5'UTR:T201C, 5'UTR:C203T, 5'UTR:C222T, 5'UTR:C241T, ORF1ab:G1738T, ORF1ab:C3037T, ORF1ab:C3140T, ORF1ab:C10029T, ORF1ab:C10954T, ORF1ab:A11117G, ORF1ab:C12789T, ORF1ab:C14408T, ORF1ab:T19839C, ORF1ab:A19974G, S:C22995A, S:A23403G, S:C23604A, S:A23756G, N:G28881A, N:G28882A. N:G28883C. N:C29197T.	11	N:R203K, N:G204R, ORF1a:P959S, ORF1a:T3255I, ORF1a:I3618V, ORF1a:T4175I, ORF1b:P314L, S:T478K, S:D614G, S:P681H, S:T732A,
hCoV-19/Mexico/PUE-InDRE-IBT-158/2020	EPI_ISL_1302380	In process	20B	B.1.1.519	22	5'UTR:C203T, 5'UTR:C222T, 5'UTR:C241T, ORF1ab:G942A, ORF1ab:C3037T, ORF1ab:C3140T, ORF1ab:T7984C, ORF1ab:C10029T, ORF1ab:C10277T, ORF1ab:C10954T, ORF1ab:A11117G, ORF1ab:C11124T, ORF1ab:C12789T, ORF1ab:C14408T, ORF1ab:T19839C, S:C22995A, S:A23403G, S:C23604A, S:A23756G, N:G28881A, N:G28882A, N:G28883C. N:C29197T.	14	N:R203K, N:G204R, ORF1a:R226K, ORF1a:P959S, ORF1a:T3255I, ORF1a:L3338F, ORF1a:I3618V, ORF1a:A3620V, ORF1a:T4175I, ORF1b:P314L, S:T478K, S:D614G, S:P681H, S:T732A,
hCoV-19/Mexico/PUE-InDRE-IBT-161/2020	EPI_ISL_1302342	In process	20B	B.1.1.222	20	5'UTR:C241T, ORF1ab:G2764T, ORF1ab:C3037T, ORF1ab:G7756T, ORF1ab:C8293T, ORF1ab:C12663T, ORF1ab:C14408T, ORF1ab:G14772T, ORF1ab:T19839C, ORF1ab:T20976C, S:C22987T, S:A23403G, S:A23756G, S:G25135T, ORF3a:C25572T, ORF3a:G25742T, ORF6:A27354G, N:C28292A, N:G28881A, N:G28882A, N:G28883C,	11	N:Q7K, N:R203K, N:G204R, ORF1a:K2497N, ORF1a:A4133V, ORF1b:P314L, ORF1b:Q435H, ORF3a:S117I, S:D614G, S:T732A, S:K1191N,
hCoV-19/Mexico/PUE-InDRE-IBT-162/2020	EPI_ISL_1302283	In process	20B	B.1.1.519	21	5'UTR:T201C, 5'UTR:C203T, 5'UTR:C222T, 5'UTR:C241T, ORF1ab:G1738T, ORF1ab:C3037T, ORF1ab:C3140T, ORF1ab:C10029T, ORF1ab:C10954T, ORF1ab:A11117G, ORF1ab:C12789T, ORF1ab:C14408T, ORF1ab:T19839C, ORF1ab:A19974G, S:C22995A, S:A23403G, S:C23604A, S:A23756G, N:G28881A, N:G28882A. N:G28883C. N:C29197T.	11	N:R203K, N:G204R, ORF1a:P959S, ORF1a:T3255I, ORF1a:I3618V, ORF1a:T4175I, ORF1b:P314L, S:T478K, S:D614G, S:P681H, S:T732A,
hCoV-19/Mexico/PUE-InDRE-IBT-163/2020	EPI_ISL_1302335	In process	20A	B.1	18	5'UTR:C241T, ORF1ab:A2294C, ORF1ab:C3037T, ORF1ab:C4084T, ORF1ab:C5646A, ORF1ab:C7600T, ORF1ab:C10647T, ORF1ab:G11083T, ORF1ab:C14408T, ORF1ab:C15277T, ORF1ab:C15810T, ORF1ab:T16456C, S:A23403G, ORF3a:C25517T, ORF3a:G25720T, M:C27143T, ORF8:C28253T, N:C28854T, 3'UTR:G29742T,	11	N:S194L, ORF1a:T677P, ORF1a:T1794K, ORF1a:T3461I, ORF1a:L3606F, ORF1b:P314L, ORF1b:H604Y, ORF1b:S997P, ORF3a:P42L, ORF3a:A110S, S:D614G,
hCoV-19/Mexico/PUE-InDRE-IBT-164/2020	EPI_ISL_1302188	In process	20B	B.1.1.519	21	5'UTR:T201C, 5'UTR:C203T, 5'UTR:C222T, 5'UTR:C241T, ORF1ab:G1738T, ORF1ab:C3037T, ORF1ab:C3140T, ORF1ab:C10029T, ORF1ab:C10954T, ORF1ab:A11117G, ORF1ab:C12789T, ORF1ab:C14408T, ORF1ab:T19839C, ORF1ab:A19974G, S:C22995A, S:A23403G, S:C23604A, S:A23756G, N:G28881A, N:G28882A. N:G28883C. N:C29197T.	11	N:R203K, N:G204R, ORF1a:P959S, ORF1a:T3255I, ORF1a:I3618V, ORF1a:T4175I, ORF1b:P314L, S:T478K, S:D614G, S:P681H, S:T732A,
hCoV-19/Mexico/PUE-InDRE-IBT-165/2021	EPI_ISL_1302371	In process	20A	B.1.551	7	5'UTR:C241T, ORF1ab:C3037T, ORF1ab:C3738T, ORF1ab:C14408T, S:A23403G, S:C23604G, N:C28854T, N:C29466T,	6	N:S194L, N:A398V, ORF1a:P1158L, ORF1b:P314L, S:D614G, S:P681R,

hCoV-19/Mexico/PUE-InDRE-IBT-166/2020	EPI_ISL_1302212	In process	20B	B.1.1.519	23	5'UTR:T201C, 5'UTR:C203T, 5'UTR:C222T, 5'UTR:C241T, ORF1ab:G1738T, ORF1ab:C3037T, ORF1ab:C3140T, ORF1ab:C10029T, ORF1ab:C10954T, ORF1ab:A11117G, ORF1ab:C12789T, ORF1ab:C14408T, ORF1ab:C15660A, ORF1ab:T19839C, ORF1ab:A19974G, S:C22995A, S:A23403G, S:C23604A, S:A23756G, S:C25294T, N:G28881A, N:G28882A. N:G28883C. N:C29197T.	12	N:R203K, N:G204R, ORF1a:P959S, ORF1a:T3255I, ORF1a:I3618V, ORF1a:T4175I, ORF1b:P314L, ORF1b:D731E, S:T478K, S:D614G, S:P681H, S:T732A,	
hCoV-19/Mexico/PUE-InDRE-IBT-167/2020	EPI_ISL_1302321	In process	20B	B.1.1.519	22	5'UTR:C203T, 5'UTR:C222T, 5'UTR:C241T, ORF1ab:C3037T, ORF1ab:C3140T, ORF1ab:G3549A, ORF1ab:C10029T, ORF1ab:C10954T, ORF1ab:A11117G, ORF1ab:C12789T, ORF1ab:C14408T, ORF1ab:T19839C, S:C22995A, S:A23403G, S:C23604A, S:A23756G, ORF3a:T25520C, ORF3a:C25904T, ORF8:T27904C, N:G28881A, N:G28882A, N:G28883C, N:C29197T,	15	N:R203K, N:G204R, ORF1a:P959S, ORF1a:G1095E, ORF1a:T3255I, ORF1a:I3618V, ORF1a:T4175I, ORF1b:P314L, ORF3a:F43S, ORF3a:S171L, ORF8:L4P, S:T478K, S:D614G, S:P681H, S:T732A,	
hCoV-19/Mexico/PUE-InDRE-IBT-168/2020	EPI_ISL_1302213	In process	20B	B.1.1.519	23	5'UTR:T201C, 5'UTR:C203T, 5'UTR:C222T, 5'UTR:C241T, ORF1ab:G1738T, ORF1ab:C3037T, ORF1ab:C3140T, ORF1ab:G3641A, ORF1ab:C7932T, ORF1ab:C10029T, ORF1ab:C10954T, ORF1ab:A11117G, ORF1ab:C12789T, ORF1ab:C14408T, ORF1ab:T19839C, ORF1ab:A19974G, S:C22995A, S:A23403G, S:C23604A, S:A23756G, N:G28881A, N:G28882A, N:G28883C. N:C29197T.	13	N:R203K, N:G204R, ORF1a:P959S, ORF1a:E1126K, ORF1a:S2556L, ORF1a:T3255I, ORF1a:I3618V, ORF1a:T4175I, ORF1b:P314L, S:T478K, S:D614G, S:P681H, S:T732A,	
hCoV-19/Mexico/PUE-InDRE-IBT-170/2020	EPI_ISL_1302333	In process	20B	B.1.1.519	21	5'UTR:T201C, 5'UTR:C203T, 5'UTR:C222T, 5'UTR:C241T, ORF1ab:G1738T, ORF1ab:C3037T, ORF1ab:C3140T, ORF1ab:C10029T, ORF1ab:C10954T, ORF1ab:A11117G, ORF1ab:C12789T, ORF1ab:C14408T, ORF1ab:T19839C, ORF1ab:A19974G, S:C22995A, S:A23403G, S:C23604A, S:A23756G, N:G28881A, N:G28882A. N:G28883C. N:C29197T.	11	N:R203K, N:G204R, ORF1a:P959S, ORF1a:T3255I, ORF1a:I3618V, ORF1a:T4175I, ORF1b:P314L, S:T478K, S:D614G, S:P681H, S:T732A,	
hCoV-19/Mexico/PUE-InDRE-IBT-171/2020	EPI_ISL_1302262	In process	20B	B.1.1.222	19	5'UTR:C241T, ORF1ab:G347A, ORF1ab:G1544T, ORF1ab:G2764T, ORF1ab:C3037T, ORF1ab:C10029T, ORF1ab:C14408T, ORF1ab:C19011A, ORF1ab:T19839C, S:C21707T, S:A23403G, S:A23756G, ORF3a:G25912T, ORF8:A27921G, ORF8:G28001T, N:C28791T, N:G28881A, N:G28882A, N:G28883C, 3'UTR:G29744A,	13	N:A173V, N:R203K, N:G204R, ORF1a:V28I, ORF1a:A427S, ORF1a:T3255I, ORF1b:P314L, ORF1b:D1848E, ORF3a:G174C, ORF8:I10V, S:H49Y, S:D614G, S:T732A,	S:21980-21982
hCoV-19/Mexico/PUE-InDRE-IBT-172/2020	EPI_ISL_1302343	In process	20B	B.1.1.222	19	5'UTR:C241T, ORF1ab:G347A, ORF1ab:G1544T, ORF1ab:G2764T, ORF1ab:C3037T, ORF1ab:C10029T, ORF1ab:C14408T, ORF1ab:C19011A, ORF1ab:T19839C, S:C21707T, S:A23403G, S:A23756G, ORF3a:G25912T, ORF8:A27921G, ORF8:G28001T, N:C28791T, N:G28881A, N:G28882A, N:G28883C, 3'UTR:G29744A,	13	N:A173V, N:R203K, N:G204R, ORF1a:V28I, ORF1a:A427S, ORF1a:T3255I, ORF1b:P314L, ORF1b:D1848E, ORF3a:G174C, ORF8:I10V, S:H49Y, S:D614G, S:T732A,	
hCoV-19/Mexico/PUE-InDRE-IBT-173/2020	EPI_ISL_1302214	In process	20B	B.1.1.222	19	5'UTR:C241T, ORF1ab:C3037T, ORF1ab:C4832T, ORF1ab:C10029T, ORF1ab:A11556G, ORF1ab:C14408T, ORF1ab:G17019T, ORF1ab:C19011A, ORF1ab:G19086T, ORF1ab:C19554T, ORF1ab:T19839C, S:A23403G, S:A23756G, ORF3a:G25912T, ORF8:A27921G, ORF8:G28001T, N:G28337T, N:G28881A, N:G28882A, N:G28883C.	14	N:D22Y, N:R203K, N:G204R, ORF1a:T3255I, ORF1a:E3764G, ORF1b:P314L, ORF1b:E1184D, ORF1b:D1848E, ORF1b:K1873N, ORF3a:G174C, ORF8:I10V, ORF9b:Q18H, S:D614G, S:T732A,	

hCoV-19/Mexico/PUE-InDRE-IBT-174/2020	EPI_ISL_1302322	In process	20B	B.1.1.519	23	5'UTR:C203T, 5'UTR:C222T, 5'UTR:C241T, ORF1ab:C3037T, ORF1ab:C3140T, ORF1ab:G3549A, ORF1ab:C10029T, ORF1ab:C10954T, ORF1ab:A11117G, ORF1ab:C12789T, ORF1ab:C14408T, ORF1ab:T19839C, ORF1ab:G21424A, S:C22995A, S:A23403G, S:G23429T, S:C23604A, S:G23608T, S:A23756G, ORF8:T27904C, N:G28881A, N:G28882A, N:G28883C, N:C29197T.	15	N:R203K, N:G204R, ORF1a:P959S, ORF1a:G1095E, ORF1a:T3255I, ORF1a:I3618V, ORF1a:T4175I, ORF1b:P314L, ORF1b:G2653S, ORF8:L4P, S:T478K, S:D614G, S:A623S, S:P681H, S:T732A,
hCoV-19/Mexico/PUE-InDRE-IBT-175/2020	EPI_ISL_1302323	In process	20B	B.1.1.519	24	5'UTR:C203T, 5'UTR:C222T, 5'UTR:C241T, ORF1ab:C2094T, ORF1ab:C3037T, ORF1ab:C3140T, ORF1ab:G6741A, ORF1ab:C10029T, ORF1ab:C10954T, ORF1ab:A11117G, ORF1ab:A11830G, ORF1ab:C12789T, ORF1ab:C14408T, ORF1ab:G15921T, ORF1ab:T19839C, ORF1ab:A20055G, S:C22995A, S:A23403G, S:C23604A, S:A23756G, N:G28881A, N:G28882A, N:G28883C, N:C29197T, N:G29468T,	14	N:R203K, N:G204R, N:D399Y, ORF1a:S610L, ORF1a:P959S, ORF1a:R2159Q, ORF1a:T3255I, ORF1a:I3618V, ORF1a:T4175I, ORF1b:P314L, S:T478K, S:D614G, S:P681H, S:T732A,
hCoV-19/Mexico/PUE-InDRE-IBT-176/2020	EPI_ISL_1302388	In process	20B	B.1.1.222	18	5'UTR:C241T, ORF1ab:C2676T, ORF1ab:C3037T, ORF1ab:C6026T, ORF1ab:C6983T, ORF1ab:C8947T, ORF1ab:A14170G, ORF1ab:C14408T, ORF1ab:C16887T, ORF1ab:T19839C, S:A23403G, S:G23611T, S:A23756G, N:G28881A, N:G28882A, N:G28883C, 3'UTR:A29735T, 3'UTR:G29755T, 3'UTR:G29810T.	8	N:R203K, N:G204R, ORF1a:P804L, ORF1a:P1921S, ORF1b:I235V, ORF1b:P314L, S:D614G, S:T732A,
hCoV-19/Mexico/PUE-InDRE-IBT-177/2020	EPI_ISL_1302189	In process	20C	B.1.503	28	5'UTR:C241T, ORF1ab:C920T, ORF1ab:C1059T, ORF1ab:C3037T, ORF1ab:T3595C, ORF1ab:C6843T, ORF1ab:C7749T, ORF1ab:A10783G, ORF1ab:A11117G, ORF1ab:C11916T, ORF1ab:C14408T, ORF1ab:C15273T, ORF1ab:C16323T, ORF1ab:C18998T, ORF1ab:T19905C, S:G22577A, S:G22708T, S:A22948G, S:A23403G, S:C24157T, S:G24794T, ORF3a:G25563T, ORF3a:C25587T, ORF3a:G25947C, ORF8:T27977C, N:G28851T, N:C29370T, N:G29402T, N:G29540A	15	N:S193I, N:T366I, N:D377Y, ORF1a:T265I, ORF1a:S2193F, ORF1a:T2495I, ORF1a:I3618V, ORF1a:S3884L, ORF1b:P314L, ORF1b:A1844V, ORF3a:Q57H, ORF3a:Q185H, S:G339S, S:D614G, S:A1078S,
hCoV-19/Mexico/PUE-InDRE-IBT-178/2020	EPI_ISL_1302154	In process	20B	B.1.1.519	21	5'UTR:C203T, 5'UTR:C222T, 5'UTR:C241T, ORF1ab:A3019G, ORF1ab:C3037T, ORF1ab:C3140T, ORF1ab:G3549A, ORF1ab:C10029T, ORF1ab:C10954T, ORF1ab:A11117G, ORF1ab:C12789T, ORF1ab:C14408T, ORF1ab:T19839C, S:C22995A, S:A23403G, S:C23604A, S:A23756G, ORF8:T27904C, N:G28881A, N:G28882A, N:G28883C, N:C29197T,	13	N:R203K, N:G204R, ORF1a:P959S, ORF1a:G1095E, ORF1a:T3255I, ORF1a:I3618V, ORF1a:T4175I, ORF1b:P314L, ORF8:L4P, S:T478K, S:D614G, S:P681H, S:T732A,
hCoV-19/Mexico/PUE-InDRE-IBT-179/2020	EPI_ISL_1302309	In process	20A	B.1.400	14	5'UTR:C241T, ORF1ab:C3037T, ORF1ab:C4345T, ORF1ab:C10156T, ORF1ab:C11339T, ORF1ab:C13458T, ORF1ab:C14408T, ORF1ab:G18286A, ORF1ab:A19782T, ORF1ab:G19969A, S:C21575T, S:T21773G, S:A23403G, N:C28854T, N:A29437G,	8	N:S194L, ORF1a:S4398L, ORF1b:P314L, ORF1b:V1607I, ORF1b:A2168T, S:L5F, S:S71A, S:D614G,
hCoV-19/Mexico/PUE-InDRE-IBT-180/2020	EPI_ISL_1302279	In process	20B	B.1.1.222	20	5'UTR:C241T, ORF1ab:C1387T, ORF1ab:C3037T, ORF1ab:C3593T, ORF1ab:C4582T, ORF1ab:C7967T, ORF1ab:C10029T, ORF1ab:C13264T, ORF1ab:C14408T, ORF1ab:T19839C, S:A23403G, S:A23756G, ORF3a:G25912T, ORF7a:C27630T, ORF7b:A27756G, ORF8:A27921G, ORF8:G28001T, N:G28881A, N:G28882A, N:G28883C, N:A29295T,	12	N:R203K, N:G204R, N:D341V, ORF1a:L1110F, ORF1a:P2568S, ORF1a:T3255I, ORF1b:P314L, ORF3a:G174C, ORF7b:M1V, ORF8:I10V, S:D614G, S:T732A,

hCoV-19/Mexico/PUE-InDRE-IBT-181/2020	EPI_ISL_1302331	In process	20B	B.1.1.222	16	5'UTR:C241T, ORF1ab:G1312A, ORF1ab:T1623C, ORF1ab:C3037T, ORF1ab:C10029T, ORF1ab:C14408T, ORF1ab:T19839C, ORF1ab:G20024A, S:A23403G, S:A23756G, ORF3a:G25912T, ORF7b:A27756G, ORF8:A27921G, ORF8:G28001T, N:G28881A, N:G28882A, N:G28883C,	11	N:R203K, N:G204R, ORF1a:I453T, ORF1a:T3255I, ORF1b:P314L, ORF1b:R2186K, ORF3a:G174C, ORF7b:M1V, ORF8:I10V, S:D614G, S:T732A,	3'UTR:29746-29762,
hCoV-19/Mexico/PUE-InDRE-IBT-182/2020	EPI_ISL_1302286	In process	20B	B.1.1.519	22	5'UTR:C203T, 5'UTR:C222T, 5'UTR:C241T, 5'UTR:A257G, ORF1ab:A3019G, ORF1ab:C3037T, ORF1ab:C3140T, ORF1ab:G3549A, ORF1ab:C10029T, ORF1ab:C10954T, ORF1ab:A11117G, ORF1ab:C12789T, ORF1ab:C14408T, ORF1ab:T19839C, S:C22995A, S:A23403G, S:C23604A, S:A23756G, ORF8:T27904C, N:G28881A, N:G28882A, N:G28883C, N:C29197T,	13	N:R203K, N:G204R, ORF1a:P959S, ORF1a:G1095E, ORF1a:T3255I, ORF1a:I3618V, ORF1a:T4175I, ORF1b:P314L, ORF8:L4P, S:T478K, S:D614G, S:P681H, S:T732A,	
hCoV-19/Mexico/PUE-InDRE-IBT-183/2020	EPI_ISL_1302215	In process	20A	B.1.397	13	5'UTR:C241T, ORF1ab:C337T, ORF1ab:G1135T, ORF1ab:C3037T, ORF1ab:C4893T, ORF1ab:C10156T, ORF1ab:C14262A, ORF1ab:C14408T, ORF1ab:G20995T, S:A23403G, S:C25047T, ORF3a:G25687T, N:G28280T, N:C28854T,	10	N:D3Y, N:S194L, ORF1a:K290N, ORF1a:T1543I, ORF1b:D265E, ORF1b:P314L, ORF1b:G2510C, ORF3a:A99S, S:D614G, S:P1162L,	
hCoV-19/Mexico/PUE-InDRE-IBT-184/2020	EPI_ISL_1302398	In process	20B	B.1.1.244	28	5'UTR:C241T, ORF1ab:C823T, ORF1ab:G845A, ORF1ab:C1912T, ORF1ab:C3037T, ORF1ab:T6667A, ORF1ab:C12412T, ORF1ab:C12786T, ORF1ab:A12831C, ORF1ab:T14313C, ORF1ab:C14408T, ORF1ab:A18507C, ORF1ab:A19005G, ORF1ab:C20823T, S:G21785C, S:G22331A, S:A23403G, S:G25340T, ORF3a:G25538T, ORF3a:G26006A, ORF7a:G27676C, N:G28373T, N:T28393G, N:G28881A, N:G28882A, N:G28883C, N:C29149T, N:G29402T, 3'UTR:G29690T,	16	N:G34W, N:R203K, N:G204R, N:D377Y, ORF1a:D194N, ORF1a:T4174I, ORF1a:Q4189P, ORF1b:P314L, ORF3a:G49V, ORF3a:S205N, ORF7a:E95Q, ORF9b:V37G, S:G75R, S:G257S, S:D614G, S:D1260Y,	
hCoV-19/Mexico/PUE-InDRE-IBT-200/2020	EPI_ISL_1302181	In process	20B	B.1.1.519	22	5'UTR:T201C, 5'UTR:C203T, 5'UTR:C241T, ORF1ab:G1738T, ORF1ab:C3037T, ORF1ab:C3140T, ORF1ab:A8658G, ORF1ab:C10029T, ORF1ab:C10954T, ORF1ab:A11117G, ORF1ab:C12789T, ORF1ab:C14408T, ORF1ab:T19839C, ORF1ab:A19974G, S:C22995A, S:A23403G, S:C23604A, S:A23756G, N:G28362A, N:G28881A, N:G28882A, N:G28883C, N:C29197T,	14	N:G30E, N:R203K, N:G204R, ORF1a:P959S, ORF1a:K2798R, ORF1a:T3255I, ORF1a:I3618V, ORF1a:T4175I, ORF1b:P314L, ORF9b:E27K, S:T478K, S:D614G, S:P681H, S:T732A,	5'UTR:222,
hCoV-19/Mexico/PUE-InDRE-IBT-202/2020	EPI_ISL_1302216	In process	20B	B.1.1.222	20	5'UTR:C241T, ORF1ab:G2764T, ORF1ab:C3037T, ORF1ab:G7756T, ORF1ab:C8293T, ORF1ab:C12663T, ORF1ab:C14408T, ORF1ab:G14772T, ORF1ab:T19839C, ORF1ab:T20976C, S:C22987T, S:A23403G, S:A23756G, S:G25135T, ORF3a:C25572T, ORF3a:G25742T, ORF6:A27354G, N:C28292A, N:G28881A, N:G28882A, N:G28883C,	11	N:Q7K, N:R203K, N:G204R, ORF1a:K2497N, ORF1a:A4133V, ORF1b:P314L, ORF1b:Q435H, ORF3a:S117I, S:D614G, S:T732A, S:K1191N,	
hCoV-19/Mexico/PUE-InDRE-IBT-203/2020	EPI_ISL_1302289	In process	20A	B.1.397	13	5'UTR:C241T, ORF1ab:C337T, ORF1ab:G1135T, ORF1ab:C3037T, ORF1ab:C4893T, ORF1ab:C10156T, ORF1ab:C14408T, ORF1ab:G20995T, S:C22624T, S:A23403G, ORF3a:G25687T, N:G28280T, N:C28854T, N:C29535T,	8	N:D3Y, N:S194L, ORF1a:K290N, ORF1a:T1543I, ORF1b:P314L, ORF1b:G2510C, ORF3a:A99S, S:D614G,	
hCoV-19/Mexico/PUE-InDRE-IBT-204/2020	EPI_ISL_1302217	In process	20B	B.1.1.519	24	5'UTR:C203T, 5'UTR:C222T, 5'UTR:C241T, ORF1ab:C3037T, ORF1ab:C3140T, ORF1ab:C10029T, ORF1ab:C10954T, ORF1ab:A11117G, ORF1ab:G11365T, ORF1ab:C11747T, ORF1ab:C12789T, ORF1ab:C14408T, ORF1ab:T19839C, ORF1ab:C21058T, S:C21575T, S:C22995A, S:A23403G, S:C23604A, S:A23756G, N:G28881A, N:G28882A, N:G28883C, N:C29197T, N:G29527T, ORF10:T29624C,	14	N:R203K, N:G204R, N:Q418H, ORF1a:P959S, ORF1a:T3255I, ORF1a:I3618V, ORF1a:T4175I, ORF1b:P314L, ORF1b:P2531S, S:L5F, S:T478K, S:D614G, S:P681H, S:T732A,	

hCoV-19/Mexico/PUE-InDRE-IBT-205/2020	EPI_ISL_1302218	In process	20B	B.1.1.519	21	5'UTR:C203T, 5'UTR:C222T, 5'UTR:C241T, ORF1ab:C3037T, ORF1ab:C3140T, ORF1ab:C10029T, ORF1ab:C10954T, ORF1ab:A11117G, ORF1ab:C12789T, ORF1ab:C14408T, ORF1ab:C18647T, ORF1ab:T19839C, S:C22995A, S:A23403G, S:C23604A, S:A23756G, ORF8:T27904C, ORF8:C28087T, N:G28881A, N:G28882A, N:G28883C, N:C29197T,	14	N:R203K, N:G204R, ORF1a:P959S, ORF1a:T3255I, ORF1a:I3618V, ORF1a:T4175I, ORF1b:P314L, ORF1b:P1727L, ORF8:L4P, ORF8:A65V, S:T478K, S:D614G, S:P681H, S:T732A,
hCoV-19/Mexico/PUE-InDRE-IBT-207/2020	EPI_ISL_1302344	In process	20B	B.1.1.519	22	5'UTR:C203T, 5'UTR:C222T, 5'UTR:C241T, ORF1ab:G942A, ORF1ab:C3037T, ORF1ab:C3140T, ORF1ab:T7984C, ORF1ab:C10029T, ORF1ab:C10277T, ORF1ab:C10954T, ORF1ab:A11117G, ORF1ab:C11124T, ORF1ab:C12789T, ORF1ab:C14408T, ORF1ab:T19839C, S:C22995A, S:A23403G, S:C23604A, S:A23756G, N:G28881A, N:G28882A, N:G28883C, N:C29197T,	14	N:R203K, N:G204R, ORF1a:R226K, ORF1a:P959S, ORF1a:T3255I, ORF1a:L3338F, ORF1a:I3618V, ORF1a:A3620V, ORF1a:T4175I, ORF1b:P314L, S:T478K, S:D614G, S:P681H, S:T732A,
hCoV-19/Mexico/PUE-InDRE-IBT-208/2020	EPI_ISL_1302176	In process	20A	B.1.243	18	5'UTR:C241T, ORF1ab:C1387T, ORF1ab:C3037T, ORF1ab:G5657T, ORF1ab:C7113T, ORF1ab:T13354C, ORF1ab:G13812T, ORF1ab:C14408T, S:A23403G, S:G23593C, S:T24076C, S:C24337T, S:C24370T, S:G25266T, ORF3a:G25644T, ORF3a:G25947T, M:T26669C, M:G26690T, N:C28854T,	9	N:S194L, ORF1a:V1798L, ORF1a:T2283I, ORF1b:M115I, ORF1b:P314L, ORF3a:Q185H, S:D614G, S:Q677H, S:C1235F,
hCoV-19/Mexico/PUE-InDRE-IBT-209/2020	EPI_ISL_1302254	In process	20A	B.1.397	13	5'UTR:C241T, ORF1ab:C337T, ORF1ab:G1135T, ORF1ab:C3037T, ORF1ab:C4893T, ORF1ab:A5440G, ORF1ab:C10156T, ORF1ab:C14262A, ORF1ab:C14408T, ORF1ab:G20995T, S:A23403G, ORF3a:G25687T, N:G28280T, N:C28854T,	9	N:D3Y, N:S194L, ORF1a:K290N, ORF1a:T1543I, ORF1b:D265E, ORF1b:P314L, ORF1b:G2510C, ORF3a:A99S, S:D614G,
hCoV-19/Mexico/PUE-InDRE-IBT-217/2020	EPI_ISL_1302296	In process	20B	B.1.1.519	22	5'UTR:C203T, 5'UTR:C222T, 5'UTR:C241T, ORF1ab:G942A, ORF1ab:C3037T, ORF1ab:C3140T, ORF1ab:T7984C, ORF1ab:C10029T, ORF1ab:C10277T, ORF1ab:C10954T, ORF1ab:A11117G, ORF1ab:C11124T, ORF1ab:C12789T, ORF1ab:C14408T, ORF1ab:T19839C, S:C22995A, S:A23403G, S:C23604A, S:A23756G, N:G28881A, N:G28882A, N:G28883C, N:C29197T,	14	N:R203K, N:G204R, ORF1a:R226K, ORF1a:P959S, ORF1a:T3255I, ORF1a:L3338F, ORF1a:I3618V, ORF1a:A3620V, ORF1a:T4175I, ORF1b:P314L, S:T478K, S:D614G, S:P681H, S:T732A,
hCoV-19/Mexico/PUE-INER-IMSS-00115/2021	EPI_ISL_1279398	In process	20B	B.1.1.519	22	5'UTR:C203T, 5'UTR:C222T, 5'UTR:C241T, ORF1ab:C3037T, ORF1ab:C3140T, ORF1ab:C10029T, ORF1ab:C10954T, ORF1ab:A11117G, ORF1ab:G11365T, ORF1ab:C12789T, ORF1ab:C14408T, ORF1ab:T19839C, ORF1ab:C21306T, S:C22995A, S:A23403G, S:C23604A, S:A23756G, N:G28881A, N:G28882A, N:G28883C, N:C29197T, N:G29527T, 3'UTR:G29706T,	12	N:R203K, N:G204R, N:Q418H, ORF1a:P959S, ORF1a:T3255I, ORF1a:I3618V, ORF1a:T4175I, ORF1b:P314L, S:T478K, S:D614G, S:P681H, S:T732A,
hCoV-19/Mexico/PUE-INER-IMSS-00116/2021	EPI_ISL_1279399	In process	20B	B.1.1.519	27	5'UTR:T201C, 5'UTR:C203T, 5'UTR:C222T, 5'UTR:C241T, ORF1ab:G1738T, ORF1ab:C3037T, ORF1ab:C3140T, ORF1ab:G4184A, ORF1ab:C10029T, ORF1ab:C10954T, ORF1ab:A11117G, ORF1ab:C12789T, ORF1ab:C14408T, ORF1ab:C19170T, ORF1ab:T19839C, ORF1ab:A19974G, ORF1ab:G20578T, ORF1ab:C21306T, S:C22995A, S:A23403G, S:C23604A, S:A23756G, S:C24023T, N:G28655T, N:G28881A, N:G28882A, N:G28883C, N:C29197T	14	N:D128Y, N:R203K, N:G204R, ORF1a:P959S, ORF1a:G1307S, ORF1a:T3255I, ORF1a:I3618V, ORF1a:T4175I, ORF1b:P314L, ORF1b:V2371L, S:T478K, S:D614G, S:P681H, S:T732A,

hCoV-19/Mexico/PUE-INER-IMSS-00117/2021	EPI_ISL_1279400	In process	20B	B.1.1.519	24	5'UTR:T201C, 5'UTR:C203T, 5'UTR:C222T, 5'UTR:C241T, ORF1ab:G1738T, ORF1ab:C3037T, ORF1ab:C3140T, ORF1ab:C10029T, ORF1ab:C10954T, ORF1ab:A11117G, ORF1ab:C12789T, ORF1ab:C14408T, ORF1ab:G14999T, ORF1ab:T19839C, ORF1ab:A19974G, ORF1ab:C21306T, S:C22995A, S:A23403G, S:C23604A, S:A23756G, ORF3a:T25496C, N:G28881A, N:G28882A, N:G28883C, N:C29197T,	13	N:R203K, N:G204R, ORF1a:P959S, ORF1a:T3255I, ORF1a:I3618V, ORF1a:T4175I, ORF1b:P314L, ORF1b:S511I, ORF3a:I35T, S:T478K, S:D614G, S:P681H, S:T732A,
hCoV-19/Mexico/PUE-INER-IMSS-00118/2021	EPI_ISL_1279401	In process	20B	B.1.1.519	26	5'UTR:C203T, 5'UTR:C222T, 5'UTR:C241T, ORF1ab:G625T, ORF1ab:C3037T, ORF1ab:C3140T, ORF1ab:C10029T, ORF1ab:C10954T, ORF1ab:A11117G, ORF1ab:C12789T, ORF1ab:G13334A, ORF1ab:C13554T, ORF1ab:C14408T, ORF1ab:T19839C, ORF1ab:C21306T, S:G22131T, S:C22995A, S:C23029T, S:A23403G, S:C23604A, S:A23756G, S:T24352C, ORF7a:G27659T, N:G28881A, N:G28882A, N:G28883C, N:C29197T,	15	N:R203K, N:G204R, ORF1a:K120N, ORF1a:P959S, ORF1a:T3255I, ORF1a:I3618V, ORF1a:T4175I, ORF1a:A4357T, ORF1b:P314L, ORF7a:R89I, S:R190M, S:T478K, S:D614G, S:P681H, S:T732A,
hCoV-19/Mexico/PUE-INER-IMSS-00119/2021	EPI_ISL_1279402	In process	20B	B.1.1.222	21	5'UTR:C241T, ORF1ab:A744G, ORF1ab:C3037T, ORF1ab:A3888G, ORF1ab:T4585C, ORF1ab:C7834T, ORF1ab:C10029T, ORF1ab:C14408T, ORF1ab:C19011A, ORF1ab:T19839C, S:G21800A, S:G22021T, S:A23403G, S:A23756G, ORF3a:T25458C, ORF3a:C25583T, ORF3a:G25912T, ORF8:A27921G, ORF8:G28001T, N:G28881A, N:G28882A, N:G28883C,	14	N:R203K, N:G204R, ORF1a:N160S, ORF1a:K1208R, ORF1a:T3255I, ORF1b:P314L, ORF1b:D1848E, ORF3a:T64I, ORF3a:G174C, ORF8:I10V, S:D80N, S:M153I, S:D614G, S:T732A,
hCoV-19/Mexico/PUE-INER-IMSS-00120/2021	EPI_ISL_1279268	In process	20B	B.1.1.519	29	5'UTR:T201C, 5'UTR:C203T, 5'UTR:C222T, 5'UTR:C241T, ORF1ab:G1738T, ORF1ab:G1772T, ORF1ab:C3037T, ORF1ab:C3140T, ORF1ab:C7600T, ORF1ab:C10029T, ORF1ab:C10954T, ORF1ab:A11117G, ORF1ab:C12789T, ORF1ab:C14408T, ORF1ab:T18091C, ORF1ab:T19839C, ORF1ab:A19974G, ORF1ab:C21306T, S:C22995A, S:A23403G, S:C23604A, S:A23756G, ORF3a:G25444T, M:C26776T, ORF8:C27945T, N:G28881A, N:G28882A, N:G28883C, N:C29197T, N:C29420T,	16	M:A85V, N:R203K, N:G204R, N:P383S, ORF1a:V503F, ORF1a:P959S, ORF1a:T3255I, ORF1a:I3618V, ORF1a:T4175I, ORF1b:P314L, ORF3a:G18C, ORF8:Q18*, S:T478K, S:D614G, S:P681H, S:T732A,
hCoV-19/Mexico/PUE-INER-IMSS-00121/2021	EPI_ISL_1279403	In process	20B	B.1.1.519	25	5'UTR:C203T, 5'UTR:C222T, 5'UTR:C241T, ORF1ab:C3037T, ORF1ab:C3140T, ORF1ab:C10029T, ORF1ab:C10954T, ORF1ab:A11117G, ORF1ab:G11365T, ORF1ab:C12789T, ORF1ab:C14408T, ORF1ab:C16376T, ORF1ab:A18887G, ORF1ab:T19839C, ORF1ab:C21306T, S:G22104T, S:C22450T, S:C22995A, S:A23403G, S:C23604A, S:A23756G, N:G28881A, N:G28882A, N:G28883C, N:C29197T, N:G29527T,	15	N:R203K, N:G204R, N:Q418H, ORF1a:P959S, ORF1a:T3255I, ORF1a:I3618V, ORF1a:T4175I, ORF1b:P314L, ORF1b:P970L, ORF1b:H1807R, S:G181V, S:T478K, S:D614G, S:P681H, S:T732A,
hCoV-19/Mexico/PUE-INER-IMSS-00122/2021	EPI_ISL_1279404	In process	20B	B.1.1.519	26	5'UTR:C203T, 5'UTR:C222T, 5'UTR:C241T, ORF1ab:G625T, ORF1ab:C3037T, ORF1ab:C3140T, ORF1ab:C10029T, ORF1ab:C10954T, ORF1ab:A11117G, ORF1ab:C12789T, ORF1ab:G13334A, ORF1ab:C13554T, ORF1ab:C14408T, ORF1ab:T19839C, ORF1ab:C21306T, S:G22131T, S:C22995A, S:C23029T, S:A23403G, S:C23604A, S:A23756G, S:T24352C, ORF7a:G27659T, N:G28881A, N:G28882A, N:G28883C, N:C29197T,	15	N:R203K, N:G204R, ORF1a:K120N, ORF1a:P959S, ORF1a:T3255I, ORF1a:I3618V, ORF1a:T4175I, ORF1a:A4357T, ORF1b:P314L, ORF7a:R89I, S:R190M, S:T478K, S:D614G, S:P681H, S:T732A,

hCoV-19/Mexico/PUE-INER-IMSS-00123/2021	EPI_ISL_1279405	In process	20B	B.1.1.519	23	5'UTR:T201C, 5'UTR:C203T, 5'UTR:C222T, 5'UTR:C241T, ORF1ab:G1738T, ORF1ab:C3037T, ORF1ab:C3140T, ORF1ab:C10029T, ORF1ab:C10954T, ORF1ab:A11117G, ORF1ab:C12789T, ORF1ab:C14408T, ORF1ab:T19839C, ORF1ab:A19974G, ORF1ab:T20256C, ORF1ab:C21306T, S:C22995A, S:A23403G, S:C23604A, S:A23756G, N:G28881A, N:G28882A. N:G28883C. N:C29197T.	11	N:R203K, N:G204R, ORF1a:P959S, ORF1a:T3255I, ORF1a:I3618V, ORF1a:T4175I, ORF1b:P314L, S:T478K, S:D614G, S:P681H, S:T732A,	
hCoV-19/Mexico/PUE-INER-IMSS-00124/2021	EPI_ISL_1279406	In process	20B	B.1.1.222	30	5'UTR:C241T, ORF1ab:G2687A, ORF1ab:C3037T, ORF1ab:C6449T, ORF1ab:G6563A, ORF1ab:C6786T, ORF1ab:C9491T, ORF1ab:T10819C, ORF1ab:A12644G, ORF1ab:C14408T, ORF1ab:T17208C, ORF1ab:A17946T, ORF1ab:C19402T, ORF1ab:T19839C, S:C21846T, S:T22888C, S:A23403G, S:A23756G, S:C24034T, S:C25207T, ORF3a:C25708T, ORF3a:C25791T, M:G26690T, ORF8:C28253T, ORF8:A28272G, N:A28522G, N:G28881A, N:G28882A, N:G28883C, N:G29383A, 3'UTR:G29751T,	15	N:R203K, N:G204R, ORF1a:V808I, ORF1a:L2062F, ORF1a:V2100I, ORF1a:T2174I, ORF1a:H3076Y, ORF1a:I4127V, ORF1b:P314L, ORF1b:H1979Y, ORF3a:L106F, ORF9b:K80R, S:T95I, S:D614G, S:T732A,	
hCoV-19/Mexico/PUE-INER-IMSS-00125/2021	EPI_ISL_1287768	In process	20B	B.1.1.222	19	5'UTR:C241T, ORF1ab:C3037T, ORF1ab:C6285T, ORF1ab:G7405T, ORF1ab:C9962T, ORF1ab:C12025T, ORF1ab:A13498G, ORF1ab:C14408T, ORF1ab:C15579T, ORF1ab:C18312T, ORF1ab:C19032T, ORF1ab:T19839C, S:G22331A, S:A23403G, S:A23756G, S:G25273T, N:G28881A, N:G28882A, N:G28883C, N:C28948T,	11	N:R203K, N:G204R, ORF1a:T2007I, ORF1a:M2380I, ORF1a:H3233Y, ORF1b:T11A, ORF1b:P314L, S:G257S, S:D614G, S:T732A, S:M1237I,	ORF7b:27879-27891, ORF8:27915-28225,
hCoV-19/Mexico/PUE-INER-IMSS-00126/2021	EPI_ISL_1279407	In process	20B	B.1.1.519	30	5'UTR:T201C, 5'UTR:C203T, 5'UTR:C222T, 5'UTR:C241T, ORF1ab:G1634A, ORF1ab:G1738T, ORF1ab:C3037T, ORF1ab:C3140T, ORF1ab:C4543T, ORF1ab:G9658T, ORF1ab:C10029T, ORF1ab:C10954T, ORF1ab:A11117G, ORF1ab:C12789T, ORF1ab:G14268T, ORF1ab:C14408T, ORF1ab:T15030C, ORF1ab:C17012T, ORF1ab:T19839C, ORF1ab:A19974G, ORF1ab:C21306T, S:C22995A, S:A23403G, S:C23604A, S:A23756G, ORF8:A28175G, N:G28881A, N:G28882A, N:G28883C, N:C29197T, 3'UTR:G29701A,	14	N:R203K, N:G204R, ORF1a:E457K, ORF1a:P959S, ORF1a:M3131I, ORF1a:T3255I, ORF1a:I3618V, ORF1a:T4175I, ORF1b:P314L, ORF1b:S1182L, S:T478K, S:D614G, S:P681H, S:T732A,	
hCoV-19/Mexico/PUE-INER-IMSS-00127/2021	EPI_ISL_1279408	In process	20B	B.1.1.519	29	5'UTR:T201C, 5'UTR:C203T, 5'UTR:C222T, 5'UTR:C241T, ORF1ab:G1738T, ORF1ab:C3037T, ORF1ab:C3140T, ORF1ab:C9943T, ORF1ab:C10029T, ORF1ab:C10954T, ORF1ab:A11117G, ORF1ab:C12789T, ORF1ab:C14408T, ORF1ab:C15928T, ORF1ab:C16887T, ORF1ab:T19839C, ORF1ab:A19974G, ORF1ab:C21306T, S:C22995A, S:A23403G, S:C23604A, S:A23756G, ORF3a:G25500A, ORF3a:G25617A, ORF8:A28175G, N:C28453T, N:G28881A, N:G28882A, N:G28883C, N:C29197T,	13	N:R203K, N:G204R, ORF1a:P959S, ORF1a:T3255I, ORF1a:I3618V, ORF1a:T4175I, ORF1b:P314L, ORF1b:P821S, ORF9b:A57V, S:T478K, S:D614G, S:P681H, S:T732A,	
hCoV-19/Mexico/PUE-INER-IMSS-00128/2021	EPI_ISL_1279409	In process	20B	B.1.1.519	29	5'UTR:T201C, 5'UTR:C203T, 5'UTR:C222T, 5'UTR:C241T, ORF1ab:G1738T, ORF1ab:C3037T, ORF1ab:C3140T, ORF1ab:C9943T, ORF1ab:C10029T, ORF1ab:C10954T, ORF1ab:A11117G, ORF1ab:C12789T, ORF1ab:C14408T, ORF1ab:C15928T, ORF1ab:C17304T, ORF1ab:T19839C, ORF1ab:A19974G, ORF1ab:C21306T, S:C22995A, S:A23403G, S:C23604A, S:A23756G, ORF3a:G25500A, ORF3a:G25617A, ORF8:A28175G, N:C28453T, N:G28881A, N:G28882A, N:G28883C, N:C29197T,	13	N:R203K, N:G204R, ORF1a:P959S, ORF1a:T3255I, ORF1a:I3618V, ORF1a:T4175I, ORF1b:P314L, ORF1b:P821S, ORF9b:A57V, S:T478K, S:D614G, S:P681H, S:T732A,	

hCoV-19/Mexico/PUE-INER-IMSS-00129/2021	EPI_ISL_1279410	In process	20B	B.1.1.519	23	5'UTR:C203T, 5'UTR:C222T, 5'UTR:C241T, ORF1ab:A3019G, ORF1ab:C3037T, ORF1ab:C3140T, ORF1ab:G3549A, ORF1ab:C6327T, ORF1ab:C10029T, ORF1ab:C10954T, ORF1ab:A11117G, ORF1ab:C12789T, ORF1ab:C14408T, ORF1ab:T19839C, ORF1ab:C21306T, S:C22995A, S:A23403G, S:C23604A, S:A23756G, ORF8:T27904C, N:G28881A, N:G28882A, N:G28883C, N:C29197T,	14	N:R203K, N:G204R, ORF1a:P959S, ORF1a:G1095E, ORF1a:T2021I, ORF1a:T3255I, ORF1a:I3618V, ORF1a:T4175I, ORF1b:P314L, ORF8:L4P, S:T478K, S:D614G, S:P681H, S:T732A,
hCoV-19/Mexico/PUE-INER-IMSS-00130/2021	EPI_ISL_1279411	In process	20B	B.1.1.519	27	5'UTR:C203T, 5'UTR:C222T, 5'UTR:C241T, ORF1ab:C3037T, ORF1ab:C3140T, ORF1ab:C6445T, ORF1ab:C10029T, ORF1ab:C10954T, ORF1ab:A11117G, ORF1ab:C11824T, ORF1ab:G12385A, ORF1ab:C12473T, ORF1ab:C12789T, ORF1ab:C14408T, ORF1ab:T19839C, ORF1ab:G20995T, ORF1ab:C21306T, S:C22995A, S:A23403G, S:C23604A, S:A23756G, M:G26526A, M:C26882T, N:G28881A, N:G28882A, N:G28883C, N:C29197T, N:G29527T	14	M:A2T, N:R203K, N:G204R, N:Q418H, ORF1a:P959S, ORF1a:T3255I, ORF1a:I3618V, ORF1a:T4175I, ORF1b:P314L, ORF1b:G2510C, S:T478K, S:D614G, S:P681H, S:T732A,
hCoV-19/Mexico/PUE-INER-IMSS-00131/2021	EPI_ISL_1279412	In process	20B	B.1.1.519	27	5'UTR:C203T, 5'UTR:C222T, 5'UTR:C241T, ORF1ab:C3037T, ORF1ab:C3140T, ORF1ab:C6445T, ORF1ab:C10029T, ORF1ab:C10954T, ORF1ab:A11117G, ORF1ab:C11824T, ORF1ab:G12385A, ORF1ab:C12473T, ORF1ab:C12789T, ORF1ab:C14408T, ORF1ab:T19839C, ORF1ab:G20995T, ORF1ab:C21306T, S:C22995A, S:A23403G, S:C23604A, S:A23756G, M:G26526A, M:C26882T, N:G28881A, N:G28882A, N:G28883C, N:C29197T, N:G29527T	14	M:A2T, N:R203K, N:G204R, N:Q418H, ORF1a:P959S, ORF1a:T3255I, ORF1a:I3618V, ORF1a:T4175I, ORF1b:P314L, ORF1b:G2510C, S:T478K, S:D614G, S:P681H, S:T732A,
hCoV-19/Mexico/PUE-INER-IMSS-00132/2021	EPI_ISL_1279413	In process	20B	B.1.1.519	28	5'UTR:C203T, 5'UTR:C222T, 5'UTR:C241T, ORF1ab:G2144T, ORF1ab:C3037T, ORF1ab:C3140T, ORF1ab:T3745C, ORF1ab:C10029T, ORF1ab:C10954T, ORF1ab:A11117G, ORF1ab:C12651T, ORF1ab:C12789T, ORF1ab:C14408T, ORF1ab:G18972T, ORF1ab:T19839C, ORF1ab:C21077T, ORF1ab:C21306T, S:C22995A, S:A23403G, S:C23604A, S:A23756G, ORF3a:C25844T, E:G26389T, M:C26527T, N:G28881A, N:G28882A, N:G28883C, N:C29197T, 3'UTR:G29734T,	18	E:V49L, M:A2V, N:R203K, N:G204R, ORF1a:V627F, ORF1a:P959S, ORF1a:T3255I, ORF1a:I3618V, ORF1a:T4129I, ORF1a:T4175I, ORF1b:P314L, ORF1b:K1835N, ORF1b:T2537I, ORF3a:T151I, S:T478K, S:D614G, S:P681H, S:T732A,
hCoV-19/Mexico/PUE-INER-IMSS-00133/2021	EPI_ISL_1279414	In process	20B	B.1.1.519	24	5'UTR:T201C, 5'UTR:C203T, 5'UTR:C222T, 5'UTR:C241T, ORF1ab:G1738T, ORF1ab:C3037T, ORF1ab:C3140T, ORF1ab:C3768T, ORF1ab:C10029T, ORF1ab:C10954T, ORF1ab:A11117G, ORF1ab:C12789T, ORF1ab:C14408T, ORF1ab:T19839C, ORF1ab:A19974G, ORF1ab:C21306T, S:C22995A, S:A23403G, S:C23604A, S:A23756G, ORF8:A28175G, N:G28881A, N:G28882A, N:G28883C, N:C29197T,	12	N:R203K, N:G204R, ORF1a:P959S, ORF1a:T1168I, ORF1a:T3255I, ORF1a:I3618V, ORF1a:T4175I, ORF1b:P314L, S:T478K, S:D614G, S:P681H, S:T732A,
hCoV-19/Mexico/PUE-INER-IMSS-00134/2021	EPI_ISL_1279415	In process	20B	B.1.1.519	26	5'UTR:C203T, 5'UTR:C222T, 5'UTR:C241T, ORF1ab:C1449T, ORF1ab:C3037T, ORF1ab:C3140T, ORF1ab:C10029T, ORF1ab:C10183T, ORF1ab:C10954T, ORF1ab:A11117G, ORF1ab:C12789T, ORF1ab:C14408T, ORF1ab:G16853T, ORF1ab:T19839C, ORF1ab:C21306T, S:C22995A, S:A23403G, S:C23604A, S:A23756G, M:C26882T, M:A26927G, ORF8:T27904C, ORF8:G28195T, N:G28881A, N:G28882A, N:G28883C, N:C29197T,	15	N:R203K, N:G204R, ORF1a:T395I, ORF1a:P959S, ORF1a:T3255I, ORF1a:I3618V, ORF1a:T4175I, ORF1b:P314L, ORF1b:G1129V, ORF8:L4P, ORF8:R101L, S:T478K, S:D614G, S:P681H, S:T732A,

hCoV-19/Mexico/PUE-INER-IMSS-00135/2021	EPI_ISL_1279289	In process	20B	B.1.1.222	24	5'UTR:C241T, ORF1ab:C1415T, ORF1ab:C3037T, ORF1ab:A8967G, ORF1ab:C10029T, ORF1ab:C13423T, ORF1ab:C14408T, ORF1ab:C15738T, ORF1ab:G17721T, ORF1ab:T19839C, S:A23403G, S:A23756G, ORF3a:G25567A, ORF3a:T25569A, ORF3a:T25570A, ORF3a:G25912T, ORF3a:A26108G, ORF6:T27296C, ORF7a:G27670T, ORF8:A27921G, ORF8:G28001T, N:G28881A, N:G28882A, N:G28883C, N:G29405C,	16	N:R203K, N:G204R, N:E378Q, ORF1a:L384F, ORF1a:K2901R, ORF1a:T3255I, ORF1b:P314L, ORF3a:A59T, ORF3a:S60T, ORF3a:G174C, ORF3a:E239G, ORF6:I32T, ORF7a:V93F, ORF8:I10V, S:D614G, S:T732A,	3'UTR:29722-29723
hCoV-19/Mexico/PUE-INER-IMSS-00136/2021	EPI_ISL_1279416	In process	20B	B.1.1.519	28	5'UTR:T201C, 5'UTR:C203T, 5'UTR:C222T, 5'UTR:C241T, ORF1ab:C346T, ORF1ab:G1738T, ORF1ab:C3037T, ORF1ab:C3140T, ORF1ab:G3753A, ORF1ab:C4158T, ORF1ab:C10029T, ORF1ab:C10954T, ORF1ab:A11117G, ORF1ab:C12789T, ORF1ab:C13957T, ORF1ab:C14408T, ORF1ab:G18020A, ORF1ab:T19839C, ORF1ab:A19974G, ORF1ab:C21306T, S:C22995A, S:A23403G, S:C23604A, S:A23756G, S:A24854G, N:G28881A, N:G28882A, N:G28883C, N:C29197T	16	N:R203K, N:G204R, ORF1a:P959S, ORF1a:R1163K, ORF1a:A1298V, ORF1a:T3255I, ORF1a:I3618V, ORF1a:T4175I, ORF1b:R164C, ORF1b:P314L, ORF1b:R1518K, S:T478K, S:D614G, S:P681H, S:T732A, S:N1098D,	
hCoV-19/Mexico/PUE-INER-IMSS-00137/2021	EPI_ISL_1287769	In process	20B	B.1.1.222	17	5'UTR:C241T, ORF1ab:G881A, ORF1ab:C3037T, ORF1ab:G3231T, ORF1ab:C6285T, ORF1ab:T7638A, ORF1ab:A13498G, ORF1ab:C14408T, ORF1ab:C15579T, ORF1ab:T19839C, S:G22021T, S:G22331A, S:T22510C, S:A23403G, S:A23756G, N:G28881A, N:G28882A, N:G28883C,	12	N:R203K, N:G204R, ORF1a:A206T, ORF1a:G989V, ORF1a:T2007I, ORF1a:F2458Y, ORF1b:T11A, ORF1b:P314L, S:M153I, S:G257S, S:D614G, S:T732A,	ORF7b:27879-27891, ORF8:27915-28225,
hCoV-19/Mexico/PUE-INER-IMSS-00138/2021	EPI_ISL_1279417	In process	20B	B.1.1.519	28	5'UTR:C203T, 5'UTR:C222T, 5'UTR:C241T, ORF1ab:C2708A, ORF1ab:C3037T, ORF1ab:C3140T, ORF1ab:G6271T, ORF1ab:C6541T, ORF1ab:C10029T, ORF1ab:C10954T, ORF1ab:A11117G, ORF1ab:G11365T, ORF1ab:C12789T, ORF1ab:C14408T, ORF1ab:T16357C, ORF1ab:T19839C, ORF1ab:C21306T, S:T22849C, S:C22995A, S:A23403G, S:C23604A, S:C23709T, S:A23756G, N:C28775T, N:G28881A, N:G28882A, N:G28883C, N:C29197T, N:G29527T,	15	N:P168S, N:R203K, N:G204R, N:Q418H, ORF1a:L815I, ORF1a:P959S, ORF1a:T3255I, ORF1a:I3618V, ORF1a:T4175I, ORF1b:P314L, S:T478K, S:D614G, S:P681H, S:T716I, S:T732A,	
hCoV-19/Mexico/PUE-INER-IMSS-00139/2021	EPI_ISL_1279418	In process	20B	B.1.1.519	23	5'UTR:C203T, 5'UTR:C222T, 5'UTR:C241T, ORF1ab:C2445T, ORF1ab:C3037T, ORF1ab:C3140T, ORF1ab:C10029T, ORF1ab:C10954T, ORF1ab:A11117G, ORF1ab:C12789T, ORF1ab:C14408T, ORF1ab:T19839C, ORF1ab:C19938T, ORF1ab:C21306T, S:C22995A, S:A23403G, S:C23604A, S:A23756G, S:C23997T, S:G25012A, N:G28881A, N:G28882A, N:G28883C, N:C29197T,	13	N:R203K, N:G204R, ORF1a:T727I, ORF1a:P959S, ORF1a:T3255I, ORF1a:I3618V, ORF1a:T4175I, ORF1b:P314L, S:T478K, S:D614G, S:P681H, S:T732A, S:P812L,	
hCoV-19/Mexico/PUE-INER-IMSS-00140/2021	EPI_ISL_1279419	In process	20B	B.1.1.222	24	5'UTR:C241T, ORF1ab:C3037T, ORF1ab:T5860C, ORF1ab:C6504T, ORF1ab:G7387A, ORF1ab:C9430T, ORF1ab:G11087A, ORF1ab:C12789T, ORF1ab:C14408T, ORF1ab:T14787C, ORF1ab:C16887T, ORF1ab:T19839C, S:T21802C, S:A23403G, S:A23756G, ORF3a:G25793A, M:G26951T, ORF7a:C27679T, ORF8:G28167A, N:C28344T, N:G28881A, N:G28882A, N:G28883C, N:A29190T, 3'UTR:G29751T,	14	N:T24I, N:R203K, N:G204R, N:Q306L, ORF1a:A2080V, ORF1a:M2374I, ORF1a:E3608K, ORF1a:T4175I, ORF1b:P314L, ORF3a:R134H, ORF7a:L96F, ORF8:E92K, S:D614G, S:T732A,	

hCoV-19/Mexico/PUE-INER-IMSS-00141/2021	EPI_ISL_1279420	In process	20B	B.1.1.519	24	5'UTR:C203T, 5'UTR:C222T, 5'UTR:C241T, ORF1ab:C2445T, ORF1ab:C3037T, ORF1ab:C3140T, ORF1ab:C10029T, ORF1ab:C10954T, ORF1ab:C11008T, ORF1ab:A11117G, ORF1ab:A12458G, ORF1ab:C12789T, ORF1ab:C14408T, ORF1ab:T19839C, ORF1ab:C19938T, ORF1ab:C21306T, S:C22995A, S:A23403G, S:C23604A, S:A23756G, S:C23997T, N:G28881A, N:G28882A, N:G28883C, N:C29197T,	14	N:R203K, N:G204R, ORF1a:T727I, ORF1a:P959S, ORF1a:T3255I, ORF1a:I3618V, ORF1a:T4065A, ORF1a:T4175I, ORF1b:P314L, S:T478K, S:D614G, S:P681H, S:T732A, S:P812L,
hCoV-19/Mexico/PUE-INER-IMSS-00142/2021	EPI_ISL_1279421	In process	20B	B.1.1.519	24	5'UTR:C203T, 5'UTR:C222T, 5'UTR:C241T, ORF1ab:C3037T, ORF1ab:C3140T, ORF1ab:A3801G, ORF1ab:C6843T, ORF1ab:C10029T, ORF1ab:C10954T, ORF1ab:A11117G, ORF1ab:C12789T, ORF1ab:C14408T, ORF1ab:C16092T, ORF1ab:T19839C, ORF1ab:C20402T, ORF1ab:C21306T, S:C22995A, S:A23403G, S:C23604A, S:A23756G, ORF3a:G25906T, N:G28881A, N:G28882A, N:G28883C, N:C29197T,	15	N:R203K, N:G204R, ORF1a:P959S, ORF1a:D1179G, ORF1a:S2193F, ORF1a:T3255I, ORF1a:I3618V, ORF1a:T4175I, ORF1b:P314L, ORF1b:S2312L, ORF3a:G172C, S:T478K, S:D614G, S:P681H, S:T732A,
hCoV-19/Mexico/PUE-INER-IMSS-00143/2021	EPI_ISL_1279422	In process	20B	B.1.1.519	27	5'UTR:A156G, 5'UTR:T201C, 5'UTR:C203T, 5'UTR:C222T, 5'UTR:C241T, ORF1ab:G1738T, ORF1ab:C3037T, ORF1ab:C3140T, ORF1ab:C10029T, ORF1ab:C10954T, ORF1ab:A11117G, ORF1ab:C12789T, ORF1ab:C14408T, ORF1ab:C19170T, ORF1ab:T19839C, ORF1ab:A19974G, ORF1ab:C20320T, ORF1ab:C21306T, S:C22995A, S:A23403G, S:C23604A, S:A23756G, M:C27005T, ORF7a:C27532T, N:G28881A, N:G28882A, N:G28883C, N:C29197T,	13	N:R203K, N:G204R, ORF1a:P959S, ORF1a:T3255I, ORF1a:I3618V, ORF1a:T4175I, ORF1b:P314L, ORF1b:H2285Y, ORF7a:H47Y, S:T478K, S:D614G, S:P681H, S:T732A,
hCoV-19/Mexico/PUE-INER-IMSS-00144/2021	EPI_ISL_1279423	In process	20B	B.1.1.519	26	5'UTR:T201C, 5'UTR:C203T, 5'UTR:C222T, 5'UTR:C241T, ORF1ab:G1738T, ORF1ab:C3037T, ORF1ab:C3140T, ORF1ab:C5812T, ORF1ab:C10029T, ORF1ab:T10891C, ORF1ab:C10954T, ORF1ab:A11117G, ORF1ab:C12789T, ORF1ab:C14408T, ORF1ab:C17746T, ORF1ab:T19839C, ORF1ab:A19974G, ORF1ab:C21306T, S:C22995A, S:A23403G, S:C23604A, S:A23756G, ORF3a:C25585T, N:G28881A, N:G28882A, N:G28883C, N:C29197T,	13	N:R203K, N:G204R, ORF1a:P959S, ORF1a:T3255I, ORF1a:I3618V, ORF1a:T4175I, ORF1b:P314L, ORF1b:P1427S, ORF3a:L65F, S:T478K, S:D614G, S:P681H, S:T732A,
hCoV-19/Mexico/PUE-INER-IMSS-00145/2021	EPI_ISL_1279424	In process	20B	B.1.1.519	24	5'UTR:C203T, 5'UTR:C222T, 5'UTR:C241T, ORF1ab:C2445T, ORF1ab:C3037T, ORF1ab:C3140T, ORF1ab:C10029T, ORF1ab:C10954T, ORF1ab:A11117G, ORF1ab:A12458G, ORF1ab:C12789T, ORF1ab:C14408T, ORF1ab:T19839C, ORF1ab:C19938T, ORF1ab:C20930T, ORF1ab:C21306T, S:C22995A, S:A23403G, S:C23604A, S:A23756G, S:C23997T, N:G28881A, N:G28882A, N:G28883C, N:C29197T,	15	N:R203K, N:G204R, ORF1a:T727I, ORF1a:P959S, ORF1a:T3255I, ORF1a:I3618V, ORF1a:T4065A, ORF1a:T4175I, ORF1b:P314L, ORF1b:T2488M, S:T478K, S:D614G, S:P681H, S:T732A, S:P812L,
hCoV-19/Mexico/PUE-INER-IMSS-00146/2021	EPI_ISL_1279425	In process	20B	B.1.1.519	24	5'UTR:C203T, 5'UTR:C222T, 5'UTR:C241T, ORF1ab:C2445T, ORF1ab:C3037T, ORF1ab:C3140T, ORF1ab:C10029T, ORF1ab:C10954T, ORF1ab:C11008T, ORF1ab:A11117G, ORF1ab:A12458G, ORF1ab:C12789T, ORF1ab:C14408T, ORF1ab:T19839C, ORF1ab:C19938T, ORF1ab:C21306T, S:C22995A, S:A23403G, S:C23604A, S:A23756G, S:C23997T, N:G28881A, N:G28882A, N:G28883C, N:C29197T,	14	N:R203K, N:G204R, ORF1a:T727I, ORF1a:P959S, ORF1a:T3255I, ORF1a:I3618V, ORF1a:T4065A, ORF1a:T4175I, ORF1b:P314L, S:T478K, S:D614G, S:P681H, S:T732A, S:P812L,

hCoV-19/Mexico/PUE-INER-IMSS-00147/2021	EPI_ISL_1279426	In process	20B	B.1.1.222	17	5'UTR:C241T, ORF1ab:C3037T, ORF1ab:C6336T, ORF1ab:C10029T, ORF1ab:C14408T, ORF1ab:C19011A, ORF1ab:T19839C, ORF1ab:C21145T, S:C21627T, S:A23403G, S:A23756G, ORF3a:G25912T, ORF8:A27921G, ORF8:G28001T, N:G28881A, N:G28882A, N:G28883C, N:C29363T,	13	N:R203K, N:G204R, N:P364S, ORF1a:S2024L, ORF1a:T3255I, ORF1b:P314L, ORF1b:D1848E, ORF1b:L2560F, ORF3a:G174C, ORF8:I10V, S:T22I, S:D614G, S:T732A,	
hCoV-19/Mexico/PUE-INER-IMSS-00148/2021	EPI_ISL_1287770	In process	20B	B.1.1.222	19	5'UTR:C241T, ORF1ab:C280T, ORF1ab:G881A, ORF1ab:C3037T, ORF1ab:G3231T, ORF1ab:C6285T, ORF1ab:T7638A, ORF1ab:A13498G, ORF1ab:C14408T, ORF1ab:C15579T, ORF1ab:C17502T, ORF1ab:T19839C, S:G22021T, S:G22331A, S:T22510C, S:A23403G, S:A23756G, N:G28881A, N:G28882A, N:G28883C,	12	N:R203K, N:G204R, ORF1a:A206T, ORF1a:G989V, ORF1a:T2007I, ORF1a:F2458Y, ORF1b:T11A, ORF1b:P314L, S:M153I, S:G257S, S:D614G, S:T732A,	ORF7b:27879-27891, ORF8:27915-28225,
hCoV-19/Mexico/PUE-INER-IMSS-00149/2021	EPI_ISL_1279427	In process	20B	B.1.1.519	26	5'UTR:C203T, 5'UTR:C222T, 5'UTR:C241T, ORF1ab:C3037T, ORF1ab:C3140T, ORF1ab:C10029T, ORF1ab:C10183T, ORF1ab:C10954T, ORF1ab:A11117G, ORF1ab:C12789T, ORF1ab:C14408T, ORF1ab:G16853T, ORF1ab:T19839C, ORF1ab:C21306T, S:C21780T, S:C22995A, S:A23403G, S:C23604A, S:A23756G, M:C26882T, M:A26927G, ORF8:T27904C, ORF8:G28195T, N:G28881A, N:G28882A, N:G28883C, N:C29197T,	15	N:R203K, N:G204R, ORF1a:P959S, ORF1a:T3255I, ORF1a:I3618V, ORF1a:T4175I, ORF1b:P314L, ORF1b:G1129V, ORF8:L4P, ORF8:R101L, S:T73I, S:T478K, S:D614G, S:P681H, S:T732A,	
hCoV-19/Mexico/PUE-INER-IMSS-00150/2021	EPI_ISL_1279428	In process	20B	B.1.1.519	28	5'UTR:A166G, 5'UTR:C203T, 5'UTR:C222T, 5'UTR:C241T, ORF1ab:T671C, ORF1ab:C1377T, ORF1ab:T1971C, ORF1ab:C2847T, ORF1ab:C3037T, ORF1ab:C3140T, ORF1ab:C10029T, ORF1ab:C10954T, ORF1ab:A11117G, ORF1ab:C12789T, ORF1ab:C14408T, ORF1ab:T19839C, ORF1ab:C21306T, S:C22747T, S:C22995A, S:A23403G, S:C23604A, S:A23756G, N:T28705C, N:A28761G, N:G28881A, N:G28882A, N:G28883C, N:C29197T, N:C29358T,	17	N:Q163R, N:R203K, N:G204R, N:T362I, ORF1a:Y136H, ORF1a:P371L, ORF1a:I569T, ORF1a:T861I, ORF1a:P959S, ORF1a:T3255I, ORF1a:I3618V, ORF1a:T4175I, ORF1b:P314L, S:T478K, S:D614G, S:P681H, S:T732A,	
hCoV-19/Mexico/PUE-INER-IMSS-00151/2021	EPI_ISL_1279429	In process	20B	B.1.1.519	22	5'UTR:C203T, 5'UTR:C222T, 5'UTR:C241T, ORF1ab:C3037T, ORF1ab:C3140T, ORF1ab:C10029T, ORF1ab:C10954T, ORF1ab:G11083T, ORF1ab:A11117G, ORF1ab:A11814G, ORF1ab:C12789T, ORF1ab:C14408T, ORF1ab:T19839C, ORF1ab:C21306T, S:C22995A, S:A23403G, S:C23604A, S:A23756G, ORF3a:C25528T, N:G28881A, N:G28882A, N:G28883C, N:C29197T,	14	N:R203K, N:G204R, ORF1a:P959S, ORF1a:T3255I, ORF1a:L3606F, ORF1a:I3618V, ORF1a:K3850R, ORF1a:T4175I, ORF1b:P314L, ORF3a:L46F, S:T478K, S:D614G, S:P681H, S:T732A,	
hCoV-19/Mexico/PUE-INER-IMSS-00152/2021	EPI_ISL_1279430	In process	20B	B.1.1.519	26	5'UTR:T201C, 5'UTR:C203T, 5'UTR:C222T, 5'UTR:C241T, ORF1ab:G1738T, ORF1ab:C3037T, ORF1ab:C3140T, ORF1ab:G3259T, ORF1ab:C10029T, ORF1ab:C10954T, ORF1ab:A11117G, ORF1ab:C12789T, ORF1ab:C14408T, ORF1ab:T19839C, ORF1ab:A19974G, ORF1ab:A21231G, ORF1ab:C21306T, S:C21638T, S:C22995A, S:A23403G, S:C23604A, S:A23756G, ORF3a:G25855T, N:G28881A, N:G28882A, N:G28883C, N:C29197T,	14	N:R203K, N:G204R, ORF1a:P959S, ORF1a:Q998H, ORF1a:T3255I, ORF1a:I3618V, ORF1a:T4175I, ORF1b:P314L, ORF3a:D155Y, S:P26S, S:T478K, S:D614G, S:P681H, S:T732A,	
hCoV-19/Mexico/PUE-INER-IMSS-00153/2021	EPI_ISL_1279431	In process	20B	B.1.1.519	24	5'UTR:T201C, 5'UTR:C203T, 5'UTR:C222T, 5'UTR:C241T, ORF1ab:G1738T, ORF1ab:C3037T, ORF1ab:C3140T, ORF1ab:C10029T, ORF1ab:C10954T, ORF1ab:A11117G, ORF1ab:C12789T, ORF1ab:C14408T, ORF1ab:G14559T, ORF1ab:T19839C, ORF1ab:A19974T, ORF1ab:C21306T, S:C22995A, S:A23403G, S:C23604A, S:A23756G, ORF3a:G25699A, N:G28881A, N:G28882A, N:G28883C, N:C29197T,	12	N:R203K, N:G204R, ORF1a:P959S, ORF1a:T3255I, ORF1a:I3618V, ORF1a:T4175I, ORF1b:P314L, ORF3a:A103T, S:T478K, S:D614G, S:P681H, S:T732A,	

hCoV-19/Mexico/PUE-INER-IMSS-00154/2021	EPI_ISL_1279432	In process	20B	B.1.1.519	29	5'UTR:C203T, 5'UTR:C222T, 5'UTR:C241T, ORF1ab:A866G, ORF1ab:C1009T, ORF1ab:C3037T, ORF1ab:C3140T, ORF1ab:C5183T, ORF1ab:T9088A, ORF1ab:C10029T, ORF1ab:C10954T, ORF1ab:A11117G, ORF1ab:C11916T, ORF1ab:C12789T, ORF1ab:C14408T, ORF1ab:C19662T, ORF1ab:T19839C, ORF1ab:C21306T, S:C21789T, S:C22995A, S:A23403G, S:C23604A, S:A23756G, ORF8:T27904C, ORF8:A27947G, N:G28747T, N:G28881A, N:G28882A, N:G28883C, N:C29197T,	16	N:R203K, N:G204R, ORF1a:I201V, ORF1a:P959S, ORF1a:P1640S, ORF1a:T3255I, ORF1a:I3618V, ORF1a:S3884L, ORF1a:T4175I, ORF1b:P314L, ORF8:L4P, S:T76I, S:T478K, S:D614G, S:P681H, S:T732A,
hCoV-19/Mexico/PUE-INER-IMSS-00155/2021	EPI_ISL_1279274	In process	20B	B.1.1.519	23	5'UTR:C203T, 5'UTR:C222T, 5'UTR:C241T, ORF1ab:C3037T, ORF1ab:C3140T, ORF1ab:G7037T, ORF1ab:C10029T, ORF1ab:C10954T, ORF1ab:A11117G, ORF1ab:C12789T, ORF1ab:C14408T, ORF1ab:T19839C, ORF1ab:C19944T, ORF1ab:C21306T, S:C22995A, S:A23403G, S:C23604A, S:A23756G, ORF3a:C25782T, ORF7a:C27661T, N:G28881A, N:G28882A, N:G28883C, N:C29197T,	13	N:R203K, N:G204R, ORF1a:P959S, ORF1a:G2258C, ORF1a:T3255I, ORF1a:I3618V, ORF1a:T4175I, ORF1b:P314L, ORF7a:Q90*, S:T478K, S:D614G, S:P681H, S:T732A,
hCoV-19/Mexico/PUE-INER-IMSS-00156/2021	EPI_ISL_1279433	In process	20B	B.1.1.519	29	5'UTR:C203T, 5'UTR:C222T, 5'UTR:C241T, ORF1ab:G362A, ORF1ab:C1342T, ORF1ab:C3037T, ORF1ab:C3140T, ORF1ab:G3692T, ORF1ab:G3871T, ORF1ab:C6573T, ORF1ab:C10029T, ORF1ab:C10954T, ORF1ab:A11117G, ORF1ab:C11824A, ORF1ab:C12756T, ORF1ab:C12789T, ORF1ab:C14408T, ORF1ab:T18488C, ORF1ab:T19839C, ORF1ab:C21306T, S:C22995A, S:A23403G, S:C23604A, S:A23756G, M:A26924G, ORF8:T27904C, N:G28881A, N:G28882A, N:G28883C, N:C29197T,	18	N:R203K, N:G204R, ORF1a:D33N, ORF1a:P959S, ORF1a:V1143F, ORF1a:K1202N, ORF1a:S2103F, ORF1a:T3255I, ORF1a:I3618V, ORF1a:T4164I, ORF1a:T4175I, ORF1b:P314L, ORF1b:I1674T, ORF8:L4P, S:T478K, S:D614G, S:P681H, S:T732A,
hCoV-19/Mexico/PUE-INER-IMSS-00157/2021	EPI_ISL_1279434	In process	20B	B.1.1.519	26	5'UTR:C203T, 5'UTR:C222T, 5'UTR:C241T, ORF1ab:C335T, ORF1ab:C3037T, ORF1ab:C3140T, ORF1ab:G7037T, ORF1ab:C9430T, ORF1ab:C10029T, ORF1ab:C10954T, ORF1ab:A11117G, ORF1ab:G12769A, ORF1ab:C12789T, ORF1ab:C13944T, ORF1ab:C14408T, ORF1ab:T19839C, ORF1ab:C21306T, S:C22995A, S:A23403G, S:C23604A, S:A23756G, S:G24638T, ORF3a:C25782T, N:G28881A, N:G28882A, N:G28883C, N:C29197T,	14	N:R203K, N:G204R, ORF1a:R24C, ORF1a:P959S, ORF1a:G2258C, ORF1a:T3255I, ORF1a:I3618V, ORF1a:T4175I, ORF1b:P314L, S:T478K, S:D614G, S:P681H, S:T732A, S:A1026S,
hCoV-19/Mexico/PUE-INER-IMSS-00158/2021	EPI_ISL_1279435	In process	20B	B.1.1.519	26	5'UTR:C203T, 5'UTR:C222T, 5'UTR:C241T, ORF1ab:A3019G, ORF1ab:C3037T, ORF1ab:C3140T, ORF1ab:C3487T, ORF1ab:G3549A, ORF1ab:C5730A, ORF1ab:C10029T, ORF1ab:C10954T, ORF1ab:A11117G, ORF1ab:C12789T, ORF1ab:C13671A, ORF1ab:C14408T, ORF1ab:T19839C, ORF1ab:C21306T, S:C22995A, S:A23403G, S:C23604A, S:A23756G, S:G25226A, ORF8:T27904C, N:G28881A, N:G28882A, N:G28883C, N:C29197T,	16	N:R203K, N:G204R, ORF1a:P959S, ORF1a:G1095E, ORF1a:T1822N, ORF1a:T3255I, ORF1a:I3618V, ORF1a:T4175I, ORF1b:F68L, ORF1b:P314L, ORF8:L4P, S:T478K, S:D614G, S:P681H, S:T732A, S:A1222T,
hCoV-19/Mexico/PUE-INER-IMSS-00159/2021	EPI_ISL_1279436	In process	20B	B.1.1.519	23	5'UTR:C203T, 5'UTR:C222T, 5'UTR:C241T, ORF1ab:C584T, ORF1ab:C2445T, ORF1ab:C3037T, ORF1ab:C3140T, ORF1ab:C10029T, ORF1ab:C10954T, ORF1ab:A11117G, ORF1ab:C12789T, ORF1ab:C14408T, ORF1ab:T19839C, ORF1ab:C19938T, ORF1ab:C21306T, S:C22995A, S:A23403G, S:C23604A, S:A23756G, S:C23997T, N:G28881A, N:G28882A, N:G28883C, N:C29197T,	14	N:R203K, N:G204R, ORF1a:L107F, ORF1a:T727I, ORF1a:P959S, ORF1a:T3255I, ORF1a:I3618V, ORF1a:T4175I, ORF1b:P314L, S:T478K, S:D614G, S:P681H, S:T732A, S:P812L,

hCoV-19/Mexico/PUE-INER-IMSS-00160/2021	EPI_ISL_1279437	In process	20B	B.1.1.519	26	5'UTR:T201C, 5'UTR:C203T, 5'UTR:C222T, 5'UTR:C241T, ORF1ab:G1738T, ORF1ab:C3037T, ORF1ab:C3140T, ORF1ab:C10029T, ORF1ab:C10954T, ORF1ab:A11117G, ORF1ab:G11335T, ORF1ab:C12789T, ORF1ab:C14408T, ORF1ab:T19839C, ORF1ab:A19974G, ORF1ab:C21306T, S:C22995A, S:A23403G, S:C23604A, S:A23756G, M:T26726C, M:T26972C, ORF8:A28175G, N:G28881A, N:G28882A, N:G28883C, N:C29197T,	11	N:R203K, N:G204R, ORF1a:P959S, ORF1a:T3255I, ORF1a:I3618V, ORF1a:T4175I, ORF1b:P314L, S:T478K, S:D614G, S:P681H, S:T732A,
hCoV-19/Mexico/PUE-INER-IMSS-00161/2021	EPI_ISL_1279438	In process	20B	B.1.1.519	27	5'UTR:C203T, 5'UTR:C222T, 5'UTR:C241T, ORF1ab:C3037T, ORF1ab:C3140T, ORF1ab:T4339C, ORF1ab:C4890T, ORF1ab:C6539T, ORF1ab:C9943T, ORF1ab:C10029T, ORF1ab:C10954T, ORF1ab:A11117G, ORF1ab:C12789T, ORF1ab:C14408T, ORF1ab:T19839C, ORF1ab:C21306T, S:C22995A, S:A23403G, S:C23410T, S:C23604A, S:A23756G, ORF3a:G25906T, ORF3a:A25983T, ORF8:C28253T, N:G28881A, N:G28882A, N:G28883C, N:C29197T,	14	N:R203K, N:G204R, ORF1a:P959S, ORF1a:T1542I, ORF1a:H2092Y, ORF1a:T3255I, ORF1a:I3618V, ORF1a:T4175I, ORF1b:P314L, ORF3a:G172C, S:T478K, S:D614G, S:P681H, S:T732A,
hCoV-19/Mexico/PUE-INER-IMSS-00282/2021	EPI_ISL_1279538	In process	20B	B.1.1.519	26	5'UTR:T201C, 5'UTR:C203T, 5'UTR:C222T, 5'UTR:C241T, ORF1ab:T277C, ORF1ab:G1738T, ORF1ab:C3037T, ORF1ab:C3140T, ORF1ab:C5192T, ORF1ab:G7829T, ORF1ab:C10029T, ORF1ab:C10954T, ORF1ab:A11117G, ORF1ab:C12789T, ORF1ab:C14408T, ORF1ab:T19839C, ORF1ab:A19974G, ORF1ab:C21306T, S:C22995A, S:A23403G, S:C23604A, S:A23756G, ORF6:T27342C, N:G28881A, N:G28882A, N:G28883C, N:C29197T,	12	N:R203K, N:G204R, ORF1a:P959S, ORF1a:V2522F, ORF1a:T3255I, ORF1a:I3618V, ORF1a:T4175I, ORF1b:P314L, S:T478K, S:D614G, S:P681H, S:T732A,
hCoV-19/Mexico/QUE-IBT-IMSS-228/2021	EPI_ISL_1288421	In process	20B	B.1.1.519	28	5'UTR:C203T, 5'UTR:C222T, 5'UTR:C241T, ORF1ab:C3037T, ORF1ab:C3140T, ORF1ab:C5646T, ORF1ab:T8305C, ORF1ab:C10029T, ORF1ab:C10369T, ORF1ab:C10833T, ORF1ab:C10954T, ORF1ab:A11117G, ORF1ab:C12789T, ORF1ab:C13262T, ORF1ab:C14408T, ORF1ab:A15864G, ORF1ab:A17205G, ORF1ab:T19839C, ORF1ab:C21306T, S:G22094C, S:C22995A, S:A23403G, S:C23604A, S:A23756G, N:G28881A, N:G28882A, N:G28883C, N:C29046T, N:C29197T,	16	N:R203K, N:G204R, N:P258L, ORF1a:P959S, ORF1a:T1794I, ORF1a:T3255I, ORF1a:A3523V, ORF1a:I3618V, ORF1a:T4175I, ORF1a:H4333Y, ORF1b:P314L, S:D178H, S:T478K, S:D614G, S:P681H, S:T732A,
hCoV-19/Mexico/QUE-IBT-IMSS-229/2021	EPI_ISL_1288422	In process	20B	B.1.1.519	29	5'UTR:T201C, 5'UTR:C203T, 5'UTR:C222T, 5'UTR:C241T, ORF1ab:C936T, ORF1ab:G1738T, ORF1ab:C3037T, ORF1ab:C3140T, ORF1ab:C10029T, ORF1ab:C10954T, ORF1ab:A11117G, ORF1ab:C12789T, ORF1ab:G13762A, ORF1ab:C14408T, ORF1ab:T19839C, ORF1ab:A19974G, ORF1ab:C21306T, S:T21835C, S:C22995A, S:A23403G, S:C23604A, S:A23756G, ORF3a:A25524C, M:C26801T, N:G28881A, N:G28882A, N:G28883C, N:G28968T, N:C29197T, N:G29227T,	14	N:R203K, N:G204R, N:S232I, ORF1a:T224I, ORF1a:P959S, ORF1a:T3255I, ORF1a:I3618V, ORF1a:T4175I, ORF1b:G99S, ORF1b:P314L, S:T478K, S:D614G, S:P681H, S:T732A,

hCoV-19/Mexico/QUE-IBT-IMSS-374/2021	EPI_ISL_1288202	In process	20B	B.1.1.519	27	5'UTR:C203T, 5'UTR:C222T, 5'UTR:C241T, ORF1ab:C1710T, ORF1ab:C1909T, ORF1ab:C3037T, ORF1ab:C3140T, ORF1ab:G3145A, ORF1ab:C4206T, ORF1ab:C6286T, ORF1ab:C10029T, ORF1ab:C10954T, ORF1ab:A11117G, ORF1ab:C12789T, ORF1ab:C14119T, ORF1ab:C14408T, ORF1ab:T19839C, ORF1ab:C21306T, S:C22995A, S:A23403G, S:C23604A, S:A23756G, S:G24095T, N:G28881A, N:G28882A, N:G28883C, N:C29197T, N:G29527T,	16	N:R203K, N:G204R, N:Q418H, ORF1a:A482V, ORF1a:P959S, ORF1a:A1314V, ORF1a:T3255I, ORF1a:I3618V, ORF1a:T4175I, ORF1b:P218S, ORF1b:P314L, S:T478K, S:D614G, S:P681H, S:T732A, S:A845S,	
hCoV-19/Mexico/QUE-IBT-IMSS-375/2021	EPI_ISL_1288203	In process	20B	B.1.1.519	25	5'UTR:C203T, 5'UTR:A223T, 5'UTR:C241T, ORF1ab:C3037T, ORF1ab:C3140T, ORF1ab:T9456G, ORF1ab:C10029T, ORF1ab:C10954T, ORF1ab:A11117G, ORF1ab:C11200T, ORF1ab:C12789T, ORF1ab:C14408T, ORF1ab:C15720T, ORF1ab:C18647T, ORF1ab:T19839C, ORF1ab:C21306T, S:C22995A, S:A23403G, S:C23604A, S:A23756G, S:C24919T, ORF3a:C25521T, N:G28881A, N:G28882A, N:G28883C, N:C29197T,	13	N:R203K, N:G204R, ORF1a:P959S, ORF1a:F3064C, ORF1a:T3255I, ORF1a:I3618V, ORF1a:T4175I, ORF1b:P314L, ORF1b:P1727L, S:T478K, S:D614G, S:P681H, S:T732A,	5'UTR:2<br>22,
hCoV-19/Mexico/QUE-IBT-IMSS-376/2021	EPI_ISL_1288204	In process	20B	B.1.1.519	28	5'UTR:C203T, 5'UTR:C222T, 5'UTR:C241T, ORF1ab:C1342T, ORF1ab:C3037T, ORF1ab:C3140T, ORF1ab:G3692T, ORF1ab:G3871T, ORF1ab:C3874T, ORF1ab:A4638G, ORF1ab:C10029T, ORF1ab:C10954T, ORF1ab:A11117G, ORF1ab:C11824A, ORF1ab:C12789T, ORF1ab:C14408T, ORF1ab:C17410T, ORF1ab:T19839C, ORF1ab:C21306T, S:C21952T, S:C22995A, S:A23403G, S:C23604A, S:A23756G, ORF8:T27904C, N:G28881A, N:G28882A, N:G28883C, N:C29197T,	16	N:R203K, N:G204R, ORF1a:P959S, ORF1a:V1143F, ORF1a:K1202N, ORF1a:N1458S, ORF1a:T3255I, ORF1a:I3618V, ORF1a:T4175I, ORF1b:P314L, ORF1b:R1315C, ORF8:L4P, S:T478K, S:D614G, S:P681H, S:T732A,	
hCoV-19/Mexico/QUE-IBT-IMSS-436/2020	EPI_ISL_1301626	In process	20A	B.1	4	5'UTR:C241T, ORF1ab:C3037T, ORF1ab:C4582T, ORF1ab:C14408T, S:A23403G,	2	ORF1b:P314L, S:D614G,	
hCoV-19/Mexico/QUE-IBT-IMSS-507/2020	EPI_ISL_1301673	In process	20A	B.1.111	8	5'UTR:C241T, ORF1ab:C3037T, ORF1ab:C10507T, ORF1ab:C14408T, ORF1ab:A18405T, ORF1ab:C18877T, S:A23403G, ORF3a:G25563T, ORF3a:A25934C,	4	ORF1b:P314L, ORF3a:Q57H, ORF3a:E181A, S:D614G,	
hCoV-19/Mexico/QUE-IBT-IMSS-510/2020	EPI_ISL_1301675	In process	20A	B.1.111	9	5'UTR:C241T, ORF1ab:C3037T, ORF1ab:C10507T, ORF1ab:G11745A, ORF1ab:C14408T, ORF1ab:A18405T, ORF1ab:C18877T, S:A23403G, ORF3a:G25563T, ORF3a:A25934C,	5	ORF1a:G3827E, ORF1b:P314L, ORF3a:Q57H, ORF3a:E181A, S:D614G,	
hCoV-19/Mexico/QUE-IBT-IMSS-516/2021	EPI_ISL_1302282	In process	20B	B.1.1.519	28	5'UTR:T201C, 5'UTR:C203T, 5'UTR:C222T, 5'UTR:C241T, ORF1ab:C936T, ORF1ab:G1738T, ORF1ab:C3037T, ORF1ab:C3140T, ORF1ab:A9194G, ORF1ab:C10029T, ORF1ab:C10954T, ORF1ab:A11117G, ORF1ab:C12789T, ORF1ab:C14408T, ORF1ab:T19839C, ORF1ab:A19974G, S:T21835C, S:C22995A, S:A23403G, S:C23604A, S:A23756G, ORF3a:A25524C, M:C26801T, N:G28881A, N:G28882A, N:G28883C, N:G28968T, N:C29197T, N:G29227T,	14	N:R203K, N:G204R, N:S232I, ORF1a:T224I, ORF1a:P959S, ORF1a:T2977A, ORF1a:T3255I, ORF1a:I3618V, ORF1a:T4175I, ORF1b:P314L, S:T478K, S:D614G, S:P681H, S:T732A,	
hCoV-19/Mexico/QUE-IBT-IMSS-517/2021	EPI_ISL_1302401	In process	20B	B.1.1.519	24	5'UTR:T201C, 5'UTR:C203T, 5'UTR:C222T, 5'UTR:C241T, ORF1ab:G1738T, ORF1ab:C3037T, ORF1ab:C3140T, ORF1ab:C10029T, ORF1ab:C10954T, ORF1ab:A11117G, ORF1ab:C12789T, ORF1ab:C14408T, ORF1ab:T19839C, ORF1ab:A19974G, ORF1ab:C21057T, S:C22995A, S:A23403G, S:C23604A, S:A23756G, ORF3a:C25782T, M:C27143T, N:G28881A, N:G28882A, N:G28883C, N:C29197T,	11	N:R203K, N:G204R, ORF1a:P959S, ORF1a:T3255I, ORF1a:I3618V, ORF1a:T4175I, ORF1b:P314L, S:T478K, S:D614G, S:P681H, S:T732A,	

hCoV-19/Mexico/QUE-InDRE-IBT-53/2020	EPI_ISL_1301541	In process	20A	B.1.243	13	5'UTR:C241T, ORF1ab:G806A, ORF1ab:C3037T, ORF1ab:T9316C, ORF1ab:G11083T, ORF1ab:G12176A, ORF1ab:C14408T, ORF1ab:C17373T, S:A23403G, S:T23407C, S:T24076C, S:C24082T, N:C28854T, N:G29543T,	6	N:S194L, ORF1a:A181T, ORF1a:L3606F, ORF1a:G3971S, ORF1b:P314L, S:D614G,
hCoV-19/Mexico/QUE-InDRE-IBT-54/2020	EPI_ISL_1301566	In process	20A	B.1	10	5'UTR:C241T, ORF1ab:C3037T, ORF1ab:A6248C, ORF1ab:T12835C, ORF1ab:C14408T, ORF1ab:G15327T, ORF1ab:C17010T, ORF1ab:A20268G, S:A23403G, ORF10:C29642T, 3'UTR:C29708T,	4	ORF1a:N1995H, ORF1b:P314L, ORF1b:M620I, S:D614G,
hCoV-19/Mexico/QUE-INER-IMSS-00105/2021	EPI_ISL_1279389	In process	20B	B.1.1.519	25	5'UTR:C203T, 5'UTR:C222T, 5'UTR:C241T, ORF1ab:C3037T, ORF1ab:C3140T, ORF1ab:C6196T, ORF1ab:C10029T, ORF1ab:C10954T, ORF1ab:A11117G, ORF1ab:C12789T, ORF1ab:C14408T, ORF1ab:C18508T, ORF1ab:T19839C, ORF1ab:C21306T, S:C22995A, S:C23191T, S:A23403G, S:C23604A, S:A23756G, S:C24865T, ORF8:T27904C, ORF8:C28087T, N:G28881A, N:G28882A, N:G28883C, N:C29197T,	14	N:R203K, N:G204R, ORF1a:P959S, ORF1a:T3255I, ORF1a:I3618V, ORF1a:T4175I, ORF1b:P314L, ORF1b:L1681F, ORF8:L4P, ORF8:A65V, S:T478K, S:D614G, S:P681H, S:T732A,
hCoV-19/Mexico/QUE-INER-IMSS-00108/2021	EPI_ISL_1279392	In process	20B	B.1.1.519	23	5'UTR:C203T, 5'UTR:C222T, 5'UTR:C241T, ORF1ab:G960A, ORF1ab:C1889T, ORF1ab:C3037T, ORF1ab:C3140T, ORF1ab:C10029T, ORF1ab:C10954T, ORF1ab:A11117G, ORF1ab:C12789T, ORF1ab:C14220T, ORF1ab:C14408T, ORF1ab:T19839C, ORF1ab:C21306T, S:C22995A, S:A23403G, S:C23604A, S:A23756G, M:G26718T, N:G28881A, N:G28882A, N:G28883C, N:C29197T,	14	M:V66L, N:R203K, N:G204R, ORF1a:R232H, ORF1a:R542C, ORF1a:P959S, ORF1a:T3255I, ORF1a:I3618V, ORF1a:T4175I, ORF1b:P314L, S:T478K, S:D614G, S:P681H, S:T732A,
hCoV-19/Mexico/QUE-INER-IMSS-00109/2021	EPI_ISL_1279393	In process	21C (Epsilon)	B.1.429	29	5'UTR:C241T, ORF1ab:C593T, ORF1ab:C1059T, ORF1ab:C2395T, ORF1ab:T2597C, ORF1ab:C3037T, ORF1ab:G7038T, ORF1ab:C8947T, ORF1ab:T9022C, ORF1ab:C10641T, ORF1ab:C12100T, ORF1ab:A12878G, ORF1ab:C14408T, ORF1ab:G17014T, ORF1ab:C19374T, S:G21600T, S:G22018T, S:T22917G, S:A23403G, S:C23613T, S:T24349C, ORF3a:G25563T, ORF3a:C25734T, M:C26681T, ORF6:G27281T, ORF7a:C27741T, ORF7b:G27890T, ORF8:A28272T, N:C28887T, N:C29362T,	15	N:T205I, ORF1a:H110Y, ORF1a:T265I, ORF1a:G2258V, ORF1a:T3459M, ORF1a:I4205V, ORF1b:P314L, ORF1b:D1183Y, ORF3a:Q57H, ORF6:W27L, S:S13I, S:W152C, S:L452R, S:D614G, S:A684V,
hCoV-19/Mexico/QUE-INER-IMSS-00110/2021	EPI_ISL_1279394	In process	20B	B.1.1.519	22	5'UTR:C203T, 5'UTR:C222T, 5'UTR:C241T, ORF1ab:C292T, ORF1ab:C3037T, ORF1ab:C3140T, ORF1ab:C3294T, ORF1ab:C10029T, ORF1ab:C10954T, ORF1ab:A11117G, ORF1ab:C12789T, ORF1ab:C13667T, ORF1ab:C14408T, ORF1ab:T19839C, ORF1ab:C21306T, S:C22995A, S:A23403G, S:C23604A, S:A23756G, N:G28881A, N:G28882A, N:G28883C, N:C29197T,	13	N:R203K, N:G204R, ORF1a:P959S, ORF1a:P1010L, ORF1a:T3255I, ORF1a:I3618V, ORF1a:T4175I, ORF1b:T67I, ORF1b:P314L, S:T478K, S:D614G, S:P681H, S:T732A,
hCoV-19/Mexico/QUE-INER-IMSS-00111/2021	EPI_ISL_1279395	In process	20B	B.1.1.519	30	5'UTR:C203T, 5'UTR:C222T, 5'UTR:C241T, ORF1ab:C1342T, ORF1ab:C3037T, ORF1ab:C3140T, ORF1ab:G3692T, ORF1ab:G3871T, ORF1ab:C3874T, ORF1ab:G4720T, ORF1ab:C9165T, ORF1ab:C10029T, ORF1ab:C10954T, ORF1ab:A11117G, ORF1ab:C11824A, ORF1ab:C12789T, ORF1ab:C14408T, ORF1ab:C14805T, ORF1ab:T19839C, ORF1ab:C21306T, S:C21952T, S:C22995A, S:A23403G, S:C23604A, S:A23756G, M:C26895T, ORF8:T27904C, N:G28881A, N:G28882A, N:G28883C, N:C29197T,	16	M:H125Y, N:R203K, N:G204R, ORF1a:P959S, ORF1a:V1143F, ORF1a:K1202N, ORF1a:T2967I, ORF1a:T3255I, ORF1a:I3618V, ORF1a:T4175I, ORF1b:P314L, ORF8:L4P, S:T478K, S:D614G, S:P681H, S:T732A,

hCoV-19/Mexico/QUE-INER-IMSS-00112/2021	EPI_ISL_1279263	In process	20B	B.1.1.519	29	5'UTR:T201C, 5'UTR:C203T, 5'UTR:C222T, 5'UTR:C241T, ORF1ab:G1738T, ORF1ab:C3037T, ORF1ab:C3140T, ORF1ab:C5284T, ORF1ab:C10029T, ORF1ab:C10954T, ORF1ab:A11117G, ORF1ab:C12789T, ORF1ab:C13761T, ORF1ab:C14408T, ORF1ab:T19839C, ORF1ab:A19974G, ORF1ab:C21306T, ORF1ab:C21370T, S:C21614T, S:C22995A, S:A23403G, S:C23604A, S:A23756G, ORF3a:C25553T, ORF8:G28077T, N:C28737T, N:G28881A, N:G28882A, N:G28883C, N:C29197T	16	N:A155V, N:R203K, N:G204R, ORF1a:P959S, ORF1a:T3255I, ORF1a:I3618V, ORF1a:T4175I, ORF1b:P314L, ORF1b:Q2635*, ORF3a:A54V, ORF8:V62L, S:L18F, S:T478K, S:D614G, S:P681H, S:T732A,
hCoV-19/Mexico/ROO-IBT-IMSS-422/2021	EPI_ISL_1288243	In process	20B	B.1.1.519	23	5'UTR:C203T, 5'UTR:C222T, 5'UTR:C241T, ORF1ab:C3037T, ORF1ab:C3140T, ORF1ab:C10029T, ORF1ab:C10954T, ORF1ab:A11117G, ORF1ab:C12789T, ORF1ab:G13571T, ORF1ab:C14408T, ORF1ab:A17871G, ORF1ab:T19839C, ORF1ab:C21306T, S:C22995A, S:A23403G, S:C23604A, S:A23756G, M:C26985T, ORF8:G28089A, N:G28881A, N:G28882A, N:G28883C, N:C29197T,	15	M:H155Y, N:R203K, N:G204R, ORF1a:P959S, ORF1a:T3255I, ORF1a:I3618V, ORF1a:T4175I, ORF1b:G35V, ORF1b:P314L, ORF1b:I1468M, ORF8:G66S, S:T478K, S:D614G, S:P681H, S:T732A,
hCoV-19/Mexico/ROO-IBT-IMSS-427/2020	EPI_ISL_1301498	In process	20A	B.1.415	7	5'UTR:C241T, ORF1ab:C3037T, ORF1ab:G6266A, ORF1ab:C8655T, ORF1ab:C14408T, ORF1ab:G15732T, ORF1ab:T19143C, S:A23403G,	4	ORF1a:A2001T, ORF1a:S2797F, ORF1b:P314L, S:D614G,
hCoV-19/Mexico/ROO-IBT-IMSS-432/2020	EPI_ISL_1301623	In process	20B	B.1.1	12	5'UTR:C241T, ORF1ab:C3037T, ORF1ab:C14408T, S:A23403G, ORF8:A27914G, ORF8:A27959G, ORF8:A28173G, ORF8:A28247G, N:A28296G, N:A28304G, N:G28881A, N:G28882A, N:G28883C,	8	N:N8S, N:N11D, N:R203K, N:G204R, ORF1b:P314L, ORF8:K94E, ORF9b:I5V, S:D614G,
hCoV-19/Mexico/ROO-IBT-IMSS-433/2020	EPI_ISL_1301624	In process	20B	B.1.1	14	5'UTR:C241T, ORF1ab:C3037T, ORF1ab:A8848G, ORF1ab:C14408T, S:A23403G, S:G25082T, ORF8:A27914G, ORF8:A27959G, ORF8:A28173G, ORF8:A28247G, N:A28296G, N:A28304G, N:G28881A, N:G28882A, N:G28883C,	9	N:N8S, N:N11D, N:R203K, N:G204R, ORF1b:P314L, ORF8:K94E, ORF9b:I5V, S:D614G, S:A1174S,
hCoV-19/Mexico/ROO-IBT-IMSS-434/2020	EPI_ISL_1301473	In process	20A	B.1.1	4	5'UTR:C241T, ORF1ab:C3037T, ORF1ab:C14408T, S:A23403G, ORF8:A27926G,	2	ORF1b:P314L, S:D614G,
hCoV-19/Mexico/ROO-IBT-IMSS-435/2020	EPI_ISL_1301625	In process	20C	B.1	5	5'UTR:C241T, ORF1ab:C1059T, ORF1ab:C3037T, ORF1ab:C14408T, S:A23403G, ORF3a:G25563T,	4	ORF1a:T265I, ORF1b:P314L, ORF3a:Q57H, S:D614G,
hCoV-19/Mexico/ROO-InDRE-IBT-10/2020	EPI_ISL_1301493	In process	20A	B.1	7	5'UTR:C241T, ORF1ab:C3037T, ORF1ab:C14408T, ORF1ab:C18877T, ORF1ab:C20233T, S:A23403G, ORF3a:G25563T, E:C26456A,	5	E:P71H, ORF1b:P314L, ORF1b:P2256S, ORF3a:Q57H, S:D614G,
hCoV-19/Mexico/ROO-InDRE-IBT-11/2020	EPI_ISL_1301696	In process	20B	B.1.1.329	13	5'UTR:C241T, ORF1ab:A2132C, ORF1ab:G2755T, ORF1ab:C3037T, ORF1ab:A3445G, ORF1ab:A10323G, ORF1ab:G10360A, ORF1ab:C14408T, ORF1ab:C17076T, ORF1ab:G18651T, S:A23403G, N:G28881A, N:G28882A, N:G28883C,	7	N:R203K, N:G204R, ORF1a:K623Q, ORF1a:K3353R, ORF1b:P314L, ORF1b:E1728D, S:D614G,
hCoV-19/Mexico/ROO-InDRE-IBT-12/2020	EPI_ISL_1301486	In process	20A	B.1.189	10	5'UTR:C241T, ORF1ab:C3037T, ORF1ab:C3987T, ORF1ab:C4582T, ORF1ab:G6404T, ORF1ab:C8655T, ORF1ab:C9207T, ORF1ab:C14408T, S:A23403G, S:T25123C, N:G28378T,	7	ORF1a:T1241I, ORF1a:V2047F, ORF1a:S2797F, ORF1a:S2981F, ORF1b:P314L, ORF9b:R32L, S:D614G,
hCoV-19/Mexico/ROO-InDRE-IBT-13/2020	EPI_ISL_1301526	In process	20A	B.1.160	6	5'UTR:C241T, ORF1ab:C3037T, ORF1ab:C14408T, S:G22992A, S:C22995T, S:A23403G, M:C26768T,	4	ORF1b:P314L, S:S477N, S:T478I, S:D614G,
hCoV-19/Mexico/ROO-InDRE-IBT-14/2020	EPI_ISL_1301505	In process	20B	B.1.1	11	5'UTR:G61T, 5'UTR:C241T, ORF1ab:C3037T, ORF1ab:G10265A, ORF1ab:C14408T, S:A23403G, ORF3a:C25517T, ORF3a:C25791T, ORF7a:C27418A, N:G28881A, N:G28882A, N:G28883C,	7	N:R203K, N:G204R, ORF1a:G3334S, ORF1b:P314L, ORF3a:P42L, ORF7a:L9M, S:D614G,
hCoV-19/Mexico/ROO-INER-IMSS-00164/2021	EPI_ISL_1279440	In process	20B	B.1.1.519	21	5'UTR:C203T, 5'UTR:C222T, 5'UTR:C241T, ORF1ab:C3037T, ORF1ab:C3140T, ORF1ab:C10029T, ORF1ab:C10954T, ORF1ab:A11117G, ORF1ab:C12789T, ORF1ab:C14408T, ORF1ab:T19839C, ORF1ab:C21306T, S:C22995A, S:A23403G, S:C23604A, S:A23756G, M:C26985T, ORF8:G28089A, N:G28881A, N:G28882A, N:G28883C, N:C29197T,	13	M:H155Y, N:R203K, N:G204R, ORF1a:P959S, ORF1a:T3255I, ORF1a:I3618V, ORF1a:T4175I, ORF1b:P314L, ORF8:G66S, S:T478K, S:D614G, S:P681H, S:T732A,

hCoV-19/Mexico/ROO-INER-IMSS-00359/2021	EPI_ISL_1279604	In process	20B	B.1.1.519	28	5'UTR:T201C, 5'UTR:C203T, 5'UTR:C222T, 5'UTR:C241T, ORF1ab:T277C, ORF1ab:C832T, ORF1ab:G1738T, ORF1ab:C3037T, ORF1ab:C3140T, ORF1ab:G7829T, ORF1ab:C10029T, ORF1ab:C10954T, ORF1ab:A11117G, ORF1ab:C12789T, ORF1ab:C14408T, ORF1ab:C17977T, ORF1ab:T19839C, ORF1ab:C19884T, ORF1ab:A19974G, ORF1ab:C21306T, S:T22897A, S:C22995A, S:A23403G, S:C23604A, S:A23756G, N:G28881A, N:G28882A, N:G28883C, N:C29197T	13	N:R203K, N:G204R, ORF1a:P959S, ORF1a:V2522F, ORF1a:T3255I, ORF1a:I3618V, ORF1a:T4175I, ORF1b:P314L, ORF1b:L1504F, S:T478K, S:D614G, S:P681H, S:T732A,
hCoV-19/Mexico/ROO-INER-IMSS-00366/2021	EPI_ISL_1279611	In process	21C (Epsilon)	B.1.427	29	5'UTR:C222T, 5'UTR:C241T, ORF1ab:C503T, ORF1ab:C1059T, ORF1ab:C1263T, ORF1ab:C3037T, ORF1ab:C3817T, ORF1ab:G9738C, ORF1ab:C9967T, ORF1ab:C13019T, ORF1ab:G13713A, ORF1ab:C14408T, ORF1ab:C16394T, ORF1ab:G17014T, ORF1ab:C17430T, ORF1ab:G18498T, S:G21600T, S:G22018T, S:G22335T, S:C22597T, S:T22917G, S:G23126T, S:A23403G, ORF3a:G25563T, M:C26681T, ORF7b:G27882A, ORF8:C28087T, ORF8:A28272T, N:C28887T, N:C29362T,	18	N:T205I, ORF1a:P80S, ORF1a:T265I, ORF1a:T333M, ORF1a:S3158T, ORF1b:P314L, ORF1b:P976L, ORF1b:D1183Y, ORF1b:M1677I, ORF3a:Q57H, ORF7b:A43T, ORF8:A65V, S:S13I, S:W152C, S:W258L, S:L452R, S:A522S, S:D614G,
hCoV-19/Mexico/ROO-INER-IMSS-00367/2021	EPI_ISL_1279612	In process	20B	B.1.1.519	23	5'UTR:C203T, 5'UTR:C222T, 5'UTR:C241T, ORF1ab:C3037T, ORF1ab:C3140T, ORF1ab:C3653T, ORF1ab:C10029T, ORF1ab:C10954T, ORF1ab:A11117G, ORF1ab:C12789T, ORF1ab:C13297T, ORF1ab:C14408T, ORF1ab:T19839C, ORF1ab:G20102T, ORF1ab:C21306T, S:C21998T, S:C22995A, S:A23403G, S:C23604A, S:A23756G, N:G28881A, N:G28882A, N:G28883C, N:C29197T,	14	N:R203K, N:G204R, ORF1a:P959S, ORF1a:L1130F, ORF1a:T3255I, ORF1a:I3618V, ORF1a:T4175I, ORF1b:P314L, ORF1b:S2212I, S:H146Y, S:T478K, S:D614G, S:P681H, S:T732A,
hCoV-19/Mexico/SIN-IBT-IMSS-431/2020	EPI_ISL_1301462	In process	20A	B.1.609	7	5'UTR:C241T, ORF1ab:C3037T, ORF1ab:C4582T, ORF1ab:C8175T, ORF1ab:G10201T, ORF1ab:C14408T, S:G21901A, S:A23403G,	4	ORF1a:A2637V, ORF1a:M3312I, ORF1b:P314L, S:D614G,
hCoV-19/Mexico/SIN-InDRE-IBT-23/2020	EPI_ISL_1301563	In process	20B	B.1.1.344	14	5'UTR:C241T, ORF1ab:C3037T, ORF1ab:T8002C, ORF1ab:T9172C, ORF1ab:A10948G, ORF1ab:C14119T, ORF1ab:C14408T, ORF1ab:T16950C, S:G22363T, S:A23403G, M:G26730T, N:G28881A, N:G28882A, N:G28883C, 3'UTR:G29773T,	6	M:V70F, N:R203K, N:G204R, ORF1b:P218S, ORF1b:P314L, S:D614G,
hCoV-19/Mexico/SIN-InDRE-IBT-24/2020	EPI_ISL_1301543	In process	20A	B.1.609	7	5'UTR:C241T, ORF1ab:C3037T, ORF1ab:C4582T, ORF1ab:C8175T, ORF1ab:G14398T, ORF1ab:C14408T, S:C22033T, S:A23403G,	4	ORF1a:A2637V, ORF1b:V311L, ORF1b:P314L, S:D614G,
hCoV-19/Mexico/SIN-InDRE-IBT-25/2020	EPI_ISL_1301459	In process	20A	B.1.243	8	5'UTR:C241T, ORF1ab:C3037T, ORF1ab:C14408T, ORF1ab:C16887T, ORF1ab:C18118A, ORF1ab:T19176C, S:A23403G, S:T24076C, N:C28854T,	4	N:S194L, ORF1b:P314L, ORF1b:L1551I, S:D614G,
hCoV-19/Mexico/SIN-InDRE-IBT-26/2020	EPI_ISL_1301544	In process	20A	B.1.609	11	5'UTR:C241T, ORF1ab:C3037T, ORF1ab:C4582T, ORF1ab:C8175T, ORF1ab:C14408T, ORF1ab:G16912T, S:C21855T, S:A23403G, S:C24374T, ORF3a:C25511T, ORF3a:C25587T, N:G29449T,	7	ORF1a:A2637V, ORF1b:P314L, ORF1b:V1149L, ORF3a:S40L, S:S98F, S:D614G, S:L938F,
hCoV-19/Mexico/SIN-InDRE-IBT-27/2020	EPI_ISL_1301699	In process	20A	B.1.609	9	5'UTR:C241T, ORF1ab:C3037T, ORF1ab:C4582T, ORF1ab:C8175T, ORF1ab:C14408T, ORF1ab:C17123T, ORF1ab:G18255T, ORF1ab:A20268G, S:A23403G, S:G23948T,	6	ORF1a:A2637V, ORF1b:P314L, ORF1b:A1219V, ORF1b:M1596I, S:D614G, S:D796Y,
hCoV-19/Mexico/SIN-InDRE-IBT-88/2020	EPI_ISL_1301573	In process	20A	B.1	9	5'UTR:C241T, ORF1ab:C584T, ORF1ab:C1348T, ORF1ab:C3037T, ORF1ab:C14408T, ORF1ab:A20268G, S:G21724T, S:A23403G, N:C28854T, 3'UTR:T29758C,	5	N:S194L, ORF1a:L107F, ORF1b:P314L, S:L54F, S:D614G,

hCoV-19/Mexico/SLP-IBT-IMSS-287/2021	EPI_ISL_1288473	In process	20B	B.1.1.519	27	5'UTR:C203T, 5'UTR:C222T, 5'UTR:C241T, ORF1ab:C3037T, ORF1ab:C3140T, ORF1ab:C9120T, ORF1ab:C10029T, ORF1ab:C10954T, ORF1ab:A11117G, ORF1ab:C12789T, ORF1ab:C14408T, ORF1ab:G19816T, ORF1ab:T19839C, ORF1ab:C21077T, ORF1ab:C21306T, S:C22995A, S:A23403G, S:C23604A, S:A23756G, S:T24217C, S:C24378T, ORF3a:G25906T, ORF3a:C26177T, ORF8:C28253T, N:G28881A, N:G28882A, N:G28883C, N:C29197T,	17	N:R203K, N:G204R, ORF1a:P959S, ORF1a:T2952I, ORF1a:T3255I, ORF1a:I3618V, ORF1a:T4175I, ORF1b:P314L, ORF1b:V2117L, ORF1b:T2537I, ORF3a:G172C, ORF3a:P262L, S:T478K, S:D614G, S:P681H, S:T732A, S:S939F,
hCoV-19/Mexico/SLP-IBT-IMSS-288/2021	EPI_ISL_1288474	In process	20B	B.1.1.519	24	5'UTR:C203T, 5'UTR:C222T, 5'UTR:C241T, ORF1ab:C3037T, ORF1ab:C3140T, ORF1ab:G3692T, ORF1ab:G3871T, ORF1ab:C10029T, ORF1ab:C10954T, ORF1ab:A11117G, ORF1ab:C11824A, ORF1ab:C12789T, ORF1ab:C14408T, ORF1ab:T19839C, ORF1ab:C21306T, S:C22995A, S:A23403G, S:C23604A, S:A23756G, ORF3a:G25456C, ORF8:T27904C, N:G28881A, N:G28882A, N:G28883C, N:C29197T,	15	N:R203K, N:G204R, ORF1a:P959S, ORF1a:V1143F, ORF1a:K1202N, ORF1a:T3255I, ORF1a:I3618V, ORF1a:T4175I, ORF1b:P314L, ORF3a:D22H, ORF8:L4P, S:T478K, S:D614G, S:P681H, S:T732A,
hCoV-19/Mexico/SLP-IBT-IMSS-289/2021	EPI_ISL_1288475	In process	20B	B.1.1.519	20	5'UTR:C203T, 5'UTR:C222T, 5'UTR:C241T, ORF1ab:C3037T, ORF1ab:C3140T, ORF1ab:C10029T, ORF1ab:C10954T, ORF1ab:A11117G, ORF1ab:C12789T, ORF1ab:C14408T, ORF1ab:T19839C, ORF1ab:C21306T, S:C22995A, S:A23403G, S:C23604A, S:A23756G, N:G28881A, N:G28882A, N:G28883C, N:A29053T, N:C29197T,	12	N:R203K, N:G204R, N:Q260H, ORF1a:P959S, ORF1a:T3255I, ORF1a:I3618V, ORF1a:T4175I, ORF1b:P314L, S:T478K, S:D614G, S:P681H, S:T732A,
hCoV-19/Mexico/SLP-IBT-IMSS-292/2021	EPI_ISL_1288183	In process	20A	B.1.609	22	5'UTR:C241T, ORF1ab:G529T, ORF1ab:T1094C, ORF1ab:C3037T, ORF1ab:C3130T, ORF1ab:T4092C, ORF1ab:C4582T, ORF1ab:C6896T, ORF1ab:C7105A, ORF1ab:A8887G, ORF1ab:C13629T, ORF1ab:C14184T, ORF1ab:C14408T, ORF1ab:G14622A, ORF1ab:C18687T, ORF1ab:A20268G, S:C21614T, S:A23403G, S:G23608T, M:C26873T, N:G28845A, N:G28871A, ORF10:C29614T,	6	N:R191H, N:G200S, ORF1a:I1276T, ORF1b:P314L, S:L18F, S:D614G,
hCoV-19/Mexico/SLP-IBT-IMSS-303/2021	EPI_ISL_1288184	In process	20B	B.1.1.519	26	5'UTR:C203T, 5'UTR:C222T, 5'UTR:C241T, ORF1ab:A866G, ORF1ab:C1009T, ORF1ab:C3037T, ORF1ab:C3140T, ORF1ab:T3733C, ORF1ab:C5183T, ORF1ab:C10029T, ORF1ab:C10954T, ORF1ab:A11117G, ORF1ab:C11916T, ORF1ab:G12223T, ORF1ab:C12789T, ORF1ab:C14408T, ORF1ab:T19839C, ORF1ab:C21306T, S:C22995A, S:A23403G, S:C23604A, S:A23756G, ORF8:T27904C, N:G28881A, N:G28882A, N:G28883C, N:C29197T,	15	N:R203K, N:G204R, ORF1a:I201V, ORF1a:P959S, ORF1a:P1640S, ORF1a:T3255I, ORF1a:I3618V, ORF1a:S3884L, ORF1a:T4175I, ORF1b:P314L, ORF8:L4P, S:T478K, S:D614G, S:P681H, S:T732A,
hCoV-19/Mexico/SLP-IBT-IMSS-314/2021	EPI_ISL_1288185	In process	20B	B.1.1.519	27	5'UTR:T201C, 5'UTR:C203T, 5'UTR:C222T, 5'UTR:C241T, ORF1ab:G1738T, ORF1ab:C3037T, ORF1ab:C3140T, ORF1ab:C6636T, ORF1ab:C10029T, ORF1ab:C10954T, ORF1ab:A11117G, ORF1ab:C12789T, ORF1ab:G13576T, ORF1ab:C14408T, ORF1ab:G14559T, ORF1ab:T19839C, ORF1ab:A19974G, ORF1ab:C21306T, S:C22995A, S:A23403G, S:C23604A, S:A23756G, ORF3a:G25699A, N:G28881A, N:G28882A, N:G28883C, N:T28921C, N:C29197T	14	N:R203K, N:G204R, ORF1a:P959S, ORF1a:T2124I, ORF1a:T3255I, ORF1a:I3618V, ORF1a:T4175I, ORF1b:A37S, ORF1b:P314L, ORF3a:A103T, S:T478K, S:D614G, S:P681H, S:T732A,

hCoV-19/Mexico/SLP-IBT-IMSS-325/2021	EPI_ISL_1288186	In process	20B	B.1.1.519	26	5'UTR:C203T, 5'UTR:C222T, 5'UTR:C241T, ORF1ab:C3037T, ORF1ab:C3140T, ORF1ab:G3692T, ORF1ab:G3871T, ORF1ab:C8016T, ORF1ab:C10029T, ORF1ab:C10954T, ORF1ab:A11117G, ORF1ab:C11824A, ORF1ab:C12789T, ORF1ab:C14408T, ORF1ab:T19839C, ORF1ab:C21306T, S:C22995A, S:A23403G, S:C23604A, S:A23756G, ORF3a:C25469T, ORF8:T27904C, N:C28697T, N:G28881A, N:G28882A, N:G28883C, N:C29197T,	17	N:P142S, N:R203K, N:G204R, ORF1a:P959S, ORF1a:V1143F, ORF1a:K1202N, ORF1a:A2584V, ORF1a:T3255I, ORF1a:I3618V, ORF1a:T4175I, ORF1b:P314L, ORF3a:S26L, ORF8:L4P, S:T478K, S:D614G, S:P681H, S:T732A,
hCoV-19/Mexico/SLP-IBT-IMSS-336/2021	EPI_ISL_1288187	In process	20B	B.1.1.519	26	5'UTR:C203T, 5'UTR:C222T, 5'UTR:C241T, ORF1ab:C3037T, ORF1ab:C3140T, ORF1ab:C6541T, ORF1ab:C10029T, ORF1ab:C10954T, ORF1ab:A11117G, ORF1ab:C11200T, ORF1ab:C12789T, ORF1ab:C14408T, ORF1ab:T19839C, ORF1ab:C21306T, S:A22023C, S:C22995A, S:A23403G, S:G23593T, S:C23604A, S:A23756G, ORF3a:A26030G, ORF8:T27904C, N:G28376A, N:G28881A, N:G28882A, N:G28883C, N:C29197T,	16	N:A35T, N:R203K, N:G204R, ORF1a:P959S, ORF1a:T3255I, ORF1a:I3618V, ORF1a:T4175I, ORF1b:P314L, ORF3a:Q213R, ORF8:L4P, S:E154A, S:T478K, S:D614G, S:Q677H, S:P681H, S:T732A,
hCoV-19/Mexico/SLP-IBT-IMSS-347/2021	EPI_ISL_1288188	In process	20B	B.1.1.519	26	5'UTR:C203T, 5'UTR:C222T, 5'UTR:C241T, ORF1ab:T559C, ORF1ab:C3037T, ORF1ab:C3140T, ORF1ab:T3745C, ORF1ab:C10029T, ORF1ab:C10954T, ORF1ab:A11117G, ORF1ab:C12789T, ORF1ab:C14408T, ORF1ab:T19839C, ORF1ab:C21306T, S:C22088T, S:C22995A, S:A23403G, S:C23604A, S:A23756G, S:C25162T, ORF3a:C25844T, ORF7a:C27741T, N:G28881A, N:G28882A, N:G28883C, N:C29197T, ORF10:C29627T,	13	N:R203K, N:G204R, ORF1a:P959S, ORF1a:T3255I, ORF1a:I3618V, ORF1a:T4175I, ORF1b:P314L, ORF3a:T151I, S:L176F, S:T478K, S:D614G, S:P681H, S:T732A,
hCoV-19/Mexico/SLP-IBT-IMSS-559/2021	EPI_ISL_1302153	In process	20B	B.1.1.222	15	5'UTR:C241T, ORF1ab:C3037T, ORF1ab:C5622T, ORF1ab:C6285T, ORF1ab:C13297T, ORF1ab:A13498G, ORF1ab:C14408T, ORF1ab:T19839C, S:G22331A, S:A23403G, S:A23756G, M:T26749C, N:G28881A, N:G28882A, N:G28883C, N:C29153A,	11	M:I76T, N:R203K, N:G204R, N:Q294K, ORF1a:P1786L, ORF1a:T2007I, ORF1b:T11A, ORF1b:P314L, S:G257S, S:D614G, S:T732A,
hCoV-19/Mexico/SLP-IBT-IMSS-560/2021	EPI_ISL_1302161	In process	20B	B.1.1.519	27	5'UTR:T201C, 5'UTR:C203T, 5'UTR:C222T, 5'UTR:C241T, ORF1ab:G1738T, ORF1ab:G1958A, ORF1ab:C2227T, ORF1ab:C3037T, ORF1ab:C3140T, ORF1ab:C6843T, ORF1ab:C10029T, ORF1ab:C10954T, ORF1ab:A11117G, ORF1ab:C12789T, ORF1ab:C14408T, ORF1ab:T19839C, ORF1ab:C19973T, ORF1ab:A19974G, S:G21746C, S:C22995A, S:C23604A, S:A23756G, S:T24175C, ORF7a:G27505C, N:G28881A, N:G28882A, N:G28883C, N:C29197T,	15	N:R203K, N:G204R, ORF1a:A565T, ORF1a:P959S, ORF1a:S2193F, ORF1a:T3255I, ORF1a:I3618V, ORF1a:T4175I, ORF1b:P314L, ORF1b:P2169L, ORF7a:G38R, S:V62L, S:T478K, S:P681H, S:T732A,
hCoV-19/Mexico/SLP-IBT-IMSS-561/2021	EPI_ISL_1302311	In process	20B	B.1.1.519	24	5'UTR:C203T, 5'UTR:C222T, 5'UTR:C241T, ORF1ab:C1710T, ORF1ab:C3037T, ORF1ab:C3140T, ORF1ab:G3145A, ORF1ab:C10029T, ORF1ab:C10954T, ORF1ab:A11117G, ORF1ab:C12789T, ORF1ab:C14408T, ORF1ab:T19839C, S:C21637T, S:C22995A, S:A23403G, S:C23604A, S:A23756G, ORF8:G28188T, N:G28881A, N:G28882A, N:G28883C, N:C29197T, N:G29440T, N:G29527T,	15	N:R203K, N:G204R, N:Q389H, N:Q418H, ORF1a:A482V, ORF1a:P959S, ORF1a:T3255I, ORF1a:I3618V, ORF1a:T4175I, ORF1b:P314L, ORF8:V99L, S:T478K, S:D614G, S:P681H, S:T732A,
hCoV-19/Mexico/SLP-IBT-IMSS-562/2021	EPI_ISL_1302352	In process	20B	B.1.1.519	23	5'UTR:C203T, 5'UTR:C222T, 5'UTR:C241T, ORF1ab:C3037T, ORF1ab:C3140T, ORF1ab:C10029T, ORF1ab:C10954T, ORF1ab:A11117G, ORF1ab:C12789T, ORF1ab:C14408T, ORF1ab:T19839C, S:C22995A, S:A23403G, S:C23604A, S:A23756G, S:C24795T, M:C27002T, N:A28877T, N:G28878C, N:G28881A, N:G28882A, N:G28883C, N:C29197T, ORF10:T29661C,	12	N:R203K, N:G204R, ORF1a:P959S, ORF1a:T3255I, ORF1a:I3618V, ORF1a:T4175I, ORF1b:P314L, S:T478K, S:D614G, S:P681H, S:T732A, S:A1078V,

hCoV-19/Mexico/SLP-IBT-IMSS-578/2021	EPI_ISL_1302362	In process	20B	B.1.1.519	25	5'UTR:C203T, 5'UTR:C222T, 5'UTR:C241T, ORF1ab:C1889T, ORF1ab:C1943T, ORF1ab:C3037T, ORF1ab:C3140T, ORF1ab:C4832T, ORF1ab:C10029T, ORF1ab:C10954T, ORF1ab:A11117G, ORF1ab:C12789T, ORF1ab:C14408T, ORF1ab:T14565C, ORF1ab:G15906T, ORF1ab:T19839C, S:C22995A, S:A23403G, S:C23604A, S:A23756G, ORF3a:G25522A, ORF7b:T27863C, N:G28881A, N:G28882A, N:G28883C, N:C29197T,	15	N:R203K, N:G204R, ORF1a:R542C, ORF1a:R560C, ORF1a:P959S, ORF1a:T3255I, ORF1a:I3618V, ORF1a:T4175I, ORF1b:P314L, ORF1b:Q813H, ORF3a:G44R, S:T478K, S:D614G, S:P681H, S:T732A,	
hCoV-19/Mexico/SLP-IBT-IMSS-579/2021	EPI_ISL_1302239	In process	20A	B.1	13	5'UTR:C241T, ORF1ab:C556T, ORF1ab:C1190T, ORF1ab:C3037T, ORF1ab:A3405G, ORF1ab:C14408T, ORF1ab:G16741A, ORF1ab:T17283C, ORF1ab:C19884T, S:C21952T, S:A23403G, ORF3a:G25879T, ORF8:C28087T, N:C28854T,	8	N:S194L, ORF1a:P309S, ORF1a:E1047G, ORF1b:P314L, ORF1b:V1092I, ORF3a:V163L, ORF8:A65V, S:D614G,	ORF1ab: 9860-9862
hCoV-19/Mexico/SLP-IBT-IMSS-580/2021	EPI_ISL_1302318	In process	20B	B.1.1.519	26	5'UTR:C203T, 5'UTR:C222T, 5'UTR:C241T, ORF1ab:C3037T, ORF1ab:C3140T, ORF1ab:G3692T, ORF1ab:G3871T, ORF1ab:C10029T, ORF1ab:C10954T, ORF1ab:A11117G, ORF1ab:C11824A, ORF1ab:C12789T, ORF1ab:C14120T, ORF1ab:C14408T, ORF1ab:C14768T, ORF1ab:G18498T, ORF1ab:T19839C, S:C22995A, S:A23403G, S:C23604A, S:A23756G, ORF7a:G27604T, ORF8:T27904C, N:G28881A, N:G28882A, N:G28883C, N:C29197T	18	N:R203K, N:G204R, ORF1a:P959S, ORF1a:V1143F, ORF1a:K1202N, ORF1a:T3255I, ORF1a:I3618V, ORF1a:T4175I, ORF1b:P218L, ORF1b:P314L, ORF1b:A434V, ORF1b:M1677I, ORF7a:V71L, ORF8:L4P, S:T478K, S:D614G, S:P681H, S:T732A,	
hCoV-19/Mexico/SLP-IBT-IMSS-581/2021	EPI_ISL_1302285	In process	20B	B.1.1.519	24	5'UTR:C203T, 5'UTR:C222T, 5'UTR:C241T, ORF1ab:C3037T, ORF1ab:C3140T, ORF1ab:G3692T, ORF1ab:G3871T, ORF1ab:C10029T, ORF1ab:C10954T, ORF1ab:A11117G, ORF1ab:C11824A, ORF1ab:C12789T, ORF1ab:C14408T, ORF1ab:T19839C, S:C22995A, S:A23403G, S:C23604A, S:A23756G, ORF3a:G25456C, E:G26428T, ORF8:T27904C, N:G28881A, N:G28882A, N:G28883C, N:C29197T,	16	E:V62F, N:R203K, N:G204R, ORF1a:P959S, ORF1a:V1143F, ORF1a:K1202N, ORF1a:T3255I, ORF1a:I3618V, ORF1a:T4175I, ORF1b:P314L, ORF3a:D22H, ORF8:L4P, S:T478K, S:D614G, S:P681H, S:T732A,	
hCoV-19/Mexico/SLP-IBT-IMSS-582/2021	EPI_ISL_1302367	In process	20B	B.1.1.519	23	5'UTR:C203T, 5'UTR:C222T, 5'UTR:C241T, ORF1ab:C1889T, ORF1ab:C1943T, ORF1ab:C3037T, ORF1ab:C3140T, ORF1ab:C10029T, ORF1ab:C10954T, ORF1ab:A11117G, ORF1ab:C12789T, ORF1ab:C14408T, ORF1ab:T14565C, ORF1ab:G15906T, ORF1ab:T19839C, S:C22995A, S:A23403G, S:C23604A, S:A23756G, ORF7a:C27643T, N:G28881A, N:G28882A, N:G28883C, N:C29197T,	15	N:R203K, N:G204R, ORF1a:R542C, ORF1a:R560C, ORF1a:P959S, ORF1a:T3255I, ORF1a:I3618V, ORF1a:T4175I, ORF1b:P314L, ORF1b:Q813H, ORF7a:P84S, S:T478K, S:D614G, S:P681H, S:T732A,	
hCoV-19/Mexico/SLP-IBT-IMSS-583/2021	EPI_ISL_1302353	In process	20B	B.1.1.519	24	5'UTR:C203T, 5'UTR:C222T, 5'UTR:C241T, ORF1ab:C3037T, ORF1ab:C3140T, ORF1ab:G3692T, ORF1ab:G3871T, ORF1ab:C9561T, ORF1ab:C10029T, ORF1ab:C10954T, ORF1ab:A11117G, ORF1ab:C11824A, ORF1ab:C12008T, ORF1ab:C12789T, ORF1ab:C14408T, ORF1ab:T19839C, S:C22995A, S:A23403G, S:C23604A, S:A23756G, ORF8:T27904C, N:G28881A, N:G28882A, N:G28883C, N:C29197T,	16	N:R203K, N:G204R, ORF1a:P959S, ORF1a:V1143F, ORF1a:K1202N, ORF1a:S3099L, ORF1a:T3255I, ORF1a:I3618V, ORF1a:L3915F, ORF1a:T4175I, ORF1b:P314L, ORF8:L4P, S:T478K, S:D614G, S:P681H, S:T732A,	

hCoV-19/Mexico/SLP-IBT-IMSS-584/2021	EPI_ISL_1302237	In process	20B	B.1.1.519	24	5'UTR:C203T, 5'UTR:C222T, 5'UTR:C241T, ORF1ab:C3037T, ORF1ab:C3140T, ORF1ab:G3692T, ORF1ab:G3871T, ORF1ab:C10029T, ORF1ab:C10954T, ORF1ab:A11117G, ORF1ab:C11824A, ORF1ab:C12789T, ORF1ab:C14408T, ORF1ab:C16608T, ORF1ab:T19839C, ORF1ab:C21431T, S:C22995A, S:A23403G, S:C23604A, S:A23756G, ORF8:T27904C, N:G28881A, N:G28882A, N:G28883C, N:C29197T,	15	N:R203K, N:G204R, ORF1a:P959S, ORF1a:V1143F, ORF1a:K1202N, ORF1a:T3255I, ORF1a:I3618V, ORF1a:T4175I, ORF1b:P314L, ORF1b:A2655V, ORF8:L4P, S:T478K, S:D614G, S:P681H, S:T732A,	
hCoV-19/Mexico/SLP-IBT-IMSS-585/2021	EPI_ISL_1302332	In process	20B	B.1.1.519	22	5'UTR:C203T, 5'UTR:C222T, 5'UTR:C241T, ORF1ab:C3037T, ORF1ab:C3140T, ORF1ab:G3692T, ORF1ab:G3871T, ORF1ab:C10029T, ORF1ab:C10954T, ORF1ab:A11117G, ORF1ab:C11824A, ORF1ab:C12789T, ORF1ab:C14408T, ORF1ab:T19839C, S:C22995A, S:A23403G, S:C23604A, S:A23756G, ORF8:T27904C, N:G28881A, N:G28882A, N:G28883C, N:C29197T,	14	N:R203K, N:G204R, ORF1a:P959S, ORF1a:V1143F, ORF1a:K1202N, ORF1a:T3255I, ORF1a:I3618V, ORF1a:T4175I, ORF1b:P314L, ORF8:L4P, S:T478K, S:D614G, S:P681H, S:T732A,	
hCoV-19/Mexico/SLP-IBT-IMSS-62/2021	EPI_ISL_1288278	In process	20A	B.1.239	11	5'UTR:C241T, ORF1ab:C3037T, ORF1ab:G5326T, ORF1ab:A5584G, ORF1ab:G9211T, ORF1ab:G12479A, ORF1ab:C14408T, ORF1ab:G16741A, ORF1ab:A20268G, S:A23403G, ORF7b:T27818C, N:C28854T,	7	N:S194L, ORF1a:E1687D, ORF1a:E2982D, ORF1a:V4072I, ORF1b:P314L, ORF1b:V1092I, S:D614G,	
hCoV-19/Mexico/SLP-IBT-IMSS-63/2021	EPI_ISL_1288279	In process	20A	B.1.396	21	5'UTR:C140T, 5'UTR:C241T, ORF1ab:C1191T, ORF1ab:A1565G, ORF1ab:C3037T, ORF1ab:C3096T, ORF1ab:A4220G, ORF1ab:C9521A, ORF1ab:C12623T, ORF1ab:C12747T, ORF1ab:C14408T, ORF1ab:C17304T, ORF1ab:A20268G, S:A23403G, S:C24138T, S:C24904T, ORF7a:C27654T, ORF7b:C27893A, N:C28854T, N:G29422T, N:C29541T, ORF10:A29567G,	11	N:S194L, ORF1a:P309L, ORF1a:N434D, ORF1a:S944L, ORF1a:K1319E, ORF1a:L3086I, ORF1a:P4120S, ORF1a:T4161I, ORF1b:P314L, S:D614G, S:T859I,	
hCoV-19/Mexico/SLP-IBT-IMSS-64/2021	EPI_ISL_1288280	In process	20A	B.1.551	18	5'UTR:C241T, ORF1ab:C3037T, ORF1ab:C3096T, ORF1ab:C3738T, ORF1ab:C9996T, ORF1ab:C14408T, ORF1ab:C16111T, ORF1ab:A17841G, ORF1ab:G19635A, ORF1ab:A20268G, S:A23403G, S:C23604G, S:G24821T, ORF3a:G25785T, ORF8:G27987A, N:G28690T, N:C28854T, N:C29370T, 3'UTR:G29751C,	12	N:L139F, N:S194L, N:T366I, ORF1a:S944L, ORF1a:P1158L, ORF1a:S3244L, ORF1b:P314L, ORF3a:W131C, ORF8:V32I, S:D614G, S:P681R, S:A1087S,	
hCoV-19/Mexico/SLP-IBT-IMSS-65/2021	EPI_ISL_1288281	In process	20A	B.1	15	5'UTR:C241T, ORF1ab:C556T, ORF1ab:C1190T, ORF1ab:C3037T, ORF1ab:C3903T, ORF1ab:C14408T, ORF1ab:C15324T, ORF1ab:G16741A, ORF1ab:C19380T, ORF1ab:T19584C, ORF1ab:C19884T, ORF1ab:A20268G, S:C21952T, S:A23403G, M:C26625T, N:C28854T,	6	N:S194L, ORF1a:P309S, ORF1a:P1213L, ORF1b:P314L, ORF1b:V1092I, S:D614G,	ORF1ab: 9860-9862
hCoV-19/Mexico/SLP-IBT-IMSS-66/2021	EPI_ISL_1288282	In process	20B	B.1.1.222	18	5'UTR:C241T, ORF1ab:C379T, ORF1ab:C3037T, ORF1ab:A6672G, ORF1ab:C14408T, ORF1ab:C16323T, ORF1ab:G17721T, ORF1ab:T19839C, ORF1ab:G20238T, ORF1ab:A20418G, S:A23403G, S:A23756G, ORF3a:G25500T, M:A26579G, N:G28881A, N:G28882A, N:G28883C, ORF10:G29579A, 3'UTR:G29751T,	7	N:R203K, N:G204R, ORF1a:D2136G, ORF1b:P314L, ORF1b:R2257S, S:D614G, S:T732A,	S:21992-21994,
hCoV-19/Mexico/SLP-IBT-IMSS-67/2021	EPI_ISL_1288283	In process	20A	B.1.396	25	5'UTR:C241T, ORF1ab:C1191T, ORF1ab:C2091T, ORF1ab:C3037T, ORF1ab:C4901T, ORF1ab:C6151T, ORF1ab:C6896T, ORF1ab:T9124C, ORF1ab:C9438T, ORF1ab:C9521A, ORF1ab:G9928T, ORF1ab:G10996T, ORF1ab:C14408T, ORF1ab:A15204G, ORF1ab:T17065C, ORF1ab:A20268G, S:C21575T, S:A21634G, S:A23403G, S:C24904T, S:G25249T, ORF3a:A25602G, ORF3a:G25644T, N:C28854T, N:G29422T, ORF10:A29567G,	12	N:S194L, ORF1a:P309L, ORF1a:T609I, ORF1a:T3058I, ORF1a:L3086I, ORF1a:M3221I, ORF1a:K3577N, ORF1b:P314L, ORF1b:Y1200H, S:L5F, S:D614G, S:M1229I,	

hCoV-19/Mexico/SLP-IBT-IMSS-68/2021	EPI_ISL_1288284	In process	20B	B.1.1.519	23	5'UTR:C203T, 5'UTR:C222T, 5'UTR:C241T, ORF1ab:C3037T, ORF1ab:C3140T, ORF1ab:G3692T, ORF1ab:G3871T, ORF1ab:C10029T, ORF1ab:C10954T, ORF1ab:A11117G, ORF1ab:C11824A, ORF1ab:C12789T, ORF1ab:C14408T, ORF1ab:T19839C, ORF1ab:C21306T, S:C22995A, S:A23403G, S:C23604A, S:A23756G, ORF8:T27904C, N:G28881A, N:G28882A, N:G28883C, N:C29197T,	14	N:R203K, N:G204R, ORF1a:P959S, ORF1a:V1143F, ORF1a:K1202N, ORF1a:T3255I, ORF1a:I3618V, ORF1a:T4175I, ORF1b:P314L, ORF8:L4P, S:T478K, S:D614G, S:P681H, S:T732A,
hCoV-19/Mexico/SLP-IBT-IMSS-69/2021	EPI_ISL_1288285	In process	20B	B.1.1.519	27	5'UTR:C203T, 5'UTR:C222T, 5'UTR:C241T, ORF1ab:C3037T, ORF1ab:C3140T, ORF1ab:G3692T, ORF1ab:G3871T, ORF1ab:T7057C, ORF1ab:C10029T, ORF1ab:C10954T, ORF1ab:A11117G, ORF1ab:C11824A, ORF1ab:C12789T, ORF1ab:C14408T, ORF1ab:T19839C, ORF1ab:C21306T, S:C22000T, S:C22995A, S:A23403G, S:C23604A, S:A23756G, S:T25319A, ORF8:T27904C, N:G28881A, N:G28882A, N:G28883C, N:C29197T, ORF10:C29642T,	15	N:R203K, N:G204R, ORF1a:P959S, ORF1a:V1143F, ORF1a:K1202N, ORF1a:T3255I, ORF1a:I3618V, ORF1a:T4175I, ORF1b:P314L, ORF8:L4P, S:T478K, S:D614G, S:P681H, S:T732A, S:C1253S,
hCoV-19/Mexico/SLP-IBT-IMSS-71/2021	EPI_ISL_1288286	In process	20B	B.1.1.519	19	5'UTR:C203T, 5'UTR:C222T, 5'UTR:C241T, ORF1ab:C3037T, ORF1ab:C3140T, ORF1ab:C10029T, ORF1ab:C10954T, ORF1ab:A11117G, ORF1ab:C14408T, ORF1ab:T19839C, ORF1ab:C21306T, S:C22995A, S:A23403G, S:C23604A, S:A23756G, N:G28881A, N:G28882A, N:G28883C, N:C29197T, N:G29527T,	11	N:R203K, N:G204R, N:Q418H, ORF1a:P959S, ORF1a:T3255I, ORF1a:I3618V, ORF1b:P314L, S:T478K, S:D614G, S:P681H, S:T732A,
hCoV-19/Mexico/SLP-IBT-IMSS-72/2021	EPI_ISL_1288287	In process	20G	B.1.2	19	5'UTR:C241T, ORF1ab:C1059T, ORF1ab:C3037T, ORF1ab:C10319T, ORF1ab:C10726T, ORF1ab:C14408T, ORF1ab:A18424G, ORF1ab:C18664T, ORF1ab:C19881T, ORF1ab:C21304T, S:A23403G, S:C24865T, ORF3a:G25563T, ORF3a:G25907T, ORF7a:G27604A, ORF8:C27964T, ORF8:C28253T, ORF8:A28254C, N:C28472T, N:C28869T,	13	N:P67S, N:P199L, ORF1a:T265I, ORF1a:L3352F, ORF1b:P314L, ORF1b:N1653D, ORF1b:R2613C, ORF3a:Q57H, ORF3a:G172V, ORF7a:V71I, ORF8:S24L, ORF8:I121L, S:D614G,
hCoV-19/Mexico/SLP-IBT-IMSS-73/2021	EPI_ISL_1288288	In process	20B	B.1.1.519	25	5'UTR:C203T, 5'UTR:C222T, 5'UTR:C241T, ORF1ab:C3037T, ORF1ab:C3140T, ORF1ab:G3692T, ORF1ab:G3871T, ORF1ab:T3982C, ORF1ab:C10029T, ORF1ab:C10954T, ORF1ab:A11117G, ORF1ab:C11824A, ORF1ab:C12789T, ORF1ab:C14408T, ORF1ab:C16049T, ORF1ab:T19839C, ORF1ab:C21306T, S:C22995A, S:A23403G, S:C23604A, S:A23756G, ORF8:T27904C, N:G28881A, N:G28882A, N:G28883C, N:C29197T,	15	N:R203K, N:G204R, ORF1a:P959S, ORF1a:V1143F, ORF1a:K1202N, ORF1a:T3255I, ORF1a:I3618V, ORF1a:T4175I, ORF1b:P314L, ORF1b:T861I, ORF8:L4P, S:T478K, S:D614G, S:P681H, S:T732A,
hCoV-19/Mexico/SLP-IBT-IMSS-74/2021	EPI_ISL_1288289	In process	20B	B.1.1.519	30	5'UTR:T201C, 5'UTR:C203T, 5'UTR:C222T, 5'UTR:C241T, ORF1ab:G1738T, ORF1ab:C3037T, ORF1ab:C3140T, ORF1ab:C4551T, ORF1ab:C6187T, ORF1ab:C7600T, ORF1ab:C9474T, ORF1ab:C10029T, ORF1ab:C10954T, ORF1ab:G11083T, ORF1ab:A11117G, ORF1ab:C12789T, ORF1ab:C14408T, ORF1ab:T19839C, ORF1ab:A19974G, ORF1ab:C21306T, S:C21742T, S:T22174C, S:C22995A, S:A23403G, S:C23604A, S:A23756G, S:G25354T, N:G28881A, N:G28882A, N:G28883C, N:C29197T,	14	N:R203K, N:G204R, ORF1a:P959S, ORF1a:T1429I, ORF1a:A3070V, ORF1a:T3255I, ORF1a:L3606F, ORF1a:I3618V, ORF1a:T4175I, ORF1b:P314L, S:T478K, S:D614G, S:P681H, S:T732A,

hCoV-19/Mexico/SLP-IBT-IMSS-75/2021	EPI_ISL_1288290	In process	20B	B.1.1.519	25	5'UTR:C203T, 5'UTR:C222T, 5'UTR:C241T, ORF1ab:C3037T, ORF1ab:C3140T, ORF1ab:G3692T, ORF1ab:G3871T, ORF1ab:C10029T, ORF1ab:C10954T, ORF1ab:A11117G, ORF1ab:C11824A, ORF1ab:C12789T, ORF1ab:C14408T, ORF1ab:T16503C, ORF1ab:T19839C, ORF1ab:C21306T, S:C21846T, S:C22995A, S:A23403G, S:C23604A, S:A23756G, ORF8:T27904C, N:G28881A, N:G28882A, N:G28883C, N:C29197T,	15	N:R203K, N:G204R, ORF1a:P959S, ORF1a:V1143F, ORF1a:K1202N, ORF1a:T3255I, ORF1a:I3618V, ORF1a:T4175I, ORF1b:P314L, ORF8:L4P, S:T95I, S:T478K, S:D614G, S:P681H, S:T732A,	
hCoV-19/Mexico/SLP-IBT-IMSS-76/2021	EPI_ISL_1288291	In process	20B	B.1.1.519	26	5'UTR:C203T, 5'UTR:C222T, 5'UTR:C241T, ORF1ab:T559C, ORF1ab:C3037T, ORF1ab:C3140T, ORF1ab:T3745C, ORF1ab:C10029T, ORF1ab:C10954T, ORF1ab:A11117G, ORF1ab:C12789T, ORF1ab:C14408T, ORF1ab:T19839C, ORF1ab:C21306T, S:C22088T, S:C22995A, S:A23403G, S:C23604A, S:A23756G, S:C25162T, ORF3a:C25844T, ORF7a:C27741T, N:G28881A, N:G28882A, N:G28883C, N:C29197T, ORF10:C29627T,	13	N:R203K, N:G204R, ORF1a:P959S, ORF1a:T3255I, ORF1a:I3618V, ORF1a:T4175I, ORF1b:P314L, ORF3a:T151I, S:L176F, S:T478K, S:D614G, S:P681H, S:T732A,	
hCoV-19/Mexico/SLP-IBT-IMSS-77/2021	EPI_ISL_1288292	In process	20B	B.1.1.519	28	5'UTR:T201C, 5'UTR:C203T, 5'UTR:C222T, 5'UTR:C241T, ORF1ab:G1738T, ORF1ab:C3037T, ORF1ab:C3140T, ORF1ab:G3753A, ORF1ab:T7237C, ORF1ab:C10029T, ORF1ab:C10954T, ORF1ab:G11083T, ORF1ab:A11117G, ORF1ab:C12789T, ORF1ab:C13115T, ORF1ab:C14408T, ORF1ab:G18020A, ORF1ab:T19839C, ORF1ab:A19974G, ORF1ab:C21306T, S:C22995A, S:A23403G, S:C23604A, S:A23756G, ORF7a:C27509T, N:G28881A, N:G28882A, N:G28883C, N:C29197T,	15	N:R203K, N:G204R, ORF1a:P959S, ORF1a:R1163K, ORF1a:T3255I, ORF1a:L3606F, ORF1a:I3618V, ORF1a:T4175I, ORF1b:P314L, ORF1b:R1518K, ORF7a:T39I, S:T478K, S:D614G, S:P681H, S:T732A,	
hCoV-19/Mexico/SLP-IBT-IMSS-78/2021	EPI_ISL_1288293	In process	20B	B.1.1.519	31	5'UTR:T201C, 5'UTR:C203T, 5'UTR:C222T, 5'UTR:C241T, ORF1ab:G1738T, ORF1ab:G2659T, ORF1ab:C3037T, ORF1ab:C3140T, ORF1ab:C10029T, ORF1ab:C10954T, ORF1ab:G11083T, ORF1ab:C11094T, ORF1ab:A11117G, ORF1ab:C12789T, ORF1ab:C14408T, ORF1ab:T19839C, ORF1ab:A19974G, ORF1ab:C21306T, S:C21742T, S:T22174C, S:C22995A, S:A23403G, S:C23604A, S:A23756G, S:G25354T, E:C26461T, ORF8:C27972T, N:C28708T, N:G28881A, N:G28882A, N:G28883C, N:C29197T,	16	E:L73F, N:R203K, N:G204R, ORF1a:K798N, ORF1a:P959S, ORF1a:T3255I, ORF1a:L3606F, ORF1a:A3610V, ORF1a:I3618V, ORF1a:T4175I, ORF1b:P314L, ORF8:Q27*, S:T478K, S:D614G, S:P681H, S:T732A,	
hCoV-19/Mexico/SLP-InDRE-IBT-40/2020	EPI_ISL_1301679	In process	20A	B.1	6	5'UTR:C241T, ORF1ab:T1237C, ORF1ab:A1755C, ORF1ab:C3037T, ORF1ab:C14408T, S:A23403G, 3'UTR:G29773T,	3	ORF1a:K497T, ORF1b:P314L, S:D614G,	
hCoV-19/Mexico/SLP-InDRE-IBT-41/2020	EPI_ISL_1301680	In process	20A	B.1	5	5'UTR:C241T, ORF1ab:T1237C, ORF1ab:C3037T, ORF1ab:C14408T, S:A23403G, 3'UTR:G29773T,	2	ORF1b:P314L, S:D614G,	
hCoV-19/Mexico/SLP-InDRE-IBT-50/2020	EPI_ISL_1301528	In process	20A	B.1.609	7	5'UTR:C241T, ORF1ab:C3037T, ORF1ab:C4582T, ORF1ab:C14408T, ORF1ab:A20268G, S:A23403G, ORF3a:G25563T, N:G28378T,	4	ORF1b:P314L, ORF3a:Q57H, ORF9b:R32L, S:D614G,	
hCoV-19/Mexico/SLP-INER-IMSS-00162/2021	EPI_ISL_1279286	In process	20B	B.1.1.519	25	5'UTR:C203T, 5'UTR:C222T, 5'UTR:C241T, ORF1ab:C3037T, ORF1ab:C3140T, ORF1ab:G7037T, ORF1ab:C10029T, ORF1ab:C10954T, ORF1ab:A11117G, ORF1ab:C12789T, ORF1ab:C14408T, ORF1ab:C17977T, ORF1ab:T19839C, ORF1ab:T20452C, ORF1ab:A20935G, ORF1ab:C21306T, S:C22995A, S:A23403G, S:C23604A, S:A23756G, S:C24967T, ORF3a:C25782T, N:G28881A, N:G28882A, N:G28883C, N:C29197T,	15	N:R203K, N:G204R, ORF1a:P959S, ORF1a:G2258C, ORF1a:T3255I, ORF1a:I3618V, ORF1a:T4175I, ORF1b:P314L, ORF1b:L1504F, ORF1b:Y2329H, ORF1b:T2490A, S:T478K, S:D614G, S:P681H, S:T732A,	3'UTR:29819-29821,

hCoV-19/Mexico/SLP-INER-IMSS-00163/2021	EPI_ISL_1279439	In process	20A	B.1.561	23	5'UTR:C241T, ORF1ab:G1685A, ORF1ab:T1891C, ORF1ab:G2035T, ORF1ab:G2516T, ORF1ab:C3037T, ORF1ab:A5780G, ORF1ab:A5999G, ORF1ab:C9541T, ORF1ab:A10323G, ORF1ab:C10798T, ORF1ab:C11866T, ORF1ab:C14408T, ORF1ab:G16917T, ORF1ab:C18693T, ORF1ab:A20268G, S:C21575T, S:G21974T, S:A23403G, ORF3a:T25577C, M:C26735T, ORF7a:C27615T, N:C28854T, N:G28975T,	13	N:S194L, N:M234I, ORF1a:A474T, ORF1a:L590F, ORF1a:V751L, ORF1a:I1839V, ORF1a:I1912V, ORF1a:K3353R, ORF1b:P314L, ORF3a:I62T, S:L5F, S:D138Y, S:D614G,
hCoV-19/Mexico/SLP-INER-IMSS-00168/2021	EPI_ISL_1279444	In process	20B	B.1.1.519	24	5'UTR:C203T, 5'UTR:C222T, 5'UTR:C241T, ORF1ab:G2809T, ORF1ab:C3037T, ORF1ab:C3140T, ORF1ab:T3745C, ORF1ab:C9803T, ORF1ab:C10029T, ORF1ab:C10954T, ORF1ab:A11117G, ORF1ab:C12789T, ORF1ab:C14408T, ORF1ab:C14708T, ORF1ab:T19839C, ORF1ab:C21306T, S:C22995A, S:A23403G, S:C23604A, S:A23756G, ORF3a:C25844T, N:G28881A, N:G28882A, N:G28883C, N:C29197T,	14	N:R203K, N:G204R, ORF1a:R848S, ORF1a:P959S, ORF1a:T3255I, ORF1a:I3618V, ORF1a:T4175I, ORF1b:P314L, ORF1b:A414V, ORF3a:T151I, S:T478K, S:D614G, S:P681H, S:T732A,
hCoV-19/Mexico/SLP-INER-IMSS-00169/2021	EPI_ISL_1279283	In process		B.1.243	0	,	1	,
hCoV-19/Mexico/SLP-INER-IMSS-00170/2021	EPI_ISL_1279269	In process	20B	B.1.1.519	26	5'UTR:C203T, 5'UTR:C222T, 5'UTR:C241T, ORF1ab:C3037T, ORF1ab:C3140T, ORF1ab:C9979T, ORF1ab:C10029T, ORF1ab:C10954T, ORF1ab:A11117G, ORF1ab:C12789T, ORF1ab:C14408T, ORF1ab:G15921T, ORF1ab:A17971G, ORF1ab:T19839C, ORF1ab:C21306T, S:C22995A, S:A23403G, S:C23604A, S:C23635T, S:A23756G, S:A23870G, ORF7a:A27609G, ORF8:C27945T, N:G28881A, N:G28882A, N:G28883C, N:C29197T,	14	N:R203K, N:G204R, ORF1a:P959S, ORF1a:T3255I, ORF1a:I3618V, ORF1a:T4175I, ORF1b:P314L, ORF1b:R1502G, ORF8:Q18*, S:T478K, S:D614G, S:P681H, S:T732A, S:I770V,
hCoV-19/Mexico/SLP-INER-IMSS-00171/2021	EPI_ISL_1279445	In process	20B	B.1.1.519	24	5'UTR:C203T, 5'UTR:C222T, 5'UTR:C241T, ORF1ab:C3037T, ORF1ab:C3140T, ORF1ab:G3692T, ORF1ab:G3871T, ORF1ab:C10029T, ORF1ab:C10954T, ORF1ab:A11117G, ORF1ab:C11824A, ORF1ab:C12789T, ORF1ab:C14408T, ORF1ab:T19839C, ORF1ab:C21306T, ORF1ab:C21431T, S:C22995A, S:A23403G, S:C23604A, S:A23756G, ORF8:T27904C, N:G28881A, N:G28882A, N:G28883C, N:C29197T,	15	N:R203K, N:G204R, ORF1a:P959S, ORF1a:V1143F, ORF1a:K1202N, ORF1a:T3255I, ORF1a:I3618V, ORF1a:T4175I, ORF1b:P314L, ORF1b:A2655V, ORF8:L4P, S:T478K, S:D614G, S:P681H, S:T732A,
hCoV-19/Mexico/SLP-INER-IMSS-00172/2021	EPI_ISL_1279446	In process	20A	B.1.551	16	5'UTR:C241T, ORF1ab:G2458T, ORF1ab:C3037T, ORF1ab:C3738T, ORF1ab:C6997T, ORF1ab:C14408T, ORF1ab:C16954T, ORF1ab:A17841G, ORF1ab:A20268G, S:A23403G, S:C23604G, M:T26573C, ORF7a:G27566T, ORF8:G27987A, N:G28690T, N:C28854T, 3'UTR:G29751C,	9	N:L139F, N:S194L, ORF1a:M731I, ORF1a:P1158L, ORF1b:P314L, ORF7a:C58F, ORF8:V32I, S:D614G, S:P681R,
hCoV-19/Mexico/SLP-INER-IMSS-00173/2021	EPI_ISL_1279447	In process	20B	B.1.1.519	27	5'UTR:T201C, 5'UTR:C203T, 5'UTR:C222T, 5'UTR:C241T, ORF1ab:C936T, ORF1ab:G1738T, ORF1ab:C3037T, ORF1ab:C3140T, ORF1ab:C10029T, ORF1ab:C10954T, ORF1ab:A11117G, ORF1ab:C12789T, ORF1ab:C13994T, ORF1ab:C14408T, ORF1ab:T19839C, ORF1ab:A19974G, ORF1ab:C21306T, S:C22995A, S:A23403G, S:C23604A, S:A23756G, S:C24795T, E:C26270T, N:G28881A, N:G28882A, N:G28883C, N:C29197T, N:G29227T,	15	E:T9I, N:R203K, N:G204R, ORF1a:T224I, ORF1a:P959S, ORF1a:T3255I, ORF1a:I3618V, ORF1a:T4175I, ORF1b:A176V, ORF1b:P314L, S:T478K, S:D614G, S:P681H, S:T732A, S:A1078V,

hCoV-19/Mexico/SLP-INER-IMSS-00218/2021	EPI_ISL_1279484	In process	20B	B.1.1.519	25	5'UTR:C203T, 5'UTR:C222T, 5'UTR:C241T, ORF1ab:C1102T, ORF1ab:C1707T, ORF1ab:C2334T, ORF1ab:C3037T, ORF1ab:C3140T, ORF1ab:C10029T, ORF1ab:C10954T, ORF1ab:A11117G, ORF1ab:C12789T, ORF1ab:C14408T, ORF1ab:T19839C, ORF1ab:G19872A, ORF1ab:C21306T, S:C22995A, S:A23403G, S:C23604A, S:A23756G, M:G26526A, N:G28881A, N:G28882A, N:G28883C, N:C29197T, N:G29527T,	15	M:A2T, N:R203K, N:G204R, N:Q418H, ORF1a:S481F, ORF1a:A690V, ORF1a:P959S, ORF1a:T3255I, ORF1a:I3618V, ORF1a:T4175I, ORF1b:P314L, S:T478K, S:D614G, S:P681H, S:T732A,	
hCoV-19/Mexico/SLP-INER-IMSS-00331/2021	EPI_ISL_1279579	In process	20A	B.1.241	19	5'UTR:C241T, ORF1ab:C2650T, ORF1ab:C3037T, ORF1ab:G6884A, ORF1ab:C9110T, ORF1ab:C11575T, ORF1ab:C14408T, ORF1ab:C14805T, ORF1ab:A16840C, ORF1ab:C17518T, ORF1ab:C19029T, ORF1ab:A20268G, S:C21676T, S:T22222C, S:A23403G, ORF6:C27213T, ORF7b:C27804A, ORF8:C28115T. N:G28364C. N:C28854T.	10	N:E31Q, N:S194L, ORF1a:G2207S, ORF1a:R2949C, ORF1b:P314L, ORF1b:K1125Q, ORF1b:L1351F, ORF7b:L17M, ORF9b:E27D, S:D614G,	
hCoV-19/Mexico/SLP-INER-IMSS-00336/2021	EPI_ISL_1279584	In process	20B	B.1.1.519	27	5'UTR:C203T, 5'UTR:C222T, 5'UTR:C241T, ORF1ab:C1968T, ORF1ab:C3037T, ORF1ab:C3140T, ORF1ab:G3692T, ORF1ab:G3871T, ORF1ab:C10029T, ORF1ab:C10954T, ORF1ab:A11117G, ORF1ab:C11824A, ORF1ab:C12789T, ORF1ab:C13517T, ORF1ab:C14408T, ORF1ab:T19839C, ORF1ab:C21306T, S:C22995A, S:A23403G, S:C23604A, S:A23756G, ORF8:T27904C, N:G28881A, N:G28882A, N:G28883C, N:C29197T, 3'UTR:C29722T, 3'UTR:C29738G,	16	N:R203K, N:G204R, ORF1a:T568I, ORF1a:P959S, ORF1a:V1143F, ORF1a:K1202N, ORF1a:T3255I, ORF1a:I3618V, ORF1a:T4175I, ORF1b:T17I, ORF1b:P314L, ORF8:L4P, S:T478K, S:D614G, S:P681H, S:T732A,	
hCoV-19/Mexico/SLP-INER-IMSS-00337/2021	EPI_ISL_1279299	In process	20B	B.1.1.519	27	5'UTR:C203T, 5'UTR:C222T, 5'UTR:C241T, ORF1ab:T851C, ORF1ab:G2632T, ORF1ab:C3037T, ORF1ab:C3140T, ORF1ab:G3692T, ORF1ab:G3871T, ORF1ab:C10029T, ORF1ab:C10954T, ORF1ab:A11117G, ORF1ab:C11824A, ORF1ab:C12789T, ORF1ab:C13368T, ORF1ab:C14408T, ORF1ab:T19839C, ORF1ab:C21306T, S:C22995A, S:A23403G, S:C23604A, S:A23756G, ORF8:T27904C, N:C28473T, N:G28881A, N:G28882A, N:G28883C, N:C29197T,	19	N:P67L, N:R203K, N:G204R, ORF1a:Y196H, ORF1a:M789I, ORF1a:P959S, ORF1a:V1143F, ORF1a:K1202N, ORF1a:T3255I, ORF1a:I3618V, ORF1a:T4175I, ORF1a:T4368I, ORF1b:P314L, ORF8:L4P, ORF9b:L64F, S:T478K, S:D614G, S:P681H, S:T732A,	3'UTR:29755-29766
hCoV-19/Mexico/SON-InDRE-IBT-106/2020	EPI_ISL_1301513	In process	20C	B.1	9	5'UTR:C241T, ORF1ab:T833C, ORF1ab:C1059T, ORF1ab:C3037T, ORF1ab:G3231T, ORF1ab:C14408T, ORF1ab:C19524T, S:A23403G, ORF3a:G25563T, ORF8:C27964T,	7	ORF1a:F190L, ORF1a:T265I, ORF1a:G989V, ORF1b:P314L, ORF3a:Q57H, ORF8:S24L, S:D614G,	
hCoV-19/Mexico/SON-InDRE-IBT-90/2020	EPI_ISL_1301464	In process	20A	B.1.609	8	5'UTR:C241T, ORF1ab:C3037T, ORF1ab:C4582T, ORF1ab:C8175T, ORF1ab:C8655T, ORF1ab:C9451T, ORF1ab:C14408T, S:A23403G, M:G26995T,	5	M:R158L, ORF1a:A2637V, ORF1a:S2797F, ORF1b:P314L, S:D614G,	
hCoV-19/Mexico/SON-InDRE-IBT-91/2020	EPI_ISL_1301565	In process	20A	B.1.232	8	5'UTR:C241T, ORF1ab:C1385T, ORF1ab:C3037T, ORF1ab:C14408T, ORF1ab:A20268G, S:A23403G, ORF8:G27987T, N:G28727T, N:C28854T,	6	N:A152S, N:S194L, ORF1a:H374Y, ORF1b:P314L, ORF8:V32L, S:D614G,	
hCoV-19/Mexico/SON-InDRE-IBT-92/2020	EPI_ISL_1301517	In process	20A	B.1	7	5'UTR:C241T, ORF1ab:C3037T, ORF1ab:C10448T, ORF1ab:C14408T, ORF1ab:C18246T, S:A23403G, N:C28854T, 3'UTR:C29769T,	4	N:S194L, ORF1a:P3395S, ORF1b:P314L, S:D614G,	
hCoV-19/Mexico/SON-InDRE-IBT-93/2020	EPI_ISL_1301706	In process	20A	B.1.609	7	5'UTR:C241T, ORF1ab:C3037T, ORF1ab:C4582T, ORF1ab:C14408T, ORF1ab:A20268G, S:A23403G, ORF6:C27247T, N:C28724T,	3	N:P151S, ORF1b:P314L, S:D614G,	
hCoV-19/Mexico/TAB-IBT-IMSS-441/2020	EPI_ISL_1301628	In process	20C	B.1	7	5'UTR:C241T, ORF1ab:C1059T, ORF1ab:C3037T, ORF1ab:C7165T, ORF1ab:C14408T, S:A23403G, ORF3a:G25563T, 3'UTR:G29751C,	4	ORF1a:T265I, ORF1b:P314L, ORF3a:Q57H, S:D614G,	
hCoV-19/Mexico/TAB-IBT-IMSS-442/2020	EPI_ISL_1301629	In process	20C	B.1.446	10	5'UTR:C241T, ORF1ab:C1059T, ORF1ab:C3037T, ORF1ab:C7165T, ORF1ab:C8655T, ORF1ab:C10313T, ORF1ab:C14408T, S:A23403G, ORF3a:G25563T, N:C29095T, 3'UTR:G29751C,	6	ORF1a:T265I, ORF1a:S2797F, ORF1a:L3350F, ORF1b:P314L, ORF3a:Q57H, S:D614G,	

hCoV-19/Mexico/TAB-InDRE-IBT-28/2020	EPI_ISL_1301700	In process	20C	B.1	9	5'UTR:C241T, ORF1ab:C1059T, ORF1ab:C3037T, ORF1ab:C11407A, ORF1ab:C14408T, ORF1ab:T14949C, ORF1ab:A20755C, S:A23403G, ORF3a:G25563T, N:G28899T,	6	N:R209I, ORF1a:T265I, ORF1b:P314L, ORF1b:S2430R, ORF3a:Q57H, S:D614G,	
hCoV-19/Mexico/TAB-InDRE-IBT-29/2020	EPI_ISL_1301444	In process	20C	B.1	6	5'UTR:C241T, ORF1ab:C1059T, ORF1ab:C3037T, ORF1ab:C7165T, ORF1ab:C14408T, S:A23403G, ORF3a:G25563T,	4	ORF1a:T265I, ORF1b:P314L, ORF3a:Q57H, S:D614G,	
hCoV-19/Mexico/TAB-InDRE-IBT-30/2020	EPI_ISL_1301701	In process	20B	B.1.1	11	5'UTR:C241T, ORF1ab:C3037T, ORF1ab:A10323G, ORF1ab:C14408T, ORF1ab:T19839C, S:A23403G, S:C24865T, N:A28877T, N:G28878C, N:G28881A, N:G28882A, N:G28883C,	5	N:R203K, N:G204R, ORF1a:K3353R, ORF1b:P314L, S:D614G,	
hCoV-19/Mexico/TAB-InDRE-IBT-31/2020	EPI_ISL_1301537	In process	20C	B.1	9	5'UTR:C241T, ORF1ab:C1059T, ORF1ab:C3037T, ORF1ab:A9204G, ORF1ab:G10201T, ORF1ab:C14408T, ORF1ab:C18601T, S:A23403G, ORF3a:G25563T, N:C29466T,	8	N:A398V, ORF1a:T265I, ORF1a:D2980G, ORF1a:M3312I, ORF1b:P314L, ORF1b:H1712Y, ORF3a:Q57H, S:D614G,	
hCoV-19/Mexico/TAM_LANGEBIO_IMSS_0001/202	EPI_ISL_1351416	In process	20G	B.1.2	24	5'UTR:C241T, ORF1ab:C1059T, ORF1ab:G2632T, ORF1ab:C3037T, ORF1ab:T7348A, ORF1ab:A7702G, ORF1ab:G8083A, ORF1ab:C10319T, ORF1ab:C13119T, ORF1ab:C14408T, ORF1ab:C14805T, ORF1ab:A16856G, ORF1ab:C18131T, ORF1ab:A18424G, ORF1ab:A21203G, ORF1ab:C21304T, S:A23403G, S:G23593T, ORF3a:T25502C, ORF3a:G25563T, ORF3a:G25907T, ORF8:C27964T, ORF8:C28093T, N:C28472T, N:C28869T,	21	N:P67S, N:P199L, ORF1a:T265I, ORF1a:M789I, ORF1a:N2361K, ORF1a:M2606I, ORF1a:L3352F, ORF1a:A4285V, ORF1b:P314L, ORF1b:D1130G, ORF1b:T1555I, ORF1b:N1653D, ORF1b:K2579R, ORF1b:R2613C, ORF3a:I37T, ORF3a:Q57H, ORF3a:G172V, ORF8:S24L, ORF8:S67F, S:D614G, S:Q677H,	
hCoV-19/Mexico/TAM_LANGEBIO_IMSS_0002/202	EPI_ISL_1351418	In process	20G	B.1.2	26	5'UTR:C241T, ORF1ab:G428A, ORF1ab:C1059T, ORF1ab:T1492C, ORF1ab:G1942T, ORF1ab:C3037T, ORF1ab:G8083A, ORF1ab:A9085G, ORF1ab:C10319T, ORF1ab:T12645C, ORF1ab:A13338G, ORF1ab:C14408T, ORF1ab:C14805T, ORF1ab:C16887T, ORF1ab:C16952T, ORF1ab:A18424G, ORF1ab:C21304T, S:A23403G, S:G23593T, ORF3a:G25563T, ORF3a:G25593A, ORF3a:G25907T, ORF3a:T25928C, ORF8:C27964T, N:C28472T, N:C28869T, N:G29402T,	19	N:P67S, N:P199L, N:D377Y, ORF1a:E55K, ORF1a:T265I, ORF1a:M2606I, ORF1a:L3352F, ORF1a:I4127T, ORF1a:N4358S, ORF1b:P314L, ORF1b:T1162I, ORF1b:N1653D, ORF1b:R2613C, ORF3a:Q57H, ORF3a:G172V, ORF3a:I179T, ORF8:S24L, S:D614G, S:Q677H,	
hCoV-19/Mexico/TAM_LANGEBIO_IMSS_0003/202	EPI_ISL_1351420	In process	20B	B.1.1.519	25	5'UTR:C203T, 5'UTR:A223T, 5'UTR:C241T, ORF1ab:C3037T, ORF1ab:C3140T, ORF1ab:G5992A, ORF1ab:C10029T, ORF1ab:A10323G, ORF1ab:C10954T, ORF1ab:A11117G, ORF1ab:G11365T, ORF1ab:C12789T, ORF1ab:C14408T, ORF1ab:T19839C, ORF1ab:C21306T, S:G22894A, S:C22995A, S:A23403G, S:C23604A, S:A23756G, S:T24364C, N:G28881A, N:G28882A, N:G28883C, N:C29197T, N:G29527T,	13	N:R203K, N:G204R, N:Q418H, ORF1a:P959S, ORF1a:T3255I, ORF1a:K3353R, ORF1a:I3618V, ORF1a:T4175I, ORF1b:P314L, S:T478K, S:D614G, S:P681H, S:T732A,	5'UTR:2 22,
hCoV-19/Mexico/TAM_LANGEBIO_IMSS_0004/202	EPI_ISL_1351422	In process	20B	B.1.1.519	26	5'UTR:C203T, 5'UTR:C222T, 5'UTR:C241T, ORF1ab:C3037T, ORF1ab:C3140T, ORF1ab:C6027T, ORF1ab:C10029T, ORF1ab:C10834A, ORF1ab:C10954T, ORF1ab:A11117G, ORF1ab:G11365T, ORF1ab:C12789T, ORF1ab:C14408T, ORF1ab:G17122T, ORF1ab:T19839C, ORF1ab:C21306T, S:C22995A, S:A23403G, S:C23604A, S:A23756G, ORF3a:G25909A, ORF8:A28084T, N:G28881A, N:G28882A, N:G28883C, N:C29197T, N:G29527T,	16	N:R203K, N:G204R, N:Q418H, ORF1a:P959S, ORF1a:P1921L, ORF1a:T3255I, ORF1a:I3618V, ORF1a:T4175I, ORF1b:P314L, ORF1b:A1219S, ORF3a:D173N, ORF8:E64V, S:T478K, S:D614G, S:P681H, S:T732A,	

hCoV-19/Mexico/TAM_LANGEBIO_IMSS_0006/202	EPI_ISL_1351424	In process	20B	B.1.1.519	23	5'UTR:A223T, 5'UTR:C241T, ORF1ab:G2118T, ORF1ab:C3037T, ORF1ab:C3140T, ORF1ab:G5992A, ORF1ab:C10029T, ORF1ab:A10323G, ORF1ab:C10954T, ORF1ab:A11117G, ORF1ab:G11365T, ORF1ab:C12789T, ORF1ab:C14408T, ORF1ab:T19839C, ORF1ab:C21306T, S:C22995A, S:A23403G, S:C23604A, S:A23756G, N:G28881A, N:G28882A, N:G28883C, N:C29197T, N:G29527T,	13	N:R203K, N:G204R, N:Q418H, ORF1a:G618V, ORF1a:P959S, ORF1a:T3255I, ORF1a:K3353R, ORF1a:I3618V, ORF1a:T4175I, ORF1b:P314L, S:D614G, S:P681H, S:T732A,	5'UTR:2 22,
hCoV-19/Mexico/TAM_LANGEBIO_IMSS_0007/202	EPI_ISL_1351426	In process	20B	B.1.1	16	5'UTR:C241T, ORF1ab:T1481C, ORF1ab:C3037T, ORF1ab:C8829T, ORF1ab:C11450A, ORF1ab:C14408T, ORF1ab:C17004T, ORF1ab:C18747T, S:A23403G, S:G24842A, S:A24902G, ORF3a:G25720T, N:G28881A, N:G28882A, N:G28883C, 3'UTR:A29700G, 3'UTR:G29779T.	10	N:R203K, N:G204R, ORF1a:F406L, ORF1a:A2855V, ORF1a:Q3729K, ORF1b:P314L, ORF3a:A110S, S:D614G, S:V1094I, S:I1114V,	
hCoV-19/Mexico/TAM_LANGEBIO_IMSS_0008/202	EPI_ISL_1351428	In process	20G	B.1.596	30	5'UTR:C241T, ORF1ab:C1059T, ORF1ab:G1261A, ORF1ab:C1853T, ORF1ab:G1890T, ORF1ab:C3037T, ORF1ab:G6536T, ORF1ab:T8830A, ORF1ab:G9300A, ORF1ab:C10319T, ORF1ab:C12025T, ORF1ab:T12645C, ORF1ab:C12676T, ORF1ab:C14408T, ORF1ab:G15380T, ORF1ab:T17865A, ORF1ab:A18424G, ORF1ab:C21304T, S:A21647T, S:A23403G, ORF3a:G25563T, ORF3a:G25907T, E:G26439T, ORF7a:T27484C, ORF8:C27964T, N:C28472T, N:C28869T, N:C29085T, 3'UTR:T29760C,ORF1ab:T17863A, ORF1ab:A17864T,	19	N:P67S, N:P199L, N:T271I, ORF1a:T265I, ORF1a:R542L, ORF1a:G2091C, ORF1a:R3012K, ORF1a:L3352F, ORF1a:I4127T, ORF1b:P314L, ORF1b:S638I, ORF1b:N1653D, ORF1b:R2613C, ORF3a:Q57H, ORF3a:G172V, ORF8:S24L, S:T29S, S:D614G,ORF1b:Y1466I,	ORF1ab: 3332- 3343
hCoV-19/Mexico/TAM_LANGEBIO_IMSS_0009/202	EPI_ISL_1351430	In process	20A	B.1.243	21	ORF1ab:A2071G, ORF1ab:C2749T, ORF1ab:C3037T, ORF1ab:G3875T, ORF1ab:C7420T, ORF1ab:C9165T, ORF1ab:C10741T, ORF1ab:A11474G, ORF1ab:T12645C, ORF1ab:C14408T, ORF1ab:G18040T, ORF1ab:C19097T, ORF1ab:A19137G, ORF1ab:A20268G, S:A23403G, S:C23604A, S:T24076C, S:G25244T, N:C28854T, N:C29119T, ORF10:G29587T, 3'UTR:T29710C,	11	N:S194L, ORF1a:A1204S, ORF1a:T2967I, ORF1a:I3737V, ORF1a:I4127T, ORF1b:P314L, ORF1b:A1525S, ORF1b:A1877V, S:D614G, S:P681H, S:V1228L,	
hCoV-19/Mexico/TAM_LANGEBIO_IMSS_0010/202	EPI_ISL_1351433	In process	20B	B.1.1.519	20	ORF1ab:G2118T, ORF1ab:C3037T, ORF1ab:C3140T, ORF1ab:C10029T, ORF1ab:C10789T, ORF1ab:C10954T, ORF1ab:A11117G, ORF1ab:C12789T, ORF1ab:C14408T, ORF1ab:C16308T, ORF1ab:T19839C, ORF1ab:C21306T, S:A23403G, S:C23604A, S:A23756G, ORF3a:C25658T, N:G28881A, N:G28882A, N:G28883C, N:C29197T, N:G29527T,	13	N:R203K, N:G204R, N:Q418H, ORF1a:G618V, ORF1a:P959S, ORF1a:T3255I, ORF1a:I3618V, ORF1a:T4175I, ORF1b:P314L, ORF3a:T89I, S:D614G, S:P681H, S:T732A,	
hCoV-19/Mexico/TAM_LANGEBIO_IMSS_0011/202	EPI_ISL_1351435	In process	20A	B.1	21	5'UTR:C241T, ORF1ab:C1190T, ORF1ab:G2782T, ORF1ab:C3037T, ORF1ab:A3061G, ORF1ab:C10449T, ORF1ab:T12645C, ORF1ab:C12747T, ORF1ab:C13168T, ORF1ab:C14408T, ORF1ab:T14560C, ORF1ab:C16173T, ORF1ab:T16731C, ORF1ab:G16741A, ORF1ab:A20268G, S:C21575T, S:A23403G, S:G23465T, ORF3a:G25955T, M:C26681T, N:C28854T, N:G28871A,	13	N:S194L, N:G200S, ORF1a:P309S, ORF1a:P3395L, ORF1a:I4127T, ORF1a:T4161I, ORF1b:P314L, ORF1b:Y365H, ORF1b:V1092I, ORF3a:G188V, S:L5F, S:D614G, S:V635F,	
hCoV-19/Mexico/TAM_LANGEBIO_IMSS_0012/202	EPI_ISL_1351437	In process	20B	B.1.1.222	20	ORF1ab:C706T, ORF1ab:A1123G, ORF1ab:C2252A, ORF1ab:C3037T, ORF1ab:C10029T, ORF1ab:T12645C, ORF1ab:A14223G, ORF1ab:C14408T, ORF1ab:G14707A, ORF1ab:C15279T, ORF1ab:G19816T, ORF1ab:T19839C, S:A23403G, S:A23756G, ORF3a:G25912T, ORF7b:A27756G, ORF8:A27921G, ORF8:G28001T, N:G28881A, N:G28882A, N:G28883C,	13	N:R203K, N:G204R, ORF1a:Q663K, ORF1a:T3255I, ORF1a:I4127T, ORF1b:P314L, ORF1b:A414T, ORF1b:V2117L, ORF3a:G174C, ORF7b:M1V, ORF8:I10V, S:D614G, S:T732A,	

hCoV-19/Mexico/TAM_LANGEBIO_IMSS_0013/202	EPI_ISL_1351439	In process	20G	B.1.2	26	5'UTR:C241T, ORF1ab:C1059T, ORF1ab:C3037T, ORF1ab:C7600T, ORF1ab:G8844T, ORF1ab:C10138T, ORF1ab:C10319T, ORF1ab:T12645C, ORF1ab:C14408T, ORF1ab:C17678T, ORF1ab:A18424G, ORF1ab:C19151T, ORF1ab:G19525T, ORF1ab:A19526G, ORF1ab:C21304T, S:A23403G, S:G25088T, ORF3a:G25444A, ORF3a:G25563T, ORF3a:C25714T, ORF3a:G25907T, M:G26951T, ORF7a:G27670T, ORF8:C27964T, N:C28472T, N:C28869T, N:A29005G,	20	N:P67S, N:P199L, ORF1a:T265I, ORF1a:R2860I, ORF1a:L3352F, ORF1a:I4127T, ORF1b:P314L, ORF1b:T1404M, ORF1b:N1653D, ORF1b:A1895V, ORF1b:D2020C, ORF1b:R2613C, ORF3a:G18S, ORF3a:Q57H, ORF3a:L108F, ORF3a:G172V, ORF7a:V93F, ORF8:S24L, S:D614G, S:V1176F,
hCoV-19/Mexico/TAM_LANGEBIO_IMSS_0023/202	EPI_ISL_1351465	In process	20B	B.1.1.519	25	5'UTR:T201C, 5'UTR:C203T, 5'UTR:C222T, 5'UTR:C241T, ORF1ab:G1274T, ORF1ab:G1738T, ORF1ab:C3037T, ORF1ab:C3140T, ORF1ab:C7504T, ORF1ab:C10029T, ORF1ab:C10954T, ORF1ab:A11117G, ORF1ab:C12789T, ORF1ab:C13115T, ORF1ab:C14408T, ORF1ab:A19974G, ORF1ab:C21306T, S:C22995A, S:A23403G, S:C23604A, S:A23756G, S:C24130T, N:G28881A, N:G28882A, N:G28883C, N:C29197T,	12	N:R203K, N:G204R, ORF1a:V337F, ORF1a:P959S, ORF1a:T3255I, ORF1a:I3618V, ORF1a:T4175I, ORF1b:P314L, S:T478K, S:D614G, S:P681H, S:T732A,
hCoV-19/Mexico/TAM_LANGEBIO_IMSS_0024/202	EPI_ISL_1351468	In process	20B	B.1.1.519	25	5'UTR:T201C, 5'UTR:C203T, 5'UTR:C222T, 5'UTR:C241T, ORF1ab:G1274T, ORF1ab:G1738T, ORF1ab:C3037T, ORF1ab:C3140T, ORF1ab:C7504T, ORF1ab:C10029T, ORF1ab:C10954T, ORF1ab:A11117G, ORF1ab:C12789T, ORF1ab:C13115T, ORF1ab:C14408T, ORF1ab:A19974G, ORF1ab:C21306T, S:C22995A, S:A23403G, S:C23604A, S:A23756G, S:C24130T, N:G28881A, N:G28882A, N:G28883C, N:C29197T,	12	N:R203K, N:G204R, ORF1a:V337F, ORF1a:P959S, ORF1a:T3255I, ORF1a:I3618V, ORF1a:T4175I, ORF1b:P314L, S:T478K, S:D614G, S:P681H, S:T732A,
hCoV-19/Mexico/TAM_LANGEBIO_IMSS_0025/202	EPI_ISL_1351471	In process	20B	B.1.1	23	5'UTR:C241T, ORF1ab:T1711C, ORF1ab:C3037T, ORF1ab:C4320T, ORF1ab:C6040T, ORF1ab:G6362A, ORF1ab:G9802T, ORF1ab:A11451G, ORF1ab:C12076T, ORF1ab:C14408T, ORF1ab:G17325T, ORF1ab:C17795T, ORF1ab:C20762T, S:A22765G, S:G22992A, S:A23403G, S:G23522C, ORF3a:G25855T, ORF7a:T27597A, ORF7a:G27598A, ORF7a:C27679T, N:G28881A, N:G28882A, N:G28883C,	15	N:R203K, N:G204R, ORF1a:A1352V, ORF1a:A2033T, ORF1a:Q3729R, ORF1b:P314L, ORF1b:L1286F, ORF1b:A1443V, ORF1b:T2432I, ORF3a:D155Y, ORF7a:D69N, ORF7a:L96F, S:S477N, S:D614G, S:E654Q,
hCoV-19/Mexico/TAM_LANGEBIO_IMSS_0026/202	EPI_ISL_1351474	In process	20B	B.1.1.519	27	5'UTR:T201C, 5'UTR:C203T, 5'UTR:C222T, 5'UTR:C241T, ORF1ab:C936T, ORF1ab:G1738T, ORF1ab:T2489C, ORF1ab:C3037T, ORF1ab:C3140T, ORF1ab:C10029T, ORF1ab:C10954T, ORF1ab:A11117G, ORF1ab:C12789T, ORF1ab:C14408T, ORF1ab:T16692C, ORF1ab:A19974G, ORF1ab:C21306T, S:C22995A, S:A23403G, S:C23604A, S:A23756G, ORF7a:A27426G, N:G28881A, N:G28882A, N:G28883C, N:C29197T, N:G29227T, N:G29321T,	13	N:R203K, N:G204R, N:V350F, ORF1a:T224I, ORF1a:P959S, ORF1a:T3255I, ORF1a:I3618V, ORF1a:T4175I, ORF1b:P314L, S:T478K, S:D614G, S:P681H, S:T732A,
hCoV-19/Mexico/TAM_LANGEBIO_IMSS_0027/202	EPI_ISL_1351477	In process	20B	B.1.1.519	20	5'UTR:C203T, 5'UTR:C222T, 5'UTR:C241T, ORF1ab:C3037T, ORF1ab:C3140T, ORF1ab:C10029T, ORF1ab:C10954T, ORF1ab:A11117G, ORF1ab:C12789T, ORF1ab:C14408T, ORF1ab:T19839C, ORF1ab:C21306T, S:C22995A, S:A23403G, S:C23604A, S:A23756G, M:C26985T, N:G28881A, N:G28882A, N:G28883C, N:C29197T,	12	M:H155Y, N:R203K, N:G204R, ORF1a:P959S, ORF1a:T3255I, ORF1a:I3618V, ORF1a:T4175I, ORF1b:P314L, S:T478K, S:D614G, S:P681H, S:T732A,

hCoV-19/Mexico/TAM_LANGEBIO_IMSS_0028/202	EPI_ISL_1351480	In process	20B	B.1.1.519	26	5'UTR:T201C, 5'UTR:C203T, 5'UTR:C222T, 5'UTR:C241T, ORF1ab:G1274T, ORF1ab:G1738T, ORF1ab:C3037T, ORF1ab:C3140T, ORF1ab:C7504T, ORF1ab:C10029T, ORF1ab:C10626T, ORF1ab:C10954T, ORF1ab:A11117G, ORF1ab:C12789T, ORF1ab:C13115T, ORF1ab:C14408T, ORF1ab:A19974G, ORF1ab:C21306T, S:C22995A, S:A23403G, S:C23604A, S:A23756G, S:C24130T, N:G28881A, N:G28882A, N:G28883C, N:C29197T,	13	N:R203K, N:G204R, ORF1a:V337F, ORF1a:P959S, ORF1a:T3255I, ORF1a:A3454V, ORF1a:I3618V, ORF1a:T4175I, ORF1b:P314L, S:T478K, S:D614G, S:P681H, S:T732A,	
hCoV-19/Mexico/TAM_LANGEBIO_IMSS_0029/202	EPI_ISL_1351483	In process	20B	B.1.1.519	28	5'UTR:T201C, 5'UTR:C203T, 5'UTR:C222T, 5'UTR:C241T, ORF1ab:G1274T, ORF1ab:G1738T, ORF1ab:C3037T, ORF1ab:C3140T, ORF1ab:C6198T, ORF1ab:C7504T, ORF1ab:C10029T, ORF1ab:C10954T, ORF1ab:A11117G, ORF1ab:C12789T, ORF1ab:C13115T, ORF1ab:C14408T, ORF1ab:G17562T, ORF1ab:T19839C, ORF1ab:A19974G, ORF1ab:C21306T, S:C22995A, S:A23403G, S:C23604A, S:A23756G, M:G27008T, N:G28881A, N:G28882A, N:G28883C, N:C29197T	14	M:K162N, N:R203K, N:G204R, ORF1a:V337F, ORF1a:P959S, ORF1a:S1978F, ORF1a:T3255I, ORF1a:I3618V, ORF1a:T4175I, ORF1b:P314L, S:T478K, S:D614G, S:P681H, S:T732A,	
hCoV-19/Mexico/TAM_LANGEBIO_IMSS_0030/202	EPI_ISL_1351486	In process	20B	B.1.1.519	26	5'UTR:T201C, 5'UTR:C203T, 5'UTR:C222T, 5'UTR:C241T, ORF1ab:G1274T, ORF1ab:G1738T, ORF1ab:C3037T, ORF1ab:C3140T, ORF1ab:C7504T, ORF1ab:C7564T, ORF1ab:C10029T, ORF1ab:C10954T, ORF1ab:A11117G, ORF1ab:C12789T, ORF1ab:C13115T, ORF1ab:C14408T, ORF1ab:T19839C, ORF1ab:A19974G, ORF1ab:C21306T, S:C22995A, S:A23403G, S:C23604A, S:A23756G, N:G28881A, N:G28882A, N:G28883C, N:C29197T,	12	N:R203K, N:G204R, ORF1a:V337F, ORF1a:P959S, ORF1a:T3255I, ORF1a:I3618V, ORF1a:T4175I, ORF1b:P314L, S:T478K, S:D614G, S:P681H, S:T732A,	
hCoV-19/Mexico/TAM_LANGEBIO_IMSS_0031/202	EPI_ISL_1351489	In process	20B	B.1.1.519	27	5'UTR:C203T, 5'UTR:C222T, 5'UTR:C241T, ORF1ab:C3037T, ORF1ab:C3140T, ORF1ab:C10029T, ORF1ab:C10954T, ORF1ab:A11117G, ORF1ab:C12789T, ORF1ab:C13297T, ORF1ab:C14408T, ORF1ab:C16877T, ORF1ab:T19839C, ORF1ab:C19983T, ORF1ab:G20102T, ORF1ab:C21306T, S:C22995A, S:A23403G, S:C23604A, S:A23756G, ORF6:C27230T, ORF8:T28082A, N:A28359G, N:T28711A, N:G28881A, N:G28882A, N:G28883C, N:C29197T,	17	N:N29S, N:R203K, N:G204R, ORF1a:P959S, ORF1a:T3255I, ORF1a:I3618V, ORF1a:T4175I, ORF1b:P314L, ORF1b:T1137I, ORF1b:S2212I, ORF6:T10I, ORF8:D63E, ORF9b:M26V, S:T478K, S:D614G, S:P681H, S:T732A,	
hCoV-19/Mexico/TAM_LANGEBIO_IMSS_0032/202	EPI_ISL_1351492	In process	20B	B.1.1.222	13	5'UTR:C241T, ORF1ab:C3037T, ORF1ab:C10029T, ORF1ab:C14408T, S:C22482T, S:A23403G, S:A23756G, ORF3a:G25912T, ORF7b:A27756G, ORF8:A27921T, ORF8:G28001T, N:G28881A, N:G28882A, N:G28883C,	10	N:R203K, N:G204R, ORF1a:T3255I, ORF1b:P314L, ORF3a:G174C, ORF7b:M1V, ORF8:I10F, S:T307I, S:D614G, S:T732A,	3'UTR:29749-29762,
hCoV-19/Mexico/TAM_LANGEBIO_IMSS_0033/202	EPI_ISL_1351494	In process	20B	B.1.1.222	19	5'UTR:C241T, ORF1ab:C3037T, ORF1ab:G4300T, ORF1ab:A13810G, ORF1ab:C14408T, ORF1ab:G16591A, ORF1ab:G17193T, ORF1ab:T19839C, ORF1ab:A21551T, S:C21637T, S:A23403G, S:A23756G, ORF3a:G25726T, ORF8:G27916T, N:G28881A, N:G28882A, N:G28883C, N:G29260T, ORF10:G29648T, 3'UTR:C29835T,	11	N:R203K, N:G204R, ORF1b:M115V, ORF1b:P314L, ORF1b:D1042N, ORF1b:E1242D, ORF1b:N2695I, ORF3a:V112F, ORF8:G8V, S:D614G, S:T732A,	
hCoV-19/Mexico/TAM_LANGEBIO_IMSS_0034/202	EPI_ISL_1351498	In process	20B	B.1.1.222	19	5'UTR:C241T, ORF1ab:C3037T, ORF1ab:G4300T, ORF1ab:A13810G, ORF1ab:C14408T, ORF1ab:G16591A, ORF1ab:G17193T, ORF1ab:T19839C, ORF1ab:A21551T, S:C21637T, S:A23403G, S:A23756G, ORF3a:G25726T, ORF8:G27916T, N:G28881A, N:G28882A, N:G28883C, N:G29260T, ORF10:G29648T, 3'UTR:C29835T,	11	N:R203K, N:G204R, ORF1b:M115V, ORF1b:P314L, ORF1b:D1042N, ORF1b:E1242D, ORF1b:N2695I, ORF3a:V112F, ORF8:G8V, S:D614G, S:T732A,	

hCoV-19/Mexico/TAM_LANGEBIO_IMSS_0036/202	EPI_ISL_1351503	In process	21C (Epsilon)	B.1.427	21	5'UTR:C241T, ORF1ab:C1059T, ORF1ab:C3037T, ORF1ab:G6717T, ORF1ab:C7869T, ORF1ab:G9738C, ORF1ab:C12651T, ORF1ab:G13713A, ORF1ab:C14408T, ORF1ab:C16394T, ORF1ab:G17014T, S:G21600T, S:G22018T, S:T22917G, S:A23403G, ORF3a:G25563T, M:C26681T, ORF8:A28272T, N:G28487A, N:G28875T, N:C28887T, N:C29362T,	16	N:V72I, N:S201I, N:T205I, ORF1a:T265I, ORF1a:S2151I, ORF1a:S2535L, ORF1a:S3158T, ORF1a:T4129I, ORF1b:P314L, ORF1b:P976L, ORF1b:D1183Y, ORF3a:Q57H, S:S13I, S:W152C, S:L452R, S:D614G,
hCoV-19/Mexico/TAM_LANGEBIO_IMSS_0040/202	EPI_ISL_1351514	In process	21C (Epsilon)	B.1.427	25	5'UTR:C241T, ORF1ab:C1059T, ORF1ab:C3037T, ORF1ab:C3817T, ORF1ab:C5284T, ORF1ab:C7165T, ORF1ab:G9738C, ORF1ab:A9922G, ORF1ab:C13019T, ORF1ab:G13713A, ORF1ab:C14408T, ORF1ab:C16394T, ORF1ab:G17014T, ORF1ab:A20715T, S:G21600T, S:G22018T, S:G22335T, S:C22597T, S:T22917G, S:A23403G, ORF3a:G25563T, M:C26681T, ORF8:C28087T, ORF8:A28272T, N:C28887T, N:C29362T	14	N:T205I, ORF1a:T265I, ORF1a:S3158T, ORF1b:P314L, ORF1b:P976L, ORF1b:D1183Y, ORF1b:R2416S, ORF3a:Q57H, ORF8:A65V, S:S13I, S:W152C, S:W258L, S:L452R, S:D614G,
hCoV-19/Mexico/TAM_LANGEBIO_IMSS_0043/202	EPI_ISL_1351523	In process	20B	B.1.1.519	23	5'UTR:C203T, 5'UTR:G204T, 5'UTR:C222T, 5'UTR:C241T, ORF1ab:C3037T, ORF1ab:C3140T, ORF1ab:G7027T, ORF1ab:C10029T, ORF1ab:C10954T, ORF1ab:A11117G, ORF1ab:C12789T, ORF1ab:C14408T, ORF1ab:T19839C, ORF1ab:C21306T, S:C22995A, S:A23403G, S:C23604A, S:C23635T, S:A23756G, S:C25207T, N:G28881A, N:G28882A, N:G28883C, N:C29197T,	12	N:R203K, N:G204R, ORF1a:P959S, ORF1a:M2254I, ORF1a:T3255I, ORF1a:I3618V, ORF1a:T4175I, ORF1b:P314L, S:T478K, S:D614G, S:P681H, S:T732A,
hCoV-19/Mexico/TAM_LANGEBIO_IMSS_0051/202	EPI_ISL_1351547	In process	20B	B.1.1.519	27	5'UTR:C203T, 5'UTR:C222T, 5'UTR:C241T, ORF1ab:C3037T, ORF1ab:C3140T, ORF1ab:C5628A, ORF1ab:C10029T, ORF1ab:C10954T, ORF1ab:A11117G, ORF1ab:A11991G, ORF1ab:C12789T, ORF1ab:C14408T, ORF1ab:C14931T, ORF1ab:T15726A, ORF1ab:T19839C, ORF1ab:G20275T, ORF1ab:C21306T, S:C22995A, S:A23403G, S:C23604A, S:A23756G, M:C26833T, ORF8:T27968C, N:G28881A, N:G28882A, N:G28883C, N:C29197T, N:G29527T	16	M:A104V, N:R203K, N:G204R, N:Q418H, ORF1a:P959S, ORF1a:T1788K, ORF1a:T3255I, ORF1a:I3618V, ORF1a:E3909G, ORF1a:T4175I, ORF1b:P314L, ORF1b:D2270Y, S:T478K, S:D614G, S:P681H, S:T732A,
hCoV-19/Mexico/TAM_LANGEBIO_IMSS_0052/202	EPI_ISL_1351549	In process	21C (Epsilon)	B.1.427	24	5'UTR:C241T, ORF1ab:C1059T, ORF1ab:C1684T, ORF1ab:T1851C, ORF1ab:C2232T, ORF1ab:C3037T, ORF1ab:C3619A, ORF1ab:C5693T, ORF1ab:G9738C, ORF1ab:G13713A, ORF1ab:C14408T, ORF1ab:C16394T, ORF1ab:G17014T, S:G21600T, S:G22018T, S:T22917G, S:A23403G, S:C25317T, ORF3a:T25467C, ORF3a:G25563T, ORF3a:T26066C, M:C26681T, ORF8:A28272T, N:C28887T, N:C29362T,	16	N:T205I, ORF1a:T265I, ORF1a:I529T, ORF1a:A656V, ORF1a:P1810S, ORF1a:S3158T, ORF1b:P314L, ORF1b:P976L, ORF1b:D1183Y, ORF3a:Q57H, ORF3a:V225A, S:S13I, S:W152C, S:L452R, S:D614G, S:S1252F,
hCoV-19/Mexico/TAM_LANGEBIO_IMSS_0053/202	EPI_ISL_1351552	In process	20G	B.1.2	23	5'UTR:C241T, ORF1ab:C1059T, ORF1ab:C3037T, ORF1ab:T7348A, ORF1ab:G8083A, ORF1ab:C10319T, ORF1ab:C13119T, ORF1ab:C14408T, ORF1ab:C14805T, ORF1ab:C15981T, ORF1ab:A17709G, ORF1ab:C18131T, ORF1ab:A18424G, ORF1ab:A21203G, ORF1ab:C21304T, S:C22006T, S:A23403G, S:C25006T, ORF3a:G25563T, ORF3a:G25907T, M:C27092T, ORF8:C27964T, N:C28472T, N:C28869T,	16	N:P67S, N:P199L, ORF1a:T265I, ORF1a:N2361K, ORF1a:M2606I, ORF1a:L3352F, ORF1a:A4285V, ORF1b:P314L, ORF1b:T1555I, ORF1b:N1653D, ORF1b:K2579R, ORF1b:R2613C, ORF3a:Q57H, ORF3a:G172V, ORF8:S24L, S:D614G,

hCoV-19/Mexico/TAM_LANGEBIO_IMSS_0131/202[illegible]	EPI_ISL_1351793	In process	20A	B.1.404	27	5'UTR:C241T, ORF1ab:C2156T, ORF1ab:C2470T, ORF1ab:C3037T, ORF1ab:G5572T, ORF1ab:C6310T, ORF1ab:C6543T, ORF1ab:G7042T, ORF1ab:C9979T, ORF1ab:A10323G, ORF1ab:A12644G, ORF1ab:C14408T, ORF1ab:T16743C, ORF1ab:T16993C, ORF1ab:C18395T, ORF1ab:G18462A, ORF1ab:C18555T, ORF1ab:C18885T, ORF1ab:G19086T, ORF1ab:A20268G, S:C21614T, S:G22992A, S:A23403G, S:C25006T, ORF3a:C25667T, ORF6:G27214A, ORF7a:C27450T, N:C28854T,	16	N:S194L, ORF1a:L631F, ORF1a:M1769I, ORF1a:T2093I, ORF1a:M2259I, ORF1a:K3353R, ORF1a:I4127V, ORF1b:P314L, ORF1b:Y1176H, ORF1b:A1643V, ORF1b:K1873N, ORF3a:S92L, ORF6:V5I, S:L18F, S:S477N, S:D614G,	
hCoV-19/Mexico/TAM-INER-IMSS-00191/2021	EPI_ISL_1279462	In process	20G	B.1.596	25	5'UTR:C241T, ORF1ab:C1059T, ORF1ab:C3037T, ORF1ab:C4832T, ORF1ab:C6402T, ORF1ab:C8139T, ORF1ab:C9559T, ORF1ab:C10319T, ORF1ab:A11451G, ORF1ab:C14408T, ORF1ab:A18424G, ORF1ab:C19593T, ORF1ab:C21304T, S:C21811A, S:C21952T, S:A23403G, S:A23592C, ORF3a:G25563T, ORF3a:G25907T, ORF8:C27964T, ORF8:T28255C, N:C28472T, N:C28869T, N:T29194C, N:T29377A, 3'UTR:C29733T,	16	N:P67S, N:P199L, ORF1a:T265I, ORF1a:P2046L, ORF1a:S2625F, ORF1a:L3352F, ORF1a:Q3729R, ORF1b:P314L, ORF1b:N1653D, ORF1b:R2613C, ORF3a:Q57H, ORF3a:G172V, ORF8:S24L, ORF8:I121T, S:D614G, S:Q677P,	
hCoV-19/Mexico/TAM-INER-IMSS-00200/2021	EPI_ISL_1279264	In process	20A	B.1.396	22	5'UTR:C241T, ORF1ab:C1191T, ORF1ab:G1458A, ORF1ab:C3037T, ORF1ab:C9521A, ORF1ab:C14408T, ORF1ab:C15848T, ORF1ab:C16349T, ORF1ab:C18183T, ORF1ab:A20268G, ORF1ab:C20790T, S:C23275T, S:A23403G, S:C24700T, S:C24904T, ORF3a:G25879T, M:T26609C, ORF7a:T27687C, ORF8:G28027A, N:C28854T, N:G29422T, ORF10:A29567G, 3'UTR:G29706C,	11	N:S194L, ORF1a:P309L, ORF1a:R398H, ORF1a:L3086I, ORF1b:P314L, ORF1b:T794I, ORF1b:S961L, ORF3a:V163L, ORF8:W45*, S:D614G,ORF8:I121*,	ORF8:28254-28256,
hCoV-19/Mexico/TAM-INER-IMSS-00208/2021	EPI_ISL_1279475	In process	21C (Epsilon)	B.1.429	24	5'UTR:C241T, ORF1ab:C1059T, ORF1ab:C2395T, ORF1ab:T2597C, ORF1ab:C3037T, ORF1ab:T6993C, ORF1ab:C8947T, ORF1ab:C12100T, ORF1ab:A12878G, ORF1ab:C14408T, ORF1ab:G17014T, S:G21600T, S:G22018T, S:T22917G, S:T23155C, S:A23403G, S:T24349C, ORF3a:G25563T, M:C26681T, ORF7b:G27890T, ORF8:G28191T, ORF8:A28272T, N:C28887T, N:G28975T. N:C29362T.	13	N:T205I, N:M234I, ORF1a:T265I, ORF1a:L2243S, ORF1a:I4205V, ORF1b:P314L, ORF1b:D1183Y, ORF3a:Q57H, ORF8:V100L, S:S13I, S:W152C, S:L452R, S:D614G,	
hCoV-19/Mexico/TAM-INER-IMSS-00209/2021	EPI_ISL_1279476	In process	20B	B.1.1.519	27	5'UTR:C203T, 5'UTR:C222T, 5'UTR:C241T, ORF1ab:C3037T, ORF1ab:C3140T, ORF1ab:C10029T, ORF1ab:C10954T, ORF1ab:A11117G, ORF1ab:C12789T, ORF1ab:C13019T, ORF1ab:C13720A, ORF1ab:C14408T, ORF1ab:G14829A, ORF1ab:T19378C, ORF1ab:T19839C, ORF1ab:C21306T, S:C22995A, S:A23403G, S:C23604A, S:A23756G, S:C25317T, M:G26775T, ORF8:T27904C, ORF8:C28087T, N:G28881A, N:G28882A, N:G28883C, N:C29197T,	18	M:A85S, N:R203K, N:G204R, ORF1a:P959S, ORF1a:T3255I, ORF1a:I3618V, ORF1a:T4175I, ORF1b:P85T, ORF1b:P314L, ORF1b:M454I, ORF1b:Y1971H, ORF8:L4P, ORF8:A65V, S:T478K, S:D614G, S:P681H, S:T732A, S:S1252F,	
hCoV-19/Mexico/TAM-INER-IMSS-00227/2021	EPI_ISL_1279492	In process	20B	B.1.1.519	25	5'UTR:C203T, 5'UTR:C222T, 5'UTR:C241T, ORF1ab:C3037T, ORF1ab:C3140T, ORF1ab:C10029T, ORF1ab:C10954T, ORF1ab:A11117G, ORF1ab:G11365T, ORF1ab:C12789T, ORF1ab:C13372T, ORF1ab:C14408T, ORF1ab:T19839C, ORF1ab:C21306T, S:C22995A, S:A23403G, S:C23604A, S:A23756G, S:C24418T, ORF8:T28062C, N:C28315T, N:G28881A, N:G28882A, N:G28883C, N:C29197T, N:G29527T,	13	N:R203K, N:G204R, N:Q418H, ORF1a:P959S, ORF1a:T3255I, ORF1a:I3618V, ORF1a:T4175I, ORF1b:P314L, ORF9b:A11V, S:T478K, S:D614G, S:P681H, S:T732A,	

hCoV-19/Mexico/TAM-INER-IMSS-00254/2021	EPI_ISL_1279516	In process	20B	B.1.1.519	23	5'UTR:C203T, 5'UTR:C222T, 5'UTR:C241T, ORF1ab:C3037T, ORF1ab:C3140T, ORF1ab:C10029T, ORF1ab:C10789T, ORF1ab:C10954T, ORF1ab:A11117G, ORF1ab:C12789T, ORF1ab:C14408T, ORF1ab:C16308T, ORF1ab:T19839C, ORF1ab:C21306T, S:C22995A, S:A23403G, S:C23604A, S:A23756G, ORF3a:C25658T, N:G28881A, N:G28882A, N:G28883C, N:C29197T, N:G29527T,	13	N:R203K, N:G204R, N:Q418H, ORF1a:P959S, ORF1a:T3255I, ORF1a:I3618V, ORF1a:T4175I, ORF1b:P314L, ORF3a:T89I, S:T478K, S:D614G, S:P681H, S:T732A,	
hCoV-19/Mexico/TAM-INER-IMSS-00255/2021	EPI_ISL_1279517	In process	20G	B.1.2	21	5'UTR:C241T, ORF1ab:C683T, ORF1ab:C1059T, ORF1ab:C1060T, ORF1ab:G2782T, ORF1ab:C3037T, ORF1ab:A6223G, ORF1ab:C10319T, ORF1ab:C14408T, ORF1ab:G17334T, ORF1ab:A17521G, ORF1ab:A18424G, ORF1ab:C21304T, S:G21724T, S:A23403G, ORF3a:G25563T, ORF3a:G25907T, ORF3a:C26058T, ORF8:C27964T, N:C28472T, N:C28869T, N:A29499T,	14	N:P67S, N:P199L, N:Q409L, ORF1a:T265I, ORF1a:L3352F, ORF1b:P314L, ORF1b:M1352V, ORF1b:N1653D, ORF1b:R2613C, ORF3a:Q57H, ORF3a:G172V, ORF8:S24L, S:L54F, S:D614G,	
hCoV-19/Mexico/TAM-INER-IMSS-00259/2021	EPI_ISL_1279521	In process	20B	B.1.1.519	22	5'UTR:C203T, 5'UTR:C222T, 5'UTR:C241T, ORF1ab:C3037T, ORF1ab:C3140T, ORF1ab:C10029T, ORF1ab:C10789T, ORF1ab:C10954T, ORF1ab:A11117G, ORF1ab:C12789T, ORF1ab:C14408T, ORF1ab:T19839C, ORF1ab:C21306T, S:C22995A, S:A23403G, S:C23604A, S:A23756G, ORF3a:C25658T, N:G28881A, N:G28882A, N:G28883C, N:C29197T, N:G29527T,	13	N:R203K, N:G204R, N:Q418H, ORF1a:P959S, ORF1a:T3255I, ORF1a:I3618V, ORF1a:T4175I, ORF1b:P314L, ORF3a:T89I, S:T478K, S:D614G, S:P681H, S:T732A,	
hCoV-19/Mexico/TAM-INER-IMSS-00268/2021	EPI_ISL_1279527	In process	20A	B.1.243	18	5'UTR:C241T, ORF1ab:G2830T, ORF1ab:C3037T, ORF1ab:C3096T, ORF1ab:C4113T, ORF1ab:G9203A, ORF1ab:C10741T, ORF1ab:C14408T, ORF1ab:C19097T, ORF1ab:A19137G, ORF1ab:A20268G, ORF1ab:C20759T, S:C21627T, S:A23403G, S:C23604A, S:T24076C, N:C28854T, 3'UTR:T29710C, 3'UTR:C29733T,	11	N:S194L, ORF1a:E855D, ORF1a:S944L, ORF1a:A1283V, ORF1a:D2980N, ORF1b:P314L, ORF1b:A1877V, ORF1b:A2431V, S:T22I, S:D614G, S:P681H,	
hCoV-19/Mexico/TAM-INER-IMSS-00276/2021	EPI_ISL_1279534	In process	20B	B.1.1.519	22	5'UTR:C203T, 5'UTR:C222T, 5'UTR:C241T, ORF1ab:C3037T, ORF1ab:C3140T, ORF1ab:C10029T, ORF1ab:C10789T, ORF1ab:C10954T, ORF1ab:A11117G, ORF1ab:C12789T, ORF1ab:C14408T, ORF1ab:T19839C, ORF1ab:C21306T, S:C22995A, S:A23403G, S:C23604A, S:A23756G, ORF3a:C25658T, N:G28881A, N:G28882A, N:G28883C, N:C29197T, N:G29527T,	13	N:R203K, N:G204R, N:Q418H, ORF1a:P959S, ORF1a:T3255I, ORF1a:I3618V, ORF1a:T4175I, ORF1b:P314L, ORF3a:T89I, S:T478K, S:D614G, S:P681H, S:T732A,	
hCoV-19/Mexico/TAM-INER-IMSS-00277/2021	EPI_ISL_1279535	In process	20B	B.1.1.519	26	5'UTR:C203T, 5'UTR:C222T, 5'UTR:C241T, ORF1ab:C1385T, ORF1ab:C3037T, ORF1ab:C3140T, ORF1ab:C4276T, ORF1ab:T6574C, ORF1ab:C10029T, ORF1ab:C10954T, ORF1ab:A11117G, ORF1ab:C12789T, ORF1ab:C14408T, ORF1ab:C15738T, ORF1ab:G18090A, ORF1ab:T19839C, ORF1ab:C20629T, ORF1ab:C21306T, S:C22995A, S:A23403G, S:C23604A, S:A23756G, ORF3a:G25471T, N:G28881A, N:G28882A, N:G28883C, N:C29197T,	14	N:R203K, N:G204R, ORF1a:H374Y, ORF1a:P959S, ORF1a:T3255I, ORF1a:I3618V, ORF1a:T4175I, ORF1b:P314L, ORF1b:H2388Y, ORF3a:D27Y, S:T478K, S:D614G, S:P681H, S:T732A,	
hCoV-19/Mexico/TAM-INER-IMSS-00278/2021	EPI_ISL_1287773	In process	20B	B.1.1.222	20	5'UTR:C241T, ORF1ab:G872A, ORF1ab:C3037T, ORF1ab:C6285T, ORF1ab:G7273A, ORF1ab:A13498G, ORF1ab:G14118A, ORF1ab:C14408T, ORF1ab:C17750T, ORF1ab:C18377T, ORF1ab:G18412T, ORF1ab:T19839C, S:G22331A, S:A23403G, S:A23756G, ORF3a:T25851G, ORF7a:G27478T, N:G28881A, N:G28882A, N:G28883C, ORF10:T29623C,	14	N:R203K, N:G204R, ORF1a:D203N, ORF1a:T2007I, ORF1b:T11A, ORF1b:P314L, ORF1b:A1428V, ORF1b:T1637I, ORF1b:V1649F, ORF3a:C153W, ORF7a:V29L, S:G257S, S:D614G, S:T732A,	ORF7b:27879-27891, ORF8:27915-28225,

hCoV-19/Mexico/TLA-IBT-IMSS-137/2021	EPI_ISL_1288153	In process	20B	B.1.1.519	25	5'UTR:T201C, 5'UTR:C203T, 5'UTR:C222T, 5'UTR:C241T, ORF1ab:C745T, ORF1ab:G1738T, ORF1ab:C3037T, ORF1ab:C3140T, ORF1ab:C10029T, ORF1ab:C10954T, ORF1ab:A11117G, ORF1ab:C12789T, ORF1ab:C14408T, ORF1ab:T19839C, ORF1ab:A19974G, ORF1ab:C21306T, S:C22995A, S:A23403G, S:C23604A, S:A23756G, ORF3a:C26060T, ORF6:C27213T, N:G28881A, N:G28882A, N:G28883C, N:C29197T,	12	N:R203K, N:G204R, ORF1a:P959S, ORF1a:T3255I, ORF1a:I3618V, ORF1a:T4175I, ORF1b:P314L, ORF3a:T223I, S:T478K, S:D614G, S:P681H, S:T732A,
hCoV-19/Mexico/TLA-IBT-IMSS-253/2021	EPI_ISL_1288441	In process	20B	B.1.1.519	25	5'UTR:T201C, 5'UTR:C203T, 5'UTR:C222T, 5'UTR:C241T, ORF1ab:A1004G, ORF1ab:G1738T, ORF1ab:C3037T, ORF1ab:C3140T, ORF1ab:G3753A, ORF1ab:C10029T, ORF1ab:C10954T, ORF1ab:A11117G, ORF1ab:C12789T, ORF1ab:C14408T, ORF1ab:G18020A, ORF1ab:T19839C, ORF1ab:A19974G, ORF1ab:C21306T, S:C22995A, S:A23403G, S:C23604A, S:A23756G, N:G28881A, N:G28882A, N:G28883C, N:C29197T,	14	N:R203K, N:G204R, ORF1a:K247E, ORF1a:P959S, ORF1a:R1163K, ORF1a:T3255I, ORF1a:I3618V, ORF1a:T4175I, ORF1b:P314L, ORF1b:R1518K, S:T478K, S:D614G, S:P681H, S:T732A,
hCoV-19/Mexico/TLA-IBT-IMSS-31/2021	EPI_ISL_1288250	In process	20B	B.1.1.519	18	5'UTR:C203T, 5'UTR:C222T, 5'UTR:C241T, ORF1ab:C3037T, ORF1ab:C3140T, ORF1ab:C10029T, ORF1ab:C10954T, ORF1ab:A11117G, ORF1ab:C11124T, ORF1ab:C14408T, ORF1ab:T19839C, S:C22995A, S:A23403G, S:C23604A, S:A23756G, N:G28881A, N:G28882A, N:G28883C, N:C29197T,	11	N:R203K, N:G204R, ORF1a:P959S, ORF1a:T3255I, ORF1a:I3618V, ORF1a:A3620V, ORF1b:P314L, S:T478K, S:D614G, S:P681H, S:T732A,
hCoV-19/Mexico/TLA-IBT-IMSS-32/2021	EPI_ISL_1288251	In process	20B	B.1.1.519	27	5'UTR:T201C, 5'UTR:C203T, 5'UTR:C222T, 5'UTR:C241T, ORF1ab:C745T, ORF1ab:G1738T, ORF1ab:A2903G, ORF1ab:C3037T, ORF1ab:C3140T, ORF1ab:G3671A, ORF1ab:C3695T, ORF1ab:C10029T, ORF1ab:C10543T, ORF1ab:C10954T, ORF1ab:A11117G, ORF1ab:C12789T, ORF1ab:C14408T, ORF1ab:T19839C, ORF1ab:A19974G, ORF1ab:C21306T, S:C22995A, S:A23403G, S:C23604A, S:A23756G, N:G28881A, N:G28882A, N:G28883C, N:C29197T,	13	N:R203K, N:G204R, ORF1a:I880V, ORF1a:P959S, ORF1a:E1136K, ORF1a:T3255I, ORF1a:I3618V, ORF1a:T4175I, ORF1b:P314L, S:T478K, S:D614G, S:P681H, S:T732A,
hCoV-19/Mexico/TLA-IBT-IMSS-33/2021	EPI_ISL_1288252	In process	20B	B.1.1.519	27	5'UTR:T201C, 5'UTR:C203T, 5'UTR:C222T, 5'UTR:C241T, ORF1ab:C745T, ORF1ab:G1738T, ORF1ab:A2903G, ORF1ab:C3037T, ORF1ab:C3140T, ORF1ab:G3671A, ORF1ab:C3695T, ORF1ab:C10029T, ORF1ab:C10543T, ORF1ab:C10954T, ORF1ab:A11117G, ORF1ab:C12789T, ORF1ab:C14408T, ORF1ab:T19839C, ORF1ab:A19974G, ORF1ab:C21306T, S:C22995A, S:A23403G, S:C23604A, S:A23756G, N:G28881A, N:G28882A, N:G28883C, N:C29197T,	13	N:R203K, N:G204R, ORF1a:I880V, ORF1a:P959S, ORF1a:E1136K, ORF1a:T3255I, ORF1a:I3618V, ORF1a:T4175I, ORF1b:P314L, S:T478K, S:D614G, S:P681H, S:T732A,
hCoV-19/Mexico/TLA-IBT-IMSS-34/2021	EPI_ISL_1288253	In process	20B	B.1.1.519	26	5'UTR:T201C, 5'UTR:C203T, 5'UTR:C222T, 5'UTR:C241T, ORF1ab:G1738T, ORF1ab:C3037T, ORF1ab:C3140T, ORF1ab:C7279T, ORF1ab:C10029T, ORF1ab:C10954T, ORF1ab:A11117G, ORF1ab:C12789T, ORF1ab:C14408T, ORF1ab:T14711C, ORF1ab:T19839C, ORF1ab:A19974G, ORF1ab:G20788A, ORF1ab:C21306T, S:C22995A, S:A23403G, S:C23604A, S:A23756G, M:G26828T, N:G28881A, N:G28882A, N:G28883C, N:C29197T,	13	N:R203K, N:G204R, ORF1a:P959S, ORF1a:T3255I, ORF1a:I3618V, ORF1a:T4175I, ORF1b:P314L, ORF1b:V415A, ORF1b:V2441I, S:T478K, S:D614G, S:P681H, S:T732A,
hCoV-19/Mexico/TLA-IBT-IMSS-429/2020	EPI_ISL_1301622	In process	20C	B.1	9	5'UTR:C241T, ORF1ab:C1059T, ORF1ab:C3037T, ORF1ab:C5822T, ORF1ab:G11083T, ORF1ab:C14408T, ORF1ab:C19813T, S:A23403G, ORF3a:G25563T, M:C26645T,	7	ORF1a:T265I, ORF1a:L1853F, ORF1a:L3606F, ORF1b:P314L, ORF1b:P2116S, ORF3a:Q57H, S:D614G,
hCoV-19/Mexico/TLA-IBT-IMSS-440/2020	EPI_ISL_1301627	In process	20C	B.1	7	5'UTR:C241T, ORF1ab:C1059T, ORF1ab:C3037T, ORF1ab:C11916T, ORF1ab:C12043T, ORF1ab:C14408T, S:A23403G, ORF3a:G25563T,	5	ORF1a:T265I, ORF1a:S3884L, ORF1b:P314L, ORF3a:Q57H, S:D614G,

hCoV-19/Mexico/TLA-IBT-IMSS-444/2020	EPI_ISL_1301467	In process	20C	B.1	6	5'UTR:C241T, ORF1ab:C1059T, ORF1ab:C3037T, ORF1ab:C4878T, ORF1ab:C14408T, S:A23403G, ORF3a:G25563T,	5	ORF1a:T265I, ORF1a:T1538I, ORF1b:P314L, ORF3a:Q57H, S:D614G,	
hCoV-19/Mexico/TLA-IBT-IMSS-448/2020	EPI_ISL_1301484	In process	20B	B.1.1.322	11	5'UTR:C241T, ORF1ab:C3037T, ORF1ab:C8655T, ORF1ab:C14408T, ORF1ab:T19839C, S:A23403G, S:A23756G, N:A28877T, N:G28878C, N:G28881A, N:G28882A, N:G28883C,	6	N:R203K, N:G204R, ORF1a:S2797F, ORF1b:P314L, S:D614G, S:T732A,	
hCoV-19/Mexico/TLA-InDRE-IBT-16/2020	EPI_ISL_1301540	In process	20A	B.1.609	9	5'UTR:C241T, ORF1ab:C3037T, ORF1ab:C4582T, ORF1ab:C4633T, ORF1ab:C8175T, ORF1ab:T8263A, ORF1ab:C14408T, S:C23029T, S:A23403G, S:C24370T,	3	ORF1a:A2637V, ORF1b:P314L, S:D614G,	
hCoV-19/Mexico/TLA-InDRE-IBT-68/2020	EPI_ISL_1301556	In process	20A	B.1.243	8	5'UTR:C241T, ORF1ab:C3037T, ORF1ab:C14408T, S:G22335T, S:A23403G, S:T24076C, M:G26526T, ORF7a:T27462C, N:C28854T,	5	M:A2S, N:S194L, ORF1b:P314L, S:W258L, S:D614G,	
hCoV-19/Mexico/TLA-InDRE-IBT-69/2020	EPI_ISL_1301557	In process	20B	B.1.1.222	11	5'UTR:C241T, ORF1ab:C3037T, ORF1ab:C8809T, ORF1ab:C12973T, ORF1ab:C14408T, ORF1ab:T19839C, S:A23403G, S:A23756G, N:G28881A, N:G28882A, N:G28883C, ORF10:C29614T,	5	N:R203K, N:G204R, ORF1b:P314L, S:D614G, S:T732A,	
hCoV-19/Mexico/TLA-InDRE-IBT-70/2020	EPI_ISL_1301546	In process	20A	B.1	7	5'UTR:C241T, ORF1ab:C3037T, ORF1ab:C4582T, ORF1ab:T12232C, ORF1ab:C14408T, S:A23403G, M:G26828T, N:C28344T,	3	N:T24I, ORF1b:P314L, S:D614G,	
hCoV-19/Mexico/TLA-InDRE-IBT-71/2020	EPI_ISL_1301558	In process	20C	B.1	14	5'UTR:C241T, ORF1ab:C1059T, ORF1ab:C3037T, ORF1ab:C11916T, ORF1ab:A12577G, ORF1ab:C14408T, ORF1ab:C18998T, ORF1ab:C19884T, S:G22708T, S:A23403G, S:C24157T, S:G24794T, ORF3a:G25563T, N:G28851T, N:G29540A,	8	N:S193I, ORF1a:T265I, ORF1a:S3884L, ORF1b:P314L, ORF1b:A1844V, ORF3a:Q57H, S:D614G, S:A1078S,	
hCoV-19/Mexico/TLA-InDRE-IBT-72/2020	EPI_ISL_1301691	In process	20B	B.1.1	8	5'UTR:C241T, ORF1ab:C3037T, ORF1ab:G11083T, ORF1ab:C14408T, ORF1ab:T19839C, S:A23403G, N:G28881A, N:G28882A, N:G28883C,	5	N:R203K, N:G204R, ORF1a:L3606F, ORF1b:P314L, S:D614G,	
hCoV-19/Mexico/TLA-InDRE-IBT-73/2020	EPI_ISL_1301692	In process	20A	B.1.189	8	5'UTR:C241T, ORF1ab:C3037T, ORF1ab:C4582T, ORF1ab:G6404T, ORF1ab:C14408T, ORF1ab:A20268G, S:A23403G, S:T25123C, N:G28378T,	4	ORF1a:V2047F, ORF1b:P314L, ORF9b:R32L, S:D614G,	
hCoV-19/Mexico/TLA-InDRE-IBT-74/2020	EPI_ISL_1301542	In process	20A	B.1.609	5	5'UTR:C241T, ORF1ab:C3037T, ORF1ab:C4582T, ORF1ab:C13119T, ORF1ab:C14408T, S:A23403G,	3	ORF1a:A4285V, ORF1b:P314L, S:D614G,	
hCoV-19/Mexico/TLA-InDRE-IBT-75/2020	EPI_ISL_1301531	In process	20A	B.1.1	9	5'UTR:C241T, ORF1ab:T1237C, ORF1ab:C3037T, ORF1ab:C14408T, ORF1ab:T19839C, S:G22021T, S:A23403G, ORF8:T28026C, N:G29254T, 3'UTR:G29773T,	4	ORF1b:P314L, ORF8:W45R, S:M153I, S:D614G,	
hCoV-19/Mexico/TLA-InDRE-IBT-76/2020	EPI_ISL_1301693	In process	20A	B.1	10	5'UTR:C241T, ORF1ab:C3037T, ORF1ab:C7488T, ORF1ab:C12970T, ORF1ab:C14408T, ORF1ab:G16122T, ORF1ab:T16906C, ORF1ab:A20268G, ORF1ab:G21372T, S:G21989T, S:A23403G,	7	ORF1a:T2408I, ORF1b:P314L, ORF1b:E885D, ORF1b:Y1147H, ORF1b:Q2635H, S:V143F, S:D614G,	
hCoV-19/Mexico/TLA-INER-IMSS-00283/2021	EPI_ISL_1287775	In process	20B	B.1.1.519	28	5'UTR:T201C, 5'UTR:C203T, 5'UTR:C222T, 5'UTR:C241T, ORF1ab:G1738T, ORF1ab:C3037T, ORF1ab:C3140T, ORF1ab:C4999T, ORF1ab:C10029T, ORF1ab:C10626T, ORF1ab:C10954T, ORF1ab:A11117G, ORF1ab:C12733T, ORF1ab:C12789T, ORF1ab:C14408T, ORF1ab:G14559T, ORF1ab:T19839C, ORF1ab:A19974G, ORF1ab:C21306T, S:C22995A, S:A23403G, S:C23604A, S:A23756G, ORF3a:G25699A, ORF7a:C27493T, N:G28881A, N:G28882A, N:G28883C, N:C29197T,	13	N:R203K, N:G204R, ORF1a:P959S, ORF1a:T3255I, ORF1a:A3454V, ORF1a:I3618V, ORF1a:T4175I, ORF1b:P314L, ORF3a:A103T, S:T478K, S:D614G, S:P681H, S:T732A,	ORF7a:27619-27628

hCoV-19/Mexico/TLA-INER-IMSS-00284/2021	EPI_ISL_1279539	In process	20B	B.1.1.519	28	5'UTR:C203T, 5'UTR:C222T, 5'UTR:C241T, ORF1ab:C3037T, ORF1ab:C3140T, ORF1ab:G3692T, ORF1ab:G3871T, ORF1ab:C6429A, ORF1ab:C9491T, ORF1ab:C10029T, ORF1ab:C10954T, ORF1ab:A11117G, ORF1ab:C11824A, ORF1ab:C12789T, ORF1ab:C14408T, ORF1ab:T19839C, ORF1ab:C21304T, ORF1ab:C21306T, S:C22995A, S:A23403G, S:C23604A, S:A23756G, S:C23758T, ORF8:T27904C, ORF8:G28077A, N:G28881A, N:G28882A, N:G28883C, N:C29197T,	18	N:R203K, N:G204R, ORF1a:P959S, ORF1a:V1143F, ORF1a:K1202N, ORF1a:P2055H, ORF1a:H3076Y, ORF1a:T3255I, ORF1a:I3618V, ORF1a:T4175I, ORF1b:P314L, ORF1b:R2613C, ORF8:L4P, ORF8:V62M, S:T478K, S:D614G, S:P681H, S:T732A,	
hCoV-19/Mexico/TLA-INER-IMSS-00285/2021	EPI_ISL_1279540	In process	20B	B.1.1.519	25	5'UTR:C203T, 5'UTR:C222T, 5'UTR:C241T, ORF1ab:A866G, ORF1ab:C1009T, ORF1ab:C3037T, ORF1ab:C3140T, ORF1ab:C5183T, ORF1ab:C10029T, ORF1ab:C10954T, ORF1ab:A11117G, ORF1ab:C11916T, ORF1ab:G12223T, ORF1ab:C12789T, ORF1ab:C14408T, ORF1ab:T19839C, ORF1ab:C21306T, S:C22995A, S:A23403G, S:C23604A, S:A23756G, ORF8:T27904C, N:G28881A, N:G28882A, N:G28883C, N:C29197T,	15	N:R203K, N:G204R, ORF1a:I201V, ORF1a:P959S, ORF1a:P1640S, ORF1a:T3255I, ORF1a:I3618V, ORF1a:S3884L, ORF1a:T4175I, ORF1b:P314L, ORF8:L4P, S:T478K, S:D614G, S:P681H, S:T732A,	
hCoV-19/Mexico/VER-IBT-IMSS-100/2021	EPI_ISL_1288311	In process	20B	B.1.1.519	23	5'UTR:C203T, 5'UTR:C222T, 5'UTR:C241T, ORF1ab:C3037T, ORF1ab:C3140T, ORF1ab:C10029T, ORF1ab:C10954T, ORF1ab:A11117G, ORF1ab:C12789T, ORF1ab:C14408T, ORF1ab:G15921T, ORF1ab:A17971G, ORF1ab:T19839C, ORF1ab:C21306T, S:C22995A, S:A23403G, S:C23604A, S:C23635T, S:A23756G, M:G26828T, N:G28881A, N:G28882A, N:G28883C, N:C29197T,	12	N:R203K, N:G204R, ORF1a:P959S, ORF1a:T3255I, ORF1a:I3618V, ORF1a:T4175I, ORF1b:P314L, ORF1b:R1502G, S:T478K, S:D614G, S:P681H, S:T732A,	S:21992-21994,
hCoV-19/Mexico/VER-IBT-IMSS-105/2021	EPI_ISL_1288315	In process	20B	B.1.1.519	25	5'UTR:C203T, 5'UTR:C222T, 5'UTR:C241T, ORF1ab:T689C, ORF1ab:C3037T, ORF1ab:C3140T, ORF1ab:C3874T, ORF1ab:A3881T, ORF1ab:C10029T, ORF1ab:C10954T, ORF1ab:A11117G, ORF1ab:G11365T, ORF1ab:C12789T, ORF1ab:C14408T, ORF1ab:T19839C, ORF1ab:A20245G, ORF1ab:C21306T, S:C22995A, S:A23403G, S:C23604A, S:A23756G, N:G28881A, N:G28882A, N:G28883C, N:C29197T, N:G29527T,	15	N:R203K, N:G204R, N:Q418H, ORF1a:S142P, ORF1a:P959S, ORF1a:I1206F, ORF1a:T3255I, ORF1a:I3618V, ORF1a:T4175I, ORF1b:P314L, ORF1b:M2260V, S:T478K, S:D614G, S:P681H, S:T732A,	
hCoV-19/Mexico/VER-IBT-IMSS-110/2021	EPI_ISL_1288319	In process	20B	B.1.1.519	27	5'UTR:T201C, 5'UTR:C203T, 5'UTR:G210T, 5'UTR:C222T, 5'UTR:C241T, ORF1ab:G1738T, ORF1ab:C3037T, ORF1ab:C3140T, ORF1ab:T3913A, ORF1ab:C10029T, ORF1ab:C10954T, ORF1ab:A11117G, ORF1ab:C12789T, ORF1ab:C13384T, ORF1ab:C14408T, ORF1ab:C17339T, ORF1ab:T19839C, ORF1ab:A19974G, ORF1ab:T20859C, ORF1ab:C21306T, S:C22995A, S:A23403G, S:C23604A, S:A23756G, N:G28881A, N:G28882A, N:G28883C, N:C29197T	12	N:R203K, N:G204R, ORF1a:P959S, ORF1a:T3255I, ORF1a:I3618V, ORF1a:T4175I, ORF1b:P314L, ORF1b:A1291V, S:T478K, S:D614G, S:P681H, S:T732A,	
hCoV-19/Mexico/VER-IBT-IMSS-111/2021	EPI_ISL_1288320	In process	20B	B.1	17	5'UTR:C241T, ORF1ab:G2305T, ORF1ab:G2755T, ORF1ab:C3037T, ORF1ab:A6985T, ORF1ab:G8102T, ORF1ab:C9319T, ORF1ab:C12412T, ORF1ab:T14313C, ORF1ab:C14408T, ORF1ab:C18657T, ORF1ab:C20016T, ORF1ab:C20178T, S:C23127T, S:A23403G, ORF3a:C25613T, ORF3a:G25912T, N:T29317C,	7	ORF1a:K680N, ORF1a:V2613F, ORF1b:P314L, ORF3a:S74F, ORF3a:G174C, S:A522V, S:D614G,	N:28880-28897

hCoV-19/Mexico/VER-IBT-IMSS-112/2021	EPI_ISL_1288321	In process	20B	B.1.1.519	27	5'UTR:T201C, 5'UTR:C203T, 5'UTR:G210T, 5'UTR:C222T, 5'UTR:C241T, ORF1ab:G1738T, ORF1ab:C3037T, ORF1ab:C3140T, ORF1ab:T3913A, ORF1ab:C10029T, ORF1ab:C10954T, ORF1ab:A11117G, ORF1ab:C12789T, ORF1ab:C13384T, ORF1ab:C14408T, ORF1ab:C17339T, ORF1ab:T19839C, ORF1ab:A19974G, ORF1ab:T20859C, ORF1ab:C21306T, S:C22995A, S:A23403G, S:C23604A, S:A23756G, N:G28881A, N:G28882A, N:G28883C, N:C29197T,	12	N:R203K, N:G204R, ORF1a:P959S, ORF1a:T3255I, ORF1a:I3618V, ORF1a:T4175I, ORF1b:P314L, ORF1b:A1291V, S:T478K, S:D614G, S:P681H, S:T732A,
hCoV-19/Mexico/VER-IBT-IMSS-113/2021	EPI_ISL_1288322	In process	20B	B.1.1.519	27	5'UTR:C203T, 5'UTR:C222T, 5'UTR:C241T, ORF1ab:T2484C, ORF1ab:C3037T, ORF1ab:C3140T, ORF1ab:A3581G, ORF1ab:C8334T, ORF1ab:C8655T, ORF1ab:C9169T, ORF1ab:C10029T, ORF1ab:C10954T, ORF1ab:A11117G, ORF1ab:C14408T, ORF1ab:T18151C, ORF1ab:T19839C, ORF1ab:G20002T, ORF1ab:C21306T, S:C22995A, S:A23403G, S:C23604A, S:A23756G, M:C26882T, ORF8:T27904C, N:G28881A, N:G28882A, N:G28883C, N:C29197T,	16	N:R203K, N:G204R, ORF1a:I740T, ORF1a:P959S, ORF1a:S1106G, ORF1a:A2690V, ORF1a:S2797F, ORF1a:T3255I, ORF1a:I3618V, ORF1b:P314L, ORF1b:D2179Y, ORF8:L4P, S:T478K, S:D614G, S:P681H, S:T732A,
hCoV-19/Mexico/VER-IBT-IMSS-115/2021	EPI_ISL_1288323	In process	20B	B.1.1.519	28	5'UTR:T201C, 5'UTR:C203T, 5'UTR:C222T, 5'UTR:C241T, ORF1ab:C745T, ORF1ab:G1738T, ORF1ab:C3037T, ORF1ab:C3140T, ORF1ab:A5212G, ORF1ab:C6541T, ORF1ab:C10029T, ORF1ab:C10954T, ORF1ab:A11117G, ORF1ab:C12789T, ORF1ab:C14408T, ORF1ab:G15850C, ORF1ab:T19839C, ORF1ab:A19974G, ORF1ab:C21306T, S:G21724T, S:C22995A, S:A23403G, S:C23604A, S:A23756G, ORF8:G28077T, N:G28881A, N:G28882A, N:G28883C, N:C29197T,	14	N:R203K, N:G204R, ORF1a:P959S, ORF1a:T3255I, ORF1a:I3618V, ORF1a:T4175I, ORF1b:P314L, ORF1b:D795H, ORF8:V62L, S:L54F, S:T478K, S:D614G, S:P681H, S:T732A,
hCoV-19/Mexico/VER-IBT-IMSS-116/2021	EPI_ISL_1288324	In process	20B	B.1.1.519	26	5'UTR:C203T, 5'UTR:C222T, 5'UTR:C241T, ORF1ab:T689C, ORF1ab:C3037T, ORF1ab:C3140T, ORF1ab:C3874T, ORF1ab:A3881T, ORF1ab:C10029T, ORF1ab:C10954T, ORF1ab:A11117G, ORF1ab:G11365T, ORF1ab:C12789T, ORF1ab:C14408T, ORF1ab:G18021A, ORF1ab:T19839C, ORF1ab:A20245G, ORF1ab:C21306T, S:C22995A, S:A23403G, S:C23604A, S:A23756G, N:G28881A, N:G28882A, N:G28883C, N:C29197T, N:G29527T,	15	N:R203K, N:G204R, N:Q418H, ORF1a:S142P, ORF1a:P959S, ORF1a:I1206F, ORF1a:T3255I, ORF1a:I3618V, ORF1a:T4175I, ORF1b:P314L, ORF1b:M2260V, S:T478K, S:D614G, S:P681H, S:T732A,
hCoV-19/Mexico/VER-IBT-IMSS-117/2021	EPI_ISL_1288325	In process	20B	B.1.1.519	23	5'UTR:C203T, 5'UTR:C222T, 5'UTR:C241T, ORF1ab:C3037T, ORF1ab:C3140T, ORF1ab:C10029T, ORF1ab:C10954T, ORF1ab:A11117G, ORF1ab:C12789T, ORF1ab:C14408T, ORF1ab:G17149A, ORF1ab:T19839C, ORF1ab:C21306T, S:C22995A, S:A23403G, S:C23604A, S:A23756G, ORF3a:C25658T, ORF3a:C26111T, N:G28881A, N:G28882A, N:G28883C, N:C29197T, N:G29315C,	15	N:R203K, N:G204R, N:D348H, ORF1a:P959S, ORF1a:T3255I, ORF1a:I3618V, ORF1a:T4175I, ORF1b:P314L, ORF1b:V1228M, ORF3a:T89I, ORF3a:P240L, S:T478K, S:D614G, S:P681H, S:T732A,
hCoV-19/Mexico/VER-IBT-IMSS-150/2021	EPI_ISL_1288352	In process	20B	B.1.1.519	23	5'UTR:C203T, 5'UTR:C222T, 5'UTR:C241T, ORF1ab:C3037T, ORF1ab:C3140T, ORF1ab:C10029T, ORF1ab:T10264C, ORF1ab:A10323G, ORF1ab:C10954T, ORF1ab:A11117G, ORF1ab:C12789T, ORF1ab:C14408T, ORF1ab:C15960T, ORF1ab:T19839C, ORF1ab:C21306T, S:C22995A, S:A23403G, S:C23604A, S:A23756G, S:C24023T, N:G28881A, N:G28882A, N:G28883C, N:C29197T,	12	N:R203K, N:G204R, ORF1a:P959S, ORF1a:T3255I, ORF1a:K3353R, ORF1a:I3618V, ORF1a:T4175I, ORF1b:P314L, S:T478K, S:D614G, S:P681H, S:T732A,

hCoV-19/Mexico/VER-IBT-IMSS-151/2021	EPI_ISL_1288353	In process	20A	B.1.243	18	5'UTR:C241T, ORF1ab:G806A, ORF1ab:G872A, ORF1ab:A1291G, ORF1ab:T2584A, ORF1ab:C3037T, ORF1ab:G3638T, ORF1ab:C5140A, ORF1ab:T7341C, ORF1ab:C14408T, ORF1ab:G16636T, ORF1ab:A20268G, ORF1ab:G21123T, S:A23403G, S:G23587C, S:T24076C, ORF3a:C25844T, N:C28854T, N:G29543T,	11	N:S194L, ORF1a:A181T, ORF1a:D203N, ORF1a:G1125C, ORF1a:D1625E, ORF1a:I2359T, ORF1b:P314L, ORF1b:A1057S, ORF3a:T151I, S:D614G, S:Q675H,	
hCoV-19/Mexico/VER-IBT-IMSS-153/2021	EPI_ISL_1288355	In process	20B	B.1.1.519	25	5'UTR:C203T, 5'UTR:C222T, 5'UTR:C241T, ORF1ab:C3037T, ORF1ab:C3140T, ORF1ab:T3745C, ORF1ab:C10029T, ORF1ab:C10954T, ORF1ab:A11117G, ORF1ab:C12789T, ORF1ab:C14408T, ORF1ab:T19839C, ORF1ab:C21306T, S:C22264T, S:A22525G, S:C22995A, S:C23191T, S:A23403G, S:C23604A, S:G23612A, S:A23756G, ORF3a:C25844T, N:G28881A, N:G28882A, N:G28883C, N:C29197T,	13	N:R203K, N:G204R, ORF1a:P959S, ORF1a:T3255I, ORF1a:I3618V, ORF1a:T4175I, ORF1b:P314L, ORF3a:T151I, S:T478K, S:D614G, S:P681H, S:A684T, S:T732A,	
hCoV-19/Mexico/VER-IBT-IMSS-156/2021	EPI_ISL_1288358	In process	20B	B.1.1.519	26	5'UTR:C203T, 5'UTR:C222T, 5'UTR:C241T, ORF1ab:C3037T, ORF1ab:C3140T, ORF1ab:G9190T, ORF1ab:C10029T, ORF1ab:C10954T, ORF1ab:A11117G, ORF1ab:C12789T, ORF1ab:C14408T, ORF1ab:G18020A, ORF1ab:T19839C, ORF1ab:C21306T, S:C22995A, S:A23403G, S:C23604A, S:A23756G, ORF7a:T27560A, ORF8:T27904C, ORF8:C28087T, N:G28881A, N:G28882A, N:G28883C, N:G28884T, N:C29037T, N:C29197T,	16	N:R203K, N:G204L, N:S255F, ORF1a:P959S, ORF1a:T3255I, ORF1a:I3618V, ORF1a:T4175I, ORF1b:P314L, ORF1b:R1518K, ORF7a:L56Q, ORF8:L4P, ORF8:A65V, S:T478K, S:D614G, S:P681H, S:T732A,	
hCoV-19/Mexico/VER-IBT-IMSS-157/2021	EPI_ISL_1288359	In process	20B	B.1.1.519	24	5'UTR:C203T, 5'UTR:C222T, 5'UTR:C241T, ORF1ab:C3037T, ORF1ab:C3140T, ORF1ab:C5055T, ORF1ab:C6720T, ORF1ab:G8768T, ORF1ab:C8969T, ORF1ab:C10029T, ORF1ab:C10954T, ORF1ab:A11117G, ORF1ab:C12789T, ORF1ab:C14408T, ORF1ab:T19839C, ORF1ab:C21306T, S:C22995A, S:A23403G, S:C23520T, S:C23604A, S:A23756G, N:G28881A, N:G28882A, N:G28883C, N:C29197T,	16	N:R203K, N:G204R, ORF1a:P959S, ORF1a:T1597I, ORF1a:T2152I, ORF1a:D2835Y, ORF1a:L2902F, ORF1a:T3255I, ORF1a:I3618V, ORF1a:T4175I, ORF1b:P314L, S:T478K, S:D614G, S:A653V, S:P681H, S:T732A,	
hCoV-19/Mexico/VER-IBT-IMSS-158/2021	EPI_ISL_1288360	In process	20B	B.1.1.519	23	5'UTR:C203T, 5'UTR:C222T, 5'UTR:C241T, ORF1ab:C3037T, ORF1ab:C3140T, ORF1ab:G7027T, ORF1ab:C10029T, ORF1ab:C10954T, ORF1ab:A11117G, ORF1ab:C12789T, ORF1ab:C14408T, ORF1ab:C16323T, ORF1ab:C16375T, ORF1ab:T19839C, ORF1ab:C21306T, S:C22995A, S:A23403G, S:C23604A, S:A23756G, S:C25207T, N:G28881A, N:G28882A, N:G28883C, N:C29197T,	13	N:R203K, N:G204R, ORF1a:P959S, ORF1a:M2254I, ORF1a:T3255I, ORF1a:I3618V, ORF1a:T4175I, ORF1b:P314L, ORF1b:P970S, S:T478K, S:D614G, S:P681H, S:T732A,	
hCoV-19/Mexico/VER-IBT-IMSS-160/2021	EPI_ISL_1288361	In process	20A	B.1	21	5'UTR:C241T, ORF1ab:C2455T, ORF1ab:C3037T, ORF1ab:A3904T, ORF1ab:A11430G, ORF1ab:C14408T, ORF1ab:A16534G, ORF1ab:T17673G, ORF1ab:G20060T, ORF1ab:A20268G, S:C21618T, S:C21707T, S:C22338G, S:T22912G, S:G22992A, S:A23403G, M:C26681T, M:G26709A, ORF7a:C27494T, ORF8:A28273T. N:G28514T. N:G28917T.	15	M:A63T, N:D81Y, N:G215V, ORF1a:Y3722C, ORF1b:P314L, ORF1b:S1023G, ORF1b:S2198I, ORF7a:P34L, ORF9b:Q77H, S:T19I, S:H49Y, S:T259R, S:N450K, S:S477N, S:D614G,	S:21983-21994,
hCoV-19/Mexico/VER-IBT-IMSS-162/2021	EPI_ISL_1288363	In process	20B	B.1.1.519	22	5'UTR:C203T, 5'UTR:C222T, 5'UTR:C241T, ORF1ab:C3037T, ORF1ab:C3140T, ORF1ab:C5812T, ORF1ab:C10029T, ORF1ab:G10523C, ORF1ab:C10954T, ORF1ab:A11117G, ORF1ab:C12789T, ORF1ab:C14408T, ORF1ab:T19839C, ORF1ab:C21306T, S:C22995A, S:A23403G, S:C23604A, S:A23756G, N:G28881A, N:G28882A, N:G28883C, N:C29197T, N:C29555T,	12	N:R203K, N:G204R, ORF1a:P959S, ORF1a:T3255I, ORF1a:V3420L, ORF1a:I3618V, ORF1a:T4175I, ORF1b:P314L, S:T478K, S:D614G, S:P681H, S:T732A,	

hCoV-19/Mexico/VER-IBT-IMSS-163/2021	EPI_ISL_1288364	In process	20B	B.1.1.519	24	5'UTR:C203T, 5'UTR:C222T, 5'UTR:C241T, ORF1ab:C3037T, ORF1ab:C3140T, ORF1ab:C4206T, ORF1ab:C5142T, ORF1ab:C10029T, ORF1ab:C10954T, ORF1ab:A11117G, ORF1ab:C12789T, ORF1ab:T13905C, ORF1ab:C14408T, ORF1ab:T19839C, ORF1ab:C21306T, S:C22995A, S:A23403G, S:C23604A, S:A23756G, ORF3a:G25906T, ORF3a:C25916T, N:G28881A, N:G28882A, N:G28883C, N:C29197T,	15	N:R203K, N:G204R, ORF1a:P959S, ORF1a:A1314V, ORF1a:T1626I, ORF1a:T3255I, ORF1a:I3618V, ORF1a:T4175I, ORF1b:P314L, ORF3a:G172C, ORF3a:T175I, S:T478K, S:D614G, S:P681H, S:T732A,
hCoV-19/Mexico/VER-IBT-IMSS-255/2021	EPI_ISL_1288444	In process	20B	B.1.1.519	24	5'UTR:C203T, 5'UTR:C222T, 5'UTR:C241T, ORF1ab:C3037T, ORF1ab:C3140T, ORF1ab:T4922A, ORF1ab:T5287C, ORF1ab:C10029T, ORF1ab:C10954T, ORF1ab:A11117G, ORF1ab:C12789T, ORF1ab:C14408T, ORF1ab:T19839C, ORF1ab:C21058T, ORF1ab:C21306T, S:C22995A, S:A23403G, S:C23604A, S:A23756G, ORF8:T27904C, ORF8:C28087T, N:G28881A, N:G28882A, N:G28883C, N:C29197T,	15	N:R203K, N:G204R, ORF1a:P959S, ORF1a:F1553I, ORF1a:T3255I, ORF1a:I3618V, ORF1a:T4175I, ORF1b:P314L, ORF1b:P2531S, ORF8:L4P, ORF8:A65V, S:T478K, S:D614G, S:P681H, S:T732A,
hCoV-19/Mexico/VER-IBT-IMSS-256/2021	EPI_ISL_1288445	In process	20B	B.1.1.519	25	5'UTR:C203T, 5'UTR:C222T, 5'UTR:C241T, ORF1ab:C3037T, ORF1ab:C3140T, ORF1ab:C5575T, ORF1ab:G7027T, ORF1ab:C10029T, ORF1ab:C10954T, ORF1ab:A11117G, ORF1ab:C12789T, ORF1ab:C14391T, ORF1ab:C14408T, ORF1ab:T16590C, ORF1ab:T19839C, ORF1ab:G20668A, ORF1ab:C21306T, S:C22995A, S:A23403G, S:C23604A, S:A23756G, S:C25207T, N:G28881A, N:G28882A, N:G28883C, N:C29197T,	13	N:R203K, N:G204R, ORF1a:P959S, ORF1a:M2254I, ORF1a:T3255I, ORF1a:I3618V, ORF1a:T4175I, ORF1b:P314L, ORF1b:A2401T, S:T478K, S:D614G, S:P681H, S:T732A,
hCoV-19/Mexico/VER-IBT-IMSS-257/2021	EPI_ISL_1288446	In process	20B	B.1.1.519	22	5'UTR:C203T, 5'UTR:C222T, 5'UTR:C241T, ORF1ab:C3037T, ORF1ab:C3140T, ORF1ab:C10029T, ORF1ab:C10954T, ORF1ab:A11117G, ORF1ab:C12789T, ORF1ab:C14408T, ORF1ab:T15726A, ORF1ab:T19839C, ORF1ab:C21306T, S:C22995A, S:A23403G, S:C23604A, S:A23756G, M:C26833T, N:G28881A, N:G28882A, N:G28883C, N:C29197T, N:G29527T,	13	M:A104V, N:R203K, N:G204R, N:Q418H, ORF1a:P959S, ORF1a:T3255I, ORF1a:I3618V, ORF1a:T4175I, ORF1b:P314L, S:T478K, S:D614G, S:P681H, S:T732A,
hCoV-19/Mexico/VER-IBT-IMSS-258/2021	EPI_ISL_1288447	In process	20B	B.1.1.519	22	5'UTR:C203T, 5'UTR:C222T, 5'UTR:C241T, ORF1ab:C3037T, ORF1ab:C3140T, ORF1ab:C10029T, ORF1ab:C10954T, ORF1ab:A11117G, ORF1ab:C12789T, ORF1ab:C14408T, ORF1ab:T15726A, ORF1ab:T19839C, ORF1ab:C21306T, S:C22995A, S:A23403G, S:C23604A, S:A23756G, M:C26833T, N:G28881A, N:G28882A, N:G28883C, N:C29197T, N:G29527T,	13	M:A104V, N:R203K, N:G204R, N:Q418H, ORF1a:P959S, ORF1a:T3255I, ORF1a:I3618V, ORF1a:T4175I, ORF1b:P314L, S:T478K, S:D614G, S:P681H, S:T732A,
hCoV-19/Mexico/VER-IBT-IMSS-259/2021	EPI_ISL_1288448	In process	20B	B.1.1.519	28	5'UTR:C203T, 5'UTR:C222T, 5'UTR:C241T, ORF1ab:G1719A, ORF1ab:C3037T, ORF1ab:C3140T, ORF1ab:T3313C, ORF1ab:C10029T, ORF1ab:C10954T, ORF1ab:A11117G, ORF1ab:C12789T, ORF1ab:C14408T, ORF1ab:C15600T, ORF1ab:T17142G, ORF1ab:C19602T, ORF1ab:T19839C, ORF1ab:C21306T, S:C22995A, S:C23275T, S:A23403G, S:C23604A, S:A23756G, ORF3a:G25906T, ORF8:C28253T, ORF8:A28254C, N:G28881A, N:G28882A, N:G28883C, N:C29197T,	14	N:R203K, N:G204R, ORF1a:S485N, ORF1a:P959S, ORF1a:T3255I, ORF1a:I3618V, ORF1a:T4175I, ORF1b:P314L, ORF3a:G172C, ORF8:I121L, S:T478K, S:D614G, S:P681H, S:T732A,
hCoV-19/Mexico/VER-IBT-IMSS-261/2021	EPI_ISL_1288449	In process	20B	B.1	15	5'UTR:C222T, 5'UTR:C241T, ORF1ab:C3037T, ORF1ab:C3140T, ORF1ab:C10029T, ORF1ab:C10954T, ORF1ab:C14408T, ORF1ab:T19839C, ORF1ab:C21306T, S:T22565C, S:C22995A, S:A23403G, S:C23604A, S:C23635T, S:A23756G, N:C29197T,	7	ORF1a:P959S, ORF1a:T3255I, ORF1b:P314L, S:T478K, S:D614G, S:P681H, S:T732A,

hCoV-19/Mexico/VER-IBT-IMSS-262/2021	EPI_ISL_1288450	In process	20B	B.1.1.519	27	5'UTR:C203T, 5'UTR:C222T, 5'UTR:C241T, ORF1ab:G942A, ORF1ab:C3037T, ORF1ab:C3140T, ORF1ab:T7984C, ORF1ab:C10029T, ORF1ab:C10277T, ORF1ab:C10954T, ORF1ab:A11117G, ORF1ab:C11124T, ORF1ab:C11956T, ORF1ab:C12789T, ORF1ab:C14408T, ORF1ab:T19839C, ORF1ab:A20676G, ORF1ab:C21306T, S:C22995A, S:A23403G, S:C23604A, S:A23756G, S:C25350A, M:T26552C, N:G28881A, N:G28882A, N:G28883C, N:C29197T	15	N:R203K, N:G204R, ORF1a:R226K, ORF1a:P959S, ORF1a:T3255I, ORF1a:L3338F, ORF1a:I3618V, ORF1a:A3620V, ORF1a:T4175I, ORF1b:P314L, S:T478K, S:D614G, S:P681H, S:T732A, S:P1263Q,
hCoV-19/Mexico/VER-IBT-IMSS-263/2021	EPI_ISL_1288451	In process	20B	B.1.1.519	29	5'UTR:T201C, 5'UTR:C203T, 5'UTR:C222T, 5'UTR:C241T, ORF1ab:G1738T, ORF1ab:C3037T, ORF1ab:C3140T, ORF1ab:G5861A, ORF1ab:C10029T, ORF1ab:C10030T, ORF1ab:C10954T, ORF1ab:C11094T, ORF1ab:A11117G, ORF1ab:C12789T, ORF1ab:C14408T, ORF1ab:T16584C, ORF1ab:T19839C, ORF1ab:A19974G, ORF1ab:C21306T, S:G22203T, S:C22995A, S:A23403G, S:C23604A, S:A23756G, ORF3a:C25667T, ORF7a:A27726G, N:G28881A, N:G28882A, N:G28883C, N:C29197T	15	N:R203K, N:G204R, ORF1a:P959S, ORF1a:V1866I, ORF1a:T3255I, ORF1a:A3610V, ORF1a:I3618V, ORF1a:T4175I, ORF1b:P314L, ORF3a:S92L, S:R214L, S:T478K, S:D614G, S:P681H, S:T732A,
hCoV-19/Mexico/VER-IBT-IMSS-264/2021	EPI_ISL_1288452	In process	20A	B.1.243	32	5'UTR:C241T, ORF1ab:G806A, ORF1ab:C1551T, ORF1ab:C3037T, ORF1ab:G4207T, ORF1ab:C5140A, ORF1ab:A6223G, ORF1ab:G6337A, ORF1ab:C7299T, ORF1ab:C12855T, ORF1ab:C14408T, ORF1ab:T14799C, ORF1ab:C16915T, ORF1ab:G17050A, ORF1ab:G17280T, ORF1ab:G18255T, ORF1ab:A18801G, ORF1ab:T19149C, ORF1ab:A20268G, S:C21721T, S:A23403G, S:C23525T, S:G23587C, S:C23896T, S:T24076C, ORF3a:A25881G, ORF3a:T25899C, ORF8:G28068T, N:G28325T, N:C28854T, N:G29543T, N:G29553T, 3'UTR:C29762T,	15	N:G18C, N:S194L, ORF1a:A181T, ORF1a:A429V, ORF1a:D1625E, ORF1a:A2345V, ORF1a:P4197L, ORF1b:P314L, ORF1b:V1195I, ORF1b:M1596I, ORF8:E59*, ORF9b:L14F, S:D614G, S:H655Y, S:Q675H,
hCoV-19/Mexico/VER-IBT-IMSS-266/2021	EPI_ISL_1288454	In process	20B	B.1.1.519	22	5'UTR:C203T, 5'UTR:C222T, 5'UTR:C241T, ORF1ab:C3037T, ORF1ab:C3140T, ORF1ab:C6213T, ORF1ab:C10029T, ORF1ab:C10954T, ORF1ab:A11117G, ORF1ab:T11187C, ORF1ab:C12789T, ORF1ab:C14408T, ORF1ab:T19839C, ORF1ab:C21306T, S:C22747T, S:C22995A, S:A23403G, S:C23604A, S:A23756G, N:G28881A, N:G28882A, N:G28883C, N:C29197T,	13	N:R203K, N:G204R, ORF1a:P959S, ORF1a:A1983V, ORF1a:T3255I, ORF1a:I3618V, ORF1a:L3641S, ORF1a:T4175I, ORF1b:P314L, S:T478K, S:D614G, S:P681H, S:T732A,
hCoV-19/Mexico/VER-IBT-IMSS-267/2021	EPI_ISL_1288455	In process	20B	B.1.1.519	26	5'UTR:C203T, 5'UTR:C222T, 5'UTR:C241T, ORF1ab:T689C, ORF1ab:C3037T, ORF1ab:C3140T, ORF1ab:C3874T, ORF1ab:A3881T, ORF1ab:C10029T, ORF1ab:C10954T, ORF1ab:A11117G, ORF1ab:G11365T, ORF1ab:C12789T, ORF1ab:C14408T, ORF1ab:G18021A, ORF1ab:T19839C, ORF1ab:A20245G, ORF1ab:C21306T, S:C22995A, S:A23403G, S:C23604A, S:A23756G, N:G28881A, N:G28882A, N:G28883C, N:C29197T, N:G29527T,	15	N:R203K, N:G204R, N:Q418H, ORF1a:S142P, ORF1a:P959S, ORF1a:I1206F, ORF1a:T3255I, ORF1a:I3618V, ORF1a:T4175I, ORF1b:P314L, ORF1b:M2260V, S:T478K, S:D614G, S:P681H, S:T732A,
hCoV-19/Mexico/VER-IBT-IMSS-268/2021	EPI_ISL_1288456	In process	20B	B.1.1.519	23	5'UTR:C203T, 5'UTR:C222T, 5'UTR:C241T, ORF1ab:C3037T, ORF1ab:C3140T, ORF1ab:A6393C, ORF1ab:C10029T, ORF1ab:C10954T, ORF1ab:A11117G, ORF1ab:G12565T, ORF1ab:C12789T, ORF1ab:C14408T, ORF1ab:T19839C, ORF1ab:C21306T, S:C22995A, S:A23403G, S:C23604A, S:A23756G, N:G28817C, N:G28881A, N:G28882A, N:G28883C, N:C29095T, N:C29197T.	14	N:A182P, N:R203K, N:G204R, ORF1a:P959S, ORF1a:D2043A, ORF1a:T3255I, ORF1a:I3618V, ORF1a:Q4100H, ORF1a:T4175I, ORF1b:P314L, S:T478K, S:D614G, S:P681H, S:T732A,

hCoV-19/Mexico/VER-IBT-IMSS-269/2021	EPI_ISL_1288457	In process	20B	B.1.1.519	22	5'UTR:C203T, 5'UTR:C222T, 5'UTR:C241T, ORF1ab:C3037T, ORF1ab:C3140T, ORF1ab:C10029T, ORF1ab:G10271A, ORF1ab:C10954T, ORF1ab:A11117G, ORF1ab:C12789T, ORF1ab:C14408T, ORF1ab:T19839C, ORF1ab:C21306T, S:C22995A, S:A23403G, S:C23604A, S:A23756G, N:G28881A, N:G28882A, N:G28883C, N:C29197T, N:A29413T, N:G29527T,	14	N:R203K, N:G204R, N:Q380H, N:Q418H, ORF1a:P959S, ORF1a:T3255I, ORF1a:V3336I, ORF1a:I3618V, ORF1a:T4175I, ORF1b:P314L, S:T478K, S:D614G, S:P681H, S:T732A,
hCoV-19/Mexico/VER-IBT-IMSS-270/2021	EPI_ISL_1288458	In process	20B	B.1.1.519	27	5'UTR:T201C, 5'UTR:C203T, 5'UTR:C222T, 5'UTR:C241T, ORF1ab:C936T, ORF1ab:G1738T, ORF1ab:C3037T, ORF1ab:C3140T, ORF1ab:G8762A, ORF1ab:C10029T, ORF1ab:C10954T, ORF1ab:A11117G, ORF1ab:C12789T, ORF1ab:C13458T, ORF1ab:C14408T, ORF1ab:T19839C, ORF1ab:A19974G, ORF1ab:C21306T, S:C22995A, S:A23403G, S:C23604A, S:A23756G, N:G28881A, N:G28882A, N:G28883C, N:C29197T, N:G29227T, 3'UTR:A29752G,	14	N:R203K, N:G204R, ORF1a:T224I, ORF1a:P959S, ORF1a:D2833N, ORF1a:T3255I, ORF1a:I3618V, ORF1a:T4175I, ORF1a:S4398L, ORF1b:P314L, S:T478K, S:D614G, S:P681H, S:T732A,
hCoV-19/Mexico/VER-IBT-IMSS-272/2021	EPI_ISL_1288459	In process	20B	B.1.1.222	20	5'UTR:C241T, ORF1ab:C583T, ORF1ab:C865T, ORF1ab:G1738T, ORF1ab:C3037T, ORF1ab:C3140T, ORF1ab:A6985T, ORF1ab:C9319T, ORF1ab:C12412T, ORF1ab:C14408T, ORF1ab:C16948T, S:A23403G, S:C23604A, S:A23756G, S:T24052C, S:G24620T, ORF8:G28237T, N:G28881A, N:G28882A, N:G28883C, N:C29197T,	10	N:R203K, N:G204R, ORF1a:P959S, ORF1b:P314L, ORF1b:P1161S, ORF8:R115L, S:D614G, S:P681H, S:T732A, S:A1020S,
hCoV-19/Mexico/VER-IBT-IMSS-273/2021	EPI_ISL_1288460	In process	20B	B.1.1.519	24	5'UTR:C203T, 5'UTR:C222T, 5'UTR:C241T, ORF1ab:C843A, ORF1ab:C3037T, ORF1ab:C3140T, ORF1ab:T3745C, ORF1ab:C10029T, ORF1ab:C10954T, ORF1ab:A11117G, ORF1ab:C12789T, ORF1ab:C14408T, ORF1ab:C15738T, ORF1ab:T19839C, ORF1ab:C21306T, S:C22995A, S:A23403G, S:C23604A, S:A23756G, ORF3a:C25844T, N:C28849T, N:G28881A, N:G28882A, N:G28883C, N:C29197T,	13	N:R203K, N:G204R, ORF1a:P193H, ORF1a:P959S, ORF1a:T3255I, ORF1a:I3618V, ORF1a:T4175I, ORF1b:P314L, ORF3a:T151I, S:T478K, S:D614G, S:P681H, S:T732A,
hCoV-19/Mexico/VER-IBT-IMSS-274/2021	EPI_ISL_1288461	In process	20B	B.1.1.519	22	5'UTR:C203T, 5'UTR:C222T, 5'UTR:C241T, ORF1ab:C3037T, ORF1ab:C3140T, ORF1ab:G7037T, ORF1ab:C10029T, ORF1ab:C10954T, ORF1ab:A11117G, ORF1ab:C12789T, ORF1ab:C14408T, ORF1ab:T19839C, ORF1ab:C21306T, S:C22995A, S:A23403G, S:C23604A, S:A23756G, ORF3a:C25782T, N:G28881A, N:G28882A, N:G28883C, N:C29197T, N:G29508A,	13	N:R203K, N:G204R, N:S412N, ORF1a:P959S, ORF1a:G2258C, ORF1a:T3255I, ORF1a:I3618V, ORF1a:T4175I, ORF1b:P314L, S:T478K, S:D614G, S:P681H, S:T732A,
hCoV-19/Mexico/VER-IBT-IMSS-275/2021	EPI_ISL_1288462	In process	20B	B.1.1.519	25	5'UTR:C203T, 5'UTR:C222T, 5'UTR:C241T, ORF1ab:C3037T, ORF1ab:C3140T, ORF1ab:C4246T, ORF1ab:C10029T, ORF1ab:C10954T, ORF1ab:A11117G, ORF1ab:C12789T, ORF1ab:C14408T, ORF1ab:T19839C, ORF1ab:C21306T, S:G21867C, S:C22181T, S:C22995A, S:A23403G, S:C23604A, S:A23756G, M:G26526A, ORF8:T28098C, N:G28881A, N:G28882A, N:G28883C, N:C29197T, N:G29527T,	16	M:A2T, N:R203K, N:G204R, N:Q418H, ORF1a:P959S, ORF1a:T3255I, ORF1a:I3618V, ORF1a:T4175I, ORF1b:P314L, ORF8:S69P, S:R102T, S:H207Y, S:T478K, S:D614G, S:P681H, S:T732A,
hCoV-19/Mexico/VER-IBT-IMSS-276/2021	EPI_ISL_1288463	In process	20B	B.1.1.519	24	5'UTR:T201C, 5'UTR:C203T, 5'UTR:C222T, 5'UTR:C241T, ORF1ab:T277C, ORF1ab:G1738T, ORF1ab:C3037T, ORF1ab:C3140T, ORF1ab:G7829T, ORF1ab:C10029T, ORF1ab:C10954T, ORF1ab:A11117G, ORF1ab:C12789T, ORF1ab:C14408T, ORF1ab:T19839C, ORF1ab:A19974G, ORF1ab:C21306T, S:C22995A, S:A23403G, S:C23604A, S:A23756G, N:G28881A, N:G28882A, N:G28883C, N:C29197T,	12	N:R203K, N:G204R, ORF1a:P959S, ORF1a:V2522F, ORF1a:T3255I, ORF1a:I3618V, ORF1a:T4175I, ORF1b:P314L, S:T478K, S:D614G, S:P681H, S:T732A,

hCoV-19/Mexico/VER-IBT-IMSS-277/2021	EPI_ISL_1288464	In process	20B	B.1.1.519	22	5'UTR:C203T, 5'UTR:C222T, 5'UTR:C241T, ORF1ab:C3037T, ORF1ab:C3140T, ORF1ab:C9615G, ORF1ab:C10029T, ORF1ab:C10954T, ORF1ab:A11117G, ORF1ab:C12789T, ORF1ab:C14408T, ORF1ab:T19839C, ORF1ab:C21306T, S:C22995A, S:A23403G, S:C23604A, S:A23756G, ORF8:T27904C, ORF8:C28087T, N:G28881A, N:G28882A, N:G28883C, N:C29197T,	14	N:R203K, N:G204R, ORF1a:P959S, ORF1a:T3117S, ORF1a:T3255I, ORF1a:I3618V, ORF1a:T4175I, ORF1b:P314L, ORF8:L4P, ORF8:A65V, S:T478K, S:D614G, S:P681H, S:T732A,	
hCoV-19/Mexico/VER-IBT-IMSS-278/2021	EPI_ISL_1288465	In process	20B	B.1.1.519	22	5'UTR:T201C, 5'UTR:C203T, 5'UTR:C222T, 5'UTR:C241T, ORF1ab:T277C, ORF1ab:C3037T, ORF1ab:C3140T, ORF1ab:C10029T, ORF1ab:C10954T, ORF1ab:A11117G, ORF1ab:C12789T, ORF1ab:C14408T, ORF1ab:T19839C, ORF1ab:A19974G, ORF1ab:C21306T, S:C22995A, S:A23403G, S:C23604A, S:A23756G, N:G28881A, N:G28882A, N:G28883C, N:C29197T,	11	N:R203K, N:G204R, ORF1a:P959S, ORF1a:T3255I, ORF1a:I3618V, ORF1a:T4175I, ORF1b:P314L, S:T478K, S:D614G, S:P681H, S:T732A,	
hCoV-19/Mexico/VER-IBT-IMSS-279/2021	EPI_ISL_1288466	In process	20B	B.1.1.432	22	5'UTR:C241T, ORF1ab:G1306T, ORF1ab:C3037T, ORF1ab:A6985T, ORF1ab:C9319T, ORF1ab:G11064T, ORF1ab:C12412T, ORF1ab:T14313C, ORF1ab:G14371T, ORF1ab:C14408T, ORF1ab:G14874T, ORF1ab:C16948T, ORF1ab:C21077T, S:C22570T, S:A23403G, S:T24052C, S:G24620T, ORF3a:C25613T, ORF3a:G25912T, N:G28881A, N:G28882A, N:G28883C, N:T29317C,	13	N:R203K, N:G204R, ORF1a:E347D, ORF1a:W3600L, ORF1b:A302S, ORF1b:P314L, ORF1b:K469N, ORF1b:P1161S, ORF1b:T2537I, ORF3a:S74F, ORF3a:G174C, S:D614G, S:A1020S,	
hCoV-19/Mexico/VER-IBT-IMSS-280/2021	EPI_ISL_1288467	In process	20B	B.1.1.432	19	5'UTR:C241T, ORF1ab:G1306T, ORF1ab:C3037T, ORF1ab:A6985T, ORF1ab:C9319T, ORF1ab:G11064T, ORF1ab:C12412T, ORF1ab:T14313C, ORF1ab:G14371T, ORF1ab:C14408T, ORF1ab:C16948T, S:C22570T, S:A23403G, S:T24052C, S:G24620T, ORF3a:C25613T, N:G28881A, N:G28882A, N:G28883C, N:T29317C,	10	N:R203K, N:G204R, ORF1a:E347D, ORF1a:W3600L, ORF1b:A302S, ORF1b:P314L, ORF1b:P1161S, ORF3a:S74F, S:D614G, S:A1020S,	
hCoV-19/Mexico/VER-IBT-IMSS-281/2021	EPI_ISL_1288468	In process	20B	B.1.1.519	24	5'UTR:C203T, 5'UTR:C222T, 5'UTR:C241T, ORF1ab:C3037T, ORF1ab:C3140T, ORF1ab:C8756T, ORF1ab:C10029T, ORF1ab:C10954T, ORF1ab:A11117G, ORF1ab:C12789T, ORF1ab:C14408T, ORF1ab:G15921T, ORF1ab:A17971G, ORF1ab:T19839C, ORF1ab:C21306T, S:C22995A, S:A23403G, S:C23604A, S:C23635T, S:A23756G, M:G26828T, N:G28881A, N:G28882A, N:G28883C, N:C29197T,	13	N:R203K, N:G204R, ORF1a:P959S, ORF1a:H2831Y, ORF1a:T3255I, ORF1a:I3618V, ORF1a:T4175I, ORF1b:P314L, ORF1b:R1502G, S:T478K, S:D614G, S:P681H, S:T732A,	S:21992-21994,
hCoV-19/Mexico/VER-IBT-IMSS-283/2021	EPI_ISL_1288469	In process	20B	B.1.1.519	26	5'UTR:C203T, 5'UTR:C222T, 5'UTR:C241T, ORF1ab:C3037T, ORF1ab:C3140T, ORF1ab:C10029T, ORF1ab:C10954T, ORF1ab:A11117G, ORF1ab:C12789T, ORF1ab:C14408T, ORF1ab:G15921T, ORF1ab:C16260T, ORF1ab:C17913T, ORF1ab:A17971G, ORF1ab:G18859T, ORF1ab:T19839C, ORF1ab:C21306T, S:C22995A, S:A23403G, S:C23604A, S:C23635T, S:A23756G, M:G26828T, N:G28881A, N:G28882A, N:G28883C, N:C29197T,	13	N:R203K, N:G204R, ORF1a:P959S, ORF1a:T3255I, ORF1a:I3618V, ORF1a:T4175I, ORF1b:P314L, ORF1b:R1502G, ORF1b:A1798S, S:T478K, S:D614G, S:P681H, S:T732A,	S:21992-21994,
hCoV-19/Mexico/VER-IBT-IMSS-352/2021	EPI_ISL_1288529	In process	20B	B.1.1.432	25	5'UTR:C241T, ORF1ab:C2245T, ORF1ab:G2282A, ORF1ab:A2618G, ORF1ab:C3037T, ORF1ab:C3695T, ORF1ab:C4206T, ORF1ab:G5194T, ORF1ab:A6985T, ORF1ab:T8503C, ORF1ab:C9319T, ORF1ab:A10081G, ORF1ab:C12412T, ORF1ab:T14313C, ORF1ab:C14408T, ORF1ab:C14802T, S:A23403G, S:G24781T, ORF3a:C25613T, ORF3a:G25912T, ORF6:T27308C, ORF8:C27925T, N:G28881A, N:G28882A, N:G28883C, N:T29317C,	12	N:R203K, N:G204R, ORF1a:E673K, ORF1a:I785V, ORF1a:A1314V, ORF1b:P314L, ORF3a:S74F, ORF3a:G174C, ORF6:I36T, ORF8:T11I, S:D614G, S:K1073N,	ORF1ab: 509-523,

hCoV-19/Mexico/VER-IBT-IMSS-353/2021	EPI_ISL_1288530	In process	20B	B.1.1.519	22	5'UTR:C203T, 5'UTR:C222T, 5'UTR:C241T, ORF1ab:C3037T, ORF1ab:C3140T, ORF1ab:C10029T, ORF1ab:C10954T, ORF1ab:A11117G, ORF1ab:C12789T, ORF1ab:C14408T, ORF1ab:G19684T, ORF1ab:T19839C, ORF1ab:C21306T, S:C22264T, S:C22995A, S:A23403G, S:C23604A, S:A23756G, S:C24381T, N:G28881A, N:G28882A, N:G28883C, N:C29197T,	13	N:R203K, N:G204R, ORF1a:P959S, ORF1a:T3255I, ORF1a:I3618V, ORF1a:T4175I, ORF1b:P314L, ORF1b:V2073L, S:T478K, S:D614G, S:P681H, S:T732A, S:S940F,	
hCoV-19/Mexico/VER-IBT-IMSS-354/2021	EPI_ISL_1288531	In process	20B	B.1.1.519	25	5'UTR:C203T, 5'UTR:C222T, 5'UTR:C241T, ORF1ab:C1385T, ORF1ab:C3037T, ORF1ab:C3140T, ORF1ab:C10029T, ORF1ab:C10954T, ORF1ab:A11117G, ORF1ab:C11124T, ORF1ab:A14033G, ORF1ab:C14408T, ORF1ab:T19839C, ORF1ab:C21306T, S:C22995A, S:A23403G, S:C23604A, S:A23756G, ORF8:T27904C, ORF8:C28087T, N:G28881A, N:G28882A, N:G28883C, N:G28975T, N:C29197T, 3'UTR:G29747T,	16	N:R203K, N:G204R, N:M234I, ORF1a:H374Y, ORF1a:P959S, ORF1a:T3255I, ORF1a:I3618V, ORF1a:A3620V, ORF1b:N189S, ORF1b:P314L, ORF8:L4P, ORF8:A65V, S:T478K, S:D614G, S:P681H, S:T732A,	
hCoV-19/Mexico/VER-IBT-IMSS-360/2021	EPI_ISL_1288442	In process	20B	B.1	22	5'UTR:C241T, ORF1ab:C2121T, ORF1ab:G2305T, ORF1ab:C3037T, ORF1ab:A6985T, ORF1ab:G8102T, ORF1ab:C9319T, ORF1ab:C10369T, ORF1ab:C12412T, ORF1ab:T14313C, ORF1ab:C14408T, ORF1ab:G16853T, ORF1ab:C18657T, ORF1ab:A19020G, ORF1ab:C20016T, ORF1ab:C20178T, S:C22227T, S:A23403G, ORF3a:C25613T, ORF3a:G25912T, ORF7a:G27506T, N:G29033T, N:T29317C,	11	N:A254S, ORF1a:T619I, ORF1a:K680N, ORF1a:V2613F, ORF1b:P314L, ORF1b:G1129V, ORF3a:S74F, ORF3a:G174C, ORF7a:G38V, S:A222V, S:D614G,	N:28880-28897
hCoV-19/Mexico/VER-IBT-IMSS-418/2021	EPI_ISL_1288239	In process	20B	B.1.1.519	29	5'UTR:T201C, 5'UTR:C203T, 5'UTR:C222T, 5'UTR:C241T, ORF1ab:G1738T, ORF1ab:C2119T, ORF1ab:C3037T, ORF1ab:C3140T, ORF1ab:C10029T, ORF1ab:C10954T, ORF1ab:A11117G, ORF1ab:C12789T, ORF1ab:C14408T, ORF1ab:T19839C, ORF1ab:A19974G, ORF1ab:C21306T, S:C22995A, S:A23403G, S:C23604A, S:C23683T, S:A23756G, S:A23764G, ORF3a:C26110T, M:G26718T, N:C28844A, N:G28881A, N:G28882A, N:G28883C, N:C29197T, 3'UTR:G29690T	14	M:V66L, N:R191S, N:R203K, N:G204R, ORF1a:P959S, ORF1a:T3255I, ORF1a:I3618V, ORF1a:T4175I, ORF1b:P314L, ORF3a:P240S, S:T478K, S:D614G, S:P681H, S:T732A,	
hCoV-19/Mexico/VER-IBT-IMSS-419/2021	EPI_ISL_1288240	In process	20B	B.1.1.222	20	5'UTR:C241T, ORF1ab:G1696T, ORF1ab:C1758T, ORF1ab:C2252A, ORF1ab:C3037T, ORF1ab:C10029T, ORF1ab:C13680T, ORF1ab:C14408T, ORF1ab:C15279T, ORF1ab:G19816T, ORF1ab:T19839C, S:A23403G, S:A23756G, ORF3a:G25912T, ORF7b:A27756G, ORF8:A27921G, ORF8:G28001T, N:G28881A, N:G28882A, N:G28883C, N:G29315C,	14	N:R203K, N:G204R, N:D348H, ORF1a:L477F, ORF1a:A498V, ORF1a:Q663K, ORF1a:T3255I, ORF1b:P314L, ORF1b:V2117L, ORF3a:G174C, ORF7b:M1V, ORF8:I10V, S:D614G, S:T732A,	
hCoV-19/Mexico/VER-IBT-IMSS-420/2021	EPI_ISL_1288241	In process	20B	B.1.1.222	18	5'UTR:C241T, ORF1ab:C3037T, ORF1ab:C5826T, ORF1ab:G6576T, ORF1ab:T10288A, ORF1ab:C11674T, ORF1ab:C14408T, ORF1ab:T19839C, S:A23403G, S:A23756G, M:A26612T, M:C27131T, ORF6:C27230T, ORF7a:T27401C, N:C28292A, N:C28453T, N:G28881A, N:G28882A, N:G28883C,	11	N:Q7K, N:R203K, N:G204R, ORF1a:T1854I, ORF1a:S2104I, ORF1b:P314L, ORF6:T10I, ORF7a:I3T, ORF9b:A57V, S:D614G, S:T732A,	

hCoV-19/Mexico/VER-IBT-IMSS-421/2021	EPI_ISL_1288242	In process	20B	B.1.1.519	30	5'UTR:T201C, 5'UTR:C203T, 5'UTR:C222T, 5'UTR:C241T, ORF1ab:G1738T, ORF1ab:C3037T, ORF1ab:C3140T, ORF1ab:G3753A, ORF1ab:C4456T, ORF1ab:C7318T, ORF1ab:C10029T, ORF1ab:C10954T, ORF1ab:A11117G, ORF1ab:C12789T, ORF1ab:C14408T, ORF1ab:T17328C, ORF1ab:G18020A, ORF1ab:T19839C, ORF1ab:A19974G, ORF1ab:C20178T, ORF1ab:C21306T, S:C21721T, S:C22995A, S:A23403G, S:C23604A, S:A23756G, ORF3a:G25445T, N:G28881A, N:G28882A, N:G28883C, N:C29197T,	14	N:R203K, N:G204R, ORF1a:P959S, ORF1a:R1163K, ORF1a:T3255I, ORF1a:I3618V, ORF1a:T4175I, ORF1b:P314L, ORF1b:R1518K, ORF3a:G18V, S:T478K, S:D614G, S:P681H, S:T732A,	
hCoV-19/Mexico/VER-IBT-IMSS-423/2021	EPI_ISL_1288244	In process	20A	B.1	25	5'UTR:C241T, ORF1ab:A2010G, ORF1ab:C3037T, ORF1ab:A3904T, ORF1ab:C7124T, ORF1ab:A11430G, ORF1ab:C14408T, ORF1ab:C16289T, ORF1ab:A16534G, ORF1ab:T17673G, ORF1ab:G20060T, ORF1ab:A20268G, S:C21618T, S:C21707T, S:C22338G, S:T22912G, S:G22992A, S:A23403G, ORF3a:C25493T, ORF3a:C25517T, M:C26681T, M:G26709A, ORF7a:C27494T, ORF8:A28273T, N:G28514T, N:G28917T,	20	M:A63T, N:D81Y, N:G215V, ORF1a:D582G, ORF1a:P2287S, ORF1a:Y3722C, ORF1b:P314L, ORF1b:A941V, ORF1b:S1023G, ORF1b:S2198I, ORF3a:T34M, ORF3a:P42L, ORF7a:P34L, ORF9b:Q77H, S:T19I, S:H49Y, S:T259R, S:N450K, S:S477N, S:D614G,	S:21983-21994,
hCoV-19/Mexico/VER-IBT-IMSS-424/2021	EPI_ISL_1288245	In process	20A	B.1	25	5'UTR:C241T, ORF1ab:A2010G, ORF1ab:C3037T, ORF1ab:A3904T, ORF1ab:C7124T, ORF1ab:A11430G, ORF1ab:C14408T, ORF1ab:C16289T, ORF1ab:A16534G, ORF1ab:T17673G, ORF1ab:G20060T, ORF1ab:A20268G, S:C21618T, S:C21707T, S:C22338G, S:T22912G, S:G22992A, S:A23403G, ORF3a:C25493T, ORF3a:C25517T, M:C26681T, M:G26709A, ORF7a:C27494T, ORF8:A28273T, N:G28514T, N:G28917T,	20	M:A63T, N:D81Y, N:G215V, ORF1a:D582G, ORF1a:P2287S, ORF1a:Y3722C, ORF1b:P314L, ORF1b:A941V, ORF1b:S1023G, ORF1b:S2198I, ORF3a:T34M, ORF3a:P42L, ORF7a:P34L, ORF9b:Q77H, S:T19I, S:H49Y, S:T259R, S:N450K, S:S477N, S:D614G,	S:21983-21994,
hCoV-19/Mexico/VER-IBT-IMSS-511/2020	EPI_ISL_1301676	In process	20B	B.1.1.432	11	5'UTR:C241T, ORF1ab:C3037T, ORF1ab:A6985T, ORF1ab:C9319T, ORF1ab:C12412T, ORF1ab:T14313C, ORF1ab:C14408T, S:A23403G, ORF3a:C25613T, N:G28881A, N:G28882A, N:G28883C,	5	N:R203K, N:G204R, ORF1b:P314L, ORF3a:S74F, S:D614G,	
hCoV-19/Mexico/VER-IBT-IMSS-512/2020	EPI_ISL_1301677	In process	20C	B.1	8	5'UTR:C241T, ORF1ab:C1059T, ORF1ab:C3037T, ORF1ab:C11124T, ORF1ab:C14408T, ORF1ab:C18601T, S:A23403G, ORF3a:G25563T, N:G29179T,	6	ORF1a:T265I, ORF1a:A3620V, ORF1b:P314L, ORF1b:H1712Y, ORF3a:Q57H, S:D614G,	
hCoV-19/Mexico/VER-IBT-IMSS-513/2020	EPI_ISL_1301678	In process	20A	B.1	6	5'UTR:C241T, ORF1ab:C3037T, ORF1ab:G11417T, ORF1ab:G13617T, ORF1ab:C14408T, S:A23403G, S:G24781T,	5	ORF1a:V3718F, ORF1b:K50N, ORF1b:P314L, S:D614G, S:K1073N,	
hCoV-19/Mexico/VER-IBT-IMSS-527/2021	EPI_ISL_1302238	In process	20B	B.1.1.519	25	5'UTR:C203T, 5'UTR:C222T, 5'UTR:C241T, ORF1ab:C3037T, ORF1ab:C3140T, ORF1ab:C9810T, ORF1ab:T9937C, ORF1ab:C10029T, ORF1ab:C10332T, ORF1ab:C10954T, ORF1ab:A11117G, ORF1ab:C12789T, ORF1ab:C14408T, ORF1ab:T19839C, ORF1ab:C21077T, S:C22995A, S:A23403G, S:C23604A, S:A23756G, ORF3a:G25906T, ORF8:C28253T, N:G28881A, N:G28882A, N:G28883C, N:C29197T, N:C29311T,	15	N:R203K, N:G204R, ORF1a:P959S, ORF1a:T3182I, ORF1a:T3255I, ORF1a:T3356I, ORF1a:I3618V, ORF1a:T4175I, ORF1b:P314L, ORF1b:T2537I, ORF3a:G172C, S:T478K, S:D614G, S:P681H, S:T732A,	
hCoV-19/Mexico/VER-IBT-IMSS-554/2021	EPI_ISL_1302305	In process	20A	B.1.36.10	21	5'UTR:C241T, ORF1ab:C745T, ORF1ab:C3037T, ORF1ab:G4280A, ORF1ab:C5239T, ORF1ab:A5995G, ORF1ab:C6573T, ORF1ab:A6821G, ORF1ab:G7059T, ORF1ab:A7384G, ORF1ab:A11117G, ORF1ab:C12068T, ORF1ab:A13898C, ORF1ab:C14408T, ORF1ab:A17056G, ORF1ab:G19962T, S:G23120T, S:A23403G, N:C28546T, N:C28854T, 3'UTR:G29692T, 3'UTR:C29774T,	12	N:S194L, ORF1a:V1339I, ORF1a:S2103F, ORF1a:T2186A, ORF1a:G2265V, ORF1a:I3618V, ORF1b:D144A, ORF1b:P314L, ORF1b:M1197V, ORF9b:P88L, S:A520S, S:D614G,	ORF8:27942-27944

hCoV-19/Mexico/VER-IBT-IMSS-555/2021	EPI_ISL_1302260	In process	20B	B.1.1.519	28	5'UTR:C203T, 5'UTR:C222T, 5'UTR:C241T, ORF1ab:C3037T, ORF1ab:C3140T, ORF1ab:C5140T, ORF1ab:C8140T, ORF1ab:C10029T, ORF1ab:C10954T, ORF1ab:A11117G, ORF1ab:C12789T, ORF1ab:C14184T, ORF1ab:C14262T, ORF1ab:C14408T, ORF1ab:C14931T, ORF1ab:T15726A, ORF1ab:C17733T, ORF1ab:T19839C, ORF1ab:G21538T, S:C22995A, S:A23403G, S:C23604A, S:A23756G, M:C26833T, N:G28881A, N:G28882A, N:G28883C, N:C29197T, N:G29527T,	14	M:A104V, N:R203K, N:G204R, N:Q418H, ORF1a:P959S, ORF1a:T3255I, ORF1a:I3618V, ORF1a:T4175I, ORF1b:P314L, ORF1b:V2691F, S:T478K, S:D614G, S:P681H, S:T732A,	
hCoV-19/Mexico/VER-IBT-IMSS-556/2021	EPI_ISL_1302341	In process	20B	B.1.1.519	23	5'UTR:C203T, 5'UTR:C222T, 5'UTR:C241T, ORF1ab:C3037T, ORF1ab:C3140T, ORF1ab:C10029T, ORF1ab:C10954T, ORF1ab:A11117G, ORF1ab:C12789T, ORF1ab:C14408T, ORF1ab:G15921T, ORF1ab:A17971G, ORF1ab:T19839C, S:C22995A, S:A23403G, S:C23604A, S:C23635T, S:A23756G, M:G26828T, ORF6:T27384C, N:G28881A, N:G28882A, N:G28883C, N:C29197T,	12	N:R203K, N:G204R, ORF1a:P959S, ORF1a:T3255I, ORF1a:I3618V, ORF1a:T4175I, ORF1b:P314L, ORF1b:R1502G, S:T478K, S:D614G, S:P681H, S:T732A,	S:21992-21994,
hCoV-19/Mexico/VER-IBT-IMSS-557/2021	EPI_ISL_1302354	In process	20A	B.1.551	12	5'UTR:C241T, ORF1ab:G2180T, ORF1ab:C3037T, ORF1ab:C3738T, ORF1ab:C14408T, ORF1ab:G19525T, ORF1ab:G19891T, S:A23403G, S:C23604G, S:A23980G, ORF3a:G25456C, N:C28854T, N:C29466T,	10	N:S194L, N:A398V, ORF1a:V639L, ORF1a:P1158L, ORF1b:P314L, ORF1b:D2020Y, ORF1b:D2142Y, ORF3a:D22H, S:D614G, S:P681R,	
hCoV-19/Mexico/VER-IBT-IMSS-79/2021	EPI_ISL_1288294	In process	20B	B.1.1.519	25	5'UTR:C203T, 5'UTR:C222T, 5'UTR:C241T, ORF1ab:C958T, ORF1ab:C3037T, ORF1ab:C3140T, ORF1ab:C10029T, ORF1ab:C10954T, ORF1ab:A11117G, ORF1ab:C12789T, ORF1ab:C14408T, ORF1ab:C18814T, ORF1ab:T19839C, ORF1ab:C21306T, S:C22995A, S:A23403G, S:C23604A, S:A23756G, ORF8:T27904C, ORF8:C28087T, N:C28512T, N:G28881A, N:G28882A, N:G28883C, N:C29167T, N:C29197T,	15	N:P80L, N:R203K, N:G204R, ORF1a:P959S, ORF1a:T3255I, ORF1a:I3618V, ORF1a:T4175I, ORF1b:P314L, ORF8:L4P, ORF8:A65V, ORF9b:Q77*, S:T478K, S:D614G, S:P681H, S:T732A,	
hCoV-19/Mexico/VER-IBT-IMSS-80/2021	EPI_ISL_1288295	In process	20B	B.1.1.519	26	5'UTR:C203T, 5'UTR:C222T, 5'UTR:C241T, ORF1ab:C958T, ORF1ab:C3037T, ORF1ab:C3140T, ORF1ab:C10029T, ORF1ab:C10954T, ORF1ab:A11117G, ORF1ab:C12789T, ORF1ab:C14408T, ORF1ab:C18814T, ORF1ab:T19839C, ORF1ab:C21306T, S:C22995A, S:A23403G, S:C23604A, S:C23634T, S:A23756G, ORF8:T27904C, ORF8:C28087T, N:C28512T, N:G28881A, N:G28882A, N:G28883C, N:C29167T, N:C29197T,	16	N:P80L, N:R203K, N:G204R, ORF1a:P959S, ORF1a:T3255I, ORF1a:I3618V, ORF1a:T4175I, ORF1b:P314L, ORF8:L4P, ORF8:A65V, ORF9b:Q77*, S:T478K, S:D614G, S:P681H, S:S691F, S:T732A,	
hCoV-19/Mexico/VER-IBT-IMSS-82/2021	EPI_ISL_1288296	In process	20B	B.1.1.519	26	5'UTR:G61A, 5'UTR:C203T, 5'UTR:C222T, 5'UTR:C241T, ORF1ab:G529T, ORF1ab:C1238A, ORF1ab:C3037T, ORF1ab:C3140T, ORF1ab:C10029T, ORF1ab:C10954T, ORF1ab:A11117G, ORF1ab:C12789T, ORF1ab:C14408T, ORF1ab:C17076T, ORF1ab:T19839C, ORF1ab:C21306T, S:C22995A, S:A23403G, S:C23604A, S:A23756G, S:G24933T, S:G25183T, N:A28477G, N:G28881A, N:G28882A, N:G28883C, N:C29197T,	15	N:R203K, N:G204R, ORF1a:H325N, ORF1a:P959S, ORF1a:T3255I, ORF1a:I3618V, ORF1a:T4175I, ORF1b:P314L, ORF9b:E65G, S:T478K, S:D614G, S:P681H, S:T732A, S:G1124V, S:E1207D,	
hCoV-19/Mexico/VER-IBT-IMSS-83/2021	EPI_ISL_1288297	In process	20C	B.1	26	5'UTR:C241T, ORF1ab:C1059T, ORF1ab:G1820A, ORF1ab:C3037T, ORF1ab:C3318T, ORF1ab:A6851C, ORF1ab:C7528T, ORF1ab:T8704C, ORF1ab:C9120T, ORF1ab:C9870T, ORF1ab:C11005A, ORF1ab:C12854T, ORF1ab:C13458T, ORF1ab:G13617A, ORF1ab:C14408T, ORF1ab:G14559A, ORF1ab:C14676T, S:T23042C, S:A23063T, S:A23403G, S:C23604A, S:G24893A, S:G25249T, ORF3a:G25563T, ORF8:C27923T, ORF8:A28272T, N:G28975A,	26	N:M234I, ORF1a:T265I, ORF1a:G519S, ORF1a:P1018L, ORF1a:T2196P, ORF1a:T2952I, ORF1a:T3202M, ORF1a:H3580Q, ORF1a:P4197S, ORF1a:S4398L, ORF1b:P314L, ORF3a:Q57H, S:S494P, S:N501Y, S:D614G, S:P681H, S:E1111K, S:M1229I,ORF8:L4M, ORF8:V5K, ORF8:A15V, ORF8:H17L, ORF8:Q18G, ORF8:E19I, ORF8:C20I, ORF8:S21T,	ORF8:27927-27956

hCoV-19/Mexico/VER-IBT-IMSS-84/2021	EPI_ISL_1288298	In process	20B	B.1.1.222	26	5'UTR:C241T, ORF1ab:C593T, ORF1ab:A1558G, ORF1ab:C3037T, ORF1ab:C3743T, ORF1ab:A3916G, ORF1ab:A5452G, ORF1ab:C6543T, ORF1ab:C6936T, ORF1ab:C8606T, ORF1ab:T9304G, ORF1ab:G11222T, ORF1ab:C14216T, ORF1ab:C14408T, ORF1ab:T19839C, S:C21614T, S:G22026A, S:G22104T, S:A22765T, S:A23403G, S:A23756G, ORF3a:C25587T, ORF3a:C25904T, M:C26534T, N:G28881A, N:G28882A, N:G28883C,	17	N:R203K, N:G204R, ORF1a:H110Y, ORF1a:I431M, ORF1a:H1160Y, ORF1a:T2093I, ORF1a:S2224F, ORF1a:L2781F, ORF1a:V3653F, ORF1b:T250I, ORF1b:P314L, ORF3a:S171L, S:L18F, S:S155N, S:G181V, S:D614G, S:T732A,	
hCoV-19/Mexico/VER-IBT-IMSS-85/2021	EPI_ISL_1288299	In process	20B	B.1.1.519	24	5'UTR:C203T, 5'UTR:C222T, 5'UTR:C241T, ORF1ab:C3037T, ORF1ab:C3140T, ORF1ab:T4922A, ORF1ab:T5287C, ORF1ab:C10029T, ORF1ab:C10954T, ORF1ab:A11117G, ORF1ab:C12789T, ORF1ab:C14408T, ORF1ab:T19839C, ORF1ab:C21058T, ORF1ab:C21306T, S:C22995A, S:A23403G, S:C23604A, S:A23756G, ORF8:T27904C, ORF8:C28087T, N:G28881A, N:G28882A, N:G28883C, N:C29197T,	15	N:R203K, N:G204R, ORF1a:P959S, ORF1a:F1553I, ORF1a:T3255I, ORF1a:I3618V, ORF1a:T4175I, ORF1b:P314L, ORF1b:P2531S, ORF8:L4P, ORF8:A65V, S:T478K, S:D614G, S:P681H, S:T732A,	
hCoV-19/Mexico/VER-IBT-IMSS-86/2021	EPI_ISL_1288300	In process	20B	B.1	24	5'UTR:C203T, 5'UTR:C222T, 5'UTR:C241T, ORF1ab:C3037T, ORF1ab:C3140T, ORF1ab:C10029T, ORF1ab:C10319T, ORF1ab:C10954T, ORF1ab:A11117G, ORF1ab:C12789T, ORF1ab:C14408T, ORF1ab:T19839C, ORF1ab:C21306T, S:C21637T, S:C22995A, S:A23403G, S:A23586T, S:C23604A, S:A23756G, ORF8:T27904C, ORF8:C28087T, N:G28881A, N:G28882A, N:G28883C, N:C29197T,	15	N:R203K, N:G204R, ORF1a:P959S, ORF1a:T3255I, ORF1a:L3352F, ORF1a:I3618V, ORF1a:T4175I, ORF1b:P314L, ORF8:L4P, ORF8:A65V, S:T478K, S:D614G, S:Q675L, S:P681H, S:T732A,	
hCoV-19/Mexico/VER-IBT-IMSS-87/2021	EPI_ISL_1288301	In process	20B	B.1	24	5'UTR:C203T, 5'UTR:C222T, 5'UTR:C241T, ORF1ab:C3037T, ORF1ab:C3140T, ORF1ab:C10029T, ORF1ab:C10319T, ORF1ab:C10954T, ORF1ab:A11117G, ORF1ab:C12789T, ORF1ab:C14408T, ORF1ab:T19839C, ORF1ab:C21306T, S:C21637T, S:C22995A, S:A23403G, S:A23586T, S:C23604A, S:A23756G, ORF8:T27904C, ORF8:C28087T, N:G28881A, N:G28882A, N:G28883C, N:C29197T,	15	N:R203K, N:G204R, ORF1a:P959S, ORF1a:T3255I, ORF1a:L3352F, ORF1a:I3618V, ORF1a:T4175I, ORF1b:P314L, ORF8:L4P, ORF8:A65V, S:T478K, S:D614G, S:Q675L, S:P681H, S:T732A,	
hCoV-19/Mexico/VER-IBT-IMSS-88/2021	EPI_ISL_1288302	In process	21C (Epsilon)	B.1.427	27	5'UTR:C241T, ORF1ab:C1059T, ORF1ab:C2455T, ORF1ab:C2607T, ORF1ab:C3037T, ORF1ab:G3072A, ORF1ab:C3817T, ORF1ab:A5236T, ORF1ab:C6310T, ORF1ab:G9738C, ORF1ab:T10795C, ORF1ab:C13019T, ORF1ab:G13713A, ORF1ab:C14408T, ORF1ab:A15325G, ORF1ab:C16394T, ORF1ab:G17014T, S:G21600T, S:G22018T, S:C22597T, S:T22917G, S:A23403G, ORF3a:G25563T, M:C26681T, ORF8:C28087T, ORF8:A28272T, N:C28887T, N:C29362T,	17	N:T205I, ORF1a:T265I, ORF1a:T781I, ORF1a:C936Y, ORF1a:K1657N, ORF1a:S3158T, ORF1b:P314L, ORF1b:M620V, ORF1b:P976L, ORF1b:D1183Y, ORF3a:Q57H, ORF3a:N257D, ORF8:A65V, S:S13I, S:W152C, S:L452R, S:D614G,	ORF3a:26159-26161,
hCoV-19/Mexico/VER-IBT-IMSS-89/2021	EPI_ISL_1288303	In process	20B	B.1.1.519	26	5'UTR:T201C, 5'UTR:C203T, 5'UTR:C222T, 5'UTR:C241T, ORF1ab:G1738T, ORF1ab:C3037T, ORF1ab:C3140T, ORF1ab:A3690G, ORF1ab:C4090T, ORF1ab:C10029T, ORF1ab:C10954T, ORF1ab:A11117G, ORF1ab:C12789T, ORF1ab:C14408T, ORF1ab:T19839C, ORF1ab:A19974G, ORF1ab:C21306T, S:A22194G, S:C22995A, S:A23403G, S:G23501T, S:C23604A, S:A23756G, N:G28881A, N:G28882A, N:G28883C, N:C29197T,	14	N:R203K, N:G204R, ORF1a:P959S, ORF1a:E1142G, ORF1a:T3255I, ORF1a:I3618V, ORF1a:T4175I, ORF1b:P314L, S:N211S, S:T478K, S:D614G, S:A647S, S:P681H, S:T732A,	

hCoV-19/Mexico/VER-IBT-IMSS-90/2021	EPI_ISL_1288304	In process	20B	B.1.1.519	23	5'UTR:T201C, 5'UTR:C203T, 5'UTR:C222T, 5'UTR:C241T, ORF1ab:G1738T, ORF1ab:C3037T, ORF1ab:C3140T, ORF1ab:C10029T, ORF1ab:C10954T, ORF1ab:A11117G, ORF1ab:T11170C, ORF1ab:C12789T, ORF1ab:C14408T, ORF1ab:T19839C, ORF1ab:A19974G, ORF1ab:C21306T, S:C22995A, S:A23403G, S:C23604A, S:A23756G, N:G28881A, N:G28882A, N:G28883C, N:C29197T,	11	N:R203K, N:G204R, ORF1a:P959S, ORF1a:T3255I, ORF1a:I3618V, ORF1a:T4175I, ORF1b:P314L, S:T478K, S:D614G, S:P681H, S:T732A,
hCoV-19/Mexico/VER-IBT-IMSS-91/2021	EPI_ISL_1288305	In process	20B	B.1.1.519	28	5'UTR:C203T, 5'UTR:C222T, 5'UTR:C241T, ORF1ab:C3037T, ORF1ab:C3140T, ORF1ab:G3549A, ORF1ab:C9474T, ORF1ab:C10029T, ORF1ab:C10954T, ORF1ab:A11117G, ORF1ab:C12789T, ORF1ab:C13247T, ORF1ab:G14118A, ORF1ab:C14408T, ORF1ab:C15525T, ORF1ab:T19839C, ORF1ab:C21306T, S:C22995A, S:A23403G, S:C23604A, S:A23756G, M:G26526T, M:A27159G, ORF6:A27270G, ORF8:T27904C, N:G28881A, N:G28882A, N:G28883C, N:C29197T,	16	M:A2S, M:S213G, N:R203K, N:G204R, ORF1a:P959S, ORF1a:G1095E, ORF1a:A3070V, ORF1a:T3255I, ORF1a:I3618V, ORF1a:T4175I, ORF1b:P314L, ORF8:L4P, S:T478K, S:D614G, S:P681H, S:T732A,
hCoV-19/Mexico/VER-IBT-IMSS-93/2021	EPI_ISL_1288306	In process	20B	B.1.1.519	22	5'UTR:C203T, 5'UTR:C222T, 5'UTR:C241T, ORF1ab:C3037T, ORF1ab:C3140T, ORF1ab:C6258T, ORF1ab:C10029T, ORF1ab:C10954T, ORF1ab:A11117G, ORF1ab:C12789T, ORF1ab:C14408T, ORF1ab:T19839C, ORF1ab:C21306T, S:C22995A, S:A23403G, S:C23604A, S:A23756G, S:G25166C, S:C25276T, N:G28881A, N:G28882A, N:G28883C, N:C29197T,	13	N:R203K, N:G204R, ORF1a:P959S, ORF1a:T1998I, ORF1a:T3255I, ORF1a:I3618V, ORF1a:T4175I, ORF1b:P314L, S:T478K, S:D614G, S:P681H, S:T732A, S:E1202Q,
hCoV-19/Mexico/VER-InDRE-IBT-160/2020	EPI_ISL_1302219	In process	20A	B.1.243	19	5'UTR:C241T, ORF1ab:C1387T, ORF1ab:C3037T, ORF1ab:T3802C, ORF1ab:G5657T, ORF1ab:C7113T, ORF1ab:T13354C, ORF1ab:G13812T, ORF1ab:C14408T, S:A23403G, S:G23593C, S:T24076C, S:C24337T, S:C24370T, S:G25266T, ORF3a:G25644T, ORF3a:G25947T, M:T26669C, M:G26690T, N:C28854T,	9	N:S194L, ORF1a:V1798L, ORF1a:T2283I, ORF1b:M115I, ORF1b:P314L, ORF3a:Q185H, S:D614G, S:Q677H, S:C1235F,
hCoV-19/Mexico/VER-InDRE-IBT-96/2020	EPI_ISL_1301514	In process	20A	B.1	7	5'UTR:C241T, ORF1ab:C3037T, ORF1ab:C10448T, ORF1ab:C14408T, ORF1ab:C18246T, S:A23403G, N:C28854T, 3'UTR:C29769T,	4	N:S194L, ORF1a:P3395S, ORF1b:P314L, S:D614G,
hCoV-19/Mexico/VER-INER-IMSS-00174/2021	EPI_ISL_1279448	In process	20B	B.1.1.519	22	5'UTR:C203T, 5'UTR:C222T, 5'UTR:C241T, ORF1ab:C2942T, ORF1ab:C3037T, ORF1ab:C3140T, ORF1ab:C10029T, ORF1ab:C10954T, ORF1ab:A11117G, ORF1ab:C12789T, ORF1ab:C14408T, ORF1ab:G18090A, ORF1ab:C19763T, ORF1ab:T19839C, ORF1ab:C21306T, S:C22995A, S:A23403G, S:C23604A, S:A23756G, N:G28881A, N:G28882A, N:G28883C, N:C29197T,	12	N:R203K, N:G204R, ORF1a:P959S, ORF1a:T3255I, ORF1a:I3618V, ORF1a:T4175I, ORF1b:P314L, ORF1b:T2099I, S:T478K, S:D614G, S:P681H, S:T732A,
hCoV-19/Mexico/VER-INER-IMSS-00175/2021	EPI_ISL_1279449	In process	20B	B.1.1.519	25	5'UTR:C203T, 5'UTR:C222T, 5'UTR:C241T, ORF1ab:C3037T, ORF1ab:C3140T, ORF1ab:G3692T, ORF1ab:C5869T, ORF1ab:C10029T, ORF1ab:C10954T, ORF1ab:A11117G, ORF1ab:C12789T, ORF1ab:C14408T, ORF1ab:T19839C, ORF1ab:C21306T, S:C22987T, S:C22995A, S:A23403G, S:C23604A, S:A23756G, ORF7a:G27754T, ORF8:T27904C, ORF8:C28087T, N:G28881A, N:G28882A, N:G28883C, N:C29197T,	15	N:R203K, N:G204R, ORF1a:P959S, ORF1a:V1143F, ORF1a:T3255I, ORF1a:I3618V, ORF1a:T4175I, ORF1b:P314L, ORF7a:E121*, ORF8:L4P, ORF8:A65V, S:T478K, S:D614G, S:P681H, S:T732A,

hCoV-19/Mexico/VER-INER-IMSS-00176/2021	EPI_ISL_1279450	In process	20B	B.1.1.519	25	5'UTR:C203T, 5'UTR:C222T, 5'UTR:C241T, ORF1ab:A270C, ORF1ab:C3037T, ORF1ab:C3140T, ORF1ab:G3692T, ORF1ab:G3871T, ORF1ab:C6429A, ORF1ab:C10029T, ORF1ab:C10954T, ORF1ab:A11117G, ORF1ab:C11824A, ORF1ab:C12789T, ORF1ab:C14408T, ORF1ab:T19839C, ORF1ab:C21306T, S:C22995A, S:A23403G, S:C23604A, S:A23756G, ORF8:T27904C, N:G28881A, N:G28882A, N:G28883C, N:C29197T,	16	N:R203K, N:G204R, ORF1a:E2A, ORF1a:P959S, ORF1a:V1143F, ORF1a:K1202N, ORF1a:P2055H, ORF1a:T3255I, ORF1a:I3618V, ORF1a:T4175I, ORF1b:P314L, ORF8:L4P, S:T478K, S:D614G, S:P681H, S:T732A,	
hCoV-19/Mexico/VER-INER-IMSS-00286/2021	EPI_ISL_1279541	In process	20B	B.1.1.519	23	5'UTR:C203T, 5'UTR:C222T, 5'UTR:C241T, ORF1ab:C3037T, ORF1ab:C3140T, ORF1ab:T8758C, ORF1ab:C10029T, ORF1ab:C10954T, ORF1ab:A11117G, ORF1ab:C12789T, ORF1ab:C14408T, ORF1ab:T19839C, ORF1ab:C21306T, S:C22995A, S:A23403G, S:C23604A, S:A23756G, ORF3a:C26124T, N:G28881A, N:G28882A, N:G28883C, N:C29197T, N:A29413T, N:G29527T,	13	N:R203K, N:G204R, N:Q380H, N:Q418H, ORF1a:P959S, ORF1a:T3255I, ORF1a:I3618V, ORF1a:T4175I, ORF1b:P314L, S:T478K, S:D614G, S:P681H, S:T732A,	
hCoV-19/Mexico/VER-INER-IMSS-00287/2021	EPI_ISL_1279542	In process	20B	B.1.1.519	27	5'UTR:C203T, 5'UTR:C222T, 5'UTR:C241T, ORF1ab:G1264T, ORF1ab:C3037T, ORF1ab:C3140T, ORF1ab:C4246T, ORF1ab:G7071T, ORF1ab:C10029T, ORF1ab:C10954T, ORF1ab:A11117G, ORF1ab:C12789T, ORF1ab:C14408T, ORF1ab:T19839C, ORF1ab:C21306T, S:G21867C, S:C22995A, S:A23403G, S:C23604A, S:A23756G, M:G26526A, ORF8:T28098C, N:C28706T, N:G28881A, N:G28882A, N:G28883C, N:C29197T, N:G29527T,	17	M:A2T, N:H145Y, N:R203K, N:G204R, N:Q418H, ORF1a:P959S, ORF1a:G2269V, ORF1a:T3255I, ORF1a:I3618V, ORF1a:T4175I, ORF1b:P314L, ORF8:S69P, S:R102T, S:T478K, S:D614G, S:P681H, S:T732A,	
hCoV-19/Mexico/VER-INER-IMSS-00288/2021	EPI_ISL_1279543	In process	20A	B.1.397	22	5'UTR:C241T, ORF1ab:C583T, ORF1ab:G1135T, ORF1ab:C1288T, ORF1ab:T1873C, ORF1ab:T2377C, ORF1ab:C2881T, ORF1ab:C3037T, ORF1ab:C6286T, ORF1ab:C6941T, ORF1ab:C9592T, ORF1ab:C14408T, ORF1ab:G15906T, ORF1ab:C19402T, ORF1ab:A20268G, S:A23403G, S:G24356T, ORF3a:G25855T, N:G28280T, N:C28441T, N:C28854T, 3'UTR:C29733T, 3'UTR:C29743T,	10	N:D3Y, N:S194L, ORF1a:K290N, ORF1b:P314L, ORF1b:Q813H, ORF1b:H1979Y, ORF3a:D155Y, ORF9b:S53L, S:D614G, S:G932C,	
hCoV-19/Mexico/VER-INER-IMSS-00289/2021	EPI_ISL_1287776	In process	20B	B.1.1.222	13	5'UTR:C241T, ORF1ab:C3037T, ORF1ab:C6285T, ORF1ab:A10791G, ORF1ab:A13498G, ORF1ab:C14408T, ORF1ab:T19839C, S:G22331A, S:A23403G, S:A23756G, ORF3a:G25906T, N:G28881A, N:G28882A, N:G28883C,	10	N:R203K, N:G204R, ORF1a:T2007I, ORF1a:H3509R, ORF1b:T11A, ORF1b:P314L, ORF3a:G172C, S:G257S, S:D614G, S:T732A,	ORF7b:27879-27891, ORF8:27915-28225,
hCoV-19/Mexico/VER-INER-IMSS-00290/2021	EPI_ISL_1279544	In process	20B	B.1.1.519	27	5'UTR:C203T, 5'UTR:C222T, 5'UTR:C241T, ORF1ab:T689C, ORF1ab:C3037T, ORF1ab:C3140T, ORF1ab:C3874T, ORF1ab:A3881T, ORF1ab:C10029T, ORF1ab:C10954T, ORF1ab:A11117G, ORF1ab:G11365T, ORF1ab:C12789T, ORF1ab:C14408T, ORF1ab:G18021A, ORF1ab:T19839C, ORF1ab:A20245G, ORF1ab:C21306T, S:C22995A, S:A23403G, S:C23604A, S:A23756G, N:G28881A, N:G28882A, N:G28883C, N:C29197T, N:G29527T, N:G29543C,	15	N:R203K, N:G204R, N:Q418H, ORF1a:S142P, ORF1a:P959S, ORF1a:I1206F, ORF1a:T3255I, ORF1a:I3618V, ORF1a:T4175I, ORF1b:P314L, ORF1b:M2260V, S:T478K, S:D614G, S:P681H, S:T732A,	

hCoV-19/Mexico/VER-INER-IMSS-00291/2021	EPI_ISL_1279545	In process	20B	B.1.1.519	27	5'UTR:C203T, 5'UTR:C222T, 5'UTR:C241T, ORF1ab:T689C, ORF1ab:C3037T, ORF1ab:C3140T, ORF1ab:C3874T, ORF1ab:A3881T, ORF1ab:C10029T, ORF1ab:C10954T, ORF1ab:A11117G, ORF1ab:G11365T, ORF1ab:C12789T, ORF1ab:C14408T, ORF1ab:G18021A, ORF1ab:T19839C, ORF1ab:A20245G, ORF1ab:C21306T, S:C22995A, S:A23403G, S:C23604A, S:A23756G, N:G28881A, N:G28882A, N:G28883C, N:C29197T, N:G29527T, N:G29543C,	15	N:R203K, N:G204R, N:Q418H, ORF1a:S142P, ORF1a:P959S, ORF1a:I1206F, ORF1a:T3255I, ORF1a:I3618V, ORF1a:T4175I, ORF1b:P314L, ORF1b:M2260V, S:T478K, S:D614G, S:P681H, S:T732A,	
hCoV-19/Mexico/VER-INER-IMSS-00292/2021	EPI_ISL_1279288	In process	20B	B.1.1.222	24	5'UTR:C241T, ORF1ab:C2121T, ORF1ab:C3037T, ORF1ab:G4300T, ORF1ab:C9598T, ORF1ab:C10741T, ORF1ab:C14408T, ORF1ab:C16323T, ORF1ab:G18106T, ORF1ab:T19839C, S:C21676T, S:A23403G, S:A23756G, S:G24368C, ORF3a:G25429T, ORF3a:C25702T, E:C26252T, ORF8:G27916T, ORF8:C27944T, ORF8:G28001T, N:C28291T, N:G28646A, N:G28881A, N:G28882A, N:G28883C,	15	E:S3L, N:A125T, N:R203K, N:G204R, N:R209G, ORF1a:T619I, ORF1b:P314L, ORF1b:A1547S, ORF3a:V13L, ORF3a:P104S, ORF8:G8V, ORF9b:P3L, S:D614G, S:T732A, S:D936H,	N:28896-28898,
hCoV-19/Mexico/VER-INER-IMSS-00293/2021	EPI_ISL_1279546	In process	20B	B.1.1.519	24	5'UTR:T201C, 5'UTR:C203T, 5'UTR:C222T, 5'UTR:C241T, ORF1ab:G1738T, ORF1ab:C3037T, ORF1ab:C3140T, ORF1ab:C3768T, ORF1ab:C10029T, ORF1ab:C10954T, ORF1ab:A11117G, ORF1ab:C12789T, ORF1ab:C14408T, ORF1ab:T19839C, ORF1ab:A19974G, ORF1ab:C21306T, S:C22995A, S:A23403G, S:C23604A, S:A23756G, ORF8:A28175G, N:G28881A, N:G28882A, N:G28883C, N:C29197T,	12	N:R203K, N:G204R, ORF1a:P959S, ORF1a:T1168I, ORF1a:T3255I, ORF1a:I3618V, ORF1a:T4175I, ORF1b:P314L, S:T478K, S:D614G, S:P681H, S:T732A,	
hCoV-19/Mexico/VER-INER-IMSS-00294/2021	EPI_ISL_1279547	In process	20B	B.1.1.519	25	5'UTR:C106T, 5'UTR:C203T, 5'UTR:C222T, 5'UTR:C241T, ORF1ab:C3037T, ORF1ab:C3140T, ORF1ab:T3745C, ORF1ab:C10029T, ORF1ab:C10954T, ORF1ab:A11117G, ORF1ab:C12789T, ORF1ab:C14408T, ORF1ab:A17615G, ORF1ab:T19839C, ORF1ab:C21306T, S:C22995A, S:A23403G, S:C23604A, S:A23756G, ORF3a:C25844T, E:G26389T, M:C26527T, N:G28881A, N:G28882A, N:G28883C, N:C29197T,	15	E:V49L, M:A2V, N:R203K, N:G204R, ORF1a:P959S, ORF1a:T3255I, ORF1a:I3618V, ORF1a:T4175I, ORF1b:P314L, ORF1b:K1383R, ORF3a:T151I, S:T478K, S:D614G, S:P681H, S:T732A,	
hCoV-19/Mexico/VER-INER-IMSS-00295/2021	EPI_ISL_1279548	In process	20B	B.1.1.519	25	5'UTR:C203T, 5'UTR:C222T, 5'UTR:C241T, ORF1ab:C3037T, ORF1ab:C3140T, ORF1ab:T3745C, ORF1ab:C10029T, ORF1ab:C10954T, ORF1ab:A11117G, ORF1ab:C12789T, ORF1ab:C12932T, ORF1ab:C14408T, ORF1ab:T19839C, ORF1ab:C21306T, S:A22525G, S:C22995A, S:C23191T, S:A23403G, S:C23604A, S:A23756G, ORF3a:C25844T, ORF7b:C27879T, N:G28881A, N:G28882A, N:G28883C, N:C29197T,	14	N:R203K, N:G204R, ORF1a:P959S, ORF1a:T3255I, ORF1a:I3618V, ORF1a:T4175I, ORF1a:P4223S, ORF1b:P314L, ORF3a:T151I, ORF7b:H42Y, S:T478K, S:D614G, S:P681H, S:T732A,	
hCoV-19/Mexico/VER-INER-IMSS-00296/2021	EPI_ISL_1279549	In process	20B	B.1.1.432	24	5'UTR:C241T, ORF1ab:G860A, ORF1ab:C1205A, ORF1ab:C3037T, ORF1ab:C4276T, ORF1ab:A6956T, ORF1ab:T6958C, ORF1ab:A6985T, ORF1ab:A9204G, ORF1ab:C9319T, ORF1ab:G9856T, ORF1ab:C12412T, ORF1ab:T12688C, ORF1ab:T14313C, ORF1ab:C14408T, ORF1ab:A15596G, S:A23403G, ORF3a:C25613T, ORF3a:G25912T, ORF8:G28166A, N:G28881A, N:G28882A, N:G28883C, N:C28948T, N:T29317C,	11	N:R203K, N:G204R, ORF1a:E199K, ORF1a:Q314K, ORF1a:I2231F, ORF1a:D2980G, ORF1b:P314L, ORF1b:Y710C, ORF3a:S74F, ORF3a:G174C, S:D614G,	
hCoV-19/Mexico/VER-INER-IMSS-00297/2021	EPI_ISL_1279550	In process	20A	B.1.397	15	5'UTR:C241T, ORF1ab:G1135T, ORF1ab:C3037T, ORF1ab:C3768T, ORF1ab:G5273A, ORF1ab:G11146T, ORF1ab:C14408T, ORF1ab:T19485C, ORF1ab:A19892G, ORF1ab:A20268G, ORF1ab:T20349C, ORF1ab:G20995T, S:A23403G, ORF8:G28166A, N:G28280T, N:C28854T,	10	N:D3Y, N:S194L, ORF1a:K290N, ORF1a:T1168I, ORF1a:A1670T, ORF1a:M3627I, ORF1b:P314L, ORF1b:D2142G, ORF1b:G2510C, S:D614G,	

hCoV-19/Mexico/VER-INER-IMSS-00298/2021	EPI_ISL_1279551	In process	20B	B.1.1.519	24	5'UTR:T201C, 5'UTR:C203T, 5'UTR:C222T, 5'UTR:C241T, ORF1ab:T277C, ORF1ab:G1738T, ORF1ab:C3037T, ORF1ab:C3140T, ORF1ab:G7829T, ORF1ab:C10029T, ORF1ab:C10954T, ORF1ab:A11117G, ORF1ab:C12789T, ORF1ab:C14408T, ORF1ab:T19839C, ORF1ab:A19974G, ORF1ab:C21306T, S:C22995A, S:A23403G, S:C23604A, S:A23756G, N:G28881A, N:G28882A, N:G28883C, N:C29197T,	12	N:R203K, N:G204R, ORF1a:P959S, ORF1a:V2522F, ORF1a:T3255I, ORF1a:I3618V, ORF1a:T4175I, ORF1b:P314L, S:T478K, S:D614G, S:P681H, S:T732A,
hCoV-19/Mexico/VER-INER-IMSS-00299/2021	EPI_ISL_1279552	In process	20B	B.1.1.519	28	5'UTR:A4T, 5'UTR:C203T, 5'UTR:C222T, 5'UTR:C241T, ORF1ab:G942A, ORF1ab:C3037T, ORF1ab:C3140T, ORF1ab:T7984C, ORF1ab:C10029T, ORF1ab:C10277T, ORF1ab:C10954T, ORF1ab:A11117G, ORF1ab:C11124T, ORF1ab:C12789T, ORF1ab:C14408T, ORF1ab:T19839C, ORF1ab:A20676G, ORF1ab:C21306T, S:C22995A, S:A23403G, S:C23604A, S:A23756G, S:C25350A, E:C26464T, M:T26552C, N:G28881A, N:G28882A, N:G28883C, N:C29197T	15	N:R203K, N:G204R, ORF1a:R226K, ORF1a:P959S, ORF1a:T3255I, ORF1a:L3338F, ORF1a:I3618V, ORF1a:A3620V, ORF1a:T4175I, ORF1b:P314L, S:T478K, S:D614G, S:P681H, S:T732A, S:P1263Q,
hCoV-19/Mexico/VER-INER-IMSS-00300/2021	EPI_ISL_1279553	In process	20B	B.1.1.519	26	5'UTR:C203T, 5'UTR:C222T, 5'UTR:C241T, ORF1ab:T689C, ORF1ab:C3037T, ORF1ab:C3140T, ORF1ab:C3874T, ORF1ab:A3881T, ORF1ab:C10029T, ORF1ab:C10954T, ORF1ab:A11117G, ORF1ab:G11365T, ORF1ab:C12789T, ORF1ab:C14408T, ORF1ab:G18021A, ORF1ab:T19839C, ORF1ab:A20245G, ORF1ab:C21306T, S:C22995A, S:A23403G, S:C23604A, S:A23756G, N:G28881A, N:G28882A, N:G28883C, N:C29197T, N:G29527T,	15	N:R203K, N:G204R, N:Q418H, ORF1a:S142P, ORF1a:P959S, ORF1a:I1206F, ORF1a:T3255I, ORF1a:I3618V, ORF1a:T4175I, ORF1b:P314L, ORF1b:M2260V, S:T478K, S:D614G, S:P681H, S:T732A,
hCoV-19/Mexico/VER-INER-IMSS-00301/2021	EPI_ISL_1279554	In process	20B	B.1.1.519	28	5'UTR:C203T, 5'UTR:C222T, 5'UTR:C241T, ORF1ab:T689C, ORF1ab:C3037T, ORF1ab:C3140T, ORF1ab:C3874T, ORF1ab:A3881T, ORF1ab:C10029T, ORF1ab:C10954T, ORF1ab:A11117G, ORF1ab:G11365T, ORF1ab:C12789T, ORF1ab:C14408T, ORF1ab:G18021A, ORF1ab:T19839C, ORF1ab:A20245G, ORF1ab:C21306T, S:C22995A, S:T23010C, S:A23403G, S:C23604A, S:A23756G, M:G26814T, N:G28881A, N:G28882A, N:G28883C, N:C29197T, N:G29527T	17	M:A98S, N:R203K, N:G204R, N:Q418H, ORF1a:S142P, ORF1a:P959S, ORF1a:I1206F, ORF1a:T3255I, ORF1a:I3618V, ORF1a:T4175I, ORF1b:P314L, ORF1b:M2260V, S:T478K, S:V483A, S:D614G, S:P681H, S:T732A,
hCoV-19/Mexico/VER-INER-IMSS-00302/2021	EPI_ISL_1279555	In process	20B	B.1.1.519	22	5'UTR:C203T, 5'UTR:C222T, 5'UTR:C241T, ORF1ab:C3037T, ORF1ab:C3140T, ORF1ab:C10029T, ORF1ab:G10271A, ORF1ab:C10954T, ORF1ab:A11117G, ORF1ab:C12789T, ORF1ab:C14408T, ORF1ab:T19839C, ORF1ab:C21306T, S:C22995A, S:A23403G, S:C23604A, S:A23756G, N:G28881A, N:G28882A, N:G28883C, N:C29197T, N:A29413T, N:G29527T,	14	N:R203K, N:G204R, N:Q380H, N:Q418H, ORF1a:P959S, ORF1a:T3255I, ORF1a:V3336I, ORF1a:I3618V, ORF1a:T4175I, ORF1b:P314L, S:T478K, S:D614G, S:P681H, S:T732A,
hCoV-19/Mexico/VER-INER-IMSS-00303/2021	EPI_ISL_1279556	In process	20B	B.1.1.519	27	5'UTR:C203T, 5'UTR:C222T, 5'UTR:C241T, ORF1ab:T689C, ORF1ab:C3037T, ORF1ab:C3140T, ORF1ab:C3874T, ORF1ab:A3881T, ORF1ab:C10029T, ORF1ab:C10954T, ORF1ab:A11117G, ORF1ab:G11365T, ORF1ab:C12789T, ORF1ab:C14408T, ORF1ab:G18021A, ORF1ab:T19839C, ORF1ab:A20245G, ORF1ab:C21306T, S:C22995A, S:A23403G, S:C23604A, S:A23756G, N:G28881A, N:G28882A, N:G28883C, N:C29197T, N:G29527T, N:G29543C,	15	N:R203K, N:G204R, N:Q418H, ORF1a:S142P, ORF1a:P959S, ORF1a:I1206F, ORF1a:T3255I, ORF1a:I3618V, ORF1a:T4175I, ORF1b:P314L, ORF1b:M2260V, S:T478K, S:D614G, S:P681H, S:T732A,

hCoV-19/Mexico/VER-INER-IMSS-00311/2021	EPI_ISL_1279564	In process	20B	B.1.1.519	26	5'UTR:C203T, 5'UTR:C222T, 5'UTR:C241T, ORF1ab:A3019G, ORF1ab:C3037T, ORF1ab:C3140T, ORF1ab:G3549A, ORF1ab:C4320T, ORF1ab:T5218C, ORF1ab:C10029T, ORF1ab:C10954T, ORF1ab:A11117G, ORF1ab:C12789T, ORF1ab:C14408T, ORF1ab:T19839C, ORF1ab:C21306T, S:C22995A, S:A23403G, S:C23604A, S:A23756G, S:G24095T, S:T24903C, ORF8:T27904C, N:G28881A, N:G28882A, N:G28883C, N:C29197T,	16	N:R203K, N:G204R, ORF1a:P959S, ORF1a:G1095E, ORF1a:A1352V, ORF1a:T3255I, ORF1a:I3618V, ORF1a:T4175I, ORF1b:P314L, ORF8:L4P, S:T478K, S:D614G, S:P681H, S:T732A, S:A845S, S:I1114T,	
hCoV-19/Mexico/VER-INER-IMSS-00313/2021	EPI_ISL_1279296	In process	20A	B.1	25	5'UTR:C241T, ORF1ab:A2010G, ORF1ab:C3037T, ORF1ab:A3904T, ORF1ab:C9344T, ORF1ab:C11020T, ORF1ab:A11430G, ORF1ab:C14408T, ORF1ab:C15960T, ORF1ab:A16534G, ORF1ab:T17673G, ORF1ab:G20060T, ORF1ab:A20268G, S:C21618T, S:C21707T, S:C22338G, S:T22912G, S:G22992A, S:A23403G, M:C26681T, M:G26709A, M:C26728T, ORF7a:C27494T, ORF8:A28273T, N:G28514T, N:G28917T,	18	M:A63T, M:A69V, N:D81Y, N:G215V, ORF1a:D582G, ORF1a:L3027F, ORF1a:Y3722C, ORF1b:P314L, ORF1b:S1023G, ORF1b:S2198I, ORF7a:P34L, ORF9b:Q77H, S:T19I, S:H49Y, S:T259R, S:N450K, S:S477N, S:D614G,	S:21983-21994, ORF3a:26155-26157,
hCoV-19/Mexico/VER-INER-IMSS-00314/2021	EPI_ISL_1279566	In process	20B	B.1.1.519	27	5'UTR:T201C, 5'UTR:C203T, 5'UTR:C222T, 5'UTR:C241T, ORF1ab:G1738T, ORF1ab:C3037T, ORF1ab:C3140T, ORF1ab:C10029T, ORF1ab:C10954T, ORF1ab:A11117G, ORF1ab:C12789T, ORF1ab:C14408T, ORF1ab:C16092T, ORF1ab:T19839C, ORF1ab:A19974G, ORF1ab:C21306T, S:G22241T, S:C22995A, S:A23403G, S:C23604A, S:A23756G, ORF3a:C25553T, E:C26408T, N:G28881A, N:G28882A, N:G28883C, N:C29197T, 3'UTR:G29755T,	14	E:S55F, N:R203K, N:G204R, ORF1a:P959S, ORF1a:T3255I, ORF1a:I3618V, ORF1a:T4175I, ORF1b:P314L, ORF3a:A54V, S:V227L, S:T478K, S:D614G, S:P681H, S:T732A,	
hCoV-19/Mexico/VER-INER-IMSS-00315/2021	EPI_ISL_1279567	In process	20B	B.1.1.519	28	5'UTR:T201C, 5'UTR:C203T, 5'UTR:C222T, 5'UTR:C241T, ORF1ab:C936T, ORF1ab:G1738T, ORF1ab:G2447T, ORF1ab:C3037T, ORF1ab:C3140T, ORF1ab:C9611T, ORF1ab:C10029T, ORF1ab:C10954T, ORF1ab:A11117G, ORF1ab:T11458C, ORF1ab:C12789T, ORF1ab:C14408T, ORF1ab:T19839C, ORF1ab:A19974G, ORF1ab:C21306T, S:C22995A, S:A23403G, S:C23604A, S:A23756G, S:T25316C, N:G28881A, N:G28882A, N:G28883C, N:C29197T, N:G29227T	15	N:R203K, N:G204R, ORF1a:T224I, ORF1a:G728C, ORF1a:P959S, ORF1a:L3116F, ORF1a:T3255I, ORF1a:I3618V, ORF1a:T4175I, ORF1b:P314L, S:T478K, S:D614G, S:P681H, S:T732A, S:S1252P,	
hCoV-19/Mexico/VER-INER-IMSS-00316/2021	EPI_ISL_1279568	In process	20B	B.1.1.519	27	5'UTR:C203T, 5'UTR:C222T, 5'UTR:C241T, ORF1ab:C3037T, ORF1ab:C3140T, ORF1ab:C4002T, ORF1ab:C10029T, ORF1ab:C10954T, ORF1ab:A11117G, ORF1ab:C12789T, ORF1ab:T13345C, ORF1ab:C14408T, ORF1ab:C18264T, ORF1ab:C18283T, ORF1ab:T19839C, ORF1ab:C21306T, S:C22088T, S:C22995A, S:A23403G, S:C23604A, S:A23756G, E:A26435G, N:G28881A, N:G28882A, N:G28883C, N:C29197T, N:G29527T, 3'UTR:C29741T,	16	E:N64S, N:R203K, N:G204R, N:Q418H, ORF1a:P959S, ORF1a:T1246I, ORF1a:T3255I, ORF1a:I3618V, ORF1a:T4175I, ORF1b:P314L, ORF1b:H1606Y, S:L176F, S:T478K, S:D614G, S:P681H, S:T732A,	
hCoV-19/Mexico/VER-INER-IMSS-00317/2021	EPI_ISL_1279569	In process	20B	B.1.1.519	26	5'UTR:C203T, 5'UTR:C222T, 5'UTR:C241T, ORF1ab:C3037T, ORF1ab:C3140T, ORF1ab:C6629T, ORF1ab:C7926T, ORF1ab:T9070C, ORF1ab:C10029T, ORF1ab:C10954T, ORF1ab:A11117G, ORF1ab:C11173T, ORF1ab:C12789T, ORF1ab:C14408T, ORF1ab:T19839C, ORF1ab:C21306T, S:C21575T, S:C22995A, S:A23403G, S:C23604A, S:A23756G, N:G28337T, N:G28881A, N:G28882A, N:G28883C, N:C29197T, N:G29527T,	17	N:D22Y, N:R203K, N:G204R, N:Q418H, ORF1a:P959S, ORF1a:L2122F, ORF1a:A2554V, ORF1a:T3255I, ORF1a:I3618V, ORF1a:T4175I, ORF1b:P314L, ORF9b:Q18H, S:L5F, S:T478K, S:D614G, S:P681H, S:T732A,	

hCoV-19/Mexico/VER-INER-IMSS-00318/2021	EPI_ISL_1279277	In process	20A	B.1	12	5'UTR:C241T, ORF1ab:G1548A, ORF1ab:G2764T, ORF1ab:C3037T, ORF1ab:T3973C, ORF1ab:T9508C, ORF1ab:C14408T, ORF1ab:G16647T, ORF1ab:A20268G, S:A21626G, S:A23403G, S:A24190G, S:G25314T,	5	ORF1a:S428N, ORF1b:P314L, S:T22A, S:D614G, S:G1251V,	ORF1ab: 11288-11296,
hCoV-19/Mexico/VER-INER-IMSS-00319/2021	EPI_ISL_1279278	In process	20A	B.1	12	5'UTR:C241T, ORF1ab:G1548A, ORF1ab:G2764T, ORF1ab:C3037T, ORF1ab:T3973C, ORF1ab:T9508C, ORF1ab:C14408T, ORF1ab:G16647T, ORF1ab:A20268G, S:A21626G, S:A23403G, S:A24190G, S:G25314T,	5	ORF1a:S428N, ORF1b:P314L, S:T22A, S:D614G, S:G1251V,	ORF1ab: 11288-11296,
hCoV-19/Mexico/VER-INER-IMSS-00345/2021	EPI_ISL_1279591	In process	20B	B.1.1.519	27	5'UTR:T201C, 5'UTR:C203T, 5'UTR:C222T, 5'UTR:C241T, ORF1ab:G1738T, ORF1ab:C3037T, ORF1ab:C3140T, ORF1ab:G3753A, ORF1ab:C10029T, ORF1ab:C10954T, ORF1ab:A11117G, ORF1ab:C12789T, ORF1ab:C14408T, ORF1ab:G18020A, ORF1ab:T19839C, ORF1ab:A19974G, ORF1ab:C21306T, S:C22995A, S:A23403G, S:C23604A, S:A23756G, ORF3a:C26058T, ORF7a:T27671C, ORF8:C27998T, N:G28881A, N:G28882A, N:G28883C, N:C29197T,	14	N:R203K, N:G204R, ORF1a:P959S, ORF1a:R1163K, ORF1a:T3255I, ORF1a:I3618V, ORF1a:T4175I, ORF1b:P314L, ORF1b:R1518K, ORF7a:V93A, S:T478K, S:D614G, S:P681H, S:T732A,	
hCoV-19/Mexico/VER-INER-IMSS-00346/2021	EPI_ISL_1279592	In process	20B	B.1.1.519	22	5'UTR:C203T, 5'UTR:C222T, 5'UTR:C241T, ORF1ab:C3037T, ORF1ab:C3140T, ORF1ab:C10029T, ORF1ab:C10954T, ORF1ab:A11117G, ORF1ab:C12789T, ORF1ab:T13905C, ORF1ab:C14408T, ORF1ab:T19839C, ORF1ab:C21306T, S:C22995A, S:A23403G, S:C23604A, S:A23756G, ORF3a:G25906T, ORF3a:C25916T, N:G28881A, N:G28882A, N:G28883C, N:C29197T,	13	N:R203K, N:G204R, ORF1a:P959S, ORF1a:T3255I, ORF1a:I3618V, ORF1a:T4175I, ORF1b:P314L, ORF3a:G172C, ORF3a:T175I, S:T478K, S:D614G, S:P681H, S:T732A,	
hCoV-19/Mexico/VER-INER-IMSS-00347/2021	EPI_ISL_1279593	In process	20B	B.1.1.519	28	5'UTR:C203T, 5'UTR:C222T, 5'UTR:C241T, ORF1ab:C3037T, ORF1ab:C3140T, ORF1ab:T3454C, ORF1ab:C9559T, ORF1ab:A9587G, ORF1ab:C10029T, ORF1ab:C10954T, ORF1ab:A11117G, ORF1ab:C12789T, ORF1ab:C13945T, ORF1ab:C14408T, ORF1ab:C14670T, ORF1ab:C14724T, ORF1ab:A16427G, ORF1ab:T19839C, ORF1ab:C21306T, S:T22207C, S:C22995A, S:A23403G, S:C23604A, S:A23756G, ORF7a:C27434T, N:G28881A, N:G28882A, N:G28883C, N:C29197T,	15	N:R203K, N:G204R, ORF1a:P959S, ORF1a:I3108V, ORF1a:T3255I, ORF1a:I3618V, ORF1a:T4175I, ORF1b:P160S, ORF1b:P314L, ORF1b:Y987C, ORF7a:T14I, S:T478K, S:D614G, S:P681H, S:T732A,	
hCoV-19/Mexico/VER-INER-IMSS-00348/2021	EPI_ISL_1279594	In process	20B	B.1.1.519	26	5'UTR:C203T, 5'UTR:C222T, 5'UTR:C241T, ORF1ab:A3019G, ORF1ab:C3037T, ORF1ab:C3140T, ORF1ab:G3549A, ORF1ab:C4320T, ORF1ab:T5218C, ORF1ab:C10029T, ORF1ab:C10954T, ORF1ab:A11117G, ORF1ab:C12789T, ORF1ab:C14408T, ORF1ab:T19839C, ORF1ab:C21306T, S:C22995A, S:A23403G, S:C23604A, S:A23756G, S:G24095T, S:T24903C, ORF8:T27904C, N:G28881A, N:G28882A, N:G28883C, N:C29197T,	16	N:R203K, N:G204R, ORF1a:P959S, ORF1a:G1095E, ORF1a:A1352V, ORF1a:T3255I, ORF1a:I3618V, ORF1a:T4175I, ORF1b:P314L, ORF8:L4P, S:T478K, S:D614G, S:P681H, S:T732A, S:A845S, S:I1114T,	
hCoV-19/Mexico/VER-INER-IMSS-00353/2021	EPI_ISL_1279599	In process	20B	B.1.1.519	27	5'UTR:T201C, 5'UTR:C203T, 5'UTR:C222T, 5'UTR:C241T, ORF1ab:T277C, ORF1ab:C601T, ORF1ab:G1738T, ORF1ab:C3037T, ORF1ab:C3140T, ORF1ab:G7829T, ORF1ab:C10029T, ORF1ab:C10954T, ORF1ab:A11117G, ORF1ab:C12789T, ORF1ab:C13721T, ORF1ab:C14408T, ORF1ab:T19839C, ORF1ab:A19974G, ORF1ab:C21306T, S:C22995A, S:A23403G, S:C23604A, S:A23756G, ORF7a:G27659T, N:G28881A, N:G28882A, N:G28883C, N:C29197T,	14	N:R203K, N:G204R, ORF1a:P959S, ORF1a:V2522F, ORF1a:T3255I, ORF1a:I3618V, ORF1a:T4175I, ORF1b:P85L, ORF1b:P314L, ORF7a:R89I, S:T478K, S:D614G, S:P681H, S:T732A,	

hCoV-19/Mexico/VER-INER-IMSS-00354/2021	EPI_ISL_1279293	In process	20B	B.1.1.519	26	5'UTR:C20T, 5'UTR:T201C, 5'UTR:C203T, 5'UTR:C222T, 5'UTR:C241T, ORF1ab:C745T, ORF1ab:G1738T, ORF1ab:C3037T, ORF1ab:C3140T, ORF1ab:C3411T, ORF1ab:C10029T, ORF1ab:C10954T, ORF1ab:A11117G, ORF1ab:C12789T, ORF1ab:C12952T, ORF1ab:C14408T, ORF1ab:T19839C, ORF1ab:A19974G, ORF1ab:C21306T, S:C22995A, S:A23403G, S:C23604A, S:A23756G, N:G28881A, N:G28882A, N:G28883C, N:C29197T,	12	N:R203K, N:G204R, ORF1a:P959S, ORF1a:A1049V, ORF1a:T3255I, ORF1a:I3618V, ORF1a:T4175I, ORF1b:P314L, S:T478K, S:D614G, S:P681H, S:T732A,	ORF7a:2 7704- 27721,
hCoV-19/Mexico/VER-INER-IMSS-00357/2021	EPI_ISL_1279602	In process	20B	B.1.1.519	24	5'UTR:C203T, 5'UTR:C222T, 5'UTR:C241T, ORF1ab:C3037T, ORF1ab:C3140T, ORF1ab:G3231T, ORF1ab:T3745C, ORF1ab:C10029T, ORF1ab:C10954T, ORF1ab:G11083T, ORF1ab:A11117G, ORF1ab:C12789T, ORF1ab:C14408T, ORF1ab:T19839C, ORF1ab:C21306T, S:C22995A, S:A23403G, S:C23604A, S:A23756G, ORF3a:C25844T, ORF3a:A26105C, N:G28881A, N:G28882A, N:G28883C, N:C29197T,	15	N:R203K, N:G204R, ORF1a:P959S, ORF1a:G989V, ORF1a:T3255I, ORF1a:L3606F, ORF1a:I3618V, ORF1a:T4175I, ORF1b:P314L, ORF3a:T151I, ORF3a:D238A, S:T478K, S:D614G, S:P681H, S:T732A,	
hCoV-19/Mexico/VER-INER-IMSS-00358/2021	EPI_ISL_1279603	In process	20B	B.1.1.519	25	5'UTR:C203T, 5'UTR:C222T, 5'UTR:C241T, ORF1ab:C3037T, ORF1ab:C3140T, ORF1ab:G3231T, ORF1ab:T3745C, ORF1ab:C9592T, ORF1ab:C10029T, ORF1ab:C10954T, ORF1ab:G11083T, ORF1ab:A11117G, ORF1ab:C12789T, ORF1ab:C14408T, ORF1ab:T19839C, ORF1ab:C21306T, S:C22995A, S:A23403G, S:C23604A, S:A23756G, ORF3a:C25844T, ORF3a:A26105C, N:G28881A, N:G28882A, N:G28883C, N:C29197T,	15	N:R203K, N:G204R, ORF1a:P959S, ORF1a:G989V, ORF1a:T3255I, ORF1a:L3606F, ORF1a:I3618V, ORF1a:T4175I, ORF1b:P314L, ORF3a:T151I, ORF3a:D238A, S:T478K, S:D614G, S:P681H, S:T732A,	
hCoV-19/Mexico/YUC-InDRE-IBT-135/2020	EPI_ISL_1301591	In process	20C	B.1	11	5'UTR:C241T, ORF1ab:C1059T, ORF1ab:C3037T, ORF1ab:G3216A, ORF1ab:C5339T, ORF1ab:C14408T, ORF1ab:T14949C, ORF1ab:G17847A, S:A23403G, S:C24382T, ORF3a:G25563T, N:G28899T,	7	N:R209I, ORF1a:T265I, ORF1a:S984N, ORF1a:P1692S, ORF1b:P314L, ORF3a:Q57H, S:D614G,	
hCoV-19/Mexico/YUC-InDRE-IBT-64/2020	EPI_ISL_1301461	In process	20A	B.1	8	5'UTR:C241T, ORF1ab:C3037T, ORF1ab:C6573T, ORF1ab:C14408T, ORF1ab:C18877T, S:A23403G, ORF3a:G25563T, E:C26456A, N:C29415T,	6	E:P71H, N:A381V, ORF1a:S2103F, ORF1b:P314L, ORF3a:Q57H, S:D614G,	
hCoV-19/Mexico/YUC-InDRE-IBT-8/2020	EPI_ISL_1301449	In process	20A	B.1	8	5'UTR:C241T, ORF1ab:G1942T, ORF1ab:C3037T, ORF1ab:G8726T, ORF1ab:C14408T, S:C23248T, S:A23403G, S:G24328T, 3'UTR:G29773T,	4	ORF1a:A2821S, ORF1b:P314L, S:D614G, S:L922F,	
hCoV-19/Mexico/YUC-InDRE-IBT-9/2020	EPI_ISL_1301479	In process	20A	B.1.1	6	5'UTR:C241T, ORF1ab:C337T, ORF1ab:C3037T, ORF1ab:C7231T, ORF1ab:C13458T, ORF1ab:C14408T, S:A23403G,	3	ORF1a:S4398L, ORF1b:P314L, S:D614G,	
hCoV-19/Mexico/YUC-INER-IMSS-00360/2021	EPI_ISL_1279605	In process	20B	B.1.1.519	26	5'UTR:T201C, 5'UTR:C203T, 5'UTR:C222T, 5'UTR:C241T, ORF1ab:G1274T, ORF1ab:G1738T, ORF1ab:C3037T, ORF1ab:C3140T, ORF1ab:C7164T, ORF1ab:C7504T, ORF1ab:C10029T, ORF1ab:C10954T, ORF1ab:A11117G, ORF1ab:C12789T, ORF1ab:C13115T, ORF1ab:C14408T, ORF1ab:T19839C, ORF1ab:A19974G, ORF1ab:C21306T, S:C22995A, S:A23403G, S:C23604A, S:A23756G, N:G28881A, N:G28882A, N:G28883C, N:C29197T,	13	N:R203K, N:G204R, ORF1a:V337F, ORF1a:P959S, ORF1a:T2300I, ORF1a:T3255I, ORF1a:I3618V, ORF1a:T4175I, ORF1b:P314L, S:T478K, S:D614G, S:P681H, S:T732A,	

hCoV-19/Mexico/YUC-INER-IMSS-00361/2021	EPI_ISL_1279606	In process	20B	B.1.1.519	27	5'UTR:T201C, 5'UTR:C203T, 5'UTR:C222T, 5'UTR:C241T, ORF1ab:G1738T, ORF1ab:C3037T, ORF1ab:C3140T, ORF1ab:C10029T, ORF1ab:C10954T, ORF1ab:A11117G, ORF1ab:C12789T, ORF1ab:T12955C, ORF1ab:G13385A, ORF1ab:C14408T, ORF1ab:C19806T, ORF1ab:T19839C, ORF1ab:A19974G, ORF1ab:C21306T, S:C22995A, S:A23403G, S:C23604A, S:A23756G, E:T26436C, N:A28765C, N:G28881A, N:G28882A, N:G28883C, N:C29197T	12	N:R203K, N:G204R, ORF1a:P959S, ORF1a:T3255I, ORF1a:I3618V, ORF1a:T4175I, ORF1a:G4374S, ORF1b:P314L, S:T478K, S:D614G, S:P681H, S:T732A,
hCoV-19/Mexico/YUC-INER-IMSS-00362/2021	EPI_ISL_1279607	In process	20B	B.1.1	29	5'UTR:G174T, 5'UTR:C222T, 5'UTR:C241T, ORF1ab:C835T, ORF1ab:C2156T, ORF1ab:C3037T, ORF1ab:C4920T, ORF1ab:C6781T, ORF1ab:G6865T, ORF1ab:C14408T, ORF1ab:C15045A, ORF1ab:A15779G, ORF1ab:G15957T, ORF1ab:C16457T, ORF1ab:A17637G, ORF1ab:T18784C, ORF1ab:T19839C, ORF1ab:G20060T, S:T21616C, S:A23403G, S:A23756G, S:C24897T, ORF3a:G26063T, ORF6:T27299C, ORF7b:T27757C, ORF7b:A27888T, N:G28881A, N:G28882A, N:G28883C, N:C29077T,	17	N:R203K, N:G204R, ORF1a:L631F, ORF1a:T1552I, ORF1a:K2200N, ORF1b:P314L, ORF1b:K771R, ORF1b:S997L, ORF1b:F1773L, ORF1b:S2198I, ORF3a:G224V, ORF6:I33T, ORF7a:*122R, ORF7b:M1T, S:D614G, S:T732A, S:P1112L,
hCoV-19/Mexico/YUC-INER-IMSS-00363/2021	EPI_ISL_1279608	In process	20B	B.1.1.519	30	5'UTR:T201C, 5'UTR:C203T, 5'UTR:C222T, 5'UTR:C241T, ORF1ab:G1274T, ORF1ab:G1738T, ORF1ab:C3037T, ORF1ab:C3140T, ORF1ab:C7504T, ORF1ab:C10029T, ORF1ab:A10579T, ORF1ab:C10954T, ORF1ab:A11117G, ORF1ab:A11755G, ORF1ab:C12789T, ORF1ab:C13115T, ORF1ab:C14408T, ORF1ab:T19839C, ORF1ab:A19974G, ORF1ab:C21306T, S:C22995A, S:A23403G, S:C23604A, S:A23756G, ORF3a:C26176T, ORF7b:G27870C, N:C28770T, N:G28881A, N:G28882A, N:G28883C, N:C29197T,	15	N:T166I, N:R203K, N:G204R, ORF1a:V337F, ORF1a:P959S, ORF1a:T3255I, ORF1a:I3618V, ORF1a:T4175I, ORF1b:P314L, ORF3a:P262S, ORF7b:E39Q, S:T478K, S:D614G, S:P681H, S:T732A,
hCoV-19/Mexico/YUC-INER-IMSS-00364/2021	EPI_ISL_1279609	In process	20B	B.1.1.519	26	5'UTR:T201C, 5'UTR:C203T, 5'UTR:C222T, 5'UTR:C241T, ORF1ab:G1274T, ORF1ab:G1738T, ORF1ab:C3037T, ORF1ab:C3140T, ORF1ab:C7504T, ORF1ab:C10029T, ORF1ab:C10954T, ORF1ab:A11117G, ORF1ab:C12789T, ORF1ab:C13115T, ORF1ab:C14408T, ORF1ab:T19839C, ORF1ab:A19974G, ORF1ab:C21306T, S:C22995A, S:A23403G, S:C23604A, S:A23756G, S:C24130T, N:G28881A, N:G28882A, N:G28883C, N:C29197T,	12	N:R203K, N:G204R, ORF1a:V337F, ORF1a:P959S, ORF1a:T3255I, ORF1a:I3618V, ORF1a:T4175I, ORF1b:P314L, S:T478K, S:D614G, S:P681H, S:T732A,
hCoV-19/Mexico/YUC-INER-IMSS-00365/2021	EPI_ISL_1279610	In process	20B	B.1.1.519	26	5'UTR:T201C, 5'UTR:C203T, 5'UTR:C222T, 5'UTR:C241T, ORF1ab:G1274T, ORF1ab:G1738T, ORF1ab:C3037T, ORF1ab:C3140T, ORF1ab:C7504T, ORF1ab:C10029T, ORF1ab:C10954T, ORF1ab:A11117G, ORF1ab:C12789T, ORF1ab:C13115T, ORF1ab:C14408T, ORF1ab:T19839C, ORF1ab:A19974G, ORF1ab:C21306T, S:C22995A, S:A23403G, S:C23604A, S:A23756G, S:C24130T, N:G28881A, N:G28882A, N:G28883C, N:C29197T,	12	N:R203K, N:G204R, ORF1a:V337F, ORF1a:P959S, ORF1a:T3255I, ORF1a:I3618V, ORF1a:T4175I, ORF1b:P314L, S:T478K, S:D614G, S:P681H, S:T732A,
hCoV-19/Mexico/YUC-INER-IMSS-00370/2021	EPI_ISL_1279613	In process	20B	B.1.1.519	27	5'UTR:T201C, 5'UTR:C203T, 5'UTR:C222T, 5'UTR:C241T, ORF1ab:G1274T, ORF1ab:G1738T, ORF1ab:C3037T, ORF1ab:C3140T, ORF1ab:C7504T, ORF1ab:C10029T, ORF1ab:C10954T, ORF1ab:A11117G, ORF1ab:C12789T, ORF1ab:C13115T, ORF1ab:C14408T, ORF1ab:T19839C, ORF1ab:A19974G, ORF1ab:C21306T, S:C22995A, S:A23403G, S:C23604A, S:A23756G, S:C24130T, ORF3a:G25913A, N:G28881A, N:G28882A, N:G28883C, N:C29197T	13	N:R203K, N:G204R, ORF1a:V337F, ORF1a:P959S, ORF1a:T3255I, ORF1a:I3618V, ORF1a:T4175I, ORF1b:P314L, ORF3a:G174D, S:T478K, S:D614G, S:P681H, S:T732A,

hCoV-19/Mexico/ZAC_INER_IMSS_00676/2021	EPI_ISL_2091132	In process	20G	B.1.2	21	5'UTR:C241T, ORF1ab:C1059T, ORF1ab:C3037T, ORF1ab:G3354A, ORF1ab:A3430G, ORF1ab:C3817T, ORF1ab:G8264T, ORF1ab:C10319T, ORF1ab:C14408T, ORF1ab:A18294G, ORF1ab:A18424G, ORF1ab:G18538T, ORF1ab:C21304T, S:C21952T, S:A23403G, ORF3a:G25563T, ORF3a:G25855C, ORF3a:G25907T, ORF3a:C26060T, ORF8:C27964T, N:C28472T, N:C28869T.	16	N:P67S, N:P199L, ORF1a:T265I, ORF1a:G1030D, ORF1a:G2667C, ORF1a:L3352F, ORF1b:P314L, ORF1b:N1653D, ORF1b:V1691L, ORF1b:R2613C, ORF3a:Q57H, ORF3a:D155H, ORF3a:G172V, ORF3a:T223I, ORF8:S24L, S:D614G,
hCoV-19/Mexico/ZAC_LANGEBIO_IMSS_0262/202	EPI_ISL_1381257	In process	20B	B.1.1.222	22	5'UTR:C241T, ORF1ab:C3037T, ORF1ab:C3792T, ORF1ab:A8848C, ORF1ab:C9430T, ORF1ab:C10029T, ORF1ab:C10039T, ORF1ab:T13289C, ORF1ab:C13818T, ORF1ab:C14408T, ORF1ab:G19009A, ORF1ab:T19839C, ORF1ab:G21159T, S:A23403G, S:A23756G, ORF3a:C25701T, ORF3a:G25912T, ORF7b:A27756G, ORF8:A27921G, ORF8:G28001T, N:G28881A, N:G28882A, N:G28883C,	13	N:R203K, N:G204R, ORF1a:A1176V, ORF1a:E2861D, ORF1a:T3255I, ORF1a:F4342L, ORF1b:P314L, ORF1b:D1848N, ORF3a:G174C, ORF7b:M1V, ORF8:I10V, S:D614G, S:T732A,
hCoV-19/Mexico/ZAC-InDRE-IBT-49/2020	EPI_ISL_1301519	In process	19B	A.3	16	ORF1ab:T490A, ORF1ab:C3177T, ORF1ab:C6040T, ORF1ab:C8950T, ORF1ab:T9642C, ORF1ab:G12478A, ORF1ab:T18736C, ORF1ab:C19662T, ORF1ab:T19713C, S:T22246G, S:C24034T, M:T26729C, ORF8:G28077C, ORF8:T28144C, N:C28896G, N:C29451T, 3'UTR:A29700G.	10	N:A208G, N:T393I, ORF1a:D75E, ORF1a:P971L, ORF1a:I3126T, ORF1a:M4071I, ORF1b:F1757L, ORF8:V62L, ORF8:L84S, S:D228E,
hCoV-19/Mexico/ZAC-InDRE-IBT-51/2020	EPI_ISL_1301469	In process	20A	B.1	5	5'UTR:C241T, ORF1ab:C774A, ORF1ab:C1059T, ORF1ab:C3037T, ORF1ab:C14408T, S:A23403G,	4	ORF1a:T170N, ORF1a:T265I, ORF1b:P314L, S:D614G,
hCoV-19/Mexico/ZAC-InDRE-IBT-52/2020	EPI_ISL_1301491	In process	20C	B.1.446	9	5'UTR:C241T, ORF1ab:C1059T, ORF1ab:C3037T, ORF1ab:G3304T, ORF1ab:G4852T, ORF1ab:C14408T, S:C21658T, S:C22452T, S:A23403G, ORF3a:G25563T,	7	ORF1a:T265I, ORF1a:E1013D, ORF1a:K1529N, ORF1b:P314L, ORF3a:Q57H, S:S297L, S:D614G,
hCoV-19/Mexico/ZAC-InDRE-IBT-55/2020	EPI_ISL_1301688	In process	20C	B.1	11	5'UTR:C241T, ORF1ab:C1059T, ORF1ab:C3037T, ORF1ab:C14408T, ORF1ab:C15120T, S:C21614T, S:G22021A, S:A23403G, ORF3a:G25563T, ORF3a:C26228T, ORF8:G28003T, N:A29158G,	7	ORF1a:T265I, ORF1b:P314L, ORF3a:Q57H, ORF8:C37F, S:L18F, S:M153I, S:D614G,
hCoV-19/Mexico/ZAC-InDRE-IBT-56/2020	EPI_ISL_1301689	In process	20B	B.1.1.222	10	5'UTR:C241T, ORF1ab:C3037T, ORF1ab:C3140T, ORF1ab:C14408T, ORF1ab:T19839C, S:G22021T, S:A23403G, S:A23756G, N:G28881A, N:G28882A, N:G28883C,	7	N:R203K, N:G204R, ORF1a:P959S, ORF1b:P314L, S:M153I, S:D614G, S:T732A,
hCoV-19/Mexico/ZAC-InDRE-IBT-57/2020	EPI_ISL_1301690	In process	20C	B.1.366	9	5'UTR:G204A, 5'UTR:C241T, ORF1ab:C1059T, ORF1ab:C3037T, ORF1ab:T8041C, ORF1ab:C14408T, ORF1ab:G19816T, S:G22225T, S:A23403G, ORF3a:G25563T,	5	ORF1a:T265I, ORF1b:P314L, ORF1b:V2117L, ORF3a:Q57H, S:D614G,
hCoV-19/Mexico/ZAC-InDRE-IBT-59/2020	EPI_ISL_1301483	In process	20A	B.1	9	5'UTR:C241T, ORF1ab:G520T, ORF1ab:C3037T, ORF1ab:C4582T, ORF1ab:C13436T, ORF1ab:C14408T, ORF1ab:C18981T, ORF1ab:T20571C, S:A23403G, 3'UTR:G29779T,	4	ORF1a:M85I, ORF1a:L4391F, ORF1b:P314L, S:D614G,
hCoV-19/Mexico/ZAC-INER-IMSS-00001/2021	EPI_ISL_1279304	In process	21C (Epsilon)	B.1.429	24	5'UTR:C241T, ORF1ab:C1059T, ORF1ab:C2395T, ORF1ab:T2597C, ORF1ab:C3037T, ORF1ab:C3885T, ORF1ab:C5622T, ORF1ab:C8947T, ORF1ab:C12100T, ORF1ab:A12878G, ORF1ab:C14408T, ORF1ab:G17014T, S:G21600T, S:C21638T, S:G22018T, S:T22917G, S:A23403G, S:T24349C, ORF3a:G25563T, M:C26681T, ORF7b:G27890T, ORF8:A28272T, N:C28887T, N:C29362T, N:G29468A,	14	N:T205I, N:D399N, ORF1a:T265I, ORF1a:P1207L, ORF1a:P1786L, ORF1a:I4205V, ORF1b:P314L, ORF1b:D1183Y, ORF3a:Q57H, S:S13I, S:P26S, S:W152C, S:L452R, S:D614G,
hCoV-19/Mexico/ZAC-INER-IMSS-00002/2021	EPI_ISL_1279305	In process	20G	B.1.2	20	5'UTR:C241T, ORF1ab:C1059T, ORF1ab:C3037T, ORF1ab:C9803T, ORF1ab:C10319T, ORF1ab:C14408T, ORF1ab:A18424G, ORF1ab:G18583T, ORF1ab:C19524T, ORF1ab:C19881T, ORF1ab:G20098T, ORF1ab:C21304T, S:A22534C, S:A23403G, S:G23755T, ORF3a:G25563T, ORF3a:G25907T, ORF7a:C27684T, ORF8:C27964T, N:C28472T, N:C28869T.	15	N:P67S, N:P199L, ORF1a:T265I, ORF1a:L3352F, ORF1b:P314L, ORF1b:N1653D, ORF1b:V1706L, ORF1b:A2211S, ORF1b:R2613C, ORF3a:Q57H, ORF3a:G172V, ORF8:S24L, S:E324D, S:D614G, S:M731I,

hCoV-19/Mexico/ZAC-INER-IMSS-00003/2021	EPI_ISL_1279306	In process	20G	B.1.2	18	5'UTR:C241T, ORF1ab:C1059T, ORF1ab:C1204T, ORF1ab:C3037T, ORF1ab:G8264T, ORF1ab:C10319T, ORF1ab:C14408T, ORF1ab:A18424G, ORF1ab:C18501T, ORF1ab:C18647T, ORF1ab:C21304T, S:A23403G, S:C23533T, ORF3a:G25563T, ORF3a:G25907T, ORF3a:C26060T, ORF8:C27964T, N:C28472T, N:C28869T,	14	N:P67S, N:P199L, ORF1a:T265I, ORF1a:G2667C, ORF1a:L3352F, ORF1b:P314L, ORF1b:N1653D, ORF1b:P1727L, ORF1b:R2613C, ORF3a:Q57H, ORF3a:G172V, ORF3a:T223I, ORF8:S24L, S:D614G,
hCoV-19/Mexico/ZAC-INER-IMSS-00004/2021	EPI_ISL_1279307	In process	20A	B.1.561	20	5'UTR:C241T, ORF1ab:G1685A, ORF1ab:G2516T, ORF1ab:C3037T, ORF1ab:C5144T, ORF1ab:A5999G, ORF1ab:A10323G, ORF1ab:C10798T, ORF1ab:C11866T, ORF1ab:C14408T, ORF1ab:G16917T, ORF1ab:C17703T, ORF1ab:C18693T, ORF1ab:A20268G, S:G21974T, S:A23403G, ORF3a:T25577C, ORF8:C28087T, N:C28728T, N:C28854T, N:G28975T,	12	N:A152V, N:S194L, N:M234I, ORF1a:A474T, ORF1a:V751L, ORF1a:I1912V, ORF1a:K3353R, ORF1b:P314L, ORF3a:I62T, ORF8:A65V, S:D138Y, S:D614G,
hCoV-19/Mexico/ZAC-INER-IMSS-00005/2021	EPI_ISL_1279308	In process	20B	B.1.1.222	23	5'UTR:C241T, ORF1ab:T1438C, ORF1ab:G1821T, ORF1ab:C3037T, ORF1ab:C4543T, ORF1ab:C5183T, ORF1ab:G9890A, ORF1ab:G13900T, ORF1ab:C14408T, ORF1ab:G15372T, ORF1ab:C17502T, ORF1ab:C18395T, ORF1ab:C19024T, ORF1ab:G19086A, ORF1ab:T19839C, S:G22017T, S:A23403G, S:C23683T, S:A23756G, S:G24095T, ORF3a:A25573G, N:G28881A, N:G28882A, N:G28883C,	14	N:R203K, N:G204R, ORF1a:G519V, ORF1a:P1640S, ORF1a:A3209T, ORF1b:D145Y, ORF1b:P314L, ORF1b:A1643V, ORF1b:L1853F, ORF3a:K61E, S:W152L, S:D614G, S:T732A, S:A845S,
hCoV-19/Mexico/ZAC-INER-IMSS-00006/2021	EPI_ISL_1279309	In process	20A	B.1.243	15	5'UTR:C241T, ORF1ab:G1820A, ORF1ab:A3008G, ORF1ab:C3037T, ORF1ab:C4252T, ORF1ab:C10741T, ORF1ab:C14408T, ORF1ab:A20268G, S:A23403G, S:C23604A, S:T24076C, M:C26873T, ORF8:T27901C, ORF8:T28140C, N:C28854T, 3'UTR:T29710C,	8	N:S194L, ORF1a:G519S, ORF1a:K915E, ORF1b:P314L, ORF8:F3S, ORF8:C83R, S:D614G, S:P681H,
hCoV-19/Mexico/ZAC-INER-IMSS-00007/2021	EPI_ISL_1279310	In process	20B	B.1.1.222	22	5'UTR:C241T, ORF1ab:C3037T, ORF1ab:C3792T, ORF1ab:A6693G, ORF1ab:T7402C, ORF1ab:A8848C, ORF1ab:C9430T, ORF1ab:C10029T, ORF1ab:C10039T, ORF1ab:C13818T, ORF1ab:C14408T, ORF1ab:G19009A, ORF1ab:T19839C, ORF1ab:G21159T, S:A23403G, S:A23756G, ORF3a:G25912T, ORF7b:A27756G, ORF8:A27921G, ORF8:G28001T, N:G28881A, N:G28882A, N:G28883C,	13	N:R203K, N:G204R, ORF1a:A1176V, ORF1a:K2143R, ORF1a:E2861D, ORF1a:T3255I, ORF1b:P314L, ORF1b:D1848N, ORF3a:G174C, ORF7b:M1V, ORF8:I10V, S:D614G, S:T732A,
hCoV-19/Mexico/ZAC-INER-IMSS-00008/2021	EPI_ISL_1279311	In process	20G	B.1.2	20	5'UTR:C241T, ORF1ab:C1059T, ORF1ab:C3037T, ORF1ab:C9803T, ORF1ab:C10319T, ORF1ab:C12528T, ORF1ab:C14408T, ORF1ab:A18424G, ORF1ab:G18583T, ORF1ab:C19524T, ORF1ab:C19881T, ORF1ab:G20098T, ORF1ab:C21304T, S:A22534C, S:A23403G, ORF3a:G25563T, ORF3a:C25658T, ORF3a:G25907T, ORF8:C27964T, N:C28472T, N:C28869T,	16	N:P67S, N:P199L, ORF1a:T265I, ORF1a:L3352F, ORF1a:T4088I, ORF1b:P314L, ORF1b:N1653D, ORF1b:V1706L, ORF1b:A2211S, ORF1b:R2613C, ORF3a:Q57H, ORF3a:T89I, ORF3a:G172V, ORF8:S24L, S:E324D, S:D614G,
hCoV-19/Mexico/ZAC-INER-IMSS-00009/2021	EPI_ISL_1279312	In process	20B	B.1.1.519	28	5'UTR:T201C, 5'UTR:C203T, 5'UTR:C222T, 5'UTR:C241T, ORF1ab:G1738T, ORF1ab:C3037T, ORF1ab:C3140T, ORF1ab:C10029T, ORF1ab:C10954T, ORF1ab:A11117G, ORF1ab:C12789T, ORF1ab:G13576T, ORF1ab:C14408T, ORF1ab:G14559T, ORF1ab:T19839C, ORF1ab:C19862T, ORF1ab:A19974G, ORF1ab:C21306T, S:C22995A, S:A23403G, S:C23604A, S:A23756G, ORF3a:G25699A, M:C26681T, N:G28881A, N:G28882A, N:G28883C, N:T28921C, N:C29197T	14	N:R203K, N:G204R, ORF1a:P959S, ORF1a:T3255I, ORF1a:I3618V, ORF1a:T4175I, ORF1b:A37S, ORF1b:P314L, ORF1b:A2132V, ORF3a:A103T, S:T478K, S:D614G, S:P681H, S:T732A,

hCoV-19/Mexico/ZAC-INER-IMSS-00010/2021	EPI_ISL_1279281	In process	20G	B.1.596	16	ORF1ab:C1059T, ORF1ab:C3037T, ORF1ab:G3152T, ORF1ab:T6634C, ORF1ab:G9389A, ORF1ab:C10319T, ORF1ab:C14408T, ORF1ab:G16677A, ORF1ab:A18424G, ORF1ab:C21304T, S:C21707T, S:A23403G, ORF3a:G25563T, ORF3a:G25907T, ORF8:C27964T, N:C28472T. N:C28869T.	14	N:P67S, N:P199L, ORF1a:T265I, ORF1a:G963C, ORF1a:D3042N, ORF1a:L3352F, ORF1b:P314L, ORF1b:N1653D, ORF1b:R2613C, ORF3a:Q57H, ORF3a:G172V, ORF8:S24L, S:H49Y, S:D614G,	5'UTR:194-248
hCoV-19/Mexico/ZAC-INER-IMSS-00011/2021	EPI_ISL_1279313	In process	20B	B.1.1.519	27	5'UTR:T201C, 5'UTR:C203T, 5'UTR:C222T, 5'UTR:C241T, ORF1ab:C936T, ORF1ab:G1738T, ORF1ab:C2710T, ORF1ab:C3037T, ORF1ab:C3140T, ORF1ab:C10029T, ORF1ab:C10954T, ORF1ab:A11117G, ORF1ab:C12789T, ORF1ab:C14408T, ORF1ab:T19839C, ORF1ab:A19974G, ORF1ab:C21306T, S:C22995A, S:A23403G, S:C23604A, S:A23756G, ORF3a:A25524C, E:T26446C, N:G28881A, N:G28882A, N:G28883C, N:C29197T, N:G29227T,	13	E:S68P, N:R203K, N:G204R, ORF1a:T224I, ORF1a:P959S, ORF1a:T3255I, ORF1a:I3618V, ORF1a:T4175I, ORF1b:P314L, S:T478K, S:D614G, S:P681H, S:T732A,	
hCoV-19/Mexico/ZAC-INER-IMSS-00012/2021	EPI_ISL_1279314	In process	20A	B.1.243	16	5'UTR:C241T, ORF1ab:C3037T, ORF1ab:C6539T, ORF1ab:T7534C, ORF1ab:A12544T, ORF1ab:C14408T, ORF1ab:G16269A, ORF1ab:C16362T, ORF1ab:A20268G, S:A23403G, S:T24076C, S:G25062T, ORF3a:G25654T, ORF6:C27219T, ORF7b:A27784G, N:C28311T, N:C28854T.	9	N:P13L, N:S194L, ORF1a:H2092Y, ORF1b:P314L, ORF3a:V88L, ORF7b:Y10C, ORF9b:P10S, S:D614G, S:G1167V,	
hCoV-19/Mexico/ZAC-INER-IMSS-00013/2021	EPI_ISL_1279315	In process	21C (Epsilon)	B.1.427	31	5'UTR:C241T, ORF1ab:C1059T, ORF1ab:C2607T, ORF1ab:C3037T, ORF1ab:C3817T, ORF1ab:G7312T, ORF1ab:C7600T, ORF1ab:C9561T, ORF1ab:G9738C, ORF1ab:C13019T, ORF1ab:G13713A, ORF1ab:G14354A, ORF1ab:C14408T, ORF1ab:C16394T, ORF1ab:G17014T, ORF1ab:A17271G, ORF1ab:C18441T, ORF1ab:G19816T, ORF1ab:C20436T, S:G21600T, S:G22018T, S:C22597T, S:T22917G, S:A23403G, ORF3a:G25563T, M:C26681T, ORF8:C28087T, ORF8:A28272T, N:C28887T, N:G29260T, N:C29362T, 3'UTR:G29759T,	17	N:T205I, ORF1a:T265I, ORF1a:T781I, ORF1a:L2349F, ORF1a:S3099L, ORF1a:S3158T, ORF1b:R296K, ORF1b:P314L, ORF1b:P976L, ORF1b:D1183Y, ORF1b:V2117L, ORF3a:Q57H, ORF8:A65V, S:S13I, S:W152C, S:L452R, S:D614G,	
hCoV-19/Mexico/ZAC-INER-IMSS-00014/2021	EPI_ISL_1279316	In process	20A	B.1.241	21	5'UTR:C241T, ORF1ab:C3037T, ORF1ab:A5648C, ORF1ab:G6205A, ORF1ab:A6529G, ORF1ab:G6884A, ORF1ab:G10870T, ORF1ab:C11575T, ORF1ab:G12797A, ORF1ab:C13957T, ORF1ab:C14408T, ORF1ab:C15720T, ORF1ab:A16840C, ORF1ab:C19029T, ORF1ab:G19117T, ORF1ab:T19209C, ORF1ab:A20268G, S:T22222C, S:T22618A, S:A23403G, ORF8:C28115T, N:C28854T,	9	N:S194L, ORF1a:K1795Q, ORF1a:G2207S, ORF1a:G4178S, ORF1b:R164C, ORF1b:P314L, ORF1b:K1125Q, ORF1b:A1884S, S:D614G,	
hCoV-19/Mexico/ZAC-INER-IMSS-00015/2021	EPI_ISL_1279317	In process	20B	B.1.1.519	27	5'UTR:C203T, 5'UTR:C222T, 5'UTR:C241T, ORF1ab:A866G, ORF1ab:C1009T, ORF1ab:C3037T, ORF1ab:C3140T, ORF1ab:T3814C, ORF1ab:C5183T, ORF1ab:C10029T, ORF1ab:C10954T, ORF1ab:A11117G, ORF1ab:C11916T, ORF1ab:G12223T, ORF1ab:C12789T, ORF1ab:C14408T, ORF1ab:G16968A, ORF1ab:T19839C, ORF1ab:C21306T, S:C22995A, S:A23403G, S:C23604A, S:A23756G, ORF8:T27904C, N:G28881A, N:G28882A, N:G28883C, N:C29197T,	15	N:R203K, N:G204R, ORF1a:I201V, ORF1a:P959S, ORF1a:P1640S, ORF1a:T3255I, ORF1a:I3618V, ORF1a:S3884L, ORF1a:T4175I, ORF1b:P314L, ORF8:L4P, S:T478K, S:D614G, S:P681H, S:T732A,	
hCoV-19/Mexico/ZAC-INER-IMSS-00016/2021	EPI_ISL_1279318	In process	20G	B.1.596	17	5'UTR:C241T, ORF1ab:C1059T, ORF1ab:C3037T, ORF1ab:G3152T, ORF1ab:T6634C, ORF1ab:G9389A, ORF1ab:C10319T, ORF1ab:C14408T, ORF1ab:G16677A, ORF1ab:A18424G, ORF1ab:C21304T, S:C21707T, S:A23403G, ORF3a:G25563T, ORF3a:G25907T, ORF8:C27964T, N:C28472T. N:C28869T.	14	N:P67S, N:P199L, ORF1a:T265I, ORF1a:G963C, ORF1a:D3042N, ORF1a:L3352F, ORF1b:P314L, ORF1b:N1653D, ORF1b:R2613C, ORF3a:Q57H, ORF3a:G172V, ORF8:S24L, S:H49Y, S:D614G,	

hCoV-19/Mexico/ZAC-INER-IMSS-00017/2021	EPI_ISL_1279319	In process	20G	B.1.2	25	5'UTR:C241T, ORF1ab:C1059T, ORF1ab:C2037T, ORF1ab:C3037T, ORF1ab:C3157T, ORF1ab:C9303T, ORF1ab:C9724T, ORF1ab:C10319T, ORF1ab:G10642T, ORF1ab:G12167A, ORF1ab:T13995G, ORF1ab:C14408T, ORF1ab:A18424G, ORF1ab:G20060T, ORF1ab:C21304T, S:A22036C, S:A23403G, ORF3a:G25563T, ORF3a:G25907T, ORF6:C27247T, ORF7a:C27625T, ORF7a:G27632T, ORF8:C27964T, N:C28472T, N:C28507T, N:C28869T,	19	N:P67S, N:P199L, ORF1a:T265I, ORF1a:A591V, ORF1a:S3013F, ORF1a:L3352F, ORF1a:V3968I, ORF1b:P314L, ORF1b:N1653D, ORF1b:S2198I, ORF1b:R2613C, ORF3a:Q57H, ORF3a:G172V, ORF7a:R78C, ORF7a:R80I, ORF8:S24L, ORF9b:A75V, S:R158S, S:D614G,
hCoV-19/Mexico/ZAC-INER-IMSS-00018/2021	EPI_ISL_1279320	In process	20B	B.1.1.519	25	5'UTR:C203T, 5'UTR:C222T, 5'UTR:C241T, ORF1ab:C3037T, ORF1ab:C3140T, ORF1ab:C6070T, ORF1ab:C10029T, ORF1ab:A10829C, ORF1ab:C10954T, ORF1ab:A11117G, ORF1ab:G12292A, ORF1ab:C12789T, ORF1ab:C14408T, ORF1ab:T19839C, ORF1ab:C21306T, S:C22377A, S:C22995A, S:A23403G, S:C23604A, S:A23756G, ORF8:T27904C, ORF8:C28087T, N:G28881A, N:G28882A, N:G28883C, N:C29197T,	16	N:R203K, N:G204R, ORF1a:P959S, ORF1a:T3255I, ORF1a:I3522L, ORF1a:I3618V, ORF1a:M4009I, ORF1a:T4175I, ORF1b:P314L, ORF8:L4P, ORF8:A65V, S:P272H, S:T478K, S:D614G, S:P681H, S:T732A,
hCoV-19/Mexico/ZAC-INER-IMSS-00019/2021	EPI_ISL_1279321	In process	20G	B.1.596	20	5'UTR:C241T, ORF1ab:C1059T, ORF1ab:C3037T, ORF1ab:C10319T, ORF1ab:C10369T, ORF1ab:A11451G, ORF1ab:C14408T, ORF1ab:A18424G, ORF1ab:C20703T, ORF1ab:C21304T, S:C21811A, S:A23403G, S:A23592C, ORF3a:G25563T, ORF3a:G25907T, ORF8:C27964T, N:C28472T, N:C28869T, N:T29194C, N:T29377A, 3'UTR:C29762T,	13	N:P67S, N:P199L, ORF1a:T265I, ORF1a:L3352F, ORF1a:Q3729R, ORF1b:P314L, ORF1b:N1653D, ORF1b:R2613C, ORF3a:Q57H, ORF3a:G172V, ORF8:S24L, S:D614G, S:Q677P,
hCoV-19/Mexico/ZAC-INER-IMSS-00020/2021	EPI_ISL_1279322	In process	21C (Epsilon)	B.1.429	26	5'UTR:C241T, ORF1ab:C1059T, ORF1ab:C1113T, ORF1ab:C2395T, ORF1ab:T2597C, ORF1ab:C3037T, ORF1ab:C6525T, ORF1ab:C8947T, ORF1ab:C12100T, ORF1ab:A12878G, ORF1ab:C14408T, ORF1ab:G17014T, ORF1ab:A19476G, S:G21600T, S:C21638T, S:G22018T, S:T22917G, S:A23403G, S:C24130T, S:T24349C, S:G25166C, ORF3a:G25563T, M:C26681T, ORF7b:G27890T, ORF8:A28272T, N:C28887T, N:C29362T,	14	N:T205I, ORF1a:T265I, ORF1a:T283I, ORF1a:T2087I, ORF1a:I4205V, ORF1b:P314L, ORF1b:D1183Y, ORF3a:Q57H, S:S13I, S:P26S, S:W152C, S:L452R, S:D614G, S:E1202Q,
hCoV-19/Mexico/ZAC-INER-IMSS-00021/2021	EPI_ISL_1279323	In process	20G	B.1.2	20	5'UTR:C241T, ORF1ab:C1059T, ORF1ab:C3037T, ORF1ab:G8083A, ORF1ab:C9521T, ORF1ab:C10319T, ORF1ab:C10369T, ORF1ab:C12488T, ORF1ab:C14408T, ORF1ab:C14805T, ORF1ab:A18424G, ORF1ab:C21304T, S:A23403G, ORF3a:G25563T, ORF3a:G25907T, ORF7a:G27441T, ORF7a:C27654T, ORF8:C27964T, N:C28472T, N:C28869T, N:G29402T,	16	N:P67S, N:P199L, N:D377Y, ORF1a:T265I, ORF1a:M2606I, ORF1a:L3086F, ORF1a:L3352F, ORF1a:P4075S, ORF1b:P314L, ORF1b:N1653D, ORF1b:R2613C, ORF3a:Q57H, ORF3a:G172V, ORF7a:E16D, ORF8:S24L, S:D614G,
hCoV-19/Mexico/ZAC-INER-IMSS-00022/2021	EPI_ISL_1279324	In process	20G	B.1.2	19	5'UTR:C241T, ORF1ab:C913T, ORF1ab:C3037T, ORF1ab:C3267T, ORF1ab:G8083A, ORF1ab:C8673T, ORF1ab:C10319T, ORF1ab:C12488T, ORF1ab:C14408T, ORF1ab:C14805T, ORF1ab:T17305C, ORF1ab:A18424G, ORF1ab:C21304T, S:A23403G, ORF3a:G25563T, ORF3a:G25907T, ORF8:C27964T, N:C28472T, N:C28869T, N:G29402T,	16	N:P67S, N:P199L, N:D377Y, ORF1a:T1001I, ORF1a:M2606I, ORF1a:S2803L, ORF1a:L3352F, ORF1a:P4075S, ORF1b:P314L, ORF1b:F1280L, ORF1b:N1653D, ORF1b:R2613C, ORF3a:Q57H, ORF3a:G172V, ORF8:S24L, S:D614G,

hCoV-19/Mexico/ZAC-INER-IMSS-00023/2021	EPI_ISL_1287761	In process	20I (Alpha, V1)	B.1.1.7	34	5'UTR:C241T, ORF1ab:C913T, ORF1ab:C3037T, ORF1ab:C3267T, ORF1ab:C5388A, ORF1ab:C5986T, ORF1ab:G6362A, ORF1ab:T6954C, ORF1ab:C12970T, ORF1ab:C13176T, ORF1ab:C14408T, ORF1ab:C14676T, ORF1ab:C15279T, ORF1ab:T16176C, ORF1ab:C17977T, ORF1ab:A20013G, S:A23063T, S:C23271A, S:A23403G, S:C23604A, S:C23709T, S:C24023T, S:C24054T, S:T24506G, S:G24914C, ORF8:C27972T, ORF8:G28048T, ORF8:A28111G, N:G28280C, N:A28281T, N:T28282A, N:G28881A, N:G28882A, N:G28883C, N:C28977T,	19	N:D3L, N:R203K, N:G204R, N:S235F, ORF1a:T1001I, ORF1a:A1708D, ORF1a:A2033T, ORF1a:I2230T, ORF1a:T4304I, ORF1b:P314L, ORF1b:L1504F, S:N501Y, S:A570D, S:D614G, S:P681H, S:T716I, S:A831V, S:S982A, S:D1118H,	ORF1ab: 11288-11296, S:21765-21770, S:21992-21994, ORF8:28254, ORF8:28271,
hCoV-19/Mexico/ZAC-INER-IMSS-00024/2021	EPI_ISL_1279325	In process	20B	B.1.1.519	27	5'UTR:C203T, 5'UTR:C222T, 5'UTR:C241T, ORF1ab:C620A, ORF1ab:C788T, ORF1ab:C1889T, ORF1ab:C2973T, ORF1ab:C3037T, ORF1ab:C3140T, ORF1ab:C10029T, ORF1ab:C10954T, ORF1ab:A11117G, ORF1ab:C12789T, ORF1ab:C14178T, ORF1ab:C14408T, ORF1ab:G18186T, ORF1ab:A18366G, ORF1ab:T19839C, ORF1ab:C21306T, S:C21595T, S:C22995A, S:A23403G, S:C23604A, S:A23756G, N:G28881A, N:G28882A, N:G28883C, N:C29197T,	16	N:R203K, N:G204R, ORF1a:R119S, ORF1a:R175C, ORF1a:R542C, ORF1a:A903V, ORF1a:P959S, ORF1a:T3255I, ORF1a:I3618V, ORF1a:T4175I, ORF1b:P314L, ORF1b:M1573I, S:T478K, S:D614G, S:P681H, S:T732A,	
hCoV-19/Mexico/ZAC-INER-IMSS-00025/2021	EPI_ISL_1279271	In process	20B	B.1.1.517	19	5'UTR:C241T, ORF1ab:C337T, ORF1ab:C815T, ORF1ab:C3037T, ORF1ab:C4582T, ORF1ab:G9190T, ORF1ab:C14408T, ORF1ab:C18388T, ORF1ab:G18756A, ORF1ab:T19839C, ORF1ab:G19891T, S:T23332C, S:A23403G, S:A23756G, ORF8:A27994G, ORF8:G28083T, N:G28881A, N:G28882A, N:G28883C, N:G29179T,	9	N:R203K, N:G204R, ORF1a:R184C, ORF1b:P314L, ORF1b:D2142Y, ORF8:D34G, ORF8:E64*, S:D614G, S:T732A,	
hCoV-19/Mexico/ZAC-INER-IMSS-00026/2021	EPI_ISL_1279265	In process	20I (Alpha, V1)	B.1.1.7	34	5'UTR:C241T, ORF1ab:C913T, ORF1ab:C3037T, ORF1ab:C3267T, ORF1ab:C5388A, ORF1ab:C5986T, ORF1ab:G6362A, ORF1ab:T6954C, ORF1ab:C12970T, ORF1ab:C13176T, ORF1ab:C14408T, ORF1ab:C14676T, ORF1ab:C15279T, ORF1ab:T16176C, ORF1ab:T17214C, ORF1ab:C17977T, S:A23063T, S:C23271A, S:A23403G, S:C23604A, S:C23709T, S:C24023T, S:C24054T, S:T24506G, S:G24914C, ORF8:C27972T, ORF8:G28048T, ORF8:A28111G, N:G28280C, N:A28281T, N:T28282A, N:G28881A, N:G28882A, N:G28883C, N:C28977T,	22	N:D3L, N:R203K, N:G204R, N:S235F, ORF1a:T1001I, ORF1a:A1708D, ORF1a:A2033T, ORF1a:I2230T, ORF1a:T4304I, ORF1b:P314L, ORF1b:L1504F, ORF8:Q27*, ORF8:R52I, ORF8:Y73C, S:N501Y, S:A570D, S:D614G, S:P681H, S:T716I, S:A831V, S:S982A, S:D1118H,	ORF1ab: 11288-11296, S:21765-21770, S:21992-21994, ORF8:28271,
hCoV-19/Mexico/ZAC-INER-IMSS-00027/2021	EPI_ISL_1279326	In process	20B	B.1.1.519	25	5'UTR:T201C, 5'UTR:C203T, 5'UTR:C222T, 5'UTR:C241T, ORF1ab:G1738T, ORF1ab:C3037T, ORF1ab:C3140T, ORF1ab:C10029T, ORF1ab:C10954T, ORF1ab:A11117G, ORF1ab:C12789T, ORF1ab:C14408T, ORF1ab:T19839C, ORF1ab:A19974G, ORF1ab:C21306T, S:C22995A, S:A23403G, S:C23604A, S:A23756G, ORF3a:C25782T, N:G28881A, N:G28882A, N:G28883C, N:C29197T, N:C29253T, 3'UTR:G29742T,	12	N:R203K, N:G204R, N:S327L, ORF1a:P959S, ORF1a:T3255I, ORF1a:I3618V, ORF1a:T4175I, ORF1b:P314L, S:T478K, S:D614G, S:P681H, S:T732A,	

hCoV-19/Mexico/ZAC-INER-IMSS-00028/2021	EPI_ISL_1279327	In process	20B	B.1.1.519	27	5'UTR:C203T, 5'UTR:C222T, 5'UTR:C241T, ORF1ab:C3037T, ORF1ab:C3140T, ORF1ab:C5301T, ORF1ab:C10029T, ORF1ab:C10954T, ORF1ab:A11117G, ORF1ab:G11365T, ORF1ab:C12789T, ORF1ab:C14408T, ORF1ab:C15720T, ORF1ab:T19839C, ORF1ab:C21077T, ORF1ab:C21306T, S:G22331A, S:C22995A, S:A23403G, S:C23604A, S:A23756G, ORF8:T27986C, N:G28881A, N:G28882A, N:G28883C, N:C29197T, N:G29527T, 3'UTR:G29751T,	15	N:R203K, N:G204R, N:Q418H, ORF1a:P959S, ORF1a:A1679V, ORF1a:T3255I, ORF1a:I3618V, ORF1a:T4175I, ORF1b:P314L, ORF1b:T2537I, S:G257S, S:T478K, S:D614G, S:P681H, S:T732A,
hCoV-19/Mexico/ZAC-INER-IMSS-00029/2021	EPI_ISL_1279328	In process	20A	B.1.239	19	5'UTR:C241T, ORF1ab:A749G, ORF1ab:C3037T, ORF1ab:A6208G, ORF1ab:T6409A, ORF1ab:G11417T, ORF1ab:C13548T, ORF1ab:C14408T, ORF1ab:G16741A, ORF1ab:C18312T, ORF1ab:G19101T, ORF1ab:A20268G, S:G22094A, S:A23403G, ORF3a:C25521T, M:C26894T, M:C27005T, N:G28300T. N:C28311T. N:C28854T.	12	N:Q9H, N:P13L, N:S194L, ORF1a:N162D, ORF1a:V3718F, ORF1b:P314L, ORF1b:V1092I, ORF1b:Q1878H, ORF9b:S6I, ORF9b:P10S, S:D178N, S:D614G,
hCoV-19/Mexico/ZAC-INER-IMSS-00030/2021	EPI_ISL_1279329	In process	20B	B.1.1.519	26	5'UTR:C203T, 5'UTR:C222T, 5'UTR:C241T, ORF1ab:C1912T, ORF1ab:G2639A, ORF1ab:C3037T, ORF1ab:C3140T, ORF1ab:C10029T, ORF1ab:C10954T, ORF1ab:A11117G, ORF1ab:C12789T, ORF1ab:C14408T, ORF1ab:G15921T, ORF1ab:C18508T, ORF1ab:T18947C, ORF1ab:T19839C, ORF1ab:C21306T, S:C21762T, S:G22484T, S:C22995A, S:A23403G, S:C23604A, S:A23756G, N:G28881A, N:G28882A, N:G28883C, N:C29197T,	16	N:R203K, N:G204R, ORF1a:E792K, ORF1a:P959S, ORF1a:T3255I, ORF1a:I3618V, ORF1a:T4175I, ORF1b:P314L, ORF1b:L1681F, ORF1b:L1827P, S:A67V, S:V308L, S:T478K, S:D614G, S:P681H, S:T732A,
hCoV-19/Mexico/ZAC-INER-IMSS-00031/2021	EPI_ISL_1279330	In process	21C (Epsilon)	B.1.429	29	5'UTR:C241T, ORF1ab:C1059T, ORF1ab:C2395T, ORF1ab:T2597C, ORF1ab:C3037T, ORF1ab:C8947T, ORF1ab:C12100T, ORF1ab:A12878G, ORF1ab:C14408T, ORF1ab:G17014T, ORF1ab:C18086T, ORF1ab:C18162T, ORF1ab:C19572T, S:G21600T, S:G22018T, S:T22917G, S:T23155C, S:A23403G, S:T24349C, S:C24784T, S:C25267T, ORF3a:G25563T, M:C26681T, ORF7b:G27890T, ORF8:G28191T, ORF8:A28272T, N:G28559A, N:C28887T, N:G28975T, N:C29362T	14	N:G96S, N:T205I, N:M234I, ORF1a:T265I, ORF1a:I4205V, ORF1b:P314L, ORF1b:D1183Y, ORF1b:T1540I, ORF3a:Q57H, ORF8:V100L, S:S13I, S:W152C, S:L452R, S:D614G,
hCoV-19/Mexico/ZAC-INER-IMSS-00032/2021	EPI_ISL_1279331	In process	20B	B.1.1	16	5'UTR:C241T, ORF1ab:G625T, ORF1ab:C1188T, ORF1ab:C3037T, ORF1ab:G4180T, ORF1ab:G8039T, ORF1ab:T9172C, ORF1ab:T11932C, ORF1ab:T12805C, ORF1ab:C14408T, ORF1ab:G20464A, S:A23403G, ORF3a:C26060T, ORF6:C27213T, N:G28881A, N:G28882A, N:G28883C,	10	N:R203K, N:G204R, ORF1a:K120N, ORF1a:S308L, ORF1a:K1305N, ORF1a:D2592Y, ORF1b:P314L, ORF1b:D2333N, ORF3a:T223I, S:D614G,
hCoV-19/Mexico/ZAC-INER-IMSS-00033/2021	EPI_ISL_1279332	In process	20B	B.1.1.519	29	5'UTR:T201C, 5'UTR:C203T, 5'UTR:C222T, 5'UTR:C241T, ORF1ab:C936T, ORF1ab:G1738T, ORF1ab:C3037T, ORF1ab:C3140T, ORF1ab:C6500T, ORF1ab:C10029T, ORF1ab:C10954T, ORF1ab:A11117G, ORF1ab:C12789T, ORF1ab:C14408T, ORF1ab:A17577G, ORF1ab:T19161A, ORF1ab:T19839C, ORF1ab:A19974G, ORF1ab:C21306T, S:C22995A, S:A23403G, S:C23604A, S:A23756G, ORF3a:C25916T, N:A28477C, N:G28881A, N:G28882A, N:G28883C, N:C29197T, N:G29227T,	15	N:R203K, N:G204R, ORF1a:T224I, ORF1a:P959S, ORF1a:P2079S, ORF1a:T3255I, ORF1a:I3618V, ORF1a:T4175I, ORF1b:P314L, ORF3a:T175I, ORF9b:E65A, S:T478K, S:D614G, S:P681H, S:T732A,

hCoV-19/Mexico/ZAC-INER-IMSS-00034/2021	EPI_ISL_1279333	In process	20B	B.1.1.519	29	5'UTR:T201C, 5'UTR:C203T, 5'UTR:C222T, 5'UTR:C241T, ORF1ab:G1738T, ORF1ab:C3037T, ORF1ab:C3140T, ORF1ab:C10029T, ORF1ab:C10834T, ORF1ab:C10954T, ORF1ab:A11117G, ORF1ab:C12789T, ORF1ab:G13576T, ORF1ab:C14408T, ORF1ab:G14559T, ORF1ab:T19839C, ORF1ab:C19862T, ORF1ab:A19974G, ORF1ab:C21306T, S:C22995A, S:A23403G, S:C23604A, S:A23756G, ORF3a:G25699A, M:C26681T, N:G28881A, N:G28882A, N:G28883C, N:T28921C, N:C29197T	14	N:R203K, N:G204R, ORF1a:P959S, ORF1a:T3255I, ORF1a:I3618V, ORF1a:T4175I, ORF1b:A37S, ORF1b:P314L, ORF1b:A2132V, ORF3a:A103T, S:T478K, S:D614G, S:P681H, S:T732A,
hCoV-19/Mexico/ZAC-INER-IMSS-00035/2021	EPI_ISL_1279334	In process	20A	B.1.561	16	5'UTR:C241T, ORF1ab:C1427T, ORF1ab:G2516T, ORF1ab:C3037T, ORF1ab:A5999G, ORF1ab:A10323G, ORF1ab:C14408T, ORF1ab:C18693T, ORF1ab:A20268G, S:G21974T, S:A23403G, ORF3a:T25577C, M:C26681T, N:C28854T, N:G28975T, N:C29095T, 3'UTR:C29754T,	10	N:S194L, N:M234I, ORF1a:H388Y, ORF1a:V751L, ORF1a:I1912V, ORF1a:K3353R, ORF1b:P314L, ORF3a:I62T, S:D138Y, S:D614G,
hCoV-19/Mexico/ZAC-INER-IMSS-00036/2021	EPI_ISL_1279335	In process	21C (Epsilon)	B.1.429	26	5'UTR:C241T, ORF1ab:C1059T, ORF1ab:C2395T, ORF1ab:T2597C, ORF1ab:C3037T, ORF1ab:C4410T, ORF1ab:G7100A, ORF1ab:T7114G, ORF1ab:C8947T, ORF1ab:C12100T, ORF1ab:A12878G, ORF1ab:C14408T, ORF1ab:G17014T, S:G21600T, S:C21638T, S:G22018T, S:T22917G, S:A23403G, S:T24283C, S:T24349C, ORF3a:G25563T, M:C26681T, ORF7b:G27890T, ORF8:A28272T, N:G28321T, N:C28887T, N:C29362T,	14	N:T205I, ORF1a:T265I, ORF1a:A1382V, ORF1a:A2279T, ORF1a:I4205V, ORF1b:P314L, ORF1b:D1183Y, ORF3a:Q57H, ORF9b:R13L, S:S13I, S:P26S, S:W152C, S:L452R, S:D614G,
hCoV-19/Mexico/ZAC-INER-IMSS-00037/2021	EPI_ISL_1279336	In process	20G	B.1.2	23	5'UTR:C241T, ORF1ab:C1059T, ORF1ab:C3037T, ORF1ab:C3634T, ORF1ab:C4586T, ORF1ab:G4656A, ORF1ab:T6778C, ORF1ab:G8083A, ORF1ab:C10319T, ORF1ab:C14408T, ORF1ab:C14805T, ORF1ab:G15906T, ORF1ab:T17268C, ORF1ab:A18424G, ORF1ab:C21304T, S:A23403G, ORF3a:G25563T, ORF3a:G25621T, ORF3a:G25907T, ORF6:G27390T, ORF7a:G27613A, ORF8:C27964T, N:C28472T, N:C28869T,	16	N:P67S, N:P199L, ORF1a:T265I, ORF1a:R1464Q, ORF1a:M2606I, ORF1a:L3352F, ORF1b:P314L, ORF1b:Q813H, ORF1b:N1653D, ORF1b:R2613C, ORF3a:Q57H, ORF3a:V77F, ORF3a:G172V, ORF7a:V74I, ORF8:S24L, S:D614G,
hCoV-19/Mexico/ZAC-INER-IMSS-00038/2021	EPI_ISL_1279337	In process	20B	B.1.1.222	21	5'UTR:C241T, ORF1ab:C835T, ORF1ab:C3037T, ORF1ab:C6781T, ORF1ab:C8090T, ORF1ab:C9286T, ORF1ab:C11416T, ORF1ab:C14408T, ORF1ab:T19839C, S:T22679C, S:A23403G, S:A23756G, S:C24865G, S:G24928T, ORF3a:A25761G, ORF3a:C25771T, ORF3a:G26063T, ORF6:A27373G, ORF7b:A27888T, N:G28881A, N:G28882A, N:G28883C,	12	N:R203K, N:G204R, ORF1a:L2609F, ORF1b:P314L, ORF3a:I123M, ORF3a:L127F, ORF3a:G224V, ORF6:M58V, S:S373P, S:D614G, S:T732A, S:H1101Q,
hCoV-19/Mexico/ZAC-INER-IMSS-00039/2021	EPI_ISL_1279338	In process	20G	B.1.2	25	5'UTR:C241T, ORF1ab:C1059T, ORF1ab:C1457T, ORF1ab:T2158C, ORF1ab:C3037T, ORF1ab:T8495G, ORF1ab:C8964T, ORF1ab:G9355T, ORF1ab:T9690C, ORF1ab:C10319T, ORF1ab:C14408T, ORF1ab:A18424G, ORF1ab:C21304T, S:A23403G, ORF3a:G25483A, ORF3a:G25563T, ORF3a:G25907T, ORF3a:G26109T, M:C26735T, M:C26936T, M:G26951T, M:A27068G, ORF8:C27964T, N:C28472T, N:C28869T, N:A29403G,	19	N:P67S, N:P199L, N:D377G, ORF1a:T265I, ORF1a:R398C, ORF1a:C2744G, ORF1a:S2900L, ORF1a:M3030I, ORF1a:I3142T, ORF1a:L3352F, ORF1b:P314L, ORF1b:N1653D, ORF1b:R2613C, ORF3a:A31T, ORF3a:Q57H, ORF3a:G172V, ORF3a:E239D, ORF8:S24L, S:D614G,
hCoV-19/Mexico/ZAC-INER-IMSS-00040/2021	EPI_ISL_1279339	In process	20B	B.1.1	15	5'UTR:C241T, ORF1ab:G625T, ORF1ab:C1188T, ORF1ab:C3037T, ORF1ab:G4180T, ORF1ab:T9172C, ORF1ab:T11932C, ORF1ab:T12805C, ORF1ab:C14408T, ORF1ab:G20464A, S:A23403G, ORF3a:C26060T, ORF6:C27213T, N:G28881A, N:G28882A, N:G28883C,	9	N:R203K, N:G204R, ORF1a:K120N, ORF1a:S308L, ORF1a:K1305N, ORF1b:P314L, ORF1b:D2333N, ORF3a:T223I, S:D614G,

hCoV-19/Mexico/ZAC-INER-IMSS-00041/2021	EPI_ISL_1279340	In process	20B	B.1.1.519	27	5'UTR:T201C, 5'UTR:C203T, 5'UTR:C222T, 5'UTR:C241T, ORF1ab:C936T, ORF1ab:G1738T, ORF1ab:G1747A, ORF1ab:C3037T, ORF1ab:C3140T, ORF1ab:C6500T, ORF1ab:C10029T, ORF1ab:C10954T, ORF1ab:A11117G, ORF1ab:C12789T, ORF1ab:C14408T, ORF1ab:G18040T, ORF1ab:T19839C, ORF1ab:A19974G, ORF1ab:C21306T, S:C22995A, S:A23403G, S:C23604A, S:A23756G, N:G28881A, N:G28882A, N:G28883C, N:C29197T, N:G29227T,	14	N:R203K, N:G204R, ORF1a:T224I, ORF1a:P959S, ORF1a:P2079S, ORF1a:T3255I, ORF1a:I3618V, ORF1a:T4175I, ORF1b:P314L, ORF1b:A1525S, S:T478K, S:D614G, S:P681H, S:T732A,
hCoV-19/Mexico/ZAC-INER-IMSS-00042/2021	EPI_ISL_1279341	In process	20B	B.1.1.519	25	5'UTR:T201C, 5'UTR:C203T, 5'UTR:C222T, 5'UTR:C241T, ORF1ab:G1738T, ORF1ab:C3037T, ORF1ab:C3140T, ORF1ab:C10029T, ORF1ab:C10954T, ORF1ab:A11117G, ORF1ab:C12789T, ORF1ab:C14408T, ORF1ab:T19839C, ORF1ab:A19974G, ORF1ab:C21306T, S:C22995A, S:A23403G, S:C23604A, S:A23756G, ORF3a:C25782T, N:G28881A, N:G28882A, N:G28883C, N:C29197T, N:C29253T, 3'UTR:G29742T,	12	N:R203K, N:G204R, N:S327L, ORF1a:P959S, ORF1a:T3255I, ORF1a:I3618V, ORF1a:T4175I, ORF1b:P314L, S:T478K, S:D614G, S:P681H, S:T732A,
hCoV-19/Mexico/ZAC-INER-IMSS-00043/2021	EPI_ISL_1279342	In process	20B	B.1.1.519	22	5'UTR:C203T, 5'UTR:C222T, 5'UTR:C241T, ORF1ab:C3037T, ORF1ab:C3140T, ORF1ab:G6943T, ORF1ab:C10029T, ORF1ab:C10954T, ORF1ab:A11117G, ORF1ab:C12789T, ORF1ab:C14408T, ORF1ab:T19839C, ORF1ab:C21306T, S:C22747T, S:C22995A, S:A23403G, S:C23604A, S:A23756G, ORF8:T28160C, N:G28881A, N:G28882A, N:G28883C, N:C29197T,	11	N:R203K, N:G204R, ORF1a:P959S, ORF1a:T3255I, ORF1a:I3618V, ORF1a:T4175I, ORF1b:P314L, S:T478K, S:D614G, S:P681H, S:T732A,
hCoV-19/Mexico/ZAC-INER-IMSS-00044/2021	EPI_ISL_1279343	In process	20B	B.1.1.519	25	5'UTR:C203T, 5'UTR:C222T, 5'UTR:C241T, ORF1ab:C1912T, ORF1ab:G2639A, ORF1ab:C3037T, ORF1ab:C3140T, ORF1ab:C10029T, ORF1ab:C10954T, ORF1ab:A11117G, ORF1ab:C12789T, ORF1ab:C14408T, ORF1ab:G15921T, ORF1ab:C18508T, ORF1ab:T18947C, ORF1ab:T19839C, ORF1ab:C21306T, S:C21762T, S:C22995A, S:A23403G, S:C23604A, S:A23756G, N:G28881A, N:G28882A, N:G28883C, N:C29197T,	15	N:R203K, N:G204R, ORF1a:E792K, ORF1a:P959S, ORF1a:T3255I, ORF1a:I3618V, ORF1a:T4175I, ORF1b:P314L, ORF1b:L1681F, ORF1b:L1827P, S:A67V, S:T478K, S:D614G, S:P681H, S:T732A,
hCoV-19/Mexico/ZAC-INER-IMSS-00045/2021	EPI_ISL_1279344	In process	20A	B.1.243	19	5'UTR:C241T, ORF1ab:C346T, ORF1ab:C3037T, ORF1ab:G4201T, ORF1ab:T9508C, ORF1ab:C10741T, ORF1ab:C14408T, ORF1ab:C15714T, ORF1ab:G19542T, ORF1ab:A19543G, ORF1ab:A19676G, ORF1ab:A20268G, S:G22992A, S:A23403G, S:C23604A, S:T24076C, S:A24774T, N:C28854T, N:G29266A, 3'UTR:T29710C,	10	N:S194L, ORF1a:M1312I, ORF1b:P314L, ORF1b:M2025I, ORF1b:I2026V, ORF1b:Q2070R, S:S477N, S:D614G, S:P681H, S:Q1071L,
hCoV-19/Mexico/ZAC-INER-IMSS-00046/2021	EPI_ISL_1279345	In process	20G	B.1.596	23	5'UTR:C241T, ORF1ab:C1059T, ORF1ab:C3037T, ORF1ab:C5147T, ORF1ab:A6672G, ORF1ab:C10319T, ORF1ab:A11451G, ORF1ab:G12163A, ORF1ab:C14408T, ORF1ab:G17058T, ORF1ab:A18424G, ORF1ab:A21137G, ORF1ab:C21304T, S:C21811A, S:A23403G, S:A23592C, ORF3a:G25563T, ORF3a:G25907T, ORF8:C27964T, N:C28472T, N:C28869T, N:G28903T, N:T29194C, N:T29377A,	18	N:P67S, N:P199L, N:M210I, ORF1a:T265I, ORF1a:R1628C, ORF1a:D2136G, ORF1a:L3352F, ORF1a:Q3729R, ORF1b:P314L, ORF1b:M1197I, ORF1b:N1653D, ORF1b:K2557R, ORF1b:R2613C, ORF3a:Q57H, ORF3a:G172V, ORF8:S24L, S:D614G, S:Q677P,

hCoV-19/Mexico/ZAC-INER-IMSS-00047/2021	EPI_ISL_1279346	In process	20B	B.1.1.519	27	5'UTR:T201C, 5'UTR:C203T, 5'UTR:C222T, 5'UTR:C241T, ORF1ab:C936T, ORF1ab:G1738T, ORF1ab:C3037T, ORF1ab:C3140T, ORF1ab:T4579A, ORF1ab:C6500T, ORF1ab:C10029T, ORF1ab:C10954T, ORF1ab:A11117G, ORF1ab:C12789T, ORF1ab:C14408T, ORF1ab:G15906T, ORF1ab:T19839C, ORF1ab:A19974G, ORF1ab:C21306T, S:C22995A, S:A23403G, S:C23604A, S:A23756G, N:G28881A, N:G28882A, N:G28883C, N:C29197T, N:G29227T,	14	N:R203K, N:G204R, ORF1a:T224I, ORF1a:P959S, ORF1a:P2079S, ORF1a:T3255I, ORF1a:I3618V, ORF1a:T4175I, ORF1b:P314L, ORF1b:Q813H, S:T478K, S:D614G, S:P681H, S:T732A,	
hCoV-19/Mexico/ZAC-INER-IMSS-00048/2021	EPI_ISL_1279347	In process	20B	B.1.1.519	27	5'UTR:T201C, 5'UTR:C203T, 5'UTR:C222T, 5'UTR:C241T, ORF1ab:C936T, ORF1ab:G1738T, ORF1ab:C3037T, ORF1ab:C3140T, ORF1ab:C6500T, ORF1ab:C10029T, ORF1ab:C10954T, ORF1ab:A11117G, ORF1ab:C12789T, ORF1ab:C14408T, ORF1ab:T19161A, ORF1ab:T19839C, ORF1ab:A19974G, ORF1ab:C21306T, S:C22995A, S:A23403G, S:C23604A, S:A23756G, N:A28477C, N:G28881A, N:G28882A, N:G28883C, N:C29197T, N:G29227T,	14	N:R203K, N:G204R, ORF1a:T224I, ORF1a:P959S, ORF1a:P2079S, ORF1a:T3255I, ORF1a:I3618V, ORF1a:T4175I, ORF1b:P314L, ORF9b:E65A, S:T478K, S:D614G, S:P681H, S:T732A,	
hCoV-19/Mexico/ZAC-INER-IMSS-00049/2021	EPI_ISL_1279348	In process	21C (Epsilon)	B.1.427	26	5'UTR:C222T, 5'UTR:C241T, ORF1ab:C503T, ORF1ab:C1059T, ORF1ab:C3037T, ORF1ab:C3817T, ORF1ab:G9738C, ORF1ab:C9967T, ORF1ab:C13019T, ORF1ab:G13713A, ORF1ab:C14408T, ORF1ab:C16394T, ORF1ab:G17014T, S:G21600T, S:G22018T, S:G22335T, S:C22597T, S:T22917G, S:A23403G, ORF3a:G25563T, M:C26681T, ORF7b:G27882A, ORF8:C28087T, ORF8:A28272T, N:C28887T, N:C29362T, 3'UTR:C29686T,	15	N:T205I, ORF1a:P80S, ORF1a:T265I, ORF1a:S3158T, ORF1b:P314L, ORF1b:P976L, ORF1b:D1183Y, ORF3a:Q57H, ORF7b:A43T, ORF8:A65V, S:S13I, S:W152C, S:W258L, S:L452R, S:D614G,	
hCoV-19/Mexico/ZAC-INER-IMSS-00050/2021	EPI_ISL_1287762	In process	20I (Alpha, V1)	B.1.1.7	34	5'UTR:C241T, ORF1ab:C913T, ORF1ab:C3037T, ORF1ab:C3267T, ORF1ab:C5388A, ORF1ab:C5986T, ORF1ab:G6362A, ORF1ab:T6954C, ORF1ab:C12970T, ORF1ab:C13176T, ORF1ab:C14408T, ORF1ab:C14676T, ORF1ab:C15279T, ORF1ab:T16176C, ORF1ab:C17977T, ORF1ab:A20013G, S:A23063T, S:C23271A, S:A23403G, S:C23604A, S:C23709T, S:C24023T, S:C24054T, S:T24506G, S:G24914C, ORF8:C27972T, ORF8:G28048T, ORF8:A28111G, N:G28280C, N:A28281T, N:T28282A, N:G28881A, N:G28882A, N:G28883C, N:C28977T,	19	N:D3L, N:R203K, N:G204R, N:S235F, ORF1a:T1001I, ORF1a:A1708D, ORF1a:A2033T, ORF1a:I2230T, ORF1a:T4304I, ORF1b:P314L, ORF1b:L1504F, S:N501Y, S:A570D, S:D614G, S:P681H, S:T716I, S:A831V, S:S982A, S:D1118H,	ORF1ab: 11288-11296, S:21765-21770, S:21992-21994, ORF8:28254, ORF8:28271,
hCoV-19/Mexico/ZAC-INER-IMSS-00051/2021	EPI_ISL_1279349	In process	20B	B.1.1.519	24	5'UTR:C203T, 5'UTR:C222T, 5'UTR:C241T, ORF1ab:C2675T, ORF1ab:C3037T, ORF1ab:C3140T, ORF1ab:C10029T, ORF1ab:C10954T, ORF1ab:A11117G, ORF1ab:C12439T, ORF1ab:C12789T, ORF1ab:C14408T, ORF1ab:G18340A, ORF1ab:G19816A, ORF1ab:T19839C, ORF1ab:C21306T, S:C22995A, S:A23403G, S:C23604A, S:A23756G, S:G24193T, N:G28881A, N:G28882A, N:G28883C, N:C29197T,	14	N:R203K, N:G204R, ORF1a:P804S, ORF1a:P959S, ORF1a:T3255I, ORF1a:I3618V, ORF1a:T4175I, ORF1b:P314L, ORF1b:V1625I, ORF1b:V2117I, S:T478K, S:D614G, S:P681H, S:T732A,	
hCoV-19/Mexico/ZAC-INER-IMSS-00052/2021	EPI_ISL_1279279	In process	20A	B.1.609	16	5'UTR:C241T, ORF1ab:C3037T, ORF1ab:C4582T, ORF1ab:C4780T, ORF1ab:C6968T, ORF1ab:G10262A, ORF1ab:C13119T, ORF1ab:T13788C, ORF1ab:G14250T, ORF1ab:C14408T, ORF1ab:C16883T, ORF1ab:A20036G, ORF1ab:A20268G, S:T22207C, S:A23403G. ORF3a:G25720T. N:C28854T.	9	N:S194L, ORF1a:A3333T, ORF1a:A4285V, ORF1b:L261F, ORF1b:P314L, ORF1b:T1139I, ORF1b:N2190S, ORF3a:A110S, S:D614G,	S:21765-21770,

hCoV-19/Mexico/ZAC-INER-IMSS-00053/2021	EPI_ISL_1279350	In process	20A	B.1.239	19	5'UTR:C241T, ORF1ab:A749G, ORF1ab:C3037T, ORF1ab:A6208G, ORF1ab:T6409A, ORF1ab:G11417T, ORF1ab:C13548T, ORF1ab:C14408T, ORF1ab:G16741A, ORF1ab:C18312T, ORF1ab:G19101T, ORF1ab:A20268G, S:G22094A, S:A23403G, S:A25235G, ORF3a:C25521T, M:C26894T, M:C27005T, N:C28311T, N:C28854T,	11	N:P13L, N:S194L, ORF1a:N162D, ORF1a:V3718F, ORF1b:P314L, ORF1b:V1092I, ORF1b:Q1878H, ORF9b:P10S, S:D178N, S:D614G, S:I1225V,	
hCoV-19/Mexico/ZAC-INER-IMSS-00054/2021	EPI_ISL_1279280	In process	20A	B.1.609	18	5'UTR:C241T, ORF1ab:C3037T, ORF1ab:C4582T, ORF1ab:C4780T, ORF1ab:C6968T, ORF1ab:A9109G, ORF1ab:C13119T, ORF1ab:T13788C, ORF1ab:G14250T, ORF1ab:C14408T, ORF1ab:C14790T, ORF1ab:A20036G, ORF1ab:A20268G, S:T22207C, S:A23403G, ORF3a:G25593T, ORF3a:G25720T, M:C26625T, N:C28854T,	8	N:S194L, ORF1a:A4285V, ORF1b:L261F, ORF1b:P314L, ORF1b:N2190S, ORF3a:K67N, ORF3a:A110S, S:D614G,	S:21765-21770,
hCoV-19/Mexico/ZAC-INER-IMSS-00055/2021	EPI_ISL_1279295	In process	20B	B.1.1.519	25	5'UTR:C203T, 5'UTR:C222T, 5'UTR:C241T, ORF1ab:A866G, ORF1ab:C1009T, ORF1ab:C3037T, ORF1ab:C3140T, ORF1ab:C5183T, ORF1ab:A6616G, ORF1ab:C10029T, ORF1ab:C10954T, ORF1ab:A11117G, ORF1ab:C11916T, ORF1ab:C12789T, ORF1ab:C14408T, ORF1ab:T19839C, ORF1ab:C21306T, S:C22995A, S:A23403G, S:C23604A, S:A23756G, ORF8:T27904C, N:G28881A, N:G28882A, N:G28883C, N:C29197T,	15	N:R203K, N:G204R, ORF1a:I201V, ORF1a:P959S, ORF1a:P1640S, ORF1a:T3255I, ORF1a:I3618V, ORF1a:S3884L, ORF1a:T4175I, ORF1b:P314L, ORF8:L4P, S:T478K, S:D614G, S:P681H, S:T732A,	ORF1ab: 3260-3274
hCoV-19/Mexico/ZAC-INER-IMSS-00056/2021	EPI_ISL_1279351	In process	21C (Epsilon)	B.1.429	33	5'UTR:C241T, ORF1ab:C1059T, ORF1ab:A1816T, ORF1ab:C2395T, ORF1ab:T2597C, ORF1ab:C3037T, ORF1ab:G3728A, ORF1ab:C5748A, ORF1ab:C5986T, ORF1ab:A7678G, ORF1ab:C8917T, ORF1ab:C8947T, ORF1ab:C11962T, ORF1ab:C12100T, ORF1ab:A12878G, ORF1ab:C14408T, ORF1ab:G17014T, ORF1ab:G17019T, ORF1ab:G17722T, S:G21600T, S:G22018T, S:C22227T, S:T22917G, S:A23403G, S:T24349C, ORF3a:G25563T, ORF3a:G25677T, ORF3a:C26176T, E:G26389T, M:C26681T, ORF7b:G27890T, ORF8:A28272T, N:C28887T, N:C29362T,	19	E:V49L, N:T205I, ORF1a:T265I, ORF1a:K517N, ORF1a:G1155S, ORF1a:T1828N, ORF1a:I4205V, ORF1b:P314L, ORF1b:D1183Y, ORF1b:E1184D, ORF1b:V1419L, ORF3a:Q57H, ORF3a:L95F, ORF3a:P262S, S:S13I, S:W152C, S:A222V, S:L452R, S:D614G,	
hCoV-19/Mexico/ZAC-INER-IMSS-00057/2021	EPI_ISL_1279352	In process	21C (Epsilon)	B.1.429	25	5'UTR:C241T, ORF1ab:C1059T, ORF1ab:C1489T, ORF1ab:C2395T, ORF1ab:T2597C, ORF1ab:C3037T, ORF1ab:C8947T, ORF1ab:G11083T, ORF1ab:C12100T, ORF1ab:A12878G, ORF1ab:C14408T, ORF1ab:G17014T, ORF1ab:C21365T, S:G21600T, S:G22018T, S:G22344T, S:T22917G, S:A23403G, S:T24349C, S:C24374T, ORF3a:G25563T, M:C26681T, ORF7b:G27890T, ORF8:A28272T, N:C28887T, N:C29362T,	14	N:T205I, ORF1a:T265I, ORF1a:L3606F, ORF1a:I4205V, ORF1b:P314L, ORF1b:D1183Y, ORF1b:P2633L, ORF3a:Q57H, S:S13I, S:W152C, S:G261V, S:L452R, S:D614G, S:L938F,	
hCoV-19/Mexico/ZAC-INER-IMSS-00058/2021	EPI_ISL_1279270	In process	20B	B.1.1.222	23	5'UTR:C203T, 5'UTR:C241T, ORF1ab:C3037T, ORF1ab:G3403T, ORF1ab:A4681G, ORF1ab:T5218C, ORF1ab:C12525T, ORF1ab:C14408T, ORF1ab:C14913T, ORF1ab:A15426G, ORF1ab:C16575T, ORF1ab:T19839C, S:A23403G, S:A23756G, S:G25061A, M:G26660T, ORF7a:G27626T, ORF8:G28041T, N:G28300T, N:G28871T, N:G28881A, N:G28882A, N:G28883C, N:G29449T,	13	M:L46F, N:Q9H, N:G200C, N:R203K, N:G204R, ORF1a:T4087I, ORF1b:P314L, ORF7a:R78L, ORF8:G50*, ORF9b:S6I, S:D614G, S:T732A, S:G1167S,	

hCoV-19/Mexico/ZAC-INER-IMSS-00059/2021	EPI_ISL_1287763	In process	20I (Alpha, V1)	B.1.1.7	35	5'UTR:C241T, ORF1ab:T356C, ORF1ab:C913T, ORF1ab:C3037T, ORF1ab:C3267T, ORF1ab:C5388A, ORF1ab:C5986T, ORF1ab:G6362A, ORF1ab:T6954C, ORF1ab:C12970T, ORF1ab:C13176T, ORF1ab:C14408T, ORF1ab:C14676T, ORF1ab:C15279T, ORF1ab:T16176C, ORF1ab:C17977T, ORF1ab:A20013G, S:A23063T, S:C23271A, S:A23403G, S:C23604A, S:C23709T, S:C24023T, S:C24054T, S:T24506G, S:G24914C, ORF8:C27972T, ORF8:G28048T, ORF8:A28111G, N:G28280C, N:A28281T, N:T28282A, N:G28881A, N:G28882A, N:G28883C, N:C28977T,	14	N:D3L, N:R203K, N:G204R, N:S235F, ORF1b:P314L, ORF1b:L1504F, S:N501Y, S:A570D, S:D614G, S:P681H, S:T716I, S:A831V, S:S982A, S:D1118H,	ORF1ab: 11288-11296, S:21765-21770, S:21992-21994, ORF8:28254, ORF8:28271,ORF1ab:7709
hCoV-19/Mexico/ZAC-INER-IMSS-00060/2021	EPI_ISL_1279353	In process	20B	B.1.1.519	25	5'UTR:C203T, 5'UTR:C222T, 5'UTR:C241T, ORF1ab:G942A, ORF1ab:C3037T, ORF1ab:C3140T, ORF1ab:C6196A, ORF1ab:C8146T, ORF1ab:C10029T, ORF1ab:C10954T, ORF1ab:A11117G, ORF1ab:C11124T, ORF1ab:C12789T, ORF1ab:C14408T, ORF1ab:T19839C, ORF1ab:G20062T, ORF1ab:A20676G, ORF1ab:C21306T, S:C22995A, S:A23403G, S:C23604A, S:A23756G, N:G28881A, N:G28882A, N:G28883C, N:C29197T,	14	N:R203K, N:G204R, ORF1a:R226K, ORF1a:P959S, ORF1a:T3255I, ORF1a:I3618V, ORF1a:A3620V, ORF1a:T4175I, ORF1b:P314L, ORF1b:V2199F, S:T478K, S:D614G, S:P681H, S:T732A,	
hCoV-19/Mexico/ZAC-INER-IMSS-00061/2021	EPI_ISL_1279354	In process	20B	B.1.1.519	26	5'UTR:T201C, 5'UTR:C203T, 5'UTR:C222T, 5'UTR:C241T, ORF1ab:C936T, ORF1ab:C1380T, ORF1ab:G1738T, ORF1ab:C3037T, ORF1ab:C3140T, ORF1ab:C10029T, ORF1ab:C10954T, ORF1ab:A11117G, ORF1ab:C12789T, ORF1ab:C14408T, ORF1ab:T14883C, ORF1ab:T19839C, ORF1ab:A19974G, ORF1ab:C21306T, S:C22995A, S:A23403G, S:C23604A, S:A23756G, N:G28881A, N:G28882A, N:G28883C, N:C29197T, N:G29227T,	13	N:R203K, N:G204R, ORF1a:T224I, ORF1a:A372V, ORF1a:P959S, ORF1a:T3255I, ORF1a:I3618V, ORF1a:T4175I, ORF1b:P314L, S:T478K, S:D614G, S:P681H, S:T732A,	
hCoV-19/Mexico/ZAC-INER-IMSS-00062/2021	EPI_ISL_1279355	In process	20B	B.1.1.222	19	5'UTR:C241T, ORF1ab:C527T, ORF1ab:C835T, ORF1ab:C2062T, ORF1ab:C3037T, ORF1ab:C6781T, ORF1ab:C8090T, ORF1ab:C11416T, ORF1ab:C13862T, ORF1ab:C14408T, ORF1ab:A17737G, ORF1ab:T19839C, S:T23371C, S:A23403G, S:A23756G, ORF3a:G26063T, ORF7b:A27888T, N:G28881A, N:G28882A, N:G28883C,	9	N:R203K, N:G204R, ORF1a:L2609F, ORF1b:T132I, ORF1b:P314L, ORF1b:T1424A, ORF3a:G224V, S:D614G, S:T732A,	
hCoV-19/Mexico/ZAC-INER-IMSS-00063/2021	EPI_ISL_1279356	In process	20B	B.1.1.519	24	5'UTR:C106T, 5'UTR:C203T, 5'UTR:C222T, 5'UTR:C241T, ORF1ab:C3037T, ORF1ab:C3140T, ORF1ab:G9130T, ORF1ab:C10029T, ORF1ab:C10954T, ORF1ab:A11117G, ORF1ab:C12789T, ORF1ab:C14408T, ORF1ab:T19839C, ORF1ab:G20398A, ORF1ab:C21306T, S:C22995A, S:A23403G, S:C23604A, S:A23756G, N:G28881A, N:G28882A, N:G28883C, N:C29197T, N:A29413T, N:G29527T,	14	N:R203K, N:G204R, N:Q380H, N:Q418H, ORF1a:P959S, ORF1a:T3255I, ORF1a:I3618V, ORF1a:T4175I, ORF1b:P314L, ORF1b:E2311K, S:T478K, S:D614G, S:P681H, S:T732A,	
hCoV-19/Mexico/ZAC-INER-IMSS-00064/2021	EPI_ISL_1279357	In process	20A	B.1.561	16	5'UTR:C241T, ORF1ab:C1427T, ORF1ab:G2516T, ORF1ab:C3037T, ORF1ab:A5999G, ORF1ab:A10323G, ORF1ab:C14408T, ORF1ab:C18693T, ORF1ab:A20268G, S:G21974T, S:A23403G, ORF3a:T25577C, M:C26681T, N:C28854T, N:G28975T, N:C29095T, 3'UTR:C29754T,	10	N:S194L, N:M234I, ORF1a:H388Y, ORF1a:V751L, ORF1a:I1912V, ORF1a:K3353R, ORF1b:P314L, ORF3a:I62T, S:D138Y, S:D614G,	

hCoV-19/Mexico/ZAC-INER-IMSS-00065/2021	EPI_ISL_1279358	In process	21C (Epsilon)	B.1.429	27	5'UTR:C241T, ORF1ab:C1059T, ORF1ab:C1489T, ORF1ab:C2395T, ORF1ab:T2597C, ORF1ab:C3037T, ORF1ab:G3259A, ORF1ab:C8947T, ORF1ab:G11083T, ORF1ab:C12100T, ORF1ab:A12878G, ORF1ab:C14408T, ORF1ab:G17014T, ORF1ab:C21365T, S:G21600T, S:G22018T, S:G22344T, S:T22917G, S:A23403G, S:T24349C, S:C24374T, ORF3a:G25563T, M:C26681T, ORF7b:G27890T, ORF8:A28272T, N:C28887T, N:C29362T, N:G29543T,	14	N:T205I, ORF1a:T265I, ORF1a:L3606F, ORF1a:I4205V, ORF1b:P314L, ORF1b:D1183Y, ORF1b:P2633L, ORF3a:Q57H, S:S13I, S:W152C, S:G261V, S:L452R, S:D614G, S:L938F,	
hCoV-19/Mexico/ZAC-INER-IMSS-00066/2021	EPI_ISL_1279359	In process	20A	B.1.404	20	5'UTR:C241T, ORF1ab:C823T, ORF1ab:C1349A, ORF1ab:G2492A, ORF1ab:C3037T, ORF1ab:T3733G, ORF1ab:G5572T, ORF1ab:G7042T, ORF1ab:A10323G, ORF1ab:G10631A, ORF1ab:A12644G, ORF1ab:C14408T, ORF1ab:T16743C, ORF1ab:C19017T, ORF1ab:G19086T, ORF1ab:A20268G, S:G22992A, S:A23403G, ORF3a:C25782T, ORF3a:G25996T, N:C28854T,	13	N:S194L, ORF1a:Q362K, ORF1a:E743K, ORF1a:M1769I, ORF1a:M2259I, ORF1a:K3353R, ORF1a:A3456T, ORF1a:I4127V, ORF1b:P314L, ORF1b:K1873N, ORF3a:V202L, S:S477N, S:D614G,	
hCoV-19/Mexico/ZAC-INER-IMSS-00067/2021	EPI_ISL_1279360	In process	20B	B.1.1.519	28	5'UTR:C203T, 5'UTR:C222T, 5'UTR:C241T, ORF1ab:C3037T, ORF1ab:C3140T, ORF1ab:T3745C, ORF1ab:C10029T, ORF1ab:C10507T, ORF1ab:C10954T, ORF1ab:A11117G, ORF1ab:C12789T, ORF1ab:G12999A, ORF1ab:C14134T, ORF1ab:C14408T, ORF1ab:T19839C, ORF1ab:C20404T, ORF1ab:C21306T, S:T22729C, S:C22995A, S:A23403G, S:C23604A, S:A23756G, ORF3a:C25844T, ORF7b:C27769T, N:C28849T, N:G28881A, N:G28882A, N:G28883C, N:C29197T,	16	N:R203K, N:G204R, ORF1a:P959S, ORF1a:T3255I, ORF1a:I3618V, ORF1a:T4175I, ORF1a:S4245N, ORF1b:P223S, ORF1b:P314L, ORF1b:P2313S, ORF3a:T151I, ORF7b:S5L, S:T478K, S:D614G, S:P681H, S:T732A,	
hCoV-19/Mexico/ZAC-INER-IMSS-00068/2021	EPI_ISL_1279300	In process	20B	B.1.1	21	5'UTR:C241T, ORF1ab:C1441T, ORF1ab:A1558G, ORF1ab:C3037T, ORF1ab:G3901A, ORF1ab:A4778G, ORF1ab:C6539T, ORF1ab:C10440T, ORF1ab:C10833T, ORF1ab:C14408T, ORF1ab:C18486T, ORF1ab:T19839C, S:A23403G, S:A23756G, ORF3a:G25906T, ORF6:C27213T, N:G28881A, N:G28882A, N:G28883C, N:C29296T, N:G29473A, ORF10:A29591G,	11	N:R203K, N:G204R, ORF1a:I431M, ORF1a:I1505V, ORF1a:H2092Y, ORF1a:A3392V, ORF1a:A3523V, ORF1b:P314L, ORF3a:G172C, S:D614G, S:T732A,	3'UTR:29750-29759,
hCoV-19/Mexico/ZAC-INER-IMSS-00069/2021	EPI_ISL_1279361	In process	20B	B.1.1.519	26	5'UTR:C203T, 5'UTR:C222T, 5'UTR:C241T, ORF1ab:C1912T, ORF1ab:G2639A, ORF1ab:C3037T, ORF1ab:C3140T, ORF1ab:G9441A, ORF1ab:C10029T, ORF1ab:C10954T, ORF1ab:A11117G, ORF1ab:C12789T, ORF1ab:C14408T, ORF1ab:G15921T, ORF1ab:C18508T, ORF1ab:T18947C, ORF1ab:T19839C, ORF1ab:C21306T, S:C21762T, S:C22995A, S:A23403G, S:C23604A, S:A23756G, N:G28881A, N:G28882A, N:G28883C, N:C29197T,	16	N:R203K, N:G204R, ORF1a:E792K, ORF1a:P959S, ORF1a:C3059Y, ORF1a:T3255I, ORF1a:I3618V, ORF1a:T4175I, ORF1b:P314L, ORF1b:L1681F, ORF1b:L1827P, S:A67V, S:T478K, S:D614G, S:P681H, S:T732A,	
hCoV-19/Mexico/ZAC-INER-IMSS-00070/2021	EPI_ISL_1279362	In process	20B	B.1.1.519	27	5'UTR:C203T, 5'UTR:C222T, 5'UTR:C241T, ORF1ab:C1912T, ORF1ab:G2639A, ORF1ab:C3037T, ORF1ab:C3140T, ORF1ab:C10029T, ORF1ab:C10954T, ORF1ab:A11117G, ORF1ab:C12789T, ORF1ab:C14408T, ORF1ab:G15921T, ORF1ab:A16067G, ORF1ab:C18508T, ORF1ab:T18947C, ORF1ab:T19839C, ORF1ab:C21306T, S:C21762T, S:C22995A, S:A23403G, S:C23604A, S:A23756G, ORF7a:G27598C, N:G28881A, N:G28882A, N:G28883C, N:C29197T,	17	N:R203K, N:G204R, ORF1a:E792K, ORF1a:P959S, ORF1a:T3255I, ORF1a:I3618V, ORF1a:T4175I, ORF1b:P314L, ORF1b:E867G, ORF1b:L1681F, ORF1b:L1827P, ORF7a:D69H, S:A67V, S:T478K, S:D614G, S:P681H, S:T732A,	

hCoV-19/Mexico/ZAC-INER-IMSS-00071/2021	EPI_ISL_1279363	In process	20B	B.1.1.519	27	5'UTR:C203T, 5'UTR:C222T, 5'UTR:C241T, ORF1ab:C620A, ORF1ab:C788T, ORF1ab:C1889T, ORF1ab:C2973T, ORF1ab:C3037T, ORF1ab:C3140T, ORF1ab:C10029T, ORF1ab:C10954T, ORF1ab:A11117G, ORF1ab:C12789T, ORF1ab:C14178T, ORF1ab:C14408T, ORF1ab:G18186T, ORF1ab:A18366G, ORF1ab:T19839C, ORF1ab:C21306T, S:C21595T, S:C22995A, S:A23403G, S:C23604A, S:A23756G, N:G28881A, N:G28882A, N:G28883C, N:C29197T,	16	N:R203K, N:G204R, ORF1a:R119S, ORF1a:R175C, ORF1a:R542C, ORF1a:A903V, ORF1a:P959S, ORF1a:T3255I, ORF1a:I3618V, ORF1a:T4175I, ORF1b:P314L, ORF1b:M1573I, S:T478K, S:D614G, S:P681H, S:T732A,
hCoV-19/Mexico/ZAC-INER-IMSS-00072/2021	EPI_ISL_1279364	In process	20A	B.1.239	19	5'UTR:C241T, ORF1ab:A749G, ORF1ab:C3037T, ORF1ab:A6208G, ORF1ab:T6409A, ORF1ab:G11417T, ORF1ab:C13548T, ORF1ab:C14408T, ORF1ab:G16741A, ORF1ab:C18312T, ORF1ab:G19101T, ORF1ab:A20268G, S:G22094A, S:A23403G, S:A25235G, ORF3a:C25521T, M:C26894T, M:C27005T, N:C28311T, N:C28854T,	11	N:P13L, N:S194L, ORF1a:N162D, ORF1a:V3718F, ORF1b:P314L, ORF1b:V1092I, ORF1b:Q1878H, ORF9b:P10S, S:D178N, S:D614G, S:I1225V,
hCoV-19/Mexico/ZAC-INER-IMSS-00073/2021	EPI_ISL_1279365	In process	20G	B.1.2	21	5'UTR:C241T, ORF1ab:C1059T, ORF1ab:C1931A, ORF1ab:G2309A, ORF1ab:C3037T, ORF1ab:C10319T, ORF1ab:C12115T, ORF1ab:C14408T, ORF1ab:G14587T, ORF1ab:A18424G, ORF1ab:G20060T, ORF1ab:T20346C, ORF1ab:C21304T, S:A23403G, ORF3a:G25563T, ORF3a:C25904T, ORF3a:G25907T, ORF8:C27964T, N:C28472T, N:C28869T, N:G29425C, 3'UTR:G29751T,	17	N:P67S, N:P199L, N:Q384H, ORF1a:T265I, ORF1a:Q556K, ORF1a:V682I, ORF1a:L3352F, ORF1b:P314L, ORF1b:A374S, ORF1b:N1653D, ORF1b:S2198I, ORF1b:R2613C, ORF3a:Q57H, ORF3a:S171L, ORF3a:G172V, ORF8:S24L, S:D614G,
hCoV-19/Mexico/ZAC-INER-IMSS-00074/2021	EPI_ISL_1279366	In process	20A	B.1.551	15	5'UTR:C241T, ORF1ab:C3037T, ORF1ab:C3738T, ORF1ab:C4084T, ORF1ab:C7945T, ORF1ab:C14408T, ORF1ab:G17601A, ORF1ab:C17934T, ORF1ab:A20268G, S:A23403G, S:C23604G, ORF7a:C27752T, N:G28628T, N:C28854T, N:G29254T, 3'UTR:G29742T,	7	N:A119S, N:S194L, ORF1a:P1158L, ORF1b:P314L, ORF7a:T120I, S:D614G, S:P681R,
hCoV-19/Mexico/ZAC-INER-IMSS-00075/2021	EPI_ISL_1279272	In process	20B	B.1.1.517	21	5'UTR:C207T, 5'UTR:C241T, ORF1ab:C337T, ORF1ab:C815T, ORF1ab:C3037T, ORF1ab:C4582T, ORF1ab:G9190T, ORF1ab:C14408T, ORF1ab:C18388T, ORF1ab:G18756A, ORF1ab:T19839C, ORF1ab:G19891T, S:T23332C, S:A23403G, S:A23756G, ORF8:A27994G, ORF8:G28083T, N:G28881A, N:G28882A, N:G28883C, N:C28915T, N:G29179T,	9	N:R203K, N:G204R, ORF1a:R184C, ORF1b:P314L, ORF1b:D2142Y, ORF8:D34G, ORF8:E64*, S:D614G, S:T732A,
hCoV-19/Mexico/ZAC-INER-IMSS-00076/2021	EPI_ISL_1279367	In process	20B	B.1.1.519	26	5'UTR:C203T, 5'UTR:C222T, 5'UTR:C241T, ORF1ab:C3037T, ORF1ab:C3140T, ORF1ab:C6406T, ORF1ab:C7528T, ORF1ab:C8950T, ORF1ab:C10029T, ORF1ab:C10954T, ORF1ab:A11117G, ORF1ab:C12789T, ORF1ab:C14408T, ORF1ab:T19839C, ORF1ab:A19959G, ORF1ab:C20178T, ORF1ab:C21306T, S:C22995A, S:A23403G, S:C23604A, S:A23756G, M:G26526A, N:G28881A, N:G28882A, N:G28883C, N:C29197T, N:G29527T,	13	M:A2T, N:R203K, N:G204R, N:Q418H, ORF1a:P959S, ORF1a:T3255I, ORF1a:I3618V, ORF1a:T4175I, ORF1b:P314L, S:T478K, S:D614G, S:P681H, S:T732A,
hCoV-19/Mexico/ZAC-INER-IMSS-00077/2021	EPI_ISL_1279273	In process		B.1.243	0	,	1	,

hCoV-19/Mexico/ZAC-INER-IMSS-00078/2021	EPI_ISL_1287764	In process	20I (Alpha, V1)	B.1.1.7	34	5'UTR:C241T, ORF1ab:C913T, ORF1ab:C3037T, ORF1ab:C3267T, ORF1ab:G4960T, ORF1ab:C5140A, ORF1ab:C6027T, ORF1ab:A8413G, ORF1ab:C12970T, ORF1ab:C13176T, ORF1ab:C14408T, ORF1ab:C14676T, ORF1ab:C15279T, ORF1ab:T16176C, ORF1ab:C17977T, ORF1ab:A20013G, S:A23063T, S:C23271A, S:A23403G, S:C23604A, S:C23709T, S:C24023T, S:C24054T, S:T24506G, S:G24914C, ORF8:C27972T, ORF8:G28048T, ORF8:A28111G, N:G28280C, N:A28281T, N:T28282A, N:G28881A, N:G28882A, N:G28883C, N:C28977T,	18	N:D3L, N:R203K, N:G204R, N:S235F, ORF1a:T1001I, ORF1a:D1625E, ORF1a:P1921L, ORF1a:T4304I, ORF1b:P314L, ORF1b:L1504F, S:N501Y, S:A570D, S:D614G, S:P681H, S:T716I, S:A831V, S:S982A, S:D1118H,	ORF1ab: 11288-11296, S:21765-21770, S:21992-21994, ORF8:28254, ORF8:28271,
hCoV-19/Mexico/ZAC-INER-IMSS-00079/2021	EPI_ISL_1287765	In process	20I (Alpha, V1)	B.1.1.7	34	5'UTR:C241T, ORF1ab:C913T, ORF1ab:C3037T, ORF1ab:C3267T, ORF1ab:C5388A, ORF1ab:C5986T, ORF1ab:G6362A, ORF1ab:T6954C, ORF1ab:C12970T, ORF1ab:C13176T, ORF1ab:C14408T, ORF1ab:C14676T, ORF1ab:C15279T, ORF1ab:T16176C, ORF1ab:C17977T, ORF1ab:A20013G, S:A23063T, S:C23271A, S:A23403G, S:C23604A, S:C23709T, S:C24023T, S:C24054T, S:T24506G, S:G24914C, ORF8:C27972T, ORF8:G28048T, ORF8:A28111G, N:G28280C, N:A28281T, N:T28282A, N:G28881A, N:G28882A, N:G28883C, N:C28977T,	19	N:D3L, N:R203K, N:G204R, N:S235F, ORF1a:T1001I, ORF1a:A1708D, ORF1a:A2033T, ORF1a:I2230T, ORF1a:T4304I, ORF1b:P314L, ORF1b:L1504F, S:N501Y, S:A570D, S:D614G, S:P681H, S:T716I, S:A831V, S:S982A, S:D1118H,	ORF1ab: 11288-11296, S:21765-21770, S:21992-21994, ORF8:28254, ORF8:28271,
hCoV-19/Mexico/ZAC-INER-IMSS-00080/2021	EPI_ISL_1279368	In process	20B	B.1.1.519	26	5'UTR:T201C, 5'UTR:C203T, 5'UTR:C222T, 5'UTR:C241T, ORF1ab:C936T, ORF1ab:G1738T, ORF1ab:G1747A, ORF1ab:C3037T, ORF1ab:C3140T, ORF1ab:C6500T, ORF1ab:C10029T, ORF1ab:C10954T, ORF1ab:A11117G, ORF1ab:C12789T, ORF1ab:C14408T, ORF1ab:T19839C, ORF1ab:A19974G, ORF1ab:C21306T, S:C22995A, S:A23403G, S:C23604A, S:A23756G, N:G28881A, N:G28882A, N:G28883C, N:C29197T, N:G29227T,	13	N:R203K, N:G204R, ORF1a:T224I, ORF1a:P959S, ORF1a:P2079S, ORF1a:T3255I, ORF1a:I3618V, ORF1a:T4175I, ORF1b:P314L, S:T478K, S:D614G, S:P681H, S:T732A,	
hCoV-19/Mexico/ZAC-INER-IMSS-00081/2021	EPI_ISL_1287766	In process	20B	B.1.1	20	5'UTR:C241T, ORF1ab:C1419T, ORF1ab:C3037T, ORF1ab:A6390G, ORF1ab:C7039T, ORF1ab:T7049C, ORF1ab:C7165T, ORF1ab:G8548A, ORF1ab:C10186T, ORF1ab:T10676C, ORF1ab:C14408T, ORF1ab:G14772T, ORF1ab:T19839C, S:C21622T, S:A23403G, S:A23756G, S:C25000T, N:G28881A, N:G28882A, N:G28883C, 3'UTR:G29779T,	9	N:R203K, N:G204R, ORF1a:A385V, ORF1a:E2042G, ORF1a:Y2262H, ORF1b:P314L, ORF1b:Q435H, S:D614G, S:T732A,	ORF8:28254,
hCoV-19/Mexico/ZAC-INER-IMSS-00082/2021	EPI_ISL_1279369	In process	20A	B.1.241	24	5'UTR:C241T, ORF1ab:T649C, ORF1ab:C3037T, ORF1ab:A5648C, ORF1ab:G6205A, ORF1ab:A6529G, ORF1ab:G6884A, ORF1ab:G7393T, ORF1ab:G10870T, ORF1ab:C11575T, ORF1ab:C11900A, ORF1ab:C13957T, ORF1ab:C14408T, ORF1ab:C15720T, ORF1ab:A16840C, ORF1ab:C19029T, ORF1ab:G19117T, ORF1ab:T19209C, ORF1ab:A20268G, S:T22222C, S:T22618A, S:A23403G, ORF3a:C25936T, ORF8:C28115T, N:C28854T	10	N:S194L, ORF1a:K1795Q, ORF1a:G2207S, ORF1a:L3879I, ORF1b:R164C, ORF1b:P314L, ORF1b:K1125Q, ORF1b:A1884S, ORF3a:H182Y, S:D614G,	

hCoV-19/Mexico/ZAC-INER-IMSS-00083/2021	EPI_ISL_1279370	In process	20A	B.1	19	5'UTR:C241T, ORF1ab:A2368T, ORF1ab:C3037T, ORF1ab:C6762T, ORF1ab:C7843T, ORF1ab:G10870T, ORF1ab:G11083T, ORF1ab:T12118G, ORF1ab:C14408T, ORF1ab:G16065A, ORF1ab:T17142G, ORF1ab:C19011T, ORF1ab:G19648T, ORF1ab:A20268G, S:C21614T, S:T21698G, S:A23403G, ORF3a:G26062T, N:C28854T, 3'UTR:C29730T,	10	N:S194L, ORF1a:K701N, ORF1a:T2166I, ORF1a:L3606F, ORF1b:P314L, ORF1b:V2061L, ORF3a:G224C, S:L18F, S:S46A, S:D614G,	
hCoV-19/Mexico/ZAC-INER-IMSS-00084/2021	EPI_ISL_1279371	In process	20G	B.1.2	19	5'UTR:C241T, ORF1ab:C1059T, ORF1ab:G1729A, ORF1ab:C3037T, ORF1ab:C9803T, ORF1ab:C10319T, ORF1ab:C14408T, ORF1ab:A18424G, ORF1ab:G18583T, ORF1ab:C19524T, ORF1ab:C19881T, ORF1ab:G20098T, ORF1ab:C21304T, S:A22534C, S:A23403G, ORF3a:G25563T, ORF3a:G25907T, ORF8:C27964T, N:C28472T. N:C28869T.	14	N:P67S, N:P199L, ORF1a:T265I, ORF1a:L3352F, ORF1b:P314L, ORF1b:N1653D, ORF1b:V1706L, ORF1b:A2211S, ORF1b:R2613C, ORF3a:Q57H, ORF3a:G172V, ORF8:S24L, S:E324D, S:D614G,	
hCoV-19/Mexico/ZAC-INER-IMSS-00085/2021	EPI_ISL_1279372	In process	20B	B.1.1.519	28	5'UTR:T201C, 5'UTR:C203T, 5'UTR:C222T, 5'UTR:C241T, ORF1ab:C936T, ORF1ab:G1738T, ORF1ab:G1747A, ORF1ab:C3037T, ORF1ab:C3140T, ORF1ab:C6500T, ORF1ab:T6800C, ORF1ab:C10029T, ORF1ab:C10954T, ORF1ab:A11117G, ORF1ab:C12789T, ORF1ab:C14408T, ORF1ab:C14898T, ORF1ab:T19839C, ORF1ab:A19974G, ORF1ab:C21306T, S:C22995A, S:A23403G, S:C23604A, S:A23756G, N:G28881A, N:G28882A, N:G28883C, N:C29197T, N:G29227T,	13	N:R203K, N:G204R, ORF1a:T224I, ORF1a:P959S, ORF1a:P2079S, ORF1a:T3255I, ORF1a:I3618V, ORF1a:T4175I, ORF1b:P314L, S:T478K, S:D614G, S:P681H, S:T732A,	
hCoV-19/Mexico/ZAC-INER-IMSS-00086/2021	EPI_ISL_1287767	In process	20I (Alpha, V1)	B.1.1.7	33	5'UTR:C241T, ORF1ab:C913T, ORF1ab:C3037T, ORF1ab:C3267T, ORF1ab:C5388A, ORF1ab:C5986T, ORF1ab:G6362A, ORF1ab:T6954C, ORF1ab:C12970T, ORF1ab:C13176T, ORF1ab:C14408T, ORF1ab:C14676T, ORF1ab:C15279T, ORF1ab:T16176C, ORF1ab:C17977T, S:A23063T, S:C23271A, S:A23403G, S:C23604A, S:C23709T, S:C24023T, S:C24054T, S:T24506G, S:G24914C, ORF8:C27972T, ORF8:G28048T, ORF8:A28111G, N:G28280C, N:A28281T, N:T28282A, N:G28881A, N:G28882A, N:G28883C, N:C28977T,	19	N:D3L, N:R203K, N:G204R, N:S235F, ORF1a:T1001I, ORF1a:A1708D, ORF1a:A2033T, ORF1a:I2230T, ORF1a:T4304I, ORF1b:P314L, ORF1b:L1504F, S:N501Y, S:A570D, S:D614G, S:P681H, S:T716I, S:A831V, S:S982A, S:D1118H,	ORF1ab: 11288-11296, S:21765-21770, S:21992-21994, ORF8:28254, ORF8:28271,
hCoV-19/Mexico/ZAC-INER-IMSS-00087/2021	EPI_ISL_1279373	In process	20B	B.1.1.519	25	5'UTR:T201C, 5'UTR:C203T, 5'UTR:C222T, 5'UTR:C241T, ORF1ab:C936T, ORF1ab:G1738T, ORF1ab:C3037T, ORF1ab:C3140T, ORF1ab:C10029T, ORF1ab:C10954T, ORF1ab:A11117G, ORF1ab:C12789T, ORF1ab:C14408T, ORF1ab:T14883C, ORF1ab:T19839C, ORF1ab:A19974G, ORF1ab:C21306T, S:C22995A, S:A23403G, S:C23604A, S:A23756G, N:G28881A, N:G28882A, N:G28883C, N:C29197T, N:G29227T,	12	N:R203K, N:G204R, ORF1a:T224I, ORF1a:P959S, ORF1a:T3255I, ORF1a:I3618V, ORF1a:T4175I, ORF1b:P314L, S:T478K, S:D614G, S:P681H, S:T732A,	
hCoV-19/Mexico/ZAC-INER-IMSS-00088/2021	EPI_ISL_1279301	In process	20B	B.1.1	23	5'UTR:C241T, ORF1ab:C1441T, ORF1ab:A1558G, ORF1ab:C3037T, ORF1ab:G3901A, ORF1ab:A4778G, ORF1ab:C6539T, ORF1ab:C10440T, ORF1ab:C10833T, ORF1ab:G13503T, ORF1ab:C14408T, ORF1ab:C18486T, ORF1ab:T19839C, S:C21618T, S:A23403G, S:A23756G, ORF3a:G25906T, ORF6:C27213T, N:G28881A, N:G28882A, N:G28883C, N:C29296T, N:G29473A, ORF10:A29591G,	12	N:R203K, N:G204R, ORF1a:I431M, ORF1a:I1505V, ORF1a:H2092Y, ORF1a:A3392V, ORF1a:A3523V, ORF1b:P314L, ORF3a:G172C, S:T19I, S:D614G, S:T732A,	3'UTR:29750-29759,

hCoV-19/Mexico/ZAC-INER-IMSS-00089/2021	EPI_ISL_1279374	In process	20A	B.1.561	26	5'UTR:C241T, ORF1ab:C683T, ORF1ab:G1685A, ORF1ab:G2516T, ORF1ab:C3037T, ORF1ab:C4795T, ORF1ab:A5999G, ORF1ab:C9534T, ORF1ab:A10323G, ORF1ab:C10798T, ORF1ab:C11866T, ORF1ab:C14408T, ORF1ab:C14583T, ORF1ab:G16917T, ORF1ab:C18032T, ORF1ab:C18693T, ORF1ab:A20268G, S:G21974T, S:C23277T, S:A23403G, ORF3a:C25549T, ORF3a:T25577C, M:C26586T, M:C26894T, M:A27007G, N:C28854T, N:G28975T	15	M:K162R, N:S194L, N:M234I, ORF1a:A474T, ORF1a:V751L, ORF1a:I1912V, ORF1a:T3090I, ORF1a:K3353R, ORF1b:P314L, ORF1b:T1522I, ORF3a:L53F, ORF3a:I62T, S:D138Y, S:T572I, S:D614G,
hCoV-19/Mexico/ZAC-INER-IMSS-00090/2021	EPI_ISL_1279375	In process	20B	B.1.1.519	27	5'UTR:C203T, 5'UTR:C222T, 5'UTR:C241T, ORF1ab:C1288T, ORF1ab:C3037T, ORF1ab:C3140T, ORF1ab:T3745C, ORF1ab:C10029T, ORF1ab:C10954T, ORF1ab:A11117G, ORF1ab:C12789T, ORF1ab:G12999A, ORF1ab:C14134T, ORF1ab:C14408T, ORF1ab:T19839C, ORF1ab:C20404T, ORF1ab:C21306T, S:T22729C, S:C22995A, S:A23403G, S:C23604A, S:A23756G, ORF3a:C25844T, N:C28849T, N:G28881A, N:G28882A, N:G28883C, N:C29197T	15	N:R203K, N:G204R, ORF1a:P959S, ORF1a:T3255I, ORF1a:I3618V, ORF1a:T4175I, ORF1a:S4245N, ORF1b:P223S, ORF1b:P314L, ORF1b:P2313S, ORF3a:T151I, S:T478K, S:D614G, S:P681H, S:T732A,
hCoV-19/Mexico/ZAC-INER-IMSS-00091/2021	EPI_ISL_1279376	In process	20A	B.1.400	19	5'UTR:C241T, ORF1ab:C3037T, ORF1ab:C3096T, ORF1ab:T5137C, ORF1ab:C5497T, ORF1ab:C5822T, ORF1ab:C14408T, ORF1ab:C14790T, ORF1ab:A18600G, ORF1ab:G19174A, ORF1ab:G19969A, ORF1ab:A20268G, S:A23403G, ORF3a:C25521T, ORF8:T28082G, ORF8:C28153T, N:C28854T, N:C29167T, N:A29437G, 3'UTR:T29698C,	9	N:S194L, ORF1a:S944L, ORF1a:L1853F, ORF1b:P314L, ORF1b:D1903N, ORF1b:A2168T, ORF8:D63E, ORF8:T87I, S:D614G,
hCoV-19/Mexico/ZAC-INER-IMSS-00092/2021	EPI_ISL_1279377	In process	20A	B.1.561	19	5'UTR:C241T, ORF1ab:G2516T, ORF1ab:C3037T, ORF1ab:C5055T, ORF1ab:A5999G, ORF1ab:A10323G, ORF1ab:C14408T, ORF1ab:C18693T, ORF1ab:A20268G, S:G21974T, S:A23403G, S:C24138T, S:T25316G, ORF3a:T25577C, M:C26681T, N:C28854T, N:G28975T, N:C29095T, N:C29144T, 3'UTR:C29754T,	12	N:S194L, N:M234I, ORF1a:V751L, ORF1a:T1597I, ORF1a:I1912V, ORF1a:K3353R, ORF1b:P314L, ORF3a:I62T, S:D138Y, S:D614G, S:T859I, S:S1252A,
hCoV-19/Mexico/ZAC-INER-IMSS-00093/2021	EPI_ISL_1279378	In process	20A	B.1.243	15	5'UTR:C241T, ORF1ab:G806A, ORF1ab:T2584A, ORF1ab:C2675T, ORF1ab:C3037T, ORF1ab:C5140A, ORF1ab:G7790A, ORF1ab:C14408T, ORF1ab:A16471C, ORF1ab:A20268G, S:A23403G, S:G23587C, S:T24076C, ORF3a:C25844T, N:C28854T, N:G29543T,	10	N:S194L, ORF1a:A181T, ORF1a:P804S, ORF1a:D1625E, ORF1a:G2509S, ORF1b:P314L, ORF1b:I1002L, ORF3a:T151I, S:D614G, S:Q675H,
hCoV-19/Mexico/ZAC-INER-IMSS-00094/2021	EPI_ISL_1279379	In process	20A	B.1.404	20	5'UTR:C241T, ORF1ab:C823T, ORF1ab:C1349A, ORF1ab:G2492A, ORF1ab:C3037T, ORF1ab:T3733G, ORF1ab:G5572T, ORF1ab:G7042T, ORF1ab:A10323G, ORF1ab:G10631A, ORF1ab:A12644G, ORF1ab:C14408T, ORF1ab:T16743C, ORF1ab:C19017T, ORF1ab:G19086T, ORF1ab:A20268G, S:G22992A, S:A23403G, ORF3a:C25782T, ORF3a:G25996T, N:C28854T	13	N:S194L, ORF1a:Q362K, ORF1a:E743K, ORF1a:M1769I, ORF1a:M2259I, ORF1a:K3353R, ORF1a:A3456T, ORF1a:I4127V, ORF1b:P314L, ORF1b:K1873N, ORF3a:V202L, S:S477N, S:D614G,
hCoV-19/Mexico/ZAC-INER-IMSS-00095/2021	EPI_ISL_1279380	In process	20G	B.1.2	21	5'UTR:C241T, ORF1ab:C1059T, ORF1ab:C1931A, ORF1ab:C3037T, ORF1ab:C3425T, ORF1ab:C7113T, ORF1ab:C10319T, ORF1ab:C12115T, ORF1ab:C14408T, ORF1ab:A18424G, ORF1ab:G20060T, ORF1ab:T20346C, ORF1ab:C21304T, S:C21590T, S:A23403G, ORF3a:G25563T, ORF3a:C25904T, ORF3a:G25907T, ORF8:C27964T, N:C28472T, N:C28869T, 3'UTR:G29751T,	16	N:P67S, N:P199L, ORF1a:T265I, ORF1a:Q556K, ORF1a:P1054S, ORF1a:T2283I, ORF1a:L3352F, ORF1b:P314L, ORF1b:N1653D, ORF1b:S2198I, ORF1b:R2613C, ORF3a:Q57H, ORF3a:S171L, ORF3a:G172V, ORF8:S24L, S:D614G,

hCoV-19/Mexico/ZAC-INER-IMSS-00096/2021	EPI_ISL_1279381	In process	20B	B.1.1.519	27	5'UTR:C203T, 5'UTR:C222T, 5'UTR:C241T, ORF1ab:C3037T, ORF1ab:C3140T, ORF1ab:G3371A, ORF1ab:C4999T, ORF1ab:C10029T, ORF1ab:C10450T, ORF1ab:C10954T, ORF1ab:A11117G, ORF1ab:C12789T, ORF1ab:C14408T, ORF1ab:T19839C, ORF1ab:C20451T, ORF1ab:C21306T, S:C22995A, S:A23403G, S:C23604A, S:A23756G, ORF3a:C25916T, M:G26526T, N:G28881A, N:G28882A, N:G28883C, N:G29000T, N:C29197T, N:G29527T	16	M:A2S, N:R203K, N:G204R, N:G243C, N:Q418H, ORF1a:P959S, ORF1a:D1036N, ORF1a:T3255I, ORF1a:I3618V, ORF1a:T4175I, ORF1b:P314L, ORF3a:T175I, S:T478K, S:D614G, S:P681H, S:T732A,
hCoV-19/Mexico/ZAC-INER-IMSS-00097/2021	EPI_ISL_1279382	In process	20A	B.1.243	15	5'UTR:C241T, ORF1ab:G806A, ORF1ab:T2584A, ORF1ab:C2675T, ORF1ab:C3037T, ORF1ab:C5140A, ORF1ab:C11202T, ORF1ab:C14408T, ORF1ab:A16471C, ORF1ab:A20268G, S:A23403G, S:G23587C, S:T24076C, ORF3a:C25844T, N:C28854T, N:G29543T,	10	N:S194L, ORF1a:A181T, ORF1a:P804S, ORF1a:D1625E, ORF1a:T3646I, ORF1b:P314L, ORF1b:I1002L, ORF3a:T151I, S:D614G, S:Q675H,
hCoV-19/Mexico/AGS-InDRE_150/2020	EPI_ISL_913953		20A	B.1.396	17	5'UTR:C241T, ORF1ab:C1191T, ORF1ab:C3037T, ORF1ab:G4960T, ORF1ab:C5183T, ORF1ab:C9521A, ORF1ab:T11443A, ORF1ab:C14408T, ORF1ab:G17562T, ORF1ab:A20129C, ORF1ab:A20268G, S:A23403G, S:C24904T, ORF7a:C27654T, ORF7b:C27893A, N:C28854T, N:G29422T, ORF10:A29567G,	7	N:S194L, ORF1a:P309L, ORF1a:P1640S, ORF1a:L3086I, ORF1b:P314L, ORF1b:E2221A, S:D614G,
hCoV-19/Mexico/AGS-InDRE_151/2020	EPI_ISL_913954		20B	B.1.1.512	17	5'UTR:C241T, ORF1ab:C280T, ORF1ab:C2925T, ORF1ab:C3037T, ORF1ab:A3075G, ORF1ab:T5926C, ORF1ab:G10265A, ORF1ab:C14408T, S:A23403G, S:G23593C, S:G24794T, S:A25256T, M:C26681T, N:G28881A, N:G28882A, N:G28883C, N:G29332T, ORF10:C29640T,	10	N:R203K, N:G204R, ORF1a:S887F, ORF1a:E937G, ORF1a:G3334S, ORF1b:P314L, S:D614G, S:Q677H, S:A1078S, S:I1232F,
hCoV-19/Mexico/AGS-InDRE-78/2020	EPI_ISL_658863		20B	B.1.1.222	16	5'UTR:C241T, ORF1ab:C3037T, ORF1ab:G3403T, ORF1ab:A4681G, ORF1ab:T5218C, ORF1ab:C12525T, ORF1ab:C14408T, ORF1ab:C14913T, ORF1ab:T19839C, S:C21904T, S:A23403G, S:A23756G, N:G28881A, N:G28882A, N:G28883C, N:G29449T, 3'UTR:G29868A,	6	N:R203K, N:G204R, ORF1a:T4087I, ORF1b:P314L, S:D614G, S:T732A,
hCoV-19/Mexico/AGS-InDRE-79/2020	EPI_ISL_658865		20A	B.1.396	17	5'UTR:C241T, ORF1ab:C1076T, ORF1ab:C1191T, ORF1ab:C3037T, ORF1ab:G4960T, ORF1ab:C8290T, ORF1ab:C9521A, ORF1ab:C14408T, ORF1ab:A20129C, ORF1ab:A20268G, S:A23403G, S:C24904T, S:G25250T, ORF7a:C27654T, ORF7b:C27893A, N:C28854T, N:G29422T, ORF10:A29567G,	8	N:S194L, ORF1a:P271S, ORF1a:P309L, ORF1a:L3086I, ORF1b:P314L, ORF1b:E2221A, S:D614G, S:V1230L,
hCoV-19/Mexico/AGS-InDRE-80/2020	EPI_ISL_658868		20B	B.1.1.222	17	5'UTR:C241T, ORF1ab:C3037T, ORF1ab:G3403T, ORF1ab:A4681G, ORF1ab:T5218C, ORF1ab:C12525T, ORF1ab:C14408T, ORF1ab:C14913T, ORF1ab:T19839C, S:C21904T, S:A23403G, S:A23756G, N:G28881A, N:G28882A, N:G28883C, N:G29449T, 3'UTR:C29870T, 3'UTR:A29872C,	6	N:R203K, N:G204R, ORF1a:T4087I, ORF1b:P314L, S:D614G, S:T732A,
hCoV-19/Mexico/AGS-InDRE-81/2020	EPI_ISL_658870		20A	B.1.404	15	5'UTR:C241T, ORF1ab:C3037T, ORF1ab:G7042T, ORF1ab:A10323G, ORF1ab:G10396T, ORF1ab:A12644G, ORF1ab:C14408T, ORF1ab:T16743C, ORF1ab:G19086T, ORF1ab:A20268G, S:G22992A, S:A23403G, S:G25273T, ORF3a:G26020A, ORF7a:G27441T, N:C28854T,	11	N:S194L, ORF1a:M2259I, ORF1a:K3353R, ORF1a:I4127V, ORF1b:P314L, ORF1b:K1873N, ORF3a:D210N, ORF7a:E16D, S:S477N, S:D614G, S:M1237I,
hCoV-19/Mexico/AGS-InDRE-82/2020	EPI_ISL_658873		20B	B.1.1.222	15	5'UTR:C241T, ORF1ab:C3037T, ORF1ab:G3403T, ORF1ab:T5218C, ORF1ab:C12525T, ORF1ab:C14408T, ORF1ab:C19151T, ORF1ab:T19839C, ORF1ab:A19890G, S:A23403G, S:A23756G, S:T25021C, N:G28881A, N:G28882A, N:G28883C, N:G29449T,	7	N:R203K, N:G204R, ORF1a:T4087I, ORF1b:P314L, ORF1b:A1895V, S:D614G, S:T732A,

hCoV-19/Mexico/AGS-InDRE-83/2020	EPI_ISL_658875	20G	B.1.2	19	5'UTR:C241T, ORF1ab:C1059T, ORF1ab:C3037T, ORF1ab:C10319T, ORF1ab:C14408T, ORF1ab:A18424G, ORF1ab:G20060T, ORF1ab:T20346C, S:A23403G, ORF3a:G25563T, ORF3a:G25907T, ORF8:C27964T, N:A28343G, N:C28472T, N:C28677T, N:C28869T, 3'UTR:G29862A, 3'UTR:C29870A, 3'UTR:A29872T, 3'UTR:A29873T,	13	N:T24A, N:P67S, N:T135I, N:P199L, ORF1a:T265I, ORF1a:L3352F, ORF1b:P314L, ORF1b:N1653D, ORF1b:S2198I, ORF3a:Q57H, ORF3a:G172V, ORF8:S24L, S:D614G,	3'UTR:29867,
hCoV-19/Mexico/AGS-InDRE-85/2020	EPI_ISL_658880	20A	B.1.404	21	5'UTR:C21T, 5'UTR:C241T, ORF1ab:C3037T, ORF1ab:G7042T, ORF1ab:G10157T, ORF1ab:A10323G, ORF1ab:C12484T, ORF1ab:A12644G, ORF1ab:C14408T, ORF1ab:T16743C, ORF1ab:G19086T, ORF1ab:G19962T, ORF1ab:A20268G, S:C22858T, S:G22992A, S:A23403G, S:G23608C, ORF3a:C26176T, N:C28854T, N:C29167T, ORF10:A29622G, 3'UTR:A29885G,	10	N:S194L, ORF1a:M2259I, ORF1a:V3298L, ORF1a:K3353R, ORF1a:I4127V, ORF1b:P314L, ORF1b:K1873N, ORF3a:P262S, S:S477N, S:D614G,	
hCoV-19/Mexico/AGS-InDRE-86/2020	EPI_ISL_658883	20A	B.1	15	5'UTR:C241T, ORF1ab:G1729A, ORF1ab:C2416T, ORF1ab:C3037T, ORF1ab:C3411T, ORF1ab:C14408T, ORF1ab:C16616T, S:A23403G, S:T23684G, ORF3a:G25563T, E:T26424C, M:C26885T, ORF6:C27263T, ORF7a:C27603T, N:C28887T, N:T29192C,	9	N:T205I, N:F307L, ORF1a:A1049V, ORF1b:P314L, ORF1b:T1050I, ORF3a:Q57H, ORF6:T21I, S:D614G, S:S708A,	
hCoV-19/Mexico/AGS-InDRE-89/2020	EPI_ISL_658891	20G	B.1.2	16	5'UTR:C241T, ORF1ab:C1059T, ORF1ab:C3037T, ORF1ab:C10319T, ORF1ab:G11083T, ORF1ab:C14408T, ORF1ab:C16887T, ORF1ab:A18424G, ORF1ab:G20060T, ORF1ab:C21304T, S:A23403G, ORF3a:G25563T, ORF3a:G25907T, ORF6:C27247T, ORF8:C27964T, N:C28472T, N:C28869T,	13	N:P67S, N:P199L, ORF1a:T265I, ORF1a:L3352F, ORF1a:L3606F, ORF1b:P314L, ORF1b:N1653D, ORF1b:S2198I, ORF1b:R2613C, ORF3a:Q57H, ORF3a:G172V, ORF8:S24L, S:D614G,	
hCoV-19/Mexico/AGU-InDRE_193/2020	EPI_ISL_933670	20A	B.1.561	16	5'UTR:C241T, ORF1ab:C1519T, ORF1ab:G2516T, ORF1ab:C3037T, ORF1ab:A5999G, ORF1ab:A10323G, ORF1ab:C14408T, ORF1ab:C18693T, ORF1ab:A20268G, S:G21974T, S:A23403G, ORF3a:T25577C, M:C26681T, N:C28854T, N:G28975T, N:C29095T, 3'UTR:C29754T,	9	N:S194L, N:M234I, ORF1a:V751L, ORF1a:I1912V, ORF1a:K3353R, ORF1b:P314L, ORF3a:I62T, S:D138Y, S:D614G,	
hCoV-19/Mexico/AGU-InDRE_194/2020	EPI_ISL_933671	20A	B.1.404	15	5'UTR:C241T, ORF1ab:C3037T, ORF1ab:G7042T, ORF1ab:A10323G, ORF1ab:G10396T, ORF1ab:A12336G, ORF1ab:A12644G, ORF1ab:C14408T, ORF1ab:T16743C, ORF1ab:G19086T, ORF1ab:A20268G, S:G22992A, S:A23403G, S:C24904T, ORF3a:G26020A, N:C28854T,	10	N:S194L, ORF1a:M2259I, ORF1a:K3353R, ORF1a:K4024R, ORF1a:I4127V, ORF1b:P314L, ORF1b:K1873N, ORF3a:D210N, S:S477N, S:D614G,	
hCoV-19/Mexico/AGU-InDRE_195/2020	EPI_ISL_933672	20A	B.1.561	17	5'UTR:C241T, ORF1ab:G2516T, ORF1ab:C3037T, ORF1ab:A5999G, ORF1ab:A10323G, ORF1ab:C14408T, ORF1ab:C18693T, ORF1ab:A20268G, ORF1ab:C21017T, S:G21974T, S:A23403G, ORF3a:T25577C, M:C26681T, N:G28361A, N:C28854T, N:G28975T, N:C29095T, 3'UTR:C29754T,	12	N:G30R, N:S194L, N:M234I, ORF1a:V751L, ORF1a:I1912V, ORF1a:K3353R, ORF1b:P314L, ORF1b:T2517I, ORF3a:I62T, ORF9b:M26I, S:D138Y, S:D614G,	
hCoV-19/Mexico/AGU-InDRE_196/2020	EPI_ISL_933673	20B	B.1.1	18	5'UTR:C241T, ORF1ab:C1191T, ORF1ab:C2094T, ORF1ab:C3037T, ORF1ab:A3379G, ORF1ab:C14408T, ORF1ab:A16725G, ORF1ab:G17572T, ORF1ab:C19454T, ORF1ab:T19839C, S:C21614T, S:C21846T, S:G23285T, S:A23403G, ORF3a:G25599T, ORF7a:C27603T, N:G28881A, N:G28882A, N:G28883C,	12	N:R203K, N:G204R, ORF1a:P309L, ORF1a:S610L, ORF1b:P314L, ORF1b:A1369S, ORF1b:T1996M, ORF3a:W69C, S:L18F, S:T95I, S:A575S, S:D614G,	

hCoV-19/Mexico/AGU-InDRE_231/2020	EPI_ISL_933708	20G	B.1.2	22	5'UTR:G105T, 5'UTR:C241T, ORF1ab:C1059T, ORF1ab:C3037T, ORF1ab:C10319T, ORF1ab:C14408T, ORF1ab:A18424G, ORF1ab:C19011T, ORF1ab:G20060T, ORF1ab:T20346C, ORF1ab:C21110T, ORF1ab:C21304T, S:C22482T, S:A23403G, ORF3a:G25563T, ORF3a:G25907T, ORF3a:C26013T, ORF8:C27964T, N:A28343G, N:C28472T, N:C28677T, N:C28869T, N:G29427C,	17	N:T24A, N:P67S, N:T135I, N:P199L, N:R385T, ORF1a:T265I, ORF1a:L3352F, ORF1b:P314L, ORF1b:N1653D, ORF1b:S2198I, ORF1b:T2548I, ORF1b:R2613C, ORF3a:Q57H, ORF3a:G172V, ORF8:S24L, S:T307I, S:D614G,
hCoV-19/Mexico/AGU-InDRE_232/2020	EPI_ISL_933709	20B	B.1.1.222	17	5'UTR:C241T, ORF1ab:C2509T, ORF1ab:C3037T, ORF1ab:C6285T, ORF1ab:C10226T, ORF1ab:G11083T, ORF1ab:A13498G, ORF1ab:C14408T, ORF1ab:T19839C, S:G22484T, S:A23403G, S:A23756G, N:G28881A, N:G28882A, N:G28883C, 3'UTR:G29692T, 3'UTR:G29742T, 3'UTR:G29773T,	10	N:R203K, N:G204R, ORF1a:T2007I, ORF1a:L3321F, ORF1a:L3606F, ORF1b:T11A, ORF1b:P314L, S:V308L, S:D614G, S:T732A,
hCoV-19/Mexico/AGU-InDRE_334/2020	EPI_ISL_1060722	20A	B.1.396	17	5'UTR:C241T, ORF1ab:C1191T, ORF1ab:C3037T, ORF1ab:G4960T, ORF1ab:C5183T, ORF1ab:C9521A, ORF1ab:T11443A, ORF1ab:C14408T, ORF1ab:G17562T, ORF1ab:A20129C, ORF1ab:A20268G, S:A23403G, S:C24904T, ORF7a:C27654T, ORF7b:C27893A, N:C28854T, N:G29422T, ORF10:A29567G,	7	N:S194L, ORF1a:P309L, ORF1a:P1640S, ORF1a:L3086I, ORF1b:P314L, ORF1b:E2221A, S:D614G,
hCoV-19/Mexico/AGU-InDRE_351/2020	EPI_ISL_1060688	20A	B.1	12	ORF1ab:C556T, ORF1ab:C1190T, ORF1ab:C3037T, ORF1ab:C14408T, ORF1ab:C19884T, ORF1ab:G20578T, S:C21952T, S:A23403G, S:T24371G, N:C28854T, N:C29272T, 3'UTR:C29870A, 3'UTR:A29871C,	6	N:S194L, ORF1a:P309S, ORF1b:P314L, ORF1b:V2371L, S:D614G, S:S937A,
hCoV-19/Mexico/AGU-InDRE_353/2020	EPI_ISL_1060689	20G	B.1.2	15	ORF1ab:C1059T, ORF1ab:C1884T, ORF1ab:G2243A, ORF1ab:C3037T, ORF1ab:C4093T, ORF1ab:C5055T, ORF1ab:C10319T, ORF1ab:G11222T, ORF1ab:C14408T, ORF1ab:G20060T, ORF1ab:C21304T, S:A23403G, ORF3a:G25563T, ORF8:C27964T, N:C28472T, N:C28869T,	14	N:P67S, N:P199L, ORF1a:T265I, ORF1a:A540V, ORF1a:V660I, ORF1a:T1597I, ORF1a:L3352F, ORF1a:V3653F, ORF1b:P314L, ORF1b:S2198I, ORF1b:R2613C, ORF3a:Q57H, ORF8:S24L, S:D614G,
hCoV-19/Mexico/AGU-InDRE_354/2020	EPI_ISL_1060725	20A	B.1.404	14	ORF1ab:G2492A, ORF1ab:C3037T, ORF1ab:T3733G, ORF1ab:G5572T, ORF1ab:A10323G, ORF1ab:A12644G, ORF1ab:C14408T, ORF1ab:T16743C, ORF1ab:G19086T, ORF1ab:A20268G, S:G22992A, S:A23403G, ORF3a:C25782T, ORF7a:T27447C, N:C28854T,	9	N:S194L, ORF1a:E743K, ORF1a:M1769I, ORF1a:K3353R, ORF1a:I4127V, ORF1b:P314L, ORF1b:K1873N, S:S477N, S:D614G,
hCoV-19/Mexico/AGU-InDRE_357/2020	EPI_ISL_1060723	20B	B.1.1.222	12	ORF1ab:G1264T, ORF1ab:C3037T, ORF1ab:T5218C, ORF1ab:T5293C, ORF1ab:C10582T, ORF1ab:C11941T, ORF1ab:C12525T, ORF1ab:C14408T, S:A23403G, S:A23756G, N:G28881A, N:G28882A, N:G28883C,	6	N:R203K, N:G204R, ORF1a:T4087I, ORF1b:P314L, S:D614G, S:T732A,
hCoV-19/Mexico/AGU-InDRE_F11762B_S1084/202	EPI_ISL_1516777	20B	B.1.1.512	14	5'UTR:C241T, ORF1ab:C2925T, ORF1ab:C3037T, ORF1ab:C6843T, ORF1ab:G10265A, ORF1ab:C14408T, S:A23403G, S:G23593C, S:G24794T, S:A25256T, S:C25276T, ORF7b:A27854G, N:G28881A, N:G28882A, N:G28883C,	10	N:R203K, N:G204R, ORF1a:S887F, ORF1a:S2193F, ORF1a:G3334S, ORF1b:P314L, S:D614G, S:Q677H, S:A1078S, S:I1232F,
hCoV-19/Mexico/AGU-InDRE_F11763B_S1085/202	EPI_ISL_1516778	20A	B.1.609	15	5'UTR:C241T, ORF1ab:C2485T, ORF1ab:C3037T, ORF1ab:C4582T, ORF1ab:T6712C, ORF1ab:C8175T, ORF1ab:G11417T, ORF1ab:T14173C, ORF1ab:C14408T, ORF1ab:G17808A, ORF1ab:A20268G, S:A23403G, S:A24874G, ORF3a:G25644T, N:G28739T, N:C28887T,	6	N:A156S, N:T205I, ORF1a:A2637V, ORF1a:V3718F, ORF1b:P314L, S:D614G,

hCoV-19/Mexico/AGU-InDRE_F11764B_S1086/202	EPI_ISL_1516779	20B	B.1.1.322	21	5'UTR:C241T, ORF1ab:G2867T, ORF1ab:C3037T, ORF1ab:G6337T, ORF1ab:C13517T, ORF1ab:C14408T, ORF1ab:C17502T, ORF1ab:G17658T, ORF1ab:C18744T, ORF1ab:C19470T, ORF1ab:T19839C, S:A23403G, S:A23756G, S:T24352C, S:G24620T, N:G28373T, N:A28877T, N:G28878C, N:G28881A, N:G28882A, N:G28883C, 3'UTR:G29690A,	10	N:G34W, N:R203K, N:G204R, ORF1a:V868L, ORF1b:T17I, ORF1b:P314L, ORF1b:M1397I, S:D614G, S:T732A, S:A1020S,	
hCoV-19/Mexico/AGU-InDRE_F11765B_S1087/202	EPI_ISL_1516780	20A	B.1.609	11	5'UTR:C241T, ORF1ab:C3037T, ORF1ab:A3752T, ORF1ab:C4582T, ORF1ab:C13119T, ORF1ab:G14250T, ORF1ab:C14408T, ORF1ab:A20268G, S:C22006T, S:A23403G, N:C28854T, N:C29253T,	7	N:S194L, N:S327L, ORF1a:R1163*, ORF1a:A4285V, ORF1b:L261F, ORF1b:P314L, S:D614G,	
hCoV-19/Mexico/AGU-InDRE_F11767B_S1089/202	EPI_ISL_1516781	20A	B.1.396	18	5'UTR:C241T, ORF1ab:C1191T, ORF1ab:C3037T, ORF1ab:G4960T, ORF1ab:C9521A, ORF1ab:C14408T, ORF1ab:A20129C, ORF1ab:A20268G, S:G21796T, S:C21952T, S:A23403G, S:A23416T, S:C24904T, ORF7a:C27654T, ORF7b:C27893A, ORF8:C28054T, N:C28854T, N:G29422T, ORF10:A29567G,	8	N:S194L, ORF1a:P309L, ORF1a:L3086I, ORF1b:P314L, ORF1b:E2221A, ORF8:S54L, S:R78S, S:D614G,	3'UTR:29858-29866
hCoV-19/Mexico/AGU-InDRE_F11768B_S1090/202	EPI_ISL_1516782	20A	B.1.580	13	5'UTR:C241T, ORF1ab:C1190T, ORF1ab:C3037T, ORF1ab:C10450T, ORF1ab:T12523C, ORF1ab:C14408T, ORF1ab:C14599T, ORF1ab:G18412T, ORF1ab:A20268G, ORF1ab:G21185C, S:A23403G, S:G25266T, ORF3a:C25521A, N:C28854T,	8	N:S194L, ORF1a:P309S, ORF1b:P314L, ORF1b:V1649F, ORF1b:W2573S, ORF3a:F43L, S:D614G, S:C1235F,	ORF3a:25531-25533
hCoV-19/Mexico/AGU-InDRE_F11769B_S1091/202	EPI_ISL_1516783	20B	B.1.1	20	5'UTR:C241T, ORF1ab:C1191T, ORF1ab:T2053A, ORF1ab:C2094T, ORF1ab:C3037T, ORF1ab:A3379G, ORF1ab:C14408T, ORF1ab:A16725G, ORF1ab:G17572T, ORF1ab:C19454T, ORF1ab:T19839C, S:C21614T, S:C21846T, S:G23285T, S:A23403G, ORF3a:G25599T, ORF7a:C27584T, ORF7a:C27603T, N:G28881A, N:G28882A, N:G28883C,	13	N:R203K, N:G204R, ORF1a:P309L, ORF1a:S610L, ORF1b:P314L, ORF1b:A1369S, ORF1b:T1996M, ORF3a:W69C, ORF7a:A64V, S:L18F, S:T95I, S:A575S, S:D614G,	
hCoV-19/Mexico/AGU-InDRE_F11770B_S1092/202	EPI_ISL_1516784	20A	B.1.243	16	5'UTR:C241T, ORF1ab:C3037T, ORF1ab:A3183G, ORF1ab:C5575T, ORF1ab:G9802T, ORF1ab:C10556T, ORF1ab:A14021G, ORF1ab:C14408T, ORF1ab:G18028T, ORF1ab:A20268G, ORF1ab:C20483T, S:C23191T, S:A23403G, S:T24076C, N:C28854T, ORF10:C29614T, 3'UTR:G29745T,	8	N:S194L, ORF1a:E973G, ORF1a:P3431S, ORF1b:D185G, ORF1b:P314L, ORF1b:A1521S, ORF1b:S2339F, S:D614G,	
hCoV-19/Mexico/AGU-InDRE_F11771B_S1093/202	EPI_ISL_1516785	20A	B.1.404	21	5'UTR:C241T, ORF1ab:C1824T, ORF1ab:C3037T, ORF1ab:G7042T, ORF1ab:A10323G, ORF1ab:A12644G, ORF1ab:C14408T, ORF1ab:C16308T, ORF1ab:T16743C, ORF1ab:G19086T, ORF1ab:G19549T, ORF1ab:G19816T, ORF1ab:A20268G, S:A21949G, S:G22992A, S:A23403G, ORF3a:C25578T, M:A27156G, ORF6:C27213T, N:C28657T, N:C28854T, 3'UTR:G29862T,	12	M:S212G, N:S194L, ORF1a:A520V, ORF1a:M2259I, ORF1a:K3353R, ORF1a:I4127V, ORF1b:P314L, ORF1b:K1873N, ORF1b:A2028S, ORF1b:V2117L, S:S477N, S:D614G,	3'UTR:29858
hCoV-19/Mexico/AGU-InDRE_F11772B_S1094/202	EPI_ISL_1516786	20A	B.1.404	16	5'UTR:C241T, ORF1ab:C3037T, ORF1ab:G4972A, ORF1ab:G7042T, ORF1ab:A10323G, ORF1ab:A12644G, ORF1ab:C14408T, ORF1ab:T16743C, ORF1ab:T18954C, ORF1ab:G19086T, ORF1ab:A20268G, S:G22992A, S:A23403G, ORF7b:T27824C, N:C28854T, N:C29095T, 3'UTR:A29863T,	8	N:S194L, ORF1a:M2259I, ORF1a:K3353R, ORF1a:I4127V, ORF1b:P314L, ORF1b:K1873N, S:S477N, S:D614G,	3'UTR:29854-29858
hCoV-19/Mexico/AGU-InDRE-55/2020	EPI_ISL_576258	20A	B.1.558	13	5'UTR:A107C, 5'UTR:C241T, ORF1ab:C1288T, ORF1ab:C3037T, ORF1ab:A9614G, ORF1ab:C11962T, ORF1ab:C14408T, ORF1ab:C19662T, ORF1ab:A20268G, S:A23403G, M:C26688T, ORF7a:C27643T, N:C28854T, N:G29315T,	6	N:S194L, N:D348Y, ORF1a:T3117A, ORF1b:P314L, ORF7a:P84S, S:D614G,	

hCoV-19/Mexico/AGU-InDRE-58/2020	EPI_ISL_576261	20D	C.23	14	5'UTR:C241T, ORF1ab:C3037T, ORF1ab:C4002T, ORF1ab:C7165A, ORF1ab:G10097A, ORF1ab:C13536T, ORF1ab:C14408T, ORF1ab:C15240T, ORF1ab:C16092T, S:A23403G, S:C23731T, ORF3a:G25481T, N:G28881A, N:G28882A, N:G28883C,	7	N:R203K, N:G204R, ORF1a:T1246I, ORF1a:G3278S, ORF1b:P314L, ORF3a:R30L, S:D614G,
hCoV-19/Mexico/BC-InDRE_134/2020	EPI_ISL_913937	20A	B.1.243	16	5'UTR:C241T, ORF1ab:C2232T, ORF1ab:T2266C, ORF1ab:C3037T, ORF1ab:C6070T, ORF1ab:C9592T, ORF1ab:C14408T, ORF1ab:C15660T, ORF1ab:C17733T, ORF1ab:C18395T, ORF1ab:A20268G, ORF1ab:C20759T, S:C21707T, S:A23403G, S:T24076C, ORF7a:C27625T, N:C28854T,	8	N:S194L, ORF1a:A656V, ORF1b:P314L, ORF1b:A1643V, ORF1b:A2431V, ORF7a:R78C, S:H49Y, S:D614G,
hCoV-19/Mexico/BC-InDRE_139/2020	EPI_ISL_913942	20A	B.1.551	18	5'UTR:C241T, ORF1ab:C3037T, ORF1ab:C3738T, ORF1ab:C8836T, ORF1ab:C9924T, ORF1ab:G10631A, ORF1ab:C14408T, ORF1ab:A20268G, S:C22323T, S:C23277T, S:A23403G, S:C23604G, S:G23868T, ORF3a:C26058T, ORF3a:G26152A, ORF7a:T27534C, N:C28849T, N:C28854T, N:C29466T,	12	N:S194L, N:A398V, ORF1a:P1158L, ORF1a:A3220V, ORF1a:A3456T, ORF1b:P314L, ORF3a:G254R, S:S254F, S:T572I, S:D614G, S:P681R, S:G769V,
hCoV-19/Mexico/BC-InDRE_140/2020	EPI_ISL_913943	20A	B.1.561	21	5'UTR:C241T, ORF1ab:C527T, ORF1ab:G2516T, ORF1ab:C3037T, ORF1ab:C3510T, ORF1ab:A4636G, ORF1ab:A5999G, ORF1ab:A10323G, ORF1ab:C10798T, ORF1ab:C11866T, ORF1ab:C14408T, ORF1ab:C15738T, ORF1ab:G16917T, ORF1ab:C18693T, ORF1ab:A20268G, S:G21974T, S:A23403G, S:C24378T, ORF3a:T25577C, ORF3a:C26124T, N:C28854T, N:G28975T,	11	N:S194L, N:M234I, ORF1a:V751L, ORF1a:A1082V, ORF1a:I1912V, ORF1a:K3353R, ORF1b:P314L, ORF3a:I62T, S:D138Y, S:D614G, S:S939F,
hCoV-19/Mexico/BC-InDRE-44/2020	EPI_ISL_516608	20A	B.1	10	5'UTR:C241T, ORF1ab:C3037T, ORF1ab:A3298C, ORF1ab:C6310T, ORF1ab:G13793A, ORF1ab:C14408T, ORF1ab:C17339T, ORF1ab:A20268G, S:C21575T, S:A23403G, N:C28854T,	7	N:S194L, ORF1a:Q1011H, ORF1b:R109H, ORF1b:P314L, ORF1b:A1291V, S:L5F, S:D614G,
hCoV-19/Mexico/BCN-ALSR-1466/2020	EPI_ISL_496339	20A	B.1	8	5'UTR:C241T, ORF1ab:C3037T, ORF1ab:T5260A, ORF1ab:C14408T, ORF1ab:A20268G, ORF1ab:A20288G, S:A23403G, S:A24862G, N:C28854T,	4	N:S194L, ORF1b:P314L, ORF1b:E2274G, S:D614G,
hCoV-19/Mexico/BCN-ALSR-1470/2020	EPI_ISL_496340	20A	B.1	5	5'UTR:C241T, ORF1ab:C3037T, ORF1ab:C14408T, ORF1ab:A20268G, S:A23403G, N:C28854T,	3	N:S194L, ORF1b:P314L, S:D614G,
hCoV-19/Mexico/BCN-ALSR-1471/2020	EPI_ISL_496341	20A	B.1	5	5'UTR:C241T, ORF1ab:C3037T, ORF1ab:C14408T, ORF1ab:A20268G, S:A23403G, N:C28854T,	3	N:S194L, ORF1b:P314L, S:D614G,
hCoV-19/Mexico/BCN-ALSR-1473/2020	EPI_ISL_496342	20A	B.1	6	5'UTR:C241T, ORF1ab:C3037T, ORF1ab:C14408T, ORF1ab:A20268G, S:G21786T, S:A23403G, N:C28854T,	4	N:S194L, ORF1b:P314L, S:G75V, S:D614G,
hCoV-19/Mexico/BCN-ALSR-1474/2020	EPI_ISL_496343	20A	B.1	8	5'UTR:C241T, ORF1ab:C3037T, ORF1ab:C7720T, ORF1ab:C14408T, ORF1ab:G17427T, ORF1ab:G18028T, ORF1ab:A20268G, S:A23403G, N:C28854T,	4	N:S194L, ORF1b:P314L, ORF1b:A1521S, S:D614G,
hCoV-19/Mexico/BCN-ALSR-1475/2020	EPI_ISL_496344	20A	B.1	10	5'UTR:C241T, ORF1ab:C3037T, ORF1ab:C5173T, ORF1ab:C10202T, ORF1ab:C14408T, ORF1ab:G17427T, ORF1ab:G18028T, ORF1ab:A20268G, S:A23403G, N:G28321T, N:C28854T,	6	N:S194L, ORF1a:L3313F, ORF1b:P314L, ORF1b:A1521S, ORF9b:R13L, S:D614G,
hCoV-19/Mexico/BCN-ALSR-1476/2020	EPI_ISL_496345	20A	B.1	10	5'UTR:C241T, ORF1ab:C3037T, ORF1ab:C7720T, ORF1ab:C9719A, ORF1ab:C14408T, ORF1ab:G17427T, ORF1ab:G18028T, ORF1ab:C18395T, ORF1ab:A20268G, S:A23403G, N:C28854T,	6	N:S194L, ORF1a:H3152N, ORF1b:P314L, ORF1b:A1521S, ORF1b:A1643V, S:D614G,
hCoV-19/Mexico/BCN-ALSR-1477/2020	EPI_ISL_496346	20B	B.1.1	8	5'UTR:C241T, ORF1ab:C3037T, ORF1ab:C14396T, ORF1ab:C14408T, ORF1ab:C18807T, S:A23403G, N:G28881A, N:G28882A, N:G28883C,	5	N:R203K, N:G204R, ORF1b:T310I, ORF1b:P314L, S:D614G,
hCoV-19/Mexico/BCN-ALSR-1478/2020	EPI_ISL_496347	20A	B.1	7	5'UTR:C241T, ORF1ab:C3037T, ORF1ab:C14408T, ORF1ab:A20268G, S:A23403G, S:A24292G, M:C26681T, N:C28854T,	3	N:S194L, ORF1b:P314L, S:D614G,

hCoV-19/Mexico/BCN-ALSR-1479/2020	EPI_ISL_496348	20A	B.1	5	5'UTR:C241T, ORF1ab:C3037T, ORF1ab:C14408T, ORF1ab:A20268G, S:A23403G, N:C28854T,	3	N:S194L, ORF1b:P314L, S:D614G,
hCoV-19/Mexico/BCN-ALSR-1485/2020	EPI_ISL_496349	20A	B.1.241	7	5'UTR:C241T, ORF1ab:C3037T, ORF1ab:C7768T, ORF1ab:C11575T, ORF1ab:C14408T, ORF1ab:A20268G, S:A23403G, N:C28854T,	3	N:S194L, ORF1b:P314L, S:D614G,
hCoV-19/Mexico/BCN-ALSR-1486/2020	EPI_ISL_496350	20A	B.1.245	8	5'UTR:C241T, ORF1ab:A1096G, ORF1ab:C3037T, ORF1ab:C14408T, ORF1ab:G14874T, ORF1ab:C18877T, S:A23403G, ORF3a:G25563T, 3'UTR:C29870A,	4	ORF1b:P314L, ORF1b:K469N, ORF3a:Q57H, S:D614G,
hCoV-19/Mexico/BCN-ALSR-1690/2020	EPI_ISL_496351	20A	B.1	9	5'UTR:C241T, ORF1ab:C3037T, ORF1ab:C14408T, ORF1ab:A20268G, ORF1ab:C21260T, S:A23403G, S:G24399T, ORF7b:C27883T, N:C28603T, N:C28854T,	6	N:S194L, ORF1b:P314L, ORF1b:S2598L, ORF7b:A43V, S:D614G, S:G946V,
hCoV-19/Mexico/BCN-ALSR-1691/2020	EPI_ISL_496352	20A	B.1.397	10	5'UTR:C241T, ORF1ab:G1135T, ORF1ab:C3037T, ORF1ab:C8655T, ORF1ab:C13774T, ORF1ab:C14408T, ORF1ab:C18115T, ORF1ab:A20268G, S:A23403G, S:G25049T, N:C28854T,	8	N:S194L, ORF1a:K290N, ORF1a:S2797F, ORF1b:P103S, ORF1b:P314L, ORF1b:H1550Y, S:D614G, S:D1163Y,
hCoV-19/Mexico/BCN-ALSR-1692/2020	EPI_ISL_496353	20A	B.1	7	5'UTR:C241T, ORF1ab:C346T, ORF1ab:C3037T, ORF1ab:C10332T, ORF1ab:C14408T, ORF1ab:A20268G, S:A23403G, N:C28854T,	4	N:S194L, ORF1a:T3356I, ORF1b:P314L, S:D614G,
hCoV-19/Mexico/BCN-ALSR-1693/2020	EPI_ISL_496354	20A	B.1	5	5'UTR:C241T, ORF1ab:C3037T, ORF1ab:C14408T, ORF1ab:A20268G, S:A23403G, N:C28854T,	3	N:S194L, ORF1b:P314L, S:D614G,
hCoV-19/Mexico/BCN-ALSR-1694/2020	EPI_ISL_496355	20A	B.1	7	5'UTR:C241T, ORF1ab:C3037T, ORF1ab:C4423T, ORF1ab:C14408T, ORF1ab:A19508C, ORF1ab:A20268G, S:A23403G, N:C28854T,	4	N:S194L, ORF1b:P314L, ORF1b:E2014A, S:D614G,
hCoV-19/Mexico/BCN-ALSR-1695/2020	EPI_ISL_496356	20A	B.1	7	5'UTR:C241T, ORF1ab:C3037T, ORF1ab:C14408T, ORF1ab:A19385C, ORF1ab:A20268G, S:A23403G, N:C28854T, N:C29167T,	4	N:S194L, ORF1b:P314L, ORF1b:D1973A, S:D614G,
hCoV-19/Mexico/BCN-ALSR-1696/2020	EPI_ISL_496357	20A	B.1.241	8	5'UTR:C241T, ORF1ab:C3037T, ORF1ab:T7942A, ORF1ab:C11575T, ORF1ab:C14408T, ORF1ab:C19029T, ORF1ab:A20268G, S:A23403G, N:C28854T,	3	N:S194L, ORF1b:P314L, S:D614G,
hCoV-19/Mexico/BCN-ALSR-1697/2020	EPI_ISL_496358	20A	B.1	7	5'UTR:C241T, ORF1ab:C3037T, ORF1ab:C14408T, ORF1ab:T17928C, ORF1ab:A20268G, S:C22000T, S:A23403G, N:C28854T,	3	N:S194L, ORF1b:P314L, S:D614G,
hCoV-19/Mexico/BCN-ALSR-1698/2020	EPI_ISL_496359	20A	B.1	6	5'UTR:C241T, ORF1ab:C3037T, ORF1ab:C14408T, ORF1ab:A20268G, S:C22208T, S:A23403G, N:C28854T,	4	N:S194L, ORF1b:P314L, S:L216F, S:D614G,
hCoV-19/Mexico/BCN-ALSR-1699/2020	EPI_ISL_496360	20A	B.1.245	7	5'UTR:C241T, ORF1ab:A1096G, ORF1ab:C3037T, ORF1ab:C14408T, ORF1ab:G14874T, ORF1ab:C18877T, S:A23403G, ORF3a:G25563T,	4	ORF1b:P314L, ORF1b:K469N, ORF3a:Q57H, S:D614G,
hCoV-19/Mexico/BCN-ALSR-1700/2020	EPI_ISL_496361	20A	B.1	7	5'UTR:C241T, ORF1ab:C3037T, ORF1ab:C9438T, ORF1ab:C14408T, ORF1ab:A20268G, S:C21772T, S:A23403G, N:C28854T,	4	N:S194L, ORF1a:T3058I, ORF1b:P314L, S:D614G,
hCoV-19/Mexico/BCN-ALSR-1701/2020	EPI_ISL_496362	20A	B.1.558	7	5'UTR:C241T, ORF1ab:C1288T, ORF1ab:C3037T, ORF1ab:C11575T, ORF1ab:C14408T, ORF1ab:A20268G, S:A23403G, N:C28854T,	3	N:S194L, ORF1b:P314L, S:D614G,
hCoV-19/Mexico/BCN-ALSR-1702/2020	EPI_ISL_496363	20A	B.1.241	7	5'UTR:C241T, ORF1ab:C3037T, ORF1ab:C7768T, ORF1ab:C11575T, ORF1ab:C14408T, ORF1ab:A20268G, S:A23403G, N:C28854T,	3	N:S194L, ORF1b:P314L, S:D614G,
hCoV-19/Mexico/BCN-ALSR-1703/2020	EPI_ISL_496364	20A	B.1	6	5'UTR:C241T, ORF1ab:C3037T, ORF1ab:C14408T, ORF1ab:A20268G, S:A23403G, N:C28854T, 3'UTR:G29715T,	3	N:S194L, ORF1b:P314L, S:D614G,
hCoV-19/Mexico/BCN-ALSR-1704/2020	EPI_ISL_496365	20A	B.1	5	5'UTR:C241T, ORF1ab:C3037T, ORF1ab:C14408T, ORF1ab:A20268G, S:A23403G, N:C28854T,	3	N:S194L, ORF1b:P314L, S:D614G,
hCoV-19/Mexico/BCN-ALSR-1705/2020	EPI_ISL_496366	20A	B.1	6	5'UTR:C241T, ORF1ab:C3037T, ORF1ab:C14408T, ORF1ab:A20268G, S:A23403G, N:C28854T, 3'UTR:G29715T,	3	N:S194L, ORF1b:P314L, S:D614G,
hCoV-19/Mexico/BCN-ALSR-1706/2020	EPI_ISL_496367	20A	B.1	5	5'UTR:C241T, ORF1ab:C3037T, ORF1ab:C14408T, ORF1ab:A20268G, S:A23403G, N:C28854T,	3	N:S194L, ORF1b:P314L, S:D614G,
hCoV-19/Mexico/BCN-ALSR-1707/2020	EPI_ISL_496368	20C	B.1	7	5'UTR:C241T, ORF1ab:C1059T, ORF1ab:C3037T, ORF1ab:C14408T, ORF1ab:G17014T, S:A23403G, ORF3a:G25563T, ORF7a:C27494T,	6	ORF1a:T265I, ORF1b:P314L, ORF1b:D1183Y, ORF3a:Q57H, ORF7a:P34L, S:D614G,

hCoV-19/Mexico/BCN-ALSR-1708/2020	EPI_ISL_496369	20A	B.1.241	9	5'UTR:C241T, ORF1ab:C3037T, ORF1ab:A8805C, ORF1ab:C11575T, ORF1ab:C14408T, ORF1ab:C16887T, ORF1ab:C19029T, ORF1ab:A20268G, S:A23403G, N:C28854T,	4	N:S194L, ORF1a:N2847T, ORF1b:P314L, S:D614G,
hCoV-19/Mexico/BCN-ALSR-1709/2020	EPI_ISL_496370	20C	B.1.267	6	5'UTR:C241T, ORF1ab:C1059T, ORF1ab:C3037T, ORF1ab:A8252G, ORF1ab:C14408T, S:A23403G, ORF3a:G25563T,	5	ORF1a:T265I, ORF1a:I2663V, ORF1b:P314L, ORF3a:Q57H, S:D614G,
hCoV-19/Mexico/BCN-ALSR-1710/2020	EPI_ISL_496371	20C	B.1	5	5'UTR:C241T, ORF1ab:C1059T, ORF1ab:C3037T, ORF1ab:C14408T, S:A23403G, ORF3a:G25563T,	4	ORF1a:T265I, ORF1b:P314L, ORF3a:Q57H, S:D614G,
hCoV-19/Mexico/BCN-ALSR-1711/2020	EPI_ISL_496372	20C	B.1	5	5'UTR:C241T, ORF1ab:C1059T, ORF1ab:C3037T, ORF1ab:C14408T, S:A23403G, ORF3a:G25563T,	4	ORF1a:T265I, ORF1b:P314L, ORF3a:Q57H, S:D614G,
hCoV-19/Mexico/BCN-ALSR-1712/2020	EPI_ISL_496373	20A	B.1	5	5'UTR:C241T, ORF1ab:C3037T, ORF1ab:C14408T, ORF1ab:A20268G, S:A23403G, N:C28854T,	3	N:S194L, ORF1b:P314L, S:D614G,
hCoV-19/Mexico/BCN-ALSR-1713/2020	EPI_ISL_496374	20A	B.1	5	5'UTR:C241T, ORF1ab:C3037T, ORF1ab:C14408T, ORF1ab:A20268G, S:A23403G, N:C28854T,	3	N:S194L, ORF1b:P314L, S:D614G,
hCoV-19/Mexico/BCN-ALSR-1714/2020	EPI_ISL_496375	20A	B.1.241	9	5'UTR:C241T, ORF1ab:C3037T, ORF1ab:C7768T, ORF1ab:C11575T, ORF1ab:C14408T, ORF1ab:C17470T, ORF1ab:G19962T, ORF1ab:A20268G, S:A23403G, N:C28854T,	3	N:S194L, ORF1b:P314L, S:D614G,
hCoV-19/Mexico/BCN-ALSR-1715/2020	EPI_ISL_496376	20A	B.1.241	10	5'UTR:C241T, ORF1ab:C3037T, ORF1ab:C7768T, ORF1ab:C11575T, ORF1ab:C14408T, ORF1ab:G14653T, ORF1ab:G19072T, ORF1ab:A20268G, S:A23403G, ORF3a:C25721T, N:C28854T,	6	N:S194L, ORF1b:P314L, ORF1b:V396F, ORF1b:D1869Y, ORF3a:A110V, S:D614G,
hCoV-19/Mexico/BCN-ALSR-2444/2020	EPI_ISL_635479	20A	B.1	9	5'UTR:C241T, ORF1ab:C3037T, ORF1ab:A3327G, ORF1ab:C11195T, ORF1ab:C14408T, ORF1ab:G17014T, S:A23403G, ORF3a:G25563T, ORF3a:C25658T, N:T29322G,	8	N:V350G, ORF1a:Q1021R, ORF1a:L3644F, ORF1b:P314L, ORF1b:D1183Y, ORF3a:Q57H, ORF3a:T89I, S:D614G,
hCoV-19/Mexico/BCN-ALSR-2446/2020	EPI_ISL_635480	20A	B.1.241	7	5'UTR:C241T, ORF1ab:C3037T, ORF1ab:C11575T, ORF1ab:C14408T, ORF1ab:C19029T, ORF1ab:A20268G, S:A23403G, N:C28854T,	3	N:S194L, ORF1b:P314L, S:D614G,
hCoV-19/Mexico/BCN-ALSR-2447/2020	EPI_ISL_635481	20A	B.1.397	8	5'UTR:C241T, ORF1ab:G1135T, ORF1ab:C1480T, ORF1ab:C3037T, ORF1ab:C14408T, ORF1ab:A20268G, S:A23403G, N:C28854T, ORF10:T29595C,	4	N:S194L, ORF1a:K290N, ORF1b:P314L, S:D614G,
hCoV-19/Mexico/BCN-ALSR-2448/2020	EPI_ISL_635482	20A	B.1	7	5'UTR:C241T, ORF1ab:C3037T, ORF1ab:C14408T, ORF1ab:A20268G, S:C22502A, S:A23403G, ORF7a:A27533G, N:C28854T,	5	N:S194L, ORF1b:P314L, ORF7a:H47R, S:Q314K, S:D614G,
hCoV-19/Mexico/BCN-ALSR-2451/2020	EPI_ISL_635483	20A	B.1	10	5'UTR:C241T, ORF1ab:C3037T, ORF1ab:C14408T, ORF1ab:C14481T, ORF1ab:C15081T, ORF1ab:A20268G, S:A23403G, S:G24547A, M:C26534T, N:C28706T, N:C28854T,	4	N:H145Y, N:S194L, ORF1b:P314L, S:D614G,
hCoV-19/Mexico/BCN-ALSR-2452/2020	EPI_ISL_635484	20C	B.1	8	5'UTR:C241T, ORF1ab:C1059T, ORF1ab:C3037T, ORF1ab:C7162T, ORF1ab:C14408T, ORF1ab:G17014T, S:A23403G, ORF3a:G25563T, M:G26951T,	5	ORF1a:T265I, ORF1b:P314L, ORF1b:D1183Y, ORF3a:Q57H, S:D614G,
hCoV-19/Mexico/BCN-ALSR-2457/2020	EPI_ISL_635485	20A	B.1.241	11	5'UTR:C241T, ORF1ab:C3037T, ORF1ab:C8320T, ORF1ab:C11575T, ORF1ab:C14408T, ORF1ab:C19029T, ORF1ab:T19355C, ORF1ab:A20268G, S:A23403G, ORF8:G28178T, N:C28854T, ORF10:T29595C,	5	N:S194L, ORF1b:P314L, ORF1b:L1963S, ORF8:L95F, S:D614G,
hCoV-19/Mexico/BCN-ALSR-2462/2020	EPI_ISL_635486	20A	B.1	6	5'UTR:C241T, ORF1ab:C3037T, ORF1ab:C14408T, ORF1ab:A20268G, S:A23403G, ORF8:C27903T, N:C28854T,	4	N:S194L, ORF1b:P314L, ORF8:L4F, S:D614G,
hCoV-19/Mexico/BCN-ALSR-2466/2020	EPI_ISL_635487	20A	B.1	11	5'UTR:C241T, ORF1ab:G1495A, ORF1ab:C3037T, ORF1ab:A4571G, ORF1ab:C5175T, ORF1ab:C12484T, ORF1ab:C14408T, ORF1ab:A20268G, ORF1ab:G21193T, S:C21627T, S:A23403G, N:C28854T,	7	N:S194L, ORF1a:N1436D, ORF1a:T1637I, ORF1b:P314L, ORF1b:D2576Y, S:T22I, S:D614G,
hCoV-19/Mexico/BCN-ALSR-2468/2020	EPI_ISL_635488	20A	B.1.558	9	5'UTR:C241T, ORF1ab:C1288T, ORF1ab:C11575T, ORF1ab:C14408T, ORF1ab:A20268G, S:A23403G, N:C28854T, N:G29254T, N:T29326C, ORF10:T29595C,	3	N:S194L, ORF1b:P314L, S:D614G,
hCoV-19/Mexico/BCN-ALSR-2475/2020	EPI_ISL_635489	20A	B.1	7	5'UTR:C241T, ORF1ab:C3037T, ORF1ab:C12781T, ORF1ab:C14408T, ORF1ab:A20268G, S:A23403G, N:C28854T, ORF10:T29595C,	3	N:S194L, ORF1b:P314L, S:D614G,

hCoV-19/Mexico/BCN-ALSR-2477/2020	EPI_ISL_635490	20A	B.1	7	5'UTR:C241T, ORF1ab:C3037T, ORF1ab:C14408T, ORF1ab:A20268G, S:A23403G, S:G25145C, S:G25244T, N:C28854T,	5	N:S194L, ORF1b:P314L, S:D614G, S:E1195Q, S:V1228L,	
hCoV-19/Mexico/BCN-ALSR-2479/2020	EPI_ISL_635491	20A	B.1	6	5'UTR:C241T, ORF1ab:C3037T, ORF1ab:C14408T, ORF1ab:A20268G, S:A23403G, ORF8:C27903T, N:C28854T,	4	N:S194L, ORF1b:P314L, ORF8:L4F, S:D614G,	
hCoV-19/Mexico/BCN-ALSR-2480/2020	EPI_ISL_635492	20A	B.1.399	7	5'UTR:C241T, ORF1ab:C3037T, ORF1ab:C14408T, ORF1ab:G18397A, ORF1ab:A20268G, S:A23403G, N:C28854T, N:C28887T,	5	N:S194L, N:T205I, ORF1b:P314L, ORF1b:V1644I, S:D614G,	
hCoV-19/Mexico/BCN-ALSR-2481/2020	EPI_ISL_635493	20A	B.1.609	12	5'UTR:C241T, ORF1ab:C3037T, ORF1ab:C3393T, ORF1ab:C4582T, ORF1ab:C8175T, ORF1ab:C14408T, ORF1ab:C14757T, ORF1ab:C16230A, ORF1ab:C17934T, ORF1ab:A20268G, ORF1ab:G20756T, S:A23403G, N:C28775T,	6	N:P168S, ORF1a:A1043V, ORF1a:A2637V, ORF1b:P314L, ORF1b:S2430I, S:D614G,	
hCoV-19/Mexico/BCN-ALSR-2483/2020	EPI_ISL_635494	20C	B.1	8	5'UTR:C241T, ORF1ab:C1059T, ORF1ab:C3037T, ORF1ab:C14408T, ORF1ab:C16762T, ORF1ab:C16954T, ORF1ab:C21058T, S:A23403G, ORF3a:G25563T,	6	ORF1a:T265I, ORF1b:P314L, ORF1b:L1099F, ORF1b:P2531S, ORF3a:Q57H, S:D614G,	
hCoV-19/Mexico/BCN-ALSR-2484/2020	EPI_ISL_635495	20A	B.1.243	6	5'UTR:C241T, ORF1ab:C3037T, ORF1ab:C14408T, ORF1ab:A20268G, S:A23403G, S:T24076C, N:C28854T,	3	N:S194L, ORF1b:P314L, S:D614G,	
hCoV-19/Mexico/BCN-ALSR-2485/2020	EPI_ISL_635496	20A	B.1	8	5'UTR:C241T, ORF1ab:C3037T, ORF1ab:C14408T, ORF1ab:A20268G, S:T23371C, S:A23403G, ORF3a:G26172T, N:C28674T, N:C28854T,	5	N:A134V, N:S194L, ORF1b:P314L, ORF3a:M260I, S:D614G,	
hCoV-19/Mexico/BCN-ALSR-2487/2020	EPI_ISL_635497	20A	B.1.558	9	5'UTR:C241T, ORF1ab:C1288T, ORF1ab:C3037T, ORF1ab:C11575T, ORF1ab:C14408T, ORF1ab:T15054C, ORF1ab:C18115T, ORF1ab:A20268G, S:A23403G, N:C28854T,	4	N:S194L, ORF1b:P314L, ORF1b:H1550Y, S:D614G,	
hCoV-19/Mexico/BCN-ALSR-2489/2020	EPI_ISL_635498	20A	B.1.243	9	5'UTR:C241T, ORF1ab:C3037T, ORF1ab:A9553G, ORF1ab:C14408T, ORF1ab:A20268G, S:A23403G, S:T24076C, S:C24824T, ORF3a:T25548C, N:C28854T,	4	N:S194L, ORF1b:P314L, S:D614G, S:H1088Y,	
hCoV-19/Mexico/BCN-ALSR-2490/2020	EPI_ISL_635499	20A	B.1	8	5'UTR:C241T, ORF1ab:C3037T, ORF1ab:C5467T, ORF1ab:G6094T, ORF1ab:C14408T, ORF1ab:A20268G, S:A23403G, ORF6:G27364T, N:C28854T,	5	N:S194L, ORF1a:Q1943H, ORF1b:P314L, ORF6:E55*, S:D614G,	
hCoV-19/Mexico/BCN-ALSR-2491/2020	EPI_ISL_635500	20A	B.1.243	6	5'UTR:C241T, ORF1ab:C3037T, ORF1ab:C14408T, ORF1ab:A20268G, S:A23403G, S:T24076C, N:C28854T,	3	N:S194L, ORF1b:P314L, S:D614G,	
hCoV-19/Mexico/BCN-ALSR-2492/2020	EPI_ISL_635501	20A	B.1	9	5'UTR:C180T, 5'UTR:C241T, ORF1ab:C715T, ORF1ab:C3037T, ORF1ab:G12832T, ORF1ab:C14408T, ORF1ab:C15924T, ORF1ab:A20268G, S:A23403G, N:C28854T,	4	N:S194L, ORF1a:Q4189H, ORF1b:P314L, S:D614G,	
hCoV-19/Mexico/BCN-ALSR-2493/2020	EPI_ISL_635502	20A	B.1	10	5'UTR:C241T, ORF1ab:C2939T, ORF1ab:C3037T, ORF1ab:C6312T, ORF1ab:C14408T, ORF1ab:A20268G, S:C21627T, S:A23403G, N:T28630A, N:C28854T, N:C28887T,	7	N:S194L, N:T205I, ORF1a:P892S, ORF1a:T2016I, ORF1b:P314L, S:T22I, S:D614G,	
hCoV-19/Mexico/BCN-ALSR-2494/2020	EPI_ISL_635503	20B	B.1.1	8	5'UTR:C241T, ORF1ab:C1473T, ORF1ab:C3037T, ORF1ab:C14408T, S:A23403G, N:G28881A, N:G28882A, N:G28883C, N:G29473A,	5	N:R203K, N:G204R, ORF1a:T403I, ORF1b:P314L, S:D614G,	3'UTR:29724-29766
hCoV-19/Mexico/BCN-ALSR-2495/2020	EPI_ISL_635504	20A	B.1	9	5'UTR:C241T, ORF1ab:C817T, ORF1ab:C3037T, ORF1ab:C11530T, ORF1ab:C14408T, ORF1ab:A20268G, S:T22195C, S:A23403G, N:C28854T, N:G28939T,	3	N:S194L, ORF1b:P314L, S:D614G,	
hCoV-19/Mexico/BCN-ALSR-2496/2020	EPI_ISL_635505	20A	B.1	8	5'UTR:C241T, ORF1ab:C2509T, ORF1ab:C3037T, ORF1ab:C14408T, ORF1ab:A20268G, ORF1ab:C20762T, S:A23403G, N:C28720T, N:C28854T,	4	N:S194L, ORF1b:P314L, ORF1b:T2432I, S:D614G,	
hCoV-19/Mexico/BCN-ALSR-2497/2020	EPI_ISL_635506	20A	B.1	7	5'UTR:C241T, ORF1ab:C3037T, ORF1ab:C14408T, ORF1ab:A20268G, ORF1ab:G20356T, S:A23403G, ORF3a:C25777T, N:C28854T,	5	N:S194L, ORF1b:P314L, ORF1b:G2297C, ORF3a:L129F, S:D614G,	
hCoV-19/Mexico/BCN-ALSR-2499/2020	EPI_ISL_635507	20A	B.1	7	5'UTR:G210T, 5'UTR:C241T, ORF1ab:C3037T, ORF1ab:C14408T, ORF1ab:A20268G, S:A22786G, S:A23403G, N:C28854T,	3	N:S194L, ORF1b:P314L, S:D614G,	

hCoV-19/Mexico/BCN-ALSR-2500/2020	EPI_ISL_635508	20A	B.1	9	5'UTR:C241T, ORF1ab:C3037T, ORF1ab:C12484T, ORF1ab:C14408T, ORF1ab:A20268G, ORF1ab:G21193T, S:C21627T, S:A23403G, ORF7a:G27415A, N:C28854T,	6	N:S194L, ORF1b:P314L, ORF1b:D2576Y, ORF7a:A8T, S:T22I, S:D614G,
hCoV-19/Mexico/BCN-ALSR-2502/2020	EPI_ISL_635509	20C	B.1.369	9	5'UTR:C241T, ORF1ab:C1059T, ORF1ab:C1973T, ORF1ab:C3037T, ORF1ab:C14408T, ORF1ab:G17014T, ORF1ab:C17135T, S:A23403G, ORF3a:G25563T, N:C28821A,	7	N:S183Y, ORF1a:T265I, ORF1b:P314L, ORF1b:D1183Y, ORF1b:P1223L, ORF3a:Q57H, S:D614G,
hCoV-19/Mexico/BCN-ALSR-2504/2020	EPI_ISL_635510	20A	B.1	7	5'UTR:C241T, ORF1ab:C3037T, ORF1ab:C14408T, ORF1ab:A20268G, ORF1ab:G20438T, S:G22021T, S:A23403G, N:C28854T,	5	N:S194L, ORF1b:P314L, ORF1b:S2324I, S:M153I, S:D614G,
hCoV-19/Mexico/BCN-ALSR-2506/2020	EPI_ISL_635511	20A	B.1	9	5'UTR:C241T, ORF1ab:C3037T, ORF1ab:C4586T, ORF1ab:C12484T, ORF1ab:C14408T, ORF1ab:A20268G, ORF1ab:G21193T, S:C21627T, S:A23403G, N:C28854T,	5	N:S194L, ORF1b:P314L, ORF1b:D2576Y, S:T22I, S:D614G,
hCoV-19/Mexico/BCN-ALSR-2507/2020	EPI_ISL_635512	20A	B.1.243	9	5'UTR:C241T, ORF1ab:C1911T, ORF1ab:C3037T, ORF1ab:C14408T, ORF1ab:C16017T, ORF1ab:A20268G, S:A23403G, S:T24076C, E:T26445C, N:C28854T,	4	N:S194L, ORF1a:S549F, ORF1b:P314L, S:D614G,
hCoV-19/Mexico/BCN-ALSR-2508/2020	EPI_ISL_635513	20A	B.1.241	9	5'UTR:C241T, ORF1ab:G1401A, ORF1ab:C3037T, ORF1ab:C7768T, ORF1ab:C9924T, ORF1ab:C11575T, ORF1ab:C14408T, ORF1ab:A20268G, S:A23403G, N:C28854T,	5	N:S194L, ORF1a:G379E, ORF1a:A3220V, ORF1b:P314L, S:D614G,
hCoV-19/Mexico/BCN-ALSR-2509/2020	EPI_ISL_635514	20A	B.1.243	12	5'UTR:C241T, ORF1ab:A1386G, ORF1ab:C3037T, ORF1ab:T8764C, ORF1ab:C9430T, ORF1ab:C9679T, ORF1ab:C10156T, ORF1ab:C14408T, ORF1ab:A20268G, S:A23403G, S:T24076C, ORF8:G28198T, N:C28854T,	5	N:S194L, ORF1a:H374R, ORF1b:P314L, ORF8:C102F, S:D614G,
hCoV-19/Mexico/BCN-ALSR-2510/2020	EPI_ISL_635515	20A	B.1	7	5'UTR:C241T, ORF1ab:C3037T, ORF1ab:C14408T, ORF1ab:A20268G, S:A23403G, ORF8:C27903T, N:C28854T, N:T29377A,	4	N:S194L, ORF1b:P314L, ORF8:L4F, S:D614G,
hCoV-19/Mexico/BCN-ALSR-2511/2020	EPI_ISL_635516	20A	B.1	13	5'UTR:C241T, ORF1ab:C478T, ORF1ab:G1135T, ORF1ab:C3037T, ORF1ab:G6850A, ORF1ab:A12064G, ORF1ab:C14408T, ORF1ab:T19765C, ORF1ab:A20268G, ORF1ab:C20759T, S:A21661C, S:A23403G, S:T25316C, N:C28854T,	6	N:S194L, ORF1a:K290N, ORF1b:P314L, ORF1b:A2431V, S:D614G, S:S1252P,
hCoV-19/Mexico/BCN-ALSR-2512/2020	EPI_ISL_635517	20A	B.1	7	5'UTR:C241T, ORF1ab:C2137T, ORF1ab:C3037T, ORF1ab:A12307G, ORF1ab:C14408T, ORF1ab:A20268G, S:A23403G, N:C28854T,	3	N:S194L, ORF1b:P314L, S:D614G,
hCoV-19/Mexico/BCN-ALSR-2513/2020	EPI_ISL_635518	20A	B.1.243	9	5'UTR:C241T, ORF1ab:C3037T, ORF1ab:C14408T, ORF1ab:A20268G, S:G22132T, S:A23403G, S:T24076C, S:C24099T, E:T26304C, N:C28854T,	5	N:S194L, ORF1b:P314L, S:R190S, S:D614G, S:A846V,
hCoV-19/Mexico/BCN-ALSR-2514/2020	EPI_ISL_635519	20A	B.1	10	5'UTR:C241T, ORF1ab:C1190T, ORF1ab:C3037T, ORF1ab:C6310T, ORF1ab:C9430T, ORF1ab:C14408T, ORF1ab:G18859T, ORF1ab:A20268G, S:G22217A, S:A23403G, N:C28854T,	6	N:S194L, ORF1a:P309S, ORF1b:P314L, ORF1b:A1798S, S:G219S, S:D614G,
hCoV-19/Mexico/BCN-ALSR-2515/2020	EPI_ISL_635520	20A	B.1	7	5'UTR:C241T, ORF1ab:G2632T, ORF1ab:C3037T, ORF1ab:G5425T, ORF1ab:C14408T, ORF1ab:A20268G, S:A23403G, ORF8:C28093T,	5	ORF1a:M789I, ORF1a:K1720N, ORF1b:P314L, ORF8:S67F, S:D614G,
hCoV-19/Mexico/BCN-ALSR-2516/2020	EPI_ISL_635521	20A	B.1	6	5'UTR:C241T, ORF1ab:G2229A, ORF1ab:C3037T, ORF1ab:C14408T, ORF1ab:A20268G, S:A23403G, N:C28854T,	4	N:S194L, ORF1a:C655Y, ORF1b:P314L, S:D614G,
hCoV-19/Mexico/BCN-ALSR-2517/2020	EPI_ISL_635522	20A	B.1	9	5'UTR:C241T, ORF1ab:C3037T, ORF1ab:C9042T, ORF1ab:C11620T, ORF1ab:C14408T, ORF1ab:A20268G, S:A23403G, N:C28854T, N:G29009A, N:C29065T,	5	N:S194L, N:V246I, ORF1a:S2926F, ORF1b:P314L, S:D614G,
hCoV-19/Mexico/BCN-ALSR-2519/2020	EPI_ISL_635523	20A	B.1	9	5'UTR:C241T, ORF1ab:G2309T, ORF1ab:C3037T, ORF1ab:C14408T, ORF1ab:A19137G, ORF1ab:A20268G, ORF1ab:G21123T, S:C22642T, S:A23403G, N:C28854T,	4	N:S194L, ORF1a:V682L, ORF1b:P314L, S:D614G,
hCoV-19/Mexico/BCN-ALSR-2520/2020	EPI_ISL_635524	20A	B.1.241	11	5'UTR:C241T, ORF1ab:A2473G, ORF1ab:C3037T, ORF1ab:C7768T, ORF1ab:C11575T, ORF1ab:T14154C, ORF1ab:C14408T, ORF1ab:A20268G, ORF1ab:C20759T, S:A23403G, ORF3a:G25500A, N:C28854T,	4	N:S194L, ORF1b:P314L, ORF1b:A2431V, S:D614G,

hCoV-19/Mexico/BCN-ALSR-2521/2020	EPI_ISL_635525	20A	B.1.243	10	5'UTR:C241T, ORF1ab:G331A, ORF1ab:C3037T, ORF1ab:C14408T, ORF1ab:T14910C, ORF1ab:A16052G, ORF1ab:A20268G, S:A23403G, S:T24076C, ORF3a:G25690T, N:C28854T,	5	N:S194L, ORF1b:P314L, ORF1b:K862R, ORF3a:G100C, S:D614G,
hCoV-19/Mexico/BCN-ALSR-2522/2020	EPI_ISL_635526	20A	B.1.243	10	5'UTR:C241T, ORF1ab:C3037T, ORF1ab:C10319T, ORF1ab:C14408T, ORF1ab:C15546T, ORF1ab:C16329T, ORF1ab:A20268G, S:A23403G, S:T24076C, N:C28854T, N:G29227T,	4	N:S194L, ORF1a:L3352F, ORF1b:P314L, S:D614G,
hCoV-19/Mexico/BCN-ALSR-2523/2020	EPI_ISL_635527	20A	B.1	13	5'UTR:C241T, ORF1ab:C3037T, ORF1ab:C8389T, ORF1ab:C10277T, ORF1ab:C11455T, ORF1ab:C12403T, ORF1ab:C14408T, ORF1ab:C17518T, ORF1ab:C18877T, S:G22240T, S:C22323T, S:A23403G, S:G24399T, ORF3a:G25563T,	8	ORF1a:L3338F, ORF1b:P314L, ORF1b:L1351F, ORF3a:Q57H, S:L226F, S:S254F, S:D614G, S:G946V,
hCoV-19/Mexico/BCN-ALSR-2524/2020	EPI_ISL_635528	20A	B.1.243	6	5'UTR:C241T, ORF1ab:C3037T, ORF1ab:C14408T, ORF1ab:A20268G, S:A23403G, S:T24076C, N:C28854T,	3	N:S194L, ORF1b:P314L, S:D614G,
hCoV-19/Mexico/BCN-ALSR-2525/2020	EPI_ISL_635529	20A	B.1.399	10	5'UTR:C241T, ORF1ab:C3037T, ORF1ab:G6410A, ORF1ab:G8017T, ORF1ab:C9711T, ORF1ab:C14408T, ORF1ab:C20104T, ORF1ab:A20268G, S:A23403G, N:C28854T, N:C28887T,	7	N:S194L, N:T205I, ORF1a:E2049K, ORF1a:S3149F, ORF1b:P314L, ORF1b:L2213F, S:D614G,
hCoV-19/Mexico/BCN-ALSR-2526/2020	EPI_ISL_635530	20A	B.1.243	8	5'UTR:C241T, ORF1ab:C3037T, ORF1ab:C14408T, ORF1ab:G19645T, ORF1ab:A20268G, S:A23403G, S:T24076C, N:G28460T, N:C28854T,	6	N:D63Y, N:S194L, ORF1b:P314L, ORF1b:V2060F, ORF9b:K59N, S:D614G,
hCoV-19/Mexico/BCN-ALSR-2527/2020	EPI_ISL_635531	20A	B.1.243	8	ORF1ab:C3037T, ORF1ab:C14408T, ORF1ab:T14910C, ORF1ab:A16052G, ORF1ab:A20268G, S:A23403G, S:T24076C, ORF3a:G25690T, N:C28854T,	5	N:S194L, ORF1b:P314L, ORF1b:K862R, ORF3a:G100C, S:D614G,
hCoV-19/Mexico/BCN-ALSR-2528/2020	EPI_ISL_635532	20A	B.1	8	5'UTR:C241T, ORF1ab:C2094T, ORF1ab:C3037T, ORF1ab:G10396T, ORF1ab:C14408T, ORF1ab:A20268G, S:A23403G, N:C28854T, 3'UTR:G29759A,	4	N:S194L, ORF1a:S610L, ORF1b:P314L, S:D614G,
hCoV-19/Mexico/BCN-ALSR-2529/2020	EPI_ISL_635533	20A	B.1	11	5'UTR:C241T, ORF1ab:C3037T, ORF1ab:C4586T, ORF1ab:C9344T, ORF1ab:C12484T, ORF1ab:C14408T, ORF1ab:A20268G, ORF1ab:G21193T, S:C21627T, S:A23403G, ORF3a:C25721T, N:C28854T,	7	N:S194L, ORF1a:L3027F, ORF1b:P314L, ORF1b:D2576Y, ORF3a:A110V, S:T22I, S:D614G,
hCoV-19/Mexico/BCN-ALSR-2530/2020	EPI_ISL_635534	20A	B.1	10	5'UTR:C241T, ORF1ab:C2137T, ORF1ab:C3037T, ORF1ab:C7979T, ORF1ab:A12307G, ORF1ab:C14408T, ORF1ab:C14649T, ORF1ab:A20268G, ORF1ab:G20683T, S:A23403G, N:C28854T,	4	N:S194L, ORF1b:P314L, ORF1b:V2406F, S:D614G,
hCoV-19/Mexico/BCN-ALSR-2531/2020	EPI_ISL_635535	20A	B.1.399	10	5'UTR:C241T, ORF1ab:C3037T, ORF1ab:G6410A, ORF1ab:G8017T, ORF1ab:C9711T, ORF1ab:C14408T, ORF1ab:C20104T, ORF1ab:A20268G, S:A23403G, N:C28854T, N:C28887T,	7	N:S194L, N:T205I, ORF1a:E2049K, ORF1a:S3149F, ORF1b:P314L, ORF1b:L2213F, S:D614G,
hCoV-19/Mexico/BCN-ALSR-2533/2020	EPI_ISL_635536	20B	B.1.1	14	5'UTR:C241T, ORF1ab:C313T, ORF1ab:C1392T, ORF1ab:C3037T, ORF1ab:G8618A, ORF1ab:C12459T, ORF1ab:A13278G, ORF1ab:C14408T, S:A23403G, S:G24040T, ORF3a:G25650T, E:C26456G, N:G28881A, N:G28882A, N:G28883C,	10	E:P71R, N:R203K, N:G204R, ORF1a:S376L, ORF1a:A2785T, ORF1a:T4065I, ORF1a:N4338S, ORF1b:P314L, ORF3a:L86F, S:D614G,
hCoV-19/Mexico/BCN-ALSR-2534/2020	EPI_ISL_635537	20A	B.1.243	11	5'UTR:C241T, ORF1ab:C3037T, ORF1ab:C4331T, ORF1ab:G9652A, ORF1ab:C14408T, ORF1ab:C18395T, ORF1ab:C19763T, ORF1ab:A20268G, S:T22543C, S:A23403G, S:T24076C, N:C28854T,	6	N:S194L, ORF1a:M3129I, ORF1b:P314L, ORF1b:A1643V, ORF1b:T2099I, S:D614G,
hCoV-19/Mexico/BCN-ALSR-2535/2020	EPI_ISL_635538	20A	B.1	11	5'UTR:C241T, ORF1ab:C3037T, ORF1ab:A4571G, ORF1ab:C5175T, ORF1ab:A6528G, ORF1ab:C12484T, ORF1ab:C14408T, ORF1ab:A20268G, ORF1ab:G21193T, S:C21627T, S:A23403G, N:C28854T,	8	N:S194L, ORF1a:N1436D, ORF1a:T1637I, ORF1a:E2088G, ORF1b:P314L, ORF1b:D2576Y, S:T22I, S:D614G,

hCoV-19/Mexico/BCN-ALSR-2536/2020	EPI_ISL_635539	20A	B.1.229	10	5'UTR:C241T, ORF1ab:C1302T, ORF1ab:C3037T, ORF1ab:G7798T, ORF1ab:C14408T, ORF1ab:A20268G, S:A23403G, ORF3a:A25589T, ORF3a:T25754C, E:T26427G, N:C28854T,	7	N:S194L, ORF1a:T346I, ORF1a:K2511N, ORF1b:P314L, ORF3a:K66I, ORF3a:V121A, S:D614G,	
hCoV-19/Mexico/BCN-ALSR-2537/2020	EPI_ISL_635540	20A	B.1	5	5'UTR:C241T, ORF1ab:C3037T, ORF1ab:C14408T, ORF1ab:A20268G, S:A23403G, N:C28854T,	3	N:S194L, ORF1b:P314L, S:D614G,	
hCoV-19/Mexico/BCN-ALSR-2538/2020	EPI_ISL_635541	20B	B.1.1.285	11	5'UTR:C241T, ORF1ab:C313T, ORF1ab:C823T, ORF1ab:C3037T, ORF1ab:A3344G, ORF1ab:C14408T, S:A23403G, ORF3a:G25660T, N:G28321T, N:G28881A, N:G28882A, N:G28883C,	7	N:R203K, N:G204R, ORF1a:S1027G, ORF1b:P314L, ORF3a:V90F, ORF9b:R13L, S:D614G,	
hCoV-19/Mexico/BCN-ALSR-2539/2020	EPI_ISL_635542	20A	B.1	8	5'UTR:C241T, ORF1ab:C2508A, ORF1ab:C3037T, ORF1ab:C14408T, ORF1ab:A20268G, S:A23403G, M:G26558A, N:C28854T, N:C29358T,	5	N:S194L, N:T362I, ORF1a:P748H, ORF1b:P314L, S:D614G,	
hCoV-19/Mexico/BCN-ALSR-2540/2020	EPI_ISL_635543	20A	B.1.239	9	5'UTR:C241T, ORF1ab:C3037T, ORF1ab:C10036T, ORF1ab:C14408T, ORF1ab:G16245T, ORF1ab:G16741A, ORF1ab:A19779G, ORF1ab:A20268G, S:A23403G, N:C28854T,	4	N:S194L, ORF1b:P314L, ORF1b:V1092I, S:D614G,	
hCoV-19/Mexico/BCN-ALSR-2541/2020	EPI_ISL_635544	20A	B.1.400	10	5'UTR:C241T, ORF1ab:G1590T, ORF1ab:C3037T, ORF1ab:C6781T, ORF1ab:C14408T, ORF1ab:G18186T, ORF1ab:G19969A, ORF1ab:A20268G, S:A23403G, N:C28854T, N:G29179T,	6	N:S194L, ORF1a:G442V, ORF1b:P314L, ORF1b:M1573I, ORF1b:A2168T, S:D614G,	
hCoV-19/Mexico/BCN-ALSR-2542/2020	EPI_ISL_635545	20A	B.1.399	6	5'UTR:C241T, ORF1ab:C3037T, ORF1ab:C14408T, ORF1ab:A20268G, S:A23403G, N:C28854T, N:C28887T,	4	N:S194L, N:T205I, ORF1b:P314L, S:D614G,	
hCoV-19/Mexico/BCN-ALSR-2543/2020	EPI_ISL_635546	20A	B.1	8	5'UTR:C241T, ORF1ab:C3037T, ORF1ab:C6385T, ORF1ab:C10755T, ORF1ab:C14408T, ORF1ab:A20268G, S:A23403G, N:C28453T, N:C28854T,	5	N:S194L, ORF1a:A3497V, ORF1b:P314L, ORF9b:A57V, S:D614G,	
hCoV-19/Mexico/BCN-ALSR-2544/2020	EPI_ISL_635547	20A	B.1	9	5'UTR:C241T, ORF1ab:C817T, ORF1ab:C3037T, ORF1ab:C3738T, ORF1ab:C14120T, ORF1ab:C14408T, ORF1ab:A20268G, S:A23403G, S:C24517T, N:C28854T,	5	N:S194L, ORF1a:P1158L, ORF1b:P218L, ORF1b:P314L, S:D614G,	
hCoV-19/Mexico/BCN-ALSR-2545/2020	EPI_ISL_635548	20A	B.1	9	5'UTR:C241T, ORF1ab:C3037T, ORF1ab:T3658C, ORF1ab:C4668T, ORF1ab:C14408T, ORF1ab:A20268G, S:A23403G, M:C26681T, N:C28854T, N:T29302C,	4	N:S194L, ORF1a:S1468F, ORF1b:P314L, S:D614G,	
hCoV-19/Mexico/BCN-ALSR-2546/2020	EPI_ISL_635549	20A	B.1	15	5'UTR:C241T, ORF1ab:G347A, ORF1ab:C3037T, ORF1ab:C4423T, ORF1ab:A11947G, ORF1ab:C14408T, ORF1ab:C16393T, ORF1ab:G16917T, ORF1ab:A20268G, ORF1ab:C21139T, S:A23403G, S:G23465A, S:G23608T, ORF3a:G25855C, ORF7a:C27513T, N:C28854T,	7	N:S194L, ORF1a:V28I, ORF1b:P314L, ORF1b:P976S, ORF3a:D155H, S:D614G, S:V635I,	
hCoV-19/Mexico/BCN-ALSR-2547/2020	EPI_ISL_635550	20A	B.1.243	10	5'UTR:C241T, ORF1ab:C3037T, ORF1ab:G5555A, ORF1ab:A12124T, ORF1ab:C14408T, ORF1ab:A20268G, S:A23403G, S:T24076C, ORF7b:T27803C, N:C28854T, 3'UTR:T29840G,	4	N:S194L, ORF1a:G1764S, ORF1b:P314L, S:D614G,	
hCoV-19/Mexico/BCN-ALSR-2548/2020	EPI_ISL_635551	20A	B.1.400	10	5'UTR:C241T, ORF1ab:G1590T, ORF1ab:C3037T, ORF1ab:C6781T, ORF1ab:C14408T, ORF1ab:G18186T, ORF1ab:G19969A, ORF1ab:A20268G, S:A23403G, N:C28854T, N:G29179T,	6	N:S194L, ORF1a:G442V, ORF1b:P314L, ORF1b:M1573I, ORF1b:A2168T, S:D614G,	
hCoV-19/Mexico/BCN-ALSR-2549/2020	EPI_ISL_635552	20A	B.1.243	8	5'UTR:C241T, ORF1ab:C3037T, ORF1ab:A5097G, ORF1ab:C14408T, ORF1ab:A20268G, S:A23403G, S:T24076C, ORF3a:C26028T, N:C28854T,	5	N:S194L, ORF1a:N1611S, ORF1b:P314L, S:D614G,ORF7a:F63H,	ORF7a:27579-27581
hCoV-19/Mexico/BCN-ALSR-2550/2020	EPI_ISL_635553	20A	B.1	9	5'UTR:C241T, ORF1ab:C2710T, ORF1ab:C3037T, ORF1ab:C8016T, ORF1ab:C14408T, ORF1ab:A20268G, S:A23403G, ORF3a:G25906T, N:G28703T, N:C28854T,	6	N:D144Y, N:S194L, ORF1a:A2584V, ORF1b:P314L, ORF3a:G172C, S:D614G,	
hCoV-19/Mexico/BCN-ALSR-2551/2020	EPI_ISL_635554	20A	B.1.241	12	5'UTR:C241T, ORF1ab:C3037T, ORF1ab:C6428T, ORF1ab:C11575T, ORF1ab:C14408T, ORF1ab:C19029T, ORF1ab:A19683C, ORF1ab:A20268G, ORF1ab:C21306T, S:C22088T, S:A23403G, N:C28854T, 3'UTR:G29747A,	6	N:S194L, ORF1a:P2055S, ORF1b:P314L, ORF1b:E2072D, S:L176F, S:D614G,	

hCoV-19/Mexico/BCN-ALSR-2552/2020	EPI_ISL_635555	20A	B.1.241	11	5'UTR:C241T, ORF1ab:A2473G, ORF1ab:C3037T, ORF1ab:C7768T, ORF1ab:C11575T, ORF1ab:T14154C, ORF1ab:C14408T, ORF1ab:A20268G, ORF1ab:C20759T, S:A23403G, ORF3a:G25500A, N:C28854T,	4	N:S194L, ORF1b:P314L, ORF1b:A2431V, S:D614G,
hCoV-19/Mexico/BCN-ALSR-2553/2020	EPI_ISL_635556	20A	B.1	11	5'UTR:C241T, ORF1ab:C3037T, ORF1ab:C4999T, ORF1ab:G9805T, ORF1ab:C10702T, ORF1ab:C14408T, ORF1ab:G18782T, ORF1ab:A20268G, S:A23403G, ORF8:G28178T, N:C28854T, N:C29061T,	6	N:S194L, N:T263I, ORF1b:P314L, ORF1b:G1772V, ORF8:L95F, S:D614G,
hCoV-19/Mexico/BCN-ALSR-2554/2020	EPI_ISL_635557	20A	B.1.243	11	5'UTR:C241T, ORF1ab:C2044T, ORF1ab:C3037T, ORF1ab:C14408T, ORF1ab:T14910C, ORF1ab:A16052G, ORF1ab:A20268G, ORF1ab:G21538T, S:A23403G, S:T24076C, ORF3a:G25690T, N:C28854T,	6	N:S194L, ORF1b:P314L, ORF1b:K862R, ORF1b:V2691F, ORF3a:G100C, S:D614G,
hCoV-19/Mexico/BCN-ALSR-2555/2020	EPI_ISL_635558	20A	B.1	9	5'UTR:C241T, ORF1ab:C1420T, ORF1ab:C3037T, ORF1ab:G13348T, ORF1ab:C14408T, ORF1ab:C17375T, ORF1ab:A20268G, S:A23403G, S:T24187C, N:C28854T,	4	N:S194L, ORF1b:P314L, ORF1b:T1303I, S:D614G,
hCoV-19/Mexico/BCN-ALSR-2556/2020	EPI_ISL_635559	20A	B.1.243	9	5'UTR:C241T, ORF1ab:C3037T, ORF1ab:C5884T, ORF1ab:C11455T, ORF1ab:C14408T, ORF1ab:A20268G, S:A23403G, S:T24076C, N:C28854T, N:A29403G,	4	N:S194L, N:D377G, ORF1b:P314L, S:D614G,
hCoV-19/Mexico/BCN-ALSR-2557/2020	EPI_ISL_635560	20A	B.1	6	5'UTR:C241T, ORF1ab:C3037T, ORF1ab:C12513T, ORF1ab:C14408T, ORF1ab:A20268G, S:A23403G, N:C28854T,	4	N:S194L, ORF1a:T4083M, ORF1b:P314L, S:D614G,
hCoV-19/Mexico/BCN-ALSR-2558/2020	EPI_ISL_635561	20A	B.1.243	8	5'UTR:C241T, ORF1ab:C3037T, ORF1ab:C14408T, ORF1ab:A20268G, S:A23403G, S:T24076C, S:C24559T, E:G26262A, N:C28854T,	3	N:S194L, ORF1b:P314L, S:D614G,
hCoV-19/Mexico/BCN-ALSR-2559/2020	EPI_ISL_635562	20B	B.1.1	9	5'UTR:C241T, ORF1ab:C3037T, ORF1ab:C14408T, ORF1ab:G15957T, S:G21770T, S:A23403G, M:T26960C, N:G28881A, N:G28882A, N:G28883C,	5	N:R203K, N:G204R, ORF1b:P314L, S:V70F, S:D614G,
hCoV-19/Mexico/BCN-ALSR-2560/2020	EPI_ISL_635563	20A	B.1.243	8	5'UTR:C241T, ORF1ab:C3037T, ORF1ab:C14408T, ORF1ab:A20268G, S:A23403G, S:T24076C, ORF3a:T25536A, N:C28854T, N:G28985T,	4	N:S194L, N:G238C, ORF1b:P314L, S:D614G,
hCoV-19/Mexico/BCN-ALSR-2561/2020	EPI_ISL_635564	20A	B.1	8	5'UTR:C241T, ORF1ab:C3037T, ORF1ab:G10523C, ORF1ab:G11042T, ORF1ab:C14408T, ORF1ab:G18651T, ORF1ab:A20268G, S:A23403G, N:C28854T,	6	N:S194L, ORF1a:V3420L, ORF1a:V3593F, ORF1b:P314L, ORF1b:E1728D, S:D614G,
hCoV-19/Mexico/BCN-ALSR-2562/2020	EPI_ISL_635565	20A	B.1.609	13	5'UTR:C241T, ORF1ab:T1088C, ORF1ab:C1684T, ORF1ab:C2704T, ORF1ab:C2881A, ORF1ab:C3037T, ORF1ab:C4582T, ORF1ab:C14408T, ORF1ab:C17440T, ORF1ab:A20268G, S:C21575T, S:C21621T, S:A23403G, M:G26727T,	7	M:A69S, ORF1a:F275L, ORF1b:P314L, ORF1b:P1325S, S:L5F, S:T20I, S:D614G,
hCoV-19/Mexico/BCN-ALSR-2563/2020	EPI_ISL_635566	20A	B.1	7	5'UTR:C241T, ORF1ab:G1954T, ORF1ab:C3037T, ORF1ab:C14408T, ORF1ab:C14649T, ORF1ab:A20268G, S:A23403G, N:C28854T,	4	N:S194L, ORF1a:Q563H, ORF1b:P314L, S:D614G,
hCoV-19/Mexico/BCN-ALSR-2565/2020	EPI_ISL_635567	20C	B.1	11	5'UTR:C71A, 5'UTR:C241T, ORF1ab:C1059T, ORF1ab:C3037T, ORF1ab:C5301T, ORF1ab:C14408T, S:A23403G, S:C23683T, ORF3a:G25563T, ORF7a:G27709T, N:C29546T, 3'UTR:G29742T,	6	ORF1a:T265I, ORF1a:A1679V, ORF1b:P314L, ORF3a:Q57H, ORF7a:A106S, S:D614G,
hCoV-19/Mexico/BCN-ALSR-2566/2020	EPI_ISL_635568	20A	B.1	9	5'UTR:C241T, ORF1ab:C2881T, ORF1ab:C3037T, ORF1ab:A3090T, ORF1ab:C14408T, ORF1ab:A20268G, S:A23403G, ORF3a:C25708T, N:T28723C, N:C28854T,	5	N:S194L, ORF1a:E942V, ORF1b:P314L, ORF3a:L106F, S:D614G,
hCoV-19/Mexico/BCN-ALSR-2568/2020	EPI_ISL_635569	20A	B.1	5	5'UTR:C241T, ORF1ab:C3037T, ORF1ab:C14408T, ORF1ab:A20268G, S:A23403G, N:C28854T,	3	N:S194L, ORF1b:P314L, S:D614G,
hCoV-19/Mexico/BCN-ALSR-2569/2020	EPI_ISL_635570	20A	B.1.243	11	5'UTR:C241T, ORF1ab:C3037T, ORF1ab:C14408T, ORF1ab:C16915T, ORF1ab:G16945A, ORF1ab:C17436T, ORF1ab:A20268G, S:A23403G, S:T24076C, M:C26801G, N:G28690T, N:C28854T,	5	N:L139F, N:S194L, ORF1b:P314L, ORF1b:A1160T, S:D614G,
hCoV-19/Mexico/BCN-ALSR-2570/2020	EPI_ISL_635571	20A	B.1.243	10	5'UTR:C241T, ORF1ab:G598T, ORF1ab:C3037T, ORF1ab:A7986G, ORF1ab:C14408T, ORF1ab:A18468C, ORF1ab:A20268G, S:A23403G, S:T24076C, N:C28854T, N:G29140T,	5	N:S194L, N:Q289H, ORF1a:Q2574R, ORF1b:P314L, S:D614G,

hCoV-19/Mexico/BCN-ALSR-2571/2020	EPI_ISL_635572	20A	B.1	8	5'UTR:C241T, ORF1ab:C3037T, ORF1ab:G3549A, ORF1ab:G10759T, ORF1ab:C14408T, ORF1ab:A20268G, S:A23403G, N:C28854T, 3'UTR:C29718T,	5	N:S194L, ORF1a:G1095E, ORF1a:M3498I, ORF1b:P314L, S:D614G,
hCoV-19/Mexico/BCN-ALSR-2572/2020	EPI_ISL_635573	20B	B.1.1.8	11	5'UTR:C241T, ORF1ab:C2462A, ORF1ab:C3037T, ORF1ab:G12160A, ORF1ab:C12488T, ORF1ab:C14408T, S:A23403G, ORF3a:C25528T, M:G27084A, N:G28881A, N:G28882A, N:G28883C,	8	M:A188T, N:R203K, N:G204R, ORF1a:L733I, ORF1a:P4075S, ORF1b:P314L, ORF3a:L46F, S:D614G,
hCoV-19/Mexico/BCN-ALSR-2573/2020	EPI_ISL_635574	20A	B.1	10	5'UTR:C241T, ORF1ab:A2806C, ORF1ab:C3037T, ORF1ab:G6443T, ORF1ab:A12229G, ORF1ab:C14408T, ORF1ab:G18782T, ORF1ab:A20268G, S:T23371C, S:A23403G, N:C28854T,	6	N:S194L, ORF1a:E847D, ORF1a:D2060Y, ORF1b:P314L, ORF1b:G1772V, S:D614G,
hCoV-19/Mexico/BCN-ALSR-2574/2020	EPI_ISL_635575	20A	B.1	10	5'UTR:C241T, ORF1ab:A2806C, ORF1ab:C3037T, ORF1ab:G6443T, ORF1ab:A12229G, ORF1ab:C14408T, ORF1ab:G18782T, ORF1ab:A20268G, S:T23371C, S:A23403G, N:C28854T,	6	N:S194L, ORF1a:E847D, ORF1a:D2060Y, ORF1b:P314L, ORF1b:G1772V, S:D614G,
hCoV-19/Mexico/BCN-ALSR-2576/2020	EPI_ISL_635576	20A	B.1.241	13	5'UTR:C241T, ORF1ab:C3037T, ORF1ab:C6786T, ORF1ab:C7768T, ORF1ab:T8634C, ORF1ab:C11575T, ORF1ab:C14408T, ORF1ab:T14907C, ORF1ab:G18148T, ORF1ab:A20268G, S:A23403G, N:C28344T, N:C28677T, N:C28854T,	8	N:T24I, N:T135I, N:S194L, ORF1a:T2174I, ORF1a:I2790T, ORF1b:P314L, ORF1b:G1561C, S:D614G,
hCoV-19/Mexico/BCN-ALSR-3618/2020	EPI_ISL_636113	20A	B.1	7	5'UTR:C241T, ORF1ab:C3037T, ORF1ab:C6286T, ORF1ab:C14408T, ORF1ab:A20268G, S:A23403G, S:G25244T, N:C28854T,	4	N:S194L, ORF1b:P314L, S:D614G, S:V1228L,
hCoV-19/Mexico/BCN-ALSR-3619/2020	EPI_ISL_636114	20A	B.1	7	5'UTR:C241T, ORF1ab:C3037T, ORF1ab:C6286T, ORF1ab:C14408T, ORF1ab:A20268G, S:A23403G, S:G25244T, N:C28854T,	4	N:S194L, ORF1b:P314L, S:D614G, S:V1228L,
hCoV-19/Mexico/BCN-ALSR-3622/2020	EPI_ISL_636115	20A	B.1	8	5'UTR:C241T, ORF1ab:C3037T, ORF1ab:C11094T, ORF1ab:C14408T, ORF1ab:A20268G, ORF1ab:A21136G, S:A23403G, S:C25169T, N:C28854T,	6	N:S194L, ORF1a:A3610V, ORF1b:P314L, ORF1b:K2557E, S:D614G, S:L1203F,
hCoV-19/Mexico/BCN-ALSR-3624/2020	EPI_ISL_636116	20A	B.1.243	12	5'UTR:C241T, ORF1ab:T2218G, ORF1ab:C3037T, ORF1ab:A8072G, ORF1ab:C14408T, ORF1ab:C19813T, ORF1ab:A20268G, S:A23403G, S:T24076C, ORF3a:G25523T, ORF7a:A27433G, N:C28854T, N:C29218T,	8	N:S194L, ORF1a:F651L, ORF1a:N2603D, ORF1b:P314L, ORF1b:P2116S, ORF3a:G44V, ORF7a:T14A, S:D614G,
hCoV-19/Mexico/BCN-ALSR-3630/2020	EPI_ISL_636117	20A	B.1.243	12	5'UTR:T42A, 5'UTR:C241T, ORF1ab:C3037T, ORF1ab:A3691C, ORF1ab:C10156T, ORF1ab:C11606T, ORF1ab:C14408T, ORF1ab:A20268G, S:A23403G, S:T24076C, N:C28854T, 3'UTR:G29736T, 3'UTR:C29738T,	4	N:S194L, ORF1a:E1142D, ORF1b:P314L, S:D614G,
hCoV-19/Mexico/BCN-ALSR-3634/2020	EPI_ISL_636118	20A	B.1.558	11	5'UTR:C241T, ORF1ab:C1288T, ORF1ab:C3037T, ORF1ab:C7423T, ORF1ab:C11575T, ORF1ab:C14408T, ORF1ab:G19101T, ORF1ab:A20268G, S:A23403G, S:G23405T, N:G28514C, N:C28854T,	7	N:D81H, N:S194L, ORF1b:P314L, ORF1b:Q1878H, ORF9b:Q77H, S:D614G, S:V615F,
hCoV-19/Mexico/BCN-ALSR-3636/2020	EPI_ISL_636119	20A	B.1.400	7	5'UTR:C241T, ORF1ab:C3037T, ORF1ab:C9096T, ORF1ab:C14408T, ORF1ab:G19969A, ORF1ab:A20268G, S:A23403G, N:C28854T,	5	N:S194L, ORF1a:A2944V, ORF1b:P314L, ORF1b:A2168T, S:D614G,
hCoV-19/Mexico/BCN-ALSR-3639/2020	EPI_ISL_636120	20B	B.1.1	16	5'UTR:C241T, ORF1ab:A1096G, ORF1ab:G1820A, ORF1ab:C3037T, ORF1ab:G10106T, ORF1ab:C14408T, ORF1ab:G14874T, ORF1ab:G15850A, ORF1ab:T18485C, ORF1ab:C18526T, ORF1ab:C18877T, S:G21724T, S:A23403G, ORF3a:G25563T, N:G28881A, N:G28882A. N:G28883C.	12	N:R203K, N:G204R, ORF1a:G519S, ORF1a:V3281L, ORF1b:P314L, ORF1b:K469N, ORF1b:D795N, ORF1b:L1673P, ORF1b:R1687C, ORF3a:Q57H, S:L54F, S:D614G,
hCoV-19/Mexico/BCN-ALSR-4762/2020	EPI_ISL_730127	20A	B.1.232	15	5'UTR:C241T, ORF1ab:C3037T, ORF1ab:T5804C, ORF1ab:C5849T, ORF1ab:C6285T, ORF1ab:C6706T, ORF1ab:C8247T, ORF1ab:C14408T, ORF1ab:C16092T, ORF1ab:A20268G, S:A23403G, E:G26325A, ORF8:G27987T, N:C28291T, N:C28854T, 3'UTR:G29825T,	9	N:S194L, ORF1a:C1847R, ORF1a:P1862S, ORF1a:T2007I, ORF1a:S2661F, ORF1b:P314L, ORF8:V32L, ORF9b:P3L, S:D614G,

hCoV-19/Mexico/BCN-ALSR-4766/2020	EPI_ISL_730128	20A	B.1.232	15	5'UTR:C241T, ORF1ab:C3037T, ORF1ab:T5804C, ORF1ab:C5849T, ORF1ab:C6285T, ORF1ab:C6706T, ORF1ab:C8247T, ORF1ab:C14408T, ORF1ab:C16092T, ORF1ab:A20268G, S:A23403G, E:G26325A, ORF8:G27987T, N:C28291T, N:C28854T, 3'UTR:G29825T,	9	N:S194L, ORF1a:C1847R, ORF1a:P1862S, ORF1a:T2007I, ORF1a:S2661F, ORF1b:P314L, ORF8:V32L, ORF9b:P3L, S:D614G,	
hCoV-19/Mexico/BCN-ALSR-4768/2020	EPI_ISL_730129	20A	B.1	11	5'UTR:C222T, 5'UTR:C241T, ORF1ab:C3037T, ORF1ab:A12498G, ORF1ab:G14400T, ORF1ab:C14408T, ORF1ab:C19269T, ORF1ab:C20233T, ORF1ab:A20268G, S:A23403G, ORF7b:C27828T, N:C28854T,	6	N:S194L, ORF1a:N4078S, ORF1b:P314L, ORF1b:P2256S, ORF7b:L25F, S:D614G,	
hCoV-19/Mexico/BCN-ALSR-4770/2020	EPI_ISL_730130	20A	B.1	6	5'UTR:C241T, ORF1ab:C3037T, ORF1ab:C14408T, ORF1ab:A20268G, S:A23403G, N:C28854T, 3'UTR:C29718T,	3	N:S194L, ORF1b:P314L, S:D614G,	
hCoV-19/Mexico/BCN-ALSR-4771/2020	EPI_ISL_730131	20B	B.1.1.222	13	5'UTR:C241T, ORF1ab:C1960T, ORF1ab:C3037T, ORF1ab:C14408T, ORF1ab:A16076G, ORF1ab:T19839C, S:A23403G, S:A23756G, ORF3a:C25521T, ORF3a:C25614T, N:G28881A, N:G28882A, N:G28883C, N:C28887T,	7	N:R203K, N:G204R, N:T205I, ORF1b:P314L, ORF1b:D870G, S:D614G, S:T732A,	
hCoV-19/Mexico/BCN-ALSR-4772/2020	EPI_ISL_730132	20G	B.1.2	15	5'UTR:C241T, ORF1ab:C1059T, ORF1ab:C3037T, ORF1ab:G8083A, ORF1ab:C10319T, ORF1ab:C14408T, ORF1ab:C14805T, ORF1ab:A18424G, ORF1ab:C21304T, S:A23403G, ORF3a:T25430C, ORF3a:G25563T, ORF3a:G25907T, ORF8:C27964T, N:C28472T, N:C28869T,	13	N:P67S, N:P199L, ORF1a:T265I, ORF1a:M2606I, ORF1a:L3352F, ORF1b:P314L, ORF1b:N1653D, ORF1b:R2613C, ORF3a:V13A, ORF3a:Q57H, ORF3a:G172V, ORF8:S24L, S:D614G,	
hCoV-19/Mexico/BCN-ALSR-4774/2020	EPI_ISL_730133	20A	B.1	6	5'UTR:C241T, ORF1ab:C3037T, ORF1ab:C14408T, ORF1ab:A20268G, S:A23403G, N:C28854T, 3'UTR:C29718T,	3	N:S194L, ORF1b:P314L, S:D614G,	
hCoV-19/Mexico/BCN-ALSR-4775/2020	EPI_ISL_730134	20C	B.1.324	12	5'UTR:C151T, 5'UTR:C241T, ORF1ab:C1059T, ORF1ab:C3037T, ORF1ab:C7528T, ORF1ab:G9479T, ORF1ab:C12885T, ORF1ab:C14408T, ORF1ab:T15087C, S:C22480T, S:A23403G, ORF3a:G25563T, N:C28890T,	7	N:S206F, ORF1a:T265I, ORF1a:G3072C, ORF1a:T4207I, ORF1b:P314L, ORF3a:Q57H, S:D614G,	
hCoV-19/Mexico/BCN-ALSR-4777/2020	EPI_ISL_730135	20B	B.1.1.93	17	5'UTR:C241T, ORF1ab:C2701T, ORF1ab:C3037T, ORF1ab:C3369T, ORF1ab:C4672T, ORF1ab:G5629T, ORF1ab:C10626T, ORF1ab:G11743T, ORF1ab:C14408T, ORF1ab:C18457T, S:A22105T, S:G22225T, S:A23403G, ORF3a:C25528T, ORF3a:C25603T, N:G28881A, N:G28882A, N:G28883C,	10	N:R203K, N:G204R, ORF1a:T1035I, ORF1a:A3454V, ORF1a:Q3826H, ORF1b:P314L, ORF1b:P1664S, ORF3a:L46F, S:D614G,N:M210L,	3'UTR:29726,N:28890-28901
hCoV-19/Mexico/BCN-ALSR-4778/2020	EPI_ISL_730136	20A	B.1.243	12	5'UTR:C241T, ORF1ab:C3037T, ORF1ab:C14408T, ORF1ab:C16877T, ORF1ab:G17050A, ORF1ab:A20268G, ORF1ab:C20759T, S:G21786T, S:A23403G, S:T24076C, ORF3a:G25699T, ORF7a:G27583T, N:C28854T,	9	N:S194L, ORF1b:P314L, ORF1b:T1137I, ORF1b:V1195I, ORF1b:A2431V, ORF3a:A103S, ORF7a:A64S, S:G75V, S:D614G,	
hCoV-19/Mexico/BCN-ALSR-4888/2020	EPI_ISL_730197	20C	B.1.595	14	5'UTR:C241T, ORF1ab:C1059T, ORF1ab:C1593T, ORF1ab:C3037T, ORF1ab:T5468C, ORF1ab:C10319T, ORF1ab:C10977T, ORF1ab:C11195T, ORF1ab:C14408T, ORF1ab:C16694T, S:A21863G, S:A23403G, S:C23506T, ORF3a:G25563T, ORF8:C27964T,	11	ORF1a:T265I, ORF1a:S443F, ORF1a:L3352F, ORF1a:A3571V, ORF1a:L3644F, ORF1b:P314L, ORF1b:T1076I, ORF3a:Q57H, ORF8:S24L, S:I101V, S:D614G,	
hCoV-19/Mexico/BCN-ALSR-4893/2020	EPI_ISL_730198	20B	B.1.1.128	12	5'UTR:T224C, 5'UTR:C241T, ORF1ab:C3037T, ORF1ab:C6781T, ORF1ab:C8626T, ORF1ab:C11916T, ORF1ab:C14408T, ORF1ab:C19185T, S:C21622T, S:A23403G, N:G28881A, N:G28882A, N:G28883C,	5	N:R203K, N:G204R, ORF1a:S3884L, ORF1b:P314L, S:D614G,	
hCoV-19/Mexico/BCN-ALSR-4894/2020	EPI_ISL_730199	20A	B.1.243	12	5'UTR:C241T, ORF1ab:C3037T, ORF1ab:T7258C, ORF1ab:G11365A, ORF1ab:T13102C, ORF1ab:C14408T, ORF1ab:T14814C, ORF1ab:C16308T, ORF1ab:G18266T, ORF1ab:A20268G, S:A23403G, S:T24076C, N:C28854T,	4	N:S194L, ORF1b:P314L, ORF1b:R1600L, S:D614G,	

hCoV-19/Mexico/BCN-ALSR-4896/2020	EPI_ISL_730200	20A	B.1	10	5'UTR:C241T, ORF1ab:G420T, ORF1ab:C3037T, ORF1ab:C14408T, ORF1ab:C14937T, ORF1ab:A20268G, ORF1ab:G21423A, S:C22450T, S:A23403G, N:C28320T, N:C28854T,	6	N:T16M, N:S194L, ORF1a:G52V, ORF1b:P314L, ORF9b:R13C, S:D614G,
hCoV-19/Mexico/BCN-ALSR-4898/2020	EPI_ISL_730201	20A	B.1.561	15	5'UTR:C241T, ORF1ab:G2516T, ORF1ab:C3037T, ORF1ab:A5999G, ORF1ab:A10323G, ORF1ab:C10798T, ORF1ab:C11866T, ORF1ab:C14408T, ORF1ab:C18693T, ORF1ab:A20268G, S:G21974T, S:A23403G, ORF3a:T25577C, ORF3a:G25740T, N:C28854T, N:G28975T,	10	N:S194L, N:M234I, ORF1a:V751L, ORF1a:I1912V, ORF1a:K3353R, ORF1b:P314L, ORF3a:I62T, ORF3a:Q116H, S:D138Y, S:D614G,
hCoV-19/Mexico/BCN-ALSR-4899/2020	EPI_ISL_730202	20A	B.1.561	15	5'UTR:C241T, ORF1ab:G2516T, ORF1ab:C3037T, ORF1ab:A5999G, ORF1ab:A10323G, ORF1ab:C10798T, ORF1ab:C11866T, ORF1ab:C14408T, ORF1ab:C18693T, ORF1ab:A20268G, S:G21974T, S:A23403G, ORF3a:T25577C, ORF3a:G25740T, N:C28854T, N:G28975T,	10	N:S194L, N:M234I, ORF1a:V751L, ORF1a:I1912V, ORF1a:K3353R, ORF1b:P314L, ORF3a:I62T, ORF3a:Q116H, S:D138Y, S:D614G,
hCoV-19/Mexico/BCN-ALSR-4900/2020	EPI_ISL_730203	20A	B.1.561	15	5'UTR:C241T, ORF1ab:G2516T, ORF1ab:C3037T, ORF1ab:A5999G, ORF1ab:A10323G, ORF1ab:C10798T, ORF1ab:C11866T, ORF1ab:C14408T, ORF1ab:C18693T, ORF1ab:A20268G, S:G21974T, S:A23403G, ORF3a:T25577C, ORF3a:G25740T, N:C28854T, N:G28975T,	10	N:S194L, N:M234I, ORF1a:V751L, ORF1a:I1912V, ORF1a:K3353R, ORF1b:P314L, ORF3a:I62T, ORF3a:Q116H, S:D138Y, S:D614G,
hCoV-19/Mexico/BCN-ALSR-4902/2020	EPI_ISL_730204	20A	B.1.561	15	5'UTR:C241T, ORF1ab:G2516T, ORF1ab:C3037T, ORF1ab:A5999G, ORF1ab:A10323G, ORF1ab:C10798T, ORF1ab:C11866T, ORF1ab:C14408T, ORF1ab:C18693T, ORF1ab:A20268G, S:G21974T, S:A23403G, ORF3a:T25577C, ORF3a:G25740T, N:C28854T, N:G28975T,	10	N:S194L, N:M234I, ORF1a:V751L, ORF1a:I1912V, ORF1a:K3353R, ORF1b:P314L, ORF3a:I62T, ORF3a:Q116H, S:D138Y, S:D614G,
hCoV-19/Mexico/BCN-ALSR-4903/2020	EPI_ISL_730205	20A	B.1	9	5'UTR:C241T, ORF1ab:C3037T, ORF1ab:G3122A, ORF1ab:G12188T, ORF1ab:C14408T, ORF1ab:A20268G, ORF1ab:A21562G, S:A23403G, N:G28307A, N:C28854T,	7	N:A12T, N:S194L, ORF1a:D953N, ORF1a:V3975F, ORF1b:P314L, ORF9b:M8I, S:D614G,
hCoV-19/Mexico/BCN-ALSR-4904/2020	EPI_ISL_730206	20A	B.1	9	5'UTR:C241T, ORF1ab:C3037T, ORF1ab:G3122A, ORF1ab:G12188T, ORF1ab:C14408T, ORF1ab:A20268G, ORF1ab:A21562G, S:A23403G, N:G28307A, N:C28854T,	7	N:A12T, N:S194L, ORF1a:D953N, ORF1a:V3975F, ORF1b:P314L, ORF9b:M8I, S:D614G,
hCoV-19/Mexico/BCN-ALSR-4905/2020	EPI_ISL_730207	20A	B.1	8	5'UTR:C241T, ORF1ab:C3037T, ORF1ab:G3122A, ORF1ab:G12188T, ORF1ab:C14408T, ORF1ab:A20268G, S:A23403G, N:G28307A, N:C28854T,	7	N:A12T, N:S194L, ORF1a:D953N, ORF1a:V3975F, ORF1b:P314L, ORF9b:M8I, S:D614G,
hCoV-19/Mexico/BCN-ALSR-4910/2020	EPI_ISL_730208	20A	B.1.561	15	5'UTR:C241T, ORF1ab:G2516T, ORF1ab:C3037T, ORF1ab:A5999G, ORF1ab:A10323G, ORF1ab:C10798T, ORF1ab:C11866T, ORF1ab:C14408T, ORF1ab:C18693T, ORF1ab:A20268G, S:G21974T, S:A23403G, ORF3a:T25577C, ORF3a:G25740T, N:C28854T, N:G28975T,	10	N:S194L, N:M234I, ORF1a:V751L, ORF1a:I1912V, ORF1a:K3353R, ORF1b:P314L, ORF3a:I62T, ORF3a:Q116H, S:D138Y, S:D614G,
hCoV-19/Mexico/BCN-ALSR-4917/2020	EPI_ISL_730209	20A	B.1.561	17	5'UTR:C241T, ORF1ab:G2516T, ORF1ab:C3037T, ORF1ab:A5999G, ORF1ab:A10323G, ORF1ab:C10798T, ORF1ab:C11866T, ORF1ab:C13378T, ORF1ab:C14408T, ORF1ab:C18693T, ORF1ab:C19164T, ORF1ab:A20268G, S:G21974T, S:A23403G, ORF3a:T25577C, N:G28300T, N:C28854T, N:G28975T,	11	N:Q9H, N:S194L, N:M234I, ORF1a:V751L, ORF1a:I1912V, ORF1a:K3353R, ORF1b:P314L, ORF3a:I62T, ORF9b:S6I, S:D138Y, S:D614G,
hCoV-19/Mexico/BCN-ALSR-4918/2020	EPI_ISL_730210	20A	B.1.241	11	5'UTR:C241T, ORF1ab:C3037T, ORF1ab:A7358G, ORF1ab:C7768T, ORF1ab:C11575T, ORF1ab:C14408T, ORF1ab:C16887T, ORF1ab:A20268G, ORF1ab:C20677T, S:G21830T, S:A23403G, N:C28854T,	6	N:S194L, ORF1a:M2365V, ORF1b:P314L, ORF1b:P2404S, S:V90F, S:D614G,

hCoV-19/Mexico/BCN-ALSR-4920/2020	EPI_ISL_730211	20A	B.1	10	5'UTR:C241T, ORF1ab:T1237C, ORF1ab:C3037T, ORF1ab:C8950T, ORF1ab:C14408T, ORF1ab:C16329T, ORF1ab:T18099C, S:A23403G, N:G28655T, N:C28887T, 3'UTR:G29773T,	4	N:D128Y, N:T205I, ORF1b:P314L, S:D614G,
hCoV-19/Mexico/BCN-ALSR-4922/2020	EPI_ISL_730212	20A	B.1	8	5'UTR:C241T, ORF1ab:C3037T, ORF1ab:G3122A, ORF1ab:G12188T, ORF1ab:C14408T, ORF1ab:A20268G, S:A23403G, N:G28307A, N:C28854T,	7	N:A12T, N:S194L, ORF1a:D953N, ORF1a:V3975F, ORF1b:P314L, ORF9b:M8I, S:D614G,
hCoV-19/Mexico/BCN-ALSR-4923/2020	EPI_ISL_730213	20A	B.1.243	18	5'UTR:C241T, ORF1ab:G806A, ORF1ab:C3037T, ORF1ab:C4832T, ORF1ab:T6868C, ORF1ab:C7869T, ORF1ab:G11083T, ORF1ab:G12176A, ORF1ab:C12400T, ORF1ab:C14408T, ORF1ab:C14599T, ORF1ab:C17373T, ORF1ab:A20268G, S:A23403G, S:T23407C, S:T24076C, ORF7a:C27752T, N:C28854T, N:G29543T,	8	N:S194L, ORF1a:A181T, ORF1a:S2535L, ORF1a:L3606F, ORF1a:G3971S, ORF1b:P314L, ORF7a:T120I, S:D614G,
hCoV-19/Mexico/BCN-ALSR-4924/2020	EPI_ISL_730214	20A	B.1.232	10	5'UTR:C241T, ORF1ab:C3037T, ORF1ab:A4107G, ORF1ab:C13665T, ORF1ab:C14408T, ORF1ab:A20268G, S:T22246C, S:A23403G, ORF3a:G26062T, ORF8:G27987T, N:C28854T,	6	N:S194L, ORF1a:K1281R, ORF1b:P314L, ORF3a:G224C, ORF8:V32L, S:D614G,
hCoV-19/Mexico/BCN-ALSR-4926/2020	EPI_ISL_730215	20A	B.1.397	9	5'UTR:C241T, ORF1ab:G1135T, ORF1ab:C3037T, ORF1ab:C6593T, ORF1ab:C12559A, ORF1ab:C14408T, ORF1ab:A20268G, S:A23403G, M:A26864T, N:C28854T,	5	N:S194L, ORF1a:K290N, ORF1a:P2110S, ORF1b:P314L, S:D614G,
hCoV-19/Mexico/BCN-ALSR-4927/2020	EPI_ISL_730216	20A	B.1.397	9	5'UTR:C241T, ORF1ab:G1135T, ORF1ab:C3037T, ORF1ab:C6593T, ORF1ab:C12559A, ORF1ab:C14408T, ORF1ab:A20268G, S:A23403G, M:A26864T, N:C28854T,	5	N:S194L, ORF1a:K290N, ORF1a:P2110S, ORF1b:P314L, S:D614G,
hCoV-19/Mexico/BCN-ALSR-4928/2020	EPI_ISL_730217	20A	B.1.397	10	5'UTR:C241T, ORF1ab:G1135T, ORF1ab:C3037T, ORF1ab:C6593T, ORF1ab:C12294A, ORF1ab:C12559A, ORF1ab:C14408T, ORF1ab:A20268G, S:A23403G, M:A26864T, N:C28854T,	6	N:S194L, ORF1a:K290N, ORF1a:P2110S, ORF1a:T4010N, ORF1b:P314L, S:D614G,
hCoV-19/Mexico/BCN-ALSR-4929/2020	EPI_ISL_730218	20A	B.1.397	9	5'UTR:C241T, ORF1ab:G1135T, ORF1ab:C3037T, ORF1ab:C6593T, ORF1ab:C12559A, ORF1ab:C14408T, ORF1ab:A20268G, S:A23403G, M:A26864T, N:C28854T,	5	N:S194L, ORF1a:K290N, ORF1a:P2110S, ORF1b:P314L, S:D614G,
hCoV-19/Mexico/BCN-ALSR-4934/2020	EPI_ISL_730219	20A	B.1	10	5'UTR:C241T, ORF1ab:C683T, ORF1ab:C3037T, ORF1ab:A8165G, ORF1ab:C14408T, ORF1ab:A20268G, S:A22101C, S:A23403G, ORF3a:G25906T, N:C28854T, N:T28927C,	6	N:S194L, ORF1a:I2634V, ORF1b:P314L, ORF3a:G172C, S:E180A, S:D614G,
hCoV-19/Mexico/BCN-ALSR-4935/2020	EPI_ISL_730220	20B	B.1.1.222	16	5'UTR:C241T, ORF1ab:T2533C, ORF1ab:C2773T, ORF1ab:C3037T, ORF1ab:C13795A, ORF1ab:C14408T, ORF1ab:A17015G, ORF1ab:C17135T, ORF1ab:T19839C, ORF1ab:A20294G, S:C21805T, S:A23403G, S:A23756G, N:G28851C, N:G28881A, N:G28882A, N:G28883C,	10	N:S193T, N:R203K, N:G204R, ORF1b:L110I, ORF1b:P314L, ORF1b:D1183G, ORF1b:P1223L, ORF1b:Y2276C, S:D614G, S:T732A,
hCoV-19/Mexico/BCN-ALSR-4937/2020	EPI_ISL_730221	20A	B.1.397	10	5'UTR:C241T, ORF1ab:G1135T, ORF1ab:C3037T, ORF1ab:C6593T, ORF1ab:A8121G, ORF1ab:C12559A, ORF1ab:C14408T, ORF1ab:A20268G, S:A23403G, M:A26864T, N:C28854T,	6	N:S194L, ORF1a:K290N, ORF1a:P2110S, ORF1a:E2619G, ORF1b:P314L, S:D614G,
hCoV-19/Mexico/BCN-ALSR-4942/2020	EPI_ISL_730222	20A	B.1.243	21	5'UTR:C241T, ORF1ab:C3037T, ORF1ab:C7113T, ORF1ab:C7948T, ORF1ab:G8653T, ORF1ab:T13354C, ORF1ab:C14408T, ORF1ab:A20268G, S:T22795A, S:T23159C, S:A23403G, S:T24076C, S:C24337T, S:G25266T, ORF3a:G25459T, ORF3a:T25525A, ORF3a:G25757T, ORF3a:A26072G, M:T26669C, M:G26690T, N:C28854T, N:C29095T,	10	N:S194L, ORF1a:T2283I, ORF1a:M2796I, ORF1b:P314L, ORF3a:A23S, ORF3a:W45R, ORF3a:R122I, ORF3a:H227R, S:D614G, S:C1235F,

hCoV-19/Mexico/BCN-ALSR-4943/2020	EPI_ISL_730223	20A	B.1.243	22	5'UTR:C241T, ORF1ab:C3037T, ORF1ab:C7113T, ORF1ab:C7948T, ORF1ab:G8653T, ORF1ab:T13354C, ORF1ab:C14408T, ORF1ab:A20268G, S:T22795A, S:T23159C, S:A23403G, S:T24076C, S:C24337T, S:G25266T, ORF3a:G25459T, ORF3a:T25525A, ORF3a:G25757T, ORF3a:A26072G, M:T26669C, M:G26690T, N:C28854T, N:C29095T, N:G29543T,	10	N:S194L, ORF1a:T2283I, ORF1a:M2796I, ORF1b:P314L, ORF3a:A23S, ORF3a:W45R, ORF3a:R122I, ORF3a:H227R, S:D614G, S:C1235F,
hCoV-19/Mexico/BCN-ALSR-4946/2020	EPI_ISL_730224	20A	B.1.561	18	5'UTR:C241T, ORF1ab:G2516T, ORF1ab:C3037T, ORF1ab:A5999G, ORF1ab:C7119T, ORF1ab:C7279T, ORF1ab:A10323G, ORF1ab:C10798T, ORF1ab:C11020T, ORF1ab:C11866T, ORF1ab:C14408T, ORF1ab:C18693T, ORF1ab:A19963G, ORF1ab:A20268G, S:G21974T, S:A23403G, ORF3a:T25577C, N:C28854T, N:G28975T.	11	N:S194L, N:M234I, ORF1a:V751L, ORF1a:I1912V, ORF1a:S2285F, ORF1a:K3353R, ORF1b:P314L, ORF1b:I2166V, ORF3a:I62T, S:D138Y, S:D614G,
hCoV-19/Mexico/BCN-ALSR-4947/2020	EPI_ISL_730225	20A	B.1.561	17	5'UTR:C241T, ORF1ab:G2516T, ORF1ab:C3037T, ORF1ab:A5999G, ORF1ab:C7279T, ORF1ab:A10323G, ORF1ab:C10798T, ORF1ab:C11020T, ORF1ab:C11866T, ORF1ab:C14408T, ORF1ab:C18693T, ORF1ab:A19963G, ORF1ab:A20268G, S:G21974T, S:A23403G, ORF3a:T25577C, N:C28854T, N:G28975T.	10	N:S194L, N:M234I, ORF1a:V751L, ORF1a:I1912V, ORF1a:K3353R, ORF1b:P314L, ORF1b:I2166V, ORF3a:I62T, S:D138Y, S:D614G,
hCoV-19/Mexico/BCN-ALSR-4951/2020	EPI_ISL_730226	20B	B.1.1.432	23	5'UTR:C241T, ORF1ab:G319A, ORF1ab:C679T, ORF1ab:C1884T, ORF1ab:G2864A, ORF1ab:C3037T, ORF1ab:A5511G, ORF1ab:A6985T, ORF1ab:G8179T, ORF1ab:C9319T, ORF1ab:T10717C, ORF1ab:C12412T, ORF1ab:T14313C, ORF1ab:C14408T, ORF1ab:C17528T, ORF1ab:C19032T, S:A23403G, ORF3a:C25460T, ORF3a:C25613T, ORF3a:G25912T, N:G28881A, N:G28882A, N:G28883C, N:T29317C,	11	N:R203K, N:G204R, ORF1a:A540V, ORF1a:E867K, ORF1a:N1749S, ORF1b:P314L, ORF1b:T1354I, ORF3a:A23V, ORF3a:S74F, ORF3a:G174C, S:D614G,
hCoV-19/Mexico/BCN-ALSR-4952/2020	EPI_ISL_730227	20A	B.1	10	5'UTR:C186T, 5'UTR:C241T, ORF1ab:C3037T, ORF1ab:C8266T, ORF1ab:C14408T, ORF1ab:A20268G, S:A23403G, S:A24913G, N:C28854T, N:C29095T, 3'UTR:C29718T,	3	N:S194L, ORF1b:P314L, S:D614G,
hCoV-19/Mexico/BCN-ALSR-4953/2020	EPI_ISL_730228	20A	B.1	11	5'UTR:C241T, ORF1ab:C3037T, ORF1ab:C3874T, ORF1ab:A9328G, ORF1ab:C14262T, ORF1ab:C14408T, ORF1ab:C18129T, ORF1ab:A20268G, S:G21724T, S:A23403G, N:C28854T, ORF10:A29647G,	4	N:S194L, ORF1b:P314L, S:L54F, S:D614G,
hCoV-19/Mexico/BCN-ALSR-5603/2020	EPI_ISL_878212	21C (Epsilon)	B.1.427	21	5'UTR:C241T, ORF1ab:C1059T, ORF1ab:T1851C, ORF1ab:C3037T, ORF1ab:C3619A, ORF1ab:G9738C, ORF1ab:G13713A, ORF1ab:C14408T, ORF1ab:C16394T, ORF1ab:G17014T, S:G21600T, S:G22018T, S:T22917G, S:A23403G, S:C25317T, ORF3a:T25467C, ORF3a:G25563T, ORF3a:T26066C, M:C26681T, ORF8:A28272T, N:C28887T, N:C29362T,	14	N:T205I, ORF1a:T265I, ORF1a:I529T, ORF1a:S3158T, ORF1b:P314L, ORF1b:P976L, ORF1b:D1183Y, ORF3a:Q57H, ORF3a:V225A, S:S13I, S:W152C, S:L452R, S:D614G, S:S1252F,
hCoV-19/Mexico/BCN-ALSR-5615/2020	EPI_ISL_878226	21C (Epsilon)	B.1.427	20	5'UTR:C241T, ORF1ab:C1059T, ORF1ab:T1851C, ORF1ab:C3037T, ORF1ab:C3619A, ORF1ab:G9738C, ORF1ab:G13713A, ORF1ab:C14408T, ORF1ab:C16394T, ORF1ab:G17014T, S:G21600T, S:G22018T, S:T22917G, S:A23403G, S:C25317T, ORF3a:T25467C, ORF3a:G25563T, ORF3a:T26066C, M:C26681T, ORF8:A28272T, N:C28887T,	14	N:T205I, ORF1a:T265I, ORF1a:I529T, ORF1a:S3158T, ORF1b:P314L, ORF1b:P976L, ORF1b:D1183Y, ORF3a:Q57H, ORF3a:V225A, S:S13I, S:W152C, S:L452R, S:D614G, S:S1252F,

hCoV-19/Mexico/BCN-ALSR-5655/2020	EPI_ISL_878300	20A	B.1.232	19	5'UTR:C241T, ORF1ab:C398T, ORF1ab:C3037T, ORF1ab:T5804C, ORF1ab:C5849T, ORF1ab:C6285T, ORF1ab:C6706T, ORF1ab:C14408T, ORF1ab:C16092T, ORF1ab:A20268G, S:T22917G, S:A23403G, ORF3a:G25720T, E:G26325A, ORF8:G27987T, N:C28291T, N:T28600C, N:C28854T, N:G29474T, 3'UTR:G29825T,	12	N:S194L, N:D401Y, ORF1a:H45Y, ORF1a:C1847R, ORF1a:P1862S, ORF1a:T2007I, ORF1b:P314L, ORF3a:A110S, ORF8:V32L, ORF9b:P3L, S:L452R, S:D614G,
hCoV-19/Mexico/BCN-ALSR-5708/2020	EPI_ISL_878367	21C (Epsilon)	B.1.429	21	5'UTR:C241T, ORF1ab:C1059T, ORF1ab:C2395T, ORF1ab:T2597C, ORF1ab:C3037T, ORF1ab:C8947T, ORF1ab:C12100T, ORF1ab:A12878G, ORF1ab:C14408T, ORF1ab:G17014T, S:G21600T, S:G22018T, S:T22917G, S:A23403G, S:T24349C, ORF3a:G25563T, M:C26681T, ORF7b:G27890T, ORF8:G28001T, ORF8:A28272T, N:C28887T, N:C29362T,	10	N:T205I, ORF1a:T265I, ORF1a:I4205V, ORF1b:P314L, ORF1b:D1183Y, ORF3a:Q57H, S:S13I, S:W152C, S:L452R, S:D614G,
hCoV-19/Mexico/BCN-ALSR-5717/2020	EPI_ISL_878375	20A	B.1	15	5'UTR:G208T, 5'UTR:C241T, ORF1ab:C3037T, ORF1ab:G4185C, ORF1ab:C14408T, ORF1ab:A20268G, ORF1ab:G20709T, S:T21775C, S:A23403G, S:G23522A, S:C24106T, S:C24181T, S:C25162A, S:C25163A, M:A26927G, N:C28854T,	7	N:S194L, ORF1a:G1307A, ORF1b:P314L, ORF1b:M2414I, S:D614G, S:E654K, S:Q1201K,
hCoV-19/Mexico/BCN-ALSR-5732/2020	EPI_ISL_878390	20A	B.1.232	18	5'UTR:C241T, ORF1ab:C3037T, ORF1ab:T5804C, ORF1ab:C5849T, ORF1ab:C6285T, ORF1ab:C6706T, ORF1ab:C14408T, ORF1ab:T14961C, ORF1ab:C16092T, ORF1ab:T17979C, ORF1ab:A20268G, S:A23403G, ORF3a:G25720T, E:G26325A, ORF8:G27987T, N:C28291T, N:C28854T, N:C29541T, 3'UTR:G29825T,	9	N:S194L, ORF1a:C1847R, ORF1a:P1862S, ORF1a:T2007I, ORF1b:P314L, ORF3a:A110S, ORF8:V32L, ORF9b:P3L, S:D614G,
hCoV-19/Mexico/BCN-ALSR-5748/2020	EPI_ISL_878400	20A	B.1.551	18	5'UTR:C241T, ORF1ab:C3037T, ORF1ab:C3738T, ORF1ab:C5784T, ORF1ab:A7444G, ORF1ab:C8836T, ORF1ab:G10631A, ORF1ab:C14408T, ORF1ab:A20268G, ORF1ab:A20929G, S:C22323T, S:A23403G, S:C23604G, S:G23868T, ORF3a:C26058T, ORF7a:T27534C, ORF7a:G27561T, N:C28854T, N:C29466T,	11	N:S194L, N:A398V, ORF1a:P1158L, ORF1a:T1840I, ORF1a:A3456T, ORF1b:P314L, ORF1b:T2488A, S:S254F, S:D614G, S:P681R, S:G769V,
hCoV-19/Mexico/BCN-ALSR-5749/2020	EPI_ISL_878401	20A	B.1.561	20	5'UTR:C241T, ORF1ab:C527T, ORF1ab:G2516T, ORF1ab:C3037T, ORF1ab:C3510T, ORF1ab:A4636G, ORF1ab:A5999G, ORF1ab:A10323G, ORF1ab:C10798T, ORF1ab:C11866T, ORF1ab:C14408T, ORF1ab:G16917T, ORF1ab:C18693T, ORF1ab:A20268G, S:G21974T, S:A23403G, S:C24378T, ORF3a:T25577C, N:C28854T, N:G28975T, 3'UTR:C29870A,	11	N:S194L, N:M234I, ORF1a:V751L, ORF1a:A1082V, ORF1a:I1912V, ORF1a:K3353R, ORF1b:P314L, ORF3a:I62T, S:D138Y, S:D614G, S:S939F,
hCoV-19/Mexico/BCN-ALSR-5753/2020	EPI_ISL_878404	20A	B.1.609	12	5'UTR:C241T, ORF1ab:C1684T, ORF1ab:C3037T, ORF1ab:C4582T, ORF1ab:C7173T, ORF1ab:C8175T, ORF1ab:C14408T, ORF1ab:G18445C, ORF1ab:A20268G, S:A23403G, ORF8:A27938T, N:G28541T, N:C28932T,	8	N:A90S, N:A220V, ORF1a:S2303F, ORF1a:A2637V, ORF1b:P314L, ORF1b:V1660L, ORF9b:E86D, S:D614G,
hCoV-19/Mexico/BCN-ALSR-5757/2020	EPI_ISL_878408	20A	B.1.561	19	5'UTR:C241T, ORF1ab:G2516T, ORF1ab:C2937T, ORF1ab:C3037T, ORF1ab:A5999G, ORF1ab:C9714T, ORF1ab:A10323G, ORF1ab:C11572T, ORF1ab:A13267T, ORF1ab:C14408T, ORF1ab:G16741T, ORF1ab:C18693T, ORF1ab:A20268G, S:G21974T, S:A23403G, ORF3a:T25577C, N:T28600C, N:C28854T, N:G28975T. N:G29000T.	13	N:S194L, N:M234I, N:G243C, ORF1a:V751L, ORF1a:T891I, ORF1a:I1912V, ORF1a:T3150I, ORF1a:K3353R, ORF1b:P314L, ORF1b:V1092F, ORF3a:I62T, S:D138Y, S:D614G,

hCoV-19/Mexico/BCN-ALSR-5773/2020	EPI_ISL_878437	20A	B.1	18	5'UTR:C241T, ORF1ab:C3037T, ORF1ab:G3047A, ORF1ab:C4931T, ORF1ab:G5023T, ORF1ab:G6323C, ORF1ab:C10537T, ORF1ab:T10792C, ORF1ab:C12005T, ORF1ab:C14408T, ORF1ab:A14533C, ORF1ab:A17861G, ORF1ab:C19145T, ORF1ab:G19180T, ORF1ab:A20268G, S:A22375G, S:A23403G, S:T23776C. N:C28854T.	10	N:S194L, ORF1a:D928N, ORF1a:L1556F, ORF1a:M1586I, ORF1a:E2020Q, ORF1b:P314L, ORF1b:D1465G, ORF1b:S1893F, ORF1b:V1905L, S:D614G,	
hCoV-19/Mexico/BCN-ALSR-6000/2020	EPI_ISL_879950	20C	B.1.612	17	5'UTR:C241T, ORF1ab:C1059T, ORF1ab:C2710T, ORF1ab:C3037T, ORF1ab:C3885T, ORF1ab:G7936T, ORF1ab:A10323G, ORF1ab:C12115T, ORF1ab:C13957T, ORF1ab:C14408T, ORF1ab:T15276A, ORF1ab:T21462A, S:C21575T, S:A23403G, S:A24010G, ORF3a:G25563T, N:G28842T, 3'UTR:G29810T,	10	N:S190I, ORF1a:T265I, ORF1a:P1207L, ORF1a:K3353R, ORF1b:R164C, ORF1b:P314L, ORF1b:N2665K, ORF3a:Q57H, S:L5F, S:D614G,	
hCoV-19/Mexico/BCN-ALSR-6012/2020	EPI_ISL_879977	21C (Epsilon)	B.1.427	25	5'UTR:C241T, ORF1ab:C1059T, ORF1ab:G2458T, ORF1ab:C3037T, ORF1ab:C5365T, ORF1ab:A8425T, ORF1ab:C9286T, ORF1ab:C9443T, ORF1ab:G9738C, ORF1ab:G13713A, ORF1ab:C14408T, ORF1ab:C16394T, ORF1ab:G17014T, S:G21600T, S:C21904T, S:G22018T, S:G22468T, S:T22835C, S:T22917G, S:A23403G, ORF3a:G25563T, M:C26681T, ORF8:A28272T, N:C28887T, N:C29362T, 3'UTR:G29755T,	13	N:T205I, ORF1a:T265I, ORF1a:M731I, ORF1a:L3060F, ORF1a:S3158T, ORF1b:P314L, ORF1b:P976L, ORF1b:D1183Y, ORF3a:Q57H, S:S13I, S:W152C, S:L452R, S:D614G,	
hCoV-19/Mexico/BCN-ALSR-6339/2021	EPI_ISL_962662	20A	B.1.243	18	5'UTR:C241T, ORF1ab:G806A, ORF1ab:C2044T, ORF1ab:T2584A, ORF1ab:C3037T, ORF1ab:A4905G, ORF1ab:C5140A, ORF1ab:C14408T, ORF1ab:G16647T, ORF1ab:A20268G, S:T22016A, S:A23403G, S:G23587C, S:T24076C, ORF3a:C25844T, ORF7a:C27476T, N:C28854T, N:C29095T, N:G29543T,	10	N:S194L, ORF1a:A181T, ORF1a:D1547G, ORF1a:D1625E, ORF1b:P314L, ORF3a:T151I, ORF7a:T28I, S:W152R, S:D614G, S:Q675H,	
hCoV-19/Mexico/BCN-ALSR-6340/2021	EPI_ISL_962663	21C (Epsilon)	B.1.429	25	5'UTR:C241T, ORF1ab:C1059T, ORF1ab:C1457T, ORF1ab:C2395T, ORF1ab:T2597C, ORF1ab:C3037T, ORF1ab:C6840T, ORF1ab:C8947T, ORF1ab:C12100T, ORF1ab:A12878G, ORF1ab:C14408T, ORF1ab:G17014T, ORF1ab:C17898T, S:G21600T, S:G22018T, S:T22917G, S:G23069T, S:A23403G, S:T24349C, ORF3a:G25563T, M:C26681T, ORF7b:G27890T, ORF8:A28272T, N:C28887T, N:C29095T, N:C29362T,	13	N:T205I, ORF1a:T265I, ORF1a:R398C, ORF1a:A2192V, ORF1a:I4205V, ORF1b:P314L, ORF1b:D1183Y, ORF3a:Q57H, S:S13I, S:W152C, S:L452R, S:V503F, S:D614G,	ORF1ab: 509-523,
hCoV-19/Mexico/BCN-ALSR-6577/2021	EPI_ISL_1081416	21C (Epsilon)	B.1.427	26	5'UTR:C241T, ORF1ab:C1059T, ORF1ab:T1851C, ORF1ab:C2232T, ORF1ab:C3037T, ORF1ab:C3619A, ORF1ab:G4510A, ORF1ab:C4795T, ORF1ab:G9738C, ORF1ab:G13713A, ORF1ab:C14408T, ORF1ab:C16394T, ORF1ab:G17014T, S:G21600T, S:G22018T, S:T22917G, S:A23403G, S:C25317T, ORF3a:T25467C, ORF3a:G25563T, ORF3a:T26066C, M:C26681T, M:C26895T, ORF8:A28272T, N:A28804G, N:C28887T, N:C29362T,	16	M:H125Y, N:T205I, ORF1a:T265I, ORF1a:I529T, ORF1a:A656V, ORF1a:S3158T, ORF1b:P314L, ORF1b:P976L, ORF1b:D1183Y, ORF3a:Q57H, ORF3a:V225A, S:S13I, S:W152C, S:L452R, S:D614G, S:S1252F,	
hCoV-19/Mexico/BCN-ALSR-6584/2020	EPI_ISL_1081423	21C (Epsilon)	B.1.427	23	5'UTR:C241T, ORF1ab:C1059T, ORF1ab:C3037T, ORF1ab:C3817T, ORF1ab:G9738C, ORF1ab:C13019T, ORF1ab:G13713A, ORF1ab:T14190C, ORF1ab:C14408T, ORF1ab:C16394T, ORF1ab:G17014T, S:G21600T, S:G22018T, S:G22335T, S:C22597T, S:T22917G, S:A23403G, ORF3a:C25521T, ORF3a:G25563T, M:C26681T, ORF8:C28087T, ORF8:A28272T, N:C28887T, N:C29362T,	13	N:T205I, ORF1a:T265I, ORF1a:S3158T, ORF1b:P314L, ORF1b:P976L, ORF1b:D1183Y, ORF3a:Q57H, ORF8:A65V, S:S13I, S:W152C, S:W258L, S:L452R, S:D614G,	S:21944-21946

hCoV-19/Mexico/BCN-ALSR-6585/2020	EPI_ISL_1081424	21C (Epsilon)	B.1.427	18	5'UTR:C241T, ORF1ab:C1059T, ORF1ab:C2485T, ORF1ab:C3037T, ORF1ab:G9738C, ORF1ab:G13713A, ORF1ab:C14408T, ORF1ab:C16394T, ORF1ab:G17014T, ORF1ab:T17859C, ORF1ab:G20931T, S:G21600T, S:G22018T, S:T22917G, S:A23403G, ORF3a:G25563T, M:C26681T, ORF8:A28272T, N:C28887T,	11	N:T205I, ORF1a:T265I, ORF1a:S3158T, ORF1b:P314L, ORF1b:P976L, ORF1b:D1183Y, ORF3a:Q57H, S:S13I, S:W152C, S:L452R, S:D614G,
hCoV-19/Mexico/BCN-ALSR-6586/2020	EPI_ISL_1081425	21C (Epsilon)	B.1.429	22	5'UTR:C241T, ORF1ab:C1059T, ORF1ab:C2395T, ORF1ab:T2597C, ORF1ab:C3037T, ORF1ab:C8947T, ORF1ab:C12100T, ORF1ab:A12878G, ORF1ab:C14408T, ORF1ab:G17014T, ORF1ab:G19542T, S:G21600T, S:G22018T, S:T22917G, S:A23403G, S:T24349C, ORF3a:G25563T, M:C26681T, ORF7b:G27890T, ORF8:G28001T, ORF8:A28272T, N:C28887T, ORF10:C29640T,	11	N:T205I, ORF1a:T265I, ORF1a:I4205V, ORF1b:P314L, ORF1b:D1183Y, ORF1b:M2025I, ORF3a:Q57H, S:S13I, S:W152C, S:L452R, S:D614G,
hCoV-19/Mexico/BCN-ALSR-6587/2020	EPI_ISL_1081426	21C (Epsilon)	B.1.427	18	5'UTR:C241T, ORF1ab:C1059T, ORF1ab:C3037T, ORF1ab:G9738C, ORF1ab:G13713A, ORF1ab:C14408T, ORF1ab:C16394T, ORF1ab:G17014T, ORF1ab:G20931T, ORF1ab:G21451T, S:G21600T, S:G22018T, S:T22917G, S:A23403G, ORF3a:G25563T, M:C26681T, ORF8:A28272T, N:G28514T, N:C28887T,	14	N:D81Y, N:T205I, ORF1a:T265I, ORF1a:S3158T, ORF1b:P314L, ORF1b:P976L, ORF1b:D1183Y, ORF1b:G2662C, ORF3a:Q57H, ORF9b:Q77H, S:S13I, S:W152C, S:L452R, S:D614G,
hCoV-19/Mexico/BCN-ALSR-6588/2020	EPI_ISL_1081427	20A	B.1.232	26	5'UTR:C241T, ORF1ab:G960T, ORF1ab:C3037T, ORF1ab:T5804C, ORF1ab:C5849T, ORF1ab:C5986T, ORF1ab:C6285T, ORF1ab:C6701T, ORF1ab:C6706T, ORF1ab:G9473A, ORF1ab:C14408T, ORF1ab:G14511A, ORF1ab:A15564G, ORF1ab:C16092T, ORF1ab:C17678T, ORF1ab:A20268G, S:G21724T, S:A23403G, ORF3a:G25720T, ORF3a:C25820T, E:G26325A, ORF6:C27335T, ORF8:G27987T, N:C28291T, N:C28854T, N:C29268T, 3'UTR:G29825T,	17	N:S194L, N:T332I, ORF1a:R232L, ORF1a:C1847R, ORF1a:P1862S, ORF1a:T2007I, ORF1a:L2146F, ORF1a:A3070T, ORF1b:P314L, ORF1b:T1404M, ORF3a:A110S, ORF3a:A143V, ORF6:T45I, ORF8:V32L, ORF9b:P3L, S:L54F, S:D614G,
hCoV-19/Mexico/BCN-ALSR-6589/2020	EPI_ISL_1081428	20A	B.1.551	14	5'UTR:C241T, ORF1ab:C3037T, ORF1ab:C3738T, ORF1ab:C8836T, ORF1ab:G10631A, ORF1ab:C14408T, ORF1ab:A20268G, S:C22323T, S:A23403G, S:C23604G, S:G23868T, ORF3a:C26058T, ORF7a:T27534C, N:C28854T, N:C29466T,	9	N:S194L, N:A398V, ORF1a:P1158L, ORF1a:A3456T, ORF1b:P314L, S:S254F, S:D614G, S:P681R, S:G769V,
hCoV-19/Mexico/BCN-ALSR-6590/2020	EPI_ISL_1081429	21C (Epsilon)	B.1.429	23	5'UTR:C241T, ORF1ab:C1059T, ORF1ab:C2395T, ORF1ab:T2597C, ORF1ab:C3037T, ORF1ab:C8947T, ORF1ab:C12100T, ORF1ab:A12878G, ORF1ab:C14408T, ORF1ab:G17014T, ORF1ab:G19542T, S:G21600T, S:G22018T, S:T22917G, S:T23031C, S:A23403G, S:T24349C, ORF3a:G25563T, M:C26681T, ORF7b:G27890T, ORF8:G28001T, ORF8:A28272T, N:C28887T, ORF10:C29640T,	12	N:T205I, ORF1a:T265I, ORF1a:I4205V, ORF1b:P314L, ORF1b:D1183Y, ORF1b:M2025I, ORF3a:Q57H, S:S13I, S:W152C, S:L452R, S:F490S, S:D614G,
hCoV-19/Mexico/BCN-ALSR-6591/2020	EPI_ISL_1081430	20A	B.1.243	15	5'UTR:C241T, ORF1ab:G806A, ORF1ab:T2584A, ORF1ab:C3037T, ORF1ab:C5140A, ORF1ab:A6378C, ORF1ab:C10543T, ORF1ab:C14408T, ORF1ab:G15327T, ORF1ab:A20268G, S:A23403G, S:G23587C, S:T24076C, ORF3a:C25844T, N:C28854T, N:G29543T,	9	N:S194L, ORF1a:A181T, ORF1a:D1625E, ORF1a:N2038T, ORF1b:P314L, ORF1b:M620I, ORF3a:T151I, S:D614G, S:Q675H,
hCoV-19/Mexico/BCN-ALSR-6592/2020	EPI_ISL_1081431	21C (Epsilon)	B.1.427	23	5'UTR:C241T, ORF1ab:C1059T, ORF1ab:C3037T, ORF1ab:C7043T, ORF1ab:G9738C, ORF1ab:C10748T, ORF1ab:G13713A, ORF1ab:C14408T, ORF1ab:C14937T, ORF1ab:C16394T, ORF1ab:G17014T, S:G21600T, S:G22018T, S:T22917G, S:A23403G, S:C24715T, ORF3a:G25563T, M:C26681T, ORF8:A28272T, N:C28310T, N:C28887T, N:A29188T, N:A29257T, N:C29362T,	14	N:P13S, N:T205I, ORF1a:T265I, ORF1a:P2260S, ORF1a:S3158T, ORF1a:L3495F, ORF1b:P314L, ORF1b:P976L, ORF1b:D1183Y, ORF3a:Q57H, S:S13I, S:W152C, S:L452R, S:D614G,

hCoV-19/Mexico/BCN-ALSR-6593/2020	EPI_ISL_1081432	21C (Epsilon)	B.1.427	26	5'UTR:C241T, ORF1ab:C1059T, ORF1ab:T1851C, ORF1ab:C2232T, ORF1ab:C3037T, ORF1ab:C3425T, ORF1ab:C3619A, ORF1ab:G4510A, ORF1ab:G8084A, ORF1ab:G9738C, ORF1ab:C13421T, ORF1ab:G13713A, ORF1ab:C14408T, ORF1ab:C16394T, ORF1ab:G17014T, S:G21600T, S:G22018T, S:T22917G, S:A23403G, S:C25317T, ORF3a:T25467C, ORF3a:G25563T, ORF3a:T26066C, M:C26681T, ORF8:A28272T, N:A28804G, N:C28887T,	18	N:T205I, ORF1a:T265I, ORF1a:I529T, ORF1a:A656V, ORF1a:P1054S, ORF1a:E2607K, ORF1a:S3158T, ORF1a:L4386F, ORF1b:P314L, ORF1b:P976L, ORF1b:D1183Y, ORF3a:Q57H, ORF3a:V225A, S:S13I, S:W152C, S:L452R, S:D614G, S:S1252F,
hCoV-19/Mexico/BCN-ALSR-6594/2020	EPI_ISL_1081433	20A	B.1.396	14	5'UTR:C241T, ORF1ab:G1126T, ORF1ab:C1191T, ORF1ab:C3037T, ORF1ab:A3205G, ORF1ab:G9479T, ORF1ab:C9521A, ORF1ab:C14408T, ORF1ab:A20268G, S:A23403G, S:C24904T, N:G28378T, N:C28854T, N:G29422T, ORF10:A29567G,	8	N:S194L, ORF1a:R287S, ORF1a:P309L, ORF1a:G3072C, ORF1a:L3086I, ORF1b:P314L, ORF9b:R32L, S:D614G,
hCoV-19/Mexico/BCN-ALSR-6595/2020	EPI_ISL_1081434	20A	B.1	17	5'UTR:C241T, 5'UTR:A257C, ORF1ab:C1594T, ORF1ab:C2401T, ORF1ab:C3037T, ORF1ab:G5305T, ORF1ab:C8016A, ORF1ab:C14408T, ORF1ab:C16338T, ORF1ab:A17153G, ORF1ab:G18674A, ORF1ab:A20268G, S:G21974T, S:A23403G, S:C24130T, ORF3a:G25500A, N:C28310T, N:C28854T,	9	N:P13S, N:S194L, ORF1a:L1680F, ORF1a:A2584E, ORF1b:P314L, ORF1b:Y1229C, ORF1b:R1736K, S:D138Y, S:D614G,
hCoV-19/Mexico/BCN-ALSR-6596/2020	EPI_ISL_1081435	20A	B.1.561	24	5'UTR:C241T, ORF1ab:C593T, ORF1ab:G2516T, ORF1ab:C2937T, ORF1ab:C3037T, ORF1ab:C5777T, ORF1ab:A5999G, ORF1ab:C7819T, ORF1ab:C9714T, ORF1ab:A10323G, ORF1ab:A13267T, ORF1ab:C14408T, ORF1ab:A16704C, ORF1ab:G16741T, ORF1ab:C18693T, ORF1ab:A20268G, S:G21974T, S:C22188T, S:A23403G, ORF3a:T25577C, ORF8:C28093T, N:C28473A, N:T28600C, N:C28854T, N:G28975T,	19	N:P67H, N:S194L, N:M234I, ORF1a:H110Y, ORF1a:V751L, ORF1a:T891I, ORF1a:H1838Y, ORF1a:I1912V, ORF1a:T3150I, ORF1a:K3353R, ORF1b:P314L, ORF1b:E1079D, ORF1b:V1092F, ORF3a:I62T, ORF8:S67F, ORF9b:L64I, S:D138Y, S:P209L, S:D614G,
hCoV-19/Mexico/BCN-ALSR-6597/2020	EPI_ISL_1081436	20A	B.1.336	15	5'UTR:C241T, ORF1ab:C857T, ORF1ab:C3037T, ORF1ab:G3871T, ORF1ab:C6501T, ORF1ab:C9967T, ORF1ab:G11083T, ORF1ab:C14408T, ORF1ab:C18129T, ORF1ab:C19586T, ORF1ab:A20268G, S:G21724T, S:G22992A, S:A23403G, N:C28854T, ORF10:A29647G,	10	N:S194L, ORF1a:L198F, ORF1a:K1202N, ORF1a:P2079L, ORF1a:L3606F, ORF1b:P314L, ORF1b:T2040I, S:L54F, S:S477N, S:D614G,
hCoV-19/Mexico/BCN-ALSR-6598/2020	EPI_ISL_1081437	21C (Epsilon)	B.1.427	17	5'UTR:C241T, ORF1ab:C1059T, ORF1ab:C3037T, ORF1ab:C6145T, ORF1ab:G9738C, ORF1ab:G13713A, ORF1ab:C14408T, ORF1ab:C16394T, ORF1ab:G17014T, ORF1ab:C18946T, S:G21600T, S:G22018T, S:T22917G, S:A23403G, ORF3a:G25563T, M:C26681T, ORF8:A28272T, N:C28887T,	11	N:T205I, ORF1a:T265I, ORF1a:S3158T, ORF1b:P314L, ORF1b:P976L, ORF1b:D1183Y, ORF3a:Q57H, S:S13I, S:W152C, S:L452R, S:D614G,
hCoV-19/Mexico/BCN-ALSR-6599/2020	EPI_ISL_1081438	20A	B.1	11	5'UTR:C241T, ORF1ab:T1271C, ORF1ab:C3037T, ORF1ab:C7749T, ORF1ab:A8547G, ORF1ab:C12400T, ORF1ab:C14408T, ORF1ab:T16383C, ORF1ab:A19611T, ORF1ab:A20268G, S:A23403G, N:C28854T,	6	N:S194L, ORF1a:F336L, ORF1a:T2495I, ORF1a:K2761R, ORF1b:P314L, S:D614G,
hCoV-19/Mexico/BCN-ALSR-6600/2020	EPI_ISL_1081439	20A	B.1.243	14	5'UTR:C241T, ORF1ab:C2232T, ORF1ab:C3037T, ORF1ab:C14408T, ORF1ab:C15660T, ORF1ab:C18395T, ORF1ab:A20268G, ORF1ab:T20375G, ORF1ab:C20759T, S:A23403G, S:T24076C, S:G24883T, ORF7a:G27415A, ORF7a:T27622C, N:C28854T,	9	N:S194L, ORF1a:A656V, ORF1b:P314L, ORF1b:A1643V, ORF1b:I2303S, ORF1b:A2431V, ORF7a:A8T, S:D614G, S:R1107S,

hCoV-19/Mexico/BCN-ALSR-6601/2020	EPI_ISL_1081440	21C (Epsilon)	B.1.427	27	5'UTR:C241T, ORF1ab:C1059T, ORF1ab:T1851C, ORF1ab:C2232T, ORF1ab:C3037T, ORF1ab:C3425T, ORF1ab:C3619A, ORF1ab:G4510A, ORF1ab:G8084A, ORF1ab:G8179T, ORF1ab:G9738C, ORF1ab:G13713A, ORF1ab:C14408T, ORF1ab:C16394T, ORF1ab:G17014T, S:G21600T, S:G22018T, S:T22917G, S:A23403G, S:C25317T, ORF3a:T25467C, ORF3a:G25563T, ORF3a:T26066C, M:C26681T, ORF6:G27261T, ORF8:A28272T, N:A28804G, N:C28887T,	18	N:T205I, ORF1a:T265I, ORF1a:I529T, ORF1a:A656V, ORF1a:P1054S, ORF1a:E2607K, ORF1a:S3158T, ORF1b:P314L, ORF1b:P976L, ORF1b:D1183Y, ORF3a:Q57H, ORF3a:V225A, ORF6:R20S, S:S13I, S:W152C, S:L452R, S:D614G, S:S1252F,
hCoV-19/Mexico/BCN-ALSR-6602/2020	EPI_ISL_1081441	20G	B.1.2	20	5'UTR:C241T, ORF1ab:C1059T, ORF1ab:T2698C, ORF1ab:C3037T, ORF1ab:C6429T, ORF1ab:C10319T, ORF1ab:C11572T, ORF1ab:C14408T, ORF1ab:A18424G, ORF1ab:C21304T, S:T22207C, S:G22225T, S:A23403G, ORF3a:G25563T, ORF3a:G25907T, ORF8:C27964T, N:C28472T, N:T28723C, N:C28869T, ORF10:T29588C, 3'UTR:G29711T,	12	N:P67S, N:P199L, ORF1a:T265I, ORF1a:P2055L, ORF1a:L3352F, ORF1b:P314L, ORF1b:N1653D, ORF1b:R2613C, ORF3a:Q57H, ORF3a:G172V, ORF8:S24L, S:D614G,
hCoV-19/Mexico/BCN-ALSR-6603/2020	EPI_ISL_1081442	21C (Epsilon)	B.1.427	20	5'UTR:C241T, ORF1ab:C1059T, ORF1ab:C3037T, ORF1ab:G9738C, ORF1ab:G13713A, ORF1ab:C14408T, ORF1ab:C15540T, ORF1ab:C16394T, ORF1ab:G17014T, ORF1ab:G18382A, ORF1ab:G20931T, S:G21600T, S:G22018T, S:T22917G, S:A23403G, ORF3a:G25563T, M:C26681T, M:G26832T, ORF8:C28253T, ORF8:A28272T, N:C28887T,	13	M:A104S, N:T205I, ORF1a:T265I, ORF1a:S3158T, ORF1b:P314L, ORF1b:P976L, ORF1b:D1183Y, ORF1b:V1639I, ORF3a:Q57H, S:S13I, S:W152C, S:L452R, S:D614G,
hCoV-19/Mexico/BCN-ALSR-6604/2020	EPI_ISL_1081443	20C	B.1.577	22	5'UTR:C241T, ORF1ab:C1059T, ORF1ab:T1482A, ORF1ab:C3037T, ORF1ab:C7834T, ORF1ab:C10030T, ORF1ab:C10319T, ORF1ab:A11782G, ORF1ab:G13348C, ORF1ab:A14010G, ORF1ab:C14408T, ORF1ab:C16694T, ORF1ab:T17407C, ORF1ab:C21057T, S:A23403G, S:A25105G, S:G25234A, ORF3a:C25521T, ORF3a:G25563T, ORF3a:C25626A, ORF8:C27964T, N:C28892T, N:C29249T,	11	N:P207S, N:P326S, ORF1a:T265I, ORF1a:F406Y, ORF1a:L3352F, ORF1b:P314L, ORF1b:T1076I, ORF3a:Q57H, ORF3a:H78Q, ORF8:S24L, S:D614G,
hCoV-19/Mexico/BCN-ALSR-6605/2020	EPI_ISL_1081444	21C (Epsilon)	B.1.429	22	5'UTR:C241T, ORF1ab:C1059T, ORF1ab:C2395T, ORF1ab:T2597C, ORF1ab:C3037T, ORF1ab:C8947T, ORF1ab:C12100T, ORF1ab:A12878G, ORF1ab:C14408T, ORF1ab:G17014T, ORF1ab:G19542T, S:G21600T, S:G22018T, S:T22917G, S:A23403G, S:T24349C, ORF3a:G25563T, M:C26681T, ORF7b:G27890T, ORF8:G28001T, ORF8:A28272T, N:C28887T, ORF10:C29640T,	11	N:T205I, ORF1a:T265I, ORF1a:I4205V, ORF1b:P314L, ORF1b:D1183Y, ORF1b:M2025I, ORF3a:Q57H, S:S13I, S:W152C, S:L452R, S:D614G,
hCoV-19/Mexico/BCN-ALSR-6606/2020	EPI_ISL_1081445	21C (Epsilon)	B.1.427	21	5'UTR:C241T, ORF1ab:C1059T, ORF1ab:C3037T, ORF1ab:C3117T, ORF1ab:T8641C, ORF1ab:G9738C, ORF1ab:A10323G, ORF1ab:G13713A, ORF1ab:C14408T, ORF1ab:C16394T, ORF1ab:G17014T, ORF1ab:G18859T, S:G21600T, S:G22018T, S:T22917G, S:A23403G, ORF3a:G25563T, M:C26681T, ORF8:G28198T, ORF8:A28272T, N:C28887T, N:C29362T,	15	N:T205I, ORF1a:T265I, ORF1a:T951I, ORF1a:S3158T, ORF1a:K3353R, ORF1b:P314L, ORF1b:P976L, ORF1b:D1183Y, ORF1b:A1798S, ORF3a:Q57H, ORF8:C102F, S:S13I, S:W152C, S:L452R, S:D614G,
hCoV-19/Mexico/BCN-ALSR-6607/2020	EPI_ISL_1081446	21C (Epsilon)	B.1.427	23	5'UTR:C241T, ORF1ab:C1059T, ORF1ab:T1851C, ORF1ab:C3037T, ORF1ab:G9738C, ORF1ab:C10833T, ORF1ab:G13713A, ORF1ab:C14408T, ORF1ab:C16394T, ORF1ab:G17014T, ORF1ab:C20930T, S:G21600T, S:G21985T, S:G22018T, S:T22917G, S:A23403G, S:C25317T, ORF3a:T25467C, ORF3a:G25563T, M:C26681T, ORF7b:A27892C, ORF8:A28272T, N:C28887T, N:C29353T,	16	N:T205I, ORF1a:T265I, ORF1a:I529T, ORF1a:S3158T, ORF1a:A3523V, ORF1b:P314L, ORF1b:P976L, ORF1b:D1183Y, ORF1b:T2488M, ORF3a:Q57H, S:S13I, S:L141F, S:W152C, S:L452R, S:D614G, S:S1252F,

hCoV-19/Mexico/BCN-ALSR-6608/2020	EPI_ISL_1081447	20A	B.1.232	21	5'UTR:C241T, ORF1ab:C3037T, ORF1ab:C5654T, ORF1ab:T5804C, ORF1ab:C5849T, ORF1ab:C6285T, ORF1ab:C6706T, ORF1ab:C14408T, ORF1ab:C16092T, ORF1ab:C17134T, ORF1ab:A20268G, S:C21812T, S:T22917G, S:A23403G, ORF3a:G25720T, E:G26325A, ORF8:G27987T, N:C28291T, N:T28600C, N:C28854T, N:G29474T, 3'UTR:G29825T,	12	N:S194L, N:D401Y, ORF1a:C1847R, ORF1a:P1862S, ORF1a:T2007I, ORF1b:P314L, ORF1b:P1223S, ORF3a:A110S, ORF8:V32L, ORF9b:P3L, S:L452R, S:D614G,	
hCoV-19/Mexico/BCN-ALSR-6609/2020	EPI_ISL_1081448	20A	B.1.232	20	5'UTR:C241T, ORF1ab:C3037T, ORF1ab:T5804C, ORF1ab:C5849T, ORF1ab:C6285T, ORF1ab:C6706T, ORF1ab:G8017T, ORF1ab:C14408T, ORF1ab:C16092T, ORF1ab:C19164T, ORF1ab:A20268G, ORF1ab:C20703T, S:A23403G, S:G23587T, ORF3a:G25720T, E:G26325A, ORF7a:A27520G, ORF8:G27987T, N:C28291T, N:C28854T, 3'UTR:G29825T,	11	N:S194L, ORF1a:C1847R, ORF1a:P1862S, ORF1a:T2007I, ORF1b:P314L, ORF3a:A110S, ORF7a:N43D, ORF8:V32L, ORF9b:P3L, S:D614G, S:Q675H,	S:21578-21580
hCoV-19/Mexico/BCN-ALSR-6610/2020	EPI_ISL_1081449	20A	B.1.561	22	5'UTR:C241T, ORF1ab:C527T, ORF1ab:G2516T, ORF1ab:C3037T, ORF1ab:C3510T, ORF1ab:A4636G, ORF1ab:C5365T, ORF1ab:A5999G, ORF1ab:A10323G, ORF1ab:C10798T, ORF1ab:C11866T, ORF1ab:C14408T, ORF1ab:G16917T, ORF1ab:C18693T, ORF1ab:A20268G, ORF1ab:C20389T, S:G21974T, S:A23403G, S:C24378T, ORF3a:T25577C, N:C28854T, N:G28975T, N:C29409T,	13	N:S194L, N:M234I, N:T379I, ORF1a:V751L, ORF1a:A1082V, ORF1a:I1912V, ORF1a:K3353R, ORF1b:P314L, ORF1b:R2308C, ORF3a:I62T, S:D138Y, S:D614G, S:S939F,	
hCoV-19/Mexico/BCN-ALSR-6611/2020	EPI_ISL_1081450	20B	B.1.1.432	36	5'UTR:C241T, ORF1ab:C1437T, ORF1ab:C3037T, ORF1ab:G3122T, ORF1ab:A3350G, ORF1ab:A4411G, ORF1ab:C6285T, ORF1ab:C6449T, ORF1ab:A6985T, ORF1ab:G7037T, ORF1ab:C7834T, ORF1ab:C8139T, ORF1ab:C9319T, ORF1ab:C11956T, ORF1ab:C12412T, ORF1ab:C12784T, ORF1ab:C13862T, ORF1ab:T14313C, ORF1ab:C14408T, ORF1ab:G18816C, ORF1ab:G20995T, ORF1ab:C21304A, ORF1ab:G21305A, S:C21627T, S:C21642T, S:C21812T, S:G22319A, S:A23403G, S:G24368T, ORF3a:C25613T, ORF3a:G25687T, ORF3a:G25912T, ORF3a:G26062T, N:G28881A, N:G28882A, N:G28883C, N:T29317C,	22	N:R203K, N:G204R, ORF1a:S391F, ORF1a:D953Y, ORF1a:S1029G, ORF1a:T2007I, ORF1a:L2062F, ORF1a:G2258C, ORF1a:S2625F, ORF1b:T132I, ORF1b:P314L, ORF1b:G2510C, ORF1b:R2613N, ORF3a:S74F, ORF3a:A99S, ORF3a:G174C, ORF3a:G224C, S:T22I, S:A27V, S:D253N, S:D614G, S:D936Y,	
hCoV-19/Mexico/BCN-ALSR-6612/2020	EPI_ISL_1081451	20G	B.1.2	21	5'UTR:C241T, ORF1ab:C683T, ORF1ab:C1059T, ORF1ab:C3037T, ORF1ab:G4232T, ORF1ab:C5497T, ORF1ab:C8752T, ORF1ab:G9928T, ORF1ab:C10319T, ORF1ab:C11620T, ORF1ab:C14408T, ORF1ab:A18424G, ORF1ab:C21304T, S:A23403G, ORF3a:G25563T, ORF3a:G25907T, ORF7a:T27687C, ORF8:C27964T, N:C28472T, N:C28869T, N:C29284T, N:G29422T,	13	N:P67S, N:P199L, ORF1a:T265I, ORF1a:D1323Y, ORF1a:M3221I, ORF1a:L3352F, ORF1b:P314L, ORF1b:N1653D, ORF1b:R2613C, ORF3a:Q57H, ORF3a:G172V, ORF8:S24L, S:D614G,	
hCoV-19/Mexico/BCN-ALSR-6613/2020	EPI_ISL_1081452	21C (Epsilon)	B.1.429	23	5'UTR:C241T, ORF1ab:A535G, ORF1ab:C1059T, ORF1ab:C2395T, ORF1ab:A2406G, ORF1ab:T2597C, ORF1ab:C2902T, ORF1ab:C3037T, ORF1ab:A5780G, ORF1ab:C7528T, ORF1ab:A12878G, ORF1ab:C14408T, ORF1ab:G15452T, ORF1ab:G17014T, S:G21600T, S:G22018T, S:T22917G, S:A23403G, S:T24349C, ORF3a:G25563T, M:C26681T, ORF7b:G27890T, ORF8:A28272T, N:C28887T,	13	N:T205I, ORF1a:T265I, ORF1a:K714R, ORF1a:I1839V, ORF1a:I4205V, ORF1b:P314L, ORF1b:G662V, ORF1b:D1183Y, ORF3a:Q57H, S:S13I, S:W152C, S:L452R, S:D614G,	

hCoV-19/Mexico/BCN-ALSR-6614/2020	EPI_ISL_1081453	20A	B.1.561	23	5'UTR:C241T, ORF1ab:C936T, ORF1ab:G2516T, ORF1ab:C3037T, ORF1ab:A5999G, ORF1ab:A10323G, ORF1ab:T10371G, ORF1ab:C10798T, ORF1ab:C11866T, ORF1ab:C12473T, ORF1ab:C13378T, ORF1ab:C14408T, ORF1ab:C18086T, ORF1ab:C18693T, ORF1ab:C19164T, ORF1ab:A20268G, S:G21974T, S:A23403G, S:C24031T, ORF3a:C25521T, ORF3a:T25577C, ORF8:C28087T, N:C28854T, N:G28975T,	13	N:S194L, N:M234I, ORF1a:T224I, ORF1a:V751L, ORF1a:I1912V, ORF1a:K3353R, ORF1a:I3369S, ORF1b:P314L, ORF1b:T1540I, ORF3a:I62T, ORF8:A65V, S:D138Y, S:D614G,
hCoV-19/Mexico/BCN-ALSR-6616/2020	EPI_ISL_1081454	21C (Epsilon)	B.1.427	22	5'UTR:C241T, ORF1ab:C1059T, ORF1ab:T1851C, ORF1ab:C2232T, ORF1ab:C3037T, ORF1ab:C3619A, ORF1ab:G9738C, ORF1ab:G13713A, ORF1ab:C14408T, ORF1ab:C16394T, ORF1ab:G17014T, S:G21600T, S:G22018T, S:T22917G, S:A23403G, S:C25317T, ORF3a:T25467C, ORF3a:G25563T, ORF3a:T26066C, M:C26681T, ORF8:A28272T, N:C28887T, N:C29362T,	15	N:T205I, ORF1a:T265I, ORF1a:I529T, ORF1a:A656V, ORF1a:S3158T, ORF1b:P314L, ORF1b:P976L, ORF1b:D1183Y, ORF3a:Q57H, ORF3a:V225A, S:S13I, S:W152C, S:L452R, S:D614G, S:S1252F,
hCoV-19/Mexico/BCN-ALSR-6617/2020	EPI_ISL_1081455	20A	B.1.232	23	5'UTR:C241T, ORF1ab:C3037T, ORF1ab:C5493A, ORF1ab:T5804C, ORF1ab:C5849T, ORF1ab:C6285T, ORF1ab:C6706T, ORF1ab:A8020G, ORF1ab:C14408T, ORF1ab:C16092T, ORF1ab:A20268G, S:G21778A, S:T22917G, S:A23403G, ORF3a:C25460T, ORF3a:G25720T, E:G26325A, ORF8:G27987T, ORF8:C28005T, N:C28291T, N:T28600C, N:C28854T, N:G29474T, 3'UTR:G29825T,	14	N:S194L, N:D401Y, ORF1a:S1743Y, ORF1a:C1847R, ORF1a:P1862S, ORF1a:T2007I, ORF1b:P314L, ORF3a:A23V, ORF3a:A110S, ORF8:V32L, ORF8:P38S, ORF9b:P3L, S:L452R, S:D614G,
hCoV-19/Mexico/BCN-ALSR-6618/2020	EPI_ISL_1081456	20G	B.1.2	19	5'UTR:C241T, ORF1ab:C1059T, ORF1ab:C3037T, ORF1ab:G8264T, ORF1ab:C10319T, ORF1ab:C14408T, ORF1ab:T15754G, ORF1ab:A18424G, ORF1ab:C21304T, S:A23403G, S:C23533T, S:C24382T, ORF3a:G25563T, ORF3a:G25907T, ORF3a:C26060T, ORF3a:G26167T, M:C26642T, ORF8:C27964T, N:C28472T, N:C28869T,	15	N:P67S, N:P199L, ORF1a:T265I, ORF1a:G2667C, ORF1a:L3352F, ORF1b:P314L, ORF1b:S763A, ORF1b:N1653D, ORF1b:R2613C, ORF3a:Q57H, ORF3a:G172V, ORF3a:T223I, ORF3a:V259L, ORF8:S24L, S:D614G,
hCoV-19/Mexico/BCN-ALSR-6637/2020	EPI_ISL_1081473	21C (Epsilon)	B.1.427	18	5'UTR:C241T, ORF1ab:C823T, ORF1ab:C1059T, ORF1ab:C3037T, ORF1ab:G9738C, ORF1ab:G13713A, ORF1ab:C14408T, ORF1ab:C16394T, ORF1ab:G17014T, S:G21600T, S:G22018T, S:T22917G, S:A23403G, ORF3a:G25563T, M:C26681T, ORF7a:G27637A, ORF8:A28272T, N:G28703C, N:C28887T,	13	N:D144H, N:T205I, ORF1a:T265I, ORF1a:S3158T, ORF1b:P314L, ORF1b:P976L, ORF1b:D1183Y, ORF3a:Q57H, ORF7a:V82I, S:S13I, S:W152C, S:L452R, S:D614G,
hCoV-19/Mexico/BCN-ALSR-6638/2020	EPI_ISL_1081474	21C (Epsilon)	B.1.427	18	5'UTR:C241T, ORF1ab:C1059T, ORF1ab:C2485T, ORF1ab:C3037T, ORF1ab:G9738C, ORF1ab:G13713A, ORF1ab:C14408T, ORF1ab:C16394T, ORF1ab:G17014T, ORF1ab:G20931T, S:G21600T, S:G22018T, S:T22917G, S:A23403G, ORF3a:G25563T, M:C26681T, ORF8:A28272T, N:C28887T, N:C29362T,	11	N:T205I, ORF1a:T265I, ORF1a:S3158T, ORF1b:P314L, ORF1b:P976L, ORF1b:D1183Y, ORF3a:Q57H, S:S13I, S:W152C, S:L452R, S:D614G,
hCoV-19/Mexico/BCN-ALSR-6639/2020	EPI_ISL_1081475	21C (Epsilon)	B.1.427	18	5'UTR:C241T, ORF1ab:C1059T, ORF1ab:C3037T, ORF1ab:G9738C, ORF1ab:C10349T, ORF1ab:G13713A, ORF1ab:C14408T, ORF1ab:C16394T, ORF1ab:G17014T, ORF1ab:G20931T, S:G21600T, S:G22018T, S:T22917G, S:A23403G, ORF3a:G25563T, ORF3a:C25714T, M:C26681T, ORF8:A28272T, N:C28887T,	13	N:T205I, ORF1a:T265I, ORF1a:S3158T, ORF1a:P3362S, ORF1b:P314L, ORF1b:P976L, ORF1b:D1183Y, ORF3a:Q57H, ORF3a:L108F, S:S13I, S:W152C, S:L452R, S:D614G,

hCoV-19/Mexico/BCN-ALSR-6640/2020	EPI_ISL_1081476	20A	B.1.551	24	5'UTR:C241T, ORF1ab:C527T, ORF1ab:C3037T, ORF1ab:C3317T, ORF1ab:C3738T, ORF1ab:C8836T, ORF1ab:G10631A, ORF1ab:C12473T, ORF1ab:C14408T, ORF1ab:A20268G, ORF1ab:T20833G, S:G21724T, S:C22323T, S:A23403G, S:C23604G, S:G23868T, ORF3a:C25728T, ORF3a:C26058T, M:C27046T, ORF7a:T27534C, ORF7a:C27630T, ORF7b:A27848G, N:C28310T, N:C28854T, N:C29466T,	14	M:T175M, N:P13S, N:S194L, N:A398V, ORF1a:P1018S, ORF1a:P1158L, ORF1a:A3456T, ORF1b:P314L, ORF1b:L2456V, S:L54F, S:S254F, S:D614G, S:P681R, S:G769V,
hCoV-19/Mexico/BCN-ALSR-6641/2020	EPI_ISL_1081477	21C (Epsilon)	B.1.427	22	5'UTR:C241T, ORF1ab:C1059T, ORF1ab:T1851C, ORF1ab:C2232T, ORF1ab:C3037T, ORF1ab:C3619A, ORF1ab:G9738C, ORF1ab:G13713A, ORF1ab:C14408T, ORF1ab:C16394T, ORF1ab:G17014T, S:G21600T, S:G22018T, S:T22917G, S:A23403G, S:C25317T, ORF3a:T25467C, ORF3a:G25563T, ORF3a:T26066C, M:C26681T, ORF8:A28272T, N:C28887T, N:C29362T,	15	N:T205I, ORF1a:T265I, ORF1a:I529T, ORF1a:A656V, ORF1a:S3158T, ORF1b:P314L, ORF1b:P976L, ORF1b:D1183Y, ORF3a:Q57H, ORF3a:V225A, S:S13I, S:W152C, S:L452R, S:D614G, S:S1252F,
hCoV-19/Mexico/BCN-ALSR-6642/2020	EPI_ISL_1081478	20B	B.1.1.519	27	5'UTR:T201C, 5'UTR:C203T, 5'UTR:C222T, 5'UTR:C241T, ORF1ab:C936T, ORF1ab:G1738T, ORF1ab:C3037T, ORF1ab:C3140T, ORF1ab:C10029T, ORF1ab:C10954T, ORF1ab:A11117G, ORF1ab:C12116T, ORF1ab:C12789T, ORF1ab:G14371T, ORF1ab:C14408T, ORF1ab:T19839C, ORF1ab:A19974G, ORF1ab:C21306T, S:C22995A, S:A23403G, S:C23604A, S:A23756G, M:T26609C, N:G28881A, N:G28882A, N:G28883C, N:C29197T, N:G29227T,	14	N:R203K, N:G204R, ORF1a:T224I, ORF1a:P959S, ORF1a:T3255I, ORF1a:I3618V, ORF1a:L3951F, ORF1a:T4175I, ORF1b:A302S, ORF1b:P314L, S:T478K, S:D614G, S:P681H, S:T732A,
hCoV-19/Mexico/BCN-ALSR-6643/2020	EPI_ISL_1081479	20A	B.1.232	21	5'UTR:C241T, ORF1ab:C3037T, ORF1ab:C3602T, ORF1ab:T5804C, ORF1ab:C5849T, ORF1ab:C6285T, ORF1ab:C6706T, ORF1ab:C14408T, ORF1ab:C16092T, ORF1ab:A20268G, S:T22917G, S:A23403G, ORF3a:C25561G, ORF3a:G25720T, E:G26325A, ORF8:G27987T, N:C28291T, N:G28460T, N:T28600C, N:C28854T, N:G29474T, 3'UTR:G29825T,	15	N:D63Y, N:S194L, N:D401Y, ORF1a:H1113Y, ORF1a:C1847R, ORF1a:P1862S, ORF1a:T2007I, ORF1b:P314L, ORF3a:Q57E, ORF3a:A110S, ORF8:V32L, ORF9b:P3L, ORF9b:K59N, S:L452R, S:D614G,
hCoV-19/Mexico/BCN-ALSR-6644/2020	EPI_ISL_1081480	20A	B.1.232	21	5'UTR:C241T, ORF1ab:C3037T, ORF1ab:C3602T, ORF1ab:T5804C, ORF1ab:C5849T, ORF1ab:C6285T, ORF1ab:C6706T, ORF1ab:C14408T, ORF1ab:C16092T, ORF1ab:A20268G, S:T22917G, S:A23403G, ORF3a:C25561G, ORF3a:G25720T, E:G26325A, ORF8:G27987T, N:C28291T, N:G28460T, N:T28600C, N:C28854T, N:G29474T, 3'UTR:G29825T,	15	N:D63Y, N:S194L, N:D401Y, ORF1a:H1113Y, ORF1a:C1847R, ORF1a:P1862S, ORF1a:T2007I, ORF1b:P314L, ORF3a:Q57E, ORF3a:A110S, ORF8:V32L, ORF9b:P3L, ORF9b:K59N, S:L452R, S:D614G,
hCoV-19/Mexico/BCN-ALSR-6645/2020	EPI_ISL_1081481	20G	B.1.2	24	5'UTR:C241T, ORF1ab:C1059T, ORF1ab:G1245A, ORF1ab:C3037T, ORF1ab:T4165C, ORF1ab:G9890T, ORF1ab:G9928T, ORF1ab:C10319T, ORF1ab:C13368T, ORF1ab:C14408T, ORF1ab:G16852T, ORF1ab:C18167T, ORF1ab:A18424G, ORF1ab:C21304T, S:T21698G, S:A23403G, ORF3a:G25563T, ORF3a:A25768C, ORF3a:G25907T, ORF7a:G27506T, ORF8:C27964T, N:C28472T, N:C28869T, N:G29422T, ORF10:C29614T,	19	N:P67S, N:P199L, ORF1a:T265I, ORF1a:G327D, ORF1a:A3209S, ORF1a:M3221I, ORF1a:L3352F, ORF1a:T4368I, ORF1b:P314L, ORF1b:G1129C, ORF1b:P1567L, ORF1b:N1653D, ORF1b:R2613C, ORF3a:Q57H, ORF3a:G172V, ORF7a:G38V, ORF8:S24L, S:S46A, S:D614G,

hCoV-19/Mexico/BCN-ALSR-6646/2020	EPI_ISL_1081482	20B	B.1.1.316	21	5'UTR:C241T, ORF1ab:C3037T, ORF1ab:C3411T, ORF1ab:G3871T, ORF1ab:C4999T, ORF1ab:A5040G, ORF1ab:A5350C, ORF1ab:G9203T, ORF1ab:A12519G, ORF1ab:G14371T, ORF1ab:C14408T, ORF1ab:T19839C, S:C21757T, S:A23403G, S:G23593C, S:A23756T, S:G25166C, E:G26262A, N:G28881A, N:G28882A, N:G28883C, 3'UTR:G29751T,	13	N:R203K, N:G204R, ORF1a:A1049V, ORF1a:K1202N, ORF1a:Q1592R, ORF1a:D2980Y, ORF1a:D4085G, ORF1b:A302S, ORF1b:P314L, S:D614G, S:Q677H, S:T732S, S:E1202Q,
hCoV-19/Mexico/BCN-ALSR-6647/2020	EPI_ISL_1081483	20B	B.1.1.316	21	5'UTR:C241T, ORF1ab:C3037T, ORF1ab:C3411T, ORF1ab:G3871T, ORF1ab:C4999T, ORF1ab:A5040G, ORF1ab:A5350C, ORF1ab:G9203T, ORF1ab:A12519G, ORF1ab:G14371T, ORF1ab:C14408T, ORF1ab:T19839C, S:C21757T, S:A23403G, S:G23593C, S:A23756T, S:G25166C, E:G26262A, N:G28881A, N:G28882A, N:G28883C, 3'UTR:G29751T,	13	N:R203K, N:G204R, ORF1a:A1049V, ORF1a:K1202N, ORF1a:Q1592R, ORF1a:D2980Y, ORF1a:D4085G, ORF1b:A302S, ORF1b:P314L, S:D614G, S:Q677H, S:T732S, S:E1202Q,
hCoV-19/Mexico/BCN-ALSR-6648/2020	EPI_ISL_1081484	20A	B.1.232	19	5'UTR:C241T, ORF1ab:C3037T, ORF1ab:T5804C, ORF1ab:C5849T, ORF1ab:C6285T, ORF1ab:C6706T, ORF1ab:A14339G, ORF1ab:C14408T, ORF1ab:C16092T, ORF1ab:A20268G, S:G22299C, S:A23403G, ORF3a:G25720T, E:G26325A, ORF8:G27987T, N:C28291T, N:T28600C, N:C28854T, N:G29474T, 3'UTR:G29825T,	12	N:S194L, N:D401Y, ORF1a:C1847R, ORF1a:P1862S, ORF1a:T2007I, ORF1b:N291S, ORF1b:P314L, ORF3a:A110S, ORF8:V32L, ORF9b:P3L, S:R246T, S:D614G,
hCoV-19/Mexico/BCN-ALSR-6649/2020	EPI_ISL_1081485	20A	B.1.551	19	5'UTR:C241T, ORF1ab:C3037T, ORF1ab:C3738T, ORF1ab:G7027A, ORF1ab:C8836T, ORF1ab:G10631A, ORF1ab:G11083T, ORF1ab:C11460T, ORF1ab:C14408T, ORF1ab:C14724T, ORF1ab:A20268G, S:C22323T, S:A23403G, S:C23604G, S:G23868T, ORF3a:C26058T, ORF7a:T27534C, N:C28854T, N:C29269A. N:C29466T.	12	N:S194L, N:A398V, ORF1a:P1158L, ORF1a:M2254I, ORF1a:A3456T, ORF1a:L3606F, ORF1a:S3732F, ORF1b:P314L, S:S254F, S:D614G, S:P681R, S:G769V,
hCoV-19/Mexico/BCN-ALSR-6650/2020	EPI_ISL_1081486	20G	B.1.2	26	5'UTR:C241T, ORF1ab:G491A, ORF1ab:C1059T, ORF1ab:A1139C, ORF1ab:T1936C, ORF1ab:C3037T, ORF1ab:C7303T, ORF1ab:G8264T, ORF1ab:C10319T, ORF1ab:C12068T, ORF1ab:C14408T, ORF1ab:C17747T, ORF1ab:A18424G, ORF1ab:T19389G, ORF1ab:C21304T, S:C21637T, S:A23403G, S:C23533T, ORF3a:G25563T, ORF3a:G25907T, ORF3a:C26060T, ORF8:C27964T, N:C28472T, N:C28869T, 3'UTR:C29708T, 3'UTR:G29779T, 3'UTR:G29781T,	17	N:P67S, N:P199L, ORF1a:A76T, ORF1a:T265I, ORF1a:K292Q, ORF1a:G2667C, ORF1a:L3352F, ORF1b:P314L, ORF1b:P1427L, ORF1b:N1653D, ORF1b:S1974R, ORF1b:R2613C, ORF3a:Q57H, ORF3a:G172V, ORF3a:T223I, ORF8:S24L, S:D614G,
hCoV-19/Mexico/BCN-ALSR-6651/2020	EPI_ISL_1081487	20A	B.1	14	5'UTR:C241T, ORF1ab:T1271C, ORF1ab:C2644T, ORF1ab:C3037T, ORF1ab:C7749T, ORF1ab:A8547G, ORF1ab:C12400T, ORF1ab:C14408T, ORF1ab:T16383C, ORF1ab:A19611T, ORF1ab:C20178T, ORF1ab:A20268G, S:A23403G, N:C28854T, N:T28912C,	6	N:S194L, ORF1a:F336L, ORF1a:T2495I, ORF1a:K2761R, ORF1b:P314L, S:D614G,
hCoV-19/Mexico/BCN-ALSR-6652/2020	EPI_ISL_1081488	20B	B.1.1.432	32	5'UTR:C241T, ORF1ab:T2034C, ORF1ab:C3037T, ORF1ab:G3122T, ORF1ab:A3350G, ORF1ab:A4987T, ORF1ab:C6449T, ORF1ab:A6985T, ORF1ab:G7037T, ORF1ab:C7834T, ORF1ab:C8139T, ORF1ab:C9319T, ORF1ab:C11956T, ORF1ab:C12412T, ORF1ab:C12784T, ORF1ab:T14313C, ORF1ab:C14408T, ORF1ab:C18176T, ORF1ab:G20995T, ORF1ab:C21304A, ORF1ab:G21305A, S:C21812T, S:T22867G, S:A23403G, S:G24368T, ORF3a:C25613T, ORF3a:G25912T, ORF3a:G26062T, ORF6:T27384C, N:G28881A, N:G28882A, N:G28883C, N:T29317C,	17	N:R203K, N:G204R, ORF1a:L590S, ORF1a:D953Y, ORF1a:S1029G, ORF1a:L2062F, ORF1a:G2258C, ORF1a:S2625F, ORF1b:P314L, ORF1b:P1570L, ORF1b:G2510C, ORF1b:R2613N, ORF3a:S74F, ORF3a:G174C, ORF3a:G224C, S:D614G, S:D936Y,

hCoV-19/Mexico/BCN-ALSR-6653/2020	EPI_ISL_1081489	20A	B.1.232	18	5'UTR:C241T, ORF1ab:C3037T, ORF1ab:T5804C, ORF1ab:C5849T, ORF1ab:C6285T, ORF1ab:C6706T, ORF1ab:C14408T, ORF1ab:C16092T, ORF1ab:A20268G, S:T22917G, S:A23403G, ORF3a:G25720T, E:G26325A, ORF8:G27987T, N:C28291T, N:T28600C, N:C28854T, N:C29253T, N:G29474T,	12	N:S194L, N:S327L, N:D401Y, ORF1a:C1847R, ORF1a:P1862S, ORF1a:T2007I, ORF1b:P314L, ORF3a:A110S, ORF8:V32L, ORF9b:P3L, S:L452R, S:D614G,	
hCoV-19/Mexico/BCN-ALSR-6654/2020	EPI_ISL_1081490	20G	B.1.2	22	5'UTR:C241T, ORF1ab:C1059T, ORF1ab:C2094T, ORF1ab:C3037T, ORF1ab:G3892T, ORF1ab:G8083A, ORF1ab:C10319T, ORF1ab:T11545C, ORF1ab:C14408T, ORF1ab:C14805T, ORF1ab:A15792C, ORF1ab:C17634A, ORF1ab:A18424G, ORF1ab:C21304T, S:C23086T, S:A23403G, S:T24427C, ORF3a:G25563T, ORF3a:G25907T, ORF8:C27964T, N:G28346A, N:C28472T, N:C28869T,	16	N:G25S, N:P67S, N:P199L, ORF1a:T265I, ORF1a:S610L, ORF1a:E1209D, ORF1a:M2606I, ORF1a:L3352F, ORF1b:P314L, ORF1b:D1389E, ORF1b:N1653D, ORF1b:R2613C, ORF3a:Q57H, ORF3a:G172V, ORF8:S24L, S:D614G,	
hCoV-19/Mexico/BCN-ALSR-6655/2020	EPI_ISL_1081491	20B	B.1.1.519	25	5'UTR:C203T, 5'UTR:C222T, 5'UTR:C241T, ORF1ab:C3037T, ORF1ab:C3140T, ORF1ab:C10029T, ORF1ab:C10954T, ORF1ab:A11117G, ORF1ab:C12789T, ORF1ab:G13126T, ORF1ab:C14408T, ORF1ab:C17436T, ORF1ab:G18090A, ORF1ab:T19839C, ORF1ab:C21306T, S:C21691T, S:C22995A, S:A23403G, S:C23604A, S:A23756G, M:G26951T, N:G28881A, N:G28882A, N:G28883C, N:G28899A, N:C29197T,	12	N:R203K, N:G204R, N:R209K, ORF1a:P959S, ORF1a:T3255I, ORF1a:I3618V, ORF1a:T4175I, ORF1b:P314L, S:T478K, S:D614G, S:P681H, S:T732A,	
hCoV-19/Mexico/BCN-ALSR-6657/2020	EPI_ISL_1081492	20A	B.1	5	5'UTR:C241T, ORF1ab:C3037T, ORF1ab:C14408T, S:T22917G, S:A23403G, ORF3a:G25563T,	4	ORF1b:P314L, ORF3a:Q57H, S:L452R, S:D614G,	
hCoV-19/Mexico/BCN-ALSR-6658/2020	EPI_ISL_1081493	20A	B.1.561	29	5'UTR:C241T, ORF1ab:C936T, ORF1ab:A2385G, ORF1ab:G2516T, ORF1ab:C3037T, ORF1ab:A5999G, ORF1ab:A10323G, ORF1ab:T10371G, ORF1ab:C10798T, ORF1ab:C11866T, ORF1ab:C12357T, ORF1ab:C13115T, ORF1ab:C13378T, ORF1ab:C14408T, ORF1ab:C18693T, ORF1ab:C19164T, ORF1ab:A20268G, ORF1ab:G20931T, S:C21575T, S:G21974T, S:A23403G, S:C23635T, S:C24031T, ORF3a:C25487T, ORF3a:C25521T, ORF3a:T25577C, ORF7a:T27511G, ORF8:C28087T, N:C28854T, N:G28975T,	17	N:S194L, N:M234I, ORF1a:T224I, ORF1a:E707G, ORF1a:V751L, ORF1a:I1912V, ORF1a:K3353R, ORF1a:I3369S, ORF1a:T4031I, ORF1b:P314L, ORF3a:T32I, ORF3a:I62T, ORF7a:Y40D, ORF8:A65V, S:L5F, S:D138Y, S:D614G,	
hCoV-19/Mexico/BCN-ALSR-6659/2020	EPI_ISL_1081494	20A	B.1.232	21	5'UTR:C241T, ORF1ab:C3037T, ORF1ab:T5804C, ORF1ab:C5849T, ORF1ab:C6285T, ORF1ab:C6706T, ORF1ab:C12412T, ORF1ab:C14408T, ORF1ab:C16092T, ORF1ab:A20268G, ORF1ab:A20481G, S:T22917G, S:C23059T, S:A23403G, ORF3a:G25440T, ORF3a:G25720T, E:G26325A, ORF8:G27987T, N:C28291T, N:T28600C, N:C28854T, N:G29474T,	12	N:S194L, N:D401Y, ORF1a:C1847R, ORF1a:P1862S, ORF1a:T2007I, ORF1b:P314L, ORF3a:K16N, ORF3a:A110S, ORF8:V32L, ORF9b:P3L, S:L452R, S:D614G,	
hCoV-19/Mexico/BCN-ALSR-6660/2020	EPI_ISL_1081495	20C	B.1.375	20	5'UTR:C241T, ORF1ab:C1059T, ORF1ab:T1171C, ORF1ab:C1670T, ORF1ab:C3037T, ORF1ab:A5747G, ORF1ab:C8299T, ORF1ab:C8917T, ORF1ab:C10789T, ORF1ab:C14408T, ORF1ab:C16349T, ORF1ab:G17259T, ORF1ab:G20126T, S:A23403G, S:G24007A, S:T25268A, ORF3a:C25413T, ORF3a:G25563T, ORF3a:C25844T, M:A26664G, N:C28887T,	13	M:I48V, N:T205I, ORF1a:T265I, ORF1a:L469F, ORF1a:T1828A, ORF1b:P314L, ORF1b:S961L, ORF1b:E1264D, ORF1b:G2220V, ORF3a:Q57H, ORF3a:T151I, S:D614G, S:C1236S,	S:21765-21770,

hCoV-19/Mexico/BCN-ALSR-6671/2021	EPI_ISL_1081506	20A	B.1.232	22	5'UTR:C241T, ORF1ab:T1965C, ORF1ab:C3037T, ORF1ab:T5804C, ORF1ab:C5849T, ORF1ab:C6285T, ORF1ab:C6706T, ORF1ab:T7434A, ORF1ab:C13176T, ORF1ab:C14408T, ORF1ab:C16092T, ORF1ab:A20268G, S:T22917G, S:A23403G, ORF3a:G25720T, ORF3a:C26204T, E:G26325A, ORF8:G27987T, N:C28291T, N:T28600C, N:C28854T, N:G29474T, 3'UTR:G29825T,	15	N:S194L, N:D401Y, ORF1a:I567T, ORF1a:C1847R, ORF1a:P1862S, ORF1a:T2007I, ORF1a:F2390Y, ORF1a:T4304I, ORF1b:P314L, ORF3a:A110S, ORF3a:T271I, ORF8:V32L, ORF9b:P3L, S:L452R, S:D614G,
hCoV-19/Mexico/BCN-ALSR-6709/2021	EPI_ISL_1081544	21C (Epsilon)	B.1.429	27	5'UTR:C241T, ORF1ab:C1059T, ORF1ab:C2395T, ORF1ab:T2597C, ORF1ab:C3037T, ORF1ab:C8947T, ORF1ab:C9803T, ORF1ab:C10604T, ORF1ab:C10641T, ORF1ab:C12100T, ORF1ab:A12878G, ORF1ab:C14408T, ORF1ab:G15550A, ORF1ab:C16523T, ORF1ab:G17014T, ORF1ab:A19182G, S:G21600T, S:G22018T, S:C22227T, S:T22917G, S:A23403G, S:T24349C, ORF3a:G25563T, M:C26681T, ORF7b:G27890T, ORF8:A28272T, N:C28887T, N:C29362T	15	N:T205I, ORF1a:T265I, ORF1a:P3447S, ORF1a:T3459M, ORF1a:I4205V, ORF1b:P314L, ORF1b:V695I, ORF1b:T1019I, ORF1b:D1183Y, ORF3a:Q57H, S:S13I, S:W152C, S:A222V, S:L452R, S:D614G,
hCoV-19/Mexico/BCN-ALSR-7152/2021	EPI_ISL_1185929	20A	B.1.243	19	5'UTR:C241T, ORF1ab:G806A, ORF1ab:C2143T, ORF1ab:T2584A, ORF1ab:C3037T, ORF1ab:A3207G, ORF1ab:C3874T, ORF1ab:C5140A, ORF1ab:C14408T, ORF1ab:C17012T, ORF1ab:C18654T, ORF1ab:A20268G, S:C22550T, S:A23403G, S:G23587C, S:T24076C, ORF3a:G25775T, ORF3a:C25844T, N:C28854T, N:G29543T,	11	N:S194L, ORF1a:A181T, ORF1a:D981G, ORF1a:D1625E, ORF1b:P314L, ORF1b:S1182L, ORF3a:W128L, ORF3a:T151I, S:P330S, S:D614G, S:Q675H,
hCoV-19/Mexico/BCN-ALSR-7154/2021	EPI_ISL_1185931	21C (Epsilon)	B.1.427	23	5'UTR:C241T, ORF1ab:C1059T, ORF1ab:T1851C, ORF1ab:C2232T, ORF1ab:C3037T, ORF1ab:C3619A, ORF1ab:G9738C, ORF1ab:G13713A, ORF1ab:C14408T, ORF1ab:C16394T, ORF1ab:G17014T, S:G21600T, S:G22018T, S:T22917G, S:A23403G, S:C25317T, ORF3a:T25467C, ORF3a:G25563T, ORF3a:T26066C, M:C26681T, ORF8:A28272T, N:C28887T, N:C29160T, N:C29362T,	16	N:T205I, N:T296I, ORF1a:T265I, ORF1a:I529T, ORF1a:A656V, ORF1a:S3158T, ORF1b:P314L, ORF1b:P976L, ORF1b:D1183Y, ORF3a:Q57H, ORF3a:V225A, S:S13I, S:W152C, S:L452R, S:D614G, S:S1252F,
hCoV-19/Mexico/BCN-ALSR-7158/2021	EPI_ISL_1185934	21C (Epsilon)	B.1.427	24	5'UTR:C241T, ORF1ab:C1059T, ORF1ab:T1851C, ORF1ab:C2232T, ORF1ab:C3037T, ORF1ab:C3619A, ORF1ab:G4510A, ORF1ab:G9738C, ORF1ab:G13713A, ORF1ab:C14408T, ORF1ab:C16394T, ORF1ab:G17014T, S:G21600T, S:G22018T, S:T22917G, S:A23403G, S:C25317T, ORF3a:T25467C, ORF3a:G25563T, ORF3a:T26066C, M:C26681T, ORF8:A28272T, N:A28804G, N:C28887T, N:C29362T,	15	N:T205I, ORF1a:T265I, ORF1a:I529T, ORF1a:A656V, ORF1a:S3158T, ORF1b:P314L, ORF1b:P976L, ORF1b:D1183Y, ORF3a:Q57H, ORF3a:V225A, S:S13I, S:W152C, S:L452R, S:D614G, S:S1252F,
hCoV-19/Mexico/BCN-ALSR-7159/2021	EPI_ISL_1185935	20A	B.1.232	19	5'UTR:C241T, ORF1ab:C3037T, ORF1ab:T5804C, ORF1ab:C5849T, ORF1ab:C6285T, ORF1ab:C6706T, ORF1ab:C14408T, ORF1ab:C16092T, ORF1ab:A20268G, S:T22917G, S:A23403G, ORF3a:G25720T, E:G26325A, ORF7b:C27849T, ORF8:G27987T, N:C28291T, N:T28600C, N:C28854T, N:G29474T, 3'UTR:G29825T,	12	N:S194L, N:D401Y, ORF1a:C1847R, ORF1a:P1862S, ORF1a:T2007I, ORF1b:P314L, ORF3a:A110S, ORF7b:L32F, ORF8:V32L, ORF9b:P3L, S:L452R, S:D614G,
hCoV-19/Mexico/BCN-ALSR-7160/2021	EPI_ISL_1185936	20G	B.1.2	23	5'UTR:G61T, 5'UTR:C241T, ORF1ab:C1059T, ORF1ab:T1927C, ORF1ab:C3037T, ORF1ab:A3076G, ORF1ab:C8293T, ORF1ab:C10319T, ORF1ab:C14408T, ORF1ab:A18424G, ORF1ab:G18583T, ORF1ab:C21304T, S:C21742T, S:A22255T, S:G22332A, S:A23403G, ORF3a:G25563T, ORF3a:G25907T, ORF8:C27964T, N:C28472T, N:C28869T, N:C29555T, ORF10:A29643G, 3'UTR:G29688T,	13	N:P67S, N:P199L, ORF1a:T265I, ORF1a:L3352F, ORF1b:P314L, ORF1b:N1653D, ORF1b:V1706L, ORF1b:R2613C, ORF3a:Q57H, ORF3a:G172V, ORF8:S24L, S:G257D, S:D614G,

hCoV-19/Mexico/BCN-ALSR-8341/2020	EPI_ISL_1531807	20A	B.1.609	9	5'UTR:C241T, ORF1ab:C888A, ORF1ab:C1302T, ORF1ab:C3037T, ORF1ab:C4582T, ORF1ab:C4586T, ORF1ab:C8175T, ORF1ab:C14408T, ORF1ab:A20268G, S:A23403G,	5	ORF1a:A208D, ORF1a:T346I, ORF1a:A2637V, ORF1b:P314L, S:D614G,
hCoV-19/Mexico/BCN-ALSR-8342/2020	EPI_ISL_1531808	20A	B.1.243	9	5'UTR:C241T, ORF1ab:C3037T, ORF1ab:C12473T, ORF1ab:C14408T, ORF1ab:C18167T, ORF1ab:A20268G, S:A23403G, S:T24076C, M:C26645T, N:C28854T,	4	N:S194L, ORF1b:P314L, ORF1b:P1567L, S:D614G,
hCoV-19/Mexico/BCN-ALSR-8343/2020	EPI_ISL_1531809	20A	B.1	8	5'UTR:C241T, ORF1ab:C1076T, ORF1ab:C3037T, ORF1ab:A5227C, ORF1ab:C6310T, ORF1ab:C14408T, ORF1ab:A20268G, S:A23403G, N:C28854T,	5	N:S194L, ORF1a:P271S, ORF1a:K1654N, ORF1b:P314L, S:D614G,
hCoV-19/Mexico/BCN-ALSR-8344/2020	EPI_ISL_1531810	20A	B.1	9	5'UTR:C241T, ORF1ab:C3037T, ORF1ab:C12484T, ORF1ab:C14408T, ORF1ab:A20268G, ORF1ab:G21193T, S:C21627T, S:A23403G, ORF3a:C25469T, N:C28854T,	6	N:S194L, ORF1b:P314L, ORF1b:D2576Y, ORF3a:S26L, S:T22I, S:D614G,
hCoV-19/Mexico/BCN-ALSR-8345/2020	EPI_ISL_1531811	20A	B.1	8	5'UTR:C241T, 5'UTR:A253G, ORF1ab:C3037T, ORF1ab:C14408T, ORF1ab:G14559T, ORF1ab:A20268G, S:A23403G, S:A23720G, N:C28854T,	4	N:S194L, ORF1b:P314L, S:D614G, S:I720V,
hCoV-19/Mexico/BCN-ALSR-8346/2020	EPI_ISL_1531812	20A	B.1.243	7	5'UTR:C241T, ORF1ab:C3037T, ORF1ab:C14408T, ORF1ab:A20268G, S:C21575T, S:A23403G, S:T24076C, N:C28854T,	4	N:S194L, ORF1b:P314L, S:L5F, S:D614G,
hCoV-19/Mexico/BCN-ALSR-8347/2020	EPI_ISL_1531813	20A	B.1	7	5'UTR:C241T, ORF1ab:C3037T, ORF1ab:C7732T, ORF1ab:C14408T, ORF1ab:C17474T, ORF1ab:A20268G, S:A23403G, N:C28854T,	4	N:S194L, ORF1b:P314L, ORF1b:T1336I, S:D614G,
hCoV-19/Mexico/BCN-ALSR-8348/2020	EPI_ISL_1531814	20A	B.1	7	5'UTR:C241T, ORF1ab:A1815G, ORF1ab:C3037T, ORF1ab:C14408T, ORF1ab:A20268G, S:A23403G, N:C28854T, 3'UTR:C29724T,	4	N:S194L, ORF1a:K517R, ORF1b:P314L, S:D614G,
hCoV-19/Mexico/BCN-ALSR-8349/2020	EPI_ISL_1531815	20A	B.1.241	8	5'UTR:C241T, ORF1ab:C3037T, ORF1ab:C7768T, ORF1ab:C11575T, ORF1ab:C14408T, ORF1ab:A20268G, S:A23403G, ORF7a:C27494T, N:C28854T,	4	N:S194L, ORF1b:P314L, ORF7a:P34L, S:D614G,
hCoV-19/Mexico/BCN-ALSR-8351/2020	EPI_ISL_1531816	20C	B.1.369	8	5'UTR:C241T, ORF1ab:C1059T, ORF1ab:C3037T, ORF1ab:C14408T, ORF1ab:C16260T, S:A23403G, ORF3a:G25563T, ORF7a:C27654T, N:C28821A,	5	N:S183Y, ORF1a:T265I, ORF1b:P314L, ORF3a:Q57H, S:D614G,
hCoV-19/Mexico/BCN-ALSR-8352/2020	EPI_ISL_1531817	20C	B.1	10	5'UTR:C241T, ORF1ab:C1059T, ORF1ab:C3037T, ORF1ab:G7798T, ORF1ab:C9811T, ORF1ab:C12534T, ORF1ab:C14408T, ORF1ab:G17562T, S:A23403G, ORF3a:G25563T, N:G28899T,	7	N:R209I, ORF1a:T265I, ORF1a:K2511N, ORF1a:T4090I, ORF1b:P314L, ORF3a:Q57H, S:D614G,
hCoV-19/Mexico/BCN-ALSR-8353/2020	EPI_ISL_1531818	20B	B.1.1.344	17	5'UTR:C241T, ORF1ab:C3037T, ORF1ab:C6636T, ORF1ab:A6750G, ORF1ab:T9172C, ORF1ab:A10948G, ORF1ab:C13019T, ORF1ab:G14118T, ORF1ab:C14408T, ORF1ab:T16950C, S:G22335T, S:A23403G, S:G23593T, ORF3a:G25816C, M:G26730T, N:G28881A, N:G28882A, N:G28883C,	10	M:V70F, N:R203K, N:G204R, ORF1a:T2124I, ORF1a:N2162S, ORF1b:P314L, ORF3a:D142H, S:W258L, S:D614G, S:Q677H,
hCoV-19/Mexico/BCN-ALSR-8354/2020	EPI_ISL_1531819	20A	B.1.561	14	5'UTR:C241T, ORF1ab:G2516T, ORF1ab:C3037T, ORF1ab:A5999G, ORF1ab:A10323G, ORF1ab:C10798T, ORF1ab:C11866T, ORF1ab:C14408T, ORF1ab:C18693T, ORF1ab:A20268G, S:G21974T, S:A23403G, ORF3a:T25577C, N:C28854T, N:G28975T,	9	N:S194L, N:M234I, ORF1a:V751L, ORF1a:I1912V, ORF1a:K3353R, ORF1b:P314L, ORF3a:I62T, S:D138Y, S:D614G,
hCoV-19/Mexico/BCN-ALSR-8356/2020	EPI_ISL_1531820	20A	B.1.561	13	5'UTR:C241T, ORF1ab:G2516T, ORF1ab:C3037T, ORF1ab:A5999G, ORF1ab:A10323G, ORF1ab:C10798T, ORF1ab:C11866T, ORF1ab:C14408T, ORF1ab:C18693T, ORF1ab:A20268G, S:A23403G, ORF3a:T25577C, N:C28854T, N:G28975T,	8	N:S194L, N:M234I, ORF1a:V751L, ORF1a:I1912V, ORF1a:K3353R, ORF1b:P314L, ORF3a:I62T, S:D614G,
hCoV-19/Mexico/BCN-ALSR-8357/2020	EPI_ISL_1531821	20A	B.1.399	11	5'UTR:C241T, ORF1ab:C3037T, ORF1ab:A4104G, ORF1ab:G6410A, ORF1ab:G8017T, ORF1ab:C9711T, ORF1ab:C14408T, ORF1ab:C20104T, ORF1ab:A20268G, S:A23403G, N:C28854T, N:C28887T,	8	N:S194L, N:T205I, ORF1a:K1280R, ORF1a:E2049K, ORF1a:S3149F, ORF1b:P314L, ORF1b:L2213F, S:D614G,

hCoV-19/Mexico/BCN-ALSR-8358/2020	EPI_ISL_1531822	20A	B.1	11	5'UTR:C241T, ORF1ab:C337T, ORF1ab:C3037T, ORF1ab:C3903T, ORF1ab:C6633T, ORF1ab:C14408T, ORF1ab:A20268G, S:A23403G, S:C25046T, ORF8:A28249G, N:C28854T, N:A29350G,	7	N:S194L, ORF1a:P1213L, ORF1a:A2123V, ORF1b:P314L, ORF8:D119G, S:D614G, S:P1162S,	
hCoV-19/Mexico/BCN-ALSR-8359/2020	EPI_ISL_1531823	20A	B.1.561	17	5'UTR:C241T, ORF1ab:G2516T, ORF1ab:C3037T, ORF1ab:A5999G, ORF1ab:A10323G, ORF1ab:C11202T, ORF1ab:C14408T, ORF1ab:C18693T, ORF1ab:A20268G, S:C21614T, S:G21974T, S:A23403G, S:C23758T, ORF3a:T25577C, ORF3a:C26192T, N:C28657T, N:C28854T, N:G28975T,	12	N:S194L, N:M234I, ORF1a:V751L, ORF1a:I1912V, ORF1a:K3353R, ORF1a:T3646I, ORF1b:P314L, ORF3a:I62T, ORF3a:P267L, S:L18F, S:D138Y, S:D614G,	
hCoV-19/Mexico/BCN-ALSR-8360/2020	EPI_ISL_1531824	20A	B.1.556	11	5'UTR:C241T, ORF1ab:C3037T, ORF1ab:G5326T, ORF1ab:C14408T, ORF1ab:C14821A, ORF1ab:C18060A, ORF1ab:A19112C, ORF1ab:A20268G, ORF1ab:A21203C, S:A23403G, N:C28854T, N:G29374T,	8	N:S194L, N:E367D, ORF1a:E1687D, ORF1b:P314L, ORF1b:P452T, ORF1b:D1882A, ORF1b:K2579T, S:D614G,	
hCoV-19/Mexico/BCN-ALSR-8361/2020	EPI_ISL_1531825	20A	B.1.241	11	5'UTR:C241T, ORF1ab:C1420T, ORF1ab:C3037T, ORF1ab:G7960T, ORF1ab:C11575T, ORF1ab:C14408T, ORF1ab:C19029T, ORF1ab:A20268G, S:A23403G, S:C23525T, N:C28854T, N:A29544G,	5	N:S194L, ORF1a:M2565I, ORF1b:P314L, S:D614G, S:H655Y,	S:21765-21770,
hCoV-19/Mexico/BCN-ALSR-8362/2020	EPI_ISL_1531826	20A	B.1	9	5'UTR:C241T, ORF1ab:A2923G, ORF1ab:C3037T, ORF1ab:G11335T, ORF1ab:C12513T, ORF1ab:C14408T, ORF1ab:A20268G, S:A23403G, S:G25311T, N:C28854T,	5	N:S194L, ORF1a:T4083M, ORF1b:P314L, S:D614G, S:C1250F,	
hCoV-19/Mexico/BCN-ALSR-8363/2020	EPI_ISL_1531827	20A	B.1.609	13	5'UTR:C241T, ORF1ab:C3037T, ORF1ab:A3647G, ORF1ab:C4582T, ORF1ab:C6578T, ORF1ab:C8175T, ORF1ab:A8845G, ORF1ab:G11306A, ORF1ab:C14408T, ORF1ab:C17934T, ORF1ab:T18309C, ORF1ab:C19718T, ORF1ab:A20268G, S:A23403G,	7	ORF1a:I1128V, ORF1a:L2105F, ORF1a:A2637V, ORF1a:D3681N, ORF1b:P314L, ORF1b:T2084I, S:D614G,	
hCoV-19/Mexico/BCN-ALSR-8364/2020	EPI_ISL_1531828	20A	B.1.243	16	5'UTR:C21T, 5'UTR:C241T, ORF1ab:C3037T, ORF1ab:C9223T, ORF1ab:G14346A, ORF1ab:C14408T, ORF1ab:G15906T, ORF1ab:A19321G, ORF1ab:A20268G, ORF1ab:A20304G, ORF1ab:A21258G, S:G22132T, S:A23403G, S:T24076C, E:T26304C, ORF7a:C27741T, N:C28854T,	6	N:S194L, ORF1b:P314L, ORF1b:Q813H, ORF1b:T1952A, S:R190S, S:D614G,	
hCoV-19/Mexico/BCN-ALSR-8365/2020	EPI_ISL_1531829	20A	B.1.396	13	5'UTR:C241T, ORF1ab:C1191T, ORF1ab:C3037T, ORF1ab:C6336T, ORF1ab:C9521A, ORF1ab:C14408T, ORF1ab:A20268G, S:C22414T, S:A23403G, S:C24904T, M:A26986T, N:C28854T, N:G29422T, ORF10:A29567G,	7	M:H155L, N:S194L, ORF1a:P309L, ORF1a:S2024L, ORF1a:L3086I, ORF1b:P314L, S:D614G,	
hCoV-19/Mexico/BCN-ALSR-8366/2020	EPI_ISL_1531830	20A	B.1	11	5'UTR:C241T, ORF1ab:T409C, ORF1ab:C2509T, ORF1ab:C3037T, ORF1ab:G12070T, ORF1ab:C14408T, ORF1ab:A18390G, ORF1ab:A20268G, ORF1ab:C21008T, S:A23403G, N:C28603T, N:C28854T,	4	N:S194L, ORF1b:P314L, ORF1b:T2514I, S:D614G,	
hCoV-19/Mexico/BCN-ALSR-8367/2020	EPI_ISL_1531831	20A	B.1	7	5'UTR:C241T, ORF1ab:C3037T, ORF1ab:C14408T, ORF1ab:T17793C, ORF1ab:A20268G, S:A23403G, S:T23527C, N:C28854T,	3	N:S194L, ORF1b:P314L, S:D614G,	
hCoV-19/Mexico/BCN-ALSR-8368/2020	EPI_ISL_1531832	20A	B.1.241	16	5'UTR:C241T, ORF1ab:C3037T, ORF1ab:C6786T, ORF1ab:C7768T, ORF1ab:T8634C, ORF1ab:C11575T, ORF1ab:C14408T, ORF1ab:T14907C, ORF1ab:C16523T, ORF1ab:G18148T, ORF1ab:G18225T, ORF1ab:A20268G, S:A22528T, S:A23403G, N:C28344T, N:C28677T, N:C28854T,	10	N:T24I, N:T135I, N:S194L, ORF1a:T2174I, ORF1a:I2790T, ORF1b:P314L, ORF1b:T1019I, ORF1b:G1561C, ORF1b:M1586I, S:D614G,	
hCoV-19/Mexico/BCN-ALSR-8369/2020	EPI_ISL_1531833	20A	B.1	11	5'UTR:C241T, ORF1ab:C3037T, ORF1ab:A11201G, ORF1ab:C14323T, ORF1ab:C14408T, ORF1ab:T17793C, ORF1ab:A20268G, S:A23403G, S:T23527C, M:G26730T, ORF8:A28113T, N:C28854T,	7	M:V70F, N:S194L, ORF1a:T3646A, ORF1b:H286Y, ORF1b:P314L, ORF8:I74F, S:D614G,	

hCoV-19/Mexico/BCN-ALSR-8370/2020	EPI_ISL_1531834	20A	B.1.241	15	5'UTR:C241T, ORF1ab:C3037T, ORF1ab:C6786T, ORF1ab:C7768T, ORF1ab:T8634C, ORF1ab:C11575T, ORF1ab:C14408T, ORF1ab:T14907C, ORF1ab:G18148T, ORF1ab:G18225T, ORF1ab:A20268G, S:A22528T, S:A23403G, N:C28344T, N:C28677T, N:C28854T,	9	N:T24I, N:T135I, N:S194L, ORF1a:T2174I, ORF1a:I2790T, ORF1b:P314L, ORF1b:G1561C, ORF1b:M1586I, S:D614G,	
hCoV-19/Mexico/BCN-ALSR-8371/2020	EPI_ISL_1531835	20A	B.1.397	15	5'UTR:C241T, ORF1ab:G1135T, ORF1ab:C3037T, ORF1ab:C5144T, ORF1ab:T8323C, ORF1ab:C14408T, ORF1ab:T15030C, ORF1ab:C15830A, ORF1ab:A19079C, ORF1ab:A20268G, S:C23029T, S:A23403G, M:G26828T, N:C28854T, N:G29090T, 3'UTR:G29759T,	7	N:S194L, N:A273S, ORF1a:K290N, ORF1b:P314L, ORF1b:A788E, ORF1b:E1871A, S:D614G,	
hCoV-19/Mexico/BCN-ALSR-8372/2020	EPI_ISL_1531836	20A	B.1.232	14	5'UTR:C241T, ORF1ab:C1684T, ORF1ab:C3037T, ORF1ab:T5804C, ORF1ab:C5849T, ORF1ab:C6285T, ORF1ab:C6706T, ORF1ab:C14408T, ORF1ab:C16092T, ORF1ab:A20268G, S:A23403G, E:G26325A, ORF8:G27987T, N:C28291T, N:C28854T,	8	N:S194L, ORF1a:C1847R, ORF1a:P1862S, ORF1a:T2007I, ORF1b:P314L, ORF8:V32L, ORF9b:P3L, S:D614G,	
hCoV-19/Mexico/BCN-ALSR-8373/2020	EPI_ISL_1531837	20A	B.1.241	15	5'UTR:C241T, ORF1ab:G713T, ORF1ab:C3037T, ORF1ab:C3736T, ORF1ab:C6786T, ORF1ab:C7768T, ORF1ab:T8634C, ORF1ab:C11575T, ORF1ab:C14408T, ORF1ab:T14907C, ORF1ab:G18148T, ORF1ab:A20268G, S:A23403G, N:C28344T, N:C28677T, N:C28854T,	9	N:T24I, N:T135I, N:S194L, ORF1a:G150C, ORF1a:T2174I, ORF1a:I2790T, ORF1b:P314L, ORF1b:G1561C, S:D614G,	3'UTR:29739-29748
hCoV-19/Mexico/BCN-ALSR-8374/2020	EPI_ISL_1531838	20A	B.1	8	5'UTR:C241T, ORF1ab:C2652T, ORF1ab:C3037T, ORF1ab:C5512T, ORF1ab:C14408T, ORF1ab:A20268G, S:A23403G, N:C28854T, 3'UTR:C29718T,	4	N:S194L, ORF1a:T796I, ORF1b:P314L, S:D614G,	
hCoV-19/Mexico/BCN-ALSR-8375/2020	EPI_ISL_1531839	20A	B.1	11	5'UTR:C241T, ORF1ab:A2775G, ORF1ab:C3037T, ORF1ab:C14408T, ORF1ab:G18756T, ORF1ab:A20268G, S:A23403G, S:G23868T, ORF3a:C25889T, N:A28293G, N:C28854T, N:A29350G,	8	N:Q7R, N:S194L, ORF1a:K837R, ORF1b:P314L, ORF3a:S166L, ORF9b:K4E, S:D614G, S:G769V,	
hCoV-19/Mexico/BCN-ALSR-8376/2020	EPI_ISL_1531840	20C	B.1.369	12	5'UTR:C241T, ORF1ab:C1059T, ORF1ab:G2272T, ORF1ab:C3037T, ORF1ab:G8003T, ORF1ab:C12116T, ORF1ab:C14408T, ORF1ab:C16260T, ORF1ab:C20762T, S:A23403G, ORF3a:G25563T, N:C28821A, 3'UTR:C29679T,	9	N:S183Y, ORF1a:T265I, ORF1a:K669N, ORF1a:V2580F, ORF1a:L3951F, ORF1b:P314L, ORF1b:T2432I, ORF3a:Q57H, S:D614G,	
hCoV-19/Mexico/BCN-ALSR-8377/2020	EPI_ISL_1531841	20A	B.1.561	15	5'UTR:C241T, ORF1ab:G2516T, ORF1ab:C3037T, ORF1ab:G3998A, ORF1ab:A5999G, ORF1ab:A10323G, ORF1ab:C10798T, ORF1ab:C11866T, ORF1ab:C14408T, ORF1ab:C18693T, ORF1ab:A20268G, S:G21974T, S:A23403G, ORF3a:T25577C, N:C28854T, N:G28975T,	10	N:S194L, N:M234I, ORF1a:V751L, ORF1a:E1245K, ORF1a:I1912V, ORF1a:K3353R, ORF1b:P314L, ORF3a:I62T, S:D138Y, S:D614G,	
hCoV-19/Mexico/BCN-ALSR-8378/2020	EPI_ISL_1531842	20A	B.1	8	5'UTR:T221C, 5'UTR:C241T, ORF1ab:C3037T, ORF1ab:T3438C, ORF1ab:C14408T, ORF1ab:C17004T, ORF1ab:A20268G, S:A23403G, N:C28854T,	4	N:S194L, ORF1a:V1058A, ORF1b:P314L, S:D614G,	
hCoV-19/Mexico/BCN-ALSR-8379/2020	EPI_ISL_1531843	20A	B.1.400	9	5'UTR:C241T, ORF1ab:C3037T, ORF1ab:T8779C, ORF1ab:C14408T, ORF1ab:G19969A, ORF1ab:A20268G, S:A23403G, S:G24305A, S:C25153T, N:C28854T,	5	N:S194L, ORF1b:P314L, ORF1b:A2168T, S:D614G, S:V915I,	
hCoV-19/Mexico/BCN-ALSR-8380/2020	EPI_ISL_1531844	20C	B.1.369	11	5'UTR:C241T, ORF1ab:C715T, ORF1ab:C1059T, ORF1ab:C3037T, ORF1ab:C14408T, ORF1ab:C16260T, S:A23403G, S:T24721C, ORF3a:G25563T, ORF7a:C27476T, N:C28821A, N:G29140T,	7	N:S183Y, N:Q289H, ORF1a:T265I, ORF1b:P314L, ORF3a:Q57H, ORF7a:T28I, S:D614G,	
hCoV-19/Mexico/BCN-ALSR-8381/2020	EPI_ISL_1531845	20A	B.1.243	10	5'UTR:C241T, ORF1ab:C3037T, ORF1ab:C14408T, ORF1ab:G14428T, ORF1ab:C18747T, ORF1ab:A20268G, S:A23403G, S:T24076C, S:C24559T, E:G26262A, N:C28854T,	4	N:S194L, ORF1b:P314L, ORF1b:V321L, S:D614G,	

hCoV-19/Mexico/BCN-ALSR-8382/2020	EPI_ISL_1531846	20A	B.1	11	5'UTR:C241T, ORF1ab:C2037T, ORF1ab:G2528A, ORF1ab:A2806C, ORF1ab:C3037T, ORF1ab:C14408T, ORF1ab:C17439T, ORF1ab:A20268G, S:A23403G, ORF6:C27371T, N:C28657T, N:C28854T,	7	N:S194L, ORF1a:A591V, ORF1a:E755K, ORF1a:E847D, ORF1b:P314L, ORF6:P57L, S:D614G,	
hCoV-19/Mexico/BCN-ALSR-8383/2020	EPI_ISL_1531847	20A	B.1.241	15	5'UTR:C241T, ORF1ab:G713T, ORF1ab:C3037T, ORF1ab:C3736T, ORF1ab:C6786T, ORF1ab:C7768T, ORF1ab:T8634C, ORF1ab:C11575T, ORF1ab:C14408T, ORF1ab:T14907C, ORF1ab:G18148T, ORF1ab:A20268G, S:A23403G, N:C28344T, N:C28677T, N:C28854T,	9	N:T24I, N:T135I, N:S194L, ORF1a:G150C, ORF1a:T2174I, ORF1a:I2790T, ORF1b:P314L, ORF1b:G1561C, S:D614G,	3'UTR:29739-29748
hCoV-19/Mexico/BCN-ALSR-8384/2020	EPI_ISL_1531848	20A	B.1.609	13	5'UTR:C241T, ORF1ab:C3037T, ORF1ab:C4455T, ORF1ab:C4582T, ORF1ab:C8175T, ORF1ab:C12374T, ORF1ab:C14408T, ORF1ab:C16575T, ORF1ab:A20075G, ORF1ab:A20268G, S:G21786T, S:A23403G, ORF7a:C27707T, N:C28603T,	8	ORF1a:A1397V, ORF1a:A2637V, ORF1a:L4037F, ORF1b:P314L, ORF1b:Q2203R, ORF7a:A105V, S:G75V, S:D614G,	
hCoV-19/Mexico/BCN-ALSR-8385/2020	EPI_ISL_1531849	20B	B.1.1	12	5'UTR:C241T, ORF1ab:C1593T, ORF1ab:G2281A, ORF1ab:C3037T, ORF1ab:T8899A, ORF1ab:C14408T, ORF1ab:T19839C, S:A23403G, ORF7a:T27687C, N:G28881A, N:G28882A, N:G28883C, N:G28884T,	6	N:R203K, N:G204L, ORF1a:S443F, ORF1a:N2878K, ORF1b:P314L, S:D614G,	
hCoV-19/Mexico/BCN-ALSR-8386/2020	EPI_ISL_1531850	20A	B.1	10	5'UTR:C241T, ORF1ab:C3037T, ORF1ab:T4690C, ORF1ab:C5892T, ORF1ab:C14408T, ORF1ab:T17793C, ORF1ab:A20268G, S:A23403G, S:T23527C, M:C26542T, N:C28854T,	5	M:T7I, N:S194L, ORF1a:T1876I, ORF1b:P314L, S:D614G,	
hCoV-19/Mexico/BCN-ALSR-8387/2020	EPI_ISL_1531851	20A	B.1.561	19	5'UTR:C241T, ORF1ab:T2148C, ORF1ab:G2516T, ORF1ab:C3037T, ORF1ab:C4543T, ORF1ab:A5999G, ORF1ab:C7851T, ORF1ab:A10323G, ORF1ab:C10798T, ORF1ab:C11866T, ORF1ab:C14408T, ORF1ab:C18693T, ORF1ab:A20268G, S:G21974T, S:A23403G, ORF3a:T25577C, M:G26769A, ORF7b:G27827T, N:C28854T, N:G28975T,	13	M:A83T, N:S194L, N:M234I, ORF1a:L628P, ORF1a:V751L, ORF1a:I1912V, ORF1a:A2529V, ORF1a:K3353R, ORF1b:P314L, ORF3a:I62T, ORF7b:M24I, S:D138Y, S:D614G,	
hCoV-19/Mexico/BCN-ALSR-8388/2020	EPI_ISL_1531852	20A	B.1.241	15	5'UTR:C241T, ORF1ab:G713T, ORF1ab:C3037T, ORF1ab:C3736T, ORF1ab:C6786T, ORF1ab:C7768T, ORF1ab:T8634C, ORF1ab:C11575T, ORF1ab:C14408T, ORF1ab:T14907C, ORF1ab:G18148T, ORF1ab:A20268G, S:A23403G, N:C28344T, N:C28677T, N:C28854T,	9	N:T24I, N:T135I, N:S194L, ORF1a:G150C, ORF1a:T2174I, ORF1a:I2790T, ORF1b:P314L, ORF1b:G1561C, S:D614G,	3'UTR:29739-29748
hCoV-19/Mexico/BCN-ALSR-8389/2020	EPI_ISL_1531853	20A	B.1.245	15	5'UTR:C241T, ORF1ab:A1096G, ORF1ab:G1976T, ORF1ab:C3037T, ORF1ab:C6494A, ORF1ab:C10349T, ORF1ab:C14408T, ORF1ab:G14874T, ORF1ab:C18877T, ORF1ab:G21485T, S:A22320C, S:C22624T, S:A23403G, ORF3a:G25563T, ORF7b:C27765T, N:C28887T,	11	N:T205I, ORF1a:D571Y, ORF1a:L2077I, ORF1a:P3362S, ORF1b:P314L, ORF1b:K469N, ORF1b:S2673I, ORF3a:Q57H, ORF7b:L4F, S:D253A, S:D614G,	
hCoV-19/Mexico/BCN-ALSR-8390/2020	EPI_ISL_1531854	20A	B.1.243	18	5'UTR:C241T, ORF1ab:C1594T, ORF1ab:C3037T, ORF1ab:C7113T, ORF1ab:T13354C, ORF1ab:C14408T, ORF1ab:T15682C, ORF1ab:A20268G, S:A23403G, S:T24076C, S:C24337T, S:G25266T, ORF3a:G25459T, ORF3a:G25757T, ORF3a:A26072G, M:T26669C, M:G26690T, N:C28854T, N:C29095T,	9	N:S194L, ORF1a:T2283I, ORF1b:P314L, ORF1b:Y739H, ORF3a:A23S, ORF3a:R122I, ORF3a:H227R, S:D614G, S:C1235F,	
hCoV-19/Mexico/BCN-ALSR-8391/2020	EPI_ISL_1531855	20A	B.1	14	5'UTR:T209C, 5'UTR:C241T, ORF1ab:C829T, ORF1ab:G2599T, ORF1ab:C3037T, ORF1ab:G3206T, ORF1ab:A7863G, ORF1ab:C14408T, ORF1ab:G14653T, ORF1ab:C18981T, ORF1ab:A20268G, S:A23403G, N:C28732T, N:C28854T, N:C29171T,	8	N:S194L, N:H300Y, ORF1a:L778F, ORF1a:D981Y, ORF1a:K2533R, ORF1b:P314L, ORF1b:V396F, S:D614G,	

hCoV-19/Mexico/BCN-ALSR-8392/2020	EPI_ISL_1531856	20A	B.1	15	5'UTR:C241T, ORF1ab:G625T, ORF1ab:C3037T, ORF1ab:T6376C, ORF1ab:C14408T, ORF1ab:C16626T, ORF1ab:A19020G, ORF1ab:C20016T, ORF1ab:A20268G, S:C21575T, S:C23114T, S:A23403G, ORF3a:G25947T, ORF8:G28079T, N:C28854T, 3'UTR:A29756G,	6	N:S194L, ORF1a:K120N, ORF1b:P314L, ORF3a:Q185H, S:L5F, S:D614G,	
hCoV-19/Mexico/BCN-ALSR-8393/2020	EPI_ISL_1531857	20A	B.1.241	18	5'UTR:C241T, ORF1ab:G713T, ORF1ab:C3037T, ORF1ab:C3736T, ORF1ab:C6786T, ORF1ab:C7768T, ORF1ab:T8634C, ORF1ab:C11575T, ORF1ab:C14408T, ORF1ab:T14907C, ORF1ab:G18148T, ORF1ab:A20268G, S:A23403G, N:C28344T, N:C28677T, N:C28854T, 3'UTR:A29746T, 3'UTR:T29760A, 3'UTR:G29864A,	9	N:T24I, N:T135I, N:S194L, ORF1a:G150C, ORF1a:T2174I, ORF1a:I2790T, ORF1b:P314L, ORF1b:G1561C, S:D614G,	3'UTR:29867-29870,3'UTR:29738, 3'UTR:29751-29755
hCoV-19/Mexico/BCN-ALSR-8394/2020	EPI_ISL_1531858	20A	B.1	15	5'UTR:C241T, ORF1ab:C2037T, ORF1ab:G2528A, ORF1ab:A2806C, ORF1ab:C3037T, ORF1ab:C3619T, ORF1ab:A5064G, ORF1ab:G6446T, ORF1ab:C14408T, ORF1ab:C17375T, ORF1ab:C17439T, ORF1ab:G20145T, ORF1ab:A20268G, S:A23403G, N:C28657T, N:C28854T,	10	N:S194L, ORF1a:A591V, ORF1a:E755K, ORF1a:E847D, ORF1a:D1600G, ORF1a:V2061F, ORF1b:P314L, ORF1b:T1303I, ORF1b:Q2226H, S:D614G,	
hCoV-19/Mexico/BCN-ALSR-8395/2020	EPI_ISL_1531859	20A	B.1.241	16	5'UTR:C241T, ORF1ab:G713T, ORF1ab:C3037T, ORF1ab:C3736T, ORF1ab:C6786T, ORF1ab:C7768T, ORF1ab:T8634C, ORF1ab:C11575T, ORF1ab:C11620T, ORF1ab:C14408T, ORF1ab:T14907C, ORF1ab:G18148T, ORF1ab:A20268G, S:A23403G, N:C28344T, N:C28677T, N:C28854T,	9	N:T24I, N:T135I, N:S194L, ORF1a:G150C, ORF1a:T2174I, ORF1a:I2790T, ORF1b:P314L, ORF1b:G1561C, S:D614G,	3'UTR:29739-29748
hCoV-19/Mexico/BCN-ALSR-8396/2020	EPI_ISL_1531860	20C	B.1	15	5'UTR:C241T, ORF1ab:C1059T, ORF1ab:C3037T, ORF1ab:C3903T, ORF1ab:C9565T, ORF1ab:C14408T, ORF1ab:C19524T, ORF1ab:A20236G, S:G22021A, S:A22069G, S:A23403G, ORF3a:G25563T, ORF7a:G27467A, N:C28868T, N:C28887T, 3'UTR:C29754T,	10	N:P199S, N:T205I, ORF1a:T265I, ORF1a:P1213L, ORF1b:P314L, ORF1b:R2257G, ORF3a:Q57H, ORF7a:R25K, S:M153I, S:D614G,	
hCoV-19/Mexico/BCN-ALSR-8397/2020	EPI_ISL_1531861	20A	B.1.400	9	5'UTR:C241T, ORF1ab:C3037T, ORF1ab:T8779C, ORF1ab:C14408T, ORF1ab:G19969A, ORF1ab:A20268G, S:A23403G, S:G24305A, S:C25153T, N:C28854T,	5	N:S194L, ORF1b:P314L, ORF1b:A2168T, S:D614G, S:V915I,	
hCoV-19/Mexico/BCN-ALSR-8398/2020	EPI_ISL_1531862	20A	B.1.561	15	5'UTR:C241T, ORF1ab:C2485T, ORF1ab:G2516T, ORF1ab:C3037T, ORF1ab:A5999G, ORF1ab:A10323G, ORF1ab:C10798T, ORF1ab:C11866T, ORF1ab:C14408T, ORF1ab:C18693T, ORF1ab:A20268G, S:G21974T, S:A23403G, ORF3a:T25577C, N:C28854T, N:G28975T,	9	N:S194L, N:M234I, ORF1a:V751L, ORF1a:I1912V, ORF1a:K3353R, ORF1b:P314L, ORF3a:I62T, S:D138Y, S:D614G,	
hCoV-19/Mexico/BCN-ALSR-8399/2020	EPI_ISL_1531863	20A	B.1.243	12	5'UTR:C241T, ORF1ab:C442T, ORF1ab:C3037T, ORF1ab:G5378A, ORF1ab:A13276T, ORF1ab:C14408T, ORF1ab:A20268G, S:A23403G, S:T24076C, ORF3a:C25728T, ORF3a:C25797T, ORF8:A27914G, N:C28854T,	4	N:S194L, ORF1a:G1705S, ORF1b:P314L, S:D614G,	
hCoV-19/Mexico/BCN-ALSR-8400/2020	EPI_ISL_1531864	20C	B.1.369	12	5'UTR:C241T, ORF1ab:C715T, ORF1ab:C1059T, ORF1ab:C3037T, ORF1ab:C9474T, ORF1ab:C14408T, ORF1ab:C16260T, S:A23403G, S:T24721C, ORF3a:G25563T, ORF7a:C27476T, N:C28821A, N:G29140T,	8	N:S183Y, N:Q289H, ORF1a:T265I, ORF1a:A3070V, ORF1b:P314L, ORF3a:Q57H, ORF7a:T28I, S:D614G,	
hCoV-19/Mexico/BCN-ALSR-8401/2020	EPI_ISL_1531865	20A	B.1.241	13	5'UTR:C241T, ORF1ab:C3037T, ORF1ab:C6786T, ORF1ab:C7768T, ORF1ab:T8634C, ORF1ab:C11575T, ORF1ab:C14408T, ORF1ab:T14907C, ORF1ab:G18148T, ORF1ab:A20268G, S:A23403G, N:C28344T, N:C28677T, N:C28854T,	8	N:T24I, N:T135I, N:S194L, ORF1a:T2174I, ORF1a:I2790T, ORF1b:P314L, ORF1b:G1561C, S:D614G,	

hCoV-19/Mexico/BCN-ALSR-8402/2020	EPI_ISL_1531866	20B	B.1.1.8	13	5'UTR:C241T, ORF1ab:C3037T, ORF1ab:C9988T, ORF1ab:G11417T, ORF1ab:C11747T, ORF1ab:C12488T, ORF1ab:C14408T, S:G21785C, S:G22017T, S:A23403G, ORF3a:C25528T, N:G28881A, N:G28882A, N:G28883C,	9	N:R203K, N:G204R, ORF1a:V3718F, ORF1a:P4075S, ORF1b:P314L, ORF3a:L46F, S:G75R, S:W152L, S:D614G,	
hCoV-19/Mexico/BCN-ALSR-8403/2020	EPI_ISL_1531867	20A	B.1.232	13	5'UTR:C241T, ORF1ab:C3037T, ORF1ab:T5804C, ORF1ab:C5849T, ORF1ab:C6285T, ORF1ab:C6706T, ORF1ab:C14408T, ORF1ab:C16092T, ORF1ab:A20268G, S:A23403G, E:G26325A, ORF8:G27987T, N:C28291T, N:C28854T,	8	N:S194L, ORF1a:C1847R, ORF1a:P1862S, ORF1a:T2007I, ORF1b:P314L, ORF8:V32L, ORF9b:P3L, S:D614G,	
hCoV-19/Mexico/BCN-ALSR-8404/2020	EPI_ISL_1531868	20A	B.1	10	5'UTR:C241T, ORF1ab:C3037T, ORF1ab:C10228T, ORF1ab:C14262T, ORF1ab:C14408T, ORF1ab:C18129T, ORF1ab:A20268G, S:G21724T, S:A23403G, N:C28854T, ORF10:A29647G,	4	N:S194L, ORF1b:P314L, S:L54F, S:D614G,	
hCoV-19/Mexico/BCN-ALSR-8405/2020	EPI_ISL_1531869	20B	B.1.1.432	16	5'UTR:C241T, ORF1ab:C3037T, ORF1ab:A6985T, ORF1ab:C9319T, ORF1ab:C10605T, ORF1ab:T10654C, ORF1ab:C12412T, ORF1ab:T14313C, ORF1ab:C14408T, ORF1ab:C16887T, S:A23403G, ORF3a:C25613T, ORF3a:G25912T, N:G28881A, N:G28882A, N:G28883C, N:T29317C,	7	N:R203K, N:G204R, ORF1a:P3447L, ORF1b:P314L, ORF3a:S74F, ORF3a:G174C, S:D614G,	
hCoV-19/Mexico/BCN-ALSR-8406/2020	EPI_ISL_1531870	20A	B.1.241	15	5'UTR:C241T, ORF1ab:G713T, ORF1ab:C3037T, ORF1ab:C3736T, ORF1ab:C6786T, ORF1ab:C7768T, ORF1ab:T8634C, ORF1ab:C11575T, ORF1ab:C14408T, ORF1ab:T14907C, ORF1ab:G18148T, ORF1ab:A20268G, S:A23403G, N:C28344T, N:C28677T, N:C28854T,	9	N:T24I, N:T135I, N:S194L, ORF1a:G150C, ORF1a:T2174I, ORF1a:I2790T, ORF1b:P314L, ORF1b:G1561C, S:D614G,	3'UTR:29739-29748
hCoV-19/Mexico/BCN-ALSR-8407/2020	EPI_ISL_1531871	20A	B.1.241	16	5'UTR:C241T, ORF1ab:C3037T, ORF1ab:C6786T, ORF1ab:C7768T, ORF1ab:T8634C, ORF1ab:C11575T, ORF1ab:C14408T, ORF1ab:T14907C, ORF1ab:G18148T, ORF1ab:G18225T, ORF1ab:A20268G, S:A22528T, S:A23403G, ORF7b:C27883T, N:C28344T, N:C28677T, N:C28854T,	10	N:T24I, N:T135I, N:S194L, ORF1a:T2174I, ORF1a:I2790T, ORF1b:P314L, ORF1b:G1561C, ORF1b:M1586I, ORF7b:A43V, S:D614G,	
hCoV-19/Mexico/BCN-ALSR-8408/2020	EPI_ISL_1531872	20A	B.1	10	5'UTR:A129G, 5'UTR:C241T, ORF1ab:C3037T, ORF1ab:T9157C, ORF1ab:C14408T, ORF1ab:G19684T, ORF1ab:A20268G, ORF1ab:C20483T, S:A23403G, ORF6:C27294T, N:C28854T,	5	N:S194L, ORF1b:P314L, ORF1b:V2073L, ORF1b:S2339F, S:D614G,	
hCoV-19/Mexico/BCN-ALSR-8409/2020	EPI_ISL_1531873	20A	B.1.241	15	5'UTR:C241T, ORF1ab:G713T, ORF1ab:C3037T, ORF1ab:C3736T, ORF1ab:C6786T, ORF1ab:C7768T, ORF1ab:T8634C, ORF1ab:C11575T, ORF1ab:C14408T, ORF1ab:T14907C, ORF1ab:G18148T, ORF1ab:A20268G, S:A23403G, N:C28344T, N:C28677T, N:C28854T,	9	N:T24I, N:T135I, N:S194L, ORF1a:G150C, ORF1a:T2174I, ORF1a:I2790T, ORF1b:P314L, ORF1b:G1561C, S:D614G,	3'UTR:29739-29748
hCoV-19/Mexico/BCN-ALSR-8410/2020	EPI_ISL_1531874	20A	B.1	9	5'UTR:C241T, ORF1ab:C1437T, ORF1ab:C3037T, ORF1ab:G7756T, ORF1ab:T11032A, ORF1ab:C14408T, ORF1ab:A20268G, S:A21766C, S:A23403G, N:C28854T,	5	N:S194L, ORF1a:S391F, ORF1a:K2497N, ORF1b:P314L, S:D614G,	
hCoV-19/Mexico/BCN-ALSR-8411/2020	EPI_ISL_1531875	20A	B.1.241	18	5'UTR:C241T, ORF1ab:G713T, ORF1ab:C3037T, ORF1ab:C3736T, ORF1ab:C6786T, ORF1ab:C7768T, ORF1ab:T8634C, ORF1ab:G11083T, ORF1ab:C11575T, ORF1ab:C14178T, ORF1ab:C14408T, ORF1ab:G14712T, ORF1ab:T14907C, ORF1ab:G18148T, ORF1ab:A20268G, S:A23403G, N:C28344T, N:C28677T, N:C28854T,	10	N:T24I, N:T135I, N:S194L, ORF1a:G150C, ORF1a:T2174I, ORF1a:I2790T, ORF1a:L3606F, ORF1b:P314L, ORF1b:G1561C, S:D614G,	3'UTR:29739-29748
hCoV-19/Mexico/BCN-ALSR-8412/2020	EPI_ISL_1531876	20A	B.1.243	18	5'UTR:C241T, ORF1ab:G2516T, ORF1ab:C3037T, ORF1ab:C7113T, ORF1ab:T13354C, ORF1ab:C14408T, ORF1ab:A20268G, S:T23159C, S:A23403G, S:T24076C, S:C24337T, S:G25266T, ORF3a:G25459T, ORF3a:G25757T, ORF3a:A26072G, M:T26669C, M:G26690T, N:C28854T, N:C29095T,	9	N:S194L, ORF1a:V751L, ORF1a:T2283I, ORF1b:P314L, ORF3a:A23S, ORF3a:R122I, ORF3a:H227R, S:D614G, S:C1235F,	

hCoV-19/Mexico/BCN-ALSR-8413/2020	EPI_ISL_1531877	20A	B.1.397	10	5'UTR:C241T, ORF1ab:G1135T, ORF1ab:A2444G, ORF1ab:C3037T, ORF1ab:C6593T, ORF1ab:C12559A, ORF1ab:C14408T, ORF1ab:A20268G, S:A23403G, M:A26864T, N:C28854T,	6	N:S194L, ORF1a:K290N, ORF1a:T727A, ORF1a:P2110S, ORF1b:P314L, S:D614G,
hCoV-19/Mexico/BCN-ALSR-8414/2020	EPI_ISL_1531878	20A	B.1.561	16	5'UTR:C241T, ORF1ab:G2516T, ORF1ab:C3037T, ORF1ab:A5999G, ORF1ab:T6892G, ORF1ab:A10323G, ORF1ab:G12188T, ORF1ab:A13267T, ORF1ab:C14408T, ORF1ab:C18693T, ORF1ab:A20268G, S:G21974T, S:A23403G, ORF3a:T25577C, ORF3a:C25665T, N:C28854T, N:G28975T,	11	N:S194L, N:M234I, ORF1a:V751L, ORF1a:I1912V, ORF1a:F2209L, ORF1a:K3353R, ORF1a:V3975F, ORF1b:P314L, ORF3a:I62T, S:D138Y, S:D614G,
hCoV-19/Mexico/BCN-ALSR-8415/2020	EPI_ISL_1531879	20A	B.1.400	14	5'UTR:C241T, ORF1ab:G1126T, ORF1ab:C3037T, ORF1ab:G13835T, ORF1ab:C14408T, ORF1ab:A16417G, ORF1ab:G19009T, ORF1ab:G19735T, ORF1ab:G19969A, ORF1ab:A20268G, ORF1ab:G21204T, S:A23403G, S:C24334T, ORF3a:C25731T, N:C28854T,	10	N:S194L, ORF1a:R287S, ORF1b:R123M, ORF1b:P314L, ORF1b:T984A, ORF1b:D1848Y, ORF1b:D2090Y, ORF1b:A2168T, ORF1b:K2579N, S:D614G,
hCoV-19/Mexico/BCN-ALSR-8416/2020	EPI_ISL_1531880	20A	B.1.561	21	5'UTR:C241T, ORF1ab:C936T, ORF1ab:G2516T, ORF1ab:C3037T, ORF1ab:A5999G, ORF1ab:A10323G, ORF1ab:C10798T, ORF1ab:C11866T, ORF1ab:C13378T, ORF1ab:T14211C, ORF1ab:C14408T, ORF1ab:C18693T, ORF1ab:C19164T, ORF1ab:A20268G, S:G21974T, S:A23403G, S:C24031T, ORF3a:C25521T, ORF3a:T25577C, ORF6:C27223T, N:C28854T, N:G28975T,	11	N:S194L, N:M234I, ORF1a:T224I, ORF1a:V751L, ORF1a:I1912V, ORF1a:K3353R, ORF1b:P314L, ORF3a:I62T, ORF6:Q8*, S:D138Y, S:D614G,
hCoV-19/Mexico/BCN-ALSR-8417/2020	EPI_ISL_1531881	20A	B.1.241	13	5'UTR:C241T, ORF1ab:C3037T, ORF1ab:C6786T, ORF1ab:C7768T, ORF1ab:T8634C, ORF1ab:C11575T, ORF1ab:C14408T, ORF1ab:T14907C, ORF1ab:G18148T, ORF1ab:A20268G, S:A23403G, N:C28344T, N:C28677T, N:C28854T,	8	N:T24I, N:T135I, N:S194L, ORF1a:T2174I, ORF1a:I2790T, ORF1b:P314L, ORF1b:G1561C, S:D614G,
hCoV-19/Mexico/BCN-ALSR-8418/2020	EPI_ISL_1531882	20A	B.1.232	16	5'UTR:C241T, ORF1ab:T2998C, ORF1ab:C3037T, ORF1ab:T5804C, ORF1ab:C5849T, ORF1ab:C6285T, ORF1ab:C6706T, ORF1ab:C14408T, ORF1ab:C16092T, ORF1ab:A20268G, S:A23403G, S:G25244T, S:G25249T, E:G26325A, ORF8:G27987T, N:C28291T, N:C28854T,	10	N:S194L, ORF1a:C1847R, ORF1a:P1862S, ORF1a:T2007I, ORF1b:P314L, ORF8:V32L, ORF9b:P3L, S:D614G, S:V1228L, S:M1229I,
hCoV-19/Mexico/BCN-ALSR-8419/2020	EPI_ISL_1531883	20A	B.1.232	14	5'UTR:C241T, ORF1ab:C3037T, ORF1ab:T5804C, ORF1ab:C5849T, ORF1ab:C6285T, ORF1ab:C6706T, ORF1ab:C14408T, ORF1ab:C16092T, ORF1ab:A20268G, S:A23403G, E:G26325A, M:C26873T, ORF8:G27987T, N:C28291T, N:C28854T,	8	N:S194L, ORF1a:C1847R, ORF1a:P1862S, ORF1a:T2007I, ORF1b:P314L, ORF8:V32L, ORF9b:P3L, S:D614G,
hCoV-19/Mexico/BCN-ALSR-8420/2020	EPI_ISL_1531884	20A	B.1	9	5'UTR:C241T, ORF1ab:C1437T, ORF1ab:C3037T, ORF1ab:T11032A, ORF1ab:C14408T, ORF1ab:A20268G, S:A21766C, S:A23403G, N:C28854T, ORF10:C29640T,	4	N:S194L, ORF1a:S391F, ORF1b:P314L, S:D614G,
hCoV-19/Mexico/BCN-ALSR-8421/2020	EPI_ISL_1531885	20A	B.1.399	12	5'UTR:C241T, ORF1ab:G1738T, ORF1ab:C3037T, ORF1ab:C5497T, ORF1ab:C9246T, ORF1ab:C14408T, ORF1ab:A20268G, S:G22706T, S:C23248T, S:A23403G, S:G24193T, N:C28854T, N:C28887T,	6	N:S194L, N:T205I, ORF1a:A2994V, ORF1b:P314L, S:V382L, S:D614G,
hCoV-19/Mexico/BCN-ALSR-8422/2020	EPI_ISL_1531886	20A	B.1	13	5'UTR:C241T, ORF1ab:C842T, ORF1ab:C3037T, ORF1ab:G6056T, ORF1ab:C9246T, ORF1ab:C14408T, ORF1ab:C18129T, ORF1ab:C18508T, ORF1ab:A20268G, S:G21724T, S:A23403G, N:C28846T, N:C28854T, ORF10:A29647G,	8	N:S194L, ORF1a:P193S, ORF1a:V1931L, ORF1a:A2994V, ORF1b:P314L, ORF1b:L1681F, S:L54F, S:D614G,

hCoV-19/Mexico/BCN-ALSR-8423/2020	EPI_ISL_1531887	20A	B.1.561	16	5'UTR:C241T, ORF1ab:G2516T, ORF1ab:C3037T, ORF1ab:A5999G, ORF1ab:A10323G, ORF1ab:C14408T, ORF1ab:C18693T, ORF1ab:A20268G, S:G21608T, S:C21614T, S:G21974T, S:A23403G, S:C23758T, ORF3a:T25577C, ORF3a:C26192T, N:C28854T, N:G28975T,	12	N:S194L, N:M234I, ORF1a:V751L, ORF1a:I1912V, ORF1a:K3353R, ORF1b:P314L, ORF3a:I62T, ORF3a:P267L, S:V16F, S:L18F, S:D138Y, S:D614G,
hCoV-19/Mexico/BCN-ALSR-8424/2020	EPI_ISL_1531888	20A	B.1.400	12	5'UTR:C241T, ORF1ab:C2536T, ORF1ab:C3037T, ORF1ab:C5822T, ORF1ab:T8779C, ORF1ab:C14408T, ORF1ab:G19969A, ORF1ab:A20268G, S:G23401T, S:A23403G, S:G24305A, S:C25153T, N:C28854T,	7	N:S194L, ORF1a:L1853F, ORF1b:P314L, ORF1b:A2168T, S:Q613H, S:D614G, S:V915I,
hCoV-19/Mexico/BCN-ALSR-8425/2020	EPI_ISL_1531889	20A	B.1.399	13	5'UTR:C21T, 5'UTR:C241T, ORF1ab:C3037T, ORF1ab:G6410A, ORF1ab:G8017T, ORF1ab:C9711T, ORF1ab:C14408T, ORF1ab:C15240T, ORF1ab:C20104T, ORF1ab:A20268G, S:A23403G, N:C28854T, N:C28887T, ORF10:T29559C,	7	N:S194L, N:T205I, ORF1a:E2049K, ORF1a:S3149F, ORF1b:P314L, ORF1b:L2213F, S:D614G,
hCoV-19/Mexico/BCN-ALSR-8426/2020	EPI_ISL_1531890	20A	B.1.609	18	5'UTR:C241T, ORF1ab:C3037T, ORF1ab:C4582T, ORF1ab:T12877C, ORF1ab:C13119T, ORF1ab:C14408T, ORF1ab:C16041T, ORF1ab:C16726T, ORF1ab:T20012C, ORF1ab:A20268G, S:A23403G, S:C24138T, S:C24382T, ORF3a:G25907C, E:G26391T, ORF7b:C27765T, ORF8:G28141T, N:C28887T, ORF10:C29666T,	10	N:T205I, ORF1a:A4285V, ORF1b:P314L, ORF1b:H1087Y, ORF1b:V2182A, ORF3a:G172A, ORF7b:L4F, ORF8:C83F, S:D614G, S:T859I,
hCoV-19/Mexico/BCN-ALSR-8427/2020	EPI_ISL_1531891	20A	B.1.400	12	5'UTR:C241T, ORF1ab:C1385T, ORF1ab:C3037T, ORF1ab:C4276T, ORF1ab:C11224T, ORF1ab:C14408T, ORF1ab:C15960T, ORF1ab:G19969A, ORF1ab:A20268G, S:A23403G, ORF7b:G27798T, N:G28851T, N:C28854T,	7	N:S193I, N:S194L, ORF1a:H374Y, ORF1b:P314L, ORF1b:A2168T, ORF7b:A15S, S:D614G,
hCoV-19/Mexico/BCN-ALSR-8428/2020	EPI_ISL_1531892	20A	B.1.561	18	5'UTR:C241T, ORF1ab:G2516T, ORF1ab:C2925T, ORF1ab:C3037T, ORF1ab:C4543T, ORF1ab:A5999G, ORF1ab:C7851T, ORF1ab:A10323G, ORF1ab:C10798T, ORF1ab:C11866T, ORF1ab:C14408T, ORF1ab:C18693T, ORF1ab:A20268G, S:G21974T, S:A23403G, ORF3a:T25577C, ORF7b:G27827T, N:C28854T, N:G28975T,	12	N:S194L, N:M234I, ORF1a:V751L, ORF1a:S887F, ORF1a:I1912V, ORF1a:A2529V, ORF1a:K3353R, ORF1b:P314L, ORF3a:I62T, ORF7b:M24I, S:D138Y, S:D614G,
hCoV-19/Mexico/BCN-ALSR-8429/2020	EPI_ISL_1531893	20B	B.1.1.222	19	5'UTR:A215G, 5'UTR:C241T, ORF1ab:T2533C, ORF1ab:C2773T, ORF1ab:C3037T, ORF1ab:T9304C, ORF1ab:C13795A, ORF1ab:C14408T, ORF1ab:A17015G, ORF1ab:C17135T, ORF1ab:T19839C, ORF1ab:A20294G, S:C21805T, S:A23403G, S:A23756G, N:C28399T, N:G28851C, N:G28881A, N:G28882A, N:G28883C,	11	N:S193T, N:R203K, N:G204R, ORF1b:L110I, ORF1b:P314L, ORF1b:D1183G, ORF1b:P1223L, ORF1b:Y2276C, ORF9b:P39L, S:D614G, S:T732A,
hCoV-19/Mexico/BCN-ALSR-8430/2020	EPI_ISL_1531894	20A	B.1.561	15	5'UTR:C241T, ORF1ab:G2516T, ORF1ab:C3037T, ORF1ab:A5999G, ORF1ab:A10323G, ORF1ab:C14408T, ORF1ab:C18693T, ORF1ab:A20268G, S:G21974T, S:A23403G, S:C23664T, S:C23758T, S:G23792A, ORF3a:T25577C, N:C28854T, N:G28975T,	11	N:S194L, N:M234I, ORF1a:V751L, ORF1a:I1912V, ORF1a:K3353R, ORF1b:P314L, ORF3a:I62T, S:D138Y, S:D614G, S:A701V, S:G744S,
hCoV-19/Mexico/BCN-ALSR-8431/2020	EPI_ISL_1531895	20B	B.1.1.432	31	5'UTR:C241T, ORF1ab:C3037T, ORF1ab:G3122T, ORF1ab:A3350G, ORF1ab:C6449T, ORF1ab:A6985T, ORF1ab:G7037T, ORF1ab:C7834T, ORF1ab:C8139T, ORF1ab:C9319T, ORF1ab:C11779T, ORF1ab:C11956T, ORF1ab:C12412T, ORF1ab:C12784T, ORF1ab:T14313C, ORF1ab:G14316C, ORF1ab:C14408T, ORF1ab:G15380T, ORF1ab:G17278T, ORF1ab:G20995T, ORF1ab:C21304A, ORF1ab:G21305A, S:C21812T, S:A23403G, S:G24368T, ORF3a:C25613T, ORF3a:G25912T, ORF3a:G26062T, N:G28881A, N:G28882A, N:G28883C, N:T29317C,	18	N:R203K, N:G204R, ORF1a:D953Y, ORF1a:S1029G, ORF1a:L2062F, ORF1a:G2258C, ORF1a:S2625F, ORF1b:Q283H, ORF1b:P314L, ORF1b:S638I, ORF1b:V1271L, ORF1b:G2510C, ORF1b:R2613N, ORF3a:S74F, ORF3a:G174C, ORF3a:G224C, S:D614G, S:D936Y,

hCoV-19/Mexico/BCN-ALSR-8432/2020	EPI_ISL_1531896	20A	B.1.243	15	5'UTR:C21T, 5'UTR:C241T, ORF1ab:C3037T, ORF1ab:C8025T, ORF1ab:C9223T, ORF1ab:G14346A, ORF1ab:C14408T, ORF1ab:G15906T, ORF1ab:A20268G, ORF1ab:A21258G, S:G22132T, S:A23403G, S:T24076C, E:T26304C, ORF7a:C27741T, N:C28854T,	6	N:S194L, ORF1a:A2587V, ORF1b:P314L, ORF1b:Q813H, S:R190S, S:D614G,
hCoV-19/Mexico/BCN-ALSR-8433/2020	EPI_ISL_1531897	20A	B.1	13	5'UTR:G208T, 5'UTR:C241T, ORF1ab:C3037T, ORF1ab:C14408T, ORF1ab:A20268G, ORF1ab:G20709T, S:A23403G, S:G23522A, S:C24106T, S:C24181T, S:C25162A, S:C25163A, M:A26927G, N:C28854T,	6	N:S194L, ORF1b:P314L, ORF1b:M2414I, S:D614G, S:E654K, S:Q1201K,
hCoV-19/Mexico/BCN-ALSR-8434/2020	EPI_ISL_1531898	20A	B.1	11	5'UTR:C241T, ORF1ab:C2836T, ORF1ab:C3037T, ORF1ab:C10228T, ORF1ab:C14262T, ORF1ab:C14408T, ORF1ab:C18129T, ORF1ab:A20268G, S:G21724T, S:A23403G, N:C28854T, ORF10:A29647G,	4	N:S194L, ORF1b:P314L, S:L54F, S:D614G,
hCoV-19/Mexico/BCN-ALSR-8435/2020	EPI_ISL_1531899	20A	B.1.561	20	5'UTR:C241T, ORF1ab:C936T, ORF1ab:G2516T, ORF1ab:C3037T, ORF1ab:A5999G, ORF1ab:A10323G, ORF1ab:C10798T, ORF1ab:C11866T, ORF1ab:C13378T, ORF1ab:C14408T, ORF1ab:C18693T, ORF1ab:C19164T, ORF1ab:A20268G, S:G21974T, S:A23403G, S:C24031T, ORF3a:C25521T, ORF3a:T25577C, ORF6:C27223T, N:C28854T, N:G28975T,	11	N:S194L, N:M234I, ORF1a:T224I, ORF1a:V751L, ORF1a:I1912V, ORF1a:K3353R, ORF1b:P314L, ORF3a:I62T, ORF6:Q8*, S:D138Y, S:D614G,
hCoV-19/Mexico/BCN-ALSR-8436/2020	EPI_ISL_1531900	20A	B.1	14	5'UTR:T209C, 5'UTR:C241T, ORF1ab:C829T, ORF1ab:G2599T, ORF1ab:C3037T, ORF1ab:G3206T, ORF1ab:A7863G, ORF1ab:C14408T, ORF1ab:G14653T, ORF1ab:C18981T, ORF1ab:A20268G, S:A23403G, N:C28732T, N:C28854T, N:C29171T,	8	N:S194L, N:H300Y, ORF1a:L778F, ORF1a:D981Y, ORF1a:K2533R, ORF1b:P314L, ORF1b:V396F, S:D614G,
hCoV-19/Mexico/BCN-ALSR-8437/2020	EPI_ISL_1531901	20A	B.1	13	5'UTR:C241T, ORF1ab:C2037T, ORF1ab:G2528A, ORF1ab:A2806C, ORF1ab:C3037T, ORF1ab:C8311T, ORF1ab:C14408T, ORF1ab:C17439T, ORF1ab:A20268G, ORF1ab:C20930T, S:A23403G, ORF6:C27371T, N:C28657T, N:C28854T,	8	N:S194L, ORF1a:A591V, ORF1a:E755K, ORF1a:E847D, ORF1b:P314L, ORF1b:T2488M, ORF6:P57L, S:D614G,
hCoV-19/Mexico/BCN-ALSR-8438/2020	EPI_ISL_1531902	21C (Epsilon)	B.1.427	17	5'UTR:C241T, ORF1ab:C1059T, ORF1ab:C3037T, ORF1ab:C4683T, ORF1ab:C6120T, ORF1ab:G9738C, ORF1ab:G13713A, ORF1ab:C14408T, ORF1ab:G17014T, S:G21600T, S:G22018T, S:T22917G, S:A23403G, ORF3a:G25563T, M:C26681T, ORF8:A28272T, N:C28887T, N:C29362T,	12	N:T205I, ORF1a:T265I, ORF1a:A1473V, ORF1a:S1952L, ORF1a:S3158T, ORF1b:P314L, ORF1b:D1183Y, ORF3a:Q57H, S:S13I, S:W152C, S:L452R, S:D614G,
hCoV-19/Mexico/BCN-ALSR-8439/2020	EPI_ISL_1531903	20A	B.1.561	18	5'UTR:C241T, ORF1ab:T1432C, ORF1ab:G2516T, ORF1ab:C3037T, ORF1ab:G4486T, ORF1ab:A5999G, ORF1ab:T7357G, ORF1ab:A10323G, ORF1ab:C10798T, ORF1ab:C11866T, ORF1ab:C14408T, ORF1ab:C18693T, ORF1ab:A20268G, ORF1ab:A20830G, S:G21974T, S:A23403G, ORF3a:T25577C, N:C28854T, N:G28975T,	11	N:S194L, N:M234I, ORF1a:V751L, ORF1a:K1407N, ORF1a:I1912V, ORF1a:K3353R, ORF1b:P314L, ORF1b:T2455A, ORF3a:I62T, S:D138Y, S:D614G,
hCoV-19/Mexico/BCN-ALSR-8440/2020	EPI_ISL_1531904	20A	B.1.561	17	5'UTR:C241T, ORF1ab:G2516T, ORF1ab:C3037T, ORF1ab:G4486T, ORF1ab:C5812T, ORF1ab:A5999G, ORF1ab:T7357G, ORF1ab:A10323G, ORF1ab:C10798T, ORF1ab:C11866T, ORF1ab:C14408T, ORF1ab:C18693T, ORF1ab:A20268G, S:G21974T, S:A23403G, ORF3a:T25577C, N:C28854T, N:G28975T,	10	N:S194L, N:M234I, ORF1a:V751L, ORF1a:K1407N, ORF1a:I1912V, ORF1a:K3353R, ORF1b:P314L, ORF3a:I62T, S:D138Y, S:D614G,

hCoV-19/Mexico/BCN-ALSR-8441/2020	EPI_ISL_1531905	20A	B.1.561	20	5'UTR:C241T, ORF1ab:C1282T, ORF1ab:G2516T, ORF1ab:C3037T, ORF1ab:A5999G, ORF1ab:A10323G, ORF1ab:C10798T, ORF1ab:C11200T, ORF1ab:C11866T, ORF1ab:C13378T, ORF1ab:C14408T, ORF1ab:C18693T, ORF1ab:C19164T, ORF1ab:A20268G, S:G21974T, S:A23403G, ORF3a:T25577C, N:G28300T, N:C28854T, N:G28975T, N:C29200T,	11	N:Q9H, N:S194L, N:M234I, ORF1a:V751L, ORF1a:I1912V, ORF1a:K3353R, ORF1b:P314L, ORF3a:I62T, ORF9b:S6I, S:D138Y, S:D614G,
hCoV-19/Mexico/BCN-ALSR-8442/2020	EPI_ISL_1531906	20A	B.1	14	5'UTR:G208T, 5'UTR:C241T, ORF1ab:C3037T, ORF1ab:G4185C, ORF1ab:C14408T, ORF1ab:A20268G, ORF1ab:G20709T, S:A23403G, S:G23522A, S:C24106T, S:C24181T, S:C25162A, S:C25163A, M:A26927G, N:C28854T,	7	N:S194L, ORF1a:G1307A, ORF1b:P314L, ORF1b:M2414I, S:D614G, S:E654K, S:Q1201K,
hCoV-19/Mexico/BCN-ALSR-8443/2020	EPI_ISL_1531907	20A	B.1.232	16	5'UTR:C241T, ORF1ab:C1684T, ORF1ab:T1847C, ORF1ab:C3037T, ORF1ab:T5804C, ORF1ab:C5849T, ORF1ab:C6285T, ORF1ab:C6706T, ORF1ab:C14408T, ORF1ab:C16092T, ORF1ab:T19449A, ORF1ab:A20268G, S:A23403G, E:G26325A, ORF8:G27987T, N:C28291T, N:C28854T,	9	N:S194L, ORF1a:S528P, ORF1a:C1847R, ORF1a:P1862S, ORF1a:T2007I, ORF1b:P314L, ORF8:V32L, ORF9b:P3L, S:D614G,
hCoV-19/Mexico/BCN-ALSR-8444/2020	EPI_ISL_1531908	20A	B.1	10	5'UTR:C241T, ORF1ab:C3037T, ORF1ab:C14408T, ORF1ab:C18129T, ORF1ab:C19586T, ORF1ab:A20268G, S:G21724T, S:G22992A, S:A23403G, N:C28854T, ORF10:A29647G,	6	N:S194L, ORF1b:P314L, ORF1b:T2040I, S:L54F, S:S477N, S:D614G,
hCoV-19/Mexico/BCN-ALSR-8445/2020	EPI_ISL_1531909	20A	B.1	14	5'UTR:G208T, 5'UTR:C241T, ORF1ab:C3037T, ORF1ab:G4185C, ORF1ab:C14408T, ORF1ab:A20268G, ORF1ab:G20709T, S:A23403G, S:G23522A, S:C24106T, S:C24181T, S:C25162A, S:C25163A, M:A26927G, N:C28854T,	7	N:S194L, ORF1a:G1307A, ORF1b:P314L, ORF1b:M2414I, S:D614G, S:E654K, S:Q1201K,
hCoV-19/Mexico/BCN-ALSR-8446/2020	EPI_ISL_1531910	20A	B.1.561	15	5'UTR:C241T, ORF1ab:G2516T, ORF1ab:C3037T, ORF1ab:A5999G, ORF1ab:A10323G, ORF1ab:C14408T, ORF1ab:C18693T, ORF1ab:A20268G, S:G21974T, S:A23403G, S:C23664T, S:C23758T, S:G23792A, ORF3a:T25577C, N:C28854T, N:G28975T,	11	N:S194L, N:M234I, ORF1a:V751L, ORF1a:I1912V, ORF1a:K3353R, ORF1b:P314L, ORF3a:I62T, S:D138Y, S:D614G, S:A701V, S:G744S,
hCoV-19/Mexico/BCN-ALSR-8447/2020	EPI_ISL_1531911	20A	B.1	15	5'UTR:G208T, 5'UTR:C241T, ORF1ab:C3037T, ORF1ab:G4185C, ORF1ab:C14408T, ORF1ab:A20268G, ORF1ab:G20709T, S:A23403G, S:G23522A, S:C24106T, S:C24181T, S:C25162A, S:C25163A, ORF3a:T25729C, M:A26927G, N:C28854T,	8	N:S194L, ORF1a:G1307A, ORF1b:P314L, ORF1b:M2414I, ORF3a:Y113H, S:D614G, S:E654K, S:Q1201K,
hCoV-19/Mexico/BCN-ALSR-8448/2020	EPI_ISL_1531912	20A	B.1.232	17	5'UTR:C241T, ORF1ab:C3037T, ORF1ab:T5804C, ORF1ab:C5849T, ORF1ab:C6285T, ORF1ab:C6706T, ORF1ab:C7164T, ORF1ab:C14408T, ORF1ab:C16092T, ORF1ab:A20268G, S:A23403G, S:C24099T, S:G24998C, ORF3a:A26226G, E:G26325A, ORF8:G27987T, N:C28291T, N:C28854T,	11	N:S194L, ORF1a:C1847R, ORF1a:P1862S, ORF1a:T2007I, ORF1a:T2300I, ORF1b:P314L, ORF8:V32L, ORF9b:P3L, S:D614G, S:A846V, S:D1146H,
hCoV-19/Mexico/BCN-ALSR-8450/2020	EPI_ISL_1531913	20A	B.1	10	5'UTR:C241T, ORF1ab:A2806C, ORF1ab:C3037T, ORF1ab:C14408T, ORF1ab:T14619C, ORF1ab:A20268G, S:A23403G, S:C23422T, N:C28854T, ORF10:A29621G, 3'UTR:G29757T,	4	N:S194L, ORF1a:E847D, ORF1b:P314L, S:D614G,
hCoV-19/Mexico/BCN-ALSR-8451/2020	EPI_ISL_1531914	20A	B.1.243	20	5'UTR:C241T, ORF1ab:A696G, ORF1ab:C3037T, ORF1ab:C7113T, ORF1ab:C11139T, ORF1ab:T13354C, ORF1ab:C14408T, ORF1ab:A20268G, S:T23159C, S:A23403G, S:T24076C, S:C24337T, S:G25266T, ORF3a:G25459T, ORF3a:G25757T, ORF3a:A26072G, M:T26669C, M:G26690T, M:A26847G, N:C28854T, N:C29095T,	11	M:M109V, N:S194L, ORF1a:D144G, ORF1a:T2283I, ORF1a:A3625V, ORF1b:P314L, ORF3a:A23S, ORF3a:R122I, ORF3a:H227R, S:D614G, S:C1235F,
hCoV-19/Mexico/BCN-ALSR-8452/2020	EPI_ISL_1531915	20A	B.1	14	5'UTR:C21T, 5'UTR:C241T, ORF1ab:C1997T, ORF1ab:A2406G, ORF1ab:C2695T, ORF1ab:C3037T, ORF1ab:C13554T, ORF1ab:C14408T, ORF1ab:C18656T, ORF1ab:A20268G, S:C21727T, S:A23403G, ORF3a:T26007C, N:C28854T, N:A29110G,	5	N:S194L, ORF1a:K714R, ORF1b:P314L, ORF1b:T1730I, S:D614G,

hCoV-19/Mexico/BCN-ALSR-8453/2020	EPI_ISL_1531916	20A	B.1	14	5'UTR:G208T, 5'UTR:C241T, ORF1ab:C3037T, ORF1ab:G4185C, ORF1ab:C14408T, ORF1ab:A20268G, ORF1ab:G20709T, S:A23403G, S:G23522A, S:C24106T, S:C24181T, S:C25162A, S:C25163A, M:A26927G, N:C28854T,	7	N:S194L, ORF1a:G1307A, ORF1b:P314L, ORF1b:M2414I, S:D614G, S:E654K, S:Q1201K,
hCoV-19/Mexico/BCN-ALSR-8454/2020	EPI_ISL_1531917	20A	B.1.243	23	5'UTR:T221C, 5'UTR:C241T, ORF1ab:C1915T, ORF1ab:G2001A, ORF1ab:C3037T, ORF1ab:C7113T, ORF1ab:C10448T, ORF1ab:C11637A, ORF1ab:T13354C, ORF1ab:C14408T, ORF1ab:G19735C, ORF1ab:A20268G, S:A23403G, S:T24076C, S:C24337T, S:G25266T, ORF3a:G25459T, ORF3a:G25757T, ORF3a:A26072G, M:T26669C, M:G26690T, N:C28744T, N:C28854T, N:C29095T,	12	N:S194L, ORF1a:R579K, ORF1a:T2283I, ORF1a:P3395S, ORF1a:T3791N, ORF1b:P314L, ORF1b:D2090H, ORF3a:A23S, ORF3a:R122I, ORF3a:H227R, S:D614G, S:C1235F,
hCoV-19/Mexico/BCN-ALSR-8455/2020	EPI_ISL_1531918	20A	B.1.561	17	5'UTR:C241T, ORF1ab:G2516T, ORF1ab:C3037T, ORF1ab:G4486T, ORF1ab:A5999G, ORF1ab:T7357G, ORF1ab:A10323G, ORF1ab:C10798T, ORF1ab:C11866T, ORF1ab:C14408T, ORF1ab:C18693T, ORF1ab:A20268G, S:G21974T, S:A23403G, ORF3a:T25577C, N:G28357A, N:C28854T, N:G28975T,	11	N:S194L, N:M234I, ORF1a:V751L, ORF1a:K1407N, ORF1a:I1912V, ORF1a:K3353R, ORF1b:P314L, ORF3a:I62T, ORF9b:R25K, S:D138Y, S:D614G,
hCoV-19/Mexico/BCN-ALSR-8456/2020	EPI_ISL_1531919	20A	B.1	14	5'UTR:G208T, 5'UTR:C241T, ORF1ab:C3037T, ORF1ab:C11001T, ORF1ab:C14408T, ORF1ab:A20268G, ORF1ab:G20709T, S:A23403G, S:G23522A, S:C24106T, S:C24181T, S:C25162A, S:C25163A, M:A26927G, N:C28854T,	7	N:S194L, ORF1a:T3579I, ORF1b:P314L, ORF1b:M2414I, S:D614G, S:E654K, S:Q1201K,
hCoV-19/Mexico/BCN-ALSR-8457/2020	EPI_ISL_1531920	20A	B.1	13	5'UTR:G185T, 5'UTR:C241T, ORF1ab:C1489T, ORF1ab:G1738T, ORF1ab:C3037T, ORF1ab:C3096T, ORF1ab:T9440A, ORF1ab:A11140T, ORF1ab:C14408T, ORF1ab:A20268G, S:C23248T, S:A23403G, ORF7a:C27752T, N:G28899T,	6	N:R209I, ORF1a:S944L, ORF1a:C3059S, ORF1b:P314L, ORF7a:T120I, S:D614G,
hCoV-19/Mexico/BCN-ALSR-8458/2020	EPI_ISL_1531921	20A	B.1	14	5'UTR:G208T, 5'UTR:C241T, ORF1ab:C3037T, ORF1ab:G4185C, ORF1ab:C14408T, ORF1ab:A20268G, ORF1ab:G20709T, S:A23403G, S:G23522A, S:C24106T, S:C24181T, S:C25162A, S:C25163A, M:A26927G, N:C28854T,	7	N:S194L, ORF1a:G1307A, ORF1b:P314L, ORF1b:M2414I, S:D614G, S:E654K, S:Q1201K,
hCoV-19/Mexico/BCN-ALSR-8459/2020	EPI_ISL_1531922	20A	B.1	14	5'UTR:G208T, 5'UTR:C241T, ORF1ab:C3037T, ORF1ab:G4185C, ORF1ab:C14408T, ORF1ab:A20268G, ORF1ab:G20709T, S:A23403G, S:G23522A, S:C24106T, S:C24181T, S:C25162A, S:C25163A, M:A26927G, N:C28854T,	7	N:S194L, ORF1a:G1307A, ORF1b:P314L, ORF1b:M2414I, S:D614G, S:E654K, S:Q1201K,
hCoV-19/Mexico/BCN-ALSR-8460/2020	EPI_ISL_1531923	20A	B.1	10	5'UTR:C241T, ORF1ab:C3037T, ORF1ab:C14408T, ORF1ab:C18129T, ORF1ab:C19586T, ORF1ab:A20268G, S:G21724T, S:G22992A, S:A23403G, N:C28854T, ORF10:A29647G,	6	N:S194L, ORF1b:P314L, ORF1b:T2040I, S:L54F, S:S477N, S:D614G,
hCoV-19/Mexico/BCN-ALSR-8461/2020	EPI_ISL_1531924	20A	B.1	10	5'UTR:C241T, ORF1ab:C3037T, ORF1ab:C14408T, ORF1ab:C18129T, ORF1ab:C19586T, ORF1ab:A20268G, S:G21724T, S:G22992A, S:A23403G, N:C28854T, ORF10:A29647G,	6	N:S194L, ORF1b:P314L, ORF1b:T2040I, S:L54F, S:S477N, S:D614G,
hCoV-19/Mexico/BCN-ALSR-8462/2020	EPI_ISL_1531925	20A	B.1.232	16	5'UTR:C241T, ORF1ab:C1684T, ORF1ab:T1847C, ORF1ab:C3037T, ORF1ab:T5804C, ORF1ab:C5849T, ORF1ab:C6285T, ORF1ab:C6706T, ORF1ab:C14408T, ORF1ab:C16092T, ORF1ab:T19449A, ORF1ab:A20268G, S:A23403G, E:G26325A, ORF8:G27987T, N:C28291T, N:C28854T,	9	N:S194L, ORF1a:S528P, ORF1a:C1847R, ORF1a:P1862S, ORF1a:T2007I, ORF1b:P314L, ORF8:V32L, ORF9b:P3L, S:D614G,
hCoV-19/Mexico/BCN-ALSR-8463/2020	EPI_ISL_1531926	20B	B.1.1.403	12	5'UTR:C241T, ORF1ab:C3037T, ORF1ab:C7423T, ORF1ab:T10107C, ORF1ab:C11967T, ORF1ab:C14408T, S:A23403G, S:C23683T, ORF7b:A27868T, N:G28881A, N:G28882A, N:G28883C, 3'UTR:A29700G,	7	N:R203K, N:G204R, ORF1a:V3281A, ORF1a:A3901V, ORF1b:P314L, ORF7b:N38I, S:D614G,

hCoV-19/Mexico/BCN-ALSR-8464/2020	EPI_ISL_1531927	20A	B.1.561	17	5'UTR:C241T, ORF1ab:G2516T, ORF1ab:C3037T, ORF1ab:G4486T, ORF1ab:A5999G, ORF1ab:T7357G, ORF1ab:A10323G, ORF1ab:C10798T, ORF1ab:C11866T, ORF1ab:C14408T, ORF1ab:C18693T, ORF1ab:A19575G, ORF1ab:A20268G, S:G21974T, S:A23403G, ORF3a:T25577C, N:C28854T, N:G28975T,	10	N:S194L, N:M234I, ORF1a:V751L, ORF1a:K1407N, ORF1a:I1912V, ORF1a:K3353R, ORF1b:P314L, ORF3a:I62T, S:D138Y, S:D614G,
hCoV-19/Mexico/BCN-ALSR-8465/2020	EPI_ISL_1531928	20A	B.1.551	17	5'UTR:C241T, ORF1ab:C3037T, ORF1ab:C3738T, ORF1ab:G5206T, ORF1ab:G7027A, ORF1ab:C8836T, ORF1ab:G10631A, ORF1ab:C14408T, ORF1ab:A20268G, S:C21597T, S:C22323T, S:A23403G, S:C23604G, S:G23868T, ORF3a:C26058T, ORF7a:T27534C, N:C28854T, N:C29466T.	12	N:S194L, N:A398V, ORF1a:P1158L, ORF1a:M1647I, ORF1a:M2254I, ORF1a:A3456T, ORF1b:P314L, S:S12F, S:S254F, S:D614G, S:P681R, S:G769V,
hCoV-19/Mexico/BCN-ALSR-8466/2020	EPI_ISL_1531929	20A	B.1.551	16	5'UTR:C241T, ORF1ab:C3037T, ORF1ab:C3738T, ORF1ab:C8836T, ORF1ab:G10631A, ORF1ab:C14408T, ORF1ab:T18783C, ORF1ab:A20268G, S:C22323T, S:A23403G, S:C23604G, S:G23868T, S:T24892C, ORF3a:C26058T, ORF7a:T27534C, N:C28854T, N:C29466T.	9	N:S194L, N:A398V, ORF1a:P1158L, ORF1a:A3456T, ORF1b:P314L, S:S254F, S:D614G, S:P681R, S:G769V,
hCoV-19/Mexico/BCN-ALSR-8467/2020	EPI_ISL_1531930	20A	B.1.243	14	5'UTR:C21T, 5'UTR:C241T, ORF1ab:A1154G, ORF1ab:T1798C, ORF1ab:C3037T, ORF1ab:G7756T, ORF1ab:G11083T, ORF1ab:C12970T, ORF1ab:C14408T, ORF1ab:A20268G, ORF1ab:G21123T, S:A23403G, S:T24076C, ORF3a:T25496A, N:C28854T,	7	N:S194L, ORF1a:M297V, ORF1a:K2497N, ORF1a:L3606F, ORF1b:P314L, ORF3a:I35K, S:D614G,
hCoV-19/Mexico/BCN-ALSR-8468/2020	EPI_ISL_1531931	20B	B.1.1.432	20	5'UTR:C241T, ORF1ab:C629T, ORF1ab:G2293T, ORF1ab:C3037T, ORF1ab:A6985T, ORF1ab:T8857C, ORF1ab:C9319T, ORF1ab:C12412T, ORF1ab:T14313C, ORF1ab:C14408T, ORF1ab:C18698T, S:A23403G, ORF3a:C25613T, ORF3a:G25912T, ORF3a:A26050C, M:G26690T, N:G28881A, N:G28882A, N:G28883C, N:C29272T, N:T29317C.	10	N:R203K, N:G204R, ORF1a:L122F, ORF1a:Q676H, ORF1b:P314L, ORF1b:A1744V, ORF3a:S74F, ORF3a:G174C, ORF3a:S220R, S:D614G,
hCoV-19/Mexico/BCN-ALSR-8469/2020	EPI_ISL_1531932	20A	B.1.561	21	5'UTR:C241T, ORF1ab:C936T, ORF1ab:G2516T, ORF1ab:C3037T, ORF1ab:A5999G, ORF1ab:A10323G, ORF1ab:T10371G, ORF1ab:C10798T, ORF1ab:C11866T, ORF1ab:C13378T, ORF1ab:C14408T, ORF1ab:C18693T, ORF1ab:C19164T, ORF1ab:A20268G, S:G21974T, S:A23403G, S:C24031T, ORF3a:C25521T, ORF3a:T25577C, ORF8:C28087T, N:C28854T, N:G28975T.	12	N:S194L, N:M234I, ORF1a:T224I, ORF1a:V751L, ORF1a:I1912V, ORF1a:K3353R, ORF1a:I3369S, ORF1b:P314L, ORF3a:I62T, ORF8:A65V, S:D138Y, S:D614G,
hCoV-19/Mexico/BCN-ALSR-8470/2020	EPI_ISL_1531933	20A	B.1.561	15	5'UTR:C241T, ORF1ab:G2516T, ORF1ab:C3037T, ORF1ab:A5999G, ORF1ab:A10323G, ORF1ab:C14408T, ORF1ab:C18693T, ORF1ab:A20268G, S:G21974T, S:A23403G, S:C23664T, S:C23758T, S:G23792A, ORF3a:T25577C, N:C28854T, N:G28975T.	11	N:S194L, N:M234I, ORF1a:V751L, ORF1a:I1912V, ORF1a:K3353R, ORF1b:P314L, ORF3a:I62T, S:D138Y, S:D614G, S:A701V, S:G744S,
hCoV-19/Mexico/BCN-ALSR-8471/2020	EPI_ISL_1531934	20A	B.1.232	18	5'UTR:C241T, ORF1ab:C1684T, ORF1ab:C3037T, ORF1ab:C5407T, ORF1ab:T5804C, ORF1ab:C5849T, ORF1ab:C6285T, ORF1ab:C6706T, ORF1ab:C14408T, ORF1ab:C14599T, ORF1ab:C16092T, ORF1ab:A20268G, S:A23403G, S:A24118T, ORF3a:C25469T, E:G26325A, ORF8:G27987T, N:C28291T, N:C28854T,	9	N:S194L, ORF1a:C1847R, ORF1a:P1862S, ORF1a:T2007I, ORF1b:P314L, ORF3a:S26L, ORF8:V32L, ORF9b:P3L, S:D614G,
hCoV-19/Mexico/BCN-ALSR-8472/2020	EPI_ISL_1531935	20A	B.1.561	16	5'UTR:C241T, ORF1ab:G2516T, ORF1ab:C3037T, ORF1ab:G4486T, ORF1ab:A5999G, ORF1ab:T7357G, ORF1ab:A10323G, ORF1ab:C10798T, ORF1ab:C11866T, ORF1ab:C14408T, ORF1ab:C18693T, ORF1ab:A20268G, S:G21974T, S:A23403G, ORF3a:T25577C, N:C28854T, N:G28975T.	10	N:S194L, N:M234I, ORF1a:V751L, ORF1a:K1407N, ORF1a:I1912V, ORF1a:K3353R, ORF1b:P314L, ORF3a:I62T, S:D138Y, S:D614G,

hCoV-19/Mexico/BCN-ALSR-8473/2020	EPI_ISL_1531936	20A	B.1.232	17	5'UTR:C241T, ORF1ab:C1684T, ORF1ab:C3037T, ORF1ab:T5804C, ORF1ab:C5849T, ORF1ab:C6285T, ORF1ab:T6522A, ORF1ab:C6706T, ORF1ab:C14408T, ORF1ab:C15714T, ORF1ab:C16092T, ORF1ab:A20268G, S:A23403G, E:G26325A, M:G26976T, ORF8:G27987T, N:C28291T, N:C28854T,	10	M:A152S, N:S194L, ORF1a:C1847R, ORF1a:P1862S, ORF1a:T2007I, ORF1a:I2086N, ORF1b:P314L, ORF8:V32L, ORF9b:P3L, S:D614G,	
hCoV-19/Mexico/BCN-ALSR-8474/2020	EPI_ISL_1531937	20A	B.1.561	16	5'UTR:C241T, ORF1ab:G2516T, ORF1ab:C3037T, ORF1ab:G4486T, ORF1ab:A5999G, ORF1ab:T7357G, ORF1ab:A10323G, ORF1ab:C10798T, ORF1ab:C11866T, ORF1ab:C14408T, ORF1ab:C18693T, ORF1ab:A20268G, S:G21974T, S:A23403G, ORF3a:T25577C, N:C28854T, N:G28975T.	10	N:S194L, N:M234I, ORF1a:V751L, ORF1a:K1407N, ORF1a:I1912V, ORF1a:K3353R, ORF1b:P314L, ORF3a:I62T, S:D138Y, S:D614G,	
hCoV-19/Mexico/BCN-ALSR-8475/2020	EPI_ISL_1531938	20B	B.1.1.222	15	5'UTR:C241T, ORF1ab:C1426T, ORF1ab:G2458T, ORF1ab:G2891A, ORF1ab:C3037T, ORF1ab:C8772T, ORF1ab:C14408T, ORF1ab:T19839C, S:C23266T, S:A23403G, S:G23593C, S:A23756G, S:A24069T, N:G28881A, N:G28882A, N:G28883C,	10	N:R203K, N:G204R, ORF1a:M731I, ORF1a:A876T, ORF1a:T2836I, ORF1b:P314L, S:D614G, S:Q677H, S:T732A, S:Q836L,	
hCoV-19/Mexico/BCN-ALSR-8476/2020	EPI_ISL_1531939	20A	B.1.232	19	5'UTR:C241T, ORF1ab:C1884T, ORF1ab:C3037T, ORF1ab:C3619T, ORF1ab:T5804C, ORF1ab:C5849T, ORF1ab:C6285T, ORF1ab:C6706T, ORF1ab:C14408T, ORF1ab:C16092T, ORF1ab:A20268G, S:A23403G, ORF3a:G25720T, E:G26325A, ORF7a:G27651T, ORF7b:C27804T, ORF8:G27987T, N:C28291T, N:C28854T, 3'UTR:G29825T,	10	N:S194L, ORF1a:A540V, ORF1a:C1847R, ORF1a:P1862S, ORF1a:T2007I, ORF1b:P314L, ORF3a:A110S, ORF8:V32L, ORF9b:P3L, S:D614G,	
hCoV-19/Mexico/BCN-ALSR-8477/2020	EPI_ISL_1531940	20A	B.1.241	21	5'UTR:C241T, ORF1ab:C3037T, ORF1ab:C7768T, ORF1ab:C10029T, ORF1ab:C11173T, ORF1ab:G11521T, ORF1ab:C11575T, ORF1ab:C14408T, ORF1ab:C18747T, ORF1ab:C19290T, ORF1ab:A20055G, ORF1ab:A20268G, S:A23403G, S:G23593T, S:C24337T, ORF3a:C25693T, ORF7a:C27707T, ORF8:A28271T, N:C28550A, N:C28854T, N:C29375T, N:C29421T.	11	N:S194L, N:P368S, N:P383L, ORF1a:T3255I, ORF1a:M3752I, ORF1b:P314L, ORF3a:L101F, ORF7a:A105V, ORF9b:D89E, S:D614G, S:Q677H,	ORF1ab: 11288-11296, ORF8:28242-28253,
hCoV-19/Mexico/BCN-ALSR-8478/2020	EPI_ISL_1531941	20A	B.1.561	23	5'UTR:C241T, ORF1ab:C1403T, ORF1ab:G2516T, ORF1ab:C3037T, ORF1ab:A5999G, ORF1ab:A10323G, ORF1ab:C10798T, ORF1ab:C11866T, ORF1ab:C13378T, ORF1ab:C14408T, ORF1ab:C18570T, ORF1ab:C18693T, ORF1ab:C19164T, ORF1ab:A20268G, S:C21622T, S:G21974T, S:A23403G, S:C24319T, ORF3a:T25577C, N:G28300T, N:C28354T, N:C28854T, N:G28975T, N:G29468T.	14	N:Q9H, N:S194L, N:M234I, N:D399Y, ORF1a:P380S, ORF1a:V751L, ORF1a:I1912V, ORF1a:K3353R, ORF1b:P314L, ORF3a:I62T, ORF9b:S6I, ORF9b:T24I, S:D138Y, S:D614G,	
hCoV-19/Mexico/BCN-CICESE-BC2/2020	EPI_ISL_747242	20A	B.1	13	5'UTR:C241T, ORF1ab:C2113T, ORF1ab:C3037T, ORF1ab:C10189T, ORF1ab:C14408T, ORF1ab:C17502T, ORF1ab:C20234A, ORF1ab:A20268G, S:C22033A, S:T23257C, S:A23403G, ORF7a:C27596T, N:C28854T, 3'UTR:C29774T.	6	N:S194L, ORF1b:P314L, ORF1b:P2256H, ORF7a:P68L, S:F157L, S:D614G,	
hCoV-19/Mexico/BCN-CICESE-BC5/2020	EPI_ISL_747243	20A	B.1.397	9	5'UTR:C241T, ORF1ab:G1135T, ORF1ab:A1226G, ORF1ab:C1376T, ORF1ab:C3037T, ORF1ab:G3403T, ORF1ab:C14408T, ORF1ab:A20268G, S:A23403G, N:C28854T.	6	N:S194L, ORF1a:K290N, ORF1a:M321V, ORF1a:P371S, ORF1b:P314L, S:D614G,	
hCoV-19/Mexico/BCN-InDRE_256/2020	EPI_ISL_1054984	20B	B.1.1.222	19	5'UTR:C241T, ORF1ab:G1268A, ORF1ab:A2745C, ORF1ab:C3037T, ORF1ab:C5986T, ORF1ab:A13288G, ORF1ab:C14408T, ORF1ab:G14772T, ORF1ab:G17562T, ORF1ab:T19839C, ORF1ab:G20208T, S:A23403G, S:A23756G, S:C25046T, ORF3a:G26056T, N:G28881A, N:G28882A, N:G28883C, N:C29409T, 3'UTR:G29773T.	12	N:R203K, N:G204R, N:T379I, ORF1a:D335N, ORF1a:D827A, ORF1b:P314L, ORF1b:Q435H, ORF1b:Q2247H, ORF3a:D222Y, S:D614G, S:T732A, S:P1162S,	

hCoV-19/Mexico/BCN-InDRE_32/2020	EPI_ISL_493347	20A	B.1	8	5'UTR:C241T, ORF1ab:G1727T, ORF1ab:C3037T, ORF1ab:C14408T, ORF1ab:T18026C, ORF1ab:A20268G, S:A23403G, ORF3a:C25710G, N:C28854T,	5	N:S194L, ORF1a:V488L, ORF1b:P314L, ORF1b:V1520A, S:D614G,	ORF3a:25855,
hCoV-19/Mexico/BCN-InDRE_335/2020	EPI_ISL_1060726	20A	B.1.551	15	ORF1ab:C3037T, ORF1ab:C3738T, ORF1ab:C8836T, ORF1ab:G10631A, ORF1ab:C14408T, ORF1ab:C17410T, ORF1ab:A20268G, S:C22323T, S:A23403G, S:C23604G, S:G23868T, ORF3a:C26058T, ORF7a:T27534C, N:C28854T, N:C29466T, 3'UTR:G29734C,	10	N:S194L, N:A398V, ORF1a:P1158L, ORF1a:A3456T, ORF1b:P314L, ORF1b:R1315C, S:S254F, S:D614G, S:P681R, S:G769V,	
hCoV-19/Mexico/BCN-InDRE_447/2021	EPI_ISL_1168525	20A	B.1.243	15	5'UTR:C241T, ORF1ab:G806A, ORF1ab:C1913T, ORF1ab:T2584A, ORF1ab:C3037T, ORF1ab:C5140A, ORF1ab:G7059A, ORF1ab:C14408T, ORF1ab:A20268G, S:A23403G, S:G23587C, S:T24076C, ORF3a:C25844T, N:C28854T, N:C28887T, N:G29543T,	10	N:S194L, N:T205I, ORF1a:A181T, ORF1a:R550C, ORF1a:D1625E, ORF1a:G2265D, ORF1b:P314L, ORF3a:T151I, S:D614G, S:Q675H,	
hCoV-19/Mexico/BCN-InDRE_F10696_S693/2021	EPI_ISL_1365658	20B	B.1.1.222	12	5'UTR:C241T, ORF1ab:C3037T, ORF1ab:C6285T, ORF1ab:A13498G, ORF1ab:C14408T, ORF1ab:T19839C, S:A23403G, S:A23756G, ORF3a:G25527T, E:C26322T, N:G28881A, N:G28882A, N:G28883C,	8	N:R203K, N:G204R, ORF1a:T2007I, ORF1b:T11A, ORF1b:P314L, ORF3a:W45C, S:D614G, S:T732A,	
hCoV-19/Mexico/BCN-InDRE_F10697_S694/2021	EPI_ISL_1365659	20B	B.1.1.222	12	5'UTR:C241T, ORF1ab:C3037T, ORF1ab:C6285T, ORF1ab:A13498G, ORF1ab:C14408T, ORF1ab:T19839C, S:A23403G, S:A23756G, ORF3a:G25527T, E:C26322T, N:G28881A, N:G28882A, N:G28883C,	8	N:R203K, N:G204R, ORF1a:T2007I, ORF1b:T11A, ORF1b:P314L, ORF3a:W45C, S:D614G, S:T732A,	
hCoV-19/Mexico/BCN-InDRE_F10699_S695/2021	EPI_ISL_1365660	20B	B.1.1.432	24	5'UTR:C241T, ORF1ab:A1103G, ORF1ab:G1599T, ORF1ab:G2144T, ORF1ab:C3037T, ORF1ab:A3183G, ORF1ab:G4960T, ORF1ab:A6985T, ORF1ab:C9319T, ORF1ab:A10323G, ORF1ab:C11653T, ORF1ab:C12412T, ORF1ab:G14181C, ORF1ab:T14313C, ORF1ab:C14408T, ORF1ab:C14724T, S:A23403G, ORF3a:G25912T, M:G26828T, N:G28321A, N:G28371T, N:G28881A, N:G28882A, N:G28883C, N:T29317C,	14	N:S33I, N:R203K, N:G204R, ORF1a:I280V, ORF1a:G445V, ORF1a:V627F, ORF1a:E973G, ORF1a:K3353R, ORF1b:L238F, ORF1b:P314L, ORF3a:G174C, ORF9b:R13H, ORF9b:V30L, S:D614G,	
hCoV-19/Mexico/BCN-InDRE_F10713_S697/2021	EPI_ISL_1365662	20A	B.1	27	5'UTR:C241T, ORF1ab:C2299T, ORF1ab:C3037T, ORF1ab:C7768T, ORF1ab:C10029T, ORF1ab:C11173T, ORF1ab:G11521T, ORF1ab:C11575T, ORF1ab:C13536T, ORF1ab:C14408T, ORF1ab:T17067C, ORF1ab:C18747T, ORF1ab:C19290T, ORF1ab:A20055G, ORF1ab:A20268G, S:G21850T, S:A23403G, S:G23593T, S:C24034T, S:C24337T, ORF3a:C25433T, ORF3a:C25693T, ORF7a:C27707T, ORF8:A28271T, N:C28550A, N:C28854T, N:C29375T, N:C29421T	13	N:S194L, N:P368S, N:P383L, ORF1a:T3255I, ORF1a:M3752I, ORF1b:P314L, ORF3a:T14I, ORF3a:L101F, ORF7a:A105V, ORF9b:D89E, S:E96D, S:D614G, S:Q677H,	ORF1ab:11288-11296, ORF8:28242-28253,
hCoV-19/Mexico/BCN-InDRE_F11110_S849/2021	EPI_ISL_1424050	20B	B.1.1.519	28	5'UTR:C203T, 5'UTR:C222T, 5'UTR:C241T, ORF1ab:C583T, ORF1ab:G1738T, ORF1ab:C3037T, ORF1ab:C3140T, ORF1ab:A3278C, ORF1ab:C10029T, ORF1ab:C10954T, ORF1ab:A11117G, ORF1ab:C12789T, ORF1ab:G13576T, ORF1ab:C14408T, ORF1ab:C19011A, ORF1ab:T19839C, ORF1ab:A19974G, ORF1ab:G20995T, ORF1ab:C21306T, S:C22995A, S:A23403G, S:C23604A, S:A23756G, ORF3a:G25699A, M:T26861C, N:G28881A, N:G28882A, N:G28883C, N:C29197T,	16	N:R203K, N:G204R, ORF1a:P959S, ORF1a:I1005L, ORF1a:T3255I, ORF1a:I3618V, ORF1a:T4175I, ORF1b:A37S, ORF1b:P314L, ORF1b:D1848E, ORF1b:G2510C, ORF3a:A103T, S:T478K, S:D614G, S:P681H, S:T732A,	
hCoV-19/Mexico/BCN-InDRE-45/2020	EPI_ISL_516609	20A	B.1.243	9	5'UTR:C241T, ORF1ab:C3037T, ORF1ab:C11074T, ORF1ab:C14408T, ORF1ab:A16052G, ORF1ab:A20268G, S:A23403G, S:T24076C, ORF3a:G25690T, N:C28854T,	5	N:S194L, ORF1b:P314L, ORF1b:K862R, ORF3a:G100C, S:D614G,	

hCoV-19/Mexico/BCN-InDRE-50/2020	EPI_ISL_516610	20A	B.1.400	9	5'UTR:C241T, ORF1ab:G1590T, ORF1ab:C3037T, ORF1ab:C6781T, ORF1ab:C14408T, ORF1ab:G19969A, ORF1ab:A20268G, S:A23403G, N:C28854T, N:G29179T,	5	N:S194L, ORF1a:G442V, ORF1b:P314L, ORF1b:A2168T, S:D614G,
hCoV-19/Mexico/BCN-InDRE-51/2020	EPI_ISL_516611	20A	B.1	8	5'UTR:C241T, ORF1ab:C3037T, ORF1ab:G5668A, ORF1ab:C5884T, ORF1ab:C14408T, ORF1ab:A20268G, S:A23403G, S:A23583T, N:C28854T,	4	N:S194L, ORF1b:P314L, S:D614G, S:Y674F,
hCoV-19/Mexico/BCN-SEARCH-7360/2021	EPI_ISL_1295671	20B	B.1.1.519	30	5'UTR:T201C, 5'UTR:C203T, 5'UTR:C222T, 5'UTR:C241T, ORF1ab:C936T, ORF1ab:G1738T, ORF1ab:C3037T, ORF1ab:C3140T, ORF1ab:C7521T, ORF1ab:C10029T, ORF1ab:C10954T, ORF1ab:A11117G, ORF1ab:A12174G, ORF1ab:C12357T, ORF1ab:C12789T, ORF1ab:C14408T, ORF1ab:T19839C, ORF1ab:A19974G, ORF1ab:C21306T, S:G21778A, S:G22094A, S:C22995A, S:A23403G, S:C23604A, S:A23756G, N:G28378C, N:G28881A, N:G28882A, N:G28883C, N:C29197T, N:G29227T,	17	N:R203K, N:G204R, ORF1a:T224I, ORF1a:P959S, ORF1a:T2419I, ORF1a:T3255I, ORF1a:I3618V, ORF1a:N3970S, ORF1a:T4031I, ORF1a:T4175I, ORF1b:P314L, ORF9b:R32P, S:D178N, S:T478K, S:D614G, S:P681H, S:T732A,
hCoV-19/Mexico/BCN-SEARCH-7370/2021	EPI_ISL_1295676	20B	B.1.1.519	29	5'UTR:C203T, 5'UTR:C222T, 5'UTR:C241T, ORF1ab:G569A, ORF1ab:C3037T, ORF1ab:C3140T, ORF1ab:C3743T, ORF1ab:T8104C, ORF1ab:C10029T, ORF1ab:A10829C, ORF1ab:C10954T, ORF1ab:A11117G, ORF1ab:C12789T, ORF1ab:C13329T, ORF1ab:C14408T, ORF1ab:T16857C, ORF1ab:T19839C, ORF1ab:C21306T, S:C22995A, S:A23403G, S:C23604A, S:A23756G, ORF8:T27904C, ORF8:C28087T, N:G28881A, N:G28882A, N:G28883C, N:C29197T, 3'UTR:T29681C, 3'UTR:A29700G,	17	N:R203K, N:G204R, ORF1a:E102K, ORF1a:P959S, ORF1a:H1160Y, ORF1a:T3255I, ORF1a:I3522L, ORF1a:I3618V, ORF1a:T4175I, ORF1a:T4355I, ORF1b:P314L, ORF8:L4P, ORF8:A65V, S:T478K, S:D614G, S:P681H, S:T732A,
hCoV-19/Mexico/BCN-SEARCH-7391/2021	EPI_ISL_1295691	20G	B.1.2	16	5'UTR:C241T, ORF1ab:C1059T, ORF1ab:T1947G, ORF1ab:C3037T, ORF1ab:C9207T, ORF1ab:C10319T, ORF1ab:G13618A, ORF1ab:C14408T, ORF1ab:A18424G, ORF1ab:C21304T, S:A23403G, ORF3a:G25563T, ORF3a:G25907T, M:A26896G, ORF8:C27964T, N:C28472T, N:C28869T,	15	M:H125R, N:P67S, N:P199L, ORF1a:T265I, ORF1a:V561G, ORF1a:S2981F, ORF1a:L3352F, ORF1b:D51N, ORF1b:P314L, ORF1b:N1653D, ORF1b:R2613C, ORF3a:Q57H, ORF3a:G172V, ORF8:S24L, S:D614G,
hCoV-19/Mexico/BCN-SEARCH-7441/2021	EPI_ISL_1295730	21C (Epsilon)	B.1.427	25	5'UTR:C241T, ORF1ab:C1059T, ORF1ab:T1851C, ORF1ab:C2232T, ORF1ab:C3037T, ORF1ab:C3619A, ORF1ab:G9738C, ORF1ab:G13713A, ORF1ab:A13884G, ORF1ab:T13885A, ORF1ab:C14408T, ORF1ab:C16394T, ORF1ab:G17014T, S:G21600T, S:G22018T, S:T22917G, S:A23403G, S:C25317T, ORF3a:T25467C, ORF3a:G25563T, ORF3a:T26066C, M:C26681T, ORF8:A28272T, N:C28344T, N:C28887T, N:C29362T,	17	N:T24I, N:T205I, ORF1a:T265I, ORF1a:I529T, ORF1a:A656V, ORF1a:S3158T, ORF1b:Y140N, ORF1b:P314L, ORF1b:P976L, ORF1b:D1183Y, ORF3a:Q57H, ORF3a:V225A, S:S13I, S:W152C, S:L452R, S:D614G, S:S1252F,
hCoV-19/Mexico/BCN-SEARCH-7475/2021	EPI_ISL_1295760	20A	B.1.232	22	5'UTR:C241T, ORF1ab:C3037T, ORF1ab:C4795T, ORF1ab:T5804C, ORF1ab:C5849T, ORF1ab:C6285T, ORF1ab:C6706T, ORF1ab:C13721A, ORF1ab:C14408T, ORF1ab:C16092T, ORF1ab:A20268G, S:T22917G, S:A23403G, S:G24926T, ORF3a:C25561G, ORF3a:G25720T, E:G26325A, ORF8:G27987T, N:C28291T, N:T28600C, N:C28854T, N:G29474T, 3'UTR:G29825T,	14	N:S194L, N:D401Y, ORF1a:C1847R, ORF1a:P1862S, ORF1a:T2007I, ORF1b:P85Q, ORF1b:P314L, ORF3a:Q57E, ORF3a:A110S, ORF8:V32L, ORF9b:P3L, S:L452R, S:D614G, S:V1122L,

hCoV-19/Mexico/BCN-SEARCH-7499/2021	EPI_ISL_1295779	21C (Epsilon)	B.1.427	21	5'UTR:C241T, ORF1ab:C1059T, ORF1ab:C1218T, ORF1ab:C3037T, ORF1ab:T3730C, ORF1ab:G9738C, ORF1ab:C12183T, ORF1ab:G13713A, ORF1ab:C14408T, ORF1ab:C16394T, ORF1ab:G17014T, ORF1ab:A19782G, ORF1ab:G20931T, S:G21600T, S:G22018T, S:T22917G, S:A23403G, ORF3a:G25563T, M:C26681T, ORF8:A28272T, N:C28887T, N:C29362T,	13	N:T205I, ORF1a:T265I, ORF1a:S318L, ORF1a:S3158T, ORF1a:S3973F, ORF1b:P314L, ORF1b:P976L, ORF1b:D1183Y, ORF3a:Q57H, S:S13I, S:W152C, S:L452R, S:D614G,	
hCoV-19/Mexico/BCN-SEARCH-7577/2021	EPI_ISL_1295837	20G	B.1.2	25	5'UTR:C241T, ORF1ab:C1059T, ORF1ab:C3037T, ORF1ab:G8861A, ORF1ab:C10319T, ORF1ab:C11572T, ORF1ab:C11878T, ORF1ab:C12484T, ORF1ab:G13897T, ORF1ab:C14408T, ORF1ab:A16011G, ORF1ab:C18060T, ORF1ab:A18424G, ORF1ab:C19955A, ORF1ab:G20962T, ORF1ab:C21304T, S:C21614T, S:A23403G, S:G23611T, ORF3a:G25563T, ORF3a:G25907T, ORF3a:A26041T, ORF8:C27964T, N:G28460A, N:C28472T, N:C28869T,	18	N:D63N, N:P67S, N:P199L, ORF1a:T265I, ORF1a:V2866M, ORF1a:L3352F, ORF1b:D144Y, ORF1b:P314L, ORF1b:N1653D, ORF1b:T2163N, ORF1b:D2499Y, ORF1b:R2613C, ORF3a:Q57H, ORF3a:G172V, ORF3a:T217S, ORF8:S24L, S:L18F, S:D614G,	
hCoV-19/Mexico/BCN-SEARCH-7585/2021	EPI_ISL_1295843	21C (Epsilon)	B.1.427	23	5'UTR:C241T, ORF1ab:C1059T, ORF1ab:T1851C, ORF1ab:C2232T, ORF1ab:C3037T, ORF1ab:C3619A, ORF1ab:G9738C, ORF1ab:G13713A, ORF1ab:C14408T, ORF1ab:C16394T, ORF1ab:G17014T, S:G21600T, S:G22018T, S:T22917G, S:A23403G, S:C25317T, ORF3a:T25467C, ORF3a:G25563T, ORF3a:T26066C, M:C26681T, ORF8:A28272T, N:C28887T, N:C29160T, N:C29362T,	16	N:T205I, N:T296I, ORF1a:T265I, ORF1a:I529T, ORF1a:A656V, ORF1a:S3158T, ORF1b:P314L, ORF1b:P976L, ORF1b:D1183Y, ORF3a:Q57H, ORF3a:V225A, S:S13I, S:W152C, S:L452R, S:D614G, S:S1252F,	
hCoV-19/Mexico/BCN-SEARCH-9176/2020	EPI_ISL_1794925	20A	B.1.241	21	5'UTR:C241T, ORF1ab:C3037T, ORF1ab:C7768T, ORF1ab:C10015T, ORF1ab:C10029T, ORF1ab:C11173T, ORF1ab:C11575T, ORF1ab:C14408T, ORF1ab:C18747T, ORF1ab:C19290T, ORF1ab:A20055G, ORF1ab:A20268G, S:A23403G, S:G23593T, ORF3a:C25693T, ORF7a:C27707T, ORF8:A28271T, N:C28550A, N:C28854T, N:C29375T, N:C29421T, 3'UTR:C29870T,	10	N:S194L, N:P368S, N:P383L, ORF1a:T3255I, ORF1b:P314L, ORF3a:L101F, ORF7a:A105V, ORF9b:D89E, S:D614G, S:Q677H,	ORF1ab: 11288-11296, ORF8:28242-28253,
hCoV-19/Mexico/BCS-InDRE_133/2020	EPI_ISL_913936	20A	B.1.565	15	5'UTR:C241T, ORF1ab:A696C, ORF1ab:C878T, ORF1ab:C3037T, ORF1ab:G3871T, ORF1ab:T3931C, ORF1ab:C6285T, ORF1ab:C6936T, ORF1ab:C14016T, ORF1ab:C14408T, ORF1ab:C15324T, ORF1ab:C19524T, ORF1ab:C20429T, S:A23403G, ORF3a:G25855C, N:C28977T,	9	N:S235F, ORF1a:D144A, ORF1a:K1202N, ORF1a:T2007I, ORF1a:S2224F, ORF1b:P314L, ORF1b:P2321L, ORF3a:D155H, S:D614G,	
hCoV-19/Mexico/BCS-InDRE_201/2020	EPI_ISL_933678	20A	B.1.234	14	5'UTR:C241T, ORF1ab:C3037T, ORF1ab:G3947T, ORF1ab:C6033T, ORF1ab:A6441G, ORF1ab:C8140T, ORF1ab:A9204G, ORF1ab:C14408T, ORF1ab:A20268G, S:A23403G, S:G23587T, N:C28854T, N:G29384T, N:C29445T, 3'UTR:C29803A,	10	N:S194L, N:D371Y, N:T391I, ORF1a:D1228Y, ORF1a:A1923V, ORF1a:K2059R, ORF1a:D2980G, ORF1b:P314L, S:D614G, S:Q675H,	
hCoV-19/Mexico/BCS-InDRE_30/2020	EPI_ISL_493345	20A	B.1	5	5'UTR:C241T, ORF1ab:C3037T, ORF1ab:C14408T, ORF1ab:A20268G, S:A23403G, N:C28854T,	3	N:S194L, ORF1b:P314L, S:D614G,	
hCoV-19/Mexico/BCS-InDRE_31/2020	EPI_ISL_493346	20A	B.1	6	5'UTR:C241T, ORF1ab:C3037T, ORF1ab:C14408T, ORF1ab:A20268G, S:A23403G, ORF3a:G25767T, N:C28854T,	4	N:S194L, ORF1b:P314L, ORF3a:M125I, S:D614G,	
hCoV-19/Mexico/BCS-InDRE_326/2020	EPI_ISL_1060747	20A	B.1	11	5'UTR:C241T, ORF1ab:T1177C, ORF1ab:C3037T, ORF1ab:T4183A, ORF1ab:C14408T, ORF1ab:A20268G, ORF1ab:C20544T, S:A21659G, S:C22264T, S:A23403G, N:C28854T, 3'UTR:G29692T,	4	N:S194L, ORF1b:P314L, S:T33A, S:D614G,	
hCoV-19/Mexico/BCS-InDRE_327/2020	EPI_ISL_1060751	20A	B.1	11	5'UTR:C241T, ORF1ab:T1177C, ORF1ab:C3037T, ORF1ab:T4183A, ORF1ab:C14408T, ORF1ab:A20268G, ORF1ab:C20544T, S:A21659G, S:C22264T, S:A23403G, N:C28854T, 3'UTR:G29692T,	4	N:S194L, ORF1b:P314L, S:T33A, S:D614G,	

hCoV-19/Mexico/BCS-InDRE_337/2020	EPI_ISL_1060748	20A	B.1.610	4	5'UTR:C241T, ORF1ab:C3037T, ORF1ab:C14408T, ORF1ab:A20268G, S:A23403G,	2	ORF1b:P314L, S:D614G,
hCoV-19/Mexico/BCS-InDRE_340/2020	EPI_ISL_1060749	20B	B.1.1	7	5'UTR:C241T, ORF1ab:C3037T, ORF1ab:C14408T, ORF1ab:T19839C, S:A23403G, N:G28881A, N:G28882A, N:G28883C,	4	N:R203K, N:G204R, ORF1b:P314L, S:D614G,
hCoV-19/Mexico/BCS-InDRE_349/2020	EPI_ISL_1060724	20A	B.1.565	17	ORF1ab:C678T, ORF1ab:A696C, ORF1ab:C878T, ORF1ab:C3037T, ORF1ab:G3871T, ORF1ab:T3931C, ORF1ab:C6285T, ORF1ab:C6936T, ORF1ab:G8117A, ORF1ab:C14016T, ORF1ab:C14408T, ORF1ab:C15324T, ORF1ab:C19524T, ORF1ab:C20429T, S:A23403G, ORF3a:G25855C, ORF8:A28119G, N:C28977T,	12	N:S235F, ORF1a:A138V, ORF1a:D144A, ORF1a:K1202N, ORF1a:T2007I, ORF1a:S2224F, ORF1a:A2618T, ORF1b:P314L, ORF1b:P2321L, ORF3a:D155H, ORF8:I76V, S:D614G,
hCoV-19/Mexico/BCS-InDRE_350/2020	EPI_ISL_1060681	20B	B.1.1.222	10	ORF1ab:C3037T, ORF1ab:G12261A, ORF1ab:A13498G, ORF1ab:C14408T, S:G22331A, S:A23403G, S:A23756G, N:G28881A, N:G28882A, N:G28883C, ORF10:A29567C,	8	N:R203K, N:G204R, ORF1a:R3999H, ORF1b:T11A, ORF1b:P314L, S:G257S, S:D614G, S:T732A,
hCoV-19/Mexico/BCS-InDRE_352/2020	EPI_ISL_1060705	21C (Epsilon)	B.1.429	18	ORF1ab:C1059T, ORF1ab:G2354A, ORF1ab:C2395T, ORF1ab:G2747T, ORF1ab:C3037T, ORF1ab:C8947T, ORF1ab:C12100T, ORF1ab:A12878G, ORF1ab:C14408T, ORF1ab:G17014T, S:T22917G, S:A23403G, S:T24349C, ORF3a:G25563T, M:C26681T, M:C27012T, ORF7b:G27890T, ORF8:A28272T, N:C28887T,	10	N:T205I, ORF1a:T265I, ORF1a:G697R, ORF1a:D828Y, ORF1a:I4205V, ORF1b:P314L, ORF1b:D1183Y, ORF3a:Q57H, S:L452R, S:D614G,
hCoV-19/Mexico/BCS-InDRE_368/2020	EPI_ISL_1060720	21C (Epsilon)	B.1.427	19	5'UTR:T78G, 5'UTR:C241T, ORF1ab:C1059T, ORF1ab:C1601T, ORF1ab:T1851C, ORF1ab:C3037T, ORF1ab:G9738C, ORF1ab:G13713A, ORF1ab:C14408T, ORF1ab:C16394T, ORF1ab:G17014T, S:G22346T, S:T22917G, S:A23403G, ORF3a:T25467C, ORF3a:G25563T, M:C26681T, ORF8:A28272T, N:C28887T, N:G29081T,	13	N:T205I, N:V270L, ORF1a:T265I, ORF1a:L446F, ORF1a:I529T, ORF1a:S3158T, ORF1b:P314L, ORF1b:P976L, ORF1b:D1183Y, ORF3a:Q57H, S:A262S, S:L452R, S:D614G,
hCoV-19/Mexico/BCS-InDRE_369/2020	EPI_ISL_1060703	20B	B.1.1.432	18	5'UTR:T179C, ORF1ab:C3037T, ORF1ab:T5494C, ORF1ab:A8466G, ORF1ab:C9319T, ORF1ab:C12412T, ORF1ab:G14290T, ORF1ab:T14313C, ORF1ab:C14408T, ORF1ab:T19710C, S:A23403G, ORF3a:C25613T, ORF3a:G26062T, N:G28881A, N:G28882A, N:G28883C, N:C29077T, N:T29317C, 3'UTR:G29744T,	8	N:R203K, N:G204R, ORF1a:K2734R, ORF1b:D275Y, ORF1b:P314L, ORF3a:S74F, ORF3a:G224C, S:D614G,
hCoV-19/Mexico/BCS-InDRE_468/2021	EPI_ISL_1168538	20B	B.1.1.519	26	5'UTR:T201C, 5'UTR:C203T, 5'UTR:C222T, 5'UTR:C241T, ORF1ab:C583T, ORF1ab:G1738T, ORF1ab:C3037T, ORF1ab:C3140T, ORF1ab:C10029T, ORF1ab:C10954T, ORF1ab:A11117G, ORF1ab:G13576T, ORF1ab:C14408T, ORF1ab:T19839C, ORF1ab:A19974G, ORF1ab:G21438T, S:C22995A, S:A23403G, S:C23604A, S:A23756G, ORF3a:G25699A, M:T26861C, ORF6:T27384C, N:G28881A, N:G28882A, N:G28883C, N:C29197T,	13	N:R203K, N:G204R, ORF1a:P959S, ORF1a:T3255I, ORF1a:I3618V, ORF1b:A37S, ORF1b:P314L, ORF1b:M2657I, ORF3a:A103T, S:T478K, S:D614G, S:P681H, S:T732A,
hCoV-19/Mexico/BCS-InDRE_469/2021	EPI_ISL_1168539	20A	B.1.232	19	5'UTR:C241T, ORF1ab:C643T, ORF1ab:C829T, ORF1ab:C1594T, ORF1ab:G2293T, ORF1ab:C3037T, ORF1ab:C7564T, ORF1ab:C11758T, ORF1ab:C14408T, ORF1ab:A20268G, ORF1ab:C21145T, ORF1ab:C21304T, S:G22599A, S:A23403G, S:G23593T, S:C25282T, ORF3a:G25690T, M:C26681T, ORF8:G27987T, N:C28854T,	10	N:S194L, ORF1a:Q676H, ORF1b:P314L, ORF1b:L2560F, ORF1b:R2613C, ORF3a:G100C, ORF8:V32L, S:R346K, S:D614G, S:Q677H,
hCoV-19/Mexico/BCS-InDRE_470/2021	EPI_ISL_1168540	21C (Epsilon)	B.1.429	20	5'UTR:C241T, ORF1ab:C1059T, ORF1ab:C2395T, ORF1ab:T2597C, ORF1ab:C3037T, ORF1ab:C8947T, ORF1ab:C10641T, ORF1ab:C12100T, ORF1ab:A12878G, ORF1ab:C14408T, ORF1ab:G17014T, S:G21600T, S:G22018T, S:T22917G, S:A23403G, S:T24349C, ORF3a:G25563T, M:C26681T, ORF7b:G27890T, ORF8:A28272T. N:C28887T.	11	N:T205I, ORF1a:T265I, ORF1a:T3459M, ORF1a:I4205V, ORF1b:P314L, ORF1b:D1183Y, ORF3a:Q57H, S:S13I, S:W152C, S:L452R, S:D614G,

hCoV-19/Mexico/BCS-InDRE_471/2021	EPI_ISL_1168541	20A	B.1.243	27	5'UTR:C241T, ORF1ab:C274T, ORF1ab:G806A, ORF1ab:T2584A, ORF1ab:C2675T, ORF1ab:C3037T, ORF1ab:C5140A, ORF1ab:C6027T, ORF1ab:C6099T, ORF1ab:C6696T, ORF1ab:C8140T, ORF1ab:C9165T, ORF1ab:C10336T, ORF1ab:G11083T, ORF1ab:C12008T, ORF1ab:C14408T, ORF1ab:C18486T, ORF1ab:A20268G, S:C21648T, S:C22227T, S:G23120T, S:A23403G, S:C23536T, S:G23587C, S:T24076C, E:C26455T, N:C28854T, N:G29543T,	17	E:P71S, N:S194L, ORF1a:A181T, ORF1a:P804S, ORF1a:D1625E, ORF1a:P1921L, ORF1a:T1945I, ORF1a:P2144L, ORF1a:T2967I, ORF1a:L3606F, ORF1a:L3915F, ORF1b:P314L, S:T29I, S:A222V, S:A520S, S:D614G, S:Q675H,
hCoV-19/Mexico/BCS-InDRE_472/2021	EPI_ISL_1168542	20A	B.1.232	19	5'UTR:C241T, ORF1ab:C643T, ORF1ab:C829T, ORF1ab:C1594T, ORF1ab:G2293T, ORF1ab:C3037T, ORF1ab:C7564T, ORF1ab:C11758T, ORF1ab:C14408T, ORF1ab:A20268G, ORF1ab:C21145T, ORF1ab:C21304T, S:G22599A, S:A23403G, S:G23593T, S:C25282T, ORF3a:G25690T, M:C26681T, ORF8:G27987T, N:C28854T.	10	N:S194L, ORF1a:Q676H, ORF1b:P314L, ORF1b:L2560F, ORF1b:R2613C, ORF3a:G100C, ORF8:V32L, S:R346K, S:D614G, S:Q677H,
hCoV-19/Mexico/BCS-InDRE_F10688_S591/2021	EPI_ISL_1337370	21C (Epsilon)	B.1.429	28	5'UTR:C241T, ORF1ab:C1059T, ORF1ab:C1912T, ORF1ab:C2395T, ORF1ab:T2597C, ORF1ab:C3037T, ORF1ab:C4683T, ORF1ab:A6799G, ORF1ab:C8947T, ORF1ab:T11049G, ORF1ab:T11632A, ORF1ab:G11634T, ORF1ab:C12100T, ORF1ab:A12878G, ORF1ab:C14408T, ORF1ab:C15738T, ORF1ab:G17014T, S:G21600T, S:G22018T, S:T22917G, S:T22965C, S:A23403G, S:T24349C, ORF3a:G25563T, M:C26681T, ORF7b:G27890T, ORF8:A28272T, N:C28887T, N:C29362T,	15	N:T205I, ORF1a:T265I, ORF1a:A1473V, ORF1a:V3595G, ORF1a:F3789L, ORF1a:C3790F, ORF1a:I4205V, ORF1b:P314L, ORF1b:D1183Y, ORF3a:Q57H, S:S13I, S:W152C, S:L452R, S:I468T, S:D614G,
hCoV-19/Mexico/BCS-InDRE_F10702_S592/2021	EPI_ISL_1337371	21C (Epsilon)	B.1.427	20	5'UTR:C241T, ORF1ab:C1059T, ORF1ab:T1195C, ORF1ab:C3037T, ORF1ab:G9738C, ORF1ab:G13713A, ORF1ab:C14408T, ORF1ab:C16394T, ORF1ab:G17014T, ORF1ab:G17964T, ORF1ab:T21326C, S:G21600T, S:G22018T, S:T22917G, S:A23403G, ORF3a:G25563T, M:C26681T, ORF8:G28178T, ORF8:A28272T. N:C28887T. N:C29362T.	14	N:T205I, ORF1a:T265I, ORF1a:S3158T, ORF1b:P314L, ORF1b:P976L, ORF1b:D1183Y, ORF1b:M1499I, ORF1b:V2620A, ORF3a:Q57H, ORF8:L95F, S:S13I, S:W152C, S:L452R, S:D614G,
hCoV-19/Mexico/BCS-InDRE_F10703_S593/2021	EPI_ISL_1337373	21C (Epsilon)	B.1.429	30	5'UTR:C241T, ORF1ab:C1059T, ORF1ab:C2395T, ORF1ab:T2597C, ORF1ab:C3037T, ORF1ab:G4162T, ORF1ab:C8947T, ORF1ab:C12100T, ORF1ab:A12878G, ORF1ab:C14408T, ORF1ab:A15596G, ORF1ab:G17014T, ORF1ab:G19872T, ORF1ab:T20277C, S:G21600T, S:G22018T, S:T22917G, S:T23155C, S:A23403G, S:T24349C, S:G25177T, ORF3a:G25563T, M:C26681T, ORF6:T27231C, ORF7b:G27890T, ORF8:G28191T, ORF8:A28272T, N:C28887T, N:G28975T, N:C29362T, 3'UTR:G29773T,	14	N:T205I, N:M234I, ORF1a:T265I, ORF1a:I4205V, ORF1b:P314L, ORF1b:Y710C, ORF1b:D1183Y, ORF3a:Q57H, ORF8:V100L, S:S13I, S:W152C, S:L452R, S:D614G, S:K1205N,
hCoV-19/Mexico/BCS-InDRE_F10705_S594/2021	EPI_ISL_1337374	21C (Epsilon)	B.1.427	22	5'UTR:C241T, ORF1ab:C1059T, ORF1ab:C3037T, ORF1ab:C3096T, ORF1ab:G9738C, ORF1ab:G13713A, ORF1ab:C14408T, ORF1ab:C16394T, ORF1ab:G17014T, ORF1ab:G17964T, ORF1ab:C18526T, ORF1ab:T21326C, S:G21600T, S:G22018T, S:T22917G, S:A23403G, ORF3a:G25563T, M:C26681T, ORF7b:G27870T, ORF8:G28178T, ORF8:A28272T. N:C28887T. N:C29362T.	17	N:T205I, ORF1a:T265I, ORF1a:S944L, ORF1a:S3158T, ORF1b:P314L, ORF1b:P976L, ORF1b:D1183Y, ORF1b:M1499I, ORF1b:R1687C, ORF1b:V2620A, ORF3a:Q57H, ORF7b:E39*, ORF8:L95F, S:S13I, S:W152C, S:L452R, S:D614G,

hCoV-19/Mexico/BCS-InDRE_F11109_S606/2021	EPI_ISL_1337392	21C (Epsilon)	B.1.427	21	5'UTR:C241T, ORF1ab:C1059T, ORF1ab:T1195C, ORF1ab:C3037T, ORF1ab:G9738C, ORF1ab:G13713A, ORF1ab:C14408T, ORF1ab:C16394T, ORF1ab:G17014T, ORF1ab:G17964T, ORF1ab:G18315A, ORF1ab:T21326C, S:G21600T, S:G22018T, S:T22917G, S:A23403G, ORF3a:G25563T, M:C26681T, ORF8:G28178T, ORF8:A28272T, N:C28887T, N:C29362T,	14	N:T205I, ORF1a:T265I, ORF1a:S3158T, ORF1b:P314L, ORF1b:P976L, ORF1b:D1183Y, ORF1b:M1499I, ORF1b:V2620A, ORF3a:Q57H, ORF8:L95F, S:S13I, S:W152C, S:L452R, S:D614G,
hCoV-19/Mexico/BCS-InDRE_F11116_S607/2021	EPI_ISL_1337393	21C (Epsilon)	B.1.427	21	5'UTR:C241T, ORF1ab:C1059T, ORF1ab:C3037T, ORF1ab:C3096T, ORF1ab:G9738C, ORF1ab:G13713A, ORF1ab:C14408T, ORF1ab:C16394T, ORF1ab:G17014T, ORF1ab:G17964T, ORF1ab:C18526T, ORF1ab:T21326C, S:G21600T, S:G22018T, S:T22917G, S:A23403G, ORF3a:G25563T, M:C26681T, ORF8:G28178T, ORF8:A28272T, N:C28887T, N:C29362T,	16	N:T205I, ORF1a:T265I, ORF1a:S944L, ORF1a:S3158T, ORF1b:P314L, ORF1b:P976L, ORF1b:D1183Y, ORF1b:M1499I, ORF1b:R1687C, ORF1b:V2620A, ORF3a:Q57H, ORF8:L95F, S:S13I, S:W152C, S:L452R, S:D614G,
hCoV-19/Mexico/BCS-InDRE-25/2020	EPI_ISL_493340	20A	B.1.1.334	5	5'UTR:C241T, ORF1ab:C3037T, ORF1ab:C14408T, ORF1ab:A20268G, ORF1ab:C20474T, S:A23403G,	3	ORF1b:P314L, ORF1b:T2336I, S:D614G,
hCoV-19/Mexico/CAM-InDRE_543/2021	EPI_ISL_1168613	20A	B.1	21	5'UTR:C241T, ORF1ab:G1820A, ORF1ab:C3037T, ORF1ab:C3429T, ORF1ab:C3457T, ORF1ab:A5894G, ORF1ab:C6388T, ORF1ab:C6487T, ORF1ab:G8536A, ORF1ab:G9928T, ORF1ab:G11540T, ORF1ab:C14408T, ORF1ab:C16575T, ORF1ab:G19677T, ORF1ab:A20268G, ORF1ab:C20762T, S:C21707T, S:A23403G, M:C26894T, M:G27074A, N:C28854T, N:A29112T,	12	N:S194L, N:E280V, ORF1a:G519S, ORF1a:T1055I, ORF1a:I1877V, ORF1a:M3221I, ORF1a:V3759F, ORF1b:P314L, ORF1b:Q2070H, ORF1b:T2432I, S:H49Y, S:D614G,
hCoV-19/Mexico/CAM-InDRE_544/2021	EPI_ISL_1168614	20B	B.1.1.519	24	5'UTR:T201C, 5'UTR:C203T, 5'UTR:C222T, 5'UTR:C241T, ORF1ab:C745T, ORF1ab:G1738T, ORF1ab:C3037T, ORF1ab:C3140T, ORF1ab:C10029T, ORF1ab:C10954T, ORF1ab:A11117G, ORF1ab:C12789T, ORF1ab:C14408T, ORF1ab:C17135T, ORF1ab:T19839C, ORF1ab:A19974G, ORF1ab:C21306T, S:C22995A, S:A23403G, S:C23604A, S:A23756G, N:G28881A, N:G28882A, N:G28883C, N:C29197T,	12	N:R203K, N:G204R, ORF1a:P959S, ORF1a:T3255I, ORF1a:I3618V, ORF1a:T4175I, ORF1b:P314L, ORF1b:P1223L, S:T478K, S:D614G, S:P681H, S:T732A,
hCoV-19/Mexico/CAM-InDRE_F10557_S813/2021	EPI_ISL_1424015	20B	B.1.1.222	23	5'UTR:C241T, ORF1ab:C3037T, ORF1ab:C4897T, ORF1ab:C6402T, ORF1ab:C9665T, ORF1ab:C10029T, ORF1ab:C14408T, ORF1ab:C19011A, ORF1ab:T19839C, ORF1ab:A21137G, S:C21846T, S:T23008C, S:A23403G, S:C23415T, S:A23756G, ORF3a:G25912T, ORF3a:C26058T, ORF3a:A26115C, M:C27059T, ORF8:A27921G, ORF8:G28001T, N:G28881A, N:G28882A, N:G28883C,	15	N:R203K, N:G204R, ORF1a:P2046L, ORF1a:P3134S, ORF1a:T3255I, ORF1b:P314L, ORF1b:D1848E, ORF1b:K2557R, ORF3a:G174C, ORF3a:E241D, ORF8:I10V, S:T95I, S:D614G, S:T618I, S:T732A,
hCoV-19/Mexico/CAM-InDRE_F10946_S753/2021	EPI_ISL_1400299	20B	B.1.1.519	24	5'UTR:T201C, 5'UTR:C203T, 5'UTR:C222T, 5'UTR:C241T, ORF1ab:G1738T, ORF1ab:C3037T, ORF1ab:C3140T, ORF1ab:T6661C, ORF1ab:T9179C, ORF1ab:C9207T, ORF1ab:C10029T, ORF1ab:C10954T, ORF1ab:A11117G, ORF1ab:C12789T, ORF1ab:C14408T, ORF1ab:T19839C, ORF1ab:A19974G, S:C22995A, S:A23403G, S:C23604A, S:A23756G, N:G28881A, N:G28882A, N:G28883C, N:C29197T,	13	N:R203K, N:G204R, ORF1a:P959S, ORF1a:S2972P, ORF1a:S2981F, ORF1a:T3255I, ORF1a:I3618V, ORF1a:T4175I, ORF1b:P314L, S:T478K, S:D614G, S:P681H, S:T732A,
hCoV-19/Mexico/CAM-InDRE-62/2020	EPI_ISL_576265	20A	B.1	16	5'UTR:C241T, ORF1ab:G940A, ORF1ab:C3037T, ORF1ab:C6648T, ORF1ab:T8188C, ORF1ab:C14408T, ORF1ab:G15666A, ORF1ab:A19298C, ORF1ab:T19301C, ORF1ab:A19302T, ORF1ab:A20268G, S:A23403G, S:A24002G, ORF7b:G27798T, N:C28831T, N:C28854T, 3'UTR:C29774T,	8	N:S194L, ORF1a:A2128V, ORF1b:P314L, ORF1b:Y1944S, ORF1b:V1945A, ORF7b:A15S, S:D614G, S:K814E,

hCoV-19/Mexico/CAM-InDRE-63/2020	EPI_ISL_576266	20D	C.23	17	5'UTR:C241T, ORF1ab:C3037T, ORF1ab:C4002T, ORF1ab:G4180T, ORF1ab:C4784T, ORF1ab:G10097A, ORF1ab:C13536T, ORF1ab:C14408T, ORF1ab:C15240T, ORF1ab:C17766T, S:A23403G, S:C23731T, S:C24865T, ORF3a:G25770T, N:G28881A, N:G28882A, N:G28883C, N:C29311T,	9	N:R203K, N:G204R, ORF1a:T1246I, ORF1a:K1305N, ORF1a:L1507F, ORF1a:G3278S, ORF1b:P314L, ORF3a:R126S, S:D614G,
hCoV-19/Mexico/CAM-InDRE-64/2020	EPI_ISL_576267	20C	B.1	14	5'UTR:C241T, ORF1ab:G625T, ORF1ab:C1059T, ORF1ab:C3037T, ORF1ab:C3738T, ORF1ab:C5348A, ORF1ab:G5432A, ORF1ab:C14408T, ORF1ab:T14949C, ORF1ab:G19312A, ORF1ab:T19460A, ORF1ab:A20755C, S:A23403G, ORF3a:G25563T, N:G28899T,	12	N:R209I, ORF1a:K120N, ORF1a:T265I, ORF1a:P1158L, ORF1a:L1695I, ORF1a:G1723S, ORF1b:P314L, ORF1b:A1949T, ORF1b:I1998K, ORF1b:S2430R, ORF3a:Q57H, S:D614G,
hCoV-19/Mexico/CHH-InDRE_325/2020	EPI_ISL_1060762	20B	B.1.1	11	ORF1ab:C498T, ORF1ab:T667C, ORF1ab:C3037T, ORF1ab:C5826T, ORF1ab:C14408T, ORF1ab:C18788T, ORF1ab:T19839C, S:A23403G, ORF3a:C25626T, N:G28881A, N:G28882A, N:G28883C,	7	N:R203K, N:G204R, ORF1a:T78I, ORF1a:T1854I, ORF1b:P314L, ORF1b:T1774I, S:D614G,
hCoV-19/Mexico/CHH-InDRE_366/2020	EPI_ISL_1060678	20A	B.1.189	18	ORF1ab:G1306T, ORF1ab:A1964G, ORF1ab:T2116A, ORF1ab:C3037T, ORF1ab:A3940G, ORF1ab:C4582T, ORF1ab:G6404T, ORF1ab:C9724T, ORF1ab:C14408T, ORF1ab:C17010T, ORF1ab:A20268G, ORF1ab:C21304A, ORF1ab:G21305A, S:A23403G, S:G23593T, S:C23604A, ORF3a:T25689C, ORF7b:C27879T, N:G28881T,	11	N:R203M, ORF1a:E347D, ORF1a:I567V, ORF1a:F617L, ORF1a:V2047F, ORF1b:P314L, ORF1b:R2613N, ORF7b:H42Y, S:D614G, S:Q677H, S:P681H,
hCoV-19/Mexico/CHH-InDRE_F11007_S878/2021	EPI_ISL_1405905	20A	B.1.609	12	5'UTR:C241T, ORF1ab:C3037T, ORF1ab:C3743T, ORF1ab:C4582T, ORF1ab:C9724T, ORF1ab:C14408T, ORF1ab:A20268G, S:A23403G, S:G23593T, S:C23604A, ORF3a:G25726T, ORF7b:C27879T, N:G28881T,	8	N:R203M, ORF1a:H1160Y, ORF1b:P314L, ORF3a:V112F, ORF7b:H42Y, S:D614G, S:Q677H, S:P681H,
hCoV-19/Mexico/CHH-InDRE_F11009_S866/2021	EPI_ISL_1399271	20G	B.1.2	21	5'UTR:C241T, ORF1ab:C1059T, ORF1ab:C2536T, ORF1ab:C2662T, ORF1ab:C3037T, ORF1ab:C5869T, ORF1ab:G7936T, ORF1ab:G8083A, ORF1ab:C10319T, ORF1ab:C14408T, ORF1ab:C14805T, ORF1ab:A18424G, ORF1ab:C21304T, S:A23403G, ORF3a:G25563T, ORF3a:G25907T, ORF8:C27964T, N:C28472T, N:C28869T, N:A29320C, N:G29402T, 3'UTR:A29752G,	14	N:P67S, N:P199L, N:Q349H, N:D377Y, ORF1a:T265I, ORF1a:M2606I, ORF1a:L3352F, ORF1b:P314L, ORF1b:N1653D, ORF1b:R2613C, ORF3a:Q57H, ORF3a:G172V, ORF8:S24L, S:D614G,
hCoV-19/Mexico/CHH-InDRE_F11012_S819/2021	EPI_ISL_1424021	20B	B.1.1.519	25	5'UTR:C203T, 5'UTR:C222T, 5'UTR:C241T, ORF1ab:C2005T, ORF1ab:C3037T, ORF1ab:C3045T, ORF1ab:C3140T, ORF1ab:T3745C, ORF1ab:C7296T, ORF1ab:C10029T, ORF1ab:C10954T, ORF1ab:A11117G, ORF1ab:C12789T, ORF1ab:C14408T, ORF1ab:T19839C, ORF1ab:C21306T, S:C22995A, S:A23403G, S:C23604A, S:A23756G, ORF3a:C25844T, N:G28307A, N:G28881A, N:G28882A, N:G28883C, N:C29197T,	16	N:A12T, N:R203K, N:G204R, ORF1a:P927L, ORF1a:P959S, ORF1a:A2344V, ORF1a:T3255I, ORF1a:I3618V, ORF1a:T4175I, ORF1b:P314L, ORF3a:T151I, ORF9b:M8I, S:T478K, S:D614G, S:P681H, S:T732A,
hCoV-19/Mexico/CHH-InDRE-43/2020	EPI_ISL_516613	20C	B.1.567	17	5'UTR:C241T, ORF1ab:C1059T, ORF1ab:C3037T, ORF1ab:C3768T, ORF1ab:G6421T, ORF1ab:T6640A, ORF1ab:C8739T, ORF1ab:C10319T, ORF1ab:C11575T, ORF1ab:C14408T, ORF1ab:C17502T, ORF1ab:A20262G, ORF1ab:G21305A, S:A23403G, ORF3a:G25563T, ORF8:C27964T, ORF8:C28087T, N:C28868T,	12	N:P199S, ORF1a:T265I, ORF1a:T1168I, ORF1a:H2125Q, ORF1a:T2825I, ORF1a:L3352F, ORF1b:P314L, ORF1b:R2613H, ORF3a:Q57H, ORF8:S24L, ORF8:A65V, S:D614G,
hCoV-19/Mexico/CHH-InDRE-46/2020	EPI_ISL_516614	20A	B.1.609	13	5'UTR:C241T, ORF1ab:A1881G, ORF1ab:C3037T, ORF1ab:C4582T, ORF1ab:C6402T, ORF1ab:C9532T, ORF1ab:C10833T, ORF1ab:C13944T, ORF1ab:C14408T, ORF1ab:C14931T, ORF1ab:C16954T, ORF1ab:A20268G, ORF1ab:G20931T, S:A23403G,	5	ORF1a:E539G, ORF1a:P2046L, ORF1a:A3523V, ORF1b:P314L, S:D614G,

hCoV-19/Mexico/CHH-InDRE-47/2020	EPI_ISL_516615	20C	B.1	12	5'UTR:C241T, ORF1ab:T582C, ORF1ab:C1059T, ORF1ab:C3037T, ORF1ab:C8733T, ORF1ab:C10279T, ORF1ab:A14246G, ORF1ab:C14408T, ORF1ab:C17747T, S:G21898T, S:A23403G, ORF3a:G25563T, ORF7a:A27456G,	8	ORF1a:V106A, ORF1a:T265I, ORF1a:T2823I, ORF1b:D260G, ORF1b:P314L, ORF1b:P1427L, ORF3a:Q57H, S:D614G,
hCoV-19/Mexico/CHH-InDRE-48/2020	EPI_ISL_516616	20A	B.1.609	13	5'UTR:C241T, ORF1ab:A1881G, ORF1ab:C3037T, ORF1ab:C4582T, ORF1ab:C6402T, ORF1ab:C9532T, ORF1ab:C10833T, ORF1ab:C13944T, ORF1ab:C14408T, ORF1ab:C14931T, ORF1ab:C16954T, ORF1ab:A20268G, ORF1ab:G20931T, S:A23403G,	5	ORF1a:E539G, ORF1a:P2046L, ORF1a:A3523V, ORF1b:P314L, S:D614G,
hCoV-19/Mexico/CHH-InDRE-49/2020	EPI_ISL_516617	20A	B.1.609	12	5'UTR:C241T, ORF1ab:G2265A, ORF1ab:C3037T, ORF1ab:C4582T, ORF1ab:C4993T, ORF1ab:C11941T, ORF1ab:C14408T, ORF1ab:T18228C, ORF1ab:A20268G, S:A23403G, ORF3a:C25688T, M:C26735T, 3'UTR:C29733T,	4	ORF1a:C667Y, ORF1b:P314L, ORF3a:A99V, S:D614G,
hCoV-19/Mexico/CHP-InDRE_107/2020	EPI_ISL_913911	20A	B.1	11	5'UTR:C241T, ORF1ab:A2806C, ORF1ab:C3037T, ORF1ab:T13857C, ORF1ab:C14408T, ORF1ab:A20268G, S:A23403G, ORF3a:G25477T, ORF3a:G25947T, ORF7a:C27418T, N:C28854T, N:C29171T,	7	N:S194L, N:H300Y, ORF1a:E847D, ORF1b:P314L, ORF3a:V29F, ORF3a:Q185H, S:D614G,
hCoV-19/Mexico/CHP-InDRE_108/2020	EPI_ISL_913912	20A	B.1	11	5'UTR:T209C, 5'UTR:C241T, ORF1ab:C3037T, ORF1ab:G11616A, ORF1ab:C14408T, ORF1ab:G14653T, ORF1ab:G19348T, ORF1ab:A20268G, S:A23403G, ORF3a:C25517G, ORF3a:G25644T, N:C28854T,	7	N:S194L, ORF1a:C3784Y, ORF1b:P314L, ORF1b:V396F, ORF1b:V1961F, ORF3a:P42R, S:D614G,
hCoV-19/Mexico/CHP-InDRE_131/2020	EPI_ISL_913934	20A	B.1	13	5'UTR:C241T, ORF1ab:G1820A, ORF1ab:C3037T, ORF1ab:C3429T, ORF1ab:C3457T, ORF1ab:A5894G, ORF1ab:G8536A, ORF1ab:G9928T, ORF1ab:G11540T, ORF1ab:C14408T, ORF1ab:C16575T, ORF1ab:A20268G, S:A23403G, N:C28854T,	8	N:S194L, ORF1a:G519S, ORF1a:T1055I, ORF1a:I1877V, ORF1a:M3221I, ORF1a:V3759F, ORF1b:P314L, S:D614G,
hCoV-19/Mexico/CHP-InDRE_132/2020	EPI_ISL_913935	20B	B.1	18	5'UTR:C241T, ORF1ab:C1913T, ORF1ab:C3037T, ORF1ab:C3040T, ORF1ab:C10456T, ORF1ab:A14406C, ORF1ab:C14408T, ORF1ab:G15906T, ORF1ab:G19684T, ORF1ab:T19839C, ORF1ab:C20148T, S:G22484T, S:A23403G, S:C24370T, ORF7a:G27478T, N:G28881A, N:G28882A, N:G28883C, N:G29422A,	9	N:R203K, N:G204R, ORF1a:R550C, ORF1b:P314L, ORF1b:Q813H, ORF1b:V2073L, ORF7a:V29L, S:V308L, S:D614G,
hCoV-19/Mexico/CHP-InDRE_168/2020	EPI_ISL_913969	20A	B.1	13	5'UTR:C241T, ORF1ab:C3037T, ORF1ab:T4183A, ORF1ab:T6640C, ORF1ab:C9438T, ORF1ab:G11083T, ORF1ab:A12869G, ORF1ab:A13868G, ORF1ab:C14408T, ORF1ab:C17762T, ORF1ab:C20544T, S:C22264T, S:A23403G, N:C28854T,	8	N:S194L, ORF1a:T3058I, ORF1a:L3606F, ORF1a:T4202A, ORF1b:K134R, ORF1b:P314L, ORF1b:A1432V, S:D614G,
hCoV-19/Mexico/CHP-InDRE_264/2020	EPI_ISL_1054962	20A	B.1	8	5'UTR:C241T, ORF1ab:C925T, ORF1ab:C3037T, ORF1ab:G9773A, ORF1ab:T12069A, ORF1ab:C14408T, ORF1ab:A20268G, S:A23403G, N:C28854T,	5	N:S194L, ORF1a:V3170I, ORF1a:L3935Q, ORF1b:P314L, S:D614G,
hCoV-19/Mexico/CHP-InDRE_265/2020	EPI_ISL_1054963	20B	B.1.1	15	5'UTR:C241T, ORF1ab:C3037T, ORF1ab:C12412T, ORF1ab:T14313C, ORF1ab:C14408T, ORF1ab:C14649T, ORF1ab:C17550T, ORF1ab:G19656A, ORF1ab:A21137G, S:A23403G, S:T24565C, S:T24691C, N:C28310T, N:G28881A, N:G28882A, N:G28883C,	6	N:P13S, N:R203K, N:G204R, ORF1b:P314L, ORF1b:K2557R, S:D614G,
hCoV-19/Mexico/CHP-InDRE_396/2020	EPI_ISL_1054973	20B	B.1.1	7	5'UTR:C241T, ORF1ab:C3037T, ORF1ab:C14408T, ORF1ab:T19839C, S:A23403G, N:G28881A, N:G28882A, N:G28883C,	4	N:R203K, N:G204R, ORF1b:P314L, S:D614G,
hCoV-19/Mexico/CHP-InDRE_397/2020	EPI_ISL_1054974	20A	B.1.243	13	5'UTR:C241T, ORF1ab:G806A, ORF1ab:C3037T, ORF1ab:C8818T, ORF1ab:C14408T, ORF1ab:A14955G, ORF1ab:A20268G, S:C21707T, S:A23403G, S:T24076C, ORF3a:C25511T, N:C28854T, N:G29543T, 3'UTR:C29739T,	6	N:S194L, ORF1a:A181T, ORF1b:P314L, ORF3a:S40L, S:H49Y, S:D614G,

hCoV-19/Mexico/CHP-InDRE_443/2021	EPI_ISL_1168521	20B	B.1.1.519	25	5'UTR:T201C, 5'UTR:C203T, 5'UTR:C222T, 5'UTR:C241T, ORF1ab:T277C, ORF1ab:G1738T, ORF1ab:C3037T, ORF1ab:C3140T, ORF1ab:G7829T, ORF1ab:C10029T, ORF1ab:C10954T, ORF1ab:A11117G, ORF1ab:C12789T, ORF1ab:C14408T, ORF1ab:T16449C, ORF1ab:T19839C, ORF1ab:A19974G, ORF1ab:C21306T, S:C22995A, S:A23403G, S:C23604A, S:A23756G, N:G28881A, N:G28882A, N:G28883C, N:C29197T,	12	N:R203K, N:G204R, ORF1a:P959S, ORF1a:V2522F, ORF1a:T3255I, ORF1a:I3618V, ORF1a:T4175I, ORF1b:P314L, S:T478K, S:D614G, S:P681H, S:T732A,
hCoV-19/Mexico/CHP-InDRE_444/2021	EPI_ISL_1168522	20B	B.1.1.519	24	5'UTR:C203T, 5'UTR:C222T, 5'UTR:C241T, ORF1ab:C3037T, ORF1ab:C3140T, ORF1ab:T3745C, ORF1ab:C10029T, ORF1ab:C10954T, ORF1ab:A11117G, ORF1ab:C12789T, ORF1ab:C14408T, ORF1ab:T14817C, ORF1ab:T19839C, ORF1ab:C21306T, S:C22995A, S:A23403G, S:C23604A, S:A23756G, S:G25012A, ORF3a:C25844T, E:G26439T, N:G28881A, N:G28882A, N:G28883C, N:C29197T,	12	N:R203K, N:G204R, ORF1a:P959S, ORF1a:T3255I, ORF1a:I3618V, ORF1a:T4175I, ORF1b:P314L, ORF3a:T151I, S:T478K, S:D614G, S:P681H, S:T732A,
hCoV-19/Mexico/CHP-InDRE_445/2021	EPI_ISL_1168523	20B	B.1.1.519	24	5'UTR:T201C, 5'UTR:C203T, 5'UTR:C222T, 5'UTR:C241T, ORF1ab:T277C, ORF1ab:G1738T, ORF1ab:C3037T, ORF1ab:C3140T, ORF1ab:G7829T, ORF1ab:C10029T, ORF1ab:C10954T, ORF1ab:A11117G, ORF1ab:C12789T, ORF1ab:C14408T, ORF1ab:T19839C, ORF1ab:A19974G, ORF1ab:C21306T, S:C22995A, S:A23403G, S:C23604A, S:A23756G, N:G28881A, N:G28882A, N:G28883C, N:C29197T,	12	N:R203K, N:G204R, ORF1a:P959S, ORF1a:V2522F, ORF1a:T3255I, ORF1a:I3618V, ORF1a:T4175I, ORF1b:P314L, S:T478K, S:D614G, S:P681H, S:T732A,
hCoV-19/Mexico/CHP-InDRE_F10422_S811/2021	EPI_ISL_1424013	20B	B.1.1.222	23	5'UTR:C241T, ORF1ab:C1758T, ORF1ab:G2086T, ORF1ab:C3037T, ORF1ab:G4205A, ORF1ab:C8770T, ORF1ab:C10029T, ORF1ab:C14408T, ORF1ab:C15279T, ORF1ab:C19164T, ORF1ab:T19425C, ORF1ab:G19816T, ORF1ab:T19839C, S:A23403G, S:A23756G, ORF3a:G25912T, ORF7b:A27756G, ORF8:A27921G, ORF8:G28001T, N:G28881A, N:G28882A, N:G28883C, N:G28895T, N:G29402T,	15	N:R203K, N:G204R, N:A208S, N:D377Y, ORF1a:A498V, ORF1a:Q607H, ORF1a:A1314T, ORF1a:T3255I, ORF1b:P314L, ORF1b:V2117L, ORF3a:G174C, ORF7b:M1V, ORF8:I10V, S:D614G, S:T732A,
hCoV-19/Mexico/CHP-InDRE-23/2020	EPI_ISL_493338	20A	B.1	6	5'UTR:C241T, ORF1ab:C3037T, ORF1ab:C4582T, ORF1ab:G14580T, ORF1ab:A20268G, S:C21762T, S:A23403G,	3	ORF1b:M371I, S:A67V, S:D614G,
hCoV-19/Mexico/CMX-INCMNSZ_COV121371/202(	EPI_ISL_1502816	20B	B.1.1.222	18	5'UTR:C241T, ORF1ab:C3037T, ORF1ab:C3140T, ORF1ab:T6335A, ORF1ab:A10323G, ORF1ab:A10333G, ORF1ab:C14408T, ORF1ab:C16349T, ORF1ab:A17013G, ORF1ab:T19839C, S:A22681T, S:A23403G, S:A23756G, ORF7a:A27474T, ORF8:G27996T, N:G28881A, N:G28882A, N:G28883C, 3'UTR:C29857T,	10	N:R203K, N:G204R, ORF1a:P959S, ORF1a:S2024T, ORF1a:K3353R, ORF1b:P314L, ORF1b:S961L, ORF8:D35Y, S:D614G, S:T732A,
hCoV-19/Mexico/CMX-INCMNSZ_COV145587/2021	EPI_ISL_1502817	20B	B.1.1.222	26	5'UTR:A1C, 5'UTR:T2C, 5'UTR:C241T, ORF1ab:T400C, ORF1ab:T682C, ORF1ab:C3037T, ORF1ab:T5860C, ORF1ab:G7387A, ORF1ab:C9430T, ORF1ab:G11087A, ORF1ab:C14408T, ORF1ab:T14787C, ORF1ab:C18431T, ORF1ab:T19839C, S:G23236T, S:A23403G, S:A23756G, M:G26951T, ORF8:G28167A, N:C28344T, N:G28881A, N:G28882A, N:G28883C, N:A29190T, N:A29334G, 3'UTR:G29751T, 3'UTR:G29828A,	13	N:T24I, N:R203K, N:G204R, N:Q306L, N:N354S, ORF1a:M2374I, ORF1a:E3608K, ORF1b:P314L, ORF1b:T1655I, ORF8:E92K, S:K558N, S:D614G, S:T732A,

hCoV-19/Mexico/CMX-INCMNSZ_COV149517/2021	EPI_ISL_1502814	20B	B.1.1.432	26	5'UTR:T2C, 5'UTR:T3C, 5'UTR:C241T, ORF1ab:G1312T, ORF1ab:A2761G, ORF1ab:C3037T, ORF1ab:A6985T, ORF1ab:C8092T, ORF1ab:C9319T, ORF1ab:G9526T, ORF1ab:C12412T, ORF1ab:T14313C, ORF1ab:C14408T, ORF1ab:C16208T, S:C21846T, S:G22201T, S:A23403G, ORF3a:A25524G, ORF3a:C25613T, ORF3a:C25824T, ORF3a:G26062T, N:C28603T, N:G28881A, N:G28882A, N:G28883C, N:T29317C,5'UTR:A1G,	10	N:R203K, N:G204R, ORF1a:L349F, ORF1a:M3087I, ORF1b:P314L, ORF1b:A914V, ORF3a:S74F, ORF3a:G224C, S:T95I, S:D614G,
hCoV-19/Mexico/CMX-INCMNSZ_COV152225/2021	EPI_ISL_1503143	20B	B.1.1.519	25	5'UTR:A4G, 5'UTR:A5G, 5'UTR:C203T, 5'UTR:C222T, 5'UTR:C241T, ORF1ab:C3037T, ORF1ab:C3140T, ORF1ab:C10029T, ORF1ab:C10440T, ORF1ab:C10954T, ORF1ab:A11117G, ORF1ab:C12789T, ORF1ab:C14408T, ORF1ab:G15594T, ORF1ab:G15921T, ORF1ab:T19839C, ORF1ab:C21306T, S:C22995A, S:A23403G, S:C23604A, S:A23756G, N:G28881A, N:G28882A, N:G28883C, N:C29197T, 3'UTR:A29850T,	13	N:R203K, N:G204R, ORF1a:P959S, ORF1a:T3255I, ORF1a:A3392V, ORF1a:I3618V, ORF1a:T4175I, ORF1b:P314L, ORF1b:K709N, S:T478K, S:D614G, S:P681H, S:T732A,
hCoV-19/Mexico/CMX-INCMNSZ_COV159238/2021	EPI_ISL_1502815	20B	B.1.1.519	27	5'UTR:C203T, 5'UTR:C222T, 5'UTR:C241T, ORF1ab:C3037T, ORF1ab:C3140T, ORF1ab:C9166T, ORF1ab:C9763T, ORF1ab:C10029T, ORF1ab:C10954T, ORF1ab:A11117G, ORF1ab:C12789T, ORF1ab:T13769G, ORF1ab:C14408T, ORF1ab:C16887T, ORF1ab:T19839C, ORF1ab:A21062G, ORF1ab:C21306T, S:C22995A, S:A23403G, S:C23604A, S:A23756G, N:G28881A, N:G28882A, N:G28883C, N:C29197T, N:C29466T, 3'UTR:C29769T,5'UTR:T2A	14	N:R203K, N:G204R, N:A398V, ORF1a:P959S, ORF1a:T3255I, ORF1a:I3618V, ORF1a:T4175I, ORF1b:M101R, ORF1b:P314L, ORF1b:K2532R, S:T478K, S:D614G, S:P681H, S:T732A,
hCoV-19/Mexico/CMX-INCMNSZ-01/2020	EPI_ISL_426361	19B	A.1	7	ORF1ab:C8782T, ORF1ab:C17747T, ORF1ab:A17858G, ORF1ab:C18060T, S:C21707T, S:C23422T, S:A24694T, ORF8:T28144C,	4	ORF1b:P1427L, ORF1b:Y1464C, ORF8:L84S, S:H49Y,
hCoV-19/Mexico/CMX-INCMNSZ-02/2020	EPI_ISL_426362	19B	A.2	7	ORF1ab:C8782T, ORF1ab:T9477A, ORF1ab:C14805T, S:C23280T, ORF3a:G25979T, ORF8:T28144C, N:C28657T, N:C28863T,	5	N:S197L, ORF1a:F3071Y, ORF3a:G196V, ORF8:L84S, S:T573I,
hCoV-19/Mexico/CMX-INCMNSZ-03/2020	EPI_ISL_426363	19B	A.1	7	ORF1ab:C8782T, ORF1ab:C17747T, ORF1ab:A17858G, ORF1ab:C18060T, S:C21707T, S:C23422T, S:A24694T, ORF8:T28144C,	4	ORF1b:P1427L, ORF1b:Y1464C, ORF8:L84S, S:H49Y,
hCoV-19/Mexico/CMX-INCMNSZ-04/2020	EPI_ISL_426364	19B	A.1	7	ORF1ab:C8782T, ORF1ab:C17747T, ORF1ab:A17858G, ORF1ab:C18060T, S:C21707T, S:C23422T, S:A24694T, ORF8:T28144C,	4	ORF1b:P1427L, ORF1b:Y1464C, ORF8:L84S, S:H49Y,
hCoV-19/Mexico/CMX-INCMNSZ-05/2020	EPI_ISL_426365	20A	B.1	7	5'UTR:C241T, ORF1ab:C1387T, ORF1ab:C3037T, ORF1ab:C14408T, ORF1ab:A20268G, S:A23403G, S:G25337T, 3'UTR:G29734C,	3	ORF1b:P314L, S:D614G, S:D1259Y,
hCoV-19/Mexico/CMX-InDRE_03/2020	EPI_ISL_424667	19B	A.5	3	ORF1ab:C8782T, ORF1ab:C17470T, ORF3a:C26088T, ORF8:T28144C,	1	ORF8:L84S,
hCoV-19/Mexico/CMX-InDRE_12/2020	EPI_ISL_455436	20A	B.1	7	5'UTR:C241T, ORF1ab:C1387T, ORF1ab:C3037T, ORF1ab:C14408T, ORF1ab:A20268G, S:A23403G, S:G25337T, 3'UTR:G29734C,	3	ORF1b:P314L, S:D614G, S:D1259Y,
hCoV-19/Mexico/CMX-InDRE_15/2020	EPI_ISL_455439	19B	A.1	7	ORF1ab:C8782T, ORF1ab:C17747T, ORF1ab:A17858G, ORF1ab:C18060T, S:C21707T, S:C23422T, S:A24694T, ORF8:T28144C,	4	ORF1b:P1427L, ORF1b:Y1464C, ORF8:L84S, S:H49Y,
hCoV-19/Mexico/CMX-InDRE_154/2020	EPI_ISL_914879	20B	B.1.1	12	5'UTR:C241T, ORF1ab:C2973T, ORF1ab:C3037T, ORF1ab:C4990T, ORF1ab:C14408T, ORF1ab:T19839C, ORF1ab:C19862T, S:A23403G, ORF8:G28077T, N:G28774T, N:G28881A, N:G28882A, N:G28883C,	8	N:L167F, N:R203K, N:G204R, ORF1a:A903V, ORF1b:P314L, ORF1b:A2132V, ORF8:V62L, S:D614G,

hCoV-19/Mexico/CMX-InDRE_160/2020	EPI_ISL_913962	20B	B.1.1.519	22	5'UTR:C203T, 5'UTR:C222T, 5'UTR:C241T, ORF1ab:G1738T, ORF1ab:A2166G, ORF1ab:C3037T, ORF1ab:C3140T, ORF1ab:C10029T, ORF1ab:C10954T, ORF1ab:A11117G, ORF1ab:C12789T, ORF1ab:C14408T, ORF1ab:T19839C, ORF1ab:A19974G, ORF1ab:C21306T, S:C22995A, S:A23403G, S:C23604A, S:A23756G, N:G28881A, N:G28882A, N:G28883C, N:C29197T,	12	N:R203K, N:G204R, ORF1a:K634R, ORF1a:P959S, ORF1a:T3255I, ORF1a:I3618V, ORF1a:T4175I, ORF1b:P314L, S:T478K, S:D614G, S:P681H, S:T732A,
hCoV-19/Mexico/CMX-InDRE_166/2020	EPI_ISL_913967	20B	B.1.1.222	11	5'UTR:C241T, ORF1ab:C3037T, ORF1ab:T10665C, ORF1ab:C14408T, ORF1ab:G19009A, ORF1ab:T19839C, S:A23403G, S:A23756G, N:G28881A, N:G28882A, N:G28883C, N:C29144T,	7	N:R203K, N:G204R, ORF1a:V3467A, ORF1b:P314L, ORF1b:D1848N, S:D614G, S:T732A,
hCoV-19/Mexico/CMX-InDRE_178/2020	EPI_ISL_913979	20B	B.1.1.519	24	5'UTR:T201C, 5'UTR:C203T, 5'UTR:C222T, 5'UTR:C241T, ORF1ab:C745T, ORF1ab:G1738T, ORF1ab:C3037T, ORF1ab:C3140T, ORF1ab:C10029T, ORF1ab:C10954T, ORF1ab:A11117G, ORF1ab:C12789T, ORF1ab:C14408T, ORF1ab:T19839C, ORF1ab:A19974G, ORF1ab:C21306T, S:C22995A, S:A23403G, S:C23604A, S:A23756G, ORF8:G28086A, N:G28881A, N:G28882A, N:G28883C, N:C29197T,	12	N:R203K, N:G204R, ORF1a:P959S, ORF1a:T3255I, ORF1a:I3618V, ORF1a:T4175I, ORF1b:P314L, ORF8:A65T, S:T478K, S:D614G, S:P681H, S:T732A,
hCoV-19/Mexico/CMX-InDRE_179/2020	EPI_ISL_913980	20B	B.1.1.519	24	5'UTR:C203T, 5'UTR:C222T, 5'UTR:C241T, ORF1ab:T1303A, ORF1ab:C3037T, ORF1ab:C3140T, ORF1ab:A7857G, ORF1ab:C10029T, ORF1ab:C10954T, ORF1ab:A11117G, ORF1ab:C12789T, ORF1ab:C14408T, ORF1ab:G15627T, ORF1ab:T19839C, ORF1ab:C20132T, ORF1ab:C21306T, S:C22995A, S:A23403G, S:C23604A, S:A23756G, N:G28881A, N:G28882A, N:G28883C, N:C29197T, ORF10:T29661C,	14	N:R203K, N:G204R, ORF1a:P959S, ORF1a:N2531S, ORF1a:T3255I, ORF1a:I3618V, ORF1a:T4175I, ORF1b:P314L, ORF1b:E720D, ORF1b:A2222V, S:T478K, S:D614G, S:P681H, S:T732A,
hCoV-19/Mexico/CMX-InDRE_184/2021	EPI_ISL_933661	20B	B.1.1.222	19	5'UTR:C241T, ORF1ab:G1721A, ORF1ab:A2969G, ORF1ab:C3037T, ORF1ab:C10029T, ORF1ab:C14408T, ORF1ab:C19011A, ORF1ab:A19308G, ORF1ab:T19839C, ORF1ab:A21137G, S:A23403G, S:C23415T, S:A23756G, ORF3a:G25912T, ORF3a:A26115C, ORF8:A27921G, ORF8:G28001T, N:G28881A, N:G28882A, N:G28883C,	14	N:R203K, N:G204R, ORF1a:A486T, ORF1a:M902V, ORF1a:T3255I, ORF1b:P314L, ORF1b:D1848E, ORF1b:K2557R, ORF3a:G174C, ORF3a:E241D, ORF8:I10V, S:D614G, S:T618I, S:T732A,
hCoV-19/Mexico/CMX-InDRE_185/2021	EPI_ISL_933662	20B	B.1.1.222	20	5'UTR:C241T, ORF1ab:G347A, ORF1ab:G1544T, ORF1ab:G2764T, ORF1ab:C3037T, ORF1ab:C10029T, ORF1ab:C14408T, ORF1ab:C19011A, ORF1ab:T19839C, S:C21707T, S:G22661C, S:A23403G, S:A23756G, ORF3a:G25912T, ORF8:A27921G, ORF8:G28001T, N:C28791T, N:G28881A, N:G28882A, N:G28883C, 3'UTR:G29744A,	14	N:A173V, N:R203K, N:G204R, ORF1a:V28I, ORF1a:A427S, ORF1a:T3255I, ORF1b:P314L, ORF1b:D1848E, ORF3a:G174C, ORF8:I10V, S:H49Y, S:V367L, S:D614G, S:T732A,
hCoV-19/Mexico/CMX-InDRE_186/2021	EPI_ISL_933663	20B	B.1.1.222	16	5'UTR:C241T, ORF1ab:C3037T, ORF1ab:C10029T, ORF1ab:C14408T, ORF1ab:C17470T, ORF1ab:T19839C, S:C22978T, S:A23403G, S:A23756G, ORF3a:G25912T, ORF3a:C26195T, ORF7b:A27756G, ORF8:A27921G, ORF8:G28001T, N:G28881A, N:G28882A, N:G28883C,	10	N:R203K, N:G204R, ORF1a:T3255I, ORF1b:P314L, ORF3a:G174C, ORF3a:T268M, ORF7b:M1V, ORF8:I10V, S:D614G, S:T732A,
hCoV-19/Mexico/CMX-InDRE_188/2020	EPI_ISL_933665	20A	B.1	7	5'UTR:C241T, ORF1ab:C3037T, ORF1ab:T14313C, ORF1ab:C14408T, ORF1ab:C14688T, S:A23403G, ORF8:A28272T, N:C28854T,	3	N:S194L, ORF1b:P314L, S:D614G,
hCoV-19/Mexico/CMX-InDRE_197/2020	EPI_ISL_933674	20A	B.1	14	5'UTR:C241T, ORF1ab:C2070T, ORF1ab:C3037T, ORF1ab:C10537T, ORF1ab:C14408T, ORF1ab:C16230A, ORF1ab:A17861G, ORF1ab:T18087C, ORF1ab:G19677T, ORF1ab:A20268G, S:A23403G, ORF3a:G25855T, ORF8:G27916A, ORF8:C27925A, N:C28854T,	9	N:S194L, ORF1a:T602I, ORF1b:P314L, ORF1b:D1465G, ORF1b:Q2070H, ORF3a:D155Y, ORF8:G8E, ORF8:T11K, S:D614G,

hCoV-19/Mexico/CMX-InDRE_198/2020	EPI_ISL_933675	20A	B.1	14	5'UTR:C241T, ORF1ab:C2070T, ORF1ab:C3037T, ORF1ab:C10537T, ORF1ab:C14408T, ORF1ab:C16230A, ORF1ab:A17861G, ORF1ab:T18087C, ORF1ab:G19677T, ORF1ab:A20268G, S:A23403G, ORF3a:G25855T, ORF8:G27916A, ORF8:C27925A, N:C28854T,	9	N:S194L, ORF1a:T602I, ORF1b:P314L, ORF1b:D1465G, ORF1b:Q2070H, ORF3a:D155Y, ORF8:G8E, ORF8:T11K, S:D614G,
hCoV-19/Mexico/CMX-InDRE_199/2020	EPI_ISL_933676	20B	B.1.1.222	14	5'UTR:C241T, ORF1ab:C3037T, ORF1ab:C7749T, ORF1ab:A13498G, ORF1ab:C14408T, ORF1ab:C16726T, ORF1ab:T19839C, S:A23403G, S:A23756G, ORF3a:G26113T, N:G28559T, N:G28881A, N:G28882A, N:G28883C, N:G28907A,	11	N:G96C, N:R203K, N:G204R, N:G212S, ORF1a:T2495I, ORF1b:T11A, ORF1b:P314L, ORF1b:H1087Y, ORF3a:E241*, S:D614G, S:T732A,
hCoV-19/Mexico/CMX-InDRE_202/2020	EPI_ISL_933679	20A	B.1	11	5'UTR:C241T, ORF1ab:C3037T, ORF1ab:C3587T, ORF1ab:A6133G, ORF1ab:T6945C, ORF1ab:C14408T, ORF1ab:C16887T, ORF1ab:A20268G, S:C21575T, S:A23403G, N:C28854T, 3'UTR:G29734T,	6	N:S194L, ORF1a:H1108Y, ORF1a:I2227T, ORF1b:P314L, S:L5F, S:D614G,
hCoV-19/Mexico/CMX-InDRE_203/2021	EPI_ISL_933680	20B	B.1.1.519	26	5'UTR:C203T, 5'UTR:C222T, 5'UTR:C241T, ORF1ab:T1303A, ORF1ab:C2091T, ORF1ab:C3037T, ORF1ab:C3140T, ORF1ab:G6986T, ORF1ab:C7764T, ORF1ab:C10029T, ORF1ab:C10954T, ORF1ab:A11117G, ORF1ab:C12786T, ORF1ab:C12789T, ORF1ab:C14408T, ORF1ab:T19839C, ORF1ab:C21306T, S:C22995A, S:A23403G, S:C23604A, S:A23756G, ORF7b:G27758T, N:G28881A, N:G28882A, N:G28883C, N:C29197T, ORF10:T29661C,	17	N:R203K, N:G204R, ORF1a:T609I, ORF1a:P959S, ORF1a:G2241C, ORF1a:S2500F, ORF1a:T3255I, ORF1a:I3618V, ORF1a:T4174I, ORF1a:T4175I, ORF1b:P314L, ORF7a:*122L, ORF7b:M1I, S:T478K, S:D614G, S:P681H, S:T732A,
hCoV-19/Mexico/CMX-InDRE_204/2021	EPI_ISL_933681	20B	B.1.1.519	24	5'UTR:T201C, 5'UTR:C203T, 5'UTR:C222T, 5'UTR:C241T, ORF1ab:G1738T, ORF1ab:C3037T, ORF1ab:C3140T, ORF1ab:C4456T, ORF1ab:C10029T, ORF1ab:C10954T, ORF1ab:A11117G, ORF1ab:G12261A, ORF1ab:C12789T, ORF1ab:C14408T, ORF1ab:T19839C, ORF1ab:A19974G, ORF1ab:C21306T, S:C22995A, S:A23403G, S:C23604A, S:A23756G, N:G28881A, N:G28882A, N:G28883C, N:C29197T,	12	N:R203K, N:G204R, ORF1a:P959S, ORF1a:T3255I, ORF1a:I3618V, ORF1a:R3999H, ORF1a:T4175I, ORF1b:P314L, S:T478K, S:D614G, S:P681H, S:T732A,
hCoV-19/Mexico/CMX-InDRE_205/2021	EPI_ISL_933682	20B	B.1.1.519	24	5'UTR:T201C, 5'UTR:C203T, 5'UTR:C222T, 5'UTR:C241T, ORF1ab:G1738T, ORF1ab:C3037T, ORF1ab:C3140T, ORF1ab:C4456T, ORF1ab:C10029T, ORF1ab:C10954T, ORF1ab:A11117G, ORF1ab:G12261A, ORF1ab:C12789T, ORF1ab:C14408T, ORF1ab:T19839C, ORF1ab:A19974G, ORF1ab:C21306T, S:C22995A, S:A23403G, S:C23604A, S:A23756G, N:G28881A, N:G28882A, N:G28883C, N:C29197T,	12	N:R203K, N:G204R, ORF1a:P959S, ORF1a:T3255I, ORF1a:I3618V, ORF1a:R3999H, ORF1a:T4175I, ORF1b:P314L, S:T478K, S:D614G, S:P681H, S:T732A,
hCoV-19/Mexico/CMX-InDRE_206/2021	EPI_ISL_933683	20B	B.1.1.519	26	5'UTR:C203T, 5'UTR:C222T, 5'UTR:C241T, ORF1ab:C3037T, ORF1ab:C3140T, ORF1ab:G3692T, ORF1ab:G3871T, ORF1ab:A6397G, ORF1ab:C10029T, ORF1ab:C10755T, ORF1ab:C10954T, ORF1ab:A11117G, ORF1ab:C11824A, ORF1ab:C12789T, ORF1ab:C14408T, ORF1ab:T19839C, ORF1ab:C21306T, S:C22995A, S:A23403G, S:C23604A, S:T23629C, S:A23756G, ORF8:T27904C, N:G28881A, N:G28882A, N:G28883C, N:C29197T,	15	N:R203K, N:G204R, ORF1a:P959S, ORF1a:V1143F, ORF1a:K1202N, ORF1a:T3255I, ORF1a:A3497V, ORF1a:I3618V, ORF1a:T4175I, ORF1b:P314L, ORF8:L4P, S:T478K, S:D614G, S:P681H, S:T732A,
hCoV-19/Mexico/CMX-InDRE_207/2021	EPI_ISL_933684	20B	B.1.1.519	23	5'UTR:C203T, 5'UTR:C222T, 5'UTR:C241T, ORF1ab:C3037T, ORF1ab:C3140T, ORF1ab:C10029T, ORF1ab:C10954T, ORF1ab:A11117G, ORF1ab:C12789T, ORF1ab:C14408T, ORF1ab:T19839C, ORF1ab:C21306T, S:C22995A, S:A23403G, S:C23604A, S:A23756G, ORF8:T27904C, ORF8:C28087T, ORF8:C28253T, N:C28603T, N:G28881A, N:G28882A, N:G28883C, N:C29197T,	13	N:R203K, N:G204R, ORF1a:P959S, ORF1a:T3255I, ORF1a:I3618V, ORF1a:T4175I, ORF1b:P314L, ORF8:L4P, ORF8:A65V, S:T478K, S:D614G, S:P681H, S:T732A,

hCoV-19/Mexico/CMX-InDRE_208/2021	EPI_ISL_933685	20B	B.1.1.519	19	5'UTR:C222T, 5'UTR:C241T, ORF1ab:C3037T, ORF1ab:C3140T, ORF1ab:C10029T, ORF1ab:C10954T, ORF1ab:A11117G, ORF1ab:C12789T, ORF1ab:C14408T, ORF1ab:C15720T, ORF1ab:T19839C, ORF1ab:C21306T, S:C22995A, S:A23403G, S:C23604A, S:A23756G, N:G28881A, N:G28882A, N:G28883C. N:C29197T.	11	N:R203K, N:G204R, ORF1a:P959S, ORF1a:T3255I, ORF1a:I3618V, ORF1a:T4175I, ORF1b:P314L, S:T478K, S:D614G, S:P681H, S:T732A,
hCoV-19/Mexico/CMX-InDRE_209/2021	EPI_ISL_933686	20B	B.1.1.519	22	5'UTR:C203T, 5'UTR:C222T, 5'UTR:C241T, ORF1ab:C3037T, ORF1ab:C3140T, ORF1ab:C10029T, ORF1ab:C10954T, ORF1ab:A11117G, ORF1ab:C12789T, ORF1ab:C14408T, ORF1ab:T19839C, ORF1ab:C21306T, S:C22995A, S:A23403G, S:C23604A, S:A23756G, ORF8:C27923T, N:C28748T, N:G28881A, N:G28882A, N:G28883C, N:C29197T, N:G29527T,	12	N:R203K, N:G204R, N:Q418H, ORF1a:P959S, ORF1a:T3255I, ORF1a:I3618V, ORF1a:T4175I, ORF1b:P314L, S:T478K, S:D614G, S:P681H, S:T732A,
hCoV-19/Mexico/CMX-InDRE_210/2021	EPI_ISL_933687	20B	B.1.1.519	29	5'UTR:C203T, 5'UTR:C222T, 5'UTR:C241T, ORF1ab:C1213T, ORF1ab:C1342T, ORF1ab:C3037T, ORF1ab:C3140T, ORF1ab:G3692T, ORF1ab:G3871T, ORF1ab:C10029T, ORF1ab:C10954T, ORF1ab:A11117G, ORF1ab:C11824A, ORF1ab:C12789T, ORF1ab:C14408T, ORF1ab:C18395T, ORF1ab:T19839C, ORF1ab:C21306T, S:C21952T, S:C22995A, S:A23403G, S:C23604A, S:A23756G, M:T26987C, ORF8:T27904C, N:C28732T, N:G28881A, N:G28882A, N:G28883C, N:C29197T,	15	N:R203K, N:G204R, ORF1a:P959S, ORF1a:V1143F, ORF1a:K1202N, ORF1a:T3255I, ORF1a:I3618V, ORF1a:T4175I, ORF1b:P314L, ORF1b:A1643V, ORF8:L4P, S:T478K, S:D614G, S:P681H, S:T732A,
hCoV-19/Mexico/CMX-InDRE_211/2021	EPI_ISL_933688	20B	B.1.1.519	32	5'UTR:C203T, 5'UTR:C222T, 5'UTR:C241T, ORF1ab:C1213T, ORF1ab:C1342T, ORF1ab:C3037T, ORF1ab:C3140T, ORF1ab:G3692T, ORF1ab:G3871T, ORF1ab:C10029T, ORF1ab:C10954T, ORF1ab:A11117G, ORF1ab:C11824A, ORF1ab:C12789T, ORF1ab:C14408T, ORF1ab:C18395T, ORF1ab:G19109T, ORF1ab:T19839C, ORF1ab:C21306T, S:C21952T, S:C22995A, S:A23403G, S:C23604A, S:A23756G, M:T26987C, ORF8:T27904C, N:C28391T, N:C28677T, N:C28732T, N:G28881A, N:G28882A, N:G28883C, N:C29197T,	18	N:R40C, N:T135I, N:R203K, N:G204R, ORF1a:P959S, ORF1a:V1143F, ORF1a:K1202N, ORF1a:T3255I, ORF1a:I3618V, ORF1a:T4175I, ORF1b:P314L, ORF1b:A1643V, ORF1b:S1881I, ORF8:L4P, S:T478K, S:D614G, S:P681H, S:T732A,
hCoV-19/Mexico/CMX-InDRE_212/2021	EPI_ISL_933689	20B	B.1.1.519	24	5'UTR:T201C, 5'UTR:C203T, 5'UTR:C222T, 5'UTR:C241T, ORF1ab:C745T, ORF1ab:G1738T, ORF1ab:C3037T, ORF1ab:C3140T, ORF1ab:C10029T, ORF1ab:C10954T, ORF1ab:A11117G, ORF1ab:C12789T, ORF1ab:C14408T, ORF1ab:C19586T, ORF1ab:T19839C, ORF1ab:A19974G, ORF1ab:C21306T, S:C22995A, S:A23403G, S:C23604A, S:A23756G, N:G28881A, N:G28882A, N:G28883C, N:C29197T,	12	N:R203K, N:G204R, ORF1a:P959S, ORF1a:T3255I, ORF1a:I3618V, ORF1a:T4175I, ORF1b:P314L, ORF1b:T2040I, S:T478K, S:D614G, S:P681H, S:T732A,
hCoV-19/Mexico/CMX-InDRE_213/2021	EPI_ISL_933690	20B	B.1.1.519	29	5'UTR:C203T, 5'UTR:C222T, 5'UTR:C241T, ORF1ab:A866G, ORF1ab:C3037T, ORF1ab:C3140T, ORF1ab:A3801G, ORF1ab:C5183T, ORF1ab:C5184T, ORF1ab:C10029T, ORF1ab:C10954T, ORF1ab:A11117G, ORF1ab:C11916T, ORF1ab:C12789T, ORF1ab:C14408T, ORF1ab:A18277G, ORF1ab:T19839C, ORF1ab:C21306T, S:C22995A, S:A23403G, S:C23604A, S:A23756G, S:T25265A, M:G26589T, ORF8:T27904C, N:G28747T, N:G28881A, N:G28882A, N:G28883C, N:C29197T,	19	M:V23L, N:R203K, N:G204R, ORF1a:I201V, ORF1a:P959S, ORF1a:D1179G, ORF1a:P1640F, ORF1a:T3255I, ORF1a:I3618V, ORF1a:S3884L, ORF1a:T4175I, ORF1b:P314L, ORF1b:I1604V, ORF8:L4P, S:T478K, S:D614G, S:P681H, S:T732A, S:C1235S,

hCoV-19/Mexico/CMX-InDRE_214/2021	EPI_ISL_933691	20B	B.1.1.519	23	5'UTR:C203T, 5'UTR:C222T, 5'UTR:C241T, ORF1ab:C3037T, ORF1ab:C3140T, ORF1ab:T4339C, ORF1ab:C10029T, ORF1ab:C10954T, ORF1ab:A11117G, ORF1ab:C12789T, ORF1ab:C14408T, ORF1ab:T19839C, ORF1ab:C21306T, S:C22995A, S:A23403G, S:C23604A, S:A23756G, ORF3a:T25535C, ORF3a:G25906T, ORF8:C28253T, N:G28881A, N:G28882A, N:G28883C, N:C29197T,	13	N:R203K, N:G204R, ORF1a:P959S, ORF1a:T3255I, ORF1a:I3618V, ORF1a:T4175I, ORF1b:P314L, ORF3a:V48A, ORF3a:G172C, S:T478K, S:D614G, S:P681H, S:T732A,
hCoV-19/Mexico/CMX-InDRE_216/2020	EPI_ISL_933693	20B	B.1.1.519	19	5'UTR:C203T, 5'UTR:C222T, 5'UTR:C241T, ORF1ab:C3037T, ORF1ab:C3140T, ORF1ab:C10029T, ORF1ab:C10954T, ORF1ab:A11117G, ORF1ab:C12789T, ORF1ab:C14408T, ORF1ab:T19839C, ORF1ab:C21306T, S:C22995A, S:A23403G, S:C23604A, S:A23756G, N:G28881A, N:G28882A, N:G28883C, N:C29197T,	11	N:R203K, N:G204R, ORF1a:P959S, ORF1a:T3255I, ORF1a:I3618V, ORF1a:T4175I, ORF1b:P314L, S:T478K, S:D614G, S:P681H, S:T732A,
hCoV-19/Mexico/CMX-InDRE_217/2020	EPI_ISL_933694	20B	B.1.1.519	26	5'UTR:C203T, 5'UTR:C222T, 5'UTR:C241T, ORF1ab:C815T, ORF1ab:C3037T, ORF1ab:C3140T, ORF1ab:C6997T, ORF1ab:C10029T, ORF1ab:C10954T, ORF1ab:A11117G, ORF1ab:C12789T, ORF1ab:C13819A, ORF1ab:T14133C, ORF1ab:C14408T, ORF1ab:T19839C, ORF1ab:C21306T, S:C22747T, S:C22995A, S:A23403G, S:C23604A, S:A23756G, ORF8:C27911T, N:C28846T, N:G28881A, N:G28882A, N:G28883C, N:C29197T,	13	N:R203K, N:G204R, ORF1a:R184C, ORF1a:P959S, ORF1a:T3255I, ORF1a:I3618V, ORF1a:T4175I, ORF1b:L118I, ORF1b:P314L, S:T478K, S:D614G, S:P681H, S:T732A,
hCoV-19/Mexico/CMX-InDRE_218/2020	EPI_ISL_933695	20B	B.1.1.519	21	5'UTR:C203T, 5'UTR:C222T, 5'UTR:C241T, ORF1ab:C3037T, ORF1ab:C3140T, ORF1ab:C6027T, ORF1ab:C10029T, ORF1ab:C10954T, ORF1ab:A11117G, ORF1ab:C12789T, ORF1ab:C14408T, ORF1ab:T19839C, ORF1ab:C21306T, S:C22995A, S:A23403G, S:C23604A, S:A23756G, ORF3a:C25528T, N:G28881A, N:G28882A, N:G28883C, N:C29197T,	13	N:R203K, N:G204R, ORF1a:P959S, ORF1a:P1921L, ORF1a:T3255I, ORF1a:I3618V, ORF1a:T4175I, ORF1b:P314L, ORF3a:L46F, S:T478K, S:D614G, S:P681H, S:T732A,
hCoV-19/Mexico/CMX-InDRE_219/2020	EPI_ISL_933696	20B	B.1.1.519	24	5'UTR:C203T, 5'UTR:C222T, 5'UTR:C241T, ORF1ab:C275T, ORF1ab:C3037T, ORF1ab:C3140T, ORF1ab:G3692T, ORF1ab:G3871T, ORF1ab:C10029T, ORF1ab:C10954T, ORF1ab:A11117G, ORF1ab:C11824A, ORF1ab:C12789T, ORF1ab:C14408T, ORF1ab:T19839C, ORF1ab:C21306T, S:C22995A, S:A23403G, S:C23604A, S:A23756G, ORF8:T27904C, N:G28881A, N:G28882A, N:G28883C, N:C29197T,	15	N:R203K, N:G204R, ORF1a:L4F, ORF1a:P959S, ORF1a:V1143F, ORF1a:K1202N, ORF1a:T3255I, ORF1a:I3618V, ORF1a:T4175I, ORF1b:P314L, ORF8:L4P, S:T478K, S:D614G, S:P681H, S:T732A,
hCoV-19/Mexico/CMX-InDRE_220/2020	EPI_ISL_933697	20B	B.1.1.519	24	5'UTR:C203T, 5'UTR:C222T, 5'UTR:C241T, ORF1ab:C275T, ORF1ab:C3037T, ORF1ab:C3140T, ORF1ab:G3692T, ORF1ab:G3871T, ORF1ab:C10029T, ORF1ab:C10954T, ORF1ab:A11117G, ORF1ab:C11824A, ORF1ab:C12789T, ORF1ab:C14408T, ORF1ab:T19839C, ORF1ab:C21306T, S:C22995A, S:A23403G, S:C23604A, S:A23756G, ORF8:T27904C, N:G28881A, N:G28882A, N:G28883C, N:C29197T,	15	N:R203K, N:G204R, ORF1a:L4F, ORF1a:P959S, ORF1a:V1143F, ORF1a:K1202N, ORF1a:T3255I, ORF1a:I3618V, ORF1a:T4175I, ORF1b:P314L, ORF8:L4P, S:T478K, S:D614G, S:P681H, S:T732A,

hCoV-19/Mexico/CMX-InDRE_221/2020	EPI_ISL_933698	20B	B.1.1.519	27	5'UTR:C203T, 5'UTR:C222T, 5'UTR:C241T, ORF1ab:A866G, ORF1ab:C3037T, ORF1ab:C3140T, ORF1ab:G3567A, ORF1ab:C5183T, ORF1ab:C9166T, ORF1ab:C10029T, ORF1ab:C10954T, ORF1ab:A11117G, ORF1ab:C11916T, ORF1ab:C12036T, ORF1ab:G12223T, ORF1ab:C12789T, ORF1ab:C14408T, ORF1ab:T19839C, ORF1ab:C21306T, S:C22995A, S:A23403G, S:C23604A, S:A23756G, ORF8:T27904C, N:G28881A, N:G28882A, N:G28883C, N:C29197T,	17	N:R203K, N:G204R, ORF1a:I201V, ORF1a:P959S, ORF1a:G1101D, ORF1a:P1640S, ORF1a:T3255I, ORF1a:I3618V, ORF1a:S3884L, ORF1a:A3924V, ORF1a:T4175I, ORF1b:P314L, ORF8:L4P, S:T478K, S:D614G, S:P681H, S:T732A,
hCoV-19/Mexico/CMX-InDRE_222/2020	EPI_ISL_933699	20B	B.1.1.519	24	5'UTR:C203T, 5'UTR:C222T, 5'UTR:C241T, ORF1ab:C900T, ORF1ab:C3037T, ORF1ab:C3140T, ORF1ab:C10029T, ORF1ab:C10954T, ORF1ab:A11117G, ORF1ab:C12789T, ORF1ab:G14118T, ORF1ab:C14408T, ORF1ab:A19831G, ORF1ab:T19839C, ORF1ab:C21306T, S:C22995A, S:A23403G, S:C23604A, S:A23756G, ORF3a:G25429T, ORF8:T27904C, N:G28881A, N:G28882A, N:G28883C, N:C29197T,	15	N:R203K, N:G204R, ORF1a:S212L, ORF1a:P959S, ORF1a:T3255I, ORF1a:I3618V, ORF1a:T4175I, ORF1b:P314L, ORF1b:I2122V, ORF3a:V13L, ORF8:L4P, S:T478K, S:D614G, S:P681H, S:T732A,
hCoV-19/Mexico/CMX-InDRE_223/2020	EPI_ISL_933700	20B	B.1.1.519	21	5'UTR:C203T, 5'UTR:C222T, 5'UTR:C241T, ORF1ab:C3037T, ORF1ab:C3140T, ORF1ab:C10029T, ORF1ab:C10954T, ORF1ab:A11117G, ORF1ab:C12789T, ORF1ab:C14408T, ORF1ab:T19839C, ORF1ab:C21306T, S:C22995A, S:A23403G, S:C23604A, S:A23756G, S:C25282T, ORF8:T27904C, N:G28881A, N:G28882A, N:G28883C, N:C29197T,	12	N:R203K, N:G204R, ORF1a:P959S, ORF1a:T3255I, ORF1a:I3618V, ORF1a:T4175I, ORF1b:P314L, ORF8:L4P, S:T478K, S:D614G, S:P681H, S:T732A,
hCoV-19/Mexico/CMX-InDRE_224/2020	EPI_ISL_933701	20B	B.1.1.519	24	5'UTR:T201C, 5'UTR:C203T, 5'UTR:C222T, 5'UTR:C241T, ORF1ab:G1738T, ORF1ab:C3037T, ORF1ab:C3140T, ORF1ab:C3602T, ORF1ab:C7504T, ORF1ab:C10029T, ORF1ab:C10954T, ORF1ab:A11117G, ORF1ab:C12789T, ORF1ab:C14408T, ORF1ab:T19839C, ORF1ab:A19974G, ORF1ab:C21306T, S:C22995A, S:A23403G, S:C23604A, S:A23756G, N:G28881A, N:G28882A, N:G28883C, N:C29197T,	12	N:R203K, N:G204R, ORF1a:P959S, ORF1a:H1113Y, ORF1a:T3255I, ORF1a:I3618V, ORF1a:T4175I, ORF1b:P314L, S:T478K, S:D614G, S:P681H, S:T732A,
hCoV-19/Mexico/CMX-InDRE_225/2020	EPI_ISL_933702	20B	B.1.1.519	24	5'UTR:C203T, 5'UTR:C222T, 5'UTR:C241T, ORF1ab:C3037T, ORF1ab:C3140T, ORF1ab:C9080T, ORF1ab:C10029T, ORF1ab:C10954T, ORF1ab:A11117G, ORF1ab:C11455T, ORF1ab:C12789T, ORF1ab:C12890T, ORF1ab:C14408T, ORF1ab:G15679T, ORF1ab:T19839C, ORF1ab:C21306T, S:C22995A, S:A23403G, S:C23604A, S:A23756G, N:G28881A, N:G28882A, N:G28883C, N:C29197T, N:G29315C,	13	N:R203K, N:G204R, N:D348H, ORF1a:P959S, ORF1a:T3255I, ORF1a:I3618V, ORF1a:T4175I, ORF1b:P314L, ORF1b:A738S, S:T478K, S:D614G, S:P681H, S:T732A,
hCoV-19/Mexico/CMX-InDRE_227/2021	EPI_ISL_933704	20B	B.1	12	5'UTR:C241T, ORF1ab:C3037T, ORF1ab:C3140T, ORF1ab:C5147T, ORF1ab:G8536T, ORF1ab:C10954T, ORF1ab:A11117G, ORF1ab:C14408T, ORF1ab:T19839C, ORF1ab:A20472C, S:A23403G, ORF7b:A27888T, N:C29197T,	7	ORF1a:P959S, ORF1a:R1628C, ORF1a:K2757N, ORF1a:I3618V, ORF1b:P314L, ORF1b:Q2335H, S:D614G,

hCoV-19/Mexico/CMX-InDRE_229/2020	EPI_ISL_933706	20B	B.1.1.222	26	5'UTR:G219T, 5'UTR:C241T, ORF1ab:T950C, ORF1ab:G1181T, ORF1ab:C2091T, ORF1ab:C3037T, ORF1ab:T7342C, ORF1ab:C8293T, ORF1ab:A10323G, ORF1ab:C14408T, ORF1ab:G14772T, ORF1ab:T14956C, ORF1ab:C15600T, ORF1ab:C17333T, ORF1ab:T19839C, ORF1ab:T20976C, S:A23403G, S:A23756G, S:G25135T, ORF3a:G25690T, ORF3a:C25904T, M:C26681T, N:C28292A, N:G28881A, N:G28882A, N:G28883C, 3'UTR:G29766T	16	N:Q7K, N:R203K, N:G204R, ORF1a:Y229H, ORF1a:V306F, ORF1a:T609I, ORF1a:K3353R, ORF1b:P314L, ORF1b:Q435H, ORF1b:F497L, ORF1b:T1289M, ORF3a:G100C, ORF3a:S171L, S:D614G, S:T732A, S:K1191N,	
hCoV-19/Mexico/CMX-InDRE_230/2020	EPI_ISL_933707	20B	B.1.1.519	24	5'UTR:C203T, 5'UTR:C222T, 5'UTR:C241T, ORF1ab:C683T, ORF1ab:C1191A, ORF1ab:A2010C, ORF1ab:C3037T, ORF1ab:C3140T, ORF1ab:C10029T, ORF1ab:C10954T, ORF1ab:A11117G, ORF1ab:C12789T, ORF1ab:C14408T, ORF1ab:T19839C, ORF1ab:C21306T, S:C22995A, S:A23403G, S:C23604A, S:A23756G, N:G28881A, N:G28882A, N:G28883C, N:C29197T, N:G29315C, N:C29421A,	15	N:R203K, N:G204R, N:D348H, N:P383Q, ORF1a:P309Q, ORF1a:D582A, ORF1a:P959S, ORF1a:T3255I, ORF1a:I3618V, ORF1a:T4175I, ORF1b:P314L, S:T478K, S:D614G, S:P681H, S:T732A,	
hCoV-19/Mexico/CMX-InDRE_233/2020	EPI_ISL_933710	20B	B.1.1	18	5'UTR:C241T, ORF1ab:C3037T, ORF1ab:C3267T, ORF1ab:C3768T, ORF1ab:T10495C, ORF1ab:G10705T, ORF1ab:A12250G, ORF1ab:C12459T, ORF1ab:C14184T, ORF1ab:C14408T, ORF1ab:C17172T, ORF1ab:G17280T, S:A23403G, S:C23604A, ORF7a:C27476T, ORF7a:C27679T, N:G28881A, N:G28882A, N:G28883C,	12	N:R203K, N:G204R, ORF1a:T1001I, ORF1a:E1024K, ORF1a:T1168I, ORF1a:R3480S, ORF1a:T4065I, ORF1b:P314L, ORF7a:T28I, ORF7a:L96F, S:D614G, S:P681H,	ORF1ab: 3333-3335,
hCoV-19/Mexico/CMX-InDRE_234/2020	EPI_ISL_933711	21C (Epsilon)	B.1.429	21	5'UTR:C241T, ORF1ab:C1059T, ORF1ab:C1489T, ORF1ab:C2395T, ORF1ab:C2450T, ORF1ab:T2597C, ORF1ab:C3037T, ORF1ab:C8947T, ORF1ab:C12100T, ORF1ab:A12878G, ORF1ab:C14408T, ORF1ab:G17014T, S:G21600T, S:G22018T, S:T22917G, S:A23403G, S:T24349C, ORF3a:G25563T, M:C26681T, ORF7b:G27890T, ORF8:A28272T, N:C28887T,	10	N:T205I, ORF1a:T265I, ORF1a:I4205V, ORF1b:P314L, ORF1b:D1183Y, ORF3a:Q57H, S:S13I, S:W152C, S:L452R, S:D614G,	
hCoV-19/Mexico/CMX-InDRE_235/2020	EPI_ISL_933712	21C (Epsilon)	B.1.429	27	5'UTR:C241T, ORF1ab:C1059T, ORF1ab:C2395T, ORF1ab:C2453T, ORF1ab:T2597C, ORF1ab:C3037T, ORF1ab:G6865T, ORF1ab:C8947T, ORF1ab:T11038C, ORF1ab:C12100T, ORF1ab:C12459T, ORF1ab:A12878G, ORF1ab:C14408T, ORF1ab:G17014T, ORF1ab:A19476G, S:G21600T, S:C21638T, S:G22018T, S:T22917G, S:A23403G, S:T24349C, ORF3a:G25563T, ORF3a:G25785T, M:C26681T, ORF7b:G27890T, ORF8:G27987T, ORF8:A28272T, N:C28887T,	16	N:T205I, ORF1a:T265I, ORF1a:L730F, ORF1a:K2200N, ORF1a:T4065I, ORF1a:I4205V, ORF1b:P314L, ORF1b:D1183Y, ORF3a:Q57H, ORF3a:W131C, ORF8:V32L, S:S13I, S:P26S, S:W152C, S:L452R, S:D614G,	
hCoV-19/Mexico/CMX-InDRE_242/2021	EPI_ISL_942928	20B	B.1.1.519	25	5'UTR:C203T, 5'UTR:C222T, 5'UTR:C241T, ORF1ab:C3037T, ORF1ab:C3140T, ORF1ab:C10029T, ORF1ab:C10954T, ORF1ab:A11117G, ORF1ab:T11569C, ORF1ab:C12789T, ORF1ab:G13661A, ORF1ab:C14408T, ORF1ab:G19542A, ORF1ab:T19839C, ORF1ab:A20180G, ORF1ab:C21306T, S:C22995A, S:A23403G, S:G23593T, S:C23604A, S:A23756G, N:G28881A, N:G28882A, N:G28883C, N:C29197T, N:C29546T,	15	N:R203K, N:G204R, ORF1a:P959S, ORF1a:T3255I, ORF1a:I3618V, ORF1a:T4175I, ORF1b:R65K, ORF1b:P314L, ORF1b:M2025I, ORF1b:Q2238R, S:T478K, S:D614G, S:Q677H, S:P681H, S:T732A,	
hCoV-19/Mexico/CMX-InDRE_247/2021	EPI_ISL_1060715	20A	B.1.243	16	5'UTR:C241T, ORF1ab:G806A, ORF1ab:C3037T, ORF1ab:C6429T, ORF1ab:G7393T, ORF1ab:T9588G, ORF1ab:C14408T, ORF1ab:C17373T, ORF1ab:C18166T, ORF1ab:T19301C, ORF1ab:A20268G, S:A23403G, S:T24076C, ORF8:C28253T, N:C28854T, N:G29315T, N:G29543T,	9	N:S194L, N:D348Y, ORF1a:A181T, ORF1a:P2055L, ORF1a:I3108S, ORF1b:P314L, ORF1b:P1567S, ORF1b:V1945A, S:D614G,	

hCoV-19/Mexico/CMX-InDRE_248/2021	EPI_ISL_1060714	20B	B.1.1.519	28	5'UTR:C203T, 5'UTR:C222T, 5'UTR:C241T, ORF1ab:C683T, ORF1ab:C1191A, ORF1ab:C3037T, ORF1ab:C3140T, ORF1ab:C4276T, ORF1ab:C8937T, ORF1ab:C10029T, ORF1ab:C10954T, ORF1ab:A11117G, ORF1ab:C12789T, ORF1ab:C14408T, ORF1ab:T19839C, ORF1ab:T20600C, ORF1ab:C21306T, S:C22995A, S:A23403G, S:C23604A, S:A23756G, S:A25336C, S:G25337C, N:G28881A, N:G28882A, N:G28883C, N:C29197T, N:G29315C, N:C29421A,	18	N:R203K, N:G204R, N:D348H, N:P383Q, ORF1a:P309Q, ORF1a:P959S, ORF1a:A2891V, ORF1a:T3255I, ORF1a:I3618V, ORF1a:T4175I, ORF1b:P314L, ORF1b:I2378T, S:T478K, S:D614G, S:P681H, S:T732A, S:E1258D, S:D1259H,
hCoV-19/Mexico/CMX-InDRE_249/2021	EPI_ISL_1060716	20B	B.1.1.519	28	5'UTR:T201C, 5'UTR:C203T, 5'UTR:C222T, 5'UTR:C241T, ORF1ab:C745T, ORF1ab:G1738T, ORF1ab:G1999T, ORF1ab:C3037T, ORF1ab:C3140T, ORF1ab:C10029T, ORF1ab:C10954T, ORF1ab:A11117G, ORF1ab:C12789T, ORF1ab:C14408T, ORF1ab:C18526T, ORF1ab:T19839C, ORF1ab:A19974G, ORF1ab:C21306T, S:C22995A, S:A23403G, S:C23604A, S:A23756G, S:A25336C, S:G25337C, N:G28881A, N:G28882A, N:G28883C, N:G29027T, N:C29197T	15	N:R203K, N:G204R, N:A252S, ORF1a:P959S, ORF1a:T3255I, ORF1a:I3618V, ORF1a:T4175I, ORF1b:P314L, ORF1b:R1687C, S:T478K, S:D614G, S:P681H, S:T732A, S:E1258D, S:D1259H,
hCoV-19/Mexico/CMX-InDRE_255/2020	EPI_ISL_1054983	20A	B.1	5	5'UTR:C241T, ORF1ab:C3037T, ORF1ab:C14408T, ORF1ab:A20268G, S:A23403G, N:C28854T,	3	N:S194L, ORF1b:P314L, S:D614G,
hCoV-19/Mexico/CMX-InDRE_268/2020	EPI_ISL_1054960	20B	B.1.1	6	5'UTR:C241T, ORF1ab:C3037T, ORF1ab:C14408T, S:A23403G, N:G28881A, N:G28882A, N:G28883C,	4	N:R203K, N:G204R, ORF1b:P314L, S:D614G,
hCoV-19/Mexico/CMX-InDRE_275/2020	EPI_ISL_1054947	20B	B.1.1.244	11	5'UTR:C241T, ORF1ab:C3037T, ORF1ab:C12412T, ORF1ab:A12820G, ORF1ab:T14313C, ORF1ab:C14407T, ORF1ab:C14408T, ORF1ab:A19960G, S:A23403G, N:G28881A, N:G28882A, N:G28883C,	5	N:R203K, N:G204R, ORF1b:P314F, ORF1b:T2165A, S:D614G,
hCoV-19/Mexico/CMX-InDRE_283/2020	EPI_ISL_1054944	20A	B.1	7	5'UTR:C241T, ORF1ab:C3037T, ORF1ab:T5911C, ORF1ab:C14408T, ORF1ab:A20268G, S:A23403G, ORF8:C28253T, N:C28854T,	3	N:S194L, ORF1b:P314L, S:D614G,
hCoV-19/Mexico/CMX-InDRE_286/2020	EPI_ISL_1054940	20B	B.1.1.222	13	5'UTR:C241T, ORF1ab:G1148T, ORF1ab:C3037T, ORF1ab:A5596G, ORF1ab:A7468T, ORF1ab:C14408T, ORF1ab:T19839C, S:A23403G, S:A23756G, ORF3a:C25549T, ORF3a:C25777T, N:G28881A, N:G28882A, N:G28883C,	8	N:R203K, N:G204R, ORF1a:G295C, ORF1b:P314L, ORF3a:L53F, ORF3a:L129F, S:D614G, S:T732A,
hCoV-19/Mexico/CMX-InDRE_303/2020	EPI_ISL_1060735	20A	B.1	11	5'UTR:C241T, ORF1ab:C1392T, ORF1ab:C3037T, ORF1ab:T6602C, ORF1ab:C11102T, ORF1ab:C14408T, ORF1ab:C18115T, ORF1ab:A20268G, S:A23403G, S:A25109G, ORF8:C27903T, N:C28854T,	8	N:S194L, ORF1a:S376L, ORF1a:P3613S, ORF1b:P314L, ORF1b:H1550Y, ORF8:L4F, S:D614G, S:I1183V,
hCoV-19/Mexico/CMX-InDRE_312/2021	EPI_ISL_1060717	20B	B.1.1.519	27	5'UTR:C203T, 5'UTR:C222T, 5'UTR:C241T, ORF1ab:T937C, ORF1ab:C1342T, ORF1ab:C3037T, ORF1ab:C3140T, ORF1ab:G3692T, ORF1ab:G3871T, ORF1ab:C10029T, ORF1ab:C10954T, ORF1ab:A11117G, ORF1ab:C11824A, ORF1ab:C12789T, ORF1ab:C14408T, ORF1ab:G17302T, ORF1ab:T19839C, ORF1ab:C21306T, S:C22995A, S:A23403G, S:C23604A, S:A23756G, S:C24034T, ORF8:T27904C, N:G28881A, N:G28882A, N:G28883C, N:C29197T,	15	N:R203K, N:G204R, ORF1a:P959S, ORF1a:V1143F, ORF1a:K1202N, ORF1a:T3255I, ORF1a:I3618V, ORF1a:T4175I, ORF1b:P314L, ORF1b:V1279F, ORF8:L4P, S:T478K, S:D614G, S:P681H, S:T732A,
hCoV-19/Mexico/CMX-InDRE_331/2020	EPI_ISL_1060719	20A	B.1.396	15	ORF1ab:C1191T, ORF1ab:C3037T, ORF1ab:C9521A, ORF1ab:C14408T, ORF1ab:A20268G, S:A23403G, S:C24904T, S:A25255T, ORF3a:C26060T, ORF7a:T27484C, N:C28854T, N:G28985A, N:G29422T, N:T29515C, N:C29541T, ORF10:A29567G.	7	N:S194L, N:G238S, ORF1a:P309L, ORF1a:L3086I, ORF1b:P314L, ORF3a:T223I, S:D614G,

hCoV-19/Mexico/CMX-InDRE_451/2021	EPI_ISL_1219711	20B	B.1.1.519	27	5'UTR:C203T, 5'UTR:C222T, 5'UTR:C241T, ORF1ab:C643T, ORF1ab:C3037T, ORF1ab:C3140T, ORF1ab:C5183T, ORF1ab:C10029T, ORF1ab:C10954T, ORF1ab:A11117G, ORF1ab:C12789T, ORF1ab:C14408T, ORF1ab:T19839C, ORF1ab:C21306T, S:A22003G, S:C22995A, S:A23403G, S:C23604A, S:A23756G, ORF3a:C25624T, M:C26768G, ORF7b:G27758A, ORF7b:T27760A, ORF8:T27904C, N:G28881A, N:G28882A, N:G28883C, N:C29197T,	17	M:I82M, N:R203K, N:G204R, ORF1a:P959S, ORF1a:P1640S, ORF1a:T3255I, ORF1a:I3618V, ORF1a:T4175I, ORF1b:P314L, ORF3a:H78Y, ORF7b:M1I, ORF7b:I2N, ORF8:L4P, S:T478K, S:D614G, S:P681H, S:T732A,
hCoV-19/Mexico/CMX-InDRE_452/2021	EPI_ISL_1219712	20B	B.1.1.519	28	5'UTR:C203T, 5'UTR:C222T, 5'UTR:C241T, ORF1ab:C3037T, ORF1ab:C3140T, ORF1ab:C4069T, ORF1ab:C4276T, ORF1ab:T8634C, ORF1ab:C10029T, ORF1ab:C10954T, ORF1ab:A11117G, ORF1ab:C12789T, ORF1ab:C14408T, ORF1ab:T19839C, ORF1ab:T20385C, ORF1ab:C21306T, S:C22995A, S:A23403G, S:C23604A, S:A23756G, S:C24130T, ORF3a:G26020T, M:A26528T, M:G26951C, N:G28881A, N:G28882A, N:G28883C, N:C29197T, 3'UTR:G29810T,	13	N:R203K, N:G204R, ORF1a:P959S, ORF1a:I2790T, ORF1a:T3255I, ORF1a:I3618V, ORF1a:T4175I, ORF1b:P314L, ORF3a:D210Y, S:T478K, S:D614G, S:P681H, S:T732A,
hCoV-19/Mexico/CMX-InDRE_F10682_S692/2021	EPI_ISL_1365657	20B	B.1.1.519	23	5'UTR:C203T, 5'UTR:C222T, 5'UTR:C241T, ORF1ab:C2638T, ORF1ab:C3037T, ORF1ab:C3140T, ORF1ab:T9412C, ORF1ab:C9438T, ORF1ab:C10029T, ORF1ab:C10954T, ORF1ab:A11117G, ORF1ab:C12789T, ORF1ab:C14408T, ORF1ab:T19839C, ORF1ab:C21306T, S:C22747T, S:C22995A, S:A23403G, S:C23604A, S:A23756G, N:G28881A, N:G28882A, N:G28883C, N:C29197T,	12	N:R203K, N:G204R, ORF1a:P959S, ORF1a:T3058I, ORF1a:T3255I, ORF1a:I3618V, ORF1a:T4175I, ORF1b:P314L, S:T478K, S:D614G, S:P681H, S:T732A,
hCoV-19/Mexico/CMX-InDRE_F10721_S738/2021	EPI_ISL_1366687	20B	B.1.1.519	24	5'UTR:T201C, 5'UTR:C203T, 5'UTR:C222T, 5'UTR:C241T, ORF1ab:G1738T, ORF1ab:C3037T, ORF1ab:C3140T, ORF1ab:A9104T, ORF1ab:C10029T, ORF1ab:C10954T, ORF1ab:A11117G, ORF1ab:C12789T, ORF1ab:C14408T, ORF1ab:T19839C, ORF1ab:A19974G, ORF1ab:C21306T, S:C22995A, S:A23403G, S:C23604A, S:A23756G, N:G28881A, N:G28882A, N:G28883C, N:C29197T, 3'UTR:G29692T,	12	N:R203K, N:G204R, ORF1a:P959S, ORF1a:S2947C, ORF1a:T3255I, ORF1a:I3618V, ORF1a:T4175I, ORF1b:P314L, S:T478K, S:D614G, S:P681H, S:T732A,
hCoV-19/Mexico/CMX-InDRE_F10723_S740/2021	EPI_ISL_1366689	20B	B.1.1.519	19	5'UTR:C203T, 5'UTR:C222T, 5'UTR:C241T, ORF1ab:C3037T, ORF1ab:C3140T, ORF1ab:C10029T, ORF1ab:C10954T, ORF1ab:A11117G, ORF1ab:C12789T, ORF1ab:C14408T, ORF1ab:T19839C, ORF1ab:C21306T, S:C22995A, S:A23403G, S:C23604A, S:A23756G, N:G28881A, N:G28882A, N:G28883C, N:C29197T,	11	N:R203K, N:G204R, ORF1a:P959S, ORF1a:T3255I, ORF1a:I3618V, ORF1a:T4175I, ORF1b:P314L, S:T478K, S:D614G, S:P681H, S:T732A,
hCoV-19/Mexico/CMX-InDRE_F10724_S739/2021	EPI_ISL_1366688	20B	B.1.1.519	20	5'UTR:C203T, 5'UTR:C222T, 5'UTR:C241T, ORF1ab:C3037T, ORF1ab:C3140T, ORF1ab:C10029T, ORF1ab:C10954T, ORF1ab:A11117G, ORF1ab:C12789T, ORF1ab:C14408T, ORF1ab:C17586T, ORF1ab:T19839C, ORF1ab:C21306T, S:C22995A, S:A23403G, S:C23604A, S:A23756G, N:G28881A, N:G28882A, N:G28883C, N:C29197T,	11	N:R203K, N:G204R, ORF1a:P959S, ORF1a:T3255I, ORF1a:I3618V, ORF1a:T4175I, ORF1b:P314L, S:T478K, S:D614G, S:P681H, S:T732A,
hCoV-19/Mexico/CMX-InDRE_F11120_S850/2021	EPI_ISL_1424051	20B	B.1.1.222	17	5'UTR:C241T, ORF1ab:C3037T, ORF1ab:A6693G, ORF1ab:A8094G, ORF1ab:C9430T, ORF1ab:C10029T, ORF1ab:C10039T, ORF1ab:C14408T, ORF1ab:T19839C, S:A23403G, S:A23756G, ORF3a:G25912T, ORF7b:A27756G, ORF8:A27921G, ORF8:G28001T, N:G28881A, N:G28882A, N:G28883C,	11	N:R203K, N:G204R, ORF1a:K2143R, ORF1a:K2610R, ORF1a:T3255I, ORF1b:P314L, ORF3a:G174C, ORF7b:M1V, ORF8:I10V, S:D614G, S:T732A,

hCoV-19/Mexico/CMX-InDRE_F11121_S877/2021	EPI_ISL_1399281	20B	B.1.1.519	29	5'UTR:C203T, 5'UTR:A223T, 5'UTR:C241T, ORF1ab:A866G, ORF1ab:C3037T, ORF1ab:C3140T, ORF1ab:C5183T, ORF1ab:C5748T, ORF1ab:C10029T, ORF1ab:C10954T, ORF1ab:A11117G, ORF1ab:C11916T, ORF1ab:C12789T, ORF1ab:C13730T, ORF1ab:C14408T, ORF1ab:T19839C, ORF1ab:C21306T, S:C22995A, S:A23403G, S:C23604A, S:A23756G, E:C26499T, ORF8:T27904C, N:G28747T, N:A28877T, N:G28878C, N:G28881A, N:G28882A, N:G28883C, N:C29197T,	17	N:R203K, N:G204R, ORF1a:I201V, ORF1a:P959S, ORF1a:P1640S, ORF1a:T1828I, ORF1a:T3255I, ORF1a:I3618V, ORF1a:S3884L, ORF1a:T4175I, ORF1b:A88V, ORF1b:P314L, ORF8:L4P, S:T478K, S:D614G, S:P681H, S:T732A,	5'UTR:2 22,
hCoV-19/Mexico/CMX-InDRE_F11123_S851/2021	EPI_ISL_1424052	20B	B.1.1.519	23	5'UTR:C203T, 5'UTR:C222T, 5'UTR:C241T, ORF1ab:C3037T, ORF1ab:C3140T, ORF1ab:C10029T, ORF1ab:C10954T, ORF1ab:A11117G, ORF1ab:C12789T, ORF1ab:C14408T, ORF1ab:G15921T, ORF1ab:C16329T, ORF1ab:T19839C, ORF1ab:A20055G, ORF1ab:C21306T, S:A21993T, S:C22995A, S:A23403G, S:C23604A, S:A23756G, N:G28881A, N:G28882A, N:G28883C, N:C29197T,	12	N:R203K, N:G204R, ORF1a:P959S, ORF1a:T3255I, ORF1a:I3618V, ORF1a:T4175I, ORF1b:P314L, S:Y144F, S:T478K, S:D614G, S:P681H, S:T732A,	
hCoV-19/Mexico/CMX-InDRE_F11124_S852/2021	EPI_ISL_1424053	20B	B.1.1.519	25	5'UTR:T201C, 5'UTR:C203T, 5'UTR:C222T, 5'UTR:C241T, ORF1ab:G1738T, ORF1ab:C3037T, ORF1ab:C3140T, ORF1ab:A3363T, ORF1ab:C10029T, ORF1ab:C10954T, ORF1ab:A11117G, ORF1ab:C12789T, ORF1ab:C13168T, ORF1ab:C14408T, ORF1ab:T19839C, ORF1ab:A19974G, ORF1ab:C21306T, S:C22995A, S:A23403G, S:C23604A, S:A23756G, ORF3a:C25791T, N:G28881A, N:G28882A, N:G28883C, N:C29197T,	12	N:R203K, N:G204R, ORF1a:P959S, ORF1a:K1033I, ORF1a:T3255I, ORF1a:I3618V, ORF1a:T4175I, ORF1b:P314L, S:T478K, S:D614G, S:P681H, S:T732A,	
hCoV-19/Mexico/CMX-InDRE_F115B_S615/2021	EPI_ISL_1340620	20B	B.1.1.519	30	5'UTR:T201C, 5'UTR:C203T, 5'UTR:C222T, 5'UTR:C241T, ORF1ab:A873G, ORF1ab:G1738T, ORF1ab:C3037T, ORF1ab:C3140T, ORF1ab:G5063A, ORF1ab:T7033C, ORF1ab:C8389T, ORF1ab:C10029T, ORF1ab:C10954T, ORF1ab:A11117G, ORF1ab:C12789T, ORF1ab:C14408T, ORF1ab:G19006T, ORF1ab:T19839C, ORF1ab:A19974G, ORF1ab:G20788A, ORF1ab:C21306T, S:G21604T, S:C22995A, S:A23403G, S:C23604A, S:A23756G, S:C24621T, N:G28881A, N:G28882A, N:G28883C, N:C29197T,	17	N:R203K, N:G204R, ORF1a:D203G, ORF1a:P959S, ORF1a:D1600N, ORF1a:T3255I, ORF1a:I3618V, ORF1a:T4175I, ORF1b:P314L, ORF1b:A1847S, ORF1b:V2441I, S:Q14H, S:T478K, S:D614G, S:P681H, S:T732A, S:A1020V,	
hCoV-19/Mexico/CMX-InDRE_F116B_S616/2021	EPI_ISL_1340621	20B	B.1.1.519	29	5'UTR:G174T, 5'UTR:T201C, 5'UTR:C203T, 5'UTR:C222T, 5'UTR:C241T, ORF1ab:C745T, ORF1ab:G1738T, ORF1ab:C1780T, ORF1ab:C3037T, ORF1ab:C3140T, ORF1ab:C9532T, ORF1ab:C10029T, ORF1ab:C10954T, ORF1ab:A11117G, ORF1ab:C12789T, ORF1ab:C14408T, ORF1ab:T19839C, ORF1ab:A19974G, ORF1ab:C21306T, S:C22995A, S:A23403G, S:C23604A, S:A23756G, ORF3a:G25489T, ORF8:G28077T, N:G28881A, N:G28882A, N:G28883C, N:C29197T, 3'UTR:C29836T,	13	N:R203K, N:G204R, ORF1a:P959S, ORF1a:T3255I, ORF1a:I3618V, ORF1a:T4175I, ORF1b:P314L, ORF3a:A33S, ORF8:V62L, S:T478K, S:D614G, S:P681H, S:T732A,	
hCoV-19/Mexico/CMX-InDRE_F117B_S617/2021	EPI_ISL_1340623	20B	B.1.1.519	26	5'UTR:C203T, 5'UTR:C222T, 5'UTR:C241T, ORF1ab:C3037T, ORF1ab:C3140T, ORF1ab:C10029T, ORF1ab:C10954T, ORF1ab:A11117G, ORF1ab:C12068T, ORF1ab:C12789T, ORF1ab:C13592A, ORF1ab:C14408T, ORF1ab:C16616T, ORF1ab:T19839C, ORF1ab:C20148T, ORF1ab:C21077T, ORF1ab:C21306T, S:C22995A, S:A23403G, S:C23604A, S:A23756G, ORF3a:G25906T, ORF8:C28253T, N:G28881A, N:G28882A, N:G28883C, N:C29197T	15	N:R203K, N:G204R, ORF1a:P959S, ORF1a:T3255I, ORF1a:I3618V, ORF1a:T4175I, ORF1b:T42N, ORF1b:P314L, ORF1b:T1050I, ORF1b:T2537I, ORF3a:G172C, S:T478K, S:D614G, S:P681H, S:T732A,	

hCoV-19/Mexico/CMX-InDRE_F93B_S648/2021	EPI_ISL_1359063	20B	B.1.1.517	26	5'UTR:C241T, ORF1ab:C3037T, ORF1ab:G9653A, ORF1ab:C10029T, ORF1ab:C10712T, ORF1ab:C14408T, ORF1ab:T19023C, ORF1ab:T19839C, ORF1ab:G19891T, ORF1ab:C20930T, ORF1ab:T21398C, S:C21658A, S:A22231G, S:G22936T, S:A23403G, S:A23756G, ORF3a:G25912T, ORF3a:G26172T, ORF7a:T27438C, ORF7b:A27756G, ORF8:A27921G, ORF8:G28001T, ORF8:C28011T, N:G28881A, N:G28882A, N:G28883C, 3'UTR:G29692T,	18	N:R203K, N:G204R, ORF1a:V3130I, ORF1a:T3255I, ORF1a:L3483F, ORF1b:P314L, ORF1b:D2142Y, ORF1b:T2488M, ORF1b:M2644T, ORF3a:G174C, ORF3a:M260I, ORF7b:M1V, ORF8:I10V, ORF8:H40Y, S:F32L, S:K458N, S:D614G, S:T732A,
hCoV-19/Mexico/CMX-InDRE_F95D_S614/2021	EPI_ISL_1340613	20B	B.1.1.519	28	5'UTR:C203T, 5'UTR:C222T, 5'UTR:C241T, ORF1ab:C1342T, ORF1ab:C3037T, ORF1ab:C3140T, ORF1ab:G3692T, ORF1ab:G3871T, ORF1ab:C5079T, ORF1ab:C10029T, ORF1ab:C10954T, ORF1ab:A11117G, ORF1ab:C11824A, ORF1ab:C12789T, ORF1ab:C14408T, ORF1ab:A14691G, ORF1ab:C16017T, ORF1ab:T19839C, ORF1ab:C21306T, S:C22995A, S:A23403G, S:C23604A, S:A23756G, M:C26527T, ORF8:T27904C, N:G28881A, N:G28882A, N:G28883C, N:C29197T,	16	M:A2V, N:R203K, N:G204R, ORF1a:P959S, ORF1a:V1143F, ORF1a:K1202N, ORF1a:T1605I, ORF1a:T3255I, ORF1a:I3618V, ORF1a:T4175I, ORF1b:P314L, ORF8:L4P, S:T478K, S:D614G, S:P681H, S:T732A,
hCoV-19/Mexico/CMX-InDRE_F97I_S613/2021	EPI_ISL_1340629	20B	B.1.1.519	25	5'UTR:C203T, 5'UTR:C222T, 5'UTR:C241T, ORF1ab:A1115G, ORF1ab:C3037T, ORF1ab:C3140T, ORF1ab:C10029T, ORF1ab:C10954T, ORF1ab:A11117G, ORF1ab:C12789T, ORF1ab:C14408T, ORF1ab:T19839C, ORF1ab:C20823T, ORF1ab:C21077T, ORF1ab:C21306T, S:C21911T, S:C22995A, S:A23403G, S:C23604A, S:A23756G, ORF8:T27904C, ORF8:C28087T, N:G28881A, N:G28882A, N:G28883C, N:C29197T,	15	N:R203K, N:G204R, ORF1a:I284V, ORF1a:P959S, ORF1a:T3255I, ORF1a:I3618V, ORF1a:T4175I, ORF1b:P314L, ORF1b:T2537I, ORF8:L4P, ORF8:A65V, S:T478K, S:D614G, S:P681H, S:T732A,
hCoV-19/Mexico/CMX-InDRE-01/2020	EPI_ISL_412972	20B	B.1.1	6	5'UTR:C241T, ORF1ab:C3037T, ORF1ab:C14408T, S:A23403G, N:G28881A, N:G28882A, N:G28883C,	4	N:R203K, N:G204R, ORF1b:P314L, S:D614G,
hCoV-19/Mexico/CMX-InDRE-06/2020	EPI_ISL_424673	19B	A.1	8	ORF1ab:C936T, ORF1ab:C8782T, ORF1ab:G11083T, ORF1ab:C17747T, ORF1ab:A17858G, ORF1ab:C18060T, S:A24694T, ORF8:T28144C, N:G28812T,	6	N:S180I, ORF1a:T224I, ORF1a:L3606F, ORF1b:P1427L, ORF1b:Y1464C, ORF8:L84S,
hCoV-19/Mexico/CMX-InDRE-07/2020	EPI_ISL_452139	20B	B.1.1	6	5'UTR:C241T, ORF1ab:C3037T, ORF1ab:C14408T, S:A23403G, N:G28881A, N:G28882A, N:G28883C,	4	N:R203K, N:G204R, ORF1b:P314L, S:D614G,
hCoV-19/Mexico/CMX-InDRE-08/2020	EPI_ISL_452141	20B	B.1.1	8	5'UTR:C241T, ORF1ab:C3037T, ORF1ab:C14408T, ORF1ab:T18736C, S:A23403G, M:C27046T, N:G28881A, N:G28882A, N:G28883C,	6	M:T175M, N:R203K, N:G204R, ORF1b:P314L, ORF1b:F1757L, S:D614G,
hCoV-19/Mexico/CMX-InDRE-101/2020	EPI_ISL_872091	20A	B.1	6	5'UTR:C241T, ORF1ab:A2205G, ORF1ab:C3037T, ORF1ab:T3493C, ORF1ab:C14408T, ORF1ab:A20268G, S:A23403G,	3	ORF1a:E647G, ORF1b:P314L, S:D614G,
hCoV-19/Mexico/CMX-InDRE-13/2020	EPI_ISL_455437	20A	B.1.609	7	5'UTR:C241T, ORF1ab:C3037T, ORF1ab:C4582T, ORF1ab:C13860T, ORF1ab:C14408T, ORF1ab:G19101T, ORF1ab:A20268G, S:A23403G,	3	ORF1b:P314L, ORF1b:Q1878H, S:D614G,
hCoV-19/Mexico/CMX-InDRE-96/2020	EPI_ISL_861420	20A	B.1.609	7	5'UTR:C241T, ORF1ab:C3037T, ORF1ab:C4582T, ORF1ab:T6023C, ORF1ab:C13119T, ORF1ab:C14408T, ORF1ab:A20268G, S:A23403G,	4	ORF1a:Y1920H, ORF1a:A4285V, ORF1b:P314L, S:D614G,
hCoV-19/Mexico/CMX-InDRE-98/2020	EPI_ISL_872088	20A	B.1	6	5'UTR:C241T, ORF1ab:C1489T, ORF1ab:C2094T, ORF1ab:C3037T, ORF1ab:C14408T, ORF1ab:A20268G, S:A23403G,	3	ORF1a:S610L, ORF1b:P314L, S:D614G,
hCoV-19/Mexico/CMX-INER-0006/2020	EPI_ISL_837608	20A	B.1.609	5	5'UTR:C241T, ORF1ab:C3037T, ORF1ab:C4582T, ORF1ab:C14408T, ORF1ab:A20268G, S:A23403G,	2	ORF1b:P314L, S:D614G,
hCoV-19/Mexico/CMX-INER-0007/2020	EPI_ISL_837609	20C	B.1	5	5'UTR:C241T, ORF1ab:C1059T, ORF1ab:C3037T, ORF1ab:C14408T, S:A23403G, ORF3a:G25563T,	4	ORF1a:T265I, ORF1b:P314L, ORF3a:Q57H, S:D614G,
hCoV-19/Mexico/CMX-INER-0008/2020	EPI_ISL_837610	20B	B.1.1	7	5'UTR:C241T, ORF1ab:C3037T, ORF1ab:C14408T, ORF1ab:T19839C, S:A23403G, N:G28881A, N:G28882A, N:G28883C,	4	N:R203K, N:G204R, ORF1b:P314L, S:D614G,
hCoV-19/Mexico/CMX-INER-0009/2020	EPI_ISL_837611	20A	B.1.609	5	5'UTR:C241T, ORF1ab:C3037T, ORF1ab:C4582T, ORF1ab:C14408T, ORF1ab:A20268G, S:A23403G,	2	ORF1b:P314L, S:D614G,

hCoV-19/Mexico/CMX-INER-0010/2020	EPI_ISL_837612	20A	B.1	8	5'UTR:C241T, ORF1ab:C3037T, ORF1ab:C5090T, ORF1ab:C8605T, ORF1ab:C14408T, ORF1ab:A20268G, S:A23403G, M:C26895T, 3'UTR:G29734C,	4	M:H125Y, ORF1a:P1609S, ORF1b:P314L, S:D614G,
hCoV-19/Mexico/CMX-INER-0011/2020	EPI_ISL_837613	20B	B.1.1	8	5'UTR:C241T, ORF1ab:C2399T, ORF1ab:C3037T, ORF1ab:C14408T, ORF1ab:C18032T, S:A23403G, N:G28881A, N:G28882A, N:G28883C,	6	N:R203K, N:G204R, ORF1a:H712Y, ORF1b:P314L, ORF1b:T1522I, S:D614G,
hCoV-19/Mexico/CMX-INER-0012/2020	EPI_ISL_837614	20A	B.1.609	6	5'UTR:C241T, ORF1ab:C3037T, ORF1ab:C4582T, ORF1ab:C14408T, ORF1ab:A20268G, S:A23403G, ORF3a:G26167T,	3	ORF1b:P314L, ORF3a:V259L, S:D614G,
hCoV-19/Mexico/CMX-INER-0013/2020	EPI_ISL_837615	20A	B.1	6	5'UTR:C241T, ORF1ab:C3037T, ORF1ab:C14408T, ORF1ab:A20268G, S:A23403G, M:C26882T, 3'UTR:G29751C,	2	ORF1b:P314L, S:D614G,
hCoV-19/Mexico/CMX-INER-0014/2020	EPI_ISL_837616	19B	A.5	3	ORF1ab:C8782T, ORF1ab:C17470T, ORF3a:C26088T, ORF8:T28144C,	1	ORF8:L84S,
hCoV-19/Mexico/CMX-INER-0015/2020	EPI_ISL_837617	20A	B.1.609	5	5'UTR:C241T, ORF1ab:C3037T, ORF1ab:C4582T, ORF1ab:C14408T, ORF1ab:A20268G, S:A23403G,	2	ORF1b:P314L, S:D614G,
hCoV-19/Mexico/CMX-INER-0016/2020	EPI_ISL_837618	20A	B.1.609	7	5'UTR:C241T, ORF1ab:C3037T, ORF1ab:C4582T, ORF1ab:C14408T, ORF1ab:A20268G, ORF1ab:C21440T, S:A23403G, S:G23593T,	4	ORF1b:P314L, ORF1b:S2658F, S:D614G, S:Q677H,
hCoV-19/Mexico/CMX-INER-0017/2020	EPI_ISL_837619	20A	B.1.609	8	5'UTR:C241T, ORF1ab:C3037T, ORF1ab:C4582T, ORF1ab:C11102T, ORF1ab:C14408T, ORF1ab:G16065A, ORF1ab:A18504C, ORF1ab:A20268G, S:A23403G,	4	ORF1a:P3613S, ORF1b:P314L, ORF1b:K1679N, S:D614G,
hCoV-19/Mexico/CMX-INER-0018/2020	EPI_ISL_837620	20A	B.1.609	6	5'UTR:C241T, ORF1ab:C3037T, ORF1ab:C4582T, ORF1ab:G12832T, ORF1ab:C14408T, ORF1ab:A20268G, S:A23403G,	3	ORF1a:Q4189H, ORF1b:P314L, S:D614G,
hCoV-19/Mexico/CMX-INER-0019/2020	EPI_ISL_837621	19B	A.5	4	ORF1ab:C8782T, ORF1ab:C11776T, ORF1ab:C17470T, ORF3a:C26088T, ORF8:T28144C,	1	ORF8:L84S,
hCoV-19/Mexico/CMX-INER-0020/2020	EPI_ISL_837622	20A	B.1.609	7	5'UTR:C241T, ORF1ab:C3037T, ORF1ab:C4582T, ORF1ab:A13318G, ORF1ab:C13860T, ORF1ab:C14408T, ORF1ab:A20268G, S:A23403G,	2	ORF1b:P314L, S:D614G,
hCoV-19/Mexico/CMX-INER-0021/2020	EPI_ISL_837623	20B	B.1.1	7	5'UTR:C241T, ORF1ab:C3037T, ORF1ab:C14408T, ORF1ab:T19839C, S:A23403G, N:G28881A, N:G28882A, N:G28883C,	4	N:R203K, N:G204R, ORF1b:P314L, S:D614G,
hCoV-19/Mexico/CMX-INER-0022/2020	EPI_ISL_837624	20A	B.1	6	5'UTR:C241T, ORF1ab:C3037T, ORF1ab:C10277T, ORF1ab:C14408T, ORF1ab:C18877T, S:A23403G, ORF3a:G25563T,	4	ORF1a:L3338F, ORF1b:P314L, ORF3a:Q57H, S:D614G,
hCoV-19/Mexico/CMX-INER-0023/2020	EPI_ISL_837625	20B	B.1.1	7	5'UTR:C241T, ORF1ab:C3037T, ORF1ab:C14408T, ORF1ab:T19839C, S:A23403G, N:G28881A, N:G28882A, N:G28883C,	4	N:R203K, N:G204R, ORF1b:P314L, S:D614G,
hCoV-19/Mexico/CMX-INER-0024/2020	EPI_ISL_837626	20C	B.1	6	5'UTR:C241T, ORF1ab:C1059T, ORF1ab:C3037T, ORF1ab:G6352A, ORF1ab:C14408T, S:A23403G, ORF3a:G25563T,	4	ORF1a:T265I, ORF1b:P314L, ORF3a:Q57H, S:D614G,
hCoV-19/Mexico/CMX-INER-0025/2020	EPI_ISL_837627	20A	B.1.609	7	5'UTR:C241T, ORF1ab:C3037T, ORF1ab:C4582T, ORF1ab:C12439T, ORF1ab:C13860T, ORF1ab:C14408T, ORF1ab:A20268G, S:A23403G,	2	ORF1b:P314L, S:D614G,
hCoV-19/Mexico/CMX-INER-0026/2020	EPI_ISL_837628	20B	B.1.1.222	8	5'UTR:C241T, ORF1ab:C3037T, ORF1ab:C14408T, ORF1ab:T19839C, S:A23403G, S:A23756G, N:G28881A, N:G28882A, N:G28883C,	5	N:R203K, N:G204R, ORF1b:P314L, S:D614G, S:T732A,
hCoV-19/Mexico/CMX-INER-0027/2020	EPI_ISL_837629	20A	B.1	6	5'UTR:C241T, ORF1ab:C3037T, ORF1ab:C14408T, ORF1ab:C16178T, ORF1ab:A16687G, ORF1ab:A20268G, S:A23403G,	4	ORF1b:P314L, ORF1b:S904L, ORF1b:I1074V, S:D614G,
hCoV-19/Mexico/CMX-INER-0028/2020	EPI_ISL_837630	20A	B.1.609	6	5'UTR:C241T, ORF1ab:C3037T, ORF1ab:C4582T, ORF1ab:C14408T, ORF1ab:A20268G, S:G22340T, S:A23403G,	3	ORF1b:P314L, S:A260S, S:D614G,
hCoV-19/Mexico/CMX-INER-0029/2020	EPI_ISL_837631	20A	B.1.610	4	5'UTR:C241T, ORF1ab:C3037T, ORF1ab:C14408T, ORF1ab:A20268G, S:A23403G,	2	ORF1b:P314L, S:D614G,
hCoV-19/Mexico/CMX-INER-0030/2020	EPI_ISL_837632	20A	B.1	6	5'UTR:C241T, ORF1ab:C3037T, ORF1ab:C10277T, ORF1ab:C14408T, ORF1ab:C18877T, S:A23403G, ORF3a:G25563T,	4	ORF1a:L3338F, ORF1b:P314L, ORF3a:Q57H, S:D614G,
hCoV-19/Mexico/CMX-INER-0031/2020	EPI_ISL_837633	20A	B.1.609	6	5'UTR:C241T, ORF1ab:C3037T, ORF1ab:C4582T, ORF1ab:C14184T, ORF1ab:C14408T, ORF1ab:A20268G, S:A23403G,	2	ORF1b:P314L, S:D614G,
hCoV-19/Mexico/CMX-INER-0032/2020	EPI_ISL_837634	20A	B.1.609	5	5'UTR:C241T, ORF1ab:C3037T, ORF1ab:C4582T, ORF1ab:C14408T, ORF1ab:A20268G, S:A23403G,	2	ORF1b:P314L, S:D614G,

hCoV-19/Mexico/CMX-INER-0033/2020	EPI_ISL_837635	20A	B.1	5	5'UTR:C241T, ORF1ab:C3037T, ORF1ab:C14408T, ORF1ab:A20268G, S:C22009T, S:A23403G,	2	ORF1b:P314L, S:D614G,	
hCoV-19/Mexico/CMX-INER-0034/2020	EPI_ISL_837636	20A	B.1.243	9	5'UTR:C241T, ORF1ab:G806A, ORF1ab:T2584A, ORF1ab:C3037T, ORF1ab:C14408T, ORF1ab:A20268G, S:A23403G, S:T24076C, N:C28854T, N:G29543T,	4	N:S194L, ORF1a:A181T, ORF1b:P314L, S:D614G,	
hCoV-19/Mexico/CMX-INER-0035/2020	EPI_ISL_837637	20C	B.1	6	5'UTR:C241T, ORF1ab:C1059T, ORF1ab:C3037T, ORF1ab:T12181G, ORF1ab:C14408T, S:A23403G, ORF3a:G25563T,	5	ORF1a:T265I, ORF1a:D3972E, ORF1b:P314L, ORF3a:Q57H, S:D614G,	
hCoV-19/Mexico/CMX-INER-0036/2020	EPI_ISL_837638	20A	B.1.610	4	5'UTR:C241T, ORF1ab:C3037T, ORF1ab:C14408T, ORF1ab:A20268G, S:A23403G,	2	ORF1b:P314L, S:D614G,	
hCoV-19/Mexico/CMX-INER-0037/2020	EPI_ISL_837639	20A	B.1.609	6	5'UTR:C241T, ORF1ab:C3037T, ORF1ab:C4582T, ORF1ab:C13860T, ORF1ab:C14408T, ORF1ab:A20268G, S:A23403G,	2	ORF1b:P314L, S:D614G,	
hCoV-19/Mexico/CMX-INER-0038/2020	EPI_ISL_837640	20A	B.1	5	5'UTR:C241T, ORF1ab:C3037T, ORF1ab:G9671A, ORF1ab:C14408T, ORF1ab:A20268G, S:A23403G,	3	ORF1a:V3136I, ORF1b:P314L, S:D614G,	
hCoV-19/Mexico/CMX-INER-0039/2020	EPI_ISL_837641	20B	B.1.1	8	5'UTR:C241T, ORF1ab:C3037T, ORF1ab:G10265A, ORF1ab:C14408T, S:A23403G, ORF3a:A26009G, N:G28881A, N:G28882A, N:G28883C,	6	N:R203K, N:G204R, ORF1a:G3334S, ORF1b:P314L, ORF3a:Y206C, S:D614G,	
hCoV-19/Mexico/CMX-INER-0040/2020	EPI_ISL_837606	20A	B.1	6	5'UTR:C241T, ORF1ab:C3037T, ORF1ab:C14408T, ORF1ab:C18877T, S:A23403G, ORF3a:G25563T, ORF7a:C27434T,	4	ORF1b:P314L, ORF3a:Q57H, ORF7a:T14I, S:D614G,	ORF1ab: 515-520,
hCoV-19/Mexico/CMX-INER-0041/2020	EPI_ISL_837642	20A	B.1.610	4	5'UTR:C241T, ORF1ab:C3037T, ORF1ab:C14408T, ORF1ab:A20268G, S:A23403G,	2	ORF1b:P314L, S:D614G,	
hCoV-19/Mexico/CMX-INER-0042/2020	EPI_ISL_837643	20B	B.1.1	8	5'UTR:C241T, ORF1ab:C3037T, ORF1ab:C13019T, ORF1ab:C14408T, ORF1ab:T19839C, S:A23403G, N:G28881A, N:G28882A, N:G28883C,	4	N:R203K, N:G204R, ORF1b:P314L, S:D614G,	
hCoV-19/Mexico/CMX-INER-0043/2020	EPI_ISL_837644	20C	B.1	6	5'UTR:C241T, ORF1ab:C1059T, ORF1ab:C3037T, ORF1ab:C14408T, S:A23403G, ORF3a:G25494A, ORF3a:G25563T,	4	ORF1a:T265I, ORF1b:P314L, ORF3a:Q57H, S:D614G,	
hCoV-19/Mexico/CMX-INER-0044/2020	EPI_ISL_837645	20A	B.1.609	7	5'UTR:C241T, ORF1ab:C3037T, ORF1ab:C4582T, ORF1ab:T6023C, ORF1ab:C13119T, ORF1ab:C14408T, ORF1ab:A20268G, S:A23403G,	4	ORF1a:Y1920H, ORF1a:A4285V, ORF1b:P314L, S:D614G,	
hCoV-19/Mexico/CMX-INER-0045/2020	EPI_ISL_837646	20B	B.1.1	8	5'UTR:C241T, ORF1ab:C3037T, ORF1ab:C3411T, ORF1ab:C14408T, ORF1ab:T19839C, S:A23403G, N:G28881A, N:G28882A, N:G28883C,	5	N:R203K, N:G204R, ORF1a:A1049V, ORF1b:P314L, S:D614G,	
hCoV-19/Mexico/CMX-INER-0046/2020	EPI_ISL_837647	20A	B.1.609	8	5'UTR:C241T, ORF1ab:C3037T, ORF1ab:T4092C, ORF1ab:C4582T, ORF1ab:C14184T, ORF1ab:C14408T, ORF1ab:A20268G, S:A23403G, M:C26873T,	3	ORF1a:I1276T, ORF1b:P314L, S:D614G,	
hCoV-19/Mexico/CMX-INER-0047/2020	EPI_ISL_837648	20B	B.1.1	8	5'UTR:C241T, ORF1ab:C3037T, ORF1ab:C14408T, ORF1ab:C21058T, S:G21795T, S:A23403G, N:G28881A, N:G28882A, N:G28883C,	6	N:R203K, N:G204R, ORF1b:P314L, ORF1b:P2531S, S:R78M, S:D614G,	
hCoV-19/Mexico/CMX-INER-0048/2020	EPI_ISL_837649	20B	B.1.1	9	5'UTR:C241T, ORF1ab:T1476C, ORF1ab:C2399T, ORF1ab:C3037T, ORF1ab:C13994T, ORF1ab:C14408T, S:A23403G, N:G28881A, N:G28882A, N:G28883C,	7	N:R203K, N:G204R, ORF1a:I404T, ORF1a:H712Y, ORF1b:A176V, ORF1b:P314L, S:D614G,	
hCoV-19/Mexico/CMX-INER-0049/2020	EPI_ISL_837650	20A	B.1.189	8	5'UTR:C241T, ORF1ab:C3037T, ORF1ab:C4582T, ORF1ab:G6404T, ORF1ab:C14408T, ORF1ab:A20268G, S:A23403G, S:T25123C, N:G28378T,	4	ORF1a:V2047F, ORF1b:P314L, ORF9b:R32L, S:D614G,	
hCoV-19/Mexico/CMX-INER-0050/2020	EPI_ISL_837651	20B	B.1.1	7	5'UTR:C241T, ORF1ab:C3037T, ORF1ab:C7165T, ORF1ab:C14408T, S:A23403G, N:G28881A, N:G28882A, N:G28883C,	4	N:R203K, N:G204R, ORF1b:P314L, S:D614G,	
hCoV-19/Mexico/CMX-INER-0051/2020	EPI_ISL_837604	20A	B.1.189	9	5'UTR:C241T, ORF1ab:C3037T, ORF1ab:C4582T, ORF1ab:G6404T, ORF1ab:C14408T, ORF1ab:A20268G, S:A23403G, S:T25123C, ORF7a:G27676T, N:G28378T,	5	ORF1a:V2047F, ORF1b:P314L, ORF7a:E95*, ORF9b:R32L, S:D614G,	
hCoV-19/Mexico/CMX-INER-0052/2020	EPI_ISL_837652	20B	B.1.1	11	5'UTR:C241T, 5'UTR:G246T, ORF1ab:C3037T, ORF1ab:T10150C, ORF1ab:C14408T, ORF1ab:T18353C, ORF1ab:T19839C, S:A23403G, M:G26727T, N:G28881A, N:G28882A, N:G28883C,	6	M:A69S, N:R203K, N:G204R, ORF1b:P314L, ORF1b:L1629S, S:D614G,	
hCoV-19/Mexico/CMX-INER-0053/2020	EPI_ISL_837653	20A	B.1	6	5'UTR:C241T, ORF1ab:C3037T, ORF1ab:G8861A, ORF1ab:C14408T, ORF1ab:C16178T, ORF1ab:A20268G, S:A23403G,	4	ORF1a:V2866M, ORF1b:P314L, ORF1b:S904L, S:D614G,	

hCoV-19/Mexico/CMX-INER-0054/2020	EPI_ISL_837654	20A	B.1	7	5'UTR:C241T, ORF1ab:A1350G, ORF1ab:C3037T, ORF1ab:G9479T, ORF1ab:C14408T, ORF1ab:A20268G, S:A23403G, 3'UTR:C29738T,	4	ORF1a:Q362R, ORF1a:G3072C, ORF1b:P314L, S:D614G,
hCoV-19/Mexico/CMX-INER-0055/2020	EPI_ISL_837655	20A	B.1	6	5'UTR:C241T, ORF1ab:C449T, ORF1ab:C3037T, ORF1ab:C14408T, ORF1ab:A20268G, S:G22532C, S:A23403G,	4	ORF1a:P62S, ORF1b:P314L, S:E324Q, S:D614G,
hCoV-19/Mexico/CMX-INER-0056/2020	EPI_ISL_837600	20A	B.1	6	5'UTR:C241T, ORF1ab:C1489T, ORF1ab:C3037T, ORF1ab:C14408T, ORF1ab:A20268G, S:C23248T, S:A23403G,	2	ORF1b:P314L, S:D614G,
hCoV-19/Mexico/CMX-INER-0057/2020	EPI_ISL_837656	20A	B.1	8	5'UTR:C241T, ORF1ab:C3037T, ORF1ab:C3773T, ORF1ab:C4582T, ORF1ab:C14408T, ORF1ab:A20268G, ORF1ab:C20825T, S:A23403G, ORF6:T27384C,	4	ORF1a:R1170C, ORF1b:P314L, ORF1b:T2453I, S:D614G,
hCoV-19/Mexico/CMX-INER-0058/2020	EPI_ISL_837657	20A	B.1.609	8	5'UTR:C241T, ORF1ab:C3037T, ORF1ab:C4582T, ORF1ab:C14408T, ORF1ab:A20268G, S:A23403G, ORF3a:T25533A, N:G28541T, N:T28862C,	5	N:A90S, N:S197P, ORF1b:P314L, ORF9b:E86D, S:D614G,
hCoV-19/Mexico/CMX-INER-0059/2020	EPI_ISL_837658	20A	B.1.609	7	5'UTR:C241T, ORF1ab:C3037T, ORF1ab:C4582T, ORF1ab:T6023C, ORF1ab:C13119T, ORF1ab:C14408T, ORF1ab:A20268G, S:A23403G,	4	ORF1a:Y1920H, ORF1a:A4285V, ORF1b:P314L, S:D614G,
hCoV-19/Mexico/CMX-INER-0060/2020	EPI_ISL_837659	20A	B.1.609	7	5'UTR:C241T, ORF1ab:C3037T, ORF1ab:C4582T, ORF1ab:T6023C, ORF1ab:C13119T, ORF1ab:C14408T, ORF1ab:A20268G, S:A23403G,	4	ORF1a:Y1920H, ORF1a:A4285V, ORF1b:P314L, S:D614G,
hCoV-19/Mexico/CMX-INER-0061/2020	EPI_ISL_837660	20C	B.1	7	5'UTR:C241T, ORF1ab:C1059T, ORF1ab:C3037T, ORF1ab:C14408T, S:A23403G, S:G24368T, ORF3a:G25563T, M:C26873T,	5	ORF1a:T265I, ORF1b:P314L, ORF3a:Q57H, S:D614G, S:D936Y,
hCoV-19/Mexico/CMX-INER-0062/2020	EPI_ISL_837661	20C	B.1	8	5'UTR:C241T, ORF1ab:A687G, ORF1ab:C1059T, ORF1ab:C3037T, ORF1ab:C14408T, ORF1ab:G20057A, S:A23403G, S:G24368T, ORF3a:G25563T,	7	ORF1a:K141R, ORF1a:T265I, ORF1b:P314L, ORF1b:G2197D, ORF3a:Q57H, S:D614G, S:D936Y,
hCoV-19/Mexico/CMX-INER-0063/2020	EPI_ISL_837662	20C	B.1	6	5'UTR:C241T, ORF1ab:C1059T, ORF1ab:C3037T, ORF1ab:C14408T, S:A23403G, S:G24368T, ORF3a:G25563T,	5	ORF1a:T265I, ORF1b:P314L, ORF3a:Q57H, S:D614G, S:D936Y,
hCoV-19/Mexico/CMX-INER-0064/2020	EPI_ISL_837663	20A	B.1	6	5'UTR:C241T, ORF1ab:C3037T, ORF1ab:C14408T, ORF1ab:G18769A, ORF1ab:A20268G, S:C21910T, S:A23403G,	3	ORF1b:P314L, ORF1b:V1768I, S:D614G,
hCoV-19/Mexico/CMX-INER-0065/2020	EPI_ISL_837664	20A	B.1.189	9	5'UTR:C241T, ORF1ab:C3037T, ORF1ab:C4582T, ORF1ab:G6404T, ORF1ab:A13768G, ORF1ab:C14408T, ORF1ab:A20268G, S:A23403G, S:T25123C, N:G28378T,	5	ORF1a:V2047F, ORF1b:M101V, ORF1b:P314L, ORF9b:R32L, S:D614G,
hCoV-19/Mexico/CMX-INER-0066/2020	EPI_ISL_837665	20A	B.1.609	6	5'UTR:C241T, ORF1ab:C3037T, ORF1ab:C4582T, ORF1ab:C13860T, ORF1ab:C14408T, ORF1ab:A20268G, S:A23403G,	2	ORF1b:P314L, S:D614G,
hCoV-19/Mexico/CMX-INER-0067/2020	EPI_ISL_837666	20C	B.1	8	5'UTR:C241T, ORF1ab:A687G, ORF1ab:C1059T, ORF1ab:C3037T, ORF1ab:C14408T, ORF1ab:G20057A, S:A23403G, S:G24368T, ORF3a:G25563T,	7	ORF1a:K141R, ORF1a:T265I, ORF1b:P314L, ORF1b:G2197D, ORF3a:Q57H, S:D614G, S:D936Y,
hCoV-19/Mexico/CMX-INER-0068/2020	EPI_ISL_837667	20A	B.1.609	9	5'UTR:C241T, ORF1ab:C3037T, ORF1ab:C4582T, ORF1ab:G9479T, ORF1ab:C13860T, ORF1ab:C14408T, ORF1ab:A20268G, S:A23403G, ORF3a:C25889T, N:C29253T,	5	N:S327L, ORF1a:G3072C, ORF1b:P314L, ORF3a:S166L, S:D614G,
hCoV-19/Mexico/CMX-INER-0069/2020	EPI_ISL_837668	20A	B.1	8	5'UTR:C241T, ORF1ab:C3037T, ORF1ab:C4010T, ORF1ab:A6248C, ORF1ab:T12835C, ORF1ab:C14408T, ORF1ab:A20268G, S:A23403G, 3'UTR:C29708T,	4	ORF1a:L1249F, ORF1a:N1995H, ORF1b:P314L, S:D614G,
hCoV-19/Mexico/CMX-INER-0070/2020	EPI_ISL_837669	20B	B.1.1.222	9	5'UTR:C241T, ORF1ab:C3037T, ORF1ab:C14408T, ORF1ab:T19839C, S:A23403G, S:A23756G, N:G28881A, N:G28882A, N:G28883C, N:G29402T,	6	N:R203K, N:G204R, N:D377Y, ORF1b:P314L, S:D614G, S:T732A,
hCoV-19/Mexico/CMX-INER-0071/2020	EPI_ISL_837670	20A	B.1.189	8	5'UTR:C241T, ORF1ab:C3037T, ORF1ab:C4582T, ORF1ab:G6404T, ORF1ab:C14408T, ORF1ab:A20268G, S:A23403G, S:T25123C, N:G28378T,	4	ORF1a:V2047F, ORF1b:P314L, ORF9b:R32L, S:D614G,
hCoV-19/Mexico/CMX-INER-0072/2020	EPI_ISL_837671	20A	B.1.609	6	5'UTR:C241T, ORF1ab:C3037T, ORF1ab:C4582T, ORF1ab:C13119T, ORF1ab:C14408T, ORF1ab:A20268G, S:A23403G,	3	ORF1a:A4285V, ORF1b:P314L, S:D614G,
hCoV-19/Mexico/CMX-INER-0073/2020	EPI_ISL_837672	20A	B.1.609	5	5'UTR:C241T, ORF1ab:C3037T, ORF1ab:C4582T, ORF1ab:C14408T, ORF1ab:A20268G, S:A23403G,	2	ORF1b:P314L, S:D614G,

hCoV-19/Mexico/CMX-INER-0074/2020	EPI_ISL_837673	20A	B.1.609	6	5'UTR:C241T, ORF1ab:C3037T, ORF1ab:C4582T, ORF1ab:C13860T, ORF1ab:C14408T, ORF1ab:A20268G, S:A23403G,	2	ORF1b:P314L, S:D614G,
hCoV-19/Mexico/CMX-INER-0075/2020	EPI_ISL_837605	20A	B.1.189	10	5'UTR:C241T, ORF1ab:C3037T, ORF1ab:C4582T, ORF1ab:G6404T, ORF1ab:C14408T, ORF1ab:A20268G, S:C22680T, S:A23403G, S:T25123C, ORF7a:G27676T, N:G28378T,	6	ORF1a:V2047F, ORF1b:P314L, ORF7a:E95*, ORF9b:R32L, S:S373L, S:D614G,
hCoV-19/Mexico/CMX-INER-0076/2020	EPI_ISL_837674	20A	B.1	6	5'UTR:C241T, ORF1ab:C3037T, ORF1ab:C14408T, ORF1ab:C18877T, S:A23403G, ORF3a:G25563T, ORF8:T28157C,	3	ORF1b:P314L, ORF3a:Q57H, S:D614G,
hCoV-19/Mexico/CMX-INER-0077/2020	EPI_ISL_837675	20A	B.1.609	8	5'UTR:C241T, ORF1ab:C3037T, ORF1ab:C4582T, ORF1ab:C13119T, ORF1ab:C14408T, ORF1ab:A20268G, S:A23403G, N:T29452C, N:C29485T,	3	ORF1a:A4285V, ORF1b:P314L, S:D614G,
hCoV-19/Mexico/CMX-INER-0078/2020	EPI_ISL_837676	20A	B.1	5	5'UTR:C241T, ORF1ab:C3037T, ORF1ab:C14408T, ORF1ab:A20268G, S:G21989T, S:A23403G,	3	ORF1b:P314L, S:V143F, S:D614G,
hCoV-19/Mexico/CMX-INER-0079/2020	EPI_ISL_837677	20C	B.1	6	5'UTR:C241T, ORF1ab:C1059T, ORF1ab:C3037T, ORF1ab:C14408T, S:A23403G, S:G24368T, ORF3a:G25563T,	5	ORF1a:T265I, ORF1b:P314L, ORF3a:Q57H, S:D614G, S:D936Y,
hCoV-19/Mexico/CMX-INER-0080/2020	EPI_ISL_837678	20A	B.1.609	7	5'UTR:C241T, ORF1ab:C3037T, ORF1ab:C4582T, ORF1ab:C13119T, ORF1ab:C14408T, ORF1ab:A20268G, S:A23403G, S:G25364A,	4	ORF1a:A4285V, ORF1b:P314L, S:D614G, S:V1268I,
hCoV-19/Mexico/CMX-INER-0081/2020	EPI_ISL_837679	20A	B.1	8	5'UTR:C241T, ORF1ab:C3037T, ORF1ab:C4010T, ORF1ab:A6248C, ORF1ab:T12835C, ORF1ab:C14408T, ORF1ab:A20268G, S:A23403G, 3'UTR:C29708T,	4	ORF1a:L1249F, ORF1a:N1995H, ORF1b:P314L, S:D614G,
hCoV-19/Mexico/CMX-INER-0082/2020	EPI_ISL_837680	20A	B.1.609	8	5'UTR:C241T, ORF1ab:C3037T, ORF1ab:C4582T, ORF1ab:G13441T, ORF1ab:C14408T, ORF1ab:A20268G, S:A23403G, ORF3a:C26176T, 3'UTR:G29706T,	4	ORF1a:Q4392H, ORF1b:P314L, ORF3a:P262S, S:D614G,
hCoV-19/Mexico/CMX-INER-0083/2020	EPI_ISL_837681	20B	B.1.1.222	9	5'UTR:C241T, ORF1ab:C2676T, ORF1ab:C3037T, ORF1ab:C14408T, ORF1ab:T19839C, S:A23403G, S:A23756G, N:G28881A, N:G28882A, N:G28883C,	6	N:R203K, N:G204R, ORF1a:P804L, ORF1b:P314L, S:D614G, S:T732A,
hCoV-19/Mexico/CMX-INER-0084/2020	EPI_ISL_837682	20A	B.1.609	7	5'UTR:C241T, ORF1ab:C3037T, ORF1ab:C4582T, ORF1ab:C13119T, ORF1ab:C14408T, ORF1ab:A20268G, S:A23403G, N:C29274T,	4	N:T334I, ORF1a:A4285V, ORF1b:P314L, S:D614G,
hCoV-19/Mexico/CMX-INER-0085/2020	EPI_ISL_837683	20B	B.1.1	9	5'UTR:C241T, ORF1ab:C3037T, ORF1ab:C14408T, ORF1ab:T19839C, ORF1ab:A21323T, S:A23403G, M:G26526T, N:G28881A, N:G28882A, N:G28883C,	6	M:A2S, N:R203K, N:G204R, ORF1b:P314L, ORF1b:Y2619F, S:D614G,
hCoV-19/Mexico/CMX-INER-0086/2020	EPI_ISL_837684	20A	B.1.609	6	5'UTR:C241T, ORF1ab:C3037T, ORF1ab:C4582T, ORF1ab:C13119T, ORF1ab:C14408T, ORF1ab:A20268G, S:A23403G,	3	ORF1a:A4285V, ORF1b:P314L, S:D614G,
hCoV-19/Mexico/CMX-INER-0087/2020	EPI_ISL_837685	20B	B.1.1	9	5'UTR:C241T, ORF1ab:C3037T, ORF1ab:G9805T, ORF1ab:C14408T, ORF1ab:T19839C, S:A23403G, S:G24095T, N:G28881A, N:G28882A, N:G28883C,	5	N:R203K, N:G204R, ORF1b:P314L, S:D614G, S:A845S,
hCoV-19/Mexico/CMX-INER-0088/2020	EPI_ISL_837686	20B	B.1.1.222	9	5'UTR:C241T, ORF1ab:C3037T, ORF1ab:T5419C, ORF1ab:C14408T, ORF1ab:T19839C, S:A23403G, S:A23756G, N:G28881A, N:G28882A, N:G28883C,	5	N:R203K, N:G204R, ORF1b:P314L, S:D614G, S:T732A,
hCoV-19/Mexico/CMX-INER-0089/2020	EPI_ISL_837687	20A	B.1.609	7	5'UTR:C241T, ORF1ab:C3037T, ORF1ab:C4582T, ORF1ab:C14408T, ORF1ab:A20268G, ORF1ab:C21440T, S:A23403G, S:G23593T,	4	ORF1b:P314L, ORF1b:S2658F, S:D614G, S:Q677H,
hCoV-19/Mexico/CMX-INER-0090/2020	EPI_ISL_837688	20A	B.1.609	11	5'UTR:C241T, ORF1ab:C1594T, ORF1ab:C3037T, ORF1ab:C4582T, ORF1ab:C8326T, ORF1ab:C8772T, ORF1ab:C13119T, ORF1ab:C14408T, ORF1ab:A20268G, S:A23403G, S:G23608T, ORF7b:C27765T,	5	ORF1a:T2836I, ORF1a:A4285V, ORF1b:P314L, ORF7b:L4F, S:D614G,
hCoV-19/Mexico/CMX-INER-0091/2020	EPI_ISL_837689	20A	B.1.189	8	5'UTR:C241T, ORF1ab:C3037T, ORF1ab:C4582T, ORF1ab:G6404T, ORF1ab:C14408T, ORF1ab:A20268G, S:A23403G, S:T25123C, N:G28378T,	4	ORF1a:V2047F, ORF1b:P314L, ORF9b:R32L, S:D614G,
hCoV-19/Mexico/CMX-INER-0092/2020	EPI_ISL_837690	20B	B.1.1	7	5'UTR:C241T, ORF1ab:T2432C, ORF1ab:C3037T, ORF1ab:C14408T, S:A23403G, N:G28881A, N:G28882A, N:G28883C,	5	N:R203K, N:G204R, ORF1a:S723P, ORF1b:P314L, S:D614G,
hCoV-19/Mexico/CMX-INER-0093/2020	EPI_ISL_837691	20A	B.1.609	8	5'UTR:C241T, ORF1ab:C3037T, ORF1ab:C4582T, ORF1ab:C5007T, ORF1ab:T5911C, ORF1ab:C13119T, ORF1ab:C14408T, ORF1ab:A20268G, S:A23403G,	4	ORF1a:T1581M, ORF1a:A4285V, ORF1b:P314L, S:D614G,

hCoV-19/Mexico/CMX-INER-0094/2020	EPI_ISL_837692	20B	B.1.1	12	5'UTR:G219T, 5'UTR:C241T, ORF1ab:G1135T, ORF1ab:A2024G, ORF1ab:C2695T, ORF1ab:C3037T, ORF1ab:C14408T, ORF1ab:T19839C, S:A23403G, N:G28881A, N:G28882A, N:G28883C, N:C29171T,	7	N:R203K, N:G204R, N:H300Y, ORF1a:K290N, ORF1a:T587A, ORF1b:P314L, S:D614G,
hCoV-19/Mexico/CMX-INER-0095/2020	EPI_ISL_837693	20B	B.1.1	9	5'UTR:C241T, ORF1ab:G1135T, ORF1ab:A2024G, ORF1ab:C3037T, ORF1ab:C14408T, ORF1ab:T19839C, S:A23403G, N:G28881A, N:G28882A, N:G28883C,	6	N:R203K, N:G204R, ORF1a:K290N, ORF1a:T587A, ORF1b:P314L, S:D614G,
hCoV-19/Mexico/CMX-INER-0096/2020	EPI_ISL_837694	20A	B.1.609	14	5'UTR:G124A, 5'UTR:C241T, ORF1ab:C3037T, ORF1ab:C4582T, ORF1ab:C7005T, ORF1ab:C7945T, ORF1ab:C12400T, ORF1ab:C13860T, ORF1ab:C14408T, ORF1ab:A20268G, ORF1ab:C20936T, S:G23126T, S:A23403G, ORF3a:C25731T, N:G29473A,	5	ORF1a:T2247I, ORF1b:P314L, ORF1b:T2490M, S:A522S, S:D614G,
hCoV-19/Mexico/CMX-INER-0097/2020	EPI_ISL_837695	20A	B.1.609	13	5'UTR:C241T, ORF1ab:C3037T, ORF1ab:T3886C, ORF1ab:C4582T, ORF1ab:C5467T, ORF1ab:C9996T, ORF1ab:C14177T, ORF1ab:C14408T, ORF1ab:G16843T, ORF1ab:T19590C, ORF1ab:A20268G, ORF1ab:T20757C, S:A23403G, ORF6:C27294T,	5	ORF1a:S3244L, ORF1b:T237I, ORF1b:P314L, ORF1b:G1126C, S:D614G,
hCoV-19/Mexico/CMX-INER-0098/2020	EPI_ISL_837696	20A	B.1.609	6	5'UTR:C241T, ORF1ab:C3037T, ORF1ab:C4582T, ORF1ab:C13119T, ORF1ab:C14408T, ORF1ab:A20268G, S:A23403G,	3	ORF1a:A4285V, ORF1b:P314L, S:D614G,
hCoV-19/Mexico/CMX-INER-0099/2020	EPI_ISL_837697	20B	B.1.1	10	5'UTR:C241T, ORF1ab:C1514T, ORF1ab:C2399T, ORF1ab:C3037T, ORF1ab:C9615T, ORF1ab:C14408T, ORF1ab:G18255T, S:A23403G, N:G28881A, N:G28882A, N:G28883C,	8	N:R203K, N:G204R, ORF1a:H417Y, ORF1a:H712Y, ORF1a:T3117I, ORF1b:P314L, ORF1b:M1596I, S:D614G,
hCoV-19/Mexico/CMX-INER-01/2020	EPI_ISL_424345	20A	B.1	6	5'UTR:C241T, ORF1ab:C823T, ORF1ab:C3037T, ORF1ab:C14408T, ORF1ab:A20268G, S:A23403G, 3'UTR:G29734C,	2	ORF1b:P314L, S:D614G,
hCoV-19/Mexico/CMX-INER-0100/2020	EPI_ISL_837698	20A	B.1.609	9	5'UTR:C241T, ORF1ab:C3037T, ORF1ab:C4582T, ORF1ab:C5812T, ORF1ab:C11572T, ORF1ab:C13860T, ORF1ab:C14408T, ORF1ab:G20006T, ORF1ab:A20268G, S:A23403G,	3	ORF1b:P314L, ORF1b:G2180V, S:D614G,
hCoV-19/Mexico/CMX-INER-0101/2020	EPI_ISL_837699	20B	B.1.1.222	9	5'UTR:C241T, ORF1ab:C3037T, ORF1ab:C14408T, ORF1ab:T19839C, ORF1ab:G20208T, S:A23403G, S:A23756G, N:G28881A, N:G28882A, N:G28883C,	6	N:R203K, N:G204R, ORF1b:P314L, ORF1b:Q2247H, S:D614G, S:T732A,
hCoV-19/Mexico/CMX-INER-0102/2020	EPI_ISL_837700	20B	B.1.1.222	9	5'UTR:C241T, ORF1ab:C3037T, ORF1ab:C14408T, ORF1ab:T19839C, S:A23403G, S:A23756G, ORF8:G28001T, N:G28881A, N:G28882A, N:G28883C,	5	N:R203K, N:G204R, ORF1b:P314L, S:D614G, S:T732A,
hCoV-19/Mexico/CMX-INER-0103/2020	EPI_ISL_837701	20B	B.1.1	11	5'UTR:C241T, ORF1ab:C1514T, ORF1ab:C2399T, ORF1ab:C3037T, ORF1ab:G4720T, ORF1ab:C9615T, ORF1ab:C14408T, ORF1ab:G18255T, S:A23403G, N:G28881A, N:G28882A, N:G28883C,	8	N:R203K, N:G204R, ORF1a:H417Y, ORF1a:H712Y, ORF1a:T3117I, ORF1b:P314L, ORF1b:M1596I, S:D614G,
hCoV-19/Mexico/CMX-INER-0104/2020	EPI_ISL_837702	20A	B.1.609	9	5'UTR:C241T, ORF1ab:C3037T, ORF1ab:C4582T, ORF1ab:T6023C, ORF1ab:C13119T, ORF1ab:C14408T, ORF1ab:A20268G, S:A23403G, S:G23426T, S:C24374T,	6	ORF1a:Y1920H, ORF1a:A4285V, ORF1b:P314L, S:D614G, S:V622F, S:L938F,
hCoV-19/Mexico/CMX-INER-0105/2020	EPI_ISL_837703	20A	B.1.189	9	5'UTR:C241T, ORF1ab:C3037T, ORF1ab:C4582T, ORF1ab:G6404T, ORF1ab:C10401T, ORF1ab:C14408T, ORF1ab:A20268G, S:A23403G, S:T25123C, N:G28378T,	5	ORF1a:V2047F, ORF1a:A3379V, ORF1b:P314L, ORF9b:R32L, S:D614G,
hCoV-19/Mexico/CMX-INER-0106/2020	EPI_ISL_837704	20B	B.1.1	9	5'UTR:C241T, ORF1ab:C3037T, ORF1ab:T10501C, ORF1ab:C14408T, ORF1ab:C17977T, ORF1ab:T19839C, S:A23403G, N:G28881A, N:G28882A, N:G28883C,	5	N:R203K, N:G204R, ORF1b:P314L, ORF1b:L1504F, S:D614G,
hCoV-19/Mexico/CMX-INER-0107/2020	EPI_ISL_837705	20A	B.1.609	8	5'UTR:C241T, ORF1ab:C3037T, ORF1ab:C4582T, ORF1ab:C13119T, ORF1ab:C14408T, ORF1ab:G19518A, ORF1ab:A20268G, S:A23403G, S:C23718T,	4	ORF1a:A4285V, ORF1b:P314L, S:D614G, S:T719I,
hCoV-19/Mexico/CMX-INER-0108/2020	EPI_ISL_837706	20A	B.1	6	5'UTR:C241T, ORF1ab:C1489T, ORF1ab:C3037T, ORF1ab:C14408T, ORF1ab:A20268G, S:C23248T, S:A23403G,	2	ORF1b:P314L, S:D614G,

hCoV-19/Mexico/CMX-INER-0109/2020	EPI_ISL_837707	20A	B.1	9	5'UTR:C241T, ORF1ab:C449T, ORF1ab:C3037T, ORF1ab:A4916G, ORF1ab:C12651T, ORF1ab:C14408T, ORF1ab:A20268G, S:A23403G, ORF8:G28079T, N:C29541T,	5	ORF1a:P62S, ORF1a:I1551V, ORF1a:T4129I, ORF1b:P314L, S:D614G,	
hCoV-19/Mexico/CMX-INER-0110/2020	EPI_ISL_837708	20C	B.1	8	5'UTR:C241T, ORF1ab:C1059T, ORF1ab:C3037T, ORF1ab:C14408T, S:A23403G, S:G24368T, ORF3a:G25563T, N:C28748T, N:G29414T,	6	N:A381S, ORF1a:T265I, ORF1b:P314L, ORF3a:Q57H, S:D614G, S:D936Y,	
hCoV-19/Mexico/CMX-INER-0111/2020	EPI_ISL_837709	20A	B.1.609	12	5'UTR:C241T, ORF1ab:C337T, ORF1ab:G1181T, ORF1ab:C3037T, ORF1ab:T4092C, ORF1ab:A4518G, ORF1ab:C4582T, ORF1ab:C7165T, ORF1ab:C14184T, ORF1ab:C14408T, ORF1ab:A20268G, S:A23403G, N:C28311T,	7	N:P13L, ORF1a:V306F, ORF1a:I1276T, ORF1a:Y1418C, ORF1b:P314L, ORF9b:P10S, S:D614G,	
hCoV-19/Mexico/CMX-INER-0112/2020	EPI_ISL_837710	20A	B.1.609	8	5'UTR:C241T, ORF1ab:C3037T, ORF1ab:C4582T, ORF1ab:C13119T, ORF1ab:T13270C, ORF1ab:C14408T, ORF1ab:A20268G, S:A23403G, S:C23530T,	3	ORF1a:A4285V, ORF1b:P314L, S:D614G,	
hCoV-19/Mexico/CMX-INER-0113/2020	EPI_ISL_837711	20A	B.1.189	9	5'UTR:C241T, ORF1ab:C3037T, ORF1ab:C4582T, ORF1ab:G6404T, ORF1ab:C10207T, ORF1ab:C14408T, ORF1ab:A20268G, S:A23403G, S:T25123C, N:G28378T,	4	ORF1a:V2047F, ORF1b:P314L, ORF9b:R32L, S:D614G,	
hCoV-19/Mexico/CMX-INER-0114/2020	EPI_ISL_837712	20B	B.1.1	10	5'UTR:C241T, ORF1ab:C3037T, ORF1ab:C6941T, ORF1ab:G7996T, ORF1ab:C14408T, ORF1ab:T19839C, S:A23403G, S:G25055T, N:G28881A, N:G28882A, N:G28883C,	5	N:R203K, N:G204R, ORF1b:P314L, S:D614G, S:D1165Y,	
hCoV-19/Mexico/CMX-INER-0115/2020	EPI_ISL_837713	20A	B.1.609	9	5'UTR:C241T, ORF1ab:C3037T, ORF1ab:C4582T, ORF1ab:C13119T, ORF1ab:C14408T, ORF1ab:A20268G, S:A23403G, S:A23614C, ORF3a:T26008C, ORF3a:G26101T,	5	ORF1a:A4285V, ORF1b:P314L, ORF3a:Y206H, ORF3a:V237F, S:D614G,	
hCoV-19/Mexico/CMX-INER-0116/2020	EPI_ISL_843191	20C	B.1	8	5'UTR:C241T, ORF1ab:C1059T, ORF1ab:C3037T, ORF1ab:C11152T, ORF1ab:C14408T, S:A23403G, ORF3a:G25563T, N:C28887T, 3'UTR:C29750T,	5	N:T205I, ORF1a:T265I, ORF1b:P314L, ORF3a:Q57H, S:D614G,	ORF7b:27792-27793,
hCoV-19/Mexico/CMX-INER-0117/2020	EPI_ISL_837714	20A	B.1.189	10	5'UTR:C241T, ORF1ab:A2932G, ORF1ab:C3037T, ORF1ab:C4582T, ORF1ab:T5386A, ORF1ab:G6404T, ORF1ab:C14408T, ORF1ab:A20268G, S:A23403G, S:T25123C, N:G28378T,	4	ORF1a:V2047F, ORF1b:P314L, ORF9b:R32L, S:D614G,	
hCoV-19/Mexico/CMX-INER-0118/2020	EPI_ISL_837715	20B	B.1.1.222	13	5'UTR:C241T, ORF1ab:C3037T, ORF1ab:A3181G, ORF1ab:C5826T, ORF1ab:C14408T, ORF1ab:C16726T, ORF1ab:T19839C, ORF1ab:G21055A, S:A23403G, S:A23756G, M:A26612T, N:G28881A, N:G28882A, N:G28883C,	8	N:R203K, N:G204R, ORF1a:T1854I, ORF1b:P314L, ORF1b:H1087Y, ORF1b:D2530N, S:D614G, S:T732A,	
hCoV-19/Mexico/CMX-INER-0119/2020	EPI_ISL_837716	20B	B.1.1	12	5'UTR:C241T, ORF1ab:C3037T, ORF1ab:A6393G, ORF1ab:C14408T, ORF1ab:T19839C, ORF1ab:T20346A, ORF1ab:G21468T, S:A23403G, S:G23593T, ORF8:C28087T, N:G28881A, N:G28882A, N:G28883C,	9	N:R203K, N:G204R, ORF1a:D2043G, ORF1b:P314L, ORF1b:H2293Q, ORF1b:M2667I, ORF8:A65V, S:D614G, S:Q677H,	
hCoV-19/Mexico/CMX-INER-0120/2020	EPI_ISL_837717	20B	B.1.1.222	10	5'UTR:C241T, ORF1ab:C3037T, ORF1ab:C10279T, ORF1ab:C14408T, ORF1ab:T19839C, S:A23403G, S:A23756G, ORF7a:G27497T, N:G28881A, N:G28882A, N:G28883C,	6	N:R203K, N:G204R, ORF1b:P314L, ORF7a:C35F, S:D614G, S:T732A,	
hCoV-19/Mexico/CMX-INER-0121/2020	EPI_ISL_837718	20A	B.1.609	7	5'UTR:C241T, ORF1ab:C3037T, ORF1ab:C4582T, ORF1ab:G11761T, ORF1ab:C14408T, ORF1ab:T18042C, ORF1ab:A20268G, S:A23403G,	3	ORF1a:K3832N, ORF1b:P314L, S:D614G,	
hCoV-19/Mexico/CMX-INER-0122/2020	EPI_ISL_837719	20B	B.1.1.222	11	5'UTR:C241T, ORF1ab:C3037T, ORF1ab:C14408T, ORF1ab:T19839C, S:C21846T, S:A23403G, S:A23756G, M:C26681T, M:C26895T, N:G28881A, N:G28882A, N:G28883C,	7	M:H125Y, N:R203K, N:G204R, ORF1b:P314L, S:T95I, S:D614G, S:T732A,	
hCoV-19/Mexico/CMX-INER-0123/2020	EPI_ISL_837720	20B	B.1.1	10	5'UTR:C241T, ORF1ab:C3037T, ORF1ab:A3908G, ORF1ab:T8221C, ORF1ab:G9526T, ORF1ab:C14408T, ORF1ab:T19839C, S:A23403G, N:G28881A, N:G28882A, N:G28883C,	6	N:R203K, N:G204R, ORF1a:I1215V, ORF1a:M3087I, ORF1b:P314L, S:D614G,	

hCoV-19/Mexico/CMX-INER-0124/2020	EPI_ISL_837721	20B	B.1.1.222	14	5'UTR:C241T, ORF1ab:C3037T, ORF1ab:A9949G, ORF1ab:G10907A, ORF1ab:C13994T, ORF1ab:C14408T, ORF1ab:G18803T, ORF1ab:T19839C, S:A23403G, S:A23756G, ORF3a:C25571T, N:G28881A, N:G28882A, N:G28883C, 3'UTR:G29751T,	9	N:R203K, N:G204R, ORF1a:A3548T, ORF1b:A176V, ORF1b:P314L, ORF1b:S1779I, ORF3a:S60F, S:D614G, S:T732A,	
hCoV-19/Mexico/CMX-INER-0125/2020	EPI_ISL_837722	20A	B.1	11	5'UTR:C241T, ORF1ab:C3037T, ORF1ab:C7488T, ORF1ab:C14408T, ORF1ab:G19999T, ORF1ab:A20268G, S:C21575T, S:A23403G, S:T24076C, ORF3a:G25785T, ORF3a:C25904T, N:C28854T,	8	N:S194L, ORF1a:T2408I, ORF1b:P314L, ORF1b:V2178F, ORF3a:W131C, ORF3a:S171L, S:L5F, S:D614G,	
hCoV-19/Mexico/CMX-INER-0126/2020	EPI_ISL_837723	20B	B.1.1.222	9	5'UTR:C241T, ORF1ab:C3037T, ORF1ab:C14408T, ORF1ab:T19839C, S:A23403G, S:A23756G, N:G28881A, N:G28882A, N:G28883C, 3'UTR:G29751T,	5	N:R203K, N:G204R, ORF1b:P314L, S:D614G, S:T732A,	
hCoV-19/Mexico/CMX-INER-0127/2020	EPI_ISL_837724	20C	B.1.319	10	5'UTR:C241T, ORF1ab:C1059T, ORF1ab:C3037T, ORF1ab:C5102T, ORF1ab:C8986T, ORF1ab:C10851T, ORF1ab:C14408T, S:A23403G, ORF3a:G25563T, ORF3a:C25916T, N:G29227T,	7	ORF1a:T265I, ORF1a:H1613Y, ORF1a:A3529V, ORF1b:P314L, ORF3a:Q57H, ORF3a:T175I, S:D614G,	
hCoV-19/Mexico/CMX-INER-0128/2020	EPI_ISL_837725	20A	B.1.609	7	5'UTR:C241T, ORF1ab:C3037T, ORF1ab:C4582T, ORF1ab:C13119T, ORF1ab:C14408T, ORF1ab:C18167T, ORF1ab:A20268G, S:A23403G,	4	ORF1a:A4285V, ORF1b:P314L, ORF1b:P1567L, S:D614G,	
hCoV-19/Mexico/CMX-INER-0129/2020	EPI_ISL_837726	20B	B.1.1.222	15	5'UTR:C241T, ORF1ab:C3037T, ORF1ab:T4615G, ORF1ab:C10449T, ORF1ab:C13035T, ORF1ab:C14408T, ORF1ab:T19839C, S:A23403G, S:A23756G, ORF3a:G25494T, ORF3a:C25515T, M:A26612T, ORF7a:C27549T, N:G28881A, N:G28882A, N:G28883C,	7	N:R203K, N:G204R, ORF1a:P3395L, ORF1a:A4257V, ORF1b:P314L, S:D614G, S:T732A,	
hCoV-19/Mexico/CMX-INER-0130/2020	EPI_ISL_837727	20A	B.1.189	11	5'UTR:C241T, ORF1ab:C3037T, ORF1ab:C4582T, ORF1ab:G6404T, ORF1ab:C14408T, ORF1ab:A20268G, S:A23403G, S:T25123C, ORF3a:G25644T, ORF8:C28087T, N:G28378T, N:C28887T,	6	N:T205I, ORF1a:V2047F, ORF1b:P314L, ORF8:A65V, ORF9b:R32L, S:D614G,	
hCoV-19/Mexico/CMX-INER-0131/2020	EPI_ISL_837728	20B	B.1.1.222	13	5'UTR:C241T, ORF1ab:C3037T, ORF1ab:G3871T, ORF1ab:G4148T, ORF1ab:C8334T, ORF1ab:A8894G, ORF1ab:C14408T, ORF1ab:T19839C, S:T22324C, S:A23403G, S:A23756G, N:G28881A, N:G28882A, N:G28883C,	9	N:R203K, N:G204R, ORF1a:K1202N, ORF1a:V1295F, ORF1a:A2690V, ORF1a:T2877A, ORF1b:P314L, S:D614G, S:T732A,	
hCoV-19/Mexico/CMX-INER-0132/2020	EPI_ISL_837729	20B	B.1.1.222	12	5'UTR:C241T, ORF1ab:C913T, ORF1ab:C3037T, ORF1ab:T8800C, ORF1ab:C14408T, ORF1ab:T19839C, S:A23403G, S:A23756G, ORF3a:C26079T, ORF8:G28001T, N:G28881A, N:G28882A, N:G28883C,	5	N:R203K, N:G204R, ORF1b:P314L, S:D614G, S:T732A,	
hCoV-19/Mexico/CMX-INER-0133/2020	EPI_ISL_837730	20B	B.1.1.222	9	5'UTR:C241T, ORF1ab:C3037T, ORF1ab:C14408T, ORF1ab:T19839C, S:A23403G, S:A23756G, N:G28881A, N:G28882A, N:G28883C, 3'UTR:G29751T,	5	N:R203K, N:G204R, ORF1b:P314L, S:D614G, S:T732A,	
hCoV-19/Mexico/CMX-INER-0134/2020	EPI_ISL_837731	20A	B.1.189	9	5'UTR:C241T, ORF1ab:C3037T, ORF1ab:C4582T, ORF1ab:C4927T, ORF1ab:G6404T, ORF1ab:C14408T, ORF1ab:A20268G, S:A23403G, S:T25123C, N:G28378T,	4	ORF1a:V2047F, ORF1b:P314L, ORF9b:R32L, S:D614G,	
hCoV-19/Mexico/CMX-INER-0135/2020	EPI_ISL_837732	20B	B.1.1	11	5'UTR:C241T, ORF1ab:C920T, ORF1ab:C3037T, ORF1ab:G10265A, ORF1ab:C14408T, S:C21648T, S:A23403G, N:A28877T, N:G28878C, N:G28881A, N:G28882A, N:G28883C,	6	N:R203K, N:G204R, ORF1a:G3334S, ORF1b:P314L, S:T29I, S:D614G,	
hCoV-19/Mexico/CMX-INER-0136/2020	EPI_ISL_837733	20B	B.1.1	11	5'UTR:C241T, ORF1ab:C3037T, ORF1ab:C8208T, ORF1ab:C8964T, ORF1ab:C14408T, ORF1ab:T19839C, ORF1ab:A20447G, S:C21855T, S:A23403G, N:G28881A, N:G28882A, N:G28883C,	8	N:R203K, N:G204R, ORF1a:T2648I, ORF1a:S2900L, ORF1b:P314L, ORF1b:K2327R, S:S98F, S:D614G,	
hCoV-19/Mexico/CMX-INER-0137/2020	EPI_ISL_837734	20B	B.1.1.222	8	5'UTR:C241T, ORF1ab:C3037T, ORF1ab:C14408T, ORF1ab:T19839C, S:A23403G, S:A23756G, N:G28881A, N:G28882A, N:G28883C,	5	N:R203K, N:G204R, ORF1b:P314L, S:D614G, S:T732A,	
hCoV-19/Mexico/CMX-INER-0138/2020	EPI_ISL_837607	20A	B.1.609	10	5'UTR:C241T, ORF1ab:C3037T, ORF1ab:C4582T, ORF1ab:T6023C, ORF1ab:C9924T, ORF1ab:G11083T, ORF1ab:C13119T, ORF1ab:C14408T, ORF1ab:A20268G, S:A23403G, S:C24442T,	7	ORF1a:E1024K, ORF1a:Y1920H, ORF1a:A3220V, ORF1a:L3606F, ORF1a:A4285V, ORF1b:P314L, S:D614G,	ORF1ab: 3333-3335,

hCoV-19/Mexico/CMX-INER-0139/2020	EPI_ISL_837735	20B	B.1.1.222	10	5'UTR:C241T, ORF1ab:C3037T, ORF1ab:T3667C, ORF1ab:C14408T, ORF1ab:T19839C, S:A23403G, S:A23756G, ORF3a:G25775T, N:G28881A, N:G28882A, N:G28883C,	6	N:R203K, N:G204R, ORF1b:P314L, ORF3a:W128L, S:D614G, S:T732A,
hCoV-19/Mexico/CMX-INER-0140/2020	EPI_ISL_837736	20A	B.1.609	15	5'UTR:C241T, ORF1ab:C3037T, ORF1ab:G3880C, ORF1ab:C4582T, ORF1ab:T6023C, ORF1ab:C13119T, ORF1ab:A14246T, ORF1ab:C14408T, ORF1ab:A15442T, ORF1ab:A20268G, S:A23403G, S:G24794T, ORF8:C27925T, N:G28817T, N:C28854T, 3'UTR:G29781T,	11	N:A182S, N:S194L, ORF1a:E1205D, ORF1a:Y1920H, ORF1a:A4285V, ORF1b:D260V, ORF1b:P314L, ORF1b:M659L, ORF8:T11I, S:D614G, S:A1078S,
hCoV-19/Mexico/CMX-INER-0141/2020	EPI_ISL_837737	20A	B.1.609	12	5'UTR:C241T, ORF1ab:C412T, ORF1ab:C3037T, ORF1ab:C4582T, ORF1ab:A8374T, ORF1ab:C9561T, ORF1ab:C14408T, ORF1ab:G15452T, ORF1ab:G19816T, ORF1ab:A20268G, S:A23403G, S:C24378T, ORF8:C27920T,	6	ORF1a:S3099L, ORF1b:P314L, ORF1b:G662V, ORF1b:V2117L, S:D614G, S:S939F,
hCoV-19/Mexico/CMX-INER-0142/2020	EPI_ISL_837738	20B	B.1.1.222	11	5'UTR:C241T, ORF1ab:C3037T, ORF1ab:T3667C, ORF1ab:A9162G, ORF1ab:C14408T, ORF1ab:T19839C, S:A23403G, S:A23756G, ORF3a:G25775T, N:G28881A, N:G28882A, N:G28883C,	7	N:R203K, N:G204R, ORF1a:N2966S, ORF1b:P314L, ORF3a:W128L, S:D614G, S:T732A,
hCoV-19/Mexico/CMX-INER-0143/2020	EPI_ISL_837739	20A	B.1.609	10	5'UTR:C241T, ORF1ab:C3037T, ORF1ab:G3473A, ORF1ab:C4582T, ORF1ab:C13119T, ORF1ab:C14408T, ORF1ab:G19518A, ORF1ab:A20268G, S:A23403G, S:A23734T, ORF6:C27219T,	4	ORF1a:G1070S, ORF1a:A4285V, ORF1b:P314L, S:D614G,
hCoV-19/Mexico/CMX-INER-0144/2020	EPI_ISL_837740	20A	B.1.243	12	5'UTR:C241T, ORF1ab:G806A, ORF1ab:C3037T, ORF1ab:T5965C, ORF1ab:C6629T, ORF1ab:C14408T, ORF1ab:C16575T, ORF1ab:C17373T, ORF1ab:A20268G, S:A23403G, S:T24076C, N:C28854T, N:G29543T,	5	N:S194L, ORF1a:A181T, ORF1a:L2122F, ORF1b:P314L, S:D614G,
hCoV-19/Mexico/CMX-INER-0145/2020	EPI_ISL_837741	20B	B.1.1.222	12	5'UTR:C241T, ORF1ab:C1912T, ORF1ab:C3037T, ORF1ab:C3884T, ORF1ab:G7798T, ORF1ab:C14408T, ORF1ab:T19839C, S:A23403G, S:A23756G, ORF8:T28199C, N:G28881A, N:G28882A, N:G28883C,	7	N:R203K, N:G204R, ORF1a:P1207S, ORF1a:K2511N, ORF1b:P314L, S:D614G, S:T732A,
hCoV-19/Mexico/CMX-INER-0146/2020	EPI_ISL_837742	20B	B.1.1.222	11	5'UTR:C241T, ORF1ab:C3037T, ORF1ab:C6636T, ORF1ab:C14408T, ORF1ab:G14857T, ORF1ab:T19839C, S:A23403G, S:A23756G, N:G28881A, N:G28882A, N:G28883C, 3'UTR:G29751T,	7	N:R203K, N:G204R, ORF1a:T2124I, ORF1b:P314L, ORF1b:V464F, S:D614G, S:T732A,
hCoV-19/Mexico/CMX-INER-0147/2020	EPI_ISL_837743	20B	B.1.1.222	13	5'UTR:C241T, ORF1ab:T2533C, ORF1ab:C3037T, ORF1ab:G3328T, ORF1ab:C9166A, ORF1ab:C10252T, ORF1ab:T12865C, ORF1ab:C14408T, ORF1ab:T19839C, S:A23403G, S:A23756G, N:G28881A, N:G28882A, N:G28883C,	6	N:R203K, N:G204R, ORF1a:Q1021H, ORF1b:P314L, S:D614G, S:T732A,
hCoV-19/Mexico/CMX-INER-0148/2020	EPI_ISL_837744	20B	B.1.1.222	15	5'UTR:C241T, ORF1ab:C311T, ORF1ab:C3037T, ORF1ab:C8299T, ORF1ab:T12056A, ORF1ab:C14408T, ORF1ab:G18225A, ORF1ab:C18591T, ORF1ab:T19839C, S:G22205T, S:A23403G, S:A23756G, ORF7a:C27654T, N:G28881A, N:G28882A, N:G28883C,	9	N:R203K, N:G204R, ORF1a:L16F, ORF1a:C3931S, ORF1b:P314L, ORF1b:M1586I, S:D215Y, S:D614G, S:T732A,
hCoV-19/Mexico/CMX-INER-0149/2020	EPI_ISL_837745	20B	B.1.1.222	9	5'UTR:C241T, ORF1ab:C3037T, ORF1ab:C14408T, ORF1ab:G14772T, ORF1ab:T19839C, S:A23403G, S:A23756G, N:G28881A, N:G28882A, N:G28883C,	6	N:R203K, N:G204R, ORF1b:P314L, ORF1b:Q435H, S:D614G, S:T732A,
hCoV-19/Mexico/CMX-INER-0150/2020	EPI_ISL_837746	20B	B.1.1.222	14	5'UTR:C241T, ORF1ab:C3037T, ORF1ab:G7042T, ORF1ab:C7267T, ORF1ab:C10741T, ORF1ab:C14408T, ORF1ab:G18040T, ORF1ab:T19839C, ORF1ab:G20808T, S:A23403G, S:A23756G, ORF8:C27944T, N:G28881A, N:G28882A, N:G28883C,	7	N:R203K, N:G204R, ORF1a:M2259I, ORF1b:P314L, ORF1b:A1525S, S:D614G, S:T732A,
hCoV-19/Mexico/CMX-INER-0151/2020	EPI_ISL_837747	20A	B.1.609	8	5'UTR:C241T, ORF1ab:C3037T, ORF1ab:T3043C, ORF1ab:C4582T, ORF1ab:C10296T, ORF1ab:C13119T, ORF1ab:C14408T, ORF1ab:A20268G, S:A23403G,	4	ORF1a:S3344F, ORF1a:A4285V, ORF1b:P314L, S:D614G,

hCoV-19/Mexico/CMX-INER-0152/2020	EPI_ISL_837748	20A	B.1	11	5'UTR:C241T, ORF1ab:C1489T, ORF1ab:C3037T, ORF1ab:A12180G, ORF1ab:C14408T, ORF1ab:C18261A, ORF1ab:A20268G, S:A21645T, S:C23248T, S:C23380T, S:A23403G, S:G24764C,	5	ORF1a:D3972G, ORF1b:P314L, S:Y28F, S:D614G, S:V1068L,
hCoV-19/Mexico/CMX-INER-0153/2020	EPI_ISL_837749	20B	B.1.1.222	9	5'UTR:C241T, ORF1ab:C3037T, ORF1ab:A5163G, ORF1ab:C14408T, ORF1ab:T19839C, S:A23403G, S:A23756G, N:G28881A, N:G28882A, N:G28883C,	6	N:R203K, N:G204R, ORF1a:E1633G, ORF1b:P314L, S:D614G, S:T732A,
hCoV-19/Mexico/CMX-INER-0154/2020	EPI_ISL_837750	20B	B.1.1.222	14	5'UTR:C241T, ORF1ab:C1912T, ORF1ab:C3037T, ORF1ab:C3884T, ORF1ab:C8208T, ORF1ab:T9628C, ORF1ab:C14408T, ORF1ab:T19839C, ORF1ab:G20238T, S:A23403G, S:A23756G, ORF8:T28199C, N:G28881A, N:G28882A, N:G28883C,	8	N:R203K, N:G204R, ORF1a:P1207S, ORF1a:T2648I, ORF1b:P314L, ORF1b:R2257S, S:D614G, S:T732A,
hCoV-19/Mexico/CMX-INER-0155/2020	EPI_ISL_837751	20B	B.1.1.518	10	5'UTR:C241T, ORF1ab:C1878T, ORF1ab:C3037T, ORF1ab:C14408T, ORF1ab:A16725G, ORF1ab:T19839C, S:T22399C, S:A23403G, N:G28881A, N:G28882A, N:G28883C,	5	N:R203K, N:G204R, ORF1a:S538L, ORF1b:P314L, S:D614G,
hCoV-19/Mexico/CMX-INER-0156/2020	EPI_ISL_837752	20B	B.1.1	11	5'UTR:C241T, ORF1ab:C3037T, ORF1ab:C3140T, ORF1ab:C5055T, ORF1ab:C8208T, ORF1ab:C14408T, ORF1ab:T19839C, S:C21855T, S:A23403G, N:G28881A, N:G28882A, N:G28883C,	8	N:R203K, N:G204R, ORF1a:P959S, ORF1a:T1597I, ORF1a:T2648I, ORF1b:P314L, S:S98F, S:D614G,
hCoV-19/Mexico/CMX-INER-0157/2020	EPI_ISL_837753	20B	B.1.1.222	8	5'UTR:C241T, ORF1ab:C3037T, ORF1ab:C14408T, ORF1ab:T19839C, S:A23403G, S:A23756G, N:G28881A, N:G28882A, N:G28883C,	5	N:R203K, N:G204R, ORF1b:P314L, S:D614G, S:T732A,
hCoV-19/Mexico/CMX-INER-0158/2020	EPI_ISL_837754	20B	B.1.1.222	11	5'UTR:C241T, ORF1ab:C3037T, ORF1ab:A13833G, ORF1ab:C14408T, ORF1ab:T19839C, S:T21877C, S:A23403G, S:A23756G, N:G28881A, N:G28882A, N:G28883C, N:A29143G,	5	N:R203K, N:G204R, ORF1b:P314L, S:D614G, S:T732A,
hCoV-19/Mexico/CMX-INER-0159/2020	EPI_ISL_837755	20B	B.1.1	9	5'UTR:C241T, ORF1ab:G1148T, ORF1ab:G2534T, ORF1ab:C3037T, ORF1ab:C14408T, ORF1ab:T19839C, S:A23403G, N:G28881A, N:G28882A, N:G28883C,	6	N:R203K, N:G204R, ORF1a:G295C, ORF1a:V757F, ORF1b:P314L, S:D614G,
hCoV-19/Mexico/CMX-INER-0160/2020	EPI_ISL_837756	20C	B.1	9	5'UTR:C241T, ORF1ab:C1059T, ORF1ab:T2904C, ORF1ab:C3037T, ORF1ab:C13366T, ORF1ab:C14408T, S:A22798T, S:A23403G, ORF3a:G25563T, 3'UTR:C29708T,	5	ORF1a:T265I, ORF1a:I880T, ORF1b:P314L, ORF3a:Q57H, S:D614G,
hCoV-19/Mexico/CMX-INER-0161/2020	EPI_ISL_837757	20A	B.1	10	5'UTR:C241T, ORF1ab:C3037T, ORF1ab:T3648G, ORF1ab:G4510T, ORF1ab:T6427C, ORF1ab:C14408T, ORF1ab:A20268G, S:A23403G, N:C28647T, N:C28854T, 3'UTR:C29696T,	5	N:A125V, N:S194L, ORF1a:I1128S, ORF1b:P314L, S:D614G,
hCoV-19/Mexico/CMX-INER-0162/2020	EPI_ISL_837758	20B	B.1.1	13	5'UTR:C241T, ORF1ab:C1545T, ORF1ab:C3037T, ORF1ab:C4276T, ORF1ab:G9479T, ORF1ab:G12575T, ORF1ab:C13115T, ORF1ab:C14408T, ORF1ab:C15738T, ORF1ab:T19839C, S:A23403G, N:G28881A, N:G28882A. N:G28883C.	7	N:R203K, N:G204R, ORF1a:A427V, ORF1a:G3072C, ORF1a:A4104S, ORF1b:P314L, S:D614G,
hCoV-19/Mexico/CMX-INER-0163/2020	EPI_ISL_837759	20B	B.1.1	12	5'UTR:C241T, ORF1ab:C3037T, ORF1ab:C4897T, ORF1ab:G10265A, ORF1ab:G11540T, ORF1ab:C14408T, S:C22088T, S:A23403G, ORF3a:C25626T, N:G28703T, N:G28881A, N:G28882A, N:G28883C,	8	N:D144Y, N:R203K, N:G204R, ORF1a:G3334S, ORF1a:V3759F, ORF1b:P314L, S:L176F, S:D614G,
hCoV-19/Mexico/CMX-INER-0164/2020	EPI_ISL_837760	20B	B.1.1.222	15	5'UTR:C241T, ORF1ab:C3037T, ORF1ab:C13458T, ORF1ab:C14408T, ORF1ab:T19839C, ORF1ab:T21363C, S:A23403G, S:A23756G, ORF3a:C25515T, M:A26612T, ORF7a:T27652C, N:A28715G, N:G28881A, N:G28882A, N:G28883C, N:G29348A,	9	N:T148A, N:R203K, N:G204R, N:A359T, ORF1a:S4398L, ORF1b:P314L, ORF7a:F87L, S:D614G, S:T732A,
hCoV-19/Mexico/CMX-INER-0165/2020	EPI_ISL_837761	20A	B.1.609	8	5'UTR:C241T, ORF1ab:C3037T, ORF1ab:C4582T, ORF1ab:C9891T, ORF1ab:C13119T, ORF1ab:G14023A, ORF1ab:C14408T, ORF1ab:A20268G, S:A23403G,	5	ORF1a:A3209V, ORF1a:A4285V, ORF1b:A186T, ORF1b:P314L, S:D614G,
hCoV-19/Mexico/CMX-INER-0166/2020	EPI_ISL_837762	20A	B.1	11	5'UTR:C241T, ORF1ab:C3037T, ORF1ab:G3863A, ORF1ab:C14408T, ORF1ab:C18377T, ORF1ab:A20268G, S:T22328C, S:A23403G, M:C26681T, ORF6:T27384C, N:G28899T, N:G29260T,	6	N:R209I, ORF1a:E1200K, ORF1b:P314L, ORF1b:T1637I, S:S256P, S:D614G,

hCoV-19/Mexico/CMX-INER-0167/2020	EPI_ISL_837763	20B	B.1.1.322	20	5'UTR:C241T, ORF1ab:C3037T, ORF1ab:G3728T, ORF1ab:T5218C, ORF1ab:C9163T, ORF1ab:C9491T, ORF1ab:C10908T, ORF1ab:C14408T, ORF1ab:C16887T, ORF1ab:T19839C, S:C22675T, S:A23403G, S:A23756G, ORF3a:G26062T, N:A28877T, N:G28878C, N:G28881A, N:G28882A, N:G28883C, ORF10:T29588C, 3'UTR:A29700G,	9	N:R203K, N:G204R, ORF1a:G1155C, ORF1a:H3076Y, ORF1a:A3548V, ORF1b:P314L, ORF3a:G224C, S:D614G, S:T732A,
hCoV-19/Mexico/CMX-INER-0168/2020	EPI_ISL_837764	20B	B.1.1.222	11	5'UTR:C241T, ORF1ab:G3004T, ORF1ab:C3037T, ORF1ab:G7027T, ORF1ab:C14408T, ORF1ab:T19839C, S:A23403G, S:A23756G, M:G26775T, N:G28881A, N:G28882A, N:G28883C,	8	M:A85S, N:R203K, N:G204R, ORF1a:E913D, ORF1a:M2254I, ORF1b:P314L, S:D614G, S:T732A,
hCoV-19/Mexico/CMX-INER-0169/2020	EPI_ISL_837765	20B	B.1.1.222	10	5'UTR:C241T, ORF1ab:C3037T, ORF1ab:G8414A, ORF1ab:C12119T, ORF1ab:C14408T, ORF1ab:T19839C, S:A23403G, S:A23756G, N:G28881A, N:G28882A, N:G28883C,	7	N:R203K, N:G204R, ORF1a:D2717N, ORF1a:P3952S, ORF1b:P314L, S:D614G, S:T732A,
hCoV-19/Mexico/CMX-INER-0170/2020	EPI_ISL_837766	20B	B.1.1.222	12	5'UTR:C241T, ORF1ab:C3037T, ORF1ab:G5194T, ORF1ab:A13833G, ORF1ab:C14408T, ORF1ab:T19839C, S:T21877C, S:A23403G, S:A23756G, ORF7b:T27817C, N:G28881A, N:G28882A, N:G28883C,	6	N:R203K, N:G204R, ORF1b:P314L, ORF7b:V21A, S:D614G, S:T732A,
hCoV-19/Mexico/CMX-INER-0171/2020	EPI_ISL_837767	20B	B.1.1.322	15	5'UTR:C241T, ORF1ab:T1847C, ORF1ab:C3037T, ORF1ab:G9190T, ORF1ab:C14408T, ORF1ab:T19839C, S:A23403G, S:A23756G, ORF3a:A26097G, N:G28460A, N:A28877T, N:G28878C, N:G28881A, N:G28882A, N:G28883C, N:C29144T,	7	N:D63N, N:R203K, N:G204R, ORF1a:S528P, ORF1b:P314L, S:D614G, S:T732A,
hCoV-19/Mexico/CMX-INER-0172/2020	EPI_ISL_837768	20B	B.1.1	12	5'UTR:C241T, ORF1ab:C1150T, ORF1ab:C3037T, ORF1ab:C3622T, ORF1ab:T10150C, ORF1ab:C14408T, ORF1ab:T19839C, S:C21762T, S:A23403G, N:G28881A, N:G28882A, N:G28883C, 3'UTR:G29747C,	5	N:R203K, N:G204R, ORF1b:P314L, S:A67V, S:D614G,
hCoV-19/Mexico/CMX-INER-0173/2020	EPI_ISL_837769	20A	B.1	9	5'UTR:C241T, ORF1ab:C2607G, ORF1ab:C3037T, ORF1ab:C14408T, ORF1ab:A20268G, ORF1ab:C20806T, S:C23230T, S:A23403G, N:C28854T, N:G29554T,	4	N:S194L, ORF1a:T781R, ORF1b:P314L, S:D614G,
hCoV-19/Mexico/CMX-INER-0174/2020	EPI_ISL_837770	20B	B.1.1.222	10	5'UTR:C241T, ORF1ab:C3037T, ORF1ab:A6613G, ORF1ab:C14408T, ORF1ab:T19839C, ORF1ab:T21153C, S:A23403G, S:A23756G, N:G28881A, N:G28882A, N:G28883C,	5	N:R203K, N:G204R, ORF1b:P314L, S:D614G, S:T732A,
hCoV-19/Mexico/CMX-INER-0175/2020	EPI_ISL_837771	20A	B.1.558	10	5'UTR:C241T, ORF1ab:C1288T, ORF1ab:C3037T, ORF1ab:G11114A, ORF1ab:C14408T, ORF1ab:C19662T, ORF1ab:A20268G, S:A23403G, S:G24666T, N:C28854T, ORF10:C29642T,	5	N:S194L, ORF1a:G3617S, ORF1b:P314L, S:D614G, S:G1035V,
hCoV-19/Mexico/CMX-INER-0176/2020	EPI_ISL_837772	20A	B.1	7	5'UTR:C241T, ORF1ab:G2900A, ORF1ab:C3037T, ORF1ab:C14408T, ORF1ab:A20268G, S:A23403G, M:C26801T, N:C28854T,	4	N:S194L, ORF1a:V879I, ORF1b:P314L, S:D614G,
hCoV-19/Mexico/CMX-INER-0177/2020	EPI_ISL_837773	20A	B.1	11	5'UTR:C241T, ORF1ab:A1192T, ORF1ab:C2940T, ORF1ab:C3037T, ORF1ab:C14408T, ORF1ab:G17427T, ORF1ab:A19611T, ORF1ab:A20268G, S:G22708C, S:A23403G, N:C28854T, N:C29362T,	4	N:S194L, ORF1a:P892L, ORF1b:P314L, S:D614G,
hCoV-19/Mexico/CMX-INER-0178/2020	EPI_ISL_837774	20B	B.1.1.222	13	5'UTR:C241T, ORF1ab:C3037T, ORF1ab:C3736T, ORF1ab:A6716G, ORF1ab:T7835C, ORF1ab:C7926T, ORF1ab:C14408T, ORF1ab:T19839C, S:A23403G, S:A23756G, N:G28881A, N:G28882A, N:G28883C, N:G29140T,	8	N:R203K, N:G204R, N:Q289H, ORF1a:S2151G, ORF1a:A2554V, ORF1b:P314L, S:D614G, S:T732A,
hCoV-19/Mexico/CMX-INER-0179/2020	EPI_ISL_837775	20B	B.1.1	14	5'UTR:C241T, ORF1ab:G376T, ORF1ab:A2479G, ORF1ab:C3037T, ORF1ab:T3746G, ORF1ab:A11024G, ORF1ab:G12223A, ORF1ab:C14408T, ORF1ab:T19839C, S:A23403G, S:G25135T, ORF3a:G25879T, N:G28881A, N:G28882A, N:G28883C,	9	N:R203K, N:G204R, ORF1a:E37D, ORF1a:S1161A, ORF1a:I3587V, ORF1b:P314L, ORF3a:V163L, S:D614G, S:K1191N,

hCoV-19/Mexico/CMX-INER-0180/2020	EPI_ISL_837776	20A	B.1.243	11	5'UTR:C241T, ORF1ab:C3037T, ORF1ab:G4399T, ORF1ab:C8668A, ORF1ab:C9565T, ORF1ab:C12676T, ORF1ab:C14408T, ORF1ab:A20268G, S:A23403G, S:T24076C, N:A28468G, N:C28854T,	6	N:S194L, ORF1a:M1378I, ORF1a:D2801E, ORF1b:P314L, ORF9b:N62S, S:D614G,
hCoV-19/Mexico/CMX-INER-0181/2020	EPI_ISL_837777	20B	B.1.1.222	15	5'UTR:C241T, ORF1ab:C3037T, ORF1ab:C5184T, ORF1ab:C14408T, ORF1ab:G14678C, ORF1ab:C16049T, ORF1ab:G17469T, ORF1ab:T19839C, S:A23403G, S:A23756G, S:C24381T, ORF7a:T27404C, N:G28881A, N:G28882A, N:G28883C, 3'UTR:G29751T,	11	N:R203K, N:G204R, ORF1a:P1640L, ORF1b:P314L, ORF1b:G404A, ORF1b:T861I, ORF1b:L1334F, ORF7a:I4T, S:D614G, S:T732A, S:S940F,
hCoV-19/Mexico/CMX-INER-0182/2020	EPI_ISL_837778	20B	B.1.1.222	12	5'UTR:C241T, ORF1ab:C3037T, ORF1ab:A6613G, ORF1ab:C14408T, ORF1ab:T19839C, ORF1ab:T21153C, S:T23278C, S:A23403G, S:C23533T, S:A23756G, N:G28881A, N:G28882A, N:G28883C,	5	N:R203K, N:G204R, ORF1b:P314L, S:D614G, S:T732A,
hCoV-19/Mexico/CMX-INER-0183/2020	EPI_ISL_837779	20B	B.1.1	14	5'UTR:C241T, ORF1ab:G376T, ORF1ab:C2062T, ORF1ab:C3037T, ORF1ab:C9611T, ORF1ab:A11024G, ORF1ab:C11530T, ORF1ab:C14408T, ORF1ab:C14649T, ORF1ab:T19839C, S:A23403G, ORF3a:G25456T, N:G28881A, N:G28882A, N:G28883C,	8	N:R203K, N:G204R, ORF1a:E37D, ORF1a:L3116F, ORF1a:I3587V, ORF1b:P314L, ORF3a:D22Y, S:D614G,
hCoV-19/Mexico/CMX-INER-0184/2020	EPI_ISL_837780	20B	B.1.1	12	5'UTR:C241T, ORF1ab:C3037T, ORF1ab:C11020T, ORF1ab:C13019T, ORF1ab:T13764C, ORF1ab:C14408T, ORF1ab:T19839C, S:A23403G, N:C28603T, N:G28881A, N:G28882A, N:G28883C, N:C29421T,	5	N:R203K, N:G204R, N:P383L, ORF1b:P314L, S:D614G,
hCoV-19/Mexico/CMX-INER-0185/2020	EPI_ISL_837781	20B	B.1.1.222	18	5'UTR:A57G, 5'UTR:C241T, ORF1ab:G2272T, ORF1ab:C3037T, ORF1ab:C5051T, ORF1ab:T11032C, ORF1ab:G11243T, ORF1ab:C14408T, ORF1ab:C16049T, ORF1ab:A19751T, ORF1ab:A19752T, ORF1ab:T19839C, S:A23403G, S:A23756G, ORF3a:G25775T, N:G28881A, N:G28882A, N:G28883C, 3'UTR:A29697T,	11	N:R203K, N:G204R, ORF1a:K669N, ORF1a:P1596S, ORF1a:V3660L, ORF1b:P314L, ORF1b:T861I, ORF1b:E2095V, ORF3a:W128L, S:D614G, S:T732A,
hCoV-19/Mexico/CMX-INER-0186/2020	EPI_ISL_837782	20B	B.1.1.222	13	5'UTR:C241T, ORF1ab:T2533C, ORF1ab:C3037T, ORF1ab:G4504T, ORF1ab:T11428C, ORF1ab:C14408T, ORF1ab:G14653T, ORF1ab:T19839C, S:A23403G, S:A23756G, N:G28881A, N:G28882A, N:G28883C, 3'UTR:G29868A,	7	N:R203K, N:G204R, ORF1a:E1413D, ORF1b:P314L, ORF1b:V396F, S:D614G, S:T732A,
hCoV-19/Mexico/CMX-INER-0187/2020	EPI_ISL_837783	20B	B.1.1.222	16	5'UTR:C241T, ORF1ab:C2842T, ORF1ab:C3037T, ORF1ab:C6539T, ORF1ab:G9136T, ORF1ab:G12261A, ORF1ab:C14408T, ORF1ab:C17333T, ORF1ab:T19839C, ORF1ab:C20544T, S:C21575T, S:A21611G, S:A23403G, S:A23756G, N:G28881A, N:G28882A, N:G28883C,	11	N:R203K, N:G204R, ORF1a:H2092Y, ORF1a:M2957I, ORF1a:R3999H, ORF1b:P314L, ORF1b:T1289M, S:L5F, S:N17D, S:D614G, S:T732A,
hCoV-19/Mexico/CMX-INER-0188/2020	EPI_ISL_837784	20A	B.1	10	5'UTR:C241T, ORF1ab:G2900A, ORF1ab:C3037T, ORF1ab:T10795G, ORF1ab:C14408T, ORF1ab:A20268G, S:A23403G, E:G26389C, M:C26801T, N:C28854T, N:C29535T,	5	E:V49L, N:S194L, ORF1a:V879I, ORF1b:P314L, S:D614G,
hCoV-19/Mexico/CMX-INER-0189/2020	EPI_ISL_837785	20B	B.1.1.512	14	5'UTR:C20T, 5'UTR:C241T, ORF1ab:C2453T, ORF1ab:C3037T, ORF1ab:A6616G, ORF1ab:G10265A, ORF1ab:G10870T, ORF1ab:C14408T, ORF1ab:C15579T, S:A23403G, S:G24794T, S:A25256T, N:G28881A, N:G28882A, N:G28883C,	8	N:R203K, N:G204R, ORF1a:L730F, ORF1a:G3334S, ORF1b:P314L, S:D614G, S:A1078S, S:I1232F,
hCoV-19/Mexico/CMX-INER-0190/2020	EPI_ISL_837786	20G	B.1.2	14	5'UTR:C241T, ORF1ab:C1059T, ORF1ab:C3037T, ORF1ab:G9479A, ORF1ab:C10319T, ORF1ab:C14408T, ORF1ab:A18424G, ORF1ab:C21304T, S:C22313T, S:A23403G, ORF3a:G25563T, ORF3a:G25907T, ORF8:C27964T, N:C28472T, N:C28869T,	13	N:P67S, N:P199L, ORF1a:T265I, ORF1a:G3072S, ORF1a:L3352F, ORF1b:P314L, ORF1b:N1653D, ORF1b:R2613C, ORF3a:Q57H, ORF3a:G172V, ORF8:S24L, S:P251S, S:D614G,

hCoV-19/Mexico/CMX-INER-0191/2020	EPI_ISL_837787	20B	B.1.1.222	16	5'UTR:C21T, 5'UTR:C241T, ORF1ab:C1419T, ORF1ab:C3037T, ORF1ab:G9203A, ORF1ab:C14408T, ORF1ab:G19009A, ORF1ab:T19839C, ORF1ab:T20631C, S:G21777A, S:A23403G, S:A23756G, ORF6:C27263T, N:G28881A, N:G28882A, N:G28883C, N:A29487G,	11	N:R203K, N:G204R, N:K405R, ORF1a:A385V, ORF1a:D2980N, ORF1b:P314L, ORF1b:D1848N, ORF6:T21I, S:G72E, S:D614G, S:T732A,
hCoV-19/Mexico/CMX-INER-0192/2020	EPI_ISL_837788	20B	B.1.1.222	14	5'UTR:C241T, ORF1ab:C2189T, ORF1ab:C3037T, ORF1ab:C7051T, ORF1ab:C10507T, ORF1ab:C14408T, ORF1ab:T19839C, S:C21575T, S:C22792T, S:A23403G, S:A23756G, N:G28881A, N:G28882A, N:G28883C, N:A29526G,	8	N:R203K, N:G204R, N:Q418R, ORF1a:L642F, ORF1b:P314L, S:L5F, S:D614G, S:T732A,
hCoV-19/Mexico/CMX-INER-0193/2020	EPI_ISL_837789	20B	B.1.1.222	16	5'UTR:C21T, 5'UTR:C241T, ORF1ab:C1419T, ORF1ab:C3037T, ORF1ab:G9203A, ORF1ab:C14408T, ORF1ab:G19009A, ORF1ab:T19839C, ORF1ab:T20631C, S:G21777A, S:A23403G, S:A23756G, ORF6:C27263T, N:G28881A, N:G28882A, N:G28883C, N:A29487G,	11	N:R203K, N:G204R, N:K405R, ORF1a:A385V, ORF1a:D2980N, ORF1b:P314L, ORF1b:D1848N, ORF6:T21I, S:G72E, S:D614G, S:T732A,
hCoV-19/Mexico/CMX-INER-0194/2020	EPI_ISL_837790	20A	B.1	7	5'UTR:C241T, ORF1ab:C3037T, ORF1ab:C10537T, ORF1ab:C14408T, ORF1ab:A17861G, ORF1ab:A20268G, S:A23403G, N:C28854T,	4	N:S194L, ORF1b:P314L, ORF1b:D1465G, S:D614G,
hCoV-19/Mexico/CMX-INER-0195/2020	EPI_ISL_837791	20B	B.1.1	14	5'UTR:C241T, ORF1ab:C1912T, ORF1ab:C3037T, ORF1ab:G11801A, ORF1ab:C12789T, ORF1ab:C14408T, ORF1ab:T17208C, ORF1ab:T19839C, ORF1ab:G21520T, S:T22328C, S:A23403G, ORF7b:C27889T, N:G28881A, N:G28882A, N:G28883C,	8	N:R203K, N:G204R, ORF1a:G3846S, ORF1a:T4175I, ORF1b:P314L, ORF1b:V2685F, S:S256P, S:D614G,
hCoV-19/Mexico/CMX-INER-0196/2020	EPI_ISL_837792	20B	B.1.1	13	5'UTR:C241T, ORF1ab:T1476C, ORF1ab:C2399T, ORF1ab:C3037T, ORF1ab:T6714G, ORF1ab:C10626T, ORF1ab:C13994T, ORF1ab:C14408T, S:A23403G, M:G26951T, N:G28881A, N:G28882A, N:G28883C, N:G29543T,	9	N:R203K, N:G204R, ORF1a:I404T, ORF1a:H712Y, ORF1a:V2150G, ORF1a:A3454V, ORF1b:A176V, ORF1b:P314L, S:D614G,
hCoV-19/Mexico/CMX-INER-0197/2020	EPI_ISL_837601	20B	B.1.1	13	5'UTR:C241T, ORF1ab:G1126T, ORF1ab:C3037T, ORF1ab:C4752T, ORF1ab:G5554T, ORF1ab:C14408T, ORF1ab:T19839C, S:C22323T, S:A23403G, S:T24756C, ORF3a:C26060T, N:G28881A, N:G28882A, N:G28883C,	10	N:R203K, N:G204R, ORF1a:R287S, ORF1a:T1496I, ORF1a:K1763N, ORF1b:P314L, ORF3a:T223I, S:S254F, S:D614G, S:V1065A,
hCoV-19/Mexico/CMX-INER-0198/2020	EPI_ISL_837793	20B	B.1.1.222	13	5'UTR:C241T, ORF1ab:C3037T, ORF1ab:G3685T, ORF1ab:G4285T, ORF1ab:C14408T, ORF1ab:T19839C, ORF1ab:G20006T, S:G22567A, S:A23403G, S:C23683T, S:A23756G, N:G28881A, N:G28882A, N:G28883C,	8	N:R203K, N:G204R, ORF1a:Q1140H, ORF1a:E1340D, ORF1b:P314L, ORF1b:G2180V, S:D614G, S:T732A,
hCoV-19/Mexico/CMX-INER-0199/2020	EPI_ISL_837794	20B	B.1.1.222	14	5'UTR:C241T, ORF1ab:T2533C, ORF1ab:C3037T, ORF1ab:C14214T, ORF1ab:G14229T, ORF1ab:C14408T, ORF1ab:T19839C, S:A23403G, S:A23756G, ORF3a:G25606T, ORF3a:G25981T, N:G28881A, N:G28882A, N:G28883C, 3'UTR:G29810T,	8	N:R203K, N:G204R, ORF1b:K254N, ORF1b:P314L, ORF3a:A72S, ORF3a:V197L, S:D614G, S:T732A,
hCoV-19/Mexico/CMX-INER-02/2020	EPI_ISL_424348	20A	B.1	8	5'UTR:C241T, ORF1ab:C3037T, ORF1ab:C12406T, ORF1ab:C14408T, ORF1ab:A20268G, S:A23403G, S:C25156T, S:T25159G, ORF3a:T25716A,	3	ORF1b:P314L, S:D614G, S:D1199E,
hCoV-19/Mexico/CMX-INER-0200/2020	EPI_ISL_837795	20B	B.1.1.222	10	5'UTR:C241T, ORF1ab:C3037T, ORF1ab:A10564G, ORF1ab:C14408T, ORF1ab:T19839C, S:A23403G, S:A23756G, ORF3a:G25489T, N:G28881A, N:G28882A, N:G28883C,	6	N:R203K, N:G204R, ORF1b:P314L, ORF3a:A33S, S:D614G, S:T732A,
hCoV-19/Mexico/CMX-INER-0201/2020	EPI_ISL_837796	20A	B.1	12	5'UTR:C241T, ORF1ab:C3037T, ORF1ab:T6069C, ORF1ab:C14408T, ORF1ab:A16973G, ORF1ab:A20268G, S:G22017T, S:A23403G, E:C26456T, ORF7a:G27703T, N:C28706T, N:C28854T, 3'UTR:G29751C,	9	E:P71L, N:H145Y, N:S194L, ORF1a:I1935T, ORF1b:P314L, ORF1b:Y1169C, ORF7a:V104F, S:W152L, S:D614G,
hCoV-19/Mexico/CMX-INER-0202/2020	EPI_ISL_837797	20B	B.1.1.222	12	5'UTR:C241T, ORF1ab:C3037T, ORF1ab:C5826T, ORF1ab:A5989T, ORF1ab:C14408T, ORF1ab:T19839C, S:C21646T, S:A23403G, S:A23756G, M:A26612T, N:G28881A, N:G28882A, N:G28883C,	6	N:R203K, N:G204R, ORF1a:T1854I, ORF1b:P314L, S:D614G, S:T732A,

hCoV-19/Mexico/CMX-INER-0203/2020	EPI_ISL_837798	20B	B.1.1.222	12	5'UTR:C241T, ORF1ab:G1748A, ORF1ab:T2264G, ORF1ab:C3037T, ORF1ab:G6647A, ORF1ab:G11083T, ORF1ab:C14408T, ORF1ab:T19839C, S:A23403G, S:A23756G, N:G28881A, N:G28882A, N:G28883C,	9	N:R203K, N:G204R, ORF1a:D495N, ORF1a:C667G, ORF1a:A2128T, ORF1a:L3606F, ORF1b:P314L, S:D614G, S:T732A,	
hCoV-19/Mexico/CMX-INER-0204/2020	EPI_ISL_837799	20B	B.1.1	18	5'UTR:C241T, ORF1ab:C1912T, ORF1ab:C3037T, ORF1ab:T7738C, ORF1ab:G11801A, ORF1ab:C12789T, ORF1ab:C14408T, ORF1ab:T17208C, ORF1ab:T19839C, ORF1ab:G21520T, S:T22328C, S:A23403G, S:C23709T, ORF3a:T25863C, ORF7b:C27889T, N:G28881A, N:G28882A, N:G28883C, N:G29300T,	10	N:R203K, N:G204R, N:D343Y, ORF1a:G3846S, ORF1a:T4175I, ORF1b:P314L, ORF1b:V2685F, S:S256P, S:D614G, S:T716I,	
hCoV-19/Mexico/CMX-INER-0205/2020	EPI_ISL_843192	20B	B.1.1.222	16	5'UTR:C241T, ORF1ab:C335T, ORF1ab:C3037T, ORF1ab:A7795G, ORF1ab:A13712G, ORF1ab:C14408T, ORF1ab:C17004T, ORF1ab:T17160G, ORF1ab:T19839C, S:A23403G, S:A23588G, S:A23756G, ORF8:C28054A, ORF8:G28198T, N:G28881A, N:G28882A, N:G28883C,	8	N:R203K, N:G204R, ORF1a:R24C, ORF1b:K82R, ORF1b:P314L, S:D614G, S:T676A, S:T732A,	ORF8:28 253,
hCoV-19/Mexico/CMX-INER-0206/2020	EPI_ISL_837800	20A	B.1	10	5'UTR:C241T, ORF1ab:C3037T, ORF1ab:C6648T, ORF1ab:C10537T, ORF1ab:C14408T, ORF1ab:A17861G, ORF1ab:A20268G, S:A23403G, ORF6:A27383G, N:C28854T, N:C29530T,	6	N:S194L, ORF1a:A2128V, ORF1b:P314L, ORF1b:D1465G, ORF6:D61G, S:D614G,	
hCoV-19/Mexico/CMX-INER-0207/2020	EPI_ISL_837801	20A	B.1	13	5'UTR:C241T, ORF1ab:C3037T, ORF1ab:T6069C, ORF1ab:G13617A, ORF1ab:C14408T, ORF1ab:G19750C, ORF1ab:A20268G, S:G22017T, S:A23403G, ORF7a:G27703T, N:C28706T, N:C28854T, 3'UTR:G29751C, 3'UTR:G29781C,	8	N:H145Y, N:S194L, ORF1a:I1935T, ORF1b:P314L, ORF1b:E2095Q, ORF7a:V104F, S:W152L, S:D614G,	
hCoV-19/Mexico/CMX-INER-0208/2020	EPI_ISL_837802	20A	B.1.551	10	5'UTR:C241T, ORF1ab:C3037T, ORF1ab:C3738T, ORF1ab:C14408T, ORF1ab:A17841G, ORF1ab:A20268G, S:A23403G, S:C23604G, N:G28690T, N:C28854T, 3'UTR:G29751C,	6	N:L139F, N:S194L, ORF1a:P1158L, ORF1b:P314L, S:D614G, S:P681R,	
hCoV-19/Mexico/CMX-INER-0209/2020	EPI_ISL_837803	20B	B.1.1.222	12	5'UTR:C241T, ORF1ab:C2156T, ORF1ab:C3037T, ORF1ab:C14408T, ORF1ab:T19839C, S:A23403G, S:A23476T, S:A23756G, M:C26681T, ORF8:G28085T, N:G28881A, N:G28882A, N:G28883C,	7	N:R203K, N:G204R, ORF1a:L631F, ORF1b:P314L, ORF8:E64D, S:D614G, S:T732A,	
hCoV-19/Mexico/CMX-INER-0210/2020	EPI_ISL_837804	20C	B.1.499	16	5'UTR:C241T, ORF1ab:C774T, ORF1ab:C1059T, ORF1ab:C3037T, ORF1ab:A3058G, ORF1ab:C5694T, ORF1ab:C11916T, ORF1ab:C14408T, ORF1ab:C18998T, S:A23403G, ORF3a:C25487T, ORF3a:G25563T, N:C28863T, N:C28948T, N:G29032T, N:G29540A, 3'UTR:G29736T,	11	N:S197L, N:E253D, ORF1a:T170I, ORF1a:T265I, ORF1a:P1810L, ORF1a:S3884L, ORF1b:P314L, ORF1b:A1844V, ORF3a:T32I, ORF3a:Q57H, S:D614G,	
hCoV-19/Mexico/CMX-INER-0211/2020	EPI_ISL_837805	20B	B.1.1	12	5'UTR:C241T, ORF1ab:C3037T, ORF1ab:C14408T, ORF1ab:G15672T, ORF1ab:A16538G, S:A22330G, S:G22346T, S:A23403G, ORF8:C28253T, N:G28881A, N:G28882A, N:G28883C, 3'UTR:C29686T,	7	N:R203K, N:G204R, ORF1b:P314L, ORF1b:E735D, ORF1b:D1024G, S:A262S, S:D614G,	
hCoV-19/Mexico/CMX-INER-0212/2020	EPI_ISL_837806	20B	B.1.1.322	16	5'UTR:G61T, 5'UTR:C241T, ORF1ab:C3037T, ORF1ab:G3638T, ORF1ab:T5218C, ORF1ab:C14408T, ORF1ab:T19839C, S:T22729C, S:A23403G, S:A23756G, ORF3a:C26028T, N:C28603T, N:A28877T, N:G28878C, N:G28881A, N:G28882A, N:G28883C,	6	N:R203K, N:G204R, ORF1a:G1125C, ORF1b:P314L, S:D614G, S:T732A,	
hCoV-19/Mexico/CMX-INER-0213/2020	EPI_ISL_837603	20A	B.1	13	5'UTR:C241T, ORF1ab:A2806C, ORF1ab:C3037T, ORF1ab:C8664T, ORF1ab:C10755T, ORF1ab:C14408T, ORF1ab:A18706G, ORF1ab:A20268G, S:A23403G, S:C23613T, ORF3a:G25687T, ORF3a:A25900G, ORF7a:C27673T, N:C28854T,	11	N:S194L, ORF1a:E847D, ORF1a:T2800I, ORF1a:A3497V, ORF1b:P314L, ORF1b:T1747A, ORF3a:A99S, ORF3a:T170A, ORF7a:Q94*, S:D614G, S:A684V,	
hCoV-19/Mexico/CMX-INER-0214/2020	EPI_ISL_837807	20A	B.1	13	5'UTR:T209C, 5'UTR:C241T, ORF1ab:G2599T, ORF1ab:C3037T, ORF1ab:C5221T, ORF1ab:A7863G, ORF1ab:C14408T, ORF1ab:G14653T, ORF1ab:C18981T, ORF1ab:A20268G, S:A23403G, N:C28732T, N:C28854T, N:C29171T,	7	N:S194L, N:H300Y, ORF1a:L778F, ORF1a:K2533R, ORF1b:P314L, ORF1b:V396F, S:D614G,	

hCoV-19/Mexico/CMX-INER-0215/2020	EPI_ISL_837808	20A	B.1	13	5'UTR:T209C, 5'UTR:C241T, ORF1ab:G2599T, ORF1ab:C3037T, ORF1ab:C5221T, ORF1ab:A7863G, ORF1ab:C14408T, ORF1ab:G14653T, ORF1ab:C18981T, ORF1ab:A20268G, S:A23403G, N:C28732T, N:C28854T, N:C29171T,	7	N:S194L, N:H300Y, ORF1a:L778F, ORF1a:K2533R, ORF1b:P314L, ORF1b:V396F, S:D614G,
hCoV-19/Mexico/CMX-INER-0216/2020	EPI_ISL_837809	20A	B.1.243	10	5'UTR:C241T, ORF1ab:C3037T, ORF1ab:A5885G, ORF1ab:G13126T, ORF1ab:C14408T, ORF1ab:C16338T, ORF1ab:A20268G, S:A23403G, S:T24076C, S:C24904T, N:C28854T,	4	N:S194L, ORF1a:T1874A, ORF1b:P314L, S:D614G,
hCoV-19/Mexico/CMX-INER-0217/2020	EPI_ISL_837602	20B	B.1.1	14	5'UTR:C241T, ORF1ab:T1476C, ORF1ab:C2399T, ORF1ab:C3037T, ORF1ab:G8131T, ORF1ab:C14408T, ORF1ab:G15260T, ORF1ab:G17425A, ORF1ab:A20511G, S:A23403G, ORF8:C28054G, ORF8:G28077T, N:G28881A, N:G28882A, N:G28883C,	11	N:R203K, N:G204R, ORF1a:I404T, ORF1a:H712Y, ORF1a:K2622N, ORF1b:P314L, ORF1b:S598I, ORF1b:V1320M, ORF8:S54*, ORF8:V62L, S:D614G,
hCoV-19/Mexico/CMX-INER-0218/2020	EPI_ISL_837810	20B	B.1.1.512	16	5'UTR:C241T, ORF1ab:A691C, ORF1ab:C3037T, ORF1ab:G10265A, ORF1ab:C11235T, ORF1ab:G11417T, ORF1ab:C14408T, ORF1ab:G16014A, ORF1ab:A16474G, S:C21614T, S:A23403G, S:G24794T, S:A25256T, N:G28881A, N:G28882A, N:G28883C, N:G29449T,	11	N:R203K, N:G204R, ORF1a:G3334S, ORF1a:A3657V, ORF1a:V3718F, ORF1b:P314L, ORF1b:S1003G, S:L18F, S:D614G, S:A1078S, S:I1232F,
hCoV-19/Mexico/CMX-INER-0219/2020	EPI_ISL_837811	20A	B.1.415	11	5'UTR:C241T, ORF1ab:C3037T, ORF1ab:G8371A, ORF1ab:T13902C, ORF1ab:C14408T, ORF1ab:G15732T, ORF1ab:G18028T, ORF1ab:T19143C, ORF1ab:A20268G, S:A23403G, N:G28851T, N:C29358T,	5	N:S193I, N:T362I, ORF1b:P314L, ORF1b:A1521S, S:D614G,
hCoV-19/Mexico/CMX-INER-0220/2020	EPI_ISL_837812	20B	B.1.1.432	19	5'UTR:C241T, ORF1ab:C3037T, ORF1ab:C3695T, ORF1ab:G5194T, ORF1ab:A6659G, ORF1ab:A6985T, ORF1ab:G7936T, ORF1ab:C9319T, ORF1ab:C11941T, ORF1ab:C12412T, ORF1ab:T14313C, ORF1ab:C14408T, S:A23403G, S:C24374T, ORF3a:C25613T, ORF3a:G25912T, N:G28881A, N:G28882A, N:G28883C, N:T29317C,	8	N:R203K, N:G204R, ORF1a:S2132G, ORF1b:P314L, ORF3a:S74F, ORF3a:G174C, S:D614G, S:L938F,
hCoV-19/Mexico/CMX-INER-0221/2020	EPI_ISL_837813	20A	B.1	8	5'UTR:C241T, ORF1ab:C3037T, ORF1ab:C10537T, ORF1ab:C14408T, ORF1ab:A17861G, ORF1ab:A20268G, S:A23403G, ORF7a:G27517A, N:C28854T,	5	N:S194L, ORF1b:P314L, ORF1b:D1465G, ORF7a:G42S, S:D614G,
hCoV-19/Mexico/CMX-INER-0222/2020	EPI_ISL_837814	20A	B.1.551	11	5'UTR:C241T, ORF1ab:C3037T, ORF1ab:C3738T, ORF1ab:C9438T, ORF1ab:C12924T, ORF1ab:C14408T, ORF1ab:A17841G, ORF1ab:A20268G, S:A23403G, S:C23604G, S:C24502T, N:C28854T,	7	N:S194L, ORF1a:P1158L, ORF1a:T3058I, ORF1a:P4220L, ORF1b:P314L, S:D614G, S:P681R,
hCoV-19/Mexico/CMX-INER-04/2020	EPI_ISL_424626	20A	B.1.201	6	5'UTR:C241T, ORF1ab:C2940T, ORF1ab:C3037T, ORF1ab:T6842A, ORF1ab:C14408T, S:A23403G, ORF3a:T26232C,	4	ORF1a:P892L, ORF1a:S2193T, ORF1b:P314L, S:D614G,
hCoV-19/Mexico/CMX-INER-05/2020	EPI_ISL_424627	19B	A.1	9	ORF1ab:C8782T, ORF1ab:C17747T, ORF1ab:A17858G, ORF1ab:C18060T, S:C21707T, S:C23422T, S:A24694T, ORF7a:G27709T, ORF8:T28144C, 3'UTR:T29867C,	5	ORF1b:P1427L, ORF1b:Y1464C, ORF7a:A106S, ORF8:L84S, S:H49Y,
hCoV-19/Mexico/CMX-INER-INMEGEN-00001/2021	EPI_ISL_1406722	20B	B.1.1.519	29	5'UTR:C203T, 5'UTR:C222T, 5'UTR:C241T, ORF1ab:C936T, ORF1ab:C3037T, ORF1ab:C3140T, ORF1ab:C4423T, ORF1ab:C5183T, ORF1ab:T7045C, ORF1ab:C10029T, ORF1ab:C10954T, ORF1ab:A11117G, ORF1ab:C12789T, ORF1ab:C14408T, ORF1ab:T19839C, ORF1ab:G21204T, ORF1ab:C21306T, S:C21622T, S:C22995A, S:A23403G, S:C23604A, S:A23756G, S:C23997T, S:G25135T, ORF3a:T25969C, ORF8:T27904C, N:G28881A, N:G28882A, N:G28883C, N:C29197T	18	N:R203K, N:G204R, ORF1a:T224I, ORF1a:P959S, ORF1a:P1640S, ORF1a:T3255I, ORF1a:I3618V, ORF1a:T4175I, ORF1b:P314L, ORF1b:K2579N, ORF3a:W193R, ORF8:L4P, S:T478K, S:D614G, S:P681H, S:T732A, S:P812L, S:K1191N,

hCoV-19/Mexico/CMX-INER-INMEGEN-00002/2021 EPI_ISL_1406723	20B	B.1.1.519	28	5'UTR:C203T, 5'UTR:C222T, 5'UTR:C241T, ORF1ab:C936T, ORF1ab:C3037T, ORF1ab:C3140T, ORF1ab:C4423T, ORF1ab:C7772T, ORF1ab:C10029T, ORF1ab:C10954T, ORF1ab:A11117G, ORF1ab:C12789T, ORF1ab:C14408T, ORF1ab:A18956G, ORF1ab:T19839C, ORF1ab:G21204T, ORF1ab:C21306T, S:C21622T, S:C22995A, S:A23403G, S:C23604A, S:A23756G, S:G25135T, ORF3a:T25969C, ORF8:T27904C, N:G28881A, N:G28882A, N:G28883C, N:C29197T,	18	N:R203K, N:G204R, ORF1a:T224I, ORF1a:P959S, ORF1a:L2503F, ORF1a:T3255I, ORF1a:I3618V, ORF1a:T4175I, ORF1b:P314L, ORF1b:N1830S, ORF1b:K2579N, ORF3a:W193R, ORF8:L4P, S:T478K, S:D614G, S:P681H, S:T732A, S:K1191N,	
hCoV-19/Mexico/CMX-INER-INMEGEN-00003/2021 EPI_ISL_1406724	20B	B.1.1.519	25	5'UTR:T201C, 5'UTR:C203T, 5'UTR:C222T, 5'UTR:C241T, ORF1ab:G1738T, ORF1ab:C3037T, ORF1ab:C3140T, ORF1ab:C3602T, ORF1ab:T4579A, ORF1ab:C7504T, ORF1ab:C10029T, ORF1ab:C10954T, ORF1ab:A11117G, ORF1ab:C12789T, ORF1ab:C14408T, ORF1ab:T19839C, ORF1ab:A19974G, ORF1ab:C21306T, S:C22995A, S:A23403G, S:C23604A, S:A23756G, N:G28881A, N:G28882A, N:G28883C, N:C29197T,	12	N:R203K, N:G204R, ORF1a:P959S, ORF1a:H1113Y, ORF1a:T3255I, ORF1a:I3618V, ORF1a:T4175I, ORF1b:P314L, S:T478K, S:D614G, S:P681H, S:T732A,	
hCoV-19/Mexico/CMX-INER-INMEGEN-00004/2021 EPI_ISL_1423994	20B	B.1.1.519	24	5'UTR:T201C, 5'UTR:C203T, 5'UTR:C222T, 5'UTR:C241T, ORF1ab:C745T, ORF1ab:G1738T, ORF1ab:C3037T, ORF1ab:C3140T, ORF1ab:C10029T, ORF1ab:C10954T, ORF1ab:A11117G, ORF1ab:C12789T, ORF1ab:C14408T, ORF1ab:T19839C, ORF1ab:A19974G, ORF1ab:C21306T, S:C22995A, S:A23403G, S:C23604A, S:A23756G, ORF8:G28089T, N:G28881A, N:G28882A, N:G28883C, N:C29197T,	11	N:R203K, N:G204R, ORF1a:P959S, ORF1a:T3255I, ORF1a:I3618V, ORF1a:T4175I, ORF8:G66C, S:T478K, S:D614G, S:P681H, S:T732A,	ORF1ab: 20407-20408
hCoV-19/Mexico/CMX-INER-INMEGEN-00005/2021 EPI_ISL_1406725	20B	B.1.1.519	27	5'UTR:C203T, 5'UTR:C222T, 5'UTR:C241T, ORF1ab:C3037T, ORF1ab:C3140T, ORF1ab:T3745C, ORF1ab:A9935G, ORF1ab:C10029T, ORF1ab:C10954T, ORF1ab:A11117G, ORF1ab:C12789T, ORF1ab:C14408T, ORF1ab:A17277C, ORF1ab:C19803A, ORF1ab:T19839C, ORF1ab:C21306T, S:C21575T, S:C22995A, S:A23403G, S:T23470C, S:C23604A, S:A23756G, ORF3a:C25844T, N:G28881A, N:G28882A, N:G28883C, N:C29197T, N:C29541T,	15	N:R203K, N:G204R, ORF1a:P959S, ORF1a:T3224A, ORF1a:T3255I, ORF1a:I3618V, ORF1a:T4175I, ORF1b:P314L, ORF1b:K1270N, ORF3a:T151I, S:L5F, S:T478K, S:D614G, S:P681H, S:T732A,	
hCoV-19/Mexico/CMX-INER-INMEGEN-00006/2021 EPI_ISL_1406726	20B	B.1.1.519	24	5'UTR:C203T, 5'UTR:C222T, 5'UTR:C241T, ORF1ab:C3037T, ORF1ab:C3140T, ORF1ab:C10029T, ORF1ab:C10954T, ORF1ab:A11117G, ORF1ab:C12789T, ORF1ab:C14408T, ORF1ab:G16943T, ORF1ab:T19839C, ORF1ab:C21306T, S:C22995A, S:A23403G, S:C23604A, S:A23756G, S:A24727G, ORF7a:C27630T, ORF8:T27904C, ORF8:C28087T, N:G28881A, N:G28882A, N:G28883C, N:C29197T,	14	N:R203K, N:G204R, ORF1a:P959S, ORF1a:T3255I, ORF1a:I3618V, ORF1a:T4175I, ORF1b:P314L, ORF1b:S1159I, ORF8:L4P, ORF8:A65V, S:T478K, S:D614G, S:P681H, S:T732A,	
hCoV-19/Mexico/CMX-INER-INMEGEN-00007/2021 EPI_ISL_1423995	20B	B.1.1.519	27	5'UTR:C203T, 5'UTR:C222T, 5'UTR:C241T, ORF1ab:T469C, ORF1ab:C3037T, ORF1ab:C3140T, ORF1ab:G7027T, ORF1ab:C8320T, ORF1ab:C10029T, ORF1ab:C10954T, ORF1ab:A11117G, ORF1ab:C12439T, ORF1ab:C12789T, ORF1ab:C14408T, ORF1ab:C15279T, ORF1ab:A18390G, ORF1ab:T19839C, ORF1ab:C21306T, S:C22995A, S:A23403G, S:C23604A, S:A23756G, S:G24445T, S:C25207T, N:G28881A, N:G28882A, N:G28883C, N:C29197T,	12	N:R203K, N:G204R, ORF1a:P959S, ORF1a:M2254I, ORF1a:T3255I, ORF1a:I3618V, ORF1a:T4175I, ORF1b:P314L, S:T478K, S:D614G, S:P681H, S:T732A,	ORF7a:27571-27635

hCoV-19/Mexico/CMX-INER-INMEGEN-00008/2021 EPI_ISL_1406727	20B	B.1.1.519	29	5'UTR:C203T, 5'UTR:A223T, 5'UTR:C241T, ORF1ab:A866G, ORF1ab:C1009T, ORF1ab:C3037T, ORF1ab:C3140T, ORF1ab:C5183T, ORF1ab:C5748T, ORF1ab:C10029T, ORF1ab:C10954T, ORF1ab:A11117G, ORF1ab:C11916T, ORF1ab:C12789T, ORF1ab:C13730T, ORF1ab:C14408T, ORF1ab:T19839C, ORF1ab:C21306T, S:C22995A, S:A23403G, S:C23604A, S:A23756G, ORF8:T27904C, N:G28747T, N:A28877T, N:G28878C, N:G28881A, N:G28882A, N:G28883C, N:C29197T,	17	N:R203K, N:G204R, ORF1a:I201V, ORF1a:P959S, ORF1a:P1640S, ORF1a:T1828I, ORF1a:T3255I, ORF1a:I3618V, ORF1a:S3884L, ORF1a:T4175I, ORF1b:A88V, ORF1b:P314L, ORF8:L4P, S:T478K, S:D614G, S:P681H, S:T732A,	5'UTR:2 22,
hCoV-19/Mexico/CMX-INER-INMEGEN-00009/2021 EPI_ISL_1406728	20B	B.1.1.519	29	5'UTR:T201C, 5'UTR:C203T, 5'UTR:C222T, 5'UTR:C241T, ORF1ab:G1738T, ORF1ab:G1782A, ORF1ab:C3037T, ORF1ab:C3140T, ORF1ab:C3602T, ORF1ab:C7504T, ORF1ab:C10029T, ORF1ab:C10954T, ORF1ab:A11117G, ORF1ab:T12568A, ORF1ab:C12789T, ORF1ab:C14408T, ORF1ab:T19839C, ORF1ab:A19974G, ORF1ab:C21306T, S:T21570G, S:C22995A, S:C23280T, S:A23403G, S:C23604A, S:A23756G, S:A23851T, N:G28881A, N:G28882A, N:G28883C, N:C29197T,	16	N:R203K, N:G204R, ORF1a:C506Y, ORF1a:P959S, ORF1a:H1113Y, ORF1a:T3255I, ORF1a:I3618V, ORF1a:T4175I, ORF1b:P314L, S:V3G, S:T478K, S:T573I, S:D614G, S:P681H, S:T732A, S:L763F,	
hCoV-19/Mexico/CMX-INER-INMEGEN-00010/2021 EPI_ISL_1406729	20B	B.1.1.519	26	5'UTR:C203T, 5'UTR:C222T, 5'UTR:C241T, ORF1ab:A1367G, ORF1ab:G2447T, ORF1ab:C3037T, ORF1ab:C3140T, ORF1ab:C6541T, ORF1ab:C10029T, ORF1ab:C10954T, ORF1ab:A11117G, ORF1ab:C12789T, ORF1ab:C14408T, ORF1ab:T19839C, ORF1ab:C20133T, ORF1ab:C21306T, S:A22023C, S:C22995A, S:A23403G, S:C23604A, S:A23756G, ORF6:G27240T, ORF8:T27904C, N:G28881A, N:G28882A, N:G28883C, N:C29197T,	16	N:R203K, N:G204R, ORF1a:I368V, ORF1a:G728C, ORF1a:P959S, ORF1a:T3255I, ORF1a:I3618V, ORF1a:T4175I, ORF1b:P314L, ORF6:E13D, ORF8:L4P, S:E154A, S:T478K, S:D614G, S:P681H, S:T732A,	
hCoV-19/Mexico/CMX-INER-INMEGEN-00011/2021 EPI_ISL_1406730	20B	B.1.1.519	27	5'UTR:T201C, 5'UTR:C203T, 5'UTR:C222T, 5'UTR:C241T, ORF1ab:G1738T, ORF1ab:C3037T, ORF1ab:C3140T, ORF1ab:G3753A, ORF1ab:A6459G, ORF1ab:A9280G, ORF1ab:C10029T, ORF1ab:C10954T, ORF1ab:A11117G, ORF1ab:C12789T, ORF1ab:C14408T, ORF1ab:A16026G, ORF1ab:G18020A, ORF1ab:T19839C, ORF1ab:A19974G, ORF1ab:C21306T, S:C22995A, S:A23403G, S:C23604A, S:A23756G, N:G28881A, N:G28882A, N:G28883C, N:C29197T,	14	N:R203K, N:G204R, ORF1a:P959S, ORF1a:R1163K, ORF1a:N2065S, ORF1a:T3255I, ORF1a:I3618V, ORF1a:T4175I, ORF1b:P314L, ORF1b:R1518K, S:T478K, S:D614G, S:P681H, S:T732A,	
hCoV-19/Mexico/CMX-INER-INMEGEN-00012/2021 EPI_ISL_1406731	20B	B.1.1.519	24	5'UTR:T201C, 5'UTR:C203T, 5'UTR:C222T, 5'UTR:C241T, ORF1ab:G1738T, ORF1ab:C3037T, ORF1ab:C3140T, ORF1ab:C10029T, ORF1ab:G10610A, ORF1ab:C10954T, ORF1ab:A11117G, ORF1ab:C12789T, ORF1ab:C14408T, ORF1ab:T19839C, ORF1ab:A19974G, ORF1ab:C21306T, S:C22995A, S:A23403G, S:C23604A, S:A23756G, ORF3a:C25553T, N:G28881A, N:G28882A, N:G28883C, N:C29197T,	13	N:R203K, N:G204R, ORF1a:P959S, ORF1a:T3255I, ORF1a:V3449I, ORF1a:I3618V, ORF1a:T4175I, ORF1b:P314L, ORF3a:A54V, S:T478K, S:D614G, S:P681H, S:T732A,	

hCoV-19/Mexico/CMX-INER-INMEGEN-00013/2021 EPI_ISL_1406732	20B	B.1.1.519	28	5'UTR:T201C, 5'UTR:C203T, 5'UTR:C222T, 5'UTR:C241T, ORF1ab:C745T, ORF1ab:G1738T, ORF1ab:C3037T, ORF1ab:C3140T, ORF1ab:G6254A, ORF1ab:C10029T, ORF1ab:C10954T, ORF1ab:A11117G, ORF1ab:A11887G, ORF1ab:C12789T, ORF1ab:C14408T, ORF1ab:A18566G, ORF1ab:T19839C, ORF1ab:A19974G, ORF1ab:C21306T, S:C22995A, S:A23403G, S:C23604A, S:A23756G, ORF8:G28077T, ORF8:G28178T, N:G28881A, N:G28882A, N:G28883C, N:C29197T,	15	N:R203K, N:G204R, ORF1a:P959S, ORF1a:A1997T, ORF1a:T3255I, ORF1a:I3618V, ORF1a:T4175I, ORF1b:P314L, ORF1b:N1700S, ORF8:V62L, ORF8:L95F, S:T478K, S:D614G, S:P681H, S:T732A,	
hCoV-19/Mexico/CMX-INER-INMEGEN-00036/2021 EPI_ISL_1406751	20B	B.1.1.519	25	5'UTR:C203T, 5'UTR:C222T, 5'UTR:C241T, ORF1ab:C3037T, ORF1ab:C3140T, ORF1ab:C10029T, ORF1ab:C10954T, ORF1ab:A11117G, ORF1ab:C12789T, ORF1ab:A12841G, ORF1ab:C14408T, ORF1ab:G15921T, ORF1ab:A17971G, ORF1ab:T19839C, ORF1ab:C21306T, S:A22034G, S:C22082T, S:C22995A, S:A23403G, S:C23604A, S:C23635T, S:A23756G, N:G28881A, N:G28882A, N:G28883C, N:C29197T,	14	N:R203K, N:G204R, ORF1a:P959S, ORF1a:T3255I, ORF1a:I3618V, ORF1a:T4175I, ORF1b:P314L, ORF1b:R1502G, S:R158G, S:P174S, S:T478K, S:D614G, S:P681H, S:T732A,	
hCoV-19/Mexico/CMX-INER-INMEGEN-00037/2021 EPI_ISL_1406752	20B	B.1.1.519	29	5'UTR:C203T, 5'UTR:C222T, 5'UTR:C241T, ORF1ab:A1367G, ORF1ab:C3037T, ORF1ab:C3140T, ORF1ab:C6541T, ORF1ab:C10029T, ORF1ab:C10954T, ORF1ab:G11083T, ORF1ab:A11117G, ORF1ab:C12789T, ORF1ab:C14408T, ORF1ab:T19839C, ORF1ab:C20133T, ORF1ab:T20424C, ORF1ab:C20428A, ORF1ab:T20432C, ORF1ab:G20433A, ORF1ab:C21306T, S:A22023C, S:C22995A, S:A23403G, S:C23604A, S:A23756G, ORF8:T27904C, N:G28881A, N:G28882A, N:G28883C, N:C29197T,	17	N:R203K, N:G204R, ORF1a:I368V, ORF1a:P959S, ORF1a:T3255I, ORF1a:L3606F, ORF1a:I3618V, ORF1a:T4175I, ORF1b:P314L, ORF1b:P2321T, ORF1b:M2322T, ORF8:L4P, S:E154A, S:T478K, S:D614G, S:P681H, S:T732A,	
hCoV-19/Mexico/CMX-INER-INMEGEN-00038/2021 EPI_ISL_1406753	20B	B.1.1.519	29	5'UTR:C203T, 5'UTR:A223T, 5'UTR:C241T, ORF1ab:C2455T, ORF1ab:C3037T, ORF1ab:C3140T, ORF1ab:T4732C, ORF1ab:A6098C, ORF1ab:C8047T, ORF1ab:C10029T, ORF1ab:C10954T, ORF1ab:A11117G, ORF1ab:G11365T, ORF1ab:C12789T, ORF1ab:C13384T, ORF1ab:C14408T, ORF1ab:T15978C, ORF1ab:T19839C, ORF1ab:C21306T, S:C21575T, S:C22995A, S:A23403G, S:C23604A, S:A23756G, N:C28507T, N:G28881A, N:G28882A, N:G28883C, N:C29197T, N:G29527T,	15	N:R203K, N:G204R, N:Q418H, ORF1a:P959S, ORF1a:T1945P, ORF1a:T3255I, ORF1a:I3618V, ORF1a:T4175I, ORF1b:P314L, ORF9b:A75V, S:L5F, S:T478K, S:D614G, S:P681H, S:T732A,	5'UTR:2 22,
hCoV-19/Mexico/CMX-INER-INMEGEN-00039/2021 EPI_ISL_1406754	20B	B.1.1.519	30	5'UTR:T201C, 5'UTR:C203T, 5'UTR:C222T, 5'UTR:C241T, ORF1ab:C745T, ORF1ab:G1738T, ORF1ab:C3037T, ORF1ab:C3140T, ORF1ab:C10029T, ORF1ab:C10954T, ORF1ab:G11083T, ORF1ab:A11117G, ORF1ab:A11887G, ORF1ab:C12789T, ORF1ab:C14408T, ORF1ab:C16376T, ORF1ab:C19032T, ORF1ab:T19839C, ORF1ab:A19974G, ORF1ab:C21306T, S:C22995A, S:A23403G, S:C23604A, S:A23756G, ORF8:G28077T, ORF8:G28178T, N:G28881A, N:G28882A, N:G28883C, N:C29197T, N:C29272T	15	N:R203K, N:G204R, ORF1a:P959S, ORF1a:T3255I, ORF1a:L3606F, ORF1a:I3618V, ORF1a:T4175I, ORF1b:P314L, ORF1b:P970L, ORF8:V62L, ORF8:L95F, S:T478K, S:D614G, S:P681H, S:T732A,	

hCoV-19/Mexico/CMX-INER-INMEGEN-00040/2021 EPI_ISL_1406755	20B	B.1.1.519	29	5'UTR:C203T, 5'UTR:C222T, 5'UTR:C241T, ORF1ab:C3037T, ORF1ab:C3140T, ORF1ab:G3692T, ORF1ab:G3871T, ORF1ab:A4501G, ORF1ab:C9520T, ORF1ab:C10029T, ORF1ab:C10626T, ORF1ab:C10954T, ORF1ab:A11117G, ORF1ab:C11824A, ORF1ab:C12789T, ORF1ab:C14408T, ORF1ab:C19011T, ORF1ab:T19839C, ORF1ab:C21306T, S:C22995A, S:A23403G, S:C23604A, S:A23756G, ORF3a:A25582T, M:A26751G, ORF8:T27904C, N:G28881A, N:G28882A, N:G28883C, N:C29197T,	17	M:T77A, N:R203K, N:G204R, ORF1a:P959S, ORF1a:V1143F, ORF1a:K1202N, ORF1a:T3255I, ORF1a:A3454V, ORF1a:I3618V, ORF1a:T4175I, ORF1b:P314L, ORF3a:T64S, ORF8:L4P, S:T478K, S:D614G, S:P681H, S:T732A,	
hCoV-19/Mexico/CMX-INER-INMEGEN-00041/2021 EPI_ISL_1406756	20B	B.1.1.519	24	5'UTR:C203T, 5'UTR:C222T, 5'UTR:C241T, ORF1ab:C3037T, ORF1ab:C3140T, ORF1ab:C6726T, ORF1ab:T8341C, ORF1ab:C9532T, ORF1ab:C10029T, ORF1ab:C10954T, ORF1ab:A11117G, ORF1ab:C12789T, ORF1ab:C14408T, ORF1ab:T19839C, ORF1ab:C19983T, ORF1ab:C21306T, S:C22995A, S:A23403G, S:C23604A, S:A23756G, N:G28881A, N:G28882A, N:G28883C, N:C29197T, N:G29527T,	13	N:R203K, N:G204R, N:Q418H, ORF1a:P959S, ORF1a:T2154I, ORF1a:T3255I, ORF1a:I3618V, ORF1a:T4175I, ORF1b:P314L, S:T478K, S:D614G, S:P681H, S:T732A,	
hCoV-19/Mexico/CMX-INER-INMEGEN-00042/2021 EPI_ISL_1424000	20B	B.1.1.222	22	5'UTR:C241T, ORF1ab:C3037T, ORF1ab:C4897T, ORF1ab:C5259T, ORF1ab:C10029T, ORF1ab:A11430G, ORF1ab:C14408T, ORF1ab:C19011A, ORF1ab:T19839C, ORF1ab:A21137G, S:C21575T, S:A23403G, S:A23756G, ORF3a:G25912T, ORF3a:A26115C, ORF7a:C27643T, ORF8:A27921G, ORF8:G28001T, ORF8:A28096G, N:G28881A, N:G28882A, N:G28883C, ORF10:C29640T,	16	N:R203K, N:G204R, ORF1a:T1665I, ORF1a:T3255I, ORF1a:Y3722C, ORF1b:P314L, ORF1b:D1848E, ORF1b:K2557R, ORF3a:G174C, ORF3a:E241D, ORF7a:P84S, ORF8:I10V, ORF8:K68R, S:L5F, S:D614G, S:T732A,	ORF6:27 313
hCoV-19/Mexico/CMX-INER-INMEGEN-00043/2021 EPI_ISL_1406757	20B	B.1.1.519	28	5'UTR:T201C, 5'UTR:C203T, 5'UTR:C222T, 5'UTR:C241T, ORF1ab:C745T, ORF1ab:G1738T, ORF1ab:C3037T, ORF1ab:C3140T, ORF1ab:A8893G, ORF1ab:C10029T, ORF1ab:C10954T, ORF1ab:A11117G, ORF1ab:C12789T, ORF1ab:C14408T, ORF1ab:T15195A, ORF1ab:T19839C, ORF1ab:A19974G, ORF1ab:C21306T, S:C22995A, S:A23403G, S:C23604A, S:A23756G, ORF3a:C26032T, ORF8:C28253T, N:C28770T, N:G28881A, N:G28882A, N:G28883C, N:C29197T	12	N:T166I, N:R203K, N:G204R, ORF1a:P959S, ORF1a:T3255I, ORF1a:I3618V, ORF1a:T4175I, ORF1b:P314L, S:T478K, S:D614G, S:P681H, S:T732A,	
hCoV-19/Mexico/CMX-INER-INMEGEN-00044/2021 EPI_ISL_1406758	20B	B.1.1.519	28	5'UTR:T201C, 5'UTR:C203T, 5'UTR:C222T, 5'UTR:C241T, ORF1ab:G1738T, ORF1ab:C2892T, ORF1ab:C3037T, ORF1ab:C3140T, ORF1ab:A5845C, ORF1ab:C6026T, ORF1ab:C10029T, ORF1ab:C10954T, ORF1ab:A11117G, ORF1ab:C12789T, ORF1ab:C14408T, ORF1ab:T19839C, ORF1ab:A19974G, ORF1ab:C21306T, S:C22995A, S:A23403G, S:C23604A, S:A23756G, ORF7b:C27804T, N:G28881A, N:G28882A, N:G28883C, N:C29197T, N:C29421T, ORF10:C29627T,	15	N:R203K, N:G204R, N:P383L, ORF1a:A876V, ORF1a:P959S, ORF1a:K1860N, ORF1a:P1921S, ORF1a:T3255I, ORF1a:I3618V, ORF1a:T4175I, ORF1b:P314L, S:T478K, S:D614G, S:P681H, S:T732A,	
hCoV-19/Mexico/CMX-INER-INMEGEN-00045/2021 EPI_ISL_1406759	20B	B.1.1.519	27	5'UTR:C203T, 5'UTR:C222T, 5'UTR:C241T, ORF1ab:C3037T, ORF1ab:C3140T, ORF1ab:C8752T, ORF1ab:C10029T, ORF1ab:C10954T, ORF1ab:A11117G, ORF1ab:C12789T, ORF1ab:A14199T, ORF1ab:C14408T, ORF1ab:C15081T, ORF1ab:G19272T, ORF1ab:T19839C, ORF1ab:C21306T, S:C22995A, S:A23403G, S:C23604A, S:A23756G, S:A24727G, S:C25318T, ORF8:T27904C, ORF8:C28087T, N:G28881A, N:G28882A, N:G28883C, N:C29197T,	14	N:R203K, N:G204R, ORF1a:P959S, ORF1a:T3255I, ORF1a:I3618V, ORF1a:T4175I, ORF1b:P314L, ORF1b:L1935F, ORF8:L4P, ORF8:A65V, S:T478K, S:D614G, S:P681H, S:T732A,	

hCoV-19/Mexico/CMX-INER-INMEGEN-00046/2021 EPI_ISL_1406760	20B	B.1.1.519	24	5'UTR:T201C, 5'UTR:C203T, 5'UTR:C222T, 5'UTR:C241T, ORF1ab:G1738T, ORF1ab:C3037T, ORF1ab:C3140T, ORF1ab:C10029T, ORF1ab:C10954T, ORF1ab:A11117G, ORF1ab:C12484T, ORF1ab:C12789T, ORF1ab:C14408T, ORF1ab:T19839C, ORF1ab:A19974G, ORF1ab:C21306T, S:C22995A, S:A23403G, S:C23604A, S:A23756G, ORF3a:C25553T, N:G28881A, N:G28882A, N:G28883C, N:C29197T,	12	N:R203K, N:G204R, ORF1a:P959S, ORF1a:T3255I, ORF1a:I3618V, ORF1a:T4175I, ORF1b:P314L, ORF3a:A54V, S:T478K, S:D614G, S:P681H, S:T732A,	
hCoV-19/Mexico/CMX-INER-INMEGEN-00047/2021 EPI_ISL_1406761	20B	B.1.1.519	28	5'UTR:T201C, 5'UTR:C203T, 5'UTR:C222T, 5'UTR:C241T, ORF1ab:G1738T, ORF1ab:C3037T, ORF1ab:C3140T, ORF1ab:C10029T, ORF1ab:C10332T, ORF1ab:C10954T, ORF1ab:A11117G, ORF1ab:C12789T, ORF1ab:A13711G, ORF1ab:C14408T, ORF1ab:C14724T, ORF1ab:T19839C, ORF1ab:A19974G, ORF1ab:C19983T, ORF1ab:C21306T, S:C22995A, S:G23285T, S:A23403G, S:C23604A, S:A23756G, ORF3a:C25517T, N:G28881A, N:G28882A, N:G28883C, N:C29197T,	15	N:R203K, N:G204R, ORF1a:P959S, ORF1a:T3255I, ORF1a:T3356I, ORF1a:I3618V, ORF1a:T4175I, ORF1b:K82E, ORF1b:P314L, ORF3a:P42L, S:T478K, S:A575S, S:D614G, S:P681H, S:T732A,	
hCoV-19/Mexico/CMX-INER-INMEGEN-00048/2021 EPI_ISL_1406762	20B	B.1.1.519	26	5'UTR:C203T, 5'UTR:C222T, 5'UTR:C241T, ORF1ab:C3037T, ORF1ab:C3140T, ORF1ab:C7002T, ORF1ab:C10029T, ORF1ab:C10954T, ORF1ab:A11117G, ORF1ab:T12349A, ORF1ab:C12789T, ORF1ab:C14408T, ORF1ab:T16314C, ORF1ab:T19839C, ORF1ab:C21306T, S:C22995A, S:A23403G, S:C23604A, S:A23756G, M:C26826T, N:G28378T, N:G28881A, N:G28882A, N:G28883C, N:C29197T, N:G29527T, 3'UTR:G29747T,	14	N:R203K, N:G204R, N:Q418H, ORF1a:P959S, ORF1a:S2246L, ORF1a:T3255I, ORF1a:I3618V, ORF1a:T4175I, ORF1b:P314L, ORF9b:R32L, S:T478K, S:D614G, S:P681H, S:T732A,	
hCoV-19/Mexico/CMX-INER-INMEGEN-00049/2021 EPI_ISL_1424001	20B	B.1.1.519	25	5'UTR:C203T, 5'UTR:C222T, 5'UTR:C241T, ORF1ab:C3037T, ORF1ab:C3140T, ORF1ab:T5983C, ORF1ab:T9061C, ORF1ab:C10029T, ORF1ab:C10954T, ORF1ab:A11117G, ORF1ab:C12789T, ORF1ab:C14318T, ORF1ab:C14408T, ORF1ab:T19839C, ORF1ab:C20429T, ORF1ab:C21306T, S:C22995A, S:A23403G, S:C23604A, S:A23756G, ORF7a:A27674T, N:G28881A, N:G28882A, N:G28883C, N:C29197T, N:G29527T,	14	N:R203K, N:G204R, N:Q418H, ORF1a:P959S, ORF1a:T3255I, ORF1a:I3618V, ORF1a:T4175I, ORF1b:T284I, ORF1b:P314L, ORF1b:P2321L, S:T478K, S:D614G, S:P681H, S:T732A,	ORF7a:27676,
hCoV-19/Mexico/CMX-INER-INMEGEN-00050/2021 EPI_ISL_1406763	20B	B.1.1.519	26	5'UTR:C203T, 5'UTR:C222T, 5'UTR:C241T, ORF1ab:C3037T, ORF1ab:C3140T, ORF1ab:A3684G, ORF1ab:C6541T, ORF1ab:C10029T, ORF1ab:C10138T, ORF1ab:C10954T, ORF1ab:A11117G, ORF1ab:C12789T, ORF1ab:C14408T, ORF1ab:T19839C, ORF1ab:C21306T, S:A22023T, S:C22995A, S:A23403G, S:C23604A, S:A23756G, M:G26754T, ORF8:T27904C, N:G28881A, N:G28882A, N:G28883C, N:C29197T, N:C29555T,	15	M:G78C, N:R203K, N:G204R, ORF1a:P959S, ORF1a:Q1140R, ORF1a:T3255I, ORF1a:I3618V, ORF1a:T4175I, ORF1b:P314L, ORF8:L4P, S:E154V, S:T478K, S:D614G, S:P681H, S:T732A,	
hCoV-19/Mexico/CMX-INER-INMEGEN-00051/2021 EPI_ISL_1406764	20B	B.1.1.519	27	5'UTR:C203T, 5'UTR:C222T, 5'UTR:C241T, ORF1ab:C3037T, ORF1ab:C3140T, ORF1ab:A3684G, ORF1ab:C6541T, ORF1ab:C10029T, ORF1ab:C10138T, ORF1ab:C10954T, ORF1ab:A11117G, ORF1ab:C12789T, ORF1ab:C14408T, ORF1ab:A15434G, ORF1ab:T19839C, ORF1ab:C21306T, S:A22023T, S:C22995A, S:A23403G, S:C23604A, S:A23756G, M:G26754T, ORF8:T27904C, N:G28881A, N:G28882A, N:G28883C, N:C29197T, N:C29555T,	16	M:G78C, N:R203K, N:G204R, ORF1a:P959S, ORF1a:Q1140R, ORF1a:T3255I, ORF1a:I3618V, ORF1a:T4175I, ORF1b:P314L, ORF1b:E656G, ORF8:L4P, S:E154V, S:T478K, S:D614G, S:P681H, S:T732A,	

hCoV-19/Mexico/CMX-INER-INMEGEN-00052/2021 EPI_ISL_1406765	20B	B.1.1.519	28	5'UTR:T201C, 5'UTR:C203T, 5'UTR:C222T, 5'UTR:C241T, ORF1ab:C745T, ORF1ab:G1738T, ORF1ab:C1959T, ORF1ab:C3037T, ORF1ab:C3140T, ORF1ab:A7094G, ORF1ab:C10029T, ORF1ab:C10954T, ORF1ab:A11117G, ORF1ab:C12789T, ORF1ab:C14408T, ORF1ab:C17213T, ORF1ab:T17505C, ORF1ab:T19839C, ORF1ab:A19974G, ORF1ab:C20629T, ORF1ab:C21306T, S:C22995A, S:A23403G, S:C23604A, S:A23756G, N:G28881A, N:G28882A, N:G28883C, N:C29197T,	15	N:R203K, N:G204R, ORF1a:A565V, ORF1a:P959S, ORF1a:T2277A, ORF1a:T3255I, ORF1a:I3618V, ORF1a:T4175I, ORF1b:P314L, ORF1b:P1249L, ORF1b:H2388Y, S:T478K, S:D614G, S:P681H, S:T732A,
hCoV-19/Mexico/CMX-INER-INMEGEN-00053/2021 EPI_ISL_1406766	20B	B.1.1.519	27	5'UTR:C203T, 5'UTR:C222T, 5'UTR:C241T, ORF1ab:C3037T, ORF1ab:C3140T, ORF1ab:T3745C, ORF1ab:T4579A, ORF1ab:G7272A, ORF1ab:G9211A, ORF1ab:A9266G, ORF1ab:C10029T, ORF1ab:C10954T, ORF1ab:A11117G, ORF1ab:C12789T, ORF1ab:C14408T, ORF1ab:T19839C, ORF1ab:C21306T, S:C22995A, S:A23403G, S:C23422T, S:C23604A, S:A23756G, ORF3a:C25844T, N:G28881A, N:G28882A, N:G28883C, N:C29197T, N:C29284T,	14	N:R203K, N:G204R, ORF1a:P959S, ORF1a:R2336K, ORF1a:S3001G, ORF1a:T3255I, ORF1a:I3618V, ORF1a:T4175I, ORF1b:P314L, ORF3a:T151I, S:T478K, S:D614G, S:P681H, S:T732A,
hCoV-19/Mexico/CMX-INER-INMEGEN-00054/2021 EPI_ISL_1406767	20B	B.1.1.519	25	5'UTR:C203T, 5'UTR:C222T, 5'UTR:C241T, ORF1ab:G1942A, ORF1ab:C3037T, ORF1ab:C3140T, ORF1ab:A3581G, ORF1ab:C10029T, ORF1ab:C10954T, ORF1ab:A11117G, ORF1ab:C12789T, ORF1ab:C14408T, ORF1ab:G18546T, ORF1ab:T19839C, ORF1ab:G20002T, ORF1ab:C21306T, S:C22995A, S:A23403G, S:C23604A, S:A23756G, M:C26882T, ORF8:T27904C, N:G28881A, N:G28882A, N:G28883C, N:C29197T,	15	N:R203K, N:G204R, ORF1a:P959S, ORF1a:S1106G, ORF1a:T3255I, ORF1a:I3618V, ORF1a:T4175I, ORF1b:P314L, ORF1b:M1693I, ORF1b:D2179Y, ORF8:L4P, S:T478K, S:D614G, S:P681H, S:T732A,
hCoV-19/Mexico/CMX-INER-INMEGEN-00055/2021 EPI_ISL_1406768	20B	B.1.1.519	26	5'UTR:T201C, 5'UTR:C203T, 5'UTR:C222T, 5'UTR:C241T, ORF1ab:C745T, ORF1ab:T809C, ORF1ab:G1738T, ORF1ab:C3037T, ORF1ab:C3140T, ORF1ab:T3700C, ORF1ab:C10029T, ORF1ab:C10954T, ORF1ab:A11117G, ORF1ab:C12789T, ORF1ab:C14408T, ORF1ab:T19839C, ORF1ab:A19974G, ORF1ab:C21306T, S:C22995A, S:A23403G, S:C23604A, S:A23756G, N:G28881A, N:G28882A, N:G28883C, N:C29197T, 3'UTR:G29692T,	12	N:R203K, N:G204R, ORF1a:Y182H, ORF1a:P959S, ORF1a:T3255I, ORF1a:I3618V, ORF1a:T4175I, ORF1b:P314L, S:T478K, S:D614G, S:P681H, S:T732A,
hCoV-19/Mexico/CMX-INER-INMEGEN-00056/2021 EPI_ISL_1406769	20B	B.1.1.519	23	5'UTR:C203T, 5'UTR:C222T, 5'UTR:C241T, ORF1ab:C3037T, ORF1ab:C3140T, ORF1ab:C10029T, ORF1ab:C10954T, ORF1ab:A11117G, ORF1ab:C12789T, ORF1ab:C14408T, ORF1ab:C19602T, ORF1ab:T19839C, ORF1ab:C21306T, S:C22995A, S:A23403G, S:C23604A, S:A23756G, ORF3a:G25775T, ORF3a:G25906T, ORF8:C28253T, N:G28881A, N:G28882A, N:G28883C, N:C29197T,	13	N:R203K, N:G204R, ORF1a:P959S, ORF1a:T3255I, ORF1a:I3618V, ORF1a:T4175I, ORF1b:P314L, ORF3a:W128L, ORF3a:G172C, S:T478K, S:D614G, S:P681H, S:T732A,
hCoV-19/Mexico/CMX-INER-INMEGEN-00057/2021 EPI_ISL_1406770	20B	B.1.1.519	27	5'UTR:C203T, 5'UTR:C222T, 5'UTR:C241T, ORF1ab:C683T, ORF1ab:C1191A, ORF1ab:C3037T, ORF1ab:C3140T, ORF1ab:A5410G, ORF1ab:C10029T, ORF1ab:C10954T, ORF1ab:A11117G, ORF1ab:C12789T, ORF1ab:C13620T, ORF1ab:C14408T, ORF1ab:T19839C, ORF1ab:C21306T, S:C22995A, S:A23403G, S:C23604A, S:A23756G, M:C26954T, N:C28290T, N:G28881A, N:G28882A, N:G28883C, N:C29197T, N:G29315C, N:C29421A,	16	N:P6L, N:R203K, N:G204R, N:D348H, N:P383Q, ORF1a:P309Q, ORF1a:P959S, ORF1a:T3255I, ORF1a:I3618V, ORF1a:T4175I, ORF1b:P314L, ORF9b:P3S, S:T478K, S:D614G, S:P681H, S:T732A,

hCoV-19/Mexico/CMX-INER-INMEGEN-00058/2021 EPI_ISL_1406771	20B	B.1.1.519	26	5'UTR:C203T, 5'UTR:C222T, 5'UTR:C241T, ORF1ab:C3037T, ORF1ab:C3140T, ORF1ab:C8293T, ORF1ab:C9120T, ORF1ab:C10029T, ORF1ab:C10954T, ORF1ab:A11117G, ORF1ab:C12789T, ORF1ab:C14408T, ORF1ab:T19839C, ORF1ab:C21077T, ORF1ab:C21306T, S:C22995A, S:A23403G, S:C23604A, S:A23756G, ORF3a:G25906T, M:C27119T, ORF8:C28253T, N:G28881A, N:G28882A, N:G28883C, N:C29171T, N:C29197T,	15	N:R203K, N:G204R, N:H300Y, ORF1a:P959S, ORF1a:T2952I, ORF1a:T3255I, ORF1a:I3618V, ORF1a:T4175I, ORF1b:P314L, ORF1b:T2537I, ORF3a:G172C, S:T478K, S:D614G, S:P681H, S:T732A,	
hCoV-19/Mexico/CMX-INER-INMEGEN-00059/2021 EPI_ISL_1406772	20B	B.1.1.519	29	5'UTR:C203T, 5'UTR:A223T, 5'UTR:C241T, ORF1ab:C2455T, ORF1ab:C3037T, ORF1ab:C3140T, ORF1ab:C8047T, ORF1ab:C8772T, ORF1ab:C10029T, ORF1ab:C10954T, ORF1ab:A11117G, ORF1ab:G11365T, ORF1ab:C12789T, ORF1ab:C13384T, ORF1ab:C14408T, ORF1ab:T15978C, ORF1ab:T19839C, ORF1ab:C21306T, S:C21575T, S:C22995A, S:T23032C, S:A23403G, S:C23604A, S:A23756G, N:C28507T, N:G28881A, N:G28882A, N:G28883C, N:C29197T, N:G29527T,	15	N:R203K, N:G204R, N:Q418H, ORF1a:P959S, ORF1a:T2836I, ORF1a:T3255I, ORF1a:I3618V, ORF1a:T4175I, ORF1b:P314L, ORF9b:A75V, S:L5F, S:T478K, S:D614G, S:P681H, S:T732A,	5'UTR:2 22,
hCoV-19/Mexico/CMX-INER-INMEGEN-00060/2021 EPI_ISL_1406773	20B	B.1.1.519	29	5'UTR:T201C, 5'UTR:C203T, 5'UTR:C222T, 5'UTR:C241T, ORF1ab:C1684T, ORF1ab:G1738T, ORF1ab:C3037T, ORF1ab:C3140T, ORF1ab:G9388A, ORF1ab:C10029T, ORF1ab:C10954T, ORF1ab:A11117G, ORF1ab:A12088G, ORF1ab:C12789T, ORF1ab:A13278G, ORF1ab:C14408T, ORF1ab:T19839C, ORF1ab:A19974G, ORF1ab:C21306T, S:C22995A, S:G23302A, S:A23403G, S:C23604A, S:A23756G, ORF3a:C25484T, N:G28881A, N:G28882A, N:G28883C, N:C29197T, N:G29315C,	14	N:R203K, N:G204R, N:D348H, ORF1a:P959S, ORF1a:T3255I, ORF1a:I3618V, ORF1a:T4175I, ORF1a:N4338S, ORF1b:P314L, ORF3a:A31V, S:T478K, S:D614G, S:P681H, S:T732A,	
hCoV-19/Mexico/CMX-INER-INMEGEN-00061/2021 EPI_ISL_1406774	20B	B.1.1.519	24	5'UTR:C203T, 5'UTR:C222T, 5'UTR:C241T, ORF1ab:C3037T, ORF1ab:C3140T, ORF1ab:G7027T, ORF1ab:C10029T, ORF1ab:C10954T, ORF1ab:A11117G, ORF1ab:C11620T, ORF1ab:C12789T, ORF1ab:C13883T, ORF1ab:C14408T, ORF1ab:T19839C, ORF1ab:C21306T, S:C22995A, S:A23403G, S:C23604A, S:A23756G, S:C25207T, ORF8:A28211C, N:G28881A, N:G28882A, N:G28883C, N:C29197T,	14	N:R203K, N:G204R, ORF1a:P959S, ORF1a:M2254I, ORF1a:T3255I, ORF1a:I3618V, ORF1a:T4175I, ORF1b:T139I, ORF1b:P314L, ORF8:E106D, S:T478K, S:D614G, S:P681H, S:T732A,	
hCoV-19/Mexico/CMX-INER-INMEGEN-00062/2021 EPI_ISL_1406775	20B	B.1.1.519	25	5'UTR:C203T, 5'UTR:C222T, 5'UTR:C241T, ORF1ab:C3037T, ORF1ab:C3140T, ORF1ab:G9335C, ORF1ab:C10029T, ORF1ab:C10954T, ORF1ab:A11117G, ORF1ab:C12789T, ORF1ab:C14408T, ORF1ab:T19839C, ORF1ab:C21306T, S:T21580G, S:C22323T, S:C22995A, S:A23403G, S:C23604A, S:A23756G, ORF3a:C26029A, ORF8:T27904C, N:G28881A, N:G28882A, N:G28883C, N:C29197T, ORF10:C29659T,	15	N:R203K, N:G204R, ORF1a:P959S, ORF1a:V3024L, ORF1a:T3255I, ORF1a:I3618V, ORF1a:T4175I, ORF1b:P314L, ORF3a:Q213K, ORF8:L4P, S:S254F, S:T478K, S:D614G, S:P681H, S:T732A,	
hCoV-19/Mexico/CMX-INER-INMEGEN-00063/2021 EPI_ISL_1406776	20B	B.1.1.519	28	5'UTR:C203T, 5'UTR:C222T, 5'UTR:C241T, ORF1ab:C3037T, ORF1ab:C3140T, ORF1ab:A4764G, ORF1ab:C10029T, ORF1ab:C10954T, ORF1ab:A11117G, ORF1ab:C12789T, ORF1ab:T12994A, ORF1ab:C14408T, ORF1ab:C17485T, ORF1ab:G18255A, ORF1ab:G18498T, ORF1ab:T19839C, ORF1ab:C21306T, S:C21575T, S:T21826C, S:C22995A, S:A23403G, S:C23604A, S:A23756G, N:C28311T, N:G28881A, N:G28882A, N:G28883C, N:C29197T, N:G29527T	18	N:P13L, N:R203K, N:G204R, N:Q418H, ORF1a:P959S, ORF1a:H1500R, ORF1a:T3255I, ORF1a:I3618V, ORF1a:T4175I, ORF1b:P314L, ORF1b:M1596I, ORF1b:M1677I, ORF9b:P10S, S:L5F, S:T478K, S:D614G, S:P681H, S:T732A,	

hCoV-19/Mexico/CMX-INER-INMEGEN-00065/2021 EPI_ISL_1824419	20B	B.1.1.519	27	5'UTR:C203T, 5'UTR:C222T, 5'UTR:C241T, ORF1ab:C3037T, ORF1ab:C3140T, ORF1ab:C10029T, ORF1ab:C10954T, ORF1ab:A11117G, ORF1ab:C12789T, ORF1ab:C13138T, ORF1ab:C14408T, ORF1ab:C17550T, ORF1ab:C19320T, ORF1ab:T19839C, ORF1ab:C21306T, S:C22995A, S:A23403G, S:C23604A, S:A23756G, S:A24162G, ORF7a:T27484C, ORF7b:G27798T, N:A28753G, N:G28881A, N:G28882A, N:G28883C, N:C29197T, N:G29527T	14	N:R203K, N:G204R, N:Q418H, ORF1a:P959S, ORF1a:T3255I, ORF1a:I3618V, ORF1a:T4175I, ORF1b:P314L, ORF7b:A15S, S:T478K, S:D614G, S:P681H, S:T732A, S:D867G,
hCoV-19/Mexico/CMX-INER-INMEGEN-00066/2021 EPI_ISL_1824420	20B	B.1.1.519	30	5'UTR:T201C, 5'UTR:C203T, 5'UTR:C222T, 5'UTR:C241T, ORF1ab:C745T, ORF1ab:G1738T, ORF1ab:C3037T, ORF1ab:C3140T, ORF1ab:C10029T, ORF1ab:C10954T, ORF1ab:A11117G, ORF1ab:A11887G, ORF1ab:C12789T, ORF1ab:C14408T, ORF1ab:C16376T, ORF1ab:T19839C, ORF1ab:A19974G, ORF1ab:C21306T, S:C21575T, S:C22995A, S:A23403G, S:C23604A, S:A23756G, S:T25272C, ORF8:G28077T, ORF8:G28178T, N:G28881A, N:G28882A, N:G28883C, N:C29197T, N:C29272T,	16	N:R203K, N:G204R, ORF1a:P959S, ORF1a:T3255I, ORF1a:I3618V, ORF1a:T4175I, ORF1b:P314L, ORF1b:P970L, ORF8:V62L, ORF8:L95F, S:L5F, S:T478K, S:D614G, S:P681H, S:T732A, S:M1237T,
hCoV-19/Mexico/CMX-INER-INMEGEN-00067/2021 EPI_ISL_1824421	20B	B.1.1.519	26	5'UTR:C203T, 5'UTR:C222T, 5'UTR:C241T, ORF1ab:C3037T, ORF1ab:C3140T, ORF1ab:T6667C, ORF1ab:C7869T, ORF1ab:C10029T, ORF1ab:C10954T, ORF1ab:A11117G, ORF1ab:C12789T, ORF1ab:C14408T, ORF1ab:T19839C, ORF1ab:C21077T, ORF1ab:C21306T, S:C22088T, S:C22995A, S:A23403G, S:C23604A, S:A23756G, ORF3a:G25906T, E:C26270T, ORF8:C28253T, N:G28881A, N:G28882A, N:G28883C, N:C29197T,	16	E:T9I, N:R203K, N:G204R, ORF1a:P959S, ORF1a:S2535L, ORF1a:T3255I, ORF1a:I3618V, ORF1a:T4175I, ORF1b:P314L, ORF1b:T2537I, ORF3a:G172C, S:L176F, S:T478K, S:D614G, S:P681H, S:T732A,
hCoV-19/Mexico/CMX-INER-INMEGEN-00068/2021 EPI_ISL_1824422	20B	B.1.1.519	24	5'UTR:C203T, 5'UTR:C222T, 5'UTR:C241T, ORF1ab:A298G, ORF1ab:C3037T, ORF1ab:C3140T, ORF1ab:T4035G, ORF1ab:A6393C, ORF1ab:C10029T, ORF1ab:C10954T, ORF1ab:A11117G, ORF1ab:C12789T, ORF1ab:C14408T, ORF1ab:T19839C, ORF1ab:C21306T, S:C22995A, S:T23084C, S:A23403G, S:C23604A, S:A23756G, N:G28881A, N:G28882A, N:G28883C, N:C29095T, N:C29197T,	14	N:R203K, N:G204R, ORF1a:P959S, ORF1a:I1257S, ORF1a:D2043A, ORF1a:T3255I, ORF1a:I3618V, ORF1a:T4175I, ORF1b:P314L, S:T478K, S:Y508H, S:D614G, S:P681H, S:T732A,
hCoV-19/Mexico/CMX-INER-INMEGEN-00069/2021 EPI_ISL_1824423	20B	B.1.1.519	27	5'UTR:C203T, 5'UTR:C222T, 5'UTR:C241T, ORF1ab:G1681T, ORF1ab:C3037T, ORF1ab:C3140T, ORF1ab:T4339C, ORF1ab:C10029T, ORF1ab:C10954T, ORF1ab:A11117G, ORF1ab:C12789T, ORF1ab:C14408T, ORF1ab:C15525T, ORF1ab:G15906T, ORF1ab:C18508T, ORF1ab:T19839C, ORF1ab:C21306T, S:C22995A, S:A23403G, S:C23604A, S:A23756G, ORF3a:G25906T, ORF8:C28253T, N:G28851T, N:G28881A, N:G28882A, N:G28883C, N:C29197T	16	N:S193I, N:R203K, N:G204R, ORF1a:E472D, ORF1a:P959S, ORF1a:T3255I, ORF1a:I3618V, ORF1a:T4175I, ORF1b:P314L, ORF1b:Q813H, ORF1b:L1681F, ORF3a:G172C, S:T478K, S:D614G, S:P681H, S:T732A,
hCoV-19/Mexico/CMX-INER-INMEGEN-00070/2021 EPI_ISL_1824424	20B	B.1.1.519	26	5'UTR:C203T, 5'UTR:C222T, 5'UTR:C241T, ORF1ab:A1677G, ORF1ab:C3037T, ORF1ab:C3140T, ORF1ab:G7320A, ORF1ab:C10029T, ORF1ab:C10954T, ORF1ab:A11117G, ORF1ab:C12789T, ORF1ab:C14408T, ORF1ab:T19839C, ORF1ab:C21306T, S:C21844T, S:C22281T, S:C22995A, S:A23403G, S:C23604A, S:A23756G, ORF3a:G25429A, ORF8:T27904C, ORF8:C28087T, N:G28881A, N:G28882A, N:G28883C, N:C29197T,	17	N:R203K, N:G204R, ORF1a:E471G, ORF1a:P959S, ORF1a:S2352N, ORF1a:T3255I, ORF1a:I3618V, ORF1a:T4175I, ORF1b:P314L, ORF3a:V13I, ORF8:L4P, ORF8:A65V, S:T240I, S:T478K, S:D614G, S:P681H, S:T732A,

hCoV-19/Mexico/CMX-INER-INMEGEN-00071/2021 EPI_ISL_1824425	20B	B.1.1.519	26	5'UTR:C203T, 5'UTR:C222T, 5'UTR:C241T, ORF1ab:C3037T, ORF1ab:C3140T, ORF1ab:C10029T, ORF1ab:C10954T, ORF1ab:A11117G, ORF1ab:C12789T, ORF1ab:A12841G, ORF1ab:C14408T, ORF1ab:G15921T, ORF1ab:A17971G, ORF1ab:T19839C, ORF1ab:C21306T, S:A22034G, S:C22082T, S:C22995A, S:A23403G, S:C23604A, S:C23635T, S:A23756G, E:C26447T, N:G28881A, N:G28882A, N:G28883C, N:C29197T,	15	E:S68F, N:R203K, N:G204R, ORF1a:P959S, ORF1a:T3255I, ORF1a:I3618V, ORF1a:T4175I, ORF1b:P314L, ORF1b:R1502G, S:R158G, S:P174S, S:T478K, S:D614G, S:P681H, S:T732A,	
hCoV-19/Mexico/CMX-INER-INMEGEN-00072/2021 EPI_ISL_1824426	20B	B.1.1.519	24	5'UTR:C203T, 5'UTR:C222T, 5'UTR:C225T, 5'UTR:C241T, ORF1ab:C3037T, ORF1ab:C3140T, ORF1ab:G7037T, ORF1ab:C9893T, ORF1ab:C10029T, ORF1ab:C10954T, ORF1ab:A11117G, ORF1ab:C12789T, ORF1ab:C14408T, ORF1ab:T19839C, ORF1ab:C21306T, S:G21786T, S:C22995A, S:A23403G, S:C23604A, S:A23756G, ORF3a:C25782T, N:G28881A, N:G28882A, N:G28883C, N:C29197T,	14	N:R203K, N:G204R, ORF1a:P959S, ORF1a:G2258C, ORF1a:L3210F, ORF1a:T3255I, ORF1a:I3618V, ORF1a:T4175I, ORF1b:P314L, S:G75V, S:T478K, S:D614G, S:P681H, S:T732A,	
hCoV-19/Mexico/CMX-INER-INMEGEN-00073/2021 EPI_ISL_1824427	20B	B.1.1.519	29	5'UTR:C203T, 5'UTR:C222T, 5'UTR:C241T, ORF1ab:C2299T, ORF1ab:C3037T, ORF1ab:C3140T, ORF1ab:C5392T, ORF1ab:C6706T, ORF1ab:C10029T, ORF1ab:C10228T, ORF1ab:C10954T, ORF1ab:A11117G, ORF1ab:G11365T, ORF1ab:C12789T, ORF1ab:C14408T, ORF1ab:T19839C, ORF1ab:C21058T, ORF1ab:C21306T, S:C21575T, S:C22995A, S:A23403G, S:C23604A, S:A23756G, ORF3a:C25521T, N:G28881A, N:G28882A, N:G28883C, N:C29197T, N:G29527T, 3'UTR:C29733T,	14	N:R203K, N:G204R, N:Q418H, ORF1a:P959S, ORF1a:T3255I, ORF1a:I3618V, ORF1a:T4175I, ORF1b:P314L, ORF1b:P2531S, S:L5F, S:T478K, S:D614G, S:P681H, S:T732A,	
hCoV-19/Mexico/CMX-INER-INMEGEN-00074/2021 EPI_ISL_1970838	20B	B.1.1.519	25	5'UTR:C203T, 5'UTR:C222T, 5'UTR:C241T, ORF1ab:C2536T, ORF1ab:C3037T, ORF1ab:C3140T, ORF1ab:T4339C, ORF1ab:C6720T, ORF1ab:C10029T, ORF1ab:C10954T, ORF1ab:A11117G, ORF1ab:C12789T, ORF1ab:C14408T, ORF1ab:T19839C, ORF1ab:C21306T, S:C22995A, S:A23403G, S:C23604A, S:A23756G, ORF3a:G25906T, ORF7a:C27684T, ORF8:C28253T, N:G28881A, N:G28882A, N:G28883C, N:C29197T,	13	N:R203K, N:G204R, ORF1a:P959S, ORF1a:T2152I, ORF1a:T3255I, ORF1a:I3618V, ORF1a:T4175I, ORF1b:P314L, ORF3a:G172C, S:T478K, S:D614G, S:P681H, S:T732A,	ORF7a:27555-27624
hCoV-19/Mexico/CMX-INER-INMEGEN-00075/2021 EPI_ISL_1824428	20B	B.1.1.519	26	5'UTR:C203T, 5'UTR:C222T, 5'UTR:C241T, ORF1ab:C1613T, ORF1ab:C2434T, ORF1ab:C3037T, ORF1ab:C3140T, ORF1ab:C6027T, ORF1ab:C10029T, ORF1ab:C10954T, ORF1ab:A11117G, ORF1ab:C12473T, ORF1ab:C12789T, ORF1ab:C14408T, ORF1ab:T19839C, ORF1ab:C21306T, S:C22995A, S:A23403G, S:C23604A, S:A23756G, ORF3a:C25528T, ORF3a:G25563C, N:G28881A, N:G28882A, N:G28883C, N:C29197T, ORF10:C29627T,	15	N:R203K, N:G204R, ORF1a:L450F, ORF1a:P959S, ORF1a:P1921L, ORF1a:T3255I, ORF1a:I3618V, ORF1a:T4175I, ORF1b:P314L, ORF3a:L46F, ORF3a:Q57H, S:T478K, S:D614G, S:P681H, S:T732A,	
hCoV-19/Mexico/CMX-INER-INMEGEN-00076/2021 EPI_ISL_1824429	20B	B.1.1.519	25	5'UTR:T201C, 5'UTR:C203T, 5'UTR:C222T, 5'UTR:C241T, ORF1ab:C936T, ORF1ab:G1738T, ORF1ab:C3037T, ORF1ab:C3140T, ORF1ab:A8825G, ORF1ab:C10029T, ORF1ab:C10954T, ORF1ab:A11117G, ORF1ab:C12789T, ORF1ab:C14408T, ORF1ab:T19839C, ORF1ab:A19974G, ORF1ab:C21306T, S:C22995A, S:A23403G, S:C23604A, S:A23756G, N:G28881A, N:G28882A, N:G28883C, N:C29197T, N:G29227T,	13	N:R203K, N:G204R, ORF1a:T224I, ORF1a:P959S, ORF1a:I2854V, ORF1a:T3255I, ORF1a:I3618V, ORF1a:T4175I, ORF1b:P314L, S:T478K, S:D614G, S:P681H, S:T732A,	

hCoV-19/Mexico/CMX-INER-INMEGEN-00092/2021 EPI_ISL_1824442	20B	B.1.1.519	28	5'UTR:C203T, 5'UTR:C222T, 5'UTR:C241T, ORF1ab:C3037T, ORF1ab:C3140T, ORF1ab:C5869T, ORF1ab:C6568T, ORF1ab:C10029T, ORF1ab:C10954T, ORF1ab:A11117G, ORF1ab:C12789T, ORF1ab:C14408T, ORF1ab:C15194T, ORF1ab:T19839C, ORF1ab:G19872T, ORF1ab:G21204A, ORF1ab:C21306T, S:C22995A, S:A23403G, S:C23604A, S:A23756G, ORF7a:G27601T, ORF8:T27904C, ORF8:G27987T, ORF8:C28087T, N:G28881A, N:G28882A, N:G28883C, N:C29197T,	16	N:R203K, N:G204R, ORF1a:P959S, ORF1a:T3255I, ORF1a:I3618V, ORF1a:T4175I, ORF1b:P314L, ORF1b:A576V, ORF7a:G70C, ORF8:L4P, ORF8:V32L, ORF8:A65V, S:T478K, S:D614G, S:P681H, S:T732A,	
hCoV-19/Mexico/CMX-INER-INMEGEN-00093/2021 EPI_ISL_1824443	20B	B.1.1.519	28	5'UTR:T201C, 5'UTR:C203T, 5'UTR:C222T, 5'UTR:C241T, ORF1ab:C745T, ORF1ab:G1738T, ORF1ab:C3037T, ORF1ab:C3140T, ORF1ab:A8893G, ORF1ab:C10029T, ORF1ab:C10954T, ORF1ab:A11117G, ORF1ab:C12789T, ORF1ab:C14408T, ORF1ab:T15195A, ORF1ab:T19839C, ORF1ab:A19974G, ORF1ab:C21306T, S:C22995A, S:A23403G, S:C23604A, S:A23756G, ORF3a:C26032T, ORF8:C28253T, N:C28770T, N:G28881A, N:G28882A, N:G28883C, N:C29197T	12	N:T166I, N:R203K, N:G204R, ORF1a:P959S, ORF1a:T3255I, ORF1a:I3618V, ORF1a:T4175I, ORF1b:P314L, S:T478K, S:D614G, S:P681H, S:T732A,	
hCoV-19/Mexico/CMX-INER-INMEGEN-00094/2021 EPI_ISL_1824444	20B	B.1.1.519	28	5'UTR:T201C, 5'UTR:C203T, 5'UTR:C222T, 5'UTR:C241T, ORF1ab:G1738T, ORF1ab:C2892T, ORF1ab:C3037T, ORF1ab:C3140T, ORF1ab:A5845C, ORF1ab:C6026T, ORF1ab:C10029T, ORF1ab:C10954T, ORF1ab:A11117G, ORF1ab:C12789T, ORF1ab:C14408T, ORF1ab:T19839C, ORF1ab:A19974G, ORF1ab:C21306T, S:C22995A, S:A23403G, S:C23604A, S:A23756G, ORF7b:C27804T, N:G28881A, N:G28882A, N:G28883C, N:C29197T, N:C29421T, ORF10:C29627T,	15	N:R203K, N:G204R, N:P383L, ORF1a:A876V, ORF1a:P959S, ORF1a:K1860N, ORF1a:P1921S, ORF1a:T3255I, ORF1a:I3618V, ORF1a:T4175I, ORF1b:P314L, S:T478K, S:D614G, S:P681H, S:T732A,	
hCoV-19/Mexico/CMX-INER-INMEGEN-00095/2021 EPI_ISL_1970842	20B	B.1.1.222	18	5'UTR:C241T, ORF1ab:C3037T, ORF1ab:C7749T, ORF1ab:C8637T, ORF1ab:C12068T, ORF1ab:A13498G, ORF1ab:C14408T, ORF1ab:T19839C, S:G22331A, S:C22591T, S:G23285T, S:A23403G, S:G23593T, S:A23756G, ORF3a:C26124T, N:G28881A, N:G28882A, N:G28883C, N:G28907A,	12	N:R203K, N:G204R, N:G212S, ORF1a:T2495I, ORF1a:T2791I, ORF1b:T11A, ORF1b:P314L, S:G257S, S:A575S, S:D614G, S:Q677H, S:T732A,	ORF7b:27879-27891, ORF8:27915-28225,
hCoV-19/Mexico/CMX-INER-INMEGEN-00096/2021 EPI_ISL_1970843	20B	B.1.1.222	18	5'UTR:C241T, ORF1ab:C3037T, ORF1ab:C7749T, ORF1ab:C8637T, ORF1ab:C12068T, ORF1ab:A13498G, ORF1ab:C14408T, ORF1ab:T19839C, S:G22331A, S:C22591T, S:G23285T, S:A23403G, S:G23593T, S:A23756G, ORF3a:C26124T, N:G28881A, N:G28882A, N:G28883C, N:G28907A,	12	N:R203K, N:G204R, N:G212S, ORF1a:T2495I, ORF1a:T2791I, ORF1b:T11A, ORF1b:P314L, S:G257S, S:A575S, S:D614G, S:Q677H, S:T732A,	ORF7b:27879-27891, ORF8:27915-28225,
hCoV-19/Mexico/CMX-INER-INMEGEN-00097/2021 EPI_ISL_1970844	20B	B.1.1.222	18	5'UTR:C241T, ORF1ab:C3037T, ORF1ab:C7749T, ORF1ab:C8637T, ORF1ab:C12068T, ORF1ab:A13498G, ORF1ab:C14408T, ORF1ab:T19839C, S:G22331A, S:C22591T, S:G23285T, S:A23403G, S:G23593T, S:A23756G, ORF3a:C26124T, N:G28881A, N:G28882A, N:G28883C, N:G28907A,	12	N:R203K, N:G204R, N:G212S, ORF1a:T2495I, ORF1a:T2791I, ORF1b:T11A, ORF1b:P314L, S:G257S, S:A575S, S:D614G, S:Q677H, S:T732A,	ORF7b:27879-27891, ORF8:27915-28225,
hCoV-19/Mexico/CMX-INER-INMEGEN-00098/2021 EPI_ISL_1970845	20B	B.1.1.222	18	5'UTR:C241T, ORF1ab:C3037T, ORF1ab:C7749T, ORF1ab:C8637T, ORF1ab:C12068T, ORF1ab:A13498G, ORF1ab:C14408T, ORF1ab:T19839C, S:G22331A, S:C22591T, S:G23285T, S:A23403G, S:G23593T, S:A23756G, ORF3a:C26124T, N:G28881A, N:G28882A, N:G28883C, N:G28907A,	12	N:R203K, N:G204R, N:G212S, ORF1a:T2495I, ORF1a:T2791I, ORF1b:T11A, ORF1b:P314L, S:G257S, S:A575S, S:D614G, S:Q677H, S:T732A,	ORF7b:27879-27891, ORF8:27915-28225,

hCoV-19/Mexico/CMX-INER-INMEGEN-00099/2021 EPI_ISL_1824445	20B	B.1.1.519	29	5'UTR:C203T, 5'UTR:C222T, 5'UTR:C241T, ORF1ab:C3037T, ORF1ab:C3140T, ORF1ab:T3745C, ORF1ab:A3879G, ORF1ab:C8016T, ORF1ab:C10029T, ORF1ab:C10954T, ORF1ab:A11117G, ORF1ab:G11291A, ORF1ab:T12182A, ORF1ab:C12473T, ORF1ab:C12789T, ORF1ab:C14408T, ORF1ab:T19839C, ORF1ab:C21306T, S:C22995A, S:A23403G, S:C23604A, S:A23756G, ORF3a:C25844T, ORF7a:G27709T, N:C28849T, N:G28881A, N:G28882A, N:G28883C, N:C29197T, N:A29419C	18	N:R203K, N:G204R, N:L382F, ORF1a:P959S, ORF1a:E1205G, ORF1a:A2584V, ORF1a:T3255I, ORF1a:I3618V, ORF1a:G3676S, ORF1a:S3973T, ORF1a:T4175I, ORF1b:P314L, ORF3a:T151I, ORF7a:A106S, S:T478K, S:D614G, S:P681H, S:T732A,
hCoV-19/Mexico/CMX-INER-INMEGEN-00100/2021 EPI_ISL_1824446	20B	B.1.1.519	27	5'UTR:C203T, 5'UTR:C222T, 5'UTR:C241T, ORF1ab:C3037T, ORF1ab:C3140T, ORF1ab:C8752T, ORF1ab:C10029T, ORF1ab:C10954T, ORF1ab:A11117G, ORF1ab:C12789T, ORF1ab:A14199T, ORF1ab:C14408T, ORF1ab:C15081T, ORF1ab:G19272T, ORF1ab:T19839C, ORF1ab:C21306T, S:C22995A, S:A23403G, S:C23604A, S:A23756G, S:A24727G, S:C25318T, ORF8:T27904C, ORF8:C28087T, N:G28881A, N:G28882A, N:G28883C, N:C29197T,	14	N:R203K, N:G204R, ORF1a:P959S, ORF1a:T3255I, ORF1a:I3618V, ORF1a:T4175I, ORF1b:P314L, ORF1b:L1935F, ORF8:L4P, ORF8:A65V, S:T478K, S:D614G, S:P681H, S:T732A,
hCoV-19/Mexico/CMX-INER-INMEGEN-00101/2021 EPI_ISL_1824447	20B	B.1.1.519	25	5'UTR:C203T, 5'UTR:C222T, 5'UTR:C241T, ORF1ab:C3037T, ORF1ab:C3140T, ORF1ab:C4246T, ORF1ab:C10029T, ORF1ab:C10954T, ORF1ab:A11117G, ORF1ab:C12789T, ORF1ab:C14408T, ORF1ab:T19839C, ORF1ab:C21306T, S:G21867C, S:C22181T, S:C22995A, S:A23403G, S:C23604A, S:A23756G, M:G26526A, ORF8:T28098C, N:G28881A, N:G28882A, N:G28883C, N:C29197T, N:G29527T,	16	M:A2T, N:R203K, N:G204R, N:Q418H, ORF1a:P959S, ORF1a:T3255I, ORF1a:I3618V, ORF1a:T4175I, ORF1b:P314L, ORF8:S69P, S:R102T, S:H207Y, S:T478K, S:D614G, S:P681H, S:T732A,
hCoV-19/Mexico/CMX-INER-INMEGEN-00102/2021 EPI_ISL_1824448	20B	B.1.1.519	26	5'UTR:T201C, 5'UTR:C203T, 5'UTR:C222T, 5'UTR:C241T, ORF1ab:C584T, ORF1ab:G1738T, ORF1ab:C3037T, ORF1ab:C3140T, ORF1ab:C10029T, ORF1ab:G10610A, ORF1ab:C10954T, ORF1ab:A11117G, ORF1ab:C12789T, ORF1ab:C14408T, ORF1ab:A17993G, ORF1ab:T19839C, ORF1ab:A19974G, ORF1ab:C21306T, S:C22995A, S:A23403G, S:C23604A, S:A23756G, ORF3a:C25553T, N:G28881A, N:G28882A, N:G28883C, N:C29197T,	15	N:R203K, N:G204R, ORF1a:L107F, ORF1a:P959S, ORF1a:T3255I, ORF1a:V3449I, ORF1a:I3618V, ORF1a:T4175I, ORF1b:P314L, ORF1b:Q1509R, ORF3a:A54V, S:T478K, S:D614G, S:P681H, S:T732A,
hCoV-19/Mexico/CMX-INER-INMEGEN-00103/2021 EPI_ISL_1824449	20B	B.1.1.519	32	5'UTR:T201C, 5'UTR:C203T, 5'UTR:C222T, 5'UTR:C241T, ORF1ab:C936T, ORF1ab:G1738T, ORF1ab:C3037T, ORF1ab:C3140T, ORF1ab:A3210G, ORF1ab:T9089G, ORF1ab:C10029T, ORF1ab:C10954T, ORF1ab:A11117G, ORF1ab:C12525T, ORF1ab:C12789T, ORF1ab:C14408T, ORF1ab:G14677A, ORF1ab:T16342C, ORF1ab:G17302T, ORF1ab:T19839C, ORF1ab:A19974G, ORF1ab:C21306T, S:C22995A, S:A23403G, S:C23604A, S:A23756G, S:C23896T, ORF3a:C25721T, N:G28881A, N:G28882A, N:G28883C, N:C29197T, N:G29227T	19	N:R203K, N:G204R, ORF1a:T224I, ORF1a:P959S, ORF1a:D982G, ORF1a:S2942A, ORF1a:T3255I, ORF1a:I3618V, ORF1a:T4087I, ORF1a:T4175I, ORF1b:P314L, ORF1b:G404S, ORF1b:S959P, ORF1b:V1279F, ORF3a:A110V, S:T478K, S:D614G, S:P681H, S:T732A,

hCoV-19/Mexico/CMX-INER-INMEGEN-00104/2021	EPI_ISL_1824450	20B	B.1.1.519	30	5'UTR:T201C, 5'UTR:C203T, 5'UTR:C222T, 5'UTR:C241T, ORF1ab:C936T, ORF1ab:G1738T, ORF1ab:C3037T, ORF1ab:C3140T, ORF1ab:T7106C, ORF1ab:C10029T, ORF1ab:C10954T, ORF1ab:A11117G, ORF1ab:C12789T, ORF1ab:C14408T, ORF1ab:G18067T, ORF1ab:T19839C, ORF1ab:A19974G, ORF1ab:C21306T, S:C22995A, S:A23403G, S:C23604A, S:A23756G, ORF3a:G25429T, ORF3a:C25626T, ORF7b:C27769T, N:G28881A, N:G28882A, N:G28883C, N:C29197T, N:G29227T, N:C29311T	16	N:R203K, N:G204R, ORF1a:T224I, ORF1a:P959S, ORF1a:Y2281H, ORF1a:T3255I, ORF1a:I3618V, ORF1a:T4175I, ORF1b:P314L, ORF1b:D1534Y, ORF3a:V13L, ORF7b:S5L, S:T478K, S:D614G, S:P681H, S:T732A,	
hCoV-19/Mexico/CMX-INER-SC239/2021	EPI_ISL_1040927	20B	B.1.1.519	26	5'UTR:C203T, 5'UTR:C222T, 5'UTR:C241T, ORF1ab:C3037T, ORF1ab:C3140T, ORF1ab:T8104C, ORF1ab:C10029T, ORF1ab:A10829C, ORF1ab:C10954T, ORF1ab:A11117G, ORF1ab:C11152T, ORF1ab:C12789T, ORF1ab:C14408T, ORF1ab:A17585G, ORF1ab:T19839C, ORF1ab:T19935A, S:C22995A, S:A23403G, S:C23604A, S:A23756G, ORF3a:G25540A, ORF8:T27904C, ORF8:C28087T, N:G28881A, N:G28882A, N:G28883C, N:C29197T,	16	N:R203K, N:G204R, ORF1a:P959S, ORF1a:T3255I, ORF1a:I3522L, ORF1a:I3618V, ORF1a:T4175I, ORF1b:P314L, ORF1b:D1373G, ORF3a:V50I, ORF8:L4P, ORF8:A65V, S:T478K, S:D614G, S:P681H, S:T732A,	5'UTR:3 8-42
hCoV-19/Mexico/CMX-INER-SC480/2021	EPI_ISL_1672849	20B	B.1.1.519	30	5'UTR:A29C, 5'UTR:C203T, 5'UTR:C222T, 5'UTR:C241T, ORF1ab:A5019T, ORF1ab:C5020G, ORF1ab:C5544A, ORF1ab:C7819T, ORF1ab:G8351T, ORF1ab:A8358C, ORF1ab:C10029T, ORF1ab:C10954T, ORF1ab:A11117G, ORF1ab:A12180G, ORF1ab:C12789T, ORF1ab:C14408T, ORF1ab:T16211G, ORF1ab:C17135T, ORF1ab:T19299G, ORF1ab:T19839C, ORF1ab:C21306T, S:C22995A, S:A23403G, S:C23604A, S:A23756G, N:G28881A, N:G28882A, N:G28883C, N:C29077T, N:C29197T, N:C29495A	17	N:R203K, N:G204R, N:Q408K, ORF1a:D1585V, ORF1a:A2696S, ORF1a:T3255I, ORF1a:I3618V, ORF1a:D3972G, ORF1a:T4175I, ORF1b:P314L, ORF1b:M915R, ORF1b:P1223L, ORF1b:Y1944*, S:T478K, S:D614G, S:P681H, S:T732A,	
hCoV-19/Mexico/CMX-INER-SC483/2021	EPI_ISL_1672864	20B	B.1.1.519	43	5'UTR:T201C, 5'UTR:C203T, 5'UTR:C222T, 5'UTR:C241T, ORF1ab:G1738T, ORF1ab:C2925T, ORF1ab:A2966T, ORF1ab:C3037T, ORF1ab:A4426G, ORF1ab:T4427G, ORF1ab:A4429G, ORF1ab:A4430C, ORF1ab:C4434T, ORF1ab:G5516A, ORF1ab:T7375C, ORF1ab:C10029T, ORF1ab:C10954T, ORF1ab:A11117G, ORF1ab:C12789T, ORF1ab:T13878A, ORF1ab:C14408T, ORF1ab:T16211G, ORF1ab:G17427T, ORF1ab:T19299G, ORF1ab:T19839C, ORF1ab:A19974G, ORF1ab:C21306T, S:C22995A, S:A23403G, S:C23604A, S:A23756G, S:T23776G, S:T23787C, S:T23788C, S:G24390C, ORF3a:C25460T, ORF3a:C25553T, M:C26768T, ORF7b:C27864T, ORF8:C28253T, N:G28881A, N:G28882A, N:G28883C, N:C29197T,	24	N:R203K, N:G204R, ORF1a:S887F, ORF1a:S901C, ORF1a:L1388V, ORF1a:M1389L, ORF1a:P1390L, ORF1a:V1751M, ORF1a:T3255I, ORF1a:I3618V, ORF1a:T4175I, ORF1b:P314L, ORF1b:M915R, ORF1b:Y1944*, ORF3a:A23V, ORF3a:A54V, ORF7b:H37Y, S:T478K, S:D614G, S:P681H, S:T732A, S:C738W, S:I742T, S:S943T,	

hCoV-19/Mexico/CMX-INER-SC486/2021	EPI_ISL_1672865	20B	B.1.1.519	67	5'UTR:T201C, 5'UTR:C203T, 5'UTR:C222T, 5'UTR:C241T, ORF1ab:T727A, ORF1ab:A729G, ORF1ab:A730T, ORF1ab:A732C, ORF1ab:T734G, ORF1ab:C745T, ORF1ab:G1738T, ORF1ab:T2904G, ORF1ab:C3037T, ORF1ab:C3040T, ORF1ab:C3041T, ORF1ab:G3119A, ORF1ab:C5140T, ORF1ab:T5185A, ORF1ab:T5266A, ORF1ab:T5292C, ORF1ab:G5387A, ORF1ab:T5389A, ORF1ab:C5997T, ORF1ab:C6078T, ORF1ab:T6142C, ORF1ab:C8344G, ORF1ab:A8348T, ORF1ab:G8351T, ORF1ab:G8353T, ORF1ab:C10029T, ORF1ab:C10954T, ORF1ab:A11117G, ORF1ab:A11887G, ORF1ab:C12789T, ORF1ab:C14408T, ORF1ab:T16211G, ORF1ab:A16214G, ORF1ab:G17064C, ORF1ab:A18253C, ORF1ab:T19299G, ORF1ab:A19548T, ORF1ab:T19839C, ORF1ab:T19940A, ORF1ab:A19974G, ORF1ab:C21306T, S:C22347G, S:A22359G, S:G22361A, S:T22378C, S:C22388T, S:T22389C, S:C22995A, S:C23191T, S:A23403G, S:C23525G, S:C23604A, S:A23756G, S:T23788C, ORF3a:T25674G, ORF3a:T25675G, ORF3a:T25829A, ORF8:G28077T, ORF8:G28178T, N:G28881A, N:G28882A, N:G28883C, N:C29197T, N:C29272T,	39	N:R203K, N:G204R, ORF1a:Y154*, ORF1a:E155G, ORF1a:D156A, ORF1a:F157V, ORF1a:I880R, ORF1a:P926S, ORF1a:E952K, ORF1a:L1676P, ORF1a:A1708T, ORF1a:P1911L, ORF1a:A1938V, ORF1a:D2693E, ORF1a:S2695C, ORF1a:A2696S, ORF1a:T3255I, ORF1a:I3618V, ORF1a:T4175I, ORF1b:P314L, ORF1b:M915R, ORF1b:Y916C, ORF1b:K1199N, ORF1b:M1596L, ORF1b:Y1944*, ORF1b:I2158K, ORF3a:L95V, ORF3a:F146Y, ORF8:V62L, ORF8:L95F, S:A262G, S:Y266C, S:V267M, S:L276S, S:T478K, S:D614G, S:H655D, S:P681H, S:T732A,	
hCoV-19/Mexico/CMX-INER-SC498/2021	EPI_ISL_1672866	20B	B.1.1.519	31	5'UTR:C203T, 5'UTR:C222T, 5'UTR:C241T, ORF1ab:C475T, ORF1ab:A3039G, ORF1ab:C3044T, ORF1ab:A3138C, ORF1ab:A5459T, ORF1ab:C8344G, ORF1ab:G8861A, ORF1ab:C10029T, ORF1ab:C10954T, ORF1ab:A11117G, ORF1ab:C12701T, ORF1ab:C12789T, ORF1ab:C14408T, ORF1ab:G14831T, ORF1ab:T16211G, ORF1ab:G16322C, ORF1ab:C16323T, ORF1ab:T19299G, ORF1ab:T19839C, ORF1ab:C21306T, S:C22995A, S:A23403G, S:C23604A, S:A23756G, N:C28854T, N:G28881A, N:G28882A, N:G28883C, N:C29197T,	21	N:S194L, N:R203K, N:G204R, ORF1a:Y925C, ORF1a:K958T, ORF1a:M1732L, ORF1a:D2693E, ORF1a:V2866M, ORF1a:T3255I, ORF1a:I3618V, ORF1a:P4146S, ORF1a:T4175I, ORF1b:P314L, ORF1b:C455F, ORF1b:M915R, ORF1b:C952S, ORF1b:Y1944*, S:T478K, S:D614G, S:P681H, S:T732A,	
hCoV-19/Mexico/CMX-INMEGEN-01/2020	EPI_ISL_522872	20C	B.1	11	5'UTR:C241T, ORF1ab:C1059T, ORF1ab:C3037T, ORF1ab:C4331T, ORF1ab:C11152T, ORF1ab:C14408T, ORF1ab:C18118T, S:A23403G, ORF3a:G25563T, N:C28887T, ORF10:C29614T, 3'UTR:C29750T,	6	N:T205I, ORF1a:T265I, ORF1b:P314L, ORF1b:L1551F, ORF3a:Q57H, S:D614G,	ORF7b:27792-27793,
hCoV-19/Mexico/CMX-INMEGEN-01-01-01/2020	EPI_ISL_1005656	20B	B.1.1.222	17	5'UTR:C241T, ORF1ab:C2061T, ORF1ab:C3037T, ORF1ab:C11824T, ORF1ab:A12527G, ORF1ab:G13723T, ORF1ab:C14408T, ORF1ab:C15960T, ORF1ab:G17395A, ORF1ab:T19839C, S:G22249T, S:A23403G, S:A23756G, ORF8:G28083T, N:G28881A, N:G28882A, N:G28883C, N:A28996G,	11	N:R203K, N:G204R, ORF1a:A599V, ORF1a:T4088A, ORF1b:A86S, ORF1b:P314L, ORF1b:V1310I, ORF8:E64*, S:L229F, S:D614G, S:T732A,	S:21765-21770,
hCoV-19/Mexico/CMX-INMEGEN-01-01-02/2020	EPI_ISL_1005657	20B	B.1.1.222	15	5'UTR:C241T, ORF1ab:C2061T, ORF1ab:C3037T, ORF1ab:C11824T, ORF1ab:G13723T, ORF1ab:C14408T, ORF1ab:G17395A, ORF1ab:C18457T, ORF1ab:T19839C, S:A23403G, S:A23756G, ORF8:G28083T, N:G28881A, N:G28882A, N:G28883C, N:A28996G,	10	N:R203K, N:G204R, ORF1a:A599V, ORF1b:A86S, ORF1b:P314L, ORF1b:V1310I, ORF1b:P1664S, ORF8:E64*, S:D614G, S:T732A,	S:21765-21770,
hCoV-19/Mexico/CMX-INMEGEN-01-01-03/2020	EPI_ISL_1005658	20B	B.1.1.222	16	5'UTR:C241T, ORF1ab:C2061T, ORF1ab:C3037T, ORF1ab:C11824T, ORF1ab:G13723T, ORF1ab:C14220T, ORF1ab:C14408T, ORF1ab:C15960T, ORF1ab:G17395A, ORF1ab:T19839C, S:A23403G, S:A23756G, ORF8:G28083T, N:G28881A, N:G28882A, N:G28883C, N:A28996G,	9	N:R203K, N:G204R, ORF1a:A599V, ORF1b:A86S, ORF1b:P314L, ORF1b:V1310I, ORF8:E64*, S:D614G, S:T732A,	S:21765-21770,

hCoV-19/Mexico/CMX-INMEGEN-01-01-04/2020	EPI_ISL_1005659	20B	B.1.1.222	15	5'UTR:C241T, ORF1ab:C2061T, ORF1ab:C3037T, ORF1ab:C11824T, ORF1ab:G13723T, ORF1ab:C14408T, ORF1ab:C15960T, ORF1ab:G17395A, ORF1ab:T19839C, S:A23403G, S:A23756G, ORF8:G28083T, N:G28881A, N:G28882A, N:G28883C, N:A28996G,	9	N:R203K, N:G204R, ORF1a:A599V, ORF1b:A86S, ORF1b:P314L, ORF1b:V1310I, ORF8:E64*, S:D614G, S:T732A,	S:21765-21770,
hCoV-19/Mexico/CMX-INMEGEN-01-01-07/2020	EPI_ISL_1005660	20B	B.1.1.222	15	5'UTR:C241T, ORF1ab:C2061T, ORF1ab:C3037T, ORF1ab:C11824T, ORF1ab:G13723T, ORF1ab:C14408T, ORF1ab:C15960T, ORF1ab:G17395A, ORF1ab:T19839C, S:A23403G, S:A23756G, ORF8:G28083T, N:G28881A, N:G28882A, N:G28883C, N:A28996G,	9	N:R203K, N:G204R, ORF1a:A599V, ORF1b:A86S, ORF1b:P314L, ORF1b:V1310I, ORF8:E64*, S:D614G, S:T732A,	S:21765-21770,
hCoV-19/Mexico/CMX-INMEGEN-01-01-11/2020	EPI_ISL_1005661	20B	B.1.1.519	22	5'UTR:C203T, 5'UTR:C222T, 5'UTR:C241T, ORF1ab:C3037T, ORF1ab:C3140T, ORF1ab:C10029T, ORF1ab:C10954T, ORF1ab:A11117G, ORF1ab:C12789T, ORF1ab:G13571T, ORF1ab:C14408T, ORF1ab:G17562T, ORF1ab:T19839C, ORF1ab:C21306T, S:C22995A, S:A23403G, S:C23604A, S:A23756G, M:G26824A, N:G28881A, N:G28882A, N:G28883C, N:C29197T,	13	M:R101K, N:R203K, N:G204R, ORF1a:P959S, ORF1a:T3255I, ORF1a:I3618V, ORF1a:T4175I, ORF1b:G35V, ORF1b:P314L, S:T478K, S:D614G, S:P681H, S:T732A,	
hCoV-19/Mexico/CMX-INMEGEN-01-01-12/2020	EPI_ISL_1005662	20B	B.1.1	20	5'UTR:C190T, 5'UTR:C241T, ORF1ab:C3037T, ORF1ab:C9967T, ORF1ab:C10440T, ORF1ab:G11083T, ORF1ab:G12734A, ORF1ab:C14408T, ORF1ab:T19839C, ORF1ab:C20759T, S:G22487C, S:A23403G, S:C23604A, ORF3a:C25521T, ORF3a:G25522A, ORF7b:G27890A, N:G28881A, N:G28882A, N:G28883C, 3'UTR:C29733T, 3'UTR:G29742T,	11	N:R203K, N:G204R, ORF1a:A3392V, ORF1a:L3606F, ORF1a:G4157S, ORF1b:P314L, ORF1b:A2431V, ORF3a:G44R, S:E309Q, S:D614G, S:P681H,	
hCoV-19/Mexico/CMX-INMEGEN-01-01-13/2020	EPI_ISL_1005663	20B	B.1.1.519	23	5'UTR:C203T, 5'UTR:C222T, 5'UTR:C241T, ORF1ab:C3037T, ORF1ab:C3140T, ORF1ab:T9070C, ORF1ab:C10029T, ORF1ab:C10954T, ORF1ab:A11117G, ORF1ab:C12789T, ORF1ab:C14184T, ORF1ab:C14408T, ORF1ab:T19839C, ORF1ab:C21306T, S:C22995A, S:A23403G, S:C23604A, S:A23756G, N:G28881A, N:G28882A, N:G28883C, N:C29197T, N:A29433G, N:G29527T,	13	N:R203K, N:G204R, N:K387R, N:Q418H, ORF1a:P959S, ORF1a:T3255I, ORF1a:I3618V, ORF1a:T4175I, ORF1b:P314L, S:T478K, S:D614G, S:P681H, S:T732A,	
hCoV-19/Mexico/CMX-INMEGEN-01-01-14/2020	EPI_ISL_1005664	20B	B.1.1.222	18	5'UTR:C241T, ORF1ab:C3037T, ORF1ab:C4897T, ORF1ab:C5259T, ORF1ab:C10029T, ORF1ab:A11430G, ORF1ab:C14408T, ORF1ab:C19011A, ORF1ab:T19839C, ORF1ab:A21137G, S:A23403G, S:A23756G, ORF3a:G25912T, ORF3a:A26115C, ORF8:A27921G, ORF8:G28001T, N:G28881A, N:G28882A, N:G28883C,	13	N:R203K, N:G204R, ORF1a:T1665I, ORF1a:T3255I, ORF1a:Y3722C, ORF1b:P314L, ORF1b:D1848E, ORF1b:K2557R, ORF3a:G174C, ORF3a:E241D, ORF8:I10V, S:D614G, S:T732A,	
hCoV-19/Mexico/CMX-INMEGEN-01-01-15/2020	EPI_ISL_1005665	20B	B.1.1.222	15	5'UTR:C241T, ORF1ab:C865T, ORF1ab:G1401A, ORF1ab:C3037T, ORF1ab:C10029T, ORF1ab:C14408T, ORF1ab:T19839C, S:A23403G, S:A23756G, ORF3a:G25912T, ORF7b:A27756G, ORF8:A27921G, ORF8:G28001T, N:G28881A, N:G28882A, N:G28883C,	10	N:R203K, N:G204R, ORF1a:G379E, ORF1a:T3255I, ORF1b:P314L, ORF3a:G174C, ORF7b:M1V, ORF8:I10V, S:D614G, S:T732A,	3'UTR:29746-29762,
hCoV-19/Mexico/CMX-INMEGEN-01-01-16/2020	EPI_ISL_1005666	20B	B.1.1.519	23	5'UTR:C203T, 5'UTR:C222T, 5'UTR:C241T, ORF1ab:C3037T, ORF1ab:C3140T, ORF1ab:C10029T, ORF1ab:C10954T, ORF1ab:A11117G, ORF1ab:T12027C, ORF1ab:C12789T, ORF1ab:C14408T, ORF1ab:T19839C, ORF1ab:C21306T, S:C21707T, S:C22995A, S:A23403G, S:C23604A, S:A23756G, M:A26748G, N:G28881A, N:G28882A, N:G28883C, N:C29197T, N:G29527T,	15	M:I76V, N:R203K, N:G204R, N:Q418H, ORF1a:P959S, ORF1a:T3255I, ORF1a:I3618V, ORF1a:M3921T, ORF1a:T4175I, ORF1b:P314L, S:H49Y, S:T478K, S:D614G, S:P681H, S:T732A,	

hCoV-19/Mexico/CMX-INMEGEN-01-01-17/2020	EPI_ISL_1005667	20B	B.1.1.519	20	5'UTR:C203T, 5'UTR:C222T, 5'UTR:C241T, ORF1ab:C3037T, ORF1ab:C3140T, ORF1ab:C10029T, ORF1ab:C10954T, ORF1ab:A11117G, ORF1ab:C12789T, ORF1ab:C14408T, ORF1ab:T19839C, ORF1ab:C21306T, S:C22995A, S:A23403G, S:C23604A, S:A23756G, N:G28881A, N:G28882A, N:G28883C, N:C29197T, N:G29527T,	12	N:R203K, N:G204R, N:Q418H, ORF1a:P959S, ORF1a:T3255I, ORF1a:I3618V, ORF1a:T4175I, ORF1b:P314L, S:T478K, S:D614G, S:P681H, S:T732A,
hCoV-19/Mexico/CMX-INMEGEN-01-01-18/2020	EPI_ISL_1005668	20A	B.1.396	23	5'UTR:C241T, ORF1ab:C1063T, ORF1ab:C1191T, ORF1ab:C3037T, ORF1ab:C6318T, ORF1ab:C7420T, ORF1ab:C9521A, ORF1ab:G10793T, ORF1ab:G14187A, ORF1ab:C14408T, ORF1ab:A15861G, ORF1ab:A19002G, ORF1ab:A20268G, S:A23403G, S:C24904T, ORF6:C27297T, ORF7a:G27676C, ORF8:C27970T, ORF8:C28087T, N:G28378T, N:C28854T, N:G29422T, ORF10:A29567G, 3'UTR:G29706T,	11	N:S194L, ORF1a:P309L, ORF1a:P2018L, ORF1a:L3086I, ORF1a:V3510F, ORF1b:P314L, ORF7a:E95Q, ORF8:T26I, ORF8:A65V, ORF9b:R32L, S:D614G,
hCoV-19/Mexico/CMX-INMEGEN-01-01-20/2020	EPI_ISL_1005669	20B	B.1.1.519	23	5'UTR:T201C, 5'UTR:C203T, 5'UTR:C222T, 5'UTR:C241T, ORF1ab:G1738T, ORF1ab:C3037T, ORF1ab:C3140T, ORF1ab:C10029T, ORF1ab:C10954T, ORF1ab:A11117G, ORF1ab:C12789T, ORF1ab:C14408T, ORF1ab:T19839C, ORF1ab:A19974G, ORF1ab:C21306T, S:C22995A, S:A23403G, S:C23604A, S:A23756G, ORF8:A28175G, N:G28881A, N:G28882A, N:G28883C, N:C29197T,	11	N:R203K, N:G204R, ORF1a:P959S, ORF1a:T3255I, ORF1a:I3618V, ORF1a:T4175I, ORF1b:P314L, S:T478K, S:D614G, S:P681H, S:T732A,
hCoV-19/Mexico/CMX-INMEGEN-01-01-21/2021	EPI_ISL_1005670	20B	B.1.1.222	20	5'UTR:C241T, ORF1ab:C1471T, ORF1ab:C3037T, ORF1ab:C4206T, ORF1ab:C9967T, ORF1ab:C10029T, ORF1ab:A11556G, ORF1ab:C14408T, ORF1ab:G17019T, ORF1ab:C19011A, ORF1ab:G19086T, ORF1ab:C19554T, ORF1ab:T19839C, S:A23403G, S:A23756G, ORF3a:G25912T, ORF8:A27921G, ORF8:G28001T, N:G28881A, N:G28882A, N:G28883C,	13	N:R203K, N:G204R, ORF1a:A1314V, ORF1a:T3255I, ORF1a:E3764G, ORF1b:P314L, ORF1b:E1184D, ORF1b:D1848E, ORF1b:K1873N, ORF3a:G174C, ORF8:I10V, S:D614G, S:T732A,
hCoV-19/Mexico/CMX-INMEGEN-01-01-22/2020	EPI_ISL_1005671	20B	B.1.1.432	26	5'UTR:C241T, ORF1ab:C3037T, ORF1ab:G3122T, ORF1ab:G6368A, ORF1ab:A6985T, ORF1ab:C9319T, ORF1ab:C10369T, ORF1ab:C12412T, ORF1ab:C12784T, ORF1ab:T14313C, ORF1ab:C14408T, ORF1ab:T16542C, ORF1ab:G20098T, S:G21770T, S:C22127T, S:A23403G, S:C24382T, ORF3a:C25613T, ORF3a:G25912T, N:G28881A, N:G28882A, N:G28883C, N:C28948T, N:C29149T, N:C29167T, N:T29317C, ORF10:C29585T,	11	N:R203K, N:G204R, ORF1a:D953Y, ORF1a:G2035R, ORF1b:P314L, ORF1b:A2211S, ORF3a:S74F, ORF3a:G174C, S:V70F, S:L189F, S:D614G,
hCoV-19/Mexico/CMX-INMEGEN-01-01-23/2021	EPI_ISL_1005672	20B	B.1.1.432	20	5'UTR:C241T, ORF1ab:A1732G, ORF1ab:C3037T, ORF1ab:C3695T, ORF1ab:G5194T, ORF1ab:A6659G, ORF1ab:A6985T, ORF1ab:C9319T, ORF1ab:C11379T, ORF1ab:C12412T, ORF1ab:T14313C, ORF1ab:C14408T, S:A23403G, ORF3a:C25613T, ORF3a:G25912T, ORF8:G28077T, N:C28744T, N:G28881A, N:G28882A, N:G28883C, N:T29317C,	9	N:R203K, N:G204R, ORF1a:S2132G, ORF1a:A3705V, ORF1b:P314L, ORF3a:S74F, ORF3a:G174C, ORF8:V62L, S:D614G,
hCoV-19/Mexico/CMX-INMEGEN-01-01-24/2020	EPI_ISL_1005673	20B	B.1.1.519	23	5'UTR:T201C, 5'UTR:C203T, 5'UTR:C222T, 5'UTR:C241T, ORF1ab:G1738T, ORF1ab:C3037T, ORF1ab:C3140T, ORF1ab:C10029T, ORF1ab:C10954T, ORF1ab:A11117G, ORF1ab:C12194T, ORF1ab:C12789T, ORF1ab:C14408T, ORF1ab:T19839C, ORF1ab:A19974G, ORF1ab:C21306T, S:C22995A, S:A23403G, S:C23604A, S:A23756G, N:G28881A, N:G28882A, N:G28883C, N:C29197T,	12	N:R203K, N:G204R, ORF1a:P959S, ORF1a:T3255I, ORF1a:I3618V, ORF1a:L3977F, ORF1a:T4175I, ORF1b:P314L, S:T478K, S:D614G, S:P681H, S:T732A,

hCoV-19/Mexico/CMX-INMEGEN-01-02-01/2020	EPI_ISL_985160	20B	B.1.1.519	24	5'UTR:C203T, 5'UTR:C222T, 5'UTR:C241T, ORF1ab:C3037T, ORF1ab:C3140T, ORF1ab:G7037T, ORF1ab:C10029T, ORF1ab:C10954T, ORF1ab:A11117G, ORF1ab:C12789T, ORF1ab:C14408T, ORF1ab:C15699T, ORF1ab:T19839C, ORF1ab:C21306T, S:C22530T, S:C22995A, S:A23403G, S:C23604A, S:A23756G, ORF3a:C25782T, N:G28881A, N:G28882A, N:G28883C, N:A28898G, N:C29197T,	14	N:R203K, N:G204R, N:R209G, ORF1a:P959S, ORF1a:G2258C, ORF1a:T3255I, ORF1a:I3618V, ORF1a:T4175I, ORF1b:P314L, S:T323I, S:T478K, S:D614G, S:P681H, S:T732A,	
hCoV-19/Mexico/CMX-INMEGEN-01-02-02/2020	EPI_ISL_985161	20B	B.1.1.519	22	5'UTR:C203T, 5'UTR:C222T, 5'UTR:C241T, ORF1ab:C3037T, ORF1ab:C3140T, ORF1ab:C3311T, ORF1ab:A8031G, ORF1ab:C10029T, ORF1ab:C10954T, ORF1ab:A11117G, ORF1ab:C12789T, ORF1ab:C14408T, ORF1ab:T19839C, ORF1ab:C21306T, S:C22687T, S:C22995A, S:A23403G, S:C23604A, S:A23756G, N:G28881A, N:G28882A, N:G28883C, N:C29197T,	13	N:R203K, N:G204R, ORF1a:P959S, ORF1a:L1016F, ORF1a:K2589R, ORF1a:T3255I, ORF1a:I3618V, ORF1a:T4175I, ORF1b:P314L, S:T478K, S:D614G, S:P681H, S:T732A,	
hCoV-19/Mexico/CMX-INMEGEN-01-02-04/2021	EPI_ISL_985151	20B	B.1.1.519	25	5'UTR:C203T, 5'UTR:C222T, 5'UTR:C241T, ORF1ab:G443A, ORF1ab:C3037T, ORF1ab:C3140T, ORF1ab:C5869T, ORF1ab:C10029T, ORF1ab:C10954T, ORF1ab:A11117G, ORF1ab:C12789T, ORF1ab:C14408T, ORF1ab:T19839C, ORF1ab:C21306T, S:C22995A, S:A23403G, S:C23604A, S:A23756G, ORF3a:G25595A, ORF8:T27904C, ORF8:C28087T, N:G28881A, N:G28882A, N:G28883C, N:C29197T, N:T29461C,	15	N:R203K, N:G204R, ORF1a:V60I, ORF1a:P959S, ORF1a:T3255I, ORF1a:I3618V, ORF1a:T4175I, ORF1b:P314L, ORF3a:R68K, ORF8:L4P, ORF8:A65V, S:T478K, S:D614G, S:P681H, S:T732A,	
hCoV-19/Mexico/CMX-INMEGEN-01-02-05/2020	EPI_ISL_985167	20B	B.1.1.222	19	5'UTR:C241T, ORF1ab:C3037T, ORF1ab:C6487T, ORF1ab:C7834T, ORF1ab:C10029T, ORF1ab:C14408T, ORF1ab:T18471C, ORF1ab:C19011A, ORF1ab:T19839C, S:G22021T, S:G22256T, S:A23403G, S:A23756G, ORF3a:G25912T, ORF8:A27921G, ORF8:G28001T, N:G28881A, N:G28882A, N:G28883C, N:C29077T,	11	N:R203K, N:G204R, ORF1a:T3255I, ORF1b:P314L, ORF1b:D1848E, ORF3a:G174C, ORF8:I10V, S:M153I, S:G232C, S:D614G, S:T732A,	
hCoV-19/Mexico/CMX-INMEGEN-01-02-07/2020	EPI_ISL_985157	20B	B.1.1.519	21	5'UTR:C203T, 5'UTR:C222T, 5'UTR:C241T, ORF1ab:C3037T, ORF1ab:C3140T, ORF1ab:C10029T, ORF1ab:C10954T, ORF1ab:A11117G, ORF1ab:C12789T, ORF1ab:C14408T, ORF1ab:T19839C, ORF1ab:C21306T, S:C22995A, S:A23403G, S:C23604A, S:A23756G, ORF8:T27904C, ORF8:C28087T, N:G28881A, N:G28882A, N:G28883C, N:C29197T,	13	N:R203K, N:G204R, ORF1a:P959S, ORF1a:T3255I, ORF1a:I3618V, ORF1a:T4175I, ORF1b:P314L, ORF8:L4P, ORF8:A65V, S:T478K, S:D614G, S:P681H, S:T732A,	
hCoV-19/Mexico/CMX-INMEGEN-01-02-08/2020	EPI_ISL_985166	20B	B.1.1	20	5'UTR:C190T, 5'UTR:C241T, ORF1ab:A2761G, ORF1ab:G3004T, ORF1ab:C3037T, ORF1ab:C3990T, ORF1ab:C8299T, ORF1ab:C10507T, ORF1ab:C10789T, ORF1ab:C14408T, ORF1ab:T19839C, S:A23403G, S:G23426A, S:C23604A, ORF3a:G25522A, ORF3a:T25580C, ORF7b:G27890A, N:G28881A, N:G28882A, N:G28883C, 3'UTR:G29759T,	10	N:R203K, N:G204R, ORF1a:E913D, ORF1a:T1242I, ORF1b:P314L, ORF3a:G44R, ORF3a:I63T, S:D614G, S:V622I, S:P681H,	3'UTR:29747-29757
hCoV-19/Mexico/CMX-INMEGEN-01-02-09/2020	EPI_ISL_985162	20B	B.1.1.519	24	5'UTR:C203T, 5'UTR:C222T, 5'UTR:C241T, ORF1ab:C3037T, ORF1ab:C3140T, ORF1ab:G3351A, ORF1ab:C8299T, ORF1ab:C10029T, ORF1ab:C10954T, ORF1ab:A11117G, ORF1ab:C12789T, ORF1ab:C14408T, ORF1ab:G15451A, ORF1ab:T19839C, ORF1ab:C21306T, S:C22995A, S:A23403G, S:C23604A, S:A23756G, M:C26885T, N:G28881A, N:G28882A, N:G28883C, N:C29197T, N:G29527T,	14	N:R203K, N:G204R, N:Q418H, ORF1a:P959S, ORF1a:S1029N, ORF1a:T3255I, ORF1a:I3618V, ORF1a:T4175I, ORF1b:P314L, ORF1b:G662S, S:T478K, S:D614G, S:P681H, S:T732A,	

hCoV-19/Mexico/CMX-INMEGEN-01-02-10/2020	EPI_ISL_985163	20B	B.1.1.519	27	5'UTR:T201C, 5'UTR:C203T, 5'UTR:C222T, 5'UTR:C241T, ORF1ab:C936T, ORF1ab:G1738T, ORF1ab:C3037T, ORF1ab:C3140T, ORF1ab:C10029T, ORF1ab:C10954T, ORF1ab:A11117G, ORF1ab:C12789T, ORF1ab:C14408T, ORF1ab:T19839C, ORF1ab:A19974G, ORF1ab:C21306T, S:T21835C, S:C22995A, S:A23403G, S:C23604A, S:A23756G, ORF3a:A25524C, ORF3a:G25599T, N:G28881A, N:G28882A, N:G28883C, N:C29197T, N:G29227T,	13	N:R203K, N:G204R, ORF1a:T224I, ORF1a:P959S, ORF1a:T3255I, ORF1a:I3618V, ORF1a:T4175I, ORF1b:P314L, ORF3a:W69C, S:T478K, S:D614G, S:P681H, S:T732A,
hCoV-19/Mexico/CMX-INMEGEN-01-02-12/2020	EPI_ISL_985164	20B	B.1.1.519	21	5'UTR:C203T, 5'UTR:C222T, 5'UTR:C241T, ORF1ab:C3037T, ORF1ab:C3140T, ORF1ab:C10029T, ORF1ab:C10954T, ORF1ab:A11117G, ORF1ab:C12789T, ORF1ab:C14408T, ORF1ab:T19839C, ORF1ab:C21306T, S:C22995A, S:C23248T, S:A23403G, S:C23604A, S:A23756G, ORF7b:G27806A, N:G28881A, N:G28882A, N:G28883C, N:C29197T,	11	N:R203K, N:G204R, ORF1a:P959S, ORF1a:T3255I, ORF1a:I3618V, ORF1a:T4175I, ORF1b:P314L, S:T478K, S:D614G, S:P681H, S:T732A,
hCoV-19/Mexico/CMX-INMEGEN-01-02-13/2020	EPI_ISL_985165	20B	B.1.1.519	28	5'UTR:C203T, 5'UTR:C222T, 5'UTR:C241T, ORF1ab:C3037T, ORF1ab:C3140T, ORF1ab:A3684G, ORF1ab:A4316T, ORF1ab:C6541T, ORF1ab:C10029T, ORF1ab:C10954T, ORF1ab:A11117G, ORF1ab:C12789T, ORF1ab:C14408T, ORF1ab:C15240T, ORF1ab:C18929T, ORF1ab:T19839C, ORF1ab:C21306T, S:A22023T, S:C22995A, S:C23380T, S:A23403G, S:C23604A, S:A23756G, ORF8:T27904C, N:G28881A, N:G28882A, N:G28883C, N:C29197T, N:G29374T,	17	N:R203K, N:G204R, N:E367D, ORF1a:P959S, ORF1a:Q1140R, ORF1a:S1351C, ORF1a:T3255I, ORF1a:I3618V, ORF1a:T4175I, ORF1b:P314L, ORF1b:P1821L, ORF8:L4P, S:E154V, S:T478K, S:D614G, S:P681H, S:T732A,
hCoV-19/Mexico/CMX-INMEGEN-01-02-14/2020	EPI_ISL_985158	20B	B.1.1.519	22	5'UTR:C203T, 5'UTR:C222T, 5'UTR:C241T, ORF1ab:C3037T, ORF1ab:C3140T, ORF1ab:C10029T, ORF1ab:C10954T, ORF1ab:A11117G, ORF1ab:C12789T, ORF1ab:C14408T, ORF1ab:T15726A, ORF1ab:T19839C, ORF1ab:C21306T, S:C22995A, S:A23403G, S:C23604A, S:A23756G, M:C26833T, N:G28881A, N:G28882A, N:G28883C, N:C29197T, N:G29527T,	13	M:A104V, N:R203K, N:G204R, N:Q418H, ORF1a:P959S, ORF1a:T3255I, ORF1a:I3618V, ORF1a:T4175I, ORF1b:P314L, S:T478K, S:D614G, S:P681H, S:T732A,
hCoV-19/Mexico/CMX-INMEGEN-01-02-15/2020	EPI_ISL_985159	20B	B.1.1.519	25	5'UTR:C203T, 5'UTR:C222T, 5'UTR:C241T, ORF1ab:C3037T, ORF1ab:C3140T, ORF1ab:C10029T, ORF1ab:C10954T, ORF1ab:A11117G, ORF1ab:C12789T, ORF1ab:C14408T, ORF1ab:G17278T, ORF1ab:T19839C, ORF1ab:G21073A, ORF1ab:C21306T, S:C21575T, S:C22995A, S:A23403G, S:C23604A, S:A23756G, S:C24919T, S:C25292T, N:G28881A, N:G28882A, N:G28883C, N:C29197T, N:G29402T,	16	N:R203K, N:G204R, N:D377Y, ORF1a:P959S, ORF1a:T3255I, ORF1a:I3618V, ORF1a:T4175I, ORF1b:P314L, ORF1b:V1271L, ORF1b:V2536I, S:L5F, S:T478K, S:D614G, S:P681H, S:T732A, S:L1244F,
hCoV-19/Mexico/CMX-INMEGEN-01-02-16/2021	EPI_ISL_985149	20B	B.1.1.519	25	5'UTR:T201C, 5'UTR:C203T, 5'UTR:C222T, 5'UTR:C241T, ORF1ab:C936T, ORF1ab:G1738T, ORF1ab:C3037T, ORF1ab:C3140T, ORF1ab:C10029T, ORF1ab:C10954T, ORF1ab:A11117G, ORF1ab:C12789T, ORF1ab:C14408T, ORF1ab:T19839C, ORF1ab:A19974G, ORF1ab:C21306T, S:C22995A, S:A23403G, S:C23604A, S:A23756G, ORF3a:A25524C, N:G28881A, N:G28882A, N:G28883C, N:C29197T, N:G29227T,	12	N:R203K, N:G204R, ORF1a:T224I, ORF1a:P959S, ORF1a:T3255I, ORF1a:I3618V, ORF1a:T4175I, ORF1b:P314L, S:T478K, S:D614G, S:P681H, S:T732A,

hCoV-19/Mexico/CMX-INMEGEN-01-02-18/2021	EPI_ISL_985153	20B	B.1.1.519	25	5'UTR:C203T, 5'UTR:C222T, 5'UTR:C241T, ORF1ab:C3037T, ORF1ab:C3140T, ORF1ab:C10029T, ORF1ab:C10954T, ORF1ab:A11117G, ORF1ab:G11365T, ORF1ab:C12789T, ORF1ab:C14408T, ORF1ab:G15957T, ORF1ab:T19839C, ORF1ab:C21058T, ORF1ab:C21306T, S:C22995A, S:A23403G, S:G23501T, S:C23604A, S:A23756G, S:G24926T, N:G28881A, N:G28882A, N:G28883C, N:C29197T, N:G29527T,	15	N:R203K, N:G204R, N:Q418H, ORF1a:P959S, ORF1a:T3255I, ORF1a:I3618V, ORF1a:T4175I, ORF1b:P314L, ORF1b:P2531S, S:T478K, S:D614G, S:A647S, S:P681H, S:T732A, S:V1122L,	ORF7b:27792-27800,
hCoV-19/Mexico/CMX-INMEGEN-01-02-19/2021	EPI_ISL_985154	20B	B.1.1.519	24	5'UTR:C203T, 5'UTR:C222T, 5'UTR:C241T, ORF1ab:C3037T, ORF1ab:C3140T, ORF1ab:T3745C, ORF1ab:C6706T, ORF1ab:C7113T, ORF1ab:C10029T, ORF1ab:C10954T, ORF1ab:A11117G, ORF1ab:C12789T, ORF1ab:C14408T, ORF1ab:T19839C, ORF1ab:C21306T, S:C22995A, S:A23403G, S:C23604A, S:A23756G, ORF3a:C25844T, N:G28881A, N:G28882A, N:G28883C, N:C29197T, N:G29392T,	14	N:R203K, N:G204R, N:K373N, ORF1a:P959S, ORF1a:T2283I, ORF1a:T3255I, ORF1a:I3618V, ORF1a:T4175I, ORF1b:P314L, ORF3a:T151I, S:T478K, S:D614G, S:P681H, S:T732A,	
hCoV-19/Mexico/CMX-INMEGEN-01-02-20/2021	EPI_ISL_985155	20B	B.1.1.519	28	5'UTR:T201C, 5'UTR:C203T, 5'UTR:C222T, 5'UTR:C241T, ORF1ab:C936T, ORF1ab:G1738T, ORF1ab:C3037T, ORF1ab:C3140T, ORF1ab:C10029T, ORF1ab:C10252T, ORF1ab:C10954T, ORF1ab:A11117G, ORF1ab:C12789T, ORF1ab:C14408T, ORF1ab:G17014T, ORF1ab:T19839C, ORF1ab:A19974G, ORF1ab:C21306T, S:C22995A, S:A23403G, S:C23604A, S:A23756G, ORF7a:T27720C, N:C28411T, N:G28881A, N:G28882A, N:G28883C, N:C29197T, N:G29227T,	14	N:R203K, N:G204R, ORF1a:T224I, ORF1a:P959S, ORF1a:T3255I, ORF1a:I3618V, ORF1a:T4175I, ORF1b:P314L, ORF1b:D1183Y, ORF9b:P43L, S:T478K, S:D614G, S:P681H, S:T732A,	
hCoV-19/Mexico/CMX-INMEGEN-01-02-21/2021	EPI_ISL_985152	20B	B.1.1.222	20	5'UTR:C241T, ORF1ab:C2252A, ORF1ab:C3037T, ORF1ab:T5929A, ORF1ab:A7949T, ORF1ab:C10029T, ORF1ab:C14408T, ORF1ab:C15279T, ORF1ab:G19816T, ORF1ab:T19839C, ORF1ab:C20936T, S:A23403G, S:A23756G, ORF3a:G25912T, ORF7b:A27756G, ORF8:A27921G, ORF8:G28001T, N:G28881A, N:G28882A, N:G28883C, 3'UTR:C29741T,	13	N:R203K, N:G204R, ORF1a:Q663K, ORF1a:S2562C, ORF1a:T3255I, ORF1b:P314L, ORF1b:V2117L, ORF1b:T2490M, ORF3a:G174C, ORF7b:M1V, ORF8:I10V, S:D614G, S:T732A,	
hCoV-19/Mexico/CMX-INMEGEN-01-02-22/2021	EPI_ISL_985150	20B	B.1.1.222	17	5'UTR:C241T, ORF1ab:C3037T, ORF1ab:C4002T, ORF1ab:C10029T, ORF1ab:C14408T, ORF1ab:T19839C, ORF1ab:C19868T, S:A23403G, S:A23756G, ORF3a:G25912T, ORF7a:A27690T, ORF7b:A27756G, ORF8:A27921G, ORF8:G28001T, ORF8:G28079T, N:G28881A, N:G28882A, N:G28883C,	11	N:R203K, N:G204R, ORF1a:T1246I, ORF1a:T3255I, ORF1b:P314L, ORF1b:T2134I, ORF3a:G174C, ORF7b:M1V, ORF8:I10V, S:D614G, S:T732A,	3'UTR:29746-29762,
hCoV-19/Mexico/CMX-INMEGEN-01-02-23/2021	EPI_ISL_985156	20A	B.1.36.31	24	5'UTR:C241T, ORF1ab:C1722T, ORF1ab:C2836T, ORF1ab:C3037T, ORF1ab:G6485T, ORF1ab:A8512G, ORF1ab:T9877C, ORF1ab:G11222T, ORF1ab:C12616T, ORF1ab:C14408T, ORF1ab:C15738T, ORF1ab:C18877T, S:A21868T, S:C22227T, S:C22444T, S:A23403G, ORF3a:C25463T, ORF3a:G25563T, ORF3a:C25916T, M:C26735T, ORF7a:G27561T, ORF8:C28253T, N:C28854T, N:C29095T, 3'UTR:C29754A,	11	N:S194L, ORF1a:A486V, ORF1a:D2074Y, ORF1a:V3653F, ORF1b:P314L, ORF3a:T24I, ORF3a:Q57H, ORF3a:T175I, S:R102S, S:A222V, S:D614G,	
hCoV-19/Mexico/CMX-INMEGEN-01-03-01/2021	EPI_ISL_944611	20B	B.1.1.222	19	5'UTR:C241T, ORF1ab:C835T, ORF1ab:C3037T, ORF1ab:C6781T, ORF1ab:C8090T, ORF1ab:C11109T, ORF1ab:C11416T, ORF1ab:C14408T, ORF1ab:A15334G, ORF1ab:G15448T, ORF1ab:T15519G, ORF1ab:T19839C, S:G22017T, S:A23403G, S:A23756G, ORF3a:G26063T, ORF7b:A27888T, N:G28881A, N:G28882A, N:G28883C,	11	N:R203K, N:G204R, ORF1a:L2609F, ORF1a:A3615V, ORF1b:P314L, ORF1b:I623V, ORF1b:G661C, ORF3a:G224V, S:W152L, S:D614G, S:T732A,	

hCoV-19/Mexico/CMX-INMEGEN-01-03-02/2021	EPI_ISL_944612	20B	B.1.1.222	19	5'UTR:C241T, ORF1ab:C835T, ORF1ab:C3037T, ORF1ab:C6781T, ORF1ab:C8090T, ORF1ab:C11109T, ORF1ab:C11416T, ORF1ab:C14408T, ORF1ab:A15334G, ORF1ab:G15448T, ORF1ab:T15519G, ORF1ab:T19839C, S:G22017T, S:A23403G, S:A23756G, ORF3a:G26063T, ORF7b:A27888T, N:G28881A, N:G28882A, N:G28883C,	11	N:R203K, N:G204R, ORF1a:L2609F, ORF1a:A3615V, ORF1b:P314L, ORF1b:I623V, ORF1b:G661C, ORF3a:G224V, S:W152L, S:D614G, S:T732A,
hCoV-19/Mexico/CMX-INMEGEN-01-03-03/2021	EPI_ISL_944613	20B	B.1.1.519	29	5'UTR:C203T, 5'UTR:C222T, 5'UTR:C241T, ORF1ab:C335T, ORF1ab:C3037T, ORF1ab:C3140T, ORF1ab:C10029T, ORF1ab:C10335T, ORF1ab:C10954T, ORF1ab:G11083T, ORF1ab:A11117G, ORF1ab:C12789T, ORF1ab:C14408T, ORF1ab:C15368T, ORF1ab:A15864G, ORF1ab:C18647T, ORF1ab:T19839C, ORF1ab:A19888C, ORF1ab:C21306T, S:C21646T, S:C22995A, S:A23403G, S:C23604A, S:A23756G, S:G24781T, S:G25311T, N:G28881A, N:G28882A, N:G28883C, N:C29197T,	18	N:R203K, N:G204R, ORF1a:R24C, ORF1a:P959S, ORF1a:T3255I, ORF1a:A3357V, ORF1a:L3606F, ORF1a:I3618V, ORF1a:T4175I, ORF1b:P314L, ORF1b:T634I, ORF1b:P1727L, S:T478K, S:D614G, S:P681H, S:T732A, S:K1073N, S:C1250F,
hCoV-19/Mexico/CMX-INMEGEN-01-03-04/2021	EPI_ISL_944614	20B	B.1.1.519	22	5'UTR:C203T, 5'UTR:C222T, 5'UTR:C241T, ORF1ab:C3037T, ORF1ab:C3140T, ORF1ab:C10029T, ORF1ab:C10954T, ORF1ab:A11117G, ORF1ab:C12789T, ORF1ab:C13329T, ORF1ab:C14408T, ORF1ab:T19839C, ORF1ab:C21306T, S:C22995A, S:A23403G, S:C23604A, S:A23756G, S:G24926T, ORF3a:G26199T, N:G28881A, N:G28882A, N:G28883C, N:C29197T,	13	N:R203K, N:G204R, ORF1a:P959S, ORF1a:T3255I, ORF1a:I3618V, ORF1a:T4175I, ORF1a:T4355I, ORF1b:P314L, S:T478K, S:D614G, S:P681H, S:T732A, S:V1122L,
hCoV-19/Mexico/CMX-INMEGEN-01-03-05/2021	EPI_ISL_944615	20A	B.1.609	18	5'UTR:C241T, ORF1ab:C3037T, ORF1ab:C3315T, ORF1ab:C4582T, ORF1ab:A6264G, ORF1ab:C8320T, ORF1ab:C9996T, ORF1ab:G11327T, ORF1ab:A12095G, ORF1ab:C14178T, ORF1ab:C14408T, ORF1ab:G19891T, ORF1ab:A20268G, S:A23403G, S:A25359G, ORF3a:T25837C, ORF6:T27280C, N:G28916T, N:A29487G,	13	N:G215C, N:K405R, ORF1a:T1017I, ORF1a:K2000R, ORF1a:S3244L, ORF1a:A3688S, ORF1a:I3944V, ORF1b:P314L, ORF1b:D2142Y, ORF3a:W149R, ORF6:W27R, S:D614G, S:K1266R,
hCoV-19/Mexico/CMX-INMEGEN-01-03-06/2021	EPI_ISL_944616	20B	B.1.1.519	25	5'UTR:T201C, 5'UTR:C203T, 5'UTR:C222T, 5'UTR:C241T, ORF1ab:G1738T, ORF1ab:C3037T, ORF1ab:C3140T, ORF1ab:C3602T, ORF1ab:C7504T, ORF1ab:C10029T, ORF1ab:C10954T, ORF1ab:A11117G, ORF1ab:C12763T, ORF1ab:C12789T, ORF1ab:C14408T, ORF1ab:T19839C, ORF1ab:A19974G, ORF1ab:C21306T, S:C22995A, S:A23403G, S:C23604A, S:A23756G, N:G28881A, N:G28882A, N:G28883C, N:C29197T,	12	N:R203K, N:G204R, ORF1a:P959S, ORF1a:H1113Y, ORF1a:T3255I, ORF1a:I3618V, ORF1a:T4175I, ORF1b:P314L, S:T478K, S:D614G, S:P681H, S:T732A,
hCoV-19/Mexico/CMX-INMEGEN-01-03-07/2021	EPI_ISL_944617	20B	B.1.1.519	23	5'UTR:C203T, 5'UTR:C222T, 5'UTR:C241T, ORF1ab:C3037T, ORF1ab:C3140T, ORF1ab:G6894A, ORF1ab:C10029T, ORF1ab:C10954T, ORF1ab:A11117G, ORF1ab:G11365T, ORF1ab:C12789T, ORF1ab:C14408T, ORF1ab:T19839C, ORF1ab:C21306T, S:C22995A, S:A23403G, S:C23604A, S:A23756G, ORF7a:C27513T, N:G28881A, N:G28882A, N:G28883C, N:C29197T, N:G29527T,	13	N:R203K, N:G204R, N:Q418H, ORF1a:P959S, ORF1a:C2210Y, ORF1a:T3255I, ORF1a:I3618V, ORF1a:T4175I, ORF1b:P314L, S:T478K, S:D614G, S:P681H, S:T732A,
hCoV-19/Mexico/CMX-INMEGEN-01-03-08/2021	EPI_ISL_944618	20B	B.1.1.519	23	5'UTR:C203T, 5'UTR:C222T, 5'UTR:C241T, ORF1ab:C3037T, ORF1ab:C3140T, ORF1ab:A3684G, ORF1ab:C6541T, ORF1ab:C10029T, ORF1ab:C10954T, ORF1ab:A11117G, ORF1ab:C12789T, ORF1ab:C14408T, ORF1ab:T19839C, ORF1ab:C21306T, S:A22023T, S:C22995A, S:A23403G, S:C23604A, S:A23756G, ORF8:T27904C, N:G28881A, N:G28882A, N:G28883C, N:C29197T,	14	N:R203K, N:G204R, ORF1a:P959S, ORF1a:Q1140R, ORF1a:T3255I, ORF1a:I3618V, ORF1a:T4175I, ORF1b:P314L, ORF8:L4P, S:E154V, S:T478K, S:D614G, S:P681H, S:T732A,

hCoV-19/Mexico/CMX-INMEGEN-01-03-09/2021	EPI_ISL_944619	20B	B.1.1.519	29	5'UTR:C203T, 5'UTR:C222T, 5'UTR:C241T, ORF1ab:C1342T, ORF1ab:C2450T, ORF1ab:C2695T, ORF1ab:C3037T, ORF1ab:C3140T, ORF1ab:G3692T, ORF1ab:G3871T, ORF1ab:C9448T, ORF1ab:C10029T, ORF1ab:C10954T, ORF1ab:A11117G, ORF1ab:C11824A, ORF1ab:C12789T, ORF1ab:C14408T, ORF1ab:T19839C, ORF1ab:C21306T, S:C22995A, S:A23403G, S:C23604A, S:A23756G, ORF8:T27904C, N:G28881A, N:G28882A, N:G28883C, N:C28905T, N:C29197T, N:G29266C,	16	N:R203K, N:G204R, N:A211V, N:L331F, ORF1a:P959S, ORF1a:V1143F, ORF1a:K1202N, ORF1a:T3255I, ORF1a:I3618V, ORF1a:T4175I, ORF1b:P314L, ORF8:L4P, S:T478K, S:D614G, S:P681H, S:T732A,
hCoV-19/Mexico/CMX-INMEGEN-01-03-10/2021	EPI_ISL_944620	20B	B.1.1.222	23	5'UTR:C241T, ORF1ab:C3037T, ORF1ab:C4897T, ORF1ab:C5259T, ORF1ab:C10029T, ORF1ab:G11083T, ORF1ab:A11430G, ORF1ab:G12769T, ORF1ab:C13862T, ORF1ab:C14408T, ORF1ab:C19011A, ORF1ab:T19839C, ORF1ab:A21137G, S:A23403G, S:A23756G, ORF3a:G25912T, ORF3a:A26115C, ORF6:A27362C, ORF8:A27921G, ORF8:G28001T, N:C28849T, N:G28881A, N:G28882A, N:G28883C,	16	N:R203K, N:G204R, ORF1a:T1665I, ORF1a:T3255I, ORF1a:L3606F, ORF1a:Y3722C, ORF1b:T132I, ORF1b:P314L, ORF1b:D1848E, ORF1b:K2557R, ORF3a:G174C, ORF3a:E241D, ORF6:E54A, ORF8:I10V, S:D614G, S:T732A,
hCoV-19/Mexico/CMX-INMEGEN-01-03-11/2021	EPI_ISL_944621	20B	B.1.1.519	30	5'UTR:C203T, 5'UTR:C222T, 5'UTR:C241T, ORF1ab:A2923G, ORF1ab:C3037T, ORF1ab:C3140T, ORF1ab:G3692T, ORF1ab:G3871T, ORF1ab:C5575T, ORF1ab:C9520T, ORF1ab:C10029T, ORF1ab:C10954T, ORF1ab:A11117G, ORF1ab:C11824A, ORF1ab:C12789T, ORF1ab:C14408T, ORF1ab:C14676T, ORF1ab:T19839C, ORF1ab:C21306T, S:C22995A, S:A23403G, S:C23604A, S:A23756G, S:G24193C, ORF3a:G25690T, ORF7b:C27804T, ORF8:T27904C, N:G28881A, N:G28882A, N:G28883C, N:C29197T,	15	N:R203K, N:G204R, ORF1a:P959S, ORF1a:V1143F, ORF1a:K1202N, ORF1a:T3255I, ORF1a:I3618V, ORF1a:T4175I, ORF1b:P314L, ORF3a:G100C, ORF8:L4P, S:T478K, S:D614G, S:P681H, S:T732A,
hCoV-19/Mexico/CMX-INMEGEN-01-03-12/2021	EPI_ISL_944622	20B	B.1.1.519	25	5'UTR:C203T, 5'UTR:C222T, 5'UTR:C241T, ORF1ab:C1513T, ORF1ab:A2497T, ORF1ab:C3037T, ORF1ab:C3140T, ORF1ab:T3745C, ORF1ab:C8344T, ORF1ab:C10029T, ORF1ab:C10954T, ORF1ab:A11117G, ORF1ab:C12789T, ORF1ab:C14408T, ORF1ab:T17877A, ORF1ab:T19839C, ORF1ab:C21306T, S:C22995A, S:A23403G, S:C23604A, S:A23756G, ORF3a:C25844T, N:G28881A, N:G28882A, N:G28883C, N:C29197T,	12	N:R203K, N:G204R, ORF1a:P959S, ORF1a:T3255I, ORF1a:I3618V, ORF1a:T4175I, ORF1b:P314L, ORF3a:T151I, S:T478K, S:D614G, S:P681H, S:T732A,
hCoV-19/Mexico/CMX-INMEGEN-01-03-13/2021	EPI_ISL_944623	20B	B.1.1.519	23	5'UTR:C203T, 5'UTR:C222T, 5'UTR:C241T, ORF1ab:C3037T, ORF1ab:C3140T, ORF1ab:G3403T, ORF1ab:C5183T, ORF1ab:C10029T, ORF1ab:C10954T, ORF1ab:A11117G, ORF1ab:C12789T, ORF1ab:C14408T, ORF1ab:T19839C, ORF1ab:C21306T, S:C22995A, S:A23403G, S:C23604A, S:A23756G, ORF3a:C25513T, ORF8:T27904C, N:G28881A, N:G28882A, N:G28883C, N:C29197T,	14	N:R203K, N:G204R, ORF1a:P959S, ORF1a:P1640S, ORF1a:T3255I, ORF1a:I3618V, ORF1a:T4175I, ORF1b:P314L, ORF3a:L41F, ORF8:L4P, S:T478K, S:D614G, S:P681H, S:T732A,
hCoV-19/Mexico/CMX-INMEGEN-01-03-14/2021	EPI_ISL_944624	20B	B.1.1.519	24	5'UTR:C203T, 5'UTR:C222T, 5'UTR:C241T, ORF1ab:C3037T, ORF1ab:C3140T, ORF1ab:C10029T, ORF1ab:C10954T, ORF1ab:A11117G, ORF1ab:C11575T, ORF1ab:C12789T, ORF1ab:C14408T, ORF1ab:G16117T, ORF1ab:T16863C, ORF1ab:T19839C, ORF1ab:T19980C, ORF1ab:C21306T, S:C22995A, S:A23403G, S:C23604A, S:A23756G, N:G28881A, N:G28882A, N:G28883C, N:C29197T, 3'UTR:C29754T,	12	N:R203K, N:G204R, ORF1a:P959S, ORF1a:T3255I, ORF1a:I3618V, ORF1a:T4175I, ORF1b:P314L, ORF1b:D884Y, S:T478K, S:D614G, S:P681H, S:T732A,

hCoV-19/Mexico/CMX-INMEGEN-01-03-15/2021	EPI_ISL_944625	20B	B.1.1.519	23	5'UTR:C203T, 5'UTR:C222T, 5'UTR:C241T, ORF1ab:C3037T, ORF1ab:C3140T, ORF1ab:C6781T, ORF1ab:C10029T, ORF1ab:C10776T, ORF1ab:C10954T, ORF1ab:A11117G, ORF1ab:C12789T, ORF1ab:T13905C, ORF1ab:C14408T, ORF1ab:T19839C, ORF1ab:C21306T, S:C22995A, S:A23403G, S:C23604A, S:A23756G, ORF3a:G25906T, N:G28881A, N:G28882A, N:G28883C, N:C29197T,	13	N:R203K, N:G204R, ORF1a:P959S, ORF1a:T3255I, ORF1a:P3504L, ORF1a:I3618V, ORF1a:T4175I, ORF1b:P314L, ORF3a:G172C, S:T478K, S:D614G, S:P681H, S:T732A,
hCoV-19/Mexico/CMX-INMEGEN-01-03-16/2021	EPI_ISL_944626	20B	B.1.1.519	24	5'UTR:C203T, 5'UTR:C222T, 5'UTR:C241T, ORF1ab:C3037T, ORF1ab:C3140T, ORF1ab:C10029T, ORF1ab:C10954T, ORF1ab:A11117G, ORF1ab:C11575T, ORF1ab:C12789T, ORF1ab:C13019T, ORF1ab:G13249A, ORF1ab:C14408T, ORF1ab:A16388C, ORF1ab:T19839C, ORF1ab:C21306T, S:C21691T, S:C22995A, S:A23403G, S:C23604A, S:A23756G, N:G28881A, N:G28882A, N:G28883C, N:C29197T,	12	N:R203K, N:G204R, ORF1a:P959S, ORF1a:T3255I, ORF1a:I3618V, ORF1a:T4175I, ORF1b:P314L, ORF1b:N974T, S:T478K, S:D614G, S:P681H, S:T732A,
hCoV-19/Mexico/CMX-INMEGEN-01-03-17/2021	EPI_ISL_944627	20B	B.1.1.519	23	5'UTR:C203T, 5'UTR:C222T, 5'UTR:C241T, ORF1ab:C3037T, ORF1ab:C3140T, ORF1ab:C10029T, ORF1ab:C10954T, ORF1ab:A11117G, ORF1ab:C11124T, ORF1ab:C12789T, ORF1ab:C14408T, ORF1ab:C14724T, ORF1ab:T19839C, ORF1ab:C21306T, S:C22995A, S:A23403G, S:C23604A, S:A23756G, ORF7a:G27670T, N:G28378T, N:G28881A, N:G28882A, N:G28883C, N:C29197T,	14	N:R203K, N:G204R, ORF1a:P959S, ORF1a:T3255I, ORF1a:I3618V, ORF1a:A3620V, ORF1a:T4175I, ORF1b:P314L, ORF7a:V93F, ORF9b:R32L, S:T478K, S:D614G, S:P681H, S:T732A,
hCoV-19/Mexico/CMX-INMEGEN-01-03-18/2021	EPI_ISL_944628	20B	B.1.1.519	27	5'UTR:T201C, 5'UTR:C203T, 5'UTR:C222T, 5'UTR:C241T, ORF1ab:G1738T, ORF1ab:C3037T, ORF1ab:C3140T, ORF1ab:C7420T, ORF1ab:C10029T, ORF1ab:C10954T, ORF1ab:A11117G, ORF1ab:C12789T, ORF1ab:C14408T, ORF1ab:C15240T, ORF1ab:T19839C, ORF1ab:A19974G, ORF1ab:A21127G, ORF1ab:C21306T, S:G21770T, S:C22995A, S:A23403G, S:C23604A, S:A23756G, N:G28881A, N:G28882A, N:G28883C, N:C29197T, N:C29249T,	14	N:R203K, N:G204R, N:P326S, ORF1a:P959S, ORF1a:T3255I, ORF1a:I3618V, ORF1a:T4175I, ORF1b:P314L, ORF1b:I2554V, S:V70F, S:T478K, S:D614G, S:P681H, S:T732A,
hCoV-19/Mexico/CMX-INMEGEN-01-03-19/2021	EPI_ISL_944629	20B	B.1.1.519	25	5'UTR:C203T, 5'UTR:C222T, 5'UTR:C241T, ORF1ab:C1441T, ORF1ab:C3037T, ORF1ab:C3140T, ORF1ab:T6061C, ORF1ab:C10029T, ORF1ab:C10954T, ORF1ab:A11117G, ORF1ab:C12789T, ORF1ab:C12815T, ORF1ab:C14408T, ORF1ab:G15372T, ORF1ab:T19839C, ORF1ab:C21306T, S:C22995A, S:A23403G, S:C23604A, S:A23756G, ORF3a:C25714T, ORF8:T27904C, N:G28881A, N:G28882A, N:G28883C, N:C29197T,	13	N:R203K, N:G204R, ORF1a:P959S, ORF1a:T3255I, ORF1a:I3618V, ORF1a:T4175I, ORF1b:P314L, ORF3a:L108F, ORF8:L4P, S:T478K, S:D614G, S:P681H, S:T732A,
hCoV-19/Mexico/CMX-INMEGEN-01-03-20/2021	EPI_ISL_944630	20B	B.1.1.519	26	5'UTR:C203T, 5'UTR:C222T, 5'UTR:C241T, ORF1ab:C1342T, ORF1ab:C3037T, ORF1ab:C3140T, ORF1ab:G3692T, ORF1ab:G3871T, ORF1ab:C5284T, ORF1ab:C7299T, ORF1ab:C10029T, ORF1ab:C10954T, ORF1ab:A11117G, ORF1ab:C11824A, ORF1ab:C12789T, ORF1ab:C14408T, ORF1ab:T19839C, ORF1ab:C21306T, S:C22995A, S:A23403G, S:C23604A, S:A23756G, ORF8:T27904C, N:G28881A, N:G28882A, N:G28883C, N:C29197T,	15	N:R203K, N:G204R, ORF1a:P959S, ORF1a:V1143F, ORF1a:K1202N, ORF1a:A2345V, ORF1a:T3255I, ORF1a:I3618V, ORF1a:T4175I, ORF1b:P314L, ORF8:L4P, S:T478K, S:D614G, S:P681H, S:T732A,

hCoV-19/Mexico/CMX-INMEGEN-01-03-21/2021	EPI_ISL_944631	20B	B.1.1.519	28	5'UTR:T201C, 5'UTR:C203T, 5'UTR:C222T, 5'UTR:C241T, ORF1ab:G1738T, ORF1ab:C3037T, ORF1ab:C3140T, ORF1ab:C10029T, ORF1ab:G10617A, ORF1ab:C10954T, ORF1ab:A11117G, ORF1ab:C12789T, ORF1ab:T13336C, ORF1ab:A14328G, ORF1ab:C14408T, ORF1ab:G14559T, ORF1ab:T19839C, ORF1ab:A19974G, ORF1ab:C21306T, S:C22995A, S:A23403G, S:C23604A, S:A23756G, ORF3a:G25699A, M:C26882T, N:G28881A, N:G28882A, N:G28883C, N:C29197T	13	N:R203K, N:G204R, ORF1a:P959S, ORF1a:T3255I, ORF1a:R3451K, ORF1a:I3618V, ORF1a:T4175I, ORF1b:P314L, ORF3a:A103T, S:T478K, S:D614G, S:P681H, S:T732A,
hCoV-19/Mexico/CMX-INMEGEN-01-03-22/2021	EPI_ISL_944632	20B	B.1.1.519	20	5'UTR:C203T, 5'UTR:C222T, 5'UTR:C241T, ORF1ab:C3037T, ORF1ab:C3140T, ORF1ab:C10029T, ORF1ab:C10954T, ORF1ab:A11117G, ORF1ab:C12789T, ORF1ab:C14408T, ORF1ab:T19839C, ORF1ab:C21306T, S:C22995A, S:A23403G, S:C23604A, S:A23756G, N:G28881A, N:G28882A, N:G28883C, N:C29197T, N:G29527T,	12	N:R203K, N:G204R, N:Q418H, ORF1a:P959S, ORF1a:T3255I, ORF1a:I3618V, ORF1a:T4175I, ORF1b:P314L, S:T478K, S:D614G, S:P681H, S:T732A,
hCoV-19/Mexico/CMX-INMEGEN-01-03-23/2021	EPI_ISL_944633	20B	B.1.1.519	24	5'UTR:C203T, 5'UTR:C222T, 5'UTR:C241T, ORF1ab:C3037T, ORF1ab:C3140T, ORF1ab:T3745C, ORF1ab:C10029T, ORF1ab:C10954T, ORF1ab:A11117G, ORF1ab:C12789T, ORF1ab:C14408T, ORF1ab:C17373T, ORF1ab:G17808T, ORF1ab:T19839C, ORF1ab:C21306T, S:C22995A, S:A23403G, S:C23604A, S:A23756G, ORF3a:C25844T, N:C28849T, N:G28881A, N:G28882A, N:G28883C, N:C29197T,	13	N:R203K, N:G204R, ORF1a:P959S, ORF1a:T3255I, ORF1a:I3618V, ORF1a:T4175I, ORF1b:P314L, ORF1b:K1447N, ORF3a:T151I, S:T478K, S:D614G, S:P681H, S:T732A,
hCoV-19/Mexico/CMX-INMEGEN-01-04-02/2021	EPI_ISL_1040564	20B	B.1.1	20	5'UTR:C190T, 5'UTR:C241T, ORF1ab:G3004T, ORF1ab:C3037T, ORF1ab:A13974T, ORF1ab:C14408T, ORF1ab:C17733T, ORF1ab:T19839C, ORF1ab:G21255C, S:A23403G, S:G23426A, S:C23604A, S:A25093G, ORF3a:G25522A, ORF3a:T25580C, ORF7b:G27890A, N:C28606T, N:G28881A, N:G28882A, N:G28883C, 3'UTR:G29747T,	10	N:R203K, N:G204R, ORF1a:E913D, ORF1b:L169F, ORF1b:P314L, ORF3a:G44R, ORF3a:I63T, S:D614G, S:V622I, S:P681H,
hCoV-19/Mexico/CMX-INMEGEN-01-04-04/2021	EPI_ISL_1040573	20B	B.1.1.519	26	5'UTR:C203T, 5'UTR:C222T, 5'UTR:C241T, ORF1ab:T1303A, ORF1ab:C3037T, ORF1ab:C3140T, ORF1ab:C6781T, ORF1ab:C10029T, ORF1ab:C10954T, ORF1ab:A11117G, ORF1ab:C12789T, ORF1ab:C14184T, ORF1ab:C14408T, ORF1ab:C17678T, ORF1ab:T19839C, ORF1ab:C21306T, S:C21780T, S:C22995A, S:A23403G, S:C23604A, S:A23756G, N:G28881A, N:G28882A, N:G28883C, N:C29197T, N:C29420T, ORF10:T29661C,	14	N:R203K, N:G204R, N:P383S, ORF1a:P959S, ORF1a:T3255I, ORF1a:I3618V, ORF1a:T4175I, ORF1b:P314L, ORF1b:T1404M, S:T73I, S:T478K, S:D614G, S:P681H, S:T732A,
hCoV-19/Mexico/CMX-INMEGEN-01-04-05/2021	EPI_ISL_1040566	20B	B.1.1.519	26	5'UTR:C203T, 5'UTR:C222T, 5'UTR:C241T, ORF1ab:A866G, ORF1ab:C1009T, ORF1ab:C3037T, ORF1ab:C3140T, ORF1ab:C5183T, ORF1ab:C10029T, ORF1ab:C10954T, ORF1ab:A11117G, ORF1ab:C11916T, ORF1ab:C12789T, ORF1ab:C14408T, ORF1ab:T19839C, ORF1ab:C21306T, S:G22992T, S:C22995A, S:A23403G, S:C23604A, S:A23756G, ORF8:T27904C, N:G28747T, N:G28881A, N:G28882A, N:G28883C, N:C29197T,	16	N:R203K, N:G204R, ORF1a:I201V, ORF1a:P959S, ORF1a:P1640S, ORF1a:T3255I, ORF1a:I3618V, ORF1a:S3884L, ORF1a:T4175I, ORF1b:P314L, ORF8:L4P, S:S477I, S:T478K, S:D614G, S:P681H, S:T732A,

hCoV-19/Mexico/CMX-INMEGEN-01-04-06/2021	EPI_ISL_1040567	20B	B.1.1.519	30	5'UTR:T201C, 5'UTR:C203T, 5'UTR:C222T, 5'UTR:C241T, 5'UTR:G242T, ORF1ab:C936T, ORF1ab:G1738T, ORF1ab:C3037T, ORF1ab:C3140T, ORF1ab:C10029T, ORF1ab:C10954T, ORF1ab:A11117G, ORF1ab:C11575T, ORF1ab:C12789T, ORF1ab:C13105T, ORF1ab:C14408T, ORF1ab:T19839C, ORF1ab:A19974G, ORF1ab:C21306T, S:C22978T, S:C22995A, S:A23403G, S:C23604A, S:A23756G, S:G24697T, N:G28881A, N:G28882A, N:G28883C, N:C29197T, N:G29227T, N:G29300C,	14	N:R203K, N:G204R, N:D343H, ORF1a:T224I, ORF1a:P959S, ORF1a:T3255I, ORF1a:I3618V, ORF1a:T4175I, ORF1b:P314L, S:T478K, S:D614G, S:P681H, S:T732A, S:K1045N,	
hCoV-19/Mexico/CMX-INMEGEN-01-04-07/2021	EPI_ISL_1040574	20B	B.1.1.519	22	5'UTR:C203T, 5'UTR:C222T, 5'UTR:C241T, ORF1ab:C3037T, ORF1ab:C3140T, ORF1ab:C5298T, ORF1ab:C10029T, ORF1ab:C10954T, ORF1ab:A11117G, ORF1ab:C12789T, ORF1ab:C14408T, ORF1ab:T19839C, ORF1ab:C21306T, S:C21742T, S:C22995A, S:A23403G, S:C23604A, S:A23756G, N:G28881A, N:G28882A, N:G28883C, N:C29197T, N:G29527T,	13	N:R203K, N:G204R, N:Q418H, ORF1a:P959S, ORF1a:T1678I, ORF1a:T3255I, ORF1a:I3618V, ORF1a:T4175I, ORF1b:P314L, S:T478K, S:D614G, S:P681H, S:T732A,	
hCoV-19/Mexico/CMX-INMEGEN-01-04-08/2021	EPI_ISL_1040568	20B	B.1.1.519	29	5'UTR:C203T, 5'UTR:C222T, 5'UTR:C241T, ORF1ab:A866G, ORF1ab:C1009T, ORF1ab:C3037T, ORF1ab:C3140T, ORF1ab:C5183T, ORF1ab:C10029T, ORF1ab:C10954T, ORF1ab:A11117G, ORF1ab:C11916T, ORF1ab:C12789T, ORF1ab:G13201A, ORF1ab:C13730T, ORF1ab:C14408T, ORF1ab:C15720T, ORF1ab:T19839C, ORF1ab:C21306T, S:C22995A, S:A23403G, S:C23604A, S:A23756G, ORF8:T27904C, N:G28747T, N:G28881A, N:G28882A, N:G28883C, N:G28975T, N:C29197T,	17	N:R203K, N:G204R, N:M234I, ORF1a:I201V, ORF1a:P959S, ORF1a:P1640S, ORF1a:T3255I, ORF1a:I3618V, ORF1a:S3884L, ORF1a:T4175I, ORF1b:A88V, ORF1b:P314L, ORF8:L4P, S:T478K, S:D614G, S:P681H, S:T732A,	
hCoV-19/Mexico/CMX-INMEGEN-01-04-09/2021	EPI_ISL_1040569	20B	B.1.1.222	24	5'UTR:C241T, ORF1ab:C335T, ORF1ab:C3037T, ORF1ab:A3557G, ORF1ab:C10449T, ORF1ab:C11747T, ORF1ab:C12970T, ORF1ab:C14408T, ORF1ab:C14805T, ORF1ab:C19554T, ORF1ab:T19839C, S:C21646T, S:G22344C, S:G23262A, S:A23403G, S:G23587T, S:A23756G, ORF3a:C26151T, ORF7a:T27722C, N:G28881A, N:G28882A, N:G28883C, N:G28903T, N:C29200T, ORF10:T29577G,	13	N:R203K, N:G204R, N:M210I, ORF1a:R24C, ORF1a:K1098E, ORF1a:P3395L, ORF1b:P314L, ORF7a:I110T, S:G261A, S:R567K, S:D614G, S:Q675H, S:T732A,	
hCoV-19/Mexico/CMX-INMEGEN-01-04-10/2021	EPI_ISL_1040575	20B	B.1.1.519	24	5'UTR:C203T, 5'UTR:C222T, 5'UTR:C241T, ORF1ab:C3037T, ORF1ab:C3140T, ORF1ab:C5646T, ORF1ab:C7355T, ORF1ab:C10029T, ORF1ab:C10954T, ORF1ab:A11117G, ORF1ab:C11322T, ORF1ab:C12789T, ORF1ab:C14408T, ORF1ab:A15864G, ORF1ab:A17205G, ORF1ab:T19839C, ORF1ab:C21306T, S:C22995A, S:A23403G, S:C23604A, S:A23756G, N:G28881A, N:G28882A, N:G28883C, N:C29197T,	14	N:R203K, N:G204R, ORF1a:P959S, ORF1a:T1794I, ORF1a:L2364F, ORF1a:T3255I, ORF1a:I3618V, ORF1a:A3686V, ORF1a:T4175I, ORF1b:P314L, S:T478K, S:D614G, S:P681H, S:T732A,	
hCoV-19/Mexico/CMX-INMEGEN-01-04-11/2021	EPI_ISL_1040560	20B	B.1.1.519	26	5'UTR:C203T, 5'UTR:A223T, 5'UTR:C241T, ORF1ab:C3037T, ORF1ab:C3140T, ORF1ab:A6276G, ORF1ab:T9456G, ORF1ab:C10029T, ORF1ab:C10954T, ORF1ab:A11117G, ORF1ab:C12789T, ORF1ab:C14408T, ORF1ab:C15720T, ORF1ab:T19839C, ORF1ab:C21306T, S:C22995A, S:A23403G, S:C23604A, S:A23756G, S:C24919T, ORF3a:C25521T, N:C28866T, N:G28881A, N:G28882A, N:G28883C, N:C29197T, 3'UTR:C29769T,	14	N:T198I, N:R203K, N:G204R, ORF1a:P959S, ORF1a:K2004R, ORF1a:F3064C, ORF1a:T3255I, ORF1a:I3618V, ORF1a:T4175I, ORF1b:P314L, S:T478K, S:D614G, S:P681H, S:T732A,	5'UTR:2 22,

hCoV-19/Mexico/CMX-INMEGEN-01-04-12/2021	EPI_ISL_1040562	20B	B.1.1.519	23	5'UTR:C203T, 5'UTR:C222T, 5'UTR:C241T, ORF1ab:C1960T, ORF1ab:C3037T, ORF1ab:C3140T, ORF1ab:G7037T, ORF1ab:C10029T, ORF1ab:C10954T, ORF1ab:A11117G, ORF1ab:C12789T, ORF1ab:C14408T, ORF1ab:T19839C, ORF1ab:C21306T, S:C22995A, S:A23403G, S:C23604A, S:A23756G, S:C24099T, ORF3a:C25782T, N:G28881A, N:G28882A, N:G28883C, N:C29197T,	13	N:R203K, N:G204R, ORF1a:P959S, ORF1a:G2258C, ORF1a:T3255I, ORF1a:I3618V, ORF1a:T4175I, ORF1b:P314L, S:T478K, S:D614G, S:P681H, S:T732A, S:A846V,
hCoV-19/Mexico/CMX-INMEGEN-01-04-13/2021	EPI_ISL_1040565	20B	B.1.1.519	27	5'UTR:T201C, 5'UTR:C203T, 5'UTR:C222T, 5'UTR:C241T, ORF1ab:C936T, ORF1ab:C1498T, ORF1ab:G1738T, ORF1ab:C3037T, ORF1ab:C3140T, ORF1ab:C10029T, ORF1ab:C10954T, ORF1ab:A11117G, ORF1ab:C12789T, ORF1ab:G14371T, ORF1ab:C14408T, ORF1ab:T19839C, ORF1ab:A19974G, ORF1ab:C21306T, S:C22995A, S:A23403G, S:C23604A, S:A23756G, M:T26609C, N:G28881A, N:G28882A, N:G28883C, N:C29197T, N:G29227T,	13	N:R203K, N:G204R, ORF1a:T224I, ORF1a:P959S, ORF1a:T3255I, ORF1a:I3618V, ORF1a:T4175I, ORF1b:A302S, ORF1b:P314L, S:T478K, S:D614G, S:P681H, S:T732A,
hCoV-19/Mexico/CMX-INMEGEN-01-04-14/2021	EPI_ISL_1040570	20B	B.1.1.519	23	5'UTR:C203T, 5'UTR:C222T, 5'UTR:C241T, ORF1ab:C3037T, ORF1ab:C3140T, ORF1ab:C10029T, ORF1ab:C10954T, ORF1ab:A11117G, ORF1ab:C12789T, ORF1ab:C14408T, ORF1ab:T19839C, ORF1ab:C21077T, ORF1ab:C21306T, S:C22995A, S:A23403G, S:C23604A, S:A23756G, ORF3a:G25906T, ORF8:C28253T, N:G28881A, N:G28882A, N:G28883C, N:C29197T, 3'UTR:A29767G,	13	N:R203K, N:G204R, ORF1a:P959S, ORF1a:T3255I, ORF1a:I3618V, ORF1a:T4175I, ORF1b:P314L, ORF1b:T2537I, ORF3a:G172C, S:T478K, S:D614G, S:P681H, S:T732A,
hCoV-19/Mexico/CMX-INMEGEN-01-04-15/2021	EPI_ISL_1040561	20B	B.1.1.519	25	5'UTR:C203T, 5'UTR:C222T, 5'UTR:C241T, ORF1ab:C1191A, ORF1ab:C3037T, ORF1ab:C3140T, ORF1ab:C10029T, ORF1ab:C10954T, ORF1ab:A11117G, ORF1ab:C12789T, ORF1ab:C14408T, ORF1ab:T19839C, ORF1ab:T20246A, ORF1ab:C21219T, ORF1ab:C21306T, S:G22436T, S:C22995A, S:A23403G, S:C23604A, S:A23756G, N:G28881A, N:G28882A, N:G28883C, N:C29197T, N:G29315C, N:C29421A,	16	N:R203K, N:G204R, N:D348H, N:P383Q, ORF1a:P309Q, ORF1a:P959S, ORF1a:T3255I, ORF1a:I3618V, ORF1a:T4175I, ORF1b:P314L, ORF1b:M2260K, S:A292S, S:T478K, S:D614G, S:P681H, S:T732A,
hCoV-19/Mexico/CMX-INMEGEN-01-04-16/2021	EPI_ISL_1040563	20B	B.1.1.519	23	5'UTR:C203T, 5'UTR:C222T, 5'UTR:C241T, ORF1ab:C3037T, ORF1ab:C3140T, ORF1ab:C10029T, ORF1ab:C10954T, ORF1ab:A11117G, ORF1ab:G11365T, ORF1ab:C12789T, ORF1ab:G14268T, ORF1ab:C14408T, ORF1ab:T19839C, ORF1ab:C21306T, S:C22995A, S:A23403G, S:C23604A, S:A23756G, ORF3a:C25613T, N:G28881A, N:G28882A, N:G28883C, N:C29197T, N:G29527T,	13	N:R203K, N:G204R, N:Q418H, ORF1a:P959S, ORF1a:T3255I, ORF1a:I3618V, ORF1a:T4175I, ORF1b:P314L, ORF3a:S74F, S:T478K, S:D614G, S:P681H, S:T732A,
hCoV-19/Mexico/CMX-INMEGEN-01-04-17/2021	EPI_ISL_1040571	20B	B.1.1.519	28	5'UTR:C203T, 5'UTR:C222T, 5'UTR:C241T, ORF1ab:C1513T, ORF1ab:G1738T, ORF1ab:C3037T, ORF1ab:C3140T, ORF1ab:C10029T, ORF1ab:C10954T, ORF1ab:A11117G, ORF1ab:C12789T, ORF1ab:G14371T, ORF1ab:C14408T, ORF1ab:C17795T, ORF1ab:T17877A, ORF1ab:T19839C, ORF1ab:A19974G, ORF1ab:C21306T, S:C22995A, S:A23403G, S:C23604A, S:A23756G, ORF3a:C25844T, M:T26609C, N:G28881A, N:G28882A, N:G28883C, N:C29197T, N:G29227T,	14	N:R203K, N:G204R, ORF1a:P959S, ORF1a:T3255I, ORF1a:I3618V, ORF1a:T4175I, ORF1b:A302S, ORF1b:P314L, ORF1b:A1443V, ORF3a:T151I, S:T478K, S:D614G, S:P681H, S:T732A,

hCoV-19/Mexico/CMX-INMEGEN-01-04-18/2021	EPI_ISL_1040572	20B	B.1.1.322	19	5'UTR:C190T, 5'UTR:C241T, ORF1ab:C3037T, ORF1ab:C3140T, ORF1ab:C10954T, ORF1ab:C14408T, ORF1ab:T19839C, ORF1ab:C21306T, S:C22995A, S:A23403G, S:G23426A, S:C23604A, S:A23756G, N:A28877T, N:G28878C, N:G28881A, N:G28882A, N:G28883C, N:C29197T, 3'UTR:A29767G,	9	N:R203K, N:G204R, ORF1a:P959S, ORF1b:P314L, S:T478K, S:D614G, S:V622I, S:P681H, S:T732A,
hCoV-19/Mexico/CMX-INMEGEN-01-04-19/2021	EPI_ISL_1040576	20B	B.1.1.519	28	5'UTR:T201C, 5'UTR:C203T, 5'UTR:C222T, 5'UTR:C241T, ORF1ab:C280T, ORF1ab:C1191A, ORF1ab:C3037T, ORF1ab:C3140T, ORF1ab:C7391T, ORF1ab:C8240T, ORF1ab:C10029T, ORF1ab:C10954T, ORF1ab:A11117G, ORF1ab:C12789T, ORF1ab:C14408T, ORF1ab:T19839C, ORF1ab:C21219T, ORF1ab:C21306T, S:T21633C, S:G22436T, S:C22995A, S:A23403G, S:C23604A, S:A23756G, N:G28881A, N:G28882A, N:G28883C, N:C29197T, N:G29315C	17	N:R203K, N:G204R, N:D348H, ORF1a:P309Q, ORF1a:P959S, ORF1a:P2376S, ORF1a:H2659Y, ORF1a:T3255I, ORF1a:I3618V, ORF1a:T4175I, ORF1b:P314L, S:L24S, S:A292S, S:T478K, S:D614G, S:P681H, S:T732A,
hCoV-19/Mexico/CMX-INMEGEN-01-04-20/2021	EPI_ISL_1040577	20B	B.1.1.519	22	5'UTR:C203T, 5'UTR:C222T, 5'UTR:C241T, ORF1ab:C3037T, ORF1ab:C3140T, ORF1ab:A8833T, ORF1ab:C10029T, ORF1ab:C10954T, ORF1ab:A11117G, ORF1ab:C12789T, ORF1ab:G14268T, ORF1ab:C14408T, ORF1ab:T19839C, ORF1ab:C21306T, S:C22995A, S:A23403G, S:C23604A, S:A23756G, ORF3a:C25613T, N:G28881A, N:G28882A, N:G28883C, N:C29197T,	12	N:R203K, N:G204R, ORF1a:P959S, ORF1a:T3255I, ORF1a:I3618V, ORF1a:T4175I, ORF1b:P314L, ORF3a:S74F, S:T478K, S:D614G, S:P681H, S:T732A,
hCoV-19/Mexico/CMX-INMEGEN-01-05-01/2021	EPI_ISL_1040591	20B	B.1.1.519	22	5'UTR:C203T, 5'UTR:C222T, 5'UTR:C241T, ORF1ab:C3037T, ORF1ab:C3140T, ORF1ab:G7037T, ORF1ab:T9004C, ORF1ab:C10029T, ORF1ab:C10954T, ORF1ab:A11117G, ORF1ab:C12789T, ORF1ab:C14408T, ORF1ab:T19839C, ORF1ab:C21306T, S:C22995A, S:A23403G, S:C23604A, S:A23756G, ORF3a:C25782T, N:G28881A, N:G28882A, N:G28883C, N:C29197T,	12	N:R203K, N:G204R, ORF1a:P959S, ORF1a:G2258C, ORF1a:T3255I, ORF1a:I3618V, ORF1a:T4175I, ORF1b:P314L, S:T478K, S:D614G, S:P681H, S:T732A,
hCoV-19/Mexico/CMX-INMEGEN-01-05-02/2021	EPI_ISL_1040593	20B	B.1.1.519	23	5'UTR:C203T, 5'UTR:C222T, 5'UTR:C241T, ORF1ab:C3037T, ORF1ab:C3140T, ORF1ab:C7420T, ORF1ab:G8017T, ORF1ab:C10029T, ORF1ab:C10954T, ORF1ab:A11117G, ORF1ab:C12789T, ORF1ab:C14408T, ORF1ab:C18508A, ORF1ab:T19839C, ORF1ab:C21306T, S:C22995A, S:A23403G, S:C23604A, S:G23628T, S:A23756G, N:G28881A, N:G28882A, N:G28883C, N:C29197T,	13	N:R203K, N:G204R, ORF1a:P959S, ORF1a:T3255I, ORF1a:I3618V, ORF1a:T4175I, ORF1b:P314L, ORF1b:L1681I, S:T478K, S:D614G, S:P681H, S:S689I, S:T732A,
hCoV-19/Mexico/CMX-INMEGEN-01-05-03/2021	EPI_ISL_1040594	20B	B.1.1.519	23	5'UTR:T201C, 5'UTR:C203T, 5'UTR:C222T, 5'UTR:C241T, ORF1ab:G1738T, ORF1ab:C3037T, ORF1ab:C3140T, ORF1ab:C10029T, ORF1ab:C10954T, ORF1ab:A11117G, ORF1ab:C12789T, ORF1ab:C13384T, ORF1ab:C14408T, ORF1ab:T19839C, ORF1ab:A19974G, ORF1ab:C21306T, S:C22995A, S:A23403G, S:C23604A, S:A23756G, N:G28881A, N:G28882A, N:G28883C, N:C29197T,	11	N:R203K, N:G204R, ORF1a:P959S, ORF1a:T3255I, ORF1a:I3618V, ORF1a:T4175I, ORF1b:P314L, S:T478K, S:D614G, S:P681H, S:T732A,
hCoV-19/Mexico/CMX-INMEGEN-01-05-04/2021	EPI_ISL_1040592	20B	B.1.1.519	26	5'UTR:T201C, 5'UTR:C203T, 5'UTR:C222T, 5'UTR:C241T, ORF1ab:G1738T, ORF1ab:T2984C, ORF1ab:C3037T, ORF1ab:C3140T, ORF1ab:C10029T, ORF1ab:C10954T, ORF1ab:A11117G, ORF1ab:C12789T, ORF1ab:C14408T, ORF1ab:C18646T, ORF1ab:A19453G, ORF1ab:T19839C, ORF1ab:A19974G, ORF1ab:C21306T, S:C22995A, S:A23403G, S:C23604A, S:A23756G, S:G25337T, N:G28881A, N:G28882A, N:G28883C, N:C29197T,	14	N:R203K, N:G204R, ORF1a:P959S, ORF1a:T3255I, ORF1a:I3618V, ORF1a:T4175I, ORF1b:P314L, ORF1b:P1727S, ORF1b:T1996A, S:T478K, S:D614G, S:P681H, S:T732A, S:D1259Y,

hCoV-19/Mexico/CMX-INMEGEN-01-05-05/2021	EPI_ISL_1040587	20B	B.1.1.519	25	5'UTR:C203T, 5'UTR:C222T, 5'UTR:C241T, ORF1ab:C3037T, ORF1ab:C3140T, ORF1ab:C3425T, ORF1ab:T3745C, ORF1ab:C9810T, ORF1ab:C10029T, ORF1ab:C10954T, ORF1ab:A11117G, ORF1ab:C12789T, ORF1ab:C14408T, ORF1ab:T19839C, ORF1ab:C21306T, S:C22591T, S:C22995A, S:A23403G, S:C23604A, S:A23756G, ORF3a:C25844T, N:C28789T, N:G28881A, N:G28882A, N:G28883C, N:C29197T,	14	N:R203K, N:G204R, ORF1a:P959S, ORF1a:P1054S, ORF1a:T3182I, ORF1a:T3255I, ORF1a:I3618V, ORF1a:T4175I, ORF1b:P314L, ORF3a:T151I, S:T478K, S:D614G, S:P681H, S:T732A,	
hCoV-19/Mexico/CMX-INMEGEN-01-05-06/2021	EPI_ISL_1040578	20B	B.1.1.519	22	5'UTR:C203T, 5'UTR:C222T, 5'UTR:C241T, ORF1ab:C3037T, ORF1ab:C3140T, ORF1ab:C10029T, ORF1ab:C10954T, ORF1ab:A11117G, ORF1ab:G11365T, ORF1ab:C12789T, ORF1ab:C14408T, ORF1ab:T19839C, ORF1ab:C21306T, S:C22995A, S:A23403G, S:C23604A, S:A23756G, N:T28861C, N:G28881A, N:G28882A, N:G28883C, N:C29197T, N:G29527T,	12	N:R203K, N:G204R, N:Q418H, ORF1a:P959S, ORF1a:T3255I, ORF1a:I3618V, ORF1a:T4175I, ORF1b:P314L, S:T478K, S:D614G, S:P681H, S:T732A,	
hCoV-19/Mexico/CMX-INMEGEN-01-05-07/2021	EPI_ISL_1040580	20B	B.1.1.519	24	5'UTR:T201C, 5'UTR:C203T, 5'UTR:C222T, 5'UTR:C241T, ORF1ab:C745T, ORF1ab:G1738T, ORF1ab:C2189T, ORF1ab:C3037T, ORF1ab:C3140T, ORF1ab:C10029T, ORF1ab:C10954T, ORF1ab:A11117G, ORF1ab:C12789T, ORF1ab:C14408T, ORF1ab:T19839C, ORF1ab:A19974G, ORF1ab:C21306T, S:C22995A, S:A23403G, S:C23604A, S:A23756G, N:G28881A, N:G28882A, N:G28883C, N:C29197T,	12	N:R203K, N:G204R, ORF1a:L642F, ORF1a:P959S, ORF1a:T3255I, ORF1a:I3618V, ORF1a:T4175I, ORF1b:P314L, S:T478K, S:D614G, S:P681H, S:T732A,	3'UTR:29738-29748,
hCoV-19/Mexico/CMX-INMEGEN-01-05-08/2021	EPI_ISL_1040584	20B	B.1.1.519	23	5'UTR:C203T, 5'UTR:C222T, 5'UTR:C241T, ORF1ab:C3037T, ORF1ab:C3140T, ORF1ab:T5983C, ORF1ab:C10029T, ORF1ab:A10855T, ORF1ab:C10954T, ORF1ab:A11117G, ORF1ab:C12789T, ORF1ab:C14408T, ORF1ab:T19839C, ORF1ab:C20429T, ORF1ab:C21306T, S:C22995A, S:A23403G, S:C23604A, S:A23756G, N:G28881A, N:G28882A, N:G28883C, N:C29197T, N:G29527T,	13	N:R203K, N:G204R, N:Q418H, ORF1a:P959S, ORF1a:T3255I, ORF1a:I3618V, ORF1a:T4175I, ORF1b:P314L, ORF1b:P2321L, S:T478K, S:D614G, S:P681H, S:T732A,	
hCoV-19/Mexico/CMX-INMEGEN-01-05-09/2021	EPI_ISL_1040595	20B	B.1.1.519	22	5'UTR:C203T, 5'UTR:C222T, 5'UTR:C241T, ORF1ab:C3037T, ORF1ab:C3140T, ORF1ab:C5183T, ORF1ab:C10029T, ORF1ab:C10954T, ORF1ab:A11117G, ORF1ab:C12789T, ORF1ab:C14408T, ORF1ab:T19839C, ORF1ab:C21306T, S:C22995A, S:A23403G, S:C23604A, S:A23756G, ORF3a:C25513T, ORF8:T27904C, N:G28881A, N:G28882A, N:G28883C, N:C29197T,	14	N:R203K, N:G204R, ORF1a:P959S, ORF1a:P1640S, ORF1a:T3255I, ORF1a:I3618V, ORF1a:T4175I, ORF1b:P314L, ORF3a:L41F, ORF8:L4P, S:T478K, S:D614G, S:P681H, S:T732A,	
hCoV-19/Mexico/CMX-INMEGEN-01-05-10/2021	EPI_ISL_1040596	20A	B.1.400	17	5'UTR:C241T, ORF1ab:G376T, ORF1ab:C3037T, ORF1ab:C4345T, ORF1ab:C10156T, ORF1ab:C11339T, ORF1ab:C11671T, ORF1ab:C14408T, ORF1ab:G18286A, ORF1ab:A19782T, ORF1ab:G19969A, ORF1ab:A20268G, S:A23403G, ORF3a:C26195T, M:C26936T, N:C28854T, N:A29437G, 3'UTR:C29774T,	7	N:S194L, ORF1a:E37D, ORF1b:P314L, ORF1b:V1607I, ORF1b:A2168T, ORF3a:T268M, S:D614G,	
hCoV-19/Mexico/CMX-INMEGEN-01-05-11/2021	EPI_ISL_1040585	20B	B.1.1.519	26	5'UTR:T201C, 5'UTR:C203T, 5'UTR:C222T, 5'UTR:C241T, ORF1ab:G1738T, ORF1ab:T2593C, ORF1ab:C3037T, ORF1ab:C3140T, ORF1ab:C9442T, ORF1ab:C10029T, ORF1ab:G10283A, ORF1ab:C10954T, ORF1ab:A11117G, ORF1ab:C12789T, ORF1ab:C14408T, ORF1ab:T19839C, ORF1ab:A19974G, ORF1ab:C21306T, S:C22995A, S:A23403G, S:C23604A, S:A23756G, N:G28881A, N:G28882A, N:G28883C, N:C29197T, 3'UTR:G29737T,	12	N:R203K, N:G204R, ORF1a:P959S, ORF1a:T3255I, ORF1a:V3340I, ORF1a:I3618V, ORF1a:T4175I, ORF1b:P314L, S:T478K, S:D614G, S:P681H, S:T732A,	

hCoV-19/Mexico/CMX-INMEGEN-01-05-12/2021	EPI_ISL_1040586	20B	B.1.1.519	22	5'UTR:C203T, 5'UTR:C222T, 5'UTR:C241T, ORF1ab:C3037T, ORF1ab:C3140T, ORF1ab:C5183T, ORF1ab:C10029T, ORF1ab:C10954T, ORF1ab:A11117G, ORF1ab:C12789T, ORF1ab:C14408T, ORF1ab:T19839C, ORF1ab:C21306T, S:C22995A, S:A23403G, S:C23604A, S:A23756G, ORF3a:C25513T, ORF8:T27904C, N:G28881A, N:G28882A, N:G28883C, N:C29197T.	14	N:R203K, N:G204R, ORF1a:P959S, ORF1a:P1640S, ORF1a:T3255I, ORF1a:I3618V, ORF1a:T4175I, ORF1b:P314L, ORF3a:L41F, ORF8:L4P, S:T478K, S:D614G, S:P681H, S:T732A,	
hCoV-19/Mexico/CMX-INMEGEN-01-05-13/2021	EPI_ISL_1040579	20B	B.1.1.222	14	5'UTR:C241T, ORF1ab:C3037T, ORF1ab:C5654T, ORF1ab:C10029T, ORF1ab:C14408T, ORF1ab:T19839C, S:A23403G, S:A23756G, ORF3a:G25912T, ORF7b:A27756G, ORF8:A27921G, ORF8:G28001T, N:G28881A, N:G28882A, N:G28883C,	9	N:R203K, N:G204R, ORF1a:T3255I, ORF1b:P314L, ORF3a:G174C, ORF7b:M1V, ORF8:I10V, S:D614G, S:T732A,	3'UTR:2 9746- 29762,
hCoV-19/Mexico/CMX-INMEGEN-01-05-14/2021	EPI_ISL_1040588	20B	B.1.1.519	25	5'UTR:C203T, 5'UTR:C222T, 5'UTR:C241T, ORF1ab:C3037T, ORF1ab:C3140T, ORF1ab:T3745C, ORF1ab:A6390G, ORF1ab:C10029T, ORF1ab:A10323G, ORF1ab:C10954T, ORF1ab:A11117G, ORF1ab:C12789T, ORF1ab:C14408T, ORF1ab:A15423G, ORF1ab:T19839C, ORF1ab:A20052G, ORF1ab:C21306T, S:C22995A, S:A23403G, S:C23604A, S:A23756G, ORF3a:C25844T, N:G28881A, N:G28882A, N:G28883C, N:C29197T,	14	N:R203K, N:G204R, ORF1a:P959S, ORF1a:E2042G, ORF1a:T3255I, ORF1a:K3353R, ORF1a:I3618V, ORF1a:T4175I, ORF1b:P314L, ORF3a:T151I, S:T478K, S:D614G, S:P681H, S:T732A,	
hCoV-19/Mexico/CMX-INMEGEN-01-05-15/2021	EPI_ISL_1040589	20B	B.1.1.519	26	5'UTR:C203T, 5'UTR:C222T, 5'UTR:C241T, ORF1ab:C3037T, ORF1ab:C3140T, ORF1ab:C10029T, ORF1ab:A10630G, ORF1ab:C10954T, ORF1ab:A11117G, ORF1ab:C12789T, ORF1ab:C14408T, ORF1ab:T19839C, ORF1ab:C21306T, S:C22995A, S:A23403G, S:C23604A, S:A23756G, S:G24914T, S:C25254T, ORF3a:C25642T, ORF3a:C25782T, ORF3a:G26063T, N:G28881A, N:G28882A, N:G28883C, N:C29197T, N:G29402T.	15	N:R203K, N:G204R, N:D377Y, ORF1a:P959S, ORF1a:T3255I, ORF1a:I3618V, ORF1a:T4175I, ORF1b:P314L, ORF3a:G224V, S:T478K, S:D614G, S:P681H, S:T732A, S:D1118Y, S:T1231I,	
hCoV-19/Mexico/CMX-INMEGEN-01-05-16/2021	EPI_ISL_1040590	20B	B.1.1.519	24	5'UTR:C203T, 5'UTR:C222T, 5'UTR:C241T, ORF1ab:C3037T, ORF1ab:C3140T, ORF1ab:G7037T, ORF1ab:C10029T, ORF1ab:C10954T, ORF1ab:A11117G, ORF1ab:C12789T, ORF1ab:C13860T, ORF1ab:C14408T, ORF1ab:C19560A, ORF1ab:T19839C, ORF1ab:C21306T, S:A22528G, S:C22995A, S:A23403G, S:C23604A, S:A23756G, ORF3a:C25782T, N:G28881A, N:G28882A, N:G28883C, N:C29197T.	13	N:R203K, N:G204R, ORF1a:P959S, ORF1a:G2258C, ORF1a:T3255I, ORF1a:I3618V, ORF1a:T4175I, ORF1b:P314L, ORF1b:S2031R, S:T478K, S:D614G, S:P681H, S:T732A,	
hCoV-19/Mexico/CMX-INMEGEN-01-05-17/2021	EPI_ISL_1040598	20B	B.1.1.519	25	5'UTR:T201C, 5'UTR:C203T, 5'UTR:C222T, 5'UTR:C241T, ORF1ab:C745T, ORF1ab:G1738T, ORF1ab:C3037T, ORF1ab:C3140T, ORF1ab:G5276A, ORF1ab:C10029T, ORF1ab:C10954T, ORF1ab:A11117G, ORF1ab:C12789T, ORF1ab:C14408T, ORF1ab:T19839C, ORF1ab:A19974G, ORF1ab:G21123T, ORF1ab:C21306T, S:C22995A, S:A23403G, S:C23604A, S:A23756G, N:G28881A, N:G28882A, N:G28883C, N:C29197T.	12	N:R203K, N:G204R, ORF1a:P959S, ORF1a:D1671N, ORF1a:T3255I, ORF1a:I3618V, ORF1a:T4175I, ORF1b:P314L, S:T478K, S:D614G, S:P681H, S:T732A,	
hCoV-19/Mexico/CMX-INMEGEN-01-05-18/2021	EPI_ISL_1040597	20B	B.1.1.519	25	5'UTR:T201C, 5'UTR:C203T, 5'UTR:C222T, 5'UTR:C241T, ORF1ab:G1738T, ORF1ab:C3037T, ORF1ab:C3140T, ORF1ab:G3753A, ORF1ab:C10029T, ORF1ab:C10954T, ORF1ab:A11117G, ORF1ab:C12789T, ORF1ab:C14408T, ORF1ab:G18020A, ORF1ab:T19839C, ORF1ab:A19974G, ORF1ab:C21306T, S:T22573G, S:C22995A, S:A23403G, S:C23604A, S:A23756G, N:G28881A, N:G28882A, N:G28883C, N:C29197T.	13	N:R203K, N:G204R, ORF1a:P959S, ORF1a:R1163K, ORF1a:T3255I, ORF1a:I3618V, ORF1a:T4175I, ORF1b:P314L, ORF1b:R1518K, S:T478K, S:D614G, S:P681H, S:T732A,	

hCoV-19/Mexico/CMX-INMEGEN-01-05-19/2021	EPI_ISL_1040581	20B	B.1.1.519	23	5'UTR:C203T, 5'UTR:C222T, 5'UTR:C241T, ORF1ab:C3037T, ORF1ab:C3140T, ORF1ab:C10029T, ORF1ab:C10078T, ORF1ab:C10954T, ORF1ab:A11117G, ORF1ab:G11365T, ORF1ab:C12789T, ORF1ab:G14350T, ORF1ab:C14408T, ORF1ab:T19839C, ORF1ab:C21306T, S:C22995A, S:A23403G, S:C23604A, S:A23756G, N:G28881A, N:G28882A, N:G28883C, N:C29197T, N:G29527T,	13	N:R203K, N:G204R, N:Q418H, ORF1a:P959S, ORF1a:T3255I, ORF1a:I3618V, ORF1a:T4175I, ORF1b:D295Y, ORF1b:P314L, S:T478K, S:D614G, S:P681H, S:T732A,
hCoV-19/Mexico/CMX-INMEGEN-01-05-20/2021	EPI_ISL_1040582	20B	B.1.1.519	26	5'UTR:C203T, 5'UTR:C222T, 5'UTR:C241T, ORF1ab:C3037T, ORF1ab:C3140T, ORF1ab:C5183T, ORF1ab:C7869T, ORF1ab:C10029T, ORF1ab:C10954T, ORF1ab:A11117G, ORF1ab:C12789T, ORF1ab:C13862T, ORF1ab:C14408T, ORF1ab:T19839C, ORF1ab:C21306T, S:C21575T, S:C22995A, S:A23403G, S:C23604A, S:A23756G, S:G24834A, M:C26681T, ORF8:T27904C, N:G28881A, N:G28882A, N:G28883C, N:C29197T,	17	N:R203K, N:G204R, ORF1a:P959S, ORF1a:P1640S, ORF1a:S2535L, ORF1a:T3255I, ORF1a:I3618V, ORF1a:T4175I, ORF1b:T132I, ORF1b:P314L, ORF8:L4P, S:L5F, S:T478K, S:D614G, S:P681H, S:T732A, S:R1091H,
hCoV-19/Mexico/CMX-INMEGEN-01-05-21/2021	EPI_ISL_1040599	20B	B.1.1	23	5'UTR:C203T, 5'UTR:C222T, 5'UTR:C241T, ORF1ab:T565C, ORF1ab:C1613T, ORF1ab:C3037T, ORF1ab:C3140T, ORF1ab:G6901T, ORF1ab:C10029T, ORF1ab:C10954T, ORF1ab:A11117G, ORF1ab:C12789T, ORF1ab:C12896T, ORF1ab:C14408T, ORF1ab:T19839C, ORF1ab:C21306T, S:C22995A, S:A23403G, S:C23604A, S:A23756T, N:G28881A, N:G28882A, N:G28883C, N:C29197T,	14	N:R203K, N:G204R, ORF1a:L450F, ORF1a:P959S, ORF1a:E2212D, ORF1a:T3255I, ORF1a:I3618V, ORF1a:T4175I, ORF1a:P4211S, ORF1b:P314L, S:T478K, S:D614G, S:P681H, S:T732S,
hCoV-19/Mexico/CMX-INMEGEN-01-05-23/2021	EPI_ISL_1040583	20B	B.1.1.519	22	5'UTR:C203T, 5'UTR:C222T, 5'UTR:C241T, ORF1ab:C3037T, ORF1ab:C3140T, ORF1ab:C10029T, ORF1ab:C10954T, ORF1ab:A11117G, ORF1ab:C12789T, ORF1ab:C14408T, ORF1ab:T19839C, ORF1ab:C21306T, S:C22995A, S:A23403G, S:C23604A, S:A23756G, ORF8:T27919C, N:C28435T, N:G28881A, N:G28882A, N:G28883C, N:C29197T, ORF10:G29645T,	13	N:R203K, N:G204R, ORF1a:P959S, ORF1a:T3255I, ORF1a:I3618V, ORF1a:T4175I, ORF1b:P314L, ORF8:I9T, ORF9b:P51L, S:T478K, S:D614G, S:P681H, S:T732A,
hCoV-19/Mexico/CMX-INMEGEN-02/2020	EPI_ISL_522873	20B	B.1.1	15	5'UTR:C241T, ORF1ab:C3037T, ORF1ab:C3140T, ORF1ab:G6865T, ORF1ab:C7125A, ORF1ab:C14408T, ORF1ab:T14769C, ORF1ab:T19839C, ORF1ab:C20436T, S:C21727T, S:A23403G, S:A23756G, N:G28881A, N:G28882A, N:G28883C, N:G29227T,	8	N:R203K, N:G204R, ORF1a:P959S, ORF1a:K2200N, ORF1a:P2287H, ORF1b:P314L, S:D614G, S:T732A,
hCoV-19/Mexico/CMX-INMEGEN-02-01-01/2021	EPI_ISL_1040618	20B	B.1.1.222	22	5'UTR:C241T, ORF1ab:C3037T, ORF1ab:C4897T, ORF1ab:C5259T, ORF1ab:C6255T, ORF1ab:C6936T, ORF1ab:C10029T, ORF1ab:A11430G, ORF1ab:C14408T, ORF1ab:C19011A, ORF1ab:T19839C, ORF1ab:A21137G, S:A23403G, S:A23756G, ORF3a:C25603T, ORF3a:G25912T, ORF3a:A26115C, ORF8:A27921G, ORF8:G28001T, N:G28881A, N:G28882A, N:G28883C, 3'UTR:A29700G,	15	N:R203K, N:G204R, ORF1a:T1665I, ORF1a:A1997V, ORF1a:S2224F, ORF1a:T3255I, ORF1a:Y3722C, ORF1b:P314L, ORF1b:D1848E, ORF1b:K2557R, ORF3a:G174C, ORF3a:E241D, ORF8:I10V, S:D614G, S:T732A,
hCoV-19/Mexico/CMX-INMEGEN-02-01-02/2021	EPI_ISL_1040621	20B	B.1.1.519	25	5'UTR:C203T, 5'UTR:C222T, 5'UTR:C241T, ORF1ab:C3037T, ORF1ab:C3140T, ORF1ab:G6894A, ORF1ab:C10029T, ORF1ab:C10954T, ORF1ab:A11117G, ORF1ab:G11365T, ORF1ab:C12789T, ORF1ab:C14408T, ORF1ab:T19839C, ORF1ab:C21306T, S:C22995A, S:A23403G, S:C23604A, S:A23756G, ORF3a:G25440T, ORF7a:C27524T, ORF7a:A27525G, N:G28881A, N:G28882A, N:G28883C, N:C29197T, N:G29527T,	15	N:R203K, N:G204R, N:Q418H, ORF1a:P959S, ORF1a:C2210Y, ORF1a:T3255I, ORF1a:I3618V, ORF1a:T4175I, ORF1b:P314L, ORF3a:K16N, ORF7a:S44L, S:T478K, S:D614G, S:P681H, S:T732A,

hCoV-19/Mexico/CMX-INMEGEN-02-01-03/2021	EPI_ISL_1040622	20B	B.1.1.519	27	5'UTR:T201C, 5'UTR:C203T, 5'UTR:C222T, 5'UTR:C241T, ORF1ab:C1190T, ORF1ab:G1738T, ORF1ab:C3037T, ORF1ab:C3140T, ORF1ab:C10029T, ORF1ab:C10954T, ORF1ab:A11117G, ORF1ab:C12789T, ORF1ab:C14178T, ORF1ab:C14408T, ORF1ab:T19839C, ORF1ab:A19974G, ORF1ab:C21306T, S:C22995A, S:A23403G, S:C23604A, S:A23756G, E:A26435C, ORF8:A28175G, N:G28881A, N:G28882A, N:G28883C, N:C29197T, N:C29311T,	13	E:N64T, N:R203K, N:G204R, ORF1a:P309S, ORF1a:P959S, ORF1a:T3255I, ORF1a:I3618V, ORF1a:T4175I, ORF1b:P314L, S:T478K, S:D614G, S:P681H, S:T732A,	
hCoV-19/Mexico/CMX-INMEGEN-02-01-04/2021	EPI_ISL_1040604	20B	B.1.1.222	16	5'UTR:C241T, ORF1ab:C3037T, ORF1ab:T8682C, ORF1ab:C10029T, ORF1ab:C14408T, ORF1ab:T19839C, ORF1ab:C20402T, S:A23403G, S:A23756G, ORF3a:G25912T, ORF3a:C26195T, ORF7b:A27756G, ORF8:A27921G, ORF8:G28001T, N:G28881A, N:G28882A, N:G28883C,	12	N:R203K, N:G204R, ORF1a:I2806T, ORF1a:T3255I, ORF1b:P314L, ORF1b:S2312L, ORF3a:G174C, ORF3a:T268M, ORF7b:M1V, ORF8:I10V, S:D614G, S:T732A,	3'UTR:29746-29762,
hCoV-19/Mexico/CMX-INMEGEN-02-01-05/2021	EPI_ISL_1040611	20B	B.1.1.222	22	5'UTR:C241T, ORF1ab:C337T, ORF1ab:C3037T, ORF1ab:A12875G, ORF1ab:C13517T, ORF1ab:A13712G, ORF1ab:C14408T, ORF1ab:T15078C, ORF1ab:G16647T, ORF1ab:T17160G, ORF1ab:T18291C, ORF1ab:C19186T, ORF1ab:T19839C, ORF1ab:C21552T, S:A23403G, S:A23588G, S:A23756G, M:C26527T, M:C26964A, N:G28881A, N:G28882A, N:G28883C, N:T29206C,	11	M:A2V, M:H148N, N:R203K, N:G204R, ORF1a:T4204A, ORF1b:T17I, ORF1b:K82R, ORF1b:P314L, S:D614G, S:T676A, S:T732A,	
hCoV-19/Mexico/CMX-INMEGEN-02-01-06/2021	EPI_ISL_1040612	20B	B.1.1.222	22	5'UTR:C241T, ORF1ab:C337T, ORF1ab:C3037T, ORF1ab:A12875G, ORF1ab:C13517T, ORF1ab:A13712G, ORF1ab:C14408T, ORF1ab:T15078C, ORF1ab:G16647T, ORF1ab:T17160G, ORF1ab:T18291C, ORF1ab:C19186T, ORF1ab:T19839C, ORF1ab:C21552T, S:A23403G, S:A23588G, S:A23756G, M:C26527T, M:C26964A, N:G28881A, N:G28882A, N:G28883C, N:T29206C,	11	M:A2V, M:H148N, N:R203K, N:G204R, ORF1a:T4204A, ORF1b:T17I, ORF1b:K82R, ORF1b:P314L, S:D614G, S:T676A, S:T732A,	
hCoV-19/Mexico/CMX-INMEGEN-02-01-07/2021	EPI_ISL_1040608	20G	B.1.2	26	5'UTR:C241T, ORF1ab:C503T, ORF1ab:C1059T, ORF1ab:C1684T, ORF1ab:C3037T, ORF1ab:A6862G, ORF1ab:G8083A, ORF1ab:T8395C, ORF1ab:C10319T, ORF1ab:A13204T, ORF1ab:A13758T, ORF1ab:C14408T, ORF1ab:C14805T, ORF1ab:A18424G, ORF1ab:C18788T, ORF1ab:T20409C, ORF1ab:C21304T, S:A23403G, ORF3a:G25563T, ORF3a:G25907T, ORF6:G27382C, ORF6:A27383T, ORF6:T27384C, ORF8:C27964T, N:A28294T, N:C28472T, N:C28869T,	18	N:Q7H, N:P67S, N:P199L, ORF1a:P80S, ORF1a:T265I, ORF1a:M2606I, ORF1a:L3352F, ORF1a:E4313D, ORF1b:P314L, ORF1b:N1653D, ORF1b:T1774I, ORF1b:R2613C, ORF3a:Q57H, ORF3a:G172V, ORF6:D61L, ORF8:S24L, ORF9b:K4I, S:D614G,	
hCoV-19/Mexico/CMX-INMEGEN-02-01-08/2021	EPI_ISL_1040617	20B	B.1.1.519	21	5'UTR:C203T, 5'UTR:C222T, 5'UTR:C241T, ORF1ab:C3037T, ORF1ab:C3140T, ORF1ab:C10029T, ORF1ab:C10954T, ORF1ab:A11117G, ORF1ab:C12789T, ORF1ab:C14408T, ORF1ab:T19839C, ORF1ab:C21306T, S:C21911T, S:C22995A, S:A23403G, S:C23604A, S:A23756G, ORF8:T27904C, N:G28881A, N:G28882A, N:G28883C, N:C29197T,	12	N:R203K, N:G204R, ORF1a:P959S, ORF1a:T3255I, ORF1a:I3618V, ORF1a:T4175I, ORF1b:P314L, ORF8:L4P, S:T478K, S:D614G, S:P681H, S:T732A,	
hCoV-19/Mexico/CMX-INMEGEN-02-01-09/2021	EPI_ISL_1040619	20B	B.1.1.519	22	5'UTR:T201C, 5'UTR:C203T, 5'UTR:C222T, 5'UTR:C241T, ORF1ab:G1738T, ORF1ab:C3037T, ORF1ab:C3140T, ORF1ab:C10029T, ORF1ab:C10954T, ORF1ab:A11117G, ORF1ab:C12789T, ORF1ab:C14408T, ORF1ab:T19839C, ORF1ab:A19974G, ORF1ab:C21306T, S:C22995A, S:A23403G, S:C23604A, S:A23756G, N:G28881A, N:G28882A, N:G28883C, N:C29197T,	11	N:R203K, N:G204R, ORF1a:P959S, ORF1a:T3255I, ORF1a:I3618V, ORF1a:T4175I, ORF1b:P314L, S:T478K, S:D614G, S:P681H, S:T732A,	

hCoV-19/Mexico/CMX-INMEGEN-02-01-10/2021	EPI_ISL_1040613	20B	B.1.1.519	25	5'UTR:T201C, 5'UTR:C203T, 5'UTR:C222T, 5'UTR:C241T, ORF1ab:G1738T, ORF1ab:C3037T, ORF1ab:C3140T, ORF1ab:G6710T, ORF1ab:C10029T, ORF1ab:C10954T, ORF1ab:A11117G, ORF1ab:C12789T, ORF1ab:C14408T, ORF1ab:T19839C, ORF1ab:A19974G, ORF1ab:C21306T, S:C22413T, S:C22995A, S:A23403G, S:C23604A, S:A23756G, ORF7a:A27489G, N:G28881A, N:G28882A, N:G28883C, N:C29197T,	13	N:R203K, N:G204R, ORF1a:P959S, ORF1a:V2149F, ORF1a:T3255I, ORF1a:I3618V, ORF1a:T4175I, ORF1b:P314L, S:T284I, S:T478K, S:D614G, S:P681H, S:T732A,
hCoV-19/Mexico/CMX-INMEGEN-02-01-11/2021	EPI_ISL_1040614	20B	B.1.1.519	25	5'UTR:T201C, 5'UTR:C203T, 5'UTR:C222T, 5'UTR:C241T, ORF1ab:G1738T, ORF1ab:C3037T, ORF1ab:C3140T, ORF1ab:G6710T, ORF1ab:C10029T, ORF1ab:C10954T, ORF1ab:A11117G, ORF1ab:C12789T, ORF1ab:C14408T, ORF1ab:T19839C, ORF1ab:A19974G, ORF1ab:C21306T, S:C22413T, S:C22995A, S:A23403G, S:C23604A, S:A23756G, ORF7a:A27489G, N:G28881A, N:G28882A, N:G28883C, N:C29197T,	13	N:R203K, N:G204R, ORF1a:P959S, ORF1a:V2149F, ORF1a:T3255I, ORF1a:I3618V, ORF1a:T4175I, ORF1b:P314L, S:T284I, S:T478K, S:D614G, S:P681H, S:T732A,
hCoV-19/Mexico/CMX-INMEGEN-02-01-12/2021	EPI_ISL_1040615	20A	B.1.232	24	5'UTR:C241T, ORF1ab:C829T, ORF1ab:C3037T, ORF1ab:T4816C, ORF1ab:G8184A, ORF1ab:C11758T, ORF1ab:C12459T, ORF1ab:C14408T, ORF1ab:A15124C, ORF1ab:C17550T, ORF1ab:G20056T, ORF1ab:A20268G, ORF1ab:C21304T, S:T22195C, S:G22599A, S:A23403G, S:G23593T, ORF3a:G25690T, ORF3a:G25726T, M:C26681T, ORF7a:C27434T, ORF8:G27987T, ORF8:C28087T, N:A28365G, N:C28854T,	17	N:E31G, N:S194L, ORF1a:G2640E, ORF1a:T4065I, ORF1b:P314L, ORF1b:I553L, ORF1b:G2197C, ORF1b:R2613C, ORF3a:G100C, ORF3a:V112F, ORF7a:T14I, ORF8:V32L, ORF8:A65V, ORF9b:N28D, S:R346K, S:D614G, S:Q677H,
hCoV-19/Mexico/CMX-INMEGEN-02-01-13/2021	EPI_ISL_1040609	20B	B.1.1.519	24	5'UTR:C203T, 5'UTR:C222T, 5'UTR:C241T, ORF1ab:C3037T, ORF1ab:C3140T, ORF1ab:C5869T, ORF1ab:C10029T, ORF1ab:C10954T, ORF1ab:A11117G, ORF1ab:C11173T, ORF1ab:C12789T, ORF1ab:C14408T, ORF1ab:T19839C, ORF1ab:C21306T, S:C22995A, S:A23403G, S:C23604A, S:A23756G, ORF3a:C25688T, ORF8:T27904C, ORF8:C28087T, N:G28881A, N:G28882A, N:G28883C, N:C29197T,	14	N:R203K, N:G204R, ORF1a:P959S, ORF1a:T3255I, ORF1a:I3618V, ORF1a:T4175I, ORF1b:P314L, ORF3a:A99V, ORF8:L4P, ORF8:A65V, S:T478K, S:D614G, S:P681H, S:T732A,
hCoV-19/Mexico/CMX-INMEGEN-02-01-14/2021	EPI_ISL_1040606	20B	B.1.1.519	26	5'UTR:T201C, 5'UTR:C203T, 5'UTR:C222T, 5'UTR:C241T, ORF1ab:G1738T, ORF1ab:C3037T, ORF1ab:C3140T, ORF1ab:C10029T, ORF1ab:C10954T, ORF1ab:A11117G, ORF1ab:C12789T, ORF1ab:C14408T, ORF1ab:G14559T, ORF1ab:T19839C, ORF1ab:A19974G, ORF1ab:C21306T, S:C22995A, S:A23403G, S:C23604A, S:A23756G, ORF3a:G25699A, ORF7b:C27807T, N:G28881A, N:G28882A, N:G28883C, N:C29197T, 3'UTR:C29754T,	12	N:R203K, N:G204R, ORF1a:P959S, ORF1a:T3255I, ORF1a:I3618V, ORF1a:T4175I, ORF1b:P314L, ORF3a:A103T, S:T478K, S:D614G, S:P681H, S:T732A,
hCoV-19/Mexico/CMX-INMEGEN-02-01-15/2021	EPI_ISL_1040607	20B	B.1.1.519	27	5'UTR:C203T, 5'UTR:C222T, 5'UTR:C241T, ORF1ab:A866G, ORF1ab:C1009T, ORF1ab:C3037T, ORF1ab:C3140T, ORF1ab:A3801G, ORF1ab:C5183T, ORF1ab:C10029T, ORF1ab:C10954T, ORF1ab:A11117G, ORF1ab:C11916T, ORF1ab:C12789T, ORF1ab:C14408T, ORF1ab:A18277G, ORF1ab:T19839C, ORF1ab:C21306T, S:C22995A, S:A23403G, S:C23604A, S:A23756G, ORF8:T27904C, N:G28747T, N:G28881A, N:G28882A, N:G28883C, N:C29197T,	17	N:R203K, N:G204R, ORF1a:I201V, ORF1a:P959S, ORF1a:D1179G, ORF1a:P1640S, ORF1a:T3255I, ORF1a:I3618V, ORF1a:S3884L, ORF1a:T4175I, ORF1b:P314L, ORF1b:I1604V, ORF8:L4P, S:T478K, S:D614G, S:P681H, S:T732A,

hCoV-19/Mexico/CMX-INMEGEN-02-01-16/2021	EPI_ISL_1040605	20B	B.1.1.519	25	5'UTR:T201C, 5'UTR:C203T, 5'UTR:C222T, 5'UTR:C241T, ORF1ab:G1738T, ORF1ab:C3037T, ORF1ab:C3140T, ORF1ab:C3602T, ORF1ab:C7504T, ORF1ab:C10029T, ORF1ab:C10954T, ORF1ab:A11117G, ORF1ab:C12789T, ORF1ab:C14408T, ORF1ab:T19839C, ORF1ab:A19974G, ORF1ab:C21306T, S:T21925C, S:C22995A, S:A23403G, S:C23604A, S:A23756G, N:G28881A, N:G28882A, N:G28883C, N:C29197T,	12	N:R203K, N:G204R, ORF1a:P959S, ORF1a:H1113Y, ORF1a:T3255I, ORF1a:I3618V, ORF1a:T4175I, ORF1b:P314L, S:T478K, S:D614G, S:P681H, S:T732A,
hCoV-19/Mexico/CMX-INMEGEN-02-01-17/2021	EPI_ISL_1040600	20A	B.1.243	19	5'UTR:C241T, ORF1ab:G806A, ORF1ab:G872A, ORF1ab:A1291G, ORF1ab:T2584A, ORF1ab:C3037T, ORF1ab:G3638T, ORF1ab:C5140A, ORF1ab:T7341C, ORF1ab:C13366T, ORF1ab:C14408T, ORF1ab:A20268G, ORF1ab:G21123T, S:A23403G, S:G23587C, S:T24076C, ORF3a:C25844T, ORF7b:G27870T, N:C28854T, N:G29543T,	11	N:S194L, ORF1a:A181T, ORF1a:D203N, ORF1a:G1125C, ORF1a:D1625E, ORF1a:I2359T, ORF1b:P314L, ORF3a:T151I, ORF7b:E39*, S:D614G, S:Q675H,
hCoV-19/Mexico/CMX-INMEGEN-02-01-18/2021	EPI_ISL_1040620	20B	B.1.1.519	26	5'UTR:C203T, 5'UTR:C222T, 5'UTR:C241T, ORF1ab:C3037T, ORF1ab:C3140T, ORF1ab:T3745C, ORF1ab:A7880G, ORF1ab:C9810T, ORF1ab:C10029T, ORF1ab:C10626T, ORF1ab:C10954T, ORF1ab:A11117G, ORF1ab:C12789T, ORF1ab:C14408T, ORF1ab:T19839C, ORF1ab:C21306T, S:C22591T, S:C22995A, S:A23403G, S:C23604A, S:A23756G, ORF3a:C25844T, N:C28789T, N:G28881A, N:G28882A, N:G28883C, N:C29197T,	15	N:R203K, N:G204R, ORF1a:P959S, ORF1a:N2539D, ORF1a:T3182I, ORF1a:T3255I, ORF1a:A3454V, ORF1a:I3618V, ORF1a:T4175I, ORF1b:P314L, ORF3a:T151I, S:T478K, S:D614G, S:P681H, S:T732A,
hCoV-19/Mexico/CMX-INMEGEN-02-01-20/2021	EPI_ISL_1040602	20B	B.1.1.519	25	5'UTR:C203T, 5'UTR:C222T, 5'UTR:C241T, ORF1ab:C3037T, ORF1ab:C3140T, ORF1ab:T3745C, ORF1ab:C10029T, ORF1ab:C10954T, ORF1ab:A11117G, ORF1ab:C12789T, ORF1ab:C14408T, ORF1ab:T19839C, ORF1ab:G20940T, ORF1ab:C21306T, S:C22995A, S:A23403G, S:C23604A, S:A23756G, ORF3a:G25459T, ORF3a:C25844T, ORF6:C27223T, ORF7b:C27847T, N:G28881A, N:G28882A, N:G28883C, N:C29197T,	15	N:R203K, N:G204R, ORF1a:P959S, ORF1a:T3255I, ORF1a:I3618V, ORF1a:T4175I, ORF1b:P314L, ORF3a:A23S, ORF3a:T151I, ORF6:Q8*, ORF7b:S31L, S:T478K, S:D614G, S:P681H, S:T732A,
hCoV-19/Mexico/CMX-INMEGEN-02-01-21/2021	EPI_ISL_1040610	20B	B.1.1.519	23	5'UTR:C203T, 5'UTR:C222T, 5'UTR:C241T, ORF1ab:C3037T, ORF1ab:C3140T, ORF1ab:C10029T, ORF1ab:C10954T, ORF1ab:C11001T, ORF1ab:A11117G, ORF1ab:C12789T, ORF1ab:C14408T, ORF1ab:G15921T, ORF1ab:T19839C, ORF1ab:C21306T, S:C22227T, S:C22995A, S:A23403G, S:C23604A, S:A23756G, N:G28881A, N:G28882A, N:G28883C, N:C29197T, 3'UTR:C29721T,	13	N:R203K, N:G204R, ORF1a:P959S, ORF1a:T3255I, ORF1a:T3579I, ORF1a:I3618V, ORF1a:T4175I, ORF1b:P314L, S:A222V, S:T478K, S:D614G, S:P681H, S:T732A,
hCoV-19/Mexico/CMX-INMEGEN-02-01-22/2021	EPI_ISL_1040601	20B	B.1.1.519	26	5'UTR:C203T, 5'UTR:C222T, 5'UTR:C241T, ORF1ab:C3037T, ORF1ab:C3140T, ORF1ab:T3745C, ORF1ab:T7984C, ORF1ab:C10029T, ORF1ab:C10341T, ORF1ab:C10954T, ORF1ab:A11117G, ORF1ab:C12789T, ORF1ab:C14408T, ORF1ab:T19839C, ORF1ab:C21306T, S:C21648T, S:C22995A, S:A23403G, S:C23604A, S:A23756G, ORF3a:G25660T, ORF3a:C25844T, ORF7a:C27641A, N:G28881A, N:G28882A, N:G28883C, N:C29197T,	16	N:R203K, N:G204R, ORF1a:P959S, ORF1a:T3255I, ORF1a:P3359L, ORF1a:I3618V, ORF1a:T4175I, ORF1b:P314L, ORF3a:V90F, ORF3a:T151I, ORF7a:S83*, S:T29I, S:T478K, S:D614G, S:P681H, S:T732A,

hCoV-19/Mexico/CMX-INMEGEN-02-01-23/2021	EPI_ISL_1040616	20B	B.1.1.519	22	5'UTR:C203T, 5'UTR:C222T, 5'UTR:C241T, ORF1ab:C1302T, ORF1ab:C3037T, ORF1ab:C3140T, ORF1ab:C10029T, ORF1ab:C10954T, ORF1ab:A11117G, ORF1ab:G11365T, ORF1ab:C12789T, ORF1ab:C14408T, ORF1ab:T19839C, ORF1ab:C21306T, S:C22995A, S:A23403G, S:C23604A, S:A23756G, N:G28881A, N:G28882A, N:G28883C, N:C29197T, N:G29527T,	13	N:R203K, N:G204R, N:Q418H, ORF1a:T346I, ORF1a:P959S, ORF1a:T3255I, ORF1a:I3618V, ORF1a:T4175I, ORF1b:P314L, S:T478K, S:D614G, S:P681H, S:T732A,	
hCoV-19/Mexico/CMX-INMEGEN-02-02-01/2021	EPI_ISL_1040554	20B	B.1.1.519	26	5'UTR:C203T, 5'UTR:A223T, 5'UTR:C241T, ORF1ab:C3037T, ORF1ab:C3140T, ORF1ab:C3393T, ORF1ab:T9456G, ORF1ab:C10029T, ORF1ab:C10954T, ORF1ab:A11117G, ORF1ab:C12789T, ORF1ab:C14408T, ORF1ab:C15720T, ORF1ab:T19839C, ORF1ab:C21306T, ORF1ab:A21550C, S:T22192C, S:C22995A, S:A23403G, S:C23604A, S:A23756G, S:C24919T, ORF3a:C25521T, N:G28881A, N:G28882A, N:G28883C, N:C29197T,	15	N:R203K, N:G204R, ORF1a:P959S, ORF1a:A1043V, ORF1a:F3064C, ORF1a:T3255I, ORF1a:I3618V, ORF1a:T4175I, ORF1b:P314L, ORF1b:N2694H, ORF1b:N2695*, S:T478K, S:D614G, S:P681H, S:T732A,	5'UTR:222, ORF1ab:21547-21549,
hCoV-19/Mexico/CMX-INMEGEN-02-02-02/2021	EPI_ISL_1040555	20B	B.1.1.519	28	5'UTR:C203T, 5'UTR:C222T, 5'UTR:C241T, ORF1ab:C678T, ORF1ab:A866G, ORF1ab:C1009T, ORF1ab:C3037T, ORF1ab:C3140T, ORF1ab:C5183T, ORF1ab:C10029T, ORF1ab:C10954T, ORF1ab:A11117G, ORF1ab:C11916T, ORF1ab:C12789T, ORF1ab:C14408T, ORF1ab:C19662T, ORF1ab:T19839C, ORF1ab:C21306T, S:C22995A, S:A23403G, S:C23604A, S:A23756G, ORF3a:C25416T, ORF8:T27904C, N:G28747T, N:G28881A, N:G28882A, N:G28883C, N:C29197T,	16	N:R203K, N:G204R, ORF1a:A138V, ORF1a:I201V, ORF1a:P959S, ORF1a:P1640S, ORF1a:T3255I, ORF1a:I3618V, ORF1a:S3884L, ORF1a:T4175I, ORF1b:P314L, ORF8:L4P, S:T478K, S:D614G, S:P681H, S:T732A,	
hCoV-19/Mexico/CMX-INMEGEN-02-02-03/2021	EPI_ISL_1040548	20A	B.1	18	5'UTR:C241T, ORF1ab:G625T, ORF1ab:C2902T, ORF1ab:C3037T, ORF1ab:C10537T, ORF1ab:C14408T, ORF1ab:A17861G, ORF1ab:C18744T, ORF1ab:C19610T, ORF1ab:A20181G, ORF1ab:A20268G, S:C22414T, S:C22450T, S:A23403G, S:G24933T, ORF3a:C25578T, ORF3a:G25606A, ORF3a:C25916T, N:C28854T,	9	N:S194L, ORF1a:K120N, ORF1b:P314L, ORF1b:D1465G, ORF1b:T2048I, ORF3a:A72T, ORF3a:T175I, S:D614G, S:G1124V,	
hCoV-19/Mexico/CMX-INMEGEN-02-02-04/2021	EPI_ISL_1040551	20B	B.1.1.222	23	5'UTR:C241T, ORF1ab:C3037T, ORF1ab:T5218C, ORF1ab:G9652T, ORF1ab:A12290G, ORF1ab:C13366T, ORF1ab:C14408T, ORF1ab:C14585T, ORF1ab:T19839C, S:C21648T, S:T22897A, S:A23403G, S:A23756G, S:C24718T, ORF3a:A25882G, ORF6:C27389T, N:C28603T, N:C28660T, N:C28863T, N:G28881A, N:G28882A, N:G28883C, N:G29449T, 3'UTR:G29690T,	11	N:S197L, N:R203K, N:G204R, ORF1a:M3129I, ORF1a:M4009V, ORF1b:P314L, ORF1b:A373V, ORF3a:T164A, S:T29I, S:D614G, S:T732A,	
hCoV-19/Mexico/CMX-INMEGEN-02-02-05/2021	EPI_ISL_1040547	20B	B.1.1.519	27	5'UTR:T201C, 5'UTR:C203T, 5'UTR:C222T, 5'UTR:C241T, ORF1ab:C936T, ORF1ab:G1738T, ORF1ab:C3037T, ORF1ab:C3140T, ORF1ab:A7003G, ORF1ab:C10029T, ORF1ab:C10450T, ORF1ab:C10954T, ORF1ab:A11117G, ORF1ab:C12789T, ORF1ab:C14408T, ORF1ab:T19839C, ORF1ab:A19974G, ORF1ab:C21306T, S:C22995A, S:A23403G, S:C23604A, S:A23756G, ORF6:C27213T, N:G28881A, N:G28882A, N:G28883C, N:C29197T, N:G29227T,	12	N:R203K, N:G204R, ORF1a:T224I, ORF1a:P959S, ORF1a:T3255I, ORF1a:I3618V, ORF1a:T4175I, ORF1b:P314L, S:T478K, S:D614G, S:P681H, S:T732A,	
hCoV-19/Mexico/CMX-INMEGEN-02-02-06/2021	EPI_ISL_1040541	20B	B.1.1.519	22	5'UTR:C203T, 5'UTR:C222T, 5'UTR:C241T, ORF1ab:C3037T, ORF1ab:C3140T, ORF1ab:C7355T, ORF1ab:C10029T, ORF1ab:C10954T, ORF1ab:A11117G, ORF1ab:C12789T, ORF1ab:C14408T, ORF1ab:C15656T, ORF1ab:C19017T, ORF1ab:T19839C, ORF1ab:C21306T, S:C22995A, S:A23403G, S:C23604A, S:A23756G, N:G28881A, N:G28882A, N:G28883C, N:C29197T,	13	N:R203K, N:G204R, ORF1a:P959S, ORF1a:L2364F, ORF1a:T3255I, ORF1a:I3618V, ORF1a:T4175I, ORF1b:P314L, ORF1b:T730I, S:T478K, S:D614G, S:P681H, S:T732A,	

hCoV-19/Mexico/CMX-INMEGEN-02-02-07/2021	EPI_ISL_1040540	20B	B.1.1.519	28	5'UTR:C203T, 5'UTR:C222T, 5'UTR:C241T, ORF1ab:A866G, ORF1ab:C1009T, ORF1ab:C3037T, ORF1ab:C3140T, ORF1ab:C5183T, ORF1ab:C10029T, ORF1ab:C10954T, ORF1ab:A11117G, ORF1ab:C11916T, ORF1ab:C12789T, ORF1ab:C12853T, ORF1ab:C14408T, ORF1ab:C19662T, ORF1ab:T19839C, ORF1ab:C21306T, S:C22995A, S:A23403G, S:C23604A, S:A23756G, M:T27113C, ORF8:T27904C, N:G28747T, N:G28881A, N:G28882A, N:G28883C, N:C29197T,	15	N:R203K, N:G204R, ORF1a:I201V, ORF1a:P959S, ORF1a:P1640S, ORF1a:T3255I, ORF1a:I3618V, ORF1a:S3884L, ORF1a:T4175I, ORF1b:P314L, ORF8:L4P, S:T478K, S:D614G, S:P681H, S:T732A,
hCoV-19/Mexico/CMX-INMEGEN-02-02-08/2021	EPI_ISL_1040535	20B	B.1.1.519	26	5'UTR:T201C, 5'UTR:C203T, 5'UTR:C222T, 5'UTR:C241T, ORF1ab:G1738T, ORF1ab:C3037T, ORF1ab:C3140T, ORF1ab:C4999T, ORF1ab:C10029T, ORF1ab:C10954T, ORF1ab:A11117G, ORF1ab:C12733T, ORF1ab:C12789T, ORF1ab:C14408T, ORF1ab:G14559T, ORF1ab:T19839C, ORF1ab:A19974G, ORF1ab:C21306T, S:C22995A, S:A23403G, S:C23604A, S:A23756G, ORF3a:G25699A, N:G28881A, N:G28882A, N:G28883C, N:C29197T,	12	N:R203K, N:G204R, ORF1a:P959S, ORF1a:T3255I, ORF1a:I3618V, ORF1a:T4175I, ORF1b:P314L, ORF3a:A103T, S:T478K, S:D614G, S:P681H, S:T732A,
hCoV-19/Mexico/CMX-INMEGEN-02-02-09/2021	EPI_ISL_1040542	20B	B.1.1.519	23	5'UTR:C203T, 5'UTR:C222T, 5'UTR:C241T, ORF1ab:C3037T, ORF1ab:C3140T, ORF1ab:T3745C, ORF1ab:C10029T, ORF1ab:C10954T, ORF1ab:A11117G, ORF1ab:C12789T, ORF1ab:C14408T, ORF1ab:T19839C, ORF1ab:C21306T, S:C22995A, S:A23403G, S:C23604A, S:A23756G, ORF3a:G25696C, ORF3a:C25844T, N:C28849T, N:G28881A, N:G28882A. N:G28883C. N:C29197T.	13	N:R203K, N:G204R, ORF1a:P959S, ORF1a:T3255I, ORF1a:I3618V, ORF1a:T4175I, ORF1b:P314L, ORF3a:E102Q, ORF3a:T151I, S:T478K, S:D614G, S:P681H, S:T732A,
hCoV-19/Mexico/CMX-INMEGEN-02-02-10/2021	EPI_ISL_1040536	20B	B.1	25	5'UTR:C203T, 5'UTR:C222T, 5'UTR:C241T, ORF1ab:C3037T, ORF1ab:C3140T, ORF1ab:C10029T, ORF1ab:C10319T, ORF1ab:C10954T, ORF1ab:A11117G, ORF1ab:C12789T, ORF1ab:C14408T, ORF1ab:C16887T, ORF1ab:T19839C, ORF1ab:C21306T, S:C21637T, S:C22995A, S:A23403G, S:A23586T, S:C23604A, S:A23756G, ORF8:T27904C, ORF8:C28087T, N:G28881A, N:G28882A, N:G28883C, N:C29197T,	15	N:R203K, N:G204R, ORF1a:P959S, ORF1a:T3255I, ORF1a:L3352F, ORF1a:I3618V, ORF1a:T4175I, ORF1b:P314L, ORF8:L4P, ORF8:A65V, S:T478K, S:D614G, S:Q675L, S:P681H, S:T732A,
hCoV-19/Mexico/CMX-INMEGEN-02-02-12/2021	EPI_ISL_1040552	20B	B.1.1.519	25	5'UTR:C203T, 5'UTR:C222T, 5'UTR:C241T, ORF1ab:C3037T, ORF1ab:C3140T, ORF1ab:G5953T, ORF1ab:G9526A, ORF1ab:C10029T, ORF1ab:C10954T, ORF1ab:A11117G, ORF1ab:G11365T, ORF1ab:C12789T, ORF1ab:C14408T, ORF1ab:C17125T, ORF1ab:T19839C, ORF1ab:C21306T, S:C22995A, S:A23403G, S:C23604A, S:A23756G, ORF8:C27903T, N:G28881A, N:G28882A, N:G28883C, N:C29197T, N:G29527T,	16	N:R203K, N:G204R, N:Q418H, ORF1a:P959S, ORF1a:L1896F, ORF1a:M3087I, ORF1a:T3255I, ORF1a:I3618V, ORF1a:T4175I, ORF1b:P314L, ORF1b:L1220F, ORF8:L4F, S:T478K, S:D614G, S:P681H, S:T732A,
hCoV-19/Mexico/CMX-INMEGEN-02-02-13/2021	EPI_ISL_1040549	20B	B.1.1.519	24	5'UTR:C203T, 5'UTR:C222T, 5'UTR:C241T, ORF1ab:C3037T, ORF1ab:C3140T, ORF1ab:C10029T, ORF1ab:C10954T, ORF1ab:A11117G, ORF1ab:C12789T, ORF1ab:C12973T, ORF1ab:C14408T, ORF1ab:T19839C, ORF1ab:C21306T, S:C22995A, S:A23403G, S:C23604A, S:A23756G, S:G24914T, ORF3a:C25782T, ORF3a:G26063T, N:C28606T, N:G28881A. N:G28882A. N:G28883C. N:C29197T.	13	N:R203K, N:G204R, ORF1a:P959S, ORF1a:T3255I, ORF1a:I3618V, ORF1a:T4175I, ORF1b:P314L, ORF3a:G224V, S:T478K, S:D614G, S:P681H, S:T732A, S:D1118Y,

hCoV-19/Mexico/CMX-INMEGEN-02-02-14/2021	EPI_ISL_1040544	20B	B.1.1.519	23	5'UTR:C203T, 5'UTR:C222T, 5'UTR:C241T, ORF1ab:C3037T, ORF1ab:C3140T, ORF1ab:C10029T, ORF1ab:C10954T, ORF1ab:A11117G, ORF1ab:C12789T, ORF1ab:C14408T, ORF1ab:G15921T, ORF1ab:A17971G, ORF1ab:T19839C, ORF1ab:C21306T, S:C22995A, S:A23403G, S:C23604A, S:C23635T, S:A23756G, N:G28621T, N:G28881A, N:G28882A, N:G28883C, N:C29197T,	12	N:R203K, N:G204R, ORF1a:P959S, ORF1a:T3255I, ORF1a:I3618V, ORF1a:T4175I, ORF1b:P314L, ORF1b:R1502G, S:T478K, S:D614G, S:P681H, S:T732A,
hCoV-19/Mexico/CMX-INMEGEN-02-02-15/2021	EPI_ISL_1040537	20B	B.1.1.519	26	5'UTR:T201C, 5'UTR:C203T, 5'UTR:C222T, 5'UTR:C241T, ORF1ab:C936T, ORF1ab:G1439T, ORF1ab:G1738T, ORF1ab:C3037T, ORF1ab:C3140T, ORF1ab:C10029T, ORF1ab:C10954T, ORF1ab:A11117G, ORF1ab:C12789T, ORF1ab:C14408T, ORF1ab:T19839C, ORF1ab:A19974G, ORF1ab:C21306T, S:C22995A, S:A23403G, S:C23604A, S:A23756G, ORF8:G28077T, N:G28881A, N:G28882A, N:G28883C, N:C29197T, N:G29227T,	14	N:R203K, N:G204R, ORF1a:T224I, ORF1a:G392C, ORF1a:P959S, ORF1a:T3255I, ORF1a:I3618V, ORF1a:T4175I, ORF1b:P314L, ORF8:V62L, S:T478K, S:D614G, S:P681H, S:T732A,
hCoV-19/Mexico/CMX-INMEGEN-02-02-16/2021	EPI_ISL_1040538	20B	B.1.1.519	28	5'UTR:C203T, 5'UTR:C222T, 5'UTR:C241T, ORF1ab:C857T, ORF1ab:C2695T, ORF1ab:C3037T, ORF1ab:C3140T, ORF1ab:C3267T, ORF1ab:T4339C, ORF1ab:C6539T, ORF1ab:C10029T, ORF1ab:C10954T, ORF1ab:T11112C, ORF1ab:A11117G, ORF1ab:C12789T, ORF1ab:C14408T, ORF1ab:G16957T, ORF1ab:T19839C, ORF1ab:C21306T, S:C22995A, S:A23403G, S:C23604A, S:A23756G, ORF3a:G25906T, ORF8:C28253T, N:G28881A, N:G28882A, N:G28883C, N:C29197T,	17	N:R203K, N:G204R, ORF1a:L198F, ORF1a:P959S, ORF1a:T1001I, ORF1a:H2092Y, ORF1a:T3255I, ORF1a:M3616T, ORF1a:I3618V, ORF1a:T4175I, ORF1b:P314L, ORF1b:V1164L, ORF3a:G172C, S:T478K, S:D614G, S:P681H, S:T732A,
hCoV-19/Mexico/CMX-INMEGEN-02-02-17/2021	EPI_ISL_1040556	20B	B.1.1.519	29	5'UTR:C203T, 5'UTR:C222T, 5'UTR:C241T, ORF1ab:C3037T, ORF1ab:C3140T, ORF1ab:C5869T, ORF1ab:C6568T, ORF1ab:C10029T, ORF1ab:C10954T, ORF1ab:A11117G, ORF1ab:C12789T, ORF1ab:A13237G, ORF1ab:C14408T, ORF1ab:A18093G, ORF1ab:C18568T, ORF1ab:T19839C, ORF1ab:G20937A, ORF1ab:G21204A, ORF1ab:C21306T, S:C22995A, S:A23403G, S:C23604A, S:A23756G, ORF8:T27904C, ORF8:G27987T, ORF8:C28087T, N:G28881A, N:G28882A, N:G28883C, N:C29197T	15	N:R203K, N:G204R, ORF1a:P959S, ORF1a:T3255I, ORF1a:I3618V, ORF1a:T4175I, ORF1b:P314L, ORF1b:L1701F, ORF8:L4P, ORF8:V32L, ORF8:A65V, S:T478K, S:D614G, S:P681H, S:T732A,
hCoV-19/Mexico/CMX-INMEGEN-02-02-18/2021	EPI_ISL_1040545	20B	B.1.1.222	24	5'UTR:C241T, ORF1ab:C835T, ORF1ab:T928C, ORF1ab:C3037T, ORF1ab:C3093T, ORF1ab:C6781T, ORF1ab:C8090T, ORF1ab:C9979T, ORF1ab:C11109T, ORF1ab:C11322T, ORF1ab:C11416T, ORF1ab:G12256T, ORF1ab:C14408T, ORF1ab:C18060T, ORF1ab:C18267T, ORF1ab:T19839C, S:G22017T, S:T22954C, S:A23403G, S:A23756G, ORF3a:G26063T, ORF7b:A27888T, N:G28881A, N:G28882A, N:G28883C,	12	N:R203K, N:G204R, ORF1a:P943L, ORF1a:L2609F, ORF1a:A3615V, ORF1a:A3686V, ORF1a:M3997I, ORF1b:P314L, ORF3a:G224V, S:W152L, S:D614G, S:T732A,
hCoV-19/Mexico/CMX-INMEGEN-02-02-19/2021	EPI_ISL_1040553	20B	B.1.1.519	25	5'UTR:C203T, 5'UTR:C222T, 5'UTR:C241T, ORF1ab:C3037T, ORF1ab:C3140T, ORF1ab:C10029T, ORF1ab:C10954T, ORF1ab:A11117G, ORF1ab:C12789T, ORF1ab:C14408T, ORF1ab:C14877T, ORF1ab:C18647T, ORF1ab:T19839C, ORF1ab:C21306T, S:C22995A, S:A23403G, S:C23604A, S:A23756G, S:G23821A, ORF7a:A27745G, ORF8:T27904C, ORF8:C28087T, N:G28881A, N:G28882A, N:G28883C, N:C29197T,	15	N:R203K, N:G204R, ORF1a:P959S, ORF1a:T3255I, ORF1a:I3618V, ORF1a:T4175I, ORF1b:P314L, ORF1b:P1727L, ORF7a:R118G, ORF8:L4P, ORF8:A65V, S:T478K, S:D614G, S:P681H, S:T732A,

hCoV-19/Mexico/CMX-INMEGEN-02-02-20/2021	EPI_ISL_1040550	20B	B.1.1.519	24	5'UTR:C203T, 5'UTR:C222T, 5'UTR:C241T, ORF1ab:C3037T, ORF1ab:C3140T, ORF1ab:G3692T, ORF1ab:G3871T, ORF1ab:C6429A, ORF1ab:C10029T, ORF1ab:C10954T, ORF1ab:A11117G, ORF1ab:C11824A, ORF1ab:C12789T, ORF1ab:C14408T, ORF1ab:T19839C, ORF1ab:C21306T, S:C22995A, S:A23403G, S:C23604A, S:A23756G, ORF8:T27904C, N:G28881A, N:G28882A, N:G28883C, N:C29197T,	15	N:R203K, N:G204R, ORF1a:P959S, ORF1a:V1143F, ORF1a:K1202N, ORF1a:P2055H, ORF1a:T3255I, ORF1a:I3618V, ORF1a:T4175I, ORF1b:P314L, ORF8:L4P, S:T478K, S:D614G, S:P681H, S:T732A,
hCoV-19/Mexico/CMX-INMEGEN-02-02-21/2021	EPI_ISL_1040539	20B	B.1.1.519	28	5'UTR:C203T, 5'UTR:C222T, 5'UTR:C241T, ORF1ab:C337T, ORF1ab:A866G, ORF1ab:C1009T, ORF1ab:A2854G, ORF1ab:C3037T, ORF1ab:C3140T, ORF1ab:C5183T, ORF1ab:C10029T, ORF1ab:C10954T, ORF1ab:A11117G, ORF1ab:C11916T, ORF1ab:C12789T, ORF1ab:G13249A, ORF1ab:C14408T, ORF1ab:T19839C, ORF1ab:C21306T, S:C22995A, S:A23403G, S:C23604A, S:A23756G, ORF3a:C26204T, ORF8:T27904C, N:G28881A, N:G28882A, N:G28883C, N:C29197T,	16	N:R203K, N:G204R, ORF1a:I201V, ORF1a:P959S, ORF1a:P1640S, ORF1a:T3255I, ORF1a:I3618V, ORF1a:S3884L, ORF1a:T4175I, ORF1b:P314L, ORF3a:T271I, ORF8:L4P, S:T478K, S:D614G, S:P681H, S:T732A,
hCoV-19/Mexico/CMX-INMEGEN-02-02-22/2021	EPI_ISL_1040546	20B	B.1.1.519	28	5'UTR:C203T, 5'UTR:C222T, 5'UTR:C241T, ORF1ab:G343T, ORF1ab:T597A, ORF1ab:C3037T, ORF1ab:C3140T, ORF1ab:C4931T, ORF1ab:T6490C, ORF1ab:C10029T, ORF1ab:C10954T, ORF1ab:A11117G, ORF1ab:C12789T, ORF1ab:C14408T, ORF1ab:T19839C, ORF1ab:C21077T, ORF1ab:A21137G, ORF1ab:C21306T, S:C22995A, S:A23403G, S:C23604A, S:A23756G, ORF3a:G25906T, ORF8:C28253T, N:C28391T, N:G28881A, N:G28882A, N:G28883C, N:C29197T	17	N:R40C, N:R203K, N:G204R, ORF1a:V111E, ORF1a:P959S, ORF1a:L1556F, ORF1a:T3255I, ORF1a:I3618V, ORF1a:T4175I, ORF1b:P314L, ORF1b:T2537I, ORF1b:K2557R, ORF3a:G172C, S:T478K, S:D614G, S:P681H, S:T732A,
hCoV-19/Mexico/CMX-INMEGEN-02-02-23/2021	EPI_ISL_1040543	20B	B.1.1.519	24	5'UTR:C203T, 5'UTR:C222T, 5'UTR:C241T, ORF1ab:C3037T, ORF1ab:C3140T, ORF1ab:C6541T, ORF1ab:C10029T, ORF1ab:C10954T, ORF1ab:A11117G, ORF1ab:C12789T, ORF1ab:C14408T, ORF1ab:G15921T, ORF1ab:T19839C, ORF1ab:C21306T, S:C21998T, S:C22227T, S:C22995A, S:A23403G, S:C23604A, S:A23756G, N:C28775T, N:G28881A, N:G28882A, N:G28883C, N:C29197T,	14	N:P168S, N:R203K, N:G204R, ORF1a:P959S, ORF1a:T3255I, ORF1a:I3618V, ORF1a:T4175I, ORF1b:P314L, S:H146Y, S:A222V, S:T478K, S:D614G, S:P681H, S:T732A,
hCoV-19/Mexico/CMX-INMEGEN-02-03-01/2021	EPI_ISL_1040637	20B	B.1.1.519	24	5'UTR:C203T, 5'UTR:C222T, 5'UTR:C241T, ORF1ab:G2534T, ORF1ab:C3037T, ORF1ab:C3140T, ORF1ab:C7860T, ORF1ab:C10029T, ORF1ab:C10954T, ORF1ab:A11117G, ORF1ab:C12789T, ORF1ab:C13356T, ORF1ab:C14408T, ORF1ab:T19839C, ORF1ab:C21306T, S:C22995A, S:A23403G, S:C23604A, S:A23756G, ORF7a:C27707T, ORF8:T28067G, N:G28881A, N:G28882A, N:G28883C, N:C29197T,	16	N:R203K, N:G204R, ORF1a:V757F, ORF1a:P959S, ORF1a:T2532I, ORF1a:T3255I, ORF1a:I3618V, ORF1a:T4175I, ORF1a:T4364I, ORF1b:P314L, ORF7a:A105V, ORF8:I58M, S:T478K, S:D614G, S:P681H, S:T732A,
hCoV-19/Mexico/CMX-INMEGEN-02-03-02/2021	EPI_ISL_1040627	20B	B.1.1.519	27	5'UTR:T201C, 5'UTR:C203T, 5'UTR:C222T, 5'UTR:C241T, ORF1ab:G731A, ORF1ab:G1738T, ORF1ab:C3037T, ORF1ab:C3140T, ORF1ab:C3602T, ORF1ab:G6166A, ORF1ab:A6790G, ORF1ab:C7504T, ORF1ab:C10029T, ORF1ab:C10954T, ORF1ab:A11117G, ORF1ab:C12789T, ORF1ab:C14408T, ORF1ab:T19839C, ORF1ab:A19974G, ORF1ab:C21306T, S:C22995A, S:A23403G, S:C23604A, S:A23756G, N:G28881A, N:G28882A, N:G28883C, N:C29197T,	13	N:R203K, N:G204R, ORF1a:D156N, ORF1a:P959S, ORF1a:H1113Y, ORF1a:T3255I, ORF1a:I3618V, ORF1a:T4175I, ORF1b:P314L, S:T478K, S:D614G, S:P681H, S:T732A,

hCoV-19/Mexico/CMX-INMEGEN-02-03-03/2021	EPI_ISL_1040628	20B	B.1.1.519	27	5'UTR:T201C, 5'UTR:C203T, 5'UTR:C222T, 5'UTR:C241T, ORF1ab:G731A, ORF1ab:G1738T, ORF1ab:C3037T, ORF1ab:C3140T, ORF1ab:C3602T, ORF1ab:G6166A, ORF1ab:A6790G, ORF1ab:C7504T, ORF1ab:C10029T, ORF1ab:C10954T, ORF1ab:A11117G, ORF1ab:C12789T, ORF1ab:C14408T, ORF1ab:T19839C, ORF1ab:A19974G, ORF1ab:C21306T, S:C22995A, S:A23403G, S:C23604A, S:A23756G, N:G28881A, N:G28882A, N:G28883C, N:C29197T,	13	N:R203K, N:G204R, ORF1a:D156N, ORF1a:P959S, ORF1a:H1113Y, ORF1a:T3255I, ORF1a:I3618V, ORF1a:T4175I, ORF1b:P314L, S:T478K, S:D614G, S:P681H, S:T732A,
hCoV-19/Mexico/CMX-INMEGEN-02-03-04/2021	EPI_ISL_1040629	20B	B.1.1.519	27	5'UTR:T201C, 5'UTR:C203T, 5'UTR:C222T, 5'UTR:C241T, ORF1ab:G731A, ORF1ab:G1738T, ORF1ab:C3037T, ORF1ab:C3140T, ORF1ab:C3602T, ORF1ab:G6166A, ORF1ab:A6790G, ORF1ab:C7504T, ORF1ab:C10029T, ORF1ab:C10954T, ORF1ab:A11117G, ORF1ab:C12789T, ORF1ab:C14408T, ORF1ab:T19839C, ORF1ab:A19974G, ORF1ab:C21306T, S:C22995A, S:A23403G, S:C23604A, S:A23756G, N:G28881A, N:G28882A, N:G28883C, N:C29197T,	13	N:R203K, N:G204R, ORF1a:D156N, ORF1a:P959S, ORF1a:H1113Y, ORF1a:T3255I, ORF1a:I3618V, ORF1a:T4175I, ORF1b:P314L, S:T478K, S:D614G, S:P681H, S:T732A,
hCoV-19/Mexico/CMX-INMEGEN-02-03-05/2021	EPI_ISL_1040640	20B	B.1.1.519	23	5'UTR:C203T, 5'UTR:C222T, 5'UTR:C241T, ORF1ab:C3037T, ORF1ab:C3140T, ORF1ab:A3867G, ORF1ab:C6690T, ORF1ab:C10029T, ORF1ab:C10954T, ORF1ab:A11117G, ORF1ab:C11575T, ORF1ab:A11600G, ORF1ab:C12789T, ORF1ab:C14408T, ORF1ab:T19839C, ORF1ab:C21306T, S:C22995A, S:A23403G, S:C23604A, S:A23756G, N:G28881A, N:G28882A, N:G28883C, N:C29197T,	14	N:R203K, N:G204R, ORF1a:P959S, ORF1a:Q1201R, ORF1a:A2142V, ORF1a:T3255I, ORF1a:I3618V, ORF1a:I3779V, ORF1a:T4175I, ORF1b:P314L, S:T478K, S:D614G, S:P681H, S:T732A,
hCoV-19/Mexico/CMX-INMEGEN-02-03-06/2021	EPI_ISL_1040638	20B	B.1.1.519	21	5'UTR:C203T, 5'UTR:C222T, 5'UTR:C241T, ORF1ab:C3037T, ORF1ab:C3140T, ORF1ab:G7037T, ORF1ab:C10029T, ORF1ab:C10954T, ORF1ab:A11117G, ORF1ab:C12789T, ORF1ab:C14408T, ORF1ab:T19839C, ORF1ab:C21306T, S:C22995A, S:A23403G, S:C23604A, S:A23756G, ORF3a:C25782T, N:G28881A, N:G28882A, N:G28883C, N:C29197T,	12	N:R203K, N:G204R, ORF1a:P959S, ORF1a:G2258C, ORF1a:T3255I, ORF1a:I3618V, ORF1a:T4175I, ORF1b:P314L, S:T478K, S:D614G, S:P681H, S:T732A,
hCoV-19/Mexico/CMX-INMEGEN-02-03-08/2021	EPI_ISL_1040631	20B	B.1.1.519	28	5'UTR:T201C, 5'UTR:C203T, 5'UTR:C222T, 5'UTR:C241T, ORF1ab:C478T, ORF1ab:C745T, ORF1ab:G1738T, ORF1ab:C3037T, ORF1ab:C3140T, ORF1ab:A6488G, ORF1ab:G7133A, ORF1ab:C10029T, ORF1ab:C10954T, ORF1ab:A11117G, ORF1ab:C12789T, ORF1ab:C14408T, ORF1ab:T19839C, ORF1ab:A19974G, ORF1ab:C21306T, S:C22995A, S:A23403G, S:C23604A, S:A23756G, ORF3a:C25886T, N:G28881A, N:G28882A, N:G28883C, N:C29197T, 3'UTR:C29675T,	14	N:R203K, N:G204R, ORF1a:P959S, ORF1a:I2075V, ORF1a:V2290I, ORF1a:T3255I, ORF1a:I3618V, ORF1a:T4175I, ORF1b:P314L, ORF3a:S165F, S:T478K, S:D614G, S:P681H, S:T732A,
hCoV-19/Mexico/CMX-INMEGEN-02-03-09/2021	EPI_ISL_1040623	20B	B.1.1.519	21	5'UTR:C203T, 5'UTR:C222T, 5'UTR:C241T, ORF1ab:C3037T, ORF1ab:C3140T, ORF1ab:C10029T, ORF1ab:C10954T, ORF1ab:A11117G, ORF1ab:C12789T, ORF1ab:C13761T, ORF1ab:C14408T, ORF1ab:T19839C, ORF1ab:C21306T, S:C22995A, S:A23403G, S:C23604A, S:A23756G, N:G28881A, N:G28882A, N:G28883C, N:C29197T, ORF10:C29625T,	11	N:R203K, N:G204R, ORF1a:P959S, ORF1a:T3255I, ORF1a:I3618V, ORF1a:T4175I, ORF1b:P314L, S:T478K, S:D614G, S:P681H, S:T732A,

hCoV-19/Mexico/CMX-INMEGEN-02-03-10/2021	EPI_ISL_1040630	20B	B.1.1.519	23	5'UTR:C203T, 5'UTR:C222T, 5'UTR:C241T, ORF1ab:C3037T, ORF1ab:C3140T, ORF1ab:T3745C, ORF1ab:C8311T, ORF1ab:C10029T, ORF1ab:C10954T, ORF1ab:A11117G, ORF1ab:C12789T, ORF1ab:C14408T, ORF1ab:T19839C, ORF1ab:C21306T, S:C22995A, S:A23403G, S:C23604A, S:A23756G, ORF3a:C25844T, N:C28849T, N:G28881A, N:G28882A, N:G28883C, N:C29197T,	12	N:R203K, N:G204R, ORF1a:P959S, ORF1a:T3255I, ORF1a:I3618V, ORF1a:T4175I, ORF1b:P314L, ORF3a:T151I, S:T478K, S:D614G, S:P681H, S:T732A,	
hCoV-19/Mexico/CMX-INMEGEN-02-03-11/2021	EPI_ISL_1040625	20B	B.1.1.519	25	5'UTR:C203T, 5'UTR:C222T, 5'UTR:C241T, ORF1ab:C3037T, ORF1ab:C3140T, ORF1ab:C5646T, ORF1ab:G7027T, ORF1ab:C10029T, ORF1ab:C10954T, ORF1ab:A11117G, ORF1ab:C12789T, ORF1ab:C14408T, ORF1ab:A15864G, ORF1ab:A17205G, ORF1ab:C18441T, ORF1ab:T19839C, ORF1ab:C21306T, S:C22995A, S:A23403G, S:C23604A, S:A23756G, E:C26461T, N:G28881A, N:G28882A, N:G28883C, N:C29197T,	14	E:L73F, N:R203K, N:G204R, ORF1a:P959S, ORF1a:T1794I, ORF1a:M2254I, ORF1a:T3255I, ORF1a:I3618V, ORF1a:T4175I, ORF1b:P314L, S:T478K, S:D614G, S:P681H, S:T732A,	
hCoV-19/Mexico/CMX-INMEGEN-02-03-12/2021	EPI_ISL_1040626	20B	B.1.1.519	25	5'UTR:T201C, 5'UTR:C203T, 5'UTR:C222T, 5'UTR:C241T, ORF1ab:C745T, ORF1ab:G1738T, ORF1ab:C3037T, ORF1ab:C3140T, ORF1ab:C10029T, ORF1ab:T10084C, ORF1ab:A10904G, ORF1ab:C10954T, ORF1ab:A11117G, ORF1ab:C12789T, ORF1ab:C14408T, ORF1ab:T19839C, ORF1ab:A19974G, ORF1ab:C21306T, S:C22995A, S:A23403G, S:C23604A, S:A23756G, N:G28881A, N:G28882A, N:G28883C, N:C29197T,	12	N:R203K, N:G204R, ORF1a:P959S, ORF1a:T3255I, ORF1a:S3547G, ORF1a:I3618V, ORF1a:T4175I, ORF1b:P314L, S:T478K, S:D614G, S:P681H, S:T732A,	
hCoV-19/Mexico/CMX-INMEGEN-02-03-14/2021	EPI_ISL_1040634	20B	B.1.1.519	29	5'UTR:C203T, 5'UTR:C222T, 5'UTR:C241T, ORF1ab:A272G, ORF1ab:C3037T, ORF1ab:C3140T, ORF1ab:A3262G, ORF1ab:C6070T, ORF1ab:C10029T, ORF1ab:C10954T, ORF1ab:A11117G, ORF1ab:C12789T, ORF1ab:C14408T, ORF1ab:G15921T, ORF1ab:A17971G, ORF1ab:G18859T, ORF1ab:T19839C, ORF1ab:C20629T, ORF1ab:C21306T, S:C22995A, S:A23403G, S:C23604A, S:C23635T, S:A23756G, ORF7a:C27406T, N:G28881A, N:G28882A, N:G28883C, N:C29197T, N:G29425T,	17	N:R203K, N:G204R, N:Q384H, ORF1a:S3G, ORF1a:P959S, ORF1a:T3255I, ORF1a:I3618V, ORF1a:T4175I, ORF1b:P314L, ORF1b:R1502G, ORF1b:A1798S, ORF1b:H2388Y, ORF7a:L5F, S:T478K, S:D614G, S:P681H, S:T732A,	
hCoV-19/Mexico/CMX-INMEGEN-02-03-15/2021	EPI_ISL_1040624	20B	B.1.1.519	23	5'UTR:C203T, 5'UTR:A223T, 5'UTR:C241T, ORF1ab:C3037T, ORF1ab:C3140T, ORF1ab:C10029T, ORF1ab:C10954T, ORF1ab:A11117G, ORF1ab:C12789T, ORF1ab:A13455G, ORF1ab:C14408T, ORF1ab:G15921T, ORF1ab:T19839C, ORF1ab:C21306T, S:A22771G, S:C22995A, S:A23403G, S:C23604A, S:A23756G, S:T24589C, N:G28881A, N:G28882A, N:G28883C, N:C29197T,	12	N:R203K, N:G204R, ORF1a:P959S, ORF1a:T3255I, ORF1a:I3618V, ORF1a:T4175I, ORF1a:Q4397R, ORF1b:P314L, S:T478K, S:D614G, S:P681H, S:T732A,	5'UTR:2 22,
hCoV-19/Mexico/CMX-INMEGEN-02-03-16/2021	EPI_ISL_1040639	20B	B.1.1.519	31	5'UTR:C203T, 5'UTR:C222T, 5'UTR:C241T, ORF1ab:A866G, ORF1ab:C1009T, ORF1ab:C3037T, ORF1ab:C3140T, ORF1ab:T4030C, ORF1ab:C5183T, ORF1ab:G9802T, ORF1ab:C10029T, ORF1ab:C10954T, ORF1ab:A11117G, ORF1ab:C11916T, ORF1ab:C12789T, ORF1ab:C14408T, ORF1ab:C17977T, ORF1ab:C19662T, ORF1ab:T19839C, ORF1ab:C21306T, S:C22995A, S:A23403G, S:C23604A, S:A23756G, ORF3a:G25477T, ORF3a:C25669T, ORF8:T27904C, N:G28747T, N:G28881A, N:G28882A, N:G28883C, N:C29197T,	18	N:R203K, N:G204R, ORF1a:I201V, ORF1a:P959S, ORF1a:P1640S, ORF1a:T3255I, ORF1a:I3618V, ORF1a:S3884L, ORF1a:T4175I, ORF1b:P314L, ORF1b:L1504F, ORF3a:V29F, ORF3a:H93Y, ORF8:L4P, S:T478K, S:D614G, S:P681H, S:T732A,	

hCoV-19/Mexico/CMX-INMEGEN-02-03-17/2021	EPI_ISL_1040641	20B	B.1.1.519	27	5'UTR:T201C, 5'UTR:C203T, 5'UTR:C222T, 5'UTR:C241T, ORF1ab:G1738T, ORF1ab:C3037T, ORF1ab:C3140T, ORF1ab:C3768T, ORF1ab:C10029T, ORF1ab:C10954T, ORF1ab:A11117G, ORF1ab:C12789T, ORF1ab:C14408T, ORF1ab:C16457T, ORF1ab:T19839C, ORF1ab:A19974G, ORF1ab:C21306T, S:C21575T, S:C22995A, S:A23235G, S:A23403G, S:C23604A, S:A23756G, N:G28881A, N:G28882A, N:G28883C, N:C29197T, N:G29260T,	15	N:R203K, N:G204R, ORF1a:P959S, ORF1a:T1168I, ORF1a:T3255I, ORF1a:I3618V, ORF1a:T4175I, ORF1b:P314L, ORF1b:S997L, S:L5F, S:T478K, S:K558R, S:D614G, S:P681H, S:T732A,
hCoV-19/Mexico/CMX-INMEGEN-02-03-18/2021	EPI_ISL_1040643	20B	B.1.1.519	25	5'UTR:C203T, 5'UTR:C222T, 5'UTR:C241T, ORF1ab:C3037T, ORF1ab:C3140T, ORF1ab:T3745C, ORF1ab:C10029T, ORF1ab:C10078T, ORF1ab:C10954T, ORF1ab:A11117G, ORF1ab:C12789T, ORF1ab:C14408T, ORF1ab:T19839C, ORF1ab:C21306T, S:C22995A, S:A23403G, S:C23604A, S:A23756G, ORF3a:G25459T, ORF3a:C25844T, ORF7b:C27847T, N:G28881A, N:G28882A, N:G28883C, N:G28975T, N:C29197T,	15	N:R203K, N:G204R, N:M234I, ORF1a:P959S, ORF1a:T3255I, ORF1a:I3618V, ORF1a:T4175I, ORF1b:P314L, ORF3a:A23S, ORF3a:T151I, ORF7b:S31L, S:T478K, S:D614G, S:P681H, S:T732A,
hCoV-19/Mexico/CMX-INMEGEN-02-03-19/2021	EPI_ISL_1040642	20B	B.1.1.519	22	5'UTR:C203T, 5'UTR:C222T, 5'UTR:C241T, ORF1ab:C3037T, ORF1ab:C3140T, ORF1ab:G7037T, ORF1ab:C10029T, ORF1ab:C10954T, ORF1ab:A11117G, ORF1ab:C12789T, ORF1ab:C14408T, ORF1ab:T19839C, ORF1ab:C21306T, S:C22995A, S:A23403G, S:C23604A, S:A23756G, ORF3a:C25782T, N:G28881A, N:G28882A, N:G28883C, N:C29197T, N:A29221C,	12	N:R203K, N:G204R, ORF1a:P959S, ORF1a:G2258C, ORF1a:T3255I, ORF1a:I3618V, ORF1a:T4175I, ORF1b:P314L, S:T478K, S:D614G, S:P681H, S:T732A,
hCoV-19/Mexico/CMX-INMEGEN-02-03-20/2021	EPI_ISL_1040635	20B	B.1.1.519	27	5'UTR:C203T, 5'UTR:C222T, 5'UTR:C241T, ORF1ab:C2695T, ORF1ab:C3037T, ORF1ab:C3140T, ORF1ab:C3267T, ORF1ab:T4339C, ORF1ab:C6539T, ORF1ab:C10029T, ORF1ab:C10954T, ORF1ab:T11112C, ORF1ab:A11117G, ORF1ab:C12789T, ORF1ab:C14408T, ORF1ab:G16957T, ORF1ab:T19839C, ORF1ab:C21306T, S:C22995A, S:A23403G, S:C23604A, S:A23756G, ORF3a:G25906T, ORF8:C28253T, N:G28881A, N:G28882A, N:G28883C, N:C29197T,	16	N:R203K, N:G204R, ORF1a:P959S, ORF1a:T1001I, ORF1a:H2092Y, ORF1a:T3255I, ORF1a:M3616T, ORF1a:I3618V, ORF1a:T4175I, ORF1b:P314L, ORF1b:V1164L, ORF3a:G172C, S:T478K, S:D614G, S:P681H, S:T732A,
hCoV-19/Mexico/CMX-INMEGEN-02-03-21/2021	EPI_ISL_1040636	20B	B.1.1.519	23	5'UTR:C203T, 5'UTR:C222T, 5'UTR:C241T, ORF1ab:C3037T, ORF1ab:C3140T, ORF1ab:C5869T, ORF1ab:C10029T, ORF1ab:C10954T, ORF1ab:A11117G, ORF1ab:C12789T, ORF1ab:C14408T, ORF1ab:T19839C, ORF1ab:C21306T, S:C22995A, S:A23403G, S:C23604A, S:A23756G, ORF3a:C25688T, ORF8:T27904C, ORF8:C28087T, N:G28881A, N:G28882A, N:G28883C, N:C29197T,	14	N:R203K, N:G204R, ORF1a:P959S, ORF1a:T3255I, ORF1a:I3618V, ORF1a:T4175I, ORF1b:P314L, ORF3a:A99V, ORF8:L4P, ORF8:A65V, S:T478K, S:D614G, S:P681H, S:T732A,
hCoV-19/Mexico/CMX-INMEGEN-02-03-22/2021	EPI_ISL_1040632	20B	B.1.1.519	25	5'UTR:C203T, 5'UTR:C222T, 5'UTR:C241T, ORF1ab:C3037T, ORF1ab:C3140T, ORF1ab:C5869T, ORF1ab:C6568T, ORF1ab:C10029T, ORF1ab:C10954T, ORF1ab:A11117G, ORF1ab:C12789T, ORF1ab:C14408T, ORF1ab:T19839C, ORF1ab:G21204A, ORF1ab:C21306T, S:C22995A, S:A23403G, S:C23604A, S:A23756G, ORF8:T27904C, ORF8:G27987T, ORF8:C28087T, N:G28881A, N:G28882A, N:G28883C, N:C29197T,	14	N:R203K, N:G204R, ORF1a:P959S, ORF1a:T3255I, ORF1a:I3618V, ORF1a:T4175I, ORF1b:P314L, ORF8:L4P, ORF8:V32L, ORF8:A65V, S:T478K, S:D614G, S:P681H, S:T732A,

hCoV-19/Mexico/CMX-INMEGEN-02-03-23/2021	EPI_ISL_1040633	20B	B.1.1.519	25	5'UTR:C203T, 5'UTR:C222T, 5'UTR:C241T, ORF1ab:C3037T, ORF1ab:C3140T, ORF1ab:G4561A, ORF1ab:C7926T, ORF1ab:T9070C, ORF1ab:C10029T, ORF1ab:C10954T, ORF1ab:A11117G, ORF1ab:C11173T, ORF1ab:C12789T, ORF1ab:C14408T, ORF1ab:T19839C, ORF1ab:C21306T, S:C22995A, S:A23403G, S:C23604A, S:A23756G, N:G28337T, N:G28881A, N:G28882A, N:G28883C, N:C29197T, N:G29527T,	15	N:D22Y, N:R203K, N:G204R, N:Q418H, ORF1a:P959S, ORF1a:A2554V, ORF1a:T3255I, ORF1a:I3618V, ORF1a:T4175I, ORF1b:P314L, ORF9b:Q18H, S:T478K, S:D614G, S:P681H, S:T732A,	
hCoV-19/Mexico/CMX-INMEGEN-02-04-01/2021	EPI_ISL_1055017	20B	B.1.1.519	27	5'UTR:T201C, 5'UTR:C203T, 5'UTR:C222T, 5'UTR:C241T, ORF1ab:C936T, ORF1ab:G1738T, ORF1ab:C3037T, ORF1ab:C3140T, ORF1ab:A6371C, ORF1ab:C10029T, ORF1ab:C10954T, ORF1ab:A11117G, ORF1ab:C12789T, ORF1ab:C14408T, ORF1ab:T19839C, ORF1ab:A19974G, ORF1ab:C21306T, S:G22100A, S:C22995A, S:A23403G, S:C23604A, S:A23756G, N:C28311T, N:G28881A, N:G28882A, N:G28883C, N:C29197T, N:G29227T,	16	N:P13L, N:R203K, N:G204R, ORF1a:T224I, ORF1a:P959S, ORF1a:M2036L, ORF1a:T3255I, ORF1a:I3618V, ORF1a:T4175I, ORF1b:P314L, ORF9b:P10S, S:E180K, S:T478K, S:D614G, S:P681H, S:T732A,	
hCoV-19/Mexico/CMX-INMEGEN-02-04-02/2021	EPI_ISL_1055009	20B	B.1.1.519	22	5'UTR:C203T, 5'UTR:C222T, 5'UTR:C241T, ORF1ab:C3037T, ORF1ab:C3140T, ORF1ab:G5704T, ORF1ab:C10029T, ORF1ab:C10954T, ORF1ab:A11117G, ORF1ab:C12789T, ORF1ab:C14408T, ORF1ab:T19839C, ORF1ab:C21306T, S:C21742T, S:C22995A, S:A23403G, S:C23604A, S:A23756G, N:G28881A, N:G28882A, N:G28883C, N:C29197T, N:G29527T,	13	N:R203K, N:G204R, N:Q418H, ORF1a:P959S, ORF1a:Q1813H, ORF1a:T3255I, ORF1a:I3618V, ORF1a:T4175I, ORF1b:P314L, S:T478K, S:D614G, S:P681H, S:T732A,	
hCoV-19/Mexico/CMX-INMEGEN-02-04-03/2021	EPI_ISL_1055010	20B	B.1.1.519	26	5'UTR:C203T, 5'UTR:C222T, 5'UTR:C241T, ORF1ab:C3037T, ORF1ab:C3140T, ORF1ab:C10029T, ORF1ab:A10630G, ORF1ab:C10954T, ORF1ab:A11117G, ORF1ab:C12789T, ORF1ab:C14408T, ORF1ab:T19839C, ORF1ab:C21306T, S:C22995A, S:A23403G, S:C23604A, S:A23756G, S:G24914T, S:C25254T, ORF3a:C25642T, ORF3a:C25782T, ORF3a:G26063T, N:G28881A, N:G28882A, N:G28883C, N:C29197T, N:G29402T,	15	N:R203K, N:G204R, N:D377Y, ORF1a:P959S, ORF1a:T3255I, ORF1a:I3618V, ORF1a:T4175I, ORF1b:P314L, ORF3a:G224V, S:T478K, S:D614G, S:P681H, S:T732A, S:D1118Y, S:T1231I,	
hCoV-19/Mexico/CMX-INMEGEN-02-04-04/2021	EPI_ISL_1055011	20B	B.1.1.519	23	5'UTR:C203T, 5'UTR:C222T, 5'UTR:C241T, ORF1ab:C3037T, ORF1ab:C3140T, ORF1ab:A3988T, ORF1ab:G9479T, ORF1ab:C10029T, ORF1ab:C10954T, ORF1ab:A11117G, ORF1ab:G11365T, ORF1ab:C12789T, ORF1ab:C14408T, ORF1ab:T19839C, ORF1ab:C21306T, S:C22995A, S:A23403G, S:C23604A, S:A23756G, N:G28881A, N:G28882A, N:G28883C, N:C29197T, N:G29527T,	13	N:R203K, N:G204R, N:Q418H, ORF1a:P959S, ORF1a:G3072C, ORF1a:T3255I, ORF1a:I3618V, ORF1a:T4175I, ORF1b:P314L, S:T478K, S:D614G, S:P681H, S:T732A,	
hCoV-19/Mexico/CMX-INMEGEN-02-04-05/2021	EPI_ISL_1055023	20B	B.1.1.222	18	5'UTR:C241T, ORF1ab:C3037T, ORF1ab:T3295C, ORF1ab:C10165T, ORF1ab:G12514T, ORF1ab:A13498G, ORF1ab:C14408T, ORF1ab:G15372A, ORF1ab:T19839C, ORF1ab:T20777C, S:G22331A, S:A23403G, S:C23635G, S:A23756G, N:C28311T, N:G28881A, N:G28882A, N:G28883C, N:G29440T,	11	N:P13L, N:R203K, N:G204R, N:Q389H, ORF1b:T11A, ORF1b:P314L, ORF1b:I2437T, ORF9b:P10S, S:G257S, S:D614G, S:T732A,	3'UTR:29750-29759,
hCoV-19/Mexico/CMX-INMEGEN-02-04-06/2021	EPI_ISL_1055021	20B	B.1.1.519	23	5'UTR:C203T, 5'UTR:C222T, 5'UTR:C241T, ORF1ab:C1710T, ORF1ab:C3037T, ORF1ab:C3140T, ORF1ab:G3145A, ORF1ab:A6484G, ORF1ab:C10029T, ORF1ab:C10954T, ORF1ab:A11117G, ORF1ab:C12789T, ORF1ab:C14408T, ORF1ab:T19839C, ORF1ab:C21306T, S:C22995A, S:A23403G, S:C23604A, S:A23756G, N:G28881A, N:G28882A, N:G28883C, N:C29197T, N:G29527T,	13	N:R203K, N:G204R, N:Q418H, ORF1a:A482V, ORF1a:P959S, ORF1a:T3255I, ORF1a:I3618V, ORF1a:T4175I, ORF1b:P314L, S:T478K, S:D614G, S:P681H, S:T732A,	

hCoV-19/Mexico/CMX-INMEGEN-02-04-07/2021	EPI_ISL_1055024	20B	B.1.1.519	27	5'UTR:C203T, 5'UTR:C222T, 5'UTR:C241T, ORF1ab:C3037T, ORF1ab:C3140T, ORF1ab:T3547A, ORF1ab:C7594T, ORF1ab:C7728T, ORF1ab:C10029T, ORF1ab:C10954T, ORF1ab:A11117G, ORF1ab:C12789T, ORF1ab:C14408T, ORF1ab:T15726A, ORF1ab:A17674G, ORF1ab:T19839C, ORF1ab:C21306T, S:C22995A, S:A23403G, S:C23604A, S:A23756G, M:C26833T, ORF6:C27389T, N:G28881A, N:G28882A, N:G28883C, N:C29197T, N:G29527T,	16	M:A104V, N:R203K, N:G204R, N:Q418H, ORF1a:P959S, ORF1a:N1094K, ORF1a:S2488F, ORF1a:T3255I, ORF1a:I3618V, ORF1a:T4175I, ORF1b:P314L, ORF1b:I1403V, S:T478K, S:D614G, S:P681H, S:T732A,
hCoV-19/Mexico/CMX-INMEGEN-02-04-08/2021	EPI_ISL_1055025	20B	B.1.1.519	26	5'UTR:C203T, 5'UTR:C222T, 5'UTR:C241T, ORF1ab:C3037T, ORF1ab:C3140T, ORF1ab:T3547A, ORF1ab:C7728T, ORF1ab:C10029T, ORF1ab:C10954T, ORF1ab:A11117G, ORF1ab:C12789T, ORF1ab:C14408T, ORF1ab:T15726A, ORF1ab:A17674G, ORF1ab:T19839C, ORF1ab:C21306T, S:C22995A, S:A23403G, S:C23604A, S:A23756G, M:C26833T, ORF6:C27389T, N:G28881A, N:G28882A, N:G28883C, N:C29197T, N:G29527T,	16	M:A104V, N:R203K, N:G204R, N:Q418H, ORF1a:P959S, ORF1a:N1094K, ORF1a:S2488F, ORF1a:T3255I, ORF1a:I3618V, ORF1a:T4175I, ORF1b:P314L, ORF1b:I1403V, S:T478K, S:D614G, S:P681H, S:T732A,
hCoV-19/Mexico/CMX-INMEGEN-02-04-09/2021	EPI_ISL_1055022	20B	B.1.1.519	27	5'UTR:C203T, 5'UTR:C222T, 5'UTR:C241T, ORF1ab:C3037T, ORF1ab:C3140T, ORF1ab:C3593T, ORF1ab:C9700T, ORF1ab:C10029T, ORF1ab:C10954T, ORF1ab:A11117G, ORF1ab:C11455T, ORF1ab:C12789T, ORF1ab:C12853T, ORF1ab:C14408T, ORF1ab:C16887T, ORF1ab:T19839C, ORF1ab:C21306T, S:C22995A, S:A23403G, S:C23604A, S:A23756G, S:A24727G, ORF8:T27904C, ORF8:C28087T, N:G28881A, N:G28882A, N:G28883C, N:C29197T	14	N:R203K, N:G204R, ORF1a:P959S, ORF1a:L1110F, ORF1a:T3255I, ORF1a:I3618V, ORF1a:T4175I, ORF1b:P314L, ORF8:L4P, ORF8:A65V, S:T478K, S:D614G, S:P681H, S:T732A,
hCoV-19/Mexico/CMX-INMEGEN-02-04-10/2021	EPI_ISL_1055018	20B	B.1.1.222	21	5'UTR:C241T, ORF1ab:C3037T, ORF1ab:C10029T, ORF1ab:T11187C, ORF1ab:C14408T, ORF1ab:G17721T, ORF1ab:C18788T, ORF1ab:T19839C, ORF1ab:T20955C, S:A23403G, S:G23522C, S:A23756G, ORF3a:G25567A, ORF3a:T25569A, ORF3a:T25570A, ORF3a:G25912T, ORF3a:A26108G, ORF8:A27921G, ORF8:G28001T, N:G28881A, N:G28882A, N:G28883C,	14	N:R203K, N:G204R, ORF1a:T3255I, ORF1a:L3641S, ORF1b:P314L, ORF1b:T1774I, ORF3a:A59T, ORF3a:S60T, ORF3a:G174C, ORF3a:E239G, ORF8:I10V, S:D614G, S:E654Q, S:T732A,
hCoV-19/Mexico/CMX-INMEGEN-02-04-11/2021	EPI_ISL_1055019	20B	B.1.1.519	27	5'UTR:C203T, 5'UTR:C222T, 5'UTR:C241T, ORF1ab:C815T, ORF1ab:C3037T, ORF1ab:C3140T, ORF1ab:A3581G, ORF1ab:C10029T, ORF1ab:C10954T, ORF1ab:A11117G, ORF1ab:C12789T, ORF1ab:C14408T, ORF1ab:T18151C, ORF1ab:T19839C, ORF1ab:G20002T, ORF1ab:C21306T, S:C22995A, S:A23403G, S:C23604A, S:A23756G, S:G25361A, ORF3a:C25658T, M:C26882T, ORF8:T27904C, N:G28881A, N:G28882A, N:G28883C, N:C29197T,	17	N:R203K, N:G204R, ORF1a:R184C, ORF1a:P959S, ORF1a:S1106G, ORF1a:T3255I, ORF1a:I3618V, ORF1a:T4175I, ORF1b:P314L, ORF1b:D2179Y, ORF3a:T89I, ORF8:L4P, S:T478K, S:D614G, S:P681H, S:T732A, S:G1267R,
hCoV-19/Mexico/CMX-INMEGEN-02-04-12/2021	EPI_ISL_1055012	20B	B.1.1.519	24	5'UTR:C203T, 5'UTR:C222T, 5'UTR:C241T, ORF1ab:C3037T, ORF1ab:C3140T, ORF1ab:C10029T, ORF1ab:C10954T, ORF1ab:A11117G, ORF1ab:C12789T, ORF1ab:C14408T, ORF1ab:C17550T, ORF1ab:C19320T, ORF1ab:T19839C, ORF1ab:C21306T, S:C22995A, S:A23403G, S:C23604A, S:A23756G, ORF7b:G27798T, N:A28753G, N:G28881A, N:G28882A, N:G28883C, N:C29197T, N:G29527T,	13	N:R203K, N:G204R, N:Q418H, ORF1a:P959S, ORF1a:T3255I, ORF1a:I3618V, ORF1a:T4175I, ORF1b:P314L, ORF7b:A15S, S:T478K, S:D614G, S:P681H, S:T732A,

hCoV-19/Mexico/CMX-INMEGEN-02-04-13/2021	EPI_ISL_1055013	20B	B.1.1.519	24	5'UTR:C203T, 5'UTR:C222T, 5'UTR:C241T, ORF1ab:C3037T, ORF1ab:C3140T, ORF1ab:C10029T, ORF1ab:C10954T, ORF1ab:A11117G, ORF1ab:C12789T, ORF1ab:C14408T, ORF1ab:C17550T, ORF1ab:C19320T, ORF1ab:T19839C, ORF1ab:C21306T, S:C22995A, S:A23403G, S:C23604A, S:A23756G, ORF7b:G27798T, N:A28753G, N:G28881A, N:G28882A, N:G28883C, N:C29197T, N:G29527T,	13	N:R203K, N:G204R, N:Q418H, ORF1a:P959S, ORF1a:T3255I, ORF1a:I3618V, ORF1a:T4175I, ORF1b:P314L, ORF7b:A15S, S:T478K, S:D614G, S:P681H, S:T732A,	
hCoV-19/Mexico/CMX-INMEGEN-02-04-14/2021	EPI_ISL_1055014	20B	B.1.1.519	30	5'UTR:T201C, 5'UTR:C203T, 5'UTR:C222T, 5'UTR:C241T, ORF1ab:G1738T, ORF1ab:C3037T, ORF1ab:C3140T, ORF1ab:C10029T, ORF1ab:C10954T, ORF1ab:A11117G, ORF1ab:C12789T, ORF1ab:C14408T, ORF1ab:C15324T, ORF1ab:C18086T, ORF1ab:T19839C, ORF1ab:A19974G, ORF1ab:T21294A, ORF1ab:G21295A, ORF1ab:G21296A, ORF1ab:C21306T, S:C22995A, S:A23403G, S:C23604A, S:A23756G, S:G25244T, M:C26527T, ORF7a:C27427T, N:G28881A, N:G28882A, N:G28883C, N:C29197T	16	M:A2V, N:R203K, N:G204R, ORF1a:P959S, ORF1a:T3255I, ORF1a:I3618V, ORF1a:T4175I, ORF1b:P314L, ORF1b:T1540I, ORF1b:G2610N, ORF7a:L12F, S:T478K, S:D614G, S:P681H, S:T732A, S:V1228L,	
hCoV-19/Mexico/CMX-INMEGEN-02-04-15/2021	EPI_ISL_1055006	20B	B.1	25	5'UTR:C203T, 5'UTR:C222T, 5'UTR:C241T, ORF1ab:C3037T, ORF1ab:C3140T, ORF1ab:C10029T, ORF1ab:C10319T, ORF1ab:C10954T, ORF1ab:A11117G, ORF1ab:C12789T, ORF1ab:C14408T, ORF1ab:T19839C, ORF1ab:C21008T, ORF1ab:C21306T, S:C21637T, S:C22995A, S:A23403G, S:A23586T, S:C23604A, S:A23756G, ORF8:T27904C, ORF8:C28087T, N:G28881A, N:G28882A, N:G28883C, N:C29197T,	16	N:R203K, N:G204R, ORF1a:P959S, ORF1a:T3255I, ORF1a:L3352F, ORF1a:I3618V, ORF1a:T4175I, ORF1b:P314L, ORF1b:T2514I, ORF8:L4P, ORF8:A65V, S:T478K, S:D614G, S:Q675L, S:P681H, S:T732A,	
hCoV-19/Mexico/CMX-INMEGEN-02-04-16/2021	EPI_ISL_1055007	20B	B.1.1.519	23	5'UTR:C203T, 5'UTR:C222T, 5'UTR:C241T, ORF1ab:A2056G, ORF1ab:C3037T, ORF1ab:C3140T, ORF1ab:C10029T, ORF1ab:C10954T, ORF1ab:A11117G, ORF1ab:C12789T, ORF1ab:C14408T, ORF1ab:G15921T, ORF1ab:C16111T, ORF1ab:T19839C, ORF1ab:C21306T, ORF1ab:G21468T, S:C22995A, S:A23403G, S:C23604A, S:A23756G, N:G28881A, N:G28882A, N:G28883C, N:C29197T,	12	N:R203K, N:G204R, ORF1a:P959S, ORF1a:T3255I, ORF1a:I3618V, ORF1a:T4175I, ORF1b:P314L, ORF1b:M2667I, S:T478K, S:D614G, S:P681H, S:T732A,	
hCoV-19/Mexico/CMX-INMEGEN-02-04-17/2021	EPI_ISL_1055005	20B	B.1.1.519	25	5'UTR:C203T, 5'UTR:C222T, 5'UTR:C241T, ORF1ab:C3037T, ORF1ab:C3140T, ORF1ab:C10029T, ORF1ab:C10954T, ORF1ab:A11117G, ORF1ab:C12789T, ORF1ab:G13514T, ORF1ab:C14408T, ORF1ab:C19602T, ORF1ab:T19839C, ORF1ab:C21306T, S:C22995A, S:A23403G, S:C23604A, S:A23756G, ORF3a:G25775T, ORF3a:G25906T, ORF8:G28209T, ORF8:C28253T, N:G28881A, N:G28882A, N:G28883C, N:C29197T,	15	N:R203K, N:G204R, ORF1a:P959S, ORF1a:T3255I, ORF1a:I3618V, ORF1a:T4175I, ORF1b:G16V, ORF1b:P314L, ORF3a:W128L, ORF3a:G172C, ORF8:E106*, S:T478K, S:D614G, S:P681H, S:T732A,	
hCoV-19/Mexico/CMX-INMEGEN-02-04-18/2021	EPI_ISL_1058046	20B	B.1.1.519	29	5'UTR:T201C, 5'UTR:C203T, 5'UTR:C222T, 5'UTR:C241T, ORF1ab:C936T, ORF1ab:G1738T, ORF1ab:T2587C, ORF1ab:A3021T, ORF1ab:C3037T, ORF1ab:C3140T, ORF1ab:C10029T, ORF1ab:C10954T, ORF1ab:A11117G, ORF1ab:C12789T, ORF1ab:C14408T, ORF1ab:T19839C, ORF1ab:A19974G, ORF1ab:C21306T, S:C22995A, S:C23191T, S:A23324G, S:A23403G, S:C23604A, S:A23756G, N:G28881A, N:G28882A, N:G28883C, N:C29197T, N:C29200T, N:G29227T	14	N:R203K, N:G204R, ORF1a:T224I, ORF1a:H919L, ORF1a:P959S, ORF1a:T3255I, ORF1a:I3618V, ORF1a:T4175I, ORF1b:P314L, S:T478K, S:T588A, S:D614G, S:P681H, S:T732A,	ORF6:27207-27208

hCoV-19/Mexico/CMX-INMEGEN-02-04-19/2021	EPI_ISL_1055008	20B	B.1.1.519	25	5'UTR:T201C, 5'UTR:C203T, 5'UTR:C222T, 5'UTR:C241T, ORF1ab:C745T, ORF1ab:G1738T, ORF1ab:C3037T, ORF1ab:C3140T, ORF1ab:C10029T, ORF1ab:T10084C, ORF1ab:A10904G, ORF1ab:C10954T, ORF1ab:A11117G, ORF1ab:C12789T, ORF1ab:C14408T, ORF1ab:T19839C, ORF1ab:A19974G, ORF1ab:C21306T, S:C22995A, S:A23403G, S:C23604A, S:A23756G, N:G28881A, N:G28882A, N:G28883C, N:C29197T,	12	N:R203K, N:G204R, ORF1a:P959S, ORF1a:T3255I, ORF1a:S3547G, ORF1a:I3618V, ORF1a:T4175I, ORF1b:P314L, S:T478K, S:D614G, S:P681H, S:T732A,	
hCoV-19/Mexico/CMX-INMEGEN-02-04-20/2021	EPI_ISL_1055020	20B	B.1.1.519	30	5'UTR:T201C, 5'UTR:C203T, 5'UTR:C222T, 5'UTR:C241T, ORF1ab:C1218T, ORF1ab:G1738T, ORF1ab:C3037T, ORF1ab:C3140T, ORF1ab:G4907A, ORF1ab:C5693T, ORF1ab:C10029T, ORF1ab:C10954T, ORF1ab:A11117G, ORF1ab:C11514T, ORF1ab:C12789T, ORF1ab:C13517T, ORF1ab:C14408T, ORF1ab:C18457T, ORF1ab:T19839C, ORF1ab:A19974G, ORF1ab:C21306T, S:C21621T, S:C22995A, S:A23403G, S:C23604A, S:A23756G, N:G28881A, N:G28882A, N:G28883C, N:C29197T, 3'UTR:G29800A,	18	N:R203K, N:G204R, ORF1a:S318L, ORF1a:P959S, ORF1a:G1548S, ORF1a:P1810S, ORF1a:T3255I, ORF1a:I3618V, ORF1a:T3750I, ORF1a:T4175I, ORF1b:T17I, ORF1b:P314L, ORF1b:P1664S, S:T20I, S:T478K, S:D614G, S:P681H, S:T732A,	
hCoV-19/Mexico/CMX-INMEGEN-02-04-21/2021	EPI_ISL_1055015	20B	B.1.1.519	28	5'UTR:T201C, 5'UTR:C203T, 5'UTR:C222T, 5'UTR:C241T, ORF1ab:C936T, ORF1ab:G1738T, ORF1ab:C3037T, ORF1ab:C3140T, ORF1ab:C10029T, ORF1ab:C10954T, ORF1ab:A11117G, ORF1ab:C12789T, ORF1ab:C14408T, ORF1ab:C16293T, ORF1ab:T19839C, ORF1ab:A19974G, ORF1ab:T20958A, ORF1ab:C21306T, S:C22995A, S:A23403G, S:C23604A, S:A23756G, E:C26455T, N:G28514C, N:G28881A, N:G28882A, N:G28883C, N:C29197T, N:G29227T	15	E:P71S, N:D81H, N:R203K, N:G204R, ORF1a:T224I, ORF1a:P959S, ORF1a:T3255I, ORF1a:I3618V, ORF1a:T4175I, ORF1b:P314L, ORF9b:Q77H, S:T478K, S:D614G, S:P681H, S:T732A,	
hCoV-19/Mexico/CMX-INMEGEN-02-04-22/2021	EPI_ISL_1055016	20B	B.1.1.519	25	5'UTR:C203T, 5'UTR:C222T, 5'UTR:C241T, ORF1ab:C2675T, ORF1ab:C3037T, ORF1ab:C3140T, ORF1ab:T3745C, ORF1ab:G3824A, ORF1ab:C10029T, ORF1ab:C10954T, ORF1ab:A11117G, ORF1ab:G12067T, ORF1ab:C12789T, ORF1ab:C14408T, ORF1ab:T19839C, ORF1ab:C21306T, S:C22995A, S:A23403G, S:C23604A, S:A23756G, ORF3a:C25844T, N:C28876T, N:G28881A, N:G28882A, N:G28883C, N:C29197T,	15	N:R203K, N:G204R, ORF1a:P804S, ORF1a:P959S, ORF1a:V1187I, ORF1a:T3255I, ORF1a:I3618V, ORF1a:M3934I, ORF1a:T4175I, ORF1b:P314L, ORF3a:T151I, S:T478K, S:D614G, S:P681H, S:T732A,	
hCoV-19/Mexico/CMX-INMEGEN-02-05-04/2020	EPI_ISL_1060612	20B	B.1.1.222	17	5'UTR:C241T, ORF1ab:C2061T, ORF1ab:C3037T, ORF1ab:C11824T, ORF1ab:G13723T, ORF1ab:C14408T, ORF1ab:C15960T, ORF1ab:G17395A, ORF1ab:T19839C, S:A23403G, S:A23756G, ORF6:T27266C, ORF8:G28083T, N:G28881A, N:G28882A, N:G28883C, N:A28996G, N:G29332T,	10	N:R203K, N:G204R, ORF1a:A599V, ORF1b:A86S, ORF1b:P314L, ORF1b:V1310I, ORF6:F22S, ORF8:E64*, S:D614G, S:T732A,	S:21765-21770,
hCoV-19/Mexico/CMX-INMEGEN-02-05-05/2020	EPI_ISL_1060613	20A	B.1.258.21	22	5'UTR:C241T, ORF1ab:C527T, ORF1ab:C609T, ORF1ab:C3037T, ORF1ab:C7093T, ORF1ab:T7767C, ORF1ab:C8047T, ORF1ab:C8956T, ORF1ab:G12988T, ORF1ab:A14116G, ORF1ab:C14408T, ORF1ab:G15598A, ORF1ab:C17104T, ORF1ab:G18028T, ORF1ab:A20268G, ORF1ab:C20451T, S:C22879A, S:A23403G, S:T24910C, M:A26555C, M:T26972C, ORF7b:C27800A, 3'UTR:G29734C,	11	M:E11D, ORF1a:P115L, ORF1a:I2501T, ORF1a:M4241I, ORF1b:T217A, ORF1b:P314L, ORF1b:V711I, ORF1b:H1213Y, ORF1b:A1521S, S:N439K, S:D614G,	S:21765-21770,

hCoV-19/Mexico/CMX-INMEGEN-02-05-06/2020	EPI_ISL_1060615	20B	B.1.1.222	17	5'UTR:C241T, ORF1ab:C2061T, ORF1ab:C3037T, ORF1ab:C11824T, ORF1ab:G13723T, ORF1ab:C14408T, ORF1ab:C15960T, ORF1ab:G17395A, ORF1ab:T19839C, S:A23403G, S:A23756G, M:C26681T, ORF7a:G27478T, ORF8:G28083T, N:G28881A, N:G28882A, N:G28883C, N:A28996G.	10	N:R203K, N:G204R, ORF1a:A599V, ORF1b:A86S, ORF1b:P314L, ORF1b:V1310I, ORF7a:V29L, ORF8:E64*, S:D614G, S:T732A,	S:21765-21770,
hCoV-19/Mexico/CMX-INMEGEN-02-05-07/2020	EPI_ISL_1075657	20C	B.1.375	16	5'UTR:C241T, ORF1ab:C1059T, ORF1ab:T1171C, ORF1ab:C3037T, ORF1ab:A5747G, ORF1ab:C10789T, ORF1ab:G14028T, ORF1ab:C14408T, ORF1ab:G17259T, ORF1ab:C21431T, S:A23403G, S:G24007A, ORF3a:G25563T, ORF3a:C25844T, M:A26664G, N:C28887T, N:G29081T,	10	M:I48V, N:T205I, N:V270L, ORF1b:M187I, ORF1b:P314L, ORF1b:E1264D, ORF1b:A2655V, ORF3a:Q57H, ORF3a:T151I, S:D614G,	ORF1ab:11083, S:21765-21770,
hCoV-19/Mexico/CMX-INMEGEN-02-05-08/2020	EPI_ISL_1060614	20B	B.1.1.222	15	5'UTR:C241T, ORF1ab:C2061T, ORF1ab:C3037T, ORF1ab:C11824T, ORF1ab:G13723T, ORF1ab:C14408T, ORF1ab:C15960T, ORF1ab:G17395A, ORF1ab:T19839C, S:A23403G, S:A23756G, ORF8:G28083T, N:G28881A, N:G28882A, N:G28883C. N:A28996G.	9	N:R203K, N:G204R, ORF1a:A599V, ORF1b:A86S, ORF1b:P314L, ORF1b:V1310I, ORF8:E64*, S:D614G, S:T732A,	S:21765-21770,
hCoV-19/Mexico/CMX-INMEGEN-02-05-09/2021	EPI_ISL_1075267	20B	B.1.1.222	17	5'UTR:C241T, ORF1ab:C2061T, ORF1ab:C3037T, ORF1ab:C11824T, ORF1ab:G13723T, ORF1ab:C14408T, ORF1ab:C15960T, ORF1ab:G17395A, ORF1ab:T19839C, ORF1ab:G21255C, S:G23108C, S:A23403G, S:A23756G, ORF8:G28083T, N:G28881A, N:G28882A, N:G28883C, N:A28996G,	10	N:R203K, N:G204R, ORF1a:A599V, ORF1b:A86S, ORF1b:P314L, ORF1b:V1310I, ORF8:E64*, S:E516Q, S:D614G, S:T732A,	S:21765-21770, ORF3a:26158-26161,
hCoV-19/Mexico/CMX-INMEGEN-02-05-10/2021	EPI_ISL_1060601	20B	B.1.1.519	23	5'UTR:C203T, 5'UTR:A223T, 5'UTR:C241T, ORF1ab:C3037T, ORF1ab:C3140T, ORF1ab:C10029T, ORF1ab:C10954T, ORF1ab:A11117G, ORF1ab:C12789T, ORF1ab:A13455G, ORF1ab:C14408T, ORF1ab:G15921T, ORF1ab:T19839C, ORF1ab:C21306T, S:A22771G, S:C22995A, S:A23403G, S:C23604A, S:A23756G, S:T24589C, N:G28881A, N:G28882A, N:G28883C, N:C29197T.	12	N:R203K, N:G204R, ORF1a:P959S, ORF1a:T3255I, ORF1a:I3618V, ORF1a:T4175I, ORF1a:Q4397R, ORF1b:P314L, S:T478K, S:D614G, S:P681H, S:T732A,	5'UTR:222,
hCoV-19/Mexico/CMX-INMEGEN-02-05-11/2021	EPI_ISL_1060602	20B	B.1.1.519	22	5'UTR:C203T, 5'UTR:C222T, 5'UTR:C241T, ORF1ab:C3037T, ORF1ab:C3140T, ORF1ab:G7037T, ORF1ab:T9004C, ORF1ab:C10029T, ORF1ab:C10954T, ORF1ab:A11117G, ORF1ab:C12789T, ORF1ab:C14408T, ORF1ab:T19839C, ORF1ab:C21306T, S:C22995A, S:A23403G, S:C23604A, S:A23756G, ORF3a:C25782T, N:G28881A, N:G28882A, N:G28883C. N:C29197T.	12	N:R203K, N:G204R, ORF1a:P959S, ORF1a:G2258C, ORF1a:T3255I, ORF1a:I3618V, ORF1a:T4175I, ORF1b:P314L, S:T478K, S:D614G, S:P681H, S:T732A,	
hCoV-19/Mexico/CMX-INMEGEN-02-05-12/2021	EPI_ISL_1060603	20B	B.1.1.519	26	5'UTR:T201C, 5'UTR:C203T, 5'UTR:C222T, 5'UTR:C241T, ORF1ab:G1738T, ORF1ab:C3037T, ORF1ab:C3140T, ORF1ab:C4780T, ORF1ab:C10029T, ORF1ab:T10135C, ORF1ab:C10954T, ORF1ab:A11117G, ORF1ab:G12734T, ORF1ab:C12789T, ORF1ab:C14408T, ORF1ab:C17333T, ORF1ab:T19839C, ORF1ab:A19974G, ORF1ab:C21306T, S:C22995A, S:A23403G, S:C23604A, S:A23756G, N:G28881A, N:G28882A, N:G28883C, N:C29197T,	13	N:R203K, N:G204R, ORF1a:P959S, ORF1a:T3255I, ORF1a:I3618V, ORF1a:G4157C, ORF1a:T4175I, ORF1b:P314L, ORF1b:T1289M, S:T478K, S:D614G, S:P681H, S:T732A,	
hCoV-19/Mexico/CMX-INMEGEN-02-05-13/2021	EPI_ISL_1060600	20B	B.1.1.222	20	5'UTR:C241T, ORF1ab:C3037T, ORF1ab:T4813C, ORF1ab:G5138T, ORF1ab:T5479C, ORF1ab:A10323G, ORF1ab:A13498G, ORF1ab:C14408T, ORF1ab:C14741T, ORF1ab:C15540T, ORF1ab:G19542A, ORF1ab:T19839C, S:G22331A, S:C22680T, S:A23403G, S:A23756G, S:G24193T, S:G25177T, N:G28881A. N:G28882A. N:G28883C.	13	N:R203K, N:G204R, ORF1a:D1625Y, ORF1a:K3353R, ORF1b:T11A, ORF1b:P314L, ORF1b:S425F, ORF1b:M2025I, S:G257S, S:S373L, S:D614G, S:T732A, S:K1205N,	3'UTR:29767-29776

hCoV-19/Mexico/CMX-INMEGEN-02-05-14/2021	EPI_ISL_1060604	20B	B.1.1.519	27	5'UTR:T201C, 5'UTR:C203T, 5'UTR:C222T, 5'UTR:C241T, ORF1ab:G1738T, ORF1ab:C1878T, ORF1ab:C3037T, ORF1ab:C3140T, ORF1ab:G3753A, ORF1ab:C5812T, ORF1ab:C10029T, ORF1ab:C10954T, ORF1ab:A11117G, ORF1ab:C12789T, ORF1ab:C13115T, ORF1ab:C14408T, ORF1ab:G18020A, ORF1ab:T19839C, ORF1ab:A19974G, ORF1ab:C21306T, S:C22995A, S:A23403G, S:C23604A, S:A23756G, N:G28881A, N:G28882A, N:G28883C, N:C29197T	14	N:R203K, N:G204R, ORF1a:S538L, ORF1a:P959S, ORF1a:R1163K, ORF1a:T3255I, ORF1a:I3618V, ORF1a:T4175I, ORF1b:P314L, ORF1b:R1518K, S:T478K, S:D614G, S:P681H, S:T732A,
hCoV-19/Mexico/CMX-INMEGEN-02-05-15/2021	EPI_ISL_1060605	20B	B.1.1.519	24	5'UTR:C203T, 5'UTR:C222T, 5'UTR:C241T, ORF1ab:C3037T, ORF1ab:C3140T, ORF1ab:C10029T, ORF1ab:C10954T, ORF1ab:A11117G, ORF1ab:C12455T, ORF1ab:C12789T, ORF1ab:C14408T, ORF1ab:G15921T, ORF1ab:A18036G, ORF1ab:T18909C, ORF1ab:T19839C, ORF1ab:C21306T, S:C22088T, S:C22995A, S:A23403G, S:C23604A, S:A23756G, N:G28881A, N:G28882A, N:G28883C, N:C29197T,	13	N:R203K, N:G204R, ORF1a:P959S, ORF1a:T3255I, ORF1a:I3618V, ORF1a:L4064F, ORF1a:T4175I, ORF1b:P314L, S:L176F, S:T478K, S:D614G, S:P681H, S:T732A,
hCoV-19/Mexico/CMX-INMEGEN-02-05-16/2021	EPI_ISL_1060606	20B	B.1.1.519	26	5'UTR:G94T, 5'UTR:C203T, 5'UTR:C222T, 5'UTR:C241T, ORF1ab:C3037T, ORF1ab:C3140T, ORF1ab:C10029T, ORF1ab:C10954T, ORF1ab:A11117G, ORF1ab:C12455T, ORF1ab:C12789T, ORF1ab:C14408T, ORF1ab:A15831T, ORF1ab:G15921T, ORF1ab:A18036G, ORF1ab:T18909C, ORF1ab:T19839C, ORF1ab:C21306T, S:C22088T, S:C22995A, S:A23403G, S:C23604A, S:A23756G, N:G28881A, N:G28882A, N:G28883C, N:C29197T,	13	N:R203K, N:G204R, ORF1a:P959S, ORF1a:T3255I, ORF1a:I3618V, ORF1a:L4064F, ORF1a:T4175I, ORF1b:P314L, S:L176F, S:T478K, S:D614G, S:P681H, S:T732A,
hCoV-19/Mexico/CMX-INMEGEN-02-05-17/2021	EPI_ISL_1060607	20B	B.1.1.519	28	5'UTR:T201C, 5'UTR:C203T, 5'UTR:C222T, 5'UTR:C241T, ORF1ab:C936T, ORF1ab:G1738T, ORF1ab:C3037T, ORF1ab:C3140T, ORF1ab:C10029T, ORF1ab:C10954T, ORF1ab:G11071T, ORF1ab:G11083T, ORF1ab:A11117G, ORF1ab:C12789T, ORF1ab:C14408T, ORF1ab:T16905C, ORF1ab:T18699C, ORF1ab:T19839C, ORF1ab:A19974G, ORF1ab:C21306T, S:C22995A, S:A23403G, S:C23604A, S:A23756G, N:G28881A, N:G28882A, N:G28883C, N:C29197T, N:G29227T	14	N:R203K, N:G204R, ORF1a:T224I, ORF1a:P959S, ORF1a:T3255I, ORF1a:L3602F, ORF1a:L3606F, ORF1a:I3618V, ORF1a:T4175I, ORF1b:P314L, S:T478K, S:D614G, S:P681H, S:T732A,
hCoV-19/Mexico/CMX-INMEGEN-02-05-18/2021	EPI_ISL_1060608	20B	B.1.1.519	26	5'UTR:C203T, 5'UTR:C222T, 5'UTR:C241T, ORF1ab:C2536A, ORF1ab:C3037T, ORF1ab:C3140T, ORF1ab:A3481T, ORF1ab:T3745C, ORF1ab:T4075A, ORF1ab:C10029T, ORF1ab:C10954T, ORF1ab:A11117G, ORF1ab:C12789T, ORF1ab:G13822A, ORF1ab:C14408T, ORF1ab:T19839C, ORF1ab:C21306T, S:C22995A, S:A23403G, S:C23604A, S:A23756G, ORF3a:C25844T, N:C28849T, N:G28881A, N:G28882A, N:G28883C, N:C29197T,	13	N:R203K, N:G204R, ORF1a:P959S, ORF1a:T3255I, ORF1a:I3618V, ORF1a:T4175I, ORF1b:V119I, ORF1b:P314L, ORF3a:T151I, S:T478K, S:D614G, S:P681H, S:T732A,
hCoV-19/Mexico/CMX-INMEGEN-02-05-19/2021	EPI_ISL_1060598	20B	B.1.1.519	23	5'UTR:C203T, 5'UTR:C222T, 5'UTR:C241T, ORF1ab:C3037T, ORF1ab:C3140T, ORF1ab:C10029T, ORF1ab:C10954T, ORF1ab:A11117G, ORF1ab:G12111A, ORF1ab:C12789T, ORF1ab:C14318T, ORF1ab:C14408T, ORF1ab:G15921T, ORF1ab:G19518T, ORF1ab:T19839C, ORF1ab:C21306T, S:C22995A, S:A23403G, S:C23604A, S:A23756G, N:G28881A, N:G28882A, N:G28883C, N:C29197T,	14	N:R203K, N:G204R, ORF1a:P959S, ORF1a:T3255I, ORF1a:I3618V, ORF1a:S3949N, ORF1a:T4175I, ORF1b:T284I, ORF1b:P314L, ORF1b:L2017F, S:T478K, S:D614G, S:P681H, S:T732A,

hCoV-19/Mexico/CMX-INMEGEN-02-05-20/2021	EPI_ISL_1060599	20B	B.1.1.519	22	5'UTR:C203T, 5'UTR:C222T, 5'UTR:C241T, ORF1ab:C3037T, ORF1ab:C3140T, ORF1ab:C10029T, ORF1ab:C10954T, ORF1ab:A11117G, ORF1ab:G11365T, ORF1ab:C12789T, ORF1ab:C14408T, ORF1ab:T19839C, ORF1ab:C21058T, ORF1ab:C21306T, S:C22995A, S:A23403G, S:C23604A, S:A23756G, N:G28881A, N:G28882A, N:G28883C, N:C29197T, N:G29527T,	13	N:R203K, N:G204R, N:Q418H, ORF1a:P959S, ORF1a:T3255I, ORF1a:I3618V, ORF1a:T4175I, ORF1b:P314L, ORF1b:P2531S, S:T478K, S:D614G, S:P681H, S:T732A,	
hCoV-19/Mexico/CMX-INMEGEN-02-05-21/2021	EPI_ISL_1060609	20B	B.1.1.519	27	5'UTR:C203T, 5'UTR:C222T, 5'UTR:C241T, ORF1ab:C920T, ORF1ab:C3037T, ORF1ab:C3140T, ORF1ab:G3692T, ORF1ab:G3871T, ORF1ab:A8446G, ORF1ab:C10029T, ORF1ab:C10954T, ORF1ab:A11117G, ORF1ab:C11824A, ORF1ab:C12789T, ORF1ab:C14408T, ORF1ab:C15120T, ORF1ab:C18171T, ORF1ab:T19839C, ORF1ab:C21306T, S:C22995A, S:A23403G, S:C23604A, S:A23756G, ORF8:T27904C, N:G28881A, N:G28882A, N:G28883C, N:C29197T,	14	N:R203K, N:G204R, ORF1a:P959S, ORF1a:V1143F, ORF1a:K1202N, ORF1a:T3255I, ORF1a:I3618V, ORF1a:T4175I, ORF1b:P314L, ORF8:L4P, S:T478K, S:D614G, S:P681H, S:T732A,	
hCoV-19/Mexico/CMX-INMEGEN-02-05-22/2021	EPI_ISL_1060611	20B	B.1.1.519	23	5'UTR:C203T, 5'UTR:C222T, 5'UTR:C241T, ORF1ab:C3037T, ORF1ab:C3140T, ORF1ab:C10029T, ORF1ab:C10954T, ORF1ab:A11117G, ORF1ab:C12789T, ORF1ab:C14408T, ORF1ab:T19839C, ORF1ab:C21306T, S:C22995A, S:A23403G, S:C23604A, S:A23756G, ORF7a:G27670T, ORF8:C28253T, N:G28881A, N:G28882A, N:G28883C, N:C29095T, N:C29197T, N:G29254T,	12	N:R203K, N:G204R, ORF1a:P959S, ORF1a:T3255I, ORF1a:I3618V, ORF1a:T4175I, ORF1b:P314L, ORF7a:V93F, S:T478K, S:D614G, S:P681H, S:T732A,	
hCoV-19/Mexico/CMX-INMEGEN-02-05-23/2021	EPI_ISL_1060610	20B	B.1.1.519	25	5'UTR:C203T, 5'UTR:C222T, 5'UTR:C241T, ORF1ab:C683T, ORF1ab:C1191A, ORF1ab:C3037T, ORF1ab:C3140T, ORF1ab:C10029T, ORF1ab:C10954T, ORF1ab:A11117G, ORF1ab:C12789T, ORF1ab:C14408T, ORF1ab:C18747T, ORF1ab:T19839C, ORF1ab:C21306T, S:C22995A, S:A23403G, S:C23604A, S:A23756G, S:T23849C, N:G28881A, N:G28882A, N:G28883C, N:C29197T, N:G29315C, N:C29421A,	14	N:R203K, N:G204R, N:D348H, N:P383Q, ORF1a:P309Q, ORF1a:P959S, ORF1a:T3255I, ORF1a:I3618V, ORF1a:T4175I, ORF1b:P314L, S:T478K, S:D614G, S:P681H, S:T732A,	
hCoV-19/Mexico/CMX-INMEGEN-02-06-01/2021	EPI_ISL_1080439	20B	B.1.1.222	21	5'UTR:C241T, ORF1ab:C3037T, ORF1ab:C10029T, ORF1ab:T11187C, ORF1ab:C14408T, ORF1ab:G17721T, ORF1ab:C18788T, ORF1ab:T19839C, ORF1ab:T20955C, S:A23403G, S:G23522C, S:A23756G, ORF3a:G25567A, ORF3a:T25569A, ORF3a:T25570A, ORF3a:G25912T, ORF3a:A26108G, ORF8:A27921G, ORF8:G28001T, N:G28881A, N:G28882A, N:G28883C,	14	N:R203K, N:G204R, ORF1a:T3255I, ORF1a:L3641S, ORF1b:P314L, ORF1b:T1774I, ORF3a:A59T, ORF3a:S60T, ORF3a:G174C, ORF3a:E239G, ORF8:I10V, S:D614G, S:E654Q, S:T732A,	
hCoV-19/Mexico/CMX-INMEGEN-02-06-02/2021	EPI_ISL_1080438	20B	B.1.1.222	18	5'UTR:C147T, 5'UTR:C241T, ORF1ab:C2232T, ORF1ab:C3037T, ORF1ab:C5654T, ORF1ab:C8320T, ORF1ab:C10029T, ORF1ab:A14255G, ORF1ab:C14408T, ORF1ab:T19839C, S:A23403G, S:A23756G, ORF3a:G25912T, ORF7b:A27756G, ORF8:A27921G, ORF8:G28001T, N:G28881A, N:G28882A, N:G28883C,	11	N:R203K, N:G204R, ORF1a:A656V, ORF1a:T3255I, ORF1b:K263R, ORF1b:P314L, ORF3a:G174C, ORF7b:M1V, ORF8:I10V, S:D614G, S:T732A,	3'UTR:29746-29762,
hCoV-19/Mexico/CMX-INMEGEN-02-06-03/2021	EPI_ISL_1080440	20B	B.1.1.519	24	5'UTR:C203T, 5'UTR:C222T, 5'UTR:C241T, ORF1ab:C3037T, ORF1ab:C3140T, ORF1ab:C10029T, ORF1ab:C10954T, ORF1ab:A11117G, ORF1ab:C12789T, ORF1ab:C12933T, ORF1ab:C14408T, ORF1ab:G15921T, ORF1ab:T19839C, ORF1ab:T20484C, ORF1ab:C21306T, S:C22995A, S:A23403G, S:C23604A, S:A23756G, ORF3a:C25658T, ORF7a:C27509T, N:G28881A, N:G28882A, N:G28883C, N:C29197T,	14	N:R203K, N:G204R, ORF1a:P959S, ORF1a:T3255I, ORF1a:I3618V, ORF1a:T4175I, ORF1a:P4223L, ORF1b:P314L, ORF3a:T89I, ORF7a:T39I, S:T478K, S:D614G, S:P681H, S:T732A,	

hCoV-19/Mexico/CMX-INMEGEN-02-06-04/2021	EPI_ISL_1080436	20B	B.1.1.519	26	5'UTR:C203T, 5'UTR:C222T, 5'UTR:C241T, ORF1ab:C3037T, ORF1ab:C3140T, ORF1ab:T3745C, ORF1ab:T7984C, ORF1ab:C9133T, ORF1ab:C10029T, ORF1ab:C10341T, ORF1ab:C10954T, ORF1ab:A11117G, ORF1ab:C12789T, ORF1ab:C14408T, ORF1ab:T19839C, ORF1ab:C21306T, S:C22995A, S:A23403G, S:C23601T, S:C23604A, S:A23756G, ORF3a:C25844T, ORF7a:C27641A, N:G28881A, N:G28882A, N:G28883C, N:C29197T,	15	N:R203K, N:G204R, ORF1a:P959S, ORF1a:T3255I, ORF1a:P3359L, ORF1a:I3618V, ORF1a:T4175I, ORF1b:P314L, ORF3a:T151I, ORF7a:S83*, S:T478K, S:D614G, S:S680F, S:P681H, S:T732A,
hCoV-19/Mexico/CMX-INMEGEN-02-06-05/2021	EPI_ISL_1080441	20B	B.1.1.519	30	5'UTR:T201C, 5'UTR:C203T, 5'UTR:C222T, 5'UTR:C241T, ORF1ab:C936T, ORF1ab:G1738T, ORF1ab:C3037T, ORF1ab:C3140T, ORF1ab:G9890A, ORF1ab:C10029T, ORF1ab:C10954T, ORF1ab:A11117G, ORF1ab:C11325T, ORF1ab:C12789T, ORF1ab:C13072T, ORF1ab:C14408T, ORF1ab:T16342C, ORF1ab:T19839C, ORF1ab:A19974G, ORF1ab:C21306T, S:C22995A, S:A23403G, S:C23604A, S:A23756G, S:C23896T, M:C26571T, N:G28881A, N:G28882A, N:G28883C, N:C29197T, N:G29227T,	16	M:L17F, N:R203K, N:G204R, ORF1a:T224I, ORF1a:P959S, ORF1a:A3209T, ORF1a:T3255I, ORF1a:I3618V, ORF1a:S3687L, ORF1a:T4175I, ORF1b:P314L, ORF1b:S959P, S:T478K, S:D614G, S:P681H, S:T732A,
hCoV-19/Mexico/CMX-INMEGEN-02-06-06/2021	EPI_ISL_1080442	20A	B.1.243	19	5'UTR:C241T, ORF1ab:C3037T, ORF1ab:G5555A, ORF1ab:G11417T, ORF1ab:A12124T, ORF1ab:C13487T, ORF1ab:C14408T, ORF1ab:A14733G, ORF1ab:A17799G, ORF1ab:A20268G, S:C21595T, S:A23403G, S:C23604A, S:T24076C, ORF3a:T25479C, M:T27138C, N:G28300T, N:C28854T, N:C29409T, N:G29473A.	10	N:Q9H, N:S194L, N:T379I, ORF1a:G1764S, ORF1a:V3718F, ORF1b:A7V, ORF1b:P314L, ORF9b:S6I, S:D614G, S:P681H,
hCoV-19/Mexico/CMX-INMEGEN-02-06-07/2021	EPI_ISL_1080443	20B	B.1.1.519	24	5'UTR:C203T, 5'UTR:C222T, 5'UTR:C241T, ORF1ab:C3037T, ORF1ab:C3140T, ORF1ab:C4832T, ORF1ab:G7037T, ORF1ab:T9004C, ORF1ab:C10029T, ORF1ab:C10954T, ORF1ab:A11117G, ORF1ab:C12789T, ORF1ab:C14408T, ORF1ab:T19839C, ORF1ab:C21306T, S:C22995A, S:A23403G, S:C23604A, S:G23608C, S:A23756G, ORF3a:C25782T, N:G28881A, N:G28882A, N:G28883C, N:C29197T,	12	N:R203K, N:G204R, ORF1a:P959S, ORF1a:G2258C, ORF1a:T3255I, ORF1a:I3618V, ORF1a:T4175I, ORF1b:P314L, S:T478K, S:D614G, S:P681H, S:T732A,
hCoV-19/Mexico/CMX-INMEGEN-02-06-08/2021	EPI_ISL_1080444	20B	B.1.1.519	18	5'UTR:C203T, 5'UTR:C222T, 5'UTR:C241T, ORF1ab:C3037T, ORF1ab:C3140T, ORF1ab:C10029T, ORF1ab:C10954T, ORF1ab:A11117G, ORF1ab:C12789T, ORF1ab:C14408T, ORF1ab:T19839C, S:C22995A, S:A23403G, S:C23604A, S:A23756G, N:G28881A, N:G28882A, N:G28883C. N:C29197T.	11	N:R203K, N:G204R, ORF1a:P959S, ORF1a:T3255I, ORF1a:I3618V, ORF1a:T4175I, ORF1b:P314L, S:T478K, S:D614G, S:P681H, S:T732A,
hCoV-19/Mexico/CMX-INMEGEN-02-06-09/2021	EPI_ISL_1080445	20B	B.1.1.519	27	5'UTR:C203T, 5'UTR:C222T, 5'UTR:C241T, ORF1ab:C3037T, ORF1ab:C3140T, ORF1ab:G3250T, ORF1ab:T3745C, ORF1ab:C10029T, ORF1ab:C10039T, ORF1ab:C10954T, ORF1ab:A11117G, ORF1ab:C12789T, ORF1ab:C14408T, ORF1ab:G17259T, ORF1ab:T19839C, ORF1ab:C21306T, S:T22729C, S:C22995A, S:A23403G, S:C23422T, S:C23604A, S:A23756G, ORF3a:C25844T, ORF7a:A27636T, N:G28881A, N:G28882A, N:G28883C, N:C29197T,	14	N:R203K, N:G204R, ORF1a:P959S, ORF1a:E995D, ORF1a:T3255I, ORF1a:I3618V, ORF1a:T4175I, ORF1b:P314L, ORF1b:E1264D, ORF3a:T151I, S:T478K, S:D614G, S:P681H, S:T732A,
hCoV-19/Mexico/CMX-INMEGEN-02-06-10/2021	EPI_ISL_1080446	20A	B.1	16	5'UTR:C241T, ORF1ab:C1380T, ORF1ab:C3037T, ORF1ab:G6341T, ORF1ab:C6401T, ORF1ab:A8080T, ORF1ab:G10396T, ORF1ab:C10833T, ORF1ab:C14408T, ORF1ab:A20268G, S:C22591T, S:A23403G, S:T23599G, S:C23638T, N:G28817T, N:C28854T, N:C29421T,	10	N:A182S, N:S194L, N:P383L, ORF1a:A372V, ORF1a:D2026Y, ORF1a:P2046S, ORF1a:A3523V, ORF1b:P314L, S:D614G, S:N679K,

hCoV-19/Mexico/CMX-INMEGEN-02-06-11/2021	EPI_ISL_1080447	20B	B.1.1.519	28	5'UTR:C203T, 5'UTR:C222T, 5'UTR:C241T, ORF1ab:C936T, ORF1ab:C3037T, ORF1ab:C3140T, ORF1ab:C4423T, ORF1ab:C5183T, ORF1ab:T7045C, ORF1ab:C10029T, ORF1ab:C10954T, ORF1ab:A11117G, ORF1ab:C12789T, ORF1ab:C14408T, ORF1ab:T19839C, ORF1ab:G21204T, ORF1ab:C21306T, S:C21622T, S:C22995A, S:A23403G, S:C23604A, S:A23756G, S:G25135T, ORF3a:T25969C, ORF8:T27904C, N:G28881A, N:G28882A, N:G28883C, N:C29197T,	17	N:R203K, N:G204R, ORF1a:T224I, ORF1a:P959S, ORF1a:P1640S, ORF1a:T3255I, ORF1a:I3618V, ORF1a:T4175I, ORF1b:P314L, ORF1b:K2579N, ORF3a:W193R, ORF8:L4P, S:T478K, S:D614G, S:P681H, S:T732A, S:K1191N,	
hCoV-19/Mexico/CMX-INMEGEN-02-06-12/2021	EPI_ISL_1080433	20B	B.1.1.519	25	5'UTR:C203T, 5'UTR:C222T, 5'UTR:C241T, ORF1ab:C3037T, ORF1ab:C3140T, ORF1ab:C3738T, ORF1ab:C10029T, ORF1ab:C10954T, ORF1ab:A11117G, ORF1ab:G11365T, ORF1ab:C12789T, ORF1ab:C14408T, ORF1ab:T19839C, ORF1ab:C21306T, S:C22995A, S:A23403G, S:C23595T, S:C23604A, S:A23756G, M:G26840T, ORF8:T28031C, N:G28881A, N:G28882A, N:G28883C, N:C29197T, N:G29527T,	14	N:R203K, N:G204R, N:Q418H, ORF1a:P959S, ORF1a:P1158L, ORF1a:T3255I, ORF1a:I3618V, ORF1a:T4175I, ORF1b:P314L, S:T478K, S:D614G, S:T678I, S:P681H, S:T732A,	S:21992-21994,
hCoV-19/Mexico/CMX-INMEGEN-02-06-13/2021	EPI_ISL_1080434	20B	B.1.1.519	25	5'UTR:C203T, 5'UTR:C222T, 5'UTR:C241T, ORF1ab:C3037T, ORF1ab:C3140T, ORF1ab:C3738T, ORF1ab:C10029T, ORF1ab:C10954T, ORF1ab:A11117G, ORF1ab:G11365T, ORF1ab:C12789T, ORF1ab:C14408T, ORF1ab:T19839C, ORF1ab:C21306T, S:C22995A, S:A23403G, S:C23595T, S:C23604A, S:A23756G, M:G26840T, ORF8:T28031C, N:G28881A, N:G28882A, N:G28883C, N:C29197T, N:G29527T,	14	N:R203K, N:G204R, N:Q418H, ORF1a:P959S, ORF1a:P1158L, ORF1a:T3255I, ORF1a:I3618V, ORF1a:T4175I, ORF1b:P314L, S:T478K, S:D614G, S:T678I, S:P681H, S:T732A,	S:21992-21994,
hCoV-19/Mexico/CMX-INMEGEN-02-06-14/2021	EPI_ISL_1080448	20B	B.1.1.519	23	5'UTR:C203T, 5'UTR:C222T, 5'UTR:C241T, ORF1ab:C3037T, ORF1ab:C3140T, ORF1ab:C10029T, ORF1ab:C10954T, ORF1ab:A11117G, ORF1ab:C12789T, ORF1ab:C14408T, ORF1ab:G15921T, ORF1ab:T19839C, ORF1ab:A20055G, ORF1ab:C21306T, S:C22995A, S:A23403G, S:C23604A, S:A23756G, ORF3a:C25810T, ORF6:T27384C, N:G28881A, N:G28882A, N:G28883C, N:C29197T,	12	N:R203K, N:G204R, ORF1a:P959S, ORF1a:T3255I, ORF1a:I3618V, ORF1a:T4175I, ORF1b:P314L, ORF3a:L140F, S:T478K, S:D614G, S:P681H, S:T732A,	
hCoV-19/Mexico/CMX-INMEGEN-02-06-15/2021	EPI_ISL_1080449	20B	B.1.1.519	23	5'UTR:C203T, 5'UTR:C222T, 5'UTR:C241T, ORF1ab:C3037T, ORF1ab:C3140T, ORF1ab:C9120T, ORF1ab:C10029T, ORF1ab:C10954T, ORF1ab:A11117G, ORF1ab:C12789T, ORF1ab:C14408T, ORF1ab:T19839C, ORF1ab:C21077T, ORF1ab:C21306T, S:C22995A, S:A23403G, S:C23604A, S:A23756G, ORF3a:G25906T, ORF8:C28253T, N:G28881A, N:G28882A, N:G28883C, N:C29197T,	14	N:R203K, N:G204R, ORF1a:P959S, ORF1a:T2952I, ORF1a:T3255I, ORF1a:I3618V, ORF1a:T4175I, ORF1b:P314L, ORF1b:T2537I, ORF3a:G172C, S:T478K, S:D614G, S:P681H, S:T732A,	
hCoV-19/Mexico/CMX-INMEGEN-02-06-16/2021	EPI_ISL_1080450	20B	B.1.1.519	24	5'UTR:C203T, 5'UTR:C222T, 5'UTR:C241T, ORF1ab:T1877C, ORF1ab:C3037T, ORF1ab:C3140T, ORF1ab:C5427T, ORF1ab:C6267T, ORF1ab:C10029T, ORF1ab:C10954T, ORF1ab:A11117G, ORF1ab:C12789T, ORF1ab:C14408T, ORF1ab:T19839C, ORF1ab:T20455C, ORF1ab:C21306T, S:C22995A, S:A23403G, S:C23604A, S:A23756G, N:G28881A, N:G28882A, N:G28883C, N:C29197T, N:G29527T,	16	N:R203K, N:G204R, N:Q418H, ORF1a:S538P, ORF1a:P959S, ORF1a:T1721I, ORF1a:A2001V, ORF1a:T3255I, ORF1a:I3618V, ORF1a:T4175I, ORF1b:P314L, ORF1b:F2330L, S:T478K, S:D614G, S:P681H, S:T732A,	

hCoV-19/Mexico/CMX-INMEGEN-02-06-17/2021	EPI_ISL_1080435	20B	B.1.1.519	21	5'UTR:C203T, 5'UTR:C222T, 5'UTR:C241T, ORF1ab:C3037T, ORF1ab:C3140T, ORF1ab:C10029T, ORF1ab:C10954T, ORF1ab:A11117G, ORF1ab:C12789T, ORF1ab:C13761T, ORF1ab:C14408T, ORF1ab:T19839C, ORF1ab:C21306T, S:C22995A, S:A23403G, S:C23604A, S:A23756G, N:G28881A, N:G28882A, N:G28883C, N:C29197T, ORF10:C29625T,	11	N:R203K, N:G204R, ORF1a:P959S, ORF1a:T3255I, ORF1a:I3618V, ORF1a:T4175I, ORF1b:P314L, S:T478K, S:D614G, S:P681H, S:T732A,	
hCoV-19/Mexico/CMX-INMEGEN-02-06-18/2021	EPI_ISL_1080451	20B	B.1.1.519	26	5'UTR:A161G, 5'UTR:C203T, 5'UTR:C222T, 5'UTR:C241T, ORF1ab:C3037T, ORF1ab:C3140T, ORF1ab:G3955T, ORF1ab:C6539T, ORF1ab:G7463A, ORF1ab:G9805T, ORF1ab:C10728T, ORF1ab:C10954T, ORF1ab:A11117G, ORF1ab:C12789T, ORF1ab:C14408T, ORF1ab:G16075T, ORF1ab:T19839C, ORF1ab:C21306T, S:C22995A, S:A23403G, S:C23604A, S:A23756G, ORF8:C27999T, N:G28881A, N:G28882A, N:G28883C, N:C29197T,	16	N:R203K, N:G204R, ORF1a:P959S, ORF1a:K1230N, ORF1a:H2092Y, ORF1a:V2400I, ORF1a:T3488I, ORF1a:I3618V, ORF1a:T4175I, ORF1b:P314L, ORF1b:D870Y, ORF8:P36S, S:T478K, S:D614G, S:P681H, S:T732A,	
hCoV-19/Mexico/CMX-INMEGEN-02-06-19/2021	EPI_ISL_1080452	20B	B.1.1.519	27	5'UTR:C203T, 5'UTR:C222T, 5'UTR:C241T, ORF1ab:C1457T, ORF1ab:C3037T, ORF1ab:C3140T, ORF1ab:T3745C, ORF1ab:C10029T, ORF1ab:T10843C, ORF1ab:C10954T, ORF1ab:A11117G, ORF1ab:C12789T, ORF1ab:C14408T, ORF1ab:T19839C, ORF1ab:C20629T, ORF1ab:C21306T, S:C22995A, S:A23403G, S:C23604A, S:A23756G, S:T24377A, ORF3a:C25844T, E:G26389T, M:C26527T, N:G28881A, N:G28882A, N:G28883C, N:C29197T,	17	E:V49L, M:A2V, N:R203K, N:G204R, ORF1a:R398C, ORF1a:P959S, ORF1a:T3255I, ORF1a:I3618V, ORF1a:T4175I, ORF1b:P314L, ORF1b:H2388Y, ORF3a:T151I, S:T478K, S:D614G, S:P681H, S:T732A, S:S939T,	
hCoV-19/Mexico/CMX-INMEGEN-02-06-20/2021	EPI_ISL_1085421	20B	B.1.1.453	15	5'UTR:C241T, ORF1ab:C3037T, ORF1ab:C8172T, ORF1ab:C11514T, ORF1ab:C14408T, ORF1ab:G18781T, ORF1ab:T19839C, ORF1ab:T21240C, ORF1ab:G21372T, S:A23403G, S:C24466A, ORF3a:G25489T, ORF3a:C26029A, N:G28881A, N:G28882A, N:G28883C,	10	N:R203K, N:G204R, ORF1a:A2636V, ORF1a:T3750I, ORF1b:P314L, ORF1b:G1772C, ORF1b:Q2635H, ORF3a:A33S, ORF3a:Q213K, S:D614G,	ORF8:28 253,
hCoV-19/Mexico/CMX-INMEGEN-02-06-21/2021	EPI_ISL_1080437	20B	B.1.1.519	30	5'UTR:T201C, 5'UTR:C203T, 5'UTR:C222T, 5'UTR:C241T, ORF1ab:C745T, ORF1ab:G1738T, ORF1ab:C3037T, ORF1ab:C3140T, ORF1ab:C4776T, ORF1ab:C6196T, ORF1ab:C10029T, ORF1ab:C10954T, ORF1ab:A11117G, ORF1ab:C12789T, ORF1ab:C14408T, ORF1ab:T16587C, ORF1ab:T19839C, ORF1ab:A19974G, ORF1ab:C21306T, S:C22995A, S:A23403G, S:C23604A, S:A23756G, ORF3a:C26110T, ORF3a:G26152T, N:A28877T, N:G28878C, N:G28881A, N:G28882A, N:G28883C, N:C29197T	14	N:R203K, N:G204R, ORF1a:P959S, ORF1a:T1504I, ORF1a:T3255I, ORF1a:I3618V, ORF1a:T4175I, ORF1b:P314L, ORF3a:P240S, ORF3a:G254*, S:T478K, S:D614G, S:P681H, S:T732A,	
hCoV-19/Mexico/CMX-INMEGEN-02-06-23/2021	EPI_ISL_1080453	20B	B.1.1.519	27	5'UTR:C203T, 5'UTR:C222T, 5'UTR:C241T, ORF1ab:C3037T, ORF1ab:C3140T, ORF1ab:C9661T, ORF1ab:C10029T, ORF1ab:C10954T, ORF1ab:A11117G, ORF1ab:C12473T, ORF1ab:C12789T, ORF1ab:C14408T, ORF1ab:G15253A, ORF1ab:A18282G, ORF1ab:C19602T, ORF1ab:T19839C, ORF1ab:C21306T, S:C22995A, S:A23403G, S:C23604A, S:A23756G, ORF3a:G25906T, ORF8:C28253T, N:G28881A, N:G28882A, N:G28883C, N:C29197T, 3'UTR:G29777T,	13	N:R203K, N:G204R, ORF1a:P959S, ORF1a:T3255I, ORF1a:I3618V, ORF1a:T4175I, ORF1b:P314L, ORF1b:V596I, ORF3a:G172C, S:T478K, S:D614G, S:P681H, S:T732A,	

hCoV-19/Mexico/CMX-INMEGEN-02-07-01/2021	EPI_ISL_1081152	20B	B.1.1.519	22	5'UTR:C203T, 5'UTR:C222T, 5'UTR:C241T, ORF1ab:T1303A, ORF1ab:C3037T, ORF1ab:C3140T, ORF1ab:C10029T, ORF1ab:C10954T, ORF1ab:A11117G, ORF1ab:C12786T, ORF1ab:C12789T, ORF1ab:C14408T, ORF1ab:T19839C, ORF1ab:C21306T, S:C22995A, S:A23403G, S:C23604A, S:A23756G, N:G28881A, N:G28882A, N:G28883C, N:C29197T, ORF10:T29661C,	12	N:R203K, N:G204R, ORF1a:P959S, ORF1a:T3255I, ORF1a:I3618V, ORF1a:T4174I, ORF1a:T4175I, ORF1b:P314L, S:T478K, S:D614G, S:P681H, S:T732A,	
hCoV-19/Mexico/CMX-INMEGEN-02-07-02/2021	EPI_ISL_1081153	21C (Epsilon)	B.1.429	25	5'UTR:C241T, ORF1ab:C1059T, ORF1ab:C2395T, ORF1ab:T2597C, ORF1ab:C3037T, ORF1ab:C4890T, ORF1ab:C8947T, ORF1ab:C9996T, ORF1ab:C12100T, ORF1ab:A12878G, ORF1ab:C14408T, ORF1ab:G17014T, S:G21600T, S:C21638T, S:G22018T, S:T22917G, S:A23403G, S:T24349C, ORF3a:G25563T, M:C26681T, ORF7a:C27612T, ORF7b:C27881T, ORF7b:G27890T, ORF8:A28272T, N:C28887T, N:C29362T,	13	N:T205I, ORF1a:T265I, ORF1a:T1542I, ORF1a:S3244L, ORF1a:I4205V, ORF1b:P314L, ORF1b:D1183Y, ORF3a:Q57H, S:S13I, S:P26S, S:W152C, S:L452R, S:D614G,	
hCoV-19/Mexico/CMX-INMEGEN-02-07-03/2021	EPI_ISL_1081154	20B	B.1.1.519	23	5'UTR:C203T, 5'UTR:C222T, 5'UTR:C241T, ORF1ab:C3037T, ORF1ab:C3140T, ORF1ab:C10029T, ORF1ab:C10954T, ORF1ab:A11117G, ORF1ab:G11365T, ORF1ab:C12789T, ORF1ab:C14408T, ORF1ab:C17004T, ORF1ab:T19839C, ORF1ab:C21306T, S:C22995A, S:A23403G, S:C23604A, S:A23756G, M:C26625T, N:G28881A, N:G28882A, N:G28883C, N:C29197T, N:G29527T,	12	N:R203K, N:G204R, N:Q418H, ORF1a:P959S, ORF1a:T3255I, ORF1a:I3618V, ORF1a:T4175I, ORF1b:P314L, S:T478K, S:D614G, S:P681H, S:T732A,	
hCoV-19/Mexico/CMX-INMEGEN-02-07-04/2021	EPI_ISL_1081155	20B	B.1.1.519	24	5'UTR:C203T, 5'UTR:C222T, 5'UTR:C241T, ORF1ab:C3037T, ORF1ab:C3140T, ORF1ab:C3961T, ORF1ab:C8092T, ORF1ab:C10029T, ORF1ab:C10954T, ORF1ab:A11117G, ORF1ab:C12789T, ORF1ab:C14408T, ORF1ab:G15921T, ORF1ab:T19839C, ORF1ab:C21306T, S:C22227T, S:C22995A, S:A23403G, S:C23604A, S:A23756G, N:G28881A, N:G28882A, N:G28883C, N:C29197T, N:G29527A,	12	N:R203K, N:G204R, ORF1a:P959S, ORF1a:T3255I, ORF1a:I3618V, ORF1a:T4175I, ORF1b:P314L, S:A222V, S:T478K, S:D614G, S:P681H, S:T732A,	
hCoV-19/Mexico/CMX-INMEGEN-02-07-05/2021	EPI_ISL_1095493	20B	B.1.1.519	23	5'UTR:C203T, 5'UTR:C222T, 5'UTR:C241T, ORF1ab:C3037T, ORF1ab:C3140T, ORF1ab:C6690T, ORF1ab:C10029T, ORF1ab:C10954T, ORF1ab:A11117G, ORF1ab:C11575T, ORF1ab:A11600G, ORF1ab:C12789T, ORF1ab:C14408T, ORF1ab:T19839C, ORF1ab:C21306T, S:C22995A, S:A23403G, S:C23604A, S:A23756G, ORF3a:G25996T, N:G28881A, N:G28882A, N:G28883C, N:C29197T,	14	N:R203K, N:G204R, ORF1a:P959S, ORF1a:A2142V, ORF1a:T3255I, ORF1a:I3618V, ORF1a:I3779V, ORF1a:T4175I, ORF1b:P314L, ORF3a:V202L, S:T478K, S:D614G, S:P681H, S:T732A,	ORF8:28254,
hCoV-19/Mexico/CMX-INMEGEN-02-07-06/2021	EPI_ISL_1095494	20B	B.1.1.519	23	5'UTR:C203T, 5'UTR:C222T, 5'UTR:C241T, ORF1ab:C3037T, ORF1ab:C3140T, ORF1ab:C6690T, ORF1ab:C10029T, ORF1ab:C10954T, ORF1ab:A11117G, ORF1ab:C11575T, ORF1ab:A11600G, ORF1ab:C12789T, ORF1ab:C14408T, ORF1ab:T19839C, ORF1ab:C21306T, S:C22995A, S:A23403G, S:C23604A, S:A23756G, ORF3a:G25996T, N:G28881A, N:G28882A, N:G28883C, N:C29197T,	14	N:R203K, N:G204R, ORF1a:P959S, ORF1a:A2142V, ORF1a:T3255I, ORF1a:I3618V, ORF1a:I3779V, ORF1a:T4175I, ORF1b:P314L, ORF3a:V202L, S:T478K, S:D614G, S:P681H, S:T732A,	ORF8:28254,
hCoV-19/Mexico/CMX-INMEGEN-02-07-07/2021	EPI_ISL_1081162	20B	B.1.1.519	23	5'UTR:T201C, 5'UTR:C203T, 5'UTR:C222T, 5'UTR:C241T, ORF1ab:G1738T, ORF1ab:C3037T, ORF1ab:C3140T, ORF1ab:C10029T, ORF1ab:G10283A, ORF1ab:C10954T, ORF1ab:A11117G, ORF1ab:C12789T, ORF1ab:C14408T, ORF1ab:T19839C, ORF1ab:A19974G, ORF1ab:C21306T, S:C22995A, S:A23403G, S:C23604A, S:A23756G, N:G28881A, N:G28882A, N:G28883C, N:C29197T,	12	N:R203K, N:G204R, ORF1a:P959S, ORF1a:T3255I, ORF1a:V3340I, ORF1a:I3618V, ORF1a:T4175I, ORF1b:P314L, S:T478K, S:D614G, S:P681H, S:T732A,	

hCoV-19/Mexico/CMX-INMEGEN-02-07-08/2021	EPI_ISL_1081163	20B	B.1.1.519	27	5'UTR:C203T, 5'UTR:C222T, 5'UTR:C241T, ORF1ab:C3037T, ORF1ab:C3140T, ORF1ab:T3745C, ORF1ab:C7083T, ORF1ab:G9176A, ORF1ab:C10029T, ORF1ab:C10954T, ORF1ab:A11117G, ORF1ab:C12789T, ORF1ab:C14134T, ORF1ab:C14408T, ORF1ab:T19839C, ORF1ab:C21306T, S:T22729C, S:C22995A, S:A23403G, S:C23604A, S:A23756G, ORF3a:C25844T, N:C28849T, N:G28881A, N:G28882A, N:G28883C, N:G28904T, N:C29197T,	16	N:R203K, N:G204R, N:A211S, ORF1a:P959S, ORF1a:S2273F, ORF1a:G2971S, ORF1a:T3255I, ORF1a:I3618V, ORF1a:T4175I, ORF1b:P223S, ORF1b:P314L, ORF3a:T151I, S:T478K, S:D614G, S:P681H, S:T732A,
hCoV-19/Mexico/CMX-INMEGEN-02-07-09/2021	EPI_ISL_1081165	20B	B.1.1.519	26	5'UTR:T201C, 5'UTR:C203T, 5'UTR:C222T, 5'UTR:C241T, ORF1ab:C745T, ORF1ab:G1738T, ORF1ab:C2003T, ORF1ab:C3037T, ORF1ab:C3140T, ORF1ab:C3784T, ORF1ab:C10029T, ORF1ab:C10954T, ORF1ab:A11117G, ORF1ab:C12789T, ORF1ab:C14408T, ORF1ab:T19839C, ORF1ab:A19974G, ORF1ab:C21306T, S:C22995A, S:A23403G, S:C23604A, S:A23756G, N:G28881A, N:G28882A, N:G28883C, N:C29197T, 3'UTR:G29688A,	12	N:R203K, N:G204R, ORF1a:L580F, ORF1a:P959S, ORF1a:T3255I, ORF1a:I3618V, ORF1a:T4175I, ORF1b:P314L, S:T478K, S:D614G, S:P681H, S:T732A,
hCoV-19/Mexico/CMX-INMEGEN-02-07-10/2021	EPI_ISL_1081164	20B	B.1.1.519	22	5'UTR:C203T, 5'UTR:C222T, 5'UTR:C241T, ORF1ab:C3037T, ORF1ab:C3140T, ORF1ab:C10029T, ORF1ab:C10954T, ORF1ab:A11117G, ORF1ab:C12789T, ORF1ab:C14408T, ORF1ab:G15921T, ORF1ab:C16111T, ORF1ab:T19839C, ORF1ab:C21306T, ORF1ab:G21468T, S:C22995A, S:A23403G, S:C23604A, S:A23756G, N:G28881A, N:G28882A, N:G28883C. N:C29197T.	12	N:R203K, N:G204R, ORF1a:P959S, ORF1a:T3255I, ORF1a:I3618V, ORF1a:T4175I, ORF1b:P314L, ORF1b:M2667I, S:T478K, S:D614G, S:P681H, S:T732A,
hCoV-19/Mexico/CMX-INMEGEN-02-07-11/2021	EPI_ISL_1081166	20B	B.1.1.519	26	5'UTR:C203T, 5'UTR:C222T, 5'UTR:C241T, ORF1ab:C3037T, ORF1ab:C3140T, ORF1ab:C10029T, ORF1ab:C10954T, ORF1ab:A11117G, ORF1ab:C12789T, ORF1ab:C14408T, ORF1ab:G14767A, ORF1ab:C14931T, ORF1ab:T15726A, ORF1ab:C18452T, ORF1ab:T19839C, ORF1ab:C21306T, S:C22995A, S:A23403G, S:C23604A, S:A23756G, M:C26833T, ORF6:C27208T, N:G28881A, N:G28882A, N:G28883C, N:C29197T, N:G29527T,	16	M:A104V, N:R203K, N:G204R, N:Q418H, ORF1a:P959S, ORF1a:T3255I, ORF1a:I3618V, ORF1a:T4175I, ORF1b:P314L, ORF1b:A434T, ORF1b:A1662V, ORF6:H3Y, S:T478K, S:D614G, S:P681H, S:T732A,
hCoV-19/Mexico/CMX-INMEGEN-02-07-12/2021	EPI_ISL_1081167	20B	B.1.1.519	23	5'UTR:C203T, 5'UTR:C222T, 5'UTR:C241T, ORF1ab:C3037T, ORF1ab:C3140T, ORF1ab:T3454C, ORF1ab:C9559T, ORF1ab:C10029T, ORF1ab:C10954T, ORF1ab:A11117G, ORF1ab:C12789T, ORF1ab:C14408T, ORF1ab:C14724T, ORF1ab:T19839C, ORF1ab:C21306T, S:C22995A, S:A23403G, S:C23604A, S:A23756G, ORF7a:C27434T, N:G28881A, N:G28882A, N:G28883C, N:C29197T,	12	N:R203K, N:G204R, ORF1a:P959S, ORF1a:T3255I, ORF1a:I3618V, ORF1a:T4175I, ORF1b:P314L, ORF7a:T14I, S:T478K, S:D614G, S:P681H, S:T732A,
hCoV-19/Mexico/CMX-INMEGEN-02-07-13/2021	EPI_ISL_1081156	20B	B.1.1.519	17	5'UTR:C203T, 5'UTR:C222T, 5'UTR:C241T, ORF1ab:C3037T, ORF1ab:C10029T, ORF1ab:A11117G, ORF1ab:C12789T, ORF1ab:C14408T, ORF1ab:T19839C, ORF1ab:C21306T, S:T22204C, S:C22995A, S:A23403G, S:A23756G, N:G28881A, N:G28882A, N:G28883C. N:C29197T.	9	N:R203K, N:G204R, ORF1a:T3255I, ORF1a:I3618V, ORF1a:T4175I, ORF1b:P314L, S:T478K, S:D614G, S:T732A,

hCoV-19/Mexico/CMX-INMEGEN-02-07-16/2021	EPI_ISL_1095495	20B	B.1.1.519	27	5'UTR:C203T, 5'UTR:C222T, 5'UTR:C241T, ORF1ab:C1191T, ORF1ab:C3037T, ORF1ab:C3140T, ORF1ab:C4002T, ORF1ab:C10029T, ORF1ab:C10954T, ORF1ab:A11117G, ORF1ab:C12789T, ORF1ab:C14408T, ORF1ab:A15596G, ORF1ab:T19839C, ORF1ab:C20178T, ORF1ab:C21057T, ORF1ab:C21306T, S:C22995A, S:A23403G, S:C23604A, S:A23756G, S:G24914T, ORF3a:C25782T, ORF3a:G26063T, N:G28881A, N:G28882A, N:G28883C, N:C29197T,	16	N:R203K, N:G204R, ORF1a:P309L, ORF1a:P959S, ORF1a:T1246I, ORF1a:T3255I, ORF1a:I3618V, ORF1a:T4175I, ORF1b:P314L, ORF1b:Y710C, ORF3a:G224V, S:T478K, S:D614G, S:P681H, S:T732A, S:D1118Y,	ORF7b:27794-27797,
hCoV-19/Mexico/CMX-INMEGEN-02-07-17/2021	EPI_ISL_1081158	20B	B.1.1.519	26	5'UTR:T201C, 5'UTR:C203T, 5'UTR:C222T, 5'UTR:C241T, ORF1ab:G1738T, ORF1ab:C3037T, ORF1ab:C3140T, ORF1ab:A3675G, ORF1ab:C10029T, ORF1ab:C10954T, ORF1ab:A11117G, ORF1ab:C12789T, ORF1ab:C14408T, ORF1ab:C18348T, ORF1ab:T19839C, ORF1ab:A19974G, ORF1ab:C21306T, S:T22204C, S:C22450T, S:C22995A, S:A23403G, S:C23604A, S:A23756G, N:G28881A, N:G28882A, N:G28883C, N:C29197T,	12	N:R203K, N:G204R, ORF1a:P959S, ORF1a:N1137S, ORF1a:T3255I, ORF1a:I3618V, ORF1a:T4175I, ORF1b:P314L, S:T478K, S:D614G, S:P681H, S:T732A,	
hCoV-19/Mexico/CMX-INMEGEN-02-07-18/2021	EPI_ISL_1081159	20B	B.1.1.519	23	5'UTR:C203T, 5'UTR:C222T, 5'UTR:C241T, ORF1ab:C3037T, ORF1ab:C3140T, ORF1ab:C10029T, ORF1ab:C10954T, ORF1ab:A11117G, ORF1ab:C11886T, ORF1ab:C12789T, ORF1ab:C14408T, ORF1ab:C18252T, ORF1ab:T19839C, ORF1ab:C21306T, S:C22995A, S:A23403G, S:C23604A, S:A23756G, N:T28705C, N:G28881A, N:G28882A, N:G28883C, N:C29197T, N:G29254T,	12	N:R203K, N:G204R, ORF1a:P959S, ORF1a:T3255I, ORF1a:I3618V, ORF1a:S3874L, ORF1a:T4175I, ORF1b:P314L, S:T478K, S:D614G, S:P681H, S:T732A,	
hCoV-19/Mexico/CMX-INMEGEN-02-07-19/2021	EPI_ISL_1081160	20B	B.1.1.519	27	5'UTR:C203T, 5'UTR:C222T, 5'UTR:C241T, ORF1ab:C683T, ORF1ab:A964G, ORF1ab:C3037T, ORF1ab:C3140T, ORF1ab:C5183T, ORF1ab:A6315G, ORF1ab:C10029T, ORF1ab:C10954T, ORF1ab:A11117G, ORF1ab:C12789T, ORF1ab:C14408T, ORF1ab:T19839C, ORF1ab:G20014T, ORF1ab:C21306T, S:A21599G, S:C22995A, S:A23403G, S:C23604A, S:A23756G, ORF3a:C25513T, ORF8:T27904C, N:G28881A, N:G28882A, N:G28883C, N:C29197T,	17	N:R203K, N:G204R, ORF1a:P959S, ORF1a:P1640S, ORF1a:K2017R, ORF1a:T3255I, ORF1a:I3618V, ORF1a:T4175I, ORF1b:P314L, ORF1b:D2183Y, ORF3a:L41F, ORF8:L4P, S:S13G, S:T478K, S:D614G, S:P681H, S:T732A,	
hCoV-19/Mexico/CMX-INMEGEN-02-07-20/2021	EPI_ISL_1081161	20B	B.1.1.519	23	5'UTR:C203T, 5'UTR:C222T, 5'UTR:C241T, ORF1ab:C3037T, ORF1ab:C3140T, ORF1ab:C9120T, ORF1ab:C10029T, ORF1ab:C10954T, ORF1ab:A11117G, ORF1ab:C12789T, ORF1ab:C14408T, ORF1ab:T19839C, ORF1ab:C21077T, ORF1ab:C21306T, S:C22995A, S:A23403G, S:C23604A, S:A23756G, ORF3a:G25906T, ORF8:C28253T, N:G28881A, N:G28882A, N:G28883C, N:C29197T,	14	N:R203K, N:G204R, ORF1a:P959S, ORF1a:T2952I, ORF1a:T3255I, ORF1a:I3618V, ORF1a:T4175I, ORF1b:P314L, ORF1b:T2537I, ORF3a:G172C, S:T478K, S:D614G, S:P681H, S:T732A,	
hCoV-19/Mexico/CMX-INMEGEN-02-07-22/2021	EPI_ISL_1081168	20B	B.1.1.519	24	5'UTR:C203T, 5'UTR:C222T, 5'UTR:C241T, ORF1ab:C3037T, ORF1ab:C3140T, ORF1ab:G3351A, ORF1ab:C8299T, ORF1ab:C10029T, ORF1ab:C10954T, ORF1ab:A11117G, ORF1ab:C12789T, ORF1ab:C14408T, ORF1ab:T19839C, ORF1ab:C21306T, S:C22995A, S:A23403G, S:C23604A, S:A23756G, M:G26779T, M:C26885T, N:G28881A, N:G28882A, N:G28883C, N:C29197T, N:G29527T,	14	M:C86F, N:R203K, N:G204R, N:Q418H, ORF1a:P959S, ORF1a:S1029N, ORF1a:T3255I, ORF1a:I3618V, ORF1a:T4175I, ORF1b:P314L, S:T478K, S:D614G, S:P681H, S:T732A,	

hCoV-19/Mexico/CMX-INMEGEN-02-07-23/2021	EPI_ISL_1081169	20B	B.1.1.519	22	5'UTR:C203T, 5'UTR:C222T, 5'UTR:C241T, ORF1ab:C988T, ORF1ab:C3037T, ORF1ab:C3140T, ORF1ab:G7037T, ORF1ab:C10029T, ORF1ab:C10954T, ORF1ab:A11117G, ORF1ab:C12789T, ORF1ab:C14408T, ORF1ab:T19839C, ORF1ab:C21306T, S:C22995A, S:A23403G, S:C23604A, S:A23756G, ORF3a:C25782T, N:G28881A, N:G28882A, N:G28883C, N:C29197T,	12	N:R203K, N:G204R, ORF1a:P959S, ORF1a:G2258C, ORF1a:T3255I, ORF1a:I3618V, ORF1a:T4175I, ORF1b:P314L, S:T478K, S:D614G, S:P681H, S:T732A,
hCoV-19/Mexico/CMX-INMEGEN-02-09-02/2021	EPI_ISL_1095508	20B	B.1.1.519	26	5'UTR:C203T, 5'UTR:C222T, 5'UTR:C241T, ORF1ab:C3037T, ORF1ab:C3096T, ORF1ab:C3140T, ORF1ab:T3745C, ORF1ab:C5467T, ORF1ab:C9491T, ORF1ab:G9705T, ORF1ab:C10029T, ORF1ab:C10954T, ORF1ab:A11117G, ORF1ab:C12789T, ORF1ab:C14408T, ORF1ab:C17766T, ORF1ab:T19839C, ORF1ab:C21306T, S:C22995A, S:A23403G, S:C23604A, S:A23756G, ORF3a:C25844T, N:G28881A, N:G28882A, N:G28883C, N:C29197T,	15	N:R203K, N:G204R, ORF1a:S944L, ORF1a:P959S, ORF1a:H3076Y, ORF1a:C3147F, ORF1a:T3255I, ORF1a:I3618V, ORF1a:T4175I, ORF1b:P314L, ORF3a:T151I, S:T478K, S:D614G, S:P681H, S:T732A,
hCoV-19/Mexico/CMX-INMEGEN-02-09-03/2021	EPI_ISL_1095509	20B	B.1.1.519	24	5'UTR:C203T, 5'UTR:C222T, 5'UTR:C241T, ORF1ab:C3037T, ORF1ab:C3140T, ORF1ab:C7011T, ORF1ab:C10029T, ORF1ab:C10954T, ORF1ab:A11117G, ORF1ab:C12789T, ORF1ab:C14408T, ORF1ab:G15243T, ORF1ab:G18225T, ORF1ab:T19839C, ORF1ab:C21306T, S:C22995A, S:A23403G, S:C23520T, S:C23604A, S:A23756G, M:G26951T, N:G28881A, N:G28882A, N:G28883C, N:C29197T,	15	N:R203K, N:G204R, ORF1a:P959S, ORF1a:A2249V, ORF1a:T3255I, ORF1a:I3618V, ORF1a:T4175I, ORF1b:P314L, ORF1b:M592I, ORF1b:M1586I, S:T478K, S:D614G, S:A653V, S:P681H, S:T732A,
hCoV-19/Mexico/CMX-INMEGEN-02-09-04/2021	EPI_ISL_1095510	20B	B.1.1.519	24	5'UTR:C203T, 5'UTR:C222T, 5'UTR:C241T, ORF1ab:C3037T, ORF1ab:C3140T, ORF1ab:C7011T, ORF1ab:C10029T, ORF1ab:C10954T, ORF1ab:A11117G, ORF1ab:C12789T, ORF1ab:C14408T, ORF1ab:G15243T, ORF1ab:G18225T, ORF1ab:T19839C, ORF1ab:C21306T, S:C22995A, S:A23403G, S:C23520T, S:C23604A, S:A23756G, M:G26951T, N:G28881A, N:G28882A, N:G28883C, N:C29197T,	15	N:R203K, N:G204R, ORF1a:P959S, ORF1a:A2249V, ORF1a:T3255I, ORF1a:I3618V, ORF1a:T4175I, ORF1b:P314L, ORF1b:M592I, ORF1b:M1586I, S:T478K, S:D614G, S:A653V, S:P681H, S:T732A,
hCoV-19/Mexico/CMX-INMEGEN-02-09-05/2021	EPI_ISL_1095496	20B	B.1.1.519	21	5'UTR:C203T, 5'UTR:C222T, 5'UTR:C241T, ORF1ab:C3037T, ORF1ab:C3140T, ORF1ab:C10029T, ORF1ab:C10165T, ORF1ab:C10954T, ORF1ab:A11117G, ORF1ab:C12789T, ORF1ab:C14408T, ORF1ab:T19839C, ORF1ab:C21306T, S:C22995A, S:A23403G, S:C23604A, S:A23756G, ORF8:C27945T, N:G28881A, N:G28882A, N:G28883C, N:C29197T,	12	N:R203K, N:G204R, ORF1a:P959S, ORF1a:T3255I, ORF1a:I3618V, ORF1a:T4175I, ORF1b:P314L, ORF8:Q18*, S:T478K, S:D614G, S:P681H, S:T732A,
hCoV-19/Mexico/CMX-INMEGEN-02-09-06/2021	EPI_ISL_1095511	20B	B.1.1.519	23	5'UTR:C203T, 5'UTR:C222T, 5'UTR:C241T, ORF1ab:C683T, ORF1ab:C1191A, ORF1ab:C3037T, ORF1ab:C3140T, ORF1ab:C10029T, ORF1ab:C10954T, ORF1ab:A11117G, ORF1ab:C12789T, ORF1ab:C14408T, ORF1ab:T19839C, ORF1ab:C21306T, S:C22995A, S:A23403G, S:C23604A, S:A23756G, N:G28881A, N:G28882A, N:G28883C, N:C29197T, N:G29315C, N:C29421A,	14	N:R203K, N:G204R, N:D348H, N:P383Q, ORF1a:P309Q, ORF1a:P959S, ORF1a:T3255I, ORF1a:I3618V, ORF1a:T4175I, ORF1b:P314L, S:T478K, S:D614G, S:P681H, S:T732A,
hCoV-19/Mexico/CMX-INMEGEN-02-09-07/2021	EPI_ISL_1095512	20B	B.1.1.222	17	5'UTR:C241T, ORF1ab:C3037T, ORF1ab:C10029T, ORF1ab:C10279T, ORF1ab:C14408T, ORF1ab:C19011A, ORF1ab:T19839C, ORF1ab:C20956T, S:G21761T, S:T22965C, S:A23403G, S:A23756G, ORF3a:G25912T, ORF8:A27921G, ORF8:G28001T, N:G28881A, N:G28882A, N:G28883C,	12	N:R203K, N:G204R, ORF1a:T3255I, ORF1b:P314L, ORF1b:D1848E, ORF1b:L2497F, ORF3a:G174C, ORF8:I10V, S:A67S, S:I468T, S:D614G, S:T732A,

hCoV-19/Mexico/CMX-INMEGEN-02-09-08/2021	EPI_ISL_1095497	20B	B.1.1.519	26	5'UTR:C203T, 5'UTR:C222T, 5'UTR:C241T, ORF1ab:T950C, ORF1ab:C3037T, ORF1ab:C3140T, ORF1ab:T9585C, ORF1ab:C10029T, ORF1ab:C10954T, ORF1ab:A11117G, ORF1ab:C11124T, ORF1ab:C12488T, ORF1ab:C12789T, ORF1ab:C14408T, ORF1ab:C19602T, ORF1ab:T19839C, ORF1ab:C21306T, S:C22995A, S:A23403G, S:C23604A, S:A23756G, ORF3a:G25906T, ORF8:C28253T, N:G28881A, N:G28882A, N:G28883C, N:C29197T	16	N:R203K, N:G204R, ORF1a:Y229H, ORF1a:P959S, ORF1a:V3107A, ORF1a:T3255I, ORF1a:I3618V, ORF1a:A3620V, ORF1a:P4075S, ORF1a:T4175I, ORF1b:P314L, ORF3a:G172C, S:T478K, S:D614G, S:P681H, S:T732A,
hCoV-19/Mexico/CMX-INMEGEN-02-09-09/2021	EPI_ISL_1095498	20B	B.1.1.519	23	5'UTR:C203T, 5'UTR:C222T, 5'UTR:C241T, ORF1ab:G806A, ORF1ab:C3037T, ORF1ab:C3140T, ORF1ab:C3176T, ORF1ab:G3549A, ORF1ab:C10029T, ORF1ab:C10954T, ORF1ab:A11117G, ORF1ab:C12789T, ORF1ab:C14408T, ORF1ab:T19839C, ORF1ab:C21306T, S:C22995A, S:A23403G, S:C23604A, S:A23756G, ORF8:T27904C, N:G28881A, N:G28882A, N:G28883C, N:C29197T,	15	N:R203K, N:G204R, ORF1a:A181T, ORF1a:P959S, ORF1a:P971S, ORF1a:G1095E, ORF1a:T3255I, ORF1a:I3618V, ORF1a:T4175I, ORF1b:P314L, ORF8:L4P, S:T478K, S:D614G, S:P681H, S:T732A,
hCoV-19/Mexico/CMX-INMEGEN-02-09-11/2021	EPI_ISL_1095499	20B	B.1.1.519	24	5'UTR:C203T, 5'UTR:C222T, 5'UTR:C241T, ORF1ab:C3037T, ORF1ab:C3140T, ORF1ab:C10029T, ORF1ab:C10277T, ORF1ab:C10954T, ORF1ab:A11117G, ORF1ab:C12225T, ORF1ab:C12789T, ORF1ab:C14408T, ORF1ab:T19839C, ORF1ab:C21306T, S:G22778C, S:C22995A, S:A23403G, S:C23604A, S:A23756G, N:G28881A, N:G28882A, N:G28883C, N:C29197T, N:G29527T, 3'UTR:G29717T,	15	N:R203K, N:G204R, N:Q418H, ORF1a:P959S, ORF1a:T3255I, ORF1a:L3338F, ORF1a:I3618V, ORF1a:A3987V, ORF1a:T4175I, ORF1b:P314L, S:E406Q, S:T478K, S:D614G, S:P681H, S:T732A,
hCoV-19/Mexico/CMX-INMEGEN-02-09-12/2021	EPI_ISL_1095500	20B	B.1.1.519	24	5'UTR:C203T, 5'UTR:C222T, 5'UTR:C241T, ORF1ab:C3037T, ORF1ab:C3140T, ORF1ab:C10029T, ORF1ab:C10277T, ORF1ab:C10954T, ORF1ab:A11117G, ORF1ab:C12225T, ORF1ab:C12789T, ORF1ab:C14408T, ORF1ab:T19839C, ORF1ab:C21306T, S:G22778C, S:C22995A, S:A23403G, S:C23604A, S:A23756G, N:G28881A, N:G28882A, N:G28883C, N:C29197T, N:G29527T, 3'UTR:G29717T,	15	N:R203K, N:G204R, N:Q418H, ORF1a:P959S, ORF1a:T3255I, ORF1a:L3338F, ORF1a:I3618V, ORF1a:A3987V, ORF1a:T4175I, ORF1b:P314L, S:E406Q, S:T478K, S:D614G, S:P681H, S:T732A,
hCoV-19/Mexico/CMX-INMEGEN-02-09-13/2021	EPI_ISL_1095501	20B	B.1.1.519	24	5'UTR:C203T, 5'UTR:C222T, 5'UTR:C241T, ORF1ab:C3037T, ORF1ab:C3140T, ORF1ab:C10029T, ORF1ab:C10277T, ORF1ab:C10954T, ORF1ab:A11117G, ORF1ab:C12225T, ORF1ab:C12789T, ORF1ab:C14408T, ORF1ab:T19839C, ORF1ab:C21306T, S:G22778C, S:C22995A, S:A23403G, S:C23604A, S:A23756G, N:G28881A, N:G28882A, N:G28883C, N:C29197T, N:G29527T, 3'UTR:G29717T,	15	N:R203K, N:G204R, N:Q418H, ORF1a:P959S, ORF1a:T3255I, ORF1a:L3338F, ORF1a:I3618V, ORF1a:A3987V, ORF1a:T4175I, ORF1b:P314L, S:E406Q, S:T478K, S:D614G, S:P681H, S:T732A,
hCoV-19/Mexico/CMX-INMEGEN-02-09-14/2021	EPI_ISL_1095502	20B	B.1.1.519	28	5'UTR:T201C, 5'UTR:C203T, 5'UTR:C222T, 5'UTR:C241T, ORF1ab:C745T, ORF1ab:G1738T, ORF1ab:C3037T, ORF1ab:C3140T, ORF1ab:C10029T, ORF1ab:C10954T, ORF1ab:A11117G, ORF1ab:C11962T, ORF1ab:C12789T, ORF1ab:C14408T, ORF1ab:C14724T, ORF1ab:T19839C, ORF1ab:A19974G, ORF1ab:C21306T, S:C22995A, S:A23403G, S:C23604A, S:A23756G, S:G25218T, ORF8:G28086A, N:G28881A, N:G28882A, N:G28883C, N:C29197T, ORF10:A29571T	13	N:R203K, N:G204R, ORF1a:P959S, ORF1a:T3255I, ORF1a:I3618V, ORF1a:T4175I, ORF1b:P314L, ORF8:A65T, S:T478K, S:D614G, S:P681H, S:T732A, S:G1219V,

hCoV-19/Mexico/CMX-INMEGEN-02-09-15/2021	EPI_ISL_1095503	20B	B.1.1.519	23	5'UTR:C203T, 5'UTR:C222T, 5'UTR:C241T, ORF1ab:C3037T, ORF1ab:C3140T, ORF1ab:C7869T, ORF1ab:C10029T, ORF1ab:C10954T, ORF1ab:A11117G, ORF1ab:C12789T, ORF1ab:C14408T, ORF1ab:T19839C, ORF1ab:C21077T, ORF1ab:C21306T, ORF1ab:A21366T, S:C22995A, S:A23403G, S:C23604A, S:A23756G, ORF6:A27391C, N:G28881A, N:G28882A, N:G28883C, N:C29197T,	13	N:R203K, N:G204R, ORF1a:P959S, ORF1a:S2535L, ORF1a:T3255I, ORF1a:I3618V, ORF1a:T4175I, ORF1b:P314L, ORF1b:T2537I, S:T478K, S:D614G, S:P681H, S:T732A,
hCoV-19/Mexico/CMX-INMEGEN-02-09-16/2021	EPI_ISL_1095504	20B	B.1.1.519	28	5'UTR:T201C, 5'UTR:C203T, 5'UTR:C222T, 5'UTR:C241T, ORF1ab:G1738T, ORF1ab:C2892T, ORF1ab:C3037T, ORF1ab:C3140T, ORF1ab:A5845C, ORF1ab:C6026T, ORF1ab:C10029T, ORF1ab:C10954T, ORF1ab:A11117G, ORF1ab:C12789T, ORF1ab:C14408T, ORF1ab:T19839C, ORF1ab:A19974G, ORF1ab:C21306T, S:C22995A, S:A23403G, S:C23604A, S:A23756G, ORF3a:T25577C, ORF7b:C27804T, N:G28881A, N:G28882A, N:G28883C, N:C29197T, ORF10:C29627T,	15	N:R203K, N:G204R, ORF1a:A876V, ORF1a:P959S, ORF1a:K1860N, ORF1a:P1921S, ORF1a:T3255I, ORF1a:I3618V, ORF1a:T4175I, ORF1b:P314L, ORF3a:I62T, S:T478K, S:D614G, S:P681H, S:T732A,
hCoV-19/Mexico/CMX-INMEGEN-02-09-17/2021	EPI_ISL_1095505	20B	B.1.1.519	23	5'UTR:C203T, 5'UTR:C222T, 5'UTR:C241T, ORF1ab:C3037T, ORF1ab:C3140T, ORF1ab:C10029T, ORF1ab:C10954T, ORF1ab:A11117G, ORF1ab:C12789T, ORF1ab:C14408T, ORF1ab:G15921T, ORF1ab:A17971G, ORF1ab:T19839C, ORF1ab:C21306T, S:G22785T, S:C22995A, S:A23403G, S:C23604A, S:C23635T, S:A23756G, N:G28881A, N:G28882A, N:G28883C, N:C29197T.	13	N:R203K, N:G204R, ORF1a:P959S, ORF1a:T3255I, ORF1a:I3618V, ORF1a:T4175I, ORF1b:P314L, ORF1b:R1502G, S:R408I, S:T478K, S:D614G, S:P681H, S:T732A,
hCoV-19/Mexico/CMX-INMEGEN-02-09-18/2020	EPI_ISL_1095516	20B	B.1.1.519	26	5'UTR:C203T, 5'UTR:C222T, 5'UTR:C241T, ORF1ab:G942A, ORF1ab:C3037T, ORF1ab:C3140T, ORF1ab:C8090T, ORF1ab:C10029T, ORF1ab:C10954T, ORF1ab:A11117G, ORF1ab:C11124T, ORF1ab:C12789T, ORF1ab:C14408T, ORF1ab:T19839C, ORF1ab:A20676G, ORF1ab:C21306T, S:C22995A, S:C23029T, S:A23403G, S:C23604A, S:A23756G, ORF8:C27942T, N:C28511T, N:G28881A, N:G28882A, N:G28883C, N:C29197T,	16	N:P80S, N:R203K, N:G204R, ORF1a:R226K, ORF1a:P959S, ORF1a:L2609F, ORF1a:T3255I, ORF1a:I3618V, ORF1a:A3620V, ORF1a:T4175I, ORF1b:P314L, ORF8:H17Y, S:T478K, S:D614G, S:P681H, S:T732A,
hCoV-19/Mexico/CMX-INMEGEN-02-09-19/2021	EPI_ISL_1095506	20B	B.1.1.519	22	5'UTR:C203T, 5'UTR:C222T, 5'UTR:C241T, ORF1ab:C3037T, ORF1ab:C3140T, ORF1ab:C6406T, ORF1ab:G7027T, ORF1ab:C10029T, ORF1ab:C10954T, ORF1ab:A11117G, ORF1ab:C12789T, ORF1ab:C14408T, ORF1ab:T19839C, ORF1ab:C21306T, S:C22995A, S:A23403G, S:C23604A, S:A23756G, S:C25207T, N:G28881A, N:G28882A, N:G28883C, N:C29197T,	12	N:R203K, N:G204R, ORF1a:P959S, ORF1a:M2254I, ORF1a:T3255I, ORF1a:I3618V, ORF1a:T4175I, ORF1b:P314L, S:T478K, S:D614G, S:P681H, S:T732A,
hCoV-19/Mexico/CMX-INMEGEN-02-09-20/2021	EPI_ISL_1095507	20B	B.1.1.519	28	5'UTR:C203T, 5'UTR:C222T, 5'UTR:C241T, ORF1ab:C832T, ORF1ab:C3037T, ORF1ab:C3140T, ORF1ab:T5911C, ORF1ab:A6393C, ORF1ab:C10029T, ORF1ab:C10954T, ORF1ab:A11117G, ORF1ab:C12789T, ORF1ab:C14408T, ORF1ab:C15251T, ORF1ab:C17977T, ORF1ab:T19839C, ORF1ab:C21306T, S:C22995A, S:A23403G, S:C23604A, S:A23756G, ORF3a:C25624T, ORF6:T27384C, N:G28703C, N:G28881A, N:G28882A, N:G28883C, N:C29095T, N:C29197T	16	N:D144H, N:R203K, N:G204R, ORF1a:P959S, ORF1a:D2043A, ORF1a:T3255I, ORF1a:I3618V, ORF1a:T4175I, ORF1b:P314L, ORF1b:T595I, ORF1b:L1504F, ORF3a:H78Y, S:T478K, S:D614G, S:P681H, S:T732A,

hCoV-19/Mexico/CMX-INMEGEN-02-09-21/2021	EPI_ISL_1095513	20B	B.1.1.519	25	5'UTR:C203T, 5'UTR:C222T, 5'UTR:C241T, ORF1ab:C3037T, ORF1ab:C3140T, ORF1ab:T3745C, ORF1ab:C4570T, ORF1ab:A6284G, ORF1ab:C10029T, ORF1ab:C10954T, ORF1ab:A11117G, ORF1ab:C11514T, ORF1ab:C12789T, ORF1ab:C14408T, ORF1ab:G16075T, ORF1ab:T19839C, ORF1ab:C21306T, S:C22995A, S:A23403G, S:C23604A, S:A23756G, ORF3a:C25844T, N:G28881A, N:G28882A, N:G28883C, N:C29197T,	15	N:R203K, N:G204R, ORF1a:P959S, ORF1a:T2007A, ORF1a:T3255I, ORF1a:I3618V, ORF1a:T3750I, ORF1a:T4175I, ORF1b:P314L, ORF1b:D870Y, ORF3a:T151I, S:T478K, S:D614G, S:P681H, S:T732A,
hCoV-19/Mexico/CMX-INMEGEN-02-09-22/2021	EPI_ISL_1095514	20B	B.1.1.519	28	5'UTR:C203T, 5'UTR:C222T, 5'UTR:C241T, ORF1ab:G2035T, ORF1ab:C2842T, ORF1ab:C3037T, ORF1ab:C3140T, ORF1ab:G3692T, ORF1ab:G3871T, ORF1ab:T7436C, ORF1ab:C10029T, ORF1ab:C10954T, ORF1ab:A11117G, ORF1ab:C11824A, ORF1ab:C12789T, ORF1ab:C14408T, ORF1ab:T19839C, ORF1ab:C20759T, ORF1ab:C21306T, S:C22995A, S:A23403G, S:C23604A, S:A23756G, ORF7b:G27877T, ORF8:T27904C, N:G28881A, N:G28882A, N:G28883C, N:C29197T,	18	N:R203K, N:G204R, ORF1a:L590F, ORF1a:P959S, ORF1a:V1143F, ORF1a:K1202N, ORF1a:Y2391H, ORF1a:T3255I, ORF1a:I3618V, ORF1a:T4175I, ORF1b:P314L, ORF1b:A2431V, ORF7b:C41F, ORF8:L4P, S:T478K, S:D614G, S:P681H, S:T732A,
hCoV-19/Mexico/CMX-INMEGEN-02-09-23/2021	EPI_ISL_1095515	20B	B.1.1.519	27	5'UTR:C203T, 5'UTR:C222T, 5'UTR:C241T, ORF1ab:C920T, ORF1ab:C3037T, ORF1ab:C3140T, ORF1ab:G3692T, ORF1ab:G3871T, ORF1ab:A8446G, ORF1ab:C10029T, ORF1ab:C10954T, ORF1ab:A11117G, ORF1ab:C11824A, ORF1ab:C12789T, ORF1ab:C14408T, ORF1ab:C15120T, ORF1ab:C18171T, ORF1ab:T19839C, ORF1ab:C21306T, S:C22995A, S:A23403G, S:C23604A, S:A23756G, ORF8:T27904C, N:G28881A, N:G28882A, N:G28883C, N:C29197T,	14	N:R203K, N:G204R, ORF1a:P959S, ORF1a:V1143F, ORF1a:K1202N, ORF1a:T3255I, ORF1a:I3618V, ORF1a:T4175I, ORF1b:P314L, ORF8:L4P, S:T478K, S:D614G, S:P681H, S:T732A,
hCoV-19/Mexico/CMX-INMEGEN-02-10-01/2021	EPI_ISL_1137462	20B	B.1.1.519	24	5'UTR:C203T, 5'UTR:C222T, 5'UTR:C241T, ORF1ab:C3037T, ORF1ab:C3140T, ORF1ab:T6667C, ORF1ab:C10029T, ORF1ab:C10954T, ORF1ab:A11117G, ORF1ab:C12789T, ORF1ab:C14408T, ORF1ab:T19839C, ORF1ab:C21077T, ORF1ab:C21306T, S:C22995A, S:A23403G, S:C23604A, S:A23756G, ORF3a:G25906T, E:C26270T, ORF8:C28253T, N:G28881A, N:G28882A, N:G28883C, N:C29197T,	14	E:T9I, N:R203K, N:G204R, ORF1a:P959S, ORF1a:T3255I, ORF1a:I3618V, ORF1a:T4175I, ORF1b:P314L, ORF1b:T2537I, ORF3a:G172C, S:T478K, S:D614G, S:P681H, S:T732A,
hCoV-19/Mexico/CMX-INMEGEN-02-10-02/2021	EPI_ISL_1137463	20B	B.1.1.519	22	5'UTR:C203T, 5'UTR:C222T, 5'UTR:C241T, ORF1ab:C3037T, ORF1ab:C3140T, ORF1ab:C10029T, ORF1ab:C10207T, ORF1ab:C10954T, ORF1ab:A11117G, ORF1ab:C12789T, ORF1ab:C14408T, ORF1ab:G19666T, ORF1ab:T19839C, ORF1ab:C21306T, S:C22995A, S:A23403G, S:C23604A, S:A23756G, ORF8:T27904C, N:G28881A, N:G28882A, N:G28883C, N:C29197T,	13	N:R203K, N:G204R, ORF1a:P959S, ORF1a:T3255I, ORF1a:I3618V, ORF1a:T4175I, ORF1b:P314L, ORF1b:D2067Y, ORF8:L4P, S:T478K, S:D614G, S:P681H, S:T732A,
hCoV-19/Mexico/CMX-INMEGEN-02-10-03/2021	EPI_ISL_1137464	20B	B.1.1.519	25	5'UTR:C203T, 5'UTR:C222T, 5'UTR:C241T, ORF1ab:C3037G, ORF1ab:C3140T, ORF1ab:T3745C, ORF1ab:C10029T, ORF1ab:C10954T, ORF1ab:A11117G, ORF1ab:T11587C, ORF1ab:C12789T, ORF1ab:C14408T, ORF1ab:C18493T, ORF1ab:T19839C, ORF1ab:C21306T, S:C22995A, S:A23403G, S:C23604A, S:A23756G, S:G24781T, ORF3a:C25844T, N:G28881A, N:G28882A, N:G28883C, N:C29197T, ORF10:C29670T,	15	N:R203K, N:G204R, ORF1a:F924L, ORF1a:P959S, ORF1a:T3255I, ORF1a:I3618V, ORF1a:T4175I, ORF1b:P314L, ORF1b:L1676F, ORF3a:T151I, S:T478K, S:D614G, S:P681H, S:T732A, S:K1073N,

hCoV-19/Mexico/CMX-INMEGEN-02-10-04/2021	EPI_ISL_1137460	20B	B.1.1.519	30	5'UTR:T201C, 5'UTR:C203T, 5'UTR:C222T, 5'UTR:C241T, ORF1ab:C745T, ORF1ab:G1738T, ORF1ab:C3037T, ORF1ab:C3140T, ORF1ab:C4776T, ORF1ab:C6196T, ORF1ab:C10029T, ORF1ab:C10954T, ORF1ab:A11117G, ORF1ab:C12789T, ORF1ab:C14408T, ORF1ab:T16587C, ORF1ab:T19839C, ORF1ab:A19974G, ORF1ab:C21306T, S:C22995A, S:A23403G, S:C23604A, S:A23756G, ORF3a:C26110T, ORF3a:G26152T, N:A28877T, N:G28878C, N:G28881A, N:G28882A, N:G28883C, N:C29197T	14	N:R203K, N:G204R, ORF1a:P959S, ORF1a:T1504I, ORF1a:T3255I, ORF1a:I3618V, ORF1a:T4175I, ORF1b:P314L, ORF3a:P240S, ORF3a:G254*, S:T478K, S:D614G, S:P681H, S:T732A,
hCoV-19/Mexico/CMX-INMEGEN-02-10-06/2021	EPI_ISL_1137465	20B	B.1.1.519	27	5'UTR:C203T, 5'UTR:C222T, 5'UTR:C241T, ORF1ab:C3037T, ORF1ab:C3140T, ORF1ab:C9661T, ORF1ab:C10029T, ORF1ab:C10954T, ORF1ab:A11117G, ORF1ab:C12473T, ORF1ab:C12789T, ORF1ab:C14408T, ORF1ab:G15253A, ORF1ab:A18282G, ORF1ab:C19602T, ORF1ab:T19839C, ORF1ab:C21306T, S:C22995A, S:A23403G, S:C23604A, S:A23756G, ORF3a:G25906T, ORF8:C28253T, N:G28881A, N:G28882A, N:G28883C, N:C29197T, 3'UTR:G29777T,	13	N:R203K, N:G204R, ORF1a:P959S, ORF1a:T3255I, ORF1a:I3618V, ORF1a:T4175I, ORF1b:P314L, ORF1b:V596I, ORF3a:G172C, S:T478K, S:D614G, S:P681H, S:T732A,
hCoV-19/Mexico/CMX-INMEGEN-02-10-07/2021	EPI_ISL_1137466	20B	B.1.1.519	27	5'UTR:C203T, 5'UTR:C222T, 5'UTR:C241T, ORF1ab:C3037T, ORF1ab:C3140T, ORF1ab:C9661T, ORF1ab:C10029T, ORF1ab:C10954T, ORF1ab:A11117G, ORF1ab:C12473T, ORF1ab:C12789T, ORF1ab:C14408T, ORF1ab:G15253A, ORF1ab:A18282G, ORF1ab:C19602T, ORF1ab:T19839C, ORF1ab:C21306T, S:C22995A, S:A23403G, S:C23604A, S:A23756G, ORF3a:G25906T, ORF8:C28253T, N:G28881A, N:G28882A, N:G28883C, N:C29197T, 3'UTR:G29777T,	13	N:R203K, N:G204R, ORF1a:P959S, ORF1a:T3255I, ORF1a:I3618V, ORF1a:T4175I, ORF1b:P314L, ORF1b:V596I, ORF3a:G172C, S:T478K, S:D614G, S:P681H, S:T732A,
hCoV-19/Mexico/CMX-INMEGEN-02-10-08/2021	EPI_ISL_1137467	20B	B.1.1.222	21	5'UTR:C241T, ORF1ab:T1099C, ORF1ab:C1427T, ORF1ab:T1523G, ORF1ab:C3037T, ORF1ab:A6693G, ORF1ab:T7402C, ORF1ab:C9430T, ORF1ab:C10029T, ORF1ab:C14408T, ORF1ab:T19839C, S:A23403G, S:A23756G, ORF3a:G25912T, ORF7b:A27756G, ORF8:A27921G, ORF8:G28001T, N:C28313T, N:G28881A, N:G28882A, N:G28883C, ORF10:C29627T,	13	N:R14C, N:R203K, N:G204R, ORF1a:H388Y, ORF1a:C420G, ORF1a:K2143R, ORF1a:T3255I, ORF1b:P314L, ORF3a:G174C, ORF7b:M1V, ORF8:I10V, S:D614G, S:T732A,
hCoV-19/Mexico/CMX-INMEGEN-02-10-09/2021	EPI_ISL_1137468	20B	B.1.1.519	26	5'UTR:T201C, 5'UTR:C203T, 5'UTR:C222T, 5'UTR:C241T, ORF1ab:G1738T, ORF1ab:C2470T, ORF1ab:C3037T, ORF1ab:C3140T, ORF1ab:A4085G, ORF1ab:C9438T, ORF1ab:C10029T, ORF1ab:C10954T, ORF1ab:A11117G, ORF1ab:C12789T, ORF1ab:C14408T, ORF1ab:A16355G, ORF1ab:T19839C, ORF1ab:A19974G, ORF1ab:C21306T, S:C22995A, S:A23403G, S:C23604A, S:A23756G, N:G28881A, N:G28882A, N:G28883C, N:C29197T,	14	N:R203K, N:G204R, ORF1a:P959S, ORF1a:I1274V, ORF1a:T3058I, ORF1a:T3255I, ORF1a:I3618V, ORF1a:T4175I, ORF1b:P314L, ORF1b:K963R, S:T478K, S:D614G, S:P681H, S:T732A,
hCoV-19/Mexico/CMX-INMEGEN-02-10-10/2021	EPI_ISL_1137469	20B	B.1.1.519	24	5'UTR:C203T, 5'UTR:C222T, 5'UTR:C241T, ORF1ab:C3037T, ORF1ab:C3140T, ORF1ab:G5008T, ORF1ab:C10029T, ORF1ab:C10954T, ORF1ab:A11117G, ORF1ab:G11365T, ORF1ab:C12789T, ORF1ab:C14408T, ORF1ab:G14500T, ORF1ab:T19839C, ORF1ab:C21306T, S:C22995A, S:A23403G, S:C23604A, S:A23756G, N:G28881A, N:G28882A, N:G28883C, N:C28887T, N:C29197T, N:G29527T,	14	N:R203K, N:G204R, N:T205I, N:Q418H, ORF1a:P959S, ORF1a:T3255I, ORF1a:I3618V, ORF1a:T4175I, ORF1b:P314L, ORF1b:V345L, S:T478K, S:D614G, S:P681H, S:T732A,

hCoV-19/Mexico/CMX-INMEGEN-02-10-11/2021	EPI_ISL_1137470	20B	B.1.1.519	26	5'UTR:C203T, 5'UTR:C222T, 5'UTR:C241T, ORF1ab:C3037T, ORF1ab:C3140T, ORF1ab:C4582T, ORF1ab:T6552C, ORF1ab:C6627T, ORF1ab:C10029T, ORF1ab:C10954T, ORF1ab:A11117G, ORF1ab:C12789T, ORF1ab:C14408T, ORF1ab:G15921T, ORF1ab:G18984A, ORF1ab:T19839C, ORF1ab:C21306T, S:T22501C, S:C22995A, S:A23403G, S:C23604A, S:A23756G, N:G28881A, N:G28882A, N:G28883C, N:C29077T, N:C29197T,	14	N:R203K, N:G204R, ORF1a:P959S, ORF1a:M2096T, ORF1a:T2121I, ORF1a:T3255I, ORF1a:I3618V, ORF1a:T4175I, ORF1b:P314L, ORF1b:M1839I, S:T478K, S:D614G, S:P681H, S:T732A,	
hCoV-19/Mexico/CMX-INMEGEN-02-10-12/2021	EPI_ISL_1137471	20B	B.1.1.519	26	5'UTR:C203T, 5'UTR:C222T, 5'UTR:C241T, ORF1ab:C3037T, ORF1ab:C3140T, ORF1ab:A3684G, ORF1ab:C6145T, ORF1ab:C6541T, ORF1ab:C10029T, ORF1ab:C10954T, ORF1ab:A11117G, ORF1ab:C12789T, ORF1ab:C14408T, ORF1ab:C18252T, ORF1ab:T19839C, ORF1ab:C21306T, S:A22023T, S:C22995A, S:A23403G, S:C23604A, S:A23756G, ORF8:T27904C, N:G28881A, N:G28882A, N:G28883C, N:C29197T, N:G29374T,	15	N:R203K, N:G204R, N:E367D, ORF1a:P959S, ORF1a:Q1140R, ORF1a:T3255I, ORF1a:I3618V, ORF1a:T4175I, ORF1b:P314L, ORF8:L4P, S:E154V, S:T478K, S:D614G, S:P681H, S:T732A,	
hCoV-19/Mexico/CMX-INMEGEN-02-10-13/2021	EPI_ISL_1137472	20B	B.1.1.519	24	5'UTR:C203T, 5'UTR:C222T, 5'UTR:C241T, ORF1ab:C1404T, ORF1ab:C1968T, ORF1ab:C3037T, ORF1ab:C3140T, ORF1ab:T3745C, ORF1ab:C10029T, ORF1ab:C10036T, ORF1ab:C10954T, ORF1ab:A11117G, ORF1ab:C12789T, ORF1ab:C14408T, ORF1ab:T19839C, ORF1ab:C21306T, S:C22995A, S:A23403G, S:C23604A, S:A23756G, ORF3a:C25844T, N:G28881A, N:G28882A, N:G28883C, N:C29197T,	14	N:R203K, N:G204R, ORF1a:P380L, ORF1a:T568I, ORF1a:P959S, ORF1a:T3255I, ORF1a:I3618V, ORF1a:T4175I, ORF1b:P314L, ORF3a:T151I, S:T478K, S:D614G, S:P681H, S:T732A,	
hCoV-19/Mexico/CMX-INMEGEN-02-10-14/2021	EPI_ISL_1137461	20B	B.1.1.519	27	5'UTR:C203T, 5'UTR:C222T, 5'UTR:C241T, ORF1ab:C1342T, ORF1ab:C3037T, ORF1ab:C3140T, ORF1ab:G3692T, ORF1ab:G3871T, ORF1ab:C5284T, ORF1ab:C10029T, ORF1ab:T10357C, ORF1ab:C10954T, ORF1ab:A11117G, ORF1ab:C11824A, ORF1ab:C12789T, ORF1ab:C14408T, ORF1ab:T19839C, ORF1ab:C21306T, S:C22995A, S:A23403G, S:C23604A, S:A23756G, ORF8:G28198T, N:G28881A, N:G28882A, N:G28883C, N:C29197T, N:C29367T,	15	N:R203K, N:G204R, N:P365L, ORF1a:P959S, ORF1a:V1143F, ORF1a:K1202N, ORF1a:T3255I, ORF1a:I3618V, ORF1a:T4175I, ORF1b:P314L, ORF8:C102F, S:T478K, S:D614G, S:P681H, S:T732A,	ORF7b:27792-27794,
hCoV-19/Mexico/CMX-INMEGEN-02-10-15/2021	EPI_ISL_1137473	20B	B.1.1.519	30	5'UTR:C203T, 5'UTR:C222T, 5'UTR:C241T, ORF1ab:A866G, ORF1ab:C1009T, ORF1ab:C3037T, ORF1ab:C3140T, ORF1ab:A3801G, ORF1ab:C5183T, ORF1ab:C10029T, ORF1ab:C10954T, ORF1ab:A11117G, ORF1ab:C11916T, ORF1ab:C12789T, ORF1ab:C14408T, ORF1ab:C16596T, ORF1ab:A18277G, ORF1ab:T19839C, ORF1ab:C21306T, S:G22813T, S:C22995A, S:G23069C, S:A23403G, S:C23604A, S:A23756G, ORF8:T27904C, N:G28747T, N:G28881A, N:G28882A, N:G28883C, N:C29197T,	19	N:R203K, N:G204R, ORF1a:I201V, ORF1a:P959S, ORF1a:D1179G, ORF1a:P1640S, ORF1a:T3255I, ORF1a:I3618V, ORF1a:S3884L, ORF1a:T4175I, ORF1b:P314L, ORF1b:I1604V, ORF8:L4P, S:K417N, S:T478K, S:V503L, S:D614G, S:P681H, S:T732A,	
hCoV-19/Mexico/CMX-INMEGEN-02-10-16/2021	EPI_ISL_1137474	20B	B.1.1.519	23	5'UTR:C203T, 5'UTR:C222T, 5'UTR:C241T, ORF1ab:C3037T, ORF1ab:C3140T, ORF1ab:C10029T, ORF1ab:C10954T, ORF1ab:A11117G, ORF1ab:C12789T, ORF1ab:C14408T, ORF1ab:T19839C, ORF1ab:C20133T, ORF1ab:C21306T, S:C22995A, S:A23403G, S:C23604A, S:A23756G, ORF3a:T26061A, ORF8:T27904C, ORF8:C28087T, N:G28881A, N:G28882A, N:G28883C, N:C29197T,	13	N:R203K, N:G204R, ORF1a:P959S, ORF1a:T3255I, ORF1a:I3618V, ORF1a:T4175I, ORF1b:P314L, ORF8:L4P, ORF8:A65V, S:T478K, S:D614G, S:P681H, S:T732A,	

ıCoV-19/Mexico/CMX-INMEGEN-02-10-17-GRO/202	EPI_ISL_1158060	20A	B.1.610	7	5'UTR:C241T, ORF1ab:C2110T, ORF1ab:C3037T, ORF1ab:C14408T, ORF1ab:A18694G, ORF1ab:A20268G, S:A23403G, S:C24748T,	3	ORF1b:P314L, ORF1b:T1743A, S:D614G,	ORF7a:27607-27617
ıCoV-19/Mexico/CMX-INMEGEN-02-10-18-GRO/202	EPI_ISL_1158061	20A	B.1.610	7	5'UTR:C241T, ORF1ab:C2110T, ORF1ab:C3037T, ORF1ab:C14408T, ORF1ab:A18694G, ORF1ab:A20268G, S:A23403G, S:C24748T,	3	ORF1b:P314L, ORF1b:T1743A, S:D614G,	ORF7a:27607-27617
ıCoV-19/Mexico/CMX-INMEGEN-02-10-19-GRO/202	EPI_ISL_1137475	20A	B.1.609	9	5'UTR:C241T, ORF1ab:T325C, ORF1ab:C3037T, ORF1ab:T3334G, ORF1ab:C4582T, ORF1ab:C13119T, ORF1ab:C14408T, ORF1ab:A20268G, S:G23311C, S:A23403G,	5	ORF1a:I1023M, ORF1a:A4285V, ORF1b:P314L, S:E583D, S:D614G,	
ıCoV-19/Mexico/CMX-INMEGEN-02-10-20-GRO/202	EPI_ISL_1158062	20A	B.1.610	7	5'UTR:C241T, ORF1ab:C2110T, ORF1ab:C3037T, ORF1ab:C14408T, ORF1ab:A18694G, ORF1ab:A20268G, S:A23403G, S:C24748T,	3	ORF1b:P314L, ORF1b:T1743A, S:D614G,	ORF7a:27607-27617
ıCoV-19/Mexico/CMX-INMEGEN-02-10-21-GRO/202	EPI_ISL_1137476	20A	B.1.610	10	5'UTR:C241T, ORF1ab:C414T, ORF1ab:C3037T, ORF1ab:C7081T, ORF1ab:C7926T, ORF1ab:C10198T, ORF1ab:C14408T, ORF1ab:A20268G, S:A23403G, ORF7a:C27741T, N:C28905T,	5	N:A211V, ORF1a:T50I, ORF1a:A2554V, ORF1b:P314L, S:D614G,	
ıCoV-19/Mexico/CMX-INMEGEN-02-10-22-GRO/202	EPI_ISL_1137459	20A	B.1.243	20	5'UTR:C241T, ORF1ab:C274T, ORF1ab:G806A, ORF1ab:T1783C, ORF1ab:T2584A, ORF1ab:C3037T, ORF1ab:C5140A, ORF1ab:T5255C, ORF1ab:C6027T, ORF1ab:T7122C, ORF1ab:C14408T, ORF1ab:C17410T, ORF1ab:C18486T, ORF1ab:A20268G, S:A23403G, S:G23587C, S:T24076C, E:C26455T, N:C28854T, N:G29543T, 3'UTR:G29747T,	10	E:P71S, N:S194L, ORF1a:A181T, ORF1a:D1625E, ORF1a:P1921L, ORF1a:I2286T, ORF1b:P314L, ORF1b:R1315C, S:D614G, S:Q675H,	
ıCoV-19/Mexico/CMX-INMEGEN-02-10-23-GRO/202	EPI_ISL_1137477	20A	B.1	17	5'UTR:C241T, ORF1ab:C1380T, ORF1ab:C3037T, ORF1ab:G5230T, ORF1ab:G6341T, ORF1ab:T8395G, ORF1ab:T12283C, ORF1ab:C14408T, ORF1ab:A20268G, S:C22591T, S:A23403G, ORF3a:G25757T, M:C26882T, N:C28849T, N:C28854T, N:C29095T, N:C29311T, 3'UTR:G29734C,	7	N:S194L, ORF1a:A372V, ORF1a:K1655N, ORF1a:D2026Y, ORF1b:P314L, ORF3a:R122I, S:D614G,	
hCoV-19/Mexico/CMX-INMEGEN-02-11-01/2021	EPI_ISL_1117353	20B	B.1.1.519	26	5'UTR:C203T, 5'UTR:C222T, 5'UTR:C241T, ORF1ab:C2094T, ORF1ab:C3037T, ORF1ab:C3140T, ORF1ab:G3328T, ORF1ab:C5079T, ORF1ab:A7888T, ORF1ab:C10029T, ORF1ab:C10954T, ORF1ab:A11117G, ORF1ab:C12789T, ORF1ab:C14408T, ORF1ab:C17678T, ORF1ab:T19839C, ORF1ab:C21306T, S:C22995A, S:A23403G, S:C23604A, S:A23756G, E:G26329T, ORF6:G27204T, N:G28881A, N:G28882A, N:G28883C, N:C29197T,	17	E:V29F, N:R203K, N:G204R, ORF1a:S610L, ORF1a:P959S, ORF1a:Q1021H, ORF1a:T1605I, ORF1a:T3255I, ORF1a:I3618V, ORF1a:T4175I, ORF1b:P314L, ORF1b:T1404M, ORF6:M1I, S:T478K, S:D614G, S:P681H, S:T732A,	
hCoV-19/Mexico/CMX-INMEGEN-02-11-02/2021	EPI_ISL_1117354	20B	B.1.1.519	29	5'UTR:T201C, 5'UTR:C203T, 5'UTR:C222T, 5'UTR:C241T, ORF1ab:C1710T, ORF1ab:G1738T, ORF1ab:C3037T, ORF1ab:C3140T, ORF1ab:C5869T, ORF1ab:C10029T, ORF1ab:C10954T, ORF1ab:A11117G, ORF1ab:C12789T, ORF1ab:C14408T, ORF1ab:C14724T, ORF1ab:A17192C, ORF1ab:T19839C, ORF1ab:A19974G, ORF1ab:C21306T, S:C22995A, S:A23403G, S:C23604A, S:A23756G, N:A28742G, N:G28881A, N:G28882A, N:G28883C, N:C29197T, N:C29250T, ORF10:C29627T,	15	N:I157V, N:R203K, N:G204R, N:P326L, ORF1a:A482V, ORF1a:P959S, ORF1a:T3255I, ORF1a:I3618V, ORF1a:T4175I, ORF1b:P314L, ORF1b:E1242A, S:T478K, S:D614G, S:P681H, S:T732A,	

hCoV-19/Mexico/CMX-INMEGEN-02-11-03/2021	EPI_ISL_1117355	20B	B.1.1.519	29	5'UTR:T201C, 5'UTR:C203T, 5'UTR:C222T, 5'UTR:C241T, ORF1ab:C1710T, ORF1ab:G1738T, ORF1ab:C3037T, ORF1ab:C3140T, ORF1ab:C5869T, ORF1ab:C10029T, ORF1ab:C10954T, ORF1ab:A11117G, ORF1ab:C12789T, ORF1ab:C14408T, ORF1ab:C14724T, ORF1ab:A17192C, ORF1ab:T19839C, ORF1ab:A19974G, ORF1ab:C21306T, S:C22995A, S:A23403G, S:C23604A, S:A23756G, N:A28742G, N:G28881A, N:G28882A, N:G28883C, N:C29197T, N:C29250T, ORF10:C29627T,	15	N:I157V, N:R203K, N:G204R, N:P326L, ORF1a:A482V, ORF1a:P959S, ORF1a:T3255I, ORF1a:I3618V, ORF1a:T4175I, ORF1b:P314L, ORF1b:E1242A, S:T478K, S:D614G, S:P681H, S:T732A,	
hCoV-19/Mexico/CMX-INMEGEN-02-11-04/2021	EPI_ISL_1117356	20B	B.1.1.519	24	5'UTR:C203T, 5'UTR:C222T, 5'UTR:C241T, ORF1ab:C1404T, ORF1ab:C1968T, ORF1ab:C3037T, ORF1ab:C3140T, ORF1ab:T3745C, ORF1ab:C10029T, ORF1ab:C10036T, ORF1ab:C10954T, ORF1ab:A11117G, ORF1ab:C12789T, ORF1ab:C14408T, ORF1ab:T19839C, ORF1ab:C21306T, S:C22995A, S:A23403G, S:C23604A, S:A23756G, ORF3a:C25844T, N:G28881A, N:G28882A, N:G28883C, N:C29197T,	14	N:R203K, N:G204R, ORF1a:P380L, ORF1a:T568I, ORF1a:P959S, ORF1a:T3255I, ORF1a:I3618V, ORF1a:T4175I, ORF1b:P314L, ORF3a:T151I, S:T478K, S:D614G, S:P681H, S:T732A,	
hCoV-19/Mexico/CMX-INMEGEN-02-11-05/2021	EPI_ISL_1117357	20B	B.1.1.222	24	5'UTR:C241T, ORF1ab:C335T, ORF1ab:C3037T, ORF1ab:T8053C, ORF1ab:C8668T, ORF1ab:C9120T, ORF1ab:C9615T, ORF1ab:C14408T, ORF1ab:C14802T, ORF1ab:C15540T, ORF1ab:G18677A, ORF1ab:T19839C, ORF1ab:C21178T, ORF1ab:G21334T, S:A23403G, S:C23718G, S:A23756G, ORF3a:G25906T, ORF7a:C27741T, ORF8:C28253T, N:G28881A, N:G28882A, N:G28883C, N:G29260T, 3'UTR:C29686T,	13	N:R203K, N:G204R, ORF1a:R24C, ORF1a:T2952I, ORF1a:T3117I, ORF1b:P314L, ORF1b:R1737H, ORF1b:H2571Y, ORF1b:A2623S, ORF3a:G172C, S:D614G, S:T719S, S:T732A,	
hCoV-19/Mexico/CMX-INMEGEN-02-11-06/2021	EPI_ISL_1117358	20B	B.1.1.222	19	5'UTR:C241T, ORF1ab:C1346A, ORF1ab:C1884T, ORF1ab:C3037T, ORF1ab:C4824T, ORF1ab:C7749T, ORF1ab:C8637T, ORF1ab:A12605G, ORF1ab:A13498G, ORF1ab:C14408T, ORF1ab:T19839C, S:G22331A, S:A23403G, S:A23756G, ORF3a:C26042T, M:C26681T, N:G28881A, N:G28882A, N:G28883C, N:G28907A,	15	N:R203K, N:G204R, N:G212S, ORF1a:P361T, ORF1a:A540V, ORF1a:S1520F, ORF1a:T2495I, ORF1a:T2791I, ORF1a:I4114V, ORF1b:T11A, ORF1b:P314L, ORF3a:T217I, S:G257S, S:D614G, S:T732A,	
hCoV-19/Mexico/CMX-INMEGEN-02-11-07/2021	EPI_ISL_1117359	20B	B.1.1.519	24	5'UTR:C203T, 5'UTR:C222T, 5'UTR:C241T, ORF1ab:C3037T, ORF1ab:C3140T, ORF1ab:C10029T, ORF1ab:C10954T, ORF1ab:A11117G, ORF1ab:C12789T, ORF1ab:C14408T, ORF1ab:G15921T, ORF1ab:T19839C, ORF1ab:T20664C, ORF1ab:C21306T, S:C22227T, S:C22995A, S:A23403G, S:C23604A, S:A23756G, ORF8:T27977C, N:G28881A, N:G28882A, N:G28883C, N:C29197T, N:G29527A,	12	N:R203K, N:G204R, ORF1a:P959S, ORF1a:T3255I, ORF1a:I3618V, ORF1a:T4175I, ORF1b:P314L, S:A222V, S:T478K, S:D614G, S:P681H, S:T732A,	
hCoV-19/Mexico/CMX-INMEGEN-02-11-08/2021	EPI_ISL_1117360	20B	B.1.1.519	21	5'UTR:C222T, 5'UTR:C241T, ORF1ab:C3037T, ORF1ab:C3140T, ORF1ab:G3468A, ORF1ab:C10029T, ORF1ab:C10954T, ORF1ab:A11117G, ORF1ab:C12789T, ORF1ab:C14408T, ORF1ab:T19839C, ORF1ab:C21306T, S:C22747T, S:C22995A, S:A23403G, S:C23604A, S:A23756G, ORF3a:C26111T, N:G28881A, N:G28882A, N:G28883C, N:C29197T,	13	N:R203K, N:G204R, ORF1a:P959S, ORF1a:G1068E, ORF1a:T3255I, ORF1a:I3618V, ORF1a:T4175I, ORF1b:P314L, ORF3a:P240L, S:T478K, S:D614G, S:P681H, S:T732A,	5'UTR:203,

hCoV-19/Mexico/CMX-INMEGEN-02-11-09/2021	EPI_ISL_1117361	20B	B.1.1.519	24	5'UTR:T201C, 5'UTR:C203T, 5'UTR:C222T, 5'UTR:C241T, ORF1ab:G1738T, ORF1ab:C3037T, ORF1ab:C3140T, ORF1ab:G5665A, ORF1ab:C10029T, ORF1ab:C10954T, ORF1ab:A11117G, ORF1ab:C12789T, ORF1ab:C14408T, ORF1ab:T19839C, ORF1ab:A19974G, ORF1ab:C21306T, S:C22995A, S:A23403G, S:C23604A, S:A23756G, N:G28881A, N:G28882A, N:G28883C, N:C29197T, ORF10:C29627T,	11	N:R203K, N:G204R, ORF1a:P959S, ORF1a:T3255I, ORF1a:I3618V, ORF1a:T4175I, ORF1b:P314L, S:T478K, S:D614G, S:P681H, S:T732A,	
hCoV-19/Mexico/CMX-INMEGEN-02-11-10/2021	EPI_ISL_1117362	20B	B.1.1.519	27	5'UTR:T201C, 5'UTR:C203T, 5'UTR:C222T, 5'UTR:C241T, ORF1ab:C745T, ORF1ab:G1738T, ORF1ab:C3037T, ORF1ab:C3140T, ORF1ab:C10029T, ORF1ab:C10954T, ORF1ab:A11117G, ORF1ab:C12789T, ORF1ab:C14408T, ORF1ab:T19839C, ORF1ab:A19974G, ORF1ab:C21306T, S:C22995A, S:C23248T, S:A23403G, S:C23604A, S:A23756G, S:G25311T, ORF8:G28077T, N:G28881A, N:G28882A, N:G28883C, N:G28895T, N:C29197T,	14	N:R203K, N:G204R, N:A208S, ORF1a:P959S, ORF1a:T3255I, ORF1a:I3618V, ORF1a:T4175I, ORF1b:P314L, ORF8:V62L, S:T478K, S:D614G, S:P681H, S:T732A, S:C1250F,	
hCoV-19/Mexico/CMX-INMEGEN-02-11-11/2021	EPI_ISL_1117363	20B	B.1.1.519	24	5'UTR:C203T, 5'UTR:C222T, 5'UTR:C241T, ORF1ab:C3037T, ORF1ab:C3140T, ORF1ab:T3745C, ORF1ab:A6390G, ORF1ab:C10029T, ORF1ab:C10954T, ORF1ab:A11117G, ORF1ab:C12789T, ORF1ab:C14408T, ORF1ab:C17862T, ORF1ab:C18555T, ORF1ab:T19839C, ORF1ab:C21306T, S:C22995A, S:A23403G, S:C23604A, S:A23756G, ORF3a:C25844T, N:G28881A, N:G28882A, N:G28883C, N:C29197T,	13	N:R203K, N:G204R, ORF1a:P959S, ORF1a:E2042G, ORF1a:T3255I, ORF1a:I3618V, ORF1a:T4175I, ORF1b:P314L, ORF3a:T151I, S:T478K, S:D614G, S:P681H, S:T732A,	
hCoV-19/Mexico/CMX-INMEGEN-02-11-12/2021	EPI_ISL_1117364	20B	B.1.1.222	25	5'UTR:C241T, ORF1ab:C3037T, ORF1ab:T5338C, ORF1ab:A6693G, ORF1ab:T7402C, ORF1ab:C9430T, ORF1ab:C9474T, ORF1ab:C10029T, ORF1ab:C10078T, ORF1ab:G11930A, ORF1ab:C14408T, ORF1ab:T19839C, S:A23403G, S:A23756G, ORF3a:G25912T, M:C27145T, ORF6:C27389T, ORF7b:A27756G, ORF8:A27921G, ORF8:G28001T, N:C28708T, N:G28881A, N:G28882A, N:G28883C, N:T29458C, N:C29503T,	13	M:T208I, N:R203K, N:G204R, ORF1a:K2143R, ORF1a:A3070V, ORF1a:T3255I, ORF1a:A3889T, ORF1b:P314L, ORF3a:G174C, ORF7b:M1V, ORF8:I10V, S:D614G, S:T732A,	ORF1ab: 518-520,
hCoV-19/Mexico/CMX-INMEGEN-02-11-13/2021	EPI_ISL_1117365	20B	B.1.1.519	26	5'UTR:C203T, 5'UTR:C222T, 5'UTR:C241T, ORF1ab:C2675T, ORF1ab:C3037T, ORF1ab:C3140T, ORF1ab:C10029T, ORF1ab:C10954T, ORF1ab:A11117G, ORF1ab:C12789T, ORF1ab:C14408T, ORF1ab:T17995C, ORF1ab:T19839C, ORF1ab:C21306T, S:T22328C, S:C22995A, S:A23403G, S:C23604A, S:A23756G, M:C26882T, ORF8:T27904C, N:G28881A, N:G28882A, N:G28883C, N:G29162C, N:C29197T, 3'UTR:G29825T,	16	N:R203K, N:G204R, N:D297H, ORF1a:P804S, ORF1a:P959S, ORF1a:T3255I, ORF1a:I3618V, ORF1a:T4175I, ORF1b:P314L, ORF1b:F1510L, ORF8:L4P, S:S256P, S:T478K, S:D614G, S:P681H, S:T732A,	
hCoV-19/Mexico/CMX-INMEGEN-02-11-14/2021	EPI_ISL_1117366	20A	B.1.241	24	5'UTR:C241T, ORF1ab:C337T, ORF1ab:C3037T, ORF1ab:C7768T, ORF1ab:A8285G, ORF1ab:C10029T, ORF1ab:C11173T, ORF1ab:G11521T, ORF1ab:C11575T, ORF1ab:C14408T, ORF1ab:C18747T, ORF1ab:C19290T, ORF1ab:A20055G, ORF1ab:A20268G, S:A23403G, S:G23593T, S:C24337T, ORF3a:C25693T, ORF3a:C26125T, ORF7a:C27707T, ORF8:A28271T, N:C28550A, N:C28854T, N:C29375T, N:C29421T,	13	N:S194L, N:P368S, N:P383L, ORF1a:M2674V, ORF1a:T3255I, ORF1a:M3752I, ORF1b:P314L, ORF3a:L101F, ORF3a:Q245*, ORF7a:A105V, ORF9b:D89E, S:D614G, S:Q677H,	ORF1ab: 11288-11296, ORF8:28242-28253,

hCoV-19/Mexico/CMX-INMEGEN-02-11-15/2021	EPI_ISL_1117367	20B	B.1.1.519	25	5'UTR:C203T, 5'UTR:C222T, 5'UTR:C241T, ORF1ab:C3037T, ORF1ab:C3140T, ORF1ab:T3745C, ORF1ab:C10029T, ORF1ab:C10954T, ORF1ab:A11117G, ORF1ab:C12789T, ORF1ab:A13992G, ORF1ab:C14408T, ORF1ab:T19839C, ORF1ab:C21306T, S:C22995A, S:A23403G, S:C23604A, S:A23756G, S:C24904T, ORF3a:C25844T, ORF3a:G25922T, ORF3a:C26028T, N:G28881A, N:G28882A, N:G28883C, N:C29197T,	13	N:R203K, N:G204R, ORF1a:P959S, ORF1a:T3255I, ORF1a:I3618V, ORF1a:T4175I, ORF1b:P314L, ORF3a:T151I, ORF3a:S177I, S:T478K, S:D614G, S:P681H, S:T732A,	
hCoV-19/Mexico/CMX-INMEGEN-02-11-16/2021	EPI_ISL_1117368	20B	B.1.1.519	25	5'UTR:C203T, 5'UTR:C222T, 5'UTR:C241T, ORF1ab:C3037T, ORF1ab:C3140T, ORF1ab:C10029T, ORF1ab:C10954T, ORF1ab:A11117G, ORF1ab:C12789T, ORF1ab:C14408T, ORF1ab:C15081T, ORF1ab:T19839C, ORF1ab:C21306T, S:C22995A, S:A23403G, S:C23604A, S:A23756G, S:A24727G, S:C25318T, ORF8:T27904C, ORF8:C28087T, ORF8:A28129G, N:G28881A, N:G28882A, N:G28883C, N:C29197T,	14	N:R203K, N:G204R, ORF1a:P959S, ORF1a:T3255I, ORF1a:I3618V, ORF1a:T4175I, ORF1b:P314L, ORF8:L4P, ORF8:A65V, ORF8:Y79C, S:T478K, S:D614G, S:P681H, S:T732A,	
hCoV-19/Mexico/CMX-INMEGEN-02-11-17/2021	EPI_ISL_1117369	20B	B.1.1.519	25	5'UTR:C203T, 5'UTR:C222T, 5'UTR:C241T, ORF1ab:C3037T, ORF1ab:C3140T, ORF1ab:C10029T, ORF1ab:C10954T, ORF1ab:A11117G, ORF1ab:C12789T, ORF1ab:C14408T, ORF1ab:C15081T, ORF1ab:T19839C, ORF1ab:C21306T, S:C22995A, S:A23403G, S:C23604A, S:A23756G, S:A24727G, S:C25318T, ORF8:T27904C, ORF8:C28087T, ORF8:A28129G, N:G28881A, N:G28882A, N:G28883C, N:C29197T,	14	N:R203K, N:G204R, ORF1a:P959S, ORF1a:T3255I, ORF1a:I3618V, ORF1a:T4175I, ORF1b:P314L, ORF8:L4P, ORF8:A65V, ORF8:Y79C, S:T478K, S:D614G, S:P681H, S:T732A,	
hCoV-19/Mexico/CMX-INMEGEN-02-11-18/2021	EPI_ISL_1117370	20B	B.1.1.519	26	5'UTR:T201C, 5'UTR:C203T, 5'UTR:C222T, 5'UTR:C241T, ORF1ab:C745T, ORF1ab:G1738T, ORF1ab:A2903G, ORF1ab:C3037T, ORF1ab:C3140T, ORF1ab:C9893T, ORF1ab:C10029T, ORF1ab:C10954T, ORF1ab:A11117G, ORF1ab:C12789T, ORF1ab:C14408T, ORF1ab:T19839C, ORF1ab:A19974G, ORF1ab:C21306T, S:C21575T, S:C22995A, S:A23403G, S:C23604A, S:A23756G, N:G28881A, N:G28882A, N:G28883C, N:C29197T,	14	N:R203K, N:G204R, ORF1a:I880V, ORF1a:P959S, ORF1a:L3210F, ORF1a:T3255I, ORF1a:I3618V, ORF1a:T4175I, ORF1b:P314L, S:L5F, S:T478K, S:D614G, S:P681H, S:T732A,	
hCoV-19/Mexico/CMX-INMEGEN-02-11-19/2021	EPI_ISL_1117371	20B	B.1.1.519	25	5'UTR:T201C, 5'UTR:C203T, 5'UTR:C222T, 5'UTR:C241T, ORF1ab:C936T, ORF1ab:G1738T, ORF1ab:C3037T, ORF1ab:C3140T, ORF1ab:C10029T, ORF1ab:C10954T, ORF1ab:A11117G, ORF1ab:C12789T, ORF1ab:C14408T, ORF1ab:T19839C, ORF1ab:A19974G, ORF1ab:C21306T, S:C22995A, S:A23403G, S:C23604A, S:A23756G, ORF6:C27294T, N:G28881A, N:G28882A, N:G28883C, N:C29197T, N:G29227T,	13	N:R203K, N:G204R, ORF1a:M85V, ORF1a:T224I, ORF1a:P959S, ORF1a:T3255I, ORF1a:I3618V, ORF1a:T4175I, ORF1b:P314L, S:T478K, S:D614G, S:P681H, S:T732A,	ORF1ab: 510-518,
hCoV-19/Mexico/CMX-INMEGEN-02-11-20/2021	EPI_ISL_1117372	20B	B.1.1.519	23	5'UTR:C203T, 5'UTR:C222T, 5'UTR:C241T, ORF1ab:C3037T, ORF1ab:C3140T, ORF1ab:C4276T, ORF1ab:C10029T, ORF1ab:C10954T, ORF1ab:A11117G, ORF1ab:C12789T, ORF1ab:C14408T, ORF1ab:T19839C, ORF1ab:C21306T, S:C21742T, S:C22995A, S:A23403G, S:C23604A, S:A23756G, ORF3a:G26020T, M:A26528T, N:G28881A, N:G28882A, N:G28883C, N:C29197T,	12	N:R203K, N:G204R, ORF1a:P959S, ORF1a:T3255I, ORF1a:I3618V, ORF1a:T4175I, ORF1b:P314L, ORF3a:D210Y, S:T478K, S:D614G, S:P681H, S:T732A,	

hCoV-19/Mexico/CMX-INMEGEN-02-11-21/2021	EPI_ISL_1117373	20A	B.1.243	19	5'UTR:C241T, ORF1ab:C274T, ORF1ab:G806A, ORF1ab:T2584A, ORF1ab:C3037T, ORF1ab:C3874T, ORF1ab:C5140A, ORF1ab:C6027T, ORF1ab:C14408T, ORF1ab:C18486T, ORF1ab:A20268G, S:G23120T, S:A23403G, S:G23587C, S:T24076C, ORF3a:C25549T, E:C26455T, N:C28854T, N:G29254A, N:G29543T,	10	E:P71S, N:S194L, ORF1a:A181T, ORF1a:D1625E, ORF1a:P1921L, ORF1b:P314L, ORF3a:L53F, S:A520S, S:D614G, S:Q675H,	
hCoV-19/Mexico/CMX-INMEGEN-02-11-22/2021	EPI_ISL_1117374	20B	B.1.1.519	22	5'UTR:C203T, 5'UTR:C222T, 5'UTR:C241T, ORF1ab:C3037T, ORF1ab:C3140T, ORF1ab:C10029T, ORF1ab:C10954T, ORF1ab:A11117G, ORF1ab:C12789T, ORF1ab:C14408T, ORF1ab:T19839C, ORF1ab:A20112T, ORF1ab:C21306T, S:C22995A, S:A23403G, S:C23604A, S:A23756G, N:G28881A, N:G28882A, N:G28883C, N:C29095T, N:C29197T, 3'UTR:C29738T,	11	N:R203K, N:G204R, ORF1a:P959S, ORF1a:T3255I, ORF1a:I3618V, ORF1a:T4175I, ORF1b:P314L, S:T478K, S:D614G, S:P681H, S:T732A,	
hCoV-19/Mexico/CMX-INMEGEN-02-11-23/2021	EPI_ISL_1117375	20B	B.1.1.519	27	5'UTR:C203T, 5'UTR:C222T, 5'UTR:C241T, ORF1ab:C3037T, ORF1ab:C3140T, ORF1ab:A4485G, ORF1ab:C6541T, ORF1ab:C9348T, ORF1ab:C10029T, ORF1ab:C10954T, ORF1ab:A11117G, ORF1ab:C12789T, ORF1ab:C14408T, ORF1ab:T19839C, ORF1ab:C21306T, S:A22023C, S:C22995A, S:C23029T, S:A23403G, S:C23604A, S:A23756G, M:C26681T, ORF8:T27904C, N:G28881A, N:G28882A, N:G28883C, N:C29197T, ORF10:C29659T,	15	N:R203K, N:G204R, ORF1a:P959S, ORF1a:K1407R, ORF1a:T3028I, ORF1a:T3255I, ORF1a:I3618V, ORF1a:T4175I, ORF1b:P314L, ORF8:L4P, S:E154A, S:T478K, S:D614G, S:P681H, S:T732A,	
hCoV-19/Mexico/CMX-INMEGEN-02-12-01/2021	EPI_ISL_1120878	20B	B.1.1.519	25	5'UTR:C203T, 5'UTR:C222T, 5'UTR:C241T, ORF1ab:C3037T, ORF1ab:C3140T, ORF1ab:C10029T, ORF1ab:C10954T, ORF1ab:A11117G, ORF1ab:C12789T, ORF1ab:A12841G, ORF1ab:C14408T, ORF1ab:G15921T, ORF1ab:A17971G, ORF1ab:T19839C, ORF1ab:C21306T, S:A22034G, S:C22082T, S:C22995A, S:A23403G, S:C23604A, S:C23635T, S:A23756G, N:G28881A, N:G28882A, N:G28883C, N:C29197T,	14	N:R203K, N:G204R, ORF1a:P959S, ORF1a:T3255I, ORF1a:I3618V, ORF1a:T4175I, ORF1b:P314L, ORF1b:R1502G, S:R158G, S:P174S, S:T478K, S:D614G, S:P681H, S:T732A,	
hCoV-19/Mexico/CMX-INMEGEN-02-12-02/2021	EPI_ISL_1120879	20B	B.1.1.519	24	5'UTR:C203T, 5'UTR:C222T, 5'UTR:C241T, ORF1ab:C1912T, ORF1ab:C3037T, ORF1ab:C3140T, ORF1ab:A3291G, ORF1ab:C10029T, ORF1ab:C10954T, ORF1ab:A11117G, ORF1ab:C12789T, ORF1ab:C14408T, ORF1ab:T19839C, ORF1ab:C21077T, ORF1ab:C21306T, S:C22995A, S:A23403G, S:C23604A, S:A23756G, ORF3a:G25906T, ORF8:C28253T, N:G28881A, N:G28882A, N:G28883C, N:C29197T,	14	N:R203K, N:G204R, ORF1a:P959S, ORF1a:Q1009R, ORF1a:T3255I, ORF1a:I3618V, ORF1a:T4175I, ORF1b:P314L, ORF1b:T2537I, ORF3a:G172C, S:T478K, S:D614G, S:P681H, S:T732A,	
hCoV-19/Mexico/CMX-INMEGEN-02-12-03/2021	EPI_ISL_1120880	20A	B.1.551	15	5'UTR:C241T, ORF1ab:C3037T, ORF1ab:C3738T, ORF1ab:C5140T, ORF1ab:C6840T, ORF1ab:T7717A, ORF1ab:C10377T, ORF1ab:C14408T, ORF1ab:C15546T, ORF1ab:C16733T, ORF1ab:A20268G, S:A23403G, S:C23604G, ORF3a:G25522A, N:C28854T, N:C29466T,	10	N:S194L, N:A398V, ORF1a:P1158L, ORF1a:A2192V, ORF1a:P3371L, ORF1b:P314L, ORF1b:S1089L, ORF3a:G44R, S:D614G, S:P681R,	3'UTR:29738-29748,
hCoV-19/Mexico/CMX-INMEGEN-02-12-04/2021	EPI_ISL_1120881	20B	B.1.1.519	27	5'UTR:C203T, 5'UTR:C222T, 5'UTR:C241T, ORF1ab:A866G, ORF1ab:C1009T, ORF1ab:C3037T, ORF1ab:C3140T, ORF1ab:C5183T, ORF1ab:C10029T, ORF1ab:C10954T, ORF1ab:A11117G, ORF1ab:C11916T, ORF1ab:C12789T, ORF1ab:C14408T, ORF1ab:G17608T, ORF1ab:T19839C, ORF1ab:C21306T, S:C22995A, S:A23403G, S:C23604A, S:A23756G, ORF8:T27904C, ORF8:C28054T, N:G28747T, N:G28881A, N:G28882A, N:G28883C, N:C29197T,	17	N:R203K, N:G204R, ORF1a:I201V, ORF1a:P959S, ORF1a:P1640S, ORF1a:T3255I, ORF1a:I3618V, ORF1a:S3884L, ORF1a:T4175I, ORF1b:P314L, ORF1b:D1381Y, ORF8:L4P, ORF8:S54L, S:T478K, S:D614G, S:P681H, S:T732A,	

hCoV-19/Mexico/CMX-INMEGEN-02-12-05/2021	EPI_ISL_1120882	20B	B.1.1.519	23	5'UTR:C203T, 5'UTR:C222T, 5'UTR:C241T, ORF1ab:G529T, ORF1ab:C3037T, ORF1ab:C3140T, ORF1ab:G5044A, ORF1ab:C10029T, ORF1ab:C10954T, ORF1ab:A11117G, ORF1ab:C12789T, ORF1ab:C14408T, ORF1ab:T17009C, ORF1ab:T19839C, ORF1ab:C21306T, S:C22995A, S:A23403G, S:C23604A, S:A23756G, N:G28881A, N:G28882A, N:G28883C, N:C29197T, N:G29260T,	12	N:R203K, N:G204R, ORF1a:P959S, ORF1a:T3255I, ORF1a:I3618V, ORF1a:T4175I, ORF1b:P314L, ORF1b:I1181T, S:T478K, S:D614G, S:P681H, S:T732A,
hCoV-19/Mexico/CMX-INMEGEN-02-12-06/2021	EPI_ISL_1120883	20B	B.1.1.519	26	5'UTR:C203T, 5'UTR:C222T, 5'UTR:C241T, ORF1ab:C3037T, ORF1ab:C3140T, ORF1ab:G3692T, ORF1ab:G3871T, ORF1ab:C4158T, ORF1ab:C9520T, ORF1ab:C10029T, ORF1ab:C10954T, ORF1ab:A11117G, ORF1ab:C11824A, ORF1ab:C12789T, ORF1ab:C14408T, ORF1ab:C18747T, ORF1ab:T19839C, ORF1ab:C21306T, S:C22995A, S:A23403G, S:C23604A, S:A23756G, ORF8:T27904C, N:G28881A, N:G28882A, N:G28883C, N:C29197T,	15	N:R203K, N:G204R, ORF1a:P959S, ORF1a:V1143F, ORF1a:K1202N, ORF1a:A1298V, ORF1a:T3255I, ORF1a:I3618V, ORF1a:T4175I, ORF1b:P314L, ORF8:L4P, S:T478K, S:D614G, S:P681H, S:T732A,
hCoV-19/Mexico/CMX-INMEGEN-02-12-07/2021	EPI_ISL_1120884	20B	B.1.1.519	24	5'UTR:C203T, 5'UTR:C222T, 5'UTR:C241T, ORF1ab:C3037T, ORF1ab:C3140T, ORF1ab:C10029T, ORF1ab:C10954T, ORF1ab:A11117G, ORF1ab:C12789T, ORF1ab:C14408T, ORF1ab:T19839C, ORF1ab:C21077T, ORF1ab:C21306T, S:C22995A, S:A23403G, S:G23545T, S:C23604A, S:A23756G, ORF3a:G25906T, ORF8:C28253T, N:G28881A, N:G28882A, N:G28883C, N:C29197T, 3'UTR:G29766T,	14	N:R203K, N:G204R, ORF1a:P959S, ORF1a:T3255I, ORF1a:I3618V, ORF1a:T4175I, ORF1b:P314L, ORF1b:T2537I, ORF3a:G172C, S:T478K, S:D614G, S:E661D, S:P681H, S:T732A,
hCoV-19/Mexico/CMX-INMEGEN-02-12-08/2021	EPI_ISL_1120885	20B	B.1.1.519	23	5'UTR:C203T, 5'UTR:C222T, 5'UTR:C241T, ORF1ab:C3037T, ORF1ab:C3140T, ORF1ab:C10029T, ORF1ab:T10076C, ORF1ab:C10954T, ORF1ab:A11117G, ORF1ab:C12789T, ORF1ab:C14408T, ORF1ab:G15921T, ORF1ab:C16111T, ORF1ab:T19839C, ORF1ab:C21306T, ORF1ab:G21468T, S:C22995A, S:A23403G, S:C23604A, S:A23756G, N:G28881A, N:G28882A, N:G28883C, N:C29197T,	13	N:R203K, N:G204R, ORF1a:P959S, ORF1a:T3255I, ORF1a:F3271L, ORF1a:I3618V, ORF1a:T4175I, ORF1b:P314L, ORF1b:M2667I, S:T478K, S:D614G, S:P681H, S:T732A,
hCoV-19/Mexico/CMX-INMEGEN-02-12-09/2021	EPI_ISL_1120886	20B	B.1.1.519	25	5'UTR:C162T, 5'UTR:C203T, 5'UTR:C222T, 5'UTR:C241T, ORF1ab:C466T, ORF1ab:C676T, ORF1ab:C3037T, ORF1ab:C3140T, ORF1ab:C5869T, ORF1ab:C10029T, ORF1ab:C10954T, ORF1ab:A11117G, ORF1ab:C12789T, ORF1ab:C14408T, ORF1ab:T19839C, ORF1ab:C21306T, S:C22995A, S:A23403G, S:C23604A, S:A23756G, ORF8:T27904C, ORF8:C28087T, N:G28881A, N:G28882A, N:G28883C, N:C29197T,	13	N:R203K, N:G204R, ORF1a:P959S, ORF1a:T3255I, ORF1a:I3618V, ORF1a:T4175I, ORF1b:P314L, ORF8:L4P, ORF8:A65V, S:T478K, S:D614G, S:P681H, S:T732A,
hCoV-19/Mexico/CMX-INMEGEN-02-12-10/2021	EPI_ISL_1120887	20B	B.1.1.519	28	5'UTR:C203T, 5'UTR:C222T, 5'UTR:C241T, ORF1ab:A866G, ORF1ab:C1009T, ORF1ab:C3037T, ORF1ab:C3140T, ORF1ab:G3860A, ORF1ab:C5183T, ORF1ab:C10029T, ORF1ab:C10954T, ORF1ab:A11117G, ORF1ab:C11916T, ORF1ab:G12223T, ORF1ab:C12789T, ORF1ab:C14408T, ORF1ab:T19839C, ORF1ab:C21306T, S:C21595T, S:C22995A, S:A23403G, S:C23604A, S:A23756G, ORF8:T27904C, ORF8:A28165C, N:G28881A, N:G28882A, N:G28883C, N:C29197T,	17	N:R203K, N:G204R, ORF1a:I201V, ORF1a:P959S, ORF1a:V1199I, ORF1a:P1640S, ORF1a:T3255I, ORF1a:I3618V, ORF1a:S3884L, ORF1a:T4175I, ORF1b:P314L, ORF8:L4P, ORF8:Q91P, S:T478K, S:D614G, S:P681H, S:T732A,

hCoV-19/Mexico/CMX-INMEGEN-02-12-11/2021	EPI_ISL_1120888	20B	B.1.1.519	25	5'UTR:T201C, 5'UTR:C203T, 5'UTR:C222T, 5'UTR:C241T, ORF1ab:G1738T, ORF1ab:C3037T, ORF1ab:C3140T, ORF1ab:C10029T, ORF1ab:G10610A, ORF1ab:C10954T, ORF1ab:A11117G, ORF1ab:C12789T, ORF1ab:C14408T, ORF1ab:T19839C, ORF1ab:A19974G, ORF1ab:C21306T, S:C22995A, S:A23403G, S:C23604A, S:A23756G, ORF3a:C25553T, M:C26885T, N:G28881A, N:G28882A, N:G28883C, N:C29197T,	13	N:R203K, N:G204R, ORF1a:P959S, ORF1a:T3255I, ORF1a:V3449I, ORF1a:I3618V, ORF1a:T4175I, ORF1b:P314L, ORF3a:A54V, S:T478K, S:D614G, S:P681H, S:T732A,	
hCoV-19/Mexico/CMX-INMEGEN-02-12-13/2021	EPI_ISL_1120889	20B	B.1.1.519	27	5'UTR:T201C, 5'UTR:C203T, 5'UTR:C222T, 5'UTR:C241T, ORF1ab:G1738T, ORF1ab:C2462T, ORF1ab:C3037T, ORF1ab:C3140T, ORF1ab:T9160A, ORF1ab:C10029T, ORF1ab:C10954T, ORF1ab:A11117G, ORF1ab:C12789T, ORF1ab:C14408T, ORF1ab:T19839C, ORF1ab:A19974G, ORF1ab:C21306T, S:C22995A, S:A23403G, S:C23604A, S:A23756G, S:T25279C, ORF3a:C25553T, N:G28300T, N:G28881A, N:G28882A, N:G28883C, N:C29197T,	14	N:Q9H, N:R203K, N:G204R, ORF1a:P959S, ORF1a:T3255I, ORF1a:I3618V, ORF1a:T4175I, ORF1b:P314L, ORF3a:A54V, ORF9b:S6I, S:T478K, S:D614G, S:P681H, S:T732A,	
hCoV-19/Mexico/CMX-INMEGEN-02-12-14/2021	EPI_ISL_1133103	20B	B.1.1.519	24	5'UTR:C203T, 5'UTR:C222T, 5'UTR:C241T, ORF1ab:C3037T, ORF1ab:C3140T, ORF1ab:C6690T, ORF1ab:C10029T, ORF1ab:C10954T, ORF1ab:A11117G, ORF1ab:C11575T, ORF1ab:A11600G, ORF1ab:C12789T, ORF1ab:C14408T, ORF1ab:T19839C, ORF1ab:C21306T, S:C22995A, S:A23403G, S:C23604A, S:A23756G, ORF3a:G25996T, M:G26653T, N:G28881A, N:G28882A, N:G28883C, N:C29197T,	15	M:R44M, N:R203K, N:G204R, ORF1a:P959S, ORF1a:A2142V, ORF1a:T3255I, ORF1a:I3618V, ORF1a:I3779V, ORF1a:T4175I, ORF1b:P314L, ORF3a:V202L, S:T478K, S:D614G, S:P681H, S:T732A,	ORF8:28 254,
hCoV-19/Mexico/CMX-INMEGEN-02-12-15/2021	EPI_ISL_1120890	20B	B.1.1.519	25	5'UTR:T201C, 5'UTR:C203T, 5'UTR:C222T, 5'UTR:C241T, ORF1ab:G1738T, ORF1ab:C3037T, ORF1ab:C3140T, ORF1ab:T9172C, ORF1ab:C10029T, ORF1ab:C10954T, ORF1ab:A11117G, ORF1ab:C12789T, ORF1ab:C14408T, ORF1ab:T19839C, ORF1ab:A19974G, ORF1ab:C21306T, S:C22995A, S:A23403G, S:C23520T, S:C23604A, S:A23756G, ORF8:T27988C, N:G28881A, N:G28882A, N:G28883C, N:C29197T,	13	N:R203K, N:G204R, ORF1a:P959S, ORF1a:T3255I, ORF1a:I3618V, ORF1a:T4175I, ORF1b:P314L, ORF8:V32A, S:T478K, S:D614G, S:A653V, S:P681H, S:T732A,	
hCoV-19/Mexico/CMX-INMEGEN-02-12-19/2021	EPI_ISL_1120891	20B	B.1.1.519	25	5'UTR:T201C, 5'UTR:C203T, 5'UTR:C222T, 5'UTR:C241T, ORF1ab:G1738T, ORF1ab:C3037T, ORF1ab:C3140T, ORF1ab:C10029T, ORF1ab:G10610A, ORF1ab:C10954T, ORF1ab:A11117G, ORF1ab:C12789T, ORF1ab:C14408T, ORF1ab:T19839C, ORF1ab:A19974G, ORF1ab:C21306T, S:C22995A, S:A23403G, S:C23604A, S:A23756G, ORF3a:C25553T, M:C26885T, N:G28881A, N:G28882A, N:G28883C, N:C29197T,	13	N:R203K, N:G204R, ORF1a:P959S, ORF1a:T3255I, ORF1a:V3449I, ORF1a:I3618V, ORF1a:T4175I, ORF1b:P314L, ORF3a:A54V, S:T478K, S:D614G, S:P681H, S:T732A,	
hCoV-19/Mexico/CMX-INMEGEN-02-12-20/2021	EPI_ISL_1120892	20B	B.1.1.222	20	5'UTR:C241T, ORF1ab:C664T, ORF1ab:A1137G, ORF1ab:A1138T, ORF1ab:C2334T, ORF1ab:C2976T, ORF1ab:C3037T, ORF1ab:T8815C, ORF1ab:A13498G, ORF1ab:C14408T, ORF1ab:C18744T, ORF1ab:G19072T, ORF1ab:T19839C, S:G22331A, S:A23403G, S:A23756G, M:C26964A, N:G28881A, N:G28882A, N:G28883C, 3'UTR:G29798T,	12	M:H148N, N:R203K, N:G204R, ORF1a:K291S, ORF1a:A690V, ORF1a:T904I, ORF1b:T11A, ORF1b:P314L, ORF1b:D1869Y, S:G257S, S:D614G, S:T732A,	

hCoV-19/Mexico/CMX-INMEGEN-02-12-21/2021	EPI_ISL_1120893	20B	B.1.1.519	23	5'UTR:C203T, 5'UTR:C222T, 5'UTR:C241T, ORF1ab:A1740G, ORF1ab:C3037T, ORF1ab:C3140T, ORF1ab:C10029T, ORF1ab:C10954T, ORF1ab:A11117G, ORF1ab:G12769T, ORF1ab:C12789T, ORF1ab:C14408T, ORF1ab:T19839C, ORF1ab:C21306T, S:C22995A, S:A23403G, S:C23604A, S:A23756G, M:G26526A, N:G28881A, N:G28882A, N:G28883C, N:C29197T, N:G29527T,	14	M:A2T, N:R203K, N:G204R, N:Q418H, ORF1a:K492R, ORF1a:P959S, ORF1a:T3255I, ORF1a:I3618V, ORF1a:T4175I, ORF1b:P314L, S:T478K, S:D614G, S:P681H, S:T732A,	
hCoV-19/Mexico/CMX-INMEGEN-02-12-22/2021	EPI_ISL_1120894	20B	B.1.1.519	25	5'UTR:C203T, 5'UTR:A223T, 5'UTR:C241T, ORF1ab:C1377T, ORF1ab:T1971C, ORF1ab:C3037T, ORF1ab:C3140T, ORF1ab:C4406T, ORF1ab:C10029T, ORF1ab:C10954T, ORF1ab:A11117G, ORF1ab:C12789T, ORF1ab:C14408T, ORF1ab:T19839C, ORF1ab:G20632A, ORF1ab:C21306T, S:C22747T, S:C22995A, S:A23403G, S:C23604A, S:A23756G, N:A28761G, N:G28881A, N:G28882A, N:G28883C, N:C29197T,	16	N:Q163R, N:R203K, N:G204R, ORF1a:P371L, ORF1a:I569T, ORF1a:P959S, ORF1a:H1381Y, ORF1a:T3255I, ORF1a:I3618V, ORF1a:T4175I, ORF1b:P314L, ORF1b:V2389I, S:T478K, S:D614G, S:P681H, S:T732A,	5'UTR:2 22,
hCoV-19/Mexico/CMX-INMEGEN-02-12-23/2021	EPI_ISL_1120895	20B	B.1.1.519	27	5'UTR:C203T, 5'UTR:C222T, 5'UTR:C241T, ORF1ab:C3037T, ORF1ab:C3140T, ORF1ab:G3246T, ORF1ab:C10029T, ORF1ab:C10954T, ORF1ab:A11117G, ORF1ab:C12789T, ORF1ab:C14408T, ORF1ab:G16377A, ORF1ab:A18294G, ORF1ab:T19839C, ORF1ab:C21306T, S:C22995A, S:A23403G, S:C23604A, S:A23756G, ORF3a:A25596G, ORF3a:C25771T, E:G26439T, M:C26907T, M:C27143T, N:G28881A, N:G28882A, N:G28883C, N:C29197T	13	N:R203K, N:G204R, ORF1a:P959S, ORF1a:S994I, ORF1a:T3255I, ORF1a:I3618V, ORF1a:T4175I, ORF1b:P314L, ORF3a:L127F, S:T478K, S:D614G, S:P681H, S:T732A,	
hCoV-19/Mexico/CMX-INMEGEN-02-12-24/2021	EPI_ISL_1120896	20B	B.1.1.519	21	5'UTR:C203T, 5'UTR:C222T, 5'UTR:C241T, ORF1ab:C3037T, ORF1ab:C3140T, ORF1ab:T4786C, ORF1ab:C10029T, ORF1ab:C10954T, ORF1ab:A11117G, ORF1ab:C11575T, ORF1ab:C12789T, ORF1ab:C14408T, ORF1ab:T19839C, ORF1ab:C21306T, S:C22995A, S:A23403G, S:C23604A, S:A23756G, N:G28881A, N:G28882A, N:G28883C, N:C29197T,	11	N:R203K, N:G204R, ORF1a:P959S, ORF1a:T3255I, ORF1a:I3618V, ORF1a:T4175I, ORF1b:P314L, S:T478K, S:D614G, S:P681H, S:T732A,	
hCoV-19/Mexico/CMX-INMEGEN-03/2020	EPI_ISL_522874	20B	B.1.1.518	11	5'UTR:C241T, ORF1ab:C3037T, ORF1ab:C14408T, ORF1ab:A16725G, ORF1ab:T18417C, ORF1ab:G18984T, ORF1ab:T19839C, ORF1ab:C20844T, S:A23403G, N:G28881A, N:G28882A, N:G28883C,	5	N:R203K, N:G204R, ORF1b:P314L, ORF1b:M1839I, S:D614G,	
hCoV-19/Mexico/CMX-INMEGEN-03-01-01/2021	EPI_ISL_1168634	20B	B.1.1.519	21	5'UTR:C203T, 5'UTR:C222T, 5'UTR:C241T, ORF1ab:C3037T, ORF1ab:C3140T, ORF1ab:C10029T, ORF1ab:C10165T, ORF1ab:C10954T, ORF1ab:A11117G, ORF1ab:C12789T, ORF1ab:C14408T, ORF1ab:T19839C, ORF1ab:C21306T, S:C22995A, S:A23403G, S:C23604A, S:A23756G, ORF8:C27945T, N:G28881A, N:G28882A, N:G28883C, N:C29197T,	12	N:R203K, N:G204R, ORF1a:P959S, ORF1a:T3255I, ORF1a:I3618V, ORF1a:T4175I, ORF1b:P314L, ORF8:Q18*, S:T478K, S:D614G, S:P681H, S:T732A,	
hCoV-19/Mexico/CMX-INMEGEN-03-01-02/2021	EPI_ISL_1168635	20B	B.1.1.519	26	5'UTR:C203T, 5'UTR:C222T, 5'UTR:C241T, ORF1ab:C3037T, ORF1ab:C3140T, ORF1ab:T3745C, ORF1ab:C10029T, ORF1ab:A10323G, ORF1ab:C10954T, ORF1ab:A11117G, ORF1ab:C12789T, ORF1ab:A13992G, ORF1ab:C14408T, ORF1ab:T19839C, ORF1ab:C21306T, ORF1ab:G21523A, S:C22995A, S:A23403G, S:C23604A, S:A23756G, ORF3a:C25844T, ORF3a:G25922T, ORF3a:C26028T, N:G28881A, N:G28882A, N:G28883C, N:C29197T,	15	N:R203K, N:G204R, ORF1a:P959S, ORF1a:T3255I, ORF1a:K3353R, ORF1a:I3618V, ORF1a:T4175I, ORF1b:P314L, ORF1b:V2686I, ORF3a:T151I, ORF3a:S177I, S:T478K, S:D614G, S:P681H, S:T732A,	

hCoV-19/Mexico/CMX-INMEGEN-03-01-03/2021	EPI_ISL_1168636	20B	B.1.1.519	28	5'UTR:T201C, 5'UTR:C203T, 5'UTR:C222T, 5'UTR:C241T, ORF1ab:G1738T, ORF1ab:C3037T, ORF1ab:C3140T, ORF1ab:C10029T, ORF1ab:C10954T, ORF1ab:A11117G, ORF1ab:C11461T, ORF1ab:C12789T, ORF1ab:C14408T, ORF1ab:C14937A, ORF1ab:A15257G, ORF1ab:A17148G, ORF1ab:T19839C, ORF1ab:A19974G, ORF1ab:C21306T, S:C21575T, S:C22995A, S:A23403G, S:C23604A, S:A23756G, ORF3a:G25440T, N:G28881A, N:G28882A, N:G28883C, N:C29197T	16	N:R203K, N:G204R, ORF1a:P959S, ORF1a:T3255I, ORF1a:I3618V, ORF1a:T4175I, ORF1b:P314L, ORF1b:D490E, ORF1b:Y597C, ORF1b:I1227M, ORF3a:K16N, S:L5F, S:T478K, S:D614G, S:P681H, S:T732A,
hCoV-19/Mexico/CMX-INMEGEN-03-01-04/2021	EPI_ISL_1168637	20B	B.1.1.519	26	5'UTR:C203T, 5'UTR:C222T, 5'UTR:C241T, ORF1ab:A866G, ORF1ab:C1009T, ORF1ab:C3037T, ORF1ab:C3140T, ORF1ab:C5183T, ORF1ab:C10029T, ORF1ab:C10954T, ORF1ab:A11117G, ORF1ab:C11916T, ORF1ab:C12789T, ORF1ab:C14408T, ORF1ab:T19839C, ORF1ab:C21306T, S:C22995A, S:A23403G, S:C23604A, S:A23756G, ORF8:T27904C, ORF8:G28178A, N:G28747T, N:G28881A, N:G28882A, N:G28883C, N:C29197T,	15	N:R203K, N:G204R, ORF1a:I201V, ORF1a:P959S, ORF1a:P1640S, ORF1a:T3255I, ORF1a:I3618V, ORF1a:S3884L, ORF1a:T4175I, ORF1b:P314L, ORF8:L4P, S:T478K, S:D614G, S:P681H, S:T732A,
hCoV-19/Mexico/CMX-INMEGEN-03-01-05/2021	EPI_ISL_1168638	20B	B.1.1.519	24	5'UTR:C203T, 5'UTR:C222T, 5'UTR:C241T, ORF1ab:C3037T, ORF1ab:C3140T, ORF1ab:C5274T, ORF1ab:C10029T, ORF1ab:C10954T, ORF1ab:A11117G, ORF1ab:C12789T, ORF1ab:C14408T, ORF1ab:G17278T, ORF1ab:T18561C, ORF1ab:T19839C, ORF1ab:C21306T, S:C22995A, S:A23403G, S:C23604A, S:A23756G, S:C24919T, N:G28881A, N:G28882A, N:G28883C, N:C29197T, N:G29402T,	14	N:R203K, N:G204R, N:D377Y, ORF1a:P959S, ORF1a:A1670V, ORF1a:T3255I, ORF1a:I3618V, ORF1a:T4175I, ORF1b:P314L, ORF1b:V1271L, S:T478K, S:D614G, S:P681H, S:T732A,
hCoV-19/Mexico/CMX-INMEGEN-03-01-06/2021	EPI_ISL_1168639	20B	B.1.1.519	25	5'UTR:T201C, 5'UTR:C203T, 5'UTR:C222T, 5'UTR:C241T, ORF1ab:G1738T, ORF1ab:C3037T, ORF1ab:C3140T, ORF1ab:C10029T, ORF1ab:C10954T, ORF1ab:A11117G, ORF1ab:C12789T, ORF1ab:C14408T, ORF1ab:T19839C, ORF1ab:C19862T, ORF1ab:A19974G, ORF1ab:C21306T, S:C22995A, S:A23403G, S:C23604A, S:A23756G, S:G24821T, ORF3a:G26152A, N:G28881A, N:G28882A, N:G28883C, N:C29197T,	14	N:R203K, N:G204R, ORF1a:P959S, ORF1a:T3255I, ORF1a:I3618V, ORF1a:T4175I, ORF1b:P314L, ORF1b:A2132V, ORF3a:G254R, S:T478K, S:D614G, S:P681H, S:T732A, S:A1087S,
hCoV-19/Mexico/CMX-INMEGEN-03-01-07/2021	EPI_ISL_1168640	20B	B.1.1.519	25	5'UTR:T201C, 5'UTR:C203T, 5'UTR:C222T, 5'UTR:C241T, ORF1ab:G1738T, ORF1ab:C3037T, ORF1ab:C3140T, ORF1ab:C10029T, ORF1ab:C10954T, ORF1ab:A11117G, ORF1ab:C12789T, ORF1ab:C14408T, ORF1ab:C18526T, ORF1ab:T19839C, ORF1ab:A19974G, ORF1ab:C21306T, S:C22995A, S:A23403G, S:C23604A, S:A23756G, M:C26607T, N:G28881A, N:G28882A, N:G28883C, N:C29197T, 3'UTR:C29738T,	13	M:L29F, N:R203K, N:G204R, ORF1a:P959S, ORF1a:T3255I, ORF1a:I3618V, ORF1a:T4175I, ORF1b:P314L, ORF1b:R1687C, S:T478K, S:D614G, S:P681H, S:T732A,
hCoV-19/Mexico/CMX-INMEGEN-03-01-08/2021	EPI_ISL_1168641	20B	B.1.1.519	22	5'UTR:C203T, 5'UTR:C222T, 5'UTR:C241T, ORF1ab:C3037T, ORF1ab:C3140T, ORF1ab:C10029T, ORF1ab:C10954T, ORF1ab:A11117G, ORF1ab:C12789T, ORF1ab:C14408T, ORF1ab:G18090A, ORF1ab:T19839C, ORF1ab:C21306T, S:C22995A, S:A23403G, S:C23604A, S:A23756G, ORF7b:C27874T, ORF8:G28079T, N:G28881A, N:G28882A, N:G28883C, N:C29197T.	12	N:R203K, N:G204R, ORF1a:P959S, ORF1a:T3255I, ORF1a:I3618V, ORF1a:T4175I, ORF1b:P314L, ORF7b:T40I, S:T478K, S:D614G, S:P681H, S:T732A,

hCoV-19/Mexico/CMX-INMEGEN-03-01-09/2021	EPI_ISL_1168642	20B	B.1.1.519	20	5'UTR:C203T, 5'UTR:C222T, 5'UTR:C241T, ORF1ab:C3037T, ORF1ab:C3140T, ORF1ab:C10029T, ORF1ab:C10954T, ORF1ab:A11117G, ORF1ab:C12789T, ORF1ab:C14408T, ORF1ab:T19839C, ORF1ab:C21306T, S:C22995A, S:A23403G, S:C23604A, S:A23756G, N:G28881A, N:G28882A, N:G28883C, N:C29197T, N:C29555T,	11	N:R203K, N:G204R, ORF1a:P959S, ORF1a:T3255I, ORF1a:I3618V, ORF1a:T4175I, ORF1b:P314L, S:T478K, S:D614G, S:P681H, S:T732A,
hCoV-19/Mexico/CMX-INMEGEN-03-01-10/2021	EPI_ISL_1168643	20B	B.1.1.519	19	5'UTR:C203T, 5'UTR:C222T, 5'UTR:C241T, ORF1ab:C3037T, ORF1ab:C3140T, ORF1ab:C10029T, ORF1ab:C10954T, ORF1ab:A11117G, ORF1ab:C12789T, ORF1ab:C14408T, ORF1ab:T19839C, ORF1ab:C21306T, S:C22995A, S:A23403G, S:C23604A, S:A23756G, N:G28881A, N:G28882A, N:G28883C, N:C29197T,	11	N:R203K, N:G204R, ORF1a:P959S, ORF1a:T3255I, ORF1a:I3618V, ORF1a:T4175I, ORF1b:P314L, S:T478K, S:D614G, S:P681H, S:T732A,
hCoV-19/Mexico/CMX-INMEGEN-03-01-11/2021	EPI_ISL_1168644	20B	B.1.1.519	21	5'UTR:C203T, 5'UTR:C222T, 5'UTR:C241T, ORF1ab:C3037T, ORF1ab:C3140T, ORF1ab:C10029T, ORF1ab:C10954T, ORF1ab:A11117G, ORF1ab:C12789T, ORF1ab:C14408T, ORF1ab:T19839C, ORF1ab:C21306T, S:C22995A, S:A23403G, S:C23604A, S:A23756G, S:G25302T, N:G28881A, N:G28882A, N:G28883C, N:C29197T, N:G29527T,	13	N:R203K, N:G204R, N:Q418H, ORF1a:P959S, ORF1a:T3255I, ORF1a:I3618V, ORF1a:T4175I, ORF1b:P314L, S:T478K, S:D614G, S:P681H, S:T732A, S:C1247F,
hCoV-19/Mexico/CMX-INMEGEN-03-01-12/2021	EPI_ISL_1168645	20B	B.1.1.519	22	5'UTR:T201C, 5'UTR:C203T, 5'UTR:C222T, 5'UTR:C241T, ORF1ab:G1738T, ORF1ab:C3037T, ORF1ab:C3140T, ORF1ab:C10029T, ORF1ab:C10954T, ORF1ab:A11117G, ORF1ab:C12789T, ORF1ab:C14408T, ORF1ab:T19839C, ORF1ab:A19974G, ORF1ab:C21306T, S:C22995A, S:A23403G, S:C23604A, S:A23756G, N:G28881A, N:G28882A, N:G28883C, N:C29197T,	11	N:R203K, N:G204R, ORF1a:P959S, ORF1a:T3255I, ORF1a:I3618V, ORF1a:T4175I, ORF1b:P314L, S:T478K, S:D614G, S:P681H, S:T732A,
hCoV-19/Mexico/CMX-INMEGEN-03-01-17/2021	EPI_ISL_1168646	20B	B.1.1.519	26	5'UTR:C203T, 5'UTR:C222T, 5'UTR:C241T, ORF1ab:C3037T, ORF1ab:C3140T, ORF1ab:G3692T, ORF1ab:G3871T, ORF1ab:A5638G, ORF1ab:C10029T, ORF1ab:C10954T, ORF1ab:A11117G, ORF1ab:C11824A, ORF1ab:C12789T, ORF1ab:C14408T, ORF1ab:G18781A, ORF1ab:T19839C, ORF1ab:C21306T, S:C22995A, S:A23403G, S:C23604A, S:C23673T, S:A23756G, ORF8:T27904C, N:G28881A, N:G28882A, N:G28883C, N:C29197T,	16	N:R203K, N:G204R, ORF1a:P959S, ORF1a:V1143F, ORF1a:K1202N, ORF1a:T3255I, ORF1a:I3618V, ORF1a:T4175I, ORF1b:P314L, ORF1b:G1772S, ORF8:L4P, S:T478K, S:D614G, S:P681H, S:S704L, S:T732A,
hCoV-19/Mexico/CMX-INMEGEN-03-01-18/2021	EPI_ISL_1168647	20B	B.1.1.519	24	5'UTR:T201C, 5'UTR:C203T, 5'UTR:C222T, 5'UTR:C241T, ORF1ab:G1738T, ORF1ab:C3037T, ORF1ab:C3140T, ORF1ab:C3602T, ORF1ab:C7504T, ORF1ab:C10029T, ORF1ab:C10954T, ORF1ab:A11117G, ORF1ab:C12789T, ORF1ab:C14408T, ORF1ab:T19839C, ORF1ab:A19974G, ORF1ab:C21306T, S:C22995A, S:A23403G, S:C23604A, S:A23756G, N:G28881A, N:G28882A, N:G28883C, N:C29197T,	12	N:R203K, N:G204R, ORF1a:P959S, ORF1a:H1113Y, ORF1a:T3255I, ORF1a:I3618V, ORF1a:T4175I, ORF1b:P314L, S:T478K, S:D614G, S:P681H, S:T732A,
hCoV-19/Mexico/CMX-INMEGEN-03-01-19/2021	EPI_ISL_1168648	20B	B.1.1.519	29	5'UTR:C203T, 5'UTR:C222T, 5'UTR:C241T, ORF1ab:C3037T, ORF1ab:C3140T, ORF1ab:G6446T, ORF1ab:C8937T, ORF1ab:C9661T, ORF1ab:C10029T, ORF1ab:C10954T, ORF1ab:A11117G, ORF1ab:G11417T, ORF1ab:C12789T, ORF1ab:A14240G, ORF1ab:C14408T, ORF1ab:G15253A, ORF1ab:A18282G, ORF1ab:C19602T, ORF1ab:T19839C, ORF1ab:C21306T, S:C22995A, S:A23403G, S:C23604A, S:A23756G, ORF3a:G25906T, ORF8:C28253T, N:G28881A, N:G28882A, N:G28883C, N:C29197T,	17	N:R203K, N:G204R, ORF1a:P959S, ORF1a:V2061F, ORF1a:A2891V, ORF1a:T3255I, ORF1a:I3618V, ORF1a:V3718F, ORF1a:T4175I, ORF1b:K258R, ORF1b:P314L, ORF1b:V596I, ORF3a:G172C, S:T478K, S:D614G, S:P681H, S:T732A,

hCoV-19/Mexico/CMX-INMEGEN-03-01-20/2021	EPI_ISL_1168649	20B	B.1.1.519	36	5'UTR:C203T, 5'UTR:C222T, 5'UTR:C241T, ORF1ab:A866G, ORF1ab:C1009T, ORF1ab:G1186A, ORF1ab:C3037T, ORF1ab:C3140T, ORF1ab:C3330T, ORF1ab:C5183T, ORF1ab:C6113T, ORF1ab:C9474T, ORF1ab:C9611T, ORF1ab:C10029T, ORF1ab:C10954T, ORF1ab:A11117G, ORF1ab:C11779A, ORF1ab:C11916T, ORF1ab:G12223T, ORF1ab:C12789T, ORF1ab:C14408T, ORF1ab:T19839C, ORF1ab:A20541G, ORF1ab:C21306T, S:C22995A, S:A23403G, S:C23604A, S:A23756G, S:C24130T, S:C24904T, ORF3a:C25642T, E:T26488C, ORF8:T27904C, N:G28881A, N:G28882A, N:G28883C, N:C29197T	20	N:R203K, N:G204R, ORF1a:I201V, ORF1a:P959S, ORF1a:T1022I, ORF1a:P1640S, ORF1a:P1950S, ORF1a:A3070V, ORF1a:L3116F, ORF1a:T3255I, ORF1a:I3618V, ORF1a:F3838L, ORF1a:S3884L, ORF1a:T4175I, ORF1b:P314L, ORF8:L4P, S:T478K, S:D614G, S:P681H, S:T732A,
hCoV-19/Mexico/CMX-INMEGEN-03-01-21/2021	EPI_ISL_1168650	20B	B.1.1.519	27	5'UTR:T201C, 5'UTR:C203T, 5'UTR:C222T, 5'UTR:C241T, ORF1ab:G1738T, ORF1ab:C3037T, ORF1ab:C3140T, ORF1ab:C6070T, ORF1ab:C7874T, ORF1ab:C10029T, ORF1ab:C10340T, ORF1ab:C10954T, ORF1ab:A11117G, ORF1ab:C12789T, ORF1ab:C14408T, ORF1ab:T19839C, ORF1ab:A19974G, ORF1ab:A21137G, ORF1ab:A21171G, ORF1ab:C21306T, S:C22995A, S:A23403G, S:C23604A, S:A23756G, N:G28881A, N:G28882A, N:G28883C, N:C29197T	15	N:R203K, N:G204R, ORF1a:P959S, ORF1a:P2537S, ORF1a:T3255I, ORF1a:P3359S, ORF1a:I3618V, ORF1a:T4175I, ORF1b:P314L, ORF1b:K2557R, ORF1b:I2568M, S:T478K, S:D614G, S:P681H, S:T732A,
hCoV-19/Mexico/CMX-INMEGEN-03-01-22/2021	EPI_ISL_1168651	20B	B.1.1.519	24	5'UTR:C203T, 5'UTR:C222T, 5'UTR:C241T, ORF1ab:C3037T, ORF1ab:C3140T, ORF1ab:G7027T, ORF1ab:C10029T, ORF1ab:C10954T, ORF1ab:A11117G, ORF1ab:C11620T, ORF1ab:C12789T, ORF1ab:C13883T, ORF1ab:C14408T, ORF1ab:T19839C, ORF1ab:C21306T, S:C22995A, S:A23403G, S:C23604A, S:A23756G, S:C25207T, ORF8:A28211C, N:G28881A, N:G28882A, N:G28883C, N:C29197T,	14	N:R203K, N:G204R, ORF1a:P959S, ORF1a:M2254I, ORF1a:T3255I, ORF1a:I3618V, ORF1a:T4175I, ORF1b:T139I, ORF1b:P314L, ORF8:E106D, S:T478K, S:D614G, S:P681H, S:T732A,
hCoV-19/Mexico/CMX-INMEGEN-03-01-23/2021	EPI_ISL_1168652	20B	B.1.1.519	25	5'UTR:C203T, 5'UTR:C222T, 5'UTR:C241T, ORF1ab:C3037T, ORF1ab:C3140T, ORF1ab:C3884T, ORF1ab:C6040T, ORF1ab:C10029T, ORF1ab:C10954T, ORF1ab:A11117G, ORF1ab:C12789T, ORF1ab:T13593C, ORF1ab:C14408T, ORF1ab:G15921T, ORF1ab:T19839C, ORF1ab:C20825T, ORF1ab:C21306T, S:C22995A, S:A23403G, S:C23604A, S:A23756G, S:G24757T, N:G28881A, N:G28882A, N:G28883C, N:C29197T,	13	N:R203K, N:G204R, ORF1a:P959S, ORF1a:P1207S, ORF1a:T3255I, ORF1a:I3618V, ORF1a:T4175I, ORF1b:P314L, ORF1b:T2453I, S:T478K, S:D614G, S:P681H, S:T732A,
hCoV-19/Mexico/CMX-INMEGEN-03-02-01/2021	EPI_ISL_1181700	20B	B.1.1.519	22	5'UTR:C203T, 5'UTR:C222T, 5'UTR:C241T, ORF1ab:C3037T, ORF1ab:C3140T, ORF1ab:C8092T, ORF1ab:C10029T, ORF1ab:C10954T, ORF1ab:A11117G, ORF1ab:C12789T, ORF1ab:C14408T, ORF1ab:T19839C, ORF1ab:C21306T, S:C22995A, S:A23403G, S:C23604A, S:A23756G, ORF8:T27904C, ORF8:C28087T, N:G28881A, N:G28882A, N:G28883C, N:C29197T,	13	N:R203K, N:G204R, ORF1a:P959S, ORF1a:T3255I, ORF1a:I3618V, ORF1a:T4175I, ORF1b:P314L, ORF8:L4P, ORF8:A65V, S:T478K, S:D614G, S:P681H, S:T732A,
hCoV-19/Mexico/CMX-INMEGEN-03-02-02/2021	EPI_ISL_1181701	20B	B.1.1.519	25	5'UTR:T201C, 5'UTR:C203T, 5'UTR:C222T, 5'UTR:C241T, ORF1ab:G1738T, ORF1ab:C3037T, ORF1ab:C3140T, ORF1ab:C3602T, ORF1ab:C7504T, ORF1ab:C10029T, ORF1ab:A10329G, ORF1ab:C10954T, ORF1ab:A11117G, ORF1ab:C12789T, ORF1ab:C14408T, ORF1ab:T19839C, ORF1ab:A19974G, ORF1ab:C21306T, S:C22995A, S:A23403G, S:C23604A, S:A23756G, N:G28881A, N:G28882A, N:G28883C, N:C29197T,	13	N:R203K, N:G204R, ORF1a:P959S, ORF1a:H1113Y, ORF1a:T3255I, ORF1a:D3355G, ORF1a:I3618V, ORF1a:T4175I, ORF1b:P314L, S:T478K, S:D614G, S:P681H, S:T732A,

hCoV-19/Mexico/CMX-INMEGEN-03-02-04/2021	EPI_ISL_1181702	20B	B.1.1.519	26	5'UTR:C203T, 5'UTR:C222T, 5'UTR:C241T, ORF1ab:G943T, ORF1ab:C3037T, ORF1ab:C3140T, ORF1ab:A3274G, ORF1ab:G3955T, ORF1ab:C5526T, ORF1ab:G5942A, ORF1ab:C10029T, ORF1ab:C10954T, ORF1ab:A11117G, ORF1ab:C12789T, ORF1ab:C14408T, ORF1ab:T19839C, ORF1ab:C21306T, S:C22995A, S:A23403G, S:C23604A, S:A23756G, ORF3a:G25947T, M:G26951C, N:G28881A, N:G28882A, N:G28883C, N:C29197T,	16	N:R203K, N:G204R, ORF1a:R226S, ORF1a:P959S, ORF1a:K1230N, ORF1a:T1754I, ORF1a:D1893N, ORF1a:T3255I, ORF1a:I3618V, ORF1a:T4175I, ORF1b:P314L, ORF3a:Q185H, S:T478K, S:D614G, S:P681H, S:T732A,	
hCoV-19/Mexico/CMX-INMEGEN-03-02-05/2021	EPI_ISL_1181703	20B	B.1.1.519	24	5'UTR:C203T, 5'UTR:C222T, 5'UTR:C241T, ORF1ab:C3037T, ORF1ab:A3090C, ORF1ab:C3140T, ORF1ab:T3745C, ORF1ab:C10029T, ORF1ab:C10954T, ORF1ab:A11117G, ORF1ab:C12789T, ORF1ab:C14408T, ORF1ab:T19839C, ORF1ab:C21306T, S:C22995A, S:A23403G, S:C23604A, S:A23756G, ORF3a:C25844T, E:G26389T, M:C26527T, N:G28881A, N:G28882A, N:G28883C, N:C29197T,	15	E:V49L, M:A2V, N:R203K, N:G204R, ORF1a:E942A, ORF1a:P959S, ORF1a:T3255I, ORF1a:I3618V, ORF1a:T4175I, ORF1b:P314L, ORF3a:T151I, S:T478K, S:D614G, S:P681H, S:T732A,	
hCoV-19/Mexico/CMX-INMEGEN-03-02-06/2021	EPI_ISL_1181704	20B	B.1.1.519	24	5'UTR:T201C, 5'UTR:C203T, 5'UTR:C222T, 5'UTR:C241T, ORF1ab:G1738T, ORF1ab:C3037T, ORF1ab:C3140T, ORF1ab:C10029T, ORF1ab:G10610A, ORF1ab:C10954T, ORF1ab:A11117G, ORF1ab:C12789T, ORF1ab:C14408T, ORF1ab:T19839C, ORF1ab:A19974G, ORF1ab:C21306T, S:C22995A, S:A23403G, S:C23604A, S:A23756G, ORF3a:C25553T, N:G28881A, N:G28882A, N:G28883C, N:C29197T,	13	N:R203K, N:G204R, ORF1a:P959S, ORF1a:T3255I, ORF1a:V3449I, ORF1a:I3618V, ORF1a:T4175I, ORF1b:P314L, ORF3a:A54V, S:T478K, S:D614G, S:P681H, S:T732A,	
hCoV-19/Mexico/CMX-INMEGEN-03-02-07/2021	EPI_ISL_1181705	20B	B.1.1.519	29	5'UTR:T201C, 5'UTR:C203T, 5'UTR:C222T, 5'UTR:C241T, ORF1ab:G1738T, ORF1ab:C3037T, ORF1ab:C3140T, ORF1ab:G6894T, ORF1ab:C7819T, ORF1ab:C10029T, ORF1ab:C10954T, ORF1ab:A11117G, ORF1ab:C12789T, ORF1ab:C14408T, ORF1ab:G15652T, ORF1ab:G15732T, ORF1ab:T19839C, ORF1ab:A19974G, ORF1ab:C20480T, ORF1ab:C21306T, S:C21721T, S:C22995A, S:A23403G, S:C23604A, S:A23756G, ORF3a:C25553T, N:G28881A, N:G28882A, N:G28883C, N:C29197T,	15	N:R203K, N:G204R, ORF1a:P959S, ORF1a:C2210F, ORF1a:T3255I, ORF1a:I3618V, ORF1a:T4175I, ORF1b:P314L, ORF1b:D729Y, ORF1b:S2338L, ORF3a:A54V, S:T478K, S:D614G, S:P681H, S:T732A,	
hCoV-19/Mexico/CMX-INMEGEN-03-02-08/2021	EPI_ISL_1181706	20B	B.1.1.222	15	5'UTR:C241T, ORF1ab:C2005T, ORF1ab:C3037T, ORF1ab:C10029T, ORF1ab:C14408T, ORF1ab:C18652T, ORF1ab:C19610T, ORF1ab:T19839C, ORF1ab:C19875T, S:A23403G, S:A23756G, ORF3a:G25912T, ORF7b:A27756G, N:G28881A, N:G28882A, N:G28883C,	10	N:R203K, N:G204R, ORF1a:T3255I, ORF1b:P314L, ORF1b:R1729C, ORF1b:T2048I, ORF3a:G174C, ORF7b:M1V, S:D614G, S:T732A,	3'UTR:29746-29762,
hCoV-19/Mexico/CMX-INMEGEN-03-02-10/2021	EPI_ISL_1181707	20B	B.1.1.519	30	5'UTR:C203T, 5'UTR:C222T, 5'UTR:C241T, ORF1ab:A866G, ORF1ab:C1009T, ORF1ab:C3037T, ORF1ab:C3140T, ORF1ab:A3801G, ORF1ab:C5183T, ORF1ab:C10029T, ORF1ab:C10954T, ORF1ab:A11117G, ORF1ab:T11343C, ORF1ab:C11916T, ORF1ab:C12789T, ORF1ab:C14408T, ORF1ab:C16596T, ORF1ab:A18277G, ORF1ab:T19839C, ORF1ab:C21306T, S:C22995A, S:G23069C, S:A23403G, S:C23604A, S:A23756G, ORF8:T27904C, N:G28747T, N:G28881A, N:G28882A, N:G28883C, N:C29197T,	19	N:R203K, N:G204R, ORF1a:I201V, ORF1a:P959S, ORF1a:D1179G, ORF1a:P1640S, ORF1a:T3255I, ORF1a:I3618V, ORF1a:I3693T, ORF1a:S3884L, ORF1a:T4175I, ORF1b:P314L, ORF1b:I1604V, ORF8:L4P, S:T478K, S:V503L, S:D614G, S:P681H, S:T732A,	

hCoV-19/Mexico/CMX-INMEGEN-03-02-11/2021	EPI_ISL_1181708	20B	B.1	24	5'UTR:C203T, 5'UTR:C222T, 5'UTR:C241T, ORF1ab:C3037T, ORF1ab:C3140T, ORF1ab:C10029T, ORF1ab:C10319T, ORF1ab:C10954T, ORF1ab:A11117G, ORF1ab:C12789T, ORF1ab:C14408T, ORF1ab:T19839C, ORF1ab:C21306T, S:C21637T, S:C22995A, S:A23403G, S:A23586T, S:C23604A, S:A23756G, ORF8:T27904C, ORF8:C28087T, N:G28881A, N:G28882A, N:G28883C, N:C29197T,	15	N:R203K, N:G204R, ORF1a:P959S, ORF1a:T3255I, ORF1a:L3352F, ORF1a:I3618V, ORF1a:T4175I, ORF1b:P314L, ORF8:L4P, ORF8:A65V, S:T478K, S:D614G, S:Q675L, S:P681H, S:T732A,	
hCoV-19/Mexico/CMX-INMEGEN-03-02-12/2021	EPI_ISL_1181709	20B	B.1.1.519	27	5'UTR:C203T, 5'UTR:C222T, 5'UTR:C241T, ORF1ab:C3037T, ORF1ab:C3140T, ORF1ab:C9661T, ORF1ab:C10029T, ORF1ab:C10954T, ORF1ab:A11117G, ORF1ab:C12473T, ORF1ab:C12789T, ORF1ab:C14408T, ORF1ab:G15253A, ORF1ab:A18282G, ORF1ab:C19602T, ORF1ab:T19839C, ORF1ab:C21306T, S:C22995A, S:A23403G, S:C23604A, S:A23756G, ORF3a:G25906T, ORF8:C28253T, N:G28881A, N:G28882A, N:G28883C, N:C29197T, 3'UTR:G29777T,	13	N:R203K, N:G204R, ORF1a:P959S, ORF1a:T3255I, ORF1a:I3618V, ORF1a:T4175I, ORF1b:P314L, ORF1b:V596I, ORF3a:G172C, S:T478K, S:D614G, S:P681H, S:T732A,	
hCoV-19/Mexico/CMX-INMEGEN-03-02-13/2021	EPI_ISL_1181710	20B	B.1.1.519	25	5'UTR:T201C, 5'UTR:C203T, 5'UTR:C222T, 5'UTR:C241T, ORF1ab:G1738T, ORF1ab:C3037T, ORF1ab:C3140T, ORF1ab:C10029T, ORF1ab:G10610A, ORF1ab:C10954T, ORF1ab:A11117G, ORF1ab:C12789T, ORF1ab:C14408T, ORF1ab:T19839C, ORF1ab:A19974G, ORF1ab:C21306T, S:C22995A, S:A23403G, S:C23604A, S:A23756G, ORF3a:C25553T, N:C28758T, N:G28881A, N:G28882A, N:G28883C, N:C29197T,	14	N:P162L, N:R203K, N:G204R, ORF1a:P959S, ORF1a:T3255I, ORF1a:V3449I, ORF1a:I3618V, ORF1a:T4175I, ORF1b:P314L, ORF3a:A54V, S:T478K, S:D614G, S:P681H, S:T732A,	
hCoV-19/Mexico/CMX-INMEGEN-03-02-14/2021	EPI_ISL_1181711	20B	B.1.1.519	25	5'UTR:C203T, 5'UTR:G210T, 5'UTR:C222T, 5'UTR:C241T, ORF1ab:C3037T, ORF1ab:C3140T, ORF1ab:C10029T, ORF1ab:A10323G, ORF1ab:C10954T, ORF1ab:A10973T, ORF1ab:A11117G, ORF1ab:C12789T, ORF1ab:C14408T, ORF1ab:T19839C, ORF1ab:C21306T, S:C22995A, S:A23403G, S:C23604A, S:A23756G, ORF3a:G25593A, M:C26882T, ORF8:T27904C, N:G28881A, N:G28882A, N:G28883C, N:C29197T,	14	N:R203K, N:G204R, ORF1a:P959S, ORF1a:T3255I, ORF1a:K3353R, ORF1a:S3570C, ORF1a:I3618V, ORF1a:T4175I, ORF1b:P314L, ORF8:L4P, S:T478K, S:D614G, S:P681H, S:T732A,	
hCoV-19/Mexico/CMX-INMEGEN-03-02-15/2021	EPI_ISL_1181712	20B	B.1.1.519	28	5'UTR:T201C, 5'UTR:C203T, 5'UTR:C222T, 5'UTR:C241T, ORF1ab:G1738T, ORF1ab:C3037T, ORF1ab:C3140T, ORF1ab:C10029T, ORF1ab:C10332T, ORF1ab:C10954T, ORF1ab:A11117G, ORF1ab:C12789T, ORF1ab:A13711G, ORF1ab:C14408T, ORF1ab:C14724T, ORF1ab:T19839C, ORF1ab:A19974G, ORF1ab:C19983T, ORF1ab:C21306T, S:C22995A, S:G23285T, S:A23403G, S:C23604A, S:A23756G, ORF3a:C25517T, N:G28881A, N:G28882A, N:G28883C, N:C29197T,	15	N:R203K, N:G204R, ORF1a:P959S, ORF1a:T3255I, ORF1a:T3356I, ORF1a:I3618V, ORF1a:T4175I, ORF1b:K82E, ORF1b:P314L, ORF3a:P42L, S:T478K, S:A575S, S:D614G, S:P681H, S:T732A,	
hCoV-19/Mexico/CMX-INMEGEN-03-02-16/2021	EPI_ISL_1181713	20B	B.1.1.519	28	5'UTR:C203T, 5'UTR:C222T, 5'UTR:C241T, ORF1ab:C2716T, ORF1ab:C3037T, ORF1ab:C3140T, ORF1ab:C10029T, ORF1ab:C10954T, ORF1ab:A11117G, ORF1ab:A12719G, ORF1ab:C12789T, ORF1ab:C14408T, ORF1ab:C15738T, ORF1ab:G15921T, ORF1ab:A17971G, ORF1ab:T19839C, ORF1ab:T19866C, ORF1ab:C21306T, S:C22995A, S:A23403G, S:C23604A, S:C23635T, S:A23756G, ORF3a:G25593C, M:G26828T, N:G28881A, N:G28882A, N:G28883C, N:C29197T,	14	N:R203K, N:G204R, ORF1a:P959S, ORF1a:T3255I, ORF1a:I3618V, ORF1a:M4152V, ORF1a:T4175I, ORF1b:P314L, ORF1b:R1502G, ORF3a:K67N, S:T478K, S:D614G, S:P681H, S:T732A,	S:21992-21994,

hCoV-19/Mexico/CMX-INMEGEN-03-02-17/2021	EPI_ISL_1181714	20B	B.1.1.519	25	5'UTR:C203T, 5'UTR:C222T, 5'UTR:C241T, ORF1ab:C3037T, ORF1ab:C3045T, ORF1ab:C3140T, ORF1ab:G3351A, ORF1ab:C10029T, ORF1ab:C10954T, ORF1ab:A11117G, ORF1ab:C12789T, ORF1ab:C14408T, ORF1ab:T19839C, ORF1ab:C21306T, S:C21811T, S:C22995A, S:A23403G, S:C23604A, S:A23756G, M:C26885T, N:G28881A, N:G28882A, N:G28883C, N:C29197T, N:G29527T, 3'UTR:G29810T,	14	N:R203K, N:G204R, N:Q418H, ORF1a:P927L, ORF1a:P959S, ORF1a:S1029N, ORF1a:T3255I, ORF1a:I3618V, ORF1a:T4175I, ORF1b:P314L, S:T478K, S:D614G, S:P681H, S:T732A,
hCoV-19/Mexico/CMX-INMEGEN-03-02-18/2021	EPI_ISL_1181715	20B	B.1.1.519	23	5'UTR:C203T, 5'UTR:C222T, 5'UTR:C241T, ORF1ab:C3037T, ORF1ab:C3140T, ORF1ab:C10029T, ORF1ab:C10954T, ORF1ab:A11117G, ORF1ab:C12789T, ORF1ab:C13072T, ORF1ab:C14408T, ORF1ab:T19839C, ORF1ab:C21306T, S:C22995A, S:A23403G, S:C23604A, S:A23756G, M:C26936T, ORF8:T27904C, ORF8:C28087T, N:G28881A, N:G28882A, N:G28883C, N:C29197T,	13	N:R203K, N:G204R, ORF1a:P959S, ORF1a:T3255I, ORF1a:I3618V, ORF1a:T4175I, ORF1b:P314L, ORF8:L4P, ORF8:A65V, S:T478K, S:D614G, S:P681H, S:T732A,
hCoV-19/Mexico/CMX-INMEGEN-03-02-19/2021	EPI_ISL_1181716	20B	B.1.1.519	29	5'UTR:T201C, 5'UTR:C203T, 5'UTR:C222T, 5'UTR:C241T, ORF1ab:G1738T, ORF1ab:C2892T, ORF1ab:C3037T, ORF1ab:C3140T, ORF1ab:A5845C, ORF1ab:C6026T, ORF1ab:C10029T, ORF1ab:C10954T, ORF1ab:A11117G, ORF1ab:C12789T, ORF1ab:C14408T, ORF1ab:T19839C, ORF1ab:A19974G, ORF1ab:C21306T, S:C22995A, S:A23403G, S:C23604A, S:A23756G, ORF7b:C27804T, N:G28881A, N:G28882A, N:G28883C, N:C29197T, N:C29421T, ORF10:C29627T, 3'UTR:C29708T,	15	N:R203K, N:G204R, N:P383L, ORF1a:A876V, ORF1a:P959S, ORF1a:K1860N, ORF1a:P1921S, ORF1a:T3255I, ORF1a:I3618V, ORF1a:T4175I, ORF1b:P314L, S:T478K, S:D614G, S:P681H, S:T732A,
hCoV-19/Mexico/CMX-INMEGEN-03-02-20/2021	EPI_ISL_1181717	20B	B.1.1.519	28	5'UTR:G167T, 5'UTR:C203T, 5'UTR:C222T, 5'UTR:C241T, ORF1ab:T950C, ORF1ab:T1099C, ORF1ab:C3037T, ORF1ab:C3140T, ORF1ab:T9585C, ORF1ab:C10029T, ORF1ab:C10954T, ORF1ab:A11117G, ORF1ab:C12488T, ORF1ab:T12496C, ORF1ab:C12789T, ORF1ab:C14408T, ORF1ab:C19602T, ORF1ab:T19839C, ORF1ab:C21306T, S:C22995A, S:A23403G, S:C23604A, S:A23756G, ORF3a:G25906T, ORF8:C28253T, N:G28881A, N:G28882A, N:G28883C, N:C29197T,	15	N:R203K, N:G204R, ORF1a:Y229H, ORF1a:P959S, ORF1a:V3107A, ORF1a:T3255I, ORF1a:I3618V, ORF1a:P4075S, ORF1a:T4175I, ORF1b:P314L, ORF3a:G172C, S:T478K, S:D614G, S:P681H, S:T732A,
hCoV-19/Mexico/CMX-INMEGEN-03-02-21/2021	EPI_ISL_1181718	20B	B.1.1.519	24	5'UTR:C203T, 5'UTR:C222T, 5'UTR:C241T, ORF1ab:T1062C, ORF1ab:C3037T, ORF1ab:C3140T, ORF1ab:T3745C, ORF1ab:C4456T, ORF1ab:C10029T, ORF1ab:C10954T, ORF1ab:A11117G, ORF1ab:C12789T, ORF1ab:C14408T, ORF1ab:T19839C, ORF1ab:C21306T, S:C22995A, S:A23403G, S:C23604A, S:A23756G, ORF3a:C25844T, N:C28849T, N:G28881A, N:G28882A, N:G28883C, N:C29197T,	13	N:R203K, N:G204R, ORF1a:F266S, ORF1a:P959S, ORF1a:T3255I, ORF1a:I3618V, ORF1a:T4175I, ORF1b:P314L, ORF3a:T151I, S:T478K, S:D614G, S:P681H, S:T732A,
hCoV-19/Mexico/CMX-INMEGEN-03-02-22/2021	EPI_ISL_1181719	20B	B.1.1.519	25	5'UTR:T201C, 5'UTR:C203T, 5'UTR:C222T, 5'UTR:C241T, ORF1ab:C936T, ORF1ab:G1306C, ORF1ab:G1738T, ORF1ab:C3037T, ORF1ab:C3140T, ORF1ab:C10029T, ORF1ab:C10954T, ORF1ab:A11117G, ORF1ab:C12789T, ORF1ab:C14408T, ORF1ab:T19839C, ORF1ab:A19974G, ORF1ab:C21306T, S:C22995A, S:A23403G, S:C23604A, S:A23756G, N:G28881A, N:G28882A, N:G28883C, N:C29197T, N:G29227T,	13	N:R203K, N:G204R, ORF1a:T224I, ORF1a:E347D, ORF1a:P959S, ORF1a:T3255I, ORF1a:I3618V, ORF1a:T4175I, ORF1b:P314L, S:T478K, S:D614G, S:P681H, S:T732A,

hCoV-19/Mexico/CMX-INMEGEN-03-02-23/2021	EPI_ISL_1193558	20B	B.1.1.519	25	5'UTR:C203T, 5'UTR:C222T, 5'UTR:C241T, ORF1ab:C3037T, ORF1ab:C3140T, ORF1ab:T5983C, ORF1ab:T9061C, ORF1ab:C10029T, ORF1ab:C10954T, ORF1ab:A11117G, ORF1ab:C12789T, ORF1ab:C14318T, ORF1ab:C14408T, ORF1ab:T19839C, ORF1ab:C20429T, ORF1ab:C21306T, S:C22995A, S:A23403G, S:C23604A, S:A23756G, ORF7a:A27674T, N:G28881A, N:G28882A, N:G28883C, N:C29197T, N:G29527T,	14	N:R203K, N:G204R, N:Q418H, ORF1a:P959S, ORF1a:T3255I, ORF1a:I3618V, ORF1a:T4175I, ORF1b:T284I, ORF1b:P314L, ORF1b:P2321L, S:T478K, S:D614G, S:P681H, S:T732A,	ORF7a:27676,
hCoV-19/Mexico/CMX-INMEGEN-03-03-01/2021	EPI_ISL_1298451	20B	B.1.1.519	25	5'UTR:C203T, 5'UTR:C222T, 5'UTR:C241T, ORF1ab:A642C, ORF1ab:C683T, ORF1ab:C3037T, ORF1ab:C3140T, ORF1ab:C4815T, ORF1ab:G7037T, ORF1ab:C10029T, ORF1ab:C10954T, ORF1ab:A11117G, ORF1ab:C12789T, ORF1ab:C14408T, ORF1ab:T19839C, ORF1ab:C21306T, S:C22995A, S:A23403G, S:C23604A, S:A23756G, ORF3a:C25782T, N:G28881A, N:G28882A, N:G28883C, N:C29197T, ORF10:A29596G,	14	N:R203K, N:G204R, ORF1a:N126T, ORF1a:P959S, ORF1a:S1517F, ORF1a:G2258C, ORF1a:T3255I, ORF1a:I3618V, ORF1a:T4175I, ORF1b:P314L, S:T478K, S:D614G, S:P681H, S:T732A,	
hCoV-19/Mexico/CMX-INMEGEN-03-03-02/2021	EPI_ISL_1298452	20B	B.1.1.519	29	5'UTR:C203T, 5'UTR:C222T, 5'UTR:C241T, ORF1ab:G443A, ORF1ab:T628C, ORF1ab:C3037T, ORF1ab:C3140T, ORF1ab:C5869T, ORF1ab:C6538T, ORF1ab:C10029T, ORF1ab:C10954T, ORF1ab:A11117G, ORF1ab:C12789T, ORF1ab:C14408T, ORF1ab:T17931C, ORF1ab:T19839C, ORF1ab:C21306T, S:C22995A, S:A23403G, S:C23604A, S:A23756G, ORF3a:G25595A, M:C27026T, ORF8:T27904C, ORF8:C28087T, N:G28881A, N:G28882A, N:G28883C, N:C29197T, N:T29461C,	15	N:R203K, N:G204R, ORF1a:V60I, ORF1a:P959S, ORF1a:T3255I, ORF1a:I3618V, ORF1a:T4175I, ORF1b:P314L, ORF3a:R68K, ORF8:L4P, ORF8:A65V, S:T478K, S:D614G, S:P681H, S:T732A,	
hCoV-19/Mexico/CMX-INMEGEN-03-03-03/2021	EPI_ISL_1298453	20B	B.1.1.222	21	5'UTR:C241T, ORF1ab:C1346A, ORF1ab:C3037T, ORF1ab:C3159T, ORF1ab:C4099T, ORF1ab:G4561A, ORF1ab:C7749T, ORF1ab:A8052G, ORF1ab:C8637T, ORF1ab:A13498G, ORF1ab:C14408T, ORF1ab:G15438T, ORF1ab:T19839C, S:G22331A, S:C22624T, S:A23403G, S:A23756G, ORF3a:C25710T, N:G28881A, N:G28882A, N:G28883C, N:G28907A,	14	N:R203K, N:G204R, N:G212S, ORF1a:P361T, ORF1a:T965I, ORF1a:T2495I, ORF1a:N2596S, ORF1a:T2791I, ORF1b:T11A, ORF1b:P314L, ORF1b:M657I, S:G257S, S:D614G, S:T732A,	
hCoV-19/Mexico/CMX-INMEGEN-03-03-04/2021	EPI_ISL_1298454	20B	B.1.1.519	28	5'UTR:C203T, 5'UTR:C222T, 5'UTR:C241T, ORF1ab:C398T, ORF1ab:C3037T, ORF1ab:C3140T, ORF1ab:C5183T, ORF1ab:A5336T, ORF1ab:C10029T, ORF1ab:C10954T, ORF1ab:A11117G, ORF1ab:C11195T, ORF1ab:C12789T, ORF1ab:T12934C, ORF1ab:C14408T, ORF1ab:T17067C, ORF1ab:T19839C, ORF1ab:C21306T, S:C22480T, S:C22995A, S:A23403G, S:C23604A, S:A23756G, ORF3a:G25555A, ORF8:T27904C, N:G28881A, N:G28882A, N:G28883C, N:C29197T	17	N:R203K, N:G204R, ORF1a:H45Y, ORF1a:P959S, ORF1a:P1640S, ORF1a:N1691Y, ORF1a:T3255I, ORF1a:I3618V, ORF1a:L3644F, ORF1a:T4175I, ORF1b:P314L, ORF3a:V55I, ORF8:L4P, S:T478K, S:D614G, S:P681H, S:T732A,	
hCoV-19/Mexico/CMX-INMEGEN-03-03-05/2021	EPI_ISL_1298455	20B	B.1.1.519	28	5'UTR:C203T, 5'UTR:C222T, 5'UTR:C241T, ORF1ab:G2407T, ORF1ab:C3037T, ORF1ab:C3140T, ORF1ab:C5974T, ORF1ab:G7037T, ORF1ab:C10029T, ORF1ab:C10954T, ORF1ab:A11117G, ORF1ab:C12789T, ORF1ab:C13944T, ORF1ab:C14408T, ORF1ab:T19839C, ORF1ab:C21306T, S:C22995A, S:C23191T, S:A23403G, S:C23604A, S:A23756G, S:C23854T, ORF3a:C25782T, ORF7a:C27549T, ORF8:G28223T, N:G28881A, N:G28882A, N:G28883C, N:C29197T,	14	N:R203K, N:G204R, ORF1a:K714N, ORF1a:P959S, ORF1a:G2258C, ORF1a:T3255I, ORF1a:I3618V, ORF1a:T4175I, ORF1b:P314L, ORF8:E110D, S:T478K, S:D614G, S:P681H, S:T732A,	

hCoV-19/Mexico/CMX-INMEGEN-03-03-06/2021	EPI_ISL_1298456	20B	B.1.1.519	25	5'UTR:C203T, 5'UTR:G204T, 5'UTR:C222T, 5'UTR:C241T, ORF1ab:C3037T, ORF1ab:C3140T, ORF1ab:C6636T, ORF1ab:G7684A, ORF1ab:C10029T, ORF1ab:C10954T, ORF1ab:A11117G, ORF1ab:C12789T, ORF1ab:C14408T, ORF1ab:T19839C, ORF1ab:C21306T, S:A22004G, S:C22995A, S:A23403G, S:C23604A, S:A23756G, ORF3a:C25721T, N:G28881A, N:G28882A, N:G28883C, N:C29197T, N:G29527T,	15	N:R203K, N:G204R, N:Q418H, ORF1a:P959S, ORF1a:T2124I, ORF1a:T3255I, ORF1a:I3618V, ORF1a:T4175I, ORF1b:P314L, ORF3a:A110V, S:N148D, S:T478K, S:D614G, S:P681H, S:T732A,
hCoV-19/Mexico/CMX-INMEGEN-03-03-07/2021	EPI_ISL_1298457	20B	B.1.1.519	23	5'UTR:C203T, 5'UTR:C222T, 5'UTR:C241T, ORF1ab:C3037T, ORF1ab:C3140T, ORF1ab:A3274G, ORF1ab:G3955T, ORF1ab:G5942A, ORF1ab:C10029T, ORF1ab:C10954T, ORF1ab:A11117G, ORF1ab:C12789T, ORF1ab:C14408T, ORF1ab:T19839C, ORF1ab:C21306T, S:C22995A, S:A23403G, S:C23604A, S:A23756G, ORF3a:G25947T, N:G28881A, N:G28882A, N:G28883C, N:C29197T,	14	N:R203K, N:G204R, ORF1a:P959S, ORF1a:K1230N, ORF1a:D1893N, ORF1a:T3255I, ORF1a:I3618V, ORF1a:T4175I, ORF1b:P314L, ORF3a:Q185H, S:T478K, S:D614G, S:P681H, S:T732A,
hCoV-19/Mexico/CMX-INMEGEN-03-03-08/2021	EPI_ISL_1298458	20B	B.1.1.519	24	5'UTR:C203T, 5'UTR:C222T, 5'UTR:C241T, ORF1ab:C3037T, ORF1ab:C3140T, ORF1ab:G5008A, ORF1ab:C10029T, ORF1ab:C10954T, ORF1ab:A11117G, ORF1ab:C12789T, ORF1ab:C14408T, ORF1ab:T19839C, ORF1ab:C21306T, S:C22995A, S:A23403G, S:C23604A, S:A23756G, S:A24727G, ORF3a:C26078T, ORF8:T27904C, ORF8:C28087T, N:G28881A, N:G28882A, N:G28883C, N:C29197T,	14	N:R203K, N:G204R, ORF1a:P959S, ORF1a:T3255I, ORF1a:I3618V, ORF1a:T4175I, ORF1b:P314L, ORF3a:T229I, ORF8:L4P, ORF8:A65V, S:T478K, S:D614G, S:P681H, S:T732A,
hCoV-19/Mexico/CMX-INMEGEN-03-03-09/2021	EPI_ISL_1298459	20B	B.1.1.519	22	5'UTR:C203T, 5'UTR:C222T, 5'UTR:C241T, ORF1ab:C3037T, ORF1ab:C3140T, ORF1ab:C10029T, ORF1ab:C10954T, ORF1ab:A11117G, ORF1ab:C12789T, ORF1ab:C14120T, ORF1ab:C14408T, ORF1ab:T19839C, ORF1ab:C21306T, S:G21793T, S:C22747T, S:C22995A, S:A23403G, S:C23604A, S:A23756G, N:G28881A, N:G28882A, N:G28883C, N:C29197T,	13	N:R203K, N:G204R, ORF1a:P959S, ORF1a:T3255I, ORF1a:I3618V, ORF1a:T4175I, ORF1b:P218L, ORF1b:P314L, S:K77N, S:T478K, S:D614G, S:P681H, S:T732A,
hCoV-19/Mexico/CMX-INMEGEN-03-03-10/2021	EPI_ISL_1298460	20B	B.1.1.519	25	5'UTR:C203T, 5'UTR:C222T, 5'UTR:C241T, ORF1ab:C3037T, ORF1ab:C3140T, ORF1ab:A6084C, ORF1ab:C10029T, ORF1ab:C10954T, ORF1ab:A11117G, ORF1ab:C11575T, ORF1ab:G12202T, ORF1ab:C12789T, ORF1ab:C14408T, ORF1ab:C17550T, ORF1ab:T19839C, ORF1ab:C21306T, S:C22120A, S:C22995A, S:A23403G, S:C23604A, S:A23756G, S:A25369G, N:G28881A, N:G28882A, N:G28883C, N:C29197T,	14	N:R203K, N:G204R, ORF1a:P959S, ORF1a:D1940A, ORF1a:T3255I, ORF1a:I3618V, ORF1a:K3979N, ORF1a:T4175I, ORF1b:P314L, S:F186L, S:T478K, S:D614G, S:P681H, S:T732A,
hCoV-19/Mexico/CMX-INMEGEN-03-03-11/2021	EPI_ISL_1298461	20B	B.1.1.519	26	5'UTR:C203T, 5'UTR:C222T, 5'UTR:C241T, ORF1ab:C3037T, ORF1ab:C3140T, ORF1ab:C5869T, ORF1ab:C10029T, ORF1ab:C10954T, ORF1ab:A11117G, ORF1ab:C12789T, ORF1ab:C14408T, ORF1ab:T19215C, ORF1ab:C19813T, ORF1ab:T19839C, ORF1ab:C21306T, S:C22995A, S:A23403G, S:C23604A, S:A23756G, S:G25217T, ORF6:A27330G, ORF8:T27904C, ORF8:C28087T, N:G28881A, N:G28882A, N:G28883C, N:C29197T,	15	N:R203K, N:G204R, ORF1a:P959S, ORF1a:T3255I, ORF1a:I3618V, ORF1a:T4175I, ORF1b:P314L, ORF1b:P2116S, ORF8:L4P, ORF8:A65V, S:T478K, S:D614G, S:P681H, S:T732A, S:G1219C,
hCoV-19/Mexico/CMX-INMEGEN-03-03-12/2021	EPI_ISL_1298462	20B	B.1.1.28	22	5'UTR:C241T, ORF1ab:C3037T, ORF1ab:G4925T, ORF1ab:G9856T, ORF1ab:G11230T, ORF1ab:C11750T, ORF1ab:T12877C, ORF1ab:C14408T, ORF1ab:T19839C, S:C22450T, S:A23403G, S:G23593T, S:T24643C, S:G25088T, S:G25337T, ORF7a:C27610T, ORF8:C27945T, N:C28310T, N:A28841G, N:G28881A, N:G28882A, N:G28883C, 3'UTR:C29738T,	14	N:P13S, N:S190G, N:R203K, N:G204R, ORF1a:D1554Y, ORF1a:M3655I, ORF1a:L3829F, ORF1b:P314L, ORF7a:H73Y, ORF8:Q18*, S:D614G, S:Q677H, S:V1176F, S:D1259Y,

hCoV-19/Mexico/CMX-INMEGEN-03-03-13/2021	EPI_ISL_1298463	20B	B.1.1.519	22	5'UTR:C203T, 5'UTR:C222T, 5'UTR:C241T, ORF1ab:C3037T, ORF1ab:C3140T, ORF1ab:C6027T, ORF1ab:C10029T, ORF1ab:C10954T, ORF1ab:A11117G, ORF1ab:C12473T, ORF1ab:C12789T, ORF1ab:C14408T, ORF1ab:T19839C, ORF1ab:C21306T, S:C22995A, S:A23403G, S:C23604A, S:A23756G, ORF3a:C25528T, N:G28881A, N:G28882A, N:G28883C, N:C29197T,	13	N:R203K, N:G204R, ORF1a:P959S, ORF1a:P1921L, ORF1a:T3255I, ORF1a:I3618V, ORF1a:T4175I, ORF1b:P314L, ORF3a:L46F, S:T478K, S:D614G, S:P681H, S:T732A,
hCoV-19/Mexico/CMX-INMEGEN-03-03-14/2021	EPI_ISL_1298464	20B	B.1.1.519	28	5'UTR:T201C, 5'UTR:C203T, 5'UTR:C222T, 5'UTR:C241T, ORF1ab:C745T, ORF1ab:G1738T, ORF1ab:C3037T, ORF1ab:C3140T, ORF1ab:C10029T, ORF1ab:C10954T, ORF1ab:A11117G, ORF1ab:C12789T, ORF1ab:C14408T, ORF1ab:T19839C, ORF1ab:A19974G, ORF1ab:C21306T, S:C21575T, S:C22995A, S:A23403G, S:C23604A, S:A23756G, M:G27074A, ORF8:G28167A, N:T28759C, N:G28881A, N:G28882A, N:G28883C, N:C29197T, ORF10:C29585T,	13	N:R203K, N:G204R, ORF1a:P959S, ORF1a:T3255I, ORF1a:I3618V, ORF1a:T4175I, ORF1b:P314L, ORF8:E92K, S:L5F, S:T478K, S:D614G, S:P681H, S:T732A,
hCoV-19/Mexico/CMX-INMEGEN-03-03-15/2021	EPI_ISL_1298465	20B	B.1.1.519	27	5'UTR:C203T, 5'UTR:C222T, 5'UTR:C241T, ORF1ab:G1820A, ORF1ab:C3037T, ORF1ab:C3140T, ORF1ab:C10029T, ORF1ab:C10954T, ORF1ab:A11117G, ORF1ab:G11365T, ORF1ab:C12789T, ORF1ab:G14335T, ORF1ab:C14408T, ORF1ab:T19839C, ORF1ab:C21306T, S:C22995A, S:A23403G, S:C23604A, S:A23756G, S:G24755T, ORF3a:T25536A, ORF3a:G25690T, N:G28881A, N:G28882A, N:G28883C, N:C29197T, N:G29527T, 3'UTR:C29733T,	16	N:R203K, N:G204R, N:Q418H, ORF1a:G519S, ORF1a:P959S, ORF1a:T3255I, ORF1a:I3618V, ORF1a:T4175I, ORF1b:V290F, ORF1b:P314L, ORF3a:G100C, S:T478K, S:D614G, S:P681H, S:T732A, S:V1065L,
hCoV-19/Mexico/CMX-INMEGEN-03-03-16/2021	EPI_ISL_1298466	20B	B.1.1.519	21	5'UTR:C203T, 5'UTR:C222T, 5'UTR:C241T, ORF1ab:C3037T, ORF1ab:C3140T, ORF1ab:A6393C, ORF1ab:C10029T, ORF1ab:C10954T, ORF1ab:A11117G, ORF1ab:C12789T, ORF1ab:C14408T, ORF1ab:T19839C, ORF1ab:C21306T, S:C22995A, S:A23403G, S:C23604A, S:A23756G, N:G28881A, N:G28882A, N:G28883C, N:C29095T, N:C29197T,	12	N:R203K, N:G204R, ORF1a:P959S, ORF1a:D2043A, ORF1a:T3255I, ORF1a:I3618V, ORF1a:T4175I, ORF1b:P314L, S:T478K, S:D614G, S:P681H, S:T732A,
hCoV-19/Mexico/CMX-INMEGEN-03-03-17/2021	EPI_ISL_1298467	20B	B.1.1.519	23	5'UTR:C203T, 5'UTR:C222T, 5'UTR:C241T, ORF1ab:C1263T, ORF1ab:C3037T, ORF1ab:C3140T, ORF1ab:C10029T, ORF1ab:C10954T, ORF1ab:A11117G, ORF1ab:G11365T, ORF1ab:C12789T, ORF1ab:C14408T, ORF1ab:C14786T, ORF1ab:T19839C, ORF1ab:C21306T, S:C22995A, S:A23403G, S:C23604A, S:A23756G, N:G28881A, N:G28882A, N:G28883C, N:C29197T, N:G29527T,	14	N:R203K, N:G204R, N:Q418H, ORF1a:T333M, ORF1a:P959S, ORF1a:T3255I, ORF1a:I3618V, ORF1a:T4175I, ORF1b:P314L, ORF1b:A440V, S:T478K, S:D614G, S:P681H, S:T732A,
hCoV-19/Mexico/CMX-INMEGEN-03-03-18/2021	EPI_ISL_1298468	20B	B.1.1.519	27	5'UTR:C203T, 5'UTR:C222T, 5'UTR:C241T, ORF1ab:C3037T, ORF1ab:C3140T, ORF1ab:C8293T, ORF1ab:C9120T, ORF1ab:C10029T, ORF1ab:C10954T, ORF1ab:A11117G, ORF1ab:C12789T, ORF1ab:C14408T, ORF1ab:T19839C, ORF1ab:C21057T, ORF1ab:C21077T, ORF1ab:C21306T, S:C22995A, S:A23403G, S:C23604A, S:A23756G, ORF3a:G25906T, M:C27119T, ORF8:C28253T, N:G28881A, N:G28882A, N:G28883C, N:C29171T, N:C29197T,	15	N:R203K, N:G204R, N:H300Y, ORF1a:P959S, ORF1a:T2952I, ORF1a:T3255I, ORF1a:I3618V, ORF1a:T4175I, ORF1b:P314L, ORF1b:T2537I, ORF3a:G172C, S:T478K, S:D614G, S:P681H, S:T732A,

hCoV-19/Mexico/CMX-INMEGEN-03-03-19/2021	EPI_ISL_1298469	20B	B.1.1.519	30	5'UTR:C186T, 5'UTR:T201C, 5'UTR:C203T, 5'UTR:C222T, 5'UTR:C241T, ORF1ab:C745T, ORF1ab:G1738T, ORF1ab:C3037T, ORF1ab:C3140T, ORF1ab:A5563C, ORF1ab:A8893G, ORF1ab:C10029T, ORF1ab:C10954T, ORF1ab:A11117G, ORF1ab:C12789T, ORF1ab:C14408T, ORF1ab:T15195A, ORF1ab:T19839C, ORF1ab:A19974G, ORF1ab:C21306T, S:C22995A, S:A23403G, S:C23604A, S:A23756G, ORF7a:A27674G, ORF8:C28253T, N:C28770T, N:G28881A, N:G28882A, N:G28883C, N:C29197T	14	N:T166I, N:R203K, N:G204R, ORF1a:P959S, ORF1a:E1766D, ORF1a:T3255I, ORF1a:I3618V, ORF1a:T4175I, ORF1b:P314L, ORF7a:Q94R, S:T478K, S:D614G, S:P681H, S:T732A,
hCoV-19/Mexico/CMX-INMEGEN-03-03-20/2021	EPI_ISL_1298470	20B	B.1.1.519	28	5'UTR:C203T, 5'UTR:C222T, 5'UTR:C241T, ORF1ab:T617C, ORF1ab:C3037T, ORF1ab:C3140T, ORF1ab:T3926C, ORF1ab:C5183T, ORF1ab:A5336T, ORF1ab:C10029T, ORF1ab:C10954T, ORF1ab:A11117G, ORF1ab:C12789T, ORF1ab:C14408T, ORF1ab:C18555T, ORF1ab:G18972T, ORF1ab:T19839C, ORF1ab:C21306T, S:C22663T, S:C22995A, S:A23403G, S:C23604A, S:A23756G, ORF8:T27904C, ORF8:T28091C, N:G28881A, N:G28882A, N:G28883C, N:C29197T	17	N:R203K, N:G204R, ORF1a:Y118H, ORF1a:P959S, ORF1a:S1221P, ORF1a:P1640S, ORF1a:N1691Y, ORF1a:T3255I, ORF1a:I3618V, ORF1a:T4175I, ORF1b:P314L, ORF1b:K1835N, ORF8:L4P, S:T478K, S:D614G, S:P681H, S:T732A,
hCoV-19/Mexico/CMX-INMEGEN-03-03-21/2021	EPI_ISL_1298471	20B	B.1.1.519	25	5'UTR:T201C, 5'UTR:C203T, 5'UTR:C222T, 5'UTR:C241T, ORF1ab:C745T, ORF1ab:G1738T, ORF1ab:A2903G, ORF1ab:C3037T, ORF1ab:C3140T, ORF1ab:C3736T, ORF1ab:C10029T, ORF1ab:C10954T, ORF1ab:A11117G, ORF1ab:C12789T, ORF1ab:C14408T, ORF1ab:T19839C, ORF1ab:A19974G, ORF1ab:C21306T, S:C22995A, S:A23403G, S:C23604A, S:A23756G, N:G28881A, N:G28882A, N:G28883C, N:C29197T,	12	N:R203K, N:G204R, ORF1a:I880V, ORF1a:P959S, ORF1a:T3255I, ORF1a:I3618V, ORF1a:T4175I, ORF1b:P314L, S:T478K, S:D614G, S:P681H, S:T732A,
hCoV-19/Mexico/CMX-INMEGEN-03-03-22/2021	EPI_ISL_1298472	20B	B.1.1.519	28	5'UTR:C203T, 5'UTR:C222T, 5'UTR:C241T, ORF1ab:C3037T, ORF1ab:A3048G, ORF1ab:C3140T, ORF1ab:C5144T, ORF1ab:C10029T, ORF1ab:C10954T, ORF1ab:A11117G, ORF1ab:C12754T, ORF1ab:C12789T, ORF1ab:G12817A, ORF1ab:C14408T, ORF1ab:T15375C, ORF1ab:G17721T, ORF1ab:T19839C, ORF1ab:C21306T, S:C22995A, S:A23403G, S:C23604A, S:A23756G, ORF3a:C25714T, ORF8:T27904C, ORF8:C27944T, N:G28881A, N:G28882A, N:G28883C, N:C29197T,	14	N:R203K, N:G204R, ORF1a:D928G, ORF1a:P959S, ORF1a:T3255I, ORF1a:I3618V, ORF1a:T4175I, ORF1b:P314L, ORF3a:L108F, ORF8:L4P, S:T478K, S:D614G, S:P681H, S:T732A,
hCoV-19/Mexico/CMX-INMEGEN-03-03-23/2021	EPI_ISL_1298473	20B	B.1.1.519	24	5'UTR:C203T, 5'UTR:C222T, 5'UTR:C241T, ORF1ab:T1090C, ORF1ab:A3019G, ORF1ab:C3037T, ORF1ab:C3140T, ORF1ab:G3549A, ORF1ab:C8090A, ORF1ab:C10029T, ORF1ab:C10954T, ORF1ab:A11117G, ORF1ab:C12789T, ORF1ab:C14408T, ORF1ab:T19839C, ORF1ab:C21306T, S:C22995A, S:A23403G, S:C23604A, S:A23756G, ORF8:T27904C, N:G28881A, N:G28882A, N:G28883C, N:C29197T,	14	N:R203K, N:G204R, ORF1a:P959S, ORF1a:G1095E, ORF1a:L2609I, ORF1a:T3255I, ORF1a:I3618V, ORF1a:T4175I, ORF1b:P314L, ORF8:L4P, S:T478K, S:D614G, S:P681H, S:T732A,
hCoV-19/Mexico/CMX-INMEGEN-03-04-01/2021	EPI_ISL_1205203	20B	B.1.1.519	22	5'UTR:C203T, 5'UTR:C222T, 5'UTR:C241T, ORF1ab:C3037T, ORF1ab:C3140T, ORF1ab:C10029T, ORF1ab:C10954T, ORF1ab:A11117G, ORF1ab:G11365T, ORF1ab:C12789T, ORF1ab:C14408T, ORF1ab:T19839C, ORF1ab:C21306T, S:C22995A, S:A23403G, S:C23604A, S:A23756G, M:G26840T, N:G28881A, N:G28882A, N:G28883C, N:C29197T, N:G29527T,	12	N:R203K, N:G204R, N:Q418H, ORF1a:P959S, ORF1a:T3255I, ORF1a:I3618V, ORF1a:T4175I, ORF1b:P314L, S:T478K, S:D614G, S:P681H, S:T732A,

hCoV-19/Mexico/CMX-INMEGEN-03-04-02/2021	EPI_ISL_1205204	20B	B.1.1.519	25	5'UTR:C203T, 5'UTR:C222T, 5'UTR:C241T, ORF1ab:C3037T, ORF1ab:A3090C, ORF1ab:C3140T, ORF1ab:T3745C, ORF1ab:C10029T, ORF1ab:C10954T, ORF1ab:A11117G, ORF1ab:C12789T, ORF1ab:C14408T, ORF1ab:T19839C, ORF1ab:C21306T, S:C22995A, S:A23403G, S:C23565T, S:C23604A, S:A23756G, ORF3a:C25844T, E:G26389T, M:C26527T, N:G28881A, N:G28882A, N:G28883C, N:C29197T,	16	E:V49L, M:A2V, N:R203K, N:G204R, ORF1a:E942A, ORF1a:P959S, ORF1a:T3255I, ORF1a:I3618V, ORF1a:T4175I, ORF1b:P314L, ORF3a:T151I, S:T478K, S:D614G, S:A668V, S:P681H, S:T732A,	3'UTR:29738-29758
hCoV-19/Mexico/CMX-INMEGEN-03-04-03/2021	EPI_ISL_1205205	20B	B.1.1.519	25	5'UTR:C203T, 5'UTR:C222T, 5'UTR:C241T, ORF1ab:C3037T, ORF1ab:C3140T, ORF1ab:C5183T, ORF1ab:G5194T, ORF1ab:T7045C, ORF1ab:C10029T, ORF1ab:C10954T, ORF1ab:A11117G, ORF1ab:C12789T, ORF1ab:G12794A, ORF1ab:C14408T, ORF1ab:T19839C, ORF1ab:C21306T, S:C22995A, S:A23403G, S:C23604A, S:A23756G, S:G25135T, ORF8:T27904C, N:G28881A, N:G28882A, N:G28883C, N:C29197T,	15	N:R203K, N:G204R, ORF1a:P959S, ORF1a:P1640S, ORF1a:T3255I, ORF1a:I3618V, ORF1a:T4175I, ORF1a:G4177R, ORF1b:P314L, ORF8:L4P, S:T478K, S:D614G, S:P681H, S:T732A, S:K1191N,	
hCoV-19/Mexico/CMX-INMEGEN-03-04-04/2021	EPI_ISL_1205206	20B	B.1.1.519	24	5'UTR:C203T, 5'UTR:C222T, 5'UTR:C241T, ORF1ab:T1090C, ORF1ab:A3019G, ORF1ab:C3037T, ORF1ab:C3140T, ORF1ab:G3549A, ORF1ab:C8090A, ORF1ab:C10029T, ORF1ab:C10954T, ORF1ab:A11117G, ORF1ab:C12789T, ORF1ab:C14408T, ORF1ab:T19839C, ORF1ab:C21306T, S:C22995A, S:A23403G, S:C23604A, S:A23756G, ORF8:T27904C, N:G28881A, N:G28882A, N:G28883C, N:C29197T,	14	N:R203K, N:G204R, ORF1a:P959S, ORF1a:G1095E, ORF1a:L2609I, ORF1a:T3255I, ORF1a:I3618V, ORF1a:T4175I, ORF1b:P314L, ORF8:L4P, S:T478K, S:D614G, S:P681H, S:T732A,	
hCoV-19/Mexico/CMX-INMEGEN-03-04-05/2021	EPI_ISL_1205207	20B	B.1.1.519	25	5'UTR:C203T, 5'UTR:C222T, 5'UTR:C241T, ORF1ab:C3037T, ORF1ab:A3090C, ORF1ab:C3140T, ORF1ab:T3745C, ORF1ab:C10029T, ORF1ab:C10954T, ORF1ab:A11117G, ORF1ab:C12789T, ORF1ab:C14408T, ORF1ab:T19839C, ORF1ab:C21306T, S:C22995A, S:A23403G, S:C23565T, S:C23604A, S:A23756G, ORF3a:C25844T, E:G26389T, M:C26527T, N:G28881A, N:G28882A, N:G28883C, N:C29197T,	16	E:V49L, M:A2V, N:R203K, N:G204R, ORF1a:E942A, ORF1a:P959S, ORF1a:T3255I, ORF1a:I3618V, ORF1a:T4175I, ORF1b:P314L, ORF3a:T151I, S:T478K, S:D614G, S:A668V, S:P681H, S:T732A,	3'UTR:29738-29758
hCoV-19/Mexico/CMX-INMEGEN-03-04-06/2021	EPI_ISL_1205208	20B	B.1.1.519	24	5'UTR:C203T, 5'UTR:C222T, 5'UTR:C241T, ORF1ab:G1508A, ORF1ab:C3037T, ORF1ab:C3140T, ORF1ab:C10029T, ORF1ab:C10954T, ORF1ab:A11117G, ORF1ab:C12789T, ORF1ab:C14408T, ORF1ab:T15203C, ORF1ab:T19839C, ORF1ab:C21306T, ORF1ab:G21538T, S:C22995A, S:A23403G, S:C23604A, S:A23756G, S:T24202C, ORF8:T28255G, N:G28881A, N:G28882A, N:G28883C, N:C29197T,	15	N:R203K, N:G204R, ORF1a:G415S, ORF1a:P959S, ORF1a:T3255I, ORF1a:I3618V, ORF1a:T4175I, ORF1b:P314L, ORF1b:V579A, ORF1b:V2691F, ORF8:I121S, S:T478K, S:D614G, S:P681H, S:T732A,	
hCoV-19/Mexico/CMX-INMEGEN-03-04-07/2021	EPI_ISL_1205209	20B	B.1.1.519	30	5'UTR:C203T, 5'UTR:C222T, 5'UTR:C241T, ORF1ab:C1427T, ORF1ab:C3037T, ORF1ab:C3140T, ORF1ab:G3692T, ORF1ab:G3871T, ORF1ab:C4158T, ORF1ab:G8861A, ORF1ab:C9520T, ORF1ab:C10029T, ORF1ab:C10954T, ORF1ab:A11117G, ORF1ab:C11824A, ORF1ab:C12789T, ORF1ab:C14408T, ORF1ab:G17280C, ORF1ab:C18747T, ORF1ab:T19839C, ORF1ab:C21306T, S:C22995A, S:A23403G, S:C23604A, S:A23756G, ORF8:T27904C, ORF8:G27967T, N:G28881A, N:G28882A, N:G28883C, N:C29197T,	18	N:R203K, N:G204R, ORF1a:H388Y, ORF1a:P959S, ORF1a:V1143F, ORF1a:K1202N, ORF1a:A1298V, ORF1a:V2866M, ORF1a:T3255I, ORF1a:I3618V, ORF1a:T4175I, ORF1b:P314L, ORF8:L4P, ORF8:C25F, S:T478K, S:D614G, S:P681H, S:T732A,	

hCoV-19/Mexico/CMX-INMEGEN-03-04-08/2021	EPI_ISL_1205210	20B	B.1.1.519	27	5'UTR:T201C, 5'UTR:C203T, 5'UTR:C222T, 5'UTR:C241T, ORF1ab:G1738T, ORF1ab:C3037T, ORF1ab:C3140T, ORF1ab:G3753A, ORF1ab:A6459G, ORF1ab:A9280G, ORF1ab:C10029T, ORF1ab:C10954T, ORF1ab:A11117G, ORF1ab:C12789T, ORF1ab:C14408T, ORF1ab:A16026G, ORF1ab:G18020A, ORF1ab:T19839C, ORF1ab:A19974G, ORF1ab:C21306T, S:C22995A, S:A23403G, S:C23604A, S:A23756G, N:G28881A, N:G28882A, N:G28883C, N:C29197T,	14	N:R203K, N:G204R, ORF1a:P959S, ORF1a:R1163K, ORF1a:N2065S, ORF1a:T3255I, ORF1a:I3618V, ORF1a:T4175I, ORF1b:P314L, ORF1b:R1518K, S:T478K, S:D614G, S:P681H, S:T732A,
hCoV-19/Mexico/CMX-INMEGEN-03-04-09/2021	EPI_ISL_1205211	20B	B.1.1.519	20	5'UTR:C203T, 5'UTR:C222T, 5'UTR:A223T, 5'UTR:C241T, ORF1ab:C3037T, ORF1ab:C3140T, ORF1ab:C10029T, ORF1ab:C10954T, ORF1ab:A11117G, ORF1ab:C12789T, ORF1ab:C14408T, ORF1ab:T19839C, ORF1ab:C21306T, S:C22995A, S:A23403G, S:C23604A, S:A23756G, N:G28881A, N:G28882A, N:G28883C, N:C29197T,	11	N:R203K, N:G204R, ORF1a:P959S, ORF1a:T3255I, ORF1a:I3618V, ORF1a:T4175I, ORF1b:P314L, S:T478K, S:D614G, S:P681H, S:T732A,
hCoV-19/Mexico/CMX-INMEGEN-03-04-10/2021	EPI_ISL_1205212	20B	B.1.1.519	21	5'UTR:C203T, 5'UTR:C222T, 5'UTR:C241T, ORF1ab:C3037T, ORF1ab:C3140T, ORF1ab:C8950T, ORF1ab:C10029T, ORF1ab:C10954T, ORF1ab:A11117G, ORF1ab:C12789T, ORF1ab:C14408T, ORF1ab:T19839C, ORF1ab:C21306T, S:C22995A, S:A23403G, S:C23604A, S:A23756G, N:G28881A, N:G28882A, N:G28883C, N:C29197T, N:G29527T,	12	N:R203K, N:G204R, N:Q418H, ORF1a:P959S, ORF1a:T3255I, ORF1a:I3618V, ORF1a:T4175I, ORF1b:P314L, S:T478K, S:D614G, S:P681H, S:T732A,
hCoV-19/Mexico/CMX-INMEGEN-03-04-11/2021	EPI_ISL_1205213	20B	B.1.1.519	19	5'UTR:C203T, 5'UTR:C222T, 5'UTR:C241T, ORF1ab:C3037T, ORF1ab:C3140T, ORF1ab:C10029T, ORF1ab:C10954T, ORF1ab:A11117G, ORF1ab:C12789T, ORF1ab:C14408T, ORF1ab:T19839C, ORF1ab:C21306T, S:C22995A, S:A23403G, S:C23604A, S:A23756G, N:G28881A, N:G28882A, N:G28883C, N:C29197T,	11	N:R203K, N:G204R, ORF1a:P959S, ORF1a:T3255I, ORF1a:I3618V, ORF1a:T4175I, ORF1b:P314L, S:T478K, S:D614G, S:P681H, S:T732A,
hCoV-19/Mexico/CMX-INMEGEN-03-04-12/2021	EPI_ISL_1205214	20B	B.1.1.222	9	5'UTR:C241T, ORF1ab:C3037T, ORF1ab:C10029T, ORF1ab:C14408T, ORF1ab:T19839C, S:A23403G, S:A23756G, N:G28881A, N:G28882A, N:G28883C,	6	N:R203K, N:G204R, ORF1a:T3255I, ORF1b:P314L, S:D614G, S:T732A,
hCoV-19/Mexico/CMX-INMEGEN-03-04-13/2021	EPI_ISL_1205215	20B	B.1.1.519	19	5'UTR:C203T, 5'UTR:C222T, 5'UTR:C241T, ORF1ab:C3037T, ORF1ab:C3140T, ORF1ab:C10029T, ORF1ab:C10954T, ORF1ab:A11117G, ORF1ab:C12789T, ORF1ab:C14408T, ORF1ab:T19839C, ORF1ab:C21306T, S:C22995A, S:A23403G, S:C23604A, S:A23756G, N:G28881A, N:G28882A, N:G28883C, N:C29197T,	11	N:R203K, N:G204R, ORF1a:P959S, ORF1a:T3255I, ORF1a:I3618V, ORF1a:T4175I, ORF1b:P314L, S:T478K, S:D614G, S:P681H, S:T732A,
hCoV-19/Mexico/CMX-INMEGEN-03-04-14/2021	EPI_ISL_1205216	20B	B.1.1.519	20	5'UTR:C203T, 5'UTR:C222T, 5'UTR:C241T, ORF1ab:C3037T, ORF1ab:C3140T, ORF1ab:C10029T, ORF1ab:C10954T, ORF1ab:A11117G, ORF1ab:C12789T, ORF1ab:C14408T, ORF1ab:T19839C, ORF1ab:C21306T, S:C22995A, S:A23403G, S:C23604A, S:A23756G, S:A24727G, N:G28881A, N:G28882A, N:G28883C, N:C29197T,	11	N:R203K, N:G204R, ORF1a:P959S, ORF1a:T3255I, ORF1a:I3618V, ORF1a:T4175I, ORF1b:P314L, S:T478K, S:D614G, S:P681H, S:T732A,
hCoV-19/Mexico/CMX-INMEGEN-03-04-15/2021	EPI_ISL_1205217	20B	B.1.1.519	20	5'UTR:C203T, 5'UTR:C222T, 5'UTR:C241T, ORF1ab:C3037T, ORF1ab:C3140T, ORF1ab:C10029T, ORF1ab:C10954T, ORF1ab:A11117G, ORF1ab:C12789T, ORF1ab:C14408T, ORF1ab:T19839C, ORF1ab:C21306T, S:C22995A, S:A23403G, S:C23604A, S:A23756G, S:A24727G, N:G28881A, N:G28882A, N:G28883C, N:C29197T,	11	N:R203K, N:G204R, ORF1a:P959S, ORF1a:T3255I, ORF1a:I3618V, ORF1a:T4175I, ORF1b:P314L, S:T478K, S:D614G, S:P681H, S:T732A,

hCoV-19/Mexico/CMX-INMEGEN-03-04-16/2021	EPI_ISL_1205218	20B	B.1.1.222	18	5'UTR:C241T, ORF1ab:C2005T, ORF1ab:C3037T, ORF1ab:C10029T, ORF1ab:C12778T, ORF1ab:C14408T, ORF1ab:G15243T, ORF1ab:C18652T, ORF1ab:C19610T, ORF1ab:T19839C, ORF1ab:C19875T, S:A21623G, S:A23403G, S:A23756G, ORF3a:G25912T, ORF7b:A27756G, N:G28881A, N:G28882A, N:G28883C,	12	N:R203K, N:G204R, ORF1a:T3255I, ORF1b:P314L, ORF1b:M592I, ORF1b:R1729C, ORF1b:T2048I, ORF3a:G174C, ORF7b:M1V, S:R21G, S:D614G, S:T732A,	3'UTR:29746-29762,
hCoV-19/Mexico/CMX-INMEGEN-03-05-01/2021	EPI_ISL_1262702	20B	B.1.1.519	27	5'UTR:C203T, 5'UTR:C222T, 5'UTR:C241T, ORF1ab:C3037T, ORF1ab:C3140T, ORF1ab:T3745C, ORF1ab:C10029T, ORF1ab:C10252T, ORF1ab:C10954T, ORF1ab:A11117G, ORF1ab:C12651T, ORF1ab:C12789T, ORF1ab:C14408T, ORF1ab:G15908A, ORF1ab:G16897A, ORF1ab:T19839C, ORF1ab:C21306T, S:G22199T, S:C22995A, S:A23403G, S:C23604A, S:A23756G, ORF3a:C25463T, ORF3a:C25844T, N:G28881A, N:G28882A, N:G28883C, N:C29197T,	17	N:R203K, N:G204R, ORF1a:P959S, ORF1a:T3255I, ORF1a:I3618V, ORF1a:T4129I, ORF1a:T4175I, ORF1b:P314L, ORF1b:G814D, ORF1b:V1144I, ORF3a:T24I, ORF3a:T151I, S:V213L, S:T478K, S:D614G, S:P681H, S:T732A,	
hCoV-19/Mexico/CMX-INMEGEN-03-05-02/2021	EPI_ISL_1262703	20B	B.1.1.519	26	5'UTR:C203T, 5'UTR:C222T, 5'UTR:C241T, ORF1ab:C3037T, ORF1ab:C3140T, ORF1ab:T3745C, ORF1ab:C10029T, ORF1ab:C10954T, ORF1ab:A11117G, ORF1ab:C12242T, ORF1ab:C12789T, ORF1ab:C14408T, ORF1ab:C18395T, ORF1ab:A19751G, ORF1ab:T19839C, ORF1ab:C21306T, S:C22995A, S:A23403G, S:C23604A, S:A23756G, S:A24162G, ORF3a:C25844T, N:C28849T, N:G28881A, N:G28882A, N:G28883C, N:C29197T,	16	N:R203K, N:G204R, ORF1a:P959S, ORF1a:T3255I, ORF1a:I3618V, ORF1a:R3993C, ORF1a:T4175I, ORF1b:P314L, ORF1b:A1643V, ORF1b:E2095G, ORF3a:T151I, S:T478K, S:D614G, S:P681H, S:T732A, S:D867G,	
hCoV-19/Mexico/CMX-INMEGEN-03-05-03/2021	EPI_ISL_1262704	20B	B.1.1.519	29	5'UTR:T201C, 5'UTR:C203T, 5'UTR:C222T, 5'UTR:C241T, ORF1ab:C936T, ORF1ab:G1738T, ORF1ab:C2470A, ORF1ab:C3037T, ORF1ab:C3140T, ORF1ab:T5035C, ORF1ab:C10029T, ORF1ab:C10954T, ORF1ab:A11117G, ORF1ab:G12031T, ORF1ab:C12789T, ORF1ab:C14408T, ORF1ab:C19017T, ORF1ab:T19839C, ORF1ab:A19974G, ORF1ab:C21306T, S:C22995A, S:A23403G, S:C23604A, S:A23756G, ORF3a:G25785T, N:G28881A, N:G28882A, N:G28883C, N:C29197T, N:G29227T,	14	N:R203K, N:G204R, ORF1a:T224I, ORF1a:P959S, ORF1a:T3255I, ORF1a:I3618V, ORF1a:Q3922H, ORF1a:T4175I, ORF1b:P314L, ORF3a:W131C, S:T478K, S:D614G, S:P681H, S:T732A,	
hCoV-19/Mexico/CMX-INMEGEN-03-05-04/2021	EPI_ISL_1262705	20B	B.1.1.519	25	5'UTR:T201C, 5'UTR:C203T, 5'UTR:C222T, 5'UTR:C241T, ORF1ab:C745T, ORF1ab:G1738T, ORF1ab:C3037T, ORF1ab:C3140T, ORF1ab:C7081T, ORF1ab:C10029T, ORF1ab:C10954T, ORF1ab:A11117G, ORF1ab:C12789T, ORF1ab:C14408T, ORF1ab:T19839C, ORF1ab:A19974G, ORF1ab:C21306T, S:C22995A, S:A23403G, S:C23604A, S:A23756G, ORF3a:T25706C, N:G28881A, N:G28882A, N:G28883C, N:C29197T,	12	N:R203K, N:G204R, ORF1a:P959S, ORF1a:T3255I, ORF1a:I3618V, ORF1a:T4175I, ORF1b:P314L, ORF3a:F105S, S:T478K, S:D614G, S:P681H, S:T732A,	
hCoV-19/Mexico/CMX-INMEGEN-03-05-05/2021	EPI_ISL_1262706	20B	B.1.1.519	22	5'UTR:C203T, 5'UTR:C222T, 5'UTR:C241T, ORF1ab:C3037T, ORF1ab:C3140T, ORF1ab:C10029T, ORF1ab:C10954T, ORF1ab:A11117G, ORF1ab:C12789T, ORF1ab:A13098G, ORF1ab:C14408T, ORF1ab:T19839C, ORF1ab:C21306T, S:C22995A, S:A23403G, S:C23604A, S:A23756G, ORF8:T27904C, ORF8:C28087T, N:G28881A, N:G28882A, N:G28883C, N:C29197T,	14	N:R203K, N:G204R, ORF1a:P959S, ORF1a:T3255I, ORF1a:I3618V, ORF1a:T4175I, ORF1a:K4278R, ORF1b:P314L, ORF8:L4P, ORF8:A65V, S:T478K, S:D614G, S:P681H, S:T732A,	

hCoV-19/Mexico/CMX-INMEGEN-03-05-07/2021	EPI_ISL_1262707	20B	B.1.1.519	26	5'UTR:C203T, 5'UTR:C222T, 5'UTR:C241T, ORF1ab:A1367G, ORF1ab:G2447T, ORF1ab:C3037T, ORF1ab:C3140T, ORF1ab:C6541T, ORF1ab:C10029T, ORF1ab:C10954T, ORF1ab:A11117G, ORF1ab:C12789T, ORF1ab:C14408T, ORF1ab:T19839C, ORF1ab:C20133T, ORF1ab:C21306T, S:A22023C, S:C22995A, S:A23403G, S:C23604A, S:A23756G, ORF6:G27240T, ORF8:T27904C, N:G28881A, N:G28882A, N:G28883C, N:C29197T,	16	N:R203K, N:G204R, ORF1a:I368V, ORF1a:G728C, ORF1a:P959S, ORF1a:T3255I, ORF1a:I3618V, ORF1a:T4175I, ORF1b:P314L, ORF6:E13D, ORF8:L4P, S:E154A, S:T478K, S:D614G, S:P681H, S:T732A,	
hCoV-19/Mexico/CMX-INMEGEN-03-05-08/2021	EPI_ISL_1262708	20B	B.1.1.519	22	5'UTR:C203T, 5'UTR:C222T, 5'UTR:C241T, ORF1ab:C3037T, ORF1ab:C3140T, ORF1ab:T9723C, ORF1ab:C10029T, ORF1ab:C10954T, ORF1ab:A11117G, ORF1ab:G11417T, ORF1ab:C12789T, ORF1ab:C14408T, ORF1ab:G15957T, ORF1ab:T19839C, ORF1ab:C21306T, S:C22995A, S:A23403G, S:C23604A, S:A23756G, N:G28881A, N:G28882A, N:G28883C, N:C29197T,	13	N:R203K, N:G204R, ORF1a:P959S, ORF1a:F3153S, ORF1a:T3255I, ORF1a:I3618V, ORF1a:V3718F, ORF1a:T4175I, ORF1b:P314L, S:T478K, S:D614G, S:P681H, S:T732A,	
hCoV-19/Mexico/CMX-INMEGEN-03-05-09/2021	EPI_ISL_1262709	20B	B.1.1.519	26	5'UTR:C203T, 5'UTR:C222T, 5'UTR:C241T, ORF1ab:C3037T, ORF1ab:C3140T, ORF1ab:T3745C, ORF1ab:C10029T, ORF1ab:C10252T, ORF1ab:C10954T, ORF1ab:A11117G, ORF1ab:C12651T, ORF1ab:C12789T, ORF1ab:C14408T, ORF1ab:G15908A, ORF1ab:G16897A, ORF1ab:T19839C, ORF1ab:C21306T, S:G22199T, S:C22995A, S:A23403G, S:C23604A, S:A23756G, ORF3a:C25844T, N:G28881A, N:G28882A, N:G28883C, N:C29197T,	16	N:R203K, N:G204R, ORF1a:P959S, ORF1a:T3255I, ORF1a:I3618V, ORF1a:T4129I, ORF1a:T4175I, ORF1b:P314L, ORF1b:G814D, ORF1b:V1144I, ORF3a:T151I, S:V213L, S:T478K, S:D614G, S:P681H, S:T732A,	
hCoV-19/Mexico/CMX-INMEGEN-03-05-10/2021	EPI_ISL_1262710	20B	B.1.1.519	25	5'UTR:C203T, 5'UTR:C222T, 5'UTR:C241T, ORF1ab:C3037T, ORF1ab:C3140T, ORF1ab:C10029T, ORF1ab:C10954T, ORF1ab:A11117G, ORF1ab:C12789T, ORF1ab:A12841G, ORF1ab:C14408T, ORF1ab:G15921T, ORF1ab:A17971G, ORF1ab:T19839C, ORF1ab:C21306T, S:A22034G, S:C22082T, S:C22995A, S:A23403G, S:C23604A, S:C23635T, S:A23756G, N:G28881A, N:G28882A, N:G28883C, N:C29197T,	14	N:R203K, N:G204R, ORF1a:P959S, ORF1a:T3255I, ORF1a:I3618V, ORF1a:T4175I, ORF1b:P314L, ORF1b:R1502G, S:R158G, S:P174S, S:T478K, S:D614G, S:P681H, S:T732A,	
hCoV-19/Mexico/CMX-INMEGEN-03-05-11/2021	EPI_ISL_1262642	20B	B.1.1.519	27	5'UTR:C203T, 5'UTR:A223T, 5'UTR:C241T, ORF1ab:C2455T, ORF1ab:C3037T, ORF1ab:C3140T, ORF1ab:C8047T, ORF1ab:C10029T, ORF1ab:C10954T, ORF1ab:A11117G, ORF1ab:G11365T, ORF1ab:C12789T, ORF1ab:C13384T, ORF1ab:C14408T, ORF1ab:T15978C, ORF1ab:T19839C, ORF1ab:C21306T, S:C21575T, S:C22995A, S:A23403G, S:C23604A, S:A23756G, N:C28507T, N:G28881A, N:G28882A, N:G28883C, N:C29197T, N:G29527T,	14	N:R203K, N:G204R, N:Q418H, ORF1a:P959S, ORF1a:T3255I, ORF1a:I3618V, ORF1a:T4175I, ORF1b:P314L, ORF9b:A75V, S:L5F, S:T478K, S:D614G, S:P681H, S:T732A,	5'UTR:2 22,
hCoV-19/Mexico/CMX-INMEGEN-03-05-12/2021	EPI_ISL_1262711	20B	B.1.1.519	22	5'UTR:C203T, 5'UTR:C222T, 5'UTR:C241T, ORF1ab:C3037T, ORF1ab:C3140T, ORF1ab:G7037T, ORF1ab:C10029T, ORF1ab:C10954T, ORF1ab:A11117G, ORF1ab:C12789T, ORF1ab:C14408T, ORF1ab:T19839C, ORF1ab:C21306T, S:G21786T, S:C22995A, S:A23403G, S:C23604A, S:A23756G, ORF3a:C25782T, N:G28881A, N:G28882A, N:G28883C, N:C29197T,	13	N:R203K, N:G204R, ORF1a:P959S, ORF1a:G2258C, ORF1a:T3255I, ORF1a:I3618V, ORF1a:T4175I, ORF1b:P314L, S:G75V, S:T478K, S:D614G, S:P681H, S:T732A,	

hCoV-19/Mexico/CMX-INMEGEN-03-05-13/2021	EPI_ISL_1262712	20B	B.1.1.519	27	5'UTR:C203T, 5'UTR:C222T, 5'UTR:C241T, ORF1ab:C1912T, ORF1ab:T2950C, ORF1ab:C3037T, ORF1ab:C3140T, ORF1ab:C3736T, ORF1ab:G8881A, ORF1ab:C10029T, ORF1ab:C10954T, ORF1ab:A11117G, ORF1ab:C12789T, ORF1ab:C14408T, ORF1ab:A15189G, ORF1ab:T19839C, ORF1ab:C21306T, S:C22995A, S:A23403G, S:C23604A, S:A23756G, S:T24892C, N:G28881A, N:G28882A, N:G28883C, N:C29095T, N:C29197T, N:G29527T,	12	N:R203K, N:G204R, N:Q418H, ORF1a:P959S, ORF1a:T3255I, ORF1a:I3618V, ORF1a:T4175I, ORF1b:P314L, S:T478K, S:D614G, S:P681H, S:T732A,	
hCoV-19/Mexico/CMX-INMEGEN-03-05-14/2021	EPI_ISL_1262713	20B	B.1.1.519	29	5'UTR:C203T, 5'UTR:C222T, 5'UTR:C241T, ORF1ab:T1066C, ORF1ab:C2106T, ORF1ab:C3037T, ORF1ab:C3140T, ORF1ab:C4752T, ORF1ab:C6701T, ORF1ab:C10029T, ORF1ab:C10954T, ORF1ab:A11117G, ORF1ab:G12111A, ORF1ab:C12789T, ORF1ab:C14408T, ORF1ab:G15921T, ORF1ab:G19518T, ORF1ab:T19839C, ORF1ab:C21306T, ORF1ab:A21405T, S:C21575T, S:C22995A, S:A23403G, S:C23604A, S:A23756G, M:G26795T, N:G28881A, N:G28882A, N:G28883C, N:C29197T,	19	M:M91I, N:R203K, N:G204R, ORF1a:T614I, ORF1a:P959S, ORF1a:T1496I, ORF1a:L2146F, ORF1a:T3255I, ORF1a:I3618V, ORF1a:S3949N, ORF1a:T4175I, ORF1b:P314L, ORF1b:L2017F, ORF1b:K2646N, S:L5F, S:T478K, S:D614G, S:P681H, S:T732A,	
hCoV-19/Mexico/CMX-INMEGEN-03-05-15/2021	EPI_ISL_1262714	20B	B.1.1.519	26	5'UTR:C203T, 5'UTR:C222T, 5'UTR:C241T, ORF1ab:C3037T, ORF1ab:C3140T, ORF1ab:T3745C, ORF1ab:C10029T, ORF1ab:C10252T, ORF1ab:C10954T, ORF1ab:A11117G, ORF1ab:C12651T, ORF1ab:C12789T, ORF1ab:C14408T, ORF1ab:G15908A, ORF1ab:G16897A, ORF1ab:T19839C, ORF1ab:C21306T, S:G22199T, S:C22995A, S:A23403G, S:C23604A, S:A23756G, ORF3a:C25844T, N:G28881A, N:G28882A, N:G28883C, N:C29197T,	16	N:R203K, N:G204R, ORF1a:P959S, ORF1a:T3255I, ORF1a:I3618V, ORF1a:T4129I, ORF1a:T4175I, ORF1b:P314L, ORF1b:G814D, ORF1b:V1144I, ORF3a:T151I, S:V213L, S:T478K, S:D614G, S:P681H, S:T732A,	
hCoV-19/Mexico/CMX-INMEGEN-03-05-16/2021	EPI_ISL_1300425	20B	B.1.1.519	23	5'UTR:C203T, 5'UTR:C222T, 5'UTR:C241T, ORF1ab:T1012C, ORF1ab:C3037T, ORF1ab:C3140T, ORF1ab:C8140T, ORF1ab:C10029T, ORF1ab:C10954T, ORF1ab:G11083T, ORF1ab:A11117G, ORF1ab:C12789T, ORF1ab:C14408T, ORF1ab:T19839C, ORF1ab:C21306T, S:C22995A, S:A23403G, S:C23604A, S:A23756G, N:G28881A, N:G28882A, N:G28883C, N:C29197T, N:G29527T,	13	N:R203K, N:G204R, N:Q418H, ORF1a:P959S, ORF1a:T3255I, ORF1a:L3606F, ORF1a:I3618V, ORF1a:T4175I, ORF1b:P314L, S:T478K, S:D614G, S:P681H, S:T732A,	ORF8:28254,
hCoV-19/Mexico/CMX-INMEGEN-03-05-17/2021	EPI_ISL_1262693	20B	B.1.1.519	25	5'UTR:T201C, 5'UTR:C203T, 5'UTR:C222T, 5'UTR:C241T, ORF1ab:G1738T, ORF1ab:C3037T, ORF1ab:C3140T, ORF1ab:A3363T, ORF1ab:C10029T, ORF1ab:C10954T, ORF1ab:A11117G, ORF1ab:C12789T, ORF1ab:C13168T, ORF1ab:C14408T, ORF1ab:T19839C, ORF1ab:A19974G, ORF1ab:C21306T, S:C22995A, S:A23403G, S:C23604A, S:A23756G, ORF3a:C25791T, N:G28881A, N:G28882A, N:G28883C, N:C29197T,	12	N:R203K, N:G204R, ORF1a:P959S, ORF1a:K1033I, ORF1a:T3255I, ORF1a:I3618V, ORF1a:T4175I, ORF1b:P314L, S:T478K, S:D614G, S:P681H, S:T732A,	
hCoV-19/Mexico/CMX-INMEGEN-03-05-18/2021	EPI_ISL_1262715	20B	B.1.1.519	24	5'UTR:C203T, 5'UTR:C222T, 5'UTR:C241T, ORF1ab:C3037T, ORF1ab:C3140T, ORF1ab:C5183T, ORF1ab:C10029T, ORF1ab:C10954T, ORF1ab:A11117G, ORF1ab:C11775T, ORF1ab:C12789T, ORF1ab:C14408T, ORF1ab:T19839C, ORF1ab:C21306T, S:C22995A, S:A23403G, S:C23604A, S:A23756G, ORF3a:C25513T, ORF7a:G27708A, ORF8:T27904C, N:G28881A, N:G28882A, N:G28883C, N:C29197T,	15	N:R203K, N:G204R, ORF1a:P959S, ORF1a:P1640S, ORF1a:T3255I, ORF1a:I3618V, ORF1a:A3837V, ORF1a:T4175I, ORF1b:P314L, ORF3a:L41F, ORF8:L4P, S:T478K, S:D614G, S:P681H, S:T732A,	

hCoV-19/Mexico/CMX-INMEGEN-03-05-19/2021	EPI_ISL_1262716	20B	B.1.1.519	27	5'UTR:T201C, 5'UTR:C203T, 5'UTR:C222T, 5'UTR:C241T, ORF1ab:G1738T, ORF1ab:C3037T, ORF1ab:C3140T, ORF1ab:G5665A, ORF1ab:C5849T, ORF1ab:C10029T, ORF1ab:C10954T, ORF1ab:A11117G, ORF1ab:C12241T, ORF1ab:C12789T, ORF1ab:C14408T, ORF1ab:T19839C, ORF1ab:A19974G, ORF1ab:C21306T, S:C22995A, S:A23403G, S:C23604A, S:A23756G, N:G28690A, N:G28881A, N:G28882A, N:G28883C, N:C29197T, ORF10:C29627T,	12	N:R203K, N:G204R, ORF1a:P959S, ORF1a:P1862S, ORF1a:T3255I, ORF1a:I3618V, ORF1a:T4175I, ORF1b:P314L, S:T478K, S:D614G, S:P681H, S:T732A,
hCoV-19/Mexico/CMX-INMEGEN-03-05-21/2021	EPI_ISL_1262717	20B	B.1.1.519	26	5'UTR:T201C, 5'UTR:C203T, 5'UTR:C222T, 5'UTR:C241T, ORF1ab:G1738T, ORF1ab:C3037T, ORF1ab:C3140T, ORF1ab:A6097G, ORF1ab:C10029T, ORF1ab:C10252T, ORF1ab:C10954T, ORF1ab:A11117G, ORF1ab:C12789T, ORF1ab:C14408T, ORF1ab:T19839C, ORF1ab:A19974G, ORF1ab:C21306T, S:C22995A, S:A23403G, S:C23604A, S:A23756G, ORF3a:C25553T, ORF7b:G27861T, N:G28881A, N:G28882A, N:G28883C, N:C29197T,	13	N:R203K, N:G204R, ORF1a:P959S, ORF1a:T3255I, ORF1a:I3618V, ORF1a:T4175I, ORF1b:P314L, ORF3a:A54V, ORF7b:D36Y, S:T478K, S:D614G, S:P681H, S:T732A,
hCoV-19/Mexico/CMX-INMEGEN-03-05-22/2021	EPI_ISL_1262694	20B	B.1.1.519	24	5'UTR:C203T, 5'UTR:C222T, 5'UTR:C241T, ORF1ab:C3037T, ORF1ab:C3140T, ORF1ab:T9456G, ORF1ab:C10029T, ORF1ab:C10954T, ORF1ab:A11117G, ORF1ab:C12789T, ORF1ab:C14408T, ORF1ab:T14429C, ORF1ab:C17518T, ORF1ab:T19839C, ORF1ab:C21306T, S:C22995A, S:A23403G, S:C23604A, S:A23756G, S:C24919T, ORF3a:T25496C, N:G28881A, N:G28882A, N:G28883C, N:C29197T,	15	N:R203K, N:G204R, ORF1a:P959S, ORF1a:F3064C, ORF1a:T3255I, ORF1a:I3618V, ORF1a:T4175I, ORF1b:P314L, ORF1b:V321A, ORF1b:L1351F, ORF3a:I35T, S:T478K, S:D614G, S:P681H, S:T732A,
hCoV-19/Mexico/CMX-INMEGEN-03-05-26/2021	EPI_ISL_1262695	20B	B.1.1.519	26	5'UTR:C203T, 5'UTR:C222T, 5'UTR:C241T, ORF1ab:C3037T, ORF1ab:C3140T, ORF1ab:G4510T, ORF1ab:A8010C, ORF1ab:C10029T, ORF1ab:C10954T, ORF1ab:A11117G, ORF1ab:C12789T, ORF1ab:C14408T, ORF1ab:G15921T, ORF1ab:G17706A, ORF1ab:A17971G, ORF1ab:T19839C, ORF1ab:C21306T, S:C22986T, S:C22995A, S:A23403G, S:C23604A, S:C23635T, S:A23756G, N:G28881A, N:G28882A, N:G28883C, N:C29197T,	14	N:R203K, N:G204R, ORF1a:P959S, ORF1a:D2582A, ORF1a:T3255I, ORF1a:I3618V, ORF1a:T4175I, ORF1b:P314L, ORF1b:R1502G, S:A475V, S:T478K, S:D614G, S:P681H, S:T732A,
hCoV-19/Mexico/CMX-INMEGEN-03-05-27/2021	EPI_ISL_1262696	20B	B.1.1.519	24	5'UTR:C203T, 5'UTR:C222T, 5'UTR:C241T, ORF1ab:C3037T, ORF1ab:C3140T, ORF1ab:T3745C, ORF1ab:C9638T, ORF1ab:C10029T, ORF1ab:C10954T, ORF1ab:A11117G, ORF1ab:C12789T, ORF1ab:C14408T, ORF1ab:G17211T, ORF1ab:T19839C, ORF1ab:C21306T, S:C22995A, S:A23403G, S:C23604A, S:A23756G, ORF3a:C25844T, N:G28881A, N:G28882A, N:G28883C, N:C29197T, N:G29422T,	14	N:R203K, N:G204R, ORF1a:P959S, ORF1a:H3125Y, ORF1a:T3255I, ORF1a:I3618V, ORF1a:T4175I, ORF1b:P314L, ORF1b:L1248F, ORF3a:T151I, S:T478K, S:D614G, S:P681H, S:T732A,
hCoV-19/Mexico/CMX-INMEGEN-03-05-28/2021	EPI_ISL_1262697	20B	B.1.1.519	26	5'UTR:C203T, 5'UTR:C222T, 5'UTR:C241T, ORF1ab:C3037T, ORF1ab:A3081G, ORF1ab:C3140T, ORF1ab:C3923T, ORF1ab:C5646T, ORF1ab:C10029T, ORF1ab:C10954T, ORF1ab:A11117G, ORF1ab:C12789T, ORF1ab:C14408T, ORF1ab:A15864G, ORF1ab:A17205G, ORF1ab:T19839C, ORF1ab:C21306T, S:C22995A, S:A23403G, S:C23604A, S:A23756G, ORF7a:C27641T, N:G28881A, N:G28882A, N:G28883C, N:C29197T, N:G29431T,	16	N:R203K, N:G204R, N:Q386H, ORF1a:E939G, ORF1a:P959S, ORF1a:P1220S, ORF1a:T1794I, ORF1a:T3255I, ORF1a:I3618V, ORF1a:T4175I, ORF1b:P314L, ORF7a:S83L, S:T478K, S:D614G, S:P681H, S:T732A,

hCoV-19/Mexico/CMX-INMEGEN-03-05-30/2021	EPI_ISL_1262698	20B	B.1.1.519	26	5'UTR:C203T, 5'UTR:C222T, 5'UTR:C241T, ORF1ab:C3037T, ORF1ab:C3140T, ORF1ab:T3745C, ORF1ab:C10029T, ORF1ab:C10954T, ORF1ab:A11117G, ORF1ab:C12242T, ORF1ab:C12789T, ORF1ab:C14408T, ORF1ab:C18395T, ORF1ab:A19751G, ORF1ab:T19839C, ORF1ab:C21306T, S:C22995A, S:A23403G, S:C23604A, S:A23756G, S:A24162G, ORF3a:C25844T, N:C28849T, N:G28881A, N:G28882A, N:G28883C, N:C29197T,	16	N:R203K, N:G204R, ORF1a:P959S, ORF1a:T3255I, ORF1a:I3618V, ORF1a:R3993C, ORF1a:T4175I, ORF1b:P314L, ORF1b:A1643V, ORF1b:E2095G, ORF3a:T151I, S:T478K, S:D614G, S:P681H, S:T732A, S:D867G,
hCoV-19/Mexico/CMX-INMEGEN-03-05-33/2021	EPI_ISL_1262699	20B	B.1.1.519	32	5'UTR:C203T, 5'UTR:C222T, 5'UTR:C241T, ORF1ab:T1303A, ORF1ab:T1417A, ORF1ab:C3037T, ORF1ab:C3140T, ORF1ab:C5221T, ORF1ab:C6027T, ORF1ab:C10029T, ORF1ab:C10954T, ORF1ab:A11117G, ORF1ab:C12786T, ORF1ab:C12789T, ORF1ab:C13665T, ORF1ab:C14408T, ORF1ab:G16065A, ORF1ab:T19839C, ORF1ab:G20377A, ORF1ab:C21306T, S:C22995A, S:A23403G, S:C23604A, S:A23756G, ORF3a:C25511T, ORF8:C28054T, ORF8:C28185A, N:G28881A, N:G28882A, N:G28883C, N:C29197T, ORF10:T29661C, 3'UTR:G29745A,	16	N:R203K, N:G204R, ORF1a:P959S, ORF1a:P1921L, ORF1a:T3255I, ORF1a:I3618V, ORF1a:T4174I, ORF1a:T4175I, ORF1b:P314L, ORF1b:G2304R, ORF3a:S40L, ORF8:S54L, S:T478K, S:D614G, S:P681H, S:T732A,
hCoV-19/Mexico/CMX-INMEGEN-03-05-35/2021	EPI_ISL_1262686	20B	B.1.1.519	25	5'UTR:C203T, 5'UTR:C222T, 5'UTR:C241T, ORF1ab:C3037T, ORF1ab:C3140T, ORF1ab:C3787T, ORF1ab:A5128G, ORF1ab:C10029T, ORF1ab:C10954T, ORF1ab:A11117G, ORF1ab:C12225T, ORF1ab:C12789T, ORF1ab:C14408T, ORF1ab:T19839C, ORF1ab:C21306T, S:G22335T, S:G22778C, S:C22995A, S:A23403G, S:C23604A, S:A23756G, N:G28881A, N:G28882A, N:G28883C, N:C29197T, N:G29527T,	15	N:R203K, N:G204R, N:Q418H, ORF1a:P959S, ORF1a:T3255I, ORF1a:I3618V, ORF1a:A3987V, ORF1a:T4175I, ORF1b:P314L, S:W258L, S:E406Q, S:T478K, S:D614G, S:P681H, S:T732A,
hCoV-19/Mexico/CMX-INMEGEN-03-05-36/2021	EPI_ISL_1262700	20B	B.1.1.519	30	5'UTR:T201C, 5'UTR:C203T, 5'UTR:C222T, 5'UTR:C241T, ORF1ab:G1738T, ORF1ab:C2197T, ORF1ab:C3037T, ORF1ab:C3140T, ORF1ab:C6114T, ORF1ab:G7829T, ORF1ab:C10029T, ORF1ab:C10954T, ORF1ab:C11074T, ORF1ab:A11117G, ORF1ab:C12789T, ORF1ab:C14408T, ORF1ab:G14854T, ORF1ab:C15951T, ORF1ab:T19839C, ORF1ab:A19974G, ORF1ab:C21306T, S:C22995A, S:A23403G, S:C23604A, S:A23756G, ORF3a:C25546T, N:G28881A, N:G28882A, N:G28883C, N:C29022T, N:C29197T,	16	N:R203K, N:G204R, N:S250F, ORF1a:P959S, ORF1a:P1950L, ORF1a:V2522F, ORF1a:T3255I, ORF1a:I3618V, ORF1a:T4175I, ORF1b:P314L, ORF1b:V463L, ORF3a:L52F, S:T478K, S:D614G, S:P681H, S:T732A,
hCoV-19/Mexico/CMX-INMEGEN-03-05-37/2021	EPI_ISL_1262638	20B	B.1.1.519	30	5'UTR:C203T, 5'UTR:C222T, 5'UTR:C241T, ORF1ab:C3037T, ORF1ab:C3140T, ORF1ab:G3692T, ORF1ab:C10029T, ORF1ab:C10954T, ORF1ab:A11117G, ORF1ab:C12789T, ORF1ab:C13515T, ORF1ab:C14408T, ORF1ab:C15952T, ORF1ab:T16993C, ORF1ab:T19839C, ORF1ab:C21145T, ORF1ab:C21306T, S:G22014T, S:C22995A, S:A23403G, S:T23443C, S:C23604A, S:A23756G, S:T24903C, S:G25314T, M:C26936T, ORF8:A28095T, N:G28881A, N:G28882A, N:G28883C, N:C29197T,	18	N:R203K, N:G204R, ORF1a:P959S, ORF1a:V1143F, ORF1a:T3255I, ORF1a:I3618V, ORF1a:T4175I, ORF1b:P314L, ORF1b:Y1176H, ORF1b:L2560F, ORF8:K68*, S:S151I, S:T478K, S:D614G, S:P681H, S:T732A, S:I1114T, S:G1251V,

hCoV-19/Mexico/CMX-INMEGEN-03-05-38/2021	EPI_ISL_1262687	20B	B.1.1.519	24	5'UTR:C203T, 5'UTR:C222T, 5'UTR:C241T, ORF1ab:C1480T, ORF1ab:G1659T, ORF1ab:C3037T, ORF1ab:C3140T, ORF1ab:C10029T, ORF1ab:C10954T, ORF1ab:A11117G, ORF1ab:C12789T, ORF1ab:C14408T, ORF1ab:C14838T, ORF1ab:T19839C, ORF1ab:C21306T, S:C22000T, S:C22995A, S:A23403G, S:C23604A, S:A23756G, M:C26912T, N:G28881A, N:G28882A, N:G28883C, N:C29197T,	12	N:R203K, N:G204R, ORF1a:G465V, ORF1a:P959S, ORF1a:T3255I, ORF1a:I3618V, ORF1a:T4175I, ORF1b:P314L, S:T478K, S:D614G, S:P681H, S:T732A,
hCoV-19/Mexico/CMX-INMEGEN-03-05-39/2021	EPI_ISL_1262682	20B	B.1.1.519	24	5'UTR:C203T, 5'UTR:C222T, 5'UTR:C241T, ORF1ab:C3037T, ORF1ab:C3140T, ORF1ab:G3340T, ORF1ab:C5183T, ORF1ab:C9985T, ORF1ab:C10029T, ORF1ab:C10954T, ORF1ab:A11117G, ORF1ab:C12789T, ORF1ab:C14408T, ORF1ab:T19839C, ORF1ab:C21306T, S:C22995A, S:A23403G, S:C23604A, S:A23756G, N:C28411T, N:G28881A, N:G28882A, N:G28883C, N:C29197T, N:G29527T,	14	N:R203K, N:G204R, N:Q418H, ORF1a:P959S, ORF1a:P1640S, ORF1a:T3255I, ORF1a:I3618V, ORF1a:T4175I, ORF1b:P314L, ORF9b:P43L, S:T478K, S:D614G, S:P681H, S:T732A,
hCoV-19/Mexico/CMX-INMEGEN-03-05-40/2021	EPI_ISL_1262701	20B	B.1.1.519	27	5'UTR:C203T, 5'UTR:C222T, 5'UTR:C241T, ORF1ab:C3037T, ORF1ab:C3140T, ORF1ab:C10029T, ORF1ab:C10954T, ORF1ab:A11117G, ORF1ab:G12111A, ORF1ab:C12789T, ORF1ab:C14408T, ORF1ab:G15921T, ORF1ab:G19518T, ORF1ab:C19524T, ORF1ab:T19839C, ORF1ab:C21306T, S:C22995A, S:A23403G, S:C23604A, S:A23756G, M:C26912T, N:T28306A, N:G28881A, N:G28882A, N:G28883C, N:C29197T, N:C29323T, 3'UTR:C29732G,	15	N:N11K, N:R203K, N:G204R, ORF1a:P959S, ORF1a:T3255I, ORF1a:I3618V, ORF1a:S3949N, ORF1a:T4175I, ORF1b:P314L, ORF1b:L2017F, ORF9b:M8K, S:T478K, S:D614G, S:P681H, S:T732A,
hCoV-19/Mexico/CMX-INMEGEN-03-05-41/2021	EPI_ISL_1262688	20B	B.1.1.519	26	5'UTR:T201C, 5'UTR:C203T, 5'UTR:C222T, 5'UTR:C241T, ORF1ab:G1738T, ORF1ab:C3037T, ORF1ab:C3140T, ORF1ab:C4752T, ORF1ab:C4780T, ORF1ab:C10029T, ORF1ab:T10135C, ORF1ab:C10954T, ORF1ab:A11117G, ORF1ab:C12789T, ORF1ab:C14408T, ORF1ab:T19839C, ORF1ab:A19974G, ORF1ab:C21306T, S:C22995A, S:A23403G, S:C23604A, S:A23756G, N:G28881A, N:G28882A, N:G28883C, N:C29197T, N:G29449A,	12	N:R203K, N:G204R, ORF1a:P959S, ORF1a:T1496I, ORF1a:T3255I, ORF1a:I3618V, ORF1a:T4175I, ORF1b:P314L, S:T478K, S:D614G, S:P681H, S:T732A,
hCoV-19/Mexico/CMX-INMEGEN-03-05-43/2021	EPI_ISL_1262631	20B	B.1.1.519	27	5'UTR:C203T, 5'UTR:C222T, 5'UTR:C241T, ORF1ab:C3037T, ORF1ab:C3140T, ORF1ab:G4510A, ORF1ab:G6894A, ORF1ab:C7011T, ORF1ab:C10029T, ORF1ab:C10252T, ORF1ab:C10954T, ORF1ab:A11117G, ORF1ab:G11365T, ORF1ab:C12789T, ORF1ab:C14408T, ORF1ab:C18917T, ORF1ab:T19839C, ORF1ab:C21306T, S:C22995A, S:A23403G, S:C23604A, S:A23756G, M:A27140G, N:G28881A, N:G28882A, N:G28883C, N:C29197T, N:G29527T	15	N:R203K, N:G204R, N:Q418H, ORF1a:P959S, ORF1a:C2210Y, ORF1a:A2249V, ORF1a:T3255I, ORF1a:I3618V, ORF1a:T4175I, ORF1b:P314L, ORF1b:T1817I, S:T478K, S:D614G, S:P681H, S:T732A,
hCoV-19/Mexico/CMX-INMEGEN-03-05-47/2021	EPI_ISL_1262689	20B	B.1.1.519	29	5'UTR:C203T, 5'UTR:C222T, 5'UTR:C241T, ORF1ab:C3037T, ORF1ab:C3140T, ORF1ab:T3745C, ORF1ab:C9907T, ORF1ab:C10029T, ORF1ab:C10954T, ORF1ab:A11117G, ORF1ab:A12259G, ORF1ab:C12789T, ORF1ab:C14408T, ORF1ab:C18877T, ORF1ab:T19839C, ORF1ab:C21306T, S:C22432T, S:C22987T, S:C22995A, S:A23403G, S:C23604A, S:A23756G, ORF3a:C25844T, N:C28849T, N:G28881A, N:G28882A, N:G28883C, N:G29179T, N:C29197T, 3'UTR:G29810A,	12	N:R203K, N:G204R, ORF1a:P959S, ORF1a:T3255I, ORF1a:I3618V, ORF1a:T4175I, ORF1b:P314L, ORF3a:T151I, S:T478K, S:D614G, S:P681H, S:T732A,

hCoV-19/Mexico/CMX-INMEGEN-03-05-48/2021	EPI_ISL_1262690	20B	B.1.1.519	23	5'UTR:C203T, 5'UTR:C222T, 5'UTR:C241T, ORF1ab:C3037T, ORF1ab:C3140T, ORF1ab:C10029T, ORF1ab:C10954T, ORF1ab:A11117G, ORF1ab:C11575T, ORF1ab:C12789T, ORF1ab:C14408T, ORF1ab:T19839C, ORF1ab:C21306T, S:C22995A, S:A23403G, S:C23604A, S:C23709T, S:A23756G, ORF3a:C25658T, E:T26442A, N:G28881A, N:G28882A, N:G28883C, N:C29197T,	14	E:N66K, N:R203K, N:G204R, ORF1a:P959S, ORF1a:T3255I, ORF1a:I3618V, ORF1a:T4175I, ORF1b:P314L, ORF3a:T89I, S:T478K, S:D614G, S:P681H, S:T716I, S:T732A,	
hCoV-19/Mexico/CMX-INMEGEN-03-05-49/2021	EPI_ISL_1262683	20B	B.1.1.519	27	5'UTR:C203T, 5'UTR:C222T, 5'UTR:C241T, ORF1ab:C3037T, ORF1ab:C3140T, ORF1ab:C5512T, ORF1ab:C6318T, ORF1ab:C10029T, ORF1ab:C10954T, ORF1ab:G10996T, ORF1ab:A11117G, ORF1ab:C11575T, ORF1ab:C12789T, ORF1ab:C14408T, ORF1ab:T16863C, ORF1ab:G18838T, ORF1ab:T19839C, ORF1ab:C21306T, S:C21575T, S:C22995A, S:A23403G, S:C23604A, S:A23756G, N:G28881A, N:G28882A, N:G28883C, N:C29197T, 3'UTR:C29754T,	15	N:R203K, N:G204R, ORF1a:P959S, ORF1a:P2018L, ORF1a:T3255I, ORF1a:K3577N, ORF1a:I3618V, ORF1a:T4175I, ORF1b:P314L, ORF1b:A1791S, S:L5F, S:T478K, S:D614G, S:P681H, S:T732A,	
hCoV-19/Mexico/CMX-INMEGEN-03-05-50/2021	EPI_ISL_1310811	20B	B.1.1.519	23	5'UTR:C203T, 5'UTR:C222T, 5'UTR:C241T, ORF1ab:C3037T, ORF1ab:C3140T, ORF1ab:C10029T, ORF1ab:C10954T, ORF1ab:A11117G, ORF1ab:C12789T, ORF1ab:C14408T, ORF1ab:A19671G, ORF1ab:T19839C, ORF1ab:C21306T, S:C22995A, S:A23403G, S:C23604A, S:A23756G, ORF7b:G27877T, N:G28362T, N:G28881A, N:G28882A, N:G28883C, N:C29197T, 3'UTR:A29700G,	11	ORF1a:P959S, ORF1a:T3255I, ORF1a:I3618V, ORF1a:T4175I, ORF1b:P314L, ORF7b:C41F, ORF9b:E27*, S:T478K, S:D614G, S:P681H, S:T732A,	N:29441-29448
hCoV-19/Mexico/CMX-INMEGEN-03-05-51/2021	EPI_ISL_1262684	20B	B.1.1.519	27	5'UTR:T201C, 5'UTR:C203T, 5'UTR:C222T, 5'UTR:C241T, ORF1ab:G1738T, ORF1ab:C3037T, ORF1ab:C3140T, ORF1ab:G3753A, ORF1ab:A6459G, ORF1ab:A9280G, ORF1ab:C10029T, ORF1ab:C10954T, ORF1ab:A11117G, ORF1ab:C12789T, ORF1ab:C14408T, ORF1ab:A16026G, ORF1ab:G18020A, ORF1ab:T19839C, ORF1ab:A19974G, ORF1ab:C21306T, S:C22995A, S:A23403G, S:C23604A, S:A23756G, N:G28881A, N:G28882A, N:G28883C, N:C29197T,	14	N:R203K, N:G204R, ORF1a:P959S, ORF1a:R1163K, ORF1a:N2065S, ORF1a:T3255I, ORF1a:I3618V, ORF1a:T4175I, ORF1b:P314L, ORF1b:R1518K, S:T478K, S:D614G, S:P681H, S:T732A,	
hCoV-19/Mexico/CMX-INMEGEN-03-05-52/2021	EPI_ISL_1262685	20B	B.1.1.222	24	5'UTR:C203T, 5'UTR:C222T, 5'UTR:C241T, ORF1ab:C3037T, ORF1ab:C3140T, ORF1ab:G4148A, ORF1ab:C5622T, ORF1ab:C10029T, ORF1ab:C10954T, ORF1ab:A11117G, ORF1ab:C12789T, ORF1ab:T13560C, ORF1ab:C14408T, ORF1ab:T19839C, ORF1ab:C21306T, S:C22747T, S:C22995A, S:A23403G, S:C23604A, S:A23756G, N:G28881A, N:G28882A, N:G28883C, N:C29197T, N:C29311T,	13	N:R203K, N:G204R, ORF1a:P959S, ORF1a:V1295I, ORF1a:P1786L, ORF1a:T3255I, ORF1a:I3618V, ORF1a:T4175I, ORF1b:P314L, S:T478K, S:D614G, S:P681H, S:T732A,	
hCoV-19/Mexico/CMX-INMEGEN-03-05-69/2021	EPI_ISL_1262680	20B	B.1.1.519	29	5'UTR:C203T, 5'UTR:C222T, 5'UTR:C241T, ORF1ab:C3037T, ORF1ab:C3140T, ORF1ab:G3692T, ORF1ab:G3871T, ORF1ab:C5812T, ORF1ab:A8446G, ORF1ab:C10029T, ORF1ab:C10789T, ORF1ab:C10954T, ORF1ab:A11117G, ORF1ab:C11824A, ORF1ab:C12789T, ORF1ab:C14408T, ORF1ab:T19839C, ORF1ab:C21306T, S:C22995A, S:A23403G, S:C23604A, S:A23756G, S:C24442T, ORF7a:A27540G, ORF8:T27904C, N:G28361A, N:G28881A, N:G28882A, N:G28883C, N:C29197T,	16	N:G30R, N:R203K, N:G204R, ORF1a:P959S, ORF1a:V1143F, ORF1a:K1202N, ORF1a:T3255I, ORF1a:I3618V, ORF1a:T4175I, ORF1b:P314L, ORF8:L4P, ORF9b:M26I, S:T478K, S:D614G, S:P681H, S:T732A,	

hCoV-19/Mexico/CMX-INMEGEN-03-05-72/2021	EPI_ISL_1262681	20B	B.1.1.519	25	5'UTR:C203T, 5'UTR:C222T, 5'UTR:C241T, ORF1ab:C3037T, ORF1ab:C3140T, ORF1ab:G3246T, ORF1ab:C10029T, ORF1ab:C10954T, ORF1ab:A11117G, ORF1ab:C12789T, ORF1ab:C14408T, ORF1ab:A18294G, ORF1ab:T19839C, ORF1ab:C21306T, S:C22995A, S:A23403G, S:C23604A, S:A23756G, ORF3a:A25596G, ORF3a:C25771T, E:G26439T, M:C26907T, N:G28881A, N:G28882A, N:G28883C, N:C29197T,	13	N:R203K, N:G204R, ORF1a:P959S, ORF1a:S994I, ORF1a:T3255I, ORF1a:I3618V, ORF1a:T4175I, ORF1b:P314L, ORF3a:L127F, S:T478K, S:D614G, S:P681H, S:T732A,	
hCoV-19/Mexico/CMX-INMEGEN-03-05-74/2021	EPI_ISL_1262691	20B	B.1.1.519	22	5'UTR:C203T, 5'UTR:C222T, 5'UTR:C241T, ORF1ab:C3037T, ORF1ab:C3140T, ORF1ab:C10029T, ORF1ab:C10456T, ORF1ab:C10954T, ORF1ab:A11117G, ORF1ab:C12789T, ORF1ab:C14408T, ORF1ab:C16332T, ORF1ab:T19839C, ORF1ab:C21306T, S:C22995A, S:A23403G, S:C23604A, S:A23756G, N:G28881A, N:G28882A, N:G28883C, N:C29197T, N:G29527T,	12	N:R203K, N:G204R, N:Q418H, ORF1a:P959S, ORF1a:T3255I, ORF1a:I3618V, ORF1a:T4175I, ORF1b:P314L, S:T478K, S:D614G, S:P681H, S:T732A,	
hCoV-19/Mexico/CMX-INMEGEN-03-05-89/2021	EPI_ISL_1262692	20B	B.1.1.519	28	5'UTR:C203T, 5'UTR:C222T, 5'UTR:C241T, ORF1ab:C3037T, ORF1ab:C3140T, ORF1ab:G7037T, ORF1ab:A9568G, ORF1ab:C10029T, ORF1ab:C10954T, ORF1ab:A11117G, ORF1ab:C12171T, ORF1ab:C12789T, ORF1ab:C13944T, ORF1ab:C14408T, ORF1ab:C15755A, ORF1ab:T19839C, ORF1ab:A21194C, ORF1ab:C21306T, S:C22995A, S:A23403G, S:C23604A, S:A23756G, ORF3a:C25782T, ORF7b:G27798T, N:G28881A, N:G28882A, N:G28883C, N:G29179T, N:C29197T,	16	N:R203K, N:G204R, ORF1a:P959S, ORF1a:G2258C, ORF1a:T3255I, ORF1a:I3618V, ORF1a:A3969V, ORF1a:T4175I, ORF1b:P314L, ORF1b:S763Y, ORF1b:D2576A, ORF7b:A15S, S:T478K, S:D614G, S:P681H, S:T732A,	
hCoV-19/Mexico/CMX-INMEGEN-03-06-12/2021	EPI_ISL_1265902	20B	B.1.1.519	29	5'UTR:C203T, 5'UTR:A223T, 5'UTR:C241T, ORF1ab:A866G, ORF1ab:C1009T, ORF1ab:C3037T, ORF1ab:C3140T, ORF1ab:C5183T, ORF1ab:C5748T, ORF1ab:C10029T, ORF1ab:C10954T, ORF1ab:A11117G, ORF1ab:C11916T, ORF1ab:C12789T, ORF1ab:C13730T, ORF1ab:C14408T, ORF1ab:T19839C, ORF1ab:C21306T, S:C22995A, S:A23403G, S:C23604A, S:A23756G, ORF8:T27904C, N:G28747T, N:A28877T, N:G28878C, N:G28881A, N:G28882A, N:G28883C, N:C29197T,	17	N:R203K, N:G204R, ORF1a:I201V, ORF1a:P959S, ORF1a:P1640S, ORF1a:T1828I, ORF1a:T3255I, ORF1a:I3618V, ORF1a:S3884L, ORF1a:T4175I, ORF1b:A88V, ORF1b:P314L, ORF8:L4P, S:T478K, S:D614G, S:P681H, S:T732A,	5'UTR:2 22,
hCoV-19/Mexico/CMX-INMEGEN-03-06-13/2021	EPI_ISL_1265903	20B	B.1.1.519	24	5'UTR:C203T, 5'UTR:C222T, 5'UTR:C241T, ORF1ab:C3037T, ORF1ab:C3140T, ORF1ab:C6706T, ORF1ab:C8660T, ORF1ab:C8950T, ORF1ab:C10029T, ORF1ab:C10954T, ORF1ab:A11117G, ORF1ab:C12789T, ORF1ab:C14408T, ORF1ab:T19839C, ORF1ab:C21306T, S:C22995A, S:A23403G, S:C23604A, S:A23756G, M:C26895T, N:G28881A, N:G28882A, N:G28883C, N:C29197T, N:G29527T,	14	M:H125Y, N:R203K, N:G204R, N:Q418H, ORF1a:P959S, ORF1a:H2799Y, ORF1a:T3255I, ORF1a:I3618V, ORF1a:T4175I, ORF1b:P314L, S:T478K, S:D614G, S:P681H, S:T732A,	
hCoV-19/Mexico/CMX-INMEGEN-03-06-14/2021	EPI_ISL_1265904	20B	B.1.1.519	27	5'UTR:C203T, 5'UTR:C222T, 5'UTR:C241T, ORF1ab:T469C, ORF1ab:C3037T, ORF1ab:C3140T, ORF1ab:G7027T, ORF1ab:C8320T, ORF1ab:C10029T, ORF1ab:C10954T, ORF1ab:A11117G, ORF1ab:C12439T, ORF1ab:C12789T, ORF1ab:C14408T, ORF1ab:C15279T, ORF1ab:A18390G, ORF1ab:T19839C, ORF1ab:C21306T, S:C22995A, S:A23403G, S:C23604A, S:A23756G, S:G24445T, S:C25207T, N:G28881A, N:G28882A, N:G28883C, N:C29197T,	12	N:R203K, N:G204R, ORF1a:P959S, ORF1a:M2254I, ORF1a:T3255I, ORF1a:I3618V, ORF1a:T4175I, ORF1b:P314L, S:T478K, S:D614G, S:P681H, S:T732A,	

hCoV-19/Mexico/CMX-INMEGEN-03-06-15/2021	EPI_ISL_1265905	20B	B.1.1.222	23	5'UTR:C241T, ORF1ab:C2925T, ORF1ab:C3037T, ORF1ab:C3792T, ORF1ab:C6449T, ORF1ab:A6693G, ORF1ab:T7402C, ORF1ab:C9430T, ORF1ab:C10029T, ORF1ab:C10039T, ORF1ab:C14408T, ORF1ab:G19009A, ORF1ab:T19839C, ORF1ab:G21159T, S:A23403G, S:A23756G, ORF3a:G25912T, ORF7b:A27756G, ORF8:A27921G, ORF8:G28001T, N:G28881A, N:G28882A, N:G28883C, N:C29149T,	14	N:R203K, N:G204R, ORF1a:S887F, ORF1a:A1176V, ORF1a:L2062F, ORF1a:K2143R, ORF1a:T3255I, ORF1b:P314L, ORF1b:D1848N, ORF3a:G174C, ORF7b:M1V, ORF8:I10V, S:D614G, S:T732A,
hCoV-19/Mexico/CMX-INMEGEN-03-06-16/2021	EPI_ISL_1265906	20B	B.1.1.519	23	5'UTR:C203T, 5'UTR:C222T, 5'UTR:C241T, ORF1ab:C3037T, ORF1ab:C3140T, ORF1ab:C10029T, ORF1ab:C10954T, ORF1ab:A11117G, ORF1ab:C12789T, ORF1ab:C14408T, ORF1ab:G15921T, ORF1ab:A17971G, ORF1ab:T19839C, ORF1ab:C21306T, S:C22995A, S:A23403G, S:C23604A, S:C23635T, S:A23756G, M:C26885T, N:G28881A, N:G28882A, N:G28883C, N:C29197T,	12	N:R203K, N:G204R, ORF1a:P959S, ORF1a:T3255I, ORF1a:I3618V, ORF1a:T4175I, ORF1b:P314L, ORF1b:R1502G, S:T478K, S:D614G, S:P681H, S:T732A,
hCoV-19/Mexico/CMX-INMEGEN-03-06-17/2021	EPI_ISL_1265907	20B	B.1.1.519	24	5'UTR:C203T, 5'UTR:C222T, 5'UTR:C241T, ORF1ab:C3037T, ORF1ab:C3140T, ORF1ab:C10029T, ORF1ab:C10954T, ORF1ab:A11117G, ORF1ab:C12789T, ORF1ab:C14408T, ORF1ab:G16943T, ORF1ab:T19839C, ORF1ab:C21306T, S:C22995A, S:A23403G, S:C23604A, S:A23756G, S:A24727G, ORF7a:C27630T, ORF8:T27904C, ORF8:C28087T, N:G28881A, N:G28882A, N:G28883C, N:C29197T,	14	N:R203K, N:G204R, ORF1a:P959S, ORF1a:T3255I, ORF1a:I3618V, ORF1a:T4175I, ORF1b:P314L, ORF1b:S1159I, ORF8:L4P, ORF8:A65V, S:T478K, S:D614G, S:P681H, S:T732A,
hCoV-19/Mexico/CMX-INMEGEN-03-06-18/2021	EPI_ISL_1265908	20B	B.1.1.519	24	5'UTR:C203T, 5'UTR:C222T, 5'UTR:C241T, ORF1ab:C3037T, ORF1ab:C3140T, ORF1ab:C10029T, ORF1ab:C10954T, ORF1ab:A11117G, ORF1ab:C12789T, ORF1ab:C14408T, ORF1ab:G16943T, ORF1ab:T19839C, ORF1ab:C21306T, S:C22995A, S:A23403G, S:C23604A, S:A23756G, S:A24727G, ORF7a:C27630T, ORF8:T27904C, ORF8:C28087T, N:G28881A, N:G28882A, N:G28883C, N:C29197T,	14	N:R203K, N:G204R, ORF1a:P959S, ORF1a:T3255I, ORF1a:I3618V, ORF1a:T4175I, ORF1b:P314L, ORF1b:S1159I, ORF8:L4P, ORF8:A65V, S:T478K, S:D614G, S:P681H, S:T732A,
hCoV-19/Mexico/CMX-INMEGEN-03-08-01/2021	EPI_ISL_1315479	20B	B.1.1.519	24	5'UTR:C203T, 5'UTR:C222T, 5'UTR:C241T, ORF1ab:C3037T, ORF1ab:C3140T, ORF1ab:C10029T, ORF1ab:C10954T, ORF1ab:A11117G, ORF1ab:C12789T, ORF1ab:C14408T, ORF1ab:G16943T, ORF1ab:T19839C, ORF1ab:C21306T, S:C22995A, S:A23403G, S:C23604A, S:A23756G, S:A24727G, ORF7a:C27630T, ORF8:T27904C, ORF8:C28087T, N:G28881A, N:G28882A, N:G28883C, N:C29197T,	14	N:R203K, N:G204R, ORF1a:P959S, ORF1a:T3255I, ORF1a:I3618V, ORF1a:T4175I, ORF1b:P314L, ORF1b:S1159I, ORF8:L4P, ORF8:A65V, S:T478K, S:D614G, S:P681H, S:T732A,
hCoV-19/Mexico/CMX-INMEGEN-03-08-10/2021	EPI_ISL_1315487	20B	B.1.1.519	27	5'UTR:T201C, 5'UTR:C203T, 5'UTR:C222T, 5'UTR:C241T, ORF1ab:G1738T, ORF1ab:C3037T, ORF1ab:C3140T, ORF1ab:G3753A, ORF1ab:A6459G, ORF1ab:A9280G, ORF1ab:C10029T, ORF1ab:C10954T, ORF1ab:A11117G, ORF1ab:C12789T, ORF1ab:C14408T, ORF1ab:A16026G, ORF1ab:G18020A, ORF1ab:T19839C, ORF1ab:A19974G, ORF1ab:C21306T, S:C22995A, S:A23403G, S:C23604A, S:A23756G, N:G28881A, N:G28882A, N:G28883C, N:C29197T	14	N:R203K, N:G204R, ORF1a:P959S, ORF1a:R1163K, ORF1a:N2065S, ORF1a:T3255I, ORF1a:I3618V, ORF1a:T4175I, ORF1b:P314L, ORF1b:R1518K, S:T478K, S:D614G, S:P681H, S:T732A,

hCoV-19/Mexico/CMX-INMEGEN-03-08-15/2021	EPI_ISL_1315492	20B	B.1.1.519	25	5'UTR:C203T, 5'UTR:C222T, 5'UTR:C241T, ORF1ab:C3037T, ORF1ab:C3140T, ORF1ab:T3454C, ORF1ab:T4339C, ORF1ab:C10029T, ORF1ab:C10279T, ORF1ab:C10954T, ORF1ab:A11117G, ORF1ab:C12789T, ORF1ab:C14408T, ORF1ab:T19839C, ORF1ab:C21306T, S:C22995A, S:A23403G, S:C23604A, S:A23756G, S:A25341C, ORF3a:G25906T, ORF8:C28253T, N:G28881A, N:G28882A, N:G28883C, N:C29197T,	13	N:R203K, N:G204R, ORF1a:P959S, ORF1a:T3255I, ORF1a:I3618V, ORF1a:T4175I, ORF1b:P314L, ORF3a:G172C, S:T478K, S:D614G, S:P681H, S:T732A, S:D1260A,	
hCoV-19/Mexico/CMX-INMEGEN-03-08-16/2021	EPI_ISL_1315493	20B	B.1.1.519	31	5'UTR:T201C, 5'UTR:C203T, 5'UTR:C222T, 5'UTR:C241T, ORF1ab:C745T, ORF1ab:G1738T, ORF1ab:T2266G, ORF1ab:C3037T, ORF1ab:C3140T, ORF1ab:C3737T, ORF1ab:A8893G, ORF1ab:C10029T, ORF1ab:C10954T, ORF1ab:A11117G, ORF1ab:C12789T, ORF1ab:C14408T, ORF1ab:T15195A, ORF1ab:C15981T, ORF1ab:T19839C, ORF1ab:A19974G, ORF1ab:C21306T, S:C22498T, S:C22995A, S:A23403G, S:C23604A, S:A23756G, ORF8:C28253T, N:G28881A, N:G28882A, N:G28883C, N:C29095T, N:C29197T,	13	N:R203K, N:G204R, ORF1a:C667W, ORF1a:P959S, ORF1a:P1158S, ORF1a:T3255I, ORF1a:I3618V, ORF1a:T4175I, ORF1b:P314L, S:T478K, S:D614G, S:P681H, S:T732A,	
hCoV-19/Mexico/CMX-INMEGEN-03-08-47/2021	EPI_ISL_1315522	20B	B.1.1.519	30	5'UTR:T201C, 5'UTR:C203T, 5'UTR:C222T, 5'UTR:C241T, ORF1ab:C745T, ORF1ab:G1738T, ORF1ab:C3037T, ORF1ab:C3140T, ORF1ab:T4804C, ORF1ab:C6040T, ORF1ab:C10029T, ORF1ab:C10954T, ORF1ab:A11117G, ORF1ab:C12789T, ORF1ab:C14408T, ORF1ab:T19839C, ORF1ab:A19974G, ORF1ab:C21306T, S:C21575T, S:C22995A, S:A23403G, S:C23604A, S:A23756G, M:G27074A, ORF8:G28167A, N:T28759C, N:G28881A, N:G28882A, N:G28883C, N:C29197T, ORF10:C29585T,	13	N:R203K, N:G204R, ORF1a:P959S, ORF1a:T3255I, ORF1a:I3618V, ORF1a:T4175I, ORF1b:P314L, ORF8:E92K, S:L5F, S:T478K, S:D614G, S:P681H, S:T732A,	
hCoV-19/Mexico/CMX-INMEGEN-03-09-76/2021	EPI_ISL_1406126	20B	B.1.1.519	22	5'UTR:C203T, 5'UTR:C222T, 5'UTR:C241T, ORF1ab:C3037T, ORF1ab:C3140T, ORF1ab:C3311T, ORF1ab:A5116G, ORF1ab:C5621T, ORF1ab:A8031G, ORF1ab:C10029T, ORF1ab:C10954T, ORF1ab:A11117G, ORF1ab:C12789T, ORF1ab:C14408T, ORF1ab:T19839C, S:C22995A, S:A23403G, S:C23604A, S:A23756G, N:G28881A, N:G28882A, N:G28883C, N:C29197T,	14	N:R203K, N:G204R, ORF1a:P959S, ORF1a:L1016F, ORF1a:P1786S, ORF1a:K2589R, ORF1a:T3255I, ORF1a:I3618V, ORF1a:T4175I, ORF1b:P314L, S:T478K, S:D614G, S:P681H, S:T732A,	
hCoV-19/Mexico/CMX-INMEGEN-03-09-77/2021	EPI_ISL_1406127	20B	B.1.1.519	30	5'UTR:C203T, 5'UTR:A223T, 5'UTR:C241T, ORF1ab:A866G, ORF1ab:C1009T, ORF1ab:C3037T, ORF1ab:C3140T, ORF1ab:C5183T, ORF1ab:C5748T, ORF1ab:C10029T, ORF1ab:C10954T, ORF1ab:A11117G, ORF1ab:C11916T, ORF1ab:C12789T, ORF1ab:C13730T, ORF1ab:C14408T, ORF1ab:T19839C, S:C21595T, S:C22995A, S:A23403G, S:C23604A, S:A23756G, S:T24664C, ORF8:T27904C, N:G28747T, N:A28877T, N:G28878C, N:G28881A, N:G28882A, N:G28883C, N:C29197T,	17	N:R203K, N:G204R, ORF1a:I201V, ORF1a:P959S, ORF1a:P1640S, ORF1a:T1828I, ORF1a:T3255I, ORF1a:I3618V, ORF1a:S3884L, ORF1a:T4175I, ORF1b:A88V, ORF1b:P314L, ORF8:L4P, S:T478K, S:D614G, S:P681H, S:T732A,	5'UTR:222,

hCoV-19/Mexico/CMX-INMEGEN-03-09-79/2021	EPI_ISL_1406128	20B	B.1.1.519	29	5'UTR:T201C, 5'UTR:C203T, 5'UTR:C222T, 5'UTR:C241T, ORF1ab:C745T, ORF1ab:G1738T, ORF1ab:C3037T, ORF1ab:C3140T, ORF1ab:C10029T, ORF1ab:C10954T, ORF1ab:G11083T, ORF1ab:A11117G, ORF1ab:A11887G, ORF1ab:C12789T, ORF1ab:C14408T, ORF1ab:C16376T, ORF1ab:C19032T, ORF1ab:T19839C, ORF1ab:A19974G, S:C22995A, S:A23403G, S:C23604A, S:A23756G, ORF8:G28077T, ORF8:G28178T, N:G28881A, N:G28882A, N:G28883C, N:C29197T, N:C29272T,	15	N:R203K, N:G204R, ORF1a:P959S, ORF1a:T3255I, ORF1a:L3606F, ORF1a:I3618V, ORF1a:T4175I, ORF1b:P314L, ORF1b:P970L, ORF8:V62L, ORF8:L95F, S:T478K, S:D614G, S:P681H, S:T732A,	
hCoV-19/Mexico/CMX-INMEGEN-03-09-80/2021	EPI_ISL_1406129	20B	B.1.1.519	26	5'UTR:T201C, 5'UTR:C203T, 5'UTR:C222T, 5'UTR:C241T, ORF1ab:C745T, ORF1ab:C1077T, ORF1ab:G1738T, ORF1ab:C3037T, ORF1ab:C3140T, ORF1ab:G8720A, ORF1ab:C10029T, ORF1ab:C10954T, ORF1ab:A11117G, ORF1ab:C12789T, ORF1ab:C14408T, ORF1ab:G15957A, ORF1ab:T19839C, S:C22995A, S:A23403G, S:C23604A, S:C23683T, S:A23756G, N:G28375T, N:G28881A, N:G28882A, N:G28883C, N:C29197T,	14	N:R203K, N:G204R, ORF1a:P271L, ORF1a:P959S, ORF1a:D2819N, ORF1a:T3255I, ORF1a:I3618V, ORF1a:T4175I, ORF1b:P314L, ORF9b:G31V, S:T478K, S:D614G, S:P681H, S:T732A,	
hCoV-19/Mexico/CMX-INMEGEN-03-09-81/2021	EPI_ISL_1406130	20B	B.1.1.519	25	5'UTR:C203T, 5'UTR:C222T, 5'UTR:C241T, ORF1ab:C1613T, ORF1ab:C2434T, ORF1ab:C3037T, ORF1ab:C3140T, ORF1ab:C6027T, ORF1ab:C10029T, ORF1ab:C10954T, ORF1ab:A11117G, ORF1ab:C12473T, ORF1ab:C12789T, ORF1ab:C14408T, ORF1ab:T19839C, S:C22995A, S:A23403G, S:C23604A, S:A23756G, ORF3a:C25528T, ORF3a:G25563C, N:G28881A, N:G28882A, N:G28883C, N:C29197T, ORF10:C29627T,	15	N:R203K, N:G204R, ORF1a:L450F, ORF1a:P959S, ORF1a:P1921L, ORF1a:T3255I, ORF1a:I3618V, ORF1a:T4175I, ORF1b:P314L, ORF3a:L46F, ORF3a:Q57H, S:T478K, S:D614G, S:P681H, S:T732A,	
hCoV-19/Mexico/CMX-INMEGEN-03-09-82/2021	EPI_ISL_1406131	20B	B.1.1.519	31	5'UTR:C203T, 5'UTR:C222T, 5'UTR:C241T, ORF1ab:C3037T, ORF1ab:C3140T, ORF1ab:G3692T, ORF1ab:G3871T, ORF1ab:C4158T, ORF1ab:C9520T, ORF1ab:C10029T, ORF1ab:C10954T, ORF1ab:A11117G, ORF1ab:C11824A, ORF1ab:C12789T, ORF1ab:C14408T, ORF1ab:T14892C, ORF1ab:A16463G, ORF1ab:C18747T, ORF1ab:T19839C, ORF1ab:C21306T, ORF1ab:C21409T, S:C22995A, S:A23403G, S:C23604A, S:A23756G, ORF8:T27904C, N:G28321T, N:G28373T, N:G28881A, N:G28882A, N:G28883C, N:C29197T,	19	N:G34W, N:R203K, N:G204R, ORF1a:P959S, ORF1a:V1143F, ORF1a:K1202N, ORF1a:A1298V, ORF1a:T3255I, ORF1a:I3618V, ORF1a:T4175I, ORF1b:P314L, ORF1b:K999R, ORF1b:P2648S, ORF8:L4P, ORF9b:R13L, S:T478K, S:D614G, S:P681H, S:T732A,	ORF3a:26158-26161,
hCoV-19/Mexico/CMX-INMEGEN-03-09-83/2021	EPI_ISL_1406132	20B	B.1.1.519	24	5'UTR:T201C, 5'UTR:C203T, 5'UTR:C222T, 5'UTR:C241T, ORF1ab:C745T, ORF1ab:C3037T, ORF1ab:C3140T, ORF1ab:C6117T, ORF1ab:C10029T, ORF1ab:C10954T, ORF1ab:A11117G, ORF1ab:C12789T, ORF1ab:C14408T, ORF1ab:T19839C, S:C22995A, S:A23403G, S:C23604A, S:A23756G, ORF3a:A25590T, ORF3a:C26201T, N:G28881A, N:G28882A, N:G28883C, N:G28975T, N:C29197T,	15	N:R203K, N:G204R, N:M234I, ORF1a:P959S, ORF1a:A1951V, ORF1a:T3255I, ORF1a:I3618V, ORF1a:T4175I, ORF1b:P314L, ORF3a:K66N, ORF3a:T270I, S:T478K, S:D614G, S:P681H, S:T732A,	
hCoV-19/Mexico/CMX-INMEGEN-03-09-84/2021	EPI_ISL_1406133	20B	B.1.1.519	23	5'UTR:C203T, 5'UTR:C222T, 5'UTR:C241T, ORF1ab:C3037T, ORF1ab:C3140T, ORF1ab:C10029T, ORF1ab:C10954T, ORF1ab:A11117G, ORF1ab:C12789T, ORF1ab:C14408T, ORF1ab:T19839C, S:C22995A, S:C23248T, S:A23403G, S:C23604A, S:A23756G, S:G25313A, N:C28748T, N:T28867C, N:G28881A, N:G28882A, N:G28883C, N:C29197T, N:G29527T,	13	N:R203K, N:G204R, N:Q418H, ORF1a:P959S, ORF1a:T3255I, ORF1a:I3618V, ORF1a:T4175I, ORF1b:P314L, S:T478K, S:D614G, S:P681H, S:T732A, S:G1251R,	

hCoV-19/Mexico/CMX-INMEGEN-03-09-85/2021	EPI_ISL_1406134	20B	B.1.1.519	30	5'UTR:C203T, 5'UTR:C222T, 5'UTR:C241T, ORF1ab:C3037T, ORF1ab:C3140T, ORF1ab:G3692T, ORF1ab:G3871T, ORF1ab:G6653T, ORF1ab:T8200C, ORF1ab:C9520T, ORF1ab:C10029T, ORF1ab:C10450T, ORF1ab:C10954T, ORF1ab:A11117G, ORF1ab:C11824A, ORF1ab:C12789T, ORF1ab:C14408T, ORF1ab:T19839C, S:C22444T, S:C22995A, S:C23248T, S:A23403G, S:C23604A, S:A23756G, ORF3a:A25601T, ORF3a:G25690T, ORF8:T27904C, N:G28881A, N:G28882A, N:G28883C, N:C29197T,	17	N:R203K, N:G204R, ORF1a:P959S, ORF1a:V1143F, ORF1a:K1202N, ORF1a:V2130F, ORF1a:T3255I, ORF1a:I3618V, ORF1a:T4175I, ORF1b:P314L, ORF3a:Q70L, ORF3a:G100C, ORF8:L4P, S:T478K, S:D614G, S:P681H, S:T732A,
hCoV-19/Mexico/CMX-INMEGEN-03-11-01/2021	EPI_ISL_1591460	20B	B.1.1.519	28	5'UTR:T201C, 5'UTR:C203T, 5'UTR:C222T, 5'UTR:C241T, ORF1ab:G1738T, ORF1ab:C2892T, ORF1ab:C3037T, ORF1ab:C3140T, ORF1ab:A5845C, ORF1ab:C6026T, ORF1ab:C10029T, ORF1ab:C10954T, ORF1ab:A11117G, ORF1ab:C12789T, ORF1ab:C14408T, ORF1ab:T19839C, ORF1ab:A19974G, ORF1ab:C21306T, S:C22995A, S:A23403G, S:C23604A, S:A23756G, ORF7b:C27804T, N:G28881A, N:G28882A, N:G28883C, N:C29197T, N:C29421T, ORF10:C29627T,	15	N:R203K, N:G204R, N:P383L, ORF1a:A876V, ORF1a:P959S, ORF1a:K1860N, ORF1a:P1921S, ORF1a:T3255I, ORF1a:I3618V, ORF1a:T4175I, ORF1b:P314L, S:T478K, S:D614G, S:P681H, S:T732A,
hCoV-19/Mexico/CMX-INMEGEN-03-11-02/2021	EPI_ISL_1591461	20B	B.1.1.519	26	5'UTR:T201C, 5'UTR:C203T, 5'UTR:C222T, 5'UTR:C241T, ORF1ab:C745T, ORF1ab:G1738T, ORF1ab:C3037T, ORF1ab:C3140T, ORF1ab:C10029T, ORF1ab:C10954T, ORF1ab:A11117G, ORF1ab:C12789T, ORF1ab:C14408T, ORF1ab:T19839C, ORF1ab:A19974G, ORF1ab:T21000C, ORF1ab:C21306T, S:C22995A, S:A23403G, S:C23604A, S:A23756G, S:G25049T, ORF3a:C26111T, N:G28881A, N:G28882A, N:G28883C, N:C29197T,	13	N:R203K, N:G204R, ORF1a:P959S, ORF1a:T3255I, ORF1a:I3618V, ORF1a:T4175I, ORF1b:P314L, ORF3a:P240L, S:T478K, S:D614G, S:P681H, S:T732A, S:D1163Y,
hCoV-19/Mexico/CMX-INMEGEN-03-11-03/2021	EPI_ISL_1591462	20B	B.1.1.519	29	5'UTR:G174T, 5'UTR:T201C, 5'UTR:C203T, 5'UTR:C222T, 5'UTR:C241T, ORF1ab:C745T, ORF1ab:G1738T, ORF1ab:C1780T, ORF1ab:C3037T, ORF1ab:C3140T, ORF1ab:C9532T, ORF1ab:C10029T, ORF1ab:C10954T, ORF1ab:A11117G, ORF1ab:C12789T, ORF1ab:C14408T, ORF1ab:T19839C, ORF1ab:A19974G, ORF1ab:C21306T, S:C22995A, S:A23403G, S:C23604A, S:A23756G, ORF3a:G25489T, ORF8:G28077T, N:G28881A, N:G28882A, N:G28883C, N:C29197T, 3'UTR:C29836T,	13	N:R203K, N:G204R, ORF1a:P959S, ORF1a:T3255I, ORF1a:I3618V, ORF1a:T4175I, ORF1b:P314L, ORF3a:A33S, ORF8:V62L, S:T478K, S:D614G, S:P681H, S:T732A,
hCoV-19/Mexico/CMX-INMEGEN-03-11-04/2021	EPI_ISL_1591463	20B	B.1.1.519	24	5'UTR:C203T, 5'UTR:C222T, 5'UTR:C241T, ORF1ab:C3037T, ORF1ab:C3140T, ORF1ab:G9407A, ORF1ab:C10029T, ORF1ab:C10954T, ORF1ab:A11117G, ORF1ab:C12789T, ORF1ab:C14408T, ORF1ab:T19839C, ORF1ab:C20628T, ORF1ab:C21306T, S:C22995A, S:C23086T, S:A23403G, S:C23604A, S:A23756G, ORF3a:C25782T, ORF6:C27294T, N:G28881A, N:G28882A, N:G28883C, N:C29197T,	12	N:R203K, N:G204R, ORF1a:P959S, ORF1a:V3048I, ORF1a:T3255I, ORF1a:I3618V, ORF1a:T4175I, ORF1b:P314L, S:T478K, S:D614G, S:P681H, S:T732A,

hCoV-19/Mexico/CMX-INMEGEN-03-11-08/2021	EPI_ISL_1591467	20B	B.1.1.519	27	5'UTR:A129G, 5'UTR:C203T, 5'UTR:C222T, 5'UTR:C241T, ORF1ab:C3037T, ORF1ab:C3140T, ORF1ab:T3745C, ORF1ab:A8842G, ORF1ab:C10029T, ORF1ab:G10870T, ORF1ab:C10954T, ORF1ab:A11117G, ORF1ab:C12789T, ORF1ab:C14408T, ORF1ab:C16726T, ORF1ab:T19839C, ORF1ab:C21306T, S:C22995A, S:A23403G, S:C23604A, S:A23756G, ORF3a:C25844T, N:A28537C, N:G28881A, N:G28882A, N:G28883C, N:C29197T, 3'UTR:G29773T	14	N:R203K, N:G204R, ORF1a:P959S, ORF1a:T3255I, ORF1a:I3618V, ORF1a:T4175I, ORF1b:P314L, ORF1b:H1087Y, ORF3a:T151I, ORF9b:E85A, S:T478K, S:D614G, S:P681H, S:T732A,	S:25331-25333,
hCoV-19/Mexico/CMX-INMEGEN-03-11-09/2021	EPI_ISL_1591468	20B	B.1.1.519	28	5'UTR:C203T, 5'UTR:C222T, 5'UTR:C241T, ORF1ab:C3037T, ORF1ab:C3140T, ORF1ab:C5869T, ORF1ab:C6568T, ORF1ab:C10029T, ORF1ab:C10954T, ORF1ab:A11117G, ORF1ab:C12789T, ORF1ab:C14408T, ORF1ab:C15194T, ORF1ab:T19839C, ORF1ab:G19872T, ORF1ab:G21204A, ORF1ab:C21306T, S:C22995A, S:A23403G, S:C23604A, S:A23756G, ORF7a:G27601T, ORF8:T27904C, ORF8:G27987T, ORF8:C28087T, N:G28881A, N:G28882A, N:G28883C, N:C29197T,	16	N:R203K, N:G204R, ORF1a:P959S, ORF1a:T3255I, ORF1a:I3618V, ORF1a:T4175I, ORF1b:P314L, ORF1b:A576V, ORF7a:G70C, ORF8:L4P, ORF8:V32L, ORF8:A65V, S:T478K, S:D614G, S:P681H, S:T732A,	
hCoV-19/Mexico/CMX-INMEGEN-03-11-10/2021	EPI_ISL_1591469	20B	B.1.1.222	25	5'UTR:C241T, ORF1ab:C593T, ORF1ab:A1558G, ORF1ab:C3037T, ORF1ab:A3916G, ORF1ab:A5452G, ORF1ab:C6543T, ORF1ab:C8606T, ORF1ab:G11222T, ORF1ab:C14216T, ORF1ab:C14408T, ORF1ab:T19839C, S:C21614T, S:G21624T, S:G22026A, S:G22104T, S:A22765T, S:A23403G, S:A23756G, ORF3a:C25587T, ORF3a:C25904T, ORF8:C28253T, N:G28881A, N:G28882A, N:G28883C, 3'UTR:G29777T,	16	N:R203K, N:G204R, ORF1a:H110Y, ORF1a:I431M, ORF1a:T2093I, ORF1a:L2781F, ORF1a:V3653F, ORF1b:T250I, ORF1b:P314L, ORF3a:S171L, S:L18F, S:R21I, S:S155N, S:G181V, S:D614G, S:T732A,	
hCoV-19/Mexico/CMX-INMEGEN-03-11-11/2021	EPI_ISL_1591470	20B	B.1.1.519	24	5'UTR:C203T, 5'UTR:C222T, 5'UTR:C241T, ORF1ab:C3037T, ORF1ab:C3140T, ORF1ab:T3745C, ORF1ab:A9266G, ORF1ab:C10029T, ORF1ab:C10954T, ORF1ab:A11117G, ORF1ab:C12789T, ORF1ab:C14408T, ORF1ab:T19839C, ORF1ab:C21306T, S:C22995A, S:A23403G, S:C23422T, S:C23604A, S:A23756G, ORF3a:C25844T, N:G28881A, N:G28882A, N:G28883C, N:C29197T, N:C29284T,	13	N:R203K, N:G204R, ORF1a:P959S, ORF1a:S3001G, ORF1a:T3255I, ORF1a:I3618V, ORF1a:T4175I, ORF1b:P314L, ORF3a:T151I, S:T478K, S:D614G, S:P681H, S:T732A,	
hCoV-19/Mexico/CMX-INMEGEN-03-11-12/2021	EPI_ISL_1591471	20B	B.1.1.519	26	5'UTR:T201C, 5'UTR:C203T, 5'UTR:C222T, 5'UTR:C241T, ORF1ab:G1738T, ORF1ab:C3037T, ORF1ab:C3140T, ORF1ab:A3363T, ORF1ab:C10029T, ORF1ab:C10954T, ORF1ab:A11117G, ORF1ab:C12789T, ORF1ab:C13168T, ORF1ab:C14408T, ORF1ab:G14414T, ORF1ab:T19839C, ORF1ab:A19974G, ORF1ab:C21306T, S:C22995A, S:A23403G, S:C23604A, S:A23756G, ORF3a:C25791T, N:G28881A, N:G28882A, N:G28883C, N:C29197T,	13	N:R203K, N:G204R, ORF1a:P959S, ORF1a:K1033I, ORF1a:T3255I, ORF1a:I3618V, ORF1a:T4175I, ORF1b:P314L, ORF1b:S316I, S:T478K, S:D614G, S:P681H, S:T732A,	
hCoV-19/Mexico/CMX-INMEGEN-03-11-16/2021	EPI_ISL_1591475	20B	B.1.1.519	24	5'UTR:C203T, 5'UTR:C222T, 5'UTR:C241T, ORF1ab:G1085T, ORF1ab:C2509T, ORF1ab:C3037T, ORF1ab:C3140T, ORF1ab:C10029T, ORF1ab:C10954T, ORF1ab:A11117G, ORF1ab:G11365T, ORF1ab:C12789T, ORF1ab:C14408T, ORF1ab:T19839C, ORF1ab:C21306T, S:C22995A, S:A23403G, S:C23604A, S:A23756G, ORF7a:G27676T, N:G28881A, N:G28882A, N:G28883C, N:C29197T, N:G29527T,	14	N:R203K, N:G204R, N:Q418H, ORF1a:V274L, ORF1a:P959S, ORF1a:T3255I, ORF1a:I3618V, ORF1a:T4175I, ORF1b:P314L, ORF7a:E95*, S:T478K, S:D614G, S:P681H, S:T732A,	

hCoV-19/Mexico/CMX-INMEGEN-03-11-17/2021	EPI_ISL_1591476	20B	B.1.1.519	26	5'UTR:C203T, 5'UTR:C222T, 5'UTR:C241T, ORF1ab:C593T, ORF1ab:C3037T, ORF1ab:C3140T, ORF1ab:C10029T, ORF1ab:C10954T, ORF1ab:A11117G, ORF1ab:C12789T, ORF1ab:C13115T, ORF1ab:T13593C, ORF1ab:C14408T, ORF1ab:G15921T, ORF1ab:C19089T, ORF1ab:T19839C, ORF1ab:T20028C, ORF1ab:C21306T, S:C22995A, S:A23403G, S:C23604A, S:A23756G, S:G24757T, N:G28881A, N:G28882A, N:G28883C, N:C29197T,	12	N:R203K, N:G204R, ORF1a:H110Y, ORF1a:P959S, ORF1a:T3255I, ORF1a:I3618V, ORF1a:T4175I, ORF1b:P314L, S:T478K, S:D614G, S:P681H, S:T732A,
hCoV-19/Mexico/CMX-INMEGEN-03-11-18/2021	EPI_ISL_1591477	20B	B.1.1.519	32	5'UTR:T201C, 5'UTR:C203T, 5'UTR:C222T, 5'UTR:C241T, ORF1ab:C936T, ORF1ab:G1738T, ORF1ab:C3037T, ORF1ab:C3140T, ORF1ab:A3210G, ORF1ab:T9089G, ORF1ab:C10029T, ORF1ab:C10954T, ORF1ab:A11117G, ORF1ab:C12525T, ORF1ab:C12789T, ORF1ab:C14408T, ORF1ab:G14677A, ORF1ab:T16342C, ORF1ab:G17302T, ORF1ab:T19839C, ORF1ab:A19974G, ORF1ab:C21306T, S:C22995A, S:A23403G, S:C23604A, S:A23756G, S:C23896T, ORF3a:C25721T, N:G28881A, N:G28882A, N:G28883C, N:C29197T, N:G29227T,	19	N:R203K, N:G204R, ORF1a:T224I, ORF1a:P959S, ORF1a:D982G, ORF1a:S2942A, ORF1a:T3255I, ORF1a:I3618V, ORF1a:T4087I, ORF1a:T4175I, ORF1b:P314L, ORF1b:G404S, ORF1b:S959P, ORF1b:V1279F, ORF3a:A110V, S:T478K, S:D614G, S:P681H, S:T732A,
hCoV-19/Mexico/CMX-INMEGEN-03-11-19/2021	EPI_ISL_1591478	20B	B.1.1.519	26	5'UTR:C203T, 5'UTR:C222T, 5'UTR:C241T, ORF1ab:C3037T, ORF1ab:C3140T, ORF1ab:T3745C, ORF1ab:C4815T, ORF1ab:C10029T, ORF1ab:C10954T, ORF1ab:A11117G, ORF1ab:C12789T, ORF1ab:C14408T, ORF1ab:T19839C, ORF1ab:C20759T, ORF1ab:C21306T, S:T22372C, S:A23403G, S:C23604A, S:A23756G, S:G24095T, ORF3a:C25844T, ORF3a:C26111A, N:C28849T, N:G28881A, N:G28882A, N:G28883C, N:C29197T,	15	N:R203K, N:G204R, ORF1a:P959S, ORF1a:S1517F, ORF1a:T3255I, ORF1a:I3618V, ORF1a:T4175I, ORF1b:P314L, ORF1b:A2431V, ORF3a:T151I, ORF3a:P240H, S:D614G, S:P681H, S:T732A, S:A845S,
hCoV-19/Mexico/CMX-INMEGEN-03-11-26/2021	EPI_ISL_1591483	20B	B.1.1.519	26	5'UTR:T201C, 5'UTR:C203T, 5'UTR:C222T, 5'UTR:C241T, ORF1ab:C584T, ORF1ab:G1738T, ORF1ab:C3037T, ORF1ab:C3140T, ORF1ab:C10029T, ORF1ab:G10610A, ORF1ab:C10954T, ORF1ab:A11117G, ORF1ab:C12789T, ORF1ab:C14408T, ORF1ab:A17993G, ORF1ab:T19839C, ORF1ab:A19974G, ORF1ab:C21306T, S:C22995A, S:A23403G, S:C23604A, S:A23756G, ORF3a:C25553T, N:G28881A, N:G28882A, N:G28883C, N:C29197T,	15	N:R203K, N:G204R, ORF1a:L107F, ORF1a:P959S, ORF1a:T3255I, ORF1a:V3449I, ORF1a:I3618V, ORF1a:T4175I, ORF1b:P314L, ORF1b:Q1509R, ORF3a:A54V, S:T478K, S:D614G, S:P681H, S:T732A,
hCoV-19/Mexico/CMX-INMEGEN-03-11-27/2021	EPI_ISL_1591484	20B	B.1.1.519	22	5'UTR:C203T, 5'UTR:C222T, 5'UTR:C241T, ORF1ab:C3037T, ORF1ab:C3140T, ORF1ab:C4999T, ORF1ab:C10029T, ORF1ab:C10954T, ORF1ab:A11117G, ORF1ab:C12789T, ORF1ab:C14408T, ORF1ab:C15579T, ORF1ab:T19839C, ORF1ab:C21306T, S:C22995A, S:A23403G, S:C23604A, S:A23756G, N:G28881A, N:G28882A, N:G28883C, N:C29197T, N:G29527T,	12	N:R203K, N:G204R, N:Q418H, ORF1a:P959S, ORF1a:T3255I, ORF1a:I3618V, ORF1a:T4175I, ORF1b:P314L, S:T478K, S:D614G, S:P681H, S:T732A,
hCoV-19/Mexico/CMX-INMEGEN-03-11-32/2021	EPI_ISL_1591489	20B	B.1.1.519	26	5'UTR:C203T, 5'UTR:C222T, 5'UTR:C241T, ORF1ab:C3037T, ORF1ab:C3140T, ORF1ab:C8293T, ORF1ab:C9120T, ORF1ab:C10029T, ORF1ab:C10954T, ORF1ab:A11117G, ORF1ab:C12789T, ORF1ab:C14408T, ORF1ab:T19839C, ORF1ab:C21077T, ORF1ab:C21306T, S:C22995A, S:A23403G, S:C23604A, S:A23756G, ORF3a:G25906T, M:C27119T, ORF8:C28253T, N:G28881A, N:G28882A, N:G28883C, N:C29171T, N:C29197T,	15	N:R203K, N:G204R, N:H300Y, ORF1a:P959S, ORF1a:T2952I, ORF1a:T3255I, ORF1a:I3618V, ORF1a:T4175I, ORF1b:P314L, ORF1b:T2537I, ORF3a:G172C, S:T478K, S:D614G, S:P681H, S:T732A,

hCoV-19/Mexico/CMX-INMEGEN-03-11-34/2021	EPI_ISL_1591490	20B	B.1.1.519	23	5'UTR:C203T, 5'UTR:C222T, 5'UTR:C241T, ORF1ab:G2281A, ORF1ab:C3037T, ORF1ab:C3140T, ORF1ab:C3311T, ORF1ab:A8031G, ORF1ab:C10029T, ORF1ab:C10954T, ORF1ab:A11117G, ORF1ab:C12789T, ORF1ab:C14408T, ORF1ab:T19839C, S:C21998T, S:C22995A, S:A23403G, S:C23604A, S:A23756G, ORF3a:C25714T, N:G28881A, N:G28882A, N:G28883C, N:C29197T,	15	N:R203K, N:G204R, ORF1a:P959S, ORF1a:L1016F, ORF1a:K2589R, ORF1a:T3255I, ORF1a:I3618V, ORF1a:T4175I, ORF1b:P314L, ORF3a:L108F, S:H146Y, S:T478K, S:D614G, S:P681H, S:T732A,	
hCoV-19/Mexico/CMX-INMEGEN-03-11-35/2021	EPI_ISL_1591491	20B	B.1.1.519	26	5'UTR:C203T, 5'UTR:C222T, 5'UTR:C241T, ORF1ab:C1909T, ORF1ab:C3037T, ORF1ab:C3140T, ORF1ab:A4027G, ORF1ab:C10029T, ORF1ab:C10456T, ORF1ab:C10954T, ORF1ab:A11117G, ORF1ab:G11365T, ORF1ab:C12789T, ORF1ab:C14408T, ORF1ab:C14925T, ORF1ab:T19839C, ORF1ab:C21306T, S:C22995A, S:C23230T, S:A23403G, S:C23604A, S:A23756G, N:G28881A, N:G28882A, N:G28883C, N:C29197T, N:G29527T,	12	N:R203K, N:G204R, N:Q418H, ORF1a:P959S, ORF1a:T3255I, ORF1a:I3618V, ORF1a:T4175I, ORF1b:P314L, S:T478K, S:D614G, S:P681H, S:T732A,	
hCoV-19/Mexico/CMX-INMEGEN-03-11-40/2021	EPI_ISL_1591495	20B	B.1.1.519	25	5'UTR:C203T, 5'UTR:C222T, 5'UTR:C241T, ORF1ab:G1942A, ORF1ab:C3037T, ORF1ab:C3140T, ORF1ab:A3581G, ORF1ab:C10029T, ORF1ab:C10954T, ORF1ab:A11117G, ORF1ab:C12789T, ORF1ab:C14408T, ORF1ab:G18546T, ORF1ab:T19839C, ORF1ab:G20002T, ORF1ab:C21306T, S:C22995A, S:A23403G, S:C23604A, S:A23756G, M:C26882T, ORF8:T27904C, N:G28881A, N:G28882A, N:G28883C, N:C29197T,	15	N:R203K, N:G204R, ORF1a:P959S, ORF1a:S1106G, ORF1a:T3255I, ORF1a:I3618V, ORF1a:T4175I, ORF1b:P314L, ORF1b:M1693I, ORF1b:D2179Y, ORF8:L4P, S:T478K, S:D614G, S:P681H, S:T732A,	
hCoV-19/Mexico/CMX-INMEGEN-03-11-42/2021	EPI_ISL_1591496	20B	B.1.1.519	24	5'UTR:C203T, 5'UTR:C222T, 5'UTR:C241T, ORF1ab:C3037T, ORF1ab:C3140T, ORF1ab:C10029T, ORF1ab:C10954T, ORF1ab:A11117G, ORF1ab:A12088G, ORF1ab:C12789T, ORF1ab:C14408T, ORF1ab:T19839C, ORF1ab:A20637G, ORF1ab:C21306T, S:C22995A, S:A23403G, S:C23604A, S:A23756G, ORF3a:G25459T, ORF3a:A25756C, N:G28881A, N:G28882A, N:G28883C, N:C29197T, 3'UTR:G29715T,	12	N:R203K, N:G204R, ORF1a:P959S, ORF1a:T3255I, ORF1a:I3618V, ORF1a:T4175I, ORF1b:P314L, ORF3a:A23S, S:T478K, S:D614G, S:P681H, S:T732A,	
hCoV-19/Mexico/CMX-INMEGEN-03-11-43/2021	EPI_ISL_1591497	20B	B.1.1.519	27	5'UTR:C203T, 5'UTR:A223T, 5'UTR:C241T, ORF1ab:C2455T, ORF1ab:C3037T, ORF1ab:C3140T, ORF1ab:C8047T, ORF1ab:C8772T, ORF1ab:C10029T, ORF1ab:C10954T, ORF1ab:A11117G, ORF1ab:G11365T, ORF1ab:C12789T, ORF1ab:C14408T, ORF1ab:T15978C, ORF1ab:T19839C, ORF1ab:C21306T, S:C21575T, S:C22995A, S:A23403G, S:C23604A, S:A23756G, N:C28507T, N:G28881A, N:G28882A, N:G28883C, N:C29197T, N:G29527T,	15	N:R203K, N:G204R, N:Q418H, ORF1a:P959S, ORF1a:T2836I, ORF1a:T3255I, ORF1a:I3618V, ORF1a:T4175I, ORF1b:P314L, ORF9b:A75V, S:L5F, S:T478K, S:D614G, S:P681H, S:T732A,	5'UTR:2 22,
hCoV-19/Mexico/CMX-INMEGEN-03-11-49/2021	EPI_ISL_1591503	20B	B.1.1.519	29	5'UTR:C203T, 5'UTR:C222T, 5'UTR:C241T, ORF1ab:C3037T, ORF1ab:C3140T, ORF1ab:T3745C, ORF1ab:A3879G, ORF1ab:C8016T, ORF1ab:C10029T, ORF1ab:C10954T, ORF1ab:A11117G, ORF1ab:G11291A, ORF1ab:T12182A, ORF1ab:C12473T, ORF1ab:C12789T, ORF1ab:C14408T, ORF1ab:T19839C, ORF1ab:C21306T, S:C22995A, S:A23403G, S:C23604A, S:A23756G, ORF3a:C25844T, ORF7a:G27709T, N:C28849T, N:G28881A, N:G28882A, N:G28883C, N:C29197T, N:A29419C	18	N:R203K, N:G204R, N:L382F, ORF1a:P959S, ORF1a:E1205G, ORF1a:A2584V, ORF1a:T3255I, ORF1a:I3618V, ORF1a:G3676S, ORF1a:S3973T, ORF1a:T4175I, ORF1b:P314L, ORF3a:T151I, ORF7a:A106S, S:T478K, S:D614G, S:P681H, S:T732A,	

hCoV-19/Mexico/CMX-INMEGEN-03-11-50/2021	EPI_ISL_1591504	20B	B.1.1.222	25	5'UTR:C241T, ORF1ab:C593T, ORF1ab:A1558G, ORF1ab:C3037T, ORF1ab:A3916G, ORF1ab:A5452G, ORF1ab:C6543T, ORF1ab:C8606T, ORF1ab:G11222T, ORF1ab:C14216T, ORF1ab:C14408T, ORF1ab:T19839C, S:C21614T, S:G21624T, S:G22026A, S:G22104T, S:A22765T, S:A23403G, S:A23756G, ORF3a:C25587T, ORF3a:C25904T, ORF8:C28253T, N:G28881A, N:G28882A, N:G28883C, 3'UTR:G29777T,	16	N:R203K, N:G204R, ORF1a:H110Y, ORF1a:I431M, ORF1a:T2093I, ORF1a:L2781F, ORF1a:V3653F, ORF1b:T250I, ORF1b:P314L, ORF3a:S171L, S:L18F, S:R21I, S:S155N, S:G181V, S:D614G, S:T732A,
hCoV-19/Mexico/CMX-INMEGEN-03-11-51/2021	EPI_ISL_1591505	20B	B.1.1.519	29	5'UTR:T201C, 5'UTR:C203T, 5'UTR:C222T, 5'UTR:C241T, ORF1ab:C1684T, ORF1ab:G1738T, ORF1ab:C3037T, ORF1ab:C3140T, ORF1ab:G9388A, ORF1ab:C10029T, ORF1ab:C10954T, ORF1ab:A11117G, ORF1ab:A12088G, ORF1ab:C12789T, ORF1ab:A13278G, ORF1ab:C14408T, ORF1ab:T19839C, ORF1ab:A19974G, ORF1ab:C21306T, S:C22995A, S:G23302A, S:A23403G, S:C23604A, S:A23756G, ORF3a:C25484T, N:G28881A, N:G28882A, N:G28883C, N:C29197T, N:G29315C,	14	N:R203K, N:G204R, N:D348H, ORF1a:P959S, ORF1a:T3255I, ORF1a:I3618V, ORF1a:T4175I, ORF1a:N4338S, ORF1b:P314L, ORF3a:A31V, S:T478K, S:D614G, S:P681H, S:T732A,
hCoV-19/Mexico/CMX-INMEGEN-03-11-57/2021	EPI_ISL_1591511	20B	B.1.1.519	26	5'UTR:C203T, 5'UTR:C222T, 5'UTR:C241T, ORF1ab:C3037T, ORF1ab:C3140T, ORF1ab:C4893T, ORF1ab:C5183T, ORF1ab:T7045C, ORF1ab:C9891T, ORF1ab:C10029T, ORF1ab:C10954T, ORF1ab:A11117G, ORF1ab:C12789T, ORF1ab:C14408T, ORF1ab:T19839C, ORF1ab:C21306T, S:C22995A, S:A23403G, S:C23604A, S:A23756G, S:A24014G, S:G25135T, ORF8:T27904C, N:G28881A, N:G28882A, N:G28883C, N:C29197T,	17	N:R203K, N:G204R, ORF1a:P959S, ORF1a:T1543I, ORF1a:P1640S, ORF1a:A3209V, ORF1a:T3255I, ORF1a:I3618V, ORF1a:T4175I, ORF1b:P314L, ORF8:L4P, S:T478K, S:D614G, S:P681H, S:T732A, S:I818V, S:K1191N,
hCoV-19/Mexico/CMX-INMEGEN-03-11-58/2021	EPI_ISL_1591512	20B	B.1.1.222	21	5'UTR:C241T, ORF1ab:C835T, ORF1ab:C3037T, ORF1ab:C4406T, ORF1ab:C6781T, ORF1ab:C8090T, ORF1ab:C11109T, ORF1ab:C11416T, ORF1ab:G11634T, ORF1ab:T14307C, ORF1ab:C14408T, ORF1ab:C16323T, ORF1ab:C17550T, ORF1ab:T19839C, S:G22017T, S:A23403G, S:A23756G, ORF3a:G26063T, ORF7b:A27888T, N:G28881A, N:G28882A, N:G28883C,	11	N:R203K, N:G204R, ORF1a:H1381Y, ORF1a:L2609F, ORF1a:A3615V, ORF1a:C3790F, ORF1b:P314L, ORF3a:G224V, S:W152L, S:D614G, S:T732A,
hCoV-19/Mexico/CMX-INMEGEN-03-11-59/2021	EPI_ISL_1591513	20B	B.1.1.519	23	5'UTR:C203T, 5'UTR:C222T, 5'UTR:C241T, ORF1ab:G1085T, ORF1ab:C2509T, ORF1ab:C3037T, ORF1ab:C3140T, ORF1ab:C10029T, ORF1ab:C10954T, ORF1ab:A11117G, ORF1ab:G11365T, ORF1ab:C12789T, ORF1ab:C14408T, ORF1ab:T19839C, ORF1ab:C21306T, S:C22995A, S:A23403G, S:C23604A, S:A23756G, N:G28881A, N:G28882A, N:G28883C, N:C29197T, N:G29527T,	13	N:R203K, N:G204R, N:Q418H, ORF1a:V274L, ORF1a:P959S, ORF1a:T3255I, ORF1a:I3618V, ORF1a:T4175I, ORF1b:P314L, S:T478K, S:D614G, S:P681H, S:T732A,
hCoV-19/Mexico/CMX-INMEGEN-03-11-66/2021	EPI_ISL_1591519	20B	B.1.1.222	27	5'UTR:C241T, ORF1ab:C3037T, ORF1ab:C4897T, ORF1ab:C5259T, ORF1ab:C9474T, ORF1ab:C10029T, ORF1ab:G11083T, ORF1ab:A11430G, ORF1ab:G12769T, ORF1ab:C13862T, ORF1ab:C14408T, ORF1ab:G15586A, ORF1ab:C17436T, ORF1ab:C19011A, ORF1ab:T19839C, ORF1ab:A21137G, S:A23403G, S:A23756G, ORF3a:G25912T, ORF3a:A26115C, ORF6:A27362C, ORF7a:G27506T, ORF8:A27921G, ORF8:G28001T, N:C28849T, N:G28881A, N:G28882A, N:G28883C,	19	N:R203K, N:G204R, ORF1a:T1665I, ORF1a:A3070V, ORF1a:T3255I, ORF1a:L3606F, ORF1a:Y3722C, ORF1b:T132I, ORF1b:P314L, ORF1b:A707T, ORF1b:D1848E, ORF1b:K2557R, ORF3a:G174C, ORF3a:E241D, ORF6:E54A, ORF7a:G38V, ORF8:I10V, S:D614G, S:T732A,

hCoV-19/Mexico/CMX-INMEGEN-03-11-67/2020	EPI_ISL_1591520	20B	B.1.1.519	26	5'UTR:T201C, 5'UTR:C203T, 5'UTR:C222T, 5'UTR:C241T, ORF1ab:C745T, ORF1ab:T809C, ORF1ab:G1738T, ORF1ab:C3037T, ORF1ab:C3140T, ORF1ab:T3700C, ORF1ab:C10029T, ORF1ab:C10954T, ORF1ab:A11117G, ORF1ab:C12789T, ORF1ab:C14408T, ORF1ab:T19839C, ORF1ab:A19974G, ORF1ab:C21306T, S:C21721T, S:A23403G, S:C23604A, S:A23756G, N:G28881A, N:G28882A, N:G28883C, N:C29197T, 3'UTR:G29692T,	11	N:R203K, N:G204R, ORF1a:Y182H, ORF1a:P959S, ORF1a:T3255I, ORF1a:I3618V, ORF1a:T4175I, ORF1b:P314L, S:D614G, S:P681H, S:T732A,
hCoV-19/Mexico/CMX-INMEGEN-03-11-73/2021	EPI_ISL_1591524	20B	B.1.1.519	27	5'UTR:C203T, 5'UTR:C222T, 5'UTR:C241T, ORF1ab:C3037T, ORF1ab:C3140T, ORF1ab:C8752T, ORF1ab:C10029T, ORF1ab:C10954T, ORF1ab:A11117G, ORF1ab:C12789T, ORF1ab:A14199T, ORF1ab:C14408T, ORF1ab:C15081T, ORF1ab:G19272T, ORF1ab:T19839C, ORF1ab:C21306T, S:C22995A, S:A23403G, S:C23604A, S:A23756G, S:A24727G, S:C25318T, ORF8:T27904C, ORF8:C28087T, N:G28881A, N:G28882A, N:G28883C, N:C29197T,	14	N:R203K, N:G204R, ORF1a:P959S, ORF1a:T3255I, ORF1a:I3618V, ORF1a:T4175I, ORF1b:P314L, ORF1b:L1935F, ORF8:L4P, ORF8:A65V, S:T478K, S:D614G, S:P681H, S:T732A,
hCoV-19/Mexico/CMX-INMEGEN-03-11-74/2021	EPI_ISL_1591525	20B	B.1.1.519	30	5'UTR:T201C, 5'UTR:C203T, 5'UTR:C222T, 5'UTR:C241T, ORF1ab:C936T, ORF1ab:G1738T, ORF1ab:C3037T, ORF1ab:C3140T, ORF1ab:T7106C, ORF1ab:C10029T, ORF1ab:C10954T, ORF1ab:A11117G, ORF1ab:C12789T, ORF1ab:C14408T, ORF1ab:G18067T, ORF1ab:T19839C, ORF1ab:A19974G, ORF1ab:C21306T, S:C22995A, S:A23403G, S:C23604A, S:A23756G, ORF3a:G25429T, ORF3a:C25626T, ORF7b:C27769T, N:G28881A, N:G28882A, N:G28883C, N:C29197T, N:G29227T, N:C29311T	16	N:R203K, N:G204R, ORF1a:T224I, ORF1a:P959S, ORF1a:Y2281H, ORF1a:T3255I, ORF1a:I3618V, ORF1a:T4175I, ORF1b:P314L, ORF1b:D1534Y, ORF3a:V13L, ORF7b:S5L, S:T478K, S:D614G, S:P681H, S:T732A,
hCoV-19/Mexico/CMX-INMEGEN-03-11-81/2021	EPI_ISL_1591530	20B	B.1.1.519	25	5'UTR:C203T, 5'UTR:C222T, 5'UTR:C241T, ORF1ab:C3037T, ORF1ab:C3140T, ORF1ab:C4246T, ORF1ab:C10029T, ORF1ab:C10954T, ORF1ab:A11117G, ORF1ab:C12789T, ORF1ab:C14408T, ORF1ab:T19839C, ORF1ab:C21306T, S:G21867C, S:C22181T, S:C22995A, S:A23403G, S:C23604A, S:A23756G, M:G26526A, ORF8:T28098C, N:G28881A, N:G28882A, N:G28883C, N:C29197T, N:G29527T,	16	M:A2T, N:R203K, N:G204R, N:Q418H, ORF1a:P959S, ORF1a:T3255I, ORF1a:I3618V, ORF1a:T4175I, ORF1b:P314L, ORF8:S69P, S:R102T, S:H207Y, S:T478K, S:D614G, S:P681H, S:T732A,
hCoV-19/Mexico/CMX-INMEGEN-03-11-82/2021	EPI_ISL_1591531	20B	B.1.1.519	26	5'UTR:T201C, 5'UTR:C203T, 5'UTR:C222T, 5'UTR:C241T, ORF1ab:C745T, ORF1ab:G1738T, ORF1ab:C3037T, ORF1ab:C3140T, ORF1ab:C10029T, ORF1ab:C10954T, ORF1ab:A11117G, ORF1ab:C12789T, ORF1ab:C14408T, ORF1ab:A17861G, ORF1ab:T19839C, ORF1ab:A19974G, ORF1ab:C21306T, S:C22995A, S:A23403G, S:C23604A, S:A23756G, S:C24099T, ORF8:G28077T, N:G28881A, N:G28882A, N:G28883C, N:C29197T,	14	N:R203K, N:G204R, ORF1a:P959S, ORF1a:T3255I, ORF1a:I3618V, ORF1a:T4175I, ORF1b:P314L, ORF1b:D1465G, ORF8:V62L, S:T478K, S:D614G, S:P681H, S:T732A, S:A846V,
hCoV-19/Mexico/CMX-INMEGEN-03-11-83/2021	EPI_ISL_1591532	20B	B.1.1.519	21	5'UTR:C203T, 5'UTR:C222T, 5'UTR:C241T, ORF1ab:C3037T, ORF1ab:C3140T, ORF1ab:T3745C, ORF1ab:C10029T, ORF1ab:C10954T, ORF1ab:A11117G, ORF1ab:C12789T, ORF1ab:C14408T, ORF1ab:T19839C, ORF1ab:C21306T, S:C22995A, S:A23403G, S:C23604A, S:A23756G, ORF3a:C25844T, N:G28881A, N:G28882A, N:G28883C, N:C29197T,	12	N:R203K, N:G204R, ORF1a:P959S, ORF1a:T3255I, ORF1a:I3618V, ORF1a:T4175I, ORF1b:P314L, ORF3a:T151I, S:T478K, S:D614G, S:P681H, S:T732A,

hCoV-19/Mexico/CMX-INMEGEN-03-11-87/2021	EPI_ISL_1591536	20B	B.1.1.519	24	5'UTR:C203T, 5'UTR:C222T, 5'UTR:C241T, ORF1ab:C3037T, ORF1ab:C3140T, ORF1ab:C10029T, ORF1ab:C10954T, ORF1ab:A11117G, ORF1ab:C12789T, ORF1ab:C14408T, ORF1ab:G15921T, ORF1ab:T19839C, ORF1ab:C21306T, S:C22227T, S:C22995A, S:A23403G, S:C23604A, S:A23756G, ORF3a:G26109T, ORF8:T27977C, N:G28881A, N:G28882A, N:G28883C, N:C29197T, N:G29527A,	13	N:R203K, N:G204R, ORF1a:P959S, ORF1a:T3255I, ORF1a:I3618V, ORF1a:T4175I, ORF1b:P314L, ORF3a:E239D, S:A222V, S:T478K, S:D614G, S:P681H, S:T732A,
hCoV-19/Mexico/CMX-INMEGEN-03-11-89/2021	EPI_ISL_1591537	20B	B.1.1.519	27	5'UTR:T201C, 5'UTR:C203T, 5'UTR:C222T, 5'UTR:C241T, ORF1ab:C745T, ORF1ab:G1738T, ORF1ab:C3037T, ORF1ab:C3140T, ORF1ab:G9646T, ORF1ab:C10029T, ORF1ab:C10954T, ORF1ab:A11117G, ORF1ab:C12789T, ORF1ab:C14408T, ORF1ab:T19839C, ORF1ab:A19974G, ORF1ab:T21000C, ORF1ab:C21306T, S:C22995A, S:A23403G, S:C23604A, S:A23756G, S:G25049T, ORF3a:C26111T, N:G28881A, N:G28882A, N:G28883C, N:C29197T,	14	N:R203K, N:G204R, ORF1a:P959S, ORF1a:Q3127H, ORF1a:T3255I, ORF1a:I3618V, ORF1a:T4175I, ORF1b:P314L, ORF3a:P240L, S:T478K, S:D614G, S:P681H, S:T732A, S:D1163Y,
hCoV-19/Mexico/CMX-INMEGEN-03-11-91/2021	EPI_ISL_1591539	20B	B.1.1.519	21	5'UTR:C203T, 5'UTR:C222T, 5'UTR:C241T, ORF1ab:C3037T, ORF1ab:C3140T, ORF1ab:C10029T, ORF1ab:C10954T, ORF1ab:A11117G, ORF1ab:C12789T, ORF1ab:C14408T, ORF1ab:T19839C, ORF1ab:C21306T, S:C21575T, S:C22995A, S:A23403G, S:C23604A, S:A23756G, S:G25012T, N:G28881A, N:G28882A, N:G28883C, N:C29197T,	13	N:R203K, N:G204R, ORF1a:P959S, ORF1a:T3255I, ORF1a:I3618V, ORF1a:T4175I, ORF1b:P314L, S:L5F, S:T478K, S:D614G, S:P681H, S:T732A, S:E1150D,
hCoV-19/Mexico/CMX-INMEGEN-04/2020	EPI_ISL_522875	20B	B.1.1.222	10	5'UTR:C241T, ORF1ab:C3037T, ORF1ab:G11083T, ORF1ab:C14408T, ORF1ab:T19839C, ORF1ab:C20178T, S:A23403G, S:A23756G, N:G28881A, N:G28882A, N:G28883C,	6	N:R203K, N:G204R, ORF1a:L3606F, ORF1b:P314L, S:D614G, S:T732A,
hCoV-19/Mexico/CMX-INMEGEN-04-04-1/2021	EPI_ISL_1628520	20B	B.1.1.519	24	5'UTR:C203T, 5'UTR:C222T, 5'UTR:C241T, ORF1ab:C3037T, ORF1ab:C3140T, ORF1ab:A6084C, ORF1ab:C10029T, ORF1ab:C10954T, ORF1ab:A11117G, ORF1ab:C11575T, ORF1ab:C12789T, ORF1ab:C14408T, ORF1ab:C17550T, ORF1ab:T19839C, ORF1ab:C21306T, S:C22995A, S:A23403G, S:C23604A, S:A23756G, S:A25369G, M:T26908A, N:G28881A, N:G28882A, N:G28883C, N:C29197T,	13	M:L129Q, N:R203K, N:G204R, ORF1a:P959S, ORF1a:D1940A, ORF1a:T3255I, ORF1a:I3618V, ORF1a:T4175I, ORF1b:P314L, S:T478K, S:D614G, S:P681H, S:T732A,
hCoV-19/Mexico/CMX-INMEGEN-04-04-18/2021	EPI_ISL_1628534	20B	B.1.1.519	25	5'UTR:C222T, ORF1ab:C3037T, ORF1ab:C3140T, ORF1ab:A4764G, ORF1ab:C10029T, ORF1ab:C10954T, ORF1ab:A11117G, ORF1ab:C12789T, ORF1ab:T12994A, ORF1ab:C14408T, ORF1ab:C17485T, ORF1ab:G18255A, ORF1ab:G18498T, ORF1ab:T19839C, ORF1ab:C21306T, S:C21575T, S:T21826C, S:A23403G, S:C23604A, S:A23756G, N:C28311T, N:G28881A, N:G28882A, N:G28883C, N:C29197T, N:G29527T,	17	N:P13L, N:R203K, N:G204R, N:Q418H, ORF1a:P959S, ORF1a:H1500R, ORF1a:T3255I, ORF1a:I3618V, ORF1a:T4175I, ORF1b:P314L, ORF1b:M1596I, ORF1b:M1677I, ORF9b:P10S, S:L5F, S:D614G, S:P681H, S:T732A,
hCoV-19/Mexico/CMX-INMEGEN-04-04-2/2021	EPI_ISL_1628521	20B	B.1	19	5'UTR:C241T, ORF1ab:C3037T, ORF1ab:C5646T, ORF1ab:C10029T, ORF1ab:C14408T, ORF1ab:T15294C, ORF1ab:C15738T, ORF1ab:G17721T, ORF1ab:C19421T, ORF1ab:T19839C, S:A23403G, S:A23756G, ORF3a:G25567A, ORF3a:T25569A, ORF3a:T25570A, ORF3a:G25912T, ORF3a:A26108G, ORF7a:G27670T, ORF8:A27921G, ORF8:G28001T,	12	ORF1a:T1794I, ORF1a:T3255I, ORF1b:P314L, ORF1b:S1985L, ORF3a:A59T, ORF3a:S60T, ORF3a:G174C, ORF3a:E239G, ORF7a:V93F, ORF8:I10V, S:D614G, S:T732A,

hCoV-19/Mexico/CMX-INMEGEN-04-04-25/2021	EPI_ISL_1628541	20B	B.1.1.519	22	5'UTR:C203T, 5'UTR:C222T, 5'UTR:C241T, ORF1ab:G654A, ORF1ab:C3037T, ORF1ab:C3140T, ORF1ab:C4999T, ORF1ab:C10029T, ORF1ab:C10954T, ORF1ab:A11117G, ORF1ab:C12789T, ORF1ab:C14408T, ORF1ab:C15579T, ORF1ab:T19839C, ORF1ab:C21306T, S:A23403G, S:C23604A, S:A23756G, N:G28881A, N:G28882A, N:G28883C, N:C29197T, N:G29527T,	12	N:R203K, N:G204R, N:Q418H, ORF1a:G130E, ORF1a:P959S, ORF1a:T3255I, ORF1a:I3618V, ORF1a:T4175I, ORF1b:P314L, S:D614G, S:P681H, S:T732A,	
hCoV-19/Mexico/CMX-INMEGEN-04-04-3/2021	EPI_ISL_1628522	20A	B.1.234	16	5'UTR:C241T, ORF1ab:G2258A, ORF1ab:C3037T, ORF1ab:A6441G, ORF1ab:C8140T, ORF1ab:A9204G, ORF1ab:C10030T, ORF1ab:C11704T, ORF1ab:G13762A, ORF1ab:C14408T, ORF1ab:C19602T, ORF1ab:A20268G, S:C22388T, S:A23403G, N:C28854T, N:C29077T, N:C29445T,	8	N:S194L, N:T391I, ORF1a:V665I, ORF1a:K2059R, ORF1a:D2980G, ORF1b:G99S, ORF1b:P314L, S:D614G,	
hCoV-19/Mexico/CMX-INMEGEN-04-04-33/2021	EPI_ISL_1628548	20B	B.1.1.519	25	5'UTR:C222T, ORF1ab:G1738T, ORF1ab:C3037T, ORF1ab:C3140T, ORF1ab:A3481C, ORF1ab:G3753A, ORF1ab:A9272G, ORF1ab:C10029T, ORF1ab:C10954T, ORF1ab:A11117G, ORF1ab:C12789T, ORF1ab:C14408T, ORF1ab:A16026G, ORF1ab:G18020A, ORF1ab:T19839C, ORF1ab:A19974G, ORF1ab:C21306T, S:A23403G, S:C23604A, S:A23756G, ORF7a:C27649T, ORF8:G28048T, N:G28881A, N:G28882A, N:G28883C, N:C29197T,	13	N:R203K, N:G204R, ORF1a:P959S, ORF1a:R1163K, ORF1a:T3255I, ORF1a:I3618V, ORF1a:T4175I, ORF1b:P314L, ORF1b:R1518K, ORF8:R52I, S:D614G, S:P681H, S:T732A,	5'UTR:76-77,
hCoV-19/Mexico/CMX-INMEGEN-04-04-34/2021	EPI_ISL_1628549	20B	B.1.1.519	31	5'UTR:T201C, 5'UTR:C203T, 5'UTR:C222T, 5'UTR:C241T, ORF1ab:C745T, ORF1ab:G1738T, ORF1ab:G2035T, ORF1ab:C3037T, ORF1ab:C3140T, ORF1ab:C8247T, ORF1ab:C10029T, ORF1ab:C10954T, ORF1ab:G11083T, ORF1ab:A11117G, ORF1ab:A11887G, ORF1ab:C12789T, ORF1ab:C13274T, ORF1ab:C14408T, ORF1ab:C16376T, ORF1ab:T19839C, ORF1ab:A19974G, ORF1ab:C21306T, S:A23403G, S:C23604A, S:A23756G, ORF8:G28077T, ORF8:G28178T, N:G28881A, N:G28882A, N:G28883C, N:C29197T, N:C29272T	17	N:R203K, N:G204R, ORF1a:L590F, ORF1a:P959S, ORF1a:S2661F, ORF1a:T3255I, ORF1a:L3606F, ORF1a:I3618V, ORF1a:T4175I, ORF1a:P4337S, ORF1b:P314L, ORF1b:P970L, ORF8:V62L, ORF8:L95F, S:D614G, S:P681H, S:T732A,	
hCoV-19/Mexico/CMX-INMEGEN-04-04-42/2021	EPI_ISL_1628556	20B	B.1.1.519	26	5'UTR:T201C, 5'UTR:C203T, 5'UTR:C222T, 5'UTR:C241T, ORF1ab:G1738T, ORF1ab:C3037T, ORF1ab:C3140T, ORF1ab:A3363T, ORF1ab:C10029T, ORF1ab:C10954T, ORF1ab:A11117G, ORF1ab:C12789T, ORF1ab:C13168T, ORF1ab:C14408T, ORF1ab:G14414T, ORF1ab:T19839C, ORF1ab:A19974G, ORF1ab:C21306T, S:C22995A, S:A23403G, S:C23604A, S:A23756G, ORF3a:C25791T, N:G28881A, N:G28882A, N:G28883C, N:C29197T,	13	N:R203K, N:G204R, ORF1a:P959S, ORF1a:K1033I, ORF1a:T3255I, ORF1a:I3618V, ORF1a:T4175I, ORF1b:P314L, ORF1b:S316I, S:T478K, S:D614G, S:P681H, S:T732A,	
hCoV-19/Mexico/CMX-INMEGEN-04-04-49/2021	EPI_ISL_1628560	20B	B.1.1.519	28	5'UTR:C203T, 5'UTR:C222T, 5'UTR:C241T, ORF1ab:C3037T, ORF1ab:C3140T, ORF1ab:T9456G, ORF1ab:C9559T, ORF1ab:C10029T, ORF1ab:C10747T, ORF1ab:C10954T, ORF1ab:A11117G, ORF1ab:C12789T, ORF1ab:C14408T, ORF1ab:C15738T, ORF1ab:T16587C, ORF1ab:T18688C, ORF1ab:A19667G, ORF1ab:T19839C, ORF1ab:C21306T, S:C22995A, S:A23403G, S:C23604A, S:A23756G, M:C26882T, ORF8:T27904C, N:G28881A, N:G28882A, N:G28883C, N:C29197T,	15	N:R203K, N:G204R, ORF1a:P959S, ORF1a:F3064C, ORF1a:T3255I, ORF1a:I3618V, ORF1a:T4175I, ORF1b:P314L, ORF1b:F1741L, ORF1b:D2067G, ORF8:L4P, S:T478K, S:D614G, S:P681H, S:T732A,	

hCoV-19/Mexico/CMX-INMEGEN-04-04-52/2021	EPI_ISL_1628563	20B	B.1.1.519	28	5'UTR:T201C, 5'UTR:C203T, 5'UTR:C222T, 5'UTR:C241T, ORF1ab:A810G, ORF1ab:C936T, ORF1ab:G1738T, ORF1ab:C3037T, ORF1ab:C3140T, ORF1ab:C10029T, ORF1ab:C10954T, ORF1ab:A11117G, ORF1ab:C12789T, ORF1ab:C14408T, ORF1ab:T19839C, ORF1ab:A19974G, ORF1ab:C21306T, S:C22995A, S:A23403G, S:C23604A, S:A23756G, S:G25354T, ORF3a:C25626T, N:G28817T, N:G28881A, N:G28882A, N:G28883C, N:C29197T, N:G29227T,	14	N:A182S, N:R203K, N:G204R, ORF1a:Y182C, ORF1a:T224I, ORF1a:P959S, ORF1a:T3255I, ORF1a:I3618V, ORF1a:T4175I, ORF1b:P314L, S:T478K, S:D614G, S:P681H, S:T732A,	
hCoV-19/Mexico/CMX-INMEGEN-04-04-57/2021	EPI_ISL_1628566	20B	B.1.1.519	25	5'UTR:C203T, 5'UTR:C222T, 5'UTR:C241T, ORF1ab:C3037T, ORF1ab:C3140T, ORF1ab:G3692T, ORF1ab:G3871T, ORF1ab:C10029T, ORF1ab:C10954T, ORF1ab:A11117G, ORF1ab:C11824A, ORF1ab:C12439T, ORF1ab:C12789T, ORF1ab:C14408T, ORF1ab:A14612G, ORF1ab:T19839C, ORF1ab:C21306T, S:A23403G, S:C23604A, S:A23756G, ORF8:T27904C, N:G28881A, N:G28882A, N:G28883C, N:C29160T, N:C29197T,	15	N:R203K, N:G204R, N:T296I, ORF1a:P959S, ORF1a:V1143F, ORF1a:K1202N, ORF1a:T3255I, ORF1a:I3618V, ORF1a:T4175I, ORF1b:P314L, ORF1b:K382R, ORF8:L4P, S:D614G, S:P681H, S:T732A,	
hCoV-19/Mexico/CMX-INMEGEN-04-04-66/2021	EPI_ISL_1857295	20B	B.1.1.519	26	5'UTR:C203T, 5'UTR:C222T, 5'UTR:C241T, ORF1ab:C3037T, ORF1ab:C3140T, ORF1ab:T3745C, ORF1ab:A9193G, ORF1ab:C10029T, ORF1ab:C10954T, ORF1ab:A11117G, ORF1ab:C12789T, ORF1ab:C14408T, ORF1ab:C16376T, ORF1ab:T16419G, ORF1ab:T19839C, ORF1ab:C21302T, ORF1ab:C21306T, S:A23403G, S:C23604A, S:A23756G, S:T25304A, ORF3a:C25844T, N:G28881A, N:G28882A, N:G28883C, N:C29197T, 3'UTR:G29744A	13	N:R203K, N:G204R, ORF1a:P959S, ORF1a:T3255I, ORF1a:I3618V, ORF1a:T4175I, ORF1b:P314L, ORF1b:P970L, ORF1b:P2612L, S:D614G, S:P681H, S:T732A, S:C1248S,	ORF3a:26158-26161,
hCoV-19/Mexico/CMX-INMEGEN-04-04-73/2021	EPI_ISL_1628579	20B	B.1.1.519	28	5'UTR:C203T, 5'UTR:C222T, 5'UTR:C241T, ORF1ab:A866G, ORF1ab:C1009T, ORF1ab:C3037T, ORF1ab:C3140T, ORF1ab:G4133A, ORF1ab:C5183T, ORF1ab:C10029T, ORF1ab:C10954T, ORF1ab:A11117G, ORF1ab:C11916T, ORF1ab:C12789T, ORF1ab:C14408T, ORF1ab:T19839C, ORF1ab:C21306T, S:C21575T, S:A23403G, S:C23604A, S:A23756G, ORF3a:G25879T, E:C26408T, ORF6:T27328C, ORF8:T27904C, N:G28881A, N:G28882A, N:G28883C, N:C29197T,	19	E:S55F, N:R203K, N:G204R, ORF1a:I201V, ORF1a:P959S, ORF1a:V1290I, ORF1a:P1640S, ORF1a:T3255I, ORF1a:I3618V, ORF1a:S3884L, ORF1a:T4175I, ORF1b:P314L, ORF3a:V163L, ORF6:S43P, ORF8:L4P, S:L5F, S:D614G, S:P681H, S:T732A,	
hCoV-19/Mexico/CMX-INMEGEN-04-04-74/2021	EPI_ISL_1628580	20B	B.1.1.222	28	5'UTR:C186T, 5'UTR:C203T, 5'UTR:C222T, 5'UTR:C241T, ORF1ab:C3037T, ORF1ab:C3140T, ORF1ab:C3736T, ORF1ab:C10029T, ORF1ab:C10954T, ORF1ab:C11020T, ORF1ab:C12789T, ORF1ab:C14408T, ORF1ab:C14790T, ORF1ab:C15240T, ORF1ab:T19839C, ORF1ab:C21306T, S:A23403G, S:C23604A, S:A23756G, S:C24023T, ORF3a:A25799C, M:C26681T, ORF8:G28001T, N:C28677T, N:G28881A, N:G28882A, N:G28883C, N:C29197T, N:G29527T	12	N:T135I, N:R203K, N:G204R, N:Q418H, ORF1a:P959S, ORF1a:T3255I, ORF1a:T4175I, ORF1b:P314L, ORF3a:K136T, S:D614G, S:P681H, S:T732A,	
hCoV-19/Mexico/CMX-INMEGEN-04-04-89/2021	EPI_ISL_1628590	20B	B.1.1.519	27	5'UTR:A129G, 5'UTR:C203T, 5'UTR:C222T, 5'UTR:C241T, ORF1ab:C3037T, ORF1ab:C3140T, ORF1ab:T3745C, ORF1ab:A8842G, ORF1ab:C10029T, ORF1ab:G10870T, ORF1ab:C10954T, ORF1ab:G11083T, ORF1ab:A11117G, ORF1ab:C12789T, ORF1ab:C14408T, ORF1ab:T19839C, ORF1ab:C21306T, S:A23403G, S:C23604A, S:A23756G, ORF3a:C25844T, N:C28473T, N:A28537C, N:G28881A, N:G28882A, N:G28883C, N:C29197T, 3'UTR:G29773T	15	N:P67L, N:R203K, N:G204R, ORF1a:P959S, ORF1a:T3255I, ORF1a:L3606F, ORF1a:I3618V, ORF1a:T4175I, ORF1b:P314L, ORF3a:T151I, ORF9b:L64F, ORF9b:E85A, S:D614G, S:P681H, S:T732A,	S:25331-25333,5'UTR:76-78

hCoV-19/Mexico/CMX-INMEGEN-04-04-90/2021	EPI_ISL_1628591	20B	B.1.1.519	24	5'UTR:C241T, ORF1ab:C745T, ORF1ab:G1738T, ORF1ab:C3037T, ORF1ab:C3140T, ORF1ab:G6254A, ORF1ab:C10029T, ORF1ab:C10954T, ORF1ab:A11117G, ORF1ab:A11887G, ORF1ab:C12789T, ORF1ab:C14408T, ORF1ab:T19839C, ORF1ab:A19974G, ORF1ab:C21306T, S:C22995A, S:A23403G, S:C23604A, S:A23756G, ORF8:G28077T, ORF8:G28178T, N:G28881A, N:G28882A, N:G28883C, N:C29197T,	14	N:R203K, N:G204R, ORF1a:P959S, ORF1a:A1997T, ORF1a:T3255I, ORF1a:I3618V, ORF1a:T4175I, ORF1b:P314L, ORF8:V62L, ORF8:L95F, S:T478K, S:D614G, S:P681H, S:T732A,
hCoV-19/Mexico/CMX-INMEGEN-05/2020	EPI_ISL_522876	20A	B.1	12	5'UTR:C241T, ORF1ab:C3037T, ORF1ab:C6538T, ORF1ab:C9924T, ORF1ab:G11083T, ORF1ab:C14408T, ORF1ab:T15348C, ORF1ab:C16308T, ORF1ab:A20268G, ORF1ab:G21123T, S:G23285T, S:A23403G, N:C29311T,	5	ORF1a:A3220V, ORF1a:L3606F, ORF1b:P314L, S:A575S, S:D614G,
hCoV-19/Mexico/CMX-INMEGEN-06/2020	EPI_ISL_522877	20A	B.1.243	17	5'UTR:C241T, ORF1ab:G806A, ORF1ab:A1428G, ORF1ab:C2106T, ORF1ab:C3037T, ORF1ab:A4050G, ORF1ab:C4331T, ORF1ab:G11083T, ORF1ab:C14408T, ORF1ab:C17373T, ORF1ab:A20268G, S:G22604T, S:A23403G, S:T24076C, ORF8:C28121A, N:G28460T, N:C28854T, N:G29543T,	11	N:D63Y, N:S194L, ORF1a:A181T, ORF1a:H388R, ORF1a:T614I, ORF1a:N1262S, ORF1a:L3606F, ORF1b:P314L, ORF9b:K59N, S:A348S, S:D614G,
hCoV-19/Mexico/CMX-INMEGEN-07/2020	EPI_ISL_522878	20B	B.1.1	11	5'UTR:C241T, ORF1ab:C3037T, ORF1ab:C10039T, ORF1ab:C10207T, ORF1ab:C11878T, ORF1ab:C13487T, ORF1ab:G13714A, ORF1ab:C14408T, S:A23403G, N:G28881A, N:G28882A, N:G28883C,	6	N:R203K, N:G204R, ORF1b:A7V, ORF1b:D83N, ORF1b:P314L, S:D614G,
hCoV-19/Mexico/CMX-INMEGEN-08/2020	EPI_ISL_522879	20A	B.1	7	5'UTR:C241T, ORF1ab:C3037T, ORF1ab:C14408T, ORF1ab:A20268G, S:C23029T, S:A23403G, ORF8:C28253T, N:C28854T,	3	N:S194L, ORF1b:P314L, S:D614G,
hCoV-19/Mexico/CMX-INMEGEN-09/2020	EPI_ISL_522880	20A	B.1.243	15	5'UTR:C241T, ORF1ab:G806A, ORF1ab:C3037T, ORF1ab:C6941T, ORF1ab:G13723T, ORF1ab:C14408T, ORF1ab:T16074C, ORF1ab:C19366T, ORF1ab:A20268G, ORF1ab:A21070G, S:A23403G, S:T24076C, ORF7a:T27653C, N:C28854T, N:G29543T, 3'UTR:A29824G,	8	N:S194L, ORF1a:A181T, ORF1b:A86S, ORF1b:P314L, ORF1b:P1967S, ORF1b:N2535D, ORF7a:F87S, S:D614G,
hCoV-19/Mexico/CMX-INMEGEN-10/2020	EPI_ISL_522940	20A	B.1.243	10	5'UTR:C241T, ORF1ab:C3037T, ORF1ab:C10252T, ORF1ab:C10834A, ORF1ab:C14408T, ORF1ab:A20268G, S:A22155C, S:A23403G, S:T24076C, ORF3a:G25599T, N:C28854T,	5	N:S194L, ORF1b:P314L, ORF3a:W69C, S:D198A, S:D614G,
hCoV-19/Mexico/CMX-INMEGEN-11/2020	EPI_ISL_522941	20C	B.1	16	5'UTR:C241T, ORF1ab:C1059T, ORF1ab:G2272A, ORF1ab:C3037T, ORF1ab:C7431T, ORF1ab:C11916T, ORF1ab:C14408T, ORF1ab:C18998T, ORF1ab:A19805G, S:G22708T, S:A23403G, S:C24157T, S:G24794T, ORF3a:G25563T, ORF8:C27911T, N:G28851T, N:G29540A,	10	N:S193I, ORF1a:T265I, ORF1a:S2389L, ORF1a:S3884L, ORF1b:P314L, ORF1b:A1844V, ORF1b:N2113S, ORF3a:Q57H, S:D614G, S:A1078S,
hCoV-19/Mexico/CMX-INMEGEN-11-01/2020	EPI_ISL_930783	20B	B.1.1	16	5'UTR:C190T, 5'UTR:C241T, ORF1ab:C3037T, ORF1ab:C9603T, ORF1ab:C14408T, ORF1ab:G14767T, ORF1ab:T19839C, S:A23403G, S:C23604A, ORF3a:G25522A, ORF3a:C25867T, M:T27138C, ORF7b:G27890A, N:G28881A, N:G28882A, N:G28883C, 3'UTR:G29742T,	9	N:R203K, N:G204R, ORF1a:T3113I, ORF1b:P314L, ORF1b:A434S, ORF3a:G44R, ORF3a:P159S, S:D614G, S:P681H,
hCoV-19/Mexico/CMX-INMEGEN-11-02/2020	EPI_ISL_933512	20B	B.1.1.222	18	5'UTR:C241T, ORF1ab:C835T, ORF1ab:C3037T, ORF1ab:C6781T, ORF1ab:C8090T, ORF1ab:C11416T, ORF1ab:A13947C, ORF1ab:C14408T, ORF1ab:G15672T, ORF1ab:A17340G, ORF1ab:T19839C, S:A23403G, S:C23557T, S:A23756G, ORF3a:G26063T, ORF7b:A27888T, N:G28881A, N:G28882A, N:G28883C,	8	N:R203K, N:G204R, ORF1a:L2609F, ORF1b:P314L, ORF1b:E735D, ORF3a:G224V, S:D614G, S:T732A,

hCoV-19/Mexico/CMX-INMEGEN-11-03/2020	EPI_ISL_933513	20A	B.1.243	20	5'UTR:C241T, ORF1ab:G806A, ORF1ab:T2584A, ORF1ab:C3037T, ORF1ab:C5140A, ORF1ab:C6027T, ORF1ab:G11417T, ORF1ab:C14408T, ORF1ab:C16394T, ORF1ab:C18486T, ORF1ab:C19524T, ORF1ab:C19875A, ORF1ab:A20268G, ORF1ab:C21057T, S:C21998T, S:A23403G, S:G23587C, S:T24076C, E:C26455T, N:C28854T, N:G29543T,	11	E:P71S, N:S194L, ORF1a:A181T, ORF1a:D1625E, ORF1a:P1921L, ORF1a:V3718F, ORF1b:P314L, ORF1b:P976L, S:H146Y, S:D614G, S:Q675H,
hCoV-19/Mexico/CMX-INMEGEN-11-04/2020	EPI_ISL_933514	20B	B.1.1.222	18	5'UTR:C241T, ORF1ab:C3037T, ORF1ab:C5974T, ORF1ab:C6636T, ORF1ab:A10864G, ORF1ab:C14408T, ORF1ab:G14772T, ORF1ab:T19839C, ORF1ab:C20178T, S:C22879T, S:A23403G, S:A23756G, ORF7a:T27484C, N:G28881A, N:G28882A, N:G28883C, N:A29040G, N:C29386A, ORF10:T29652C,	9	N:R203K, N:G204R, N:K256R, N:D371E, ORF1a:T2124I, ORF1b:P314L, ORF1b:Q435H, S:D614G, S:T732A,
hCoV-19/Mexico/CMX-INMEGEN-11-05/2020	EPI_ISL_933515	20B	B.1.1	11	5'UTR:C241T, ORF1ab:C3037T, ORF1ab:A9204G, ORF1ab:C14408T, ORF1ab:T19839C, S:C22033A, S:A23403G, S:A25192T, ORF3a:G25687A, N:G28881A, N:G28882A, N:G28883C,	7	N:R203K, N:G204R, ORF1a:D2980G, ORF1b:P314L, ORF3a:A99T, S:F157L, S:D614G,
hCoV-19/Mexico/CMX-INMEGEN-11-06/2020	EPI_ISL_933516	20B	B.1.1.222	16	ORF1ab:G2035T, ORF1ab:C3037T, ORF1ab:C3884T, ORF1ab:C4686T, ORF1ab:C6196T, ORF1ab:C14408T, ORF1ab:C18981T, ORF1ab:T19839C, S:C22480T, S:A23403G, S:C23655T, S:A23756G, ORF8:G27916A, ORF8:G28083T, N:G28881A, N:G28882A, N:G28883C,	11	N:R203K, N:G204R, ORF1a:L590F, ORF1a:P1207S, ORF1a:T1474I, ORF1b:P314L, ORF8:G8E, ORF8:E64*, S:D614G, S:S698L, S:T732A,
hCoV-19/Mexico/CMX-INMEGEN-11-07/2020	EPI_ISL_933517	20A	B.1	12	5'UTR:C241T, ORF1ab:G1820A, ORF1ab:C2695T, ORF1ab:C3037T, ORF1ab:T9508C, ORF1ab:C10605T, ORF1ab:C14408T, ORF1ab:A16474G, S:C22432T, S:A23403G, S:C23664T, ORF3a:C26060T, ORF8:C27945T,	8	ORF1a:G519S, ORF1a:P3447L, ORF1b:P314L, ORF1b:S1003G, ORF3a:T223I, ORF8:Q18*, S:D614G, S:A701V,
hCoV-19/Mexico/CMX-INMEGEN-11-08/2020	EPI_ISL_933518	20B	B.1.1.222	21	5'UTR:C241T, ORF1ab:C593T, ORF1ab:A1558G, ORF1ab:C3037T, ORF1ab:A3916G, ORF1ab:A6360C, ORF1ab:C6543T, ORF1ab:C8606T, ORF1ab:G11222T, ORF1ab:C14216T, ORF1ab:C14408T, ORF1ab:T19839C, S:C21614T, S:G22026A, S:G22104T, S:A23403G, S:A23756G, ORF3a:C25587T, ORF3a:C25904T, N:G28881A, N:G28882A, N:G28883C,	16	N:R203K, N:G204R, ORF1a:H110Y, ORF1a:I431M, ORF1a:D2032A, ORF1a:T2093I, ORF1a:L2781F, ORF1a:V3653F, ORF1b:T250I, ORF1b:P314L, ORF3a:S171L, S:L18F, S:S155N, S:G181V, S:D614G, S:T732A,
hCoV-19/Mexico/CMX-INMEGEN-11-09/2020	EPI_ISL_933519	20A	B.1	12	5'UTR:C241T, ORF1ab:C3037T, ORF1ab:C10537T, ORF1ab:T13027C, ORF1ab:C13620T, ORF1ab:C14408T, ORF1ab:A17861G, ORF1ab:A20268G, ORF1ab:G21159A, S:A23403G, M:C26858T, M:T27089C, N:C28854T,	4	N:S194L, ORF1b:P314L, ORF1b:D1465G, S:D614G,
hCoV-19/Mexico/CMX-INMEGEN-11-11/2020	EPI_ISL_933520	20B	B.1.1.519	24	5'UTR:C203T, 5'UTR:C222T, 5'UTR:C241T, ORF1ab:C1342T, ORF1ab:C3037T, ORF1ab:C3140T, ORF1ab:G3692T, ORF1ab:G3871T, ORF1ab:C10029T, ORF1ab:C10954T, ORF1ab:A11117G, ORF1ab:C11824A, ORF1ab:C12789T, ORF1ab:C14408T, ORF1ab:T19839C, ORF1ab:C21306T, S:C22995A, S:A23403G, S:C23604A, S:A23756G, ORF8:T27904C, N:G28881A, N:G28882A, N:G28883C, N:C29197T,	14	N:R203K, N:G204R, ORF1a:P959S, ORF1a:V1143F, ORF1a:K1202N, ORF1a:T3255I, ORF1a:I3618V, ORF1a:T4175I, ORF1b:P314L, ORF8:L4P, S:T478K, S:D614G, S:P681H, S:T732A,
hCoV-19/Mexico/CMX-INMEGEN-11-12/2020	EPI_ISL_933521	20B	B.1	26	5'UTR:C203T, 5'UTR:C222T, 5'UTR:C241T, ORF1ab:C3037T, ORF1ab:C3140T, ORF1ab:G3692T, ORF1ab:G3871T, ORF1ab:A8446G, ORF1ab:C10029T, ORF1ab:C10319T, ORF1ab:C10954T, ORF1ab:A11117G, ORF1ab:C11824A, ORF1ab:C12789T, ORF1ab:C14408T, ORF1ab:T19839C, ORF1ab:C21306T, S:C22995A, S:A23403G, S:C23604A, S:A23756G, ORF8:T27904C, N:G28881A, N:G28882A, N:G28883C, N:C29197T, 3'UTR:G29690T,	15	N:R203K, N:G204R, ORF1a:P959S, ORF1a:V1143F, ORF1a:K1202N, ORF1a:T3255I, ORF1a:L3352F, ORF1a:I3618V, ORF1a:T4175I, ORF1b:P314L, ORF8:L4P, S:T478K, S:D614G, S:P681H, S:T732A,

hCoV-19/Mexico/CMX-INMEGEN-11-13/2020	EPI_ISL_933522	20B	B.1.1.519	23	5'UTR:C203T, 5'UTR:C222T, 5'UTR:C241T, ORF1ab:A2219G, ORF1ab:C3037T, ORF1ab:C3140T, ORF1ab:C10029T, ORF1ab:C10954T, ORF1ab:A11117G, ORF1ab:C12789T, ORF1ab:C14408T, ORF1ab:T19839C, ORF1ab:C21306T, S:C22995A, S:A23403G, S:C23604A, S:A23756G, S:G24914T, ORF3a:C25782T, ORF3a:G26063T, N:G28881A, N:G28882A, N:G28883C, N:C29197T,	14	N:R203K, N:G204R, ORF1a:I652V, ORF1a:P959S, ORF1a:T3255I, ORF1a:I3618V, ORF1a:T4175I, ORF1b:P314L, ORF3a:G224V, S:T478K, S:D614G, S:P681H, S:T732A, S:D1118Y,
hCoV-19/Mexico/CMX-INMEGEN-11-14/2020	EPI_ISL_933523	20A	B.1.189	20	5'UTR:C241T, ORF1ab:G373A, ORF1ab:C3037T, ORF1ab:C3903T, ORF1ab:C4582T, ORF1ab:G6404T, ORF1ab:C6762T, ORF1ab:C14408T, ORF1ab:C14818T, ORF1ab:C15450T, ORF1ab:T17262C, ORF1ab:T19956A, ORF1ab:A20268G, S:G21974T, S:A23403G, S:C23987T, S:T25123C, N:G28378T, N:C28657T, N:C28887T, N:G29179T,	9	N:T205I, ORF1a:P1213L, ORF1a:V2047F, ORF1a:T2166I, ORF1b:P314L, ORF9b:R32L, S:D138Y, S:D614G, S:P809S,
hCoV-19/Mexico/CMX-INMEGEN-11-15/2020	EPI_ISL_933524	20A	B.1.189	20	5'UTR:C241T, ORF1ab:G373A, ORF1ab:C3037T, ORF1ab:C3903T, ORF1ab:C4582T, ORF1ab:G6404T, ORF1ab:C6762T, ORF1ab:C14408T, ORF1ab:C14818T, ORF1ab:C15450T, ORF1ab:T17262C, ORF1ab:T19956A, ORF1ab:A20268G, S:G21974T, S:A23403G, S:C23987T, S:T25123C, N:G28378T, N:C28657T, N:C28887T, N:G29179T,	9	N:T205I, ORF1a:P1213L, ORF1a:V2047F, ORF1a:T2166I, ORF1b:P314L, ORF9b:R32L, S:D138Y, S:D614G, S:P809S,
hCoV-19/Mexico/CMX-INMEGEN-11-16/2020	EPI_ISL_933525	20A	B.1.243	24	5'UTR:C241T, ORF1ab:C274T, ORF1ab:G806A, ORF1ab:T1783C, ORF1ab:T2584A, ORF1ab:C3037T, ORF1ab:C3602T, ORF1ab:G4146A, ORF1ab:C5140A, ORF1ab:T5255C, ORF1ab:C6027T, ORF1ab:C6633T, ORF1ab:G12191T, ORF1ab:C14408T, ORF1ab:C14599G, ORF1ab:G16647T, ORF1ab:C18486T, ORF1ab:A20268G, S:A23403G, S:G23587C, S:T24076C, E:C26455T, N:C28854T, N:G29543T, ORF10:C29627T,	13	E:P71S, N:S194L, ORF1a:A181T, ORF1a:H1113Y, ORF1a:G1294D, ORF1a:D1625E, ORF1a:P1921L, ORF1a:A2123V, ORF1a:V3976F, ORF1b:P314L, ORF1b:L378V, S:D614G, S:Q675H,
hCoV-19/Mexico/CMX-INMEGEN-11-17/2020	EPI_ISL_933526	20B	B.1.1.222	21	5'UTR:C241T, ORF1ab:T2014C, ORF1ab:C3037T, ORF1ab:G4207T, ORF1ab:T10299C, ORF1ab:C10525T, ORF1ab:G11417T, ORF1ab:C14189T, ORF1ab:C14408T, ORF1ab:G14772T, ORF1ab:C16887T, ORF1ab:G18651T, ORF1ab:T19839C, ORF1ab:A20696G, S:G22216T, S:A23403G, S:A23756G, ORF7a:T27722C, N:G28881A, N:G28882A, N:G28883C, ORF10:T29652C,	13	N:R203K, N:G204R, ORF1a:M3345T, ORF1a:V3718F, ORF1b:A241V, ORF1b:P314L, ORF1b:Q435H, ORF1b:E1728D, ORF1b:N2410S, ORF7a:I110T, S:Q218H, S:D614G, S:T732A,
hCoV-19/Mexico/CMX-INMEGEN-11-18/2020	EPI_ISL_933527	20A	B.1	21	5'UTR:G210T, 5'UTR:C241T, ORF1ab:C3037T, ORF1ab:C4423T, ORF1ab:G11417T, ORF1ab:C12068T, ORF1ab:G14268T, ORF1ab:C14408T, ORF1ab:C15141T, ORF1ab:C16393T, ORF1ab:G16917T, ORF1ab:A20268G, ORF1ab:A20636C, ORF1ab:C21139T, S:A23403G, S:G23465A, S:G23608T, S:C23987T, ORF3a:G25489T, ORF3a:G25855C, ORF7a:C27513T, N:C28854T,	10	N:S194L, ORF1a:V3718F, ORF1b:P314L, ORF1b:P976S, ORF1b:E2390A, ORF3a:A33S, ORF3a:D155H, S:D614G, S:V635I, S:P809S,
hCoV-19/Mexico/CMX-INMEGEN-11-19/2020	EPI_ISL_933528	20B	B.1.1.222	14	5'UTR:C241T, ORF1ab:T670C, ORF1ab:T1623C, ORF1ab:C3037T, ORF1ab:C8140T, ORF1ab:C8326T, ORF1ab:C14408T, ORF1ab:T19839C, S:G22021T, S:A23403G, S:A23756G, M:C27143T, N:G28881A, N:G28882A, N:G28883C,	7	N:R203K, N:G204R, ORF1a:I453T, ORF1b:P314L, S:M153I, S:D614G, S:T732A,

hCoV-19/Mexico/CMX-INMEGEN-11-20/2020	EPI_ISL_933529	20G	B.1.2	20	5'UTR:C241T, ORF1ab:C1059T, ORF1ab:C1911T, ORF1ab:C1973T, ORF1ab:C3037T, ORF1ab:C10319T, ORF1ab:C14408T, ORF1ab:A18424G, ORF1ab:C20801T, ORF1ab:C21304T, ORF1ab:A21390G, S:C22591T, S:G23311T, S:A23403G, ORF3a:G25563T, ORF3a:G25907T, M:C26681T, ORF8:C27964T, ORF8:C28253T, N:C28472T. N:C28869T.	14	N:P67S, N:P199L, ORF1a:T265I, ORF1a:S549F, ORF1a:L3352F, ORF1b:P314L, ORF1b:N1653D, ORF1b:T2445I, ORF1b:R2613C, ORF3a:Q57H, ORF3a:G172V, ORF8:S24L, S:E583D, S:D614G,	
hCoV-19/Mexico/CMX-INMEGEN-11-21/2020	EPI_ISL_933530	20B	B.1.1.222	22	5'UTR:C241T, ORF1ab:C3037T, ORF1ab:G3242T, ORF1ab:C3510T, ORF1ab:C4980A, ORF1ab:G7495T, ORF1ab:C9344T, ORF1ab:C13297T, ORF1ab:C13517T, ORF1ab:C14408T, ORF1ab:T14472G, ORF1ab:G15906T, ORF1ab:C16699T, ORF1ab:T17065C, ORF1ab:G18186T, ORF1ab:T19839C, S:A23403G, S:A23756G, ORF8:C28253T, N:G28881A, N:G28882A, N:G28883C, 3'UTR:G29747C,	15	N:R203K, N:G204R, ORF1a:G993C, ORF1a:A1082V, ORF1a:T1572K, ORF1a:M2410I, ORF1a:L3027F, ORF1b:T17I, ORF1b:P314L, ORF1b:Q813H, ORF1b:R1078C, ORF1b:Y1200H, ORF1b:M1573I, S:D614G, S:T732A,	
hCoV-19/Mexico/CMX-INMEGEN-11-22/2020	EPI_ISL_933531	20B	B.1.1.519	22	5'UTR:T201C, 5'UTR:C203T, 5'UTR:C222T, 5'UTR:C241T, ORF1ab:G1738T, ORF1ab:C3037T, ORF1ab:C3140T, ORF1ab:C10029T, ORF1ab:C10954T, ORF1ab:A11117G, ORF1ab:C12789T, ORF1ab:C14408T, ORF1ab:T19839C, ORF1ab:A19974G, ORF1ab:C21306T, S:C22995A, S:A23403G, S:C23604A, S:A23756G, N:G28881A, N:G28882A, N:G28883C, N:C29197T.	11	N:R203K, N:G204R, ORF1a:P959S, ORF1a:T3255I, ORF1a:I3618V, ORF1a:T4175I, ORF1b:P314L, S:T478K, S:D614G, S:P681H, S:T732A,	
hCoV-19/Mexico/CMX-INMEGEN-12/2020	EPI_ISL_522942	19B	A	2	ORF1ab:C8782T, ORF1ab:C18060T, ORF8:T28144C,	1	ORF8:L84S,	
hCoV-19/Mexico/CMX-INMEGEN-12-03-01/2020	EPI_ISL_955194	20A	B.1	14	5'UTR:C241T, ORF1ab:C1380T, ORF1ab:C3037T, ORF1ab:G6341T, ORF1ab:G10396T, ORF1ab:C14408T, ORF1ab:A20268G, S:C22591T, S:T23042C, S:A23403G, S:T23599G, S:C23638T, N:G28817T, N:C28854T, N:C29421T,	9	N:A182S, N:S194L, N:P383L, ORF1a:A372V, ORF1a:D2026Y, ORF1b:P314L, S:S494P, S:D614G, S:N679K,	
hCoV-19/Mexico/CMX-INMEGEN-12-03-02/2020	EPI_ISL_955195	20B	B.1.1.222	22	5'UTR:C241T, ORF1ab:T2998C, ORF1ab:C3037T, ORF1ab:A4228T, ORF1ab:G9871T, ORF1ab:C14408T, ORF1ab:G16246T, ORF1ab:T17023C, ORF1ab:T19839C, ORF1ab:G20853T, S:C21622T, S:A23403G, S:A23756G, S:G25249C, ORF3a:C25626T, ORF3a:C25904T, M:C26681T, ORF7b:T27817C, N:A28480G, N:G28881A, N:G28882A, N:G28883C, N:G29402T,	13	N:R203K, N:G204R, N:D377Y, ORF1b:P314L, ORF1b:A927S, ORF1b:S1186P, ORF1b:M2462I, ORF3a:S171L, ORF7b:V21A, ORF9b:D66G, S:D614G, S:T732A, S:M1229I,	
hCoV-19/Mexico/CMX-INMEGEN-12-03-04/2020	EPI_ISL_955196	20B	B.1.1.519	21	5'UTR:C203T, 5'UTR:C222T, 5'UTR:C241T, ORF1ab:C3037T, ORF1ab:C3140T, ORF1ab:C10029T, ORF1ab:C10954T, ORF1ab:A11117G, ORF1ab:C12789T, ORF1ab:C14408T, ORF1ab:A15864G, ORF1ab:T19839C, ORF1ab:C21306T, S:C22995A, S:A23403G, S:C23604A, S:A23756G, N:G28881A, N:G28882A, N:G28883C, N:C29197T, ORF10:C29592T,	11	N:R203K, N:G204R, ORF1a:P959S, ORF1a:T3255I, ORF1a:I3618V, ORF1a:T4175I, ORF1b:P314L, S:T478K, S:D614G, S:P681H, S:T732A,	
hCoV-19/Mexico/CMX-INMEGEN-12-03-05/2020	EPI_ISL_955192	20B	B.1.1.28	20	5'UTR:C241T, ORF1ab:C925T, ORF1ab:C2644T, ORF1ab:C3037T, ORF1ab:G4925T, ORF1ab:A10081G, ORF1ab:G11230T, ORF1ab:C14408T, ORF1ab:T19839C, S:C22450T, S:A23403G, S:G23593T, S:T24643C, S:G25088T, ORF7a:G27754C, ORF8:C27945T, N:A28841G, N:G28881A, N:G28882A, N:G28883C, 3'UTR:C29738T.	12	N:S190G, N:R203K, N:G204R, ORF1a:D1554Y, ORF1a:M3655I, ORF1b:P314L, ORF7a:E121Q, ORF8:Q18*, S:D614G, S:Q677H, S:V1176F,ORF1a:L2039I,	ORF1ab: 6372-6380
hCoV-19/Mexico/CMX-INMEGEN-12-03-06/2020	EPI_ISL_955197	20A	B.1.396	16	5'UTR:C241T, ORF1ab:G1126T, ORF1ab:C1191T, ORF1ab:C3037T, ORF1ab:A3205G, ORF1ab:A3297G, ORF1ab:T8634C, ORF1ab:C9521A, ORF1ab:C14408T, ORF1ab:A20268G, S:A23403G, S:C24904T, N:G28378T, N:C28854T, N:G29422T, N:C29485T, ORF10:A29567G,	9	N:S194L, ORF1a:R287S, ORF1a:P309L, ORF1a:Q1011R, ORF1a:I2790T, ORF1a:L3086I, ORF1b:P314L, ORF9b:R32L, S:D614G,	

hCoV-19/Mexico/CMX-INMEGEN-12-03-07/2020	EPI_ISL_955198	20A	B.1.243	16	5'UTR:T232G, 5'UTR:C241T, ORF1ab:G1522T, ORF1ab:C3037T, ORF1ab:G3433A, ORF1ab:C7869T, ORF1ab:C10277T, ORF1ab:G12239A, ORF1ab:C14408T, ORF1ab:C15660T, ORF1ab:G16647T, ORF1ab:A20268G, S:A23403G, S:T24076C, ORF3a:G25621T, N:C28854T, 3'UTR:G29759T,	8	N:S194L, ORF1a:K419N, ORF1a:S2535L, ORF1a:L3338F, ORF1a:D3992N, ORF1b:P314L, ORF3a:V77F, S:D614G,
hCoV-19/Mexico/CMX-INMEGEN-12-03-08/2020	EPI_ISL_955199	20B	B.1.1.222	17	5'UTR:C241T, ORF1ab:C3037T, ORF1ab:C5497T, ORF1ab:T8053C, ORF1ab:C9120T, ORF1ab:C9223T, ORF1ab:C9438T, ORF1ab:C9615T, ORF1ab:C14408T, ORF1ab:T19839C, S:A23403G, S:A23756G, ORF7a:T27597C, ORF7a:C27741T, N:C28311T, N:G28881A, N:G28882A, N:G28883C,	10	N:P13L, N:R203K, N:G204R, ORF1a:T2952I, ORF1a:T3058I, ORF1a:T3117I, ORF1b:P314L, ORF9b:P10S, S:D614G, S:T732A,
hCoV-19/Mexico/CMX-INMEGEN-12-03-09/2020	EPI_ISL_955200	20B	B.1.1.222	14	5'UTR:C241T, ORF1ab:T1623C, ORF1ab:C3037T, ORF1ab:C8140T, ORF1ab:C8326T, ORF1ab:C9430T, ORF1ab:C14408T, ORF1ab:T19839C, S:G22021T, S:A23403G, S:A23756G, ORF7a:C27741T, N:G28881A, N:G28882A, N:G28883C,	7	N:R203K, N:G204R, ORF1a:I453T, ORF1b:P314L, S:M153I, S:D614G, S:T732A,
hCoV-19/Mexico/CMX-INMEGEN-12-03-10/2020	EPI_ISL_955201	20B	B.1.1.518	19	5'UTR:C241T, ORF1ab:C2878T, ORF1ab:C3037T, ORF1ab:A6453G, ORF1ab:A9019G, ORF1ab:C10156T, ORF1ab:G12322A, ORF1ab:C14408T, ORF1ab:A16725G, ORF1ab:T19839C, ORF1ab:C20703T, ORF1ab:A21137G, S:A23403G, ORF3a:G25552T, N:G28378T, N:A28482G, N:G28881A, N:G28882A, N:G28883C, N:C29409T,	11	N:Q70R, N:R203K, N:G204R, N:T379I, ORF1a:E2063G, ORF1b:P314L, ORF1b:K2557R, ORF3a:A54S, ORF9b:R32L, ORF9b:K67E, S:D614G,
hCoV-19/Mexico/CMX-INMEGEN-12-03-11/2020	EPI_ISL_955202	20A	B.1	11	5'UTR:C241T, ORF1ab:T1237C, ORF1ab:C3037T, ORF1ab:C3768T, ORF1ab:C14408T, ORF1ab:C16329T, ORF1ab:C20844A, S:A23403G, S:G23587T, N:G28655T, N:C28887T, 3'UTR:G29773T,	6	N:D128Y, N:T205I, ORF1a:T1168I, ORF1b:P314L, S:D614G, S:Q675H,
hCoV-19/Mexico/CMX-INMEGEN-12-03-12/2020	EPI_ISL_955193	20B	B.1.1.222	19	5'UTR:C241T, ORF1ab:C3037T, ORF1ab:C4893T, ORF1ab:T5218C, ORF1ab:C7104T, ORF1ab:G7393T, ORF1ab:G8179T, ORF1ab:C14408T, ORF1ab:T19839C, S:T21886C, S:A23403G, S:A23503T, S:A23756G, S:C23758T, S:C23929T, ORF3a:C26028T, ORF8:C27945T, N:G28881A, N:G28882A, N:G28883C,	8	N:R203K, N:G204R, ORF1a:T1543I, ORF1a:T2280I, ORF1b:P314L, ORF8:Q18*, S:D614G, S:T732A,
hCoV-19/Mexico/CMX-INMEGEN-12-03-13/2020	EPI_ISL_955203	20B	B.1.1.519	24	5'UTR:T201C, 5'UTR:C203T, 5'UTR:C222T, 5'UTR:C241T, ORF1ab:G1738T, ORF1ab:C3037T, ORF1ab:C3140T, ORF1ab:C10029T, ORF1ab:C10954T, ORF1ab:A11117G, ORF1ab:C12789T, ORF1ab:C14408T, ORF1ab:G14559T, ORF1ab:T19839C, ORF1ab:A19974G, ORF1ab:C21306T, S:C22995A, S:A23403G, S:C23604A, S:A23756G, ORF3a:G25699A, N:G28881A, N:G28882A, N:G28883C, N:C29197T,	12	N:R203K, N:G204R, ORF1a:P959S, ORF1a:T3255I, ORF1a:I3618V, ORF1a:T4175I, ORF1b:P314L, ORF3a:A103T, S:T478K, S:D614G, S:P681H, S:T732A,
hCoV-19/Mexico/CMX-INMEGEN-12-03-14/2020	EPI_ISL_955204	20A	B.1	14	5'UTR:C241T, ORF1ab:C1380T, ORF1ab:C3037T, ORF1ab:G6341T, ORF1ab:G10396T, ORF1ab:C14408T, ORF1ab:A20268G, S:C22591T, S:T23042C, S:A23403G, S:T23599G, S:C23638T, N:G28817T, N:C28854T, N:C29421T,	9	N:A182S, N:S194L, N:P383L, ORF1a:A372V, ORF1a:D2026Y, ORF1b:P314L, S:S494P, S:D614G, S:N679K,
hCoV-19/Mexico/CMX-INMEGEN-12-03-15/2020	EPI_ISL_955205	20B	B.1.1.222	14	5'UTR:C241T, ORF1ab:T1623C, ORF1ab:C3037T, ORF1ab:C8140T, ORF1ab:C8326T, ORF1ab:C9430T, ORF1ab:C14408T, ORF1ab:T19839C, S:G22021T, S:A23403G, S:A23756G, ORF7a:C27741T, N:G28881A, N:G28882A, N:G28883C,	7	N:R203K, N:G204R, ORF1a:I453T, ORF1b:P314L, S:M153I, S:D614G, S:T732A,

hCoV-19/Mexico/CMX-INMEGEN-12-03-16/2020	EPI_ISL_955206	21C (Epsilon)	B.1.429	24	5'UTR:C241T, ORF1ab:C1059T, ORF1ab:C2395T, ORF1ab:C2453T, ORF1ab:T2597C, ORF1ab:C3037T, ORF1ab:C8947T, ORF1ab:T11038C, ORF1ab:C12100T, ORF1ab:A12878G, ORF1ab:C14408T, ORF1ab:G17014T, S:G21600T, S:C21638T, S:G22018T, S:T22917G, S:A23403G, S:T24349C, ORF3a:G25563T, M:C26681T, ORF7b:G27890T, ORF8:G27987T, ORF8:A28272T, N:C28887T, N:C29362T,	13	N:T205I, ORF1a:T265I, ORF1a:L730F, ORF1a:I4205V, ORF1b:P314L, ORF1b:D1183Y, ORF3a:Q57H, ORF8:V32L, S:S13I, S:P26S, S:W152C, S:L452R, S:D614G,
hCoV-19/Mexico/CMX-INMEGEN-12-03-17/2020	EPI_ISL_955207	20A	B.1	25	5'UTR:C241T, ORF1ab:T724C, ORF1ab:C1059T, ORF1ab:C1489T, ORF1ab:C3037T, ORF1ab:A3132G, ORF1ab:G5194T, ORF1ab:C12919T, ORF1ab:A13733G, ORF1ab:C14408T, ORF1ab:C16568T, ORF1ab:T17268C, ORF1ab:G19414T, ORF1ab:A20268G, S:C21575T, S:C23248T, S:A23403G, ORF3a:C25452T, ORF3a:C25658T, ORF3a:C25777T, ORF3a:C26110T, ORF7b:T27826A, ORF8:G28109T, N:C28717T, N:G28851A, N:T28921C,	14	N:S193N, ORF1a:T265I, ORF1a:Q956R, ORF1b:K89R, ORF1b:P314L, ORF1b:T1034I, ORF1b:V1983L, ORF3a:T89I, ORF3a:L129F, ORF3a:P240S, ORF7b:M24K, ORF8:Q72H, S:L5F, S:D614G,
hCoV-19/Mexico/CMX-INMEGEN-12-03-20/2020	EPI_ISL_955189	20B	B.1.1.222	18	5'UTR:C241T, ORF1ab:C3037T, ORF1ab:T3295C, ORF1ab:C6558T, ORF1ab:C10165T, ORF1ab:G12514T, ORF1ab:A13498G, ORF1ab:G13617T, ORF1ab:C14408T, ORF1ab:C15960T, ORF1ab:C16391A, ORF1ab:T19839C, S:G22331A, S:A23403G, S:A23756G, ORF3a:T25865C, N:G28881A, N:G28882A. N:G28883C.	11	N:R203K, N:G204R, ORF1a:A2098V, ORF1b:T11A, ORF1b:K50N, ORF1b:P314L, ORF1b:A975D, ORF3a:I158T, S:G257S, S:D614G, S:T732A,
hCoV-19/Mexico/CMX-INMEGEN-12-03-21/2020	EPI_ISL_955190	20B	B.1.1.222	13	5'UTR:C241T, ORF1ab:C3037T, ORF1ab:C7749T, ORF1ab:C8637T, ORF1ab:A13498G, ORF1ab:C14408T, ORF1ab:T19839C, S:G22331A, S:A23403G, S:A23756G, N:G28881A, N:G28882A, N:G28883C, N:G28907A,	10	N:R203K, N:G204R, N:G212S, ORF1a:T2495I, ORF1a:T2791I, ORF1b:T11A, ORF1b:P314L, S:G257S, S:D614G, S:T732A,
hCoV-19/Mexico/CMX-INMEGEN-12-03-22/2020	EPI_ISL_955191	20B	B.1.1.222	19	5'UTR:C241T, ORF1ab:C3037T, ORF1ab:T3295C, ORF1ab:C4009T, ORF1ab:C9611T, ORF1ab:C10165T, ORF1ab:G12514T, ORF1ab:A13498G, ORF1ab:C14408T, ORF1ab:C15960T, ORF1ab:T19839C, S:G22331A, S:A23403G, S:A23756G, S:C24865T, ORF3a:T25865C, N:G28881A, N:G28882A, N:G28883C, N:C29046T,	10	N:R203K, N:G204R, N:P258L, ORF1a:L3116F, ORF1b:T11A, ORF1b:P314L, ORF3a:I158T, S:G257S, S:D614G, S:T732A,
hCoV-19/Mexico/CMX-INMEGEN-12-03-23/2020	EPI_ISL_955208	20B	B.1.1.519	22	5'UTR:C203T, 5'UTR:C222T, 5'UTR:C241T, ORF1ab:C3037T, ORF1ab:C3140T, ORF1ab:C10029T, ORF1ab:C10954T, ORF1ab:A11117G, ORF1ab:C12789T, ORF1ab:C14408T, ORF1ab:G15921T, ORF1ab:A17971G, ORF1ab:T19839C, ORF1ab:C21306T, S:C22995A, S:A23403G, S:C23604A, S:C23635T, S:A23756G, N:G28881A, N:G28882A, N:G28883C, N:C29197T,	12	N:R203K, N:G204R, ORF1a:P959S, ORF1a:T3255I, ORF1a:I3618V, ORF1a:T4175I, ORF1b:P314L, ORF1b:R1502G, S:T478K, S:D614G, S:P681H, S:T732A,
hCoV-19/Mexico/CMX-INMEGEN-12-03-24/2020	EPI_ISL_955209	20A	B.1.396	19	5'UTR:C241T, ORF1ab:C1191T, ORF1ab:C3037T, ORF1ab:G5012T, ORF1ab:G6176A, ORF1ab:C9521A, ORF1ab:C13368T, ORF1ab:G13993T, ORF1ab:C14184T, ORF1ab:C14408T, ORF1ab:A20268G, S:A23403G, S:C24904T, ORF3a:A25806C, ORF3a:C26151A, ORF7a:C27406T, N:C28854T, N:G29422T, N:G29440T, ORF10:A29567G.	11	N:S194L, N:Q389H, ORF1a:P309L, ORF1a:V1583F, ORF1a:D1971N, ORF1a:L3086I, ORF1a:T4368I, ORF1b:A176S, ORF1b:P314L, ORF7a:L5F, S:D614G,

hCoV-19/Mexico/CMX-INMEGEN-12-04-01/2020	EPI_ISL_1005559	20B	B.1.1.519	22	5'UTR:C186T, 5'UTR:C203T, 5'UTR:C222T, 5'UTR:C241T, ORF1ab:C3037T, ORF1ab:C3140T, ORF1ab:C10029T, ORF1ab:C10954T, ORF1ab:A11117G, ORF1ab:G11234T, ORF1ab:C11379T, ORF1ab:C12789T, ORF1ab:C14408T, ORF1ab:T19839C, ORF1ab:C21306T, S:C22995A, S:A23403G, S:C23604A, S:A23756G, N:G28881A, N:G28882A, N:G28883C. N:C29197T.	13	N:R203K, N:G204R, ORF1a:P959S, ORF1a:T3255I, ORF1a:I3618V, ORF1a:A3657S, ORF1a:A3705V, ORF1a:T4175I, ORF1b:P314L, S:T478K, S:D614G, S:P681H, S:T732A,
hCoV-19/Mexico/CMX-INMEGEN-12-04-02/2020	EPI_ISL_1005560	20B	B.1.1.432	26	5'UTR:C241T, ORF1ab:G420A, ORF1ab:G2305T, ORF1ab:A2437T, ORF1ab:C3037T, ORF1ab:A6985T, ORF1ab:C9319T, ORF1ab:C12412T, ORF1ab:G13763T, ORF1ab:A13949G, ORF1ab:T14313C, ORF1ab:C14408T, ORF1ab:C16995T, ORF1ab:C20016T, S:C23114T, S:A23403G, ORF3a:C25613T, ORF3a:G25785T, ORF3a:G25912T, ORF3a:G26152T, ORF8:A27976T, ORF8:C28054T, N:G28881A, N:G28882A, N:G28883C, N:T29317C, N:G29543C,	15	N:R203K, N:G204R, ORF1a:G52D, ORF1a:K680N, ORF1a:R724S, ORF1b:G99V, ORF1b:D161G, ORF1b:P314L, ORF3a:S74F, ORF3a:W131C, ORF3a:G174C, ORF3a:G254*, ORF8:H28L, ORF8:S54L, S:D614G,
hCoV-19/Mexico/CMX-INMEGEN-12-04-03/2020	EPI_ISL_1005561	20B	B.1.1.432	24	5'UTR:C241T, ORF1ab:G2305T, ORF1ab:A2437T, ORF1ab:C3037T, ORF1ab:A6985T, ORF1ab:C9319T, ORF1ab:C12412T, ORF1ab:A13949G, ORF1ab:T14313C, ORF1ab:C14408T, ORF1ab:C16995T, ORF1ab:C20016T, S:C23114T, S:A23403G, ORF3a:C25613T, ORF3a:G25785T, ORF3a:G25912T, ORF3a:G26152T, ORF8:A27976T, ORF8:C28054T, N:G28881A, N:G28882A, N:G28883C, N:T29317C, N:G29543C,	13	N:R203K, N:G204R, ORF1a:K680N, ORF1a:R724S, ORF1b:D161G, ORF1b:P314L, ORF3a:S74F, ORF3a:W131C, ORF3a:G174C, ORF3a:G254*, ORF8:H28L, ORF8:S54L, S:D614G,
hCoV-19/Mexico/CMX-INMEGEN-12-04-04/2020	EPI_ISL_1005562	20B	B.1.1.222	16	5'UTR:C241T, ORF1ab:A1366G, ORF1ab:C3037T, ORF1ab:C4543T, ORF1ab:T5028A, ORF1ab:C9319T, ORF1ab:C14408T, ORF1ab:T19839C, S:A23403G, S:A23756G, ORF7a:C27434T, ORF8:T27939C, N:G28881A, N:G28882A, N:G28883C, ORF10:A29631G, 3'UTR:G29706A,	8	N:R203K, N:G204R, ORF1a:M1588K, ORF1b:P314L, ORF7a:T14I, ORF8:F16L, S:D614G, S:T732A,
hCoV-19/Mexico/CMX-INMEGEN-12-04-05/2020	EPI_ISL_1005563	20B	B.1.1.519	25	5'UTR:C203T, 5'UTR:C222T, 5'UTR:C241T, ORF1ab:C3037T, ORF1ab:C3140T, ORF1ab:C6336T, ORF1ab:C7162T, ORF1ab:C10029T, ORF1ab:C10954T, ORF1ab:A11117G, ORF1ab:C12789T, ORF1ab:G13249A, ORF1ab:C14408T, ORF1ab:T19839C, ORF1ab:C21306T, S:C22995A, S:A23403G, S:C23604A, S:A23756G, ORF3a:C25782T, N:G28395A, N:C28657T, N:G28881A, N:G28882A, N:G28883C, N:C29197T,	14	N:R41Q, N:R203K, N:G204R, ORF1a:P959S, ORF1a:S2024L, ORF1a:T3255I, ORF1a:I3618V, ORF1a:T4175I, ORF1b:P314L, ORF9b:G38S, S:T478K, S:D614G, S:P681H, S:T732A,
hCoV-19/Mexico/CMX-INMEGEN-12-04-06/2020	EPI_ISL_1005564	20B	B.1.1.222	19	5'UTR:C241T, ORF1ab:C3037T, ORF1ab:C4897T, ORF1ab:C5259T, ORF1ab:C10029T, ORF1ab:T11168C, ORF1ab:A11430G, ORF1ab:C14408T, ORF1ab:C19011A, ORF1ab:T19839C, ORF1ab:A21137G, S:A23403G, S:A23756G, ORF3a:G25912T, ORF3a:A26115C, ORF8:A27921G, ORF8:G28001T, N:G28881A, N:G28882A. N:G28883C.	14	N:R203K, N:G204R, ORF1a:T1665I, ORF1a:T3255I, ORF1a:F3635L, ORF1a:Y3722C, ORF1b:P314L, ORF1b:D1848E, ORF1b:K2557R, ORF3a:G174C, ORF3a:E241D, ORF8:I10V, S:D614G, S:T732A,
hCoV-19/Mexico/CMX-INMEGEN-12-04-07/2020	EPI_ISL_1005565	20B	B.1.1.519	26	5'UTR:C203T, 5'UTR:C222T, 5'UTR:C241T, ORF1ab:A866G, ORF1ab:C1009T, ORF1ab:C3037T, ORF1ab:C3140T, ORF1ab:C5183T, ORF1ab:C10029T, ORF1ab:C10954T, ORF1ab:A11117G, ORF1ab:C11758A, ORF1ab:C11916T, ORF1ab:C12789T, ORF1ab:C14408T, ORF1ab:T19839C, ORF1ab:C21306T, S:C22995A, S:A23403G, S:C23604A, S:A23756G, ORF8:T27904C, N:G28747T, N:G28881A, N:G28882A, N:G28883C, N:C29197T,	15	N:R203K, N:G204R, ORF1a:I201V, ORF1a:P959S, ORF1a:P1640S, ORF1a:T3255I, ORF1a:I3618V, ORF1a:S3884L, ORF1a:T4175I, ORF1b:P314L, ORF8:L4P, S:T478K, S:D614G, S:P681H, S:T732A,

hCoV-19/Mexico/CMX-INMEGEN-12-04-08/2020	EPI_ISL_1005566	20B	B.1.1.316	19	5'UTR:C241T, ORF1ab:T856C, ORF1ab:G1976T, ORF1ab:C3037T, ORF1ab:C6286A, ORF1ab:G7675T, ORF1ab:C14408T, ORF1ab:C18508T, ORF1ab:T19839C, ORF1ab:G21372T, S:C21757T, S:A23403G, S:G23593C, S:A23756T, ORF7a:A27578G, N:G28881A, N:G28882A, N:G28883C, ORF10:C29614T, 3'UTR:G29751T,	10	N:R203K, N:G204R, ORF1a:D571Y, ORF1b:P314L, ORF1b:L1681F, ORF1b:Q2635H, ORF7a:Q62R, S:D614G, S:Q677H, S:T732S,	
hCoV-19/Mexico/CMX-INMEGEN-12-04-09/2020	EPI_ISL_1005567	20G	B.1.2	22	5'UTR:C241T, ORF1ab:C1059T, ORF1ab:C3037T, ORF1ab:C10319T, ORF1ab:G11083T, ORF1ab:C13517A, ORF1ab:C14408T, ORF1ab:G18255T, ORF1ab:A18424G, ORF1ab:T19516C, ORF1ab:C20468T, ORF1ab:C21304T, S:C22264T, S:T23042C, S:A23403G, S:C23453T, S:G23868T, S:G25019T, ORF3a:G25563T, ORF3a:G25907T, ORF8:C27964T, N:C28472T. N:C28869T.	19	N:P67S, N:P199L, ORF1a:T265I, ORF1a:L3352F, ORF1a:L3606F, ORF1b:T17N, ORF1b:P314L, ORF1b:M1596I, ORF1b:N1653D, ORF1b:A2334V, ORF1b:R2613C, ORF3a:Q57H, ORF3a:G172V, ORF8:S24L, S:S494P, S:D614G, S:P631S, S:G769V, S:D1153Y,	3'UTR:29759-29777
hCoV-19/Mexico/CMX-INMEGEN-12-04-10/2020	EPI_ISL_1005568	20B	B.1.1.519	20	5'UTR:C203T, 5'UTR:C222T, 5'UTR:C241T, ORF1ab:C3037T, ORF1ab:C3140T, ORF1ab:C10029T, ORF1ab:C10954T, ORF1ab:A11117G, ORF1ab:C12789T, ORF1ab:C14408T, ORF1ab:T19839C, ORF1ab:C21306T, S:C22995A, S:A23403G, S:C23604A, S:A23756G, N:G28881A, N:G28882A, N:G28883C, N:C29197T, N:G29527T,	12	N:R203K, N:G204R, N:Q418H, ORF1a:P959S, ORF1a:T3255I, ORF1a:I3618V, ORF1a:T4175I, ORF1b:P314L, S:T478K, S:D614G, S:P681H, S:T732A,	
hCoV-19/Mexico/CMX-INMEGEN-12-04-11/2020	EPI_ISL_1005569	20B	B.1.1.222	12	5'UTR:C241T, ORF1ab:C3037T, ORF1ab:C14408T, ORF1ab:G16246T, ORF1ab:T17023C, ORF1ab:T19839C, S:A23403G, S:A23756G, ORF3a:C25904T, N:G28881A, N:G28882A, N:G28883C, N:G29402T,	9	N:R203K, N:G204R, N:D377Y, ORF1b:P314L, ORF1b:A927S, ORF1b:S1186P, ORF3a:S171L, S:D614G, S:T732A,	
hCoV-19/Mexico/CMX-INMEGEN-12-04-12/2020	EPI_ISL_1005570	20A	B.1	9	5'UTR:C241T, ORF1ab:C1426T, ORF1ab:C3037T, ORF1ab:C14408T, ORF1ab:A20268G, S:G21724T, S:A23403G, S:C24064T, ORF3a:C25925T, N:G29044A,	4	ORF1b:P314L, ORF3a:P178L, S:L54F, S:D614G,	
hCoV-19/Mexico/CMX-INMEGEN-12-04-13/2020	EPI_ISL_1005571	20B	B.1.1.519	22	5'UTR:C203T, 5'UTR:C222T, 5'UTR:C241T, ORF1ab:C3037T, ORF1ab:C3140T, ORF1ab:T3745C, ORF1ab:C10029T, ORF1ab:C10954T, ORF1ab:A11117G, ORF1ab:C12789T, ORF1ab:C14408T, ORF1ab:T19839C, ORF1ab:C21306T, S:C22995A, S:A23403G, S:C23604A, S:A23756G, S:C24370T, ORF3a:C25844T, N:G28881A, N:G28882A, N:G28883C. N:C29197T.	12	N:R203K, N:G204R, ORF1a:P959S, ORF1a:T3255I, ORF1a:I3618V, ORF1a:T4175I, ORF1b:P314L, ORF3a:T151I, S:T478K, S:D614G, S:P681H, S:T732A,	
hCoV-19/Mexico/CMX-INMEGEN-12-04-14/2020	EPI_ISL_1005572	20B	B.1.1.519	21	5'UTR:C203T, 5'UTR:C222T, 5'UTR:C241T, ORF1ab:C3037T, ORF1ab:C3140T, ORF1ab:C10029T, ORF1ab:C10954T, ORF1ab:A11117G, ORF1ab:C11575T, ORF1ab:C12789T, ORF1ab:C14408T, ORF1ab:C19269T, ORF1ab:T19839C, ORF1ab:C21306T, S:C22995A, S:A23403G, S:C23604A, S:A23756G, N:G28881A, N:G28882A, N:G28883C, N:C29197T,	11	N:R203K, N:G204R, ORF1a:P959S, ORF1a:T3255I, ORF1a:I3618V, ORF1a:T4175I, ORF1b:P314L, S:T478K, S:D614G, S:P681H, S:T732A,	
hCoV-19/Mexico/CMX-INMEGEN-12-04-15/2020	EPI_ISL_1005573	20B	B.1.1.222	16	5'UTR:C241T, ORF1ab:C1387T, ORF1ab:C3037T, ORF1ab:C3593T, ORF1ab:C4582T, ORF1ab:C10029T, ORF1ab:C14408T, ORF1ab:T19839C, S:A23403G, S:A23756G, ORF3a:G25912T, ORF7b:A27756G, ORF8:A27921G, ORF8:G28001T, N:G28881A, N:G28882A, N:G28883C,	10	N:R203K, N:G204R, ORF1a:L1110F, ORF1a:T3255I, ORF1b:P314L, ORF3a:G174C, ORF7b:M1V, ORF8:I10V, S:D614G, S:T732A,	
hCoV-19/Mexico/CMX-INMEGEN-12-04-16/2020	EPI_ISL_1005574	20B	B.1.1.222	16	5'UTR:C241T, ORF1ab:C1387T, ORF1ab:C3037T, ORF1ab:C3593T, ORF1ab:C4582T, ORF1ab:C10029T, ORF1ab:C14408T, ORF1ab:T19839C, S:A23403G, S:A23756G, ORF3a:G25912T, ORF7b:A27756G, ORF8:A27921G, ORF8:G28001T, N:G28881A, N:G28882A, N:G28883C,	10	N:R203K, N:G204R, ORF1a:L1110F, ORF1a:T3255I, ORF1b:P314L, ORF3a:G174C, ORF7b:M1V, ORF8:I10V, S:D614G, S:T732A,	

hCoV-19/Mexico/CMX-INMEGEN-12-04-17/2020	EPI_ISL_1005575	20B	B.1.1.519	23	5'UTR:T201C, 5'UTR:C203T, 5'UTR:C222T, 5'UTR:C241T, ORF1ab:C745T, ORF1ab:G1738T, ORF1ab:C3037T, ORF1ab:C3140T, ORF1ab:C10029T, ORF1ab:C10954T, ORF1ab:A11117G, ORF1ab:C12789T, ORF1ab:C14408T, ORF1ab:T19839C, ORF1ab:A19974G, ORF1ab:C21306T, S:C22995A, S:A23403G, S:C23604A, S:A23756G, N:G28881A, N:G28882A, N:G28883C, N:C29197T,	11	N:R203K, N:G204R, ORF1a:P959S, ORF1a:T3255I, ORF1a:I3618V, ORF1a:T4175I, ORF1b:P314L, S:T478K, S:D614G, S:P681H, S:T732A,
hCoV-19/Mexico/CMX-INMEGEN-12-04-18/2020	EPI_ISL_1005576	20B	B.1.1.222	24	5'UTR:C241T, ORF1ab:C2252A, ORF1ab:C3037T, ORF1ab:G5194T, ORF1ab:G9237A, ORF1ab:C10029T, ORF1ab:C14408T, ORF1ab:C15279T, ORF1ab:C16887T, ORF1ab:G19816T, ORF1ab:T19839C, ORF1ab:C20930T, S:C23243T, S:A23403G, S:A23756G, ORF3a:G25912T, M:G26538A, M:C26607T, M:G26730C, ORF7b:A27756G, ORF8:A27921G, ORF8:G28001T, N:G28881A, N:G28882A, N:G28883C,	17	M:G6S, M:L29F, M:V70L, N:R203K, N:G204R, ORF1a:Q663K, ORF1a:R2991K, ORF1a:T3255I, ORF1b:P314L, ORF1b:V2117L, ORF1b:T2488M, ORF3a:G174C, ORF7b:M1V, ORF8:I10V, S:P561S, S:D614G, S:T732A,
hCoV-19/Mexico/CMX-INMEGEN-12-04-19/2020	EPI_ISL_1005577	20B	B.1.1.519	23	5'UTR:C203T, 5'UTR:C222T, 5'UTR:C241T, ORF1ab:C3037T, ORF1ab:C3140T, ORF1ab:C10029T, ORF1ab:C10954T, ORF1ab:A11117G, ORF1ab:C12789T, ORF1ab:C14408T, ORF1ab:T15726A, ORF1ab:T19839C, ORF1ab:C21306T, S:C22995A, S:A23403G, S:T23470C, S:C23604A, S:A23756G, M:C26833T, N:G28881A, N:G28882A, N:G28883C, N:C29197T, N:G29527T,	13	M:A104V, N:R203K, N:G204R, N:Q418H, ORF1a:P959S, ORF1a:T3255I, ORF1a:I3618V, ORF1a:T4175I, ORF1b:P314L, S:T478K, S:D614G, S:P681H, S:T732A,
hCoV-19/Mexico/CMX-INMEGEN-12-04-20/2020	EPI_ISL_1005578	20B	B.1.1.519	23	5'UTR:C203T, 5'UTR:C222T, 5'UTR:C241T, ORF1ab:C3037T, ORF1ab:C3140T, ORF1ab:T3745C, ORF1ab:C10029T, ORF1ab:C10954T, ORF1ab:A11117G, ORF1ab:C12789T, ORF1ab:C14408T, ORF1ab:T19839C, ORF1ab:C21306T, S:T22951C, S:C22995A, S:A23403G, S:C23604A, S:A23756G, ORF3a:C25844T, N:C28310T, N:G28881A, N:G28882A, N:G28883C, N:C29197T,	13	N:P13S, N:R203K, N:G204R, ORF1a:P959S, ORF1a:T3255I, ORF1a:I3618V, ORF1a:T4175I, ORF1b:P314L, ORF3a:T151I, S:T478K, S:D614G, S:P681H, S:T732A,
hCoV-19/Mexico/CMX-INMEGEN-12-04-21/2020	EPI_ISL_1005579	20B	B.1.1.519	22	5'UTR:C203T, 5'UTR:C222T, 5'UTR:C241T, ORF1ab:C3037T, ORF1ab:C3140T, ORF1ab:C10029T, ORF1ab:C10954T, ORF1ab:A11117G, ORF1ab:G11365T, ORF1ab:C12789T, ORF1ab:C14408T, ORF1ab:T19839C, ORF1ab:C21058T, ORF1ab:C21306T, S:C22995A, S:A23403G, S:C23604A, S:A23756G, N:G28881A, N:G28882A, N:G28883C, N:C29197T, N:G29527T,	13	N:R203K, N:G204R, N:Q418H, ORF1a:P959S, ORF1a:T3255I, ORF1a:I3618V, ORF1a:T4175I, ORF1b:P314L, ORF1b:P2531S, S:T478K, S:D614G, S:P681H, S:T732A,
hCoV-19/Mexico/CMX-INMEGEN-12-04-22/2020	EPI_ISL_1005580	20B	B.1.1.519	23	5'UTR:C203T, 5'UTR:C222T, 5'UTR:C241T, ORF1ab:C683T, ORF1ab:C1191A, ORF1ab:C3037T, ORF1ab:C3140T, ORF1ab:C10029T, ORF1ab:C10954T, ORF1ab:A11117G, ORF1ab:C12789T, ORF1ab:C14408T, ORF1ab:T19839C, ORF1ab:C21306T, S:C22995A, S:A23403G, S:C23604A, S:A23756G, N:G28881A, N:G28882A, N:G28883C, N:C29197T, N:G29315C, N:C29421A,	14	N:R203K, N:G204R, N:D348H, N:P383Q, ORF1a:P309Q, ORF1a:P959S, ORF1a:T3255I, ORF1a:I3618V, ORF1a:T4175I, ORF1b:P314L, S:T478K, S:D614G, S:P681H, S:T732A,
hCoV-19/Mexico/CMX-INMEGEN-12-04-23/2020	EPI_ISL_1005581	20B	B.1.1.519	23	5'UTR:C203T, 5'UTR:C222T, 5'UTR:C241T, ORF1ab:C3037T, ORF1ab:C3140T, ORF1ab:C5646T, ORF1ab:C10029T, ORF1ab:C10954T, ORF1ab:A11117G, ORF1ab:C12789T, ORF1ab:C14408T, ORF1ab:A15864G, ORF1ab:A17205G, ORF1ab:T19839C, ORF1ab:C21306T, S:C22995A, S:A23403G, S:C23604A, S:C23625T, S:A23756G, N:G28881A, N:G28882A, N:G28883C, N:C29197T,	13	N:R203K, N:G204R, ORF1a:P959S, ORF1a:T1794I, ORF1a:T3255I, ORF1a:I3618V, ORF1a:T4175I, ORF1b:P314L, S:T478K, S:D614G, S:P681H, S:A688V, S:T732A,

hCoV-19/Mexico/CMX-INMEGEN-12-04-24/2020	EPI_ISL_1005582	20B	B.1.1.519	24	5'UTR:C203T, 5'UTR:C222T, 5'UTR:C241T, ORF1ab:C3037T, ORF1ab:C3140T, ORF1ab:C10029T, ORF1ab:C10954T, ORF1ab:A11117G, ORF1ab:T11569C, ORF1ab:C12789T, ORF1ab:G13661A, ORF1ab:C14408T, ORF1ab:G19542A, ORF1ab:T19839C, ORF1ab:A20180G, ORF1ab:C21306T, S:C22995A, S:A23403G, S:G23593T, S:C23604A, S:A23756G, N:G28881A, N:G28882A, N:G28883C, N:C29197T,	15	N:R203K, N:G204R, ORF1a:P959S, ORF1a:T3255I, ORF1a:I3618V, ORF1a:T4175I, ORF1b:R65K, ORF1b:P314L, ORF1b:M2025I, ORF1b:Q2238R, S:T478K, S:D614G, S:Q677H, S:P681H, S:T732A,
hCoV-19/Mexico/CMX-INMEGEN-13/2020	EPI_ISL_522978	20B	B.1.1	12	5'UTR:C241T, ORF1ab:C3037T, ORF1ab:C14408T, ORF1ab:C16289T, ORF1ab:A16878G, ORF1ab:A16947G, ORF1ab:C17333T, ORF1ab:C18788T, S:A23403G, ORF3a:C25517A, N:G28881A, N:G28882A, N:G28883C,	8	N:R203K, N:G204R, ORF1b:P314L, ORF1b:A941V, ORF1b:T1289M, ORF1b:T1774I, ORF3a:P42H, S:D614G,
hCoV-19/Mexico/CMX-INMEGEN-14/2020	EPI_ISL_522979	20B	B.1.1.344	23	5'UTR:C241T, ORF1ab:C2508T, ORF1ab:C3037T, ORF1ab:G5515T, ORF1ab:C5748T, ORF1ab:A8130G, ORF1ab:C8240T, ORF1ab:T9172C, ORF1ab:G9479T, ORF1ab:A10948G, ORF1ab:C14408T, ORF1ab:T16950C, ORF1ab:G18255T, S:A23403G, S:G23608T, ORF3a:A25575C, M:G26730T, ORF7a:C27513T, N:A28877T, N:G28878C, N:G28881A, N:G28882A, N:G28883C, 3'UTR:C29769T,	12	M:V70F, N:R203K, N:G204R, ORF1a:P748L, ORF1a:T1828I, ORF1a:K2622R, ORF1a:H2659Y, ORF1a:G3072C, ORF1b:P314L, ORF1b:M1596I, ORF3a:K61N, S:D614G,
hCoV-19/Mexico/CMX-INMEGEN-15/2020	EPI_ISL_522980	20C	B.1.369	13	5'UTR:C241T, ORF1ab:C1059T, ORF1ab:C2395T, ORF1ab:C3037T, ORF1ab:G3259T, ORF1ab:C14408T, ORF1ab:C16260T, ORF1ab:C16289T, S:A23403G, S:G24751T, ORF3a:G25563T, ORF3a:G25740T, ORF8:C28057T, N:C28821A,	10	N:S183Y, ORF1a:T265I, ORF1a:Q998H, ORF1b:P314L, ORF1b:A941V, ORF3a:Q57H, ORF3a:Q116H, ORF8:A55V, S:D614G, S:L1063F,
hCoV-19/Mexico/CMX-INMEGEN-16/2020	EPI_ISL_522981	20B	B.1.1	13	5'UTR:C241T, ORF1ab:T1476C, ORF1ab:C2399T, ORF1ab:C3037T, ORF1ab:C5301T, ORF1ab:C14408T, ORF1ab:G18225T, S:C22624T, S:A23403G, S:C23854T, N:G28881A, N:G28882A, N:G28883C, 3'UTR:G29734T,	8	N:R203K, N:G204R, ORF1a:I404T, ORF1a:H712Y, ORF1a:A1679V, ORF1b:P314L, ORF1b:M1586I, S:D614G,
hCoV-19/Mexico/CMX-INMEGEN-17/2020	EPI_ISL_522982	20A	B.1	9	5'UTR:C241T, ORF1ab:G2900A, ORF1ab:C3037T, ORF1ab:C14408T, ORF1ab:G15760T, ORF1ab:T16209C, ORF1ab:A20268G, S:A23403G, M:C26801T, N:C28854T,	5	N:S194L, ORF1a:V879I, ORF1b:P314L, ORF1b:G765C, S:D614G,
hCoV-19/Mexico/CMX-INMEGEN-18/2020	EPI_ISL_522983	20A	B.1	12	5'UTR:C241T, ORF1ab:C346T, ORF1ab:A570G, ORF1ab:C3037T, ORF1ab:G3566T, ORF1ab:A8031G, ORF1ab:C14408T, ORF1ab:G19086T, ORF1ab:A20268G, S:A23403G, S:C23709T, N:G28851T, N:C28854T,	9	N:S193I, N:S194L, ORF1a:E102G, ORF1a:G1101C, ORF1a:K2589R, ORF1b:P314L, ORF1b:K1873N, S:D614G, S:T716I,
hCoV-19/Mexico/CMX-INMEGEN-19/2020	EPI_ISL_522984	20B	B.1.1.222	17	5'UTR:C241T, ORF1ab:G837T, ORF1ab:C3037T, ORF1ab:G4201T, ORF1ab:C5784T, ORF1ab:A6716G, ORF1ab:T7835C, ORF1ab:C12008T, ORF1ab:C14408T, ORF1ab:C19017T, ORF1ab:A19137G, ORF1ab:T19839C, S:C21575T, S:A23403G, S:A23756G, N:G28881A, N:G28882A, N:G28883C,	11	N:R203K, N:G204R, ORF1a:C191F, ORF1a:M1312I, ORF1a:T1840I, ORF1a:S2151G, ORF1a:L3915F, ORF1b:P314L, S:L5F, S:D614G, S:T732A,
hCoV-19/Mexico/CMX-INMEGEN-20/2020	EPI_ISL_522985	20B	B.1.1.222	11	5'UTR:C241T, ORF1ab:C3037T, ORF1ab:C7573T, ORF1ab:C8092T, ORF1ab:G12070T, ORF1ab:C14408T, ORF1ab:T19839C, S:A23403G, S:A23756G, N:G28881A, N:G28882A, N:G28883C,	5	N:R203K, N:G204R, ORF1b:P314L, S:D614G, S:T732A,
hCoV-19/Mexico/CMX-INMEGEN-21/2020	EPI_ISL_522986	20B	B.1.1.222	13	5'UTR:C241T, ORF1ab:C3037T, ORF1ab:G4201T, ORF1ab:A6716G, ORF1ab:T7835C, ORF1ab:C12008T, ORF1ab:C14408T, ORF1ab:C19017T, ORF1ab:T19839C, S:A23403G, S:A23756G, N:G28881A, N:G28882A, N:G28883C,	8	N:R203K, N:G204R, ORF1a:M1312I, ORF1a:S2151G, ORF1a:L3915F, ORF1b:P314L, S:D614G, S:T732A,
hCoV-19/Mexico/CMX-INMEGEN-22/2020	EPI_ISL_523500	20B	B.1.1.322	13	5'UTR:C241T, ORF1ab:C3037T, ORF1ab:C8950T, ORF1ab:G12080A, ORF1ab:C14408T, ORF1ab:T19839C, S:A23403G, S:A23756G, N:A28877T, N:G28878C, N:G28881A, N:G28882A, N:G28883C, N:G29516A,	7	N:R203K, N:G204R, N:D415N, ORF1a:A3939T, ORF1b:P314L, S:D614G, S:T732A,

hCoV-19/Mexico/COA-InDRE_355/2020	EPI_ISL_1060686	20A	B.1.397	11	ORF1ab:G1135T, ORF1ab:C1385T, ORF1ab:C3037T, ORF1ab:C9365T, ORF1ab:C13665T, ORF1ab:C14408T, S:C22323T, S:G22604T, S:A23403G, S:A25050T, ORF8:C28093T, N:C28854T,	9	N:S194L, ORF1a:K290N, ORF1a:H374Y, ORF1b:P314L, ORF8:S67F, S:S254F, S:A348S, S:D614G, S:D1163V,
hCoV-19/Mexico/COA-InDRE_361/2020	EPI_ISL_1060676	20B	B.1.1	10	ORF1ab:C3037T, ORF1ab:G9434T, ORF1ab:G13726A, ORF1ab:C14408T, ORF1ab:C19554T, S:A23403G, S:G23587C, ORF7b:T27817G, N:G28881A, N:G28882A, N:G28883C,	8	N:R203K, N:G204R, ORF1a:V3057L, ORF1b:V87I, ORF1b:P314L, ORF7b:V21G, S:D614G, S:Q675H,
hCoV-19/Mexico/COA-InDRE_365/2020	EPI_ISL_1060706	20A	B.1.396	23	ORF1ab:G376T, ORF1ab:C1191T, ORF1ab:C1471T, ORF1ab:C3037T, ORF1ab:G8536A, ORF1ab:C9521A, ORF1ab:C10969T, ORF1ab:A12095G, ORF1ab:G14187A, ORF1ab:C14408T, ORF1ab:C18395T, ORF1ab:C19170T, ORF1ab:A20268G, S:C21859T, S:A23403G, S:C24904T, ORF3a:G25947C, N:C28677T, N:C28854T, N:G29422T, ORF10:A29567G, ORF10:C29627T, 3'UTR:G29703T, 3'UTR:T29867A,	10	N:T135I, N:S194L, ORF1a:E37D, ORF1a:P309L, ORF1a:L3086I, ORF1a:I3944V, ORF1b:P314L, ORF1b:A1643V, ORF3a:Q185H, S:D614G,
hCoV-19/Mexico/COA-InDRE_40/2020	EPI_ISL_516620	20B	B.1.1	11	5'UTR:C106T, 5'UTR:C241T, ORF1ab:G2809T, ORF1ab:C3037T, ORF1ab:C5869T, ORF1ab:C14408T, ORF1ab:C18828T, S:A23403G, ORF3a:C26042T, N:G28881A, N:G28882A, N:G28883C,	6	N:R203K, N:G204R, ORF1a:R848S, ORF1b:P314L, ORF3a:T217I, S:D614G,
hCoV-19/Mexico/COA-InDRE_406/2021	EPI_ISL_1168484	20G	B.1.2	18	5'UTR:C241T, ORF1ab:C1059T, ORF1ab:C3037T, ORF1ab:G3340T, ORF1ab:T4579A, ORF1ab:C10319T, ORF1ab:C10790T, ORF1ab:C14408T, ORF1ab:C15328T, ORF1ab:A18424G, ORF1ab:C20402T, ORF1ab:C21304T, S:A23403G, ORF3a:G25563T, ORF3a:G25907T, ORF8:C27964T, N:G28378T, N:C28472T, N:C28869T,	15	N:P67S, N:P199L, ORF1a:T265I, ORF1a:L3352F, ORF1a:H3509Y, ORF1b:P314L, ORF1b:L621F, ORF1b:N1653D, ORF1b:S2312L, ORF1b:R2613C, ORF3a:Q57H, ORF3a:G172V, ORF8:S24L, ORF9b:R32L, S:D614G,
hCoV-19/Mexico/COA-InDRE_407/2021	EPI_ISL_1168485	20A	B.1.558	21	5'UTR:C241T, ORF1ab:C344T, ORF1ab:C556T, ORF1ab:C1288T, ORF1ab:G2086T, ORF1ab:C3037T, ORF1ab:G6369A, ORF1ab:C6638T, ORF1ab:T8746A, ORF1ab:C11339T, ORF1ab:G11417T, ORF1ab:C12439T, ORF1ab:C14408T, ORF1ab:C17004T, ORF1ab:C19662T, ORF1ab:A20268G, ORF1ab:G20962T, S:A23403G, ORF3a:C26060T, ORF7a:C27630T, ORF8:G27915T, N:C28854T,	12	N:S194L, ORF1a:L27F, ORF1a:Q607H, ORF1a:G2035E, ORF1a:H2125Y, ORF1a:F2827L, ORF1a:V3718F, ORF1b:P314L, ORF1b:D2499Y, ORF3a:T223I, ORF8:G8*, S:D614G,
hCoV-19/Mexico/COA-InDRE_408/2021	EPI_ISL_1168486	20A	B.1.558	22	5'UTR:C241T, ORF1ab:C344T, ORF1ab:C556T, ORF1ab:C1288T, ORF1ab:G2086T, ORF1ab:C3037T, ORF1ab:G6369A, ORF1ab:C6638T, ORF1ab:T8746A, ORF1ab:C11339T, ORF1ab:G11417T, ORF1ab:C12439T, ORF1ab:T12591A, ORF1ab:C14408T, ORF1ab:C17004T, ORF1ab:C19662T, ORF1ab:A20268G, ORF1ab:G20962T, S:A23403G, ORF3a:C26060T, ORF7a:C27630T, ORF8:G27915T, N:C28854T,	13	N:S194L, ORF1a:L27F, ORF1a:Q607H, ORF1a:G2035E, ORF1a:H2125Y, ORF1a:F2827L, ORF1a:V3718F, ORF1a:V4109D, ORF1b:P314L, ORF1b:D2499Y, ORF3a:T223I, ORF8:G8*, S:D614G,
hCoV-19/Mexico/COA-InDRE_409/2021	EPI_ISL_1168487	20A	B.1.243	16	5'UTR:C241T, ORF1ab:C1404T, ORF1ab:C3037T, ORF1ab:C4010T, ORF1ab:C5284T, ORF1ab:C7162T, ORF1ab:C10741T, ORF1ab:C14408T, ORF1ab:C19097T, ORF1ab:A19137G, ORF1ab:A19167G, ORF1ab:A20268G, S:A23403G, S:C23604A, S:T24076C, N:C28854T, 3'UTR:T29710C,	7	N:S194L, ORF1a:P380L, ORF1a:L1249F, ORF1b:P314L, ORF1b:A1877V, S:D614G, S:P681H,

hCoV-19/Mexico/COA-InDRE_410/2021	EPI_ISL_1168488	20B	B.1.1.519	25	5'UTR:T201C, 5'UTR:C203T, 5'UTR:C222T, 5'UTR:C241T, ORF1ab:C1218T, ORF1ab:G1738T, ORF1ab:C3037T, ORF1ab:C3140T, ORF1ab:A8848T, ORF1ab:C10029T, ORF1ab:C10954T, ORF1ab:A11117G, ORF1ab:C12789T, ORF1ab:C14408T, ORF1ab:G15908T, ORF1ab:C19610T, ORF1ab:T19839C, S:C21621T, S:C22995A, S:A23403G, S:C23604A, S:A23756G, N:G28881A, N:G28882A, N:G28883C, N:C29197T,	16	N:R203K, N:G204R, ORF1a:S318L, ORF1a:P959S, ORF1a:E2861D, ORF1a:T3255I, ORF1a:I3618V, ORF1a:T4175I, ORF1b:P314L, ORF1b:G814V, ORF1b:T2048I, S:T20I, S:T478K, S:D614G, S:P681H, S:T732A,
hCoV-19/Mexico/COA-InDRE_411/2021	EPI_ISL_1168489	20G	B.1.2	14	5'UTR:C241T, ORF1ab:C1059T, ORF1ab:G2494A, ORF1ab:C3037T, ORF1ab:C10319T, ORF1ab:C14408T, ORF1ab:A18424G, ORF1ab:G20060T, ORF1ab:C21304T, S:A23403G, ORF3a:G25563T, ORF3a:G25907T, ORF8:C27964T, N:C28472T, N:C28869T,	12	N:P67S, N:P199L, ORF1a:T265I, ORF1a:L3352F, ORF1b:P314L, ORF1b:N1653D, ORF1b:S2198I, ORF1b:R2613C, ORF3a:Q57H, ORF3a:G172V, ORF8:S24L, S:D614G,
hCoV-19/Mexico/COA-InDRE_412/2021	EPI_ISL_1168490	20B	B.1.1.519	27	5'UTR:T201C, 5'UTR:C203T, 5'UTR:C222T, 5'UTR:C241T, ORF1ab:C1218T, ORF1ab:G1738T, ORF1ab:C3037T, ORF1ab:C3140T, ORF1ab:C4002T, ORF1ab:G4907A, ORF1ab:C7389T, ORF1ab:C10029T, ORF1ab:C10954T, ORF1ab:A11117G, ORF1ab:C12789T, ORF1ab:C14408T, ORF1ab:T19839C, ORF1ab:A19974G, ORF1ab:C21306T, S:C21621T, S:C22995A, S:A23403G, S:C23604A, S:A23756G, N:G28881A, N:G28882A, N:G28883C, N:C29197T,	16	N:R203K, N:G204R, ORF1a:S318L, ORF1a:P959S, ORF1a:T1246I, ORF1a:G1548S, ORF1a:A2375V, ORF1a:T3255I, ORF1a:I3618V, ORF1a:T4175I, ORF1b:P314L, S:T20I, S:T478K, S:D614G, S:P681H, S:T732A,
hCoV-19/Mexico/COA-InDRE_413/2021	EPI_ISL_1168491	20A	B.1.243	18	5'UTR:C241T, ORF1ab:C3037T, ORF1ab:C7162T, ORF1ab:C8318A, ORF1ab:A10323G, ORF1ab:C14408T, ORF1ab:C16173T, ORF1ab:A17969G, ORF1ab:C19097T, ORF1ab:A19137G, ORF1ab:C19263T, ORF1ab:A20268G, S:T22228A, S:A23403G, S:C23604A, S:T24076C, E:C26340T, N:C28854T, 3'UTR:T29710C,	8	N:S194L, ORF1a:P2685T, ORF1a:K3353R, ORF1b:P314L, ORF1b:D1501G, ORF1b:A1877V, S:D614G, S:P681H,
hCoV-19/Mexico/COA-InDRE_483/2021	EPI_ISL_1168553	20B	B.1.1.316	21	5'UTR:C241T, ORF1ab:G2830A, ORF1ab:A2866G, ORF1ab:C3037T, ORF1ab:C3411T, ORF1ab:C3602T, ORF1ab:G3871T, ORF1ab:A5040G, ORF1ab:G7693A, ORF1ab:C8772T, ORF1ab:G11083T, ORF1ab:A12519G, ORF1ab:C14408T, ORF1ab:T19839C, S:C21757T, S:A23403G, S:G23593C, S:A23756T, N:G28881A, N:G28882A, N:G28883C, 3'UTR:G29751T,	13	N:R203K, N:G204R, ORF1a:A1049V, ORF1a:H1113Y, ORF1a:K1202N, ORF1a:Q1592R, ORF1a:T2836I, ORF1a:L3606F, ORF1a:D4085G, ORF1b:P314L, S:D614G, S:Q677H, S:T732S,
hCoV-19/Mexico/COA-InDRE_484/2021	EPI_ISL_1168554	20B	B.1.1.222	19	5'UTR:C241T, ORF1ab:C3037T, ORF1ab:G4399T, ORF1ab:C6449T, ORF1ab:C6896T, ORF1ab:T13474A, ORF1ab:A13712G, ORF1ab:C14408T, ORF1ab:C19011T, ORF1ab:T19839C, ORF1ab:G19858T, ORF1ab:A21037T, S:A23403G, S:A23756G, ORF8:G28079T, N:G28882A, N:G28883C, N:C29085T, N:C29409T, 3'UTR:G29692T,	12	N:G204R, N:T271I, N:T379I, ORF1a:M1378I, ORF1a:L2062F, ORF1b:C3S, ORF1b:K82R, ORF1b:P314L, ORF1b:A2131S, ORF1b:I2524F, S:D614G, S:T732A,
hCoV-19/Mexico/COA-InDRE_485/2021	EPI_ISL_1168555	21C (Epsilon)	B.1.427	23	5'UTR:C241T, ORF1ab:C1059T, ORF1ab:C2607T, ORF1ab:C3037T, ORF1ab:C3817T, ORF1ab:A7374G, ORF1ab:G9738C, ORF1ab:G13713A, ORF1ab:C14408T, ORF1ab:C16394T, ORF1ab:G17014T, ORF1ab:C20436T, S:G21600T, S:G22018T, S:C22597T, S:A23403G, ORF3a:G25563T, M:C26681T, ORF6:A27241G, ORF8:C28087T, ORF8:A28272T, N:C28887T, N:C29362T, 3'UTR:G29759T,	14	N:T205I, ORF1a:T265I, ORF1a:T781I, ORF1a:N2370S, ORF1a:S3158T, ORF1b:P314L, ORF1b:P976L, ORF1b:D1183Y, ORF3a:Q57H, ORF6:I14V, ORF8:A65V, S:S13I, S:W152C, S:D614G,
hCoV-19/Mexico/COA-InDRE_486/2021	EPI_ISL_1168556	20A	B.1	11	5'UTR:C241T, ORF1ab:C1060T, ORF1ab:C3037T, ORF1ab:A5010G, ORF1ab:C7165T, ORF1ab:C11620T, ORF1ab:C14408T, ORF1ab:T16062G, ORF1ab:A20268G, S:G21974C, S:A23403G, ORF8:C27903T,	6	ORF1a:Q1582R, ORF1b:P314L, ORF1b:N865K, ORF8:L4F, S:D138H, S:D614G,

hCoV-19/Mexico/COA-InDRE_487/2021	EPI_ISL_1168557	20A	B.1	13	5'UTR:C241T, ORF1ab:C2156T, ORF1ab:C3037T, ORF1ab:C4158T, ORF1ab:C6636T, ORF1ab:G10759T, ORF1ab:C11173T, ORF1ab:C14408T, ORF1ab:C18138T, ORF1ab:A20268G, S:A23403G, S:G23593T, ORF3a:C25513A, N:C28854T,	9	N:S194L, ORF1a:L631F, ORF1a:A1298V, ORF1a:T2124I, ORF1a:M3498I, ORF1b:P314L, ORF3a:L41I, S:D614G, S:Q677H,
hCoV-19/Mexico/COA-InDRE_488/2021	EPI_ISL_1168558	20A	B.1	21	5'UTR:C241T, ORF1ab:C344T, ORF1ab:C1288T, ORF1ab:G2072A, ORF1ab:C3037T, ORF1ab:G6369A, ORF1ab:C9165T, ORF1ab:C10474T, ORF1ab:C11339T, ORF1ab:G11417T, ORF1ab:C12439T, ORF1ab:C14408T, ORF1ab:C16650T, ORF1ab:C17004T, ORF1ab:C19662T, ORF1ab:A20268G, ORF1ab:G20962T, S:A23403G, ORF3a:C26060T, ORF7a:C27630T, N:C28603T, N:C28854T,	10	N:S194L, ORF1a:L27F, ORF1a:G603S, ORF1a:G2035E, ORF1a:T2967I, ORF1a:V3718F, ORF1b:P314L, ORF1b:D2499Y, ORF3a:T223I, S:D614G,
hCoV-19/Mexico/COA-InDRE_489/2021	EPI_ISL_1168559	20A	B.1	12	5'UTR:C241T, ORF1ab:C3037T, ORF1ab:C4158T, ORF1ab:C6636T, ORF1ab:G10759T, ORF1ab:C11173T, ORF1ab:C14408T, ORF1ab:A20268G, S:A23403G, S:G23593T, S:G25352T, ORF3a:C25513A, N:C28854T,	9	N:S194L, ORF1a:A1298V, ORF1a:T2124I, ORF1a:M3498I, ORF1b:P314L, ORF3a:L41I, S:D614G, S:Q677H, S:V1264L,
hCoV-19/Mexico/COA-InDRE_490/2021	EPI_ISL_1168560	20A	B.1	11	5'UTR:C241T, ORF1ab:C3037T, ORF1ab:G10759T, ORF1ab:C13297T, ORF1ab:C14408T, ORF1ab:A20268G, S:A23403G, S:G23593T, ORF3a:G25423C, ORF3a:G25906T, M:C27002T, N:C28854T,	7	N:S194L, ORF1a:M3498I, ORF1b:P314L, ORF3a:G11R, ORF3a:G172C, S:D614G, S:Q677H,
hCoV-19/Mexico/COA-InDRE_491/2021	EPI_ISL_1168561	20A	B.1.232	18	5'UTR:C241T, ORF1ab:C829T, ORF1ab:G2293T, ORF1ab:C3037T, ORF1ab:G6894T, ORF1ab:A7786C, ORF1ab:C10893T, ORF1ab:C11758T, ORF1ab:C14408T, ORF1ab:A20268G, S:G22599A, S:A23403G, S:G23593T, ORF3a:G25471T, ORF3a:G25621A, ORF3a:G25690T, M:C26681T, ORF8:G27987T, N:C28854T,	13	N:S194L, ORF1a:Q676H, ORF1a:C2210F, ORF1a:K2507N, ORF1a:T3543I, ORF1b:P314L, ORF3a:D27Y, ORF3a:V77I, ORF3a:G100C, ORF8:V32L, S:R346K, S:D614G, S:Q677H,
hCoV-19/Mexico/COA-InDRE_492/2021	EPI_ISL_1168562	20A	B.1.232	19	5'UTR:C241T, ORF1ab:C829T, ORF1ab:G2293T, ORF1ab:C3037T, ORF1ab:G6894T, ORF1ab:A7786C, ORF1ab:C10893T, ORF1ab:C11758T, ORF1ab:C14408T, ORF1ab:A20268G, ORF1ab:C21304T, S:G22599A, S:A23403G, S:G23593T, ORF3a:G25471T, ORF3a:G25621A, ORF3a:G25690T, M:C26681T, ORF8:G27987T, N:C28854T,	14	N:S194L, ORF1a:Q676H, ORF1a:C2210F, ORF1a:K2507N, ORF1a:T3543I, ORF1b:P314L, ORF1b:R2613C, ORF3a:D27Y, ORF3a:V77I, ORF3a:G100C, ORF8:V32L, S:R346K, S:D614G, S:Q677H,
hCoV-19/Mexico/COA-InDRE_493/2021	EPI_ISL_1168563	20A	B.1	13	5'UTR:C241T, ORF1ab:C3037T, ORF1ab:G10759T, ORF1ab:C14376T, ORF1ab:C14408T, ORF1ab:A20268G, S:A23403G, S:G23593T, ORF3a:G25906T, M:C27002T, ORF6:G27382C, ORF6:A27383T, ORF6:T27384C, N:C28854T,	7	N:S194L, ORF1a:M3498I, ORF1b:P314L, ORF3a:G172C, ORF6:D61L, S:D614G, S:Q677H,
hCoV-19/Mexico/COA-InDRE_494/2021	EPI_ISL_1168564	21C (Epsilon)	B.1.429	28	5'UTR:C241T, ORF1ab:C1059T, ORF1ab:C2395T, ORF1ab:T2597C, ORF1ab:C3037T, ORF1ab:C8947T, ORF1ab:T10831A, ORF1ab:C12100T, ORF1ab:A12878G, ORF1ab:C14408T, ORF1ab:G17014T, S:G21600T, S:T21908C, S:G22018T, S:T22917G, S:A23403G, S:T24349C, ORF3a:G25563T, E:G26506T, M:C26681T, ORF7b:G27890T, ORF8:G27987C, ORF8:G28191T, ORF8:A28272T, N:C28887T, N:G28975T, N:G29179T, N:C29362T, 3'UTR:C29784T,	14	N:T205I, N:M234I, ORF1a:T265I, ORF1a:I4205V, ORF1b:P314L, ORF1b:D1183Y, ORF3a:Q57H, ORF8:V32L, ORF8:V100L, S:S13I, S:S116P, S:W152C, S:L452R, S:D614G,
hCoV-19/Mexico/COA-InDRE_495/2021	EPI_ISL_1168565	20B	B.1.1.222	14	5'UTR:C241T, ORF1ab:C3037T, ORF1ab:T10339C, ORF1ab:C14408T, ORF1ab:C14676T, ORF1ab:T19839C, S:G21985A, S:A23403G, S:A23756G, S:T24499C, ORF8:G28167A, N:G28881A, N:G28882A, N:G28883C, N:A29110G,	6	N:R203K, N:G204R, ORF1b:P314L, ORF8:E92K, S:D614G, S:T732A,

hCoV-19/Mexico/COA-InDRE_496/2021	EPI_ISL_1168566	20A	B.1.243	13	5'UTR:C241T, ORF1ab:C1469T, ORF1ab:C3037T, ORF1ab:C7162T, ORF1ab:C12809T, ORF1ab:C14408T, ORF1ab:C19097T, ORF1ab:A19137G, ORF1ab:A20268G, S:A23403G, S:C23604A, S:T24076C, N:C28854T, 3'UTR:T29710C,	7	N:S194L, ORF1a:R402C, ORF1a:L4182F, ORF1b:P314L, ORF1b:A1877V, S:D614G, S:P681H,
hCoV-19/Mexico/COA-InDRE_497/2021	EPI_ISL_1168567	20B	B.1.1.519	19	5'UTR:C203T, 5'UTR:C222T, 5'UTR:C241T, ORF1ab:C3037T, ORF1ab:C3140T, ORF1ab:C10029T, ORF1ab:C10954T, ORF1ab:A11117G, ORF1ab:C12789T, ORF1ab:C14408T, ORF1ab:T19839C, ORF1ab:C21306T, S:C22995A, S:A23403G, S:C23604A, S:A23756G, N:G28881A, N:G28882A, N:G28883C, N:C29197T,	11	N:R203K, N:G204R, ORF1a:P959S, ORF1a:T3255I, ORF1a:I3618V, ORF1a:T4175I, ORF1b:P314L, S:T478K, S:D614G, S:P681H, S:T732A,
hCoV-19/Mexico/COA-InDRE_498/2021	EPI_ISL_1168568	20A	B.1	13	5'UTR:C241T, ORF1ab:C1060T, ORF1ab:C3037T, ORF1ab:A5010G, ORF1ab:C7165T, ORF1ab:C11620T, ORF1ab:C14408T, ORF1ab:T16062G, ORF1ab:A20268G, S:G21974C, S:A23403G, M:C26822T, ORF8:C27903T, N:C28854T,	7	N:S194L, ORF1a:Q1582R, ORF1b:P314L, ORF1b:N865K, ORF8:L4F, S:D138H, S:D614G,
hCoV-19/Mexico/COA-InDRE_499/2021	EPI_ISL_1168569	20A	B.1.487	21	5'UTR:C241T, ORF1ab:C344T, ORF1ab:C1288T, ORF1ab:C1734T, ORF1ab:C3037T, ORF1ab:C4832T, ORF1ab:G6373T, ORF1ab:T8151C, ORF1ab:C9391T, ORF1ab:G11287T, ORF1ab:C12439T, ORF1ab:T13641C, ORF1ab:C14408T, ORF1ab:C19154T, ORF1ab:C19662T, ORF1ab:A20268G, ORF1ab:C20930T, ORF1ab:G20962T, S:A23403G, ORF3a:C26060T, ORF7a:C27630T, N:C28854T,	12	N:S194L, ORF1a:L27F, ORF1a:T490I, ORF1a:M2036I, ORF1a:V2629A, ORF1a:L3674F, ORF1b:P314L, ORF1b:T1896I, ORF1b:T2488M, ORF1b:D2499Y, ORF3a:T223I, S:D614G,
hCoV-19/Mexico/COA-InDRE_505/2021	EPI_ISL_1168575	20A	B.1	12	5'UTR:C241T, ORF1ab:C3037T, ORF1ab:C8127T, ORF1ab:C9893T, ORF1ab:G10759T, ORF1ab:C14408T, ORF1ab:A20268G, S:A23403G, S:G23593T, S:T24664A, N:C28290T, N:C28854T, N:C29445T,	10	N:P6L, N:S194L, N:T391I, ORF1a:A2621V, ORF1a:L3210F, ORF1a:M3498I, ORF1b:P314L, ORF9b:P3S, S:D614G, S:Q677H,
hCoV-19/Mexico/COA-InDRE_506/2021	EPI_ISL_1168576	20G	B.1.2	19	5'UTR:C241T, ORF1ab:C1059T, ORF1ab:C3037T, ORF1ab:G3216T, ORF1ab:A6575G, ORF1ab:G8083A, ORF1ab:T8953C, ORF1ab:C10319T, ORF1ab:C14408T, ORF1ab:C14805T, ORF1ab:C16551T, ORF1ab:A18424G, S:C21707T, S:A23403G, ORF3a:G25563T, ORF3a:G25907T, ORF8:C27964T, N:C28472T, N:A28581G, N:C28869T,	15	N:P67S, N:D103G, N:P199L, ORF1a:T265I, ORF1a:S984I, ORF1a:S2104G, ORF1a:M2606I, ORF1a:L3352F, ORF1b:P314L, ORF1b:N1653D, ORF3a:Q57H, ORF3a:G172V, ORF8:S24L, S:H49Y, S:D614G,
hCoV-19/Mexico/COA-InDRE_507/2021	EPI_ISL_1168577	20G	B.1.2	22	5'UTR:C241T, ORF1ab:C1059T, ORF1ab:C1419T, ORF1ab:C3037T, ORF1ab:A3235G, ORF1ab:C10319T, ORF1ab:C12815T, ORF1ab:C14408T, ORF1ab:G15760A, ORF1ab:T17142G, ORF1ab:A18424G, S:G22484T, S:A23403G, S:G25354T, ORF3a:G25563T, ORF3a:G25907T, ORF3a:T25999C, ORF7a:G27651T, ORF8:T27922C, ORF8:C27964T, N:C28472T, N:C28869T, N:A29424T,	15	N:P67S, N:P199L, N:Q384L, ORF1a:T265I, ORF1a:A385V, ORF1a:L3352F, ORF1b:P314L, ORF1b:G765S, ORF1b:N1653D, ORF3a:Q57H, ORF3a:G172V, ORF8:I10T, ORF8:S24L, S:V308L, S:D614G,
hCoV-19/Mexico/COA-InDRE_508/2021	EPI_ISL_1168578	20B	B.1.1	20	5'UTR:C190T, 5'UTR:C241T, ORF1ab:A2761G, ORF1ab:G3004T, ORF1ab:C3037T, ORF1ab:C3990T, ORF1ab:C8299T, ORF1ab:C14408T, ORF1ab:T19839C, S:A23403G, S:G23426A, S:C23604A, ORF3a:G25522A, ORF3a:T25580C, ORF7a:C27689T, ORF7b:G27890A, ORF8:T27912G, N:G28881A, N:G28882A, N:G28883C, N:C29323T,	12	N:R203K, N:G204R, ORF1a:E913D, ORF1a:T1242I, ORF1b:P314L, ORF3a:G44R, ORF3a:I63T, ORF7a:P99L, ORF8:L7V, S:D614G, S:V622I, S:P681H,

hCoV-19/Mexico/COA-InDRE_509/2021	EPI_ISL_1168579	20A	B.1.243	23	5'UTR:C241T, ORF1ab:C274T, ORF1ab:G806A, ORF1ab:T2584A, ORF1ab:G2776T, ORF1ab:C3037T, ORF1ab:G4162T, ORF1ab:C5140A, ORF1ab:C6027T, ORF1ab:C8964T, ORF1ab:C9693T, ORF1ab:C14408T, ORF1ab:T15144C, ORF1ab:G16647T, ORF1ab:C18486T, ORF1ab:A20268G, S:G23120T, S:A23403G, S:G23587C, S:T24076C, E:C26455T, ORF6:T27360A, N:C28854T, N:G29543T,	13	E:P71S, N:S194L, ORF1a:A181T, ORF1a:K837N, ORF1a:D1625E, ORF1a:P1921L, ORF1a:S2900L, ORF1a:A3143V, ORF1b:P314L, ORF6:D53E, S:A520S, S:D614G, S:Q675H,
hCoV-19/Mexico/COA-InDRE_510/2021	EPI_ISL_1168580	20B	B.1.1	20	5'UTR:C241T, ORF1ab:C1170A, ORF1ab:C3037T, ORF1ab:T4579A, ORF1ab:A8358G, ORF1ab:C12473T, ORF1ab:C14120T, ORF1ab:C14408T, ORF1ab:T14853C, ORF1ab:T19839C, S:C21595T, S:G21800T, S:T22156C, S:A23403G, S:C25292T, ORF7b:C27807T, N:A28792T, N:G28881A, N:G28882A, N:G28883C, N:G28903T,	10	N:R203K, N:G204R, N:M210I, ORF1a:S302Y, ORF1a:H2698R, ORF1b:P218L, ORF1b:P314L, S:D80Y, S:D614G, S:L1244F,
hCoV-19/Mexico/COA-InDRE_511/2021	EPI_ISL_1168581	20B	B.1.1.519	25	5'UTR:T201C, 5'UTR:C203T, 5'UTR:C222T, 5'UTR:C241T, ORF1ab:C313T, ORF1ab:G428A, ORF1ab:C1218T, ORF1ab:G1738T, ORF1ab:C3037T, ORF1ab:C3140T, ORF1ab:G4907A, ORF1ab:C10029T, ORF1ab:C10954T, ORF1ab:A11117G, ORF1ab:C12789T, ORF1ab:C14408T, ORF1ab:T19839C, S:C21621T, S:C22995A, S:A23403G, S:C23604A, S:A23756G, N:G28881A, N:G28882A, N:G28883C, N:C29197T,	15	N:R203K, N:G204R, ORF1a:E55K, ORF1a:S318L, ORF1a:P959S, ORF1a:G1548S, ORF1a:T3255I, ORF1a:I3618V, ORF1a:T4175I, ORF1b:P314L, S:T20I, S:T478K, S:D614G, S:P681H, S:T732A,
hCoV-19/Mexico/COA-InDRE_512/2021	EPI_ISL_1168582	20A	B.1.558	25	5'UTR:C241T, ORF1ab:C1288T, ORF1ab:G1306A, ORF1ab:G2305T, ORF1ab:C2902T, ORF1ab:C3037T, ORF1ab:G6476A, ORF1ab:C10335T, ORF1ab:G10705T, ORF1ab:C10712T, ORF1ab:C11575T, ORF1ab:T12751G, ORF1ab:C14408T, ORF1ab:C18060T, ORF1ab:A19782T, ORF1ab:T19896C, ORF1ab:A20268G, S:G22147A, S:A23403G, S:C23664T, ORF3a:G25455T, ORF3a:G25770T, ORF3a:T25807C, ORF3a:G26109T, N:C28854T, N:C29541T	12	N:S194L, ORF1a:K680N, ORF1a:V2071I, ORF1a:A3357V, ORF1a:R3480S, ORF1a:L3483F, ORF1b:P314L, ORF3a:K21N, ORF3a:R126S, ORF3a:E239D, S:D614G, S:A701V,
hCoV-19/Mexico/COA-InDRE_513/2021	EPI_ISL_1168583	20G	B.1.596	19	5'UTR:C241T, ORF1ab:C1059T, ORF1ab:C3037T, ORF1ab:C5628T, ORF1ab:C10319T, ORF1ab:A11451G, ORF1ab:T11809C, ORF1ab:G13754A, ORF1ab:C14408T, ORF1ab:A18424G, S:C21811A, S:A23403G, S:A23592C, ORF3a:G25563T, ORF3a:G25907T, ORF8:C27964T, N:C28472T, N:C28869T, N:T29194C, N:T29377A.	14	N:P67S, N:P199L, ORF1a:T265I, ORF1a:T1788M, ORF1a:L3352F, ORF1a:Q3729R, ORF1b:R96K, ORF1b:P314L, ORF1b:N1653D, ORF3a:Q57H, ORF3a:G172V, ORF8:S24L, S:D614G, S:Q677P,
hCoV-19/Mexico/COA-InDRE_514/2021	EPI_ISL_1168584	20A	B.1	3	5'UTR:C241T, ORF1ab:C3037T, ORF1ab:C14408T, S:A23403G,	2	ORF1b:P314L, S:D614G,
hCoV-19/Mexico/COA-InDRE_515/2021	EPI_ISL_1168585	20A	B.1.36.10	5	5'UTR:C241T, ORF1ab:C3037T, ORF1ab:C14408T, S:A23403G, N:C28854T, N:C28928T,	4	N:S194L, N:L219F, ORF1b:P314L, S:D614G,
hCoV-19/Mexico/COA-InDRE_516/2021	EPI_ISL_1168586	20A	B.1	5	5'UTR:C241T, ORF1ab:C3037T, ORF1ab:C14408T, S:A23403G, ORF3a:T26084A, N:C29034T,	4	N:A254V, ORF1b:P314L, ORF3a:F231Y, S:D614G,
hCoV-19/Mexico/COA-InDRE_517/2021	EPI_ISL_1168587	20A	B.1	6	5'UTR:C241T, ORF1ab:C3037T, ORF1ab:C14408T, S:T23020C, S:A23403G, ORF3a:A26137C, ORF3a:C26195G,	4	ORF1b:P314L, ORF3a:I249L, ORF3a:T268R, S:D614G,
hCoV-19/Mexico/COA-InDRE_F10752_S698/2021	EPI_ISL_1365663	20A	B.1.243	17	5'UTR:C241T, ORF1ab:G545A, ORF1ab:A2658T, ORF1ab:C2933T, ORF1ab:C3037T, ORF1ab:T3733C, ORF1ab:C4543T, ORF1ab:C12880T, ORF1ab:C14408T, ORF1ab:C19097T, ORF1ab:A19137G, ORF1ab:A20268G, S:A23403G, S:C23604A, S:T24076C, ORF3a:C25513T, N:C28854T, 3'UTR:T29710C,	9	N:S194L, ORF1a:G94S, ORF1a:K798M, ORF1a:L890F, ORF1b:P314L, ORF1b:A1877V, ORF3a:L41F, S:D614G, S:P681H,

hCoV-19/Mexico/COA-InDRE_F10757_S699/2021	EPI_ISL_1365664	20A	B.1.396	23	5'UTR:C241T, ORF1ab:C643T, ORF1ab:C1077T, ORF1ab:C1191T, ORF1ab:C2044T, ORF1ab:C3037T, ORF1ab:C6070T, ORF1ab:C9118T, ORF1ab:C9521A, ORF1ab:C10604T, ORF1ab:C10626T, ORF1ab:G12202A, ORF1ab:C14408T, ORF1ab:T18900C, ORF1ab:A20268G, S:A23403G, S:C24904T, ORF8:T28136C, N:C28725T, N:C28854T, N:G28907A, N:G29422T, ORF10:A29558G, ORF10:A29567G,	10	N:P151L, N:S194L, N:G212S, ORF1a:P271L, ORF1a:P309L, ORF1a:L3086I, ORF1a:P3447S, ORF1a:A3454V, ORF1b:P314L, S:D614G,
hCoV-19/Mexico/COA-InDRE_F10759_S700/2021	EPI_ISL_1365665	20A	B.1.1	29	5'UTR:C241T, ORF1ab:C1288T, ORF1ab:G2305T, ORF1ab:C2902T, ORF1ab:C3037T, ORF1ab:G6476A, ORF1ab:C9943T, ORF1ab:C10335T, ORF1ab:T12751G, ORF1ab:C14408T, ORF1ab:G14712T, ORF1ab:G17523T, ORF1ab:C18060T, ORF1ab:T19896C, ORF1ab:T20157C, ORF1ab:A20268G, ORF1ab:G21204T, S:G22147A, S:G22487C, S:A23403G, S:C23664T, S:G25062T, ORF3a:G25455T, ORF3a:G25770T, ORF3a:T25807C, ORF3a:G26109T, E:C26333T, M:G26727T, N:C28854T, N:G28985T,	17	E:T30I, M:A69S, N:S194L, N:G238C, ORF1a:K680N, ORF1a:V2071I, ORF1a:A3357V, ORF1b:P314L, ORF1b:M1352I, ORF1b:K2579N, ORF3a:K21N, ORF3a:R126S, ORF3a:E239D, S:E309Q, S:D614G, S:A701V, S:G1167V,
hCoV-19/Mexico/COA-InDRE_F10764_S701/2021	EPI_ISL_1365666	20B	B.1.1.222	19	5'UTR:C241T, ORF1ab:C3037T, ORF1ab:A6693G, ORF1ab:C9430T, ORF1ab:C10029T, ORF1ab:C10039T, ORF1ab:T12532C, ORF1ab:C14408T, ORF1ab:G19009A, ORF1ab:T19839C, S:A23403G, S:A23756G, ORF7b:A27756G, ORF8:A27921G, ORF8:C27923T, ORF8:G28001T, N:G28881A, N:G28882A, N:G28883C, N:C29253T.	11	N:R203K, N:G204R, N:S327L, ORF1a:K2143R, ORF1a:T3255I, ORF1b:P314L, ORF1b:D1848N, ORF7b:M1V, ORF8:I10V, S:D614G, S:T732A,
hCoV-19/Mexico/COA-InDRE_F10766_S702/2021	EPI_ISL_1365667	20A	B.1.558	22	5'UTR:C241T, ORF1ab:C344T, ORF1ab:C1288T, ORF1ab:A2006G, ORF1ab:C3037T, ORF1ab:A4305G, ORF1ab:C9223T, ORF1ab:A9726G, ORF1ab:G11083T, ORF1ab:C12439T, ORF1ab:C13297T, ORF1ab:C14408T, ORF1ab:C19662T, ORF1ab:A20268G, ORF1ab:G20962T, S:C21646T, S:A23403G, ORF3a:C26060T, ORF7a:C27630T, ORF7a:A27724G, ORF8:G28086T, N:G28727T, N:C28854T,	13	N:A152S, N:S194L, ORF1a:L27F, ORF1a:I581V, ORF1a:K1347R, ORF1a:Y3154C, ORF1a:L3606F, ORF1b:P314L, ORF1b:D2499Y, ORF3a:T223I, ORF7a:T111A, ORF8:A65S, S:D614G,
hCoV-19/Mexico/COA-InDRE_F11070_S842/2021	EPI_ISL_1424044	20B	B.1.1.222	19	5'UTR:C241T, ORF1ab:G2764T, ORF1ab:C3037T, ORF1ab:C9924T, ORF1ab:A11595G, ORF1ab:C14408T, ORF1ab:T14551C, ORF1ab:G14712T, ORF1ab:G15530T, ORF1ab:C16733T, ORF1ab:A19752G, ORF1ab:T19839C, S:C21711T, S:A23403G, S:G23587T, S:A23756G, N:G28881A, N:G28882A, N:G28883C. 3'UTR:C29835T.	11	N:R203K, N:G204R, ORF1a:A3220V, ORF1a:Q3777R, ORF1b:P314L, ORF1b:C688F, ORF1b:S1089L, S:S50L, S:D614G, S:Q675H, S:T732A,
hCoV-19/Mexico/COA-InDRE_F11071_S873/2021	EPI_ISL_1399278	20G	B.1.2	23	5'UTR:C241T, ORF1ab:C1059T, ORF1ab:C2508T, ORF1ab:C3037T, ORF1ab:G4444T, ORF1ab:C10319T, ORF1ab:G11417T, ORF1ab:C14408T, ORF1ab:C16887T, ORF1ab:G17478A, ORF1ab:C17676T, ORF1ab:A18424G, ORF1ab:C19560T, ORF1ab:C21304T, S:C21614T, S:A23403G, S:G23900C, ORF3a:G25563T, ORF3a:G25907T, ORF8:C27942T, ORF8:C27964T, N:C28472T, N:G28541T, N:C28869T,	18	N:P67S, N:A90S, N:P199L, ORF1a:T265I, ORF1a:P748L, ORF1a:L3352F, ORF1a:V3718F, ORF1b:P314L, ORF1b:N1653D, ORF1b:R2613C, ORF3a:Q57H, ORF3a:G172V, ORF8:H17Y, ORF8:S24L, ORF9b:E86D, S:L18F, S:D614G, S:E780Q,
hCoV-19/Mexico/COA-InDRE_F11072_S843/2021	EPI_ISL_1424045	20B	B.1.1.222	20	5'UTR:C241T, ORF1ab:C1288T, ORF1ab:C3037T, ORF1ab:C5497T, ORF1ab:A5661G, ORF1ab:A7385C, ORF1ab:T8053C, ORF1ab:C9120T, ORF1ab:C9615T, ORF1ab:C14408T, ORF1ab:T19839C, ORF1ab:T21534C, S:A23403G, S:A23756G, ORF3a:C25413T, ORF7a:T27597C, ORF7a:C27741T, N:G28881A, N:G28882A, N:G28883C, 3'UTR:G29692T,	9	N:R203K, N:G204R, ORF1a:Q1799R, ORF1a:M2374L, ORF1a:T2952I, ORF1a:T3117I, ORF1b:P314L, S:D614G, S:T732A,

hCoV-19/Mexico/COA-InDRE-36/2020	EPI_ISL_516618	20B	B.1.1	9	5'UTR:C241T, ORF1ab:G2809T, ORF1ab:C3037T, ORF1ab:G6503A, ORF1ab:C6906T, ORF1ab:C14408T, S:A23403G, N:G28881A, N:G28882A, N:G28883C,	7	N:R203K, N:G204R, ORF1a:R848S, ORF1a:A2080T, ORF1a:S2214L, ORF1b:P314L, S:D614G,
hCoV-19/Mexico/COA-InDRE-37/2020	EPI_ISL_516619	20B	B.1.1	11	5'UTR:C241T, ORF1ab:G2809T, ORF1ab:C3037T, ORF1ab:G6503A, ORF1ab:C6906T, ORF1ab:G8084C, ORF1ab:A8086T, ORF1ab:C14408T, S:A23403G, N:G28881A, N:G28882A, N:G28883C,	8	N:R203K, N:G204R, ORF1a:R848S, ORF1a:A2080T, ORF1a:S2214L, ORF1a:E2607H, ORF1b:P314L, S:D614G,
hCoV-19/Mexico/COL-InDRE_346/2020	EPI_ISL_1060760	20B	B.1.1.244	11	5'UTR:C241T, ORF1ab:C3037T, ORF1ab:C12412T, ORF1ab:A12820G, ORF1ab:T14313C, ORF1ab:C14407T, ORF1ab:C14408T, ORF1ab:A19960G, S:A23403G, N:G28881A, N:G28882A, N:G28883C,	5	N:R203K, N:G204R, ORF1b:P314F, ORF1b:T2165A, S:D614G,
hCoV-19/Mexico/COL-InDRE_348/2020	EPI_ISL_1060757	20A	B.1	9	5'UTR:C241T, ORF1ab:A2497G, ORF1ab:C3037T, ORF1ab:G5230T, ORF1ab:C14408T, ORF1ab:T16413C, ORF1ab:A20268G, S:A23403G, N:C28854T, ORF10:C29640T,	4	N:S194L, ORF1a:K1655N, ORF1b:P314L, S:D614G,
hCoV-19/Mexico/COL-InDRE_394/2020	EPI_ISL_1054988	20C	B.1	12	5'UTR:C241T, ORF1ab:C1059T, ORF1ab:C3037T, ORF1ab:C7267T, ORF1ab:C11152T, ORF1ab:C14408T, S:G21724T, S:A23403G, ORF3a:G25563T, ORF7a:A27675C, N:C28887T, ORF10:C29614T, 3'UTR:C29750T,	7	N:T205I, ORF1a:T265I, ORF1b:P314L, ORF3a:Q57H, ORF7a:Q94H, S:L54F, S:D614G,
hCoV-19/Mexico/COL-InDRE_545/2021	EPI_ISL_1168615	20B	B.1.1.519	26	5'UTR:C203T, 5'UTR:C222T, 5'UTR:C241T, ORF1ab:G942A, ORF1ab:C3037T, ORF1ab:C3140T, ORF1ab:C10029T, ORF1ab:C10954T, ORF1ab:A11117G, ORF1ab:C11124T, ORF1ab:C12789T, ORF1ab:C14408T, ORF1ab:C14649T, ORF1ab:T19839C, ORF1ab:A20676G, ORF1ab:C21306T, S:T22514A, S:T22515A, S:C22995A, S:A23403G, S:C23604A, S:A23756G, N:G28881A, N:G28882A, N:G28883C, N:C29197T, 3'UTR:G29862T,	14	N:R203K, N:G204R, ORF1a:R226K, ORF1a:P959S, ORF1a:T3255I, ORF1a:I3618V, ORF1a:A3620V, ORF1a:T4175I, ORF1b:P314L, S:F318N, S:T478K, S:D614G, S:P681H, S:T732A,
hCoV-19/Mexico/COL-InDRE_546/2021	EPI_ISL_1168616	20B	B.1.1.222	21	5'UTR:C241T, ORF1ab:C3037T, ORF1ab:C3393T, ORF1ab:G4300T, ORF1ab:G10354T, ORF1ab:C12073T, ORF1ab:C14408T, ORF1ab:G15372T, ORF1ab:C18647T, ORF1ab:T19839C, ORF1ab:A21551T, S:C21637T, S:G21974C, S:A23403G, S:A23756G, ORF3a:G25726T, N:G28881A, N:G28882A, N:G28883C, N:G29260T, ORF10:G29648T, 3'UTR:C29835T,	11	N:R203K, N:G204R, ORF1a:A1043V, ORF1a:K3363N, ORF1b:P314L, ORF1b:P1727L, ORF1b:N2695I, ORF3a:V112F, S:D138H, S:D614G, S:T732A,
hCoV-19/Mexico/COL-InDRE_547/2021	EPI_ISL_1168617	20G	B.1.2	19	5'UTR:C241T, ORF1ab:A969T, ORF1ab:C1059T, ORF1ab:C3037T, ORF1ab:C10319T, ORF1ab:C14408T, ORF1ab:G17562T, ORF1ab:A18424G, ORF1ab:G19962T, ORF1ab:G20060T, ORF1ab:C21304T, S:C21575T, S:A23403G, ORF3a:G25563T, ORF3a:G25907T, ORF6:C27247T, ORF8:C27964T, N:C28472T, N:C28810T. N:C28869T.	14	N:P67S, N:P199L, ORF1a:E235V, ORF1a:T265I, ORF1a:L3352F, ORF1b:P314L, ORF1b:N1653D, ORF1b:S2198I, ORF1b:R2613C, ORF3a:Q57H, ORF3a:G172V, ORF8:S24L, S:L5F, S:D614G,
hCoV-19/Mexico/COL-InDRE_548/2021	EPI_ISL_1168618	20B	B.1.1.316	26	5'UTR:C241T, ORF1ab:A2303G, ORF1ab:C3037T, ORF1ab:C3411T, ORF1ab:G3871T, ORF1ab:A5040G, ORF1ab:C8947T, ORF1ab:A12519G, ORF1ab:C14408T, ORF1ab:T16539C, ORF1ab:C19269T, ORF1ab:T19839C, S:C21757T, S:A23403G, S:T23581C, S:G23593C, S:A23756T, S:G24893C, ORF3a:C25782T, ORF3a:G25912T, N:G28881A, N:G28882A, N:G28883C, N:C29272T, 3'UTR:G29751T, 3'UTR:G29862T, 3'UTR:A29866C,	13	N:R203K, N:G204R, ORF1a:K680E, ORF1a:A1049V, ORF1a:K1202N, ORF1a:Q1592R, ORF1a:D4085G, ORF1b:P314L, ORF3a:G174C, S:D614G, S:Q677H, S:T732S, S:E1111Q,

hCoV-19/Mexico/COL-InDRE_F11125_S608/2021	EPI_ISL_1337395	21C (Epsilon)	B.1.429	25	5'UTR:C206T, 5'UTR:C241T, ORF1ab:C1059T, ORF1ab:C2395T, ORF1ab:T2597C, ORF1ab:C3037T, ORF1ab:G3518T, ORF1ab:C8947T, ORF1ab:C12100T, ORF1ab:A12878G, ORF1ab:C14408T, ORF1ab:G17014T, S:G21600T, S:G22018T, S:T22917G, S:T23155C, S:A23403G, S:T24349C, ORF3a:G25563T, M:C26681T, ORF7b:G27890T, ORF8:G28191T, ORF8:A28272T, N:C28887T, N:G28975T, N:C29362T,	13	N:T205I, N:M234I, ORF1a:T265I, ORF1a:V1085F, ORF1a:I4205V, ORF1b:P314L, ORF1b:D1183Y, ORF3a:Q57H, ORF8:V100L, S:S13I, S:W152C, S:L452R, S:D614G,	
hCoV-19/Mexico/COL-InDRE-75/2020	EPI_ISL_576278	20A	B.1.396	18	5'UTR:C241T, ORF1ab:C1191T, ORF1ab:C3037T, ORF1ab:C9521A, ORF1ab:C10116T, ORF1ab:C10128T, ORF1ab:C14408T, ORF1ab:A14434C, ORF1ab:A20268G, S:A23403G, S:C24904T, ORF3a:A25602G, ORF3a:G26113C, M:C26801T, ORF6:C27247T, N:C28854T, N:G28907A, N:G29422T. ORF10:A29567G.	10	N:S194L, N:G212S, ORF1a:P309L, ORF1a:L3086I, ORF1a:T3284I, ORF1a:T3288I, ORF1b:P314L, ORF1b:K323Q, ORF3a:E241Q, S:D614G,	
hCoV-19/Mexico/COL-InDRE-76/2020	EPI_ISL_576279	20A	B.1.558	10	5'UTR:C241T, ORF1ab:C1288T, ORF1ab:C3037T, ORF1ab:C11575T, ORF1ab:C14408T, ORF1ab:C18060T, ORF1ab:A20268G, S:G22487C, S:A23403G, N:C28854T, ORF10:G29593A,	4	N:S194L, ORF1b:P314L, S:E309Q, S:D614G,	
hCoV-19/Mexico/DUR-InDRE-09/2020	EPI_ISL_455432	19B	A.1	8	ORF1ab:C8782T, ORF1ab:A16467G, ORF1ab:C17747T, ORF1ab:A17858G, ORF1ab:C18060T, S:T22896C, S:C23185T, ORF8:T28144C, N:C29200T,	4	ORF1b:P1427L, ORF1b:Y1464C, ORF8:L84S, S:V445A,	
hCoV-19/Mexico/GRO-InDRE_120/2020	EPI_ISL_913924	20B	B.1.1.322	18	5'UTR:C241T, ORF1ab:C676T, ORF1ab:C3037T, ORF1ab:C5284T, ORF1ab:C14408T, ORF1ab:T19839C, ORF1ab:C21216T, ORF1ab:C21306T, S:A23403G, S:A23756G, S:C24642T, ORF8:C28253T, N:A28877T, N:G28878C, N:G28881A, N:G28882A, N:G28883C, N:A29020G, N:C29144T.	6	N:R203K, N:G204R, ORF1b:P314L, S:D614G, S:T732A, S:T1027I,	
hCoV-19/Mexico/GRO-InDRE_136/2020	EPI_ISL_913939	20B	B.1.1.222	18	5'UTR:C241T, ORF1ab:C3037T, ORF1ab:C7834T, ORF1ab:C10029T, ORF1ab:T10879C, ORF1ab:C14408T, ORF1ab:C19011A, ORF1ab:T19839C, S:G22021T, S:A23403G, S:A23756G, ORF3a:G25912T, M:C26873T, ORF8:A27921G, ORF8:G28001T, N:G28881A, N:G28882A. N:G28883C. 3'UTR:G29688T.	10	N:R203K, N:G204R, ORF1a:T3255I, ORF1b:P314L, ORF1b:D1848E, ORF3a:G174C, ORF8:I10V, S:M153I, S:D614G, S:T732A,	
hCoV-19/Mexico/GRO-InDRE_146/2020	EPI_ISL_913949	20B	B.1.1.222	18	5'UTR:C241T, ORF1ab:C3037T, ORF1ab:G5206T, ORF1ab:C5526T, ORF1ab:C9502T, ORF1ab:A13512G, ORF1ab:C14408T, ORF1ab:T19839C, ORF1ab:G19947T, S:C23266T, S:A23403G, S:G23593C, S:A23756G, S:A24069T, ORF7a:C27625T, N:G28881A, N:G28882A, N:G28883C. N:G29392T.	12	N:R203K, N:G204R, N:K373N, ORF1a:M1647I, ORF1a:T1754I, ORF1b:P314L, ORF1b:K2160N, ORF7a:R78C, S:D614G, S:Q677H, S:T732A, S:Q836L,	
hCoV-19/Mexico/GRO-InDRE_159/2020	EPI_ISL_913961	20B	B.1.1.222	23	5'UTR:C241T, ORF1ab:A1136G, ORF1ab:C2973T, ORF1ab:C3037T, ORF1ab:C3903T, ORF1ab:T8053C, ORF1ab:C9120T, ORF1ab:C9615T, ORF1ab:A13032G, ORF1ab:C14408T, ORF1ab:C14802T, ORF1ab:C19185T, ORF1ab:T19839C, S:A23403G, S:A23756G, ORF7a:T27597C, ORF7a:C27741T, ORF7b:T27815A, ORF8:C28253T, N:G28881A, N:G28882A, N:G28883C, N:A29299G. N:G29348T.	12	N:R203K, N:G204R, N:A359S, ORF1a:K291E, ORF1a:A903V, ORF1a:P1213L, ORF1a:T2952I, ORF1a:T3117I, ORF1a:N4256S, ORF1b:P314L, S:D614G, S:T732A,	
hCoV-19/Mexico/GRO-InDRE_163/2020	EPI_ISL_913964	20A	B.1	15	5'UTR:C241T, ORF1ab:C556T, ORF1ab:C1190T, ORF1ab:C3037T, ORF1ab:T9982C, ORF1ab:C14408T, ORF1ab:G16741A, ORF1ab:C19763T, ORF1ab:C19884T, ORF1ab:A20268G, S:C21952T, S:A23403G, N:C28606T, N:C28854T, 3'UTR:C29730A, 3'UTR:G29766T,	6	N:S194L, ORF1a:P309S, ORF1b:P314L, ORF1b:V1092I, ORF1b:T2099I, S:D614G,	ORF1ab: 9860-9862

hCoV-19/Mexico/GRO-InDRE_173/2020	EPI_ISL_913974	20B	B.1.1.519	25	5'UTR:C203T, 5'UTR:C222T, 5'UTR:C241T, ORF1ab:C3037T, ORF1ab:C3140T, ORF1ab:G3692T, ORF1ab:G3871T, ORF1ab:C10029T, ORF1ab:C10954T, ORF1ab:A11117G, ORF1ab:C11824A, ORF1ab:C12789T, ORF1ab:C14408T, ORF1ab:C19718T, ORF1ab:T19839C, ORF1ab:C21306T, S:C22995A, S:A23403G, S:C23604A, S:A23756G, ORF7a:G27746T, ORF8:T27904C, N:G28881A, N:G28882A, N:G28883C, N:C29197T,	16	N:R203K, N:G204R, ORF1a:P959S, ORF1a:V1143F, ORF1a:K1202N, ORF1a:T3255I, ORF1a:I3618V, ORF1a:T4175I, ORF1b:P314L, ORF1b:T2084I, ORF7a:R118I, ORF8:L4P, S:T478K, S:D614G, S:P681H, S:T732A,	
hCoV-19/Mexico/GRO-InDRE_276/2020	EPI_ISL_1054958	20B	B.1.1	11	5'UTR:C241T, ORF1ab:C3037T, ORF1ab:A6510G, ORF1ab:G7936T, ORF1ab:A11860G, ORF1ab:C14408T, S:A23403G, ORF7a:G27598A, ORF7a:C27679T, N:G28881A, N:G28882A, N:G28883C,	7	N:R203K, N:G204R, ORF1a:N2082S, ORF1b:P314L, ORF7a:D69N, ORF7a:L96F, S:D614G,	
hCoV-19/Mexico/GRO-InDRE_284/2020	EPI_ISL_1054946	20C	B.1	13	5'UTR:C241T, ORF1ab:C1059T, ORF1ab:G2808T, ORF1ab:C3037T, ORF1ab:C9365T, ORF1ab:C14408T, ORF1ab:C19545T, S:G21950C, S:G21956C, S:C23275T, S:A23403G, ORF3a:A25431G, ORF3a:G25563T, M:G26730T,	8	M:V70F, ORF1a:T265I, ORF1a:R848M, ORF1b:P314L, ORF3a:Q57H, S:V130L, S:E132Q, S:D614G,	
hCoV-19/Mexico/GRO-InDRE_332/2020	EPI_ISL_1060683	20B	B.1.1.432	20	5'UTR:C241T, ORF1ab:C3037T, ORF1ab:G5461T, ORF1ab:C5512T, ORF1ab:C6525T, ORF1ab:C9319T, ORF1ab:C12412T, ORF1ab:T14313C, ORF1ab:C14408T, ORF1ab:C15738T, ORF1ab:A19899G, S:A23403G, S:C23439T, S:T24856C, ORF3a:C25613T, N:G28881A, N:G28882A, N:G28883C, N:T29317C, N:G29332T, ORF10:G29645A.	8	N:R203K, N:G204R, ORF1a:M1732I, ORF1a:T2087I, ORF1b:P314L, ORF3a:S74F, S:D614G, S:A626V,	
hCoV-19/Mexico/GRO-InDRE_384/2020	EPI_ISL_1054991	20A	B.1.397	14	5'UTR:C241T, ORF1ab:G1135T, ORF1ab:C3037T, ORF1ab:A3867T, ORF1ab:C8208T, ORF1ab:C10015T, ORF1ab:C10641T, ORF1ab:C14408T, ORF1ab:A20268G, S:C21952T, S:A23403G, S:A25050T, ORF3a:C25469T, N:C28310T, N:C28854T,	10	N:P13S, N:S194L, ORF1a:K290N, ORF1a:Q1201L, ORF1a:T2648I, ORF1a:T3459M, ORF1b:P314L, ORF3a:S26L, S:D614G, S:D1163V,	
hCoV-19/Mexico/GRO-InDRE_385/2020	EPI_ISL_1054992	20B	B.1.1.222	11	5'UTR:C241T, ORF1ab:C3037T, ORF1ab:C14408T, ORF1ab:T19839C, S:A23403G, S:A23756G, ORF3a:G25429T, ORF8:G27916T, ORF8:C27944T, N:G28881A, N:G28882A, N:G28883C,	7	N:R203K, N:G204R, ORF1b:P314L, ORF3a:V13L, ORF8:G8V, S:D614G, S:T732A,	
hCoV-19/Mexico/GRO-InDRE_F11063_S841/2021	EPI_ISL_1424043	20B	B.1.1.519	26	5'UTR:C203T, 5'UTR:C222T, 5'UTR:C241T, ORF1ab:C3037T, ORF1ab:C3140T, ORF1ab:G3692T, ORF1ab:G3871T, ORF1ab:C5884T, ORF1ab:T9070C, ORF1ab:C10029T, ORF1ab:C10954T, ORF1ab:A11117G, ORF1ab:C11824A, ORF1ab:C12789T, ORF1ab:C14408T, ORF1ab:C15613T, ORF1ab:T19839C, ORF1ab:C21306T, S:C22995A, S:A23403G, S:C23604A, S:A23756G, ORF8:T27904C, N:G28881A, N:G28882A, N:G28883C, N:C29197T,	15	N:R203K, N:G204R, ORF1a:P959S, ORF1a:V1143F, ORF1a:K1202N, ORF1a:T3255I, ORF1a:I3618V, ORF1a:T4175I, ORF1b:P314L, ORF1b:H716Y, ORF8:L4P, S:T478K, S:D614G, S:P681H, S:T732A,	
hCoV-19/Mexico/GRO-InDRE_F48G_S568/2021	EPI_ISL_1334381	20I (Alpha, V1)	B.1.1.7	31	5'UTR:C241T, ORF1ab:C913T, ORF1ab:C3037T, ORF1ab:C3267T, ORF1ab:C5388A, ORF1ab:C5986T, ORF1ab:T6954C, ORF1ab:C14408T, ORF1ab:C14676T, ORF1ab:C15279T, ORF1ab:T16176C, ORF1ab:A17615G, ORF1ab:C18032T, S:A23063T, S:C23271A, S:A23403G, S:C23604A, S:C23709T, S:T24506G, S:G24914C, M:G26730C, ORF8:C27972T, ORF8:G28048T, ORF8:A28111G, N:G28280C, N:A28281T, N:T28282A, N:C28603T, N:G28881A, N:G28882A, N:G28883C, N:C28977T,	21	M:V70L, N:D3L, N:R203K, N:G204R, N:S235F, ORF1a:T1001I, ORF1a:A1708D, ORF1a:I2230T, ORF1b:P314L, ORF1b:K1383R, ORF1b:T1522I, ORF8:Q27*, ORF8:R52I, ORF8:Y73C, S:N501Y, S:A570D, S:D614G, S:P681H, S:T716I, S:S982A, S:D1118H,	ORF1ab: 11288-11296, S:21765-21770, S:21992-21994, ORF8:28271,

hCoV-19/Mexico/GRO-InDRE-10/2020	EPI_ISL_455434	20C	B.1	6	5'UTR:C241T, ORF1ab:C1059T, ORF1ab:C3037T, ORF1ab:C14408T, ORF1ab:A20755C, S:A23403G, ORF3a:G25563T,	5	ORF1a:T265I, ORF1b:P314L, ORF1b:S2430R, ORF3a:Q57H, S:D614G,
hCoV-19/Mexico/GRO-InDRE-24/2020	EPI_ISL_493339	20A	B.1	4	5'UTR:C241T, ORF1ab:C414T, ORF1ab:C3037T, ORF1ab:C14408T, S:A23403G,	3	ORF1a:T50I, ORF1b:P314L, S:D614G,
hCoV-19/Mexico/GRO-InDRE-91/2020	EPI_ISL_658896	20A	B.1.609	12	5'UTR:C241T, ORF1ab:C3037T, ORF1ab:G3994T, ORF1ab:C4582T, ORF1ab:C5482T, ORF1ab:C13860T, ORF1ab:C14408T, ORF1ab:G15202T, ORF1ab:G15982T, ORF1ab:C19955T, ORF1ab:A20268G, S:A23403G, 3'UTR:C29738T,	5	ORF1b:P314L, ORF1b:V579L, ORF1b:V839L, ORF1b:T2163I, S:D614G,
hCoV-19/Mexico/GRO-InDRE-92/2020	EPI_ISL_658899	20A	B.1.609	13	5'UTR:C241T, ORF1ab:C3037T, ORF1ab:G3994T, ORF1ab:C4582T, ORF1ab:C5482T, ORF1ab:C13860T, ORF1ab:C14408T, ORF1ab:G15202T, ORF1ab:G15982T, ORF1ab:G16564T, ORF1ab:C19955T, ORF1ab:A20268G, S:A23403G, 3'UTR:C29738T,	6	ORF1b:P314L, ORF1b:V579L, ORF1b:V839L, ORF1b:A1033S, ORF1b:T2163I, S:D614G,
hCoV-19/Mexico/GRO-InDRE-93/2020	EPI_ISL_658901	20B	B.1.1	12	5'UTR:T11G, 5'UTR:C241T, ORF1ab:C3037T, ORF1ab:T4330C, ORF1ab:A6520T, ORF1ab:T6522A, ORF1ab:C14408T, ORF1ab:T19839C, ORF1ab:G21544A, S:A23403G, N:G28881A, N:G28882A, N:G28883C,	7	N:R203K, N:G204R, ORF1a:K2085N, ORF1a:I2086N, ORF1b:P314L, ORF1b:V2693I, S:D614G,
hCoV-19/Mexico/GTO-InDRE_115/2020	EPI_ISL_913919	20A	B.1.189	10	5'UTR:C241T, ORF1ab:C3037T, ORF1ab:C4582T, ORF1ab:G6404T, ORF1ab:C8109T, ORF1ab:C14408T, ORF1ab:A20268G, S:A23403G, S:T25123C, ORF8:G28044A, N:G28378T,	6	ORF1a:V2047F, ORF1a:T2615I, ORF1b:P314L, ORF8:A51T, ORF9b:R32L, S:D614G,
hCoV-19/Mexico/GTO-InDRE_142/2020	EPI_ISL_913945	20B	B.1.1.222	12	5'UTR:C241T, ORF1ab:C3037T, ORF1ab:C6285T, ORF1ab:A13498G, ORF1ab:C14408T, ORF1ab:T19839C, S:A23403G, S:A23756G, ORF3a:G25447T, ORF3a:A25449T, N:G28881A, N:G28882A, N:G28883C,	8	N:R203K, N:G204R, ORF1a:T2007I, ORF1b:T11A, ORF1b:P314L, ORF3a:E19Y, S:D614G, S:T732A,
hCoV-19/Mexico/GTO-InDRE_180/2020	EPI_ISL_913981	20B	B.1.1.71	20	5'UTR:C241T, ORF1ab:C3037T, ORF1ab:C3267T, ORF1ab:G6710T, ORF1ab:C7162T, ORF1ab:A10716G, ORF1ab:T11638C, ORF1ab:C11775T, ORF1ab:C14408T, S:C21691T, S:G21898T, S:A23403G, ORF3a:C25667T, ORF7b:G27806T, N:G28881A, N:G28882A, N:G28883C, 3'UTR:C29708T, 3'UTR:C29733T, 3'UTR:G29736T, 3'UTR:C29774T,	9	N:R203K, N:G204R, ORF1a:T1001I, ORF1a:V2149F, ORF1a:N3484S, ORF1a:A3837V, ORF1b:P314L, ORF3a:S92L, S:D614G,
hCoV-19/Mexico/GUA-06_LA_FB_05_0178/2020	EPI_ISL_1238787	20A	B.1.243	15	5'UTR:C241T, ORF1ab:C3037T, ORF1ab:C7113T, ORF1ab:T13354C, ORF1ab:C14408T, ORF1ab:C18115T, ORF1ab:A20268G, ORF1ab:C20574T, S:A23403G, S:T24076C, S:C24337T, S:G25266T, M:T26669C, M:G26690T, ORF7a:A27398T, N:C28854T,	7	N:S194L, ORF1a:T2283I, ORF1b:P314L, ORF1b:H1550Y, ORF7a:K2I, S:D614G, S:C1235F,
hCoV-19/Mexico/GUA-09_LA_FB_05_0249/2020	EPI_ISL_1238788	20A	B.1.243	16	5'UTR:C241T, ORF1ab:C3037T, ORF1ab:C7113T, ORF1ab:T13354C, ORF1ab:C14408T, ORF1ab:C18115T, ORF1ab:A20268G, ORF1ab:C20574T, S:A23403G, S:T24076C, S:C24337T, S:G25266T, S:A25377T, M:T26669C, M:G26690T, ORF7a:A27398T, N:C28854T,	8	N:S194L, ORF1a:T2283I, ORF1b:P314L, ORF1b:H1550Y, ORF7a:K2I, S:D614G, S:C1235F, S:Y1272F,
hCoV-19/Mexico/GUA-14_LA_FB_05_0336/2020	EPI_ISL_1238789	20B	B.1.1	17	5'UTR:C241T, ORF1ab:C3037T, ORF1ab:G6337T, ORF1ab:C13517T, ORF1ab:C14408T, ORF1ab:C17502T, ORF1ab:G17658T, ORF1ab:T19839C, S:A23403G, S:T24352C, S:G24620T, N:G28373T, N:A28877T, N:G28878C, N:G28881A, N:G28882A, N:G28883C, N:G29425T,	9	N:G34W, N:R203K, N:G204R, N:Q384H, ORF1b:T17I, ORF1b:P314L, ORF1b:M1397I, S:D614G, S:A1020S,
hCoV-19/Mexico/GUA-20_LA_FB_05_0345/2020	EPI_ISL_1238790	20B	B.1.1	11	5'UTR:T10A, 5'UTR:T13A, 5'UTR:C241T, ORF1ab:C3037T, ORF1ab:C14408T, S:A23403G, N:A28877T, N:G28878C, N:G28881A, N:G28882A, N:G28883C, 3'UTR:C29870A,	4	N:R203K, N:G204R, ORF1b:P314L, S:D614G,

hCoV-19/Mexico/GUA-26_LA_FB_05_0353/2020	EPI_ISL_1238791	20B	B.1.1.322	18	5'UTR:C241T, ORF1ab:C3037T, ORF1ab:G6337T, ORF1ab:C11589A, ORF1ab:C13517T, ORF1ab:C14408T, ORF1ab:C17502T, ORF1ab:G17658T, ORF1ab:T19839C, S:A23403G, S:A23756G, S:T24352C, S:G24620T, N:G28373T, N:A28877T, N:G28878C, N:G28881A, N:G28882A, N:G28883C.	10	N:G34W, N:R203K, N:G204R, ORF1a:T3775K, ORF1b:T17I, ORF1b:P314L, ORF1b:M1397I, S:D614G, S:T732A, S:A1020S,
hCoV-19/Mexico/GUA-31_LA_FB_05_0401/2020	EPI_ISL_1238792	20B	B.1.1	12	5'UTR:C241T, ORF1ab:C3037T, ORF1ab:C13517T, ORF1ab:C14408T, ORF1ab:G17658T, S:A23403G, S:T24352C, S:G24620T, N:A28877T, N:G28878C, N:G28881A, N:G28882A, N:G28883C,	7	N:R203K, N:G204R, ORF1b:T17I, ORF1b:P314L, ORF1b:M1397I, S:D614G, S:A1020S,
hCoV-19/Mexico/GUA-35_LA_FB_05_0403/2020	EPI_ISL_1238793	20B	B.1.1.322	18	5'UTR:C241T, ORF1ab:C3037T, ORF1ab:G6337T, ORF1ab:C11589A, ORF1ab:C13517T, ORF1ab:C14408T, ORF1ab:C17502T, ORF1ab:G17658T, ORF1ab:T19839C, S:A23403G, S:A23756G, S:T24352C, S:G24620T, N:G28373T, N:A28877T, N:G28878C, N:G28881A, N:G28882A, N:G28883C.	10	N:G34W, N:R203K, N:G204R, ORF1a:T3775K, ORF1b:T17I, ORF1b:P314L, ORF1b:M1397I, S:D614G, S:T732A, S:A1020S,
hCoV-19/Mexico/GUA-InDRE_250/2021	EPI_ISL_1060692	20C	B.1	13	5'UTR:C241T, ORF1ab:C1059T, ORF1ab:G2577T, ORF1ab:C3037T, ORF1ab:T4639C, ORF1ab:C9693T, ORF1ab:C14408T, ORF1ab:C16575T, ORF1ab:C18131T, ORF1ab:T20322C, S:T22138C, S:A23403G, ORF3a:G25563T, N:C28892T,	8	N:P207S, ORF1a:T265I, ORF1a:S771I, ORF1a:A3143V, ORF1b:P314L, ORF1b:T1555I, ORF3a:Q57H, S:D614G,
hCoV-19/Mexico/GUA-InDRE_251/2021	EPI_ISL_1060718	20G	B.1.2	19	5'UTR:C241T, ORF1ab:C1059T, ORF1ab:C3037T, ORF1ab:G4444T, ORF1ab:C10319T, ORF1ab:G11417T, ORF1ab:C14408T, ORF1ab:G17478A, ORF1ab:A18424G, ORF1ab:C21304T, S:C21614T, S:A23403G, S:G23900C, S:C24130T, ORF3a:G25563T, ORF3a:G25907T, ORF8:C27942T, ORF8:C27964T, N:C28472T. N:C28869T.	15	N:P67S, N:P199L, ORF1a:T265I, ORF1a:L3352F, ORF1a:V3718F, ORF1b:P314L, ORF1b:N1653D, ORF1b:R2613C, ORF3a:Q57H, ORF3a:G172V, ORF8:H17Y, ORF8:S24L, S:L18F, S:D614G, S:E780Q,
hCoV-19/Mexico/GUA-InDRE_252/2021	EPI_ISL_1060704	20B	B.1.1.222	19	5'UTR:C241T, ORF1ab:T1169C, ORF1ab:G2599A, ORF1ab:C3037T, ORF1ab:G3122T, ORF1ab:C4927T, ORF1ab:C14408T, ORF1ab:C14491T, ORF1ab:A15468G, ORF1ab:T19839C, S:G21641T, S:A22474G, S:G23236T, S:A23403G, S:A23756G, ORF3a:G26062A, ORF8:T27900C, N:G28881A, N:G28882A, N:G28883C,	11	N:R203K, N:G204R, ORF1a:S302P, ORF1a:D953Y, ORF1b:P314L, ORF3a:G224S, ORF8:F3L, S:A27S, S:K558N, S:D614G, S:T732A,
hCoV-19/Mexico/GUA-InDRE_277/2020	EPI_ISL_1054952	20A	B.1	9	5'UTR:C241T, ORF1ab:A2497G, ORF1ab:C3037T, ORF1ab:G5230T, ORF1ab:C14408T, ORF1ab:T16413C, ORF1ab:A20268G, S:A23403G, N:C28854T, ORF10:C29640T,	4	N:S194L, ORF1a:K1655N, ORF1b:P314L, S:D614G,
hCoV-19/Mexico/GUA-InDRE_296/2020	EPI_ISL_1060728	20B	B.1.1.432	16	5'UTR:C241T, ORF1ab:C3037T, ORF1ab:C3695T, ORF1ab:A6985T, ORF1ab:C9319T, ORF1ab:C12412T, ORF1ab:T14313C, ORF1ab:C14408T, ORF1ab:C15017T, S:C21952T, S:A23403G, ORF3a:C25613T, N:C28697T, N:G28881A, N:G28882A. N:G28883C. N:T29317C.	7	N:P142S, N:R203K, N:G204R, ORF1b:P314L, ORF1b:A517V, ORF3a:S74F, S:D614G,
hCoV-19/Mexico/GUA-InDRE_298/2020	EPI_ISL_1060746	20A	B.1.128	8	5'UTR:C241T, ORF1ab:C3037T, ORF1ab:C8293T, ORF1ab:G11335T, ORF1ab:C12919T, ORF1ab:C14408T, ORF1ab:A20268G, S:A23403G, N:C28854T,	3	N:S194L, ORF1b:P314L, S:D614G,
hCoV-19/Mexico/GUA-InDRE_299/2020	EPI_ISL_1060742	20B	B.1.1.322	12	5'UTR:C241T, ORF1ab:C3037T, ORF1ab:C14408T, ORF1ab:T19839C, ORF1ab:C21306T, S:A23403G, S:A23756G, N:A28877T, N:G28878C, N:G28881A, N:G28882A, N:G28883C, N:C29144T,	5	N:R203K, N:G204R, ORF1b:P314L, S:D614G, S:T732A,
hCoV-19/Mexico/GUA-InDRE_306/2020	EPI_ISL_1060741	20A	B.1	12	5'UTR:C241T, ORF1ab:C1758T, ORF1ab:G2324A, ORF1ab:C3037T, ORF1ab:C4927T, ORF1ab:C6636T, ORF1ab:C12513T, ORF1ab:C14408T, ORF1ab:G15982T, ORF1ab:A20268G, S:A23403G, N:C28775T, N:C28854T,	9	N:P168S, N:S194L, ORF1a:A498V, ORF1a:A687T, ORF1a:T2124I, ORF1a:T4083M, ORF1b:P314L, ORF1b:V839L, S:D614G,

hCoV-19/Mexico/GUA-InDRE_307/2020	EPI_ISL_1060738	20A	B.1	11	5'UTR:C241T, ORF1ab:C3037T, ORF1ab:C14408T, ORF1ab:A20268G, S:A23403G, ORF7a:A27486G, ORF7a:A27489G, ORF7a:A27547G, ORF7a:A27548G, ORF7a:A27558G, ORF7a:A27571G, N:C28854T,	5	N:S194L, ORF1b:P314L, ORF7a:N52G, ORF7a:S60G, S:D614G,
hCoV-19/Mexico/GUA-InDRE_314/2020	EPI_ISL_1060698	20A	B.1.243	8	ORF1ab:C3037T, ORF1ab:G7756T, ORF1ab:G9190T, ORF1ab:C14408T, ORF1ab:A20244C, ORF1ab:A20268G, S:A23403G, S:T24076C, N:C28854T,	5	N:S194L, ORF1a:K2497N, ORF1b:P314L, ORF1b:Q2259H, S:D614G,
hCoV-19/Mexico/GUA-InDRE_338/2020	EPI_ISL_1060682	20A	B.1.609	9	ORF1ab:C3037T, ORF1ab:C4582T, ORF1ab:C4920T, ORF1ab:G11083T, ORF1ab:C13119T, ORF1ab:C14408T, ORF1ab:T15366C, ORF1ab:A20268G, S:A23403G, ORF3a:G26062T,	6	ORF1a:T1552I, ORF1a:L3606F, ORF1a:A4285V, ORF1b:P314L, ORF3a:G224C, S:D614G,
hCoV-19/Mexico/GUA-InDRE_383/2020	EPI_ISL_1054986	20A	B.1.397	14	5'UTR:C241T, ORF1ab:G1135T, ORF1ab:C3037T, ORF1ab:A3867T, ORF1ab:C8208T, ORF1ab:C10015T, ORF1ab:C10641T, ORF1ab:C14408T, ORF1ab:A20268G, S:C21952T, S:A23403G, S:A25050T, ORF3a:C25469T, N:C28310T, N:C28854T,	10	N:P13S, N:S194L, ORF1a:K290N, ORF1a:Q1201L, ORF1a:T2648I, ORF1a:T3459M, ORF1b:P314L, ORF3a:S26L, S:D614G, S:D1163V,
hCoV-19/Mexico/GUA-InDRE_395/2020	EPI_ISL_1054970	20B	B.1.1.222	15	5'UTR:C241T, ORF1ab:C3037T, ORF1ab:G7560A, ORF1ab:G8695A, ORF1ab:C13551T, ORF1ab:C14408T, ORF1ab:T19839C, S:A23403G, S:A23756G, ORF7a:C27673T, N:G28881A, N:G28882A, N:G28883C, N:G29468T, 3'UTR:C29738T, 3'UTR:G29781T,	8	N:R203K, N:G204R, N:D399Y, ORF1a:R2432K, ORF1b:P314L, ORF7a:Q94*, S:D614G, S:T732A,
hCoV-19/Mexico/GUA-InDRE_F10333_S628/2021	EPI_ISL_1340643	20B	B.1.1.519	22	5'UTR:C203T, 5'UTR:C222T, 5'UTR:C241T, ORF1ab:C3037T, ORF1ab:C3140T, ORF1ab:C10029T, ORF1ab:C10954T, ORF1ab:A11117G, ORF1ab:C12789T, ORF1ab:C13335T, ORF1ab:C14408T, ORF1ab:T19839C, ORF1ab:C21306T, S:C22995A, S:A23403G, S:C23604A, S:A23756G, S:A25108C, M:C26607T, N:G28881A, N:G28882A, N:G28883C, N:C29197T,	14	M:L29F, N:R203K, N:G204R, ORF1a:P959S, ORF1a:T3255I, ORF1a:I3618V, ORF1a:T4175I, ORF1a:A4357V, ORF1b:P314L, S:T478K, S:D614G, S:P681H, S:T732A, S:E1182D,
hCoV-19/Mexico/GUA-InDRE_F10341_S587/2021	EPI_ISL_1337363	21C (Epsilon)	B.1.429	30	5'UTR:C241T, ORF1ab:C1059T, ORF1ab:A1966T, ORF1ab:C2395T, ORF1ab:T2597C, ORF1ab:C3037T, ORF1ab:G3307A, ORF1ab:C8947T, ORF1ab:C10408T, ORF1ab:T10831A, ORF1ab:C12100T, ORF1ab:A12878G, ORF1ab:C14408T, ORF1ab:G17014T, ORF1ab:G17721T, ORF1ab:A19515G, S:G21600T, S:G22018T, S:T22917G, S:A23403G, S:T24349C, ORF3a:G25563T, M:C26681T, ORF7b:G27890T, ORF8:G27987C, ORF8:G28191T, ORF8:A28272T, N:C28887T, N:G28975T, N:C29171T, N:C29362T,	15	N:T205I, N:M234I, N:H300Y, ORF1a:T265I, ORF1a:M1014I, ORF1a:I4205V, ORF1b:P314L, ORF1b:D1183Y, ORF3a:Q57H, ORF8:V32L, ORF8:V100L, S:S13I, S:W152C, S:L452R, S:D614G,
hCoV-19/Mexico/GUA-InDRE_F10342_S808/2021	EPI_ISL_1424010	20B	B.1.1.222	20	5'UTR:C241T, ORF1ab:C3037T, ORF1ab:C6285T, ORF1ab:C6501T, ORF1ab:A8203G, ORF1ab:C10189T, ORF1ab:T11077C, ORF1ab:A13498G, ORF1ab:C14408T, ORF1ab:T19839C, S:A22116C, S:A23403G, S:A23756G, M:C27119T, ORF8:A28175G, N:C28290T, N:C28651T, N:G28881A, N:G28882A, N:G28883C, N:G28922T,	12	N:P6L, N:R203K, N:G204R, N:A217S, ORF1a:T2007I, ORF1a:P2079L, ORF1b:T11A, ORF1b:P314L, ORF9b:P3S, S:N185T, S:D614G, S:T732A,
hCoV-19/Mexico/GUA-InDRE_F10343_S809/2021	EPI_ISL_1424011	20B	B.1.1.519	27	5'UTR:T201C, 5'UTR:C203T, 5'UTR:C222T, 5'UTR:C241T, ORF1ab:G1738T, ORF1ab:C3037T, ORF1ab:C3140T, ORF1ab:G3340T, ORF1ab:G4207A, ORF1ab:C10029T, ORF1ab:C10954T, ORF1ab:A11117G, ORF1ab:C12789T, ORF1ab:C14408T, ORF1ab:G17562T, ORF1ab:T19839C, ORF1ab:A19974G, ORF1ab:C21306T, S:C22450T, S:C22995A, S:A23403G, S:C23604A, S:A23756G, ORF3a:C25791T, N:G28881A, N:G28882A, N:G28883C, N:C29197T	11	N:R203K, N:G204R, ORF1a:P959S, ORF1a:T3255I, ORF1a:I3618V, ORF1a:T4175I, ORF1b:P314L, S:T478K, S:D614G, S:P681H, S:T732A,

hCoV-19/Mexico/GUA-InDRE_F10344_S810/2021	EPI_ISL_1424012	20B	B.1.1.222	16	5'UTR:C241T, ORF1ab:C3037T, ORF1ab:T5386C, ORF1ab:C6285T, ORF1ab:A8203G, ORF1ab:A10880T, ORF1ab:A13498G, ORF1ab:C14408T, ORF1ab:T14598C, ORF1ab:T19839C, S:A22116C, S:A23403G, S:A23756G, M:C27119T, N:G28881A, N:G28882A, N:G28883C,	9	N:R203K, N:G204R, ORF1a:T2007I, ORF1a:M3539L, ORF1b:T11A, ORF1b:P314L, S:N185T, S:D614G, S:T732A,
hCoV-19/Mexico/GUA-InDRE_F10345_S629/2021	EPI_ISL_1340644	20B	B.1.1.519	26	5'UTR:T201C, 5'UTR:C203T, 5'UTR:C222T, 5'UTR:C241T, ORF1ab:C936T, ORF1ab:G1738T, ORF1ab:C3037T, ORF1ab:G3122T, ORF1ab:C3140T, ORF1ab:C10029T, ORF1ab:C10954T, ORF1ab:A11117G, ORF1ab:C12789T, ORF1ab:C14408T, ORF1ab:T19839C, ORF1ab:A19974G, ORF1ab:C21306T, S:C22995A, S:A23403G, S:C23604A, S:A23756G, N:G28881A, N:G28882A, N:G28883C, N:C29197T, N:G29227T, N:G29557T.	13	N:R203K, N:G204R, ORF1a:T224I, ORF1a:D953Y, ORF1a:P959S, ORF1a:T3255I, ORF1a:I3618V, ORF1a:T4175I, ORF1b:P314L, S:T478K, S:D614G, S:P681H, S:T732A,
hCoV-19/Mexico/GUA-InDRE_F10347_S630/2021	EPI_ISL_1340646	20B	B.1.1.519	24	5'UTR:C203T, 5'UTR:C222T, 5'UTR:C241T, ORF1ab:C3037T, ORF1ab:C3140T, ORF1ab:C3444T, ORF1ab:C10029T, ORF1ab:C10954T, ORF1ab:A11117G, ORF1ab:C12789T, ORF1ab:C14408T, ORF1ab:G17058T, ORF1ab:T19839C, ORF1ab:C21306T, S:A22101G, S:C22995A, S:A23403G, S:C23604A, S:A23756G, N:G28326T, N:G28881A, N:G28882A, N:G28883C, N:C29197T, N:G29234A,	17	N:G18V, N:R203K, N:G204R, N:G321S, ORF1a:P959S, ORF1a:A1060V, ORF1a:T3255I, ORF1a:I3618V, ORF1a:T4175I, ORF1b:P314L, ORF1b:M1197I, ORF9b:V15L, S:E180G, S:T478K, S:D614G, S:P681H, S:T732A,
hCoV-19/Mexico/GUA-InDRE_F10348_S631/2021	EPI_ISL_1340624	20B	B.1.1.519	23	5'UTR:C203T, 5'UTR:C222T, 5'UTR:C241T, ORF1ab:C3037T, ORF1ab:C3140T, ORF1ab:C10029T, ORF1ab:C10954T, ORF1ab:A11117G, ORF1ab:C12789T, ORF1ab:C13860T, ORF1ab:C14408T, ORF1ab:T19839C, ORF1ab:C21306T, S:C22995A, S:A23403G, S:C23604A, S:A23756G, S:A25108C, ORF3a:C25413T, M:G26648A, N:G28881A, N:G28882A, N:G28883C, N:C29197T.	12	N:R203K, N:G204R, ORF1a:P959S, ORF1a:T3255I, ORF1a:I3618V, ORF1a:T4175I, ORF1b:P314L, S:T478K, S:D614G, S:P681H, S:T732A, S:E1182D,
hCoV-19/Mexico/GUA-InDRE_F10349_S632/2021	EPI_ISL_1340630	20B	B.1.1.519	23	5'UTR:C203T, 5'UTR:C222T, 5'UTR:C241T, ORF1ab:C3037T, ORF1ab:C3140T, ORF1ab:A3818G, ORF1ab:C10029T, ORF1ab:C10954T, ORF1ab:A11117G, ORF1ab:G12163T, ORF1ab:C12789T, ORF1ab:C14408T, ORF1ab:T19839C, ORF1ab:C21306T, S:T21610C, S:C22995A, S:A23403G, S:C23604A, S:A23756G, N:C28863T, N:G28881A, N:G28882A, N:G28883C, N:C29197T.	14	N:S197L, N:R203K, N:G204R, ORF1a:P959S, ORF1a:K1185E, ORF1a:T3255I, ORF1a:I3618V, ORF1a:Q3966H, ORF1a:T4175I, ORF1b:P314L, S:T478K, S:D614G, S:P681H, S:T732A,
hCoV-19/Mexico/GUA-InDRE_F10350_S588/2021	EPI_ISL_1337365	21C (Epsilon)	B.1.429	21	5'UTR:C241T, ORF1ab:C1059T, ORF1ab:C2395T, ORF1ab:T2597C, ORF1ab:A2645C, ORF1ab:C3037T, ORF1ab:C8947T, ORF1ab:C12100T, ORF1ab:A12878G, ORF1ab:C14408T, ORF1ab:G17014T, S:G21600T, S:G22018T, S:T22917G, S:A23403G, S:T24349C, ORF3a:G25563T, M:C26681T, ORF7b:G27890T, ORF8:A28272T, N:C28887T, N:C29362T.	11	N:T205I, ORF1a:T265I, ORF1a:K794Q, ORF1a:I4205V, ORF1b:P314L, ORF1b:D1183Y, ORF3a:Q57H, S:S13I, S:W152C, S:L452R, S:D614G,
hCoV-19/Mexico/GUA-InDRE_F10351_S854/2021	EPI_ISL_1399260	20A	B.1.551	19	5'UTR:C241T, ORF1ab:C2455T, ORF1ab:C3037T, ORF1ab:C3738T, ORF1ab:C8722T, ORF1ab:G9968T, ORF1ab:C10029T, ORF1ab:C11036T, ORF1ab:C14408T, ORF1ab:A18861T, ORF1ab:C19524T, ORF1ab:A20268G, S:C21621T, S:A23403G, S:C23604G, S:C25276T, ORF7b:C27847T, N:C28706T, N:C28854T, N:C29466T.	12	N:H145Y, N:S194L, N:A398V, ORF1a:P1158L, ORF1a:A3235S, ORF1a:T3255I, ORF1a:L3591F, ORF1b:P314L, ORF7b:S31L, S:T20I, S:D614G, S:P681R,

hCoV-19/Mexico/GUA-InDRE_F10352_S655/2021	EPI_ISL_1359070	20A	B.1	23	5'UTR:C241T, ORF1ab:C1288T, ORF1ab:A1389C, ORF1ab:C2445T, ORF1ab:G2782T, ORF1ab:C3037T, ORF1ab:C3096T, ORF1ab:C5746T, ORF1ab:A5999G, ORF1ab:T6631C, ORF1ab:C10279T, ORF1ab:C14408T, ORF1ab:C18060T, ORF1ab:A19387G, ORF1ab:T19791G, ORF1ab:A20268G, S:C21707T, S:A23403G, ORF3a:G25437T, N:G28280T, N:C28854T, N:G28899T, N:C29065A, N:G29402T,	13	N:D3Y, N:S194L, N:R209I, N:D377Y, ORF1a:N375T, ORF1a:T727I, ORF1a:S944L, ORF1a:I1912V, ORF1b:P314L, ORF1b:S1974G, ORF3a:L15F, S:H49Y, S:D614G,
hCoV-19/Mexico/GUA-InDRE_F10353_S633/2021	EPI_ISL_1340608	20B	B.1.1.519	25	5'UTR:C203T, 5'UTR:C222T, 5'UTR:C241T, ORF1ab:C2262T, ORF1ab:C3037T, ORF1ab:C3140T, ORF1ab:G3918A, ORF1ab:C10029T, ORF1ab:C10954T, ORF1ab:A11117G, ORF1ab:C12789T, ORF1ab:C14408T, ORF1ab:C14805T, ORF1ab:T19839C, ORF1ab:C21306T, S:C22995A, S:A23403G, S:C23604A, S:A23756G, S:A25108C, N:G28881A, N:G28882A, N:G28883C, N:C29197T, N:A29282G, N:C29421T,	16	N:R203K, N:G204R, N:I337V, N:P383L, ORF1a:T666I, ORF1a:P959S, ORF1a:S1218N, ORF1a:T3255I, ORF1a:I3618V, ORF1a:T4175I, ORF1b:P314L, S:T478K, S:D614G, S:P681H, S:T732A, S:E1182D,
hCoV-19/Mexico/GUA-InDRE_F10355_S856/2021	EPI_ISL_1399261	20B	B.1.1.519	22	5'UTR:C203T, 5'UTR:C222T, 5'UTR:C241T, ORF1ab:C3037T, ORF1ab:C3140T, ORF1ab:C10029T, ORF1ab:C10954T, ORF1ab:A11117G, ORF1ab:C12789T, ORF1ab:G13370T, ORF1ab:C14408T, ORF1ab:G15372T, ORF1ab:T19839C, ORF1ab:C21306T, S:C22995A, S:A23403G, S:C23604A, S:A23756G, S:C24099T, N:G28881A, N:G28882A, N:G28883C, N:C29197T,	13	N:R203K, N:G204R, ORF1a:P959S, ORF1a:T3255I, ORF1a:I3618V, ORF1a:T4175I, ORF1a:V4369F, ORF1b:P314L, S:T478K, S:D614G, S:P681H, S:T732A, S:A846V,
hCoV-19/Mexico/GUA-InDRE_F10356_S656/2021	EPI_ISL_1359071	20A	B.1.126	17	5'UTR:C241T, ORF1ab:C683T, ORF1ab:T3011C, ORF1ab:C3037T, ORF1ab:G5704T, ORF1ab:G6865T, ORF1ab:G10193A, ORF1ab:C14408T, ORF1ab:T17772C, ORF1ab:G20102T, S:G22205T, S:C23124T, S:A23403G, S:G23611T, ORF3a:G25598T, ORF3a:G25617T, ORF7b:T27869C, N:T28853G,	11	N:S194A, ORF1a:Q1813H, ORF1a:K2200N, ORF1a:E3310K, ORF1b:P314L, ORF1b:S2212I, ORF3a:W69L, ORF3a:K75N, S:D215Y, S:P521L, S:D614G,
hCoV-19/Mexico/GUA-InDRE_F10357_S634/2021	EPI_ISL_1340614	20B	B.1.1.519	25	5'UTR:C203T, 5'UTR:C222T, 5'UTR:A223T, 5'UTR:C241T, ORF1ab:C3037T, ORF1ab:C3140T, ORF1ab:C7125T, ORF1ab:C10029T, ORF1ab:C10954T, ORF1ab:A11117G, ORF1ab:C12789T, ORF1ab:C14408T, ORF1ab:G15921T, ORF1ab:G17893T, ORF1ab:T19839C, ORF1ab:C21306T, S:C22995A, S:A23403G, S:C23604A, S:A23756G, ORF8:T28110C, N:C28737T, N:G28881A, N:G28882A, N:G28883C, N:C29197T,	15	N:A155V, N:R203K, N:G204R, ORF1a:P959S, ORF1a:P2287L, ORF1a:T3255I, ORF1a:I3618V, ORF1a:T4175I, ORF1b:P314L, ORF1b:A1476S, ORF8:Y73H, S:T478K, S:D614G, S:P681H, S:T732A,
hCoV-19/Mexico/GUA-InDRE_F10358_S635/2021	EPI_ISL_1340626	20B	B.1.1.519	25	5'UTR:C203T, 5'UTR:C222T, 5'UTR:C241T, ORF1ab:G942A, ORF1ab:C3037T, ORF1ab:C3140T, ORF1ab:C8140T, ORF1ab:C10029T, ORF1ab:C10954T, ORF1ab:A11117G, ORF1ab:C11124T, ORF1ab:C12789T, ORF1ab:C14408T, ORF1ab:G18379T, ORF1ab:T19839C, ORF1ab:A20676G, ORF1ab:C21306T, S:C22995A, S:A23403G, S:C23604A, S:A23756G, M:T27035C, N:G28881A, N:G28882A, N:G28883C, N:C29197T,	14	N:R203K, N:G204R, ORF1a:R226K, ORF1a:P959S, ORF1a:T3255I, ORF1a:I3618V, ORF1a:A3620V, ORF1a:T4175I, ORF1b:P314L, ORF1b:G1638C, S:T478K, S:D614G, S:P681H, S:T732A,
hCoV-19/Mexico/GUA-InDRE_F10359_S657/2021	EPI_ISL_1359072	20B	B.1.1.517	25	5'UTR:C241T, ORF1ab:C3037T, ORF1ab:C7164T, ORF1ab:C14408T, ORF1ab:C16887T, ORF1ab:G17014T, ORF1ab:G18340A, ORF1ab:C18388T, ORF1ab:G19788A, ORF1ab:T19839C, ORF1ab:G19891T, S:T23332C, S:A23403G, S:A23756G, S:T23950C, ORF8:A27994G, ORF8:G28083T, ORF8:T28208C, N:C28603T, N:G28881A, N:G28882A, N:G28883C, N:A28983G, N:C29144T, N:G29543T, 3'UTR:C29774A,	12	N:R203K, N:G204R, N:K237R, ORF1a:T2300I, ORF1b:P314L, ORF1b:D1183Y, ORF1b:V1625I, ORF1b:D2142Y, ORF8:D34G, ORF8:E64*, S:D614G, S:T732A,

hCoV-19/Mexico/GUA-InDRE_F10360_S658/2021	EPI_ISL_1359073	20B	B.1.1.517	25	5'UTR:C241T, ORF1ab:C3037T, ORF1ab:C7164T, ORF1ab:C14408T, ORF1ab:C16887T, ORF1ab:G17014T, ORF1ab:G18340A, ORF1ab:C18388T, ORF1ab:G19788A, ORF1ab:T19839C, ORF1ab:G19891T, S:T23332C, S:A23403G, S:A23756G, S:T23950C, ORF8:A27994G, ORF8:G28083T, ORF8:T28208C, N:C28603T, N:G28881A, N:G28882A, N:G28883C, N:A28983G, N:C29144T, N:G29543T, 3'UTR:C29774A,	12	N:R203K, N:G204R, N:K237R, ORF1a:T2300I, ORF1b:P314L, ORF1b:D1183Y, ORF1b:V1625I, ORF1b:D2142Y, ORF8:D34G, ORF8:E64*, S:D614G, S:T732A,
hCoV-19/Mexico/GUA-InDRE_F10361_S659/2021	EPI_ISL_1359074	20B	B.1.1.222	22	5'UTR:C241T, ORF1ab:C3037T, ORF1ab:G3403T, ORF1ab:T5218C, ORF1ab:C6031T, ORF1ab:C12525T, ORF1ab:C12924T, ORF1ab:C14408T, ORF1ab:C14790T, ORF1ab:A19182G, ORF1ab:T19839C, S:A23403G, S:T23689C, S:A23756G, S:G24794T, ORF3a:G26153T, ORF6:C27294T, N:G28300T, N:G28881A, N:G28882A, N:G28883C, N:G28975T, N:G29449T,	12	N:Q9H, N:R203K, N:G204R, N:M234I, ORF1a:T4087I, ORF1a:P4220L, ORF1b:P314L, ORF3a:G254V, ORF9b:S6I, S:D614G, S:T732A, S:A1078S,
hCoV-19/Mexico/GUA-InDRE_F10362_S636/2021	EPI_ISL_1340610	20B	B.1.1.519	25	5'UTR:C203T, 5'UTR:C222T, 5'UTR:C241T, ORF1ab:A535T, ORF1ab:C3037T, ORF1ab:C3140T, ORF1ab:C10029T, ORF1ab:C10198T, ORF1ab:C10954T, ORF1ab:A11117G, ORF1ab:C12789T, ORF1ab:C14408T, ORF1ab:C15212T, ORF1ab:G18028T, ORF1ab:T19839C, ORF1ab:C21306T, S:C22995A, S:A23403G, S:C23604A, S:A23756G, N:G28881A, N:G28882A, N:G28883C, N:C29197T, ORF10:C29666A, 3'UTR:G29751C,	13	N:R203K, N:G204R, ORF1a:P959S, ORF1a:T3255I, ORF1a:I3618V, ORF1a:T4175I, ORF1b:P314L, ORF1b:T582I, ORF1b:A1521S, S:T478K, S:D614G, S:P681H, S:T732A,
hCoV-19/Mexico/GUA-InDRE_F10364_S660/2021	EPI_ISL_1359075	20B	B.1.1.222	20	5'UTR:C241T, ORF1ab:T1169C, ORF1ab:C3037T, ORF1ab:G3122T, ORF1ab:C14408T, ORF1ab:C14491T, ORF1ab:T19839C, ORF1ab:C20759T, S:G21641T, S:A23403G, S:A23756G, S:G23868T, S:T24469C, ORF3a:G25644T, ORF3a:G26062A, ORF8:T27900C, N:G28881A, N:G28882A, N:G28883C, 3'UTR:G29736T, 3'UTR:C29784T,	12	N:R203K, N:G204R, ORF1a:S302P, ORF1a:D953Y, ORF1b:P314L, ORF1b:A2431V, ORF3a:G224S, ORF8:F3L, S:A27S, S:D614G, S:T732A, S:G769V,
hCoV-19/Mexico/GUA-InDRE_F10366_S661/2021	EPI_ISL_1359076	20A	B.1	24	5'UTR:C241T, ORF1ab:C1288T, ORF1ab:A1389C, ORF1ab:C2445T, ORF1ab:G2782T, ORF1ab:C3037T, ORF1ab:C3096T, ORF1ab:C5746T, ORF1ab:A5999G, ORF1ab:T6631C, ORF1ab:A13625G, ORF1ab:C14408T, ORF1ab:G16206T, ORF1ab:C18060T, ORF1ab:A19387G, ORF1ab:C19718T, ORF1ab:T19791G, ORF1ab:A20268G, S:A23403G, ORF3a:G25437T, ORF3a:G25555T, N:G28280T, N:C28854T, N:G28899T, N:G29402T,	16	N:D3Y, N:S194L, N:R209I, N:D377Y, ORF1a:N375T, ORF1a:T727I, ORF1a:S944L, ORF1a:I1912V, ORF1b:D53G, ORF1b:P314L, ORF1b:E913D, ORF1b:S1974G, ORF1b:T2084I, ORF3a:L15F, ORF3a:V55F, S:D614G,
hCoV-19/Mexico/GUA-InDRE_F10367_S637/2021	EPI_ISL_1340616	20B	B.1.1.519	21	5'UTR:C203T, 5'UTR:C222T, 5'UTR:C241T, ORF1ab:C3037T, ORF1ab:C3140T, ORF1ab:C10029T, ORF1ab:C10954T, ORF1ab:A11117G, ORF1ab:C12789T, ORF1ab:C14408T, ORF1ab:T19839C, ORF1ab:C21306T, S:C22995A, S:A23403G, S:C23604A, S:A23756G, N:G28560A, N:G28881A, N:G28882A, N:G28883C, N:C29197T, ORF10:C29625T,	13	N:G96D, N:R203K, N:G204R, ORF1a:P959S, ORF1a:T3255I, ORF1a:I3618V, ORF1a:T4175I, ORF1b:P314L, ORF9b:V93M, S:T478K, S:D614G, S:P681H, S:T732A,
hCoV-19/Mexico/GUA-InDRE_F10376_S662/2021	EPI_ISL_1359077	20B	B.1.1.519	25	5'UTR:C203T, 5'UTR:C222T, 5'UTR:C241T, ORF1ab:C3037T, ORF1ab:C3140T, ORF1ab:T4111G, ORF1ab:C7162T, ORF1ab:C10029T, ORF1ab:C10954T, ORF1ab:A11117G, ORF1ab:C14408T, ORF1ab:G17944T, ORF1ab:T19839C, ORF1ab:C21306T, ORF1ab:T21384C, S:C22995A, S:A23403G, S:C23604A, S:A23756G, ORF3a:C25528T, N:G28812T, N:G28881A, N:G28882A, N:G28883C, N:C28887T, N:C29197T,	15	N:S180I, N:R203K, N:G204R, N:T205I, ORF1a:P959S, ORF1a:D1282E, ORF1a:T3255I, ORF1a:I3618V, ORF1b:P314L, ORF1b:V1493L, ORF3a:L46F, S:T478K, S:D614G, S:P681H, S:T732A,

hCoV-19/Mexico/GUA-InDRE_F10377_S663/2021	EPI_ISL_1359078	20A	B.1.561	19	5'UTR:C222T, 5'UTR:C241T, ORF1ab:C527T, ORF1ab:C3037T, ORF1ab:A4636G, ORF1ab:A5999G, ORF1ab:A10323G, ORF1ab:C10798T, ORF1ab:C11866T, ORF1ab:C14408T, ORF1ab:G16917T, ORF1ab:C18693T, ORF1ab:A20268G, S:C21614T, S:A23403G, S:C24378T, ORF3a:T25577C, N:C28854T, N:G28975T. N:C29144T.	9	N:S194L, N:M234I, ORF1a:I1912V, ORF1a:K3353R, ORF1b:P314L, ORF3a:I62T, S:L18F, S:D614G, S:S939F,
hCoV-19/Mexico/GUA-InDRE_F10378_S664/2021	EPI_ISL_1359079	20B	B.1.1	22	5'UTR:C190T, 5'UTR:C241T, ORF1ab:A2761G, ORF1ab:G3004T, ORF1ab:C3037T, ORF1ab:C3990T, ORF1ab:C8299T, ORF1ab:C14408T, ORF1ab:G17790T, ORF1ab:T19839C, S:C22792T, S:A23403G, S:G23426A, S:C23604A, S:C25096T, ORF3a:G25522A, ORF3a:T25580C, ORF3a:G25641T, ORF7a:C27697A, ORF7b:G27890A, N:G28881A, N:G28882A, N:G28883C,	13	N:R203K, N:G204R, ORF1a:E913D, ORF1a:T1242I, ORF1b:P314L, ORF1b:Q1441H, ORF3a:G44R, ORF3a:I63T, ORF3a:L83F, ORF7a:L102I, S:D614G, S:V622I, S:P681H,
hCoV-19/Mexico/GUA-InDRE_F10379_S638/2021	EPI_ISL_1340647	20B	B.1.1.519	25	5'UTR:T201C, 5'UTR:C203T, 5'UTR:C222T, 5'UTR:C241T, ORF1ab:C936T, ORF1ab:G1738T, ORF1ab:C3037T, ORF1ab:G3122T, ORF1ab:C3140T, ORF1ab:C10029T, ORF1ab:C10954T, ORF1ab:A11117G, ORF1ab:C12789T, ORF1ab:C14408T, ORF1ab:T19839C, ORF1ab:A19974G, ORF1ab:C21306T, S:C22995A, S:A23403G, S:C23604A, S:A23756G, N:G28881A, N:G28882A, N:G28883C, N:C29197T, N:G29227T,	13	N:R203K, N:G204R, ORF1a:T224I, ORF1a:D953Y, ORF1a:P959S, ORF1a:T3255I, ORF1a:I3618V, ORF1a:T4175I, ORF1b:P314L, S:T478K, S:D614G, S:P681H, S:T732A,
hCoV-19/Mexico/GUA-InDRE_F10380_S639/2021	EPI_ISL_1340648	20B	B.1.1.519	30	5'UTR:T201C, 5'UTR:C203T, 5'UTR:C222T, 5'UTR:C241T, ORF1ab:C1684T, ORF1ab:G1738T, ORF1ab:C2973T, ORF1ab:C3037T, ORF1ab:C3140T, ORF1ab:G9388A, ORF1ab:C10029T, ORF1ab:C10954T, ORF1ab:A11117G, ORF1ab:A12088G, ORF1ab:C12789T, ORF1ab:C14408T, ORF1ab:T19839C, ORF1ab:A19974G, ORF1ab:C21306T, S:C22995A, S:A23403G, S:C23604A, S:A23756G, ORF3a:C25484T, ORF7a:G27441T, N:G28881A, N:G28882A, N:G28883C, N:C29197T, N:G29315C, N:C29546T	15	N:R203K, N:G204R, N:D348H, ORF1a:A903V, ORF1a:P959S, ORF1a:T3255I, ORF1a:I3618V, ORF1a:T4175I, ORF1b:P314L, ORF3a:A31V, ORF7a:E16D, S:T478K, S:D614G, S:P681H, S:T732A,
hCoV-19/Mexico/GUA-InDRE_F10381_S640/2021	EPI_ISL_1340650	20B	B.1.1.519	20	5'UTR:C203T, 5'UTR:C222T, 5'UTR:C241T, ORF1ab:C3037T, ORF1ab:C3140T, ORF1ab:G8581A, ORF1ab:C10029T, ORF1ab:C10954T, ORF1ab:A11117G, ORF1ab:C12789T, ORF1ab:C14408T, ORF1ab:T19839C, ORF1ab:C21306T, S:C22995A, S:A23403G, S:C23604A, S:A23756G, N:G28881A, N:G28882A, N:G28883C. N:C29197T.	11	N:R203K, N:G204R, ORF1a:P959S, ORF1a:T3255I, ORF1a:I3618V, ORF1a:T4175I, ORF1b:P314L, S:T478K, S:D614G, S:P681H, S:T732A,
hCoV-19/Mexico/GUA-InDRE_F10382_S641/2021	EPI_ISL_1340631	20B	B.1.1.519	29	5'UTR:T201C, 5'UTR:C203T, 5'UTR:C222T, 5'UTR:C241T, ORF1ab:C936T, ORF1ab:G1738T, ORF1ab:C3037T, ORF1ab:C3140T, ORF1ab:C6395T, ORF1ab:C9112T, ORF1ab:C10029T, ORF1ab:G10523C, ORF1ab:C10954T, ORF1ab:A11117G, ORF1ab:C12789T, ORF1ab:C14408T, ORF1ab:T19839C, ORF1ab:A19974G, ORF1ab:C21306T, S:C22995A, S:A23403G, S:C23604A, S:A23756G, S:T24529C, ORF3a:C25824A, N:G28881A, N:G28882A, N:G28883C, N:C29197T, N:G29227T,	14	N:R203K, N:G204R, ORF1a:T224I, ORF1a:P959S, ORF1a:T3255I, ORF1a:V3420L, ORF1a:I3618V, ORF1a:T4175I, ORF1b:P314L, ORF3a:N144K, S:T478K, S:D614G, S:P681H, S:T732A,
hCoV-19/Mexico/GUA-InDRE_F10383_S665/2021	EPI_ISL_1359080	20B	B.1.1.222	14	5'UTR:C241T, ORF1ab:C3037T, ORF1ab:C10029T, ORF1ab:C14408T, ORF1ab:T19839C, S:A23403G, S:A23756G, M:C26985T, ORF7b:A27756G, ORF8:A27921G, ORF8:G28001T, ORF8:G28086T, N:G28881A, N:G28882A, N:G28883C,	10	M:H155Y, N:R203K, N:G204R, ORF1a:T3255I, ORF1b:P314L, ORF7b:M1V, ORF8:I10V, ORF8:A65S, S:D614G, S:T732A,

hCoV-19/Mexico/GUA-InDRE_F10384_S666/2021	EPI_ISL_1359081	20B	B.1.1.222	18	5'UTR:C241T, ORF1ab:C829T, ORF1ab:A2207G, ORF1ab:T2533C, ORF1ab:C3037T, ORF1ab:C6582T, ORF1ab:C8175T, ORF1ab:C14408T, ORF1ab:T19839C, ORF1ab:C20703T, S:A23403G, S:A23756G, S:C25156T, ORF3a:C25603T, ORF8:C27920T, N:G28655T, N:G28881A, N:G28882A, N:G28883C,	9	N:D128Y, N:R203K, N:G204R, ORF1a:I648V, ORF1a:T2106I, ORF1a:A2637V, ORF1b:P314L, S:D614G, S:T732A,
hCoV-19/Mexico/GUA-InDRE_F10385_S667/2021	EPI_ISL_1359082	20B	B.1.1.519	24	5'UTR:C203T, 5'UTR:C222T, 5'UTR:C241T, ORF1ab:A1236G, ORF1ab:C3037T, ORF1ab:C3140T, ORF1ab:C10029T, ORF1ab:C10954T, ORF1ab:A11117G, ORF1ab:C14408T, ORF1ab:T19839C, ORF1ab:C21306T, S:C22995A, S:A23403G, S:C23604A, S:A23756G, S:G25217T, ORF8:T27904C, N:A28877T, N:G28878C, N:G28881A, N:G28882A, N:G28883C, N:C29197T, 3'UTR:C29722T,	13	N:R203K, N:G204R, ORF1a:D324G, ORF1a:P959S, ORF1a:T3255I, ORF1a:I3618V, ORF1b:P314L, ORF8:L4P, S:T478K, S:D614G, S:P681H, S:T732A, S:G1219C,
hCoV-19/Mexico/GUA-InDRE_F10386_S668/2021	EPI_ISL_1359083	20B	B.1.1.222	25	5'UTR:C241T, ORF1ab:C3037T, ORF1ab:G3403T, ORF1ab:A4501G, ORF1ab:T5218C, ORF1ab:A12498G, ORF1ab:C12525T, ORF1ab:C14408T, ORF1ab:C14790T, ORF1ab:T19839C, S:A23403G, S:C23673T, S:T23689C, S:A23756G, S:G24794T, S:C25047T, ORF3a:G26153T, M:G26526T, ORF6:C27294T, N:G28300T, N:C28399T, N:G28881A, N:G28882A, N:G28883C, N:G28975T, N:G29449T,	16	M:A2S, N:Q9H, N:R203K, N:G204R, N:M234I, ORF1a:N4078S, ORF1a:T4087I, ORF1b:P314L, ORF3a:G254V, ORF9b:S6I, ORF9b:P39L, S:D614G, S:S704L, S:T732A, S:A1078S, S:P1162L,
hCoV-19/Mexico/GUA-InDRE_F10387_S642/2021	EPI_ISL_1340633	20B	B.1.1.519	29	5'UTR:T201C, 5'UTR:C203T, 5'UTR:C222T, 5'UTR:C241T, ORF1ab:C936T, ORF1ab:G1738T, ORF1ab:A2491G, ORF1ab:C3037T, ORF1ab:G3122T, ORF1ab:C3140T, ORF1ab:C10029T, ORF1ab:C10954T, ORF1ab:A11117G, ORF1ab:C12789T, ORF1ab:C14408T, ORF1ab:T19527C, ORF1ab:T19839C, ORF1ab:A19974G, ORF1ab:C21306T, S:C21575T, S:C22995A, S:A23403G, S:C23604A, S:A23756G, N:G28881A, N:G28882A, N:G28883C, N:C29197T, N:G29227T, ORF10:C29659T,	14	N:R203K, N:G204R, ORF1a:T224I, ORF1a:D953Y, ORF1a:P959S, ORF1a:T3255I, ORF1a:I3618V, ORF1a:T4175I, ORF1b:P314L, S:L5F, S:T478K, S:D614G, S:P681H, S:T732A,
hCoV-19/Mexico/GUA-InDRE_F10388_S669/2021	EPI_ISL_1359084	20A	B.1	21	5'UTR:C241T, ORF1ab:C274T, ORF1ab:G542A, ORF1ab:G806A, ORF1ab:C3037T, ORF1ab:C5140A, ORF1ab:C6027T, ORF1ab:A8413G, ORF1ab:C8441A, ORF1ab:C9943T, ORF1ab:A10323G, ORF1ab:C11663T, ORF1ab:C14408T, ORF1ab:C18486T, ORF1ab:T19866C, ORF1ab:A20268G, S:G23120T, S:A23403G, S:G23587C, E:C26455T, N:C28854T, N:G29543T,	12	E:P71S, N:S194L, ORF1a:E93K, ORF1a:A181T, ORF1a:D1625E, ORF1a:P1921L, ORF1a:K3353R, ORF1a:L3800F, ORF1b:P314L, S:A520S, S:D614G, S:Q675H,
hCoV-19/Mexico/GUA-InDRE_F10389_S670/2021	EPI_ISL_1359085	20A	B.1.243	19	5'UTR:C241T, ORF1ab:C601T, ORF1ab:C3037T, ORF1ab:A10767G, ORF1ab:G11048T, ORF1ab:C14408T, ORF1ab:G17320T, ORF1ab:C19097T, ORF1ab:A19137G, ORF1ab:A20268G, ORF1ab:C21145T, S:C21911T, S:A23403G, S:C23604A, S:T24076C, ORF3a:C25469T, N:G28280T, N:C28854T, N:C29555T, 3'UTR:T29710C,	11	N:D3Y, N:S194L, ORF1a:N3501S, ORF1a:V3595F, ORF1b:P314L, ORF1b:A1285S, ORF1b:A1877V, ORF1b:L2560F, ORF3a:S26L, S:D614G, S:P681H,
hCoV-19/Mexico/GUA-InDRE_F10390_S589/2021	EPI_ISL_1337367	21C (Epsilon)	B.1.429	26	5'UTR:C66T, 5'UTR:C241T, ORF1ab:C1059T, ORF1ab:C2395T, ORF1ab:T2597C, ORF1ab:C3037T, ORF1ab:C7528T, ORF1ab:C8947T, ORF1ab:C12043T, ORF1ab:C12100T, ORF1ab:A12878G, ORF1ab:C14408T, ORF1ab:G14874T, ORF1ab:G17014T, ORF1ab:C20462T, S:G21600T, S:G22018T, S:T22917G, S:A23403G, S:T24349C, ORF3a:G25563T, ORF3a:C26044G, M:C26681T, ORF7b:G27890T, ORF8:A28272T, N:C28887T, N:C29362T,	13	N:T205I, ORF1a:T265I, ORF1a:I4205V, ORF1b:P314L, ORF1b:K469N, ORF1b:D1183Y, ORF1b:T2332I, ORF3a:Q57H, ORF3a:Q218E, S:S13I, S:W152C, S:L452R, S:D614G,

hCoV-19/Mexico/GUA-InDRE_F10391_S643/2021	EPI_ISL_1340627	20B	B.1.1.519	30	5'UTR:T201C, 5'UTR:C203T, 5'UTR:C222T, 5'UTR:C241T, ORF1ab:G376A, ORF1ab:G1738T, ORF1ab:C3037T, ORF1ab:C3140T, ORF1ab:C3602T, ORF1ab:G4255T, ORF1ab:C7504T, ORF1ab:C7749T, ORF1ab:C10029T, ORF1ab:C10954T, ORF1ab:A11117G, ORF1ab:C12789T, ORF1ab:C14408T, ORF1ab:T15427C, ORF1ab:T19839C, ORF1ab:A19974G, ORF1ab:C21306T, S:C22995A, S:A23403G, S:C23604A, S:A23756G, ORF3a:G25440T, M:G26951T, N:G28881A, N:G28882A, N:G28883C, N:C29197T,	14	N:R203K, N:G204R, ORF1a:P959S, ORF1a:H1113Y, ORF1a:T2495I, ORF1a:T3255I, ORF1a:I3618V, ORF1a:T4175I, ORF1b:P314L, ORF3a:K16N, S:T478K, S:D614G, S:P681H, S:T732A,
hCoV-19/Mexico/GUA-InDRE_F10392_S671/2021	EPI_ISL_1359086	20B	B.1.1.519	21	5'UTR:C203T, 5'UTR:C222T, 5'UTR:C241T, ORF1ab:A2317G, ORF1ab:C3037T, ORF1ab:C3140T, ORF1ab:G5018T, ORF1ab:C10029T, ORF1ab:C10954T, ORF1ab:A11117G, ORF1ab:C14408T, ORF1ab:T19839C, ORF1ab:A20207G, ORF1ab:C21306T, S:C22995A, S:A23403G, S:C23604A, S:A23756G, N:G28881A, N:G28882A, N:G28883C, N:C29197T,	12	N:R203K, N:G204R, ORF1a:P959S, ORF1a:D1585Y, ORF1a:T3255I, ORF1a:I3618V, ORF1b:P314L, ORF1b:Q2247R, S:T478K, S:D614G, S:P681H, S:T732A,
hCoV-19/Mexico/GUA-InDRE_F10393_S672/2021	EPI_ISL_1359087	20B	B.1.1.222	25	5'UTR:C241T, ORF1ab:C3037T, ORF1ab:G3403T, ORF1ab:A4501G, ORF1ab:T5218C, ORF1ab:A12498G, ORF1ab:C12525T, ORF1ab:C14408T, ORF1ab:C14790T, ORF1ab:T19839C, S:A23403G, S:C23673T, S:T23689C, S:A23756G, S:G24794T, S:C25047T, ORF3a:G26153T, M:G26526T, ORF6:C27294T, N:G28300T, N:C28399T, N:G28881A, N:G28882A, N:G28883C, N:G28975T, N:G29449T,	16	M:A2S, N:Q9H, N:R203K, N:G204R, N:M234I, ORF1a:N4078S, ORF1a:T4087I, ORF1b:P314L, ORF3a:G254V, ORF9b:S6I, ORF9b:P39L, S:D614G, S:S704L, S:T732A, S:A1078S, S:P1162L,
hCoV-19/Mexico/GUA-InDRE_F10395_S644/2021	EPI_ISL_1340617	20B	B.1.1.519	20	5'UTR:C203T, 5'UTR:C222T, 5'UTR:C241T, ORF1ab:C3037T, ORF1ab:C3140T, ORF1ab:G3549A, ORF1ab:C10029T, ORF1ab:C10954T, ORF1ab:A11117G, ORF1ab:C12789T, ORF1ab:C14408T, ORF1ab:T19839C, ORF1ab:C21306T, S:C22995A, S:A23403G, S:C23604A, S:A23756G, N:G28881A, N:G28882A, N:G28883C, N:C29197T,	12	N:R203K, N:G204R, ORF1a:P959S, ORF1a:G1095E, ORF1a:T3255I, ORF1a:I3618V, ORF1a:T4175I, ORF1b:P314L, S:T478K, S:D614G, S:P681H, S:T732A,
hCoV-19/Mexico/GUA-InDRE_F10816_S751/2021	EPI_ISL_1366700	20B	B.1.1.519	21	5'UTR:C203T, 5'UTR:C222T, 5'UTR:C241T, ORF1ab:C3037T, ORF1ab:C3140T, ORF1ab:A9409G, ORF1ab:C10029T, ORF1ab:C10954T, ORF1ab:A11117G, ORF1ab:C12789T, ORF1ab:C14408T, ORF1ab:T19839C, ORF1ab:C21306T, S:C22995A, S:A23403G, S:C23604A, S:A23756G, N:G28881A, N:G28882A, N:G28883C, N:C29197T, N:G29527T,	12	N:R203K, N:G204R, N:Q418H, ORF1a:P959S, ORF1a:T3255I, ORF1a:I3618V, ORF1a:T4175I, ORF1b:P314L, S:T478K, S:D614G, S:P681H, S:T732A,
hCoV-19/Mexico/GUA-InDRE_F10817_S710/2021	EPI_ISL_1365675	20C	B.1	24	5'UTR:C241T, ORF1ab:C1059T, ORF1ab:C1593T, ORF1ab:A2131T, ORF1ab:A2437G, ORF1ab:G2599T, ORF1ab:C3037T, ORF1ab:T3049C, ORF1ab:C3927T, ORF1ab:A3963G, ORF1ab:G7756T, ORF1ab:C7764T, ORF1ab:C8281T, ORF1ab:C14408T, ORF1ab:C14724T, ORF1ab:C18167T, S:G23311A, S:A23403G, S:G25340T, ORF3a:G25563T, ORF3a:C25844T, ORF8:A27918G, N:C28866T, N:G29227T, N:G29374A,	16	N:T198I, ORF1a:T265I, ORF1a:S443F, ORF1a:E622D, ORF1a:L778F, ORF1a:S1221L, ORF1a:K1233R, ORF1a:K2497N, ORF1a:S2500F, ORF1b:P314L, ORF1b:P1567L, ORF3a:Q57H, ORF3a:T151I, ORF8:I9V, S:D614G, S:D1260Y,
hCoV-19/Mexico/GUA-InDRE_F10818_S804/2021	EPI_ISL_1400349	20B	B.1.1.222	20	5'UTR:C241T, ORF1ab:C3037T, ORF1ab:A6693G, ORF1ab:T7402C, ORF1ab:A8848C, ORF1ab:C9430T, ORF1ab:C10029T, ORF1ab:C10039T, ORF1ab:C13818T, ORF1ab:C14408T, ORF1ab:G19009A, ORF1ab:T19839C, S:A23403G, S:A23756G, ORF3a:G25912T, ORF7b:A27756G, ORF8:A27921G, ORF8:G28001T, N:G28881A, N:G28882A, N:G28883C,	12	N:R203K, N:G204R, ORF1a:K2143R, ORF1a:E2861D, ORF1a:T3255I, ORF1b:P314L, ORF1b:D1848N, ORF3a:G174C, ORF7b:M1V, ORF8:I10V, S:D614G, S:T732A,

hCoV-19/Mexico/GUA-InDRE_F10819_S803/2021	EPI_ISL_1400348	20B	B.1.1.222	20	5'UTR:C241T, ORF1ab:C3037T, ORF1ab:C4897T, ORF1ab:C10029T, ORF1ab:A11430G, ORF1ab:C12781T, ORF1ab:C14408T, ORF1ab:G17658T, ORF1ab:C19011A, ORF1ab:T19839C, ORF1ab:A21137G, ORF1ab:G21255T, S:A23403G, S:A23756G, ORF3a:G25912T, ORF3a:A26115C, ORF8:A27921G, ORF8:G28001T, N:G28881A, N:G28882A, N:G28883C,	13	N:R203K, N:G204R, ORF1a:T3255I, ORF1a:Y3722C, ORF1b:P314L, ORF1b:M1397I, ORF1b:D1848E, ORF1b:K2557R, ORF3a:G174C, ORF3a:E241D, ORF8:I10V, S:D614G, S:T732A,
hCoV-19/Mexico/GUA-InDRE_F10820_S802/2021	EPI_ISL_1400347	20B	B.1.1.519	28	5'UTR:T201C, 5'UTR:C203T, 5'UTR:C222T, 5'UTR:C241T, ORF1ab:G1738T, ORF1ab:C3037T, ORF1ab:C3140T, ORF1ab:C5183T, ORF1ab:C10029T, ORF1ab:C10954T, ORF1ab:A11117G, ORF1ab:C12789T, ORF1ab:C14408T, ORF1ab:T16584C, ORF1ab:C17502T, ORF1ab:T19839C, ORF1ab:A19974G, ORF1ab:C21306T, S:G22203T, S:C22995A, S:A23403G, S:C23604A, S:A23756G, ORF7a:A27726G, N:G28881A, N:G28882A, N:G28883C, N:C29095T, N:C29197T,	13	N:R203K, N:G204R, ORF1a:P959S, ORF1a:P1640S, ORF1a:T3255I, ORF1a:I3618V, ORF1a:T4175I, ORF1b:P314L, S:R214L, S:T478K, S:D614G, S:P681H, S:T732A,
hCoV-19/Mexico/GUA-InDRE_F10821_S801/2021	EPI_ISL_1400346	20B	B.1.1.519	23	5'UTR:C203T, 5'UTR:C222T, 5'UTR:C241T, ORF1ab:C3037T, ORF1ab:C3140T, ORF1ab:C10029T, ORF1ab:C10954T, ORF1ab:A11117G, ORF1ab:G11245T, ORF1ab:C11866T, ORF1ab:C12789T, ORF1ab:C14408T, ORF1ab:G15921T, ORF1ab:T19839C, ORF1ab:C21306T, S:C22995A, S:A23403G, S:C23604A, S:A23756G, ORF3a:G25784T, N:G28881A, N:G28882A, N:G28883C, N:C29197T,	12	N:R203K, N:G204R, ORF1a:P959S, ORF1a:T3255I, ORF1a:I3618V, ORF1a:T4175I, ORF1b:P314L, ORF3a:W131L, S:T478K, S:D614G, S:P681H, S:T732A,
hCoV-19/Mexico/GUA-InDRE_F10822_S603/2021	EPI_ISL_1337387	21C (Epsilon)	B.1.429	25	5'UTR:C241T, ORF1ab:C1059T, ORF1ab:C2395T, ORF1ab:T2597C, ORF1ab:C3037T, ORF1ab:C8316T, ORF1ab:T8649G, ORF1ab:C8947T, ORF1ab:G11029T, ORF1ab:C12100T, ORF1ab:A12878G, ORF1ab:C14408T, ORF1ab:G17014T, S:G21600T, S:G22018T, S:T22917G, S:C22987T, S:A23403G, S:T24349C, S:C24904T, ORF3a:G25563T, M:C26681T, ORF7b:G27890T, ORF8:A28272T, N:C28887T, N:C29362T,	13	N:T205I, ORF1a:T265I, ORF1a:T2684I, ORF1a:V2795G, ORF1a:L3588F, ORF1a:I4205V, ORF1b:P314L, ORF1b:D1183Y, ORF3a:Q57H, S:S13I, S:W152C, S:L452R, S:D614G,
hCoV-19/Mexico/GUA-InDRE_F10823_S800/2021	EPI_ISL_1400345	20B	B.1.1.519	22	5'UTR:C203T, 5'UTR:C222T, 5'UTR:C241T, ORF1ab:C3037T, ORF1ab:C3140T, ORF1ab:G3242T, ORF1ab:C10029T, ORF1ab:C10954T, ORF1ab:A11117G, ORF1ab:C12789T, ORF1ab:C14408T, ORF1ab:T19839C, ORF1ab:C21306T, S:C22995A, S:A23403G, S:C23604A, S:A23756G, ORF3a:C25703T, N:G28881A, N:G28882A, N:G28883C, N:C29197T, N:G29527T,	14	N:R203K, N:G204R, N:Q418H, ORF1a:P959S, ORF1a:G993C, ORF1a:T3255I, ORF1a:I3618V, ORF1a:T4175I, ORF1b:P314L, ORF3a:P104L, S:T478K, S:D614G, S:P681H, S:T732A,
hCoV-19/Mexico/GUA-InDRE_F10824_S799/2021	EPI_ISL_1400344	20B	B.1.1.519	25	5'UTR:C203T, 5'UTR:C222T, 5'UTR:C241T, ORF1ab:T1227C, ORF1ab:C3037T, ORF1ab:C3140T, ORF1ab:C10029T, ORF1ab:C10954T, ORF1ab:A11117G, ORF1ab:C12789T, ORF1ab:C14408T, ORF1ab:G16206T, ORF1ab:C17172T, ORF1ab:T19839C, ORF1ab:C21306T, S:C22995A, S:A23403G, S:C23604A, S:A23756G, S:G23873T, S:A25108C, N:G28881A, N:G28882A, N:G28883C, N:G28904T, N:C29197T,	16	N:R203K, N:G204R, N:A211S, ORF1a:M321T, ORF1a:P959S, ORF1a:T3255I, ORF1a:I3618V, ORF1a:T4175I, ORF1b:P314L, ORF1b:E913D, S:T478K, S:D614G, S:P681H, S:T732A, S:A771S, S:E1182D,

hCoV-19/Mexico/GUA-InDRE_F10825_S798/2021	EPI_ISL_1400343	20B	B.1.1.519	29	5'UTR:C203T, 5'UTR:C222T, 5'UTR:C241T, ORF1ab:C1342T, ORF1ab:C3037T, ORF1ab:C3140T, ORF1ab:G3692T, ORF1ab:G3871T, ORF1ab:C4423T, ORF1ab:C10029T, ORF1ab:C10954T, ORF1ab:A11117G, ORF1ab:C11824A, ORF1ab:C12789T, ORF1ab:C14408T, ORF1ab:C16017T, ORF1ab:T19839C, ORF1ab:C21306T, S:C22995A, S:A23403G, S:C23604A, S:A23756G, ORF3a:G25855T, ORF8:T27904C, N:C28724T, N:G28881A, N:G28882A, N:G28883C, N:C29197T, N:G29254T,	16	N:P151S, N:R203K, N:G204R, ORF1a:P959S, ORF1a:V1143F, ORF1a:K1202N, ORF1a:T3255I, ORF1a:I3618V, ORF1a:T4175I, ORF1b:P314L, ORF3a:D155Y, ORF8:L4P, S:T478K, S:D614G, S:P681H, S:T732A,
hCoV-19/Mexico/GUA-InDRE_F10826_S894/2021	EPI_ISL_1405915	20A	B.1	14	5'UTR:C241T, ORF1ab:C1824T, ORF1ab:C3037T, ORF1ab:C3096T, ORF1ab:C7299T, ORF1ab:C10456T, ORF1ab:A11595G, ORF1ab:C14408T, ORF1ab:C18796T, ORF1ab:A20268G, ORF1ab:C20628T, S:A23403G, ORF3a:C26060T, N:C28854T. 3'UTR:G29810T.	8	N:S194L, ORF1a:A520V, ORF1a:S944L, ORF1a:A2345V, ORF1a:Q3777R, ORF1b:P314L, ORF3a:T223I, S:D614G,
hCoV-19/Mexico/GUA-InDRE_F10827_S797/2021	EPI_ISL_1400342	20B	B.1.1.519	33	5'UTR:T201C, 5'UTR:C203T, 5'UTR:C222T, 5'UTR:C241T, ORF1ab:C829T, ORF1ab:C936T, ORF1ab:G1738T, ORF1ab:C3037T, ORF1ab:C3140T, ORF1ab:C9112T, ORF1ab:C10029T, ORF1ab:G10523C, ORF1ab:G10870T, ORF1ab:C10954T, ORF1ab:A11117G, ORF1ab:C12789T, ORF1ab:C14408T, ORF1ab:C18877T, ORF1ab:T19839C, ORF1ab:A19974G, ORF1ab:C21306T, S:G22051T, S:T22822C, S:C22995A, S:A23403G, S:C23604A, S:A23756G, S:C24502T, S:C25276T, N:G28881A, N:G28882A, N:G28883C, N:C29197T, N:G29227T,	13	N:R203K, N:G204R, ORF1a:T224I, ORF1a:P959S, ORF1a:T3255I, ORF1a:V3420L, ORF1a:I3618V, ORF1a:T4175I, ORF1b:P314L, S:T478K, S:D614G, S:P681H, S:T732A,
hCoV-19/Mexico/GUA-InDRE_F10829_S796/2021	EPI_ISL_1400341	20B	B.1.1.519	23	5'UTR:C203T, 5'UTR:C222T, 5'UTR:C241T, ORF1ab:C1191T, ORF1ab:C3037T, ORF1ab:C3140T, ORF1ab:C3444T, ORF1ab:C10029T, ORF1ab:C10954T, ORF1ab:A11117G, ORF1ab:C12789T, ORF1ab:C14408T, ORF1ab:G17058T, ORF1ab:T19839C, ORF1ab:C21306T, S:C22995A, S:A23403G, S:C23604A, S:A23756G, ORF3a:C25460T, N:G28881A. N:G28882A. N:G28883C. N:C29197T.	15	N:R203K, N:G204R, ORF1a:P309L, ORF1a:P959S, ORF1a:A1060V, ORF1a:T3255I, ORF1a:I3618V, ORF1a:T4175I, ORF1b:P314L, ORF1b:M1197I, ORF3a:A23V, S:T478K, S:D614G, S:P681H, S:T732A,
hCoV-19/Mexico/GUA-InDRE_F10830_S602/2021	EPI_ISL_1337386	21C (Epsilon)	B.1.429	28	5'UTR:C241T, ORF1ab:C1059T, ORF1ab:C1427T, ORF1ab:C2395T, ORF1ab:T2597C, ORF1ab:C3037T, ORF1ab:C8947T, ORF1ab:C12100T, ORF1ab:A12878G, ORF1ab:C14408T, ORF1ab:G17014T, ORF1ab:C18877T, S:G21600T, S:G22018T, S:T22917G, S:T23155C, S:A23403G, S:T24349C, ORF3a:G25563T, M:C26681T, ORF7b:G27758A, ORF7b:T27760A, ORF7b:G27890T, ORF8:G28191T, ORF8:A28272T, N:G28628T, N:C28887T, N:G28975T, N:C29362T,	16	N:A119S, N:T205I, N:M234I, ORF1a:T265I, ORF1a:H388Y, ORF1a:I4205V, ORF1b:P314L, ORF1b:D1183Y, ORF3a:Q57H, ORF7b:M1I, ORF7b:I2N, ORF8:V100L, S:S13I, S:W152C, S:L452R, S:D614G,
hCoV-19/Mexico/GUA-InDRE_F10831_S795/2021	EPI_ISL_1400340	20B	B.1.1.222	16	5'UTR:C241T, ORF1ab:C3037T, ORF1ab:C6285T, ORF1ab:G6374T, ORF1ab:C11514T, ORF1ab:A13498G, ORF1ab:C14408T, ORF1ab:C19263T, ORF1ab:T19839C, ORF1ab:C20474T, S:A23403G, S:T23596C, S:A23756G, M:C27119T, N:G28881A, N:G28882A, N:G28883C.	10	N:R203K, N:G204R, ORF1a:T2007I, ORF1a:D2037Y, ORF1a:T3750I, ORF1b:T11A, ORF1b:P314L, ORF1b:T2336I, S:D614G, S:T732A,
hCoV-19/Mexico/GUA-InDRE_F10832_S893/2021	EPI_ISL_1405914	20A	B.1	19	5'UTR:C241T, ORF1ab:C2340A, ORF1ab:C3037T, ORF1ab:C4563T, ORF1ab:G4712A, ORF1ab:T6667C, ORF1ab:C11020T, ORF1ab:C14408T, ORF1ab:G14712T, ORF1ab:C18555T, ORF1ab:C19151T, ORF1ab:A20268G, ORF1ab:A21137G, S:C21575T, S:G21800T, S:G23120T, S:A23403G, M:C26549T, N:G28373T, N:C28854T.	12	N:G34W, N:S194L, ORF1a:S692Y, ORF1a:S1433L, ORF1a:V1483I, ORF1b:P314L, ORF1b:A1895V, ORF1b:K2557R, S:L5F, S:D80Y, S:A520S, S:D614G,

hCoV-19/Mexico/GUA-InDRE_F10833_S574/2021	EPI_ISL_1334386	20I (Alpha, V1)	B.1.1.7	34	5'UTR:C241T, ORF1ab:C913T, ORF1ab:C3037T, ORF1ab:C3267T, ORF1ab:C5388A, ORF1ab:C5986T, ORF1ab:T6954C, ORF1ab:C9693T, ORF1ab:C14408T, ORF1ab:C14676T, ORF1ab:C15279T, ORF1ab:T16176C, S:C22716A, S:T22717C, S:T23042C, S:A23063T, S:C23271A, S:A23403G, S:C23604A, S:C23709T, S:T24506G, S:G24914C, S:G25218T, ORF3a:C25553T, ORF7a:C27615T, ORF8:C27972T, ORF8:G28048T, ORF8:A28111G, N:G28280C, N:A28281T, N:T28282A, N:G28881A, N:G28882A, N:G28883C, N:C28977T,	23	N:D3L, N:R203K, N:G204R, N:S235F, ORF1a:T1001I, ORF1a:A1708D, ORF1a:I2230T, ORF1a:A3143V, ORF1b:P314L, ORF3a:A54V, ORF8:Q27*, ORF8:R52I, ORF8:Y73C, S:T385N, S:S494P, S:N501Y, S:A570D, S:D614G, S:P681H, S:T716I, S:S982A, S:D1118H, S:G1219V,	ORF1ab: 11288-11296, S:21765-21770, S:21992-21994, ORF8:28271,
hCoV-19/Mexico/GUA-InDRE_F10834_S794/2021	EPI_ISL_1400339	20B	B.1.1.519	24	5'UTR:C203T, 5'UTR:C222T, 5'UTR:C241T, ORF1ab:C3037T, ORF1ab:C3140T, ORF1ab:C5175T, ORF1ab:C10029T, ORF1ab:C10954T, ORF1ab:A11117G, ORF1ab:G11417T, ORF1ab:C12789T, ORF1ab:C14408T, ORF1ab:T19839C, ORF1ab:C21306T, S:G22616T, S:C22995A, S:A23403G, S:C23604A, S:A23756G, M:C26645T, ORF8:T28250C, N:G28881A, N:G28882A, N:G28883C, N:C29197T,	14	N:R203K, N:G204R, ORF1a:P959S, ORF1a:T1637I, ORF1a:T3255I, ORF1a:I3618V, ORF1a:V3718F, ORF1a:T4175I, ORF1b:P314L, S:A352S, S:T478K, S:D614G, S:P681H, S:T732A,	
hCoV-19/Mexico/GUA-InDRE_F10835_S793/2021	EPI_ISL_1400338	20B	B.1.1.519	26	5'UTR:C203T, 5'UTR:C222T, 5'UTR:C241T, ORF1ab:A1816G, ORF1ab:C1913T, ORF1ab:C2232T, ORF1ab:C3037T, ORF1ab:C3140T, ORF1ab:A3818G, ORF1ab:A9970T, ORF1ab:C10029T, ORF1ab:C10954T, ORF1ab:G11083T, ORF1ab:A11117G, ORF1ab:C12073T, ORF1ab:C12789T, ORF1ab:C14408T, ORF1ab:T19839C, ORF1ab:C21306T, S:C22995A, S:A23403G, S:C23604A, S:A23756G, N:G28881A, N:G28882A, N:G28883C, N:C29197T,	15	N:R203K, N:G204R, ORF1a:R550C, ORF1a:A656V, ORF1a:P959S, ORF1a:K1185E, ORF1a:T3255I, ORF1a:L3606F, ORF1a:I3618V, ORF1a:T4175I, ORF1b:P314L, S:T478K, S:D614G, S:P681H, S:T732A,	
hCoV-19/Mexico/GUA-InDRE_F10837_S792/2021	EPI_ISL_1400337	20B	B.1.1.519	27	5'UTR:C203T, 5'UTR:C222T, 5'UTR:C241T, ORF1ab:C3037T, ORF1ab:C3140T, ORF1ab:C4002T, ORF1ab:T8662C, ORF1ab:C10029T, ORF1ab:C10954T, ORF1ab:A11117G, ORF1ab:C12789T, ORF1ab:C14408T, ORF1ab:C18828T, ORF1ab:C19152T, ORF1ab:T19839C, ORF1ab:C21306T, S:C22995A, S:A23403G, S:C23604A, S:A23756G, ORF3a:C25731T, ORF8:T27904C, ORF8:C28087T, ORF8:C28253T, N:G28881A, N:G28882A, N:G28883C, N:C29197T	14	N:R203K, N:G204R, ORF1a:P959S, ORF1a:T1246I, ORF1a:T3255I, ORF1a:I3618V, ORF1a:T4175I, ORF1b:P314L, ORF8:L4P, ORF8:A65V, S:T478K, S:D614G, S:P681H, S:T732A,	
hCoV-19/Mexico/GUA-InDRE_F10838_S601/2021	EPI_ISL_1337384	21C (Epsilon)	B.1.429	25	5'UTR:C241T, ORF1ab:C1059T, ORF1ab:A1832G, ORF1ab:C2395T, ORF1ab:T2597C, ORF1ab:C3037T, ORF1ab:C8947T, ORF1ab:C12100T, ORF1ab:A12878G, ORF1ab:C14408T, ORF1ab:G17014T, S:G21600T, S:G22018T, S:T22917G, S:A23403G, S:G23426T, S:G24095T, S:T24349C, ORF3a:G25563T, M:C26625T, M:C26681T, ORF7a:C27406T, ORF7b:G27890T, ORF8:A28272T, N:C28887T, N:C29362T,	14	N:T205I, ORF1a:T265I, ORF1a:I523V, ORF1a:I4205V, ORF1b:P314L, ORF1b:D1183Y, ORF3a:Q57H, ORF7a:L5F, S:S13I, S:W152C, S:L452R, S:D614G, S:V622F, S:A845S,	

hCoV-19/Mexico/GUA-InDRE_F10839_S758/2021	EPI_ISL_1400304	20B	B.1.1.519	25	5'UTR:C203T, 5'UTR:C222T, 5'UTR:C241T, ORF1ab:C1722T, ORF1ab:C3037T, ORF1ab:C3140T, ORF1ab:C6665T, ORF1ab:C10029T, ORF1ab:C10954T, ORF1ab:A11117G, ORF1ab:C12025T, ORF1ab:C12789T, ORF1ab:C14408T, ORF1ab:G15921T, ORF1ab:T19839C, ORF1ab:C21306T, ORF1ab:G21468T, S:C22995A, S:A23403G, S:C23604A, S:A23756G, ORF3a:G25526T, N:G28881A, N:G28882A, N:G28883C, N:C29197T,	15	N:R203K, N:G204R, ORF1a:A486V, ORF1a:P959S, ORF1a:P2134S, ORF1a:T3255I, ORF1a:I3618V, ORF1a:T4175I, ORF1b:P314L, ORF1b:M2667I, ORF3a:W45L, S:T478K, S:D614G, S:P681H, S:T732A,
hCoV-19/Mexico/GUA-InDRE_F10840_S791/2021	EPI_ISL_1400336	20B	B.1.1.519	25	5'UTR:T201C, 5'UTR:C203T, 5'UTR:C222T, 5'UTR:C241T, ORF1ab:C745T, ORF1ab:G1738T, ORF1ab:C3037T, ORF1ab:C3140T, ORF1ab:C9532T, ORF1ab:C10029T, ORF1ab:C10954T, ORF1ab:A11117G, ORF1ab:C12789T, ORF1ab:C14408T, ORF1ab:T19839C, ORF1ab:A19974G, ORF1ab:C21306T, S:C22995A, S:A23403G, S:C23604A, S:A23756G, ORF8:G28077T, N:G28881A, N:G28882A, N:G28883C, N:C29197T,	12	N:R203K, N:G204R, ORF1a:P959S, ORF1a:T3255I, ORF1a:I3618V, ORF1a:T4175I, ORF1b:P314L, ORF8:V62L, S:T478K, S:D614G, S:P681H, S:T732A,
hCoV-19/Mexico/GUA-InDRE_F10841_S790/2021	EPI_ISL_1400335	20B	B.1.1.519	23	5'UTR:C203T, 5'UTR:C222T, 5'UTR:C241T, ORF1ab:C3037T, ORF1ab:C3140T, ORF1ab:C10029T, ORF1ab:C10954T, ORF1ab:A11117G, ORF1ab:C12789T, ORF1ab:C14408T, ORF1ab:T16863C, ORF1ab:C18714T, ORF1ab:T19839C, ORF1ab:C21306T, S:C22995A, S:A23403G, S:C23604A, S:A23756G, N:G28881A, N:G28882A, N:G28883C, N:C29197T, N:G29477T, 3'UTR:C29754T,	12	N:R203K, N:G204R, N:D402Y, ORF1a:P959S, ORF1a:T3255I, ORF1a:I3618V, ORF1a:T4175I, ORF1b:P314L, S:T478K, S:D614G, S:P681H, S:T732A,
hCoV-19/Mexico/GUA-InDRE_F10842_S789/2021	EPI_ISL_1400334	20B	B.1.1.519	23	5'UTR:C203T, 5'UTR:C222T, 5'UTR:C241T, ORF1ab:C3037T, ORF1ab:C3140T, ORF1ab:G7027T, ORF1ab:C7926T, ORF1ab:C10029T, ORF1ab:C10954T, ORF1ab:A11117G, ORF1ab:C12789T, ORF1ab:C14408T, ORF1ab:A19407G, ORF1ab:T19839C, ORF1ab:C21306T, S:C22995A, S:A23403G, S:C23604A, S:A23756G, S:C25207T, N:G28881A, N:G28882A, N:G28883C, N:C29197T,	13	N:R203K, N:G204R, ORF1a:P959S, ORF1a:M2254I, ORF1a:A2554V, ORF1a:T3255I, ORF1a:I3618V, ORF1a:T4175I, ORF1b:P314L, S:T478K, S:D614G, S:P681H, S:T732A,
hCoV-19/Mexico/GUA-InDRE_F10843_S788/2021	EPI_ISL_1400333	20B	B.1.1.519	23	5'UTR:C203T, 5'UTR:C222T, 5'UTR:C241T, ORF1ab:C3037T, ORF1ab:C3140T, ORF1ab:G7027T, ORF1ab:C7926T, ORF1ab:C10029T, ORF1ab:C10954T, ORF1ab:A11117G, ORF1ab:C12789T, ORF1ab:C14408T, ORF1ab:A19407G, ORF1ab:T19839C, ORF1ab:C21306T, S:C22995A, S:A23403G, S:C23604A, S:A23756G, S:C25207T, N:G28881A, N:G28882A, N:G28883C, N:C29197T,	13	N:R203K, N:G204R, ORF1a:P959S, ORF1a:M2254I, ORF1a:A2554V, ORF1a:T3255I, ORF1a:I3618V, ORF1a:T4175I, ORF1b:P314L, S:T478K, S:D614G, S:P681H, S:T732A,
hCoV-19/Mexico/GUA-InDRE_F10844_S892/2021	EPI_ISL_1405912	20G	B.1.2	21	5'UTR:C241T, ORF1ab:C1059T, ORF1ab:C3037T, ORF1ab:C4965T, ORF1ab:C7945T, ORF1ab:G9928T, ORF1ab:C10319T, ORF1ab:T12619C, ORF1ab:C14408T, ORF1ab:A15002G, ORF1ab:A18424G, ORF1ab:C21304T, S:C22264T, S:A23403G, ORF3a:G25563T, ORF3a:G25907T, ORF3a:C26060T, M:A26952G, ORF8:C27964T, N:C28472T, N:G28817T, N:C28869T,	17	M:I144V, N:P67S, N:A182S, N:P199L, ORF1a:T265I, ORF1a:T1567I, ORF1a:M3221I, ORF1a:L3352F, ORF1b:P314L, ORF1b:Y512C, ORF1b:N1653D, ORF1b:R2613C, ORF3a:Q57H, ORF3a:G172V, ORF3a:T223I, ORF8:S24L, S:D614G,
hCoV-19/Mexico/GUA-InDRE_F10845_S787/2021	EPI_ISL_1400332	20B	B.1.1.222	20	5'UTR:C241T, ORF1ab:C3037T, ORF1ab:G11540T, ORF1ab:C14408T, ORF1ab:C14898T, ORF1ab:G17523T, ORF1ab:T19839C, ORF1ab:A21137G, S:G21800T, S:C22987T, S:A23403G, S:C23557T, S:A23756G, ORF3a:C25714T, ORF3a:G25775T, ORF7a:C27476T, ORF8:C28045T, N:G28881A, N:G28882A, N:G28883C, N:G29402T.	14	N:R203K, N:G204R, N:D377Y, ORF1a:V3759F, ORF1b:P314L, ORF1b:M1352I, ORF1b:K2557R, ORF3a:L108F, ORF3a:W128L, ORF7a:T28I, ORF8:A51V, S:D80Y, S:D614G, S:T732A,

hCoV-19/Mexico/GUA-InDRE_F10846_S786/2021	EPI_ISL_1400331	20B	B.1.1.519	26	5'UTR:C203T, 5'UTR:C222T, 5'UTR:C241T, ORF1ab:G2447T, ORF1ab:C3037T, ORF1ab:C3140T, ORF1ab:G3692T, ORF1ab:G3871T, ORF1ab:C10029T, ORF1ab:C10954T, ORF1ab:A11117G, ORF1ab:C11824A, ORF1ab:C12789T, ORF1ab:C14408T, ORF1ab:T19839C, ORF1ab:C21431T, S:T22913C, S:C22995A, S:A23403G, S:C23604A, S:A23756G, ORF8:T27904C, N:G28881A, N:G28882A, N:G28883C, N:C29197T, N:G29227T,	17	N:R203K, N:G204R, ORF1a:G728C, ORF1a:P959S, ORF1a:V1143F, ORF1a:K1202N, ORF1a:T3255I, ORF1a:I3618V, ORF1a:T4175I, ORF1b:P314L, ORF1b:A2655V, ORF8:L4P, S:Y451H, S:T478K, S:D614G, S:P681H, S:T732A,
hCoV-19/Mexico/GUA-InDRE_F10847_S785/2021	EPI_ISL_1400330	20B	B.1.1.519	20	5'UTR:C203T, 5'UTR:C222T, 5'UTR:C241T, ORF1ab:C3037T, ORF1ab:C3140T, ORF1ab:C5175T, ORF1ab:C10029T, ORF1ab:C10954T, ORF1ab:A11117G, ORF1ab:C12789T, ORF1ab:C14408T, ORF1ab:T19839C, S:C22995A, S:A23403G, S:C23604A, S:A23756G, ORF8:T28250C, N:G28881A, N:G28882A, N:G28883C, N:C29197T,	12	N:R203K, N:G204R, ORF1a:P959S, ORF1a:T1637I, ORF1a:T3255I, ORF1a:I3618V, ORF1a:T4175I, ORF1b:P314L, S:T478K, S:D614G, S:P681H, S:T732A,
hCoV-19/Mexico/GUA-InDRE_F10848_S784/2021	EPI_ISL_1400329	20B	B.1.1.519	34	5'UTR:T201C, 5'UTR:C203T, 5'UTR:C222T, 5'UTR:C241T, ORF1ab:T409C, ORF1ab:C829T, ORF1ab:C936T, ORF1ab:G1738T, ORF1ab:C3037T, ORF1ab:C3140T, ORF1ab:C4048T, ORF1ab:C9112T, ORF1ab:C10029T, ORF1ab:G10523C, ORF1ab:C10954T, ORF1ab:A11117G, ORF1ab:C12789T, ORF1ab:C14408T, ORF1ab:A18085G, ORF1ab:C18877T, ORF1ab:T19839C, ORF1ab:A19974G, ORF1ab:C21306T, S:G22051T, S:C22995A, S:A23403G, S:C23604A, S:A23756G, S:C25276T, ORF8:C28253T, N:G28881A, N:G28882A, N:G28883C, N:C29197T, N:G29227T	14	N:R203K, N:G204R, ORF1a:T224I, ORF1a:P959S, ORF1a:T3255I, ORF1a:V3420L, ORF1a:I3618V, ORF1a:T4175I, ORF1b:P314L, ORF1b:T1540A, S:T478K, S:D614G, S:P681H, S:T732A,
hCoV-19/Mexico/GUA-InDRE_F10849_S891/2021	EPI_ISL_1405910	20G	B.1.2	29	5'UTR:C241T, ORF1ab:C683T, ORF1ab:C1059T, ORF1ab:C3037T, ORF1ab:C4575T, ORF1ab:C5497T, ORF1ab:G9928T, ORF1ab:C10319T, ORF1ab:C11962T, ORF1ab:C14408T, ORF1ab:C15352T, ORF1ab:C17491T, ORF1ab:A18424G, ORF1ab:C19274T, ORF1ab:C21304T, S:C23114T, S:A23403G, ORF3a:G25563T, ORF3a:C25672T, ORF3a:G25907T, ORF3a:C25916T, M:T26777C, M:G26828T, M:C27145T, ORF7a:T27687C, ORF8:C27964T, N:C28472T, N:C28869T, N:G29402T, N:G29422T,	20	M:T208I, N:P67S, N:P199L, N:D377Y, ORF1a:T265I, ORF1a:T1437I, ORF1a:M3221I, ORF1a:L3352F, ORF1b:P314L, ORF1b:L629F, ORF1b:P1342S, ORF1b:N1653D, ORF1b:P1936L, ORF1b:R2613C, ORF3a:Q57H, ORF3a:L94F, ORF3a:G172V, ORF3a:T175I, ORF8:S24L, S:D614G,
hCoV-19/Mexico/GUA-InDRE_F10850_S890/2021	EPI_ISL_1405909	20G	B.1.2	29	5'UTR:C241T, ORF1ab:C683T, ORF1ab:C1059T, ORF1ab:C3037T, ORF1ab:C4575T, ORF1ab:C5497T, ORF1ab:G9928T, ORF1ab:C10319T, ORF1ab:C11962T, ORF1ab:C14408T, ORF1ab:C15352T, ORF1ab:C17491T, ORF1ab:A18424G, ORF1ab:C19274T, ORF1ab:C21304T, S:C23114T, S:A23403G, ORF3a:G25563T, ORF3a:C25672T, ORF3a:G25907T, ORF3a:C25916T, M:T26777C, M:G26828T, M:C27145T, ORF7a:T27687C, ORF8:C27964T, N:C28472T, N:C28869T, N:G29402T, N:G29422T,	20	M:T208I, N:P67S, N:P199L, N:D377Y, ORF1a:T265I, ORF1a:T1437I, ORF1a:M3221I, ORF1a:L3352F, ORF1b:P314L, ORF1b:L629F, ORF1b:P1342S, ORF1b:N1653D, ORF1b:P1936L, ORF1b:R2613C, ORF3a:Q57H, ORF3a:L94F, ORF3a:G172V, ORF3a:T175I, ORF8:S24L, S:D614G,

hCoV-19/Mexico/GUA-InDRE_F10851_S600/2021	EPI_ISL_1337383	21C (Epsilon)	B.1.429	34	5'UTR:C241T, ORF1ab:G526T, ORF1ab:C1059T, ORF1ab:C2395T, ORF1ab:T2597C, ORF1ab:C3037T, ORF1ab:G5008T, ORF1ab:C8947T, ORF1ab:C9515T, ORF1ab:T10831A, ORF1ab:G11083T, ORF1ab:C12100T, ORF1ab:A12878G, ORF1ab:C13536T, ORF1ab:C14408T, ORF1ab:G17014T, ORF1ab:T17353C, ORF1ab:A19515G, ORF1ab:C21058T, S:G21600T, S:G22018T, S:T22917G, S:A23403G, S:T24349C, ORF3a:G25563T, M:C26681T, ORF7b:G27890T, ORF8:G27987C, ORF8:A28158G, ORF8:G28191T, ORF8:A28272T, N:C28887T, N:G28975T, N:C29362T, 3'UTR:C29743T,	18	N:T205I, N:M234I, ORF1a:E87D, ORF1a:T265I, ORF1a:L3606F, ORF1a:I4205V, ORF1b:P314L, ORF1b:D1183Y, ORF1b:F1296L, ORF1b:P2531S, ORF3a:Q57H, ORF8:V32L, ORF8:N89D, ORF8:V100L, S:S13I, S:W152C, S:L452R, S:D614G,
hCoV-19/Mexico/GUA-InDRE_F10852_S599/2021	EPI_ISL_1337382	21C (Epsilon)	B.1.429	28	5'UTR:C241T, ORF1ab:C1059T, ORF1ab:G1463A, ORF1ab:C2395T, ORF1ab:T2597C, ORF1ab:C3037T, ORF1ab:G3955T, ORF1ab:C8090T, ORF1ab:C8947T, ORF1ab:C10641T, ORF1ab:C12100T, ORF1ab:A12878G, ORF1ab:C14408T, ORF1ab:G17014T, S:G21600T, S:G22018T, S:T22917G, S:A23403G, S:T24349C, S:C25231T, ORF3a:G25563T, ORF3a:C25714T, E:A26487G, E:C26522T, M:C26681T, ORF7b:G27890T, ORF8:A28272T, N:C28887T, N:C29362T,	15	N:T205I, ORF1a:T265I, ORF1a:G400S, ORF1a:K1230N, ORF1a:L2609F, ORF1a:T3459M, ORF1a:I4205V, ORF1b:P314L, ORF1b:D1183Y, ORF3a:Q57H, ORF3a:L108F, S:S13I, S:W152C, S:L452R, S:D614G,
hCoV-19/Mexico/GUA-InDRE_F10853_S598/2021	EPI_ISL_1337380	21C (Epsilon)	B.1.429	27	5'UTR:C241T, ORF1ab:C1059T, ORF1ab:C2395T, ORF1ab:T2597C, ORF1ab:C3037T, ORF1ab:A6677G, ORF1ab:C8947T, ORF1ab:C9430T, ORF1ab:C12100T, ORF1ab:G12131T, ORF1ab:A12878G, ORF1ab:C14408T, ORF1ab:G17014T, ORF1ab:G19419A, ORF1ab:T20028C, S:G21600T, S:G22018T, S:T22917G, S:A23403G, S:T24349C, ORF3a:G25563T, ORF3a:T26100C, M:C26681T, ORF7b:G27890T, ORF8:A28272T, N:C28887T, N:C29362T, N:G29477C,	13	N:T205I, N:D402H, ORF1a:T265I, ORF1a:I2138V, ORF1a:A3956S, ORF1a:I4205V, ORF1b:P314L, ORF1b:D1183Y, ORF3a:Q57H, S:S13I, S:W152C, S:L452R, S:D614G,
hCoV-19/Mexico/GUA-InDRE_F10854_S783/2021	EPI_ISL_1400328	20B	B.1.1.519	19	5'UTR:C203T, 5'UTR:C222T, 5'UTR:C241T, ORF1ab:C3037T, ORF1ab:C3140T, ORF1ab:C10029T, ORF1ab:C10954T, ORF1ab:A11117G, ORF1ab:C12789T, ORF1ab:C14408T, ORF1ab:T19839C, ORF1ab:C21306T, S:C22995A, S:A23403G, S:C23604A, S:A23756G, N:G28881A, N:G28882A, N:G28883C, N:C29197T,	11	N:R203K, N:G204R, ORF1a:P959S, ORF1a:T3255I, ORF1a:I3618V, ORF1a:T4175I, ORF1b:P314L, S:T478K, S:D614G, S:P681H, S:T732A,
hCoV-19/Mexico/GUA-InDRE_F10855_S782/2021	EPI_ISL_1400327	20B	B.1.1.222	26	5'UTR:T201C, 5'UTR:C203T, 5'UTR:C222T, 5'UTR:A223T, 5'UTR:C241T, ORF1ab:G1738T, ORF1ab:C3037T, ORF1ab:C3140T, ORF1ab:C3447T, ORF1ab:C10029T, ORF1ab:C10765T, ORF1ab:C10954T, ORF1ab:C12789T, ORF1ab:C14408T, ORF1ab:C17125T, ORF1ab:T19839C, ORF1ab:A19974G, ORF1ab:C21306T, S:C22995A, S:A23403G, S:C23604A, S:A23756G, N:G28881A, N:G28882A, N:G28883C, N:G29140T, N:C29197T,	13	N:R203K, N:G204R, N:Q289H, ORF1a:P959S, ORF1a:A1061V, ORF1a:T3255I, ORF1a:T4175I, ORF1b:P314L, ORF1b:L1220F, S:T478K, S:D614G, S:P681H, S:T732A,
hCoV-19/Mexico/GUA-InDRE_F10856_S781/2021	EPI_ISL_1400326	20B	B.1.1.222	27	5'UTR:C241T, ORF1ab:C1191T, ORF1ab:C3037T, ORF1ab:G3403T, ORF1ab:T5218C, ORF1ab:A5533G, ORF1ab:C11339T, ORF1ab:C12525T, ORF1ab:G13201T, ORF1ab:C13887T, ORF1ab:C14408T, ORF1ab:T14592C, ORF1ab:C14790T, ORF1ab:C15714T, ORF1ab:T19839C, S:A23403G, S:T23689C, S:A23756G, S:G24794T, ORF3a:G25913T, ORF3a:G26153T, ORF6:C27294T, N:G28300T, N:G28881A, N:G28882A, N:G28883C, N:G28975T, N:G29449T	13	N:Q9H, N:R203K, N:G204R, N:M234I, ORF1a:P309L, ORF1a:T4087I, ORF1b:P314L, ORF3a:G174V, ORF3a:G254V, ORF9b:S6I, S:D614G, S:T732A, S:A1078S,

hCoV-19/Mexico/GUA-InDRE_F10857_S780/2021	EPI_ISL_1400325	20B	B.1.1.222	25	5'UTR:C241T, ORF1ab:C1191T, ORF1ab:C3037T, ORF1ab:G3403T, ORF1ab:T5218C, ORF1ab:A5533G, ORF1ab:C12525T, ORF1ab:C14408T, ORF1ab:T14592C, ORF1ab:C14790T, ORF1ab:T19839C, S:A23403G, S:T23689C, S:A23756G, S:G24794T, ORF3a:G25913T, ORF3a:G26153T, ORF3a:G26196T, ORF6:C27294T, N:G28300T, N:G28798A, N:G28881A, N:G28882A, N:G28883C, N:G28975T, N:G29449T,	13	N:Q9H, N:R203K, N:G204R, N:M234I, ORF1a:P309L, ORF1a:T4087I, ORF1b:P314L, ORF3a:G174V, ORF3a:G254V, ORF9b:S6I, S:D614G, S:T732A, S:A1078S,
hCoV-19/Mexico/GUA-InDRE_F10858_S889/2021	EPI_ISL_1405907	20A	B.1.243	22	5'UTR:C241T, ORF1ab:C274T, ORF1ab:G806A, ORF1ab:T2584A, ORF1ab:C3037T, ORF1ab:C5140A, ORF1ab:C6027T, ORF1ab:A12759G, ORF1ab:C14408T, ORF1ab:T17106C, ORF1ab:C18486T, ORF1ab:T19797C, ORF1ab:A20268G, S:G21786T, S:G23120T, S:A23403G, S:G23587C, S:T24076C, E:C26455T, M:C27128T, N:C28854T, N:T29351C, N:G29543T,	12	E:P71S, N:S194L, N:Y360H, ORF1a:A181T, ORF1a:D1625E, ORF1a:P1921L, ORF1a:D4165G, ORF1b:P314L, S:G75V, S:A520S, S:D614G, S:Q675H,
hCoV-19/Mexico/GUA-InDRE_F10859_S888/2021	EPI_ISL_1405906	20A	B.1	24	5'UTR:C241T, ORF1ab:C3037T, ORF1ab:C3042T, ORF1ab:A3904T, ORF1ab:C6883T, ORF1ab:C10714T, ORF1ab:C11050T, ORF1ab:A11430G, ORF1ab:C14408T, ORF1ab:A16534G, ORF1ab:T17673G, ORF1ab:G20060T, ORF1ab:A20268G, S:C21618T, S:C21707T, S:T22912G, S:G22992A, S:A23403G, M:C26681T, M:G26709A, ORF7a:C27494T, ORF8:A28273T, N:G28514T, N:G28917T, 3'UTR:G29745A,	15	M:A63T, N:D81Y, N:G215V, ORF1a:P926L, ORF1a:Y3722C, ORF1b:P314L, ORF1b:S1023G, ORF1b:S2198I, ORF7a:P34L, ORF9b:Q77H, S:T19I, S:H49Y, S:N450K, S:S477N, S:D614G,
hCoV-19/Mexico/GUA-InDRE_F10860_S779/2021	EPI_ISL_1400324	20B	B.1.1.519	25	5'UTR:C203T, 5'UTR:G210T, 5'UTR:C222T, 5'UTR:C241T, ORF1ab:C1762T, ORF1ab:C3037T, ORF1ab:C3140T, ORF1ab:G6352T, ORF1ab:C10029T, ORF1ab:A10286G, ORF1ab:C10954T, ORF1ab:A11117G, ORF1ab:C12789T, ORF1ab:C14408T, ORF1ab:T19839C, ORF1ab:C20762T, ORF1ab:C21306T, S:C22995A, S:A23403G, S:C23604A, S:A23756G, ORF7a:C27707T, N:G28881A, N:G28882A, N:G28883C, N:C29197T,	15	N:R203K, N:G204R, ORF1a:P959S, ORF1a:K2029N, ORF1a:T3255I, ORF1a:I3341V, ORF1a:I3618V, ORF1a:T4175I, ORF1b:P314L, ORF1b:T2432I, ORF7a:A105V, S:T478K, S:D614G, S:P681H, S:T732A,
hCoV-19/Mexico/GUA-InDRE_F10861_S597/2021	EPI_ISL_1337379	21C (Epsilon)	B.1.427	24	5'UTR:C241T, ORF1ab:C1059T, ORF1ab:C3037T, ORF1ab:C3817T, ORF1ab:C8895T, ORF1ab:G9738C, ORF1ab:C13019T, ORF1ab:G13713A, ORF1ab:C14408T, ORF1ab:C16394T, ORF1ab:C17010T, ORF1ab:G17014T, ORF1ab:C18246T, S:G21600T, S:G22018T, S:C22597T, S:T22917G, S:A23403G, ORF3a:G25563T, E:T26436G, M:C26681T, ORF8:C28087T, ORF8:A28272T, N:C28887T, N:C29362T,	14	E:N64K, N:T205I, ORF1a:T265I, ORF1a:T2877I, ORF1a:S3158T, ORF1b:P314L, ORF1b:P976L, ORF1b:D1183Y, ORF3a:Q57H, ORF8:A65V, S:S13I, S:W152C, S:L452R, S:D614G,
hCoV-19/Mexico/GUA-InDRE_F10862_S596/2021	EPI_ISL_1337377	21C (Epsilon)	B.1.427	23	5'UTR:C241T, ORF1ab:C1059T, ORF1ab:C3037T, ORF1ab:C3817T, ORF1ab:C8895T, ORF1ab:G9738C, ORF1ab:C13019T, ORF1ab:G13713A, ORF1ab:C14408T, ORF1ab:C16394T, ORF1ab:C17010T, ORF1ab:G17014T, ORF1ab:C18246T, S:G21600T, S:G22018T, S:C22597T, S:T22917G, S:A23403G, ORF3a:G25563T, E:T26436G, M:C26681T, ORF8:A28272T, N:C28887T, N:C29362T,	13	E:N64K, N:T205I, ORF1a:T265I, ORF1a:T2877I, ORF1a:S3158T, ORF1b:P314L, ORF1b:P976L, ORF1b:D1183Y, ORF3a:Q57H, S:S13I, S:W152C, S:L452R, S:D614G,

hCoV-19/Mexico/GUA-InDRE_F10863_S778/2021	EPI_ISL_1400323	20B	B.1.1.519	29	5'UTR:C203T, 5'UTR:C222T, 5'UTR:C241T, ORF1ab:T2116C, ORF1ab:C3037T, ORF1ab:C3140T, ORF1ab:C6636T, ORF1ab:C9226T, ORF1ab:C10029T, ORF1ab:C10954T, ORF1ab:A11117G, ORF1ab:C12789T, ORF1ab:C14408T, ORF1ab:C14838T, ORF1ab:C16733T, ORF1ab:G18040T, ORF1ab:T19839C, ORF1ab:G20635C, ORF1ab:C21306T, S:C22995A, S:A23403G, S:C23604A, S:A23756G, ORF3a:G26062T, M:C26912T, N:G28881A, N:G28882A, N:G28883C, N:C29197T, N:C29545T,	16	N:R203K, N:G204R, ORF1a:P959S, ORF1a:T2124I, ORF1a:T3255I, ORF1a:I3618V, ORF1a:T4175I, ORF1b:P314L, ORF1b:S1089L, ORF1b:A1525S, ORF1b:E2390Q, ORF3a:G224C, S:T478K, S:D614G, S:P681H, S:T732A,
hCoV-19/Mexico/GUA-InDRE_F10864_S777/2021	EPI_ISL_1400322	20B	B.1.1.519	29	5'UTR:C203T, 5'UTR:C222T, 5'UTR:C241T, ORF1ab:T2116C, ORF1ab:C3037T, ORF1ab:C3140T, ORF1ab:C6636T, ORF1ab:C9226T, ORF1ab:C10029T, ORF1ab:C10954T, ORF1ab:A11117G, ORF1ab:C12789T, ORF1ab:C14408T, ORF1ab:C14838T, ORF1ab:C16733T, ORF1ab:G18040T, ORF1ab:T19839C, ORF1ab:G20635C, ORF1ab:C21306T, S:C22995A, S:A23403G, S:C23604A, S:A23756G, ORF3a:G26062T, M:C26912T, N:G28881A, N:G28882A, N:G28883C, N:C29197T, N:C29545T,	16	N:R203K, N:G204R, ORF1a:P959S, ORF1a:T2124I, ORF1a:T3255I, ORF1a:I3618V, ORF1a:T4175I, ORF1b:P314L, ORF1b:S1089L, ORF1b:A1525S, ORF1b:E2390Q, ORF3a:G224C, S:T478K, S:D614G, S:P681H, S:T732A,
hCoV-19/Mexico/GUA-InDRE_F10865_S776/2021	EPI_ISL_1400321	20B	B.1.1.519	28	5'UTR:C203T, 5'UTR:C222T, 5'UTR:C241T, ORF1ab:C745T, ORF1ab:C3037T, ORF1ab:C3140T, ORF1ab:C3311T, ORF1ab:A8031G, ORF1ab:C9857T, ORF1ab:C10029T, ORF1ab:G10220A, ORF1ab:C10954T, ORF1ab:A11117G, ORF1ab:T11541G, ORF1ab:C12789T, ORF1ab:C14408T, ORF1ab:C14763T, ORF1ab:C18440T, ORF1ab:T19839C, ORF1ab:C21306T, S:C22995A, S:A23403G, S:G23587T, S:C23604A, S:A23756G, N:G28881A, N:G28882A, N:G28883C, N:C29197T	17	N:R203K, N:G204R, ORF1a:P959S, ORF1a:L1016F, ORF1a:K2589R, ORF1a:T3255I, ORF1a:D3319N, ORF1a:I3618V, ORF1a:V3759G, ORF1a:T4175I, ORF1b:P314L, ORF1b:S1658F, S:T478K, S:D614G, S:Q675H, S:P681H, S:T732A,
hCoV-19/Mexico/GUA-InDRE_F10866_S887/2021	EPI_ISL_1405904	20B	B.1.1.519	23	5'UTR:C203T, 5'UTR:C222T, 5'UTR:C241T, ORF1ab:C3037T, ORF1ab:C3140T, ORF1ab:G7037T, ORF1ab:T9880C, ORF1ab:C10029T, ORF1ab:C10954T, ORF1ab:A11117G, ORF1ab:C12789T, ORF1ab:C14408T, ORF1ab:T19839C, ORF1ab:C21306T, S:C22995A, S:A23403G, S:C23604A, S:A23756G, ORF3a:C25782T, N:G28878T, N:G28881A, N:G28882A, N:G28883C, N:C29197T,	13	N:S202I, N:R203K, N:G204R, ORF1a:P959S, ORF1a:G2258C, ORF1a:T3255I, ORF1a:I3618V, ORF1a:T4175I, ORF1b:P314L, S:T478K, S:D614G, S:P681H, S:T732A,
hCoV-19/Mexico/GUA-InDRE_F10867_S775/2021	EPI_ISL_1400320	20B	B.1.1.519	24	5'UTR:C203T, 5'UTR:C222T, 5'UTR:C241T, ORF1ab:G787T, ORF1ab:C1909T, ORF1ab:C3037T, ORF1ab:C3140T, ORF1ab:C10029T, ORF1ab:C10954T, ORF1ab:A11117G, ORF1ab:C12789T, ORF1ab:C14408T, ORF1ab:T16863C, ORF1ab:T19839C, ORF1ab:C21306T, S:C22995A, S:A23403G, S:C23604A, S:A23756G, M:T27134C, N:G28881A, N:G28882A, N:G28883C, N:C29197T, 3'UTR:C29754T,	12	N:R203K, N:G204R, ORF1a:M174I, ORF1a:P959S, ORF1a:T3255I, ORF1a:I3618V, ORF1a:T4175I, ORF1b:P314L, S:T478K, S:D614G, S:P681H, S:T732A,
hCoV-19/Mexico/GUA-InDRE_F10868_S595/2021	EPI_ISL_1337376	21C (Epsilon)	B.1.429	27	5'UTR:C241T, ORF1ab:C1059T, ORF1ab:C2395T, ORF1ab:T2597C, ORF1ab:C3037T, ORF1ab:C8947T, ORF1ab:C12100T, ORF1ab:A12878G, ORF1ab:C14408T, ORF1ab:C15981T, ORF1ab:G17014T, ORF1ab:C17304T, ORF1ab:A20262G, S:G21600T, S:G22018T, S:T22702C, S:T22917G, S:A23403G, S:T24349C, ORF3a:C25433T, ORF3a:C25521T, ORF3a:G25563T, ORF3a:C26151A, M:C26681T, ORF7b:G27890T, ORF8:A28272T, N:C28887T, N:C29362T,	11	N:T205I, ORF1a:T265I, ORF1a:I4205V, ORF1b:P314L, ORF1b:D1183Y, ORF3a:T14I, ORF3a:Q57H, S:S13I, S:W152C, S:L452R, S:D614G,

hCoV-19/Mexico/GUA-InDRE_F10869_S774/2021	EPI_ISL_1400319	20B	B.1.1.519	24	5'UTR:C203T, 5'UTR:C222T, 5'UTR:C241T, ORF1ab:C346T, ORF1ab:C3037T, ORF1ab:C3140T, ORF1ab:C10029T, ORF1ab:C10954T, ORF1ab:T11112C, ORF1ab:A11117G, ORF1ab:C12789T, ORF1ab:C14408T, ORF1ab:C16750T, ORF1ab:T19839C, ORF1ab:C21306T, S:G21600T, S:C22995A, S:A23403G, S:C23604A, S:A23756G, N:G28881A, N:G28882A, N:G28883C, N:C29197T, N:A29334G,	15	N:R203K, N:G204R, N:N354S, ORF1a:P959S, ORF1a:T3255I, ORF1a:M3616T, ORF1a:I3618V, ORF1a:T4175I, ORF1b:P314L, ORF1b:P1095S, S:S13I, S:T478K, S:D614G, S:P681H, S:T732A,
hCoV-19/Mexico/GUA-InDRE_F10870_S773/2021	EPI_ISL_1400318	20B	B.1.1.519	23	5'UTR:C203T, 5'UTR:C222T, 5'UTR:C241T, ORF1ab:C3037T, ORF1ab:C3140T, ORF1ab:G7027T, ORF1ab:C7926T, ORF1ab:C10029T, ORF1ab:C10954T, ORF1ab:A11117G, ORF1ab:C12789T, ORF1ab:C14408T, ORF1ab:A19407G, ORF1ab:T19839C, ORF1ab:C21306T, S:C22995A, S:A23403G, S:C23604A, S:A23756G, S:C25207T, N:G28881A, N:G28882A, N:G28883C, N:C29197T,	13	N:R203K, N:G204R, ORF1a:P959S, ORF1a:M2254I, ORF1a:A2554V, ORF1a:T3255I, ORF1a:I3618V, ORF1a:T4175I, ORF1b:P314L, S:T478K, S:D614G, S:P681H, S:T732A,
hCoV-19/Mexico/GUA-InDRE_F10871_S886/2021	EPI_ISL_1405903	20B	B.1.1.432	23	5'UTR:C241T, ORF1ab:C3037T, ORF1ab:G3122T, ORF1ab:A6985T, ORF1ab:C9319T, ORF1ab:C10369T, ORF1ab:C12412T, ORF1ab:C13994T, ORF1ab:T14313C, ORF1ab:C14408T, ORF1ab:C16575T, ORF1ab:C20844T, S:G21770T, S:C22127T, S:A23403G, S:C24382T, N:G28881A, N:G28882A, N:G28883C, N:C28948T, N:C29149T, N:C29167T, N:T29317C, ORF10:C29585T,	8	N:R203K, N:G204R, ORF1a:D953Y, ORF1b:A176V, ORF1b:P314L, S:V70F, S:L189F, S:D614G,
hCoV-19/Mexico/GUA-InDRE_F10876_S772/2021	EPI_ISL_1400317	20B	B.1.1.519	22	5'UTR:C203T, 5'UTR:C222T, 5'UTR:C241T, ORF1ab:C3037T, ORF1ab:C3140T, ORF1ab:C7047T, ORF1ab:C10029T, ORF1ab:C10954T, ORF1ab:A11117G, ORF1ab:C12789T, ORF1ab:C14408T, ORF1ab:T19839C, ORF1ab:C21306T, S:C22995A, S:A23403G, S:C23604A, S:A23756G, ORF8:T27995C, N:G28817T, N:G28881A, N:G28882A, N:G28883C, N:C29197T,	13	N:A182S, N:R203K, N:G204R, ORF1a:P959S, ORF1a:S2261F, ORF1a:T3255I, ORF1a:I3618V, ORF1a:T4175I, ORF1b:P314L, S:T478K, S:D614G, S:P681H, S:T732A,
hCoV-19/Mexico/GUA-InDRE_F10877_S771/2021	EPI_ISL_1400316	20B	B.1.1.222	20	5'UTR:C241T, ORF1ab:C3037T, ORF1ab:C6285T, ORF1ab:G7360T, ORF1ab:A8203G, ORF1ab:C13423T, ORF1ab:A13498G, ORF1ab:C14408T, ORF1ab:A18141G, ORF1ab:T19839C, ORF1ab:T21357C, S:C22088A, S:A22116C, S:A23403G, S:A23756G, ORF3a:A25501C, M:C27119T, N:G28655T, N:G28881A, N:G28882A, N:G28883C,	12	N:D128Y, N:R203K, N:G204R, ORF1a:T2007I, ORF1a:M2365I, ORF1b:T11A, ORF1b:P314L, ORF3a:I37L, S:L176I, S:N185T, S:D614G, S:T732A,
hCoV-19/Mexico/GUA-InDRE_F10878_S770/2021	EPI_ISL_1400315	20B	B.1.1.519	24	5'UTR:C203T, 5'UTR:C222T, 5'UTR:C241T, ORF1ab:C3037T, ORF1ab:C3140T, ORF1ab:A3255G, ORF1ab:C6384T, ORF1ab:C10029T, ORF1ab:C10954T, ORF1ab:G11083T, ORF1ab:A11117G, ORF1ab:G11365T, ORF1ab:C12789T, ORF1ab:C14408T, ORF1ab:T19839C, ORF1ab:C21306T, S:C22995A, S:A23403G, S:C23604A, S:A23756G, N:G28881A, N:G28882A, N:G28883C, N:C29197T, N:G29527T,	15	N:R203K, N:G204R, N:Q418H, ORF1a:P959S, ORF1a:N997S, ORF1a:A2040V, ORF1a:T3255I, ORF1a:L3606F, ORF1a:I3618V, ORF1a:T4175I, ORF1b:P314L, S:T478K, S:D614G, S:P681H, S:T732A,
hCoV-19/Mexico/GUA-InDRE_F10879_S885/2021	EPI_ISL_1405902	20G	B.1.2	18	5'UTR:C241T, ORF1ab:C1059T, ORF1ab:C3037T, ORF1ab:C6990T, ORF1ab:G8083A, ORF1ab:C10319T, ORF1ab:C14408T, ORF1ab:C14805T, ORF1ab:A18424G, ORF1ab:G19999T, ORF1ab:C21304T, S:A23403G, ORF3a:G25563T, ORF3a:G25907T, ORF8:C27964T, N:C28472T, N:G28703T, N:C28869T, N:G29402T,	16	N:P67S, N:D144Y, N:P199L, N:D377Y, ORF1a:T265I, ORF1a:S2242F, ORF1a:M2606I, ORF1a:L3352F, ORF1b:P314L, ORF1b:N1653D, ORF1b:V2178F, ORF1b:R2613C, ORF3a:Q57H, ORF3a:G172V, ORF8:S24L, S:D614G,

hCoV-19/Mexico/GUA-InDRE_F10880_S884/2021	EPI_ISL_1405901	21C (Epsilon)	B.1.427	24	5'UTR:T224C, 5'UTR:C241T, ORF1ab:C1059T, ORF1ab:C3037T, ORF1ab:A3061G, ORF1ab:C4582T, ORF1ab:G9738C, ORF1ab:C10369T, ORF1ab:C11345T, ORF1ab:G13713A, ORF1ab:C14408T, ORF1ab:C16394T, ORF1ab:G17014T, ORF1ab:C18189T, ORF1ab:C18501T, S:G21600T, S:G22018T, S:T22917G, S:A23403G, ORF3a:G25563T, M:C26681T, ORF7a:C27688T, ORF8:A28272T, N:C28887T, 3'UTR:G29764T,	13	N:T205I, ORF1a:T265I, ORF1a:S3158T, ORF1a:L3694F, ORF1b:P314L, ORF1b:P976L, ORF1b:D1183Y, ORF3a:Q57H, ORF7a:P99S, S:S13I, S:W152C, S:L452R, S:D614G,
hCoV-19/Mexico/GUA-InDRE_F10881_S883/2021	EPI_ISL_1405900	21C (Epsilon)	B.1.427	24	5'UTR:T224C, 5'UTR:C241T, ORF1ab:C1059T, ORF1ab:C3037T, ORF1ab:A3061G, ORF1ab:C4582T, ORF1ab:G9738C, ORF1ab:C10369T, ORF1ab:C11345T, ORF1ab:G13713A, ORF1ab:C14408T, ORF1ab:C16394T, ORF1ab:G17014T, ORF1ab:C18189T, ORF1ab:C18501T, S:G21600T, S:G22018T, S:T22917G, S:A23403G, ORF3a:G25563T, M:C26681T, ORF7a:C27688T, ORF8:A28272T, N:C28887T, 3'UTR:G29764T,	13	N:T205I, ORF1a:T265I, ORF1a:S3158T, ORF1a:L3694F, ORF1b:P314L, ORF1b:P976L, ORF1b:D1183Y, ORF3a:Q57H, ORF7a:P99S, S:S13I, S:W152C, S:L452R, S:D614G,
hCoV-19/Mexico/GUA-InDRE_F10882_S769/2021	EPI_ISL_1400314	20B	B.1.1.519	20	5'UTR:C203T, 5'UTR:C222T, 5'UTR:C241T, ORF1ab:C3037T, ORF1ab:C3140T, ORF1ab:C10029T, ORF1ab:C10954T, ORF1ab:A11117G, ORF1ab:C12789T, ORF1ab:C13761T, ORF1ab:C14408T, ORF1ab:T19839C, ORF1ab:C21306T, S:C22995A, S:A23403G, S:C23604A, S:A23756G, N:G28881A, N:G28882A, N:G28883C, N:C29197T,	11	N:R203K, N:G204R, ORF1a:P959S, ORF1a:T3255I, ORF1a:I3618V, ORF1a:T4175I, ORF1b:P314L, S:T478K, S:D614G, S:P681H, S:T732A,
hCoV-19/Mexico/GUA-InDRE_F84G_S609/2021	EPI_ISL_1340611	20B	B.1.1.519	25	5'UTR:C203T, 5'UTR:C222T, 5'UTR:C241T, ORF1ab:G2447T, ORF1ab:C3037T, ORF1ab:C3140T, ORF1ab:G3692T, ORF1ab:G3871T, ORF1ab:C10029T, ORF1ab:C10954T, ORF1ab:A11117G, ORF1ab:C11824A, ORF1ab:C12789T, ORF1ab:C14408T, ORF1ab:T19839C, ORF1ab:C21306T, S:T22913C, S:C22995A, S:A23403G, S:C23604A, S:A23756G, ORF8:T27904C, N:G28881A, N:G28882A, N:G28883C, N:C29197T,	16	N:R203K, N:G204R, ORF1a:G728C, ORF1a:P959S, ORF1a:V1143F, ORF1a:K1202N, ORF1a:T3255I, ORF1a:I3618V, ORF1a:T4175I, ORF1b:P314L, ORF8:L4P, S:Y451H, S:T478K, S:D614G, S:P681H, S:T732A,
hCoV-19/Mexico/GUA-InDRE-19/2020	EPI_ISL_493334	20C	B.1	6	5'UTR:C241T, ORF1ab:C1059T, ORF1ab:C3037T, ORF1ab:C11916T, ORF1ab:C14408T, S:A23403G, ORF3a:G25563T,	5	ORF1a:T265I, ORF1a:S3884L, ORF1b:P314L, ORF3a:Q57H, S:D614G,
hCoV-19/Mexico/GUA-InDRE-20/2020	EPI_ISL_493335	20C	B.1	7	5'UTR:C241T, ORF1ab:C1059T, ORF1ab:C3037T, ORF1ab:C6070T, ORF1ab:C14408T, S:A23403G, ORF3a:G25563T, ORF8:G27996T,	5	ORF1a:T265I, ORF1b:P314L, ORF3a:Q57H, ORF8:D35Y, S:D614G,
hCoV-19/Mexico/HGO-InDRE_123/2020	EPI_ISL_913927	20A	B.1	16	5'UTR:C241T, ORF1ab:A2806C, ORF1ab:C3037T, ORF1ab:G3496A, ORF1ab:G4504A, ORF1ab:A10323G, ORF1ab:T10992C, ORF1ab:C12400T, ORF1ab:C14408T, ORF1ab:A14457G, ORF1ab:T17160C, ORF1ab:A20268G, S:G22051T, S:A23403G, ORF3a:A25472G, ORF8:G28038T, N:C28854T,	8	N:S194L, ORF1a:E847D, ORF1a:K3353R, ORF1a:I3576T, ORF1b:P314L, ORF3a:D27G, ORF8:V49L, S:D614G,
hCoV-19/Mexico/HGO-InDRE_148/2020	EPI_ISL_913951	20B	B.1.1	16	5'UTR:C241T, ORF1ab:C313T, ORF1ab:C823T, ORF1ab:C3037T, ORF1ab:C3903T, ORF1ab:G6320A, ORF1ab:C9474T, ORF1ab:C11074T, ORF1ab:G11083T, ORF1ab:C14408T, ORF1ab:A20640G, S:C21707T, S:A23403G, N:T28516C, N:G28881A, N:G28882A, N:G28883C,	10	N:R203K, N:G204R, ORF1a:P1213L, ORF1a:V2019I, ORF1a:A3070V, ORF1a:L3606F, ORF1b:P314L, ORF9b:M78T, S:H49Y, S:D614G,
hCoV-19/Mexico/HGO-InDRE_156/2020	EPI_ISL_913958	20C	B.1.582	13	5'UTR:C241T, ORF1ab:T580C, ORF1ab:A939G, ORF1ab:C1059T, ORF1ab:C3037T, ORF1ab:A10323G, ORF1ab:C14408T, ORF1ab:T15276A, S:A23403G, ORF3a:G25563T, E:T26510C, ORF7a:A27579G, ORF7a:C27679T, N:G28842T,	8	N:S190I, ORF1a:K225R, ORF1a:T265I, ORF1a:K3353R, ORF1b:P314L, ORF3a:Q57H, ORF7a:L96F, S:D614G,

hCoV-19/Mexico/HGO-InDRE_164/2020	EPI_ISL_913965	20B	B.1.1.519	20	5'UTR:C203T, 5'UTR:C222T, 5'UTR:C241T, ORF1ab:C3037T, ORF1ab:C3140T, ORF1ab:C10029T, ORF1ab:C10954T, ORF1ab:A11117G, ORF1ab:C12789T, ORF1ab:C14408T, ORF1ab:T19839C, ORF1ab:C21306T, S:C22995A, S:A23403G, S:C23604A, S:A23756G, N:G28881A, N:G28882A, N:G28883C, N:C29197T, N:G29315C,	12	N:R203K, N:G204R, N:D348H, ORF1a:P959S, ORF1a:T3255I, ORF1a:I3618V, ORF1a:T4175I, ORF1b:P314L, S:T478K, S:D614G, S:P681H, S:T732A,
hCoV-19/Mexico/HGO-InDRE_181/2020	EPI_ISL_913982	20B	B.1.1.519	22	5'UTR:C203T, 5'UTR:C222T, 5'UTR:C241T, ORF1ab:C3037T, ORF1ab:C3140T, ORF1ab:C5886T, ORF1ab:C10029T, ORF1ab:A10829C, ORF1ab:C10954T, ORF1ab:A11117G, ORF1ab:C12789T, ORF1ab:C14408T, ORF1ab:T19839C, ORF1ab:C21306T, S:C22995A, S:A23403G, S:C23604A, S:A23756G, ORF8:T27904C, N:G28881A, N:G28882A, N:G28883C, N:C29197T,	14	N:R203K, N:G204R, ORF1a:P959S, ORF1a:T1874I, ORF1a:T3255I, ORF1a:I3522L, ORF1a:I3618V, ORF1a:T4175I, ORF1b:P314L, ORF8:L4P, S:T478K, S:D614G, S:P681H, S:T732A,
hCoV-19/Mexico/HGO-InDRE_183/2020	EPI_ISL_913984	20B	B.1.1.519	16	5'UTR:C203T, 5'UTR:C222T, 5'UTR:C241T, ORF1ab:C3037T, ORF1ab:C3140T, ORF1ab:C10029T, ORF1ab:C10954T, ORF1ab:A11117G, ORF1ab:C12789T, ORF1ab:C14408T, ORF1ab:T19839C, S:A23403G, ORF8:T27904C, N:G28881A, N:G28882A, N:G28883C, N:C29197T,	9	N:R203K, N:G204R, ORF1a:P959S, ORF1a:T3255I, ORF1a:I3618V, ORF1a:T4175I, ORF1b:P314L, ORF8:L4P, S:D614G,
hCoV-19/Mexico/HID-InDRE_161/2020	EPI_ISL_914880	20A	B.1.229	12	5'UTR:C241T, ORF1ab:C3037T, ORF1ab:G8863T, ORF1ab:C10456T, ORF1ab:C14408T, ORF1ab:A17609G, ORF1ab:C18796T, ORF1ab:A20268G, S:A23403G, S:C24609T, ORF3a:C26060T, M:C26894T, N:C28854T,	6	N:S194L, ORF1b:P314L, ORF1b:D1381G, ORF3a:T223I, S:D614G, S:A1016V,
hCoV-19/Mexico/HID-InDRE_271/2020	EPI_ISL_1054950	20B	B.1.1	8	5'UTR:C241T, ORF1ab:G2265T, ORF1ab:C3037T, ORF1ab:C14408T, ORF1ab:T19839C, S:A23403G, N:G28881A, N:G28882A, N:G28883C,	5	N:R203K, N:G204R, ORF1a:C667F, ORF1b:P314L, S:D614G,
hCoV-19/Mexico/HID-InDRE_290/2020	EPI_ISL_1060732	20A	B.1.610	8	5'UTR:C241T, ORF1ab:C3037T, ORF1ab:G11417T, ORF1ab:C14408T, ORF1ab:T16899C, ORF1ab:A20268G, S:A23403G, N:T29107C, N:C29485T,	3	ORF1a:V3718F, ORF1b:P314L, S:D614G,
hCoV-19/Mexico/HID-InDRE_291/2020	EPI_ISL_1060731	20A	B.1.609	9	5'UTR:C241T, ORF1ab:C920T, ORF1ab:C2749T, ORF1ab:C3037T, ORF1ab:C4582T, ORF1ab:C14408T, ORF1ab:A20268G, S:A23403G, ORF3a:G26062T, N:G28916T,	4	N:G215C, ORF1b:P314L, ORF3a:G224C, S:D614G,
hCoV-19/Mexico/HID-InDRE_292/2020	EPI_ISL_1060733	20A	B.1.189	8	5'UTR:C241T, ORF1ab:C3037T, ORF1ab:C14408T, ORF1ab:C16948T, ORF1ab:A20268G, S:A23403G, N:G28378T, N:C28854T, 3'UTR:G29745T,	5	N:S194L, ORF1b:P314L, ORF1b:P1161S, ORF9b:R32L, S:D614G,
hCoV-19/Mexico/HID-InDRE_293/2020	EPI_ISL_1060734	20A	B.1.189	8	5'UTR:C241T, ORF1ab:C3037T, ORF1ab:C14408T, ORF1ab:C16948T, ORF1ab:A20268G, S:A23403G, N:G28378T, N:C28854T, 3'UTR:G29745T,	5	N:S194L, ORF1b:P314L, ORF1b:P1161S, ORF9b:R32L, S:D614G,
hCoV-19/Mexico/HID-InDRE_297/2020	EPI_ISL_1060727	20A	B.1.94	13	5'UTR:C241T, ORF1ab:C1314T, ORF1ab:C3037T, ORF1ab:A3964G, ORF1ab:C7420T, ORF1ab:C14408T, ORF1ab:G17014T, ORF1ab:C17999T, ORF1ab:T18477C, ORF1ab:C20468T, S:T22016C, S:A23403G, M:A26939T. N:C28332T.	9	N:P20L, ORF1a:T350I, ORF1b:P314L, ORF1b:D1183Y, ORF1b:T1511I, ORF1b:A2334V, ORF9b:P17S, S:W152R, S:D614G,
hCoV-19/Mexico/HID-InDRE_304/2020	EPI_ISL_1060736	20B	B.1.1	7	5'UTR:C241T, ORF1ab:C3037T, ORF1ab:C14408T, S:A23403G, ORF3a:C25463T, N:G28881A, N:G28882A, N:G28883C,	5	N:R203K, N:G204R, ORF1b:P314L, ORF3a:T24I, S:D614G,
hCoV-19/Mexico/HID-InDRE_309/2020	EPI_ISL_1060700	20A	B.1.243	9	5'UTR:C241T, ORF1ab:C3037T, ORF1ab:C14408T, ORF1ab:C15746T, ORF1ab:A20268G, S:A23403G, S:T24076C, S:T24793A, M:C26801T, N:C28854T,	4	N:S194L, ORF1b:P314L, ORF1b:T760I, S:D614G,
hCoV-19/Mexico/HID-InDRE_311/2020	EPI_ISL_1060684	20B	B.1.1	6	ORF1ab:C3037T, ORF1ab:C13487T, ORF1ab:C14408T, S:A23403G, N:G28881A, N:G28882A, N:G28883C,	5	N:R203K, N:G204R, ORF1b:A7V, ORF1b:P314L, S:D614G,
hCoV-19/Mexico/HID-InDRE_386/2020	EPI_ISL_1054987	20B	B.1.1.222	8	5'UTR:C241T, ORF1ab:C3037T, ORF1ab:C14408T, ORF1ab:T19839C, S:A23403G, S:A23756G, N:G28881A, N:G28882A, N:G28883C,	5	N:R203K, N:G204R, ORF1b:P314L, S:D614G, S:T732A,

hCoV-19/Mexico/HID-InDRE_459/2021	EPI_ISL_1168529	20B	B.1.1.222	16	5'UTR:C241T, ORF1ab:G1542T, ORF1ab:G2458T, ORF1ab:C3037T, ORF1ab:C7749T, ORF1ab:A13498G, ORF1ab:C14265T, ORF1ab:C14408T, ORF1ab:A18543G, ORF1ab:C19024A, ORF1ab:T19839C, S:A23403G, S:A23756G, ORF3a:C25521T, N:G28881A, N:G28882A, N:G28883C,	10	N:R203K, N:G204R, ORF1a:R426L, ORF1a:M731I, ORF1a:T2495I, ORF1b:T11A, ORF1b:P314L, ORF1b:L1853I, S:D614G, S:T732A,
hCoV-19/Mexico/HID-InDRE_460/2021	EPI_ISL_1168530	20B	B.1.1.222	17	5'UTR:C241T, ORF1ab:C3037T, ORF1ab:G3085T, ORF1ab:G3871T, ORF1ab:G12988T, ORF1ab:C14408T, ORF1ab:T19839C, S:G22225T, S:A23403G, S:A23756G, S:C23833A, ORF3a:G25906T, ORF8:G27916T, N:G28881A, N:G28882A, N:G28883C, N:G29179A, N:C29445T,	11	N:R203K, N:G204R, N:T391I, ORF1a:E940D, ORF1a:K1202N, ORF1a:M4241I, ORF1b:P314L, ORF3a:G172C, ORF8:G8V, S:D614G, S:T732A,
hCoV-19/Mexico/HID-InDRE_461/2021	EPI_ISL_1168531	20B	B.1.1.519	28	5'UTR:T201C, 5'UTR:C203T, 5'UTR:C222T, 5'UTR:C241T, ORF1ab:C936T, ORF1ab:G1738T, ORF1ab:C3037T, ORF1ab:C3140T, ORF1ab:C8626T, ORF1ab:C10029T, ORF1ab:C10954T, ORF1ab:A11117G, ORF1ab:C12789T, ORF1ab:C14408T, ORF1ab:C17104T, ORF1ab:G19657A, ORF1ab:T19839C, ORF1ab:A19974G, ORF1ab:C21306T, S:C22995A, S:A23403G, S:C23604A, S:A23756G, ORF3a:C25626T, N:G28881A, N:G28882A, N:G28883C, N:C29197T, N:G29227T,	14	N:R203K, N:G204R, ORF1a:T224I, ORF1a:P959S, ORF1a:T3255I, ORF1a:I3618V, ORF1a:T4175I, ORF1b:P314L, ORF1b:H1213Y, ORF1b:G2064R, S:T478K, S:D614G, S:P681H, S:T732A,
hCoV-19/Mexico/HID-InDRE_481/2021	EPI_ISL_1168551	20B	B.1.1.222	20	5'UTR:C241T, ORF1ab:C3037T, ORF1ab:C3177T, ORF1ab:G11417T, ORF1ab:C14220T, ORF1ab:C14408T, ORF1ab:G16245A, ORF1ab:T16976C, ORF1ab:C18714T, ORF1ab:G19086T, ORF1ab:T19839C, ORF1ab:A21526G, S:A23403G, S:A23756G, S:T24346C, ORF3a:G25429T, ORF3a:C25771T, ORF8:C27944T, N:G28881A, N:G28882A, N:G28883C,	12	N:R203K, N:G204R, ORF1a:P971L, ORF1a:V3718F, ORF1b:P314L, ORF1b:V1170A, ORF1b:K1873N, ORF1b:I2687V, ORF3a:V13L, ORF3a:L127F, S:D614G, S:T732A,
hCoV-19/Mexico/HID-InDRE_518/2021	EPI_ISL_1168588	20B	B.1.1.222	20	5'UTR:C241T, ORF1ab:C835T, ORF1ab:C3037T, ORF1ab:G3947T, ORF1ab:C4406T, ORF1ab:C6781T, ORF1ab:C8090T, ORF1ab:C11109T, ORF1ab:C11416T, ORF1ab:C14408T, ORF1ab:T19839C, S:G22017T, S:A23403G, S:A23756G, ORF3a:G26063T, ORF7b:A27888T, N:G28881A, N:G28882A, N:G28883C, 3'UTR:G29862T, 3'UTR:A29866C,	11	N:R203K, N:G204R, ORF1a:D1228Y, ORF1a:H1381Y, ORF1a:L2609F, ORF1a:A3615V, ORF1b:P314L, ORF3a:G224V, S:W152L, S:D614G, S:T732A,
hCoV-19/Mexico/HID-InDRE_519/2021	EPI_ISL_1168589	20B	B.1.1.222	19	5'UTR:C241T, ORF1ab:C3037T, ORF1ab:C10029T, ORF1ab:C11109T, ORF1ab:C14408T, ORF1ab:T19839C, S:T22990C, S:A23403G, S:A23756G, S:A24241G, S:T24352C, ORF3a:G25912T, ORF3a:T26007A, ORF7b:A27756G, ORF7b:C27879T, ORF8:A27921G, ORF8:G28001T, N:G28881A, N:G28882A, N:G28883C,	12	N:R203K, N:G204R, ORF1a:T3255I, ORF1a:A3615V, ORF1b:P314L, ORF3a:G174C, ORF3a:S205R, ORF7b:M1V, ORF7b:H42Y, ORF8:I10V, S:D614G, S:T732A,
hCoV-19/Mexico/HID-InDRE_520/2021	EPI_ISL_1168590	20B	B.1.1.519	26	5'UTR:C203T, 5'UTR:C222T, 5'UTR:C241T, ORF1ab:T1937C, ORF1ab:C3037T, ORF1ab:C3140T, ORF1ab:C4878T, ORF1ab:C10029T, ORF1ab:C10954T, ORF1ab:A11117G, ORF1ab:C12789T, ORF1ab:C14408T, ORF1ab:T19839C, ORF1ab:C21306T, S:C22995A, S:A23403G, S:C23604A, S:A23756G, ORF7a:C27679T, ORF7b:G27798T, N:G28881A, N:G28882A, N:G28883C, N:C29197T, N:G29527T, 3'UTR:G29862T, 3'UTR:A29866C,	16	N:R203K, N:G204R, N:Q418H, ORF1a:S558P, ORF1a:P959S, ORF1a:T1538I, ORF1a:T3255I, ORF1a:I3618V, ORF1a:T4175I, ORF1b:P314L, ORF7a:L96F, ORF7b:A15S, S:T478K, S:D614G, S:P681H, S:T732A,

hCoV-19/Mexico/HID-InDRE_521/2021	EPI_ISL_1168591	20B	B.1.1.519	28	5'UTR:T201C, 5'UTR:C203T, 5'UTR:C222T, 5'UTR:C241T, ORF1ab:C936T, ORF1ab:G1738T, ORF1ab:C3037T, ORF1ab:C3140T, ORF1ab:C10029T, ORF1ab:C10954T, ORF1ab:A11117G, ORF1ab:C12789T, ORF1ab:C14408T, ORF1ab:T19839C, S:T22386C, S:C22945A, S:C22995A, S:A23403G, S:C23604A, S:A23756G, ORF3a:G26113C, ORF7a:G27572C, N:G28881A, N:G28882A, N:G28883C, N:G29027T, N:C29197T, N:G29227T, 3'UTR:G29862T,	16	N:R203K, N:G204R, N:A252S, ORF1a:T224I, ORF1a:P959S, ORF1a:T3255I, ORF1a:I3618V, ORF1a:T4175I, ORF1b:P314L, ORF3a:E241Q, ORF7a:S60T, S:F275S, S:T478K, S:D614G, S:P681H, S:T732A,
hCoV-19/Mexico/HID-InDRE_F10298_S626/2021	EPI_ISL_1340640	20B	B.1.1.519	24	5'UTR:C203T, 5'UTR:C222T, 5'UTR:C241T, ORF1ab:C3037T, ORF1ab:C3140T, ORF1ab:G7037T, ORF1ab:C10029T, ORF1ab:C10954T, ORF1ab:A11117G, ORF1ab:C12789T, ORF1ab:C14408T, ORF1ab:C15240T, ORF1ab:T19839C, ORF1ab:C21306T, S:C22995A, S:A23403G, S:C23601A, S:C23604A, S:A23756G, S:A24820G, ORF3a:C25782T, N:G28881A, N:G28882A, N:G28883C, N:C29197T,	13	N:R203K, N:G204R, ORF1a:P959S, ORF1a:G2258C, ORF1a:T3255I, ORF1a:I3618V, ORF1a:T4175I, ORF1b:P314L, S:T478K, S:D614G, S:S680Y, S:P681H, S:T732A,
hCoV-19/Mexico/HID-InDRE_F10300_S806/2021	EPI_ISL_1424008	20B	B.1.1.519	28	5'UTR:C203T, 5'UTR:C222T, 5'UTR:C241T, ORF1ab:T1357C, ORF1ab:C3037T, ORF1ab:C3140T, ORF1ab:C6258T, ORF1ab:C8818T, ORF1ab:C10029T, ORF1ab:C10507T, ORF1ab:C10954T, ORF1ab:A11117G, ORF1ab:C12789T, ORF1ab:C14408T, ORF1ab:G14638T, ORF1ab:C15928T, ORF1ab:G17964A, ORF1ab:T19839C, ORF1ab:C21306T, S:C22995A, S:A23403G, S:C23604A, S:A23756G, S:A24433G, ORF3a:G25785T, N:G28881A, N:G28882A, N:G28883C, N:C29197T,	16	N:R203K, N:G204R, ORF1a:P959S, ORF1a:T1998I, ORF1a:T3255I, ORF1a:I3618V, ORF1a:T4175I, ORF1b:P314L, ORF1b:A391S, ORF1b:P821S, ORF1b:M1499I, ORF3a:W131C, S:T478K, S:D614G, S:P681H, S:T732A,
hCoV-19/Mexico/HID-InDRE_F10306_S627/2021	EPI_ISL_1340642	20B	B.1.1.519	25	5'UTR:T201C, 5'UTR:C203T, 5'UTR:C222T, 5'UTR:C241T, ORF1ab:C745T, ORF1ab:G1738T, ORF1ab:C3037T, ORF1ab:C3140T, ORF1ab:G6352T, ORF1ab:C10029T, ORF1ab:C10954T, ORF1ab:A11117G, ORF1ab:C12789T, ORF1ab:C14408T, ORF1ab:T19839C, ORF1ab:A19974G, ORF1ab:G20464T, ORF1ab:C21306T, S:C22995A, S:A23403G, S:C23604A, S:A23756G, N:G28881A, N:G28882A, N:G28883C, N:C29197T,	13	N:R203K, N:G204R, ORF1a:P959S, ORF1a:K2029N, ORF1a:T3255I, ORF1a:I3618V, ORF1a:T4175I, ORF1b:P314L, ORF1b:D2333Y, S:T478K, S:D614G, S:P681H, S:T732A,
hCoV-19/Mexico/HID-InDRE_F10309_S807/2021	EPI_ISL_1424009	20B	B.1.1.519	28	5'UTR:C203T, 5'UTR:C222T, 5'UTR:C241T, ORF1ab:G945T, ORF1ab:C3037T, ORF1ab:C3140T, ORF1ab:C6636T, ORF1ab:C10029T, ORF1ab:C10954T, ORF1ab:A11117G, ORF1ab:C12789T, ORF1ab:C14408T, ORF1ab:C14838T, ORF1ab:C16733T, ORF1ab:C16750T, ORF1ab:T19839C, ORF1ab:G20635C, ORF1ab:C21306T, S:C22995A, S:A23403G, S:C23604A, S:A23756G, ORF3a:C25416T, ORF3a:G26062T, M:C26912T, N:G28881A, N:G28882A, N:G28883C, N:C29197T	17	N:R203K, N:G204R, ORF1a:G227V, ORF1a:P959S, ORF1a:T2124I, ORF1a:T3255I, ORF1a:I3618V, ORF1a:T4175I, ORF1b:P314L, ORF1b:S1089L, ORF1b:P1095S, ORF1b:E2390Q, ORF3a:G224C, S:T478K, S:D614G, S:P681H, S:T732A,
hCoV-19/Mexico/HID-InDRE_F10781_S741/2021	EPI_ISL_1366690	20B	B.1.1.519	28	5'UTR:T201C, 5'UTR:C203T, 5'UTR:C222T, 5'UTR:C241T, ORF1ab:C1684T, ORF1ab:G1738T, ORF1ab:C3037T, ORF1ab:C3140T, ORF1ab:T9709C, ORF1ab:C10029T, ORF1ab:C10954T, ORF1ab:A11117G, ORF1ab:A12088G, ORF1ab:C12789T, ORF1ab:C14408T, ORF1ab:T19839C, ORF1ab:A19974G, ORF1ab:C21306T, S:C22995A, S:A23403G, S:C23604A, S:A23756G, ORF3a:C25484T, E:T26445G, N:G28881A, N:G28882A, N:G28883C, N:C29197T, N:G29315C	13	N:R203K, N:G204R, N:D348H, ORF1a:P959S, ORF1a:T3255I, ORF1a:I3618V, ORF1a:T4175I, ORF1b:P314L, ORF3a:A31V, S:T478K, S:D614G, S:P681H, S:T732A,

hCoV-19/Mexico/HID-InDRE_F10786_S742/2021	EPI_ISL_1366691	20B	B.1.1.519	27	5'UTR:T201C, 5'UTR:C203T, 5'UTR:C222T, 5'UTR:C241T, ORF1ab:C745T, ORF1ab:G1738T, ORF1ab:C3037T, ORF1ab:C3140T, ORF1ab:G6254A, ORF1ab:C10029T, ORF1ab:C10954T, ORF1ab:A11117G, ORF1ab:C12789T, ORF1ab:C14408T, ORF1ab:T19839C, ORF1ab:A19974G, ORF1ab:C21306T, S:C22995A, S:A23403G, S:C23410T, S:C23604A, S:A23756G, ORF8:G28077T, ORF8:G28178T, N:G28881A, N:G28882A, N:G28883C, N:C29197T,	14	N:R203K, N:G204R, ORF1a:P959S, ORF1a:A1997T, ORF1a:T3255I, ORF1a:I3618V, ORF1a:T4175I, ORF1b:P314L, ORF8:V62L, ORF8:L95F, S:T478K, S:D614G, S:P681H, S:T732A,	
hCoV-19/Mexico/HID-InDRE_F10789_S743/2021	EPI_ISL_1366692	20B	B.1.1.519	32	5'UTR:T201C, 5'UTR:C203T, 5'UTR:C222T, 5'UTR:C241T, ORF1ab:C745T, ORF1ab:G1738T, ORF1ab:C2544T, ORF1ab:C3037T, ORF1ab:C3140T, ORF1ab:C5281T, ORF1ab:G6254A, ORF1ab:C7267T, ORF1ab:C10029T, ORF1ab:C10954T, ORF1ab:A11117G, ORF1ab:C12789T, ORF1ab:C14408T, ORF1ab:T19839C, ORF1ab:A19974G, ORF1ab:C21306T, S:G22918T, S:C22995A, S:A23403G, S:C23604A, S:A23756G, ORF8:G28077T, ORF8:G28178T, N:A28477G, N:C28849T, N:G28881A, N:G28882A, N:G28883C, N:C29197T,	16	N:R203K, N:G204R, ORF1a:T760I, ORF1a:P959S, ORF1a:A1997T, ORF1a:T3255I, ORF1a:I3618V, ORF1a:T4175I, ORF1b:P314L, ORF8:V62L, ORF8:L95F, ORF9b:E65G, S:T478K, S:D614G, S:P681H, S:T732A,	
hCoV-19/Mexico/HID-InDRE-54/2020	EPI_ISL_576257	20B	B.1.1	14	5'UTR:C241T, ORF1ab:C3037T, ORF1ab:G4720T, ORF1ab:G7393T, ORF1ab:C9559T, ORF1ab:C14408T, ORF1ab:A18473G, ORF1ab:G19525A, ORF1ab:T19839C, S:A23403G, S:A24369G, ORF8:T27995C, N:G28881A, N:G28882A, N:G28883C,	7	N:R203K, N:G204R, ORF1b:P314L, ORF1b:Q1669R, ORF1b:D2020N, S:D614G, S:D936G,	
hCoV-19/Mexico/HID-InDRE-65/2020	EPI_ISL_576268	20B	B.1.1.222	18	5'UTR:C241T, ORF1ab:G1048A, ORF1ab:G2494T, ORF1ab:C3037T, ORF1ab:G5036A, ORF1ab:T5394G, ORF1ab:C12924T, ORF1ab:C14408T, ORF1ab:T19839C, ORF1ab:G21123T, S:A23403G, S:A23756G, M:C26735T, ORF7a:C27732T, ORF8:C28076T, N:G28690T, N:G28881A, N:G28882A, N:G28883C.	10	N:L139F, N:R203K, N:G204R, ORF1a:E743D, ORF1a:G1591R, ORF1a:F1710C, ORF1a:P4220L, ORF1b:P314L, S:D614G, S:T732A,	
hCoV-19/Mexico/JAL-InDRE_245/2021	EPI_ISL_1008714	20J (Gamma, V3	P.1	35	5'UTR:C241T, ORF1ab:T733C, ORF1ab:C2749T, ORF1ab:C3037T, ORF1ab:C3828T, ORF1ab:A5648C, ORF1ab:C5730T, ORF1ab:A6319G, ORF1ab:A6613G, ORF1ab:C12778T, ORF1ab:C13860T, ORF1ab:C14408T, ORF1ab:G17259T, ORF1ab:C18131T, S:C21614T, S:C21621A, S:C21638T, S:G21974T, S:G22132T, S:A22812C, S:G23012A, S:A23063T, S:A23403G, S:C23525T, S:C24642T, S:G25088T, ORF3a:T26149C, ORF8:G28167A, N:C28512G, N:A28877T, N:G28878C, N:G28881A, N:G28882A, N:G28883C, 3'UTR:T29834A, 3'UTR:T29858A,	24	N:P80R, N:R203K, N:G204R, ORF1a:S1188L, ORF1a:K1795Q, ORF1a:T1822I, ORF1b:P314L, ORF1b:E1264D, ORF1b:T1555I, ORF3a:S253P, ORF8:E92K, ORF9b:Q77E, S:L18F, S:T20N, S:P26S, S:D138Y, S:R190S, S:K417T, S:E484K, S:N501Y, S:D614G, S:H655Y, S:T1027I, S:V1176F,	ORF1ab: 11288-11296,
hCoV-19/Mexico/JAL-InDRE_313/2020	EPI_ISL_1060739	20A	B.1.609	14	5'UTR:C241T, ORF1ab:C3037T, ORF1ab:C4582T, ORF1ab:T6348G, ORF1ab:C7728T, ORF1ab:A10219C, ORF1ab:A10323G, ORF1ab:C13119T, ORF1ab:C14408T, ORF1ab:A18354G, ORF1ab:A20268G, S:A23403G, ORF3a:G25677T. ORF3a:G25947T. N:G28939T.	9	ORF1a:L2028R, ORF1a:S2488F, ORF1a:E3318D, ORF1a:K3353R, ORF1a:A4285V, ORF1b:P314L, ORF3a:L95F, ORF3a:Q185H, S:D614G,	
hCoV-19/Mexico/JAL-InDRE_371/2021	EPI_ISL_1093145	20B	P.2	23	5'UTR:C100T, 5'UTR:C241T, ORF1ab:C3037T, ORF1ab:T3477C, ORF1ab:T3766C, ORF1ab:C5694T, ORF1ab:C9711T, ORF1ab:T10667G, ORF1ab:C11824T, ORF1ab:C12053T, ORF1ab:A12964G, ORF1ab:C14408T, ORF1ab:G19101T, ORF1ab:A19578G, S:G23012A, S:A23403G, S:G25088T, ORF8:C28253T, N:G28628T, N:G28881A, N:G28882A, N:G28883C, N:G28975T, 3'UTR:C29754T,	14	N:A119S, N:R203K, N:G204R, N:M234I, ORF1a:V1071A, ORF1a:P1810L, ORF1a:S3149F, ORF1a:L3468V, ORF1a:L3930F, ORF1b:P314L, ORF1b:Q1878H, S:E484K, S:D614G, S:V1176F,	

hCoV-19/Mexico/JAL-InDRE_372/2021	EPI_ISL_1093146	20B	P.2	24	5'UTR:C100T, 5'UTR:C241T, ORF1ab:C3037T, ORF1ab:T3477C, ORF1ab:T3766C, ORF1ab:C5694T, ORF1ab:C9711T, ORF1ab:T10667G, ORF1ab:C11824T, ORF1ab:C12053T, ORF1ab:C12076T, ORF1ab:A12964G, ORF1ab:C14408T, ORF1ab:C17410T, ORF1ab:A19578G, S:G23012A, S:A23403G, S:G25088T, ORF8:C28253T, N:G28628T, N:G28881A, N:G28882A, N:G28883C, N:G28975T, 3'UTR:C29754T,	14	N:A119S, N:R203K, N:G204R, N:M234I, ORF1a:V1071A, ORF1a:P1810L, ORF1a:S3149F, ORF1a:L3468V, ORF1a:L3930F, ORF1b:P314L, ORF1b:R1315C, S:E484K, S:D614G, S:V1176F,
hCoV-19/Mexico/JAL-InDRE_373/2021	EPI_ISL_1093147	20B	P.2	24	5'UTR:C100T, 5'UTR:C241T, ORF1ab:C3037T, ORF1ab:T3477C, ORF1ab:T3766C, ORF1ab:C5694T, ORF1ab:C9711T, ORF1ab:T10667G, ORF1ab:C11824T, ORF1ab:C12053T, ORF1ab:A12964G, ORF1ab:C14408T, ORF1ab:C15720T, ORF1ab:A19578G, S:G22131T, S:G23012A, S:A23403G, S:G25088T, ORF8:C28253T, N:G28628T, N:G28881A, N:G28882A, N:G28883C, N:G28975T, 3'UTR:C29754T,	14	N:A119S, N:R203K, N:G204R, N:M234I, ORF1a:V1071A, ORF1a:P1810L, ORF1a:S3149F, ORF1a:L3468V, ORF1a:L3930F, ORF1b:P314L, S:R190M, S:E484K, S:D614G, S:V1176F,
hCoV-19/Mexico/JAL-InDRE_419/2021	EPI_ISL_1168497	20A	B.1.243	25	5'UTR:C193T, 5'UTR:C241T, ORF1ab:C274T, ORF1ab:T606C, ORF1ab:G806A, ORF1ab:T2584A, ORF1ab:C3037T, ORF1ab:C3874T, ORF1ab:C5140A, ORF1ab:C6027T, ORF1ab:G6476T, ORF1ab:G11596T, ORF1ab:C13255T, ORF1ab:C13620T, ORF1ab:C14408T, ORF1ab:C18486T, ORF1ab:A20268G, S:C22413T, S:G23120T, S:A23403G, S:G23587C, S:T24076C, ORF3a:C25549T, E:C26455T, N:C28854T, N:G29543T,	14	E:P71S, N:S194L, ORF1a:I114T, ORF1a:A181T, ORF1a:D1625E, ORF1a:P1921L, ORF1a:V2071F, ORF1a:Q3777H, ORF1b:P314L, ORF3a:L53F, S:T284I, S:A520S, S:D614G, S:Q675H,
hCoV-19/Mexico/JAL-InDRE_420/2021	EPI_ISL_1168498	20A	B.1	10	5'UTR:G61T, 5'UTR:C241T, ORF1ab:C3037T, ORF1ab:C14408T, ORF1ab:G20033A, ORF1ab:A20268G, S:A23403G, S:A24913G, N:C28854T, N:C29095T, 3'UTR:C29718T,	4	N:S194L, ORF1b:P314L, ORF1b:R2189H, S:D614G,
hCoV-19/Mexico/JAL-InDRE_421/2021	EPI_ISL_1168499	20B	B.1.1.519	22	5'UTR:C203T, 5'UTR:C222T, 5'UTR:C241T, ORF1ab:G2195T, ORF1ab:C3037T, ORF1ab:C3140T, ORF1ab:C10029T, ORF1ab:C10954T, ORF1ab:A11117G, ORF1ab:G12514T, ORF1ab:C12789T, ORF1ab:C14408T, ORF1ab:T19839C, ORF1ab:C20629T, ORF1ab:C21306T, S:C22995A, S:A23403G, S:C23604A, S:A23756G, N:G28881A, N:G28882A, N:G28883C, N:C29197T,	13	N:R203K, N:G204R, ORF1a:D644Y, ORF1a:P959S, ORF1a:T3255I, ORF1a:I3618V, ORF1a:T4175I, ORF1b:P314L, ORF1b:H2388Y, S:T478K, S:D614G, S:P681H, S:T732A,
hCoV-19/Mexico/JAL-InDRE_422/2021	EPI_ISL_1168500	20A	B.1.239	14	5'UTR:C241T, ORF1ab:C3037T, ORF1ab:C12651T, ORF1ab:C13620T, ORF1ab:C13994T, ORF1ab:C14408T, ORF1ab:G16741A, ORF1ab:A20268G, S:C21575T, S:A23403G, S:C24138T, ORF3a:T25519C, ORF3a:C25904T, N:G28337A, N:C28854T,	11	N:D22N, N:S194L, ORF1a:T4129I, ORF1b:A176V, ORF1b:P314L, ORF1b:V1092I, ORF3a:F43L, ORF3a:S171L, S:L5F, S:D614G, S:T859I,
hCoV-19/Mexico/JAL-InDRE_423/2021	EPI_ISL_1168501	21C (Epsilon)	B.1.429	25	5'UTR:C241T, ORF1ab:C1059T, ORF1ab:C2395T, ORF1ab:T2597C, ORF1ab:C3037T, ORF1ab:C8543T, ORF1ab:T8599C, ORF1ab:C8947T, ORF1ab:C12100T, ORF1ab:A12878G, ORF1ab:C14408T, ORF1ab:G17014T, S:G21600T, S:C21638T, S:G22018T, S:A22206G, S:T22917G, S:A23403G, S:T24283C, S:T24349C, ORF3a:G25563T, M:C26681T, ORF7b:G27890T, ORF8:A28272T, N:C28887T, N:C29362T,	13	N:T205I, ORF1a:T265I, ORF1a:L2760F, ORF1a:I4205V, ORF1b:P314L, ORF1b:D1183Y, ORF3a:Q57H, S:S13I, S:P26S, S:W152C, S:D215G, S:L452R, S:D614G,

hCoV-19/Mexico/JAL-InDRE_424/2021	EPI_ISL_1168502	20A	B.1.243	24	5'UTR:C241T, ORF1ab:C274T, ORF1ab:G806A, ORF1ab:G1946T, ORF1ab:T2584A, ORF1ab:C3037T, ORF1ab:C5140A, ORF1ab:C6027T, ORF1ab:C7528T, ORF1ab:G8013C, ORF1ab:G13843T, ORF1ab:C14408T, ORF1ab:A17256T, ORF1ab:C18377T, ORF1ab:C18486T, ORF1ab:A20268G, S:G23120T, S:G23282T, S:C23320T, S:A23403G, S:G23587C, S:T24076C, E:C26455T, N:C28854T, N:G29543T,	14	E:P71S, N:S194L, ORF1a:A181T, ORF1a:V561F, ORF1a:D1625E, ORF1a:P1921L, ORF1a:S2583T, ORF1b:D126Y, ORF1b:P314L, ORF1b:T1637I, S:A520S, S:D574Y, S:D614G, S:Q675H,
hCoV-19/Mexico/JAL-InDRE_425/2021	EPI_ISL_1168503	20A	B.1	16	5'UTR:C230T, 5'UTR:C241T, ORF1ab:C2706T, ORF1ab:C3037T, ORF1ab:C14408T, ORF1ab:C14724T, ORF1ab:C16375T, ORF1ab:G19723T, ORF1ab:G19947C, ORF1ab:A20268G, ORF1ab:A21137G, S:G21800T, S:C22482T, S:G23120T, S:A23403G, ORF3a:G25455A, N:C28854T,	11	N:S194L, ORF1a:T814I, ORF1b:P314L, ORF1b:P970S, ORF1b:V2086F, ORF1b:K2160N, ORF1b:K2557R, S:D80Y, S:T307I, S:A520S, S:D614G,
hCoV-19/Mexico/JAL-InDRE_426/2021	EPI_ISL_1168504	20B	B.1.1.519	23	5'UTR:C203T, 5'UTR:C222T, 5'UTR:C241T, ORF1ab:C3037T, ORF1ab:C3140T, ORF1ab:C3311T, ORF1ab:A8031G, ORF1ab:C10029T, ORF1ab:C10954T, ORF1ab:A11117G, ORF1ab:A11782G, ORF1ab:C12789T, ORF1ab:C14408T, ORF1ab:T19839C, ORF1ab:C21306T, S:C22995A, S:A23403G, S:C23604A, S:A23756G, E:C26270T, N:G28881A, N:G28882A, N:G28883C, N:C29197T,	14	E:T9I, N:R203K, N:G204R, ORF1a:P959S, ORF1a:L1016F, ORF1a:K2589R, ORF1a:T3255I, ORF1a:I3618V, ORF1a:T4175I, ORF1b:P314L, S:T478K, S:D614G, S:P681H, S:T732A,
hCoV-19/Mexico/JAL-InDRE_427/2021	EPI_ISL_1168505	21C (Epsilon)	B.1.427	25	5'UTR:C241T, ORF1ab:C1059T, ORF1ab:C2607T, ORF1ab:C3037T, ORF1ab:C3817T, ORF1ab:C5301T, ORF1ab:C7772T, ORF1ab:G9738C, ORF1ab:C9985T, ORF1ab:C13019T, ORF1ab:G13713A, ORF1ab:C14408T, ORF1ab:C16394T, ORF1ab:G17014T, ORF1ab:C18747T, S:G21600T, S:G22018T, S:C22597T, S:T22917G, S:A23403G, ORF3a:G25563T, M:C26681T, ORF8:C28087T, ORF8:A28272T, N:C28887T, N:C29362T,	15	N:T205I, ORF1a:T265I, ORF1a:T781I, ORF1a:A1679V, ORF1a:L2503F, ORF1a:S3158T, ORF1b:P314L, ORF1b:P976L, ORF1b:D1183Y, ORF3a:Q57H, ORF8:A65V, S:S13I, S:W152C, S:L452R, S:D614G,
hCoV-19/Mexico/JAL-InDRE_428/2021	EPI_ISL_1168506	20B	B.1.1	14	5'UTR:C241T, ORF1ab:A3027G, ORF1ab:C3037T, ORF1ab:C5301T, ORF1ab:C6696T, ORF1ab:A8244G, ORF1ab:C11020T, ORF1ab:C14408T, S:A23403G, ORF8:C27970T, ORF8:G28077A, N:G28881A, N:G28882A, N:G28883C, N:G29516A,	11	N:R203K, N:G204R, N:D415N, ORF1a:Y921C, ORF1a:A1679V, ORF1a:P2144L, ORF1a:Q2660R, ORF1b:P314L, ORF8:T26I, ORF8:V62M, S:D614G,
hCoV-19/Mexico/JAL-InDRE_429/2021	EPI_ISL_1168507	20A	B.1.243	18	5'UTR:C241T, ORF1ab:G806A, ORF1ab:T2584A, ORF1ab:C3037T, ORF1ab:C5140A, ORF1ab:A10323G, ORF1ab:G10846T, ORF1ab:C14408T, ORF1ab:C19113T, ORF1ab:T19149C, ORF1ab:A20268G, S:A23403G, S:G23587C, S:T24076C, ORF3a:T25899C, ORF3a:G26062T, ORF7b:G27827T, N:C28854T, N:G29543T,	10	N:S194L, ORF1a:A181T, ORF1a:D1625E, ORF1a:K3353R, ORF1a:M3527I, ORF1b:P314L, ORF3a:G224C, ORF7b:M24I, S:D614G, S:Q675H,
hCoV-19/Mexico/JAL-InDRE_430/2021	EPI_ISL_1168508	20A	B.1.243	21	5'UTR:C241T, ORF1ab:C274T, ORF1ab:G806A, ORF1ab:T1344C, ORF1ab:T2584A, ORF1ab:C3037T, ORF1ab:C5140A, ORF1ab:C6027T, ORF1ab:C7119T, ORF1ab:C14408T, ORF1ab:T14619C, ORF1ab:G15530T, ORF1ab:C18486T, ORF1ab:A20268G, S:G23120T, S:A23403G, S:G23587C, S:T24076C, E:C26455T, N:C28854T, N:G29543T, 3'UTR:T29834C,	12	E:P71S, N:S194L, ORF1a:A181T, ORF1a:L360S, ORF1a:D1625E, ORF1a:P1921L, ORF1a:S2285F, ORF1b:P314L, ORF1b:C688F, S:A520S, S:D614G, S:Q675H,

hCoV-19/Mexico/JAL-InDRE_431/2021	EPI_ISL_1168509	20B	B.1.1.519	24	5'UTR:T201C, 5'UTR:C203T, 5'UTR:C222T, 5'UTR:C241T, ORF1ab:G1738T, ORF1ab:C3037T, ORF1ab:C3140T, ORF1ab:C10029T, ORF1ab:C10954T, ORF1ab:A11117G, ORF1ab:C12789T, ORF1ab:C14408T, ORF1ab:T19839C, ORF1ab:A19974G, ORF1ab:G20434A, ORF1ab:G20578T, ORF1ab:C21306T, S:C22995A, S:A23403G, S:C23604A, S:A23756G, N:G28881A, N:G28882A, N:G28883C, N:C29197T.	13	N:R203K, N:G204R, ORF1a:P959S, ORF1a:T3255I, ORF1a:I3618V, ORF1a:T4175I, ORF1b:P314L, ORF1b:D2323N, ORF1b:V2371L, S:T478K, S:D614G, S:P681H, S:T732A,
hCoV-19/Mexico/JAL-InDRE_432/2021	EPI_ISL_1168510	20A	B.1.239	14	5'UTR:C241T, ORF1ab:C3037T, ORF1ab:C8481T, ORF1ab:C13620T, ORF1ab:C13994T, ORF1ab:C14408T, ORF1ab:G16741A, ORF1ab:A20268G, S:A23403G, ORF3a:T25852C, ORF3a:C25904T, ORF7a:C27600T, N:C28854T, N:G28985T, 3'UTR:T29710A.	9	N:S194L, N:G238C, ORF1a:P2739L, ORF1b:A176V, ORF1b:P314L, ORF1b:V1092I, ORF3a:Y154H, ORF3a:S171L, S:D614G,
hCoV-19/Mexico/JAL-InDRE_433/2021	EPI_ISL_1168511	20A	B.1.243	19	5'UTR:C241T, ORF1ab:G806A, ORF1ab:T2584A, ORF1ab:C3037T, ORF1ab:G3880T, ORF1ab:C5140A, ORF1ab:C12132T, ORF1ab:C14408T, ORF1ab:C15738T, ORF1ab:C16658T, ORF1ab:C16952T, ORF1ab:A20268G, S:C21855T, S:A23403G, S:G23587C, S:T24076C, ORF3a:C25844T, M:C27002T, N:C28854T, N:G29543T.	12	N:S194L, ORF1a:A181T, ORF1a:E1205D, ORF1a:D1625E, ORF1a:A3956V, ORF1b:P314L, ORF1b:T1064I, ORF1b:T1162I, ORF3a:T151I, S:S98F, S:D614G, S:Q675H,
hCoV-19/Mexico/JAL-InDRE_434/2021	EPI_ISL_1168512	20G	B.1.2	22	5'UTR:C241T, ORF1ab:C1059T, ORF1ab:C3037T, ORF1ab:C8140T, ORF1ab:G9928T, ORF1ab:C10319T, ORF1ab:C14408T, ORF1ab:C15960T, ORF1ab:C18167T, ORF1ab:A18424G, ORF1ab:C21304T, S:C21646T, S:A23403G, S:C24068A, ORF3a:G25563T, ORF3a:G25855T, ORF3a:G25907T, M:C27002T, ORF8:C27945T, ORF8:C27964T, ORF8:C28011G, N:C28472T, N:C28869T.	17	N:P67S, N:P199L, ORF1a:T265I, ORF1a:M3221I, ORF1a:L3352F, ORF1b:P314L, ORF1b:P1567L, ORF1b:N1653D, ORF1b:R2613C, ORF3a:Q57H, ORF3a:D155Y, ORF3a:G172V, ORF8:Q18*, ORF8:S24L, ORF8:H40D, S:D614G, S:Q836K,
hCoV-19/Mexico/JAL-InDRE_435/2021	EPI_ISL_1168513	21C (Epsilon)	B.1.427	21	5'UTR:C241T, ORF1ab:C1059T, ORF1ab:C3037T, ORF1ab:G9738C, ORF1ab:G12769T, ORF1ab:G13304T, ORF1ab:G13713A, ORF1ab:C14408T, ORF1ab:C14845T, ORF1ab:C16394T, ORF1ab:G17014T, ORF1ab:C17104T, S:G21600T, S:G22018T, S:T22917G, S:A23403G, ORF3a:G25563T, M:C26681T, ORF8:A28272T, N:C28887T, N:C29218T, N:C29362T.	13	N:T205I, ORF1a:T265I, ORF1a:S3158T, ORF1a:G4347C, ORF1b:P314L, ORF1b:P976L, ORF1b:D1183Y, ORF1b:H1213Y, ORF3a:Q57H, S:S13I, S:W152C, S:L452R, S:D614G,
hCoV-19/Mexico/JAL-InDRE_436/2021	EPI_ISL_1168514	20G	B.1.2	20	5'UTR:C241T, ORF1ab:C1059T, ORF1ab:G2732A, ORF1ab:C3037T, ORF1ab:G3340T, ORF1ab:C6633T, ORF1ab:G8368A, ORF1ab:C9502T, ORF1ab:C10319T, ORF1ab:C14408T, ORF1ab:C15328T, ORF1ab:C16536T, ORF1ab:A18424G, ORF1ab:C21304T, S:A23403G, ORF3a:G25563T, ORF3a:G25907T, ORF3a:C26204T, ORF8:C27964T, N:C28472T, N:C28869T,	15	N:P67S, N:P199L, ORF1a:T265I, ORF1a:V823I, ORF1a:A2123V, ORF1a:L3352F, ORF1b:P314L, ORF1b:L621F, ORF1b:N1653D, ORF1b:R2613C, ORF3a:Q57H, ORF3a:G172V, ORF3a:T271I, ORF8:S24L, S:D614G,
hCoV-19/Mexico/JAL-InDRE_437/2021	EPI_ISL_1168515	20A	B.1.243	18	5'UTR:C241T, ORF1ab:G806A, ORF1ab:T2584A, ORF1ab:C3037T, ORF1ab:C5140A, ORF1ab:A10323G, ORF1ab:G10846T, ORF1ab:C14408T, ORF1ab:C19113T, ORF1ab:T19149C, ORF1ab:A20268G, S:A23403G, S:G23587C, S:T24076C, ORF3a:T25899C, ORF3a:G26062T, ORF7b:G27827T, N:C28854T, N:G29543T.	10	N:S194L, ORF1a:A181T, ORF1a:D1625E, ORF1a:K3353R, ORF1a:M3527I, ORF1b:P314L, ORF3a:G224C, ORF7b:M24I, S:D614G, S:Q675H,

hCoV-19/Mexico/JAL-InDRE_438/2021	EPI_ISL_1168516	21C (Epsilon)	B.1.427	23	5'UTR:C241T, ORF1ab:C1059T, ORF1ab:C3037T, ORF1ab:C5812T, ORF1ab:G9738C, ORF1ab:G13713A, ORF1ab:C14408T, ORF1ab:C15540T, ORF1ab:C16394T, ORF1ab:G17014T, ORF1ab:G20931T, S:G21600T, S:G22018T, S:T22917G, S:A23403G, S:C23929T, ORF3a:G25563T, M:C26681T, M:G26918T, ORF8:C28253T, ORF8:A28272T, N:C28887T, N:C29109A, N:C29362T,	12	N:T205I, N:P279Q, ORF1a:T265I, ORF1a:S3158T, ORF1b:P314L, ORF1b:P976L, ORF1b:D1183Y, ORF3a:Q57H, S:S13I, S:W152C, S:L452R, S:D614G,	
hCoV-19/Mexico/JAL-InDRE_439/2021	EPI_ISL_1168517	20A	B.1.243	22	5'UTR:C241T, ORF1ab:C274T, ORF1ab:G806A, ORF1ab:T2584A, ORF1ab:C3037T, ORF1ab:C5140A, ORF1ab:C6027T, ORF1ab:C10336T, ORF1ab:C12008T, ORF1ab:A14041G, ORF1ab:C14408T, ORF1ab:G16365T, ORF1ab:A18298G, ORF1ab:C18486T, ORF1ab:A20268G, S:G23120T, S:A23403G, S:C23536T, S:G23587C, S:T24076C, E:C26455T, N:C28854T, N:G29543T,	13	E:P71S, N:S194L, ORF1a:A181T, ORF1a:D1625E, ORF1a:P1921L, ORF1a:L3915F, ORF1b:I192V, ORF1b:P314L, ORF1b:L966F, ORF1b:I1611V, S:A520S, S:D614G, S:Q675H,	
hCoV-19/Mexico/JAL-InDRE_440/2021	EPI_ISL_1168518	21C (Epsilon)	B.1.427	25	5'UTR:C147T, 5'UTR:C241T, ORF1ab:C1059T, ORF1ab:C2607T, ORF1ab:C3037T, ORF1ab:C3817T, ORF1ab:G9738C, ORF1ab:C11776T, ORF1ab:C13019T, ORF1ab:G13713A, ORF1ab:C14408T, ORF1ab:G15594T, ORF1ab:C16394T, ORF1ab:G17014T, S:G21600T, S:G22018T, S:C22597T, S:T22917G, S:A23403G, ORF3a:G25563T, M:C26681T, ORF8:C27972T, ORF8:C28087T, ORF8:A28272T, N:C28887T, N:C29362T,	15	N:T205I, ORF1a:T265I, ORF1a:T781I, ORF1a:S3158T, ORF1b:P314L, ORF1b:K709N, ORF1b:P976L, ORF1b:D1183Y, ORF3a:Q57H, ORF8:Q27*, ORF8:A65V, S:S13I, S:W152C, S:L452R, S:D614G,	3'UTR:29762-29766
hCoV-19/Mexico/JAL-InDRE_441/2021	EPI_ISL_1168519	20A	B.1.243	22	5'UTR:C241T, ORF1ab:C274T, ORF1ab:G625T, ORF1ab:G806A, ORF1ab:T2584A, ORF1ab:C3037T, ORF1ab:C5140A, ORF1ab:C5974T, ORF1ab:C6027T, ORF1ab:T11121G, ORF1ab:T13006A, ORF1ab:C13432T, ORF1ab:C14408T, ORF1ab:C18486T, ORF1ab:C19524T, ORF1ab:A20268G, S:G23120T, S:A23403G, S:G23587C, S:T24076C, E:C26455T, N:C28854T, N:G29543T,	11	E:P71S, N:S194L, ORF1a:K120N, ORF1a:A181T, ORF1a:D1625E, ORF1a:P1921L, ORF1a:I3619S, ORF1b:P314L, S:A520S, S:D614G, S:Q675H,	
hCoV-19/Mexico/JAL-InDRE_442/2021	EPI_ISL_1168520	20B	B.1.1.222	24	5'UTR:C241T, ORF1ab:C3037T, ORF1ab:C6786T, ORF1ab:C10252T, ORF1ab:T10819C, ORF1ab:C12513T, ORF1ab:C14408T, ORF1ab:T17208C, ORF1ab:A17946T, ORF1ab:T19839C, S:C21846T, S:A23403G, S:A23756G, S:C24034T, ORF3a:C25708T, M:G26720T, M:G26774T, ORF8:C28253T, ORF8:A28272G, N:A28522G, N:G28881A, N:G28882A, N:G28883C, N:G29383A, 3'UTR:G29751T,	11	M:M84I, N:R203K, N:G204R, ORF1a:T2174I, ORF1a:T4083M, ORF1b:P314L, ORF3a:L106F, ORF9b:K80R, S:T95I, S:D614G, S:T732A,	
hCoV-19/Mexico/JAL-InDRE_482/2021	EPI_ISL_1168552	20G	B.1.2	21	5'UTR:C241T, ORF1ab:T517C, ORF1ab:C1059T, ORF1ab:C3037T, ORF1ab:T4684C, ORF1ab:C10319T, ORF1ab:C10833T, ORF1ab:G13094T, ORF1ab:T13216C, ORF1ab:G14187A, ORF1ab:C14408T, ORF1ab:A18424G, ORF1ab:C21304T, ORF1ab:A21390G, S:C21805T, S:A23403G, S:G25249T, ORF3a:G25563T, ORF3a:G25907T, ORF8:C27964T, N:C28472T, N:C28869T,	14	N:P67S, N:P199L, ORF1a:T265I, ORF1a:L3352F, ORF1a:A3523V, ORF1a:A4277S, ORF1b:P314L, ORF1b:N1653D, ORF1b:R2613C, ORF3a:Q57H, ORF3a:G172V, ORF8:S24L, S:D614G, S:M1229I,	
hCoV-19/Mexico/JAL-InDRE_549/2021	EPI_ISL_1168619	20A	B.1.243	18	5'UTR:C241T, ORF1ab:G806A, ORF1ab:T2584A, ORF1ab:C3037T, ORF1ab:A3076T, ORF1ab:G4790A, ORF1ab:C5140A, ORF1ab:C6388T, ORF1ab:C8299T, ORF1ab:T10526G, ORF1ab:C14408T, ORF1ab:A20268G, S:A23403G, S:G23587C, S:T24076C, S:G24257T, ORF3a:C25844T, N:C28854T, N:G29543T,	11	N:S194L, ORF1a:A181T, ORF1a:E937D, ORF1a:G1509S, ORF1a:D1625E, ORF1a:S3421A, ORF1b:P314L, ORF3a:T151I, S:D614G, S:Q675H, S:A899S,	

hCoV-19/Mexico/JAL-InDRE_550/2021	EPI_ISL_1168620	20A	B.1.243	27	5'UTR:C241T, ORF1ab:C274T, ORF1ab:G806A, ORF1ab:G1505A, ORF1ab:G2086A, ORF1ab:T2584A, ORF1ab:C3037T, ORF1ab:C3738T, ORF1ab:C5140A, ORF1ab:C6027T, ORF1ab:G13812T, ORF1ab:A14236G, ORF1ab:C14408T, ORF1ab:C15240T, ORF1ab:C18486T, ORF1ab:A20268G, S:T21598A, S:G23120T, S:A23403G, S:G23587C, S:T24076C, ORF3a:G25538T, E:C26455T, ORF8:C28138A, N:C28854T, N:G29543T, 3'UTR:G29862T, 3'UTR:A29866C,	14	E:P71S, N:S194L, ORF1a:A181T, ORF1a:V414I, ORF1a:P1158L, ORF1a:D1625E, ORF1a:P1921L, ORF1b:M115I, ORF1b:I257V, ORF1b:P314L, ORF3a:G49V, S:A520S, S:D614G, S:Q675H,
hCoV-19/Mexico/JAL-InDRE_551/2021	EPI_ISL_1168621	20A	B.1.243	20	5'UTR:C241T, ORF1ab:C274T, ORF1ab:G806A, ORF1ab:T2584A, ORF1ab:C3037T, ORF1ab:C5140A, ORF1ab:C6027T, ORF1ab:C6555T, ORF1ab:G6865T, ORF1ab:C14408T, ORF1ab:C18486T, ORF1ab:A20268G, S:G23120T, S:C23320T, S:A23403G, S:G23587C, S:T24076C, S:A24400G, E:C26455T, N:C28854T, N:G29543T,	11	E:P71S, N:S194L, ORF1a:A181T, ORF1a:D1625E, ORF1a:P1921L, ORF1a:A2097V, ORF1a:K2200N, ORF1b:P314L, S:A520S, S:D614G, S:Q675H,
hCoV-19/Mexico/JAL-InDRE_552/2021	EPI_ISL_1168622	20B	B.1.1.519	25	5'UTR:C203T, 5'UTR:C222T, 5'UTR:C241T, ORF1ab:C3037T, ORF1ab:C3140T, ORF1ab:G5012T, ORF1ab:C10029T, ORF1ab:A10829C, ORF1ab:C10954T, ORF1ab:A11117G, ORF1ab:C14408T, ORF1ab:T19839C, ORF1ab:C21306T, S:C22995A, S:A23403G, S:C23604A, S:A23756G, ORF3a:C25626T, ORF8:T27904C, ORF8:C28087T, N:G28881A, N:G28882A, N:G28883C, N:C29197T, 3'UTR:G29862T, 3'UTR:A29866C,	14	N:R203K, N:G204R, ORF1a:P959S, ORF1a:V1583F, ORF1a:T3255I, ORF1a:I3522L, ORF1a:I3618V, ORF1b:P314L, ORF8:L4P, ORF8:A65V, S:T478K, S:D614G, S:P681H, S:T732A,
hCoV-19/Mexico/JAL-InDRE_553/2021	EPI_ISL_1168623	20G	B.1.2	23	5'UTR:C241T, ORF1ab:C1059T, ORF1ab:C3037T, ORF1ab:C9430T, ORF1ab:C10319T, ORF1ab:C11003T, ORF1ab:C14408T, ORF1ab:C14670T, ORF1ab:T14808C, ORF1ab:C16329T, ORF1ab:A18424G, ORF1ab:C21304T, S:A23403G, S:C24034T, ORF3a:G25563T, ORF3a:G25907T, M:C26873T, ORF8:C27964T, N:C28472T, N:T28723C, N:C28869T, N:G29402T, 3'UTR:G29862T, 3'UTR:A29866C,	13	N:P67S, N:P199L, N:D377Y, ORF1a:T265I, ORF1a:L3352F, ORF1a:H3580Y, ORF1b:P314L, ORF1b:N1653D, ORF1b:R2613C, ORF3a:Q57H, ORF3a:G172V, ORF8:S24L, S:D614G,
hCoV-19/Mexico/JAL-InDRE_F100G_S576/2021	EPI_ISL_1324759	20B	P.2	26	5'UTR:C100T, 5'UTR:C241T, ORF1ab:C3037T, ORF1ab:T3477C, ORF1ab:T3766C, ORF1ab:C4456T, ORF1ab:C5694T, ORF1ab:C8964T, ORF1ab:C9711T, ORF1ab:T10667G, ORF1ab:C11824T, ORF1ab:C12053T, ORF1ab:C12073T, ORF1ab:A12964G, ORF1ab:C14408T, ORF1ab:A19578G, S:G23012A, S:A23403G, S:G25088T, S:C25231T, ORF8:C28253T, N:G28628T, N:G28881A, N:G28882A, N:G28883C, N:G28975T, 3'UTR:C29754T,	14	N:A119S, N:R203K, N:G204R, N:M234I, ORF1a:V1071A, ORF1a:P1810L, ORF1a:S2900L, ORF1a:S3149F, ORF1a:L3468V, ORF1a:L3930F, ORF1b:P314L, S:E484K, S:D614G, S:V1176F,
hCoV-19/Mexico/JAL-InDRE_F101G_S577/2021	EPI_ISL_1324760	20B	P.2	24	5'UTR:C100T, 5'UTR:C241T, ORF1ab:C1912T, ORF1ab:C3037T, ORF1ab:T3477C, ORF1ab:T3766C, ORF1ab:C5694T, ORF1ab:C9711T, ORF1ab:T10667G, ORF1ab:C11824T, ORF1ab:C12053T, ORF1ab:A12820G, ORF1ab:A12964G, ORF1ab:C14408T, ORF1ab:A19578G, S:G23012A, S:A23403G, S:G25088T, ORF8:C28253T, N:G28628T, N:G28881A, N:G28882A, N:G28883C, N:G28975T, 3'UTR:C29754T,	13	N:A119S, N:R203K, N:G204R, N:M234I, ORF1a:V1071A, ORF1a:P1810L, ORF1a:S3149F, ORF1a:L3468V, ORF1a:L3930F, ORF1b:P314L, S:E484K, S:D614G, S:V1176F,

hCoV-19/Mexico/JAL-InDRE_F102G_S578/2021	EPI_ISL_1324761	20B	P.2	23	5'UTR:C100T, 5'UTR:C241T, ORF1ab:C1912T, ORF1ab:C3037T, ORF1ab:T3477C, ORF1ab:T3766C, ORF1ab:C5694T, ORF1ab:C9711T, ORF1ab:T10667G, ORF1ab:C11824T, ORF1ab:C12053T, ORF1ab:A12964G, ORF1ab:C14408T, ORF1ab:A19578G, S:G23012A, S:A23403G, S:G25088T, ORF8:C28253T, N:G28628T, N:G28881A, N:G28882A, N:G28883C, N:G28975T, 3'UTR:C29754T,	13	N:A119S, N:R203K, N:G204R, N:M234I, ORF1a:V1071A, ORF1a:P1810L, ORF1a:S3149F, ORF1a:L3468V, ORF1a:L3930F, ORF1b:P314L, S:E484K, S:D614G, S:V1176F,
hCoV-19/Mexico/JAL-InDRE_F103G_S579/2021	EPI_ISL_1324762	20B	P.2	26	5'UTR:C100T, 5'UTR:C241T, ORF1ab:G1820A, ORF1ab:C3037T, ORF1ab:T3477C, ORF1ab:T3766C, ORF1ab:C5694T, ORF1ab:T6046C, ORF1ab:C9711T, ORF1ab:T10667G, ORF1ab:C11824T, ORF1ab:C12053T, ORF1ab:A12964G, ORF1ab:C14408T, ORF1ab:A19578G, S:G23012A, S:A23403G, S:G25088T, ORF3a:C26198T, ORF8:C28253T, N:G28628T, N:G28881A, N:G28882A, N:G28883C, N:G28975T, 3'UTR:C29754T, 3'UTR:C29762T,	15	N:A119S, N:R203K, N:G204R, N:M234I, ORF1a:G519S, ORF1a:V1071A, ORF1a:P1810L, ORF1a:S3149F, ORF1a:L3468V, ORF1a:L3930F, ORF1b:P314L, ORF3a:T269M, S:E484K, S:D614G, S:V1176F,
hCoV-19/Mexico/JAL-InDRE_F10432_S712/2021	EPI_ISL_1366661	20B	B.1.1.519	23	5'UTR:C203T, 5'UTR:C222T, 5'UTR:C241T, ORF1ab:C3037T, ORF1ab:C3140T, ORF1ab:T4339C, ORF1ab:C10029T, ORF1ab:C10954T, ORF1ab:A11117G, ORF1ab:C12789T, ORF1ab:C14408T, ORF1ab:T19839C, ORF1ab:C21306T, S:C22995A, S:A23403G, S:C23604A, S:A23756G, ORF3a:G25906T, ORF7a:G27651T, ORF8:C28253T, N:G28881A, N:G28882A, N:G28883C, N:C29197T,	12	N:R203K, N:G204R, ORF1a:P959S, ORF1a:T3255I, ORF1a:I3618V, ORF1a:T4175I, ORF1b:P314L, ORF3a:G172C, S:T478K, S:D614G, S:P681H, S:T732A,
hCoV-19/Mexico/JAL-InDRE_F10438_S713/2021	EPI_ISL_1366662	20B	B.1.1.519	27	5'UTR:C203T, 5'UTR:C222T, 5'UTR:C241T, ORF1ab:C3037T, ORF1ab:C3140T, ORF1ab:C10029T, ORF1ab:C10954T, ORF1ab:A11117G, ORF1ab:G11365T, ORF1ab:T11475C, ORF1ab:C12789T, ORF1ab:C14408T, ORF1ab:T19839C, ORF1ab:C21077T, ORF1ab:C21306T, S:C22995A, S:A23403G, S:C23604A, S:C23683T, S:A23756G, S:G24368C, M:C26919G, N:G28881A, N:G28882A, N:G28883C, N:C29197T, N:C29509T, N:G29527T,	16	M:L133V, N:R203K, N:G204R, N:Q418H, ORF1a:P959S, ORF1a:T3255I, ORF1a:I3618V, ORF1a:I3737T, ORF1a:T4175I, ORF1b:P314L, ORF1b:T2537I, S:T478K, S:D614G, S:P681H, S:T732A, S:D936H,
hCoV-19/Mexico/JAL-InDRE_F104G_S580/2021	EPI_ISL_1324763	20B	P.2	24	5'UTR:C100T, 5'UTR:C241T, ORF1ab:C3037T, ORF1ab:T3477C, ORF1ab:T3766C, ORF1ab:C5694T, ORF1ab:C9711T, ORF1ab:T10667G, ORF1ab:C11824T, ORF1ab:C12053T, ORF1ab:A12964G, ORF1ab:C14408T, ORF1ab:C16428T, ORF1ab:A19578G, S:G23012A, S:A23403G, S:G25088T, ORF8:C28253T, N:G28628T, N:G28881A, N:G28882A, N:G28883C, N:G28975T, 3'UTR:C29733T, 3'UTR:C29754T,	13	N:A119S, N:R203K, N:G204R, N:M234I, ORF1a:V1071A, ORF1a:P1810L, ORF1a:S3149F, ORF1a:L3468V, ORF1a:L3930F, ORF1b:P314L, S:E484K, S:D614G, S:V1176F,
hCoV-19/Mexico/JAL-InDRE_F105G_S581/2021	EPI_ISL_1324764	20B	P.2	24	5'UTR:C100T, 5'UTR:C241T, ORF1ab:C3037T, ORF1ab:T3477C, ORF1ab:T3766C, ORF1ab:C4993T, ORF1ab:C5694T, ORF1ab:C9711T, ORF1ab:T10667G, ORF1ab:C11824T, ORF1ab:C12053T, ORF1ab:A12964G, ORF1ab:C14408T, ORF1ab:A19578G, S:G23012A, S:A23403G, S:G25088T, ORF8:C28253T, N:G28628T, N:G28881A, N:G28882A, N:G28883C, N:G28975T, 3'UTR:C29721A, 3'UTR:C29754T,	13	N:A119S, N:R203K, N:G204R, N:M234I, ORF1a:V1071A, ORF1a:P1810L, ORF1a:S3149F, ORF1a:L3468V, ORF1a:L3930F, ORF1b:P314L, S:E484K, S:D614G, S:V1176F,

hCoV-19/Mexico/JAL-InDRE_F106G_S582/2021	EPI_ISL_1324765	20B	P.2	23	5'UTR:C100T, 5'UTR:C241T, ORF1ab:C1441T, ORF1ab:C3037T, ORF1ab:T3477C, ORF1ab:T3766C, ORF1ab:C5694T, ORF1ab:C9711T, ORF1ab:T10667G, ORF1ab:C11824T, ORF1ab:C12053T, ORF1ab:A12964G, ORF1ab:C14408T, ORF1ab:A19578G, S:G23012A, S:A23403G, S:G25088T, ORF8:C28253T, N:G28628T, N:G28881A, N:G28882A, N:G28883C, N:G28975T, 3'UTR:C29754T,	13	N:A119S, N:R203K, N:G204R, N:M234I, ORF1a:V1071A, ORF1a:P1810L, ORF1a:S3149F, ORF1a:L3468V, ORF1a:L3930F, ORF1b:P314L, S:E484K, S:D614G, S:V1176F,
hCoV-19/Mexico/JAL-InDRE_F107G_S583/2021	EPI_ISL_1324766	20B	P.2	23	5'UTR:C100T, 5'UTR:C241T, ORF1ab:C3037T, ORF1ab:T3477C, ORF1ab:T3766C, ORF1ab:C5694T, ORF1ab:C9711T, ORF1ab:T10667G, ORF1ab:C11824T, ORF1ab:C12053T, ORF1ab:A12964G, ORF1ab:C14408T, ORF1ab:A19578G, S:G23012A, S:A23403G, S:G25088T, ORF8:C28253T, N:G28436T, N:G28628T, N:G28881A, N:G28882A, N:G28883C, N:G28975T, 3'UTR:C29754T,	14	N:A55S, N:A119S, N:R203K, N:G204R, N:M234I, ORF1a:V1071A, ORF1a:P1810L, ORF1a:S3149F, ORF1a:L3468V, ORF1a:L3930F, ORF1b:P314L, S:E484K, S:D614G, S:V1176F,
hCoV-19/Mexico/JAL-InDRE_F10947_S865/2021	EPI_ISL_1399270	20A	B.1.243	21	5'UTR:C241T, ORF1ab:C274T, ORF1ab:G806A, ORF1ab:C2003T, ORF1ab:T2584A, ORF1ab:C3037T, ORF1ab:C5140A, ORF1ab:C5826T, ORF1ab:C6027T, ORF1ab:C14408T, ORF1ab:C18486T, ORF1ab:A20268G, S:C21622T, S:G23120T, S:A23403G, S:G23587C, S:T23605C, S:T24076C, E:C26455T, N:G28378T, N:C28854T, N:G29543T,	12	E:P71S, N:S194L, ORF1a:A181T, ORF1a:L580F, ORF1a:D1625E, ORF1a:T1854I, ORF1a:P1921L, ORF1b:P314L, ORF9b:R32L, S:A520S, S:D614G, S:Q675H,
hCoV-19/Mexico/JAL-InDRE_F10948_S752/2021	EPI_ISL_1400298	20B	B.1.1.519	22	5'UTR:C203T, 5'UTR:C222T, 5'UTR:C241T, ORF1ab:C3037T, ORF1ab:C3140T, ORF1ab:C10029T, ORF1ab:C10714T, ORF1ab:C10954T, ORF1ab:A11117G, ORF1ab:C12789T, ORF1ab:C14408T, ORF1ab:T19839C, ORF1ab:C21306T, S:C21622T, S:C22995A, S:A23403G, S:C23604A, S:A23756G, N:G28881A, N:G28882A, N:G28883C, N:C29197T, N:C29555T,	11	N:R203K, N:G204R, ORF1a:P959S, ORF1a:T3255I, ORF1a:I3618V, ORF1a:T4175I, ORF1b:P314L, S:T478K, S:D614G, S:P681H, S:T732A,
hCoV-19/Mexico/JAL-InDRE_F10954_S881/2021	EPI_ISL_1405913	20A	B.1.243	25	5'UTR:C241T, ORF1ab:C274T, ORF1ab:G806A, ORF1ab:T2584A, ORF1ab:C3037T, ORF1ab:C5140A, ORF1ab:C5167T, ORF1ab:C6027T, ORF1ab:C9165T, ORF1ab:C10336T, ORF1ab:G11083T, ORF1ab:C12008T, ORF1ab:C14408T, ORF1ab:C18486T, ORF1ab:A20268G, S:C21575T, S:C22227T, S:G23120T, S:A23403G, S:C23536T, S:G23587C, S:T24076C, E:C26455T, N:C28531T, N:C28854T, N:G29543T,	15	E:P71S, N:S194L, ORF1a:A181T, ORF1a:D1625E, ORF1a:P1921L, ORF1a:T2967I, ORF1a:L3606F, ORF1a:L3915F, ORF1b:P314L, ORF9b:T83I, S:L5F, S:A222V, S:A520S, S:D614G, S:Q675H,
hCoV-19/Mexico/JAL-InDRE_F10958_S880/2021	EPI_ISL_1405911	20B	B.1.1.519	27	5'UTR:T201C, 5'UTR:C203T, 5'UTR:C222T, 5'UTR:C241T, ORF1ab:C936T, ORF1ab:G1738T, ORF1ab:C3037T, ORF1ab:C3140T, ORF1ab:A9760G, ORF1ab:C10029T, ORF1ab:C10954T, ORF1ab:A11117G, ORF1ab:C12789T, ORF1ab:C14408T, ORF1ab:G15349A, ORF1ab:A19974G, ORF1ab:T21183C, ORF1ab:C21306T, S:C22995A, S:A23403G, S:C23604A, S:A23756G, N:G28881A, N:G28882A, N:G28883C, N:C29167T, N:C29197T, N:G29227T,	13	N:R203K, N:G204R, ORF1a:T224I, ORF1a:P959S, ORF1a:T3255I, ORF1a:I3618V, ORF1a:T4175I, ORF1b:P314L, ORF1b:V628I, S:T478K, S:D614G, S:P681H, S:T732A,
hCoV-19/Mexico/JAL-InDRE_F10959_S879/2021	EPI_ISL_1405908	20A	B.1.239	15	5'UTR:C241T, ORF1ab:A372G, ORF1ab:C2667T, ORF1ab:C3037T, ORF1ab:G6008A, ORF1ab:G11417T, ORF1ab:C13548T, ORF1ab:C14408T, ORF1ab:G16741A, ORF1ab:A20268G, ORF1ab:T20600C, S:G22094A, S:A23403G, S:C24023T, N:C28311T, N:C28854T,	12	N:P13L, N:S194L, ORF1a:E36G, ORF1a:A801V, ORF1a:V1915I, ORF1a:V3718F, ORF1b:P314L, ORF1b:V1092I, ORF1b:I2378T, ORF9b:P10S, S:D178N, S:D614G,

hCoV-19/Mexico/JAL-InDRE_F99G_S575/2021	EPI_ISL_1324758	20B	P.2	25	5'UTR:C100T, 5'UTR:C241T, ORF1ab:C3037T, ORF1ab:T3477C, ORF1ab:C3583T, ORF1ab:T3766C, ORF1ab:C5694T, ORF1ab:C9711T, ORF1ab:C9891T, ORF1ab:T10667G, ORF1ab:C11824T, ORF1ab:C12053T, ORF1ab:A12964G, ORF1ab:C14408T, ORF1ab:A19578G, S:G23012A, S:A23403G, S:G25088T, ORF7a:A27599T, ORF8:C28253T, N:G28628T, N:G28881A, N:G28882A, N:G28883C, N:G28975T, 3'UTR:C29754T,	15	N:A119S, N:R203K, N:G204R, N:M234I, ORF1a:V1071A, ORF1a:P1810L, ORF1a:S3149F, ORF1a:A3209V, ORF1a:L3468V, ORF1a:L3930F, ORF1b:P314L, ORF7a:D69V, S:E484K, S:D614G, S:V1176F,
hCoV-19/Mexico/JAL-InDRE-11/2020	EPI_ISL_455435	19A	B	0	3'UTR:G29711T,	1	,
hCoV-19/Mexico/JAL-InDRE-16/2020	EPI_ISL_455454	19A	B	2	ORF1ab:T2586C, ORF1ab:T13572G, 3'UTR:G29711T,	1	ORF1a:V774A,
hCoV-19/Mexico/JAL-InDRE-F11737-S1182/2021	EPI_ISL_1558828	20B	P.2	26	5'UTR:C100T, 5'UTR:C241T, ORF1ab:T433A, ORF1ab:C3037T, ORF1ab:T3477C, ORF1ab:T3766C, ORF1ab:C5694T, ORF1ab:C9711T, ORF1ab:T10667G, ORF1ab:C11824T, ORF1ab:C12053T, ORF1ab:A12964G, ORF1ab:C14408T, ORF1ab:G17800T, ORF1ab:A19578G, S:C22208T, S:G23012A, S:A23403G, S:G25088T, ORF8:C28253T, N:G28628T, N:G28881A, N:G28882A, N:G28883C, N:G28975T, 3'UTR:C29754T, 3'UTR:C29762T,	15	N:A119S, N:R203K, N:G204R, N:M234I, ORF1a:V1071A, ORF1a:P1810L, ORF1a:S3149F, ORF1a:L3468V, ORF1a:L3930F, ORF1b:P314L, ORF1b:A1445S, S:L216F, S:E484K, S:D614G, S:V1176F,
hCoV-19/Mexico/JAL-InDRE-F11738-S1183/2021	EPI_ISL_1558829	20B	B.1.1.28	27	5'UTR:C100T, 5'UTR:C241T, ORF1ab:C3037T, ORF1ab:T3477C, ORF1ab:T3766C, ORF1ab:C3903T, ORF1ab:C5694T, ORF1ab:C9711T, ORF1ab:C9962T, ORF1ab:T10667G, ORF1ab:C11824T, ORF1ab:C12053T, ORF1ab:A12964G, ORF1ab:C14408T, ORF1ab:A15267G, ORF1ab:A19578G, S:C21575T, S:A23403G, S:G25088T, ORF8:C28253T, N:G28628T, N:C28706T, N:G28881A, N:G28882A, N:G28883C, N:G28975T, 3'UTR:C29733T, 3'UTR:C29754T,	16	N:A119S, N:H145Y, N:R203K, N:G204R, N:M234I, ORF1a:V1071A, ORF1a:P1213L, ORF1a:P1810L, ORF1a:S3149F, ORF1a:H3233Y, ORF1a:L3468V, ORF1a:L3930F, ORF1b:P314L, S:L5F, S:D614G, S:V1176F,
hCoV-19/Mexico/JAL-InDRE-F11741-S1184/2021	EPI_ISL_1558830	20B	P.2	26	5'UTR:C100T, 5'UTR:C241T, ORF1ab:C3037T, ORF1ab:T3477C, ORF1ab:T3766C, ORF1ab:C3903T, ORF1ab:C5694T, ORF1ab:C9711T, ORF1ab:T10667G, ORF1ab:C11824T, ORF1ab:C12053T, ORF1ab:A12964G, ORF1ab:C14408T, ORF1ab:A19578G, S:C21575T, S:G23012A, S:A23403G, S:G25088T, ORF8:C28253T, N:G28628T, N:C28706T, N:G28881A, N:G28882A, N:G28883C, N:G28975T, 3'UTR:C29733T, 3'UTR:C29754T,	16	N:A119S, N:H145Y, N:R203K, N:G204R, N:M234I, ORF1a:V1071A, ORF1a:P1213L, ORF1a:P1810L, ORF1a:S3149F, ORF1a:L3468V, ORF1a:L3930F, ORF1b:P314L, S:L5F, S:E484K, S:D614G, S:V1176F,
hCoV-19/Mexico/JAL-InDRE-F11742-S1185/2021	EPI_ISL_1558831	20B	P.2	27	5'UTR:C100T, 5'UTR:A156G, 5'UTR:C241T, ORF1ab:C807T, ORF1ab:C3037T, ORF1ab:T3477C, ORF1ab:T3766C, ORF1ab:C5694T, ORF1ab:C9711T, ORF1ab:T10667G, ORF1ab:C11824T, ORF1ab:C12053T, ORF1ab:A12964G, ORF1ab:C14408T, ORF1ab:C17999T, ORF1ab:A19578G, S:C22450T, S:G23012A, S:A23403G, S:G25088T, ORF8:C28253T, N:G28628T, N:G28881A, N:G28882A, N:G28883C, N:G28975T, N:C29144T, 3'UTR:C29754T,	15	N:A119S, N:R203K, N:G204R, N:M234I, ORF1a:A181V, ORF1a:V1071A, ORF1a:P1810L, ORF1a:S3149F, ORF1a:L3468V, ORF1a:L3930F, ORF1b:P314L, ORF1b:T1511I, S:E484K, S:D614G, S:V1176F,

hCoV-19/Mexico/JAL-InDRE-F11743-S1186/2021	EPI_ISL_1558832	20B	P.2	26	5'UTR:C100T, 5'UTR:C241T, ORF1ab:C3037T, ORF1ab:T3477C, ORF1ab:T3766C, ORF1ab:C3903T, ORF1ab:C5694T, ORF1ab:C9711T, ORF1ab:T10667G, ORF1ab:C11824T, ORF1ab:C12053T, ORF1ab:A12964G, ORF1ab:C14408T, ORF1ab:A19578G, S:C21575T, S:G23012A, S:A23403G, S:G25088T, ORF8:C28253T, N:G28628T, N:C28706T, N:G28881A, N:G28882A, N:G28883C, N:G28975T, 3'UTR:C29733T, 3'UTR:C29754T,	16	N:A119S, N:H145Y, N:R203K, N:G204R, N:M234I, ORF1a:V1071A, ORF1a:P1213L, ORF1a:P1810L, ORF1a:S3149F, ORF1a:L3468V, ORF1a:L3930F, ORF1b:P314L, S:L5F, S:E484K, S:D614G, S:V1176F,
hCoV-19/Mexico/JAL-InDRE-F11744-S913/2021	EPI_ISL_1558833	20B	P.2	26	5'UTR:C100T, 5'UTR:C241T, ORF1ab:C3037T, ORF1ab:T3477C, ORF1ab:T3766C, ORF1ab:C3903T, ORF1ab:C5694T, ORF1ab:C9711T, ORF1ab:T10667G, ORF1ab:C11824T, ORF1ab:C12053T, ORF1ab:A12964G, ORF1ab:C14408T, ORF1ab:A19578G, S:C21575T, S:G23012A, S:A23403G, S:G25088T, ORF8:C28253T, N:G28628T, N:C28706T, N:G28881A, N:G28882A, N:G28883C, N:G28975T, 3'UTR:C29733T, 3'UTR:C29754T,	16	N:A119S, N:H145Y, N:R203K, N:G204R, N:M234I, ORF1a:V1071A, ORF1a:P1213L, ORF1a:P1810L, ORF1a:S3149F, ORF1a:L3468V, ORF1a:L3930F, ORF1b:P314L, S:L5F, S:E484K, S:D614G, S:V1176F,
hCoV-19/Mexico/JAL-LaDEER-133706/2021	EPI_ISL_1360407	20B	P.2	23	5'UTR:C100T, 5'UTR:C241T, ORF1ab:C3037T, ORF1ab:T3477C, ORF1ab:T3766C, ORF1ab:C5694T, ORF1ab:C9711T, ORF1ab:T10667G, ORF1ab:C11824T, ORF1ab:C12053T, ORF1ab:A12964G, ORF1ab:C14408T, ORF1ab:A19578G, S:G23012A, S:A23403G, S:G25088T, ORF8:C28253T, N:G28436T, N:G28628T, N:G28881A, N:G28882A, N:G28883C, N:G28975T, 3'UTR:C29754T.	14	N:A55S, N:A119S, N:R203K, N:G204R, N:M234I, ORF1a:V1071A, ORF1a:P1810L, ORF1a:S3149F, ORF1a:L3468V, ORF1a:L3930F, ORF1b:P314L, S:E484K, S:D614G, S:V1176F,
hCoV-19/Mexico/JAL-LaDEER-139093/2021	EPI_ISL_1360412	20B	P.2	23	5'UTR:C100T, 5'UTR:C241T, ORF1ab:C3037T, ORF1ab:T3477C, ORF1ab:T3766C, ORF1ab:C5694T, ORF1ab:C9711T, ORF1ab:T10667G, ORF1ab:C11824T, ORF1ab:C12053T, ORF1ab:A12964G, ORF1ab:C14408T, ORF1ab:G19101T, ORF1ab:A19578G, S:G23012A, S:A23403G, S:G25088T, ORF8:C28253T, N:G28628T, N:G28881A, N:G28882A, N:G28883C, N:G28975T, 3'UTR:C29754T,	14	N:A119S, N:R203K, N:G204R, N:M234I, ORF1a:V1071A, ORF1a:P1810L, ORF1a:S3149F, ORF1a:L3468V, ORF1a:L3930F, ORF1b:P314L, ORF1b:Q1878H, S:E484K, S:D614G, S:V1176F,
hCoV-19/Mexico/JAL-LaDEER-145340/2021	EPI_ISL_1360408	20B	P.2	23	5'UTR:C100T, 5'UTR:C241T, ORF1ab:C1441T, ORF1ab:C3037T, ORF1ab:T3477C, ORF1ab:T3766C, ORF1ab:C5694T, ORF1ab:C9711T, ORF1ab:T10667G, ORF1ab:C11824T, ORF1ab:C12053T, ORF1ab:A12964G, ORF1ab:C14408T, ORF1ab:A19578G, S:G23012A, S:A23403G, S:G25088T, ORF8:C28253T, N:G28628T, N:G28881A, N:G28882A, N:G28883C, N:G28975T, 3'UTR:C29754T,	13	N:A119S, N:R203K, N:G204R, N:M234I, ORF1a:V1071A, ORF1a:P1810L, ORF1a:S3149F, ORF1a:L3468V, ORF1a:L3930F, ORF1b:P314L, S:E484K, S:D614G, S:V1176F,
hCoV-19/Mexico/JAL-LaDEER-145365/2021	EPI_ISL_1360409	20B	P.2	23	5'UTR:C100T, 5'UTR:C241T, ORF1ab:C1441T, ORF1ab:C3037T, ORF1ab:T3477C, ORF1ab:T3766C, ORF1ab:C5694T, ORF1ab:C9711T, ORF1ab:T10667G, ORF1ab:C11824T, ORF1ab:C12053T, ORF1ab:A12964G, ORF1ab:C14408T, ORF1ab:A19578G, S:G23012A, S:A23403G, S:G25088T, ORF8:C28253T, N:G28628T, N:G28881A, N:G28882A, N:G28883C, N:G28975T, 3'UTR:C29754T,	13	N:A119S, N:R203K, N:G204R, N:M234I, ORF1a:V1071A, ORF1a:P1810L, ORF1a:S3149F, ORF1a:L3468V, ORF1a:L3930F, ORF1b:P314L, S:E484K, S:D614G, S:V1176F,

hCoV-19/Mexico/JAL-LaDEER-147248/2021	EPI_ISL_1360410	20B	P.2	26	5'UTR:C100T, 5'UTR:C241T, ORF1ab:C3037T, ORF1ab:T3477C, ORF1ab:T3766C, ORF1ab:C4993T, ORF1ab:C5694T, ORF1ab:C9711T, ORF1ab:T10667G, ORF1ab:C11824T, ORF1ab:C12053T, ORF1ab:A12964G, ORF1ab:C14408T, ORF1ab:A19578G, S:G23012A, S:A23403G, S:G25088T, ORF8:C28253T, N:G28628T, N:G28881A, N:G28882A, N:G28883C, N:G28975T, 3'UTR:C29721A, 3'UTR:C29754T, 3'UTR:T29867A, 3'UTR:C29870A,	13	N:A119S, N:R203K, N:G204R, N:M234I, ORF1a:V1071A, ORF1a:P1810L, ORF1a:S3149F, ORF1a:L3468V, ORF1a:L3930F, ORF1b:P314L, S:E484K, S:D614G, S:V1176F,
hCoV-19/Mexico/JAL-LaDEER-E39931/2021	EPI_ISL_1360411	20B	P.2	26	5'UTR:C100T, 5'UTR:C241T, ORF1ab:C3037T, ORF1ab:T3477C, ORF1ab:T3766C, ORF1ab:C5694T, ORF1ab:C9711T, ORF1ab:T10667G, ORF1ab:C11824T, ORF1ab:C12053T, ORF1ab:A12964G, ORF1ab:C14408T, ORF1ab:C16428T, ORF1ab:A19578G, S:G23012A, S:A23403G, S:A24148G, S:G25088T, ORF8:C28253T, N:G28628T, N:G28881A, N:G28882A, N:G28883C, N:G28975T, 3'UTR:C29733T, 3'UTR:C29754T, 3'UTR:C29870A,	13	N:A119S, N:R203K, N:G204R, N:M234I, ORF1a:V1071A, ORF1a:P1810L, ORF1a:S3149F, ORF1a:L3468V, ORF1a:L3930F, ORF1b:P314L, S:E484K, S:D614G, S:V1176F,
hCoV-19/Mexico/JAL-UANL-001/2020	EPI_ISL_1092361	20B	B.1.1	11	5'UTR:C241T, ORF1ab:C313T, ORF1ab:C3037T, ORF1ab:C9532T, ORF1ab:C12357T, ORF1ab:C14408T, ORF1ab:G16377T, S:A23403G, ORF3a:G25855T, N:G28881A, N:G28882A, N:G28883C,	6	N:R203K, N:G204R, ORF1a:T4031I, ORF1b:P314L, ORF3a:D155Y, S:D614G,
hCoV-19/Mexico/JAL-UANL-002/2020	EPI_ISL_1091261	20B	B.1.1	11	5'UTR:C241T, ORF1ab:C313T, ORF1ab:C1150T, ORF1ab:C3037T, ORF1ab:C6361T, ORF1ab:C12880T, ORF1ab:A13611G, ORF1ab:C14408T, S:A23403G, N:G28881A, N:G28882A, N:G28883C,	4	N:R203K, N:G204R, ORF1b:P314L, S:D614G,
hCoV-19/Mexico/MEX-InDRE_121/2020	EPI_ISL_913925	20B	B.1.1.222	17	5'UTR:C241T, ORF1ab:C1419T, ORF1ab:C3037T, ORF1ab:G9203A, ORF1ab:C11747T, ORF1ab:C14408T, ORF1ab:C15579T, ORF1ab:C16017T, ORF1ab:G19009A, ORF1ab:T19839C, ORF1ab:T20631C, S:A23403G, S:A23756G, ORF6:C27263T, N:G28881A, N:G28882A, N:G28883C, N:A29487G,	10	N:R203K, N:G204R, N:K405R, ORF1a:A385V, ORF1a:D2980N, ORF1b:P314L, ORF1b:D1848N, ORF6:T21I, S:D614G, S:T732A,
hCoV-19/Mexico/MEX-InDRE_124/2020	EPI_ISL_913928	20B	B.1.1.222	17	5'UTR:C140T, 5'UTR:C241T, ORF1ab:C2594T, ORF1ab:C3037T, ORF1ab:T5218C, ORF1ab:G11333T, ORF1ab:C14408T, ORF1ab:T19839C, ORF1ab:G20679T, ORF1ab:C20938T, S:A23403G, S:A23756G, S:C24745T, ORF3a:C26028T, N:G28881A, N:G28882A, N:G28883C, N:G29554A,	7	N:R203K, N:G204R, ORF1a:P777S, ORF1a:V3690L, ORF1b:P314L, S:D614G, S:T732A,
hCoV-19/Mexico/MEX-InDRE_138/2020	EPI_ISL_913941	20B	B.1.1	14	5'UTR:C241T, ORF1ab:A623G, ORF1ab:C2937T, ORF1ab:C3037T, ORF1ab:C14408T, ORF1ab:C15714T, ORF1ab:C18714T, ORF1ab:G19549T, S:A23403G, S:C23410T, ORF7b:C27874T, ORF8:T28105G, N:G28881A, N:G28882A, N:G28883C,	9	N:R203K, N:G204R, ORF1a:K120E, ORF1a:T891I, ORF1b:P314L, ORF1b:A2028S, ORF7b:T40I, ORF8:I71S, S:D614G,
hCoV-19/Mexico/MEX-InDRE_170/2020	EPI_ISL_913971	20B	B.1.1.519	20	5'UTR:C203T, 5'UTR:C222T, 5'UTR:C241T, ORF1ab:C3037T, ORF1ab:C3140T, ORF1ab:C10029T, ORF1ab:C10954T, ORF1ab:A11117G, ORF1ab:C12789T, ORF1ab:C14408T, ORF1ab:T19839C, ORF1ab:C21306T, S:C21575T, S:C22995A, S:A23403G, S:C23604A, S:A23756G, N:G28881A, N:G28882A, N:G28883C, N:C29197T,	12	N:R203K, N:G204R, ORF1a:P959S, ORF1a:T3255I, ORF1a:I3618V, ORF1a:T4175I, ORF1b:P314L, S:L5F, S:T478K, S:D614G, S:P681H, S:T732A,

hCoV-19/Mexico/MEX-InDRE_177/2020	EPI_ISL_913978	20B	B.1.1.519	21	5'UTR:C203T, 5'UTR:C222T, 5'UTR:C241T, ORF1ab:C3037T, ORF1ab:C3140T, ORF1ab:G7037T, ORF1ab:C10029T, ORF1ab:C10954T, ORF1ab:A11117G, ORF1ab:C12789T, ORF1ab:C14408T, ORF1ab:T19839C, ORF1ab:C21306T, S:C22995A, S:A23403G, S:C23604A, S:A23756G, ORF3a:C25782T, N:G28881A, N:G28882A, N:G28883C, N:C29197T,	12	N:R203K, N:G204R, ORF1a:P959S, ORF1a:G2258C, ORF1a:T3255I, ORF1a:I3618V, ORF1a:T4175I, ORF1b:P314L, S:T478K, S:D614G, S:P681H, S:T732A,
hCoV-19/Mexico/MEX-InDRE_182/2020	EPI_ISL_913983	20B	B.1.1.519	28	5'UTR:C203T, 5'UTR:C222T, 5'UTR:C241T, ORF1ab:C1342T, ORF1ab:C1377T, ORF1ab:C3037T, ORF1ab:C3140T, ORF1ab:G3692T, ORF1ab:G3871T, ORF1ab:C6573T, ORF1ab:C10029T, ORF1ab:C10954T, ORF1ab:A11117G, ORF1ab:C11824A, ORF1ab:C12789T, ORF1ab:C14408T, ORF1ab:T18488C, ORF1ab:G19684T, ORF1ab:T19839C, ORF1ab:C21306T, S:C22995A, S:A23403G, S:C23604A, S:A23756G, ORF8:T27904C, N:G28881A, N:G28882A, N:G28883C, N:C29197T,	18	N:R203K, N:G204R, ORF1a:P371L, ORF1a:P959S, ORF1a:V1143F, ORF1a:K1202N, ORF1a:S2103F, ORF1a:T3255I, ORF1a:I3618V, ORF1a:T4175I, ORF1b:P314L, ORF1b:I1674T, ORF1b:V2073L, ORF8:L4P, S:T478K, S:D614G, S:P681H, S:T732A,
hCoV-19/Mexico/MEX-InDRE_200/2020	EPI_ISL_933677	20B	B.1.1.222	16	5'UTR:C241T, ORF1ab:A1065G, ORF1ab:C3037T, ORF1ab:C8092T, ORF1ab:G12070T, ORF1ab:G13007A, ORF1ab:G13459T, ORF1ab:C14408T, ORF1ab:C15240T, ORF1ab:T19839C, ORF1ab:A21137G, S:A23403G, S:A23756G, S:C23758T, N:G28881A, N:G28882A, N:G28883C,	8	N:R203K, N:G204R, ORF1a:N267S, ORF1a:A4248T, ORF1b:P314L, ORF1b:K2557R, S:D614G, S:T732A,
hCoV-19/Mexico/MEX-InDRE_215/2021	EPI_ISL_933692	20B	B.1.1.519	28	5'UTR:C203T, 5'UTR:C222T, 5'UTR:C241T, ORF1ab:C1342T, ORF1ab:C2334T, ORF1ab:C3037T, ORF1ab:C3140T, ORF1ab:G3692T, ORF1ab:G3871T, ORF1ab:G5150A, ORF1ab:C10029T, ORF1ab:C10954T, ORF1ab:A11117G, ORF1ab:C11824A, ORF1ab:C12789T, ORF1ab:C14408T, ORF1ab:T19839C, ORF1ab:G21123T, ORF1ab:C21306T, S:C22995A, S:A23403G, S:C23604A, S:A23756G, ORF8:T27904C, ORF8:C28087T, N:G28881A, N:G28882A, N:G28883C, N:C29197T,	17	N:R203K, N:G204R, ORF1a:A690V, ORF1a:P959S, ORF1a:V1143F, ORF1a:K1202N, ORF1a:V1629I, ORF1a:T3255I, ORF1a:I3618V, ORF1a:T4175I, ORF1b:P314L, ORF8:L4P, ORF8:A65V, S:T478K, S:D614G, S:P681H, S:T732A,
hCoV-19/Mexico/MEX-InDRE_226/2020	EPI_ISL_933703	20B	B.1.1.519	22	5'UTR:C203T, 5'UTR:C222T, 5'UTR:C241T, ORF1ab:C3037T, ORF1ab:C3140T, ORF1ab:T9456G, ORF1ab:C10029T, ORF1ab:C10954T, ORF1ab:A11117G, ORF1ab:C12789T, ORF1ab:C14408T, ORF1ab:T19839C, ORF1ab:C21306T, S:C22995A, S:A23403G, S:C23604A, S:A23756G, S:C24919T, ORF3a:T25496C, N:G28881A, N:G28882A, N:G28883C, N:C29197T,	13	N:R203K, N:G204R, ORF1a:P959S, ORF1a:F3064C, ORF1a:T3255I, ORF1a:I3618V, ORF1a:T4175I, ORF1b:P314L, ORF3a:I35T, S:T478K, S:D614G, S:P681H, S:T732A,
hCoV-19/Mexico/MEX-InDRE_228/2021	EPI_ISL_933705	20B	B.1.1.222	28	5'UTR:C241T, ORF1ab:C593T, ORF1ab:A1558G, ORF1ab:C3037T, ORF1ab:A3916G, ORF1ab:C6543T, ORF1ab:C8606T, ORF1ab:A9149G, ORF1ab:A10840G, ORF1ab:G11222T, ORF1ab:C11344T, ORF1ab:C14216T, ORF1ab:C14408T, ORF1ab:T19839C, S:C21614T, S:G22026A, S:G22104T, S:A23403G, S:A23756G, ORF3a:C25587T, ORF3a:C25904T, ORF7a:T27586C, ORF8:C28153T, N:G28378A, N:G28881A, N:G28882A, N:G28883C, N:C29253T, N:C29353T,	20	N:R203K, N:G204R, N:S327L, ORF1a:H110Y, ORF1a:I431M, ORF1a:T2093I, ORF1a:L2781F, ORF1a:I2962V, ORF1a:V3653F, ORF1b:T250I, ORF1b:P314L, ORF3a:S171L, ORF7a:F65L, ORF8:T87I, ORF9b:R32H, S:L18F, S:S155N, S:G181V, S:D614G, S:T732A,
hCoV-19/Mexico/MEX-InDRE_259/2020	EPI_ISL_1054977	20B	B.1.1	6	5'UTR:C241T, ORF1ab:C3037T, ORF1ab:C14408T, S:A23403G, N:G28881A, N:G28882A, N:G28883C,	4	N:R203K, N:G204R, ORF1b:P314L, S:D614G,

hCoV-19/Mexico/MEX-InDRE_263/2020	EPI_ISL_1054964	20B	B.1.1	14	5'UTR:C241T, ORF1ab:T1476C, ORF1ab:C2399T, ORF1ab:C3037T, ORF1ab:A8051G, ORF1ab:C10189T, ORF1ab:C13994T, ORF1ab:C14408T, S:A23403G, ORF3a:G25526T, ORF3a:G25563C, N:C28854T, N:G28881A, N:G28882A, N:G28883C,	11	N:S194L, N:R203K, N:G204R, ORF1a:I404T, ORF1a:H712Y, ORF1a:N2596D, ORF1b:A176V, ORF1b:P314L, ORF3a:W45L, ORF3a:Q57H, S:D614G,
hCoV-19/Mexico/MEX-InDRE_274/2020	EPI_ISL_1054954	20B	B.1.1	11	5'UTR:C241T, ORF1ab:C3037T, ORF1ab:A6510G, ORF1ab:G7936T, ORF1ab:A11860G, ORF1ab:C14408T, S:A23403G, ORF7a:G27598A, ORF7a:C27679T, N:G28881A, N:G28882A, N:G28883C,	7	N:R203K, N:G204R, ORF1a:N2082S, ORF1b:P314L, ORF7a:D69N, ORF7a:L96F, S:D614G,
hCoV-19/Mexico/MEX-InDRE_278/2020	EPI_ISL_1054953	20B	B.1.1	10	5'UTR:C241T, ORF1ab:C3037T, ORF1ab:C8208T, ORF1ab:C13487A, ORF1ab:C14408T, S:A23403G, ORF6:C27247T, N:G28881A, N:G28882A, N:G28883C, N:G29140T,	7	N:R203K, N:G204R, N:Q289H, ORF1a:T2648I, ORF1b:A7E, ORF1b:P314L, S:D614G,
hCoV-19/Mexico/MEX-InDRE_282/2020	EPI_ISL_1054949	20B	B.1.1.222	14	5'UTR:C241T, ORF1ab:C3037T, ORF1ab:G3403T, ORF1ab:T5218C, ORF1ab:A6035G, ORF1ab:G9130T, ORF1ab:C12525T, ORF1ab:C14408T, ORF1ab:T19839C, S:A23403G, S:A23756G, N:G28881A, N:G28882A, N:G28883C, N:G29449T,	7	N:R203K, N:G204R, ORF1a:S1924G, ORF1a:T4087I, ORF1b:P314L, S:D614G, S:T732A,
hCoV-19/Mexico/MEX-InDRE_302/2020	EPI_ISL_1060743	20B	B.1.1.432	14	5'UTR:C241T, ORF1ab:C3037T, ORF1ab:G3122T, ORF1ab:C3773T, ORF1ab:A6985T, ORF1ab:C9319T, ORF1ab:C12412T, ORF1ab:T14313C, ORF1ab:C14408T, S:A23403G, N:G28881A, N:G28882A, N:G28883C, N:T29317C, N:C29420T,	7	N:R203K, N:G204R, N:P383S, ORF1a:D953Y, ORF1a:R1170C, ORF1b:P314L, S:D614G,
hCoV-19/Mexico/MEX-InDRE_318/2020	EPI_ISL_1060758	20B	B.1.1.222	7	ORF1ab:C3037T, ORF1ab:C14408T, ORF1ab:T19839C, S:A23403G, S:A23756G, N:G28881A, N:G28882A, N:G28883C,	5	N:R203K, N:G204R, ORF1b:P314L, S:D614G, S:T732A,
hCoV-19/Mexico/MEX-InDRE_324/2020	EPI_ISL_1060752	20B	B.1.1.518	18	5'UTR:C241T, ORF1ab:C3037T, ORF1ab:A6453G, ORF1ab:A9019G, ORF1ab:C10156T, ORF1ab:G12322A, ORF1ab:C14408T, ORF1ab:A16725G, ORF1ab:T19839C, ORF1ab:C20703T, S:A23403G, ORF3a:G25552T, M:C27059T, ORF8:C28102T, N:G28378T, N:A28482G, N:G28881A, N:G28882A, N:G28883C,	10	N:Q70R, N:R203K, N:G204R, ORF1a:E2063G, ORF1b:P314L, ORF3a:A54S, ORF8:P70L, ORF9b:R32L, ORF9b:K67E, S:D614G,
hCoV-19/Mexico/MEX-InDRE_467/2021	EPI_ISL_1168537	20B	B.1.1.519	23	5'UTR:T201C, 5'UTR:C203T, 5'UTR:C222T, 5'UTR:C241T, ORF1ab:G1738T, ORF1ab:C3037T, ORF1ab:C3140T, ORF1ab:G7829T, ORF1ab:C10029T, ORF1ab:C10954T, ORF1ab:A11117G, ORF1ab:C12789T, ORF1ab:C14408T, ORF1ab:T19839C, ORF1ab:A19974G, ORF1ab:C21306T, S:C22995A, S:A23403G, S:C23604A, S:A23756G, N:G28881A, N:G28882A, N:G28883C, N:C29197T,	12	N:R203K, N:G204R, ORF1a:P959S, ORF1a:V2522F, ORF1a:T3255I, ORF1a:I3618V, ORF1a:T4175I, ORF1b:P314L, S:T478K, S:D614G, S:P681H, S:T732A,
hCoV-19/Mexico/MEX-InDRE_542/2021	EPI_ISL_1168612	20B	B.1.1.519	25	5'UTR:C203T, 5'UTR:C222T, 5'UTR:C241T, ORF1ab:C3037T, ORF1ab:C3140T, ORF1ab:C6627T, ORF1ab:C10029T, ORF1ab:C10954T, ORF1ab:A11117G, ORF1ab:C12115T, ORF1ab:C12789T, ORF1ab:C14408T, ORF1ab:A16980G, ORF1ab:T19839C, ORF1ab:C21306T, S:C22995A, S:A23403G, S:C23604A, S:A23756G, E:G26262A, ORF8:T27904C, ORF8:C28087T, N:G28881A, N:G28882A, N:G28883C, N:C29197T,	14	N:R203K, N:G204R, ORF1a:P959S, ORF1a:T2121I, ORF1a:T3255I, ORF1a:I3618V, ORF1a:T4175I, ORF1b:P314L, ORF8:L4P, ORF8:A65V, S:T478K, S:D614G, S:P681H, S:T732A,
hCoV-19/Mexico/MIC-InDRE_191/2020	EPI_ISL_933668	20B	B.1.1.222	15	5'UTR:C241T, ORF1ab:C3037T, ORF1ab:C5826T, ORF1ab:G6576T, ORF1ab:C12513T, ORF1ab:C14408T, ORF1ab:T19839C, ORF1ab:G19872T, S:A23403G, S:A23756G, M:A26612T, M:C27131T, N:C28292A, N:G28881A, N:G28882A, N:G28883C,	9	N:Q7K, N:R203K, N:G204R, ORF1a:T1854I, ORF1a:S2104I, ORF1a:T4083M, ORF1b:P314L, S:D614G, S:T732A,
hCoV-19/Mexico/MIC-InDRE_390/2020	EPI_ISL_1054993	20A	B.1.243	13	5'UTR:C241T, ORF1ab:G806A, ORF1ab:C3037T, ORF1ab:C9870T, ORF1ab:C14408T, ORF1ab:C14724T, ORF1ab:C15960T, ORF1ab:G17019T, ORF1ab:A20268G, S:C21575T, S:A23403G, S:T24076C, N:C28854T, N:G29543T,	7	N:S194L, ORF1a:A181T, ORF1a:T3202M, ORF1b:P314L, ORF1b:E1184D, S:L5F, S:D614G,

hCoV-19/Mexico/MIC-InDRE_F10346_S853/2021	EPI_ISL_1399259	20B	B.1.1.519	21	5'UTR:C203T, 5'UTR:C222T, 5'UTR:C241T, ORF1ab:C3037T, ORF1ab:C3140T, ORF1ab:C10029T, ORF1ab:C10954T, ORF1ab:A11117G, ORF1ab:C12789T, ORF1ab:C14408T, ORF1ab:T19839C, ORF1ab:C21306T, S:G22918T, S:C22995A, S:A23403G, S:C23604A, S:A23756G, N:C28695T, N:G28881A, N:G28882A, N:G28883C, N:C29197T.	12	N:T141I, N:R203K, N:G204R, ORF1a:P959S, ORF1a:T3255I, ORF1a:I3618V, ORF1a:T4175I, ORF1b:P314L, S:T478K, S:D614G, S:P681H, S:T732A,
hCoV-19/Mexico/MIC-InDRE-68/2020	EPI_ISL_576271	20G	B.1.2	21	5'UTR:C241T, ORF1ab:C1059T, ORF1ab:A2854G, ORF1ab:C3037T, ORF1ab:G5008T, ORF1ab:C10319T, ORF1ab:C14408T, ORF1ab:A18424G, ORF1ab:G18546T, ORF1ab:A19298C, ORF1ab:T19301C, ORF1ab:A19302T, ORF1ab:C21304T, S:A22623G, S:A23403G, ORF3a:G25563T, ORF3a:G25907T, ORF8:C27964T, N:C28472T, N:C28869T, N:G29260T, N:A29499T,	16	N:P67S, N:P199L, N:Q409L, ORF1a:T265I, ORF1a:L3352F, ORF1b:P314L, ORF1b:N1653D, ORF1b:M1693I, ORF1b:Y1944S, ORF1b:V1945A, ORF1b:R2613C, ORF3a:Q57H, ORF3a:G172V, ORF8:S24L, S:N354S, S:D614G,
hCoV-19/Mexico/MIC-InDRE-73/2020	EPI_ISL_576276	20A	B.1	11	5'UTR:C241T, ORF1ab:A2497G, ORF1ab:C3037T, ORF1ab:A10323G, ORF1ab:C14408T, ORF1ab:A20268G, ORF1ab:T20385C, S:T22191C, S:A23403G, S:G23663T, E:C26447T, N:C28854T,	7	E:S68F, N:S194L, ORF1a:K3353R, ORF1b:P314L, S:I210T, S:D614G, S:A701S,
hCoV-19/Mexico/MIC-InDRE-74/2020	EPI_ISL_576277	20A	B.1.189	15	5'UTR:C241T, ORF1ab:C3037T, ORF1ab:G4124T, ORF1ab:C4582T, ORF1ab:G5830T, ORF1ab:G6404T, ORF1ab:G7294A, ORF1ab:G8179T, ORF1ab:C14408T, ORF1ab:A20268G, S:A23403G, S:T25123C, N:G28378T, N:T28382C, N:C28969T, N:G28985A,	8	N:S37P, N:G238S, ORF1a:V1287L, ORF1a:K1855N, ORF1a:V2047F, ORF1b:P314L, ORF9b:R32L, S:D614G,
hCoV-19/Mexico/MOR-InDRE_113/2020	EPI_ISL_913917	20B	B.1.1	11	5'UTR:C241T, ORF1ab:G1135T, ORF1ab:A2024G, ORF1ab:C3037T, ORF1ab:A12820G, ORF1ab:C14408T, ORF1ab:G18733A, ORF1ab:T19839C, S:A23403G, N:G28881A, N:G28882A, N:G28883C,	7	N:R203K, N:G204R, ORF1a:K290N, ORF1a:T587A, ORF1b:P314L, ORF1b:G1756R, S:D614G,
hCoV-19/Mexico/MOR-InDRE_118/2020	EPI_ISL_913922	20A	B.1	13	5'UTR:C241T, ORF1ab:C874T, ORF1ab:C2334T, ORF1ab:C3037T, ORF1ab:A5689G, ORF1ab:A5780C, ORF1ab:C10594T, ORF1ab:C14408T, ORF1ab:A20268G, S:A23403G, ORF7a:C27549T, ORF8:T28251G, ORF8:C28253T, N:C28854T,	6	N:S194L, ORF1a:A690V, ORF1a:I1839L, ORF1b:P314L, ORF8:F120V, S:D614G,
hCoV-19/Mexico/MOR-InDRE_137/2020	EPI_ISL_913940	20A	B.1.551	13	5'UTR:C241T, ORF1ab:C3037T, ORF1ab:C3738T, ORF1ab:C6310T, ORF1ab:C7945T, ORF1ab:G14118A, ORF1ab:C14408T, ORF1ab:A20268G, S:A23403G, S:C23604G, N:G28628T, N:C28854T, N:G29254T, 3'UTR:G29742T,	6	N:A119S, N:S194L, ORF1a:P1158L, ORF1b:P314L, S:D614G, S:P681R,
hCoV-19/Mexico/MOR-InDRE_155/2020	EPI_ISL_913957	20B	B.1.1	22	5'UTR:C190T, 5'UTR:C241T, ORF1ab:A1633G, ORF1ab:G3004T, ORF1ab:C3037T, ORF1ab:C3177T, ORF1ab:C4776A, ORF1ab:C11916T, ORF1ab:C14408T, ORF1ab:T19839C, S:A23403G, S:G23426A, S:C23604A, ORF3a:G25522A, ORF3a:T25580C, ORF7b:G27890A, N:C28310T, N:G28314T, N:G28881A, N:G28882A, N:G28883C, N:G29254T, 3'UTR:G29747T,	15	N:P13S, N:R14L, N:R203K, N:G204R, ORF1a:E913D, ORF1a:P971L, ORF1a:T1504N, ORF1a:S3884L, ORF1b:P314L, ORF3a:G44R, ORF3a:I63T, ORF9b:A11S, S:D614G, S:V622I, S:P681H,
hCoV-19/Mexico/MOR-InDRE_172/2020	EPI_ISL_913973	20B	B.1.1.519	21	5'UTR:C203T, 5'UTR:C222T, 5'UTR:C241T, ORF1ab:C3037T, ORF1ab:C3140T, ORF1ab:A4666G, ORF1ab:C10029T, ORF1ab:C10954T, ORF1ab:A11117G, ORF1ab:C12789T, ORF1ab:C14408T, ORF1ab:T19839C, ORF1ab:C21306T, S:C21575T, S:C22995A, S:A23403G, S:C23604A, S:A23756G, N:G28881A, N:G28882A. N:G28883C. N:C29197T.	12	N:R203K, N:G204R, ORF1a:P959S, ORF1a:T3255I, ORF1a:I3618V, ORF1a:T4175I, ORF1b:P314L, S:L5F, S:T478K, S:D614G, S:P681H, S:T732A,

hCoV-19/Mexico/MOR-InDRE_175/2020	EPI_ISL_913976	20G	B.1.2	21	5'UTR:C241T, ORF1ab:C1059T, ORF1ab:C3037T, ORF1ab:C10319T, ORF1ab:G13094T, ORF1ab:T13216C, ORF1ab:G14187A, ORF1ab:C14408T, ORF1ab:A18424G, ORF1ab:A20913G, ORF1ab:C21304T, ORF1ab:A21390G, S:A23403G, ORF3a:G25563T, ORF3a:G25793T, ORF3a:G25907T, M:C26873T, ORF7a:C27600T, ORF8:C27964T, N:C28472T, N:C28869T. N:C29363T.	14	N:P67S, N:P199L, N:P364S, ORF1a:T265I, ORF1a:L3352F, ORF1a:A4277S, ORF1b:P314L, ORF1b:N1653D, ORF1b:R2613C, ORF3a:Q57H, ORF3a:R134L, ORF3a:G172V, ORF8:S24L, S:D614G,
hCoV-19/Mexico/MOR-InDRE_254/2020	EPI_ISL_1054995	20B	B.1.1	11	5'UTR:C241T, ORF1ab:C3037T, ORF1ab:C4965T, ORF1ab:C7165T, ORF1ab:C14408T, ORF1ab:G17334T, S:A23403G, N:G28881A, N:G28882A, N:G28883C, N:G29374A, 3'UTR:T29760A,	5	N:R203K, N:G204R, ORF1a:T1567I, ORF1b:P314L, S:D614G,
hCoV-19/Mexico/MOR-InDRE_270/2020	EPI_ISL_1054961	20A	B.1	9	5'UTR:C241T, ORF1ab:G2782T, ORF1ab:C3037T, ORF1ab:C14408T, ORF1ab:C19862T, ORF1ab:A20268G, S:T23335C, S:A23403G, ORF3a:C25452T, N:C28887T,	4	N:T205I, ORF1b:P314L, ORF1b:A2132V, S:D614G,
hCoV-19/Mexico/MOR-InDRE_280/2020	EPI_ISL_1054956	20A	B.1.610	4	5'UTR:C241T, ORF1ab:C3037T, ORF1ab:C14408T, ORF1ab:A20268G, S:A23403G,	2	ORF1b:P314L, S:D614G,
hCoV-19/Mexico/MOR-InDRE_285/2020	EPI_ISL_1054945	20B	B.1.1	14	5'UTR:C241T, ORF1ab:C1387T, ORF1ab:C3037T, ORF1ab:A4143G, ORF1ab:C10868T, ORF1ab:C14408T, ORF1ab:C16480T, ORF1ab:C19185T, ORF1ab:T19839C, S:C22214G, S:A23403G, N:G28881A, N:G28882A, N:G28883C, N:G29392T,	8	N:R203K, N:G204R, N:K373N, ORF1a:E1293G, ORF1b:P314L, ORF1b:P1005S, S:Q218E, S:D614G,
hCoV-19/Mexico/MOR-InDRE_342/2020	EPI_ISL_1060679	20B	B.1.1.222	15	ORF1ab:C1758T, ORF1ab:C2252A, ORF1ab:C3037T, ORF1ab:C10029T, ORF1ab:C14408T, ORF1ab:C15279T, S:A23403G, S:A23756G, S:G24219T, S:G24220T, ORF7b:A27756G, ORF8:A27921G, ORF8:G28001T, N:G28881A, N:G28882A, N:G28883C,	11	N:R203K, N:G204R, ORF1a:A498V, ORF1a:Q663K, ORF1a:T3255I, ORF1b:P314L, ORF7b:M1V, ORF8:I10V, S:D614G, S:T732A, S:W886F,
hCoV-19/Mexico/MOR-InDRE_359/2020	EPI_ISL_1060754	20B	B.1.1	15	5'UTR:C241T, ORF1ab:C1387T, ORF1ab:C3037T, ORF1ab:A4143G, ORF1ab:C10868T, ORF1ab:C14408T, ORF1ab:C16480T, ORF1ab:C19185T, ORF1ab:T19839C, S:C22214G, S:A23403G, ORF3a:G25907T, N:G28881A, N:G28882A, N:G28883C, N:G29392T,	9	N:R203K, N:G204R, N:K373N, ORF1a:E1293G, ORF1b:P314L, ORF1b:P1005S, ORF3a:G172V, S:Q218E, S:D614G,
hCoV-19/Mexico/MOR-InDRE_448/2021	EPI_ISL_1168526	20B	B.1.1.519	25	5'UTR:C203T, 5'UTR:C222T, 5'UTR:C241T, ORF1ab:G2632T, ORF1ab:C3037T, ORF1ab:C3140T, ORF1ab:A3684G, ORF1ab:C8344T, ORF1ab:C10029T, ORF1ab:C10954T, ORF1ab:A11117G, ORF1ab:C12789T, ORF1ab:C14408T, ORF1ab:T19839C, ORF1ab:C20543T, ORF1ab:C21306T, S:A22023C, S:C22995A, S:A23403G, S:C23604A, S:A23756G, ORF8:T27904C, N:G28881A, N:G28882A, N:G28883C, N:C29197T,	16	N:R203K, N:G204R, ORF1a:M789I, ORF1a:P959S, ORF1a:Q1140R, ORF1a:T3255I, ORF1a:I3618V, ORF1a:T4175I, ORF1b:P314L, ORF1b:S2359F, ORF8:L4P, S:E154A, S:T478K, S:D614G, S:P681H, S:T732A,
hCoV-19/Mexico/MOR-InDRE_462/2021	EPI_ISL_1168532	20B	B.1.1.519	24	5'UTR:C203T, 5'UTR:C222T, 5'UTR:C241T, ORF1ab:C3037T, ORF1ab:C3140T, ORF1ab:C4320T, ORF1ab:C10029T, ORF1ab:C10954T, ORF1ab:A11117G, ORF1ab:C12789T, ORF1ab:C14408T, ORF1ab:T19839C, ORF1ab:G20069T, ORF1ab:C20133T, ORF1ab:C21306T, S:A22023C, S:C22995A, S:A23403G, S:C23604A, S:A23756G, ORF8:T27904C, N:G28881A, N:G28882A, N:G28883C, N:C29197T,	15	N:R203K, N:G204R, ORF1a:P959S, ORF1a:A1352V, ORF1a:T3255I, ORF1a:I3618V, ORF1a:T4175I, ORF1b:P314L, ORF1b:G2201V, ORF8:L4P, S:E154A, S:T478K, S:D614G, S:P681H, S:T732A,

hCoV-19/Mexico/MOR-InDRE_463/2021	EPI_ISL_1168533	20G	B.1.2	21	5'UTR:C241T, ORF1ab:C1059T, ORF1ab:C3037T, ORF1ab:G3340T, ORF1ab:C10319T, ORF1ab:C14408T, ORF1ab:T17166C, ORF1ab:A18424G, ORF1ab:G20060T, ORF1ab:G20578T, ORF1ab:C21304T, S:T22585C, S:A23403G, ORF3a:G25563T, ORF3a:G25907T, M:A26945G, ORF6:C27247T, ORF8:C27964T, N:C28472T, N:C28869T, N:G29402T, 3'UTR:C29733A,	14	N:P67S, N:P199L, N:D377Y, ORF1a:T265I, ORF1a:L3352F, ORF1b:P314L, ORF1b:N1653D, ORF1b:S2198I, ORF1b:V2371L, ORF1b:R2613C, ORF3a:Q57H, ORF3a:G172V, ORF8:S24L, S:D614G,
hCoV-19/Mexico/MOR-InDRE_464/2021	EPI_ISL_1168534	20B	B.1.1.222	16	5'UTR:C241T, ORF1ab:C3037T, ORF1ab:G3085T, ORF1ab:G3871T, ORF1ab:G12988T, ORF1ab:C14408T, ORF1ab:T19839C, S:G22225T, S:A23403G, S:A23756G, ORF3a:G25906T, ORF8:G27916T, N:G28881A, N:G28882A, N:G28883C, N:G29179A, N:C29445T,	11	N:R203K, N:G204R, N:T391I, ORF1a:E940D, ORF1a:K1202N, ORF1a:M4241I, ORF1b:P314L, ORF3a:G172C, ORF8:G8V, S:D614G, S:T732A,
hCoV-19/Mexico/MOR-InDRE_465/2021	EPI_ISL_1168535	20B	B.1.1.519	24	5'UTR:T201C, 5'UTR:C203T, 5'UTR:C222T, 5'UTR:C241T, ORF1ab:G1738T, ORF1ab:C3037T, ORF1ab:C3140T, ORF1ab:C10029T, ORF1ab:C10954T, ORF1ab:A11117G, ORF1ab:C12789T, ORF1ab:C14408T, ORF1ab:C18086T, ORF1ab:T19839C, ORF1ab:A19974G, ORF1ab:C21306T, S:C22995A, S:A23403G, S:C23604A, S:A23756G, ORF3a:G25563C, N:G28881A, N:G28882A, N:G28883C, N:C29197T,	13	N:R203K, N:G204R, ORF1a:P959S, ORF1a:T3255I, ORF1a:I3618V, ORF1a:T4175I, ORF1b:P314L, ORF1b:T1540I, ORF3a:Q57H, S:T478K, S:D614G, S:P681H, S:T732A,
hCoV-19/Mexico/MOR-InDRE_466/2021	EPI_ISL_1168536	20B	B.1.1.222	18	5'UTR:C241T, ORF1ab:C3037T, ORF1ab:C3393T, ORF1ab:G4300T, ORF1ab:C14408T, ORF1ab:T17497C, ORF1ab:T19839C, ORF1ab:A21551T, S:C21637T, S:G21974C, S:A23403G, S:A23756G, N:C28278A, N:G28881A, N:G28882A, N:G28883C, N:G29260T, ORF10:G29648T, 3'UTR:C29835T,	10	N:S2Y, N:R203K, N:G204R, ORF1a:A1043V, ORF1b:P314L, ORF1b:Y1344H, ORF1b:N2695I, S:D138H, S:D614G, S:T732A,
hCoV-19/Mexico/MOR-InDRE_522/2021	EPI_ISL_1168592	20B	B.1	18	5'UTR:C241T, ORF1ab:C3037T, ORF1ab:G3971T, ORF1ab:T6602C, ORF1ab:C10029T, ORF1ab:C10954T, ORF1ab:C14408T, ORF1ab:C15925T, ORF1ab:T19839C, ORF1ab:C21306T, ORF1ab:A21366T, S:A23403G, S:C23604A, S:A23756G, S:G25249T, E:A26492T, N:G28881A, N:G28882A, N:C29197T,	9	N:R203K, ORF1a:V1236F, ORF1a:T3255I, ORF1b:P314L, ORF1b:L820F, S:D614G, S:P681H, S:T732A, S:M1229I,
hCoV-19/Mexico/MOR-InDRE_523/2021	EPI_ISL_1168593	20B	B.1.1.519	27	5'UTR:T201C, 5'UTR:C203T, 5'UTR:C222T, 5'UTR:C241T, ORF1ab:G1738T, ORF1ab:C3037T, ORF1ab:C3140T, ORF1ab:C10029T, ORF1ab:C10954T, ORF1ab:A11117G, ORF1ab:C12789T, ORF1ab:C14408T, ORF1ab:C15324T, ORF1ab:T19839C, ORF1ab:A19974G, ORF1ab:C21306T, S:C22995A, S:A23403G, S:C23604A, S:A23756G, S:G25244T, ORF7a:C27427T, N:G28881A, N:G28882A, N:G28883C, N:C29197T, 3'UTR:G29862T, 3'UTR:A29866C	13	N:R203K, N:G204R, ORF1a:P959S, ORF1a:T3255I, ORF1a:I3618V, ORF1a:T4175I, ORF1b:P314L, ORF7a:L12F, S:T478K, S:D614G, S:P681H, S:T732A, S:V1228L,
hCoV-19/Mexico/MOR-InDRE_524/2021	EPI_ISL_1168594	20B	B.1.1.519	28	5'UTR:C203T, 5'UTR:C222T, 5'UTR:C241T, ORF1ab:C3037T, ORF1ab:C3140T, ORF1ab:C10029T, ORF1ab:C10954T, ORF1ab:A11117G, ORF1ab:C12789T, ORF1ab:C14408T, ORF1ab:T19839C, ORF1ab:A19890T, ORF1ab:A20465G, ORF1ab:C21306T, S:G22346A, S:C22995A, S:A23403G, S:C23604A, S:A23756G, S:G24914T, S:C25047T, ORF3a:C25782T, ORF3a:G26063T, ORF7a:T27555A, ORF7a:T27565A, N:G28881A, N:G28882A, N:G28883C, N:C29197T,	19	N:R203K, N:G204R, ORF1a:P959S, ORF1a:T3255I, ORF1a:I3618V, ORF1a:T4175I, ORF1b:P314L, ORF1b:R2141S, ORF1b:D2333G, ORF3a:G224V, ORF7a:F54L, ORF7a:C58S, S:A262T, S:T478K, S:D614G, S:P681H, S:T732A, S:D1118Y, S:P1162L,

hCoV-19/Mexico/MOR-InDRE_525/2021	EPI_ISL_1168595	20B	B.1.1.519	35	5'UTR:T201C, 5'UTR:C203T, 5'UTR:C222T, 5'UTR:C241T, ORF1ab:A810G, ORF1ab:C936T, ORF1ab:G1738T, ORF1ab:C3037T, ORF1ab:C3140T, ORF1ab:C10029T, ORF1ab:C10954T, ORF1ab:A11117G, ORF1ab:C12789T, ORF1ab:C14408T, ORF1ab:C16650T, ORF1ab:T19839C, ORF1ab:A19974G, ORF1ab:C21306T, S:T22507A, S:T22508A, S:T22515A, S:T22516A, S:C22995A, S:A23403G, S:C23604A, S:A23756G, S:G23948T, ORF3a:C25626T, N:G28817T, N:G28881A, N:G28882A, N:G28883C, N:C29197T, N:G29227T, 3'UTR:G29862T, 3'UTR:A29866C	17	N:A182S, N:R203K, N:G204R, ORF1a:Y182C, ORF1a:T224I, ORF1a:P959S, ORF1a:T3255I, ORF1a:I3618V, ORF1a:T4175I, ORF1b:P314L, S:S316T, S:F318*, S:T478K, S:D614G, S:P681H, S:T732A, S:D796Y,
hCoV-19/Mexico/MOR-InDRE_526/2021	EPI_ISL_1168596	20B	B.1.1.519	22	5'UTR:C203T, 5'UTR:C222T, 5'UTR:C241T, ORF1ab:C3037T, ORF1ab:C3140T, ORF1ab:C10029T, ORF1ab:C10954T, ORF1ab:A11117G, ORF1ab:C12781T, ORF1ab:C12789T, ORF1ab:C14408T, ORF1ab:T19839C, ORF1ab:C21306T, S:C21575T, S:C22995A, S:A23403G, S:C23604A, S:A23756G, N:G28881A, N:G28882A, N:G28883C, N:C29197T, N:G29224T,	13	N:R203K, N:G204R, N:M317I, ORF1a:P959S, ORF1a:T3255I, ORF1a:I3618V, ORF1a:T4175I, ORF1b:P314L, S:L5F, S:T478K, S:D614G, S:P681H, S:T732A,
hCoV-19/Mexico/MOR-InDRE_527/2021	EPI_ISL_1168597	20B	B.1.1.222	24	5'UTR:C241T, ORF1ab:C3037T, ORF1ab:A3148G, ORF1ab:C10029T, ORF1ab:C14408T, ORF1ab:C15738T, ORF1ab:G17721T, ORF1ab:T19839C, S:A22375T, S:A23403G, S:A23756G, ORF3a:G25567A, ORF3a:T25569A, ORF3a:T25570A, ORF3a:G25912T, ORF3a:A26190C, ORF7a:G27670T, ORF8:A27921G, ORF8:G28001T, N:G28881A, N:G28882A, N:G28883C, 3'UTR:A29716T, 3'UTR:G29862T, 3'UTR:A29866C,	12	N:R203K, N:G204R, ORF1a:T3255I, ORF1b:P314L, ORF3a:A59T, ORF3a:S60T, ORF3a:G174C, ORF7a:V93F, ORF8:I10V, S:Q271H, S:D614G, S:T732A,
hCoV-19/Mexico/MOR-InDRE_528/2021	EPI_ISL_1168598	20B	B.1.1.519	28	5'UTR:C203T, 5'UTR:C222T, 5'UTR:C241T, ORF1ab:C3037T, ORF1ab:C3140T, ORF1ab:C6401T, ORF1ab:C10029T, ORF1ab:C10954T, ORF1ab:A11117G, ORF1ab:C12068T, ORF1ab:C12789T, ORF1ab:C14408T, ORF1ab:G15921T, ORF1ab:T19839C, ORF1ab:C21306T, S:A22390T, S:A22394T, S:T22397A, S:A22771G, S:C22995A, S:A23403G, S:C23604A, S:A23756G, ORF3a:G25855T, N:G28881A, N:G28882A, N:G28883C, N:C29197T, 3'UTR:G29862T	15	N:R203K, N:G204R, ORF1a:P959S, ORF1a:P2046S, ORF1a:T3255I, ORF1a:I3618V, ORF1a:T4175I, ORF1b:P314L, ORF3a:D155Y, S:K278*, S:Y279N, S:T478K, S:D614G, S:P681H, S:T732A,
hCoV-19/Mexico/MOR-InDRE_529/2021	EPI_ISL_1168599	20B	B.1.1.519	24	5'UTR:C203T, 5'UTR:C222T, 5'UTR:C241T, ORF1ab:C3037T, ORF1ab:C3140T, ORF1ab:G5206T, ORF1ab:G6865T, ORF1ab:C10029T, ORF1ab:C10954T, ORF1ab:A11117G, ORF1ab:C12789T, ORF1ab:C13724T, ORF1ab:C14408T, ORF1ab:T19839C, ORF1ab:C21306T, S:A22443C, S:C22995A, S:A23403G, S:C23604A, S:A23756G, ORF8:T27904C, N:G28881A, N:G28882A, N:G28883C, N:C29197T,	16	N:R203K, N:G204R, ORF1a:P959S, ORF1a:M1647I, ORF1a:K2200N, ORF1a:T3255I, ORF1a:I3618V, ORF1a:T4175I, ORF1b:A86V, ORF1b:P314L, ORF8:L4P, S:D294A, S:T478K, S:D614G, S:P681H, S:T732A,
hCoV-19/Mexico/MOR-InDRE_530/2021	EPI_ISL_1168600	20B	B.1.1.519	24	5'UTR:C203T, 5'UTR:C222T, 5'UTR:C241T, ORF1ab:C3037T, ORF1ab:C3140T, ORF1ab:G3549A, ORF1ab:C8616T, ORF1ab:C10029T, ORF1ab:C10954T, ORF1ab:A11117G, ORF1ab:C12789T, ORF1ab:C14408T, ORF1ab:T19839C, ORF1ab:C21306T, S:A22512C, S:C22995A, S:A23403G, S:C23604A, S:A23756G, M:G26526T, ORF8:T27904C, N:G28881A, N:G28882A, N:G28883C, N:C29197T,	16	M:A2S, N:R203K, N:G204R, ORF1a:P959S, ORF1a:G1095E, ORF1a:A2784V, ORF1a:T3255I, ORF1a:I3618V, ORF1a:T4175I, ORF1b:P314L, ORF8:L4P, S:N317T, S:T478K, S:D614G, S:P681H, S:T732A,

hCoV-19/Mexico/MOR-InDRE_531/2021	EPI_ISL_1168601	20B	B.1.1.222	25	5'UTR:C241T, ORF1ab:C3037T, ORF1ab:G3085T, ORF1ab:G3871T, ORF1ab:G12988T, ORF1ab:C14408T, ORF1ab:A19321C, ORF1ab:A19535C, ORF1ab:T19839C, ORF1ab:A21526T, S:G22225T, S:A23403G, S:A23756G, ORF3a:T26145A, ORF7a:G27396T, ORF7a:A27548C, ORF7a:T27581A, ORF8:G27916T, N:G28881A, N:G28882A, N:G28883C, N:G28966T, N:A28967T, N:G28968T, N:G29179A, N:C29445T,	18	N:R203K, N:G204R, N:E231D, N:S232F, N:T391I, ORF1a:E940D, ORF1a:K1202N, ORF1a:M4241I, ORF1b:P314L, ORF1b:T1952P, ORF1b:N2023T, ORF1b:I2687F, ORF7a:M1I, ORF7a:N52T, ORF7a:F63Y, ORF8:G8V, S:D614G, S:T732A,
hCoV-19/Mexico/MOR-InDRE_532/2021	EPI_ISL_1168602	20B	B.1.1.519	37	5'UTR:T201C, 5'UTR:C203T, 5'UTR:C222T, 5'UTR:C241T, ORF1ab:C936T, ORF1ab:G1738T, ORF1ab:C3037T, ORF1ab:C3140T, ORF1ab:T9445C, ORF1ab:C10029T, ORF1ab:C10954T, ORF1ab:A11117G, ORF1ab:C12789T, ORF1ab:C14408T, ORF1ab:C14741A, ORF1ab:T19839C, ORF1ab:A19974G, ORF1ab:C21306T, S:A22443C, S:C22747T, S:C22995A, S:T23102A, S:T23104A, S:T23106A, S:C23114A, S:A23403G, S:C23604A, S:A23756G, ORF3a:C25626T, N:G28378A, N:A28381G, N:G28817T, N:G28881A, N:G28882A, N:G28883C, N:C29197T, N:G29227T, 3'UTR:G29868A,	20	N:A182S, N:R203K, N:G204R, ORF1a:T224I, ORF1a:P959S, ORF1a:T3255I, ORF1a:I3618V, ORF1a:T4175I, ORF1b:P314L, ORF1b:S425Y, ORF9b:R32H, ORF9b:D33G, S:D294A, S:T478K, S:S514T, S:F515Y, S:L518I, S:D614G, S:P681H, S:T732A,
hCoV-19/Mexico/MOR-InDRE_533/2021	EPI_ISL_1168603	20B	B.1.1.519	20	5'UTR:C241T, ORF1ab:C3037T, ORF1ab:C3140T, ORF1ab:A3591T, ORF1ab:C10029T, ORF1ab:C10954T, ORF1ab:A11117G, ORF1ab:C14408T, ORF1ab:G15921T, ORF1ab:T19839C, ORF1ab:C21306T, S:A23403G, S:C23604A, S:A23756G, N:G28857T, N:G28881A, N:G28882A, N:G28883C, N:C29197T, 3'UTR:G29862T, 3'UTR:A29866C.	11	N:R195I, N:R203K, N:G204R, ORF1a:P959S, ORF1a:N1109I, ORF1a:T3255I, ORF1a:I3618V, ORF1b:P314L, S:D614G, S:P681H, S:T732A,
hCoV-19/Mexico/MOR-InDRE_534/2021	EPI_ISL_1168604	20B	B.1.1.519	26	5'UTR:C203T, 5'UTR:C222T, 5'UTR:C241T, ORF1ab:C3037T, ORF1ab:C3140T, ORF1ab:C5748A, ORF1ab:C5833T, ORF1ab:G8179T, ORF1ab:C10029T, ORF1ab:C10207T, ORF1ab:C10954T, ORF1ab:A11117G, ORF1ab:C14408T, ORF1ab:G19666T, ORF1ab:T19839C, ORF1ab:C21306T, S:C22995A, S:A23403G, S:C23604A, S:A23756G, ORF8:T27904C, N:G28881A, N:G28882A, N:G28883C, N:C29197T, 3'UTR:G29862T, 3'UTR:A29866C,	13	N:R203K, N:G204R, ORF1a:P959S, ORF1a:T1828N, ORF1a:T3255I, ORF1a:I3618V, ORF1b:P314L, ORF1b:D2067Y, ORF8:L4P, S:T478K, S:D614G, S:P681H, S:T732A,
hCoV-19/Mexico/MOR-InDRE_F10613_S685/2021	EPI_ISL_1365650	20B	B.1.1.519	28	5'UTR:T201C, 5'UTR:C203T, 5'UTR:C222T, 5'UTR:C241T, ORF1ab:C936T, ORF1ab:G1738T, ORF1ab:C3037T, ORF1ab:C3140T, ORF1ab:A3292G, ORF1ab:C6336T, ORF1ab:C7843T, ORF1ab:C10029T, ORF1ab:C10954T, ORF1ab:A11117G, ORF1ab:C12789T, ORF1ab:C14408T, ORF1ab:G16968A, ORF1ab:T19839C, ORF1ab:A19974G, ORF1ab:G20269T, ORF1ab:C21306T, S:A23403G, S:C23604A, S:A23756G, N:G28881A, N:G28882A, N:G28883C, N:C29197T, N:G29227T,	13	N:R203K, N:G204R, ORF1a:T224I, ORF1a:P959S, ORF1a:S2024L, ORF1a:T3255I, ORF1a:I3618V, ORF1a:T4175I, ORF1b:P314L, ORF1b:A2268S, S:D614G, S:P681H, S:T732A,
hCoV-19/Mexico/MOR-InDRE_F10622_S686/2021	EPI_ISL_1365651	20B	B.1.1.222	17	5'UTR:C241T, ORF1ab:C1288T, ORF1ab:C2143T, ORF1ab:C3037T, ORF1ab:C5339T, ORF1ab:C12403T, ORF1ab:C14408T, ORF1ab:C15024T, ORF1ab:G15629T, ORF1ab:G16377T, ORF1ab:C19170T, ORF1ab:T19839C, S:A23403G, S:A23756G, ORF8:G27916T, N:G28881A, N:G28882A, N:G28883C,	8	N:R203K, N:G204R, ORF1a:P1692S, ORF1b:P314L, ORF1b:C721F, ORF8:G8V, S:D614G, S:T732A,

hCoV-19/Mexico/MOR-InDRE_F10623_S687/2021	EPI_ISL_1365652	20B	B.1.1.222	18	5'UTR:G210T, 5'UTR:C241T, ORF1ab:C3037T, ORF1ab:G3085T, ORF1ab:G3871T, ORF1ab:C5482T, ORF1ab:C12231T, ORF1ab:G12988T, ORF1ab:C14408T, ORF1ab:T19839C, S:G22225T, S:A23403G, S:A23756G, ORF3a:G25906T, ORF7b:C27828T, N:G28881A, N:G28882A, N:G28883C, N:C29445T,	12	N:R203K, N:G204R, N:T391I, ORF1a:E940D, ORF1a:K1202N, ORF1a:S3989F, ORF1a:M4241I, ORF1b:P314L, ORF3a:G172C, ORF7b:L25F, S:D614G, S:T732A,
hCoV-19/Mexico/MOR-InDRE_F10626_S728/2021	EPI_ISL_1366677	20B	B.1.1.519	27	5'UTR:C203T, 5'UTR:C222T, 5'UTR:C241T, ORF1ab:C1342T, ORF1ab:C3037T, ORF1ab:C3140T, ORF1ab:G3692T, ORF1ab:G3871T, ORF1ab:C7299T, ORF1ab:C10029T, ORF1ab:C10954T, ORF1ab:A11117G, ORF1ab:C11824A, ORF1ab:C12789T, ORF1ab:C14408T, ORF1ab:C16887T, ORF1ab:T19839C, ORF1ab:C21306T, S:C21575T, S:C22995A, S:A23403G, S:C23604A, S:A23756G, ORF8:T27904C, N:G28881A, N:G28882A, N:G28883C, N:C29197T,	16	N:R203K, N:G204R, ORF1a:P959S, ORF1a:V1143F, ORF1a:K1202N, ORF1a:A2345V, ORF1a:T3255I, ORF1a:I3618V, ORF1a:T4175I, ORF1b:P314L, ORF8:L4P, S:L5F, S:T478K, S:D614G, S:P681H, S:T732A,
hCoV-19/Mexico/MOR-InDRE_F10627_S688/2021	EPI_ISL_1365653	20B	B.1.1.222	21	5'UTR:C241T, ORF1ab:C3037T, ORF1ab:A3148G, ORF1ab:C10029T, ORF1ab:C12809T, ORF1ab:C14408T, ORF1ab:C15738T, ORF1ab:G17721T, ORF1ab:T19839C, S:A23403G, S:A23756G, ORF3a:G25567A, ORF3a:T25569A, ORF3a:T25570A, ORF3a:A26108G, ORF7a:G27670T, ORF8:A27921G, ORF8:G28001T, ORF8:G28230A, N:G28881A, N:G28882A, N:G28883C,	13	N:R203K, N:G204R, ORF1a:T3255I, ORF1a:L4182F, ORF1b:P314L, ORF3a:A59T, ORF3a:S60T, ORF3a:E239G, ORF7a:V93F, ORF8:I10V, ORF8:D113N, S:D614G, S:T732A,
hCoV-19/Mexico/MOR-InDRE_F10634_S815/2021	EPI_ISL_1424017	20B	B.1.1.519	26	5'UTR:T201C, 5'UTR:C203T, 5'UTR:C222T, 5'UTR:C241T, ORF1ab:G1738T, ORF1ab:C2189T, ORF1ab:C3037T, ORF1ab:C3140T, ORF1ab:C6665T, ORF1ab:C10029T, ORF1ab:C10954T, ORF1ab:A11117G, ORF1ab:C12789T, ORF1ab:C14408T, ORF1ab:T19839C, ORF1ab:A19974G, ORF1ab:C21306T, S:C22995A, S:A23403G, S:C23604A, S:A23756G, ORF3a:G25699A, ORF3a:C26028T, N:G28881A, N:G28882A, N:G28883C, N:C29197T,	14	N:R203K, N:G204R, ORF1a:L642F, ORF1a:P959S, ORF1a:P2134S, ORF1a:T3255I, ORF1a:I3618V, ORF1a:T4175I, ORF1b:P314L, ORF3a:A103T, S:T478K, S:D614G, S:P681H, S:T732A,
hCoV-19/Mexico/MOR-InDRE_F10635_S689/2021	EPI_ISL_1365654	20B	B.1.1.222	19	5'UTR:C241T, ORF1ab:C3037T, ORF1ab:C4099T, ORF1ab:C10029T, ORF1ab:C12932T, ORF1ab:C14408T, ORF1ab:C14790T, ORF1ab:T19839C, S:A23403G, S:A23756G, S:C24138T, S:G24794T, ORF7a:G27556T, ORF7b:A27756G, ORF8:A27921G, ORF8:G28001T, N:G28881A, N:G28882A, N:G28883C, N:G29024T,	13	N:R203K, N:G204R, N:A251S, ORF1a:T3255I, ORF1a:P4223S, ORF1b:P314L, ORF7a:A55S, ORF7b:M1V, ORF8:I10V, S:D614G, S:T732A, S:T859I, S:A1078S,
hCoV-19/Mexico/MOR-InDRE_F10650_S729/2021	EPI_ISL_1366678	20B	B.1.1.519	28	5'UTR:T201C, 5'UTR:C203T, 5'UTR:C222T, 5'UTR:C241T, ORF1ab:C1218T, ORF1ab:G1738T, ORF1ab:C3037T, ORF1ab:C3140T, ORF1ab:G4907A, ORF1ab:C10029T, ORF1ab:C10954T, ORF1ab:A11117G, ORF1ab:C12789T, ORF1ab:C13517T, ORF1ab:C14408T, ORF1ab:C18457T, ORF1ab:T19839C, ORF1ab:A19974G, ORF1ab:C21306T, S:C21621T, S:C22995A, S:A23403G, S:C23604A, S:A23756G, N:G28881A, N:G28882A, N:G28883C, N:C29197T, 3'UTR:G29800A,	16	N:R203K, N:G204R, ORF1a:S318L, ORF1a:P959S, ORF1a:G1548S, ORF1a:T3255I, ORF1a:I3618V, ORF1a:T4175I, ORF1b:T17I, ORF1b:P314L, ORF1b:P1664S, S:T20I, S:T478K, S:D614G, S:P681H, S:T732A,

hCoV-19/Mexico/MOR-InDRE_F10653_S730/2021	EPI_ISL_1366679	20B	B.1.1.519	27	5'UTR:T201C, 5'UTR:C203T, 5'UTR:C222T, 5'UTR:C241T, ORF1ab:C875T, ORF1ab:G1738T, ORF1ab:C2119T, ORF1ab:G2764T, ORF1ab:C3037T, ORF1ab:C3140T, ORF1ab:C10029T, ORF1ab:C10954T, ORF1ab:A11117G, ORF1ab:C12789T, ORF1ab:C14408T, ORF1ab:T19839C, ORF1ab:A19974G, ORF1ab:C21306T, S:C22995A, S:A23403G, S:C23604A, S:A23756G, S:A23764G, N:G28881A, N:G28882A, N:G28883C, N:C29197T, 3'UTR:G29690T,	12	N:R203K, N:G204R, ORF1a:L204F, ORF1a:P959S, ORF1a:T3255I, ORF1a:I3618V, ORF1a:T4175I, ORF1b:P314L, S:T478K, S:D614G, S:P681H, S:T732A,
hCoV-19/Mexico/MOR-InDRE_F10656_S731/2021	EPI_ISL_1366680	20B	B.1.1.519	27	5'UTR:C203T, 5'UTR:C222T, 5'UTR:C241T, ORF1ab:C3037T, ORF1ab:C3140T, ORF1ab:G3692T, ORF1ab:G3871T, ORF1ab:C10029T, ORF1ab:C10954T, ORF1ab:A11117G, ORF1ab:C11824A, ORF1ab:C12789T, ORF1ab:C14408T, ORF1ab:G17814A, ORF1ab:T19839C, ORF1ab:C21306T, S:C22995A, S:A23403G, S:C23604A, S:A23756G, ORF3a:A25591G, ORF8:T27904C, ORF8:A28065T, N:G28881A, N:G28882A, N:G28883C, N:C29197T, N:G29260T	16	N:R203K, N:G204R, ORF1a:P959S, ORF1a:V1143F, ORF1a:K1202N, ORF1a:T3255I, ORF1a:I3618V, ORF1a:T4175I, ORF1b:P314L, ORF3a:K67E, ORF8:L4P, ORF8:I58F, S:T478K, S:D614G, S:P681H, S:T732A,
hCoV-19/Mexico/MOR-InDRE_F10660_S732/2021	EPI_ISL_1366681	20B	B.1.1.519	26	5'UTR:C203T, 5'UTR:C222T, 5'UTR:C241T, ORF1ab:G1681T, ORF1ab:C3037T, ORF1ab:C3140T, ORF1ab:T4339C, ORF1ab:C10029T, ORF1ab:C10954T, ORF1ab:A11117G, ORF1ab:C12789T, ORF1ab:C14408T, ORF1ab:G15906T, ORF1ab:T19839C, ORF1ab:G21099T, ORF1ab:C21306T, S:C22624A, S:C22995A, S:A23403G, S:C23604A, S:A23756G, ORF3a:G25906T, ORF8:C28253T, N:G28881A, N:G28882A, N:G28883C, N:C29197T,	16	N:R203K, N:G204R, ORF1a:E472D, ORF1a:P959S, ORF1a:T3255I, ORF1a:I3618V, ORF1a:T4175I, ORF1b:P314L, ORF1b:Q813H, ORF1b:E2544D, ORF3a:G172C, S:N354K, S:T478K, S:D614G, S:P681H, S:T732A,
hCoV-19/Mexico/MOR-InDRE_F10663_S690/2021	EPI_ISL_1365655	20B	B.1.1.519	21	5'UTR:C203T, 5'UTR:C222T, 5'UTR:C241T, ORF1ab:C3037T, ORF1ab:C3140T, ORF1ab:T7801C, ORF1ab:C10029T, ORF1ab:C10954T, ORF1ab:A11117G, ORF1ab:C12789T, ORF1ab:C14408T, ORF1ab:T19839C, ORF1ab:C21306T, S:C22995A, S:A23403G, S:C23604A, S:A23756G, ORF3a:C25528T, N:G28881A, N:G28882A, N:G28883C, N:C29197T,	12	N:R203K, N:G204R, ORF1a:P959S, ORF1a:T3255I, ORF1a:I3618V, ORF1a:T4175I, ORF1b:P314L, ORF3a:L46F, S:T478K, S:D614G, S:P681H, S:T732A,
hCoV-19/Mexico/MOR-InDRE_F10665_S816/2021	EPI_ISL_1424018	20B	B.1	22	5'UTR:C203T, 5'UTR:C222T, 5'UTR:C241T, ORF1ab:C1267T, ORF1ab:C3037T, ORF1ab:C3140T, ORF1ab:G5018A, ORF1ab:C8956T, ORF1ab:C10029T, ORF1ab:C10954T, ORF1ab:A11117G, ORF1ab:C12789T, ORF1ab:C12853T, ORF1ab:C14408T, ORF1ab:C14520T, ORF1ab:T19839C, ORF1ab:C21306T, S:C22995A, S:A23403G, S:C23604A, S:A23756G, M:T27029C, N:C29197T,	10	ORF1a:P959S, ORF1a:D1585N, ORF1a:T3255I, ORF1a:I3618V, ORF1a:T4175I, ORF1b:P314L, S:T478K, S:D614G, S:P681H, S:T732A,
hCoV-19/Mexico/MOR-InDRE_F10908_S864/2021	EPI_ISL_1399269	20B	B.1.1.519	30	5'UTR:C203T, 5'UTR:C222T, 5'UTR:C241T, ORF1ab:C3037T, ORF1ab:C3140T, ORF1ab:C9798T, ORF1ab:C10029T, ORF1ab:C10277T, ORF1ab:C10954T, ORF1ab:A11117G, ORF1ab:C12225T, ORF1ab:G12514T, ORF1ab:C12789T, ORF1ab:C12823T, ORF1ab:C14184T, ORF1ab:C14408T, ORF1ab:G14486C, ORF1ab:A19167T, ORF1ab:T19839C, ORF1ab:C21306T, ORF1ab:C21342T, S:G22778C, S:A23403G, S:C23604A, S:A23756G, ORF3a:T25911G, N:G28881A, N:G28882A, N:G28883C, N:C29197T, N:G29527T	18	N:R203K, N:G204R, N:Q418H, ORF1a:P959S, ORF1a:A3178V, ORF1a:T3255I, ORF1a:L3338F, ORF1a:I3618V, ORF1a:A3987V, ORF1a:T4175I, ORF1b:P314L, ORF1b:R340T, ORF1b:K1900N, ORF3a:D173E, S:E406Q, S:D614G, S:P681H, S:T732A,

hCoV-19/Mexico/MOR-InDRE_F10916_S763/2021	EPI_ISL_1400309	20B	B.1.1.519	22	5'UTR:C203T, 5'UTR:C222T, 5'UTR:C241T, ORF1ab:C3037T, ORF1ab:C3140T, ORF1ab:G5425T, ORF1ab:C10029T, ORF1ab:C10954T, ORF1ab:A11117G, ORF1ab:C12789T, ORF1ab:C14408T, ORF1ab:T19839C, ORF1ab:C21145T, ORF1ab:C21306T, S:C22995A, S:A23403G, S:C23604A, S:A23756G, N:G28881A, N:G28882A, N:G28883C, N:C29197T, N:C29358T,	14	N:R203K, N:G204R, N:T362I, ORF1a:P959S, ORF1a:K1720N, ORF1a:T3255I, ORF1a:I3618V, ORF1a:T4175I, ORF1b:P314L, ORF1b:L2560F, S:T478K, S:D614G, S:P681H, S:T732A,
hCoV-19/Mexico/MOR-InDRE_F10922_S762/2021	EPI_ISL_1400308	20B	B.1.1.519	28	5'UTR:T201C, 5'UTR:C203T, 5'UTR:C222T, 5'UTR:C241T, ORF1ab:A810G, ORF1ab:C936T, ORF1ab:G1738T, ORF1ab:C3037T, ORF1ab:C3140T, ORF1ab:G3955T, ORF1ab:C10029T, ORF1ab:C10954T, ORF1ab:A11117G, ORF1ab:C12789T, ORF1ab:C14408T, ORF1ab:T19839C, ORF1ab:A19974G, ORF1ab:C21306T, S:C22995A, S:A23403G, S:C23604A, S:A23756G, ORF3a:C25626T, N:G28817T, N:G28881A, N:G28882A, N:G28883C, N:C29197T, N:G29227T	15	N:A182S, N:R203K, N:G204R, ORF1a:Y182C, ORF1a:T224I, ORF1a:P959S, ORF1a:K1230N, ORF1a:T3255I, ORF1a:I3618V, ORF1a:T4175I, ORF1b:P314L, S:T478K, S:D614G, S:P681H, S:T732A,
hCoV-19/Mexico/MOR-InDRE_F10923_S761/2021	EPI_ISL_1400307	20B	B.1.1.519	29	5'UTR:C203T, 5'UTR:C222T, 5'UTR:C241T, ORF1ab:C1513T, ORF1ab:A2497T, ORF1ab:C3037T, ORF1ab:C3140T, ORF1ab:T3745C, ORF1ab:C4582T, ORF1ab:C8344T, ORF1ab:C10029T, ORF1ab:C10954T, ORF1ab:A11117G, ORF1ab:C12789T, ORF1ab:C14408T, ORF1ab:T17877A, ORF1ab:T19839C, ORF1ab:C19862T, ORF1ab:C20703T, ORF1ab:C21306T, S:C22995A, S:A23403G, S:C23604A, S:A23756G, ORF3a:C25844T, N:G28881A, N:G28882A, N:G28883C, N:C29197T, N:G29474T,	14	N:R203K, N:G204R, N:D401Y, ORF1a:P959S, ORF1a:T3255I, ORF1a:I3618V, ORF1a:T4175I, ORF1b:P314L, ORF1b:A2132V, ORF3a:T151I, S:T478K, S:D614G, S:P681H, S:T732A,
hCoV-19/Mexico/MOR-InDRE_F10926_S760/2021	EPI_ISL_1400306	20B	B.1.1.519	24	5'UTR:C203T, 5'UTR:C222T, 5'UTR:C241T, ORF1ab:T1303A, ORF1ab:C3037T, ORF1ab:C3140T, ORF1ab:C10029T, ORF1ab:C10954T, ORF1ab:A11117G, ORF1ab:A12082T, ORF1ab:C12789T, ORF1ab:C14408T, ORF1ab:T19839C, ORF1ab:A21137G, ORF1ab:C21306T, S:G21724T, S:C22995A, S:A23403G, S:C23604A, S:A23756G, N:G28881A, N:G28882A, N:G28883C, N:C29197T, ORF10:T29661C	13	N:R203K, N:G204R, ORF1a:P959S, ORF1a:T3255I, ORF1a:I3618V, ORF1a:T4175I, ORF1b:P314L, ORF1b:K2557R, S:L54F, S:T478K, S:D614G, S:P681H, S:T732A,
hCoV-19/Mexico/MOR-InDRE_F10927_S759/2021	EPI_ISL_1400305	20B	B.1.1.519	28	5'UTR:C203T, 5'UTR:C222T, 5'UTR:C241T, ORF1ab:G1820A, ORF1ab:C2704T, ORF1ab:C3037T, ORF1ab:C3140T, ORF1ab:C10029T, ORF1ab:C10954T, ORF1ab:A11117G, ORF1ab:C12789T, ORF1ab:T13905C, ORF1ab:C14408T, ORF1ab:A19631G, ORF1ab:T19839C, ORF1ab:C21306T, S:C22995A, S:A23403G, S:C23604A, S:A23756G, S:A23942G, ORF3a:G25770T, ORF3a:G25906T, ORF3a:C25916T, N:G28881A, N:G28882A, N:G28883C, N:G29179T, N:C29197T,	17	N:R203K, N:G204R, ORF1a:G519S, ORF1a:P959S, ORF1a:T3255I, ORF1a:I3618V, ORF1a:T4175I, ORF1b:P314L, ORF1b:N2055S, ORF3a:R126S, ORF3a:G172C, ORF3a:T175I, S:T478K, S:D614G, S:P681H, S:T732A, S:I794V,
hCoV-19/Mexico/MOR-InDRE_F10930_S757/2021	EPI_ISL_1400303	20B	B.1.1.519	22	5'UTR:C203T, 5'UTR:C222T, 5'UTR:C241T, ORF1ab:C1684T, ORF1ab:C3037T, ORF1ab:C3140T, ORF1ab:T3745C, ORF1ab:A6390G, ORF1ab:C10029T, ORF1ab:C10954T, ORF1ab:A11117G, ORF1ab:C12789T, ORF1ab:C14408T, ORF1ab:T19839C, S:C22995A, S:A23403G, S:C23604A, S:A23756G, ORF3a:C25844T, N:G28881A, N:G28882A, N:G28883C, N:C29197T,	13	N:R203K, N:G204R, ORF1a:P959S, ORF1a:E2042G, ORF1a:T3255I, ORF1a:I3618V, ORF1a:T4175I, ORF1b:P314L, ORF3a:T151I, S:T478K, S:D614G, S:P681H, S:T732A,

hCoV-19/Mexico/MOR-InDRE_F10933_S756/2021	EPI_ISL_1400302	20B	B.1.1.519	21	5'UTR:C203T, 5'UTR:C222T, 5'UTR:C241T, ORF1ab:C3037T, ORF1ab:C3140T, ORF1ab:C10029T, ORF1ab:C10954T, ORF1ab:A11117G, ORF1ab:C12789T, ORF1ab:C14408T, ORF1ab:A16388C, ORF1ab:A18702T, ORF1ab:T19839C, S:C22995A, S:A23403G, S:C23604A, S:A23668G, S:A23756G, N:G28881A, N:G28882A, N:G28883C, N:C29197T,	12	N:R203K, N:G204R, ORF1a:P959S, ORF1a:T3255I, ORF1a:I3618V, ORF1a:T4175I, ORF1b:P314L, ORF1b:N974T, S:T478K, S:D614G, S:P681H, S:T732A,
hCoV-19/Mexico/MOR-InDRE_F10934_S818/2021	EPI_ISL_1424020	20B	B.1.1.519	24	5'UTR:T201C, 5'UTR:C203T, 5'UTR:C222T, 5'UTR:C241T, ORF1ab:G1738T, ORF1ab:C3037T, ORF1ab:C3140T, ORF1ab:C10029T, ORF1ab:C10954T, ORF1ab:A11117G, ORF1ab:C12789T, ORF1ab:C12970T, ORF1ab:C14408T, ORF1ab:T15696C, ORF1ab:T19839C, ORF1ab:A19974G, ORF1ab:C21306T, S:C22687T, S:A23403G, S:C23604A, S:A23756G, N:G28881A, N:G28882A, N:G28883C, N:C29197T,	10	N:R203K, N:G204R, ORF1a:P959S, ORF1a:T3255I, ORF1a:I3618V, ORF1a:T4175I, ORF1b:P314L, S:D614G, S:P681H, S:T732A,
hCoV-19/Mexico/MOR-InDRE_F10935_S755/2021	EPI_ISL_1400301	20B	B.1.1.519	29	5'UTR:C203T, 5'UTR:C222T, 5'UTR:C241T, ORF1ab:C1213T, ORF1ab:C1342T, ORF1ab:C3037T, ORF1ab:C3140T, ORF1ab:G3871T, ORF1ab:C10029T, ORF1ab:C10954T, ORF1ab:A11117G, ORF1ab:C11824A, ORF1ab:C11916T, ORF1ab:C12789T, ORF1ab:C13774T, ORF1ab:C14408T, ORF1ab:C18395T, ORF1ab:T19839C, S:C21952T, S:C22995A, S:A23403G, S:C23604A, S:A23756G, M:T26987C, ORF8:T27904C, N:G28881A, N:G28882A, N:G28883C, N:C29197T, N:G29402T,	17	N:R203K, N:G204R, N:D377Y, ORF1a:P959S, ORF1a:K1202N, ORF1a:T3255I, ORF1a:I3618V, ORF1a:S3884L, ORF1a:T4175I, ORF1b:P103S, ORF1b:P314L, ORF1b:A1643V, ORF8:L4P, S:T478K, S:D614G, S:P681H, S:T732A,
hCoV-19/Mexico/MOR-InDRE_F10942_S882/2021	EPI_ISL_1405899	20B	B.1.1.519	21	5'UTR:C203T, 5'UTR:C222T, 5'UTR:C241T, ORF1ab:G2035T, ORF1ab:C3037T, ORF1ab:C3140T, ORF1ab:C10029T, ORF1ab:C10954T, ORF1ab:A11117G, ORF1ab:C12789T, ORF1ab:C14408T, ORF1ab:T19839C, S:C22995A, S:A23403G, S:C23604A, S:A23756G, ORF3a:C25528T, ORF3a:G25906T, N:G28881A, N:G28882A, N:G28883C, N:C29197T,	14	N:R203K, N:G204R, ORF1a:L590F, ORF1a:P959S, ORF1a:T3255I, ORF1a:I3618V, ORF1a:T4175I, ORF1b:P314L, ORF3a:L46F, ORF3a:G172C, S:T478K, S:D614G, S:P681H, S:T732A,
hCoV-19/Mexico/MOR-InDRE_F10943_S754/2021	EPI_ISL_1400300	20B	B.1.1.519	25	5'UTR:C203T, 5'UTR:C222T, 5'UTR:C241T, ORF1ab:C3037T, ORF1ab:C3140T, ORF1ab:C4345T, ORF1ab:C5628T, ORF1ab:G9595T, ORF1ab:C10029T, ORF1ab:C10954T, ORF1ab:C11094T, ORF1ab:A11117G, ORF1ab:C12789T, ORF1ab:C14408T, ORF1ab:T19839C, S:C22995A, S:A23403G, S:C23604A, S:A23756G, ORF8:T27904C, N:G28827T, N:G28881A, N:G28882A, N:G28883C, N:C29197T, N:C29364T,	17	N:R185L, N:R203K, N:G204R, N:P364L, ORF1a:P959S, ORF1a:T1788M, ORF1a:L3110F, ORF1a:T3255I, ORF1a:A3610V, ORF1a:I3618V, ORF1a:T4175I, ORF1b:P314L, ORF8:L4P, S:T478K, S:D614G, S:P681H, S:T732A,
hCoV-19/Mexico/MOR-InDRE_F11061_S840/2021	EPI_ISL_1424042	20B	B.1.1.519	27	5'UTR:C203T, 5'UTR:C222T, 5'UTR:C241T, ORF1ab:G1820A, ORF1ab:C2704T, ORF1ab:C3037T, ORF1ab:C3140T, ORF1ab:C10029T, ORF1ab:C10954T, ORF1ab:A11117G, ORF1ab:C12789T, ORF1ab:T13905C, ORF1ab:C14408T, ORF1ab:A19631G, ORF1ab:T19839C, ORF1ab:C21306T, S:C22995A, S:A23403G, S:C23604A, S:A23756G, S:A23942G, ORF3a:G25906T, ORF3a:C25916T, N:G28881A, N:G28882A, N:G28883C, N:G29179T, N:C29197T,	16	N:R203K, N:G204R, ORF1a:G519S, ORF1a:P959S, ORF1a:T3255I, ORF1a:I3618V, ORF1a:T4175I, ORF1b:P314L, ORF1b:N2055S, ORF3a:G172C, ORF3a:T175I, S:T478K, S:D614G, S:P681H, S:T732A, S:I794V,
hCoV-19/Mexico/MOR-InDRE-26/2020	EPI_ISL_493341	20B	B.1.1.222	9	5'UTR:C241T, ORF1ab:T2533C, ORF1ab:C3037T, ORF1ab:C14408T, ORF1ab:T19839C, S:A23403G, S:A23756G, N:G28881A, N:G28882A, N:G28883C,	5	N:R203K, N:G204R, ORF1b:P314L, S:D614G, S:T732A,
hCoV-19/Mexico/NAY-InDRE_27/2020	EPI_ISL_493342	20A	B.1.609	8	5'UTR:C241T, ORF1ab:C3037T, ORF1ab:C4582T, ORF1ab:C8175T, ORF1ab:C14408T, ORF1ab:A20268G, S:A23403G, S:A24110C, S:G24356T,	5	ORF1a:A2637V, ORF1b:P314L, S:D614G, S:I850L, S:G932C,

hCoV-19/Mexico/NAY-InDRE_28/2020	EPI_ISL_493343	20C	B.1.320	12	5'UTR:C241T, ORF1ab:C379A, ORF1ab:C1059T, ORF1ab:T2153C, ORF1ab:C3037T, ORF1ab:G4657A, ORF1ab:C14408T, ORF1ab:T15465C, S:G21578T, S:A23403G, ORF3a:G25563T, N:C28657T, N:G28842T,	7	N:S190I, ORF1a:T265I, ORF1a:W630R, ORF1b:P314L, ORF3a:Q57H, S:V6F, S:D614G,	
hCoV-19/Mexico/NAY-InDRE_F11035_S868/2021	EPI_ISL_1399273	20A	B.1.561	21	5'UTR:C241T, ORF1ab:C936T, ORF1ab:C3037T, ORF1ab:A5999G, ORF1ab:A10323G, ORF1ab:C10449T, ORF1ab:C10519T, ORF1ab:C10798T, ORF1ab:C11866T, ORF1ab:C13378T, ORF1ab:C14408T, ORF1ab:C18693T, ORF1ab:C19164T, ORF1ab:A20268G, S:G21974T, S:A23403G, S:C24031T, ORF3a:C25521T, ORF3a:T25577C, N:C28854T, N:G28975T, ORF10:C29666T,	10	N:S194L, N:M234I, ORF1a:T224I, ORF1a:I1912V, ORF1a:K3353R, ORF1a:P3395L, ORF1b:P314L, ORF3a:I62T, S:D138Y, S:D614G,	
hCoV-19/Mexico/NAY-InDRE_F11036_S869/2021	EPI_ISL_1399274	20A	B.1.126	18	5'UTR:C241T, ORF1ab:T3011C, ORF1ab:C3037T, ORF1ab:G5704T, ORF1ab:G10193A, ORF1ab:A13455T, ORF1ab:C14408T, ORF1ab:G20102T, S:G22205T, S:A23403G, S:G23611T, S:A23894G, ORF3a:G25598T, ORF3a:G25855T, ORF3a:G25971T, ORF7b:T27869C, N:T28853G, N:C28887T, 3'UTR:G29779T,	13	N:S194A, N:T205I, ORF1a:Q1813H, ORF1a:E3310K, ORF1a:Q4397L, ORF1b:P314L, ORF1b:S2212I, ORF3a:W69L, ORF3a:D155Y, ORF3a:W193C, S:D215Y, S:D614G, S:T778A,	
hCoV-19/Mexico/NLE-InDRE_237/2021	EPI_ISL_961467	20I (Alpha, V1)	B.1.1.7	31	5'UTR:C241T, ORF1ab:C913T, ORF1ab:C3037T, ORF1ab:C3267T, ORF1ab:C5388A, ORF1ab:C5986T, ORF1ab:T6954C, ORF1ab:C14408T, ORF1ab:C14676T, ORF1ab:C15279T, ORF1ab:T16176C, ORF1ab:A17615G, ORF1ab:C18032T, S:A23063T, S:C23271A, S:A23403G, S:C23604A, S:C23709T, S:T24506G, S:G24914C, M:G26730C, ORF8:C27972T, ORF8:G28048T, ORF8:A28111G, N:G28280C, N:A28281T, N:T28282A, N:C28603T, N:G28881A, N:G28882A, N:G28883C, N:C28977T,	21	M:V70L, N:D3L, N:R203K, N:G204R, N:S235F, ORF1a:T1001I, ORF1a:A1708D, ORF1a:I2230T, ORF1b:P314L, ORF1b:K1383R, ORF1b:T1522I, ORF8:Q27*, ORF8:R52I, ORF8:Y73C, S:N501Y, S:A570D, S:D614G, S:P681H, S:T716I, S:S982A, S:D1118H,	ORF1ab: 11288-11296, S:21765-21770, S:21992-21994,
hCoV-19/Mexico/NLE-InDRE_238/2021	EPI_ISL_961468	20I (Alpha, V1)	B.1.1.7	31	5'UTR:C241T, ORF1ab:C913T, ORF1ab:C3037T, ORF1ab:C3267T, ORF1ab:C5388A, ORF1ab:C5986T, ORF1ab:T6954C, ORF1ab:C14408T, ORF1ab:C14676T, ORF1ab:C15279T, ORF1ab:T16176C, ORF1ab:A17615G, ORF1ab:C18032T, S:A23063T, S:C23271A, S:A23403G, S:C23604A, S:C23709T, S:T24506G, S:G24914C, M:G26730C, ORF8:C27972T, ORF8:G28048T, ORF8:A28111G, N:G28280C, N:A28281T, N:T28282A, N:C28603T, N:G28881A, N:G28882A, N:G28883C, N:C28977T,	21	M:V70L, N:D3L, N:R203K, N:G204R, N:S235F, ORF1a:T1001I, ORF1a:A1708D, ORF1a:I2230T, ORF1b:P314L, ORF1b:K1383R, ORF1b:T1522I, ORF8:Q27*, ORF8:R52I, ORF8:Y73C, S:N501Y, S:A570D, S:D614G, S:P681H, S:T716I, S:S982A, S:D1118H,	ORF1ab: 11288-11296, S:21765-21770, S:21992-21994,
hCoV-19/Mexico/NLE-InDRE_239/2021	EPI_ISL_942927	20A	B.1	4	5'UTR:C241T, ORF1ab:C3037T, ORF1ab:C14408T, S:A23403G, ORF7a:C27630T,	2	ORF1b:P314L, S:D614G,	
hCoV-19/Mexico/NLE-InDRE_240/2021	EPI_ISL_961469	20I (Alpha, V1)	B.1.1.7	31	5'UTR:C241T, ORF1ab:C913T, ORF1ab:C3037T, ORF1ab:C3267T, ORF1ab:C5388A, ORF1ab:C5986T, ORF1ab:T6954C, ORF1ab:C14408T, ORF1ab:C14676T, ORF1ab:C15279T, ORF1ab:T16176C, ORF1ab:A17615G, ORF1ab:C18032T, S:A23063T, S:C23271A, S:A23403G, S:C23604A, S:C23709T, S:T24506G, S:G24914C, M:G26730C, ORF8:C27972T, ORF8:G28048T, ORF8:A28111G, N:G28280C, N:A28281T, N:T28282A, N:C28603T, N:G28881A, N:G28882A, N:G28883C, N:C28977T,	21	M:V70L, N:D3L, N:R203K, N:G204R, N:S235F, ORF1a:T1001I, ORF1a:A1708D, ORF1a:I2230T, ORF1b:P314L, ORF1b:K1383R, ORF1b:T1522I, ORF8:Q27*, ORF8:R52I, ORF8:Y73C, S:N501Y, S:A570D, S:D614G, S:P681H, S:T716I, S:S982A, S:D1118H,	ORF1ab: 11288-11296, S:21765-21770, S:21992-21994,

hCoV-19/Mexico/NLE-InDRE_389/2021	EPI_ISL_1168467	20A	B.1	19	5'UTR:C241T, ORF1ab:C344T, ORF1ab:C1288T, ORF1ab:C1473T, ORF1ab:C1722T, ORF1ab:A2386G, ORF1ab:C3037T, ORF1ab:A10323G, ORF1ab:C12439T, ORF1ab:C14408T, ORF1ab:C16111T, ORF1ab:C17004T, ORF1ab:C19662T, ORF1ab:A20268G, ORF1ab:G20962T, ORF1ab:G21468T, S:A23403G, ORF3a:C26060T, ORF7a:C27630T, N:C28854T,	10	N:S194L, ORF1a:L27F, ORF1a:T403I, ORF1a:A486V, ORF1a:K3353R, ORF1b:P314L, ORF1b:D2499Y, ORF1b:M2667I, ORF3a:T223I, S:D614G,	
hCoV-19/Mexico/NLE-InDRE_390/2021	EPI_ISL_1168468	20B	B.1.1.222	20	5'UTR:C241T, ORF1ab:C3037T, ORF1ab:G3242T, ORF1ab:T4697C, ORF1ab:C4980A, ORF1ab:C9344T, ORF1ab:C13297T, ORF1ab:C13517T, ORF1ab:C14408T, ORF1ab:T14472G, ORF1ab:G15906T, ORF1ab:T17065C, ORF1ab:G18186T, ORF1ab:T19839C, S:A23403G, S:A23756G, ORF8:C28253T, N:G28881A, N:G28882A, N:G28883C, 3'UTR:G29747C,	13	N:R203K, N:G204R, ORF1a:G993C, ORF1a:S1478P, ORF1a:T1572K, ORF1a:L3027F, ORF1b:T17I, ORF1b:P314L, ORF1b:Q813H, ORF1b:Y1200H, ORF1b:M1573I, S:D614G, S:T732A,	
hCoV-19/Mexico/NLE-InDRE_391/2021	EPI_ISL_1168469	20C	B.1.595	22	5'UTR:C241T, ORF1ab:C1059T, ORF1ab:G1599T, ORF1ab:C3037T, ORF1ab:T9208A, ORF1ab:G9817A, ORF1ab:C10319T, ORF1ab:C14408T, ORF1ab:A16978G, ORF1ab:T17319C, ORF1ab:A19751G, ORF1ab:G20578T, ORF1ab:A21137G, S:A23403G, S:G23545T, S:C24621T, S:C25046T, ORF3a:G25563T, ORF3a:G25855T, E:C26455T, M:T26816C, ORF8:C27945T, ORF8:C27964T,	17	E:P71S, ORF1a:T265I, ORF1a:G445V, ORF1a:L3352F, ORF1b:P314L, ORF1b:R1171G, ORF1b:E2095G, ORF1b:V2371L, ORF1b:K2557R, ORF3a:Q57H, ORF3a:D155Y, ORF8:Q18*, ORF8:S24L, S:D614G, S:E661D, S:A1020V, S:P1162S,	
hCoV-19/Mexico/NLE-InDRE_392/2021	EPI_ISL_1168470	20B	B.1.1.519	21	5'UTR:C203T, 5'UTR:C222T, 5'UTR:C241T, ORF1ab:C3037T, ORF1ab:C3140T, ORF1ab:C10029T, ORF1ab:C10954T, ORF1ab:A11117G, ORF1ab:C12789T, ORF1ab:C14408T, ORF1ab:A17460T, ORF1ab:T19839C, ORF1ab:C21306T, S:C22995A, S:G23236T, S:A23403G, S:C23604A, S:A23756G, N:G28881A, N:G28882A, N:G28883C, N:C29197T,	12	N:R203K, N:G204R, ORF1a:P959S, ORF1a:T3255I, ORF1a:I3618V, ORF1a:T4175I, ORF1b:P314L, S:T478K, S:K558N, S:D614G, S:P681H, S:T732A,	
hCoV-19/Mexico/NLE-InDRE_393/2021	EPI_ISL_1168471	20A	B.1.243	19	5'UTR:C241T, ORF1ab:C2623T, ORF1ab:C2973T, ORF1ab:C3037T, ORF1ab:C5140T, ORF1ab:C7869T, ORF1ab:C14408T, ORF1ab:A15046C, ORF1ab:C19097T, ORF1ab:A19137G, ORF1ab:A20268G, S:A23403G, S:C23604A, S:T24076C, S:G24812T, S:G25244T, ORF3a:G25595T, N:C28854T, ORF10:G29587T, 3'UTR:T29710C,	11	N:S194L, ORF1a:A903V, ORF1a:S2535L, ORF1b:P314L, ORF1b:I527L, ORF1b:A1877V, ORF3a:R68I, S:D614G, S:P681H, S:D1084Y, S:V1228L,	
hCoV-19/Mexico/NLE-InDRE_394/2021	EPI_ISL_1168472	20A	B.1	11	5'UTR:C241T, ORF1ab:C556T, ORF1ab:C1190T, ORF1ab:C3037T, ORF1ab:C12756A, ORF1ab:C14408T, ORF1ab:G16741A, ORF1ab:C19884T, ORF1ab:A20268G, S:C21952T, S:A23403G, N:C28854T,	6	N:S194L, ORF1a:P309S, ORF1a:T4164N, ORF1b:P314L, ORF1b:V1092I, S:D614G,	ORF1ab: 9860-9862
hCoV-19/Mexico/NLE-InDRE_395/2021	EPI_ISL_1168473	20B	B.1.1.519	27	5'UTR:T201C, 5'UTR:C203T, 5'UTR:C222T, 5'UTR:C241T, ORF1ab:G1738T, ORF1ab:C3037T, ORF1ab:C3140T, ORF1ab:C3261T, ORF1ab:A5026G, ORF1ab:C5512T, ORF1ab:C10029T, ORF1ab:C10954T, ORF1ab:A11117G, ORF1ab:C12789T, ORF1ab:C13356T, ORF1ab:C14408T, ORF1ab:C18086T, ORF1ab:T19839C, ORF1ab:A19974G, ORF1ab:C21306T, S:C22995A, S:A23403G, S:C23604A, S:A23756G, N:G28881A, N:G28882A, N:G28883C, N:C29197T	14	N:R203K, N:G204R, ORF1a:P959S, ORF1a:T999I, ORF1a:T3255I, ORF1a:I3618V, ORF1a:T4175I, ORF1a:T4364I, ORF1b:P314L, ORF1b:T1540I, S:T478K, S:D614G, S:P681H, S:T732A,	

hCoV-19/Mexico/NLE-InDRE_396/2021	EPI_ISL_1168474	21C (Epsilon)	B.1.429	25	5'UTR:C241T, ORF1ab:C1059T, ORF1ab:C2395T, ORF1ab:T2597C, ORF1ab:C3037T, ORF1ab:C8947T, ORF1ab:G10523C, ORF1ab:C12100T, ORF1ab:A12878G, ORF1ab:C14408T, ORF1ab:G17014T, S:G21600T, S:G22018T, S:C22591T, S:T22917G, S:A23403G, S:T24349C, ORF3a:G25563T, M:C26681T, ORF7b:G27890T, ORF8:G28079T, ORF8:G28191T, ORF8:A28272T, N:C28887T, N:G28975T, N:C29362T,	13	N:T205I, N:M234I, ORF1a:T265I, ORF1a:V3420L, ORF1a:I4205V, ORF1b:P314L, ORF1b:D1183Y, ORF3a:Q57H, ORF8:V100L, S:S13I, S:W152C, S:L452R, S:D614G,
hCoV-19/Mexico/NLE-InDRE_397/2021	EPI_ISL_1168475	20A	B.1.558	21	5'UTR:C241T, ORF1ab:C344T, ORF1ab:C1288T, ORF1ab:C3037T, ORF1ab:G8513T, ORF1ab:C12439T, ORF1ab:C14408T, ORF1ab:C15273T, ORF1ab:G18449T, ORF1ab:C19662T, ORF1ab:A20268G, ORF1ab:G20962T, ORF1ab:G21523T, S:G22518C, S:A23403G, ORF3a:C26060T, E:T26445C, ORF7a:C27630T, N:C28332T, N:C28391T, N:C28854T, N:G29422T,	13	N:P20L, N:R40C, N:S194L, ORF1a:L27F, ORF1a:V2750F, ORF1b:P314L, ORF1b:S1661I, ORF1b:D2499Y, ORF1b:V2686F, ORF3a:T223I, ORF9b:P17S, S:R319T, S:D614G,
hCoV-19/Mexico/NLE-InDRE_398/2021	EPI_ISL_1168476	20G	B.1.2	18	5'UTR:C241T, ORF1ab:C1059T, ORF1ab:C3037T, ORF1ab:C4347T, ORF1ab:C9050T, ORF1ab:C10319T, ORF1ab:C14183T, ORF1ab:C14408T, ORF1ab:C14703T, ORF1ab:A18424G, S:A23403G, S:G24933T, ORF3a:G25563T, ORF3a:G25907T, M:G26834T, ORF7a:C27737T, ORF8:C27964T, N:C28472T, N:C28869T,	15	N:P67S, N:P199L, ORF1a:T265I, ORF1a:S1361F, ORF1a:P2929S, ORF1a:L3352F, ORF1b:T239I, ORF1b:P314L, ORF1b:N1653D, ORF3a:Q57H, ORF3a:G172V, ORF7a:T115I, ORF8:S24L, S:D614G, S:G1124V,
hCoV-19/Mexico/NLE-InDRE_399/2021	EPI_ISL_1168477	21C (Epsilon)	B.1.429	26	5'UTR:C241T, ORF1ab:C1059T, ORF1ab:C2395T, ORF1ab:T2597C, ORF1ab:C3037T, ORF1ab:G7100A, ORF1ab:C8947T, ORF1ab:C12100T, ORF1ab:G12160A, ORF1ab:C12161G, ORF1ab:G12163A, ORF1ab:A12878G, ORF1ab:C14408T, ORF1ab:G17014T, S:G21600T, S:C21638T, S:G22018T, S:T22917G, S:A23403G, S:T24283C, S:T24349C, ORF3a:G25563T, M:C26681T, ORF7b:G27890T, ORF8:A28272T, N:C28887T, N:C29362T,	13	N:T205I, ORF1a:T265I, ORF1a:A2279T, ORF1a:Q3966E, ORF1a:I4205V, ORF1b:P314L, ORF1b:D1183Y, ORF3a:Q57H, S:S13I, S:P26S, S:W152C, S:L452R, S:D614G,
hCoV-19/Mexico/NLE-InDRE_400/2021	EPI_ISL_1168478	20B	B.1.1.519	25	5'UTR:C203T, 5'UTR:C222T, 5'UTR:C241T, ORF1ab:C3037T, ORF1ab:C3140T, ORF1ab:G5539A, ORF1ab:C6286T, ORF1ab:G6894A, ORF1ab:C9611T, ORF1ab:C10029T, ORF1ab:C10954T, ORF1ab:A11117G, ORF1ab:G11365T, ORF1ab:C12789T, ORF1ab:C14408T, ORF1ab:T19839C, ORF1ab:C21306T, S:C22995A, S:A23403G, S:C23604A, S:A23756G, N:G28881A, N:G28882A, N:G28883C, N:C29197T, N:G29527T,	14	N:R203K, N:G204R, N:Q418H, ORF1a:P959S, ORF1a:C2210Y, ORF1a:L3116F, ORF1a:T3255I, ORF1a:I3618V, ORF1a:T4175I, ORF1b:P314L, S:T478K, S:D614G, S:P681H, S:T732A,
hCoV-19/Mexico/NLE-InDRE_401/2021	EPI_ISL_1168479	20A	B.1.558	22	5'UTR:C241T, ORF1ab:C344G, ORF1ab:C1288T, ORF1ab:C3037T, ORF1ab:T4111C, ORF1ab:C12439T, ORF1ab:C14408T, ORF1ab:C15273T, ORF1ab:G18449T, ORF1ab:C19662T, ORF1ab:A20268G, ORF1ab:G20962T, ORF1ab:A21436G, ORF1ab:G21523T, S:A21764T, S:T22020C, S:C22858T, S:A23403G, ORF3a:C25549T, ORF3a:C26060T, ORF7a:C27630T, N:C28854T, N:G29422T,	12	N:S194L, ORF1a:L27V, ORF1b:P314L, ORF1b:S1661I, ORF1b:D2499Y, ORF1b:M2657V, ORF1b:V2686F, ORF3a:L53F, ORF3a:T223I, S:I68L, S:M153T, S:D614G,

hCoV-19/Mexico/NLE-InDRE_402/2021	EPI_ISL_1168480	20G	B.1.2	24	5'UTR:G210T, 5'UTR:C241T, ORF1ab:C1059T, ORF1ab:C3037T, ORF1ab:T7348A, ORF1ab:C7843T, ORF1ab:G8083A, ORF1ab:C10319T, ORF1ab:C13119T, ORF1ab:A13296G, ORF1ab:C14408T, ORF1ab:C14805T, ORF1ab:C18131T, ORF1ab:A18424G, ORF1ab:A21203G, ORF1ab:C21304T, S:A23403G, ORF3a:G25563T, ORF3a:G25907T, ORF3a:C26228T, ORF7b:C27828T, ORF8:C27964T, N:C28472T, N:C28869T, 3'UTR:G29779T,	18	N:P67S, N:P199L, ORF1a:T265I, ORF1a:N2361K, ORF1a:M2606I, ORF1a:L3352F, ORF1a:A4285V, ORF1a:D4344G, ORF1b:P314L, ORF1b:T1555I, ORF1b:N1653D, ORF1b:K2579R, ORF1b:R2613C, ORF3a:Q57H, ORF3a:G172V, ORF7b:L25F, ORF8:S24L, S:D614G,
hCoV-19/Mexico/NLE-InDRE_403/2021	EPI_ISL_1168481	20B	B.1.1.519	22	5'UTR:C203T, 5'UTR:C222T, 5'UTR:C241T, ORF1ab:C3037T, ORF1ab:C3140T, ORF1ab:C3330T, ORF1ab:T4351C, ORF1ab:C10029T, ORF1ab:C10954T, ORF1ab:A11117G, ORF1ab:C12789T, ORF1ab:C14408T, ORF1ab:T19839C, ORF1ab:C21306T, S:C22995A, S:A23403G, S:C23604A, S:A23756G, ORF3a:G25775T, N:G28881A, N:G28882A, N:G28883C, N:C29197T,	13	N:R203K, N:G204R, ORF1a:P959S, ORF1a:T1022I, ORF1a:T3255I, ORF1a:I3618V, ORF1a:T4175I, ORF1b:P314L, ORF3a:W128L, S:T478K, S:D614G, S:P681H, S:T732A,
hCoV-19/Mexico/NLE-InDRE_404/2021	EPI_ISL_1168482	20A	B.1.396	14	5'UTR:C241T, ORF1ab:G1126T, ORF1ab:C1191T, ORF1ab:C3037T, ORF1ab:A3205G, ORF1ab:C9521A, ORF1ab:C14408T, ORF1ab:A20268G, S:A23403G, S:C24904T, ORF7a:G27601T, N:G28378T, N:C28854T, N:G29422T, ORF10:A29567G,	8	N:S194L, ORF1a:R287S, ORF1a:P309L, ORF1a:L3086I, ORF1b:P314L, ORF7a:G70C, ORF9b:R32L, S:D614G,
hCoV-19/Mexico/NLE-InDRE_405/2021	EPI_ISL_1168483	20G	B.1.2	23	5'UTR:C241T, ORF1ab:C1059T, ORF1ab:C1625T, ORF1ab:T2533C, ORF1ab:C3037T, ORF1ab:G3497A, ORF1ab:T3952C, ORF1ab:C6525T, ORF1ab:C10319T, ORF1ab:G11083T, ORF1ab:C14408T, ORF1ab:C15613T, ORF1ab:A18424G, ORF1ab:C21304T, S:C22624T, S:A23403G, S:G25166C, ORF3a:G25563T, ORF3a:G25907T, E:C26335T, ORF8:C27964T, N:C28472T, N:C28869T, N:A29499T,	18	N:P67S, N:P199L, N:Q409L, ORF1a:T265I, ORF1a:L454F, ORF1a:A1078T, ORF1a:T2087I, ORF1a:L3352F, ORF1a:L3606F, ORF1b:P314L, ORF1b:H716Y, ORF1b:N1653D, ORF1b:R2613C, ORF3a:Q57H, ORF3a:G172V, ORF8:S24L, S:D614G, S:E1202Q,
hCoV-19/Mexico/NLE-InDRE_F10500_S673/2021	EPI_ISL_1365639	20G	B.1.2	21	5'UTR:A161G, 5'UTR:C241T, ORF1ab:C1059T, ORF1ab:C3037T, ORF1ab:G4444T, ORF1ab:C10319T, ORF1ab:G11417T, ORF1ab:C14408T, ORF1ab:T16851C, ORF1ab:G17478A, ORF1ab:A18424G, ORF1ab:C21304T, S:C21614T, S:A23403G, S:G23900C, ORF3a:G25563T, ORF3a:G25907T, ORF8:C27942T, ORF8:C27964T, N:C28472T, N:C28869T, N:C29236T,	15	N:P67S, N:P199L, ORF1a:T265I, ORF1a:L3352F, ORF1a:V3718F, ORF1b:P314L, ORF1b:N1653D, ORF1b:R2613C, ORF3a:Q57H, ORF3a:G172V, ORF8:H17Y, ORF8:S24L, S:L18F, S:D614G, S:E780Q,
hCoV-19/Mexico/NLE-InDRE_F10501_S674/2021	EPI_ISL_1365640	20B	B.1.1.222	22	5'UTR:C241T, ORF1ab:C829T, ORF1ab:T2533C, ORF1ab:C3037T, ORF1ab:A3603G, ORF1ab:A8479G, ORF1ab:C8480T, ORF1ab:C14408T, ORF1ab:C16468T, ORF1ab:T19839C, ORF1ab:C20233T, ORF1ab:C20703T, S:C21952T, S:A23403G, S:A23756G, ORF3a:C25603T, ORF3a:G26172T, ORF7b:C27804T, ORF8:C27920T, N:G28655T, N:G28881A, N:G28882A, N:G28883C,	11	N:D128Y, N:R203K, N:G204R, ORF1a:H1113R, ORF1a:P2739S, ORF1b:P314L, ORF1b:P1001S, ORF1b:P2256S, ORF3a:M260I, S:D614G, S:T732A,
hCoV-19/Mexico/NLE-InDRE_F10502_S714/2021	EPI_ISL_1366663	20B	B.1.1.519	25	5'UTR:C203T, 5'UTR:C222T, 5'UTR:C241T, ORF1ab:C3037T, ORF1ab:C3140T, ORF1ab:C10029T, ORF1ab:C10954T, ORF1ab:A11117G, ORF1ab:C12789T, ORF1ab:C14408T, ORF1ab:C16362T, ORF1ab:T19839C, ORF1ab:C21306T, S:C22995A, S:A23403G, S:C23604A, S:A23756G, S:C24865T, ORF3a:C25571T, ORF7a:C27688T, ORF8:C28253T, N:G28881A, N:G28882A, N:G28883C, N:C29197T, N:G29527T,	14	N:R203K, N:G204R, N:Q418H, ORF1a:P959S, ORF1a:T3255I, ORF1a:I3618V, ORF1a:T4175I, ORF1b:P314L, ORF3a:S60F, ORF7a:P99S, S:T478K, S:D614G, S:P681H, S:T732A,

hCoV-19/Mexico/NLE-InDRE_F10504_S857/2021	EPI_ISL_1399262	20A	B.1.243	15	5'UTR:C241T, ORF1ab:C3037T, ORF1ab:C14408T, ORF1ab:C16338T, ORF1ab:C19097T, ORF1ab:A19137G, ORF1ab:A20268G, S:A23403G, S:C23604A, S:A23746T, S:T24076C, S:G25244T, M:C26882T, N:C28854T, ORF10:G29587T, 3'UTR:T29710C,	6	N:S194L, ORF1b:P314L, ORF1b:A1877V, S:D614G, S:P681H, S:V1228L,	
hCoV-19/Mexico/NLE-InDRE_F10506_S675/2021	EPI_ISL_1365641	20G	B.1.2	17	5'UTR:C241T, ORF1ab:C1059T, ORF1ab:C3037T, ORF1ab:C4543T, ORF1ab:C9430T, ORF1ab:C10319T, ORF1ab:C14408T, ORF1ab:C14670T, ORF1ab:T14808C, ORF1ab:A18424G, ORF1ab:C21304T, S:A23403G, ORF3a:G25563T, ORF3a:G25907T, ORF8:C27964T, N:C28472T, N:C28869T, N:G29402T,	12	N:P67S, N:P199L, N:D377Y, ORF1a:T265I, ORF1a:L3352F, ORF1b:P314L, ORF1b:N1653D, ORF1b:R2613C, ORF3a:Q57H, ORF3a:G172V, ORF8:S24L, S:D614G,	
hCoV-19/Mexico/NLE-InDRE_F10507_S676/2021	EPI_ISL_1365642	20B	B.1.1.222	20	5'UTR:C241T, ORF1ab:C1545T, ORF1ab:C3037T, ORF1ab:C4524T, ORF1ab:C5544T, ORF1ab:A6693G, ORF1ab:C9430T, ORF1ab:C10029T, ORF1ab:C10030T, ORF1ab:C14408T, ORF1ab:T19839C, S:C22747T, S:A23403G, S:A23756G, ORF3a:G25912T, ORF7b:A27756G, ORF8:A27921G, ORF8:G28001T, N:G28881A, N:G28882A, N:G28883C,	13	N:R203K, N:G204R, ORF1a:A427V, ORF1a:A1420V, ORF1a:T1760I, ORF1a:K2143R, ORF1a:T3255I, ORF1b:P314L, ORF3a:G174C, ORF7b:M1V, ORF8:I10V, S:D614G, S:T732A,	
hCoV-19/Mexico/NLE-InDRE_F10509_S858/2021	EPI_ISL_1399263	20G	B.1.2	21	5'UTR:C241T, ORF1ab:C1059T, ORF1ab:C3037T, ORF1ab:G4908A, ORF1ab:C9207T, ORF1ab:C10319T, ORF1ab:C14408T, ORF1ab:T17088A, ORF1ab:A18424G, ORF1ab:A20055G, ORF1ab:C21304T, S:C21691T, S:A23403G, ORF3a:G25555T, ORF3a:G25563T, ORF3a:C25844T, ORF3a:G25907T, ORF8:C27964T, ORF8:G27987T, N:C28472T, N:C28869T, N:G28975T,	17	N:P67S, N:P199L, N:M234I, ORF1a:T265I, ORF1a:G1548D, ORF1a:S2981F, ORF1a:L3352F, ORF1b:P314L, ORF1b:N1653D, ORF1b:R2613C, ORF3a:V55F, ORF3a:Q57H, ORF3a:T151I, ORF3a:G172V, ORF8:S24L, ORF8:V32L, S:D614G,	
hCoV-19/Mexico/NLE-InDRE_F10510_S859/2021	EPI_ISL_1399264	20B	B.1.1.316	19	5'UTR:C241T, ORF1ab:C3037T, ORF1ab:C3411T, ORF1ab:G3871T, ORF1ab:A5040G, ORF1ab:A12519G, ORF1ab:C14408T, ORF1ab:G16405A, ORF1ab:T16539C, ORF1ab:T19839C, S:C21757T, S:A23403G, S:G23593C, S:A23756T, ORF3a:C26022T, N:G28881A, N:G28882A, N:G28883C, N:C29421T, 3'UTR:G29751T.	12	N:R203K, N:G204R, N:P383L, ORF1a:A1049V, ORF1a:K1202N, ORF1a:Q1592R, ORF1a:D4085G, ORF1b:P314L, ORF1b:V980I, S:D614G, S:Q677H, S:T732S,	
hCoV-19/Mexico/NLE-InDRE_F10511_S715/2021	EPI_ISL_1366664	20B	B.1.1.519	20	5'UTR:C203T, 5'UTR:C222T, 5'UTR:C241T, ORF1ab:C3037T, ORF1ab:C3140T, ORF1ab:C10029T, ORF1ab:C10954T, ORF1ab:A11117G, ORF1ab:C12789T, ORF1ab:C14408T, ORF1ab:T19839C, ORF1ab:C21306T, S:C22995A, S:A23403G, S:C23604A, S:A23756G, ORF8:C27920T, N:G28881A, N:G28882A, N:G28883C, N:C29197T,	11	N:R203K, N:G204R, ORF1a:P959S, ORF1a:T3255I, ORF1a:I3618V, ORF1a:T4175I, ORF1b:P314L, S:T478K, S:D614G, S:P681H, S:T732A,	
hCoV-19/Mexico/NLE-InDRE_F10517_S677/2021	EPI_ISL_1365643	20A	B.1.243	13	5'UTR:C241T, ORF1ab:C3037T, ORF1ab:T3733C, ORF1ab:C14408T, ORF1ab:C19097T, ORF1ab:A19137G, ORF1ab:A20268G, S:A23403G, S:C23604A, S:T24076C, S:G24305A, ORF7a:C27527T, N:C28854T, 3'UTR:T29710C,	7	N:S194L, ORF1b:P314L, ORF1b:A1877V, ORF7a:P45L, S:D614G, S:P681H, S:V915I,	S:21992-21994,
hCoV-19/Mexico/NLE-InDRE_F10518_S678/2021	EPI_ISL_1365644	20A	B.1.243	19	5'UTR:C241T, ORF1ab:C1471T, ORF1ab:C3037T, ORF1ab:C10456T, ORF1ab:A12556G, ORF1ab:C12710T, ORF1ab:C14408T, ORF1ab:C14649T, ORF1ab:C15952T, ORF1ab:C19097T, ORF1ab:A19137G, ORF1ab:A20268G, S:C22432T, S:A23403G, S:C23604A, S:T24076C, M:C26801T, N:C28854T, N:A28972G. 3'UTR:T29710C.	5	N:S194L, ORF1b:P314L, ORF1b:A1877V, S:D614G, S:P681H,	

hCoV-19/Mexico/NLE-InDRE_F10519_S679/2021	EPI_ISL_1365645	20B	B.1.1.222	25	5'UTR:C106T, 5'UTR:C241T, ORF1ab:T1169C, ORF1ab:C1684T, ORF1ab:A2679G, ORF1ab:C3037T, ORF1ab:G3122T, ORF1ab:C13115T, ORF1ab:C14408T, ORF1ab:C14491T, ORF1ab:G14575T, ORF1ab:A17367T, ORF1ab:T19839C, S:G21641T, S:A23403G, S:A23756G, S:T24469C, ORF3a:G25471A, ORF3a:G25644T, ORF3a:G26062A, ORF7a:C27476T, ORF8:T27900C, N:G28881A, N:G28882A, N:G28883C, 3'UTR:G29736T,	14	N:R203K, N:G204R, ORF1a:S302P, ORF1a:N805S, ORF1a:D953Y, ORF1b:P314L, ORF1b:A370S, ORF3a:D27N, ORF3a:G224S, ORF7a:T28I, ORF8:F3L, S:A27S, S:D614G, S:T732A,
hCoV-19/Mexico/NLE-InDRE_F10520_S680/2021	EPI_ISL_1365646	20A	B.1.243	14	5'UTR:C241T, ORF1ab:C3037T, ORF1ab:C8208T, ORF1ab:C14408T, ORF1ab:C19097T, ORF1ab:A19137G, ORF1ab:A20268G, S:A23403G, S:C23604A, S:T24076C, ORF3a:C25427T, ORF3a:A25531G, ORF8:C27970T, N:C28854T, 3'UTR:T29710C,	9	N:S194L, ORF1a:T2648I, ORF1b:P314L, ORF1b:A1877V, ORF3a:T12I, ORF3a:I47V, ORF8:T26I, S:D614G, S:P681H,
hCoV-19/Mexico/NLE-InDRE_F10521_S681/2021	EPI_ISL_1365647	20B	B.1.1.316	19	5'UTR:C241T, ORF1ab:C3037T, ORF1ab:C3411T, ORF1ab:G3871T, ORF1ab:A5040G, ORF1ab:A12519G, ORF1ab:C14408T, ORF1ab:G16405A, ORF1ab:T16539C, ORF1ab:T19839C, S:C21757T, S:A23403G, S:G23593C, S:A23756T, ORF3a:C26022T, N:G28881A, N:G28882A, N:G28883C, N:C29421T, 3'UTR:G29751T.	12	N:R203K, N:G204R, N:P383L, ORF1a:A1049V, ORF1a:K1202N, ORF1a:Q1592R, ORF1a:D4085G, ORF1b:P314L, ORF1b:V980I, S:D614G, S:Q677H, S:T732S,
hCoV-19/Mexico/NLE-InDRE_F10522_S682/2021	EPI_ISL_1365648	20B	B.1.1.222	18	5'UTR:C241T, ORF1ab:C3037T, ORF1ab:C5544T, ORF1ab:A6693G, ORF1ab:C9430T, ORF1ab:C10029T, ORF1ab:C10030T, ORF1ab:C14408T, ORF1ab:T19839C, S:C22747T, S:A23403G, S:A23756G, ORF3a:G25912T, ORF7b:A27756G, ORF8:A27921G, ORF8:G28001T, N:G28881A, N:G28882A, N:G28883C,	11	N:R203K, N:G204R, ORF1a:T1760I, ORF1a:K2143R, ORF1a:T3255I, ORF1b:P314L, ORF3a:G174C, ORF7b:M1V, ORF8:I10V, S:D614G, S:T732A,
hCoV-19/Mexico/NLE-InDRE_F10523_S716/2021	EPI_ISL_1366665	20B	B.1.1.519	22	5'UTR:C203T, 5'UTR:C222T, 5'UTR:C241T, ORF1ab:C3037T, ORF1ab:C3140T, ORF1ab:G9087A, ORF1ab:C10029T, ORF1ab:C10954T, ORF1ab:A11117G, ORF1ab:G11365T, ORF1ab:C12789T, ORF1ab:C14408T, ORF1ab:T19839C, ORF1ab:C21306T, S:C22995A, S:A23403G, S:C23604A, S:A23756G, N:G28881A, N:G28882A, N:G28883C, N:C29197T, N:G29527T,	13	N:R203K, N:G204R, N:Q418H, ORF1a:P959S, ORF1a:G2941D, ORF1a:T3255I, ORF1a:I3618V, ORF1a:T4175I, ORF1b:P314L, S:T478K, S:D614G, S:P681H, S:T732A,
hCoV-19/Mexico/NLE-InDRE_F10524_S590/2021	EPI_ISL_1337368	21C (Epsilon)	B.1.429	31	5'UTR:C173T, 5'UTR:C241T, ORF1ab:C1059T, ORF1ab:C2395T, ORF1ab:T2597C, ORF1ab:C3037T, ORF1ab:G7100A, ORF1ab:C8947T, ORF1ab:C12100T, ORF1ab:G12160A, ORF1ab:C12161G, ORF1ab:G12163A, ORF1ab:A12878G, ORF1ab:C14408T, ORF1ab:G17014T, ORF1ab:C17135T, S:G21600T, S:C21638T, S:G22018T, S:C22088T, S:T22917G, S:A23403G, S:T24283C, S:T24349C, ORF3a:G25563T, ORF3a:A26190C, ORF3a:C26208T, M:C26681T, ORF7b:G27890T, ORF8:A28272T, N:C28887T, N:C29362T,	16	N:T205I, ORF1a:T265I, ORF1a:A2279T, ORF1a:Q3966E, ORF1a:I4205V, ORF1b:P314L, ORF1b:D1183Y, ORF1b:P1223L, ORF3a:Q57H, ORF3a:E266D, S:S13I, S:P26S, S:W152C, S:L176F, S:L452R, S:D614G,
hCoV-19/Mexico/NLE-InDRE_F10791_S703/2021	EPI_ISL_1365668	20A	B.1.243	16	5'UTR:C241T, ORF1ab:C3037T, ORF1ab:G4904T, ORF1ab:C14408T, ORF1ab:C18029T, ORF1ab:C19097T, ORF1ab:A19137G, ORF1ab:A20268G, ORF1ab:G21123T, S:A23403G, S:C23604A, S:T24076C, ORF3a:C25427T, ORF8:C27970T, N:C28854T, 3'UTR:T29710C, 3'UTR:C29837T.	9	N:S194L, ORF1a:D1547Y, ORF1b:P314L, ORF1b:A1521V, ORF1b:A1877V, ORF3a:T12I, ORF8:T26I, S:D614G, S:P681H,

hCoV-19/Mexico/NLE-InDRE_F10792_S744/2021	EPI_ISL_1366693	20B	B.1.1.519	30	5'UTR:T201C, 5'UTR:C203T, 5'UTR:C222T, 5'UTR:C241T, ORF1ab:C936T, ORF1ab:G1738T, ORF1ab:A3021T, ORF1ab:C3037T, ORF1ab:C3140T, ORF1ab:A8073G, ORF1ab:C10029T, ORF1ab:C10954T, ORF1ab:A11117G, ORF1ab:C12789T, ORF1ab:C14408T, ORF1ab:T19839C, ORF1ab:A19974G, ORF1ab:C21306T, S:C22995A, S:C23191T, S:A23324G, S:A23403G, S:C23604A, S:A23756G, N:C28838T, N:G28881A, N:G28882A, N:G28883C, N:C29197T, N:G29227T, 3'UTR:C29784T,	16	N:R189C, N:R203K, N:G204R, ORF1a:T224I, ORF1a:H919L, ORF1a:P959S, ORF1a:N2603S, ORF1a:T3255I, ORF1a:I3618V, ORF1a:T4175I, ORF1b:P314L, S:T478K, S:T588A, S:D614G, S:P681H, S:T732A,
hCoV-19/Mexico/NLE-InDRE_F10793_S745/2021	EPI_ISL_1366694	20B	B.1.1.519	24	5'UTR:T201C, 5'UTR:C203T, 5'UTR:C222T, 5'UTR:C241T, ORF1ab:G1738T, ORF1ab:C3037T, ORF1ab:C3140T, ORF1ab:C10029T, ORF1ab:C10954T, ORF1ab:A11023G, ORF1ab:A11117G, ORF1ab:C12789T, ORF1ab:C14408T, ORF1ab:A18861T, ORF1ab:T19839C, ORF1ab:A19974G, ORF1ab:C21306T, S:C22995A, S:A23403G, S:C23604A, S:A23756G, N:G28881A, N:G28882A, N:G28883C, N:C29197T,	11	N:R203K, N:G204R, ORF1a:P959S, ORF1a:T3255I, ORF1a:I3618V, ORF1a:T4175I, ORF1b:P314L, S:T478K, S:D614G, S:P681H, S:T732A,
hCoV-19/Mexico/NLE-InDRE_F10794_S746/2021	EPI_ISL_1366695	20B	B.1.1.519	23	5'UTR:C203T, 5'UTR:C222T, 5'UTR:C241T, ORF1ab:C3037T, ORF1ab:C3140T, ORF1ab:T3745C, ORF1ab:C8074T, ORF1ab:C10029T, ORF1ab:C10954T, ORF1ab:A11117G, ORF1ab:C12789T, ORF1ab:C12880T, ORF1ab:C14408T, ORF1ab:T19839C, ORF1ab:C21306T, S:C22995A, S:A23403G, S:C23604A, S:A23756G, ORF3a:C25844T, N:G28881A, N:G28882A, N:G28883C, N:C29197T,	12	N:R203K, N:G204R, ORF1a:P959S, ORF1a:T3255I, ORF1a:I3618V, ORF1a:T4175I, ORF1b:P314L, ORF3a:T151I, S:T478K, S:D614G, S:P681H, S:T732A,
hCoV-19/Mexico/NLE-InDRE_F10795_S704/2021	EPI_ISL_1365669	20B	B.1.1.316	19	5'UTR:C241T, ORF1ab:C3037T, ORF1ab:C3411T, ORF1ab:G3871T, ORF1ab:A5040G, ORF1ab:A12519G, ORF1ab:C14408T, ORF1ab:G16405A, ORF1ab:T16539C, ORF1ab:T19839C, S:C21757T, S:A23403G, S:G23593C, S:A23756T, ORF3a:C26022T, N:G28881A, N:G28882A, N:G28883C, N:C29421T, 3'UTR:G29751T,	12	N:R203K, N:G204R, N:P383L, ORF1a:A1049V, ORF1a:K1202N, ORF1a:Q1592R, ORF1a:D4085G, ORF1b:P314L, ORF1b:V980I, S:D614G, S:Q677H, S:T732S,
hCoV-19/Mexico/NLE-InDRE_F10796_S705/2021	EPI_ISL_1365670	20G	B.1.2	24	5'UTR:C241T, ORF1ab:C583T, ORF1ab:C1059T, ORF1ab:G2632T, ORF1ab:C3037T, ORF1ab:T7348A, ORF1ab:A7702G, ORF1ab:G8083A, ORF1ab:T9590C, ORF1ab:C10319T, ORF1ab:C13119T, ORF1ab:C14408T, ORF1ab:C14805T, ORF1ab:C18131T, ORF1ab:A18424G, ORF1ab:A21203G, ORF1ab:C21304T, S:A23403G, S:G23593T, ORF3a:T25502C, ORF3a:G25563T, ORF3a:G25907T, ORF8:C27964T, N:C28472T, N:C28869T,	20	N:P67S, N:P199L, ORF1a:T265I, ORF1a:M789I, ORF1a:N2361K, ORF1a:M2606I, ORF1a:Y3109H, ORF1a:L3352F, ORF1a:A4285V, ORF1b:P314L, ORF1b:T1555I, ORF1b:N1653D, ORF1b:K2579R, ORF1b:R2613C, ORF3a:I37T, ORF3a:Q57H, ORF3a:G172V, ORF8:S24L, S:D614G, S:Q677H,
hCoV-19/Mexico/NLE-InDRE_F10797_S706/2021	EPI_ISL_1365671	20G	B.1.2	24	5'UTR:C241T, ORF1ab:C583T, ORF1ab:C1059T, ORF1ab:G2632T, ORF1ab:C3037T, ORF1ab:T7348A, ORF1ab:A7702G, ORF1ab:G8083A, ORF1ab:T9590C, ORF1ab:C10319T, ORF1ab:C13119T, ORF1ab:C14408T, ORF1ab:C14805T, ORF1ab:C18131T, ORF1ab:A18424G, ORF1ab:A21203G, ORF1ab:C21304T, S:A23403G, S:G23593T, ORF3a:T25502C, ORF3a:G25563T, ORF3a:G25907T, ORF8:C27964T, N:C28472T, N:C28869T,	20	N:P67S, N:P199L, ORF1a:T265I, ORF1a:M789I, ORF1a:N2361K, ORF1a:M2606I, ORF1a:Y3109H, ORF1a:L3352F, ORF1a:A4285V, ORF1b:P314L, ORF1b:T1555I, ORF1b:N1653D, ORF1b:K2579R, ORF1b:R2613C, ORF3a:I37T, ORF3a:Q57H, ORF3a:G172V, ORF8:S24L, S:D614G, S:Q677H,

hCoV-19/Mexico/NLE-InDRE_F10799_S707/2021	EPI_ISL_1365672	20A	B.1.243	15	5'UTR:C241T, ORF1ab:C3037T, ORF1ab:A4500G, ORF1ab:C5850T, ORF1ab:C5986T, ORF1ab:C14408T, ORF1ab:C19097T, ORF1ab:A19137G, ORF1ab:A20268G, S:A23403G, S:C23604A, S:T24076C, ORF3a:C25427T, ORF8:C27970T, N:C28854T, 3'UTR:T29710C,	9	N:S194L, ORF1a:Q1412R, ORF1a:P1862L, ORF1b:P314L, ORF1b:A1877V, ORF3a:T12I, ORF8:T26I, S:D614G, S:P681H,
hCoV-19/Mexico/NLE-InDRE_F10800_S747/2021	EPI_ISL_1366696	20B	B.1.1.519	23	5'UTR:C203T, 5'UTR:C222T, 5'UTR:C241T, ORF1ab:C3037T, ORF1ab:C3140T, ORF1ab:G3692T, ORF1ab:G3871T, ORF1ab:C10029T, ORF1ab:C10954T, ORF1ab:A11117G, ORF1ab:C11824A, ORF1ab:C12789T, ORF1ab:C14408T, ORF1ab:T19839C, ORF1ab:C21306T, S:C22995A, S:A23403G, S:C23604A, S:A23756G, ORF8:T27904C, N:G28881A, N:G28882A, N:G28883C, N:C29197T,	14	N:R203K, N:G204R, ORF1a:P959S, ORF1a:V1143F, ORF1a:K1202N, ORF1a:T3255I, ORF1a:I3618V, ORF1a:T4175I, ORF1b:P314L, ORF8:L4P, S:T478K, S:D614G, S:P681H, S:T732A,
hCoV-19/Mexico/NLE-InDRE_F10801_S748/2021	EPI_ISL_1366697	20B	B.1.1.519	25	5'UTR:C203T, 5'UTR:C222T, 5'UTR:C241T, ORF1ab:C3037T, ORF1ab:C3140T, ORF1ab:T3745C, ORF1ab:C10029T, ORF1ab:C10954T, ORF1ab:A11117G, ORF1ab:C12789T, ORF1ab:C13105T, ORF1ab:C14408T, ORF1ab:C16362T, ORF1ab:T19839C, ORF1ab:C20759T, ORF1ab:C21306T, S:C22995A, S:A23403G, S:C23604A, S:A23756G, ORF3a:C25578T, ORF3a:C25844T, N:G28881A, N:G28882A, N:G28883C, N:C29197T,	13	N:R203K, N:G204R, ORF1a:P959S, ORF1a:T3255I, ORF1a:I3618V, ORF1a:T4175I, ORF1b:P314L, ORF1b:A2431V, ORF3a:T151I, S:T478K, S:D614G, S:P681H, S:T732A,
hCoV-19/Mexico/NLE-InDRE_F10802_S708/2021	EPI_ISL_1365673	20B	B.1.1.432	21	5'UTR:C241T, ORF1ab:C3037T, ORF1ab:C3695T, ORF1ab:G5194T, ORF1ab:A6985T, ORF1ab:G9242A, ORF1ab:C9319T, ORF1ab:C12412T, ORF1ab:T14313C, ORF1ab:C14408T, ORF1ab:C19185T, S:C22938T, S:A23403G, S:C23422T, ORF3a:C25844T, ORF3a:G25912T, ORF8:T27909C, N:G28881A, N:G28882A, N:G28883C, N:T29317C, N:A29533G,	9	N:R203K, N:G204R, ORF1a:E2993K, ORF1b:P314L, ORF3a:T151I, ORF3a:G174C, ORF8:F6L, S:S459F, S:D614G,
hCoV-19/Mexico/NLE-InDRE_F10803_S860/2021	EPI_ISL_1399265	20A	B.1.243	16	5'UTR:C241T, ORF1ab:C3037T, ORF1ab:C14408T, ORF1ab:C16338T, ORF1ab:C19097T, ORF1ab:A19137G, ORF1ab:C19402T, ORF1ab:A20268G, S:A23403G, S:C23604A, S:A23746T, S:T24076C, S:G25244T, E:G26428T, N:C28854T, ORF10:G29587T, 3'UTR:T29710C,	8	E:V62F, N:S194L, ORF1b:P314L, ORF1b:A1877V, ORF1b:H1979Y, S:D614G, S:P681H, S:V1228L,
hCoV-19/Mexico/NLE-InDRE_F10804_S861/2021	EPI_ISL_1399266	20A	B.1.1	24	5'UTR:C241T, ORF1ab:C1288T, ORF1ab:G2305T, ORF1ab:C3037T, ORF1ab:C5221T, ORF1ab:G8083T, ORF1ab:C10335T, ORF1ab:C12085T, ORF1ab:T12751G, ORF1ab:A13566G, ORF1ab:C14408T, ORF1ab:C18060T, ORF1ab:T19896C, ORF1ab:A20268G, S:G22147A, S:G22487C, S:C23114T, S:A23403G, S:C23664T, ORF3a:G25455T, ORF3a:G25770T, ORF3a:A25788G, ORF3a:T25807C, ORF3a:G26109T, N:C28854T,	11	N:S194L, ORF1a:K680N, ORF1a:M2606I, ORF1a:A3357V, ORF1b:P314L, ORF3a:K21N, ORF3a:R126S, ORF3a:E239D, S:E309Q, S:D614G, S:A701V,
hCoV-19/Mexico/NLE-InDRE_F10806_S862/2021	EPI_ISL_1399267	20G	B.1.2	25	5'UTR:C106T, 5'UTR:C241T, ORF1ab:C1059T, ORF1ab:C3037T, ORF1ab:T7348A, ORF1ab:G8083A, ORF1ab:C10319T, ORF1ab:C11479T, ORF1ab:C13119T, ORF1ab:C14408T, ORF1ab:C14805T, ORF1ab:C14931T, ORF1ab:C18131T, ORF1ab:A18424G, ORF1ab:A21203G, ORF1ab:C21304T, S:A23403G, S:C24642T, ORF3a:G25563T, ORF3a:G25907T, ORF3a:C26228T, E:C26256T, ORF8:C27964T, N:C28344T, N:C28472T, N:C28869T,	18	N:T24I, N:P67S, N:P199L, ORF1a:T265I, ORF1a:N2361K, ORF1a:M2606I, ORF1a:L3352F, ORF1a:A4285V, ORF1b:P314L, ORF1b:T1555I, ORF1b:N1653D, ORF1b:K2579R, ORF1b:R2613C, ORF3a:Q57H, ORF3a:G172V, ORF8:S24L, S:D614G, S:T1027I,

hCoV-19/Mexico/NLE-InDRE_F10807_S749/2021	EPI_ISL_1366698	20B	B.1.1.519	22	5'UTR:C203T, 5'UTR:C222T, 5'UTR:C241T, ORF1ab:C3037T, ORF1ab:C3140T, ORF1ab:C10029T, ORF1ab:C10954T, ORF1ab:A11117G, ORF1ab:C12789T, ORF1ab:C14408T, ORF1ab:T19839C, ORF1ab:C21306T, S:C22995A, S:A23403G, S:C23604A, S:A23756G, ORF3a:C25571T, ORF7a:C27688T, N:G28881A, N:G28882A, N:G28883C, N:C29197T, N:G29527T,	14	N:R203K, N:G204R, N:Q418H, ORF1a:P959S, ORF1a:T3255I, ORF1a:I3618V, ORF1a:T4175I, ORF1b:P314L, ORF3a:S60F, ORF7a:P99S, S:T478K, S:D614G, S:P681H, S:T732A,
hCoV-19/Mexico/NLE-InDRE_F10808_S863/2021	EPI_ISL_1399268	20C	B.1.564	22	5'UTR:C241T, ORF1ab:G806A, ORF1ab:C832T, ORF1ab:C1059T, ORF1ab:T1936C, ORF1ab:C3037T, ORF1ab:C4582T, ORF1ab:A10994G, ORF1ab:C13487T, ORF1ab:C14408T, ORF1ab:C16260T, ORF1ab:G17721T, ORF1ab:G19872T, ORF1ab:T20331C, S:C21707T, S:A23403G, S:C23557T, S:C23635T, S:C24937T, ORF3a:G25563T, M:G26690T, N:C28821A, N:T28879C,	9	N:S183Y, ORF1a:A181T, ORF1a:T265I, ORF1a:K3577E, ORF1b:A7V, ORF1b:P314L, ORF3a:Q57H, S:H49Y, S:D614G,
hCoV-19/Mexico/NLE-InDRE_F10809_S750/2021	EPI_ISL_1366699	20B	B.1.1.519	24	5'UTR:C203T, 5'UTR:C222T, 5'UTR:C241T, ORF1ab:C3037T, ORF1ab:C3140T, ORF1ab:G3351A, ORF1ab:T8593C, ORF1ab:C10029T, ORF1ab:C10954T, ORF1ab:A11117G, ORF1ab:C12789T, ORF1ab:C14408T, ORF1ab:T19839C, ORF1ab:C21306T, S:C22995A, S:A23403G, S:C23604A, S:A23756G, ORF3a:C25460T, N:G28881A, N:G28882A, N:G28883C, N:C29197T, N:G29527T, 3'UTR:C29721T,	14	N:R203K, N:G204R, N:Q418H, ORF1a:P959S, ORF1a:S1029N, ORF1a:T3255I, ORF1a:I3618V, ORF1a:T4175I, ORF1b:P314L, ORF3a:A23V, S:T478K, S:D614G, S:P681H, S:T732A,
hCoV-19/Mexico/NLE-InDRE_F10811_S709/2021	EPI_ISL_1365674	20B	B.1.1.519	27	5'UTR:C203T, 5'UTR:C222T, 5'UTR:C241T, ORF1ab:A1547G, ORF1ab:C3037T, ORF1ab:C3140T, ORF1ab:T4111G, ORF1ab:C7162T, ORF1ab:C10029T, ORF1ab:C10954T, ORF1ab:A11117G, ORF1ab:C12789T, ORF1ab:C14408T, ORF1ab:G17944T, ORF1ab:T19839C, ORF1ab:C21306T, ORF1ab:T21384C, S:C22995A, S:A23403G, S:C23604A, S:A23756G, ORF3a:C25528T, N:G28812T, N:G28881A, N:G28882A, N:G28883C, N:C28887T, N:C29197T,	17	N:S180I, N:R203K, N:G204R, N:T205I, ORF1a:S428G, ORF1a:P959S, ORF1a:D1282E, ORF1a:T3255I, ORF1a:I3618V, ORF1a:T4175I, ORF1b:P314L, ORF1b:V1493L, ORF3a:L46F, S:T478K, S:D614G, S:P681H, S:T732A,
hCoV-19/Mexico/NLE-InDRE-59/2020	EPI_ISL_576262	20B	B.1.1.344	12	5'UTR:C241T, ORF1ab:C3037T, ORF1ab:T9172C, ORF1ab:A10948G, ORF1ab:C14408T, ORF1ab:C15738T, ORF1ab:T16950C, ORF1ab:C18267T, S:A23403G, M:G26730T, N:G28881A, N:G28882A, N:G28883C,	5	M:V70F, N:R203K, N:G204R, ORF1b:P314L, S:D614G,
hCoV-19/Mexico/NLE-InDRE-60/2020	EPI_ISL_576263	20B	B.1.1	10	5'UTR:C241T, ORF1ab:C3037T, ORF1ab:T8647C, ORF1ab:C14408T, ORF1ab:A15604G, S:A23403G, S:G24794T, N:G28373T, N:G28881A, N:G28882A, N:G28883C,	7	N:G34W, N:R203K, N:G204R, ORF1b:P314L, ORF1b:N713D, S:D614G, S:A1078S,
hCoV-19/Mexico/NLE-UANL-001/2020	EPI_ISL_779169	19B	A.2	7	ORF1ab:C8782T, ORF1ab:T9477A, ORF1ab:C14805T, ORF3a:G25979T, M:C26681T, ORF8:T28144C, N:C28657T, N:C28863T,	4	N:S197L, ORF1a:F3071Y, ORF3a:G196V, ORF8:L84S,
hCoV-19/Mexico/NLE-UANL-002/2021	EPI_ISL_979339	20B	B.1.1.432	26	5'UTR:C241T, ORF1ab:T1888G, ORF1ab:G2755T, ORF1ab:C3037T, ORF1ab:G3122T, ORF1ab:G3860T, ORF1ab:A6985T, ORF1ab:C7113T, ORF1ab:C9319T, ORF1ab:C12412T, ORF1ab:C12784T, ORF1ab:T14313C, ORF1ab:C14408T, ORF1ab:A15063G, ORF1ab:G18449T, ORF1ab:C18664T, ORF1ab:T19205C, ORF1ab:T19449C, S:A23403G, S:A25165T, ORF3a:C25613T, ORF3a:G25912T, N:G28881A, N:G28882A, N:G28883C, N:T29317C, 3'UTR:C29750A,	12	N:R203K, N:G204R, ORF1a:D953Y, ORF1a:V1199F, ORF1a:T2283I, ORF1b:P314L, ORF1b:S1661I, ORF1b:V1913A, ORF3a:S74F, ORF3a:G174C, S:D614G, S:Q1201H,
hCoV-19/Mexico/NLE-UANL-003/2020	EPI_ISL_779170	20A	B.1	6	5'UTR:C241T, ORF1ab:C3037T, ORF1ab:C14408T, ORF1ab:C18877T, ORF1ab:T19968C, S:A23403G, ORF3a:G25563T,	3	ORF1b:P314L, ORF3a:Q57H, S:D614G,

hCoV-19/Mexico/NLE-UANL-003/2021	EPI_ISL_979340	20B	B.1.1.519	25	5'UTR:C203T, 5'UTR:C222T, 5'UTR:C241T, ORF1ab:C3037T, ORF1ab:C3140T, ORF1ab:C6286T, ORF1ab:G6894A, ORF1ab:C9611T, ORF1ab:C10029T, ORF1ab:C10156T, ORF1ab:C10954T, ORF1ab:A11117G, ORF1ab:G11365T, ORF1ab:C12789T, ORF1ab:C14408T, ORF1ab:T19839C, ORF1ab:C21306T, S:C22995A, S:A23403G, S:C23604A, S:A23756G, N:G28881A, N:G28882A, N:G28883C, N:C29197T, N:G29527T,	14	N:R203K, N:G204R, N:Q418H, ORF1a:P959S, ORF1a:C2210Y, ORF1a:L3116F, ORF1a:T3255I, ORF1a:I3618V, ORF1a:T4175I, ORF1b:P314L, S:T478K, S:D614G, S:P681H, S:T732A,
hCoV-19/Mexico/NLE-UANL-004/2020	EPI_ISL_779171	20A	B.1.609	7	5'UTR:C241T, ORF1ab:C3037T, ORF1ab:C4582T, ORF1ab:C14408T, ORF1ab:G19684T, ORF1ab:A20268G, S:C22713T, S:A23403G,	4	ORF1b:P314L, ORF1b:V2073L, S:P384L, S:D614G,
hCoV-19/Mexico/NLE-UANL-004/2021	EPI_ISL_979341	20B	B.1.1	17	5'UTR:C241T, ORF1ab:A1431G, ORF1ab:C1514T, ORF1ab:C3037T, ORF1ab:G3152T, ORF1ab:A7531G, ORF1ab:C12880T, ORF1ab:C13019T, ORF1ab:C14408T, ORF1ab:G19549T, S:A23403G, S:C23929T, S:G24368C, ORF3a:C25889T, M:G26951C, N:G28881A, N:G28882A, N:G28883C,	10	N:R203K, N:G204R, ORF1a:N389S, ORF1a:H417Y, ORF1a:G963C, ORF1b:P314L, ORF1b:A2028S, ORF3a:S166L, S:D614G, S:D936H,
hCoV-19/Mexico/NLE-UANL-005/2020	EPI_ISL_779172	20A	B.1	8	5'UTR:C241T, ORF1ab:A2775G, ORF1ab:C3037T, ORF1ab:C14408T, ORF1ab:A20268G, S:C21575T, S:A23403G, N:C28854T, N:A29350G,	5	N:S194L, ORF1a:K837R, ORF1b:P314L, S:L5F, S:D614G,
hCoV-19/Mexico/NLE-UANL-005/2021	EPI_ISL_979342	21C (Epsilon)	B.1.429	32	5'UTR:C173T, 5'UTR:C241T, ORF1ab:C1059T, ORF1ab:C2395T, ORF1ab:T2597C, ORF1ab:C3037T, ORF1ab:C6896T, ORF1ab:G7100A, ORF1ab:C8947T, ORF1ab:C12100T, ORF1ab:G12160A, ORF1ab:C12161G, ORF1ab:G12163A, ORF1ab:A12878G, ORF1ab:C14408T, ORF1ab:G17014T, S:G21600T, S:C21638T, S:G22018T, S:T22917G, S:A23403G, S:C23638T, S:T24283C, S:T24349C, ORF3a:G25563T, ORF3a:A26190C, ORF3a:C26208T, M:C26681T, ORF7b:G27890T, ORF8:G28198T, ORF8:A28272T, N:C28887T, N:C29362T,	15	N:T205I, ORF1a:T265I, ORF1a:A2279T, ORF1a:Q3966E, ORF1a:I4205V, ORF1b:P314L, ORF1b:D1183Y, ORF3a:Q57H, ORF3a:E266D, ORF8:C102F, S:S13I, S:P26S, S:W152C, S:L452R, S:D614G,
hCoV-19/Mexico/NLE-UANL-006/2020	EPI_ISL_779173	20C	B.1.369	9	5'UTR:C241T, ORF1ab:C1059T, ORF1ab:C3037T, ORF1ab:C14408T, ORF1ab:C16260T, ORF1ab:C18395T, S:A23403G, S:G23587T, ORF3a:G25563T, N:C28821A,	7	N:S183Y, ORF1a:T265I, ORF1b:P314L, ORF1b:A1643V, ORF3a:Q57H, S:D614G, S:Q675H,
hCoV-19/Mexico/NLE-UANL-006/2021	EPI_ISL_979343	20B	B.1.1.519	23	5'UTR:C203T, 5'UTR:C222T, 5'UTR:C241T, ORF1ab:C3037T, ORF1ab:C3140T, ORF1ab:G6894T, ORF1ab:G7027T, ORF1ab:C10029T, ORF1ab:C10954T, ORF1ab:A11117G, ORF1ab:C12789T, ORF1ab:C14408T, ORF1ab:C15763A, ORF1ab:T19839C, ORF1ab:C21306T, S:C22995A, S:A23403G, S:C23604A, S:A23756G, S:C25207T, N:G28881A, N:G28882A, N:G28883C, N:C29197T,	14	N:R203K, N:G204R, ORF1a:P959S, ORF1a:C2210F, ORF1a:M2254I, ORF1a:T3255I, ORF1a:I3618V, ORF1a:T4175I, ORF1b:P314L, ORF1b:L766I, S:T478K, S:D614G, S:P681H, S:T732A,
hCoV-19/Mexico/NLE-UANL-007/2020	EPI_ISL_779174	20B	B.1.1.344	10	5'UTR:C241T, ORF1ab:C3037T, ORF1ab:T9172C, ORF1ab:A10948G, ORF1ab:C14408T, ORF1ab:T16950C, S:A23403G, M:G26730T, N:G28881A, N:G28882A, N:G28883C,	5	M:V70F, N:R203K, N:G204R, ORF1b:P314L, S:D614G,
hCoV-19/Mexico/NLE-UANL-007/2021	EPI_ISL_979344	20A	B.1.396	19	5'UTR:C241T, ORF1ab:C475T, ORF1ab:C1191T, ORF1ab:C3037T, ORF1ab:C5654T, ORF1ab:A6705G, ORF1ab:C9521A, ORF1ab:C14408T, ORF1ab:C18464A, ORF1ab:G20208T, ORF1ab:A20268G, S:A23403G, S:C24904T, ORF3a:G25494T, ORF3a:G25540T, ORF6:T27384C, N:C28854T, N:G29422T, N:G29543T, ORF10:A29567G,	9	N:S194L, ORF1a:P309L, ORF1a:N2147S, ORF1a:L3086I, ORF1b:P314L, ORF1b:P1666H, ORF1b:Q2247H, ORF3a:V50F, S:D614G,
hCoV-19/Mexico/NLE-UANL-008/2020	EPI_ISL_779175	20B	B.1.1.344	11	5'UTR:C241T, ORF1ab:C3037T, ORF1ab:T9172C, ORF1ab:A10948G, ORF1ab:C14408T, ORF1ab:C15952T, ORF1ab:T16950C, S:A23403G, M:G26730T, N:G28881A, N:G28882A, N:G28883C,	5	M:V70F, N:R203K, N:G204R, ORF1b:P314L, S:D614G,

hCoV-19/Mexico/NLE-UANL-008/2021	EPI_ISL_979345	20A	B.1.241	23	5'UTR:C241T, ORF1ab:C3037T, ORF1ab:C3634T, ORF1ab:G4649T, ORF1ab:A5648C, ORF1ab:A6529G, ORF1ab:G6884A, ORF1ab:G8003T, ORF1ab:C11575T, ORF1ab:T12388C, ORF1ab:C14408T, ORF1ab:C15720T, ORF1ab:A16840C, ORF1ab:C17718A, ORF1ab:C19029T, ORF1ab:G19117T, ORF1ab:A20268G, S:T22222C, S:T22618A, S:A23403G, ORF7a:G27506T, ORF7a:C27673A, ORF8:C28115T, N:C28854T,	11	N:S194L, ORF1a:A1462S, ORF1a:K1795Q, ORF1a:G2207S, ORF1a:V2580F, ORF1b:P314L, ORF1b:K1125Q, ORF1b:A1884S, ORF7a:G38V, ORF7a:Q94K, S:D614G,	
hCoV-19/Mexico/NLE-UANL-009/2020	EPI_ISL_779176	20B	B.1.1.344	11	5'UTR:C241T, ORF1ab:C3037T, ORF1ab:T9172C, ORF1ab:A10948G, ORF1ab:C14408T, ORF1ab:T16950C, ORF1ab:C17304T, S:A23403G, M:G26730T, N:G28881A, N:G28882A, N:G28883C,	5	M:V70F, N:R203K, N:G204R, ORF1b:P314L, S:D614G,	
hCoV-19/Mexico/NLE-UANL-009/2021	EPI_ISL_979346	20G	B.1.2	19	5'UTR:C241T, ORF1ab:C1059T, ORF1ab:C3037T, ORF1ab:G7312A, ORF1ab:T10006C, ORF1ab:C10319T, ORF1ab:C14408T, ORF1ab:C17119T, ORF1ab:A18424G, ORF1ab:C18647T, ORF1ab:C21304T, ORF1ab:A21390G, S:C21614T, S:A23403G, ORF3a:G25563T, ORF3a:G25907T, ORF8:C27964T, N:C28472T, N:C28869T, N:C29200T,	13	N:P67S, N:P199L, ORF1a:T265I, ORF1a:L3352F, ORF1b:P314L, ORF1b:N1653D, ORF1b:P1727L, ORF1b:R2613C, ORF3a:Q57H, ORF3a:G172V, ORF8:S24L, S:L18F, S:D614G,	
hCoV-19/Mexico/NLE-UANL-010/2021	EPI_ISL_979347	20A	B.1	12	5'UTR:C241T, ORF1ab:C2662T, ORF1ab:C3037T, ORF1ab:T10480C, ORF1ab:C14408T, ORF1ab:C15951T, ORF1ab:G16377T, ORF1ab:C17410T, ORF1ab:A20268G, S:A23403G, ORF3a:T25779C, ORF8:C28253T, N:C28854T,	4	N:S194L, ORF1b:P314L, ORF1b:R1315C, S:D614G,	ORF7a:2 7571-27616
hCoV-19/Mexico/NLE-UANL-011/2021	EPI_ISL_979348	20A	B.1.243	18	5'UTR:C241T, ORF1ab:C3037T, ORF1ab:C10741T, ORF1ab:G11150A, ORF1ab:C12672T, ORF1ab:C14408T, ORF1ab:G17122T, ORF1ab:C19097T, ORF1ab:A19137G, ORF1ab:A20268G, S:A23403G, S:C23604A, S:T24076C, ORF7a:G27506T, N:G28817T, N:C28854T, N:C29218T, N:G29468T, 3'UTR:T29710C,	11	N:A182S, N:S194L, N:D399Y, ORF1a:V3629I, ORF1a:A4136V, ORF1b:P314L, ORF1b:A1219S, ORF1b:A1877V, ORF7a:G38V, S:D614G, S:P681H,	
hCoV-19/Mexico/NLE-UANL-012/2020	EPI_ISL_779177	20B	B.1.1.344	10	5'UTR:C241T, ORF1ab:C3037T, ORF1ab:T9172C, ORF1ab:A10948G, ORF1ab:C14408T, ORF1ab:T16950C, S:A23403G, M:G26730T, N:G28881A, N:G28882A, N:G28883C,	5	M:V70F, N:R203K, N:G204R, ORF1b:P314L, S:D614G,	
hCoV-19/Mexico/NLE-UANL-012/2021	EPI_ISL_979349	20A	B.1	21	5'UTR:C241T, ORF1ab:C3037T, ORF1ab:C4582T, ORF1ab:C4878T, ORF1ab:A5391G, ORF1ab:C11322T, ORF1ab:C13372T, ORF1ab:C14408T, ORF1ab:G16741T, ORF1ab:A20268G, ORF1ab:C20543T, S:C21588T, S:T22917G, S:T23030C, S:A23403G, S:C24138A, M:G26792A, ORF7a:G27478T, ORF7a:T27522G, ORF8:C27945T, ORF8:G28223T, N:G28321A,	16	ORF1a:T1538I, ORF1a:N1709S, ORF1a:A3686V, ORF1b:P314L, ORF1b:V1092F, ORF1b:S2359F, ORF7a:V29L, ORF7a:N43K, ORF8:Q18*, ORF8:E110D, ORF9b:R13H, S:P9L, S:L452R, S:F490L, S:D614G, S:T859N,	S:21992-21994,
hCoV-19/Mexico/NLE-UANL-013/2020	EPI_ISL_779178	20B	B.1.1.344	11	5'UTR:C241T, ORF1ab:C3037T, ORF1ab:T9172C, ORF1ab:A10948G, ORF1ab:C14408T, ORF1ab:T16950C, ORF1ab:C19602T, S:A23403G, M:G26730T, N:G28881A, N:G28882A, N:G28883C,	5	M:V70F, N:R203K, N:G204R, ORF1b:P314L, S:D614G,	
hCoV-19/Mexico/NLE-UANL-013/2021	EPI_ISL_979350	20B	B.1.1.432	29	5'UTR:C64T, 5'UTR:C241T, ORF1ab:C3037T, ORF1ab:C4276T, ORF1ab:A4860G, ORF1ab:C5850T, ORF1ab:A6063G, ORF1ab:C6541T, ORF1ab:A6985T, ORF1ab:C9319T, ORF1ab:G9841A, ORF1ab:C12412T, ORF1ab:C13009T, ORF1ab:T14313C, ORF1ab:C14408T, ORF1ab:A14990G, ORF1ab:G17014T, ORF1ab:C18570T, ORF1ab:C20233T, S:A23403G, ORF3a:C25613T, ORF3a:G25912T, E:C26370T, ORF6:T27384C, N:G28881A, N:G28882A, N:G28883C, N:C29311T, N:T29317C, 3'UTR:C29738T,	12	N:R203K, N:G204R, ORF1a:D1532G, ORF1a:P1862L, ORF1a:D1933G, ORF1b:P314L, ORF1b:D508G, ORF1b:D1183Y, ORF1b:P2256S, ORF3a:S74F, ORF3a:G174C, S:D614G,	

hCoV-19/Mexico/NLE-UANL-014/2020	EPI_ISL_779179	20A	B.1	12	5'UTR:C241T, ORF1ab:A2806C, ORF1ab:C3037T, ORF1ab:G11417T, ORF1ab:T13857C, ORF1ab:C14408T, ORF1ab:G15732T, ORF1ab:T20088C, ORF1ab:A20268G, ORF1ab:A20634G, S:A23403G, ORF3a:G25477T, N:C28854T,	6	N:S194L, ORF1a:E847D, ORF1a:V3718F, ORF1b:P314L, ORF3a:V29F, S:D614G,
hCoV-19/Mexico/NLE-UANL-014/2021	EPI_ISL_979351	20B	B.1.1.222	25	5'UTR:C241T, ORF1ab:C593T, ORF1ab:A1558G, ORF1ab:C3037T, ORF1ab:A3916G, ORF1ab:A5452G, ORF1ab:C6543T, ORF1ab:C8606T, ORF1ab:G11222T, ORF1ab:C14216T, ORF1ab:C14408T, ORF1ab:T19839C, S:C21614T, S:G21624T, S:G22026A, S:G22104T, S:A22765T, S:A23403G, S:A23756G, ORF3a:C25587T, ORF3a:C25904T, ORF8:C28253T, N:G28881A, N:G28882A, N:G28883C, 3'UTR:G29777T,	16	N:R203K, N:G204R, ORF1a:H110Y, ORF1a:I431M, ORF1a:T2093I, ORF1a:L2781F, ORF1a:V3653F, ORF1b:T250I, ORF1b:P314L, ORF3a:S171L, S:L18F, S:R21I, S:S155N, S:G181V, S:D614G, S:T732A,
hCoV-19/Mexico/NLE-UANL-015/2020	EPI_ISL_779180	20A	B.1	11	5'UTR:C241T, ORF1ab:C2416T, ORF1ab:C3037T, ORF1ab:C5869T, ORF1ab:C10790T, ORF1ab:C11651T, ORF1ab:C14408T, S:C22450T, S:A23403G, ORF3a:G25563T, ORF7a:C27513T, N:G28378T,	6	ORF1a:H3509Y, ORF1a:L3796F, ORF1b:P314L, ORF3a:Q57H, ORF9b:R32L, S:D614G,
hCoV-19/Mexico/NLE-UANL-015/2021	EPI_ISL_979352	20B	B.1.1.28	21	5'UTR:C241T, ORF1ab:C346T, ORF1ab:C1190T, ORF1ab:C2469T, ORF1ab:C3037T, ORF1ab:C3140T, ORF1ab:C4832T, ORF1ab:G7070T, ORF1ab:C14408T, ORF1ab:C15960T, ORF1ab:G18105T, S:A23403G, S:C24107T, S:C24378T, S:G24620T, S:G25088T, ORF7b:G27806T, ORF8:A28273T, N:T28630C, N:G28881A, N:G28882A, N:G28883C,	13	N:R203K, N:G204R, ORF1a:P309S, ORF1a:A735V, ORF1a:P959S, ORF1a:G2269C, ORF1b:P314L, ORF1b:Q1546H, S:D614G, S:L849F, S:S939F, S:A1020S, S:V1176F,
hCoV-19/Mexico/NLE-UANL-016/2021	EPI_ISL_1091241	20B	B.1.1.28	26	5'UTR:C241T, ORF1ab:C346T, ORF1ab:C1190T, ORF1ab:C2469T, ORF1ab:C3037T, ORF1ab:C3140T, ORF1ab:C4475T, ORF1ab:C4832T, ORF1ab:G7070T, ORF1ab:C14408T, ORF1ab:C15960T, ORF1ab:G18105T, ORF1ab:A21137G, S:A21651T, S:G21668C, S:C23248T, S:A23403G, S:C24378T, S:G24620T, S:G25088T, M:G26918A, ORF8:A28273T, N:T28630C, N:G28881A, N:G28882A, N:G28883C, N:C29520T,	17	N:R203K, N:G204R, N:S416L, ORF1a:P309S, ORF1a:A735V, ORF1a:P959S, ORF1a:R1404C, ORF1a:G2269C, ORF1b:P314L, ORF1b:Q1546H, ORF1b:K2557R, S:N30I, S:V36L, S:D614G, S:S939F, S:A1020S, S:V1176F,
hCoV-19/Mexico/NLE-UANL-017/2020	EPI_ISL_779181	20A	B.1	9	5'UTR:C241T, ORF1ab:A2806C, ORF1ab:C3037T, ORF1ab:G11417T, ORF1ab:T13857C, ORF1ab:C14408T, ORF1ab:A20268G, S:A23403G, ORF3a:G25477T, N:C28854T,	6	N:S194L, ORF1a:E847D, ORF1a:V3718F, ORF1b:P314L, ORF3a:V29F, S:D614G,
hCoV-19/Mexico/NLE-UANL-017/2021	EPI_ISL_1091254	20B	B.1.1.316	18	5'UTR:C241T, ORF1ab:C3037T, ORF1ab:C3411T, ORF1ab:G3871T, ORF1ab:A5040G, ORF1ab:A12519G, ORF1ab:C14408T, ORF1ab:T16539C, ORF1ab:T19839C, S:C21757T, S:A23403G, S:G23593C, S:A23756T, ORF3a:C26022T, N:G28881A, N:G28882A, N:G28883C, N:C29421T, 3'UTR:G29751T,	11	N:R203K, N:G204R, N:P383L, ORF1a:A1049V, ORF1a:K1202N, ORF1a:Q1592R, ORF1a:D4085G, ORF1b:P314L, S:D614G, S:Q677H, S:T732S,
hCoV-19/Mexico/NLE-UANL-018/2020	EPI_ISL_779182	20B	B.1.1	7	5'UTR:C241T, ORF1ab:C3037T, ORF1ab:C13329A, ORF1ab:C14408T, S:A23403G, N:G28881A, N:G28882A, N:G28883C,	5	N:R203K, N:G204R, ORF1a:T4355N, ORF1b:P314L, S:D614G,
hCoV-19/Mexico/NLE-UANL-018/2021	EPI_ISL_1091262	20A	B.1.609	17	5'UTR:C241T, ORF1ab:C3037T, ORF1ab:C4582T, ORF1ab:A5368G, ORF1ab:A8972G, ORF1ab:C11767T, ORF1ab:C14408T, ORF1ab:T19668C, ORF1ab:A20268G, ORF1ab:G21372T, S:G21786T, S:A23403G, S:C23604A, S:C23683T, S:A23898G, ORF3a:G25687T, N:G28975T, ORF10:G29645T,	9	N:M234I, ORF1a:I2903V, ORF1b:P314L, ORF1b:Q2635H, ORF3a:A99S, S:G75V, S:D614G, S:P681H, S:Q779R,
hCoV-19/Mexico/NLE-UANL-019/2020	EPI_ISL_779183	20A	B.1	6	5'UTR:C241T, ORF1ab:G1548A, ORF1ab:C3037T, ORF1ab:C14408T, ORF1ab:A20268G, S:C21622T, S:A23403G,	3	ORF1a:S428N, ORF1b:P314L, S:D614G,

hCoV-19/Mexico/NLE-UANL-019/2021	EPI_ISL_1091253	20B	B.1.1.519	20	5'UTR:C203T, 5'UTR:C222T, 5'UTR:C241T, ORF1ab:C3037T, ORF1ab:C3140T, ORF1ab:C10029T, ORF1ab:C10954T, ORF1ab:A11117G, ORF1ab:C12789T, ORF1ab:C14408T, ORF1ab:A17460T, ORF1ab:T19839C, ORF1ab:C21306T, S:C22995A, S:A23403G, S:C23604A, S:A23756G, N:G28881A, N:G28882A, N:G28883C, N:C29197T,	11	N:R203K, N:G204R, ORF1a:P959S, ORF1a:T3255I, ORF1a:I3618V, ORF1a:T4175I, ORF1b:P314L, S:T478K, S:D614G, S:P681H, S:T732A,	3'UTR:29737-29750,
hCoV-19/Mexico/NLE-UANL-020/2020	EPI_ISL_779184	20A	B.1.558	13	5'UTR:C241T, ORF1ab:C344T, ORF1ab:C1288T, ORF1ab:C3037T, ORF1ab:C12439T, ORF1ab:C14408T, ORF1ab:C17004T, ORF1ab:C19662T, ORF1ab:A20268G, ORF1ab:G20962T, S:A23403G, ORF3a:C26060T, ORF7a:C27630T, N:C28854T,	6	N:S194L, ORF1a:L27F, ORF1b:P314L, ORF1b:D2499Y, ORF3a:T223I, S:D614G,	
hCoV-19/Mexico/NLE-UANL-020/2021	EPI_ISL_1092458	20B	B.1.1.329	27	5'UTR:C241T, ORF1ab:C1758T, ORF1ab:A2132C, ORF1ab:G2324A, ORF1ab:C2706T, ORF1ab:C2710T, ORF1ab:C3037T, ORF1ab:C8266T, ORF1ab:C8326T, ORF1ab:C8841T, ORF1ab:A9364G, ORF1ab:T9370C, ORF1ab:G10106A, ORF1ab:A10323G, ORF1ab:G10360A, ORF1ab:C13281A, ORF1ab:C13424T, ORF1ab:T13954C, ORF1ab:C14408T, ORF1ab:A20985G, ORF1ab:T21117C, S:A23403G, S:C23604A, ORF7a:C27752T, N:G28881A, N:G28882A, N:G28883C, ORF10:C29614T,	15	N:R203K, N:G204R, ORF1a:A498V, ORF1a:K623Q, ORF1a:A687T, ORF1a:T814I, ORF1a:T2859I, ORF1a:V3281I, ORF1a:K3353R, ORF1a:P4339H, ORF1a:R4387C, ORF1b:P314L, ORF7a:T120I, S:D614G, S:P681H,	
hCoV-19/Mexico/NLE-UANL-021/2020	EPI_ISL_979327	20B	B.1.1	18	5'UTR:C190T, 5'UTR:C241T, ORF1ab:C1387T, ORF1ab:C2706T, ORF1ab:A2908G, ORF1ab:C3037T, ORF1ab:C12651T, ORF1ab:C14408T, ORF1ab:T19839C, S:A23403G, S:C23604A, ORF3a:G25522A, ORF7b:G27890A, ORF8:T28245G, N:G28881A, N:G28882A, N:G28883C, N:C29358T, 3'UTR:G29751C,	9	N:R203K, N:G204R, N:T362I, ORF1a:T814I, ORF1a:T4129I, ORF1b:P314L, ORF3a:G44R, S:D614G, S:P681H,	ORF8:28254,
hCoV-19/Mexico/NLE-UANL-021/2021	EPI_ISL_1091255	20A	B.1.243	16	5'UTR:C241T, ORF1ab:C1009T, ORF1ab:C3037T, ORF1ab:C10741T, ORF1ab:C14408T, ORF1ab:C14481T, ORF1ab:C19097T, ORF1ab:A19137G, ORF1ab:A20268G, S:A23403G, S:C23604A, S:T24076C, S:G24928T, S:G25244T, N:C28854T, ORF10:G29587T, 3'UTR:T29710C,	6	N:S194L, ORF1b:P314L, ORF1b:A1877V, S:D614G, S:P681H, S:V1228L,	
hCoV-19/Mexico/NLE-UANL-022/2020	EPI_ISL_779185	20A	B.1	12	5'UTR:C241T, ORF1ab:A708C, ORF1ab:C3037T, ORF1ab:T3055C, ORF1ab:A11782G, ORF1ab:C12513T, ORF1ab:C14408T, ORF1ab:G19518T, ORF1ab:A20268G, S:A23403G, ORF3a:C25886T, N:C28854T, N:G29557T,	7	N:S194L, ORF1a:E148A, ORF1a:T4083M, ORF1b:P314L, ORF1b:L2017F, ORF3a:S165F, S:D614G,	
hCoV-19/Mexico/NLE-UANL-022/2021	EPI_ISL_1091244	20A	B.1.243	16	5'UTR:C241T, ORF1ab:C3037T, ORF1ab:C3393T, ORF1ab:C4194T, ORF1ab:C10741T, ORF1ab:C14408T, ORF1ab:C16338T, ORF1ab:C19097T, ORF1ab:A19137G, ORF1ab:A20268G, S:A23403G, S:C23604A, S:T24076C, S:G25244T, N:C28854T, ORF10:G29587T, 3'UTR:T29710C,	8	N:S194L, ORF1a:A1043V, ORF1a:T1310I, ORF1b:P314L, ORF1b:A1877V, S:D614G, S:P681H, S:V1228L,	
hCoV-19/Mexico/NLE-UANL-023/2020	EPI_ISL_779186	20G	B.1.2	17	5'UTR:C241T, ORF1ab:C1059T, ORF1ab:C3037T, ORF1ab:C10319T, ORF1ab:G13094T, ORF1ab:T13216C, ORF1ab:G14187A, ORF1ab:C14408T, ORF1ab:A18424G, ORF1ab:C21304T, ORF1ab:A21390G, S:A23403G, ORF3a:G25563T, ORF3a:G25907T, ORF8:C27964T, N:C28472T, N:C28869T, N:C29363T,	13	N:P67S, N:P199L, N:P364S, ORF1a:T265I, ORF1a:L3352F, ORF1a:A4277S, ORF1b:P314L, ORF1b:N1653D, ORF1b:R2613C, ORF3a:Q57H, ORF3a:G172V, ORF8:S24L, S:D614G,	
hCoV-19/Mexico/NLE-UANL-023/2021	EPI_ISL_1091242	20A	B.1.243	17	5'UTR:C241T, ORF1ab:C3037T, ORF1ab:C3393T, ORF1ab:C4194T, ORF1ab:C8947T, ORF1ab:C10741T, ORF1ab:C14408T, ORF1ab:C16338T, ORF1ab:C19097T, ORF1ab:A19137G, ORF1ab:A20268G, S:A23403G, S:C23604A, S:T24076C, S:G25244T, N:C28854T, ORF10:G29587T, 3'UTR:T29710C,	8	N:S194L, ORF1a:A1043V, ORF1a:T1310I, ORF1b:P314L, ORF1b:A1877V, S:D614G, S:P681H, S:V1228L,	

hCoV-19/Mexico/NLE-UANL-024/2020	EPI_ISL_779187	20A	B.1.243	24	5'UTR:C241T, ORF1ab:C274T, ORF1ab:G806A, ORF1ab:G1946T, ORF1ab:T2584A, ORF1ab:C3037T, ORF1ab:C5140A, ORF1ab:C6027T, ORF1ab:A6841C, ORF1ab:A11000G, ORF1ab:C14408T, ORF1ab:C14925T, ORF1ab:C18486T, ORF1ab:A20268G, ORF1ab:C20544T, ORF1ab:C21234T, S:G23120T, S:C23320T, S:A23403G, S:G23587C, S:T24076C, S:A24400G, E:C26455T, N:C28854T, N:G29543T,	11	E:P71S, N:S194L, ORF1a:A181T, ORF1a:V561F, ORF1a:D1625E, ORF1a:P1921L, ORF1a:T3579A, ORF1b:P314L, S:A520S, S:D614G, S:Q675H,	
hCoV-19/Mexico/NLE-UANL-024/2021	EPI_ISL_1091245	20B	B.1.1	17	5'UTR:C241T, ORF1ab:C1441T, ORF1ab:A1558G, ORF1ab:C3037T, ORF1ab:A4778G, ORF1ab:C10440T, ORF1ab:C14408T, ORF1ab:C18486T, ORF1ab:T19839C, S:A23403G, S:A23756G, ORF3a:G25906T, ORF3a:C26029A, N:G28881A, N:G28882A, N:G28883C, N:G29473A, ORF10:A29591G,	10	N:R203K, N:G204R, ORF1a:I431M, ORF1a:I1505V, ORF1a:A3392V, ORF1b:P314L, ORF3a:G172C, ORF3a:Q213K, S:D614G, S:T732A,	3'UTR:29750-29759,
hCoV-19/Mexico/NLE-UANL-025/2020	EPI_ISL_979328	20A	B.1	14	5'UTR:C241T, ORF1ab:C3037T, ORF1ab:G4280A, ORF1ab:C4582T, ORF1ab:C4999T, ORF1ab:A10323G, ORF1ab:C12781T, ORF1ab:C14408T, ORF1ab:T17154C, ORF1ab:C18348T, ORF1ab:A20268G, ORF1ab:C21306T, S:A23403G, N:G28321A, N:G28881A,	6	N:R203K, ORF1a:V1339I, ORF1a:K3353R, ORF1b:P314L, ORF9b:R13H, S:D614G,	ORF8:28254,
hCoV-19/Mexico/NLE-UANL-025/2021	EPI_ISL_1091256	21C (Epsilon)	B.1.427	25	5'UTR:C106T, 5'UTR:C241T, ORF1ab:C1059T, ORF1ab:T1851C, ORF1ab:C3037T, ORF1ab:C3619A, ORF1ab:C5812T, ORF1ab:G9738C, ORF1ab:G13713A, ORF1ab:C14408T, ORF1ab:C16394T, ORF1ab:G17014T, S:G21600T, S:G22018T, S:T22521C, S:C22522T, S:T22917G, S:A23403G, S:C25317T, ORF3a:T25467C, ORF3a:G25563T, ORF3a:T26066C, M:C26681T, ORF8:A28272T, N:C28887T, N:C29362T,	15	N:T205I, ORF1a:T265I, ORF1a:I529T, ORF1a:S3158T, ORF1b:P314L, ORF1b:P976L, ORF1b:D1183Y, ORF3a:Q57H, ORF3a:V225A, S:S13I, S:W152C, S:V320A, S:L452R, S:D614G, S:S1252F,	
hCoV-19/Mexico/NLE-UANL-026/2020	EPI_ISL_779188	20A	B.1.558	26	5'UTR:C241T, ORF1ab:C1288T, ORF1ab:G2305T, ORF1ab:C2902T, ORF1ab:C3037T, ORF1ab:G6476A, ORF1ab:C10335T, ORF1ab:C10525T, ORF1ab:C11575T, ORF1ab:T12751G, ORF1ab:C13255T, ORF1ab:C14408T, ORF1ab:C14694T, ORF1ab:C18060T, ORF1ab:T19896C, ORF1ab:A20268G, S:C21575T, S:G22147A, S:G22487C, S:A23403G, S:C23664T, ORF3a:G25455T, ORF3a:G25770T, ORF3a:T25807C, ORF3a:G26109T, ORF7a:C27418T, N:C28854T,	12	N:S194L, ORF1a:K680N, ORF1a:V2071I, ORF1a:A3357V, ORF1b:P314L, ORF3a:K21N, ORF3a:R126S, ORF3a:E239D, S:L5F, S:E309Q, S:D614G, S:A701V,	
hCoV-19/Mexico/NLE-UANL-026/2021	EPI_ISL_1091257	20A	B.1.243	17	5'UTR:C241T, ORF1ab:C1457T, ORF1ab:C3037T, ORF1ab:C10741T, ORF1ab:C14408T, ORF1ab:C19097T, ORF1ab:A19137G, ORF1ab:C19813T, ORF1ab:A20268G, S:C22439T, S:A23403G, S:C23604A, S:T24076C, S:C24787T, N:G28690A, N:C28854T, N:C29545T, 3'UTR:T29710C,	8	N:S194L, ORF1a:R398C, ORF1b:P314L, ORF1b:A1877V, ORF1b:P2116S, S:L293F, S:D614G, S:P681H,	
hCoV-19/Mexico/NLE-UANL-027/2020	EPI_ISL_779189	20B	B.1.1.432	21	5'UTR:C241T, ORF1ab:C3037T, ORF1ab:T6343A, ORF1ab:A6985T, ORF1ab:G9203A, ORF1ab:C9319T, ORF1ab:C12412T, ORF1ab:T14313C, ORF1ab:C14408T, S:C21658T, S:G21974T, S:G21987A, S:A23403G, S:C23982T, ORF3a:C25613T, ORF3a:G25912T, ORF7b:C27881T, N:C28708T, N:G28881A, N:G28882A, N:G28883C, N:T29317C.	11	N:R203K, N:G204R, ORF1a:D2026E, ORF1a:D2980N, ORF1b:P314L, ORF3a:S74F, ORF3a:G174C, S:D138Y, S:G142D, S:D614G, S:P807L,	

hCoV-19/Mexico/NLE-UANL-027/2021	EPI_ISL_1091249	20C	B.1.595	21	5'UTR:C241T, ORF1ab:C1059T, ORF1ab:C3037T, ORF1ab:C4158T, ORF1ab:C7039T, ORF1ab:T8158C, ORF1ab:C10030T, ORF1ab:C10319T, ORF1ab:G10384A, ORF1ab:A11117G, ORF1ab:C12439T, ORF1ab:C13247T, ORF1ab:C14408T, ORF1ab:C16694T, ORF1ab:A18284G, S:A21717G, S:A23403G, S:G25352C, ORF3a:G25563T, ORF3a:C25904T, ORF8:C27964T, N:C28892T,	14	N:P207S, ORF1a:T265I, ORF1a:A1298V, ORF1a:L3352F, ORF1a:I3618V, ORF1b:P314L, ORF1b:T1076I, ORF1b:H1606R, ORF3a:Q57H, ORF3a:S171L, ORF8:S24L, S:Q52R, S:D614G, S:V1264L,	
hCoV-19/Mexico/NLE-UANL-028/2020	EPI_ISL_979329	20B	B.1.1.222	18	5'UTR:C241T, ORF1ab:A929G, ORF1ab:C3037T, ORF1ab:G11083T, ORF1ab:C11575T, ORF1ab:C14408T, ORF1ab:A15498G, ORF1ab:C18511T, ORF1ab:T19839C, S:C21707T, S:G22331A, S:A23403G, S:A23756G, ORF3a:A25964G, M:C26735T, ORF8:T28245G, N:G28881A, N:G28882A, N:G28883C,	11	N:R203K, N:G204R, ORF1a:I222V, ORF1a:L3606F, ORF1b:P314L, ORF1b:P1682S, ORF3a:E191G, S:H49Y, S:G257S, S:D614G, S:T732A,	ORF8:28254,
hCoV-19/Mexico/NLE-UANL-028/2021	EPI_ISL_1091243	20A	B.1.243	18	5'UTR:C241T, ORF1ab:C3037T, ORF1ab:C7267T, ORF1ab:C10714T, ORF1ab:C10741T, ORF1ab:A11298G, ORF1ab:C14408T, ORF1ab:C19097T, ORF1ab:A19137G, ORF1ab:C19269T, ORF1ab:A20268G, S:A23403G, S:C23604A, S:T24076C, ORF7a:G27516T, ORF8:C28236T, N:C28854T, N:C29541T, 3'UTR:T29710C,	8	N:S194L, ORF1a:K3678R, ORF1b:P314L, ORF1b:A1877V, ORF7a:E41D, ORF8:R115C, S:D614G, S:P681H,	
hCoV-19/Mexico/NLE-UANL-029/2020	EPI_ISL_779190	20B	B.1.1.519	25	5'UTR:T201C, 5'UTR:C203T, 5'UTR:C222T, 5'UTR:C241T, ORF1ab:C1218T, ORF1ab:G1738T, ORF1ab:C3037T, ORF1ab:C3140T, ORF1ab:G4907A, ORF1ab:C10029T, ORF1ab:C10954T, ORF1ab:A11117G, ORF1ab:C12789T, ORF1ab:C14408T, ORF1ab:T19839C, ORF1ab:A19974G, ORF1ab:C21306T, S:C21621T, S:C22995A, S:A23403G, S:C23604A, S:A23756G, N:G28881A, N:G28882A, N:G28883C, N:C29197T,	14	N:R203K, N:G204R, ORF1a:S318L, ORF1a:P959S, ORF1a:G1548S, ORF1a:T3255I, ORF1a:I3618V, ORF1a:T4175I, ORF1b:P314L, S:T20I, S:T478K, S:D614G, S:P681H, S:T732A,	
hCoV-19/Mexico/NLE-UANL-029/2021	EPI_ISL_1091246	20B	B.1.1.432	26	5'UTR:C241T, ORF1ab:C3037T, ORF1ab:G3122T, ORF1ab:A6985T, ORF1ab:C9319T, ORF1ab:C10369T, ORF1ab:C12412T, ORF1ab:C12784T, ORF1ab:T14313C, ORF1ab:C14408T, ORF1ab:C16394T, S:G21770T, S:C22127T, S:C23127T, S:A23403G, S:C24382T, ORF3a:C25613T, ORF3a:G25912T, ORF6:T27384C, N:G28881A, N:G28882A, N:G28883C, N:C28948T, N:C29149T, N:C29167T, N:T29317C, ORF10:C29585T,	11	N:R203K, N:G204R, ORF1a:D953Y, ORF1b:P314L, ORF1b:P976L, ORF3a:S74F, ORF3a:G174C, S:V70F, S:L189F, S:A522V, S:D614G,	ORF1ab: 6656-6679
hCoV-19/Mexico/NLE-UANL-030/2020	EPI_ISL_779191	20C	B.1.595	13	5'UTR:C241T, ORF1ab:T1592A, ORF1ab:C3037T, ORF1ab:A8001G, ORF1ab:C10319T, ORF1ab:G10835T, ORF1ab:C14408T, S:A23403G, ORF3a:C25427T, ORF3a:G25563T, ORF3a:C25844T, ORF3a:G25907T, ORF8:C27964T, N:A28877G,	12	N:S202G, ORF1a:S443T, ORF1a:D2579G, ORF1a:L3352F, ORF1a:V3524F, ORF1b:P314L, ORF3a:T12I, ORF3a:Q57H, ORF3a:T151I, ORF3a:G172V, ORF8:S24L, S:D614G,	
hCoV-19/Mexico/NLE-UANL-031/2020	EPI_ISL_779192	20A	B.1.243	16	5'UTR:C241T, ORF1ab:C1218T, ORF1ab:C3037T, ORF1ab:C3264T, ORF1ab:C10741T, ORF1ab:C12119T, ORF1ab:A12356G, ORF1ab:C14408T, ORF1ab:C19097T, ORF1ab:A20268G, S:A23403G, S:C23604A, S:T24076C, ORF3a:T25773C, ORF6:G27281T, N:C28854T, 3'UTR:T29710C,	10	N:S194L, ORF1a:S318L, ORF1a:T1000I, ORF1a:P3952S, ORF1a:T4031A, ORF1b:P314L, ORF1b:A1877V, ORF6:W27L, S:D614G, S:P681H,	
hCoV-19/Mexico/NLE-UANL-032/2020	EPI_ISL_779193	20A	B.1.396	12	5'UTR:C241T, ORF1ab:C1191T, ORF1ab:A2647G, ORF1ab:C3037T, ORF1ab:C8025T, ORF1ab:C9521A, ORF1ab:C14408T, ORF1ab:A20268G, S:A23403G, S:C24904T, N:C28854T, N:G29422T, ORF10:A29567G,	6	N:S194L, ORF1a:P309L, ORF1a:A2587V, ORF1a:L3086I, ORF1b:P314L, S:D614G,	

hCoV-19/Mexico/NLE-UANL-033/2020	EPI_ISL_779194	20B	B.1.1.222	22	5'UTR:C241T, ORF1ab:C1960T, ORF1ab:C3037T, ORF1ab:C5526T, ORF1ab:C5724T, ORF1ab:C10029T, ORF1ab:C14408T, ORF1ab:C15810T, ORF1ab:T18003G, ORF1ab:T19839C, ORF1ab:G21325A, S:C23127T, S:A23403G, S:C23575T, S:A23756G, S:G24236T, ORF3a:G25912T, ORF8:A27921G, ORF8:G28001T, N:C28868T, N:G28881A, N:G28882A, N:G28883C,	15	N:P199S, N:R203K, N:G204R, ORF1a:T1754I, ORF1a:T1820I, ORF1a:T3255I, ORF1b:P314L, ORF1b:S1512R, ORF1b:V2620I, ORF3a:G174C, ORF8:I10V, S:A522V, S:D614G, S:T732A, S:A892S,
hCoV-19/Mexico/NLE-UANL-034/2020	EPI_ISL_779195	20B	B.1.1.432	22	5'UTR:C241T, ORF1ab:C3037T, ORF1ab:G6872A, ORF1ab:A6985T, ORF1ab:C9319T, ORF1ab:C9561T, ORF1ab:C12412T, ORF1ab:A13683G, ORF1ab:C13730T, ORF1ab:T14313C, ORF1ab:C14408T, ORF1ab:T19000G, ORF1ab:G20274T, S:C22264T, S:A23403G, S:A24791G, ORF3a:C25613T, ORF3a:C25658T, ORF3a:T25929G, N:C28344T, N:G28881A, N:G28882A, N:G28883C,	14	N:T24I, N:R203K, N:G204R, ORF1a:V2203I, ORF1a:S3099L, ORF1b:A88V, ORF1b:P314L, ORF1b:L1845V, ORF1b:M2269I, ORF3a:S74F, ORF3a:T89I, ORF3a:I179M, S:D614G, S:T1077A,
hCoV-19/Mexico/NLE-UANL-035/2020	EPI_ISL_779196	20B	B.1.1.434	17	5'UTR:C241T, ORF1ab:C936T, ORF1ab:A2342G, ORF1ab:C3037T, ORF1ab:G3259T, ORF1ab:G5816A, ORF1ab:C11591T, ORF1ab:C14408T, ORF1ab:G17122T, ORF1ab:C18348T, ORF1ab:G20734T, S:A23403G, S:A25065G, ORF3a:A26069G, N:G28881A, N:G28882A, N:G28883C, 3'UTR:G29747T,	13	N:R203K, N:G204R, ORF1a:T224I, ORF1a:I693V, ORF1a:Q998H, ORF1a:A1851T, ORF1a:L3776F, ORF1b:P314L, ORF1b:A1219S, ORF1b:D2423Y, ORF3a:E226G, S:D614G, S:D1168G,
hCoV-19/Mexico/NLE-UANL-036/2020	EPI_ISL_779197	20A	B.1	18	5'UTR:C241T, ORF1ab:C3037T, ORF1ab:C4084T, ORF1ab:C5866T, ORF1ab:C7732T, ORF1ab:T9630C, ORF1ab:G10882T, ORF1ab:G11521T, ORF1ab:A11782G, ORF1ab:A12124T, ORF1ab:C14408T, ORF1ab:C16733T, ORF1ab:C18508T, ORF1ab:A20268G, S:A23403G, M:T26966C, ORF7a:G27506C, N:C28770T, N:C28854T,	10	N:T166I, N:S194L, ORF1a:F3122S, ORF1a:M3539I, ORF1a:M3752I, ORF1b:P314L, ORF1b:S1089L, ORF1b:L1681F, ORF7a:G38A, S:D614G,
hCoV-19/Mexico/NLE-UANL-037/2020	EPI_ISL_779198	20C	B.1.576	17	5'UTR:C241T, ORF1ab:C335T, ORF1ab:C1059T, ORF1ab:C3037T, ORF1ab:C10615T, ORF1ab:C12053T, ORF1ab:G12243T, ORF1ab:C14220T, ORF1ab:C14408T, ORF1ab:T16425G, ORF1ab:C17639T, ORF1ab:C20016T, ORF1ab:T20748C, S:G22201T, S:A23403G, ORF3a:G25563T, E:C26455T, N:C28863T,	10	E:P71S, N:S197L, ORF1a:R24C, ORF1a:T265I, ORF1a:L3930F, ORF1a:R3993L, ORF1b:P314L, ORF1b:S1391L, ORF3a:Q57H, S:D614G,
hCoV-19/Mexico/NLE-UANL-038/2020	EPI_ISL_979330	20B	B.1.1.432	24	5'UTR:C241T, ORF1ab:C3037T, ORF1ab:G3122T, ORF1ab:A6985T, ORF1ab:C9319T, ORF1ab:C10369T, ORF1ab:C12412T, ORF1ab:C12784T, ORF1ab:T14313C, ORF1ab:C14408T, S:G21770T, S:C22127T, S:C23127T, S:A23403G, S:C24382T, ORF3a:C25613T, ORF3a:G25912T, N:G28881A, N:G28882A, N:G28883C, N:C28948T, N:C29149T, N:C29167T, N:T29317C, ORF10:C29585T,	10	N:R203K, N:G204R, ORF1a:D953Y, ORF1b:P314L, ORF3a:S74F, ORF3a:G174C, S:V70F, S:L189F, S:A522V, S:D614G,
hCoV-19/Mexico/NLE-UANL-039/2020	EPI_ISL_979331	20G	B.1.2	31	5'UTR:C186T, 5'UTR:C241T, ORF1ab:C1059T, ORF1ab:G2095T, ORF1ab:C3037T, ORF1ab:C3587T, ORF1ab:C7267T, ORF1ab:C7420T, ORF1ab:G9928T, ORF1ab:C10319T, ORF1ab:T14179C, ORF1ab:C14408T, ORF1ab:G15444T, ORF1ab:C15656T, ORF1ab:G18318T, ORF1ab:A18424G, ORF1ab:C21304T, S:C21575T, S:A23403G, S:G23593C, S:G23868T, S:G24124T, ORF3a:G25563T, ORF3a:G25907T, E:A26459G, ORF8:C27964T, N:C28472T, N:C28869T, N:C29400T, N:G29405C, N:G29422T, N:G29554T,	22	E:D72G, N:P67S, N:P199L, N:A376V, N:E378Q, ORF1a:T265I, ORF1a:H1108Y, ORF1a:M3221I, ORF1a:L3352F, ORF1b:P314L, ORF1b:M659I, ORF1b:T730I, ORF1b:N1653D, ORF1b:R2613C, ORF3a:Q57H, ORF3a:G172V, ORF8:S24L, S:L5F, S:D614G, S:Q677H, S:G769V, S:K854N,

hCoV-19/Mexico/NLE-UANL-040/2020	EPI_ISL_979332	20A	B.1.558	18	5'UTR:C241T, ORF1ab:C344T, ORF1ab:C1288T, ORF1ab:A2006G, ORF1ab:C3037T, ORF1ab:C9223T, ORF1ab:G9973A, ORF1ab:C12439T, ORF1ab:C13297T, ORF1ab:C14408T, ORF1ab:C19662T, ORF1ab:A20268G, ORF1ab:G20962T, S:C21646T, S:A23403G, ORF3a:C26060T, ORF7a:C27630T, N:G28727T, N:C28854T.	8	N:A152S, N:S194L, ORF1a:L27F, ORF1a:I581V, ORF1b:P314L, ORF1b:D2499Y, ORF3a:T223I, S:D614G,	
hCoV-19/Mexico/NLE-UANL-041/2020	EPI_ISL_979333	20A	B.1.396	19	5'UTR:C241T, ORF1ab:C1191T, ORF1ab:C3037T, ORF1ab:C4009A, ORF1ab:C9521A, ORF1ab:A9634T, ORF1ab:C14184T, ORF1ab:C14408T, ORF1ab:C15240T, ORF1ab:A20268G, S:A23403G, S:C24904T, ORF3a:G25563C, M:C26895T, N:G28851A, N:C28854T, N:G29422T, ORF10:A29567G, ORF10:C29642T, 3'UTR:T29729C,	10	M:H125Y, N:S193N, N:S194L, ORF1a:P309L, ORF1a:F1248L, ORF1a:L3086I, ORF1a:L3123F, ORF1b:P314L, ORF3a:Q57H, S:D614G,	
hCoV-19/Mexico/NLE-UANL-042/2020	EPI_ISL_979334	20C	B.1.366	19	5'UTR:C241T, ORF1ab:C1059T, ORF1ab:C1572T, ORF1ab:C3037T, ORF1ab:C3117T, ORF1ab:C5944T, ORF1ab:T8041C, ORF1ab:G11417T, ORF1ab:C12970T, ORF1ab:C14408T, ORF1ab:A16052G, S:G22225T, S:A23403G, S:G23593A, ORF3a:G25563T, M:G26567T, M:C26645T, ORF8:A28069G, N:C28706T, N:C28868T,	12	M:K15N, N:H145Y, N:P199S, ORF1a:T265I, ORF1a:T436I, ORF1a:T951I, ORF1a:V3718F, ORF1b:P314L, ORF1b:K862R, ORF3a:Q57H, ORF8:E59G, S:D614G,	S:21860-21862
hCoV-19/Mexico/NLE-UANL-043/2020	EPI_ISL_979335	20A	B.1.243	16	5'UTR:C241T, ORF1ab:C1009T, ORF1ab:C1538T, ORF1ab:C3037T, ORF1ab:C10741T, ORF1ab:C14408T, ORF1ab:C19097T, ORF1ab:A19137G, ORF1ab:A20268G, S:A23403G, S:C23604A, S:T24076C, S:G24928T, S:G25244T, N:C28854T, ORF10:G29587T, 3'UTR:T29710C,	7	N:S194L, ORF1a:P425S, ORF1b:P314L, ORF1b:A1877V, S:D614G, S:P681H, S:V1228L,	
hCoV-19/Mexico/NLE-UANL-044/2020	EPI_ISL_979336	20B	B.1.1.519	26	5'UTR:C203T, 5'UTR:C222T, 5'UTR:C241T, ORF1ab:C3037T, ORF1ab:C3140T, ORF1ab:C10029T, ORF1ab:C10954T, ORF1ab:C10969T, ORF1ab:A11117G, ORF1ab:C12651T, ORF1ab:C12789T, ORF1ab:C14408T, ORF1ab:G17278T, ORF1ab:T19839C, ORF1ab:C21306T, S:C22388T, S:C22995A, S:A23403G, S:C23604A, S:A23756G, S:C24919T, ORF7a:C27688T, N:G28881A, N:G28882A, N:G28883C, N:C29197T, N:G29402T,	15	N:R203K, N:G204R, N:D377Y, ORF1a:P959S, ORF1a:T3255I, ORF1a:I3618V, ORF1a:T4129I, ORF1a:T4175I, ORF1b:P314L, ORF1b:V1271L, ORF7a:P99S, S:T478K, S:D614G, S:P681H, S:T732A,	
hCoV-19/Mexico/NLE-UANL-045/2020	EPI_ISL_979337	20A	B.1.396	18	5'UTR:C241T, ORF1ab:C475T, ORF1ab:C1191T, ORF1ab:C3037T, ORF1ab:C5654T, ORF1ab:A6705G, ORF1ab:C9521A, ORF1ab:C14408T, ORF1ab:C18464A, ORF1ab:G20208T, ORF1ab:A20268G, S:A23403G, S:C24904T, ORF3a:G25494T, ORF3a:G25540T, N:C28854T, N:G29422T, N:G29543T, ORF10:A29567G,	9	N:S194L, ORF1a:P309L, ORF1a:N2147S, ORF1a:L3086I, ORF1b:P314L, ORF1b:P1666H, ORF1b:Q2247H, ORF3a:V50F, S:D614G,	
hCoV-19/Mexico/NLE-UANL-049/2020	EPI_ISL_961792	20B	B.1.1.344	10	5'UTR:C241T, ORF1ab:C3037T, ORF1ab:T9172C, ORF1ab:A10948G, ORF1ab:C14408T, ORF1ab:T16950C, S:A23403G, M:G26730T, N:G28881A, N:G28882A, N:G28883C,	5	M:V70F, N:R203K, N:G204R, ORF1b:P314L, S:D614G,	
hCoV-19/Mexico/NLE-UANL-050/2020	EPI_ISL_961791	20B	B.1.1.344	9	5'UTR:C241T, ORF1ab:C3037T, ORF1ab:T9172C, ORF1ab:A10948G, ORF1ab:T16950C, S:A23403G, M:G26730T, N:G28881A, N:G28882A, N:G28883C,	4	M:V70F, N:R203K, N:G204R, S:D614G,	
hCoV-19/Mexico/NLE-UANL-052/2020	EPI_ISL_961785	20D	C.23	20	5'UTR:C186T, 5'UTR:C241T, ORF1ab:C3037T, ORF1ab:C4002T, ORF1ab:C7165A, ORF1ab:C8266T, ORF1ab:C9979T, ORF1ab:G10097A, ORF1ab:C11074T, ORF1ab:G11083T, ORF1ab:C13536T, ORF1ab:C14408T, ORF1ab:C15240T, ORF1ab:C16937T, S:A23403G, S:C23731T, S:C24381T, N:G28881A, N:G28882A, N:G28883C, N:C29167T,	9	N:R203K, N:G204R, ORF1a:T1246I, ORF1a:G3278S, ORF1a:L3606F, ORF1b:P314L, ORF1b:P1157L, S:D614G, S:S940F,	

hCoV-19/Mexico/NLE-UANL-053/2020	EPI_ISL_961781	20A	B.1	9	5'UTR:C241T, ORF1ab:A2806C, ORF1ab:C3037T, ORF1ab:G11417T, ORF1ab:T13857C, ORF1ab:C14408T, ORF1ab:A20268G, S:A23403G, ORF3a:G25477T, N:C28854T,	6	N:S194L, ORF1a:E847D, ORF1a:V3718F, ORF1b:P314L, ORF3a:V29F, S:D614G,	
hCoV-19/Mexico/NLE-UANL-054/2020	EPI_ISL_961777	20B	B.1.1.222	14	5'UTR:C241T, ORF1ab:C3037T, ORF1ab:C3743T, ORF1ab:G4399T, ORF1ab:C12754T, ORF1ab:A13712G, ORF1ab:C14408T, ORF1ab:C19011T, ORF1ab:T19839C, ORF1ab:G19962T, S:A23403G, S:A23756G, N:G28881A, N:G28882A, N:G28883C,	8	N:R203K, N:G204R, ORF1a:H1160Y, ORF1a:M1378I, ORF1b:K82R, ORF1b:P314L, S:D614G, S:T732A,	
hCoV-19/Mexico/NLE-UANL-055/2020	EPI_ISL_961774	20A	B.1.396	13	5'UTR:C241T, ORF1ab:C1191T, ORF1ab:C3037T, ORF1ab:G7553A, ORF1ab:C9521A, ORF1ab:C10507T, ORF1ab:C14408T, ORF1ab:A19073G, ORF1ab:A20268G, S:A23403G, S:C24904T, N:C28854T, N:G29422T, ORF10:A29567G,	7	N:S194L, ORF1a:P309L, ORF1a:V2430I, ORF1a:L3086I, ORF1b:P314L, ORF1b:D1869G, S:D614G,	
hCoV-19/Mexico/NLE-UANL-056/2020	EPI_ISL_961767	20A	B.1	16	5'UTR:C241T, ORF1ab:T523C, ORF1ab:C3037T, ORF1ab:A4571G, ORF1ab:C5175T, ORF1ab:G11083T, ORF1ab:C12484T, ORF1ab:C14408T, ORF1ab:C20259T, ORF1ab:A20268G, ORF1ab:G21193T, S:C21627T, S:A23403G, ORF3a:C25460T, ORF3a:C25669T, N:C28854T, 3'UTR:G29755T,	10	N:S194L, ORF1a:N1436D, ORF1a:T1637I, ORF1a:L3606F, ORF1b:P314L, ORF1b:D2576Y, ORF3a:A23V, ORF3a:H93Y, S:T22I, S:D614G,	
hCoV-19/Mexico/NLE-UANL-057/2020	EPI_ISL_961768	20A	B.1.396	13	5'UTR:C241T, ORF1ab:C1191T, ORF1ab:C3037T, ORF1ab:C9521A, ORF1ab:G11083T, ORF1ab:C14184T, ORF1ab:C14408T, ORF1ab:A20055G, ORF1ab:A20268G, S:A23403G, S:C24904T, N:C28854T, N:G29422T, ORF10:A29567G,	6	N:S194L, ORF1a:P309L, ORF1a:L3086I, ORF1a:L3606F, ORF1b:P314L, S:D614G,	
hCoV-19/Mexico/NLE-UANL-058/2020	EPI_ISL_961760	20B	B.1.1.344	15	5'UTR:C203T, 5'UTR:C241T, ORF1ab:C3037T, ORF1ab:C5866T, ORF1ab:T9172C, ORF1ab:C10156T, ORF1ab:A10948G, ORF1ab:C14408T, ORF1ab:T16950C, S:A23403G, S:G24199T, M:G26730T, N:G28307T, N:G28881A, N:G28882A, N:G28883C,	7	M:V70F, N:A12S, N:R203K, N:G204R, ORF1b:P314L, ORF9b:M8I, S:D614G,	
hCoV-19/Mexico/NLE-UANL-059/2020	EPI_ISL_961762	20C	B.1.366	16	5'UTR:C241T, ORF1ab:C1059T, ORF1ab:C1572T, ORF1ab:C3037T, ORF1ab:T8041C, ORF1ab:C8937T, ORF1ab:C9438T, ORF1ab:C12970T, ORF1ab:C14408T, ORF1ab:A16052G, ORF1ab:C19594A, S:G22225T, S:C22712T, S:A23403G, ORF3a:G25563T, N:C28868T, N:C28948T,	11	N:P199S, ORF1a:T265I, ORF1a:T436I, ORF1a:A2891V, ORF1a:T3058I, ORF1b:P314L, ORF1b:K862R, ORF1b:L2043I, ORF3a:Q57H, S:P384S, S:D614G,	S:21860-21862
hCoV-19/Mexico/NLE-UANL-060/2020	EPI_ISL_961763	20A	B.1.396	15	5'UTR:C241T, ORF1ab:T682C, ORF1ab:C1191T, ORF1ab:C3037T, ORF1ab:G4180A, ORF1ab:C9521A, ORF1ab:C14184T, ORF1ab:C14408T, ORF1ab:C18647T, ORF1ab:A20268G, S:C21621T, S:A23403G, S:C24904T, N:C28854T, N:G29422T, ORF10:A29567G,	7	N:S194L, ORF1a:P309L, ORF1a:L3086I, ORF1b:P314L, ORF1b:P1727L, S:T20I, S:D614G,	
hCoV-19/Mexico/NLE-UANL-062/2020	EPI_ISL_961780	20A	B.1.558	18	5'UTR:C241T, ORF1ab:C344T, ORF1ab:C1288T, ORF1ab:C1473T, ORF1ab:C1722T, ORF1ab:A2386G, ORF1ab:A2994G, ORF1ab:C3037T, ORF1ab:C12439T, ORF1ab:C14408T, ORF1ab:C16111T, ORF1ab:C17004T, ORF1ab:C19662T, ORF1ab:A20268G, ORF1ab:G20962T, S:A23403G, ORF3a:C26060T, ORF7a:C27630T, N:C28854T,	9	N:S194L, ORF1a:L27F, ORF1a:T403I, ORF1a:A486V, ORF1a:E910G, ORF1b:P314L, ORF1b:D2499Y, ORF3a:T223I, S:D614G,	
hCoV-19/Mexico/NLE-UANL-079/2020	EPI_ISL_961765	20A	B.1	11	5'UTR:C241T, ORF1ab:A2806C, ORF1ab:C3037T, ORF1ab:A10323G, ORF1ab:G11417T, ORF1ab:T13857C, ORF1ab:C14408T, ORF1ab:A20268G, S:A23403G, ORF3a:G25477T, ORF3a:G25494T, N:C28854T,	7	N:S194L, ORF1a:E847D, ORF1a:K3353R, ORF1a:V3718F, ORF1b:P314L, ORF3a:V29F, S:D614G,	

hCoV-19/Mexico/NLE-UANL-083/2020	EPI_ISL_961771	20B	B.1.1.344	15	5'UTR:C241T, ORF1ab:C547T, ORF1ab:C3037T, ORF1ab:A7240G, ORF1ab:T9172C, ORF1ab:A10948G, ORF1ab:C13860T, ORF1ab:C14408T, ORF1ab:G16377T, ORF1ab:T16950C, S:A23403G, M:G26730T, M:C27143T, N:G28881A, N:G28882A, N:G28883C,	5	M:V70F, N:R203K, N:G204R, ORF1b:P314L, S:D614G,
hCoV-19/Mexico/NLE-UANL-084/2020	EPI_ISL_961761	20B	B.1.1.344	17	5'UTR:C241T, ORF1ab:C3037T, ORF1ab:C3044T, ORF1ab:A8077G, ORF1ab:T9172C, ORF1ab:A9409G, ORF1ab:A10948G, ORF1ab:C14408T, ORF1ab:T16950C, ORF1ab:G20730T, S:A23403G, S:G24095T, M:G26730T, ORF6:A27347G, N:G28690T, N:G28881A, N:G28882A, N:G28883C,	10	M:V70F, N:L139F, N:R203K, N:G204R, ORF1a:P927S, ORF1b:P314L, ORF1b:K2421N, ORF6:Y49C, S:D614G, S:A845S,
hCoV-19/Mexico/NLE-UANL-085/2020	EPI_ISL_961773	20B	B.1.1.432	21	5'UTR:C241T, ORF1ab:G1148T, ORF1ab:C2667T, ORF1ab:C2842T, ORF1ab:C3037T, ORF1ab:G4124T, ORF1ab:G4255T, ORF1ab:C4795T, ORF1ab:C5512T, ORF1ab:A6985T, ORF1ab:C9661T, ORF1ab:G11417T, ORF1ab:C12412T, ORF1ab:T14313C, ORF1ab:C14408T, ORF1ab:C16323T, S:A23403G, ORF3a:C25613T, ORF7a:C27425T, N:G28881A, N:G28882A, N:G28883C,	10	N:R203K, N:G204R, ORF1a:G295C, ORF1a:A801V, ORF1a:V1287L, ORF1a:V3718F, ORF1b:P314L, ORF3a:S74F, ORF7a:T11I, S:D614G,
hCoV-19/Mexico/NLE-UANL-086/2020	EPI_ISL_961772	20C	B.1	11	5'UTR:C241T, ORF1ab:C583T, ORF1ab:C1059T, ORF1ab:C3037T, ORF1ab:C7318T, ORF1ab:C14408T, ORF1ab:C15470T, ORF1ab:C17285T, ORF1ab:C21058T, S:A23403G, ORF3a:G25563T, ORF8:C27964T,	8	ORF1a:T265I, ORF1b:P314L, ORF1b:P668L, ORF1b:S1273L, ORF1b:P2531S, ORF3a:Q57H, ORF8:S24L, S:D614G,
hCoV-19/Mexico/NLE-UANL-087/2020	EPI_ISL_961766	20B	B.1.1.28	13	5'UTR:C241T, ORF1ab:C3037T, ORF1ab:C4832T, ORF1ab:C6807T, ORF1ab:T14049G, ORF1ab:C14408T, ORF1ab:C19402T, S:A23403G, S:G24620T, S:G25088T, N:G28881A, N:G28882A, N:G28883C, N:G29384A,	9	N:R203K, N:G204R, N:D371N, ORF1a:T2181I, ORF1b:P314L, ORF1b:H1979Y, S:D614G, S:A1020S, S:V1176F,
hCoV-19/Mexico/NLE-UANL-088/2020	EPI_ISL_979338	20C	B.1.588	24	5'UTR:C241T, ORF1ab:C1059T, ORF1ab:C3037T, ORF1ab:G3929T, ORF1ab:G6052A, ORF1ab:G7675C, ORF1ab:C10319T, ORF1ab:C10440T, ORF1ab:A11782G, ORF1ab:T12865C, ORF1ab:C14408T, ORF1ab:C19170T, ORF1ab:G19872T, S:C21621T, S:G22201T, S:A23403G, ORF3a:G25563T, ORF3a:G25855T, ORF7b:G27758T, ORF8:C27964T, N:G28975T, N:G29254T, N:G29474T, 3'UTR:C29743T, 3'UTR:G29764T,	14	N:M234I, N:D401Y, ORF1a:T265I, ORF1a:V1222F, ORF1a:L3352F, ORF1a:A3392V, ORF1b:P314L, ORF3a:Q57H, ORF3a:D155Y, ORF7a:*122L, ORF7b:M1I, ORF8:S24L, S:T20I, S:D614G,
hCoV-19/Mexico/NLE-UANL-126/2020	EPI_ISL_648595	20A	B.1.610	5	5'UTR:C241T, ORF1ab:C3037T, ORF1ab:C14408T, ORF1ab:A20268G, S:A23403G, 3'UTR:G29734C,	2	ORF1b:P314L, S:D614G,
hCoV-19/Mexico/NLE-UANL-2088/2020	EPI_ISL_648603	20B	B.1.1.344	10	5'UTR:C241T, ORF1ab:C3037T, ORF1ab:T9172C, ORF1ab:A10948G, ORF1ab:C14408T, ORF1ab:T16950C, S:A23403G, M:G26730T, N:G28881A, N:G28882A, N:G28883C,	5	M:V70F, N:R203K, N:G204R, ORF1b:P314L, S:D614G,
hCoV-19/Mexico/NLE-UANL-2098/2020	EPI_ISL_648602	20B	B.1.1.344	10	5'UTR:C241T, ORF1ab:C3037T, ORF1ab:T9172C, ORF1ab:A10948G, ORF1ab:C14408T, ORF1ab:T16950C, S:A23403G, M:G26730T, N:G28881A, N:G28882A, N:G28883C,	5	M:V70F, N:R203K, N:G204R, ORF1b:P314L, S:D614G,
hCoV-19/Mexico/NLE-UANL-4479/2020	EPI_ISL_648607	20A	B.1	8	5'UTR:C241T, ORF1ab:G1548A, ORF1ab:C3037T, ORF1ab:C8476T, ORF1ab:T9508C, ORF1ab:C14408T, ORF1ab:A20268G, S:A23403G, S:A24190G,	3	ORF1a:S428N, ORF1b:P314L, S:D614G,
hCoV-19/Mexico/NL-InDRE_122/2020	EPI_ISL_913926	20A	B.1	9	5'UTR:C241T, ORF1ab:C3037T, ORF1ab:C10537T, ORF1ab:C14408T, ORF1ab:A17861G, ORF1ab:C18744T, ORF1ab:A20268G, S:A23403G, ORF3a:C25578T, N:C28854T,	4	N:S194L, ORF1b:P314L, ORF1b:D1465G, S:D614G,
hCoV-19/Mexico/OAX-InDRE_110/2020	EPI_ISL_913914	20A	B.1.609	12	5'UTR:C241T, ORF1ab:C3037T, ORF1ab:C4582T, ORF1ab:A10323G, ORF1ab:C13119T, ORF1ab:C14408T, ORF1ab:A20268G, S:G22205T, S:A23403G, ORF3a:G25526T, ORF7b:C27765T, N:C28887T, 3'UTR:C29774T,	8	N:T205I, ORF1a:K3353R, ORF1a:A4285V, ORF1b:P314L, ORF3a:W45L, ORF7b:L4F, S:D215Y, S:D614G,

hCoV-19/Mexico/OAX-InDRE_158/2020	EPI_ISL_913960	20C	B.1	21	5'UTR:G213T, 5'UTR:C241T, ORF1ab:C1059T, ORF1ab:C2245T, ORF1ab:C3037T, ORF1ab:G4255T, ORF1ab:C6720T, ORF1ab:C11152T, ORF1ab:G11417T, ORF1ab:C14408T, ORF1ab:C16289T, ORF1ab:C16658T, ORF1ab:T19065A, S:G21578T, S:A23403G, S:A24902G, ORF3a:G25440T, ORF3a:G25563T, ORF3a:G25593T, ORF7a:G27463T, N:C28887T, 3'UTR:C29750T.	14	N:T205I, ORF1a:T265I, ORF1a:T2152I, ORF1a:V3718F, ORF1b:P314L, ORF1b:A941V, ORF1b:T1064I, ORF3a:K16N, ORF3a:Q57H, ORF3a:K67N, ORF7a:V24F, S:V6F, S:D614G, S:I1114V,
hCoV-19/Mexico/OAX-InDRE_171/2020	EPI_ISL_913972	20B	B.1.1.519	23	5'UTR:C203T, 5'UTR:C222T, 5'UTR:C241T, ORF1ab:C3037T, ORF1ab:C3140T, ORF1ab:C3177T, ORF1ab:G7037T, ORF1ab:C10029T, ORF1ab:C10954T, ORF1ab:A11117G, ORF1ab:C12789T, ORF1ab:C14408T, ORF1ab:C19186T, ORF1ab:T19839C, ORF1ab:C21306T, S:C22995A, S:A23403G, S:C23604A, S:A23756G, ORF3a:C25782T, N:G28881A, N:G28882A, N:G28883C, N:C29197T,	13	N:R203K, N:G204R, ORF1a:P959S, ORF1a:P971L, ORF1a:G2258C, ORF1a:T3255I, ORF1a:I3618V, ORF1a:T4175I, ORF1b:P314L, S:T478K, S:D614G, S:P681H, S:T732A,
hCoV-19/Mexico/OAX-InDRE_257/2020	EPI_ISL_1054996	20B	B.1.1.198	14	5'UTR:C241T, ORF1ab:C3037T, ORF1ab:C3261T, ORF1ab:C5826T, ORF1ab:C13487T, ORF1ab:C14408T, S:A23403G, ORF3a:C25539T, ORF6:C27294T, N:C28789T, N:G28881A, N:G28882A, N:G28883C, N:A29089G, N:G29402T,	8	N:R203K, N:G204R, N:D377Y, ORF1a:T999I, ORF1a:T1854I, ORF1b:A7V, ORF1b:P314L, S:D614G,
hCoV-19/Mexico/OAX-InDRE_258/2020	EPI_ISL_1054990	20B	B.1.1	8	5'UTR:C241T, ORF1ab:C3037T, ORF1ab:G7037T, ORF1ab:C14408T, S:A23403G, N:G28881A, N:G28882A, N:G28883C, N:C29197T,	5	N:R203K, N:G204R, ORF1a:G2258C, ORF1b:P314L, S:D614G,
hCoV-19/Mexico/OAX-InDRE_262/2020	EPI_ISL_1054979	20A	B.1	16	5'UTR:C241T, ORF1ab:C1380T, ORF1ab:G2830T, ORF1ab:C3037T, ORF1ab:T4837C, ORF1ab:T5479C, ORF1ab:G6341T, ORF1ab:C6786T, ORF1ab:C7564T, ORF1ab:G8371T, ORF1ab:C14408T, ORF1ab:C17883T, ORF1ab:A20268G, S:T22465C, S:C22591T, S:A23403G, N:C28854T,	8	N:S194L, ORF1a:A372V, ORF1a:E855D, ORF1a:D2026Y, ORF1a:T2174I, ORF1a:Q2702H, ORF1b:P314L, S:D614G,
hCoV-19/Mexico/OAX-InDRE_266/2020	EPI_ISL_1054966	20B	B.1.1	15	5'UTR:C241T, ORF1ab:C3037T, ORF1ab:C12412T, ORF1ab:T14313C, ORF1ab:C14408T, ORF1ab:C16329T, ORF1ab:G19656A, ORF1ab:A21137G, S:A23403G, S:T24565C, S:T24691C, M:C26611T, N:C28310T, N:G28881A, N:G28882A, N:G28883C,	7	M:T30I, N:P13S, N:R203K, N:G204R, ORF1b:P314L, ORF1b:K2557R, S:D614G,
hCoV-19/Mexico/OAX-InDRE_267/2020	EPI_ISL_1054965	20A	B.1.405	12	5'UTR:G208T, 5'UTR:C241T, ORF1ab:C3037T, ORF1ab:C6402T, ORF1ab:C13261T, ORF1ab:C13829T, ORF1ab:C14408T, ORF1ab:A20268G, S:A23403G, S:G23522A, ORF3a:A25747T, N:G28373T, N:C28854T,	8	N:G34W, N:S194L, ORF1a:P2046L, ORF1b:A121V, ORF1b:P314L, ORF3a:N119Y, S:D614G, S:E654K,
hCoV-19/Mexico/OAX-InDRE_333/2020	EPI_ISL_1060694	20B	B.1.1.222	11	ORF1ab:C3037T, ORF1ab:C3811T, ORF1ab:C6285T, ORF1ab:C13329T, ORF1ab:A13498G, ORF1ab:C14408T, S:G22331A, S:A23403G, S:A23756G, N:G28881A, N:G28882A, N:G28883C,	9	N:R203K, N:G204R, ORF1a:T2007I, ORF1a:T4355I, ORF1b:T11A, ORF1b:P314L, S:G257S, S:D614G, S:T732A,
hCoV-19/Mexico/OAX-InDRE_347/2020	EPI_ISL_1060691	20B	B.1.1.432	21	ORF1ab:C3037T, ORF1ab:G4925T, ORF1ab:T6376C, ORF1ab:G6421T, ORF1ab:T8806C, ORF1ab:C9319T, ORF1ab:G11266T, ORF1ab:C12412T, ORF1ab:T14313C, ORF1ab:C14408T, ORF1ab:C15656T, S:A23403G, ORF3a:C25613T, ORF3a:C25721T, ORF8:C27972T, ORF8:T28033C, N:G28881A, N:G28882A, N:G28883C, N:G28985T, N:G29179T, N:T29317C,	12	N:R203K, N:G204R, N:G238C, ORF1a:D1554Y, ORF1a:L3667F, ORF1b:P314L, ORF1b:T730I, ORF3a:S74F, ORF3a:A110V, ORF8:Q27*, ORF8:I47T, S:D614G,
hCoV-19/Mexico/OAX-InDRE_399/2020	EPI_ISL_1054976	20B	B.1.1.432	18	5'UTR:C241T, ORF1ab:G1306T, ORF1ab:T2737C, ORF1ab:C3037T, ORF1ab:A6985T, ORF1ab:C9319T, ORF1ab:C12412T, ORF1ab:T14313C, ORF1ab:C14408T, ORF1ab:G14874T, S:A23403G, S:G24620T, ORF3a:G25912T, N:G28881A, N:G28882A, N:G28883C, N:G29260A, N:T29317C, 3'UTR:G29868C,	8	N:R203K, N:G204R, ORF1a:E347D, ORF1b:P314L, ORF1b:K469N, ORF3a:G174C, S:D614G, S:A1020S,

hCoV-19/Mexico/OAX-InDRE_400/2020	EPI_ISL_1054989	20B	B.1.1.222	8	5'UTR:C241T, ORF1ab:C3037T, ORF1ab:C14408T, ORF1ab:T19839C, S:A23403G, S:A23756G, N:G28881A, N:G28882A, N:G28883C,	5	N:R203K, N:G204R, ORF1b:P314L, S:D614G, S:T732A,
hCoV-19/Mexico/OAX-InDRE_536/2021	EPI_ISL_1168606	20B	B.1.1.519	26	5'UTR:C203T, 5'UTR:C222T, 5'UTR:C241T, ORF1ab:C3037T, ORF1ab:C3140T, ORF1ab:C5183T, ORF1ab:C10029T, ORF1ab:C10954T, ORF1ab:A11117G, ORF1ab:C12789T, ORF1ab:C13862T, ORF1ab:C14408T, ORF1ab:C15216T, ORF1ab:T19839C, ORF1ab:C21306T, S:C21575T, S:C22995A, S:A23403G, S:C23604A, S:A23756G, S:G24834A, ORF3a:C25571T, ORF8:T27904C, N:G28881A, N:G28882A, N:G28883C, N:C29197T,	17	N:R203K, N:G204R, ORF1a:P959S, ORF1a:P1640S, ORF1a:T3255I, ORF1a:I3618V, ORF1a:T4175I, ORF1b:T132I, ORF1b:P314L, ORF3a:S60F, ORF8:L4P, S:L5F, S:T478K, S:D614G, S:P681H, S:T732A, S:R1091H,
hCoV-19/Mexico/OAX-InDRE_537/2021	EPI_ISL_1168607	20B	B.1.1.519	27	5'UTR:C203T, 5'UTR:C222T, 5'UTR:C241T, ORF1ab:C1342T, ORF1ab:C3037T, ORF1ab:C3140T, ORF1ab:C3686T, ORF1ab:G3871T, ORF1ab:C7165T, ORF1ab:C10029T, ORF1ab:C10954T, ORF1ab:A11117G, ORF1ab:C11824A, ORF1ab:C14408T, ORF1ab:T19839C, ORF1ab:C21306T, ORF1ab:C21557T, S:A23403G, S:C23604A, S:A23756G, S:G24959T, ORF8:T27904C, N:G28881A, N:G28882A, N:G28883C, N:C29197T, 3'UTR:G29862T, 3'UTR:A29866C,	13	N:R203K, N:G204R, ORF1a:P959S, ORF1a:H1141Y, ORF1a:K1202N, ORF1a:T3255I, ORF1a:I3618V, ORF1b:P314L, ORF8:L4P, S:D614G, S:P681H, S:T732A, S:V1133F,
hCoV-19/Mexico/OAX-InDRE_538/2021	EPI_ISL_1168608	20B	B.1.1.222	12	5'UTR:C241T, ORF1ab:C3037T, ORF1ab:C8697A, ORF1ab:C14408T, ORF1ab:T19839C, S:A23403G, S:A23756G, N:G28881A, N:G28882A, N:G28883C, 3'UTR:C29784T, 3'UTR:G29862T, 3'UTR:A29866C,	6	N:R203K, N:G204R, ORF1a:A2811D, ORF1b:P314L, S:D614G, S:T732A,
hCoV-19/Mexico/OAX-InDRE_539/2021	EPI_ISL_1168609	20B	B.1.1	10	5'UTR:C241T, ORF1ab:C3037T, ORF1ab:C14408T, S:A23403G, ORF3a:G26051C, ORF8:C27972T, N:G28881A, N:G28882A, N:G28883C, N:C29545A, 3'UTR:G29862T,	6	N:R203K, N:G204R, ORF1b:P314L, ORF3a:S220T, ORF8:Q27*, S:D614G,
hCoV-19/Mexico/OAX-InDRE_541/2021	EPI_ISL_1168611	20B	B.1.1.222	16	5'UTR:C241T, ORF1ab:C3037T, ORF1ab:C7749T, ORF1ab:C8637T, ORF1ab:C14408T, ORF1ab:T19839C, S:A23403G, S:G23593T, S:A23756G, ORF8:A27899T, ORF8:T27900A, ORF8:C27903A, ORF8:G27906C, N:G28881A, N:G28882A, N:G28883C, N:G28907A,	9	N:R203K, N:G204R, N:G212S, ORF1a:T2495I, ORF1a:T2791I, ORF1b:P314L, S:D614G, S:Q677H, S:T732A,
hCoV-19/Mexico/OAX-InDRE-61/2020	EPI_ISL_576264	20A	B.1.243	11	5'UTR:C241T, ORF1ab:C1077T, ORF1ab:C3037T, ORF1ab:G7393T, ORF1ab:G11299T, ORF1ab:C14408T, ORF1ab:G19006T, ORF1ab:A20268G, S:G23012C, S:A23403G, S:T24076C, N:C28854T,	7	N:S194L, ORF1a:P271L, ORF1a:K3678N, ORF1b:P314L, ORF1b:A1847S, S:E484Q, S:D614G,
hCoV-19/Mexico/PUE-InDRE_128/2020	EPI_ISL_913931	20A	B.1	16	5'UTR:C241T, ORF1ab:C593T, ORF1ab:C3037T, ORF1ab:C4158T, ORF1ab:C6813T, ORF1ab:C14408T, ORF1ab:C14724T, ORF1ab:C15108T, ORF1ab:C19032T, ORF1ab:G19656T, ORF1ab:A20268G, S:A23403G, ORF3a:G25483T, ORF6:T27222C, N:C28854T, 3'UTR:G29751T, 3'UTR:A29767G,	8	N:S194L, ORF1a:H110Y, ORF1a:A1298V, ORF1a:T2183I, ORF1b:P314L, ORF1b:K2063N, ORF3a:A31S, S:D614G,
hCoV-19/Mexico/PUE-InDRE_129/2020	EPI_ISL_913932	20A	B.1	4	5'UTR:C241T, ORF1ab:T1711C, ORF1ab:C3037T, ORF1ab:C14408T, S:A23403G,	2	ORF1b:P314L, S:D614G,
hCoV-19/Mexico/PUE-InDRE_157/2020	EPI_ISL_913959	20B	B.1.1.519	23	5'UTR:C203T, 5'UTR:C222T, 5'UTR:C241T, ORF1ab:G1738T, ORF1ab:C3037T, ORF1ab:C3140T, ORF1ab:C10029T, ORF1ab:C10954T, ORF1ab:A11117G, ORF1ab:C12789T, ORF1ab:C14408T, ORF1ab:C16887T, ORF1ab:T19839C, ORF1ab:A19974G, ORF1ab:C21306T, S:C22995A, S:A23403G, S:C23604A, S:A23756G, ORF8:A28175G, N:G28881A, N:G28882A, N:G28883C, N:C29197T,	11	N:R203K, N:G204R, ORF1a:P959S, ORF1a:T3255I, ORF1a:I3618V, ORF1a:T4175I, ORF1b:P314L, S:T478K, S:D614G, S:P681H, S:T732A,

hCoV-19/Mexico/PUE-InDRE_190/2020	EPI_ISL_933667	20A	B.1	11	5'UTR:C241T, ORF1ab:C3037T, ORF1ab:G4201T, ORF1ab:C5544T, ORF1ab:C14408T, ORF1ab:C15738T, ORF1ab:G16741A, ORF1ab:C17474T, ORF1ab:G19670A, ORF1ab:A20268G, S:A23403G, N:C28854T,	8	N:S194L, ORF1a:M1312I, ORF1a:T1760I, ORF1b:P314L, ORF1b:V1092I, ORF1b:T1336I, ORF1b:G2068E, S:D614G,
hCoV-19/Mexico/PUE-InDRE_269/2020	EPI_ISL_1054959	20A	B.1	3	5'UTR:C241T, ORF1ab:C3037T, ORF1ab:C14408T, S:A23403G,	2	ORF1b:P314L, S:D614G,
hCoV-19/Mexico/PUE-InDRE_381/2020	EPI_ISL_1054968	20B	B.1.1.222	10	5'UTR:C241T, ORF1ab:C3037T, ORF1ab:G10870T, ORF1ab:C14408T, ORF1ab:C14790T, ORF1ab:T19839C, S:A23403G, S:A23756G, N:G28881A, N:G28882A, N:G28883C,	5	N:R203K, N:G204R, ORF1b:P314L, S:D614G, S:T732A,
hCoV-19/Mexico/PUE-InDRE_F10410_S645/2021	EPI_ISL_1340651	20B	B.1.1.519	23	5'UTR:C203T, 5'UTR:C222T, 5'UTR:C241T, ORF1ab:C1057T, ORF1ab:C3037T, ORF1ab:C3140T, ORF1ab:G3549A, ORF1ab:C10029T, ORF1ab:C10954T, ORF1ab:A11117G, ORF1ab:C12789T, ORF1ab:C14408T, ORF1ab:T19839C, ORF1ab:G20274T, ORF1ab:C21306T, S:C22995A, S:A23403G, S:C23604A, S:A23756G, ORF8:T27904C, N:G28881A, N:G28882A, N:G28883C, N:C29197T,	14	N:R203K, N:G204R, ORF1a:P959S, ORF1a:G1095E, ORF1a:T3255I, ORF1a:I3618V, ORF1a:T4175I, ORF1b:P314L, ORF1b:M2269I, ORF8:L4P, S:T478K, S:D614G, S:P681H, S:T732A,
hCoV-19/Mexico/PUE-InDRE_F10412_S646/2021	EPI_ISL_1340652	20B	B.1.1.519	26	5'UTR:C203T, 5'UTR:C222T, 5'UTR:C241T, ORF1ab:C3037T, ORF1ab:C3140T, ORF1ab:A4870G, ORF1ab:G7027T, ORF1ab:C10029T, ORF1ab:C10954T, ORF1ab:A11117G, ORF1ab:C12789T, ORF1ab:C14408T, ORF1ab:G18412T, ORF1ab:T19839C, ORF1ab:C21306T, S:G21718A, S:C22995A, S:A23403G, S:C23604A, S:A23756G, S:C25207T, ORF3a:C25466A, M:A26708G, N:G28881A, N:G28882A, N:G28883C, N:C29197T,	14	N:R203K, N:G204R, ORF1a:P959S, ORF1a:M2254I, ORF1a:T3255I, ORF1a:I3618V, ORF1a:T4175I, ORF1b:P314L, ORF1b:V1649F, ORF3a:P25H, S:T478K, S:D614G, S:P681H, S:T732A,
hCoV-19/Mexico/PUE-InDRE_F10413_S711/2021	EPI_ISL_1366660	20B	B.1.1.519	26	5'UTR:C203T, 5'UTR:C222T, 5'UTR:C241T, ORF1ab:C3037T, ORF1ab:C3140T, ORF1ab:C7869T, ORF1ab:C8092T, ORF1ab:C9080T, ORF1ab:C10029T, ORF1ab:C10954T, ORF1ab:A11117G, ORF1ab:C12312T, ORF1ab:C12789T, ORF1ab:T13593C, ORF1ab:C14408T, ORF1ab:G15921T, ORF1ab:T19839C, ORF1ab:C21306T, S:C22995A, S:A23403G, S:C23604A, S:A23756G, ORF7a:G27637T, N:G28881A, N:G28882A, N:G28883C, N:C29197T,	14	N:R203K, N:G204R, ORF1a:P959S, ORF1a:S2535L, ORF1a:T3255I, ORF1a:I3618V, ORF1a:A4016V, ORF1a:T4175I, ORF1b:P314L, ORF7a:V82F, S:T478K, S:D614G, S:P681H, S:T732A,
hCoV-19/Mexico/PUE-InDRE_F10894_S768/2021	EPI_ISL_1400313	20B	B.1.1.519	26	5'UTR:T201C, 5'UTR:C203T, 5'UTR:C222T, 5'UTR:C241T, ORF1ab:G1738T, ORF1ab:C3037T, ORF1ab:C3140T, ORF1ab:C10029T, ORF1ab:C10954T, ORF1ab:A11117G, ORF1ab:G11335T, ORF1ab:C12789T, ORF1ab:C14408T, ORF1ab:T19839C, ORF1ab:A19974G, ORF1ab:C21306T, S:C22995A, S:A23403G, S:C23604A, S:A23756G, M:T26726C, M:T26972C, ORF8:A28175G, N:G28881A, N:G28882A, N:G28883C, N:C29197T,	11	N:R203K, N:G204R, ORF1a:P959S, ORF1a:T3255I, ORF1a:I3618V, ORF1a:T4175I, ORF1b:P314L, S:T478K, S:D614G, S:P681H, S:T732A,
hCoV-19/Mexico/PUE-InDRE_F10895_S767/2021	EPI_ISL_1400312	20B	B.1.1.519	23	5'UTR:C203T, 5'UTR:C222T, 5'UTR:C241T, ORF1ab:C3037T, ORF1ab:C3140T, ORF1ab:T3745C, ORF1ab:T6644C, ORF1ab:C10029T, ORF1ab:C10954T, ORF1ab:A11117G, ORF1ab:C12789T, ORF1ab:C14408T, ORF1ab:G17544T, ORF1ab:T19839C, ORF1ab:C21306T, S:C22995A, S:A23403G, S:C23604A, S:A23756G, ORF3a:C25844T, N:G28881A, N:G28882A, N:G28883C, N:C29197T,	13	N:R203K, N:G204R, ORF1a:P959S, ORF1a:T3255I, ORF1a:I3618V, ORF1a:T4175I, ORF1b:P314L, ORF1b:M1359I, ORF3a:T151I, S:T478K, S:D614G, S:P681H, S:T732A,

hCoV-19/Mexico/PUE-InDRE_F10901_S766/2021	EPI_ISL_1400311	20B	B.1.1.222	20	5'UTR:C241T, ORF1ab:C3037T, ORF1ab:A3081G, ORF1ab:A3888G, ORF1ab:C7834T, ORF1ab:G9130T, ORF1ab:C9638T, ORF1ab:C10029T, ORF1ab:C14408T, ORF1ab:C19011A, ORF1ab:T19839C, S:G21800A, S:G22021T, S:A23403G, S:A23756G, ORF8:A27921G, ORF8:G28001T, N:G28881A, N:G28882A, N:G28883C, N:C29077T.	13	N:R203K, N:G204R, ORF1a:E939G, ORF1a:K1208R, ORF1a:H3125Y, ORF1a:T3255I, ORF1b:P314L, ORF1b:D1848E, ORF8:I10V, S:D80N, S:M153I, S:D614G, S:T732A,
hCoV-19/Mexico/PUE-InDRE_F10903_S765/2021	EPI_ISL_1400310	20B	B.1.1.519	31	5'UTR:C203T, 5'UTR:C222T, 5'UTR:C241T, ORF1ab:A866G, ORF1ab:T868C, ORF1ab:C3037T, ORF1ab:C3140T, ORF1ab:C5183T, ORF1ab:C8389T, ORF1ab:C10029T, ORF1ab:C10954T, ORF1ab:C11109T, ORF1ab:A11117G, ORF1ab:C11916T, ORF1ab:G12223T, ORF1ab:C12789T, ORF1ab:T13880C, ORF1ab:C14408T, ORF1ab:C14599T, ORF1ab:T19839C, ORF1ab:C21306T, S:C22995A, S:A23403G, S:C23604A, S:A23756G, ORF3a:G25477T, ORF8:T27904C, N:G28842T, N:G28881A, N:G28882A, N:G28883C, N:C29197T,	19	N:S190I, N:R203K, N:G204R, ORF1a:I201V, ORF1a:P959S, ORF1a:P1640S, ORF1a:T3255I, ORF1a:A3615V, ORF1a:I3618V, ORF1a:S3884L, ORF1a:T4175I, ORF1b:V138A, ORF1b:P314L, ORF3a:V29F, ORF8:L4P, S:T478K, S:D614G, S:P681H, S:T732A,
hCoV-19/Mexico/PUE-InDRE-05/2020	EPI_ISL_424672	20A	B.1	9	5'UTR:C241T, ORF1ab:A1308G, ORF1ab:C3037T, ORF1ab:C14408T, ORF1ab:C17639T, ORF1ab:A20268G, S:A23403G, ORF7a:G27506T, N:C28638T, 3'UTR:A29700G,	6	N:P122L, ORF1a:N348S, ORF1b:P314L, ORF1b:S1391L, ORF7a:G38V, S:D614G,
hCoV-19/Mexico/PUE-InDRE-17/2020	EPI_ISL_455455	20B	B.1.1	8	5'UTR:C241T, ORF1ab:C3037T, ORF1ab:C14408T, S:A23403G, M:C27046T, N:G28881A, N:G28882A, N:G28883C, N:G29224T,	6	M:T175M, N:R203K, N:G204R, N:M317I, ORF1b:P314L, S:D614G,
hCoV-19/Mexico/QRO-InDRE_143/2020	EPI_ISL_913946	20A	B.1	7	5'UTR:C241T, ORF1ab:C3037T, ORF1ab:A6133G, ORF1ab:C14408T, ORF1ab:C16887T, ORF1ab:A20268G, S:A23403G, 3'UTR:G29734T,	2	ORF1b:P314L, S:D614G,
hCoV-19/Mexico/QRO-InDRE_144/2020	EPI_ISL_913947	20B	B.1.1.222	12	5'UTR:C241T, ORF1ab:C3037T, ORF1ab:C6285T, ORF1ab:C12854A, ORF1ab:A13498G, ORF1ab:C14408T, ORF1ab:T19839C, S:A23403G, S:A23756G, ORF3a:C25714T, N:G28881A, N:G28882A, N:G28883C,	9	N:R203K, N:G204R, ORF1a:T2007I, ORF1a:P4197T, ORF1b:T11A, ORF1b:P314L, ORF3a:L108F, S:D614G, S:T732A,
hCoV-19/Mexico/QRO-InDRE-84/2020	EPI_ISL_658878	20C	B.1.369	20	5'UTR:C241T, ORF1ab:C1059T, ORF1ab:G2393A, ORF1ab:C2395T, ORF1ab:C3037T, ORF1ab:G3259T, ORF1ab:G3433T, ORF1ab:A6646G, ORF1ab:C7600T, ORF1ab:G11540T, ORF1ab:C14408T, ORF1ab:C16260T, ORF1ab:C19961T, S:G21641T, S:A23403G, S:C23481T, S:G24751T, ORF3a:G25444C, ORF3a:G25563T, N:C28821A, N:A29004T,	14	N:S183Y, N:Q244L, ORF1a:T265I, ORF1a:V710I, ORF1a:Q998H, ORF1a:V3759F, ORF1b:P314L, ORF1b:T2165M, ORF3a:G18R, ORF3a:Q57H, S:A27S, S:D614G, S:S640F, S:L1063F,
hCoV-19/Mexico/QRO-InDRE-87/2020	EPI_ISL_658886	20B	B.1.1.316	15	5'UTR:C241T, ORF1ab:C3037T, ORF1ab:C5812T, ORF1ab:C14408T, ORF1ab:T19839C, ORF1ab:C21459T, S:C21757T, S:C22480T, S:A23403G, S:A23756T, ORF3a:C25658T, N:G28881A, N:G28882A, N:G28883C, N:C29095T, 3'UTR:G29751T.	6	N:R203K, N:G204R, ORF1b:P314L, ORF3a:T89I, S:D614G, S:T732S,
hCoV-19/Mexico/QRO-InDRE-88/2020	EPI_ISL_658888	20A	B.1	13	5'UTR:C241T, ORF1ab:C3037T, ORF1ab:C4321T, ORF1ab:G5305T, ORF1ab:C13721T, ORF1ab:C14408T, ORF1ab:G17058T, ORF1ab:T17559C, ORF1ab:A20268G, S:A23403G, S:C24734T, ORF3a:C25433T, N:C28854T, ORF10:C29614T.	8	N:S194L, ORF1a:L1680F, ORF1b:P85L, ORF1b:P314L, ORF1b:M1197I, ORF3a:T14I, S:D614G, S:H1058Y,
hCoV-19/Mexico/QRO-InDRE-90/2020	EPI_ISL_658893	20B	B.1.1.222	21	5'UTR:C241T, ORF1ab:G1268A, ORF1ab:C3037T, ORF1ab:C5986T, ORF1ab:C12870T, ORF1ab:A13288G, ORF1ab:C14408T, ORF1ab:G14772T, ORF1ab:G16117T, ORF1ab:G17562T, ORF1ab:T19839C, ORF1ab:G20208T, S:A23403G, S:A23756G, S:C25046T, ORF3a:G26056T, ORF7a:A27655C, N:G28881A, N:G28882A, N:G28883C, N:C29409T, 3'UTR:G29773T.	14	N:R203K, N:G204R, N:T379I, ORF1a:D335N, ORF1a:T4202I, ORF1b:P314L, ORF1b:Q435H, ORF1b:D884Y, ORF1b:Q2247H, ORF3a:D222Y, ORF7a:I88L, S:D614G, S:T732A, S:P1162S,

hCoV-19/Mexico/QROO-InDRE-53/2020	EPI_ISL_658904	20B	B.1.1	8	5'UTR:C241T, ORF1ab:A2817G, ORF1ab:C3037T, ORF1ab:C12412T, ORF1ab:C14408T, S:A23403G, N:G28881A, N:G28882A, N:G28883C,	5	N:R203K, N:G204R, ORF1a:K851R, ORF1b:P314L, S:D614G,
hCoV-19/Mexico/QUE-InDRE_246/2021	EPI_ISL_1060697	20B	B.1.1.519	21	5'UTR:C203T, 5'UTR:C222T, 5'UTR:C241T, ORF1ab:C3037T, ORF1ab:C3140T, ORF1ab:C10029T, ORF1ab:C10954T, ORF1ab:A11117G, ORF1ab:C12789T, ORF1ab:G13205T, ORF1ab:C14408T, ORF1ab:T18688G, ORF1ab:T19839C, ORF1ab:C21306T, S:C22995A, S:A23403G, S:C23604A, S:A23756G, N:G28881A, N:G28882A, N:G28883C, N:C29197T,	13	N:R203K, N:G204R, ORF1a:P959S, ORF1a:T3255I, ORF1a:I3618V, ORF1a:T4175I, ORF1a:A4314S, ORF1b:P314L, ORF1b:F1741V, S:T478K, S:D614G, S:P681H, S:T732A,
hCoV-19/Mexico/QUE-InDRE_294/2020	EPI_ISL_1060729	20B	B.1.1	6	5'UTR:C241T, ORF1ab:C3037T, ORF1ab:C14408T, S:A23403G, N:G28881A, N:G28882A, N:G28883C,	4	N:R203K, N:G204R, ORF1b:P314L, S:D614G,
hCoV-19/Mexico/QUE-InDRE_295/2020	EPI_ISL_1060730	20A	B.1	3	5'UTR:C241T, ORF1ab:C3037T, ORF1ab:C14408T, S:A23403G,	2	ORF1b:P314L, S:D614G,
hCoV-19/Mexico/QUE-InDRE_343/2020	EPI_ISL_1060701	20B	B.1.1.222	15	5'UTR:C241T, ORF1ab:C3037T, ORF1ab:T3295C, ORF1ab:C10165T, ORF1ab:G13201A, ORF1ab:A13498G, ORF1ab:C14408T, ORF1ab:T19839C, S:G22331A, S:A23403G, S:A23756G, ORF3a:C25549T, ORF3a:G25687T, N:G28881A. N:G28882A. N:G28883C.	9	N:R203K, N:G204R, ORF1b:T11A, ORF1b:P314L, ORF3a:L53F, ORF3a:A99S, S:G257S, S:D614G, S:T732A,
hCoV-19/Mexico/QUE-InDRE_345/2020	EPI_ISL_1060696	20B	B.1.1.222	18	ORF1ab:A2466G, ORF1ab:C3037T, ORF1ab:G4144A, ORF1ab:C7162T, ORF1ab:C7851T, ORF1ab:C12068T, ORF1ab:C12809T, ORF1ab:C12885T, ORF1ab:C13458T, ORF1ab:C14408T, ORF1ab:C19029T, ORF1ab:A21405G, S:A23403G, S:A23756G, ORF3a:G25429T, ORF8:C28253T, N:G28881A, N:G28882A, N:G28883C,	11	N:R203K, N:G204R, ORF1a:K734R, ORF1a:A2529V, ORF1a:L4182F, ORF1a:T4207I, ORF1a:S4398L, ORF1b:P314L, ORF3a:V13L, S:D614G, S:T732A,
hCoV-19/Mexico/QUE-InDRE_374/2021	EPI_ISL_1168452	20G	B.1.596	25	5'UTR:C241T, ORF1ab:C1059T, ORF1ab:C3037T, ORF1ab:G4109T, ORF1ab:A8837C, ORF1ab:C10319T, ORF1ab:A11451G, ORF1ab:C14408T, ORF1ab:A18424G, ORF1ab:G18703T, ORF1ab:C20178T, ORF1ab:T20625C, ORF1ab:C21304T, S:C21590T, S:C21811A, S:A23403G, S:A23592C, S:C24904T, ORF3a:G25563T, ORF3a:G25907T, ORF8:A27924G, ORF8:C27964T, N:C28472T, N:C28869T, N:T29194C, N:T29377A.	17	N:P67S, N:P199L, ORF1a:T265I, ORF1a:D1282Y, ORF1a:I2858L, ORF1a:L3352F, ORF1a:Q3729R, ORF1b:P314L, ORF1b:N1653D, ORF1b:D1746Y, ORF1b:R2613C, ORF3a:Q57H, ORF3a:G172V, ORF8:T11A, ORF8:S24L, S:D614G, S:Q677P,
hCoV-19/Mexico/QUE-InDRE_375/2021	EPI_ISL_1168453	20B	B.1.1.519	27	5'UTR:C203T, 5'UTR:C222T, 5'UTR:C241T, ORF1ab:C3037T, ORF1ab:C3140T, ORF1ab:C5183T, ORF1ab:C10029T, ORF1ab:T10254C, ORF1ab:C10954T, ORF1ab:A11117G, ORF1ab:C11916T, ORF1ab:C12789T, ORF1ab:C13592A, ORF1ab:C14408T, ORF1ab:T19839C, ORF1ab:C21306T, S:G22661T, S:C22995A, S:A23403G, S:C23604A, S:A23756G, ORF6:C27389T, ORF8:T27904C, ORF8:G28195T, N:G28881A, N:G28882A, N:G28883C, N:C29197T,	18	N:R203K, N:G204R, ORF1a:P959S, ORF1a:P1640S, ORF1a:T3255I, ORF1a:L3330S, ORF1a:I3618V, ORF1a:S3884L, ORF1a:T4175I, ORF1b:T42N, ORF1b:P314L, ORF8:L4P, ORF8:R101L, S:V367F, S:T478K, S:D614G, S:P681H, S:T732A,
hCoV-19/Mexico/QUE-InDRE_376/2021	EPI_ISL_1168454	20B	B.1.1.519	23	5'UTR:C203T, 5'UTR:C222T, 5'UTR:C241T, ORF1ab:C3037T, ORF1ab:C3140T, ORF1ab:G5012T, ORF1ab:C10029T, ORF1ab:A10829C, ORF1ab:C10954T, ORF1ab:A11117G, ORF1ab:C12789T, ORF1ab:C14408T, ORF1ab:T19839C, ORF1ab:C21306T, S:C22995A, S:A23403G, S:C23604A, S:A23756G, ORF8:T27904C, ORF8:C28087T, N:G28881A, N:G28882A, N:G28883C, N:C29197T,	15	N:R203K, N:G204R, ORF1a:P959S, ORF1a:V1583F, ORF1a:T3255I, ORF1a:I3522L, ORF1a:I3618V, ORF1a:T4175I, ORF1b:P314L, ORF8:L4P, ORF8:A65V, S:T478K, S:D614G, S:P681H, S:T732A,

hCoV-19/Mexico/QUE-InDRE_377/2021	EPI_ISL_1168455	20B	B.1.1.519	22	5'UTR:C203T, 5'UTR:C222T, 5'UTR:C241T, ORF1ab:C3037T, ORF1ab:C3140T, ORF1ab:C6401T, ORF1ab:C7732T, ORF1ab:C10029T, ORF1ab:C10954T, ORF1ab:A11117G, ORF1ab:C12789T, ORF1ab:C14408T, ORF1ab:T19839C, ORF1ab:C21306T, S:C22747T, S:C22995A, S:A23403G, S:C23604A, S:A23756G, N:G28881A, N:G28882A, N:G28883C, N:C29197T,	12	N:R203K, N:G204R, ORF1a:P959S, ORF1a:P2046S, ORF1a:T3255I, ORF1a:I3618V, ORF1a:T4175I, ORF1b:P314L, S:T478K, S:D614G, S:P681H, S:T732A,
hCoV-19/Mexico/QUE-InDRE_378/2021	EPI_ISL_1168456	20B	B.1.1.519	27	5'UTR:T201C, 5'UTR:C203T, 5'UTR:C222T, 5'UTR:C241T, ORF1ab:C539T, ORF1ab:G1738T, ORF1ab:C3037T, ORF1ab:C3140T, ORF1ab:G5617A, ORF1ab:C10029T, ORF1ab:C10954T, ORF1ab:A11117G, ORF1ab:C12789T, ORF1ab:C14189T, ORF1ab:C14408T, ORF1ab:C15857T, ORF1ab:T18417C, ORF1ab:T19839C, ORF1ab:A19974G, ORF1ab:C21306T, S:C22995A, S:A23403G, S:C23604A, S:A23756G, N:G28881A, N:G28882A, N:G28883C, N:C29197T,	14	N:R203K, N:G204R, ORF1a:L92F, ORF1a:P959S, ORF1a:T3255I, ORF1a:I3618V, ORF1a:T4175I, ORF1b:A241V, ORF1b:P314L, ORF1b:T797I, S:T478K, S:D614G, S:P681H, S:T732A,
hCoV-19/Mexico/QUE-InDRE_379/2021	EPI_ISL_1168457	20B	B.1.1.519	20	5'UTR:C203T, 5'UTR:C222T, 5'UTR:C241T, ORF1ab:C3037T, ORF1ab:C3140T, ORF1ab:C7047T, ORF1ab:C10029T, ORF1ab:C10954T, ORF1ab:A11117G, ORF1ab:C12789T, ORF1ab:C14408T, ORF1ab:T19839C, ORF1ab:C21306T, S:C22995A, S:A23403G, S:C23604A, S:A23756G, N:G28881A, N:G28882A, N:G28883C, N:C29197T,	12	N:R203K, N:G204R, ORF1a:P959S, ORF1a:S2261F, ORF1a:T3255I, ORF1a:I3618V, ORF1a:T4175I, ORF1b:P314L, S:T478K, S:D614G, S:P681H, S:T732A,
hCoV-19/Mexico/QUE-InDRE_380/2021	EPI_ISL_1168458	20B	B.1.1.519	26	5'UTR:C203T, 5'UTR:C222T, 5'UTR:C241T, ORF1ab:C1185T, ORF1ab:C3037T, ORF1ab:C3140T, ORF1ab:A4219G, ORF1ab:C10029T, ORF1ab:C10954T, ORF1ab:A11117G, ORF1ab:C12789T, ORF1ab:C14408T, ORF1ab:T16863C, ORF1ab:T19839C, ORF1ab:C21306T, ORF1ab:G21485T, S:C22995A, S:A23403G, S:C23604A, S:A23756G, ORF3a:C26198T, N:G28881A, N:G28882A, N:G28883C, N:C29197T, N:A29552G, 3'UTR:C29754T,	14	N:R203K, N:G204R, ORF1a:A307V, ORF1a:P959S, ORF1a:T3255I, ORF1a:I3618V, ORF1a:T4175I, ORF1b:P314L, ORF1b:S2673I, ORF3a:T269M, S:T478K, S:D614G, S:P681H, S:T732A,
hCoV-19/Mexico/QUE-InDRE_381/2021	EPI_ISL_1168459	20B	B.1.1.519	21	5'UTR:C203T, 5'UTR:C222T, 5'UTR:C241T, ORF1ab:C3037T, ORF1ab:C3140T, ORF1ab:G7027T, ORF1ab:C10029T, ORF1ab:C10954T, ORF1ab:A11117G, ORF1ab:C12789T, ORF1ab:C14408T, ORF1ab:T19839C, ORF1ab:C21306T, S:C22995A, S:A23403G, S:C23604A, S:A23756G, S:C25207T, N:G28881A, N:G28882A, N:G28883C, N:C29197T,	12	N:R203K, N:G204R, ORF1a:P959S, ORF1a:M2254I, ORF1a:T3255I, ORF1a:I3618V, ORF1a:T4175I, ORF1b:P314L, S:T478K, S:D614G, S:P681H, S:T732A,
hCoV-19/Mexico/QUE-InDRE_382/2021	EPI_ISL_1168460	20G	B.1.2	21	5'UTR:C241T, ORF1ab:C683T, ORF1ab:C1059T, ORF1ab:C3037T, ORF1ab:A6705G, ORF1ab:C10319T, ORF1ab:C13262T, ORF1ab:C14408T, ORF1ab:G15243T, ORF1ab:A15489G, ORF1ab:A18424G, ORF1ab:A20400G, ORF1ab:C21304T, S:A21923G, S:A23403G, S:C23604A, ORF3a:G25563T, ORF3a:G25907T, E:C26305T, ORF8:C27964T, N:C28472T, N:C28869T,	17	E:L21F, N:P67S, N:P199L, ORF1a:T265I, ORF1a:N2147S, ORF1a:L3352F, ORF1a:H4333Y, ORF1b:P314L, ORF1b:M592I, ORF1b:N1653D, ORF1b:R2613C, ORF3a:Q57H, ORF3a:G172V, ORF8:S24L, S:N121D, S:D614G, S:P681H,
hCoV-19/Mexico/QUE-InDRE_383/2021	EPI_ISL_1168461	20A	B.1	12	5'UTR:C241T, ORF1ab:G2498A, ORF1ab:C3037T, ORF1ab:G3239T, ORF1ab:C6285T, ORF1ab:C14408T, ORF1ab:T19506C, ORF1ab:T19839C, S:G22518C, S:A23403G, S:A23756G, ORF3a:G25447T, ORF3a:A25449T,	8	ORF1a:E745K, ORF1a:D992Y, ORF1a:T2007I, ORF1b:P314L, ORF3a:E19Y, S:R319T, S:D614G, S:T732A,

hCoV-19/Mexico/QUE-InDRE_384/2021	EPI_ISL_1168462	20B	B.1.1.519	23	5'UTR:C203T, 5'UTR:C222T, 5'UTR:C241T, 5'UTR:A253G, ORF1ab:G2281A, ORF1ab:C3037T, ORF1ab:C3140T, ORF1ab:G6008A, ORF1ab:C10029T, ORF1ab:C10954T, ORF1ab:A11117G, ORF1ab:C12789T, ORF1ab:C14408T, ORF1ab:C17410T, ORF1ab:T19839C, ORF1ab:C21306T, S:C22995A, S:A23403G, S:C23604A, S:A23756G, N:G28881A, N:G28882A, N:G28883C, N:C29197T,	13	N:R203K, N:G204R, ORF1a:P959S, ORF1a:V1915I, ORF1a:T3255I, ORF1a:I3618V, ORF1a:T4175I, ORF1b:P314L, ORF1b:R1315C, S:T478K, S:D614G, S:P681H, S:T732A,
hCoV-19/Mexico/QUE-InDRE_385/2021	EPI_ISL_1168463	21C (Epsilon)	B.1.429	27	5'UTR:C241T, ORF1ab:C1059T, ORF1ab:C2395T, ORF1ab:C2453T, ORF1ab:T2597C, ORF1ab:C3037T, ORF1ab:C8123T, ORF1ab:C8947T, ORF1ab:T11038C, ORF1ab:C12100T, ORF1ab:A12878G, ORF1ab:C14408T, ORF1ab:G17014T, ORF1ab:C19032T, ORF1ab:A19476G, S:G21600T, S:C21638T, S:G22018T, S:T22917G, S:A23403G, S:T24349C, ORF3a:G25563T, M:C26681T, ORF7b:G27890T, ORF8:G27987T, ORF8:A28272T, N:C28887T, N:C29362T,	14	N:T205I, ORF1a:T265I, ORF1a:L730F, ORF1a:L2620F, ORF1a:I4205V, ORF1b:P314L, ORF1b:D1183Y, ORF3a:Q57H, ORF8:V32L, S:S13I, S:P26S, S:W152C, S:L452R, S:D614G,
hCoV-19/Mexico/QUE-InDRE_386/2021	EPI_ISL_1168464	20B	B.1.1.519	27	5'UTR:T201C, 5'UTR:C203T, 5'UTR:C222T, 5'UTR:C241T, ORF1ab:G1738T, ORF1ab:C3037T, ORF1ab:C3140T, ORF1ab:C10029T, ORF1ab:A10761G, ORF1ab:C10954T, ORF1ab:A11117G, ORF1ab:C12789T, ORF1ab:C14408T, ORF1ab:C15237T, ORF1ab:T19839C, ORF1ab:A19974G, ORF1ab:C20762T, ORF1ab:C21306T, S:C22995A, S:A23403G, S:C23604A, S:A23756G, ORF3a:G25699A, ORF7a:C27612T, N:G28881A, N:G28882A, N:G28883C, N:C29197T,	14	N:R203K, N:G204R, ORF1a:P959S, ORF1a:T3255I, ORF1a:K3499R, ORF1a:I3618V, ORF1a:T4175I, ORF1b:P314L, ORF1b:T2432I, ORF3a:A103T, S:T478K, S:D614G, S:P681H, S:T732A,
hCoV-19/Mexico/QUE-InDRE_387/2021	EPI_ISL_1168465	20B	B.1.1.519	32	5'UTR:T201C, 5'UTR:C203T, 5'UTR:C222T, 5'UTR:C241T, ORF1ab:C936T, ORF1ab:G1738T, ORF1ab:C3037T, ORF1ab:C3140T, ORF1ab:G7042T, ORF1ab:C7521T, ORF1ab:C10029T, ORF1ab:C10954T, ORF1ab:A11117G, ORF1ab:A12174G, ORF1ab:C12357T, ORF1ab:C12789T, ORF1ab:C14408T, ORF1ab:A18504G, ORF1ab:T19839C, ORF1ab:A19974G, ORF1ab:C21306T, S:C22995A, S:A23403G, S:C23604A, S:G23608T, S:A23756G, ORF3a:G26062T, ORF7a:C27493T, N:G28881A, N:G28882A, N:G28883C, N:C29197T, N:G29227T,	18	N:R203K, N:G204R, ORF1a:T224I, ORF1a:P959S, ORF1a:M2259I, ORF1a:T2419I, ORF1a:T3255I, ORF1a:I3618V, ORF1a:N3970S, ORF1a:T4031I, ORF1a:T4175I, ORF1b:P314L, ORF3a:G224C, ORF7a:P34S, S:T478K, S:D614G, S:P681H, S:T732A,
hCoV-19/Mexico/QUE-InDRE_388/2021	EPI_ISL_1168466	20B	B.1.1.222	16	5'UTR:C241T, ORF1ab:G463A, ORF1ab:C3037T, ORF1ab:C6336T, ORF1ab:C10029T, ORF1ab:C14408T, ORF1ab:C19011A, ORF1ab:T19839C, S:A23403G, S:A23756G, ORF8:A27921G, ORF8:G28001T, N:G28881A, N:G28882A, N:G28883C, N:T29161C, N:C29363T,	10	N:R203K, N:G204R, N:P364S, ORF1a:S2024L, ORF1a:T3255I, ORF1b:P314L, ORF1b:D1848E, ORF8:I10V, S:D614G, S:T732A,
hCoV-19/Mexico/QUE-InDRE_401/2020	EPI_ISL_1054971	20A	B.1	11	5'UTR:C241T, ORF1ab:T1237C, ORF1ab:C2340T, ORF1ab:C3037T, ORF1ab:C3768T, ORF1ab:C14408T, ORF1ab:C16329T, S:A23403G, S:G23587T, N:G28655T, N:C28887T, 3'UTR:G29773T,	7	N:D128Y, N:T205I, ORF1a:S692F, ORF1a:T1168I, ORF1b:P314L, S:D614G, S:Q675H,
hCoV-19/Mexico/QUE-InDRE_402/2020	EPI_ISL_1054972	20A	B.1.609	10	5'UTR:C241T, ORF1ab:C3037T, ORF1ab:C4582T, ORF1ab:C11514T, ORF1ab:C13860T, ORF1ab:C14408T, ORF1ab:G16707T, ORF1ab:A20268G, ORF1ab:C21306T, S:A23403G, S:C23604A,	4	ORF1a:T3750I, ORF1b:P314L, S:D614G, S:P681H,

hCoV-19/Mexico/QUE-InDRE_473/2021	EPI_ISL_1168543	20B	B.1.1.519	20	5'UTR:C203T, 5'UTR:C222T, 5'UTR:C241T, ORF1ab:C307T, ORF1ab:C3037T, ORF1ab:C3140T, ORF1ab:C9430T, ORF1ab:C10029T, ORF1ab:C10954T, ORF1ab:A11117G, ORF1ab:C14408T, ORF1ab:T19839C, ORF1ab:C21306T, S:C22995A, S:A23403G, S:C23604A, S:A23756G, N:G28881A, N:G28882A, N:G28883C. N:C29197T.	10	N:R203K, N:G204R, ORF1a:P959S, ORF1a:T3255I, ORF1a:I3618V, ORF1b:P314L, S:T478K, S:D614G, S:P681H, S:T732A,
hCoV-19/Mexico/QUE-InDRE_474/2021	EPI_ISL_1168544	20B	B.1	22	5'UTR:C203T, 5'UTR:C222T, 5'UTR:C241T, ORF1ab:C3037T, ORF1ab:C3140T, ORF1ab:C5219T, ORF1ab:T9070C, ORF1ab:G9425T, ORF1ab:C10029T, ORF1ab:C10954T, ORF1ab:A11117G, ORF1ab:C14408T, ORF1ab:T19839C, ORF1ab:C20233T, ORF1ab:C21306T, S:C22995A, S:A23403G, S:C23604A, S:A23756G, N:G28881A, N:G28882A, N:C29197T, N:G29527T,	13	N:R203K, N:Q418H, ORF1a:P959S, ORF1a:H1652Y, ORF1a:A3054S, ORF1a:T3255I, ORF1a:I3618V, ORF1b:P314L, ORF1b:P2256S, S:T478K, S:D614G, S:P681H, S:T732A,
hCoV-19/Mexico/QUE-InDRE_475/2021	EPI_ISL_1168545	20B	B.1.1.519	27	5'UTR:T201C, 5'UTR:C203T, 5'UTR:C222T, 5'UTR:C241T, ORF1ab:C936T, ORF1ab:G1738T, ORF1ab:C3037T, ORF1ab:C3140T, ORF1ab:C10029T, ORF1ab:C10954T, ORF1ab:A11117G, ORF1ab:C14408T, ORF1ab:T19839C, ORF1ab:A19974G, ORF1ab:C21306T, S:T21835C, S:C22995A, S:A23403G, S:C23604A, S:A23756G, ORF3a:A25524C, M:C26801T, N:G28881A, N:G28882A, N:G28883C, N:G28968T, N:C29197T, N:G29227T,	12	N:R203K, N:G204R, N:S232I, ORF1a:T224I, ORF1a:P959S, ORF1a:T3255I, ORF1a:I3618V, ORF1b:P314L, S:T478K, S:D614G, S:P681H, S:T732A,
hCoV-19/Mexico/QUE-InDRE_476/2021	EPI_ISL_1168546	20A	B.1.243	19	5'UTR:C241T, ORF1ab:C1489T, ORF1ab:C3037T, ORF1ab:C3093T, ORF1ab:A3237T, ORF1ab:G3838A, ORF1ab:G3994T, ORF1ab:G3995A, ORF1ab:C11074T, ORF1ab:C14408T, ORF1ab:C17470T, ORF1ab:A20268G, ORF1ab:C20405T, S:A22798T, S:A23403G, S:T24076C, ORF3a:G25906C, ORF3a:A26174G, M:C26801T. N:C28854T.	9	N:S194L, ORF1a:P943L, ORF1a:Q991L, ORF1a:E1244K, ORF1b:P314L, ORF1b:P2313L, ORF3a:G172R, ORF3a:E261G, S:D614G,
hCoV-19/Mexico/QUE-InDRE_477/2021	EPI_ISL_1168547	20G	B.1.2	20	5'UTR:C241T, ORF1ab:C1059T, ORF1ab:G2243A, ORF1ab:C3037T, ORF1ab:C10319T, ORF1ab:G10396T, ORF1ab:C12823T, ORF1ab:A13209C, ORF1ab:C14408T, ORF1ab:G16396T, ORF1ab:A18424G, ORF1ab:G20060T, ORF1ab:C21304T, S:G23282T, S:A23403G, ORF3a:G25563T, ORF3a:G25644T, ORF3a:G25907T, ORF8:C27964T, N:C28472T, N:C28869T.	16	N:P67S, N:P199L, ORF1a:T265I, ORF1a:V660I, ORF1a:L3352F, ORF1a:N4315T, ORF1b:P314L, ORF1b:G977C, ORF1b:N1653D, ORF1b:S2198I, ORF1b:R2613C, ORF3a:Q57H, ORF3a:G172V, ORF8:S24L, S:D574Y, S:D614G,
hCoV-19/Mexico/QUE-InDRE_478/2021	EPI_ISL_1168548	20A	B.1	11	5'UTR:C241T, ORF1ab:C1613T, ORF1ab:C5005T, ORF1ab:A11782G, ORF1ab:C14408T, ORF1ab:C16332T, ORF1ab:C18508T, ORF1ab:A20268G, ORF1ab:A20985G, S:A23403G, M:C26606T, N:C28854T.	5	N:S194L, ORF1a:L450F, ORF1b:P314L, ORF1b:L1681F, S:D614G,
hCoV-19/Mexico/QUE-InDRE_479/2021	EPI_ISL_1168549	20B	B.1.1.519	23	5'UTR:T201C, 5'UTR:C203T, 5'UTR:C222T, 5'UTR:C241T, ORF1ab:G1738T, ORF1ab:C3037T, ORF1ab:C3140T, ORF1ab:C10029T, ORF1ab:C10954T, ORF1ab:A11117G, ORF1ab:C14408T, ORF1ab:T19839C, ORF1ab:A19974G, ORF1ab:C21057T, ORF1ab:C21306T, S:C22995A, S:A23403G, S:C23604A, S:A23756G, ORF3a:C25782T, N:G28881A. N:G28882A. N:G28883C. N:C29197T.	10	N:R203K, N:G204R, ORF1a:P959S, ORF1a:T3255I, ORF1a:I3618V, ORF1b:P314L, S:T478K, S:D614G, S:P681H, S:T732A,

hCoV-19/Mexico/QUE-InDRE_480/2021	EPI_ISL_1168550	20B	B.1.1.519	26	5'UTR:C203T, 5'UTR:C222T, 5'UTR:C241T, ORF1ab:C1342T, ORF1ab:C3037T, ORF1ab:C3140T, ORF1ab:G3692T, ORF1ab:G3871T, ORF1ab:C3874T, ORF1ab:C10029T, ORF1ab:C10954T, ORF1ab:A11117G, ORF1ab:C11824A, ORF1ab:C14408T, ORF1ab:T19839C, ORF1ab:C21306T, S:C21952T, S:T22258C, S:C22995A, S:A23403G, S:C23604A, S:A23756G, ORF8:T27904C, N:G28881A, N:G28882A, N:G28883C, N:C29358T,	14	N:R203K, N:G204R, N:T362I, ORF1a:P959S, ORF1a:V1143F, ORF1a:K1202N, ORF1a:T3255I, ORF1a:I3618V, ORF1b:P314L, ORF8:L4P, S:T478K, S:D614G, S:P681H, S:T732A,
hCoV-19/Mexico/QUE-InDRE_540/2021	EPI_ISL_1168610	20A	B.1.243	20	5'UTR:C241T, ORF1ab:C1471T, ORF1ab:C3037T, ORF1ab:C10456T, ORF1ab:T12391C, ORF1ab:A12556G, ORF1ab:C12710T, ORF1ab:C14408T, ORF1ab:C15952T, ORF1ab:C19097T, ORF1ab:A19137G, ORF1ab:T19330G, ORF1ab:A19410T, ORF1ab:G19435T, ORF1ab:A20268G, S:A23403G, S:C23604A, S:T24076C, N:C28854T, N:A28972G, 3'UTR:T29710C,	8	N:S194L, ORF1b:P314L, ORF1b:A1877V, ORF1b:F1955V, ORF1b:K1981N, ORF1b:V1990L, S:D614G, S:P681H,
hCoV-19/Mexico/QUE-InDRE_554/2021	EPI_ISL_1168624	20B	B.1.1.519	27	5'UTR:T201C, 5'UTR:C203T, 5'UTR:C222T, 5'UTR:C241T, ORF1ab:C936T, ORF1ab:G1738T, ORF1ab:C2102T, ORF1ab:C3037T, ORF1ab:C3140T, ORF1ab:C10029T, ORF1ab:C10954T, ORF1ab:C10969T, ORF1ab:A11117G, ORF1ab:C12789T, ORF1ab:C14408T, ORF1ab:T19839C, ORF1ab:A19974G, ORF1ab:C21306T, S:C22995A, S:A23403G, S:C23604A, S:A23756G, S:T25375C, N:G28881A, N:G28882A, N:G28883C, N:C29197T, N:G29227T,	12	N:R203K, N:G204R, ORF1a:T224I, ORF1a:P959S, ORF1a:T3255I, ORF1a:I3618V, ORF1a:T4175I, ORF1b:P314L, S:T478K, S:D614G, S:P681H, S:T732A,
hCoV-19/Mexico/QUE-InDRE_555/2021	EPI_ISL_1168625	20G	B.1.596	24	5'UTR:C241T, ORF1ab:G598T, ORF1ab:C629T, ORF1ab:C1059T, ORF1ab:A1903G, ORF1ab:T2047C, ORF1ab:T2112C, ORF1ab:C3037T, ORF1ab:C10319T, ORF1ab:C11322T, ORF1ab:C14024T, ORF1ab:C14408T, ORF1ab:C15960T, ORF1ab:A18424G, ORF1ab:C21304T, S:A22231G, S:G22661T, S:A23403G, ORF3a:G25563T, ORF3a:G25907T, ORF3a:T26099G, E:T26479C, ORF8:C27964T, N:C28472T, N:C28869T,	17	N:P67S, N:P199L, ORF1a:L122F, ORF1a:T265I, ORF1a:I616T, ORF1a:L3352F, ORF1a:A3686V, ORF1b:A186V, ORF1b:P314L, ORF1b:N1653D, ORF1b:R2613C, ORF3a:Q57H, ORF3a:G172V, ORF3a:I236S, ORF8:S24L, S:V367F, S:D614G,
hCoV-19/Mexico/QUE-InDRE_556/2021	EPI_ISL_1168626	20B	B.1.1.519	25	5'UTR:T201C, 5'UTR:C203T, 5'UTR:C222T, 5'UTR:C241T, ORF1ab:G1738T, ORF1ab:C3037T, ORF1ab:C3140T, ORF1ab:C10029T, ORF1ab:C10954T, ORF1ab:A11117G, ORF1ab:C12789T, ORF1ab:C14408T, ORF1ab:T19839C, ORF1ab:A19974G, ORF1ab:C21057T, ORF1ab:C21306T, S:C22467A, S:C22995A, S:A23403G, S:C23604A, S:A23756G, ORF3a:C25782T, N:G28881A, N:G28882A, N:G28883C, N:C29197T,	12	N:R203K, N:G204R, ORF1a:P959S, ORF1a:T3255I, ORF1a:I3618V, ORF1a:T4175I, ORF1b:P314L, S:T302K, S:T478K, S:D614G, S:P681H, S:T732A,
hCoV-19/Mexico/QUE-InDRE_557/2021	EPI_ISL_1168627	20B	B.1.1.519	22	5'UTR:C203T, 5'UTR:C222T, 5'UTR:C241T, ORF1ab:C3037T, ORF1ab:C3140T, ORF1ab:C10029T, ORF1ab:T10363C, ORF1ab:C10954T, ORF1ab:A11117G, ORF1ab:C12789T, ORF1ab:C14408T, ORF1ab:T18495C, ORF1ab:T19839C, ORF1ab:G20167T, ORF1ab:C21306T, S:C22995A, S:A23403G, S:C23604A, S:A23756G, N:G28881A, N:G28882A, N:G28883C, N:C29197T,	12	N:R203K, N:G204R, ORF1a:P959S, ORF1a:T3255I, ORF1a:I3618V, ORF1a:T4175I, ORF1b:P314L, ORF1b:D2234Y, S:T478K, S:D614G, S:P681H, S:T732A,

hCoV-19/Mexico/QUE-InDRE_558/2021	EPI_ISL_1168628	20B	B.1.1.519	31	5'UTR:C203T, 5'UTR:C222T, 5'UTR:C241T, ORF1ab:C3037T, ORF1ab:C3140T, ORF1ab:C10029T, ORF1ab:C10954T, ORF1ab:A11117G, ORF1ab:A12585T, ORF1ab:C12789T, ORF1ab:C14408T, ORF1ab:G15921T, ORF1ab:A17971G, ORF1ab:T19839C, ORF1ab:G19997C, ORF1ab:T20166G, ORF1ab:C21306T, S:G22493T, S:T22521G, S:C22995A, S:A23403G, S:C23604A, S:C23635T, S:A23756G, S:C24337T, M:G26828T, N:G28881A, N:G28882A, N:G28883C, N:C29197T, 3'UTR:G29862T, 3'UTR:A29866C	16	N:R203K, N:G204R, ORF1a:P959S, ORF1a:T3255I, ORF1a:I3618V, ORF1a:K4107I, ORF1a:T4175I, ORF1b:P314L, ORF1b:R1502G, ORF1b:R2177T, S:G311*, S:V320G, S:T478K, S:D614G, S:P681H, S:T732A,
hCoV-19/Mexico/QUE-InDRE_559/2021	EPI_ISL_1168629	20A	B.1.396	26	5'UTR:C241T, ORF1ab:C1191T, ORF1ab:T1459G, ORF1ab:C1627T, ORF1ab:C2455T, ORF1ab:C3037T, ORF1ab:A3989C, ORF1ab:A5706G, ORF1ab:C9521A, ORF1ab:T10480C, ORF1ab:G10974A, ORF1ab:G11330T, ORF1ab:G11548C, ORF1ab:G11550C, ORF1ab:G14187A, ORF1ab:C14408T, ORF1ab:C15720T, ORF1ab:G18394T, ORF1ab:A20268G, S:A23403G, S:C24904T, N:C28854T, N:G29422T, ORF10:A29567G, ORF10:T29597C, 3'UTR:G29862T, 3'UTR:A29866C,	12	N:S194L, ORF1a:P309L, ORF1a:T1242P, ORF1a:Y1814C, ORF1a:L3086I, ORF1a:S3570N, ORF1a:V3689L, ORF1a:M3761I, ORF1a:C3762S, ORF1b:P314L, ORF1b:A1643S, S:D614G,
hCoV-19/Mexico/QUE-InDRE_560/2021	EPI_ISL_1168630	20B	B.1.1.316	19	5'UTR:C106T, 5'UTR:C222T, 5'UTR:C241T, ORF1ab:C3037T, ORF1ab:C3411T, ORF1ab:C4586T, ORF1ab:A12519G, ORF1ab:C12973T, ORF1ab:C12974T, ORF1ab:C14408T, ORF1ab:T19839C, S:C21757T, S:A23403G, S:G23593C, S:A23756T, N:G28881A, N:G28882A, N:G28883C, 3'UTR:G29737T, 3'UTR:G29751T,	8	N:R203K, N:G204R, ORF1a:A1049V, ORF1a:D4085G, ORF1b:P314L, S:D614G, S:Q677H, S:T732S,
hCoV-19/Mexico/QUE-InDRE_561/2021	EPI_ISL_1168631	20B	B.1.1.519	22	5'UTR:C203T, 5'UTR:C222T, 5'UTR:C241T, ORF1ab:C3037T, ORF1ab:C3140T, ORF1ab:C4893A, ORF1ab:C5974T, ORF1ab:C6449T, ORF1ab:C10029T, ORF1ab:C10954T, ORF1ab:A11117G, ORF1ab:C14408T, ORF1ab:G15921T, ORF1ab:T19839C, ORF1ab:C21306T, S:C22995A, S:A23403G, S:C23604A, S:A23756G, N:G28881A, N:G28882A, N:G28883C, N:C29197T,	12	N:R203K, N:G204R, ORF1a:P959S, ORF1a:T1543K, ORF1a:L2062F, ORF1a:T3255I, ORF1a:I3618V, ORF1b:P314L, S:T478K, S:D614G, S:P681H, S:T732A,
hCoV-19/Mexico/QUE-InDRE_562/2021	EPI_ISL_1168632	20B	B.1.1.519	21	5'UTR:C203T, 5'UTR:C222T, 5'UTR:C241T, ORF1ab:C3037T, ORF1ab:C3140T, ORF1ab:C3311T, ORF1ab:A8031G, ORF1ab:C10029T, ORF1ab:C10954T, ORF1ab:A11117G, ORF1ab:C14408T, ORF1ab:T19839C, ORF1ab:C21306T, S:C22995A, S:A23403G, S:C23604A, S:A23756G, N:G28881A, N:G28882A, N:G28883C, N:C29197T, 3'UTR:A29871C,	12	N:R203K, N:G204R, ORF1a:P959S, ORF1a:L1016F, ORF1a:K2589R, ORF1a:T3255I, ORF1a:I3618V, ORF1b:P314L, S:T478K, S:D614G, S:P681H, S:T732A,
hCoV-19/Mexico/QUE-InDRE_563/2021	EPI_ISL_1168633	20B	B.1.1.519	23	5'UTR:C203T, 5'UTR:C222T, 5'UTR:C241T, ORF1ab:C3037T, ORF1ab:C3140T, ORF1ab:C10029T, ORF1ab:A10558T, ORF1ab:C10954T, ORF1ab:A11117G, ORF1ab:C14408T, ORF1ab:T19839C, ORF1ab:C21306T, S:C22995A, S:A23403G, S:C23604A, S:A23756G, M:G26814T, ORF7a:C27673A, N:G28881A, N:G28882A, N:G28883C, N:G28895C, N:C29197T, 3'UTR:C29870A,	13	M:A98S, N:R203K, N:G204R, N:A208P, ORF1a:P959S, ORF1a:T3255I, ORF1a:I3618V, ORF1b:P314L, ORF7a:Q94K, S:T478K, S:D614G, S:P681H, S:T732A,

hCoV-19/Mexico/QUE-InDRE_F10573_S718/2021	EPI_ISL_1366667	20B	B.1.1.519	29	5'UTR:C203T, 5'UTR:C222T, 5'UTR:C241T, ORF1ab:C1342T, ORF1ab:C3037T, ORF1ab:C3140T, ORF1ab:G3692T, ORF1ab:G3871T, ORF1ab:C10029T, ORF1ab:C10954T, ORF1ab:A11117G, ORF1ab:C11824A, ORF1ab:G12098A, ORF1ab:C12789T, ORF1ab:C14408T, ORF1ab:C16017T, ORF1ab:T19839C, ORF1ab:C21306T, S:C22995A, S:A23403G, S:C23604A, S:A23756G, ORF3a:G25855T, ORF8:T27904C, ORF8:C28253T, N:G28881A, N:G28882A, N:G28883C, N:C29197T, 3'UTR:A29716G,	16	N:R203K, N:G204R, ORF1a:P959S, ORF1a:V1143F, ORF1a:K1202N, ORF1a:T3255I, ORF1a:I3618V, ORF1a:A3945T, ORF1a:T4175I, ORF1b:P314L, ORF3a:D155Y, ORF8:L4P, S:T478K, S:D614G, S:P681H, S:T732A,
hCoV-19/Mexico/QUE-InDRE_F10577_S719/2021	EPI_ISL_1366668	20B	B.1.1.519	28	5'UTR:C190T, 5'UTR:C203T, 5'UTR:C222T, 5'UTR:C241T, ORF1ab:A866G, ORF1ab:C3037T, ORF1ab:C3140T, ORF1ab:C5183T, ORF1ab:C5284T, ORF1ab:C10029T, ORF1ab:C10954T, ORF1ab:A11117G, ORF1ab:C11916T, ORF1ab:C12789T, ORF1ab:C14408T, ORF1ab:T19839C, ORF1ab:C21306T, S:G22241T, S:C22995A, S:G23012C, S:A23403G, S:C23604A, S:A23756G, M:G26840A, ORF8:T27904C, N:G28881A, N:G28882A, N:G28883C, N:C29197T,	17	N:R203K, N:G204R, ORF1a:I201V, ORF1a:P959S, ORF1a:P1640S, ORF1a:T3255I, ORF1a:I3618V, ORF1a:S3884L, ORF1a:T4175I, ORF1b:P314L, ORF8:L4P, S:V227L, S:T478K, S:E484Q, S:D614G, S:P681H, S:T732A,
hCoV-19/Mexico/QUE-InDRE_F10583_S720/2021	EPI_ISL_1366669	20B	B.1.1.519	23	5'UTR:C203T, 5'UTR:C222T, 5'UTR:C241T, ORF1ab:C3037T, ORF1ab:C3140T, ORF1ab:C10029T, ORF1ab:C10954T, ORF1ab:A11117G, ORF1ab:C12789T, ORF1ab:C14408T, ORF1ab:T19839C, ORF1ab:C21077T, ORF1ab:C21306T, S:C22995A, S:A23403G, S:C23604A, S:A23756G, ORF3a:G25906T, ORF8:C28253T, N:G28881A, N:G28882A, N:G28883C, N:C29197T, N:G29440T,	14	N:R203K, N:G204R, N:Q389H, ORF1a:P959S, ORF1a:T3255I, ORF1a:I3618V, ORF1a:T4175I, ORF1b:P314L, ORF1b:T2537I, ORF3a:G172C, S:T478K, S:D614G, S:P681H, S:T732A,
hCoV-19/Mexico/QUE-InDRE_F10593_S814/2021	EPI_ISL_1424016	20B	B.1.1.519	23	5'UTR:C203T, 5'UTR:C222T, 5'UTR:C241T, ORF1ab:C3037T, ORF1ab:C3140T, ORF1ab:G7027T, ORF1ab:C10029T, ORF1ab:C10954T, ORF1ab:A11117G, ORF1ab:C12789T, ORF1ab:C14408T, ORF1ab:C18486T, ORF1ab:T19839C, ORF1ab:C21306T, S:C22995A, S:A23403G, S:C23604A, S:A23756G, S:C25207T, N:C28603T, N:G28881A, N:G28882A, N:G28883C, N:C29197T,	12	N:R203K, N:G204R, ORF1a:P959S, ORF1a:M2254I, ORF1a:T3255I, ORF1a:I3618V, ORF1a:T4175I, ORF1b:P314L, S:T478K, S:D614G, S:P681H, S:T732A,
hCoV-19/Mexico/QUE-InDRE_F10598_S721/2021	EPI_ISL_1366670	20B	B.1.1.519	21	5'UTR:C203T, 5'UTR:C222T, 5'UTR:C241T, ORF1ab:G2281A, ORF1ab:C3037T, ORF1ab:C3140T, ORF1ab:G6008A, ORF1ab:C10029T, ORF1ab:C10954T, ORF1ab:A11117G, ORF1ab:C12789T, ORF1ab:C14408T, ORF1ab:T19839C, ORF1ab:C21306T, S:C22995A, S:A23403G, S:C23604A, S:A23756G, N:G28881A, N:G28882A, N:G28883C, N:C29197T,	12	N:R203K, N:G204R, ORF1a:P959S, ORF1a:V1915I, ORF1a:T3255I, ORF1a:I3618V, ORF1a:T4175I, ORF1b:P314L, S:T478K, S:D614G, S:P681H, S:T732A,
hCoV-19/Mexico/QUE-InDRE_F10599_S722/2021	EPI_ISL_1366671	20B	B.1.1.519	23	5'UTR:C203T, 5'UTR:C222T, 5'UTR:C241T, ORF1ab:C3037T, ORF1ab:C3140T, ORF1ab:C10029T, ORF1ab:C10954T, ORF1ab:A11117G, ORF1ab:C12789T, ORF1ab:C14408T, ORF1ab:T19839C, ORF1ab:C21077T, ORF1ab:C21306T, S:C22995A, S:A23403G, S:C23604A, S:A23756G, ORF3a:G25906T, ORF8:C28253T, N:G28881A, N:G28882A, N:G28883C, N:C29197T, N:G29440T,	14	N:R203K, N:G204R, N:Q389H, ORF1a:P959S, ORF1a:T3255I, ORF1a:I3618V, ORF1a:T4175I, ORF1b:P314L, ORF1b:T2537I, ORF3a:G172C, S:T478K, S:D614G, S:P681H, S:T732A,

hCoV-19/Mexico/QUE-InDRE_F10602_S723/2021	EPI_ISL_1366672	20B	B.1.1.519	22	5'UTR:C203T, 5'UTR:C222T, 5'UTR:C241T, ORF1ab:G2281A, ORF1ab:C3037T, ORF1ab:C3140T, ORF1ab:G6008A, ORF1ab:C10029T, ORF1ab:C10954T, ORF1ab:A11117G, ORF1ab:C12115T, ORF1ab:C12789T, ORF1ab:C14408T, ORF1ab:T19839C, ORF1ab:C21306T, S:C22995A, S:A23403G, S:C23604A, S:A23756G, N:G28881A, N:G28882A, N:G28883C, N:C29197T,	12	N:R203K, N:G204R, ORF1a:P959S, ORF1a:V1915I, ORF1a:T3255I, ORF1a:I3618V, ORF1a:T4175I, ORF1b:P314L, S:T478K, S:D614G, S:P681H, S:T732A,
hCoV-19/Mexico/QUE-InDRE_F10603_S724/2021	EPI_ISL_1366673	20B	B.1.1.519	25	5'UTR:C203T, 5'UTR:C222T, 5'UTR:C241T, ORF1ab:C3037T, ORF1ab:C3140T, ORF1ab:C6471A, ORF1ab:C10029T, ORF1ab:C10954T, ORF1ab:A11117G, ORF1ab:C12789T, ORF1ab:C14408T, ORF1ab:T19839C, ORF1ab:C21306T, S:C21844T, S:C22281T, S:C22995A, S:A23403G, S:C23604A, S:A23756G, ORF3a:G25429A, ORF8:T27904C, ORF8:C28087T, N:G28881A, N:G28882A, N:G28883C, N:C29197T,	16	N:R203K, N:G204R, ORF1a:P959S, ORF1a:T2069N, ORF1a:T3255I, ORF1a:I3618V, ORF1a:T4175I, ORF1b:P314L, ORF3a:V13I, ORF8:L4P, ORF8:A65V, S:T240I, S:T478K, S:D614G, S:P681H, S:T732A,
hCoV-19/Mexico/QUE-InDRE_F10607_S725/2021	EPI_ISL_1366674	20B	B.1.1.519	24	5'UTR:C203T, 5'UTR:C222T, 5'UTR:C241T, ORF1ab:C3037T, ORF1ab:C3140T, ORF1ab:C10029T, ORF1ab:C10954T, ORF1ab:A11117G, ORF1ab:C12789T, ORF1ab:C14408T, ORF1ab:T19839C, ORF1ab:C21306T, S:C21844T, S:C22281T, S:C22995A, S:A23403G, S:C23604A, S:A23756G, ORF3a:G25429A, ORF8:T27904C, ORF8:C28087T, N:G28881A, N:G28882A, N:G28883C, N:C29197T,	15	N:R203K, N:G204R, ORF1a:P959S, ORF1a:T3255I, ORF1a:I3618V, ORF1a:T4175I, ORF1b:P314L, ORF3a:V13I, ORF8:L4P, ORF8:A65V, S:T240I, S:T478K, S:D614G, S:P681H, S:T732A,
hCoV-19/Mexico/QUE-InDRE_F10608_S726/2021	EPI_ISL_1366675	20B	B.1.1.519	21	5'UTR:C203T, 5'UTR:C222T, 5'UTR:C241T, ORF1ab:G2281A, ORF1ab:C3037T, ORF1ab:C3140T, ORF1ab:G6008A, ORF1ab:C10029T, ORF1ab:C10954T, ORF1ab:A11117G, ORF1ab:C12789T, ORF1ab:C14408T, ORF1ab:T19839C, ORF1ab:C21306T, S:C22995A, S:A23403G, S:C23604A, S:A23756G, N:G28881A, N:G28882A, N:G28883C, N:C29197T,	12	N:R203K, N:G204R, ORF1a:P959S, ORF1a:V1915I, ORF1a:T3255I, ORF1a:I3618V, ORF1a:T4175I, ORF1b:P314L, S:T478K, S:D614G, S:P681H, S:T732A,
hCoV-19/Mexico/QUE-InDRE_F10614_S727/2021	EPI_ISL_1366676	20B	B.1.1.519	22	5'UTR:C203T, 5'UTR:C222T, 5'UTR:C241T, ORF1ab:C3037T, ORF1ab:C3140T, ORF1ab:C4404T, ORF1ab:C10029T, ORF1ab:C10954T, ORF1ab:A11117G, ORF1ab:C12789T, ORF1ab:C14408T, ORF1ab:T16863C, ORF1ab:T19839C, ORF1ab:C21306T, S:C22995A, S:A23403G, S:C23604A, S:A23756G, N:G28881A, N:G28882A, N:G28883C, N:C29197T, 3'UTR:C29754T,	12	N:R203K, N:G204R, ORF1a:P959S, ORF1a:A1380V, ORF1a:T3255I, ORF1a:I3618V, ORF1a:T4175I, ORF1b:P314L, S:T478K, S:D614G, S:P681H, S:T732A,
hCoV-19/Mexico/QUE-InDRE_F11013_S820/2021	EPI_ISL_1424022	20B	B.1.1.519	26	5'UTR:C203T, 5'UTR:C222T, 5'UTR:C241T, ORF1ab:C3037T, ORF1ab:C3140T, ORF1ab:C6196T, ORF1ab:C10029T, ORF1ab:C10954T, ORF1ab:A11117G, ORF1ab:C12789T, ORF1ab:C14408T, ORF1ab:T19839C, ORF1ab:C21306T, S:C22995A, S:A23403G, S:C23604A, S:A23756G, S:C24865T, M:C26885T, ORF8:T27904C, ORF8:C27920T, ORF8:C28087T, N:C28377T, N:G28881A, N:G28882A, N:G28883C, N:C29197T,	15	N:A35V, N:R203K, N:G204R, ORF1a:P959S, ORF1a:T3255I, ORF1a:I3618V, ORF1a:T4175I, ORF1b:P314L, ORF8:L4P, ORF8:A65V, ORF9b:R32C, S:T478K, S:D614G, S:P681H, S:T732A,
hCoV-19/Mexico/QUE-InDRE_F11015_S821/2021	EPI_ISL_1424023	20B	B.1.1.519	23	5'UTR:C203T, 5'UTR:C222T, 5'UTR:C241T, ORF1ab:G2273A, ORF1ab:C3037T, ORF1ab:C3140T, ORF1ab:G3871T, ORF1ab:C10029T, ORF1ab:C10954T, ORF1ab:A11117G, ORF1ab:C11824A, ORF1ab:C12789T, ORF1ab:C14408T, ORF1ab:G18651T, ORF1ab:T19839C, ORF1ab:C21306T, S:A23403G, S:C23604A, S:A23756G, ORF8:T27904C, N:G28881A, N:G28882A, N:G28883C, N:C29197T,	14	N:R203K, N:G204R, ORF1a:E670K, ORF1a:P959S, ORF1a:K1202N, ORF1a:T3255I, ORF1a:I3618V, ORF1a:T4175I, ORF1b:P314L, ORF1b:E1728D, ORF8:L4P, S:D614G, S:P681H, S:T732A,

hCoV-19/Mexico/QUE-InDRE_F11016_S830/2021	EPI_ISL_1424032	20B	B.1.1.519	26	5'UTR:C203T, 5'UTR:C222T, 5'UTR:C241T, ORF1ab:C1469T, ORF1ab:C3037T, ORF1ab:C3140T, ORF1ab:C10029T, ORF1ab:C10954T, ORF1ab:A11117G, ORF1ab:C12789T, ORF1ab:C14408T, ORF1ab:C16596T, ORF1ab:T19839C, ORF1ab:C21306T, S:C21844T, S:C22281T, S:C22995A, S:A23403G, S:C23604A, S:A23756G, ORF3a:G25429A, ORF8:T27904C, ORF8:C28087T, N:G28881A, N:G28882A, N:G28883C, N:C29197T,	16	N:R203K, N:G204R, ORF1a:R402C, ORF1a:P959S, ORF1a:T3255I, ORF1a:I3618V, ORF1a:T4175I, ORF1b:P314L, ORF3a:V13I, ORF8:L4P, ORF8:A65V, S:T240I, S:T478K, S:D614G, S:P681H, S:T732A,
hCoV-19/Mexico/QUE-InDRE_F11017_S823/2021	EPI_ISL_1424025	20B	B.1.1.222	14	5'UTR:C241T, ORF1ab:C3037T, ORF1ab:C6285T, ORF1ab:C8139A, ORF1ab:C13035T, ORF1ab:C13297T, ORF1ab:A13498G, ORF1ab:C14408T, ORF1ab:T19839C, S:A23403G, S:A23756G, N:G28881A, N:G28882A, N:G28883C, N:C29153A,	10	N:R203K, N:G204R, N:Q294K, ORF1a:T2007I, ORF1a:S2625Y, ORF1a:A4257V, ORF1b:T11A, ORF1b:P314L, S:D614G, S:T732A,
hCoV-19/Mexico/QUE-InDRE_F11019_S824/2021	EPI_ISL_1424026	20B	B.1.1.519	23	5'UTR:C203T, 5'UTR:C222T, 5'UTR:C241T, ORF1ab:C3037T, ORF1ab:C3140T, ORF1ab:T3172C, ORF1ab:C3809T, ORF1ab:T8704C, ORF1ab:C10029T, ORF1ab:C10954T, ORF1ab:A11117G, ORF1ab:C12789T, ORF1ab:C14408T, ORF1ab:G18020A, ORF1ab:T19839C, ORF1ab:C21306T, S:C22995A, S:A23403G, S:C23604A, S:A23756G, N:G28881A, N:G28882A, N:G28883C, N:C29197T,	13	N:R203K, N:G204R, ORF1a:P959S, ORF1a:L1182F, ORF1a:T3255I, ORF1a:I3618V, ORF1a:T4175I, ORF1b:P314L, ORF1b:R1518K, S:T478K, S:D614G, S:P681H, S:T732A,
hCoV-19/Mexico/QUE-InDRE_F11020_S825/2021	EPI_ISL_1424027	20B	B.1.1.519	21	5'UTR:C203T, 5'UTR:C222T, 5'UTR:C241T, ORF1ab:C3037T, ORF1ab:C3140T, ORF1ab:T3172C, ORF1ab:C3809T, ORF1ab:T8704C, ORF1ab:C10029T, ORF1ab:C10954T, ORF1ab:A11117G, ORF1ab:C12789T, ORF1ab:C14408T, ORF1ab:G18020A, ORF1ab:T19839C, S:A23403G, S:C23604A, S:A23756G, N:G28881A, N:G28882A, N:G28883C, N:C29197T,	12	N:R203K, N:G204R, ORF1a:P959S, ORF1a:L1182F, ORF1a:T3255I, ORF1a:I3618V, ORF1a:T4175I, ORF1b:P314L, ORF1b:R1518K, S:D614G, S:P681H, S:T732A,
hCoV-19/Mexico/QUE-InDRE_F11022_S826/2021	EPI_ISL_1424028	20B	B.1.1.519	21	5'UTR:C203T, 5'UTR:C222T, 5'UTR:C241T, ORF1ab:C3037T, ORF1ab:C3140T, ORF1ab:C3619T, ORF1ab:C10029T, ORF1ab:C10954T, ORF1ab:A11117G, ORF1ab:C12789T, ORF1ab:C14408T, ORF1ab:C18695T, ORF1ab:T19839C, ORF1ab:C21306T, S:C22995A, S:A23403G, S:C23604A, S:A23756G, N:G28881A, N:G28882A, N:G28883C, N:C29197T,	12	N:R203K, N:G204R, ORF1a:P959S, ORF1a:T3255I, ORF1a:I3618V, ORF1a:T4175I, ORF1b:P314L, ORF1b:T1743I, S:T478K, S:D614G, S:P681H, S:T732A,
hCoV-19/Mexico/QUE-InDRE_F11023_S827/2021	EPI_ISL_1424029	20B	B.1.1.519	21	5'UTR:C203T, 5'UTR:C222T, 5'UTR:C241T, ORF1ab:C3037T, ORF1ab:C3140T, ORF1ab:C3619T, ORF1ab:C10029T, ORF1ab:C10954T, ORF1ab:A11117G, ORF1ab:C12789T, ORF1ab:C14408T, ORF1ab:C18695T, ORF1ab:T19839C, ORF1ab:C21306T, S:C22995A, S:A23403G, S:C23604A, S:A23756G, N:G28881A, N:G28882A, N:G28883C, N:C29197T,	12	N:R203K, N:G204R, ORF1a:P959S, ORF1a:T3255I, ORF1a:I3618V, ORF1a:T4175I, ORF1b:P314L, ORF1b:T1743I, S:T478K, S:D614G, S:P681H, S:T732A,
hCoV-19/Mexico/QUE-InDRE_F11024_S828/2021	EPI_ISL_1424030	20B	B.1.1.519	29	5'UTR:C203T, 5'UTR:C222T, 5'UTR:C241T, ORF1ab:G881A, ORF1ab:C2644T, ORF1ab:C3037T, ORF1ab:C3140T, ORF1ab:C8140T, ORF1ab:C9693T, ORF1ab:C10029T, ORF1ab:C10954T, ORF1ab:A11117G, ORF1ab:C12789T, ORF1ab:C14408T, ORF1ab:T19839C, ORF1ab:G21010T, ORF1ab:C21306T, S:C22995A, S:A23403G, S:C23604A, S:A23756G, ORF6:G27382C, ORF6:A27383T, ORF6:T27384C, ORF7a:C27679T, ORF8:C28253T, N:G28881A, N:G28882A, N:G28883C, N:C29197T,	16	N:R203K, N:G204R, ORF1a:A206T, ORF1a:P959S, ORF1a:A3143V, ORF1a:T3255I, ORF1a:I3618V, ORF1a:T4175I, ORF1b:P314L, ORF1b:V2515L, ORF6:D61L, ORF7a:L96F, S:T478K, S:D614G, S:P681H, S:T732A,

hCoV-19/Mexico/QUE-InDRE_F11025_S829/2021	EPI_ISL_1424031	20B	B.1.1.519	21	5'UTR:C203T, 5'UTR:C222T, 5'UTR:C241T, ORF1ab:C3037T, ORF1ab:C3140T, ORF1ab:C3619T, ORF1ab:C10029T, ORF1ab:C10954T, ORF1ab:A11117G, ORF1ab:C12789T, ORF1ab:C14408T, ORF1ab:C18695T, ORF1ab:T19839C, ORF1ab:C21306T, S:C22995A, S:A23403G, S:C23604A, S:A23756G, N:G28881A, N:G28882A, N:G28883C, N:C29197T,	12	N:R203K, N:G204R, ORF1a:P959S, ORF1a:T3255I, ORF1a:I3618V, ORF1a:T4175I, ORF1b:P314L, ORF1b:T1743I, S:T478K, S:D614G, S:P681H, S:T732A,
hCoV-19/Mexico/QUE-InDRE_F11027_S822/2021	EPI_ISL_1424024	20B	B.1.1.519	24	5'UTR:C203T, 5'UTR:C222T, 5'UTR:C241T, ORF1ab:C3037T, ORF1ab:C3140T, ORF1ab:C10029T, ORF1ab:C10954T, ORF1ab:A11117G, ORF1ab:C12789T, ORF1ab:C14408T, ORF1ab:T19839C, ORF1ab:C21306T, S:C21844T, S:C22281T, S:C22995A, S:A23403G, S:C23604A, S:A23756G, ORF3a:G25429A, ORF8:T27904C, ORF8:C28087T, N:G28881A, N:G28882A, N:G28883C, N:C29197T,	15	N:R203K, N:G204R, ORF1a:P959S, ORF1a:T3255I, ORF1a:I3618V, ORF1a:T4175I, ORF1b:P314L, ORF3a:V13I, ORF8:L4P, ORF8:A65V, S:T240I, S:T478K, S:D614G, S:P681H, S:T732A,
hCoV-19/Mexico/QUE-InDRE_F11028_S831/2021	EPI_ISL_1424033	20B	B.1.1.519	28	5'UTR:T201C, 5'UTR:C203T, 5'UTR:C222T, 5'UTR:C241T, ORF1ab:G1738T, ORF1ab:C1912T, ORF1ab:C3037T, ORF1ab:C3140T, ORF1ab:G4162T, ORF1ab:C6401T, ORF1ab:C10029T, ORF1ab:C10954T, ORF1ab:A11117G, ORF1ab:C12789T, ORF1ab:C13515T, ORF1ab:C14408T, ORF1ab:T19839C, ORF1ab:A19974G, ORF1ab:C21057T, ORF1ab:C21306T, S:G22468T, S:C22995A, S:A23403G, S:C23604A, S:A23756G, N:G28881A, N:G28882A, N:G28883C, N:C29197T	12	N:R203K, N:G204R, ORF1a:P959S, ORF1a:P2046S, ORF1a:T3255I, ORF1a:I3618V, ORF1a:T4175I, ORF1b:P314L, S:T478K, S:D614G, S:P681H, S:T732A,
hCoV-19/Mexico/QUE-InDRE_F88B_S610/2021	EPI_ISL_1340634	20B	B.1.1.519	25	5'UTR:T201C, 5'UTR:C203T, 5'UTR:C222T, 5'UTR:C241T, ORF1ab:G1738T, ORF1ab:C3037T, ORF1ab:C3140T, ORF1ab:C9166T, ORF1ab:C9638T, ORF1ab:C10029T, ORF1ab:C10954T, ORF1ab:A11117G, ORF1ab:C12789T, ORF1ab:C14408T, ORF1ab:T19839C, ORF1ab:A19974G, ORF1ab:C21306T, S:C21742T, S:C22995A, S:A23403G, S:C23604A, S:A23756G, N:G28881A, N:G28882A, N:G28883C, N:C29197T,	12	N:R203K, N:G204R, ORF1a:P959S, ORF1a:H3125Y, ORF1a:T3255I, ORF1a:I3618V, ORF1a:T4175I, ORF1b:P314L, S:T478K, S:D614G, S:P681H, S:T732A,
hCoV-19/Mexico/QUE-InDRE_F89B_S611/2021	EPI_ISL_1340654	20B	B.1.1.519	25	5'UTR:C203T, 5'UTR:C222T, 5'UTR:C241T, ORF1ab:C3037T, ORF1ab:C3140T, ORF1ab:C10029T, ORF1ab:C10954T, ORF1ab:A11117G, ORF1ab:C12789T, ORF1ab:C14408T, ORF1ab:T19839C, ORF1ab:C21306T, S:C21844T, S:C22281T, S:C22995A, S:A23403G, S:C23604A, S:A23756G, ORF3a:G25429A, ORF8:T27904C, ORF8:C28087T, N:G28881A, N:G28882A, N:G28883C, N:C29197T, N:G29543T,	15	N:R203K, N:G204R, ORF1a:P959S, ORF1a:T3255I, ORF1a:I3618V, ORF1a:T4175I, ORF1b:P314L, ORF3a:V13I, ORF8:L4P, ORF8:A65V, S:T240I, S:T478K, S:D614G, S:P681H, S:T732A,
hCoV-19/Mexico/QUE-InDRE_F90B_S612/2021	EPI_ISL_1340619	20B	B.1.1.519	25	5'UTR:C203T, 5'UTR:C222T, 5'UTR:C241T, ORF1ab:C3037T, ORF1ab:C3140T, ORF1ab:G5953T, ORF1ab:G9526A, ORF1ab:C10029T, ORF1ab:C10954T, ORF1ab:A11117G, ORF1ab:G11365T, ORF1ab:C12789T, ORF1ab:C14408T, ORF1ab:C17125T, ORF1ab:T19839C, ORF1ab:C21306T, S:C22995A, S:A23403G, S:C23604A, S:A23756G, ORF8:C27903T, N:G28881A, N:G28882A, N:G28883C, N:C29197T, N:G29527T,	16	N:R203K, N:G204R, N:Q418H, ORF1a:P959S, ORF1a:L1896F, ORF1a:M3087I, ORF1a:T3255I, ORF1a:I3618V, ORF1a:T4175I, ORF1b:P314L, ORF1b:L1220F, ORF8:L4F, S:T478K, S:D614G, S:P681H, S:T732A,

hCoV-19/Mexico/QUE-InDRE_F9839_S618/2021	EPI_ISL_1340655	20B	B.1.1.519	29	5'UTR:T201C, 5'UTR:C203T, 5'UTR:C222T, 5'UTR:C241T, ORF1ab:C936T, ORF1ab:G1738T, ORF1ab:C3037T, ORF1ab:C3140T, ORF1ab:C9451T, ORF1ab:C10029T, ORF1ab:C10954T, ORF1ab:A11117G, ORF1ab:C12789T, ORF1ab:C14408T, ORF1ab:T19839C, ORF1ab:A19974G, ORF1ab:C21306T, S:T21835C, S:C22995A, S:A23403G, S:C23604A, S:A23756G, ORF3a:A25524C, M:C26801T, N:G28881A, N:G28882A, N:G28883C, N:G28968T, N:C29197T, N:G29227T,	13	N:R203K, N:G204R, N:S232I, ORF1a:T224I, ORF1a:P959S, ORF1a:T3255I, ORF1a:I3618V, ORF1a:T4175I, ORF1b:P314L, S:T478K, S:D614G, S:P681H, S:T732A,
hCoV-19/Mexico/QUE-InDRE_F9840_S649/2021	EPI_ISL_1359064	20B	B.1.1.222	21	5'UTR:C241T, ORF1ab:C3037T, ORF1ab:C4897T, ORF1ab:C10029T, ORF1ab:A11430G, ORF1ab:A13063G, ORF1ab:C14408T, ORF1ab:C19011A, ORF1ab:T19839C, ORF1ab:A21137G, ORF1ab:G21255T, S:G22801T, S:A23403G, S:A23756G, ORF3a:T25745C, ORF3a:G25912T, ORF3a:A26115C, ORF8:A27921G, ORF8:G28001T, N:G28881A, N:G28882A, N:G28883C,	13	N:R203K, N:G204R, ORF1a:T3255I, ORF1a:Y3722C, ORF1b:P314L, ORF1b:D1848E, ORF1b:K2557R, ORF3a:I118T, ORF3a:G174C, ORF3a:E241D, ORF8:I10V, S:D614G, S:T732A,
hCoV-19/Mexico/QUE-InDRE_F9841_S619/2021	EPI_ISL_1340657	20B	B.1.1.519	27	5'UTR:C203T, 5'UTR:C222T, 5'UTR:C241T, ORF1ab:C3037T, ORF1ab:C3140T, ORF1ab:C5183T, ORF1ab:C10029T, ORF1ab:C10954T, ORF1ab:A11117G, ORF1ab:C12789T, ORF1ab:C14408T, ORF1ab:A16266G, ORF1ab:A19462G, ORF1ab:T19839C, ORF1ab:C21306T, S:C22995A, S:A23403G, S:C23444G, S:C23604A, S:A23756G, ORF3a:T25926C, M:C26768G, ORF8:T27904C, N:G28881A, N:G28882A, N:G28883C, N:C29197T, 3'UTR:C29769T	16	M:I82M, N:R203K, N:G204R, ORF1a:P959S, ORF1a:P1640S, ORF1a:T3255I, ORF1a:I3618V, ORF1a:T4175I, ORF1b:P314L, ORF1b:T1999A, ORF8:L4P, S:T478K, S:D614G, S:Q628E, S:P681H, S:T732A,
hCoV-19/Mexico/QUE-InDRE_F9843_S620/2021	EPI_ISL_1340636	20B	B.1.1.519	22	5'UTR:C203T, 5'UTR:C222T, 5'UTR:C241T, ORF1ab:C3037T, ORF1ab:C3140T, ORF1ab:A6677G, ORF1ab:C6701T, ORF1ab:C10029T, ORF1ab:C10954T, ORF1ab:A11117G, ORF1ab:C12789T, ORF1ab:C14408T, ORF1ab:T19839C, ORF1ab:C21306T, S:C22995A, S:A23403G, S:C23604A, S:A23756G, N:G28881A, N:G28882A, N:G28883C, N:T28930A, N:C29197T,	13	N:R203K, N:G204R, ORF1a:P959S, ORF1a:I2138V, ORF1a:L2146F, ORF1a:T3255I, ORF1a:I3618V, ORF1a:T4175I, ORF1b:P314L, S:T478K, S:D614G, S:P681H, S:T732A,
hCoV-19/Mexico/QUE-InDRE_F9844_S650/2021	EPI_ISL_1359065	20B	B.1.1.222	25	5'UTR:C241T, ORF1ab:C2110T, ORF1ab:C3037T, ORF1ab:T5860C, ORF1ab:G7387A, ORF1ab:C9430T, ORF1ab:C9979T, ORF1ab:A10323G, ORF1ab:G11087A, ORF1ab:C14408T, ORF1ab:T14787C, ORF1ab:C15240T, ORF1ab:T19839C, S:A23403G, S:A23756G, ORF3a:G25687A, ORF3a:G25793A, M:G26951T, ORF7a:G27561A, ORF8:G28167A, N:C28344T, N:G28881A, N:G28882A, N:G28883C, N:A29190T, 3'UTR:G29751T,	13	N:T24I, N:R203K, N:G204R, N:Q306L, ORF1a:M2374I, ORF1a:K3353R, ORF1a:E3608K, ORF1b:P314L, ORF3a:A99T, ORF3a:R134H, ORF8:E92K, S:D614G, S:T732A,
hCoV-19/Mexico/QUE-InDRE_F9845_S585/2021	EPI_ISL_1337360	21C (Epsilon)	B.1.429	27	5'UTR:C241T, ORF1ab:C593T, ORF1ab:C1059T, ORF1ab:C2395T, ORF1ab:T2597C, ORF1ab:C3037T, ORF1ab:C8947T, ORF1ab:C10641T, ORF1ab:C12100T, ORF1ab:A12878G, ORF1ab:C14408T, ORF1ab:G17014T, ORF1ab:C19374T, S:G21600T, S:G22018T, S:T22917G, S:A23403G, S:C23613T, S:T24349C, ORF3a:G25563T, ORF3a:C25734T, M:C26681T, ORF6:G27281T, ORF7a:C27741T, ORF7b:G27890T, ORF8:A28272T, N:C28887T, N:C29362T,	14	N:T205I, ORF1a:H110Y, ORF1a:T265I, ORF1a:T3459M, ORF1a:I4205V, ORF1b:P314L, ORF1b:D1183Y, ORF3a:Q57H, ORF6:W27L, S:S13I, S:W152C, S:L452R, S:D614G, S:A684V,

hCoV-19/Mexico/QUE-InDRE_F9846_S586/2021	EPI_ISL_1337362	21C (Epsilon)	B.1.429	25	5'UTR:C241T, ORF1ab:C593T, ORF1ab:C1059T, ORF1ab:C2395T, ORF1ab:T2597C, ORF1ab:C3037T, ORF1ab:C8947T, ORF1ab:C10641T, ORF1ab:C11747T, ORF1ab:C12100T, ORF1ab:A12878G, ORF1ab:C14408T, ORF1ab:G17014T, S:G21600T, S:G22018T, S:T22917G, S:A23403G, S:T24349C, ORF3a:G25563T, M:C26681T, ORF7a:C27654T, ORF7b:G27890T, ORF8:A28272T, N:G28685T, N:C28887T, N:C29362T,	13	N:A138S, N:T205I, ORF1a:H110Y, ORF1a:T265I, ORF1a:T3459M, ORF1a:I4205V, ORF1b:P314L, ORF1b:D1183Y, ORF3a:Q57H, S:S13I, S:W152C, S:L452R, S:D614G,	
hCoV-19/Mexico/QUE-InDRE_F9847_S621/2021	EPI_ISL_1340637	20B	B.1.1.519	28	5'UTR:C203T, 5'UTR:C222T, 5'UTR:C241T, ORF1ab:C1342T, ORF1ab:C1929T, ORF1ab:C3037T, ORF1ab:C3140T, ORF1ab:C3686T, ORF1ab:G3692T, ORF1ab:G3871T, ORF1ab:C10029T, ORF1ab:C10954T, ORF1ab:A11117G, ORF1ab:C11824A, ORF1ab:C12789T, ORF1ab:C14408T, ORF1ab:T19839C, ORF1ab:C21306T, S:C22995A, S:A23403G, S:C23604A, S:A23756G, S:G24959T, ORF8:T27904C, N:G28881A, N:G28882A, N:G28883C, N:T28978C, N:C29197T,	17	N:R203K, N:G204R, ORF1a:A555V, ORF1a:P959S, ORF1a:H1141Y, ORF1a:V1143F, ORF1a:K1202N, ORF1a:T3255I, ORF1a:I3618V, ORF1a:T4175I, ORF1b:P314L, ORF8:L4P, S:T478K, S:D614G, S:P681H, S:T732A, S:V1133F,	
hCoV-19/Mexico/QUE-InDRE_F9848_S622/2021	EPI_ISL_1340639	20B	B.1.1.519	23	5'UTR:C203T, 5'UTR:C222T, 5'UTR:C241T, ORF1ab:C2644T, ORF1ab:C3037T, ORF1ab:C3140T, ORF1ab:C10029T, ORF1ab:C10954T, ORF1ab:A11117G, ORF1ab:G11365T, ORF1ab:C12789T, ORF1ab:C14408T, ORF1ab:T19839C, ORF1ab:C21306T, S:C22995A, S:A23403G, S:C23604A, S:A23756G, S:C24442T, N:G28881A, N:G28882A, N:G28883C, N:C29197T, N:G29527T,	12	N:R203K, N:G204R, N:Q418H, ORF1a:P959S, ORF1a:T3255I, ORF1a:I3618V, ORF1a:T4175I, ORF1b:P314L, S:T478K, S:D614G, S:P681H, S:T732A,	
hCoV-19/Mexico/QUE-InDRE-04/2020	EPI_ISL_424670	19B	A	2	ORF1ab:C8782T, ORF3a:C26088T, ORF8:T28144C,	1	ORF8:L84S,	
hCoV-19/Mexico/QUE-InDRE-18/2020	EPI_ISL_455456	19B	A	2	ORF1ab:C8782T, ORF3a:C26088T, ORF8:T28144C,	1	ORF8:L84S,	
hCoV-19/Mexico/ROO-InDRE_243/2020	EPI_ISL_942929	21C (Epsilon)	B.1.429	22	5'UTR:C241T, ORF1ab:C632T, ORF1ab:C1059T, ORF1ab:C2395T, ORF1ab:T2597C, ORF1ab:C3037T, ORF1ab:C5869T, ORF1ab:C8947T, ORF1ab:C12100T, ORF1ab:A12878G, ORF1ab:C13592T, ORF1ab:C14408T, ORF1ab:G17014T, S:G21600T, S:G22018T, S:T22917G, S:A23403G, S:T24349C, ORF3a:G25563T, M:C26681T, ORF7b:G27890T, ORF8:A28272T, N:C28887T,	12	N:T205I, ORF1a:L123F, ORF1a:T265I, ORF1a:I4205V, ORF1b:T42I, ORF1b:P314L, ORF1b:D1183Y, ORF3a:Q57H, S:S13I, S:W152C, S:L452R, S:D614G,	
hCoV-19/Mexico/ROO-InDRE_F10710_S696/2021	EPI_ISL_1365661	20B	B.1.1	25	5'UTR:C241T, ORF1ab:C1684T, ORF1ab:T1921C, ORF1ab:C2388T, ORF1ab:C3037T, ORF1ab:G3047T, ORF1ab:C9611T, ORF1ab:T10084C, ORF1ab:C14408T, ORF1ab:C14925T, ORF1ab:G17790T, ORF1ab:A18671G, ORF1ab:C19884T, ORF1ab:G20493A, S:A23403G, S:T24835C, ORF3a:C25710T, ORF6:G27382C, ORF6:A27383T, ORF6:T27384C, ORF7b:C27828T, N:G28881A, N:G28882A, N:G28883C, N:C29149T, 3'UTR:C29686T,	11	N:R203K, N:G204R, ORF1a:T708I, ORF1a:D928Y, ORF1a:L3116F, ORF1b:P314L, ORF1b:Q1441H, ORF1b:D1735G, ORF6:D61L, ORF7b:L25F, S:D614G,	S:21992-21994,

hCoV-19/Mexico/ROO-InDRE_F120Q_S584/2021	EPI_ISL_1324767	20J (Gamma, V3	P.1	38	5'UTR:C241T, ORF1ab:T733C, ORF1ab:C2749T, ORF1ab:C3037T, ORF1ab:C3828T, ORF1ab:A5648C, ORF1ab:A6319G, ORF1ab:A6613G, ORF1ab:A10323G, ORF1ab:C12778T, ORF1ab:C13860T, ORF1ab:C14408T, ORF1ab:G16377A, ORF1ab:C16887T, ORF1ab:G17259T, S:C21614T, S:C21621A, S:C21638T, S:G21974T, S:G22132T, S:A22812C, S:G23012A, S:A23063T, S:A23403G, S:C23525T, S:G23593C, S:C24642T, S:G25088T, ORF3a:T26149C, M:G26828T, ORF8:G28167A, N:C28512G, N:A28877T, N:G28878C, N:G28881A, N:G28882A, N:G28883C, N:C28948T, 3'UTR:T29834A,	24	N:P80R, N:R203K, N:G204R, ORF1a:S1188L, ORF1a:K1795Q, ORF1a:K3353R, ORF1b:P314L, ORF1b:E1264D, ORF3a:S253P, ORF8:E92K, ORF9b:Q77E, S:L18F, S:T20N, S:P26S, S:D138Y, S:R190S, S:K417T, S:E484K, S:N501Y, S:D614G, S:H655Y, S:Q677H, S:T1027I, S:V1176F,	ORF1ab: 11288-11296,
hCoV-19/Mexico/ROO-InDRE-102/2020	EPI_ISL_872092	20C	B.1	7	5'UTR:C40T, 5'UTR:C241T, ORF1ab:C1059T, ORF1ab:C3037T, ORF1ab:C14408T, ORF1ab:C16308T, S:A23403G, ORF3a:G25563T,	4	ORF1a:T265I, ORF1b:P314L, ORF3a:Q57H, S:D614G,	
hCoV-19/Mexico/ROO-InDRE-14/2020	EPI_ISL_455438	20A	B.1	5	5'UTR:C241T, ORF1ab:C3037T, ORF1ab:C14408T, ORF1ab:A20268G, S:A23403G, S:G25183A,	2	ORF1b:P314L, S:D614G,	
hCoV-19/Mexico/SEARCH-7817/2021	EPI_ISL_1366325	21C (Epsilon)	B.1.429	25	5'UTR:C241T, ORF1ab:C1059T, ORF1ab:C2395T, ORF1ab:T2597C, ORF1ab:C3037T, ORF1ab:C4331T, ORF1ab:G7328T, ORF1ab:C8947T, ORF1ab:C12100T, ORF1ab:A12878G, ORF1ab:C14408T, ORF1ab:T15621C, ORF1ab:G17014T, ORF1ab:G21372T, S:G21600T, S:G22018T, S:T22917G, S:A23403G, S:T24349C, ORF3a:G25563T, M:C26681T, ORF7b:G27890T, ORF8:A28272T, N:C28887T, N:C29167T, N:C29362T,	12	N:T205I, ORF1a:T265I, ORF1a:A2355S, ORF1a:I4205V, ORF1b:P314L, ORF1b:D1183Y, ORF1b:Q2635H, ORF3a:Q57H, S:S13I, S:W152C, S:L452R, S:D614G,	
hCoV-19/Mexico/SEARCH-7931/2021	EPI_ISL_1366326	20G	B.1.596	22	5'UTR:C241T, ORF1ab:C1059T, ORF1ab:C3037T, ORF1ab:C10319T, ORF1ab:A11451G, ORF1ab:C14408T, ORF1ab:A18424G, ORF1ab:C21304T, S:C21811A, S:C22591T, S:A23403G, S:A23592C, S:G24586T, ORF3a:G25563T, ORF3a:T25774C, ORF3a:G25907T, ORF7a:G27506T, ORF8:C27964T, N:C28472T, N:C28869T, N:T29194C, N:T29377A, N:C29445T,	16	N:P67S, N:P199L, N:T391I, ORF1a:T265I, ORF1a:L3352F, ORF1a:Q3729R, ORF1b:P314L, ORF1b:N1653D, ORF1b:R2613C, ORF3a:Q57H, ORF3a:W128R, ORF3a:G172V, ORF7a:G38V, ORF8:S24L, S:D614G, S:Q677P,	
hCoV-19/Mexico/SEARCH-7932/2021	EPI_ISL_1366327	20G	B.1.596	22	5'UTR:C241T, ORF1ab:C1059T, ORF1ab:C3037T, ORF1ab:C10319T, ORF1ab:A11451G, ORF1ab:C14408T, ORF1ab:A18424G, ORF1ab:C21304T, S:C21811A, S:C22591T, S:A23403G, S:A23592C, S:G24586T, ORF3a:G25563T, ORF3a:T25774C, ORF3a:G25907T, ORF7a:G27506T, ORF8:C27964T, N:C28472T, N:C28869T, N:T29194C, N:T29377A, N:C29445T,	16	N:P67S, N:P199L, N:T391I, ORF1a:T265I, ORF1a:L3352F, ORF1a:Q3729R, ORF1b:P314L, ORF1b:N1653D, ORF1b:R2613C, ORF3a:Q57H, ORF3a:W128R, ORF3a:G172V, ORF7a:G38V, ORF8:S24L, S:D614G, S:Q677P,	
hCoV-19/Mexico/SEARCH-7933/2021	EPI_ISL_1366328	21C (Epsilon)	B.1.429	24	5'UTR:C241T, ORF1ab:C1059T, ORF1ab:C2395T, ORF1ab:T2597C, ORF1ab:C3037T, ORF1ab:C8947T, ORF1ab:C10641T, ORF1ab:C12100T, ORF1ab:A12878G, ORF1ab:C14408T, ORF1ab:G17014T, ORF1ab:C17304T, S:G21600T, S:G22018T, S:T22917G, S:A23403G, S:T24349C, ORF3a:G25563T, M:C26681T, ORF7b:G27890T, ORF8:A28272T, N:C28887T, N:C29095T, N:C29362T, ORF10:G29648A,	11	N:T205I, ORF1a:T265I, ORF1a:T3459M, ORF1a:I4205V, ORF1b:P314L, ORF1b:D1183Y, ORF3a:Q57H, S:S13I, S:W152C, S:L452R, S:D614G,	
hCoV-19/Mexico/SEARCH-7934/2021	EPI_ISL_1366329	21C (Epsilon)	B.1.427	21	5'UTR:C241T, ORF1ab:C1059T, ORF1ab:A2854G, ORF1ab:C3037T, ORF1ab:T4885C, ORF1ab:G6901T, ORF1ab:G9738C, ORF1ab:G13304T, ORF1ab:G13713A, ORF1ab:C14408T, ORF1ab:C16394T, ORF1ab:G17014T, S:G21600T, S:G22018T, S:T22917G, S:A23403G, ORF3a:G25563T, M:C26681T, ORF8:A28272T, N:C28887T, N:C29218T, N:C29362T,	13	N:T205I, ORF1a:T265I, ORF1a:E2212D, ORF1a:S3158T, ORF1a:G4347C, ORF1b:P314L, ORF1b:P976L, ORF1b:D1183Y, ORF3a:Q57H, S:S13I, S:W152C, S:L452R, S:D614G,	

hCoV-19/Mexico/SEARCH-7935/2021	EPI_ISL_1366330	21C (Epsilon)	B.1.427	23	5'UTR:C241T, ORF1ab:C1059T, ORF1ab:C2485T, ORF1ab:C3037T, ORF1ab:G6349T, ORF1ab:G9738C, ORF1ab:G13713A, ORF1ab:C14408T, ORF1ab:C16394T, ORF1ab:G17014T, ORF1ab:C17402T, ORF1ab:G18397T, ORF1ab:C19554T, S:G21600T, S:G22018T, S:T22917G, S:A23403G, ORF3a:G25563T, M:C26681T, M:C26985T, M:C27143T, ORF8:A28272T, N:C28887T, N:C29362T,	14	M:H155Y, N:T205I, ORF1a:T265I, ORF1a:S3158T, ORF1b:P314L, ORF1b:P976L, ORF1b:D1183Y, ORF1b:A1312V, ORF1b:V1644L, ORF3a:Q57H, S:S13I, S:W152C, S:L452R, S:D614G,
hCoV-19/Mexico/SEARCH-7936/2021	EPI_ISL_1366331	20G	B.1.596	23	5'UTR:C241T, ORF1ab:C1059T, ORF1ab:A1378T, ORF1ab:C3037T, ORF1ab:C10319T, ORF1ab:A11451G, ORF1ab:C14408T, ORF1ab:A18424G, ORF1ab:C21304T, S:C21811A, S:C22591T, S:A23403G, S:A23592C, S:G24586T, ORF3a:G25563T, ORF3a:T25774C, ORF3a:G25907T, ORF7a:G27506T, ORF8:C27964T, N:C28472T, N:C28869T, N:T29194C, N:T29377A, N:C29445T,	16	N:P67S, N:P199L, N:T391I, ORF1a:T265I, ORF1a:L3352F, ORF1a:Q3729R, ORF1b:P314L, ORF1b:N1653D, ORF1b:R2613C, ORF3a:Q57H, ORF3a:W128R, ORF3a:G172V, ORF7a:G38V, ORF8:S24L, S:D614G, S:Q677P,
hCoV-19/Mexico/SEARCH-7937/2021	EPI_ISL_1366332	21C (Epsilon)	B.1.427	27	5'UTR:C241T, ORF1ab:C583T, ORF1ab:C1059T, ORF1ab:T1851C, ORF1ab:C2232T, ORF1ab:C3037T, ORF1ab:C3619A, ORF1ab:G4510A, ORF1ab:G9738C, ORF1ab:C10252T, ORF1ab:G13713A, ORF1ab:C14408T, ORF1ab:C16394T, ORF1ab:G17014T, ORF1ab:G19670A, S:G21600T, S:G22018T, S:T22917G, S:A23403G, S:C25317T, ORF3a:T25467C, ORF3a:G25563T, ORF3a:T26066C, M:C26681T, ORF8:A28272T, N:A28804G, N:C28887T, N:C29362T,	16	N:T205I, ORF1a:T265I, ORF1a:I529T, ORF1a:A656V, ORF1a:S3158T, ORF1b:P314L, ORF1b:P976L, ORF1b:D1183Y, ORF1b:G2068E, ORF3a:Q57H, ORF3a:V225A, S:S13I, S:W152C, S:L452R, S:D614G, S:S1252F,
hCoV-19/Mexico/SEARCH-7938/2021	EPI_ISL_1366333	21C (Epsilon)	B.1.429	24	5'UTR:C241T, ORF1ab:C1059T, ORF1ab:C2395T, ORF1ab:T2597C, ORF1ab:C3037T, ORF1ab:C8947T, ORF1ab:C12100T, ORF1ab:A12878G, ORF1ab:C14408T, ORF1ab:G17014T, S:G21600T, S:G22018T, S:G22349T, S:T22917G, S:T23155C, S:A23403G, S:T24349C, ORF3a:G25563T, M:C26681T, ORF7b:G27890T, ORF8:G28191T, ORF8:A28272T, N:C28887T, N:G28975T, N:C29362T,	13	N:T205I, N:M234I, ORF1a:T265I, ORF1a:I4205V, ORF1b:P314L, ORF1b:D1183Y, ORF3a:Q57H, ORF8:V100L, S:S13I, S:W152C, S:A263S, S:L452R, S:D614G,
hCoV-19/Mexico/SEARCH-7939/2021	EPI_ISL_1366334	21C (Epsilon)	B.1.429	27	5'UTR:C241T, ORF1ab:C593T, ORF1ab:C1059T, ORF1ab:C2395T, ORF1ab:T2597C, ORF1ab:C3037T, ORF1ab:C8947T, ORF1ab:C10641T, ORF1ab:C12100T, ORF1ab:A12878G, ORF1ab:C14408T, ORF1ab:C15960T, ORF1ab:C15981T, ORF1ab:G17014T, S:G21600T, S:C21627T, S:G22018T, S:T22917G, S:A23403G, S:A23881G, S:T24349C, ORF3a:G25563T, M:C26681T, ORF7b:G27890T, ORF8:A28272T, N:C28453T, N:C28887T, N:C29362T,	14	N:T205I, ORF1a:H110Y, ORF1a:T265I, ORF1a:T3459M, ORF1a:I4205V, ORF1b:P314L, ORF1b:D1183Y, ORF3a:Q57H, ORF9b:A57V, S:S13I, S:T22I, S:W152C, S:L452R, S:D614G,
hCoV-19/Mexico/SIN_CIAD_HJ0923/2021	EPI_ISL_1120614	20G	B.1.2	30	5'UTR:C241T, ORF1ab:C1059T, ORF1ab:G1599T, ORF1ab:C3037T, ORF1ab:C3330T, ORF1ab:A3879G, ORF1ab:C6681T, ORF1ab:T9027C, ORF1ab:G9128T, ORF1ab:C10319T, ORF1ab:C10789T, ORF1ab:G12911T, ORF1ab:C14408T, ORF1ab:A18424G, ORF1ab:T20256C, ORF1ab:T20361C, ORF1ab:C20719T, ORF1ab:C21304T, S:G23009T, S:A23403G, ORF3a:G25563T, ORF3a:G25907T, ORF8:C27964T, N:C28472T, N:C28869T,ORF1ab:G2804C, ORF1ab:T5118A, ORF1ab:A9902G, ORF1ab:C19637T, ORF1ab:A20637T, ORF1ab:T21087C,	24	N:P67S, N:P199L, ORF1a:T265I, ORF1a:G445V, ORF1a:T1022I, ORF1a:E1205G, ORF1a:A2139V, ORF1a:I2921T, ORF1a:V2955L, ORF1a:L3352F, ORF1a:V4216F, ORF1b:P314L, ORF1b:N1653D, ORF1b:R2613C, ORF3a:Q57H, ORF3a:G172V, ORF8:S24L, S:V483F, S:D614G,ORF1a:E847Q, ORF1a:F1618Y, ORF1a:K3213E, ORF1b:A2057V, ORF1b:E2390D,

hCoV-19/Mexico/SIN_CIAD_S0148/2020	EPI_ISL_1491365	20B	B.1.1.205	37	5'UTR:C241T, ORF1ab:C3140T, ORF1ab:A3645G, ORF1ab:A4759G, ORF1ab:A5172G, ORF1ab:G5539A, ORF1ab:A9162G, ORF1ab:T9172C, ORF1ab:T10120C, ORF1ab:A10464T, ORF1ab:A10948G, ORF1ab:C14408T, ORF1ab:G16402A, ORF1ab:A16403C, ORF1ab:T16406C, ORF1ab:C16407T, ORF1ab:A16641G, ORF1ab:T16950C, ORF1ab:T20200C, ORF1ab:A20849G, ORF1ab:C21057T, ORF1ab:A21548T, ORF1ab:C21549T, ORF1ab:A21550C, ORF1ab:A21551T, S:A23403G, S:T23791C, S:A24067G, S:T25001A, M:G26730T, ORF8:G28068T, N:G28881A, N:G28882A, N:G28883C, N:T29515C, ORF10:A29571G, ORF10:A29649G, 3'UTR:T29698C,	18	M:V70F, N:R203K, N:G204R, ORF1a:P959S, ORF1a:D1127G, ORF1a:H1636R, ORF1a:N2966S, ORF1a:K3400M, ORF1b:P314L, ORF1b:D979T, ORF1b:V980A, ORF1b:F2245L, ORF1b:N2461S, ORF1b:N2694I, ORF1b:N2695L, ORF8:E59*, S:D614G, S:S1147T,	
hCoV-19/Mexico/SIN_CIAD_S5894/2021	EPI_ISL_1491364	20A	B.1.234	17	5'UTR:C241T, ORF1ab:G2258A, ORF1ab:G2782T, ORF1ab:C3037T, ORF1ab:A6441G, ORF1ab:G6943T, ORF1ab:C7279T, ORF1ab:C8140T, ORF1ab:A9204G, ORF1ab:C10336T, ORF1ab:C10641T, ORF1ab:C14408T, ORF1ab:A20268G, S:C22000T, S:A23403G, N:C28657T, N:C28744T, N:C28854T,	7	N:S194L, ORF1a:V665I, ORF1a:K2059R, ORF1a:D2980G, ORF1a:T3459M, ORF1b:P314L, S:D614G,	
hCoV-19/Mexico/SIN_CIAD_S5975/2021	EPI_ISL_1660616	21C (Epsilon)	B.1.429	23	5'UTR:C241T, ORF1ab:C1059T, ORF1ab:C2395T, ORF1ab:T2597C, ORF1ab:T2860C, ORF1ab:C3037T, ORF1ab:C8947T, ORF1ab:C12100T, ORF1ab:A12878G, ORF1ab:C14408T, ORF1ab:G17014T, S:G21600T, S:G22018T, S:T22917G, S:A23403G, S:T24349C, S:C24865T, ORF3a:G25563T, M:C26681T, M:C27092T, ORF7a:G27497T, ORF7b:G27890T, ORF8:G28191T, ORF8:A28272T.	11	ORF1a:T265I, ORF1a:I4205V, ORF1b:P314L, ORF1b:D1183Y, ORF3a:Q57H, ORF7a:C35F, ORF8:V100L, S:S13I, S:W152C, S:L452R, S:D614G,	
hCoV-19/Mexico/SIN_CIAD_S5976/2021	EPI_ISL_1627079	20B	B.1.1.222	31	5'UTR:C241T, ORF1ab:C3037T, ORF1ab:A6693G, ORF1ab:C9430T, ORF1ab:C10029T, ORF1ab:C14408T, ORF1ab:T14586A, ORF1ab:T19839C, S:C22033A, S:A23403G, S:A23756G, ORF7b:A27756G, ORF8:A27921G, ORF8:G28001T, N:G28881A, N:G28882A, N:G28883C, 3'UTR:G29692A, 3'UTR:T29693A, 3'UTR:C29696A, 3'UTR:T29698A, 3'UTR:T29699A, 3'UTR:G29701A, 3'UTR:G29702A, 3'UTR:G29703A, 3'UTR:G29705A, 3'UTR:G29706A, 3'UTR:T29710A, 3'UTR:G29711A, 3'UTR:G29717A,3'UTR:C29708A, 3'UTR:T29709A,	10	N:R203K, N:G204R, ORF1a:K2143R, ORF1a:T3255I, ORF1b:P314L, ORF7b:M1V, ORF8:I10V, S:F157L, S:D614G, S:T732A,	3'UTR:29688-29691
hCoV-19/Mexico/SIN_CIAD_S6084/2021	EPI_ISL_1491363	21C (Epsilon)	B.1.427	26	5'UTR:C241T, ORF1ab:C1059T, ORF1ab:C3037T, ORF1ab:A4177G, ORF1ab:A4912G, ORF1ab:C7043T, ORF1ab:G9738C, ORF1ab:C9803T, ORF1ab:G13713A, ORF1ab:C14408T, ORF1ab:C14937T, ORF1ab:A15218G, ORF1ab:G16507A, ORF1ab:T16544A, ORF1ab:T16552C, ORF1ab:T16659A, ORF1ab:G17014T, ORF1ab:A17256G, ORF1ab:G18105A, S:G21600T, S:G22018T, S:T22917G, S:A23403G, ORF3a:G25563T, M:C26681T, ORF8:A28272T, N:C28887T,	15	N:T205I, ORF1a:T265I, ORF1a:P2260S, ORF1a:S3158T, ORF1b:P314L, ORF1b:K584R, ORF1b:G1014S, ORF1b:V1026D, ORF1b:F1029L, ORF1b:D1183Y, ORF3a:Q57H, S:S13I, S:W152C, S:L452R, S:D614G,	
hCoV-19/Mexico/SIN-CIAD-HJ1154/2021	EPI_ISL_1482628		B.1.1.519	0	,	1	,	

hCoV-19/Mexico/SIN-CIAD-S5971/2021	EPI_ISL_1482627	21C (Epsilon)	B.1.429	43	5'UTR:C241T, ORF1ab:C2395T, ORF1ab:T2597C, ORF1ab:C3037T, ORF1ab:C3602T, ORF1ab:C8597T, ORF1ab:C8947T, ORF1ab:C10165T, ORF1ab:C12100T, ORF1ab:A12878G, ORF1ab:C14408T, ORF1ab:G17014T, S:G21600T, S:G22018T, S:T22917G, S:C23029T, S:A23403G, S:T24349C, ORF3a:G25563T, ORF3a:T26064C, M:C26681T, ORF8:A28272T, N:C28887T, N:C29362T, 3'UTR:C29788A, 3'UTR:T29789A, 3'UTR:T29791A, 3'UTR:G29794T, 3'UTR:G29795T, 3'UTR:G29798A, 3'UTR:T29804A, 3'UTR:T29807A, 3'UTR:G29808A, 3'UTR:T29809A, 3'UTR:G29810A, 3'UTR:T29811A, 3'UTR:T29816A, 3'UTR:T29817A, 3'UTR:T29820A, 3'UTR:T29821A, 3'UTR:T29822A, 3'UTR:T29823A, 3'UTR:G29825A, 3'UTR:T29826A,	11	N:T205I, ORF1a:H1113Y, ORF1a:L2778F, ORF1a:I4205V, ORF1b:P314L, ORF1b:D1183Y, ORF3a:Q57H, S:S13I, S:W152C, S:L452R, S:D614G,	3'UTR:29800-29803,5'UTR:4, 3'UTR:29726-29727
hCoV-19/Mexico/SIN-InDRE_F10813_S604/2021	EPI_ISL_1337389	21C (Epsilon)	B.1.427	22	5'UTR:C241T, ORF1ab:C1059T, ORF1ab:G1148T, ORF1ab:C3037T, ORF1ab:C5512T, ORF1ab:C6402T, ORF1ab:G9738C, ORF1ab:G13459T, ORF1ab:G13713A, ORF1ab:C14408T, ORF1ab:C16394T, ORF1ab:G17014T, ORF1ab:G20931T, S:G21600T, S:G22018T, S:T22917G, S:A23403G, ORF3a:G25563T, M:C26681T, ORF8:C27972T, ORF8:A28272T, N:C28887T, N:C29362T,	14	N:T205I, ORF1a:T265I, ORF1a:G295C, ORF1a:P2046L, ORF1a:S3158T, ORF1b:P314L, ORF1b:P976L, ORF1b:D1183Y, ORF3a:Q57H, ORF8:Q27*, S:S13I, S:W152C, S:L452R, S:D614G,	
hCoV-19/Mexico/SIN-InDRE-21/2020	EPI_ISL_493336	20A	B.1.609	6	5'UTR:C241T, ORF1ab:C3037T, ORF1ab:C4582T, ORF1ab:C8175T, ORF1ab:C14408T, ORF1ab:A20268G, S:A23403G,	3	ORF1a:A2637V, ORF1b:P314L, S:D614G,	
hCoV-19/Mexico/SLP_UASLP_A002/2021	EPI_ISL_1469112	20B	B.1.1.517	21	5'UTR:T10A, 5'UTR:T13A, 5'UTR:C241T, ORF1ab:G2731A, ORF1ab:C3037T, ORF1ab:C4582T, ORF1ab:C5301T, ORF1ab:A9436T, ORF1ab:C14408T, ORF1ab:G15957T, ORF1ab:C18388T, ORF1ab:T19839C, ORF1ab:G19891T, S:T23332C, S:A23403G, S:A23756G, ORF8:A27994G, ORF8:G28083T, N:G28881A, N:G28882A, N:G28883C, ORF10:T29660C,	9	N:R203K, N:G204R, ORF1a:A1679V, ORF1b:P314L, ORF1b:D2142Y, ORF8:D34G, ORF8:E64*, S:D614G, S:T732A,	
hCoV-19/Mexico/SLP_UASLP_A004/2021	EPI_ISL_1469115	20A	B.1.241	22	5'UTR:T10A, 5'UTR:T13A, 5'UTR:C241T, ORF1ab:C823T, ORF1ab:C3037T, ORF1ab:G4133A, ORF1ab:G6884A, ORF1ab:C11575T, ORF1ab:C14408T, ORF1ab:A16840C, ORF1ab:C19029T, ORF1ab:G20102T, ORF1ab:A20268G, S:C21676T, S:T22222C, S:A23403G, S:C23634T, S:G24872T, ORF6:C27213T, ORF7b:C27804A, ORF8:C28115T, N:G28364C, N:C28854T,	12	N:E31Q, N:S194L, ORF1a:V1290I, ORF1a:G2207S, ORF1b:P314L, ORF1b:K1125Q, ORF1b:S2212I, ORF7b:L17M, ORF9b:E27D, S:D614G, S:S691F, S:V1104L,	
hCoV-19/Mexico/SLP_UASLP_A005/2021	EPI_ISL_1469116	20A	B.1	20	5'UTR:C241T, ORF1ab:T1222G, ORF1ab:C3037T, ORF1ab:A5914G, ORF1ab:C6740T, ORF1ab:C11200T, ORF1ab:C12651T, ORF1ab:C14408T, ORF1ab:C15324T, ORF1ab:G15372T, ORF1ab:C17518T, ORF1ab:C18526T, ORF1ab:C19032T, S:T22020C, S:G22240A, S:A23403G, S:G23868T, ORF3a:G25913T, ORF8:C28253T, N:G28903C, 3'UTR:T29851C,	10	N:M210I, ORF1a:R2159W, ORF1a:T4129I, ORF1b:P314L, ORF1b:L1351F, ORF1b:R1687C, ORF3a:G174V, S:M153T, S:D614G, S:G769V,	

hCoV-19/Mexico/SLP_UASLP_A007/2021	EPI_ISL_1469117	20B	B.1.1.519	30	5'UTR:T18G, 5'UTR:C19G, 5'UTR:C20G, 5'UTR:C21A, 5'UTR:T201C, 5'UTR:C203T, 5'UTR:C222T, 5'UTR:C241T, ORF1ab:G1738T, ORF1ab:C3037T, ORF1ab:C3140T, ORF1ab:C10029T, ORF1ab:C109,54T, ORF1ab:A11117G, ORF1ab:C12789T, ORF1ab:C14408T, ORF1ab:T19839C, ORF1ab:A19974G, ORF1ab:C21057T, ORF1ab:C21306T, S:C22995A, S:A23403G, S:C23604A, S:A23756G, ORF3a:C25782T, N:G28881A, N:G28882A, N:G28883C, N:C29197T,5'UTR:A12T,	11	N:R203K, N:G204R, ORF1a:P959S, ORF1a:T3255I, ORF1a:I3618V, ORF1a:T4175I, ORF1b:P314L, S:T478K, S:D614G, S:P681H, S:T732A,
hCoV-19/Mexico/SLP_UASLP_A008/2021	EPI_ISL_1469118	20A	B.1	22	5'UTR:C241T, ORF1ab:A2010G, ORF1ab:C3037T, ORF1ab:A3904T, ORF1ab:A11430G, ORF1ab:C14408T, ORF1ab:A16534G, ORF1ab:T17673G, ORF1ab:G20060T, ORF1ab:A20268G, S:C21618T, S:C21707T, S:C22338G, S:T22912G, S:G22992A, S:A23403G, M:C26681T, M:G26709A, ORF7a:C27494T, ORF8:A28273T, N:G28514T, N:G28917T,5'UTR:A12T,	18	M:A63T, N:D81Y, N:G215V, ORF1a:D582G, ORF1a:Y3722C, ORF1b:P314L, ORF1b:S1023G, ORF1b:S2198I, ORF7a:P34L, ORF9b:Q77H, S:T19I, S:H49Y, S:L141F, S:T259R, S:N450K, S:S477N, S:D614G,S:Y144N,
hCoV-19/Mexico/SLP_UASLP_A009/2021	EPI_ISL_1469119	20B	B.1.1.519	32	5'UTR:T11G, 5'UTR:T18G, 5'UTR:C19G, 5'UTR:C20G, 5'UTR:C21A, 5'UTR:C203T, 5'UTR:C222T, 5'UTR:C241T, ORF1ab:C3037T, ORF1ab:C3140T, ORF1ab:G3692T, ORF1ab:G3871T, ORF1ab:C9803T, ORF1ab:C10029T, ORF1ab:A10194G, ORF1ab:C10954T, ORF1ab:A11117G, ORF1ab:C11824A, ORF1ab:C12789T, ORF1ab:C14408T, ORF1ab:T19839C, ORF1ab:C21306T, S:C22995A, S:A23403G, S:C23604A, S:A23756G, ORF8:T27904C, N:G28881A, N:G28882A, N:G28883C, N:C29197T,5'UTR:G8T, 5'UTR:A12T,	15	N:R203K, N:G204R, ORF1a:P959S, ORF1a:V1143F, ORF1a:K1202N, ORF1a:T3255I, ORF1a:E3310G, ORF1a:I3618V, ORF1a:T4175I, ORF1b:P314L, ORF8:L4P, S:T478K, S:D614G, S:P681H, S:T732A,
hCoV-19/Mexico/SLP_UASLP_A015/2021	EPI_ISL_1469108	20B	B.1.1.512	18	5'UTR:C241T, ORF1ab:C280T, ORF1ab:C2925T, ORF1ab:C3037T, ORF1ab:C4321T, ORF1ab:C7874T, ORF1ab:C8357T, ORF1ab:G10265A, ORF1ab:C14408T, S:C21811T, S:A23403G, S:G23593C, S:G24794T, S:A25256T, ORF8:C28076T, N:G28881A, N:G28882A, N:G28883C,5'UTR:A12T,	11	N:R203K, N:G204R, ORF1a:S887F, ORF1a:P2537S, ORF1a:H2698Y, ORF1a:G3334S, ORF1b:P314L, S:D614G, S:Q677H, S:A1078S, S:I1232F,
hCoV-19/Mexico/SLP_UASLP_A018/2021	EPI_ISL_1469109	20B	B.1.1.344	21	5'UTR:C241T, ORF1ab:C547T, ORF1ab:G2879A, ORF1ab:C3037T, ORF1ab:G3431T, ORF1ab:C3583T, ORF1ab:T9172C, ORF1ab:A10948G, ORF1ab:G11335T, ORF1ab:T11431C, ORF1ab:C14408T, ORF1ab:T16950C, S:C22450T, S:A23403G, S:C23604A, M:G26730T, M:C27143T, N:T28334C, N:G28881A, N:G28882A, N:G28883C, N:G29447T.	10	M:V70F, N:S21P, N:R203K, N:G204R, N:V392L, ORF1a:A872T, ORF1a:V1056L, ORF1b:P314L, S:D614G, S:P681H,
hCoV-19/Mexico/SLP_UASLP_A019/2021	EPI_ISL_1469110	20B	B.1.1.519	27	5'UTR:C203T, 5'UTR:C222T, 5'UTR:C241T, ORF1ab:C3037T, ORF1ab:C3140T, ORF1ab:G3692T, ORF1ab:G3871T, ORF1ab:C4009T, ORF1ab:C10029T, ORF1ab:C10954T, ORF1ab:A11117G, ORF1ab:C11824A, ORF1ab:C12789T, ORF1ab:T13686G, ORF1ab:G13687T, ORF1ab:C14408T, ORF1ab:T19839C, ORF1ab:C21306T, S:G22763T, S:C22995A, S:A23403G, S:C23604A, S:A23756G, ORF8:T27904C, N:G28881A, N:G28882A, N:G28883C, N:C29197T,	17	N:R203K, N:G204R, ORF1a:P959S, ORF1a:V1143F, ORF1a:K1202N, ORF1a:T3255I, ORF1a:I3618V, ORF1a:T4175I, ORF1b:H73Q, ORF1b:E74*, ORF1b:P314L, ORF8:L4P, S:V401L, S:T478K, S:D614G, S:P681H, S:T732A,

hCoV-19/Mexico/SLP_UASLP_A020/2021	EPI_ISL_1469111	20A	B.1.241	20	5'UTR:C241T, ORF1ab:C823T, ORF1ab:C3037T, ORF1ab:G4133A, ORF1ab:G6884A, ORF1ab:C11575T, ORF1ab:C14408T, ORF1ab:A16840C, ORF1ab:C19029T, ORF1ab:G20102T, ORF1ab:A20268G, S:C21676T, S:T22222C, S:A23403G, S:C23634T, S:G24872T, ORF6:C27213T, ORF7b:C27804A, ORF8:C28115T, N:G28364C. N:C28854T.	12	N:E31Q, N:S194L, ORF1a:V1290I, ORF1a:G2207S, ORF1b:P314L, ORF1b:K1125Q, ORF1b:S2212I, ORF7b:L17M, ORF9b:E27D, S:D614G, S:S691F, S:V1104L,
hCoV-19/Mexico/SLP_UASLP_A041/2021	EPI_ISL_1469113	20A	B.1	22	5'UTR:C241T, ORF1ab:C337T, ORF1ab:G2438A, ORF1ab:C2910T, ORF1ab:C3037T, ORF1ab:C14408T, ORF1ab:C16057T, ORF1ab:C18032T, ORF1ab:T18567C, ORF1ab:G20014T, ORF1ab:A20268G, ORF1ab:C20457T, S:A23403G, S:G23593T, ORF3a:C25931T, M:C26801T, ORF8:C28253T, N:G28378T, N:G28808A, N:C28854T, 3'UTR:C29750T, 3'UTR:G29755T,5'UTR:A12T,	12	N:G179S, N:S194L, ORF1a:E725K, ORF1a:T882I, ORF1b:P314L, ORF1b:P864S, ORF1b:T1522I, ORF1b:D2183Y, ORF3a:S180F, ORF9b:R32L, S:D614G, S:Q677H,
hCoV-19/Mexico/SLP_UASLP_A047/2021	EPI_ISL_1469114	20A	B.1.396	24	5'UTR:C241T, ORF1ab:C1191T, ORF1ab:C2091T, ORF1ab:C3037T, ORF1ab:C4331T, ORF1ab:C4901T, ORF1ab:C6151T, ORF1ab:T9124C, ORF1ab:C9521A, ORF1ab:G9928T, ORF1ab:G10996T, ORF1ab:C14408T, ORF1ab:A15204G, ORF1ab:T17065C, ORF1ab:A20268G, S:A21634G, S:A23403G, S:C24904T, S:G25249T, ORF3a:A25602G, ORF3a:G25644T, N:C28854T, N:G29422T, ORF10:A29567G,5'UTR:A12T,	10	N:S194L, ORF1a:P309L, ORF1a:T609I, ORF1a:L3086I, ORF1a:M3221I, ORF1a:K3577N, ORF1b:P314L, ORF1b:Y1200H, S:D614G, S:M1229I,
'-19/Mexico/SLP-AH1COV2SS033_S22 SLP-UASLP	EPI_ISL_1500639		B.1	0	,	1	,
'-19/Mexico/SLP-AH1COV2SS035_S24 SLP-UASLP	EPI_ISL_1500866		B.1.1	0	,	1	,
hCoV-19/Mexico/SLP-InDRE_130/2020	EPI_ISL_913933	20B	B.1.1.222	16	5'UTR:C241T, ORF1ab:C829T, ORF1ab:T2533C, ORF1ab:C3037T, ORF1ab:C11575T, ORF1ab:C14408T, ORF1ab:T19839C, ORF1ab:C20703T, S:A23403G, S:A23756G, ORF3a:C25603T, ORF3a:G26227T, ORF8:C27920T, N:G28655T, N:G28881A, N:G28882A, N:G28883C,	6	N:D128Y, N:R203K, N:G204R, ORF1b:P314L, S:D614G, S:T732A,
hCoV-19/Mexico/SLP-InDRE_145/2020	EPI_ISL_913948	20A	B.1.189	13	5'UTR:C241T, ORF1ab:G881A, ORF1ab:C3037T, ORF1ab:C14408T, ORF1ab:C16948T, ORF1ab:A20268G, S:A23403G, S:G23621T, S:C25207T, M:G26814T, ORF7a:A27742C, N:G28378T, N:C28854T, 3'UTR:G29745T,	9	M:A98S, N:S194L, ORF1a:A206T, ORF1b:P314L, ORF1b:P1161S, ORF7a:K117Q, ORF9b:R32L, S:D614G, S:V687L,
hCoV-19/Mexico/SLP-InDRE_162/2020	EPI_ISL_913963	20B	B.1.1.322	18	5'UTR:C241T, ORF1ab:C3037T, ORF1ab:C5284T, ORF1ab:C7471T, ORF1ab:T14401C, ORF1ab:C14408T, ORF1ab:G18394T, ORF1ab:G19327T, ORF1ab:T19839C, ORF1ab:C21306T, S:G22201T, S:A23403G, S:A23756G, N:A28877T, N:G28878C, N:G28881A, N:G28882A. N:G28883C. N:C29144T.	8	N:R203K, N:G204R, ORF1b:F312L, ORF1b:P314L, ORF1b:A1643S, ORF1b:A1954S, S:D614G, S:T732A,
hCoV-19/Mexico/SLP-InDRE_192/2020	EPI_ISL_933669	20A	B.1	19	5'UTR:C241T, 5'UTR:A257C, ORF1ab:C1473T, ORF1ab:C1594T, ORF1ab:C3037T, ORF1ab:C3264T, ORF1ab:T3493C, ORF1ab:C7843T, ORF1ab:C14408T, ORF1ab:C14790T, ORF1ab:C15963T, ORF1ab:T18358C, ORF1ab:A20268G, S:C21859T, S:A22771G, S:G23012A, S:A23403G, N:C28854T, N:C29253T, N:C29304T,	8	N:S194L, N:S327L, N:P344L, ORF1a:T403I, ORF1a:T1000I, ORF1b:P314L, S:E484K, S:D614G,
hCoV-19/Mexico/SLP-InDRE_260/2020	EPI_ISL_1054978	20B	B.1.1.222	16	5'UTR:C241T, ORF1ab:A1291G, ORF1ab:C2721T, ORF1ab:C3037T, ORF1ab:C13584T, ORF1ab:C14408T, ORF1ab:A19020G, ORF1ab:T19839C, ORF1ab:G21123T, S:A23403G, S:A23756G, ORF3a:G25775T, M:A26945G, N:G28817T. N:G28881A. N:G28882A. N:G28883C.	8	N:A182S, N:R203K, N:G204R, ORF1a:A819V, ORF1b:P314L, ORF3a:W128L, S:D614G, S:T732A,

hCoV-19/Mexico/SLP-InDRE_288/2020	EPI_ISL_1054942	20A	B.1	15	5'UTR:C119A, 5'UTR:C241T, ORF1ab:C1288T, ORF1ab:C1380T, ORF1ab:C2445T, ORF1ab:C3037T, ORF1ab:C3096T, ORF1ab:C8730T, ORF1ab:C14408T, ORF1ab:C18060T, ORF1ab:C18828T, ORF1ab:T19791G, ORF1ab:A20268G, S:A23403G, N:C28854T, 3'UTR:C29733T,	7	N:S194L, ORF1a:A372V, ORF1a:T727I, ORF1a:S944L, ORF1a:S2822F, ORF1b:P314L, S:D614G,
hCoV-19/Mexico/SLP-InDRE_289/2020	EPI_ISL_1054943	20A	B.1.1	4	5'UTR:C241T, ORF1ab:C3037T, ORF1ab:C14408T, ORF1ab:T19839C, S:A23403G,	2	ORF1b:P314L, S:D614G,
hCoV-19/Mexico/SLP-InDRE_301/2020	EPI_ISL_1060745	20C	B.1	10	5'UTR:C241T, ORF1ab:C1059T, ORF1ab:C3037T, ORF1ab:C9907T, ORF1ab:C14408T, ORF1ab:C17125T, ORF1ab:T18144C, ORF1ab:G21468T, S:C22664T, S:A23403G, ORF3a:G25563T,	6	ORF1a:T265I, ORF1b:P314L, ORF1b:L1220F, ORF1b:M2667I, ORF3a:Q57H, S:D614G,
hCoV-19/Mexico/SLP-InDRE_315/2020	EPI_ISL_1060702	20A	B.1	9	5'UTR:C241T, ORF1ab:C3037T, ORF1ab:C3411A, ORF1ab:C14408T, ORF1ab:A20268G, S:A23403G, S:T23569C, ORF3a:C25624T, E:T26390C, N:C28854T,	6	E:V49A, N:S194L, ORF1a:A1049D, ORF1b:P314L, ORF3a:H78Y, S:D614G,
hCoV-19/Mexico/SLP-InDRE_316/2020	EPI_ISL_1060695	20B	B.1.1.222	14	ORF1ab:C3037T, ORF1ab:C14408T, ORF1ab:T19839C, ORF1ab:C20806T, S:A23403G, S:A23756G, S:G25249T, ORF3a:C25515T, ORF3a:C25671T, M:A26612T, N:G28881A, N:G28882A, N:G28883C, N:C29085T, 3'UTR:C29732T,	7	N:R203K, N:G204R, N:T271I, ORF1b:P314L, S:D614G, S:T732A, S:M1229I,
hCoV-19/Mexico/SLP-InDRE_317/2020	EPI_ISL_1060750	20A	B.1.609	11	5'UTR:C241T, ORF1ab:C1077A, ORF1ab:C3037T, ORF1ab:C4582T, ORF1ab:A7796G, ORF1ab:C14408T, ORF1ab:C17104T, ORF1ab:A20055G, ORF1ab:A20268G, ORF1ab:A21137G, S:A23403G, 3'UTR:G29825C,	6	ORF1a:P271Q, ORF1a:K2511E, ORF1b:P314L, ORF1b:H1213Y, ORF1b:K2557R, S:D614G,
hCoV-19/Mexico/SLP-InDRE_367/2020	EPI_ISL_1060690	20B	B.1.1.222	14	ORF1ab:C3037T, ORF1ab:C7749T, ORF1ab:C8637T, ORF1ab:A10323G, ORF1ab:A13498G, ORF1ab:C14408T, ORF1ab:T19839C, S:G22331A, S:A23403G, S:G23593T, S:A23756G, N:G28881A, N:G28882A, N:G28883C, N:G28907A,	12	N:R203K, N:G204R, N:G212S, ORF1a:T2495I, ORF1a:T2791I, ORF1a:K3353R, ORF1b:T11A, ORF1b:P314L, S:G257S, S:D614G, S:Q677H, S:T732A,
hCoV-19/Mexico/SLP-InDRE_370/2020	EPI_ISL_1060721	20A	B.1.558	15	5'UTR:C241T, ORF1ab:C344T, ORF1ab:C1288T, ORF1ab:C3037T, ORF1ab:G7070T, ORF1ab:C9223T, ORF1ab:C14408T, ORF1ab:C19662T, ORF1ab:A20268G, ORF1ab:G20962T, S:G21785C, S:A23403G, S:G24781T, ORF3a:C26060T, ORF7a:C27630T, N:C28854T,	9	N:S194L, ORF1a:L27F, ORF1a:G2269C, ORF1b:P314L, ORF1b:D2499Y, ORF3a:T223I, S:G75R, S:D614G, S:K1073N,
hCoV-19/Mexico/SLP-InDRE_454/2021	EPI_ISL_1219714	20A	B.1.396	20	5'UTR:C241T, ORF1ab:C1191T, ORF1ab:C3037T, ORF1ab:C3096T, ORF1ab:C5055T, ORF1ab:C8092T, ORF1ab:C9521A, ORF1ab:C14408T, ORF1ab:T16494C, ORF1ab:C17304T, ORF1ab:A20268G, S:C21682T, S:G23012A, S:A23403G, S:C24904T, ORF7a:C27654T, ORF7b:C27893A, N:C28854T, N:G29422T, N:C29541T, ORF10:A29567G,	8	N:S194L, ORF1a:P309L, ORF1a:S944L, ORF1a:T1597I, ORF1a:L3086I, ORF1b:P314L, S:E484K, S:D614G,
hCoV-19/Mexico/SLP-InDRE-69/2020	EPI_ISL_576272	20A	B.1.189	10	5'UTR:C241T, ORF1ab:C3037T, ORF1ab:T3519C, ORF1ab:G3560A, ORF1ab:C4755T, ORF1ab:C14408T, ORF1ab:C16948T, ORF1ab:A20268G, S:A23403G, N:G28378T, N:C28854T,	8	N:S194L, ORF1a:V1085A, ORF1a:V1099M, ORF1a:P1497L, ORF1b:P314L, ORF1b:P1161S, ORF9b:R32L, S:D614G,
hCoV-19/Mexico/SLP-InDRE-77/2020	EPI_ISL_660068	20A	B.1.397	22	5'UTR:C241T, ORF1ab:G1135T, ORF1ab:C1314T, ORF1ab:G2639A, ORF1ab:C3037T, ORF1ab:A3362G, ORF1ab:A3867T, ORF1ab:G5037T, ORF1ab:C5167T, ORF1ab:C8208T, ORF1ab:C10641T, ORF1ab:C11442T, ORF1ab:C14408T, ORF1ab:G19656T, ORF1ab:A20268G, S:C23240T, S:A23403G, S:A25050T, ORF3a:G25650A, E:C26455T, N:C28310T, N:C28854T, 3'UTR:G29742A,	16	E:P71S, N:P13S, N:S194L, ORF1a:K290N, ORF1a:T350I, ORF1a:E792K, ORF1a:K1033E, ORF1a:Q1201L, ORF1a:G1591V, ORF1a:T2648I, ORF1a:T3459M, ORF1a:A3726V, ORF1b:P314L, ORF1b:K2063N, S:D614G, S:D1163V,

:oV-19/Mexico/SLP-UASLP-AH1COV2SS017_S6/20	EPI_ISL_1494728	20A	B.1.396	21	5'UTR:C241T, ORF1ab:C1191T, ORF1ab:C3037T, ORF1ab:C3096T, ORF1ab:C5055T, ORF1ab:C8092T, ORF1ab:C9521A, ORF1ab:C14408T, ORF1ab:T16494C, ORF1ab:C17304T, ORF1ab:A20268G, S:C21682T, S:G23012A, S:A23403G, S:C24904T, ORF7a:C27654T, ORF7b:C27893A, N:C28854T, N:G29422T, N:C29541T, ORF10:A29567G, 3'UTR:C29686T,	8	N:S194L, ORF1a:P309L, ORF1a:S944L, ORF1a:T1597I, ORF1a:L3086I, ORF1b:P314L, S:E484K, S:D614G,	
:oV-19/Mexico/SLP-UASLP-AH1COV2SS018_S7/20	EPI_ISL_1494729	20B	B.1.1.519	28	5'UTR:C203T, 5'UTR:C222T, 5'UTR:C241T, ORF1ab:C3037T, ORF1ab:C3140T, ORF1ab:G3692T, ORF1ab:G3871T, ORF1ab:C9391T, ORF1ab:C10029T, ORF1ab:C10954T, ORF1ab:A11117G, ORF1ab:G11562T, ORF1ab:C11824A, ORF1ab:C12499T, ORF1ab:C12789T, ORF1ab:C14408T, ORF1ab:T19839C, ORF1ab:C21306T, S:C22995A, S:A23403G, S:C23604A, S:A23756G, ORF8:T27904C, N:C28348T, N:G28881A, N:G28882A, N:G28883C, N:C29197T, 3'UTR:C29870A,	16	N:R203K, N:G204R, ORF1a:P959S, ORF1a:V1143F, ORF1a:K1202N, ORF1a:T3255I, ORF1a:I3618V, ORF1a:C3766F, ORF1a:T4175I, ORF1b:P314L, ORF8:L4P, ORF9b:A22V, S:T478K, S:D614G, S:P681H, S:T732A,	
:oV-19/Mexico/SLP-UASLP-AH1COV2SS019_S8/20	EPI_ISL_1494730	20B	B.1.1.519	24	5'UTR:C203T, 5'UTR:C222T, 5'UTR:C241T, ORF1ab:C3037T, ORF1ab:C3140T, ORF1ab:A6393C, ORF1ab:C10029T, ORF1ab:C10954T, ORF1ab:A11117G, ORF1ab:C12789T, ORF1ab:C14408T, ORF1ab:T19839C, ORF1ab:C21306T, S:C22995A, S:A23403G, S:C23604A, S:A23756G, S:G24834A, N:G28881A, N:G28882A, N:G28883C, N:C29095T, N:C29197T, 3'UTR:T29867A, 3'UTR:C29870A,	13	N:R203K, N:G204R, ORF1a:P959S, ORF1a:D2043A, ORF1a:T3255I, ORF1a:I3618V, ORF1a:T4175I, ORF1b:P314L, S:T478K, S:D614G, S:P681H, S:T732A, S:R1091H,	
oV-19/Mexico/SLP-UASLP-AH1COV2SS024_S13/2(	EPI_ISL_1494674	20A	B.1.189	13	5'UTR:C241T, ORF1ab:C4582T, ORF1ab:G6404T, ORF1ab:C14408T, ORF1ab:A20268G, ORF1ab:C20703T, S:A23403G, S:G24812T, S:T25123C, ORF7a:G27676T, N:G28378T, 3'UTR:T29867A, 3'UTR:G29868A, 3'UTR:C29870A,	6	ORF1a:V2047F, ORF1b:P314L, ORF7a:E95*, ORF9b:R32L, S:D614G, S:D1084Y,	
oV-19/Mexico/SLP-UASLP-AH1COV2SS025_S14/2(	EPI_ISL_1494717	20A	B.1.189	10	5'UTR:C241T, ORF1ab:T1483A, ORF1ab:C3037T, ORF1ab:C4582T, ORF1ab:G6404T, ORF1ab:C14408T, ORF1ab:A20268G, S:A23403G, S:T25123C, ORF7a:G27676T, N:G28378T,	6	ORF1a:F406L, ORF1a:V2047F, ORF1b:P314L, ORF7a:E95*, ORF9b:R32L, S:D614G,	
oV-19/Mexico/SLP-UASLP-AH1COV2SS026_S15/2(	EPI_ISL_1494724	20C	B.1	10	5'UTR:C241T, ORF1ab:C1059T, ORF1ab:C1348T, ORF1ab:C3037T, ORF1ab:C11152T, ORF1ab:C14408T, S:A23403G, ORF3a:G25563T, N:C28887T, 3'UTR:C29750T, 3'UTR:A29792G,	5	N:T205I, ORF1a:T265I, ORF1b:P314L, ORF3a:Q57H, S:D614G,	ORF7b:27792-27793,
oV-19/Mexico/SLP-UASLP-AH1COV2SS027_S16/2(	EPI_ISL_1494725	20A	B.1.189	10	5'UTR:C241T, ORF1ab:C3037T, ORF1ab:C3425T, ORF1ab:C4582T, ORF1ab:G6404T, ORF1ab:C7834T, ORF1ab:C14408T, ORF1ab:A20268G, S:A23403G, S:T25123C, N:G28378T,	5	ORF1a:P1054S, ORF1a:V2047F, ORF1b:P314L, ORF9b:R32L, S:D614G,	
oV-19/Mexico/SLP-UASLP-AH1COV2SS028_S17/2(	EPI_ISL_1494726	20A	B.1.189	17	5'UTR:C241T, ORF1ab:C3037T, ORF1ab:G3482A, ORF1ab:G3871T, ORF1ab:G4510T, ORF1ab:C4582T, ORF1ab:G6404T, ORF1ab:C9967T, ORF1ab:A12829G, ORF1ab:C14408T, ORF1ab:C19273T, ORF1ab:A20268G, S:G22021T, S:A23403G, S:T25123C, S:C25156T, ORF7a:G27676T, N:G28378T,	9	ORF1a:G1073R, ORF1a:K1202N, ORF1a:V2047F, ORF1b:P314L, ORF1b:P1936S, ORF7a:E95*, ORF9b:R32L, S:M153I, S:D614G,	
oV-19/Mexico/SLP-UASLP-AH1COV2SS029_S18/2(	EPI_ISL_1494727	20A	B.1.610	6	5'UTR:C241T, ORF1ab:C3037T, ORF1ab:C14408T, ORF1ab:A20268G, S:A23403G, S:A23440T, N:C28677A,	3	N:T135N, ORF1b:P314L, S:D614G,	
hCoV-19/Mexico/SON-InDRE_135/2020	EPI_ISL_913938	20A	B.1	11	5'UTR:T221C, 5'UTR:C241T, ORF1ab:C3037T, ORF1ab:C3165T, ORF1ab:C5730A, ORF1ab:C14408T, ORF1ab:A20268G, S:A23403G, ORF3a:C26039T, ORF3a:G26199T, M:C26873T, N:C28854T,	6	N:S194L, ORF1a:A967V, ORF1a:T1822N, ORF1b:P314L, ORF3a:S216L, S:D614G,	

hCoV-19/Mexico/SON-InDRE_29/2020	EPI_ISL_493344	20A	B.1.558	7	5'UTR:C241T, ORF1ab:C1288T, ORF1ab:C3037T, ORF1ab:C11575T, ORF1ab:C14408T, ORF1ab:C18060T, S:A23403G, N:C28854T,	3	N:S194L, ORF1b:P314L, S:D614G,
hCoV-19/Mexico/SON-InDRE_323/2020	EPI_ISL_1060755	20A	B.1.243	16	5'UTR:C241T, ORF1ab:A2497G, ORF1ab:C3037T, ORF1ab:C7113T, ORF1ab:T13354C, ORF1ab:C14408T, ORF1ab:C19572T, ORF1ab:A20268G, ORF1ab:G20548T, ORF1ab:G20679T, S:A23403G, S:T24076C, S:C24337T, S:G25266T, M:T26669C, M:G26690T, N:C28854T,	6	N:S194L, ORF1a:T2283I, ORF1b:P314L, ORF1b:D2361Y, S:D614G, S:C1235F,
hCoV-19/Mexico/SON-InDRE_328/2020	EPI_ISL_1060756	20A	B.1	12	5'UTR:C241T, ORF1ab:C1179T, ORF1ab:T3011C, ORF1ab:C3037T, ORF1ab:C4331T, ORF1ab:C8802T, ORF1ab:C14408T, ORF1ab:C16887T, ORF1ab:A18019C, ORF1ab:C18312T, ORF1ab:A20055G, S:C21575T, S:A23403G,	5	ORF1a:P305L, ORF1a:T2846I, ORF1b:P314L, S:L5F, S:D614G,
hCoV-19/Mexico/SON-InDRE_329/2020	EPI_ISL_1060763	20A	B.1	8	5'UTR:C241T, ORF1ab:C3037T, ORF1ab:A8040G, ORF1ab:C14408T, ORF1ab:A20268G, S:A23403G, ORF8:C28253T, N:C28854T, 3'UTR:C29741T,	4	N:S194L, ORF1a:D2592G, ORF1b:P314L, S:D614G,
hCoV-19/Mexico/SON-InDRE_330/2020	EPI_ISL_1060759	20A	B.1.105	15	5'UTR:C106T, 5'UTR:C241T, ORF1ab:C3037T, ORF1ab:G6884A, ORF1ab:C14408T, ORF1ab:A16840C, ORF1ab:G18040T, ORF1ab:C19029T, ORF1ab:A20268G, S:T22222C, S:C22227T, S:A23403G, ORF6:G27214T, ORF8:C28115T, N:G28514T, N:C28854T,	10	N:D81Y, N:S194L, ORF1a:G2207S, ORF1b:P314L, ORF1b:K1125Q, ORF1b:A1525S, ORF6:V5F, ORF9b:Q77H, S:A222V, S:D614G,
hCoV-19/Mexico/SON-InDRE_336/2020	EPI_ISL_1060680	20A	B.1	13	ORF1ab:C823T, ORF1ab:C1489T, ORF1ab:G1738T, ORF1ab:C3037T, ORF1ab:C3096T, ORF1ab:T9440A, ORF1ab:A11140T, ORF1ab:G13762A, ORF1ab:C14408T, ORF1ab:C16329T, ORF1ab:A20268G, S:C23248T, S:A23403G, N:G28899T,	6	N:R209I, ORF1a:S944L, ORF1a:C3059S, ORF1b:G99S, ORF1b:P314L, S:D614G,
hCoV-19/Mexico/SON-InDRE_339/2020	EPI_ISL_1060753	20A	B.1.402	15	5'UTR:C241T, ORF1ab:C3037T, ORF1ab:C4582T, ORF1ab:C4633T, ORF1ab:G5518A, ORF1ab:C8175T, ORF1ab:C14408T, ORF1ab:C19402T, ORF1ab:A20268G, S:A23403G, S:G23587T, S:C24370T, ORF3a:G25471T, ORF8:G28085T, ORF10:G29645A, 3'UTR:G29868C,	7	ORF1a:A2637V, ORF1b:P314L, ORF1b:H1979Y, ORF3a:D27Y, ORF8:E64D, S:D614G, S:Q675H,
hCoV-19/Mexico/SON-InDRE_34/2020	EPI_ISL_493349	20A	B.1.558	8	5'UTR:C241T, ORF1ab:C1288T, ORF1ab:C3037T, ORF1ab:C11575T, ORF1ab:C14408T, ORF1ab:C18060T, ORF1ab:A20268G, S:A23403G, N:C28854T,	3	N:S194L, ORF1b:P314L, S:D614G,
hCoV-19/Mexico/SON-InDRE_35/2020	EPI_ISL_516622	20A	B.1	12	5'UTR:C241T, ORF1ab:C3037T, ORF1ab:C9438T, ORF1ab:C12756T, ORF1ab:C14408T, ORF1ab:C14625T, ORF1ab:A20268G, S:G21793A, S:G21795T, S:C22227T, S:A23403G, S:T24076C, N:C28854T,	7	N:S194L, ORF1a:T3058I, ORF1a:T4164I, ORF1b:P314L, S:R78M, S:A222V, S:D614G,
hCoV-19/Mexico/SON-InDRE_398/2020	EPI_ISL_1054975	20B	B.1.1.222	15	5'UTR:C241T, ORF1ab:C1912T, ORF1ab:C3037T, ORF1ab:C3884T, ORF1ab:T7022C, ORF1ab:C11650T, ORF1ab:C14408T, ORF1ab:G17259C, ORF1ab:T19839C, S:A23403G, S:A23756G, ORF8:T28199C, ORF8:T28234C, N:G28881A, N:G28882A, N:G28883C,	8	N:R203K, N:G204R, ORF1a:P1207S, ORF1b:P314L, ORF1b:E1264D, ORF8:V114A, S:D614G, S:T732A,
hCoV-19/Mexico/SON-InDRE_42/2020	EPI_ISL_516623	20A	B.1.243	8	5'UTR:C241T, ORF1ab:C3037T, ORF1ab:C3551T, ORF1ab:A10181G, ORF1ab:C14408T, ORF1ab:A20268G, S:A23403G, S:T24076C, N:C28854T,	5	N:S194L, ORF1a:P1096S, ORF1a:I3306V, ORF1b:P314L, S:D614G,
hCoV-19/Mexico/SON-InDRE-103/2020	EPI_ISL_872093	20A	B.1	5	5'UTR:C241T, ORF1ab:C3037T, ORF1ab:C14408T, ORF1ab:A20268G, S:A23403G, N:C28854T,	3	N:S194L, ORF1b:P314L, S:D614G,
hCoV-19/Mexico/SON-InDRE-99/2020	EPI_ISL_872089	20C	B.1.320	10	5'UTR:C241T, ORF1ab:C379A, ORF1ab:C1059T, ORF1ab:T2153C, ORF1ab:C3037T, ORF1ab:G4657A, ORF1ab:C14408T, S:G21578T, S:A23403G, ORF3a:G25563T, N:G28842T,	7	N:S190I, ORF1a:T265I, ORF1a:W630R, ORF1b:P314L, ORF3a:Q57H, S:V6F, S:D614G,

hCoV-19/Mexico/TAB-InDRE_380/2020	EPI_ISL_1054980	20A	B.1.243	13	5'UTR:C241T, ORF1ab:G806A, ORF1ab:C3037T, ORF1ab:A3674C, ORF1ab:G4951A, ORF1ab:C13708T, ORF1ab:C14408T, ORF1ab:G17964T, ORF1ab:A20268G, S:C21627T, S:A23403G, S:T24076C, N:C28854T, N:G29543T,	8	N:S194L, ORF1a:A181T, ORF1a:N1137H, ORF1b:L81F, ORF1b:P314L, ORF1b:M1499I, S:T22I, S:D614G,	
hCoV-19/Mexico/TAB-InDRE_446/2021	EPI_ISL_1168524	20B	B.1.1.222	18	5'UTR:C241T, ORF1ab:C1968T, ORF1ab:G2305T, ORF1ab:C3037T, ORF1ab:G3692T, ORF1ab:T4138C, ORF1ab:G11221T, ORF1ab:C11919T, ORF1ab:C12781T, ORF1ab:C14408T, ORF1ab:T15717C, ORF1ab:T19839C, S:C22664T, S:A23403G, S:A23756G, N:G28881A, N:G28882A, N:G28883C, 3'UTR:C29754T,	10	N:R203K, N:G204R, ORF1a:T568I, ORF1a:K680N, ORF1a:V1143F, ORF1a:M3652I, ORF1a:S3885F, ORF1b:P314L, S:D614G, S:T732A,	
hCoV-19/Mexico/TAB-InDRE-100/2020	EPI_ISL_872090	20C	B.1	6	5'UTR:C241T, ORF1ab:C1059T, ORF1ab:C3037T, ORF1ab:C7165T, ORF1ab:C14408T, S:A23403G, ORF3a:G25563T,	4	ORF1a:T265I, ORF1b:P314L, ORF3a:Q57H, S:D614G,	
hCoV-19/Mexico/TAB-InDRE-105/2020	EPI_ISL_872098	20A	B.1	5	5'UTR:C241T, ORF1ab:C3037T, ORF1ab:T6678C, ORF1ab:C14408T, ORF1ab:A20268G, S:A23403G,	3	ORF1a:I2138T, ORF1b:P314L, S:D614G,	
hCoV-19/Mexico/TAM-InDRE_119/2020	EPI_ISL_913923	20C	B.1	14	5'UTR:C241T, ORF1ab:C346T, ORF1ab:C1059T, ORF1ab:C3037T, ORF1ab:A3881G, ORF1ab:C9870T, ORF1ab:C14408T, ORF1ab:C17634T, ORF1ab:C19545T, ORF1ab:T20174C, S:G21950C, S:A23403G, ORF3a:A25431G, ORF3a:G25459T, ORF3a:G25563T,	9	ORF1a:T265I, ORF1a:I1206V, ORF1a:T3202M, ORF1b:P314L, ORF1b:V2236A, ORF3a:A23S, ORF3a:Q57H, S:V130L, S:D614G,	
hCoV-19/Mexico/TAM-InDRE_149/2020	EPI_ISL_913952	20B	B.1.1.432	26	5'UTR:C241T, ORF1ab:C3037T, ORF1ab:T4823C, ORF1ab:T6067C, ORF1ab:A6985T, ORF1ab:C9319T, ORF1ab:G10617A, ORF1ab:C12412T, ORF1ab:C12459T, ORF1ab:T14313C, ORF1ab:C14408T, ORF1ab:C15240T, ORF1ab:G15327T, ORF1ab:C15371T, S:T22070C, S:A23403G, ORF3a:C25413T, ORF3a:C25613T, ORF3a:G25912T, M:C26636T, ORF8:T27919C, N:G28881A, N:G28882A, N:G28883C, N:G29204A, N:T29317C, 3'UTR:G29759T,	14	N:R203K, N:G204R, N:A311T, ORF1a:S1520P, ORF1a:R3451K, ORF1a:T4065I, ORF1b:P314L, ORF1b:M620I, ORF1b:T635M, ORF3a:S74F, ORF3a:G174C, ORF8:I9T, S:Y170H, S:D614G,	
hCoV-19/Mexico/TAM-InDRE_165/2020	EPI_ISL_913966	20B	B.1.1.222	20	5'UTR:C241T, ORF1ab:C3037T, ORF1ab:C4795T, ORF1ab:C9559T, ORF1ab:G11417T, ORF1ab:C14408T, ORF1ab:G16245A, ORF1ab:C18714T, ORF1ab:G19086T, ORF1ab:T19839C, ORF1ab:A21526G, S:A23403G, S:A23756G, S:T24346C, ORF3a:G25429T, ORF3a:C25771T, ORF7a:T27722C, ORF8:C27944T, N:G28881A, N:G28882A, N:G28883C,	11	N:R203K, N:G204R, ORF1a:V3718F, ORF1b:P314L, ORF1b:K1873N, ORF1b:I2687V, ORF3a:V13L, ORF3a:L127F, ORF7a:I110T, S:D614G, S:T732A,	
hCoV-19/Mexico/TAM-InDRE_189/2020	EPI_ISL_933666	20A	B.1	10	5'UTR:C241T, ORF1ab:C3037T, ORF1ab:A3076C, ORF1ab:C6633T, ORF1ab:C14408T, ORF1ab:A20268G, S:A23403G, S:C25047T, ORF8:C28253T, N:G28747T, N:C28854T,	6	N:S194L, ORF1a:E937D, ORF1a:A2123V, ORF1b:P314L, S:D614G, S:P1162L,	
hCoV-19/Mexico/TAM-InDRE_236/2021	EPI_ISL_961466	20I (Alpha, V1)	B.1.1.7	31	5'UTR:C241T, ORF1ab:C913T, ORF1ab:C3037T, ORF1ab:C3267T, ORF1ab:C5388A, ORF1ab:C5986T, ORF1ab:T6954C, ORF1ab:C14408T, ORF1ab:C14676T, ORF1ab:C15279T, ORF1ab:T16176C, ORF1ab:A17615G, ORF1ab:C18032T, S:A23063T, S:C23271A, S:A23403G, S:C23604A, S:C23709T, S:T24506G, S:G24914C, M:G26730C, ORF8:C27972T, ORF8:G28048T, ORF8:A28111G, N:G28280C, N:A28281T, N:T28282A, N:C28603T, N:G28881A, N:G28882A, N:G28883C, N:C28977T,	21	M:V70L, N:D3L, N:R203K, N:G204R, N:S235F, ORF1a:T1001I, ORF1a:A1708D, ORF1a:I2230T, ORF1b:P314L, ORF1b:K1383R, ORF1b:T1522I, ORF8:Q27*, ORF8:R52I, ORF8:Y73C, S:N501Y, S:A570D, S:D614G, S:P681H, S:T716I, S:S982A, S:D1118H,	ORF1ab: 11288-11296, S:21765-21770, S:21992-21994,

hCoV-19/Mexico/TAM-InDRE_244/2021	EPI_ISL_1008713	20I (Alpha, V1)	B.1.1.7	33	5'UTR:C241T, ORF1ab:C292T, ORF1ab:C913T, ORF1ab:C3037T, ORF1ab:C3267T, ORF1ab:C5388A, ORF1ab:C5986T, ORF1ab:T6954C, ORF1ab:C14408T, ORF1ab:C14676T, ORF1ab:C15279T, ORF1ab:T16176C, S:A23063T, S:C23170T, S:C23271A, S:A23403G, S:C23604A, S:C23709T, S:T24506G, S:G24914C, ORF3a:C25626T, ORF8:C27972T, ORF8:G28048T, ORF8:A28111G, N:G28280C, N:A28281T, N:T28282A, N:A28295C, N:G28881A, N:G28882A, N:G28883C, N:C28977T, 3'UTR:G29862T, 3'UTR:A29866C,	20	N:D3L, N:N8H, N:R203K, N:G204R, N:S235F, ORF1a:T1001I, ORF1a:A1708D, ORF1a:I2230T, ORF1b:P314L, ORF8:Q27*, ORF8:R52I, ORF8:Y73C, ORF9b:K4N, S:N501Y, S:A570D, S:D614G, S:P681H, S:T716I, S:S982A, S:D1118H,	ORF1ab: 11288-11296, S:21765-21770, S:21992-21994, 3'UTR:29869,
hCoV-19/Mexico/TAM-InDRE_253/2020	EPI_ISL_1054994	20A	B.1.243	14	5'UTR:C241T, ORF1ab:G806A, ORF1ab:C2106T, ORF1ab:C3037T, ORF1ab:A4050G, ORF1ab:C4331T, ORF1ab:G11083T, ORF1ab:C14408T, ORF1ab:C17373T, ORF1ab:A20268G, S:G22604T, S:A23403G, S:T24076C, N:C28854T, N:G29543T,	8	N:S194L, ORF1a:A181T, ORF1a:T614I, ORF1a:N1262S, ORF1a:L3606F, ORF1b:P314L, S:A348S, S:D614G,	
hCoV-19/Mexico/TAM-InDRE_279/2020	EPI_ISL_1054955	20B	B.1.1	8	5'UTR:C241T, ORF1ab:C3037T, ORF1ab:C14408T, ORF1ab:T19839C, S:A23403G, N:G28881A, N:G28882A, N:G28883C, 3'UTR:G29868A,	4	N:R203K, N:G204R, ORF1b:P314L, S:D614G,	
hCoV-19/Mexico/TAM-InDRE_287/2020	EPI_ISL_1054941	20A	B.1	10	5'UTR:C241T, 5'UTR:A257C, ORF1ab:C1594T, ORF1ab:C2942T, ORF1ab:C3037T, ORF1ab:A3273C, ORF1ab:C14408T, ORF1ab:G18105T, ORF1ab:A20268G, S:A23403G, N:C28854T,	5	N:S194L, ORF1a:Q1003P, ORF1b:P314L, ORF1b:Q1546H, S:D614G,	
hCoV-19/Mexico/TAM-InDRE_300/2020	EPI_ISL_1060744	20B	B.1.1	11	5'UTR:C241T, ORF1ab:C3037T, ORF1ab:C6636T, ORF1ab:A8170G, ORF1ab:C14408T, ORF1ab:G18781T, ORF1ab:T19839C, S:A23403G, N:G28881A, N:G28882A, N:G28883C, N:C29167T,	6	N:R203K, N:G204R, ORF1a:T2124I, ORF1b:P314L, ORF1b:G1772C, S:D614G,	
hCoV-19/Mexico/TAM-InDRE_308/2020	EPI_ISL_1060737	20A	B.1.610	4	5'UTR:C241T, ORF1ab:C3037T, ORF1ab:C14408T, ORF1ab:A20268G, S:A23403G,	2	ORF1b:P314L, S:D614G,	
hCoV-19/Mexico/TAM-InDRE_344/2020	EPI_ISL_1060693	20B	B.1.1.432	18	ORF1ab:G1312T, ORF1ab:A2761G, ORF1ab:C3037T, ORF1ab:C9319T, ORF1ab:G9526T, ORF1ab:C12412T, ORF1ab:T14313C, ORF1ab:C14408T, ORF1ab:C16208T, S:A23403G, ORF3a:A25524G, ORF3a:C25613T, ORF3a:G26062T, N:C28603T, N:G28631A, N:G28881A, N:G28882A, N:G28883C, N:T29317C,	10	N:G120R, N:R203K, N:G204R, ORF1a:L349F, ORF1a:M3087I, ORF1b:P314L, ORF1b:A914V, ORF3a:S74F, ORF3a:G224C, S:D614G,	
hCoV-19/Mexico/TAM-InDRE_38/2020	EPI_ISL_516624	20C	B.1	9	5'UTR:C241T, ORF1ab:C1059T, ORF1ab:C3037T, ORF1ab:T3825C, ORF1ab:C14408T, ORF1ab:C15240T, ORF1ab:C16332T, S:G22616T, S:A23403G, ORF3a:G25563T,	6	ORF1a:T265I, ORF1a:V1187A, ORF1b:P314L, ORF3a:Q57H, S:A352S, S:D614G,	
hCoV-19/Mexico/TAM-InDRE_382/2020	EPI_ISL_1054985	20A	B.1	5	5'UTR:C241T, ORF1ab:C3037T, ORF1ab:C14408T, ORF1ab:A20268G, S:A23403G, N:C28854T,	3	N:S194L, ORF1b:P314L, S:D614G,	
hCoV-19/Mexico/TAM-InDRE_388/2020	EPI_ISL_1054981	20A	B.1	10	5'UTR:C241T, 5'UTR:A257C, ORF1ab:T1465C, ORF1ab:T2008C, ORF1ab:C3037T, ORF1ab:A13913C, ORF1ab:C14408T, ORF1ab:A20268G, S:A23403G, N:C28854T, N:G29179T,	4	N:S194L, ORF1b:N149T, ORF1b:P314L, S:D614G,	
hCoV-19/Mexico/TAM-InDRE_389/2020	EPI_ISL_1054982	20A	B.1	14	5'UTR:C241T, ORF1ab:C3037T, ORF1ab:A3058G, ORF1ab:C6347T, ORF1ab:C8407T, ORF1ab:C12400T, ORF1ab:C14408T, ORF1ab:A19611T, ORF1ab:A20268G, S:C22689T, S:A23403G, S:G24000T, E:C26461T, ORF8:C28253T, N:C28854T,	6	E:L73F, N:S194L, ORF1b:P314L, S:T376I, S:D614G, S:S813I,	
hCoV-19/Mexico/TAM-InDRE_39/2020	EPI_ISL_516625	20A	B.1	9	5'UTR:C241T, ORF1ab:C3037T, ORF1ab:C3992T, ORF1ab:C6696T, ORF1ab:C7104T, ORF1ab:C14408T, ORF1ab:A20268G, S:A23403G, ORF3a:G25996T, N:G29477T,	6	N:D402Y, ORF1a:P2144L, ORF1a:T2280I, ORF1b:P314L, ORF3a:V202L, S:D614G,	

hCoV-19/Mexico/TAM-InDRE_449/2021	EPI_ISL_1168527	20G	B.1.2	21	5'UTR:C241T, ORF1ab:A929G, ORF1ab:C1059T, ORF1ab:C3037T, ORF1ab:T7348A, ORF1ab:G8083A, ORF1ab:C10319T, ORF1ab:C13119T, ORF1ab:C14408T, ORF1ab:C14805T, ORF1ab:A15177C, ORF1ab:C18131T, ORF1ab:A18424G, ORF1ab:A19067T, ORF1ab:C21304T, S:A23403G, ORF3a:G25563T, ORF3a:G25907T, ORF8:C27964T, N:C28472T, N:C28869T, 3'UTR:C29732T,	17	N:P67S, N:P199L, ORF1a:I222V, ORF1a:T265I, ORF1a:N2361K, ORF1a:M2606I, ORF1a:L3352F, ORF1a:A4285V, ORF1b:P314L, ORF1b:T1555I, ORF1b:N1653D, ORF1b:Q1867L, ORF1b:R2613C, ORF3a:Q57H, ORF3a:G172V, ORF8:S24L, S:D614G,	
hCoV-19/Mexico/TAM-InDRE_450/2021	EPI_ISL_1168528	20B	B.1.1.432	17	5'UTR:C241T, ORF1ab:A3019G, ORF1ab:C3037T, ORF1ab:A6985T, ORF1ab:C9319T, ORF1ab:C12412T, ORF1ab:C12924T, ORF1ab:T14313C, ORF1ab:C14408T, ORF1ab:G16206T, ORF1ab:G16917T, ORF1ab:G21255C, S:A23403G, ORF3a:C25513T, N:G28881A, N:G28882A, N:G28883C, N:T29317C,	7	N:R203K, N:G204R, ORF1a:P4220L, ORF1b:P314L, ORF1b:E913D, ORF3a:L41F, S:D614G,	
hCoV-19/Mexico/TAM-InDRE_453/2021	EPI_ISL_1219713	20B	B.1.1.222	15	5'UTR:C241T, ORF1ab:C2061T, ORF1ab:C3037T, ORF1ab:C11824T, ORF1ab:G13723T, ORF1ab:C14408T, ORF1ab:G14653T, ORF1ab:G17395A, ORF1ab:T19839C, S:A23403G, S:A23756G, ORF8:G28083T, N:G28881A, N:G28882A, N:G28883C, N:A28996G,	10	N:R203K, N:G204R, ORF1a:A599V, ORF1b:A86S, ORF1b:P314L, ORF1b:V396F, ORF1b:V1310I, ORF8:E64*, S:D614G, S:T732A,	S:21765-21770,
hCoV-19/Mexico/TAM-InDRE_455/2021	EPI_ISL_1219715	20B	B.1.1.222	14	5'UTR:C241T, ORF1ab:C2061T, ORF1ab:C3037T, ORF1ab:C11824T, ORF1ab:G13723T, ORF1ab:C14408T, ORF1ab:G14653T, ORF1ab:G17395A, ORF1ab:T19839C, S:A23403G, S:A23756G, ORF8:G28083T, N:G28881A, N:G28882A, N:G28883C,	10	N:R203K, N:G204R, ORF1a:A599V, ORF1b:A86S, ORF1b:P314L, ORF1b:V396F, ORF1b:V1310I, ORF8:E64*, S:D614G, S:T732A,	S:21765-21770,
hCoV-19/Mexico/TAM-InDRE_456/2021	EPI_ISL_1219716	20B	B.1.1.222	15	5'UTR:C241T, ORF1ab:C2061T, ORF1ab:C3037T, ORF1ab:G6421T, ORF1ab:C11824T, ORF1ab:G13723T, ORF1ab:C14408T, ORF1ab:G14653T, ORF1ab:G17395A, ORF1ab:T19839C, S:A23403G, S:A23756G, ORF8:G28083T, N:G28881A, N:G28882A, N:G28883C,	10	N:R203K, N:G204R, ORF1a:A599V, ORF1b:A86S, ORF1b:P314L, ORF1b:V396F, ORF1b:V1310I, ORF8:E64*, S:D614G, S:T732A,	S:21765-21770,
hCoV-19/Mexico/TAM-InDRE_457/2021	EPI_ISL_1219717	20B	B.1.1.222	15	5'UTR:C241T, ORF1ab:C2061T, ORF1ab:C3037T, ORF1ab:G6421T, ORF1ab:C11824T, ORF1ab:G13723T, ORF1ab:C14408T, ORF1ab:G14653T, ORF1ab:G17395A, ORF1ab:T19839C, S:A23403G, S:A23756G, ORF8:G28083T, N:G28881A, N:G28882A, N:G28883C,	10	N:R203K, N:G204R, ORF1a:A599V, ORF1b:A86S, ORF1b:P314L, ORF1b:V396F, ORF1b:V1310I, ORF8:E64*, S:D614G, S:T732A,	S:21765-21770,
hCoV-19/Mexico/TAM-InDRE_458/2021	EPI_ISL_1219718	20B	B.1.1.222	16	5'UTR:C241T, ORF1ab:C2061T, ORF1ab:C3037T, ORF1ab:T8923C, ORF1ab:C11824T, ORF1ab:G13723T, ORF1ab:C14408T, ORF1ab:C15738T, ORF1ab:G17395A, ORF1ab:T19839C, S:A23403G, S:A23756G, ORF8:G28083T, N:G28881A, N:G28882A, N:G28883C, N:A28996G,	9	N:R203K, N:G204R, ORF1a:A599V, ORF1b:A86S, ORF1b:P314L, ORF1b:V1310I, ORF8:E64*, S:D614G, S:T732A,	S:21765-21770,
hCoV-19/Mexico/TAM-InDRE_F10561_S683/2021	EPI_ISL_1365649	20B	B.1.1.222	14	5'UTR:C241T, ORF1ab:C3037T, ORF1ab:C3505T, ORF1ab:A13498G, ORF1ab:C14408T, ORF1ab:C16393T, ORF1ab:T16899C, ORF1ab:T19839C, S:A23403G, S:A23756G, S:A24292G, ORF7a:T27672C, N:G28881A, N:G28882A, N:G28883C,	7	N:R203K, N:G204R, ORF1b:T11A, ORF1b:P314L, ORF1b:P976S, S:D614G, S:T732A,	

hCoV-19/Mexico/TAM-InDRE_F108L_S569/2021	EPI_ISL_1334382	20I (Alpha, V1)	B.1.1.7	32	5'UTR:C241T, ORF1ab:C292T, ORF1ab:C913T, ORF1ab:C3037T, ORF1ab:C3267T, ORF1ab:C5388A, ORF1ab:C5986T, ORF1ab:T6954C, ORF1ab:C14408T, ORF1ab:C14676T, ORF1ab:C15279T, ORF1ab:T16176C, ORF1ab:T16563A, S:A23063T, S:C23170T, S:C23271A, S:A23403G, S:C23604A, S:C23709T, S:T24506G, S:G24914C, ORF3a:C25626T, ORF8:C27972T, ORF8:G28048T, ORF8:A28111G, N:G28280C, N:A28281T, N:T28282A, N:A28295C, N:G28881A, N:G28882A, N:G28883C, N:C28977T,	20	N:D3L, N:N8H, N:R203K, N:G204R, N:S235F, ORF1a:T1001I, ORF1a:A1708D, ORF1a:I2230T, ORF1b:P314L, ORF8:Q27*, ORF8:R52I, ORF8:Y73C, ORF9b:K4N, S:N501Y, S:A570D, S:D614G, S:P681H, S:T716I, S:S982A, S:D1118H,	ORF1ab: 11288-11296, S:21765-21770, S:21992-21994, ORF8:28271,
hCoV-19/Mexico/TAM-InDRE_F11051_S870/2021	EPI_ISL_1399275	20B	B.1.1.222	20	5'UTR:C241T, ORF1ab:T562G, ORF1ab:C3037T, ORF1ab:C9438T, ORF1ab:A10323G, ORF1ab:C10755T, ORF1ab:C11345T, ORF1ab:C12741T, ORF1ab:C13743T, ORF1ab:C14408T, ORF1ab:T15684C, ORF1ab:A15759G, ORF1ab:T19839C, ORF1ab:G20275T, S:C23059T, S:A23403G, S:A23756G, ORF3a:T25623C, N:G28881A, N:G28882A, N:G28883C,	11	N:R203K, N:G204R, ORF1a:T3058I, ORF1a:K3353R, ORF1a:A3497V, ORF1a:L3694F, ORF1a:T4159I, ORF1b:P314L, ORF1b:D2270Y, S:D614G, S:T732A,	
hCoV-19/Mexico/TAM-InDRE_F11055_S871/2021	EPI_ISL_1399276	20G	B.1.2	23	5'UTR:C241T, ORF1ab:C813T, ORF1ab:C1059T, ORF1ab:C2509T, ORF1ab:C3037T, ORF1ab:T7348A, ORF1ab:G8083A, ORF1ab:C10319T, ORF1ab:C13119T, ORF1ab:C14408T, ORF1ab:C14805T, ORF1ab:C18131T, ORF1ab:A18424G, ORF1ab:A21203G, ORF1ab:C21304T, S:C21846T, S:A23403G, ORF3a:G25563T, ORF3a:G25907T, M:G26763T, ORF8:C27964T, N:C28472T, N:C28869T, 3'UTR:G29781T,	19	M:A81S, N:P67S, N:P199L, ORF1a:T183I, ORF1a:T265I, ORF1a:N2361K, ORF1a:M2606I, ORF1a:L3352F, ORF1a:A4285V, ORF1b:P314L, ORF1b:T1555I, ORF1b:N1653D, ORF1b:K2579R, ORF1b:R2613C, ORF3a:Q57H, ORF3a:G172V, ORF8:S24L, S:T95I, S:D614G,	
hCoV-19/Mexico/TAM-InDRE_F11057_S872/2021	EPI_ISL_1399277	20G	B.1.2	21	5'UTR:C241T, ORF1ab:C1059T, ORF1ab:C3037T, ORF1ab:C7224T, ORF1ab:G8264T, ORF1ab:C8917T, ORF1ab:C10319T, ORF1ab:G14244T, ORF1ab:C14408T, ORF1ab:A18424G, ORF1ab:C21304T, S:A23403G, S:C23533T, S:C23997T, ORF3a:G25563T, ORF3a:G25907T, ORF3a:C26060T, ORF8:C27942T, ORF8:C27964T, N:C28472T, N:C28603T, N:C28869T,	17	N:P67S, N:P199L, ORF1a:T265I, ORF1a:A2320V, ORF1a:G2667C, ORF1a:L3352F, ORF1b:W259C, ORF1b:P314L, ORF1b:N1653D, ORF1b:R2613C, ORF3a:Q57H, ORF3a:G172V, ORF3a:T223I, ORF8:H17Y, ORF8:S24L, S:D614G, S:P812L,	
hCoV-19/Mexico/TAM-InDRE_F11058_S837/2021	EPI_ISL_1424039	20B	B.1.1.519	25	5'UTR:C203T, 5'UTR:C222T, 5'UTR:C241T, ORF1ab:C3037T, ORF1ab:C3140T, ORF1ab:A3627G, ORF1ab:C10029T, ORF1ab:C10954T, ORF1ab:A11117G, ORF1ab:C12789T, ORF1ab:C14408T, ORF1ab:G15418T, ORF1ab:G15921T, ORF1ab:G19493A, ORF1ab:T19839C, ORF1ab:C21306T, S:C22326T, S:C22995A, S:A23403G, S:G23438T, S:C23604A, S:A23756G, N:G28881A, N:G28882A, N:G28883C, N:C29197T,	16	N:R203K, N:G204R, ORF1a:P959S, ORF1a:N1121S, ORF1a:T3255I, ORF1a:I3618V, ORF1a:T4175I, ORF1b:P314L, ORF1b:A651S, ORF1b:R2009K, S:S255F, S:T478K, S:D614G, S:A626S, S:P681H, S:T732A,	
hCoV-19/Mexico/TAM-InDRE_F11059_S838/2021	EPI_ISL_1424040	20B	B.1.1.519	23	5'UTR:C203T, 5'UTR:C222T, 5'UTR:C241T, ORF1ab:C884T, ORF1ab:C3037T, ORF1ab:C3140T, ORF1ab:C3743T, ORF1ab:C10029T, ORF1ab:C10954T, ORF1ab:A11117G, ORF1ab:C12789T, ORF1ab:C14408T, ORF1ab:T19839C, ORF1ab:C21306T, S:C22995A, S:A23403G, S:C23604A, S:A23756G, M:G26526A, N:G28881A, N:G28882A, N:G28883C, N:C29197T, N:G29527T,	15	M:A2T, N:R203K, N:G204R, N:Q418H, ORF1a:R207C, ORF1a:P959S, ORF1a:H1160Y, ORF1a:T3255I, ORF1a:I3618V, ORF1a:T4175I, ORF1b:P314L, S:T478K, S:D614G, S:P681H, S:T732A,	

hCoV-19/Mexico/TAM-InDRE_F11060_S839/2021	EPI_ISL_1424041	20B	B.1.1.519	23	5'UTR:C203T, 5'UTR:C222T, 5'UTR:C241T, ORF1ab:G598A, ORF1ab:C3037T, ORF1ab:C3140T, ORF1ab:C10029T, ORF1ab:C10954T, ORF1ab:A11117G, ORF1ab:C12789T, ORF1ab:C14408T, ORF1ab:T19839C, ORF1ab:C21306T, S:A23403G, S:C23604A, S:A23756G, ORF7a:T27504C, ORF8:T27904C, ORF8:T27907G, ORF8:G28077C, N:G28881A, N:G28882A, N:G28883C, N:C29197T,	13	N:R203K, N:G204R, ORF1a:P959S, ORF1a:T3255I, ORF1a:I3618V, ORF1a:T4175I, ORF1b:P314L, ORF8:L4P, ORF8:V5G, ORF8:V62L, S:D614G, S:P681H, S:T732A,	
hCoV-19/Mexico/TAM-InDRE_F114L_S572/2021	EPI_ISL_1334385	20I (Alpha, V1)	B.1.1.7	34	5'UTR:C241T, ORF1ab:C913T, ORF1ab:C2334T, ORF1ab:C2485T, ORF1ab:C3037T, ORF1ab:C3267T, ORF1ab:C5388A, ORF1ab:C5986T, ORF1ab:G6894T, ORF1ab:T6954C, ORF1ab:T8603C, ORF1ab:C14408T, ORF1ab:C14676T, ORF1ab:C15279T, ORF1ab:T16176C, ORF1ab:A17615G, ORF1ab:C18555T, S:A23063T, S:C23271A, S:A23403G, S:C23604A, S:C23709T, S:T24506G, S:G24914C, ORF3a:G25437T, ORF8:C27972T, ORF8:G28048T, ORF8:A28111G, N:G28280C, N:A28281T, N:T28282A, N:G28881A, N:G28882A, N:G28883C, N:C28977T,	23	N:D3L, N:R203K, N:G204R, N:S235F, ORF1a:A690V, ORF1a:T1001I, ORF1a:A1708D, ORF1a:C2210F, ORF1a:I2230T, ORF1a:F2780L, ORF1b:P314L, ORF1b:K1383R, ORF3a:L15F, ORF8:Q27*, ORF8:R52I, ORF8:Y73C, S:N501Y, S:A570D, S:D614G, S:P681H, S:T716I, S:S982A, S:D1118H,	ORF1ab: 11288-11296, S:21765-21770, S:21992-21994, ORF8:28271,
hCoV-19/Mexico/TAM-InDRE_F86L_S567/2021	EPI_ISL_1334380	20I (Alpha, V1)	B.1.1.7	35	5'UTR:C241T, ORF1ab:C292T, ORF1ab:C913T, ORF1ab:G1820A, ORF1ab:C3037T, ORF1ab:C3267T, ORF1ab:C5388A, ORF1ab:C5986T, ORF1ab:T6954C, ORF1ab:C14408T, ORF1ab:C14676T, ORF1ab:C15279T, ORF1ab:T16176C, ORF1ab:T16563A, S:T22861C, S:A23063T, S:C23170T, S:C23271A, S:A23403G, S:C23604A, S:C23709T, S:T24436C, S:T24506G, S:G24914C, ORF3a:C25626T, ORF8:C27972T, ORF8:G28048T, ORF8:A28111G, N:G28280C, N:A28281T, N:T28282A, N:A28295C, N:G28881A, N:G28882A, N:G28883C, N:C28977T,	21	N:D3L, N:N8H, N:R203K, N:G204R, N:S235F, ORF1a:G519S, ORF1a:T1001I, ORF1a:A1708D, ORF1a:I2230T, ORF1b:P314L, ORF8:Q27*, ORF8:R52I, ORF8:Y73C, ORF9b:K4N, S:N501Y, S:A570D, S:D614G, S:P681H, S:T716I, S:S982A, S:D1118H,	ORF1ab: 11288-11296, S:21765-21770, S:21992-21994, ORF8:28271,
hCoV-19/Mexico/TAM-InDRE_F87L_S647/2021	EPI_ISL_1359062	20B	B.1.1.222	14	5'UTR:C241T, ORF1ab:C2061T, ORF1ab:C3037T, ORF1ab:G6421T, ORF1ab:C11824T, ORF1ab:G13723T, ORF1ab:C14408T, ORF1ab:G14653T, ORF1ab:T19839C, S:A23403G, S:A23756G, ORF8:G28083T, N:G28881A, N:G28882A, N:G28883C,	9	N:R203K, N:G204R, ORF1a:A599V, ORF1b:A86S, ORF1b:P314L, ORF1b:V396F, ORF8:E64*, S:D614G, S:T732A,	S:21765-21770,
hCoV-19/Mexico/TAM-InDRE-94/2020	EPI_ISL_794592	20I (Alpha, V1)	B.1.1.7	32	5'UTR:C241T, ORF1ab:C913T, ORF1ab:C2110T, ORF1ab:C3037T, ORF1ab:C3267T, ORF1ab:C5388A, ORF1ab:C5986T, ORF1ab:T6954C, ORF1ab:T7984C, ORF1ab:C14120T, ORF1ab:C14408T, ORF1ab:C14676T, ORF1ab:C15279T, ORF1ab:T16176C, ORF1ab:C19390T, S:A23063T, S:C23271A, S:A23403G, S:C23604A, S:C23709T, S:T24506G, S:G24914C, ORF8:C27972T, ORF8:G28048T, ORF8:A28095T, ORF8:A28111G, N:G28280C, N:A28281T, N:T28282A, N:G28881A, N:G28882A, N:G28883C, N:C28977T,	21	N:D3L, N:R203K, N:G204R, N:S235F, ORF1a:T1001I, ORF1a:A1708D, ORF1a:I2230T, ORF1b:P218L, ORF1b:P314L, ORF1b:P1975S, ORF8:Q27*, ORF8:R52I, ORF8:K68*, ORF8:Y73C, S:N501Y, S:A570D, S:D614G, S:P681H, S:T716I, S:S982A, S:D1118H,	ORF1ab: 11288-11296, S:21765-21770, S:21992-21994,
hCoV-19/Mexico/TAM-UANL-001/2020	EPI_ISL_1091263	20A	B.1.243	15	5'UTR:C241T, ORF1ab:C3037T, ORF1ab:T8359C, ORF1ab:C10741T, ORF1ab:C14408T, ORF1ab:C19097T, ORF1ab:A20268G, S:G22100A, S:G22104T, S:A23403G, S:C23604A, S:T24076C, M:A26675G, M:G26713C, N:C28854T, 3'UTR:T29710C,	8	M:C64S, N:S194L, ORF1b:P314L, ORF1b:A1877V, S:E180K, S:G181V, S:D614G, S:P681H,	

hCoV-19/Mexico/TAM-UANL-003/2020	EPI_ISL_1091264	20A	B.1.558	16	5'UTR:C241T, ORF1ab:C344T, ORF1ab:C1288T, ORF1ab:C3037T, ORF1ab:A8115G, ORF1ab:C12439T, ORF1ab:C14408T, ORF1ab:C17004T, ORF1ab:C19662T, ORF1ab:A20268G, ORF1ab:G20962T, ORF1ab:G21255T, S:A23403G, ORF3a:C26060T, ORF7a:C27630T. N:C28854T. 3'UTR:G29688T.	7	N:S194L, ORF1a:L27F, ORF1a:E2617G, ORF1b:P314L, ORF1b:D2499Y, ORF3a:T223I, S:D614G,	
hCoV-19/Mexico/TAM-UANL-004/2020	EPI_ISL_1091265	20A	B.1.609	15	5'UTR:C241T, ORF1ab:G2144T, ORF1ab:C3037T, ORF1ab:C4582T, ORF1ab:C8106T, ORF1ab:A9848G, ORF1ab:C10039T, ORF1ab:A10874G, ORF1ab:C14408T, ORF1ab:C18795T, ORF1ab:A20268G, ORF1ab:G21204T, S:A23403G, S:C23683T, N:G28280T, N:G28975T,	9	N:D3Y, N:M234I, ORF1a:V627F, ORF1a:A2614V, ORF1a:S3195G, ORF1a:N3537D, ORF1b:P314L, ORF1b:K2579N, S:D614G,	
hCoV-19/Mexico/TAM-UANL-005/2020	EPI_ISL_1091248	20B	B.1.1	11	5'UTR:C241T, ORF1ab:C3037T, ORF1ab:C4321T, ORF1ab:C14408T, ORF1ab:G18266T, ORF1ab:T19839C, S:G21800T, S:A23403G, ORF3a:G25757T, N:G28881A, N:G28882A, N:G28883C,	7	N:R203K, N:G204R, ORF1b:P314L, ORF1b:R1600L, ORF3a:R122I, S:D80Y, S:D614G,	
hCoV-19/Mexico/TAM-UANL-006/2020	EPI_ISL_1091266	20G	B.1.2	19	5'UTR:C241T, ORF1ab:C1059T, ORF1ab:A1622G, ORF1ab:C3037T, ORF1ab:T3677C, ORF1ab:C4570T, ORF1ab:G6461A, ORF1ab:T10093C, ORF1ab:C10319T, ORF1ab:C14408T, ORF1ab:A18424G, ORF1ab:C21304T, S:G22225T, S:A23403G, ORF3a:G25563T, ORF3a:G25907T, ORF8:C27964T, N:C28472T, N:C28869T, N:C29268T,	15	N:P67S, N:P199L, N:T332I, ORF1a:T265I, ORF1a:I453V, ORF1a:F1138L, ORF1a:V2066M, ORF1a:L3352F, ORF1b:P314L, ORF1b:N1653D, ORF1b:R2613C, ORF3a:Q57H, ORF3a:G172V, ORF8:S24L, S:D614G,	
hCoV-19/Mexico/TAM-UANL-007/2020	EPI_ISL_1091267	20C	B.1	16	5'UTR:C241T, ORF1ab:C583T, ORF1ab:C1059T, ORF1ab:G3025T, ORF1ab:C3037T, ORF1ab:C4276T, ORF1ab:A6346G, ORF1ab:C7279T, ORF1ab:C10128T, ORF1ab:C14408T, ORF1ab:C15470T, ORF1ab:C17285T, ORF1ab:A19540G, S:A23403G, ORF3a:G25563T, ORF8:C27964T. N:G29032A.	10	ORF1a:T265I, ORF1a:M920I, ORF1a:T3288I, ORF1b:P314L, ORF1b:P668L, ORF1b:S1273L, ORF1b:M2025V, ORF3a:Q57H, ORF8:S24L, S:D614G,	
hCoV-19/Mexico/TAM-UANL-008/2020	EPI_ISL_1091268	20A	B.1	14	5'UTR:C241T, ORF1ab:T1592A, ORF1ab:C3037T, ORF1ab:G11083T, ORF1ab:C11681T, ORF1ab:A11782G, ORF1ab:C14408T, ORF1ab:G17562T, ORF1ab:C18508T, ORF1ab:A20268G, S:C21597T, S:A23403G, S:G24697T. N:C28854T. N:C29555T.	8	N:S194L, ORF1a:S443T, ORF1a:L3606F, ORF1b:P314L, ORF1b:L1681F, S:S12F, S:D614G, S:K1045N,	
hCoV-19/Mexico/TAM-UANL-009/2020	EPI_ISL_1091269	20A	B.1.243	15	5'UTR:C241T, ORF1ab:C3037T, ORF1ab:T9445C, ORF1ab:C14408T, ORF1ab:C17074T, ORF1ab:C17373T, ORF1ab:C18322T, ORF1ab:C19164T, ORF1ab:A20268G, S:A23403G, S:T24076C, E:T26410C, ORF6:C27303T, N:C28854T, N:C29421A. 3'UTR:C29835T.	7	E:F56L, N:S194L, N:P383Q, ORF1b:P314L, ORF1b:L1203F, ORF1b:H1619Y, S:D614G,	
hCoV-19/Mexico/TAM-UANL-010/2020	EPI_ISL_1091252	20C	B.1.366	16	5'UTR:C241T, ORF1ab:C1059T, ORF1ab:C1572T, ORF1ab:C2644T, ORF1ab:C3037T, ORF1ab:T8041C, ORF1ab:C11758T, ORF1ab:C12073T, ORF1ab:C12970T, ORF1ab:C14408T, ORF1ab:A16052G, ORF1ab:C17747T, ORF1ab:T20299C, S:G22225T, S:A23403G, ORF3a:G25563T. N:C28868T.	8	N:P199S, ORF1a:T265I, ORF1a:T436I, ORF1b:P314L, ORF1b:K862R, ORF1b:P1427L, ORF3a:Q57H, S:D614G,	S:21860-21862
hCoV-19/Mexico/TAM-UANL-011/2020	EPI_ISL_1091270	20B	B.1.1	19	5'UTR:C241T, ORF1ab:C1616T, ORF1ab:C3037T, ORF1ab:C6578T, ORF1ab:T9172C, ORF1ab:C10038T, ORF1ab:A10948G, ORF1ab:C14408T, ORF1ab:T16950C, S:C21660T, S:A23403G, S:C25006T, M:G26730T, ORF6:T27384C, ORF7b:T27826A, ORF8:T27922C, N:G28881A, N:G28882A, N:G28883C, N:C29376T,	12	M:V70F, N:R203K, N:G204R, N:P368L, ORF1a:L451F, ORF1a:L2105F, ORF1a:T3258I, ORF1b:P314L, ORF7b:M24K, ORF8:I10T, S:T33I, S:D614G,	

hCoV-19/Mexico/TAM-UANL-012/2020	EPI_ISL_1091271	20A	B.1.558	12	5'UTR:C241T, ORF1ab:C1288T, ORF1ab:G2075A, ORF1ab:C2401T, ORF1ab:C3037T, ORF1ab:G3685A, ORF1ab:C14408T, ORF1ab:G15016T, ORF1ab:C17304T, ORF1ab:C19662T, ORF1ab:A20268G, S:A23403G, N:C28854T,	5	N:S194L, ORF1a:G604S, ORF1b:P314L, ORF1b:A517S, S:D614G,
hCoV-19/Mexico/TAM-UANL-013/2020	EPI_ISL_1091272	20B	B.1.1.222	11	5'UTR:C241T, ORF1ab:C3037T, ORF1ab:C12455T, ORF1ab:C14408T, ORF1ab:T19839C, S:T22888C, S:A23403G, S:A23756G, N:G28881A, N:G28882A, N:G28883C, 3'UTR:G29751T,	6	N:R203K, N:G204R, ORF1a:L4064F, ORF1b:P314L, S:D614G, S:T732A,
hCoV-19/Mexico/TAM-UANL-014/2020	EPI_ISL_1091258	20A	B.1.609	14	5'UTR:C241T, ORF1ab:G520T, ORF1ab:C920T, ORF1ab:C2749T, ORF1ab:C3037T, ORF1ab:C4582T, ORF1ab:C5812T, ORF1ab:C14408T, ORF1ab:A20268G, S:A23403G, ORF3a:G26062T, M:C26895T, N:G28916T, N:G29511T, 3'UTR:G29777T,	7	M:H125Y, N:G215C, N:S413I, ORF1a:M85I, ORF1b:P314L, ORF3a:G224C, S:D614G,
hCoV-19/Mexico/TAM-UANL-015/2020	EPI_ISL_1091259	20A	B.1.558	22	5'UTR:C241T, ORF1ab:C1288T, ORF1ab:G2305T, ORF1ab:C2902T, ORF1ab:C3037T, ORF1ab:G6476A, ORF1ab:C10335T, ORF1ab:C11575T, ORF1ab:T12751G, ORF1ab:C14408T, ORF1ab:C18060T, ORF1ab:T19896C, ORF1ab:A20268G, ORF1ab:G21204T, S:G22147A, S:A23403G, S:C23664T, ORF3a:G25455T, ORF3a:G25770T, ORF3a:T25807C, ORF3a:G26109T, ORF3a:C26139T, N:C28854T,	11	N:S194L, ORF1a:K680N, ORF1a:V2071I, ORF1a:A3357V, ORF1b:P314L, ORF1b:K2579N, ORF3a:K21N, ORF3a:R126S, ORF3a:E239D, S:D614G, S:A701V,
hCoV-19/Mexico/TAM-UANL-017/2020	EPI_ISL_1091247	20A	B.1	11	5'UTR:C241T, ORF1ab:C934T, ORF1ab:C2371T, ORF1ab:A2806C, ORF1ab:C3037T, ORF1ab:G11417T, ORF1ab:T13857C, ORF1ab:C14408T, ORF1ab:A20268G, S:A23403G, ORF3a:G25477T, N:C28854T,	6	N:S194L, ORF1a:E847D, ORF1a:V3718F, ORF1b:P314L, ORF3a:V29F, S:D614G,
hCoV-19/Mexico/TAM-UANL-018/2020	EPI_ISL_1091273	20A	B.1.558	17	5'UTR:C241T, ORF1ab:C344T, ORF1ab:C1288T, ORF1ab:C1473T, ORF1ab:C1722T, ORF1ab:A2386G, ORF1ab:C3037T, ORF1ab:C12439T, ORF1ab:C14408T, ORF1ab:C16111T, ORF1ab:C17004T, ORF1ab:C19662T, ORF1ab:A20268G, ORF1ab:G20962T, S:A23403G, ORF3a:C26060T, ORF7a:C27630T, N:C28854T,	8	N:S194L, ORF1a:L27F, ORF1a:T403I, ORF1a:A486V, ORF1b:P314L, ORF1b:D2499Y, ORF3a:T223I, S:D614G,
hCoV-19/Mexico/TAM-UANL-019/2020	EPI_ISL_1091260	20B	B.1.1	14	5'UTR:C203T, 5'UTR:C241T, ORF1ab:C3037T, ORF1ab:C11514T, ORF1ab:C14408T, ORF1ab:C15738T, ORF1ab:G17988T, ORF1ab:C18377T, ORF1ab:G19549T, S:C21648T, S:A23403G, ORF8:C28253T, N:G28881A, N:G28882A, N:G28883C,	9	N:R203K, N:G204R, ORF1a:T3750I, ORF1b:P314L, ORF1b:K1507N, ORF1b:T1637I, ORF1b:A2028S, S:T29I, S:D614G,
hCoV-19/Mexico/TAM-UANL-020/2020	EPI_ISL_1091274	20A	B.1.558	17	5'UTR:C241T, ORF1ab:C344T, ORF1ab:C1288T, ORF1ab:C3037T, ORF1ab:G8548A, ORF1ab:C9223T, ORF1ab:C12439T, ORF1ab:C14220T, ORF1ab:C14408T, ORF1ab:C19662T, ORF1ab:A20268G, ORF1ab:G20962T, S:A23403G, ORF3a:C26060T, ORF7a:A27421C, ORF7a:C27630T, N:C28854T, N:C28957T,	7	N:S194L, ORF1a:L27F, ORF1b:P314L, ORF1b:D2499Y, ORF3a:T223I, ORF7a:I10L, S:D614G,
hCoV-19/Mexico/TLA-InDRE_109/2020	EPI_ISL_913913	20A	B.1.609	12	5'UTR:G210T, 5'UTR:C241T, ORF1ab:C3037T, ORF1ab:T3124C, ORF1ab:C4582T, ORF1ab:C14408T, ORF1ab:G17964T, ORF1ab:A20268G, S:A23403G, M:C26681T, M:C26988T, 3'UTR:G29734T, 3'UTR:G29755T,	3	ORF1b:P314L, ORF1b:M1499I, S:D614G,
hCoV-19/Mexico/TLA-InDRE_126/2020	EPI_ISL_913929	20A	B.1.243	17	5'UTR:C241T, ORF1ab:G472T, ORF1ab:A929G, ORF1ab:T2335G, ORF1ab:C2559T, ORF1ab:C3037T, ORF1ab:C9073T, ORF1ab:C11962T, ORF1ab:A11991G, ORF1ab:C14408T, ORF1ab:A20268G, ORF1ab:G20679T, S:A23403G, S:T24076C, ORF3a:C26060T, ORF6:A27310G, N:C28854T, N:G29449T,	8	N:S194L, ORF1a:I222V, ORF1a:P765L, ORF1a:E3909G, ORF1b:P314L, ORF3a:T223I, ORF6:I37V, S:D614G,

hCoV-19/Mexico/TLA-InDRE_141/2020	EPI_ISL_913944	20B	B.1.1.222	16	5'UTR:C241T, ORF1ab:C3037T, ORF1ab:C6336T, ORF1ab:C10029T, ORF1ab:C14408T, ORF1ab:C19011A, ORF1ab:T19839C, S:C21627T, S:A23403G, S:A23756G, ORF3a:G25912T, ORF8:A27921G, ORF8:G28001T, N:G28881A, N:G28882A, N:G28883C, N:C29363T,	12	N:R203K, N:G204R, N:P364S, ORF1a:S2024L, ORF1a:T3255I, ORF1b:P314L, ORF1b:D1848E, ORF3a:G174C, ORF8:I10V, S:T22I, S:D614G, S:T732A,
hCoV-19/Mexico/TLA-InDRE_147/2020	EPI_ISL_913950	20B	B.1.1.432	19	5'UTR:C241T, ORF1ab:C3037T, ORF1ab:C4540T, ORF1ab:G5461T, ORF1ab:C5512T, ORF1ab:A6985T, ORF1ab:C9319T, ORF1ab:C12412T, ORF1ab:T14313C, ORF1ab:C14408T, S:A23403G, ORF3a:C25613T, ORF3a:G25912T, N:A28877T, N:G28878C, N:G28881A, N:G28882A, N:G28883C, N:T29317C, ORF10:G29645A,	7	N:R203K, N:G204R, ORF1a:M1732I, ORF1b:P314L, ORF3a:S74F, ORF3a:G174C, S:D614G,
hCoV-19/Mexico/TLA-InDRE_153/2020	EPI_ISL_913956	20C	B.1	17	5'UTR:C241T, ORF1ab:C1059T, ORF1ab:C3037T, ORF1ab:A5501C, ORF1ab:C10507T, ORF1ab:C11916T, ORF1ab:C14408T, ORF1ab:G16564T, ORF1ab:C18998T, S:G21586T, S:G22708T, S:A23403G, S:C24157T, S:G24794T, ORF3a:G25563T, ORF8:G28085T, N:G28851T, N:G29540A,	11	N:S193I, ORF1a:T265I, ORF1a:S3884L, ORF1b:P314L, ORF1b:A1033S, ORF1b:A1844V, ORF3a:Q57H, ORF8:E64D, S:L8F, S:D614G, S:A1078S,
hCoV-19/Mexico/TLA-InDRE_174/2020	EPI_ISL_913975	20B	B.1.1.519	25	5'UTR:T201C, 5'UTR:C203T, 5'UTR:C222T, 5'UTR:C241T, ORF1ab:G1738T, ORF1ab:C3037T, ORF1ab:C3140T, ORF1ab:G3753A, ORF1ab:C10029T, ORF1ab:C10954T, ORF1ab:A11117G, ORF1ab:C12789T, ORF1ab:C14408T, ORF1ab:G18020A, ORF1ab:T19839C, ORF1ab:A19974G, ORF1ab:C21077T, ORF1ab:C21306T, S:C22995A, S:A23403G, S:C23604A, S:A23756G, N:G28881A, N:G28882A, N:G28883C, N:C29197T,	14	N:R203K, N:G204R, ORF1a:P959S, ORF1a:R1163K, ORF1a:T3255I, ORF1a:I3618V, ORF1a:T4175I, ORF1b:P314L, ORF1b:R1518K, ORF1b:T2537I, S:T478K, S:D614G, S:P681H, S:T732A,
hCoV-19/Mexico/TLA-InDRE_176/2020	EPI_ISL_913977	20B	B.1.1.519	27	5'UTR:T201C, 5'UTR:C203T, 5'UTR:C222T, 5'UTR:C241T, ORF1ab:C936T, ORF1ab:G1738T, ORF1ab:C3037T, ORF1ab:C3140T, ORF1ab:C10029T, ORF1ab:C10954T, ORF1ab:A11117G, ORF1ab:C12789T, ORF1ab:C14408T, ORF1ab:C16887T, ORF1ab:T19839C, ORF1ab:A19974G, ORF1ab:C20628T, ORF1ab:C21306T, S:C21575T, S:C22995A, S:A23403G, S:C23604A, S:A23756G, N:G28881A, N:G28882A, N:G28883C, N:C29197T, N:G29227T,	13	N:R203K, N:G204R, ORF1a:T224I, ORF1a:P959S, ORF1a:T3255I, ORF1a:I3618V, ORF1a:T4175I, ORF1b:P314L, S:L5F, S:T478K, S:D614G, S:P681H, S:T732A,
hCoV-19/Mexico/TLA-InDRE_261/2020	EPI_ISL_1054967	20A	B.1.609	10	5'UTR:C241T, ORF1ab:C3037T, ORF1ab:C4582T, ORF1ab:C14178T, ORF1ab:C14408T, ORF1ab:C15857T, ORF1ab:A20268G, S:A23403G, ORF3a:T25837C, N:G28916T, N:A29487G,	6	N:G215C, N:K405R, ORF1b:P314L, ORF1b:T797I, ORF3a:W149R, S:D614G,
hCoV-19/Mexico/TLA-InDRE_273/2020	EPI_ISL_1054951	20A	B.1	13	5'UTR:C241T, ORF1ab:C2902T, ORF1ab:C3037T, ORF1ab:C8937T, ORF1ab:C10232T, ORF1ab:C14408T, ORF1ab:C17999T, ORF1ab:A20268G, S:A23403G, S:T23986C, ORF3a:C25521T, ORF7a:A27675G, ORF8:C28253T, N:C28854T,	6	N:S194L, ORF1a:A2891V, ORF1a:R3323C, ORF1b:P314L, ORF1b:T1511I, S:D614G,
hCoV-19/Mexico/TLA-InDRE_281/2020	EPI_ISL_1054948	20A	B.1.609	13	5'UTR:C241T, ORF1ab:C3037T, ORF1ab:G3530T, ORF1ab:C4582T, ORF1ab:T6023C, ORF1ab:C6701T, ORF1ab:C13119T, ORF1ab:C14408T, ORF1ab:G17014T, ORF1ab:A20268G, S:A23403G, N:G28302C, N:G28851T, 3'UTR:A29700G,	10	N:R10P, N:S193I, ORF1a:D1089Y, ORF1a:Y1920H, ORF1a:L2146F, ORF1a:A4285V, ORF1b:P314L, ORF1b:D1183Y, ORF9b:E7Q, S:D614G,
hCoV-19/Mexico/TLA-InDRE_305/2020	EPI_ISL_1060740	20A	B.1.610	4	5'UTR:C241T, ORF1ab:C3037T, ORF1ab:C14408T, ORF1ab:A20268G, S:A23403G,	2	ORF1b:P314L, S:D614G,
hCoV-19/Mexico/TLA-InDRE_320/2020	EPI_ISL_1060685	20B	B.1.1.222	16	ORF1ab:G370T, ORF1ab:G1172T, ORF1ab:G3004T, ORF1ab:C3037T, ORF1ab:G7010A, ORF1ab:G7100A, ORF1ab:C14408T, ORF1ab:T19839C, ORF1ab:C19868T, S:A23403G, S:A23756G, ORF3a:G25996T, N:G28881A, N:G28882A, N:G28883C, N:G29315T, 3'UTR:G29751T,	12	N:R203K, N:G204R, N:D348Y, ORF1a:V303F, ORF1a:E913D, ORF1a:A2249T, ORF1a:A2279T, ORF1b:P314L, ORF1b:T2134I, ORF3a:V202L, S:D614G, S:T732A,

hCoV-19/Mexico/TLA-InDRE_391/2020	EPI_ISL_1054969	20B	B.1.1.432	14	5'UTR:C241T, ORF1ab:C3037T, ORF1ab:G3122T, ORF1ab:C3773T, ORF1ab:A6985T, ORF1ab:C9319T, ORF1ab:C12412T, ORF1ab:T14313C, ORF1ab:C14408T, S:A23403G, N:G28881A, N:G28882A, N:G28883C, N:T29317C, N:C29420T,	7	N:R203K, N:G204R, N:P383S, ORF1a:D953Y, ORF1a:R1170C, ORF1b:P314L, S:D614G,
hCoV-19/Mexico/TLA-InDRE-106/2020	EPI_ISL_872099	20A	B.1.609	7	5'UTR:C241T, ORF1ab:C3037T, ORF1ab:C4321T, ORF1ab:C4582T, ORF1ab:C13119T, ORF1ab:C14408T, ORF1ab:A20268G, S:A23403G,	3	ORF1a:A4285V, ORF1b:P314L, S:D614G,
hCoV-19/Mexico/TLA-InDRE-56/2020	EPI_ISL_576259	20B	B.1.1.222	13	5'UTR:C241T, ORF1ab:A1866T, ORF1ab:C3037T, ORF1ab:A9980C, ORF1ab:C14408T, ORF1ab:T19839C, S:A23403G, S:A23756G, S:T24490C, ORF6:T27242C, N:C28744T, N:G28881A, N:G28882A, N:G28883C,	8	N:R203K, N:G204R, ORF1a:Y534F, ORF1a:N3239H, ORF1b:P314L, ORF6:I14T, S:D614G, S:T732A,
hCoV-19/Mexico/TLA-InDRE-57/2020	EPI_ISL_576260	20B	B.1.1	9	5'UTR:C241T, ORF1ab:C3037T, ORF1ab:C14408T, ORF1ab:T19839C, S:A23403G, ORF3a:G25784C, M:G26690T, N:G28881A, N:G28882A, N:G28883C,	5	N:R203K, N:G204R, ORF1b:P314L, ORF3a:W131S, S:D614G,
hCoV-19/Mexico/TLA-InDRE-97/2020	EPI_ISL_861421	20A	B.1.609	7	5'UTR:C241T, ORF1ab:C3037T, ORF1ab:C4321T, ORF1ab:C4582T, ORF1ab:C13119T, ORF1ab:C14408T, ORF1ab:A20268G, S:A23403G,	3	ORF1a:A4285V, ORF1b:P314L, S:D614G,
hCoV-19/Mexico/VER-InDRE_111/2021	EPI_ISL_913915	20B	B.1.1.432	15	5'UTR:C241T, ORF1ab:C3037T, ORF1ab:A3940G, ORF1ab:T6343A, ORF1ab:A6985T, ORF1ab:C9319T, ORF1ab:C12412T, ORF1ab:T14313C, ORF1ab:C14408T, S:A23403G, ORF3a:C25613T, ORF3a:G25912T, N:G28881A, N:G28882A, N:G28883C, N:T29317C,	7	N:R203K, N:G204R, ORF1a:D2026E, ORF1b:P314L, ORF3a:S74F, ORF3a:G174C, S:D614G,
hCoV-19/Mexico/VER-InDRE_112/2021	EPI_ISL_913916	20B	B.1.1.222	12	5'UTR:C241T, ORF1ab:C3037T, ORF1ab:A5995G, ORF1ab:C14408T, ORF1ab:T19839C, ORF1ab:C20428T, S:A23403G, S:A23756G, N:G28881A, N:G28882A, N:G28883C, ORF10:T29656C, 3'UTR:G29751T,	6	N:R203K, N:G204R, ORF1b:P314L, ORF1b:P2321S, S:D614G, S:T732A,
hCoV-19/Mexico/VER-InDRE_116/2020	EPI_ISL_913920	20A	B.1	16	5'UTR:C241T, ORF1ab:A2699G, ORF1ab:C3037T, ORF1ab:T6871C, ORF1ab:A10286G, ORF1ab:C14408T, ORF1ab:G14829T, ORF1ab:A20268G, ORF1ab:G20623A, S:G23311T, S:A23403G, S:T23978C, ORF3a:G25855T, N:T28672A, N:C28854T, N:G28922T, 3'UTR:C29750T,	10	N:S194L, N:A217S, ORF1a:T812A, ORF1a:I3341V, ORF1b:P314L, ORF1b:M454I, ORF1b:D2386N, ORF3a:D155Y, S:E583D, S:D614G,
hCoV-19/Mexico/VER-InDRE_117/2020	EPI_ISL_913921	20B	B.1.1.432	19	5'UTR:C241T, ORF1ab:C745A, ORF1ab:C3037T, ORF1ab:G3994T, ORF1ab:C4084T, ORF1ab:A4235G, ORF1ab:A6985T, ORF1ab:C9319T, ORF1ab:C12412T, ORF1ab:T14313C, ORF1ab:C14408T, S:T22119C, S:A23403G, ORF3a:C25613T, ORF3a:C25624T, ORF3a:G25912T, N:G28881A, N:G28882A, N:G28883C, N:T29317C,	10	N:R203K, N:G204R, ORF1a:N160K, ORF1a:N1324D, ORF1b:P314L, ORF3a:S74F, ORF3a:H78Y, ORF3a:G174C, S:F186S, S:D614G,
hCoV-19/Mexico/VER-InDRE_127/2020	EPI_ISL_913930	20C	B.1	16	5'UTR:C241T, ORF1ab:C313T, ORF1ab:C1059T, ORF1ab:C3037T, ORF1ab:C11152T, ORF1ab:A12458G, ORF1ab:C14408T, ORF1ab:C16329T, S:A23403G, S:G25091T, ORF3a:G25563T, ORF3a:C25603T, M:C26885T, M:C27046T, N:C28887T, ORF10:C29614T, 3'UTR:C29750T,	8	M:T175M, N:T205I, ORF1a:T265I, ORF1a:T4065A, ORF1b:P314L, ORF3a:Q57H, S:D614G, S:V1177L,
hCoV-19/Mexico/VER-InDRE_152/2020	EPI_ISL_913955	20B	B.1.1.432	20	5'UTR:C241T, ORF1ab:C3037T, ORF1ab:C6926T, ORF1ab:A6985T, ORF1ab:C9319T, ORF1ab:C12073T, ORF1ab:C12412T, ORF1ab:C12756T, ORF1ab:T14313C, ORF1ab:C14408T, ORF1ab:T14542C, ORF1ab:T20452C, S:T22119C, S:A23403G, ORF3a:C25613T, ORF3a:C25624T, ORF3a:G25912T, N:G28881A, N:G28882A, N:G28883C, N:T29317C,	12	N:R203K, N:G204R, ORF1a:P2221S, ORF1a:T4164I, ORF1b:P314L, ORF1b:F359L, ORF1b:Y2329H, ORF3a:S74F, ORF3a:H78Y, ORF3a:G174C, S:F186S, S:D614G,

hCoV-19/Mexico/VER-InDRE_169/2020	EPI_ISL_913970	20B	B.1.1.519	24	5'UTR:C203T, 5'UTR:C222T, 5'UTR:C241T, ORF1ab:G1738T, ORF1ab:C3037T, ORF1ab:C3140T, ORF1ab:C10029T, ORF1ab:C10954T, ORF1ab:A11117G, ORF1ab:A12088G, ORF1ab:C12789T, ORF1ab:C14408T, ORF1ab:T19839C, ORF1ab:A19974G, ORF1ab:G20434T, ORF1ab:C21306T, S:C22995A, S:A23403G, S:C23604A, S:A23756G, N:G28881A, N:G28882A, N:G28883C, N:C29197T, N:G29315C,	13	N:R203K, N:G204R, N:D348H, ORF1a:P959S, ORF1a:T3255I, ORF1a:I3618V, ORF1a:T4175I, ORF1b:P314L, ORF1b:D2323Y, S:T478K, S:D614G, S:P681H, S:T732A,
hCoV-19/Mexico/VER-InDRE_272/2020	EPI_ISL_1054957	20A	B.1.610	4	5'UTR:C241T, ORF1ab:C3037T, ORF1ab:C14408T, ORF1ab:A20268G, S:A23403G,	2	ORF1b:P314L, S:D614G,
hCoV-19/Mexico/VER-InDRE_321/2020	EPI_ISL_1060761	20A	B.1.397	12	5'UTR:C241T, ORF1ab:A1110G, ORF1ab:G1135T, ORF1ab:C1288T, ORF1ab:C3037T, ORF1ab:G9479T, ORF1ab:C14195T, ORF1ab:C14408T, ORF1ab:A20268G, S:A23403G, N:G28280T, N:G28580T, N:C28854T,	9	N:D3Y, N:D103Y, N:S194L, ORF1a:K282R, ORF1a:K290N, ORF1a:G3072C, ORF1b:T243I, ORF1b:P314L, S:D614G,
hCoV-19/Mexico/VER-InDRE_322/2020	EPI_ISL_1060687	20B	B.1.1	9	ORF1ab:C3037T, ORF1ab:C14408T, ORF1ab:T19839C, ORF1ab:G19947T, S:A23403G, S:G24757T, ORF3a:T25713C, N:G28881A, N:G28882A, N:G28883C,	5	N:R203K, N:G204R, ORF1b:P314L, ORF1b:K2160N, S:D614G,
hCoV-19/Mexico/VER-InDRE_360/2020	EPI_ISL_1060677	20A	B.1	12	ORF1ab:C3037T, ORF1ab:T7711C, ORF1ab:G9188A, ORF1ab:C10376T, ORF1ab:G11083T, ORF1ab:C11941T, ORF1ab:C14408T, ORF1ab:T19989C, S:C22858T, S:A23403G, S:C24130T, N:C28854T, 3'UTR:G29779T,	6	N:S194L, ORF1a:V2975M, ORF1a:P3371S, ORF1a:L3606F, ORF1b:P314L, S:D614G,
hCoV-19/Mexico/VER-InDRE_362/2020	EPI_ISL_1060699	20B	B.1.1.222	10	ORF1ab:C3037T, ORF1ab:C14408T, ORF1ab:A21137G, S:G21800T, S:C22987T, S:A23403G, S:A23756G, M:C26681T, N:G28881A, N:G28882A, N:G28883C,	7	N:R203K, N:G204R, ORF1b:P314L, ORF1b:K2557R, S:D80Y, S:D614G, S:T732A,
hCoV-19/Mexico/VER-InDRE_363/2020	EPI_ISL_1060707	20B	B.1.1.432	24	ORF1ab:G1306T, ORF1ab:C3037T, ORF1ab:C9298T, ORF1ab:C9319T, ORF1ab:C12412T, ORF1ab:T14313C, ORF1ab:C14408T, ORF1ab:C14571T, ORF1ab:C16948T, ORF1ab:G18149T, S:T22408A, S:C22570T, S:A23403G, S:T23596C, S:G23804C, S:C24912T, ORF3a:C25613T, ORF3a:C25614T, ORF7b:G27890A, ORF8:G28221T, N:G28881A, N:G28882A, N:G28883C, N:G28985T, N:T29317C,	13	N:R203K, N:G204R, N:G238C, ORF1a:E347D, ORF1b:P314L, ORF1b:P1161S, ORF1b:G1561V, ORF3a:S74F, ORF8:E110*, S:N282K, S:D614G, S:E748Q, S:T1117I,
hCoV-19/Mexico/VER-InDRE_414/2021	EPI_ISL_1168492	20A	B.1	21	5'UTR:C241T, ORF1ab:A2010G, ORF1ab:C2416T, ORF1ab:C3037T, ORF1ab:A3904T, ORF1ab:A11430G, ORF1ab:C14408T, ORF1ab:A15177C, ORF1ab:A16534G, ORF1ab:T17673G, ORF1ab:G20060T, ORF1ab:A20268G, S:C21618T, S:C21707T, S:T22912G, S:G22992A, S:A23403G, M:C26681T, M:G26709A, ORF7a:C27494T, ORF8:A28273T, N:G28514T,	14	M:A63T, N:D81Y, ORF1a:D582G, ORF1a:Y3722C, ORF1b:P314L, ORF1b:S1023G, ORF1b:S2198I, ORF7a:P34L, ORF9b:Q77H, S:T19I, S:H49Y, S:N450K, S:S477N, S:D614G,
hCoV-19/Mexico/VER-InDRE_415/2021	EPI_ISL_1168493	20B	B.1	21	5'UTR:C203T, 5'UTR:C222T, 5'UTR:C241T, ORF1ab:C3037T, ORF1ab:C3140T, ORF1ab:C3876T, ORF1ab:C10029T, ORF1ab:C10954T, ORF1ab:A11117G, ORF1ab:T12656C, ORF1ab:C12789T, ORF1ab:C14408T, ORF1ab:T19839C, ORF1ab:C21306T, S:C22995A, S:A23403G, S:C23604A, S:A23756G, S:C24616T, ORF8:T27904C, ORF8:C28087T, N:C29197T,	12	ORF1a:P959S, ORF1a:A1204V, ORF1a:T3255I, ORF1a:I3618V, ORF1a:T4175I, ORF1b:P314L, ORF8:L4P, ORF8:A65V, S:T478K, S:D614G, S:P681H, S:T732A,
hCoV-19/Mexico/VER-InDRE_416/2021	EPI_ISL_1168494	20A	B.1.609	14	5'UTR:C241T, ORF1ab:C3037T, ORF1ab:C4582T, ORF1ab:C5339T, ORF1ab:C9996T, ORF1ab:G11327T, ORF1ab:C14178T, ORF1ab:C14408T, ORF1ab:C15857T, ORF1ab:G19891T, ORF1ab:A20268G, S:A23403G, ORF3a:T25837C, N:G28916T, N:A29487G,	10	N:G215C, N:K405R, ORF1a:P1692S, ORF1a:S3244L, ORF1a:A3688S, ORF1b:P314L, ORF1b:T797I, ORF1b:D2142Y, ORF3a:W149R, S:D614G,

hCoV-19/Mexico/VER-InDRE_417/2021	EPI_ISL_1168495	20B	B.1.1.519	20	5'UTR:C203T, 5'UTR:C222T, 5'UTR:C241T, ORF1ab:C3037T, ORF1ab:C10029T, ORF1ab:C10954T, ORF1ab:A11117G, ORF1ab:C12789T, ORF1ab:C14408T, ORF1ab:T19839C, ORF1ab:C21306T, S:C22995A, S:A23403G, S:C23604A, S:A23756G, ORF8:T27904C, ORF8:C28087T, N:G28881A, N:G28882A, N:G28883C, N:C29197T,	12	N:R203K, N:G204R, ORF1a:T3255I, ORF1a:I3618V, ORF1a:T4175I, ORF1b:P314L, ORF8:L4P, ORF8:A65V, S:T478K, S:D614G, S:P681H, S:T732A,	
hCoV-19/Mexico/VER-InDRE_418/2021	EPI_ISL_1168496	20B	B.1.1.519	23	5'UTR:C203T, 5'UTR:C222T, 5'UTR:C241T, ORF1ab:C3037T, ORF1ab:C3044T, ORF1ab:C3140T, ORF1ab:G7027T, ORF1ab:C10029T, ORF1ab:C10954T, ORF1ab:A11117G, ORF1ab:C12789T, ORF1ab:C14408T, ORF1ab:T19839C, ORF1ab:G20668A, ORF1ab:C21306T, S:C22995A, S:A23403G, S:C23604A, S:A23756G, S:C25207T, N:G28881A, N:G28882A, N:G28883C, N:C29197T,	14	N:R203K, N:G204R, ORF1a:P927S, ORF1a:P959S, ORF1a:M2254I, ORF1a:T3255I, ORF1a:I3618V, ORF1a:T4175I, ORF1b:P314L, ORF1b:A2401T, S:T478K, S:D614G, S:P681H, S:T732A,	
hCoV-19/Mexico/VER-InDRE_F10671_S817/2021	EPI_ISL_1424019	20B	B.1.1.519	22	5'UTR:C186T, 5'UTR:C203T, 5'UTR:C222T, 5'UTR:C241T, ORF1ab:C3037T, ORF1ab:C3140T, ORF1ab:C10029T, ORF1ab:C10954T, ORF1ab:A11117G, ORF1ab:G11234T, ORF1ab:C12789T, ORF1ab:C14184T, ORF1ab:C14408T, ORF1ab:T19839C, ORF1ab:C21306T, S:C22995A, S:A23403G, S:C23604A, S:A23756G, N:G28881A, N:G28882A, N:G28883C, N:C29197T,	12	N:R203K, N:G204R, ORF1a:P959S, ORF1a:T3255I, ORF1a:I3618V, ORF1a:A3657S, ORF1a:T4175I, ORF1b:P314L, S:T478K, S:D614G, S:P681H, S:T732A,	
hCoV-19/Mexico/VER-InDRE_F10672_S733/2021	EPI_ISL_1366682	20B	B.1.1.519	22	5'UTR:C203T, 5'UTR:C222T, 5'UTR:C241T, ORF1ab:C3037T, ORF1ab:C3140T, ORF1ab:T8758C, ORF1ab:C10029T, ORF1ab:C10954T, ORF1ab:A11117G, ORF1ab:C12789T, ORF1ab:C14408T, ORF1ab:T19839C, ORF1ab:C21306T, S:C22995A, S:A23403G, S:C23604A, S:A23756G, N:G28881A, N:G28882A, N:G28883C, N:C29197T, N:A29413T, N:G29527T,	13	N:R203K, N:G204R, N:Q380H, N:Q418H, ORF1a:P959S, ORF1a:T3255I, ORF1a:I3618V, ORF1a:T4175I, ORF1b:P314L, S:T478K, S:D614G, S:P681H, S:T732A,	
hCoV-19/Mexico/VER-InDRE_F10673_S734/2021	EPI_ISL_1366683	20B	B.1.1.519	25	5'UTR:C203T, 5'UTR:C222T, 5'UTR:C241T, ORF1ab:C2062T, ORF1ab:C3037T, ORF1ab:C3140T, ORF1ab:C10029T, ORF1ab:C10954T, ORF1ab:A11117G, ORF1ab:G11335T, ORF1ab:G11365T, ORF1ab:C12789T, ORF1ab:C14322T, ORF1ab:C14408T, ORF1ab:T19839C, ORF1ab:C21306T, S:C22995A, S:A23403G, S:C23604A, S:A23756G, N:G28881A, N:G28882A, N:G28883C, N:C29197T, N:C29420T, N:G29527T,	13	N:R203K, N:G204R, N:P383S, N:Q418H, ORF1a:P959S, ORF1a:T3255I, ORF1a:I3618V, ORF1a:T4175I, ORF1b:P314L, S:T478K, S:D614G, S:P681H, S:T732A,	
hCoV-19/Mexico/VER-InDRE_F10674_S735/2021	EPI_ISL_1366684	20B	B.1.1.519	22	5'UTR:T201C, 5'UTR:C203T, 5'UTR:C222T, 5'UTR:C241T, ORF1ab:G1738T, ORF1ab:C3037T, ORF1ab:C3140T, ORF1ab:C10029T, ORF1ab:C10954T, ORF1ab:A11117G, ORF1ab:C12789T, ORF1ab:C14408T, ORF1ab:T19839C, ORF1ab:A19974G, ORF1ab:C21306T, S:C22995A, S:A23403G, S:C23604A, S:A23756G, N:G28881A, N:G28882A, N:G28883C, N:C29197T,	11	N:R203K, N:G204R, ORF1a:P959S, ORF1a:T3255I, ORF1a:I3618V, ORF1a:T4175I, ORF1b:P314L, S:T478K, S:D614G, S:P681H, S:T732A,	
hCoV-19/Mexico/VER-InDRE_F10678_S691/2021	EPI_ISL_1365656	20B	B.1.1.519	26	5'UTR:C203T, 5'UTR:C222T, 5'UTR:C241T, ORF1ab:C3037T, ORF1ab:C3140T, ORF1ab:C10029T, ORF1ab:C10954T, ORF1ab:A11117G, ORF1ab:C12789T, ORF1ab:C14408T, ORF1ab:G15921T, ORF1ab:C16260T, ORF1ab:C17913T, ORF1ab:A17971G, ORF1ab:G18859T, ORF1ab:T19839C, ORF1ab:C21306T, S:C22995A, S:A23403G, S:C23604A, S:C23635T, S:A23756G, M:G26828T, N:G28881A, N:G28882A, N:G28883C, N:C29197T,	13	N:R203K, N:G204R, ORF1a:P959S, ORF1a:T3255I, ORF1a:I3618V, ORF1a:T4175I, ORF1b:P314L, ORF1b:R1502G, ORF1b:A1798S, S:T478K, S:D614G, S:P681H, S:T732A,	S:21992-21994,

hCoV-19/Mexico/VER-InDRE_F10679_S736/2021	EPI_ISL_1366685	20B	B.1.1.519	24	5'UTR:C203T, 5'UTR:C222T, 5'UTR:C241T, ORF1ab:C3037T, ORF1ab:C3140T, ORF1ab:A6613G, ORF1ab:C6990T, ORF1ab:G9973A, ORF1ab:C10029T, ORF1ab:C10954T, ORF1ab:A11117G, ORF1ab:C12789T, ORF1ab:C14408T, ORF1ab:T19839C, ORF1ab:C21306T, S:C22995A, S:A23403G, S:C23604A, S:A23756G, ORF3a:G25555T, N:G28881A, N:G28882A, N:G28883C, N:C29197T, N:G29527T,	14	N:R203K, N:G204R, N:Q418H, ORF1a:P959S, ORF1a:S2242F, ORF1a:T3255I, ORF1a:I3618V, ORF1a:T4175I, ORF1b:P314L, ORF3a:V55F, S:T478K, S:D614G, S:P681H, S:T732A,
hCoV-19/Mexico/VER-InDRE_F10681_S737/2021	EPI_ISL_1366686	20B	B.1.1.519	21	5'UTR:C203T, 5'UTR:C222T, 5'UTR:C241T, ORF1ab:C3037T, ORF1ab:C3140T, ORF1ab:C10029T, ORF1ab:C10954T, ORF1ab:A11117G, ORF1ab:C12789T, ORF1ab:C14408T, ORF1ab:T18870C, ORF1ab:T19839C, ORF1ab:C21306T, S:C22995A, S:A23403G, S:C23604A, S:A23756G, ORF8:T27904C, N:G28881A, N:G28882A, N:G28883C, N:C29197T,	12	N:R203K, N:G204R, ORF1a:P959S, ORF1a:T3255I, ORF1a:I3618V, ORF1a:T4175I, ORF1b:P314L, ORF8:L4P, S:T478K, S:D614G, S:P681H, S:T732A,
hCoV-19/Mexico/VER-InDRE_F11084_S845/2021	EPI_ISL_1424046	20B	B.1.1.519	21	5'UTR:C203T, 5'UTR:C222T, 5'UTR:C241T, ORF1ab:C3037T, ORF1ab:C3140T, ORF1ab:T9723C, ORF1ab:C10029T, ORF1ab:C10369T, ORF1ab:C10954T, ORF1ab:A11117G, ORF1ab:C12789T, ORF1ab:C14408T, ORF1ab:T19839C, ORF1ab:C21306T, S:C22995A, S:A23403G, S:C23604A, S:A23756G, N:G28881A, N:G28882A, N:G28883C, N:C29197T,	12	N:R203K, N:G204R, ORF1a:P959S, ORF1a:F3153S, ORF1a:T3255I, ORF1a:I3618V, ORF1a:T4175I, ORF1b:P314L, S:T478K, S:D614G, S:P681H, S:T732A,
hCoV-19/Mexico/VER-InDRE_F11086_S874/2021	EPI_ISL_1399279	20A	B.1.243	22	5'UTR:C241T, ORF1ab:G806A, ORF1ab:A1132C, ORF1ab:A1291G, ORF1ab:T2584A, ORF1ab:C3037T, ORF1ab:C5140A, ORF1ab:T7341C, ORF1ab:T13701C, ORF1ab:C14408T, ORF1ab:A20268G, ORF1ab:G21123T, S:C21707T, S:A23403G, S:G23587C, S:T24076C, S:G25290T, ORF3a:C25844T, ORF7b:G27758A, ORF7b:T27760A, N:C28854T, N:G29543T, 3'UTR:C29774T,	13	N:S194L, ORF1a:A181T, ORF1a:E289D, ORF1a:D1625E, ORF1a:I2359T, ORF1b:P314L, ORF3a:T151I, ORF7b:M1I, ORF7b:I2N, S:H49Y, S:D614G, S:Q675H, S:C1243F,
hCoV-19/Mexico/VER-InDRE_F11087_S846/2021	EPI_ISL_1424047	20B	B.1.1.519	26	5'UTR:T201C, 5'UTR:C203T, 5'UTR:C222T, 5'UTR:C241T, ORF1ab:C936T, ORF1ab:G1738T, ORF1ab:C3037T, ORF1ab:C3140T, ORF1ab:C10029T, ORF1ab:C10954T, ORF1ab:A11117G, ORF1ab:C12789T, ORF1ab:C14408T, ORF1ab:C16877T, ORF1ab:T19839C, ORF1ab:A19974G, ORF1ab:C21306T, S:C22995A, S:A23403G, S:C23604A, S:A23756G, N:G28881A, N:G28882A, N:G28883C, N:G28980T, N:C29197T, N:G29227T,	14	N:R203K, N:G204R, N:G236V, ORF1a:T224I, ORF1a:P959S, ORF1a:T3255I, ORF1a:I3618V, ORF1a:T4175I, ORF1b:P314L, ORF1b:T1137I, S:T478K, S:D614G, S:P681H, S:T732A,
hCoV-19/Mexico/VER-InDRE_F11090_S847/2021	EPI_ISL_1424048	20B	B.1.1.519	23	5'UTR:A129G, 5'UTR:C203T, 5'UTR:C222T, 5'UTR:C241T, ORF1ab:C629T, ORF1ab:C3037T, ORF1ab:C3140T, ORF1ab:C10029T, ORF1ab:C10954T, ORF1ab:A11117G, ORF1ab:C12789T, ORF1ab:C14408T, ORF1ab:C14621T, ORF1ab:T19839C, ORF1ab:C21306T, S:C22995A, S:A23403G, S:C23604A, S:A23756G, N:G28881A, N:G28882A, N:G28883C, N:C29197T, N:G29527T,	14	N:R203K, N:G204R, N:Q418H, ORF1a:L122F, ORF1a:P959S, ORF1a:T3255I, ORF1a:I3618V, ORF1a:T4175I, ORF1b:P314L, ORF1b:T385M, S:T478K, S:D614G, S:P681H, S:T732A,
hCoV-19/Mexico/VER-InDRE_F11093_S848/2021	EPI_ISL_1424049	20B	B.1.1.519	24	5'UTR:C203T, 5'UTR:C222T, 5'UTR:C241T, ORF1ab:A3019G, ORF1ab:C3037T, ORF1ab:C3140T, ORF1ab:G3549A, ORF1ab:T5218C, ORF1ab:C10029T, ORF1ab:C10954T, ORF1ab:A11117G, ORF1ab:C12789T, ORF1ab:C14408T, ORF1ab:T19839C, ORF1ab:C21306T, S:C22995A, S:A23403G, S:C23604A, S:A23756G, S:T24903C, ORF8:T27904C, N:G28881A, N:G28882A, N:G28883C, N:C29197T,	14	N:R203K, N:G204R, ORF1a:P959S, ORF1a:G1095E, ORF1a:T3255I, ORF1a:I3618V, ORF1a:T4175I, ORF1b:P314L, ORF8:L4P, S:T478K, S:D614G, S:P681H, S:T732A, S:I1114T,

hCoV-19/Mexico/VER-InDRE-104/2020	EPI_ISL_872097	20B	B.1.1.432	10	5'UTR:C241T, ORF1ab:C3037T, ORF1ab:A6985T, ORF1ab:C9319T, ORF1ab:C12412T, ORF1ab:T14313C, ORF1ab:C14408T, S:A23403G, N:G28881A, N:G28882A, N:G28883C,	4	N:R203K, N:G204R, ORF1b:P314L, S:D614G,
hCoV-19/Mexico/VER-InDRE-66/2020	EPI_ISL_576269	20B	B.1.1.432	19	5'UTR:C241T, ORF1ab:T1543C, ORF1ab:C3037T, ORF1ab:C4901A, ORF1ab:A6985T, ORF1ab:C9319T, ORF1ab:C12412T, ORF1ab:T14313C, ORF1ab:C14408T, ORF1ab:G17325T, ORF1ab:G18106T, S:G23120T, S:A23403G, ORF3a:C25613T, ORF3a:G25912T, ORF7a:G27506T, N:G28881A, N:G28882A, N:G28883C, N:T29317C,	11	N:R203K, N:G204R, ORF1a:L1546I, ORF1b:P314L, ORF1b:L1286F, ORF1b:A1547S, ORF3a:S74F, ORF3a:G174C, ORF7a:G38V, S:A520S, S:D614G,
hCoV-19/Mexico/VER-InDRE-67/2020	EPI_ISL_576270	20B	B.1.1.432	17	5'UTR:C241T, ORF1ab:C1063T, ORF1ab:C3037T, ORF1ab:A6985T, ORF1ab:C9319T, ORF1ab:C12412T, ORF1ab:T14313C, ORF1ab:C14408T, ORF1ab:G16700T, S:A23403G, S:A23414T, ORF3a:T25485C, ORF3a:C25613T, ORF6:C27213T, N:G28881A, N:G28882A, N:G28883C, N:T29317C,	7	N:R203K, N:G204R, ORF1b:P314L, ORF1b:R1078L, ORF3a:S74F, S:D614G, S:T618S,
hCoV-19/Mexico/YUC-InDRE_33/2020	EPI_ISL_493348	20A	B.1	7	5'UTR:C241T, ORF1ab:A1558G, ORF1ab:C3037T, ORF1ab:C6573T, ORF1ab:C14408T, ORF1ab:A20268G, S:T22192C, S:A23403G,	4	ORF1a:I431M, ORF1a:S2103F, ORF1b:P314L, S:D614G,
hCoV-19/Mexico/YUC-InDRE_500/2021	EPI_ISL_1168570	20B	B.1.1.519	25	5'UTR:T201C, 5'UTR:C203T, 5'UTR:C222T, 5'UTR:C241T, ORF1ab:C1218T, ORF1ab:G1738T, ORF1ab:C3037T, ORF1ab:C3140T, ORF1ab:G4907A, ORF1ab:C10029T, ORF1ab:C10954T, ORF1ab:A11117G, ORF1ab:C12789T, ORF1ab:C14408T, ORF1ab:T19839C, ORF1ab:A19974G, ORF1ab:C21306T, S:C21621T, S:C22995A, S:A23403G, S:C23604A, S:A23756G, N:G28881A, N:G28882A, N:G28883C, N:C29197T,	14	N:R203K, N:G204R, ORF1a:S318L, ORF1a:P959S, ORF1a:G1548S, ORF1a:T3255I, ORF1a:I3618V, ORF1a:T4175I, ORF1b:P314L, S:T20I, S:T478K, S:D614G, S:P681H, S:T732A,
hCoV-19/Mexico/YUC-InDRE_501/2021	EPI_ISL_1168571	20B	B.1.1.519	25	5'UTR:C203T, 5'UTR:C222T, 5'UTR:C241T, ORF1ab:C3037T, ORF1ab:C3140T, ORF1ab:T5159G, ORF1ab:C5812T, ORF1ab:T9757C, ORF1ab:C10029T, ORF1ab:C10954T, ORF1ab:A11117G, ORF1ab:C12789T, ORF1ab:C14408T, ORF1ab:C18747T, ORF1ab:T19839C, ORF1ab:C21306T, S:C22995A, S:A23403G, S:C23604A, S:A23756G, ORF8:C28153T, N:G28881A, N:G28882A, N:G28883C, N:C29197T, N:C29555T,	13	N:R203K, N:G204R, ORF1a:P959S, ORF1a:F1632V, ORF1a:T3255I, ORF1a:I3618V, ORF1a:T4175I, ORF1b:P314L, ORF8:T87I, S:T478K, S:D614G, S:P681H, S:T732A,
hCoV-19/Mexico/YUC-InDRE_502/2021	EPI_ISL_1168572	20B	B.1.1.222	19	5'UTR:C241T, ORF1ab:C3037T, ORF1ab:C4897T, ORF1ab:C10029T, ORF1ab:A11430G, ORF1ab:C13432T, ORF1ab:C14408T, ORF1ab:C19011A, ORF1ab:T19839C, ORF1ab:A21137G, S:A23403G, S:A23756G, S:A23813T, ORF3a:G25912T, ORF3a:A26115C, ORF8:A27921G, ORF8:G28001T, N:G28881A, N:G28882A, N:G28883C,	13	N:R203K, N:G204R, ORF1a:T3255I, ORF1a:Y3722C, ORF1b:P314L, ORF1b:D1848E, ORF1b:K2557R, ORF3a:G174C, ORF3a:E241D, ORF8:I10V, S:D614G, S:T732A, S:N751Y,
hCoV-19/Mexico/YUC-InDRE_503/2021	EPI_ISL_1168573	20B	B.1.1.519	26	5'UTR:T201C, 5'UTR:C203T, 5'UTR:C222T, 5'UTR:C241T, ORF1ab:C1218T, ORF1ab:G1738T, ORF1ab:C3037T, ORF1ab:C3140T, ORF1ab:G4907A, ORF1ab:C10954T, ORF1ab:A11117G, ORF1ab:C12789T, ORF1ab:C14408T, ORF1ab:T19839C, ORF1ab:A19974G, ORF1ab:A21089C, S:C21621T, S:C22995A, S:A23403G, S:C23604A, S:A23756G, E:C26296T, N:G28881A, N:G28882A, N:G28883C, N:G28895T, N:C29197T,	16	E:L18F, N:R203K, N:G204R, N:A208S, ORF1a:S318L, ORF1a:P959S, ORF1a:G1548S, ORF1a:I3618V, ORF1a:T4175I, ORF1b:P314L, ORF1b:D2541A, S:T20I, S:T478K, S:D614G, S:P681H, S:T732A,

hCoV-19/Mexico/YUC-InDRE_504/2021	EPI_ISL_1168574	20G	B.1.2	21	5'UTR:C241T, ORF1ab:G960A, ORF1ab:C1059T, ORF1ab:C3037T, ORF1ab:A6705G, ORF1ab:A6876G, ORF1ab:C7321T, ORF1ab:A9052G, ORF1ab:C10319T, ORF1ab:C14408T, ORF1ab:A15489G, ORF1ab:A18424G, ORF1ab:C18877T, ORF1ab:C21304T, S:T22447C, S:A23403G, S:C23604A, ORF3a:G25563T, ORF3a:G25907T, ORF8:C27964T, N:C28472T, N:C28869T.	15	N:P67S, N:P199L, ORF1a:R232H, ORF1a:T265I, ORF1a:N2147S, ORF1a:K2204R, ORF1a:L3352F, ORF1b:P314L, ORF1b:N1653D, ORF1b:R2613C, ORF3a:Q57H, ORF3a:G172V, ORF8:S24L, S:D614G, S:P681H,
hCoV-19/Mexico/YUC-InDRE_F10540_S717/2021	EPI_ISL_1366666	20B	B.1.1.519	28	5'UTR:T201C, 5'UTR:C203T, 5'UTR:C222T, 5'UTR:C241T, ORF1ab:C745T, ORF1ab:G1085T, ORF1ab:G1738T, ORF1ab:A2280G, ORF1ab:C3037T, ORF1ab:C3140T, ORF1ab:C10029T, ORF1ab:C10954T, ORF1ab:A11117G, ORF1ab:C12789T, ORF1ab:C14408T, ORF1ab:A18474G, ORF1ab:T19839C, ORF1ab:A19974G, ORF1ab:C21306T, S:C22995A, S:A23403G, S:C23604A, S:A23756G, ORF3a:G25429T, ORF3a:C25671T, N:G28881A, N:G28882A, N:G28883C, N:C29197T,	14	N:R203K, N:G204R, ORF1a:V274L, ORF1a:K672R, ORF1a:P959S, ORF1a:T3255I, ORF1a:I3618V, ORF1a:T4175I, ORF1b:P314L, ORF3a:V13L, S:T478K, S:D614G, S:P681H, S:T732A,
hCoV-19/Mexico/YUC-InDRE_F10541_S812/2021	EPI_ISL_1424014	20B	B.1.1.519	26	5'UTR:T201C, 5'UTR:C203T, 5'UTR:C222T, 5'UTR:C241T, ORF1ab:G1274T, ORF1ab:G1738T, ORF1ab:C3037T, ORF1ab:C3140T, ORF1ab:G6713T, ORF1ab:C7504T, ORF1ab:C10029T, ORF1ab:C10954T, ORF1ab:A11117G, ORF1ab:C12789T, ORF1ab:C13115T, ORF1ab:C14408T, ORF1ab:T19839C, ORF1ab:A19974G, ORF1ab:C21306T, S:C22995A, S:A23403G, S:C23604A, S:A23756G, N:G28881A, N:G28882A, N:G28883C, N:C29197T,	13	N:R203K, N:G204R, ORF1a:V337F, ORF1a:P959S, ORF1a:V2150F, ORF1a:T3255I, ORF1a:I3618V, ORF1a:T4175I, ORF1b:P314L, S:T478K, S:D614G, S:P681H, S:T732A,
hCoV-19/Mexico/YUC-InDRE_F9942_S651/2021	EPI_ISL_1359066	20G	B.1.596	20	5'UTR:C241T, ORF1ab:C1059T, ORF1ab:C3037T, ORF1ab:G3470A, ORF1ab:C3589T, ORF1ab:T8710C, ORF1ab:C10319T, ORF1ab:A11451G, ORF1ab:C14408T, ORF1ab:A18424G, ORF1ab:C21304T, S:C21811A, S:A23403G, S:A23592C, ORF3a:G25563T, ORF3a:G25907T, ORF8:C27964T, N:C28472T, N:C28869T, N:T29194C, N:T29377A.	14	N:P67S, N:P199L, ORF1a:T265I, ORF1a:G1069R, ORF1a:L3352F, ORF1a:Q3729R, ORF1b:P314L, ORF1b:N1653D, ORF1b:R2613C, ORF3a:Q57H, ORF3a:G172V, ORF8:S24L, S:D614G, S:Q677P,
hCoV-19/Mexico/YUC-InDRE_F9948_S805/2021	EPI_ISL_1424007	20B	B.1.1.519	31	5'UTR:T201C, 5'UTR:C203T, 5'UTR:C222T, 5'UTR:C241T, ORF1ab:C936T, ORF1ab:G1738T, ORF1ab:T2489C, ORF1ab:C2695T, ORF1ab:C3037T, ORF1ab:C3140T, ORF1ab:G3971T, ORF1ab:C10029T, ORF1ab:C10854T, ORF1ab:C10954T, ORF1ab:A11117G, ORF1ab:C12789T, ORF1ab:C14408T, ORF1ab:T19839C, ORF1ab:C20946T, ORF1ab:C21306T, S:C22995A, S:A23403G, S:C23604A, S:A23756G, M:C26964T, ORF7a:A27426G, N:G28881A, N:G28882A, N:G28883C, N:C29197T, N:G29227T, N:G29321T,	16	M:H148Y, N:R203K, N:G204R, N:V350F, ORF1a:T224I, ORF1a:P959S, ORF1a:V1236F, ORF1a:T3255I, ORF1a:S3530L, ORF1a:I3618V, ORF1a:T4175I, ORF1b:P314L, S:T478K, S:D614G, S:P681H, S:T732A,
hCoV-19/Mexico/YUC-InDRE_F9950_S623/2021	EPI_ISL_1340658	20B	B.1.1.519	24	5'UTR:C203T, 5'UTR:C222T, 5'UTR:C241T, ORF1ab:C3037T, ORF1ab:C3140T, ORF1ab:C10029T, ORF1ab:C10954T, ORF1ab:A11117G, ORF1ab:C12789T, ORF1ab:C13297T, ORF1ab:C14408T, ORF1ab:T19839C, ORF1ab:G20102T, ORF1ab:C21306T, S:C22995A, S:A23403G, S:C23604A, S:A23756G, ORF6:C27230T, ORF8:T28082A, N:A28359G, N:G28881A, N:G28882A, N:G28883C, N:C29197T,	16	N:N29S, N:R203K, N:G204R, ORF1a:P959S, ORF1a:T3255I, ORF1a:I3618V, ORF1a:T4175I, ORF1b:P314L, ORF1b:S2212I, ORF6:T10I, ORF8:D63E, ORF9b:M26V, S:T478K, S:D614G, S:P681H, S:T732A,

hCoV-19/Mexico/YUC-InDRE_F9951_S624/2021	EPI_ISL_1340661	20B	B.1.1.519	27	5'UTR:T201C, 5'UTR:C203T, 5'UTR:C222T, 5'UTR:C241T, ORF1ab:G1274T, ORF1ab:G1738T, ORF1ab:C3037T, ORF1ab:C3140T, ORF1ab:C7164T, ORF1ab:C7504T, ORF1ab:C10029T, ORF1ab:C10954T, ORF1ab:A11117G, ORF1ab:C12789T, ORF1ab:C13115T, ORF1ab:C14408T, ORF1ab:G17331T, ORF1ab:T19839C, ORF1ab:A19974G, ORF1ab:C21306T, S:C22995A, S:A23403G, S:C23604A, S:A23756G, N:G28881A, N:G28882A, N:G28883C, N:C29197T	14	N:R203K, N:G204R, ORF1a:V337F, ORF1a:P959S, ORF1a:T2300I, ORF1a:T3255I, ORF1a:I3618V, ORF1a:T4175I, ORF1b:P314L, ORF1b:E1288D, S:T478K, S:D614G, S:P681H, S:T732A,	
hCoV-19/Mexico/YUC-InDRE_F9953_S652/2021	EPI_ISL_1359067	20B	B.1.1.222	13	5'UTR:C241T, ORF1ab:C3037T, ORF1ab:C10029T, ORF1ab:C14408T, ORF1ab:T19839C, S:C22482T, S:A23403G, S:A23756G, ORF7b:A27756G, ORF8:A27921G, ORF8:G28001T, N:G28881A, N:G28882A, N:G28883C,	9	N:R203K, N:G204R, ORF1a:T3255I, ORF1b:P314L, ORF7b:M1V, ORF8:I10V, S:T307I, S:D614G, S:T732A,	
hCoV-19/Mexico/YUC-InDRE-95/2020	EPI_ISL_861419	20A	B.1	5	5'UTR:C241T, ORF1ab:C3037T, ORF1ab:C9120T, ORF1ab:C14408T, ORF1ab:C15324T, S:A23403G,	3	ORF1a:T2952I, ORF1b:P314L, S:D614G,	
hCoV-19/Mexico/ZAC-InDRE_F10107_S573/2021	EPI_ISL_1335757	20I (Alpha, V1)	B.1.1.7	32	5'UTR:C241T, ORF1ab:C913T, ORF1ab:C3037T, ORF1ab:C3267T, ORF1ab:C5388A, ORF1ab:C5986T, ORF1ab:G6362A, ORF1ab:T6954C, ORF1ab:C12970T, ORF1ab:C13176T, ORF1ab:C14408T, ORF1ab:C14676T, ORF1ab:C15279T, ORF1ab:T16176C, S:A23063T, S:C23271A, S:A23403G, S:C23604A, S:C23709T, S:C24023T, S:C24054T, S:T24506G, S:G24914C, ORF8:C27972T, ORF8:G28048T, ORF8:A28111G, N:G28280C, N:A28281T, N:T28282A, N:G28881A, N:G28882A, N:G28883C, N:C28977T,	18	N:D3L, N:R203K, N:G204R, N:S235F, ORF1a:T1001I, ORF1a:A1708D, ORF1a:A2033T, ORF1a:I2230T, ORF1a:T4304I, ORF1b:P314L, S:N501Y, S:A570D, S:D614G, S:P681H, S:T716I, S:A831V, S:S982A, S:D1118H,	ORF1ab: 11288-11296, S:21765-21770, S:21992-21994, ORF8:28254, ORF8:28271,
hCoV-19/Mexico/ZAC-InDRE_F10108_S625/2021	EPI_ISL_1340660	20B	B.1.1.519	26	5'UTR:C203T, 5'UTR:C222T, 5'UTR:C241T, ORF1ab:C3037T, ORF1ab:C3140T, ORF1ab:C6406T, ORF1ab:C7528T, ORF1ab:C8950T, ORF1ab:C10029T, ORF1ab:C10954T, ORF1ab:A11117G, ORF1ab:C12789T, ORF1ab:C14408T, ORF1ab:T19839C, ORF1ab:A19959G, ORF1ab:C20178T, ORF1ab:C21306T, S:C22995A, S:A23403G, S:C23604A, S:A23756G, M:G26526A, N:G28881A, N:G28882A, N:G28883C, N:C29197T, N:G29527T,	13	M:A2T, N:R203K, N:G204R, N:Q418H, ORF1a:P959S, ORF1a:T3255I, ORF1a:I3618V, ORF1a:T4175I, ORF1b:P314L, S:T478K, S:D614G, S:P681H, S:T732A,	
hCoV-19/Mexico/ZAC-InDRE_F10109_S653/2021	EPI_ISL_1359068	20A	B.1.243	17	5'UTR:C241T, ORF1ab:C346T, ORF1ab:C3037T, ORF1ab:G4201T, ORF1ab:T9508C, ORF1ab:C14408T, ORF1ab:C15714T, ORF1ab:G19542T, ORF1ab:A19543G, ORF1ab:A19676G, ORF1ab:A20268G, S:G22992A, S:A23403G, S:C23604A, S:T24076C, N:C28854T, N:G29266A, 3'UTR:T29710C,	9	N:S194L, ORF1a:M1312I, ORF1b:P314L, ORF1b:M2025I, ORF1b:I2026V, ORF1b:Q2070R, S:S477N, S:D614G, S:P681H,	
hCoV-19/Mexico/ZAC-InDRE_F10118_S654/2021	EPI_ISL_1359069	20G	B.1.596	19	5'UTR:C241T, ORF1ab:C1059T, ORF1ab:C3037T, ORF1ab:T7001A, ORF1ab:C10319T, ORF1ab:A11451G, ORF1ab:C13405T, ORF1ab:C14408T, ORF1ab:A18424G, ORF1ab:C21304T, S:C21811A, S:A23403G, S:A23592C, ORF3a:G25563T, ORF3a:G25907T, ORF8:C27964T, N:C28472T, N:C28869T, N:T29194C, N:T29377A,	14	N:P67S, N:P199L, ORF1a:T265I, ORF1a:S2246T, ORF1a:L3352F, ORF1a:Q3729R, ORF1b:P314L, ORF1b:N1653D, ORF1b:R2613C, ORF3a:Q57H, ORF3a:G172V, ORF8:S24L, S:D614G, S:Q677P,	
hCoV-19/Mexico/ZAC-InDRE-70/2020	EPI_ISL_576273	20A	B.1.243	8	5'UTR:C241T, ORF1ab:C3037T, ORF1ab:C14408T, ORF1ab:A20268G, S:A23403G, S:T24076C, S:G25062T, ORF8:C28253G, N:C28854T,	5	N:S194L, ORF1b:P314L, ORF8:F120L, S:D614G, S:G1167V,	

hCoV-19/Mexico/ZAC-InDRE-71/2020	EPI_ISL_576274	20A	B.1.243	11	5'UTR:C241T, ORF1ab:C3037T, ORF1ab:C14408T, ORF1ab:A19298C, ORF1ab:T19301C, ORF1ab:A19302T, ORF1ab:A20268G, S:A23403G, S:T24076C, S:G25062T, ORF8:C28253G, N:C28854T,	7	N:S194L, ORF1b:P314L, ORF1b:Y1944S, ORF1b:V1945A, ORF8:F120L, S:D614G, S:G1167V,
hCoV-19/Mexico/ZAC-InDRE-72/2020	EPI_ISL_576275	20B	B.1.1	9	5'UTR:C100T, 5'UTR:C241T, ORF1ab:C3037T, ORF1ab:C14408T, ORF1ab:T17454C, ORF1ab:T19839C, S:A23403G, N:G28881A, N:G28882A, N:G28883C,	4	N:R203K, N:G204R, ORF1b:P314L, S:D614G,
