# Supplementary material for: Genetic Analysis of SARS-CoV-2 Variants in Mexico during the First Year of the COVID-19 Pandemic
Source: Viruses. 2021 Oct 26;13(11):2161. doi: 10.3390/v13112161 (PMC8622467; doi:10.3390/v13112161)
Supplement: Supplementary file 1 [file viruses-13-02161-s001.zip › SupplementaryTable_S2.pdf]

Table S2. Patient demographic and clinical characteristics. The information of the 1520 genome sequences generated by us are shown in blue.

Virus name	Accession ID	Collection date	Location	Host	Gender	Patient age	Patient status	Passage	Specimen
hCoV-19/Mexico/CAM-IBT-IMSS-426/2020	EPI_ISL_1301620	18/03/2020	Campeche	Human	Female	59	Ambulatory	Original	Nasopharyngeal and oropharyngeal swab
hCoV-19/Mexico/ROO-IBT-IMSS-427/2020	EPI_ISL_1301498	25/03/2020	Quintana Roo	Human	Female	35	Ambulatory	Original	Nasopharyngeal and oropharyngeal swab
hCoV-19/Mexico/GUA-IBT-IMSS-428/2020	EPI_ISL_1301621	26/03/2020	Guanajuato	Human	Female	59	Ambulatory	Original	Nasopharyngeal and oropharyngeal swab
hCoV-19/Mexico/TLA-IBT-IMSS-429/2020	EPI_ISL_1301622	27/03/2020	Tlaxcala	Human	Female	20	Ambulatory	Original	Nasopharyngeal and oropharyngeal swab
hCoV-19/Mexico/CMX-INER-IBT-2/2020	EPI_ISL_1301718	27/03/2020	Mexico City	Human	Female	unknown	Hospitalized	Original	Pharyngeal swab
hCoV-19/Mexico/CMX-INER-IBT-1/2020	EPI_ISL_1301717	28/03/2020	Mexico City	Human	Female	unknown	Hospitalized	Original	Pharyngeal swab
hCoV-19/Mexico/CMX-INER-IBT-4/2020	EPI_ISL_1301520	29/03/2020	Mexico City	Human	Male	unknown	Hospitalized	Original	Pharyngeal swab
hCoV-19/Mexico/CMX-INER-IBT-5/2020	EPI_ISL_1301731	30/03/2020	Mexico City	Human	Male	unknown	Hospitalized	Original	Tracheal aspirate
hCoV-19/Mexico/COL-IBT-IMSS-430/2020	EPI_ISL_1301450	01/04/2020	Colima	Human	Male	54	Hospitalized	Original	Nasopharyngeal and oropharyngeal swab
hCoV-19/Mexico/CMX-IBT-IMSS-02/2020	EPI_ISL_955257	01/04/2020	Mexico City	Human	Male	31	Deceased	Original	Nasopharyngeal and oropharyngeal swab
hCoV-19/Mexico/ROO-IBT-IMSS-432/2020	EPI_ISL_1301623	02/04/2020	Quintana Roo	Human	Male	22	Ambulatory	Original	Nasopharyngeal and oropharyngeal swab
hCoV-19/Mexico/ROO-IBT-IMSS-433/2020	EPI_ISL_1301624	02/04/2020	Quintana Roo	Human	Male	25	Ambulatory	Original	Nasopharyngeal and oropharyngeal swab
hCoV-19/Mexico/CMX-INER-IBT-6/2020	EPI_ISL_1301452	02/04/2020	Mexico City	Human	Male	unknown	Hospitalized	Original	Nasopharyngeal swab
hCoV-19/Mexico/SIN-IBT-IMSS-431/2020	EPI_ISL_1301462	02/04/2020	Sinaloa	Human	Female	49	Ambulatory	Original	Nasopharyngeal and oropharyngeal swab
hCoV-19/Mexico/ROO-IBT-IMSS-435/2020	EPI_ISL_1301625	03/04/2020	Quintana Roo	Human	Female	26	Ambulatory	Original	Nasopharyngeal and oropharyngeal swab
hCoV-19/Mexico/ROO-IBT-IMSS-434/2020	EPI_ISL_1301473	03/04/2020	Quintana Roo	Human	Male	23	Hospitalized	Original	Nasopharyngeal and oropharyngeal swab
hCoV-19/Mexico/CMX-INER-IBT-8/2020	EPI_ISL_1301489	03/04/2020	Mexico City	Human	Male	unknown	Hospitalized	Original	Nasopharyngeal swab
hCoV-19/Mexico/GUA-IBT-IMSS-438/2020	EPI_ISL_1301466	04/04/2020	Guanajuato	Human	Male	35	Ambulatory	Original	Nasopharyngeal and oropharyngeal swab
hCoV-19/Mexico/CMX-INER-IBT-7/2020	EPI_ISL_1301598	05/04/2020	Mexico City	Human	Male	unknown	Hospitalized	Original	Nasopharyngeal swab
hCoV-19/Mexico/CMX-IBT-IMSS-03/2020	EPI_ISL_955256	05/04/2020	Mexico City	Human	Male	55	Hospitalized	Original	Nasopharyngeal and oropharyngeal swab
hCoV-19/Mexico/GUA-IBT-IMSS-437/2020	EPI_ISL_1301509	06/04/2020	Guanajuato	Human	Female	42	Hospitalized	Original	Nasopharyngeal and oropharyngeal swab
hCoV-19/Mexico/CHH-IBT-IMSS-439/2020	EPI_ISL_1301552	06/04/2020	Chihuahua	Human	Male	26	Ambulatory	Original	Nasopharyngeal and oropharyngeal swab
hCoV-19/Mexico/QUE-IBT-IMSS-436/2020	EPI_ISL_1301626	06/04/2020	Queretaro	Human	Male	42	Hospitalized	Original	Nasopharyngeal and oropharyngeal swab
hCoV-19/Mexico/TAB-IBT-IMSS-441/2020	EPI_ISL_1301628	06/04/2020	Tabasco	Human	Female	45	Ambulatory	Original	Nasopharyngeal and oropharyngeal swab
hCoV-19/Mexico/TAB-IBT-IMSS-442/2020	EPI_ISL_1301629	06/04/2020	Tabasco	Human	Male	49	Ambulatory	Original	Nasopharyngeal and oropharyngeal swab
hCoV-19/Mexico/BCS-IBT-IMSS-20/2020	EPI_ISL_955255	06/04/2020	Baja California Sur	Human	Male	39	Ambulatory	Original	Nasopharyngeal and oropharyngeal swab
hCoV-19/Mexico/CMX-INER-IBT-12/2020	EPI_ISL_1301605	07/04/2020	Mexico City	Human	Male	unknown	Hospitalized	Original	Nasopharyngeal swab
hCoV-19/Mexico/AGU-InDRE-IBT-42/2020	EPI_ISL_1301681	07/04/2020	Aguascalientes	Human	Male	41	Ambulatory	Original	Pharyngeal swab
hCoV-19/Mexico/BCS-IBT-IMSS-09/2020	EPI_ISL_955250	07/04/2020	Baja California Sur	Human	Female	43	Ambulatory	Original	Nasopharyngeal and oropharyngeal swab
hCoV-19/Mexico/BCS-IBT-IMSS-12/2020	EPI_ISL_955251	07/04/2020	Baja California Sur	Human	Male	48	Ambulatory	Original	Nasopharyngeal and oropharyngeal swab
hCoV-19/Mexico/BCS-IBT-IMSS-13/2020	EPI_ISL_955252	07/04/2020	Baja California Sur	Human	Female	50	Ambulatory	Original	Nasopharyngeal and oropharyngeal swab
hCoV-19/Mexico/BCS-IBT-IMSS-14/2020	EPI_ISL_955253	07/04/2020	Baja California Sur	Human	Male	37	Ambulatory	Original	Nasopharyngeal and oropharyngeal swab
hCoV-19/Mexico/CMX-IBT-IMSS-07/2020	EPI_ISL_955254	07/04/2020	Mexico City	Human	Male	49	Ambulatory	Original	Nasopharyngeal and oropharyngeal swab
hCoV-19/Mexico/TLA-IBT-IMSS-440/2020	EPI_ISL_1301627	08/04/2020	Tlaxcala	Human	Female	39	Ambulatory	Original	Nasopharyngeal and oropharyngeal swab
hCoV-19/Mexico/AGU-IBT-IMSS-446/2020	EPI_ISL_1301631	08/04/2020	Aguascalientes	Human	Male	57	Ambulatory	Original	Nasopharyngeal and oropharyngeal swab
hCoV-19/Mexico/BCS-IBT-IMSS-11/2020	EPI_ISL_955233	08/04/2020	Baja California Sur	Human	Female	33	Ambulatory	Original	Nasopharyngeal and oropharyngeal swab

hCoV-19/Mexico/BCN-IBT-IMSS-17/2020	EPI_ISL_955240	08/04/2020	Baja California	Human	Male	42	Ambulatory	Original	Nasopharyngeal and oropharyngeal swab
hCoV-19/Mexico/BCN-IBT-IMSS-18/2020	EPI_ISL_955241	08/04/2020	Baja California	Human	Male	24	Ambulatory	Original	Nasopharyngeal and oropharyngeal swab
hCoV-19/Mexico/BCS-IBT-IMSS-15/2020	EPI_ISL_955242	08/04/2020	Baja California Sur	Human	Female	41	Ambulatory	Original	Nasopharyngeal and oropharyngeal swab
hCoV-19/Mexico/BCS-IBT-IMSS-16/2020	EPI_ISL_955243	08/04/2020	Baja California Sur	Human	Female	35	Ambulatory	Original	Nasopharyngeal and oropharyngeal swab
hCoV-19/Mexico/GRO-IBT-IMSS-08/2020	EPI_ISL_955244	08/04/2020	Guerrero	Human	Male	59	Deceased	Original	Nasopharyngeal and oropharyngeal swab
hCoV-19/Mexico/GRO-IBT-IMSS-10/2020	EPI_ISL_955245	08/04/2020	Guerrero	Human	Male	32	Ambulatory	Original	Nasopharyngeal and oropharyngeal swab
hCoV-19/Mexico/MEX-IBT-IMSS-04/2020	EPI_ISL_955246	08/04/2020	State of Mexico	Human	Female	26	Ambulatory	Original	Nasopharyngeal and oropharyngeal swab
hCoV-19/Mexico/MEX-IBT-IMSS-19/2020	EPI_ISL_955247	08/04/2020	State of Mexico	Human	Male	26	Ambulatory	Original	Nasopharyngeal and oropharyngeal swab
hCoV-19/Mexico/CMX-IBT-IMSS-05/2020	EPI_ISL_955248	08/04/2020	Mexico City	Human	Male	30	Ambulatory	Original	Pharyngeal swab
hCoV-19/Mexico/CMX-IBT-IMSS-06/2020	EPI_ISL_955249	08/04/2020	Mexico City	Human	Male	22	Hospitalized	Original	Nasopharyngeal and oropharyngeal swab
hCoV-19/Mexico/AGU-InDRE-IBT-43/2020	EPI_ISL_1301682	09/04/2020	Aguascalientes	Human	Male	62	Ambulatory	Original	Pharyngeal swab
hCoV-19/Mexico/CMX-INER-IBT-10/2020	EPI_ISL_1301600	10/04/2020	Mexico City	Human	Male	unknown	Hospitalized	Original	Nasopharyngeal swab
hCoV-19/Mexico/BCN-IBT-IMSS-445/2020	EPI_ISL_1301630	10/04/2020	Baja California	Human	Male	33	Ambulatory	Original	Nasopharyngeal and oropharyngeal swab
hCoV-19/Mexico/CMX-INER-IBT-11/2020	EPI_ISL_1301490	10/04/2020	Mexico City	Human	Male	unknown	Hospitalized	Original	Nasopharyngeal swab
hCoV-19/Mexico/SLP-InDRE-IBT-40/2020	EPI_ISL_1301679	11/04/2020	San Luis Potosi	Human	Male	31	Ambulatory	Original	Pharyngeal swab
hCoV-19/Mexico/SLP-InDRE-IBT-41/2020	EPI_ISL_1301680	11/04/2020	San Luis Potosi	Human	Female	30	Ambulatory	Original	Pharyngeal swab
hCoV-19/Mexico/OAX-IBT-IMSS-443/2020	EPI_ISL_1301446	11/04/2020	Oaxaca	Human	Male	27	Ambulatory	Original	Nasopharyngeal and oropharyngeal swab
hCoV-19/Mexico/TLA-IBT-IMSS-444/2020	EPI_ISL_1301467	11/04/2020	Tlaxcala	Human	Male	66	Hospitalized	Original	Nasopharyngeal and oropharyngeal swab
hCoV-19/Mexico/AGU-IBT-IMSS-447/2020	EPI_ISL_1301525	12/04/2020	Aguascalientes	Human	Female	43	Ambulatory	Original	Nasopharyngeal and oropharyngeal swab
hCoV-19/Mexico/AGU-InDRE-IBT-44/2020	EPI_ISL_1301683	12/04/2020	Aguascalientes	Human	Male	unknown	unknown	Original	Pharyngeal swab
hCoV-19/Mexico/AGU-InDRE-IBT-45/2020	EPI_ISL_1301684	12/04/2020	Aguascalientes	Human	Male	55	Hospitalized	Original	Pharyngeal swab
hCoV-19/Mexico/AGU-InDRE-IBT-47/2020	EPI_ISL_1301686	12/04/2020	Aguascalientes	Human	Female	38	Ambulatory	Original	Pharyngeal swab
hCoV-19/Mexico/CMX-INER-IBT-13/2020	EPI_ISL_1301465	12/04/2020	Mexico City	Human	Male	unknown	Hospitalized	Original	Nasopharyngeal swab
hCoV-19/Mexico/MOR-IBT-IMSS-453/2020	EPI_ISL_1301636	13/04/2020	Morelos	Human	Female	46	Ambulatory	Original	Nasopharyngeal and oropharyngeal swab
hCoV-19/Mexico/AGU-InDRE-IBT-46/2020	EPI_ISL_1301685	13/04/2020	Aguascalientes	Human	Female	54	Ambulatory	Original	Pharyngeal swab
hCoV-19/Mexico/TLA-IBT-IMSS-448/2020	EPI_ISL_1301484	13/04/2020	Tlaxcala	Human	Female	62	Hospitalized	Original	Nasopharyngeal and oropharyngeal swab
hCoV-19/Mexico/CMX-IBT-IMSS-22/2020	EPI_ISL_955234	16/04/2020	Mexico City	Human	Female	47	Ambulatory	Original	Nasopharyngeal and oropharyngeal swab
hCoV-19/Mexico/CMX-IBT-IMSS-21/2020	EPI_ISL_955239	16/04/2020	Mexico City	Human	Male	26	Ambulatory	Original	Nasopharyngeal and oropharyngeal swab
hCoV-19/Mexico/PUE-IBT-IMSS-449/2020	EPI_ISL_1301632	17/04/2020	Puebla	Human	Male	63	Ambulatory	Original	Nasopharyngeal and oropharyngeal swab
hCoV-19/Mexico/PUE-IBT-IMSS-457/2020	EPI_ISL_1301640	17/04/2020	Puebla	Human	Female	64	Ambulatory	Original	Nasopharyngeal and oropharyngeal swab
hCoV-19/Mexico/GRO-IBT-IMSS-26/2020	EPI_ISL_955236	17/04/2020	Guerrero	Human	Female	38	Ambulatory	Original	Nasopharyngeal and oropharyngeal swab
hCoV-19/Mexico/MEX-IBT-IMSS-25/2020	EPI_ISL_955237	17/04/2020	State of Mexico	Human	Female	29	Ambulatory	Original	Nasopharyngeal and oropharyngeal swab
hCoV-19/Mexico/CMX-IBT-IMSS-24/2020	EPI_ISL_955238	17/04/2020	Mexico City	Human	Male	46	Hospitalized	Original	Nasopharyngeal and oropharyngeal swab
hCoV-19/Mexico/PUE-IBT-IMSS-450/2020	EPI_ISL_1301633	18/04/2020	Puebla	Human	Female	59	Ambulatory	Original	Nasopharyngeal and oropharyngeal swab
hCoV-19/Mexico/PUE-IBT-IMSS-451/2020	EPI_ISL_1301634	18/04/2020	Puebla	Human	Male	38	Ambulatory	Original	Nasopharyngeal and oropharyngeal swab
hCoV-19/Mexico/CMX-INER-IBT-14/2020	EPI_ISL_1301606	19/04/2020	Mexico City	Human	Male	unknown	Hospitalized	Original	Nasopharyngeal swab
hCoV-19/Mexico/PUE-IBT-IMSS-454/2020	EPI_ISL_1301637	19/04/2020	Puebla	Human	Male	52	Hospitalized	Original	Nasopharyngeal and oropharyngeal swab
hCoV-19/Mexico/CMX-IBT-IMSS-23/2020	EPI_ISL_955235	19/04/2020	Mexico City	Human	Male	27	Ambulatory	Original	Nasopharyngeal swab
hCoV-19/Mexico/CMX-INER-IBT-16/2020	EPI_ISL_1301608	20/04/2020	Mexico City	Human	Male	unknown	Hospitalized	Original	Nasopharyngeal swab
hCoV-19/Mexico/PUE-IBT-IMSS-452/2020	EPI_ISL_1301635	20/04/2020	Puebla	Human	Male	30	Ambulatory	Original	Nasopharyngeal and oropharyngeal swab
hCoV-19/Mexico/OAX-IBT-IMSS-455/2020	EPI_ISL_1301638	20/04/2020	Oaxaca	Human	Male	82	Hospitalized	Original	Nasopharyngeal and oropharyngeal swab

hCoV-19/Mexico/MIC-IBT-IMSS-459/2020	EPI_ISL_1301642	20/04/2020	Michoacan	Human	Female	25	Ambulatory	Original	Nasopharyngeal and oropharyngeal swab
hCoV-19/Mexico/CMX-INER-IBT-17/2020	EPI_ISL_1301719	20/04/2020	Mexico City	Human	Male	unknown	Hospitalized	Original	Pharyngeal swab
hCoV-19/Mexico/CMX-INER-IBT-15/2020	EPI_ISL_1301607	21/04/2020	Mexico City	Human	Female	unknown	Hospitalized	Original	Nasopharyngeal swab
hCoV-19/Mexico/CMX-INER-IBT-19/2020	EPI_ISL_1301503	22/04/2020	Mexico City	Human	Male	unknown	Hospitalized	Original	Pharyngeal swab
hCoV-19/Mexico/CMX-INER-IBT-18/2020	EPI_ISL_1301609	22/04/2020	Mexico City	Human	Male	unknown	Hospitalized	Original	Nasopharyngeal swab
hCoV-19/Mexico/PUE-IBT-IMSS-456/2020	EPI_ISL_1301639	22/04/2020	Puebla	Human	Male	42	Hospitalized	Original	Nasopharyngeal and oropharyngeal swab
hCoV-19/Mexico/COL-IBT-IMSS-458/2020	EPI_ISL_1301641	23/04/2020	Colima	Human	Male	40	Ambulatory	Original	Nasopharyngeal and oropharyngeal swab
hCoV-19/Mexico/PUE-IBT-IMSS-460/2020	EPI_ISL_1301643	23/04/2020	Puebla	Human	Female	47	Ambulatory	Original	Nasopharyngeal and oropharyngeal swab
hCoV-19/Mexico/MEX-InDRE-IBT-119/2020	EPI_ISL_1301575	24/04/2020	State of Mexico	Human	Male	54	Ambulatory	Original	Nasopharyngeal swab
hCoV-19/Mexico/COL-IBT-IMSS-461/2020	EPI_ISL_1301644	24/04/2020	Colima	Human	Female	29	Ambulatory	Original	Nasopharyngeal and oropharyngeal swab
hCoV-19/Mexico/PUE-IBT-IMSS-462/2020	EPI_ISL_1301645	24/04/2020	Puebla	Human	Male	30	Hospitalized	Original	Nasopharyngeal and oropharyngeal swab
hCoV-19/Mexico/BCN-IBT-IMSS-476/2020	EPI_ISL_1301657	24/04/2020	Baja California	Human	Male	37	Ambulatory	Original	Nasopharyngeal and oropharyngeal swab
hCoV-19/Mexico/PUE-IBT-IMSS-466/2020	EPI_ISL_1301649	25/04/2020	Puebla	Human	Male	61	Hospitalized	Original	Nasopharyngeal and oropharyngeal swab
hCoV-19/Mexico/MEX-InDRE-IBT-112/2020	EPI_ISL_1301447	25/04/2020	State of Mexico	Human	Male	46	Ambulatory	Original	Nasopharyngeal swab
hCoV-19/Mexico/BCS-IBT-IMSS-468/2020	EPI_ISL_1301593	26/04/2020	Baja California Sur	Human	Female	81	Hospitalized	Original	Nasopharyngeal swab
hCoV-19/Mexico/BCS-IBT-IMSS-469/2020	EPI_ISL_1301651	26/04/2020	Baja California Sur	Human	Female	34	Hospitalized	Original	Nasopharyngeal and oropharyngeal swab
hCoV-19/Mexico/BCS-IBT-IMSS-470/2020	EPI_ISL_1301652	26/04/2020	Baja California Sur	Human	Female	28	Ambulatory	Original	Nasopharyngeal and oropharyngeal swab
hCoV-19/Mexico/SLP-InDRE-IBT-50/2020	EPI_ISL_1301528	27/04/2020	San Luis Potosi	Human	Male	unknown	unknown	Original	Pharyngeal swab
hCoV-19/Mexico/MEX-InDRE-IBT-121/2020	EPI_ISL_1301577	27/04/2020	State of Mexico	Human	Male	67	Deceased	Original	Nasopharyngeal swab
hCoV-19/Mexico/CMX-INER-IBT-20/2020	EPI_ISL_1301610	27/04/2020	Mexico City	Human	Male	unknown	Hospitalized	Original	Nasopharyngeal swab
hCoV-19/Mexico/COL-IBT-IMSS-463/2020	EPI_ISL_1301646	27/04/2020	Colima	Human	Male	27	Ambulatory	Original	Nasopharyngeal and oropharyngeal swab
hCoV-19/Mexico/MOR-IBT-IMSS-464/2020	EPI_ISL_1301647	27/04/2020	Morelos	Human	Male	54	Ambulatory	Original	Nasopharyngeal and oropharyngeal swab
hCoV-19/Mexico/MOR-IBT-IMSS-465/2020	EPI_ISL_1301648	27/04/2020	Morelos	Human	Female	44	Ambulatory	Original	Nasopharyngeal and oropharyngeal swab
hCoV-19/Mexico/MOR-IBT-IMSS-467/2020	EPI_ISL_1301650	27/04/2020	Morelos	Human	Female	42	Ambulatory	Original	Nasopharyngeal and oropharyngeal swab
hCoV-19/Mexico/CMX-INER-IBT-22/2020	EPI_ISL_1301499	28/04/2020	Mexico City	Human	Male	unknown	Hospitalized	Original	Nasopharyngeal swab
hCoV-19/Mexico/MEX-InDRE-IBT-120/2020	EPI_ISL_1301576	28/04/2020	State of Mexico	Human	Male	unknown	Ambulatory	Original	Nasopharyngeal swab
hCoV-19/Mexico/MEX-InDRE-IBT-122/2020	EPI_ISL_1301578	28/04/2020	State of Mexico	Human	Male	52	Deceased	Original	Nasopharyngeal swab
hCoV-19/Mexico/YUC-InDRE-IBT-135/2020	EPI_ISL_1301591	28/04/2020	Yucatan	Human	Female	unknown	unknown	Original	Nasopharyngeal swab
hCoV-19/Mexico/CMX-INER-IBT-21/2020	EPI_ISL_1301611	28/04/2020	Mexico City	Human	Male	unknown	Hospitalized	Original	Nasopharyngeal swab
hCoV-19/Mexico/BCN-IBT-IMSS-474/2020	EPI_ISL_1301655	28/04/2020	Baja California	Human	Female	54	Ambulatory	Original	Nasopharyngeal and oropharyngeal swab
hCoV-19/Mexico/BCN-IBT-IMSS-475/2020	EPI_ISL_1301656	28/04/2020	Baja California	Human	Male	39	Hospitalized	Original	Nasopharyngeal and oropharyngeal swab
hCoV-19/Mexico/CMX-INER-IBT-23/2020	EPI_ISL_1301515	29/04/2020	Mexico City	Human	Male	unknown	Hospitalized	Original	Pharyngeal swab
hCoV-19/Mexico/CMX-INER-IBT-24/2020	EPI_ISL_1301612	29/04/2020	Mexico City	Human	Female	unknown	Hospitalized	Original	Nasopharyngeal swab
hCoV-19/Mexico/CMX-INER-IBT-25/2020	EPI_ISL_1301613	29/04/2020	Mexico City	Human	Female	unknown	Hospitalized	Original	Nasopharyngeal swab
hCoV-19/Mexico/MOR-IBT-IMSS-471/2020	EPI_ISL_1301653	29/04/2020	Morelos	Human	Male	40	Ambulatory	Original	Nasopharyngeal and oropharyngeal swab
hCoV-19/Mexico/BCN-IBT-IMSS-477/2020	EPI_ISL_1301658	29/04/2020	Baja California	Human	Female	42	Ambulatory	Original	Nasopharyngeal and oropharyngeal swab
hCoV-19/Mexico/BCN-IBT-IMSS-478/2020	EPI_ISL_1301659	29/04/2020	Baja California	Human	Female	34	Ambulatory	Original	Nasopharyngeal and oropharyngeal swab
hCoV-19/Mexico/ZAC-InDRE-IBT-49/2020	EPI_ISL_1301519	30/04/2020	Zacatecas	Human	Female	65	Deceased	Original	Pharyngeal swab
hCoV-19/Mexico/BCN-IBT-IMSS-479/2020	EPI_ISL_1301530	30/04/2020	Baja California	Human	Male	19	Ambulatory	Original	Nasopharyngeal and oropharyngeal swab
hCoV-19/Mexico/BCS-IBT-IMSS-473/2020	EPI_ISL_1301535	30/04/2020	Baja California Sur	Human	Male	38	Ambulatory	Original	Pharyngeal swab
hCoV-19/Mexico/CMX-INER-IBT-28/2020	EPI_ISL_1301614	30/04/2020	Mexico City	Human	Female	unknown	Hospitalized	Original	Nasopharyngeal swab

hCoV-19/Mexico/BCN-IBT-IMSS-480/2020	EPI_ISL_1301660	30/04/2020	Baja California	Human	Male	47	Ambulatory	Original	Nasopharyngeal and oropharyngeal swab
hCoV-19/Mexico/BCN-IBT-IMSS-481/2020	EPI_ISL_1301661	30/04/2020	Baja California	Human	Male	25	Ambulatory	Original	Nasopharyngeal and oropharyngeal swab
hCoV-19/Mexico/BCN-IBT-IMSS-483/2020	EPI_ISL_1301662	30/04/2020	Baja California	Human	Male	50	Hospitalized	Original	Nasopharyngeal and oropharyngeal swab
hCoV-19/Mexico/BCN-IBT-IMSS-484/2020	EPI_ISL_1301663	30/04/2020	Baja California	Human	Female	76	Hospitalized	Original	Nasopharyngeal and oropharyngeal swab
hCoV-19/Mexico/CMX-INER-IBT-29/2020	EPI_ISL_1301524	01/05/2020	Mexico City	Human	Female	unknown	Hospitalized	Original	Nasopharyngeal swab
hCoV-19/Mexico/BCN-IBT-IMSS-482/2020	EPI_ISL_1301538	01/05/2020	Baja California	Human	Female	35	Ambulatory	Original	Nasopharyngeal and oropharyngeal swab
hCoV-19/Mexico/MOR-IBT-IMSS-472/2020	EPI_ISL_1301654	01/05/2020	Morelos	Human	Female	39	Ambulatory	Original	Nasopharyngeal and oropharyngeal swab
hCoV-19/Mexico/MEX-InDRE-IBT-125/2020	EPI_ISL_1301709	01/05/2020	State of Mexico	Human	Male	74	Deceased	Original	Pharyngeal swab
hCoV-19/Mexico/BCN-IBT-IMSS-486/2020	EPI_ISL_1301665	02/05/2020	Baja California	Human	Female	30	Ambulatory	Original	Nasopharyngeal and oropharyngeal swab
hCoV-19/Mexico/BCN-IBT-IMSS-487/2020	EPI_ISL_1301666	02/05/2020	Baja California	Human	Female	37	Ambulatory	Original	Nasopharyngeal and oropharyngeal swab
hCoV-19/Mexico/ZAC-InDRE-IBT-57/2020	EPI_ISL_1301690	02/05/2020	Zacatecas	Human	Male	27	Hospitalized	Original	Pharyngeal swab
hCoV-19/Mexico/BCN-IBT-IMSS-488/2020	EPI_ISL_1301714	02/05/2020	Baja California	Human	Female	43	Ambulatory	Original	Pharyngeal swab
hCoV-19/Mexico/CMX-INER-IBT-26/2020	EPI_ISL_1301522	03/05/2020	Mexico City	Human	Male	unknown	Hospitalized	Original	Pharyngeal swab
hCoV-19/Mexico/OAX-InDRE-IBT-18/2020	EPI_ISL_1301529	03/05/2020	Oaxaca	Human	Male	63	Deceased	Original	Nasopharyngeal swab
hCoV-19/Mexico/DUR-InDRE-IBT-109/2020	EPI_ISL_1301456	03/05/2020	Durango	Human	Female	33	Ambulatory	Original	Nasopharyngeal swab
hCoV-19/Mexico/BCN-IBT-IMSS-489/2020	EPI_ISL_1301715	03/05/2020	Baja California	Human	Male	58	Ambulatory	Original	Pharyngeal swab
hCoV-19/Mexico/CMX-INER-IBT-27/2020	EPI_ISL_1301720	03/05/2020	Mexico City	Human	Male	unknown	Hospitalized	Original	Pharyngeal swab
hCoV-19/Mexico/MEX-InDRE-IBT-123/2020	EPI_ISL_1301579	04/05/2020	State of Mexico	Human	Male	38	Ambulatory	Original	Nasopharyngeal swab
hCoV-19/Mexico/CMX-InDRE-IBT-118/2020	EPI_ISL_1301580	04/05/2020	Mexico City	Human	Male	37	Ambulatory	Original	Nasopharyngeal swab
hCoV-19/Mexico/CMX-InDRE-IBT-113/2020	EPI_ISL_1301581	04/05/2020	Mexico City	Human	Male	42	Ambulatory	Original	Nasopharyngeal swab
hCoV-19/Mexico/CMX-InDRE-IBT-114/2020	EPI_ISL_1301582	04/05/2020	Mexico City	Human	Male	42	Ambulatory	Original	Nasopharyngeal swab
hCoV-19/Mexico/CMX-InDRE-IBT-115/2020	EPI_ISL_1301583	04/05/2020	Mexico City	Human	Male	31	Ambulatory	Original	Nasopharyngeal swab
hCoV-19/Mexico/MEX-InDRE-IBT-116/2020	EPI_ISL_1301584	04/05/2020	State of Mexico	Human	Female	30	Ambulatory	Original	Nasopharyngeal swab
hCoV-19/Mexico/GRO-IBT-IMSS-485/2020	EPI_ISL_1301664	04/05/2020	Guerrero	Human	Female	33	Ambulatory	Original	Nasopharyngeal and oropharyngeal swab
hCoV-19/Mexico/BCS-IBT-IMSS-491/2020	EPI_ISL_1301667	04/05/2020	Baja California Sur	Human	Female	44	Ambulatory	Original	Nasopharyngeal and oropharyngeal swab
hCoV-19/Mexico/MEX-InDRE-IBT-124/2020	EPI_ISL_1301494	04/05/2020	State of Mexico	Human	Male	40	Deceased	Original	Nasopharyngeal swab
hCoV-19/Mexico/BCS-IBT-IMSS-493/2020	EPI_ISL_1301669	05/05/2020	Baja California Sur	Human	Female	39	Ambulatory	Original	Nasopharyngeal and oropharyngeal swab
hCoV-19/Mexico/BCS-IBT-IMSS-494/2020	EPI_ISL_1301670	05/05/2020	Baja California Sur	Human	Female	25	Ambulatory	Original	Nasopharyngeal and oropharyngeal swab
hCoV-19/Mexico/CMX-INER-IBT-34/2020	EPI_ISL_1301721	05/05/2020	Mexico City	Human	Female	unknown	Hospitalized	Original	Pharyngeal swab
hCoV-19/Mexico/TLA-InDRE-IBT-16/2020	EPI_ISL_1301540	06/05/2020	Tlaxcala	Human	Male	35	Ambulatory	Original	Pharyngeal swab
hCoV-19/Mexico/CMX-INER-IBT-35/2020	EPI_ISL_1301550	06/05/2020	Mexico City	Human	Male	unknown	Hospitalized	Original	Pharyngeal swab
hCoV-19/Mexico/BCS-IBT-IMSS-490/2020	EPI_ISL_1301594	06/05/2020	Baja California Sur	Human	Male	37	Ambulatory	Original	Nasopharyngeal swab
hCoV-19/Mexico/CMX-InDRE-IBT-97/2020	EPI_ISL_1301501	07/05/2020	Mexico City	Human	Male	unknown	Deceased	Original	
hCoV-19/Mexico/CMX-INER-IBT-39/2020	EPI_ISL_1301510	07/05/2020	Mexico City	Human	Female	unknown	Hospitalized	Original	Nasopharyngeal swab
hCoV-19/Mexico/CMX-INER-IBT-37/2020	EPI_ISL_1301471	07/05/2020	Mexico City	Human	Male	unknown	Hospitalized	Original	Nasopharyngeal swab
hCoV-19/Mexico/CMX-INER-IBT-38/2020	EPI_ISL_1301488	07/05/2020	Mexico City	Human	Female	unknown	Hospitalized	Original	Nasopharyngeal swab
hCoV-19/Mexico/CMX-INER-IBT-36/2020	EPI_ISL_1301722	07/05/2020	Mexico City	Human	Male	unknown	Hospitalized	Original	Pharyngeal swab
hCoV-19/Mexico/CMX-IBT-IMSS-492/2020	EPI_ISL_1301668	08/05/2020	Mexico City	Human	Female	13	Hospitalized	Original	Nasopharyngeal and oropharyngeal swab
hCoV-19/Mexico/CAM-InDRE-IBT-48/2020	EPI_ISL_1301687	08/05/2020	Campeche	Human	Male	33	Ambulatory	Original	Pharyngeal swab
hCoV-19/Mexico/CMX-INER-IBT-40/2020	EPI_ISL_1301445	08/05/2020	Mexico City	Human	Female	unknown	Hospitalized	Original	Nasopharyngeal swab
hCoV-19/Mexico/CHH-IBT-IMSS-497/2020	EPI_ISL_1301716	08/05/2020	Chihuahua	Human	Female	23	Ambulatory	Original	Pharyngeal swab

hCoV-19/Mexico/CMX-INER-IBT-41/2020	EPI_ISL_1301533	09/05/2020	Mexico City	Human	Male	unknown	Hospitalized	Original	Pharyngeal swab
hCoV-19/Mexico/CMX-INER-IBT-44/2020	EPI_ISL_1301615	09/05/2020	Mexico City	Human	Female	unknown	Hospitalized	Original	Nasopharyngeal swab
hCoV-19/Mexico/MOR-IBT-IMSS-496/2020	EPI_ISL_1301595	11/05/2020	Morelos	Human	Male	42	Ambulatory	Original	Nasopharyngeal swab
hCoV-19/Mexico/CMX-INER-IBT-45/2020	EPI_ISL_1301616	11/05/2020	Mexico City	Human	Male	unknown	Hospitalized	Original	Nasopharyngeal swab
hCoV-19/Mexico/GUA-IBT-IMSS-498/2020	EPI_ISL_1301468	11/05/2020	Guanajuato	Human	Male	50	Hospitalized	Original	Nasopharyngeal and oropharyngeal swab
hCoV-19/Mexico/CMX-INER-IBT-46/2020	EPI_ISL_1301504	12/05/2020	Mexico City	Human	Male	unknown	Hospitalized	Original	Pharyngeal swab
hCoV-19/Mexico/BCS-IBT-IMSS-499/2020	EPI_ISL_1301596	12/05/2020	Baja California Sur	Human	Female	41	Ambulatory	Original	Nasopharyngeal swab
hCoV-19/Mexico/MOR-InDRE-IBT-7/2020	EPI_ISL_1301480	12/05/2020	Morelos	Human	Male	31	Deceased	Original	Pharyngeal swab
hCoV-19/Mexico/CMX-INER-IBT-47/2020	EPI_ISL_1301723	12/05/2020	Mexico City	Human	Female	unknown	Hospitalized	Original	Pharyngeal swab
hCoV-19/Mexico/COL-IBT-IMSS-500/2020	EPI_ISL_1301516	13/05/2020	Colima	Human	Male	76	Hospitalized	Original	Nasopharyngeal and oropharyngeal swab
hCoV-19/Mexico/CMX-INER-IBT-49/2020	EPI_ISL_1301617	13/05/2020	Mexico City	Human	Male	unknown	Hospitalized	Original	Nasopharyngeal swab
hCoV-19/Mexico/CMX-INER-IBT-48/2020	EPI_ISL_1301724	13/05/2020	Mexico City	Human	Male	unknown	Hospitalized	Original	Pharyngeal swab
hCoV-19/Mexico/CMX-INER-IBT-50/2020	EPI_ISL_1301545	14/05/2020	Mexico City	Human	Male	unknown	Hospitalized	Original	Pharyngeal swab
hCoV-19/Mexico/GRO-IBT-IMSS-502/2020	EPI_ISL_1301671	14/05/2020	Guerrero	Human	Female	49	Ambulatory	Original	Nasopharyngeal and oropharyngeal swab
hCoV-19/Mexico/COL-IBT-IMSS-501/2020	EPI_ISL_1301518	15/05/2020	Colima	Human	Male	41	Ambulatory	Original	Nasopharyngeal and oropharyngeal swab
hCoV-19/Mexico/GRO-IBT-IMSS-504/2020	EPI_ISL_1301536	15/05/2020	Guerrero	Human	Male	39	Ambulatory	Original	Nasopharyngeal and oropharyngeal swab
hCoV-19/Mexico/GRO-IBT-IMSS-503/2020	EPI_ISL_1301553	15/05/2020	Guerrero	Human	Female	31	Ambulatory	Original	Pharyngeal swab
hCoV-19/Mexico/GRO-InDRE-IBT-95/2020	EPI_ISL_1301458	15/05/2020	Guerrero	Human	Male	79	Hospitalized	Original	Pharyngeal swab
hCoV-19/Mexico/CMX-IBT-IMSS-505/2020	EPI_ISL_1301672	16/05/2020	Mexico City	Human	Female	9	Hospitalized	Original	Nasopharyngeal and oropharyngeal swab
hCoV-19/Mexico/HID-IBT-IMSS-506/2020	EPI_ISL_1301463	16/05/2020	Hidalgo	Human	Female	24	Ambulatory	Original	Nasopharyngeal and oropharyngeal swab
hCoV-19/Mexico/QUE-IBT-IMSS-507/2020	EPI_ISL_1301673	17/05/2020	Queretaro	Human	Female	30	Ambulatory	Original	Nasopharyngeal and oropharyngeal swab
hCoV-19/Mexico/VER-IBT-IMSS-512/2020	EPI_ISL_1301677	17/05/2020	Veracruz	Human	Male	41	Hospitalized	Original	Nasopharyngeal and oropharyngeal swab
hCoV-19/Mexico/VER-IBT-IMSS-513/2020	EPI_ISL_1301678	17/05/2020	Veracruz	Human	Female	68	Hospitalized	Original	Nasopharyngeal and oropharyngeal swab
hCoV-19/Mexico/CMX-INER-IBT-51/2020	EPI_ISL_1301725	17/05/2020	Mexico City	Human	Female	unknown	Hospitalized	Original	Pharyngeal swab
hCoV-19/Mexico/BCS-IBT-IMSS-509/2020	EPI_ISL_1301597	18/05/2020	Baja California Sur	Human	Female	34	Ambulatory	Original	Nasopharyngeal swab
hCoV-19/Mexico/CMX-INER-IBT-52/2020	EPI_ISL_1301618	18/05/2020	Mexico City	Human	Male	unknown	Hospitalized	Original	Nasopharyngeal swab
hCoV-19/Mexico/HID-IBT-IMSS-508/2020	EPI_ISL_1301674	18/05/2020	Hidalgo	Human	Female	41	Ambulatory	Original	Nasopharyngeal and oropharyngeal swab
hCoV-19/Mexico/QUE-IBT-IMSS-510/2020	EPI_ISL_1301675	18/05/2020	Queretaro	Human	Female	48	Ambulatory	Original	Nasopharyngeal and oropharyngeal swab
hCoV-19/Mexico/CMX-INER-IBT-53/2020	EPI_ISL_1301726	18/05/2020	Mexico City	Human	Female	unknown	Hospitalized	Original	Pharyngeal swab
hCoV-19/Mexico/VER-IBT-IMSS-511/2020	EPI_ISL_1301676	19/05/2020	Veracruz	Human	Female	66	Hospitalized	Original	Nasopharyngeal and oropharyngeal swab
hCoV-19/Mexico/GRO-InDRE-IBT-94/2020	EPI_ISL_1301487	19/05/2020	Guerrero	Human	Female	28	Hospitalized	Original	Pharyngeal swab
hCoV-19/Mexico/CMX-INER-IBT-54/2020	EPI_ISL_1301507	20/05/2020	Mexico City	Human	Female	unknown	Hospitalized	Original	Pharyngeal swab
hCoV-19/Mexico/CMX-INER-IBT-55/2020	EPI_ISL_1301551	20/05/2020	Mexico City	Human	Male	unknown	Hospitalized	Original	Pharyngeal swab
hCoV-19/Mexico/CMX-INER-IBT-56/2020	EPI_ISL_1301732	20/05/2020	Mexico City	Human	Male	unknown	Hospitalized	Original	Tracheal aspirate
hCoV-19/Mexico/CMX-INER-IBT-60/2020	EPI_ISL_1301554	22/05/2020	Mexico City	Human	Male	unknown	Hospitalized	Original	Pharyngeal swab
hCoV-19/Mexico/CMX-INER-IBT-62/2020	EPI_ISL_1301619	22/05/2020	Mexico City	Human	Female	unknown	Hospitalized	Original	Nasopharyngeal swab
hCoV-19/Mexico/CMX-INER-IBT-58/2020	EPI_ISL_1301727	22/05/2020	Mexico City	Human	Female	unknown	Hospitalized	Original	Pharyngeal swab
hCoV-19/Mexico/CMX-INER-IBT-59/2020	EPI_ISL_1301506	23/05/2020	Mexico City	Human	Female	unknown	Hospitalized	Original	Nasopharyngeal swab
hCoV-19/Mexico/CMX-INER-IBT-61/2020	EPI_ISL_1301728	23/05/2020	Mexico City	Human	Female	unknown	Hospitalized	Original	Pharyngeal swab
hCoV-19/Mexico/CMX-INER-IBT-57/2020	EPI_ISL_1301733	23/05/2020	Mexico City	Human	Female	unknown	Hospitalized	Original	Tracheal aspirate
hCoV-19/Mexico/SON-InDRE-IBT-106/2020	EPI_ISL_1301513	25/05/2020	Sonora	Human	Male	60	Ambulatory	Original	Pharyngeal swab

hCoV-19/Mexico/ZAC-InDRE-IBT-51/2020	EPI_ISL_1301469	25/05/2020	Zacatecas	Human	Male	40	Hospitalized	Original	Pharyngeal swab
hCoV-19/Mexico/ZAC-InDRE-IBT-52/2020	EPI_ISL_1301491	25/05/2020	Zacatecas	Human	Male	54	Hospitalized	Original	Pharyngeal swab
hCoV-19/Mexico/CMX-INER-IBT-63/2020	EPI_ISL_1301729	25/05/2020	Mexico City	Human	Male	unknown	Hospitalized	Original	Pharyngeal swab
hCoV-19/Mexico/CMX-INER-IBT-31/2020	EPI_ISL_1301602	26/05/2020	Mexico City	Human	Female	unknown	Hospitalized	Original	Nasopharyngeal swab
hCoV-19/Mexico/CMX-INER-IBT-32/2020	EPI_ISL_1301603	26/05/2020	Mexico City	Human	Male	unknown	Hospitalized	Original	Nasopharyngeal swab
hCoV-19/Mexico/CMX-INER-IBT-30/2020	EPI_ISL_1301472	26/05/2020	Mexico City	Human	Male	unknown	Hospitalized	Original	Tracheal aspirate
hCoV-19/Mexico/CMX-INER-IBT-33/2020	EPI_ISL_1301604	27/05/2020	Mexico City	Human	Male	unknown	Hospitalized	Original	Nasopharyngeal swab
hCoV-19/Mexico/TAB-InDRE-IBT-31/2020	EPI_ISL_1301537	29/05/2020	Tabasco	Human	Male	unknown	Hospitalized	Original	Nasopharyngeal swab
hCoV-19/Mexico/TAB-InDRE-IBT-29/2020	EPI_ISL_1301444	29/05/2020	Tabasco	Human	Male	unknown	Hospitalized	Original	Pharyngeal swab
hCoV-19/Mexico/TAB-InDRE-IBT-30/2020	EPI_ISL_1301701	29/05/2020	Tabasco	Human	Male	unknown	Hospitalized	Original	Pharyngeal swab
hCoV-19/Mexico/TAB-InDRE-IBT-28/2020	EPI_ISL_1301700	30/05/2020	Tabasco	Human	Male	unknown	Hospitalized	Original	Pharyngeal swab
hCoV-19/Mexico/MOR-InDRE-IBT-98/2020	EPI_ISL_1301478	31/05/2020	Morelos	Human	Male	26	Hospitalized	Original	Pharyngeal swab
hCoV-19/Mexico/CHH-InDRE-IBT-89/2020	EPI_ISL_1301705	31/05/2020	Chihuahua	Human	Female	38	Ambulatory	Original	Pharyngeal swab
hCoV-19/Mexico/BCN-InDRE-IBT-3/2020	EPI_ISL_1301532	01/06/2020	Baja California	Human	Female	70	Deceased	Original	Pharyngeal swab
hCoV-19/Mexico/TLA-InDRE-IBT-70/2020	EPI_ISL_1301546	01/06/2020	Tlaxcala	Human	Female	53	Hospitalized	Original	
hCoV-19/Mexico/BCS-InDRE-IBT-62/2020	EPI_ISL_1301555	01/06/2020	Baja California Sur	Human	Male	35	Hospitalized	Original	
hCoV-19/Mexico/TLA-InDRE-IBT-68/2020	EPI_ISL_1301556	01/06/2020	Tlaxcala	Human	Male	25	Ambulatory	Original	
hCoV-19/Mexico/TLA-InDRE-IBT-69/2020	EPI_ISL_1301557	01/06/2020	Tlaxcala	Human	Male	41	Ambulatory	Original	
hCoV-19/Mexico/NAY-InDRE-IBT-80/2020	EPI_ISL_1301559	01/06/2020	Nayarit	Human	Male	71	Deceased	Original	
hCoV-19/Mexico/BCS-InDRE-IBT-58/2020	EPI_ISL_1301567	01/06/2020	Baja California Sur	Human	Male	40	Hospitalized	Original	Nasopharyngeal swab
hCoV-19/Mexico/BCS-InDRE-IBT-63/2020	EPI_ISL_1301568	01/06/2020	Baja California Sur	Human	Female	44	Ambulatory	Original	Nasopharyngeal swab
hCoV-19/Mexico/SIN-InDRE-IBT-88/2020	EPI_ISL_1301573	01/06/2020	Sinaloa	Human	Female	79	Ambulatory	Original	Nasopharyngeal swab
hCoV-19/Mexico/BCN-InDRE-IBT-5/2020	EPI_ISL_1301695	01/06/2020	Baja California	Human	Female	92	Deceased	Original	Pharyngeal swab
hCoV-19/Mexico/BCS-InDRE-IBT-60/2020	EPI_ISL_1301451	01/06/2020	Baja California Sur	Human	Female	29	Ambulatory	Original	Nasopharyngeal swab
hCoV-19/Mexico/YUC-InDRE-IBT-64/2020	EPI_ISL_1301461	01/06/2020	Yucatan	Human	Female	55	Ambulatory	Original	Nasopharyngeal swab
hCoV-19/Mexico/ZAC-InDRE-IBT-59/2020	EPI_ISL_1301483	01/06/2020	Zacatecas	Human	Male	53	Ambulatory	Original	Nasopharyngeal swab
hCoV-19/Mexico/BCS-InDRE-IBT-61/2020	EPI_ISL_1301730	01/06/2020	Baja California Sur	Human	Male	32	Ambulatory	Original	Nasopharyngeal and oropharyngeal swab
hCoV-19/Mexico/OAX-InDRE-IBT-15/2020	EPI_ISL_1301512	02/06/2020	Oaxaca	Human	Female	53	Deceased	Original	Nasopharyngeal swab
hCoV-19/Mexico/BCN-InDRE-IBT-2/2020	EPI_ISL_1301521	02/06/2020	Baja California	Human	Female	25	Ambulatory	Original	Pharyngeal swab
hCoV-19/Mexico/QUE-InDRE-IBT-53/2020	EPI_ISL_1301541	02/06/2020	Queretaro	Human	Male	30	Hospitalized	Original	Pharyngeal swab
hCoV-19/Mexico/HID-InDRE-IBT-66/2020	EPI_ISL_1301549	02/06/2020	Hidalgo	Human	Male	66	Hospitalized	Original	Pharyngeal swab
hCoV-19/Mexico/NAY-InDRE-IBT-81/2020	EPI_ISL_1301560	02/06/2020	Nayarit	Human	Male	27	Hospitalized	Original	
hCoV-19/Mexico/HID-InDRE-IBT-65/2020	EPI_ISL_1301569	02/06/2020	Hidalgo	Human	Female	50	Deceased	Original	Nasopharyngeal swab
hCoV-19/Mexico/ZAC-InDRE-IBT-55/2020	EPI_ISL_1301688	02/06/2020	Zacatecas	Human	Female	36	Ambulatory	Original	Pharyngeal swab
hCoV-19/Mexico/ZAC-InDRE-IBT-56/2020	EPI_ISL_1301689	02/06/2020	Zacatecas	Human	Male	73	Deceased	Original	Pharyngeal swab
hCoV-19/Mexico/ROO-InDRE-IBT-11/2020	EPI_ISL_1301696	02/06/2020	Quintana Roo	Human	Female	55	Deceased	Original	Pharyngeal swab
hCoV-19/Mexico/ROO-InDRE-IBT-12/2020	EPI_ISL_1301486	02/06/2020	Quintana Roo	Human	Male	41	Deceased	Original	Pharyngeal swab
hCoV-19/Mexico/ROO-InDRE-IBT-10/2020	EPI_ISL_1301493	02/06/2020	Quintana Roo	Human	Female	49	Ambulatory	Original	Pharyngeal swab
hCoV-19/Mexico/BCN-InDRE-IBT-1/2020	EPI_ISL_1301495	02/06/2020	Baja California	Human	Female	unknown	Ambulatory	Original	Pharyngeal swab
hCoV-19/Mexico/COA-InDRE-IBT-105/2020	EPI_ISL_1301500	03/06/2020	Coahuila	Human	Male	unknown	Ambulatory	Original	Pharyngeal swab
hCoV-19/Mexico/ROO-InDRE-IBT-13/2020	EPI_ISL_1301526	03/06/2020	Quintana Roo	Human	Male	29	Hospitalized	Original	Pharyngeal swab

hCoV-19/Mexico/QUE-InDRE-IBT-54/2020	EPI_ISL_1301566	03/06/2020	Queretaro	Human	Female	42	Deceased	Original	Nasopharyngeal swab
hCoV-19/Mexico/NAY-InDRE-IBT-77/2020	EPI_ISL_1301694	03/06/2020	Nayarit	Human	Male	unknown	unknown	Original	Pharyngeal swab
hCoV-19/Mexico/GRO-InDRE-IBT-111/2020	EPI_ISL_1301455	03/06/2020	Guerrero	Human	unknown	unknown	Deceased	Original	
hCoV-19/Mexico/BCS-InDRE-IBT-6/2020	EPI_ISL_1301460	03/06/2020	Baja California Sur	Human	Female	45	Ambulatory	Original	Pharyngeal swab
hCoV-19/Mexico/OAX-InDRE-IBT-21/2020	EPI_ISL_1301502	04/06/2020	Oaxaca	Human	Male	71	Hospitalized	Original	Pharyngeal swab
hCoV-19/Mexico/ROO-InDRE-IBT-14/2020	EPI_ISL_1301505	04/06/2020	Quintana Roo	Human	Male	65	Deceased	Original	Pharyngeal swab
hCoV-19/Mexico/GRO-InDRE-IBT-110/2020	EPI_ISL_1301508	04/06/2020	Guerrero	Human	Female	unknown	Hospitalized	Original	Pharyngeal swab
hCoV-19/Mexico/GUA-InDRE-IBT-99/2020	EPI_ISL_1301511	04/06/2020	Guanajuato	Human	Female	77	Hospitalized	Original	Pharyngeal swab
hCoV-19/Mexico/CAM-InDRE-IBT-33/2020	EPI_ISL_1301527	04/06/2020	Campeche	Human	Female	35	Ambulatory	Original	Pharyngeal swab
hCoV-19/Mexico/NAY-InDRE-IBT-79/2020	EPI_ISL_1301534	04/06/2020	Nayarit	Human	Female	49	Ambulatory	Original	
hCoV-19/Mexico/GUA-InDRE-IBT-101/2020	EPI_ISL_1301548	04/06/2020	Guanajuato	Human	Female	17	Ambulatory	Original	Pharyngeal swab
hCoV-19/Mexico/TLA-InDRE-IBT-71/2020	EPI_ISL_1301558	04/06/2020	Tlaxcala	Human	Female	unknown	Hospitalized	Original	
hCoV-19/Mexico/CHP-InDRE-IBT-84/2020	EPI_ISL_1301570	04/06/2020	Chiapas	Human	Male	46	Deceased	Original	Nasopharyngeal swab
hCoV-19/Mexico/OAX-InDRE-IBT-17/2020	EPI_ISL_1301697	04/06/2020	Oaxaca	Human	Female	46	Ambulatory	Original	Pharyngeal swab
hCoV-19/Mexico/OAX-InDRE-IBT-19/2020	EPI_ISL_1301698	04/06/2020	Oaxaca	Human	Male	56	Hospitalized	Original	Pharyngeal swab
hCoV-19/Mexico/YUC-InDRE-IBT-8/2020	EPI_ISL_1301449	04/06/2020	Yucatan	Human	Male	24	Ambulatory	Original	Pharyngeal swab
hCoV-19/Mexico/CAM-InDRE-IBT-34/2020	EPI_ISL_1301457	04/06/2020	Campeche	Human	Male	44	Ambulatory	Original	Pharyngeal swab
hCoV-19/Mexico/BCN-InDRE-IBT-4/2020	EPI_ISL_1301470	04/06/2020	Baja California	Human	Female	83	Deceased	Original	Pharyngeal swab
hCoV-19/Mexico/HID-InDRE-IBT-67/2020	EPI_ISL_1301476	04/06/2020	Hidalgo	Human	Male	25	Ambulatory	Original	Nasopharyngeal swab
hCoV-19/Mexico/OAX-InDRE-IBT-20/2020	EPI_ISL_1301492	04/06/2020	Oaxaca	Human	Female	38	Hospitalized	Original	Pharyngeal swab
hCoV-19/Mexico/CAM-InDRE-IBT-35/2020	EPI_ISL_1301702	04/06/2020	Campeche	Human	Female	30	Ambulatory	Original	Pharyngeal swab
hCoV-19/Mexico/CAM-InDRE-IBT-36/2020	EPI_ISL_1301703	04/06/2020	Campeche	Human	Male	50	Ambulatory	Original	Pharyngeal swab
hCoV-19/Mexico/CAM-InDRE-IBT-37/2020	EPI_ISL_1301704	04/06/2020	Campeche	Human	Male	52	Ambulatory	Original	Pharyngeal swab
hCoV-19/Mexico/GUA-InDRE-IBT-100/2020	EPI_ISL_1301707	04/06/2020	Guanajuato	Human	Male	57	Ambulatory	Original	Pharyngeal swab
hCoV-19/Mexico/SIN-InDRE-IBT-24/2020	EPI_ISL_1301543	05/06/2020	Sinaloa	Human	Female	57	Hospitalized	Original	
hCoV-19/Mexico/SIN-InDRE-IBT-26/2020	EPI_ISL_1301544	05/06/2020	Sinaloa	Human	Male	49	Hospitalized	Original	
hCoV-19/Mexico/CHP-InDRE-IBT-83/2020	EPI_ISL_1301562	05/06/2020	Chiapas	Human	Male	54	Ambulatory	Original	
hCoV-19/Mexico/CHP-InDRE-IBT-85/2020	EPI_ISL_1301571	05/06/2020	Chiapas	Human	Female	71	Ambulatory	Original	Nasopharyngeal swab
hCoV-19/Mexico/SIN-InDRE-IBT-27/2020	EPI_ISL_1301699	05/06/2020	Sinaloa	Human	Male	73	Deceased	Original	Pharyngeal swab
hCoV-19/Mexico/OAX-InDRE-IBT-22/2020	EPI_ISL_1301474	05/06/2020	Oaxaca	Human	Female	29	Ambulatory	Original	
hCoV-19/Mexico/YUC-InDRE-IBT-9/2020	EPI_ISL_1301479	05/06/2020	Yucatan	Human	Female	unknown	unknown	Original	Pharyngeal swab
hCoV-19/Mexico/GUA-InDRE-IBT-102/2020	EPI_ISL_1301708	05/06/2020	Guanajuato	Human	Female	26	Ambulatory	Original	Pharyngeal swab
hCoV-19/Mexico/SON-InDRE-IBT-91/2020	EPI_ISL_1301565	06/06/2020	Sonora	Human	Male	57	Hospitalized	Original	Bronchial swab
hCoV-19/Mexico/CHP-InDRE-IBT-86/2020	EPI_ISL_1301572	06/06/2020	Chiapas	Human	Male	83	Hospitalized	Original	Nasopharyngeal swab
hCoV-19/Mexico/GRO-InDRE-IBT-117/2020	EPI_ISL_1301585	06/06/2020	Guerrero	Human	Male	47	Ambulatory	Original	Nasopharyngeal swab
hCoV-19/Mexico/TLA-InDRE-IBT-72/2020	EPI_ISL_1301691	06/06/2020	Tlaxcala	Human	Female	64	Deceased	Original	Pharyngeal swab
hCoV-19/Mexico/TLA-InDRE-IBT-73/2020	EPI_ISL_1301692	06/06/2020	Tlaxcala	Human	Male	60	Deceased	Original	Pharyngeal swab
hCoV-19/Mexico/CHP-InDRE-IBT-87/2020	EPI_ISL_1301448	06/06/2020	Chiapas	Human	Female	57	Hospitalized	Original	Nasopharyngeal swab
hCoV-19/Mexico/SON-InDRE-IBT-90/2020	EPI_ISL_1301464	06/06/2020	Sonora	Human	Female	31	Ambulatory	Original	Pharyngeal swab
hCoV-19/Mexico/GUA-InDRE-IBT-103/2020	EPI_ISL_1301485	06/06/2020	Guanajuato	Human	Male	36	Ambulatory	Original	Pharyngeal swab
hCoV-19/Mexico/SON-InDRE-IBT-93/2020	EPI_ISL_1301706	06/06/2020	Sonora	Human	Female	80	Deceased	Original	Pharyngeal swab

hCoV-19/Mexico/TLA-InDRE-IBT-74/2020	EPI_ISL_1301542	07/06/2020	Tlaxcala	Human	Female	55	Hospitalized	Original	Pharyngeal swab
hCoV-19/Mexico/NAY-InDRE-IBT-82/2020	EPI_ISL_1301561	07/06/2020	Nayarit	Human	Male	75	Hospitalized	Original	
hCoV-19/Mexico/SON-InDRE-IBT-92/2020	EPI_ISL_1301517	08/06/2020	Sonora	Human	Female	78	Ambulatory	Original	Pharyngeal swab
hCoV-19/Mexico/TLA-InDRE-IBT-75/2020	EPI_ISL_1301531	08/06/2020	Tlaxcala	Human	Male	68	Hospitalized	Original	Pharyngeal swab
hCoV-19/Mexico/GRO-InDRE-IBT-130/2020	EPI_ISL_1301547	08/06/2020	Guerrero	Human	Male	34	Ambulatory	Original	Nasopharyngeal swab
hCoV-19/Mexico/GRO-InDRE-IBT-129/2020	EPI_ISL_1301589	08/06/2020	Guerrero	Human	Male	41	Ambulatory	Original	Nasopharyngeal swab
hCoV-19/Mexico/TLA-InDRE-IBT-76/2020	EPI_ISL_1301693	08/06/2020	Tlaxcala	Human	Male	82	Ambulatory	Original	Pharyngeal swab
hCoV-19/Mexico/SIN-InDRE-IBT-25/2020	EPI_ISL_1301459	08/06/2020	Sinaloa	Human	Female	56	Deceased	Original	Nasopharyngeal swab
hCoV-19/Mexico/DUR-InDRE-IBT-108/2020	EPI_ISL_1301475	08/06/2020	Durango	Human	Female	32	Ambulatory	Original	Nasopharyngeal swab
hCoV-19/Mexico/CMX-InDRE-IBT-39/2020	EPI_ISL_1301477	09/06/2020	Mexico City	Human	Male	58	Hospitalized	Original	Pharyngeal swab
hCoV-19/Mexico/CMX-InDRE-IBT-38/2020	EPI_ISL_1301497	09/06/2020	Mexico City	Human	Male	50	Hospitalized	Original	Pharyngeal swab
hCoV-19/Mexico/GRO-InDRE-IBT-132/2020	EPI_ISL_1301523	10/06/2020	Guerrero	Human	Male	48	Ambulatory	Original	Nasopharyngeal swab
hCoV-19/Mexico/GRO-InDRE-IBT-131/2020	EPI_ISL_1301590	10/06/2020	Guerrero	Human	Female	37	Ambulatory	Original	Nasopharyngeal swab
hCoV-19/Mexico/GRO-InDRE-IBT-107/2020	EPI_ISL_1301574	25/06/2020	Guerrero	Human	Male	40	Ambulatory	Original	Nasopharyngeal swab
hCoV-19/Mexico/SIN-InDRE-IBT-23/2020	EPI_ISL_1301563	26/06/2020	Sinaloa	Human	Male	38	Ambulatory	Original	
hCoV-19/Mexico/VER-InDRE-IBT-96/2020	EPI_ISL_1301514	27/06/2020	Veracruz	Human	Female	39	Ambulatory	Original	Pharyngeal swab
hCoV-19/Mexico/CHH-InDRE-IBT-104/2020	EPI_ISL_1301481	03/07/2020	Chihuahua	Human	Male	36	Ambulatory	Original	Pharyngeal swab
hCoV-19/Mexico/COL-InDRE-IBT-136/2020	EPI_ISL_1301564	07/07/2020	Colima	Human	Female	unknown	unknown	Original	
hCoV-19/Mexico/COL-InDRE-IBT-137/2020	EPI_ISL_1301482	08/07/2020	Colima	Human	Female	unknown	unknown	Original	Pharyngeal swab
hCoV-19/Mexico/JAL-InDRE-IBT-138/2020	EPI_ISL_1301710	11/07/2020	Jalisco	Human	Female	unknown	unknown	Original	Pharyngeal swab
hCoV-19/Mexico/CMX-InDRE-IBT-126/2020	EPI_ISL_1301586	16/07/2020	Mexico City	Human	Female	unknown	Ambulatory	Original	Nasopharyngeal swab
hCoV-19/Mexico/CMX-InDRE-IBT-127/2020	EPI_ISL_1301587	20/07/2020	Mexico City	Human	Female	34	Ambulatory	Original	Nasopharyngeal swab
hCoV-19/Mexico/JAL-InDRE-IBT-142/2020	EPI_ISL_1301713	30/07/2020	Jalisco	Human	Female	75	Hospitalized	Original	Pharyngeal swab
hCoV-19/Mexico/JAL-InDRE-IBT-139/2020	EPI_ISL_1301711	01/08/2020	Jalisco	Human	Female	47	Ambulatory	Original	Pharyngeal swab
hCoV-19/Mexico/JAL-InDRE-IBT-140/2020	EPI_ISL_1301712	02/08/2020	Jalisco	Human	Female	31	Ambulatory	Original	Pharyngeal swab
hCoV-19/Mexico/JAL-InDRE-IBT-143/2020	EPI_ISL_1301592	03/08/2020	Jalisco	Human	Male	46	Ambulatory	Original	Nasopharyngeal swab
hCoV-19/Mexico/JAL-InDRE-IBT-141/2020	EPI_ISL_1301454	03/08/2020	Jalisco	Human	Male	39	Ambulatory	Original	Pharyngeal swab
hCoV-19/Mexico/CMX-INER-IBT-134/2020	EPI_ISL_1302229	04/08/2020	Mexico City	Human	Female	unknown	Hospitalized	Original	Nasopharyngeal and oropharyngeal swab
hCoV-19/Mexico/CMX-INER-IBT-135/2020	EPI_ISL_1302328	04/08/2020	Mexico City	Human	Female	unknown	Hospitalized	Original	Nasopharyngeal and oropharyngeal swab
hCoV-19/Mexico/CMX-INER-IBT-136/2020	EPI_ISL_1302280	06/08/2020	Mexico City	Human	Female	unknown	Hospitalized	Original	Nasopharyngeal and oropharyngeal swab
hCoV-19/Mexico/CMX-INER-IBT-138/2020	EPI_ISL_1302155	07/08/2020	Mexico City	Human	Male	unknown	Hospitalized	Original	Nasopharyngeal and oropharyngeal swab
hCoV-19/Mexico/CMX-INER-IBT-137/2020	EPI_ISL_1302291	08/08/2020	Mexico City	Human	Female	unknown	Hospitalized	Original	Nasopharyngeal and oropharyngeal swab
hCoV-19/Mexico/CMX-INER-IBT-139/2020	EPI_ISL_1302271	10/08/2020	Mexico City	Human	Female	unknown	Hospitalized	Original	Pharyngeal swab
hCoV-19/Mexico/CMX-InDRE-IBT-128/2020	EPI_ISL_1301588	14/08/2020	Mexico City	Human	Male	39	Ambulatory	Original	Nasopharyngeal swab
hCoV-19/Mexico/CMX-INER-IBT-140/2020	EPI_ISL_1302338	14/08/2020	Mexico City	Human	Female	unknown	Hospitalized	Original	Nasopharyngeal and oropharyngeal swab
hCoV-19/Mexico/CMX-INER-IBT-141/2020	EPI_ISL_1302160	16/08/2020	Mexico City	Human	Male	unknown	Hospitalized	Original	Nasopharyngeal and oropharyngeal swab
hCoV-19/Mexico/CMX-INER-IBT-142/2020	EPI_ISL_1302185	18/08/2020	Mexico City	Human	Female	unknown	Hospitalized	Original	Pharyngeal swab
hCoV-19/Mexico/CMX-INER-IBT-143/2020	EPI_ISL_1302312	20/08/2020	Mexico City	Human	Female	unknown	Hospitalized	Original	Pharyngeal swab
hCoV-19/Mexico/CMX-INER-IBT-144/2020	EPI_ISL_1302303	26/08/2020	Mexico City	Human	Female	unknown	Hospitalized	Original	Nasopharyngeal and oropharyngeal swab
hCoV-19/Mexico/CMX-INER-IBT-145/2020	EPI_ISL_1302277	29/08/2020	Mexico City	Human	Male	unknown	Hospitalized	Original	Nasopharyngeal and oropharyngeal swab
hCoV-19/Mexico/CMX-INER-IBT-146/2020	EPI_ISL_1302381	02/09/2020	Mexico City	Human	Male	unknown	Hospitalized	Original	Pharyngeal swab

hCoV-19/Mexico/CMX-INER-IBT-148/2020	EPI_ISL_1302205	04/09/2020	Mexico City	Human	Female	unknown	Hospitalized	Original	Pharyngeal swab
hCoV-19/Mexico/CMX-INER-IBT-147/2020	EPI_ISL_1302257	07/09/2020	Mexico City	Human	Female	unknown	Hospitalized	Original	Pharyngeal swab
hCoV-19/Mexico/CMX-INER-IBT-42/2020	EPI_ISL_1301539	08/09/2020	Mexico City	Human	Female	unknown	Hospitalized	Original	Nasopharyngeal swab
hCoV-19/Mexico/CMX-INER-IBT-43/2020	EPI_ISL_1301453	08/09/2020	Mexico City	Human	Male	unknown	Hospitalized	Original	Nasopharyngeal swab
hCoV-19/Mexico/CMX-INER-IBT-149/2020	EPI_ISL_1302206	09/09/2020	Mexico City	Human	Female	unknown	Hospitalized	Original	Pharyngeal swab
hCoV-19/Mexico/CMX-INER-IBT-150/2020	EPI_ISL_1302207	10/09/2020	Mexico City	Human	Male	unknown	Hospitalized	Original	Pharyngeal swab
hCoV-19/Mexico/CMX-INER-IBT-151/2020	EPI_ISL_1302255	15/09/2020	Mexico City	Human	Female	unknown	Hospitalized	Original	Nasopharyngeal and oropharyngeal swab
hCoV-19/Mexico/CMX-INER-IBT-152/2020	EPI_ISL_1302249	17/09/2020	Mexico City	Human	Male	unknown	Hospitalized	Original	Nasopharyngeal and oropharyngeal swab
hCoV-19/Mexico/CMX-INER-IBT-153/2020	EPI_ISL_1302319	21/09/2020	Mexico City	Human	Female	unknown	Hospitalized	Original	Nasopharyngeal and oropharyngeal swab
hCoV-19/Mexico/CMX-INER-IBT-154/2020	EPI_ISL_1302247	24/09/2020	Mexico City	Human	Male	unknown	Hospitalized	Original	Pharyngeal swab
hCoV-19/Mexico/CMX-INER-IBT-155/2020	EPI_ISL_1302180	25/09/2020	Mexico City	Human	Female	unknown	Hospitalized	Original	Nasopharyngeal and oropharyngeal swab
hCoV-19/Mexico/CMX-INER-IBT-156/2020	EPI_ISL_1302230	29/09/2020	Mexico City	Human	Male	unknown	Hospitalized	Original	Nasopharyngeal and oropharyngeal swab
hCoV-19/Mexico/CMX-INER-IBT-160/2020	EPI_ISL_1302297	30/09/2020	Mexico City	Human	Female	unknown	Hospitalized	Original	Nasopharyngeal and oropharyngeal swab
hCoV-19/Mexico/CMX-INER-IBT-159/2020	EPI_ISL_1302193	01/10/2020	Mexico City	Human	Female	unknown	Hospitalized	Original	Nasopharyngeal and oropharyngeal swab
hCoV-19/Mexico/CMX-INER-IBT-161/2020	EPI_ISL_1302231	02/10/2020	Mexico City	Human	Male	unknown	Hospitalized	Original	Nasopharyngeal and oropharyngeal swab
hCoV-19/Mexico/CMX-INER-IBT-158/2020	EPI_ISL_1302152	06/10/2020	Mexico City	Human	Male	unknown	Hospitalized	Original	Nasopharyngeal and oropharyngeal swab
hCoV-19/Mexico/CMX-INER-IBT-157/2020	EPI_ISL_1302349	07/10/2020	Mexico City	Human	Male	unknown	Hospitalized	Original	Nasopharyngeal and oropharyngeal swab
hCoV-19/Mexico/CMX-INER-IBT-162/2020	EPI_ISL_1302326	09/10/2020	Mexico City	Human	Male	unknown	Hospitalized	Original	Pharyngeal swab
hCoV-19/Mexico/CMX-INER-IBT-163/2020	EPI_ISL_1302256	14/10/2020	Mexico City	Human	Male	unknown	Hospitalized	Original	Nasopharyngeal and oropharyngeal swab
hCoV-19/Mexico/CMX-INER-IBT-168/2020	EPI_ISL_1302292	15/10/2020	Mexico City	Human	Female	unknown	Hospitalized	Original	Nasopharyngeal and oropharyngeal swab
hCoV-19/Mexico/CMX-INER-IBT-167/2020	EPI_ISL_1302194	17/10/2020	Mexico City	Human	Male	unknown	Hospitalized	Original	Nasopharyngeal and oropharyngeal swab
hCoV-19/Mexico/CMX-INER-IBT-164/2020	EPI_ISL_1302298	20/10/2020	Mexico City	Human	Male	unknown	Hospitalized	Original	Nasopharyngeal and oropharyngeal swab
hCoV-19/Mexico/CMX-INER-IBT-165/2020	EPI_ISL_1302250	23/10/2020	Mexico City	Human	Female	unknown	Hospitalized	Original	Pharyngeal swab
hCoV-19/Mexico/CMX-INER-IBT-166/2020	EPI_ISL_1302170	24/10/2020	Mexico City	Human	Male	unknown	Hospitalized	Original	Nasopharyngeal and oropharyngeal swab
hCoV-19/Mexico/CMX-INER-IBT-170/2020	EPI_ISL_1302246	26/10/2020	Mexico City	Human	Female	unknown	Hospitalized	Original	Nasopharyngeal and oropharyngeal swab
hCoV-19/Mexico/CMX-INER-IBT-171/2020	EPI_ISL_1302208	28/10/2020	Mexico City	Human	Female	unknown	Hospitalized	Original	Pharyngeal swab
hCoV-19/Mexico/CMX-INER-IBT-169/2020	EPI_ISL_1302290	01/11/2020	Mexico City	Human	Male	unknown	Hospitalized	Original	Pharyngeal swab
hCoV-19/Mexico/CMX-INER-IBT-172/2020	EPI_ISL_1302329	03/11/2020	Mexico City	Human	Male	unknown	Hospitalized	Original	Nasopharyngeal and oropharyngeal swab
hCoV-19/Mexico/CMX-INER-IBT-174/2020	EPI_ISL_1302186	04/11/2020	Mexico City	Human	Female	unknown	Hospitalized	Original	Pharyngeal swab
hCoV-19/Mexico/CMX-INER-IBT-173/2020	EPI_ISL_1302336	10/11/2020	Mexico City	Human	Male	unknown	Hospitalized	Original	Nasopharyngeal and oropharyngeal swab
hCoV-19/Mexico/CMX-INER-IBT-175/2020	EPI_ISL_1302339	12/11/2020	Mexico City	Human	Male	unknown	Hospitalized	Original	Nasopharyngeal and oropharyngeal swab
hCoV-19/Mexico/CMX-INER-IBT-66/2020	EPI_ISL_1302385	14/11/2020	Mexico City	Human	Male	unknown	Hospitalized	Original	Nasopharyngeal and oropharyngeal swab
hCoV-19/Mexico/CMX-INER-IBT-67/2020	EPI_ISL_1302263	15/11/2020	Mexico City	Human	Male	unknown	Hospitalized	Original	Nasopharyngeal and oropharyngeal swab
hCoV-19/Mexico/CMX-INER-IBT-69/2020	EPI_ISL_1302365	18/11/2020	Mexico City	Human	Female	unknown	Hospitalized	Original	Nasopharyngeal and oropharyngeal swab
hCoV-19/Mexico/CMX-INER-IBT-70/2020	EPI_ISL_1302369	18/11/2020	Mexico City	Human	Female	unknown	Hospitalized	Original	Nasopharyngeal and oropharyngeal swab
hCoV-19/Mexico/CMX-INER-IBT-72/2020	EPI_ISL_1302253	20/11/2020	Mexico City	Human	Female	unknown	Hospitalized	Original	Nasopharyngeal and oropharyngeal swab
hCoV-19/Mexico/CMX-INER-IBT-71/2020	EPI_ISL_1302301	21/11/2020	Mexico City	Human	Male	unknown	Hospitalized	Original	Nasopharyngeal and oropharyngeal swab
hCoV-19/Mexico/CMX-INER-IBT-74/2020	EPI_ISL_1302307	23/11/2020	Mexico City	Human	Male	unknown	Hospitalized	Original	Nasopharyngeal and oropharyngeal swab
hCoV-19/Mexico/CMX-INER-IBT-75/2020	EPI_ISL_1302325	23/11/2020	Mexico City	Human	Female	unknown	Hospitalized	Original	Nasopharyngeal and oropharyngeal swab
hCoV-19/Mexico/CMX-INER-IBT-77/2020	EPI_ISL_1302299	24/11/2020	Mexico City	Human	Female	unknown	Hospitalized	Original	Nasopharyngeal and oropharyngeal swab
hCoV-19/Mexico/CMX-INER-IBT-76/2020	EPI_ISL_1302357	24/11/2020	Mexico City	Human	Male	unknown	Hospitalized	Original	Nasopharyngeal and oropharyngeal swab

hCoV-19/Mexico/CMX-INER-IBT-81/2020	EPI_ISL_1302366	24/11/2020	Mexico City	Human	Male	unknown	Hospitalized	Original	Nasopharyngeal and oropharyngeal swab
hCoV-19/Mexico/CMX-INER-IBT-78/2020	EPI_ISL_1302171	26/11/2020	Mexico City	Human	Female	unknown	Hospitalized	Original	Nasopharyngeal and oropharyngeal swab
hCoV-19/Mexico/CMX-INER-IBT-80/2020	EPI_ISL_1302346	27/11/2020	Mexico City	Human	Female	unknown	Hospitalized	Original	Nasopharyngeal and oropharyngeal swab
hCoV-19/Mexico/CMX-INER-IBT-79/2020	EPI_ISL_1302159	28/11/2020	Mexico City	Human	Female	unknown	Hospitalized	Original	Nasopharyngeal and oropharyngeal swab
hCoV-19/Mexico/CMX-INER-IBT-106/2020	EPI_ISL_1302377	03/12/2020	Mexico City	Human	Female	unknown	Hospitalized	Original	Nasopharyngeal and oropharyngeal swab
hCoV-19/Mexico/CMX-INER-IBT-102/2020	EPI_ISL_1302389	03/12/2020	Mexico City	Human	Male	unknown	Hospitalized	Original	Nasopharyngeal and oropharyngeal swab
hCoV-19/Mexico/CMX-INER-IBT-107/2020	EPI_ISL_1302191	03/12/2020	Mexico City	Human	Female	unknown	Hospitalized	Original	Nasopharyngeal and oropharyngeal swab
hCoV-19/Mexico/CMX-INER-IBT-96/2020	EPI_ISL_1302234	04/12/2020	Mexico City	Human	Female	unknown	Hospitalized	Original	Nasopharyngeal and oropharyngeal swab
hCoV-19/Mexico/CMX-INER-IBT-105/2020	EPI_ISL_1302345	04/12/2020	Mexico City	Human	Male	unknown	Hospitalized	Original	Nasopharyngeal and oropharyngeal swab
hCoV-19/Mexico/CMX-INER-IBT-100/2020	EPI_ISL_1302391	04/12/2020	Mexico City	Human	Male	unknown	Hospitalized	Original	Nasopharyngeal and oropharyngeal swab
hCoV-19/Mexico/CMX-INER-IBT-94/2020	EPI_ISL_1302394	05/12/2020	Mexico City	Human	Male	unknown	Hospitalized	Original	Nasopharyngeal and oropharyngeal swab
hCoV-19/Mexico/CMX-INER-IBT-86/2020	EPI_ISL_1302302	06/12/2020	Mexico City	Human	Female	unknown	Hospitalized	Original	Nasopharyngeal and oropharyngeal swab
hCoV-19/Mexico/CMX-INER-IBT-91/2020	EPI_ISL_1302232	08/12/2020	Mexico City	Human	Female	unknown	Hospitalized	Original	Nasopharyngeal and oropharyngeal swab
hCoV-19/Mexico/CMX-INER-IBT-95/2020	EPI_ISL_1302233	08/12/2020	Mexico City	Human	Female	unknown	Hospitalized	Original	Nasopharyngeal and oropharyngeal swab
hCoV-19/Mexico/CMX-INER-IBT-83/2020	EPI_ISL_1302317	08/12/2020	Mexico City	Human	Female	unknown	Hospitalized	Original	Nasopharyngeal and oropharyngeal swab
hCoV-19/Mexico/CMX-INER-IBT-85/2020	EPI_ISL_1302320	08/12/2020	Mexico City	Human	Male	unknown	Hospitalized	Original	Nasopharyngeal and oropharyngeal swab
hCoV-19/Mexico/CMX-INER-IBT-92/2020	EPI_ISL_1302330	08/12/2020	Mexico City	Human	Male	unknown	Hospitalized	Original	Nasopharyngeal and oropharyngeal swab
hCoV-19/Mexico/CMX-INER-IBT-101/2020	EPI_ISL_1302364	08/12/2020	Mexico City	Human	Male	unknown	Hospitalized	Original	Nasopharyngeal and oropharyngeal swab
hCoV-19/Mexico/CMX-INER-IBT-103/2020	EPI_ISL_1302375	08/12/2020	Mexico City	Human	Female	unknown	Hospitalized	Original	Nasopharyngeal and oropharyngeal swab
hCoV-19/Mexico/CMX-INER-IBT-98/2020	EPI_ISL_1302383	08/12/2020	Mexico City	Human	Male	unknown	Hospitalized	Original	Nasopharyngeal and oropharyngeal swab
hCoV-19/Mexico/CMX-INER-IBT-108/2020	EPI_ISL_1302384	08/12/2020	Mexico City	Human	Female	unknown	Hospitalized	Original	Nasopharyngeal and oropharyngeal swab
hCoV-19/Mexico/CMX-INER-IBT-104/2020	EPI_ISL_1302399	08/12/2020	Mexico City	Human	Female	unknown	Hospitalized	Original	Nasopharyngeal and oropharyngeal swab
hCoV-19/Mexico/CMX-INER-IBT-89/2020	EPI_ISL_1302258	08/12/2020	Mexico City	Human	Female	unknown	Hospitalized	Original	Nasopharyngeal and oropharyngeal swab
hCoV-19/Mexico/CMX-INER-IBT-87/2020	EPI_ISL_1302308	09/12/2020	Mexico City	Human	Male	unknown	Hospitalized	Original	Nasopharyngeal and oropharyngeal swab
hCoV-19/Mexico/CMX-INER-IBT-84/2020	EPI_ISL_1302395	09/12/2020	Mexico City	Human	Female	unknown	Hospitalized	Original	Nasopharyngeal and oropharyngeal swab
hCoV-19/Mexico/CMX-IMSS_K4/2020	EPI_ISL_1096141	10/12/2020	Mexico City	Human	Male	32	Deceased	Original	Bronchioalveolar lavage
hCoV-19/Mexico/CMX-IMSS_K2/2020	EPI_ISL_1096985	10/12/2020	Mexico City	Human	Female	57	Deceased	Original	Bronchioalveolar lavage
hCoV-19/Mexico/CMX-INER-IBT-97/2020	EPI_ISL_1302304	14/12/2020	Mexico City	Human	Female	unknown	Hospitalized	Original	Nasopharyngeal and oropharyngeal swab
hCoV-19/Mexico/CMX-INER-IBT-88/2020	EPI_ISL_1302350	14/12/2020	Mexico City	Human	Male	unknown	Hospitalized	Original	Nasopharyngeal and oropharyngeal swab
hCoV-19/Mexico/PUE-InDRE-IBT-156/2020	EPI_ISL_1302211	15/12/2020	Puebla	Human	Female	36	Ambulatory	Original	Pharyngeal swab
hCoV-19/Mexico/AGU-InDRE-IBT-201/2020	EPI_ISL_1302183	17/12/2020	Aguascalientes	Human	Male	19	Ambulatory	Original	Pharyngeal swab
hCoV-19/Mexico/PUE-InDRE-IBT-205/2020	EPI_ISL_1302218	18/12/2020	Puebla	Human	Male	21	Ambulatory	Original	Pharyngeal swab
hCoV-19/Mexico/PUE-InDRE-IBT-158/2020	EPI_ISL_1302380	18/12/2020	Puebla	Human	Female	49	Ambulatory	Original	Pharyngeal swab
hCoV-19/Mexico/CMX-INER-IBT-90/2020	EPI_ISL_1302386	18/12/2020	Mexico City	Human	Female	unknown	Hospitalized	Original	Nasopharyngeal and oropharyngeal swab
hCoV-19/Mexico/PUE-InDRE-IBT-157/2020	EPI_ISL_1302174	18/12/2020	Puebla	Human	Male	38	Ambulatory	Original	
hCoV-19/Mexico/PUE-InDRE-IBT-208/2020	EPI_ISL_1302176	18/12/2020	Puebla	Human	Male	54	Ambulatory	Original	Pharyngeal swab
hCoV-19/Mexico/VER-InDRE-IBT-160/2020	EPI_ISL_1302219	19/12/2020	Veracruz	Human	Male	52	Ambulatory	Original	Pharyngeal swab
hCoV-19/Mexico/PUE-InDRE-IBT-209/2020	EPI_ISL_1302254	19/12/2020	Puebla	Human	Female	31	Ambulatory	Original	Pharyngeal swab
hCoV-19/Mexico/PUE-InDRE-IBT-161/2020	EPI_ISL_1302342	20/12/2020	Puebla	Human	Female	42	Ambulatory	Original	Pharyngeal swab
hCoV-19/Mexico/PUE-InDRE-IBT-162/2020	EPI_ISL_1302283	20/12/2020	Puebla	Human	Female	52	Ambulatory	Original	Pharyngeal swab
hCoV-19/Mexico/PUE-InDRE-IBT-204/2020	EPI_ISL_1302217	21/12/2020	Puebla	Human	Female	30	Ambulatory	Original	Pharyngeal swab

hCoV-19/Mexico/PUE-InDRE-IBT-179/2020	EPI_ISL_1302309	21/12/2020	Puebla	Human	Female	52	Ambulatory	Original	Pharyngeal swab
hCoV-19/Mexico/CHP-InDRE-IBT-196/2020	EPI_ISL_1302310	21/12/2020	Oaxaca	Human	Female	32	Ambulatory	Original	Pharyngeal swab
hCoV-19/Mexico/PUE-InDRE-IBT-181/2020	EPI_ISL_1302331	21/12/2020	Puebla	Human	Female	40	Ambulatory	Original	Pharyngeal swab
hCoV-19/Mexico/PUE-InDRE-IBT-172/2020	EPI_ISL_1302343	21/12/2020	Puebla	Human	Male	28	Ambulatory	Original	Pharyngeal swab
hCoV-19/Mexico/PUE-InDRE-IBT-184/2020	EPI_ISL_1302398	21/12/2020	Puebla	Human	Male	75	Deceased	Original	Pharyngeal swab
hCoV-19/Mexico/AGU-InDRE-IBT-210/2020	EPI_ISL_1302177	21/12/2020	Aguascalientes	Human	Female	26	Ambulatory	Original	Pharyngeal swab
hCoV-19/Mexico/PUE-InDRE-IBT-164/2020	EPI_ISL_1302188	21/12/2020	Puebla	Human	Female	34	Ambulatory	Original	Pharyngeal swab
hCoV-19/Mexico/AGU-InDRE-IBT-206/2020	EPI_ISL_1302200	21/12/2020	Aguascalientes	Human	Female	27	Ambulatory	Original	Pharyngeal swab
hCoV-19/Mexico/AGU-InDRE-IBT-211/2020	EPI_ISL_1302201	21/12/2020	Aguascalientes	Human	Male	20	Ambulatory	Original	Pharyngeal swab
hCoV-19/Mexico/PUE-InDRE-IBT-182/2020	EPI_ISL_1302286	21/12/2020	Puebla	Human	Female	23	Ambulatory	Original	Pharyngeal swab
hCoV-19/Mexico/PUE-InDRE-IBT-203/2020	EPI_ISL_1302289	21/12/2020	Puebla	Human	Male	36	Ambulatory	Original	Pharyngeal swab
hCoV-19/Mexico/PUE-InDRE-IBT-178/2020	EPI_ISL_1302154	22/12/2020	Puebla	Human	Male	45	Ambulatory	Original	Pharyngeal swab
hCoV-19/Mexico/MOR-InDRE-IBT-147/2020	EPI_ISL_1302162	22/12/2020	Morelos	Human	Male	49	Ambulatory	Original	Pharyngeal swab
hCoV-19/Mexico/PUE-InDRE-IBT-183/2020	EPI_ISL_1302215	22/12/2020	Puebla	Human	Male	43	Ambulatory	Original	Pharyngeal swab
hCoV-19/Mexico/PUE-InDRE-IBT-150/2020	EPI_ISL_1302241	22/12/2020	Puebla	Human	Male	55	Ambulatory	Original	Pharyngeal swab
hCoV-19/Mexico/PUE-InDRE-IBT-155/2020	EPI_ISL_1302242	22/12/2020	Puebla	Human	Female	43	Ambulatory	Original	Pharyngeal swab
hCoV-19/Mexico/MOR-InDRE-IBT-199/2020	EPI_ISL_1302293	22/12/2020	Morelos	Human	Male	44	Ambulatory	Original	Pharyngeal swab
hCoV-19/Mexico/PUE-InDRE-IBT-217/2020	EPI_ISL_1302296	22/12/2020	Puebla	Human	Female	23	Ambulatory	Original	Pharyngeal swab
hCoV-19/Mexico/PUE-InDRE-IBT-152/2020	EPI_ISL_1302313	22/12/2020	Puebla	Human	Male	26	Ambulatory	Original	Pharyngeal swab
hCoV-19/Mexico/PUE-InDRE-IBT-175/2020	EPI_ISL_1302323	22/12/2020	Puebla	Human	Female	47	Ambulatory	Original	Pharyngeal swab
hCoV-19/Mexico/PUE-InDRE-IBT-170/2020	EPI_ISL_1302333	22/12/2020	Puebla	Human	Female	47	Ambulatory	Original	Pharyngeal swab
hCoV-19/Mexico/AGU-InDRE-IBT-212/2020	EPI_ISL_1302337	22/12/2020	Aguascalientes	Human	Female	23	Ambulatory	Original	Pharyngeal swab
hCoV-19/Mexico/MOR-InDRE-IBT-185/2020	EPI_ISL_1302358	22/12/2020	Morelos	Human	Male	33	Ambulatory	Original	Pharyngeal swab
hCoV-19/Mexico/MOR-InDRE-IBT-148/2020	EPI_ISL_1302382	22/12/2020	Morelos	Human	Female	39	Ambulatory	Original	Pharyngeal swab
hCoV-19/Mexico/PUE-InDRE-IBT-176/2020	EPI_ISL_1302388	22/12/2020	Puebla	Human	Male	73	Ambulatory	Original	Pharyngeal swab
hCoV-19/Mexico/MOR-InDRE-IBT-191/2020	EPI_ISL_1302396	22/12/2020	Morelos	Human	Female	30	Ambulatory	Original	Pharyngeal swab
hCoV-19/Mexico/PUE-InDRE-IBT-149/2020	EPI_ISL_1302397	22/12/2020	Puebla	Human	Female	31	Ambulatory	Original	Pharyngeal swab
hCoV-19/Mexico/PUE-InDRE-IBT-200/2020	EPI_ISL_1302181	22/12/2020	Puebla	Human	Male	27	Ambulatory	Original	Pharyngeal swab
hCoV-19/Mexico/BCN-InDRE-IBT-144/2020	EPI_ISL_1302182	22/12/2020	Baja California	Human	Female	67	Ambulatory	Original	
hCoV-19/Mexico/CHP-InDRE-IBT-197/2020	EPI_ISL_1302197	22/12/2020	Oaxaca	Human	Female	41	Ambulatory	Original	Pharyngeal swab
hCoV-19/Mexico/GRO-InDRE-IBT-153/2020	EPI_ISL_1302199	22/12/2020	Guerrero	Human	Male	33	Ambulatory	Original	Pharyngeal swab
hCoV-19/Mexico/PUE-InDRE-IBT-154/2020	EPI_ISL_1302210	22/12/2020	Puebla	Human	Male	32	Ambulatory	Original	Pharyngeal swab
hCoV-19/Mexico/PUE-InDRE-IBT-166/2020	EPI_ISL_1302212	22/12/2020	Puebla	Human	Male	39	Ambulatory	Original	Pharyngeal swab
hCoV-19/Mexico/MOR-InDRE-IBT-198/2020	EPI_ISL_1302259	22/12/2020	Morelos	Human	Male	36	Ambulatory	Original	Pharyngeal swab
hCoV-19/Mexico/PUE-InDRE-IBT-171/2020	EPI_ISL_1302262	22/12/2020	Puebla	Human	Male	37	Ambulatory	Original	Pharyngeal swab
hCoV-19/Mexico/MOR-InDRE-IBT-145/2020	EPI_ISL_1302265	22/12/2020	Morelos	Human	Female	35	Ambulatory	Original	Pharyngeal swab
hCoV-19/Mexico/AGU-InDRE-IBT-215/2020	EPI_ISL_1302274	22/12/2020	Aguascalientes	Human	Female	51	Ambulatory	Original	Pharyngeal swab
hCoV-19/Mexico/MOR-InDRE-IBT-151/2020	EPI_ISL_1302276	22/12/2020	Morelos	Human	Male	54	Ambulatory	Original	Pharyngeal swab
hCoV-19/Mexico/PUE-InDRE-IBT-180/2020	EPI_ISL_1302279	22/12/2020	Puebla	Human	Male	50	Ambulatory	Original	Pharyngeal swab
hCoV-19/Mexico/CHP-InDRE-IBT-159/2020	EPI_ISL_1302287	22/12/2020	Chiapas	Human	Female	56	Deceased	Original	Pharyngeal swab
hCoV-19/Mexico/AGU-InDRE-IBT-213/2020	EPI_ISL_1302166	23/12/2020	Aguascalientes	Human	Female	56	Ambulatory	Original	Pharyngeal swab

hCoV-19/Mexico/CHP-InDRE-IBT-194/2020	EPI_ISL_1302167	23/12/2020	Chiapas	Human	Female	27	Ambulatory	Original	Pharyngeal swab
hCoV-19/Mexico/MOR-InDRE-IBT-193/2020	EPI_ISL_1302168	23/12/2020	Morelos	Human	Female	30	Ambulatory	Original	Pharyngeal swab
hCoV-19/Mexico/PUE-InDRE-IBT-168/2020	EPI_ISL_1302213	23/12/2020	Puebla	Human	Male	41	Ambulatory	Original	Pharyngeal swab
hCoV-19/Mexico/PUE-InDRE-IBT-173/2020	EPI_ISL_1302214	23/12/2020	Puebla	Human	Male	27	Ambulatory	Original	Pharyngeal swab
hCoV-19/Mexico/PUE-InDRE-IBT-202/2020	EPI_ISL_1302216	23/12/2020	Puebla	Human	Male	47	Ambulatory	Original	Pharyngeal swab
hCoV-19/Mexico/MOR-InDRE-IBT-192/2020	EPI_ISL_1302295	23/12/2020	Morelos	Human	Male	24	Ambulatory	Original	Pharyngeal swab
hCoV-19/Mexico/CHP-InDRE-IBT-195/2020	EPI_ISL_1302300	23/12/2020	Chiapas	Human	Female	39	Ambulatory	Original	Pharyngeal swab
hCoV-19/Mexico/MOR-InDRE-IBT-188/2020	EPI_ISL_1302314	23/12/2020	Morelos	Human	Male	24	Ambulatory	Original	Pharyngeal swab
hCoV-19/Mexico/PUE-InDRE-IBT-167/2020	EPI_ISL_1302321	23/12/2020	Puebla	Human	Male	57	Deceased	Original	Pharyngeal swab
hCoV-19/Mexico/PUE-InDRE-IBT-174/2020	EPI_ISL_1302322	23/12/2020	Puebla	Human	Male	38	Ambulatory	Original	Pharyngeal swab
hCoV-19/Mexico/MOR-InDRE-IBT-189/2020	EPI_ISL_1302340	23/12/2020	Morelos	Human	Female	32	Ambulatory	Original	Pharyngeal swab
hCoV-19/Mexico/MOR-InDRE-IBT-186/2020	EPI_ISL_1302363	23/12/2020	Morelos	Human	Male	37	Ambulatory	Original	Pharyngeal swab
hCoV-19/Mexico/MOR-InDRE-IBT-187/2020	EPI_ISL_1302175	23/12/2020	Morelos	Human	Male	26	Ambulatory	Original	Pharyngeal swab
hCoV-19/Mexico/AGU-InDRE-IBT-214/2020	EPI_ISL_1302184	23/12/2020	Aguascalientes	Human	Female	46	Ambulatory	Original	Pharyngeal swab
hCoV-19/Mexico/PUE-InDRE-IBT-146/2020	EPI_ISL_1302187	23/12/2020	Puebla	Human	Female	30	Ambulatory	Original	Pharyngeal swab
hCoV-19/Mexico/PUE-InDRE-IBT-177/2020	EPI_ISL_1302189	23/12/2020	Puebla	Human	Female	40	Ambulatory	Original	Pharyngeal swab
hCoV-19/Mexico/AGU-InDRE-IBT-216/2020	EPI_ISL_1302202	23/12/2020	Aguascalientes	Human	Male	24	Ambulatory	Original	Pharyngeal swab
hCoV-19/Mexico/MOR-InDRE-IBT-190/2020	EPI_ISL_1302209	23/12/2020	Morelos	Human	Female	28	Ambulatory	Original	Pharyngeal swab
hCoV-19/Mexico/CMX-INER-IBT-113/2020	EPI_ISL_1302266	23/12/2020	Mexico City	Human	Male	unknown	Hospitalized	Original	Nasopharyngeal and oropharyngeal swab
hCoV-19/Mexico/PUE-InDRE-IBT-207/2020	EPI_ISL_1302344	24/12/2020	Puebla	Human	Female	43	Ambulatory	Original	Pharyngeal swab
hCoV-19/Mexico/CMX-INER-IBT-112/2020	EPI_ISL_1302373	25/12/2020	Mexico City	Human	Male	unknown	Hospitalized	Original	Nasopharyngeal and oropharyngeal swab
hCoV-19/Mexico/PUE-InDRE-IBT-163/2020	EPI_ISL_1302335	27/12/2020	Puebla	Human	Male	60	Deceased	Original	Pharyngeal swab
hCoV-19/Mexico/CMX-INER-IBT-109/2020	EPI_ISL_1302179	28/12/2020	Mexico City	Human	Male	unknown	Hospitalized	Original	Nasopharyngeal and oropharyngeal swab
hCoV-19/Mexico/CMX-INER-IBT-111/2020	EPI_ISL_1302163	31/12/2020	Mexico City	Human	Male	unknown	Hospitalized	Original	Nasopharyngeal and oropharyngeal swab
hCoV-19/Mexico/CMX-INER-IBT-114/2020	EPI_ISL_1302400	31/12/2020	Mexico City	Human	Male	unknown	Hospitalized	Original	Nasopharyngeal and oropharyngeal swab
hCoV-19/Mexico/CMX-INER-IBT-110/2020	EPI_ISL_1302334	31/12/2020	Mexico City	Human	Male	unknown	Hospitalized	Original	Nasopharyngeal and oropharyngeal swab
hCoV-19/Mexico/CMX-INER-IBT-115/2020	EPI_ISL_1302356	31/12/2020	Mexico City	Human	Male	unknown	Hospitalized	Original	Nasopharyngeal and oropharyngeal swab
hCoV-19/Mexico/CMX-INER-IBT-116/2021	EPI_ISL_1302227	04/01/2021	Mexico City	Human	Male	unknown	Hospitalized	Original	Nasopharyngeal and oropharyngeal swab
hCoV-19/Mexico/CMX-INER-IBT-120/2021	EPI_ISL_1302228	04/01/2021	Mexico City	Human	Female	unknown	Hospitalized	Original	Nasopharyngeal and oropharyngeal swab
hCoV-19/Mexico/CMX-INER-IBT-131/2021	EPI_ISL_1302245	04/01/2021	Mexico City	Human	Female	unknown	Hospitalized	Original	Nasopharyngeal and oropharyngeal swab
hCoV-19/Mexico/CMX-INER-IBT-132/2021	EPI_ISL_1302306	04/01/2021	Mexico City	Human	Female	unknown	Hospitalized	Original	Nasopharyngeal and oropharyngeal swab
hCoV-19/Mexico/CMX-INER-IBT-121/2021	EPI_ISL_1302360	04/01/2021	Mexico City	Human	Female	unknown	Hospitalized	Original	Nasopharyngeal and oropharyngeal swab
hCoV-19/Mexico/CMX-INER-IBT-130/2021	EPI_ISL_1302368	04/01/2021	Mexico City	Human	Female	unknown	Hospitalized	Original	Nasopharyngeal and oropharyngeal swab
hCoV-19/Mexico/CMX-INER-IBT-133/2021	EPI_ISL_1302374	04/01/2021	Mexico City	Human	Female	unknown	Hospitalized	Original	Nasopharyngeal and oropharyngeal swab
hCoV-19/Mexico/CMX-INER-IBT-129/2021	EPI_ISL_1302376	04/01/2021	Mexico City	Human	Male	unknown	Hospitalized	Original	Nasopharyngeal and oropharyngeal swab
hCoV-19/Mexico/CMX-INER-IBT-124/2021	EPI_ISL_1302273	04/01/2021	Mexico City	Human	Female	unknown	Hospitalized	Original	Nasopharyngeal and oropharyngeal swab
hCoV-19/Mexico/CMX-INER-IBT-122/2021	EPI_ISL_1302278	04/01/2021	Mexico City	Human	Male	unknown	Hospitalized	Original	Nasopharyngeal and oropharyngeal swab
hCoV-19/Mexico/CMX-INER-IBT-119/2021	EPI_ISL_1302316	05/01/2021	Mexico City	Human	Male	unknown	Hospitalized	Original	Nasopharyngeal and oropharyngeal swab
hCoV-19/Mexico/CMX-INER-IBT-123/2021	EPI_ISL_1302387	05/01/2021	Mexico City	Human	Male	unknown	Hospitalized	Original	Nasopharyngeal and oropharyngeal swab
hCoV-19/Mexico/CMX-INER-IBT-127/2021	EPI_ISL_1302198	05/01/2021	Mexico City	Human	Male	unknown	Hospitalized	Original	Nasopharyngeal and oropharyngeal swab
hCoV-19/Mexico/CMX-INER-IBT-125/2021	EPI_ISL_1302267	05/01/2021	Mexico City	Human	Male	unknown	Hospitalized	Original	Nasopharyngeal and oropharyngeal swab

hCoV-19/Mexico/CMX-INER-IBT-128/2021	EPI_ISL_1302361	06/01/2021	Mexico City	Human	Female	unknown	Hospitalized	Original	Nasopharyngeal and oropharyngeal swab
hCoV-19/Mexico/CMX-INER-IBT-118/2021	EPI_ISL_1302192	06/01/2021	Mexico City	Human	Female	unknown	Hospitalized	Original	Nasopharyngeal and oropharyngeal swab
hCoV-19/Mexico/CMX-INER-IBT-117/2021	EPI_ISL_1302327	11/01/2021	Mexico City	Human	Female	unknown	Hospitalized	Original	Nasopharyngeal and oropharyngeal swab
hCoV-19/Mexico/PUE-InDRE-IBT-165/2021	EPI_ISL_1302371	14/01/2021	Puebla	Human	Male	41	Ambulatory	Original	Pharyngeal swab
hCoV-19/Mexico/MEX-IBT-IMSS-515/2021	EPI_ISL_1302236	29/01/2021	State of Mexico	Human	Male	39	Hospitalized	Original	Nasopharyngeal and oropharyngeal swab
hCoV-19/Mexico/CMX-IBT-IMSS-531/2021	EPI_ISL_1302294	29/01/2021	Mexico City	Human	Female	32	Ambulatory	Original	Nasopharyngeal and oropharyngeal swab
hCoV-19/Mexico/VER-IBT-IMSS-557/2021	EPI_ISL_1302354	29/01/2021	Veracruz	Human	Male	51	Ambulatory	Original	Pharyngeal swab
hCoV-19/Mexico/CMX-IBT-IMSS-520/2021	EPI_ISL_1302355	29/01/2021	Mexico City	Human	Female	26	Ambulatory	Original	Nasopharyngeal and oropharyngeal swab
hCoV-19/Mexico/PUE-IBT-IMSS-577/2021	EPI_ISL_1302390	29/01/2021	Puebla	Human	Female	56	Ambulatory	Original	Nasopharyngeal and oropharyngeal swab
hCoV-19/Mexico/MEX-IBT-IMSS-544/2021	EPI_ISL_1302172	29/01/2021	State of Mexico	Human	Male	72	Hospitalized	Original	Nasopharyngeal and oropharyngeal swab
hCoV-19/Mexico/CMX-IBT-IMSS-521/2021	EPI_ISL_1302284	29/01/2021	Mexico City	Human	Female	43	Ambulatory	Original	Nasopharyngeal and oropharyngeal swab
hCoV-19/Mexico/SLP-IBT-IMSS-559/2021	EPI_ISL_1302153	30/01/2021	San Luis Potosi	Human	Male	26	Ambulatory	Original	Nasopharyngeal and oropharyngeal swab
hCoV-19/Mexico/MEX-IBT-IMSS-547/2021	EPI_ISL_1302157	30/01/2021	State of Mexico	Human	Male	23	Hospitalized	Original	Pharyngeal swab
hCoV-19/Mexico/BCN-IBT-IMSS-519/2021	EPI_ISL_1302220	30/01/2021	Baja California	Human	Male	35	Ambulatory	Original	Nasopharyngeal and oropharyngeal swab
hCoV-19/Mexico/SLP-IBT-IMSS-584/2021	EPI_ISL_1302237	30/01/2021	San Luis Potosi	Human	Female	30	Ambulatory	Original	Nasopharyngeal and oropharyngeal swab
hCoV-19/Mexico/VER-IBT-IMSS-527/2021	EPI_ISL_1302238	30/01/2021	Veracruz	Human	Female	71	Hospitalized	Original	Nasopharyngeal and oropharyngeal swab
hCoV-19/Mexico/SLP-IBT-IMSS-585/2021	EPI_ISL_1302332	30/01/2021	San Luis Potosi	Human	Male	62	Hospitalized	Original	Nasopharyngeal and oropharyngeal swab
hCoV-19/Mexico/VER-IBT-IMSS-556/2021	EPI_ISL_1302341	30/01/2021	Veracruz	Human	Male	60	Hospitalized	Original	Nasopharyngeal and oropharyngeal swab
hCoV-19/Mexico/SLP-IBT-IMSS-583/2021	EPI_ISL_1302353	30/01/2021	San Luis Potosi	Human	Male	53	Hospitalized	Original	Nasopharyngeal and oropharyngeal swab
hCoV-19/Mexico/PUE-IBT-IMSS-576/2021	EPI_ISL_1302173	30/01/2021	Puebla	Human	Male	41	Ambulatory	Original	Nasopharyngeal and oropharyngeal swab
hCoV-19/Mexico/MEX-IBT-IMSS-545/2021	EPI_ISL_1302195	30/01/2021	State of Mexico	Human	Female	37	Hospitalized	Original	Nasopharyngeal and oropharyngeal swab
hCoV-19/Mexico/HID-IBT-IMSS-522/2021	EPI_ISL_1302264	30/01/2021	Hidalgo	Human	Male	61	Hospitalized	Original	Nasopharyngeal and oropharyngeal swab
hCoV-19/Mexico/SLP-IBT-IMSS-560/2021	EPI_ISL_1302161	31/01/2021	San Luis Potosi	Human	Female	25	Ambulatory	Original	Nasopharyngeal and oropharyngeal swab
hCoV-19/Mexico/MEX-IBT-IMSS-546/2021	EPI_ISL_1302169	31/01/2021	State of Mexico	Human	Female	58	Hospitalized	Original	Pharyngeal swab
hCoV-19/Mexico/QUE-IBT-IMSS-517/2021	EPI_ISL_1302401	31/01/2021	Queretaro	Human	Male	87	Hospitalized	Original	Nasopharyngeal and oropharyngeal swab
hCoV-19/Mexico/HID-IBT-IMSS-514/2021	EPI_ISL_1302235	31/01/2021	Hidalgo	Human	Female	56	Hospitalized	Original	Nasopharyngeal and oropharyngeal swab
hCoV-19/Mexico/VER-IBT-IMSS-554/2021	EPI_ISL_1302305	31/01/2021	Veracruz	Human	Male	67	Hospitalized	Original	Nasopharyngeal and oropharyngeal swab
hCoV-19/Mexico/SLP-IBT-IMSS-561/2021	EPI_ISL_1302311	31/01/2021	San Luis Potosi	Human	Male	19	Ambulatory	Original	Nasopharyngeal and oropharyngeal swab
hCoV-19/Mexico/SLP-IBT-IMSS-562/2021	EPI_ISL_1302352	31/01/2021	San Luis Potosi	Human	Female	58	Ambulatory	Original	Nasopharyngeal and oropharyngeal swab
hCoV-19/Mexico/CMX-IBT-IMSS-538/2021	EPI_ISL_1302178	31/01/2021	Mexico City	Human	Female	75	Hospitalized	Original	Nasopharyngeal and oropharyngeal swab
hCoV-19/Mexico/CMX-IBT-IMSS-523/2021	EPI_ISL_1302203	31/01/2021	Mexico City	Human	Male	71	Hospitalized	Original	Pharyngeal swab
hCoV-19/Mexico/CMX-IBT-IMSS-530/2021	EPI_ISL_1302204	31/01/2021	Mexico City	Human	Male	50	Ambulatory	Original	Pharyngeal swab
hCoV-19/Mexico/MEX-IBT-IMSS-551/2021	EPI_ISL_1302252	31/01/2021	State of Mexico	Human	Male	84	Hospitalized	Original	Pharyngeal swab
hCoV-19/Mexico/VER-IBT-IMSS-555/2021	EPI_ISL_1302260	31/01/2021	Veracruz	Human	Male	58	Hospitalized	Original	Nasopharyngeal and oropharyngeal swab
hCoV-19/Mexico/PUE-IBT-IMSS-575/2021	EPI_ISL_1302268	31/01/2021	Puebla	Human	Female	40	Ambulatory	Original	Pharyngeal swab
hCoV-19/Mexico/CMX-IBT-IMSS-528/2021	EPI_ISL_1302275	31/01/2021	Mexico City	Human	Female	56	Ambulatory	Original	Pharyngeal swab
hCoV-19/Mexico/QUE-IBT-IMSS-516/2021	EPI_ISL_1302282	31/01/2021	Queretaro	Human	Male	56	Hospitalized	Original	Nasopharyngeal and oropharyngeal swab
hCoV-19/Mexico/PUE-IBT-IMSS-574/2021	EPI_ISL_1302288	31/01/2021	Puebla	Human	Female	39	Ambulatory	Original	Pharyngeal swab
hCoV-19/Mexico/CMX-IBT-IMSS-535/2021	EPI_ISL_1302165	01/02/2021	Mexico City	Human	Male	51	Ambulatory	Original	Nasopharyngeal and oropharyngeal swab
hCoV-19/Mexico/CMX-IBT-IMSS-536/2021	EPI_ISL_1302223	01/02/2021	Mexico City	Human	Male	45	Hospitalized	Original	Nasopharyngeal and oropharyngeal swab
hCoV-19/Mexico/SLP-IBT-IMSS-579/2021	EPI_ISL_1302239	01/02/2021	San Luis Potosi	Human	Male	70	Hospitalized	Original	Nasopharyngeal and oropharyngeal swab

hCoV-19/Mexico/MEX-IBT-IMSS-542/2021	EPI_ISL_1302248	01/02/2021	State of Mexico	Human	Male	70	Hospitalized	Original	Pharyngeal swab
hCoV-19/Mexico/CMX-IBT-IMSS-533/2021	EPI_ISL_1302315	01/02/2021	Mexico City	Human	Male	61	Hospitalized	Original	Nasopharyngeal and oropharyngeal swab
hCoV-19/Mexico/SLP-IBT-IMSS-580/2021	EPI_ISL_1302318	01/02/2021	San Luis Potosi	Human	Male	40	Ambulatory	Original	Nasopharyngeal and oropharyngeal swab
hCoV-19/Mexico/CMX-IBT-IMSS-537/2021	EPI_ISL_1302347	01/02/2021	Mexico City	Human	Male	80	Hospitalized	Original	Pharyngeal swab
hCoV-19/Mexico/MEX-IBT-IMSS-543/2021	EPI_ISL_1302348	01/02/2021	State of Mexico	Human	Male	58	Hospitalized	Original	Pharyngeal swab
hCoV-19/Mexico/MEX-IBT-IMSS-549/2021	EPI_ISL_1302351	01/02/2021	State of Mexico	Human	Male	56	Hospitalized	Original	Nasopharyngeal and oropharyngeal swab
hCoV-19/Mexico/MEX-IBT-IMSS-552/2021	EPI_ISL_1302359	01/02/2021	State of Mexico	Human	Male	61	Hospitalized	Original	Nasopharyngeal and oropharyngeal swab
hCoV-19/Mexico/SLP-IBT-IMSS-578/2021	EPI_ISL_1302362	01/02/2021	San Luis Potosi	Human	Female	67	Ambulatory	Original	Nasopharyngeal and oropharyngeal swab
hCoV-19/Mexico/SLP-IBT-IMSS-582/2021	EPI_ISL_1302367	01/02/2021	San Luis Potosi	Human	Male	70	Hospitalized	Original	Nasopharyngeal and oropharyngeal swab
hCoV-19/Mexico/MEX-IBT-IMSS-541/2021	EPI_ISL_1302370	01/02/2021	State of Mexico	Human	Female	58	Hospitalized	Original	Pharyngeal swab
hCoV-19/Mexico/CMX-IBT-IMSS-534/2021	EPI_ISL_1302378	01/02/2021	Mexico City	Human	Female	49	Hospitalized	Original	Nasopharyngeal and oropharyngeal swab
hCoV-19/Mexico/MEX-IBT-IMSS-525/2021	EPI_ISL_1302196	01/02/2021	State of Mexico	Human	Female	82	Hospitalized	Original	Pharyngeal swab
hCoV-19/Mexico/MEX-IBT-IMSS-540/2021	EPI_ISL_1302251	01/02/2021	State of Mexico	Human	Male	62	Hospitalized	Original	Pharyngeal swab
hCoV-19/Mexico/CMX-IBT-IMSS-526/2021	EPI_ISL_1302261	01/02/2021	Mexico City	Human	Male	75	Hospitalized	Original	Pharyngeal swab
hCoV-19/Mexico/MEX-IBT-IMSS-550/2021	EPI_ISL_1302270	01/02/2021	State of Mexico	Human	Male	52	Hospitalized	Original	Nasopharyngeal and oropharyngeal swab
hCoV-19/Mexico/CMX-IBT-IMSS-518/2021	EPI_ISL_1302272	01/02/2021	Mexico City	Human	Female	31	Ambulatory	Original	Nasopharyngeal and oropharyngeal swab
hCoV-19/Mexico/SLP-IBT-IMSS-581/2021	EPI_ISL_1302285	01/02/2021	San Luis Potosi	Human	Male	60	Ambulatory	Original	Nasopharyngeal and oropharyngeal swab
hCoV-19/Mexico/CMX-IBT-IMSS-568/2021	EPI_ISL_1302156	02/02/2021	Mexico City	Human	Male	46	Ambulatory	Original	Nasopharyngeal and oropharyngeal swab
hCoV-19/Mexico/MEX-IBT-IMSS-553/2021	EPI_ISL_1302158	02/02/2021	State of Mexico	Human	Female	65	Hospitalized	Original	Nasopharyngeal and oropharyngeal swab
hCoV-19/Mexico/CMX-IBT-IMSS-529/2021	EPI_ISL_1302222	02/02/2021	Mexico City	Human	Female	28	Hospitalized	Original	Nasopharyngeal and oropharyngeal swab
hCoV-19/Mexico/CMX-IBT-IMSS-563/2021	EPI_ISL_1302224	02/02/2021	Mexico City	Human	Female	25	Ambulatory	Original	Nasopharyngeal and oropharyngeal swab
hCoV-19/Mexico/CMX-IBT-IMSS-571/2021	EPI_ISL_1302225	02/02/2021	Mexico City	Human	Female	27	Ambulatory	Original	Nasopharyngeal and oropharyngeal swab
hCoV-19/Mexico/CMX-IBT-IMSS-573/2021	EPI_ISL_1302226	02/02/2021	Mexico City	Human	Male	41	Ambulatory	Original	Nasopharyngeal and oropharyngeal swab
hCoV-19/Mexico/CMX-IBT-IMSS-532/2021	EPI_ISL_1302240	02/02/2021	Mexico City	Human	Male	40	Ambulatory	Original	Pharyngeal swab
hCoV-19/Mexico/CMX-IBT-IMSS-564/2021	EPI_ISL_1302243	02/02/2021	Mexico City	Human	Male	46	Ambulatory	Original	Nasopharyngeal and oropharyngeal swab
hCoV-19/Mexico/CMX-IBT-IMSS-569/2021	EPI_ISL_1302324	02/02/2021	Mexico City	Human	Female	51	Ambulatory	Original	Nasopharyngeal and oropharyngeal swab
hCoV-19/Mexico/CMX-IBT-IMSS-570/2021	EPI_ISL_1302372	02/02/2021	Mexico City	Human	Male	59	Ambulatory	Original	Nasopharyngeal and oropharyngeal swab
hCoV-19/Mexico/CMX-IBT-IMSS-565/2021	EPI_ISL_1302379	02/02/2021	Mexico City	Human	Male	39	Ambulatory	Original	Nasopharyngeal and oropharyngeal swab
hCoV-19/Mexico/MEX-IBT-IMSS-539/2021	EPI_ISL_1302393	02/02/2021	State of Mexico	Human	Female	44	Hospitalized	Original	Pharyngeal swab
hCoV-19/Mexico/CMX-IBT-IMSS-572/2021	EPI_ISL_1302190	02/02/2021	Mexico City	Human	Female	43	Ambulatory	Original	Nasopharyngeal and oropharyngeal swab
hCoV-19/Mexico/CMX-IBT-IMSS-567/2021	EPI_ISL_1302281	02/02/2021	Mexico City	Human	Female	64	Ambulatory	Original	Nasopharyngeal and oropharyngeal swab
hCoV-19/Mexico/CHH-INER-IMSS-00262/2021	EPI_ISL_1279276	03/02/2021	Chihuahua	Human	Female	67	Hospitalized	Original	Nasopharyngeal and oropharyngeal swab
hCoV-19/Mexico/CMX-IBT-IMSS-216/2021	EPI_ISL_1288411	03/02/2021	Mexico City	Human	Male	58	Hospitalized	Original	Nasopharyngeal and oropharyngeal swab
hCoV-19/Mexico/TAM-INER-IMSS-00191/2021	EPI_ISL_1279462	03/02/2021	Tamaulipas	Human	Female	67	Ambulatory	Original	Nasopharyngeal and oropharyngeal swab
hCoV-19/Mexico/CMX-IBT-IMSS-524/2021	EPI_ISL_1302221	03/02/2021	Mexico City	Human	Female	25	Ambulatory	Original	Nasopharyngeal and oropharyngeal swab
hCoV-19/Mexico/CMX-IBT-IMSS-586/2021	EPI_ISL_1302244	03/02/2021	Mexico City	Human	Male	53	Ambulatory	Original	Nasopharyngeal and oropharyngeal swab
hCoV-19/Mexico/CMX-IBT-IMSS-566/2021	EPI_ISL_1302392	03/02/2021	Mexico City	Human	Female	76	Hospitalized	Original	Nasopharyngeal and oropharyngeal swab
hCoV-19/Mexico/HID-IBT-IMSS-265/2021	EPI_ISL_1288453	05/02/2021	Hidalgo	Human	Female	73	Hospitalized	Original	Nasopharyngeal and oropharyngeal swab
hCoV-19/Mexico/ZAC-INER-IMSS-00017/2021	EPI_ISL_1279319	05/02/2021	Zacatecas	Human	Female	45	Ambulatory	Original	Nasopharyngeal and oropharyngeal swab
hCoV-19/Mexico/MEX-IBT-IMSS-214/2021	EPI_ISL_1288410	06/02/2021	State of Mexico	Human	Male	73	Hospitalized	Original	Nasopharyngeal and oropharyngeal swab
hCoV-19/Mexico/ZAC-INER-IMSS-00007/2021	EPI_ISL_1279310	06/02/2021	Zacatecas	Human	Male	82	Ambulatory	Original	Nasopharyngeal and oropharyngeal swab

hCoV-19/Mexico/MOR-IBT-IMSS-103/2021	EPI_ISL_1288167	07/02/2021	Morelos	Human	Male	70	Hospitalized	Original	Nasopharyngeal and oropharyngeal swab
hCoV-19/Mexico/GRO-IBT-IMSS-215/2021	EPI_ISL_1288178	07/02/2021	Guerrero	Human	Male	79	Hospitalized	Original	Oropharyngeal swab
hCoV-19/Mexico/ZAC-INER-IMSS-00002/2021	EPI_ISL_1279305	07/02/2021	Zacatecas	Human	Female	22	Ambulatory	Original	Nasopharyngeal and oropharyngeal swab
hCoV-19/Mexico/ZAC-INER-IMSS-00008/2021	EPI_ISL_1279311	07/02/2021	Zacatecas	Human	Female	74	Hospitalized	Original	Nasopharyngeal and oropharyngeal swab
hCoV-19/Mexico/ZAC-INER-IMSS-00010/2021	EPI_ISL_1279281	08/02/2021	Zacatecas	Human	Female	20	Ambulatory	Original	Nasopharyngeal and oropharyngeal swab
hCoV-19/Mexico/OAX-IBT-IMSS-136/2021	EPI_ISL_1288170	08/02/2021	Oaxaca	Human	Female	30	Ambulatory	Original	Nasopharyngeal and oropharyngeal swab
hCoV-19/Mexico/OAX-IBT-IMSS-148/2021	EPI_ISL_1288171	08/02/2021	Oaxaca	Human	Male	46	Ambulatory	Original	Nasopharyngeal and oropharyngeal swab
hCoV-19/Mexico/OAX-IBT-IMSS-159/2021	EPI_ISL_1288172	08/02/2021	Oaxaca	Human	Female	68	Hospitalized	Original	Nasopharyngeal and oropharyngeal swab
hCoV-19/Mexico/COA-INER-IMSS-00193/2021	EPI_ISL_1279464	08/02/2021	Coahuila	Human	Female	57	Hospitalized	Original	Nasopharyngeal and oropharyngeal swab
hCoV-19/Mexico/DUR-INER-IMSS-00194/2021	EPI_ISL_1279465	08/02/2021	Durango	Human	Female	35	Ambulatory	Original	Nasopharyngeal and oropharyngeal swab
hCoV-19/Mexico/CHH-INER-IMSS-00195/2021	EPI_ISL_1279466	08/02/2021	Chihuahua	Human	Female	30	Hospitalized	Original	Nasopharyngeal and oropharyngeal swab
hCoV-19/Mexico/DUR-INER-IMSS-00197/2021	EPI_ISL_1279468	08/02/2021	Durango	Human	Female	35	Ambulatory	Original	Nasopharyngeal and oropharyngeal swab
hCoV-19/Mexico/CHH-INER-IMSS-00219/2021	EPI_ISL_1279485	08/02/2021	Chihuahua	Human	Female	68	Hospitalized	Original	Nasopharyngeal and oropharyngeal swab
hCoV-19/Mexico/BCS-IBT-IMSS-97/2021	EPI_ISL_1288309	08/02/2021	Baja California Sur	Human	Female	37	Ambulatory	Original	Nasopharyngeal and oropharyngeal swab
hCoV-19/Mexico/BCS-IBT-IMSS-98/2021	EPI_ISL_1288310	08/02/2021	Baja California Sur	Human	Male	35	Ambulatory	Original	Nasopharyngeal and oropharyngeal swab
hCoV-19/Mexico/BCS-IBT-IMSS-101/2021	EPI_ISL_1288312	08/02/2021	Baja California Sur	Human	Female	45	Hospitalized	Original	Nasopharyngeal and oropharyngeal swab
hCoV-19/Mexico/ZAC-INER-IMSS-00001/2021	EPI_ISL_1279304	08/02/2021	Zacatecas	Human	Female	34	Ambulatory	Original	Nasopharyngeal and oropharyngeal swab
hCoV-19/Mexico/ZAC-INER-IMSS-00003/2021	EPI_ISL_1279306	08/02/2021	Zacatecas	Human	Male	66	Ambulatory	Original	Nasopharyngeal and oropharyngeal swab
hCoV-19/Mexico/ZAC-INER-IMSS-00004/2021	EPI_ISL_1279307	08/02/2021	Zacatecas	Human	Male	20	Ambulatory	Original	Nasopharyngeal and oropharyngeal swab
hCoV-19/Mexico/ZAC-INER-IMSS-00005/2021	EPI_ISL_1279308	08/02/2021	Zacatecas	Human	Female	20	Ambulatory	Original	Nasopharyngeal and oropharyngeal swab
hCoV-19/Mexico/ZAC-INER-IMSS-00006/2021	EPI_ISL_1279309	08/02/2021	Zacatecas	Human	Female	25	Ambulatory	Original	Nasopharyngeal and oropharyngeal swab
hCoV-19/Mexico/ZAC-INER-IMSS-00009/2021	EPI_ISL_1279312	08/02/2021	Zacatecas	Human	Male	54	Hospitalized	Original	Nasopharyngeal and oropharyngeal swab
hCoV-19/Mexico/MOR-IBT-IMSS-386/2021	EPI_ISL_1288213	08/02/2021	Morelos	Human	Male	76	Hospitalized	Original	Nasopharyngeal and oropharyngeal swab
hCoV-19/Mexico/MOR-IBT-IMSS-387/2021	EPI_ISL_1288214	08/02/2021	Morelos	Human	Female	79	Hospitalized	Original	Nasopharyngeal and oropharyngeal swab
hCoV-19/Mexico/MOR-IBT-IMSS-388/2021	EPI_ISL_1288215	08/02/2021	Morelos	Human	Female	67	HOSPITALIZADO	Original	Nasopharyngeal and oropharyngeal swab
hCoV-19/Mexico/PUE-IBT-IMSS-61/2021	EPI_ISL_1288277	08/02/2021	Puebla	Human	Female	69	Ambulatory	Original	Nasopharyngeal and oropharyngeal swab
hCoV-19/Mexico/VER-IBT-IMSS-80/2021	EPI_ISL_1288295	08/02/2021	Veracruz	Human	Male	66	Hospitalized	Original	Nasopharyngeal and oropharyngeal swab
hCoV-19/Mexico/AGU-IBT-IMSS-238/2021	EPI_ISL_1288154	09/02/2021	Aguascalientes	Human	Female	47	Ambulatory	Original	Nasopharyngeal and oropharyngeal swab
hCoV-19/Mexico/AGU-IBT-IMSS-348/2021	EPI_ISL_1288155	09/02/2021	Aguascalientes	Human	Female	19	Ambulatory	Original	Nasopharyngeal and oropharyngeal swab
hCoV-19/Mexico/MOR-IBT-IMSS-370/2021	EPI_ISL_1288156	09/02/2021	Morelos	Human	Male	84	Hospitalized	Original	Nasopharyngeal and oropharyngeal swab
hCoV-19/Mexico/MOR-IBT-IMSS-381/2021	EPI_ISL_1288157	09/02/2021	Morelos	Human	Female	58	Hospitalized	Original	Nasopharyngeal and oropharyngeal swab
hCoV-19/Mexico/MOR-IBT-IMSS-392/2021	EPI_ISL_1288158	09/02/2021	Morelos	Human	Male	33	Ambulatory	Original	Nasopharyngeal and oropharyngeal swab
hCoV-19/Mexico/MOR-IBT-IMSS-414/2021	EPI_ISL_1288160	09/02/2021	Morelos	Human	Male	77	Hospitalized	Original	Nasopharyngeal and oropharyngeal swab
hCoV-19/Mexico/OAX-IBT-IMSS-425/2021	EPI_ISL_1288161	09/02/2021	Oaxaca	Human	Female	37	Ambulatory	Original	Nasopharyngeal and oropharyngeal swab
hCoV-19/Mexico/OAX-IBT-IMSS-37/2021	EPI_ISL_1288162	09/02/2021	Oaxaca	Human	Male	40	Ambulatory	Original	Nasopharyngeal and oropharyngeal swab
hCoV-19/Mexico/OAX-IBT-IMSS-48/2021	EPI_ISL_1288163	09/02/2021	Oaxaca	Human	Male	53	Ambulatory	Original	Nasopharyngeal and oropharyngeal swab
hCoV-19/Mexico/OAX-IBT-IMSS-59/2021	EPI_ISL_1288164	09/02/2021	Oaxaca	Human	Female	26	Ambulatory	Original	Nasopharyngeal and oropharyngeal swab
hCoV-19/Mexico/MOR-IBT-IMSS-92/2021	EPI_ISL_1288166	09/02/2021	Morelos	Human	Female	69	Hospitalized	Original	Nasopharyngeal and oropharyngeal swab
hCoV-19/Mexico/OAX-IBT-IMSS-114/2021	EPI_ISL_1288168	09/02/2021	Oaxaca	Human	Female	22	Ambulatory	Original	Nasopharyngeal and oropharyngeal swab
hCoV-19/Mexico/OAX-IBT-IMSS-125/2021	EPI_ISL_1288169	09/02/2021	Oaxaca	Human	Male	38	Ambulatory	Original	Nasopharyngeal and oropharyngeal swab
hCoV-19/Mexico/OAX-IBT-IMSS-170/2021	EPI_ISL_1288173	09/02/2021	Oaxaca	Human	Male	30	Ambulatory	Original	Nasopharyngeal and oropharyngeal swab

hCoV-19/Mexico/OAX-IBT-IMSS-171/2021	EPI_ISL_1288174	09/02/2021	Oaxaca	Human	Female	30	Ambulatory	Original	Nasopharyngeal and oropharyngeal swab
hCoV-19/Mexico/GRO-IBT-IMSS-182/2021	EPI_ISL_1288175	09/02/2021	Guerrero	Human	Male	59	Hospitalized	Original	Nasopharyngeal and oropharyngeal swab
hCoV-19/Mexico/GRO-IBT-IMSS-193/2021	EPI_ISL_1288176	09/02/2021	Guerrero	Human	Male	36	Ambulatory	Original	Nasopharyngeal and oropharyngeal swab
hCoV-19/Mexico/GRO-IBT-IMSS-204/2021	EPI_ISL_1288177	09/02/2021	Guerrero	Human	Male	29	Ambulatory	Original	Nasopharyngeal and oropharyngeal swab
hCoV-19/Mexico/BCS-IBT-IMSS-282/2021	EPI_ISL_1288182	09/02/2021	Baja California Sur	Human	Male	36	Ambulatory	Original	Oropharyngeal swab
hCoV-19/Mexico/SLP-IBT-IMSS-303/2021	EPI_ISL_1288184	09/02/2021	San Luis Potosi	Human	Male	71	Hospitalized	Original	Nasopharyngeal and oropharyngeal swab
hCoV-19/Mexico/CMX-IBT-IMSS-217/2021	EPI_ISL_1288412	09/02/2021	Mexico City	Human	Female	67	Hospitalized	Original	Nasopharyngeal and oropharyngeal swab
hCoV-19/Mexico/NLE-INER-IMSS-00192/2021	EPI_ISL_1279463	09/02/2021	Nuevo Leon	Human	Female	0	Hospitalized	Original	Nasopharyngeal and oropharyngeal swab
hCoV-19/Mexico/DUR-INER-IMSS-00196/2021	EPI_ISL_1279467	09/02/2021	Durango	Human	Female	49	Ambulatory	Original	Nasopharyngeal and oropharyngeal swab
hCoV-19/Mexico/COA-INER-IMSS-00199/2021	EPI_ISL_1279470	09/02/2021	Coahuila	Human	Female	44	Ambulatory	Original	Nasopharyngeal and oropharyngeal swab
hCoV-19/Mexico/TAM-INER-IMSS-00208/2021	EPI_ISL_1279475	09/02/2021	Tamaulipas	Human	Male	32	Ambulatory	Original	Nasopharyngeal and oropharyngeal swab
hCoV-19/Mexico/BCS-IBT-IMSS-96/2021	EPI_ISL_1288308	09/02/2021	Baja California Sur	Human	Male	34	Ambulatory	Original	Nasopharyngeal and oropharyngeal swab
hCoV-19/Mexico/ZAC-INER-IMSS-00011/2021	EPI_ISL_1279313	09/02/2021	Zacatecas	Human	Female	49	Ambulatory	Original	Nasopharyngeal and oropharyngeal swab
hCoV-19/Mexico/ZAC-INER-IMSS-00012/2021	EPI_ISL_1279314	09/02/2021	Zacatecas	Human	Female	44	Ambulatory	Original	Nasopharyngeal and oropharyngeal swab
hCoV-19/Mexico/ZAC-INER-IMSS-00013/2021	EPI_ISL_1279315	09/02/2021	Zacatecas	Human	Male	31	Ambulatory	Original	Nasopharyngeal and oropharyngeal swab
hCoV-19/Mexico/ZAC-INER-IMSS-00014/2021	EPI_ISL_1279316	09/02/2021	Zacatecas	Human	Female	84	Hospitalized	Original	Nasopharyngeal and oropharyngeal swab
hCoV-19/Mexico/ZAC-INER-IMSS-00015/2021	EPI_ISL_1279317	09/02/2021	Zacatecas	Human	Female	66	Ambulatory	Original	Nasopharyngeal and oropharyngeal swab
hCoV-19/Mexico/ZAC-INER-IMSS-00021/2021	EPI_ISL_1279323	09/02/2021	Zacatecas	Human	Female	39	Ambulatory	Original	Nasopharyngeal and oropharyngeal swab
hCoV-19/Mexico/MOR-IBT-IMSS-389/2021	EPI_ISL_1288216	09/02/2021	Morelos	Human	Male	67	Ambulatory	Original	Nasopharyngeal and oropharyngeal swab
hCoV-19/Mexico/MOR-IBT-IMSS-390/2021	EPI_ISL_1288217	09/02/2021	Morelos	Human	Male	33	Hospitalized	Original	Nasopharyngeal and oropharyngeal swab
hCoV-19/Mexico/GRO-IBT-IMSS-394/2021	EPI_ISL_1288220	09/02/2021	Guerrero	Human	Male	46	Ambulatory	Original	Nasopharyngeal and oropharyngeal swab
hCoV-19/Mexico/GRO-IBT-IMSS-395/2021	EPI_ISL_1288221	09/02/2021	Guerrero	Human	Female	35	Ambulatory	Original	Nasopharyngeal and oropharyngeal swab
hCoV-19/Mexico/GRO-IBT-IMSS-401/2021	EPI_ISL_1288225	09/02/2021	Guerrero	Human	Male	25	Ambulatory	Original	Nasopharyngeal and oropharyngeal swab
hCoV-19/Mexico/GRO-IBT-IMSS-404/2021	EPI_ISL_1288226	09/02/2021	Guerrero	Human	Female	38	Ambulatory	Original	Nasopharyngeal and oropharyngeal swab
hCoV-19/Mexico/GRO-IBT-IMSS-405/2021	EPI_ISL_1288227	09/02/2021	Guerrero	Human	Female	70	Ambulatory	Original	Nasopharyngeal and oropharyngeal swab
hCoV-19/Mexico/GRO-IBT-IMSS-406/2021	EPI_ISL_1288228	09/02/2021	Guerrero	Human	Female	53	Ambulatory	Original	Nasopharyngeal and oropharyngeal swab
hCoV-19/Mexico/GRO-IBT-IMSS-407/2021	EPI_ISL_1288229	09/02/2021	Guerrero	Human	Male	32	Ambulatory	Original	Nasopharyngeal and oropharyngeal swab
hCoV-19/Mexico/GRO-IBT-IMSS-408/2021	EPI_ISL_1288230	09/02/2021	Guerrero	Human	Female	51	Ambulatory	Original	Nasopharyngeal and oropharyngeal swab
hCoV-19/Mexico/GRO-IBT-IMSS-409/2021	EPI_ISL_1288231	09/02/2021	Guerrero	Human	Female	41	Ambulatory	Original	Nasopharyngeal and oropharyngeal swab
hCoV-19/Mexico/GRO-IBT-IMSS-410/2021	EPI_ISL_1288232	09/02/2021	Guerrero	Human	Female	27	Ambulatory	Original	Nasopharyngeal and oropharyngeal swab
hCoV-19/Mexico/GRO-IBT-IMSS-411/2021	EPI_ISL_1288233	09/02/2021	Guerrero	Human	Female	58	Ambulatory	Original	Nasopharyngeal and oropharyngeal swab
hCoV-19/Mexico/GRO-IBT-IMSS-412/2021	EPI_ISL_1288234	09/02/2021	Guerrero	Human	Female	46	Ambulatory	Original	Nasopharyngeal and oropharyngeal swab
hCoV-19/Mexico/GRO-IBT-IMSS-413/2021	EPI_ISL_1288235	09/02/2021	Guerrero	Human	Male	71	Ambulatory	Original	Nasopharyngeal and oropharyngeal swab
hCoV-19/Mexico/GRO-IBT-IMSS-415/2021	EPI_ISL_1288236	09/02/2021	Guerrero	Human	Female	46	Ambulatory	Original	Nasopharyngeal and oropharyngeal swab
hCoV-19/Mexico/GRO-IBT-IMSS-416/2021	EPI_ISL_1288237	09/02/2021	Guerrero	Human	Male	39	Ambulatory	Original	Nasopharyngeal and oropharyngeal swab
hCoV-19/Mexico/GRO-IBT-IMSS-417/2021	EPI_ISL_1288238	09/02/2021	Guerrero	Human	Male	30	Ambulatory	Original	Nasopharyngeal and oropharyngeal swab
hCoV-19/Mexico/VER-IBT-IMSS-83/2021	EPI_ISL_1288297	09/02/2021	Veracruz	Human	Male	72	Hospitalized	Original	Nasopharyngeal and oropharyngeal swab
hCoV-19/Mexico/CHH-INER-IMSS-00220/2021	EPI_ISL_1279266	10/02/2021	Chihuahua	Human	Male	41	Hospitalized	Original	Nasopharyngeal swab
hCoV-19/Mexico/ZAC-INER-IMSS-00023/2021	EPI_ISL_1287761	10/02/2021	Zacatecas	Human	Female	59	Ambulatory	Original	Nasopharyngeal and oropharyngeal swab
hCoV-19/Mexico/TLA-IBT-IMSS-137/2021	EPI_ISL_1288153	10/02/2021	Tlaxcala	Human	Female	48	Ambulatory	Original	Nasopharyngeal and oropharyngeal swab
hCoV-19/Mexico/MOR-IBT-IMSS-403/2021	EPI_ISL_1288159	10/02/2021	Morelos	Human	Male	50	Ambulatory	Original	Nasopharyngeal and oropharyngeal swab

hCoV-19/Mexico/MOR-IBT-IMSS-81/2021	EPI_ISL_1288165	10/02/2021	Morelos	Human	Male	31	Ambulatory	Original	Nasopharyngeal and oropharyngeal swab
hCoV-19/Mexico/BCS-IBT-IMSS-226/2021	EPI_ISL_1288179	10/02/2021	Baja California Sur	Human	Male	56	Ambulatory	Original	Nasopharyngeal and oropharyngeal swab
hCoV-19/Mexico/BCS-IBT-IMSS-237/2021	EPI_ISL_1288180	10/02/2021	Baja California Sur	Human	Male	54	Ambulatory	Original	Nasopharyngeal and oropharyngeal swab
hCoV-19/Mexico/BCS-IBT-IMSS-260/2021	EPI_ISL_1288181	10/02/2021	Baja California Sur	Human	Female	42	Ambulatory	Original	Nasopharyngeal and oropharyngeal swab
hCoV-19/Mexico/SLP-IBT-IMSS-292/2021	EPI_ISL_1288183	10/02/2021	San Luis Potosi	Human	Male	59	Hospitalized	Original	Nasopharyngeal and oropharyngeal swab
hCoV-19/Mexico/SLP-IBT-IMSS-314/2021	EPI_ISL_1288185	10/02/2021	San Luis Potosi	Human	Male	57	Ambulatory	Original	Nasopharyngeal and oropharyngeal swab
hCoV-19/Mexico/SLP-IBT-IMSS-325/2021	EPI_ISL_1288186	10/02/2021	San Luis Potosi	Human	Female	30	Ambulatory	Original	Nasopharyngeal and oropharyngeal swab
hCoV-19/Mexico/SLP-IBT-IMSS-336/2021	EPI_ISL_1288187	10/02/2021	San Luis Potosi	Human	Male	21	Ambulatory	Original	Nasopharyngeal and oropharyngeal swab
hCoV-19/Mexico/SLP-IBT-IMSS-347/2021	EPI_ISL_1288188	10/02/2021	San Luis Potosi	Human	Male	32	Ambulatory	Original	Nasopharyngeal and oropharyngeal swab
hCoV-19/Mexico/CMX-IBT-IMSS-359/2021	EPI_ISL_1288189	10/02/2021	Mexico City	Human	Female	23	Ambulatory	Original	Nasopharyngeal and oropharyngeal swab
hCoV-19/Mexico/CMX-IBT-IMSS-361/2021	EPI_ISL_1288190	10/02/2021	Mexico City	Human	Female	27	Ambulatory	Original	Nasopharyngeal and oropharyngeal swab
hCoV-19/Mexico/CMX-IBT-IMSS-362/2021	EPI_ISL_1288191	10/02/2021	Mexico City	Human	Female	39	Ambulatory	Original	Nasopharyngeal and oropharyngeal swab
hCoV-19/Mexico/ROO-INER-IMSS-00359/2021	EPI_ISL_1279604	10/02/2021	Quintana Roo	Human	Male	75	Hospitalized	Original	Nasopharyngeal and oropharyngeal swab
hCoV-19/Mexico/MEX-IBT-IMSS-213/2021	EPI_ISL_1288409	10/02/2021	State of Mexico	Human	Male	28	Ambulatory	Original	Nasopharyngeal and oropharyngeal swab
hCoV-19/Mexico/BCS-IBT-IMSS-242/2021	EPI_ISL_1288431	10/02/2021	Baja California Sur	Human	Female	28	Ambulatory	Original	Nasopharyngeal and oropharyngeal swab
hCoV-19/Mexico/BCS-IBT-IMSS-243/2021	EPI_ISL_1288432	10/02/2021	Baja California Sur	Human	Female	26	Ambulatory	Original	Nasopharyngeal and oropharyngeal swab
hCoV-19/Mexico/BCS-IBT-IMSS-244/2021	EPI_ISL_1288433	10/02/2021	Baja California Sur	Human	Female	29	Ambulatory	Original	Nasopharyngeal and oropharyngeal swab
hCoV-19/Mexico/BCS-IBT-IMSS-245/2021	EPI_ISL_1288434	10/02/2021	Baja California Sur	Human	Female	27	Ambulatory	Original	Nasopharyngeal and oropharyngeal swab
hCoV-19/Mexico/ROO-INER-IMSS-00164/2021	EPI_ISL_1279440	10/02/2021	Quintana Roo	Human	Female	62	Ambulatory	Original	Nasopharyngeal and oropharyngeal swab
hCoV-19/Mexico/DUR-INER-IMSS-00198/2021	EPI_ISL_1279469	10/02/2021	Durango	Human	Male	53	Hospitalized	Original	Nasopharyngeal and oropharyngeal swab
hCoV-19/Mexico/NLE-INER-IMSS-00203/2021	EPI_ISL_1279472	10/02/2021	Nuevo Leon	Human	Female	39	Ambulatory	Original	Nasopharyngeal and oropharyngeal swab
hCoV-19/Mexico/TAM-INER-IMSS-00209/2021	EPI_ISL_1279476	10/02/2021	Tamaulipas	Human	Female	37	Ambulatory	Original	Nasopharyngeal and oropharyngeal swab
hCoV-19/Mexico/COA-INER-IMSS-00212/2021	EPI_ISL_1279478	10/02/2021	Coahuila	Human	Female	18	Ambulatory	Original	Nasopharyngeal swab
hCoV-19/Mexico/SLP-INER-IMSS-00218/2021	EPI_ISL_1279484	10/02/2021	San Luis Potosi	Human	Female	71	Ambulatory	Original	Nasopharyngeal and oropharyngeal swab
hCoV-19/Mexico/VER-IBT-IMSS-86/2021	EPI_ISL_1288300	10/02/2021	Veracruz	Human	Female	68	Ambulatory	Original	Nasopharyngeal swab
hCoV-19/Mexico/VER-IBT-IMSS-87/2021	EPI_ISL_1288301	10/02/2021	Veracruz	Human	Female	43	Ambulatory	Original	Nasopharyngeal swab
hCoV-19/Mexico/VER-IBT-IMSS-88/2021	EPI_ISL_1288302	10/02/2021	Veracruz	Human	Male	53	Ambulatory	Original	Nasopharyngeal and oropharyngeal swab
hCoV-19/Mexico/VER-IBT-IMSS-89/2021	EPI_ISL_1288303	10/02/2021	Veracruz	Human	Male	32	Ambulatory	Original	Nasopharyngeal and oropharyngeal swab
hCoV-19/Mexico/VER-IBT-IMSS-90/2021	EPI_ISL_1288304	10/02/2021	Veracruz	Human	Male	44	Ambulatory	Original	Nasopharyngeal and oropharyngeal swab
hCoV-19/Mexico/VER-IBT-IMSS-91/2021	EPI_ISL_1288305	10/02/2021	Veracruz	Human	Male	40	Ambulatory	Original	Nasopharyngeal and oropharyngeal swab
hCoV-19/Mexico/VER-IBT-IMSS-93/2021	EPI_ISL_1288306	10/02/2021	Veracruz	Human	Male	20	Ambulatory	Original	Nasopharyngeal and oropharyngeal swab
hCoV-19/Mexico/VER-IBT-IMSS-100/2021	EPI_ISL_1288311	10/02/2021	Veracruz	Human	Female	72	Hospitalized	Original	Nasopharyngeal and oropharyngeal swab
hCoV-19/Mexico/VER-IBT-IMSS-105/2021	EPI_ISL_1288315	10/02/2021	Veracruz	Human	Male	77	Ambulatory	Original	Nasopharyngeal and oropharyngeal swab
hCoV-19/Mexico/VER-IBT-IMSS-110/2021	EPI_ISL_1288319	10/02/2021	Veracruz	Human	Female	35	Ambulatory	Original	Nasopharyngeal and oropharyngeal swab
hCoV-19/Mexico/VER-IBT-IMSS-111/2021	EPI_ISL_1288320	10/02/2021	Veracruz	Human	Female	23	Ambulatory	Original	Nasopharyngeal and oropharyngeal swab
hCoV-19/Mexico/VER-IBT-IMSS-112/2021	EPI_ISL_1288321	10/02/2021	Veracruz	Human	Male	48	Ambulatory	Original	Nasopharyngeal and oropharyngeal swab
hCoV-19/Mexico/VER-IBT-IMSS-113/2021	EPI_ISL_1288322	10/02/2021	Veracruz	Human	Male	33	Ambulatory	Original	Nasopharyngeal and oropharyngeal swab
hCoV-19/Mexico/VER-IBT-IMSS-115/2021	EPI_ISL_1288323	10/02/2021	Veracruz	Human	Male	43	Ambulatory	Original	Nasopharyngeal and oropharyngeal swab
hCoV-19/Mexico/VER-IBT-IMSS-116/2021	EPI_ISL_1288324	10/02/2021	Veracruz	Human	Male	80	Ambulatory	Original	Nasopharyngeal and oropharyngeal swab
hCoV-19/Mexico/VER-IBT-IMSS-117/2021	EPI_ISL_1288325	10/02/2021	Veracruz	Human	Female	23	Ambulatory	Original	Nasopharyngeal and oropharyngeal swab
hCoV-19/Mexico/CHP-IBT-IMSS-118/2021	EPI_ISL_1288326	10/02/2021	Chiapas	Human	Male	44	Ambulatory	Original	Nasopharyngeal and oropharyngeal swab

hCoV-19/Mexico/PUE-IBT-IMSS-120/2021	EPI_ISL_1288328	10/02/2021	Puebla	Human	Female	38	Ambulatory	Original	Nasopharyngeal and oropharyngeal swab
hCoV-19/Mexico/CHP-IBT-IMSS-123/2021	EPI_ISL_1288330	10/02/2021	Chiapas	Human	Female	28	Ambulatory	Original	Nasopharyngeal and oropharyngeal swab
hCoV-19/Mexico/PUE-IBT-IMSS-126/2021	EPI_ISL_1288332	10/02/2021	Puebla	Human	Male	33	Ambulatory	Original	Nasopharyngeal and oropharyngeal swab
hCoV-19/Mexico/PUE-IBT-IMSS-129/2021	EPI_ISL_1288335	10/02/2021	Puebla	Human	Male	29	Ambulatory	Original	Nasopharyngeal and oropharyngeal swab
hCoV-19/Mexico/PUE-IBT-IMSS-130/2021	EPI_ISL_1288336	10/02/2021	Puebla	Human	Female	45	Ambulatory	Original	Nasopharyngeal and oropharyngeal swab
hCoV-19/Mexico/PUE-IBT-IMSS-138/2021	EPI_ISL_1288341	10/02/2021	Puebla	Human	Female	29	Ambulatory	Original	Nasopharyngeal and oropharyngeal swab
hCoV-19/Mexico/PUE-IBT-IMSS-139/2021	EPI_ISL_1288342	10/02/2021	Puebla	Human	Male	44	Ambulatory	Original	Oropharyngeal swab
hCoV-19/Mexico/PUE-IBT-IMSS-140/2021	EPI_ISL_1288343	10/02/2021	Puebla	Human	Male	37	Ambulatory	Original	Nasopharyngeal and oropharyngeal swab
hCoV-19/Mexico/PUE-IBT-IMSS-141/2021	EPI_ISL_1288344	10/02/2021	Puebla	Human	Female	37	Ambulatory	Original	Nasopharyngeal and oropharyngeal swab
hCoV-19/Mexico/PUE-IBT-IMSS-143/2021	EPI_ISL_1288346	10/02/2021	Puebla	Human	Female	32	Ambulatory	Original	Nasopharyngeal and oropharyngeal swab
hCoV-19/Mexico/PUE-IBT-IMSS-144/2021	EPI_ISL_1288347	10/02/2021	Puebla	Human	Male	62	Ambulatory	Original	Nasopharyngeal and oropharyngeal swab
hCoV-19/Mexico/GRO-IBT-IMSS-155/2021	EPI_ISL_1288357	10/02/2021	Guerrero	Human	Male	52	Ambulatory	Original	Oropharyngeal swab
hCoV-19/Mexico/MEX-IBT-IMSS-177/2021	EPI_ISL_1288376	10/02/2021	State of Mexico	Human	Female	44	Ambulatory	Original	Nasopharyngeal and oropharyngeal swab
hCoV-19/Mexico/MEX-IBT-IMSS-178/2021	EPI_ISL_1288377	10/02/2021	State of Mexico	Human	Male	24	Ambulatory	Original	Nasopharyngeal and oropharyngeal swab
hCoV-19/Mexico/MEX-IBT-IMSS-179/2021	EPI_ISL_1288378	10/02/2021	State of Mexico	Human	Female	18	Ambulatory	Original	Nasopharyngeal and oropharyngeal swab
hCoV-19/Mexico/MEX-IBT-IMSS-180/2021	EPI_ISL_1288379	10/02/2021	State of Mexico	Human	Female	24	Ambulatory	Original	Nasopharyngeal and oropharyngeal swab
hCoV-19/Mexico/MEX-IBT-IMSS-181/2021	EPI_ISL_1288380	10/02/2021	State of Mexico	Human	Female	28	Ambulatory	Original	Nasopharyngeal and oropharyngeal swab
hCoV-19/Mexico/MEX-IBT-IMSS-183/2021	EPI_ISL_1288381	10/02/2021	State of Mexico	Human	Male	42	Ambulatory	Original	Nasopharyngeal and oropharyngeal swab
hCoV-19/Mexico/VER-IBT-IMSS-360/2021	EPI_ISL_1288442	10/02/2021	Veracruz	Human	Male	51	Ambulatory	Original	Nasopharyngeal swab
hCoV-19/Mexico/VER-IBT-IMSS-255/2021	EPI_ISL_1288444	10/02/2021	Veracruz	Human	Female	48	Ambulatory	Original	Nasopharyngeal swab
hCoV-19/Mexico/ZAC-INER-IMSS-00016/2021	EPI_ISL_1279318	10/02/2021	Zacatecas	Human	Female	86	Hospitalized	Original	Nasopharyngeal and oropharyngeal swab
hCoV-19/Mexico/ZAC-INER-IMSS-00018/2021	EPI_ISL_1279320	10/02/2021	Zacatecas	Human	Male	40	Ambulatory	Original	Nasopharyngeal and oropharyngeal swab
hCoV-19/Mexico/ZAC-INER-IMSS-00019/2021	EPI_ISL_1279321	10/02/2021	Zacatecas	Human	Male	24	Ambulatory	Original	Nasopharyngeal and oropharyngeal swab
hCoV-19/Mexico/ZAC-INER-IMSS-00022/2021	EPI_ISL_1279324	10/02/2021	Zacatecas	Human	Female	28	Ambulatory	Original	Nasopharyngeal and oropharyngeal swab
hCoV-19/Mexico/ZAC-INER-IMSS-00032/2021	EPI_ISL_1279331	10/02/2021	Zacatecas	Human	Female	35	Ambulatory	Original	Nasopharyngeal and oropharyngeal swab
hCoV-19/Mexico/QUE-IBT-IMSS-374/2021	EPI_ISL_1288202	10/02/2021	Queretaro	Human	Female	37	Ambulatory	Original	Nasopharyngeal and oropharyngeal swab
hCoV-19/Mexico/QUE-IBT-IMSS-375/2021	EPI_ISL_1288203	10/02/2021	Queretaro	Human	Male	61	Ambulatory	Original	Nasopharyngeal and oropharyngeal swab
hCoV-19/Mexico/QUE-IBT-IMSS-376/2021	EPI_ISL_1288204	10/02/2021	Queretaro	Human	Female	65	Ambulatory	Original	Nasopharyngeal and oropharyngeal swab
hCoV-19/Mexico/MEX-IBT-IMSS-379/2021	EPI_ISL_1288207	10/02/2021	State of Mexico	Human	Female	67	Ambulatory	Original	Nasopharyngeal swab
hCoV-19/Mexico/MEX-IBT-IMSS-380/2021	EPI_ISL_1288208	10/02/2021	State of Mexico	Human	Male	6	Ambulatory	Original	Nasopharyngeal swab
hCoV-19/Mexico/MEX-IBT-IMSS-383/2021	EPI_ISL_1288210	10/02/2021	State of Mexico	Human	Male	54	Ambulatory	Original	Oropharyngeal swab
hCoV-19/Mexico/GRO-IBT-IMSS-391/2021	EPI_ISL_1288218	10/02/2021	Guerrero	Human	Male	75	Hospitalized	Original	Nasopharyngeal and oropharyngeal swab
hCoV-19/Mexico/GRO-IBT-IMSS-396/2021	EPI_ISL_1288222	10/02/2021	Guerrero	Human	Male	63	Hospitalized	Original	Nasopharyngeal and oropharyngeal swab
hCoV-19/Mexico/OAX-IBT-IMSS-398/2021	EPI_ISL_1288223	10/02/2021	Oaxaca	Human	Male	58	Ambulatory	Original	Nasopharyngeal and oropharyngeal swab
hCoV-19/Mexico/VER-IBT-IMSS-418/2021	EPI_ISL_1288239	10/02/2021	Veracruz	Human	Female	62	Hospitalized	Original	Nasopharyngeal and oropharyngeal swab
hCoV-19/Mexico/VER-IBT-IMSS-419/2021	EPI_ISL_1288240	10/02/2021	Veracruz	Human	Male	84	Hospitalized	Original	Nasopharyngeal and oropharyngeal swab
hCoV-19/Mexico/VER-IBT-IMSS-420/2021	EPI_ISL_1288241	10/02/2021	Veracruz	Human	Female	41	Ambulatory	Original	Nasopharyngeal and oropharyngeal swab
hCoV-19/Mexico/VER-IBT-IMSS-421/2021	EPI_ISL_1288242	10/02/2021	Veracruz	Human	Female	67	Ambulatory	Original	Nasopharyngeal and oropharyngeal swab
hCoV-19/Mexico/ROO-IBT-IMSS-422/2021	EPI_ISL_1288243	10/02/2021	Quintana Roo	Human	Female	61	Hospitalized	Original	Nasopharyngeal and oropharyngeal swab
hCoV-19/Mexico/VER-IBT-IMSS-423/2021	EPI_ISL_1288244	10/02/2021	Veracruz	Human	Female	21	Ambulatory	Original	Nasopharyngeal and oropharyngeal swab
hCoV-19/Mexico/VER-IBT-IMSS-424/2021	EPI_ISL_1288245	10/02/2021	Veracruz	Human	Male	19	Ambulatory	Original	Nasopharyngeal and oropharyngeal swab

hCoV-19/Mexico/MOR-IBT-IMSS-27/2021	EPI_ISL_1288246	10/02/2021	Morelos	Human	Male	46	Hospitalized	Original	Nasopharyngeal and oropharyngeal swab
hCoV-19/Mexico/PUE-IBT-IMSS-36/2021	EPI_ISL_1288255	10/02/2021	Puebla	Human	Female	9	Ambulatory	Original	Nasopharyngeal and oropharyngeal swab
hCoV-19/Mexico/PUE-IBT-IMSS-47/2021	EPI_ISL_1288265	10/02/2021	Puebla	Human	Male	58	Hospitalized	Original	Nasopharyngeal and oropharyngeal swab
hCoV-19/Mexico/PUE-IBT-IMSS-49/2021	EPI_ISL_1288266	10/02/2021	Puebla	Human	Male	74	Hospitalized	Original	Nasopharyngeal and oropharyngeal swab
hCoV-19/Mexico/PUE-IBT-IMSS-51/2021	EPI_ISL_1288268	10/02/2021	Puebla	Human	Male	68	Ambulatory	Original	Nasopharyngeal and oropharyngeal swab
hCoV-19/Mexico/PUE-IBT-IMSS-56/2021	EPI_ISL_1288273	10/02/2021	Puebla	Human	Male	47	Ambulatory	Original	Nasopharyngeal and oropharyngeal swab
hCoV-19/Mexico/PUE-IBT-IMSS-58/2021	EPI_ISL_1288275	10/02/2021	Puebla	Human	Female	38	Ambulatory	Original	Nasopharyngeal and oropharyngeal swab
hCoV-19/Mexico/PUE-IBT-IMSS-60/2021	EPI_ISL_1288276	10/02/2021	Puebla	Human	Female	67	Hospitalized	Original	Nasopharyngeal and oropharyngeal swab
hCoV-19/Mexico/SLP-IBT-IMSS-72/2021	EPI_ISL_1288287	10/02/2021	San Luis Potosi	Human	Male	78	Hospitalized	Original	Nasopharyngeal and oropharyngeal swab
hCoV-19/Mexico/SLP-IBT-IMSS-73/2021	EPI_ISL_1288288	10/02/2021	San Luis Potosi	Human	Female	76	Hospitalized	Original	Nasopharyngeal and oropharyngeal swab
hCoV-19/Mexico/SLP-IBT-IMSS-74/2021	EPI_ISL_1288289	10/02/2021	San Luis Potosi	Human	Male	42	Hospitalized	Original	Nasopharyngeal and oropharyngeal swab
hCoV-19/Mexico/SLP-IBT-IMSS-75/2021	EPI_ISL_1288290	10/02/2021	San Luis Potosi	Human	Female	39	Ambulatory	Original	Nasopharyngeal and oropharyngeal swab
hCoV-19/Mexico/SLP-IBT-IMSS-78/2021	EPI_ISL_1288293	10/02/2021	San Luis Potosi	Human	Male	34	Ambulatory	Original	Nasopharyngeal and oropharyngeal swab
hCoV-19/Mexico/VER-IBT-IMSS-79/2021	EPI_ISL_1288294	10/02/2021	Veracruz	Human	Female	62	Hospitalized	Original	Nasopharyngeal and oropharyngeal swab
hCoV-19/Mexico/VER-IBT-IMSS-82/2021	EPI_ISL_1288296	10/02/2021	Veracruz	Human	Female	47	Ambulatory	Original	Nasopharyngeal and oropharyngeal swab
hCoV-19/Mexico/VER-IBT-IMSS-84/2021	EPI_ISL_1288298	10/02/2021	Veracruz	Human	Male	22	Ambulatory	Original	Nasopharyngeal and oropharyngeal swab
hCoV-19/Mexico/VER-IBT-IMSS-85/2021	EPI_ISL_1288299	10/02/2021	Veracruz	Human	Male	55	Ambulatory	Original	Nasopharyngeal swab
hCoV-19/Mexico/TAM-INER-IMSS-00200/2021	EPI_ISL_1279264	11/02/2021	Nuevo Leon	Human	Female	38	Ambulatory	Original	Nasopharyngeal and oropharyngeal swab
hCoV-19/Mexico/ZAC-INER-IMSS-00026/2021	EPI_ISL_1279265	11/02/2021	Zacatecas	Human	Female	39	Ambulatory	Original	Nasopharyngeal and oropharyngeal swab
hCoV-19/Mexico/ZAC-INER-IMSS-00025/2021	EPI_ISL_1279271	11/02/2021	Zacatecas	Human	Male	33	Ambulatory	Original	Nasopharyngeal and oropharyngeal swab
hCoV-19/Mexico/CAM-INER-IMSS-00368/2021	EPI_ISL_1279282	11/02/2021	Campeche	Human	Male	30	Ambulatory	Original	Nasopharyngeal and oropharyngeal swab
hCoV-19/Mexico/NLE-INER-IMSS-00202/2021	EPI_ISL_1279287	11/02/2021	Nuevo Leon	Human	Male	50	Ambulatory	Original	Nasopharyngeal and oropharyngeal swab
hCoV-19/Mexico/NLE-INER-IMSS-00204/2021	EPI_ISL_1279298	11/02/2021	Nuevo Leon	Human	Female	24	Ambulatory	Original	Nasopharyngeal and oropharyngeal swab
hCoV-19/Mexico/COA-INER-IMSS-00207/2021	EPI_ISL_1287771	11/02/2021	Coahuila	Human	Male	37	Hospitalized	Original	Nasopharyngeal and oropharyngeal swab
hCoV-19/Mexico/CMX-IBT-IMSS-363/2021	EPI_ISL_1288192	11/02/2021	Mexico City	Human	Female	62	Ambulatory	Original	Nasopharyngeal and oropharyngeal swab
hCoV-19/Mexico/CMX-IBT-IMSS-367/2021	EPI_ISL_1288196	11/02/2021	Mexico City	Human	Male	39	Hospitalized	Original	Oropharyngeal swab
hCoV-19/Mexico/CMX-IBT-IMSS-368/2021	EPI_ISL_1288197	11/02/2021	Mexico City	Human	Male	74	Hospitalized	Original	Oropharyngeal swab
hCoV-19/Mexico/MEX-IBT-IMSS-369/2021	EPI_ISL_1288198	11/02/2021	State of Mexico	Human	Female	63	Hospitalized	Original	Oropharyngeal swab
hCoV-19/Mexico/MEX-IBT-IMSS-371/2021	EPI_ISL_1288199	11/02/2021	State of Mexico	Human	Male	72	Hospitalized	Original	Oropharyngeal swab
hCoV-19/Mexico/ROO-INER-IMSS-00366/2021	EPI_ISL_1279611	11/02/2021	Quintana Roo	Human	Male	31	Ambulatory	Original	Nasopharyngeal and oropharyngeal swab
hCoV-19/Mexico/ROO-INER-IMSS-00367/2021	EPI_ISL_1279612	11/02/2021	Quintana Roo	Human	Female	21	Ambulatory	Original	Nasopharyngeal and oropharyngeal swab
hCoV-19/Mexico/MEX-IBT-IMSS-203/2021	EPI_ISL_1288400	11/02/2021	State of Mexico	Human	Female	32	Ambulatory	Original	Nasopharyngeal and oropharyngeal swab
hCoV-19/Mexico/MEX-IBT-IMSS-205/2021	EPI_ISL_1288401	11/02/2021	State of Mexico	Human	Male	59	Hospitalized	Original	Nasopharyngeal and oropharyngeal swab
hCoV-19/Mexico/MEX-IBT-IMSS-206/2021	EPI_ISL_1288402	11/02/2021	State of Mexico	Human	Male	49	Ambulatory	Original	Nasopharyngeal and oropharyngeal swab
hCoV-19/Mexico/MEX-IBT-IMSS-207/2021	EPI_ISL_1288403	11/02/2021	State of Mexico	Human	Female	66	Hospitalized	Original	Nasopharyngeal and oropharyngeal swab
hCoV-19/Mexico/MEX-IBT-IMSS-208/2021	EPI_ISL_1288404	11/02/2021	State of Mexico	Human	Female	57	Ambulatory	Original	Nasopharyngeal and oropharyngeal swab
hCoV-19/Mexico/MEX-IBT-IMSS-209/2021	EPI_ISL_1288405	11/02/2021	State of Mexico	Human	Female	22	Ambulatory	Original	Nasopharyngeal and oropharyngeal swab
hCoV-19/Mexico/MEX-IBT-IMSS-210/2021	EPI_ISL_1288406	11/02/2021	State of Mexico	Human	Male	49	Ambulatory	Original	Nasopharyngeal and oropharyngeal swab
hCoV-19/Mexico/MEX-IBT-IMSS-211/2021	EPI_ISL_1288407	11/02/2021	State of Mexico	Human	Female	39	Ambulatory	Original	Nasopharyngeal and oropharyngeal swab
hCoV-19/Mexico/MEX-IBT-IMSS-212/2021	EPI_ISL_1288408	11/02/2021	State of Mexico	Human	Male	39	Ambulatory	Original	Nasopharyngeal and oropharyngeal swab
hCoV-19/Mexico/QUE-IBT-IMSS-228/2021	EPI_ISL_1288421	11/02/2021	Queretaro	Human	Female	64	Hospitalized	Original	Nasopharyngeal and oropharyngeal swab

hCoV-19/Mexico/QUE-IBT-IMSS-229/2021	EPI_ISL_1288422	11/02/2021	Queretaro	Human	Female	86	Hospitalized	Original	Nasopharyngeal and oropharyngeal swab
hCoV-19/Mexico/BCS-IBT-IMSS-230/2021	EPI_ISL_1288423	11/02/2021	Baja California Sur	Human	Female	32	Ambulatory	Original	Nasopharyngeal and oropharyngeal swab
hCoV-19/Mexico/BCS-IBT-IMSS-231/2021	EPI_ISL_1288424	11/02/2021	Baja California Sur	Human	Male	31	Ambulatory	Original	Nasopharyngeal and oropharyngeal swab
hCoV-19/Mexico/BCS-IBT-IMSS-232/2021	EPI_ISL_1288425	11/02/2021	Baja California Sur	Human	Female	32	Ambulatory	Original	Nasopharyngeal and oropharyngeal swab
hCoV-19/Mexico/BCS-IBT-IMSS-235/2021	EPI_ISL_1288427	11/02/2021	Baja California Sur	Human	Female	40	Ambulatory	Original	Nasopharyngeal and oropharyngeal swab
hCoV-19/Mexico/BCS-IBT-IMSS-236/2021	EPI_ISL_1288428	11/02/2021	Baja California Sur	Human	Male	49	Ambulatory	Original	Nasopharyngeal and oropharyngeal swab
hCoV-19/Mexico/BCS-IBT-IMSS-239/2021	EPI_ISL_1288429	11/02/2021	Baja California Sur	Human	Male	27	Ambulatory	Original	Nasopharyngeal and oropharyngeal swab
hCoV-19/Mexico/BCS-IBT-IMSS-240/2021	EPI_ISL_1288430	11/02/2021	Baja California Sur	Human	Female	33	Ambulatory	Original	Nasopharyngeal and oropharyngeal swab
hCoV-19/Mexico/CMX-IBT-IMSS-338/2021	EPI_ISL_1288517	11/02/2021	Mexico City	Human	Male	60	Hospitalized	Original	Nasopharyngeal and oropharyngeal swab
hCoV-19/Mexico/NLE-INER-IMSS-00201/2021	EPI_ISL_1279471	11/02/2021	Nuevo Leon	Human	Female	49	Hospitalized	Original	Nasopharyngeal and oropharyngeal swab
hCoV-19/Mexico/NLE-INER-IMSS-00205/2021	EPI_ISL_1279473	11/02/2021	Nuevo Leon	Human	Male	34	Ambulatory	Original	Nasopharyngeal and oropharyngeal swab
hCoV-19/Mexico/COA-INER-IMSS-00211/2021	EPI_ISL_1279477	11/02/2021	Coahuila	Human	Female	75	Ambulatory	Original	Nasopharyngeal and oropharyngeal swab
hCoV-19/Mexico/CHH-INER-IMSS-00260/2021	EPI_ISL_1279522	11/02/2021	Chihuahua	Human	Female	62	Ambulatory	Original	Pharyngeal swab
hCoV-19/Mexico/CHH-INER-IMSS-00261/2021	EPI_ISL_1279523	11/02/2021	Chihuahua	Human	Male	25	Ambulatory	Original	Nasopharyngeal and oropharyngeal swab
hCoV-19/Mexico/CHP-IBT-IMSS-95/2021	EPI_ISL_1288307	11/02/2021	Chiapas	Human	Female	47	Ambulatory	Original	Nasopharyngeal and oropharyngeal swab
hCoV-19/Mexico/BCS-IBT-IMSS-102/2021	EPI_ISL_1288313	11/02/2021	Baja California Sur	Human	Male	52	Ambulatory	Original	Nasopharyngeal and oropharyngeal swab
hCoV-19/Mexico/BCS-IBT-IMSS-104/2021	EPI_ISL_1288314	11/02/2021	Baja California Sur	Human	Female	23	Ambulatory	Original	Nasopharyngeal and oropharyngeal swab
hCoV-19/Mexico/BCS-IBT-IMSS-106/2021	EPI_ISL_1288316	11/02/2021	Baja California Sur	Human	Male	60	Ambulatory	Original	Nasopharyngeal and oropharyngeal swab
hCoV-19/Mexico/BCS-IBT-IMSS-108/2021	EPI_ISL_1288317	11/02/2021	Baja California Sur	Human	Female	46	Ambulatory	Original	Nasopharyngeal and oropharyngeal swab
hCoV-19/Mexico/BCS-IBT-IMSS-109/2021	EPI_ISL_1288318	11/02/2021	Baja California Sur	Human	Male	35	Ambulatory	Original	Nasopharyngeal and oropharyngeal swab
hCoV-19/Mexico/CHP-IBT-IMSS-119/2021	EPI_ISL_1288327	11/02/2021	Chiapas	Human	Female	57	Ambulatory	Original	Nasopharyngeal and oropharyngeal swab
hCoV-19/Mexico/CHP-IBT-IMSS-122/2021	EPI_ISL_1288329	11/02/2021	Chiapas	Human	Male	23	Ambulatory	Original	Nasopharyngeal and oropharyngeal swab
hCoV-19/Mexico/PUE-IBT-IMSS-124/2021	EPI_ISL_1288331	11/02/2021	Puebla	Human	Male	54	Ambulatory	Original	Nasopharyngeal swab
hCoV-19/Mexico/PUE-IBT-IMSS-127/2021	EPI_ISL_1288333	11/02/2021	Puebla	Human	Male	39	Ambulatory	Original	Nasopharyngeal swab
hCoV-19/Mexico/PUE-IBT-IMSS-128/2021	EPI_ISL_1288334	11/02/2021	Puebla	Human	Female	19	Ambulatory	Original	Nasopharyngeal and oropharyngeal swab
hCoV-19/Mexico/PUE-IBT-IMSS-131/2021	EPI_ISL_1288337	11/02/2021	Puebla	Human	Female	30	Ambulatory	Original	Nasopharyngeal and oropharyngeal swab
hCoV-19/Mexico/PUE-IBT-IMSS-132/2021	EPI_ISL_1288338	11/02/2021	Puebla	Human	Female	67	Ambulatory	Original	Nasopharyngeal swab
hCoV-19/Mexico/PUE-IBT-IMSS-133/2021	EPI_ISL_1288339	11/02/2021	Puebla	Human	Female	51	Ambulatory	Original	Nasopharyngeal and oropharyngeal swab
hCoV-19/Mexico/PUE-IBT-IMSS-134/2021	EPI_ISL_1288340	11/02/2021	Puebla	Human	Female	57	Ambulatory	Original	Nasopharyngeal swab
hCoV-19/Mexico/PUE-IBT-IMSS-142/2021	EPI_ISL_1288345	11/02/2021	Puebla	Human	Female	52	Ambulatory	Original	Nasopharyngeal and oropharyngeal swab
hCoV-19/Mexico/PUE-IBT-IMSS-145/2021	EPI_ISL_1288348	11/02/2021	Puebla	Human	Male	30	Ambulatory	Original	Nasopharyngeal and oropharyngeal swab
hCoV-19/Mexico/PUE-IBT-IMSS-146/2021	EPI_ISL_1288349	11/02/2021	Puebla	Human	Female	26	Ambulatory	Original	Nasopharyngeal and oropharyngeal swab
hCoV-19/Mexico/VER-IBT-IMSS-151/2021	EPI_ISL_1288353	11/02/2021	Veracruz	Human	Female	31	Ambulatory	Original	Nasopharyngeal and oropharyngeal swab
hCoV-19/Mexico/OAX-IBT-IMSS-152/2021	EPI_ISL_1288354	11/02/2021	Oaxaca	Human	Male	32	Ambulatory	Original	Nasopharyngeal and oropharyngeal swab
hCoV-19/Mexico/VER-IBT-IMSS-153/2021	EPI_ISL_1288355	11/02/2021	Veracruz	Human	Female	43	Ambulatory	Original	Nasopharyngeal and oropharyngeal swab
hCoV-19/Mexico/GRO-IBT-IMSS-154/2021	EPI_ISL_1288356	11/02/2021	Guerrero	Human	Female	60	Hospitalized	Original	Nasopharyngeal and oropharyngeal swab
hCoV-19/Mexico/VER-IBT-IMSS-158/2021	EPI_ISL_1288360	11/02/2021	Veracruz	Human	Male	31	Ambulatory	Original	Nasopharyngeal and oropharyngeal swab
hCoV-19/Mexico/VER-IBT-IMSS-160/2021	EPI_ISL_1288361	11/02/2021	Veracruz	Human	Female	48	Ambulatory	Original	Nasopharyngeal and oropharyngeal swab
hCoV-19/Mexico/GRO-IBT-IMSS-161/2021	EPI_ISL_1288362	11/02/2021	Guerrero	Human	Female	25	Ambulatory	Original	Nasopharyngeal and oropharyngeal swab
hCoV-19/Mexico/VER-IBT-IMSS-162/2021	EPI_ISL_1288363	11/02/2021	Veracruz	Human	Male	34	Ambulatory	Original	Nasopharyngeal and oropharyngeal swab
hCoV-19/Mexico/VER-IBT-IMSS-163/2021	EPI_ISL_1288364	11/02/2021	Veracruz	Human	Female	39	Ambulatory	Original	Nasopharyngeal and oropharyngeal swab

hCoV-19/Mexico/MEX-IBT-IMSS-164/2021	EPI_ISL_1288365	11/02/2021	State of Mexico	Human	Male	81	Hospitalized	Original	Nasopharyngeal and oropharyngeal swab
hCoV-19/Mexico/MEX-IBT-IMSS-165/2021	EPI_ISL_1288366	11/02/2021	State of Mexico	Human	Male	43	Hospitalized	Original	Nasopharyngeal and oropharyngeal swab
hCoV-19/Mexico/MEX-IBT-IMSS-166/2021	EPI_ISL_1288367	11/02/2021	State of Mexico	Human	Male	55	Hospitalized	Original	Nasopharyngeal and oropharyngeal swab
hCoV-19/Mexico/MEX-IBT-IMSS-167/2021	EPI_ISL_1288368	11/02/2021	State of Mexico	Human	Male	61	Hospitalized	Original	Nasopharyngeal and oropharyngeal swab
hCoV-19/Mexico/MOR-IBT-IMSS-172/2021	EPI_ISL_1288371	11/02/2021	Morelos	Human	Male	62	Hospitalized	Original	Nasopharyngeal and oropharyngeal swab
hCoV-19/Mexico/MOR-IBT-IMSS-174/2021	EPI_ISL_1288373	11/02/2021	Morelos	Human	Male	69	Hospitalized	Original	Nasopharyngeal and oropharyngeal swab
hCoV-19/Mexico/MOR-IBT-IMSS-175/2021	EPI_ISL_1288374	11/02/2021	Morelos	Human	Male	63	Hospitalized	Original	Nasopharyngeal and oropharyngeal swab
hCoV-19/Mexico/MOR-IBT-IMSS-176/2021	EPI_ISL_1288375	11/02/2021	Morelos	Human	Female	73	Ambulatory	Original	Nasopharyngeal and oropharyngeal swab
hCoV-19/Mexico/MEX-IBT-IMSS-184/2021	EPI_ISL_1288382	11/02/2021	State of Mexico	Human	Female	12	Ambulatory	Original	Nasopharyngeal and oropharyngeal swab
hCoV-19/Mexico/MEX-IBT-IMSS-185/2021	EPI_ISL_1288383	11/02/2021	State of Mexico	Human	Female	49	Ambulatory	Original	Nasopharyngeal and oropharyngeal swab
hCoV-19/Mexico/MEX-IBT-IMSS-186/2021	EPI_ISL_1288384	11/02/2021	State of Mexico	Human	Male	71	Hospitalized	Original	Nasopharyngeal and oropharyngeal swab
hCoV-19/Mexico/MEX-IBT-IMSS-188/2021	EPI_ISL_1288386	11/02/2021	State of Mexico	Human	Male	47	Ambulatory	Original	Nasopharyngeal and oropharyngeal swab
hCoV-19/Mexico/OAX-IBT-IMSS-189/2021	EPI_ISL_1288387	11/02/2021	Oaxaca	Human	Male	51	Ambulatory	Original	Nasopharyngeal and oropharyngeal swab
hCoV-19/Mexico/OAX-IBT-IMSS-190/2021	EPI_ISL_1288388	11/02/2021	Oaxaca	Human	Male	52	Ambulatory	Original	Nasopharyngeal and oropharyngeal swab
hCoV-19/Mexico/AGU-IBT-IMSS-195/2021	EPI_ISL_1288392	11/02/2021	Aguascalientes	Human	Male	32	Ambulatory	Original	Nasopharyngeal and oropharyngeal swab
hCoV-19/Mexico/AGU-IBT-IMSS-196/2021	EPI_ISL_1288393	11/02/2021	Aguascalientes	Human	Male	35	Ambulatory	Original	Nasopharyngeal and oropharyngeal swab
hCoV-19/Mexico/BCS-IBT-IMSS-233/2021	EPI_ISL_1288426	11/02/2021	Baja California Sur	Human	Female	52	Ambulatory	Original	Nasopharyngeal and oropharyngeal swab
hCoV-19/Mexico/VER-IBT-IMSS-256/2021	EPI_ISL_1288445	11/02/2021	Veracruz	Human	Male	20	Ambulatory	Original	Nasopharyngeal and oropharyngeal swab
hCoV-19/Mexico/VER-IBT-IMSS-257/2021	EPI_ISL_1288446	11/02/2021	Veracruz	Human	Female	58	Ambulatory	Original	Nasopharyngeal and oropharyngeal swab
hCoV-19/Mexico/VER-IBT-IMSS-258/2021	EPI_ISL_1288447	11/02/2021	Veracruz	Human	Male	14	Ambulatory	Original	Nasopharyngeal and oropharyngeal swab
hCoV-19/Mexico/VER-IBT-IMSS-259/2021	EPI_ISL_1288448	11/02/2021	Veracruz	Human	Male	41	Ambulatory	Original	Nasopharyngeal and oropharyngeal swab
hCoV-19/Mexico/VER-IBT-IMSS-261/2021	EPI_ISL_1288449	11/02/2021	Veracruz	Human	Male	24	Ambulatory	Original	Nasopharyngeal and oropharyngeal swab
hCoV-19/Mexico/VER-IBT-IMSS-262/2021	EPI_ISL_1288450	11/02/2021	Veracruz	Human	Male	41	Ambulatory	Original	Nasopharyngeal and oropharyngeal swab
hCoV-19/Mexico/VER-IBT-IMSS-263/2021	EPI_ISL_1288451	11/02/2021	Veracruz	Human	Female	21	Ambulatory	Original	Nasopharyngeal and oropharyngeal swab
hCoV-19/Mexico/VER-IBT-IMSS-264/2021	EPI_ISL_1288452	11/02/2021	Veracruz	Human	Male	20	Ambulatory	Original	Nasopharyngeal and oropharyngeal swab
hCoV-19/Mexico/VER-IBT-IMSS-266/2021	EPI_ISL_1288454	11/02/2021	Veracruz	Human	Male	33	Ambulatory	Original	Nasopharyngeal and oropharyngeal swab
hCoV-19/Mexico/VER-IBT-IMSS-267/2021	EPI_ISL_1288455	11/02/2021	Veracruz	Human	Female	34	Ambulatory	Original	Nasopharyngeal and oropharyngeal swab
hCoV-19/Mexico/VER-IBT-IMSS-268/2021	EPI_ISL_1288456	11/02/2021	Veracruz	Human	Female	21	Ambulatory	Original	Nasopharyngeal and oropharyngeal swab
hCoV-19/Mexico/VER-IBT-IMSS-269/2021	EPI_ISL_1288457	11/02/2021	Veracruz	Human	Male	37	Ambulatory	Original	Nasopharyngeal and oropharyngeal swab
hCoV-19/Mexico/VER-IBT-IMSS-270/2021	EPI_ISL_1288458	11/02/2021	Veracruz	Human	Male	31	Ambulatory	Original	Nasopharyngeal and oropharyngeal swab
hCoV-19/Mexico/VER-IBT-IMSS-272/2021	EPI_ISL_1288459	11/02/2021	Veracruz	Human	Female	35	Ambulatory	Original	Nasopharyngeal and oropharyngeal swab
hCoV-19/Mexico/VER-IBT-IMSS-273/2021	EPI_ISL_1288460	11/02/2021	Veracruz	Human	Male	39	Ambulatory	Original	Nasopharyngeal and oropharyngeal swab
hCoV-19/Mexico/VER-IBT-IMSS-274/2021	EPI_ISL_1288461	11/02/2021	Veracruz	Human	Male	27	Ambulatory	Original	Nasopharyngeal and oropharyngeal swab
hCoV-19/Mexico/VER-IBT-IMSS-278/2021	EPI_ISL_1288465	11/02/2021	Veracruz	Human	Female	41	Ambulatory	Original	Nasopharyngeal and oropharyngeal swab
hCoV-19/Mexico/VER-IBT-IMSS-279/2021	EPI_ISL_1288466	11/02/2021	Veracruz	Human	Female	40	Ambulatory	Original	Nasopharyngeal and oropharyngeal swab
hCoV-19/Mexico/VER-IBT-IMSS-280/2021	EPI_ISL_1288467	11/02/2021	Veracruz	Human	Female	72	Ambulatory	Original	Nasopharyngeal and oropharyngeal swab
hCoV-19/Mexico/VER-IBT-IMSS-281/2021	EPI_ISL_1288468	11/02/2021	Veracruz	Human	Female	20	Ambulatory	Original	Nasopharyngeal and oropharyngeal swab
hCoV-19/Mexico/VER-IBT-IMSS-283/2021	EPI_ISL_1288469	11/02/2021	Veracruz	Human	Male	30	Ambulatory	Original	Nasopharyngeal and oropharyngeal swab
hCoV-19/Mexico/BCS-IBT-IMSS-310/2021	EPI_ISL_1288493	11/02/2021	Baja California Sur	Human	Female	21	Ambulatory	Original	Nasopharyngeal and oropharyngeal swab
hCoV-19/Mexico/ZAC-INER-IMSS-00020/2021	EPI_ISL_1279322	11/02/2021	Zacatecas	Human	Male	66	Hospitalized	Original	Nasopharyngeal and oropharyngeal swab
hCoV-19/Mexico/ZAC-INER-IMSS-00024/2021	EPI_ISL_1279325	11/02/2021	Zacatecas	Human	Male	44	Ambulatory	Original	Nasopharyngeal and oropharyngeal swab

hCoV-19/Mexico/ZAC-INER-IMSS-00027/2021	EPI_ISL_1279326	11/02/2021	Zacatecas	Human	Male	21	Ambulatory	Original	Nasopharyngeal and oropharyngeal swab
hCoV-19/Mexico/ZAC-INER-IMSS-00028/2021	EPI_ISL_1279327	11/02/2021	Zacatecas	Human	Male	35	Ambulatory	Original	Nasopharyngeal and oropharyngeal swab
hCoV-19/Mexico/ZAC-INER-IMSS-00029/2021	EPI_ISL_1279328	11/02/2021	Zacatecas	Human	Female	18	Ambulatory	Original	Nasopharyngeal and oropharyngeal swab
hCoV-19/Mexico/ZAC-INER-IMSS-00033/2021	EPI_ISL_1279332	11/02/2021	Zacatecas	Human	Female	25	Ambulatory	Original	Nasopharyngeal and oropharyngeal swab
hCoV-19/Mexico/CMX-IBT-IMSS-372/2021	EPI_ISL_1288200	11/02/2021	Mexico City	Human	Male	69	Hospitalized	Original	Oropharyngeal swab
hCoV-19/Mexico/MEX-IBT-IMSS-377/2021	EPI_ISL_1288205	11/02/2021	State of Mexico	Human	Male	41	Ambulatory	Original	Nasopharyngeal and oropharyngeal swab
hCoV-19/Mexico/MEX-IBT-IMSS-378/2021	EPI_ISL_1288206	11/02/2021	State of Mexico	Human	Female	40	Ambulatory	Original	Nasopharyngeal and oropharyngeal swab
hCoV-19/Mexico/MEX-IBT-IMSS-382/2021	EPI_ISL_1288209	11/02/2021	State of Mexico	Human	Female	68	Ambulatory	Original	Nasopharyngeal and oropharyngeal swab
hCoV-19/Mexico/CMX-IBT-IMSS-384/2021	EPI_ISL_1288211	11/02/2021	Mexico City	Human	Male	44	Ambulatory	Original	Nasopharyngeal and oropharyngeal swab
hCoV-19/Mexico/GRO-IBT-IMSS-393/2021	EPI_ISL_1288219	11/02/2021	Guerrero	Human	Male	50	Hospitalized	Original	Nasopharyngeal and oropharyngeal swab
hCoV-19/Mexico/OAX-IBT-IMSS-399/2021	EPI_ISL_1288224	11/02/2021	Oaxaca	Human	Female	43	Ambulatory	Original	Nasopharyngeal and oropharyngeal swab
hCoV-19/Mexico/MEX-IBT-IMSS-30/2021	EPI_ISL_1288249	11/02/2021	State of Mexico	Human	Male	31	Ambulatory	Original	Nasopharyngeal and oropharyngeal swab
hCoV-19/Mexico/TLA-IBT-IMSS-31/2021	EPI_ISL_1288250	11/02/2021	Tlaxcala	Human	Male	47	Hospitalized	Original	Nasopharyngeal and oropharyngeal swab
hCoV-19/Mexico/TLA-IBT-IMSS-32/2021	EPI_ISL_1288251	11/02/2021	Tlaxcala	Human	Male	69	Hospitalized	Original	Nasopharyngeal and oropharyngeal swab
hCoV-19/Mexico/TLA-IBT-IMSS-33/2021	EPI_ISL_1288252	11/02/2021	Tlaxcala	Human	Male	48	Hospitalized	Original	Nasopharyngeal and oropharyngeal swab
hCoV-19/Mexico/PUE-IBT-IMSS-35/2021	EPI_ISL_1288254	11/02/2021	Puebla	Human	Male	58	Ambulatory	Original	Nasopharyngeal and oropharyngeal swab
hCoV-19/Mexico/PUE-IBT-IMSS-38/2021	EPI_ISL_1288256	11/02/2021	Puebla	Human	Female	54	Ambulatory	Original	Nasopharyngeal and oropharyngeal swab
hCoV-19/Mexico/PUE-IBT-IMSS-39/2021	EPI_ISL_1288257	11/02/2021	Puebla	Human	Male	45	Ambulatory	Original	Nasopharyngeal and oropharyngeal swab
hCoV-19/Mexico/PUE-IBT-IMSS-40/2021	EPI_ISL_1288258	11/02/2021	Puebla	Human	Male	44	Ambulatory	Original	Nasopharyngeal and oropharyngeal swab
hCoV-19/Mexico/PUE-IBT-IMSS-41/2021	EPI_ISL_1288259	11/02/2021	Puebla	Human	Male	50	Ambulatory	Original	Nasopharyngeal swab
hCoV-19/Mexico/PUE-IBT-IMSS-42/2021	EPI_ISL_1288260	11/02/2021	Puebla	Human	Male	58	Ambulatory	Original	Nasopharyngeal swab
hCoV-19/Mexico/PUE-IBT-IMSS-43/2021	EPI_ISL_1288261	11/02/2021	Puebla	Human	Female	41	Ambulatory	Original	Nasopharyngeal swab
hCoV-19/Mexico/PUE-IBT-IMSS-44/2021	EPI_ISL_1288262	11/02/2021	Puebla	Human	Male	57	Ambulatory	Original	Oropharyngeal swab
hCoV-19/Mexico/PUE-IBT-IMSS-45/2021	EPI_ISL_1288263	11/02/2021	Puebla	Human	Female	31	Ambulatory	Original	Nasopharyngeal swab
hCoV-19/Mexico/PUE-IBT-IMSS-46/2021	EPI_ISL_1288264	11/02/2021	Puebla	Human	Female	28	Ambulatory	Original	Nasopharyngeal swab
hCoV-19/Mexico/PUE-IBT-IMSS-50/2021	EPI_ISL_1288267	11/02/2021	Puebla	Human	Female	88	Ambulatory	Original	Nasopharyngeal swab
hCoV-19/Mexico/PUE-IBT-IMSS-52/2021	EPI_ISL_1288269	11/02/2021	Puebla	Human	Female	53	Ambulatory	Original	Oropharyngeal swab
hCoV-19/Mexico/PUE-IBT-IMSS-53/2021	EPI_ISL_1288270	11/02/2021	Puebla	Human	Female	26	Ambulatory	Original	Oropharyngeal swab
hCoV-19/Mexico/PUE-IBT-IMSS-54/2021	EPI_ISL_1288271	11/02/2021	Puebla	Human	Female	46	Ambulatory	Original	Oropharyngeal swab
hCoV-19/Mexico/PUE-IBT-IMSS-55/2021	EPI_ISL_1288272	11/02/2021	Puebla	Human	Female	34	Ambulatory	Original	Nasopharyngeal and oropharyngeal swab
hCoV-19/Mexico/PUE-IBT-IMSS-57/2021	EPI_ISL_1288274	11/02/2021	Puebla	Human	Female	33	Ambulatory	Original	Nasopharyngeal and oropharyngeal swab
hCoV-19/Mexico/SLP-IBT-IMSS-65/2021	EPI_ISL_1288281	11/02/2021	San Luis Potosi	Human	Female	24	Ambulatory	Original	Nasopharyngeal and oropharyngeal swab
hCoV-19/Mexico/SLP-IBT-IMSS-66/2021	EPI_ISL_1288282	11/02/2021	San Luis Potosi	Human	Female	22	Ambulatory	Original	Nasopharyngeal and oropharyngeal swab
hCoV-19/Mexico/SLP-IBT-IMSS-67/2021	EPI_ISL_1288283	11/02/2021	San Luis Potosi	Human	Female	36	Ambulatory	Original	Nasopharyngeal and oropharyngeal swab
hCoV-19/Mexico/SLP-IBT-IMSS-68/2021	EPI_ISL_1288284	11/02/2021	San Luis Potosi	Human	Female	46	Ambulatory	Original	Nasopharyngeal and oropharyngeal swab
hCoV-19/Mexico/ZAC_INER_IMSS_00676/2021	EPI_ISL_2091132	11/02/2021	Zacatecas	Human	Male	32	Ambulatory	Original	Nasopharyngeal and oropharyngeal swab
hCoV-19/Mexico/DUR_INER_IMSS_00680/2021	EPI_ISL_2091136	11/02/2021	Durango	Human	Male	29	Ambulatory	Original	Nasopharyngeal and oropharyngeal swab
hCoV-19/Mexico/CAM-INER-IMSS-00369/2021	EPI_ISL_1279294	12/02/2021	Campeche	Human	Female	41	Ambulatory	Original	Nasopharyngeal and oropharyngeal swab
hCoV-19/Mexico/ZAC-INER-IMSS-00081/2021	EPI_ISL_1287766	12/02/2021	Zacatecas	Human	Male	88	Hospitalized	Original	Nasopharyngeal and oropharyngeal swab
hCoV-19/Mexico/CMX-IBT-IMSS-364/2021	EPI_ISL_1288193	12/02/2021	Mexico City	Human	Female	65	Ambulatory	Original	Nasopharyngeal and oropharyngeal swab
hCoV-19/Mexico/CMX-IBT-IMSS-365/2021	EPI_ISL_1288194	12/02/2021	Mexico City	Human	Female	64	Ambulatory	Original	Nasopharyngeal and oropharyngeal swab

hCoV-19/Mexico/CMX-IBT-IMSS-366/2021	EPI_ISL_1288195	12/02/2021	Mexico City	Human	Male	75	Ambulatory	Original	Nasopharyngeal and oropharyngeal swab
hCoV-19/Mexico/YUC-INER-IMSS-00360/2021	EPI_ISL_1279605	12/02/2021	Yucatan	Human	Male	21	Ambulatory	Original	Nasopharyngeal swab
hCoV-19/Mexico/YUC-INER-IMSS-00361/2021	EPI_ISL_1279606	12/02/2021	Yucatan	Human	Male	31	Ambulatory	Original	Nasopharyngeal swab
hCoV-19/Mexico/YUC-INER-IMSS-00362/2021	EPI_ISL_1279607	12/02/2021	Yucatan	Human	Female	50	Ambulatory	Original	Nasopharyngeal and oropharyngeal swab
hCoV-19/Mexico/YUC-INER-IMSS-00363/2021	EPI_ISL_1279608	12/02/2021	Yucatan	Human	Female	33	Ambulatory	Original	Nasopharyngeal and oropharyngeal swab
hCoV-19/Mexico/CAM-INER-IMSS-00371/2021	EPI_ISL_1279614	12/02/2021	Campeche	Human	Male	34	Ambulatory	Original	Nasopharyngeal and oropharyngeal swab
hCoV-19/Mexico/AGU-IBT-IMSS-223/2021	EPI_ISL_1288417	12/02/2021	Mexico City	Human	Female	79	Hospitalized	Original	Nasopharyngeal swab
hCoV-19/Mexico/CMX-IBT-IMSS-224/2021	EPI_ISL_1288418	12/02/2021	Mexico City	Human	Female	81	Hospitalized	Original	Nasopharyngeal swab
hCoV-19/Mexico/CMX-IBT-IMSS-227/2021	EPI_ISL_1288420	12/02/2021	Mexico City	Human	Male	40	Hospitalized	Original	Oropharyngeal swab
hCoV-19/Mexico/BCS-IBT-IMSS-246/2021	EPI_ISL_1288435	12/02/2021	Baja California Sur	Human	Female	28	Ambulatory	Original	Nasopharyngeal and oropharyngeal swab
hCoV-19/Mexico/BCS-IBT-IMSS-247/2021	EPI_ISL_1288436	12/02/2021	Baja California Sur	Human	Male	24	Ambulatory	Original	Nasopharyngeal and oropharyngeal swab
hCoV-19/Mexico/BCS-IBT-IMSS-248/2021	EPI_ISL_1288437	12/02/2021	Baja California Sur	Human	Male	36	Ambulatory	Original	Nasopharyngeal and oropharyngeal swab
hCoV-19/Mexico/BCS-IBT-IMSS-250/2021	EPI_ISL_1288438	12/02/2021	Baja California Sur	Human	Male	40	Ambulatory	Original	Nasopharyngeal and oropharyngeal swab
hCoV-19/Mexico/BCS-IBT-IMSS-251/2021	EPI_ISL_1288439	12/02/2021	Baja California Sur	Human	Female	39	Ambulatory	Original	Nasopharyngeal and oropharyngeal swab
hCoV-19/Mexico/MEX-IBT-IMSS-337/2021	EPI_ISL_1288516	12/02/2021	State of Mexico	Human	Male	72	Hospitalized	Original	Oropharyngeal swab
hCoV-19/Mexico/MEX-IBT-IMSS-339/2021	EPI_ISL_1288518	12/02/2021	State of Mexico	Human	Female	26	Hospitalized	Original	Nasopharyngeal and oropharyngeal swab
hCoV-19/Mexico/CMX-IBT-IMSS-340/2021	EPI_ISL_1288519	12/02/2021	Mexico City	Human	Male	9	Ambulatory	Original	Nasopharyngeal and oropharyngeal swab
hCoV-19/Mexico/CMX-IBT-IMSS-345/2021	EPI_ISL_1288524	12/02/2021	Mexico City	Human	Male	57	Ambulatory	Original	Nasopharyngeal and oropharyngeal swab
hCoV-19/Mexico/CMX-IBT-IMSS-346/2021	EPI_ISL_1288525	12/02/2021	Mexico City	Human	Female	65	Ambulatory	Original	Nasopharyngeal and oropharyngeal swab
hCoV-19/Mexico/CMX-IBT-IMSS-349/2021	EPI_ISL_1288526	12/02/2021	Mexico City	Human	Female	47	Ambulatory	Original	Nasopharyngeal and oropharyngeal swab
hCoV-19/Mexico/CMX-IBT-IMSS-350/2021	EPI_ISL_1288527	12/02/2021	Mexico City	Human	Male	58	Ambulatory	Original	Nasopharyngeal and oropharyngeal swab
hCoV-19/Mexico/CMX-IBT-IMSS-351/2021	EPI_ISL_1288528	12/02/2021	Mexico City	Human	Female	36	Ambulatory	Original	Nasopharyngeal and oropharyngeal swab
hCoV-19/Mexico/VER-IBT-IMSS-352/2021	EPI_ISL_1288529	12/02/2021	Veracruz	Human	Female	38	Ambulatory	Original	Nasopharyngeal swab
hCoV-19/Mexico/VER-IBT-IMSS-353/2021	EPI_ISL_1288530	12/02/2021	Veracruz	Human	Female	34	Ambulatory	Original	Nasopharyngeal swab
hCoV-19/Mexico/VER-IBT-IMSS-354/2021	EPI_ISL_1288531	12/02/2021	Veracruz	Human	Male	49	Ambulatory	Original	Nasopharyngeal and oropharyngeal swab
hCoV-19/Mexico/NLE-INER-IMSS-00206/2021	EPI_ISL_1279474	12/02/2021	Nuevo Leon	Human	Male	47	Hospitalized	Original	Nasopharyngeal and oropharyngeal swab
hCoV-19/Mexico/COA-INER-IMSS-00216/2021	EPI_ISL_1279482	12/02/2021	Coahuila	Human	Male	73	Hospitalized	Original	Nasopharyngeal and oropharyngeal swab
hCoV-19/Mexico/COA-INER-IMSS-00217/2021	EPI_ISL_1279483	12/02/2021	Coahuila	Human	Male	50	Hospitalized	Original	Nasopharyngeal and oropharyngeal swab
hCoV-19/Mexico/TAM-INER-IMSS-00227/2021	EPI_ISL_1279492	12/02/2021	Tamaulipas	Human	Female	53	Ambulatory	Original	Pharyngeal swab
hCoV-19/Mexico/COA-INER-IMSS-00247/2021	EPI_ISL_1279510	12/02/2021	Coahuila	Human	Female	46	Ambulatory	Original	Nasopharyngeal and oropharyngeal swab
hCoV-19/Mexico/TAM-INER-IMSS-00277/2021	EPI_ISL_1279535	12/02/2021	Tamaulipas	Human	Female	28	Ambulatory	Original	Nasopharyngeal and oropharyngeal swab
hCoV-19/Mexico/CMX-IBT-IMSS-147/2021	EPI_ISL_1288350	12/02/2021	Mexico City	Human	Female	46	Ambulatory	Original	Nasopharyngeal and oropharyngeal swab
hCoV-19/Mexico/VER-IBT-IMSS-150/2021	EPI_ISL_1288352	12/02/2021	Veracruz	Human	Female	35	Ambulatory	Original	Nasopharyngeal and oropharyngeal swab
hCoV-19/Mexico/VER-IBT-IMSS-156/2021	EPI_ISL_1288358	12/02/2021	Veracruz	Human	Male	53	Hospitalized	Original	Nasopharyngeal and oropharyngeal swab
hCoV-19/Mexico/VER-IBT-IMSS-157/2021	EPI_ISL_1288359	12/02/2021	Veracruz	Human	Female	65	Hospitalized	Original	Nasopharyngeal and oropharyngeal swab
hCoV-19/Mexico/MEX-IBT-IMSS-169/2021	EPI_ISL_1288370	12/02/2021	State of Mexico	Human	Male	65	Hospitalized	Original	Nasopharyngeal and oropharyngeal swab
hCoV-19/Mexico/MOR-IBT-IMSS-173/2021	EPI_ISL_1288372	12/02/2021	Morelos	Human	Male	57	Hospitalized	Original	Nasopharyngeal and oropharyngeal swab
hCoV-19/Mexico/MEX-IBT-IMSS-187/2021	EPI_ISL_1288385	12/02/2021	State of Mexico	Human	Male	65	Hospitalized	Original	Nasopharyngeal and oropharyngeal swab
hCoV-19/Mexico/OAX-IBT-IMSS-191/2021	EPI_ISL_1288389	12/02/2021	Oaxaca	Human	Male	23	Ambulatory	Original	Nasopharyngeal and oropharyngeal swab
hCoV-19/Mexico/OAX-IBT-IMSS-192/2021	EPI_ISL_1288390	12/02/2021	Oaxaca	Human	Female	31	Ambulatory	Original	Nasopharyngeal and oropharyngeal swab
hCoV-19/Mexico/OAX-IBT-IMSS-194/2021	EPI_ISL_1288391	12/02/2021	Oaxaca	Human	Female	23	Ambulatory	Original	Nasopharyngeal and oropharyngeal swab

hCoV-19/Mexico/MEX-IBT-IMSS-197/2021	EPI_ISL_1288394	12/02/2021	State of Mexico	Human	Female	68	Hospitalized	Original	Nasopharyngeal and oropharyngeal swab
hCoV-19/Mexico/MEX-IBT-IMSS-198/2021	EPI_ISL_1288395	12/02/2021	State of Mexico	Human	Male	55	Hospitalized	Original	Nasopharyngeal and oropharyngeal swab
hCoV-19/Mexico/MEX-IBT-IMSS-199/2021	EPI_ISL_1288396	12/02/2021	State of Mexico	Human	Female	71	Hospitalized	Original	Nasopharyngeal and oropharyngeal swab
hCoV-19/Mexico/MEX-IBT-IMSS-200/2021	EPI_ISL_1288397	12/02/2021	State of Mexico	Human	Female	58	Hospitalized	Original	Nasopharyngeal and oropharyngeal swab
hCoV-19/Mexico/MEX-IBT-IMSS-201/2021	EPI_ISL_1288398	12/02/2021	State of Mexico	Human	Male	69	Hospitalized	Original	Nasopharyngeal and oropharyngeal swab
hCoV-19/Mexico/MEX-IBT-IMSS-202/2021	EPI_ISL_1288399	12/02/2021	State of Mexico	Human	Female	52	Hospitalized	Original	Nasopharyngeal and oropharyngeal swab
hCoV-19/Mexico/BCS-IBT-IMSS-252/2021	EPI_ISL_1288440	12/02/2021	Baja California Sur	Human	Female	36	Ambulatory	Original	Nasopharyngeal and oropharyngeal swab
hCoV-19/Mexico/TLA-IBT-IMSS-253/2021	EPI_ISL_1288441	12/02/2021	Tlaxcala	Human	Male	64	Hospitalized	Original	Nasopharyngeal and oropharyngeal swab
hCoV-19/Mexico/VER-IBT-IMSS-275/2021	EPI_ISL_1288462	12/02/2021	Veracruz	Human	Female	61	Ambulatory	Original	Nasopharyngeal and oropharyngeal swab
hCoV-19/Mexico/VER-IBT-IMSS-276/2021	EPI_ISL_1288463	12/02/2021	Veracruz	Human	Female	26	Ambulatory	Original	Nasopharyngeal and oropharyngeal swab
hCoV-19/Mexico/VER-IBT-IMSS-277/2021	EPI_ISL_1288464	12/02/2021	Veracruz	Human	Female	58	Ambulatory	Original	Nasopharyngeal and oropharyngeal swab
hCoV-19/Mexico/SLP-IBT-IMSS-287/2021	EPI_ISL_1288473	12/02/2021	San Luis Potosi	Human	Female	71	Hospitalized	Original	Nasopharyngeal and oropharyngeal swab
hCoV-19/Mexico/SLP-IBT-IMSS-289/2021	EPI_ISL_1288475	12/02/2021	San Luis Potosi	Human	Male	65	Hospitalized	Original	Nasopharyngeal and oropharyngeal swab
hCoV-19/Mexico/CMX-IBT-IMSS-291/2021	EPI_ISL_1288477	12/02/2021	Mexico City	Human	Female	44	Ambulatory	Original	Nasopharyngeal and oropharyngeal swab
hCoV-19/Mexico/CMX-IBT-IMSS-293/2021	EPI_ISL_1288478	12/02/2021	Mexico City	Human	Male	82	Hospitalized	Original	Nasopharyngeal and oropharyngeal swab
hCoV-19/Mexico/CMX-IBT-IMSS-294/2021	EPI_ISL_1288479	12/02/2021	Mexico City	Human	Female	52	Ambulatory	Original	Nasopharyngeal and oropharyngeal swab
hCoV-19/Mexico/MEX-IBT-IMSS-295/2021	EPI_ISL_1288480	12/02/2021	State of Mexico	Human	Male	41	Hospitalized	Original	Nasopharyngeal and oropharyngeal swab
hCoV-19/Mexico/GRO-IBT-IMSS-302/2021	EPI_ISL_1288487	12/02/2021	Guerrero	Human	Female	26	Ambulatory	Original	Nasopharyngeal and oropharyngeal swab
hCoV-19/Mexico/OAX-IBT-IMSS-304/2021	EPI_ISL_1288488	12/02/2021	Oaxaca	Human	Male	22	Ambulatory	Original	Nasopharyngeal and oropharyngeal swab
hCoV-19/Mexico/OAX-IBT-IMSS-305/2021	EPI_ISL_1288489	12/02/2021	Oaxaca	Human	Female	46	Ambulatory	Original	Nasopharyngeal and oropharyngeal swab
hCoV-19/Mexico/OAX-IBT-IMSS-306/2021	EPI_ISL_1288490	12/02/2021	Oaxaca	Human	Male	29	Ambulatory	Original	Nasopharyngeal and oropharyngeal swab
hCoV-19/Mexico/BCS-IBT-IMSS-308/2021	EPI_ISL_1288491	12/02/2021	Baja California Sur	Human	Male	47	Ambulatory	Original	Nasopharyngeal and oropharyngeal swab
hCoV-19/Mexico/BCS-IBT-IMSS-309/2021	EPI_ISL_1288492	12/02/2021	Baja California Sur	Human	Female	21	Ambulatory	Original	Nasopharyngeal and oropharyngeal swab
hCoV-19/Mexico/MEX-IBT-IMSS-311/2021	EPI_ISL_1288494	12/02/2021	State of Mexico	Human	Male	51	Ambulatory	Original	Nasopharyngeal and oropharyngeal swab
hCoV-19/Mexico/OAX-IBT-IMSS-316/2021	EPI_ISL_1288498	12/02/2021	Oaxaca	Human	Female	35	Ambulatory	Original	Nasopharyngeal and oropharyngeal swab
hCoV-19/Mexico/ZAC-INER-IMSS-00030/2021	EPI_ISL_1279329	12/02/2021	Zacatecas	Human	Male	24	Ambulatory	Original	Nasopharyngeal and oropharyngeal swab
hCoV-19/Mexico/ZAC-INER-IMSS-00031/2021	EPI_ISL_1279330	12/02/2021	Zacatecas	Human	Female	28	Ambulatory	Original	Nasopharyngeal and oropharyngeal swab
hCoV-19/Mexico/ZAC-INER-IMSS-00034/2021	EPI_ISL_1279333	12/02/2021	Zacatecas	Human	Male	50	Ambulatory	Original	Nasopharyngeal and oropharyngeal swab
hCoV-19/Mexico/ZAC-INER-IMSS-00035/2021	EPI_ISL_1279334	12/02/2021	Zacatecas	Human	Female	53	Ambulatory	Original	Nasopharyngeal and oropharyngeal swab
hCoV-19/Mexico/ZAC-INER-IMSS-00036/2021	EPI_ISL_1279335	12/02/2021	Zacatecas	Human	Female	72	Hospitalized	Original	Nasopharyngeal and oropharyngeal swab
hCoV-19/Mexico/ZAC-INER-IMSS-00039/2021	EPI_ISL_1279338	12/02/2021	Zacatecas	Human	Male	41	Ambulatory	Original	Nasopharyngeal and oropharyngeal swab
hCoV-19/Mexico/CMX-IBT-IMSS-373/2021	EPI_ISL_1288201	12/02/2021	Mexico City	Human	Male	58	Hospitalized	Original	Oropharyngeal swab
hCoV-19/Mexico/CMX-IBT-IMSS-385/2021	EPI_ISL_1288212	12/02/2021	Mexico City	Human	Male	68	Hospitalized	Original	Nasopharyngeal and oropharyngeal swab
hCoV-19/Mexico/HID-IBT-IMSS-28/2021	EPI_ISL_1288247	12/02/2021	Hidalgo	Human	Female	63	Hospitalized	Original	Oropharyngeal swab
hCoV-19/Mexico/MEX-IBT-IMSS-29/2021	EPI_ISL_1288248	12/02/2021	State of Mexico	Human	Male	53	Hospitalized	Original	Nasopharyngeal and oropharyngeal swab
hCoV-19/Mexico/TLA-IBT-IMSS-34/2021	EPI_ISL_1288253	12/02/2021	Tlaxcala	Human	Male	32	Ambulatory	Original	Nasopharyngeal and oropharyngeal swab
hCoV-19/Mexico/SLP-IBT-IMSS-62/2021	EPI_ISL_1288278	12/02/2021	San Luis Potosi	Human	Female	83	Hospitalized	Original	Nasopharyngeal and oropharyngeal swab
hCoV-19/Mexico/SLP-IBT-IMSS-63/2021	EPI_ISL_1288279	12/02/2021	San Luis Potosi	Human	Female	66	Hospitalized	Original	Nasopharyngeal and oropharyngeal swab
hCoV-19/Mexico/SLP-IBT-IMSS-64/2021	EPI_ISL_1288280	12/02/2021	San Luis Potosi	Human	Female	50	Ambulatory	Original	Nasopharyngeal and oropharyngeal swab
hCoV-19/Mexico/SLP-IBT-IMSS-69/2021	EPI_ISL_1288285	12/02/2021	San Luis Potosi	Human	Female	25	Ambulatory	Original	Nasopharyngeal and oropharyngeal swab
hCoV-19/Mexico/SLP-IBT-IMSS-71/2021	EPI_ISL_1288286	12/02/2021	San Luis Potosi	Human	Male	86	Ambulatory	Original	Nasopharyngeal and oropharyngeal swab

hCoV-19/Mexico/SLP-IBT-IMSS-76/2021	EPI_ISL_1288291	12/02/2021	San Luis Potosi	Human	Female	54	Ambulatory	Original	Nasopharyngeal and oropharyngeal swab
hCoV-19/Mexico/SLP-IBT-IMSS-77/2021	EPI_ISL_1288292	12/02/2021	San Luis Potosi	Human	Female	45	Ambulatory	Original	Nasopharyngeal and oropharyngeal swab
hCoV-19/Mexico/COA_INER_IMSS_00679/2021	EPI_ISL_2091135	12/02/2021	Coahuila	Human	Male	48	Hospitalized	Original	Nasopharyngeal and oropharyngeal swab
hCoV-19/Mexico/YUC-INER-IMSS-00364/2021	EPI_ISL_1279609	13/02/2021	Yucatan	Human	Female	67	Hospitalized	Original	Nasopharyngeal and oropharyngeal swab
hCoV-19/Mexico/YUC-INER-IMSS-00365/2021	EPI_ISL_1279610	13/02/2021	Yucatan	Human	Male	45	Hospitalized	Original	Nasopharyngeal and oropharyngeal swab
hCoV-19/Mexico/MEX-IBT-IMSS-218/2021	EPI_ISL_1288413	13/02/2021	State of Mexico	Human	Male	12	Ambulatory	Original	Nasopharyngeal and oropharyngeal swab
hCoV-19/Mexico/CMX-IBT-IMSS-219/2021	EPI_ISL_1288414	13/02/2021	Mexico City	Human	Male	61	Hospitalized	Original	Oropharyngeal swab
hCoV-19/Mexico/CMX-IBT-IMSS-221/2021	EPI_ISL_1288415	13/02/2021	Mexico City	Human	Female	44	Hospitalized	Original	Oropharyngeal swab
hCoV-19/Mexico/MEX-IBT-IMSS-222/2021	EPI_ISL_1288416	13/02/2021	State of Mexico	Human	Female	67	Ambulatory	Original	Oropharyngeal swab
hCoV-19/Mexico/CMX-IBT-IMSS-225/2021	EPI_ISL_1288419	13/02/2021	Mexico City	Human	Female	64	Ambulatory	Original	Oropharyngeal swab
hCoV-19/Mexico/MEX-IBT-IMSS-318/2021	EPI_ISL_1288500	13/02/2021	State of Mexico	Human	Male	62	Ambulatory	Original	Nasopharyngeal and oropharyngeal swab
hCoV-19/Mexico/MOR-IBT-IMSS-319/2021	EPI_ISL_1288501	13/02/2021	Morelos	Human	Male	65	Ambulatory	Original	Nasopharyngeal and oropharyngeal swab
hCoV-19/Mexico/MOR-IBT-IMSS-320/2021	EPI_ISL_1288502	13/02/2021	Morelos	Human	Male	72	Hospitalized	Original	Nasopharyngeal and oropharyngeal swab
hCoV-19/Mexico/MOR-IBT-IMSS-321/2021	EPI_ISL_1288503	13/02/2021	Morelos	Human	Female	65	Hospitalized	Original	Nasopharyngeal and oropharyngeal swab
hCoV-19/Mexico/MOR-IBT-IMSS-322/2021	EPI_ISL_1288504	13/02/2021	Morelos	Human	Female	67	Hospitalized	Original	Nasopharyngeal and oropharyngeal swab
hCoV-19/Mexico/MOR-IBT-IMSS-323/2021	EPI_ISL_1288505	13/02/2021	Morelos	Human	Male	52	Ambulatory	Original	Nasopharyngeal and oropharyngeal swab
hCoV-19/Mexico/MOR-IBT-IMSS-324/2021	EPI_ISL_1288506	13/02/2021	Morelos	Human	Male	54	Hospitalized	Original	Nasopharyngeal and oropharyngeal swab
hCoV-19/Mexico/CMX-IBT-IMSS-326/2021	EPI_ISL_1288507	13/02/2021	Mexico City	Human	Male	72	Hospitalized	Original	Nasopharyngeal swab
hCoV-19/Mexico/CMX-IBT-IMSS-327/2021	EPI_ISL_1288508	13/02/2021	Mexico City	Human	Female	62	Hospitalized	Original	Nasopharyngeal swab
hCoV-19/Mexico/CMX-IBT-IMSS-328/2021	EPI_ISL_1288509	13/02/2021	Mexico City	Human	Female	81	Hospitalized	Original	Nasopharyngeal swab
hCoV-19/Mexico/CMX-IBT-IMSS-329/2021	EPI_ISL_1288510	13/02/2021	Mexico City	Human	Male	39	Hospitalized	Original	Nasopharyngeal swab
hCoV-19/Mexico/CMX-IBT-IMSS-330/2021	EPI_ISL_1288511	13/02/2021	Mexico City	Human	Male	53	Hospitalized	Original	Nasopharyngeal swab
hCoV-19/Mexico/CMX-IBT-IMSS-331/2021	EPI_ISL_1288512	13/02/2021	Mexico City	Human	Male	52	Hospitalized	Original	Nasopharyngeal swab
hCoV-19/Mexico/HID-IBT-IMSS-332/2021	EPI_ISL_1288513	13/02/2021	Hidalgo	Human	Female	33	Ambulatory	Original	Oropharyngeal swab
hCoV-19/Mexico/HID-IBT-IMSS-333/2021	EPI_ISL_1288514	13/02/2021	Hidalgo	Human	Female	30	Ambulatory	Original	Oropharyngeal swab
hCoV-19/Mexico/CMX-IBT-IMSS-334/2021	EPI_ISL_1288515	13/02/2021	Mexico City	Human	Female	66	Ambulatory	Original	Nasopharyngeal and oropharyngeal swab
hCoV-19/Mexico/TAM_LANGEBIO_IMSS_0007/20	EPI_ISL_1351426	13/02/2021	Nuevo Leon	Human	Male	80	Hospitalized	Original	Pharyngeal and Nasopharyngeal swab
hCoV-19/Mexico/NLE-INER-IMSS-00213/2021	EPI_ISL_1279479	13/02/2021	Nuevo Leon	Human	Male	75	Hospitalized	Original	Nasopharyngeal and oropharyngeal swab
hCoV-19/Mexico/NLE-INER-IMSS-00214/2021	EPI_ISL_1279480	13/02/2021	Nuevo Leon	Human	Male	62	Ambulatory	Original	Nasopharyngeal and oropharyngeal swab
hCoV-19/Mexico/NLE-INER-IMSS-00215/2021	EPI_ISL_1279481	13/02/2021	Nuevo Leon	Human	Male	64	Hospitalized	Original	Nasopharyngeal and oropharyngeal swab
hCoV-19/Mexico/COA-INER-IMSS-00240/2021	EPI_ISL_1279504	13/02/2021	Coahuila	Human	Female	31	Hospitalized	Original	Nasopharyngeal and oropharyngeal swab
hCoV-19/Mexico/COA-INER-IMSS-00241/2021	EPI_ISL_1279505	13/02/2021	Coahuila	Human	Female	45	Ambulatory	Original	Nasopharyngeal and oropharyngeal swab
hCoV-19/Mexico/COA-INER-IMSS-00246/2021	EPI_ISL_1279509	13/02/2021	Coahuila	Human	Female	44	Hospitalized	Original	Nasopharyngeal and oropharyngeal swab
hCoV-19/Mexico/CHH-INER-IMSS-00251/2021	EPI_ISL_1279513	13/02/2021	Chihuahua	Human	Male	61	Hospitalized	Original	Nasopharyngeal and oropharyngeal swab
hCoV-19/Mexico/CHH-INER-IMSS-00272/2021	EPI_ISL_1279531	13/02/2021	Chihuahua	Human	Female	68	Hospitalized	Original	Nasopharyngeal and oropharyngeal swab
hCoV-19/Mexico/HID-IBT-IMSS-254/2021	EPI_ISL_1288443	13/02/2021	Hidalgo	Human	Female	57	Hospitalized	Original	Oropharyngeal swab
hCoV-19/Mexico/CMX-IBT-IMSS-284/2021	EPI_ISL_1288470	13/02/2021	Mexico City	Human	Male	62	Ambulatory	Original	Nasopharyngeal and oropharyngeal swab
hCoV-19/Mexico/SLP-IBT-IMSS-288/2021	EPI_ISL_1288474	13/02/2021	San Luis Potosi	Human	Female	68	Hospitalized	Original	Nasopharyngeal and oropharyngeal swab
hCoV-19/Mexico/CMX-IBT-IMSS-290/2021	EPI_ISL_1288476	13/02/2021	Mexico City	Human	Male	10	Ambulatory	Original	Nasopharyngeal and oropharyngeal swab
hCoV-19/Mexico/CMX-IBT-IMSS-296/2021	EPI_ISL_1288481	13/02/2021	Mexico City	Human	Male	68	Hospitalized	Original	Nasopharyngeal and oropharyngeal swab
hCoV-19/Mexico/CMX-IBT-IMSS-297/2021	EPI_ISL_1288482	13/02/2021	Mexico City	Human	Female	56	Hospitalized	Original	Nasopharyngeal and oropharyngeal swab

hCoV-19/Mexico/MEX-IBT-IMSS-299/2021	EPI_ISL_1288484	13/02/2021	State of Mexico	Human	Male	60	Hospitalized	Original	Nasopharyngeal and oropharyngeal swab
hCoV-19/Mexico/CMX-IBT-IMSS-300/2021	EPI_ISL_1288485	13/02/2021	Mexico City	Human	Male	74	Hospitalized	Original	Nasopharyngeal and oropharyngeal swab
hCoV-19/Mexico/MEX-IBT-IMSS-313/2021	EPI_ISL_1288496	13/02/2021	State of Mexico	Human	Female	79	Hospitalized	Original	Nasopharyngeal and oropharyngeal swab
hCoV-19/Mexico/CMX-IBT-IMSS-317/2021	EPI_ISL_1288499	13/02/2021	Mexico City	Human	Male	72	Hospitalized	Original	Nasopharyngeal and oropharyngeal swab
hCoV-19/Mexico/ZAC-INER-IMSS-00037/2021	EPI_ISL_1279336	13/02/2021	Zacatecas	Human	Female	54	Ambulatory	Original	Nasopharyngeal and oropharyngeal swab
hCoV-19/Mexico/ZAC-INER-IMSS-00038/2021	EPI_ISL_1279337	13/02/2021	Zacatecas	Human	Male	43	Ambulatory	Original	Nasopharyngeal and oropharyngeal swab
hCoV-19/Mexico/ZAC-INER-IMSS-00040/2021	EPI_ISL_1279339	13/02/2021	Zacatecas	Human	Female	55	Ambulatory	Original	Nasopharyngeal and oropharyngeal swab
hCoV-19/Mexico/ZAC-INER-IMSS-00041/2021	EPI_ISL_1279340	13/02/2021	Zacatecas	Human	Female	44	Ambulatory	Original	Nasopharyngeal and oropharyngeal swab
hCoV-19/Mexico/ZAC-INER-IMSS-00042/2021	EPI_ISL_1279341	13/02/2021	Zacatecas	Human	Female	35	Ambulatory	Original	Nasopharyngeal and oropharyngeal swab
hCoV-19/Mexico/ZAC-INER-IMSS-00043/2021	EPI_ISL_1279342	13/02/2021	Zacatecas	Human	Male	41	Ambulatory	Original	Nasopharyngeal and oropharyngeal swab
hCoV-19/Mexico/ZAC-INER-IMSS-00051/2021	EPI_ISL_1279349	13/02/2021	Zacatecas	Human	Female	44	Ambulatory	Original	Nasopharyngeal and oropharyngeal swab
hCoV-19/Mexico/ZAC-INER-IMSS-00052/2021	EPI_ISL_1279279	14/02/2021	Zacatecas	Human	Female	50	Ambulatory	Original	Nasopharyngeal and oropharyngeal swab
hCoV-19/Mexico/ZAC-INER-IMSS-00050/2021	EPI_ISL_1287762	14/02/2021	Zacatecas	Human	Female	92	Ambulatory	Original	Nasopharyngeal and oropharyngeal swab
hCoV-19/Mexico/YUC-INER-IMSS-00370/2021	EPI_ISL_1279613	14/02/2021	Yucatan	Human	Female	90	Hospitalized	Original	Nasopharyngeal and oropharyngeal swab
hCoV-19/Mexico/CMX-IBT-IMSS-341/2021	EPI_ISL_1288520	14/02/2021	Mexico City	Human	Female	7	Ambulatory	Original	Nasopharyngeal and oropharyngeal swab
hCoV-19/Mexico/CMX-IBT-IMSS-342/2021	EPI_ISL_1288521	14/02/2021	Mexico City	Human	Male	56	Hospitalized	Original	Oropharyngeal swab
hCoV-19/Mexico/MEX-IBT-IMSS-344/2021	EPI_ISL_1288523	14/02/2021	State of Mexico	Human	Male	67	Hospitalized	Original	Nasopharyngeal and oropharyngeal swab
hCoV-19/Mexico/CMX-IBT-IMSS-355/2021	EPI_ISL_1288532	14/02/2021	Mexico City	Human	Male	38	Hospitalized	Original	Nasopharyngeal and oropharyngeal swab
hCoV-19/Mexico/MEX-IBT-IMSS-356/2021	EPI_ISL_1288533	14/02/2021	State of Mexico	Human	Male	56	Hospitalized	Original	Nasopharyngeal and oropharyngeal swab
hCoV-19/Mexico/NLE-INER-IMSS-00221/2021	EPI_ISL_1279486	14/02/2021	Nuevo Leon	Human	Female	26	Ambulatory	Original	Nasopharyngeal and oropharyngeal swab
hCoV-19/Mexico/NLE-INER-IMSS-00222/2021	EPI_ISL_1279487	14/02/2021	Nuevo Leon	Human	Female	73	Hospitalized	Original	Nasopharyngeal swab
hCoV-19/Mexico/NLE-INER-IMSS-00223/2021	EPI_ISL_1279488	14/02/2021	Nuevo Leon	Human	Male	75	Hospitalized	Original	Nasopharyngeal and oropharyngeal swab
hCoV-19/Mexico/COA-INER-IMSS-00239/2021	EPI_ISL_1279503	14/02/2021	Coahuila	Human	Female	48	Hospitalized	Original	Nasopharyngeal and oropharyngeal swab
hCoV-19/Mexico/NLE-INER-IMSS-00279/2021	EPI_ISL_1279536	14/02/2021	Nuevo Leon	Human	Male	79	Hospitalized	Original	Nasopharyngeal and oropharyngeal swab
hCoV-19/Mexico/CMX-IBT-IMSS-285/2021	EPI_ISL_1288471	14/02/2021	Mexico City	Human	Male	77	Hospitalized	Original	Nasopharyngeal and oropharyngeal swab
hCoV-19/Mexico/MEX-IBT-IMSS-286/2021	EPI_ISL_1288472	14/02/2021	State of Mexico	Human	Male	67	Hospitalized	Original	Nasopharyngeal and oropharyngeal swab
hCoV-19/Mexico/CMX-IBT-IMSS-298/2021	EPI_ISL_1288483	14/02/2021	Mexico City	Human	Male	82	Hospitalized	Original	Nasopharyngeal and oropharyngeal swab
hCoV-19/Mexico/CMX-IBT-IMSS-301/2021	EPI_ISL_1288486	14/02/2021	Mexico City	Human	Female	58	Ambulatory	Original	Nasopharyngeal swab
hCoV-19/Mexico/MEX-IBT-IMSS-312/2021	EPI_ISL_1288495	14/02/2021	State of Mexico	Human	Female	70	Hospitalized	Original	Nasopharyngeal and oropharyngeal swab
hCoV-19/Mexico/MEX-IBT-IMSS-315/2021	EPI_ISL_1288497	14/02/2021	State of Mexico	Human	Male	66	Hospitalized	Original	Nasopharyngeal and oropharyngeal swab
hCoV-19/Mexico/ZAC-INER-IMSS-00044/2021	EPI_ISL_1279343	14/02/2021	Zacatecas	Human	Male	77	Ambulatory	Original	Nasopharyngeal and oropharyngeal swab
hCoV-19/Mexico/ZAC-INER-IMSS-00045/2021	EPI_ISL_1279344	14/02/2021	Zacatecas	Human	Female	19	Ambulatory	Original	Nasopharyngeal and oropharyngeal swab
hCoV-19/Mexico/ZAC-INER-IMSS-00046/2021	EPI_ISL_1279345	14/02/2021	Zacatecas	Human	Female	57	Ambulatory	Original	Nasopharyngeal and oropharyngeal swab
hCoV-19/Mexico/ZAC-INER-IMSS-00047/2021	EPI_ISL_1279346	14/02/2021	Zacatecas	Human	Male	59	Hospitalized	Original	Nasopharyngeal and oropharyngeal swab
hCoV-19/Mexico/ZAC-INER-IMSS-00048/2021	EPI_ISL_1279347	14/02/2021	Zacatecas	Human	Female	71	Hospitalized	Original	Nasopharyngeal and oropharyngeal swab
hCoV-19/Mexico/ZAC-INER-IMSS-00049/2021	EPI_ISL_1279348	14/02/2021	Zacatecas	Human	Female	31	Ambulatory	Original	Nasopharyngeal and oropharyngeal swab
hCoV-19/Mexico/COA-INER-IMSS-00242/2021	EPI_ISL_1279267	15/02/2021	Coahuila	Human	Male	36	Ambulatory	Original	Nasopharyngeal and oropharyngeal swab
hCoV-19/Mexico/ZAC-INER-IMSS-00058/2021	EPI_ISL_1279270	15/02/2021	Zacatecas	Human	Male	80	Hospitalized	Original	Nasopharyngeal and oropharyngeal swab
hCoV-19/Mexico/ZAC-INER-IMSS-00054/2021	EPI_ISL_1279280	15/02/2021	Zacatecas	Human	Male	49	Ambulatory	Original	Nasopharyngeal and oropharyngeal swab
hCoV-19/Mexico/ZAC-INER-IMSS-00055/2021	EPI_ISL_1279295	15/02/2021	Zacatecas	Human	Male	52	Ambulatory	Original	Nasopharyngeal and oropharyngeal swab
hCoV-19/Mexico/ZAC-INER-IMSS-00059/2021	EPI_ISL_1287763	15/02/2021	Zacatecas	Human	Male	41	Ambulatory	Original	Nasopharyngeal and oropharyngeal swab

hCoV-19/Mexico/MEX-IBT-IMSS-343/2021	EPI_ISL_1288522	15/02/2021	State of Mexico	Human	Male	67	Hospitalized	Original	Nasopharyngeal swab
hCoV-19/Mexico/VER-INER-IMSS-00174/2021	EPI_ISL_1279448	15/02/2021	Veracruz	Human	Female	81	Hospitalized	Original	Nasopharyngeal and oropharyngeal swab
hCoV-19/Mexico/NLE-INER-IMSS-00224/2021	EPI_ISL_1279489	15/02/2021	Nuevo Leon	Human	Female	54	Ambulatory	Original	Nasopharyngeal and oropharyngeal swab
hCoV-19/Mexico/NLE-INER-IMSS-00225/2021	EPI_ISL_1279490	15/02/2021	Nuevo Leon	Human	Male	27	Ambulatory	Original	Nasopharyngeal and oropharyngeal swab
hCoV-19/Mexico/NLE-INER-IMSS-00226/2021	EPI_ISL_1279491	15/02/2021	Nuevo Leon	Human	Male	29	Ambulatory	Original	Nasopharyngeal and oropharyngeal swab
hCoV-19/Mexico/DUR-INER-IMSS-00228/2021	EPI_ISL_1279493	15/02/2021	Durango	Human	Female	13	Ambulatory	Original	Nasopharyngeal and oropharyngeal swab
hCoV-19/Mexico/DUR-INER-IMSS-00229/2021	EPI_ISL_1279494	15/02/2021	Durango	Human	Female	37	Ambulatory	Original	Nasopharyngeal and oropharyngeal swab
hCoV-19/Mexico/NLE-INER-IMSS-00231/2021	EPI_ISL_1279495	15/02/2021	Nuevo Leon	Human	Female	38	Ambulatory	Original	Nasopharyngeal and oropharyngeal swab
hCoV-19/Mexico/COA-INER-IMSS-00243/2021	EPI_ISL_1279506	15/02/2021	Coahuila	Human	Female	34	Hospitalized	Original	Nasopharyngeal and oropharyngeal swab
hCoV-19/Mexico/COA-INER-IMSS-00244/2021	EPI_ISL_1279507	15/02/2021	Coahuila	Human	Male	43	Hospitalized	Original	Nasopharyngeal and oropharyngeal swab
hCoV-19/Mexico/COA-INER-IMSS-00245/2021	EPI_ISL_1279508	15/02/2021	Coahuila	Human	Female	59	Ambulatory	Original	Nasopharyngeal and oropharyngeal swab
hCoV-19/Mexico/COA-INER-IMSS-00248/2021	EPI_ISL_1279511	15/02/2021	Coahuila	Human	Male	51	Ambulatory	Original	Nasopharyngeal and oropharyngeal swab
hCoV-19/Mexico/COA-INER-IMSS-00256/2021	EPI_ISL_1279518	15/02/2021	Coahuila	Human	Male	81	Hospitalized	Original	Nasopharyngeal and oropharyngeal swab
hCoV-19/Mexico/ZAC-INER-IMSS-00068/2021	EPI_ISL_1279300	15/02/2021	Zacatecas	Human	Female	31	Ambulatory	Original	Nasopharyngeal and oropharyngeal swab
hCoV-19/Mexico/ZAC-INER-IMSS-00053/2021	EPI_ISL_1279350	15/02/2021	Zacatecas	Human	Female	28	Ambulatory	Original	Nasopharyngeal and oropharyngeal swab
hCoV-19/Mexico/ZAC-INER-IMSS-00056/2021	EPI_ISL_1279351	15/02/2021	Zacatecas	Human	Male	56	Hospitalized	Original	Nasopharyngeal and oropharyngeal swab
hCoV-19/Mexico/ZAC-INER-IMSS-00057/2021	EPI_ISL_1279352	15/02/2021	Zacatecas	Human	Female	33	Ambulatory	Original	Nasopharyngeal and oropharyngeal swab
hCoV-19/Mexico/ZAC-INER-IMSS-00060/2021	EPI_ISL_1279353	15/02/2021	Zacatecas	Human	Female	55	Ambulatory	Original	Nasopharyngeal and oropharyngeal swab
hCoV-19/Mexico/ZAC-INER-IMSS-00061/2021	EPI_ISL_1279354	15/02/2021	Zacatecas	Human	Male	43	Ambulatory	Original	Nasopharyngeal and oropharyngeal swab
hCoV-19/Mexico/ZAC-INER-IMSS-00062/2021	EPI_ISL_1279355	15/02/2021	Zacatecas	Human	Male	50	Ambulatory	Original	Nasopharyngeal and oropharyngeal swab
hCoV-19/Mexico/ZAC-INER-IMSS-00063/2021	EPI_ISL_1279356	15/02/2021	Zacatecas	Human	Male	27	Ambulatory	Original	Nasopharyngeal and oropharyngeal swab
hCoV-19/Mexico/ZAC-INER-IMSS-00064/2021	EPI_ISL_1279357	15/02/2021	Zacatecas	Human	Male	57	Ambulatory	Original	Nasopharyngeal and oropharyngeal swab
hCoV-19/Mexico/ZAC-INER-IMSS-00065/2021	EPI_ISL_1279358	15/02/2021	Zacatecas	Human	Female	25	Ambulatory	Original	Nasopharyngeal and oropharyngeal swab
hCoV-19/Mexico/ZAC-INER-IMSS-00067/2021	EPI_ISL_1279360	15/02/2021	Zacatecas	Human	Female	60	Hospitalized	Original	Nasopharyngeal and oropharyngeal swab
hCoV-19/Mexico/ZAC-INER-IMSS-00082/2021	EPI_ISL_1279369	15/02/2021	Zacatecas	Human	Male	56	Ambulatory	Original	Nasopharyngeal and oropharyngeal swab
hCoV-19/Mexico/ZAC-INER-IMSS-00075/2021	EPI_ISL_1279272	16/02/2021	Zacatecas	Human	Female	27	Ambulatory	Original	Nasopharyngeal and oropharyngeal swab
hCoV-19/Mexico/ZAC-INER-IMSS-00077/2021	EPI_ISL_1279273	16/02/2021	Zacatecas	Human	Male	19	Ambulatory	Original	Nasopharyngeal and oropharyngeal swab
hCoV-19/Mexico/NLE-INER-IMSS-00230/2021	EPI_ISL_1279285	16/02/2021	Nuevo Leon	Human	Female	59	Hospitalized	Original	Nasopharyngeal and oropharyngeal swab
hCoV-19/Mexico/ZAC-INER-IMSS-00078/2021	EPI_ISL_1287764	16/02/2021	Zacatecas	Human	Female	66	Ambulatory	Original	Nasopharyngeal and oropharyngeal swab
hCoV-19/Mexico/ZAC-INER-IMSS-00079/2021	EPI_ISL_1287765	16/02/2021	Zacatecas	Human	Male	53	Ambulatory	Original	Nasopharyngeal and oropharyngeal swab
hCoV-19/Mexico/DUR-INER-IMSS-00273/2021	EPI_ISL_1287772	16/02/2021	Durango	Human	Female	50	Ambulatory	Original	Nasopharyngeal and oropharyngeal swab
hCoV-19/Mexico/TAM-INER-IMSS-00278/2021	EPI_ISL_1287773	16/02/2021	Tamaulipas	Human	Male	64	Hospitalized	Original	Nasopharyngeal and oropharyngeal swab
hCoV-19/Mexico/ZAC-INER-IMSS-00083/2021	EPI_ISL_1279370	16/02/2021	Zacatecas	Human	Male	38	Ambulatory	Original	Nasopharyngeal and oropharyngeal swab
hCoV-19/Mexico/ZAC-INER-IMSS-00084/2021	EPI_ISL_1279371	16/02/2021	Zacatecas	Human	Male	59	Ambulatory	Original	Nasopharyngeal and oropharyngeal swab
hCoV-19/Mexico/PUE-INER-IMSS-00140/2021	EPI_ISL_1279419	16/02/2021	Puebla	Human	Female	33	Ambulatory	Original	Nasopharyngeal and oropharyngeal swab
hCoV-19/Mexico/PUE-INER-IMSS-00144/2021	EPI_ISL_1279423	16/02/2021	Puebla	Human	Female	61	Hospitalized	Original	Nasopharyngeal and oropharyngeal swab
hCoV-19/Mexico/NLE-INER-IMSS-00232/2021	EPI_ISL_1279496	16/02/2021	Nuevo Leon	Human	Female	47	Ambulatory	Original	Nasopharyngeal and oropharyngeal swab
hCoV-19/Mexico/NLE-INER-IMSS-00233/2021	EPI_ISL_1279497	16/02/2021	Nuevo Leon	Human	Female	26	Ambulatory	Original	Nasopharyngeal and oropharyngeal swab
hCoV-19/Mexico/NLE-INER-IMSS-00234/2021	EPI_ISL_1279498	16/02/2021	Nuevo Leon	Human	Male	70	Hospitalized	Original	Nasopharyngeal and oropharyngeal swab
hCoV-19/Mexico/NLE-INER-IMSS-00235/2021	EPI_ISL_1279499	16/02/2021	Nuevo Leon	Human	Female	45	Ambulatory	Original	Nasopharyngeal swab
hCoV-19/Mexico/NLE-INER-IMSS-00236/2021	EPI_ISL_1279500	16/02/2021	Nuevo Leon	Human	Female	62	Hospitalized	Original	Nasopharyngeal and oropharyngeal swab

hCoV-19/Mexico/NLE-INER-IMSS-00237/2021	EPI_ISL_1279501	16/02/2021	Nuevo Leon	Human	Male	87	Hospitalized	Original	Nasopharyngeal and oropharyngeal swab
hCoV-19/Mexico/COA-INER-IMSS-00238/2021	EPI_ISL_1279502	16/02/2021	Coahuila	Human	Female	21	Hospitalized	Original	Nasopharyngeal and oropharyngeal swab
hCoV-19/Mexico/TAM-INER-IMSS-00254/2021	EPI_ISL_1279516	16/02/2021	Tamaulipas	Human	Male	41	Ambulatory	Original	Nasopharyngeal and oropharyngeal swab
hCoV-19/Mexico/CHH-INER-IMSS-00269/2021	EPI_ISL_1279528	16/02/2021	Chihuahua	Human	Female	52	Hospitalized	Original	Nasopharyngeal and oropharyngeal swab
hCoV-19/Mexico/DUR-INER-IMSS-00275/2021	EPI_ISL_1279533	16/02/2021	Durango	Human	Female	33	Ambulatory	Original	Nasopharyngeal and oropharyngeal swab
hCoV-19/Mexico/NLE-INER-IMSS-00249/2021	EPI_ISL_1279302	16/02/2021	Nuevo Leon	Human	Male	34	Ambulatory	Original	Nasopharyngeal and oropharyngeal swab
hCoV-19/Mexico/ZAC-INER-IMSS-00066/2021	EPI_ISL_1279359	16/02/2021	Zacatecas	Human	Male	72	Hospitalized	Original	Nasopharyngeal and oropharyngeal swab
hCoV-19/Mexico/ZAC-INER-IMSS-00069/2021	EPI_ISL_1279361	16/02/2021	Zacatecas	Human	Male	43	Ambulatory	Original	Nasopharyngeal and oropharyngeal swab
hCoV-19/Mexico/ZAC-INER-IMSS-00070/2021	EPI_ISL_1279362	16/02/2021	Zacatecas	Human	Female	46	Ambulatory	Original	Nasopharyngeal and oropharyngeal swab
hCoV-19/Mexico/ZAC-INER-IMSS-00071/2021	EPI_ISL_1279363	16/02/2021	Zacatecas	Human	Female	43	Ambulatory	Original	Nasopharyngeal and oropharyngeal swab
hCoV-19/Mexico/ZAC-INER-IMSS-00072/2021	EPI_ISL_1279364	16/02/2021	Zacatecas	Human	Male	28	Ambulatory	Original	Nasopharyngeal and oropharyngeal swab
hCoV-19/Mexico/ZAC-INER-IMSS-00073/2021	EPI_ISL_1279365	16/02/2021	Zacatecas	Human	Female	44	Ambulatory	Original	Nasopharyngeal and oropharyngeal swab
hCoV-19/Mexico/ZAC-INER-IMSS-00074/2021	EPI_ISL_1279366	16/02/2021	Zacatecas	Human	Male	37	Ambulatory	Original	Nasopharyngeal and oropharyngeal swab
hCoV-19/Mexico/ZAC-INER-IMSS-00076/2021	EPI_ISL_1279367	16/02/2021	Zacatecas	Human	Female	26	Ambulatory	Original	Nasopharyngeal and oropharyngeal swab
hCoV-19/Mexico/ZAC-INER-IMSS-00080/2021	EPI_ISL_1279368	16/02/2021	Zacatecas	Human	Female	50	Ambulatory	Original	Nasopharyngeal and oropharyngeal swab
hCoV-19/Mexico/ZAC-INER-IMSS-00086/2021	EPI_ISL_1287767	17/02/2021	Zacatecas	Human	Female	38	Ambulatory	Original	Nasopharyngeal and oropharyngeal swab
hCoV-19/Mexico/PUE-INER-IMSS-00125/2021	EPI_ISL_1287768	17/02/2021	Puebla	Human	Male	25	Ambulatory	Original	Nasopharyngeal and oropharyngeal swab
hCoV-19/Mexico/ZAC-INER-IMSS-00085/2021	EPI_ISL_1279372	17/02/2021	Zacatecas	Human	Male	38	Ambulatory	Original	Nasopharyngeal and oropharyngeal swab
hCoV-19/Mexico/ZAC-INER-IMSS-00087/2021	EPI_ISL_1279373	17/02/2021	Zacatecas	Human	Male	87	Hospitalized	Original	Nasopharyngeal and oropharyngeal swab
hCoV-19/Mexico/PUE-INER-IMSS-00121/2021	EPI_ISL_1279403	17/02/2021	Puebla	Human	Female	55	Ambulatory	Original	Nasopharyngeal and oropharyngeal swab
hCoV-19/Mexico/PUE-INER-IMSS-00141/2021	EPI_ISL_1279420	17/02/2021	Puebla	Human	Male	53	Hospitalized	Original	Nasopharyngeal and oropharyngeal swab
hCoV-19/Mexico/PUE-INER-IMSS-00142/2021	EPI_ISL_1279421	17/02/2021	Puebla	Human	Male	82	Hospitalized	Original	Nasopharyngeal and oropharyngeal swab
hCoV-19/Mexico/PUE-INER-IMSS-00143/2021	EPI_ISL_1279422	17/02/2021	Puebla	Human	Male	58	Hospitalized	Original	Nasopharyngeal and oropharyngeal swab
hCoV-19/Mexico/PUE-INER-IMSS-00149/2021	EPI_ISL_1279427	17/02/2021	Puebla	Human	Female	34	Ambulatory	Original	Nasopharyngeal and oropharyngeal swab
hCoV-19/Mexico/VER-INER-IMSS-00175/2021	EPI_ISL_1279449	17/02/2021	Veracruz	Human	Male	50	Ambulatory	Original	Nasopharyngeal and oropharyngeal swab
hCoV-19/Mexico/NLE-INER-IMSS-00250/2021	EPI_ISL_1279512	17/02/2021	Nuevo Leon	Human	Male	63	Ambulatory	Original	Nasopharyngeal and oropharyngeal swab
hCoV-19/Mexico/NLE-INER-IMSS-00252/2021	EPI_ISL_1279514	17/02/2021	Nuevo Leon	Human	Female	77	Ambulatory	Original	Nasopharyngeal and oropharyngeal swab
hCoV-19/Mexico/NLE-INER-IMSS-00253/2021	EPI_ISL_1279515	17/02/2021	Nuevo Leon	Human	Male	89	Hospitalized	Original	Nasopharyngeal and oropharyngeal swab
hCoV-19/Mexico/TAM-INER-IMSS-00255/2021	EPI_ISL_1279517	17/02/2021	Tamaulipas	Human	Female	45	Ambulatory	Original	Nasopharyngeal and oropharyngeal swab
hCoV-19/Mexico/COA-INER-IMSS-00257/2021	EPI_ISL_1279519	17/02/2021	Coahuila	Human	Male	79	Hospitalized	Original	Nasopharyngeal and oropharyngeal swab
hCoV-19/Mexico/COA-INER-IMSS-00258/2021	EPI_ISL_1279520	17/02/2021	Coahuila	Human	Female	47	Hospitalized	Original	Nasopharyngeal and oropharyngeal swab
hCoV-19/Mexico/TAM-INER-IMSS-00268/2021	EPI_ISL_1279527	17/02/2021	Tamaulipas	Human	Female	44	Ambulatory	Original	Nasopharyngeal and oropharyngeal swab
hCoV-19/Mexico/DUR-INER-IMSS-00274/2021	EPI_ISL_1279532	17/02/2021	Durango	Human	Female	62	Hospitalized	Original	Nasopharyngeal and oropharyngeal swab
hCoV-19/Mexico/TAM-INER-IMSS-00276/2021	EPI_ISL_1279534	17/02/2021	Tamaulipas	Human	Female	49	Hospitalized	Original	Nasopharyngeal and oropharyngeal swab
hCoV-19/Mexico/VER-INER-IMSS-00286/2021	EPI_ISL_1279541	17/02/2021	Veracruz	Human	Male	35	Ambulatory	Original	Nasopharyngeal and oropharyngeal swab
hCoV-19/Mexico/ZAC-INER-IMSS-00088/2021	EPI_ISL_1279301	17/02/2021	Zacatecas	Human	Female	53	Ambulatory	Original	Nasopharyngeal and oropharyngeal swab
hCoV-19/Mexico/SLP-INER-IMSS-00162/2021	EPI_ISL_1279286	18/02/2021	San Luis Potosi	Human	Male	53	Hospitalized	Original	Nasopharyngeal and oropharyngeal swab
hCoV-19/Mexico/VER-INER-IMSS-00357/2021	EPI_ISL_1279602	18/02/2021	Veracruz	Human	Male	76	Hospitalized	Original	Nasopharyngeal and oropharyngeal swab
hCoV-19/Mexico/VER-INER-IMSS-00358/2021	EPI_ISL_1279603	18/02/2021	Veracruz	Human	Male	82	Hospitalized	Original	Nasopharyngeal and oropharyngeal swab
hCoV-19/Mexico/ZAC-INER-IMSS-00089/2021	EPI_ISL_1279374	18/02/2021	Zacatecas	Human	Male	26	Ambulatory	Original	Nasopharyngeal and oropharyngeal swab
hCoV-19/Mexico/ZAC-INER-IMSS-00090/2021	EPI_ISL_1279375	18/02/2021	Zacatecas	Human	Female	40	Ambulatory	Original	Nasopharyngeal and oropharyngeal swab

hCoV-19/Mexico/ZAC-INER-IMSS-00091/2021	EPI_ISL_1279376	18/02/2021	Zacatecas	Human	Female	83	Hospitalized	Original	Nasopharyngeal and oropharyngeal swab
hCoV-19/Mexico/ZAC-INER-IMSS-00092/2021	EPI_ISL_1279377	18/02/2021	Zacatecas	Human	Female	42	Ambulatory	Original	Nasopharyngeal and oropharyngeal swab
hCoV-19/Mexico/ZAC-INER-IMSS-00093/2021	EPI_ISL_1279378	18/02/2021	Zacatecas	Human	Male	40	Ambulatory	Original	Nasopharyngeal and oropharyngeal swab
hCoV-19/Mexico/ZAC-INER-IMSS-00094/2021	EPI_ISL_1279379	18/02/2021	Zacatecas	Human	Female	73	Ambulatory	Original	Nasopharyngeal and oropharyngeal swab
hCoV-19/Mexico/ZAC-INER-IMSS-00095/2021	EPI_ISL_1279380	18/02/2021	Zacatecas	Human	Female	46	Ambulatory	Original	Nasopharyngeal and oropharyngeal swab
hCoV-19/Mexico/ZAC-INER-IMSS-00096/2021	EPI_ISL_1279381	18/02/2021	Zacatecas	Human	Male	54	Ambulatory	Original	Nasopharyngeal and oropharyngeal swab
hCoV-19/Mexico/PUE-INER-IMSS-00122/2021	EPI_ISL_1279404	18/02/2021	Puebla	Human	Male	22	Ambulatory	Original	Nasopharyngeal and oropharyngeal swab
hCoV-19/Mexico/PUE-INER-IMSS-00123/2021	EPI_ISL_1279405	18/02/2021	Puebla	Human	Male	27	Ambulatory	Original	Nasopharyngeal and oropharyngeal swab
hCoV-19/Mexico/PUE-INER-IMSS-00124/2021	EPI_ISL_1279406	18/02/2021	Puebla	Human	Female	44	Ambulatory	Original	Nasopharyngeal and oropharyngeal swab
hCoV-19/Mexico/PUE-INER-IMSS-00145/2021	EPI_ISL_1279424	18/02/2021	Puebla	Human	Female	30	Ambulatory	Original	Nasopharyngeal swab
hCoV-19/Mexico/PUE-INER-IMSS-00146/2021	EPI_ISL_1279425	18/02/2021	Puebla	Human	Male	33	Ambulatory	Original	Nasopharyngeal swab
hCoV-19/Mexico/PUE-INER-IMSS-00147/2021	EPI_ISL_1279426	18/02/2021	Puebla	Human	Female	48	Ambulatory	Original	Nasopharyngeal swab
hCoV-19/Mexico/PUE-INER-IMSS-00152/2021	EPI_ISL_1279430	18/02/2021	Puebla	Human	Male	32	Ambulatory	Original	Nasopharyngeal and oropharyngeal swab
hCoV-19/Mexico/SLP-INER-IMSS-00163/2021	EPI_ISL_1279439	18/02/2021	San Luis Potosi	Human	Male	49	Hospitalized	Original	Nasopharyngeal and oropharyngeal swab
hCoV-19/Mexico/TAM-INER-IMSS-00259/2021	EPI_ISL_1279521	18/02/2021	Tamaulipas	Human	Male	27	Hospitalized	Original	Nasopharyngeal and oropharyngeal swab
hCoV-19/Mexico/NLE-INER-IMSS-00264/2021	EPI_ISL_1279524	18/02/2021	Nuevo Leon	Human	Male	72	Ambulatory	Original	Nasopharyngeal and oropharyngeal swab
hCoV-19/Mexico/DUR-INER-IMSS-00270/2021	EPI_ISL_1279529	18/02/2021	Durango	Human	Male	52	Ambulatory	Original	Nasopharyngeal and oropharyngeal swab
hCoV-19/Mexico/MOR-INER-IMSS-00307/2021	EPI_ISL_1279560	18/02/2021	Morelos	Human	Male	44	Hospitalized	Original	Nasopharyngeal and oropharyngeal swab
hCoV-19/Mexico/QUE-INER-IMSS-00112/2021	EPI_ISL_1279263	19/02/2021	Queretaro	Human	Male	55	Ambulatory	Original	Nasopharyngeal and oropharyngeal swab
hCoV-19/Mexico/PUE-INER-IMSS-00155/2021	EPI_ISL_1279274	19/02/2021	Puebla	Human	Male	33	Ambulatory	Original	Nasopharyngeal and oropharyngeal swab
hCoV-19/Mexico/NLE-INER-IMSS-00266/2021	EPI_ISL_1279275	19/02/2021	Nuevo Leon	Human	Female	69	Hospitalized	Original	Nasopharyngeal swab
hCoV-19/Mexico/NLE-INER-IMSS-00263/2021	EPI_ISL_1279284	19/02/2021	Nuevo Leon	Human	Female	55	Hospitalized	Original	Nasopharyngeal and oropharyngeal swab
hCoV-19/Mexico/VER-INER-IMSS-00292/2021	EPI_ISL_1279288	19/02/2021	Veracruz	Human	Male	55	Hospitalized	Original	Nasopharyngeal and oropharyngeal swab
hCoV-19/Mexico/PUE-INER-IMSS-00135/2021	EPI_ISL_1279289	19/02/2021	Puebla	Human	Male	34	Ambulatory	Original	Nasopharyngeal and oropharyngeal swab
hCoV-19/Mexico/PUE-INER-IMSS-00148/2021	EPI_ISL_1287770	19/02/2021	Puebla	Human	Male	44	Ambulatory	Original	Nasopharyngeal swab
hCoV-19/Mexico/VER-INER-IMSS-00289/2021	EPI_ISL_1287776	19/02/2021	Veracruz	Human	Male	46	Ambulatory	Original	Nasopharyngeal and oropharyngeal swab
hCoV-19/Mexico/TAM_LANGEBIO_IMSS_0024/20	EPI_ISL_1351468	19/02/2021	Yucatan	Human	Male	45	Ambulatory	Original	Pharyngeal and Nasopharyngeal swab
hCoV-19/Mexico/PUE-INER-IMSS-00126/2021	EPI_ISL_1279407	19/02/2021	Puebla	Human	Female	48	Ambulatory	Original	Nasopharyngeal and oropharyngeal swab
hCoV-19/Mexico/PUE-INER-IMSS-00130/2021	EPI_ISL_1279411	19/02/2021	Puebla	Human	Female	52	Ambulatory	Original	Nasopharyngeal and oropharyngeal swab
hCoV-19/Mexico/PUE-INER-IMSS-00131/2021	EPI_ISL_1279412	19/02/2021	Puebla	Human	Female	15	Ambulatory	Original	Nasopharyngeal and oropharyngeal swab
hCoV-19/Mexico/PUE-INER-IMSS-00132/2021	EPI_ISL_1279413	19/02/2021	Puebla	Human	Male	54	Ambulatory	Original	Nasopharyngeal and oropharyngeal swab
hCoV-19/Mexico/PUE-INER-IMSS-00153/2021	EPI_ISL_1279431	19/02/2021	Puebla	Human	Male	43	Ambulatory	Original	Nasopharyngeal and oropharyngeal swab
hCoV-19/Mexico/PUE-INER-IMSS-00154/2021	EPI_ISL_1279432	19/02/2021	Puebla	Human	Male	47	Ambulatory	Original	Nasopharyngeal and oropharyngeal swab
hCoV-19/Mexico/VER-INER-IMSS-00176/2021	EPI_ISL_1279450	19/02/2021	Veracruz	Human	Male	68	Hospitalized	Original	Nasopharyngeal and oropharyngeal swab
hCoV-19/Mexico/AGU-INER-IMSS-00190/2021	EPI_ISL_1279461	19/02/2021	Aguascalientes	Human	Female	36	Ambulatory	Original	Nasopharyngeal and oropharyngeal swab
hCoV-19/Mexico/NLE-INER-IMSS-00265/2021	EPI_ISL_1279525	19/02/2021	Nuevo Leon	Human	Female	57	Hospitalized	Original	Nasopharyngeal and oropharyngeal swab
hCoV-19/Mexico/NLE-INER-IMSS-00267/2021	EPI_ISL_1279526	19/02/2021	Nuevo Leon	Human	Male	70	Ambulatory	Original	Nasopharyngeal and oropharyngeal swab
hCoV-19/Mexico/DUR-INER-IMSS-00271/2021	EPI_ISL_1279530	19/02/2021	Durango	Human	Male	59	Ambulatory	Original	Nasopharyngeal and oropharyngeal swab
hCoV-19/Mexico/PUE-INER-IMSS-00282/2021	EPI_ISL_1279538	19/02/2021	Puebla	Human	Female	21	Ambulatory	Original	Nasopharyngeal and oropharyngeal swab
hCoV-19/Mexico/VER-INER-IMSS-00287/2021	EPI_ISL_1279542	19/02/2021	Veracruz	Human	Male	28	Ambulatory	Original	Nasopharyngeal and oropharyngeal swab
hCoV-19/Mexico/VER-INER-IMSS-00288/2021	EPI_ISL_1279543	19/02/2021	Veracruz	Human	Male	40	Ambulatory	Original	Nasopharyngeal and oropharyngeal swab

hCoV-19/Mexico/VER-INER-IMSS-00290/2021	EPI_ISL_1279544	19/02/2021	Veracruz	Human	Male	22	Ambulatory	Original	Nasopharyngeal and oropharyngeal swab
hCoV-19/Mexico/VER-INER-IMSS-00291/2021	EPI_ISL_1279545	19/02/2021	Veracruz	Human	Male	38	Ambulatory	Original	Nasopharyngeal and oropharyngeal swab
hCoV-19/Mexico/VER-INER-IMSS-00299/2021	EPI_ISL_1279552	19/02/2021	Veracruz	Human	Female	33	Ambulatory	Original	Nasopharyngeal and oropharyngeal swab
hCoV-19/Mexico/VER-INER-IMSS-00300/2021	EPI_ISL_1279553	19/02/2021	Veracruz	Human	Female	49	Ambulatory	Original	Nasopharyngeal and oropharyngeal swab
hCoV-19/Mexico/VER-INER-IMSS-00301/2021	EPI_ISL_1279554	19/02/2021	Veracruz	Human	Male	69	Hospitalized	Original	Nasopharyngeal and oropharyngeal swab
hCoV-19/Mexico/VER-INER-IMSS-00302/2021	EPI_ISL_1279555	19/02/2021	Veracruz	Human	Male	31	Hospitalized	Original	Nasopharyngeal and oropharyngeal swab
hCoV-19/Mexico/VER-INER-IMSS-00303/2021	EPI_ISL_1279556	19/02/2021	Veracruz	Human	Female	45	Ambulatory	Original	Nasopharyngeal and oropharyngeal swab
hCoV-19/Mexico/VER-INER-IMSS-00311/2021	EPI_ISL_1279564	19/02/2021	Veracruz	Human	Female	37	Ambulatory	Original	Nasopharyngeal and oropharyngeal swab
hCoV-19/Mexico/VER-INER-IMSS-00353/2021	EPI_ISL_1279599	19/02/2021	Veracruz	Human	Male	15	Ambulatory	Original	Nasopharyngeal and oropharyngeal swab
hCoV-19/Mexico/PUE_INER_IMSS_00678/2021	EPI_ISL_2091134	19/02/2021	Puebla	Human	Male	38	Ambulatory	Original	Nasopharyngeal and oropharyngeal swab
hCoV-19/Mexico/PUE-INER-IMSS-00120/2021	EPI_ISL_1279268	20/02/2021	Puebla	Human	Male	64	Hospitalized	Original	Nasopharyngeal and oropharyngeal swab
hCoV-19/Mexico/SLP-INER-IMSS-00170/2021	EPI_ISL_1279269	20/02/2021	San Luis Potosi	Human	Male	48	Hospitalized	Original	Nasopharyngeal and oropharyngeal swab
hCoV-19/Mexico/VER-INER-IMSS-00354/2021	EPI_ISL_1279293	20/02/2021	Veracruz	Human	Male	24	Ambulatory	Original	Pharyngeal swab
hCoV-19/Mexico/PUE-INER-IMSS-00137/2021	EPI_ISL_1287769	20/02/2021	Puebla	Human	Male	26	Ambulatory	Original	Nasopharyngeal and oropharyngeal swab
hCoV-19/Mexico/TAM_LANGEBIO_IMSS_0001/20	EPI_ISL_1351416	20/02/2021	Tamaulipas	Human	Female	90	Hospitalized	Original	Pharyngeal and Nasopharyngeal swab
hCoV-19/Mexico/ZAC-INER-IMSS-00097/2021	EPI_ISL_1279382	20/02/2021	Zacatecas	Human	Male	40	Ambulatory	Original	Nasopharyngeal and oropharyngeal swab
hCoV-19/Mexico/QUE-INER-IMSS-00105/2021	EPI_ISL_1279389	20/02/2021	Queretaro	Human	Female	63	Hospitalized	Original	Nasopharyngeal and oropharyngeal swab
hCoV-19/Mexico/QUE-INER-IMSS-00108/2021	EPI_ISL_1279392	20/02/2021	Queretaro	Human	Female	67	Hospitalized	Original	Nasopharyngeal and oropharyngeal swab
hCoV-19/Mexico/QUE-INER-IMSS-00109/2021	EPI_ISL_1279393	20/02/2021	Queretaro	Human	Female	48	Hospitalized	Original	Nasopharyngeal and oropharyngeal swab
hCoV-19/Mexico/PUE-INER-IMSS-00117/2021	EPI_ISL_1279400	20/02/2021	Puebla	Human	Male	77	Hospitalized	Original	Nasopharyngeal and oropharyngeal swab
hCoV-19/Mexico/PUE-INER-IMSS-00118/2021	EPI_ISL_1279401	20/02/2021	Puebla	Human	Male	61	Hospitalized	Original	Nasopharyngeal and oropharyngeal swab
hCoV-19/Mexico/PUE-INER-IMSS-00119/2021	EPI_ISL_1279402	20/02/2021	Puebla	Human	Male	50	Hospitalized	Original	Nasopharyngeal and oropharyngeal swab
hCoV-19/Mexico/PUE-INER-IMSS-00127/2021	EPI_ISL_1279408	20/02/2021	Puebla	Human	Female	72	Ambulatory	Original	Nasopharyngeal and oropharyngeal swab
hCoV-19/Mexico/PUE-INER-IMSS-00128/2021	EPI_ISL_1279409	20/02/2021	Puebla	Human	Male	15	Ambulatory	Original	Nasopharyngeal and oropharyngeal swab
hCoV-19/Mexico/PUE-INER-IMSS-00133/2021	EPI_ISL_1279414	20/02/2021	Puebla	Human	Male	57	Ambulatory	Original	Nasopharyngeal and oropharyngeal swab
hCoV-19/Mexico/PUE-INER-IMSS-00134/2021	EPI_ISL_1279415	20/02/2021	Puebla	Human	Female	23	Ambulatory	Original	Nasopharyngeal and oropharyngeal swab
hCoV-19/Mexico/PUE-INER-IMSS-00136/2021	EPI_ISL_1279416	20/02/2021	Puebla	Human	Male	46	Ambulatory	Original	Nasopharyngeal and oropharyngeal swab
hCoV-19/Mexico/PUE-INER-IMSS-00138/2021	EPI_ISL_1279417	20/02/2021	Puebla	Human	Male	48	Ambulatory	Original	Nasopharyngeal and oropharyngeal swab
hCoV-19/Mexico/PUE-INER-IMSS-00139/2021	EPI_ISL_1279418	20/02/2021	Puebla	Human	Male	32	Ambulatory	Original	Nasopharyngeal and oropharyngeal swab
hCoV-19/Mexico/PUE-INER-IMSS-00150/2021	EPI_ISL_1279428	20/02/2021	Puebla	Human	Male	44	Ambulatory	Original	Nasopharyngeal swab
hCoV-19/Mexico/PUE-INER-IMSS-00151/2021	EPI_ISL_1279429	20/02/2021	Puebla	Human	Female	39	Ambulatory	Original	Nasopharyngeal swab
hCoV-19/Mexico/PUE-INER-IMSS-00156/2021	EPI_ISL_1279433	20/02/2021	Puebla	Human	Male	50	Ambulatory	Original	Nasopharyngeal swab
hCoV-19/Mexico/PUE-INER-IMSS-00157/2021	EPI_ISL_1279434	20/02/2021	Puebla	Human	Male	80	Ambulatory	Original	Nasopharyngeal swab
hCoV-19/Mexico/PUE-INER-IMSS-00158/2021	EPI_ISL_1279435	20/02/2021	Puebla	Human	Female	27	Ambulatory	Original	Nasopharyngeal swab
hCoV-19/Mexico/PUE-INER-IMSS-00159/2021	EPI_ISL_1279436	20/02/2021	Puebla	Human	Male	35	Ambulatory	Original	Nasopharyngeal swab
hCoV-19/Mexico/PUE-INER-IMSS-00160/2021	EPI_ISL_1279437	20/02/2021	Puebla	Human	Male	38	Ambulatory	Original	Nasopharyngeal swab
hCoV-19/Mexico/PUE-INER-IMSS-00161/2021	EPI_ISL_1279438	20/02/2021	Puebla	Human	Male	41	Ambulatory	Original	Nasopharyngeal swab
hCoV-19/Mexico/SLP-INER-IMSS-00171/2021	EPI_ISL_1279445	20/02/2021	San Luis Potosi	Human	Male	78	Hospitalized	Original	Nasopharyngeal and oropharyngeal swab
hCoV-19/Mexico/SLP-INER-IMSS-00172/2021	EPI_ISL_1279446	20/02/2021	San Luis Potosi	Human	Male	65	Hospitalized	Original	Nasopharyngeal and oropharyngeal swab
hCoV-19/Mexico/SLP-INER-IMSS-00173/2021	EPI_ISL_1279447	20/02/2021	San Luis Potosi	Human	Female	53	Hospitalized	Original	Nasopharyngeal and oropharyngeal swab
hCoV-19/Mexico/OAX-INER-IMSS-00182/2021	EPI_ISL_1279453	20/02/2021	Oaxaca	Human	Male	57	Hospitalized	Original	Nasopharyngeal and oropharyngeal swab

hCoV-19/Mexico/OAX-INER-IMSS-00183/2021	EPI_ISL_1279454	20/02/2021	Oaxaca	Human	Female	51	Ambulatory	Original	Nasopharyngeal and oropharyngeal swab
hCoV-19/Mexico/GRO-INER-IMSS-00280/2021	EPI_ISL_1279537	20/02/2021	Guerrero	Human	Female	81	Hospitalized	Original	Nasopharyngeal and oropharyngeal swab
hCoV-19/Mexico/VER-INER-IMSS-00293/2021	EPI_ISL_1279546	20/02/2021	Veracruz	Human	Male	61	Hospitalized	Original	Nasopharyngeal and oropharyngeal swab
hCoV-19/Mexico/VER-INER-IMSS-00294/2021	EPI_ISL_1279547	20/02/2021	Veracruz	Human	Female	92	Hospitalized	Original	Nasopharyngeal and oropharyngeal swab
hCoV-19/Mexico/GRO-INER-IMSS-00304/2021	EPI_ISL_1279557	20/02/2021	Guerrero	Human	Male	47	Ambulatory	Original	Nasopharyngeal and oropharyngeal swab
hCoV-19/Mexico/PUE-INER-IMSS-00115/2021	EPI_ISL_1279398	20/02/2021	Puebla	Human	Female	85	Hospitalized	Original	Nasopharyngeal and oropharyngeal swab
hCoV-19/Mexico/PUE-INER-IMSS-00116/2021	EPI_ISL_1279399	20/02/2021	Puebla	Human	Female	60	Hospitalized	Original	Nasopharyngeal and oropharyngeal swab
hCoV-19/Mexico/SLP-INER-IMSS-00169/2021	EPI_ISL_1279283	21/02/2021	San Luis Potosi	Human	Male	84	Hospitalized	Original	Nasopharyngeal and oropharyngeal swab
hCoV-19/Mexico/GRO-INER-IMSS-00281/2021	EPI_ISL_1287774	21/02/2021	Guerrero	Human	Male	91	Hospitalized	Original	Pharyngeal swab
hCoV-19/Mexico/TAM_LANGEBIO_IMSS_0023/20	EPI_ISL_1351465	21/02/2021	Yucatan	Human	Male	75	Hospitalized	Original	Pharyngeal and Nasopharyngeal swab
hCoV-19/Mexico/TAM_LANGEBIO_IMSS_0026/20	EPI_ISL_1351474	21/02/2021	Quintana Roo	Human	Male	55	Hospitalized	Original	Pharyngeal and Nasopharyngeal swab
hCoV-19/Mexico/TAM_LANGEBIO_IMSS_0027/20	EPI_ISL_1351477	21/02/2021	Quintana Roo	Human	Female	66	Hospitalized	Original	Pharyngeal and Nasopharyngeal swab
hCoV-19/Mexico/TAM_LANGEBIO_IMSS_0029/20	EPI_ISL_1351483	21/02/2021	Quintana Roo	Human	Female	39	Hospitalized	Original	Pharyngeal and Nasopharyngeal swab
hCoV-19/Mexico/QUE-INER-IMSS-00110/2021	EPI_ISL_1279394	21/02/2021	Queretaro	Human	Male	97	Hospitalized	Original	Nasopharyngeal and oropharyngeal swab
hCoV-19/Mexico/PUE-INER-IMSS-00129/2021	EPI_ISL_1279410	21/02/2021	Puebla	Human	Male	36	Ambulatory	Original	Nasopharyngeal and oropharyngeal swab
hCoV-19/Mexico/OAX-INER-IMSS-00165/2021	EPI_ISL_1279441	21/02/2021	Oaxaca	Human	Male	48	Hospitalized	Original	Nasopharyngeal and oropharyngeal swab
hCoV-19/Mexico/OAX-INER-IMSS-00166/2021	EPI_ISL_1279442	21/02/2021	Oaxaca	Human	Female	23	Ambulatory	Original	Nasopharyngeal and oropharyngeal swab
hCoV-19/Mexico/OAX-INER-IMSS-00167/2021	EPI_ISL_1279443	21/02/2021	Oaxaca	Human	Male	28	Hospitalized	Original	Nasopharyngeal and oropharyngeal swab
hCoV-19/Mexico/SLP-INER-IMSS-00168/2021	EPI_ISL_1279444	21/02/2021	San Luis Potosi	Human	Female	50	Hospitalized	Original	Nasopharyngeal and oropharyngeal swab
hCoV-19/Mexico/VER-INER-IMSS-00295/2021	EPI_ISL_1279548	21/02/2021	Veracruz	Human	Male	83	Hospitalized	Original	Nasopharyngeal and oropharyngeal swab
hCoV-19/Mexico/VER-INER-IMSS-00296/2021	EPI_ISL_1279549	21/02/2021	Veracruz	Human	Male	86	Hospitalized	Original	Nasopharyngeal and oropharyngeal swab
hCoV-19/Mexico/VER-INER-IMSS-00297/2021	EPI_ISL_1279550	21/02/2021	Veracruz	Human	Male	61	Hospitalized	Original	Nasopharyngeal and oropharyngeal swab
hCoV-19/Mexico/VER-INER-IMSS-00298/2021	EPI_ISL_1279551	21/02/2021	Veracruz	Human	Male	40	Hospitalized	Original	Nasopharyngeal and oropharyngeal swab
hCoV-19/Mexico/MOR-INER-IMSS-00305/2021	EPI_ISL_1279558	21/02/2021	Morelos	Human	Male	83	Hospitalized	Original	Nasopharyngeal and oropharyngeal swab
hCoV-19/Mexico/QUE-INER-IMSS-00111/2021	EPI_ISL_1279395	21/02/2021	Queretaro	Human	Female	53	Hospitalized	Original	Nasopharyngeal and oropharyngeal swab
hCoV-19/Mexico/VER-INER-IMSS-00318/2021	EPI_ISL_1279277	22/02/2021	Veracruz	Human	Female	71	Hospitalized	Original	Nasopharyngeal and oropharyngeal swab
hCoV-19/Mexico/VER-INER-IMSS-00319/2021	EPI_ISL_1279278	22/02/2021	Veracruz	Human	Male	53	Hospitalized	Original	Nasopharyngeal and oropharyngeal swab
hCoV-19/Mexico/CMX-INER-IMSS-00179/2021	EPI_ISL_1279290	22/02/2021	Mexico City	Human	Female	54	Hospitalized	Original	Pharyngeal swab
hCoV-19/Mexico/CMX-INER-IMSS-00180/2021	EPI_ISL_1279291	22/02/2021	Mexico City	Human	Male	81	Hospitalized	Original	Pharyngeal swab
hCoV-19/Mexico/CMX-INER-IMSS-00181/2021	EPI_ISL_1279292	22/02/2021	Mexico City	Human	Male	77	Hospitalized	Original	Pharyngeal swab
hCoV-19/Mexico/VER-INER-IMSS-00313/2021	EPI_ISL_1279296	22/02/2021	Veracruz	Human	Male	71	Hospitalized	Original	Nasopharyngeal and oropharyngeal swab
hCoV-19/Mexico/MEX-INER-IMSS-00324/2021	EPI_ISL_1279297	22/02/2021	State of Mexico	Human	Female	85	Hospitalized	Original	Nasopharyngeal and oropharyngeal swab
hCoV-19/Mexico/TLA-INER-IMSS-00283/2021	EPI_ISL_1287775	22/02/2021	Tlaxcala	Human	Male	63	Hospitalized	Original	Nasopharyngeal and oropharyngeal swab
hCoV-19/Mexico/TAM_LANGEBIO_IMSS_0002/20	EPI_ISL_1351418	22/02/2021	Nuevo Leon	Human	Female	41	Ambulatory	Original	Pharyngeal and Nasopharyngeal swab
hCoV-19/Mexico/TAM_LANGEBIO_IMSS_0003/20	EPI_ISL_1351420	22/02/2021	Nuevo Leon	Human	Female	42	Ambulatory	Original	Pharyngeal and Nasopharyngeal swab
hCoV-19/Mexico/TAM_LANGEBIO_IMSS_0004/20	EPI_ISL_1351422	22/02/2021	Nuevo Leon	Human	Male	54	Hospitalized	Original	Pharyngeal and Nasopharyngeal swab
hCoV-19/Mexico/TAM_LANGEBIO_IMSS_0006/20	EPI_ISL_1351424	22/02/2021	Nuevo Leon	Human	Male	19	Ambulatory	Original	Pharyngeal and Nasopharyngeal swab
hCoV-19/Mexico/TAM_LANGEBIO_IMSS_0008/20	EPI_ISL_1351428	22/02/2021	Coahuila	Human	Female	28	Ambulatory	Original	Pharyngeal and Nasopharyngeal swab
hCoV-19/Mexico/TAM_LANGEBIO_IMSS_0031/20	EPI_ISL_1351489	22/02/2021	Quintana Roo	Human	Female	36	Ambulatory	Original	Pharyngeal and Nasopharyngeal swab
hCoV-19/Mexico/TAM_LANGEBIO_IMSS_0033/20	EPI_ISL_1351494	22/02/2021	Tabasco	Human	Female	37	Ambulatory	Original	Pharyngeal and Nasopharyngeal swab
hCoV-19/Mexico/TAM_LANGEBIO_IMSS_0034/20	EPI_ISL_1351498	22/02/2021	Tabasco	Human	Male	33	Ambulatory	Original	Pharyngeal and Nasopharyngeal swab

hCoV-19/Mexico/BCS_INER_IMSS_00482/2021	EPI_ISL_1595603	22/02/2021	Baja California Sur	Human	Female	72	Hospitalized	Original	Nasopharyngeal and oropharyngeal swab
hCoV-19/Mexico/MEX-INER-IMSS-00098/2021	EPI_ISL_1279383	22/02/2021	State of Mexico	Human	Male	61	Ambulatory	Original	Nasopharyngeal and oropharyngeal swab
hCoV-19/Mexico/MEX-INER-IMSS-00100/2021	EPI_ISL_1279384	22/02/2021	State of Mexico	Human	Male	77	Hospitalized	Original	Pharyngeal swab
hCoV-19/Mexico/MEX-INER-IMSS-00101/2021	EPI_ISL_1279385	22/02/2021	State of Mexico	Human	Male	24	Ambulatory	Original	Nasopharyngeal and oropharyngeal swab
hCoV-19/Mexico/CMX-INER-IMSS-00102/2021	EPI_ISL_1279386	22/02/2021	Mexico City	Human	Female	49	Ambulatory	Original	Pharyngeal swab
hCoV-19/Mexico/MEX-INER-IMSS-00103/2021	EPI_ISL_1279387	22/02/2021	State of Mexico	Human	Female	31	Hospitalized	Original	Nasopharyngeal and oropharyngeal swab
hCoV-19/Mexico/MEX-INER-IMSS-00104/2021	EPI_ISL_1279388	22/02/2021	State of Mexico	Human	Male	66	Hospitalized	Original	Nasopharyngeal and oropharyngeal swab
hCoV-19/Mexico/MEX-INER-IMSS-00106/2021	EPI_ISL_1279390	22/02/2021	State of Mexico	Human	Female	64	Hospitalized	Original	Nasopharyngeal and oropharyngeal swab
hCoV-19/Mexico/MEX-INER-IMSS-00107/2021	EPI_ISL_1279391	22/02/2021	State of Mexico	Human	Female	56	Hospitalized	Original	Nasopharyngeal and oropharyngeal swab
hCoV-19/Mexico/MOR-INER-IMSS-00177/2021	EPI_ISL_1279451	22/02/2021	Morelos	Human	Male	76	Hospitalized	Original	Nasopharyngeal and oropharyngeal swab
hCoV-19/Mexico/CMX-INER-IMSS-00178/2021	EPI_ISL_1279452	22/02/2021	Mexico City	Human	Female	33	Hospitalized	Original	Pharyngeal swab
hCoV-19/Mexico/TLA-INER-IMSS-00284/2021	EPI_ISL_1279539	22/02/2021	Tlaxcala	Human	Male	62	Hospitalized	Original	Nasopharyngeal and oropharyngeal swab
hCoV-19/Mexico/MEX-INER-IMSS-00306/2021	EPI_ISL_1279559	22/02/2021	State of Mexico	Human	Female	34	Hospitalized	Original	Nasopharyngeal and oropharyngeal swab
hCoV-19/Mexico/MEX-INER-IMSS-00308/2021	EPI_ISL_1279561	22/02/2021	State of Mexico	Human	Female	82	Ambulatory	Original	Nasopharyngeal and oropharyngeal swab
hCoV-19/Mexico/MEX-INER-IMSS-00309/2021	EPI_ISL_1279562	22/02/2021	State of Mexico	Human	Female	63	Hospitalized	Original	Nasopharyngeal and oropharyngeal swab
hCoV-19/Mexico/MEX-INER-IMSS-00310/2021	EPI_ISL_1279563	22/02/2021	State of Mexico	Human	Female	70	Hospitalized	Original	Nasopharyngeal and oropharyngeal swab
hCoV-19/Mexico/OAX-INER-IMSS-00312/2021	EPI_ISL_1279565	22/02/2021	Oaxaca	Human	Female	35	Ambulatory	Original	Nasopharyngeal and oropharyngeal swab
hCoV-19/Mexico/VER-INER-IMSS-00314/2021	EPI_ISL_1279566	22/02/2021	Veracruz	Human	Female	82	Hospitalized	Original	Nasopharyngeal and oropharyngeal swab
hCoV-19/Mexico/VER-INER-IMSS-00315/2021	EPI_ISL_1279567	22/02/2021	Veracruz	Human	Female	74	Hospitalized	Original	Nasopharyngeal and oropharyngeal swab
hCoV-19/Mexico/VER-INER-IMSS-00316/2021	EPI_ISL_1279568	22/02/2021	Veracruz	Human	Male	55	Hospitalized	Original	Nasopharyngeal and oropharyngeal swab
hCoV-19/Mexico/VER-INER-IMSS-00317/2021	EPI_ISL_1279569	22/02/2021	Veracruz	Human	Female	65	Hospitalized	Original	Nasopharyngeal and oropharyngeal swab
hCoV-19/Mexico/MEX-INER-IMSS-00321/2021	EPI_ISL_1279571	22/02/2021	State of Mexico	Human	Female	59	Ambulatory	Original	Nasopharyngeal and oropharyngeal swab
hCoV-19/Mexico/HID-INER-IMSS-00330/2021	EPI_ISL_1279578	22/02/2021	Hidalgo	Human	Female	37	Hospitalized	Original	Nasopharyngeal and oropharyngeal swab
hCoV-19/Mexico/HID-INER-IMSS-00332/2021	EPI_ISL_1279580	22/02/2021	Hidalgo	Human	Male	94	Hospitalized	Original	Nasopharyngeal and oropharyngeal swab
hCoV-19/Mexico/MOR-INER-IMSS-00333/2021	EPI_ISL_1279581	22/02/2021	Morelos	Human	Male	67	Ambulatory	Original	Nasopharyngeal and oropharyngeal swab
hCoV-19/Mexico/CMX-INER-IMSS-00334/2021	EPI_ISL_1279582	22/02/2021	Mexico City	Human	Female	37	Ambulatory	Original	Nasopharyngeal and oropharyngeal swab
hCoV-19/Mexico/MEX-INER-IMSS-00335/2021	EPI_ISL_1279583	22/02/2021	State of Mexico	Human	Male	25	Ambulatory	Original	Nasopharyngeal and oropharyngeal swab
hCoV-19/Mexico/OAX-INER-IMSS-00343/2021	EPI_ISL_1279589	22/02/2021	Oaxaca	Human	Male	62	Ambulatory	Original	Nasopharyngeal and oropharyngeal swab
hCoV-19/Mexico/OAX-INER-IMSS-00344/2021	EPI_ISL_1279590	22/02/2021	Oaxaca	Human	Female	60	Hospitalized	Original	Nasopharyngeal and oropharyngeal swab
hCoV-19/Mexico/VER-INER-IMSS-00345/2021	EPI_ISL_1279591	22/02/2021	Veracruz	Human	Male	78	Hospitalized	Original	Nasopharyngeal and oropharyngeal swab
hCoV-19/Mexico/VER-INER-IMSS-00347/2021	EPI_ISL_1279593	22/02/2021	Veracruz	Human	Female	59	Hospitalized	Original	Nasopharyngeal and oropharyngeal swab
hCoV-19/Mexico/VER-INER-IMSS-00348/2021	EPI_ISL_1279594	22/02/2021	Veracruz	Human	Male	43	Hospitalized	Original	Nasopharyngeal and oropharyngeal swab
hCoV-19/Mexico/MEX-INER-IMSS-00113/2021	EPI_ISL_1279396	22/02/2021	State of Mexico	Human	Male	50	Hospitalized	Original	Nasopharyngeal and oropharyngeal swab
hCoV-19/Mexico/HID-INER-IMSS-00114/2021	EPI_ISL_1279397	22/02/2021	Hidalgo	Human	Male	1	Ambulatory	Original	Nasopharyngeal swab
hCoV-19/Mexico/CMX_INER_IMSS_00677/2021	EPI_ISL_2091133	22/02/2021	Mexico City	Human	Male	17	Ambulatory	Original	Oropharyngeal swab
hCoV-19/Mexico/SLP-INER-IMSS-00337/2021	EPI_ISL_1279299	23/02/2021	San Luis Potosi	Human	Female	73	Hospitalized	Original	Nasopharyngeal and oropharyngeal swab
hCoV-19/Mexico/MEX-INER-IMSS-00328/2021	EPI_ISL_1287777	23/02/2021	State of Mexico	Human	Female	41	Hospitalized	Original	Nasopharyngeal and oropharyngeal swab
hCoV-19/Mexico/CMX-INER-IMSS-00355/2021	EPI_ISL_1279600	23/02/2021	Mexico City	Human	Female	62	Hospitalized	Original	Pharyngeal swab
hCoV-19/Mexico/TAM_LANGEBIO_IMSS_0011/20	EPI_ISL_1351435	23/02/2021	Chihuahua	Human	Male	66	Ambulatory	Original	Pharyngeal and Nasopharyngeal swab
hCoV-19/Mexico/TAM_LANGEBIO_IMSS_0025/20	EPI_ISL_1351471	23/02/2021	Yucatan	Human	Male	49	Hospitalized	Original	Pharyngeal and Nasopharyngeal swab
hCoV-19/Mexico/TAM_LANGEBIO_IMSS_0028/20	EPI_ISL_1351480	23/02/2021	Yucatan	Human	Male	37	Ambulatory	Original	Pharyngeal and Nasopharyngeal swab

hCoV-19/Mexico/TAM_LANGEBIO_IMSS_0131/20	EPI_ISL_1351793	23/02/2021	State of Mexico	Human	Male	46	Hospitalized	Original	Pharyngeal and Nasopharyngeal swab
hCoV-19/Mexico/CMX-INER-IMSS-00184/2021	EPI_ISL_1279455	23/02/2021	Mexico City	Human	Female	54	Hospitalized	Original	Nasopharyngeal and oropharyngeal swab
hCoV-19/Mexico/CMX-INER-IMSS-00185/2021	EPI_ISL_1279456	23/02/2021	Mexico City	Human	Male	62	Hospitalized	Original	Pharyngeal swab
hCoV-19/Mexico/CMX-INER-IMSS-00186/2021	EPI_ISL_1279457	23/02/2021	Mexico City	Human	Female	72	Hospitalized	Original	Pharyngeal swab
hCoV-19/Mexico/MEX-INER-IMSS-00187/2021	EPI_ISL_1279458	23/02/2021	State of Mexico	Human	Female	58	Ambulatory	Original	Pharyngeal swab
hCoV-19/Mexico/CMX-INER-IMSS-00188/2021	EPI_ISL_1279459	23/02/2021	Mexico City	Human	Male	39	Ambulatory	Original	Pharyngeal swab
hCoV-19/Mexico/CMX-INER-IMSS-00189/2021	EPI_ISL_1279460	23/02/2021	Mexico City	Human	Female	100	Hospitalized	Original	Pharyngeal swab
hCoV-19/Mexico/TLA-INER-IMSS-00285/2021	EPI_ISL_1279540	23/02/2021	Tlaxcala	Human	Male	79	Hospitalized	Original	Nasopharyngeal and oropharyngeal swab
hCoV-19/Mexico/MEX-INER-IMSS-00320/2021	EPI_ISL_1279570	23/02/2021	State of Mexico	Human	Female	48	Hospitalized	Original	Pharyngeal swab
hCoV-19/Mexico/MEX-INER-IMSS-00322/2021	EPI_ISL_1279572	23/02/2021	State of Mexico	Human	Female	32	Hospitalized	Original	Pharyngeal swab
hCoV-19/Mexico/MEX-INER-IMSS-00323/2021	EPI_ISL_1279573	23/02/2021	State of Mexico	Human	Male	45	Ambulatory	Original	Nasopharyngeal and oropharyngeal swab
hCoV-19/Mexico/MEX-INER-IMSS-00325/2021	EPI_ISL_1279574	23/02/2021	State of Mexico	Human	Male	27	Ambulatory	Original	Nasopharyngeal and oropharyngeal swab
hCoV-19/Mexico/MEX-INER-IMSS-00326/2021	EPI_ISL_1279575	23/02/2021	State of Mexico	Human	Male	69	Hospitalized	Original	Nasopharyngeal and oropharyngeal swab
hCoV-19/Mexico/MEX-INER-IMSS-00327/2021	EPI_ISL_1279576	23/02/2021	State of Mexico	Human	Male	48	Hospitalized	Original	Nasopharyngeal and oropharyngeal swab
hCoV-19/Mexico/MEX-INER-IMSS-00329/2021	EPI_ISL_1279577	23/02/2021	State of Mexico	Human	Male	47	Hospitalized	Original	Pharyngeal swab
hCoV-19/Mexico/SLP-INER-IMSS-00331/2021	EPI_ISL_1279579	23/02/2021	San Luis Potosi	Human	Female	69	Hospitalized	Original	Nasopharyngeal and oropharyngeal swab
hCoV-19/Mexico/SLP-INER-IMSS-00336/2021	EPI_ISL_1279584	23/02/2021	San Luis Potosi	Human	Female	40	Hospitalized	Original	Nasopharyngeal and oropharyngeal swab
hCoV-19/Mexico/MEX-INER-IMSS-00338/2021	EPI_ISL_1279585	23/02/2021	State of Mexico	Human	Male	70	Ambulatory	Original	Nasopharyngeal and oropharyngeal swab
hCoV-19/Mexico/MEX-INER-IMSS-00339/2021	EPI_ISL_1279586	23/02/2021	State of Mexico	Human	Male	52	Ambulatory	Original	Nasopharyngeal and oropharyngeal swab
hCoV-19/Mexico/VER-INER-IMSS-00346/2021	EPI_ISL_1279592	23/02/2021	Veracruz	Human	Female	37	Ambulatory	Original	Nasopharyngeal and oropharyngeal swab
hCoV-19/Mexico/MEX-INER-IMSS-00351/2021	EPI_ISL_1279597	23/02/2021	State of Mexico	Human	Female	40	Hospitalized	Original	Pharyngeal swab
hCoV-19/Mexico/PUE-IBT-IMSS-149/2021	EPI_ISL_1288351	23/02/2021	Puebla	Human	Male	18	Ambulatory	Original	Nasopharyngeal and oropharyngeal swab
hCoV-19/Mexico/MEX-INER-IMSS-00340/2021	EPI_ISL_1279303	23/02/2021	State of Mexico	Human	Male	40	Ambulatory	Original	Nasopharyngeal and oropharyngeal swab
hCoV-19/Mexico/MEX-INER-IMSS-00356/2021	EPI_ISL_1279601	24/02/2021	State of Mexico	Human	Female	49	Hospitalized	Original	Pharyngeal swab
hCoV-19/Mexico/TAM_LANGEBIO_IMSS_0009/20	EPI_ISL_1351430	24/02/2021	Nuevo Leon	Human	Female	24	Ambulatory	Original	Pharyngeal and Nasopharyngeal swab
hCoV-19/Mexico/TAM_LANGEBIO_IMSS_0030/20	EPI_ISL_1351486	24/02/2021	Yucatan	Human	Male	29	Ambulatory	Original	Pharyngeal and Nasopharyngeal swab
hCoV-19/Mexico/TAM_LANGEBIO_IMSS_0040/20	EPI_ISL_1351514	24/02/2021	Jalisco	Human	Male	67	Hospitalized	Original	Nasopharyngeal swab
hCoV-19/Mexico/CMX-INER-IMSS-00341/2021	EPI_ISL_1279587	24/02/2021	Mexico City	Human	Female	89	Hospitalized	Original	Pharyngeal swab
hCoV-19/Mexico/CMX-INER-IMSS-00342/2021	EPI_ISL_1279588	24/02/2021	Mexico City	Human	Female	43	Ambulatory	Original	Nasopharyngeal and oropharyngeal swab
hCoV-19/Mexico/CMX-INER-IMSS-00349/2021	EPI_ISL_1279595	24/02/2021	Mexico City	Human	Male	5	Ambulatory	Original	Nasopharyngeal and oropharyngeal swab
hCoV-19/Mexico/CMX-INER-IMSS-00350/2021	EPI_ISL_1279596	24/02/2021	Mexico City	Human	Female	38	Hospitalized	Original	Nasopharyngeal and oropharyngeal swab
hCoV-19/Mexico/CMX-INER-IMSS-00352/2021	EPI_ISL_1279598	24/02/2021	Mexico City	Human	Male	37	Hospitalized	Original	Nasopharyngeal and oropharyngeal swab
hCoV-19/Mexico/CMX_INER_IMSS_00681/2021	EPI_ISL_2091137	24/02/2021	Mexico City	Human	Female	33	Ambulatory	Original	Nasopharyngeal and oropharyngeal swab
hCoV-19/Mexico/TAM_LANGEBIO_IMSS_0010/20	EPI_ISL_1351433	25/02/2021	Tamaulipas	Human	Female	24	Ambulatory	Original	Pharyngeal and Nasopharyngeal swab
hCoV-19/Mexico/TAM_LANGEBIO_IMSS_0032/20	EPI_ISL_1351492	25/02/2021	Yucatan	Human	Male	75	Hospitalized	Original	Pharyngeal and Nasopharyngeal swab
hCoV-19/Mexico/CHH_INER_IMSS_00375/2021	EPI_ISL_1585387	25/02/2021	Chihuahua	Human	Male	48	Hospitalized	Original	Nasopharyngeal and oropharyngeal swab
hCoV-19/Mexico/MOR-IBT-IMSS-168/2021	EPI_ISL_1288369	25/02/2021	Morelos	Human	Male	72	Ambulatory	Original	Nasopharyngeal and oropharyngeal swab
hCoV-19/Mexico/TAM_LANGEBIO_IMSS_0012/20	EPI_ISL_1351437	26/02/2021	Durango	Human	Male	53	Ambulatory	Original	Pharyngeal and Nasopharyngeal swab
hCoV-19/Mexico/TAM_LANGEBIO_IMSS_0043/20	EPI_ISL_1351523	27/02/2021	Guanajuato	Human	Male	69	Hospitalized	Original	Pharyngeal and Nasopharyngeal swab
hCoV-19/Mexico/TAM_LANGEBIO_IMSS_0053/20	EPI_ISL_1351552	27/02/2021	Sonora	Human	Male	71	Hospitalized	Original	Pharyngeal and Nasopharyngeal swab
hCoV-19/Mexico/TAM_LANGEBIO_IMSS_0013/20	EPI_ISL_1351439	28/02/2021	Coahuila	Human	Male	52	Hospitalized	Original	Pharyngeal and Nasopharyngeal swab

hCoV-19/Mexico/TAM_LANGEBIO_IMSS_0036/20	EPI_ISL_1351503	28/02/2021	Quintana Roo	Human	Male	70	Ambulatory	Original	Pharyngeal and Nasopharyngeal swab
hCoV-19/Mexico/TAM_LANGEBIO_IMSS_0051/20	EPI_ISL_1351547	28/02/2021	Sinaloa	Human	Female	29	Ambulatory	Original	Pharyngeal and Nasopharyngeal swab
hCoV-19/Mexico/TAM_LANGEBIO_IMSS_0052/20	EPI_ISL_1351549	28/02/2021	Sonora	Human	Male	68	Hospitalized	Original	Pharyngeal swab
hCoV-19/Mexico/ZAC_LANGEBIO_IMSS_0262/20	EPI_ISL_1381257	28/02/2021	Zacatecas	Human	Male	43	Ambulatory	Original	Pharyngeal / Nasopharyngeal swab
hCoV-19/Mexico/CHH_INER_IMSS_00376/2021	EPI_ISL_1595595	28/02/2021	Chihuahua	Human	Male	41	Hospitalized	Original	Nasopharyngeal and oropharyngeal swab
hCoV-19/Mexico/AGU-InDRE-IBT-2320/NC/2020		09/04/2020	Aguascalientes	Human	Male	unknown	unknown	Original	Pharyngeal swab
hCoV-19/Mexico/AGU-InDRE-IBT-308408/NC/2020		22/12/2020	Aguascalientes	Human	Female	26	Ambulatory	Original	Pharyngeal swab
hCoV-19/Mexico/AGU-InDRE-IBT-308412/NC/2020		23/12/2020	Aguascalientes	Human	Female	36	Ambulatory	Original	Pharyngeal swab
hCoV-19/Mexico/AGU-InDRE-IBT-308472/NC/2020		22/12/2020	Aguascalientes	Human	Female	34	Ambulatory	Original	Pharyngeal swab
hCoV-19/Mexico/AGU-InDRE-IBT-308483/NC/2020		21/12/2020	Aguascalientes	Human	Female	23	Ambulatory	Original	Pharyngeal swab
hCoV-19/Mexico/AGU-InDRE-IBT-308486/NC/2020		23/12/2020	Aguascalientes	Human	Male	54	Ambulatory	Original	Pharyngeal swab
hCoV-19/Mexico/AGU-InDRE-IBT-308487/NC/2020		22/12/2020	Aguascalientes	Human	Female	23	Ambulatory	Original	Pharyngeal swab
hCoV-19/Mexico/BCN-InDRE-IBT-31425/NC/2020		16/04/2020	Baja California	Human	Female	unknown	unknown	Original	NA
hCoV-19/Mexico/CHH_LANGEBIO_IMSS_36471/NC/2021		17/02/2021	Chihuahua	Human	Female	64	Hospitalized	Original	Pharyngeal /Nasopharyngeal swab
hCoV-19/Mexico/CHH_LANGEBIO_IMSS_36483/NC/2021		20/02/2021	Chihuahua	Human	Male	69	Hospitalized	Original	Pharyngeal /Nasopharyngeal swab
hCoV-19/Mexico/CHH_LANGEBIO_IMSS_37309/NC/2021		25/02/2021	Chihuahua	Human	Male	58	Hospitalized	Original	Pharyngeal /Nasopharyngeal swab
hCoV-19/Mexico/CHH-InDRE-IBT-32583/NC/2020		03/07/2020	Chihuahua	Human	Female	50	Ambulatory	Original	Pharyngeal swab
hCoV-19/Mexico/CHP-IBT-IMSS-18228/NC/2020		20/04/2020	Chiapas	Human	Male	38	Ambulatory	Original	Pharyngeal / Nasopharyngeal swab
hCoV-19/Mexico/CHP-InDRE-IBT-24337/NC/2020		05/06/2020	Chiapas	Human	Male	75	Ambulatory	Original	NA
hCoV-19/Mexico/CHP-InDRE-IBT-308232/NC/2020		23/12/2020	Chiapas	Human	Female	48	Ambulatory	Original	Pharyngeal swab
hCoV-19/Mexico/CHP-InDRE-IBT-308281/NC/2020		23/12/2020	Chiapas	Human	Male	51	Hospitalized	Original	Pharyngeal swab
hCoV-19/Mexico/CHP-InDRE-IBT-308290/NC/2021		05/01/2021	Chiapas	Human	Female	31	Ambulatory	Original	Pharyngeal swab
hCoV-19/Mexico/CHP-InDRE-IBT-308298/NC/2020		22/12/2020	Chiapas	Human	Female	24	Ambulatory	Original	Pharyngeal swab
hCoV-19/Mexico/CHP-InDRE-IBT-308302/NC/2020		22/12/2020	Chiapas	Human	Male	35	Ambulatory	Original	Pharyngeal swab
hCoV-19/Mexico/CHP-InDRE-IBT-308303/NC/2020		21/12/2020	Chiapas	Human	Male	33	Ambulatory	Original	Pharyngeal swab
hCoV-19/Mexico/CHP-InDRE-IBT-308305/NC/2020		21/12/2020	Chiapas	Human	Female	27	Ambulatory	Original	Pharyngeal swab
hCoV-19/Mexico/CHP-InDRE-IBT-308309/NC/2020		22/12/2020	Chiapas	Human	Male	24	Ambulatory	Original	Pharyngeal swab
hCoV-19/Mexico/CMX-IBT-IMSS-46932/NC/2021		29/01/2021	Mexico City	Human	Female	75	Hospitalized	Original	Pharyngeal swab / Nasopharyngeal swab
hCoV-19/Mexico/CMX-IBT-IMSS-46934/NC/2021		29/01/2021	Mexico City	Human	Male	24	Hospitalized	Original	Pharyngeal swab / Nasopharyngeal swab
hCoV-19/Mexico/CMX-IBT-IMSS-47488/NC/2021		31/01/2021	Mexico City	Human	Male	48	Hospitalized	Original	Pharyngeal swab / Nasopharyngeal swab
hCoV-19/Mexico/CMX-IBT-IMSS-47872/NC/2021		01/02/2021	Mexico City	Human	Male	51	Hospitalized	Original	Pharyngeal swab / Nasopharyngeal swab
hCoV-19/Mexico/CMX-IBT-IMSS-47881/NC/2021		02/02/2021	Mexico City	Human	Female	73	Hospitalized	Original	Pharyngeal swab / Nasopharyngeal swab
hCoV-19/Mexico/CMX-IBT-IMSS-48574/NC/2021		02/02/2021	Mexico City	Human	Female	91	Hospitalized	Original	Pharyngeal swab / Nasopharyngeal swab
hCoV-19/Mexico/CMX-IBT-IMSS-48576/NC/2021		03/02/2021	Mexico City	Human	Male	80	Hospitalized	Original	Pharyngeal swab / Nasopharyngeal swab
hCoV-19/Mexico/CMX-IBT-IMSS-48587/NC/2021		02/02/2021	Mexico City	Human	Male	60	Hospitalized	Original	Pharyngeal swab / Nasopharyngeal swab
hCoV-19/Mexico/CMX-InDRE-IBT-37541/NC/2020		24/07/2020	Mexico City	Human	Female	74	Hospitalized	Original	Nasopharyngeal swab
hCoV-19/Mexico/CMX-INER-IBT-10/NC/2020		13/11/2020	Mexico City	Human	Male	unknown	Hospitalized	Original	Pharyngeal swab / Nasopharyngeal swab
hCoV-19/Mexico/CMX-INER-IBT-11/NC/2020		13/11/2020	Mexico City	Human	Male	unknown	Hospitalized	Original	Pharyngeal swab / Nasopharyngeal swab
hCoV-19/Mexico/CMX-INER-IBT-12/NC/2020		14/11/2020	Mexico City	Human	Male	unknown	Hospitalized	Original	Pharyngeal swab / Nasopharyngeal swab
hCoV-19/Mexico/CMX-INER-IBT-14/NC/2020		16/11/2020	Mexico City	Human	Female	unknown	Hospitalized	Original	Pharyngeal swab / Nasopharyngeal swab
hCoV-19/Mexico/CMX-INER-IBT-15/NC/2020		16/11/2020	Mexico City	Human	Female	unknown	Hospitalized	Original	Pharyngeal swab / Nasopharyngeal swab

hCoV-19/Mexico/CMX-INER-IBT-157/NC/2020	14/04/2020	Mexico City	Human	Female	unknown	Hospitalized	Original	Nasopharyngeal swab
hCoV-19/Mexico/CMX-INER-IBT-177/NC/2020	09/12/2020	Mexico City	Human	Male	unknown	Hospitalized	Original	Pharyngeal swab / Nasopharyngeal swab
hCoV-19/Mexico/CMX-INER-IBT-183/NC/2020	29/12/2020	Mexico City	Human	Male	unknown	Hospitalized	Original	Pharyngeal swab / Nasopharyngeal swab
hCoV-19/Mexico/CMX-INER-IBT-185/NC/2020	25/12/2020	Mexico City	Human	Male	unknown	Hospitalized	Original	Pharyngeal swab / Nasopharyngeal swab
hCoV-19/Mexico/CMX-INER-IBT-2/NC/2020	15/11/2020	Mexico City	Human	Female	unknown	Hospitalized	Original	Pharyngeal swab / Nasopharyngeal swab
hCoV-19/Mexico/CMX-INER-IBT-21/NC/2020	19/11/2020	Mexico City	Human	Female	unknown	Hospitalized	Original	Pharyngeal swab / Nasopharyngeal swab
hCoV-19/Mexico/CMX-INER-IBT-227/NC/2021	05/01/2021	Mexico City	Human	Female	unknown	Hospitalized	Original	Pharyngeal swab / Nasopharyngeal swab
hCoV-19/Mexico/CMX-INER-IBT-2321/NC/2020	05/08/2020	Mexico City	Human	Male	unknown	Hospitalized	Original	Pharyngeal swab / Nasopharyngeal swab
hCoV-19/Mexico/CMX-INER-IBT-238/NC/2021	05/01/2021	Mexico City	Human	Female	unknown	Hospitalized	Original	Pharyngeal swab / Nasopharyngeal swab
hCoV-19/Mexico/CMX-INER-IBT-2405/NC/2020	11/08/2020	Mexico City	Human	Female	unknown	Hospitalized	Original	Pharyngeal swab
hCoV-19/Mexico/CMX-INER-IBT-2417/NC/2020	12/08/2020	Mexico City	Human	Female	unknown	Hospitalized	Original	Pharyngeal swab
hCoV-19/Mexico/CMX-INER-IBT-242/NC/2021	04/01/2021	Mexico City	Human	Male	unknown	Hospitalized	Original	Pharyngeal swab / Nasopharyngeal swab
hCoV-19/Mexico/CMX-INER-IBT-245/NC/2020	20/04/2020	Mexico City	Human	Male	unknown	Hospitalized	Original	Tracheal aspirate
hCoV-19/Mexico/CMX-INER-IBT-245/NC/2021	14/01/2021	Mexico City	Human	Female	unknown	Hospitalized	Original	Pharyngeal swab / Nasopharyngeal swab
hCoV-19/Mexico/CMX-INER-IBT-2478/NC/2020	17/08/2020	Mexico City	Human	Male	unknown	Hospitalized	Original	Pharyngeal swab
hCoV-19/Mexico/CMX-INER-IBT-2538/NC/2020	23/08/2020	Mexico City	Human	Male	unknown	Hospitalized	Original	Pharyngeal swab
hCoV-19/Mexico/CMX-INER-IBT-2539/NC/2020	21/08/2020	Mexico City	Human	Female	unknown	Hospitalized	Original	Pharyngeal swab
hCoV-19/Mexico/CMX-INER-IBT-2646/NC/2020	28/08/2020	Mexico City	Human	Male	unknown	Hospitalized	Original	Pharyngeal swab
hCoV-19/Mexico/CMX-INER-IBT-2679/NC/2020	03/09/2020	Mexico City	Human	Male	unknown	Hospitalized	Original	Pharyngeal swab
hCoV-19/Mexico/CMX-INER-IBT-2693/NC/2020	06/09/2020	Mexico City	Human	Male	unknown	Hospitalized	Original	Pharyngeal swab / Nasopharyngeal swab
hCoV-19/Mexico/CMX-INER-IBT-2728/NC/2020	14/09/2020	Mexico City	Human	Male	unknown	Hospitalized	Original	Pharyngeal swab / Nasopharyngeal swab
hCoV-19/Mexico/CMX-INER-IBT-2748/NC/2020	22/09/2020	Mexico City	Human	Male	unknown	Hospitalized	Original	Pharyngeal swab
hCoV-19/Mexico/CMX-INER-IBT-2757/NC/2020	28/09/2020	Mexico City	Human	Male	unknown	Hospitalized	Original	Pharyngeal swab / Nasopharyngeal swab
hCoV-19/Mexico/CMX-INER-IBT-2797/NC/2020	05/10/2020	Mexico City	Human	Female	unknown	Hospitalized	Original	Pharyngeal swab / Nasopharyngeal swab
hCoV-19/Mexico/CMX-INER-IBT-2807/NC/2020	12/10/2020	Mexico City	Human	Female	unknown	Hospitalized	Original	Pharyngeal swab / Nasopharyngeal swab
hCoV-19/Mexico/CMX-INER-IBT-2839/NC/2020	30/10/2020	Mexico City	Human	Male	unknown	Hospitalized	Original	Pharyngeal swab / Nasopharyngeal swab
hCoV-19/Mexico/CMX-INER-IBT-303/NC/2020	27/04/2020	Mexico City	Human	Female	unknown	Hospitalized	Original	Nasopharyngeal swab
hCoV-19/Mexico/CMX-INER-IBT-32/NC/2020	29/11/2020	Mexico City	Human	Female	unknown	Hospitalized	Original	Pharyngeal swab / Nasopharyngeal swab
hCoV-19/Mexico/CMX-INER-IBT-338/NC/2020	29/04/2020	Mexico City	Human	Female	unknown	Hospitalized	Original	Nasopharyngeal swab
hCoV-19/Mexico/CMX-INER-IBT-34/NC/2020	29/03/2020	Mexico City	Human	Male	unknown	Hospitalized	Original	Pharyngeal swab
hCoV-19/Mexico/CMX-INER-IBT-5/NC/2020	18/11/2020	Mexico City	Human	Female	unknown	Hospitalized	Original	Pharyngeal swab / Nasopharyngeal swab
hCoV-19/Mexico/CMX-INER-IBT-6/NC/2020	19/11/2020	Mexico City	Human	Male	unknown	Hospitalized	Original	Pharyngeal swab / Nasopharyngeal swab
hCoV-19/Mexico/CMX-INER-IBT-659/NC/2020	13/05/2020	Mexico City	Human	Male	unknown	Hospitalized	Original	Nasopharyngeal swab
hCoV-19/Mexico/CMX-INER-IBT-7/NC/2020	19/11/2020	Mexico City	Human	Female	unknown	Hospitalized	Original	Pharyngeal swab / Nasopharyngeal swab
hCoV-19/Mexico/CMX-INER-IBT-710/NC/2020	14/05/2020	Mexico City	Human	Female	unknown	Hospitalized	Original	Nasopharyngeal swab
hCoV-19/Mexico/CMX-INER-IBT-8/NC/2020	28/11/2020	Mexico City	Human	Male	unknown	Hospitalized	Original	Pharyngeal swab / Nasopharyngeal swab
hCoV-19/Mexico/CMX-INER-IBT-9/NC/2020	13/11/2020	Mexico City	Human	Female	unknown	Hospitalized	Original	Pharyngeal swab / Nasopharyngeal swab
hCoV-19/Mexico/CMX-INER-IBT-90/NC/2020	07/04/2020	Mexico City	Human	Female	unknown	Hospitalized	Original	Nasopharyngeal swab
hCoV-19/Mexico/CMX-INER-IBT-975/NC/2020	25/05/2020	Mexico City	Human	Male	unknown	Hospitalized	Original	Nasopharyngeal swab
hCoV-19/Mexico/COA_LANGEBIO_IMSS_37339/NC/2021	25/02/2021	Coahuila	Human	Female	42	Hospitalized	Original	Pharyngeal /Nasopharyngeal swab
hCoV-19/Mexico/COA_LANGEBIO_IMSS_37994/NC/2021	28/02/2021	Coahuila	Human	Female	63	Hospitalized	Original	Pharyngeal /Nasopharyngeal swab

hCoV-19/Mexico/COA_LANGEBIO_IMSS_38056/NC/2021	26/02/2021	Coahuila	Human	Female	77	Hospitalized	Original	Pharyngeal /Nasopharyngeal swab
hCoV-19/Mexico/COA_LANGEBIO_IMSS_38556/NC/2021	28/02/2021	Coahuila	Human	Male	74	Hospitalized	Original	Pharyngeal /Nasopharyngeal swab
hCoV-19/Mexico/COA-InDRE-IBT-28845/NC/2020	31/05/2020	Coahuila	Human	unknown	23	Ambulatory	Original	Pharyngeal swab
hCoV-19/Mexico/COL-InDRE-IBT-20796/NC/2020	01/06/2020	Colima	Human	Male	30	Hospitalized	Original	Pharyngeal swab
hCoV-19/Mexico/COL-InDRE-IBT-39994/NC/2020	25/06/2020	Colima	Human	Female	unknown	Hospitalized	Original	Pharyngeal swab
hCoV-19/Mexico/DUR_LANGEBIO_IMSS_36866/NC/2021	24/02/2021	Durango	Human	Male	62	Hospitalized	Original	Pharyngeal /Nasopharyngeal swab
hCoV-19/Mexico/DUR_LANGEBIO_IMSS_37503/NC/2021	25/02/2021	Durango	Human	Male	45	Ambulatory	Original	Pharyngeal /Nasopharyngeal swab
hCoV-19/Mexico/DUR_LANGEBIO_IMSS_38016/NC/2021	28/02/2021	Durango	Human	Female	66	Hospitalized	Original	Pharyngeal /Nasopharyngeal swab
hCoV-19/Mexico/DUR_LANGEBIO_IMSS_38025/NC/2021	28/02/2021	Durango	Human	Female	68	Hospitalized	Original	Pharyngeal /Nasopharyngeal swab
hCoV-19/Mexico/DUR-InDRE-IBT-34067/NC/2020	01/06/2020	Durango	Human	Female	32	unknown	Original	Nasopharyngeal swab
hCoV-19/Mexico/DUR-InDRE-IBT-34216/NC/2020	08/06/2020	Durango	Human	Female	39	Ambulatory	Original	Nasopharyngeal swab
hCoV-19/Mexico/HID-InDRE-IBT-23549/NC/2020	02/06/2020	Hidalgo	Human	Male	79	Deceased	Original	Nasopharyngeal swab
hCoV-19/Mexico/HID-InDRE-IBT-23555/NC/2020	04/06/2020	Hidalgo	Human	Male	42	Deceased	Original	Nasopharyngeal swab
hCoV-19/Mexico/HID-InDRE-IBT-23556/NC/2020	04/06/2020	Hidalgo	Human	Male	29	Ambulatory	Original	Pharyngeal swab
hCoV-19/Mexico/JAL-InDRE-IBT-43035/NC/2020	30/07/2020	Jalisco	Human	Female	24	Hospitalized	Original	Nasopharyngeal swab
hCoV-19/Mexico/MEX-IBT-IMSS-46960/NC/2021	30/01/2021	State of Mexico	Human	Male	74	Hospitalized	Original	Pharyngeal swab / Nasopharyngeal swab
hCoV-19/Mexico/MEX-IBT-IMSS-46962/NC/2021	30/01/2021	State of Mexico	Human	Female	61	Hospitalized	Original	Pharyngeal swab / Nasopharyngeal swab
hCoV-19/Mexico/MEX-IBT-IMSS-48296/NC/2021	29/01/2021	State of Mexico	Human	Female	43	Hospitalized	Original	Pharyngeal swab
hCoV-19/Mexico/MEX-IBT-IMSS-48303/NC/2021	30/01/2021	State of Mexico	Human	Male	56	Hospitalized	Original	Pharyngeal swab / Nasopharyngeal swab
hCoV-19/Mexico/MEX-IBT-IMSS-48308/NC/2021	31/01/2021	State of Mexico	Human	Male	82	Hospitalized	Original	Pharyngeal swab / Nasopharyngeal swab
hCoV-19/Mexico/MEX-IBT-IMSS-48313/NC/2021	30/01/2021	State of Mexico	Human	Male	43	Hospitalized	Original	Pharyngeal swab
hCoV-19/Mexico/MEX-IBT-IMSS-48336/NC/2021	01/02/2021	State of Mexico	Human	Female	71	Hospitalized	Original	Pharyngeal swab / Nasopharyngeal swab
hCoV-19/Mexico/MOR-InDRE-IBT-307998/NC/2020	22/12/2020	Morelos	Human	Female	41	Ambulatory	Original	Pharyngeal swab
hCoV-19/Mexico/MOR-InDRE-IBT-308004/NC/2020	22/12/2020	Morelos	Human	Male	35	Ambulatory	Original	Pharyngeal swab
hCoV-19/Mexico/MOR-InDRE-IBT-308212/NC/2020	23/12/2020	Morelos	Human	Male	30	Ambulatory	Original	Pharyngeal swab
hCoV-19/Mexico/MOR-InDRE-IBT-308214/NC/2020	23/12/2020	Morelos	Human	Male	45	Ambulatory	Original	Pharyngeal swab
hCoV-19/Mexico/MOR-InDRE-IBT-308234/NC/2020	23/12/2020	Morelos	Human	Male	28	Ambulatory	Original	Pharyngeal swab
hCoV-19/Mexico/MOR-InDRE-IBT-308259/NC/2020	23/12/2020	Morelos	Human	Male	49	Ambulatory	Original	Pharyngeal swab
hCoV-19/Mexico/MOR-InDRE-IBT-308329/NC/2020	22/12/2020	Morelos	Human	Male	36	Ambulatory	Original	Pharyngeal swab
hCoV-19/Mexico/MOR-InDRE-IBT-308342/NC/2020	21/12/2020	Morelos	Human	Female	45	Ambulatory	Original	Pharyngeal swab
hCoV-19/Mexico/MOR-InDRE-IBT-308351/NC/2020	21/12/2020	Morelos	Human	Female	44	Ambulatory	Original	Pharyngeal swab
hCoV-19/Mexico/NAY-InDRE-IBT-25291/NC/2020	21/05/2020	Nayarit	Human	Male	unknown	Ambulatory	Original	Pharyngeal swab
hCoV-19/Mexico/NLE_LANGEBIO_IMSS_34985/NC/2021	21/02/2021	Nuevo Leon	Human	Male	67	Hospitalized	Original	Nasopharyngeal swab
hCoV-19/Mexico/NLE_LANGEBIO_IMSS_35136/NC/2021	22/02/2021	Nuevo Leon	Human	Male	25	Ambulatory	Original	Pharyngeal /Nasopharyngeal swab
hCoV-19/Mexico/NLE_LANGEBIO_IMSS_35312/NC/2021	21/02/2021	Nuevo Leon	Human	Female	33	Ambulatory	Original	Pharyngeal /Nasopharyngeal swab
hCoV-19/Mexico/NLE_LANGEBIO_IMSS_35857/NC/2021	23/02/2021	Nuevo Leon	Human	Female	32	Hospitalized	Original	Pharyngeal /Nasopharyngeal swab
hCoV-19/Mexico/NLE_LANGEBIO_IMSS_37009/NC/2021	25/02/2021	Nuevo Leon	Human	Male	57	Hospitalized	Original	Pharyngeal /Nasopharyngeal swab
hCoV-19/Mexico/NLE_LANGEBIO_IMSS_37061/NC/2021	26/02/2021	Nuevo Leon	Human	Male	67	Hospitalized	Original	Pharyngeal /Nasopharyngeal swab
hCoV-19/Mexico/NLE_LANGEBIO_IMSS_37456/NC/2021	26/02/2021	Nuevo Leon	Human	Female	79	Hospitalized	Original	Pharyngeal /Nasopharyngeal swab
hCoV-19/Mexico/NLE-InDRE-IBT-25241/NC/2020	04/06/2020	Nuevo Leon	Human	Female	66	Hospitalized	Original	Pharyngeal swab
hCoV-19/Mexico/NLE-InDRE-IBT-25243/NC/2020	05/06/2020	Nuevo Leon	Human	Male	51	Hospitalized	Original	Pharyngeal swab

hCoV-19/Mexico/OAX-InDRE-IBT-27468/NC/2020	03/06/2020	Oaxaca	Human	Female	69	Ambulatory	Original	Pharyngeal swab
hCoV-19/Mexico/OAX-InDRE-IBT-27475/NC/2020	04/06/2020	Oaxaca	Human	Male	unknown	unknown	Original	Pharyngeal swab
hCoV-19/Mexico/PUE_LANGEBIO_IMSS_81667/NC/2021	28/02/2021	Puebla	Human	Male	59	Hospitalized	Original	Pharyngeal /Nasopharyngeal swab
hCoV-19/Mexico/PUE-IBT-IMSS-47245/NC/2021	31/01/2021	Puebla	Human	Male	41	Hospitalized	Original	Pharyngeal swab
hCoV-19/Mexico/PUE-IBT-IMSS-48688/NC/2021	30/01/2021	Puebla	Human	Female	49	Ambulatory	Original	Pharyngeal swab
hCoV-19/Mexico/PUE-InDRE-IBT-28747/NC/2020	05/06/2020	Puebla	Human	Male	unknown	unknown	Original	Pharyngeal swab
hCoV-19/Mexico/PUE-InDRE-IBT-308002/NC/2020	23/12/2020	Puebla	Human	Male	33	Ambulatory	Original	Pharyngeal swab
hCoV-19/Mexico/PUE-InDRE-IBT-308027/NC/2020	22/12/2020	Puebla	Human	Female	27	Ambulatory	Original	Pharyngeal swab
hCoV-19/Mexico/PUE-InDRE-IBT-308032/NC/2020	22/12/2020	Puebla	Human	Male	30	Ambulatory	Original	Pharyngeal swab
hCoV-19/Mexico/PUE-InDRE-IBT-308052/NC/2020	23/12/2020	Puebla	Human	Female	28	Ambulatory	Original	Pharyngeal swab
hCoV-19/Mexico/PUE-InDRE-IBT-308062/NC/2020	21/12/2020	Puebla	Human	Female	30	Ambulatory	Original	Pharyngeal swab
hCoV-19/Mexico/PUE-InDRE-IBT-308100/NC/2020	22/12/2020	Puebla	Human	Male	58	Ambulatory	Original	Pharyngeal swab
hCoV-19/Mexico/PUE-InDRE-IBT-308102/NC/2020	23/12/2020	Puebla	Human	Male	48	Ambulatory	Original	Pharyngeal swab
hCoV-19/Mexico/PUE-InDRE-IBT-308107/NC/2020	22/12/2020	Puebla	Human	Female	40	Ambulatory	Original	Pharyngeal swab
hCoV-19/Mexico/PUE-InDRE-IBT-308128/NC/2020	21/12/2020	Puebla	Human	Male	29	Ambulatory	Original	Pharyngeal swab
hCoV-19/Mexico/PUE-InDRE-IBT-308132/NC/2020	22/12/2020	Puebla	Human	Female	34	Ambulatory	Original	Pharyngeal swab
hCoV-19/Mexico/PUE-InDRE-IBT-308135/NC/2020	23/12/2020	Puebla	Human	Male	56	Ambulatory	Original	Pharyngeal swab
hCoV-19/Mexico/PUE-InDRE-IBT-308138/NC/2020	23/12/2020	Puebla	Human	Male	32	Ambulatory	Original	Pharyngeal swab
hCoV-19/Mexico/PUE-InDRE-IBT-308154/NC/2020	22/12/2020	Puebla	Human	Female	26	Ambulatory	Original	Pharyngeal swab
hCoV-19/Mexico/PUE-InDRE-IBT-308173/NC/2020	22/12/2020	Puebla	Human	Male	53	Ambulatory	Original	Pharyngeal swab
hCoV-19/Mexico/PUE-InDRE-IBT-308186/NC/2020	25/12/2020	Puebla	Human	Male	38	Ambulatory	Original	Pharyngeal swab
hCoV-19/Mexico/PUE-InDRE-IBT-308366/NC/2020	21/12/2020	Puebla	Human	Female	58	Deceased	Original	Pharyngeal swab
hCoV-19/Mexico/PUE-InDRE-IBT-308405/NC/2020	23/12/2020	Puebla	Human	Female	33	Ambulatory	Original	Pharyngeal swab
hCoV-19/Mexico/PUE-InDRE-IBT-308415/NC/2020	23/12/2020	Puebla	Human	Male	44	Ambulatory	Original	Pharyngeal swab
hCoV-19/Mexico/PUE-InDRE-IBT-308424/NC/2020	22/12/2020	Puebla	Human	Female	22	Ambulatory	Original	Pharyngeal swab
hCoV-19/Mexico/PUE-InDRE-IBT-308426/NC/2020	18/12/2020	Puebla	Human	Male	38	Ambulatory	Original	Pharyngeal swab
hCoV-19/Mexico/PUE-InDRE-IBT-308428/NC/2020	18/12/2020	Puebla	Human	Male	20	Ambulatory	Original	Pharyngeal swab
hCoV-19/Mexico/PUE-InDRE-IBT-308441/NC/2020	17/12/2020	Puebla	Human	Male	43	Ambulatory	Original	Pharyngeal swab
hCoV-19/Mexico/PUE-InDRE-IBT-308454/NC/2020	24/12/2020	Puebla	Human	Male	32	Ambulatory	Original	Pharyngeal swab
hCoV-19/Mexico/PUE-InDRE-IBT-308466/NC/2020	22/12/2020	Puebla	Human	Male	62	Ambulatory	Original	Pharyngeal swab
hCoV-19/Mexico/QUE-InDRE-IBT-21063/NC/2020	06/06/2020	Queretaro	Human	Female	19	Ambulatory	Original	Pharyngeal swab
hCoV-19/Mexico/QUE-InDRE-IBT-21066/NC/2020	03/06/2020	Queretaro	Human	Female	55	Hospitalized	Original	Nasopharyngeal swab
hCoV-19/Mexico/QUE-InDRE-IBT-21067/NC/2020	26/05/2020	Queretaro	Human	Female	38	Ambulatory	Original	Nasopharyngeal swab
hCoV-19/Mexico/SIN-InDRE-IBT-27557/NC/2020	05/06/2020	Sinaloa	Human	Female	54	Ambulatory	Original	NA
hCoV-19/Mexico/SLP-IBT-IMSS-48517/NC/2021	02/02/2021	San Luis Potosi	Human	Female	71	Hospitalized	Original	Pharyngeal swab / Nasopharyngeal swab
hCoV-19/Mexico/SLP-IBT-IMSS-48530/NC/2021	30/01/2021	San Luis Potosi	Human	Female	48	Ambulatory	Original	Pharyngeal swab / Nasopharyngeal swab
hCoV-19/Mexico/SLP-IBT-IMSS-48798/NC/2021	01/02/2021	San Luis Potosi	Human	Male	45	Ambulatory	Original	Pharyngeal swab / Nasopharyngeal swab
hCoV-19/Mexico/SLP-IBT-IMSS-48825/NC/2021	30/01/2021	San Luis Potosi	Human	Female	75	Ambulatory	Original	Pharyngeal swab / Nasopharyngeal swab
hCoV-19/Mexico/SLP-InDRE-IBT-977/NC/2020	09/04/2020	San Luis Potosi	Human	Male	6	Ambulatory	Original	Nasopharyngeal swab
hCoV-19/Mexico/SLP-InDRE-IBT-978/NC/2020	09/04/2020	San Luis Potosi	Human	Female	33	Ambulatory	Original	Pharyngeal swab
hCoV-19/Mexico/SON_LANGEBIO_IMSS_28900/NC/2021	28/02/2021	Sonora	Human	Male	62	Hospitalized	Original	Pharyngeal swab

hCoV-19/Mexico/SON-InDRE-IBT-29407/NC/2020		07/06/2020	Sonora	Human	Male	60	Hospitalized	Original	Pharyngeal swab
hCoV-19/Mexico/SON-InDRE-IBT-29413/NC/2020		09/06/2020	Sonora	Human	Male	32	Hospitalized	Original	Pharyngeal swab
hCoV-19/Mexico/TAB-InDRE-IBT-28657/NC/2020		10/06/2020	Tabasco	Human	Female	51	Hospitalized	Original	Pharyngeal swab
hCoV-19/Mexico/TLA-InDRE-IBT-24011/NC/2020		02/06/2020	Tlaxcala	Human	Female	54	Deceased	Original	NA
hCoV-19/Mexico/TLA-InDRE-IBT-24014/NC/2020		03/06/2020	Tlaxcala	Human	Male	43	Hospitalized	Original	NA
hCoV-19/Mexico/VER-IBT-IMSS-48502/NC/2021		30/01/2021	Veracruz	Human	Male	26	Hospitalized	Original	Pharyngeal swab / Nasopharyngeal swab
hCoV-19/Mexico/YUC_LANGEBIO_IMSS_05338/NC/2021		25/02/2021	Yucatan	Human	Male	73	Hospitalized	Original	Nasopharyngeal swab
hCoV-19/Mexico/YUC-InDRE-IBT-22139/NC/2020		01/06/2020	Yucatan	Human	Male	26	Ambulatory	Original	Pharyngeal swab
hCoV-19/Mexico/CMX-InDRE-01/2020	EPI_ISL_412972	27/02/2020	Mexico City	Human	Male	35	Hospitalized	Original	Nasopharyngeal swab
hCoV-19/Mexico/CMX-InDRE-07/2020	EPI_ISL_452139	28/02/2020	State of Mexico	Human	Male	45	Released	Original	Nasopharyngeal swab
hCoV-19/Mexico/CMX-InDRE_03/2020	EPI_ISL_424667	04/03/2020	State of Mexico	Human	Male	71	unknown	Original	Thracheal Swab
hCoV-19/Mexico/QUE-InDRE-04/2020	EPI_ISL_424670	10/03/2020	Queretaro	Human	Male	43	unknown	Original	Oropharyngeal swab
hCoV-19/Mexico/CMX-INCMNSZ-02/2020	EPI_ISL_426362	10/03/2020	Mexico City	Human	Male	71	unknown	Original	Pharyngeal and Nasopharyngeal swab
hCoV-19/Mexico/CMX-InDRE-08/2020	EPI_ISL_452141	11/03/2020	State of Mexico	Human	Male	38	Released	Original	Nasopharyngeal swab
hCoV-19/Mexico/DUR-InDRE-09/2020	EPI_ISL_455432	11/03/2020	Durango	Human	Male	74	Deceased	Original	Oropharyngeal swab
hCoV-19/Mexico/CMX-InDRE_15/2020	EPI_ISL_455439	11/03/2020	Mexico City	Human	Male	39	Released	Original	Oropharyngeal swab
hCoV-19/Mexico/PUE-InDRE-05/2020	EPI_ISL_424672	11/03/2020	Puebla	Human	Male	31	unknown	Original	Oropharyngeal swab
hCoV-19/Mexico/CMX-INER-01/2020	EPI_ISL_424345	12/03/2020	Mexico City	Human	Male	42	unknown	Original	Nasopharyngeal swab
hCoV-19/Mexico/CMX-InDRE_12/2020	EPI_ISL_455436	12/03/2020	Mexico City	Human	Male	32	Released	Original	Oropharyngeal swab
hCoV-19/Mexico/QUE-InDRE-18/2020	EPI_ISL_455456	12/03/2020	Queretaro	Human	Female	44	Released	Original	
hCoV-19/Mexico/CMX-InDRE-06/2020	EPI_ISL_424673	12/03/2020	Mexico City	Human	Female	63	unknown	Original	Oropharyngeal swab
hCoV-19/Mexico/CMX-INCMNSZ-01/2020	EPI_ISL_426361	12/03/2020	Mexico City	Human	Male	32	unknown	Original	Pharyngeal and Nasopharyngeal swab
hCoV-19/Mexico/CMX-INCMNSZ-03/2020	EPI_ISL_426363	12/03/2020	Mexico City	Human	Male	38	unknown	Original	Pharyngeal and Nasopharyngeal swab
hCoV-19/Mexico/CMX-INCMNSZ-04/2020	EPI_ISL_426364	12/03/2020	Mexico City	Human	Male	70	Deceased	Original	Pharyngeal and Nasopharyngeal swab
hCoV-19/Mexico/CMX-INCMNSZ-05/2020	EPI_ISL_426365	12/03/2020	Mexico City	Human	Female	70	unknown	Original	Pharyngeal and Nasopharyngeal swab
hCoV-19/Mexico/CMX-INER-02/2020	EPI_ISL_424348	13/03/2020	Mexico City	Human	Female	55	unknown	Original	Nasopharyngeal swab
hCoV-19/Mexico/GRO-InDRE-10/2020	EPI_ISL_455434	13/03/2020	Guerrero	Human	Male	33	Released	Original	Oropharyngeal swab
hCoV-19/Mexico/ROO-InDRE-14/2020	EPI_ISL_455438	14/03/2020	Quintana Roo	Human	Male	45	Released	Original	Oropharyngeal swab
hCoV-19/Mexico/PUE-InDRE-17/2020	EPI_ISL_455455	14/03/2020	Puebla	Human	Female	32	Released	Original	Oropharyngeal swab
hCoV-19/Mexico/CMX-INER-04/2020	EPI_ISL_424626	15/03/2020	Mexico City	Human	Female	25	unknown	Original	Nasopharyngeal swab
hCoV-19/Mexico/CMX-INER-0006/2020	EPI_ISL_837608	15/03/2020	Mexico City	Human	Male	41	Deceased	Original	Tracheal aspirate
hCoV-19/Mexico/NLE-UANL-126/2020	EPI_ISL_648595	16/03/2020	Nuevo Leon	Human	Female	58	unknown	Original	
hCoV-19/Mexico/CMX-INER-05/2020	EPI_ISL_424627	16/03/2020	Mexico City	Human	Male	38	unknown	Original	Nasopharyngeal swab
hCoV-19/Mexico/NLE-UANL-001/2020	EPI_ISL_779169	16/03/2020	Nuevo Leon	Human	Female	29	unknown	Original	
hCoV-19/Mexico/JAL-InDRE-16/2020	EPI_ISL_455454	18/03/2020	Jalisco	Human	Male	58	Deceased	Original	Oropharyngeal swab
hCoV-19/Mexico/JAL-InDRE-11/2020	EPI_ISL_455435	20/03/2020	Jalisco	Human	Male	56	Deceased	Original	Oropharyngeal swab
hCoV-19/Mexico/CMX-INER-0007/2020	EPI_ISL_837609	20/03/2020	Mexico City	Human	Female	82	Deceased	Original	Oropharyngeal swab
hCoV-19/Mexico/CMX-INER-0008/2020	EPI_ISL_837610	20/03/2020	Mexico City	Human	Male	40	Deceased	Original	Oropharyngeal swab
hCoV-19/Mexico/CMX-InDRE-13/2020	EPI_ISL_455437	21/03/2020	Mexico City	Human	Male	56	Deceased	Original	
hCoV-19/Mexico/NLE-UANL-003/2020	EPI_ISL_779170	22/03/2020	Nuevo Leon	Human	Male	64	unknown	Original	
hCoV-19/Mexico/CMX-INER-0009/2020	EPI_ISL_837611	22/03/2020	Mexico City	Human	Male	65	Deceased	Original	Tracheal aspirate

hCoV-19/Mexico/CMX-INER-0010/2020	EPI_ISL_837612	22/03/2020	Mexico City	Human	Male	60	Deceased	Original	Oropharyngeal swab
hCoV-19/Mexico/CMX-INER-0011/2020	EPI_ISL_837613	22/03/2020	Mexico City	Human	Male	30	Deceased	Original	Oropharyngeal swab
hCoV-19/Mexico/CMX-INER-0012/2020	EPI_ISL_837614	24/03/2020	Mexico City	Human	Male	46	Deceased	Original	Oropharyngeal swab
hCoV-19/Mexico/CMX-INER-0013/2020	EPI_ISL_837615	24/03/2020	Mexico City	Human	Female	60	Hospitalized	Original	Oropharyngeal swab
hCoV-19/Mexico/CMX-INER-0014/2020	EPI_ISL_837616	24/03/2020	Mexico City	Human	Female	65	Deceased	Original	Oropharyngeal swab
hCoV-19/Mexico/BCN-ALSR-1712/2020	EPI_ISL_496373	25/03/2020	Baja California	Human	unknown	unknown	unknown	Original	Nasopharyngeal swab
hCoV-19/Mexico/NLE-UANL-004/2020	EPI_ISL_779171	25/03/2020	Nuevo Leon	Human	Male	67	unknown	Original	
hCoV-19/Mexico/BCN-ALSR-1710/2020	EPI_ISL_496371	26/03/2020	Baja California	Human	unknown	unknown	unknown	Original	Nasopharyngeal swab
hCoV-19/Mexico/BCN-ALSR-1711/2020	EPI_ISL_496372	26/03/2020	Baja California	Human	unknown	unknown	unknown	Original	Nasopharyngeal swab
hCoV-19/Mexico/CMX-INER-0015/2020	EPI_ISL_837617	26/03/2020	Mexico City	Human	Male	49	Deceased	Original	Tracheal aspirate
hCoV-19/Mexico/BCN-ALSR-1709/2020	EPI_ISL_496370	27/03/2020	Baja California	Human	unknown	unknown	unknown	Original	Nasopharyngeal swab
hCoV-19/Mexico/GUA-InDRE-19/2020	EPI_ISL_493334	27/03/2020	Guanajuato	Human	Female	73	Deceased	Original	Oropharyngeal swab
hCoV-19/Mexico/CMX-INER-0016/2020	EPI_ISL_837618	28/03/2020	Mexico City	Human	Female	34	Deceased	Original	Tracheal aspirate
hCoV-19/Mexico/CMX-INER-0017/2020	EPI_ISL_837619	29/03/2020	Mexico City	Human	Female	65	Hospitalized	Original	Tracheal aspirate
hCoV-19/Mexico/CMX-INER-0018/2020	EPI_ISL_837620	30/03/2020	Mexico City	Human	Male	53	Deceased	Original	Tracheal aspirate
hCoV-19/Mexico/SON-InDRE-99/2020	EPI_ISL_872089	01/04/2020	Sonora	Human	Female	71	Deceased	Original	Oropharyngeal swab
hCoV-19/Mexico/CMX-INER-0019/2020	EPI_ISL_837621	01/04/2020	Mexico City	Human	Male	50	Deceased	Original	Tracheal aspirate
hCoV-19/Mexico/CMX-INER-0020/2020	EPI_ISL_837622	01/04/2020	Mexico City	Human	Female	61	Deceased	Original	Nasopharyngeal swab
hCoV-19/Mexico/CMX-INER-0021/2020	EPI_ISL_837623	01/04/2020	Mexico City	Human	Female	83	Hospitalized	Original	Nasopharyngeal swab
hCoV-19/Mexico/GUA-InDRE-20/2020	EPI_ISL_493335	02/04/2020	Guanajuato	Human	Female	74	Deceased	Original	Oropharyngeal swab
hCoV-19/Mexico/CMX-INER-0022/2020	EPI_ISL_837624	02/04/2020	Mexico City	Human	Male	71	Released	Original	Nasopharyngeal swab
hCoV-19/Mexico/CMX-INER-0023/2020	EPI_ISL_837625	03/04/2020	Mexico City	Human	Female	54	Deceased	Original	
hCoV-19/Mexico/CMX-INER-0024/2020	EPI_ISL_837626	03/04/2020	Mexico City	Human	Male	44	Deceased	Original	Nasopharyngeal swab
hCoV-19/Mexico/TAB-InDRE-100/2020	EPI_ISL_872090	04/04/2020	Tabasco	Human	Male	38	Deceased	Original	Oropharyngeal swab
hCoV-19/Mexico/CMX-InDRE-101/2020	EPI_ISL_872091	04/04/2020	State of Mexico	Human	Male	24	Deceased	Original	Oropharyngeal swab
hCoV-19/Mexico/SON-InDRE-103/2020	EPI_ISL_872093	04/04/2020	Sonora	Human	Male	33	Deceased	Original	Oropharyngeal swab
hCoV-19/Mexico/CMX-INER-0025/2020	EPI_ISL_837627	04/04/2020	Mexico City	Human	Male	40	Hospitalized	Original	Nasopharyngeal swab
hCoV-19/Mexico/CMX-INER-0026/2020	EPI_ISL_837628	04/04/2020	Mexico City	Human	Male	34	Released	Original	Nasopharyngeal swab
hCoV-19/Mexico/CMX-INER-0027/2020	EPI_ISL_837629	06/04/2020	Mexico City	Human	Female	46	Released	Original	Nasopharyngeal swab
hCoV-19/Mexico/CMX-INER-0028/2020	EPI_ISL_837630	06/04/2020	Mexico City	Human	Male	39	Deceased	Original	Tracheal aspirate
hCoV-19/Mexico/CMX-INER-0029/2020	EPI_ISL_837631	06/04/2020	Mexico City	Human	Male	50	Hospitalized	Original	Tracheal aspirate
hCoV-19/Mexico/CMX-INER-0030/2020	EPI_ISL_837632	06/04/2020	Mexico City	Human	Male	27	Released	Original	Nasopharyngeal swab
hCoV-19/Mexico/CMX-INER-0031/2020	EPI_ISL_837633	06/04/2020	Mexico City	Human	Male	38	Hospitalized	Original	Nasopharyngeal swab
hCoV-19/Mexico/CMX-INER-0032/2020	EPI_ISL_837634	07/04/2020	Mexico City	Human	Female	45	Deceased	Original	Nasopharyngeal swab
hCoV-19/Mexico/CMX-INER-0033/2020	EPI_ISL_837635	08/04/2020	Mexico City	Human	Female	41	Deceased	Original	Nasopharyngeal swab
hCoV-19/Mexico/CMX-INER-0034/2020	EPI_ISL_837636	08/04/2020	Mexico City	Human	Female	33	Released	Original	Nasopharyngeal swab
hCoV-19/Mexico/CMX-INER-0035/2020	EPI_ISL_837637	08/04/2020	Mexico City	Human	Male	28	Released	Original	Nasopharyngeal swab
hCoV-19/Mexico/CMX-INER-0036/2020	EPI_ISL_837638	08/04/2020	Mexico City	Human	Female	66	Hospitalized	Original	Nasopharyngeal swab
hCoV-19/Mexico/CMX-INER-0037/2020	EPI_ISL_837639	08/04/2020	Mexico City	Human	Male	59	Deceased	Original	Tracheal aspirate
hCoV-19/Mexico/CMX-INER-0038/2020	EPI_ISL_837640	08/04/2020	Mexico City	Human	Female	48	Hospitalized	Original	Nasopharyngeal swab
hCoV-19/Mexico/CMX-InDRE-98/2020	EPI_ISL_872088	09/04/2020	Mexico City	Human	Male	65	Deceased	Original	Oropharyngeal swab

hCoV-19/Mexico/QROO-InDRE-53/2020	EPI_ISL_658904	10/04/2020	Quintana Roo	Human	Male	38	Released	Original	Oropharyngeal swab
hCoV-19/Mexico/YUC-InDRE-95/2020	EPI_ISL_861419	10/04/2020	Yucatan	Human	Female	38	Deceased	Original	Oropharyngeal swab
hCoV-19/Mexico/CMX-INER-0040/2020	EPI_ISL_837606	10/04/2020	Mexico City	Human	Male	43	Deceased	Original	Nasopharyngeal swab
hCoV-19/Mexico/CMX-INER-0039/2020	EPI_ISL_837641	10/04/2020	Mexico City	Human	Female	70	Deceased	Original	Tracheal aspirate
hCoV-19/Mexico/CMX-INER-0041/2020	EPI_ISL_837642	10/04/2020	Mexico City	Human	Female	36	Hospitalized	Original	Tracheal aspirate
hCoV-19/Mexico/CMX-INER-0042/2020	EPI_ISL_837643	10/04/2020	Mexico City	Human	Male	42	Deceased	Original	Tracheal aspirate
hCoV-19/Mexico/CMX-INER-0043/2020	EPI_ISL_837644	10/04/2020	Mexico City	Human	Female	46	Released	Original	Nasopharyngeal swab
hCoV-19/Mexico/CMX-INER-0044/2020	EPI_ISL_837645	10/04/2020	Mexico City	Human	Female	57	Released	Original	Nasopharyngeal swab
hCoV-19/Mexico/CMX-INER-0045/2020	EPI_ISL_837646	11/04/2020	Mexico City	Human	Male	56	Deceased	Original	Nasopharyngeal swab
hCoV-19/Mexico/ROO-InDRE-102/2020	EPI_ISL_872092	13/04/2020	Quintana Roo	Human	Male	33	Deceased	Original	Oropharyngeal swab
hCoV-19/Mexico/CMX-INER-0046/2020	EPI_ISL_837647	13/04/2020	Mexico City	Human	Male	56	Deceased	Original	Tracheal aspirate
hCoV-19/Mexico/CMX-INER-0047/2020	EPI_ISL_837648	13/04/2020	Mexico City	Human	Male	63	Deceased	Original	Nasopharyngeal swab
hCoV-19/Mexico/CMX-INER-0048/2020	EPI_ISL_837649	13/04/2020	Mexico City	Human	Male	58	Deceased	Original	Tracheal aspirate
hCoV-19/Mexico/CMX-INER-0051/2020	EPI_ISL_837604	14/04/2020	Mexico City	Human	Male	58	Deceased	Original	Tracheal aspirate
hCoV-19/Mexico/CMX-INER-0049/2020	EPI_ISL_837650	14/04/2020	Mexico City	Human	Male	33	Deceased	Original	Nasopharyngeal swab
hCoV-19/Mexico/CMX-INER-0050/2020	EPI_ISL_837651	14/04/2020	Mexico City	Human	Female	41	Hospitalized	Original	Nasopharyngeal swab
hCoV-19/Mexico/CMX-InDRE-96/2020	EPI_ISL_861420	15/04/2020	Mexico City	Human	Male	35	Deceased	Original	Oropharyngeal swab
hCoV-19/Mexico/CMX-INER-0052/2020	EPI_ISL_837652	15/04/2020	Mexico City	Human	Male	34	Hospitalized	Original	Nasopharyngeal swab
hCoV-19/Mexico/CMX-INER-0053/2020	EPI_ISL_837653	15/04/2020	Mexico City	Human	Male	64	Hospitalized	Original	Tracheal aspirate
hCoV-19/Mexico/CMX-INER-0056/2020	EPI_ISL_837600	16/04/2020	Mexico City	Human	Male	41	Deceased	Original	Nasopharyngeal swab
hCoV-19/Mexico/CMX-INER-0054/2020	EPI_ISL_837654	16/04/2020	Mexico City	Human	Male	65	Hospitalized	Original	Tracheal aspirate
hCoV-19/Mexico/CMX-INER-0055/2020	EPI_ISL_837655	16/04/2020	Mexico City	Human	Male	61	Deceased	Original	Nasopharyngeal swab
hCoV-19/Mexico/CMX-INER-0057/2020	EPI_ISL_837656	16/04/2020	Mexico City	Human	Male	47	Released	Original	Nasopharyngeal swab
hCoV-19/Mexico/CMX-INER-0058/2020	EPI_ISL_837657	16/04/2020	Mexico City	Human	Male	59	Released	Original	Nasopharyngeal swab
hCoV-19/Mexico/CMX-INER-0059/2020	EPI_ISL_837658	16/04/2020	Mexico City	Human	Male	52	Hospitalized	Original	Nasopharyngeal swab
hCoV-19/Mexico/SIN-InDRE-21/2020	EPI_ISL_493336	17/04/2020	Sinaloa	Human	Male	67	Deceased	Original	Oropharyngeal swab
hCoV-19/Mexico/CMX-INER-0060/2020	EPI_ISL_837659	17/04/2020	Mexico City	Human	Male	39	Deceased	Original	Tracheal aspirate
hCoV-19/Mexico/CMX-INER-0061/2020	EPI_ISL_837660	17/04/2020	Mexico City	Human	Male	56	Deceased	Original	Tracheal aspirate
hCoV-19/Mexico/CMX-INER-0062/2020	EPI_ISL_837661	17/04/2020	Mexico City	Human	Male	36	Hospitalized	Original	Nasopharyngeal swab
hCoV-19/Mexico/CMX-INER-0063/2020	EPI_ISL_837662	18/04/2020	Mexico City	Human	Male	52	Hospitalized	Original	Nasopharyngeal swab
hCoV-19/Mexico/CMX-INER-0064/2020	EPI_ISL_837663	18/04/2020	Mexico City	Human	Male	34	Released	Original	Nasopharyngeal swab
hCoV-19/Mexico/CMX-INER-0065/2020	EPI_ISL_837664	18/04/2020	Mexico City	Human	Female	24	Hospitalized	Original	Nasopharyngeal swab
hCoV-19/Mexico/CMX-INER-0066/2020	EPI_ISL_837665	18/04/2020	Mexico City	Human	Male	59	Hospitalized	Original	Nasopharyngeal swab
hCoV-19/Mexico/CMX-INER-0067/2020	EPI_ISL_837666	19/04/2020	Mexico City	Human	Female	53	Hospitalized	Original	Nasopharyngeal swab
hCoV-19/Mexico/CMX-INER-0068/2020	EPI_ISL_837667	19/04/2020	Mexico City	Human	Male	50	Hospitalized	Original	Nasopharyngeal swab
hCoV-19/Mexico/BCN-ALSR-1708/2020	EPI_ISL_496369	20/04/2020	Baja California	Human	unknown	unknown	unknown	Original	Nasopharyngeal swab
hCoV-19/Mexico/CMX-INER-0069/2020	EPI_ISL_837668	20/04/2020	Mexico City	Human	Female	unknown	unknown	Original	Nasopharyngeal swab
hCoV-19/Mexico/CMX-INER-0070/2020	EPI_ISL_837669	20/04/2020	Mexico City	Human	Male	53	Released	Original	Nasopharyngeal swab
hCoV-19/Mexico/BCN-ALSR-1707/2020	EPI_ISL_496368	21/04/2020	Baja California	Human	unknown	unknown	unknown	Original	Nasopharyngeal swab
hCoV-19/Mexico/CMX-INER-0071/2020	EPI_ISL_837670	21/04/2020	Mexico City	Human	Male	45	Hospitalized	Original	Tracheal aspirate
hCoV-19/Mexico/CMX-INER-0072/2020	EPI_ISL_837671	21/04/2020	Mexico City	Human	Male	41	Hospitalized	Original	Tracheal aspirate

hCoV-19/Mexico/CMX-INER-0073/2020	EPI_ISL_837672	21/04/2020	Mexico City	Human	Male	59	Deceased	Original	Nasopharyngeal swab
hCoV-19/Mexico/BCN-ALSR-1698/2020	EPI_ISL_496359	22/04/2020	Baja California	Human	unknown	unknown	unknown	Original	Nasopharyngeal swab
hCoV-19/Mexico/BCN-ALSR-1699/2020	EPI_ISL_496360	22/04/2020	Baja California	Human	unknown	unknown	unknown	Original	Nasopharyngeal swab
hCoV-19/Mexico/BCN-ALSR-1700/2020	EPI_ISL_496361	22/04/2020	Baja California	Human	unknown	unknown	unknown	Original	Nasopharyngeal swab
hCoV-19/Mexico/BCN-ALSR-1701/2020	EPI_ISL_496362	22/04/2020	Baja California	Human	unknown	unknown	unknown	Original	Nasopharyngeal swab
hCoV-19/Mexico/BCN-ALSR-1702/2020	EPI_ISL_496363	22/04/2020	Baja California	Human	unknown	unknown	unknown	Original	Nasopharyngeal swab
hCoV-19/Mexico/BCN-ALSR-1703/2020	EPI_ISL_496364	22/04/2020	Baja California	Human	unknown	unknown	unknown	Original	Nasopharyngeal swab
hCoV-19/Mexico/BCN-ALSR-1704/2020	EPI_ISL_496365	22/04/2020	Baja California	Human	unknown	unknown	unknown	Original	Nasopharyngeal swab
hCoV-19/Mexico/BCN-ALSR-1705/2020	EPI_ISL_496366	22/04/2020	Baja California	Human	unknown	unknown	unknown	Original	Nasopharyngeal swab
hCoV-19/Mexico/BCN-ALSR-1706/2020	EPI_ISL_496367	22/04/2020	Baja California	Human	unknown	unknown	unknown	Original	Nasopharyngeal swab
hCoV-19/Mexico/CMX-INER-0074/2020	EPI_ISL_837673	22/04/2020	Mexico City	Human	Male	43	Deceased	Original	Tracheal aspirate
hCoV-19/Mexico/CMX-INER-0075/2020	EPI_ISL_837605	23/04/2020	Mexico City	Human	Male	53	Deceased	Original	Tracheal aspirate
hCoV-19/Mexico/CMX-INER-0076/2020	EPI_ISL_837674	23/04/2020	Mexico City	Human	Female	38	Hospitalized	Original	Oropharyngeal swab
hCoV-19/Mexico/VER-InDRE-104/2020	EPI_ISL_872097	24/04/2020	Veracruz	Human	Male	39	Deceased	Original	Oropharyngeal swab
hCoV-19/Mexico/BCN-ALSR-1696/2020	EPI_ISL_496357	24/04/2020	Baja California	Human	unknown	unknown	unknown	Original	Nasopharyngeal swab
hCoV-19/Mexico/BCN-ALSR-1697/2020	EPI_ISL_496358	24/04/2020	Baja California	Human	unknown	unknown	unknown	Original	Nasopharyngeal swab
hCoV-19/Mexico/CMX-INER-0077/2020	EPI_ISL_837675	24/04/2020	Mexico City	Human	Male	29	Released	Original	Oropharyngeal swab
hCoV-19/Mexico/BCN-ALSR-1695/2020	EPI_ISL_496356	25/04/2020	Baja California	Human	unknown	unknown	unknown	Original	Nasopharyngeal swab
hCoV-19/Mexico/CMX-INER-0078/2020	EPI_ISL_837676	25/04/2020	Mexico City	Human	Male	45	Deceased	Original	Tracheal aspirate
hCoV-19/Mexico/CMX-INER-0079/2020	EPI_ISL_837677	25/04/2020	Mexico City	Human	Male	67	Deceased	Original	
hCoV-19/Mexico/TAB-InDRE-105/2020	EPI_ISL_872098	26/04/2020	Tabasco	Human	Male	37	Deceased	Original	Oropharyngeal swab
hCoV-19/Mexico/CMX-INER-0080/2020	EPI_ISL_837678	26/04/2020	Mexico City	Human	Male	48	Hospitalized	Original	Nasopharyngeal swab
hCoV-19/Mexico/BCN-ALSR-1691/2020	EPI_ISL_496352	27/04/2020	Baja California	Human	unknown	unknown	unknown	Original	Nasopharyngeal swab
hCoV-19/Mexico/BCN-ALSR-1692/2020	EPI_ISL_496353	27/04/2020	Baja California	Human	unknown	unknown	unknown	Original	Nasopharyngeal swab
hCoV-19/Mexico/BCN-ALSR-1693/2020	EPI_ISL_496354	27/04/2020	Baja California	Human	unknown	unknown	unknown	Original	Nasopharyngeal swab
hCoV-19/Mexico/BCN-ALSR-1694/2020	EPI_ISL_496355	27/04/2020	Baja California	Human	unknown	unknown	unknown	Original	Nasopharyngeal swab
hCoV-19/Mexico/CMX-INER-0081/2020	EPI_ISL_837679	27/04/2020	Mexico City	Human	Female	55	Hospitalized	Original	Oropharyngeal swab
hCoV-19/Mexico/BCN-ALSR-1470/2020	EPI_ISL_496340	28/04/2020	Baja California	Human	unknown	unknown	unknown	Original	Nasopharyngeal swab
hCoV-19/Mexico/BCN-ALSR-1471/2020	EPI_ISL_496341	28/04/2020	Baja California	Human	unknown	unknown	unknown	Original	Nasopharyngeal swab
hCoV-19/Mexico/BCN-ALSR-1473/2020	EPI_ISL_496342	28/04/2020	Baja California	Human	unknown	unknown	unknown	Original	Nasopharyngeal swab
hCoV-19/Mexico/BCN-ALSR-1474/2020	EPI_ISL_496343	28/04/2020	Baja California	Human	unknown	unknown	unknown	Original	Nasopharyngeal swab
hCoV-19/Mexico/BCN-ALSR-1475/2020	EPI_ISL_496344	28/04/2020	Baja California	Human	unknown	unknown	unknown	Original	Nasopharyngeal swab
hCoV-19/Mexico/BCN-ALSR-1476/2020	EPI_ISL_496345	28/04/2020	Baja California	Human	unknown	unknown	unknown	Original	Nasopharyngeal swab
hCoV-19/Mexico/BCN-ALSR-1477/2020	EPI_ISL_496346	28/04/2020	Baja California	Human	unknown	unknown	unknown	Original	Nasopharyngeal swab
hCoV-19/Mexico/BCN-ALSR-1478/2020	EPI_ISL_496347	28/04/2020	Baja California	Human	unknown	unknown	unknown	Original	Nasopharyngeal swab
hCoV-19/Mexico/BCN-ALSR-1479/2020	EPI_ISL_496348	28/04/2020	Baja California	Human	unknown	unknown	unknown	Original	Nasopharyngeal swab
hCoV-19/Mexico/BCN-ALSR-1485/2020	EPI_ISL_496349	28/04/2020	Baja California	Human	unknown	unknown	unknown	Original	Nasopharyngeal swab
hCoV-19/Mexico/BCN-ALSR-1486/2020	EPI_ISL_496350	28/04/2020	Baja California	Human	unknown	unknown	unknown	Original	Nasopharyngeal swab
hCoV-19/Mexico/BCN-ALSR-1690/2020	EPI_ISL_496351	28/04/2020	Baja California	Human	unknown	unknown	unknown	Original	Nasopharyngeal swab
hCoV-19/Mexico/NLE-UANL-005/2020	EPI_ISL_779172	28/04/2020	Nuevo Leon	Human	Male	51	unknown	Original	
hCoV-19/Mexico/BCN-ALSR-1466/2020	EPI_ISL_496339	29/04/2020	Baja California	Human	unknown	unknown	unknown	Original	Nasopharyngeal swab

hCoV-19/Mexico/BCN-ALSR-1713/2020	EPI_ISL_496374	29/04/2020	Baja California	Human	unknown	unknown	unknown	Original	Nasopharyngeal swab
hCoV-19/Mexico/BCN-ALSR-1714/2020	EPI_ISL_496375	29/04/2020	Baja California	Human	unknown	unknown	unknown	Original	Nasopharyngeal swab
hCoV-19/Mexico/BCN-ALSR-1715/2020	EPI_ISL_496376	29/04/2020	Baja California	Human	unknown	unknown	unknown	Original	Nasopharyngeal swab
hCoV-19/Mexico/CMX-INER-0082/2020	EPI_ISL_837680	29/04/2020	Mexico City	Human	Female	49	Released	Original	Oropharyngeal swab
hCoV-19/Mexico/CMX-INER-0083/2020	EPI_ISL_837681	29/04/2020	Mexico City	Human	Female	36	Released	Original	Oropharyngeal swab
hCoV-19/Mexico/CMX-INER-0084/2020	EPI_ISL_837682	29/04/2020	Mexico City	Human	Male	64	Hospitalized	Original	Nasopharyngeal swab
hCoV-19/Mexico/SLP-UASLP-AH1COV2SS024_S	EPI_ISL_1494674	30/04/2020	San Luis Potosi	Human	Male	42	Deceased	Original	
hCoV-19/Mexico/TLA-InDRE-97/2020	EPI_ISL_861421	30/04/2020	Tlaxcala	Human	Female	28	Deceased	Original	Oropharyngeal swab
hCoV-19/Mexico/TLA-InDRE-106/2020	EPI_ISL_872099	30/04/2020	Tlaxcala	Human	Male	34	Deceased	Original	Oropharyngeal swab
hCoV-19/Mexico/CMX-INER-0085/2020	EPI_ISL_837683	30/04/2020	Mexico City	Human	Male	48	Deceased	Original	Nasopharyngeal swab
hCoV-19/Mexico/CHP-InDRE-23/2020	EPI_ISL_493338	04/05/2020	Chiapas	Human	Female	68	Deceased	Original	Oropharyngeal swab
hCoV-19/Mexico/GRO-InDRE-24/2020	EPI_ISL_493339	04/05/2020	Guerrero	Human	Male	42	Live	Original	Oropharyngeal swab
hCoV-19/Mexico/CMX-INER-0086/2020	EPI_ISL_837684	04/05/2020	Mexico City	Human	Male	52	Released	Original	Oropharyngeal swab
hCoV-19/Mexico/CMX-INER-0087/2020	EPI_ISL_837685	06/05/2020	Mexico City	Human	Male	44	Released	Original	Oropharyngeal swab
hCoV-19/Mexico/CMX-INER-0088/2020	EPI_ISL_837686	07/05/2020	Mexico City	Human	Male	56	Hospitalized	Original	Tracheal aspirate
hCoV-19/Mexico/CMX-INER-0089/2020	EPI_ISL_837687	07/05/2020	Mexico City	Human	Female	61	Deceased	Original	Oropharyngeal swab
hCoV-19/Mexico/CMX-INER-0090/2020	EPI_ISL_837688	08/05/2020	Mexico City	Human	Male	26	Released	Original	Oropharyngeal swab
hCoV-19/Mexico/CMX-INER-0091/2020	EPI_ISL_837689	08/05/2020	Mexico City	Human	Male	34	Released	Original	Oropharyngeal swab
hCoV-19/Mexico/CMX-INER-0092/2020	EPI_ISL_837690	09/05/2020	Mexico City	Human	Female	45	Deceased	Original	Nasopharyngeal swab
hCoV-19/Mexico/CMX-INER-0093/2020	EPI_ISL_837691	10/05/2020	Mexico City	Human	Female	46	Hospitalized	Original	Tracheal aspirate
hCoV-19/Mexico/CMX-INER-0094/2020	EPI_ISL_837692	11/05/2020	Mexico City	Human	Male	49	Released	Original	Oropharyngeal swab
hCoV-19/Mexico/CMX-INER-0095/2020	EPI_ISL_837693	11/05/2020	Mexico City	Human	Female	44	Deceased	Original	Nasopharyngeal swab
hCoV-19/Mexico/BCN-ALSR-8341/2020	EPI_ISL_1531807	12/05/2020	Baja California	Human	unknown	unknown	unknown	Original	Nasal swab
hCoV-19/Mexico/BCN-ALSR-8342/2020	EPI_ISL_1531808	12/05/2020	Baja California	Human	unknown	unknown	unknown	Original	Nasal swab
hCoV-19/Mexico/BCN-ALSR-2444/2020	EPI_ISL_635479	13/05/2020	Baja California	Human	unknown	unknown	unknown	Original	Nasopharyngeal swab
hCoV-19/Mexico/CMX-INER-0096/2020	EPI_ISL_837694	13/05/2020	Mexico City	Human	Female	72	Deceased	Original	Nasopharyngeal swab
hCoV-19/Mexico/CMX-INMEGEN-02-10-17-GRO/2	EPI_ISL_1158060	14/05/2020	Mexico City	Human	Female	37	unknown	Original	Oropharyngeal swab
hCoV-19/Mexico/BCN-ALSR-2446/2020	EPI_ISL_635480	14/05/2020	Baja California	Human	unknown	unknown	unknown	Original	Nasopharyngeal swab
hCoV-19/Mexico/BCN-ALSR-2447/2020	EPI_ISL_635481	14/05/2020	Baja California	Human	unknown	unknown	unknown	Original	Nasopharyngeal swab
hCoV-19/Mexico/BCN-ALSR-2448/2020	EPI_ISL_635482	14/05/2020	Baja California	Human	unknown	unknown	unknown	Original	Nasopharyngeal swab
hCoV-19/Mexico/CMX-INER-0097/2020	EPI_ISL_837695	14/05/2020	Mexico City	Human	Female	39	Hospitalized	Original	Nasopharyngeal swab
hCoV-19/Mexico/CMX-INER-0098/2020	EPI_ISL_837696	14/05/2020	Mexico City	Human	Male	71	Deceased	Original	Nasopharyngeal swab
hCoV-19/Mexico/CMX-INER-0099/2020	EPI_ISL_837697	14/05/2020	Mexico City	Human	Male	32	Released	Original	Oropharyngeal swab
hCoV-19/Mexico/BCN-ALSR-8343/2020	EPI_ISL_1531809	14/05/2020	Baja California	Human	unknown	unknown	unknown	Original	Nasal swab
hCoV-19/Mexico/BCN-ALSR-8344/2020	EPI_ISL_1531810	14/05/2020	Baja California	Human	unknown	unknown	unknown	Original	Nasal swab
hCoV-19/Mexico/BCN-ALSR-2451/2020	EPI_ISL_635483	15/05/2020	Baja California	Human	unknown	unknown	unknown	Original	Nasopharyngeal swab
hCoV-19/Mexico/BCN-ALSR-2452/2020	EPI_ISL_635484	15/05/2020	Baja California	Human	unknown	unknown	unknown	Original	Nasopharyngeal swab
hCoV-19/Mexico/CMX-INER-0100/2020	EPI_ISL_837698	15/05/2020	Mexico City	Human	Male	unknown	unknown	Original	Nasopharyngeal swab
hCoV-19/Mexico/SON-InDRE_35/2020	EPI_ISL_516622	15/05/2020	Sonora	Human	Male	20	Released	Original	Oropharyngeal swab
hCoV-19/Mexico/SON-InDRE_29/2020	EPI_ISL_493344	15/05/2020	Sonora	Human	Male	61	Released	Original	Oropharyngeal swab
hCoV-19/Mexico/BCN-ALSR-8345/2020	EPI_ISL_1531811	15/05/2020	Baja California	Human	unknown	unknown	unknown	Original	Nasal swab

hCoV-19/Mexico/BCN-ALSR-2457/2020	EPI_ISL_635485	16/05/2020	Baja California	Human	unknown	unknown	unknown	Original	Nasopharyngeal swab
hCoV-19/Mexico/BCN-ALSR-8346/2020	EPI_ISL_1531812	16/05/2020	Baja California	Human	unknown	unknown	unknown	Original	Nasal swab
hCoV-19/Mexico/BCN-ALSR-8347/2020	EPI_ISL_1531813	16/05/2020	Baja California	Human	unknown	unknown	unknown	Original	Nasal swab
hCoV-19/Mexico/BCN-ALSR-8348/2020	EPI_ISL_1531814	16/05/2020	Baja California	Human	unknown	unknown	unknown	Original	Nasal swab
hCoV-19/Mexico/BCN-ALSR-8349/2020	EPI_ISL_1531815	16/05/2020	Baja California	Human	unknown	unknown	unknown	Original	Nasal swab
hCoV-19/Mexico/CMX-INMEGEN-02-10-18-GRO/2	EPI_ISL_1158061	17/05/2020	Mexico City	Human	Female	99	unknown	Original	Oropharyngeal swab
hCoV-19/Mexico/CMX-INER-0101/2020	EPI_ISL_837699	18/05/2020	Mexico City	Human	Female	53	Released	Original	Oropharyngeal swab
hCoV-19/Mexico/CMX-INER-0102/2020	EPI_ISL_837700	18/05/2020	Mexico City	Human	Male	57	Deceased	Original	Nasopharyngeal swab
hCoV-19/Mexico/NLE-UANL-006/2020	EPI_ISL_779173	18/05/2020	Nuevo Leon	Human	Male	68	unknown	Original	
hCoV-19/Mexico/BCN-ALSR-2462/2020	EPI_ISL_635486	19/05/2020	Baja California	Human	unknown	unknown	unknown	Original	Nasopharyngeal swab
hCoV-19/Mexico/BCN-ALSR-2466/2020	EPI_ISL_635487	19/05/2020	Baja California	Human	unknown	unknown	unknown	Original	Nasopharyngeal swab
hCoV-19/Mexico/CMX-INER-0103/2020	EPI_ISL_837701	19/05/2020	Mexico City	Human	Male	38	Released	Original	Oropharyngeal swab
hCoV-19/Mexico/CMX-INER-0104/2020	EPI_ISL_837702	19/05/2020	Mexico City	Human	Female	53	Released	Original	Oropharyngeal swab
hCoV-19/Mexico/CMX-INER-0105/2020	EPI_ISL_837703	19/05/2020	Mexico City	Human	Male	46	Deceased	Original	Nasopharyngeal swab
hCoV-19/Mexico/CMX-INMEGEN-02-10-19-GRO/2	EPI_ISL_1137475	19/05/2020	Mexico City	Human	Male	37	unknown	Original	Oropharyngeal swab
hCoV-19/Mexico/NLE-UANL-007/2020	EPI_ISL_779174	19/05/2020	Nuevo Leon	Human	Female	28	unknown	Original	
hCoV-19/Mexico/NLE-UANL-008/2020	EPI_ISL_779175	19/05/2020	Nuevo Leon	Human	Male	65	unknown	Original	
hCoV-19/Mexico/NLE-UANL-009/2020	EPI_ISL_779176	19/05/2020	Nuevo Leon	Human	Female	21	unknown	Original	
hCoV-19/Mexico/BCN-ALSR-2468/2020	EPI_ISL_635488	20/05/2020	Baja California	Human	unknown	unknown	unknown	Original	Nasopharyngeal swab
hCoV-19/Mexico/CMX-INER-0106/2020	EPI_ISL_837704	20/05/2020	Mexico City	Human	Female	28	Hospitalized	Original	Oropharyngeal swab
hCoV-19/Mexico/CMX-INER-0107/2020	EPI_ISL_837705	20/05/2020	Mexico City	Human	Male	74	Deceased	Original	Nasopharyngeal swab
hCoV-19/Mexico/CMX-INER-0108/2020	EPI_ISL_837706	20/05/2020	Mexico City	Human	Male	67	Hospitalized	Original	Nasopharyngeal swab
hCoV-19/Mexico/SLP-UASLP-AH1COV2SS025_S	EPI_ISL_1494717	20/05/2020	San Luis Potosi	Human	Male	42	Hospitalized	Original	
hCoV-19/Mexico/CMX-INER-0109/2020	EPI_ISL_837707	21/05/2020	Mexico City	Human	Female	38	Released	Original	Oropharyngeal swab
hCoV-19/Mexico/CMX-INER-0110/2020	EPI_ISL_837708	21/05/2020	Mexico City	Human	Male	53	Hospitalized	Original	Nasopharyngeal swab
hCoV-19/Mexico/BCN-ALSR-2475/2020	EPI_ISL_635489	22/05/2020	Baja California	Human	unknown	unknown	unknown	Original	Nasopharyngeal swab
hCoV-19/Mexico/BCN-ALSR-2477/2020	EPI_ISL_635490	22/05/2020	Baja California	Human	unknown	unknown	unknown	Original	Nasopharyngeal swab
hCoV-19/Mexico/SLP-UASLP-AH1COV2SS027_S	EPI_ISL_1494725	22/05/2020	San Luis Potosi	Human	Male	60	Hospitalized	Original	
hCoV-19/Mexico/BCN-InDRE_32/2020	EPI_ISL_493347	22/05/2020	Baja California	Human	Female	9	Released	Original	Oropharyngeal swab
hCoV-19/Mexico/NLE-UANL-2098/2020	EPI_ISL_648602	23/05/2020	Nuevo Leon	Human	Male	53	unknown	Original	
hCoV-19/Mexico/NLE-UANL-2088/2020	EPI_ISL_648603	23/05/2020	Nuevo Leon	Human	Male	44	unknown	Original	
hCoV-19/Mexico/CMX-INER-0111/2020	EPI_ISL_837709	23/05/2020	Mexico City	Human	Female	25	Released	Original	Oropharyngeal swab
hCoV-19/Mexico/CMX-INER-0112/2020	EPI_ISL_837710	23/05/2020	Mexico City	Human	Male	35	Hospitalized	Original	Tracheal aspirate
hCoV-19/Mexico/NLE-UANL-050/2020	EPI_ISL_961791	23/05/2020	Nuevo Leon	Human	Male	44	Hospitalized	Original	
hCoV-19/Mexico/NLE-UANL-049/2020	EPI_ISL_961792	23/05/2020	Nuevo Leon	Human	Female	26	Hospitalized	Original	
hCoV-19/Mexico/BCS-InDRE-25/2020	EPI_ISL_493340	23/05/2020	Baja California Sur	Human	Male	47	Deceased	Original	Oropharyngeal swab
hCoV-19/Mexico/NLE-UANL-012/2020	EPI_ISL_779177	23/05/2020	Nuevo Leon	Human	Female	21	unknown	Original	
hCoV-19/Mexico/NLE-UANL-013/2020	EPI_ISL_779178	23/05/2020	Nuevo Leon	Human	Female	42	unknown	Original	
hCoV-19/Mexico/CMX-INER-0113/2020	EPI_ISL_837711	24/05/2020	Mexico City	Human	Male	48	Released	Original	Oropharyngeal swab
hCoV-19/Mexico/CMX-INER-0114/2020	EPI_ISL_837712	25/05/2020	Mexico City	Human	Male	34	Released	Original	Oropharyngeal swab
hCoV-19/Mexico/CMX-INER-0115/2020	EPI_ISL_837713	25/05/2020	Mexico City	Human	Female	49	Released	Original	Oropharyngeal swab

hCoV-19/Mexico/CMX-INMEGEN-02-10-20-GRO/2	EPI_ISL_1158062	26/05/2020	Mexico City	Human	Male	21	unknown	Original	Oropharyngeal swab
hCoV-19/Mexico/BCN-ALSR-2479/2020	EPI_ISL_635491	26/05/2020	Baja California	Human	unknown	unknown	unknown	Original	Nasopharyngeal swab
hCoV-19/Mexico/BCN-ALSR-2480/2020	EPI_ISL_635492	26/05/2020	Baja California	Human	unknown	unknown	unknown	Original	Nasopharyngeal swab
hCoV-19/Mexico/BCN-ALSR-2481/2020	EPI_ISL_635493	26/05/2020	Baja California	Human	unknown	unknown	unknown	Original	Nasopharyngeal swab
hCoV-19/Mexico/BCN-ALSR-2483/2020	EPI_ISL_635494	26/05/2020	Baja California	Human	unknown	unknown	unknown	Original	Nasopharyngeal swab
hCoV-19/Mexico/BCN-ALSR-2484/2020	EPI_ISL_635495	26/05/2020	Baja California	Human	unknown	unknown	unknown	Original	Nasopharyngeal swab
hCoV-19/Mexico/BCN-ALSR-2485/2020	EPI_ISL_635496	26/05/2020	Baja California	Human	unknown	unknown	unknown	Original	Nasopharyngeal swab
hCoV-19/Mexico/BCN-ALSR-2487/2020	EPI_ISL_635497	26/05/2020	Baja California	Human	unknown	unknown	unknown	Original	Nasopharyngeal swab
hCoV-19/Mexico/SLP-UASLP-AH1COV2SS028_S	EPI_ISL_1494726	26/05/2020	San Luis Potosi	Human	Male	96	Hospitalized	Original	
hCoV-19/Mexico/SLP-UASLP-AH1COV2SS029_S	EPI_ISL_1494727	26/05/2020	San Luis Potosi	Human	Male	40	Released	Original	
hCoV-19/Mexico/NAY-InDRE_27/2020	EPI_ISL_493342	26/05/2020	Nayarit	Human	Female	50	Released	Original	Oropharyngeal swab
hCoV-19/Mexico/BCS-InDRE_31/2020	EPI_ISL_493346	26/05/2020	Baja California Sur	Human	Male	20	Deceased	Original	Oropharyngeal swab
hCoV-19/Mexico/CMX-INER-0117/2020	EPI_ISL_837714	27/05/2020	Mexico City	Human	Female	unknown	unknown	Original	Oropharyngeal swab
hCoV-19/Mexico/CMX-INER-0118/2020	EPI_ISL_837715	27/05/2020	Mexico City	Human	Female	40	Released	Original	Oropharyngeal swab
hCoV-19/Mexico/JAL-UANL-001/2020	EPI_ISL_1092361	27/05/2020	Jalisco	Human	Female	54	unknown	Original	
hCoV-19/Mexico/CMX-INER-0116/2020	EPI_ISL_843191	27/05/2020	Mexico City	Human	Female	42	Released	Original	Oropharyngeal swab
hCoV-19/Mexico/MOR-InDRE-26/2020	EPI_ISL_493341	27/05/2020	Morelos	Human	Male	29	Released	Original	Oropharyngeal swab
hCoV-19/Mexico/YUC-InDRE_33/2020	EPI_ISL_493348	27/05/2020	Yucatan	Human	Male	92	Deceased	Original	Oropharyngeal swab
hCoV-19/Mexico/BCN-ALSR-3634/2020	EPI_ISL_636118	27/05/2020	Baja California	Human	unknown	unknown	unknown	Original	Nasopharyngeal swab
hCoV-19/Mexico/CMX-INER-0119/2020	EPI_ISL_837716	28/05/2020	Mexico City	Human	Male	48	Released	Original	Oropharyngeal swab
hCoV-19/Mexico/CMX-INER-0120/2020	EPI_ISL_837717	28/05/2020	Mexico City	Human	Female	27	Hospitalized	Original	Oropharyngeal swab
hCoV-19/Mexico/SON-InDRE_34/2020	EPI_ISL_493349	28/05/2020	Sonora	Human	Male	83	Live	Original	Oropharyngeal swab
hCoV-19/Mexico/BCN-ALSR-3636/2020	EPI_ISL_636119	28/05/2020	Baja California	Human	unknown	unknown	unknown	Original	Nasopharyngeal swab
hCoV-19/Mexico/BCN-ALSR-2489/2020	EPI_ISL_635498	29/05/2020	Baja California	Human	unknown	unknown	unknown	Original	Nasopharyngeal swab
hCoV-19/Mexico/BCN-ALSR-2490/2020	EPI_ISL_635499	29/05/2020	Baja California	Human	unknown	unknown	unknown	Original	Nasopharyngeal swab
hCoV-19/Mexico/CMX-INER-0121/2020	EPI_ISL_837718	29/05/2020	Mexico City	Human	Female	54	Hospitalized	Original	Oropharyngeal swab
hCoV-19/Mexico/CMX-INER-0122/2020	EPI_ISL_837719	29/05/2020	Mexico City	Human	Male	60	Hospitalized	Original	Nasopharyngeal swab
hCoV-19/Mexico/CMX-INER-0123/2020	EPI_ISL_837720	29/05/2020	Mexico City	Human	Female	25	Released	Original	Oropharyngeal swab
hCoV-19/Mexico/CMX-INER-0124/2020	EPI_ISL_837721	29/05/2020	Mexico City	Human	Male	34	Released	Original	Oropharyngeal swab
hCoV-19/Mexico/CMX-INER-0125/2020	EPI_ISL_837722	29/05/2020	Mexico City	Human	Female	53	Hospitalized	Original	Nasopharyngeal swab
hCoV-19/Mexico/NAY-InDRE_28/2020	EPI_ISL_493343	29/05/2020	Nayarit	Human	Male	72	Released	Original	Oropharyngeal swab
hCoV-19/Mexico/BCS-InDRE_30/2020	EPI_ISL_493345	29/05/2020	Baja California Sur	Human	Female	73	Released	Original	Oropharyngeal swab
hCoV-19/Mexico/BCN-ALSR-3622/2020	EPI_ISL_636115	29/05/2020	Baja California	Human	unknown	unknown	unknown	Original	Nasopharyngeal swab
hCoV-19/Mexico/BCN-ALSR-2491/2020	EPI_ISL_635500	29/05/2020	Baja California	Human	unknown	unknown	unknown	Original	Nasopharyngeal swab
hCoV-19/Mexico/BCN-ALSR-2492/2020	EPI_ISL_635501	29/05/2020	Baja California	Human	unknown	unknown	unknown	Original	Nasopharyngeal swab
hCoV-19/Mexico/BCN-ALSR-2493/2020	EPI_ISL_635502	29/05/2020	Baja California	Human	unknown	unknown	unknown	Original	Nasopharyngeal swab
hCoV-19/Mexico/BCN-ALSR-2494/2020	EPI_ISL_635503	29/05/2020	Baja California	Human	unknown	unknown	unknown	Original	Nasopharyngeal swab
hCoV-19/Mexico/CMX-INER-0126/2020	EPI_ISL_837723	30/05/2020	Mexico City	Human	Female	23	Released	Original	Oropharyngeal swab
hCoV-19/Mexico/BCN-ALSR-2495/2020	EPI_ISL_635504	30/05/2020	Baja California	Human	unknown	unknown	unknown	Original	Nasopharyngeal swab
hCoV-19/Mexico/BCN-ALSR-2496/2020	EPI_ISL_635505	30/05/2020	Baja California	Human	unknown	unknown	unknown	Original	Nasopharyngeal swab
hCoV-19/Mexico/CMX-INER-0127/2020	EPI_ISL_837724	31/05/2020	Mexico City	Human	Male	57	Deceased	Original	Nasopharyngeal swab

hCoV-19/Mexico/CMX-INER-0128/2020	EPI_ISL_837725	31/05/2020	Mexico City	Human	Male	105	Deceased	Original	Nasopharyngeal swab
hCoV-19/Mexico/BCN-ALSR-2497/2020	EPI_ISL_635506	01/06/2020	Baja California	Human	unknown	unknown	unknown	Original	Nasopharyngeal swab
hCoV-19/Mexico/BCN-ALSR-2499/2020	EPI_ISL_635507	01/06/2020	Baja California	Human	unknown	unknown	unknown	Original	Nasopharyngeal swab
hCoV-19/Mexico/CMX-INER-0129/2020	EPI_ISL_837726	02/06/2020	Mexico City	Human	Male	23	Released	Original	Oropharyngeal swab
hCoV-19/Mexico/JAL-UANL-002/2020	EPI_ISL_1091261	03/06/2020	Jalisco	Human	Female	85	unknown	Original	
hCoV-19/Mexico/CMX-INER-0130/2020	EPI_ISL_837727	03/06/2020	Mexico City	Human	Male	80	Deceased	Original	Oropharyngeal swab
hCoV-19/Mexico/BCN-ALSR-3618/2020	EPI_ISL_636113	03/06/2020	Baja California	Human	unknown	unknown	unknown	Original	Nasopharyngeal swab
hCoV-19/Mexico/BCN-ALSR-2500/2020	EPI_ISL_635508	03/06/2020	Baja California	Human	unknown	unknown	unknown	Original	Nasopharyngeal swab
hCoV-19/Mexico/BCN-ALSR-2502/2020	EPI_ISL_635509	03/06/2020	Baja California	Human	unknown	unknown	unknown	Original	Nasopharyngeal swab
hCoV-19/Mexico/CMX-INER-0131/2020	EPI_ISL_837728	04/06/2020	Mexico City	Human	Male	32	Released	Original	Oropharyngeal swab
hCoV-19/Mexico/BCN-ALSR-3619/2020	EPI_ISL_636114	04/06/2020	Baja California	Human	unknown	unknown	unknown	Original	Nasopharyngeal swab
hCoV-19/Mexico/BCN-ALSR-2504/2020	EPI_ISL_635510	05/06/2020	Baja California	Human	unknown	unknown	unknown	Original	Nasopharyngeal swab
hCoV-19/Mexico/CMX-INER-0132/2020	EPI_ISL_837729	06/06/2020	Mexico City	Human	Female	47	Hospitalized	Original	Oropharyngeal swab
hCoV-19/Mexico/CMX-INER-0133/2020	EPI_ISL_837730	06/06/2020	Mexico City	Human	Male	43	Deceased	Original	Nasopharyngeal swab
hCoV-19/Mexico/SON-InDRE_42/2020	EPI_ISL_516623	06/06/2020	Sonora	Human	Male	35	Deceased	Original	Oropharyngeal swab
hCoV-19/Mexico/BCN-ALSR-2506/2020	EPI_ISL_635511	06/06/2020	Baja California	Human	unknown	unknown	unknown	Original	Nasopharyngeal swab
hCoV-19/Mexico/GRO-InDRE-91/2020	EPI_ISL_658896	08/06/2020	Guerrero	Human	Female	46	Released	Original	Oropharyngeal swab
hCoV-19/Mexico/CMX-INER-0134/2020	EPI_ISL_837731	08/06/2020	Mexico City	Human	Male	59	Deceased	Original	Tracheal aspirate
hCoV-19/Mexico/BCN-ALSR-8351/2020	EPI_ISL_1531816	08/06/2020	Baja California	Human	unknown	unknown	unknown	Original	Nasal swab
hCoV-19/Mexico/BCN-ALSR-2507/2020	EPI_ISL_635512	08/06/2020	Baja California	Human	unknown	unknown	unknown	Original	Nasopharyngeal swab
hCoV-19/Mexico/BCN-ALSR-2508/2020	EPI_ISL_635513	08/06/2020	Baja California	Human	unknown	unknown	unknown	Original	Nasopharyngeal swab
hCoV-19/Mexico/BCN-ALSR-2509/2020	EPI_ISL_635514	08/06/2020	Baja California	Human	unknown	unknown	unknown	Original	Nasopharyngeal swab
hCoV-19/Mexico/CMX-INER-0135/2020	EPI_ISL_837732	09/06/2020	Mexico City	Human	Male	46	Released	Original	Oropharyngeal swab
hCoV-19/Mexico/CMX-INER-0136/2020	EPI_ISL_837733	09/06/2020	Mexico City	Human	Male	45	Hospitalized	Original	Oropharyngeal swab
hCoV-19/Mexico/CMX-INER-0137/2020	EPI_ISL_837734	10/06/2020	Mexico City	Human	Female	51	Released	Original	Oropharyngeal swab
hCoV-19/Mexico/BCN-ALSR-8352/2020	EPI_ISL_1531817	10/06/2020	Baja California	Human	unknown	unknown	unknown	Original	Nasal swab
hCoV-19/Mexico/BCN-ALSR-2510/2020	EPI_ISL_635515	10/06/2020	Baja California	Human	unknown	unknown	unknown	Original	Nasopharyngeal swab
hCoV-19/Mexico/BCN-ALSR-3624/2020	EPI_ISL_636116	12/06/2020	Sonora	Human	unknown	unknown	unknown	Original	Nasopharyngeal swab
hCoV-19/Mexico/BCN-ALSR-3630/2020	EPI_ISL_636117	12/06/2020	Baja California	Human	unknown	unknown	unknown	Original	Nasopharyngeal swab
hCoV-19/Mexico/BCN-ALSR-3639/2020	EPI_ISL_636120	12/06/2020	Sonora	Human	unknown	unknown	unknown	Original	Nasopharyngeal swab
hCoV-19/Mexico/BCN-ALSR-2511/2020	EPI_ISL_635516	12/06/2020	Baja California	Human	unknown	unknown	unknown	Original	Nasopharyngeal swab
hCoV-19/Mexico/BCN-ALSR-2512/2020	EPI_ISL_635517	12/06/2020	Baja California	Human	unknown	unknown	unknown	Original	Nasopharyngeal swab
hCoV-19/Mexico/COA-InDRE-36/2020	EPI_ISL_516618	13/06/2020	Coahuila	Human	Male	51	Released	Original	Oropharyngeal swab
hCoV-19/Mexico/COA-InDRE-37/2020	EPI_ISL_516619	13/06/2020	Coahuila	Human	Female	32	Released	Original	Oropharyngeal swab
hCoV-19/Mexico/CMX-INER-0138/2020	EPI_ISL_837607	14/06/2020	Mexico City	Human	Female	53	Released	Original	Oropharyngeal swab
hCoV-19/Mexico/CMX-INER-0139/2020	EPI_ISL_837735	15/06/2020	Mexico City	Human	Female	32	Released	Original	Oropharyngeal swab
hCoV-19/Mexico/TAM-InDRE_39/2020	EPI_ISL_516625	15/06/2020	Tamaulipas	Human	Male	79	Deceased	Original	Oropharyngeal swab
hCoV-19/Mexico/AGU-InDRE-55/2020	EPI_ISL_576258	15/06/2020	Aguascalientes	Human	Female	67	Released	Original	Oropharyngeal swab
hCoV-19/Mexico/BCN-ALSR-2513/2020	EPI_ISL_635518	15/06/2020	Baja California	Human	unknown	unknown	unknown	Original	Nasopharyngeal swab
hCoV-19/Mexico/BCN-ALSR-2514/2020	EPI_ISL_635519	15/06/2020	Baja California	Human	unknown	unknown	unknown	Original	Nasopharyngeal swab
hCoV-19/Mexico/BCN-ALSR-2515/2020	EPI_ISL_635520	15/06/2020	Baja California	Human	unknown	unknown	unknown	Original	Nasopharyngeal swab

hCoV-19/Mexico/BCN-ALSR-2516/2020	EPI_ISL_635521	15/06/2020	Baja California	Human	unknown	unknown	unknown	Original	Nasopharyngeal swab
hCoV-19/Mexico/BCN-ALSR-2517/2020	EPI_ISL_635522	15/06/2020	Baja California	Human	unknown	unknown	unknown	Original	Nasopharyngeal swab
hCoV-19/Mexico/BCN-ALSR-2519/2020	EPI_ISL_635523	15/06/2020	Baja California	Human	unknown	unknown	unknown	Original	Nasopharyngeal swab
hCoV-19/Mexico/CMX-INER-0140/2020	EPI_ISL_837736	16/06/2020	Mexico City	Human	Male	59	Hospitalized	Original	Nasopharyngeal swab
hCoV-19/Mexico/NLE-UANL-052/2020	EPI_ISL_961785	16/06/2020	Nuevo Leon	Human	Male	24	Hospitalized	Original	
hCoV-19/Mexico/BCN-ALSR-2520/2020	EPI_ISL_635524	16/06/2020	Baja California	Human	unknown	unknown	unknown	Original	Nasopharyngeal swab
hCoV-19/Mexico/BCN-ALSR-2521/2020	EPI_ISL_635525	16/06/2020	Baja California	Human	unknown	unknown	unknown	Original	Nasopharyngeal swab
hCoV-19/Mexico/BCN-ALSR-2522/2020	EPI_ISL_635526	16/06/2020	Baja California	Human	unknown	unknown	unknown	Original	Nasopharyngeal swab
hCoV-19/Mexico/BCN-ALSR-2523/2020	EPI_ISL_635527	16/06/2020	Baja California	Human	unknown	unknown	unknown	Original	Nasopharyngeal swab
hCoV-19/Mexico/BCN-ALSR-2524/2020	EPI_ISL_635528	16/06/2020	Baja California	Human	unknown	unknown	unknown	Original	Nasopharyngeal swab
hCoV-19/Mexico/NLE-UANL-4479/2020	EPI_ISL_648607	17/06/2020	Nuevo Leon	Human	Male	56	unknown	Original	
hCoV-19/Mexico/NLE-UANL-014/2020	EPI_ISL_779179	17/06/2020	Nuevo Leon	Human	Female	56	unknown	Original	
hCoV-19/Mexico/NLE-UANL-015/2020	EPI_ISL_779180	17/06/2020	Nuevo Leon	Human	Male	33	unknown	Original	
hCoV-19/Mexico/NLE-UANL-017/2020	EPI_ISL_779181	17/06/2020	Nuevo Leon	Human	Female	33	unknown	Original	
hCoV-19/Mexico/BCN-ALSR-2525/2020	EPI_ISL_635529	17/06/2020	Baja California	Human	unknown	unknown	unknown	Original	Nasopharyngeal swab
hCoV-19/Mexico/BCN-ALSR-2526/2020	EPI_ISL_635530	17/06/2020	Baja California	Human	unknown	unknown	unknown	Original	Nasopharyngeal swab
hCoV-19/Mexico/BCN-ALSR-2527/2020	EPI_ISL_635531	17/06/2020	Baja California	Human	unknown	unknown	unknown	Original	Nasopharyngeal swab
hCoV-19/Mexico/CMX-INER-0141/2020	EPI_ISL_837737	18/06/2020	Mexico City	Human	Male	29	Hospitalized	Original	Nasopharyngeal swab
hCoV-19/Mexico/CMX-INER-0142/2020	EPI_ISL_837738	18/06/2020	Mexico City	Human	Male	29	Released	Original	
hCoV-19/Mexico/SIN_CIAD_S0148/2020	EPI_ISL_1491365	18/06/2020	Sinaloa	Human	Male	2	unknown	Original	Oropharyngeal swab
hCoV-19/Mexico/BCN-ALSR-8353/2020	EPI_ISL_1531818	18/06/2020	Baja California	Human	unknown	unknown	unknown	Original	Nasal swab
hCoV-19/Mexico/BCN-ALSR-8354/2020	EPI_ISL_1531819	18/06/2020	Baja California	Human	unknown	unknown	unknown	Original	Nasal swab
hCoV-19/Mexico/BCN-ALSR-2528/2020	EPI_ISL_635532	18/06/2020	Baja California	Human	unknown	unknown	unknown	Original	Nasopharyngeal swab
hCoV-19/Mexico/CMX-INER-0143/2020	EPI_ISL_837739	19/06/2020	Mexico City	Human	Male	45	Released	Original	Oropharyngeal swab
hCoV-19/Mexico/CMX-INER-0144/2020	EPI_ISL_837740	19/06/2020	Mexico City	Human	Male	44	Released	Original	Nasopharyngeal and oropharyngeal swab
hCoV-19/Mexico/COA-InDRE_40/2020	EPI_ISL_516620	19/06/2020	Coahuila	Human	Female	24	Released	Original	Oropharyngeal swab
hCoV-19/Mexico/TAM-InDRE_38/2020	EPI_ISL_516624	19/06/2020	Tamaulipas	Human	Male	49	Released	Original	Oropharyngeal swab
hCoV-19/Mexico/CMX-INMEGEN-02-10-21-GRO/2	EPI_ISL_1137476	19/06/2020	Mexico City	Human	Female	25	unknown	Original	Oropharyngeal swab
hCoV-19/Mexico/NLE-UANL-053/2020	EPI_ISL_961781	20/06/2020	Nuevo Leon	Human	Female	22	Hospitalized	Original	
hCoV-19/Mexico/CMX-INER-0145/2020	EPI_ISL_837741	22/06/2020	Mexico City	Human	Female	81	Hospitalized	Original	Nasopharyngeal swab
hCoV-19/Mexico/CMX-INER-0146/2020	EPI_ISL_837742	22/06/2020	Mexico City	Human	Female	30	Released	Original	Oropharyngeal swab
hCoV-19/Mexico/BCN-ALSR-2529/2020	EPI_ISL_635533	22/06/2020	Baja California	Human	unknown	unknown	unknown	Original	Nasopharyngeal swab
hCoV-19/Mexico/BCN-ALSR-2530/2020	EPI_ISL_635534	22/06/2020	Baja California	Human	unknown	unknown	unknown	Original	Nasopharyngeal swab
hCoV-19/Mexico/BCN-ALSR-2531/2020	EPI_ISL_635535	22/06/2020	Baja California	Human	unknown	unknown	unknown	Original	Nasopharyngeal swab
hCoV-19/Mexico/CMX-INER-0147/2020	EPI_ISL_837743	23/06/2020	Mexico City	Human	Male	33	Released	Original	Nasopharyngeal swab
hCoV-19/Mexico/BCN-ALSR-2533/2020	EPI_ISL_635536	23/06/2020	Baja California	Human	unknown	unknown	unknown	Original	Nasopharyngeal swab
hCoV-19/Mexico/BCN-ALSR-2534/2020	EPI_ISL_635537	23/06/2020	Baja California	Human	unknown	unknown	unknown	Original	Nasopharyngeal swab
hCoV-19/Mexico/BCN-ALSR-2535/2020	EPI_ISL_635538	23/06/2020	Baja California	Human	unknown	unknown	unknown	Original	Nasopharyngeal swab
hCoV-19/Mexico/BCN-ALSR-2536/2020	EPI_ISL_635539	23/06/2020	Baja California	Human	unknown	unknown	unknown	Original	Nasopharyngeal swab
hCoV-19/Mexico/CMX-INER-0148/2020	EPI_ISL_837744	24/06/2020	Mexico City	Human	Male	unknown	unknown	Original	Nasopharyngeal and oropharyngeal swab
hCoV-19/Mexico/CMX-INER-0149/2020	EPI_ISL_837745	24/06/2020	Mexico City	Human	Male	48	Released	Original	Nasopharyngeal and oropharyngeal swab

hCoV-19/Mexico/BCN-ALSR-8356/2020	EPI_ISL_1531820	24/06/2020	Baja California	Human	unknown	unknown	unknown	Original	Nasal swab
hCoV-19/Mexico/BCN-ALSR-8357/2020	EPI_ISL_1531821	24/06/2020	Baja California	Human	unknown	unknown	unknown	Original	Nasal swab
hCoV-19/Mexico/BCN-ALSR-2537/2020	EPI_ISL_635540	24/06/2020	Baja California	Human	unknown	unknown	unknown	Original	Nasopharyngeal swab
hCoV-19/Mexico/BCN-ALSR-2538/2020	EPI_ISL_635541	24/06/2020	Baja California	Human	unknown	unknown	unknown	Original	Nasopharyngeal swab
hCoV-19/Mexico/BCN-ALSR-2539/2020	EPI_ISL_635542	24/06/2020	Baja California	Human	unknown	unknown	unknown	Original	Nasopharyngeal swab
hCoV-19/Mexico/BCN-ALSR-2540/2020	EPI_ISL_635543	24/06/2020	Baja California	Human	unknown	unknown	unknown	Original	Nasopharyngeal swab
hCoV-19/Mexico/CMX-INER-0150/2020	EPI_ISL_837746	25/06/2020	Mexico City	Human	Female	45	Released	Original	Nasopharyngeal and oropharyngeal swab
hCoV-19/Mexico/BCN-ALSR-2541/2020	EPI_ISL_635544	25/06/2020	Baja California	Human	unknown	unknown	unknown	Original	Nasopharyngeal swab
hCoV-19/Mexico/BCN-ALSR-2542/2020	EPI_ISL_635545	25/06/2020	Baja California	Human	unknown	unknown	unknown	Original	Nasopharyngeal swab
hCoV-19/Mexico/BCN-ALSR-2543/2020	EPI_ISL_635546	25/06/2020	Baja California	Human	unknown	unknown	unknown	Original	Nasopharyngeal swab
hCoV-19/Mexico/BC-InDRE-44/2020	EPI_ISL_516608	26/06/2020	Baja California	Human	Male	33	Released	Original	Oropharyngeal swab
hCoV-19/Mexico/CHH-InDRE-43/2020	EPI_ISL_516613	26/06/2020	Chihuahua	Human	Female	22	Released	Original	Oropharyngeal swab
hCoV-19/Mexico/BCN-ALSR-2544/2020	EPI_ISL_635547	26/06/2020	Baja California	Human	unknown	unknown	unknown	Original	Nasopharyngeal swab
hCoV-19/Mexico/TAM-UANL-013/2020	EPI_ISL_1091272	27/06/2020	Tamaulipas	Human	Male	25	unknown	Original	
hCoV-19/Mexico/CMX-INER-0151/2020	EPI_ISL_837747	27/06/2020	Mexico City	Human	Male	48	Released	Original	Nasopharyngeal and oropharyngeal swab
hCoV-19/Mexico/CMX-INER-0152/2020	EPI_ISL_837748	27/06/2020	Mexico City	Human	Male	64	Deceased	Original	Nasopharyngeal and oropharyngeal swab
hCoV-19/Mexico/BCN-InDRE-45/2020	EPI_ISL_516609	27/06/2020	Baja California	Human	Male	41	Released	Original	Oropharyngeal swab
hCoV-19/Mexico/CHH-InDRE-47/2020	EPI_ISL_516615	27/06/2020	Chihuahua	Human	Male	29	Released	Original	Oropharyngeal swab
hCoV-19/Mexico/NLE-UANL-062/2020	EPI_ISL_961780	27/06/2020	Nuevo Leon	Human	Male	58	Hospitalized	Original	
hCoV-19/Mexico/BCN-ALSR-2545/2020	EPI_ISL_635548	27/06/2020	Baja California	Human	unknown	unknown	unknown	Original	Nasopharyngeal swab
hCoV-19/Mexico/CHH-InDRE-46/2020	EPI_ISL_516614	28/06/2020	Chihuahua	Human	Male	63	Released	Original	Oropharyngeal swab
hCoV-19/Mexico/BCN-ALSR-2546/2020	EPI_ISL_635549	29/06/2020	Baja California	Human	unknown	unknown	unknown	Original	Nasopharyngeal swab
hCoV-19/Mexico/BCN-ALSR-2547/2020	EPI_ISL_635550	29/06/2020	Baja California	Human	unknown	unknown	unknown	Original	Nasopharyngeal swab
hCoV-19/Mexico/CMX-INER-0153/2020	EPI_ISL_837749	30/06/2020	Mexico City	Human	Male	56	Hospitalized	Original	Nasopharyngeal and oropharyngeal swab
hCoV-19/Mexico/BCN-InDRE-50/2020	EPI_ISL_516610	30/06/2020	Baja California	Human	Male	25	Released	Original	Oropharyngeal swab
hCoV-19/Mexico/CHH-InDRE-48/2020	EPI_ISL_516616	30/06/2020	Chihuahua	Human	Female	52	Released	Original	Oropharyngeal swab
hCoV-19/Mexico/BCN-ALSR-8358/2020	EPI_ISL_1531822	30/06/2020	Baja California	Human	unknown	unknown	unknown	Original	Nasal swab
hCoV-19/Mexico/BCN-ALSR-2548/2020	EPI_ISL_635551	30/06/2020	Baja California	Human	unknown	unknown	unknown	Original	Nasopharyngeal swab
hCoV-19/Mexico/CMX-INER-0154/2020	EPI_ISL_837750	01/07/2020	Mexico City	Human	Female	61	Released	Original	Nasopharyngeal swab
hCoV-19/Mexico/CHH-InDRE-49/2020	EPI_ISL_516617	01/07/2020	Chihuahua	Human	Female	49	Released	Original	Oropharyngeal swab
hCoV-19/Mexico/BCN-ALSR-2549/2020	EPI_ISL_635552	01/07/2020	Baja California	Human	unknown	unknown	unknown	Original	Nasopharyngeal swab
hCoV-19/Mexico/BCN-ALSR-2550/2020	EPI_ISL_635553	01/07/2020	Baja California	Human	unknown	unknown	unknown	Original	Nasopharyngeal swab
hCoV-19/Mexico/BCN-ALSR-2551/2020	EPI_ISL_635554	01/07/2020	Baja California	Human	unknown	unknown	unknown	Original	Nasopharyngeal swab
hCoV-19/Mexico/CMX-INER-0155/2020	EPI_ISL_837751	02/07/2020	Mexico City	Human	Female	21	Released	Original	Oropharyngeal swab
hCoV-19/Mexico/CMX-INER-0156/2020	EPI_ISL_837752	02/07/2020	Mexico City	Human	Male	unknown	unknown	Original	Nasopharyngeal and oropharyngeal swab
hCoV-19/Mexico/NLE-UANL-054/2020	EPI_ISL_961777	02/07/2020	Nuevo Leon	Human	Male	31	Hospitalized	Original	
hCoV-19/Mexico/BCN-ALSR-2552/2020	EPI_ISL_635555	02/07/2020	Baja California	Human	unknown	unknown	unknown	Original	Nasopharyngeal swab
hCoV-19/Mexico/BCN-ALSR-2553/2020	EPI_ISL_635556	02/07/2020	Baja California	Human	unknown	unknown	unknown	Original	Nasopharyngeal swab
hCoV-19/Mexico/BCN-ALSR-4888/2020	EPI_ISL_730197	03/07/2020	Baja California	Human	unknown	unknown	unknown	Original	Nasopharyngeal swab
hCoV-19/Mexico/CMX-INER-0157/2020	EPI_ISL_837753	03/07/2020	Mexico City	Human	Male	36	Deceased	Original	Nasopharyngeal and oropharyngeal swab
hCoV-19/Mexico/CMX-INER-0158/2020	EPI_ISL_837754	03/07/2020	Mexico City	Human	Female	29	Hospitalized	Original	Oropharyngeal swab

hCoV-19/Mexico/BCN-ALSR-8360/2020	EPI_ISL_1531824	03/07/2020	Baja California	Human	unknown	unknown	unknown	Original	Nasal swab
hCoV-19/Mexico/BCN-ALSR-2554/2020	EPI_ISL_635557	03/07/2020	Baja California	Human	unknown	unknown	unknown	Original	Nasopharyngeal swab
hCoV-19/Mexico/BCN-ALSR-2555/2020	EPI_ISL_635558	03/07/2020	Baja California	Human	unknown	unknown	unknown	Original	Nasopharyngeal swab
hCoV-19/Mexico/BCN-ALSR-4893/2020	EPI_ISL_730198	04/07/2020	Baja California	Human	unknown	unknown	unknown	Original	Nasopharyngeal swab
hCoV-19/Mexico/BCN-ALSR-4894/2020	EPI_ISL_730199	04/07/2020	Baja California	Human	unknown	unknown	unknown	Original	Nasopharyngeal swab
hCoV-19/Mexico/BCN-ALSR-2556/2020	EPI_ISL_635559	04/07/2020	Baja California	Human	unknown	unknown	unknown	Original	Nasopharyngeal swab
hCoV-19/Mexico/BCN-ALSR-2557/2020	EPI_ISL_635560	04/07/2020	Baja California	Human	unknown	unknown	unknown	Original	Nasopharyngeal swab
hCoV-19/Mexico/BCN-ALSR-2558/2020	EPI_ISL_635561	04/07/2020	Baja California	Human	unknown	unknown	unknown	Original	Nasopharyngeal swab
hCoV-19/Mexico/BCN-ALSR-2559/2020	EPI_ISL_635562	04/07/2020	Baja California	Human	unknown	unknown	unknown	Original	Nasopharyngeal swab
hCoV-19/Mexico/CMX-INER-0159/2020	EPI_ISL_837755	05/07/2020	Mexico City	Human	Female	29	Released	Original	Oropharyngeal swab
hCoV-19/Mexico/BCN-InDRE-51/2020	EPI_ISL_516611	05/07/2020	Baja California	Human	Male	74	Deceased	Original	Oropharyngeal swab
hCoV-19/Mexico/NLE-UANL-018/2020	EPI_ISL_779182	05/07/2020	Nuevo Leon	Human	Male	51	unknown	Original	
hCoV-19/Mexico/NLE-UANL-019/2020	EPI_ISL_779183	05/07/2020	Nuevo Leon	Human	Male	35	unknown	Original	
hCoV-19/Mexico/NLE-UANL-020/2020	EPI_ISL_779184	05/07/2020	Nuevo Leon	Human	Female	51	unknown	Original	
hCoV-19/Mexico/CMX-INER-0160/2020	EPI_ISL_837756	06/07/2020	Mexico City	Human	Female	39	Released	Original	Nasopharyngeal and oropharyngeal swab
hCoV-19/Mexico/CMX-INER-0161/2020	EPI_ISL_837757	06/07/2020	Mexico City	Human	Female	56	Deceased	Original	Nasopharyngeal and oropharyngeal swab
hCoV-19/Mexico/ROO-InDRE_243/2020	EPI_ISL_942929	06/07/2020	Quintana Roo	Human	Male	36	Released	Original	Oropharyngeal swab
hCoV-19/Mexico/BCN-ALSR-2560/2020	EPI_ISL_635563	06/07/2020	Baja California	Human	unknown	unknown	unknown	Original	Nasopharyngeal swab
hCoV-19/Mexico/BCN-ALSR-2561/2020	EPI_ISL_635564	06/07/2020	Baja California	Human	unknown	unknown	unknown	Original	Nasopharyngeal swab
hCoV-19/Mexico/BCN-ALSR-2562/2020	EPI_ISL_635565	06/07/2020	Baja California	Human	unknown	unknown	unknown	Original	Nasopharyngeal swab
hCoV-19/Mexico/BCN-ALSR-2563/2020	EPI_ISL_635566	06/07/2020	Baja California	Human	unknown	unknown	unknown	Original	Nasopharyngeal swab
hCoV-19/Mexico/BCN-ALSR-2565/2020	EPI_ISL_635567	06/07/2020	Baja California	Human	unknown	unknown	unknown	Original	Nasopharyngeal swab
hCoV-19/Mexico/CMX-INER-0162/2020	EPI_ISL_837758	07/07/2020	Mexico City	Human	Male	57	Deceased	Original	Nasopharyngeal and oropharyngeal swab
hCoV-19/Mexico/CMX-INER-0163/2020	EPI_ISL_837759	07/07/2020	Mexico City	Human	Female	64	unknown	Original	Nasopharyngeal and oropharyngeal swab
hCoV-19/Mexico/BCN-ALSR-4896/2020	EPI_ISL_730200	07/07/2020	Baja California	Human	unknown	unknown	unknown	Original	Nasopharyngeal swab
hCoV-19/Mexico/NLE-UANL-055/2020	EPI_ISL_961774	07/07/2020	Nuevo Leon	Human	Male	81	Hospitalized	Original	
hCoV-19/Mexico/BCN-ALSR-2566/2020	EPI_ISL_635568	07/07/2020	Baja California	Human	unknown	unknown	unknown	Original	Nasopharyngeal swab
hCoV-19/Mexico/BCN-ALSR-2568/2020	EPI_ISL_635569	07/07/2020	Baja California	Human	unknown	unknown	unknown	Original	Nasopharyngeal swab
hCoV-19/Mexico/BCN-ALSR-4898/2020	EPI_ISL_730201	08/07/2020	Baja California	Human	unknown	unknown	unknown	Original	Nasopharyngeal swab
hCoV-19/Mexico/BCN-ALSR-4899/2020	EPI_ISL_730202	08/07/2020	Baja California	Human	unknown	unknown	unknown	Original	Nasopharyngeal swab
hCoV-19/Mexico/BCN-ALSR-4900/2020	EPI_ISL_730203	08/07/2020	Baja California	Human	unknown	unknown	unknown	Original	Nasopharyngeal swab
hCoV-19/Mexico/BCN-ALSR-4902/2020	EPI_ISL_730204	08/07/2020	Baja California	Human	unknown	unknown	unknown	Original	Nasopharyngeal swab
hCoV-19/Mexico/BCN-ALSR-4903/2020	EPI_ISL_730205	08/07/2020	Baja California	Human	unknown	unknown	unknown	Original	Nasopharyngeal swab
hCoV-19/Mexico/BCN-ALSR-2569/2020	EPI_ISL_635570	08/07/2020	Baja California	Human	unknown	unknown	unknown	Original	Nasopharyngeal swab
hCoV-19/Mexico/BCN-ALSR-2570/2020	EPI_ISL_635571	08/07/2020	Baja California	Human	unknown	unknown	unknown	Original	Nasopharyngeal swab
hCoV-19/Mexico/BCN-ALSR-2571/2020	EPI_ISL_635572	08/07/2020	Baja California	Human	unknown	unknown	unknown	Original	Nasopharyngeal swab
hCoV-19/Mexico/CMX-INER-0164/2020	EPI_ISL_837760	09/07/2020	Mexico City	Human	Female	65	Released	Original	Nasopharyngeal and oropharyngeal swab
hCoV-19/Mexico/CMX-INER-0165/2020	EPI_ISL_837761	10/07/2020	Mexico City	Human	Female	30	Released	Original	Nasopharyngeal and oropharyngeal swab
hCoV-19/Mexico/CMX-INER-0166/2020	EPI_ISL_837762	10/07/2020	Mexico City	Human	Male	50	Deceased	Original	Nasopharyngeal and oropharyngeal swab
hCoV-19/Mexico/BCN-ALSR-4904/2020	EPI_ISL_730206	11/07/2020	Baja California	Human	unknown	unknown	unknown	Original	Nasopharyngeal swab
hCoV-19/Mexico/BCN-ALSR-4905/2020	EPI_ISL_730207	11/07/2020	Baja California	Human	unknown	unknown	unknown	Original	Nasopharyngeal swab

hCoV-19/Mexico/NLE-UANL-085/2020	EPI_ISL_961773	11/07/2020	Nuevo Leon	Human	Male	57	Hospitalized	Original	
hCoV-19/Mexico/CMX-INER-0167/2020	EPI_ISL_837763	12/07/2020	Mexico City	Human	Female	37	Released	Original	Oropharyngeal swab
hCoV-19/Mexico/CMX-INER-0168/2020	EPI_ISL_837764	13/07/2020	Mexico City	Human	Female	64	Released	Original	Nasopharyngeal and oropharyngeal swab
hCoV-19/Mexico/CMX-INER-0169/2020	EPI_ISL_837765	13/07/2020	Mexico City	Human	Male	35	Released	Original	Nasopharyngeal and oropharyngeal swab
hCoV-19/Mexico/NLE-UANL-086/2020	EPI_ISL_961772	13/07/2020	Nuevo Leon	Human	Male	53	Hospitalized	Original	
hCoV-19/Mexico/CMX-INER-0170/2020	EPI_ISL_837766	14/07/2020	Mexico City	Human	Male	38	Released	Original	Nasopharyngeal and oropharyngeal swab
hCoV-19/Mexico/CMX-INER-0171/2020	EPI_ISL_837767	14/07/2020	Mexico City	Human	Female	68	Hospitalized	Original	Nasopharyngeal and oropharyngeal swab
hCoV-19/Mexico/CMX-INER-0172/2020	EPI_ISL_837768	15/07/2020	Mexico City	Human	Male	47	Hospitalized	Original	Tracheal aspirate
hCoV-19/Mexico/CMX-INER-0173/2020	EPI_ISL_837769	15/07/2020	Mexico City	Human	Female	31	Hospitalized	Original	Nasopharyngeal and oropharyngeal swab
hCoV-19/Mexico/NLE-UANL-083/2020	EPI_ISL_961771	15/07/2020	Nuevo Leon	Human	Male	42	Hospitalized	Original	
hCoV-19/Mexico/CMX-INER-0174/2020	EPI_ISL_837770	16/07/2020	Mexico City	Human	Female	36	Released	Original	Oropharyngeal swab
hCoV-19/Mexico/CMX-INER-0175/2020	EPI_ISL_837771	16/07/2020	Mexico City	Human	Male	27	Hospitalized	Original	Nasopharyngeal and oropharyngeal swab
hCoV-19/Mexico/BCN-ALSR-2573/2020	EPI_ISL_635574	16/07/2020	Baja California	Human	unknown	unknown	unknown	Original	Nasopharyngeal swab
hCoV-19/Mexico/BCN-ALSR-2574/2020	EPI_ISL_635575	16/07/2020	Baja California	Human	unknown	unknown	unknown	Original	Nasopharyngeal swab
hCoV-19/Mexico/CMX-INER-0176/2020	EPI_ISL_837772	17/07/2020	Mexico City	Human	Female	71	Deceased	Original	Tracheal aspirate
hCoV-19/Mexico/CMX-INER-0177/2020	EPI_ISL_837773	17/07/2020	Mexico City	Human	Female	43	Released	Original	Nasopharyngeal and oropharyngeal swab
hCoV-19/Mexico/CHP-InDRE_168/2020	EPI_ISL_913969	17/07/2020	Chiapas	Human	Female	22	unknown	Original	Oropharyngeal swab
hCoV-19/Mexico/BCN-ALSR-4910/2020	EPI_ISL_730208	17/07/2020	Baja California	Human	unknown	unknown	unknown	Original	Nasopharyngeal swab
hCoV-19/Mexico/GRO-InDRE-92/2020	EPI_ISL_658899	18/07/2020	Guerrero	Human	Female	47	Released	Original	Oropharyngeal swab
hCoV-19/Mexico/CMX-INER-0178/2020	EPI_ISL_837774	18/07/2020	Mexico City	Human	Male	30	Released	Original	Oropharyngeal swab
hCoV-19/Mexico/CMX-INER-0179/2020	EPI_ISL_837775	18/07/2020	Mexico City	Human	Male	84	Deceased	Original	Nasopharyngeal swab
hCoV-19/Mexico/MOR-InDRE_113/2020	EPI_ISL_913917	18/07/2020	Morelos	Human	Female	28	unknown	Original	Oropharyngeal swab
hCoV-19/Mexico/CMX-INER-0180/2020	EPI_ISL_837776	19/07/2020	Mexico City	Human	Female	49	Released	Original	Oropharyngeal swab
hCoV-19/Mexico/SON-InDRE_329/2020	EPI_ISL_1060763	19/07/2020	Sonora	Human	Male	56	unknown	Original	Oropharyngeal swab
hCoV-19/Mexico/NLE-UANL-056/2020	EPI_ISL_961767	19/07/2020	Nuevo Leon	Human	Male	38	Hospitalized	Original	
hCoV-19/Mexico/NLE-UANL-057/2020	EPI_ISL_961768	19/07/2020	Nuevo Leon	Human	Male	39	Hospitalized	Original	
hCoV-19/Mexico/CMX-INER-0181/2020	EPI_ISL_837777	20/07/2020	Mexico City	Human	Male	46	Hospitalized	Original	Nasopharyngeal and oropharyngeal swab
hCoV-19/Mexico/CMX-INER-0182/2020	EPI_ISL_837778	20/07/2020	Mexico City	Human	Female	24	Released	Original	Nasopharyngeal and oropharyngeal swab
hCoV-19/Mexico/BCN-ALSR-8361/2020	EPI_ISL_1531825	20/07/2020	Baja California	Human	unknown	unknown	unknown	Original	Nasal swab
hCoV-19/Mexico/BCN-ALSR-8362/2020	EPI_ISL_1531826	20/07/2020	Baja California	Human	unknown	unknown	unknown	Original	Nasal swab
hCoV-19/Mexico/BCN-ALSR-2572/2020	EPI_ISL_635573	20/07/2020	Baja California	Human	unknown	unknown	unknown	Original	Nasopharyngeal swab
hCoV-19/Mexico/CMX-INER-0183/2020	EPI_ISL_837779	21/07/2020	Mexico City	Human	Male	unknown	unknown	Original	Nasopharyngeal and oropharyngeal swab
hCoV-19/Mexico/CMX-INER-0184/2020	EPI_ISL_837780	21/07/2020	Mexico City	Human	Female	24	Released	Original	Oropharyngeal swab
hCoV-19/Mexico/CHP-InDRE_107/2020	EPI_ISL_913911	21/07/2020	Chiapas	Human	Male	43	unknown	Original	Oropharyngeal swab
hCoV-19/Mexico/CHP-InDRE_108/2020	EPI_ISL_913912	21/07/2020	Chiapas	Human	Female	34	unknown	Original	Oropharyngeal swab
hCoV-19/Mexico/NLE-UANL-087/2020	EPI_ISL_961766	21/07/2020	Nuevo Leon	Human	Male	37	Hospitalized	Original	
hCoV-19/Mexico/GTO-InDRE_115/2020	EPI_ISL_913919	22/07/2020	Guanajuato	Human	Female	32	unknown	Original	Oropharyngeal swab
hCoV-19/Mexico/CHH-InDRE_325/2020	EPI_ISL_1060762	22/07/2020	Chihuahua	Human	Male	36	Released	Original	Oropharyngeal swab
hCoV-19/Mexico/HID-InDRE-54/2020	EPI_ISL_576257	22/07/2020	Hidalgo	Human	Female	63	unknown	Original	Oropharyngeal swab
hCoV-19/Mexico/CMX-INER-0185/2020	EPI_ISL_837781	23/07/2020	Mexico City	Human	Female	31	Released	Original	Oropharyngeal swab
hCoV-19/Mexico/TLA-InDRE_109/2020	EPI_ISL_913913	23/07/2020	Tlaxcala	Human	Male	28	unknown	Original	Oropharyngeal swab

hCoV-19/Mexico/TLA-InDRE_153/2020	EPI_ISL_913956	23/07/2020	Tlaxcala	Human	Female	44	unknown	Original	Oropharyngeal swab
hCoV-19/Mexico/HID-InDRE_309/2020	EPI_ISL_1060700	23/07/2020	Hidalgo	Human	Male	52	unknown	Original	Oropharyngeal swab
hCoV-19/Mexico/SLP-InDRE_315/2020	EPI_ISL_1060702	23/07/2020	San Luis Potosi	Human	Female	3	unknown	Original	Oropharyngeal swab
hCoV-19/Mexico/VER-InDRE_321/2020	EPI_ISL_1060761	23/07/2020	Veracruz	Human	Female	28	unknown	Original	Oropharyngeal swab
hCoV-19/Mexico/CMX-InDRE_154/2020	EPI_ISL_914879	23/07/2020	Mexico City	Human	Female	63	Released	Original	Oropharyngeal swab
hCoV-19/Mexico/NLE-UANL-079/2020	EPI_ISL_961765	23/07/2020	Nuevo Leon	Human	Male	59	Hospitalized	Original	
hCoV-19/Mexico/SLP-InDRE_316/2020	EPI_ISL_1060695	23/07/2020	San Luis Potosi	Human	Male	31	unknown	Original	Oropharyngeal swab
hCoV-19/Mexico/GUA-InDRE_314/2020	EPI_ISL_1060698	23/07/2020	Guanajuato	Human	Male	41	unknown	Original	Oropharyngeal swab
hCoV-19/Mexico/SLP-AH1COV2SS033_S22 SLP-I	EPI_ISL_1500639	23/07/2020	San Luis Potosi	Human	Female	57	Hospitalized	Original	Nasopharyngeal swab
hCoV-19/Mexico/TAM-InDRE_253/2020	EPI_ISL_1054994	24/07/2020	Tamaulipas	Human	Female	50	unknown	Original	Oropharyngeal swab
hCoV-19/Mexico/MOR-InDRE_254/2020	EPI_ISL_1054995	24/07/2020	Morelos	Human	Female	83	Hospitalized	Original	Oropharyngeal swab
hCoV-19/Mexico/OAX-InDRE_257/2020	EPI_ISL_1054996	24/07/2020	Oaxaca	Human	Male	51	unknown	Original	Oropharyngeal swab
hCoV-19/Mexico/CMX-INER-0186/2020	EPI_ISL_837782	24/07/2020	Mexico City	Human	Female	55	Released	Original	Oropharyngeal swab
hCoV-19/Mexico/CMX-INER-0187/2020	EPI_ISL_837783	24/07/2020	Mexico City	Human	Female	56	Released	Original	Nasopharyngeal and oropharyngeal swab
hCoV-19/Mexico/OAX-InDRE_110/2020	EPI_ISL_913914	24/07/2020	Oaxaca	Human	Female	49	Hospitalized	Original	Oropharyngeal swab
hCoV-19/Mexico/CMX-InDRE_188/2020	EPI_ISL_933665	24/07/2020	Mexico City	Human	Female	39	unknown	Original	Oropharyngeal swab
hCoV-19/Mexico/COL-InDRE_346/2020	EPI_ISL_1060760	24/07/2020	Colima	Human	Female	29	Released	Original	Oropharyngeal swab
hCoV-19/Mexico/HID-InDRE_311/2020	EPI_ISL_1060684	24/07/2020	Hidalgo	Human	Male	58	unknown	Original	Oropharyngeal swab
hCoV-19/Mexico/VER-InDRE_322/2020	EPI_ISL_1060687	24/07/2020	Veracruz	Human	Male	69	unknown	Original	Oropharyngeal swab
hCoV-19/Mexico/BCN-ALSR-2576/2020	EPI_ISL_635576	24/07/2020	Baja California	Human	unknown	unknown	unknown	Original	Nasopharyngeal swab
hCoV-19/Mexico/MIC-InDRE_390/2020	EPI_ISL_1054993	25/07/2020	Michoacan	Human	Female	51	unknown	Original	Oropharyngeal swab
hCoV-19/Mexico/MEX-InDRE_318/2020	EPI_ISL_1060758	25/07/2020	State of Mexico	Human	Male	54	Released	Original	Oropharyngeal swab
hCoV-19/Mexico/SON-InDRE_330/2020	EPI_ISL_1060759	25/07/2020	Sonora	Human	Female	29	unknown	Original	Oropharyngeal swab
hCoV-19/Mexico/OAX-InDRE_258/2020	EPI_ISL_1054990	26/07/2020	Oaxaca	Human	Female	31	unknown	Original	Oropharyngeal swab
hCoV-19/Mexico/GRO-InDRE_384/2020	EPI_ISL_1054991	26/07/2020	Guerrero	Human	Female	41	unknown	Original	Oropharyngeal swab
hCoV-19/Mexico/GRO-InDRE_385/2020	EPI_ISL_1054992	26/07/2020	Guerrero	Human	Male	63	unknown	Original	Oropharyngeal swab
hCoV-19/Mexico/COL-InDRE_348/2020	EPI_ISL_1060757	26/07/2020	Colima	Human	Female	47	Released	Original	Oropharyngeal swab
hCoV-19/Mexico/CMX-InDRE_255/2020	EPI_ISL_1054983	27/07/2020	Mexico City	Human	Male	1	Released	Original	Oropharyngeal swab
hCoV-19/Mexico/BCN-InDRE_256/2020	EPI_ISL_1054984	27/07/2020	Baja California	Human	Female	22	unknown	Original	Oropharyngeal swab
hCoV-19/Mexico/TAM-InDRE_382/2020	EPI_ISL_1054985	27/07/2020	Tamaulipas	Human	Male	36	unknown	Original	Oropharyngeal swab
hCoV-19/Mexico/GUA-InDRE_383/2020	EPI_ISL_1054986	27/07/2020	Guanajuato	Human	Male	60	unknown	Original	Oropharyngeal swab
hCoV-19/Mexico/HID-InDRE_386/2020	EPI_ISL_1054987	27/07/2020	Hidalgo	Human	Male	54	unknown	Original	Oropharyngeal swab
hCoV-19/Mexico/COL-InDRE_394/2020	EPI_ISL_1054988	27/07/2020	Colima	Human	Female	29	Released	Original	Oropharyngeal swab
hCoV-19/Mexico/OAX-InDRE_400/2020	EPI_ISL_1054989	27/07/2020	Oaxaca	Human	Female	33	unknown	Original	Oropharyngeal swab
hCoV-19/Mexico/SLP-AH1COV2SS035_S24 SLP-I	EPI_ISL_1500866	27/07/2020	San Luis Potosi	Human	Male	57	Deceased	Original	
hCoV-19/Mexico/CMX-INER-0188/2020	EPI_ISL_837784	27/07/2020	Mexico City	Human	Female	58	Deceased	Original	Nasopharyngeal and oropharyngeal swab
hCoV-19/Mexico/CMX-INER-0189/2020	EPI_ISL_837785	27/07/2020	Mexico City	Human	Male	52	Hospitalized	Original	Nasopharyngeal and oropharyngeal swab
hCoV-19/Mexico/SON-InDRE_323/2020	EPI_ISL_1060755	27/07/2020	Sonora	Human	Male	61	unknown	Original	Oropharyngeal swab
hCoV-19/Mexico/SON-InDRE_328/2020	EPI_ISL_1060756	27/07/2020	Sonora	Human	Female	55	unknown	Original	Oropharyngeal swab
hCoV-19/Mexico/TLA-InDRE_320/2020	EPI_ISL_1060685	27/07/2020	Tlaxcala	Human	Male	30	unknown	Original	Oropharyngeal swab
hCoV-19/Mexico/TAB-InDRE_380/2020	EPI_ISL_1054980	28/07/2020	Tabasco	Human	Male	64	Released	Original	Oropharyngeal swab

hCoV-19/Mexico/TAM-InDRE_388/2020	EPI_ISL_1054981	28/07/2020	Tamaulipas	Human	Female	30	unknown	Original	Oropharyngeal swab
hCoV-19/Mexico/TAM-InDRE_389/2020	EPI_ISL_1054982	28/07/2020	Tamaulipas	Human	Female	37	unknown	Original	Oropharyngeal swab
hCoV-19/Mexico/MEX-InDRE_259/2020	EPI_ISL_1054977	28/07/2020	State of Mexico	Human	Female	41	unknown	Original	Oropharyngeal swab
hCoV-19/Mexico/SLP-InDRE_260/2020	EPI_ISL_1054978	28/07/2020	San Luis Potosi	Human	Male	58	Hospitalized	Original	Oropharyngeal swab
hCoV-19/Mexico/OAX-InDRE_262/2020	EPI_ISL_1054979	28/07/2020	Oaxaca	Human	Male	39	unknown	Original	Oropharyngeal swab
hCoV-19/Mexico/MEX-InDRE_324/2020	EPI_ISL_1060752	28/07/2020	State of Mexico	Human	Female	26	Released	Original	Oropharyngeal swab
hCoV-19/Mexico/SON-InDRE_339/2020	EPI_ISL_1060753	28/07/2020	Sonora	Human	Male	unknown	unknown	Original	Oropharyngeal swab
hCoV-19/Mexico/MOR-InDRE_359/2020	EPI_ISL_1060754	28/07/2020	Morelos	Human	Female	35	unknown	Original	Oropharyngeal swab
hCoV-19/Mexico/CMX-INER-0190/2020	EPI_ISL_837786	29/07/2020	Mexico City	Human	Male	48	Released	Original	Oropharyngeal swab
hCoV-19/Mexico/CMX-INER-0191/2020	EPI_ISL_837787	29/07/2020	Mexico City	Human	Male	38	Hospitalized	Original	Nasopharyngeal and oropharyngeal swab
hCoV-19/Mexico/CHP-InDRE_396/2020	EPI_ISL_1054973	29/07/2020	Chiapas	Human	Male	18	unknown	Original	Oropharyngeal swab
hCoV-19/Mexico/CHP-InDRE_397/2020	EPI_ISL_1054974	29/07/2020	Chiapas	Human	Female	18	unknown	Original	Oropharyngeal swab
hCoV-19/Mexico/SON-InDRE_398/2020	EPI_ISL_1054975	29/07/2020	Sonora	Human	Male	34	unknown	Original	Oropharyngeal swab
hCoV-19/Mexico/OAX-InDRE_399/2020	EPI_ISL_1054976	29/07/2020	Oaxaca	Human	Female	40	unknown	Original	Oropharyngeal swab
hCoV-19/Mexico/BCN-ALSR-8363/2020	EPI_ISL_1531827	29/07/2020	Baja California	Human	unknown	unknown	unknown	Original	Nasal swab
hCoV-19/Mexico/BCN-ALSR-8364/2020	EPI_ISL_1531828	29/07/2020	Baja California	Human	unknown	unknown	unknown	Original	Nasal swab
hCoV-19/Mexico/BCN-ALSR-8365/2020	EPI_ISL_1531829	29/07/2020	Baja California	Human	unknown	unknown	unknown	Original	Nasal swab
hCoV-19/Mexico/BCN-ALSR-8366/2020	EPI_ISL_1531830	29/07/2020	Baja California	Human	unknown	unknown	unknown	Original	Nasal swab
hCoV-19/Mexico/CMX-INER-0192/2020	EPI_ISL_837788	30/07/2020	Mexico City	Human	Male	unknown	unknown	Original	Nasopharyngeal and oropharyngeal swab
hCoV-19/Mexico/TLA-InDRE_261/2020	EPI_ISL_1054967	30/07/2020	Tlaxcala	Human	Male	25	unknown	Original	Oropharyngeal swab
hCoV-19/Mexico/PUE-InDRE_381/2020	EPI_ISL_1054968	30/07/2020	Puebla	Human	Female	46	Released	Original	Oropharyngeal swab
hCoV-19/Mexico/TLA-InDRE_391/2020	EPI_ISL_1054969	30/07/2020	Tlaxcala	Human	Female	23	unknown	Original	Oropharyngeal swab
hCoV-19/Mexico/GUA-InDRE_395/2020	EPI_ISL_1054970	30/07/2020	Guanajuato	Human	Male	41	unknown	Original	Oropharyngeal swab
hCoV-19/Mexico/QUE-InDRE_401/2020	EPI_ISL_1054971	30/07/2020	Queretaro	Human	Male	29	unknown	Original	Oropharyngeal swab
hCoV-19/Mexico/QUE-InDRE_402/2020	EPI_ISL_1054972	30/07/2020	Queretaro	Human	Male	38	unknown	Original	Oropharyngeal swab
hCoV-19/Mexico/SLP-InDRE_317/2020	EPI_ISL_1060750	30/07/2020	San Luis Potosi	Human	Female	37	unknown	Original	Oropharyngeal swab
hCoV-19/Mexico/BCS-InDRE_327/2020	EPI_ISL_1060751	30/07/2020	Baja California Sur	Human	Male	28	unknown	Original	Oropharyngeal swab
hCoV-19/Mexico/SLP-UASLP-AH1COV2SS026_S	EPI_ISL_1494724	30/07/2020	San Luis Potosi	Human	Male	70	Deceased	Original	
hCoV-19/Mexico/CMX-INER-0193/2020	EPI_ISL_837789	31/07/2020	Mexico City	Human	Male	5	Released	Original	Oropharyngeal swab
hCoV-19/Mexico/CMX-INER-0194/2020	EPI_ISL_837790	31/07/2020	Mexico City	Human	Male	78	Deceased	Original	Oropharyngeal swab
hCoV-19/Mexico/BCS-InDRE_326/2020	EPI_ISL_1060747	31/07/2020	Baja California Sur	Human	Female	41	unknown	Original	Oropharyngeal swab
hCoV-19/Mexico/BCS-InDRE_337/2020	EPI_ISL_1060748	31/07/2020	Baja California Sur	Human	Male	28	unknown	Original	Oropharyngeal swab
hCoV-19/Mexico/BCS-InDRE_340/2020	EPI_ISL_1060749	31/07/2020	Baja California Sur	Human	Male	38	unknown	Original	Oropharyngeal swab
hCoV-19/Mexico/CMX-INMEGEN-01/2020	EPI_ISL_522872	31/07/2020	Mexico City	Human	Female	38	Live	Original	Nasopharyngeal swab
hCoV-19/Mexico/CMX-INMEGEN-02/2020	EPI_ISL_522873	31/07/2020	Mexico City	Human	Male	29	Live	Original	Nasopharyngeal swab
hCoV-19/Mexico/CMX-INMEGEN-03/2020	EPI_ISL_522874	31/07/2020	Mexico City	Human	Male	60	Live	Original	Nasopharyngeal swab
hCoV-19/Mexico/CMX-INMEGEN-04/2020	EPI_ISL_522875	31/07/2020	Mexico City	Human	Female	22	Live	Original	Nasopharyngeal swab
hCoV-19/Mexico/CMX-INMEGEN-05/2020	EPI_ISL_522876	31/07/2020	Mexico City	Human	Female	92	Live	Original	Nasopharyngeal swab
hCoV-19/Mexico/CMX-INMEGEN-08/2020	EPI_ISL_522879	31/07/2020	Mexico City	Human	Male	22	Live	Original	Nasopharyngeal swab
hCoV-19/Mexico/CMX-INMEGEN-11/2020	EPI_ISL_522941	31/07/2020	Mexico City	Human	Female	9	Live	Original	Nasopharyngeal swab
hCoV-19/Mexico/GUA-InDRE_298/2020	EPI_ISL_1060746	01/08/2020	Guanajuato	Human	Female	33	unknown	Original	Oropharyngeal swab

hCoV-19/Mexico/CMX-INMEGEN-06/2020	EPI_ISL_522877	01/08/2020	Mexico City	Human	Male	45	Live	Original	Nasopharyngeal swab
hCoV-19/Mexico/CMX-INMEGEN-07/2020	EPI_ISL_522878	01/08/2020	Mexico City	Human	Male	30	Live	Original	Nasopharyngeal swab
hCoV-19/Mexico/CMX-INMEGEN-09/2020	EPI_ISL_522880	01/08/2020	Mexico City	Human	Male	25	Live	Original	Nasopharyngeal swab
hCoV-19/Mexico/CMX-INMEGEN-10/2020	EPI_ISL_522940	01/08/2020	Mexico City	Human	Male	45	Live	Original	Nasopharyngeal swab
hCoV-19/Mexico/CMX-INER-0195/2020	EPI_ISL_837791	02/08/2020	Mexico City	Human	Male	48	Deceased	Original	Nasopharyngeal and oropharyngeal swab
hCoV-19/Mexico/OAX-InDRE_266/2020	EPI_ISL_1054966	02/08/2020	Oaxaca	Human	Female	51	unknown	Original	Oropharyngeal swab
hCoV-19/Mexico/TAM-InDRE_300/2020	EPI_ISL_1060744	02/08/2020	Tamaulipas	Human	Male	31	unknown	Original	Oropharyngeal swab
hCoV-19/Mexico/SLP-InDRE_301/2020	EPI_ISL_1060745	02/08/2020	San Luis Potosi	Human	Female	24	unknown	Original	Oropharyngeal swab
hCoV-19/Mexico/BCN-ALSR-4942/2020	EPI_ISL_730222	02/08/2020	Baja California	Human	unknown	unknown	unknown	Original	Nasopharyngeal swab
hCoV-19/Mexico/BCN-ALSR-4943/2020	EPI_ISL_730223	02/08/2020	Baja California	Human	unknown	unknown	unknown	Original	Nasopharyngeal swab
hCoV-19/Mexico/BCN-ALSR-4946/2020	EPI_ISL_730224	02/08/2020	Baja California	Human	unknown	unknown	unknown	Original	Nasopharyngeal swab
hCoV-19/Mexico/BCN-ALSR-4947/2020	EPI_ISL_730225	02/08/2020	Baja California	Human	unknown	unknown	unknown	Original	Nasopharyngeal swab
hCoV-19/Mexico/BCN-ALSR-4951/2020	EPI_ISL_730226	02/08/2020	Baja California	Human	unknown	unknown	unknown	Original	Nasopharyngeal swab
hCoV-19/Mexico/BCN-ALSR-4952/2020	EPI_ISL_730227	02/08/2020	Baja California	Human	unknown	unknown	unknown	Original	Nasopharyngeal swab
hCoV-19/Mexico/BCN-ALSR-4953/2020	EPI_ISL_730228	02/08/2020	Baja California	Human	unknown	unknown	unknown	Original	Nasopharyngeal swab
hCoV-19/Mexico/CMX-INER-0196/2020	EPI_ISL_837792	03/08/2020	Mexico City	Human	Female	unknown	unknown	Original	Nasopharyngeal and oropharyngeal swab
hCoV-19/Mexico/MEX-InDRE_263/2020	EPI_ISL_1054964	03/08/2020	State of Mexico	Human	Female	48	Released	Original	Oropharyngeal swab
hCoV-19/Mexico/OAX-InDRE_267/2020	EPI_ISL_1054965	03/08/2020	Oaxaca	Human	Male	57	unknown	Original	Oropharyngeal swab
hCoV-19/Mexico/GUA-InDRE_299/2020	EPI_ISL_1060742	03/08/2020	Guanajuato	Human	Female	23	unknown	Original	Oropharyngeal swab
hCoV-19/Mexico/MEX-InDRE_302/2020	EPI_ISL_1060743	03/08/2020	State of Mexico	Human	Male	59	Hospitalized	Original	Oropharyngeal swab
hCoV-19/Mexico/BCN-ALSR-4917/2020	EPI_ISL_730209	03/08/2020	Baja California	Human	unknown	unknown	unknown	Original	Nasopharyngeal swab
hCoV-19/Mexico/CHP-InDRE_264/2020	EPI_ISL_1054962	04/08/2020	Chiapas	Human	Male	37	unknown	Original	Oropharyngeal swab
hCoV-19/Mexico/CHP-InDRE_265/2020	EPI_ISL_1054963	04/08/2020	Chiapas	Human	Male	45	unknown	Original	Oropharyngeal swab
hCoV-19/Mexico/TLA-InDRE_305/2020	EPI_ISL_1060740	04/08/2020	Tlaxcala	Human	Female	44	unknown	Original	Oropharyngeal swab
hCoV-19/Mexico/GUA-InDRE_306/2020	EPI_ISL_1060741	04/08/2020	Guanajuato	Human	Female	39	unknown	Original	Oropharyngeal swab
hCoV-19/Mexico/BCN-ALSR-8367/2020	EPI_ISL_1531831	04/08/2020	Baja California	Human	unknown	unknown	unknown	Original	Nasal swab
hCoV-19/Mexico/BCN-ALSR-8368/2020	EPI_ISL_1531832	04/08/2020	Baja California	Human	unknown	unknown	unknown	Original	Nasal swab
hCoV-19/Mexico/BCN-ALSR-8369/2020	EPI_ISL_1531833	04/08/2020	Baja California	Human	unknown	unknown	unknown	Original	Nasal swab
hCoV-19/Mexico/BCN-ALSR-8370/2020	EPI_ISL_1531834	04/08/2020	Baja California	Human	unknown	unknown	unknown	Original	Nasal swab
hCoV-19/Mexico/BCN-ALSR-8371/2020	EPI_ISL_1531835	04/08/2020	Baja California	Human	unknown	unknown	unknown	Original	Nasal swab
hCoV-19/Mexico/GUA-InDRE_307/2020	EPI_ISL_1060738	05/08/2020	Guanajuato	Human	Male	34	unknown	Original	Oropharyngeal swab
hCoV-19/Mexico/JAL-InDRE_313/2020	EPI_ISL_1060739	05/08/2020	Jalisco	Human	Male	63	unknown	Original	Oropharyngeal swab
hCoV-19/Mexico/NLE-UANL-059/2020	EPI_ISL_961762	05/08/2020	Nuevo Leon	Human	Female	18	Hospitalized	Original	
hCoV-19/Mexico/NLE-UANL-060/2020	EPI_ISL_961763	05/08/2020	Nuevo Leon	Human	Male	32	Hospitalized	Original	
hCoV-19/Mexico/BCN-ALSR-8372/2020	EPI_ISL_1531836	05/08/2020	Baja California	Human	unknown	unknown	unknown	Original	Nasal swab
hCoV-19/Mexico/BCN-ALSR-8373/2020	EPI_ISL_1531837	05/08/2020	Baja California	Human	unknown	unknown	unknown	Original	Nasal swab
hCoV-19/Mexico/CMX-INMEGEN-20/2020	EPI_ISL_522985	05/08/2020	Mexico City	Human	Female	52	Live	Original	Nasopharyngeal swab
hCoV-19/Mexico/CMX-INER-0197/2020	EPI_ISL_837601	05/08/2020	Mexico City	Human	Female	58	Released	Original	Nasopharyngeal and oropharyngeal swab
hCoV-19/Mexico/CMX-INER-0198/2020	EPI_ISL_837793	06/08/2020	Mexico City	Human	Female	23	Released	Original	Oropharyngeal swab
hCoV-19/Mexico/HID-InDRE_304/2020	EPI_ISL_1060736	06/08/2020	Hidalgo	Human	Male	63	Released	Original	Oropharyngeal swab
hCoV-19/Mexico/TAM-InDRE_308/2020	EPI_ISL_1060737	06/08/2020	Tamaulipas	Human	Female	48	unknown	Original	Oropharyngeal swab

hCoV-19/Mexico/CMX-INMEGEN-22/2020	EPI_ISL_523500	06/08/2020	Mexico City	Human	Male	39	Live	Original	Nasopharyngeal swab
hCoV-19/Mexico/NLE-UANL-084/2020	EPI_ISL_961761	06/08/2020	Nuevo Leon	Human	Female	47	Hospitalized	Original	
hCoV-19/Mexico/CMX-INMEGEN-21/2020	EPI_ISL_522986	06/08/2020	Mexico City	Human	Female	33	Live	Original	Nasopharyngeal swab
hCoV-19/Mexico/BCN-ALSR-8374/2020	EPI_ISL_1531838	06/08/2020	Baja California	Human	unknown	unknown	unknown	Original	Nasal swab
hCoV-19/Mexico/BCN-ALSR-8375/2020	EPI_ISL_1531839	06/08/2020	Baja California	Human	unknown	unknown	unknown	Original	Nasal swab
hCoV-19/Mexico/BCN-ALSR-8376/2020	EPI_ISL_1531840	06/08/2020	Baja California	Human	unknown	unknown	unknown	Original	Nasal swab
hCoV-19/Mexico/CMX-INMEGEN-12/2020	EPI_ISL_522942	06/08/2020	Mexico City	Human	Female	25	Live	Original	Nasopharyngeal swab
hCoV-19/Mexico/CMX-INMEGEN-13/2020	EPI_ISL_522978	06/08/2020	Mexico City	Human	Male	49	Live	Original	Nasopharyngeal swab
hCoV-19/Mexico/CMX-INMEGEN-14/2020	EPI_ISL_522979	06/08/2020	Mexico City	Human	Female	49	Live	Original	Nasopharyngeal swab
hCoV-19/Mexico/CMX-INMEGEN-15/2020	EPI_ISL_522980	06/08/2020	Mexico City	Human	Female	25	Live	Original	Nasopharyngeal swab
hCoV-19/Mexico/CMX-INMEGEN-16/2020	EPI_ISL_522981	06/08/2020	Mexico City	Human	Female	15	Live	Original	Nasopharyngeal swab
hCoV-19/Mexico/CMX-INMEGEN-17/2020	EPI_ISL_522982	06/08/2020	Mexico City	Human	Female	46	Live	Original	Nasopharyngeal swab
hCoV-19/Mexico/CMX-INMEGEN-18/2020	EPI_ISL_522983	06/08/2020	Mexico City	Human	Male	27	Live	Original	Nasopharyngeal swab
hCoV-19/Mexico/CMX-INMEGEN-19/2020	EPI_ISL_522984	06/08/2020	Mexico City	Human	Female	51	Live	Original	Nasopharyngeal swab
hCoV-19/Mexico/CMX-InDRE_268/2020	EPI_ISL_1054960	07/08/2020	Mexico City	Human	Male	26	Released	Original	Oropharyngeal swab
hCoV-19/Mexico/MOR-InDRE_270/2020	EPI_ISL_1054961	07/08/2020	Morelos	Human	Male	39	unknown	Original	Oropharyngeal swab
hCoV-19/Mexico/AGU-InDRE-58/2020	EPI_ISL_576261	07/08/2020	Aguascalientes	Human	Female	66	Released	Original	Oropharyngeal swab
hCoV-19/Mexico/BCN-ALSR-8377/2020	EPI_ISL_1531841	07/08/2020	Baja California	Human	unknown	unknown	unknown	Original	Nasal swab
hCoV-19/Mexico/BCN-ALSR-8378/2020	EPI_ISL_1531842	07/08/2020	Baja California	Human	unknown	unknown	unknown	Original	Nasal swab
hCoV-19/Mexico/CMX-INER-0199/2020	EPI_ISL_837794	08/08/2020	Mexico City	Human	Male	81	Deceased	Original	Nasopharyngeal and oropharyngeal swab
hCoV-19/Mexico/PUE-InDRE_269/2020	EPI_ISL_1054959	08/08/2020	Puebla	Human	Female	63	unknown	Original	Oropharyngeal swab
hCoV-19/Mexico/CMX-InDRE_303/2020	EPI_ISL_1060735	08/08/2020	Mexico City	Human	Male	47	Released	Original	Oropharyngeal swab
hCoV-19/Mexico/BCN-ALSR-4918/2020	EPI_ISL_730210	08/08/2020	Baja California	Human	unknown	unknown	unknown	Original	Nasopharyngeal swab
hCoV-19/Mexico/CMX-INER-0200/2020	EPI_ISL_837795	09/08/2020	Mexico City	Human	Male	72	Hospitalized	Original	Nasopharyngeal and oropharyngeal swab
hCoV-19/Mexico/BCN-ALSR-4920/2020	EPI_ISL_730211	09/08/2020	Baja California	Human	unknown	unknown	unknown	Original	Nasopharyngeal swab
hCoV-19/Mexico/CMX-INER-0201/2020	EPI_ISL_837796	10/08/2020	Mexico City	Human	Male	57	Hospitalized	Original	Oropharyngeal swab
hCoV-19/Mexico/CMX-INER-0202/2020	EPI_ISL_837797	10/08/2020	Mexico City	Human	Male	30	Hospitalized	Original	Nasopharyngeal and oropharyngeal swab
hCoV-19/Mexico/VER-InDRE_272/2020	EPI_ISL_1054957	10/08/2020	Veracruz	Human	Male	46	unknown	Original	Oropharyngeal swab
hCoV-19/Mexico/GRO-InDRE_276/2020	EPI_ISL_1054958	10/08/2020	Guerrero	Human	Male	48	unknown	Original	Oropharyngeal swab
hCoV-19/Mexico/CMX-INER-0203/2020	EPI_ISL_837798	11/08/2020	Mexico City	Human	Male	60	Released	Original	Oropharyngeal swab
hCoV-19/Mexico/CMX-INER-0204/2020	EPI_ISL_837799	11/08/2020	Mexico City	Human	Male	24	Released	Original	Oropharyngeal swab
hCoV-19/Mexico/MEX-InDRE_274/2020	EPI_ISL_1054954	11/08/2020	State of Mexico	Human	Female	40	unknown	Original	Oropharyngeal swab
hCoV-19/Mexico/TAM-InDRE_279/2020	EPI_ISL_1054955	11/08/2020	Tamaulipas	Human	Female	35	unknown	Original	Oropharyngeal swab
hCoV-19/Mexico/MOR-InDRE_280/2020	EPI_ISL_1054956	11/08/2020	Morelos	Human	Male	26	unknown	Original	Oropharyngeal swab
hCoV-19/Mexico/BCN-ALSR-8379/2020	EPI_ISL_1531843	11/08/2020	Baja California	Human	unknown	unknown	unknown	Original	Nasal swab
hCoV-19/Mexico/BCN-ALSR-8380/2020	EPI_ISL_1531844	11/08/2020	Baja California	Human	unknown	unknown	unknown	Original	Nasal swab
hCoV-19/Mexico/BCN-ALSR-8381/2020	EPI_ISL_1531845	11/08/2020	Baja California	Human	unknown	unknown	unknown	Original	Nasal swab
hCoV-19/Mexico/BCN-ALSR-8382/2020	EPI_ISL_1531846	11/08/2020	Baja California	Human	unknown	unknown	unknown	Original	Nasal swab
hCoV-19/Mexico/BCN-ALSR-8383/2020	EPI_ISL_1531847	11/08/2020	Baja California	Human	unknown	unknown	unknown	Original	Nasal swab
hCoV-19/Mexico/BCN-ALSR-8384/2020	EPI_ISL_1531848	11/08/2020	Baja California	Human	unknown	unknown	unknown	Original	Nasal swab
hCoV-19/Mexico/BCN-ALSR-8385/2020	EPI_ISL_1531849	11/08/2020	Baja California	Human	unknown	unknown	unknown	Original	Nasal swab

hCoV-19/Mexico/BCN-ALSR-8386/2020	EPI_ISL_1531850	11/08/2020	Baja California	Human	unknown	unknown	unknown	Original	Nasal swab
hCoV-19/Mexico/GUA-06_LA_FB_05_0178/2020	EPI_ISL_1238787	12/08/2020	Guanajuato	Human	Female	27	Released	Original	Oropharyngeal swab
hCoV-19/Mexico/HID-InDRE_271/2020	EPI_ISL_1054950	12/08/2020	Hidalgo	Human	Male	65	unknown	Original	Oropharyngeal swab
hCoV-19/Mexico/TLA-InDRE_273/2020	EPI_ISL_1054951	12/08/2020	Tlaxcala	Human	Male	20	unknown	Original	Oropharyngeal swab
hCoV-19/Mexico/GUA-InDRE_277/2020	EPI_ISL_1054952	12/08/2020	Guanajuato	Human	Male	30	unknown	Original	Oropharyngeal swab
hCoV-19/Mexico/MEX-InDRE_278/2020	EPI_ISL_1054953	12/08/2020	State of Mexico	Human	Male	43	unknown	Original	Oropharyngeal swab
hCoV-19/Mexico/NLE-UANL-058/2020	EPI_ISL_961760	12/08/2020	Nuevo Leon	Human	Female	31	Hospitalized	Original	
hCoV-19/Mexico/BCN-ALSR-8387/2020	EPI_ISL_1531851	12/08/2020	Baja California	Human	unknown	unknown	unknown	Original	Nasal swab
hCoV-19/Mexico/CMX-INER-0206/2020	EPI_ISL_837800	13/08/2020	Mexico City	Human	Male	26	Released	Original	Nasopharyngeal and oropharyngeal swab
hCoV-19/Mexico/CMX-InDRE_275/2020	EPI_ISL_1054947	13/08/2020	Mexico City	Human	Female	18	Released	Original	Oropharyngeal swab
hCoV-19/Mexico/TLA-InDRE_281/2020	EPI_ISL_1054948	13/08/2020	Tlaxcala	Human	Male	29	unknown	Original	Oropharyngeal swab
hCoV-19/Mexico/MEX-InDRE_282/2020	EPI_ISL_1054949	13/08/2020	State of Mexico	Human	Female	36	unknown	Original	Oropharyngeal swab
hCoV-19/Mexico/TLA-InDRE-56/2020	EPI_ISL_576259	13/08/2020	Tlaxcala	Human	Male	27	Released	Original	Oropharyngeal swab
hCoV-19/Mexico/TLA-InDRE-57/2020	EPI_ISL_576260	13/08/2020	Tlaxcala	Human	Male	51	Released	Original	Oropharyngeal swab
hCoV-19/Mexico/BCN-ALSR-4922/2020	EPI_ISL_730212	13/08/2020	Baja California	Human	unknown	unknown	unknown	Original	Nasopharyngeal swab
hCoV-19/Mexico/BCN-ALSR-4923/2020	EPI_ISL_730213	13/08/2020	Baja California	Human	unknown	unknown	unknown	Original	Nasopharyngeal swab
hCoV-19/Mexico/CMX-INER-0205/2020	EPI_ISL_843192	13/08/2020	Mexico City	Human	Female	81	Deceased	Original	Nasopharyngeal and oropharyngeal swab
hCoV-19/Mexico/BCN-ALSR-8388/2020	EPI_ISL_1531852	13/08/2020	Baja California	Human	unknown	unknown	unknown	Original	Nasal swab
hCoV-19/Mexico/BCN-ALSR-8389/2020	EPI_ISL_1531853	13/08/2020	Baja California	Human	unknown	unknown	unknown	Original	Nasal swab
hCoV-19/Mexico/NLE-InDRE-59/2020	EPI_ISL_576262	14/08/2020	Nuevo Leon	Human	Female	28	Released	Original	Oropharyngeal swab
hCoV-19/Mexico/NLE-InDRE-60/2020	EPI_ISL_576263	14/08/2020	Nuevo Leon	Human	Male	65	Hospitalized	Original	Oropharyngeal swab
hCoV-19/Mexico/VER-InDRE-67/2020	EPI_ISL_576270	14/08/2020	Veracruz	Human	Male	30	Released	Original	Oropharyngeal swab
hCoV-19/Mexico/ZAC-InDRE-72/2020	EPI_ISL_576275	14/08/2020	Zacatecas	Human	Male	73	Deceased	Original	Oropharyngeal swab
hCoV-19/Mexico/BCN-ALSR-8390/2020	EPI_ISL_1531854	14/08/2020	Baja California	Human	unknown	unknown	unknown	Original	Nasal swab
hCoV-19/Mexico/BCN-ALSR-8391/2020	EPI_ISL_1531855	14/08/2020	Baja California	Human	unknown	unknown	unknown	Original	Nasal swab
hCoV-19/Mexico/BCN-ALSR-8392/2020	EPI_ISL_1531856	14/08/2020	Baja California	Human	unknown	unknown	unknown	Original	Nasal swab
hCoV-19/Mexico/BCN-ALSR-8393/2020	EPI_ISL_1531857	14/08/2020	Baja California	Human	unknown	unknown	unknown	Original	Nasal swab
hCoV-19/Mexico/BCN-ALSR-8394/2020	EPI_ISL_1531858	14/08/2020	Baja California	Human	unknown	unknown	unknown	Original	Nasal swab
hCoV-19/Mexico/GRO-InDRE_284/2020	EPI_ISL_1054946	15/08/2020	Guerrero	Human	Female	23	unknown	Original	Oropharyngeal swab
hCoV-19/Mexico/VER-InDRE-66/2020	EPI_ISL_576269	16/08/2020	Veracruz	Human	Male	28	Released	Original	Oropharyngeal swab
hCoV-19/Mexico/CMX-InDRE_283/2020	EPI_ISL_1054944	17/08/2020	Mexico City	Human	Male	34	Released	Original	Oropharyngeal swab
hCoV-19/Mexico/MOR-InDRE_285/2020	EPI_ISL_1054945	17/08/2020	Morelos	Human	Male	51	unknown	Original	Oropharyngeal swab
hCoV-19/Mexico/MIC-InDRE-68/2020	EPI_ISL_576271	17/08/2020	Michoacan	Human	Male	68	Hospitalized	Original	Oropharyngeal swab
hCoV-19/Mexico/BCN-ALSR-8395/2020	EPI_ISL_1531859	17/08/2020	Baja California	Human	unknown	unknown	unknown	Original	Nasal swab
hCoV-19/Mexico/BCN-ALSR-8396/2020	EPI_ISL_1531860	17/08/2020	Baja California	Human	unknown	unknown	unknown	Original	Nasal swab
hCoV-19/Mexico/BCN-ALSR-8397/2020	EPI_ISL_1531861	17/08/2020	Baja California	Human	unknown	unknown	unknown	Original	Nasal swab
hCoV-19/Mexico/TAM-InDRE_287/2020	EPI_ISL_1054941	18/08/2020	Tamaulipas	Human	Female	47	unknown	Original	Oropharyngeal swab
hCoV-19/Mexico/SLP-InDRE_288/2020	EPI_ISL_1054942	18/08/2020	San Luis Potosi	Human	Male	39	unknown	Original	Oropharyngeal swab
hCoV-19/Mexico/SLP-InDRE_289/2020	EPI_ISL_1054943	18/08/2020	San Luis Potosi	Human	Male	36	unknown	Original	Oropharyngeal swab
hCoV-19/Mexico/HID-InDRE_293/2020	EPI_ISL_1060734	18/08/2020	Hidalgo	Human	Female	37	unknown	Original	Oropharyngeal swab
hCoV-19/Mexico/OAX-InDRE-61/2020	EPI_ISL_576264	18/08/2020	Oaxaca	Human	Female	32	Released	Original	Oropharyngeal swab

hCoV-19/Mexico/BCN-ALSR-8398/2020	EPI_ISL_1531862	18/08/2020	Baja California	Human	unknown	unknown	unknown	Original	Nasal swab
hCoV-19/Mexico/GUA-09_LA_FB_05_0249/2020	EPI_ISL_1238788	19/08/2020	Guanajuato	Human	Male	31	Released	Original	Oropharyngeal swab
hCoV-19/Mexico/CMX-INER-0207/2020	EPI_ISL_837801	19/08/2020	Mexico City	Human	Male	unknown	unknown	Original	Nasopharyngeal and oropharyngeal swab
hCoV-19/Mexico/CMX-InDRE_286/2020	EPI_ISL_1054940	19/08/2020	Mexico City	Human	Female	23	Released	Original	Oropharyngeal swab
hCoV-19/Mexico/HID-InDRE_290/2020	EPI_ISL_1060732	19/08/2020	Hidalgo	Human	Male	48	unknown	Original	Oropharyngeal swab
hCoV-19/Mexico/HID-InDRE_292/2020	EPI_ISL_1060733	19/08/2020	Hidalgo	Human	Female	58	unknown	Original	Oropharyngeal swab
hCoV-19/Mexico/BCN-ALSR-8399/2020	EPI_ISL_1531863	19/08/2020	Baja California	Human	unknown	unknown	unknown	Original	Nasal swab
hCoV-19/Mexico/CMX-INER-0208/2020	EPI_ISL_837802	20/08/2020	Mexico City	Human	Female	47	Released	Original	Oropharyngeal swab
hCoV-19/Mexico/CMX-INER-0209/2020	EPI_ISL_837803	20/08/2020	Mexico City	Human	Female	47	Released	Original	Oropharyngeal swab
hCoV-19/Mexico/CMX-INER-0210/2020	EPI_ISL_837804	20/08/2020	Mexico City	Human	Male	44	Released	Original	Oropharyngeal swab
hCoV-19/Mexico/HID-InDRE_291/2020	EPI_ISL_1060731	20/08/2020	Hidalgo	Human	Male	37	unknown	Original	Oropharyngeal swab
hCoV-19/Mexico/MIC-InDRE-73/2020	EPI_ISL_576276	20/08/2020	Michoacan	Human	Male	42	Released	Original	Oropharyngeal swab
hCoV-19/Mexico/MIC-InDRE-74/2020	EPI_ISL_576277	20/08/2020	Michoacan	Human	Female	78	Released	Original	Oropharyngeal swab
hCoV-19/Mexico/BCN-ALSR-4924/2020	EPI_ISL_730214	20/08/2020	Baja California	Human	unknown	unknown	unknown	Original	Nasopharyngeal swab
hCoV-19/Mexico/CMX-INER-0211/2020	EPI_ISL_837805	21/08/2020	Mexico City	Human	Female	39	Hospitalized	Original	Oropharyngeal swab
hCoV-19/Mexico/CAM-InDRE-62/2020	EPI_ISL_576265	21/08/2020	Campeche	Human	Male	44	Released	Original	Oropharyngeal swab
hCoV-19/Mexico/CAM-InDRE-63/2020	EPI_ISL_576266	21/08/2020	Campeche	Human	Male	60	Deceased	Original	Oropharyngeal swab
hCoV-19/Mexico/CAM-InDRE-64/2020	EPI_ISL_576267	21/08/2020	Campeche	Human	Male	64	Released	Original	Oropharyngeal swab
hCoV-19/Mexico/SLP-InDRE-69/2020	EPI_ISL_576272	21/08/2020	San Luis Potosi	Human	Male	31	Released	Original	Oropharyngeal swab
hCoV-19/Mexico/ZAC-InDRE-70/2020	EPI_ISL_576273	21/08/2020	Zacatecas	Human	Male	26	Released	Original	Oropharyngeal swab
hCoV-19/Mexico/ZAC-InDRE-71/2020	EPI_ISL_576274	21/08/2020	Zacatecas	Human	Female	25	Released	Original	Oropharyngeal swab
hCoV-19/Mexico/BCN-ALSR-8400/2020	EPI_ISL_1531864	21/08/2020	Baja California	Human	unknown	unknown	unknown	Original	Nasal swab
hCoV-19/Mexico/BCN-ALSR-8401/2020	EPI_ISL_1531865	21/08/2020	Baja California	Human	unknown	unknown	unknown	Original	Nasal swab
hCoV-19/Mexico/BCN-ALSR-8402/2020	EPI_ISL_1531866	21/08/2020	Baja California	Human	unknown	unknown	unknown	Original	Nasal swab
hCoV-19/Mexico/BCN-ALSR-4771/2020	EPI_ISL_730131	22/08/2020	Baja California	Human	unknown	unknown	unknown	Original	Nasopharyngeal swab
hCoV-19/Mexico/BCN-ALSR-4778/2020	EPI_ISL_730136	22/08/2020	Baja California	Human	unknown	unknown	unknown	Original	Nasopharyngeal swab
hCoV-19/Mexico/CMX-INER-0212/2020	EPI_ISL_837806	22/08/2020	Mexico City	Human	Female	71	Deceased	Original	Nasopharyngeal swab
hCoV-19/Mexico/BCN-ALSR-4762/2020	EPI_ISL_730127	24/08/2020	Baja California	Human	unknown	unknown	unknown	Original	Nasopharyngeal swab
hCoV-19/Mexico/BCN-ALSR-4766/2020	EPI_ISL_730128	24/08/2020	Baja California	Human	unknown	unknown	unknown	Original	Nasopharyngeal swab
hCoV-19/Mexico/BCN-ALSR-4770/2020	EPI_ISL_730130	24/08/2020	Baja California	Human	unknown	unknown	unknown	Original	Nasopharyngeal swab
hCoV-19/Mexico/COL-InDRE-75/2020	EPI_ISL_576278	24/08/2020	Colima	Human	Female	76	Deceased	Original	Oropharyngeal swab
hCoV-19/Mexico/COL-InDRE-76/2020	EPI_ISL_576279	24/08/2020	Colima	Human	Male	27	Released	Original	Oropharyngeal swab
hCoV-19/Mexico/BCN-ALSR-8403/2020	EPI_ISL_1531867	24/08/2020	Baja California	Human	unknown	unknown	unknown	Original	Nasal swab
hCoV-19/Mexico/CMX-INER-0213/2020	EPI_ISL_837603	24/08/2020	Mexico City	Human	Female	54	Released	Original	Nasopharyngeal and oropharyngeal swab
hCoV-19/Mexico/BCN-ALSR-4768/2020	EPI_ISL_730129	25/08/2020	Baja California	Human	unknown	unknown	unknown	Original	Nasopharyngeal swab
hCoV-19/Mexico/BCN-ALSR-4772/2020	EPI_ISL_730132	25/08/2020	Baja California	Human	unknown	unknown	unknown	Original	Nasopharyngeal swab
hCoV-19/Mexico/BCN-ALSR-4774/2020	EPI_ISL_730133	25/08/2020	Baja California	Human	unknown	unknown	unknown	Original	Nasopharyngeal swab
hCoV-19/Mexico/BCN-ALSR-4775/2020	EPI_ISL_730134	25/08/2020	Baja California	Human	unknown	unknown	unknown	Original	Nasopharyngeal swab
hCoV-19/Mexico/BCN-ALSR-4777/2020	EPI_ISL_730135	25/08/2020	Baja California	Human	unknown	unknown	unknown	Original	Nasopharyngeal swab
hCoV-19/Mexico/CMX-INER-0214/2020	EPI_ISL_837807	25/08/2020	Mexico City	Human	Male	82	Released	Original	Nasopharyngeal and oropharyngeal swab
hCoV-19/Mexico/CMX-INER-0215/2020	EPI_ISL_837808	25/08/2020	Mexico City	Human	Female	70	Released	Original	Nasopharyngeal and oropharyngeal swab

hCoV-19/Mexico/HID-InDRE-65/2020	EPI_ISL_576268	25/08/2020	Hidalgo	Human	Male	84	Deceased	Original	Oropharyngeal swab
hCoV-19/Mexico/BCN-ALSR-4926/2020	EPI_ISL_730215	25/08/2020	Baja California	Human	unknown	unknown	unknown	Original	Nasopharyngeal swab
hCoV-19/Mexico/BCN-ALSR-4927/2020	EPI_ISL_730216	25/08/2020	Baja California	Human	unknown	unknown	unknown	Original	Nasopharyngeal swab
hCoV-19/Mexico/BCN-ALSR-4928/2020	EPI_ISL_730217	25/08/2020	Baja California	Human	unknown	unknown	unknown	Original	Nasopharyngeal swab
hCoV-19/Mexico/BCN-ALSR-4929/2020	EPI_ISL_730218	25/08/2020	Baja California	Human	unknown	unknown	unknown	Original	Nasopharyngeal swab
hCoV-19/Mexico/GUA-14_LA_FB_05_0336/2020	EPI_ISL_1238789	26/08/2020	Guanajuato	Human	Male	29	Released	Original	Oropharyngeal swab
hCoV-19/Mexico/GUA-20_LA_FB_05_0345/2020	EPI_ISL_1238790	26/08/2020	Guanajuato	Human	Female	29	Released	Original	Oropharyngeal swab
hCoV-19/Mexico/GUA-26_LA_FB_05_0353/2020	EPI_ISL_1238791	26/08/2020	Guanajuato	Human	Female	22	Released	Original	Oropharyngeal swab
hCoV-19/Mexico/BCN-ALSR-4934/2020	EPI_ISL_730219	26/08/2020	Baja California	Human	unknown	unknown	unknown	Original	Nasopharyngeal swab
hCoV-19/Mexico/BCN-ALSR-4935/2020	EPI_ISL_730220	26/08/2020	Baja California	Human	unknown	unknown	unknown	Original	Nasopharyngeal swab
hCoV-19/Mexico/BCN-ALSR-8359/2020	EPI_ISL_1531823	26/08/2020	Baja California	Human	unknown	unknown	unknown	Original	Nasal swab
hCoV-19/Mexico/BCN-ALSR-8404/2020	EPI_ISL_1531868	26/08/2020	Baja California	Human	unknown	unknown	unknown	Original	Nasal swab
hCoV-19/Mexico/BCN-ALSR-8405/2020	EPI_ISL_1531869	26/08/2020	Baja California	Human	unknown	unknown	unknown	Original	Nasal swab
hCoV-19/Mexico/BCN-ALSR-8406/2020	EPI_ISL_1531870	26/08/2020	Baja California	Human	unknown	unknown	unknown	Original	Nasal swab
hCoV-19/Mexico/BCN-ALSR-8407/2020	EPI_ISL_1531871	26/08/2020	Baja California	Human	unknown	unknown	unknown	Original	Nasal swab
hCoV-19/Mexico/BCN-ALSR-8408/2020	EPI_ISL_1531872	26/08/2020	Baja California	Human	unknown	unknown	unknown	Original	Nasal swab
hCoV-19/Mexico/CMX-INER-0216/2020	EPI_ISL_837809	27/08/2020	Mexico City	Human	Female	82	Deceased	Original	Nasopharyngeal and oropharyngeal swab
hCoV-19/Mexico/BCN-CICESE-BC2/2020	EPI_ISL_747242	28/08/2020	Baja California	Human	Female	24	Outpatient	Vero E6 P1	Nasopharyngeal swab
hCoV-19/Mexico/CMX-INER-0217/2020	EPI_ISL_837602	28/08/2020	Mexico City	Human	Female	70	Released	Original	Nasopharyngeal and oropharyngeal swab
hCoV-19/Mexico/BCN-ALSR-4937/2020	EPI_ISL_730221	31/08/2020	Baja California	Human	unknown	unknown	unknown	Original	Nasopharyngeal swab
hCoV-19/Mexico/BCN-ALSR-8409/2020	EPI_ISL_1531873	31/08/2020	Baja California	Human	unknown	unknown	unknown	Original	Nasal swab
hCoV-19/Mexico/BCN-ALSR-8410/2020	EPI_ISL_1531874	31/08/2020	Baja California	Human	unknown	unknown	unknown	Original	Nasal swab
hCoV-19/Mexico/BCN-ALSR-8411/2020	EPI_ISL_1531875	31/08/2020	Baja California	Human	unknown	unknown	unknown	Original	Nasal swab
hCoV-19/Mexico/BCN-ALSR-8412/2020	EPI_ISL_1531876	01/09/2020	Baja California	Human	unknown	unknown	unknown	Original	Nasal swab
hCoV-19/Mexico/BCN-ALSR-8413/2020	EPI_ISL_1531877	01/09/2020	Baja California	Human	unknown	unknown	unknown	Original	Nasal swab
hCoV-19/Mexico/GUA-31_LA_FB_05_0401/2020	EPI_ISL_1238792	02/09/2020	Guanajuato	Human	Female	38	Released	Original	Oropharyngeal swab
hCoV-19/Mexico/GUA-35_LA_FB_05_0403/2020	EPI_ISL_1238793	02/09/2020	Guanajuato	Human	Male	47	Released	Original	Oropharyngeal swab
hCoV-19/Mexico/CMX-INER-0218/2020	EPI_ISL_837810	02/09/2020	Mexico City	Human	Male	38	Released	Original	Oropharyngeal swab
hCoV-19/Mexico/CMX-INER-0219/2020	EPI_ISL_837811	02/09/2020	Mexico City	Human	Female	64	Released	Original	Nasopharyngeal and oropharyngeal swab
hCoV-19/Mexico/BCN-ALSR-8414/2020	EPI_ISL_1531878	02/09/2020	Baja California	Human	unknown	unknown	unknown	Original	Nasal swab
hCoV-19/Mexico/BCN-ALSR-8415/2020	EPI_ISL_1531879	02/09/2020	Baja California	Human	unknown	unknown	unknown	Original	Nasal swab
hCoV-19/Mexico/BCN-ALSR-8416/2020	EPI_ISL_1531880	02/09/2020	Baja California	Human	unknown	unknown	unknown	Original	Nasal swab
hCoV-19/Mexico/BCN-ALSR-8417/2020	EPI_ISL_1531881	02/09/2020	Baja California	Human	unknown	unknown	unknown	Original	Nasal swab
hCoV-19/Mexico/BCN-ALSR-8418/2020	EPI_ISL_1531882	02/09/2020	Baja California	Human	unknown	unknown	unknown	Original	Nasal swab
hCoV-19/Mexico/CMX-INER-0220/2020	EPI_ISL_837812	03/09/2020	Mexico City	Human	Male	33	Hospitalized	Original	Nasopharyngeal and oropharyngeal swab
hCoV-19/Mexico/CMX-INER-0221/2020	EPI_ISL_837813	03/09/2020	Mexico City	Human	Female	unknown	unknown	Original	Nasopharyngeal and oropharyngeal swab
hCoV-19/Mexico/CMX-INER-0222/2020	EPI_ISL_837814	03/09/2020	Mexico City	Human	Male	52	Hospitalized	Original	Nasopharyngeal and oropharyngeal swab
hCoV-19/Mexico/BCN-ALSR-8419/2020	EPI_ISL_1531883	03/09/2020	Baja California	Human	unknown	unknown	unknown	Original	Nasal swab
hCoV-19/Mexico/BCN-ALSR-8420/2020	EPI_ISL_1531884	03/09/2020	Baja California	Human	unknown	unknown	unknown	Original	Nasal swab
hCoV-19/Mexico/BCN-ALSR-8421/2020	EPI_ISL_1531885	03/09/2020	Baja California	Human	unknown	unknown	unknown	Original	Nasal swab
hCoV-19/Mexico/BCN-ALSR-8422/2020	EPI_ISL_1531886	03/09/2020	Baja California	Human	unknown	unknown	unknown	Original	Nasal swab

hCoV-19/Mexico/BCN-ALSR-8423/2020	EPI_ISL_1531887	03/09/2020	Baja California	Human	unknown	unknown	unknown	Original	Nasal swab
hCoV-19/Mexico/BCN-ALSR-8424/2020	EPI_ISL_1531888	03/09/2020	Baja California	Human	unknown	unknown	unknown	Original	Nasal swab
hCoV-19/Mexico/BCN-ALSR-8425/2020	EPI_ISL_1531889	03/09/2020	Baja California	Human	unknown	unknown	unknown	Original	Nasal swab
hCoV-19/Mexico/BCN-ALSR-8426/2020	EPI_ISL_1531890	04/09/2020	Baja California	Human	unknown	unknown	unknown	Original	Nasal swab
hCoV-19/Mexico/BCN-ALSR-8427/2020	EPI_ISL_1531891	04/09/2020	Baja California	Human	unknown	unknown	unknown	Original	Nasal swab
hCoV-19/Mexico/GRO-InDRE-93/2020	EPI_ISL_658901	07/09/2020	Guerrero	Human	Female	47	Released	Original	Oropharyngeal swab
hCoV-19/Mexico/BCN-ALSR-8428/2020	EPI_ISL_1531892	07/09/2020	Baja California	Human	unknown	unknown	unknown	Original	Nasal swab
hCoV-19/Mexico/BCN-ALSR-8436/2020	EPI_ISL_1531900	08/09/2020	Baja California	Human	unknown	unknown	unknown	Original	Nasal swab
hCoV-19/Mexico/BCN-ALSR-8429/2020	EPI_ISL_1531893	08/09/2020	Baja California	Human	unknown	unknown	unknown	Original	Nasal swab
hCoV-19/Mexico/BCN-ALSR-8430/2020	EPI_ISL_1531894	08/09/2020	Baja California	Human	unknown	unknown	unknown	Original	Nasal swab
hCoV-19/Mexico/BCN-ALSR-8431/2020	EPI_ISL_1531895	08/09/2020	Baja California	Human	unknown	unknown	unknown	Original	Nasal swab
hCoV-19/Mexico/BCN-ALSR-8432/2020	EPI_ISL_1531896	08/09/2020	Baja California	Human	unknown	unknown	unknown	Original	Nasal swab
hCoV-19/Mexico/BCN-ALSR-8433/2020	EPI_ISL_1531897	08/09/2020	Baja California	Human	unknown	unknown	unknown	Original	Nasal swab
hCoV-19/Mexico/BCN-ALSR-8434/2020	EPI_ISL_1531898	08/09/2020	Baja California	Human	unknown	unknown	unknown	Original	Nasal swab
hCoV-19/Mexico/BCN-ALSR-8435/2020	EPI_ISL_1531899	08/09/2020	Baja California	Human	unknown	unknown	unknown	Original	Nasal swab
hCoV-19/Mexico/TAM-UANL-010/2020	EPI_ISL_1091252	09/09/2020	Tamaulipas	Human	Male	57	unknown	Original	
hCoV-19/Mexico/TAM-UANL-009/2020	EPI_ISL_1091269	09/09/2020	Tamaulipas	Human	Male	60	unknown	Original	
hCoV-19/Mexico/TAM-UANL-003/2020	EPI_ISL_1091264	12/09/2020	Tamaulipas	Human	Male	50	unknown	Original	
hCoV-19/Mexico/TAM-UANL-020/2020	EPI_ISL_1091274	17/09/2020	Tamaulipas	Human	Male	47	unknown	Original	
hCoV-19/Mexico/BCN-ALSR-8437/2020	EPI_ISL_1531901	17/09/2020	Baja California	Human	unknown	unknown	unknown	Original	Nasal swab
hCoV-19/Mexico/BCN-ALSR-8438/2020	EPI_ISL_1531902	17/09/2020	Baja California	Human	unknown	unknown	unknown	Original	Nasal swab
hCoV-19/Mexico/BCN-ALSR-8439/2020	EPI_ISL_1531903	17/09/2020	Baja California	Human	unknown	unknown	unknown	Original	Nasal swab
hCoV-19/Mexico/TAM-UANL-019/2020	EPI_ISL_1091260	18/09/2020	Tamaulipas	Human	Female	24	unknown	Original	
hCoV-19/Mexico/BCN-CICESE-BC5/2020	EPI_ISL_747243	21/09/2020	Baja California	Human	Male	29	Hospitalized	Vero E6 P1	Nasopharyngeal swab
hCoV-19/Mexico/BCN-ALSR-8440/2020	EPI_ISL_1531904	22/09/2020	Baja California	Human	unknown	unknown	unknown	Original	Nasal swab
hCoV-19/Mexico/BCN-ALSR-8441/2020	EPI_ISL_1531905	22/09/2020	Baja California	Human	unknown	unknown	unknown	Original	Nasal swab
hCoV-19/Mexico/BCN-ALSR-8442/2020	EPI_ISL_1531906	23/09/2020	Baja California	Human	unknown	unknown	unknown	Original	Nasal swab
hCoV-19/Mexico/BCN-ALSR-8443/2020	EPI_ISL_1531907	23/09/2020	Baja California	Human	unknown	unknown	unknown	Original	Nasal swab
hCoV-19/Mexico/BCN-ALSR-8450/2020	EPI_ISL_1531913	23/09/2020	Baja California	Human	unknown	unknown	unknown	Original	Nasal swab
hCoV-19/Mexico/BCN-ALSR-8451/2020	EPI_ISL_1531914	23/09/2020	Baja California	Human	unknown	unknown	unknown	Original	Nasal swab
hCoV-19/Mexico/BCN-ALSR-8452/2020	EPI_ISL_1531915	23/09/2020	Baja California	Human	unknown	unknown	unknown	Original	Nasal swab
hCoV-19/Mexico/BCN-ALSR-8453/2020	EPI_ISL_1531916	23/09/2020	Baja California	Human	unknown	unknown	unknown	Original	Nasal swab
hCoV-19/Mexico/BCN-ALSR-8462/2020	EPI_ISL_1531925	23/09/2020	Baja California	Human	unknown	unknown	unknown	Original	Nasal swab
hCoV-19/Mexico/BCN-ALSR-8471/2020	EPI_ISL_1531934	23/09/2020	Baja California	Human	unknown	unknown	unknown	Original	Nasal swab
hCoV-19/Mexico/BCN-ALSR-8455/2020	EPI_ISL_1531918	24/09/2020	Baja California	Human	unknown	unknown	unknown	Original	Nasal swab
hCoV-19/Mexico/BCN-ALSR-8463/2020	EPI_ISL_1531926	24/09/2020	Baja California	Human	unknown	unknown	unknown	Original	Nasal swab
hCoV-19/Mexico/BCN-ALSR-8456/2020	EPI_ISL_1531919	25/09/2020	Baja California	Human	unknown	unknown	unknown	Original	Nasal swab
hCoV-19/Mexico/BCN-ALSR-8464/2020	EPI_ISL_1531927	25/09/2020	Baja California	Human	unknown	unknown	unknown	Original	Nasal swab
hCoV-19/Mexico/BCN-ALSR-8444/2020	EPI_ISL_1531908	28/09/2020	Baja California	Human	unknown	unknown	unknown	Original	Nasal swab
hCoV-19/Mexico/BCN-ALSR-8445/2020	EPI_ISL_1531909	28/09/2020	Baja California	Human	unknown	unknown	unknown	Original	Nasal swab
hCoV-19/Mexico/BCN-ALSR-8454/2020	EPI_ISL_1531917	28/09/2020	Baja California	Human	unknown	unknown	unknown	Original	Nasal swab

hCoV-19/Mexico/BCN-ALSR-8457/2020	EPI_ISL_1531920	28/09/2020	Baja California	Human	unknown	unknown	unknown	Original	Nasal swab
hCoV-19/Mexico/BCN-ALSR-8459/2020	EPI_ISL_1531922	28/09/2020	Baja California	Human	unknown	unknown	unknown	Original	Nasal swab
hCoV-19/Mexico/BCN-ALSR-8460/2020	EPI_ISL_1531923	28/09/2020	Baja California	Human	unknown	unknown	unknown	Original	Nasal swab
hCoV-19/Mexico/TAM-UANL-011/2020	EPI_ISL_1091270	02/10/2020	Tamaulipas	Human	Male	16	unknown	Original	
hCoV-19/Mexico/BCN-ALSR-8448/2020	EPI_ISL_1531912	02/10/2020	Baja California	Human	unknown	unknown	unknown	Original	Nasal swab
hCoV-19/Mexico/BCN-ALSR-8458/2020	EPI_ISL_1531921	02/10/2020	Baja California	Human	unknown	unknown	unknown	Original	Nasal swab
hCoV-19/Mexico/BCN-ALSR-8461/2020	EPI_ISL_1531924	02/10/2020	Baja California	Human	unknown	unknown	unknown	Original	Nasal swab
hCoV-19/Mexico/TAM-UANL-012/2020	EPI_ISL_1091271	03/10/2020	Tamaulipas	Human	Male	36	unknown	Original	
hCoV-19/Mexico/TAM-UANL-017/2020	EPI_ISL_1091247	06/10/2020	Tamaulipas	Human	Male	78	unknown	Original	
hCoV-19/Mexico/BCN-ALSR-8446/2020	EPI_ISL_1531910	08/10/2020	Baja California	Human	unknown	unknown	unknown	Original	Nasal swab
hCoV-19/Mexico/BCN-ALSR-8447/2020	EPI_ISL_1531911	08/10/2020	Baja California	Human	unknown	unknown	unknown	Original	Nasal swab
hCoV-19/Mexico/AGU-InDRE_F11763B_S1085/20	EPI_ISL_1516778	10/10/2020	Aguascalientes	Human	Male	43	unknown	Original	Oropharyngeal swab
hCoV-19/Mexico/TAM-UANL-018/2020	EPI_ISL_1091273	11/10/2020	Tamaulipas	Human	Male	47	unknown	Original	
hCoV-19/Mexico/TAM-UANL-001/2020	EPI_ISL_1091263	12/10/2020	Tamaulipas	Human	Male	55	unknown	Original	
hCoV-19/Mexico/CMX-INMEGEN-02-10-23-GRO/2	EPI_ISL_1137477	12/10/2020	Mexico City	Human	Male	53	unknown	Original	Oropharyngeal swab
hCoV-19/Mexico/VER-InDRE_116/2020	EPI_ISL_913920	13/10/2020	Veracruz	Human	Female	35	unknown	Original	Oropharyngeal swab
hCoV-19/Mexico/VER-InDRE_117/2020	EPI_ISL_913921	13/10/2020	Veracruz	Human	Male	55	unknown	Original	Oropharyngeal swab
hCoV-19/Mexico/TAM-InDRE_119/2020	EPI_ISL_913923	13/10/2020	Tamaulipas	Human	Male	39	unknown	Original	Oropharyngeal swab
hCoV-19/Mexico/MOR-InDRE_118/2020	EPI_ISL_913922	14/10/2020	Morelos	Human	Male	46	unknown	Original	Oropharyngeal swab
hCoV-19/Mexico/GRO-InDRE_120/2020	EPI_ISL_913924	14/10/2020	Guerrero	Human	Female	28	unknown	Original	Oropharyngeal swab
hCoV-19/Mexico/TAM-UANL-014/2020	EPI_ISL_1091258	16/10/2020	Tamaulipas	Human	Female	31	unknown	Original	
hCoV-19/Mexico/TAM-UANL-015/2020	EPI_ISL_1091259	16/10/2020	Tamaulipas	Human	Female	55	unknown	Original	
hCoV-19/Mexico/BCN-ALSR-8470/2020	EPI_ISL_1531933	16/10/2020	Baja California	Human	unknown	unknown	unknown	Original	Nasal swab
hCoV-19/Mexico/CMX-InDRE_166/2020	EPI_ISL_913967	16/10/2020	Mexico City	Human	Male	28	Released	Original	Oropharyngeal swab
hCoV-19/Mexico/NL-InDRE_122/2020	EPI_ISL_913926	17/10/2020	Nuevo Leon	Human	Male	15	Released	Original	Oropharyngeal swab
hCoV-19/Mexico/MEX-InDRE_121/2020	EPI_ISL_913925	19/10/2020	State of Mexico	Human	Male	19	unknown	Original	Oropharyngeal swab
hCoV-19/Mexico/TAM-InDRE_189/2020	EPI_ISL_933666	19/10/2020	Tamaulipas	Human	Male	36	unknown	Original	Oropharyngeal swab
hCoV-19/Mexico/HGO-InDRE_123/2020	EPI_ISL_913927	20/10/2020	Hidalgo	Human	Female	39	Released	Original	Oropharyngeal swab
hCoV-19/Mexico/MEX-InDRE_124/2020	EPI_ISL_913928	20/10/2020	State of Mexico	Human	Male	26	Released	Original	Oropharyngeal swab
hCoV-19/Mexico/MOR-InDRE_155/2020	EPI_ISL_913957	20/10/2020	Morelos	Human	Male	60	Released	Original	Oropharyngeal swab
hCoV-19/Mexico/TAM-UANL-007/2020	EPI_ISL_1091267	21/10/2020	Tamaulipas	Human	Male	41	unknown	Original	
hCoV-19/Mexico/BCN-ALSR-8472/2020	EPI_ISL_1531935	21/10/2020	Baja California	Human	unknown	unknown	unknown	Original	Nasal swab
hCoV-19/Mexico/TLA-InDRE_126/2020	EPI_ISL_913929	21/10/2020	Tlaxcala	Human	Female	32	Released	Original	Oropharyngeal swab
hCoV-19/Mexico/VER-InDRE_127/2020	EPI_ISL_913930	21/10/2020	Veracruz	Human	Male	31	Released	Original	Oropharyngeal swab
hCoV-19/Mexico/PUE-InDRE_128/2020	EPI_ISL_913931	21/10/2020	Puebla	Human	Female	25	Released	Original	Oropharyngeal swab
hCoV-19/Mexico/PUE-InDRE_129/2020	EPI_ISL_913932	21/10/2020	Puebla	Human	Female	49	Released	Original	Oropharyngeal swab
hCoV-19/Mexico/PUE-InDRE_190/2020	EPI_ISL_933667	21/10/2020	Puebla	Human	Female	45	unknown	Original	Oropharyngeal swab
hCoV-19/Mexico/AGU-InDRE_F11767B_S1089/20	EPI_ISL_1516781	21/10/2020	Aguascalientes	Human	Male	34	unknown	Original	Oropharyngeal swab
hCoV-19/Mexico/TAM-UANL-008/2020	EPI_ISL_1091268	22/10/2020	Tamaulipas	Human	Male	22	unknown	Original	
hCoV-19/Mexico/AGU-InDRE_F11768B_S1090/20	EPI_ISL_1516782	22/10/2020	Aguascalientes	Human	unknown	unknown	unknown	Original	Oropharyngeal swab
hCoV-19/Mexico/TAM-UANL-005/2020	EPI_ISL_1091248	29/10/2020	Tamaulipas	Human	Female	35	unknown	Original	

hCoV-19/Mexico/TAM-UANL-004/2020	EPI_ISL_1091265	29/10/2020	Tamaulipas	Human	Male	57	unknown	Original	
hCoV-19/Mexico/TAM-UANL-006/2020	EPI_ISL_1091266	29/10/2020	Tamaulipas	Human	Male	38	unknown	Original	
hCoV-19/Mexico/AGS-InDRE-89/2020	EPI_ISL_658891	01/11/2020	Aguascalientes	Human	Male	37	Released	Original	Oropharyngeal swab
hCoV-19/Mexico/SLP-InDRE-77/2020	EPI_ISL_660068	01/11/2020	San Luis Potosi	Human	Male	66	Released	Original	Oropharyngeal swab
hCoV-19/Mexico/AGS-InDRE-78/2020	EPI_ISL_658863	02/11/2020	Aguascalientes	Human	Female	45	Released	Original	Oropharyngeal swab
hCoV-19/Mexico/AGS-InDRE-79/2020	EPI_ISL_658865	02/11/2020	Aguascalientes	Human	Male	28	Released	Original	Oropharyngeal swab
hCoV-19/Mexico/AGS-InDRE-80/2020	EPI_ISL_658868	02/11/2020	Aguascalientes	Human	Male	42	Released	Original	Oropharyngeal swab
hCoV-19/Mexico/AGS-InDRE-81/2020	EPI_ISL_658870	02/11/2020	Aguascalientes	Human	Female	48	Released	Original	Oropharyngeal swab
hCoV-19/Mexico/AGS-InDRE-82/2020	EPI_ISL_658873	02/11/2020	Aguascalientes	Human	Female	19	Released	Original	Oropharyngeal swab
hCoV-19/Mexico/AGS-InDRE-83/2020	EPI_ISL_658875	02/11/2020	Aguascalientes	Human	Female	26	Released	Original	Oropharyngeal swab
hCoV-19/Mexico/QRO-InDRE-84/2020	EPI_ISL_658878	02/11/2020	Queretaro	Human	Male	23	Released	Original	Oropharyngeal swab
hCoV-19/Mexico/AGS-InDRE-85/2020	EPI_ISL_658880	02/11/2020	Aguascalientes	Human	Male	41	Released	Original	Oropharyngeal swab
hCoV-19/Mexico/AGS-InDRE-86/2020	EPI_ISL_658883	02/11/2020	Aguascalientes	Human	Female	45	Released	Original	Oropharyngeal swab
hCoV-19/Mexico/QRO-InDRE-87/2020	EPI_ISL_658886	03/11/2020	Queretaro	Human	Female	47	Released	Original	Oropharyngeal swab
hCoV-19/Mexico/QRO-InDRE-88/2020	EPI_ISL_658888	03/11/2020	Queretaro	Human	Female	44	Released	Original	Oropharyngeal swab
hCoV-19/Mexico/QRO-InDRE-90/2020	EPI_ISL_658893	03/11/2020	Queretaro	Human	Female	46	Released	Original	Oropharyngeal swab
hCoV-19/Mexico/AGU-InDRE_F11765B_S1087/20	EPI_ISL_1516780	04/11/2020	Aguascalientes	Human	Female	55	unknown	Original	Oropharyngeal swab
hCoV-19/Mexico/CMX-InDRE_229/2020	EPI_ISL_933706	06/11/2020	Mexico City	Human	Female	51	unknown	Original	Oropharyngeal swab
hCoV-19/Mexico/AGU-InDRE_F11762B_S1084/20	EPI_ISL_1516777	07/11/2020	Aguascalientes	Human	Female	69	unknown	Original	Oropharyngeal swab
hCoV-19/Mexico/BCN-ALSR-8468/2020	EPI_ISL_1531931	09/11/2020	Baja California	Human	unknown	unknown	unknown	Original	Nasal swab
hCoV-19/Mexico/CMX-INMEGEN-01-01-02/2020	EPI_ISL_1005657	09/11/2020	Mexico City	Human	Male	32	unknown	Original	Oropharyngeal swab
hCoV-19/Mexico/CMX-INMEGEN-01-01-03/2020	EPI_ISL_1005658	10/11/2020	Mexico City	Human	Male	32	unknown	Original	Oropharyngeal swab
hCoV-19/Mexico/BCN-ALSR-8474/2020	EPI_ISL_1531937	11/11/2020	Baja California	Human	unknown	unknown	unknown	Original	Nasal swab
hCoV-19/Mexico/CMX-INMEGEN-03-11-67/2020	EPI_ISL_1591520	12/11/2020	Mexico City	Human	Male	16	unknown	Original	Oropharyngeal swab
hCoV-19/Mexico/CMX-INCMNSZ_COV121371/202	EPI_ISL_1502816	13/11/2020	Mexico City	Human	Female	29	Released	Original	Nasopharyngeal swab
hCoV-19/Mexico/CMX-INMEGEN-02-09-18/2020	EPI_ISL_1095516	13/11/2020	Mexico City	Human	Female	30	unknown	Original	Oropharyngeal swab
hCoV-19/Mexico/NLE-UANL-030/2020	EPI_ISL_779191	14/11/2020	Nuevo Leon	Human	Female	35	unknown	Original	
hCoV-19/Mexico/NLE-UANL-035/2020	EPI_ISL_779196	16/11/2020	Nuevo Leon	Human	Female	43	unknown	Original	
hCoV-19/Mexico/SLP-InDRE_130/2020	EPI_ISL_913933	17/11/2020	San Luis Potosi	Human	Female	43	unknown	Original	Oropharyngeal swab
hCoV-19/Mexico/AGU-InDRE_F11764B_S1086/20	EPI_ISL_1516779	17/11/2020	Aguascalientes	Human	Female	36	unknown	Original	Oropharyngeal swab
hCoV-19/Mexico/NLE-UANL-026/2020	EPI_ISL_779188	17/11/2020	Nuevo Leon	Human	Male	54	unknown	Original	
hCoV-19/Mexico/HGO-InDRE_156/2020	EPI_ISL_913958	18/11/2020	Hidalgo	Human	Male	58	unknown	Original	Oropharyngeal swab
hCoV-19/Mexico/CMX-InDRE_230/2020	EPI_ISL_933707	18/11/2020	Mexico City	Human	Female	31	unknown	Original	Oropharyngeal swab
hCoV-19/Mexico/CMX-INMEGEN-12-03-09/2020	EPI_ISL_955200	18/11/2020	Mexico City	Human	Male	53	unknown	Original	Oropharyngeal swab
hCoV-19/Mexico/CMX-INMEGEN-12-03-10/2020	EPI_ISL_955201	18/11/2020	Mexico City	Human	Female	43	unknown	Original	Oropharyngeal swab
hCoV-19/Mexico/CMX-INMEGEN-12-03-11/2020	EPI_ISL_955202	18/11/2020	Mexico City	Human	Female	61	unknown	Original	Oropharyngeal swab
hCoV-19/Mexico/CMX-INMEGEN-12-03-13/2020	EPI_ISL_955203	18/11/2020	Mexico City	Human	Female	20	unknown	Original	Oropharyngeal swab
hCoV-19/Mexico/CMX-INMEGEN-12-03-14/2020	EPI_ISL_955204	18/11/2020	Mexico City	Human	Female	21	unknown	Original	Oropharyngeal swab
hCoV-19/Mexico/CMX-INMEGEN-12-03-15/2020	EPI_ISL_955205	18/11/2020	Mexico City	Human	Male	62	unknown	Original	Oropharyngeal swab
hCoV-19/Mexico/CMX-INMEGEN-12-03-16/2020	EPI_ISL_955206	18/11/2020	Mexico City	Human	Male	30	unknown	Original	Oropharyngeal swab
hCoV-19/Mexico/NLE-UANL-031/2020	EPI_ISL_779192	18/11/2020	Nuevo Leon	Human	Male	32	unknown	Original	

hCoV-19/Mexico/CMX-INMEGEN-01-01-07/2020	EPI_ISL_1005660	18/11/2020	Mexico City	Human	Female	20	unknown	Original	Oropharyngeal swab
hCoV-19/Mexico/CMX-INMEGEN-12-03-20/2020	EPI_ISL_955189	19/11/2020	Mexico City	Human	Male	27	unknown	Original	Oropharyngeal swab
hCoV-19/Mexico/CMX-INMEGEN-12-03-21/2020	EPI_ISL_955190	19/11/2020	Mexico City	Human	Male	40	unknown	Original	Oropharyngeal swab
hCoV-19/Mexico/CMX-INMEGEN-12-03-22/2020	EPI_ISL_955191	19/11/2020	Mexico City	Human	Male	53	unknown	Original	Oropharyngeal swab
hCoV-19/Mexico/CMX-INMEGEN-12-03-01/2020	EPI_ISL_955194	19/11/2020	Mexico City	Human	Male	62	unknown	Original	Oropharyngeal swab
hCoV-19/Mexico/CMX-INMEGEN-12-03-02/2020	EPI_ISL_955195	19/11/2020	Mexico City	Human	Female	67	unknown	Original	Oropharyngeal swab
hCoV-19/Mexico/CMX-INMEGEN-12-03-06/2020	EPI_ISL_955197	19/11/2020	Mexico City	Human	Male	47	unknown	Original	Oropharyngeal swab
hCoV-19/Mexico/CMX-INMEGEN-12-03-07/2020	EPI_ISL_955198	19/11/2020	Mexico City	Human	Male	32	unknown	Original	Oropharyngeal swab
hCoV-19/Mexico/CMX-INMEGEN-12-03-08/2020	EPI_ISL_955199	19/11/2020	Mexico City	Human	Male	38	unknown	Original	Oropharyngeal swab
hCoV-19/Mexico/CMX-INMEGEN-12-03-17/2020	EPI_ISL_955207	19/11/2020	Mexico City	Human	Female	46	unknown	Original	Oropharyngeal swab
hCoV-19/Mexico/CMX-INMEGEN-12-03-23/2020	EPI_ISL_955208	19/11/2020	Mexico City	Human	Female	10	unknown	Original	Oropharyngeal swab
hCoV-19/Mexico/CMX-INMEGEN-12-03-24/2020	EPI_ISL_955209	19/11/2020	Mexico City	Human	Female	10	unknown	Original	Oropharyngeal swab
hCoV-19/Mexico/NLE-UANL-022/2020	EPI_ISL_779185	19/11/2020	Nuevo Leon	Human	Male	54	unknown	Original	
hCoV-19/Mexico/NLE-UANL-023/2020	EPI_ISL_779186	19/11/2020	Nuevo Leon	Human	Male	24	unknown	Original	
hCoV-19/Mexico/BCN-ALSR-8466/2020	EPI_ISL_1531929	20/11/2020	Baja California	Human	unknown	unknown	unknown	Original	Nasal swab
hCoV-19/Mexico/BCN-ALSR-8477/2020	EPI_ISL_1531940	20/11/2020	Baja California	Human	unknown	unknown	unknown	Original	Nasal swab
hCoV-19/Mexico/CHP-InDRE_131/2020	EPI_ISL_913934	20/11/2020	Chiapas	Human	Male	32	unknown	Original	Oropharyngeal swab
hCoV-19/Mexico/CHP-InDRE_132/2020	EPI_ISL_913935	20/11/2020	Chiapas	Human	Male	30	unknown	Original	Oropharyngeal swab
hCoV-19/Mexico/CMX-INMEGEN-11-06/2020	EPI_ISL_933516	20/11/2020	Mexico City	Human	Male	25	unknown	Original	Oropharyngeal swab
hCoV-19/Mexico/CMX-INMEGEN-11-08/2020	EPI_ISL_933518	20/11/2020	Mexico City	Human	Female	49	unknown	Original	Oropharyngeal swab
hCoV-19/Mexico/CMX-INMEGEN-12-03-05/2020	EPI_ISL_955192	20/11/2020	Mexico City	Human	Male	69	unknown	Original	Oropharyngeal swab
hCoV-19/Mexico/CMX-INMEGEN-12-03-12/2020	EPI_ISL_955193	20/11/2020	Mexico City	Human	Female	25	unknown	Original	Oropharyngeal swab
hCoV-19/Mexico/CMX-INMEGEN-12-03-04/2020	EPI_ISL_955196	20/11/2020	Mexico City	Human	Male	31	unknown	Original	Oropharyngeal swab
hCoV-19/Mexico/CMX-INMEGEN-12-04-01/2020	EPI_ISL_1005559	20/11/2020	Mexico City	Human	Male	51	unknown	Original	Oropharyngeal swab
hCoV-19/Mexico/VER-InDRE_169/2020	EPI_ISL_913970	21/11/2020	Veracruz	Human	Female	68	Released	Original	Oropharyngeal swab
hCoV-19/Mexico/MIC-InDRE_191/2020	EPI_ISL_933668	21/11/2020	Michoacan	Human	Female	56	unknown	Original	Oropharyngeal swab
hCoV-19/Mexico/NLE-UANL-037/2020	EPI_ISL_779198	21/11/2020	Nuevo Leon	Human	Female	51	unknown	Original	
hCoV-19/Mexico/BCN-ALSR-5655/2020	EPI_ISL_878300	23/11/2020	Baja California	Human	unknown	unknown	unknown	Original	Nasal swab
hCoV-19/Mexico/QUE-InDRE_294/2020	EPI_ISL_1060729	23/11/2020	Queretaro	Human	Male	22	unknown	Original	Oropharyngeal swab
hCoV-19/Mexico/QUE-InDRE_295/2020	EPI_ISL_1060730	23/11/2020	Queretaro	Human	Male	40	unknown	Original	Oropharyngeal swab
hCoV-19/Mexico/BCN-ALSR-5748/2020	EPI_ISL_878400	23/11/2020	Baja California	Human	unknown	unknown	unknown	Original	Nasal swab
hCoV-19/Mexico/BCN-ALSR-5749/2020	EPI_ISL_878401	23/11/2020	Baja California	Human	unknown	unknown	unknown	Original	Nasal swab
hCoV-19/Mexico/BCN-ALSR-5753/2020	EPI_ISL_878404	23/11/2020	Baja California	Human	unknown	unknown	unknown	Original	Nasal swab
hCoV-19/Mexico/BCN-ALSR-5757/2020	EPI_ISL_878408	23/11/2020	Baja California	Human	unknown	unknown	unknown	Original	Nasal swab
hCoV-19/Mexico/BCN-ALSR-5773/2020	EPI_ISL_878437	23/11/2020	Baja California	Human	unknown	unknown	unknown	Original	Nasal swab
hCoV-19/Mexico/CMX-INMEGEN-12-04-09/2020	EPI_ISL_1005567	23/11/2020	Mexico City	Human	Female	31	unknown	Original	Oropharyngeal swab
hCoV-19/Mexico/CMX-INMEGEN-12-04-10/2020	EPI_ISL_1005568	23/11/2020	Mexico City	Human	Female	49	unknown	Original	Oropharyngeal swab
hCoV-19/Mexico/CMX-INMEGEN-12-04-11/2020	EPI_ISL_1005569	23/11/2020	Mexico City	Human	Male	47	unknown	Original	Oropharyngeal swab
hCoV-19/Mexico/CMX-INMEGEN-12-04-12/2020	EPI_ISL_1005570	23/11/2020	Mexico City	Human	Female	29	unknown	Original	Oropharyngeal swab
hCoV-19/Mexico/BCN-ALSR-5717/2020	EPI_ISL_878375	24/11/2020	Baja California	Human	unknown	unknown	unknown	Original	Nasal swab
hCoV-19/Mexico/BCN-ALSR-5732/2020	EPI_ISL_878390	24/11/2020	Baja California	Human	unknown	unknown	unknown	Original	Nasal swab

hCoV-19/Mexico/SON-InDRE_135/2020	EPI_ISL_913938	24/11/2020	Sonora	Human	Female	47	unknown	Original	Oropharyngeal swab
hCoV-19/Mexico/GUA-InDRE_296/2020	EPI_ISL_1060728	24/11/2020	Guanajuato	Human	Female	34	unknown	Original	Oropharyngeal swab
hCoV-19/Mexico/BCN-ALSR-8467/2020	EPI_ISL_1531930	25/11/2020	Baja California	Human	unknown	unknown	unknown	Original	Nasal swab
hCoV-19/Mexico/BCN-ALSR-8473/2020	EPI_ISL_1531936	25/11/2020	Baja California	Human	unknown	unknown	unknown	Original	Nasal swab
hCoV-19/Mexico/BC-InDRE_134/2020	EPI_ISL_913937	25/11/2020	Baja California	Human	Female	57	unknown	Original	Oropharyngeal swab
hCoV-19/Mexico/PUE-InDRE_157/2020	EPI_ISL_913959	25/11/2020	Puebla	Human	Female	44	unknown	Original	Oropharyngeal swab
hCoV-19/Mexico/AGU-InDRE_F11771B_S1093/20	EPI_ISL_1516785	25/11/2020	Aguascalientes	Human	Female	unknown	unknown	Original	Oropharyngeal swab
hCoV-19/Mexico/BCN-ALSR-8465/2020	EPI_ISL_1531928	26/11/2020	Baja California	Human	unknown	unknown	unknown	Original	Nasal swab
hCoV-19/Mexico/BCN-ALSR-8476/2020	EPI_ISL_1531939	26/11/2020	Baja California	Human	unknown	unknown	unknown	Original	Nasal swab
hCoV-19/Mexico/GRO-InDRE_136/2020	EPI_ISL_913939	26/11/2020	Guerrero	Human	Male	23	unknown	Original	Oropharyngeal swab
hCoV-19/Mexico/CMX-INMEGEN-11-02/2020	EPI_ISL_933512	26/11/2020	Mexico City	Human	Male	34	unknown	Original	Oropharyngeal swab
hCoV-19/Mexico/CMX-INMEGEN-11-03/2020	EPI_ISL_933513	26/11/2020	Mexico City	Human	Female	20	unknown	Original	Oropharyngeal swab
hCoV-19/Mexico/CMX-INMEGEN-11-04/2020	EPI_ISL_933514	26/11/2020	Mexico City	Human	Female	45	unknown	Original	Oropharyngeal swab
hCoV-19/Mexico/HID-InDRE_297/2020	EPI_ISL_1060727	26/11/2020	Hidalgo	Human	Male	55	Released	Original	Oropharyngeal swab
hCoV-19/Mexico/CMX-INMEGEN-11-01/2020	EPI_ISL_930783	26/11/2020	Mexico City	Human	Male	50	unknown	Original	Oropharyngeal swab
hCoV-19/Mexico/BCN-ALSR-8469/2020	EPI_ISL_1531932	27/11/2020	Baja California	Human	unknown	unknown	unknown	Original	Nasal swab
hCoV-19/Mexico/BCN-ALSR-8475/2020	EPI_ISL_1531938	27/11/2020	Baja California	Human	unknown	unknown	unknown	Original	Nasal swab
hCoV-19/Mexico/BCS-InDRE_133/2020	EPI_ISL_913936	27/11/2020	Baja California Sur	Human	Female	21	unknown	Original	Oropharyngeal swab
hCoV-19/Mexico/MEX-InDRE_170/2020	EPI_ISL_913971	27/11/2020	State of Mexico	Human	Female	52	unknown	Original	Oropharyngeal swab
hCoV-19/Mexico/CMX-INMEGEN-11-05/2020	EPI_ISL_933515	27/11/2020	Mexico City	Human	Female	82	unknown	Original	Oropharyngeal swab
hCoV-19/Mexico/NLE-UANL-027/2020	EPI_ISL_779189	27/11/2020	Nuevo Leon	Human	Male	26	unknown	Original	
hCoV-19/Mexico/CMX-INMEGEN-11-07/2020	EPI_ISL_933517	28/11/2020	Mexico City	Human	Female	56	unknown	Original	Oropharyngeal swab
hCoV-19/Mexico/BCN-ALSR-8478/2020	EPI_ISL_1531941	30/11/2020	Baja California	Human	unknown	unknown	unknown	Original	Nasal swab
hCoV-19/Mexico/BCN-ALSR-5708/2020	EPI_ISL_878367	30/11/2020	Baja California	Human	unknown	unknown	unknown	Original	Nasal swab
hCoV-19/Mexico/CMX-INMEGEN-11-09/2020	EPI_ISL_933519	30/11/2020	Mexico City	Human	Female	38	unknown	Original	Oropharyngeal swab
hCoV-19/Mexico/CMX-INMEGEN-11-11/2020	EPI_ISL_933520	30/11/2020	Mexico City	Human	Male	21	unknown	Original	Oropharyngeal swab
hCoV-19/Mexico/CMX-INMEGEN-11-12/2020	EPI_ISL_933521	30/11/2020	Mexico City	Human	Male	25	unknown	Original	Oropharyngeal swab
hCoV-19/Mexico/CMX-INMEGEN-11-13/2020	EPI_ISL_933522	30/11/2020	Mexico City	Human	Male	33	unknown	Original	Oropharyngeal swab
hCoV-19/Mexico/CMX-INMEGEN-11-14/2020	EPI_ISL_933523	30/11/2020	Mexico City	Human	Female	40	unknown	Original	Oropharyngeal swab
hCoV-19/Mexico/CMX-INMEGEN-11-15/2020	EPI_ISL_933524	30/11/2020	Mexico City	Human	Male	15	unknown	Original	Oropharyngeal swab
hCoV-19/Mexico/CMX-INMEGEN-11-16/2020	EPI_ISL_933525	30/11/2020	Mexico City	Human	Female	57	unknown	Original	Oropharyngeal swab
hCoV-19/Mexico/CMX-INMEGEN-11-17/2020	EPI_ISL_933526	30/11/2020	Mexico City	Human	Female	38	unknown	Original	Oropharyngeal swab
hCoV-19/Mexico/CMX-INMEGEN-11-18/2020	EPI_ISL_933527	30/11/2020	Mexico City	Human	Male	35	unknown	Original	Oropharyngeal swab
hCoV-19/Mexico/CMX-INMEGEN-11-19/2020	EPI_ISL_933528	30/11/2020	Mexico City	Human	Female	70	unknown	Original	Oropharyngeal swab
hCoV-19/Mexico/CMX-INMEGEN-11-20/2020	EPI_ISL_933529	30/11/2020	Mexico City	Human	Female	36	unknown	Original	Oropharyngeal swab
hCoV-19/Mexico/CMX-INMEGEN-11-21/2020	EPI_ISL_933530	30/11/2020	Mexico City	Human	Female	63	unknown	Original	Oropharyngeal swab
hCoV-19/Mexico/CMX-INMEGEN-11-22/2020	EPI_ISL_933531	30/11/2020	Mexico City	Human	Female	88	unknown	Original	Oropharyngeal swab
hCoV-19/Mexico/BCN-ALSR-6000/2020	EPI_ISL_879950	30/11/2020	Baja California	Human	unknown	unknown	unknown	Original	Nasal swab
hCoV-19/Mexico/BCN-ALSR-6012/2020	EPI_ISL_879977	30/11/2020	Baja California	Human	unknown	unknown	unknown	Original	Nasal swab
hCoV-19/Mexico/BCN-ALSR-5603/2020	EPI_ISL_878212	01/12/2020	Baja California	Human	unknown	unknown	unknown	Original	Nasal swab
hCoV-19/Mexico/BCN-ALSR-5615/2020	EPI_ISL_878226	01/12/2020	Baja California	Human	unknown	unknown	unknown	Original	Nasal swab

hCoV-19/Mexico/TLA-InDRE_174/2020	EPI_ISL_913975	04/12/2020	Tlaxcala	Human	Female	55	unknown	Original	Oropharyngeal swab
hCoV-19/Mexico/NLE-UANL-032/2020	EPI_ISL_779193	04/12/2020	Nuevo Leon	Human	Female	60	unknown	Original	
hCoV-19/Mexico/NLE-UANL-034/2020	EPI_ISL_779195	05/12/2020	Nuevo Leon	Human	Female	69	unknown	Original	
hCoV-19/Mexico/NLE-UANL-036/2020	EPI_ISL_779197	05/12/2020	Nuevo Leon	Human	Male	59	unknown	Original	
hCoV-19/Mexico/AGU-InDRE_F11770B_S1092/20	EPI_ISL_1516784	06/12/2020	Aguascalientes	Human	Female	unknown	unknown	Original	Oropharyngeal swab
hCoV-19/Mexico/OAX-InDRE_171/2020	EPI_ISL_913972	07/12/2020	Oaxaca	Human	Male	31	unknown	Original	Oropharyngeal swab
hCoV-19/Mexico/BCN-ALSR-6637/2020	EPI_ISL_1081473	07/12/2020	Baja California	Human	unknown	unknown	unknown	Original	Nasal swab
hCoV-19/Mexico/BCN-ALSR-6638/2020	EPI_ISL_1081474	07/12/2020	Baja California	Human	unknown	unknown	unknown	Original	Nasal swab
hCoV-19/Mexico/GTO-InDRE_142/2020	EPI_ISL_913945	08/12/2020	Guanajuato	Human	Male	35	unknown	Original	Oropharyngeal swab
hCoV-19/Mexico/OAX-InDRE_158/2020	EPI_ISL_913960	08/12/2020	Oaxaca	Human	Female	29	unknown	Original	Oropharyngeal swab
hCoV-19/Mexico/BCN-ALSR-6639/2020	EPI_ISL_1081475	08/12/2020	Baja California	Human	unknown	unknown	unknown	Original	Nasal swab
hCoV-19/Mexico/BCN-ALSR-6640/2020	EPI_ISL_1081476	08/12/2020	Baja California	Human	unknown	unknown	unknown	Original	Nasal swab
hCoV-19/Mexico/SON-InDRE_336/2020	EPI_ISL_1060680	08/12/2020	Sonora	Human	Female	23	unknown	Original	Oropharyngeal swab
hCoV-19/Mexico/MOR-InDRE_137/2020	EPI_ISL_913940	09/12/2020	Morelos	Human	Female	36	unknown	Original	Oropharyngeal swab
hCoV-19/Mexico/MEX-InDRE_138/2020	EPI_ISL_913941	09/12/2020	State of Mexico	Human	Female	33	unknown	Original	Oropharyngeal swab
hCoV-19/Mexico/BC-InDRE_139/2020	EPI_ISL_913942	09/12/2020	Baja California	Human	Female	53	unknown	Original	Oropharyngeal swab
hCoV-19/Mexico/BC-InDRE_140/2020	EPI_ISL_913943	09/12/2020	Baja California	Human	Female	29	unknown	Original	Oropharyngeal swab
hCoV-19/Mexico/GRO-InDRE_159/2020	EPI_ISL_913961	09/12/2020	Guerrero	Human	Female	54	unknown	Original	Oropharyngeal swab
hCoV-19/Mexico/MOR-InDRE_172/2020	EPI_ISL_913973	09/12/2020	Morelos	Human	Male	27	unknown	Original	Oropharyngeal swab
hCoV-19/Mexico/GRO-InDRE_173/2020	EPI_ISL_913974	09/12/2020	Guerrero	Human	Male	33	unknown	Original	Oropharyngeal swab
hCoV-19/Mexico/CMX-INMEGEN-02-05-07/2020	EPI_ISL_1075657	09/12/2020	Mexico City	Human	Male	49	unknown	Original	
hCoV-19/Mexico/TLA-InDRE_176/2020	EPI_ISL_913977	10/12/2020	Tlaxcala	Human	Male	25	unknown	Original	Oropharyngeal swab
hCoV-19/Mexico/SLP-InDRE_192/2020	EPI_ISL_933669	10/12/2020	San Luis Potosi	Human	Female	64	unknown	Original	Oropharyngeal swab
hCoV-19/Mexico/CMX-INMEGEN-02-05-06/2020	EPI_ISL_1060615	10/12/2020	Mexico City	Human	Male	50	unknown	Original	Oropharyngeal swab
hCoV-19/Mexico/BCN-InDRE_335/2020	EPI_ISL_1060726	10/12/2020	Baja California	Human	Female	48	unknown	Original	Oropharyngeal swab
hCoV-19/Mexico/BCN-ALSR-6641/2020	EPI_ISL_1081477	10/12/2020	Baja California	Human	unknown	unknown	unknown	Original	Nasal swab
hCoV-19/Mexico/BCN-ALSR-6642/2020	EPI_ISL_1081478	10/12/2020	Baja California	Human	unknown	unknown	unknown	Original	Nasal swab
hCoV-19/Mexico/BCN-ALSR-6643/2020	EPI_ISL_1081479	10/12/2020	Baja California	Human	unknown	unknown	unknown	Original	Nasal swab
hCoV-19/Mexico/BCN-ALSR-6644/2020	EPI_ISL_1081480	10/12/2020	Baja California	Human	unknown	unknown	unknown	Original	Nasal swab
hCoV-19/Mexico/BCN-ALSR-6645/2020	EPI_ISL_1081481	10/12/2020	Baja California	Human	unknown	unknown	unknown	Original	Nasal swab
hCoV-19/Mexico/BCN-ALSR-6646/2020	EPI_ISL_1081482	10/12/2020	Baja California	Human	unknown	unknown	unknown	Original	Nasal swab
hCoV-19/Mexico/BCN-ALSR-6647/2020	EPI_ISL_1081483	10/12/2020	Baja California	Human	unknown	unknown	unknown	Original	Nasal swab
hCoV-19/Mexico/BCN-ALSR-6648/2020	EPI_ISL_1081484	10/12/2020	Baja California	Human	unknown	unknown	unknown	Original	Nasal swab
hCoV-19/Mexico/TLA-InDRE_141/2020	EPI_ISL_913944	11/12/2020	Tlaxcala	Human	Male	40	unknown	Original	Oropharyngeal swab
hCoV-19/Mexico/GRO-InDRE_163/2020	EPI_ISL_913964	11/12/2020	Guerrero	Human	Male	29	unknown	Original	Oropharyngeal swab
hCoV-19/Mexico/MOR-InDRE_175/2020	EPI_ISL_913976	11/12/2020	Morelos	Human	Male	36	unknown	Original	Oropharyngeal swab
hCoV-19/Mexico/CMX-INMEGEN-02-05-08/2020	EPI_ISL_1060614	11/12/2020	Mexico City	Human	Male	50	unknown	Original	Oropharyngeal swab
hCoV-19/Mexico/BCN-ALSR-6650/2020	EPI_ISL_1081486	11/12/2020	Baja California	Human	unknown	unknown	unknown	Original	Nasal swab
hCoV-19/Mexico/SLP-InDRE_145/2020	EPI_ISL_913948	12/12/2020	San Luis Potosi	Human	Female	20	unknown	Original	Oropharyngeal swab
hCoV-19/Mexico/GRO-InDRE_146/2020	EPI_ISL_913949	12/12/2020	Guerrero	Human	Male	36	unknown	Original	Oropharyngeal swab
hCoV-19/Mexico/MEX-InDRE_177/2020	EPI_ISL_913978	12/12/2020	State of Mexico	Human	Female	45	unknown	Original	Oropharyngeal swab

hCoV-19/Mexico/BCN-ALSR-6649/2020	EPI_ISL_1081485	12/12/2020	Baja California	Human	unknown	unknown	unknown	Original	Nasal swab
hCoV-19/Mexico/BCN-ALSR-6651/2020	EPI_ISL_1081487	12/12/2020	Baja California	Human	unknown	unknown	unknown	Original	Nasal swab
hCoV-19/Mexico/NLE-UANL-033/2020	EPI_ISL_779194	12/12/2020	Nuevo Leon	Human	Female	54	unknown	Original	
hCoV-19/Mexico/NLE-UANL-028/2020	EPI_ISL_979329	12/12/2020	Nuevo Leon	Human	Male	21	unknown	Original	
hCoV-19/Mexico/QRO-InDRE_143/2020	EPI_ISL_913946	13/12/2020	Queretaro	Human	Male	33	unknown	Original	Oropharyngeal swab
hCoV-19/Mexico/QRO-InDRE_144/2020	EPI_ISL_913947	13/12/2020	Queretaro	Human	Male	25	unknown	Original	Oropharyngeal swab
hCoV-19/Mexico/HGO-InDRE_148/2020	EPI_ISL_913951	13/12/2020	Hidalgo	Human	Female	26	unknown	Original	Oropharyngeal swab
hCoV-19/Mexico/SLP-InDRE_162/2020	EPI_ISL_913963	13/12/2020	San Luis Potosi	Human	Male	34	unknown	Original	Oropharyngeal swab
hCoV-19/Mexico/GTO-InDRE_180/2020	EPI_ISL_913981	13/12/2020	Guanajuato	Human	Female	52	Hospitalized	Original	Oropharyngeal swab
hCoV-19/Mexico/TLA-InDRE_147/2020	EPI_ISL_913950	14/12/2020	Tlaxcala	Human	Male	39	unknown	Original	Oropharyngeal swab
hCoV-19/Mexico/CMX-InDRE_160/2020	EPI_ISL_913962	14/12/2020	Mexico City	Human	Male	33	Released	Original	Oropharyngeal swab
hCoV-19/Mexico/CMX-InDRE_178/2020	EPI_ISL_913979	14/12/2020	Mexico City	Human	Male	38	Released	Original	Oropharyngeal swab
hCoV-19/Mexico/HGO-InDRE_181/2020	EPI_ISL_913982	14/12/2020	Hidalgo	Human	Male	38	unknown	Original	Oropharyngeal swab
hCoV-19/Mexico/HGO-InDRE_183/2020	EPI_ISL_913984	14/12/2020	Hidalgo	Human	Male	26	unknown	Original	Oropharyngeal swab
hCoV-19/Mexico/BCN-ALSR-6586/2020	EPI_ISL_1081425	14/12/2020	Baja California	Human	unknown	unknown	unknown	Original	Nasal swab
hCoV-19/Mexico/HID-InDRE_161/2020	EPI_ISL_914880	14/12/2020	Hidalgo	Human	Female	40	unknown	Original	Oropharyngeal swab
hCoV-19/Mexico/GUA-InDRE_338/2020	EPI_ISL_1060682	14/12/2020	Guanajuato	Human	Male	32	unknown	Original	Oropharyngeal swab
hCoV-19/Mexico/GRO-InDRE_332/2020	EPI_ISL_1060683	14/12/2020	Guerrero	Human	Male	29	unknown	Original	Oropharyngeal swab
hCoV-19/Mexico/OAX-InDRE_333/2020	EPI_ISL_1060694	14/12/2020	Oaxaca	Human	Female	18	unknown	Original	Oropharyngeal swab
hCoV-19/Mexico/TAM-InDRE_149/2020	EPI_ISL_913952	15/12/2020	Tamaulipas	Human	Male	26	unknown	Original	Oropharyngeal swab
hCoV-19/Mexico/HGO-InDRE_164/2020	EPI_ISL_913965	15/12/2020	Hidalgo	Human	Female	36	unknown	Original	Oropharyngeal swab
hCoV-19/Mexico/TAM-InDRE_165/2020	EPI_ISL_913966	15/12/2020	Tamaulipas	Human	Female	45	unknown	Original	Oropharyngeal swab
hCoV-19/Mexico/CMX-InDRE_179/2020	EPI_ISL_913980	15/12/2020	Mexico City	Human	Female	63	Released	Original	Oropharyngeal swab
hCoV-19/Mexico/CMX-InDRE_216/2020	EPI_ISL_933693	15/12/2020	Mexico City	Human	Female	30	unknown	Original	Oropharyngeal swab
hCoV-19/Mexico/QUE-InDRE_343/2020	EPI_ISL_1060701	15/12/2020	Queretaro	Human	Male	41	unknown	Original	Oropharyngeal swab
hCoV-19/Mexico/BCS-InDRE_352/2020	EPI_ISL_1060705	15/12/2020	Baja California Sur	Human	Male	29	unknown	Original	Oropharyngeal swab
hCoV-19/Mexico/BCS-InDRE_349/2020	EPI_ISL_1060724	15/12/2020	Baja California Sur	Human	Male	60	unknown	Original	Oropharyngeal swab
hCoV-19/Mexico/AGU-InDRE_354/2020	EPI_ISL_1060725	15/12/2020	Aguascalientes	Human	Male	63	unknown	Original	Oropharyngeal swab
hCoV-19/Mexico/BCN-ALSR-6652/2020	EPI_ISL_1081488	15/12/2020	Baja California	Human	unknown	unknown	unknown	Original	Nasal swab
hCoV-19/Mexico/BCN-ALSR-6653/2020	EPI_ISL_1081489	15/12/2020	Baja California	Human	unknown	unknown	unknown	Original	Nasal swab
hCoV-19/Mexico/BCN-ALSR-6654/2020	EPI_ISL_1081490	15/12/2020	Baja California	Human	unknown	unknown	unknown	Original	Nasal swab
hCoV-19/Mexico/BCN-ALSR-6655/2020	EPI_ISL_1081491	15/12/2020	Baja California	Human	unknown	unknown	unknown	Original	Nasal swab
hCoV-19/Mexico/MEX-InDRE_226/2020	EPI_ISL_933703	15/12/2020	State of Mexico	Human	Male	34	unknown	Original	Oropharyngeal swab
hCoV-19/Mexico/OAX-InDRE_347/2020	EPI_ISL_1060691	15/12/2020	Oaxaca	Human	Male	44	unknown	Original	Oropharyngeal swab
hCoV-19/Mexico/QUE-InDRE_345/2020	EPI_ISL_1060696	15/12/2020	Queretaro	Human	Female	70	unknown	Original	Oropharyngeal swab
hCoV-19/Mexico/AGS-InDRE_150/2020	EPI_ISL_913953	16/12/2020	Aguascalientes	Human	Male	44	unknown	Original	Oropharyngeal swab
hCoV-19/Mexico/AGS-InDRE_151/2020	EPI_ISL_913954	16/12/2020	Aguascalientes	Human	Male	20	unknown	Original	Oropharyngeal swab
hCoV-19/Mexico/AGU-InDRE_195/2020	EPI_ISL_933672	16/12/2020	Aguascalientes	Human	Female	28	unknown	Original	Oropharyngeal swab
hCoV-19/Mexico/AGU-InDRE_196/2020	EPI_ISL_933673	16/12/2020	Aguascalientes	Human	Male	37	unknown	Original	Oropharyngeal swab
hCoV-19/Mexico/AGU-InDRE_334/2020	EPI_ISL_1060722	16/12/2020	Aguascalientes	Human	Male	44	unknown	Original	Oropharyngeal swab
hCoV-19/Mexico/AGU-InDRE_357/2020	EPI_ISL_1060723	16/12/2020	Aguascalientes	Human	Female	24	unknown	Original	Oropharyngeal swab

hCoV-19/Mexico/AGU-InDRE_231/2020	EPI_ISL_933708	16/12/2020	Aguascalientes	Human	Female	37	unknown	Original	Oropharyngeal swab
hCoV-19/Mexico/AGU-InDRE_232/2020	EPI_ISL_933709	16/12/2020	Aguascalientes	Human	Female	56	unknown	Original	Oropharyngeal swab
hCoV-19/Mexico/CMX-InDRE_235/2020	EPI_ISL_933712	16/12/2020	Mexico City	Human	Female	21	unknown	Original	Oropharyngeal swab
hCoV-19/Mexico/CHH-InDRE_366/2020	EPI_ISL_1060678	16/12/2020	Chihuahua	Human	Female	45	unknown	Original	Oropharyngeal swab
hCoV-19/Mexico/MOR-InDRE_342/2020	EPI_ISL_1060679	16/12/2020	Morelos	Human	Male	33	unknown	Original	Oropharyngeal swab
hCoV-19/Mexico/BCS-InDRE_350/2020	EPI_ISL_1060681	16/12/2020	Baja California Sur	Human	Female	22	unknown	Original	Oropharyngeal swab
hCoV-19/Mexico/COA-InDRE_355/2020	EPI_ISL_1060686	16/12/2020	Coahuila	Human	Male	24	unknown	Original	Oropharyngeal swab
hCoV-19/Mexico/AGU-InDRE_353/2020	EPI_ISL_1060689	16/12/2020	Aguascalientes	Human	Female	24	unknown	Original	Oropharyngeal swab
hCoV-19/Mexico/TAM-InDRE_344/2020	EPI_ISL_1060693	16/12/2020	Tamaulipas	Human	Female	55	unknown	Original	Oropharyngeal swab
hCoV-19/Mexico/CMX-INMEGEN-01-01-01/2020	EPI_ISL_1005656	16/12/2020	Mexico City	Human	Female	59	unknown	Original	Oropharyngeal swab
hCoV-19/Mexico/CMX-INMEGEN-01-01-04/2020	EPI_ISL_1005659	16/12/2020	Mexico City	Human	Male	unknown	unknown	Original	Oropharyngeal swab
hCoV-19/Mexico/VER-InDRE_152/2020	EPI_ISL_913955	17/12/2020	Veracruz	Human	Male	25	unknown	Original	Oropharyngeal swab
hCoV-19/Mexico/MEX-InDRE_182/2020	EPI_ISL_913983	17/12/2020	State of Mexico	Human	Female	36	Released	Original	Oropharyngeal swab
hCoV-19/Mexico/AGU-InDRE_193/2020	EPI_ISL_933670	17/12/2020	Aguascalientes	Human	Male	57	unknown	Original	Oropharyngeal swab
hCoV-19/Mexico/AGU-InDRE_194/2020	EPI_ISL_933671	17/12/2020	Aguascalientes	Human	Male	26	unknown	Original	Oropharyngeal swab
hCoV-19/Mexico/CMX-InDRE_222/2020	EPI_ISL_933699	17/12/2020	Mexico City	Human	Male	58	unknown	Original	Oropharyngeal swab
hCoV-19/Mexico/COA-InDRE_365/2020	EPI_ISL_1060706	17/12/2020	Coahuila	Human	Female	45	unknown	Original	Oropharyngeal swab
hCoV-19/Mexico/VER-InDRE_363/2020	EPI_ISL_1060707	17/12/2020	Veracruz	Human	Male	60	unknown	Original	Oropharyngeal swab
hCoV-19/Mexico/BCS-InDRE_368/2020	EPI_ISL_1060720	17/12/2020	Baja California Sur	Human	Female	25	unknown	Original	Oropharyngeal swab
hCoV-19/Mexico/SLP-InDRE_370/2020	EPI_ISL_1060721	17/12/2020	San Luis Potosi	Human	Female	26	unknown	Original	Oropharyngeal swab
hCoV-19/Mexico/BCN-ALSR-6584/2020	EPI_ISL_1081423	17/12/2020	Baja California	Human	unknown	unknown	unknown	Original	Nasal swab
hCoV-19/Mexico/BCN-ALSR-6585/2020	EPI_ISL_1081424	17/12/2020	Baja California	Human	unknown	unknown	unknown	Original	Nasal swab
hCoV-19/Mexico/BCN-ALSR-6657/2020	EPI_ISL_1081492	17/12/2020	Baja California	Human	unknown	unknown	unknown	Original	Nasal swab
hCoV-19/Mexico/BCN-ALSR-6658/2020	EPI_ISL_1081493	17/12/2020	Baja California	Human	unknown	unknown	unknown	Original	Nasal swab
hCoV-19/Mexico/BCN-ALSR-6659/2020	EPI_ISL_1081494	17/12/2020	Baja California	Human	unknown	unknown	unknown	Original	Nasal swab
hCoV-19/Mexico/BCN-ALSR-6660/2020	EPI_ISL_1081495	17/12/2020	Baja California	Human	unknown	unknown	unknown	Original	Nasal swab
hCoV-19/Mexico/CMX-InDRE_223/2020	EPI_ISL_933700	17/12/2020	Mexico City	Human	Male	34	unknown	Original	Oropharyngeal swab
hCoV-19/Mexico/CMX-InDRE_233/2020	EPI_ISL_933710	17/12/2020	Mexico City	Human	Female	18	unknown	Original	Oropharyngeal swab
hCoV-19/Mexico/COA-InDRE_361/2020	EPI_ISL_1060676	17/12/2020	Coahuila	Human	Female	30	unknown	Original	Oropharyngeal swab
hCoV-19/Mexico/VER-InDRE_360/2020	EPI_ISL_1060677	17/12/2020	Veracruz	Human	Female	40	Released	Original	Oropharyngeal swab
hCoV-19/Mexico/AGU-InDRE_351/2020	EPI_ISL_1060688	17/12/2020	Aguascalientes	Human	Male	21	unknown	Original	Oropharyngeal swab
hCoV-19/Mexico/SLP-InDRE_367/2020	EPI_ISL_1060690	17/12/2020	San Luis Potosi	Human	Male	30	unknown	Original	Oropharyngeal swab
hCoV-19/Mexico/VER-InDRE_362/2020	EPI_ISL_1060699	17/12/2020	Veracruz	Human	Female	40	unknown	Original	Oropharyngeal swab
hCoV-19/Mexico/BCS-InDRE_201/2020	EPI_ISL_933678	18/12/2020	Baja California Sur	Human	Male	24	unknown	Original	Oropharyngeal swab
hCoV-19/Mexico/BCS-InDRE_369/2020	EPI_ISL_1060703	18/12/2020	Baja California Sur	Human	Female	30	unknown	Original	Oropharyngeal swab
hCoV-19/Mexico/CMX-InDRE_331/2020	EPI_ISL_1060719	18/12/2020	Mexico City	Human	Male	35	unknown	Original	Oropharyngeal swab
hCoV-19/Mexico/BCN-ALSR-6587/2020	EPI_ISL_1081426	18/12/2020	Baja California	Human	unknown	unknown	unknown	Original	Nasal swab
hCoV-19/Mexico/BCN-ALSR-6588/2020	EPI_ISL_1081427	18/12/2020	Baja California	Human	unknown	unknown	unknown	Original	Nasal swab
hCoV-19/Mexico/BCN-ALSR-6589/2020	EPI_ISL_1081428	18/12/2020	Baja California	Human	unknown	unknown	unknown	Original	Nasal swab
hCoV-19/Mexico/BCN-ALSR-6590/2020	EPI_ISL_1081429	18/12/2020	Baja California	Human	unknown	unknown	unknown	Original	Nasal swab
hCoV-19/Mexico/BCN-ALSR-6591/2020	EPI_ISL_1081430	18/12/2020	Baja California	Human	unknown	unknown	unknown	Original	Nasal swab

hCoV-19/Mexico/CMX-INMEGEN-12-04-23/2020	EPI_ISL_1005581	18/12/2020	Mexico City	Human	Male	69	unknown	Original	Oropharyngeal swab
hCoV-19/Mexico/CMX-INMEGEN-12-04-24/2020	EPI_ISL_1005582	18/12/2020	Mexico City	Human	Male	31	unknown	Original	Oropharyngeal swab
hCoV-19/Mexico/CMX-InDRE_217/2020	EPI_ISL_933694	19/12/2020	Mexico City	Human	Male	14	unknown	Original	Oropharyngeal swab
hCoV-19/Mexico/CMX-INMEGEN-02-05-05/2020	EPI_ISL_1060613	19/12/2020	Mexico City	Human	Male	31	unknown	Original	Oropharyngeal swab
hCoV-19/Mexico/BCN-ALSR-6592/2020	EPI_ISL_1081431	19/12/2020	Baja California	Human	unknown	unknown	unknown	Original	Nasal swab
hCoV-19/Mexico/BCN-ALSR-6593/2020	EPI_ISL_1081432	19/12/2020	Baja California	Human	unknown	unknown	unknown	Original	Nasal swab
hCoV-19/Mexico/BCN-ALSR-6594/2020	EPI_ISL_1081433	19/12/2020	Baja California	Human	unknown	unknown	unknown	Original	Nasal swab
hCoV-19/Mexico/BCN-ALSR-6595/2020	EPI_ISL_1081434	19/12/2020	Baja California	Human	unknown	unknown	unknown	Original	Nasal swab
hCoV-19/Mexico/BCN-ALSR-6596/2020	EPI_ISL_1081435	19/12/2020	Baja California	Human	unknown	unknown	unknown	Original	Nasal swab
hCoV-19/Mexico/CMX-InDRE_234/2020	EPI_ISL_933711	19/12/2020	Mexico City	Human	Female	51	unknown	Original	Oropharyngeal swab
hCoV-19/Mexico/NLE-UANL-024/2020	EPI_ISL_779187	19/12/2020	Nuevo Leon	Human	Male	56	unknown	Original	
hCoV-19/Mexico/NLE-UANL-029/2020	EPI_ISL_779190	19/12/2020	Nuevo Leon	Human	Male	68	unknown	Original	
hCoV-19/Mexico/NLE-UANL-021/2020	EPI_ISL_979327	19/12/2020	Nuevo Leon	Human	Male	37	unknown	Original	
hCoV-19/Mexico/NLE-UANL-025/2020	EPI_ISL_979328	19/12/2020	Nuevo Leon	Human	Male	18	unknown	Original	
hCoV-19/Mexico/CMX-InDRE_197/2020	EPI_ISL_933674	20/12/2020	Mexico City	Human	Male	34	unknown	Original	Oropharyngeal swab
hCoV-19/Mexico/CMX-InDRE_198/2020	EPI_ISL_933675	20/12/2020	Mexico City	Human	Male	77	unknown	Original	Oropharyngeal swab
hCoV-19/Mexico/CMX-InDRE_218/2020	EPI_ISL_933695	20/12/2020	Mexico City	Human	Female	30	unknown	Original	Oropharyngeal swab
hCoV-19/Mexico/CMX-InDRE_219/2020	EPI_ISL_933696	20/12/2020	Mexico City	Human	Male	59	unknown	Original	Oropharyngeal swab
hCoV-19/Mexico/CMX-InDRE_220/2020	EPI_ISL_933697	20/12/2020	Mexico City	Human	Female	49	unknown	Original	Oropharyngeal swab
hCoV-19/Mexico/CMX-INMEGEN-02-05-04/2020	EPI_ISL_1060612	20/12/2020	Mexico City	Human	Female	46	unknown	Original	Oropharyngeal swab
hCoV-19/Mexico/CMX-InDRE_224/2020	EPI_ISL_933701	20/12/2020	Mexico City	Human	Male	45	unknown	Original	Oropharyngeal swab
hCoV-19/Mexico/CMX-InDRE_225/2020	EPI_ISL_933702	20/12/2020	Mexico City	Human	Male	8	unknown	Original	Oropharyngeal swab
hCoV-19/Mexico/CMX-InDRE_199/2020	EPI_ISL_933676	21/12/2020	Mexico City	Human	Female	32	unknown	Original	Oropharyngeal swab
hCoV-19/Mexico/MEX-InDRE_200/2020	EPI_ISL_933677	21/12/2020	State of Mexico	Human	Male	32	unknown	Original	Oropharyngeal swab
hCoV-19/Mexico/CMX-InDRE_221/2020	EPI_ISL_933698	21/12/2020	Mexico City	Human	Female	46	unknown	Original	Oropharyngeal swab
hCoV-19/Mexico/BCN-ALSR-6597/2020	EPI_ISL_1081436	21/12/2020	Baja California	Human	unknown	unknown	unknown	Original	Nasal swab
hCoV-19/Mexico/BCN-ALSR-6598/2020	EPI_ISL_1081437	21/12/2020	Baja California	Human	unknown	unknown	unknown	Original	Nasal swab
hCoV-19/Mexico/BCN-ALSR-6599/2020	EPI_ISL_1081438	21/12/2020	Baja California	Human	unknown	unknown	unknown	Original	Nasal swab
hCoV-19/Mexico/BCN-ALSR-6600/2020	EPI_ISL_1081439	21/12/2020	Baja California	Human	unknown	unknown	unknown	Original	Nasal swab
hCoV-19/Mexico/BCN-ALSR-6601/2020	EPI_ISL_1081440	21/12/2020	Baja California	Human	unknown	unknown	unknown	Original	Nasal swab
hCoV-19/Mexico/BCN-ALSR-6602/2020	EPI_ISL_1081441	21/12/2020	Baja California	Human	unknown	unknown	unknown	Original	Nasal swab
hCoV-19/Mexico/BCN-ALSR-6603/2020	EPI_ISL_1081442	21/12/2020	Baja California	Human	unknown	unknown	unknown	Original	Nasal swab
hCoV-19/Mexico/BCN-ALSR-6604/2020	EPI_ISL_1081443	21/12/2020	Baja California	Human	unknown	unknown	unknown	Original	Nasal swab
hCoV-19/Mexico/CMX-INMEGEN-12-04-02/2020	EPI_ISL_1005560	21/12/2020	Mexico City	Human	Male	46	unknown	Original	Oropharyngeal swab
hCoV-19/Mexico/CMX-INMEGEN-12-04-03/2020	EPI_ISL_1005561	21/12/2020	Mexico City	Human	Female	26	unknown	Original	Oropharyngeal swab
hCoV-19/Mexico/CMX-INMEGEN-12-04-04/2020	EPI_ISL_1005562	21/12/2020	Mexico City	Human	Female	61	unknown	Original	Oropharyngeal swab
hCoV-19/Mexico/CMX-INMEGEN-12-04-05/2020	EPI_ISL_1005563	21/12/2020	Mexico City	Human	Male	20	unknown	Original	Oropharyngeal swab
hCoV-19/Mexico/CMX-INMEGEN-12-04-06/2020	EPI_ISL_1005564	21/12/2020	Mexico City	Human	Female	60	unknown	Original	Oropharyngeal swab
hCoV-19/Mexico/CMX-INMEGEN-12-04-07/2020	EPI_ISL_1005565	21/12/2020	Mexico City	Human	Female	56	unknown	Original	Oropharyngeal swab
hCoV-19/Mexico/CMX-INMEGEN-12-04-08/2020	EPI_ISL_1005566	21/12/2020	Mexico City	Human	Female	28	unknown	Original	Oropharyngeal swab
hCoV-19/Mexico/CMX-INMEGEN-12-04-21/2020	EPI_ISL_1005579	21/12/2020	Mexico City	Human	Male	41	unknown	Original	Oropharyngeal swab

hCoV-19/Mexico/CMX-INMEGEN-12-04-22/2020	EPI_ISL_1005580	21/12/2020	Mexico City	Human	Female	36	unknown	Original	Oropharyngeal swab
hCoV-19/Mexico/BCN-ALSR-6605/2020	EPI_ISL_1081444	22/12/2020	Baja California	Human	unknown	unknown	unknown	Original	Nasal swab
hCoV-19/Mexico/AGU-InDRE_F11769B_S1091/20	EPI_ISL_1516783	22/12/2020	Aguascalientes	Human	Female	28	unknown	Original	Oropharyngeal swab
hCoV-19/Mexico/CMX-INMEGEN-12-04-14/2020	EPI_ISL_1005572	22/12/2020	Mexico City	Human	Male	20	unknown	Original	Oropharyngeal swab
hCoV-19/Mexico/CMX-INMEGEN-12-04-15/2020	EPI_ISL_1005573	22/12/2020	Mexico City	Human	Female	46	unknown	Original	Oropharyngeal swab
hCoV-19/Mexico/CMX-INMEGEN-12-04-16/2020	EPI_ISL_1005574	22/12/2020	Mexico City	Human	Male	38	unknown	Original	Oropharyngeal swab
hCoV-19/Mexico/CMX-INMEGEN-12-04-20/2020	EPI_ISL_1005578	22/12/2020	Mexico City	Human	Male	47	unknown	Original	Oropharyngeal swab
hCoV-19/Mexico/BCN-ALSR-6606/2020	EPI_ISL_1081445	23/12/2020	Baja California	Human	unknown	unknown	unknown	Original	Nasal swab
hCoV-19/Mexico/BCN-ALSR-6607/2020	EPI_ISL_1081446	23/12/2020	Baja California	Human	unknown	unknown	unknown	Original	Nasal swab
hCoV-19/Mexico/BCN-ALSR-6608/2020	EPI_ISL_1081447	23/12/2020	Baja California	Human	unknown	unknown	unknown	Original	Nasal swab
hCoV-19/Mexico/BCN-ALSR-6609/2020	EPI_ISL_1081448	23/12/2020	Baja California	Human	unknown	unknown	unknown	Original	Nasal swab
hCoV-19/Mexico/BCN-ALSR-6610/2020	EPI_ISL_1081449	23/12/2020	Baja California	Human	unknown	unknown	unknown	Original	Nasal swab
hCoV-19/Mexico/BCN-ALSR-6612/2020	EPI_ISL_1081451	23/12/2020	Baja California	Human	unknown	unknown	unknown	Original	Nasal swab
hCoV-19/Mexico/CMX-INMEGEN-12-04-13/2020	EPI_ISL_1005571	23/12/2020	Mexico City	Human	Male	38	unknown	Original	Oropharyngeal swab
hCoV-19/Mexico/CMX-INMEGEN-12-04-17/2020	EPI_ISL_1005575	23/12/2020	Mexico City	Human	Male	32	unknown	Original	Oropharyngeal swab
hCoV-19/Mexico/CMX-INMEGEN-12-04-18/2020	EPI_ISL_1005576	23/12/2020	Mexico City	Human	Male	36	unknown	Original	Oropharyngeal swab
hCoV-19/Mexico/CMX-INMEGEN-12-04-19/2020	EPI_ISL_1005577	23/12/2020	Mexico City	Human	Female	5	unknown	Original	Oropharyngeal swab
hCoV-19/Mexico/NLE-UANL-038/2020	EPI_ISL_979330	24/12/2020	Nuevo Leon	Human	Female	39	unknown	Original	
hCoV-19/Mexico/NLE-UANL-039/2020	EPI_ISL_979331	25/12/2020	Nuevo Leon	Human	Female	48	unknown	Original	
hCoV-19/Mexico/CMX-INMEGEN-01-01-24/2020	EPI_ISL_1005673	26/12/2020	Mexico City	Human	Male	50	unknown	Original	Oropharyngeal swab
hCoV-19/Mexico/NLE-UANL-040/2020	EPI_ISL_979332	26/12/2020	Nuevo Leon	Human	Male	48	unknown	Original	
hCoV-19/Mexico/NLE-UANL-041/2020	EPI_ISL_979333	26/12/2020	Nuevo Leon	Human	Female	20	unknown	Original	
hCoV-19/Mexico/NLE-UANL-042/2020	EPI_ISL_979334	26/12/2020	Nuevo Leon	Human	Male	34	unknown	Original	
hCoV-19/Mexico/CMX-INMEGEN-01-01-17/2020	EPI_ISL_1005667	26/12/2020	Mexico City	Human	Female	42	unknown	Original	Oropharyngeal swab
hCoV-19/Mexico/CMX-INMEGEN-01-01-18/2020	EPI_ISL_1005668	26/12/2020	Mexico City	Human	Female	58	unknown	Original	Oropharyngeal swab
hCoV-19/Mexico/NLE-UANL-043/2020	EPI_ISL_979335	27/12/2020	Nuevo Leon	Human	Female	26	unknown	Original	
hCoV-19/Mexico/NLE-UANL-044/2020	EPI_ISL_979336	27/12/2020	Nuevo Leon	Human	Female	12	unknown	Original	
hCoV-19/Mexico/NLE-UANL-045/2020	EPI_ISL_979337	27/12/2020	Nuevo Leon	Human	Male	44	unknown	Original	
hCoV-19/Mexico/BCN-ALSR-6611/2020	EPI_ISL_1081450	28/12/2020	Baja California	Human	unknown	unknown	unknown	Original	Nasal swab
hCoV-19/Mexico/BCN-SEARCH-9176/2020	EPI_ISL_1794925	28/12/2020	Baja California	Human	unknown	unknown	unknown	Original	Nasal swab
hCoV-19/Mexico/CMX-InDRE_202/2020	EPI_ISL_933679	29/12/2020	Mexico City	Human	Male	59	unknown	Original	Oropharyngeal swab
hCoV-19/Mexico/BCN-ALSR-6613/2020	EPI_ISL_1081452	29/12/2020	Baja California	Human	unknown	unknown	unknown	Original	Nasal swab
hCoV-19/Mexico/BCN-ALSR-6614/2020	EPI_ISL_1081453	29/12/2020	Baja California	Human	unknown	unknown	unknown	Original	Nasal swab
hCoV-19/Mexico/CMX-INMEGEN-01-02-08/2020	EPI_ISL_985166	29/12/2020	Mexico City	Human	Male	94	unknown	Original	Oropharyngeal swab
hCoV-19/Mexico/CMX-INMEGEN-01-02-05/2020	EPI_ISL_985167	29/12/2020	Mexico City	Human	Male	13	unknown	Original	Oropharyngeal swab
hCoV-19/Mexico/CMX-INMEGEN-01-01-20/2020	EPI_ISL_1005669	29/12/2020	Mexico City	Human	Female	15	unknown	Original	Oropharyngeal swab
hCoV-19/Mexico/BCN-ALSR-6616/2020	EPI_ISL_1081454	30/12/2020	Baja California	Human	unknown	unknown	unknown	Original	Nasal swab
hCoV-19/Mexico/BCN-ALSR-6617/2020	EPI_ISL_1081455	30/12/2020	Baja California	Human	unknown	unknown	unknown	Original	Nasal swab
hCoV-19/Mexico/BCN-ALSR-6618/2020	EPI_ISL_1081456	30/12/2020	Baja California	Human	unknown	unknown	unknown	Original	Nasal swab
hCoV-19/Mexico/CMX-INMEGEN-01-02-01/2020	EPI_ISL_985160	30/12/2020	Mexico City	Human	Male	67	unknown	Original	Oropharyngeal swab
hCoV-19/Mexico/CMX-INMEGEN-01-02-02/2020	EPI_ISL_985161	30/12/2020	Mexico City	Human	Male	25	unknown	Original	Oropharyngeal swab

hCoV-19/Mexico/CMX-INMEGEN-01-02-09/2020	EPI_ISL_985162	30/12/2020	Mexico City	Human	Female	67	unknown	Original	Oropharyngeal swab
hCoV-19/Mexico/CMX-INMEGEN-01-02-10/2020	EPI_ISL_985163	30/12/2020	Mexico City	Human	Female	29	unknown	Original	Oropharyngeal swab
hCoV-19/Mexico/CMX-INMEGEN-01-02-12/2020	EPI_ISL_985164	30/12/2020	Mexico City	Human	Male	38	unknown	Original	Oropharyngeal swab
hCoV-19/Mexico/CMX-INMEGEN-01-02-13/2020	EPI_ISL_985165	30/12/2020	Mexico City	Human	Female	49	unknown	Original	Oropharyngeal swab
hCoV-19/Mexico/CMX-INMEGEN-01-01-22/2020	EPI_ISL_1005671	30/12/2020	Mexico City	Human	Female	28	unknown	Original	Oropharyngeal swab
hCoV-19/Mexico/CMX-INMEGEN-01-01-11/2020	EPI_ISL_1005661	30/12/2020	Mexico City	Human	Female	unknown	unknown	Original	Oropharyngeal swab
hCoV-19/Mexico/CMX-INMEGEN-01-01-12/2020	EPI_ISL_1005662	30/12/2020	Mexico City	Human	Female	49	unknown	Original	Oropharyngeal swab
hCoV-19/Mexico/CMX-INMEGEN-01-01-13/2020	EPI_ISL_1005663	30/12/2020	Mexico City	Human	Male	48	unknown	Original	Oropharyngeal swab
hCoV-19/Mexico/CMX-INMEGEN-01-01-14/2020	EPI_ISL_1005664	30/12/2020	Mexico City	Human	Female	37	unknown	Original	Oropharyngeal swab
hCoV-19/Mexico/CMX-INMEGEN-01-01-15/2020	EPI_ISL_1005665	30/12/2020	Mexico City	Human	Male	36	unknown	Original	Oropharyngeal swab
hCoV-19/Mexico/CMX-INMEGEN-01-01-16/2020	EPI_ISL_1005666	30/12/2020	Mexico City	Human	Female	34	unknown	Original	Oropharyngeal swab
hCoV-19/Mexico/TAM-InDRE-94/2020	EPI_ISL_794592	31/12/2020	Tamaulipas	Human	Male	56	Hospitalized	Original	Nasopharyngeal and oropharyngeal swab
hCoV-19/Mexico/CMX-INMEGEN-01-02-07/2020	EPI_ISL_985157	31/12/2020	Mexico City	Human	Female	34	unknown	Original	Oropharyngeal swab
hCoV-19/Mexico/CMX-INMEGEN-01-02-14/2020	EPI_ISL_985158	31/12/2020	Mexico City	Human	Female	8	unknown	Original	Oropharyngeal swab
hCoV-19/Mexico/CMX-INMEGEN-01-02-15/2020	EPI_ISL_985159	31/12/2020	Mexico City	Human	Male	16	unknown	Original	Oropharyngeal swab
hCoV-19/Mexico/NLE-UANL-088/2020	EPI_ISL_979338	31/12/2020	Nuevo Leon	Human	Male	51	unknown	Original	
hCoV-19/Mexico/CMX-INMEGEN-01-02-23/2021	EPI_ISL_985156	01/01/2021	Mexico City	Human	Female	39	unknown	Original	Oropharyngeal swab
hCoV-19/Mexico/CMX-INMEGEN-01-01-21/2021	EPI_ISL_1005670	01/01/2021	Mexico City	Human	Male	61	unknown	Original	Oropharyngeal swab
hCoV-19/Mexico/NLE-UANL-002/2021	EPI_ISL_979339	01/01/2021	Nuevo Leon	Human	Male	36	unknown	Original	
hCoV-19/Mexico/VER-InDRE_111/2021	EPI_ISL_913915	02/01/2021	Veracruz	Human	Female	56	unknown	Original	Oropharyngeal swab
hCoV-19/Mexico/VER-InDRE_112/2021	EPI_ISL_913916	02/01/2021	Veracruz	Human	Female	35	unknown	Original	Oropharyngeal swab
hCoV-19/Mexico/CMX-INMEGEN-01-02-18/2021	EPI_ISL_985153	02/01/2021	Mexico City	Human	Male	14	unknown	Original	Oropharyngeal swab
hCoV-19/Mexico/CMX-INMEGEN-01-02-19/2021	EPI_ISL_985154	02/01/2021	Mexico City	Human	Female	28	unknown	Original	Oropharyngeal swab
hCoV-19/Mexico/CMX-INMEGEN-01-02-20/2021	EPI_ISL_985155	02/01/2021	Mexico City	Human	Male	26	unknown	Original	Oropharyngeal swab
hCoV-19/Mexico/NLE-UANL-003/2021	EPI_ISL_979340	02/01/2021	Nuevo Leon	Human	Female	25	unknown	Original	
hCoV-19/Mexico/NLE-UANL-004/2021	EPI_ISL_979341	02/01/2021	Nuevo Leon	Human	Male	68	unknown	Original	
hCoV-19/Mexico/NLE-UANL-005/2021	EPI_ISL_979342	03/01/2021	Nuevo Leon	Human	Female	20	unknown	Original	
hCoV-19/Mexico/CMX-INCMNSZ_COV145587/202	EPI_ISL_1502817	04/01/2021	Mexico City	Human	Female	56	Released	Original	Nasopharyngeal swab
hCoV-19/Mexico/JAL-LaDEER-133706/2021	EPI_ISL_1360407	04/01/2021	Jalisco	Human	Female	72	Live	Original	Oropharyngeal swab
hCoV-19/Mexico/CMX-INMEGEN-01-03-04/2021	EPI_ISL_944614	04/01/2021	Mexico City	Human	Female	31	unknown	Original	Oropharyngeal swab
hCoV-19/Mexico/CMX-INMEGEN-01-02-22/2021	EPI_ISL_985150	04/01/2021	Mexico City	Human	Male	40	unknown	Original	Oropharyngeal swab
hCoV-19/Mexico/CMX-INMEGEN-01-02-04/2021	EPI_ISL_985151	04/01/2021	Mexico City	Human	Female	66	unknown	Original	Oropharyngeal swab
hCoV-19/Mexico/CMX-INMEGEN-01-02-21/2021	EPI_ISL_985152	04/01/2021	Mexico City	Human	Male	21	unknown	Original	Oropharyngeal swab
hCoV-19/Mexico/CMX-INMEGEN-01-01-23/2021	EPI_ISL_1005672	04/01/2021	Mexico City	Human	Female	26	unknown	Original	Oropharyngeal swab
hCoV-19/Mexico/CMX-INMEGEN-02-10-22-GRO/2	EPI_ISL_1137459	04/01/2021	Mexico City	Human	Male	30	unknown	Original	Oropharyngeal swab
hCoV-19/Mexico/CMX-INER-SC239/2021	EPI_ISL_1040927	05/01/2021	Mexico City	Human	Male	53	Live	Original	Oropharyngeal swab
hCoV-19/Mexico/JAL-InDRE_F107G_S583/2021	EPI_ISL_1324766	05/01/2021	Jalisco	Human	Female	71	Released	Original	Oropharyngeal swab
hCoV-19/Mexico/SLP-UASLP-AH1COV2SS019_S	EPI_ISL_1494730	05/01/2021	San Luis Potosi	Human	Female	51	Released	Original	
hCoV-19/Mexico/CMX-INMEGEN-01-02-16/2021	EPI_ISL_985149	05/01/2021	Mexico City	Human	Female	51	unknown	Original	Oropharyngeal swab
hCoV-19/Mexico/JAL-InDRE_371/2021	EPI_ISL_1093145	06/01/2021	Jalisco	Human	Male	37	unknown	Original	Oropharyngeal swab
hCoV-19/Mexico/CMX-InDRE_184/2021	EPI_ISL_933661	06/01/2021	Mexico City	Human	Female	50	unknown	Original	Oropharyngeal swab

hCoV-19/Mexico/CMX-InDRE_185/2021	EPI_ISL_933662	06/01/2021	Mexico City	Human	Female	54	unknown	Original	Oropharyngeal swab
hCoV-19/Mexico/CMX-InDRE_203/2021	EPI_ISL_933680	06/01/2021	Mexico City	Human	Male	36	unknown	Original	Oropharyngeal swab
hCoV-19/Mexico/JAL-LaDEER-139093/2021	EPI_ISL_1360412	06/01/2021	Jalisco	Human	Male	83	Live	Original	Oropharyngeal swab
hCoV-19/Mexico/GUA-InDRE_250/2021	EPI_ISL_1060692	06/01/2021	Guanajuato	Human	Female	49	unknown	Original	Oropharyngeal swab
hCoV-19/Mexico/CMX-InDRE_204/2021	EPI_ISL_933681	07/01/2021	Mexico City	Human	Female	36	unknown	Original	Oropharyngeal swab
hCoV-19/Mexico/CMX-InDRE_205/2021	EPI_ISL_933682	07/01/2021	Mexico City	Human	Male	35	unknown	Original	Oropharyngeal swab
hCoV-19/Mexico/CMX-InDRE_206/2021	EPI_ISL_933683	07/01/2021	Mexico City	Human	Male	36	unknown	Original	Oropharyngeal swab
hCoV-19/Mexico/CMX-InDRE_207/2021	EPI_ISL_933684	07/01/2021	Mexico City	Human	Male	49	unknown	Original	Oropharyngeal swab
hCoV-19/Mexico/CMX-InDRE_208/2021	EPI_ISL_933685	07/01/2021	Mexico City	Human	Female	36	unknown	Original	Oropharyngeal swab
hCoV-19/Mexico/CMX-InDRE_209/2021	EPI_ISL_933686	07/01/2021	Mexico City	Human	Male	60	unknown	Original	Oropharyngeal swab
hCoV-19/Mexico/CMX-InDRE_210/2021	EPI_ISL_933687	07/01/2021	Mexico City	Human	Female	16	unknown	Original	Oropharyngeal swab
hCoV-19/Mexico/CMX-InDRE_211/2021	EPI_ISL_933688	07/01/2021	Mexico City	Human	Female	17	unknown	Original	Oropharyngeal swab
hCoV-19/Mexico/CMX-InDRE_212/2021	EPI_ISL_933689	07/01/2021	Mexico City	Human	Female	76	unknown	Original	Oropharyngeal swab
hCoV-19/Mexico/GUA-InDRE_251/2021	EPI_ISL_1060718	07/01/2021	Guanajuato	Human	Male	27	unknown	Original	Oropharyngeal swab
hCoV-19/Mexico/CMX-InDRE_227/2021	EPI_ISL_933704	07/01/2021	Mexico City	Human	Female	27	unknown	Original	Oropharyngeal swab
hCoV-19/Mexico/CMX-InDRE_213/2021	EPI_ISL_933690	08/01/2021	Mexico City	Human	Female	46	unknown	Original	Oropharyngeal swab
hCoV-19/Mexico/CMX-InDRE_214/2021	EPI_ISL_933691	08/01/2021	Mexico City	Human	Male	58	unknown	Original	Oropharyngeal swab
hCoV-19/Mexico/MEX-InDRE_215/2021	EPI_ISL_933692	08/01/2021	State of Mexico	Human	Female	25	unknown	Original	Oropharyngeal swab
hCoV-19/Mexico/SLP-UASLP-AH1COV2SS017_S	EPI_ISL_1494728	08/01/2021	San Luis Potosi	Human	Female	59	Released	Original	
hCoV-19/Mexico/MEX-InDRE_228/2021	EPI_ISL_933705	08/01/2021	State of Mexico	Human	Female	26	unknown	Original	Oropharyngeal swab
hCoV-19/Mexico/CMX-INMEGEN-01-03-14/2021	EPI_ISL_944624	08/01/2021	Mexico City	Human	Female	21	unknown	Original	Oropharyngeal swab
hCoV-19/Mexico/CMX-INMEGEN-01-03-15/2021	EPI_ISL_944625	08/01/2021	Mexico City	Human	Female	62	unknown	Original	Oropharyngeal swab
hCoV-19/Mexico/NLE-UANL-006/2021	EPI_ISL_979343	08/01/2021	Nuevo Leon	Human	Female	41	unknown	Original	
hCoV-19/Mexico/NLE-UANL-007/2021	EPI_ISL_979344	08/01/2021	Nuevo Leon	Human	Male	51	unknown	Original	
hCoV-19/Mexico/NLE-UANL-008/2021	EPI_ISL_979345	08/01/2021	Nuevo Leon	Human	Female	30	unknown	Original	
hCoV-19/Mexico/SLP-InDRE_454/2021	EPI_ISL_1219714	08/01/2021	San Luis Potosi	Human	Female	53	Released	Original	Oropharyngeal swab
hCoV-19/Mexico/CMX-INMEGEN-03-03-03/2021	EPI_ISL_1298453	08/01/2021	Mexico City	Human	Female	28	unknown	Original	Oropharyngeal swab
hCoV-19/Mexico/CMX-InDRE_186/2021	EPI_ISL_933663	09/01/2021	Mexico City	Human	Male	56	unknown	Original	Oropharyngeal swab
hCoV-19/Mexico/NLE-UANL-009/2021	EPI_ISL_979346	09/01/2021	Nuevo Leon	Human	Male	20	unknown	Original	
hCoV-19/Mexico/NLE-UANL-010/2021	EPI_ISL_979347	09/01/2021	Nuevo Leon	Human	Male	52	unknown	Original	
hCoV-19/Mexico/NLE-UANL-011/2021	EPI_ISL_979348	09/01/2021	Nuevo Leon	Human	Male	68	unknown	Original	
hCoV-19/Mexico/NLE-UANL-012/2021	EPI_ISL_979349	09/01/2021	Nuevo Leon	Human	Male	25	unknown	Original	
hCoV-19/Mexico/NLE-UANL-013/2021	EPI_ISL_979350	09/01/2021	Nuevo Leon	Human	Male	17	unknown	Original	
hCoV-19/Mexico/CMX-INMEGEN-01-03-13/2021	EPI_ISL_944623	10/01/2021	Mexico City	Human	Female	20	unknown	Original	Oropharyngeal swab
hCoV-19/Mexico/NLE-UANL-014/2021	EPI_ISL_979351	10/01/2021	Nuevo Leon	Human	Male	49	unknown	Original	
hCoV-19/Mexico/NLE-UANL-015/2021	EPI_ISL_979352	10/01/2021	Nuevo Leon	Human	Female	47	unknown	Original	
hCoV-19/Mexico/JAL-InDRE_372/2021	EPI_ISL_1093146	11/01/2021	Jalisco	Human	Male	60	unknown	Original	Oropharyngeal swab
hCoV-19/Mexico/SLP-UASLP-AH1COV2SS018_S	EPI_ISL_1494729	11/01/2021	San Luis Potosi	Human	Male	38	Hospitalized	Original	
hCoV-19/Mexico/CMX-INMEGEN-01-03-01/2021	EPI_ISL_944611	11/01/2021	Mexico City	Human	Female	62	unknown	Original	Oropharyngeal swab
hCoV-19/Mexico/CMX-INMEGEN-01-03-02/2021	EPI_ISL_944612	11/01/2021	Mexico City	Human	Male	67	unknown	Original	Oropharyngeal swab
hCoV-19/Mexico/CMX-INMEGEN-01-03-03/2021	EPI_ISL_944613	11/01/2021	Mexico City	Human	Male	48	unknown	Original	Oropharyngeal swab

hCoV-19/Mexico/CMX-INMEGEN-01-03-05/2021	EPI_ISL_944615	11/01/2021	Mexico City	Human	Female	69	unknown	Original	Oropharyngeal swab
hCoV-19/Mexico/CMX-INMEGEN-01-03-06/2021	EPI_ISL_944616	11/01/2021	Mexico City	Human	Female	47	unknown	Original	Oropharyngeal swab
hCoV-19/Mexico/CMX-INMEGEN-01-03-09/2021	EPI_ISL_944619	11/01/2021	Mexico City	Human	Female	53	unknown	Original	Oropharyngeal swab
hCoV-19/Mexico/CMX-INMEGEN-01-03-17/2021	EPI_ISL_944627	11/01/2021	Mexico City	Human	Male	46	unknown	Original	Oropharyngeal swab
hCoV-19/Mexico/CMX-INMEGEN-01-03-18/2021	EPI_ISL_944628	11/01/2021	Mexico City	Human	Male	52	unknown	Original	Oropharyngeal swab
hCoV-19/Mexico/CMX-INMEGEN-01-03-21/2021	EPI_ISL_944631	11/01/2021	Mexico City	Human	Female	40	unknown	Original	Oropharyngeal swab
hCoV-19/Mexico/CMX-INMEGEN-01-03-23/2021	EPI_ISL_944633	11/01/2021	Mexico City	Human	Female	10	unknown	Original	Oropharyngeal swab
hCoV-19/Mexico/CMX-INMEGEN-01-03-07/2021	EPI_ISL_944617	12/01/2021	Mexico City	Human	Male	32	unknown	Original	Oropharyngeal swab
hCoV-19/Mexico/CMX-INMEGEN-01-03-12/2021	EPI_ISL_944622	12/01/2021	Mexico City	Human	Male	25	unknown	Original	Oropharyngeal swab
hCoV-19/Mexico/CMX-INMEGEN-01-03-16/2021	EPI_ISL_944626	12/01/2021	Mexico City	Human	Male	30	unknown	Original	Oropharyngeal swab
hCoV-19/Mexico/BCN-ALSR-6339/2021	EPI_ISL_962662	12/01/2021	Baja California	Human	unknown	unknown	unknown	Original	Nasal swab
hCoV-19/Mexico/BCN-ALSR-6340/2021	EPI_ISL_962663	12/01/2021	Baja California	Human	unknown	unknown	unknown	Original	Nasal swab
hCoV-19/Mexico/GUA-InDRE_252/2021	EPI_ISL_1060704	13/01/2021	Guanajuato	Human	Male	35	unknown	Original	Oropharyngeal swab
hCoV-19/Mexico/CMX-INMEGEN-01-03-19/2021	EPI_ISL_944629	13/01/2021	Mexico City	Human	Female	61	unknown	Original	Oropharyngeal swab
hCoV-19/Mexico/CMX-INMEGEN-01-03-20/2021	EPI_ISL_944630	13/01/2021	Mexico City	Human	Male	27	unknown	Original	Oropharyngeal swab
hCoV-19/Mexico/CMX-InDRE_242/2021	EPI_ISL_942928	13/01/2021	Mexico City	Human	Male	48	Released	Original	Oropharyngeal swab
hCoV-19/Mexico/TAM-InDRE_236/2021	EPI_ISL_961466	13/01/2021	Tamaulipas	Human	Female	70	Released	Original	Oropharyngeal swab
hCoV-19/Mexico/CMX-INMEGEN-01-04-15/2021	EPI_ISL_1040561	14/01/2021	Mexico City	Human	Female	74	unknown	Original	Nasopharyngeal swab
hCoV-19/Mexico/CMX-INMEGEN-01-05-17/2021	EPI_ISL_1040598	14/01/2021	Mexico City	Human	Female	83	unknown	Original	Nasopharyngeal swab
hCoV-19/Mexico/CMX-INMEGEN-01-03-08/2021	EPI_ISL_944618	14/01/2021	Mexico City	Human	Female	38	unknown	Original	Oropharyngeal swab
hCoV-19/Mexico/CMX-INMEGEN-01-03-10/2021	EPI_ISL_944620	14/01/2021	Mexico City	Human	Female	43	unknown	Original	Oropharyngeal swab
hCoV-19/Mexico/CMX-INMEGEN-01-03-11/2021	EPI_ISL_944621	14/01/2021	Mexico City	Human	Female	61	unknown	Original	Oropharyngeal swab
hCoV-19/Mexico/CMX-INMEGEN-01-03-22/2021	EPI_ISL_944632	14/01/2021	Mexico City	Human	Male	53	unknown	Original	Oropharyngeal swab
hCoV-19/Mexico/QUE-InDRE_246/2021	EPI_ISL_1060697	14/01/2021	Queretaro	Human	Female	34	unknown	Original	Oropharyngeal swab
hCoV-19/Mexico/CMX-INMEGEN-01-04-11/2021	EPI_ISL_1040560	15/01/2021	Mexico City	Human	Male	58	unknown	Original	Nasopharyngeal swab
hCoV-19/Mexico/CMX-INMEGEN-01-04-12/2021	EPI_ISL_1040562	15/01/2021	Mexico City	Human	Female	58	unknown	Original	Nasopharyngeal swab
hCoV-19/Mexico/CMX-INMEGEN-01-04-16/2021	EPI_ISL_1040563	15/01/2021	Mexico City	Human	Female	21	unknown	Original	Nasopharyngeal swab
hCoV-19/Mexico/CMX-INMEGEN-01-05-02/2021	EPI_ISL_1040593	15/01/2021	Mexico City	Human	Male	47	unknown	Original	Nasopharyngeal swab
hCoV-19/Mexico/CMX-INMEGEN-01-05-03/2021	EPI_ISL_1040594	15/01/2021	Mexico City	Human	Male	25	unknown	Original	Nasopharyngeal swab
hCoV-19/Mexico/CMX-INMEGEN-01-05-09/2021	EPI_ISL_1040595	15/01/2021	Mexico City	Human	Female	39	unknown	Original	Nasopharyngeal swab
hCoV-19/Mexico/CMX-INMEGEN-01-05-10/2021	EPI_ISL_1040596	15/01/2021	Mexico City	Human	Male	37	unknown	Original	Nasopharyngeal swab
hCoV-19/Mexico/CMX-INMEGEN-01-05-18/2021	EPI_ISL_1040597	15/01/2021	Mexico City	Human	Female	3	unknown	Original	Nasopharyngeal swab
hCoV-19/Mexico/NLE-UANL-016/2021	EPI_ISL_1091241	15/01/2021	Nuevo Leon	Human	Female	40	unknown	Original	
hCoV-19/Mexico/NLE-UANL-019/2021	EPI_ISL_1091253	15/01/2021	Nuevo Leon	Human	Male	63	unknown	Original	
hCoV-19/Mexico/NLE-UANL-017/2021	EPI_ISL_1091254	15/01/2021	Nuevo Leon	Human	Female	53	unknown	Original	
hCoV-19/Mexico/NLE-UANL-018/2021	EPI_ISL_1091262	15/01/2021	Nuevo Leon	Human	Male	29	unknown	Original	
hCoV-19/Mexico/CMX-INCMNSZ_COV149517/202	EPI_ISL_1502814	15/01/2021	Mexico City	Human	unknown	unknown	Released	Original	Nasopharyngeal swab
hCoV-19/Mexico/NLE-UANL-020/2021	EPI_ISL_1092458	15/01/2021	Nuevo Leon	Human	Male	86	unknown	Original	
hCoV-19/Mexico/COL-InDRE_545/2021	EPI_ISL_1168615	15/01/2021	Colima	Human	Female	72	Deceased	Original	Oropharyngeal swab
hCoV-19/Mexico/JAL-InDRE_549/2021	EPI_ISL_1168619	15/01/2021	Jalisco	Human	Male	26	Released	Original	Oropharyngeal swab
hCoV-19/Mexico/JAL-InDRE_550/2021	EPI_ISL_1168620	15/01/2021	Jalisco	Human	Male	78	Live	Original	Oropharyngeal swab

hCoV-19/Mexico/JAL-InDRE_551/2021	EPI_ISL_1168621	15/01/2021	Jalisco	Human	Male	62	Released	Original	Oropharyngeal swab
hCoV-19/Mexico/JAL-InDRE_552/2021	EPI_ISL_1168622	15/01/2021	Jalisco	Human	Female	70	Released	Original	Oropharyngeal swab
hCoV-19/Mexico/JAL-InDRE_553/2021	EPI_ISL_1168623	15/01/2021	Jalisco	Human	Male	26	Released	Original	Oropharyngeal swab
hCoV-19/Mexico/MOR-InDRE_462/2021	EPI_ISL_1168532	15/01/2021	Morelos	Human	Male	18	Released	Original	Oropharyngeal swab
hCoV-19/Mexico/MOR-InDRE_463/2021	EPI_ISL_1168533	15/01/2021	Morelos	Human	Male	67	Hospitalized	Original	Oropharyngeal swab
hCoV-19/Mexico/MOR-InDRE_464/2021	EPI_ISL_1168534	15/01/2021	Morelos	Human	Male	84	Released	Original	Oropharyngeal swab
hCoV-19/Mexico/MOR-InDRE_465/2021	EPI_ISL_1168535	15/01/2021	Morelos	Human	Female	54	Released	Original	Oropharyngeal swab
hCoV-19/Mexico/JAL-InDRE_482/2021	EPI_ISL_1168552	15/01/2021	Jalisco	Human	Female	33	Released	Original	Oropharyngeal swab
hCoV-19/Mexico/COA-InDRE_505/2021	EPI_ISL_1168575	15/01/2021	Coahuila	Human	Female	36	Released	Original	Oropharyngeal swab
hCoV-19/Mexico/COA-InDRE_506/2021	EPI_ISL_1168576	15/01/2021	Coahuila	Human	Male	31	Released	Original	Oropharyngeal swab
hCoV-19/Mexico/COA-InDRE_507/2021	EPI_ISL_1168577	15/01/2021	Coahuila	Human	Female	46	Released	Original	Oropharyngeal swab
hCoV-19/Mexico/COA-InDRE_508/2021	EPI_ISL_1168578	15/01/2021	Coahuila	Human	Male	63	Released	Original	Oropharyngeal swab
hCoV-19/Mexico/COA-InDRE_509/2021	EPI_ISL_1168579	15/01/2021	Coahuila	Human	Female	56	Deceased	Original	Oropharyngeal swab
hCoV-19/Mexico/COA-InDRE_510/2021	EPI_ISL_1168580	15/01/2021	Coahuila	Human	Male	68	Hospitalized	Original	Oropharyngeal swab
hCoV-19/Mexico/COA-InDRE_511/2021	EPI_ISL_1168581	15/01/2021	Coahuila	Human	Male	47	Released	Original	Oropharyngeal swab
hCoV-19/Mexico/COA-InDRE_512/2021	EPI_ISL_1168582	15/01/2021	Coahuila	Human	Male	61	Released	Original	Oropharyngeal swab
hCoV-19/Mexico/COA-InDRE_516/2021	EPI_ISL_1168586	15/01/2021	Coahuila	Human	Male	32	Released	Original	Oropharyngeal swab
hCoV-19/Mexico/COA-InDRE_517/2021	EPI_ISL_1168587	15/01/2021	Coahuila	Human	Male	71	Deceased	Original	Oropharyngeal swab
hCoV-19/Mexico/NLE-InDRE_403/2021	EPI_ISL_1168481	15/01/2021	Nuevo Leon	Human	Female	59	Released	Original	Oropharyngeal swab
hCoV-19/Mexico/VER-InDRE_416/2021	EPI_ISL_1168494	15/01/2021	Veracruz	Human	Female	52	Live	Original	Oropharyngeal swab
hCoV-19/Mexico/CMX-INMEGEN-01-04-02/2021	EPI_ISL_1040564	16/01/2021	Mexico City	Human	Male	23	unknown	Original	Nasopharyngeal swab
hCoV-19/Mexico/CMX-INMEGEN-01-05-01/2021	EPI_ISL_1040591	16/01/2021	Mexico City	Human	Female	37	unknown	Original	Nasopharyngeal swab
hCoV-19/Mexico/CMX-INMEGEN-01-05-04/2021	EPI_ISL_1040592	16/01/2021	Mexico City	Human	Female	47	unknown	Original	Nasopharyngeal swab
hCoV-19/Mexico/NLE-UANL-021/2021	EPI_ISL_1091255	16/01/2021	Nuevo Leon	Human	Female	20	unknown	Original	
hCoV-19/Mexico/SLP_UASLP_A002/2021	EPI_ISL_1469112	16/01/2021	San Luis Potosi	Human	Male	21	Released	Original	Oropharyngeal swab
hCoV-19/Mexico/HID-InDRE_460/2021	EPI_ISL_1168530	16/01/2021	Hidalgo	Human	Male	63	Live	Original	Oropharyngeal swab
hCoV-19/Mexico/HID-InDRE_461/2021	EPI_ISL_1168531	16/01/2021	Hidalgo	Human	Female	31	Live	Original	Oropharyngeal swab
hCoV-19/Mexico/MOR-InDRE_466/2021	EPI_ISL_1168536	16/01/2021	Morelos	Human	Male	67	Hospitalized	Original	Oropharyngeal swab
hCoV-19/Mexico/MEX-InDRE_467/2021	EPI_ISL_1168537	16/01/2021	State of Mexico	Human	Male	65	Hospitalized	Original	Oropharyngeal swab
hCoV-19/Mexico/BCS-InDRE_468/2021	EPI_ISL_1168538	16/01/2021	Baja California Sur	Human	Female	25	Released	Original	Oropharyngeal swab
hCoV-19/Mexico/QUE-InDRE_473/2021	EPI_ISL_1168543	16/01/2021	Queretaro	Human	Male	34	Released	Original	Oropharyngeal swab
hCoV-19/Mexico/QUE-InDRE_474/2021	EPI_ISL_1168544	16/01/2021	Queretaro	Human	Female	30	Released	Original	Oropharyngeal swab
hCoV-19/Mexico/QUE-InDRE_475/2021	EPI_ISL_1168545	16/01/2021	Queretaro	Human	Male	58	Released	Original	Oropharyngeal swab
hCoV-19/Mexico/QUE-InDRE_476/2021	EPI_ISL_1168546	16/01/2021	Queretaro	Human	Female	61	Released	Original	Oropharyngeal swab
hCoV-19/Mexico/QUE-InDRE_477/2021	EPI_ISL_1168547	16/01/2021	Queretaro	Human	Female	35	Released	Original	Oropharyngeal swab
hCoV-19/Mexico/QUE-InDRE_478/2021	EPI_ISL_1168548	16/01/2021	Queretaro	Human	Female	32	Released	Original	Oropharyngeal swab
hCoV-19/Mexico/QUE-InDRE_479/2021	EPI_ISL_1168549	16/01/2021	Queretaro	Human	Male	18	Released	Original	Oropharyngeal swab
hCoV-19/Mexico/COA-InDRE_483/2021	EPI_ISL_1168553	16/01/2021	Coahuila	Human	Male	23	Released	Original	Oropharyngeal swab
hCoV-19/Mexico/COA-InDRE_484/2021	EPI_ISL_1168554	16/01/2021	Coahuila	Human	Female	13	Released	Original	Oropharyngeal swab
hCoV-19/Mexico/COA-InDRE_485/2021	EPI_ISL_1168555	16/01/2021	Coahuila	Human	Male	73	Released	Original	Oropharyngeal swab
hCoV-19/Mexico/VER-InDRE_414/2021	EPI_ISL_1168492	16/01/2021	Veracruz	Human	Male	62	Live	Original	Oropharyngeal swab

hCoV-19/Mexico/CMX-INMEGEN-01-04-13/2021	EPI_ISL_1040565	17/01/2021	Mexico City	Human	Female	58	unknown	Original	Nasopharyngeal swab
hCoV-19/Mexico/CMX-INMEGEN-02-01-02/2021	EPI_ISL_1040621	17/01/2021	Mexico City	Human	Female	63	unknown	Original	Nasopharyngeal swab
hCoV-19/Mexico/CMX-INMEGEN-02-01-03/2021	EPI_ISL_1040622	17/01/2021	Mexico City	Human	Female	91	unknown	Original	Nasopharyngeal swab
hCoV-19/Mexico/CAM-InDRE_544/2021	EPI_ISL_1168614	17/01/2021	Campeche	Human	Male	59	Deceased	Original	Oropharyngeal swab
hCoV-19/Mexico/BCS-InDRE_469/2021	EPI_ISL_1168539	17/01/2021	Baja California Sur	Human	Male	43	Released	Original	Oropharyngeal swab
hCoV-19/Mexico/BCS-InDRE_470/2021	EPI_ISL_1168540	17/01/2021	Baja California Sur	Human	Female	78	Released	Original	Oropharyngeal swab
hCoV-19/Mexico/BCS-InDRE_471/2021	EPI_ISL_1168541	17/01/2021	Baja California Sur	Human	Male	43	Released	Original	Oropharyngeal swab
hCoV-19/Mexico/BCS-InDRE_472/2021	EPI_ISL_1168542	17/01/2021	Baja California Sur	Human	Male	84	Released	Original	Oropharyngeal swab
hCoV-19/Mexico/QUE-InDRE_480/2021	EPI_ISL_1168550	17/01/2021	Queretaro	Human	Female	51	Deceased	Original	Oropharyngeal swab
hCoV-19/Mexico/COA-InDRE_486/2021	EPI_ISL_1168556	17/01/2021	Coahuila	Human	Female	36	Released	Original	Oropharyngeal swab
hCoV-19/Mexico/COA-InDRE_487/2021	EPI_ISL_1168557	17/01/2021	Coahuila	Human	Male	26	Released	Original	Oropharyngeal swab
hCoV-19/Mexico/COA-InDRE_488/2021	EPI_ISL_1168558	17/01/2021	Coahuila	Human	Male	70	Hospitalized	Original	Oropharyngeal swab
hCoV-19/Mexico/COA-InDRE_489/2021	EPI_ISL_1168559	17/01/2021	Coahuila	Human	Female	57	Released	Original	Oropharyngeal swab
hCoV-19/Mexico/COA-InDRE_490/2021	EPI_ISL_1168560	17/01/2021	Coahuila	Human	Male	63	Deceased	Original	Oropharyngeal swab
hCoV-19/Mexico/COA-InDRE_513/2021	EPI_ISL_1168583	17/01/2021	Coahuila	Human	Female	74	Released	Original	Oropharyngeal swab
hCoV-19/Mexico/MOR-InDRE_522/2021	EPI_ISL_1168592	17/01/2021	Morelos	Human	Male	58	Released	Original	Oropharyngeal swab
hCoV-19/Mexico/NLE-InDRE_390/2021	EPI_ISL_1168468	17/01/2021	Nuevo Leon	Human	Female	52	Released	Original	Oropharyngeal swab
hCoV-19/Mexico/NLE-InDRE_394/2021	EPI_ISL_1168472	17/01/2021	Nuevo Leon	Human	Female	36	Released	Original	Oropharyngeal swab
hCoV-19/Mexico/NLE-InDRE_398/2021	EPI_ISL_1168476	17/01/2021	Nuevo Leon	Human	Male	52	Released	Original	Oropharyngeal swab
hCoV-19/Mexico/NLE-InDRE_405/2021	EPI_ISL_1168483	17/01/2021	Nuevo Leon	Human	Male	49	Released	Original	Oropharyngeal swab
hCoV-19/Mexico/JAL-InDRE_419/2021	EPI_ISL_1168497	17/01/2021	Jalisco	Human	Female	36	Released	Original	Oropharyngeal swab
hCoV-19/Mexico/CMX-INMEGEN-01-04-05/2021	EPI_ISL_1040566	18/01/2021	Mexico City	Human	Male	11	unknown	Original	Nasopharyngeal swab
hCoV-19/Mexico/CMX-INMEGEN-01-04-06/2021	EPI_ISL_1040567	18/01/2021	Mexico City	Human	Female	40	unknown	Original	Nasopharyngeal swab
hCoV-19/Mexico/CMX-INMEGEN-01-04-08/2021	EPI_ISL_1040568	18/01/2021	Mexico City	Human	Female	61	unknown	Original	Nasopharyngeal swab
hCoV-19/Mexico/CMX-INMEGEN-01-04-09/2021	EPI_ISL_1040569	18/01/2021	Mexico City	Human	Male	54	unknown	Original	Nasopharyngeal swab
hCoV-19/Mexico/CMX-INMEGEN-01-04-14/2021	EPI_ISL_1040570	18/01/2021	Mexico City	Human	Male	38	unknown	Original	Nasopharyngeal swab
hCoV-19/Mexico/CMX-INMEGEN-01-04-17/2021	EPI_ISL_1040571	18/01/2021	Mexico City	Human	Male	20	unknown	Original	Nasopharyngeal swab
hCoV-19/Mexico/CMX-INMEGEN-01-04-18/2021	EPI_ISL_1040572	18/01/2021	Mexico City	Human	Female	31	unknown	Original	Nasopharyngeal swab
hCoV-19/Mexico/CMX-INMEGEN-01-05-07/2021	EPI_ISL_1040580	18/01/2021	Mexico City	Human	Male	26	unknown	Original	Nasopharyngeal swab
hCoV-19/Mexico/CMX-INMEGEN-01-05-05/2021	EPI_ISL_1040587	18/01/2021	Mexico City	Human	Male	55	unknown	Original	Nasopharyngeal swab
hCoV-19/Mexico/CMX-INMEGEN-01-05-14/2021	EPI_ISL_1040588	18/01/2021	Mexico City	Human	Male	68	unknown	Original	Nasopharyngeal swab
hCoV-19/Mexico/CMX-INMEGEN-01-05-15/2021	EPI_ISL_1040589	18/01/2021	Mexico City	Human	Female	52	unknown	Original	Nasopharyngeal swab
hCoV-19/Mexico/CMX-INMEGEN-01-05-16/2021	EPI_ISL_1040590	18/01/2021	Mexico City	Human	Male	21	unknown	Original	Nasopharyngeal swab
hCoV-19/Mexico/JAL-InDRE_373/2021	EPI_ISL_1093147	18/01/2021	Jalisco	Human	Female	78	unknown	Original	Oropharyngeal swab
hCoV-19/Mexico/CAM-InDRE_543/2021	EPI_ISL_1168613	18/01/2021	Campeche	Human	Female	32	Live	Original	Oropharyngeal swab
hCoV-19/Mexico/COL-InDRE_546/2021	EPI_ISL_1168616	18/01/2021	Colima	Human	Male	34	Live	Original	Oropharyngeal swab
hCoV-19/Mexico/COL-InDRE_547/2021	EPI_ISL_1168617	18/01/2021	Colima	Human	Male	57	Deceased	Original	Oropharyngeal swab
hCoV-19/Mexico/COL-InDRE_548/2021	EPI_ISL_1168618	18/01/2021	Colima	Human	Male	69	Live	Original	Oropharyngeal swab
hCoV-19/Mexico/JAL-LaDEER-145365/2021	EPI_ISL_1360409	18/01/2021	Jalisco	Human	Female	19	Live	Original	Oropharyngeal swab
hCoV-19/Mexico/JAL-LaDEER-147248/2021	EPI_ISL_1360410	18/01/2021	Jalisco	Human	Female	18	Live	Original	Oropharyngeal swab
hCoV-19/Mexico/JAL-InDRE_422/2021	EPI_ISL_1168500	18/01/2021	Jalisco	Human	Female	34	Released	Original	Oropharyngeal swab

hCoV-19/Mexico/CHP-InDRE_443/2021	EPI_ISL_1168521	18/01/2021	Chiapas	Human	Female	76	Live	Original	Oropharyngeal swab
hCoV-19/Mexico/HID-InDRE_459/2021	EPI_ISL_1168529	18/01/2021	Hidalgo	Human	Male	40	Live	Original	Oropharyngeal swab
hCoV-19/Mexico/HID-InDRE_481/2021	EPI_ISL_1168551	18/01/2021	Hidalgo	Human	Female	53	Released	Original	Oropharyngeal swab
hCoV-19/Mexico/COA-InDRE_491/2021	EPI_ISL_1168561	18/01/2021	Coahuila	Human	Male	46	Released	Original	Oropharyngeal swab
hCoV-19/Mexico/COA-InDRE_492/2021	EPI_ISL_1168562	18/01/2021	Coahuila	Human	Female	25	Released	Original	Oropharyngeal swab
hCoV-19/Mexico/COA-InDRE_493/2021	EPI_ISL_1168563	18/01/2021	Coahuila	Human	Female	33	Released	Original	Oropharyngeal swab
hCoV-19/Mexico/COA-InDRE_494/2021	EPI_ISL_1168564	18/01/2021	Coahuila	Human	Male	39	Released	Original	Oropharyngeal swab
hCoV-19/Mexico/COA-InDRE_495/2021	EPI_ISL_1168565	18/01/2021	Coahuila	Human	Male	38	Released	Original	Oropharyngeal swab
hCoV-19/Mexico/COA-InDRE_496/2021	EPI_ISL_1168566	18/01/2021	Coahuila	Human	Male	62	Live	Original	Oropharyngeal swab
hCoV-19/Mexico/COA-InDRE_497/2021	EPI_ISL_1168567	18/01/2021	Coahuila	Human	Female	35	Released	Original	Oropharyngeal swab
hCoV-19/Mexico/COA-InDRE_498/2021	EPI_ISL_1168568	18/01/2021	Coahuila	Human	Male	44	Released	Original	Oropharyngeal swab
hCoV-19/Mexico/YUC-InDRE_500/2021	EPI_ISL_1168570	18/01/2021	Yucatan	Human	Female	31	Released	Original	Oropharyngeal swab
hCoV-19/Mexico/YUC-InDRE_501/2021	EPI_ISL_1168571	18/01/2021	Yucatan	Human	Female	41	Released	Original	Oropharyngeal swab
hCoV-19/Mexico/YUC-InDRE_502/2021	EPI_ISL_1168572	18/01/2021	Yucatan	Human	Female	29	Released	Original	Oropharyngeal swab
hCoV-19/Mexico/YUC-InDRE_503/2021	EPI_ISL_1168573	18/01/2021	Yucatan	Human	Male	46	Released	Original	Oropharyngeal swab
hCoV-19/Mexico/YUC-InDRE_504/2021	EPI_ISL_1168574	18/01/2021	Yucatan	Human	Male	45	Released	Original	Oropharyngeal swab
hCoV-19/Mexico/NLE-InDRE_239/2021	EPI_ISL_942927	18/01/2021	Nuevo Leon	Human	Female	15	Released	Original	Oropharyngeal swab
hCoV-19/Mexico/NLE-InDRE_391/2021	EPI_ISL_1168469	18/01/2021	Nuevo Leon	Human	Female	32	Released	Original	Oropharyngeal swab
hCoV-19/Mexico/NLE-InDRE_397/2021	EPI_ISL_1168475	18/01/2021	Nuevo Leon	Human	Male	64	Released	Original	Oropharyngeal swab
hCoV-19/Mexico/NLE-InDRE_401/2021	EPI_ISL_1168479	18/01/2021	Nuevo Leon	Human	Female	24	Released	Original	Oropharyngeal swab
hCoV-19/Mexico/NLE-InDRE_402/2021	EPI_ISL_1168480	18/01/2021	Nuevo Leon	Human	Female	32	Released	Original	Oropharyngeal swab
hCoV-19/Mexico/COA-InDRE_406/2021	EPI_ISL_1168484	18/01/2021	Coahuila	Human	Female	70	Released	Original	Oropharyngeal swab
hCoV-19/Mexico/COA-InDRE_407/2021	EPI_ISL_1168485	18/01/2021	Coahuila	Human	Male	54	Released	Original	Oropharyngeal swab
hCoV-19/Mexico/COA-InDRE_408/2021	EPI_ISL_1168486	18/01/2021	Coahuila	Human	Male	70	Released	Original	Oropharyngeal swab
hCoV-19/Mexico/COA-InDRE_409/2021	EPI_ISL_1168487	18/01/2021	Coahuila	Human	Female	17	Released	Original	Oropharyngeal swab
hCoV-19/Mexico/COA-InDRE_410/2021	EPI_ISL_1168488	18/01/2021	Coahuila	Human	Male	29	Released	Original	Oropharyngeal swab
hCoV-19/Mexico/COA-InDRE_411/2021	EPI_ISL_1168489	18/01/2021	Coahuila	Human	Male	58	Released	Original	Oropharyngeal swab
hCoV-19/Mexico/COA-InDRE_412/2021	EPI_ISL_1168490	18/01/2021	Coahuila	Human	Male	31	Deceased	Original	Oropharyngeal swab
hCoV-19/Mexico/VER-InDRE_415/2021	EPI_ISL_1168493	18/01/2021	Veracruz	Human	Female	80	Live	Original	Oropharyngeal swab
hCoV-19/Mexico/JAL-InDRE_420/2021	EPI_ISL_1168498	18/01/2021	Jalisco	Human	Male	23	Released	Original	Oropharyngeal swab
hCoV-19/Mexico/JAL-InDRE_421/2021	EPI_ISL_1168499	18/01/2021	Jalisco	Human	Female	59	Released	Original	Oropharyngeal swab
hCoV-19/Mexico/NLE-InDRE_237/2021	EPI_ISL_961467	18/01/2021	Nuevo Leon	Human	Female	48	Released	Original	Oropharyngeal swab
hCoV-19/Mexico/NLE-InDRE_238/2021	EPI_ISL_961468	18/01/2021	Nuevo Leon	Human	Male	50	Released	Original	Oropharyngeal swab
hCoV-19/Mexico/NLE-InDRE_240/2021	EPI_ISL_961469	18/01/2021	Nuevo Leon	Human	Male	18	Released	Original	Oropharyngeal swab
hCoV-19/Mexico/CMX-INMEGEN-01-04-04/2021	EPI_ISL_1040573	19/01/2021	Mexico City	Human	Male	29	unknown	Original	Nasopharyngeal swab
hCoV-19/Mexico/CMX-INMEGEN-01-04-07/2021	EPI_ISL_1040574	19/01/2021	Mexico City	Human	Male	60	unknown	Original	Nasopharyngeal swab
hCoV-19/Mexico/CMX-INMEGEN-01-05-06/2021	EPI_ISL_1040578	19/01/2021	Mexico City	Human	Male	47	unknown	Original	Nasopharyngeal swab
hCoV-19/Mexico/CMX-INMEGEN-01-05-08/2021	EPI_ISL_1040584	19/01/2021	Mexico City	Human	Female	58	unknown	Original	Nasopharyngeal swab
hCoV-19/Mexico/CMX-INMEGEN-01-05-11/2021	EPI_ISL_1040585	19/01/2021	Mexico City	Human	Male	28	unknown	Original	Nasopharyngeal swab
hCoV-19/Mexico/CMX-INMEGEN-01-05-12/2021	EPI_ISL_1040586	19/01/2021	Mexico City	Human	Female	26	unknown	Original	Nasopharyngeal swab
hCoV-19/Mexico/CMX-INMEGEN-01-05-21/2021	EPI_ISL_1040599	19/01/2021	Mexico City	Human	Male	19	unknown	Original	Nasopharyngeal swab

hCoV-19/Mexico/COL-InDRE_F11125_S608/2021	EPI_ISL_1337395	19/01/2021	Colima	Human	Male	77	Deceased	Original	Oropharyngeal swab
hCoV-19/Mexico/JAL-LaDEER-E39931/2021	EPI_ISL_1360411	19/01/2021	Jalisco	Human	Female	34	Live	Original	Oropharyngeal swab
hCoV-19/Mexico/SLP_UASLP_A004/2021	EPI_ISL_1469115	19/01/2021	San Luis Potosi	Human	Male	31	Released	Original	Oropharyngeal swab
hCoV-19/Mexico/SLP_UASLP_A005/2021	EPI_ISL_1469116	19/01/2021	San Luis Potosi	Human	Female	21	Released	Original	Oropharyngeal swab
hCoV-19/Mexico/BCN-ALSR-6671/2021	EPI_ISL_1081506	19/01/2021	Baja California	Human	unknown	unknown	unknown	Original	Nasal swab
hCoV-19/Mexico/BCN-ALSR-6709/2021	EPI_ISL_1081544	19/01/2021	Baja California	Human	unknown	unknown	unknown	Original	Nasal swab
hCoV-19/Mexico/JAL-InDRE_423/2021	EPI_ISL_1168501	19/01/2021	Jalisco	Human	Female	44	Released	Original	Oropharyngeal swab
hCoV-19/Mexico/JAL-InDRE_424/2021	EPI_ISL_1168502	19/01/2021	Jalisco	Human	Male	43	Released	Original	Oropharyngeal swab
hCoV-19/Mexico/JAL-InDRE_425/2021	EPI_ISL_1168503	19/01/2021	Jalisco	Human	Female	37	Hospitalized	Original	Oropharyngeal swab
hCoV-19/Mexico/JAL-InDRE_426/2021	EPI_ISL_1168504	19/01/2021	Jalisco	Human	Male	16	Released	Original	Oropharyngeal swab
hCoV-19/Mexico/JAL-InDRE_427/2021	EPI_ISL_1168505	19/01/2021	Jalisco	Human	Female	54	Hospitalized	Original	Oropharyngeal swab
hCoV-19/Mexico/JAL-InDRE_428/2021	EPI_ISL_1168506	19/01/2021	Jalisco	Human	Male	84	Hospitalized	Original	Oropharyngeal swab
hCoV-19/Mexico/JAL-InDRE_429/2021	EPI_ISL_1168507	19/01/2021	Jalisco	Human	Female	23	Released	Original	Oropharyngeal swab
hCoV-19/Mexico/CHP-InDRE_444/2021	EPI_ISL_1168522	19/01/2021	Chiapas	Human	Male	47	Deceased	Original	Oropharyngeal swab
hCoV-19/Mexico/COA-InDRE_499/2021	EPI_ISL_1168569	19/01/2021	Coahuila	Human	Male	84	Hospitalized	Original	Oropharyngeal swab
hCoV-19/Mexico/HID-InDRE_518/2021	EPI_ISL_1168588	19/01/2021	Hidalgo	Human	Female	46	Live	Original	Oropharyngeal swab
hCoV-19/Mexico/MOR-InDRE_523/2021	EPI_ISL_1168593	19/01/2021	Morelos	Human	Female	58	Hospitalized	Original	Oropharyngeal swab
hCoV-19/Mexico/MOR-InDRE_524/2021	EPI_ISL_1168594	19/01/2021	Morelos	Human	Male	39	Released	Original	Oropharyngeal swab
hCoV-19/Mexico/NLE-InDRE_392/2021	EPI_ISL_1168470	19/01/2021	Nuevo Leon	Human	Male	57	Released	Original	Oropharyngeal swab
hCoV-19/Mexico/NLE-InDRE_395/2021	EPI_ISL_1168473	19/01/2021	Nuevo Leon	Human	Male	22	Released	Original	Oropharyngeal swab
hCoV-19/Mexico/NLE-InDRE_396/2021	EPI_ISL_1168474	19/01/2021	Nuevo Leon	Human	Female	32	Released	Original	Oropharyngeal swab
hCoV-19/Mexico/NLE-InDRE_399/2021	EPI_ISL_1168477	19/01/2021	Nuevo Leon	Human	Female	65	Released	Original	Oropharyngeal swab
hCoV-19/Mexico/CMX-INMEGEN-02-02-01/2021	EPI_ISL_1040554	20/01/2021	Mexico City	Human	Female	63	unknown	Original	Nasopharyngeal swab
hCoV-19/Mexico/CMX-INMEGEN-02-02-02/2021	EPI_ISL_1040555	20/01/2021	Mexico City	Human	Male	32	unknown	Original	Nasopharyngeal swab
hCoV-19/Mexico/CMX-INMEGEN-02-02-17/2021	EPI_ISL_1040556	20/01/2021	Mexico City	Human	Male	42	unknown	Original	Nasopharyngeal swab
hCoV-19/Mexico/CMX-INMEGEN-01-04-10/2021	EPI_ISL_1040575	20/01/2021	Mexico City	Human	Female	23	unknown	Original	Nasopharyngeal swab
hCoV-19/Mexico/CMX-INMEGEN-01-04-19/2021	EPI_ISL_1040576	20/01/2021	Mexico City	Human	Female	56	unknown	Original	Nasopharyngeal swab
hCoV-19/Mexico/CMX-INMEGEN-01-05-13/2021	EPI_ISL_1040579	20/01/2021	Mexico City	Human	Female	41	unknown	Original	Nasopharyngeal swab
hCoV-19/Mexico/CMX-INMEGEN-01-05-19/2021	EPI_ISL_1040581	20/01/2021	Mexico City	Human	Male	27	unknown	Original	Nasopharyngeal swab
hCoV-19/Mexico/CMX-INMEGEN-01-05-20/2021	EPI_ISL_1040582	20/01/2021	Mexico City	Human	Male	68	unknown	Original	Nasopharyngeal swab
hCoV-19/Mexico/CMX-INMEGEN-01-05-23/2021	EPI_ISL_1040583	20/01/2021	Mexico City	Human	Male	42	unknown	Original	Nasopharyngeal swab
hCoV-19/Mexico/BCN-ALSR-6577/2021	EPI_ISL_1081416	20/01/2021	Baja California	Human	unknown	unknown	unknown	Original	Nasal swab
hCoV-19/Mexico/QUE-InDRE_554/2021	EPI_ISL_1168624	20/01/2021	Queretaro	Human	Male	36	Released	Original	Oropharyngeal swab
hCoV-19/Mexico/SLP_UASLP_A015/2021	EPI_ISL_1469108	20/01/2021	San Luis Potosi	Human	Female	30	Released	Original	Oropharyngeal swab
hCoV-19/Mexico/SLP_UASLP_A018/2021	EPI_ISL_1469109	20/01/2021	San Luis Potosi	Human	Male	71	Released	Original	Oropharyngeal swab
hCoV-19/Mexico/SLP_UASLP_A007/2021	EPI_ISL_1469117	20/01/2021	San Luis Potosi	Human	Female	40	Hospitalized	Original	Oropharyngeal swab
hCoV-19/Mexico/SLP_UASLP_A008/2021	EPI_ISL_1469118	20/01/2021	San Luis Potosi	Human	Female	25	Released	Original	Oropharyngeal swab
hCoV-19/Mexico/SLP_UASLP_A009/2021	EPI_ISL_1469119	20/01/2021	San Luis Potosi	Human	Female	44	Released	Original	Oropharyngeal swab
hCoV-19/Mexico/JAL-InDRE_430/2021	EPI_ISL_1168508	20/01/2021	Jalisco	Human	Male	24	Released	Original	Oropharyngeal swab
hCoV-19/Mexico/JAL-InDRE_431/2021	EPI_ISL_1168509	20/01/2021	Jalisco	Human	Male	77	Hospitalized	Original	Oropharyngeal swab
hCoV-19/Mexico/JAL-InDRE_432/2021	EPI_ISL_1168510	20/01/2021	Jalisco	Human	Male	31	Released	Original	Oropharyngeal swab

hCoV-19/Mexico/JAL-InDRE_433/2021	EPI_ISL_1168511	20/01/2021	Jalisco	Human	Female	35	Released	Original	Oropharyngeal swab
hCoV-19/Mexico/JAL-InDRE_434/2021	EPI_ISL_1168512	20/01/2021	Jalisco	Human	Female	37	Released	Original	Oropharyngeal swab
hCoV-19/Mexico/BCN-InDRE_447/2021	EPI_ISL_1168525	20/01/2021	Baja California	Human	Male	67	Deceased	Original	Oropharyngeal swab
hCoV-19/Mexico/MOR-InDRE_525/2021	EPI_ISL_1168595	20/01/2021	Morelos	Human	Male	46	Released	Original	Oropharyngeal swab
hCoV-19/Mexico/MOR-InDRE_528/2021	EPI_ISL_1168598	20/01/2021	Morelos	Human	Female	49	Released	Original	Oropharyngeal swab
hCoV-19/Mexico/OAX-InDRE_537/2021	EPI_ISL_1168607	20/01/2021	Oaxaca	Human	Male	59	Deceased	Original	Oropharyngeal swab
hCoV-19/Mexico/NLE-InDRE_393/2021	EPI_ISL_1168471	20/01/2021	Nuevo Leon	Human	Female	36	Released	Original	Oropharyngeal swab
hCoV-19/Mexico/NLE-InDRE_404/2021	EPI_ISL_1168482	20/01/2021	Nuevo Leon	Human	Female	77	Released	Original	Oropharyngeal swab
hCoV-19/Mexico/COA-InDRE_413/2021	EPI_ISL_1168491	20/01/2021	Coahuila	Human	Male	45	Released	Original	Oropharyngeal swab
hCoV-19/Mexico/VER-InDRE_417/2021	EPI_ISL_1168495	20/01/2021	Veracruz	Human	Male	45	Live	Original	Oropharyngeal swab
hCoV-19/Mexico/VER-InDRE_418/2021	EPI_ISL_1168496	20/01/2021	Veracruz	Human	Male	52	Hospitalized	Original	Oropharyngeal swab
hCoV-19/Mexico/CMX-INMEGEN-01-04-20/2021	EPI_ISL_1040577	21/01/2021	Mexico City	Human	Male	52	unknown	Original	Nasopharyngeal swab
hCoV-19/Mexico/GRO-InDRE_F48G_S568/2021	EPI_ISL_1334381	21/01/2021	Guerrero	Human	Male	53	Decease	Original	Oropharyngeal swab
hCoV-19/Mexico/JAL-LaDEER-145340/2021	EPI_ISL_1360408	21/01/2021	Jalisco	Human	Male	22	Live	Original	Oropharyngeal swab
hCoV-19/Mexico/SLP_UASLP_A019/2021	EPI_ISL_1469110	21/01/2021	San Luis Potosi	Human	Male	37	Released	Original	Oropharyngeal swab
hCoV-19/Mexico/SLP_UASLP_A020/2021	EPI_ISL_1469111	21/01/2021	San Luis Potosi	Human	Female	35	Released	Original	Oropharyngeal swab
hCoV-19/Mexico/JAL-InDRE_435/2021	EPI_ISL_1168513	21/01/2021	Jalisco	Human	Male	69	Deceased	Original	Oropharyngeal swab
hCoV-19/Mexico/JAL-InDRE_436/2021	EPI_ISL_1168514	21/01/2021	Jalisco	Human	Female	52	Released	Original	Oropharyngeal swab
hCoV-19/Mexico/JAL-InDRE_437/2021	EPI_ISL_1168515	21/01/2021	Jalisco	Human	Male	77	Hospitalized	Original	Oropharyngeal swab
hCoV-19/Mexico/TAB-InDRE_446/2021	EPI_ISL_1168524	21/01/2021	Tabasco	Human	Male	73	Hospitalized	Original	Oropharyngeal swab
hCoV-19/Mexico/HID-InDRE_519/2021	EPI_ISL_1168589	21/01/2021	Hidalgo	Human	Female	51	Live	Original	Oropharyngeal swab
hCoV-19/Mexico/HID-InDRE_520/2021	EPI_ISL_1168590	21/01/2021	Hidalgo	Human	Female	75	Live	Original	Oropharyngeal swab
hCoV-19/Mexico/MOR-InDRE_526/2021	EPI_ISL_1168596	21/01/2021	Morelos	Human	Male	44	Released	Original	Oropharyngeal swab
hCoV-19/Mexico/MOR-InDRE_527/2021	EPI_ISL_1168597	21/01/2021	Morelos	Human	Male	31	Released	Original	Oropharyngeal swab
hCoV-19/Mexico/MOR-InDRE_529/2021	EPI_ISL_1168599	21/01/2021	Morelos	Human	Female	40	Released	Original	Oropharyngeal swab
hCoV-19/Mexico/MOR-InDRE_530/2021	EPI_ISL_1168600	21/01/2021	Morelos	Human	Male	87	Released	Original	Oropharyngeal swab
hCoV-19/Mexico/MOR-InDRE_533/2021	EPI_ISL_1168603	21/01/2021	Morelos	Human	Female	77	Released	Original	Oropharyngeal swab
hCoV-19/Mexico/OAX-InDRE_536/2021	EPI_ISL_1168606	21/01/2021	Oaxaca	Human	Male	32	unknown	Original	Oropharyngeal swab
hCoV-19/Mexico/OAX-InDRE_538/2021	EPI_ISL_1168608	21/01/2021	Oaxaca	Human	Male	52	Released	Original	Oropharyngeal swab
hCoV-19/Mexico/OAX-InDRE_539/2021	EPI_ISL_1168609	21/01/2021	Oaxaca	Human	Male	83	Live	Original	Oropharyngeal swab
hCoV-19/Mexico/NLE-InDRE_389/2021	EPI_ISL_1168467	21/01/2021	Nuevo Leon	Human	Male	31	Released	Original	Oropharyngeal swab
hCoV-19/Mexico/NLE-InDRE_400/2021	EPI_ISL_1168478	21/01/2021	Nuevo Leon	Human	Male	27	Released	Original	Oropharyngeal swab
hCoV-19/Mexico/CMX-INMEGEN-02-02-04/2021	EPI_ISL_1040551	22/01/2021	Mexico City	Human	Male	38	unknown	Original	Nasopharyngeal swab
hCoV-19/Mexico/CMX-INMEGEN-02-02-12/2021	EPI_ISL_1040552	22/01/2021	Mexico City	Human	Male	25	unknown	Original	Nasopharyngeal swab
hCoV-19/Mexico/CMX-INMEGEN-02-02-19/2021	EPI_ISL_1040553	22/01/2021	Mexico City	Human	Female	51	unknown	Original	Nasopharyngeal swab
hCoV-19/Mexico/CMX-INMEGEN-02-01-09/2021	EPI_ISL_1040619	22/01/2021	Mexico City	Human	Male	41	unknown	Original	Nasopharyngeal swab
hCoV-19/Mexico/CMX-INMEGEN-02-01-18/2021	EPI_ISL_1040620	22/01/2021	Mexico City	Human	Female	59	unknown	Original	Nasopharyngeal swab
hCoV-19/Mexico/NLE-UANL-023/2021	EPI_ISL_1091242	22/01/2021	Nuevo Leon	Human	Female	69	unknown	Original	
hCoV-19/Mexico/NLE-UANL-022/2021	EPI_ISL_1091244	22/01/2021	Nuevo Leon	Human	Male	90	unknown	Original	
hCoV-19/Mexico/NLE-UANL-024/2021	EPI_ISL_1091245	22/01/2021	Nuevo Leon	Human	Female	68	unknown	Original	
hCoV-19/Mexico/NLE-UANL-027/2021	EPI_ISL_1091249	22/01/2021	Nuevo Leon	Human	Female	61	unknown	Original	

hCoV-19/Mexico/NLE-UANL-025/2021	EPI_ISL_1091256	22/01/2021	Nuevo Leon	Human	Female	60	unknown	Original	
hCoV-19/Mexico/NLE-UANL-026/2021	EPI_ISL_1091257	22/01/2021	Nuevo Leon	Human	Female	44	unknown	Original	
hCoV-19/Mexico/OAX-InDRE_541/2021	EPI_ISL_1168611	22/01/2021	Oaxaca	Human	Male	46	Released	Original	Oropharyngeal swab
hCoV-19/Mexico/MEX-InDRE_542/2021	EPI_ISL_1168612	22/01/2021	State of Mexico	Human	Male	44	Live	Original	Oropharyngeal swab
hCoV-19/Mexico/QUE-InDRE_555/2021	EPI_ISL_1168625	22/01/2021	Queretaro	Human	Female	35	Released	Original	Oropharyngeal swab
hCoV-19/Mexico/QUE-InDRE_556/2021	EPI_ISL_1168626	22/01/2021	Queretaro	Human	Female	33	Released	Original	Oropharyngeal swab
hCoV-19/Mexico/QUE-InDRE_557/2021	EPI_ISL_1168627	22/01/2021	Queretaro	Human	Female	26	Released	Original	Oropharyngeal swab
hCoV-19/Mexico/QUE-InDRE_558/2021	EPI_ISL_1168628	22/01/2021	Queretaro	Human	Male	37	Released	Original	Oropharyngeal swab
hCoV-19/Mexico/QUE-InDRE_559/2021	EPI_ISL_1168629	22/01/2021	Queretaro	Human	Female	34	Released	Original	Oropharyngeal swab
hCoV-19/Mexico/QUE-InDRE_560/2021	EPI_ISL_1168630	22/01/2021	Queretaro	Human	Female	20	Released	Original	Oropharyngeal swab
hCoV-19/Mexico/QUE-InDRE_561/2021	EPI_ISL_1168631	22/01/2021	Queretaro	Human	Male	22	Released	Original	Oropharyngeal swab
hCoV-19/Mexico/QUE-InDRE_562/2021	EPI_ISL_1168632	22/01/2021	Queretaro	Human	Male	26	Released	Original	Oropharyngeal swab
hCoV-19/Mexico/QUE-InDRE_563/2021	EPI_ISL_1168633	22/01/2021	Queretaro	Human	Male	39	Live	Original	Oropharyngeal swab
hCoV-19/Mexico/SLP_UASLP_A041/2021	EPI_ISL_1469113	22/01/2021	San Luis Potosi	Human	Female	25	Released	Original	Oropharyngeal swab
hCoV-19/Mexico/JAL-InDRE_438/2021	EPI_ISL_1168516	22/01/2021	Jalisco	Human	Female	53	Released	Original	Oropharyngeal swab
hCoV-19/Mexico/JAL-InDRE_439/2021	EPI_ISL_1168517	22/01/2021	Jalisco	Human	Male	44	Released	Original	Oropharyngeal swab
hCoV-19/Mexico/CHP-InDRE_445/2021	EPI_ISL_1168523	22/01/2021	Chiapas	Human	Male	56	Deceased	Original	Oropharyngeal swab
hCoV-19/Mexico/TAM-InDRE_449/2021	EPI_ISL_1168527	22/01/2021	Tamaulipas	Human	Male	86	Deceased	Original	Oropharyngeal swab
hCoV-19/Mexico/TAM-InDRE_450/2021	EPI_ISL_1168528	22/01/2021	Tamaulipas	Human	Male	47	Deceased	Original	Oropharyngeal swab
hCoV-19/Mexico/MOR-InDRE_531/2021	EPI_ISL_1168601	22/01/2021	Morelos	Human	Female	33	Released	Original	Oropharyngeal swab
hCoV-19/Mexico/MOR-InDRE_532/2021	EPI_ISL_1168602	22/01/2021	Morelos	Human	Male	33	Released	Original	Oropharyngeal swab
hCoV-19/Mexico/QUE-InDRE_388/2021	EPI_ISL_1168466	22/01/2021	Queretaro	Human	Female	60	Hospitalized	Original	Oropharyngeal swab
hCoV-19/Mexico/CMX-InDRE_451/2021	EPI_ISL_1219711	22/01/2021	Mexico City	Human	Male	52	Hospitalized	Original	Oropharyngeal swab
hCoV-19/Mexico/CMX-InDRE_452/2021	EPI_ISL_1219712	22/01/2021	Mexico City	Human	Male	86	Released	Original	Oropharyngeal swab
hCoV-19/Mexico/CMX-INMEGEN-02-02-03/2021	EPI_ISL_1040548	23/01/2021	Mexico City	Human	Female	24	unknown	Original	Nasopharyngeal swab
hCoV-19/Mexico/CMX-INMEGEN-02-02-13/2021	EPI_ISL_1040549	23/01/2021	Mexico City	Human	Male	70	unknown	Original	Nasopharyngeal swab
hCoV-19/Mexico/CMX-INMEGEN-02-02-20/2021	EPI_ISL_1040550	23/01/2021	Mexico City	Human	Male	11	unknown	Original	Nasopharyngeal swab
hCoV-19/Mexico/CMX-INMEGEN-02-01-17/2021	EPI_ISL_1040600	23/01/2021	Mexico City	Human	Male	52	unknown	Original	Nasopharyngeal swab
hCoV-19/Mexico/CMX-INMEGEN-02-01-20/2021	EPI_ISL_1040602	23/01/2021	Mexico City	Human	Female	29	unknown	Original	Nasopharyngeal swab
hCoV-19/Mexico/CMX-INMEGEN-02-01-01/2021	EPI_ISL_1040618	23/01/2021	Mexico City	Human	Male	51	unknown	Original	Nasopharyngeal swab
hCoV-19/Mexico/QUE-InDRE_540/2021	EPI_ISL_1168610	23/01/2021	Queretaro	Human	Female	49	Released	Original	Oropharyngeal swab
hCoV-19/Mexico/JAL-InDRE_440/2021	EPI_ISL_1168518	23/01/2021	Jalisco	Human	Male	32	Released	Original	Oropharyngeal swab
hCoV-19/Mexico/JAL-InDRE_441/2021	EPI_ISL_1168519	23/01/2021	Jalisco	Human	Female	77	Hospitalized	Original	Oropharyngeal swab
hCoV-19/Mexico/JAL-InDRE_442/2021	EPI_ISL_1168520	23/01/2021	Jalisco	Human	Male	14	Released	Original	Oropharyngeal swab
hCoV-19/Mexico/HID-InDRE_521/2021	EPI_ISL_1168591	23/01/2021	Hidalgo	Human	Female	32	Live	Original	Oropharyngeal swab
hCoV-19/Mexico/BCN-ALSR-7158/2021	EPI_ISL_1185934	23/01/2021	Baja California	Human	unknown	unknown	unknown	Original	Nasal swab
hCoV-19/Mexico/BCN-ALSR-7159/2021	EPI_ISL_1185935	23/01/2021	Baja California	Human	unknown	unknown	unknown	Original	Nasal swab
hCoV-19/Mexico/BCN-ALSR-7160/2021	EPI_ISL_1185936	23/01/2021	Baja California	Human	unknown	unknown	unknown	Original	Nasal swab
hCoV-19/Mexico/SEARCH-7931/2021	EPI_ISL_1366326	23/01/2021	Baja California	Human	unknown	unknown	unknown	Original	Nasal swab
hCoV-19/Mexico/CMX-INMEGEN-02-02-05/2021	EPI_ISL_1040547	24/01/2021	Mexico City	Human	Male	40	unknown	Original	Nasopharyngeal swab
hCoV-19/Mexico/CMX-INMEGEN-02-01-08/2021	EPI_ISL_1040617	24/01/2021	Mexico City	Human	Male	71	unknown	Original	Nasopharyngeal swab

hCoV-19/Mexico/CMX-INCMNSZ_COV152225/202	EPI_ISL_1503143	24/01/2021	Mexico City	Human	Male	80	Released	Original	Nasopharyngeal swab
hCoV-19/Mexico/SLP_UASLP_A047/2021	EPI_ISL_1469114	24/01/2021	San Luis Potosi	Human	Female	27	Released	Original	Oropharyngeal swab
hCoV-19/Mexico/QUE-InDRE_374/2021	EPI_ISL_1168452	24/01/2021	Queretaro	Human	Female	25	Released	Original	Oropharyngeal swab
hCoV-19/Mexico/QUE-InDRE_375/2021	EPI_ISL_1168453	24/01/2021	Queretaro	Human	Male	36	Released	Original	Oropharyngeal swab
hCoV-19/Mexico/QUE-InDRE_376/2021	EPI_ISL_1168454	24/01/2021	Queretaro	Human	Male	34	Released	Original	Oropharyngeal swab
hCoV-19/Mexico/QUE-InDRE_377/2021	EPI_ISL_1168455	24/01/2021	Queretaro	Human	Female	32	Released	Original	Oropharyngeal swab
hCoV-19/Mexico/QUE-InDRE_378/2021	EPI_ISL_1168456	24/01/2021	Queretaro	Human	Female	37	Released	Original	Oropharyngeal swab
hCoV-19/Mexico/QUE-InDRE_379/2021	EPI_ISL_1168457	24/01/2021	Queretaro	Human	Male	73	Deceased	Original	Oropharyngeal swab
hCoV-19/Mexico/CMX-INMEGEN-02-02-14/2021	EPI_ISL_1040544	25/01/2021	Mexico City	Human	Male	27	unknown	Original	Nasopharyngeal swab
hCoV-19/Mexico/CMX-INMEGEN-02-02-18/2021	EPI_ISL_1040545	25/01/2021	Mexico City	Human	Female	34	unknown	Original	Nasopharyngeal swab
hCoV-19/Mexico/CMX-INMEGEN-02-02-22/2021	EPI_ISL_1040546	25/01/2021	Mexico City	Human	Female	52	unknown	Original	Nasopharyngeal swab
hCoV-19/Mexico/CMX-INMEGEN-02-01-05/2021	EPI_ISL_1040611	25/01/2021	Mexico City	Human	Male	68	unknown	Original	Nasopharyngeal swab
hCoV-19/Mexico/CMX-INMEGEN-02-01-06/2021	EPI_ISL_1040612	25/01/2021	Mexico City	Human	Female	64	unknown	Original	Nasopharyngeal swab
hCoV-19/Mexico/CMX-INMEGEN-02-01-10/2021	EPI_ISL_1040613	25/01/2021	Mexico City	Human	Female	11	unknown	Original	Nasopharyngeal swab
hCoV-19/Mexico/CMX-INMEGEN-02-01-11/2021	EPI_ISL_1040614	25/01/2021	Mexico City	Human	Female	10	unknown	Original	Nasopharyngeal swab
hCoV-19/Mexico/CMX-INMEGEN-02-01-12/2021	EPI_ISL_1040615	25/01/2021	Mexico City	Human	Female	22	unknown	Original	Nasopharyngeal swab
hCoV-19/Mexico/CMX-INMEGEN-02-01-23/2021	EPI_ISL_1040616	25/01/2021	Mexico City	Human	Female	63	unknown	Original	Nasopharyngeal swab
hCoV-19/Mexico/JAL-InDRE_F105G_S581/2021	EPI_ISL_1324764	25/01/2021	Jalisco	Human	Female	18	Released	Original	Oropharyngeal swab
hCoV-19/Mexico/JAL-InDRE_F106G_S582/2021	EPI_ISL_1324765	25/01/2021	Jalisco	Human	Male	22	Released	Original	Oropharyngeal swab
hCoV-19/Mexico/MOR-InDRE_448/2021	EPI_ISL_1168526	25/01/2021	Morelos	Human	Female	38	Hospitalized	Original	Oropharyngeal swab
hCoV-19/Mexico/MOR-InDRE_534/2021	EPI_ISL_1168604	25/01/2021	Morelos	Human	Female	45	Released	Original	Oropharyngeal swab
hCoV-19/Mexico/BCN-ALSR-7152/2021	EPI_ISL_1185929	25/01/2021	Baja California	Human	unknown	unknown	unknown	Original	Nasal swab
hCoV-19/Mexico/BCN-ALSR-7154/2021	EPI_ISL_1185931	25/01/2021	Baja California	Human	unknown	unknown	unknown	Original	Nasal swab
hCoV-19/Mexico/SEARCH-7932/2021	EPI_ISL_1366327	25/01/2021	Baja California	Human	unknown	unknown	unknown	Original	Nasal swab
hCoV-19/Mexico/SEARCH-7933/2021	EPI_ISL_1366328	25/01/2021	Baja California	Human	unknown	unknown	unknown	Original	Nasal swab
hCoV-19/Mexico/SEARCH-7934/2021	EPI_ISL_1366329	25/01/2021	Baja California	Human	unknown	unknown	unknown	Original	Nasal swab
hCoV-19/Mexico/SEARCH-7935/2021	EPI_ISL_1366330	25/01/2021	Baja California	Human	unknown	unknown	unknown	Original	Nasal swab
hCoV-19/Mexico/QUE-InDRE_380/2021	EPI_ISL_1168458	25/01/2021	Queretaro	Human	Female	52	Released	Original	Oropharyngeal swab
hCoV-19/Mexico/QUE-InDRE_381/2021	EPI_ISL_1168459	25/01/2021	Queretaro	Human	Male	44	Released	Original	Oropharyngeal swab
hCoV-19/Mexico/QUE-InDRE_382/2021	EPI_ISL_1168460	25/01/2021	Queretaro	Human	Male	56	Released	Original	Oropharyngeal swab
hCoV-19/Mexico/QUE-InDRE_383/2021	EPI_ISL_1168461	25/01/2021	Queretaro	Human	Female	33	Released	Original	Oropharyngeal swab
hCoV-19/Mexico/QUE-InDRE_384/2021	EPI_ISL_1168462	25/01/2021	Queretaro	Human	Male	41	Released	Original	Oropharyngeal swab
hCoV-19/Mexico/QUE-InDRE_385/2021	EPI_ISL_1168463	25/01/2021	Queretaro	Human	Female	59	Live	Original	Oropharyngeal swab
hCoV-19/Mexico/QUE-InDRE_386/2021	EPI_ISL_1168464	25/01/2021	Queretaro	Human	Female	3	Released	Original	Oropharyngeal swab
hCoV-19/Mexico/CMX-INMEGEN-02-02-06/2021	EPI_ISL_1040541	26/01/2021	Mexico City	Human	Female	60	unknown	Original	Nasopharyngeal swab
hCoV-19/Mexico/CMX-INMEGEN-02-02-09/2021	EPI_ISL_1040542	26/01/2021	Mexico City	Human	Female	36	unknown	Original	Nasopharyngeal swab
hCoV-19/Mexico/CMX-INMEGEN-02-02-23/2021	EPI_ISL_1040543	26/01/2021	Mexico City	Human	Female	8	unknown	Original	Nasopharyngeal swab
hCoV-19/Mexico/CMX-INMEGEN-02-01-22/2021	EPI_ISL_1040601	26/01/2021	Mexico City	Human	Male	32	unknown	Original	Nasopharyngeal swab
hCoV-19/Mexico/CMX-INMEGEN-02-01-07/2021	EPI_ISL_1040608	26/01/2021	Mexico City	Human	Male	70	unknown	Original	Nasopharyngeal swab
hCoV-19/Mexico/CMX-INMEGEN-02-01-13/2021	EPI_ISL_1040609	26/01/2021	Mexico City	Human	Male	31	unknown	Original	Nasopharyngeal swab
hCoV-19/Mexico/CMX-INMEGEN-02-01-21/2021	EPI_ISL_1040610	26/01/2021	Mexico City	Human	Male	47	unknown	Original	Nasopharyngeal swab

hCoV-19/Mexico/SEARCH-7937/2021	EPI_ISL_1366332	26/01/2021	Baja California	Human	unknown	unknown	unknown	Original	Nasal swab
hCoV-19/Mexico/TAM-InDRE_244/2021	EPI_ISL_1008713	26/01/2021	Tamaulipas	Human	Female	39	Released	Original	Pharyngeal swab
hCoV-19/Mexico/QUE-InDRE_387/2021	EPI_ISL_1168465	26/01/2021	Queretaro	Human	Female	35	Released	Original	Oropharyngeal swab
hCoV-19/Mexico/CMX-INMEGEN-02-02-07/2021	EPI_ISL_1040540	27/01/2021	Mexico City	Human	Female	49	unknown	Original	Nasopharyngeal swab
hCoV-19/Mexico/CMX-INMEGEN-02-01-04/2021	EPI_ISL_1040604	27/01/2021	Mexico City	Human	Female	56	unknown	Original	Nasopharyngeal swab
hCoV-19/Mexico/CMX-INMEGEN-02-01-14/2021	EPI_ISL_1040606	27/01/2021	Mexico City	Human	Male	26	unknown	Original	Nasopharyngeal swab
hCoV-19/Mexico/CMX-INMEGEN-02-01-15/2021	EPI_ISL_1040607	27/01/2021	Mexico City	Human	Female	47	unknown	Original	Nasopharyngeal swab
hCoV-19/Mexico/CMX-INMEGEN-02-03-18/2021	EPI_ISL_1040643	27/01/2021	Mexico City	Human	Female	78	unknown	Original	Nasopharyngeal swab
hCoV-19/Mexico/CMX-InDRE_312/2021	EPI_ISL_1060717	27/01/2021	Mexico City	Human	Male	67	unknown	Original	Oropharyngeal swab
hCoV-19/Mexico/SEARCH-7936/2021	EPI_ISL_1366331	27/01/2021	Baja California	Human	unknown	unknown	unknown	Original	Nasal swab
hCoV-19/Mexico/CMX-INMEGEN-02-02-21/2021	EPI_ISL_1040539	28/01/2021	Mexico City	Human	Female	45	unknown	Original	Nasopharyngeal swab
hCoV-19/Mexico/CMX-INMEGEN-02-01-16/2021	EPI_ISL_1040605	28/01/2021	Mexico City	Human	Female	29	unknown	Original	Nasopharyngeal swab
hCoV-19/Mexico/CMX-InDRE_249/2021	EPI_ISL_1060716	28/01/2021	Mexico City	Human	Female	unknown	unknown	Original	Oropharyngeal swab
hCoV-19/Mexico/JAL-InDRE_245/2021	EPI_ISL_1008714	28/01/2021	Jalisco	Human	Female	19	Released	Orginal	Pharyngeal swab
hCoV-19/Mexico/CMX-INMEGEN-02-02-08/2021	EPI_ISL_1040535	29/01/2021	Mexico City	Human	Male	47	unknown	Original	Nasopharyngeal swab
hCoV-19/Mexico/CMX-INMEGEN-02-02-10/2021	EPI_ISL_1040536	29/01/2021	Mexico City	Human	Female	34	unknown	Original	Nasopharyngeal swab
hCoV-19/Mexico/CMX-INMEGEN-02-02-15/2021	EPI_ISL_1040537	29/01/2021	Mexico City	Human	Female	29	unknown	Original	Nasopharyngeal swab
hCoV-19/Mexico/CMX-INMEGEN-02-02-16/2021	EPI_ISL_1040538	29/01/2021	Mexico City	Human	Female	37	unknown	Original	Nasopharyngeal swab
hCoV-19/Mexico/CMX-INMEGEN-02-03-05/2021	EPI_ISL_1040640	29/01/2021	Mexico City	Human	Male	34	unknown	Original	Nasopharyngeal swab
hCoV-19/Mexico/CMX-INMEGEN-02-03-17/2021	EPI_ISL_1040641	29/01/2021	Mexico City	Human	Male	53	unknown	Original	Nasopharyngeal swab
hCoV-19/Mexico/CMX-INMEGEN-02-03-19/2021	EPI_ISL_1040642	29/01/2021	Mexico City	Human	Male	59	unknown	Original	Nasopharyngeal swab
hCoV-19/Mexico/NLE-UANL-028/2021	EPI_ISL_1091243	29/01/2021	Nuevo Leon	Human	Male	38	unknown	Original	
hCoV-19/Mexico/NLE-UANL-029/2021	EPI_ISL_1091246	29/01/2021	Nuevo Leon	Human	Female	27	unknown	Original	
hCoV-19/Mexico/CMX-INMEGEN-02-04-07/2021	EPI_ISL_1055024	29/01/2021	Mexico City	Human	Female	56	unknown	Original	Oropharyngeal swab
hCoV-19/Mexico/CMX-INMEGEN-02-04-08/2021	EPI_ISL_1055025	29/01/2021	Mexico City	Human	Male	54	unknown	Original	Oropharyngeal swab
hCoV-19/Mexico/CMX-INMEGEN-02-03-15/2021	EPI_ISL_1040624	30/01/2021	Mexico City	Human	Male	6	unknown	Original	Nasopharyngeal swab
hCoV-19/Mexico/CMX-INMEGEN-02-03-06/2021	EPI_ISL_1040638	30/01/2021	Mexico City	Human	Male	19	unknown	Original	Nasopharyngeal swab
hCoV-19/Mexico/CMX-INMEGEN-02-03-16/2021	EPI_ISL_1040639	30/01/2021	Mexico City	Human	Male	12	unknown	Original	Nasopharyngeal swab
hCoV-19/Mexico/CMX-InDRE_247/2021	EPI_ISL_1060715	30/01/2021	Mexico City	Human	Male	unknown	unknown	Original	Oropharyngeal swab
hCoV-19/Mexico/CMX-INMEGEN-02-03-01/2021	EPI_ISL_1040637	31/01/2021	Mexico City	Human	Male	53	unknown	Original	Nasopharyngeal swab
hCoV-19/Mexico/CMX-INMEGEN-02-04-05/2021	EPI_ISL_1055023	31/01/2021	Mexico City	Human	Male	30	unknown	Original	Oropharyngeal swab
hCoV-19/Mexico/CMX-INMEGEN-02-06-11/2021	EPI_ISL_1080447	01/02/2021	Mexico City	Human	Female	40	unknown	Original	Oropharyngeal swab
hCoV-19/Mexico/CMX-INMEGEN-02-05-22/2021	EPI_ISL_1060611	01/02/2021	Mexico City	Human	Female	65	unknown	Original	Oropharyngeal swab
hCoV-19/Mexico/CMX-INMEGEN-02-03-09/2021	EPI_ISL_1040623	02/02/2021	Mexico City	Human	Male	25	unknown	Original	Nasopharyngeal swab
hCoV-19/Mexico/CMX-INMEGEN-02-03-14/2021	EPI_ISL_1040634	02/02/2021	Mexico City	Human	Male	67	unknown	Original	Nasopharyngeal swab
hCoV-19/Mexico/CMX-INMEGEN-02-03-20/2021	EPI_ISL_1040635	02/02/2021	Mexico City	Human	Female	71	unknown	Original	Nasopharyngeal swab
hCoV-19/Mexico/CMX-INMEGEN-02-03-21/2021	EPI_ISL_1040636	02/02/2021	Mexico City	Human	Male	39	unknown	Original	Nasopharyngeal swab
hCoV-19/Mexico/CMX-InDRE_248/2021	EPI_ISL_1060714	02/02/2021	Mexico City	Human	Female	unknown	unknown	Original	Oropharyngeal swab
hCoV-19/Mexico/BCN-SEARCH-7577/2021	EPI_ISL_1295837	02/02/2021	Baja California	Human	unknown	unknown	unknown	Original	Nasal swab
hCoV-19/Mexico/BCN-SEARCH-7585/2021	EPI_ISL_1295843	02/02/2021	Baja California	Human	unknown	unknown	unknown	Original	Nasal swab
hCoV-19/Mexico/AGU-InDRE_F11772B_S1094/20	EPI_ISL_1516786	02/02/2021	Aguascalientes	Human	Female	13	unknown	Original	Oropharyngeal swab

hCoV-19/Mexico/CMX-INMEGEN-02-04-06/2021	EPI_ISL_1055021	02/02/2021	Mexico City	Human	Female	37	unknown	Original	Oropharyngeal swab
hCoV-19/Mexico/CMX-INMEGEN-02-04-09/2021	EPI_ISL_1055022	02/02/2021	Mexico City	Human	Male	49	unknown	Original	Oropharyngeal swab
hCoV-19/Mexico/CMX-INMEGEN-02-03-08/2021	EPI_ISL_1040631	03/02/2021	Mexico City	Human	Male	30	unknown	Original	Nasopharyngeal swab
hCoV-19/Mexico/CMX-INMEGEN-02-03-22/2021	EPI_ISL_1040632	03/02/2021	Mexico City	Human	Female	38	unknown	Original	Nasopharyngeal swab
hCoV-19/Mexico/CMX-INMEGEN-02-03-23/2021	EPI_ISL_1040633	03/02/2021	Mexico City	Human	Male	49	unknown	Original	Nasopharyngeal swab
hCoV-19/Mexico/CMX-INMEGEN-02-05-09/2021	EPI_ISL_1075267	03/02/2021	Mexico City	Human	Male	46	unknown	Original	
hCoV-19/Mexico/CMX-INMEGEN-02-06-04/2021	EPI_ISL_1080436	03/02/2021	Mexico City	Human	Male	24	unknown	Original	Oropharyngeal swab
hCoV-19/Mexico/CMX-INMEGEN-02-06-02/2021	EPI_ISL_1080438	03/02/2021	Mexico City	Human	Female	59	unknown	Original	Oropharyngeal swab
hCoV-19/Mexico/CMX-INMEGEN-02-06-03/2021	EPI_ISL_1080440	03/02/2021	Mexico City	Human	Female	41	unknown	Original	Oropharyngeal swab
hCoV-19/Mexico/CMX-INMEGEN-02-06-05/2021	EPI_ISL_1080441	03/02/2021	Mexico City	Human	Male	43	unknown	Original	Oropharyngeal swab
hCoV-19/Mexico/CMX-INMEGEN-02-06-06/2021	EPI_ISL_1080442	03/02/2021	Mexico City	Human	Male	45	unknown	Original	Oropharyngeal swab
hCoV-19/Mexico/CMX-INMEGEN-02-06-07/2021	EPI_ISL_1080443	03/02/2021	Mexico City	Human	Male	27	unknown	Original	Oropharyngeal swab
hCoV-19/Mexico/CMX-INMEGEN-02-06-08/2021	EPI_ISL_1080444	03/02/2021	Mexico City	Human	Female	54	unknown	Original	Oropharyngeal swab
hCoV-19/Mexico/CMX-INMEGEN-02-06-09/2021	EPI_ISL_1080445	03/02/2021	Mexico City	Human	Male	48	unknown	Original	Oropharyngeal swab
hCoV-19/Mexico/CMX-INMEGEN-02-05-13/2021	EPI_ISL_1060600	03/02/2021	Mexico City	Human	Female	85	unknown	Original	Oropharyngeal swab
hCoV-19/Mexico/CMX-INMEGEN-02-05-10/2021	EPI_ISL_1060601	03/02/2021	Mexico City	Human	Female	5	unknown	Original	Oropharyngeal swab
hCoV-19/Mexico/CMX-INMEGEN-02-05-11/2021	EPI_ISL_1060602	03/02/2021	Mexico City	Human	Male	24	unknown	Original	Oropharyngeal swab
hCoV-19/Mexico/CMX-INMEGEN-02-05-12/2021	EPI_ISL_1060603	03/02/2021	Mexico City	Human	Female	42	unknown	Original	Oropharyngeal swab
hCoV-19/Mexico/CMX-INMEGEN-02-05-14/2021	EPI_ISL_1060604	03/02/2021	Mexico City	Human	Male	63	unknown	Original	Oropharyngeal swab
hCoV-19/Mexico/CMX-INMEGEN-02-05-15/2021	EPI_ISL_1060605	03/02/2021	Mexico City	Human	Female	69	unknown	Original	Oropharyngeal swab
hCoV-19/Mexico/CMX-INMEGEN-02-05-16/2021	EPI_ISL_1060606	03/02/2021	Mexico City	Human	Female	45	unknown	Original	Oropharyngeal swab
hCoV-19/Mexico/CMX-INMEGEN-02-05-17/2021	EPI_ISL_1060607	03/02/2021	Mexico City	Human	Male	27	unknown	Original	Oropharyngeal swab
hCoV-19/Mexico/CMX-INMEGEN-02-05-18/2021	EPI_ISL_1060608	03/02/2021	Mexico City	Human	Male	41	unknown	Original	Oropharyngeal swab
hCoV-19/Mexico/CMX-INMEGEN-02-05-21/2021	EPI_ISL_1060609	03/02/2021	Mexico City	Human	Female	32	unknown	Original	Oropharyngeal swab
hCoV-19/Mexico/CMX-INMEGEN-02-05-23/2021	EPI_ISL_1060610	03/02/2021	Mexico City	Human	Female	50	unknown	Original	Oropharyngeal swab
hCoV-19/Mexico/SIN_CIAD_HJ0923/2021	EPI_ISL_1120614	03/02/2021	Sinaloa	Human	Female	41	.ive, symptomati	Original	Nasopharyngeal swab
hCoV-19/Mexico/SEARCH-7938/2021	EPI_ISL_1366333	03/02/2021	Baja California	Human	unknown	unknown	unknown	Original	Nasal swab
hCoV-19/Mexico/CMX-INMEGEN-02-04-01/2021	EPI_ISL_1055017	03/02/2021	Mexico City	Human	Male	29	unknown	Original	Oropharyngeal swab
hCoV-19/Mexico/CMX-INMEGEN-02-04-10/2021	EPI_ISL_1055018	03/02/2021	Mexico City	Human	Female	29	unknown	Original	Oropharyngeal swab
hCoV-19/Mexico/CMX-INMEGEN-02-04-11/2021	EPI_ISL_1055019	03/02/2021	Mexico City	Human	Female	44	unknown	Original	Oropharyngeal swab
hCoV-19/Mexico/CMX-INMEGEN-02-04-20/2021	EPI_ISL_1055020	03/02/2021	Mexico City	Human	Female	29	unknown	Original	Oropharyngeal swab
hCoV-19/Mexico/CMX-INMEGEN-02-03-02/2021	EPI_ISL_1040627	04/02/2021	Mexico City	Human	Male	41	unknown	Original	Nasopharyngeal swab
hCoV-19/Mexico/CMX-INMEGEN-02-03-03/2021	EPI_ISL_1040628	04/02/2021	Mexico City	Human	Female	19	unknown	Original	Nasopharyngeal swab
hCoV-19/Mexico/CMX-INMEGEN-02-03-04/2021	EPI_ISL_1040629	04/02/2021	Mexico City	Human	Female	45	unknown	Original	Nasopharyngeal swab
hCoV-19/Mexico/CMX-INMEGEN-02-03-10/2021	EPI_ISL_1040630	04/02/2021	Mexico City	Human	Female	77	unknown	Original	Nasopharyngeal swab
hCoV-19/Mexico/CMX-INMEGEN-02-06-12/2021	EPI_ISL_1080433	04/02/2021	Mexico City	Human	Male	14	unknown	Original	Oropharyngeal swab
hCoV-19/Mexico/CMX-INMEGEN-02-06-13/2021	EPI_ISL_1080434	04/02/2021	Mexico City	Human	Male	17	unknown	Original	Oropharyngeal swab
hCoV-19/Mexico/CMX-INMEGEN-02-06-17/2021	EPI_ISL_1080435	04/02/2021	Mexico City	Human	Female	25	unknown	Original	Oropharyngeal swab
hCoV-19/Mexico/CMX-INMEGEN-02-06-10/2021	EPI_ISL_1080446	04/02/2021	Mexico City	Human	Male	28	unknown	Original	Oropharyngeal swab
hCoV-19/Mexico/CMX-INMEGEN-02-06-14/2021	EPI_ISL_1080448	04/02/2021	Mexico City	Human	Female	34	unknown	Original	Oropharyngeal swab
hCoV-19/Mexico/CMX-INMEGEN-02-06-15/2021	EPI_ISL_1080449	04/02/2021	Mexico City	Human	Male	25	unknown	Original	Oropharyngeal swab

hCoV-19/Mexico/CMX-INMEGEN-02-06-16/2021	EPI_ISL_1080450	04/02/2021	Mexico City	Human	Female	53	unknown	Original	Oropharyngeal swab
hCoV-19/Mexico/CMX-INMEGEN-02-04-02/2021	EPI_ISL_1055009	04/02/2021	Mexico City	Human	Male	30	unknown	Original	Oropharyngeal swab
hCoV-19/Mexico/CMX-INMEGEN-02-04-03/2021	EPI_ISL_1055010	04/02/2021	Mexico City	Human	Female	33	unknown	Original	Oropharyngeal swab
hCoV-19/Mexico/CMX-INMEGEN-02-04-04/2021	EPI_ISL_1055011	04/02/2021	Mexico City	Human	Male	27	unknown	Original	Oropharyngeal swab
hCoV-19/Mexico/CMX-INMEGEN-02-04-12/2021	EPI_ISL_1055012	04/02/2021	Mexico City	Human	Male	27	unknown	Original	Oropharyngeal swab
hCoV-19/Mexico/CMX-INMEGEN-02-04-13/2021	EPI_ISL_1055013	04/02/2021	Mexico City	Human	Female	43	unknown	Original	Oropharyngeal swab
hCoV-19/Mexico/CMX-INMEGEN-02-04-14/2021	EPI_ISL_1055014	04/02/2021	Mexico City	Human	Male	51	unknown	Original	Oropharyngeal swab
hCoV-19/Mexico/CMX-INMEGEN-02-04-21/2021	EPI_ISL_1055015	04/02/2021	Mexico City	Human	Male	52	unknown	Original	Oropharyngeal swab
hCoV-19/Mexico/CMX-INMEGEN-02-04-22/2021	EPI_ISL_1055016	04/02/2021	Mexico City	Human	Female	27	unknown	Original	Oropharyngeal swab
hCoV-19/Mexico/TAM-InDRE_453/2021	EPI_ISL_1219713	04/02/2021	Tamaulipas	Human	Male	47	Released	Original	Oropharyngeal swab
hCoV-19/Mexico/CMX-INMEGEN-02-03-11/2021	EPI_ISL_1040625	05/02/2021	Mexico City	Human	Male	31	unknown	Original	Nasopharyngeal swab
hCoV-19/Mexico/CMX-INMEGEN-02-03-12/2021	EPI_ISL_1040626	05/02/2021	Mexico City	Human	Male	5	unknown	Original	Nasopharyngeal swab
hCoV-19/Mexico/CMX-INMEGEN-02-07-11/2021	EPI_ISL_1081166	05/02/2021	Mexico City	Human	Female	28	unknown	Original	Oropharyngeal swab
hCoV-19/Mexico/CMX-INMEGEN-02-07-12/2021	EPI_ISL_1081167	05/02/2021	Mexico City	Human	Female	30	unknown	Original	Oropharyngeal swab
hCoV-19/Mexico/CMX-INMEGEN-02-07-22/2021	EPI_ISL_1081168	05/02/2021	Mexico City	Human	Female	31	unknown	Original	Oropharyngeal swab
hCoV-19/Mexico/CMX-INMEGEN-02-07-23/2021	EPI_ISL_1081169	05/02/2021	Mexico City	Human	Male	27	unknown	Original	Oropharyngeal swab
hCoV-19/Mexico/CMX-INMEGEN-02-05-19/2021	EPI_ISL_1060598	05/02/2021	Mexico City	Human	Female	57	unknown	Original	Oropharyngeal swab
hCoV-19/Mexico/CMX-INMEGEN-02-05-20/2021	EPI_ISL_1060599	05/02/2021	Mexico City	Human	Male	46	unknown	Original	Oropharyngeal swab
hCoV-19/Mexico/CMX-INMEGEN-02-06-01/2021	EPI_ISL_1080439	05/02/2021	Mexico City	Human	Female	64	unknown	Original	Oropharyngeal swab
hCoV-19/Mexico/CMX-INMEGEN-02-06-18/2021	EPI_ISL_1080451	05/02/2021	Mexico City	Human	Male	7	unknown	Original	Oropharyngeal swab
hCoV-19/Mexico/CMX-INMEGEN-02-06-19/2021	EPI_ISL_1080452	05/02/2021	Mexico City	Human	Female	31	unknown	Original	Oropharyngeal swab
hCoV-19/Mexico/CMX-INMEGEN-02-09-06/2021	EPI_ISL_1095511	05/02/2021	Mexico City	Human	Male	24	unknown	Original	Oropharyngeal swab
hCoV-19/Mexico/CMX-INMEGEN-02-09-07/2021	EPI_ISL_1095512	05/02/2021	Mexico City	Human	Female	46	unknown	Original	Oropharyngeal swab
hCoV-19/Mexico/CMX-INMEGEN-02-09-21/2021	EPI_ISL_1095513	05/02/2021	Mexico City	Human	Female	31	unknown	Original	Oropharyngeal swab
hCoV-19/Mexico/CMX-INMEGEN-02-09-22/2021	EPI_ISL_1095514	05/02/2021	Mexico City	Human	Male	28	unknown	Original	Oropharyngeal swab
hCoV-19/Mexico/CMX-INMEGEN-02-09-23/2021	EPI_ISL_1095515	05/02/2021	Mexico City	Human	Female	45	unknown	Original	Oropharyngeal swab
hCoV-19/Mexico/CMX-INMEGEN-02-11-06/2021	EPI_ISL_1117358	05/02/2021	Mexico City	Human	Female	14	unknown	Original	Oropharyngeal swab
hCoV-19/Mexico/CMX-INMEGEN-02-11-14/2021	EPI_ISL_1117366	05/02/2021	Mexico City	Human	Male	31	unknown	Original	Oropharyngeal swab
hCoV-19/Mexico/CMX-INMEGEN-02-11-15/2021	EPI_ISL_1117367	05/02/2021	Mexico City	Human	Female	66	unknown	Original	Oropharyngeal swab
hCoV-19/Mexico/JAL-InDRE_F101G_S577/2021	EPI_ISL_1324760	05/02/2021	Jalisco	Human	Female	16	Released	Original	Oropharyngeal swab
hCoV-19/Mexico/JAL-InDRE_F102G_S578/2021	EPI_ISL_1324761	05/02/2021	Jalisco	Human	Male	35	Released	Original	Oropharyngeal swab
hCoV-19/Mexico/CMX-INMEGEN-02-04-18/2021	EPI_ISL_1058046	05/02/2021	Mexico City	Human	Female	51	unknown	Original	
hCoV-19/Mexico/CMX-INMEGEN-02-10-04/2021	EPI_ISL_1137460	05/02/2021	Mexico City	Human	Female	22	unknown	Original	Oropharyngeal swab
hCoV-19/Mexico/CMX-INMEGEN-02-10-09/2021	EPI_ISL_1137468	05/02/2021	Mexico City	Human	Female	24	unknown	Original	Oropharyngeal swab
hCoV-19/Mexico/CMX-INMEGEN-02-10-10/2021	EPI_ISL_1137469	05/02/2021	Mexico City	Human	Female	27	unknown	Original	Oropharyngeal swab
hCoV-19/Mexico/CMX-INMEGEN-02-10-11/2021	EPI_ISL_1137470	05/02/2021	Mexico City	Human	Male	65	unknown	Original	Oropharyngeal swab
hCoV-19/Mexico/CMX-INMEGEN-02-04-17/2021	EPI_ISL_1055005	05/02/2021	Mexico City	Human	Female	85	unknown	Original	Oropharyngeal swab
hCoV-19/Mexico/CMX-INMEGEN-02-04-15/2021	EPI_ISL_1055006	05/02/2021	Mexico City	Human	Female	54	unknown	Original	Oropharyngeal swab
hCoV-19/Mexico/CMX-INMEGEN-02-04-16/2021	EPI_ISL_1055007	05/02/2021	Mexico City	Human	Male	61	unknown	Original	Oropharyngeal swab
hCoV-19/Mexico/CMX-INMEGEN-02-04-19/2021	EPI_ISL_1055008	05/02/2021	Mexico City	Human	Female	24	unknown	Original	Oropharyngeal swab
hCoV-19/Mexico/CMX-INMEGEN-02-09-05/2021	EPI_ISL_1095496	06/02/2021	Mexico City	Human	Male	26	unknown	Original	Oropharyngeal swab

hCoV-19/Mexico/CMX-INMEGEN-02-09-03/2021	EPI_ISL_1095509	06/02/2021	Mexico City	Human	Male	44	unknown	Original	Oropharyngeal swab
hCoV-19/Mexico/CMX-INMEGEN-02-09-04/2021	EPI_ISL_1095510	06/02/2021	Mexico City	Human	Female	45	unknown	Original	Oropharyngeal swab
hCoV-19/Mexico/CMX-INMEGEN-02-11-16/2021	EPI_ISL_1117368	06/02/2021	Mexico City	Human	Male	53	unknown	Original	Oropharyngeal swab
hCoV-19/Mexico/CMX-INMEGEN-02-11-17/2021	EPI_ISL_1117369	06/02/2021	Mexico City	Human	Female	54	unknown	Original	Oropharyngeal swab
hCoV-19/Mexico/CMX-INMEGEN-02-11-18/2021	EPI_ISL_1117370	06/02/2021	Mexico City	Human	Male	52	unknown	Original	Oropharyngeal swab
hCoV-19/Mexico/CMX-INMEGEN-02-10-01/2021	EPI_ISL_1137462	06/02/2021	Mexico City	Human	Male	38	unknown	Original	Oropharyngeal swab
hCoV-19/Mexico/CMX-INMEGEN-02-10-02/2021	EPI_ISL_1137463	06/02/2021	Mexico City	Human	Male	35	unknown	Original	Oropharyngeal swab
hCoV-19/Mexico/CMX-INMEGEN-02-10-06/2021	EPI_ISL_1137465	06/02/2021	Mexico City	Human	Male	51	unknown	Original	Oropharyngeal swab
hCoV-19/Mexico/CMX-INMEGEN-02-07-05/2021	EPI_ISL_1095493	07/02/2021	Mexico City	Human	Female	29	unknown	Original	Oropharyngeal swab
hCoV-19/Mexico/CMX-INMEGEN-02-07-06/2021	EPI_ISL_1095494	07/02/2021	Mexico City	Human	Male	76	unknown	Original	Oropharyngeal swab
hCoV-19/Mexico/CMX-INMEGEN-02-09-02/2021	EPI_ISL_1095508	07/02/2021	Mexico City	Human	Male	54	unknown	Original	Oropharyngeal swab
hCoV-19/Mexico/CMX-INMEGEN-02-10-03/2021	EPI_ISL_1137464	07/02/2021	Mexico City	Human	Female	27	unknown	Original	Oropharyngeal swab
hCoV-19/Mexico/CMX-INMEGEN-02-10-07/2021	EPI_ISL_1137466	07/02/2021	Mexico City	Human	Male	18	unknown	Original	Oropharyngeal swab
hCoV-19/Mexico/CMX-INMEGEN-02-10-08/2021	EPI_ISL_1137467	07/02/2021	Mexico City	Human	Male	15	unknown	Original	Oropharyngeal swab
hCoV-19/Mexico/TAM-InDRE_456/2021	EPI_ISL_1219716	07/02/2021	Tamaulipas	Human	Female	56	Released	Original	Oropharyngeal swab
hCoV-19/Mexico/TAM-InDRE_457/2021	EPI_ISL_1219717	07/02/2021	Tamaulipas	Human	Male	58	Released	Original	Oropharyngeal swab
hCoV-19/Mexico/TAM-InDRE_458/2021	EPI_ISL_1219718	07/02/2021	Tamaulipas	Human	Male	50	Released	Original	Oropharyngeal swab
hCoV-19/Mexico/CMX-INMEGEN-02-07-09/2021	EPI_ISL_1081165	08/02/2021	Mexico City	Human	Female	47	unknown	Original	Oropharyngeal swab
hCoV-19/Mexico/CMX-INMEGEN-02-06-20/2021	EPI_ISL_1085421	08/02/2021	Mexico City	Human	Female	35	unknown	Original	
hCoV-19/Mexico/CMX-INMEGEN-02-06-21/2021	EPI_ISL_1080437	08/02/2021	Mexico City	Human	Female	22	unknown	Original	Oropharyngeal swab
hCoV-19/Mexico/CMX-INMEGEN-02-06-23/2021	EPI_ISL_1080453	08/02/2021	Mexico City	Human	Male	51	unknown	Original	Oropharyngeal swab
hCoV-19/Mexico/CMX-INMEGEN-02-09-08/2021	EPI_ISL_1095497	08/02/2021	Mexico City	Human	Female	30	unknown	Original	Oropharyngeal swab
hCoV-19/Mexico/CMX-INMEGEN-02-09-09/2021	EPI_ISL_1095498	08/02/2021	Mexico City	Human	Female	68	unknown	Original	Oropharyngeal swab
hCoV-19/Mexico/CMX-INMEGEN-02-09-11/2021	EPI_ISL_1095499	08/02/2021	Mexico City	Human	Female	23	unknown	Original	Oropharyngeal swab
hCoV-19/Mexico/CMX-INMEGEN-02-09-12/2021	EPI_ISL_1095500	08/02/2021	Mexico City	Human	Male	56	unknown	Original	Oropharyngeal swab
hCoV-19/Mexico/CMX-INMEGEN-02-09-13/2021	EPI_ISL_1095501	08/02/2021	Mexico City	Human	Female	24	unknown	Original	Oropharyngeal swab
hCoV-19/Mexico/CMX-INMEGEN-02-09-14/2021	EPI_ISL_1095502	08/02/2021	Mexico City	Human	Female	24	unknown	Original	Oropharyngeal swab
hCoV-19/Mexico/CMX-INMEGEN-02-09-15/2021	EPI_ISL_1095503	08/02/2021	Mexico City	Human	Male	25	unknown	Original	Oropharyngeal swab
hCoV-19/Mexico/CMX-INMEGEN-02-09-16/2021	EPI_ISL_1095504	08/02/2021	Mexico City	Human	Female	24	unknown	Original	Oropharyngeal swab
hCoV-19/Mexico/CMX-INMEGEN-02-09-17/2021	EPI_ISL_1095505	08/02/2021	Mexico City	Human	Female	24	unknown	Original	Oropharyngeal swab
hCoV-19/Mexico/CMX-INMEGEN-02-09-19/2021	EPI_ISL_1095506	08/02/2021	Mexico City	Human	Female	24	unknown	Original	Oropharyngeal swab
hCoV-19/Mexico/CMX-INMEGEN-02-09-20/2021	EPI_ISL_1095507	08/02/2021	Mexico City	Human	Female	24	unknown	Original	Oropharyngeal swab
hCoV-19/Mexico/CMX-INMEGEN-02-11-01/2021	EPI_ISL_1117353	08/02/2021	Mexico City	Human	Male	19	unknown	Original	Oropharyngeal swab
hCoV-19/Mexico/CMX-INMEGEN-02-11-02/2021	EPI_ISL_1117354	08/02/2021	Mexico City	Human	Female	34	unknown	Original	Oropharyngeal swab
hCoV-19/Mexico/CMX-INMEGEN-02-11-03/2021	EPI_ISL_1117355	08/02/2021	Mexico City	Human	Female	15	unknown	Original	Oropharyngeal swab
hCoV-19/Mexico/CMX-INMEGEN-02-11-09/2021	EPI_ISL_1117361	08/02/2021	Mexico City	Human	Male	48	unknown	Original	Oropharyngeal swab
hCoV-19/Mexico/CMX-INMEGEN-02-11-10/2021	EPI_ISL_1117362	08/02/2021	Mexico City	Human	Male	59	unknown	Original	Oropharyngeal swab
hCoV-19/Mexico/CMX-INMEGEN-02-11-11/2021	EPI_ISL_1117363	08/02/2021	Mexico City	Human	Male	65	unknown	Original	Oropharyngeal swab
hCoV-19/Mexico/BCN-SEARCH-7370/2021	EPI_ISL_1295676	08/02/2021	Baja California	Human	unknown	unknown	unknown	Original	Nasal swab
hCoV-19/Mexico/CMX-INMEGEN-02-10-14/2021	EPI_ISL_1137461	08/02/2021	Mexico City	Human	Male	32	unknown	Original	Oropharyngeal swab
hCoV-19/Mexico/CMX-INMEGEN-02-10-12/2021	EPI_ISL_1137471	08/02/2021	Mexico City	Human	Male	44	unknown	Original	Oropharyngeal swab

hCoV-19/Mexico/CMX-INMEGEN-02-10-13/2021	EPI_ISL_1137472	08/02/2021	Mexico City	Human	Male	48	unknown	Original	Oropharyngeal swab
hCoV-19/Mexico/CMX-INMEGEN-02-10-15/2021	EPI_ISL_1137473	08/02/2021	Mexico City	Human	Female	62	unknown	Original	Oropharyngeal swab
hCoV-19/Mexico/CMX-INMEGEN-02-10-16/2021	EPI_ISL_1137474	08/02/2021	Mexico City	Human	Female	21	unknown	Original	Oropharyngeal swab
hCoV-19/Mexico/CMX-INMEGEN-02-07-07/2021	EPI_ISL_1081162	09/02/2021	Mexico City	Human	Female	65	unknown	Original	Oropharyngeal swab
hCoV-19/Mexico/CMX-INMEGEN-02-07-08/2021	EPI_ISL_1081163	09/02/2021	Mexico City	Human	Female	75	unknown	Original	Oropharyngeal swab
hCoV-19/Mexico/CMX-INMEGEN-02-07-10/2021	EPI_ISL_1081164	09/02/2021	Mexico City	Human	Female	35	unknown	Original	Oropharyngeal swab
hCoV-19/Mexico/CMX-INMEGEN-02-11-04/2021	EPI_ISL_1117356	09/02/2021	Mexico City	Human	Male	71	unknown	Original	Oropharyngeal swab
hCoV-19/Mexico/CMX-INMEGEN-02-11-05/2021	EPI_ISL_1117357	09/02/2021	Mexico City	Human	Female	29	unknown	Original	Oropharyngeal swab
hCoV-19/Mexico/CMX-INMEGEN-02-11-07/2021	EPI_ISL_1117359	09/02/2021	Mexico City	Human	Female	64	unknown	Original	Oropharyngeal swab
hCoV-19/Mexico/CMX-INMEGEN-02-11-08/2021	EPI_ISL_1117360	09/02/2021	Mexico City	Human	Female	55	unknown	Original	Oropharyngeal swab
hCoV-19/Mexico/CMX-INMEGEN-02-11-12/2021	EPI_ISL_1117364	09/02/2021	Mexico City	Human	Male	33	unknown	Original	Oropharyngeal swab
hCoV-19/Mexico/CMX-INMEGEN-02-11-13/2021	EPI_ISL_1117365	09/02/2021	Mexico City	Human	Male	76	unknown	Original	Oropharyngeal swab
hCoV-19/Mexico/CMX-INMEGEN-02-11-20/2021	EPI_ISL_1117372	09/02/2021	Mexico City	Human	Male	39	unknown	Original	Oropharyngeal swab
hCoV-19/Mexico/CMX-INMEGEN-02-11-23/2021	EPI_ISL_1117375	09/02/2021	Mexico City	Human	Male	68	unknown	Original	Oropharyngeal swab
hCoV-19/Mexico/SIN_CIAD_S5894/2021	EPI_ISL_1491364	09/02/2021	Sinaloa	Human	Male	82	unknown	Original	Oropharyngeal swab
hCoV-19/Mexico/CMX-INMEGEN-02-07-01/2021	EPI_ISL_1081152	10/02/2021	Mexico City	Human	Female	63	unknown	Original	Oropharyngeal swab
hCoV-19/Mexico/CMX-INMEGEN-02-07-02/2021	EPI_ISL_1081153	10/02/2021	Mexico City	Human	Female	58	unknown	Original	Oropharyngeal swab
hCoV-19/Mexico/CMX-INMEGEN-02-07-03/2021	EPI_ISL_1081154	10/02/2021	Mexico City	Human	Female	68	unknown	Original	Oropharyngeal swab
hCoV-19/Mexico/CMX-INMEGEN-02-07-04/2021	EPI_ISL_1081155	10/02/2021	Mexico City	Human	Male	80	unknown	Original	Oropharyngeal swab
hCoV-19/Mexico/CMX-INMEGEN-02-07-13/2021	EPI_ISL_1081156	10/02/2021	Mexico City	Human	Female	29	unknown	Original	Oropharyngeal swab
hCoV-19/Mexico/CMX-INMEGEN-02-07-17/2021	EPI_ISL_1081158	10/02/2021	Mexico City	Human	Male	14	unknown	Original	Oropharyngeal swab
hCoV-19/Mexico/CMX-INMEGEN-02-07-18/2021	EPI_ISL_1081159	10/02/2021	Mexico City	Human	Female	28	unknown	Original	Oropharyngeal swab
hCoV-19/Mexico/CMX-INMEGEN-02-07-19/2021	EPI_ISL_1081160	10/02/2021	Mexico City	Human	Female	66	unknown	Original	Oropharyngeal swab
hCoV-19/Mexico/CMX-INMEGEN-02-07-20/2021	EPI_ISL_1081161	10/02/2021	Mexico City	Human	Male	50	unknown	Original	Oropharyngeal swab
hCoV-19/Mexico/CMX-INMEGEN-02-07-16/2021	EPI_ISL_1095495	10/02/2021	Mexico City	Human	Male	66	unknown	Original	Oropharyngeal swab
hCoV-19/Mexico/CMX-INMEGEN-02-11-19/2021	EPI_ISL_1117371	10/02/2021	Mexico City	Human	Female	57	unknown	Original	Oropharyngeal swab
hCoV-19/Mexico/CMX-INMEGEN-02-11-21/2021	EPI_ISL_1117373	10/02/2021	Mexico City	Human	Male	61	unknown	Original	Oropharyngeal swab
hCoV-19/Mexico/CMX-INMEGEN-02-11-22/2021	EPI_ISL_1117374	10/02/2021	Mexico City	Human	Female	43	unknown	Original	Oropharyngeal swab
hCoV-19/Mexico/CMX-INMEGEN-02-12-03/2021	EPI_ISL_1120880	10/02/2021	Mexico City	Human	Male	57	unknown	Original	Oropharyngeal swab
hCoV-19/Mexico/CMX-INMEGEN-02-12-04/2021	EPI_ISL_1120881	10/02/2021	Mexico City	Human	Male	39	unknown	Original	Oropharyngeal swab
hCoV-19/Mexico/CMX-INMEGEN-02-12-06/2021	EPI_ISL_1120883	10/02/2021	Mexico City	Human	Female	63	unknown	Original	Oropharyngeal swab
hCoV-19/Mexico/CMX-INMEGEN-02-12-07/2021	EPI_ISL_1120884	10/02/2021	Mexico City	Human	Female	53	unknown	Original	Oropharyngeal swab
hCoV-19/Mexico/CMX-INMEGEN-02-12-08/2021	EPI_ISL_1120885	10/02/2021	Mexico City	Human	Male	47	unknown	Original	Oropharyngeal swab
hCoV-19/Mexico/BCN-SEARCH-7360/2021	EPI_ISL_1295671	10/02/2021	Baja California	Human	unknown	unknown	unknown	Original	Nasal swab
hCoV-19/Mexico/JAL-InDRE_F103G_S579/2021	EPI_ISL_1324762	10/02/2021	Jalisco	Human	Male	34	Released	Original	Oropharyngeal swab
hCoV-19/Mexico/JAL-InDRE_F104G_S580/2021	EPI_ISL_1324763	10/02/2021	Jalisco	Human	Female	34	Released	Original	Oropharyngeal swab
hCoV-19/Mexico/CMX-INER-INMEGEN-00036/202	EPI_ISL_1406751	11/02/2021	Mexico City	Human	Male	22	unknown	Original	
hCoV-19/Mexico/CMX-INCMNSZ_COV159238/202	EPI_ISL_1502815	11/02/2021	Mexico City	Human	Male	66	Deceased	Original	Nasopharyngeal swab
hCoV-19/Mexico/CMX-INMEGEN-02-12-02/2021	EPI_ISL_1120879	11/02/2021	Mexico City	Human	Male	56	unknown	Original	Oropharyngeal swab
hCoV-19/Mexico/CMX-INMEGEN-02-12-05/2021	EPI_ISL_1120882	11/02/2021	Mexico City	Human	Male	76	unknown	Original	Oropharyngeal swab
hCoV-19/Mexico/CMX-INMEGEN-03-05-21/2021	EPI_ISL_1262717	11/02/2021	Mexico City	Human	Male	7	unknown	Original	Oropharyngeal swab

hCoV-19/Mexico/TAM-InDRE_455/2021	EPI_ISL_1219715	11/02/2021	Tamaulipas	Human	Male	1	Released	Original	Oropharyngeal swab
hCoV-19/Mexico/CMX-INMEGEN-02-12-01/2021	EPI_ISL_1120878	12/02/2021	Mexico City	Human	Female	28	unknown	Original	Oropharyngeal swab
hCoV-19/Mexico/CMX-INMEGEN-02-12-15/2021	EPI_ISL_1120890	12/02/2021	Mexico City	Human	Male	18	unknown	Original	Oropharyngeal swab
hCoV-19/Mexico/CMX-INMEGEN-02-12-14/2021	EPI_ISL_1133103	12/02/2021	Mexico City	Human	Male	15	unknown	Original	
hCoV-19/Mexico/CMX-INMEGEN-03-01-04/2021	EPI_ISL_1168637	12/02/2021	Mexico City	Human	Male	57	unknown	Original	Oropharyngeal swab
hCoV-19/Mexico/BCN-SEARCH-7391/2021	EPI_ISL_1295691	12/02/2021	Baja California	Human	unknown	unknown	unknown	Original	Nasal swab
hCoV-19/Mexico/CMX-INMEGEN-02-12-11/2021	EPI_ISL_1120888	13/02/2021	Mexico City	Human	Female	66	unknown	Original	Oropharyngeal swab
hCoV-19/Mexico/CMX-INMEGEN-02-12-13/2021	EPI_ISL_1120889	13/02/2021	Mexico City	Human	Female	21	unknown	Original	Oropharyngeal swab
hCoV-19/Mexico/CMX-INMEGEN-02-12-19/2021	EPI_ISL_1120891	13/02/2021	Mexico City	Human	Female	66	unknown	Original	Oropharyngeal swab
hCoV-19/Mexico/YUC-InDRE_F9951_S624/2021	EPI_ISL_1340661	14/02/2021	Yucatan	Human	Female	39	Released	Original	Oropharyngeal swab
hCoV-19/Mexico/ZAC-InDRE_F10107_S573/2021	EPI_ISL_1335757	15/02/2021	Zacatecas	Human	Female	40	Released	Original	Oropharyngeal swab
hCoV-19/Mexico/NLE-InDRE_F10500_S673/2021	EPI_ISL_1365639	15/02/2021	Nuevo Leon	Human	Female	28	Released	Original	Oropharyngeal swab
hCoV-19/Mexico/NLE-InDRE_F10501_S674/2021	EPI_ISL_1365640	15/02/2021	Nuevo Leon	Human	Male	62	Hospitalized	Original	Oropharyngeal swab
hCoV-19/Mexico/NLE-InDRE_F10517_S677/2021	EPI_ISL_1365643	15/02/2021	Nuevo Leon	Human	Female	71	Released	Original	Oropharyngeal swab
hCoV-19/Mexico/NLE-InDRE_F10518_S678/2021	EPI_ISL_1365644	15/02/2021	Nuevo Leon	Human	Female	46	Released	Original	Oropharyngeal swab
hCoV-19/Mexico/TAM-InDRE_F10561_S683/2021	EPI_ISL_1365649	15/02/2021	Tamaulipas	Human	Female	74	Deceased	Original	Oropharyngeal swab
hCoV-19/Mexico/MOR-InDRE_F10622_S686/2021	EPI_ISL_1365651	15/02/2021	Morelos	Human	Male	49	Released	Original	Oropharyngeal swab
hCoV-19/Mexico/MOR-InDRE_F10623_S687/2021	EPI_ISL_1365652	15/02/2021	Morelos	Human	Female	89	Released	Original	Oropharyngeal swab
hCoV-19/Mexico/VER-InDRE_F10678_S691/2021	EPI_ISL_1365656	15/02/2021	Veracruz	Human	Male	24	Released	Original	Oropharyngeal swab
hCoV-19/Mexico/ROO-InDRE_F10710_S696/2021	EPI_ISL_1365661	15/02/2021	Quintana Roo	Human	Male	38	Released	Original	Oropharyngeal swab
hCoV-19/Mexico/SIN-CIAD-S5971/2021	EPI_ISL_1482627	15/02/2021	Sinaloa	Human	Male	40	unknown	Original	Oropharyngeal swab
hCoV-19/Mexico/SIN_CIAD_S5975/2021	EPI_ISL_1660616	15/02/2021	Sinaloa	Human	Male	41	unknown	Original	Oropharyngeal swab
hCoV-19/Mexico/CMX-INMEGEN-02-12-09/2021	EPI_ISL_1120886	15/02/2021	Mexico City	Human	Female	14	unknown	Original	Oropharyngeal swab
hCoV-19/Mexico/CMX-INMEGEN-02-12-10/2021	EPI_ISL_1120887	15/02/2021	Mexico City	Human	Female	67	unknown	Original	Oropharyngeal swab
hCoV-19/Mexico/CMX-INMEGEN-02-12-20/2021	EPI_ISL_1120892	15/02/2021	Mexico City	Human	Female	38	unknown	Original	Oropharyngeal swab
hCoV-19/Mexico/CMX-INMEGEN-02-12-21/2021	EPI_ISL_1120893	15/02/2021	Mexico City	Human	Female	76	unknown	Original	Oropharyngeal swab
hCoV-19/Mexico/CMX-INMEGEN-02-12-22/2021	EPI_ISL_1120894	15/02/2021	Mexico City	Human	Male	45	unknown	Original	Oropharyngeal swab
hCoV-19/Mexico/CMX-INMEGEN-02-12-23/2021	EPI_ISL_1120895	15/02/2021	Mexico City	Human	Male	17	unknown	Original	Oropharyngeal swab
hCoV-19/Mexico/CMX-INMEGEN-02-12-24/2021	EPI_ISL_1120896	15/02/2021	Mexico City	Human	Female	28	unknown	Original	Oropharyngeal swab
hCoV-19/Mexico/CMX-INMEGEN-03-01-01/2021	EPI_ISL_1168634	15/02/2021	Mexico City	Human	Female	49	unknown	Original	Oropharyngeal swab
hCoV-19/Mexico/CMX-INMEGEN-03-01-02/2021	EPI_ISL_1168635	15/02/2021	Mexico City	Human	Male	64	unknown	Original	Oropharyngeal swab
hCoV-19/Mexico/CMX-INMEGEN-03-01-03/2021	EPI_ISL_1168636	15/02/2021	Mexico City	Human	Female	59	unknown	Original	Oropharyngeal swab
hCoV-19/Mexico/CMX-INMEGEN-03-01-05/2021	EPI_ISL_1168638	15/02/2021	Mexico City	Human	Male	66	unknown	Original	Oropharyngeal swab
hCoV-19/Mexico/CMX-INMEGEN-03-01-06/2021	EPI_ISL_1168639	15/02/2021	Mexico City	Human	Male	66	unknown	Original	Oropharyngeal swab
hCoV-19/Mexico/CMX-INMEGEN-03-01-07/2021	EPI_ISL_1168640	15/02/2021	Mexico City	Human	Female	52	unknown	Original	Oropharyngeal swab
hCoV-19/Mexico/CMX-INMEGEN-03-01-08/2021	EPI_ISL_1168641	15/02/2021	Mexico City	Human	Female	60	unknown	Original	Oropharyngeal swab
hCoV-19/Mexico/JAL-InDRE_F99G_S575/2021	EPI_ISL_1324758	15/02/2021	Jalisco	Human	Male	24	Released	Original	Oropharyngeal swab
hCoV-19/Mexico/GUA-InDRE_F10341_S587/2021	EPI_ISL_1337363	15/02/2021	Guanajuato	Human	Male	51	Released	Original	Oropharyngeal swab
hCoV-19/Mexico/NLE-InDRE_F10502_S714/2021	EPI_ISL_1366663	15/02/2021	Nuevo Leon	Human	Male	41	Hospitalized	Original	Oropharyngeal swab
hCoV-19/Mexico/MOR-InDRE_F10626_S728/2021	EPI_ISL_1366677	15/02/2021	Morelos	Human	Female	74	Released	Original	Oropharyngeal swab
hCoV-19/Mexico/CMX-InDRE_F10721_S738/2021	EPI_ISL_1366687	15/02/2021	Mexico City	Human	Male	34	Released	Original	Oropharyngeal swab

hCoV-19/Mexico/CMX-InDRE_F10724_S739/2021	EPI_ISL_1366688	15/02/2021	Mexico City	Human	Male	45	Deceased	Original	Oropharyngeal swab
hCoV-19/Mexico/CMX-INMEGEN-03-09-76/2021	EPI_ISL_1406126	15/02/2021	Mexico City	Human	Male	55	unknown	Original	Oropharyngeal swab
hCoV-19/Mexico/YUC-InDRE_F9948_S805/2021	EPI_ISL_1424007	15/02/2021	Yucatan	Human	Male	42	Released	Original	Oropharyngeal swab
hCoV-19/Mexico/COA-InDRE_514/2021	EPI_ISL_1168584	15/02/2021	Coahuila	Human	Female	48	Released	Original	Oropharyngeal swab
hCoV-19/Mexico/COA-InDRE_515/2021	EPI_ISL_1168585	15/02/2021	Coahuila	Human	Female	25	Released	Original	Oropharyngeal swab
hCoV-19/Mexico/CMX-INMEGEN-03-04-07/2021	EPI_ISL_1205209	15/02/2021	Mexico City	Human	Female	59	unknown	Original	Oropharyngeal swab
hCoV-19/Mexico/QUE-InDRE_F89B_S611/2021	EPI_ISL_1340654	15/02/2021	Queretaro	Human	Female	28	Released	Original	Oropharyngeal swab
hCoV-19/Mexico/QUE-InDRE_F9839_S618/2021	EPI_ISL_1340655	15/02/2021	Queretaro	Human	Male	58	Released	Original	Oropharyngeal swab
hCoV-19/Mexico/QUE-InDRE_F9841_S619/2021	EPI_ISL_1340657	15/02/2021	Queretaro	Human	Female	46	Released	Original	Oropharyngeal swab
hCoV-19/Mexico/YUC-InDRE_F9950_S623/2021	EPI_ISL_1340658	15/02/2021	Yucatan	Human	Female	44	Released	Original	Oropharyngeal swab
hCoV-19/Mexico/ZAC-InDRE_F10108_S625/2021	EPI_ISL_1340660	15/02/2021	Zacatecas	Human	Female	68	Released	Original	Oropharyngeal swab
hCoV-19/Mexico/NLE-InDRE_F10504_S857/2021	EPI_ISL_1399262	15/02/2021	Nuevo Leon	Human	Female	26	Released	Original	Oropharyngeal swab
hCoV-19/Mexico/JAL-InDRE-F11743-S1186/2021	EPI_ISL_1558832	15/02/2021	Jalisco	Human	Female	70	Released	Original	Oropharyngeal swab
hCoV-19/Mexico/JAL-InDRE-F11744-S913/2021	EPI_ISL_1558833	15/02/2021	Jalisco	Human	Male	69	Released	Original	Oropharyngeal swab
hCoV-19/Mexico/QUE-InDRE_F9840_S649/2021	EPI_ISL_1359064	15/02/2021	Queretaro	Human	Male	57	Released	Original	Oropharyngeal swab
hCoV-19/Mexico/YUC-InDRE_F9942_S651/2021	EPI_ISL_1359066	15/02/2021	Yucatan	Human	Male	28	Released	Original	Oropharyngeal swab
hCoV-19/Mexico/YUC-InDRE_F9953_S652/2021	EPI_ISL_1359067	15/02/2021	Yucatan	Human	Male	62	Hospitalized	Original	Oropharyngeal swab
hCoV-19/Mexico/GUA-InDRE_F10376_S662/2021	EPI_ISL_1359077	15/02/2021	Guanajuato	Human	Female	20	Released	Original	Oropharyngeal swab
hCoV-19/Mexico/GUA-InDRE_F10377_S663/2021	EPI_ISL_1359078	15/02/2021	Guanajuato	Human	Female	60	Released	Original	Oropharyngeal swab
hCoV-19/Mexico/SIN_CIAD_S5976/2021	EPI_ISL_1627079	15/02/2021	Sinaloa	Human	Male	83	unknown	Original	Oropharyngeal swab
hCoV-19/Mexico/NLE-InDRE_F10506_S675/2021	EPI_ISL_1365641	16/02/2021	Nuevo Leon	Human	Female	58	Released	Original	Oropharyngeal swab
hCoV-19/Mexico/NLE-InDRE_F10507_S676/2021	EPI_ISL_1365642	16/02/2021	Nuevo Leon	Human	Female	57	Released	Original	Oropharyngeal swab
hCoV-19/Mexico/NLE-InDRE_F10519_S679/2021	EPI_ISL_1365645	16/02/2021	Nuevo Leon	Human	Male	59	Released	Original	Oropharyngeal swab
hCoV-19/Mexico/NLE-InDRE_F10520_S680/2021	EPI_ISL_1365646	16/02/2021	Nuevo Leon	Human	Male	37	Released	Original	Oropharyngeal swab
hCoV-19/Mexico/NLE-InDRE_F10521_S681/2021	EPI_ISL_1365647	16/02/2021	Nuevo Leon	Human	Female	40	Released	Original	Oropharyngeal swab
hCoV-19/Mexico/NLE-InDRE_F10522_S682/2021	EPI_ISL_1365648	16/02/2021	Nuevo Leon	Human	Female	7	Released	Original	Oropharyngeal swab
hCoV-19/Mexico/MOR-InDRE_F10613_S685/2021	EPI_ISL_1365650	16/02/2021	Morelos	Human	Male	67	Hospitalized	Original	Oropharyngeal swab
hCoV-19/Mexico/MOR-InDRE_F10627_S688/2021	EPI_ISL_1365653	16/02/2021	Morelos	Human	Male	30	Released	Original	Oropharyngeal swab
hCoV-19/Mexico/MOR-InDRE_F10635_S689/2021	EPI_ISL_1365654	16/02/2021	Morelos	Human	Male	26	Released	Original	Oropharyngeal swab
hCoV-19/Mexico/CMX-InDRE_F10682_S692/2021	EPI_ISL_1365657	16/02/2021	Mexico City	Human	Male	74	Deceased	Original	Oropharyngeal swab
hCoV-19/Mexico/CMX-INMEGEN-03-02-10/2021	EPI_ISL_1181707	16/02/2021	Mexico City	Human	Male	38	unknown	Original	Oropharyngeal swab
hCoV-19/Mexico/JAL-InDRE_F100G_S576/2021	EPI_ISL_1324759	16/02/2021	Jalisco	Human	Female	34	Released	Original	Oropharyngeal swab
hCoV-19/Mexico/QUE-InDRE_F9845_S585/2021	EPI_ISL_1337360	16/02/2021	Queretaro	Human	Female	49	Released	Original	Oropharyngeal swab
hCoV-19/Mexico/QUE-InDRE_F9846_S586/2021	EPI_ISL_1337362	16/02/2021	Queretaro	Human	Male	43	Released	Original	Oropharyngeal swab
hCoV-19/Mexico/NLE-InDRE_F10524_S590/2021	EPI_ISL_1337368	16/02/2021	Nuevo Leon	Human	Male	31	Released	Original	Oropharyngeal swab
hCoV-19/Mexico/JAL-InDRE_F10432_S712/2021	EPI_ISL_1366661	16/02/2021	Jalisco	Human	Male	78	Released	Original	Oropharyngeal swab
hCoV-19/Mexico/NLE-InDRE_F10523_S716/2021	EPI_ISL_1366665	16/02/2021	Nuevo Leon	Human	Male	69	Released	Original	Oropharyngeal swab
hCoV-19/Mexico/QUE-InDRE_F10573_S718/2021	EPI_ISL_1366667	16/02/2021	Queretaro	Human	Male	34	Released	Original	Oropharyngeal swab
hCoV-19/Mexico/QUE-InDRE_F10577_S719/2021	EPI_ISL_1366668	16/02/2021	Queretaro	Human	Male	50	Released	Original	Oropharyngeal swab
hCoV-19/Mexico/VER-InDRE_F10679_S736/2021	EPI_ISL_1366685	16/02/2021	Veracruz	Human	Female	77	Released	Original	Oropharyngeal swab
hCoV-19/Mexico/CMX-InDRE_F10723_S740/2021	EPI_ISL_1366689	16/02/2021	Mexico City	Human	Female	62	Released	Original	Oropharyngeal swab

hCoV-19/Mexico/GUA-InDRE_F10342_S808/2021	EPI_ISL_1424010	16/02/2021	Guanajuato	Human	Male	65	Hospitalized	Original	Oropharyngeal swab
hCoV-19/Mexico/GUA-InDRE_F10343_S809/2021	EPI_ISL_1424011	16/02/2021	Guanajuato	Human	Male	57	Hospitalized	Original	Oropharyngeal swab
hCoV-19/Mexico/GUA-InDRE_F10344_S810/2021	EPI_ISL_1424012	16/02/2021	Guanajuato	Human	Male	40	Hospitalized	Original	Oropharyngeal swab
hCoV-19/Mexico/MOR-InDRE_F10634_S815/2021	EPI_ISL_1424017	16/02/2021	Morelos	Human	Female	25	Released	Original	Oropharyngeal swab
hCoV-19/Mexico/QUE-InDRE_F88B_S610/2021	EPI_ISL_1340634	16/02/2021	Queretaro	Human	Male	40	Released	Original	Oropharyngeal swab
hCoV-19/Mexico/QUE-InDRE_F9843_S620/2021	EPI_ISL_1340636	16/02/2021	Queretaro	Human	Male	24	Released	Original	Oropharyngeal swab
hCoV-19/Mexico/QUE-InDRE_F9847_S621/2021	EPI_ISL_1340637	16/02/2021	Queretaro	Human	Female	34	Released	Original	Oropharyngeal swab
hCoV-19/Mexico/QUE-InDRE_F9848_S622/2021	EPI_ISL_1340639	16/02/2021	Queretaro	Human	Female	39	Released	Original	Oropharyngeal swab
hCoV-19/Mexico/HID-InDRE_F10298_S626/2021	EPI_ISL_1340640	16/02/2021	Hidalgo	Human	Male	51	Deceased	Original	Oropharyngeal swab
hCoV-19/Mexico/HID-InDRE_F10306_S627/2021	EPI_ISL_1340642	16/02/2021	Hidalgo	Human	Male	68	Hospitalized	Original	Oropharyngeal swab
hCoV-19/Mexico/GUA-InDRE_F10333_S628/2021	EPI_ISL_1340643	16/02/2021	Guanajuato	Human	Female	43	Deceased	Original	Oropharyngeal swab
hCoV-19/Mexico/GUA-InDRE_F10345_S629/2021	EPI_ISL_1340644	16/02/2021	Guanajuato	Human	Male	52	Hospitalized	Original	Oropharyngeal swab
hCoV-19/Mexico/GUA-InDRE_F10347_S630/2021	EPI_ISL_1340646	16/02/2021	Guanajuato	Human	Female	90	Hospitalized	Original	Oropharyngeal swab
hCoV-19/Mexico/GUA-InDRE_F10379_S638/2021	EPI_ISL_1340647	16/02/2021	Guanajuato	Human	Female	18	Released	Original	Oropharyngeal swab
hCoV-19/Mexico/GUA-InDRE_F10380_S639/2021	EPI_ISL_1340648	16/02/2021	Guanajuato	Human	Male	21	Released	Original	Oropharyngeal swab
hCoV-19/Mexico/GUA-InDRE_F10381_S640/2021	EPI_ISL_1340650	16/02/2021	Guanajuato	Human	Male	33	Released	Original	Oropharyngeal swab
hCoV-19/Mexico/PUE-InDRE_F10410_S645/2021	EPI_ISL_1340651	16/02/2021	Puebla	Human	Male	49	Released	Original	Oropharyngeal swab
hCoV-19/Mexico/PUE-InDRE_F10412_S646/2021	EPI_ISL_1340652	16/02/2021	Puebla	Human	Female	63	Released	Original	Oropharyngeal swab
hCoV-19/Mexico/SEARCH-7817/2021	EPI_ISL_1366325	16/02/2021	Baja California	Human	unknown	unknown	unknown	Original	Nasal swab
hCoV-19/Mexico/SEARCH-7939/2021	EPI_ISL_1366334	16/02/2021	Baja California	Human	unknown	unknown	unknown	Original	Nasal swab
hCoV-19/Mexico/NLE-InDRE_F10509_S858/2021	EPI_ISL_1399263	16/02/2021	Nuevo Leon	Human	Female	72	Hospitalized	Original	Oropharyngeal swab
hCoV-19/Mexico/TAM-InDRE_F11051_S870/2021	EPI_ISL_1399275	16/02/2021	Tamaulipas	Human	Female	71	Deceased	Original	Oropharyngeal swab
hCoV-19/Mexico/CMX-INMEGEN-03-11-17/2021	EPI_ISL_1591476	16/02/2021	Mexico City	Human	Male	24	unknown	Original	Oropharyngeal swab
hCoV-19/Mexico/QUE-InDRE_F9844_S650/2021	EPI_ISL_1359065	16/02/2021	Queretaro	Human	Male	25	Released	Original	Oropharyngeal swab
hCoV-19/Mexico/ZAC-InDRE_F10109_S653/2021	EPI_ISL_1359068	16/02/2021	Zacatecas	Human	Male	56	Released	Original	Oropharyngeal swab
hCoV-19/Mexico/ZAC-InDRE_F10118_S654/2021	EPI_ISL_1359069	16/02/2021	Zacatecas	Human	Female	50	Hospitalized	Original	Oropharyngeal swab
hCoV-19/Mexico/GUA-InDRE_F10378_S664/2021	EPI_ISL_1359079	16/02/2021	Guanajuato	Human	Female	13	Released	Original	Oropharyngeal swab
hCoV-19/Mexico/CMX-INER-INMEGEN-00037/202	EPI_ISL_1406752	17/02/2021	Mexico City	Human	Female	57	unknown	Original	
hCoV-19/Mexico/CMX-INER-INMEGEN-00038/202	EPI_ISL_1406753	17/02/2021	Mexico City	Human	Female	49	unknown	Original	
hCoV-19/Mexico/CMX-INER-INMEGEN-00039/202	EPI_ISL_1406754	17/02/2021	Mexico City	Human	Male	40	unknown	Original	
hCoV-19/Mexico/CMX-INER-INMEGEN-00040/202	EPI_ISL_1406755	17/02/2021	Mexico City	Human	Female	18	unknown	Original	
hCoV-19/Mexico/CMX-INER-INMEGEN-00041/202	EPI_ISL_1406756	17/02/2021	Mexico City	Human	Female	43	unknown	Original	
hCoV-19/Mexico/CMX-INER-INMEGEN-00043/202	EPI_ISL_1406757	17/02/2021	Mexico City	Human	Male	34	unknown	Original	
hCoV-19/Mexico/CMX-INER-INMEGEN-00044/202	EPI_ISL_1406758	17/02/2021	Mexico City	Human	Female	36	unknown	Original	
hCoV-19/Mexico/CMX-INMEGEN-03-01-09/2021	EPI_ISL_1168642	17/02/2021	Mexico City	Human	Male	22	unknown	Original	Oropharyngeal swab
hCoV-19/Mexico/CMX-INMEGEN-03-01-10/2021	EPI_ISL_1168643	17/02/2021	Mexico City	Human	Female	46	unknown	Original	Oropharyngeal swab
hCoV-19/Mexico/CMX-INMEGEN-03-01-11/2021	EPI_ISL_1168644	17/02/2021	Mexico City	Human	Male	17	unknown	Original	Oropharyngeal swab
hCoV-19/Mexico/CMX-INMEGEN-03-02-11/2021	EPI_ISL_1181708	17/02/2021	Mexico City	Human	Female	57	unknown	Original	Oropharyngeal swab
hCoV-19/Mexico/CMX-INMEGEN-03-02-12/2021	EPI_ISL_1181709	17/02/2021	Mexico City	Human	Male	58	unknown	Original	Oropharyngeal swab
hCoV-19/Mexico/BCN-SEARCH-7441/2021	EPI_ISL_1295730	17/02/2021	Baja California	Human	unknown	unknown	unknown	Original	Nasal swab
hCoV-19/Mexico/TAM-InDRE_F86L_S567/2021	EPI_ISL_1334380	17/02/2021	Tamaulipas	Human	Male	35	Released	Original	Oropharyngeal swab

hCoV-19/Mexico/GUA-InDRE_F10390_S589/2021	EPI_ISL_1337367	17/02/2021	Guanajuato	Human	Male	24	Released	Original	Oropharyngeal swab
hCoV-19/Mexico/BCS-InDRE_F10705_S594/2021	EPI_ISL_1337374	17/02/2021	Baja California Sur	Human	Female	67	Deceased	Original	Oropharyngeal swab
hCoV-19/Mexico/NLE-InDRE_F10511_S715/2021	EPI_ISL_1366664	17/02/2021	Nuevo Leon	Human	Female	54	Released	Original	Oropharyngeal swab
hCoV-19/Mexico/QUE-InDRE_F10583_S720/2021	EPI_ISL_1366669	17/02/2021	Queretaro	Human	Female	27	Released	Original	Oropharyngeal swab
hCoV-19/Mexico/MOR-InDRE_F10650_S729/2021	EPI_ISL_1366678	17/02/2021	Morelos	Human	Male	76	Released	Original	Oropharyngeal swab
hCoV-19/Mexico/MOR-InDRE_F10656_S731/2021	EPI_ISL_1366680	17/02/2021	Morelos	Human	Male	83	Hospitalized	Original	Oropharyngeal swab
hCoV-19/Mexico/CMX-INMEGEN-03-09-77/2021	EPI_ISL_1406127	17/02/2021	Mexico City	Human	Female	57	unknown	Original	Oropharyngeal swab
hCoV-19/Mexico/CMX-INMEGEN-03-09-79/2021	EPI_ISL_1406128	17/02/2021	Mexico City	Human	Male	40	unknown	Original	Oropharyngeal swab
hCoV-19/Mexico/CMX-INMEGEN-03-09-80/2021	EPI_ISL_1406129	17/02/2021	Mexico City	Human	Female	49	unknown	Original	Oropharyngeal swab
hCoV-19/Mexico/CMX-INMEGEN-03-09-81/2021	EPI_ISL_1406130	17/02/2021	Mexico City	Human	Female	16	unknown	Original	Oropharyngeal swab
hCoV-19/Mexico/CMX-INMEGEN-03-09-82/2021	EPI_ISL_1406131	17/02/2021	Mexico City	Human	Female	18	unknown	Original	Oropharyngeal swab
hCoV-19/Mexico/CMX-INMEGEN-03-09-83/2021	EPI_ISL_1406132	17/02/2021	Mexico City	Human	Female	3	unknown	Original	Oropharyngeal swab
hCoV-19/Mexico/CMX-INMEGEN-03-09-84/2021	EPI_ISL_1406133	17/02/2021	Mexico City	Human	Female	68	unknown	Original	Oropharyngeal swab
hCoV-19/Mexico/CMX-INMEGEN-03-09-85/2021	EPI_ISL_1406134	17/02/2021	Mexico City	Human	Female	52	unknown	Original	Oropharyngeal swab
hCoV-19/Mexico/CMX-INER-INMEGEN-00042/202	EPI_ISL_1424000	17/02/2021	Mexico City	Human	Female	52	unknown	Original	
hCoV-19/Mexico/VER-InDRE_F10671_S817/2021	EPI_ISL_1424019	17/02/2021	Veracruz	Human	Female	62	Released	Original	Oropharyngeal swab
hCoV-19/Mexico/CMX-INER-SC480/2021	EPI_ISL_1672849	17/02/2021	Mexico City	Human	Male	86	Hospitalized	Original	
hCoV-19/Mexico/CMX-INER-INMEGEN-00092/202	EPI_ISL_1824442	17/02/2021	Mexico City	Human	Male	57	unknown	Original	
hCoV-19/Mexico/CMX-INER-INMEGEN-00093/202	EPI_ISL_1824443	17/02/2021	Mexico City	Human	Male	34	unknown	Original	
hCoV-19/Mexico/CMX-INER-INMEGEN-00094/202	EPI_ISL_1824444	17/02/2021	Mexico City	Human	Female	26	unknown	Original	
hCoV-19/Mexico/CMX-InDRE_F97I_S613/2021	EPI_ISL_1340629	17/02/2021	Mexico City	Human	Female	unknown	Released	Original	Oropharyngeal swab
hCoV-19/Mexico/GUA-InDRE_F10349_S632/2021	EPI_ISL_1340630	17/02/2021	Guanajuato	Human	Female	44	Hospitalized	Original	Oropharyngeal swab
hCoV-19/Mexico/GUA-InDRE_F10382_S641/2021	EPI_ISL_1340631	17/02/2021	Guanajuato	Human	Male	39	Released	Original	Oropharyngeal swab
hCoV-19/Mexico/GUA-InDRE_F10387_S642/2021	EPI_ISL_1340633	17/02/2021	Guanajuato	Human	Male	38	Released	Original	Oropharyngeal swab
hCoV-19/Mexico/MIC-InDRE_F10346_S853/2021	EPI_ISL_1399259	17/02/2021	Michoacan	Human	Male	65	Hospitalized	Original	Oropharyngeal swab
hCoV-19/Mexico/NLE-InDRE_F10510_S859/2021	EPI_ISL_1399264	17/02/2021	Nuevo Leon	Human	Female	41	Released	Original	Oropharyngeal swab
hCoV-19/Mexico/TAM-InDRE_F11055_S871/2021	EPI_ISL_1399276	17/02/2021	Tamaulipas	Human	Female	54	Released	Original	Oropharyngeal swab
hCoV-19/Mexico/CMX-INMEGEN-03-11-01/2021	EPI_ISL_1591460	17/02/2021	Mexico City	Human	Female	26	unknown	Original	Oropharyngeal swab
hCoV-19/Mexico/CMX-INMEGEN-03-11-09/2021	EPI_ISL_1591468	17/02/2021	Mexico City	Human	Male	57	unknown	Original	Oropharyngeal swab
hCoV-19/Mexico/GUA-InDRE_F10383_S665/2021	EPI_ISL_1359080	17/02/2021	Guanajuato	Human	Female	56	Released	Original	Oropharyngeal swab
hCoV-19/Mexico/GUA-InDRE_F10384_S666/2021	EPI_ISL_1359081	17/02/2021	Guanajuato	Human	Female	28	Released	Original	Oropharyngeal swab
hCoV-19/Mexico/GUA-InDRE_F10385_S667/2021	EPI_ISL_1359082	17/02/2021	Guanajuato	Human	Female	60	Released	Original	Oropharyngeal swab
hCoV-19/Mexico/GUA-InDRE_F10386_S668/2021	EPI_ISL_1359083	17/02/2021	Guanajuato	Human	Male	16	Released	Original	Oropharyngeal swab
hCoV-19/Mexico/GUA-InDRE_F10388_S669/2021	EPI_ISL_1359084	17/02/2021	Guanajuato	Human	Male	52	Released	Original	Oropharyngeal swab
hCoV-19/Mexico/GUA-InDRE_F10389_S670/2021	EPI_ISL_1359085	17/02/2021	Guanajuato	Human	Female	55	Released	Original	Oropharyngeal swab
hCoV-19/Mexico/BCN-InDRE_F10696_S693/2021	EPI_ISL_1365658	18/02/2021	Baja California	Human	Female	30	Released	Original	Oropharyngeal swab
hCoV-19/Mexico/BCN-InDRE_F10697_S694/2021	EPI_ISL_1365659	18/02/2021	Baja California	Human	Male	32	Released	Original	Oropharyngeal swab
hCoV-19/Mexico/BCN-InDRE_F10699_S695/2021	EPI_ISL_1365660	18/02/2021	Baja California	Human	Female	39	Released	Original	Oropharyngeal swab
hCoV-19/Mexico/CMX-INER-INMEGEN-00045/202	EPI_ISL_1406759	18/02/2021	Mexico City	Human	Female	45	unknown	Original	
hCoV-19/Mexico/CMX-INMEGEN-04-04-1/2021	EPI_ISL_1628520	18/02/2021	Mexico City	Human	Male	66	unknown	Original	Oropharyngeal swab
hCoV-19/Mexico/CMX-INER-INMEGEN-00095/202	EPI_ISL_1970842	18/02/2021	Mexico City	Human	Male	7	unknown	Original	

hCoV-19/Mexico/CMX-INER-INMEGEN-00096/202	EPI_ISL_1970843	18/02/2021	Mexico City	Human	Female	12	unknown	Original	
hCoV-19/Mexico/CMX-INER-INMEGEN-00097/202	EPI_ISL_1970844	18/02/2021	Mexico City	Human	Male	31	unknown	Original	
hCoV-19/Mexico/CMX-INER-INMEGEN-00098/202	EPI_ISL_1970845	18/02/2021	Mexico City	Human	Female	64	unknown	Original	
hCoV-19/Mexico/CMX-INMEGEN-03-01-12/2021	EPI_ISL_1168645	18/02/2021	Mexico City	Human	Female	49	unknown	Original	Oropharyngeal swab
hCoV-19/Mexico/CMX-INMEGEN-03-01-17/2021	EPI_ISL_1168646	18/02/2021	Mexico City	Human	Female	69	unknown	Original	Oropharyngeal swab
hCoV-19/Mexico/CMX-INMEGEN-03-01-18/2021	EPI_ISL_1168647	18/02/2021	Mexico City	Human	Male	13	unknown	Original	Oropharyngeal swab
hCoV-19/Mexico/CMX-INMEGEN-03-01-21/2021	EPI_ISL_1168650	18/02/2021	Mexico City	Human	Male	68	unknown	Original	Oropharyngeal swab
hCoV-19/Mexico/CMX-INMEGEN-03-01-22/2021	EPI_ISL_1168651	18/02/2021	Mexico City	Human	Male	53	unknown	Original	Oropharyngeal swab
hCoV-19/Mexico/CMX-INMEGEN-03-01-23/2021	EPI_ISL_1168652	18/02/2021	Mexico City	Human	Female	32	unknown	Original	Oropharyngeal swab
hCoV-19/Mexico/CMX-INMEGEN-03-02-02/2021	EPI_ISL_1181701	18/02/2021	Mexico City	Human	Male	54	unknown	Original	Oropharyngeal swab
hCoV-19/Mexico/CMX-INMEGEN-03-02-05/2021	EPI_ISL_1181703	18/02/2021	Mexico City	Human	Female	42	unknown	Original	Oropharyngeal swab
hCoV-19/Mexico/CMX-INMEGEN-03-02-06/2021	EPI_ISL_1181704	18/02/2021	Mexico City	Human	Female	41	unknown	Original	Oropharyngeal swab
hCoV-19/Mexico/BCN-SEARCH-7475/2021	EPI_ISL_1295760	18/02/2021	Baja California	Human	unknown	unknown	unknown	Original	Nasal swab
hCoV-19/Mexico/GUA-InDRE_F10350_S588/2021	EPI_ISL_1337365	18/02/2021	Guanajuato	Human	Female	68	Deceased	Original	Oropharyngeal swab
hCoV-19/Mexico/BCS-InDRE_F10688_S591/2021	EPI_ISL_1337370	18/02/2021	Baja California Sur	Human	Female	61	Released	Original	Oropharyngeal swab
hCoV-19/Mexico/BCS-InDRE_F10702_S592/2021	EPI_ISL_1337371	18/02/2021	Baja California Sur	Human	Female	38	Released	Original	Oropharyngeal swab
hCoV-19/Mexico/BCS-InDRE_F10703_S593/2021	EPI_ISL_1337373	18/02/2021	Baja California Sur	Human	Male	41	Released	Original	Oropharyngeal swab
hCoV-19/Mexico/MOR-InDRE_F10653_S730/2021	EPI_ISL_1366679	18/02/2021	Morelos	Human	Female	28	Released	Original	Oropharyngeal swab
hCoV-19/Mexico/HID-InDRE_F10309_S807/2021	EPI_ISL_1424009	18/02/2021	Hidalgo	Human	Male	84	Deceased	Original	Oropharyngeal swab
hCoV-19/Mexico/QUE-InDRE_F10593_S814/2021	EPI_ISL_1424016	18/02/2021	Queretaro	Human	Male	17	Released	Original	Oropharyngeal swab
hCoV-19/Mexico/TAM-InDRE_F11058_S837/2021	EPI_ISL_1424039	18/02/2021	Tamaulipas	Human	Female	31	Released	Original	Oropharyngeal swab
hCoV-19/Mexico/CMX-INER-SC483/2021	EPI_ISL_1672864	18/02/2021	Mexico City	Human	Male	62	Hospitalized	Original	Nasopharyngeal swab
hCoV-19/Mexico/CMX-INER-SC486/2021	EPI_ISL_1672865	18/02/2021	Mexico City	Human	Male	57	Deceased	Original	Nasopharyngeal swab
hCoV-19/Mexico/CMX-INER-INMEGEN-00099/202	EPI_ISL_1824445	18/02/2021	Mexico City	Human	Male	29	unknown	Original	
hCoV-19/Mexico/CMX-INER-INMEGEN-00100/202	EPI_ISL_1824446	18/02/2021	Mexico City	Human	Male	26	unknown	Original	
hCoV-19/Mexico/CMX-INER-INMEGEN-00101/202	EPI_ISL_1824447	18/02/2021	Mexico City	Human	Female	55	unknown	Original	
hCoV-19/Mexico/CMX-INER-INMEGEN-00102/202	EPI_ISL_1824448	18/02/2021	Mexico City	Human	Male	31	unknown	Original	
hCoV-19/Mexico/CMX-INER-INMEGEN-00103/202	EPI_ISL_1824449	18/02/2021	Mexico City	Human	Female	39	unknown	Original	
hCoV-19/Mexico/CMX-INER-INMEGEN-00104/202	EPI_ISL_1824450	18/02/2021	Mexico City	Human	Female	42	unknown	Original	
hCoV-19/Mexico/QUE-InDRE_F90B_S612/2021	EPI_ISL_1340619	18/02/2021	Queretaro	Human	Female	51	Deceased	Original	Oropharyngeal swab
hCoV-19/Mexico/CMX-InDRE_F115B_S615/2021	EPI_ISL_1340620	18/02/2021	Mexico City	Human	Male	23	Released	Original	Oropharyngeal swab
hCoV-19/Mexico/CMX-InDRE_F116B_S616/2021	EPI_ISL_1340621	18/02/2021	Mexico City	Human	Male	54	Released	Original	Oropharyngeal swab
hCoV-19/Mexico/CMX-InDRE_F117B_S617/2021	EPI_ISL_1340623	18/02/2021	Mexico City	Human	Male	44	Released	Original	Oropharyngeal swab
hCoV-19/Mexico/GUA-InDRE_F10348_S631/2021	EPI_ISL_1340624	18/02/2021	Guanajuato	Human	Male	57	Hospitalized	Original	Oropharyngeal swab
hCoV-19/Mexico/GUA-InDRE_F10358_S635/2021	EPI_ISL_1340626	18/02/2021	Guanajuato	Human	Female	33	Released	Original	Oropharyngeal swab
hCoV-19/Mexico/GUA-InDRE_F10391_S643/2021	EPI_ISL_1340627	18/02/2021	Guanajuato	Human	Female	65	Released	Original	Oropharyngeal swab
hCoV-19/Mexico/GUA-InDRE_F10351_S854/2021	EPI_ISL_1399260	18/02/2021	Guanajuato	Human	Male	77	Hospitalized	Original	Oropharyngeal swab
hCoV-19/Mexico/TAM-InDRE_F11057_S872/2021	EPI_ISL_1399277	18/02/2021	Tamaulipas	Human	Male	41	Released	Original	Oropharyngeal swab
hCoV-19/Mexico/JAL-InDRE-F11742-S1185/2021	EPI_ISL_1558831	18/02/2021	Jalisco	Human	Female	45	Released	Original	Oropharyngeal swab
hCoV-19/Mexico/CMX-INMEGEN-03-11-02/2021	EPI_ISL_1591461	18/02/2021	Mexico City	Human	Male	59	unknown	Original	Oropharyngeal swab
hCoV-19/Mexico/CMX-INMEGEN-03-11-10/2021	EPI_ISL_1591469	18/02/2021	Mexico City	Human	Female	72	unknown	Original	Oropharyngeal swab

hCoV-19/Mexico/CMX-INMEGEN-03-11-18/2021	EPI_ISL_1591477	18/02/2021	Mexico City	Human	Female	39	unknown	Original	Oropharyngeal swab
hCoV-19/Mexico/CMX-INMEGEN-03-11-26/2021	EPI_ISL_1591483	18/02/2021	Mexico City	Human	Male	31	unknown	Original	Oropharyngeal swab
hCoV-19/Mexico/GUA-InDRE_F10392_S671/2021	EPI_ISL_1359086	18/02/2021	Guanajuato	Human	Male	43	Released	Original	Oropharyngeal swab
hCoV-19/Mexico/GUA-InDRE_F10393_S672/2021	EPI_ISL_1359087	18/02/2021	Guanajuato	Human	Male	21	Released	Original	Oropharyngeal swab
hCoV-19/Mexico/CMX-INMEGEN-03-11-49/2021	EPI_ISL_1591503	18/02/2021	Mexico City	Human	Male	29	unknown	Original	Oropharyngeal swab
hCoV-19/Mexico/CMX-INMEGEN-03-11-50/2021	EPI_ISL_1591504	18/02/2021	Mexico City	Human	Female	60	unknown	Original	Oropharyngeal swab
hCoV-19/Mexico/CMX-INMEGEN-03-11-57/2021	EPI_ISL_1591511	18/02/2021	Mexico City	Human	Female	71	unknown	Original	Oropharyngeal swab
hCoV-19/Mexico/CMX-INMEGEN-03-11-73/2021	EPI_ISL_1591524	18/02/2021	Mexico City	Human	Male	26	unknown	Original	Oropharyngeal swab
hCoV-19/Mexico/CMX-INMEGEN-03-11-74/2021	EPI_ISL_1591525	18/02/2021	Mexico City	Human	Female	42	unknown	Original	Oropharyngeal swab
hCoV-19/Mexico/CMX-INMEGEN-03-11-81/2021	EPI_ISL_1591530	18/02/2021	Mexico City	Human	Male	55	unknown	Original	Oropharyngeal swab
hCoV-19/Mexico/CMX-INMEGEN-03-11-82/2021	EPI_ISL_1591531	18/02/2021	Mexico City	Human	Male	26	unknown	Original	Oropharyngeal swab
hCoV-19/Mexico/CMX-INMEGEN-03-11-87/2021	EPI_ISL_1591536	18/02/2021	Mexico City	Human	Male	23	unknown	Original	Oropharyngeal swab
hCoV-19/Mexico/CMX-INMEGEN-03-11-89/2021	EPI_ISL_1591537	18/02/2021	Mexico City	Human	Female	58	unknown	Original	Oropharyngeal swab
hCoV-19/Mexico/MOR-InDRE_F10663_S690/2021	EPI_ISL_1365655	19/02/2021	Morelos	Human	Male	71	Released	Original	Oropharyngeal swab
hCoV-19/Mexico/COA-InDRE_F10752_S698/2021	EPI_ISL_1365663	19/02/2021	Coahuila	Human	Male	28	Released	Original	Oropharyngeal swab
hCoV-19/Mexico/CMX-INER-INMEGEN-00046/202	EPI_ISL_1406760	19/02/2021	Mexico City	Human	Female	15	unknown	Original	
hCoV-19/Mexico/CMX-INER-INMEGEN-00047/202	EPI_ISL_1406761	19/02/2021	Mexico City	Human	Male	10	unknown	Original	
hCoV-19/Mexico/CMX-INER-INMEGEN-00048/202	EPI_ISL_1406762	19/02/2021	Mexico City	Human	Female	18	unknown	Original	
hCoV-19/Mexico/CMX-INER-INMEGEN-00074/202	EPI_ISL_1970838	19/02/2021	Mexico City	Human	Female	64	unknown	Original	
hCoV-19/Mexico/CMX-INMEGEN-03-01-19/2021	EPI_ISL_1168648	19/02/2021	Mexico City	Human	Male	30	unknown	Original	Oropharyngeal swab
hCoV-19/Mexico/CMX-INMEGEN-03-01-20/2021	EPI_ISL_1168649	19/02/2021	Mexico City	Human	Female	74	unknown	Original	Oropharyngeal swab
hCoV-19/Mexico/CMX-INMEGEN-03-02-01/2021	EPI_ISL_1181700	19/02/2021	Mexico City	Human	Female	49	unknown	Original	Oropharyngeal swab
hCoV-19/Mexico/CMX-INMEGEN-03-02-04/2021	EPI_ISL_1181702	19/02/2021	Mexico City	Human	Female	49	unknown	Original	Oropharyngeal swab
hCoV-19/Mexico/CMX-INMEGEN-03-02-07/2021	EPI_ISL_1181705	19/02/2021	Mexico City	Human	Male	33	unknown	Original	Oropharyngeal swab
hCoV-19/Mexico/CMX-INMEGEN-03-02-08/2021	EPI_ISL_1181706	19/02/2021	Mexico City	Human	Female	60	unknown	Original	Oropharyngeal swab
hCoV-19/Mexico/CMX-INMEGEN-03-02-13/2021	EPI_ISL_1181710	19/02/2021	Mexico City	Human	Female	29	unknown	Original	Oropharyngeal swab
hCoV-19/Mexico/CMX-INMEGEN-03-02-19/2021	EPI_ISL_1181716	19/02/2021	Mexico City	Human	Female	40	unknown	Original	Oropharyngeal swab
hCoV-19/Mexico/JAL-InDRE_F10438_S713/2021	EPI_ISL_1366662	19/02/2021	Jalisco	Human	Female	26	Released	Original	Oropharyngeal swab
hCoV-19/Mexico/YUC-InDRE_F10540_S717/2021	EPI_ISL_1366666	19/02/2021	Yucatan	Human	Female	74	Released	Original	Oropharyngeal swab
hCoV-19/Mexico/QUE-InDRE_F10598_S721/2021	EPI_ISL_1366670	19/02/2021	Queretaro	Human	Female	56	Released	Original	Oropharyngeal swab
hCoV-19/Mexico/QUE-InDRE_F10599_S722/2021	EPI_ISL_1366671	19/02/2021	Queretaro	Human	Male	58	Released	Original	Oropharyngeal swab
hCoV-19/Mexico/MOR-InDRE_F10660_S732/2021	EPI_ISL_1366681	19/02/2021	Morelos	Human	Male	17	Released	Original	Oropharyngeal swab
hCoV-19/Mexico/VER-InDRE_F10672_S733/2021	EPI_ISL_1366682	19/02/2021	Veracruz	Human	Female	75	Hospitalized	Original	Oropharyngeal swab
hCoV-19/Mexico/CHP-InDRE_F10422_S811/2021	EPI_ISL_1424013	19/02/2021	Chiapas	Human	Female	63	Hospitalized	Original	Oropharyngeal swab
hCoV-19/Mexico/CAM-InDRE_F10557_S813/2021	EPI_ISL_1424015	19/02/2021	Campeche	Human	Female	49	Hospitalized	Original	Oropharyngeal swab
hCoV-19/Mexico/TAM-InDRE_F11059_S838/2021	EPI_ISL_1424040	19/02/2021	Tamaulipas	Human	Male	36	Released	Original	Oropharyngeal swab
hCoV-19/Mexico/VER-InDRE_F11084_S845/2021	EPI_ISL_1424046	19/02/2021	Veracruz	Human	Female	70	Hospitalized	Original	Oropharyngeal swab
hCoV-19/Mexico/CMX-INER-SC498/2021	EPI_ISL_1672866	19/02/2021	Mexico City	Human	Female	67	Hospitalized	Original	Nasopharyngeal swab
hCoV-19/Mexico/CMX-INER-INMEGEN-00065/202	EPI_ISL_1824419	19/02/2021	Mexico City	Human	Male	70	unknown	Original	
hCoV-19/Mexico/CMX-INER-INMEGEN-00066/202	EPI_ISL_1824420	19/02/2021	Mexico City	Human	Male	64	unknown	Original	
hCoV-19/Mexico/CMX-INER-INMEGEN-00067/202	EPI_ISL_1824421	19/02/2021	Mexico City	Human	Female	62	unknown	Original	

hCoV-19/Mexico/CMX-INER-INMEGEN-00068/202	EPI_ISL_1824422	19/02/2021	Mexico City	Human	Male	48	unknown	Original	
hCoV-19/Mexico/CMX-INER-INMEGEN-00069/202	EPI_ISL_1824423	19/02/2021	Mexico City	Human	Male	37	unknown	Original	
hCoV-19/Mexico/CMX-INER-INMEGEN-00070/202	EPI_ISL_1824424	19/02/2021	Mexico City	Human	Male	16	unknown	Original	
hCoV-19/Mexico/CMX-INER-INMEGEN-00071/202	EPI_ISL_1824425	19/02/2021	Mexico City	Human	Female	79	unknown	Original	
hCoV-19/Mexico/CMX-INER-INMEGEN-00072/202	EPI_ISL_1824426	19/02/2021	Mexico City	Human	Female	54	unknown	Original	
hCoV-19/Mexico/CMX-INER-INMEGEN-00073/202	EPI_ISL_1824427	19/02/2021	Mexico City	Human	Female	33	unknown	Original	
hCoV-19/Mexico/CMX-INER-INMEGEN-00075/202	EPI_ISL_1824428	19/02/2021	Mexico City	Human	Female	59	unknown	Original	
hCoV-19/Mexico/CMX-INER-INMEGEN-00076/202	EPI_ISL_1824429	19/02/2021	Mexico City	Human	Male	73	unknown	Original	
hCoV-19/Mexico/GUA-InDRE_F84G_S609/2021	EPI_ISL_1340611	19/02/2021	Guanajuato	Human	Male	60	Released	Original	Oropharyngeal swab
hCoV-19/Mexico/CMX-InDRE_F95D_S614/2021	EPI_ISL_1340613	19/02/2021	Mexico City	Human	Female	53	Released	Original	Oropharyngeal swab
hCoV-19/Mexico/GUA-InDRE_F10357_S634/2021	EPI_ISL_1340614	19/02/2021	Guanajuato	Human	Male	29	Released	Original	Oropharyngeal swab
hCoV-19/Mexico/GUA-InDRE_F10367_S637/2021	EPI_ISL_1340616	19/02/2021	Guanajuato	Human	Female	17	Released	Original	Oropharyngeal swab
hCoV-19/Mexico/GUA-InDRE_F10395_S644/2021	EPI_ISL_1340617	19/02/2021	Guanajuato	Human	Male	41	Released	Original	Oropharyngeal swab
hCoV-19/Mexico/PUE-InDRE_F10903_S765/2021	EPI_ISL_1400310	19/02/2021	Puebla	Human	Male	48	Hospitalized	Original	Oropharyngeal swab
hCoV-19/Mexico/CMX-INMEGEN-03-11-34/2021	EPI_ISL_1591490	19/02/2021	Mexico City	Human	Female	50	unknown	Original	Oropharyngeal swab
hCoV-19/Mexico/CMX-INMEGEN-03-11-42/2021	EPI_ISL_1591496	19/02/2021	Mexico City	Human	Male	50	unknown	Original	Oropharyngeal swab
hCoV-19/Mexico/CMX-INMEGEN-03-03-04/2021	EPI_ISL_1298454	19/02/2021	Mexico City	Human	Female	59	unknown	Original	Oropharyngeal swab
hCoV-19/Mexico/CMX-INMEGEN-03-03-21/2021	EPI_ISL_1298471	19/02/2021	Mexico City	Human	Female	24	unknown	Original	Oropharyngeal swab
hCoV-19/Mexico/GUA-InDRE_F10352_S655/2021	EPI_ISL_1359070	19/02/2021	Guanajuato	Human	Female	72	Hospitalized	Original	Oropharyngeal swab
hCoV-19/Mexico/GUA-InDRE_F10356_S656/2021	EPI_ISL_1359071	19/02/2021	Guanajuato	Human	Male	38	Released	Original	Oropharyngeal swab
hCoV-19/Mexico/GUA-InDRE_F10359_S657/2021	EPI_ISL_1359072	19/02/2021	Guanajuato	Human	Female	35	Released	Original	Oropharyngeal swab
hCoV-19/Mexico/GUA-InDRE_F10360_S658/2021	EPI_ISL_1359073	19/02/2021	Guanajuato	Human	Female	95	Released	Original	Oropharyngeal swab
hCoV-19/Mexico/GUA-InDRE_F10361_S659/2021	EPI_ISL_1359074	19/02/2021	Guanajuato	Human	Male	40	Released	Original	Oropharyngeal swab
hCoV-19/Mexico/SIN_CIAD_S6084/2021	EPI_ISL_1491363	19/02/2021	Sinaloa	Human	Female	39	unknown	Original	Oropharyngeal swab
hCoV-19/Mexico/CMX-INMEGEN-03-11-58/2021	EPI_ISL_1591512	19/02/2021	Mexico City	Human	Female	19	unknown	Original	Oropharyngeal swab
hCoV-19/Mexico/CMX-INMEGEN-03-11-66/2021	EPI_ISL_1591519	19/02/2021	Mexico City	Human	Female	25	unknown	Original	Oropharyngeal swab
hCoV-19/Mexico/CMX-INMEGEN-03-02-14/2021	EPI_ISL_1181711	20/02/2021	Mexico City	Human	Female	20	unknown	Original	Oropharyngeal swab
hCoV-19/Mexico/CMX-INMEGEN-03-02-15/2021	EPI_ISL_1181712	20/02/2021	Mexico City	Human	Male	56	unknown	Original	Oropharyngeal swab
hCoV-19/Mexico/CMX-INMEGEN-03-02-16/2021	EPI_ISL_1181713	20/02/2021	Mexico City	Human	Male	42	unknown	Original	Oropharyngeal swab
hCoV-19/Mexico/CMX-INMEGEN-03-02-22/2021	EPI_ISL_1181719	20/02/2021	Mexico City	Human	Male	21	unknown	Original	Oropharyngeal swab
hCoV-19/Mexico/QUE-InDRE_F10602_S723/2021	EPI_ISL_1366672	20/02/2021	Queretaro	Human	Male	38	Released	Original	Oropharyngeal swab
hCoV-19/Mexico/QUE-InDRE_F10603_S724/2021	EPI_ISL_1366673	20/02/2021	Queretaro	Human	Female	49	Released	Original	Oropharyngeal swab
hCoV-19/Mexico/VER-InDRE_F10673_S734/2021	EPI_ISL_1366683	20/02/2021	Veracruz	Human	Male	45	Released	Original	Oropharyngeal swab
hCoV-19/Mexico/VER-InDRE_F10674_S735/2021	EPI_ISL_1366684	20/02/2021	Veracruz	Human	Female	70	Hospitalized	Original	Oropharyngeal swab
hCoV-19/Mexico/CMX-INER-INMEGEN-00049/202	EPI_ISL_1424001	20/02/2021	Mexico City	Human	Female	58	unknown	Original	
hCoV-19/Mexico/HID-InDRE_F10300_S806/2021	EPI_ISL_1424008	20/02/2021	Hidalgo	Human	Female	48	Released	Original	Oropharyngeal swab
hCoV-19/Mexico/GUA-InDRE_F10353_S633/2021	EPI_ISL_1340608	20/02/2021	Guanajuato	Human	Male	51	Hospitalized	Original	Oropharyngeal swab
hCoV-19/Mexico/GUA-InDRE_F10362_S636/2021	EPI_ISL_1340610	20/02/2021	Guanajuato	Human	Male	74	Released	Original	Oropharyngeal swab
hCoV-19/Mexico/GUA-InDRE_F10355_S856/2021	EPI_ISL_1399261	20/02/2021	Guanajuato	Human	Female	60	Hospitalized	Original	Oropharyngeal swab
hCoV-19/Mexico/VER-InDRE_F11086_S874/2021	EPI_ISL_1399279	20/02/2021	Veracruz	Human	Female	38	Released	Original	Oropharyngeal swab
hCoV-19/Mexico/JAL-InDRE-F11741-S1184/2021	EPI_ISL_1558830	20/02/2021	Jalisco	Human	Female	60	Released	Original	Oropharyngeal swab

hCoV-19/Mexico/CMX-INMEGEN-03-03-01/2021	EPI_ISL_1298451	20/02/2021	Mexico City	Human	Female	30	unknown	Original	Oropharyngeal swab
hCoV-19/Mexico/CMX-INMEGEN-03-03-02/2021	EPI_ISL_1298452	20/02/2021	Mexico City	Human	Male	65	unknown	Original	Oropharyngeal swab
hCoV-19/Mexico/CMX-INMEGEN-03-03-05/2021	EPI_ISL_1298455	20/02/2021	Mexico City	Human	Female	16	unknown	Original	Oropharyngeal swab
hCoV-19/Mexico/CMX-INMEGEN-03-03-06/2021	EPI_ISL_1298456	20/02/2021	Mexico City	Human	Female	69	unknown	Original	Oropharyngeal swab
hCoV-19/Mexico/CMX-INMEGEN-03-03-15/2021	EPI_ISL_1298465	20/02/2021	Mexico City	Human	Male	49	unknown	Original	Oropharyngeal swab
hCoV-19/Mexico/GUA-InDRE_F10364_S660/2021	EPI_ISL_1359075	20/02/2021	Guanajuato	Human	Male	47	Released	Original	Oropharyngeal swab
hCoV-19/Mexico/GUA-InDRE_F10366_S661/2021	EPI_ISL_1359076	20/02/2021	Guanajuato	Human	Female	50	Released	Original	Oropharyngeal swab
hCoV-19/Mexico/CMX-INER-INMEGEN-00050/202	EPI_ISL_1406763	21/02/2021	Mexico City	Human	Female	50	unknown	Original	
hCoV-19/Mexico/CMX-INER-INMEGEN-00051/202	EPI_ISL_1406764	21/02/2021	Mexico City	Human	Male	20	unknown	Original	
hCoV-19/Mexico/CMX-INMEGEN-03-02-17/2021	EPI_ISL_1181714	21/02/2021	Mexico City	Human	Female	69	unknown	Original	Oropharyngeal swab
hCoV-19/Mexico/PUE-InDRE_F10413_S711/2021	EPI_ISL_1366660	21/02/2021	Puebla	Human	Female	28	Released	Original	Oropharyngeal swab
hCoV-19/Mexico/QUE-InDRE_F10607_S725/2021	EPI_ISL_1366674	21/02/2021	Queretaro	Human	Female	29	Released	Original	Oropharyngeal swab
hCoV-19/Mexico/QUE-InDRE_F10608_S726/2021	EPI_ISL_1366675	21/02/2021	Queretaro	Human	Female	36	Hospitalized	Original	Oropharyngeal swab
hCoV-19/Mexico/VER-InDRE_F10681_S737/2021	EPI_ISL_1366686	21/02/2021	Veracruz	Human	Female	74	Released	Original	Oropharyngeal swab
hCoV-19/Mexico/JAL-InDRE_F10948_S752/2021	EPI_ISL_1400298	21/02/2021	Jalisco	Human	Female	66	Hospitalized	Original	Oropharyngeal swab
hCoV-19/Mexico/GUA-InDRE_F10831_S795/2021	EPI_ISL_1400340	21/02/2021	Guanajuato	Human	Female	81	Released	Original	Oropharyngeal swab
hCoV-19/Mexico/YUC-InDRE_F10541_S812/2021	EPI_ISL_1424014	21/02/2021	Yucatan	Human	Male	31	Released	Original	Oropharyngeal swab
hCoV-19/Mexico/MOR-InDRE_F10665_S816/2021	EPI_ISL_1424018	21/02/2021	Morelos	Human	Male	44	Hospitalized	Original	Oropharyngeal swab
hCoV-19/Mexico/MOR-InDRE_F10908_S864/2021	EPI_ISL_1399269	21/02/2021	Morelos	Human	Male	64	Hospitalized	Original	Oropharyngeal swab
hCoV-19/Mexico/JAL-InDRE_F10947_S865/2021	EPI_ISL_1399270	21/02/2021	Jalisco	Human	Male	28	Released	Original	Oropharyngeal swab
hCoV-19/Mexico/MOR-InDRE_F10916_S763/2021	EPI_ISL_1400309	21/02/2021	Morelos	Human	Female	47	Hospitalized	Original	Oropharyngeal swab
hCoV-19/Mexico/NLE-InDRE_F10799_S707/2021	EPI_ISL_1365672	22/02/2021	Nuevo Leon	Human	Female	50	Released	Original	Oropharyngeal swab
hCoV-19/Mexico/NLE-InDRE_F10802_S708/2021	EPI_ISL_1365673	22/02/2021	Nuevo Leon	Human	Male	79	Hospitalized	Original	Oropharyngeal swab
hCoV-19/Mexico/CMX-INER-INMEGEN-00052/202	EPI_ISL_1406765	22/02/2021	Mexico City	Human	Male	27	unknown	Original	
hCoV-19/Mexico/CMX-INER-INMEGEN-00053/202	EPI_ISL_1406766	22/02/2021	Mexico City	Human	Female	20	unknown	Original	
hCoV-19/Mexico/CMX-INMEGEN-04-04-52/2021	EPI_ISL_1628563	22/02/2021	Mexico City	Human	Male	22	unknown	Original	Oropharyngeal swab
hCoV-19/Mexico/CMX-INMEGEN-03-02-18/2021	EPI_ISL_1181715	22/02/2021	Mexico City	Human	Male	52	unknown	Original	Oropharyngeal swab
hCoV-19/Mexico/CMX-INMEGEN-03-02-20/2021	EPI_ISL_1181717	22/02/2021	Mexico City	Human	Female	27	unknown	Original	Oropharyngeal swab
hCoV-19/Mexico/CMX-INMEGEN-03-02-21/2021	EPI_ISL_1181718	22/02/2021	Mexico City	Human	Female	21	unknown	Original	Oropharyngeal swab
hCoV-19/Mexico/GUA-InDRE_F10833_S574/2021	EPI_ISL_1334386	22/02/2021	Guanajuato	Human	Female	48	Released	Original	Oropharyngeal swab
hCoV-19/Mexico/HID-InDRE_F10781_S741/2021	EPI_ISL_1366690	22/02/2021	Hidalgo	Human	Female	50	Deceased	Original	Oropharyngeal swab
hCoV-19/Mexico/NLE-InDRE_F10792_S744/2021	EPI_ISL_1366693	22/02/2021	Nuevo Leon	Human	Male	51	Released	Original	Oropharyngeal swab
hCoV-19/Mexico/NLE-InDRE_F10801_S748/2021	EPI_ISL_1366697	22/02/2021	Nuevo Leon	Human	Female	60	Deceased	Original	Oropharyngeal swab
hCoV-19/Mexico/GUA-InDRE_F10816_S751/2021	EPI_ISL_1366700	22/02/2021	Guanajuato	Human	Male	65	Hospitalized	Original	Oropharyngeal swab
hCoV-19/Mexico/CHH-InDRE_F11007_S878/2021	EPI_ISL_1405905	22/02/2021	Chihuahua	Human	Female	32	Released	Original	Oropharyngeal swab
hCoV-19/Mexico/GUA-InDRE_F10832_S893/2021	EPI_ISL_1405914	22/02/2021	Guanajuato	Human	Male	51	Released	Original	Oropharyngeal swab
hCoV-19/Mexico/COA-InDRE_F11070_S842/2021	EPI_ISL_1424044	22/02/2021	Coahuila	Human	Male	86	Hospitalized	Original	Oropharyngeal swab
hCoV-19/Mexico/COA-InDRE_F11072_S843/2021	EPI_ISL_1424045	22/02/2021	Coahuila	Human	Female	53	Hospitalized	Original	Oropharyngeal swab
hCoV-19/Mexico/CMX-INMEGEN-03-02-23/2021	EPI_ISL_1193558	22/02/2021	Mexico City	Human	Male	56	unknown	Original	
hCoV-19/Mexico/NLE-InDRE_F10803_S860/2021	EPI_ISL_1399265	22/02/2021	Nuevo Leon	Human	Male	39	Released	Original	Oropharyngeal swab
hCoV-19/Mexico/NLE-InDRE_F10804_S861/2021	EPI_ISL_1399266	22/02/2021	Nuevo Leon	Human	Female	36	Released	Original	Oropharyngeal swab

hCoV-19/Mexico/CHH-InDRE_F11009_S866/2021	EPI_ISL_1399271	22/02/2021	Chihuahua	Human	Female	18	Released	Original	Oropharyngeal swab
hCoV-19/Mexico/COA-InDRE_F11071_S873/2021	EPI_ISL_1399278	22/02/2021	Coahuila	Human	Male	55	Deceased	Original	Oropharyngeal swab
hCoV-19/Mexico/MOR-InDRE_F10927_S759/2021	EPI_ISL_1400305	22/02/2021	Morelos	Human	Male	46	Hospitalized	Original	Oropharyngeal swab
hCoV-19/Mexico/MOR-InDRE_F10926_S760/2021	EPI_ISL_1400306	22/02/2021	Morelos	Human	Female	33	Hospitalized	Original	Oropharyngeal swab
hCoV-19/Mexico/MOR-InDRE_F10922_S762/2021	EPI_ISL_1400308	22/02/2021	Morelos	Human	Male	45	Hospitalized	Original	Oropharyngeal swab
hCoV-19/Mexico/PUE-InDRE_F10901_S766/2021	EPI_ISL_1400311	22/02/2021	Puebla	Human	Female	36	Released	Original	Oropharyngeal swab
hCoV-19/Mexico/PUE-InDRE_F10895_S767/2021	EPI_ISL_1400312	22/02/2021	Puebla	Human	Female	86	Deceased	Original	Oropharyngeal swab
hCoV-19/Mexico/PUE-InDRE_F10894_S768/2021	EPI_ISL_1400313	22/02/2021	Puebla	Human	Male	36	Hospitalized	Original	Oropharyngeal swab
hCoV-19/Mexico/GUA-InDRE_F10854_S783/2021	EPI_ISL_1400328	22/02/2021	Guanajuato	Human	Female	57	Released	Original	Oropharyngeal swab
hCoV-19/Mexico/GUA-InDRE_F10846_S786/2021	EPI_ISL_1400331	22/02/2021	Guanajuato	Human	Female	60	Released	Original	Oropharyngeal swab
hCoV-19/Mexico/GUA-InDRE_F10835_S793/2021	EPI_ISL_1400338	22/02/2021	Guanajuato	Human	Female	53	Released	Original	Oropharyngeal swab
hCoV-19/Mexico/GUA-InDRE_F10834_S794/2021	EPI_ISL_1400339	22/02/2021	Guanajuato	Human	Female	70	Released	Original	Oropharyngeal swab
hCoV-19/Mexico/CMX-INMEGEN-03-11-03/2021	EPI_ISL_1591462	22/02/2021	Mexico City	Human	Male	21	unknown	Original	Oropharyngeal swab
hCoV-19/Mexico/CMX-INMEGEN-03-11-11/2021	EPI_ISL_1591470	22/02/2021	Mexico City	Human	Female	47	unknown	Original	Oropharyngeal swab
hCoV-19/Mexico/CMX-INMEGEN-03-11-19/2021	EPI_ISL_1591478	22/02/2021	Mexico City	Human	Female	41	unknown	Original	Oropharyngeal swab
hCoV-19/Mexico/CMX-INMEGEN-03-11-27/2021	EPI_ISL_1591484	22/02/2021	Mexico City	Human	Male	13	unknown	Original	Oropharyngeal swab
hCoV-19/Mexico/CMX-INMEGEN-03-11-32/2021	EPI_ISL_1591489	22/02/2021	Mexico City	Human	Male	52	unknown	Original	Oropharyngeal swab
hCoV-19/Mexico/CMX-INMEGEN-03-11-35/2021	EPI_ISL_1591491	22/02/2021	Mexico City	Human	Female	43	unknown	Original	Oropharyngeal swab
hCoV-19/Mexico/CMX-INMEGEN-03-11-40/2021	EPI_ISL_1591495	22/02/2021	Mexico City	Human	Male	41	unknown	Original	Oropharyngeal swab
hCoV-19/Mexico/CMX-INMEGEN-03-03-07/2021	EPI_ISL_1298457	22/02/2021	Mexico City	Human	Male	29	unknown	Original	Oropharyngeal swab
hCoV-19/Mexico/CMX-INMEGEN-03-03-08/2021	EPI_ISL_1298458	22/02/2021	Mexico City	Human	Male	68	unknown	Original	Oropharyngeal swab
hCoV-19/Mexico/CMX-INMEGEN-03-03-09/2021	EPI_ISL_1298459	22/02/2021	Mexico City	Human	Male	41	unknown	Original	Oropharyngeal swab
hCoV-19/Mexico/CMX-INMEGEN-03-03-10/2021	EPI_ISL_1298460	22/02/2021	Mexico City	Human	Male	50	unknown	Original	Oropharyngeal swab
hCoV-19/Mexico/CMX-INMEGEN-03-03-11/2021	EPI_ISL_1298461	22/02/2021	Mexico City	Human	Male	12	unknown	Original	Oropharyngeal swab
hCoV-19/Mexico/CMX-INMEGEN-03-03-12/2021	EPI_ISL_1298462	22/02/2021	Mexico City	Human	Male	43	unknown	Original	Oropharyngeal swab
hCoV-19/Mexico/CMX-INMEGEN-03-03-13/2021	EPI_ISL_1298463	22/02/2021	Mexico City	Human	Female	64	unknown	Original	Oropharyngeal swab
hCoV-19/Mexico/CMX-INMEGEN-03-03-14/2021	EPI_ISL_1298464	22/02/2021	Mexico City	Human	Female	54	unknown	Original	Oropharyngeal swab
hCoV-19/Mexico/CMX-INMEGEN-03-03-16/2021	EPI_ISL_1298466	22/02/2021	Mexico City	Human	Female	29	unknown	Original	Oropharyngeal swab
hCoV-19/Mexico/CMX-INMEGEN-03-03-18/2021	EPI_ISL_1298468	22/02/2021	Mexico City	Human	Female	52	unknown	Original	Oropharyngeal swab
hCoV-19/Mexico/CMX-INMEGEN-03-03-22/2021	EPI_ISL_1298472	22/02/2021	Mexico City	Human	Female	32	unknown	Original	Oropharyngeal swab
hCoV-19/Mexico/CMX-INMEGEN-03-05-16/2021	EPI_ISL_1300425	22/02/2021	Mexico City	Human	Female	37	unknown	Original	
hCoV-19/Mexico/TAM-InDRE_F87L_S647/2021	EPI_ISL_1359062	22/02/2021	Tamaulipas	Human	Male	57	Released	Original	Oropharyngeal swab
hCoV-19/Mexico/CMX-INMEGEN-03-11-59/2021	EPI_ISL_1591513	22/02/2021	Mexico City	Human	Male	36	unknown	Original	Oropharyngeal swab
hCoV-19/Mexico/BCN-InDRE_F10713_S697/2021	EPI_ISL_1365662	23/02/2021	Coahuila	Human	Female	61	Released	Original	Oropharyngeal swab
hCoV-19/Mexico/COA-InDRE_F10757_S699/2021	EPI_ISL_1365664	23/02/2021	Coahuila	Human	Female	61	Released	Original	Oropharyngeal swab
hCoV-19/Mexico/NLE-InDRE_F10791_S703/2021	EPI_ISL_1365668	23/02/2021	Nuevo Leon	Human	Male	22	Released	Original	Oropharyngeal swab
hCoV-19/Mexico/NLE-InDRE_F10811_S709/2021	EPI_ISL_1365674	23/02/2021	Nuevo Leon	Human	Male	37	Released	Original	Oropharyngeal swab
hCoV-19/Mexico/GUA-InDRE_F10817_S710/2021	EPI_ISL_1365675	23/02/2021	Guanajuato	Human	Female	58	Hospitalized	Original	Oropharyngeal swab
hCoV-19/Mexico/CMX-INER-INMEGEN-00054/202	EPI_ISL_1406767	23/02/2021	Mexico City	Human	Male	46	unknown	Original	
hCoV-19/Mexico/CMX-INER-INMEGEN-00055/202	EPI_ISL_1406768	23/02/2021	Mexico City	Human	Male	56	unknown	Original	
hCoV-19/Mexico/CMX-INER-INMEGEN-00056/202	EPI_ISL_1406769	23/02/2021	Mexico City	Human	Female	74	unknown	Original	

hCoV-19/Mexico/CMX-INER-INMEGEN-00057/202	EPI_ISL_1406770	23/02/2021	Mexico City	Human	Male	37	unknown	Original	
hCoV-19/Mexico/CMX-INER-INMEGEN-00058/202	EPI_ISL_1406771	23/02/2021	Mexico City	Human	Female	70	unknown	Original	
hCoV-19/Mexico/CMX-INER-INMEGEN-00059/202	EPI_ISL_1406772	23/02/2021	Mexico City	Human	Female	83	unknown	Original	
hCoV-19/Mexico/CMX-INER-INMEGEN-00060/202	EPI_ISL_1406773	23/02/2021	Mexico City	Human	Male	59	unknown	Original	
hCoV-19/Mexico/CMX-INER-INMEGEN-00061/202	EPI_ISL_1406774	23/02/2021	Mexico City	Human	Female	50	unknown	Original	
hCoV-19/Mexico/GUA-InDRE_F10838_S601/2021	EPI_ISL_1337384	23/02/2021	Guanajuato	Human	Male	72	Released	Original	Oropharyngeal swab
hCoV-19/Mexico/QUE-InDRE_F10614_S727/2021	EPI_ISL_1366676	23/02/2021	Queretaro	Human	Male	34	Released	Original	Oropharyngeal swab
hCoV-19/Mexico/HID-InDRE_F10786_S742/2021	EPI_ISL_1366691	23/02/2021	Hidalgo	Human	Female	55	Released	Original	Oropharyngeal swab
hCoV-19/Mexico/NLE-InDRE_F10793_S745/2021	EPI_ISL_1366694	23/02/2021	Nuevo Leon	Human	Female	46	Hospitalized	Original	Oropharyngeal swab
hCoV-19/Mexico/NLE-InDRE_F10800_S747/2021	EPI_ISL_1366696	23/02/2021	Nuevo Leon	Human	Male	66	Released	Original	Oropharyngeal swab
hCoV-19/Mexico/NLE-InDRE_F10807_S749/2021	EPI_ISL_1366698	23/02/2021	Nuevo Leon	Human	Male	52	Released	Original	Oropharyngeal swab
hCoV-19/Mexico/GUA-InDRE_F10821_S801/2021	EPI_ISL_1400346	23/02/2021	Guanajuato	Human	Female	59	Hospitalized	Original	Oropharyngeal swab
hCoV-19/Mexico/GUA-InDRE_F10844_S892/2021	EPI_ISL_1405912	23/02/2021	Guanajuato	Human	Male	57	Released	Original	Oropharyngeal swab
hCoV-19/Mexico/CHH-InDRE_F11012_S819/2021	EPI_ISL_1424021	23/02/2021	Chiapas	Human	Male	43	Released	Original	Oropharyngeal swab
hCoV-19/Mexico/TAM-InDRE_F11060_S839/2021	EPI_ISL_1424041	23/02/2021	Tamaulipas	Human	Male	64	Released	Original	Oropharyngeal swab
hCoV-19/Mexico/CMX-InDRE_F11120_S850/2021	EPI_ISL_1424051	23/02/2021	Mexico City	Human	Female	71	Released	Original	Oropharyngeal swab
hCoV-19/Mexico/CMX-INMEGEN-03-04-01/2021	EPI_ISL_1205203	23/02/2021	Mexico City	Human	Male	81	unknown	Original	Oropharyngeal swab
hCoV-19/Mexico/CMX-INMEGEN-03-04-03/2021	EPI_ISL_1205205	23/02/2021	Mexico City	Human	Male	19	unknown	Original	Oropharyngeal swab
hCoV-19/Mexico/CMX-INMEGEN-03-04-05/2021	EPI_ISL_1205207	23/02/2021	Mexico City	Human	Female	62	unknown	Original	Oropharyngeal swab
hCoV-19/Mexico/CMX-INMEGEN-03-04-06/2021	EPI_ISL_1205208	23/02/2021	Mexico City	Human	Male	65	unknown	Original	Oropharyngeal swab
hCoV-19/Mexico/NLE-InDRE_F10806_S862/2021	EPI_ISL_1399267	23/02/2021	Nuevo Leon	Human	Male	74	Hospitalized	Original	Oropharyngeal swab
hCoV-19/Mexico/NLE-InDRE_F10808_S863/2021	EPI_ISL_1399268	23/02/2021	Nuevo Leon	Human	Male	85	Hospitalized	Original	Oropharyngeal swab
hCoV-19/Mexico/NAY-InDRE_F11035_S868/2021	EPI_ISL_1399273	23/02/2021	Nayarit	Human	Male	75	Released	Original	Oropharyngeal swab
hCoV-19/Mexico/GUA-InDRE_F10839_S758/2021	EPI_ISL_1400304	23/02/2021	Guanajuato	Human	Female	17	Released	Original	Oropharyngeal swab
hCoV-19/Mexico/MOR-InDRE_F10923_S761/2021	EPI_ISL_1400307	23/02/2021	Morelos	Human	Male	31	Released	Original	Oropharyngeal swab
hCoV-19/Mexico/GUA-InDRE_F10855_S782/2021	EPI_ISL_1400327	23/02/2021	Guanajuato	Human	Female	38	Released	Original	Oropharyngeal swab
hCoV-19/Mexico/GUA-InDRE_F10848_S784/2021	EPI_ISL_1400329	23/02/2021	Guanajuato	Human	Male	33	Released	Original	Oropharyngeal swab
hCoV-19/Mexico/GUA-InDRE_F10845_S787/2021	EPI_ISL_1400332	23/02/2021	Guanajuato	Human	Female	42	Released	Original	Oropharyngeal swab
hCoV-19/Mexico/GUA-InDRE_F10841_S790/2021	EPI_ISL_1400335	23/02/2021	Guanajuato	Human	Female	49	Released	Original	Oropharyngeal swab
hCoV-19/Mexico/GUA-InDRE_F10840_S791/2021	EPI_ISL_1400336	23/02/2021	Guanajuato	Human	Male	46	Released	Original	Oropharyngeal swab
hCoV-19/Mexico/GUA-InDRE_F10837_S792/2021	EPI_ISL_1400337	23/02/2021	Guanajuato	Human	Female	18	Released	Original	Oropharyngeal swab
hCoV-19/Mexico/CMX-INMEGEN-03-03-17/2021	EPI_ISL_1298467	23/02/2021	Mexico City	Human	Female	59	unknown	Original	Oropharyngeal swab
hCoV-19/Mexico/CMX-INMEGEN-03-03-19/2021	EPI_ISL_1298469	23/02/2021	Mexico City	Human	Male	6	unknown	Original	Oropharyngeal swab
hCoV-19/Mexico/CMX-INMEGEN-03-03-20/2021	EPI_ISL_1298470	23/02/2021	Mexico City	Human	Female	76	unknown	Original	Oropharyngeal swab
hCoV-19/Mexico/CMX-INMEGEN-03-11-51/2021	EPI_ISL_1591505	23/02/2021	Mexico City	Human	Female	39	unknown	Original	Oropharyngeal swab
hCoV-19/Mexico/COA-InDRE_F10759_S700/2021	EPI_ISL_1365665	24/02/2021	Coahuila	Human	Male	38	Released	Original	Oropharyngeal swab
hCoV-19/Mexico/NLE-InDRE_F10795_S704/2021	EPI_ISL_1365669	24/02/2021	Nuevo Leon	Human	Female	24	Released	Original	Oropharyngeal swab
hCoV-19/Mexico/NLE-InDRE_F10796_S705/2021	EPI_ISL_1365670	24/02/2021	Nuevo Leon	Human	Female	70	Released	Original	Oropharyngeal swab
hCoV-19/Mexico/NLE-InDRE_F10797_S706/2021	EPI_ISL_1365671	24/02/2021	Nuevo Leon	Human	Female	22	Released	Original	Oropharyngeal swab
hCoV-19/Mexico/CMX-INER-INMEGEN-00001/202	EPI_ISL_1406722	24/02/2021	Mexico City	Human	Male	10	unknown	Original	
hCoV-19/Mexico/CMX-INER-INMEGEN-00002/202	EPI_ISL_1406723	24/02/2021	Mexico City	Human	Female	69	unknown	Original	

hCoV-19/Mexico/CMX-INER-INMEGEN-00003/202	EPI_ISL_1406724	24/02/2021	Mexico City	Human	Male	29	unknown	Original	
hCoV-19/Mexico/CMX-INER-INMEGEN-00062/202	EPI_ISL_1406775	24/02/2021	Mexico City	Human	Female	21	unknown	Original	
hCoV-19/Mexico/CMX-INMEGEN-04-04-25/2021	EPI_ISL_1628541	24/02/2021	Mexico City	Human	Male	41	unknown	Original	Oropharyngeal swab
hCoV-19/Mexico/CMX-INMEGEN-04-04-33/2021	EPI_ISL_1628548	24/02/2021	Mexico City	Human	Male	43	unknown	Original	Oropharyngeal swab
hCoV-19/Mexico/CMX-INMEGEN-04-04-57/2021	EPI_ISL_1628566	24/02/2021	Mexico City	Human	Male	36	unknown	Original	Oropharyngeal swab
hCoV-19/Mexico/CMX-INMEGEN-03-05-01/2021	EPI_ISL_1262702	24/02/2021	Mexico City	Human	Female	36	unknown	Original	Oropharyngeal swab
hCoV-19/Mexico/CMX-INMEGEN-03-05-02/2021	EPI_ISL_1262703	24/02/2021	Mexico City	Human	Female	15	unknown	Original	Oropharyngeal swab
hCoV-19/Mexico/CMX-INMEGEN-03-05-03/2021	EPI_ISL_1262704	24/02/2021	Mexico City	Human	Male	58	unknown	Original	Oropharyngeal swab
hCoV-19/Mexico/CMX-INMEGEN-03-05-04/2021	EPI_ISL_1262705	24/02/2021	Mexico City	Human	Male	28	unknown	Original	Oropharyngeal swab
hCoV-19/Mexico/CMX-INMEGEN-03-05-05/2021	EPI_ISL_1262706	24/02/2021	Mexico City	Human	Male	45	unknown	Original	Oropharyngeal swab
hCoV-19/Mexico/CMX-INMEGEN-03-05-07/2021	EPI_ISL_1262707	24/02/2021	Mexico City	Human	Male	50	unknown	Original	Oropharyngeal swab
hCoV-19/Mexico/CMX-INMEGEN-03-05-08/2021	EPI_ISL_1262708	24/02/2021	Mexico City	Human	Male	64	unknown	Original	Oropharyngeal swab
hCoV-19/Mexico/CMX-INMEGEN-03-05-09/2021	EPI_ISL_1262709	24/02/2021	Mexico City	Human	Male	9	unknown	Original	Oropharyngeal swab
hCoV-19/Mexico/CMX-INMEGEN-03-05-10/2021	EPI_ISL_1262710	24/02/2021	Mexico City	Human	Female	53	unknown	Original	Oropharyngeal swab
hCoV-19/Mexico/CMX-INMEGEN-03-05-12/2021	EPI_ISL_1262711	24/02/2021	Mexico City	Human	Female	58	unknown	Original	Oropharyngeal swab
hCoV-19/Mexico/CMX-INMEGEN-03-05-13/2021	EPI_ISL_1262712	24/02/2021	Mexico City	Human	Female	64	unknown	Original	Oropharyngeal swab
hCoV-19/Mexico/CMX-INMEGEN-03-05-14/2021	EPI_ISL_1262713	24/02/2021	Mexico City	Human	Male	34	unknown	Original	Oropharyngeal swab
hCoV-19/Mexico/CMX-INMEGEN-03-05-15/2021	EPI_ISL_1262714	24/02/2021	Mexico City	Human	Male	31	unknown	Original	Oropharyngeal swab
hCoV-19/Mexico/CMX-INMEGEN-03-05-18/2021	EPI_ISL_1262715	24/02/2021	Mexico City	Human	Male	35	unknown	Original	Oropharyngeal swab
hCoV-19/Mexico/CMX-INMEGEN-03-05-19/2021	EPI_ISL_1262716	24/02/2021	Mexico City	Human	Male	32	unknown	Original	Oropharyngeal swab
hCoV-19/Mexico/TAM-InDRE_F108L_S569/2021	EPI_ISL_1334382	24/02/2021	Tamaulipas	Human	Male	46	Released	Original	Oropharyngeal swab
hCoV-19/Mexico/TAM-InDRE_F114L_S572/2021	EPI_ISL_1334385	24/02/2021	Tamaulipas	Human	Male	44	Released	Original	Oropharyngeal swab
hCoV-19/Mexico/GUA-InDRE_F10853_S598/2021	EPI_ISL_1337380	24/02/2021	Guanajuato	Human	Female	39	Released	Original	Oropharyngeal swab
hCoV-19/Mexico/GUA-InDRE_F10852_S599/2021	EPI_ISL_1337382	24/02/2021	Guanajuato	Human	Female	22	Released	Original	Oropharyngeal swab
hCoV-19/Mexico/GUA-InDRE_F10851_S600/2021	EPI_ISL_1337383	24/02/2021	Guanajuato	Human	Male	33	Released	Original	Oropharyngeal swab
hCoV-19/Mexico/GUA-InDRE_F10830_S602/2021	EPI_ISL_1337386	24/02/2021	Guanajuato	Human	Male	55	Deceased	Original	Oropharyngeal swab
hCoV-19/Mexico/SIN-InDRE_F10813_S604/2021	EPI_ISL_1337389	24/02/2021	Sinaloa	Human	Male	84	Hospitalized	Original	Oropharyngeal swab
hCoV-19/Mexico/NLE-InDRE_F10794_S746/2021	EPI_ISL_1366695	24/02/2021	Nuevo Leon	Human	Female	36	Released	Original	Oropharyngeal swab
hCoV-19/Mexico/NLE-InDRE_F10809_S750/2021	EPI_ISL_1366699	24/02/2021	Nuevo Leon	Human	Female	52	Released	Original	Oropharyngeal swab
hCoV-19/Mexico/GUA-InDRE_F10820_S802/2021	EPI_ISL_1400347	24/02/2021	Guanajuato	Human	Male	78	Hospitalized	Original	Oropharyngeal swab
hCoV-19/Mexico/GUA-InDRE_F10858_S889/2021	EPI_ISL_1405907	24/02/2021	Guanajuato	Human	Male	38	Released	Original	Oropharyngeal swab
hCoV-19/Mexico/GUA-InDRE_F10850_S890/2021	EPI_ISL_1405909	24/02/2021	Guanajuato	Human	Female	21	Released	Original	Oropharyngeal swab
hCoV-19/Mexico/GUA-InDRE_F10849_S891/2021	EPI_ISL_1405910	24/02/2021	Guanajuato	Human	Female	14	Released	Original	Oropharyngeal swab
hCoV-19/Mexico/JAL-InDRE_F10958_S880/2021	EPI_ISL_1405911	24/02/2021	Jalisco	Human	Female	64	Released	Original	Oropharyngeal swab
hCoV-19/Mexico/JAL-InDRE_F10954_S881/2021	EPI_ISL_1405913	24/02/2021	Jalisco	Human	Female	48	Released	Original	Oropharyngeal swab
hCoV-19/Mexico/MOR-InDRE_F10934_S818/2021	EPI_ISL_1424020	24/02/2021	Morelos	Human	Male	53	Released	Original	Oropharyngeal swab
hCoV-19/Mexico/GRO-InDRE_F11063_S841/2021	EPI_ISL_1424043	24/02/2021	Guerrero	Human	Male	67	Deceased	Original	Oropharyngeal swab
hCoV-19/Mexico/VER-InDRE_F11087_S846/2021	EPI_ISL_1424047	24/02/2021	Veracruz	Human	Male	60	Hospitalized	Original	Oropharyngeal swab
hCoV-19/Mexico/VER-InDRE_F11090_S847/2021	EPI_ISL_1424048	24/02/2021	Veracruz	Human	Female	71	Released	Original	Oropharyngeal swab
hCoV-19/Mexico/CMX-InDRE_F11123_S851/2021	EPI_ISL_1424052	24/02/2021	Mexico City	Human	Male	57	Hospitalized	Original	Oropharyngeal swab
hCoV-19/Mexico/CMX-INMEGEN-03-04-02/2021	EPI_ISL_1205204	24/02/2021	Mexico City	Human	Female	83	unknown	Original	Oropharyngeal swab

hCoV-19/Mexico/CMX-INMEGEN-03-04-04/2021	EPI_ISL_1205206	24/02/2021	Mexico City	Human	Male	40	unknown	Original	Oropharyngeal swab
hCoV-19/Mexico/CMX-INMEGEN-03-05-11/2021	EPI_ISL_1262642	24/02/2021	Mexico City	Human	Male	30	unknown	Original	Oropharyngeal swab
hCoV-19/Mexico/MOR-InDRE_F10935_S755/2021	EPI_ISL_1400301	24/02/2021	Morelos	Human	Male	58	Released	Original	Oropharyngeal swab
hCoV-19/Mexico/MOR-InDRE_F10933_S756/2021	EPI_ISL_1400302	24/02/2021	Morelos	Human	Male	63	Hospitalized	Original	Oropharyngeal swab
hCoV-19/Mexico/MOR-InDRE_F10930_S757/2021	EPI_ISL_1400303	24/02/2021	Morelos	Human	Male	67	Hospitalized	Original	Oropharyngeal swab
hCoV-19/Mexico/GUA-InDRE_F10857_S780/2021	EPI_ISL_1400325	24/02/2021	Guanajuato	Human	Female	38	Released	Original	Oropharyngeal swab
hCoV-19/Mexico/GUA-InDRE_F10856_S781/2021	EPI_ISL_1400326	24/02/2021	Guanajuato	Human	Female	19	Released	Original	Oropharyngeal swab
hCoV-19/Mexico/GUA-InDRE_F10843_S788/2021	EPI_ISL_1400333	24/02/2021	Guanajuato	Human	Female	31	Released	Original	Oropharyngeal swab
hCoV-19/Mexico/GUA-InDRE_F10842_S789/2021	EPI_ISL_1400334	24/02/2021	Guanajuato	Human	Male	11	Released	Original	Oropharyngeal swab
hCoV-19/Mexico/CMX-INMEGEN-03-11-43/2021	EPI_ISL_1591497	24/02/2021	Mexico City	Human	Female	44	unknown	Original	Oropharyngeal swab
hCoV-19/Mexico/CMX-INMEGEN-03-03-23/2021	EPI_ISL_1298473	24/02/2021	Mexico City	Human	Male	40	unknown	Original	Oropharyngeal swab
hCoV-19/Mexico/CMX-INMEGEN-03-08-15/2021	EPI_ISL_1315492	24/02/2021	Mexico City	Human	Male	47	unknown	Original	Oropharyngeal swab
hCoV-19/Mexico/CMX-INMEGEN-03-08-16/2021	EPI_ISL_1315493	24/02/2021	Mexico City	Human	Male	73	unknown	Original	Oropharyngeal swab
hCoV-19/Mexico/CMX-InDRE_F93B_S648/2021	EPI_ISL_1359063	24/02/2021	Mexico City	Human	Female	72	Released	Original	Oropharyngeal swab
hCoV-19/Mexico/CMX-INMEGEN-03-08-01/2021	EPI_ISL_1315479	25/02/2021	Mexico City	Human	Female	58	unknown	Original	Oropharyngeal swab
hCoV-19/Mexico/COA-InDRE_F10764_S701/2021	EPI_ISL_1365666	25/02/2021	Coahuila	Human	Male	79	Deceased	Original	Oropharyngeal swab
hCoV-19/Mexico/CMX-INER-INMEGEN-00005/202	EPI_ISL_1406725	25/02/2021	Mexico City	Human	Female	36	unknown	Original	
hCoV-19/Mexico/CMX-INER-INMEGEN-00006/202	EPI_ISL_1406726	25/02/2021	Mexico City	Human	Female	35	unknown	Original	
hCoV-19/Mexico/CMX-INER-INMEGEN-00063/202	EPI_ISL_1406776	25/02/2021	Mexico City	Human	Female	39	unknown	Original	
hCoV-19/Mexico/SIN-CIAD-HJ1154/2021	EPI_ISL_1482628	25/02/2021	Sinaloa	Human	Female	62	unknown	Original	Oropharyngeal swab
hCoV-19/Mexico/CMX-INMEGEN-04-04-2/2021	EPI_ISL_1628521	25/02/2021	Mexico City	Human	Male	53	unknown	Original	Oropharyngeal swab
hCoV-19/Mexico/CMX-INMEGEN-04-04-18/2021	EPI_ISL_1628534	25/02/2021	Mexico City	Human	Male	56	unknown	Original	Oropharyngeal swab
hCoV-19/Mexico/CMX-INMEGEN-04-04-34/2021	EPI_ISL_1628549	25/02/2021	Mexico City	Human	Male	13	unknown	Original	Oropharyngeal swab
hCoV-19/Mexico/CMX-INMEGEN-04-04-49/2021	EPI_ISL_1628560	25/02/2021	Mexico City	Human	Female	22	unknown	Original	Oropharyngeal swab
hCoV-19/Mexico/CMX-INMEGEN-04-04-73/2021	EPI_ISL_1628579	25/02/2021	Mexico City	Human	Male	44	unknown	Original	Oropharyngeal swab
hCoV-19/Mexico/CMX-INMEGEN-04-04-89/2021	EPI_ISL_1628590	25/02/2021	Mexico City	Human	Male	45	unknown	Original	Oropharyngeal swab
hCoV-19/Mexico/CMX-INMEGEN-03-05-36/2021	EPI_ISL_1262700	25/02/2021	Mexico City	Human	Female	84	unknown	Original	Oropharyngeal swab
hCoV-19/Mexico/CMX-INMEGEN-03-05-40/2021	EPI_ISL_1262701	25/02/2021	Mexico City	Human	Female	46	unknown	Original	Oropharyngeal swab
hCoV-19/Mexico/CMX-INMEGEN-03-06-14/2021	EPI_ISL_1265904	25/02/2021	Mexico City	Human	Male	12	unknown	Original	Oropharyngeal swab
hCoV-19/Mexico/CMX-INMEGEN-03-06-15/2021	EPI_ISL_1265905	25/02/2021	Mexico City	Human	Male	48	unknown	Original	Oropharyngeal swab
hCoV-19/Mexico/CMX-INMEGEN-03-06-17/2021	EPI_ISL_1265907	25/02/2021	Mexico City	Human	Female	58	unknown	Original	Oropharyngeal swab
hCoV-19/Mexico/CMX-INMEGEN-03-06-18/2021	EPI_ISL_1265908	25/02/2021	Mexico City	Human	Female	35	unknown	Original	Oropharyngeal swab
hCoV-19/Mexico/GUA-InDRE_F10868_S595/2021	EPI_ISL_1337376	25/02/2021	Guanajuato	Human	Female	37	Released	Original	Oropharyngeal swab
hCoV-19/Mexico/GUA-InDRE_F10862_S596/2021	EPI_ISL_1337377	25/02/2021	Guanajuato	Human	Female	30	Released	Original	Oropharyngeal swab
hCoV-19/Mexico/GUA-InDRE_F10861_S597/2021	EPI_ISL_1337379	25/02/2021	Guanajuato	Human	Female	37	Released	Original	Oropharyngeal swab
hCoV-19/Mexico/GUA-InDRE_F10822_S603/2021	EPI_ISL_1337387	25/02/2021	Guanajuato	Human	Female	74	Hospitalized	Original	Oropharyngeal swab
hCoV-19/Mexico/GUA-InDRE_F10827_S797/2021	EPI_ISL_1400342	25/02/2021	Guanajuato	Human	Male	71	Hospitalized	Original	Oropharyngeal swab
hCoV-19/Mexico/GUA-InDRE_F10825_S798/2021	EPI_ISL_1400343	25/02/2021	Guanajuato	Human	Female	87	Deceased	Original	Oropharyngeal swab
hCoV-19/Mexico/GUA-InDRE_F10824_S799/2021	EPI_ISL_1400344	25/02/2021	Guanajuato	Human	Female	55	Deceased	Original	Oropharyngeal swab
hCoV-19/Mexico/GUA-InDRE_F10823_S800/2021	EPI_ISL_1400345	25/02/2021	Guanajuato	Human	Male	69	Hospitalized	Original	Oropharyngeal swab
hCoV-19/Mexico/GUA-InDRE_F10819_S803/2021	EPI_ISL_1400348	25/02/2021	Guanajuato	Human	Male	66	Hospitalized	Original	Oropharyngeal swab

hCoV-19/Mexico/GUA-InDRE_F10818_S804/2021	EPI_ISL_1400349	25/02/2021	Guanajuato	Human	Male	84	Hospitalized	Original	Oropharyngeal swab
hCoV-19/Mexico/MOR-InDRE_F10942_S882/2021	EPI_ISL_1405899	25/02/2021	Morelos	Human	Female	83	Hospitalized	Original	Oropharyngeal swab
hCoV-19/Mexico/GUA-InDRE_F10866_S887/2021	EPI_ISL_1405904	25/02/2021	Guanajuato	Human	Male	20	Released	Original	Oropharyngeal swab
hCoV-19/Mexico/GUA-InDRE_F10859_S888/2021	EPI_ISL_1405906	25/02/2021	Guanajuato	Human	Female	38	Released	Original	Oropharyngeal swab
hCoV-19/Mexico/JAL-InDRE_F10959_S879/2021	EPI_ISL_1405908	25/02/2021	Jalisco	Human	Male	72	Released	Original	Oropharyngeal swab
hCoV-19/Mexico/QUE-InDRE_F11013_S820/2021	EPI_ISL_1424022	25/02/2021	Queretaro	Human	Female	53	Hospitalized	Original	Oropharyngeal swab
hCoV-19/Mexico/QUE-InDRE_F11015_S821/2021	EPI_ISL_1424023	25/02/2021	Queretaro	Human	Female	39	Released	Original	Oropharyngeal swab
hCoV-19/Mexico/QUE-InDRE_F11017_S823/2021	EPI_ISL_1424025	25/02/2021	Queretaro	Human	Female	66	Released	Original	Oropharyngeal swab
hCoV-19/Mexico/QUE-InDRE_F11016_S830/2021	EPI_ISL_1424032	25/02/2021	Queretaro	Human	Male	62	Hospitalized	Original	Oropharyngeal swab
hCoV-19/Mexico/MOR-InDRE_F11061_S840/2021	EPI_ISL_1424042	25/02/2021	Morelos	Human	Male	85	Deceased	Original	Oropharyngeal swab
hCoV-19/Mexico/VER-InDRE_F11093_S848/2021	EPI_ISL_1424049	25/02/2021	Veracruz	Human	Female	50	Released	Original	Oropharyngeal swab
hCoV-19/Mexico/CMX-InDRE_F11124_S852/2021	EPI_ISL_1424053	25/02/2021	Mexico City	Human	Male	48	Hospitalized	Original	Oropharyngeal swab
hCoV-19/Mexico/CMX-INMEGEN-03-05-17/2021	EPI_ISL_1262693	25/02/2021	Mexico City	Human	Male	62	unknown	Original	Oropharyngeal swab
hCoV-19/Mexico/CMX-INMEGEN-03-05-22/2021	EPI_ISL_1262694	25/02/2021	Mexico City	Human	Male	20	unknown	Original	Oropharyngeal swab
hCoV-19/Mexico/CMX-INMEGEN-03-05-26/2021	EPI_ISL_1262695	25/02/2021	Mexico City	Human	Male	65	unknown	Original	Oropharyngeal swab
hCoV-19/Mexico/CMX-INMEGEN-03-05-27/2021	EPI_ISL_1262696	25/02/2021	Mexico City	Human	Female	39	unknown	Original	Oropharyngeal swab
hCoV-19/Mexico/CMX-INMEGEN-03-05-28/2021	EPI_ISL_1262697	25/02/2021	Mexico City	Human	Male	41	unknown	Original	Oropharyngeal swab
hCoV-19/Mexico/CMX-INMEGEN-03-05-30/2021	EPI_ISL_1262698	25/02/2021	Mexico City	Human	Male	19	unknown	Original	Oropharyngeal swab
hCoV-19/Mexico/CMX-INMEGEN-03-05-33/2021	EPI_ISL_1262699	25/02/2021	Mexico City	Human	Male	49	unknown	Original	Oropharyngeal swab
hCoV-19/Mexico/NAY-InDRE_F11036_S869/2021	EPI_ISL_1399274	25/02/2021	Nayarit	Human	Female	48	Released	Original	Oropharyngeal swab
hCoV-19/Mexico/GUA-InDRE_F10878_S770/2021	EPI_ISL_1400315	25/02/2021	Guanajuato	Human	Female	58	Released	Original	Oropharyngeal swab
hCoV-19/Mexico/GUA-InDRE_F10877_S771/2021	EPI_ISL_1400316	25/02/2021	Guanajuato	Human	Female	55	Released	Original	Oropharyngeal swab
hCoV-19/Mexico/GUA-InDRE_F10876_S772/2021	EPI_ISL_1400317	25/02/2021	Guanajuato	Human	Male	60	Released	Original	Oropharyngeal swab
hCoV-19/Mexico/GUA-InDRE_F10869_S774/2021	EPI_ISL_1400319	25/02/2021	Guanajuato	Human	Female	53	Released	Original	Oropharyngeal swab
hCoV-19/Mexico/GUA-InDRE_F10867_S775/2021	EPI_ISL_1400320	25/02/2021	Guanajuato	Human	Female	29	Released	Original	Oropharyngeal swab
hCoV-19/Mexico/GUA-InDRE_F10865_S776/2021	EPI_ISL_1400321	25/02/2021	Guanajuato	Human	Male	33	Released	Original	Oropharyngeal swab
hCoV-19/Mexico/GUA-InDRE_F10864_S777/2021	EPI_ISL_1400322	25/02/2021	Guanajuato	Human	Female	60	Released	Original	Oropharyngeal swab
hCoV-19/Mexico/GUA-InDRE_F10863_S778/2021	EPI_ISL_1400323	25/02/2021	Guanajuato	Human	Male	42	Released	Original	Oropharyngeal swab
hCoV-19/Mexico/GUA-InDRE_F10860_S779/2021	EPI_ISL_1400324	25/02/2021	Guanajuato	Human	Female	61	Released	Original	Oropharyngeal swab
hCoV-19/Mexico/CMX-INER-INMEGEN-00004/202	EPI_ISL_1423994	25/02/2021	Mexico City	Human	Female	10	unknown	Original	
hCoV-19/Mexico/CMX-INMEGEN-03-11-04/2021	EPI_ISL_1591463	25/02/2021	Mexico City	Human	Male	13	unknown	Original	Oropharyngeal swab
hCoV-19/Mexico/COA-InDRE_F10766_S702/2021	EPI_ISL_1365667	26/02/2021	Coahuila	Human	Female	67	Released	Original	Oropharyngeal swab
hCoV-19/Mexico/CMX-INER-INMEGEN-00011/202	EPI_ISL_1406730	26/02/2021	Mexico City	Human	Male	62	unknown	Original	
hCoV-19/Mexico/CMX-INMEGEN-04-04-42/2021	EPI_ISL_1628556	26/02/2021	Mexico City	Human	Female	56	unknown	Original	Oropharyngeal swab
hCoV-19/Mexico/CMX-INMEGEN-04-04-74/2021	EPI_ISL_1628580	26/02/2021	Mexico City	Human	Female	60	unknown	Original	Oropharyngeal swab
hCoV-19/Mexico/CMX-INMEGEN-03-06-13/2021	EPI_ISL_1265903	26/02/2021	Mexico City	Human	Female	47	unknown	Original	Oropharyngeal swab
hCoV-19/Mexico/CMX-INMEGEN-03-06-16/2021	EPI_ISL_1265906	26/02/2021	Mexico City	Human	Male	41	unknown	Original	Oropharyngeal swab
hCoV-19/Mexico/BCN-SEARCH-7499/2021	EPI_ISL_1295779	26/02/2021	Baja California	Human	unknown	unknown	unknown	Original	Nasal swab
hCoV-19/Mexico/CAM-InDRE_F10946_S753/2021	EPI_ISL_1400299	26/02/2021	Campeche	Human	Female	54	Hospitalized	Original	Oropharyngeal swab
hCoV-19/Mexico/GUA-InDRE_F10881_S883/2021	EPI_ISL_1405900	26/02/2021	Guanajuato	Human	Female	47	Released	Original	Oropharyngeal swab
hCoV-19/Mexico/GUA-InDRE_F10880_S884/2021	EPI_ISL_1405901	26/02/2021	Guanajuato	Human	Male	48	Released	Original	Oropharyngeal swab

hCoV-19/Mexico/GUA-InDRE_F10879_S885/2021	EPI_ISL_1405902	26/02/2021	Guanajuato	Human	Female	19	Released	Original	Oropharyngeal swab
hCoV-19/Mexico/GUA-InDRE_F10871_S886/2021	EPI_ISL_1405903	26/02/2021	Guanajuato	Human	Female	33	Released	Original	Oropharyngeal swab
hCoV-19/Mexico/GUA-InDRE_F10826_S894/2021	EPI_ISL_1405915	26/02/2021	Guanajuato	Human	Male	66	Hospitalized	Original	Oropharyngeal swab
hCoV-19/Mexico/QUE-InDRE_F11019_S824/2021	EPI_ISL_1424026	26/02/2021	Queretaro	Human	Female	36	Released	Original	Oropharyngeal swab
hCoV-19/Mexico/QUE-InDRE_F11020_S825/2021	EPI_ISL_1424027	26/02/2021	Queretaro	Human	Male	43	Released	Original	Oropharyngeal swab
hCoV-19/Mexico/QUE-InDRE_F11022_S826/2021	EPI_ISL_1424028	26/02/2021	Queretaro	Human	Female	47	Released	Original	Oropharyngeal swab
hCoV-19/Mexico/QUE-InDRE_F11023_S827/2021	EPI_ISL_1424029	26/02/2021	Queretaro	Human	Male	50	Hospitalized	Original	Oropharyngeal swab
hCoV-19/Mexico/CMX-INMEGEN-04-04-66/2021	EPI_ISL_1857295	26/02/2021	Mexico City	Human	Female	59	unknown	Original	Oropharyngeal swab
hCoV-19/Mexico/CMX-INMEGEN-03-04-08/2021	EPI_ISL_1205210	26/02/2021	Mexico City	Human	Female	24	unknown	Original	Oropharyngeal swab
hCoV-19/Mexico/CMX-INMEGEN-03-04-09/2021	EPI_ISL_1205211	26/02/2021	Mexico City	Human	Male	25	unknown	Original	Oropharyngeal swab
hCoV-19/Mexico/CMX-INMEGEN-03-04-10/2021	EPI_ISL_1205212	26/02/2021	Mexico City	Human	Female	38	unknown	Original	Oropharyngeal swab
hCoV-19/Mexico/CMX-INMEGEN-03-04-11/2021	EPI_ISL_1205213	26/02/2021	Mexico City	Human	female	41	unknown	Original	Oropharyngeal swab
hCoV-19/Mexico/CMX-INMEGEN-03-04-12/2021	EPI_ISL_1205214	26/02/2021	Mexico City	Human	male	62	unknown	Original	Oropharyngeal swab
hCoV-19/Mexico/CMX-INMEGEN-03-05-43/2021	EPI_ISL_1262631	26/02/2021	Mexico City	Human	Male	44	unknown	Original	Oropharyngeal swab
hCoV-19/Mexico/CMX-INMEGEN-03-05-37/2021	EPI_ISL_1262638	26/02/2021	Mexico City	Human	Male	49	unknown	Original	Oropharyngeal swab
hCoV-19/Mexico/CMX-INMEGEN-03-05-35/2021	EPI_ISL_1262686	26/02/2021	Mexico City	Human	Male	73	unknown	Original	Oropharyngeal swab
hCoV-19/Mexico/CMX-INMEGEN-03-05-38/2021	EPI_ISL_1262687	26/02/2021	Mexico City	Human	Male	23	unknown	Original	Oropharyngeal swab
hCoV-19/Mexico/CMX-INMEGEN-03-05-41/2021	EPI_ISL_1262688	26/02/2021	Mexico City	Human	Male	56	unknown	Original	Oropharyngeal swab
hCoV-19/Mexico/CMX-INMEGEN-03-05-47/2021	EPI_ISL_1262689	26/02/2021	Mexico City	Human	Male	50	unknown	Original	Oropharyngeal swab
hCoV-19/Mexico/CMX-INMEGEN-03-05-48/2021	EPI_ISL_1262690	26/02/2021	Mexico City	Human	Female	30	unknown	Original	Oropharyngeal swab
hCoV-19/Mexico/CMX-INMEGEN-03-05-74/2021	EPI_ISL_1262691	26/02/2021	Mexico City	Human	Male	53	unknown	Original	Oropharyngeal swab
hCoV-19/Mexico/CMX-INMEGEN-03-05-89/2021	EPI_ISL_1262692	26/02/2021	Mexico City	Human	Male	56	unknown	Original	Oropharyngeal swab
hCoV-19/Mexico/CMX-InDRE_F11121_S877/2021	EPI_ISL_1399281	26/02/2021	Mexico City	Human	Male	68	Released	Original	Oropharyngeal swab
hCoV-19/Mexico/MOR-InDRE_F10943_S754/2021	EPI_ISL_1400300	26/02/2021	Morelos	Human	Male	71	Released	Original	Oropharyngeal swab
hCoV-19/Mexico/GUA-InDRE_F10870_S773/2021	EPI_ISL_1400318	26/02/2021	Guanajuato	Human	Female	30	Released	Original	Oropharyngeal swab
hCoV-19/Mexico/CMX-INER-INMEGEN-00007/202	EPI_ISL_1423995	26/02/2021	Mexico City	Human	Male	12	unknown	Original	
hCoV-19/Mexico/CMX-INMEGEN-03-11-08/2021	EPI_ISL_1591467	26/02/2021	Mexico City	Human	Female	23	unknown	Original	Oropharyngeal swab
hCoV-19/Mexico/CMX-INMEGEN-03-11-12/2021	EPI_ISL_1591471	26/02/2021	Mexico City	Human	Female	31	unknown	Original	Oropharyngeal swab
hCoV-19/Mexico/CMX-INMEGEN-03-11-91/2021	EPI_ISL_1591539	26/02/2021	Mexico City	Human	Female	50	unknown	Original	Oropharyngeal swab
hCoV-19/Mexico/CMX-INMEGEN-03-08-10/2021	EPI_ISL_1315487	26/02/2021	Mexico City	Human	Female	24	unknown	Original	Oropharyngeal swab
hCoV-19/Mexico/CMX-INMEGEN-03-08-47/2021	EPI_ISL_1315522	26/02/2021	Mexico City	Human	Female	44	unknown	Original	Oropharyngeal swab
hCoV-19/Mexico/CMX-INMEGEN-03-11-83/2021	EPI_ISL_1591532	26/02/2021	Mexico City	Human	Female	48	unknown	Original	Oropharyngeal swab
hCoV-19/Mexico/CMX-INER-INMEGEN-00008/202	EPI_ISL_1406727	27/02/2021	Mexico City	Human	Male	55	unknown	Original	
hCoV-19/Mexico/CMX-INER-INMEGEN-00010/202	EPI_ISL_1406729	27/02/2021	Mexico City	Human	Female	38	unknown	Original	
hCoV-19/Mexico/CMX-INER-INMEGEN-00012/202	EPI_ISL_1406731	27/02/2021	Mexico City	Human	Male	68	unknown	Original	
hCoV-19/Mexico/CMX-INER-INMEGEN-00013/202	EPI_ISL_1406732	27/02/2021	Mexico City	Human	Female	30	unknown	Original	
hCoV-19/Mexico/CMX-INMEGEN-04-04-3/2021	EPI_ISL_1628522	27/02/2021	Mexico City	Human	Female	33	unknown	Original	Oropharyngeal swab
hCoV-19/Mexico/CMX-INMEGEN-04-04-90/2021	EPI_ISL_1628591	27/02/2021	Mexico City	Human	Male	54	unknown	Original	Oropharyngeal swab
hCoV-19/Mexico/CMX-INMEGEN-03-06-12/2021	EPI_ISL_1265902	27/02/2021	Mexico City	Human	Male	55	unknown	Original	Oropharyngeal swab
hCoV-19/Mexico/ROO-InDRE_F120Q_S584/2021	EPI_ISL_1324767	27/02/2021	Quintana Roo	Human	Male	38	Released	Original	Oropharyngeal swab
hCoV-19/Mexico/BCS-InDRE_F11109_S606/2021	EPI_ISL_1337392	27/02/2021	Baja California Sur	Human	Male	48	Released	Original	Oropharyngeal swab

hCoV-19/Mexico/BCS-InDRE_F11116_S607/2021	EPI_ISL_1337393	27/02/2021	Baja California Sur	Human	Female	31	Released	Original	Oropharyngeal swab
hCoV-19/Mexico/HID-InDRE_F10789_S743/2021	EPI_ISL_1366692	27/02/2021	Hidalgo	Human	Male	47	Released	Original	Oropharyngeal swab
hCoV-19/Mexico/GUA-InDRE_F10829_S796/2021	EPI_ISL_1400341	27/02/2021	Guanajuato	Human	Female	66	Hospitalized	Original	Oropharyngeal swab
hCoV-19/Mexico/QUE-InDRE_F11024_S828/2021	EPI_ISL_1424030	27/02/2021	Queretaro	Human	Male	49	Released	Original	Oropharyngeal swab
hCoV-19/Mexico/QUE-InDRE_F11025_S829/2021	EPI_ISL_1424031	27/02/2021	Queretaro	Human	Female	20	Released	Original	Oropharyngeal swab
hCoV-19/Mexico/CMX-INMEGEN-03-04-13/2021	EPI_ISL_1205215	27/02/2021	Mexico City	Human	female	38	unknown	Original	Oropharyngeal swab
hCoV-19/Mexico/CMX-INMEGEN-03-04-14/2021	EPI_ISL_1205216	27/02/2021	Mexico City	Human	male	68	unknown	Original	Oropharyngeal swab
hCoV-19/Mexico/CMX-INMEGEN-03-04-15/2021	EPI_ISL_1205217	27/02/2021	Mexico City	Human	male	11	unknown	Original	Oropharyngeal swab
hCoV-19/Mexico/CMX-INMEGEN-03-05-39/2021	EPI_ISL_1262682	27/02/2021	Mexico City	Human	Male	44	unknown	Original	Oropharyngeal swab
hCoV-19/Mexico/CMX-INMEGEN-03-05-49/2021	EPI_ISL_1262683	27/02/2021	Mexico City	Human	Male	47	unknown	Original	Oropharyngeal swab
hCoV-19/Mexico/CMX-INMEGEN-03-05-51/2021	EPI_ISL_1262684	27/02/2021	Mexico City	Human	Female	43	unknown	Original	Oropharyngeal swab
hCoV-19/Mexico/CMX-INMEGEN-03-05-52/2021	EPI_ISL_1262685	27/02/2021	Mexico City	Human	Male	36	unknown	Original	Oropharyngeal swab
hCoV-19/Mexico/CMX-INMEGEN-03-05-50/2021	EPI_ISL_1310811	27/02/2021	Mexico City	Human	Male	22	unknown	Original	
hCoV-19/Mexico/GUA-InDRE_F10882_S769/2021	EPI_ISL_1400314	27/02/2021	Guanajuato	Human	Female	27	Released	Original	Oropharyngeal swab
hCoV-19/Mexico/GUA-InDRE_F10847_S785/2021	EPI_ISL_1400330	27/02/2021	Guanajuato	Human	Female	57	Released	Original	Oropharyngeal swab
hCoV-19/Mexico/CMX-INMEGEN-03-11-16/2021	EPI_ISL_1591475	27/02/2021	Mexico City	Human	Female	29	unknown	Original	Oropharyngeal swab
hCoV-19/Mexico/CMX-INER-INMEGEN-00009/202	EPI_ISL_1406728	28/02/2021	Mexico City	Human	Male	13	unknown	Original	
hCoV-19/Mexico/QUE-InDRE_F11027_S822/2021	EPI_ISL_1424024	28/02/2021	Queretaro	Human	Female	78	Released	Original	Oropharyngeal swab
hCoV-19/Mexico/QUE-InDRE_F11028_S831/2021	EPI_ISL_1424033	28/02/2021	Queretaro	Human	Female	25	Released	Original	Oropharyngeal swab
hCoV-19/Mexico/BCN-InDRE_F11110_S849/2021	EPI_ISL_1424050	28/02/2021	Baja California	Human	Male	27	Released	Original	Oropharyngeal swab
hCoV-19/Mexico/CMX-INMEGEN-03-04-16/2021	EPI_ISL_1205218	28/02/2021	Mexico City	Human	female	33	unknown	Original	Oropharyngeal swab
hCoV-19/Mexico/CMX-INMEGEN-03-05-69/2021	EPI_ISL_1262680	28/02/2021	Mexico City	Human	Female	55	unknown	Original	Oropharyngeal swab
hCoV-19/Mexico/CMX-INMEGEN-03-05-72/2021	EPI_ISL_1262681	28/02/2021	Mexico City	Human	Male	28	unknown	Original	Oropharyngeal swab
hCoV-19/Mexico/JAL-InDRE-F11737-S1182/2021	EPI_ISL_1558828	28/02/2021	Jalisco	Human	Female	unknown	Released	Original	Oropharyngeal swab
hCoV-19/Mexico/JAL-InDRE-F11738-S1183/2021	EPI_ISL_1558829	28/02/2021	Jalisco	Human	Male	45	Released	Original	Oropharyngeal swab
