# Supplementary material for: Genetic Analysis of SARS-CoV-2 Variants in Mexico during the First Year of the COVID-19 Pandemic
Source: Viruses. 2021 Oct 26;13(11):2161. doi: 10.3390/v13112161 (PMC8622467; doi:10.3390/v13112161)
Supplement: Supplementary file 1 [file viruses-13-02161-s001.zip › SupplementaryTable_S3.pdf]

We gratefully acknowledge the following Authors from the Originating laboratories responsible for obtaining the specimens, as well as the Submitting laboratories where the genome data were generated and shared via GISAID, on which this research is based.

All Submitters of data may be contacted directly via www.gisaid.org

Authors are sorted alphabetically.

Accession ID	Originating Laboratory	Submitting Laboratory	Authors
EPI_ISL_699656, EPI_ISL_699657	1-Laboratory of Microbiology, National Reference Lab, Charles Nicolle Hospital; 2-University of Tunis ElManar, Faculty of Medicine of Tunis, LR99ES09, Tunis, Tunisia	1-Clinical and Experimental Pharmacology Lab, LR16SP02, National Center of Pharmacovigilance, University of Tunis El Manar, Tunis, Tunisia. 2-Neurodegenerative diseases and psychiatric troubles, LR18SP03, Razi Hospital, University of Tunis El Manar, Tunis, Tunisia. 3- Ministry of Health, National Observatory of New and Emerging Diseases, 1006, Tunis, Tunisia	Alia Ben Kahla; Asma Ferjani; Gaies Emna; Guedi Berrabeh; Hanen ElJebari; Ilhem Boutiba-Ben Boubaker; Jalila Ben Khelil; Maher Kharrat; Mouna Ben Sassi; Mouna Safer; Nissaf Ben Alaya; Riadh Daghfous; Riadh Gouider.; Salma Abid; Salwa Mrabet; Sameh Trabelsi; Sana Ferjani
EPI_ISL_467432	AMPATH-DBN	KRISP, KZN Research Innovation and Sequencing Platform	Chimukangara B; Giandhari J; Khan S; Lessells R; Mdlalose K; Pillay S; Tegally H; Wilkinson E; York D; de Oliveira T
EPI_ISL_542187	ASST GOM Niguarda	Dep. Of Oncology and Hemato-Oncology University of Milan	Antonio Piralla; Carlo Federico Perno; Chiara Vismara; Claudia Alteri; Elisa Matarazzo; Fausto Baldanti; Federica Giardina; Federica Novazzi; Luna Colagrossi; Maria Antonello; Massimo Puoti; Monica Tallarita; Oscar Massimiliano Epis; Roberto Fumagalli; Silvia Renica; Stefano Gaiarsa; Valentino Costabile; Valeria Cento
EPI_ISL_694552	AZ SPHL, Arizona Department of Health Services	TGen North	Ashlyn Pfeiffer; Chris French; Darrin Lemmer; Dave Engelthaler; Hayley Yaglom; Jolene Bowers; Megan Folkerts; The Arizona COVID Genomics Union (ACGU)
EPI_ISL_528937	Agenzia di Tutela della Salute di Bergamo	Istituto Zooprofilattico Sperimentale dell'Abruzzo e Molise "G.Caporale"	Ancora M; Cammà C; Curini V; Di Domenico M; Di Pasquale A; Lorusso A; Mangone I; Marcacci M; Puglia I; Rinaldi A; Savini G.
EPI_ISL_549091	Akershus University Hospital, Department for Microbiology and Infectious Disease Control	Norwegian Institute of Public Health, Department of Virology	Hilde Elshaug; Hilde Synnøve Vollan; Kamilla Heddeland Instefjord; Karoline Bragstad; Kathrine Stene-Johansen; Olav Hungnes; Rasmus Riis Kopperud
EPI_ISL_509688	Alabama Department of Public Health Bureau of Clinical Laboratories	Pathogen Discovery, Respiratory Viruses Branch, Division of Viral Diseases, Centers for Disease Control and Prevention	Anna Uehara; Clinton Paden; Haibin Wang; Jing Zhang; Krista Queen; Suxiang Tong; Yan Li; Ying Tao
EPI_ISL_528713	Alsafar - Khalifa University Abu Dhabi	Alsafar - Khalifa University Abu Dhabi	Andreas Henschel; Ernesto Damiani; Gihan Daw Elbait; Guan Tay; Habiba Alsafar; Rifat Hamoudi; Samuel Feng
EPI_ISL_527740	Area De Salud Alajuela Norte - Clinica Dr. Marcial Rodriguez	Inciensa, Instituto Costarricense de Investigación y Enseñanza en Nutrición y Salud	Adriana Godinez & Melany Calderon; Claudio Soto-Garita; Estela Cordero; Francisco Duarte; Hebleen Porras
EPI_ISL_500639, EPI_ISL_500677, EPI_ISL_509505	Area of Virology, Serology and Virology Division (SAViD), New South Wales Health Pathology Randwick	Area of Virology, Serology and Virology Division (SAViD), New South Wales Health Pathology Randwick	Rawlinson, W.
EPI_ISL_475831, EPI_ISL_475835, EPI_ISL_475866	Austrian Agency for Health and Food Safety (AGES)	Bergthaler laboratory, CeMM Research Center for Molecular Medicine of the Austrian Academy of Sciences	Alexander Lercher; Alexandra Popa; Andreas Bergthaler; Benedikt Agerer; Christoph Bock; Daniela Schmid; Dorothee von Laer; Elisabeth Puchhammer-Stoeckl; Franz Allerberger; Gregor Hörmann; Guenter Weiss; Henrique Colaco; Jakob-Wendelin Genger; Jan Laine; Judith Aberle; Kinga Rigler-Hohenwarter; Lukas Endler; Manfred Nairz; Mark Smyth; Martin Senekowitsch; Michael Schuster; Peter Hufnagl; Rainer Gattringer; Stephan Aberle; Thomas Penz; Wegene Borena
EPI_ISL_461492	B.J. Medical College and Civil hospital	Gujarat Biotechnology Research Centre	; A M Kadri; Ankit Hinsu; Apurvasinh Puvar; Chaitanya Joshi; Dinesh Kumar; Harsh Bakshi; Janvi Raval; Kamlesh J Upadhyay; Komal Patel; Labdhi Pandya; Madhvi Joshi; Maharshi Pandya; Monika Gandhi; Neelam Nathani; Nidhi Patel; Nitin Savaliya; Pinal Trivedi; Pranay Shah; Pritesh Sabara; R D Dixit; Raghawendra Kumar; Snehal Bagatharia; Tejas Shah; Zarna Patel; Zuber Saiyed
EPI_ISL_450344	Bangladesh Institute of Tropical & Infectious Diseases, COVID-19 Testing Laboratory	Basic and Applied Research on Jute Project	A S M Anwarul Huq; AMAM Zonaed Siddiki; Eaftekhar Ahmed Rana; Emdadul Mannan Emdad; Goutam Buddha Das; M A Hassan Chowdhury; Md. Monjurul Alam; Md. Nazmul Haq Rony; Md. Sabbir Hossain; Md. Samiul Haque; Md. Shahidul Islam; Md. Shakeel Ahmed; Md. Sharifur Rahman; Paritous Kumar Biswas; Rasel Ahmed; Shah Md Tamim Kabir
EPI_ISL_444022	Baylor College of Medicine	Baylor College of Medicine: HGSC	David Henke; Donna Muzny; Erin Nicholson; George Weissenberger; Ginger Metcalf; Harsha Doddapaneni; Hsu Chao; Hua Shen; Joseph F. Petrosino; Kavya Kottapalli; Kristi L. Hoffman; Matthew C. Ross; Matthew Wong; Pedro Piedra; Qingchang Meng; Richard Sucgang; Sara J.J. Cregeen; Sejal Salvi; Tulin Ayvaz; Vasanthi Avadhanula; Vipin Menon; Yimiti Meiheerguli; Zeineen Momin
EPI_ISL_529213	Beijing Institute of Microbiology and Epidemiology	Beijing Institute of Microbiology and Epidemiology	Cui, Y.; Fan; Guo, Y.; Hang; Hou, J.; Li, B.; Mi, Z.; Mu, J.; Qin, E.; Song; Teng; Wu, Y.; Xu, Z.; Yajun.; Yang, R.; Yong, Y.; Yue; Zhang, X.
EPI_ISL_755232	Biolab Diagnostic Laboratories	Andersen lab at Scripps Research	Ahmad Tibi; Amid Abdelnour with SEARCH Alliance San Diego; Issa Abu-Dayyeh; Lama Hussein; Lina Mohammad; Zein Naber
EPI_ISL_1040927, EPI_ISL_1672849, EPI_ISL_1672864, EPI_ISL_1672865, EPI_ISL_1672866	Biologia molecular de enfermedades emergentes y EPOC, Instituto Nacional de Enfermedades Respiratorias	Biologia molecular de enfermedades emergentes y EPOC, Instituto Nacional de Enfermedades Respiratorias	; Eduardo Becerril-Vargas; Hernández-Teran Alejandra; José Arturo Martínez-Orozco; Mario Mújica-Sánchez; Mejía-Nepomuceno Fidencio; Perez-Padilla Rogelio; Ramirez-Gonzalez Ernesto.; Rodríguez-Maldonado Abril; Salas-Hernández Jorge; Serna Ricardo; Vazquez-Perez Joel Armando; Wong-Arambula Claudia
EPI_ISL_511479	CH Porto - H Sto Antonio	Instituto Nacional de Saude (INSA)	Borges et al
EPI_ISL_454237	CHLO	Instituto Nacional de Saude (INSA)	Borges et al
EPI_ISL_418222	CHRU Bretonneau - Serv. Bacterio-Virol.	National Reference Center for Viruses of Respiratory Infections, Institut Pasteur, Paris	Angela Brisebarre; Etienne Simon-Lorière; Fabiana Gambaro; Flora Donati; Julien Marlet; Marion Barbet; Maud Vanpeene; Mélanie Albert; Méline Bizard; Sylvie Behillil; Sylvie van der Werf; Vincent Enouf
EPI_ISL_443266	CHU - Hôpital Cavale Blanche - Labo. de Virologie	National Reference Center for Viruses of Respiratory Infections, Institut Pasteur, Paris	Angela Brisebarre; Etienne Simon-Lorière; Flora Donati; Léa Pilorge; Marion Barbet; Maud Vanpeene; Mélanie Albert; Méline Bizard; Sylvie Behillil; Sylvie van der Werf; Vincent Enouf
EPI_ISL_644683	CHU Montpellier	CNR Virus des Infections Respiratoires - France SUD	Antonin Bal; Bruno Lina; Gregory Destras; Gwendolyne Burfin; Hadrien Règue; Laurence Josset; Martine Valette; Michel Segondy; Quentin Semanas; Vincent Foulongne
EPI_ISL_645217, EPI_ISL_666722	CHU Nantes	CNR Virus des Infections Respiratoires - France SUD	Antonin Bal; Bruno Lina; Celine Bressollette; Gregory Destras; Gwendolyne Burfin; Hadrien Règue; Laurence Josset; Louise Castain; Martine Valette; Quentin Semanas; Virginie Ferré
EPI_ISL_660712	CHU Nîmes	CNR Virus des Infections Respiratoires - France SUD	Antonin Bal; Bruno Lina; Gregory Destras; Gwendolyne Burfin; Hadrien Règue; Jean-Philippe Lavigne; Laurence Josset; Marie-Josée Carles; Martine Valette; Maxence Lotellier; Quentin Semanas; Stephan Robin
EPI_ISL_413648	CHU Sao Joao, Porto	Instituto Nacional de Saude (INSA)	Inês Costa; Joana Isidro; Joana Mendonça; João Paulo Gomes; João Tiago Guimarães; Luís Vieira; Pedro Pechirra; Raquel Guiomar; Vítor Borges
EPI_ISL_641552	CHU Toulouse	CNR Virus des Infections Respiratoires - France SUD	Antonin Bal; Bruno Lina; Gregory Destras; Gwendolyne Burfin; Hadrien Règue; Jean Michel Mansuy; Laurence Josset; Martine Valette; Quentin Semanas
EPI_ISL_535736, EPI_ISL_535741	CHUL-LABO MULTI / MICRO	Laboratoire de santé publique du Québec	Guillaume Bourque; Ioannis Ragoussis; Jesse Shapiro; Mark Lathrop and Michel Roger; Sandrine Moreira
EPI_ISL_1219714	CICSaB/UASLP/LESP SLP	Instituto de Diagnostico y Referencia Epidemiologicos (INDRE)	Abril Rodriguez-Maldonado; Andreu Comas-Garcia; Ariadna Medina-Benitez; Claudia Wong-Arambula; David Fragoso-Fonseca; Ernesto Ramirez-Gonzalez.; Gisela Barrera-Badillo; Irma Lopez-Martinez; Joaquin Quiroz-Mercado; Lucia Hernandez-Rivas; Natividad Cruz-Ortiz; Sergio Rangel-Guerrero; Tatiana Nunez-Garcia; Vanessa Rivero-Arredondo
EPI_ISL_412981	CR&WISCO GENERAL HOSPITAL	Hubei Provincial Center for Disease Control and Prevention	Bin Fang; Bo Yang; Bo Yu; Faxian Zhan; Guojun Ye; Jing Li; Junqiang Xu; Kun Cai; Linlin Liu; Xiang Li; Xiao Yu; Xixiang Huo; Yongzhong Jiang.
EPI_ISL_418235	Cabinet médical	National Reference Center for Viruses of Respiratory Infections, Institut Pasteur, Paris	Angela Brisebarre; Etienne Simon-Lorière; Flora Donati; Marion Barbet; Maud Vanpeene; Mélanie Albert; Méline Bizard; Sylvie Behillil; Sylvie van der Werf; Vincent Enouf
EPI_ISL_582321, EPI_ISL_582494	Cadham Provincial Laboratory	National Microbiology Laboratory (NML)	Anna Majer; Anneliese Landgraff; CanCOGeN's metadata curation team; Darian Hole; David Alexander; Elsie Grudeski; Gary Van Domselaar; Grace Seo; Jared Bullard; Jennifer Tanner; Kerry Dust; Madison Chapel; Morag Graham; Natalie Knox; Nathalie Bastien; Paul Van Caeseele; Philip Mabon; Public Health Agency of Canada CanCOGeN team; Rhiannon Huzarewich; Russell Mandes; Shari Tyson; Timothy Booth; Yan Li
EPI_ISL_579092	Canterbury Health Laboratories	Institute of Environmental Science and Research (ESR)	Anja Werno; Antje van der Linden; Arlo Upton; Chris Mansell; David Hammer; Dragana Drinkovic; Erasmus Smit; Gary McAuliffe; Hana Sofia Andersson; Hermes Perez; James Ussher; Jill Sherwood; Jing Wang; Joep de Ligt; Josh Freeman; Julia Howard; Juliet Elvy; Lauren Jelly; Mary DeAlmeida; Matt Blakiston; Matt Storey; Matthew Rogers; Max Bloomfield; Michael Addidle; Michelle Balm; Muhammad Faisal; Nikki Freed; Olin Silander; Sally Roberts; Sarah Jefferies; Sharmini Muttaiyah; Susan Morpeth; Susan Taylor; Timothy Blackmore; Vani Sathyendran; Veronica Playle; Virginia Hope; Xiaoyun Ren
EPI_ISL_475618, EPI_ISL_475663, EPI_ISL_475675, EPI_ISL_475680, EPI_ISL_475688	Cedars-Sinai Medical Center, Department of Pathology & Laboratory Medicine, Molecular Pathology Laboratory	Cedars-Sinai Medical Center, Molecular Pathology Laboratory of Department of Pathology & Laboratory Medicine and Genomic Core	Brian Davis; Eric Vail; Jasmine T Plummer; Jean Lopategui; Jianbo Song; John Paul Govindavari; Jong Taek Kim; Stephanie Chen; Wenjuan Zhang
EPI_ISL_522461, EPI_ISL_522468	Center for Laboratory Control of Infectious Diseases, Korea Centers for Diseases Control and Prevention	Center for Laboratory Control of Infectious Diseases, Korea Centers for Diseases Control and Prevention	Ae Kyung Park; Eunkyung Shin; Heui Man Kim; Jeong-Min Kim; Jin Sun No; Junyoung Kim; Myung Guk Han; Yoon-Seok Chung
EPI_ISL_419672	Center for Virology, Medical University of Vienna	Bergthaler laboratory, CeMM Research Center for Molecular Medicine of the Austrian Academy of Sciences	Alexander Lercher; Alexandra Popa; Andreas Bergthaler; Benedikt Agerer; Christoph Bock; Elisabeth Puchhammer-Stöckl; Henrique Colaco; Jakob-Wendelin Genger; Judith Aberle; Lukas Endler; Mark Smyth; Michael Schuster; Stephan Aberle; Thomas Penz
EPI_ISL_414497	Center of Medical Microbiology, Virology, and Hospital Hygiene, University of Duesseldorf	Center of Medical Microbiology, Virology, and Hospital Hygiene, University of Duesseldorf	Alexander Dilthey; Andreas Walker; Björn-Erik Jensen; Daniel Strelow; Detlef Kindgen-Milles; Jörg Timm; Klaus Pfeffer; Malte Kohns Vasconcelos; Marcel Andree; Ortwin Adams; Sandra Hauka; Tina Senff; Tobias Wienemann; Torsten Feldt; Torsten Houwaart
EPI_ISL_418228	Centre Hospitalier Compiègne Laboratoire de Biologie	National Reference Center for Viruses of Respiratory Infections, Institut Pasteur, Paris	Angela Brisebarre; Etienne Simon-Lorière; Flora Donati; Marion Barbet; Maud Vanpeene; Mélanie Albert; Méline Bizard; Raulin Olivia; Sylvie Behillil; Sylvie van der Werf; Vincent Enouf
EPI_ISL_416749	Centre Hospitalier de Valence	CNR Virus des Infections Respiratoires - France SUD	Alexandre; Antonin; Bal; Bouscambert-Duchamp; Brengel-Pesce; Bruno.; Cheynet; Destras; Florence; Gaymard; Gregory; Josset; Karen; Laurence; Lina; Martine; Maude; Morfin-Sherpa; Valette; Valérie
EPI_ISL_418412	Centre Hospitalier des Vals d'Ardeche	CNR Virus des Infections Respiratoires - France SUD	Alexandre Gaymard; Antonin Bal; Bruno Lina; Carine Moustaud; Florence Morfin-Sherpa; Gregory Destras; Gwendolyne Burfin; Laurence Josset; Martine Valette; Maude Bouscambert-Duchamp; Raphaelle Lamy; Solenne Brun
EPI_ISL_483968	Centre for Clinical Infection and Diagnostics	COVID-19 Genomics UK (COG-UK) Consortium	Ali Raza Awan; Chloe Fisher; Jonathan Edgeworth; Luke Snell; Penny Cliff; Rahul Batra

	Research and Genomics Innovation Unit, Guy's and St. Thomas' NHS Trust		
EPI_ISL_427674, EPI_ISL_427768	Centre for Infectious Diseases and Microbiology Public Health	NSW Health Pathology - Institute of Clinical Pathology and Medical Research; Westmead Hospital; University of Sydney	Arnott A; Bachmann N; Basile K; Byun R; Carter I; Chang S; Chen SC; Draper J; Dwyer DE for the 2019-nCoV Study Group; Eden JS; Gall M; Gray K; Holmes EC; Kok J; Lam C; Maddocks S; O'Sullivan MV; Propenko M; Rockett R; Sadsad R; Sim E; Sintchenko V; Sorrell T; Timms V
EPI_ISL_535981	Centre hospitalier Anna-Laberge	Laboratoire de santé publique du Québec	Guillaume Bourque; Ioannis Ragoussis; Jesse Shapiro; Mark Lathrop and Michel Roger on behalf of the CoVSeQ research group; Sandrine Moreira
EPI_ISL_666685	Centre hospitalier Métropole Savoie	CNR Virus des Infections Respiratoires - France SUD	Antonin Bal; Bruno Lina; Carine Dumollard; Gregory Destras; Gwendolyne Burfin; Hadrien Règue; Jérôme Grosjean; Laurence Josset; Martine Valette; Quentin Semanas
EPI_ISL_629023	Centro de Biotecnología Vegetal, Universidad Andrés Bello, Center for Genome Regulation	Center for Mathematical Modeling and Center for Genome Regulation. Santiago, Chile	Allende ML; Arriagada G; Bastias M; Bustos F; Castro E; González M; M; Maass A; Meneses C.; Montecino; Orellana A; Sanhueza D; Travisany D
EPI_ISL_1531807, EPI_ISL_1531808, EPI_ISL_1531809, EPI_ISL_1531810, EPI_ISL_1531811, EPI_ISL_1531812, EPI_ISL_1531813, EPI_ISL_1531814, EPI_ISL_1531815, EPI_ISL_1531816, EPI_ISL_1531817, EPI_ISL_1531818, EPI_ISL_1531819, EPI_ISL_1531820, EPI_ISL_1531821, EPI_ISL_1531822, EPI_ISL_1531823, EPI_ISL_1531824, EPI_ISL_1531825, EPI_ISL_1531826, EPI_ISL_1531827, EPI_ISL_1531828, EPI_ISL_1531829, EPI_ISL_1531830, EPI_ISL_1531831, EPI_ISL_1531832, EPI_ISL_1531833, EPI_ISL_1531834, EPI_ISL_1531835, EPI_ISL_1531836, EPI_ISL_1531837, EPI_ISL_1531838, EPI_ISL_1531839, EPI_ISL_1531840, EPI_ISL_1531841, EPI_ISL_1531842, EPI_ISL_1531843, EPI_ISL_1531844, EPI_ISL_1531845, EPI_ISL_1531846, EPI_ISL_1531847, EPI_ISL_1531848, EPI_ISL_1531849, EPI_ISL_1531850, EPI_ISL_1531851, EPI_ISL_1531852, EPI_ISL_1531853, EPI_ISL_1531854, EPI_ISL_1531855, EPI_ISL_1531856, EPI_ISL_1531857, EPI_ISL_1531858, EPI_ISL_1531859, EPI_ISL_1531860, EPI_ISL_1531861, EPI_ISL_1531862, EPI_ISL_1531863, EPI_ISL_1531864, EPI_ISL_1531865, EPI_ISL_1531866, EPI_ISL_1531867, EPI_ISL_1531868, EPI_ISL_1531869, EPI_ISL_1531870, EPI_ISL_1531871, EPI_ISL_1531872, EPI_ISL_1531873, EPI_ISL_1531874, EPI_ISL_1531875, EPI_ISL_1531876, EPI_ISL_1531877, EPI_ISL_1531878, EPI_ISL_1531879, EPI_ISL_1531880, EPI_ISL_1531881, EPI_ISL_1531882, EPI_ISL_1531883, EPI_ISL_1531884, EPI_ISL_1531885, EPI_ISL_1531886, EPI_ISL_1531887, EPI_ISL_1531888, EPI_ISL_1531889, EPI_ISL_1531890, EPI_ISL_1531891, EPI_ISL_1531892, EPI_ISL_1531893, EPI_ISL_1531894, EPI_ISL_1531895, EPI_ISL_1531896, EPI_ISL_1531897, EPI_ISL_1531898, EPI_ISL_1531899, EPI_ISL_1531900, EPI_ISL_1531901, EPI_ISL_1531902, EPI_ISL_1531903, EPI_ISL_1531904, EPI_ISL_1531905, EPI_ISL_1531906, EPI_ISL_1531907, EPI_ISL_1531908, EPI_ISL_1531909, EPI_ISL_1531910, EPI_ISL_1531911, EPI_ISL_1531912, EPI_ISL_1531913, EPI_ISL_1531914, EPI_ISL_1531915, EPI_ISL_1531916, EPI_ISL_1531917, EPI_ISL_1531918, EPI_ISL_1531919, EPI_ISL_1531920, EPI_ISL_1531921, EPI_ISL_1531922, EPI_ISL_1531923, EPI_ISL_1531924, EPI_ISL_1531925, EPI_ISL_1531926, EPI_ISL_1531927, EPI_ISL_1531928, EPI_ISL_1531929, EPI_ISL_1531930, EPI_ISL_1531931, EPI_ISL_1531932, EPI_ISL_1531933, EPI_ISL_1531934, EPI_ISL_1531935, EPI_ISL_1531936, EPI_ISL_1531937, EPI_ISL_1531938, EPI_ISL_1531939, EPI_ISL_1531940, EPI_ISL_1531941			
see above	Centro de Diagnostico COVID-19 UABC Tijuana	Andersen lab at Scripps Research	German Ibarra; Jonathan Vincent Baena; Jorge Luis Jimenez Niebla; Manuel Sanchez Alavez; Oscar Efren Zazueta Fierro; SEARCH Alliance San Diego with Idanya Rubi Serafin Higuera
EPI_ISL_635479, EPI_ISL_635480, EPI_ISL_635481, EPI_ISL_635482, EPI_ISL_635483, EPI_ISL_635484, EPI_ISL_635485, EPI_ISL_635486, EPI_ISL_635487, EPI_ISL_635488, EPI_ISL_635489, EPI_ISL_635490, EPI_ISL_635491, EPI_ISL_635492, EPI_ISL_635493, EPI_ISL_635494, EPI_ISL_635495, EPI_ISL_635496, EPI_ISL_635497, EPI_ISL_635498, EPI_ISL_635499, EPI_ISL_635500, EPI_ISL_635501, EPI_ISL_635502, EPI_ISL_635503, EPI_ISL_635504, EPI_ISL_635505, EPI_ISL_635506, EPI_ISL_635507, EPI_ISL_635508, EPI_ISL_635509, EPI_ISL_635510, EPI_ISL_635511, EPI_ISL_635512, EPI_ISL_635513, EPI_ISL_635514, EPI_ISL_635515, EPI_ISL_635516, EPI_ISL_635517, EPI_ISL_635518, EPI_ISL_635519, EPI_ISL_635520, EPI_ISL_635521, EPI_ISL_635522, EPI_ISL_635523, EPI_ISL_635524, EPI_ISL_635525, EPI_ISL_635526, EPI_ISL_635527, EPI_ISL_635528, EPI_ISL_635529, EPI_ISL_635530, EPI_ISL_635531, EPI_ISL_635532, EPI_ISL_635533, EPI_ISL_635534, EPI_ISL_635535, EPI_ISL_635536, EPI_ISL_635537, EPI_ISL_635538, EPI_ISL_635539, EPI_ISL_635540, EPI_ISL_635541, EPI_ISL_635542, EPI_ISL_635543, EPI_ISL_635544, EPI_ISL_635545, EPI_ISL_635546, EPI_ISL_635547, EPI_ISL_635548, EPI_ISL_635549, EPI_ISL_635550, EPI_ISL_635551, EPI_ISL_635552, EPI_ISL_635553, EPI_ISL_635554, EPI_ISL_635555, EPI_ISL_635556, EPI_ISL_635557, EPI_ISL_635558, EPI_ISL_635559, EPI_ISL_635560, EPI_ISL_635561, EPI_ISL_635562, EPI_ISL_635563, EPI_ISL_635564, EPI_ISL_635565, EPI_ISL_635566, EPI_ISL_635567, EPI_ISL_635568, EPI_ISL_635569, EPI_ISL_635570, EPI_ISL_635571, EPI_ISL_635572, EPI_ISL_635573, EPI_ISL_635574, EPI_ISL_635575, EPI_ISL_635576			
see above	Centro de Diagn√≥stico COVID-19 UABC Tijuana	Andersen lab at Scripps Research	Germán Ibarra; Jonathan Vincent Baena; Jorge Luis Jiménez Niebla; Manuel Sánchez Alavez; Oscar Efrén Zazueta Fierro; SEARCH Alliance San Diego with Idanya Rubí Serafín Higuera
EPI_ISL_1469108, EPI_ISL_1469109, EPI_ISL_1469110, EPI_ISL_1469111, EPI_ISL_1469112, EPI_ISL_1469113, EPI_ISL_1469114, EPI_ISL_1469115, EPI_ISL_1469116, EPI_ISL_1469117, EPI_ISL_1469118, EPI_ISL_1469119			
see above	Centro de Investigacion en Ciencias de la Salud y Biomedicina	CINVESTAV	Andreu Comas; Sofia Bernal
EPI_ISL_1494674, EPI_ISL_1494717	Centro de Investigación en Ciencias de la Salud y Biomedicina, U.A.S.L.P	Centro de Investigación en Ciencias de la Salud y Biomedicina, U.A.S.L.P	MD PhD Sofía Bernal Silva
EPI_ISL_1500639, EPI_ISL_1500866	Centro de Investigación en Ciencias de la Salud y Biomedicina, U.A.S.L.P.	Centro de Investigación en Ciencias de la Salud y Biomedicina, U.A.S.L.P.	MD PhD Sofía Bernal Silva
EPI_ISL_1494724, EPI_ISL_1494725, EPI_ISL_1494726, EPI_ISL_1494727, EPI_ISL_1494728, EPI_ISL_1494729, EPI_ISL_1494730			
see above	Centro de Investigación en Ciencias de la Salud y Biomedicina, U.A.S.LP.	Centro de Investigación en Ciencias de la Salud y Biomedicina, U.A.S.LP.	MD PhD Sofía Bernal Silva
EPI_ISL_450522	Centrālā Laboratorija	Latvian Biomedical Research and Study Centre	Ivars Silamiķelis; Jana Osīte; Jānis Kloviņš; Kaspars Megnis; Marta Priedīte; Monta Ustinova; Stella Lapiņa; Uga Dumpis; Vita Rovīte; Ņikita Zrelovs
EPI_ISL_729477, EPI_ISL_753918, EPI_ISL_753988, EPI_ISL_754006	Charité Universitätsmedizin Berlin, Institut für Virologie/Labor Berlin	Charité Universitätsmedizin Berlin, Institut für Virologie	Barbara Mühlemann; Christian Drosten; Julia Schneider; Jörn Beheim-Schwarzbach; Talitha Veith; Terry Jones; Victor M Corman
EPI_ISL_468071, EPI_ISL_468077	Child Health Research Foundation	Child Health Research Foundation	Afroza Akter Tanni; Hafizur Rahman; Maksuda Islam; Md Saiful Islam Sajib; Roly Malaker; Samir K Saha; Senjuti Saha; Syed Muktadir Al Sium
EPI_ISL_437912	Child Health Research Foundation	Child Health Research Lab	Maksuda Islam; Md Hafizur Rahman; Md Hasanuzzaman; Md Saiful Islam Sajib; Md Shahidul Islam; Roly Malaker; Samir K Saha; Senjuti Saha; Zabed B Ahmed
EPI_ISL_447427, EPI_ISL_447450	Clinical Microbiology Laboratory, Sheba Medical Center	Stern Lab	Stern Lab
EPI_ISL_528022, EPI_ISL_528046	Clinical Virology	Clinical Bacteriology	Adrian Egli; Alexander Gensch; Alfredo Mari; Christian Nickel; Hans Hirsch; Hans Pargger; Helena MB Seth-Smith; Julia Bielicki; Karoline Leuzinger; Kirstine K. Soegaard; Madlen Stange; Manuel Battegay; Martin Siegemund; Michael Osthoff; Michael Schweitzer; Myrta Brunner; Rita Schneider-SliFemalea; Roland Bingisser; Sarah Tschudin-Sutter; Simon Fuchs; Stefano Bassetti; Tim Roloff
EPI_ISL_605816	Clinical Virology Laboratory, Institute of Liver and Biliary Sciences	ILBS - IGIB	Abhishek Padhi; Ekta Gupta; Jaswinder Singh Maras; Reshu Agarwal; Sheetalnath Rooge; Shridhar Sivasubbu; Shvetank Sharma; Vinod Scaria
EPI_ISL_447324	Clinical Virology Laboratory, Soroka Medical Center and the Faculty of Health Sciences, Ben-Gurion University of the Negev	Stern Lab	Stern Lab
EPI_ISL_527416, EPI_ISL_527431	Colorado State University - Ebel Lab	Colorado State University - Ebel Lab	Greg Ebel et al.
EPI_ISL_632265	Communicable Disease Laboratory, Public Health Directorate	Communicable Disease Laboratory, Public Health Directorate	AlAbbas, Z.; AlHujairi, Z.; AlTaif, Z.; AlWasti, H.
EPI_ISL_474816	Complejo Hospitalario Universitario de Albacete	SeqCOVID-SPAIN consortium/IBV(CSIC)	Caridad Sainz de Baranda Camino and SeqCOVID-SPAIN consortium; Encarnacion Simarro Córdoba; Julia Lozano Serra; Lorena Robles Fonseca; Monica Parra Grandes
EPI_ISL_700441	Conville CDC wc CVC	NHLS/UCT	Arash Iranzadeh; Bruna Galvao; Carolyn Williamson; Deelan Doolabh; Diana Hardie; Houriiyah Tegally; Innocent Mudau; Kruger Marais; Lynn Tyers; Marvin Hsiao; Stephen Korsman
EPI_ISL_468389	County of San Luis Obispo Public Health Laboratory	Chan-Zuckerberg Biohub	CZB Cliahub Consortium
EPI_ISL_437044	County of Santa Clara Public Health	Chan-Zuckerberg Biohub	CZB Cliahub Consortium
EPI_ISL_436641, EPI_ISL_436672	County of Santa Clara Public Health Department	Chan-Zuckerberg Biohub	CZB Cliahub Consortium
EPI_ISL_560647	Delaware Public Health Lab	Delaware Public Health Lab	Gregory Hovan
EPI_ISL_417008, EPI_ISL_417018, EPI_ISL_515061	Department of Clinical Microbiology	GIGA Medical Genomics	Artesi Maria; Axelle Chaslain; Bontems Sébastien; Boreux Raphaël; Bours Vincent.; Cecile Meex; Celine Fombellida-Lopez; Durkin Keith; Hayette Marie-Pierre; Keith Durkin; Maria Artesi; Marie-Pierre Hayette; Meex Cécile; Melin Pierrette; Pierrette Melin; Raphael Boreux; Sebastien Bontems; Vincent Bours.
EPI_ISL_429285, EPI_ISL_452032	Department of Clinical Microbiology, Copenhagen University Hospital, Hvidovre, Kettegaard Alle 30, 2650 Hvidovre.	Albertsen lab, Department of Chemistry and Bioscience, Aalborg University, Denmark	Rasmus Kirkegaard
EPI_ISL_417187	Department of Clinical Pathology, Pamela Youde Nethersole Eastern Hospital	Department of Health Technology and Informatics, Faculty of Health and Social Science, The Hong Kong Polytechnic University	Alan Ka-Lun WU; Alex Yat-Man HO; Barry Kin-Chung WONG; David Ho-Keung SHUM; Eugene Yuk-Keung TSO; Gilman Kit-Hang SIU; Hiu-Yin LAO; Kam-Tong Yip; Kenneth Siu-Sing LEUNG; Kingsley King-Gee TAM; Kit-Man SIN; Kitty Sau-Chun FUNG; Kwok-Cheung LUNG; Lam-Kwong LEE; Man-Chun CHAN; Ming-Pan CHOI; Miranda Chong-Yee YAU; Raymond Wai-To LIU; Sandy Ka-Yee CHAU; Shea Ping YIP; Tak-Lun Que; Timothy Ting-Leung NG; Wai-Shing LEUNG; Wing Cheong YAM; Wing-Kin TO; Yuk-Yung NG
EPI_ISL_481261	Department of Emerging Infectious Diseases, Institute of Tropical Medicine, Nagasaki University	Department of Emerging Infectious Diseases, Institute of Tropical Medicine, Nagasaki University	Haruka Abe; Jiro Yasuda; Rokusuke Yoshikawa; Yuichiro Furusato
EPI_ISL_568564	Department of Infectious Diseases and Immunology, National Hospital Organization Nagoya Medical Center	Clinical Research Center, National Hospital Organization Nagoya Medical Center	Hirotaka Ode; Kazuhiro Matsuoka; Mai Kubota; Masakazu Matsuda; Mayumi Imahashi; Mikiko Mori; Nakasuji Miho; Yasumasa Iwatani; Yoshihiro Nakata; Yoshiyuki Yokomaku
EPI_ISL_644945	Department of Infectious Diseases, Keio University School of Medicine, Tokyo, Japan	Center for Medical Genetics, Keio University School of Medicine, Tokyo, Japan	Haruhiko Siomi; Hirotsugu Ishizu; Kenjiro Kosaki; Kodai Abe; Yuka Iwasaki
EPI_ISL_422407	Department of Laboratory Medicine, National Taiwan University Hospital	Microbial Genomics Core Lab, National Taiwan University Centers of Genomic and Precision Medicine	Chiao-Ling Li; Pei-Jer Chen; Shan-Chwen Chang; Shiou-Hwei Yeh; Sui-Yuan Chang; Ya-Yun Lai; You-Yu Lin
EPI_ISL_492849	Department of Medical Microbiology, Western Sussex Hospitals NHS Foundation Trust, St Richard's Hospital	Wellcome Sanger Institute for the COVID-19 Genomics UK (COG-UK) consortium	Cordelia Langford; David K. Jackson; Dominic Kwiatkowski; Ewan Harrison; Ian Johnston; John Sillitoe on behalf of the Wellcome Sanger Institute COVID-19 Surveillance Team (http://www.sanger.ac.uk/covid-team); Jonathan Lewis; Manasa Mutingwende; Michelle Erkiert; Olga Podplomyk; Paul Randell and Alex Alderton; Roberto Amato; Sarah Lowdon; Sonia Goncalves
EPI_ISL_497771, EPI_ISL_497864	Department of Microbiology, The University of Hong Kong	Department of Microbiology, The University of Hong Kong	Kelvin K.W. To; Kwok-Yung Yuen
EPI_ISL_438678, EPI_ISL_573138, EPI_ISL_612272	Department of Pathology, University of Cambridge	COVID-19 Genomics UK (COG-UK) Consortium	Aminu S. Jahun; Anna Yakovleva; Charlotte J. Houldcroft; Fahad A Khokhar; Grant Hall; Ian Goodfellow; Iliana Georgana; Laura G Caller; Luke W Meredith; M. Est√©e T√∂r√∂k; Malte Pinckert; Martin D. Curran; Myra Hosmillo; Sarah L. Caddy; Surendra Parmar; Theresa Feltwell; William L. Hamilton; Yasmin Chaudhry

EPI_ISL_438346, EPI_ISL_438362, EPI_ISL_438427, EPI_ISL_439574, EPI_ISL_439884, EPI_ISL_440287, EPI_ISL_440297, EPI_ISL_441289, EPI_ISL_442230			
see above	Department of Pathology, University of Cambridge	Wellcome Sanger Institute for the COVID-19 Genomics UK (COG-UK) consortium	Alex Alderton; Aminu S. Jahun; Anna Yakovleva; Charlotte J. Houldcroft; Cordelia Langford; David K. Jackson; Dominic Kwiatkowski; Ewan Harrison; Fahad A Khokhar; Grant Hall; Ian Goodfellow; Ian Johnston; John Sillitoe on behalf of the Wellcome Sanger Institute COVID-19 Surveillance Team (http://www.sanger.ac.uk/covid-team); Laura G Caller; Luke W Meredith; M. Estée Török; Martin D. Curran; Myra Hosmillo; Roberto Amato; Sarah L. Caddy; Sonia Goncalves; Theresa Feltwell; William L. Hamilton
EPI_ISL_418390, EPI_ISL_481647	Department of Virology and Immunology, University of Helsinki and Helsinki University Hospital, Huslab Finland	Department of Virology, Faculty of Medicine, University of Helsinki, Helsinki, Finland	Hannimari Kallio-Kokko; Harri Kangas; Jenni Virtanen; Maija Suvanto; Olli Vapalahti; Pekka Ellonen; Sari Hannula; Teemu Smura
EPI_ISL_671267, EPI_ISL_682655	Department of Virus and Microbiological Special Diagnostics, Statens Serum Institut, Copenhagen, Denmark	Albertsen Lab, Department of Chemistry and Bioscience, Aalborg University, Denmark	Danish Covid-19 Genome Consortium
EPI_ISL_437021, EPI_ISL_437647, EPI_ISL_437663	Department of Virus and Microbiological Special Diagnostics, Statens Serum Institut, Copenhagen, Denmark, Artillerivej 5, 2300 Copenahgen S	Albertsen lab, Department of Chemistry and Bioscience, Aalborg University, Denmark	Rasmus Kirkegaard
EPI_ISL_614437, EPI_ISL_614488, EPI_ISL_614557, EPI_ISL_614581, EPI_ISL_614657, EPI_ISL_615213, EPI_ISL_615467, EPI_ISL_615680, EPI_ISL_616029, EPI_ISL_616310, EPI_ISL_616503, EPI_ISL_616617, EPI_ISL_616719, EPI_ISL_616787, EPI_ISL_616855, EPI_ISL_617756, EPI_ISL_618036, EPI_ISL_618374, EPI_ISL_619978, EPI_ISL_620200, EPI_ISL_620286, EPI_ISL_620364, EPI_ISL_621648, EPI_ISL_622042, EPI_ISL_622168, EPI_ISL_622259, EPI_ISL_622332, EPI_ISL_622429, EPI_ISL_622531, EPI_ISL_622596			
see above	Department of Virus and Microbiological Special Diagnostics, Statens Serum Institut, Denmark	Albertsen lab, Department of Chemistry and Bioscience, Aalborg University, Denmark	Danish Covid-19 Genome Consortia
EPI_ISL_415648	Department of Virus and Microbiological Special diagnostics, Statens Serum Institut, Copenhagen, Denmark.	ViFU	Anders Fomsgaard; Maiken Worsoe Rosenstierne; Morten Rasmussen
EPI_ISL_666602	Dept. of Microbiology and Infection Control, Akershus University Hospital HF	Dept. of Microbiology and Infection Control, Akershus University Hospital HF	Alexander Hesselberg Løvestad; Hege Vangstein Aamot; Nina Handal; Ole Herman Ambur; Silje Bakken Jørgensen
EPI_ISL_774894	Designated Reference Institute for Chemical Measurements (DRICM)	DNA SOLUTION LTD.	Abdul Khaleque; Abu Sufian; Hasan Ul Haider; Jannatun Naima; Kazi Nadim Hasan; MSM Chowdhury; Mala Khan; Mamudul Hasan Razu; Md. Imran Khan; Mizanur Rahman; Mohammad Fazle Alam Rabbi
EPI_ISL_722872	Dipartimento di Scienze Biomediche e Oncologia Umana - Azienda Ospedaliero Universitaria Consorziale Policlinico	Istituto Zooprofilattico Sperimentale della Puglia e della Basilicata	Bianco A.; Capozzi L.; Chironna M.; Del Sambro L.; Loconsole D.; Parisi A.
EPI_ISL_504185	Discovery DNA	Discovery DNA	Alice Li; Aneal Khan; Desmond Koo; Dustin Hittel; Leo Dimnik; Marina Kerr
EPI_ISL_760151	Division of Emerging Infectious Diseases, Bureau of Infectious Diseases Diagnosis Control, Korea Disease Control and Prevention Agency	Division of Emerging Infectious Diseases, Bureau of Infectious Diseases Diagnosis Control, Korea Disease Control and Prevention Agency	Ae Kyung Park; Chaeyoung Lee; Eun-Jin Kim; Heui Man Kim; Il-Hwan Kim; Jeong-Min Kim; Namjoo Lee; Sang Hee Woo
EPI_ISL_497961, EPI_ISL_510609, EPI_ISL_526708	Division of Viral Diseases, Center for Laboratory Control of Infectious Diseases, Korea Centers for Diseases Control and Prevention	Division of Viral Diseases, Center for Laboratory Control of Infectious Diseases, Korea Centers for Diseases Control and Prevention	Heui Man Kim; Hye-Jun Jo; Jeong-Min Kim; Jun-Sub Kim; Myung Guk Han; Namjoo Lee; Sang Hee Woo; Yoon-Seok Chung
EPI_ISL_700552, EPI_ISL_700567	Dr Abdurahman CDC wc DAC	NHLS/UCT	Arash Iranzadeh; Bruna Galvao; Carolyn Williamson; Deelan Doolabh; Diana Hardie; Innocent Mudau; Kruger Marais; Lynn Tyers; Marvin Hsiao; Stephen Korsman
EPI_ISL_414530, EPI_ISL_422855, EPI_ISL_455196, EPI_ISL_460643, EPI_ISL_523127, EPI_ISL_523199, EPI_ISL_523341, EPI_ISL_523460, EPI_ISL_523523, EPI_ISL_523614, EPI_ISL_523664, EPI_ISL_632518, EPI_ISL_722800			
see above	Dutch COVID-19 response team	Erasmus Medical Center	Anne van der Linden; Annemiek van der Eijk; Aura Timen; Bas Oude Munnink; Claudia Schapendonk; Corien Swaan; Corine GeurtsvanKessel; David Nieuwenhuijse; Emmanuelle Munger; Irina Chestakova; Jeroen van Kampen; Jolanda Voermans; Madelief Mollers; Manon Haverkate; Marion Koopmans; Marjan Boter; Mark Pronk; Mart Stein; Pascal Lexmond; Reina Sikkema; Richard Molenkamp; Sandra Kengne Kamga Mobou; Stefan van Nieuwkoop; Theo Bestebroer; on behalf of the Dutch national COVID-19 response team.
EPI_ISL_454766, EPI_ISL_547557	Dutch COVID-19 response team	National Institute for Public Health and the Environment (RIVM)	Adam Meijer; AnneMarie van den Brandt; Bas van der Veer; Chantal Reusken; Dennis Schmitz; Florian Zwagemaker; Harry Vennema; Jeroen Cremer; Pieter Overduin; Sharon van den Brink; on behalf of the national COVID-19 response team
EPI_ISL_1238787, EPI_ISL_1238788, EPI_ISL_1238789, EPI_ISL_1238790, EPI_ISL_1238791, EPI_ISL_1238792, EPI_ISL_1238793			
see above	Ecological and Evolutionary Genomics. UGA-CINVESTAV	Unidad Universitaria de Secuenciación Masiva y Bioinformática (UUSMB). IBT-UNAM	Angelica Cibrian
EPI_ISL_614265	Eurofins	National Reference Center for Viruses of Respiratory Infections, Institut Pasteur, Paris	Angela Brisebarre; Camille Capel; Etienne Simon-Lorière; Marion Barbet; Maud Vanpeene; Méline Bizard; Sylvie Behillil; Sylvie van der Werf; Vincent Enouf
EPI_ISL_424855	FL Bureau of Public Health Laboratories-Tampa	Pathogen Discovery, Respiratory Viruses Branch, Division of Viral Diseases, Centers for Disease Control and Prevention	Alison S. Laufer Halpin; Anna Uehara; Christopher A. Elkins; Clinton R. Paden; Haibin Wang; Jing Zhang; Krista Queen; Mary S. Keckler; Rachel Marine; Suxiang Tong; Yan Li; Ying Tao
EPI_ISL_480862, EPI_ISL_508786, EPI_ISL_509724, EPI_ISL_512555, EPI_ISL_512564, EPI_ISL_514188, EPI_ISL_517875, EPI_ISL_568603, EPI_ISL_594313, EPI_ISL_653169			
see above	Florida Bureau of Public Health Laboratories	Florida Bureau of Public Health Laboratories	Jason Blanton; Sarah Schmedes
EPI_ISL_632010	Flushing Hospital Medical Center	New York City Public Health Laboratory	Jade Wang; et al.
EPI_ISL_469040	GMERS Medical College & Hospital	Gujarat Biotechnology Research Centre	A M Kadri; Ankit Hinsu; Apurvasinh Puvar; Chaitanya Joshi; Dinesh Kumar; Fenil Patel; Harsh Bakshi; Janvi Raval; Komal Patel; Labdhi Pandya; Madhvi Joshi; Maharshi Pandya; Meenakshi Shah; Monika Gandhi; Neena Doshi; Nidhi Patel; Nitin Savaliya; Pinal Trivedi; Pritesh Sabara; R D Dixit; Raghawendra Kumar; Snehal Bagatharia; Tejas Shah; Varsha Godbole; Zarna Patel; Zuber Saiyed
EPI_ISL_582834	Gavle klinisk mikrobiologi	The Public Health Agency of Sweden	Anna Risberg; Anna-Malin Linde; Karin Tegmark-Wisell; Maria Lind Karlberg; Mattias Haukland; Mia Brytting; Olov Svartstrom; Oskar Karlsson Lindsjo; Petra Edquist; Reza Advani; Sandra Broddesson
EPI_ISL_747243	General Hospital Laboratory	Pathogen Laboratory (BSL3), Biomedical Innovation Department, Experimental and Applied Biology Division, Scientific Research Center and High Education from Ensenada (CICESE)	Cervantes-Luevano K.; Galindo C. and Licea-Navarro A.; Martinez M.A.; Saavedra-Flores A.
EPI_ISL_746571, EPI_ISL_746824	Genetica Molecular and Subdepartamento de Virologia ISP Chile	Instituto de Salud Publica de Chile	Andres Castillo; Barbara Parra; Gisselle Barra; Jaime Lagos; Javier Tognarelli; Jorge Fernandez; Loredana Arata; Patricia Bustos; Rodrigo Fasce
EPI_ISL_428368, EPI_ISL_480248	Genomic Laboratory (GLAB) (Conjoint lab of Health Directorate of Istanbul and Istanbul Technical University)	Genomic Laboratory (GLAB), Istanbul Technical University	Arzu Irvem; Betsi Köse; Bugra Agaoglu; Elifnaz Çelik; Gizem Alkurt; Gizem Dinler Doganay; Ilker Karacan; Jale Yildiz; Levent Doganay; Mehtap Aydın; Ozlem Akgun Dogan; Tugba Kizilboga Akgun; Yasemin Kendir Demirkol
EPI_ISL_1366326, EPI_ISL_1366327, EPI_ISL_1366328, EPI_ISL_1366329, EPI_ISL_1366330, EPI_ISL_1366331, EPI_ISL_1366332, EPI_ISL_1366333, EPI_ISL_1366334			
see above	Genomica Lab Molecular, Mexico	Andersen lab at Scripps Research	Jose Horacio Reyna Verdugo; Jose Roman Chavez Mendez; Luis Alberto Rangel Gonzalez; Martin Gonzalez Ibarra; SEARCH Alliance San Diego with Jonathan Gonzalez Garcia
EPI_ISL_730197, EPI_ISL_730198, EPI_ISL_730199, EPI_ISL_730200, EPI_ISL_730201, EPI_ISL_730202, EPI_ISL_730203, EPI_ISL_730204, EPI_ISL_730205, EPI_ISL_730206, EPI_ISL_730207, EPI_ISL_730208, EPI_ISL_730209, EPI_ISL_730210, EPI_ISL_730211, EPI_ISL_730212, EPI_ISL_730213, EPI_ISL_730214, EPI_ISL_730215, EPI_ISL_730216, EPI_ISL_730217, EPI_ISL_730218, EPI_ISL_730219, EPI_ISL_730220, EPI_ISL_730221, EPI_ISL_730222, EPI_ISL_730223, EPI_ISL_730224, EPI_ISL_730225, EPI_ISL_730226, EPI_ISL_730227, EPI_ISL_730228, EPI_ISL_1081423, EPI_ISL_1081424, EPI_ISL_1081425, EPI_ISL_1081426, EPI_ISL_1081427, EPI_ISL_1081428, EPI_ISL_1081429, EPI_ISL_1081430, EPI_ISL_1081431, EPI_ISL_1081432, EPI_ISL_1081433, EPI_ISL_1081434, EPI_ISL_1081435, EPI_ISL_1081436, EPI_ISL_1081437, EPI_ISL_1081438, EPI_ISL_1081439, EPI_ISL_1081440, EPI_ISL_1081441, EPI_ISL_1081442, EPI_ISL_1081443, EPI_ISL_1081444, EPI_ISL_1081445, EPI_ISL_1081446, EPI_ISL_1081447, EPI_ISL_1081448, EPI_ISL_1081449, EPI_ISL_1081450, EPI_ISL_1081451, EPI_ISL_1081452, EPI_ISL_1081453, EPI_ISL_1081454, EPI_ISL_1081455, EPI_ISL_1081456, EPI_ISL_1081473, EPI_ISL_1081474, EPI_ISL_1081475, EPI_ISL_1081476, EPI_ISL_1081477, EPI_ISL_1081478, EPI_ISL_1081479, EPI_ISL_1081480, EPI_ISL_1081481, EPI_ISL_1081482, EPI_ISL_1081483, EPI_ISL_1081484, EPI_ISL_1081485, EPI_ISL_1081486, EPI_ISL_1081487, EPI_ISL_1081488, EPI_ISL_1081489, EPI_ISL_1081490, EPI_ISL_1081491, EPI_ISL_1081492, EPI_ISL_1081493, EPI_ISL_1081494, EPI_ISL_1081495			
see above	Genomica Lab Molecular, M√©xico	Andersen lab at Scripps Research	Jose Horacio Reyna Verdugo; Jose Roman Chavez Mendez; Luis Alberto Rangel Gonzalez; Martin Gonzalez Ibarra; SEARCH Alliance San Diego with Jonathan Gonzalez Garcia
EPI_ISL_461478	Government Medical College, Vadodara	Gujarat Biotechnology Research Centre	; A M Kadri; Ankit Hinsu; Apurvasinh Puvar; Chaitanya Joshi; Dinesh Kumar; Fenil Patel; Harsh Bakshi; Janvi Raval; Komal Patel; Labdhi Pandya; Madhvi Joshi; Maharshi Pandya; Monika Gandhi; Nidhi Patel; Nitin Savaliya; Pinal Trivedi; Pritesh Sabara; R D Dixit; R N Daveshwar; Raghawendra Kumar; Snehal Bagatharia; Tanuja Javadekar; Tejas Shah; Zarna Patel; Zuber Saiyed
EPI_ISL_698244, EPI_ISL_698610, EPI_ISL_699142	Group 42 (G42) Healthcare, Abu Dhabi, United Arab Emirates; Department of Health, The United Arab Emirates	G42 Healthcare	Ashish Koshy; Budoor Alqarni; Denghui Liu; Fang Chen; Hanif Khalak; Huanming Yang; Javier Quilez; Jian Wang; Junhua Li; Ke Liang; Long Lin; Mohammed Saifuddin Fasihuddin; Nan Qiao; Nawal Ahmed Mohamed Al Kaabi; Pauline Ogrodzki; Pei Wu; Peng Xiao; Pengjuan Liu; Rong Liu; Sally Mahmoud; Siyang Liu; Stephen S. Francis; Tao Ma; Vinay Kusuma; Walid Abbas Zaher; Weibin Liu; Wenjun He; Xavier Anton; Xin Jin; Xin Meng; Xinyu Huang; Xun Xu; Zhaorong Yuan
EPI_ISL_435056	Gujarat Biotechnology Research Centre	Gujarat Biotechnology Research Centre	Afzal Ansari; Akanksha Verma; Amit Kanani; Ankit Hinsu; Apurvasinh Puvar; Bhavesh Modi; Binita Aring; Chaitanya Joshi; Dinesh Kumar; Dipa Kinariwala; Disha Patel; Gaurishankar Shrimali; Geeta Vaghela; Janvi Raval; Kairavi Joshi; Kamlesh J Upadhyay; Madhvi Joshi; Maharshi Pandya; Monika Gandhi; Nidhi Sood; Nitin Savaliya; Pinal Trivedi; Pranay Shah; Pritesh Sabara; R D Dixit; Raghawendra Kumar; Ramesh Pandit; Snehal Bagatharia; Sonia Barve; Tejas Shah; Zuber Saiyed
EPI_ISL_422462, EPI_ISL_547628	Gundersen Molecular Diagnostics Laboratory	Kabara Cancer Research Institute	Craig S. Richmond; Paraic A. Kenny
EPI_ISL_596363	HELIX LCC	WHO National Influenza Centre Russian Federation	Andrey Komissarov; Anna Ivanova; Artem Fadeev; Daria Danilenko; Dmitry Bazhenov; Kseniya Komissarova
EPI_ISL_445349	HOSPITAL SAN JUAN DE DIOS	Instituto de Salud Publica de Chile	Alejandra Acevedo; Andrés E Castillo; Bárbara Parra; Carolina Tambley; Gabriel Leal; Jaime Lagos; Jorge Fernandez; Loredana Arata; Patricia Bustos; Paz Tapia; Rodrigo Fasce; Winston Andrade
EPI_ISL_418244	HOSPITAL UNIVERSITARIO VIRGEN DE LAS NIEVES	Instituto de Salud Carlos III	A. Monzón; F. Casas; I. Jiménez; I. Sanbonmatsu S.; Iglesias-Caballero; M. Camarero; M. Cuesta; M. González-Esguevillas; M. Molinero Calamita; M. Zaballos; P. Jiménez; S. Juliá; S. Pozo; S. Varona
EPI_ISL_640082	Heideveld Emergency Centre	NHLS/UCT	Arash Iranzadeh; Bruna Galvao; Carolyn Williamson; Deelan Doolabh; Diana Hardie; Innocent Mudau; Kruger Marais; Lynn Tyers; Marvin Hsiao; Stephen Korsman
EPI_ISL_700168	Hematopathology Laboratory, ACTREC, TMC	Hematopathology Laboratory, ACTREC, TMC	ACTREC; Hematopathology Laboratory
EPI_ISL_462479	Hospital Clinic	Instituto de Salud Carlos III	A. Monzón; F. Casas; I; I. Jiménez; Iglesias-Caballero; M. Camarero; M. Cuesta; M. González-Esguevillas; M. Molinero Calamita; M. Zaballos; M.A Marcos; P. Jiménez; S. Juliá; S. Pozo; S. Varona
EPI_ISL_1120614	Hospital Margarita Maza de Juarez	CIAD LDM-LGM	Bruno Gome-Gil; Julissa Enciso-Ibarra
EPI_ISL_1482628	Hospital Margarita Maza de Juárez	Microbial Genomics Laboratory	Alejandra Garcia-Gasca; Bruno Gomez-Gil; Daniel Fregoso-Rueda; Julissa Enciso-Ibarra

EPI_ISL_468315, EPI_ISL_515546	Hospital Municipal do Tatuape Carmino Caricchio	Instituto Adolfo Lutz, Interdiciplinary Procedures Center, Strategic Laboratory	Claudia Regina Gonçalves; Claudio Tavares Sacchi; Erica Valessa Ramos Gomes
EPI_ISL_523956	Hospital Regional de Assis	Instituto Adolfo Lutz, Interdiciplinary Procedures Center, Strategic Laboratory	Claudia Regina Gonçalves; Claudio Tavares Sacchi; Erica Valessa Ramos Gomes
EPI_ISL_491438	Hospital San Rafael de Alajuela	Inciensa, Instituto Costarricense de Investigación y Enseñanza en Nutrición y Salud	Adriana Godinez & Melany Calderon; Claudio Soto-Garita; Estela Cordero; Francisco Duarte; Hebleen Brenes
EPI_ISL_1482627, EPI_ISL_1491363, EPI_ISL_1491364, EPI_ISL_1491365, EPI_ISL_1627079, EPI_ISL_1660616	Hospital Sharp	Microbial Genomics Laboratory	Alejandra Garcia-Gasca; Bruno Gomez-Gil; Daniel Fregoso-Rueda; Julissa Enciso-Ibarra
EPI_ISL_428683, EPI_ISL_529992	Hospital Universitario 12 de Octubre	Hospital Universitario 12 de Octubre	Elias Dahdouh; Esther Viedma; Fernando Lázaro; Jesús Mingorance; Juan Carlos Galán; Julio García; Mª Dolores Folgueira; Natalia Stella; Rafael Cantón; Rafael Delgado; Raúl Recio; Sara González
EPI_ISL_467179	Hospital Universitario Araba. Vitoria-Gasteiz	SeqCOVID-SPAIN consortium/IBV(CSIC)	Amaia Aguirre Quiñonero; Andrés Canut Blasco. and SeqCOVID-SPAIN consortium; Carmen Gómez González; Marina Fernández Torres; Mª Concepción Lecaroz Agara; Mª Rosario Almela Ferrer; Silvia Hernáez Crespo
EPI_ISL_428679	Hospital Universitario La Paz	Hospital Universitario 12 de Octubre	Elias Dahdouh; Esther Viedma; Fernando Lázaro; Jesús Mingorance; Juan Carlos Galán; Julio García; Mª Dolores Folgueira; Natalia Stella; Rafael Cantón; Rafael Delgado; Raúl Recio; Sara González
EPI_ISL_530106	Hospital Universitario Ramón y Cajal	Hospital Universitario La Paz	Elias Dahdouh; Esther Viedma; Fernando Lázaro; Jesús Mingorance; Juan Carlos Galán; Julio García; Mª Dolores Folgueira; Natalia Stella; Rafael Cantón; Rafael Delgado; Raúl Recio; Sara González
EPI_ISL_474851	Hospital Universitario Virgen de las Nieves de Granada-SAS	SeqCOVID-SPAIN consortium/IBV(CSIC)	Irene Pedrosa Corral; José M. Navarro-Marí and SeqCOVID-SPAIN consortium; Mercedes Pérez Ruiz; Sara Sanbonmatsu Gámez
EPI_ISL_434772, EPI_ISL_542539, EPI_ISL_542719, EPI_ISL_542813, EPI_ISL_542815, EPI_ISL_542893, EPI_ISL_542915, EPI_ISL_543155, EPI_ISL_544842, EPI_ISL_545081, EPI_ISL_545087, EPI_ISL_545168, EPI_ISL_545169, EPI_ISL_545216, EPI_ISL_545303, EPI_ISL_545420, EPI_ISL_545446, EPI_ISL_546191			
see above	Houston Methodist Hospital	Houston Methodist Hospital	Chia-Wei Chou; Concepcion C. Cantu; Daniel Boutz; David W. Bernard; Ghazaleh Eskandari; Hakon Jonsson; Heather Hendrickson; Hoang A. T. Nguyen; Hung-Che Kuo; Ilya J. Finkelstein; J. Hunter Long; James J. Davis; Jason S. McLellan; Jimmy Gollihar; Jule Goike; Kamyab Javanmardi; Kari Stefansson; Layne Pruitt; Marcus Nguyen; Matthew Ojeda Saavedra; Maulik Shukla; Muthiah Kumaraswami; Paul A. Christensen; Prasanti Yerramilli; Randall J. Olsen; S. Wesley Long; Sishir Subedi; and James M. Musser
EPI_ISL_645018, EPI_ISL_645047	Human Genome Variation Research Group, Malopolska Centre of Biotechnology	Human Genome Variation Research Group, Malopolska Centre of Biotechnology	Botwina, P.; Branicki, W.; Dabrowska, A.; Foremny, J.; Gromowski, T.; Klajmon, A.; Kopera, K.; Kowalski, M.; Labaj; Marszalek, K.; Owczarek, K.; P.P.; Pisarek, A.; Pospiech, E.; Pyrc, K.; Sanak, M.; Swadzba, J.; Szczepanski, A.
EPI_ISL_526215	Hungarian Defence Forces Military Medical Centre	National Laboratory of Virology, Szentágothai Research Centre	Balázs Somogyi; Bálint Eszenyi; Endre Gábor Tóth; Ferenc Jakab; Gábor Kemenesi
EPI_ISL_536340	Hôpital Charles-LeMoyne	Laboratoire de santé publique du Québec	Guillaume Bourque; Ioannis Ragoussis; Jesse Shapiro; Mark Lathrop and Michel Roger on behalf of the CoVSeQ research group; Sandrine Moreira
EPI_ISL_536208, EPI_ISL_536230	Hôpital de Hull	Laboratoire de santé publique du Québec	Guillaume Bourque; Ioannis Ragoussis; Jesse Shapiro; Mark Lathrop and Michel Roger on behalf of the CoVSeQ research group; Sandrine Moreira
EPI_ISL_535744	Hôpital de Lasalle	Laboratoire de santé publique du Québec	Guillaume Bourque; Ioannis Ragoussis; Jesse Shapiro; Mark Lathrop and Michel Roger; Sandrine Moreira
EPI_ISL_455640	ICMR-National Institute of Cholera and Enteric Diseases	National Institute of Biomedical Genomics	Ananya Chatterjee; Arindam Maitra; Hasina Banu; Mamta Chawla Sarkar; Saumitra Das; Shanta Dutta; Sreedhar Chinnaswamy
EPI_ISL_410045	IL Department of Public Health Chicago Laboratory	Pathogen Discovery, Respiratory Viruses Branch, Division of Viral Diseases, Centers for Dieases Control and Prevention	Anna Uehara; Brett L. Whitaker; Brian Lynch; Clinton R. Paden; Janna' R. Murray; Jing Zhang; Krista Queen; Lijuan Wang; Senthil Kumar K. Sakthivel; Shifaq Kamili; Stephen Lindstrom; Susan I. Gerber; Suxiang Tong; Xiaoyan Lu; Yan Li; Ying Tao
EPI_ISL_424850	IL Department of Public Health Chicago Laboratory	Pathogen Discovery, Respiratory Viruses Branch, Division of Viral Diseases, Centers for Disease Control and Prevention	Alison S. Laufer Halpin; Anna Uehara; Christopher A. Elkins; Clinton R. Paden; Haibin Wang; Jing Zhang; Krista Queen; Mary S. Keckler; Rachel Marine; Suxiang Tong; Yan Li; Ying Tao
EPI_ISL_1340629	INER	Instituto de Diagnostico y Referencia Epidemiologicos (INDRE)	Abril Rodriguez-Maldonado; Ariadna Medina-Benitez; Claudia Wong-Arambula; Ernesto Ramirez-Gonzalez.; Gisela Barrera-Badillo; Irma Lopez-Martinez; Joaquin Quiroz-Mercado; Lucia Hernandez-Rivas; Natividad Cruz-Ortiz; Sergio Rangel-Guerrero; Tatiana Nunez-Garcia; Vanessa Rivero-Arredondo
EPI_ISL_493330	INMI Lazzaro Spallanzani IRCCS	INMI Lazzaro Spallanzani IRCCS	Antonino Di Caro; Barbara Bartolini; Cesare E.M. Gruber; Francesco Messina; Maria R. Capobianchi; Martina Rueca
EPI_ISL_751353, EPI_ISL_751355, EPI_ISL_751360	IRCCS Sacro Cuore Don Calabria Hospital, Department of Infectious, Tropical Diseases & Microbiology	University of Verona, Department of Biotechnology	Antonio Mori; Chiara Degli Esposti; Chiara Piubelli; Cristina Beltrami; Elena Pomari; Emanuela Cosentino; Giulia Lopatriello; Luca Marcolungo; Massimo Delledonne; Michela Deiana
EPI_ISL_1502814, EPI_ISL_1502815, EPI_ISL_1502816, EPI_ISL_1502817, EPI_ISL_1503143	Infectious Diseases Department , Instituto Nacional de Ciencias Medicas y Nutrición	Instituto Nacional de Ciencias Medicas y Nutricion Infectious Diseases	Diana Paola Davalos Martinez; Fernando Arteaga Cabello; Fernando Ledesma Barrientos; Guillermo M. Ruiz-Palacios; Luis Alberto Garcia Andrade; Luz Elena Cervantes Villar; Miriam Arciniega Fuentes; Pilar Ramos Cervantes; Violeta Ibarra Gonzalez
EPI_ISL_496339, EPI_ISL_496340, EPI_ISL_496341, EPI_ISL_496342, EPI_ISL_496343, EPI_ISL_496344, EPI_ISL_496345, EPI_ISL_496346, EPI_ISL_496347, EPI_ISL_496348, EPI_ISL_496349, EPI_ISL_496350, EPI_ISL_496351, EPI_ISL_496352, EPI_ISL_496353, EPI_ISL_496354, EPI_ISL_496355, EPI_ISL_496356, EPI_ISL_496357, EPI_ISL_496358, EPI_ISL_496359, EPI_ISL_496360, EPI_ISL_496361, EPI_ISL_496362, EPI_ISL_496363, EPI_ISL_496364, EPI_ISL_496365, EPI_ISL_496366, EPI_ISL_496367, EPI_ISL_496368, EPI_ISL_496369, EPI_ISL_496370, EPI_ISL_496371, EPI_ISL_496372, EPI_ISL_496373, EPI_ISL_496374, EPI_ISL_496375, EPI_ISL_496376			
see above	Infectolab	Andersen lab at Scripps Research	Carlos A. Cota Haros; Octavio Renteria Pacheco; SEARCH Alliance San Diego with Samuel Navarro Alvarez
EPI_ISL_418206, EPI_ISL_420078, EPI_ISL_481237	Institut Pasteur Dakar	Institut Pasteur de Dakar	Amadou Alpha Sall; Amadou Alpha Sall.; Mamadou Diop; Mamadou Malado Jallow; Marie Henriette Dior Ndione; Moussa Moise Diagne; Ndongo Dia; Ousmane Faye; Safietou Sanke
EPI_ISL_490100	Institute for Medical Research, Infectious Disease Research Centre, National Institutes of Health, Ministry of Health Malaysia	Institute for Medical Research, Infectious Disease Research Centre, National Institutes of Health, Ministry of Health Malaysia	Kalyanasundram J; Kamel K; Mohd-Zawawi Z; Suppiah J; Thayan R
EPI_ISL_602509, EPI_ISL_602518	Institute for Virology, University Hospital Essen	Center of Medical Microbiology, Virology, and Hospital Hygiene, University of Duesseldorf	Alexander Dilthey; Andreas Walker; Daniel Strelow; Jessica Nicolai; Jörg Timm; Klaus Pfeffer; Lisanna Hülse; Malte Kohns Vasconcelos; Maximilian Damagnez; Nadine Lübke; Olympia E. Anastasiou; Tobias Wienemann; Torsten Houwaart; Ulf Dittmer
EPI_ISL_723079	Institute of Medical Genetics and Applied Genomics	Institute of Medical Genetics and Applied Genomics	Angel Angelov; Caspar Gross; Daniela Bezdan; Michael Bitzer; Michael Sonnabend; Michaela Pogoda; Nicolas Casadei; Siri Göpel; Stephan Ossowski; Thomas Iftner; Tina Ganzenmüller
EPI_ISL_635260	Institute of Microbiology and Immunology, Faculty of Medicine, University of Ljubljana	Institute of Microbiology and Immunology, Faculty of Medicine, University of Ljubljana	Mario Poljak; Miša Korva; Samo Zakotnik; Tatjana Avšič – Županc; Tomaž Mark Zorec
EPI_ISL_402123	Institute of Pathogen Biology, Chinese Academy of Medical Sciences & Peking Union Medical College	Institute of Pathogen Biology, Chinese Academy of Medical Sciences & Peking Union Medical College	Chao Wu; Jianwei Wang; Lili Ren; Qi Jin; Yiwei Liu; Zhiqiang Wu; Zichun Xiang
EPI_ISL_508338, EPI_ISL_508385	Institute of Post Graduate Medical Education & Research	National Institute of Biomedical Genomics	Arindam Maitra; Aritra Biswas; Jayeeta Haldar; Monimoy Banerjee; Raja Ray; Saumitra Das
EPI_ISL_511903	Institute of Post Graduate Medical Education & Research	National Institute of Biomedical Genomics - DBT's PAN-INDIA 1000 SARS--CoV-2 RNA Genome Sequencing Consortium	Arindam Maitra; Aritra Biswas; Jayeeta Haldar; Monimoy Banerjee; Raja Ray; Saumitra Das
EPI_ISL_491175, EPI_ISL_491226	Instituto Gulbenkian de Ciência	Instituto Gulbenkian de Ciência	Cathy Paulino; Joao Sobral; João Costa; Ricardo Leite; Susana Ladeiro
EPI_ISL_426364	Instituto Nacional de Ciencias Medicas y Nutricion Salvador Zubiran	Instituto Nacional de Ciencias Medicas y Nutricion	Adnan Araiza Rodríguez; Alejandro Sánchez; Alfredo Ponce de León Garduño; Blanca Taboada; Carlos F. Arias; Carolina González Torres; Celia Boukadida; Cesar Raúl González Bonilla; Concepción Grajales Muñiz; Edgar Mendieta Condado; Eduardo Becerril Vargas; Fabiola Garcés Ayala; Fernando Ledesma Barrientos; Francisco Javier Gaytán Cervantes; Francisco Pulido; Gisela Barrera Badillo; Gloria Vázquez; Guillermo M. Ruiz-Palacios; Irma López Martínez; Joel Armando Vázquez Pérez; José Arturo Martínez Orozco; José Ernesto Ramírez González; José Esteban Muñoz Medina; Lucia Hernández Rivas; Luis Alberto García Andrade; Mario Mújica Sánchez; Pavel Isa; Pilar Ramos Cervantes; Ricardo Grande; Santiago Avila Ríos; Victor Hugo Borja Aburto; Violeta Ibarra Gonzalez
EPI_ISL_426361, EPI_ISL_426362, EPI_ISL_426363, EPI_ISL_426365	Instituto Nacional de Ciencias Medicas y Nutricion Salvador Zubiran	Instituto Nacional de Ciencias Medicas y Nutricion Salvador Zubiran	Adnan Araiza Rodríguez; Alejandro Sánchez; Alfredo Ponce de León Garduño; Blanca Taboada; Carlos F. Arias; Carolina González Torres; Celia Boukadida; Cesar Raúl González Bonilla; Concepción Grajales Muñiz; Edgar Mendieta Condado; Eduardo Becerril Vargas; Fabiola Garcés Ayala; Fernando Ledesma Barrientos; Francisco Javier Gaytán Cervantes; Francisco Pulido; Gisela Barrera Badillo; Gloria Vázquez; Guillermo M. Ruiz-Palacios; Irma López Martínez; Joel Armando Vázquez Pérez; José Arturo Martínez Orozco; José Ernesto Ramírez González; José Esteban Muñoz Medina; Lucia Hernández Rivas; Luis Alberto García Andrade; Mario Mújica Sánchez; Pavel Isa; Pilar Ramos Cervantes; Ricardo Grande; Santiago Avila Ríos; Victor Hugo Borja Aburto; Violeta Ibarra Gonzalez
EPI_ISL_424345, EPI_ISL_424348, EPI_ISL_424626, EPI_ISL_424627	Instituto Nacional de Enfermedades Respiratorias	Instituto Nacional de Enfermedades Respiratorias	Adnan Araiza Rodríguez; Alejandro Sánchez; Alfredo Ponce de León Garduño; Blanca Taboada; Carlos F. Arias.; Carolina González Torres; Celia Boukadida; Cesar Raúl González Bonilla; Concepción Grajales Muñiz; Edgar Mendieta Condado; Eduardo Becerril Vargas; Fabiola Garcés Ayala; Fernando Ledesma Barrientos; Francisco Javier Gaytán Cervantes; Francisco Pulido; Gisela Barrera Badillo; Gloria Vázquez; Guillermo M. Ruiz-Palacios; Irma López Martínez; Joel Armando Vázquez Pérez; Jorge Salas Hernández; José Arturo Martínez Orozco; José Ernesto Ramírez González; José Esteban Muñoz Medina; Lucia Hernández Rivas; Luis Alberto García Andrade; Mario Mújica Sánchez; Pavel Isa; Pilar Ramos Cervantes; Ricardo Grande; Santiago Avila Ríos; Victor Hugo Borja Aburto; Violeta Ibarra Gonzalez
EPI_ISL_412972	Instituto Nacional de Enfermedades Respiratorias	Instituto de Diagnostico y Referencia Epidemiologicos (INDRE)	Araiza-Rodriguez Adnan; Arias Carlos; Barrera-Badillo Gisela; Boukadida Celia; Garces-Ayala Fabiola; Hernandez-Rivas Lucia; Isa Pavel; Lopez Susana; Lopez-Martinez Irma; Martinez Arturo; Mendieta-Condado Edgar; Munoz-Medina Esteban; Ramirez-Gonzalez Ernesto; Rodriguez-Maldonado Abril; Sanchez Alejandro; Taboada Blanca; Vazquez-Perez Joel; Wong-Arambula Claudia
EPI_ISL_837600, EPI_ISL_837601, EPI_ISL_837602, EPI_ISL_837603, EPI_ISL_837604, EPI_ISL_837605, EPI_ISL_837606, EPI_ISL_837607, EPI_ISL_837608, EPI_ISL_837609, EPI_ISL_837610, EPI_ISL_837611, EPI_ISL_837612, EPI_ISL_837613, EPI_ISL_837614, EPI_ISL_837615, EPI_ISL_837616, EPI_ISL_837617, EPI_ISL_837618, EPI_ISL_837619, EPI_ISL_837620, EPI_ISL_837621, EPI_ISL_837622, EPI_ISL_837623, EPI_ISL_837624, EPI_ISL_837625, EPI_ISL_837626, EPI_ISL_837627, EPI_ISL_837628, EPI_ISL_837629, EPI_ISL_837630, EPI_ISL_837631, EPI_ISL_837632, EPI_ISL_837633, EPI_ISL_837634, EPI_ISL_837635, EPI_ISL_837636, EPI_ISL_837637, EPI_ISL_837638, EPI_ISL_837639, EPI_ISL_837640, EPI_ISL_837641, EPI_ISL_837642, EPI_ISL_837643, EPI_ISL_837644, EPI_ISL_837645, EPI_ISL_837646, EPI_ISL_837647, EPI_ISL_837648, EPI_ISL_837649, EPI_ISL_837650, EPI_ISL_837651, EPI_ISL_837652, EPI_ISL_837653, EPI_ISL_837654, EPI_ISL_837655, EPI_ISL_837656, EPI_ISL_837657, EPI_ISL_837658, EPI_ISL_837659, EPI_ISL_837660, EPI_ISL_837661, EPI_ISL_837662, EPI_ISL_837663, EPI_ISL_837664, EPI_ISL_837665, EPI_ISL_837666, EPI_ISL_837667, EPI_ISL_837668, EPI_ISL_837669, EPI_ISL_837670, EPI_ISL_837671, EPI_ISL_837672, EPI_ISL_837673, EPI_ISL_837674, EPI_ISL_837675, EPI_ISL_837676, EPI_ISL_837677, EPI_ISL_837678, EPI_ISL_837679, EPI_ISL_837680, EPI_ISL_837681, EPI_ISL_837682, EPI_ISL_837683, EPI_ISL_837684, EPI_ISL_837685, EPI_ISL_837686, EPI_ISL_837687, EPI_ISL_837688, EPI_ISL_837689, EPI_ISL_837690, EPI_ISL_837691, EPI_ISL_837692, EPI_ISL_837693, EPI_ISL_837694, EPI_ISL_837695, EPI_ISL_837696, EPI_ISL_837697, EPI_ISL_837698, EPI_ISL_837699, EPI_ISL_837700, EPI_ISL_837701, EPI_ISL_837702, EPI_ISL_837703, EPI_ISL_837704, EPI_ISL_837705, EPI_ISL_837706, EPI_ISL_837707, EPI_ISL_837708, EPI_ISL_837709, EPI_ISL_837710, EPI_ISL_837711, EPI_ISL_837712, EPI_ISL_837713, EPI_ISL_837714, EPI_ISL_837715, EPI_ISL_837716, EPI_ISL_837717, EPI_ISL_837718, EPI_ISL_837719, EPI_ISL_837720, EPI_ISL_837721, EPI_ISL_837722, EPI_ISL_837723, EPI_ISL_837724, EPI_ISL_837725, EPI_ISL_837726, EPI_ISL_837727, EPI_ISL_837728, EPI_ISL_837729, EPI_ISL_837730, EPI_ISL_837731, EPI_ISL_837732, EPI_ISL_837733, EPI_ISL_837734, EPI_ISL_837735, EPI_ISL_837736, EPI_ISL_837737, EPI_ISL_837738, EPI_ISL_837739, EPI_ISL_837740, EPI_ISL_837741, EPI_ISL_837742, EPI_ISL_837743, EPI_ISL_837744, EPI_ISL_837745, EPI_ISL_837746, EPI_ISL_837747, EPI_ISL_837748, EPI_ISL_837749, EPI_ISL_837750, EPI_ISL_837751, EPI_ISL_837752, EPI_ISL_837753, EPI_ISL_837754, EPI_ISL_837755, EPI_ISL_837756, EPI_ISL_837757, EPI_ISL_837758, EPI_ISL_837759, EPI_ISL_837760, EPI_ISL_837761, EPI_ISL_837762, EPI_ISL_837763, EPI_ISL_837764, EPI_ISL_837765, EPI_ISL_837766, EPI_ISL_837767, EPI_ISL_837768, EPI_ISL_837769, EPI_ISL_837770, EPI_ISL_837771, EPI_ISL_837772, EPI_ISL_837773, EPI_ISL_837774, EPI_ISL_837775, EPI_ISL_837776, EPI_ISL_837777, EPI_ISL_837778, EPI_ISL_837779, EPI_ISL_837780, EPI_ISL_837781, EPI_ISL_837782, EPI_ISL_837783, EPI_ISL_837784, EPI_ISL_837785, EPI_ISL_837786, EPI_ISL_837787, EPI_ISL_837788, EPI_ISL_837789, EPI_ISL_837790, EPI_ISL_837791,			

EPI_ISL_837792, EPI_ISL_837793, EPI_ISL_837794, EPI_ISL_837795, EPI_ISL_837796, EPI_ISL_837797, EPI_ISL_837798, EPI_ISL_837799, EPI_ISL_837800, EPI_ISL_837801, EPI_ISL_837802, EPI_ISL_837803, EPI_ISL_837804, EPI_ISL_837805, EPI_ISL_837806, EPI_ISL_837807, EPI_ISL_837808, EPI_ISL_837809, EPI_ISL_837810, EPI_ISL_837811, EPI_ISL_837812, EPI_ISL_837813, EPI_ISL_837814, EPI_ISL_843191, EPI_ISL_843192			
see above	Instituto Nacional de Enfermedades Respiratorias (INER)	Instituto Nacional de Enfermedades Respiratorias (INER)	Alejandra Hernández-Terán; Alma Rincón-Rubio; Celia Boukadida; Edgar Sevilla-Reyes; Eduardo Becerril-Vargas; Fidencio Mejía-Nepomuceno; Hector Esteban Paz-Juárez; Joel Armando Vázquez-Pérez; Jorge Salas-Hernández; José Arturo Martínez-Orozco; Margarita Matías-Florentino; Mario Mújica-Sánchez; Olivia Briceño; Santiago Ávila-Ríos
EPI_ISL_1824419, EPI_ISL_1824420, EPI_ISL_1824421, EPI_ISL_1824422, EPI_ISL_1824423, EPI_ISL_1824424, EPI_ISL_1824425, EPI_ISL_1824426, EPI_ISL_1824427, EPI_ISL_1824428, EPI_ISL_1824429, EPI_ISL_1824442, EPI_ISL_1824443, EPI_ISL_1824444, EPI_ISL_1824445, EPI_ISL_1824446, EPI_ISL_1824447, EPI_ISL_1824448, EPI_ISL_1824449, EPI_ISL_1824450, EPI_ISL_1970838, EPI_ISL_1970842, EPI_ISL_1970843, EPI_ISL_1970844, EPI_ISL_1970845			
see above	Instituto Nacional de Medicina Genomica	Centro de Investigación en Enfermedades Infecciosas (CIENI), Instituto Nacional de Enfermedades Respiratorias (INER)	Arriaga-Canon C; Avila-Rios S; Boukadida C; Cedro-Tanda A; Herrera-Montalvo LA; Hidalgo-Miranda A; Matias-Florentino M; Mendoza-Vargas A; Perez-Garcia M; Reyes-Grajeda JP; Reyes-Teran G
EPI_ISL_1406722, EPI_ISL_1406723, EPI_ISL_1406724, EPI_ISL_1406725, EPI_ISL_1406726, EPI_ISL_1406727, EPI_ISL_1406728, EPI_ISL_1406729, EPI_ISL_1406730, EPI_ISL_1406731, EPI_ISL_1406732, EPI_ISL_1406751, EPI_ISL_1406752, EPI_ISL_1406753, EPI_ISL_1406754, EPI_ISL_1406755, EPI_ISL_1406756, EPI_ISL_1406757, EPI_ISL_1406758, EPI_ISL_1406759, EPI_ISL_1406760, EPI_ISL_1406761, EPI_ISL_1406762, EPI_ISL_1406763, EPI_ISL_1406764, EPI_ISL_1406765, EPI_ISL_1406766, EPI_ISL_1406767, EPI_ISL_1406768, EPI_ISL_1406769, EPI_ISL_1406770, EPI_ISL_1406771, EPI_ISL_1406772, EPI_ISL_1406773, EPI_ISL_1406774, EPI_ISL_1406775, EPI_ISL_1406776, EPI_ISL_1423994, EPI_ISL_1423995, EPI_ISL_1424000, EPI_ISL_1424001			
see above	Instituto Nacional de Medicina Genomica	Instituto Nacional de Enfermedades Respiratorias (INER), Centro de Investigación en Enfermedades Infecciosas (CIENI)	Arriaga-Canon C; Avila-Rios S; Boukadida C; Cedro-Tanda A; Herrera-Montalvo LA; Hidalgo-Miranda A; Matias-Florentino M; Mendoza-Vargas A; Reyes-Grajeda JP; Reyes-Teran G
EPI_ISL_522873, EPI_ISL_522874, EPI_ISL_522875, EPI_ISL_522876, EPI_ISL_522877, EPI_ISL_522878, EPI_ISL_930783, EPI_ISL_933512, EPI_ISL_933513, EPI_ISL_933514, EPI_ISL_933515, EPI_ISL_933516, EPI_ISL_933517, EPI_ISL_933518, EPI_ISL_933519, EPI_ISL_933520, EPI_ISL_933521, EPI_ISL_933522, EPI_ISL_933523, EPI_ISL_933524, EPI_ISL_933525, EPI_ISL_933526, EPI_ISL_933527, EPI_ISL_933528, EPI_ISL_933529, EPI_ISL_933530, EPI_ISL_933531, EPI_ISL_944611, EPI_ISL_944612, EPI_ISL_944613, EPI_ISL_944614, EPI_ISL_944615, EPI_ISL_944616, EPI_ISL_944617, EPI_ISL_944618, EPI_ISL_944619, EPI_ISL_944620, EPI_ISL_944621, EPI_ISL_944622, EPI_ISL_944623, EPI_ISL_944624, EPI_ISL_944625, EPI_ISL_944626, EPI_ISL_944627, EPI_ISL_944628, EPI_ISL_944629, EPI_ISL_944630, EPI_ISL_944631, EPI_ISL_944632, EPI_ISL_944633, EPI_ISL_955189, EPI_ISL_955190, EPI_ISL_955191, EPI_ISL_955192, EPI_ISL_955193, EPI_ISL_955194, EPI_ISL_955195, EPI_ISL_955196, EPI_ISL_955197, EPI_ISL_955198, EPI_ISL_955199, EPI_ISL_955200, EPI_ISL_955201, EPI_ISL_955202, EPI_ISL_955203, EPI_ISL_955204, EPI_ISL_955205, EPI_ISL_955206, EPI_ISL_955207, EPI_ISL_955208, EPI_ISL_955209, EPI_ISL_985149, EPI_ISL_985150, EPI_ISL_985151, EPI_ISL_985152, EPI_ISL_985153, EPI_ISL_985154, EPI_ISL_985155, EPI_ISL_985156, EPI_ISL_985157, EPI_ISL_985158, EPI_ISL_985159, EPI_ISL_985160, EPI_ISL_985161, EPI_ISL_985162, EPI_ISL_985163, EPI_ISL_985164, EPI_ISL_985165, EPI_ISL_985166, EPI_ISL_985167, EPI_ISL_1005559, EPI_ISL_1005560, EPI_ISL_1005561, EPI_ISL_1005562, EPI_ISL_1005563, EPI_ISL_1005564, EPI_ISL_1005565, EPI_ISL_1005566, EPI_ISL_1005567, EPI_ISL_1005568, EPI_ISL_1005569, EPI_ISL_1005570, EPI_ISL_1005571, EPI_ISL_1005572, EPI_ISL_1005573, EPI_ISL_1005574, EPI_ISL_1005575, EPI_ISL_1005576, EPI_ISL_1005577, EPI_ISL_1005578, EPI_ISL_1005579, EPI_ISL_1005580, EPI_ISL_1005581, EPI_ISL_1005582, EPI_ISL_1005656, EPI_ISL_1005657, EPI_ISL_1005658, EPI_ISL_1005659, EPI_ISL_1005660, EPI_ISL_1005661, EPI_ISL_1005662, EPI_ISL_1005663, EPI_ISL_1005664, EPI_ISL_1005665, EPI_ISL_1005666, EPI_ISL_1005667, EPI_ISL_1005668, EPI_ISL_1005669, EPI_ISL_1005670, EPI_ISL_1005671, EPI_ISL_1005672, EPI_ISL_1005673, EPI_ISL_1040535, EPI_ISL_1040536, EPI_ISL_1040537, EPI_ISL_1040538, EPI_ISL_1040539, EPI_ISL_1040540, EPI_ISL_1040541, EPI_ISL_1040542, EPI_ISL_1040543, EPI_ISL_1040544, EPI_ISL_1040545, EPI_ISL_1040546, EPI_ISL_1040547, EPI_ISL_1040548, EPI_ISL_1040549, EPI_ISL_1040550, EPI_ISL_1040551, EPI_ISL_1040552, EPI_ISL_1040553, EPI_ISL_1040554, EPI_ISL_1040555, EPI_ISL_1040556, EPI_ISL_1040560, EPI_ISL_1040561, EPI_ISL_1040562, EPI_ISL_1040563, EPI_ISL_1040564, EPI_ISL_1040565, EPI_ISL_1040566, EPI_ISL_1040567, EPI_ISL_1040568, EPI_ISL_1040569, EPI_ISL_1040570, EPI_ISL_1040571, EPI_ISL_1040572, EPI_ISL_1040573, EPI_ISL_1040574, EPI_ISL_1040575, EPI_ISL_1040576, EPI_ISL_1040577, EPI_ISL_1040578, EPI_ISL_1040579, EPI_ISL_1040580, EPI_ISL_1040581, EPI_ISL_1040582, EPI_ISL_1040583, EPI_ISL_1040584, EPI_ISL_1040585, EPI_ISL_1040586, EPI_ISL_1040587, EPI_ISL_1040588, EPI_ISL_1040589, EPI_ISL_1040590, EPI_ISL_1040591, EPI_ISL_1040592, EPI_ISL_1040593, EPI_ISL_1040594, EPI_ISL_1040595, EPI_ISL_1040596, EPI_ISL_1040597, EPI_ISL_1040598, EPI_ISL_1040599, EPI_ISL_1040600, EPI_ISL_1040601, EPI_ISL_1040602, EPI_ISL_1040604, EPI_ISL_1040605, EPI_ISL_1040606, EPI_ISL_1040607, EPI_ISL_1040608, EPI_ISL_1040609, EPI_ISL_1040610, EPI_ISL_1040611, EPI_ISL_1040612, EPI_ISL_1040613, EPI_ISL_1040614, EPI_ISL_1040615, EPI_ISL_1040616, EPI_ISL_1040617, EPI_ISL_1040618, EPI_ISL_1040619, EPI_ISL_1040620, EPI_ISL_1040621, EPI_ISL_1040622, EPI_ISL_1040623, EPI_ISL_1040624, EPI_ISL_1040625, EPI_ISL_1040626, EPI_ISL_1040627, EPI_ISL_1040628, EPI_ISL_1040629, EPI_ISL_1040630, EPI_ISL_1040631, EPI_ISL_1040632, EPI_ISL_1040633, EPI_ISL_1040634, EPI_ISL_1040635, EPI_ISL_1040636, EPI_ISL_1040637, EPI_ISL_1040638, EPI_ISL_1040639, EPI_ISL_1040640, EPI_ISL_1040641, EPI_ISL_1040642, EPI_ISL_1040643, EPI_ISL_1055005, EPI_ISL_1055006, EPI_ISL_1055007, EPI_ISL_1055008, EPI_ISL_1055009, EPI_ISL_1055010, EPI_ISL_1055011, EPI_ISL_1055012, EPI_ISL_1055013, EPI_ISL_1055014, EPI_ISL_1055015, EPI_ISL_1055016, EPI_ISL_1055017, EPI_ISL_1055018, EPI_ISL_1055019, EPI_ISL_1055020, EPI_ISL_1055021, EPI_ISL_1055022, EPI_ISL_1055023, EPI_ISL_1055024, EPI_ISL_1055025, EPI_ISL_1058046, EPI_ISL_1060598, EPI_ISL_1060599, EPI_ISL_1060600, EPI_ISL_1060601, EPI_ISL_1060602, EPI_ISL_1060603, EPI_ISL_1060604, EPI_ISL_1060605, EPI_ISL_1060606, EPI_ISL_1060607, EPI_ISL_1060608, EPI_ISL_1060609, EPI_ISL_1060610, EPI_ISL_1060611, EPI_ISL_1060612, EPI_ISL_1060613, EPI_ISL_1060614, EPI_ISL_1060615, EPI_ISL_1075267, EPI_ISL_1075657, EPI_ISL_1080433, EPI_ISL_1080434, EPI_ISL_1080435, EPI_ISL_1080436, EPI_ISL_1080437, EPI_ISL_1080438, EPI_ISL_1080439, EPI_ISL_1080440, EPI_ISL_1080441, EPI_ISL_1080442, EPI_ISL_1080443, EPI_ISL_1080444, EPI_ISL_1080445, EPI_ISL_1080446, EPI_ISL_1080447, EPI_ISL_1080448, EPI_ISL_1080449, EPI_ISL_1080450, EPI_ISL_1080451, EPI_ISL_1080452, EPI_ISL_1080453, EPI_ISL_1081152, EPI_ISL_1081153, EPI_ISL_1081154, EPI_ISL_1081155, EPI_ISL_1081156, EPI_ISL_1081158, EPI_ISL_1081159, EPI_ISL_1081160, EPI_ISL_1081161, EPI_ISL_1081162, EPI_ISL_1081163, EPI_ISL_1081164, EPI_ISL_1081165, EPI_ISL_1081166, EPI_ISL_1081167, EPI_ISL_1081168, EPI_ISL_1081169, EPI_ISL_1085421, EPI_ISL_1095493, EPI_ISL_1095494, EPI_ISL_1095495, EPI_ISL_1095496, EPI_ISL_1095497, EPI_ISL_1095498, EPI_ISL_1095499, EPI_ISL_1095500, EPI_ISL_1095501, EPI_ISL_1095502, EPI_ISL_1095503, EPI_ISL_1095504, EPI_ISL_1095505, EPI_ISL_1095506, EPI_ISL_1095507, EPI_ISL_1095508, EPI_ISL_1095509, EPI_ISL_1095510, EPI_ISL_1095511, EPI_ISL_1095512, EPI_ISL_1095513, EPI_ISL_1095514, EPI_ISL_1095515, EPI_ISL_1095516, EPI_ISL_1117353, EPI_ISL_1117354, EPI_ISL_1117355, EPI_ISL_1117356, EPI_ISL_1117357, EPI_ISL_1117358, EPI_ISL_1117359, EPI_ISL_1117360, EPI_ISL_1117361, EPI_ISL_1117362, EPI_ISL_1117363, EPI_ISL_1117364, EPI_ISL_1117365, EPI_ISL_1117366, EPI_ISL_1117367, EPI_ISL_1117368, EPI_ISL_1117369, EPI_ISL_1117370, EPI_ISL_1117371, EPI_ISL_1117372, EPI_ISL_1117373, EPI_ISL_1117374, EPI_ISL_1117375, EPI_ISL_1120878, EPI_ISL_1120879, EPI_ISL_1120880, EPI_ISL_1120881, EPI_ISL_1120882, EPI_ISL_1120883, EPI_ISL_1120884, EPI_ISL_1120885, EPI_ISL_1120886, EPI_ISL_1120887, EPI_ISL_1120888, EPI_ISL_1120889, EPI_ISL_1120890, EPI_ISL_1120891, EPI_ISL_1120892, EPI_ISL_1120893, EPI_ISL_1120894, EPI_ISL_1120895, EPI_ISL_1120896, EPI_ISL_1133103, EPI_ISL_1137460, EPI_ISL_1137461, EPI_ISL_1137462, EPI_ISL_1137463, EPI_ISL_1137464, EPI_ISL_1137465, EPI_ISL_1137466, EPI_ISL_1137467, EPI_ISL_1137468, EPI_ISL_1137469, EPI_ISL_1137470, EPI_ISL_1137471, EPI_ISL_1137472, EPI_ISL_1137473, EPI_ISL_1137474, EPI_ISL_1168634, EPI_ISL_1168635, EPI_ISL_1168636, EPI_ISL_1168637, EPI_ISL_1168638, EPI_ISL_1168639, EPI_ISL_1168640, EPI_ISL_1168641, EPI_ISL_1168642, EPI_ISL_1168643, EPI_ISL_1168644, EPI_ISL_1168645, EPI_ISL_1168646, EPI_ISL_1168647, EPI_ISL_1168648, EPI_ISL_1168649, EPI_ISL_1168650, EPI_ISL_1168651, EPI_ISL_1168652, EPI_ISL_1181700, EPI_ISL_1181701, EPI_ISL_1181702, EPI_ISL_1181703, EPI_ISL_1181704, EPI_ISL_1181705, EPI_ISL_1181706, EPI_ISL_1181707, EPI_ISL_1181708, EPI_ISL_1181709, EPI_ISL_1181710, EPI_ISL_1181711, EPI_ISL_1181712, EPI_ISL_1181713, EPI_ISL_1181714, EPI_ISL_1181715, EPI_ISL_1181716, EPI_ISL_1181717, EPI_ISL_1181718, EPI_ISL_1181719, EPI_ISL_1193558, EPI_ISL_1205203, EPI_ISL_1205204, EPI_ISL_1205205, EPI_ISL_1205206, EPI_ISL_1205207, EPI_ISL_1205208, EPI_ISL_1205209, EPI_ISL_1205210, EPI_ISL_1205211, EPI_ISL_1205212, EPI_ISL_1205213, EPI_ISL_1205214, EPI_ISL_1205215, EPI_ISL_1205216, EPI_ISL_1205217, EPI_ISL_1205218, EPI_ISL_1262631, EPI_ISL_1262638, EPI_ISL_1262642, EPI_ISL_1262680, EPI_ISL_1262681, EPI_ISL_1262682, EPI_ISL_1262683, EPI_ISL_1262684, EPI_ISL_1262685, EPI_ISL_1262686, EPI_ISL_1262687, EPI_ISL_1262688, EPI_ISL_1262689, EPI_ISL_1262690, EPI_ISL_1262691, EPI_ISL_1262692, EPI_ISL_1262693, EPI_ISL_1262694, EPI_ISL_1262695, EPI_ISL_1262696, EPI_ISL_1262697, EPI_ISL_1262698, EPI_ISL_1262699, EPI_ISL_1262700, EPI_ISL_1262701, EPI_ISL_1262702, EPI_ISL_1262703, EPI_ISL_1262704, EPI_ISL_1262705, EPI_ISL_1262706, EPI_ISL_1262707, EPI_ISL_1262708, EPI_ISL_1262709, EPI_ISL_1262710, EPI_ISL_1262711, EPI_ISL_1262712, EPI_ISL_1262713, EPI_ISL_1262714, EPI_ISL_1262715, EPI_ISL_1262716, EPI_ISL_1262717, EPI_ISL_1265902, EPI_ISL_1265903, EPI_ISL_1265904, EPI_ISL_1265905, EPI_ISL_1265906, EPI_ISL_1265907, EPI_ISL_1265908, EPI_ISL_1298451, EPI_ISL_1298452, EPI_ISL_1298453, EPI_ISL_1298454, EPI_ISL_1298455, EPI_ISL_1298456, EPI_ISL_1298457, EPI_ISL_1298458, EPI_ISL_1298459, EPI_ISL_1298460, EPI_ISL_1298461, EPI_ISL_1298462, EPI_ISL_1298463, EPI_ISL_1298464, EPI_ISL_1298465, EPI_ISL_1298466, EPI_ISL_1298467, EPI_ISL_1298468, EPI_ISL_1298469, EPI_ISL_1298470, EPI_ISL_1298471, EPI_ISL_1298472, EPI_ISL_1298473, EPI_ISL_1300425, EPI_ISL_1310811, EPI_ISL_1315479, EPI_ISL_1315487, EPI_ISL_1315492, EPI_ISL_1315493, EPI_ISL_1315522, EPI_ISL_1406126, EPI_ISL_1406127, EPI_ISL_1406128, EPI_ISL_1406129, EPI_ISL_1406130, EPI_ISL_1406131, EPI_ISL_1406132, EPI_ISL_1406133, EPI_ISL_1406134, EPI_ISL_1591460, EPI_ISL_1591461, EPI_ISL_1591462, EPI_ISL_1591463, EPI_ISL_1591467, EPI_ISL_1591468, EPI_ISL_1591469, EPI_ISL_1591470, EPI_ISL_1591471, EPI_ISL_1591475, EPI_ISL_1591476, EPI_ISL_1591477, EPI_ISL_1591478, EPI_ISL_1591483, EPI_ISL_1591484, EPI_ISL_1591489, EPI_ISL_1591490, EPI_ISL_1591491, EPI_ISL_1591495, EPI_ISL_1591496, EPI_ISL_1591497, EPI_ISL_1591503, EPI_ISL_1591504, EPI_ISL_1591505, EPI_ISL_1591511, EPI_ISL_1591512, EPI_ISL_1591513, EPI_ISL_1591519, EPI_ISL_1591520, EPI_ISL_1591524, EPI_ISL_1591525, EPI_ISL_1591530, EPI_ISL_1591531, EPI_ISL_1591532, EPI_ISL_1591536, EPI_ISL_1591537, EPI_ISL_1591539, EPI_ISL_1628520, EPI_ISL_1628521, EPI_ISL_1628522, EPI_ISL_1628534, EPI_ISL_1628541, EPI_ISL_1628548, EPI_ISL_1628549, EPI_ISL_1628556, EPI_ISL_1628560, EPI_ISL_1628563, EPI_ISL_1628566, EPI_ISL_1628579, EPI_ISL_1628580, EPI_ISL_1628590, EPI_ISL_1628591, EPI_ISL_1857295			
see above	Instituto Nacional de Medicina Genomica	Instituto Nacional de Medicina Genomica	Alcaraz N; Alcaraz-Millman M; Arriaga-Canon C; Canseco Mendez JC; Cedro-Tanda A; Cisneros-Villanueva M; Frías-Jimenez E; Garcia-Cardenas FJ; Garcia-Cárdenas FJ; Gonzalez-Barrera D; Gonzalez-Woge MA; González-Woge MA; Herrera-Montalvo LA; Herrera-Montalvo LA.; Hidalgo-Miranda A; Hurtado-Cordova E; Mendoza-Vargas A; Miranda-Ortiz H; Munguia-Garza; Munguia-Garza P; Orjeda-Rodriguez M; Peñaloza-Figueroa F; Ramirez-Vega O; Rangel-DeLeon D; Reyes-Grajeda JP; Rosas-Escobar P; Sifuentes-Rojas C
EPI_ISL_522872, EPI_ISL_522879, EPI_ISL_522880, EPI_ISL_522940, EPI_ISL_522941, EPI_ISL_522942, EPI_ISL_522978, EPI_ISL_522979, EPI_ISL_522980, EPI_ISL_522981, EPI_ISL_522982, EPI_ISL_522983, EPI_ISL_522984, EPI_ISL_522985, EPI_ISL_522986, EPI_ISL_523500			
see above	Instituto Nacional de Medicina Genómica	Instituto Nacional de Medicina Genómica	Cedro-Tanda A; Cisneros-Villanueva M; Herrera-Montalvo LA; Hidalgo-Miranda A; Hurtado-Cordova E; Mendoza-Vargas A; Peñaloza-Figueroa F; Reyes-Grajeda JP
EPI_ISL_536518	Instituto Nacional de Salud	Laboratorio de Infecciones Respiratorias Agudas	David Tarazona; Dennis Carhuaricra; Eduardo Juscamayta Lopez; Faviola Valdivia Guerrero; Lenin Maturrano Hernandez; Nancy Rojas Serrano; Ronnie Gavilan Chavez
EPI_ISL_498154	Instituto Nacional de Salud, Bogotá, Colombia	Instituto Nacional de Salud, Bogotá, Colombia	Astrid C. Flórez; Carlos Andrés Durán; Carlos Franco-Muñoz; Carolina Ferro; Christian Julian Villabona-Arenas; Diana Marcela Walteros-Acero; Diego A. Álvarez-Díaz; Diego Andrés Prada; Franklin Prieto; Jonathan Reales; Jose A. Usme-Ciro; Katherine Laiton-Donato; Liz Villabona-Arenas; Marcela Mercado-Reyes; Martha Lucia Ospina Martinez; Nicolas D. Franco-Sierra; Sussy Echeverria; Zulma M. Cucunubá
EPI_ISL_424673	Instituto de Diagnostico y Referencia Epidemiologicos	Instituto de Diagnostico y Referencia Epidemiologicos	Adnan Araiza Rodríguez; Alejandro Sánchez; Alfredo Ponce de León Garduño; Blanca Taboada; Carlos F. Arias; Carolina González Torres; Celia Boukadida; Cesar Raúl González Bonilla; Concepción Grajales Muñiz; Edgar Mendieta Condado; Eduardo Becerril Vargas; Fabiola Garcés Ayala; Fernando Ledesma Barrientos; Francisco Javier Gaytán Cervantes; Francisco Pulido; Gisela Barrera Badillo; Gloria Vázquez; Guillermo M. Ruiz-Palacios; Irma López Martínez; Joel Armando Vázquez Pérez; José Arturo Martínez Orozco; José Ernesto Ramírez González; José Esteban Muñoz Medina; Lucia Hernández Rivas; Luis Alberto García Andrade; Mario Mújica Sánchez; Pavel Isa; Pilar Ramos Cervantes; Ricardo Grande; Santiago Avila Ríos; Victor Hugo Borja Aburto; Violeta Ibarra Gonzalez
EPI_ISL_455432, EPI_ISL_455434, EPI_ISL_455435, EPI_ISL_455436, EPI_ISL_455437, EPI_ISL_455438, EPI_ISL_455439, EPI_ISL_455454, EPI_ISL_455455, EPI_ISL_455456, EPI_ISL_493334, EPI_ISL_493335, EPI_ISL_493336, EPI_ISL_493338, EPI_ISL_493339, EPI_ISL_493340, EPI_ISL_493341, EPI_ISL_493342, EPI_ISL_493343, EPI_ISL_493344, EPI_ISL_493345, EPI_ISL_493346, EPI_ISL_493347, EPI_ISL_493348, EPI_ISL_493349, EPI_ISL_516608, EPI_ISL_516609, EPI_ISL_516610, EPI_ISL_516611, EPI_ISL_516613, EPI_ISL_516614, EPI_ISL_516615, EPI_ISL_516616, EPI_ISL_516617, EPI_ISL_516618, EPI_ISL_516619, EPI_ISL_516620, EPI_ISL_516622, EPI_ISL_516623, EPI_ISL_516624, EPI_ISL_516625, EPI_ISL_576257, EPI_ISL_576258, EPI_ISL_576259, EPI_ISL_576260, EPI_ISL_576261, EPI_ISL_576262, EPI_ISL_576263, EPI_ISL_576264, EPI_ISL_576265, EPI_ISL_576266, EPI_ISL_576267, EPI_ISL_576268, EPI_ISL_576269, EPI_ISL_576270, EPI_ISL_576271, EPI_ISL_576272, EPI_ISL_576273, EPI_ISL_576274, EPI_ISL_576275, EPI_ISL_576276, EPI_ISL_576277, EPI_ISL_576278, EPI_ISL_576279, EPI_ISL_658863, EPI_ISL_658865, EPI_ISL_658868, EPI_ISL_658870, EPI_ISL_658873, EPI_ISL_658875, EPI_ISL_658878, EPI_ISL_658880, EPI_ISL_658883, EPI_ISL_658886, EPI_ISL_658888, EPI_ISL_658891, EPI_ISL_658893, EPI_ISL_658896, EPI_ISL_658899, EPI_ISL_658901, EPI_ISL_658904, EPI_ISL_861419, EPI_ISL_861420, EPI_ISL_861421, EPI_ISL_872088, EPI_ISL_872089, EPI_ISL_872090, EPI_ISL_872091, EPI_ISL_872092, EPI_ISL_872093, EPI_ISL_872097, EPI_ISL_872098, EPI_ISL_872099			
see above	Instituto de Diagnostico y Referencia Epidemiologicos (INDRE)	Instituto de Diagnostico y Referencia Epidemiologicos (INDRE)	; Abril Rodriguez-Maldonado; Adnan Araiza-Rodriguez; Adnan-Araiza Rodríguez; Araiza-Rodriguez Adnan; Barrera-Badillo Gisela; Claudia Wong-Arambula; Dayanira Arellano-Suarez; Edgar Mendieta-Condado; Ernesto Ramirez-Gonzalez; Ernesto Ramirez-Gonzalez.; Fabiola Garces-Ayala; Garces-Ayala Fabiola; Garces-Ayala Fabiola. Ramirez-Gonzalez Ernesto; Garces-Ayala Fabiola. Taboada Ramírez Blanca. Ramirez-Gonzalez Ernesto; Garces-Ayala Fabiola. Taboada Ramirez Blanca. Ramirez-Gonzalez Ernesto; Garces-Ayala Fabiola. Taboada Ramírez Blanca. Ramirez-Gonzalez Ernesto; Gaudalupe Herrera-Ramirez; Gisela Barrera-Badillo; Gisela Barrera-Badillo.; Hernandez-Rivas Lucia; Irma Lopez-Martinez; Irma Lopez-Martinez.; Jazmin Galicia-Hernandez.; Linda Andarade-Sanchez; Lopez-Martinez Irma; Lopez-Martinez Irma.; Lucia Hernandez-Rivas; Marisol Galindo-Galindo; Mendieta-Condado Edgar; Natividad Cruz-Ortiz; Octavio Ruiz-Muñiz; Pamela Ramirez-Medina; Ramirez-Gonzalez Ernesto; Ramirez-Gonzalez Ernesto.; Rodriguez-Maldonado Abril; Roman Canul-Aguilar; Taboada Ramirez Blanca; Taboada Ramírez Blanca; Taboada Ramírez Blanca.; Taboada Ramírez Blanca. Ramirez-Gonzalez Ernesto; Tatiana Nunez-Garcia; Wong-Arambula Claudia; Wong-Arambula Claudia.
EPI_ISL_452139, EPI_ISL_452141	Instituto de Diagnostico y Referencia Epidemiologicos (INDRE)	Instituto de diagnóstico y Referencia Epidemiologicos (INDRE)	Araiza-Rodriguez Adnan; Barrera-Badillo Gisela; Garces-Ayala Fabiola; Hernandez-Rivas Lucia; Lopez-Martinez Irma; Lopez-Martinez Irma.; Mendieta-Condado Edgar; Ramirez-Gonzalez Ernesto; Rodriguez-Maldonado Abril; Wong-Arambula Claudia
EPI_ISL_660068	Instituto de Diagnostico y Referencia Epidemiologicos (INDRE)	Instituto de diagnóstico y Referencia Epidemiologicos (INDRE) Departamento de Virología	Abril Rodriguez-Maldonado; Claudia Wong-Arambula; Dayanira Arellano-Suarez; Ernesto Ramirez-Gonzalez.; Fabiola Garces-Ayala; Gisela Barrera-Badillo; Irma Lopez-Martinez; Lucia Hernandez-Rivas; Natividad Cruz-Ortiz; Tatiana Nunez-Garcia
EPI_ISL_1060676, EPI_ISL_1060677, EPI_ISL_1060678, EPI_ISL_1060679, EPI_ISL_1060680, EPI_ISL_1060681, EPI_ISL_1060682, EPI_ISL_1060683, EPI_ISL_1060684, EPI_ISL_1060685, EPI_ISL_1060686, EPI_ISL_1060687, EPI_ISL_1060688, EPI_ISL_1060689, EPI_ISL_1060690, EPI_ISL_1060691, EPI_ISL_1060692, EPI_ISL_1060693, EPI_ISL_1060694, EPI_ISL_1060695, EPI_ISL_1060696, EPI_ISL_1060697, EPI_ISL_1060698, EPI_ISL_1060699, EPI_ISL_1060700, EPI_ISL_1060701, EPI_ISL_1060702, EPI_ISL_1060703, EPI_ISL_1060704, EPI_ISL_1060705, EPI_ISL_1060706, EPI_ISL_1060707, EPI_ISL_1060714, EPI_ISL_1060715, EPI_ISL_1060716, EPI_ISL_1060717, EPI_ISL_1060718, EPI_ISL_1060719, EPI_ISL_1060720, EPI_ISL_1060721, EPI_ISL_1060722, EPI_ISL_1060723, EPI_ISL_1060724, EPI_ISL_1060725, EPI_ISL_1060726, EPI_ISL_1060727, EPI_ISL_1060728, EPI_ISL_1060729, EPI_ISL_1060730, EPI_ISL_1060731, EPI_ISL_1060732, EPI_ISL_1060733, EPI_ISL_1060734, EPI_ISL_1060735, EPI_ISL_1060736, EPI_ISL_1060737, EPI_ISL_1060738, EPI_ISL_1060739, EPI_ISL_1060740, EPI_ISL_1060741, EPI_ISL_1060742, EPI_ISL_1060743, EPI_ISL_1060744, EPI_ISL_1060745, EPI_ISL_1060746, EPI_ISL_1060747, EPI_ISL_1060748, EPI_ISL_1060749, EPI_ISL_1060750, EPI_ISL_1060751, EPI_ISL_1060752, EPI_ISL_1060753, EPI_ISL_1060754, EPI_ISL_1060755, EPI_ISL_1060756, EPI_ISL_1060757, EPI_ISL_1060758, EPI_ISL_1060759, EPI_ISL_1060760, EPI_ISL_1060761, EPI_ISL_1060762, EPI_ISL_1060763			
see above	Instituto de Diagnostico y Referencia Epidemiologicos (INDRE)_RNLSP	Instituto de Diagnostico y Referencia Epidemiologicos (INDRE)	Abril Rodriguez-Maldonado; Adnan Araiza-Rodriguez; Claudia Wong-Arambula; David Fragoso-Fonseca; Ernesto Ramirez-Gonzalez.; Fabiola Garces-Ayala; Gisela Barrera-Badillo; Irma Lopez-Martinez; Lucia Hernandez-Rivas; Mayra Jimenez-Morales; Nancy Munoz-Hernandez; Natividad Cruz-Ortiz; Sergio Rangel-Guerrero; Tatiana Nunez-Garcia
EPI_ISL_913911, EPI_ISL_913912, EPI_ISL_913913, EPI_ISL_913914, EPI_ISL_913915, EPI_ISL_913916, EPI_ISL_913917, EPI_ISL_913919, EPI_ISL_913920, EPI_ISL_913921, EPI_ISL_913922, EPI_ISL_913923, EPI_ISL_913924, EPI_ISL_913925, EPI_ISL_913926, EPI_ISL_913927, EPI_ISL_913928, EPI_ISL_913929, EPI_ISL_913930, EPI_ISL_913931, EPI_ISL_913932, EPI_ISL_913933, EPI_ISL_913934, EPI_ISL_913935, EPI_ISL_913936, EPI_ISL_913937, EPI_ISL_913938, EPI_ISL_913939, EPI_ISL_913940, EPI_ISL_913941, EPI_ISL_913942, EPI_ISL_913943, EPI_ISL_913944, EPI_ISL_913945, EPI_ISL_913946, EPI_ISL_913947, EPI_ISL_913948, EPI_ISL_913949, EPI_ISL_913950, EPI_ISL_913951, EPI_ISL_913952, EPI_ISL_913953, EPI_ISL_913954, EPI_ISL_913955, EPI_ISL_913956, EPI_ISL_913957, EPI_ISL_913958, EPI_ISL_913959, EPI_ISL_913960, EPI_ISL_913961, EPI_ISL_913962, EPI_ISL_913963, EPI_ISL_913964, EPI_ISL_913965, EPI_ISL_913966, EPI_ISL_913967, EPI_ISL_913969, EPI_ISL_913970, EPI_ISL_913971, EPI_ISL_913972, EPI_ISL_913973, EPI_ISL_913974, EPI_ISL_913975, EPI_ISL_913976, EPI_ISL_913977, EPI_ISL_913978, EPI_ISL_913979, EPI_ISL_913980, EPI_ISL_913981, EPI_ISL_913982, EPI_ISL_913983, EPI_ISL_913984, EPI_ISL_914879, EPI_ISL_914880, EPI_ISL_933661, EPI_ISL_933662, EPI_ISL_933663, EPI_ISL_933665, EPI_ISL_933666, EPI_ISL_933667, EPI_ISL_933668, EPI_ISL_933669, EPI_ISL_933670, EPI_ISL_933671, EPI_ISL_933672, EPI_ISL_933673, EPI_ISL_933674, EPI_ISL_933675, EPI_ISL_933676, EPI_ISL_933677, EPI_ISL_933678, EPI_ISL_933679, EPI_ISL_933680, EPI_ISL_933681, EPI_ISL_933682, EPI_ISL_933683, EPI_ISL_933684, EPI_ISL_933685, EPI_ISL_933686, EPI_ISL_933687, EPI_ISL_933688, EPI_ISL_933689, EPI_ISL_933690, EPI_ISL_933691, EPI_ISL_933692, EPI_ISL_933693, EPI_ISL_933694, EPI_ISL_933695, EPI_ISL_933696, EPI_ISL_933697, EPI_ISL_933698, EPI_ISL_933699, EPI_ISL_933700, EPI_ISL_933701, EPI_ISL_933702, EPI_ISL_933703, EPI_ISL_933704, EPI_ISL_933705, EPI_ISL_933706, EPI_ISL_933707, EPI_ISL_933708, EPI_ISL_933709, EPI_ISL_933710, EPI_ISL_933711, EPI_ISL_933712, EPI_ISL_942928, EPI_ISL_942929, EPI_ISL_1054940, EPI_ISL_1054941, EPI_ISL_1054942, EPI_ISL_1054943, EPI_ISL_1054944, EPI_ISL_1054945, EPI_ISL_1054946, EPI_ISL_1054947, EPI_ISL_1054948, EPI_ISL_1054949, EPI_ISL_1054950, EPI_ISL_1054951, EPI_ISL_1054952, EPI_ISL_1054953, EPI_ISL_1054954, EPI_ISL_1054955, EPI_ISL_1054956, EPI_ISL_1054957, EPI_ISL_1054958, EPI_ISL_1054959, EPI_ISL_1054960, EPI_ISL_1054961, EPI_ISL_1054962, EPI_ISL_1054963, EPI_ISL_1054964, EPI_ISL_1054965, EPI_ISL_1054966, EPI_ISL_1054967, EPI_ISL_1054968, EPI_ISL_1054969, EPI_ISL_1054970, EPI_ISL_1054971, EPI_ISL_1054972, EPI_ISL_1054973, EPI_ISL_1054974, EPI_ISL_1054975, EPI_ISL_1054976, EPI_ISL_1054977, EPI_ISL_1054978, EPI_ISL_1054979, EPI_ISL_1054980, EPI_ISL_1054981, EPI_ISL_1054982, EPI_ISL_1054983, EPI_ISL_1054984, EPI_ISL_1054985, EPI_ISL_1054986, EPI_ISL_1054987, EPI_ISL_1054988, EPI_ISL_1054989, EPI_ISL_1054990, EPI_ISL_1054991, EPI_ISL_1054992, EPI_ISL_1054993, EPI_ISL_1054994, EPI_ISL_1054995, EPI_ISL_1054996, EPI_ISL_1168452, EPI_ISL_1168453, EPI_ISL_1168454, EPI_ISL_1168455, EPI_ISL_1168456, EPI_ISL_1168457, EPI_ISL_1168458, EPI_ISL_1168459, EPI_ISL_1168460, EPI_ISL_1168461, EPI_ISL_1168462, EPI_ISL_1168463, EPI_ISL_1168464, EPI_ISL_1168465, EPI_ISL_1168466, EPI_ISL_1168467, EPI_ISL_1168468, EPI_ISL_1168469, EPI_ISL_1168470, EPI_ISL_1168471, EPI_ISL_1168472, EPI_ISL_1168473, EPI_ISL_1168474, EPI_ISL_1168475, EPI_ISL_1168476, EPI_ISL_1168477, EPI_ISL_1168478, EPI_ISL_1168479, EPI_ISL_1168480, EPI_ISL_1168481, EPI_ISL_1168482, EPI_ISL_1168483, EPI_ISL_1168484, EPI_ISL_1168485, EPI_ISL_1168486, EPI_ISL_1168487, EPI_ISL_1168488, EPI_ISL_1168489, EPI_ISL_1168490, EPI_ISL_1168491, EPI_ISL_1168492, EPI_ISL_1168493, EPI_ISL_1168494, EPI_ISL_1168495, EPI_ISL_1168496, EPI_ISL_1168497, EPI_ISL_1168498, EPI_ISL_1168499, EPI_ISL_1168500, EPI_ISL_1168501, EPI_ISL_1168502, EPI_ISL_1168503, EPI_ISL_1168504, EPI_ISL_1168505, EPI_ISL_1168506, EPI_ISL_1168507, EPI_ISL_1168508, EPI_ISL_1168509, EPI_ISL_1168510, EPI_ISL_1168511, EPI_ISL_1168512, EPI_ISL_1168513, EPI_ISL_1168514, EPI_ISL_1168515, EPI_ISL_1168516, EPI_ISL_1168517, EPI_ISL_1168518, EPI_ISL_1168519, EPI_ISL_1168520, EPI_ISL_1168521, EPI_ISL_1168522, EPI_ISL_1168523, EPI_ISL_1168524, EPI_ISL_1168525, EPI_ISL_1168526, EPI_ISL_1168527, EPI_ISL_1168528, EPI_ISL_1168529, EPI_ISL_1168530, EPI_ISL_1168531, EPI_ISL_1168532, EPI_ISL_1168533, EPI_ISL_1168534, EPI_ISL_1168535, EPI_ISL_1168536, EPI_ISL_1168537, EPI_ISL_1168538, EPI_ISL_1168539, EPI_ISL_1168540, EPI_ISL_1168541, EPI_ISL_1168542, EPI_ISL_1168543, EPI_ISL_1168544, EPI_ISL_1168545, EPI_ISL_1168546, EPI_ISL_1168547, EPI_ISL_1168548, EPI_ISL_1168549, EPI_ISL_1168550, EPI_ISL_1168551, EPI_ISL_1168552, EPI_ISL_1168553, EPI_ISL_1168554, EPI_ISL_1168555, EPI_ISL_1168556, EPI_ISL_1168557, EPI_ISL_1168558, EPI_ISL_1168559, EPI_ISL_1168560, EPI_ISL_1168561, EPI_ISL_1168562, EPI_ISL_1168563, EPI_ISL_1168564, EPI_ISL_1168565, EPI_ISL_1168566, EPI_ISL_1168567, EPI_ISL_1168568, EPI_ISL_1168569, EPI_ISL_1168570, EPI_ISL_1168571, EPI_ISL_1168572, EPI_ISL_1168573, EPI_ISL_1168574, EPI_ISL_1168575, EPI_ISL_1168576, EPI_ISL_1168577, EPI_ISL_1168578, EPI_ISL_1168579, EPI_ISL_1168580, EPI_ISL_1168581, EPI_ISL_1168582, EPI_ISL_1168583, EPI_ISL_1168584, EPI_ISL_1168585, EPI_ISL_1168586, EPI_ISL_1168587, EPI_ISL_1168588, EPI_ISL_1168589, EPI_ISL_1168590, EPI_ISL_1168591, EPI_ISL_1168592, EPI_ISL_1168593, EPI_ISL_1168594, EPI_ISL_1168595, EPI_ISL_1168596, EPI_ISL_1168597, EPI_ISL_1168598, EPI_ISL_1168599, EPI_ISL_1168600, EPI_ISL_1168601, EPI_ISL_1168602, EPI_ISL_1168603, EPI_ISL_1168604, EPI_ISL_1168606, EPI_ISL_1168607, EPI_ISL_1168608, EPI_ISL_1168609, EPI_ISL_1168610, EPI_ISL_1168611, EPI_ISL_1168612, EPI_ISL_1168613, EPI_ISL_1168614, EPI_ISL_1168615, EPI_ISL_1168616, EPI_ISL_1168617, EPI_ISL_1168618, EPI_ISL_1168619, EPI_ISL_1168620, EPI_ISL_1168621, EPI_ISL_1168622, EPI_ISL_1168623, EPI_ISL_1168624, EPI_ISL_1168625, EPI_ISL_1168626, EPI_ISL_1168627, EPI_ISL_1168628, EPI_ISL_1168629, EPI_ISL_1168630, EPI_ISL_1168631, EPI_ISL_1168632, EPI_ISL_1168633, EPI_ISL_1219711, EPI_ISL_1219712, EPI_ISL_1219713, EPI_ISL_1219715, EPI_ISL_1219716, EPI_ISL_1219717, EPI_ISL_1219718			
see above	Instituto de Diagnostico y Referencia Epidemiologicos INDRE_RNLSP	Instituto de Diagnostico y Referencia Epidemiologicos (INDRE)	Abril Rodriguez-Maldonado; Adnan Araiza-Rodriguez; Ariadna Medina-Benitez; Claudia Wong-Arambula; David Fragoso-Fonseca; Ernesto Ramirez-Gonzalez.; Fabiola Garces-Ayala; Gisela Barrera-Badillo; Irma Lopez-Martinez; Joaquin Quiroz-Mercado; Lucia Hernandez-Rivas; Mayra Jimenez-Morales; Nancy Munoz-Hernandez; Natividad Cruz-Ortiz; Sergio Rangel-Guerrero; Tatiana Nunez-Garcia; Vanessa Rivero-Arredondo
EPI_ISL_474971, EPI_ISL_475010, EPI_ISL_514308	Israel Central Virology laboratory	Israel Central Virology laboratory	Efrat Dahan Bucris; Ella Mendelson; Michal Mandelboim; Neta Zuckerman; Oran Erster
EPI_ISL_631571	Jamaica Hospital Medical Center	New York City Public Health Laboratory	Jade Wang; et al.
EPI_ISL_416565	Japanese Quarantine Stations	Pathogen Genomics Center, National Institute of Infectious Diseases	Hajime Kamiya; Hideki Hasegawa; Ikuyo Takayama; Kentaro Itokawa; Makoto Kuroda; Masanori Hashino; Motoi Suzuki; Rina Tanaka; Shinji Saito; Takaji Wakita; Takuri Takahashi; Takuya Yamagishi; Tomoko Kageyama; Tsuyoshi Sekizuka
EPI_ISL_457796	Johns Hopkins Hospital Department of Pathology	Johns Hopkins Hospital Department of Pathology	Amanda Ernlund; Craig Howser; Heba H. Mostafa; Kristina Zudock; Mark Hopkins; Melanie Kirsche; Michael C. Schatz; Nidia Trovao; Norah Sadowski; Oluwaseun Falade-Nwulia; Paul Morris; Peter M. Thielen; Shirlee Wohl; Srividya Ramakrishnan; Stuart C. Ray; Thomas Mehoke; Victoria Gniazdowski; Winston Timp; Yunfan Fan
EPI_ISL_455639	KRISP, KZN Research Innovation and Sequencing Platform	KRISP, KZN Research Innovation and Sequencing Platform	Chimukangara B; Deforche K; Giandhari J; Lessells R; Pillay S; Tegally H; Wilkinson E; de Oliveira T
EPI_ISL_418985, EPI_ISL_420442	KU Leuven, Clinical and Epidemiological Virology	KU Leuven, Clinical and Epidemiological Virology	Bert Vanmechelen; Joan Marti-Carreras; Piet Maes; Tony Wawina

EPI_ISL_458156, EPI_ISL_462211, EPI_ISL_462239, EPI_ISL_464065	KU Leuven, Rega Institute, Clinical and Epidemiological Virology	KU Leuven, Rega Institute, Clinical and Epidemiological Virology	Bert Vanmechelen; Joan Marti-Carerras; Piet Maes; Tony Wawina-Bokalanga
EPI_ISL_469075, EPI_ISL_510834	Karolinska Universitetslaboratoriet	The Public Health Agency of Sweden	Anna Risberg; Anna-Malin Linde; Karin Tegmark-Wisell; Maria Lind Karlberg; Mattias Haukland; Mia Brytting; Olov Svartstrom; Oskar Karlsson Lindsjo; Petra Edquist; Reza Advani; Sandra Broddesson; Shamam Muradrasoli
EPI_ISL_512816	Kenema Government Hospital, Ministry of Health and Sanitation	Kenema Government Hospital, Ministry of Health and Sanitation	Andersen, K.; Garry, R.; Goba, A.; Grant, D.; Happi, C.; Jalloh, S.; Mehta, S.; Momoh, M.; Olawoye, I.; Oluniyi, P.; Park, D.; Sandi, J.; Siddle, K.; Tomkins-Tinch, C.
EPI_ISL_489996, EPI_ISL_489997	King Fahad Medical City	King Fahad Medical City	Alghoraibi, M.; Alosaimi, B.; Enani, M.; Naeem, A.
EPI_ISL_516974	King Georges Medical University	CSIR-National Botanical Research Institute	Amita Jain; Babita Singh; Danish Nasar Khan; Hricha Mishra; Kishan Sahu; MLB Bhatt; Mehar H.Asif; Om Prakash; Priti Prasad; SK Barik; Samir V. Sawant; Shantanu Prakash; Sumit Kr. Bag; Suruchi Shukla
EPI_ISL_458037	King Institute of Preventive Medicine & Research	CSIR-Centre for Cellular and Molecular Biology	Archana Bharadwaj Siva; Dhiviya Vedagiri; Divya Gupta; Divya Tej Sowpati; G. Dhinakar Raj; G. Ravikumar; K Thangaraj; K.Kaveri; Karthik Bharadwaj Tallapaka; Krishnan Harinivas Harshan; Lamuk Zaveri; Namami Gaur; P.Padmapriya; Payel Mukherjee; Priya Singh; Purushotham Vodnala; R. P. Aravindh Babu; R.Kiruba; Rakesh K Mishra; S.Magesh; S.Sivasubramanian; S.Vennila; Sakshi Shambhavi; Santosh Kumar Kuncha; Shagufta Khan; Sofia Banu; Tulasi Nagabandi; Vishal Sah
EPI_ISL_483550	Kingdom of Bahrein Ministry of Health	Erasmus Medical Center	Amjad Ghanem Mohamed; Anne van der Linden; Bas Oude Munnink; Claudia Schapendonk; David Nieuwenhuijse; Ebrahim Shehad; Fatema; Hashmeya Al Wasti; Irina Chestakova; Marion Koopmans; Mark Pronk; Pascal Lexmond; Reina Sikkema; Richard Molenkamp; Stefan van Nieuwkoop; Theo Bestebroer; on behalf of the Dutch national COVID-19 response team.
EPI_ISL_429160	Klinisk mikrobiologi Orebro	The Public Health Agency of Sweden	Anna Risberg; Anna-Malin Linde; Karin Tegmark-Wisell; Maria Lind Karlberg; Martin Sundqvist; Olov Svartstrom; Oskar Karlsson Lindsjo; Shaman Muradrasoli
EPI_ISL_510871, EPI_ISL_676512	Klinsisk mikrobiologi Linkoping	The Public Health Agency of Sweden	Anna Risberg; Anna-Malin Linde; Department of Microbiology; Karin Tegmark-Wisell; Maria Lind Karlberg; Mattias Haukland; Mia Brytting; Olov Svartstrom; Oskar Karlsson Lindsjo; Petra Edquist; Reza Advani; Sandra Broddesson; The Public Health Agency of Sweden
EPI_ISL_424868	LA Office of Public Health Laboratories	Pathogen Discovery, Respiratory Viruses Branch, Division of Viral Diseases, Centers for Disease Control and Prevention	Alison S. Laufer Halpin; Anna Uehara; Christopher A. Elkins; Clinton R. Paden; Haibin Wang; Jing Zhang; Krista Queen; Mary S. Keckler; Rachel Marine; Suxiang Tong; Yan Li; Ying Tao
EPI_ISL_1137459, EPI_ISL_1137475, EPI_ISL_1137476, EPI_ISL_1137477, EPI_ISL_1158060, EPI_ISL_1158061, EPI_ISL_1158062			
see above	LABDIS-UAGro	Instituto Nacional de Medicina Genomica	Adolfo Roman-Roman; Alcaraz N; Azucena D. Alvarez-Diaz; Berenice Illades-Aguiar; Cedro-Tanda A; Ciresthel Bello-Rios; Cisneros-Villanueva M; Daniel Hernandez-Sotelo; Fredy O Beltran-Anaya; Gladys W. Valente-Nio; Gonzalez-Barrera D; Herrera-Montalvo LA; Hidalgo-Miranda A; Hugo A. Rodriguez-Ruiz; Jaqueline Loaeza-Loaeza; Julio C. Azucar-Hesiquio; Luis I. Basilio-Leyva.; Madian Valadez-Quinones; Marco Antonio-Leyva; Mendoza-Vargas A; Oscar del Moral-Hernandez; Ramirez-Vega O; Rangel-DeLeon D; Reyes-Grajeda JP; Roberto Dircio-Maldonado; Sifuentes-Rojas C
EPI_ISL_1516777, EPI_ISL_1516778, EPI_ISL_1516779, EPI_ISL_1516780, EPI_ISL_1516781, EPI_ISL_1516782, EPI_ISL_1516783, EPI_ISL_1516784, EPI_ISL_1516785, EPI_ISL_1516786			
see above	LESP Aguascalientes	Instituto de Diagnostico y Referencia Epidemiologicos (INDRE)	Abril Rodriguez-Maldonado; Ariadna Medina-Benitez; Claudia Wong-Arambula; Ernesto Ramirez-Gonzalez.; Gisela Barrera-Badillo; Irma Lopez-Martinez; Joaquin Quiroz-Mercado; Lucia Hernandez-Rivas; Natividad Cruz-Ortiz; Sergio Rangel-Guerrero; Tatiana Nunez-Garcia; Vanessa Rivero-Arredondo
EPI_ISL_1365658, EPI_ISL_1365659, EPI_ISL_1365660, EPI_ISL_1424050	LESP Baja California	Instituto de Diagnostico y Referencia Epidemiologicos (INDRE)	Abril Rodriguez-Maldonado; Ariadna Medina-Benitez; Claudia Wong-Arambula; Ernesto Ramirez-Gonzalez.; Gisela Barrera-Badillo; Irma Lopez-Martinez; Joaquin Quiroz-Mercado; Lucia Hernandez-Rivas; Natividad Cruz-Ortiz; Sergio Rangel-Guerrero; Tatiana Nunez-Garcia; Vanessa Rivero-Arredondo
EPI_ISL_1337370, EPI_ISL_1337371, EPI_ISL_1337373, EPI_ISL_1337374, EPI_ISL_1337392, EPI_ISL_1337393	LESP Baja California Sur	Instituto de Diagnostico y Referencia Epidemiologicos (INDRE)	Abril Rodriguez-Maldonado; Ariadna Medina-Benitez; Claudia Wong-Arambula; Ernesto Ramirez-Gonzalez.; Gisela Barrera-Badillo; Irma Lopez-Martinez; Joaquin Quiroz-Mercado; Lucia Hernandez-Rivas; Natividad Cruz-Ortiz; Sergio Rangel-Guerrero; Tatiana Nunez-Garcia; Vanessa Rivero-Arredondo
EPI_ISL_1400299, EPI_ISL_1424015	LESP Campeche	Instituto de Diagnostico y Referencia Epidemiologicos (INDRE)	Abril Rodriguez-Maldonado; Ariadna Medina-Benitez; Claudia Wong-Arambula; Ernesto Ramirez-Gonzalez.; Gisela Barrera-Badillo; Irma Lopez-Martinez; Joaquin Quiroz-Mercado; Lucia Hernandez-Rivas; Natividad Cruz-Ortiz; Sergio Rangel-Guerrero; Tatiana Nunez-Garcia; Vanessa Rivero-Arredondo
EPI_ISL_1424013, EPI_ISL_1424020	LESP Chiapas	Instituto de Diagnostico y Referencia Epidemiologicos (INDRE)	Abril Rodriguez-Maldonado; Ariadna Medina-Benitez; Claudia Wong-Arambula; Ernesto Ramirez-Gonzalez.; Gisela Barrera-Badillo; Irma Lopez-Martinez; Joaquin Quiroz-Mercado; Lucia Hernandez-Rivas; Natividad Cruz-Ortiz; Sergio Rangel-Guerrero; Tatiana Nunez-Garcia; Vanessa Rivero-Arredondo
EPI_ISL_1399271, EPI_ISL_1405905	LESP Chihuahua	Instituto de Diagnostico y Referencia Epidemiologicos (INDRE)	Abril Rodriguez-Maldonado; Ariadna Medina-Benitez; Claudia Wong-Arambula; Ernesto Ramirez-Gonzalez.; Gisela Barrera-Badillo; Irma Lopez-Martinez; Joaquin Quiroz-Mercado; Lucia Hernandez-Rivas; Natividad Cruz-Ortiz; Sergio Rangel-Guerrero; Tatiana Nunez-Garcia; Vanessa Rivero-Arredondo
EPI_ISL_1365657, EPI_ISL_1366687, EPI_ISL_1366688, EPI_ISL_1366689, EPI_ISL_1399281, EPI_ISL_1424051, EPI_ISL_1424052, EPI_ISL_1424053			
see above	LESP Ciudad de Mexico	Instituto de Diagnostico y Referencia Epidemiologicos (INDRE)	Abril Rodriguez-Maldonado; Ariadna Medina-Benitez; Claudia Wong-Arambula; Ernesto Ramirez-Gonzalez.; Gisela Barrera-Badillo; Irma Lopez-Martinez; Joaquin Quiroz-Mercado; Lucia Hernandez-Rivas; Natividad Cruz-Ortiz; Sergio Rangel-Guerrero; Tatiana Nunez-Garcia; Vanessa Rivero-Arredondo
EPI_ISL_1365662, EPI_ISL_1365663, EPI_ISL_1365664, EPI_ISL_1365665, EPI_ISL_1365666, EPI_ISL_1365667, EPI_ISL_1399278, EPI_ISL_1424044, EPI_ISL_1424045			
see above	LESP Coahuila	Instituto de Diagnostico y Referencia Epidemiologicos (INDRE)	Abril Rodriguez-Maldonado; Ariadna Medina-Benitez; Claudia Wong-Arambula; Ernesto Ramirez-Gonzalez.; Gisela Barrera-Badillo; Irma Lopez-Martinez; Joaquin Quiroz-Mercado; Lucia Hernandez-Rivas; Natividad Cruz-Ortiz; Sergio Rangel-Guerrero; Tatiana Nunez-Garcia; Vanessa Rivero-Arredondo
EPI_ISL_1337395	LESP Colima	Instituto de Diagnostico y Referencia Epidemiologicos (INDRE)	Abril Rodriguez-Maldonado; Ariadna Medina-Benitez; Claudia Wong-Arambula; Ernesto Ramirez-Gonzalez.; Gisela Barrera-Badillo; Irma Lopez-Martinez; Joaquin Quiroz-Mercado; Lucia Hernandez-Rivas; Natividad Cruz-Ortiz; Sergio Rangel-Guerrero; Tatiana Nunez-Garcia; Vanessa Rivero-Arredondo
EPI_ISL_1334386, EPI_ISL_1337363, EPI_ISL_1337365, EPI_ISL_1337367, EPI_ISL_1337376, EPI_ISL_1337377, EPI_ISL_1337379, EPI_ISL_1337380, EPI_ISL_1337382, EPI_ISL_1337383, EPI_ISL_1337384, EPI_ISL_1337386, EPI_ISL_1337387, EPI_ISL_1340608, EPI_ISL_1340610, EPI_ISL_1340611, EPI_ISL_1340614, EPI_ISL_1340616, EPI_ISL_1340617, EPI_ISL_1340624, EPI_ISL_1340626, EPI_ISL_1340627, EPI_ISL_1340630, EPI_ISL_1340631, EPI_ISL_1340633, EPI_ISL_1340643, EPI_ISL_1340644, EPI_ISL_1340646, EPI_ISL_1340647, EPI_ISL_1340648, EPI_ISL_1340650, EPI_ISL_1359070, EPI_ISL_1359071, EPI_ISL_1359072, EPI_ISL_1359073, EPI_ISL_1359074, EPI_ISL_1359075, EPI_ISL_1359076, EPI_ISL_1359077, EPI_ISL_1359078, EPI_ISL_1359079, EPI_ISL_1359080, EPI_ISL_1359081, EPI_ISL_1359082, EPI_ISL_1359083, EPI_ISL_1359084, EPI_ISL_1359085, EPI_ISL_1359086, EPI_ISL_1359087, EPI_ISL_1365675, EPI_ISL_1366700, EPI_ISL_1399260, EPI_ISL_1399261, EPI_ISL_1400304, EPI_ISL_1400314, EPI_ISL_1400315, EPI_ISL_1400316, EPI_ISL_1400317, EPI_ISL_1400318, EPI_ISL_1400319, EPI_ISL_1400320, EPI_ISL_1400321, EPI_ISL_1400322, EPI_ISL_1400323, EPI_ISL_1400324, EPI_ISL_1400325, EPI_ISL_1400326, EPI_ISL_1400327, EPI_ISL_1400328, EPI_ISL_1400329, EPI_ISL_1400330, EPI_ISL_1400331, EPI_ISL_1400332, EPI_ISL_1400333, EPI_ISL_1400334, EPI_ISL_1400335, EPI_ISL_1400336, EPI_ISL_1400337, EPI_ISL_1400338, EPI_ISL_1400339, EPI_ISL_1400340, EPI_ISL_1400341, EPI_ISL_1400342, EPI_ISL_1400343, EPI_ISL_1400344, EPI_ISL_1400345, EPI_ISL_1400346, EPI_ISL_1400347, EPI_ISL_1400348, EPI_ISL_1400349, EPI_ISL_1405900, EPI_ISL_1405901, EPI_ISL_1405902, EPI_ISL_1405903, EPI_ISL_1405904, EPI_ISL_1405906, EPI_ISL_1405907, EPI_ISL_1405909, EPI_ISL_1405910, EPI_ISL_1405912, EPI_ISL_1405914, EPI_ISL_1405915, EPI_ISL_1424010, EPI_ISL_1424011, EPI_ISL_1424012			
see above	LESP Guanajuato	Instituto de Diagnostico y Referencia Epidemiologicos (INDRE)	Abril Rodriguez-Maldonado; Ariadna Medina-Benitez; Claudia Wong-Arambula; Ernesto Ramirez-Gonzalez.; Gisela Barrera-Badillo; Irma Lopez-Martinez; Joaquin Quiroz-Mercado; Lucia Hernandez-Rivas; Natividad Cruz-Ortiz; Sergio Rangel-Guerrero; Tatiana Nunez-Garcia; Vanessa Rivero-Arredondo
EPI_ISL_1334381, EPI_ISL_1424043	LESP Guerrero	Instituto de Diagnostico y Referencia Epidemiologicos (INDRE)	Abril Rodriguez-Maldonado; Ariadna Medina-Benitez; Claudia Wong-Arambula; Ernesto Ramirez-Gonzalez.; Gisela Barrera-Badillo; Irma Lopez-Martinez; Joaquin Quiroz-Mercado; Lucia Hernandez-Rivas; Natividad Cruz-Ortiz; Sergio Rangel-Guerrero; Tatiana Nunez-Garcia; Vanessa Rivero-Arredondo
EPI_ISL_1340640, EPI_ISL_1340642, EPI_ISL_1366690, EPI_ISL_1366691, EPI_ISL_1366692, EPI_ISL_1424008, EPI_ISL_1424009			
see above	LESP Hidalgo	Instituto de Diagnostico y Referencia Epidemiologicos (INDRE)	Abril Rodriguez-Maldonado; Ariadna Medina-Benitez; Claudia Wong-Arambula; Ernesto Ramirez-Gonzalez.; Gisela Barrera-Badillo; Irma Lopez-Martinez; Joaquin Quiroz-Mercado; Lucia Hernandez-Rivas; Natividad Cruz-Ortiz; Sergio Rangel-Guerrero; Tatiana Nunez-Garcia; Vanessa Rivero-Arredondo
EPI_ISL_1366661, EPI_ISL_1366662, EPI_ISL_1399259, EPI_ISL_1399270, EPI_ISL_1400298, EPI_ISL_1405908, EPI_ISL_1405911, EPI_ISL_1405913			
see above	LESP Jalisco	Instituto de Diagnostico y Referencia Epidemiologicos (INDRE)	Abril Rodriguez-Maldonado; Ariadna Medina-Benitez; Claudia Wong-Arambula; Ernesto Ramirez-Gonzalez.; Gisela Barrera-Badillo; Irma Lopez-Martinez; Joaquin Quiroz-Mercado; Lucia Hernandez-Rivas; Natividad Cruz-Ortiz; Sergio Rangel-Guerrero; Tatiana Nunez-Garcia; Vanessa Rivero-Arredondo
EPI_ISL_1558828, EPI_ISL_1558829, EPI_ISL_1558830, EPI_ISL_1558831, EPI_ISL_1558832, EPI_ISL_1558833	LESP Jalisco/UdeG	Instituto de Diagnostico y Referencia Epidemiologicos (INDRE)	Abril Rodriguez-Maldonado; Ariadna Medina-Benitez; Claudia Wong-Arambula; Ernesto Ramirez-Gonzalez.; Gisela Barrera-Badillo; Irma Lopez-Martinez; Joaquin Quiroz-Mercado; Lucia Hernandez-Rivas; Natividad Cruz-Ortiz; Sergio Rangel-Guerrero; Tatiana Nunez-Garcia; Vanessa Rivero-Arredondo
EPI_ISL_1365650, EPI_ISL_1365651, EPI_ISL_1365652, EPI_ISL_1365653, EPI_ISL_1365654, EPI_ISL_1365655, EPI_ISL_1366677, EPI_ISL_1366678, EPI_ISL_1366679, EPI_ISL_1366680, EPI_ISL_1366681, EPI_ISL_1400300, EPI_ISL_1400301, EPI_ISL_1400302, EPI_ISL_1400303, EPI_ISL_1400305, EPI_ISL_1400306, EPI_ISL_1400307, EPI_ISL_1400308, EPI_ISL_1400309, EPI_ISL_1405899, EPI_ISL_1424017, EPI_ISL_1424019, EPI_ISL_1424042			
see above	LESP Morelos	Instituto de Diagnostico y Referencia Epidemiologicos (INDRE)	Abril Rodriguez-Maldonado; Ariadna Medina-Benitez; Claudia Wong-Arambula; Ernesto Ramirez-Gonzalez.; Gisela Barrera-Badillo; Irma Lopez-Martinez; Joaquin Quiroz-Mercado; Lucia Hernandez-Rivas; Natividad Cruz-Ortiz; Sergio Rangel-Guerrero; Tatiana Nunez-Garcia; Vanessa Rivero-Arredondo
EPI_ISL_1399273, EPI_ISL_1399274	LESP Nayarit	Instituto de Diagnostico y Referencia Epidemiologicos (INDRE)	Abril Rodriguez-Maldonado; Ariadna Medina-Benitez; Claudia Wong-Arambula; Ernesto Ramirez-Gonzalez.; Gisela Barrera-Badillo; Irma Lopez-Martinez; Joaquin Quiroz-Mercado; Lucia Hernandez-Rivas; Natividad Cruz-Ortiz; Sergio Rangel-Guerrero; Tatiana Nunez-Garcia; Vanessa Rivero-Arredondo
EPI_ISL_1337368, EPI_ISL_1365639, EPI_ISL_1365640, EPI_ISL_1365641, EPI_ISL_1365642, EPI_ISL_1365643, EPI_ISL_1365644, EPI_ISL_1365645, EPI_ISL_1365646, EPI_ISL_1365647, EPI_ISL_1365648, EPI_ISL_1365668, EPI_ISL_1365669, EPI_ISL_1365670, EPI_ISL_1365671, EPI_ISL_1365672, EPI_ISL_1365673, EPI_ISL_1365674, EPI_ISL_1366663, EPI_ISL_1366664, EPI_ISL_1366665, EPI_ISL_1366693, EPI_ISL_1366694, EPI_ISL_1366695, EPI_ISL_1366696, EPI_ISL_1366697, EPI_ISL_1366698, EPI_ISL_1366699, EPI_ISL_1399262, EPI_ISL_1399263, EPI_ISL_1399264, EPI_ISL_1399265, EPI_ISL_1399266, EPI_ISL_1399267, EPI_ISL_1399268, EPI_ISL_1399269			
see above	LESP Nuevo Leon	Instituto de Diagnostico y Referencia Epidemiologicos (INDRE)	Abril Rodriguez-Maldonado; Ariadna Medina-Benitez; Claudia Wong-Arambula; Ernesto Ramirez-Gonzalez.; Gisela Barrera-Badillo; Irma Lopez-Martinez; Joaquin Quiroz-Mercado; Lucia Hernandez-Rivas; Natividad Cruz-Ortiz; Sergio Rangel-Guerrero; Tatiana Nunez-Garcia; Vanessa Rivero-Arredondo
EPI_ISL_942927, EPI_ISL_961467, EPI_ISL_961468, EPI_ISL_961469	LESP Nuevo Leon/Grupo de Diagnostico ARIES	Instituto de Diagnostico y Referencia Epidemiologicos (INDRE)	Abril Rodriguez-Maldonado; Claudia Wong-Arambula; Ernesto Ramirez-Gonzalez.; Fabiola Garces-Ayala; Gisela Barrera-Badillo; Irma Lopez-Martinez; Lucia Hernandez-Rivas; Natividad Cruz-Ortiz; Tatiana Nunez-Garcia
EPI_ISL_1340651, EPI_ISL_1340652, EPI_ISL_1366660, EPI_ISL_1400310, EPI_ISL_1400311, EPI_ISL_1400312, EPI_ISL_1400313			
see above	LESP Puebla	Instituto de Diagnostico y Referencia Epidemiologicos (INDRE)	Abril Rodriguez-Maldonado; Ariadna Medina-Benitez; Claudia Wong-Arambula; Ernesto Ramirez-Gonzalez.; Gisela Barrera-Badillo; Irma Lopez-Martinez; Joaquin Quiroz-Mercado; Lucia Hernandez-Rivas; Natividad Cruz-Ortiz; Sergio Rangel-Guerrero; Tatiana Nunez-Garcia; Vanessa Rivero-Arredondo
EPI_ISL_1337360, EPI_ISL_1337362, EPI_ISL_1340636, EPI_ISL_1340637, EPI_ISL_1340639, EPI_ISL_1340655, EPI_ISL_1340657, EPI_ISL_1359064, EPI_ISL_1359065, EPI_ISL_1366667, EPI_ISL_1366668, EPI_ISL_1366669, EPI_ISL_1366670, EPI_ISL_1366671, EPI_ISL_1366672, EPI_ISL_1366673, EPI_ISL_1366674, EPI_ISL_1366675, EPI_ISL_1366676, EPI_ISL_1424016, EPI_ISL_1424021, EPI_ISL_1424022, EPI_ISL_1424023, EPI_ISL_1424024, EPI_ISL_1424025, EPI_ISL_1424026, EPI_ISL_1424027, EPI_ISL_1424028, EPI_ISL_1424029, EPI_ISL_1424030, EPI_ISL_1424031, EPI_ISL_1424032, EPI_ISL_1424033			

see above	LESP Queretaro	Instituto de Diagnostico y Referencia Epidemiologicos (INDRE)	Abril Rodriguez-Maldonado; Ariadna Medina-Benitez; Claudia Wong-Arambula; Ernesto Ramirez-Gonzalez.; Gisela Barrera-Badillo; Irma Lopez-Martinez; Joaquin Quiroz-Mercado; Lucia Hernandez-Rivas; Natividad Cruz-Ortiz; Sergio Rangel-Guerrero; Tatiana Nunez-Garcia; Vanessa Rivero-Arredondo
EPI_ISL_1324767, EPI_ISL_1365661	LESP Quintana Roo	Instituto de Diagnostico y Referencia Epidemiologicos (INDRE)	Abril Rodriguez-Maldonado; Ariadna Medina-Benitez; Claudia Wong-Arambula; Ernesto Ramirez-Gonzalez.; Gisela Barrera-Badillo; Irma Lopez-Martinez; Joaquin Quiroz-Mercado; Lucia Hernandez-Rivas; Natali Vega-Magana; Natividad Cruz-Ortiz; Sergio Rangel-Guerrero; Tatiana Nunez-Garcia; Vanessa Rivero-Arredondo
EPI_ISL_1337389	LESP Sinaloa	Instituto de Diagnostico y Referencia Epidemiologicos (INDRE)	Abril Rodriguez-Maldonado; Ariadna Medina-Benitez; Claudia Wong-Arambula; Ernesto Ramirez-Gonzalez.; Gisela Barrera-Badillo; Irma Lopez-Martinez; Joaquin Quiroz-Mercado; Lucia Hernandez-Rivas; Natividad Cruz-Ortiz; Sergio Rangel-Guerrero; Tatiana Nunez-Garcia; Vanessa Rivero-Arredondo
EPI_ISL_1365649, EPI_ISL_1399275, EPI_ISL_1399276, EPI_ISL_1399277, EPI_ISL_1424039, EPI_ISL_1424040, EPI_ISL_1424041			
see above	LESP Tamaulipas	Instituto de Diagnostico y Referencia Epidemiologicos (INDRE)	Abril Rodriguez-Maldonado; Ariadna Medina-Benitez; Claudia Wong-Arambula; Ernesto Ramirez-Gonzalez.; Gisela Barrera-Badillo; Irma Lopez-Martinez; Joaquin Quiroz-Mercado; Lucia Hernandez-Rivas; Natividad Cruz-Ortiz; Sergio Rangel-Guerrero; Tatiana Nunez-Garcia; Vanessa Rivero-Arredondo
EPI_ISL_1365656, EPI_ISL_1366682, EPI_ISL_1366683, EPI_ISL_1366684, EPI_ISL_1366685, EPI_ISL_1366686, EPI_ISL_1399279, EPI_ISL_1424018, EPI_ISL_1424046, EPI_ISL_1424047, EPI_ISL_1424048, EPI_ISL_1424049			
see above	LESP Veracruz	Instituto de Diagnostico y Referencia Epidemiologicos (INDRE)	Abril Rodriguez-Maldonado; Ariadna Medina-Benitez; Claudia Wong-Arambula; Ernesto Ramirez-Gonzalez.; Gisela Barrera-Badillo; Irma Lopez-Martinez; Joaquin Quiroz-Mercado; Lucia Hernandez-Rivas; Natividad Cruz-Ortiz; Sergio Rangel-Guerrero; Tatiana Nunez-Garcia; Vanessa Rivero-Arredondo
EPI_ISL_1340658, EPI_ISL_1340661, EPI_ISL_1359066, EPI_ISL_1359067, EPI_ISL_1366666, EPI_ISL_1424007, EPI_ISL_1424014			
see above	LESP Yucatan	Instituto de Diagnostico y Referencia Epidemiologicos (INDRE)	Abril Rodriguez-Maldonado; Ariadna Medina-Benitez; Claudia Wong-Arambula; Ernesto Ramirez-Gonzalez.; Gisela Barrera-Badillo; Irma Lopez-Martinez; Joaquin Quiroz-Mercado; Lucia Hernandez-Rivas; Natividad Cruz-Ortiz; Sergio Rangel-Guerrero; Tatiana Nunez-Garcia; Vanessa Rivero-Arredondo
EPI_ISL_1340660, EPI_ISL_1359068, EPI_ISL_1359069	LESP Zacatecas	Instituto de Diagnostico y Referencia Epidemiologicos (INDRE)	Abril Rodriguez-Maldonado; Ariadna Medina-Benitez; Claudia Wong-Arambula; Ernesto Ramirez-Gonzalez.; Gisela Barrera-Badillo; Irma Lopez-Martinez; Joaquin Quiroz-Mercado; Lucia Hernandez-Rivas; Natividad Cruz-Ortiz; Sergio Rangel-Guerrero; Tatiana Nunez-Garcia; Vanessa Rivero-Arredondo
EPI_ISL_1335757	LESP Zacatecas	Instituto de diagnóstico y Referencia Epidemiologicos (INDRE) Departamento de Virología	Abril Rodriguez-Maldonado; Ariadna Medina-Benitez; Claudia Wong-Arambula; Ernesto Ramirez-Gonzalez.; Gisela Barrera-Badillo; Irma Lopez-Martinez; Joaquin Quiroz-Mercado; Lucia Hernandez-Rivas; Natividad Cruz-Ortiz; Sergio Rangel-Guerrero; Tatiana Nunez-Garcia; Vanessa Rivero-Arredondo
EPI_ISL_435573, EPI_ISL_578719, EPI_ISL_578947	LSUHS Emerging Viral Threat Laboratory	Microbial Genome Sequencing Center	Abida Siddiqa; Adam Greer; Andrew D. Yurochko; Byeong-Jae Lee; Camille F. Abshire; Chan-ki Min; Christopher G. Kevil; Daniel J. Snyder; Edna Ondari; Jason M. Bodily; Jeremy P. Kamil; John A. Vanchiere; Katarzyna Zwolinska; Maarten Van Diest; Malgorzata Bienkowska-Haba; Martin J. Sapp; Md Maksudul Alam; Monica Gestal-Cartele; Paul M. Weinberger; Rona S. Scott; Vaughn S. Cooper
EPI_ISL_468743, EPI_ISL_468744	Lab voor klinische biologie	Onderzoeksgroep Virologie	Bruno Verhasselt; Hans Nauwynck; Laurens Lambrechts; Linos Vandekerckhove; Marthe Pauwels; Nick Vereecke; Sebastiaan Theuns
EPI_ISL_456210	LabPLUS	Institute of Environmental Science and Research (ESR)	Anja Werno; Antje van der Linden; Arlo Upton; Chris Mansell; David Hammer; Dragana Drinkovic; Erasmus Smit; Gary McAuliffe; Hana Sofia Andersson; James Ussher; Jill Sherwood; Joep de Ligt; Josh Freeman; Julia Howard; Juliet Elvy; Lauren Jelly; Mary DeAlmeida; Matt Blakiston; Matt Storey; Matthew Rogers; Max Bloomfield; Michael Addidle; Michelle Balm; Sally Roberts; Sarah Jefferies; Sharmini Muttaiyah; Susan Morpeth; Susan Taylor; Timothy Blackmore; Vani Sathyendran; Veronica Playle; Virginia Hope; Xiaoyun Ren
EPI_ISL_547977	LabTests	Institute of Environmental Science and Research (ESR)	Anja Werno; Antje van der Linden; Arlo Upton; Chris Mansell; David Hammer; Dragana Drinkovic; Erasmus Smit; Gary McAuliffe; Hana Sofia Andersson; Hermes Perez; James Ussher; Jill Sherwood; Jing Wang; Joep de Ligt; Josh Freeman; Julia Howard; Juliet Elvy; Lauren Jelly; Mary DeAlmeida; Matt Blakiston; Matt Storey; Matthew Rogers; Max Bloomfield; Michael Addidle; Michelle Balm; Muhammad Faisal; Nikki Freed; Olin Silander; Sally Roberts; Sarah Jefferies; Sharmini Muttaiyah; Susan Morpeth; Susan Taylor; Timothy Blackmore; Vani Sathyendran; Veronica Playle; Virginia Hope; Xiaoyun Ren
EPI_ISL_560637	Labo Analyses Med	National Reference Center for Viruses of Respiratory Infections, Institut Pasteur, Paris	Etienne Simon-Lorière; Fabiana Gambaro; Maud Vanpeene; Sylvie Behillil; Sylvie van der Werf; Vincent Enouf
EPI_ISL_476705	Labor Kneißler GmbH & Co. KG	Heinrich Pette Institute, Leibniz Institute for Experimental Virology	Adam Grundhoff; Manja Czech-Sioli; Matthias Ottinger; Melanie M. Brinkmann; Nicole Fischer; Thomas Günther
EPI_ISL_429718, EPI_ISL_434495	Laboratoire National de Sante, Microbiology, Virology	Laboratoire National de Sante, Microbiology, Epidemiology and Microbial Genomics	Anke Wienecke-Baldacchino; Ardashel Latsuzbaia; Catherine Ragimbeau; Guillaume Fournier; Jessica Tapp; Joel Mossong; Tamir Abdelrahman; Trung Nguyen Nguyen
EPI_ISL_419564	Laboratoire National de Santé, Microbiology, Virology	Laboratoire National de Santé, Microbiology, Epidemiology and Microbial Genomics	Anke Wienecke-Baldacchino; Ardashel Latsuzbaia; Catherine Ragimbeau; Guillaume Fournier; Jessica Tapp; Joel Mossong; Tamir Abdelrahman; Trung Nguyen Nguyen
EPI_ISL_660474	Laboratoire de Microbiologie CHU Souro Sanou	Centre Muraz	Abdoul-Salam Ouedraogo; Arsène Zongo; Essia Belarbi; Fabian Leendertz; Grit Schubert; Halidou Tinto; Lassana Sangaré; Soumeya Ouangraoua; Yacouba Sawadogo; Zekiba Tarnagda
EPI_ISL_414623	Laboratoire de Virologie Institut de Virologie - INSERM U 1109 Hôpitaux Universitaires de Strasbourg	National Reference Center for Viruses of Respiratory Infections, Institut Pasteur, Paris	Angela Brisebarre; Flora Donati Vincent Enouf; Marion Barbet; Maud Vanpeene; Méline Bizard; Mélnie Albert; Samira Fafi-Kremer; Sylvie Behillil; Sylvie van der Werf
EPI_ISL_463942	Laboratoire de microbiologie, Hopital de Verdun	Smith Laboratory, Centre de Recherche CHU Sainte-Justine	Ivan Pavlov; Marieke Rozendaal; Martin Smith
EPI_ISL_1340620, EPI_ISL_1340621, EPI_ISL_1340623, EPI_ISL_1359063	Laboratorio BIOMEDICA	Instituto de Diagnostico y Referencia Epidemiologicos (INDRE)	Abril Rodriguez-Maldonado; Ariadna Medina-Benitez; Claudia Wong-Arambula; Ernesto Ramirez-Gonzalez.; Gisela Barrera-Badillo; Irma Lopez-Martinez; Joaquin Quiroz-Mercado; Lucia Hernandez-Rivas; Natividad Cruz-Ortiz; Sergio Rangel-Guerrero; Tatiana Nunez-Garcia; Vanessa Rivero-Arredondo
EPI_ISL_424667	Laboratorio Estatal de Salud Publica del Estado de México	Instituto de Diagnóstico y Referencia Epidemiológicos	Adnan Araiza Rodríguez; Alejandro Sánchez; Alfredo Ponce de León Garduño; Blanca Taboada; Carlos F. Arias.; Carolina González Torres; Celia Boukadida; Cesar Raúl González Bonilla; Concepción Grajales Muñiz; Edgar Mendieta Condado; Eduardo Becerril Vargas; Fabiola Garcés Ayala; Fernando Ledesma Barrientos; Francisco Javier Gaytán Cervantes; Francisco Pulido; Gisela Barrera Badillo; Gloria Vázquez; Guillermo M. Ruiz-Palacios; Irma López Martínez; Joel Armando Vázquez Pérez; José Arturo Martínez Orozco; José Ernesto Ramírez González; José Esteban Muñoz Medina; Lucia Hernández Rivas; Luis Alberto García Andrade; Mario Mújica Sánchez; Pavel Isa; Pilar Ramos Cervantes; Ricardo Grande; Santiago Avila Ríos; Victor Hugo Borja Aburto; Violeta Ibarra Gonzalez
EPI_ISL_424672	Laboratorio Estatal de Salud Publica del Estado de Puebla	Instituto de Diagnostico y Referencia Epidemiologicos	Adnan Araiza Rodríguez; Alejandro Sánchez; Alfredo Ponce de León Garduño; Blanca Taboada; Carlos F. Arias; Carolina González Torres; Celia Boukadida; Cesar Raúl González Bonilla; Concepción Grajales Muñiz; Edgar Mendieta Condado; Eduardo Becerril Vargas; Fabiola Garcés Ayala; Fernando Ledesma Barrientos; Francisco Javier Gaytán Cervantes; Francisco Pulido; Gisela Barrera Badillo; Gloria Vázquez; Guillermo M. Ruiz-Palacios; Irma López Martínez; Joel Armando Vázquez Pérez; José Arturo Martínez Orozco; José Ernesto Ramírez González; José Esteban Muñoz Medina; Lucia Hernández Rivas; Luis Alberto García Andrade; Mario Mújica Sánchez; Pavel Isa; Pilar Ramos Cervantes; Ricardo Grande; Santiago Avila Ríos; Victor Hugo Borja Aburto; Violeta Ibarra Gonzalez
EPI_ISL_424670	Laboratorio Estatal de Salud Publica del Estado de Queretaro	Instituto de Diagnóstico y Referencia Epidemiologicos	Adnan Araiza Rodríguez; Alejandro Sánchez; Alfredo Ponce de León Garduño; Blanca Taboada; Carlos F. Arias; Carolina González Torres; Celia Boukadida; Cesar Raúl González Bonilla; Concepción Grajales Muñiz; Edgar Mendieta Condado; Eduardo Becerril Vargas; Fabiola Garcés Ayala; Fernando Ledesma Barrientos; Francisco Javier Gaytán Cervantes; Francisco Pulido; Gisela Barrera Badillo; Gloria Vázquez; Guillermo M. Ruiz-Palacios; Irma López Martínez; Joel Armando Vázquez Pérez; José Arturo Martínez Orozco; José Ernesto Ramírez González; José Esteban Muñoz Medina; Lucia Hernández Rivas; Luis Alberto García Andrade; Mario Mújica Sánchez; Pavel Isa; Pilar Ramos Cervantes; Ricardo Grande; Santiago Avila Ríos; Victor Hugo Borja Aburto; Violeta Ibarra Gonzalez
EPI_ISL_779185, EPI_ISL_779186, EPI_ISL_779187, EPI_ISL_779188, EPI_ISL_779189, EPI_ISL_779190, EPI_ISL_779191, EPI_ISL_779192, EPI_ISL_779193, EPI_ISL_779194, EPI_ISL_779195, EPI_ISL_779196, EPI_ISL_779197, EPI_ISL_779198, EPI_ISL_979327, EPI_ISL_979328, EPI_ISL_979329, EPI_ISL_979330, EPI_ISL_979331, EPI_ISL_979332, EPI_ISL_979333, EPI_ISL_979334, EPI_ISL_979335, EPI_ISL_979336, EPI_ISL_979337, EPI_ISL_979338, EPI_ISL_979339, EPI_ISL_979340, EPI_ISL_979341, EPI_ISL_979342, EPI_ISL_979343, EPI_ISL_979344, EPI_ISL_979345, EPI_ISL_979346, EPI_ISL_979347, EPI_ISL_979348, EPI_ISL_979349, EPI_ISL_979350, EPI_ISL_979351, EPI_ISL_979352, EPI_ISL_1091241, EPI_ISL_1091242, EPI_ISL_1091243, EPI_ISL_1091244, EPI_ISL_1091245, EPI_ISL_1091246, EPI_ISL_1091249, EPI_ISL_1091253, EPI_ISL_1091254, EPI_ISL_1091255, EPI_ISL_1091256, EPI_ISL_1091257, EPI_ISL_1091262, EPI_ISL_1092458			
see above	Laboratorio Estatal de Salud Pública de Nuevo León	Laboratorio de Infectología Molecular, Departamento de Bioquímica y Medicina Molecular,Facultad de Medicina - Universidad Autónoma de Nuevo León	Ana M. Rivas-Estilla; Consuelo Treviño-Garza; Daniel Arellanos-Soto; Else del Carmen García-García; Gloria A. Jasso-de-la-Peña; Kame A. Galán-Huerta; Manuel E. de-la-O-Cavazos; María F. Herrera-Saldivar; Natalia Martínez-Acuña; Roberto Montes-de-Oca; Samuel Buentello-Wong; Sonia A. Lozano-Sepúlveda
EPI_ISL_794592	Laboratorio Estatal de Salud Pública de Tamaulipas	Instituto de diagnóstico y Referencia Epidemiologicos (INDRE)	Abril Rodriguez-Maldonado; Ana Maria Cortez-Calderon; Bernardita Reyes-Berrones; Celia Alpuche-Aranda; Claudia Wong-Arambula; Ernesto Ramirez-Gonzalez.; Fabiola Garces-Ayala; Gisela Barrera-Badillo; Gloria Molina-Gamboa; Hilda del Carmen-Selvera; Hugo Lopez Gatell-Ramirez; Irma Lopez-Martinez; Jose Luis Alomia-Zegarra; Lucia Hernandez-Rivas
EPI_ISL_1091247, EPI_ISL_1091248, EPI_ISL_1091252, EPI_ISL_1091258, EPI_ISL_1091259, EPI_ISL_1091260, EPI_ISL_1091263, EPI_ISL_1091264, EPI_ISL_1091265, EPI_ISL_1091266, EPI_ISL_1091267, EPI_ISL_1091268, EPI_ISL_1091269, EPI_ISL_1091270, EPI_ISL_1091271, EPI_ISL_1091272, EPI_ISL_1091273, EPI_ISL_1091274			
see above	Laboratorio PGM	Laboratorio de Infectología Molecular, Departamento de Bioquímica y Medicina Molecular,Facultad de Medicina - Universidad Autónoma de Nuevo León	Ana M. Rivas-Estilla; Daniel Arellanos-Soto; Eduardo Garza-de-la-Peña; Gabriela Elizondo; Javier Ramos-Jimenez; Kame A. Galán-Huerta; María F. Herrera-Saldivar; Natalia Martínez-Acuña; Sonia A. Lozano-Sepúlveda
EPI_ISL_1091261, EPI_ISL_1092361	Laboratorio de Apoyo a la Vigilancia e Investigación Epidemiológica - CIBO	Laboratorio de Infectología Molecular, Departamento de Bioquímica y Medicina Molecular,Facultad de Medicina - Universidad Autónoma de Nuevo León	Alma M. Perez-Rios; Ana M. Rivas-Estilla; Blanca M. Torres; Claudia L. Charles-Niño; Daniel Arellanos-Soto; Diego A. Gonzalez-Altamirano; Hector Montoya-Fuentes; Javier Ramos-Jimenez; Jose J. Figueroa-Delgadillo; Kame A. Galán-Huerta; María F. Herrera-Saldivar; Natalia Martínez-Acuña; Rafael Soto-Alvarez; Sonia A. Lozano-Sepúlveda
EPI_ISL_1093145, EPI_ISL_1093146, EPI_ISL_1093147, EPI_ISL_1324758, EPI_ISL_1324759, EPI_ISL_1324760, EPI_ISL_1324761, EPI_ISL_1324762, EPI_ISL_1324763, EPI_ISL_1324764, EPI_ISL_1324765, EPI_ISL_1324766			
see above	Laboratorio de Diagnostico de Enfermedades Emergentes y Reemergentes	Instituto de Diagnostico y Referencia Epidemiologicos (INDRE)	Abril Rodriguez-Maldonado; Ariadna Medina-Benitez; Claudia Wong-Arambula; Ernesto Ramirez-Gonzalez.; Fabiola Garces-Ayala; Gisela Barrera-Badillo; Irma Lopez-Martinez; Joaquin Quiroz-Mercado; Lucia Hernandez-Rivas; Natali Vega-Magana; Natividad Cruz-Ortiz; Sergio Rangel-Guerrero; Tatiana Nunez-Garcia; Vanessa Rivero-Arredondo
EPI_ISL_1360407, EPI_ISL_1360408, EPI_ISL_1360409, EPI_ISL_1360410, EPI_ISL_1360411, EPI_ISL_1360412	Laboratorio de Enfermedades Emergentes y Reemergentes	Laboratorio de Enfermedades Emergentes y Reemergentes	García-Chagollan Mariel; Hernández-Bello Jorge; Muñoz-Valle José Francisco; Peña-Rodriguez Marcela; Vega-Magaña Natali; Viera-Segura Oliver
EPI_ISL_648595, EPI_ISL_648602, EPI_ISL_648603	Laboratorio de Infectología Servicio de Infectología Hospital Universitario Dr. José Eleuterio González - Universidad Autónoma de Nuevo León	Laboratorio de Infectología Molecular Departamento de Bioquímica y Medicina Molecular Facultad de Medicina - Universidad Autónoma de Nuevo León	Adrian Camacho-Ortiz; Ana M. Rivas-Estilla; Consuelo Treviño-Garza; Daniel Arellanos-Soto; Eduardo Perez-Alba; Elvira Garza-González; Kame A. Galán-Huerta; Laura Nuzzolo-Shihadeh; Manuel E. de-la-O-Cavazos; María F. Herrera-Saldivar; Natalia Martínez-Acuña; Paola Bocanegra-Ibarias; Roberto Montes-de-Oca; Samantha M. Flores-Treviño; Sonia A. Lozano-Sepúlveda
EPI_ISL_648607, EPI_ISL_779169, EPI_ISL_779170, EPI_ISL_779171, EPI_ISL_779172, EPI_ISL_779173, EPI_ISL_779174, EPI_ISL_779175, EPI_ISL_779176, EPI_ISL_779177, EPI_ISL_779178, EPI_ISL_779179, EPI_ISL_779180, EPI_ISL_779181, EPI_ISL_779182, EPI_ISL_779183, EPI_ISL_779184, EPI_ISL_961760, EPI_ISL_961761, EPI_ISL_961762, EPI_ISL_961763, EPI_ISL_961765, EPI_ISL_961766, EPI_ISL_961767, EPI_ISL_961768, EPI_ISL_961771, EPI_ISL_961772, EPI_ISL_961773, EPI_ISL_961774, EPI_ISL_961777, EPI_ISL_961780, EPI_ISL_961781, EPI_ISL_961785, EPI_ISL_961791, EPI_ISL_961792			
see above	Laboratorio de Infectología, Servicio de Infectología, Hospital Universitario Dr. José Eleuterio González - Universidad Autónoma de Nuevo León	Laboratorio de Infectología Molecular, Departamento de Bioquímica y Medicina Molecular,Facultad de Medicina - Universidad Autónoma de Nuevo León	Adrian Camacho-Ortiz; Ana M. Rivas-Estilla; Consuelo Treviño-Garza; Daniel Arellanos-Soto; Eduardo Perez-Alba; Elvira Garza-González; Kame A. Galán-Huerta; Laura Nuzzolo-Shihadeh; Manuel E. de-la-O-Cavazos; María F. Herrera-Saldivar; Natalia Martínez-Acuña; Paola Bocanegra-Ibarias; Roberto Montes-de-Oca; Samantha M. Flores-Treviño; Sonia A. Lozano-Sepúlveda

EPI_ISL_648319	Laboratorio de Investigaciones de Baney	University Hospital Basel, Clinical Bacteriology	Adrian Egli; Alfredo Mari; Bonifacio Manguire Nlavo; Carlos Cortes; Claudia Daubenberger; Diosdado Odjama Nseng Ada; Elizabeth Nyakarungu; Guillermo Garcia; Helena Seth-Smith; Madlen Stange; Maximilian Mpina; Mitoha Ondo O Ayekaba; Philip Wonder Phiri; Salome Hosch; Tim Roloff; Tobias Schindler
EPI_ISL_491461	Laboratorio de Referencia Nacional de Virus Respiratorio. Instituto Nacional de Salud Perú	Laboratorio de Referencia Nacional de Biotecnología y Biología Molecular. Instituto Nacional de Salud Perú	Carlos Padilla Rojas; Henri Bailon Calderon; Johanna Balbuena Torrez; Karolyn Vega Chozo; Marco Galarza Perez; Maribel Huaringa Nuñez; Nancy Rojas Serrano; Omar Caceres Rey; Priscila Lope Pari
EPI_ISL_514264	Laboratorio de Referencia Nacional de Virus Respiratorio. Instituto Nacional de Salud. Perú	Laboratorio de Referencia Nacional de Biotecnologia y Biologia Molecular.Instituto Nacional de Salud.Perú	Carlos Padilla Rojas; Henri Bailon Calderon; Johanna Balbuena Torrez; Karolyn Vega Chozo; Marco Galarza Perez; Maribel Huaringa Nuñez; Nancy Rojas Serrano; Omar Caceres Rey; Priscila Lope Pari
EPI_ISL_482468	Laboratorio de Referencia Nacional de Virus Respiratorios. Instituto Nacional de Salud Peru	Laboratorio de Referencia Nacional de Biotecnologia y Biologia Molecular. Instituto Nacional de Salud Peru	Carlos Padilla Rojas; Henri Bailon Calderon; Johanna Balbuena Torres; Karolyn Vega Chozo; Maribel Huaringa Nuñez; Nancy Rojas Serrano; Omar Caceres Rey; Priscila Lope Pari
EPI_ISL_430814	Laboratorio de Virología del Hospital de Niños Dr. Ricardo Gutierrez	Área de Secuenciación del Laboratorio de Virología del Hospital de Niños Dr. Ricardo Gutierrez on behalf of 'Proyecto Argentino Interinstitucional de genomica de SARS-CoV-2' (PAIS Consortium)	AS; E; Goya; Gravis; LE; Lusso; MI; MS; Mistchenko; Nabaes Jodar; Natale; S; Valinotto; Viegas, M.
EPI_ISL_1340619, EPI_ISL_1340634, EPI_ISL_1340654	Laboratorios BIMODI	Instituto de Diagnostico y Referencia Epidemiologicos (INDRE)	Abril Rodriguez-Maldonado; Ariadna Medina-Benitez; Claudia Wong-Arambula; Ernesto Ramirez-Gonzalez.; Gisela Barrera-Badillo; Irma Lopez-Martinez; Joaquin Quiroz-Mercado; Lucia Hernandez-Rivas; Natividad Cruz-Ortiz; Sergio Rangel-Guerrero; Tatiana Nunez-Garcia; Vanessa Rivero-Arredondo
EPI_ISL_1340613	Laboratorios DIAGNOMOL	Instituto de Diagnostico y Referencia Epidemiologicos (INDRE)	Abril Rodriguez-Maldonado; Ariadna Medina-Benitez; Claudia Wong-Arambula; Ernesto Ramirez-Gonzalez.; Gisela Barrera-Badillo; Irma Lopez-Martinez; Joaquin Quiroz-Mercado; Lucia Hernandez-Rivas; Natividad Cruz-Ortiz; Sergio Rangel-Guerrero; Tatiana Nunez-Garcia; Vanessa Rivero-Arredondo
EPI_ISL_1359062	Laboratorios LISTER	Instituto de Diagnostico y Referencia Epidemiologicos (INDRE)	Abril Rodriguez-Maldonado; Ariadna Medina-Benitez; Claudia Wong-Arambula; Ernesto Ramirez-Gonzalez.; Gisela Barrera-Badillo; Irma Lopez-Martinez; Joaquin Quiroz-Mercado; Lucia Hernandez-Rivas; Natividad Cruz-Ortiz; Sergio Rangel-Guerrero; Tatiana Nunez-Garcia; Vanessa Rivero-Arredondo
EPI_ISL_961466, EPI_ISL_1334380, EPI_ISL_1334382, EPI_ISL_1334385	Laboratorios Lister	Instituto de Diagnostico y Referencia Epidemiologicos (INDRE)	Abril Rodriguez-Maldonado; Ariadna Medina-Benitez; Claudia Wong-Arambula; Ernesto Ramirez-Gonzalez.; Fabiola Garces-Ayala; Gisela Barrera-Badillo; Irma Lopez-Martinez; Joaquin Quiroz-Mercado; Lucia Hernandez-Rivas; Natividad Cruz-Ortiz; Sergio Rangel-Guerrero; Tatiana Nunez-Garcia; Vanessa Rivero-Arredondo
EPI_ISL_435045	Laboratory of Applied Genetics	RSE "National Center for Biotechnology"	Alexandr Shevtsov; Asylulan Amirgazin; Ilyas Akhmetollayev; Ruslan Kalendar; Viktoriya Lutsay; Yerlan Ramanculov
EPI_ISL_434468	Laboratory of Microbiology, Medical School, National and Kapodistrian University of Athens	Laboratory of Biology, Department of Medicine, Democritus University of Thrace	Bampali, M.; Dovrolis, N.; Froukala, E.; Gatzidou, E.; Kassela K.; N. and Karakasiliotis, I.; Spanakis; Stavropoulou, A.; Tsakris, A.; Veletza, S.
EPI_ISL_451646	Laboratory of Molecular Biology, Diagnostyka sp. z o.o.	Laboratory of Recombinant Vaccines	Anna Piotrowska-Mietelska; Boguslaw Szewczyk; Krystyna Bienkowska-Szewczyk; Lukasz Rabalski; Maciej Kosinski
EPI_ISL_639639	Latvijas Infektoloģijas centrs	Latvian Biomedical Research and Study Centre	Ivars Silamiķelis; Jeļena Storoženko; Jānis Kloviņš; Kaspars Megnis; Monta Ustinova; Oksana Savicka; Tatjana Kolupajeva; Uga Dumpis; Vita Rovīte; Ņikita Zrelovs
EPI_ISL_498552	Lebanese American University	Lebanese American University	Abdallah, J.; Abi Habib, W.; El Shesheny, R.; Goldstein; J. and Kayali, G.; Mokhbat, J.; R.J.; Webby
EPI_ISL_538890, EPI_ISL_538900, EPI_ISL_538963, EPI_ISL_538976, EPI_ISL_539123, EPI_ISL_539172	Leeds Teaching Hospitals NHS Trust and Public Health England, National Infection Service (Leeds laboratory)	Wellcome Sanger Institute for the COVID-19 Genomics UK (COG-UK) consortium	Antony Hale and Alex Alderton; Cordelia Langford; David K. Jackson; Dominic Kwiatkowski; Ewan Harrison; Holli Carden; Ian Johnston; John Sillitoe on behalf of the Wellcome Sanger Institute COVID-19 Surveillance Team; Katherine L. Harper; Louissa Macfarlane-Smith; Roberto Amato; Sonia Goncalves
EPI_ISL_551318, EPI_ISL_554172, EPI_ISL_554694, EPI_ISL_556143, EPI_ISL_558199, EPI_ISL_558906, EPI_ISL_558933, EPI_ISL_558971, EPI_ISL_559053, EPI_ISL_559410, EPI_ISL_559423, EPI_ISL_559586, EPI_ISL_559611, EPI_ISL_559630, EPI_ISL_559631, EPI_ISL_567654, EPI_ISL_567792, EPI_ISL_568172, EPI_ISL_575413, EPI_ISL_580650, EPI_ISL_581444, EPI_ISL_589424, EPI_ISL_590306			
see above	Lighthouse Lab in Alderley Park	Wellcome Sanger Institute for the COVID-19 Genomics UK (COG-UK) consortium	Cordelia Langford; David K. Jackson; Dominic Kwiatkowski; Ewan Harrison; Ian Johnston; Jacquelyn Wynn; John Sillitoe on behalf of the Wellcome Sanger Institute COVID-19 Surveillance Team; John Sillitoe on behalf of the Wellcome Sanger Institute COVID-19 Surveillance Team (http://www.sanger.ac.uk/covid-team); Mairead Hyland; Roberto Amato; Sonia Goncalves; The Lighthouse Lab in Alderley Park and Alex Alderton
EPI_ISL_552051	Lighthouse Lab in Cambridge	Wellcome Sanger Institute for the COVID-19 Genomics UK (COG-UK) consortium	Cordelia Langford; David K. Jackson; Dominic Kwiatkowski; Ewan Harrison; Ian Johnston; John Sillitoe on behalf of the Wellcome Sanger Institute COVID-19 Surveillance Team; Rob Howes; Roberto Amato; Sonia Goncalves; The Lighthouse Lab in Cambridge and Alex Alderton
EPI_ISL_531685, EPI_ISL_531901, EPI_ISL_532239, EPI_ISL_533364, EPI_ISL_536972, EPI_ISL_540217, EPI_ISL_540362, EPI_ISL_549707, EPI_ISL_567431, EPI_ISL_581030, EPI_ISL_590273, EPI_ISL_590323, EPI_ISL_590381, EPI_ISL_590540, EPI_ISL_602019			
see above	Lighthouse Lab in Glasgow	Wellcome Sanger Institute for the COVID-19 Genomics UK (COG-UK) consortium	Anna Dominiczak and Alex Alderton; Carol Clugston; Cordelia Langford; David Gray; David K. Jackson; Dominic Kwiatkowski; Ewan Harrison; Harper VanSteenhouse; Ian Johnston; John Sillitoe; John Sillitoe on behalf of the Wellcome Sanger Institute COVID-19 Surveillance Team; John Sillitoe on behalf of the Wellcome Sanger Institute COVID-19 Surveillance Team (http://www.sanger.ac.uk/covid-team); Roberto Amato; Sonia Goncalves; Yumi Kasai
EPI_ISL_549868, EPI_ISL_551028, EPI_ISL_551955, EPI_ISL_552340, EPI_ISL_553185, EPI_ISL_554217, EPI_ISL_554221, EPI_ISL_557456, EPI_ISL_557984, EPI_ISL_558361			
see above	Lighthouse Lab in Milton Keynes	Wellcome Sanger Institute for the COVID-19 Genomics UK (COG-UK) consortium	Cordelia Langford; David K. Jackson; Dominic Kwiatkowski; Ewan Harrison; Ian Johnston; John Sillitoe on behalf of the Wellcome Sanger Institute COVID-19 Surveillance Team (http://www.sanger.ac.uk/covid-team); Roberto Amato; Sonia Goncalves; The Lighthouse Lab in Milton Keynes and Alex Alderton
EPI_ISL_484371	Lincolnshire Hospitals and DeepSeq Nottingham	COVID-19 Genomics UK (COG-UK) Consortium	Christopher Moore; Fei Sang; Joeseph Chappell; Johnny Debebe; Jonathan Ball; Matthew Carlisle; Matthew Loose; Nadine Holmes; Nichola Duckworth; Patrick McClure; Sarah Walsh; Tim Sloan; Victoria Wright
EPI_ISL_1008713	Lister Laboratorio de Referencia S.A. de C.V.	Instituto de diagnóstico y Referencia Epidemiologicos (INDRE) Departamento de Virología	Abril Rodriguez-Maldonado; Claudia Wong-Arambula; Ernesto Ramirez-Gonzalez.; Fabiola Garces-Ayala; Gisela Barrera-Badillo; Irma Lopez-Martinez; Lucia Hernandez-Rivas; Natividad Cruz-Ortiz; Tatiana Nunez-Garcia
EPI_ISL_603082, EPI_ISL_636843	Lithuanian University of Health Sciences Hospital, Department of Laboratory Medicine	Lithuanian University of Health Sciences, Molecular cardiology lab.	Arnoldas Pautienius; Astra Vitkauskiene; Dovydas Gecys; Ingrida Olendraitė; Kamile Tamusauskaite; Laura Pareckaite; Lukas Zemaitis; Vaiva Lesauskaite
EPI_ISL_439681, EPI_ISL_439812, EPI_ISL_472119, EPI_ISL_499705, EPI_ISL_499711, EPI_ISL_500040, EPI_ISL_517076, EPI_ISL_517236, EPI_ISL_517498			
see above	Liverpool Clinical Laboratories	COVID-19 Genomics UK (COG-UK) Consortium	A Alrezaihi; Alessandro Gerada; Alistair Darby; Angela Cowell; Anita Lucaci; Anu Chawla; Cassie Olateju; Catherine Hartley; Charlotte Nelson; Ecaterina Vamos; Elaine O'Toole; Elaine O‚ÄôToole; Eleanor G Bentley; Ghada T Shawli; Isabel García-Dorival; Isabel Garc√≠a-Dorival; James Johnson; James P Stewart; Jenifer Manson; Joanne Watts; Jones Benjamin; Jordan J Clark; Julian Hiscox; L Luu; Lucille Rainbow; M Almsaud; Margaret Hughes; Mark Whitehead; Matthew Gemmell; Miren Iturriza-Gomara; Muhannad Alruwaili; N.P Randle; Neil Swainston; PKF Gilmore; Parul Sharma; Rebekah Penrice-Randal; Rebekah Penrice-Randal¬†; Richard Eccles; Richard Gregory; Sam Haldenby; Steve Paterson; Stuart D Armstrong; Trevor Ian Robinson; Ximeng Han
EPI_ISL_477277	M Health Fairview	Minnesota Department of Health, Public Health Laboratory	Jacob Garfin; Kelly Pung; Matt Plumb; and Xiong Wang
EPI_ISL_568998, EPI_ISL_569036, EPI_ISL_569295	MEPHI, Aix Marseille University	MEPHI, Aix Marseille University	Anthony LEVASSEUR
EPI_ISL_471163, EPI_ISL_561164	MRCG at LSHTM Genomics lab	MRCG at LSHTM Genomics lab	Abdoulie Kanteh; Abdul Karim sesay; Bakary Sanyang; Jarra Manneh; Mariama Kujabi; Sesay et al
EPI_ISL_428855	MRCG at LSHTM Geomics lab	MRCG at LSHTM Genomics lab	Sesay et al
EPI_ISL_450014, EPI_ISL_450047, EPI_ISL_450092, EPI_ISL_450108	MSHS Clinical Microbiology Laboratories	MSHS Pathogen Surveillance Program	Adolfo Garcia-Sastre; Ajay Obla; Alberto Paniz-mondolfi; Ana S. Gonzalez-Reiche; Bremy Alburquerque; Emilia Sordillo; Florian Krammer; Gopi Patel; Harm van Bakel; Jayeeta Dutta; Jose Polanco; Juan Soto; Judith Aberg; Lisa Miorin; Matthew Hernandez; Melissa Gitman; Melissa Smith; Mitchell Sullivan; Randy Albrecht; Robert Sebra; Shelcie Fabre; Shwetha Sridhar Hara; Viviana Simon; Wen-chun Liu; Ying-Chih Wang; Zenab Khan
EPI_ISL_631528	Maimonides Medical Center	New York City Public Health Laboratory	Jade Wang; et al.
EPI_ISL_428348	Maison de Santé du Val d'Ormois	National Reference Center for Viruses of Respiratory Infections, Institut Pasteur, Paris	Angela Brisebarre; Etienne Simon-Lorière; Flora Donati; Marion Barbet; Maud Vanpeene; Mélanie Albert; Méline Bizard; Sylvie Behillil; Sylvie van der Werf; Vincent Enouf
EPI_ISL_460202, EPI_ISL_460207	Massachusetts General Hospital	Infectious Disease Program, Broad Institute of Harvard and MIT	A.E.; Adams, G.; Anahtar, M.; B.L.; B.W.; Bauer, M.; Birren; Branda, J.; Carter, A.; Cerrato, F.; Chaluvadi, S.; Chapman; Cusick, C.; D.J.; DeRuff, K.; Flowers, K.; Gallagher, G.; Gladden-Young, A.; Gnirke, A.; Harris, J.; J.E.; K.J.; LaRocque, R.; Lagerborg, K.; Lemieux; Lin; Loreth, C.; MacInnis; Neumann, A.; Normandin, E.; P.C.; Park; Pierce, V.; Reilly, S.; Rosenberg, E.; Rudy, M.; Ryan, E.; S.B.; Sabeti; Shaw, B.; Siddle; Slater, D.; Smole, S.; Tomkins-Tinch, C.; Turbett, S.
EPI_ISL_515832	Medical Disagnostics Services (MDS)	KRISP, KZN Research Innovation and Sequencing Platform	ChimukangaraB; Giandhari J; Khan S; Lessells R; Mdlalose K; Pillay S; Tegally H; Wilkinson E; York D; de Oliveira T
EPI_ISL_435281	Medistra Hospital Jakarta	Eijkman Institute for Molecular Biology, Ministry of Research and Technology/National Agency for Research and Innovation	Amin Soebandrio; David H Muljono; Edison Johar; Frilasita A Yudhaputri; Hidayat Trimarsanto; Khin Saw Myint; Safarina G Malik
EPI_ISL_516269	Michigan Department of Health and Human Services, Bureau of Laboratories	Michigan Department of Health and Human Services, Bureau of Laboratories	Blankenship HM; Riner D; Soehnlen MK
EPI_ISL_480333	Microbial Genomics Laboratory, Institut Pasteur de Montevideo	Microbial Genomics Laboratory, Institut Pasteur de Montevideo	Cecilia Salazar; Gonzalo Moratorio; Gregorio Iraola; Ignacio Ferrés; Marianoel Pereira; Pilar Moreno
EPI_ISL_480772	Microbiological Diagnostic Unit - Public Health Laboratory (MDU-PHL)	MDU-PHL	Sait, M.; Schultz M.; Seemann T.; Sherry, N.
EPI_ISL_447274	Microbiology laboratory, Assuta Ashdod University-Affiliated Hospital	Stern Lab	Stern Lab
EPI_ISL_613700	Microbiology, Department of Pathology, St. Bernard's Hospital, Gibraltar Health Authority	Respiratory Virus Unit, Microbiology Services Colindale, Public Health England	Charlotte Gillborn-Jones (Gibraltar); Dr Nicholas Cortes (Gibraltar); PHE Covid Sequencing Team

EPI_ISL_429873, EPI_ISL_437310	Ministry of Health Turkey	Ministry of Health Turkey	Ayşe Başak Altaş; Fatma Bayrakdar; Gülay Korukluoğlu; Selçuk Kılıç; Süleyman Yalçın; Tülin Demir; Yasemin Coşgun
EPI_ISL_417474, EPI_ISL_419395, EPI_ISL_450780, EPI_ISL_483013	Minnesota Department of Health, Public Health Laboratory	Minnesota Department of Health, Public Health Laboratory	Jacob Garfin; Jake Garfin and Xiong Wang; Matt Plumb; and Xiong Wang
EPI_ISL_435135	Mohammed Bin Rashid University of Medicine and Health Sciences	Al Jalila Genomics Center	Abdulmajeed Alkhaja; Abiola Catherine Senok; Ahmad Abou Tayoun; Alawi Alsheikh-Ali; Divinlal Harilal; Hamda Khansaheb; Hanan Al Suwaidi; Mohammed Uddin; Norbert Nowotny; Qutayba Hamid; Rabih Halwani; Rifat Hamoudi; Rupa Murthy Varghese; Sathishkumar Ramaswamy; Tom Loney; Zulfa Omar Deesi
EPI_ISL_467486, EPI_ISL_467494, EPI_ISL_487336, EPI_ISL_487338	Molecular Diagnostics Services (MDS)	KRISP, KZN Research Innovation and Sequencing Platform	Chimukangara B; Giandhari J; Khan S; Lessells R; Mdlalose K; Pillay S; Tegally H; Wilkinson E; York D; de Oliveira T
EPI_ISL_451307	Molecular Virology Unit, Fondazione IRCCS Policlinico San Matteo , Pavia	Laboratory of Virology, INMI Lazzaro Spallanzani IRCCS	Antonino Di Caro; Antonio Piralla; Barbara Bartolini; Cesare E.M. Gruber; Fausto Baldanti; Maria R. Capobianchi; Martina Rueca
EPI_ISL_486821, EPI_ISL_486822, EPI_ISL_486829	Molecular diagnostic laboratory of Federal Budget Institution of Science "Central Research Institute of Epidemiology" of The Federal Service on Customers' Rights Protection and Human Well-being Surveillance	Group of Genomics and Postgenomic Technologies of Central Research Institute of Epidemilology	Akimkin VG; Bulanenko VP; Kaptelova VV; Korneenko EV; Samoilov AE; Shipulina OY; Speranskaya AS; Tivanova EV; Valdokhina AV
EPI_ISL_614351	Molecular diagnostic unit for viral haemorrhagic fevers and emerging viruses, Bouaké CHU Laboratory	Project group Epidemology of Highly Pathogenic Microorganisms, Robert Koch-Institute	Adjaratou Traoré; Bamba Fatoumata Touré; Chantal Akoua-Koffi; Coulibaly Mbegnan; Diané Bamourou; Essia Belarbi; Etilé Anoh; Fabian Leendertz; Grit Schubert; Kra Ouffoué; Monemo Pacome; Safiatou Karidioula; Soundélé Maïté
EPI_ISL_467493, EPI_ISL_482726, EPI_ISL_487314, EPI_ISL_495527, EPI_ISL_495543, EPI_ISL_509368, EPI_ISL_602659, EPI_ISL_602894			
see above	NHLS-IALCH	KRISP, KZN Research Innovation and Sequencing Platform	Chimukangara B; Giandhari J; Khan S; Lessells R; Mdlalose K; Pillay S; Tegally H; Wilkinson E; York D; de Oliveira T
EPI_ISL_459102, EPI_ISL_469965, EPI_ISL_489620, EPI_ISL_489693, EPI_ISL_501634, EPI_ISL_524527, EPI_ISL_532876, EPI_ISL_533016			
see above	NHSGGC West of Scotland Specialist Virology Centre / MRC-University of Glasgow Centre for Virus Research	Wellcome Sanger Institute for the COVID-19 Genomics UK (COG-UK) consortium	Alasdair MacLean; Alice Broos; Ana da Silva Filipe; Antonia Ho; Cordelia Langford; Daniel Mair; David K. Jackson; David L Robertson; Dominic Kwiatkowski; Elihu Aranday-Cortes; Emma Thomson and Alex Alderton; Ewan Harrison; Ian Johnston; James Shepherd; Jenna Nichols; John Sillitoe; John Sillitoe on behalf of the Wellcome Sanger Institute COVID-19 Surveillance Team (http://www.sanger.ac.uk/covid-team); Joseph Hughes; Kathy Li; Kathy Smollett; Kirstyn Brunker; Kyriaki Nomikou; Lily Tong; Marc Niebel; Natasha Jesudason; Natasha Johnson; Patawee Asamaphan; Rajiv Shah; Richard Orton; Roberto Amato; Rory Gunson; Sarah McDonald; Sonia Goncalves; Sreenu Vattipally; Stephen Carmichael; Yasmin Parr
EPI_ISL_452211, EPI_ISL_452214, EPI_ISL_454529, EPI_ISL_454563, EPI_ISL_454569	NIV Influenza	NIV Influenza	Potdar V
EPI_ISL_452117	NJ Public Health and Environmental Laboratories	Pathogen Discovery, Respiratory Viruses Branch, Division of Viral Diseases, Centers for Disease Control and Prevention	Alison S. Laufer Halpin; Anna Montmayeur; Anna Uehara; Christopher A. Elkins; Clinton R. Paden; Haibin Wang; Jing Zhang; Krista Queen; Mary S. Keckler; Rachel Marine; Suxiang Tong; Yan Li; Ying Tao; Zachary Weiner
EPI_ISL_488505, EPI_ISL_488766, EPI_ISL_488826, EPI_ISL_492923	NU-OMICS DNA Sequencing research facility, Northumbria University	Wellcome Sanger Institute for the COVID-19 Genomics UK (COG-UK) consortium	Andrew Nelson; Brendan Payne; Chris Duncan; Clive Graham; Cordelia Langford; Darren Smith and Alex Alderton; David K. Jackson; Debra Padgett; Dominic Kwiatkowski; Edward Barton; Emma Swindells; Ewan Harrison; Garren Scott; Gary Black; Gary Eltringham; Greg Young; Ian Johnston; Jane Greenaway; Jennifer Collins; John Allan; John Sillitoe on behalf of the Wellcome Sanger Institute COVID-19 Surveillance Team (http://www.sanger.ac.uk/covid-team); Joshua Loh; Lynn Dover; Matthew Bashton; Paul Baker; Roberto Amato; Sarah Essex; Sheia Waugh; Shirelle Burton-Fanning; Sonia Goncalves; Steve Ligget; Wen Yew; Yusri Taha
EPI_ISL_436048	NYC Department of Health and Mental Hygiene	Pathogen Discovery, Respiratory Viruses Branch, Division of Viral Diseases, Centers for Disease Control and Prevention	Anna Uehara; Bettina Bankamp; Christy Harrison; Clinton R. Paden; Haibin Wang; Jasmine Padilla; Jennifer Rakeman; Jing Zhang; Justin Lee; Krista Queen; Suxiang Tong; Yan Li; Ying Tao; Zachary Weiner
EPI_ISL_418200	NYU Langone Health	Department of Pathology and Medicine, New York University School of Medicine	Adriana Heguy; Amy Rapkiewicz; Antonio Serrano; Christian Marier; Dacia Dimartino; Emily Huang; Gael Westby; George Jour; Guomiao Shen; Jared Pinnell; John Cadley; John Chen; Margaret Black; Marie Samanovic-Golden; Mark J. Mulligan; Matija Snuderl; Matthew T. Maurano; Megan Hogan; Nick Vulpescu; Paolo Cotzia; Paul Zappile; Peter Meyn; Xiaojun Feng
EPI_ISL_480042	Nagoya City Public Health Research Institute	Pathogen Genomics Center, National Institute of Infectious Diseases	Hajime Kamiya; Kentaro Itokawa; Makoto Kuroda; Masanori Hashino; Motoi Suzuki; Rina Tanaka; Shinichiro Shibata; Takuya Miki; Tsuyoshi Sekizuka
EPI_ISL_435061, EPI_ISL_435065	National Centre for Disease control (NCDC), CSIR-Institute of Genomics and Integrative Biology (CSIR-IGIB)	NCDC/CSIR-IGIB	Aarti Tewari; Anurag Agrawal; Bharathram Uppili; Bibhash Nandi; Debasis Dash; Dhirendra Kumar; Hema Gogia; Hemlata Lall; Himanshu Vashisht; Mahesh Dhar; Manju Bala; Meena Datta; Mitali Mukerji; Mohammed Faruq; Nidhi Saini; Nishu Tyagi; Partha Rakshit; Pooja Sharma; Poonam Gupta; Pramod Kumar; Prateek Singh; Preeti Madan; Priyanka Singh; Rajesh Pandey; Sandhya Kabra; Saruchi Wadhwa; Satyabrata Bag; Simrita Singh; Sujeet Singh; Uma Sharma; Varun Jaiswal; Vivekanand A
EPI_ISL_420104	National Centre for Infectious Diseases	Programme in Emerging Infectious Diseases, Duke-NUS Medical School	Barnaby E Young; Danielle E Anderson; David CB Lye; Gavin JD Smith; Jayanthi Jayakumar; Martin Linster; Yan Zhuang; Yee Sin Leo; Yvonne CF Su
EPI_ISL_416036	National Influenza Center - Instituto Adolfo Lutz	Instituto Adolfo Lutz, Interdiciplinary Procedures Center, Strategic Laboratory	Adriana Bugno; Adriano Abbud; Carlos Henrique Camargo; Claudia Regina Gonçalves; Claudio Tavares Sacchi; Daniela Bernardes Borges da Silva; Erica Valessa Ramos Gomes; Fabiana Cristina Pereira dos Santos; Maria do Carmo Sampaio Tavares Timenetsky; Simone Guadagnucci Morillo; Terezinha Maria de Paiva
EPI_ISL_498791	National Institute of Laboratory Medicine and Referral Center	Genomic Research Lab, BCSIR	A. K. M. Shamsuzzaman; Abu Sayeed Mohammad Mahmud; Asish Kumar Ghosh; Barna Goswami; Eshrar Osman; Iffat Jahan; Mahmuda Yeasmin; Md. Ahasan Habib; Md. Maruf Ahmed Molla; Md. Murshed Hasan Sarkar; Md. Saddam Hossain; Md. Salim Khan; Mohammad Samir Uzzaman; Salek Ahmed Sajib; Shahina Akter; Sheikh Md. Selim Al Din; Tanjina Akhter Banu; Tasnim Nafisa; Utpal Chandra Roy
EPI_ISL_541683, EPI_ISL_541725	National Institute of Virology, NIV Influenza	National Institute of Virology, NIV Influenza	Potdar V
EPI_ISL_512640	National Laboratory for Influenza/Virology reference laboratory, Public Health Center of the Ministry of Health of Ukraine	Respiratory Virus Unit, Microbiology Services Colindale, Public Health England	Dr. Iryna Demchyshyna; PHE Covid Sequencing Team
EPI_ISL_644791	National Microbiology Reference Laboratory	Quadram Institute Bioscience	Agnes Juru; Alexander Goredema; Ana-Victoria Gutierrez; Andrew J. Page; Andrew Tarupiwa; Barbra Murwira; Beuty Makamure; Charles Nyagupe; David Baker; Gaetan Thilliez; Gemma Kay; Gibson Mhlanga; Hlanai Gumbo; Isaac Phiri; Justin O'Grady; Leonardo de Oliveira Martins; Muchaneta Mugabe; Portia Manangazira; Robert Kingsley; Sekesai Zinyowera; Tapfumanei Mashe; Tatenda Takawira; Thanh Le Viet
EPI_ISL_435689, EPI_ISL_443210, EPI_ISL_574516	National Public Health Laboratory, National Centre for Infectious Diseases	National Public Health Laboratory, National Centre for Infectious Diseases	Chavatte Jean-Marc; Cui Lin; Lin Cui; Lin Raymond Tzer Pin; Mak Tze Minn; Octavia Sophie; Raymond Tzer Pin Lin; Sophie Octavia; Tze Minn Mak; Zhenyang Zhou
EPI_ISL_480297	National Reference Laboratory "Influenza and acute respiratory diseases"	NRL-HIV	Ivailo Alexiev; Ivan Ivanov; Ivva Philipova
EPI_ISL_435677	National Virology Reference Laboratory	National Public Health Laboratory, National Centre for Infectious Diseases	Chavatte Jean-Marc; Cui Lin; Lin Raymond Tzer Pin; Mak Tze Minn; Octavia Sophie; Taib Surita; Zaini Zainun
EPI_ISL_578309	National Virus Reference Laboratory	National Virus Reference Laboratory	Cillian F De Gascun; Gabriel Gonzalez; Jonathan Dean; Michael Carr; Suzie Coughlan
EPI_ISL_475232	Nebraska Public Health Laboratory	UNMC COVID-19 Response Team	UNMC COVID-19 Response Team
EPI_ISL_515296, EPI_ISL_515459	Nevada State Public Health Laboratory	Nevada State Public Health Laboratory	Andrew Gorzalski; Chris Laverdure; Cyprian Rossetto; David Jackson; Heather Kerwin; Joel R. Sevinsky; Natalie Crawford; Paul Hartley; Richard Tillett; Stephanie Van Hooser; Subhash C. Verma; and Mark Pandori
EPI_ISL_542086, EPI_ISL_732747	New Mexico Department of Health Scientific Laboratory	New Mexico Department of Health Scientific Laboratory	Anastacia Griego-Fisher; D'Eldra Malone; Ellie Johnson
EPI_ISL_527884	Nigeria Centre for Disease Control (NCDC)	African Centre of Excellence for Genomics of Infectious Diseases (ACEGID), Redeemer's University, Ede, Osun State, Nigeria	Oluniyi P.E. et al
EPI_ISL_534241	Norra Alvsborgs lanssjukhus	The Public Health Agency of Sweden	Anna Risberg; Anna-Malin Linde; Karin Tegmark-Wisell; Maria Lind Karlberg; Mattias Haukland; Mia Brytting; Olov Svartstrom; Oskar Karlsson Lindsjo; Petra Edquist; Reza Advani; Sandra Broddesson
EPI_ISL_573313	Northumbria University / South Tees Hospitals NHS Foundation Trust / North Cumbria Integrated Care NHS Foundation Trust / North Tees and Hartlepool NHS Foundation Trust / Newcastle Hospitals NHS Foundation Trust	COVID-19 Genomics UK (COG-UK) Consortium	Andrew Nelson; Brendan Payne; Clive Graham; Darren L Smith; Debra Padgett; Edward Barton; Emma Swindells; Garren Scott; Gary Black; Gary Eltringham; Giles S Holt; Greg R Young; Jane Greenaway; Jennifer Collins; John Allan; Joshua Loh; Lynn Dover; Matthew Bashton; Mohammad A Tariq; Paul Baker; Sarah Essex; Steve Liggett; Wen C Yew; Yusri Taha
EPI_ISL_626365, EPI_ISL_626395	Northwestern Memorial Hospital	Ozer Lab	Alan R. Hauser; Chad J. Achenbach; Chao Qi; Egon A. Ozer; Hannah H. Nam; Judd F. Hultquist; Lacy M. Simons; Lawrence J. Jennings; Michael G. Ison; Ramon Lorenzo-Redondo; Scott C. Roberts
EPI_ISL_509203, EPI_ISL_525885	OHSU Lab Services Molecular Microbiology Lab	Oregon SARS-CoV-2 Genome Sequencing Center	Alec J. Hirsch; Andrew C. Adey; Benjamin N. Bimber; Brendan L. O'Connell; Brian J. O'Roak; Daniel N. Streblow; Guang Fan; Ruth V. Nichols; Sally B. Grindstaff; William B. Messer
EPI_ISL_480119	Oita Prefectural Institute of Public Health and Environmental Science	Pathogen Genomics Center, National Institute of Infectious Diseases	Hajime Kamiya; Kentaro Itokawa; Makoto Kuroda; Mari Sasaki; Masanori Hashino; Motoi Suzuki; Rina Tanaka; Tsuyoshi Sekizuka
EPI_ISL_457974, EPI_ISL_457988, EPI_ISL_457989	Oman-NIC	Oman-NIC	Abdulla Balkhair; Ahlam Al-Amri; Aisha Al-Amri; Aisha Al-Busaidi; Amina Al Jardani; Fahad Zadjali; Fatma BaAlawi; Hamida AL Barwani; Hanan Al-kindi; Intisar Al-Shukri; Khulood Al-Mammary; Mohammed Al-Tobi; Samiha Al Kharusi; Samira Al-Maruqi; Zeyana AL-Dahmani
EPI_ISL_491155	Oman-National Influenza Center	Biotechnology & OMICs Laboratory	Abdul Latif Khan; Adil Al-Wahaibi; Adil Khan; Ahlam Al-Amri; Ahmed Al-Harrasi; Ahmed Al-Rawahi; Aisha Al-Amri; Aisha Al-Busaidi; Amina Al-Jardani; Hanan Al-Kindi; Intisar Al-Shukri; Sajjad Asaf; Samiha Al-Kharusi; Samira Al-Mahruqi; Seif Al-Abri.
EPI_ISL_569735,	Omsk Research Institute of Natural Focal	WHO National Influenza Centre Russian Federation	Aleksei Vasilenko; Andrey Komissarov; Artem Fadeev; Daria Nashatyreva; Ekaterina Gradoboeva; Ekaterina Savkina; Elena Poleshchuk; Valery Yakimenko

EPI_ISL_569738, EPI_ISL_569742	Infections		
EPI_ISL_486123, EPI_ISL_486139	Orange County Public Health Laboratory	Chan-Zuckerberg Biohub	CZB Cliahub Consortium
EPI_ISL_475129	Orebro klinisk mikrobiologi	The Public Health Agency of Sweden	Anna Risberg; Anna-Malin Linde; Karin Tegmark-Wisell; Maria Lind Karlberg; Mattias Haukland; Olov Svartstrom; Oskar Karlsson Lindsjo; Petra Edquist; Reza Advani; Sandra Broddesson; Shamam Muradrasoli
EPI_ISL_549128	Ostfold Hospital Trust - Kalnes, Centre for Laboratory Medicine, Section for gene technology and infection serology	Norwegian Institute of Public Health, Department of Virology	Hilde Elshaug; Hilde Synnøve Vollan; Kamilla Heddeland Instefjord; Karoline Bragstad; Kathrine Stene-Johansen; Olav Hungnes; Rasmus Riis Kopperud
EPI_ISL_478763, EPI_ISL_478960, EPI_ISL_534986, EPI_ISL_549450, EPI_ISL_560053, EPI_ISL_572850, EPI_ISL_573009, EPI_ISL_576573			
see above	Oxford Viromics, NDM, University of Oxford; Oxford University Hospitals; Basingstoke and North Hampshire Hospital	COVID-19 Genomics UK (COG-UK) Consortium	Alex Mobbs; Amy Trebes; Anita Justice; Catrin Moore; Christophe Fraser; David Bonsall; David Buck; Emma Wise; George Macintyre; Jessica Lynch; John Todd; Mariateresa de Cesare; Matilde Mori; Monique Andersson; Nathan Moore; Nick Cortes; Robert Shaw; Stephen Kidd; Tanya Golubchik; Timothy Peto
EPI_ISL_439979, EPI_ISL_440218, EPI_ISL_440738, EPI_ISL_442694, EPI_ISL_442736, EPI_ISL_443943, EPI_ISL_488328, EPI_ISL_488341, EPI_ISL_488359, EPI_ISL_492575			
see above	PHE South West Regional Laboratory, National Infection Service	Wellcome Sanger Institute for the COVID-19 Genomics UK (COG-UK) consortium	Alex Alderton; Barry Vipond; Cordelia Langford; David K. Jackson; Dominic Kwiatkowski; Dr Peter Muir; Ewan Harrison; Hannah Pymont; Ian Johnston; John Sillitoe on behalf of the Wellcome Sanger Institute COVID-19 Surveillance Team (http://www.sanger.ac.uk/covid-team); Rich Hopes; Roberto Amato; Sonia Goncalves; Stephanie Hutchings; and Alex Alderton
EPI_ISL_605826	PathWest Laboratory Medicine WA	PathWest Laboratory Medicine WA Microbial Surveillance Unit	PathWest Laboratory Medicine WA Microbial Surveillance Unit
EPI_ISL_513182, EPI_ISL_513235, EPI_ISL_678029, EPI_ISL_678037, EPI_ISL_678134, EPI_ISL_678195, EPI_ISL_751237			
see above	Pathogen Genomics Lab King Abdullah University of Science and Technology(KAUST)	Pathogen Genomics Lab King Abdullah University of Science and Technology(KAUST)	Abbas Al Mutair; Abdulaziz Alahmadi; Afrah Alsomali; Amanda Ooi; Amit Kumar Subudhi; Anwar Hashem; Arnab Pain; Asim Khogeer; Awad A-Omari; Fadwa Alofi; Fathia Ben Rached; Jumana Taha; Kahled Algithami; Luke Esau; Naif Almontashiri; Nashwa Al-khotani; Olga Douvropoulou; Raeece Naeem; Rahul P Salunke; Raushan Nugmanova; Samer Salih; Sara Mfarrej; Sharif Hala
EPI_ISL_747242	Pathogen Lab (BSL3), Biomedical Innovation Department, Applied and Experimental Biology Division, Scientific Research Center and High Education from Ensenada (CICESE)	Pathogen Laboratory (BSL3), Biomedical Innovation Department, Experimental and Applied Biology Division, Scientific Research Center and High Education from Ensenada (CICESE)	Cervantes-Luevano K; Galindo C and Licea-Navarro A; Martinez M; Saavedra A
EPI_ISL_574487	Programme in Emerging Infectious Diseases, Duke-NUS Medical School	National Public Health Laboratory, National Centre for Infectious Diseases	Adrian Eng Zheng Kang; Danielle E Anderson; Lin Cui; Raymond Tzer Pin Lin; Sophie Octavia; Tze Minn Mak; Zhenyang Zhou
EPI_ISL_482296, EPI_ISL_482362, EPI_ISL_482451, EPI_ISL_482455	Providence St. Joseph Health Molecular Genomics Laboratory	Providence St. Joseph Health Molecular Genomics Laboratory	Alexa K Dowdell; Brian D Piening; Carlo B Bifulco; Fred L Robinson; Mary Campbell
EPI_ISL_448293	Quadram Institute Bioscience	COVID-19 Genomics UK (COG-UK) Consortium	Alexander J Trotter; Alison E. Mather; Alp Aydin; Ana P. Tedim; Anastasia Kolyva; Andrew Bell; Andrew J. Page; Claire Stuart; Dave J. Baker; Gemma L. Kay; John Wain; Justin O'Grady; Leonardo de Oliveira Martins; Lizzie Meadows; Maria Diaz; Mark Webber; Muhammed Yasir; Nabil-Fareed Alikhan; Ngozi Elumogo; Nicholas M. Thomson; Rachael Stanley; Rachel Gilroy; Reenesh Prakash; Samir Dervisevic; Samuel Bloomfield; Steven Rudder; Thanh Le-Viet
EPI_ISL_425480, EPI_ISL_432900, EPI_ISL_432921, EPI_ISL_441892, EPI_ISL_448406, EPI_ISL_448414, EPI_ISL_453638, EPI_ISL_461931, EPI_ISL_472404			
see above	Queens Medical Centre, Clinical Microbiology Department / DeepSeq Nottingham	COVID-19 Genomics UK (COG-UK) Consortium	Christopher Moore; Fei Sang; Gemma Clark; Hannah Howson-Wells; Johnny Debebe; Jonathan Ball; Joseph Chappell; Manjinder Khakh; Matthew Carlisle; Matthew Loose; Michelle M Lister; Nadine Holmes; Patrick McClure; Theocharis Tsoleridis; Vicki M Fleming; Victoria Wright; Wendy Smith
EPI_ISL_467902, EPI_ISL_467906, EPI_ISL_467925, EPI_ISL_494543, EPI_ISL_498728, EPI_ISL_571017, EPI_ISL_571160, EPI_ISL_571354, EPI_ISL_571913, EPI_ISL_571981, EPI_ISL_572139, EPI_ISL_572150, EPI_ISL_572166, EPI_ISL_603938, EPI_ISL_604242, EPI_ISL_604284, EPI_ISL_604297, EPI_ISL_604337, EPI_ISL_604380, EPI_ISL_604546, EPI_ISL_604615, EPI_ISL_604709, EPI_ISL_604762, EPI_ISL_604962, EPI_ISL_604974			
see above	Quest Diagnostics	Quest Diagnostics	Anderson; Anderson, B.; B.P.; D.F.; Gerasimova, A.; Grover, D.; Hua, M.; K.E.; Kagan; Lacbawan, F.; Liu Y.; Livingston; Owen, R.; R.M.; R.M. and Owen, R.; Rosenthal; S.H.; Shalhout
EPI_ISL_455751	REGIONAL VRDL,ICMR-RMRC BBSR	Immunogenomics lab, Institute of Life Sciences, Bhubaneswar	Ajay Parida; Arup Ghosh; Atimukta Jha; COVID-19 team of ILS & RMRC; DBT's PAN-INDIA 1000 SARS-CoV2 RNA genome sequencing consortium; Debdutta Bhattacharya; Ghulam Hussain Syed; Jaya Singh Khastri; Jyotirmayee Turuk; Manasi Priyadarshini; Orissa COVID-19 study group; Punit Prasad; Rajeeb Swain; Rupesh Dash; Sanghamitra Pati; Shanti Senapati; Shuchi Smita; Soma Chattopadhyay; Sunil Raghav; Swati Madhulika; Tushar K. Beuria; Viplov K. Biswas
EPI_ISL_426435	RI State Health Laboratories	Pathogen Discovery, Respiratory Viruses Branch, Division of Viral Diseases, Centers for Disease Control and Prevention	Alison S. Laufer Halpin; Anna Uehara; Christopher A. Elkins; Clinton R. Paden; Haibin Wang; Jing Zhang; Krista Queen; Mary S. Keckler; Rachel Marine; Suxiang Tong; Yan Li; Ying Tao
EPI_ISL_759965	RSUD Dr. Soetomo	Institute of Tropical Disease, Universitas Airlangga	Aldise M Nastri; Gatot Soegiarto; Jezzy R Dewantari; Joni Wahyuhadi; Kazufumi Shimizu; Krisnoadi Rahardjo; Laksmi Wulandari; Maria I Lusida; Resti Yudhawati; Rima R Prasetya; Soetjipto; Yasuko Mori
EPI_ISL_441379, EPI_ISL_585236	Regional Virus Laboratory, Belfast Health and Social Care Trust	COVID-19 Genomics UK (COG-UK) Consortium	Alison Watt; Ciara Cox; Conall McCaughey; David Simpson; Derek Fairley; James McKenna; Mairead Connor; Susan Feeney; Tanya Curran; Zoltan Molnar
EPI_ISL_459389, EPI_ISL_469864, EPI_ISL_489248, EPI_ISL_489327	Regional Virus Laboratory, Belfast Health and Social Care Trust	Wellcome Sanger Institute for the COVID-19 Genomics UK (COG-UK) consortium	Alison Watt; Ciara Cox; Conall McCaughey; Cordelia Langford; David K. Jackson; David Simpson; Derek Fairley; Dominic Kwiatkowski; Ewan Harrison; Ian Johnston; James McKenna; John Sillitoe on behalf of the Wellcome Sanger Institute COVID-19 Surveillance Team (http://www.sanger.ac.uk/covid-team); Mairead Connor; Roberto Amato; Sonia Goncalves; Susan Feeney; Tanya Curran; Zoltan Molnar; and Alex Alderton
EPI_ISL_686598	Respiratory Virus Unit, Microbiology Services Colindale, Public Health England	COVID-19 Genomics UK (COG-UK) Consortium	PHE Covid Sequencing Team
EPI_ISL_412116, EPI_ISL_415144, EPI_ISL_417215, EPI_ISL_417246, EPI_ISL_417296, EPI_ISL_418668, EPI_ISL_421783, EPI_ISL_421807, EPI_ISL_421953, EPI_ISL_423383, EPI_ISL_459960, EPI_ISL_464302, EPI_ISL_464416, EPI_ISL_464549, EPI_ISL_464563, EPI_ISL_464741, EPI_ISL_464771, EPI_ISL_464779, EPI_ISL_464839, EPI_ISL_464954, EPI_ISL_465436, EPI_ISL_465991, EPI_ISL_466076, EPI_ISL_466120, EPI_ISL_466615, EPI_ISL_566064			
see above	Respiratory Virus Unit, Microbiology Services Colindale, Public Health England	Respiratory Virus Unit, Microbiology Services Colindale, Public Health England	Angie Lackenby; Joanna Ellis; Jonathan Hubb; Kirstin Edwards; Leena Bhaw; Maria Zambon; Monica Galiano; Omolola Akinbami; PHE Covid Sequencing Team; Richard Myers; Shahjahan Miah; Steven Platt; Tiina Talts; Tina Talts
EPI_ISL_511332	SYNLAB	Instituto Nacional de Saude (INSA)	Borges et al
EPI_ISL_511214, EPI_ISL_511217	SYNLAB	Instituto Nacional de Saude (INSA) and Instituto Gulbenkian de Ciencia (IGC)	Borges et al
EPI_ISL_479933	Saitama Prefectural Institute of Public Health	Pathogen Genomics Center, National Institute of Infectious Diseases	Hajime Kamiya; Hayato Ehara; Kentaro Itokawa; Makoto Kuroda; Masanori Hashino; Motoi Suzuki; Rina Tanaka; Tsuyoshi Sekizuka
EPI_ISL_467961, EPI_ISL_494714, EPI_ISL_494727, EPI_ISL_636125, EPI_ISL_878212, EPI_ISL_878226, EPI_ISL_878300, EPI_ISL_878367, EPI_ISL_878375, EPI_ISL_878390, EPI_ISL_878400, EPI_ISL_878401, EPI_ISL_878404, EPI_ISL_878408, EPI_ISL_878437, EPI_ISL_879950, EPI_ISL_879977			
see above	San Diego County Public Health Laboratory	Andersen lab at Scripps Research	Brett Austin; Jovan Shephard; SEARCH Alliance San Diego with Tracy Basler
EPI_ISL_486300	San Joaquin County Public Health Lab	Chan-Zuckerberg Biohub	CZB Cliahub Consortium
EPI_ISL_542282, EPI_ISL_542285, EPI_ISL_542321	San Matteo Hospital Pavia	Dep. Of Oncology and Hemato-Oncology University of Milan	Antonio Piralla; Carlo Federico Perno; Chiara Vismara; Claudia Alteri; Elisa Matarazzo; Fausto Baldanti; Federica Giardina; Federica Novazzi; Luna Colagrossi; Maria Antonello; Massimo Puoti; Monica Tallarita; Oscar Massimiliano Epis; Roberto Fumagalli; Silvia Renica; Stefano Gaiarsa; Valentino Costabile; Valeria Cento
EPI_ISL_435583	Santa Clara County Public Health Department	Chiu Laboratory, University of California, San Francisco	Brandon Bonin; Debra A. Wadford; Elsa Villarino; Scot Federman; Wei Gu; Xianding Deng; and Charles Y. Chiu
EPI_ISL_479800	Sapporo City Institute of Public Health	Pathogen Genomics Center, National Institute of Infectious Diseases	Asami Ohnishi; Hajime Kamiya; Kentaro Itokawa; Makoto Kuroda; Masanori Hashino; Motoi Suzuki; Rina Tanaka; Tsuyoshi Sekizuka
EPI_ISL_676592	Scientific Veterinary Institute Novi Sad	Veterinary Specialized Institute "Kraljevo", Serbia	Afonso, C.; Banovic Djeri, B.; Jankovic, M.; Jovanovic, T.; Knezevic, A.; Petrovic, T.; Sekler, M.; Tesovic, B.; Vidanovic, D.; Volkening, J.
EPI_ISL_437561, EPI_ISL_437575	Scripps Medical Laboratory	Andersen lab at Scripps Research	Ellen Stefanski; Ian Mchardy; SEARCH Alliance San Diego with Michael Quigley
EPI_ISL_430128	Seattle Flu Study	Seattle Flu Study	Chu et al
EPI_ISL_471542	Secretaria de Saude de Mogi das Cruzes	Instituto Adolfo Lutz, Interdiciplinary Procedures Center, Strategic Laboratory	Claudia Regina Gonçalves; Claudio Tavares Sacchi; Erica Valessa Ramos Gomes
EPI_ISL_469284	Service de Virologie Hôpital Saint-Louis	Laboratory Cell Biology of Viral Infection-INSERM unit 944	Ali Amara; Constance Delaugerre; Laurent Meertens; Lucie Bonnet-Madin; Maud SALMONA; Séverine Mercier-Delarue
EPI_ISL_538151	Servicio de Microbiología y Parasitología clínica. UCEIMP. Hospital Universitario Virgen del Rocío/IBIS/CSIC/US	SeqCOVID-SPAIN consortium/IBV(CSIC)	Guillermo Martín Gutiérrez; Javier Aznar Martín and SeqCOVID-SPAIN consortium; Lidia Gálvez Benitez; Verónica González Galán; Ángel Rodríguez Villodres
EPI_ISL_660288	Servicio de Microbiología, Laboratori Clínic Metropolitana Nord. Hospital Universitari Germans Trias i Pujol. Institut d'Investigació en Ciències de la Salut Germans Trias i Pujol (IGTP)	SeqCOVID-SPAIN consortium/IBV(CSIC)	Adrián Antuori; Anabel Fernández; Anna Not; Antoni E. Bordoy; Elisa Martró; Nona Romaní and SeqCOVID-SPAIN consortium
EPI_ISL_636113, EPI_ISL_636114, EPI_ISL_636115, EPI_ISL_636116, EPI_ISL_636117, EPI_ISL_636118, EPI_ISL_636119, EPI_ISL_636120, EPI_ISL_730127, EPI_ISL_730128, EPI_ISL_730129, EPI_ISL_730130, EPI_ISL_730131, EPI_ISL_730132, EPI_ISL_730133, EPI_ISL_730134, EPI_ISL_730135, EPI_ISL_730136, EPI_ISL_962662, EPI_ISL_962663, EPI_ISL_1081416, EPI_ISL_1081506, EPI_ISL_1081544, EPI_ISL_1185929, EPI_ISL_1185931, EPI_ISL_1185934, EPI_ISL_1185935, EPI_ISL_1185936, EPI_ISL_1295671, EPI_ISL_1295676, EPI_ISL_1295691, EPI_ISL_1295730, EPI_ISL_1295760, EPI_ISL_1295779, EPI_ISL_1295837, EPI_ISL_1295843, EPI_ISL_1366325, EPI_ISL_1794925			
see above	Sharp HealthCare Laboratory	Andersen lab at Scripps Research	Art Mendoza; Cathy Woerle; Jacquelyn Berumen; Liam McGinnis; Omid Bakhtar; SEARCH Alliance San Diego with Aaron Harding
EPI_ISL_582662	Sheikh Khalifa Medical City	Molecular/Surveillance lab Sheikh Khalifa Medical City	Amirtharaj Francis; Hala Imambaccus; Hiba Saud; Sahar Almarzooqi; Sajeed Abdul; Stefan Weber

EPI_ISL_696265	Sonora Quest Laboratories, Laboratory Sciences of Arizona	TGen North	Ashlyn Pfeiffer; Chris French; Darrin Lemmer; Dave Engelthaler; Hayley Yaglom; Jolene Bowers; Megan Folkerts; The Arizona COVID Genomics Union (ACGU)
EPI_ISL_469241	Special Infectious Agents Unit	Special Infectious Agents Unit	A.M.; Al-Sobahy; Azhar; E.I.; El-Kafrawy; Farraj; Hassan; N.A.; S.A.; T.L.; Tolah; Uthman
EPI_ISL_752646	State Laboratories Division, Hawaii State Department of Health	State Laboratories Division, Hawaii State Department of Health	Drew Kuwazaki; Edward Desmond; Pamela O'Brien; Razvan Sultana; Sabrina Diemert
EPI_ISL_447587	Tamil Nadu Veterinary and Animal Sciences University	CSIR-Centre for Cellular and Molecular Biology	Archana Bharadwaj Siva; Dhiviya Vedagiri; Divya Gupta; Divya Tej Sowpati; G Dhinakar Raj; G Ravi Kumar; K Kaveri; Karthik Bharadwaj Tallapaka; Krishnan Harinivas Harshan; Kumarasamy Thangaraj; Lamuk Zaveri; Namami Gaur; P Padmapriya; Payel Mukherjee; Priya Singh; Purushotham Vodnala; R Kiruba; Rakesh K Mishra; S Magesh; S Sivasubramanian; S Vennila; Sakshi Shambhavi; Santosh Kumar Kuncha; Shagufta Khan; Sofia Banu; Tulasi Nagabandi; Vishal Sah
EPI_ISL_452112	Texas DSHS Lab Services	Pathogen Discovery, Respiratory Viruses Branch, Division of Viral Diseases, Centers for Disease Control and Prevention	Alison S. Laufer Halpin; Anna Montmayeur; Anna Uehara; Christopher A. Elkins; Clinton R. Paden; Haibin Wang; Jing Zhang; Krista Queen; Mary S. Keckler; Rachel Marine; Suxiang Tong; Yan Li; Ying Tao; Zachary Weiner
EPI_ISL_672172, EPI_ISL_672264	The Ashley Laboratory, Stanford University	Chan-Zuckerberg Biohub	CZB Cliahub Consortium
EPI_ISL_577633, EPI_ISL_626573	The National Institute of Public Health	State Veterinary Institute Prague	A; D; H; J; Jirincova; L; Nagy; Novakova; Trnka; Vecerova
EPI_ISL_491093	The National Institute of Public Health	The National Institute of Public Health and State Veterinary Institute Prague	A; D; H; J; Jirincova; L; Nagy; Novakova; Trnka; Vecerova
EPI_ISL_417551, EPI_ISL_417648, EPI_ISL_417682, EPI_ISL_417688, EPI_ISL_417755, EPI_ISL_417757	The National University Hospital of Iceland	deCODE genetics	Agnar Helgason; Alma Moller; Arna B Agustsdottir; Arnaldur Gylfason; Asgeir Sigurdsson; Aslaug Jonasdottir; Berglind Eiriksdottir; Bjarni Thorbjornsson; Brynjar O Jensson; Daniel F Gudbjartsson; Droplaug N Magnusdottir; Elisabet E Gardarsdottir; Emil A Thorarensen; Gardar Sveinbjornsson; Gisli Masson; Gudmundur Georgsson; Gudmundur L Norddahl; Gudrun Sigmundsdottir; Hakon Jonsson; Hilma Holm; Ingileif Jonsdottir; Jona Saemundsdottir; Kamilla S Josefsdottir; Kari Stefansson; Karl G Kristinsson; Kjartan R Gudmundsson; Kristin E Sveinsdottir; Louise le Roux; Maney Sveinsdottir; Olafia S Gretarsdottir; Olafur T Magnusson; Pall Melsted; Patrick Sulem; Run Fridriksdottir; Thora R Gunnarsdottir; Thordur Kristjansson; Thorolfur Gudnason; Unnur Thorsteinsdottir
EPI_ISL_479894	Tokyo Metropolitan Institute of Public Health	Pathogen Genomics Center, National Institute of Infectious Diseases	Hajime Kamiya; Kenji Sadamasu; Kentaro Itokawa; Makoto Kuroda; Mami Nagashima; Masanori Hashino; Motoi Suzuki; Rina Tanaka; Takashi Chiba; Tsuyoshi Sekizuka
EPI_ISL_463970, EPI_ISL_586299, EPI_ISL_586325, EPI_ISL_755867	Toronto Invasive Bacterial Diseases Network	McMaster University	Ahmed Draia; Allison McGeer; Andrew G. McArthur; Angel Li; Emily Panousis; Hooman Derakhshani; Jalees Nasir; Kuganya Nirmalarajah; Michael Surette; Patryk Aftanas; Samira Mubareka
EPI_ISL_483194, EPI_ISL_483253, EPI_ISL_483275, EPI_ISL_483290, EPI_ISL_483339, EPI_ISL_483466, EPI_ISL_483525			
see above	UC San Diego Center for Advanced Laboratory Medicine	Andersen lab at Scripps Research	Ji H Shin; SEARCH Alliance San Diego with David Pride
EPI_ISL_537525	UCLA Pathology Clinical Microbiology Lab	Kruglyak Lab	Guo et al.
EPI_ISL_454329	ULSM - Matosinhos	Instituto Nacional de Saude (INSA)	Borges et al
EPI_ISL_424890	UT-Unified State Labs: Public Health Utah DOH	Pathogen Discovery, Respiratory Viruses Branch, Division of Viral Diseases, Centers for Disease Control and Prevention	Alison S. Laufer Halpin; Anna Uehara; Christopher A. Elkins; Clinton R. Paden; Haibin Wang; Jing Zhang; Krista Queen; Mary S. Keckler; Rachel Marine; Suxiang Tong; Yan Li; Ying Tao
EPI_ISL_416655, EPI_ISL_418895, EPI_ISL_418954, EPI_ISL_426073, EPI_ISL_477685, EPI_ISL_570029, EPI_ISL_570081, EPI_ISL_570199, EPI_ISL_570200, EPI_ISL_570345			
see above	UW Virology Lab	UW Virology Lab	Alexander Greninger; Amin Addetia; Hong Xie; Keith Jerome; Keith R Jerome; Lasata Shrestha; Meei-Li Huang; Pavitra Roychoudhury; Truong Nguyen; Victoria M Rachleff
EPI_ISL_734524, EPI_ISL_734812	UZ Leuven, National Reference Laboratory for Coronaviruses, Laboratory Medicine, Leuven, Belgium	KU Leuven, Rega Institute, Clinical and Epidemiological Virology	Bert Vanmechelen; Joan Marti-Carerras; Piet Maes; Tony Wawina-Bokalanga
EPI_ISL_615094	Umea klinisk mikrobiologi	The Public Health Agency of Sweden	Anna Risberg; Anna-Malin Linde; Karin Tegmark-Wisell; Maria Lind Karlberg; Mattias Haukland; Mia Brytting; Olov Svartstrom; Oskar Karlsson Lindsjo; Petra Edquist; Reza Advani; Sandra Broddesson
EPI_ISL_1008714	Unidad de Patología Clínica	Instituto de diagnóstico y Referencia Epidemiologicos (INDRE) Departamento de Virología	Abril Rodriguez-Maldonado; Claudia Wong-Arambula; Ernesto Ramirez-Gonzalez.; Fabiola Garces-Ayala; Gisela Barrera-Badillo; Irma Lopez-Martinez; Lucia Hernandez-Rivas; Natividad Cruz-Ortiz; Tatiana Nunez-Garcia
EPI_ISL_569991	Unity Health Toronto	Ontario Institute for Cancer Research	Bernard Lam; Felicia Vincelli; Ilinca Lungu; Jared T. Simpson; Jeremy Johns; Karel Boissinot; Larissa M. Matukas; Le Luu; Mark Downing; Paul Krzyzanowski; Philip Zuzarte; Ramzi Fattouh; Richard de Borja; Samira Mubareka; TIBDN; Trina Otterman; Wai Sum Siu; Yan Chen; Zhi Cui
EPI_ISL_437955	Universitaetsklinik für Innere Medizin II Innsbruck	Bergthaler laboratory, CeMM Research Center for Molecular Medicine of the Austrian Academy of Sciences	Alexander Lercher; Alexandra Popa; Andreas Bergthaler; Benedikt Agerer; Christoph Bock; Dorothee von Laer; Elisabeth Puchhammer-Stoeckl; Guenter Weiss; Henrique Colaco; Jakob-Wendelin Genger; Jan Laine; Judith Aberle; Lukas Endler; Manfred Nairz; Mark Smyth; Martin Senekowitsch; Michael Schuster; Stephan Aberle; Thomas Penz; Wegene Borena
EPI_ISL_665192	University College London Hospital	COVID-19 Genomics UK (COG-UK) Consortium	Catherine Houlihan; Dan Frampton; Judith Heaney; Matthew Byott; Moira Spyer and Eleni Nastouli; Stuart Kirk
EPI_ISL_775977, EPI_ISL_775992, EPI_ISL_776113	University Medical Center Hamburg Eppendorf	Heinrich Pette Institute, Leibniz Institute for Experimental Virology	Adam Grundhoff; Alexis Robitaille; Johannes Knobloch; Martin Aepfelbacher; Nicole Fischer; Thomas Günther
EPI_ISL_447936	University of Birmingham	COVID-19 Genomics UK (COG-UK) Consortium	Alex Richter; Andrew Bosworth; Andrew D Beggs; Celina M Whalley; Charlotte Poxon; Claire McMurray; Husam Osman; Joanne Stockton; Josh Quick; Kasun Wanigasooriya; Mike Kidd; Nicholas Loman; Oliver Pickles; Radoslaw Poplawski; Samuel Nicholls; Will Rowe
EPI_ISL_447888, EPI_ISL_548452	University of California, Davis	Chan-Zuckerberg Biohub	CZB Cliahub Consortium
EPI_ISL_583304, EPI_ISL_590748	University of Michigan Clinical Microbiology Laboratory	Lauring Lab, University of Michigan, Department of Microbiology and Immunology	Valesano
EPI_ISL_417514, EPI_ISL_421315, EPI_ISL_427455, EPI_ISL_428300, EPI_ISL_436572, EPI_ISL_484840, EPI_ISL_484886, EPI_ISL_509835			
see above	University of Wisconsin-Madison AIDS Vaccine Research Laboratories	University of Wisconsin-Madison AIDS Vaccine Research Laboratories	Gage Moreno; Katarina Braun; et al. AIDS Vaccine Research Laboratories
EPI_ISL_450828	Uppsala Narakut Aleris	The Public Health Agency of Sweden	Anna Risberg; Anna-Malin Linde; Annika Nilsson; Karin Tegmark-Wisell; Maria Lind Karlberg; Mia Brytting; Olov Svartstrom; Oskar Karlsson Lindsjo; Theresa Enkirch
EPI_ISL_654953	Uppsala klinisk mikrobiologi	The Public Health Agency of Sweden	Anna Risberg; Anna-Malin Linde; Karin Tegmark-Wisell; Maria Lind Karlberg; Mattias Haukland; Mia Brytting; Olov Svartstrom; Oskar Karlsson Lindsjo; Petra Edquist; Reza Advani; Sandra Broddesson
EPI_ISL_417977, EPI_ISL_430035, EPI_ISL_498632, EPI_ISL_524382, EPI_ISL_648561, EPI_ISL_648573	Utah Public Health Laboratory	Utah Public Health Laboratory	David R. Hillyard; E. Susan Slechta; Erin L. Young; Erin Young; Heidi Butz; Jeffrey B. Stevenson; Kelly Oakeson; Melanie A. Mallory; Michael T. Pyne; Salika M. Shakir; Tara Gallagher
EPI_ISL_549173	Vestfold Hospital, Toensberg Department of Microbiology	Norwegian Institute of Public Health, Department of Virology	Hilde Elshaug; Hilde Synnøve Vollan; Kamilla Heddeland Instefjord; Karoline Bragstad; Kathrine Stene-Johansen; Olav Hungnes; Rasmus Riis Kopperud
EPI_ISL_426677, EPI_ISL_426682, EPI_ISL_426784, EPI_ISL_456454, EPI_ISL_456589	Victorian Infectious Diseases Reference Laboratory (VIDRL)	Microbiological Diagnostic Unit Public Health Laboratory and Victorian Infectious Diseases Reference Laboratory, Doherty Institute	Caly L.; Druce J.; Sait, M.; Schultz M.; Seemann T.; Sherry, N.
EPI_ISL_480662, EPI_ISL_521953	Victorian Infectious Diseases Reference Laboratory (VIDRL)	VIDRL and MDU-PHL	Caly L.; Druce J.; Sait, M.; Schultz M.; Seemann T.; Sherry, N.
EPI_ISL_419753, EPI_ISL_419754, EPI_ISL_419771	Victorian Infectious Diseases Reference Laboratory (VIDRL)	Victorian Infectious Diseases Reference Laboratory and Microbiological Diagnostic Unit Public Health Laboratory, Doherty Institute	Caly L.; Druce J.; Sait, M.; Schultz M.; Seemann T.; Sherry, N.
EPI_ISL_560401	Vilnius University Hospital Santaros Klinikos, Vilnius University	Institute of Biotechnology, Life Sciences Center, Vilnius University and Thermo Fisher Scientific	Albertas Timinskas; Alma Gedvilaite; Aurelija Zvirbliene; Daniel Naumovas; Justinas Slikas; Laimonas Griskevicius; Ligita Jancioriene; Mindaugas Paulauskas
EPI_ISL_451722, EPI_ISL_486535, EPI_ISL_489977, EPI_ISL_511985, EPI_ISL_535645, EPI_ISL_541472	Viollier AG	Department of Biosystems Science and Engineering, ETH Zürich	Christian Beisel; Christiane Beckmann; Christoph Noppen; Elodie Burcklen; Ina Nissen; Ivan Topolsky; Maurice Redondo; Natascha Santacroce; Niko Beerenwinkel; Noemie Santamaria de Souza; Olivier Kobel; Pedro Ferreira; Philipp Jablonski; Sarah Nadeau; Sophie Seidel; Susana Posada-Céspedes; Tanja Stadler; Tobias Schär
EPI_ISL_434710, EPI_ISL_435156, EPI_ISL_437357	Viral Respiratory Lab, National Institute for Biomedical Research (INRB)	Pathogen Sequencing Lab, National Institute for Biomedical Research (INRB)	Adrienne Amuri Aziza; Allison Black; Amuri Aziza; Andrew Rambaut; Catherine Pratt; Eddy Kinganda-Lusamaki; Edith Nkwembe; Francisca Muyembe Mawete; Ian Goodfellow; James Hadfield; Jean-Jacques Muyembe Tamfum; Josh Quick; Kristian Andersen; Matthias Pauthner; Michael Wiley; Nick Loman; Placide Mbala-Kingebeni; Steve Ahuka-Mundeke; Trevor Bedford
EPI_ISL_485853, EPI_ISL_522817,	Virginia DCLS	Virginia DCLS	Virginia DCLS

EPI_ISL_526901			
EPI_ISL_487893, EPI_ISL_488974	Virology Department, Royal Infirmary of Edinburgh, NHS Lothian / School of Biological Sciences, University of Edinburgh	Wellcome Sanger Institute for the COVID-19 Genomics UK (COG-UK) consortium	Colquhoun R; Cordelia Langford; David K. Jackson; Dewar R; Dominic Kwiatkowski; Ewan Harrison; Hill V; Ian Johnston; Jackson B; John Sillitoe on behalf of the Wellcome Sanger Institute COVID-19 Surveillance Team (http://www.sanger.ac.uk/covid-team); McCrone JT; McHugh M; O'Toole Á; Rambaut A; Roberto Amato; Rooke S; Scher E; Sonia Goncalves; Templeton K and Alex Alderton; Yu X
EPI_ISL_425866, EPI_ISL_425870, EPI_ISL_425937, EPI_ISL_433117, EPI_ISL_439237, EPI_ISL_453122, EPI_ISL_473936			
see above	Virology Department, Royal Infirmary of Edinburgh, NHS Lothian / School of Biological Sciences, University of Edinburgh / Institute of Genetics and Molecular Medicine, University of Edinburgh	COVID-19 Genomics UK (COG-UK) Consortium	Balcaza C; Colquhoun R; Dewar R; Gallagher M; Hill V; Jackson B; McCrone JT; McHugh M; O'Toole A; O’Toole Á; O‚ÄôToole √Å; Rambaut A; Rooke S; Scher E; Templeton K; Williams TC; Yu X
EPI_ISL_420171, EPI_ISL_420289	Virology Department, Sheffield Teaching Hospitals NHS Foundation Trust	Department of Infection, Immunity and Cardiovascular Disease, The Florey Institute, The Medical School, University of Sheffield	Adri Angyal; Alex Keeley; Benjamin Lindsey; Cariad Evans; Danielle Groves; Dave Partridge; Luke Green; Matthew Parker; Matthew Wyles; Mehmet Yavuz; Mohammad Raza; Paul Parsons; Rachel Tucker; Rebecca Brown; Thushan de Silva
EPI_ISL_537248	Virology Department, Sheffield Teaching Hospitals NHS Foundation Trust / Department of Infection, Immunity and Cardiovascular Disease, The Medical School, University of Sheffield	Wellcome Sanger Institute for the COVID-19 Genomics UK (COG-UK) consortium	Adri Angyal; Alex Keeley; Benjamin Lindsey; Cariad Evans and Alex Alderton; Cordelia Langford; Danielle Groves; Dave Partridge; David K. Jackson; Dominic Kwiatkowski; Ewan Harrison; Ian Johnston; John Sillitoe on behalf of the Wellcome Sanger Institute COVID-19 Surveillance Team; Luke Green; Matthew Parker; Matthew Wyles; Mehmet Yavuz; Mohammad Raza; Paul Parsons; Rachel Tucker; Rebecca Brown; Roberto Amato; Sonia Goncalves; Thushan de Silva
EPI_ISL_432720	Virology Department, Sheffield Teaching Hospitals NHS Foundation Trust / Virology Department, Sheffield Teaching Hospitals NHS Foundation Trust	COVID-19 Genomics UK (COG-UK) Consortium	Adri Angyal; Alex Keeley; Benjamin Lindsey; Cariad Evans; Danielle Groves; Dave Partridge; Luke Green; Matthew Parker; Matthew Wyles; Mehmet Yavuz; Mohammad Raza; Paul Parsons; Rachel Tucker; Rebecca Brown; Thushan de Silva
EPI_ISL_462000, EPI_ISL_475466, EPI_ISL_490581	Virology Department, Sheffield Teaching Hospitals NHS Foundation Trust/Department of Infection, Immunity and Cardiovascular Disease, The Medical School, University of Sheffield	COVID-19 Genomics UK (COG-UK) Consortium	Adri Angyal; Alex Keeley; Benjamin Lindsey; Cariad Evans; Danielle Groves; Dave Partridge; Katie Johnson; Laura Carrilero; Luke Green; Matthew Parker; Matthew Wyles; Mehmet Yavuz; Mohammad Raza; Nikki Smith; Paul Parsons; Rachel Tucker; Rebecca Brown; Thushan de Silva
EPI_ISL_448864	Virology Laboratory, Castle Hill Hospital, Hull University Teaching Hospitals NHS Trust/Department of Infection, Immunity and Cardiovascular Disease, The Medical School, University of Sheffield	COVID-19 Genomics UK (COG-UK) Consortium	Adri Angyal; Alex Keeley; Benjamin Lindsey; Cariad Evans; Danielle Groves; Dave Partridge; Katie Johnson; Laura Carrilero; Luke Green; Matthew Parker; Matthew Wyles; Mehmet Yavuz; Mohammad Raza; Nikki Smith; Paul Parsons; Rachel Tucker; Rebecca Brown; Thushan de Silva
EPI_ISL_707943	Virology, Universitätsklinikum des Saarlandes	Epigenetics, Saarland University	Jörn Walter; Kathrin Kattler; Markus Vogelgesang; Sascha Tierling; Sigrun Smola; Stefan Lohse
EPI_ISL_427322, EPI_ISL_430084, EPI_ISL_524011, EPI_ISL_733289	WHO National Influenza Centre Russian Federation	WHO National Influenza Centre Russian Federation	Andrey Komissarov; Anna Ivanova; Artem Fadeev; Daria Danilenko; Dmitry Bazhenov; Dmitry Lioznov; Elena Nabieva; Georgii Bazykin; Ksenia Safina; Kseniya Komissarova; Mariia Sergeeva
EPI_ISL_525612	Wadsworth Center, New York State Department of Health	Wadsworth Center, New York State Department of Health	Daryl M. Lamson; Erica Lasek-Nesselquist; Jonathan Plitnick; Kirsten St. George; Matthew D. Shudt; Navjot Singh; Sara Griesemer
EPI_ISL_676821, EPI_ISL_676923, EPI_ISL_676946	Wadsworth Center, New York State Department.of Health	Wadsworth Center, New York State Department.of Health	Alexis Russel; Daryl M. Lamson; Erasmus Schneider; Erica Lasek-Nesselquist; John Kelly; Jonathan Plitnick; Kirsten St. George; Navjot Singh; Sara Griesemer
EPI_ISL_456190	Waikato Hospital	Institute of Environmental Science and Research (ESR)	Anja Werno; Antje van der Linden; Arlo Upton; Chris Mansell; David Hammer; Dragana Drinkovic; Erasmus Smit; Gary McAuliffe; Hana Sofia Andersson; James Ussher; Jill Sherwood; Joep de Ligt; Josh Freeman; Julia Howard; Juliet Elvy; Lauren Jelly; Mary DeAlmeida; Matt Blakiston; Matt Storey; Matthew Rogers; Max Bloomfield; Michael Addidle; Michelle Balm; Sally Roberts; Sarah Jefferies; Sharmini Muttaiyah; Susan Morpeth; Susan Taylor; Timothy Blackmore; Vani Sathyendran; Veronica Playle; Virginia Hope; Xiaoyun Ren
EPI_ISL_415920, EPI_ISL_420985, EPI_ISL_421004, EPI_ISL_421005, EPI_ISL_422024, EPI_ISL_422099, EPI_ISL_422151, EPI_ISL_432425, EPI_ISL_445448, EPI_ISL_445688, EPI_ISL_446423			
see above	Wales Specialist Virology Centre	Public Health Wales Microbiology Cardiff	Alec Birchley; Alexander Adams; Amy Gaskin; Bree Gatica-Wilcox; Catherine Moore; Jason Coombes; Joanne Watkins; Johnathan Evans; Laura Gifford; Lauren Gilbert; Lee Graham; Malorie Perry; Matt Bull; Matthew Bull; Nicole Pacchiarini; Sally Corden; Sara Kumziene-Summerhayes; Sara Rey; Sarah Taylor; Simon Cottrell; Simon Cottrell Sara Rey; Sophie Jones; Tom Connor
EPI_ISL_472747, EPI_ISL_472967, EPI_ISL_474318, EPI_ISL_627207	Wales Specialist Virology Centre Sequencing lab: Pathogen Genomics Unit	COVID-19 Genomics UK (COG-UK) Consortium	Alec Birchley; Alexander Adams; Amy Gaskin; Angela Marchbank; Bree Gatica-Wilcox; Catherine Moore; Jason Coombes; Joanne Watkins; Joel Southgate; Johnathan Evans; Laura Gifford; Lauren Gilbert; Lee Graham; Malorie Perry; Matthew Bull; Nicole Pacchiarini; Sally Corden; Sara Kumziene-Summerhayes; Sara Rey; Sarah Taylor; Simon Cottrell; Sophie Jones; Tom Connor
EPI_ISL_417137, EPI_ISL_434164, EPI_ISL_434241, EPI_ISL_449910, EPI_ISL_463350, EPI_ISL_463530, EPI_ISL_463605, EPI_ISL_495676, EPI_ISL_495757, EPI_ISL_496205, EPI_ISL_497205			
see above	Washington State Department of Health	Seattle Flu Study	Amanda Adler; Barry R. Lutz; Benjamin Pelle; Brian Hiatt; Caitlin R. Wolf; Chris D. Frazar; Chu et al; Chu etl al; Deborah A. Nickerson; Elisabeth Brandstetter; Geoff; Geoff Melly; Helen Y. Chu; Janet A. Englund; Jay Shendure; Jover Lee; Kairsten Fay; Kirsten Lacombe; Lea M. Starita; Mark J. Rieder; Matthew Richardson; Matthew Thompson; Melissa Truong; Michael Boeckh; Michael Famulare; Misja Ilcisin; Peter D. Han; Philip Dykema; Romesh Gautom; Scott Lindquist; Thomas R. Sibley; Trevor Bedford
EPI_ISL_425752, EPI_ISL_425759, EPI_ISL_425762, EPI_ISL_425768, EPI_ISL_425772, EPI_ISL_425797, EPI_ISL_433327, EPI_ISL_477940			
see above	West of Scotland Specialist Virology Centre, NHSGGC / MRC-University of Glasgow Centre for Virus Research	COVID-19 Genomics UK (COG-UK) Consortium	Alasdair MacLean; Alice Broos; Ana da Silva Filipe; Antonia Ho; Daniel Mair; David L Robertson; Elihu Aranday-Cortes; Emma Thomson; James Shepherd; Jenna Nichols; Joseph Hughes; Kathy Li; Kathy Smollett; Kirstyn Brunker; Kyriaki Nomikou; Lily Tong; Marc Niebel; Natasha Jesudason; Natasha Johnson; Patawee Asamaphan; Rajiv Shah; Richard Orton; Rory Gunson; Rory Gunson.; Sarah McDonald; Sreenu Vattipally; Stephen Carmichael; Yasmin Parr
EPI_ISL_422425	Zhejiang Provincial Center for Disease Control and Prevention	Zhejiang Provincial Center for Disease Control and Prevention	Yanjun Zhang; Yi Sun
EPI_ISL_437612	unknown	Faculty of Medicine	Buathong, R.; Bunprakob, S.; Ghai, S.; Joyjinda, Y.; Mungaomklang, A.; Petcharat, S.; Plipat; Prasithsirikul, W.; Rodpan, A.; Sirichan, N.; T. and Hemachudha, T.; Wacharapluesadee, S.
EPI_ISL_507017, EPI_ISL_507034	unknown	Infectious Diseases Research, King Abdullah International Medical Research Center (KAIMRC)	Alghoribi; M.F.
