# Supplementary material for: Genetic Analysis of SARS-CoV-2 Variants in Mexico during the First Year of the COVID-19 Pandemic
Source: Viruses. 2021 Oct 26;13(11):2161. doi: 10.3390/v13112161 (PMC8622467; doi:10.3390/v13112161)
Supplement: Supplementary file 1 [file viruses-13-02161-s001.zip › SupplementaryTable_S4.pdf]

**Table S4. List of nucleotide and amino acid changes of the 3915 Mexican sequences compared to the reference strain Wuhan-Hu-1.**

| Genomic position | Ancestral nucleotide | Mutated nucleotide | ORF | ORF position | gene | Gene position | Type of Substitution | Animo acid replacement | Number of sequences |
|---|---|---|---|---|---|---|---|---|---|
| 745 | c | t | ORF1ab | 480 | nsp1 | 480 | S | | 72 |
| 745 | c | a | ORF1ab | 480 | nsp1 | 480 | NS | N160K | 1 |
| 806 | g | a | ORF1ab | 541 | nsp2 | 1 | NS | A1T | 62 |
| 936 | c | t | ORF1ab | 671 | nsp2 | 131 | NS | T44I | 94 |
| 1059 | c | t | ORF1ab | 794 | nsp2 | 254 | NS | T85I | 368 |
| 1191 | c | a | ORF1ab | 926 | nsp2 | 386 | NS | P129Q | 8 |
| 1191 | c | t | ORF1ab | 926 | nsp2 | 386 | NS | P129L | 41 |
| 1288 | c | t | ORF1ab | 1023 | nsp2 | 483 | S | | 50 |
| 1738 | g | t | ORF1ab | 1473 | nsp2 | 933 | S | | 428 |
| 2395 | c | t | ORF1ab | 2130 | nsp2 | 1590 | S | | 61 |
| 2516 | g | t | ORF1ab | 2251 | nsp2 | 1711 | NS | V571L | 51 |
| 2597 | t | c | ORF1ab | 2332 | nsp2 | 1792 | S | | 57 |
| **3037** | **c** | **t** | **ORF1ab** | **2772** | **nsp3** | **318** | **S** | | **3886** |
| 3037 | c | g | ORF1ab | 2772 | nsp3 | 318 | NS | F106L | 1 |
| **3140** | **c** | **t** | **ORF1ab** | **2875** | **nsp3** | **421** | **NS** | **P141S** | **1534** |
| 3692 | g | t | ORF1ab | 3427 | nsp3 | 973 | NS | V325F | 83 |
| 3745 | t | c | ORF1ab | 3480 | nsp3 | 1026 | S | | 94 |
| 3871 | g | t | ORF1ab | 3606 | nsp3 | 1152 | NS | K384N | 104 |
| 4582 | c | t | ORF1ab | 4317 | nsp3 | 1863 | S | | 256 |
| 5140 | c | a | ORF1ab | 4875 | nsp3 | 2421 | NS | D807E | 44 |
| 5140 | c | t | ORF1ab | 4875 | nsp3 | 2421 | S | | 5 |
| 5183 | c | t | ORF1ab | 4918 | nsp3 | 2464 | NS | P822S | 69 |
| 5999 | a | g | ORF1ab | 5734 | nsp3 | 3280 | NS | I1094V | 55 |
| 6285 | c | t | ORF1ab | 6020 | nsp3 | 3566 | NS | T1189I | 63 |
| 6404 | g | t | ORF1ab | 6139 | nsp3 | 3685 | NS | V1229F | 49 |
| 6985 | a | t | ORF1ab | 6720 | nsp3 | 4266 | S | | 60 |
| 8655 | c | t | ORF1ab | 8390 | nsp4 | 101 | NS | S34F | 40 |
| 8947 | c | t | ORF1ab | 8682 | nsp4 | 393 | S | | 61 |
| 9319 | c | t | ORF1ab | 9054 | nsp4 | 765 | S | | 67 |
| 9738 | g | c | ORF1ab | 9473 | nsp4 | 1184 | NS | S395T | 60 |
| **10029** | **c** | **t** | **ORF1ab** | **9764** | **nsp4** | **1475** | **NS** | **T492I** | **1635** |
| 10319 | c | t | ORF1ab | 10054 | nsp5 | 265 | NS | L89F | 138 |
| 10323 | a | g | ORF1ab | 10058 | nsp5 | 269 | NS | K90R | 109 |
| **10954** | **c** | **t** | **ORF1ab** | **10689** | **nsp5** | **900** | **S** | | **1532** |

| | | | | | | | | | |
|---|---|---|---|---|---|---|---|---|---|
| 11083 | g | t | ORF1ab | 10818 | nsp6 | 111 | NS | L37F | 81 |
| 11083 | g | a | ORF1ab | 10818 | nsp6 | 111 | S | | 2 |
| **11117** | **a** | **g** | **ORF1ab** | **10852** | **nsp6** | **145** | **NS** | **I49V** | **1530** |
| 11365 | g | t | ORF1ab | 11100 | nsp6 | 393 | S | | 72 |
| 11365 | g | a | ORF1ab | 11100 | nsp6 | 393 | S | | 1 |
| 11417 | g | t | ORF1ab | 11152 | nsp6 | 445 | NS | V149F | 40 |
| 11575 | c | t | ORF1ab | 11310 | nsp6 | 603 | S | | 75 |
| 11824 | c | a | ORF1ab | 11559 | nsp6 | 852 | S | | 82 |
| 11824 | c | t | ORF1ab | 11559 | nsp6 | 852 | S | | 45 |
| 11866 | c | t | ORF1ab | 11601 | nsp7 | 25 | S | | 40 |
| 11916 | c | t | ORF1ab | 11651 | nsp7 | 75 | NS | Syn | 57 |
| 12100 | c | t | ORF1ab | 11835 | nsp8 | 9 | S | | 60 |
| 12412 | c | t | ORF1ab | 12147 | nsp8 | 321 | S | | 74 |
| **12789** | **c** | **t** | **ORF1ab** | **12524** | **nsp9** | **104** | **NS** | **T35I** | **1495** |
| 12878 | a | g | ORF1ab | 12613 | nsp9 | 193 | NS | I65V | 59 |
| 13119 | c | t | ORF1ab | 12854 | nsp10 | 95 | NS | A32V | 71 |
| 13498 | a | g | ORF1ab | 31 | nsp12 | 31 | NS | T11A | 62 |
| 13713 | g | a | ORF1ab | 246 | nsp12 | 246 | S | | 60 |
| 14313 | t | c | ORF1ab | 846 | nsp12 | 846 | S | | 72 |
| **14408** | **c** | **t** | **ORF1ab** | **941** | **nsp12** | **941** | **NS** | **P314L** | **3890** |
| 15921 | g | t | ORF1ab | 2454 | nsp12 | 2454 | S | | 65 |
| 16394 | c | t | ORF1ab | 2927 | nsp13 | 158 | NS | P53L | 63 |
| 17014 | g | t | ORF1ab | 3547 | nsp13 | 778 | NS | D260Y | 131 |
| 18424 | a | g | ORF1ab | 4957 | nsp14 | 385 | NS | N129D | 122 |
| 18693 | c | t | ORF1ab | 5226 | nsp14 | 654 | S | | 52 |
| 19011 | c | t | ORF1ab | 5544 | nsp14 | 972 | S | | 5 |
| 19011 | c | a | ORF1ab | 5544 | nsp14 | 972 | NS | D324E | 35 |
| **19839** | **t** | **c** | **ORF1ab** | **6372** | **nsp15** | **219** | **S** | | **2087** |
| 19974 | a | g | ORF1ab | 6507 | nsp15 | 354 | S | | 420 |
| 19974 | a | t | ORF1ab | 6507 | nsp15 | 354 | S | | 1 |
| 20268 | a | g | ORF1ab | 6801 | nsp15 | 648 | S | | 918 |
| 21304 | c | t | ORF1ab | 7837 | nsp16 | 646 | NS | R216C | 114 |
| 21304 | c | a | ORF1ab | 7837 | nsp16 | 646 | NS | R216S | 6 |
| **21306** | **c** | **t** | **ORF1ab** | **7839** | **nsp16** | **648** | **S** | | **1369** |
| 21575 | c | t | S | 13 | | | NS | L5F | 80 |
| 21600 | g | t | S | 38 | | | NS | S13I | 118 |
| 21974 | g | t | S | 412 | | | NS | D138Y | 57 |
| 21974 | g | c | S | 412 | | | NS | D138H | 4 |

| | | | | | | | |
|---|---|---|---|---|---|---|---|
| 22018 | g | t | S | 456 | NS | W152C | 117 |
| 22331 | g | a | S | 769 | NS | G257S | 53 |
| 22917 | t | g | S | 1355 | NS | L452R | 131 |
| **22995** | **c** | **a** | **S** | **1433** | **NS** | **T478K** | **1495** |
| 22995 | c | t | S | 1433 | NS | T478I | 1 |
| **23403** | **a** | **g** | **S** | **1841** | **NS** | **D614G** | **3859** |
| 23587 | g | t | S | 2025 | NS | Q675H | 14 |
| 23587 | g | c | S | 2025 | NS | Q675H | 44 |
| 23593 | g | t | S | 2031 | NS | Q677H | 41 |
| 23593 | g | c | S | 2031 | NS | Q677H | 21 |
| 23593 | g | a | S | 2031 | S | | 1 |
| **23604** | **c** | **a** | **S** | **2042** | **NS** | **P681H** | **1627** |
| 23604 | c | g | S | 2042 | NS | P681R | 24 |
| **23756** | **a** | **g** | **S** | **2194** | **NS** | **T732A** | **1981** |
| 23756 | a | t | S | 2194 | NS | T732S | 14 |
| 24076 | t | c | S | 2514 | S | | 175 |
| 24349 | t | c | S | 2787 | S | | 59 |
| 24904 | c | t | S | 3342 | S | | 41 |
| 25123 | t | c | S | 3561 | S | | 46 |
| 25563 | g | t | ORF3a | 171 | NS | Q57H | 396 |
| 25563 | g | c | ORF3a | 171 | NS | Q57H | 6 |
| 25577 | t | c | ORF3a | 185 | NS | I62T | 53 |
| 25613 | c | t | ORF3a | 221 | NS | S74F | 61 |
| 25782 | c | t | ORF3a | 390 | S | | 54 |
| 25844 | c | t | ORF3a | 452 | NS | T151I | 122 |
| 25906 | g | t | ORF3a | 514 | NS | G172C | 76 |
| 25906 | g | c | ORF3a | 514 | NS | G172R | 1 |
| 25907 | g | t | ORF3a | 515 | NS | G172V | 125 |
| 25907 | g | c | ORF3a | 515 | NS | G172A | 1 |
| 25912 | g | t | ORF3a | 520 | NS | G174C | 138 |
| 26681 | c | t | M | 159 | S | | 164 |
| 27756 | a | g | ORF7a | 363 | S | | 48 |
| 27756 | a | g | ORF7b | 1 | NS | M1V | 48 |
| 27904 | t | c | ORF8 | 11 | NS | L4P | 321 |
| 27904 | t | g | ORF8 | 11 | NS | L4R | 1 |
| 27921 | a | g | ORF8 | 28 | NS | I10V | 95 |
| 27921 | a | t | ORF8 | 28 | NS | I10F | 1 |
| 27964 | c | t | ORF8 | 71 | NS | S24L | 134 |

| | | | | | | | |
|---|---|---|---|---|---|---|---|
| 27987 | g | t | ORF8 | 94 | NS | V32L | 48 |
| 27987 | g | c | ORF8 | 94 | NS | V32L | 3 |
| 27987 | g | a | ORF8 | 94 | NS | V32I | 2 |
| 28001 | g | t | ORF8 | 108 | S | | 106 |
| 28001 | g | c | ORF8 | 108 | S | | 1 |
| 28087 | c | t | ORF8 | 194 | NS | A65V | 127 |
| 28253 | c | t | ORF8 | 360 | S | | 138 |
| 28253 | c | g | ORF8 | 360 | NS | F120L | 2 |
| 28378 | g | t | N | 105 | S | | 66 |
| 28378 | g | a | N | 105 | S | | 2 |
| 28378 | g | c | N | 105 | S | | 1 |
| 28472 | c | t | N | 199 | NS | P67S | 125 |
| 28854 | c | t | N | 581 | NS | S194L | 759 |
| 28869 | c | t | N | 596 | NS | P199L | 123 |
| **28881** | **g** | **a** | **N** | **608** | **NS** | **R203K** | **2348** |
| 28881 | g | t | N | 608 | NS | R203M | 4 |
| **28882** | **g** | **a** | **N** | **609** | **S** | | **2348** |
| **28883** | **g** | **c** | **N** | **610** | **NS** | **G204R** | **2346** |
| 28887 | c | t | N | 614 | NS | T205I | 160 |
| 28975 | g | t | N | 702 | NS | M234I | 109 |
| 28975 | g | a | N | 702 | NS | M234I | 1 |
| **29197** | **c** | **t** | **N** | **924** | **S** | | **1531** |
| 29227 | g | t | N | 954 | S | | 90 |
| 29317 | t | c | N | 1044 | S | | 61 |
| 29362 | c | t | N | 1089 | S | | 96 |
| 29402 | g | t | N | 1129 | NS | D377Y | 41 |
| 29422 | g | t | N | 1149 | S | | 44 |
| 29422 | g | a | N | 1149 | S | | 1 |
| 29527 | g | t | N | 1254 | NS | Q418H | 198 |
| 29527 | g | a | N | 1254 | S | | 4 |
| 29710 | t | c | 3UTR | 36 | Non-coding | | 40 |
| 29710 | t | a | 3UTR | 36 | Non-coding | | 1 |
| 29751 | g | t | 3UTR | 77 | Non-coding | | 39 |
| 29751 | g | c | 3UTR | 77 | Non-coding | | 15 |
| 29751 | g | a | 3UTR | 77 | Non-coding | | 1 |
