# Supplementary material for: Genetic Analysis of SARS-CoV-2 Variants in Mexico during the First Year of the COVID-19 Pandemic
Source: Viruses. 2021 Oct 26;13(11):2161. doi: 10.3390/v13112161 (PMC8622467; doi:10.3390/v13112161)
Supplement: Supplementary file 1 [file viruses-13-02161-s001.zip › SupplementaryTable_S5.pdf]

Table S5. List of variants that appears one or two times throughout the year in Mexico.

Lineage	# of global seq	Prevalence since identification	Number of Mexican sequences															
			Aguascalientes	Baja California	Baja California Sur	Campeche	Chiapas	Chihuahua	Coahuila	Colima	Durango	Guanajuato	Guerrero	Hidalgo	Jalisco	Mexico City	Michoacan	Morelos
A.2	1,486	<0.5%														1		
A.2.5	883	<0.5%																
A.3	417	<0.5%																
B.1.1.10	834	<0.5%														1		
B.1.1.128	109	<0.5%		1														
B.1.1.133	105	<0.5%																2
B.1.1.198	653	<0.5%																
B.1.1.205	189	<0.5%																
B.1.1.207	671	<0.5%																
B.1.1.285	138	<0.5%		1														
B.1.1.329	186	<0.5%																
B.1.1.334	55	<0.5%			1													
B.1.1.362	134	<0.5%						2										
B.1.1.403	28	<0.5%		1														
B.1.1.434	2,231	<0.5%																
B.1.1.453	110	<0.5%														1		
B.1.1.70	2,567	<0.5%														1		
B.1.1.71	160	<0.5%										1						
B.1.1.8	276	<0.5%		2														
B.1.1.93	34	<0.5%		1														
B.1.105	247	<0.5%																
B.1.111	741	<0.5%																
B.1.126	506	<0.5%										1						
B.1.128	1,341	<0.5%										1						
B.1.160	23,361	1%																
B.1.201	170	<0.5%														1		
B.1.229	85	<0.5%		1										1				
B.1.258.21	123	<0.5%														1		
B.1.267	49	<0.5%		1														
B.1.301	209	<0.5%											1					
B.1.319	296	<0.5%														1		
B.1.324	480	<0.5%		1														
B.1.336	312	<0.5%		1														

B.1.36.31	519	<0.5%								1
B.1.375	959	<0.5%		1						1
B.1.402	115	<0.5%								
B.1.405	54	<0.5%								
B.1.415	78	<0.5%								1
B.1.487	43	<0.5%						1		
B.1.503	260	<0.5%								
B.1.556	119	<0.5%		1						
B.1.564	1,983	<0.5%								1
B.1.565	946	<0.5%			2					
B.1.567	244	<0.5%					1			
B.1.576	697	<0.5%								
B.1.577	1,521	<0.5%		1						
B.1.578	37	<0.5%				1				
B.1.580	75	<0.5%	1							
B.1.582	1,435	<0.5%							1	
B.1.588	1,061	<0.5%								
B.1.612	615	<0.5%		1						
B.1.94	34	<0.5%							1	
D.2	12,271	1%								

Lineage	# of global seq	Prevalence since identification	Number of Mexican sequences															
			Nayarit	Nuevo Leon	Oaxaca	Puebla	Queretaro	Quintana Roo	San Luis Potosi	Sinaloa	Sonora	State of Mexico	Tabasco	Tamaulipas	Tlaxcala	Veracruz	Yucatan	Zacatecas
A.2	1486	<0.5%		1														
A.2.5	883	<0.5%		1														
A.3	417	<0.5%																1
B.1.1.10	834	<0.5%																
B.1.1.128	109	<0.5%																
B.1.1.133	105	<0.5%																
B.1.1.198	653	<0.5%			1													
B.1.1.205	189	<0.5%								1								
B.1.1.207	671	<0.5%		1														
B.1.1.285	138	<0.5%																
B.1.1.329	186	<0.5%		1				1										
B.1.1.334	55	<0.5%																
B.1.1.362	134	<0.5%																
B.1.1.403	28	<0.5%																
B.1.1.434	2231	<0.5%		1														
B.1.1.453	110	<0.5%																
B.1.1.70	2567	<0.5%																
B.1.1.71	160	<0.5%																
B.1.1.8	276	<0.5%																
B.1.1.93	34	<0.5%																
B.1.105	247	<0.5%									1							
B.1.111	741	<0.5%					2											
B.1.126	506	<0.5%	1															
B.1.128	1341	<0.5%																
B.1.160	23361	0.01						1										
B.1.201	170	<0.5%																
B.1.229	85	<0.5%																
B.1.258.21	123	<0.5%																
B.1.267	49	<0.5%																
B.1.301	209	<0.5%																
B.1.319	296	<0.5%																
B.1.324	480	<0.5%																
B.1.336	312	<0.5%																

B.1.36.31	519	<0.5%					
B.1.375	959	<0.5%					
B.1.402	115	<0.5%					1
B.1.405	54	<0.5%		1			
B.1.415	78	<0.5%				1	
B.1.487	43	<0.5%					
B.1.503	260	<0.5%			1		
B.1.556	119	<0.5%					
B.1.564	1983	<0.5%	1				
B.1.565	946	<0.5%					
B.1.567	244	<0.5%					
B.1.576	697	<0.5%	1				
B.1.577	1521	<0.5%					
B.1.578	37	<0.5%					
B.1.580	75	<0.5%					
B.1.582	1435	<0.5%					
B.1.588	1061	<0.5%	1				
B.1.612	615	<0.5%					
B.1.94	34	<0.5%					
D.2	12,271	1%	1				
