# Supplementary material for: Genetic Analysis of SARS-CoV-2 Variants in Mexico during the First Year of the COVID-19 Pandemic
Source: Viruses. 2021 Oct 26;13(11):2161. doi: 10.3390/v13112161 (PMC8622467; doi:10.3390/v13112161)
Supplement: Supplementary file 1 [file viruses-13-02161-s001.zip › SupplementaryTable_S6.pdf]

Table S6. Probational density in each federal state of Mexico

State	Poblation	Persons/km2	Region id
AGU	1,425,607	254	CN
QUE	2,368,467	203	CN
GUA	6,166,934	201	CN
SLP	2,822,255	46	CN
ZAC	1,622,138	22	CN
CMX	9,209,944	6163	CS
MEX	16,992,418	760	CS
MOR	1,971,520	404	CS
TLA	1,342,977	336	CS
PUE	6,583,278	192	CS
HID	3,082,841	148	CS
NLE	5,784,442	90	NE
TAM	3,527,735	44	NE
COA	3,146,771	21	NE
BCN	3,769,020	53	NW
SIN	3,026,943	53	NW
SON	2,944,840	16	NW
CHH	3,741,869	15	NW
DUR	1,832,650	15	NW
BCS	798,477	11	NW
VER	8,062,579	112	S
TAB	2,402,598	97	S
CHP	5,217,908	76	S
YUC	2,320,898	59	S
GRO	3,540,685	56	S
OAX	4,131,148	44	S
ROO	1,857,985	42	S
CAM	928,363	16	S
JAL	8,348,151	148	WE
COL	731,391	130	WE
MIC	4,748,846	81	WE
NAY	1,235,456	44	WE
